# Expanded diversity of pedinophytes provides a window into the evolution of the genetic code in organelles

**DOI:** 10.1371/journal.pgen.1011901

**Published:** 2025-10-22

**Authors:** Dovilė Barcytė, David Žihala, Marek Eliáš

**Affiliations:** 1 Department of Biology and Ecology, Faculty of Science, University of Ostrava, Ostrava, Czech Republic; 2 Department of Hematooncology, Faculty of Medicine, University of Ostrava, Ostrava, Czech Republic; University College Dublin, IRELAND

## Abstract

Mitochondria and plastids of various lineages exhibit genetic code alterations. However, the knowledge of the diversity and occurrence, mechanistic underpinnings, and evolutionary origins of codon reassignments in organelles remains incomplete. To address this gap, we focused on organelles of the neglected green algal class Pedinophyceae, as well as pedinophyte-derived secondary plastids of green-coloured dinoflagellates (peDinoflagellates). We isolated and characterized a novel pedinophyte, herein formally described as *Oistococcus okinawensis* gen. et sp. nov., and phenotypically documented the previously sequenced but morphologically uncharacterized strain YPF-701, herein described as *Akinorimonas japonica* gen. et sp. nov. Based on phylogenetic analyses, both new taxa were classified into the expanded family Resultomonadaceae. We sequenced the organellar genomes of *O. okinawensis*, and utilizing existing raw (meta)genomic data we assembled organellar genome sequences from other previously unexplored pedinophyte lineages. Bioinformatic analyses of the expanded set of pedinophyte organellar genomes painted a complex picture of their genetic code landscape. Concerning mitochondria, the stop-to-Trp reassignment of the UGA codon turned out to have evolved multiple times in pedinophytes, and the Arg-to-Ala reassignment of AGA/AGG codons was shown to be apomorphic for the whole order Marsupiomonadales. The latter has additionally converted UUA and UUG into termination codons, relying on specific mutations in the mtRF1a protein. All pedinophyte mitochondria seem to decode AUA as methionine rather than the standard isoleucine, and an analogous reassignment seems to be evolving also in plastids of two separate pedinophyte lineages. Finally, apart from the previously reported Ile-to-Met AUA reassignment, peDinoflagellate plastids have switched the meaning of the AGA/AGG codons from arginine to another amino acid (most likely alanine), and have modified their pRF2 protein to mediate translation termination at UUA/UCA codons. Pedinophyte(-derived) organelles present a broad spectrum of codon reassignments and provide important insights into the emergence and mechanisms of non-standard codon translation.

## Author summary

The genetic code is a highly conserved attribute of life, but the list of exceptions from the so-called standard genetic code is ever spreading to embrace additional branches of the tree of life and new forms of departures from the standard. The richest source of such exceptions are mitochondria, whereas plastids, despite their analogous origin from an endosymbiotic (cyano)bacterium, are much more modest in this regard. Here, we enrich the landscape of non-standard genetic codes by focusing on organelles of the class Pedinophyceae, a poorly know group of green algae. We describe new members of this group and expand the database of pedinophyte organellar genome sequences. By analysing these new resources, we uncover hitherto unnoticed changes in the meaning of particular codons (i.e., codon reassignments) in pedinophyte mitochondria and define the underlying molecular mechanisms. We also point to an incipient genetic code change in plastids of certain pedinophytes. Most strikingly, we demonstrate that a unique evolutionary offshoot of pedinophyte plastids, persisting as a secondary plastid in several peculiar dinoflagellates, has adopted a combination of multiple codon reassignments including a type so far known only from mitochondria, namely codons normally encoding amino acids transformed into a signal for translation termination.

## Introduction

The class Pedinophyceae is a small group of green algae within the phylum Chlorophyta. The concept of pedinophytes as a group separate from other “primitive” or early-diverging chlorophytes (i.e., “prasinophytes”) emerged from detailed ultrastructural investigations focusing on the flagellar apparatus, mitosis, and cytokinesis [1]. The class currently accommodates five genera of single-celled wall-less uniflagellate organisms classified into two orders: Pedinomonadales and Marsupiomonadales [2]. The Pedinomonadales includes the genera *Pedinomonas* and *Chlorochytridion* (both belonging to the family Pedinomonadaceae) thriving in freshwater and soil environments. In contrast, the order Marsupiomonadales embraces marine algae and contains three presently monotypic genera, *Marsupiomonas* and *Protoeuglena* grouped in the family Marsupiomonadaceae, and *Resultomonas* (originally described as *Resultor* [1]) representing the family Resultomonadaceae. Most pedinophytes are free-living, except for *Protoeuglena*, which primarily thrives as an endosymbiont of the heterotrophic dinoflagellate *Noctiluca scintillans* [3,4].

Notably, a polyphyletic assemblage of other dinoflagellates (*Lepidodinium* spp. and so-called TGD and MGD strains), collectively termed as peDinoflagellates, have incorporated pedinophytes as permanent plastids within their cells [5–7]. These secondary plastids exhibit a varying degree of reduction, with those in TGD and MGD strains having retained a residual green algal nucleus (nucleomorph), which seems to have been lost in *Lepidodinium* [8]. Interestingly, plastid phylogenomics showed that these plastids form a monophyletic group, representing a lineage more closely related to the freshwater/terrestrial Pedinomonadales than to their marine cousins Marsupiomonadales [7]. Most recently, a lineage of plastid genomes recovered from freshwater metagenomes and forming the closest known sister group to peDinoflagellate plastids was reported in a preprint [9]. Indeed, there is ample molecular evidence for the existence of pedinophyte lineages awaiting morphological investigation and formal description [2,3]. One such lineage includes the organism referred to as Pedinophyceae sp. YPF-701, for which nuclear and plastid genome sequences have been reported [6,10].

While the pedinophyte diversity remains poorly explored, the group as such has attracted interest regarding its evolutionary position among green algae. The initial phylogenetic analyses of combined plastid genome-encoded proteins surprisingly placed Pedinophyceae as a sister group of Chlorellales, disrupting thus the monophyly of the class Trebouxiophyceae [11,12]. However, this result was subsequently recognized as a phylogenetic artefact [13], and nuclear gene phylogenies strongly support Pedinophyceae as a basal lineage of the so-called core chlorophytes additionally comprised of Chlorophyceae, Trebouxiophyceae (including Chlorellales), Ulvophyceae, Bryopsidophyceae (a separate lineage not directly related to the remaining traditional ulvophytes), and Chlorodendrophyceae [2,10,14–16].

Apart from their pivotal position in the phylogenetic tree of green algae, Pedinophyceae also stand out for their unique genomic features. For example, when it comes to both the nuclear genome size and the number of genes, Pedinophyceae sp. YPF-701 occupies an intermediate position between those of most “prasinophytes” and the rest of the core Chlorophyta [10]. Meanwhile, mitochondrial genomes (mitogenomes) show various codon reassignments. Specifically, UGA normally serving as a stop codon is decoded as tryptophan in *Pedinomonas minor* UTEX LB 1350 [17], and the arginine codons AGA and AGG were suggested to be instead decoded as alanine in *Marsupiomonas* sp. NIES-1824 [18]. Whether or not these deviations from the standard genetic code extend to other pedinophyte mitogenomes has yet to be tested. It is also worth noting that the isoleucine codon AUA is reassigned to methionine in peDinoflagellate plastid genomes (plastomes) [7,19], while this departure was not reported to be shared by other fully sequenced plastomes of pedinophytes themselves [11,20]. Finally, pedinophyte organellar genomes are generally intron-free, unlike those of other core chlorophytes [21].

As part of our long-term effort to explore uncharted areas of protist diversity, we isolated a new marine alga, denoted “Okinawa strain”, which turned out to represent the class Pedinophyceae in preliminary phylogenetic analyses. This prompted us to conceive a broader study of this neglected group, focusing on their organellar genomes. Here we report on a comparative analysis of pedinophyte plastomes and mitogenomes, including the newly sequenced ones from Okinawa strain and assembled from (meta)genomic data for other representatives. This expanded dataset not only improved our understanding of the pedinophyte phylogeny, but also provided a detailed picture of organellar genome evolution in this group. Of particular importance are the newly uncovered changes in the genetic code in pedinophyte organelles, which offer valuable insights into the fundamental principles and general models of the genetic code evolution. Together with the formal description of new taxa, including two new genera and species, our study makes a major step forward in the exploration of algal biology.

## Results

### Morphological observations of the studied strains

**Fig 1:**
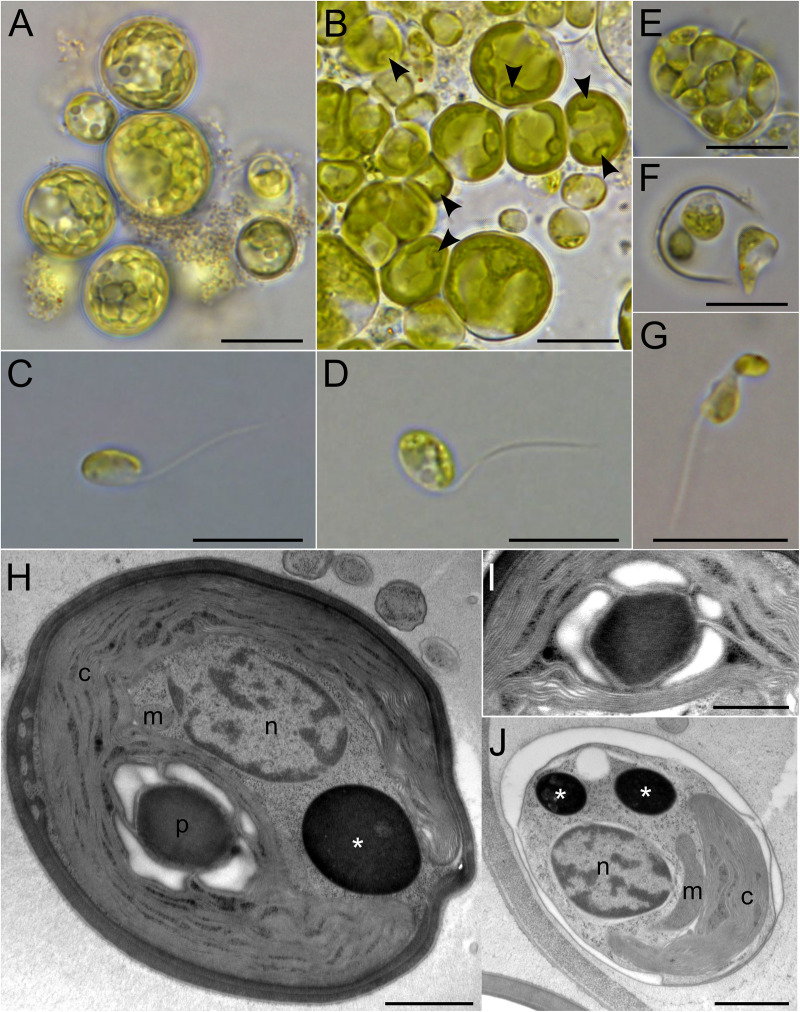
Morphology (A–G) and ultrastructure (H–J) of Okinawa strain, described as *Oistococcus okinawensis* gen. et sp. nov. (A) Ball-shaped cells grown in F/2 medium using NaNO_3_ as the nitrogen source. (B) Ball-shaped cells in F/2 medium using NH_4_Cl as the nitrogen source, demonstrating prominent pyrenoids (arrowheads). (C, D) Flagellate cells. (E) Sporangium. (F) Freshly released cells, amoeboid in shape. (G) Fusion of two cells. (H) TEM micrograph of a walled cell containing a cup-shaped chloroplast (c) with a pyrenoid (p), a single nucleus (n) and a mitochondrion (m), as well as an electron-dense body (asterisk), potentially corresponding to a lipid droplet. (I) A close-up of the pyrenoid covered by starch plates. (J) Naked cell produced within the walled sporangium. Scale bars: 10 µm (A–G), 1000 nm (G, J), 500 nm (I).

The morphological and ultrastructural characteristics of the newly established Okinawa strain were rather unlike those of the previously studied pedinophytes (Fig 1). Cultures grown in F/2-NaNO_3_ medium were dominated by ball-like cells, measuring 6.0–27.0 µm in diameter (Fig 1A). Only when transferred to fresh medium did flagellate cells emerge. However, they swam only for a very short period of time, making their documentation challenging. We propose that the “ball cells” represent a transitional stage between the flagellate and sporangium forms, potentially forming under unfavorable conditions as a survival strategy. Indeed, they contained a rather thick cell wall in contrast to the naked flagellates. Similar immotile cells with clear pyrenoids as present in Okinawa strain (Fig 1B) were considered to be young sporangia in the pedinophyte *Chlorochytridion tuberculatum* [22]. The strain appeared to favour F/2-NH_4_Cl medium with flagellates constantly emerging even for several weeks. They had an oval or disc-like shape, being 4.0–8.0 µm long and 2.5–6.5 µm wide (Fig 1C and 1D). However, upon release from the sporangium (Fig 1E), the swimmers initially adopted an amoeboid shape (Fig 1F), and remained intermingled for a while. Each cell had a single flagellum, approximately twice the length of the cell body (Fig 1C and 1D). When resting, the flagellum folded backwards enclosing one side of the cell, a characteristic feature of pedinophytes [1]. Additionally, a flagellar groove, also typical for this class, was observed (Fig 1C). While we did not see the typical jumping motion associated with *Resultomonas* [1], the flagellum did extend and then bend backwards. The flagellate cells contained a single crescent-like plastid without a clearly visible pyrenoid (Fig 1C and 1D). The plastid narrowed in the centre, creating the illusion of two separate structures (Fig 1D). The pyrenoid became apparent only in settled cells (Fig 1B). A tiny eyespot was occasionally seen in the lateral middle of the plastid of Okinawa strain swimmers (Fig 1F). A single vacuole was also observed in some cells (Fig 1D). Our observations revealed at least 12 zoospores within mature sporangia, with the possibility of even higher numbers (Fig 1E). Some of the observed sporangia might also function as gametangia, as we did observe the fusion of two differently sized cells (which were also slightly smaller than the typical swimmers), suggesting the presence of sexual reproduction (Fig 1G). After the fusion, the immotile ball-like cell was formed. Transmission electron microscopy (TEM) confirmed the presence of a single plastid with a pyrenoid covered by starch plates (Fig 1H and 1I). Furthermore, only a single mitochondrial profile was observed per cell, along with a single or several electron-dense bodies, potentially corresponding to lipid droplets (Fig 1H and 1J). Finally, TEM unambiguously revealed that both walled and naked cells were produced within the walled mother cell (Fig 1J).

**Fig 2:**
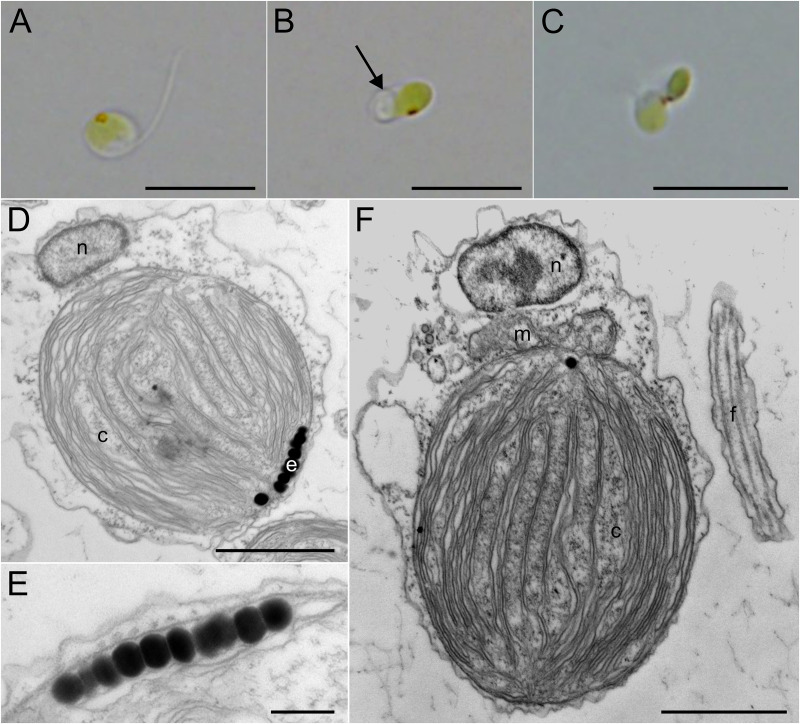
Morphology (A–C) and ultrastructure (D–F) of Pedinophyceae sp. NIES-2566, described as *Akinorimonas japonica* gen. et sp. nov. (A) A cell demonstrating the main features of the organism, including its ovoid shape, a single flagellum, and a single chloroplast with a prominent eyespot. (B) A coiled flagellum (arrow) resting on the cell surface. (C) Fusion of two unequally sized cells. (D) TEM micrograph showing a uninucleate (n) wall-less cell containing a chloroplast (c) with an eyespot (e). (E) A close-up of the eyespot, composed of a single layer of globules. (F) A detailed view of the cell and its flagellum (f). A mitochondrion (m), positioned between the nucleus and the chloroplast. Scale bars: 10 µm (A–C), 1000 nm (D, F), 200 nm (E).

Our phylogenetic analyses reported below indicate that the alga known as Pedinophyceae sp. YPF-701, which curiously was also isolated from a marine sand sample in Japan (Isonoura, Wakayama) is related to Okinawa strain. Strikingly, it has never been documented at the morphological level in the literature despite previous genomic studies of this alga [6,10]. Hence, we here fill in this omission (Fig 2). In contrast to Okinawa strain, Pedinophyceae sp. YPF-701 (= NIES-2566) appeared to favour the F/2-NaNO_3_ medium by demonstrating faster growth, and the strain did not form conspicuous ball cells in any of the two media used. The cells demonstrated a flattened round to ellipsoid shape, measuring 4.0–6.5 µm in length and 2.0–4.5 µm in width. The single, sublaterally emerging flagellum was approximately of the same length as the cell or slightly longer (Fig 2A). It tended either to coil and rest at the same cell end where it originated (Fig 2B), or to be thrown backward around the resting cell, as seen in other pedinophytes. During swimming, the flagellum trailed backwards. Each cell contained a single plate-like chloroplast with a conspicuous eyespot (Fig 2A–2C). The eyespot was composed of a single layer of closely-packed, electron-dense granules (Fig 2D and 2E). No pyrenoid was observed, even under TEM (Fig 2D and 2F). The absence of a typical green algal cell wall was also confirmed. TEM revealed only a single mitochondrial profile, consistently located between the nucleus and the chloroplast (Fig 2F). The strain reproduced by binary fission, and sexual reproduction was also observed. The latter occurred through the fusion of two differently sized cells (Fig 2C). The merged cells swam for some time before settling down.

The morphological uniqueness of Okinawa strain and Pedinophyceae sp. YPF-701, together with the results of molecular phylogenetic analyses described in the subsequent section, lead us to recognize both algae as new taxa at the genus and species level. Below we provide their formal description as *Oistococcus okinawensis* gen. et sp. nov. and *Akinorimonas japonica* gen. et sp. nov., respectively. For the sake of simplicity, we use the newly proposed taxonomic names (*O. okinawensis* and *A. japonica*) throughout the rest of the Results section.

### Updated phylogenetic scheme of Pedinophyceae

To examine the phylogenetic position of *O. okinawensis*, we used Illumina sequencing to obtain a draft genome assembly for this organism, including complete organellar genome sequences. In addition, as detailed in Material and Methods below, we supplemented our phylogenetic analyses by pedinophyte sequences obtained by exploitation of existing sequencing data. Briefly, we obtained the previously unavailable mitogenome sequence from *A. japonica*. Furthermore, we identified metagenomic samples containing sequences from a close relative of *Resultomonas moestrupii*, the sole *Resultomonas* species earlier referred to as *Resultor mikron* [1], for which only sequences of the plastidial rRNA operon and the *rbcL* gene have been reported [2,23]. By assembling these metagenomic reads we obtained complete organellar sequences from this uncultivated organism, referred here to as *Resultomonas* sp. Cadiz, together with organellar genome sequences from another pedinophyte denoted Marsupiomonadaceae sp. Cadiz. We also annotated and included in the analyses the mitogenome and plastome sequences of *Protoeuglena noctilucae*, which were released as part of the Aquatic Symbiosis Genomics Project [24]. We could not include in our study the metagenome-derived pedinophyte plastome sequences reported by Shrestha et al. [9], as most of our analyses had been finished before the respective preprint was released, and the plastome assemblies described in it had not been accessible at the time we submitted our paper for publication. Nevertheless, to improve the representation of mitogenomes from the order Pedinomonadales we searched the metagenome assembly IMGM3300027621, which yielded one of the Pedinomonadales plastomes (IMGM3300027621_BIN154) reported by Shrestha et al. [9] to be phylogenetically close to peDinoflagellate plastomes, and obtained a partial mitogenome sequence that very likely belongs to the same organism (referred to as Pedinomonadales sp. IMGM3300027621). For logistical reasons, we could include this mitogenome in only some of the analyses reported below.

**Fig 3:**
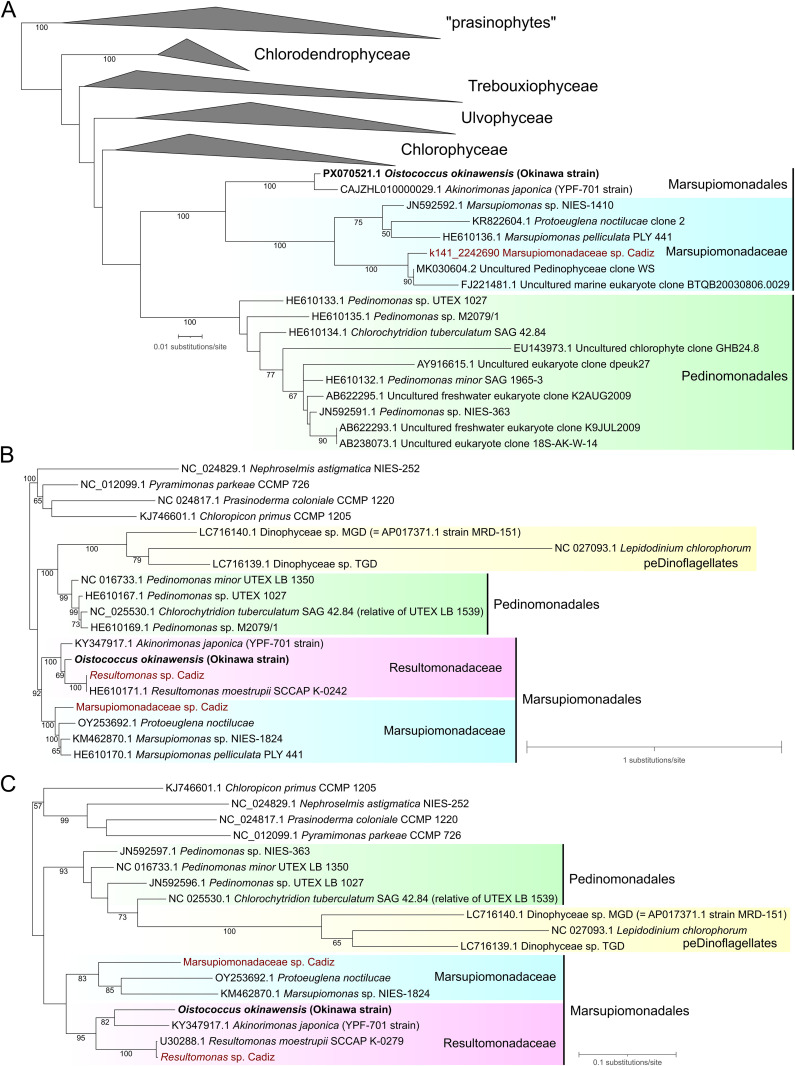
Single gene/operon phylogenetic analyses showing phylogenetic placement and closest relatives of the newly characterized Okinawa strain (*O. okinawensis*). (A) Maximum likelihood phylogenetic tree inferred from nuclear 18S rRNA gene sequences. The alignment used for the tree inference consisted of 1,800 aligned nucleotide positions, the substitution model employed was TIM3e+I + R4. Except for Pedinophyceae, other major chlorophyte clades are collapsed as triangles for simplicity. A full version of the tree is shown in S1 Fig. (B) Maximum likelihood phylogenetic tree inferred from plastid rRNA operon sequences. The alignment used for the tree inference consisted of 4,345 aligned nucleotide positions, the substitution model employed was GTR + F + R4. (C) Maximum likelihood phylogenetic tree inferred from plastid *rbcL* sequences. The alignment used for the tree inference consisted of 1,428 aligned nucleotide positions, the substitution model employed was TIM2 + F + G4 for the first, JC + I + G4 for the second, and GTR + F + I + G4 for the third codon positions. Newly obtained sequences from *O. okinawensis* are shown in bold, while those assembled metagenomically are marked in burgundy.

We investigated the phylogeny of pedinophytes using different molecular markers or their combinations. In the tree inferred from a selection of 18S rDNA sequences, including representatives of major Chlorophyta lineages with a nearly exhaustive sampling of pedinophytes, *O. okinawensis* was affiliated with full support to *A. japonica* and together with it made one of the two principal branches of a fully supported clade that we equate with the order Marsupiomonadales (Figs 3A and S1). Only one 18S rDNA sequence in our “Cadiz” metagenomic co-assembly was identified to have a pedinophyte affinity, branching closest to two other environmental DNA sequences: “Uncultured Pedinophyceae clone WS” from the subarctic White Sea [25] and “Uncultured marine eukaryote clone BTQB20030806.0029” from Quantuck bay, Long Island, USA. Together, these sequences formed a clade sister to cultivated Marsupiomonadaceae representatives. Based on this, we consider the sequence to represent the uncultivated Marsupiomonadaceae sp. Cadiz (see above), whereas the expected 18S rDNA sequence from *Resultomonas* sp. Cadiz has not been assembled. The class Pedinophyceae (Marsupiomonadales plus Pedinomonadales) was monophyletic in the tree, yet without statistical support; a result seen also in previous 18S rDNA-based phylogenies (e.g., [2]).

Parallel phylogenetic analyses based on the plastid rRNA operon and *rbcL* sequences corroborated the close relationship between *O. okinawensis* and *A. japonica*, but in these trees the two strains were additionally joined by sequences from the genus *Resultomonas* (absent in the 18S rDNA tree due to lack of data), including an authentic strain of *R. moestrupii* and the uncultivated *Resultomonas* sp. Cadiz (Fig 3B and 3C). While this broader clade was strongly supported in both trees (bootstrap values 100% and 95%, respectively), the branching order of *O. okinawensis*, *A. japonica*, and *Resultomonas* spp. differed between them, with the latter being either sister to *O. okinawensis* alone or to *O. okinawensis* and *A. japonica* combined. These alternative topologies received only moderate statistical support. Despite these discrepancies, both analyses indicate that *O. okinawensis* and the previously unassigned *A. japonica* are best classified as members of the family Resultomonadaceae.

Further insights into the pedinophyte phylogeny were obtained by employing multigene (“phylogenomic”) analyses, using concatenated protein sequences encoded by the plastid and mitochondrial genomes. For the plastome-based phylogenomic analysis we utilized 79 genes (18,812 amino acid positions in the final supermatrix) and included a broad selection of green algal taxa with a comprehensive sampling of available pedinophyte plastomes. However, to avoid their possible negative impact on the phylogenetic inference we excluded plastomes of peDinoflagellates, which were previously placed as a highly divergent sister group of Pedinomonadales [7]. As a result, we here present the first phylogenomic analysis including all formally recognized genera of pedinophytes. In addition, we included *Scourfieldia* sp. M0560/2, utilizing plastome-derived transcript sequences extracted from an existing transcriptome assembly [14]. This organism represents (nominally; no details justifying the taxonomic identification of the strain have been reported) the order Scourfieldiales, which in AlgaeBase (https://www.algaebase.org/browse/taxonomy/detail/?taxonid=90174 [26]) as well as the NCBI Taxonomic Database (https://www.ncbi.nlm.nih.gov/taxonomy, txid35430) is classified along with Pedinomonadales and Marsupiomonadales in the class Pedinophyceae, although we could not find an explicit proposal for such a placement in the literature.

**Fig 4:**
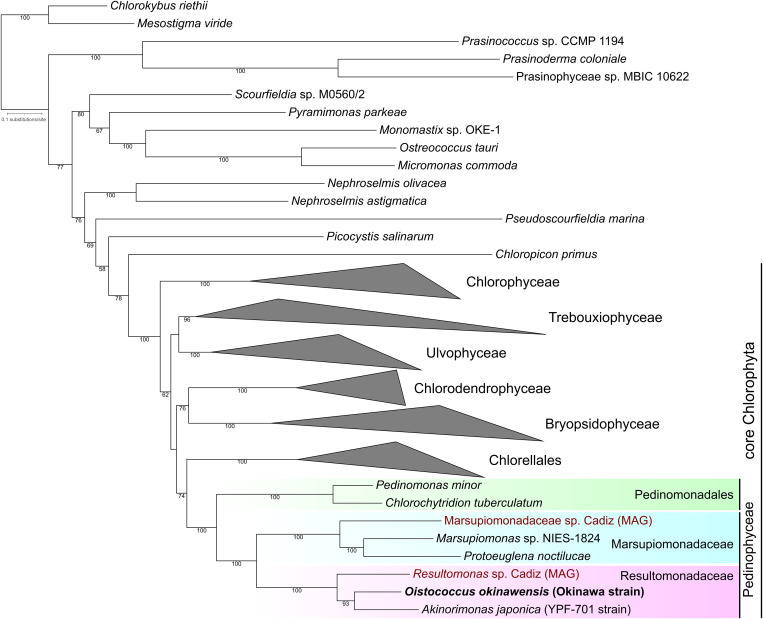
Maximum likelihood phylogenetic tree inferred from a concatenated dataset of 79 plastome-encoded proteins from major lineages of Chlorophyta. The alignment used for the tree inference consisted of 18,812 amino acid positions, the substitution model employed was Q.yeast+I + G4. Major chlorophyte non-pedinophyte clades are collapsed as triangles (a full version of the tree is available in S2 Fig). The newly obtained sequence from *O. okinawensis* is shown in bold, while those assembled metagenomically are marked in burgundy.

The resulting tree (Figs 4 and S2) was generally congruent with previous plastome-based phylogenies of green algae (e.g., [20,27]). Similar to the *rbcL* gene phylogeny, the 79-gene analysis grouped *O. okinawensis* and *A. japonica* as sister taxa, further joined by *Resultomonas* sp. Cadiz. Within the monophyletic Marsupiomonadaceae, *Prot. noctilucae* and *Marsupiomonas* sp. NIES-1824 clustered together with full support and Marsupiomonadaceae sp. Cadiz constituted a lineage sister to them (Figs 4 and S2). Marsupiomonadales and Pedinomonadales were each monophyletic and sister to each other, with all these branches fully supported. The pedinophyte clade was recovered as a sister group to the order Chlorellales, but this (only moderately supported) relationship, which disrupts the monophyly of Trebouxiophyceae, is a well-documented artefact of green algal plastome-based phylogenomics [13]. No support for the inclusion of *Scourfieldia* sp. M0560/2 into Pedinophyceae was found in our analysis. The former alga formed a separate branch outside the core Chlorophyta clade, with moderate support united with representatives of Mamiellophyceae and Pyramimonadophyceae; a result congruent with the phylogenetic position of *Scourfieldia* sp. M0560/2 as previously inferred from a supermatrix of nucleus-encoded proteins [14].

In the previously published mitogenome-based phylogenomic analysis, *Ped. minor* UTEX LB 1350 and *Marsupiomonas* sp. NIES-1824 (the only pedinophytes included) grouped together as expected [18]. However, their sisterhood was only weakly supported. Additionally, these organisms showed extremely long branches compared to other major green algal lineages, pointing to a high evolutionary divergence of their mitogenome sequences. Our taxonomically expanded mitogenome-based phylogenomic analysis utilizing 16 mitogenome-encoded proteins (4,287 amino acid positions) confirmed Marsupiomonadales as a well-defined group with the two family-level clades receiving maximal support (S3 Fig). While the branching order of Marsupiomonadaceae mimicked the other phylogenies, the branching pattern of Resultomonadaceae differed, with *O. okinawensis* placed sister to *A. japonica* and *Resultomonas* sp. Cadiz combined (S3 Fig). However, the latter relationship received only weak statistical support. Meanwhile, *Ped. minor* was grouped with full support with the uncultivated putative pedinomonadalean represented by the mitogenome sequence obtained from the metagenome assembly IMGM3300027621, but the Pedinomonadales clade was closer to Chlorophyceae than to Marsupiomonadales in the tree (S3 Fig). This aberrant topology is apparently artificial and presumably manifests a long branch attraction artefact (LBA) caused by the fact that mitochondrial sequences of Chlorophyceae, omitted in the analysis by [18], are also highly divergent.

### Evolutionary patterns of pedinophyte plastomes: A nascent Ile-to-Met AUA reassignment

The newly characterized pedinophyte plastomes all exhibit the conventional quadripartite structure including a large and a small single-copy region separated by inverted repeat regions (S4 Fig; note that this architecture is to be confirmed for Marsupiomonadaceae sp. Cadiz, but the incomplete assembly of its plastome is consistent with this structure). The major characteristics of the pedinophyte plastomes, including their size, GC content, and the number of genes of different categories, are summarized in Table 1. The respective values vary within relatively narrow confines, with *C. tuberculatum* having the largest plastome size (126,694 bp), *Marsupiomonas* sp. NIES-1824 exhibiting the highest GC content (40.34%), and members of Marsupiomonadaceae family together with *C. tuberculatum* possessing the highest number of conserved protein-coding genes among Pedinophyceae, that is 75. The differences in the repertoire of protein-coding genes specifically concern a few genes (S1 Table), including *infA* missing in Pedinomonadales and peDinoflagellates; *secG* (=*ycf47*) missing in Marsupiomonadaceae and the peDinoflagellate *Lepidodinium chlorophorum*; *cysA* and *cysT* absent in the Resultomonadaceae and peDinoflagellates; and *minD* absent in *Resultomonas* sp*.* Cadiz and peDinoflagellates. The latter gene is part of the small single-copy region in all pedinophytes (S4 Fig), but our metagenomically assembled *Resultomonas* sp*.* Cadiz sequence scaffold covering the region lacks the gene and we also could not detect it in the whole metagenome assembly, suggesting its real absence. Consistently, these five genes exhibit variation in their presence across other green algal plastomes as well [21].

**Table 1:** General features of all sequenced pedinophyte plastid genomes.

	Marsupiomonadales						Pedinomonadales	
	Resultomonadaceae			Marsupiomonadaceae			Pedinomonadaceae	
	*Oistococcus okinawensis* (Okinawa strain)	*Akinorimonas japonica* (Pedinophyceae sp. YPF-701) (KY347917.1)	*Resultomonas* sp. Cadiz	*Protoeuglena noctilucae* (OY253692.1)	Marsupiomonada-ceae sp. Cadiz	*Marsupiomonas* sp. NIES-1824 (KM462870.1)	*Pedimononas minor* UTEX LB 1350 (NC_016733.1)	*Chlorochytridion tuberculatum* SAG 42.84 (NC_025530.1)
Size (bp)	108,389	91,755	85,359	100,950	74,028 (incomplete)	94,262	98,340	126,694
LSC (bp)	85,791	65,111	66,139	71,957	–	68,185	70,398	86,619
SSC (bp)	6,008	5,648	4,806	6,059	–	6,225	6,664	7,927
Inverted repeat (bp)	8,295	10,498	7,207	11,467	–	9,926	10,639	16,074
GC content (%)	38.88	37.74	35.33	32.39	–	40.34	34.84	33.43
Conserved protein-coding genes	73	73	72	75	75	75	74	75
Non-intronic lineage-specific ORFs	4	0	0	0	0	0	5	1
tRNA genes	29	26	25	28	26	27	28	28
rRNA genes	3	3	3	3	3	3	3	3
Other non-coding RNA genes (only *ssrA*)	0	0	0	1	1	1	1	1
Number of functional genes in inverted repeats	11	13	12	9	11	13	14	13

Additional differences among pedinophyte plastomes are manifested in the presence of non-conserved ORFs, with *O. okinawensis* containing four and *Ped. minor* five such ORFs (excluding duplications in the inverted repeats in the latter). However, neither BLAST nor HHpred searches could provide any clues to the origin or function of the annotated ORFs. Introns are rare in pedinophyte plastomes, as they were found only in *C. tuberculatum* SAG 42.84 and two of the three peDinoflagellates. Pedinophyte plastomes also vary in the presence of the *ssrA* gene specifying tmRNA, a non-coding RNA that together with the SmpB protein forms the tmRNP complex that mediates rescue of stalled ribosomes [28]. Our analyses revealed that the plastid *ssrA* gene and the nucleus-encoded SmpB exhibit the expected co-occurrence pattern in pedinophytes (Table 1 and S5 Fig), with both genes present in Pedinomonadales and Marsupiomonadaceae (the presence of SmpB could not be confirmed in *Marsupiomonas* sp. NIES-1824 and Marsupiomonadaceae sp. Cadiz for the lack of relevant data), whereas no SmpB homolog could be identified in the nuclear genome data from *O. okinawensis* and *A. japonica*, consistent with the lack of *ssrA* in the Resultomonadaceae plastomes.

Differences among pedinophyte plastomes also concern their tRNA gene repertoires. For example, *trnL(caa)*, *trnS(gga)*, *trnT(ggu)*, and *trnR(ccg)* exhibit varying presence in pedinophytes (S2 Table), but the respective cognate codons (UUG, UCC, ACC, and CGG, respectively) are used by all pedinophyte plastomes (S3 Table), evidently with their standard meaning (S6A and S7 Figs and S4 Table). While the UUG codon can be decoded by the product of the *trnL(uaa)* gene, which is universal in pedinophyte plastomes (S2 Table), the UCC and ACC codons are potentially decoded by *trnS(uga)* and *trnT(ugu)* products, provided that U at the first anticodon position is unmodified, enabling the tRNAs to decode (via superwobbling [29]) all four codons in the respective codon boxes. How the arginine CGG codon is decoded in the absence of the *trnR(ccg)* gene is not clear, as there is also no *trnR(ucg)* gene that would allow for a superwobbling-based decoding mechanism, but the absence of both tRNA genes in plastomes of green algal taxa is not unprecedented [21]. The situation is unique for the *trnI(cau)* gene, which specifies a tRNA responsible for decoding the AUA codon as isoleucine in plastids. The tRNA undergoes a modification of its first anticodon position resulting in the non-standard nucleoside lysidine, at least in some taxa further converted to 2-aminovaleramididine [30,31]. The modification of cytidine to lysidine is catalysed by the product of the *tilS* (=*ycf62*) gene, i.e., tRNA(Ile)-lysidine synthase. Notably, *trnI(cau)* and *tilS* are both consistently absent from *Ped. minor* and all three Resultomonadaceae members investigated, raising the question as to how – in terms of the mechanism and the actual amino acid outcome – the AUA codon is translated in these pedinophytes.

To illuminate these questions, we considered the usage of the AUA codon in pedinophyte plastomes (S3 Table) and correlated the AUA occurrence in coding sequences with the conservation of the amino acid residues at the given positions in the encoded protein sequences. Specifically, we followed a previously developed procedure [32,33] and analysed the occurrence of AUA-encoded positions in two sets of multiple alignments of plastome-encoded proteins, one corresponding to the selection of green algal sequences employed in our phylogenomic analysis (“Chloro dataset”), and the other limited to sequences from pedinophytes and peDinoflagellates (“Pedino dataset”; technical details of the analysis are described in Material and Methods). Full results are presented in S1 Dataset, for details concerning codons of specific interest see S6 Fig. We found that pedinophytes that have retained the *trnI(cau)* and *tilS* genes all exhibit AUA primarily at conserved Ile positions (S6A Fig), consistent with the standard AUA meaning in these species, whereas the pedinophytes lacking the two genes differ. In *Ped. minor* and *Resultomonas* sp. Cadiz the AUA codon is very rare (only five instances in each case identified in the annotated coding sequences) and virtually never at positions conserved for the encoded amino acid (exception being a single conserved Met position in the Pedino dataset in the latter taxon). In *A. japonica* AUA is used only slightly more often (17 times), only two times at conserved positions in each the Chloro dataset (conserved Met and Tyr positions) and the Pedino dataset (conserved Met and Ile positions). The AUA codon usage is the highest in the *O. okinawensis* plastome, where it occurs at 62 positions, six and four of which are, respectively, conserved when assessed based on the Chloro and the Pedino dataset. Crucially, in both cases a majority of these conserved position (four and three, respectively) correspond to conserved Met positions while only one to a conserved Ile position (S6A Fig).

We note that using the common existing tools for detecting codon reassignments, FACIL (S7 Fig) and Codetta (S4 Table), did not assign any specific meaning to the AUA codon in any pedinophyte plastome. This is apparently because of the low usage of the codons, combined with the fact that both programs employ (in their default mode) a taxonomically broad selection of sequences as the reference dataset, differing thus from our taxonomically more focused and thus presumably more sensitive analysis. Altogether, we interpreted our observations as an indication that an Ile-to-Met reassignment of the AUA codon is ongoing in the *Ped. minor* and Resultomonadaceae lineages (for further details see Discussion), and we thus translated this codon as methionine for the purpose of generating protein sequences employed in the phylogenomic analysis reported in the previous section.

### Plastomes of peDinoflagellates exhibit multiple codon reassignments

**Fig 5:**
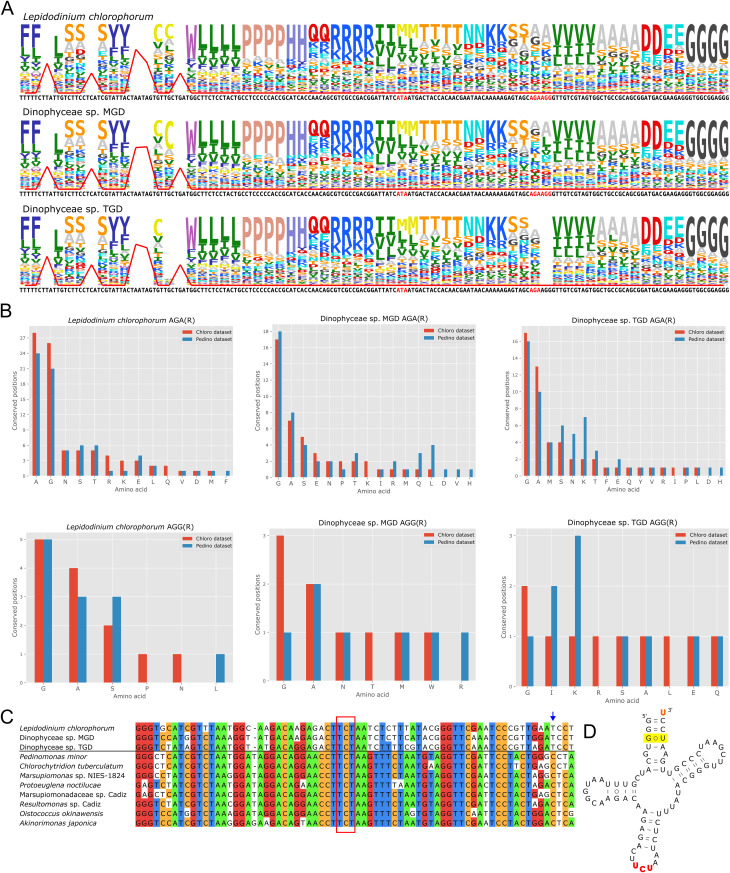
Sense-to-sense codon reassignments in peDinoflagellate plastomes. (A) FACIL outputs of plastomes indicating a non-standard meaning of the following codons: AUA (Ile-to-Met), AGA and AGG (Arg-to-Ala?), TTA (Leu-to-stop), and UCA (Ser-to-stop). The red line indicates a signal for the given codon functioning as a termination codon. (B) Plots showing the distribution of the AGA and AGG codons in each of the three studied peDinoflagellates at conserved amino acid positions (the same amino acid in at least 70% of all sequences compared) defined by multiple alignments of plastome-encoded proteins. Different bar colours represent two different comparisons based on a taxonomically broad set of chlorophyte sequences (“Chloro dataset”) and pedinophyte/peDinoflagellate sequences (“Pedino dataset”). Note that this analysis generally favours glycine over alanine as the most likely amino acid encoded by the AGA and AGG codon in peDinoflagellate plastomes. (C) Sequence alignment of genes for tRNAs with the anticodon UCU occurring in the plastomes from peDinoflagellates (top) and pedinophytes (bottom). The red box highlights the anticodon region, while the blue arrow points to the position 70 substituted with T in the peDinoflagellate sequences as expected if the tRNA is to be charged with alanine. (D) The predicted secondary structure of the *L. chlorophorum* plastidial tRNA_UCU_. The G3·U70 pair characteristic for tRNA^Ala^ is highlighted in yellow, whereas the nucleotide occupying the position 73 (U, highlighted in orange) does not fit the expected tRNA^Ala^ pattern.

While the conversion of the rarely used AUA into a methionine codon may be in its early evolutionary stage in pedinophytes, this codon is used abundantly in peDinoflagellate plastomes and has clearly been “fully” reassigned this way, providing the first case of a sense-to-sense reassignment reported from plastids [7,19]. The Ile-to-Met AUA reassignment is also supported by a strong signal in our analyses (Figs 5A and S6B and S5 Table), which unexpectedly revealed that the previous authors had missed additional codon reassignments in peDinoflagellate plastids, specifically concerning the two arginine codons AGA and AGG (collectively AGR, with “R” standing for a purine), the leucine codon UUA, and the serine codon UCA. In the case of AGR, both codons are used frequently (with AGA being more abundant; S6 Table), yet analyses with both FACIL (Fig 5A) and Codetta (S5 Table) yielded results inconsistent with the standard meaning of these codons. Specifically, neither returned arginine among the most likely amino acids being encoded by these codons and FACIL instead favoured the alternative amino acid alanine in all three peDinoflagellate species. Codetta recapitulated this result only for *L. chlorophorum* and left the meaning of both AGR codons undecided for the two other species. However, a conflicting evidence was provided by our more focused analysis relying on the Chloro and Pedino datasets, providing a stronger or at least comparably strong signal for a reassignment of these codons to glycine when compared to alanine (Fig 5B).

The peDinoflagellate plastomes specify a tRNA with the anticodon UCU (tRNA_UCU_) cognate to AGR. Phylogenetic analysis of tRNAs using Neighbor-Net is consistent with the simplest evolutionary scenario, i.e., that the peDinoflagellate tRNA_UCU_ has originated from tRNA^Arg^_UCU_ specified by the plastomes of pedinophytes (S8 Fig), but it exhibits a specific mutation that is consistent with the notion of this tRNA being charged by alanine instead of arginine. Specifically, the peDinoflagellate version has a substitution at the position 70 (following the standardized numbering of the generalized tRNA sequence) that changes the G3:C70 pair to G3·U70 (Fig 5C and 5D), which is a major tRNA^Ala^ identity determinant conserved across all domains of life [34]. Admittedly, the tRNA does not exhibit another feature characteristic to tRNA^Ala^ (but also a number of tRNAs with other amino acid specificities), that is A at the position 73. This is all the more surprising that A73 is present in the presumably ancestral tRNA^Arg^_UCU_ in the pedinophyte plastids. However, we found that the peDinoflagellate plastidial tRNA^Ala^_UGC_, i.e., a standard tRNA species decoding the standard GCN alanine box, varies at the position 73. Whereas strains MGD and TGD exhibit the expected A73, *L. chlorophorum* has U at this position like the putative tRNA^Ala^_UCU_ (S9 Fig), and no editing at this position is suggested by transcriptome data. It is thus likely that the alanyl-tRNA synthetase operating in peDinoflagellate plastids can charge tRNAs with U73. That the peDinoflagellate tRNA_UCU_ would mediate the alternative AGR decoding as glycine suggested by the Pedino datasets analysis (see above) seems much less likely, as the cognate tRNA lacks all the conserved tRNA^Gly^ identity elements (as listed by [34]), including C2:G71 and G3:C70 pairs and cytidines at the second and third anticodon position (Fig 5D).

**Fig 6:**
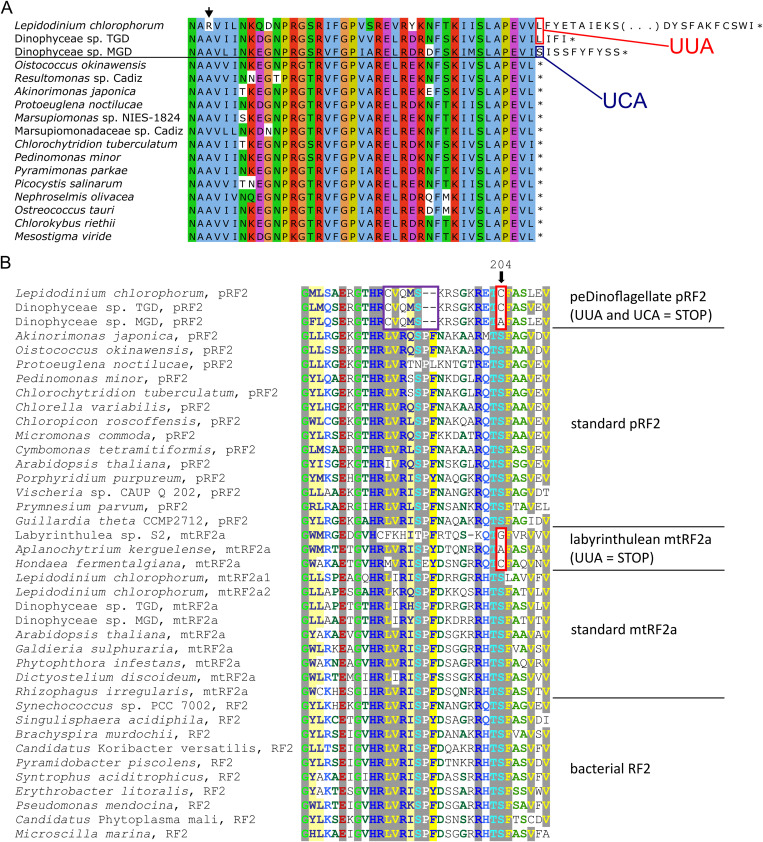
Non-standard termination codons employed in peDinoflagellate plastids. (A) An example of UUA and UCA as putative termination codons in peDinoflagellate plastid genes. The figure shows an alignment of the C-terminal region of protein sequences encoded by the *rpl14* gene, with the residues encoded by UUA or UCA (boxed in red or blue, respectively) found at positions where a termination codon is expected. Note that the peDinoflagellate sequences shown were obtained by conceptual translation of transcriptome-derived sequences (to account for RNA editing) and using the standard genetic code (expect for previously and robustly documented decoding of AUA as methionine). The black arrow points to a position in the *L. chlorophorum* sequence corresponding to an AGG codon, which in fact encodes alanine (or less likely glycine), but not arginine, in the peDinoflagellate plastid (see main text). (B) Sequence alignment of the codon-binding region of the peDinoflagellate pRF2 protein and selected homologs. The mutation of the highly conserved Ser204 residue in peDinoflagellate pRF2 and labyrinthulean mtRF2a that is supposed to mediate recognition of UUA as a termination codon [33] is highlighted by an arrow and a red box. The region in the peDinoflagellate pRF2 proteins exhibiting additional mutations including deletions is boxed in violet; this pronounced modification is hypothesized to be responsible for decoding UCA as a termination codon. Accession numbers of the sequences included are provided in S11 Fig and S7 Table.

The case of the UUA and UCA codons (collectively UYA, with “Y” standing for a pyrimidine) in peDinoflagellate plastids is even more striking. FACIL suggested both as potential termination codons (Fig 5A), and sense-to-stop reassignment of both was indeed corroborated by two observations. Crucially, when sequences of mature mRNA transcripts modified by RNA editing (see below) are considered, these codons occur exclusively at the very 3’ ends of coding sequences as defined based on the comparison with homologous genes from other plastomes (Fig 6A; other examples provided in S10 Fig). Notably, fused genes were previously claimed to be present in the peDinoflagellate plastomes [7], but all these cases stemmed from assuming that the UYA codons are read through during translation rather than signalling translation termination. Consistent with the function of UYA as termination codons, no tRNAs cognate to them, i.e., having the anticodon UAA and UGA, respectively, are specified by any peDinoflagellate plastome. UUA and UCA are used as obvious termination codons 33/32/20 and 10/11/6 times, respectively, in Dinophyceae sp. MGD/TGD/*L. chlorophorum* plastomes. In other words, over a half of the plastid protein-coding sequences end with one of the non-standard termination codons in the former two species, and in 26 out of 64 instances in *L. chlorophorum*.

Decoding of any non-standard termination codon as a translation stop signal implies specific changes in proteins known as release factors (RFs), which interact with the termination codon in the ribosomal A site and subsequently catalyse the hydrolysis of the bond linking the nascent polypeptide with the tRNA that decoded the preceding codon, releasing thus the polypeptide from the ribosome [35]. The standard configuration in organelles includes an RF pair, mtRF1a and mtRF2a in mitochondria and pRF1 and pRF2 in plastids, which are direct evolutionary descendants of RF1 and RF2 functioning in eubacteria and exhibiting a partially overlapping codon specificity, recognizing the codon pairs UAG/UAA and UAA/UGA, respectively [36]. To illuminate the molecular underpinnings of the UYA decoding as termination codons in peDinoflagellates, we searched the transcriptome data from all three representatives and identified homologs of organellar RFs (S7 Table). Phylogenetic analysis clearly distinguished both expected mitochondrion-targeted forms (mtRF1a and mtRF2a) and orthologs of the plastid-targeted pRF2, notably indicating that the peDinoflagellate pRF2 is of the pedinophyte origin (S11 Fig). In contrast, pRF1 orthologs were missing and instead a group of unrelated RF1-type proteins was identified in all three peDinoflagellates that was not directly related to the Cyanobacteria-derived pRF1 of other plastids and instead branched among RF1 proteins from the bacterial phylum Bacteroidota (S11A Fig). Two of the proteins possess a readily recognisable N-terminal signal peptide, which is characteristic for nucleus-encoded plastid-targeted proteins in peDinoflagellates [8]. A signal peptide was not predicted in the third protein (the one from TGD strain) by the tool employed, but a region predicted as a transmembrane segment was found somewhat more downstream of the very N-terminus and we assume it may function as a signal peptide, too. Hence, peDinoflagellates adopted by horizontal gene transfer a bacterial RF1 protein to serve as a functional equivalent of the standard pRF1. Crucially, while the codon-binding region of pRF1 looks normal in peDinoflagellates (S12 Fig), their pRF2 exhibits a highly modified codon-binding region compared to homologs from other plastids and bacteria (Fig 6B). The homologous mtRF2a protein in the protist group Labyrinthulea (Stramenopiles) was previously found to have mutated the invariant serine residue at the position 204 to alanine, glycine or cysteine, and this alteration was proposed to cause this protein recognize UUA as a non-standard termination codon [33]. It is then notable that the same position is mutated to cysteine or alanine in peDinoflagellate pRF2 (Fig 6B). As obvious from the alignment displayed, this protein exhibits additional substitutions of conserved amino acid residues as well as a two-residue deletion in the codon-binding domain, and we hypothesize they modify the binding properties of the protein such that it recognizes also UCA.

As already mentioned, the analyses of the codon meaning reported above took into account the fact that the primary transcripts in peDinoflagellate plastids undergo extensive substitutional editing, which changes the identity of nucleotides in the mature mRNA compared to the original genome sequence [7]. We thus analysed the occurrence of different codons in coding sequences of all three peDinoflagellates by considering the respective nucleotide sequences documented by the available transcriptome data. Editing at a particular site may not only result in a conversion of a codon into a non-synonymous one (changing thus the encoded amino acid), but may also convert a termination codon to a sense codon, restoring thus an intact coding sequence. A notable example is provided by the *rpoB* gene interrupted by the stop codon UGA in TGD strain, which led Matsuo et al. [7] to propose that the gene is pseudogenised in this alga. However, inspecting the transcriptome reads mapped to the plastome genomic sequence, we found that editing changes the first position of the codon to C, resulting in the arginine codon CGA that restores an intact protein-coding sequence. RNA editing also rationalizes the apparent presence of in-frame non-standard termination codons UUA or UCA in certain genes in the peDinoflagellate plastomes. For example, the *ycf3* gene of *L. chlorophorum* contains an in-frame UUA (21^st^ codon from the start of the coding sequence), which is however edited to CUA, ensuring that the length of the coding sequence remains unchanged. We assume editing removes also a single remaining in-frame UUA codon (in the *rpoC2* gene in Dinophyceae sp. TGD) not covered by the available transcriptome data to directly prove this.

### Updated perspective on the UGA and AGR codon reassignments in pedinophytes mitochondria

The seven complete and one partial pedinophyte mitogenomes analysed in this study more than triple the two genomes compared in the previous analysis [18], although the expansion is highly biased towards representatives of Marsupiomonadales. An overview of the general characteristics of pedinophyte mitogenomes is provided in Table 2. There is a notable difference between mitogenomes of Marsupiomonadales and that of *Ped. minor* in the GC content, with the former ranging between 38.69% and 51.40%, but dropping to only to 22.20% in the latter. The two largest genomes belong to *O. okinawensis* and *Prot. noctilucae*, both nearly twice the size of the other mitogenomes (Table 2). In *O. okinawensis*, this expansion is primarily due to the presence of an extended region representing a noncoding sequence (we ignore the spurious status of the single short non-conserved ORF annotation in this region), a segment of which is present as a long inverted repeat (S13 Fig). Interestingly, the mitogenome of *Ped. minor* exhibits a similar architectural feature, containing two longer non-coding regions separated by just a second copy of a duplicated tRNA gene [17]. The reason for the increased size of the *Prot. noctilucae* mitogenome is different, namely the presence of introns within protein-coding genes, which is unique among all analysed pedinophyte mitogenomes. Specifically, the *cox1* and *cox3* genes harbour two and one, respectively, group II introns each containing an ORF that encodes a reverse transcriptase/maturase enzyme. Noteworthy, only *O. okinawensis* and *Ped. minor* contain a group II intron in their *rnl* gene. All characterized (i.e., functionally annotated) genes in pedinophyte mitogenomes are located on the same DNA strand, but there is no apparent conservation in the gene order. The split of the *rnl* gene into two separate fragments observed in the two previously investigated pedinophyte mitogenomes [18] is conserved across the whole group. All members of Marsupiomonadales share the same complement of 17 mitochondrial protein-coding genes and compared to the *Ped. minor* mitogenome notably lack the *atp8* gene, while the mitogenome of the latter alga lacks seven genes (*atp9*, *cox2*, *cox3*, *rpl6*, *rpl14*, *rpl16*, and *rps12*) conserved in Marsupiomonadales. One to seven non-conserved ORFs are found in pedinophyte mitogenomes; their origin could not be deciphered even when using highly sensitive homology searches.

**Table 2:** General features of all sequenced pedinophyte mitochondrial genomes.

	Marsupiomonadales						Pedinomonadales	
	Resultomonadaceae			Marsupiomonadaceae			Pedinomonadaceae	
	*Oistococcus okinawensis* (Okinawa strain)	*Akinorimonas japonica* (Pedinophyceae sp. YPF-701)	*Resultomonas* sp. Cadiz	*Protoeuglena noctilucae*	Marsupiomonadaceae sp. Cadiz	*Marsupiomonas* sp. NIES-1824 (MN782006.1)	*Pedimononas minor* UTEX LB 1350 (NC_000892.1)	Pedinomonadales sp. IMGM330002762
Size (bp)	45,896	24,584	25,866	45,034	21,669	24,252	25,137	>17,235
GC (%)	38.69	46.72	48.86	38.96	41.29	51.40	22.20	22.95
tRNA genes	20	20	21	20	21	20	8	8
rRNA genes	3	3	3	3	3	2	2	2
Protein-coding genes	17	17	17	17	17	17	11	11
Non-intronic lineage-specific ORFs	7	2	2	6	1	0	0	0
Genes interrupted by introns	1 (*rnl_a*)	0	0	2	2	0	1 (*rnl_a*)	0

While *Ped. minor* has lost many mitochondrial tRNA genes [18], Marsupiomonadales have a more complete set, with 20 genes in most cases and *R. moestrupii* additionally uniquely possessing the *trnR(ucg)* gene (S8 Table). It is obvious that mitochondria of all pedinophytes rely on tRNA import from the cytoplasm, which in Marsupiomonadales includes a tRNA for decoding threonine codons and, with the exception of *R. moestrupii*, a tRNA matching the arginine-encoding CGN codon box. Notably, the number of imported tRNAs to compensate for the absence of specific tRNA genes in the mitogenome would be expected to be higher if the standard genetic code was assumed to be operational in the pedinophyte mitochondria. However, as described in the subsequent section, pedinophyte mitochondria exhibit departures from the standard genetic code that decrease the number of tRNA required for translation of mitochondrial genes.

In fact, two other cases of a non-standard codon meaning were already noted in analyses of the two previously sequenced pedinophyte mitogenomes. The first concerns the mitogenome of *Ped. minor*, where the UGA codon has been reassigned to encode tryptophan [17]. Here we show that the same codon reassignment has independently evolved in *O. okinawensis* (S14 and S15 Figs and S9 Table). Furthermore, the metagenome-derived Pedinomonadales sp. IMGM3300027621 mitogenome also clearly decodes UGA as Trp (S16 Fig), indicating that this is a feature characteristic and ancestral for the whole order Pedinomonadales. The two pedinomonadaleans and the unrelated *O. okinawensis* all lack the standard *trnW(cca)* present in the other pedinophytes and instead possess *trnW(uca)* that can read both UGG and UGA. Strikingly, the *trnW(uca)* gene in the *Ped. minor* genome was suggested not to have evolved by simple conversion of the pre-existing *trnW(cca)* gene; instead, it was proposed to have emerged from a duplicated *trnC(gca)* gene [37]. The latter notion is not corroborated by our analysis using a different methodology and a broader sequence sampling, but relationship of the pedinomonadalean tRNA gene to *trnW(cca)* genes from other species is not supported either, making the origin of this tRNA uncertain (S17 Fig). In contrast, the *trnW(uca)* gene in *O. okinawensis* has evolved from the original *trnW(cca)*, as evidenced by the high sequence similarity of all marsupiomonadalean *trnW* genes and our phylogenetic analysis (S17 Fig). The usage of the reassigned UGA in pedinophytes is frequent, the codon appearing 62 times in *Ped. minor*, 66 times in Pedinomonadales sp. IMGM330002762, and a remarkable 131 times in *O. okinawensis*, while the standard tryptophan codon, UGG, is used much less frequently (only 2, 3, and 27 times respectively; S10 Table).

The second previously known genetic code change in pedinophyte mitochondria concerns the AGR codons, which were shown to encode alanine rather than the standard arginine in *Marsupiomonas* sp. NIES-1824 [18]. Our expanded analysis of pedinophyte mitogenomes (S14–S16 Figs and S9 Table) revealed that this reassignment holds for the entire Marsupiomonadales order and hence evolved already in the common ancestor of this group. In contrast, the plesiomorphic state of the AGR codons is encountered in Pedinomonadales, with the standard meaning strongly supported for AGA in both *Ped. minor* and Pedinomonadales sp. IMGM3300027621 and indirectly implied for the rarely used AGG on the assumption of the codon being decoded by the same tRNA as AGA. The Arg-to-Ala reassignment of both AGR codons (or AGG alone) has also been reported in mitochondria of a different group of green algae, specifically certain members of Sphaeropleales (Chlorophyceae) [32,37], and as described in the previous section, the same reassignment has most likely also evolved in the plastid of peDinoflagellates.

**Fig 7:**
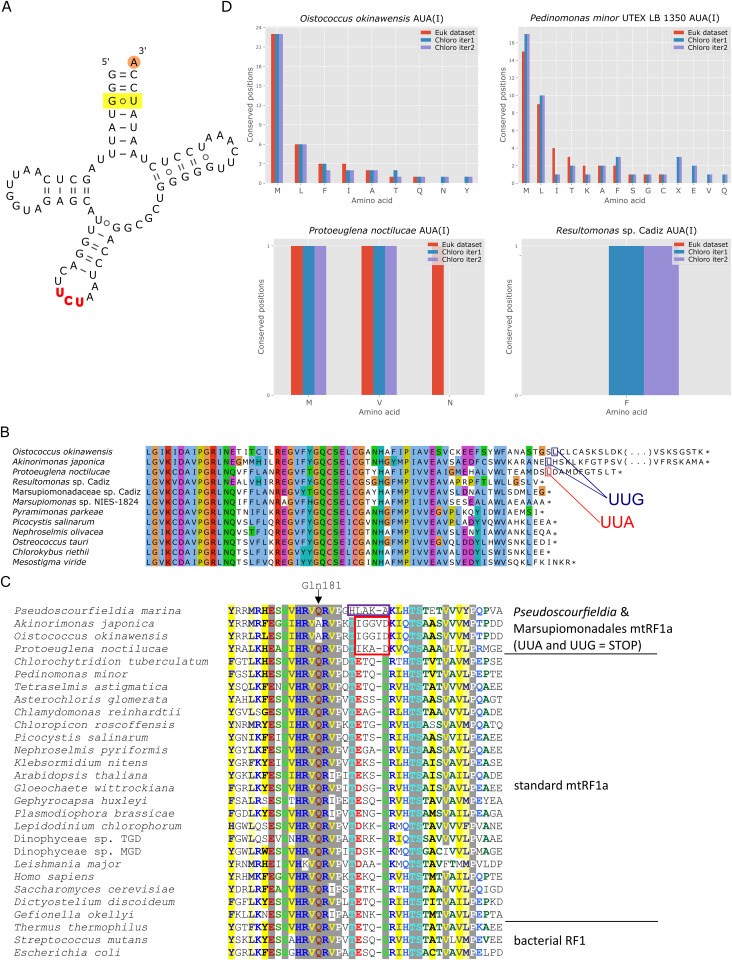
Codon reassignments in pedinophyte mitochondria. (A) The predicted secondary structure of the *O. okinawensis* mitochondrial tRNA_UCU_. The G3·U70 pair and the A73 position characteristic for tRNA^Ala^ are highlighted. The tRNA displayed represents a group of related mitochondrial tRNAs in Marsupiomonadales mediating the Arg-to-Ala reassignment of AGR codons. (B) An example of UUA and UUG as putative termination codons in mitochondrial genes of Marsupiomonadales. The figure shows an alignment of the C-terminal region of protein sequences encoded by the *cox2* gene, with the residues encoded by UUA or UUG (boxed in red or blue, respectively) found at positions where a termination codon is expected. (C) Sequence alignment of the codon-binding region of the pedinophyte mtRF1a protein and selected homologs. The region independently mutated in mtRF1a proteins from Marsupiomonadales (boxed in red) and *Pseud. marina* (Pseudoscourfieldiophyceae; boxed in purple) correlate with these taxa having independently undergone the Leu-to-stop UUR reassignment. The position of the conserved Gln181 residue mutated in mtRF1a from Resultomonadaceae is indicated by the arrow on the top (see text for further discussion). Accession numbers of the sequences included are provided in S11 Fig and S7 Table. (D) Plots showing the distribution of the AUA codon in each of the pedinophyte mitogenomes analysed in this study at conserved amino acid positions (the same amino acid in at least 70% of all sequences compared) defined by multiple alignments of mitogenome-encoded proteins. Different bar colours represent results of three different comparisons, namely using a eukaryote-wide sequence set (“Euk dataset”) and a selection of chlorophyte mitogenome sequences (“Chloro” dataset) in two iterations (“iter1” and iter2”; see Methods for further explanation).

As with the latter case, we investigated how the signal for the particular non-standard meaning of the AGR codons relates to the features of the cognate tRNA_UCU_ specified by the Marsupiomonadales mitogenomes. Sequence similarity comparisons did not suggest any specific affinity of this tRNA species to any other in pedinophyte or generally chlorophyte mitogenomes, and phylogenetic analysis with Neighbor-Net did not provide conclusive results regarding the origin of tRNA_UCU_ in Marsupiomonadales (S17 Fig). Consistent with the inferred meaning of the AGR codons, the marsupiomonadalean tRNA_UCU_ exhibits the key tRNA^Ala^ identity elements, including the G3·U70 pair and adenosine at the position 73 (Figs 7A and S18), yet it does not seem to have evolved from a tRNA^Ala^ gene. Hence, it is most parsimonious to assume that it is a transmogrified version of the *trnR(ucu)* gene that was presumably present in the ancestral pedinophyte mitogenome to specify a tRNA decoding AGR codons (in the standard way), while the *trnR(ucu)* gene was lost in Pedinomonadales (S8 Table) and these algae must import a tRNA^Arg^_UCU_ from the cytosol to secure the AGR decoding.

### Previously unnoticed codon reassignments in pedinophyte mitochondria

While the two genetic code alterations in pedinophyte mitochondria discussed so far are underpinned by tRNA gain/modifications rather than loss, we discovered additional changes in codon meaning that rationalize the absence of particular tRNA genes in pedinophyte mitogenomes. Previous analysis of the *Marsupiomonas* sp. NIES-1824 mitogenome noted the complete lack of two out of six leucine codons, UUA and UUG (collectively UUR) [18]. Strikingly, both of them do occur in the newly available Marsupiomonadales mitogenomes, but only at the 3’ end of coding sequences where a termination codon is expected (Figs 7B and S19). This is also evident from the respective FACIL outputs, which assign no amino acid to any of the UUR codons in Marsupiomonadales mitogenomes, and instead indicate they may serve as termination codons (S14 Fig). Furthermore, no gene for a tRNA cognate to UUA and/or UUG is present in any Marsupiomonadales mitogenome. In contrast, in *Ped. minor* and Pedinomonadales sp. IMGM3300027621 both codons are frequently used along the whole length of coding sequences of mitochondrial genes and clearly have the standard meaning (S14–S16 Figs). Mitogenomes of both species contain a gene, *trnL(caa)*, whose product is expected to decode UUG but not UUA. It is not clear if this implies import of tRNA^Leu^_UAA_ or whether the *trnL(caa)* product undergoes a post-transcriptional modification extending its codon specificity to UUA. At any rate, the putative Leu-to-stop reassignment of UUR happened only after the Pedinomonadales diverged from the Marsupiomonadales lineage.

Previously, the UUR codons were proposed to serve as termination signals in the unrelated green alga *Pseudoscourfieldia marina* (previously also known as *Pycnococcus provasolii*) representing the separate class Pseudoscourfieldiophyceae [38,39]. Furthermore, specific mutations in the codon-binding region of the mtRF1a protein from this species were noted that may hypothetically enable UUR binding [32]. Inspection of mtRF1a sequences from Marsupiomonadales revealed that they exhibit mutations in the same region as observed in *Pseud. marina*. In contrast, both available mtRF1a sequences from Pedinomonadales representatives appear normal in this region (Fig 7C), correlating with the standard decoding of UUR as leucine codons in the *Ped. minor* mitogenome (the information of the UUR meaning is unavailable for *C. tuberculatum*). These observations are consistent with the notion that UUR codons are – at least latently – interpreted as termination codons in the mitochondria of the whole Marsupiomonadales order.

Finally, all pedinophyte mitogenomes possess only up to two genes specifying tRNAs with the anticodon CAU, and as indicated by our analysis, they correspond to the initiator tRNA^fMet^_CAU_ and/or the elongator tRNA^Met^_CAU_ (S17 Fig). Furthermore, we did not identify any candidate for a nuclear genome-encoded tRNA(Ile)-lysidine synthase that could potentially operate in the mitochondrion and lysidinylate tRNA^Ile^_CAU_ cognate to AUA, raising the question as to how the codon is decoded in the pedinophyte mitochondria. Considering the usage (S10 Table) and the signal for the AUA meaning in pedinophyte mitogenomes (Figs 7D and S14–S16 and S9 Table), two different subgroups emerged among the seven species compared. One comprises *Ped. minor*, Pedinomonadales sp. IMGM3300027621, and *O. okinawensis* characterized by a relatively high numbers of AUA instances in mitochondrial genes (115, 140, and 87, respectively) and a strong signal for the codon encoding methionine instead of isoleucine. The remaining five pedinophytes exhibit a relatively low (27 instances in *Prot. noctilucae*) to a very low AUA usage (1–4 instances in *Resultomonas* sp. Cadiz, Marsupiomonadaceae sp. Cadiz, *Marsupiomonas* sp. NIES-1824, and *A. japonica*), and unsurprisingly the meaning of the codon in these organisms could not be unambiguously deduced from the sequence comparisons performed. Hence, at least in *Ped. minor* and *O. okinawensis*, but potentially in pedinophytes in general, AUA is most likely decoded by tRNA^Met^_CAU_, presumably thanks to a presently undefined chemical alteration of the molecule that expands its codon binding specificity from AUG to AUR.

## Discussion

### New taxa in Pedinophyceae

The main focus of this study is the evolution of the genetic code in pedinophyte organelles. Nevertheless, our results also contribute to the basic exploration of the organismal diversity of pedinophytes, and by extension of green algae in general. Most notably, the microscopic observations of the newly isolated Okinawa strain (*O. okinawensis*) reveal a new morphotype for pedinophytes, while our investigation of Pedinophyceae sp. YPF-701 (*A. japonica*) eventually provide morphological details of a pedinophyte previously subjected to genomic characterization [6,10]. In addition, by including into the analyses the first organellar genome sequences of a representative of the genus *Resultomonas* acquired by exploring metagenome data, we can now for the first time robustly assess the phylogenetic position and internal diversity of the currently monotypic family Resultomonadaceae. Our results expand the circumscription of the family by showing that the genus *Resultomonas* is related to Okinawa strain and Pedinophyceae sp. YPF-701, together constituting a clade whose members exhibit considerable phylogenetic divergence (Figs 3 and 4), distinctive genomic features (e.g., the stop-to-Trp UGA reassignment only in the mitogenome of Okinawa strain), as well as notable morphological differences.

Specifically, these algae vary in cell size, with *Resultomonas* having the smallest cell size [1]. In addition, Pedinophyceae sp. YPF-701 has a prominent eyespot, which is always clearly visible in every cell, opposing the situation in Okinawa strain (Figs 1C, 1D and 2A, 2B) and *Resultomonas* [1]. In contrast, the pyrenoid in *Resultomonas* is clearly visible even under a light microscope, whereas it is hardly distinguishable in Okinawa strain and absent in Pedinophyceae sp. YPF-701. The coiling of the flagellum on the cell (Fig 2B) was observed only in Pedinophyceae sp. YPF-701, while the conspicuous ball-like cells (Fig 1A and 1B) are formed exclusively in Okinawa strain. Neither of these features have been noted for *Resultomonas* [1]. Finally, the Okinawa strain and Pedinophyceae sp. YPF-701 also differ in their asexual reproduction, the former typically producing zoospores and aplanospores, the later multiplying by binary fission. All these details combined make a strong case for recognizing Okinawa strain and Pedinophyceae sp. YPF-701 as new taxa, each as a new species in a new genus in the family Resultomonadaceae. The formal description of *Oistococcus okinawensis* gen. et sp. nov. and *Akinorimonas japonica* gen. et sp. nov. is provided at the end of Discussion.

### Pedinophyte(-derived) organelles as a hot-spot of genetic code evolution

Alterations of organellar genetic codes are not distributed evenly across the eukaryote tree of life. Chlorophyte green algae stand out as one of the most evolutionary dynamic groups in this regard, with a rich landscape of codon reassignments and the molecular determinants behind them described before especially for chlorophyte mitochondria [32,37]. The list of genetic code alterations encountered before in chlorophyte plastids is also impressive and includes a few cases of the stop-to-Trp UGA reassignment [40–42], a single stop-to-Arg UGA reassignment ([43]; note that the same reassignment proposed by the authors also for the UAG codon does not seem to be justified by the data), a putative case of a dual (sense/stop) UGA meaning with an unclear identity of the encoded amino acid [44], and a single Ile-to-Met AUA reassignment [45], not counting the same reassignment in the *de facto* chlorophyte plastids in peDinoflagellates [7].

**Fig 8:**
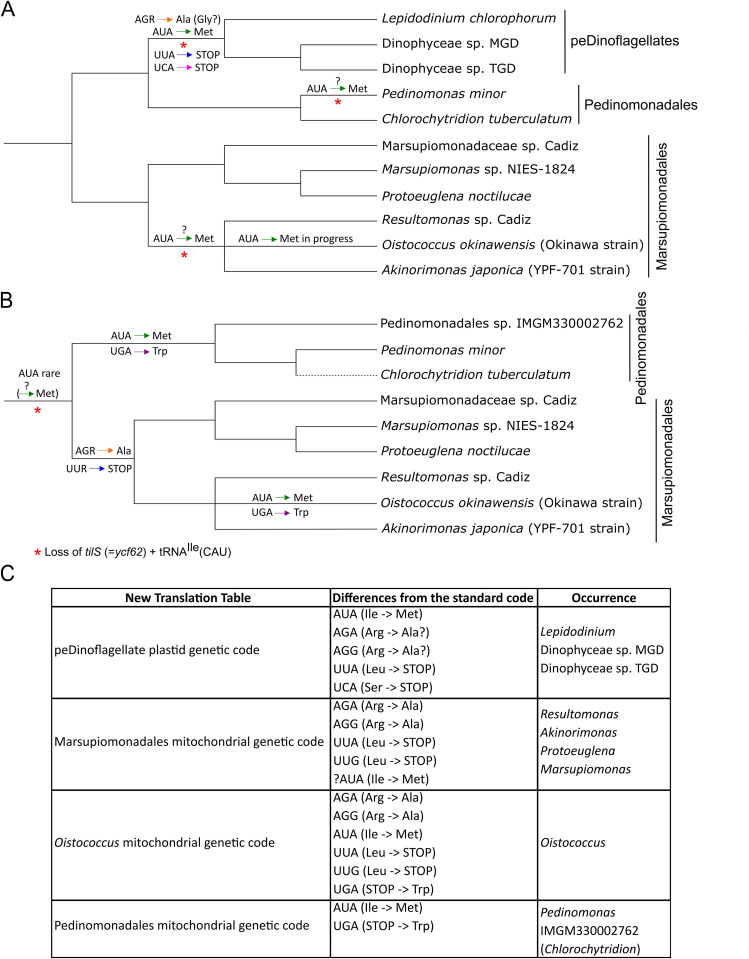
Synthetic overview of evolutionary events in the genetic code evolution in pedinophyte organelles. Genetic code changes in plastids (A) and mitochondria (B) mapped onto the consensus phylogeny of the group based on our phylogenomic analyses. Plastids of peDinoflagellate are included in the scheme as a pedinophyte plastid offshoot, placed as a sister lineage of Pedinomonadales according to previous analyses [7]. Note that the branching order indicated in panel B for the three Pedinomonadales representatives reflects the plastome-based phylogeny reported by Shrestha et al. [9], assuming that the Pedinomonadales sp. IMGM330002762 mitogenome belongs to the same organism as the plastome IMGM3300027621_BIN154. Codon reassignments are mapped to branches where they are inferred to have occurred, with a question mark above the respective arrow indicating that the reassignment is uncertain or perhaps in an initial stage. The uncertainty regarding the identity of the amino acid encoded by the AGR codons in peDinoflagellate plastids (see main text) is also indicated. (C) Novel translation tables (not yet included in the NCBI list of alternative genetic codes) required for proper conceptual translation of peDinoflagellate plastid genes and mitochondrial genes in different subsets of pedinophyte taxa. The question mark at the AUA codon in the Marsupiomonadales mitochondrial genetic code marks the uncertain meaning of this rarely used codon (perhaps primarily decoded by the near-cognate tRNA^Met^_CAU_; see main text). Note also that the validity of the newly proposed “Pedinomonadadales code” to *Chlorochytridion* needs to be tested by analysing a full mitogenome sequence to confirm AUA as a methionine codon (this is also indicated in the figure by using a dashed line for placing *C. tuberculatum* in the tree in panel B).

By expanding the sampling and analyses of organellar genomes of pedinophytes and their evolutionary derivatives, i.e., peDinoflagellate plastids, we further underscore the propensity of green algal organelles for evolutionarily unleashing their genetic codes. In fact, pedinophyte(-derived) organelles emerge as a hot-spot of genetic code evolution and substantially broaden the range of genetic code variation reported from organelles, particularly from plastids. Below we discuss the individual cases of newly documented genetic code changes, focusing on the specific evolutionary scenario that has generated them. The evolutionary events pertaining to the genetic code in pedinophyte plastids and their descendants in peDinoflagellates are schematically depicted in Fig 8A, the analogous scheme for pedinophyte mitochondria is provided as Fig 8B. We note that some of our interpretations are necessarily speculative and will have to be tested by targeted biochemical or proteomic investigations. For example, in the following discussion we assume that codon decoding in pedinophyte mitochondria may depend on tRNAs imported from the cytosol, a mechanism common in mitochondria in many other eukaryotes. In contrast, tRNA import into plastids is presently documented or anticipated only in a few land plant lineages that exhibit reduced plastidial tRNA gene sets [46,47], which is not the case of pedinophyte and peDinoflagellate plastids; hence, we consider only explanations not assuming tRNA import into these plastids.

### Parallel evolution of sense-to-sense codon reassignments in pedinophyte(-derived) organelles

Our analyses point to a possible parallel evolutionary trend that affects the plastids and mitochondria in pedinophytes and concerns the AUA codon. For pedinophyte plastomes, the simplest interpretation is that with the loss of *trnI(cau)* and *tilS* independently in the *Ped. minor* and Resultomonadaceae lineages, AUA (perhaps already used only rarely) became a *de facto* unassigned codon, inefficiently decoded by near-cognate tRNAs, primarily perhaps by the elongator tRNA^Met^_CAU_. Nevertheless, at least in the case of *O. okinawensis* the AUA codon appears to be on the way to become reassigned to methionine, hypothetically thanks to a modification of tRNA^Met^_CAU_ that increases its affinity to the codon. The strongly documented establishment of AUA as a commonly used methionine codon in plastomes of peDinoflagellates [7,19] indicates the presence of a much more efficient decoding mechanism than in *O. okinawensis*, let alone the other *trnI(cau)*/*tilS*-lacking pedinophytes. We may speculate that the pedinophyte plastid exhibits a “preadaptation”, possibly a specific feature associated with tRNA^Met^_CAU_ increasing its ability to decode the AUA codon, which makes the pedinophyte(-derived) plastid prone to losing the standard AUA decoding machinery and entering a path towards a fully-fledged Ile-to-Met reassignment of the codon. The potential uniqueness of the situation in pedinophytes/peDinoflagellates is highlighted by the fact that there is only a single other known instance of the AUA reassignment in plastids, namely in the *Chloroparvula* lineage of the green algal class Chloropicophyceae [45].

While the loss of the ancestral mechanism of AUA decoding in pedinophyte plastid has affected separately only some of the lineages within the class, an analogous change pertaining the mitochondrion seems to have occurred in an early pedinophyte evolution. The standard decoding of AUA as isoleucine in mitochondria may in principle still be achieved, mediated by an imported tRNA utilizing a lysidinylation-independent mechanism, as is the case of certain metazoan lineages [48], but our analyses paint an alternative picture (Fig 8B). Specifically, the data suggest that the pedinophyte stem lineage experienced the loss of the canonical AUA-decoding machinery accompanied with a drastic reduction of the AUA codon usage, although perhaps not its complete disappearance. Most lineages descending from the last pedinophyte common ancestor have kept the mitochondrial AUA codon abundance low and presumably translate it with the near-cognate elongator tRNA^Met^_CAU_, like we proposed for the pedinophyte plastids that independently lost the *tilS* gene and the standard AUA-decoding tRNA. However, in the lineages leading to Pedinomonadales and *O. okinawensis*, AUA has been independently adopted as a *bona fide* methionine codon thanks to a presently unknown molecular adaptation, most likely by the tRNA^Met^_CAU_, specified by the mitogenome in *O. okinawensis* but imported from the cytosol in Pedinomonadales, receiving a post-translation modification that makes it an efficient AUA decoder. At least two different alternative modifications of the mitochondrial tRNA^Met^_CAU_ underpinning AUA decoding as methionine have been characterized, both from different opisthokont models [49], but their possible occurrence in pedinophyte mitochondria cannot be inferred from purely bioinformatic analyses. It would be interesting to investigate if this hypothetical mechanism may at the same time operate in the plastid of peDinoflagellate and those pedinophytes that lack the standard AUA-cognate RNA^Ile^. Notably, the fate of the mitochondrial AUA codon in pedinophytes is probably not unprecedented among chlorophytes as a whole, as it was flagged as an emerging methionine codon also in the *Scenedesminia* phylogroup [32].

When a genetic code change is encountered in an organism being the first representative of a broader group investigated in this regard, it may stem from a recent evolutionary change that have impacted a terminal branch or may be indicative of a more broadly shared trait due to an ancient evolutionary event. The latter happens to be the case of the Arg-to-Ala reassignment of the AGR codons previously identified in a single member of the pedinophyte order Marsupiomonadales [18] and here, thanks to our expanded sampling, showed to be an apomorphy of the whole group (Fig 8B). Much more surprisingly, our analyses revealed that the same two codons have changed their meaning also in the plastids of peDinoflagellates, expanding thus the scope of genetic code changes in these poorly characterized secondary plastids beyond the previously noticed AUA reassignment [7,19]. Specifically, we provided convincing evidence that neither AGA nor AGG encode arginine in peDinoflagellate plastids and have undergone a sense-to-sense reassignment. Although investigating the occurrence of the AGR codons at conserved positions provided a mixed signal regarding the identity of the encoded amino acid, evaluating the identity elements of the cognate tRNA strongly favours its charging with alanine over the competing glycine. However, we note that decoding of AGR codons as Gly is robustly documented from mitochondria of certain metazoans, with the cognate tRNAs lacking some of the tRNA^Gly^ identity elements [50,51]. Combined, we posit that the AGR codons in peDinoflagellate plastids are translated as alanine, but call for verification of this conclusion by a proteomic analysis.

### Non-standard termination codons in peDinoflagellate plastids and pedinophyte mitochondria

An even more unexpected genetic code change in peDinoflagellate plastomes has impacted the UUA and UCA codons, which we show to have switched to signal translation termination, recording thus the first known case of a sense-to-stop reassignment outside mitochondria. Furthermore, our analyses shed additional light on the diverging evolutionary paths followed by the molecular machinery of plastidial translation. Mitochondria employing non-standard termination codons have been investigated before and found to exhibit various departures concerning their RF set. In vertebrates a novel specialized paralog of mtRF1a evolved to recognise the (normally arginine) codons AGA and AGG [52,53], while in *Scenedesminia* and in Labyrinthulea specific mutations in the codon-binding region of mtRF1a and mtRF2a, respectively, have been proposed to broaden the codon specificity of these RFs [32,33]. Our results indicate that peDinoflagellate plastids have followed the latter mechanism to allow for using the UYA codon pair to terminate translation. Indeed, the identification of the same organellar RF2 mutation in two independent lineages exhibiting the Leu-to-stop UUA reassignment, i.e., the substitution of the Ser204 residue in the labyrinthulean mtRF2a [33] and the peDinoflagellate pRF2 (this work; Fig 6B), makes a very strong case for the mutation being directly mechanistically involved in the changed UUA meaning. Regarding the mechanism of UCA decoding as a translation termination signal in peDinoflagellate plastids, there is a precedent in the Scenedesmaceae mitochondria, in which a mutation of a conserved arginine residue in mtRF1a was proposed to be involved [32]. However, this substitution seems to broaden the codon-binding specificity of the protein towards both UCA and UCG (both being termination codons in Scenedesmaceae [32]), while the latter codon has clearly retained its standard meaning (encoding serine) in peDinoflagellate plastids (Figs 5A and S6B and S5 Table). Indeed, the codon-binding region of the peDinoflagellate pRF1 looks normal (S12 Fig). Hence, we propose that UCA in peDinoflagellate plastids is decoded by pRF2, adapted to this task by the additional modifications that accompany the Ser204 substitution (Fig 6B).

Strikingly, in parallel to the discovery of the sense-to-stop codon reassignments in peDinoflagellate plastids, we found evidence that an analogous genetic code change has affected the mitochondria of Marsupiomonadales. Specifically, all Marsupiomonadales share the lack of UUA and UUG as the standard leucine codons, which is correlated with a major modification of the codon-binding domain of their mtRF1a protein (Fig 8B). Crucially, analogously altered mRF1a was previously found in the non-pedinophyte green alga *Pseud. marina* and proposed to underpin the use of UUA and UUG as termination codons in the mitochondrion of this organism [32]. Hence, we encounter another parallel association between particular RF modifications and non-standard termination codon use, this time involving mtRF1a and UUR in two different chlorophyte lineages. Such a pattern strongly supports a direct causal link between the changes in the RF and the specifically changed codon meaning. We, therefore, posit that the decoding of both UUA and UUG as termination codons in Marsupiomonadales is mediated by their modified mtRF1a proteins.

UUA and UUG being abundant standard codons in the Pedinomonadales mitochondria (see Results) are not informative on the actual evolutionary course towards their reassignment in the Marsupiomonadales stem lineage. Hence, any of the three major codon reassignment models [54] is conceivable in this case. Removal of the UUR codons from the mitogenome prior to any change in their decoding mechanism, as assumed by the codon disappearance (also called codon capture) model, may have occurred in the Marsupiomonadales stem lineage via mutating these codons to the synonymous CUR codons owing to a GC-favouring mutation bias. Indeed, the GC content of Marsupiomonadales mitogenomes is generally much higher than the GC content of the two Pedinomonadales mitogenomes available for comparison (Table 2), allowing for the possibility that the latter represents the ancestral state in Pedinophyceae as a whole and Marsupiomonadales have increased their mitogenome GC content secondarily. With the ambiguous intermediate model as an explanation for UUR reassignment in Marsupiomonadales, mtRF1a had acquired the mutations making the protein cognate to UUR before the cognate tRNA was lost, but if the two changes happened in the reverse order, the unassigned codon model was followed. In this regard it would be interesting to investigate the presently missing sequence of the mtRF1a from *Marsupiomonas* sp. NIES-1824, which does not use UUR codons at all. On the ground of parsimony reasoning we expect it exhibits the same modifications as the other Marsupiomonadales members, which would imply that it evolved from an ancestor that used UUR as termination codons and their absence in the *Marsupiomonas* sp. NIES-1824 mitogenome is secondary. More unexpected would be to find the *Marsupiomonas* sp. NIES-1824 mtRF1a having a standard structure, as this would necessitate to invoke multiple recurrent Leu-to-stop UUR reassignments in Marsupiomonadales evolution.

Regarding modifications in mtRF1a in pedinophytes, it is notable that the proteins in Resultomonadaceae additionally exhibits a substitution of the conserved Gln181 residue (Fig 7C), which is analogous to a substitution observed in mtRF1a from representatives of the green algal phylogroup *Scenedesminia* (Chlorophyceae) characterized by a stop-to-sense reassignments of the UAG codon in their mitochondria. While this substitution was hypothesized to disable the mtRF1a protein to recognize UAG in *Scenedesminia* [32], Resultomonadaceae members have retained it as a termination codon (S10 Table). It is possible that the effect of Gln181 substitution is contingent on other positions in the protein, and in the specific case of mtRF1a in Resultomonadacae it is compensated for by the other unusual mutations (substitutions and an insertion) seen in the protein (Fig 7C). Alternatively, our new observation may mean that the hypothesis that Gln181 substitution interferes with UAG binding by mtRF1a is not valid, which would necessitate looking for a different change in mtRF1a to explain the stop-to-sense UAG reassignments in *Scenedesminia*.

### The evolution of UGA decoding in pedinophyte mitochondria

Before our study it was not known whether the stop-to-Trp UGA reassignment in the mitochondrion of *Ped. minor* [17] is unique to this species among pedinophytes or whether it indicates a broader occurrence of this generally common genetic code change. Here we show that it is shared by the uncultivated Pedinomonadales sp. IMGM3300027621 (S16 Fig) and most likely also the pedinomonadalean *C. tuberculatum*, as we could infer from the few mitochondrial transcripts found in the transcriptome assembly available for this species (S20 Fig), indicating that this is a feature characteristic and ancestral for the whole order Pedinomonadales. However, the stop-to-Trp UGA reassignment is clearly polyphyletic in pedinophytes, as we here report it from the newly isolated Marsupiomonadales member *O. okinawensis* phylogenetically nested among relatives lacking this reassignment (Fig 8B). This is hardly surprising, as the same reassignment has evolved multiple times in mitochondria across the eukaryote phylogeny and may be a feature of whole major clades [36,55–58]. In green algae its occurrence is restricted to a few terminal branches. In addition to the two pedinophytes, it has been reported only from *Pseud. marina* (=*Pyc. provasolii*, see above) [38] and two unrelated representatives of the class Chlorophyceae, i.e., the genus *Jenufa* [59] and *Limnomonas spitsbergensis* [60]. Furthermore, although not explicitly mentioned in the accompanying publication [41], UGA as a Trp codon was noticed in the mitogenome of the non-photosynthetic chlorophycean Volvocales sp. NrCl902 (LC516061.1).

This sporadic recurrent UGA reassignment in the Chlorophyta phylum reflects a specific situation in this group, whose ancestor was proposed to have ceased to use UGA in mitochondrial genes and to have lost the mtRF2a protein recognizing UGA as a termination codon [36]. The reappearance of UGA with the new meaning as a tryptophan codon in distantly related chlorophytes is then an exemplar manifestation of the codon disappearance model of codon reassignment [54]. The UGA usage in mitochondria of pedinophytes other than those with the reassignment is consistent with this interpretation, as the codon is either completely missing or present at very low numbers with the apparent function of a termination codon, namely twice in *Marsupiomonas* sp. NIES-1824 (in the *cox1* and *nad4L* genes) and once in *Prot. noctilucae* (in the non-conserved *orf761*, which is not necessarily a functional gene). We confirmed the absence of a mtRF2a ortholog in all pedinophytes for which nuclear genome or transcriptome assemblies are available, and we extrapolate the protein is missing also in *Marsupiomonas* sp. NIES-1824. Notably, analogous rare occurrences of mitochondrial UGA as a termination codon in the absence of mtRF2a have been previously noticed in certain other chlorophytes.

The latter observations raise the question as to how translation termination at the UGA codon is achieved in these cases, and two alternative mechanisms of translation termination at these codons have been proposed. One might operate in the family Scenedesmaceae, hypothesized to rely on mtRF1a having its codon specificity extended by unique mutations making it more mtRF2a-like [32]. Of the available pedinophyte mtRF1a sequences only that from *C. tuberculatum* exhibits one of the mutations (unusual serine residue at the position 206; Fig 7C), but as this alga most likely has UGA as a tryptophan codon, its mRF1a is not expected to recognize UGA. This finding casts doubt on the hypothetical effect of the Ser206 residue in the mtRF1a towards its affinity to UGA. Regardless, the mtRF1a sequence from *Marsupiomonas* sp. NIES-1824, where the question of translation termination at the mitochondrial UGA is most relevant, cannot be inspected at present, so a possible modification of this protein that would make it cognate to UGA cannot be ruled out.

An alternative explanation for the occasional occurrence of UGA as a termination codon in chlorophyte mitochondria is dual targeting of the pRF2 protein to both the plastid and the mitochondrion [36]. *Marsupiomonas* sp. NIES-1824 uses UGA as a termination codon in three plastid genes (S3 Table), indicating that pRF2 is present in this alga (again, we cannot check this directly) and may thus potentially serve also in its mitochondrion. In this regard it is interesting to note that the pedinophyte plastomes are depauperate in the UGA occurrence, with the codon being completely absent or present only up to three times (S3 Table), which conforms to a pattern that has been previously noticed to be common in chlorophyte plastomes in general [36]. Two species, *A. japonica* and *Resultomonas* sp. Cadiz, do not use UGA in any of the two organelles, but pRF2 has been retained by all pedinophytes for which this could be checked by genome or transcriptome data, including *A. japonica* (S11 Fig). Why pRF2 is then preserved in *A. japonica* if mtRF1a and pRF1 are theoretically capable to secure translation termination at both the UAG and UAA codons in the mitochondria and plastids? We speculate that the answer may relate to the fact that the Marsupiomonadales mtRF1a proteins exhibit mutations in the codon-binding region that we above hypothesized to endow the protein to recognize UUR codons. It is possible that the mutations negatively affect the ability of the protein to bind UAA, i.e., the most abundant termination codon in Marsupiomonadales mitochondria, whose decoding would then depend on the imported pRF2.

### Concluding remarks

By focusing on organellar genomes of pedinophytes and their second-hand evolutionary descendants, we detected changes in the codon meaning that had been missed by previous investigations. Possibly most notable is the use of UYA codons to terminate translation in peDinoflagellate plastids, which is the first case of a sense-to-stop codon reassignment ever discovered outside mitochondria. Our analyses thus uncovered the existence of several new genetic code variants (specific combinations of codon meaning; Fig 8C) that are not covered by the current list of genetic codes maintained by the National Center for Biotechnology Information (https://www.ncbi.nlm.nih.gov/Taxonomy/Utils/wprintgc.cgi) and used for conceptual translation of nucleotide sequence data to populate protein sequence databases. A corollary of this is the fact that many pedinophyte (and peDinoflagellate) protein sequences currently stored in the databases, and thus commonly employed by researchers for various analyses, are incorrect. This problem is not new and affects mitochondrion-encoded proteins from other groups with genetic code variants not fitting the “official” list [32,33]. Hence, the community of biologists should exercise more care when deducing the amino acid sequences of organelle-encoding proteins, and a major update of the sequence database content is in order. On a more positive note, our results provide a strong new evidence for the conclusion that particular mutations in the organellar RF1-type or RF2-type release factors evolve recurrently to extend the codon-binding specificity of the protein towards codons normally encoding amino acids, providing thus the mechanistic underpinnings of specific sense-to-stop codon reassignment. Future investigations into these modified release factors, including atomistic computer simulations and possibly also wet-lab experiments, will illuminate the exact role of the candidate mutations in the changed codon-binding specificity.

**Formal description of new taxa**

**Class Pedinophyceae Moestrup 1991**

**Order Marsupiomonadales B.Marin 2012**

**Family Resultomonadaceae B.Marin 2012**

#### *Oistococcus* Barcytė and M.Eliáš gen. nov.

Description: Cells oval or disc-shaped. Flagellum single, inserted sublaterally, approximately twice the cell length. Flagellar groove present. Chloroplast single with a pyrenoid. Eyespot present in flagellated cells. Asexual reproduction by zoospores and aplanospores. Sexual reproduction by anisogamy.

Etymology: The genus name originates from OIST (Okinawa Institute of Science and Technology) to mark the stay of DB at this institution during which she collected the sand sample from which the organism was isolated. In addition, the sample was collected at the beach adjacent to OIST, adding a special significance to the name. The root -coccus refers to the ball-like cells typical for this organism.

Type species: *Oistococcus okinawensis* sp. nov.

#### *Oistococcus okinawensis* Barcytė and M.Eliáš sp. nov. (Fig 1A–1G).

Description: With the characters of the genus. Flagellate cells 4.0–8.0 µm long and 2.5–6.5 µm wide. Ball cells (including sporangia) 6.0–27 µm in diameter.

Holotype: Metabolically inactive (cryopreserved) culture at the Culture Collection of Algae of Charles University in Prague, Czech Republic (CAUP; https://botany.natur.cuni.cz/algo/caup.html) under the code CAUP-CRYO-Okinawa.

Type locality: Tancha beach, Onna village, Okinawa, Japan (26°28’9.7“N 127°49’43.3”E)

Etymology: The species epithet refers to Okinawa island (Japan) from where the organism was isolated.

#### *Akinorimonas* Barcytė and M.Eliáš gen. nov.

Description: Cells ovoid to ellipsoidal. Flagellum single, inserted sublaterally, matching the length of the cell or longer. Chloroplast single, with eyespot. Pyrenoid absent. Asexual reproduction by binary fission. Sexual reproduction by anisogamy.

Etymology: The genus is named to honour Dr. Akinori Yabuki, a distinguished protistologist who isolated strain NIES-2566, on the basis of which the type species of the genus is described. The term “monas” is included in the genus name in accord with an established tradition to use it for genera representing organism that are flagellated in their vegetative state.

Type species: *Akinorimonas japonica* sp. nov.

#### *Akinorimonas japonica* Barcytė and M.Eliáš sp. nov. (Fig 2A–2C).

Description: With the characters of the genus. Flagellate cells 4.0–6.5 µm long and 2.0–4.5 µm wide.

Holotype: The authentic strain NIES-2566 permanently cryopreserved in a metabolically inactive state in the Microbial Culture Collection at the National Institute for Environmental Studies (NIES Collection, Tsukuba, Japan).

Type locality: Isonoura Beach, Isonoura, Wakayama, Japan (34°15’32“N, 135°5’41”E).

Etymology: The species epithet refers to Japan, from where the organism originates.

## Materials and methods

### Strain origin, cultivation, microscopy, DNA isolation, and sequencing

A pedinophyte alga (Okinawa strain) was isolated from a sand sample taken in Tancha beach, Onna village, Okinawa, Japan at the end of April 2023. The established strain was cultivated in F/2 medium (using either NaNO_3_ or NH_4_Cl as a nitrogen source) at 20°C under continuous light. For morphological observations, we additionally purchased the unidentified pedinophyte NIES-2566 (= Pedinophyceae sp. YPF-701; https://mcc.nies.go.jp/). Light microscopic observations were performed using an Olympus BX53 light microscope (Tokyo, Japan). Micrographs were taken with an Olympus DP73 digital camera (Tokyo, Japan). The Olympus cellSens Imaging Software v1.6 was used to process images and obtain morphometric measurements of the cells. Specimens for TEM were prepared as detailed in Barcytė et al. [27], and grids were examined using a JEOL JEM-1011 electron microscope (Tokyo, Japan). Total genomic DNA was isolated from Okinawa strain by first grinding the centrifuged culture under liquid nitrogen and then using MasterPure Complete DNA & RNA Purification Kit (Biosearch Technologies, Hoddesdon, UK) according to the manufacturer’s instructions. The extracted DNA was sequenced with Illumina NovaSeq6000 (PE150 mode) by Eurofins Genomics (Constance, Germany).

### Assembly of organellar genomes

The sequencing reads obtained for Okinawa strain were trimmed with Trimmomatic v.0.39 [61] with the following settings: ILLUMINACLIP:2:20:10 LEADING:3 TRAILING:3 SLIDINGWINDOW:4:15 MINLEN:75. The reads were assembled with SPAdes v3.15.5 with the default settings [62]. The resulting assembly contains sequences from bacteria co-cultured with the alga, and the nuclear genome sequence is fragmented into many scaffolds, limiting the utility of the assembly for large-scale analyses of the nuclear gene repertoire. TBLASTN searches were carried out against the assembly using amino acid sequences from both plastid and mitochondrial proteins of *Ped. minor* as queries (GenBank: NC_016733.1 and NC_000892.1, respectively). Both plastome and mitogenome were assembled using NOVOPlasty v4.3.5 [63]. The *rbcL* gene extracted from the SPAdes assembly was used as a starting point (seed) for the plastome assembly, while the *nad6* gene served the same purpose for the mitogenome assembly. In both assemblies, a k‐mer of 33 was used, and organellar genomes were assembled into single, circular mapping contigs (both as two alternative, presumably co-existing variants due to the presence of inverted repeats). The assembled genome sequences were deposited at GenBank and are available under the accession numbers PX112427(plastome) and PX118532 (mitogenome).

We used PebbleScout [64] to probe the metagenomic sequence data in the Sequence Read Archive (SRA) for sequences identical to the *Resultomonas moestrupii* plastid *rbcL* gene (U30288.1). Three samples were identified (ERR4674117, ERR4674714, and ERR4674142), coming from two closely spaced localities in the shoreline zone of the Gulf of Cadiz. Given the geographic proximity of these samples we co-assembled the original metagenome sequencing reads from all three of them using MEGAHIT v1.2.9 with default settings [65]. Three main plastid scaffolds, one with the coverage twice as high as the other two (as expected from the inverted repeat region), were identified as originating from a member of the family Resultomonadaceae. This organism was denoted *Resultomonas* sp. Cadiz, based on the extremely high similarity of its *rbcL* or plastidial rRNA gene sequences to the previously published *R. moestrupii* sequences. The termini of the three scaffolds exhibited perfect overlaps and could be manually joined into a complete circular mapping sequence. Meanwhile, a complete mitochondrial genome sequence, assembled as a single scaffold and identified as originating from a member of the Resultomonadaceae based on its expected placement in the mitochondrial phylogeny, was assigned to *Resultomonas* sp. Cadiz.

The same metagenome assembly additionally contained genomic fragments of another pedinophyte, identified as a member of the family Marsupiomonadaceae based on single-gene phylogenetic analyses and denoted Marsupiomonadaceae sp. Cadiz. Similar to *Resultomonas* sp. Cadiz, we detected three plastome scaffolds from Marsupiomonadaceae sp. Cadiz in the assembly. Even though the terminal sequences of these scaffolds turned out to be chimeric, we could manually reconstruct a nearly complete plastid genome by leveraging sequence overlaps across the scaffolds and searching for the sequencing reads that bridged these pieces. The reconstructed Marsupiomonadaceae sp. Cadiz plastome sequence lacks only one of the copies of the inverted repeats, as the *psbA* and *psaC* gene sequences flanking the region remained incomplete. As for *Resultomonas* sp. Cadiz, the mitogenome of Marsupiomonadaceae sp. Cadiz (assigned to this organism based on the fact that no other mitogenome sequence from a Marsupiomonadaceae representative was identified in the total assembly) was also assembled as a single scaffold. Both the plastome and mitogenome sequences of the metagenomically assembled members of the families Resultomonadaceae and Marsupiomonadaceae are available on the Figshare repository (https://doi.org/10.6084/m9.figshare.29835647).

The mitochondrial genome sequence could not be found in the previously published genome assembly from Pedinophyceae sp. YPF-701 [10]. Hence, we downloaded the available raw Illumina (ERR6667566) and Oxford Nanopore reads (ERR6667567) deposited by Repetti et al. [10] to the SRA and assembled them using Unicycler v0.4.8 [66]. This mitogenome sequence was identified in the assembly and deposited to the GenBank under the accession number BK072070. The assembled but unannotated organellar genome sequences of *Prot. noctilucae* were downloaded from GenBank (OY253692.1 and OY253691.1). To obtain the mitogenome sequence corresponding to the uncultivated Pedinomonadales member (further referred to as Pedinomonadales sp. IMGM330002762) represented by the metagenome-derived plastome IMGM3300027621_BIN154 reported in a recent preprint [9], the respective full metagenome assembly (IMGM3300027621) was downloaded from the JGI IGM/M database (https://img.jgi.doe.gov/) and searched with TBLASTN with protein sequences encoded by the *Ped. minor* mitogenome. A single non-redundant set of scaffolds exhibiting a specific relationship to pedinophyte mitogenomes was retrieved and assembled into a single linear sequence that could not be closed into a circular-mapping sequence but includes all genes present in the *Ped. minor* mitogenome, indicating it is nearly complete.

### Genetic code analyses

To identify potential deviations from the standard codon meaning, we initially analysed the organellar genomes of pedinophytes and plastid genomes of peDinoflagellates with FACIL [67] and Codetta [68]. In addition, we employed an approach analogous to that previously used to analyse genetic code changes in mitochondria of other groups [32,33]. Briefly, all coding sequences were translated using the standard genetic code and added to the respective sets of orthologs in a dataset of green algal plastome-encoded protein sequences (“Chloro dataset”) used for the phylogenomic analysis described below (see “Phylogenetic and phylogenomic analyses”) or in a pre-existing dataset of eukaryotic mitogenome-encoded protein sequences [32]. Multiple sequence alignments were then computed and a custom Python script was used to evaluate codons based on positions where a single amino acid was present in over 70% of sequences. Next, the sequences were retranslated using specific criteria: a codon was considered potentially reassigned if it appeared in at least five conserved alignment positions, with fewer than 10% aligning to the standard amino acid. Such codon was assigned an alternative translation if the most frequent conserved amino acid appeared at least five times and was twice as common as the second most frequent. Otherwise, such codons were translated as an unknown amino acid (X). The resulting proteins were then analysed analogously, but with a smaller cohort consisting only of sequences from pedinophyte and peDinoflagellate for plastid genes (“Pedino dataset”) or only of chlorophyte sequences for mitochondrial genes. The resulting plots are presented as S1 and S2 Datasets for the plastid and mitochondrial genes, respectively. We note that the Pedinomonadales sp. IMGM330002762 mitogenome sequence was obtained only at the very late stage of our study after the iterative analysis of the codon meaning in pedinophyte mitochondria had been completed. To estimate the most likely meaning of the codons in this mitogenome, we translated the nucleotide sequences using the standard genetic code, added them to the “Chloro iter2” dataset, and analyzed the updated alignments as described above. The resulting plots for each codon in Pedinomonadales sp. IMGM330002762 are provided as S3 Dataset.

As translation and hence codon interpretation concerns mature mRNA molecules rather than gene sequences, the analyses of the genetic code in peDinoflagellate plastids had to account for RNA editing that is known to occur in these organelles and changes the nucleotide sequences of primary transcripts [7]. To this end, RNA-seq reads of *L. chlorophorum* (SRA DRR124369), Dinophyceae sp. MGD (DRR190721), and Dinophyceae sp. TGD strain (DRR190720) were mapped to the corresponding plastome sequences using BWA v0.7.17-r1188 [69] and SAMtools v1.19.2 [70]. The resulting alignments were inspected with IGV v2.4.17 [71]. Discrepancies between plastid genome sequences and mapped reads, with a focus on protein-coding regions, were manually identified and corrected. A position was considered edited only when the majority of reads supported the change. In one case (the *rpoC2* gene from Dinophyceae sp. TGD), we also corrected an apparent one-nucleotide deletion in a homopolymer track that most likely represents an error in the plastome sequence and introduces a frame-shift into the coding sequence, splitting it into two separate parts. Since some positions were either not covered by reads or covered by too few reads to reliably infer post-transcriptional base conversions, not all edited positions could be corrected. We thus note that the codon counts reported for peDinoflagellate plastomes should be regarded as approximate, and the translated sequences may not be fully accurate.

### Annotation of organellar genomes

The newly sequenced or assembled organellar genomes, and those available before but lacking a published annotation, were annotated with MFannot available online (https://megasun.bch.umontreal.ca/apps/mfannot/ [72]). For plastid genome the conventional Translation Table 11 was used, whereas for mitogenomes Translation Table 1 or Translation Table 4 was employed depending on the inferred meaning of the UGA codon in the respective mitogenome (stop codon or Trp codon, respectively). The delimitation of coding sequences (CDSs) was then curated manually, with the 5’ ends (initiation codons) adjusted based on the comparison with closest homologs, whereas in the case of mitochondrial genes in Marsupiomonadales, UUA and UUG were considered as termination codons and the 3’ ends of CDSs were redefined accordingly in relevant cases. The annotation of tRNA genes was additionally refined with the help of tRNAscan‐SE 2.0 [73], which we also used to reannotate the complements of tRNA genes in previously published genomes. The delimitation of rRNA genes was further adjusted based on results of Infernal cmscan searches against the Rfam database (https://www.ebi.ac.uk/jdispatcher/rna/infernal_cmscan). The identity of some open reading frames (ORFs) was verified using HHpred [74]. Organellar genome maps were generated using OGDRAW v1.3.1 [75].

### Phylogenetic and phylogenomic analyses

For phylogenetic analyses of the 18S rRNA gene, the respective sequences were compiled from GenBank and from our newly generated (meta)genomic assemblies for 18 pedinophyte taxa and 74 other representative green algae. In the case of pedinophytes we included sequences from all major cultured and uncultured lineages except for long-branched clones of *Protoeuglena* [3]. The sequences were aligned with the L-INS-i method implemented in MAFFT v7 [76] using the online server (https://mafft.cbrc.jp/alignment/server/). Manual trimming of the alignment retained 1,800 well-aligned positions, which were subjected to a phylogenetic analysis using IQ-TREE v2.2.5 [77]. The best-fitting model, TIM3e+I + R4, was automatically chosen by the program (i.e., using the MFP option [78]) and branch support was estimated using 100 non-parametric bootstrap replicates; the same bootstrapping method was applied to all subsequent analyses. Since 18S rDNA sequence is not available for *R. moestrupii*, we additionally carried out phylogenetic analyses of the plastid rRNA operon (16S rDNA, *trnI(gat)*, *trnA(tgc)*, 23S rDNA) and *rbcL* sequences, including the respective sequences previously reported from this species [2,23], as well as highly divergent sequences derived from the three peDinoflagellate plastomes [7]. The plastid rRNA operon and *rbcL* datasets included 19 and 18 sequences, respectively, aligned in the same manner as described above for the 18S rDNA dataset. The plastid rRNA operon alignment was trimmed employing trimAl v1.2rev59 [79] with the -automated1 mode, resulting in 4,345 aligned positions. The phylogenetic tree was inferred using GTR + F + R4 evolutionary model as automatically selected by MFP. The *rbcL* alignment was not trimmed and was directly used for phylogenetic analysis with all 1,428 aligned positions. The GTR + F + I + G4 model was selected as best-fitting the data by MFP.

In order to better understand the evolutionary relationships of our newly sequenced Okinawa strain within pedinophytes, we incorporated its plastome-encoded protein sequences into the phylogenomic analysis utilizing 79 protein sequences as originally conceived by Turmel et al. [20]. We, in fact, further expanded the datasets updated recently by Barcytė et al. [27] by including additional pedinophytes: Pedinophyceae sp. YPF-701 (KY347917.1), *Prot. noctilucae* (OY253692.1), the newly assembled MAGs of *Resultomonas* sp. Cadiz and Marsupiomonadaceae sp. Cadiz, as well as *Scourfieldia* sp. M0560/2 without a settled taxonomic affiliation [14]. Given the evidence for the AUA codon not being translated in a standard way (as isoleucine) in the plastid of *Ped. minor* and Resultomonadaceae, we conceptually translated this (rare) codon arbitrarily as methionine (considering the putative evolutionary trend toward the fully-fledged Ile-to-Met AUA reassignment). The single-gene datasets were aligned with MAFFT v7.520 using the L-INS-i method. The alignments were trimmed with trimAl using the -gappyout mode. The trimmed alignments were concatenated with FASconCAT-G v1.05 [80], resulting in 81 sequences with 18,812 aligned positions. The Q.yeast+I + G4 model was chosen as best fitting the dataset (TEST option was used for determining the evolutionary model as implemented in IQ-TREE). The phylogenetic analysis of mitochondrion-encoded protein sequences utilized conceptual translations of annotated CDSs obtained with appropriate altered genetic codes, with the combinations of non-standard codon meaning differing for different taxa according to the codon reassignments identified in them as described in the Results section. Similar to the approach used for plastome-encoded proteins, we built a supermatrix containing 4,287 aligned positions from 16 mitogenome-encoded proteins and then used IQ-TREE (with the LG + F + I + G4 evolutionary model) to infer a maximum likelihood tree. All phylogenetic trees were visualized with the Interactive Tree of Life (iTOL) v6 (https://itol.embl.de [81]).

Phylogenetic relationships of tRNA genes in plastomes and mitogenomes of interest were investigated using the Neighbor-Net phylogenetic network approach. Sequence alignments were performed with MAFFT, employing the Q-INS-I algorithm for plastome tRNAs and the G-INS-I algorithm for mitogenome tRNAs. After alignment, anticodons were deleted from the sequences, and the phylogenetic networks were constructed using SplitsTree4 [82]. Organellar homologs of release factors in pedinophytes and peDinoflagellates were identified by BLAST from available sequence resources (details in S7 Table). In the case of taxa with only genome assemblies, the exon-intron structure of the respective genes was deduced manually considering sequence conservation with homologs (only incomplete gene models could be deduced with confidence in some cases). To identify organellar RF sequences from *Lepidodinium chlorophorum*, a transcriptome assembly was obtained from available RNA-seq reads (SRA DRR124369) using Trinity-v2.15.1 [83]. The presence of targeting presequences in peDinoflagellate bacteria-derived RFs was evaluated with TargetP-2.0 [84], while the presence of predicted transmembrane segments was assessed with TMHMM-2.0 [85]. For phylogenetic analyses, previously analysed sequences of organellar RFs were selected based on literature data and further supplemented by bacterial homologs collected using BLAST searched against the nr30 protein sequence database (https://toolkit.tuebingen.mpg.de/tools/psiblast). Phylogenies were inferred from alignments created using MAFFT with the L-INS-i method and trimmed with trimAl using the -gappyout mode. The tree search was conducted using IQ-TREE with the LG + F + R10 substitution model and ultrafast bootstrapping with 20,000 replicates.

## Supporting information

S1 Fig Full version of the ML phylogenetic tree inferred from nuclear 18S rRNA gene sequences. Further details on the tree can be found in the legend to Fig 3A, which shows a simplified version of the tree.
(PDF)

S2 Fig Full version of the ML phylogenetic tree inferred from a concatenated dataset of 79 plastome-encoded protein sequences. Further details on the tree can be found in the legend to Fig 4, which shows a simplified version of the tree.
(PDF)

S3 Fig Maximum likelihood phylogenetic tree inferred from a concatenated dataset of 16 mitogenome-encoded proteins from major lineages of Chlorophyta. The alignment used for the tree inference consisted of 4,287 amino acid positions, the substitution model employed was LG + F + I + G4. Newly obtained sequences from cultured pedinophyte representatives are shown in bold, while those assembled metagenomically are marked in burgundy.
(PDF)

S4 Fig Gene maps of the newly assembled/annotated plastid genomes. Genes are represented as blocks, with those transcribed in the clockwise direction facing inward and those transcribed in the counter-clockwise direction facing outward. Different colours indicate the functional categories of the genes. The inner circle plot displays the GC content, with the thin grey line marking 50%. Segments annotated in the inner circle include: IRA and IRB (inverted repeats, also indicated by thickened regions in the outer circle) and SSC and LSC (short and long single-copy regions, respectively). Note that the plastome of Marsupiomonadaceae sp. Cadiz is incomplete and is therefore shown as linear.
(PDF)

S5 Fig Maximum likelihood phylogenetic tree of SmpB proteins. The tree includes bacterial proteins and eukaryotic nucleus-encoded homologs targeted to plastid or mitochondria. The tree inference was based on an alignment created using MAFFT with the E-INS-i method, which was subsequently trimmed with trimAl using the -automated1 mode, resulting in 153 well-aligned positions. The analysis was conducted using IQ-TREE with the LG + R7 substitution model and ultrafast bootstrapping with 10,000 replicates.
(PDF)

S6 Fig Plots showing the distribution of selected codons at conserved amino acid positions in plastomes from pedinophytes (A) and peDinoflagellates (B). Plots for all codons are provided in S1 Dataset. Note that no plot shown for the given codon and a particular species (of those analysed in this study) means that not a single codon in the respective whole genome occupied a conserved amino acid position as defined by our criteria (i.e., the same amino acid in at least 70% of all sequences compared). For further details concerning the methodology and display convention see the legend to Fig 5B.
(PDF)

S7 Fig FACIL outputs for pedinophyte plastomes analysed in this study. (PDF)

S8 Fig Neighbor-Net phylogenetic network of tRNA gene sequences from pedinophyte and peDinoflagellate plastomes. For tRNAs with the UCU anticodon, which are of specific interest in this study (see text), sequences are annotated separately for pedinophytes and peDinoflagellates.
(PDF)

S9 Fig Predicted structures of the peDinoflagellate plastidial tRNA^Ala^_UGC_. Note the unexpected nucleotide (U; highlighted) at the position 73 of the tRNA from *L. chlorophorum*.
(PDF)

S10 Fig Examples of UUA and UCA as termination codons in peDinoflagellate plastid genes. The display convention follows that one used in Fig 6A.
(PDF)

S11 Fig Phylogenetic analysis of organellar release factors in pedinophytes and peDinoflagellates. Sequences included in the analyses are indicated with the respective NCBI accession number unless stated otherwise, details on pedinophyte and peDinoflagellate sequences are provided in S7 Table. (A) Maximum likelihood phylogenetic tree inferred from selected bacterial and organellar RF1 homologs. (B) Maximum likelihood phylogenetic tree inferred from selected bacterial and organellar RF2 homologs.
(PDF)

S12 Fig Multiple sequence alignment of the codon-binding region in peDinoflagellate plastid-targeted RF1, selected bacterial homologs, and conventional pRF1 proteins. Accession numbers of the sequences included are provided in S11 Fig and S7 Table.
(PDF)

S13 Fig Gene maps of the newly assembled/annotated mitochondrial genomes. Genes are represented as blocks, with different colours indicating the functional categories of the genes. Note that all genes are located on the same strand, transcribed in the counter-clockwise direction. The inner circle plot displays the GC content, with the thin grey line marking 50%.
(PDF)

S14 Fig FACIL outputs for pedinophyte mitogenomes analysed in this study. (PDF)

S15 Fig Plots showing the distribution of selected codons in all studied pedinophyte mitogenomes at conserved amino acid positions. Plots for all codons are provided in S2 Dataset. For further details concerning the methodology and display convention see the legend to Fig 5B. (A) Evidence for UGA in *O. okinawensis* decoded as tryptophan. (B) Evidence for a reassignment of the AGR codons in Marsupiomonadales as opposed to *Ped. minor*. (C) Evidence for UUR codons having preserved their standard meaning in *Ped. minor*.
(PDF)

S16 Fig Analysis of codon meaning in the mitogenome of Pedinomonadales sp. IMGM3300027621. A – FACIL output, B – plots showing the distribution of selected codons at conserved amino acid positions. Plots for all codons are provided in S3 Dataset.
(PDF)

S17 Fig Neighbor-Net phylogenetic network of tRNA gene sequences from chlorophyte mitogenomes. (PDF)

S18 Fig Sequence alignment of the tRNA_UCU_ species specified by mitogenomes of Marsupiomonadales and selected other green algae. Note the G3·U70 pair characteristic for tRNA^Ala^ present in the tRNA sequences from Marsupiomonadales and *Tetradesmus obliquus* (Sphaeropleales), consistent with the Arg-to-Ala AGR reassignment in both taxa.
(PDF)

S19 Fig Examples of UUA and UUG as termination codons in Marsupiomonadales. The display convention follows that one used in Fig 6A.
(PDF)

S20 Fig Evidence for UGA encoding tryptophan *Chlorochytridion tuberculatum.* The figure shows alignments of protein sequences encoded by the mitogenome of *Pedinomonas minor* with homologous sequences (putative mitochondrial transcript) identified in the available transcriptome assembly from *C. tuberculatum* and conceptually translated with the standard genetic code. The asterisks highlighted in red correspond to UGA codons.
(PDF)

S1 Table Protein-coding genes (including ORFs) identified in pedinophyte plastomes. (XLSX)

S2 Table tRNAs genes identified in pedinophyte and peDinoflagellate plastomes. (XLSX)

S3 Table Codon counts for pedinophyte plastomes. Marsupiomonadaceae sp. Cadiz excluded due to an incomplete plastome sequence available.
(XLSX)

S4 Table Codetta outputs for selected codons in pedinophyte plastomes. (XLSX)

S5 Table Codetta outputs for selected codons in peDinoflagellate plastomes. (XLSX)

S6 Table Codon counts for peDinoflagellate plastomes. Be aware that the numbers may not be completely accurate due to regions of the genome where the possible effect of RNA editing to the mature mRNA sequences could not be checked due to lack of coverage in transcriptome data.
(XLSX)

S7 Table Pedinophyte and peDinoflagellate organellar release factors. (XLSX)

S8 Table tRNAs genes identified in pedinophyte mitogenomes. (XLSX)

S9 Table Codetta outputs for selected codons in pedinophyte mitogenomes. (XLSX)

S10 Table Codon counts for pedinophyte mitogenomes. (XLSX)

S1 Dataset Plots showing the distribution of codons at conserved amino acid positions in all studied pedinophyte and peDinoflagellate plastomes. Absence of a plot for a given codon in a given taxon means the codon was not present at any position deemed conserved.
(PDF)

S2 Dataset Plots showing the distribution of codons at conserved amino acid positions in all studied pedinophyte mitogenomes. Absence of a plot for a given codon in a given taxon means the codon was not present at any position deemed conserved.
(PDF)

S3 Dataset Plots showing the distribution of codons at conserved amino acid positions in the mitogenome of the uncultivated Pedinomonadales sp. IMGM330002762. Absence of a plot for a given codon means the codon was not present at any position deemed conserved.
(PDF)
