# Supplementary figures and images for: Expanded diversity of pedinophytes provides a window into the evolution of the genetic code in organelles
Source: PLoS Genet. 2025 Oct 22;21(10):e1011901. doi: 10.1371/journal.pgen.1011901 (PMC12574857; doi:10.1371/journal.pgen.1011901)

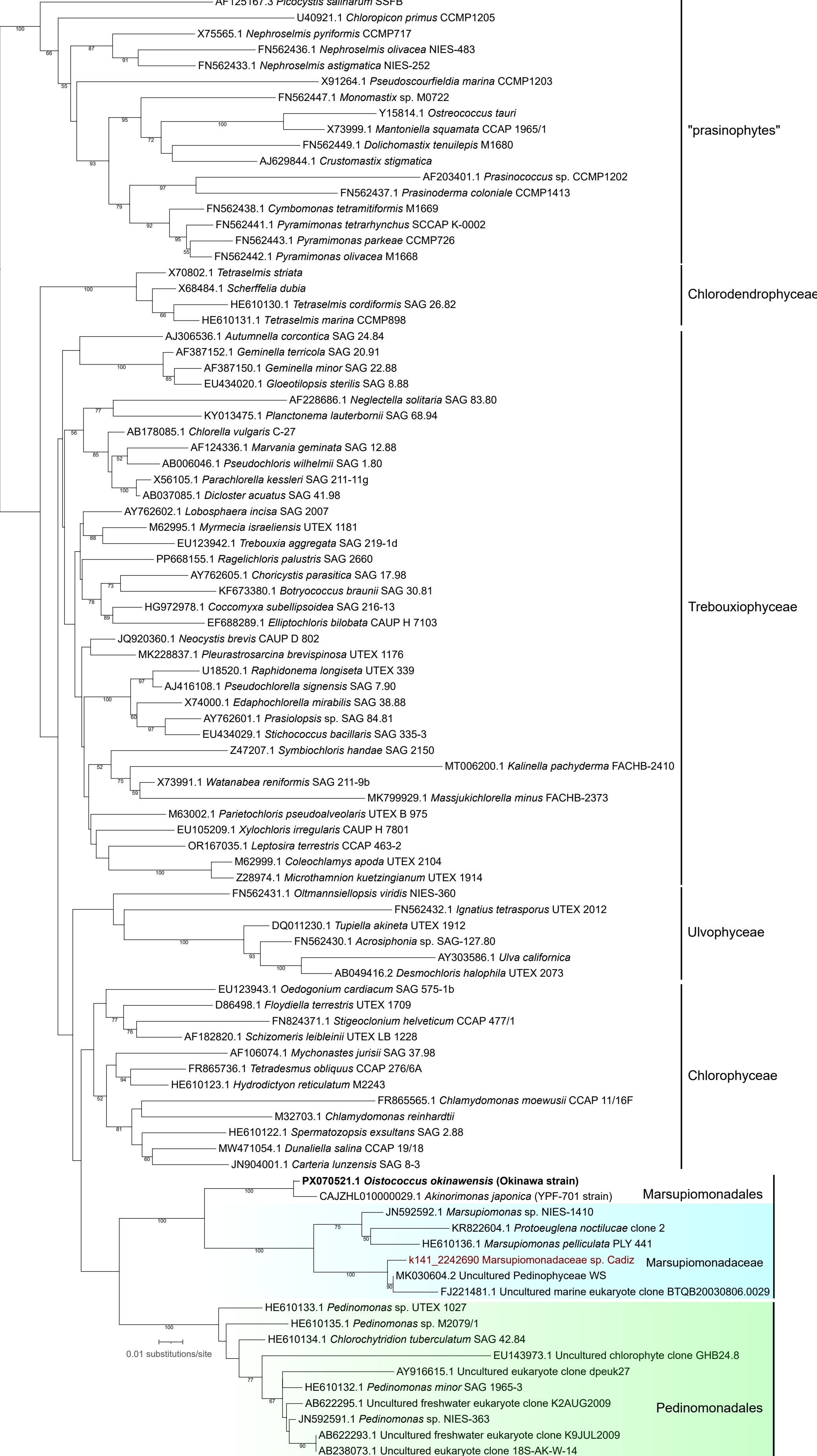

Supplement: S1 Fig — Further details on the tree can be found in the legend to Fig 3A, which shows a simplified version of the tree. (PDF) [file pgen.1011901.s001.pdf]

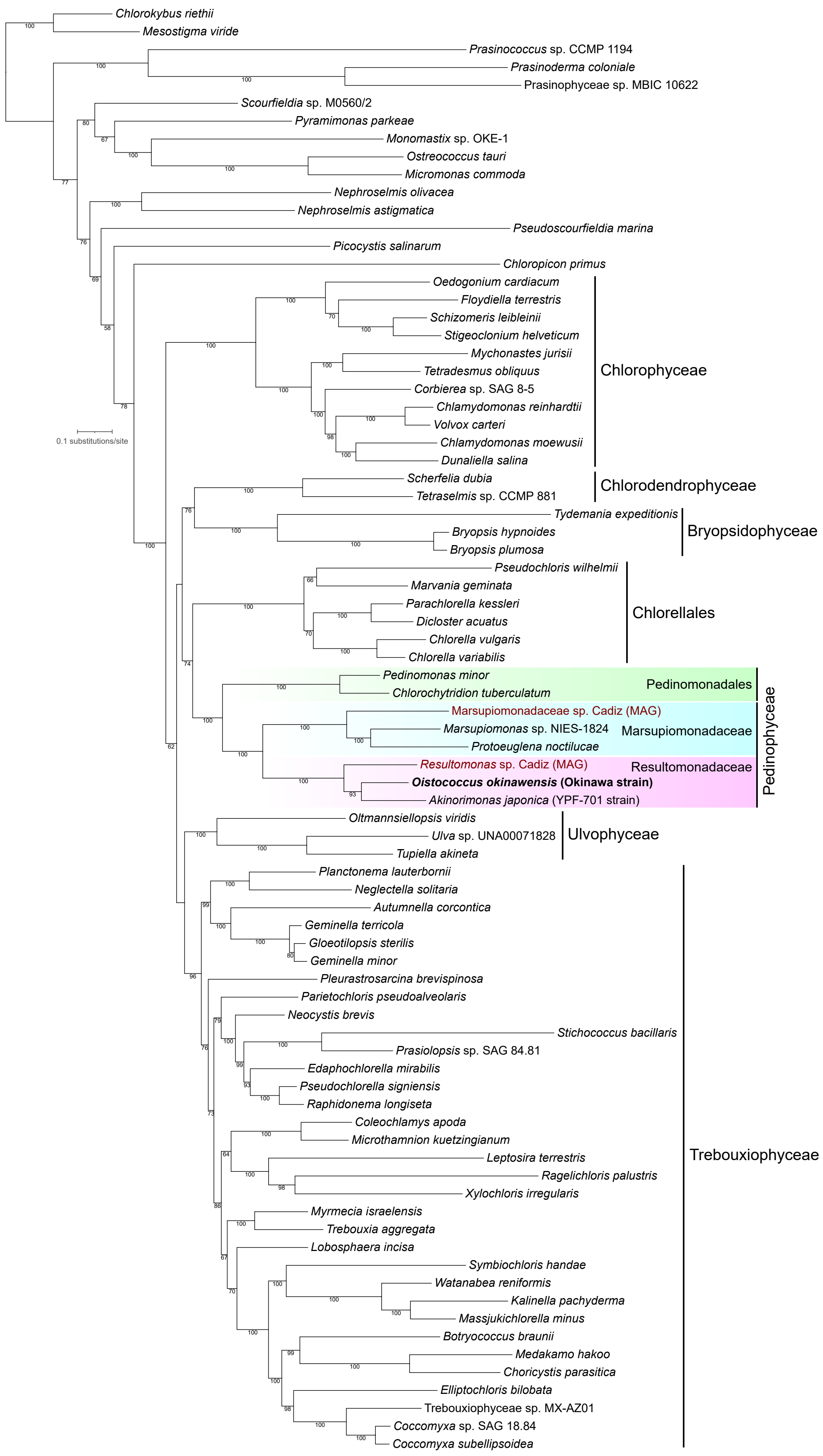

Supplement: S2 Fig — Further details on the tree can be found in the legend to Fig 4, which shows a simplified version of the tree. (PDF) [file pgen.1011901.s002.pdf]

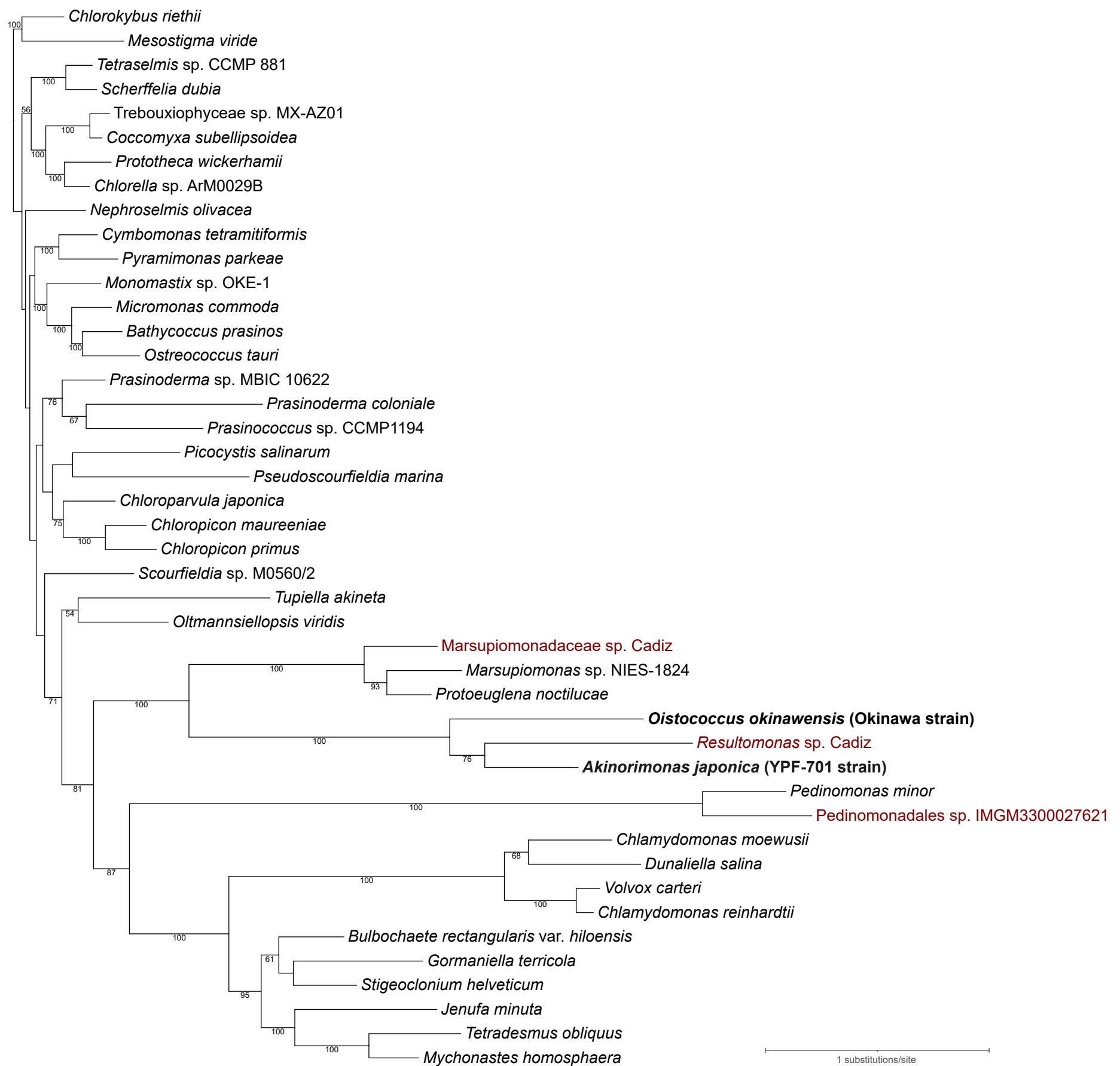

Supplement: S3 Fig — The alignment used for the tree inference consisted of 4,287 amino acid positions, the substitution model employed was LG + F + I + G4. Newly obtained sequences from cultured pedinophyte representatives are shown in bold, while those assembled metagenomically are marked in burgundy. (PDF) [file pgen.1011901.s003.pdf]

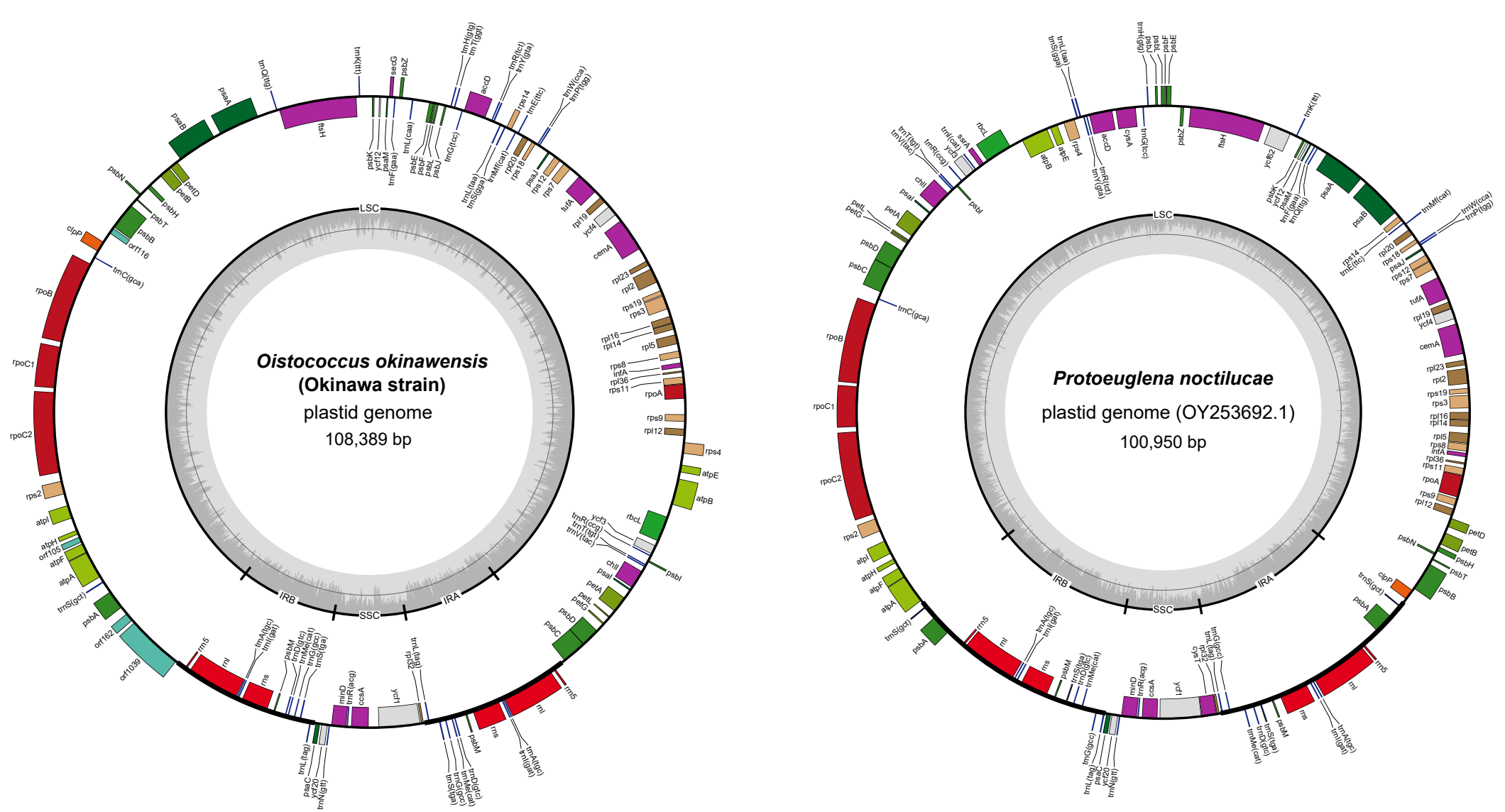

Supplement: S4 Fig — Genes are represented as blocks, with those transcribed in the clockwise direction facing inward and those transcribed in the counter-clockwise direction facing outward. Different colours indicate the functional categories of the genes. The inner circle plot displays the GC content, with the thin grey line marking 50%. Segments annotated in the inner circle include: IRA and IRB (inverted repeats, also indicated by thickened regions in the outer circle) and SSC and LSC (short and long single-copy regions, respectively). Note that the plastome of Marsupiomonadaceae sp. Cadiz is incomplete and is therefore shown as linear. (PDF) [file pgen.1011901.s004.pdf]

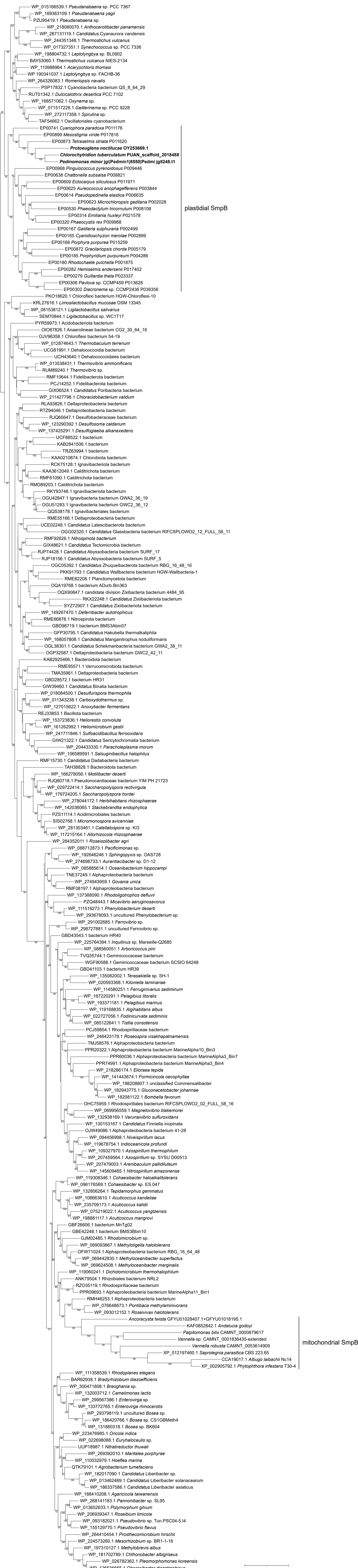

Supplement: S5 Fig — The tree includes bacterial proteins and eukaryotic nucleus-encoded homologs targeted to plastid or mitochondria. The tree inference was based on an alignment created using MAFFT with the E-INS-i method, which was subsequently trimmed with trimAl using the -automated1 mode, resulting in 153 well-aligned positions. The analysis was conducted using IQ-TREE with the LG + R7 substitution model and ultrafast bootstrapping with 10,000 replicates. (PDF) [file pgen.1011901.s005.pdf]

## UUG

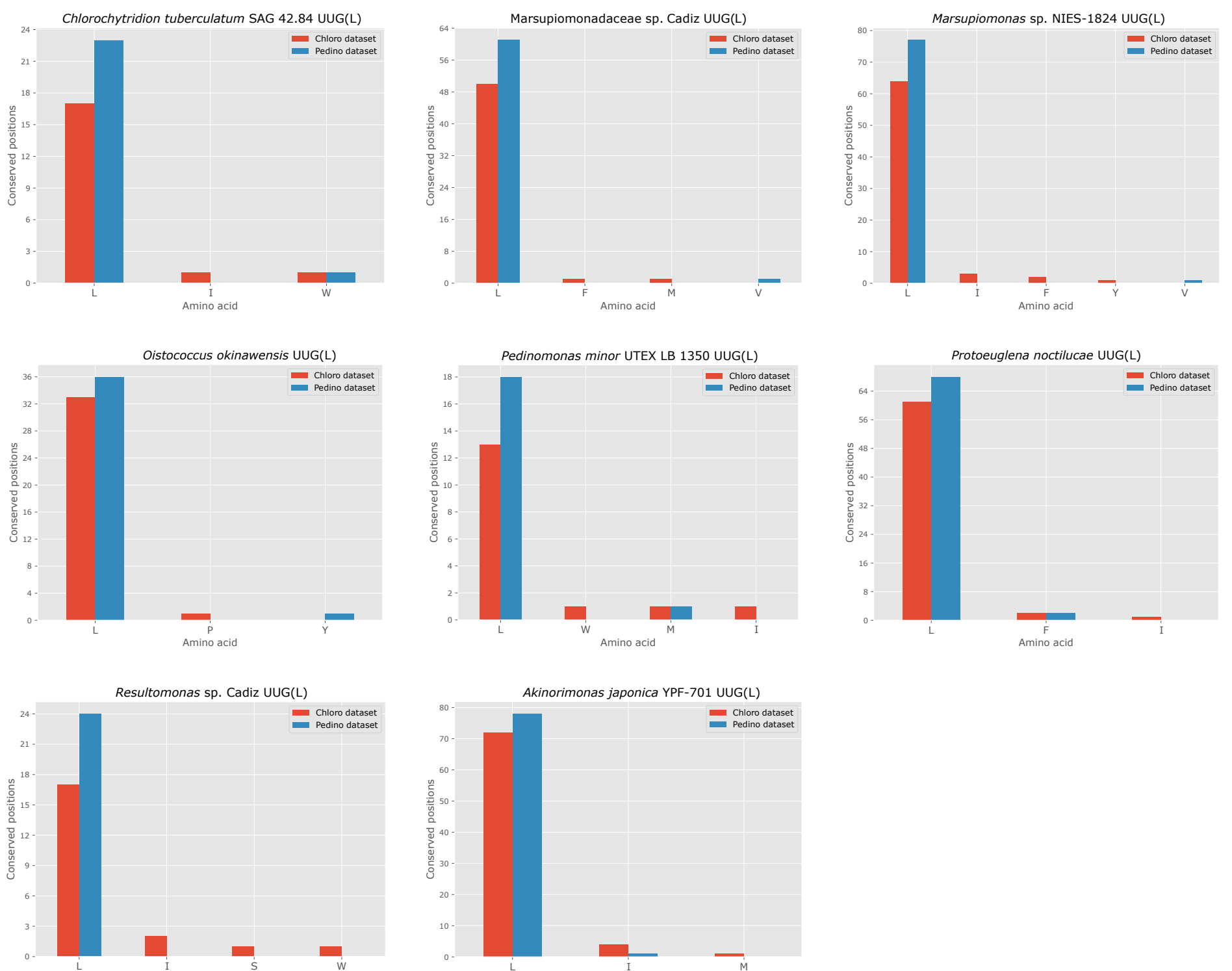

## CGG

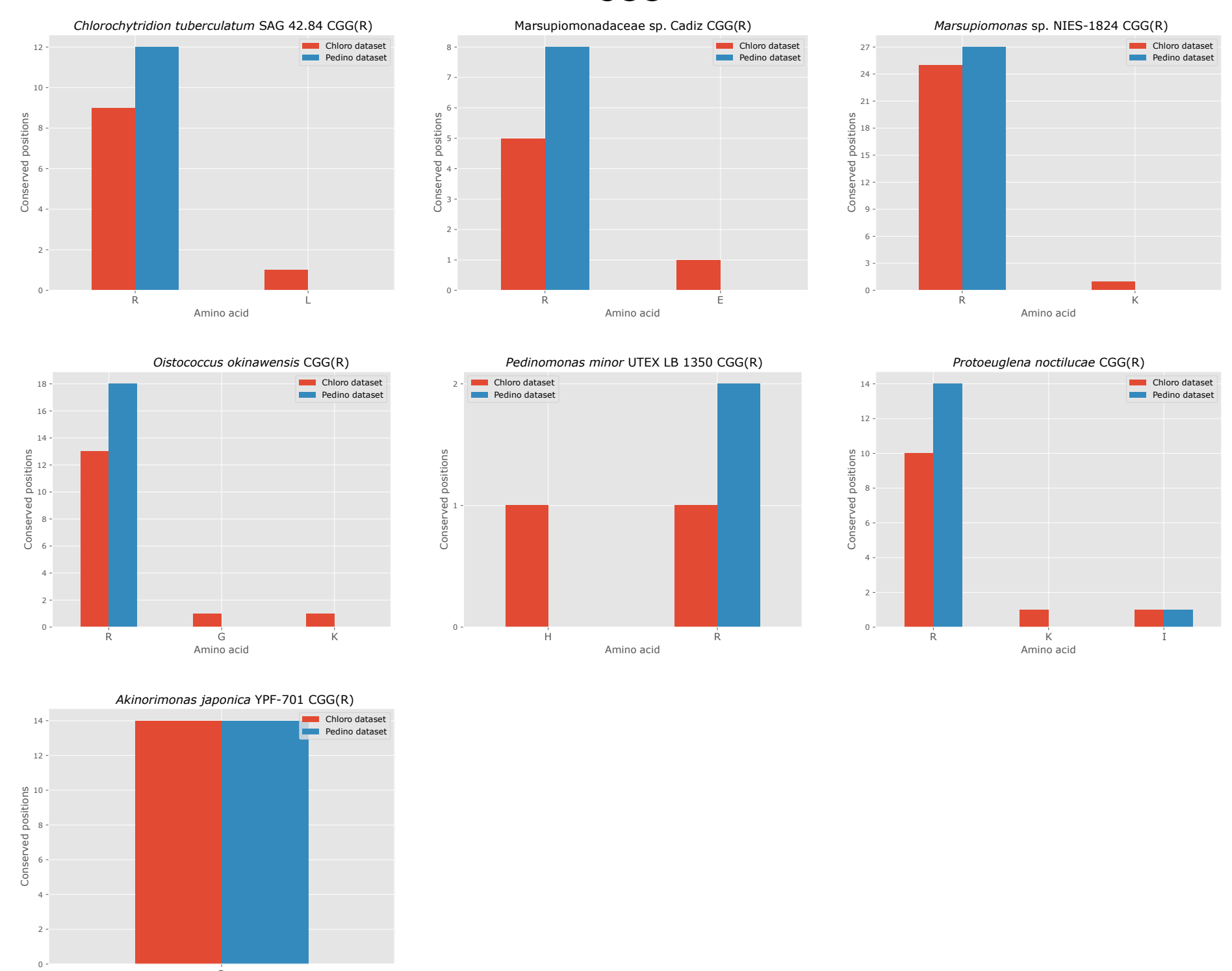

## UCC

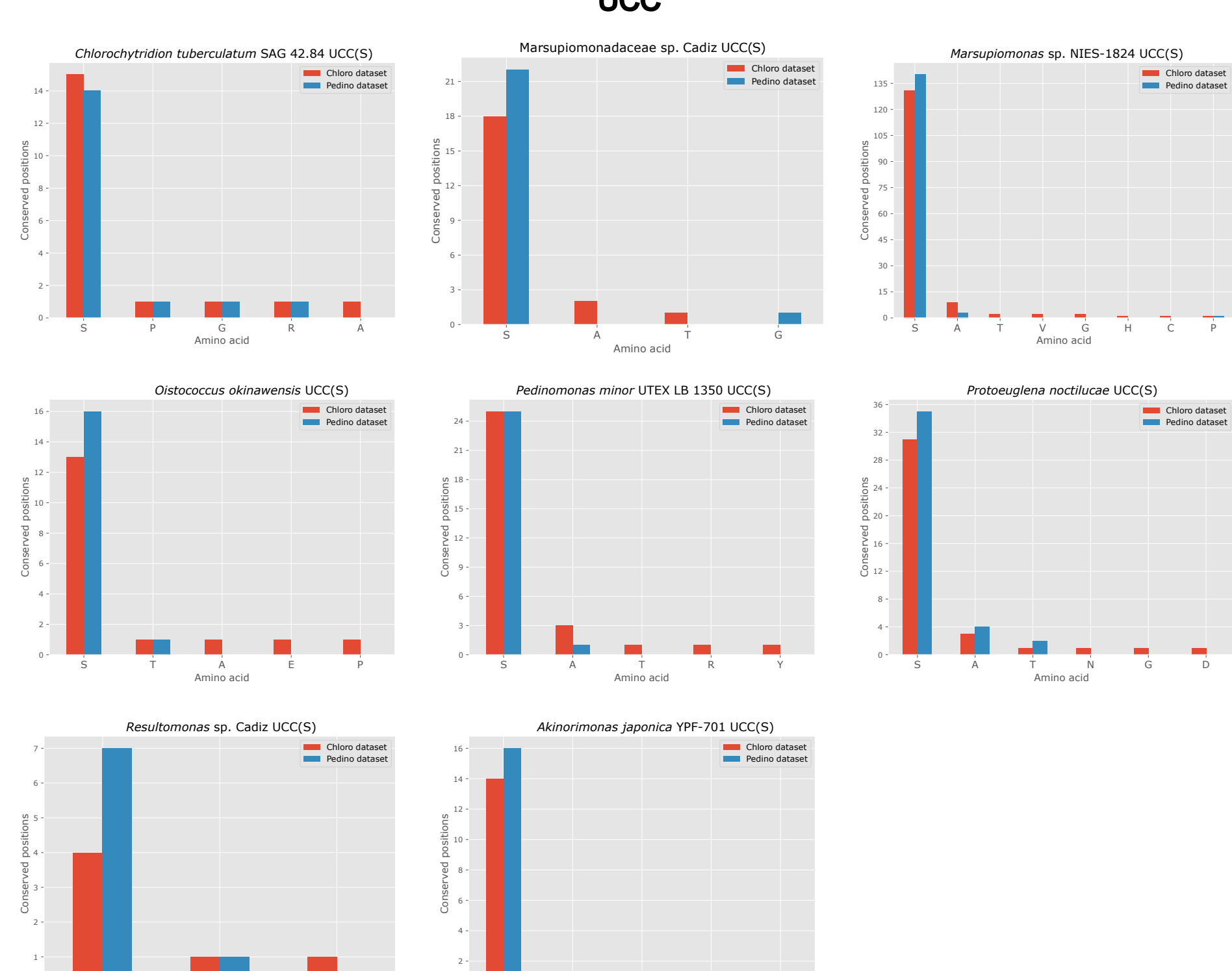

## ACC

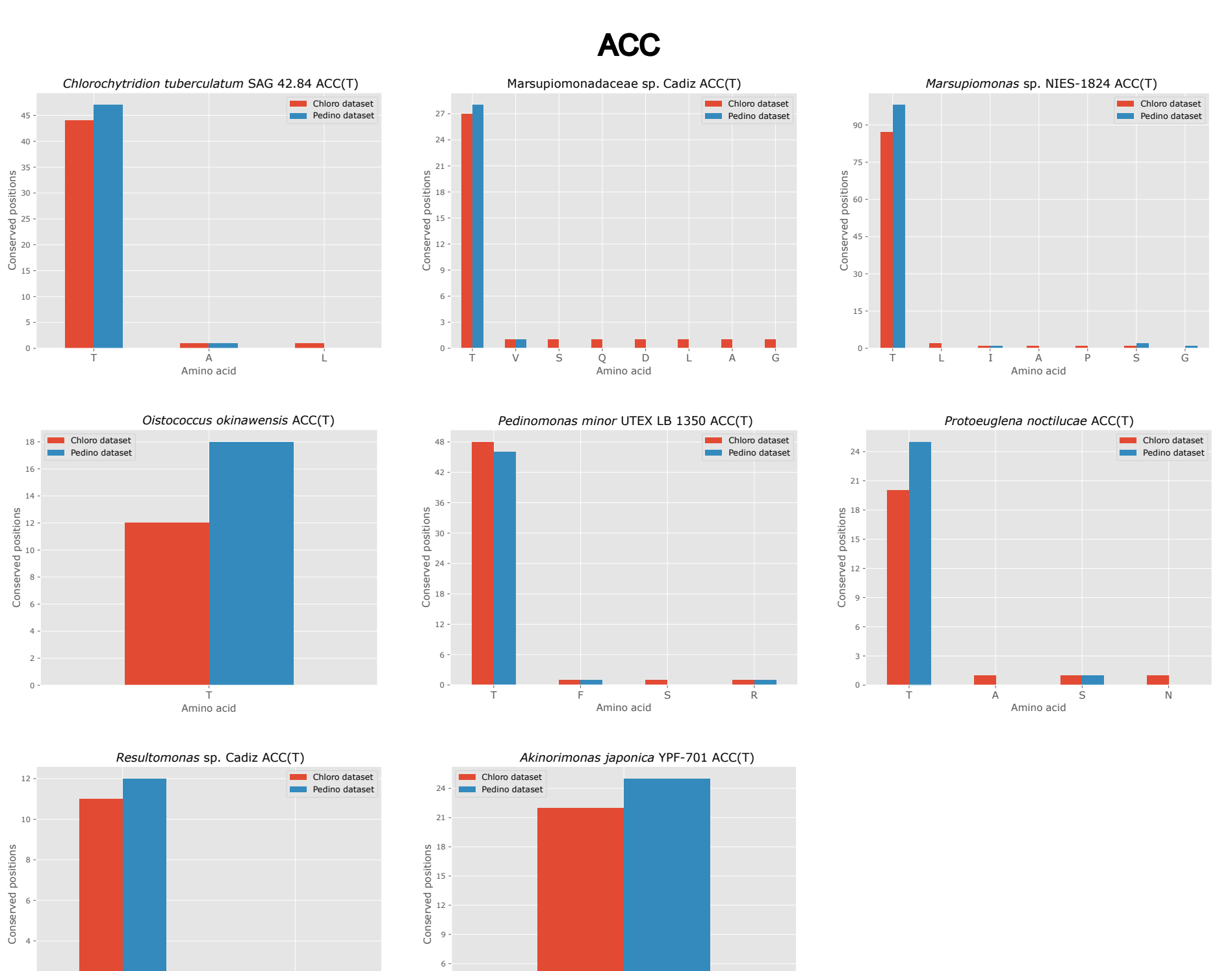

## AUA

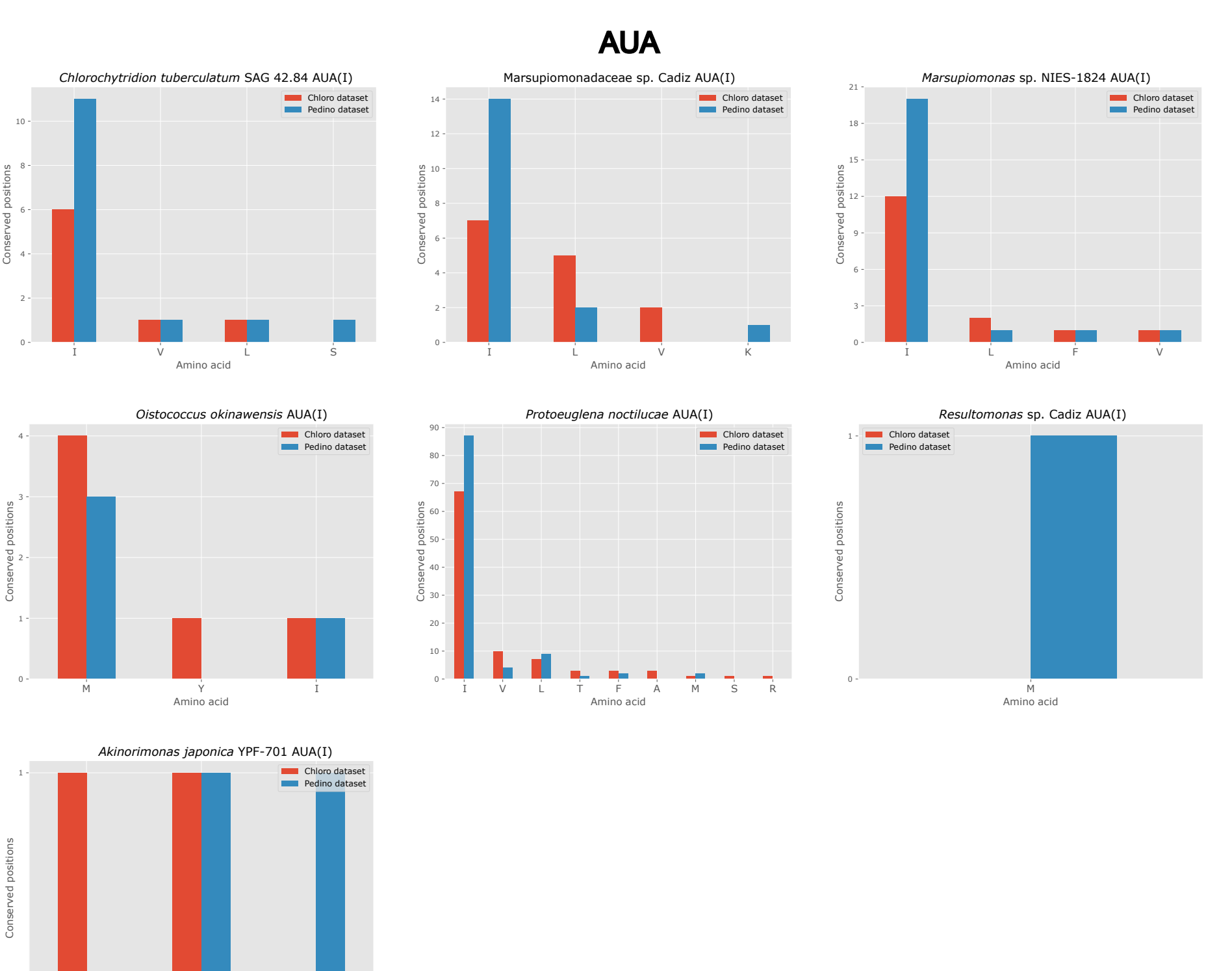

## B

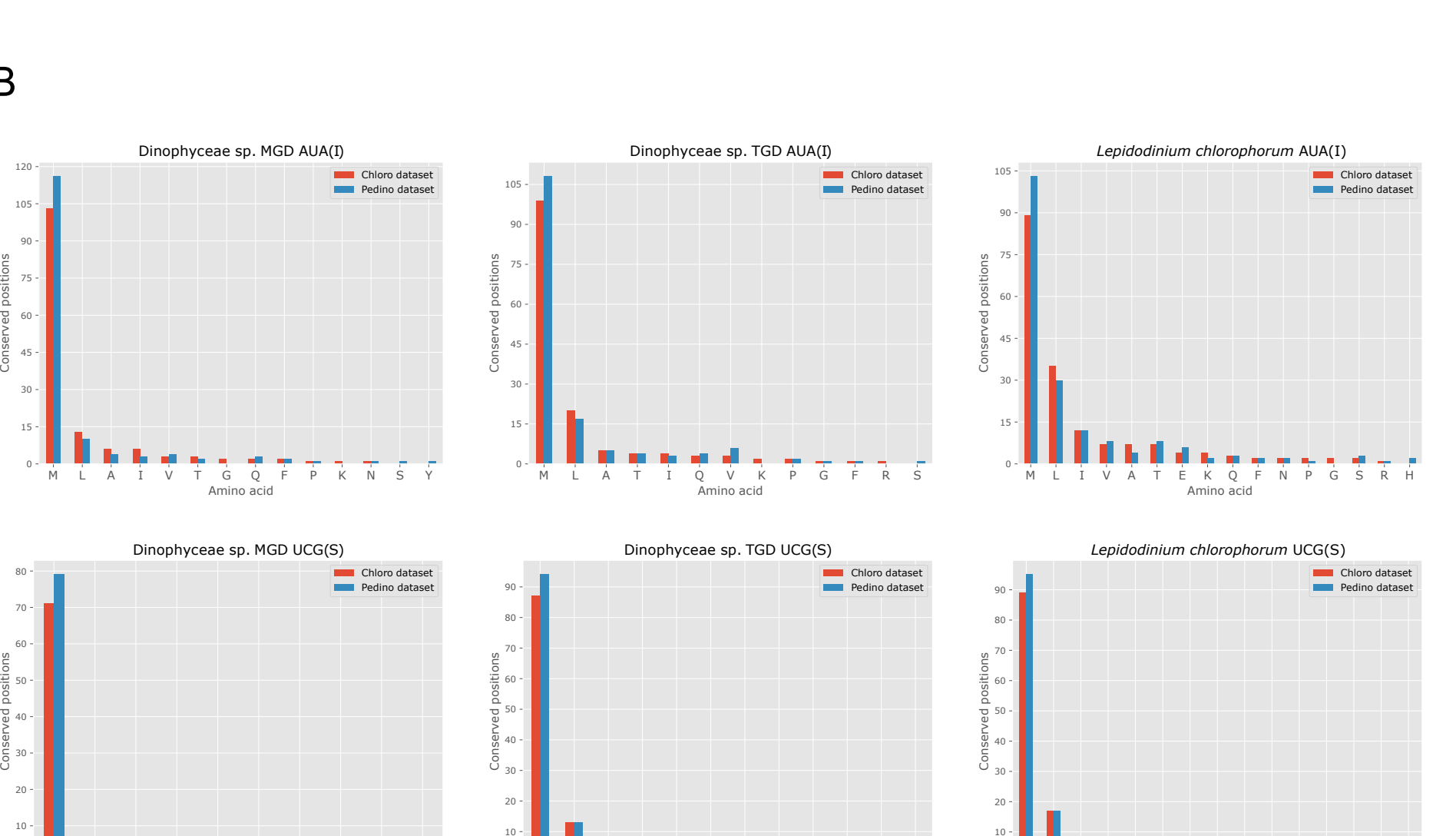

Supplement: S6 Fig — Plots for all codons are provided in S1 Dataset. Note that no plot shown for the given codon and a particular species (of those analysed in this study) means that not a single codon in the respective whole genome occupied a conserved amino acid position as defined by our criteria (i.e., the same amino acid in at least 70% of all sequences compared). For further details concerning the methodology and display convention see the legend to Fig 5B. (PDF) [file pgen.1011901.s006.pdf]

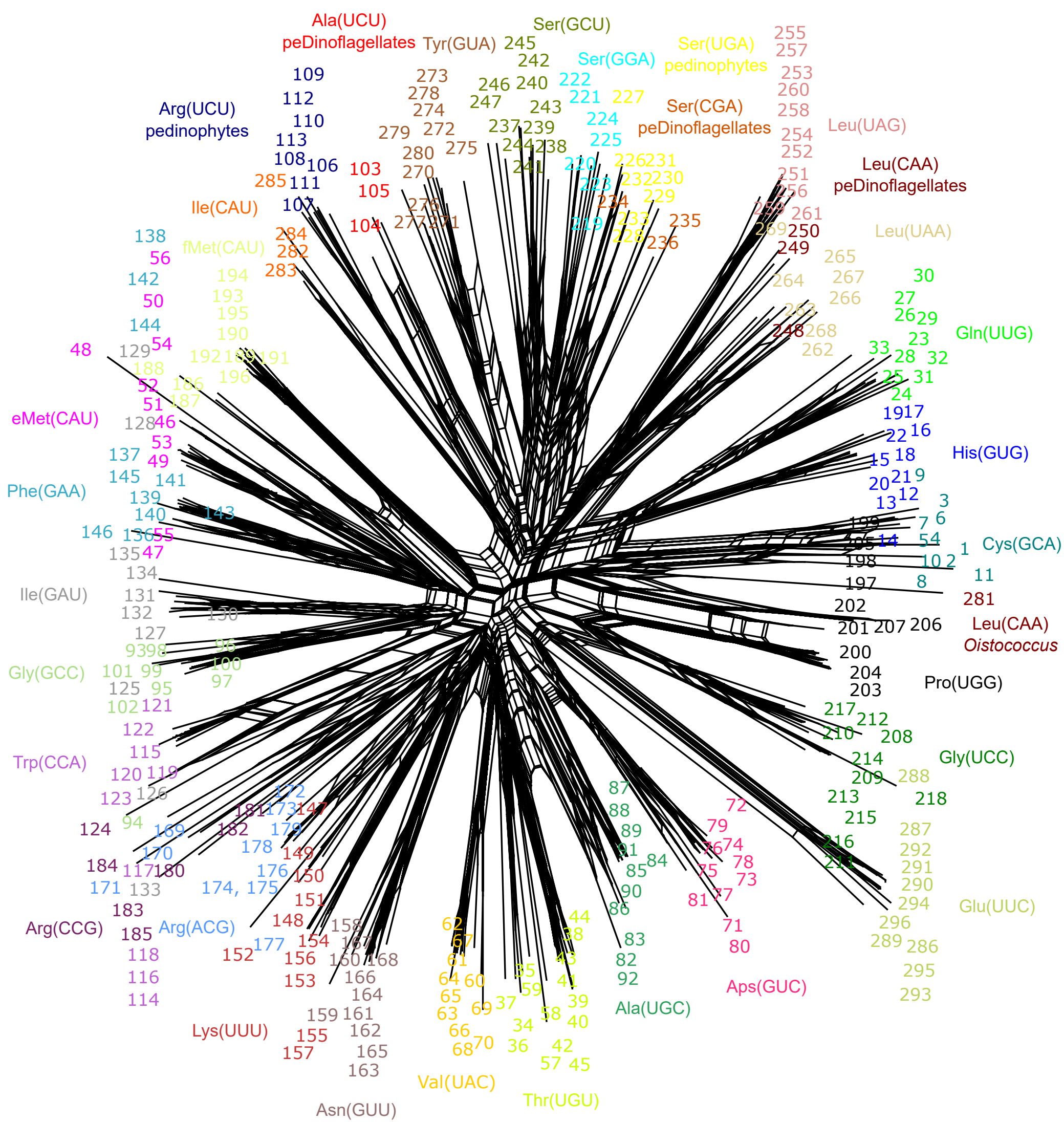

Supplement: S8 Fig — For tRNAs with the UCU anticodon, which are of specific interest in this study (see text), sequences are annotated separately for pedinophytes and peDinoflagellates. (PDF) [file pgen.1011901.s008.pdf]

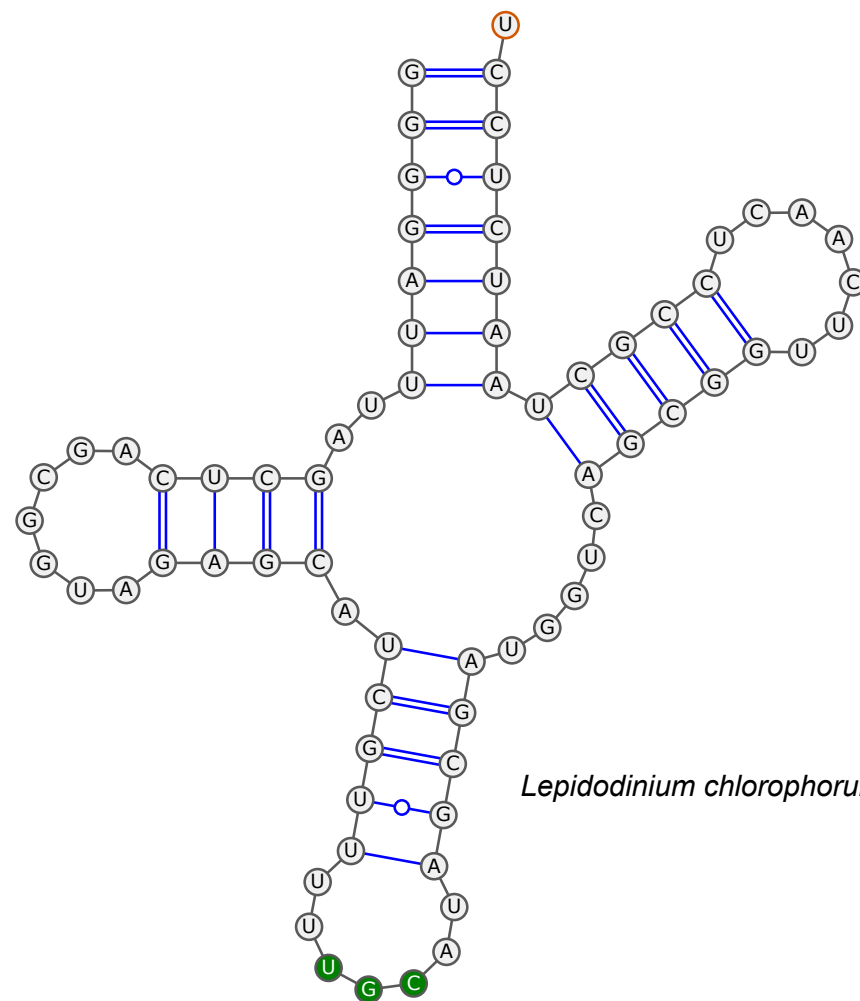

*Lepidodinium chlorophorum*

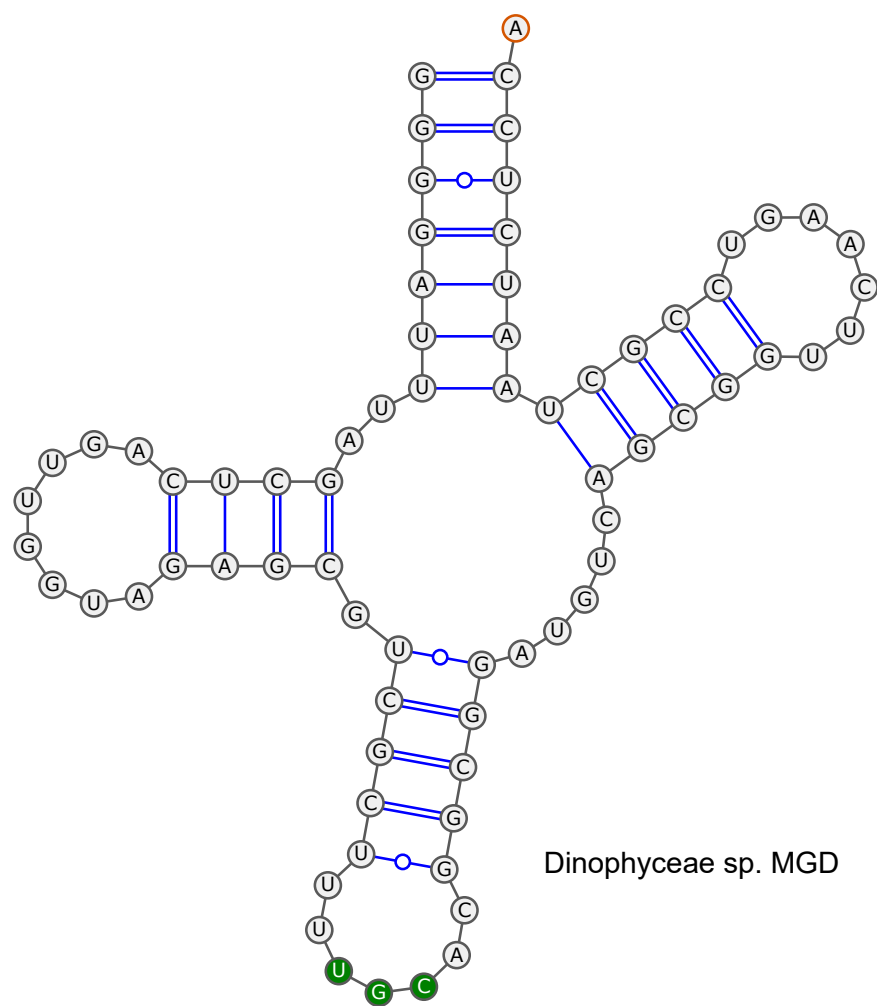

Dinophyceae sp. MGD

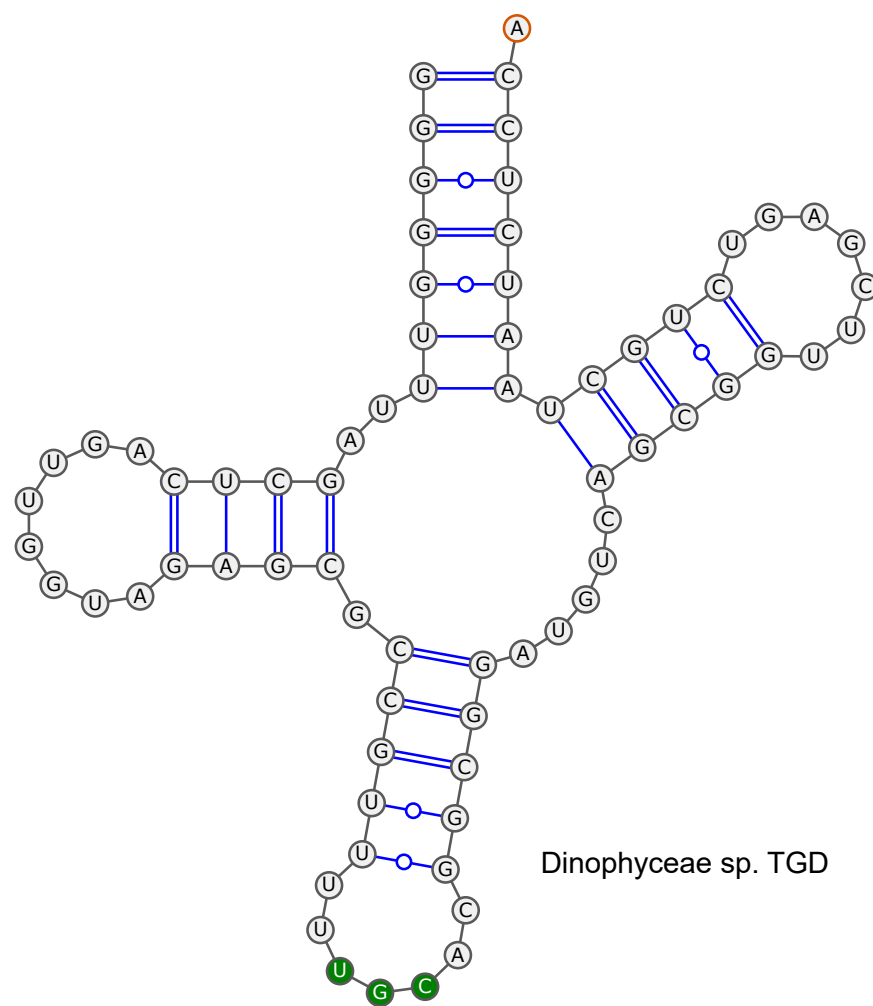

Dinophyceae sp. TGD

Supplement: S9 Fig — Note the unexpected nucleotide (U; highlighted) at the position 73 of the tRNA from L. chlorophorum. (PDF) [file pgen.1011901.s009.pdf]

rps7

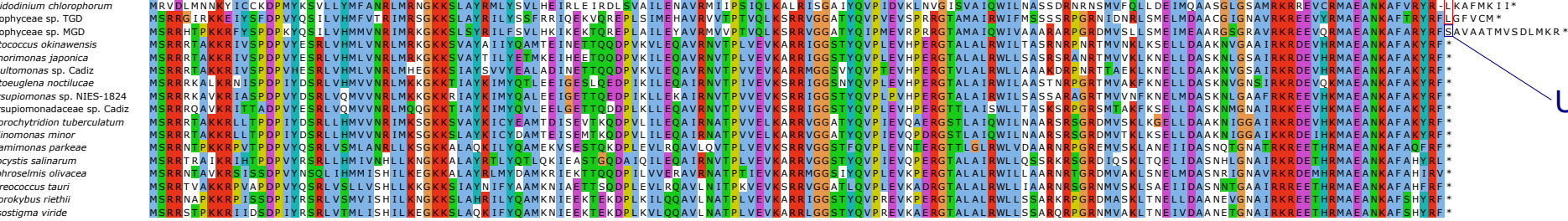

rps19

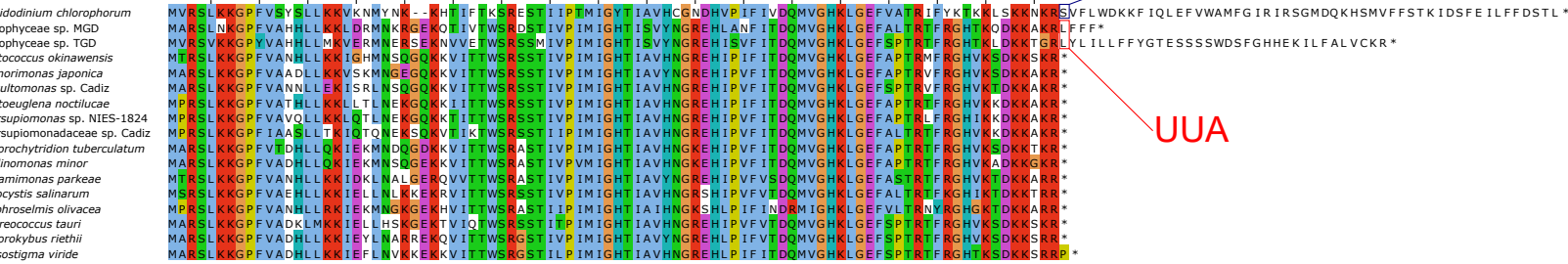

atp1

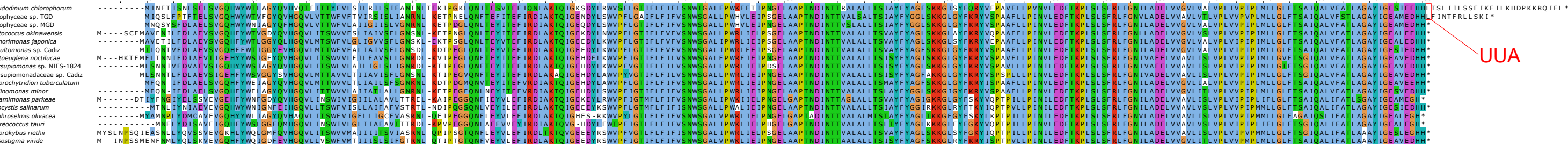

Supplement: S10 Fig — The display convention follows that one used in Fig 6A. (PDF) [file pgen.1011901.s010.pdf]

A

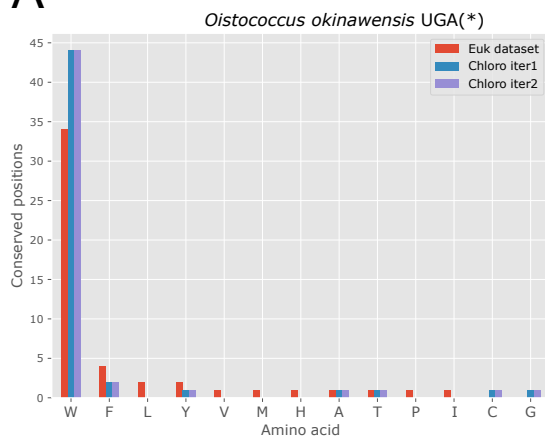

C

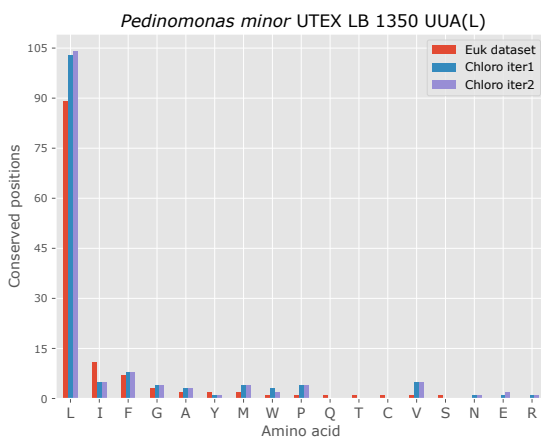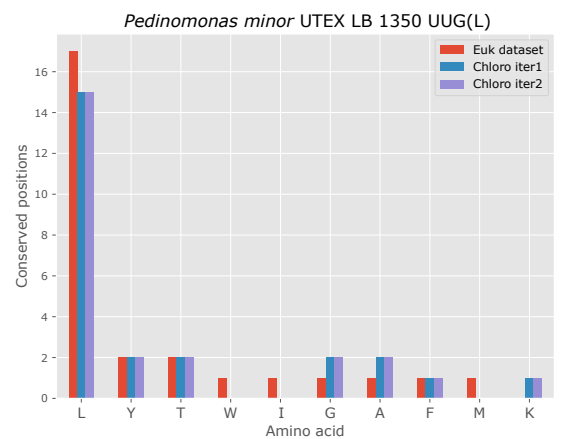

B

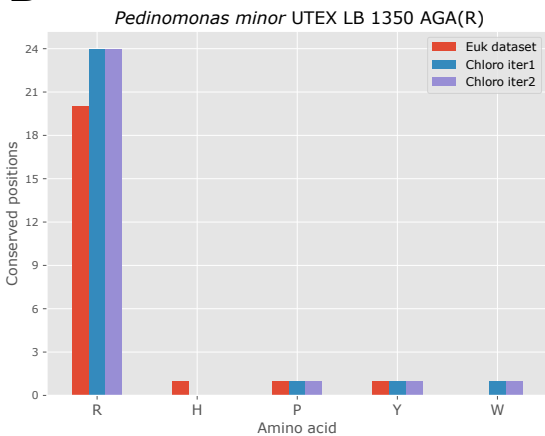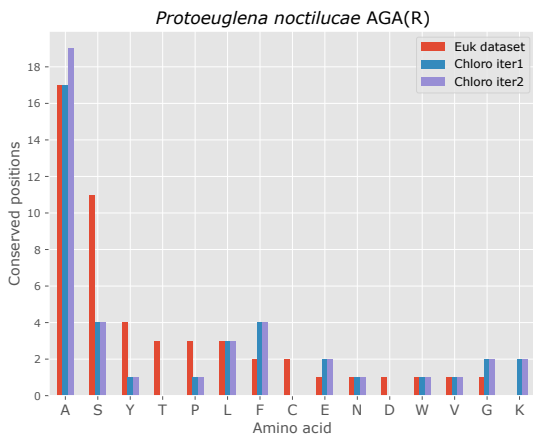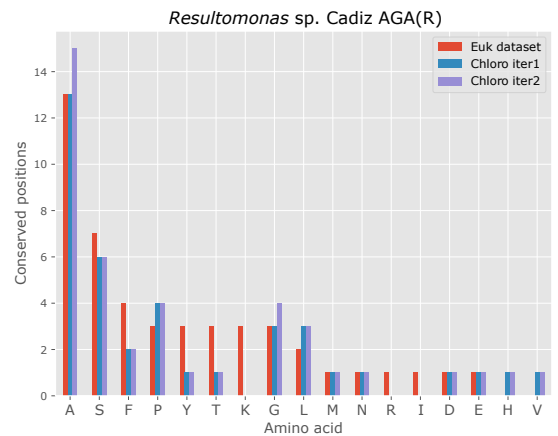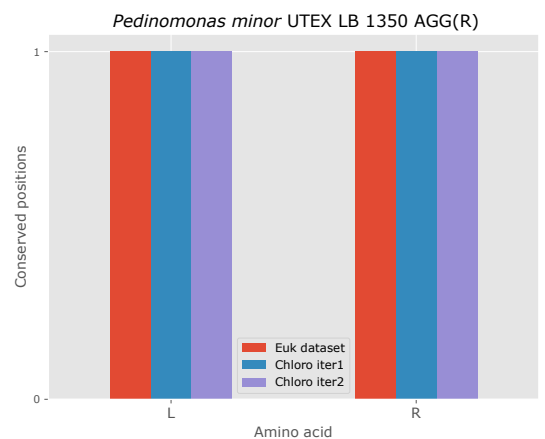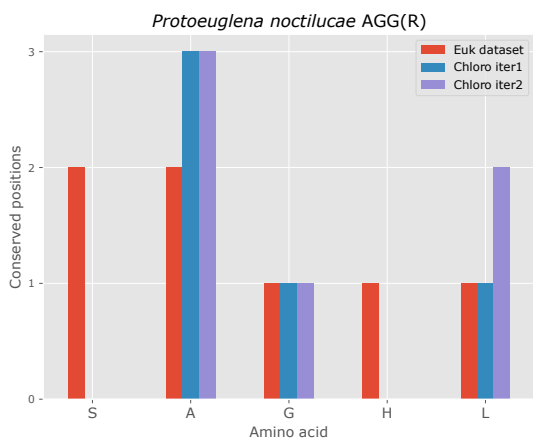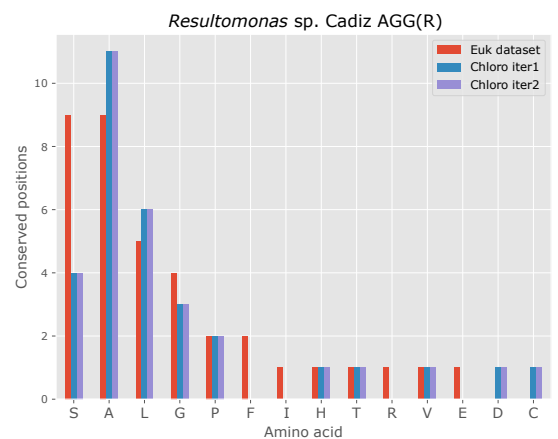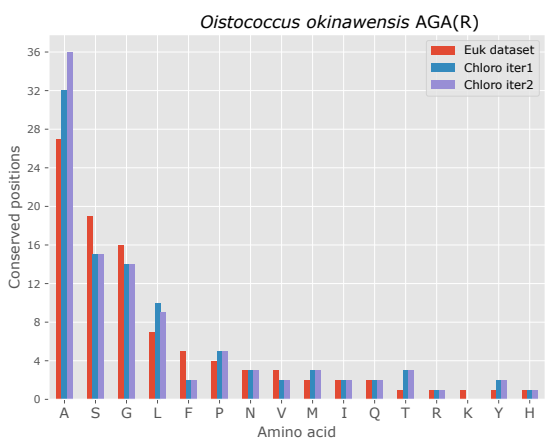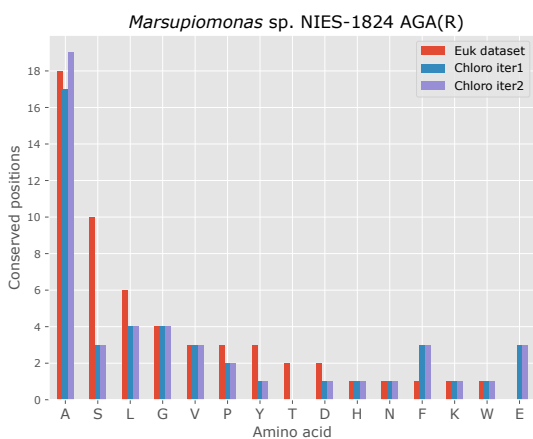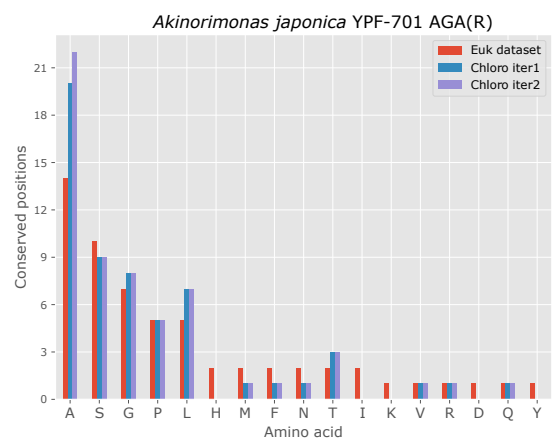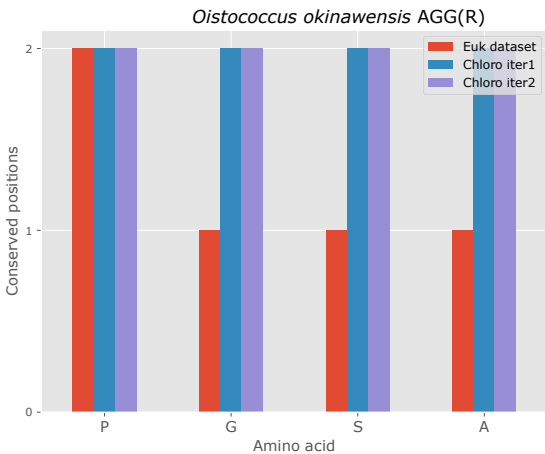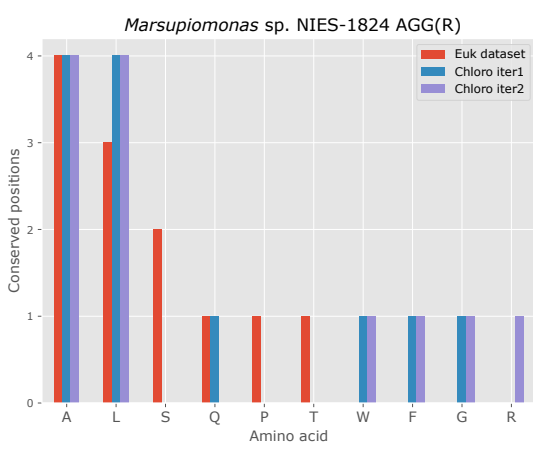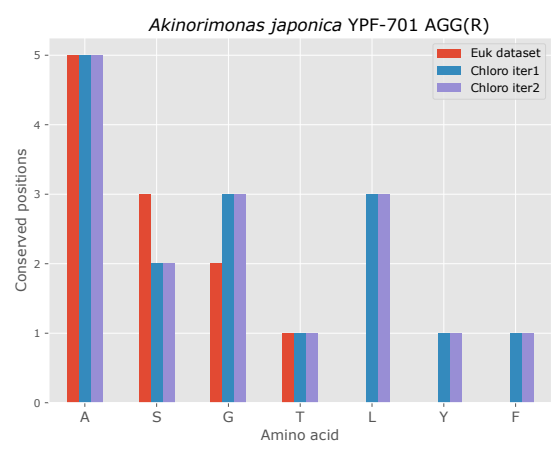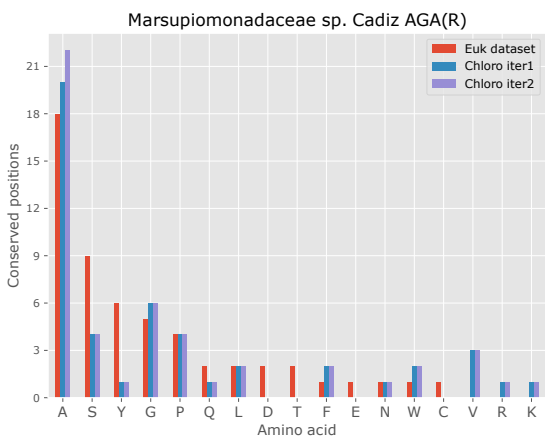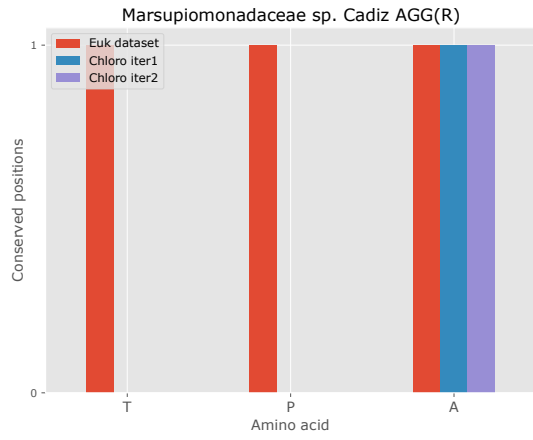

Supplement: S15 Fig — Plots for all codons are provided in S2 Dataset. For further details concerning the methodology and display convention see the legend to Fig 5B. (A) Evidence for UGA in O. okinawensis decoded as tryptophan. (B) Evidence for a reassignment of the AGR codons in Marsupiomonadales as opposed to Ped. minor. (C) Evidence for UUR codons having preserved their standard meaning in Ped. minor. (PDF) [file pgen.1011901.s015.pdf]

A

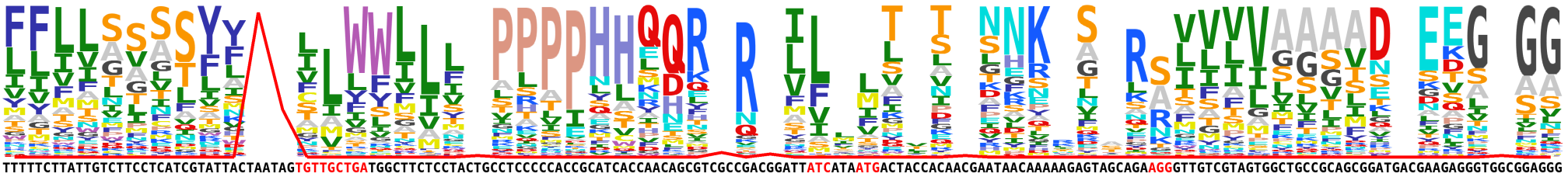

B

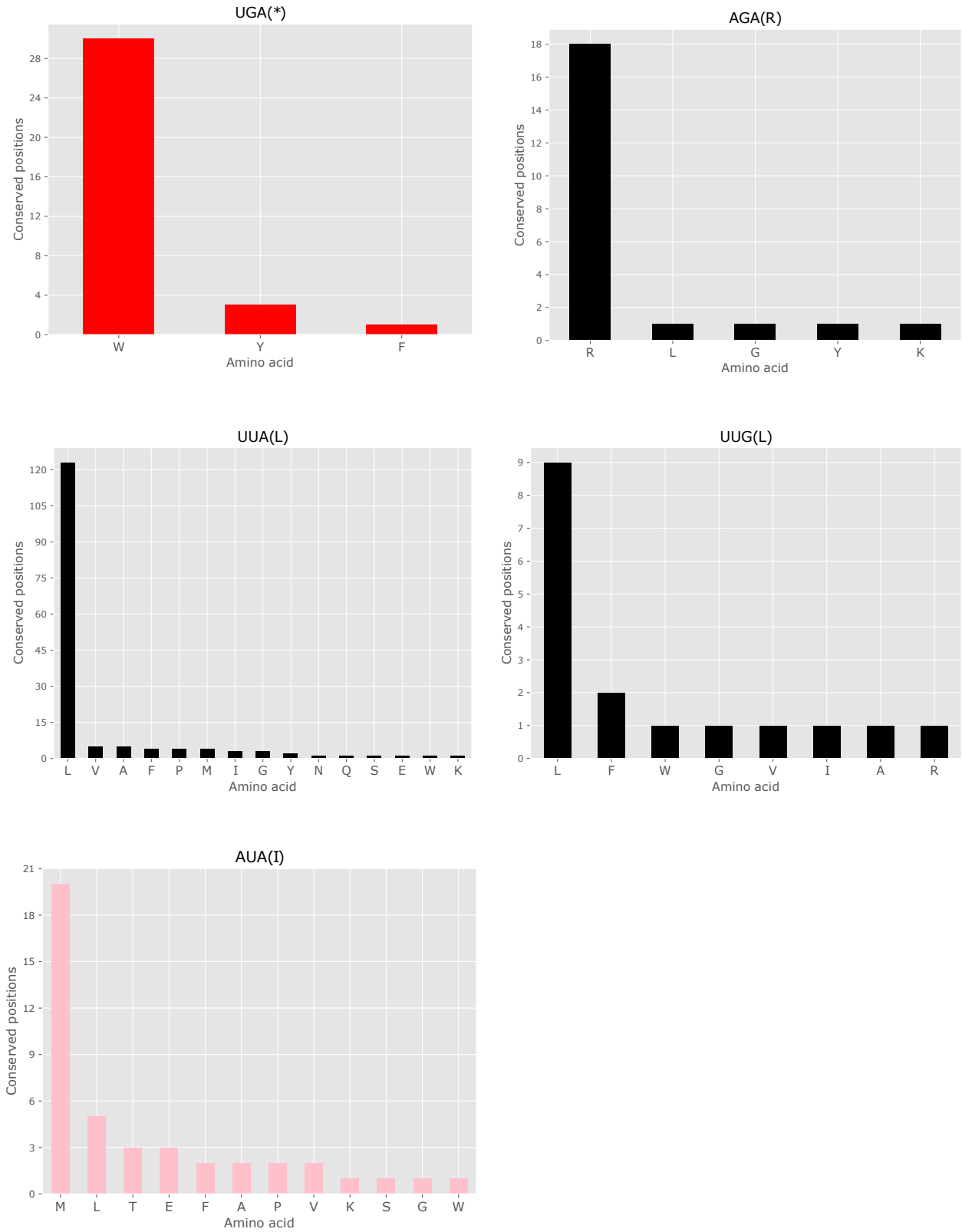

Supplement: S16 Fig — IMGM3300027621. A – FACIL output, B – plots showing the distribution of selected codons at conserved amino acid positions. Plots for all codons are provided in S3 Dataset. (PDF) [file pgen.1011901.s016.pdf]

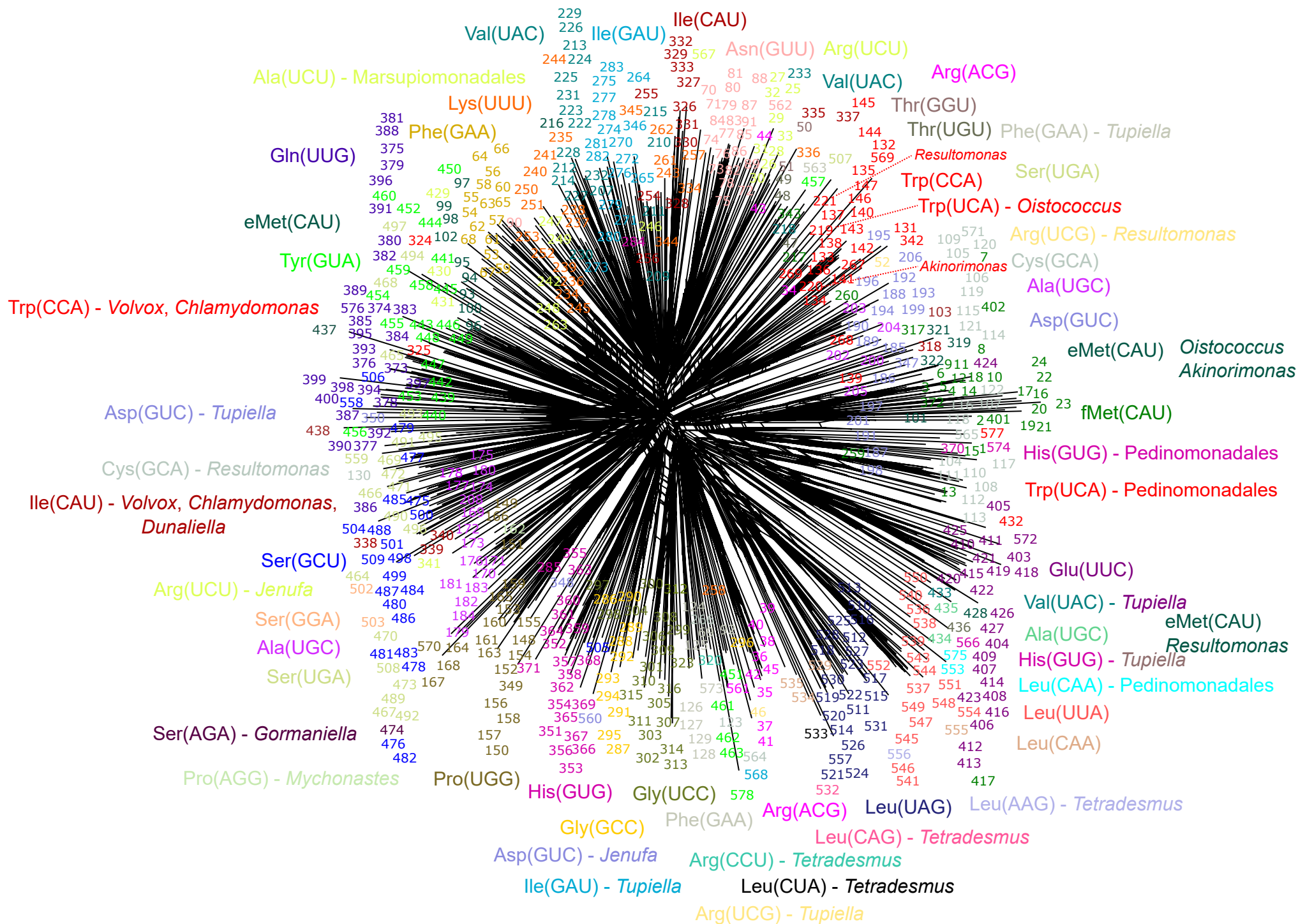

Supplement: S17 Fig — (PDF) [file pgen.1011901.s017.pdf]
