# Supplementary material for: Expanded diversity of pedinophytes provides a window into the evolution of the genetic code in organelles
Source: PLoS Genet. 2025 Oct 22;21(10):e1011901. doi: 10.1371/journal.pgen.1011901 (PMC12574857; doi:10.1371/journal.pgen.1011901)
Supplement: S12 Fig — Accession numbers of the sequences included are provided in S11 Fig and S7 Table. (PDF) [file pgen.1011901.s012.pdf]

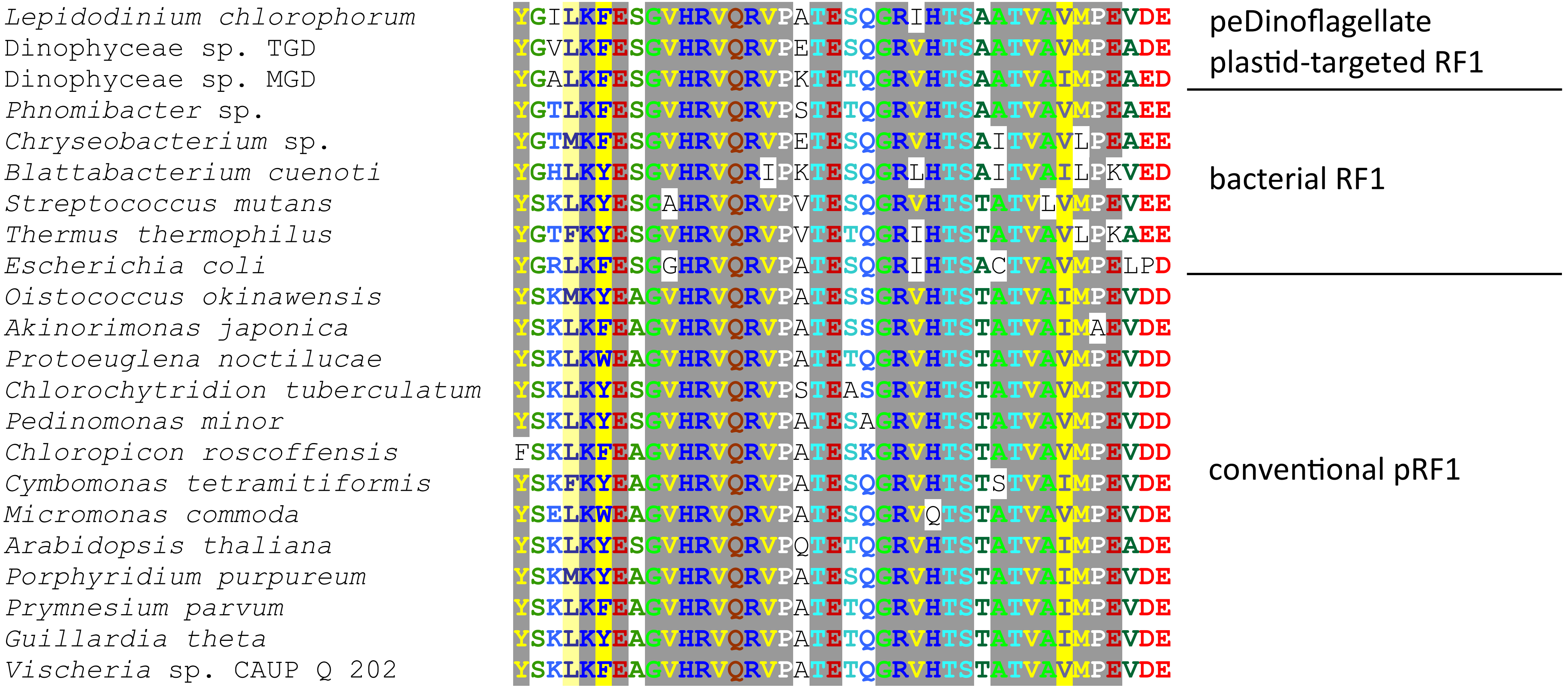

Lepidodinium chlorophorum YGILKFESGVHRVQRVPATESQGRIHTSAATVAVMPEVDE
Dinophyceae sp. TGD YGVLKFESGVHRVQRVPETESQGRVHTSAATVAVMPEADE
Dinophyceae sp. MGD YGALKFESGVHRVQRVPKTETQGRVHTSAATVAIMPEAED
Phnomibacter sp. YGTLKFESGVHRVQRVPSTETQGRVHTSAATVAVMPEAEE
Chryseobacterium sp. YGTMKFESGVHRVQRVPETESQGRVHTSAITVAVLPEAEE
Blattabacterium cuenoti YGHLKYESGVHRVQRIPKTESQGRLHTSAITVAILPKVED
Streptococcus mutans YSKLKYESGAHRVQRVPVTESQGRVHTSTATVLVMPEVEE
Thermus thermophilus YGTFKYESGVHRVQRVPVTETQGRIHTSTATVAVLPKAEE
Escherichia coli YGRLKFESGGHRVQRVPATESQGRIHTSACTVAVMPELPD
Oistococcus okinawensis YSKMKYEAGVHRVQRVPATESSGRVHTSTATVAIMPEVDD
Akinorimonas japonica YSKLKFEAGVHRVQRVPATESSGRVHTSTATVAIMAEVDE
Protoeuglena noctilucae YSKLKWEAGVHRVQRVPATETQGRVHTSTATVAVMPEVDD
Chlorochytridion tuberculatum YSKLKYESGVHRVQRVPSTEASGRVHTSTATVAVMPEVDD
Pedinomonas minor YSKLKYESGVHRVQRVPATESAGRVHTSTATVAVMPEVDD
Chloropicon roscoffensis FSKLKFEAGVHRVQRVPATESKGRVHTSTATVAVMPEVDD
Cymbomonas tetramitiformis YSKFKYEAGVHRVQRVPATESQGRVHTSTSTVAIMPEVDE
Micromonas commoda YSELKWEAGVHRVQRVPATESQGRVQTSTATVAVMPEVDE
Arabidopsis thaliana YSKLKYESGVHRVQRVPQTETQGRVHTSTATVAIMPEADE
Porphyridium purpureum YSKMKYEAGVHRVQRVPATESQGRVHTSTATVAIMPEVDE
Prymnesium parvum YSKLKFEAGVHRVQRVPATETQGRVHTSTATVAIMPEVDE
Guillardia theta YSKLKYEAGVHRVQRVPATETQGRVHTSTATVAIMPEVDE
Vischeria sp. CAUP Q 202 YSKLKFEAGVHRVQRVPATETQGRVHTSTATVAVMPEVDE
peDinoflagellate plastid-targeted RF1
bacterial RF1
conventional pRF1
