# Supplementary material for: Expanded diversity of pedinophytes provides a window into the evolution of the genetic code in organelles
Source: PLoS Genet. 2025 Oct 22;21(10):e1011901. doi: 10.1371/journal.pgen.1011901 (PMC12574857; doi:10.1371/journal.pgen.1011901)
Supplement: S13 Fig — Genes are represented as blocks, with different colours indicating the functional categories of the genes. Note that all genes are located on the same strand, transcribed in the counter-clockwise direction. The inner circle plot displays the GC content, with the thin grey line marking 50%. (PDF) [file pgen.1011901.s013.pdf]

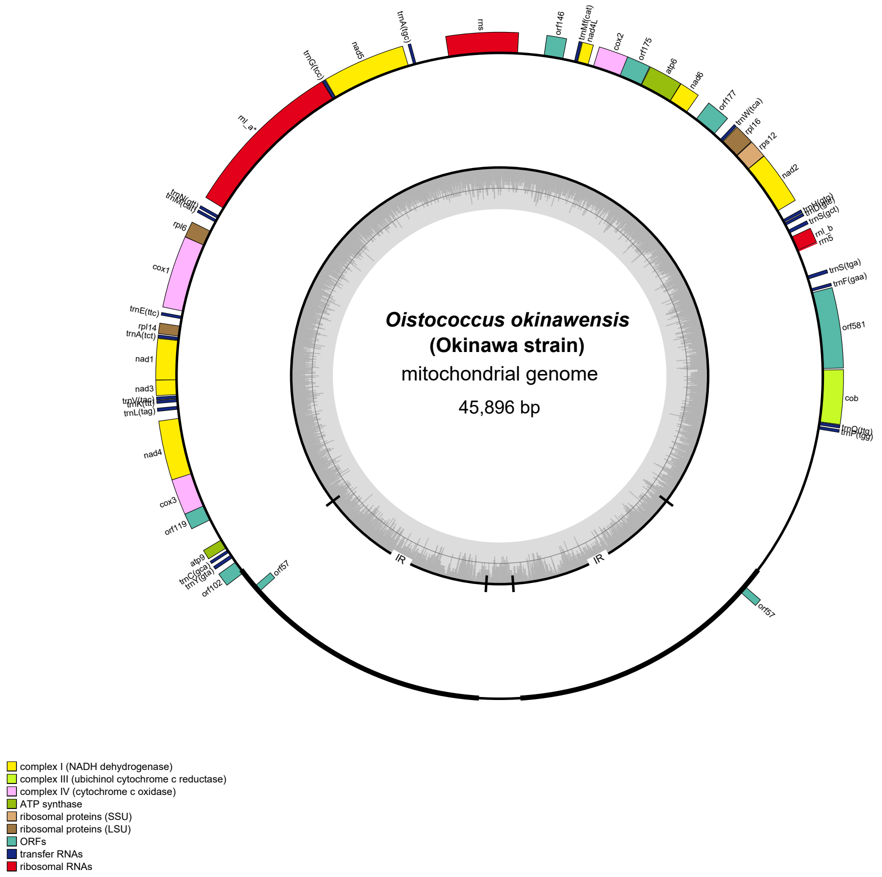
Oistococcus okinawensis
(Okinawa strain)
mitochondrial genome
45,896 bp
IR
IR
complex I (NADH dehydrogenase)
complex III (ubichinol cytochrome c reductase)
complex IV (cytochrome c oxidase)
ATP synthase
ribosomal proteins (SSU)
ribosomal proteins (LSU)
ORFs
transfer RNAs
ribosomal RNAs

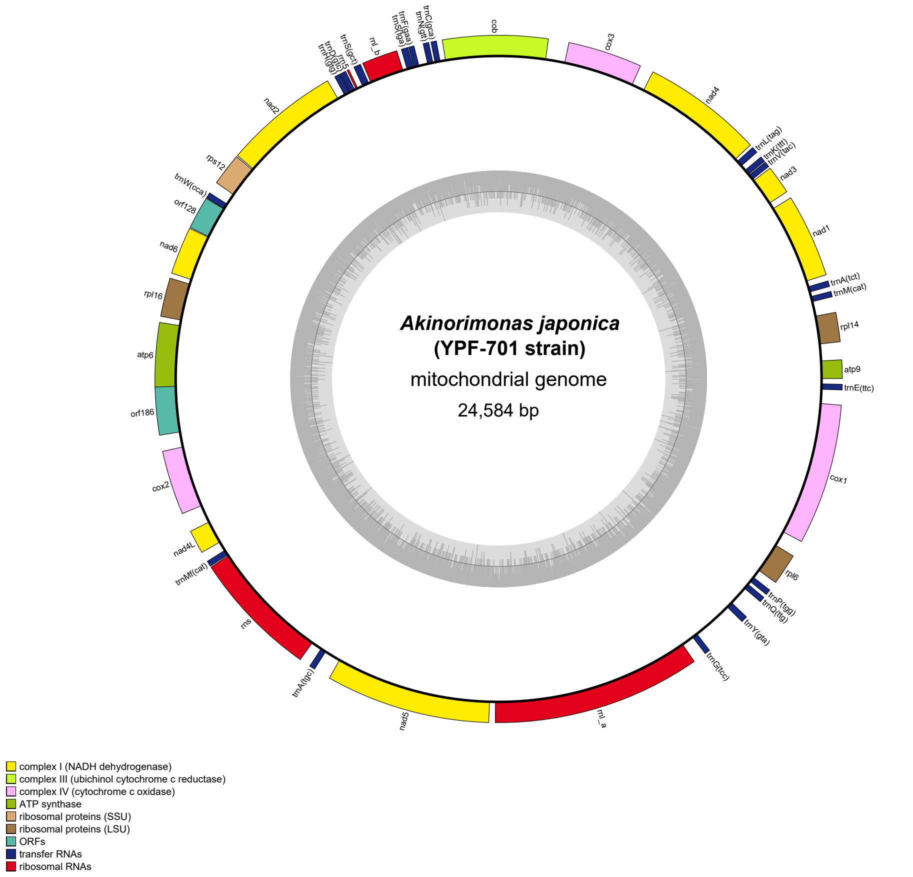
Akinorimonas japonica
(YPF-701 strain)
mitochondrial genome
24,584 bp
complex I (NADH dehydrogenase)
complex III (ubichinol cytochrome c reductase)
complex IV (cytochrome c oxidase)
ATP synthase
ribosomal proteins (SSU)
ribosomal proteins (LSU)
ORFs
transfer RNAs
ribosomal RNAs

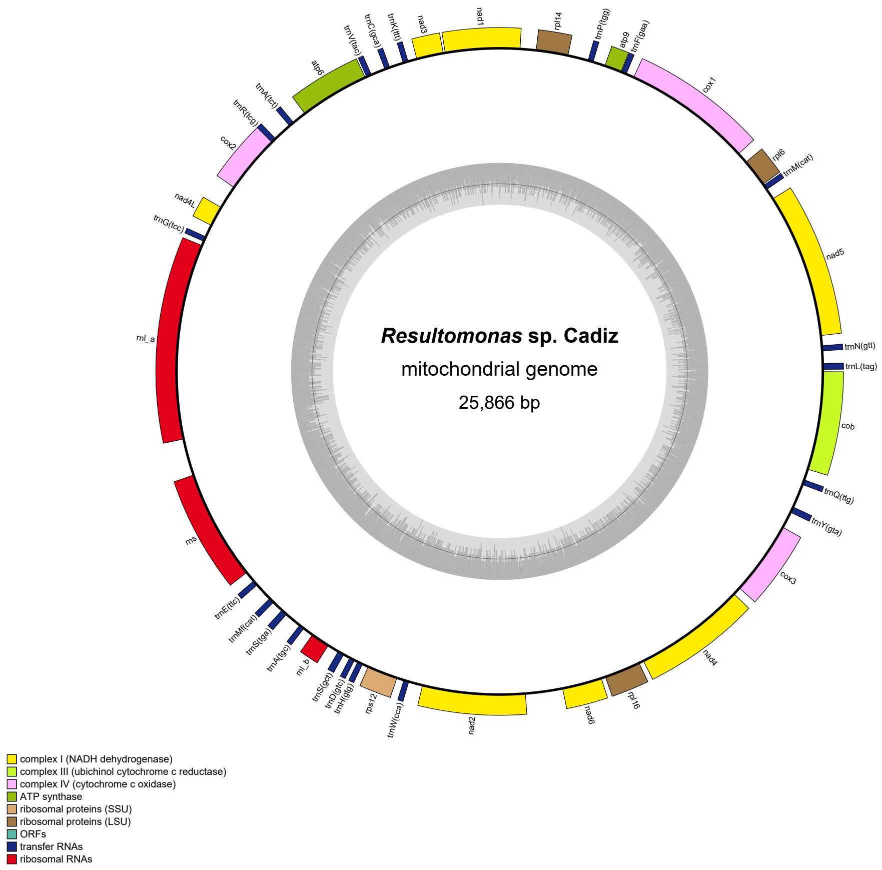
Resultomonas sp. Cadiz
mitochondrial genome
25,866 bp
complex I (NADH dehydrogenase)
complex III (ubichinol cytochrome c reductase)
complex IV (cytochrome c oxidase)
ATP synthase
ribosomal proteins (SSU)
ribosomal proteins (LSU)
ORFs
transfer RNAs
ribosomal RNAs

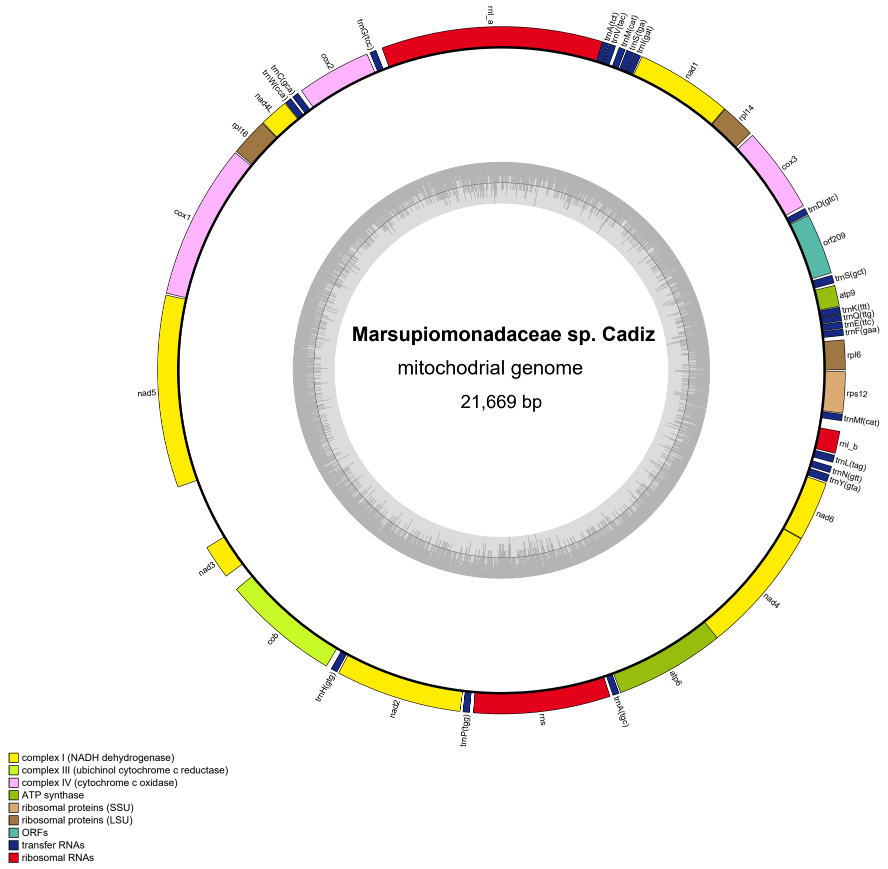
Marsupiomonadaceae sp. Cadiz
mitochodrial genome
21,669 bp
complex I (NADH dehydrogenase)
complex III (ubichinol cytochrome c reductase)
complex IV (cytochrome c oxidase)
ATP synthase
ribosomal proteins (SSU)
ribosomal proteins (LSU)
ORFs
transfer RNAs
ribosomal RNAs

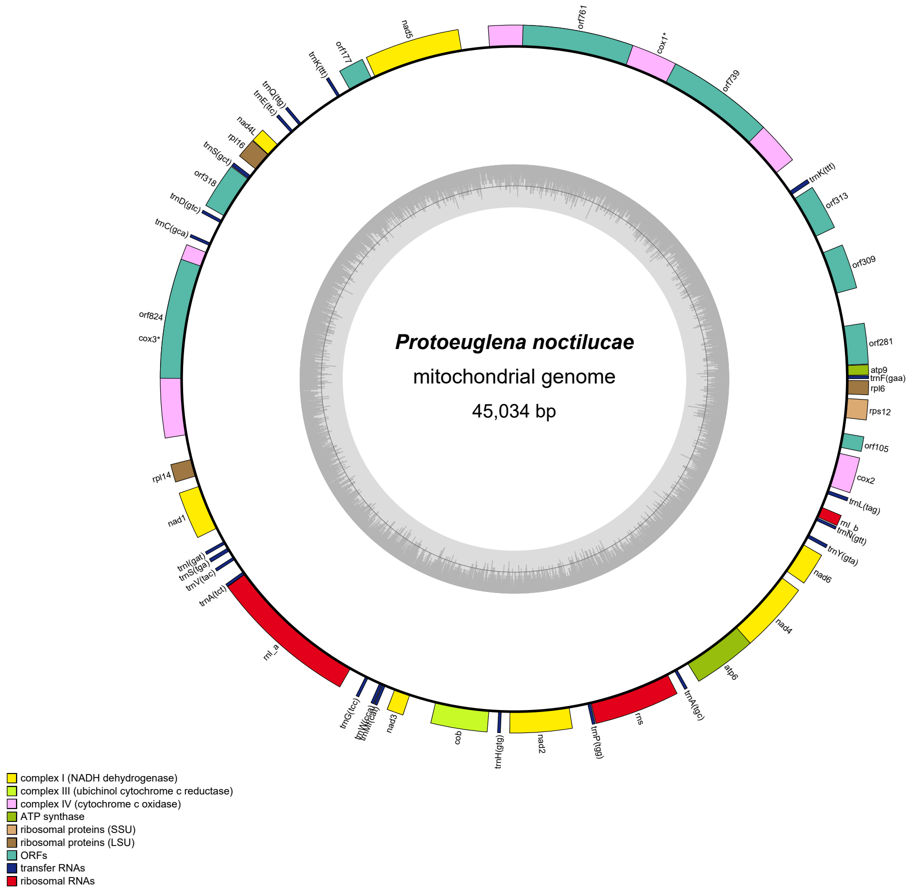
Protoeuglena noctilucae
mitochondrial genome
45,034 bp
complex I (NADH dehydrogenase)
complex III (ubichinol cytochrome c reductase)
complex IV (cytochrome c oxidase)
ATP synthase
ribosomal proteins (SSU)
ribosomal proteins (LSU)
ORFs
transfer RNAs
ribosomal RNAs

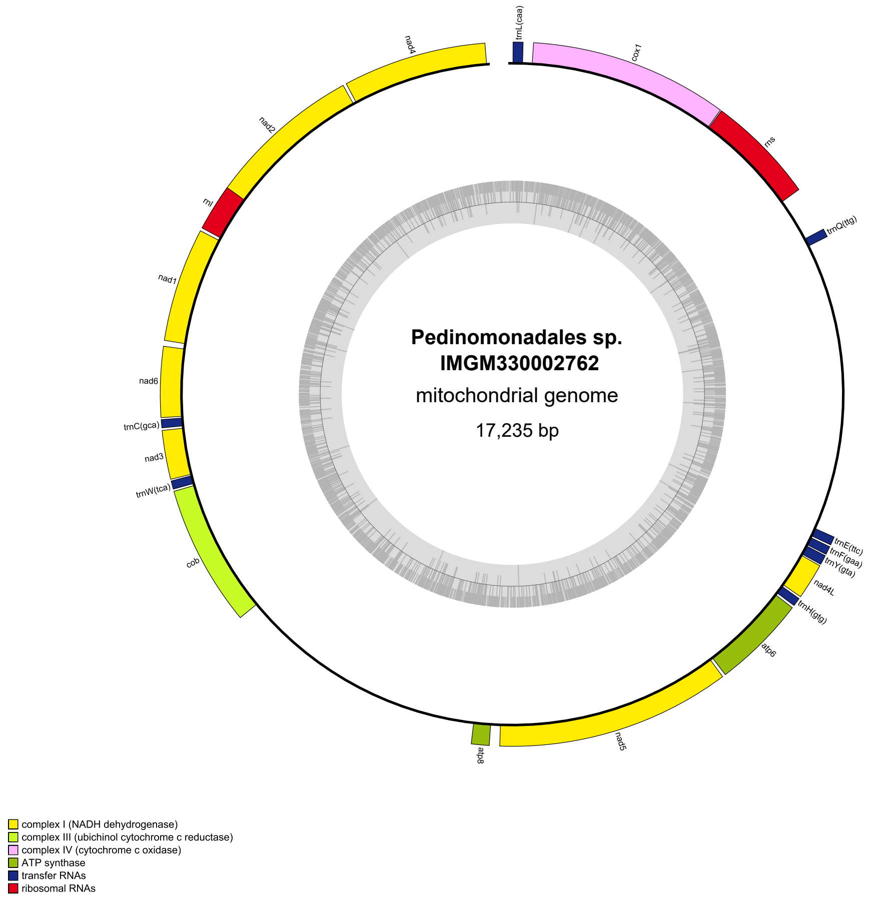
Pedinomonadales sp.
IMGM330002762
mitochondrial genome
17,235 bp
complex I (NADH dehydrogenase)
complex III (ubichinol cytochrome c reductase)
complex IV (cytochrome c oxidase)
ATP synthase
transfer RNAs
ribosomal RNAs
