# Supplementary material for: Expanded diversity of pedinophytes provides a window into the evolution of the genetic code in organelles
Source: PLoS Genet. 2025 Oct 22;21(10):e1011901. doi: 10.1371/journal.pgen.1011901 (PMC12574857; doi:10.1371/journal.pgen.1011901)
Supplement: S14 Fig — (PDF) [file pgen.1011901.s014.pdf]

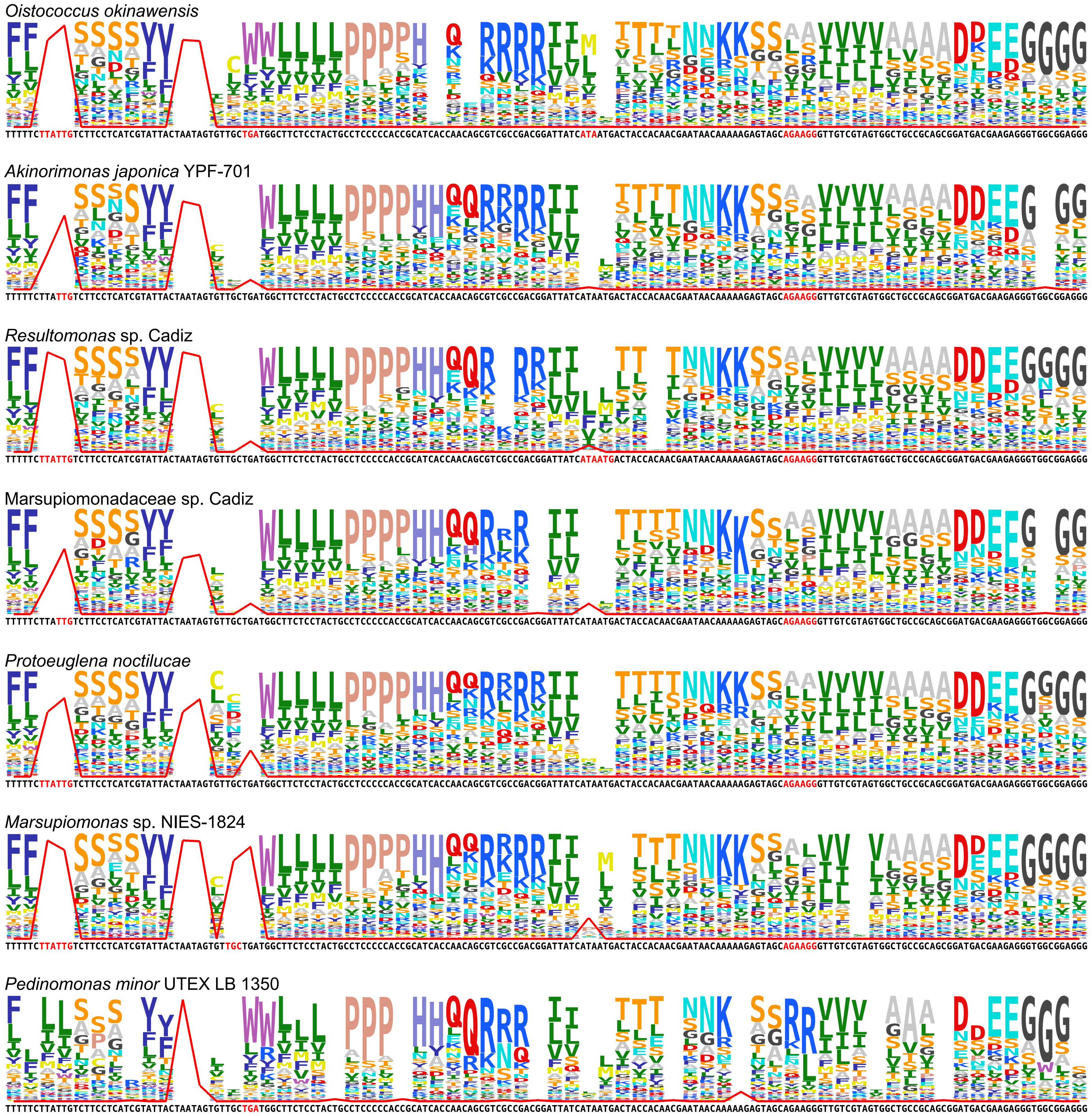

Oistococcus okinawensis
TTTTTCTTATTGTCTTCCTCATCGTATTACTAATAGTGTTGCTGATGGCTTCTCCTACTGCCTCCCCCACCGCATCACCAACAGCGTCGCCGACGGATTATCATAATGACTACCACAACGAATAACAAAAAGAGTAGCAGAAGGGTTGTCGTAGTGGCTGCCGCAGCGGATGACGAAGAGGGTGGCGGAGGG
Akinorimonas japonica YPF-701
TTTTTCTTATTGTCTTCCTCATCGTATTACTAATAGTGTTGCTGATGGCTTCTCCTACTGCCTCCCCCACCGCATCACCAACAGCGTCGCCGACGGATTATCATAATGACTACCACAACGAATAACAAAAAGAGTAGCAGAAGGGTTGTCGTAGTGGCTGCCGCAGCGGATGACGAAGAGGGTGGCGGAGGG
Resultomonas sp. Cadiz
TTTTTCTTATTGTCTTCCTCATCGTATTACTAATAGTGTTGCTGATGGCTTCTCCTACTGCCTCCCCCACCGCATCACCAACAGCGTCGCCGACGGATTATCATAATGACTACCACAACGAATAACAAAAAGAGTAGCAGAAGGGTTGTCGTAGTGGCTGCCGCAGCGGATGACGAAGAGGGTGGCGGAGGG
Marsupiomonadaceae sp. Cadiz
TTTTTCTTATTGTCTTCCTCATCGTATTACTAATAGTGTTGCTGATGGCTTCTCCTACTGCCTCCCCCACCGCATCACCAACAGCGTCGCCGACGGATTATCATAATGACTACCACAACGAATAACAAAAAGAGTAGCAGAAGGGTTGTCGTAGTGGCTGCCGCAGCGGATGACGAAGAGGGTGGCGGAGGG
Protoeuglena noctilucae
TTTTTCTTATTGTCTTCCTCATCGTATTACTAATAGTGTTGCTGATGGCTTCTCCTACTGCCTCCCCCACCGCATCACCAACAGCGTCGCCGACGGATTATCATAATGACTACCACAACGAATAACAAAAAGAGTAGCAGAAGGGTTGTCGTAGTGGCTGCCGCAGCGGATGACGAAGAGGGTGGCGGAGGG
Marsupiomonas sp. NIES-1824
TTTTTCTTATTGTCTTCCTCATCGTATTACTAATAGTGTTGCTGATGGCTTCTCCTACTGCCTCCCCCACCGCATCACCAACAGCGTCGCCGACGGATTATCATAATGACTACCACAACGAATAACAAAAAGAGTAGCAGAAGGGTTGTCGTAGTGGCTGCCGCAGCGGATGACGAAGAGGGTGGCGGAGGG
Pedinomonas minor UTEX LB 1350
TTTTTCTTATTGTCTTCCTCATCGTATTACTAATAGTGTTGCTGATGGCTTCTCCTACTGCCTCCCCCACCGCATCACCAACAGCGTCGCCGACGGATTATCATAATGACTACCACAACGAATAACAAAAAGAGTAGCAGAAGGGTTGTCGTAGTGGCTGCCGCAGCGGATGACGAAGAGGGTGGCGGAGGG
