# Supplementary material for: Expanded diversity of pedinophytes provides a window into the evolution of the genetic code in organelles
Source: PLoS Genet. 2025 Oct 22;21(10):e1011901. doi: 10.1371/journal.pgen.1011901 (PMC12574857; doi:10.1371/journal.pgen.1011901)
Supplement: S18 Fig — Note the G3·U70 pair characteristic for tRNAAla present in the tRNA sequences from Marsupiomonadales and Tetradesmus obliquus (Sphaeropleales), consistent with the Arg-to-Ala AGR reassignment in both taxa. (PDF) [file pgen.1011901.s018.pdf]

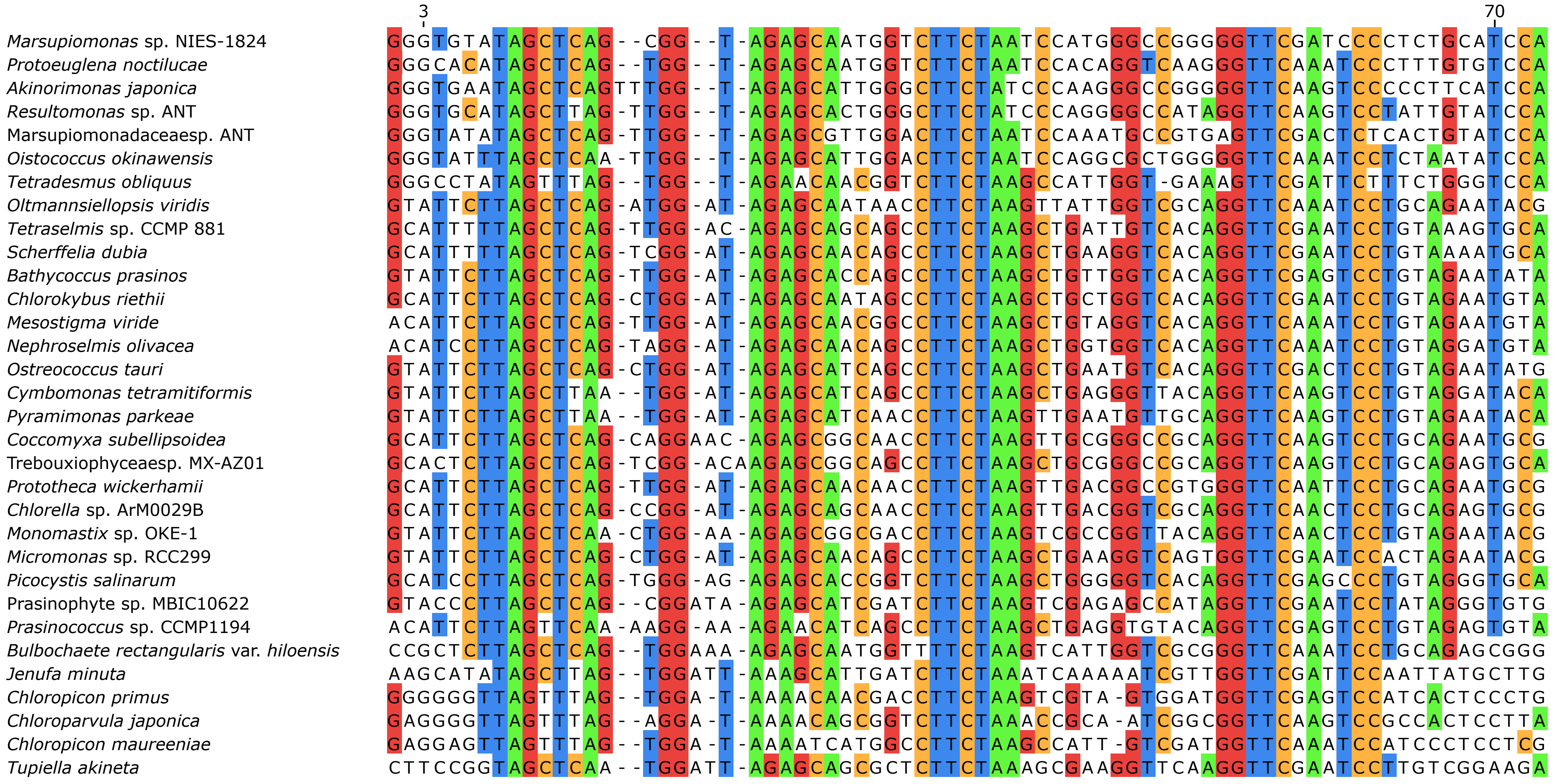

3
70
Marsupiomonas sp. NIES-1824
Protoeuglena noctilucae
Akinorimonas japonica
Resultomonas sp. ANT
Marsupiomonadaceaesp. ANT
Oistococcus okinawensis
Tetradesmus obliquus
Oltmannsiellopsis viridis
Tetraselmis sp. CCMP 881
Scherffelia dubia
Bathycoccus prasinos
Chlorokybus riethii
Mesostigma viride
Nephroselmis olivacea
Ostreococcus tauri
Cymbomonas tetramitiformis
Pyramimonas parkeae
Coccomyxa subellipsoidea
Trebouxiophyceaesp. MX-AZ01
Prototheca wickerhamii
Chlorella sp. ArM0029B
Monomastix sp. OKE-1
Micromonas sp. RCC299
Picocystis salinarum
Prasinophyte sp. MBIC10622
Prasinococcus sp. CCMP1194
Bulbochaete rectangularis var. hiloensis
Jenufa minuta
Chloropicon primus
Chloroparvula japonica
Chloropicon maureeniae
Tupiella akineta
