# Supplementary material for: Expanded diversity of pedinophytes provides a window into the evolution of the genetic code in organelles
Source: PLoS Genet. 2025 Oct 22;21(10):e1011901. doi: 10.1371/journal.pgen.1011901 (PMC12574857; doi:10.1371/journal.pgen.1011901)
Supplement: S19 Fig — The display convention follows that one used in Fig 6A. (PDF) [file pgen.1011901.s019.pdf]

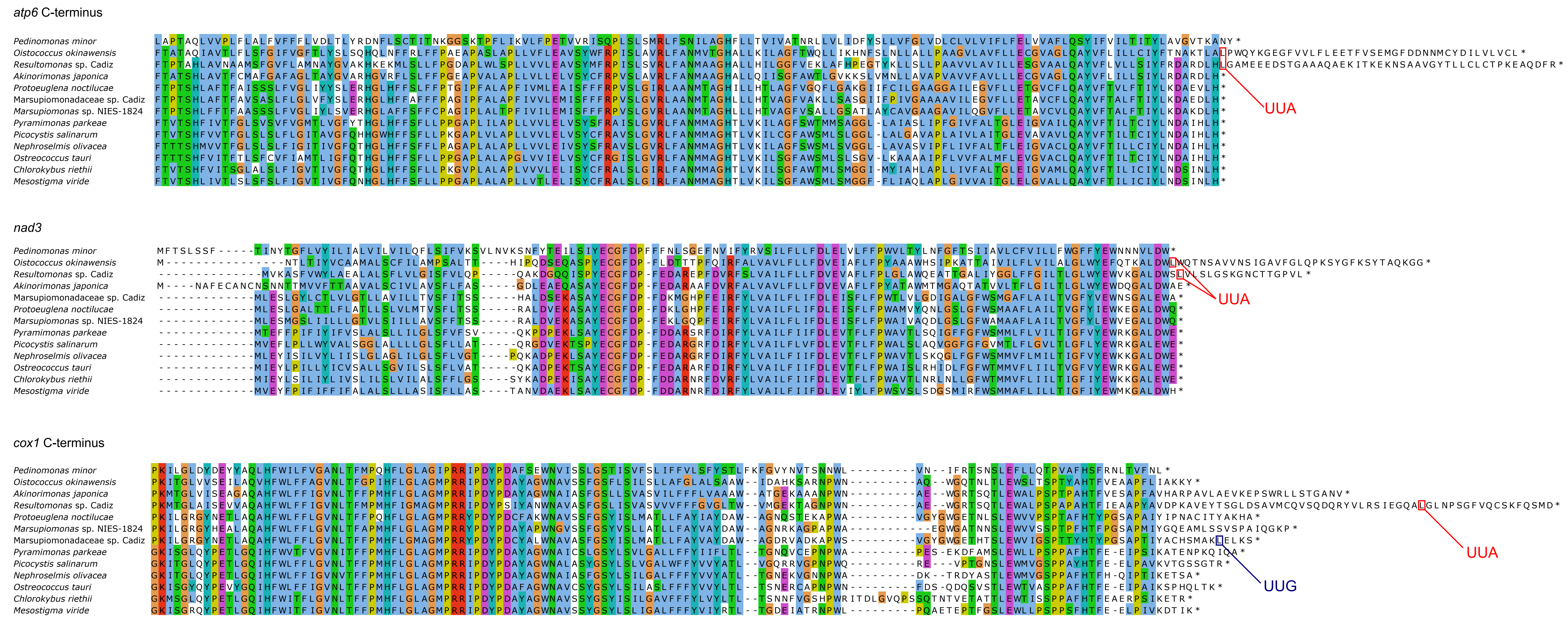
atp6 C-terminus
Pedinomonas minor
Oistococcus okinawensis
Resultomonas sp. Cadiz
Akinorimonas japonica
Protoeuglena noctilucae
Marsupiomonadaceae sp. Cadiz
Marsupiomonas sp. NIES-1824
Pyramimonas parkeae
Picocystis salinarum
Nephroselmis olivacea
Ostreococcus tauri
Chlorokybus riethii
Mesostigma viride
UUA
nad3
Pedinomonas minor
Oistococcus okinawensis
Resultomonas sp. Cadiz
Akinorimonas japonica
Marsupiomonadaceae sp. Cadiz
Protoeuglena noctilucae
Marsupiomonas sp. NIES-1824
Pyramimonas parkeae
Picocystis salinarum
Nephroselmis olivacea
Ostreococcus tauri
Chlorokybus riethii
Mesostigma viride
UUA
cox1 C-terminus
Pedinomonas minor
Oistococcus okinawensis
Akinorimonas japonica
Resultomonas sp. Cadiz
Protoeuglena noctilucae
Marsupiomonas sp. NIES-1824
Marsupiomonadaceae sp. Cadiz
Pyramimonas parkeae
Picocystis salinarum
Nephroselmis olivacea
Ostreococcus tauri
Chlorokybus riethii
Mesostigma viride
UUA
UUG
