# Supplementary material for: Expanded diversity of pedinophytes provides a window into the evolution of the genetic code in organelles
Source: PLoS Genet. 2025 Oct 22;21(10):e1011901. doi: 10.1371/journal.pgen.1011901 (PMC12574857; doi:10.1371/journal.pgen.1011901)
Supplement: S20 Fig — The figure shows alignments of protein sequences encoded by the mitogenome of Pedinomonas minor with homologous sequences (putative mitochondrial transcript) identified in the available transcriptome assembly from C. tuberculatum and conceptually translated with the standard genetic code. The asterisks highlighted in red correspond to UGA codons. (PDF) [file pgen.1011901.s020.pdf]

```
Query= lcl|NC_000892.1_prot_NP_050057.1_2 [gene=nad5]
         (619 letters)


>scaffold-PUAN-2016427-Pedinomonas_tuberculata
          Length = 452

Query: 153 FWYSKLQSGKSAIKAVFLNRIGDSFFLIALGLTFYLFGSDDLFLISSLSVFYEKQIVKYL 212
           F YSK+QSGKSAIKAVFLNRIGDSFFLIALGL FYLFGSD+LFLISSLS  Y+K I++YL
Sbjct: 451 F*YSKIQSGKSAIKAVFLNRIGDSFFLIALGLCFYLFGSDNLFLISSLSYLYDKLILEYL 272

Query: 213 IIAYLIASIAKSAQILLHVWLPDAIEAPTPVSSLLHAATLVGAGVYLLIKLSFLSLLDFD 272
           II++LIASIAKSAQI LH+WLPDAIEAPTPVSSLLHAATLVGAGVYL+IKL FLSLL+FD
Sbjct: 271 IISFLIASIAKSAQIFLHIWLPDAIEAPTPVSSLLHAATLVGAGVYLIIKLFFLSLLNFD 92

Query: 273 SSNFIIVIGILTSFLAGLIGFNQFDTKRII 302
            +NFIIVIGILTSFLAGLIGFNQFDTKRII
Sbjct: 91  LANFIIVIGILTSFLAGLIGFNQFDTKRII 2

############################

Query= lcl|NC_000892.1_prot_NP_050060.1_5 [gene=cox1]
         (523 letters)


>scaffold-PUAN-2016993-Pedinomonas_tuberculata
          Length = 505

Query: 24  AGFAGVLAVTLSLLIRLQLVATGYGWLAL------NYQFYNTIVTAHGLLIVFFLLMPAL 77
           AGF+G+LAV LSLLIRLQLV+ GYG ++       NYQ YNTI+TAHGLLIVFFLL+PAL
Sbjct: 1   AGFSGILAVILSLLIRLQLVSNGYG*MSHPNQPYGNYQLYNTIITAHGLLIVFFLLIPAL 180

Query: 78  IGGFGNWIVPVLIGSIDMAYPRLNNISFWLLPPSLLLLVGSMYIEIGAGTGWTVYPPLSL 137
           +GGFGN IVPVLIGSIDMAYPRLNN+SF L+PPSLLL+V SMYIEIGAGTG TVYPPLS
Sbjct: 181 MGGFGN*IVPVLIGSIDMAYPRLNNVSF*LIPPSLLLIVSSMYIEIGAGTG*TVYPPLSS 360

Query: 138 I--EFHSSASVDMAIFSLHVSGLSSLLGAINFIVTIFCMKTRGLSWRA 183
              + H +ASVD+AIFSLHV+G+SSLLGAINFIVTIFCMKTRGL+ RA
Sbjct: 361 FQGQAHDNASVDLAIFSLHVAGVSSLLGAINFIVTIFCMKTRGLA*RA 504


>scaffold-PUAN-2013880-Pedinomonas_tuberculata
          Length = 275

Query: 354 GFSGVLLANGGLDLLFHDTYYVVGHFHYVLSLGAIFALFSGFYYWTPKILGLDYDEYYAQ 413
           GFSGVLL+NGGLDLLFHDTYYVVGHFHYVLSLGA+FA+F+GFYY TPKILGLDYDE+Y Q
Sbjct: 3   GFSGVLLSNGGLDLLFHDTYYVVGHFHYVLSLGAVFAIFAGFYY*TPKILGLDYDEFYGQ 182

Query: 414 LHFWILFVGANLTFMPQHFLGLAGIPRRIPD 444
           LHF  LF+GANLTF+P HFLGL+G+PRRIPD
Sbjct: 183 LHF*TLFIGANLTFIPHHFLGLSGMPRRIPD 275

############################

Query= lcl|NC_000892.1_prot_NP_050066.1_11 [gene=cob]
         (374 letters)

>scaffold-PUAN-2002648-Pedinomonas_tuberculata
          Length = 893

Query: 88  FFFIVVYAHIFRAFFYSSFTYPREFVWFIGIIILFLIILTAFIGYVLPWGQISFWGATVI 147
           FFFIVVYAHIFRAF+YSSFTYPREFV FIG+IILFLII TAFIGYVLP GQISF GATVI
Sbjct: 691 FFFIVVYAHIFRAFYYSSFTYPREFV*FIGVIILFLIIATAFIGYVLP*GQISF*GATVI 512

Query: 148 TSIVTAIPLLGNDLVTWVWGGFNVDDPTLHRFFSLHYLMPFVLLGLVVVHILALHQYGSN 207
           TSIVTAIPLLGNDLV  V GGFNVDDPTLHRFFSLHYL+PFVL+GL+VVHILALHQYGSN
Sbjct: 511 TSIVTAIPLLGNDLVA*V*GGFNVDDPTLHRFFSLHYLIPFVLVGLMVVHILALHQYGSN 332

Query: 208 NPIGISIDADKVTLHPYFTIKDLAGVFLIFIFYFYFVCFEPDFLNHPDNCIPANSIKTPV 267
            PIG++IDADKV+LHPYFTIKD  G+F+IFI YFYFVCF+PDFLNHPDNCIPANSIKTPV
Sbjct: 331 TPIGLTIDADKVSLHPYFTIKD-XGIFIIFIVYFYFVCFDPDFLNHPDNCIPANSIKTPV 155

Query: 268 HIVPEWYFLVVYAILRSIPNKLLGILAILLVFVCFALLPIVYGYSSRAMLF 318
           HIVPE YFLVVYAILRSIPNKLLGILAILLVFVCFALLP+VYGYS+RA+LF
Sbjct: 154 HIVPE*YFLVVYAILRSIPNKLLGILAILLVFVCFALLPMVYGYSTRAILF 2


Query: 22  SPVIINYFWNFGSLAGVFLFIQILTGIFLAMFYIPSADAAFASVEFLMRDVNNGWFIRFM 81
           SPV INYF  FGSLAGVFL IQILTGIFLAMFYIPSADAAFASVEFL+RDVNNG  IRF+
Sbjct: 891 SPVSINYF*GFGSLAGVFLIIQILTGIFLAMFYIPSADAAFASVEFLIRDVNNG*MIRFI 712

Query: 82  HVNGASFFFIVVYAHIFRAFF 102
           H NGASFFF++++  I+   F
Sbjct: 711 HANGASFFFLLLFMLIYLELF 649
```
