# Supplementary material for: Expanded diversity of pedinophytes provides a window into the evolution of the genetic code in organelles
Source: PLoS Genet. 2025 Oct 22;21(10):e1011901. doi: 10.1371/journal.pgen.1011901 (PMC12574857; doi:10.1371/journal.pgen.1011901)
Supplement: S1 Dataset — Absence of a plot for a given codon in a given taxon means the codon was not present at any position deemed conserved. (PDF) [file pgen.1011901.s031.pdf]

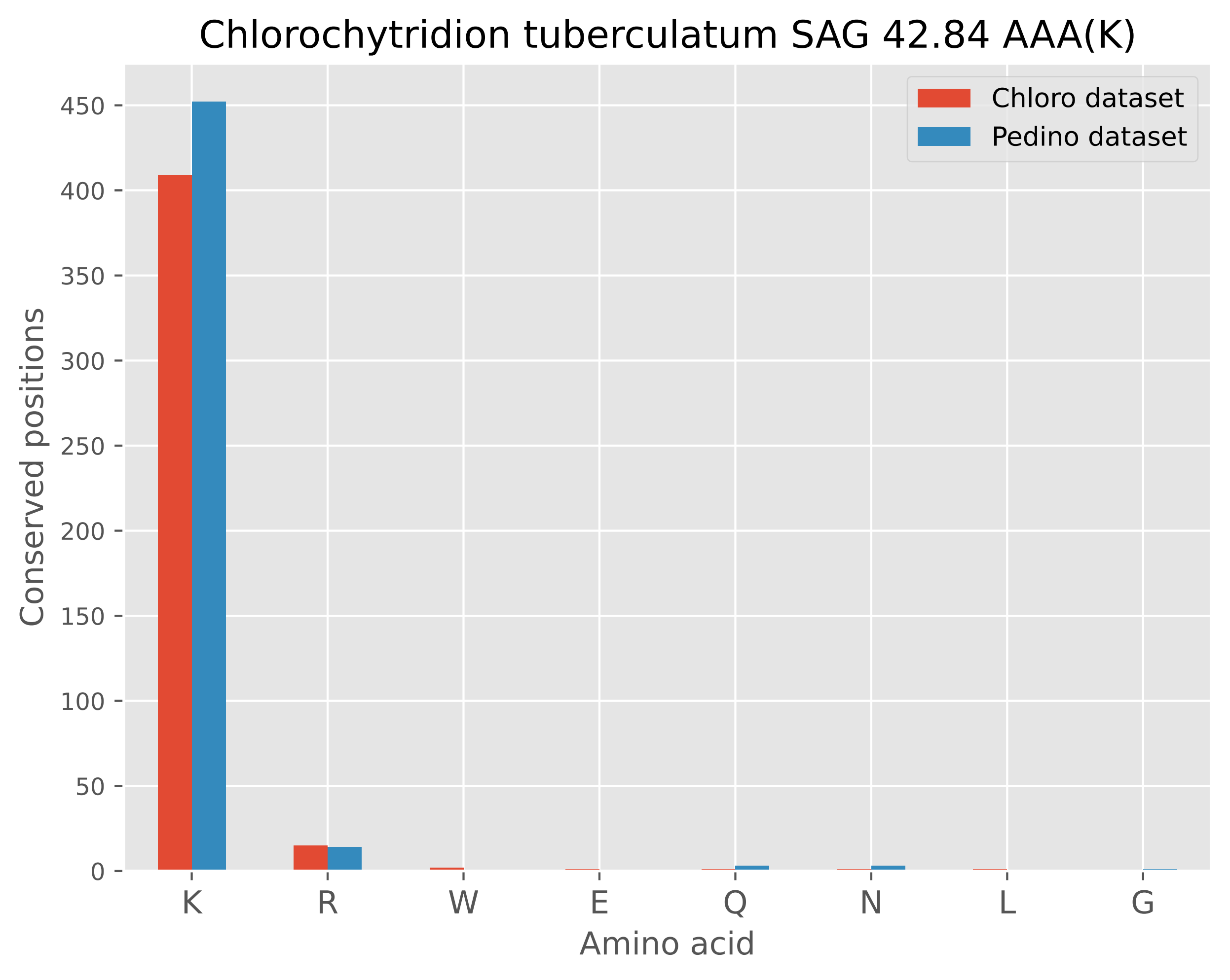
Chlorochytridion tuberculatum SAG 42.84 AAA(K)
Chloro dataset
Pedino dataset
Conserved positions
0
50
100
150
200
250
300
350
400
450
K
R
W
E
Q
N
L
G
Amino acid

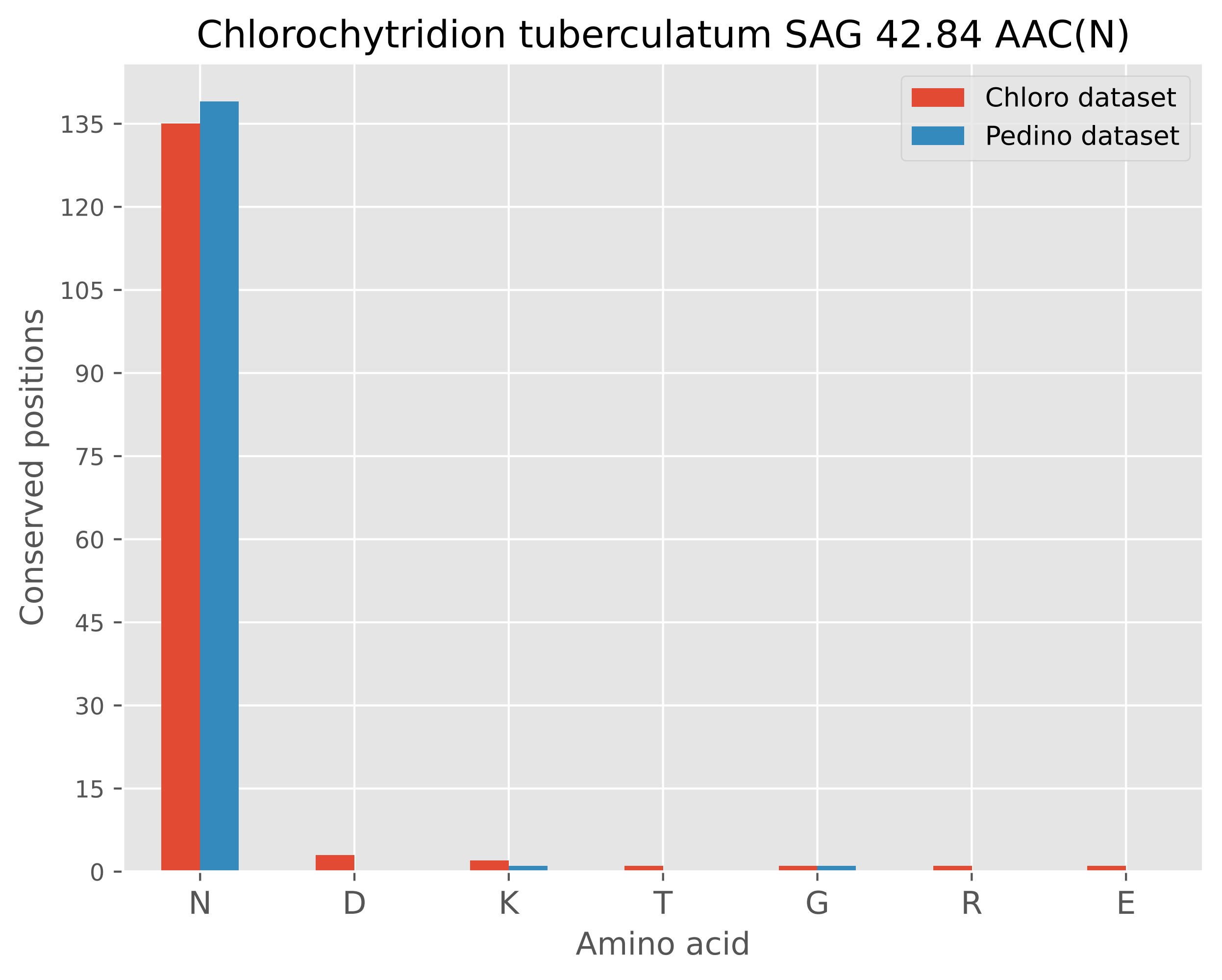
Chlorochytridion tuberculatum SAG 42.84 AAC(N)
Chloro dataset
Pedino dataset
Conserved positions
0
15
30
45
60
75
90
105
120
135
N
D
K
T
G
R
E
Amino acid

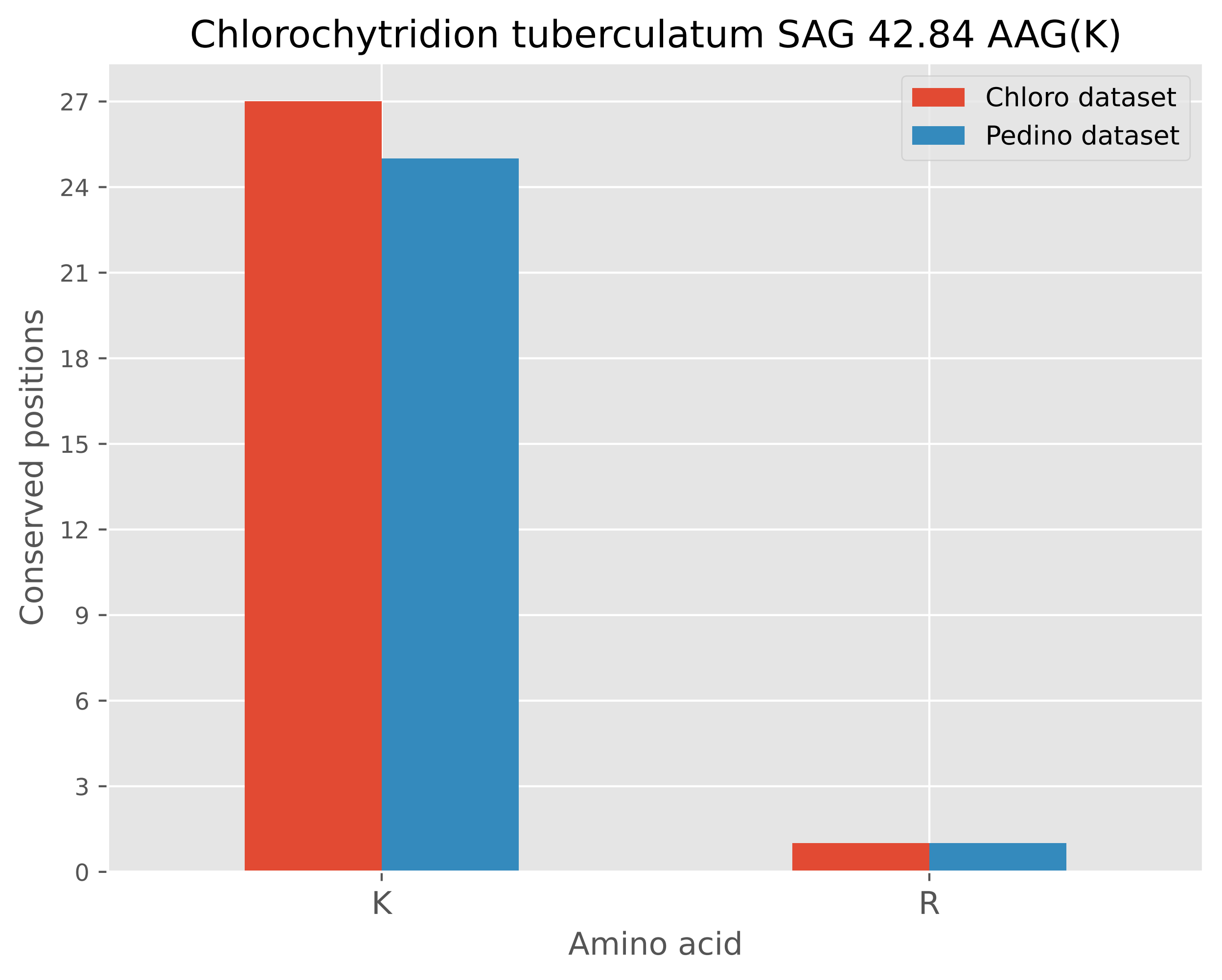
Chlorochytridion tuberculatum SAG 42.84 AAG(K)
Chloro dataset
Pedino dataset
Conserved positions
0
3
6
9
12
15
18
21
24
27
K
R
Amino acid

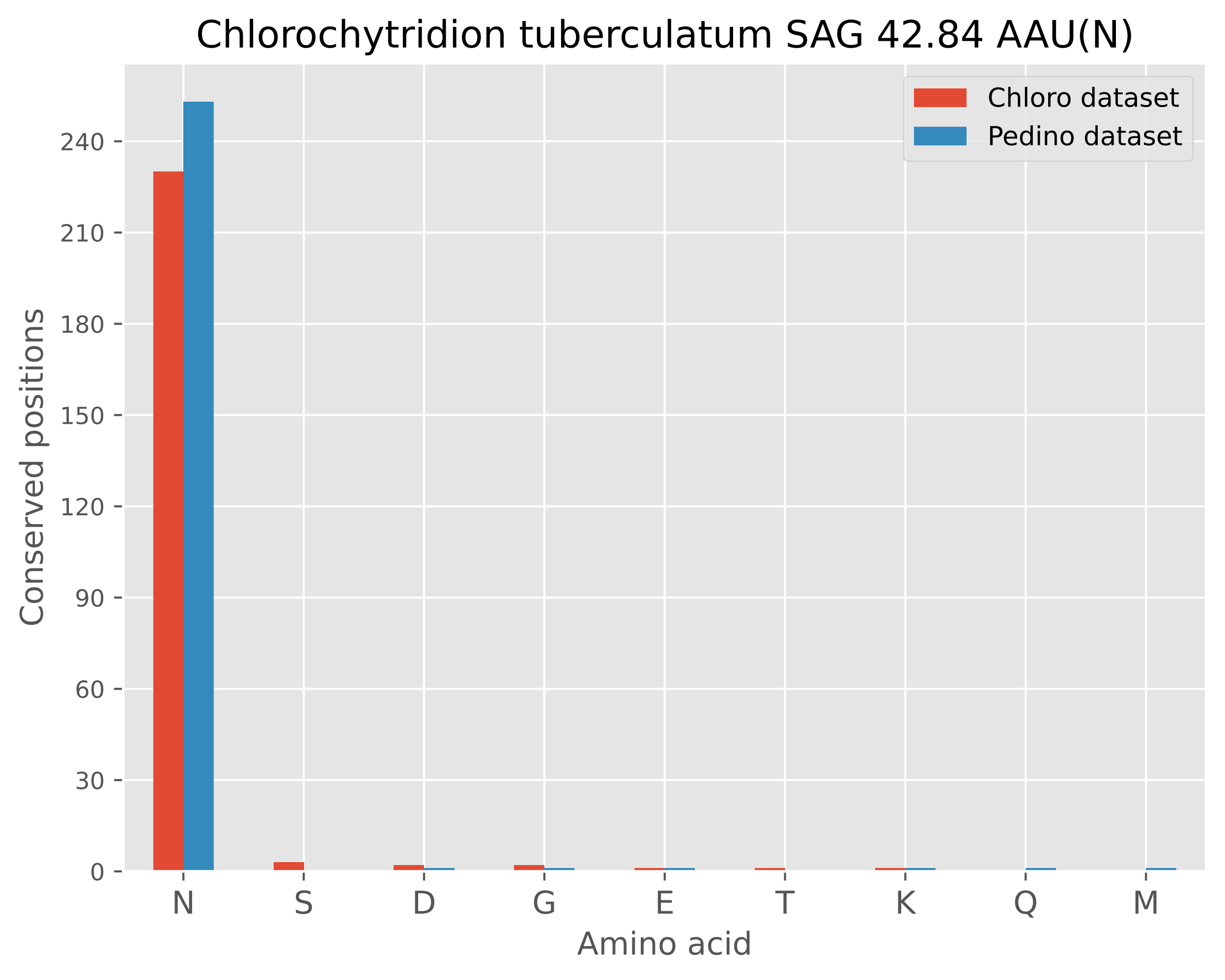

Chlorochytridion tuberculatum SAG 42.84 AAU(N)
Chloro dataset
Pedino dataset
Conserved positions
0
30
60
90
120
150
180
210
240
N
S
D
G
E
T
K
Q
M
Amino acid

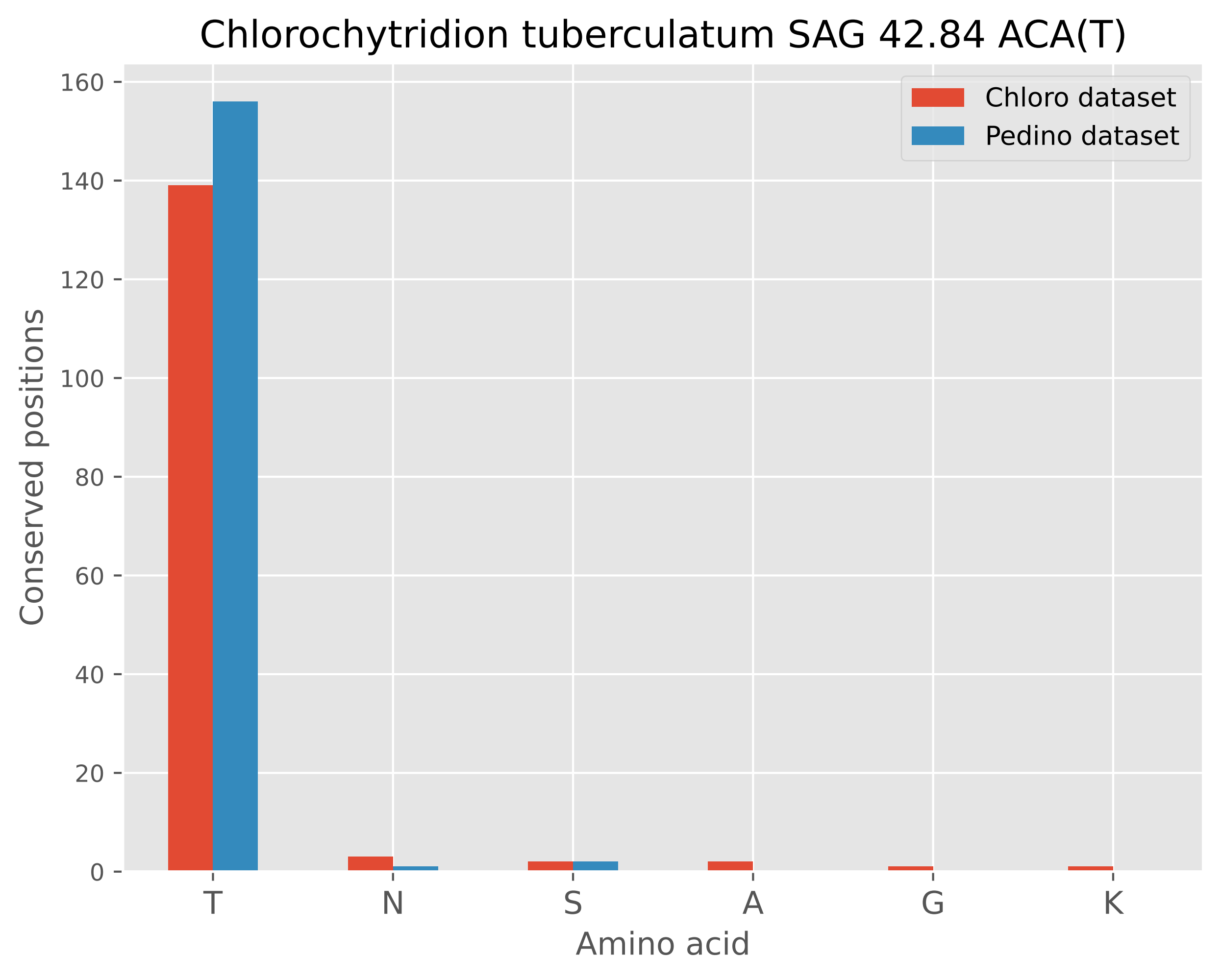

Chlorochytridion tuberculatum SAG 42.84 ACA(T)
Chloro dataset
Pedino dataset
Conserved positions
0
20
40
60
80
100
120
140
160
T
N
S
A
G
K
Amino acid

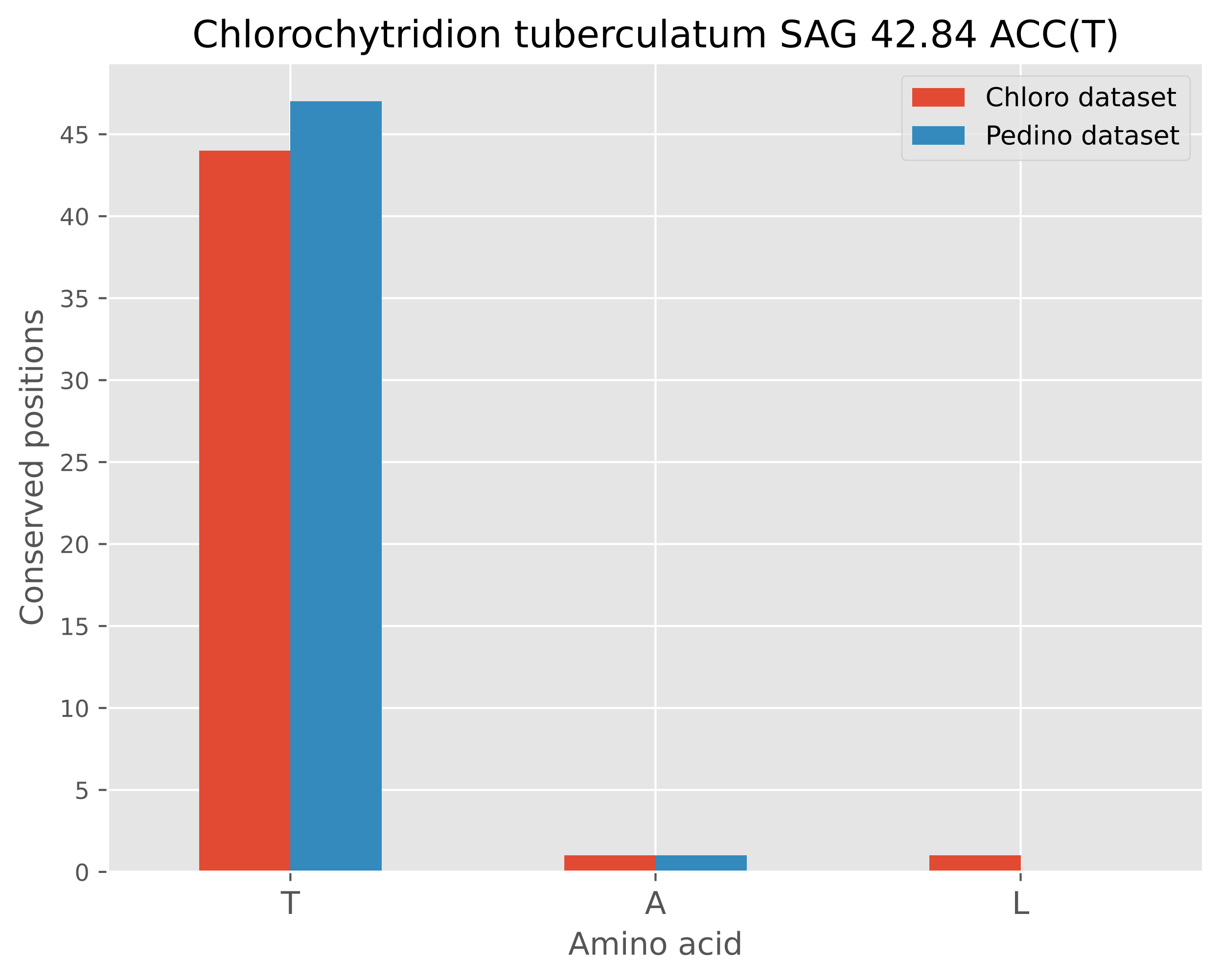
Chlorochytridion tuberculatum SAG 42.84 ACC(T)
Chloro dataset
Pedino dataset
Conserved positions
0
5
10
15
20
25
30
35
40
45
T
A
L
Amino acid

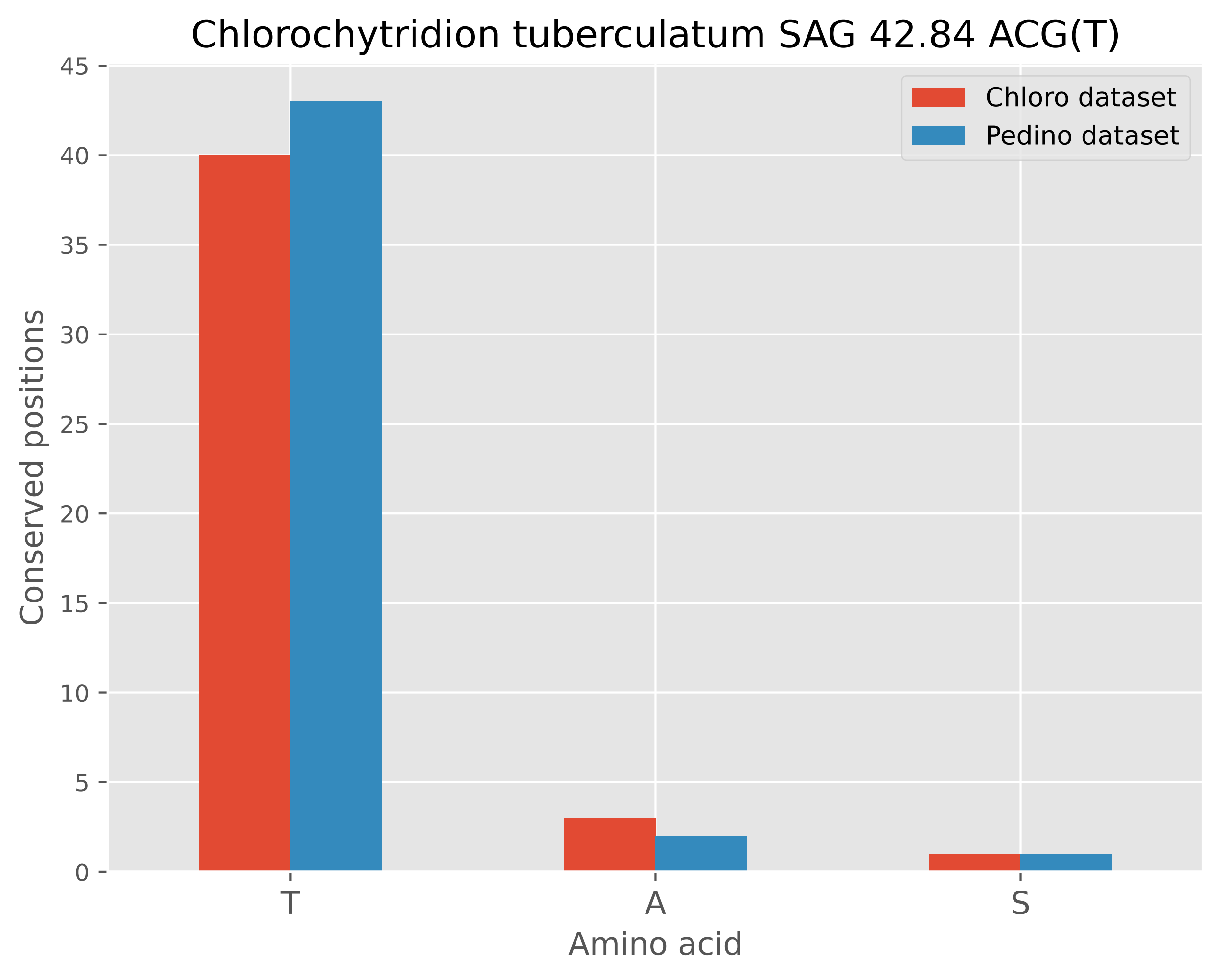

Chlorochytridion tuberculatum SAG 42.84 ACG(T)
Chloro dataset
Pedino dataset
Conserved positions
0
5
10
15
20
25
30
35
40
45
T
A
S
Amino acid

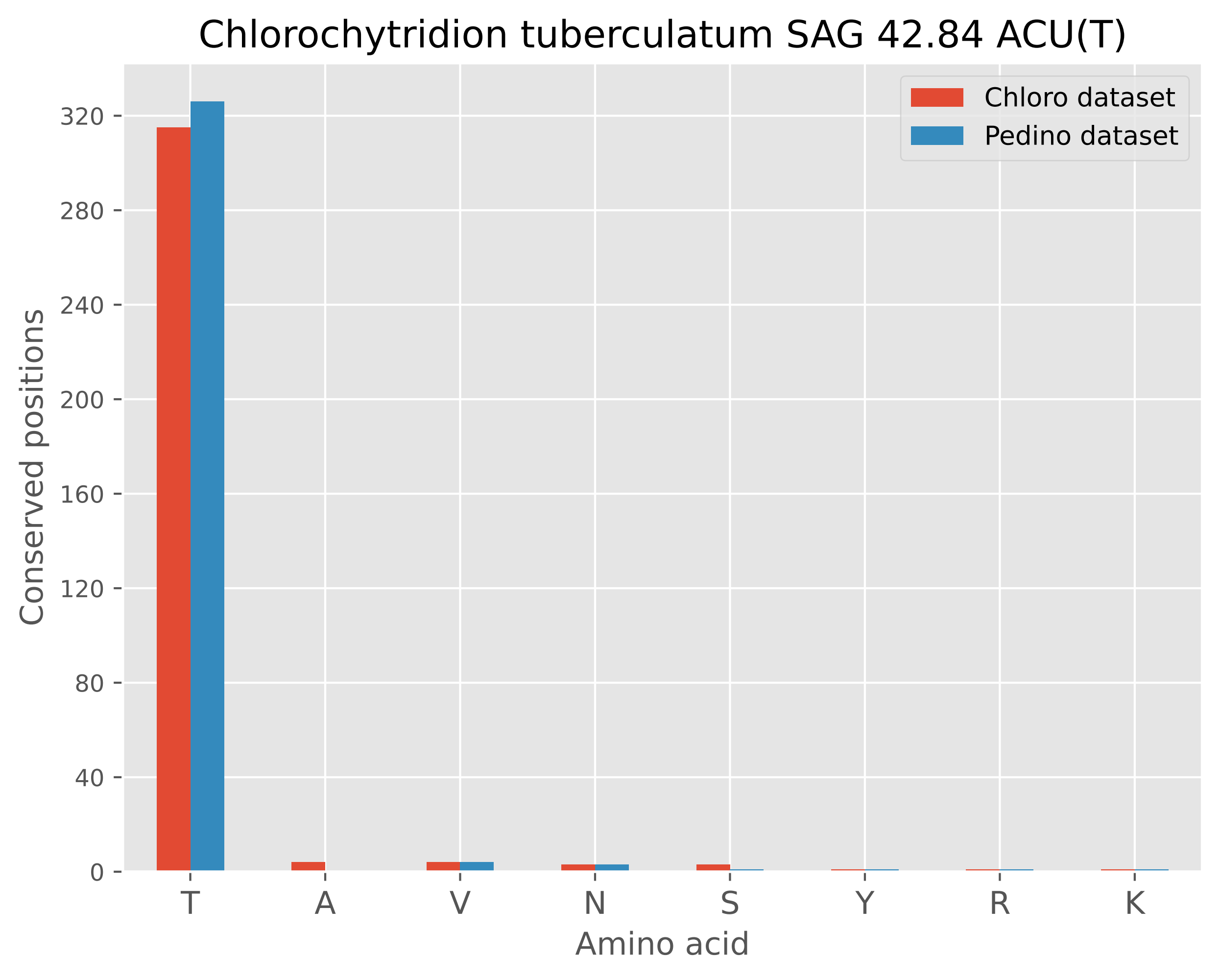

Chlorochytridion tuberculatum SAG 42.84 ACU(T)
Chloro dataset
Pedino dataset
Conserved positions
0
40
80
120
160
200
240
280
320
T
A
V
N
S
Y
R
K
Amino acid

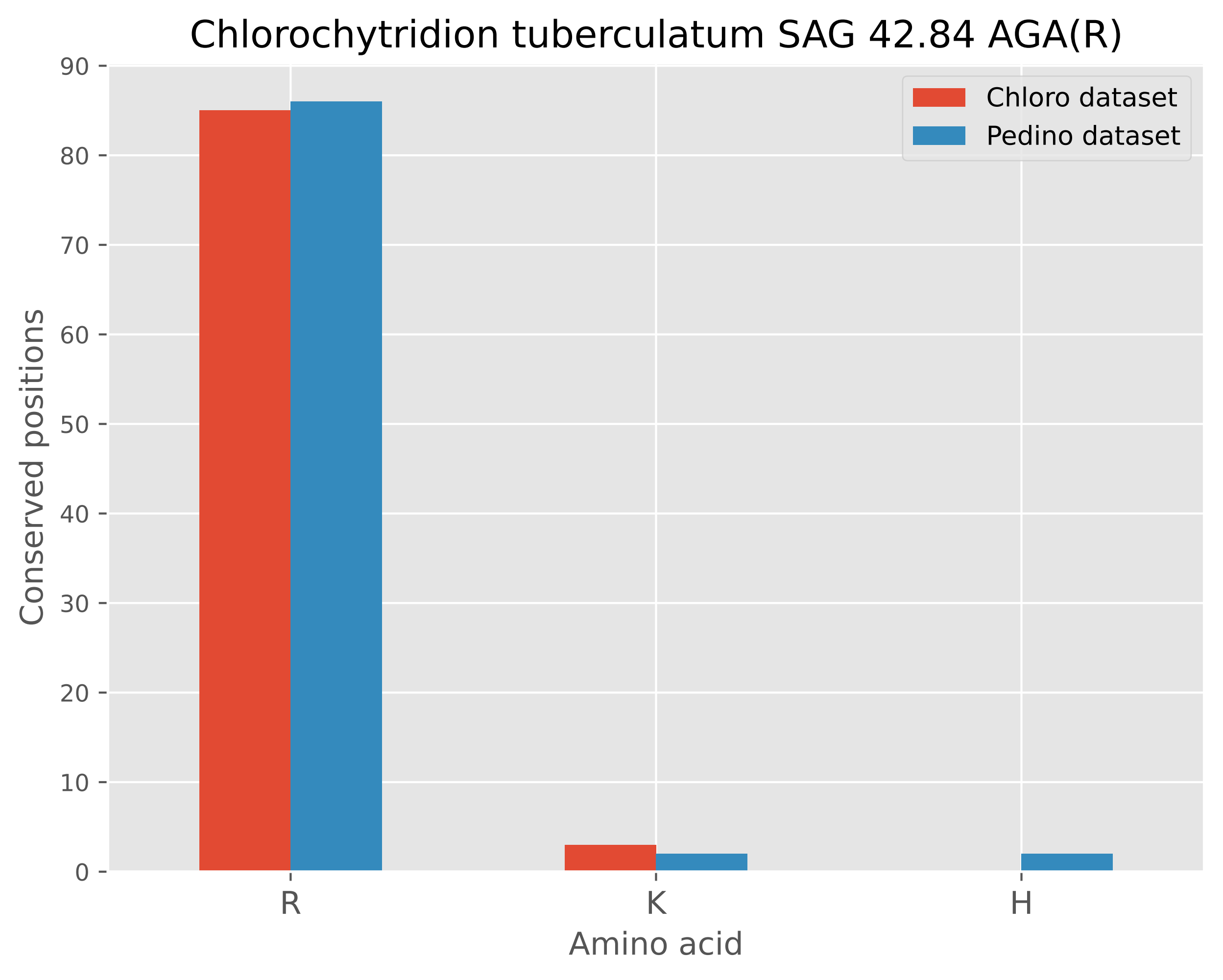
Chlorochytridion tuberculatum SAG 42.84 AGA(R)
Conserved positions
Amino acid
R
K
H
Chloro dataset
Pedino dataset
0
10
20
30
40
50
60
70
80
90

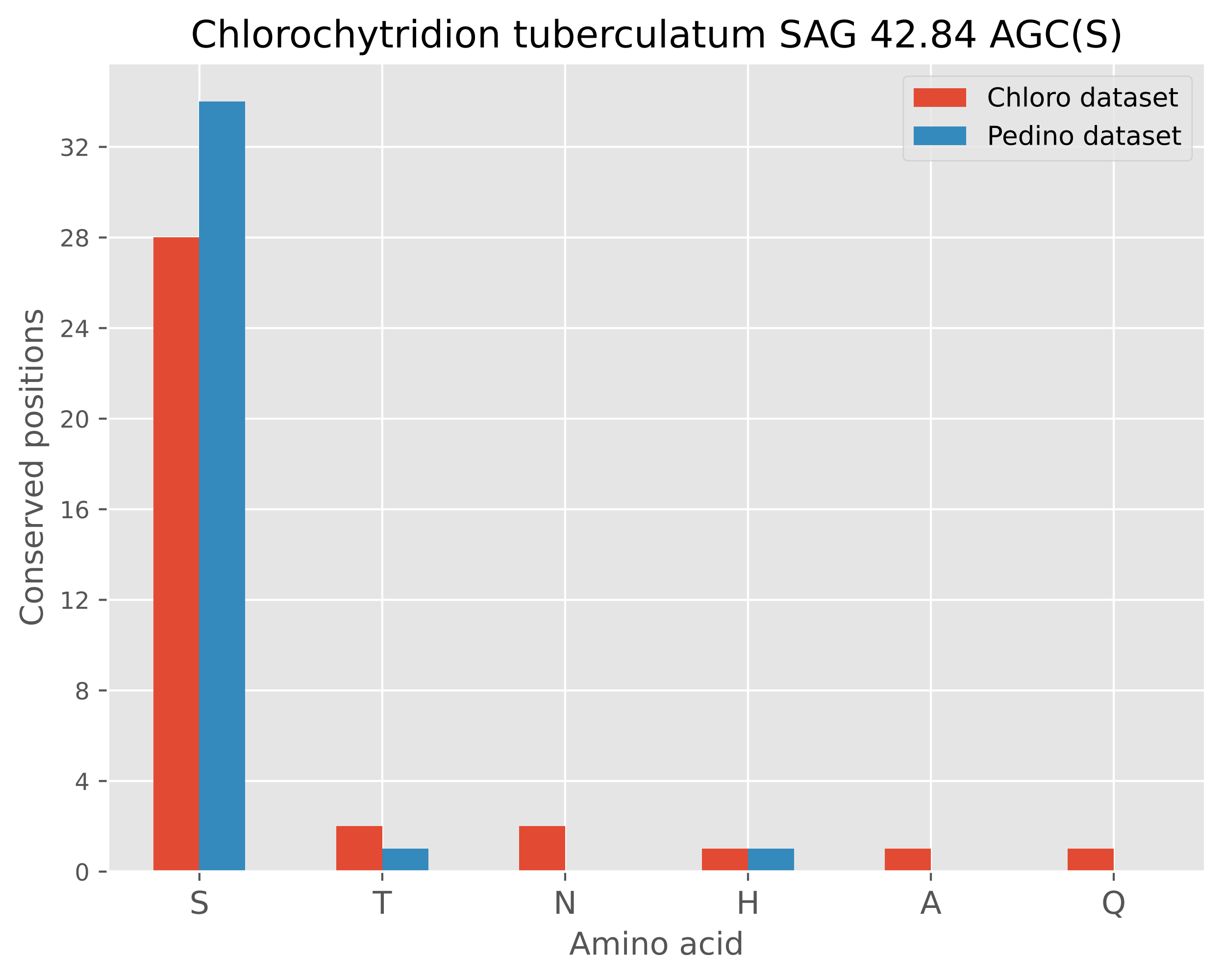

Chlorochytridion tuberculatum SAG 42.84 AGC(S)
Chloro dataset
Pedino dataset
Conserved positions
0
4
8
12
16
20
24
28
32
S
T
N
H
A
Q
Amino acid

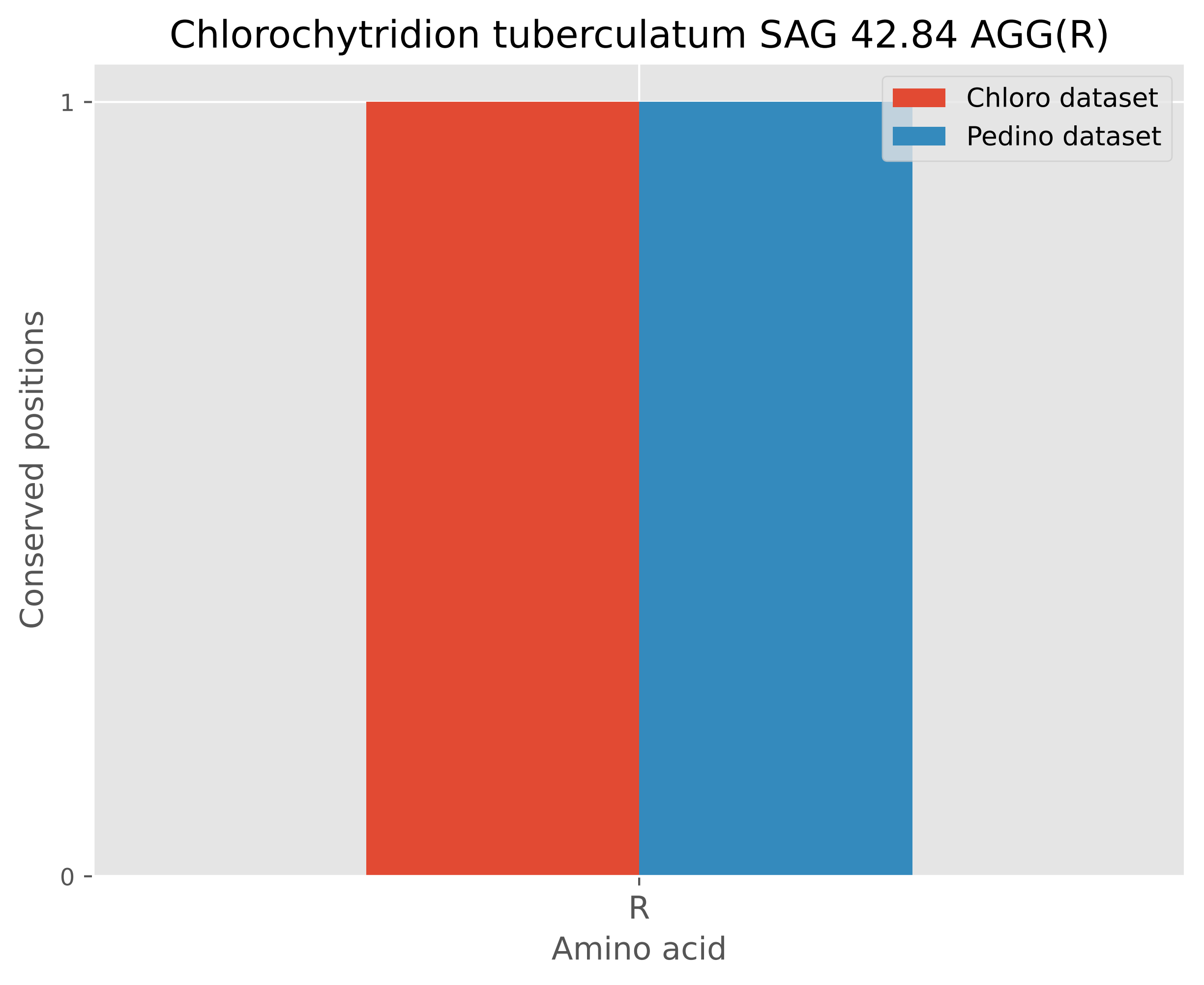
Chlorochytridion tuberculatum SAG 42.84 AGG(R)
Chloro dataset
Pedino dataset
Conserved positions
1
0
R
Amino acid

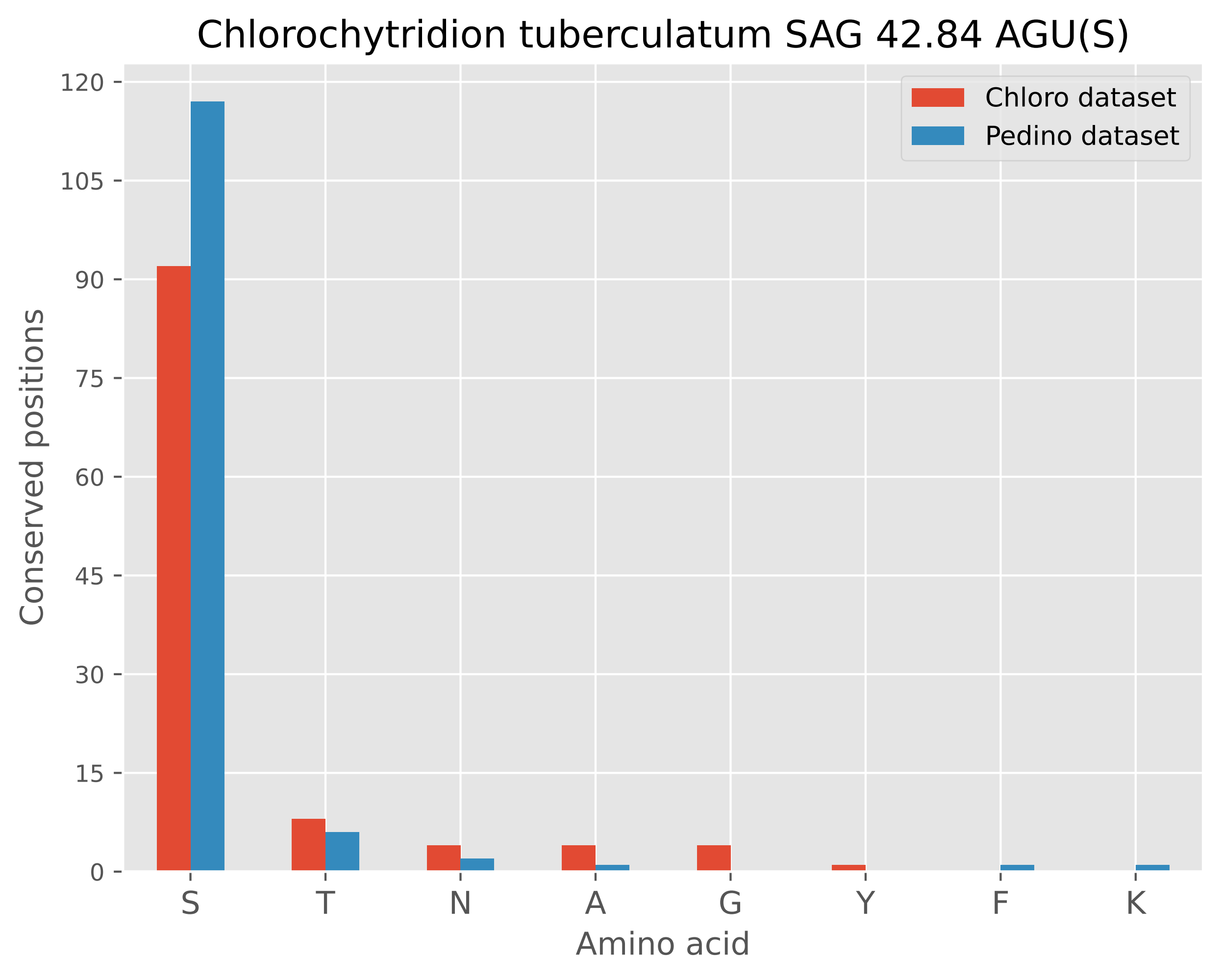
Chlorochytridion tuberculatum SAG 42.84 AGU(S)
Chloro dataset
Pedino dataset
Conserved positions
0
15
30
45
60
75
90
105
120
S
T
N
A
G
Y
F
K
Amino acid

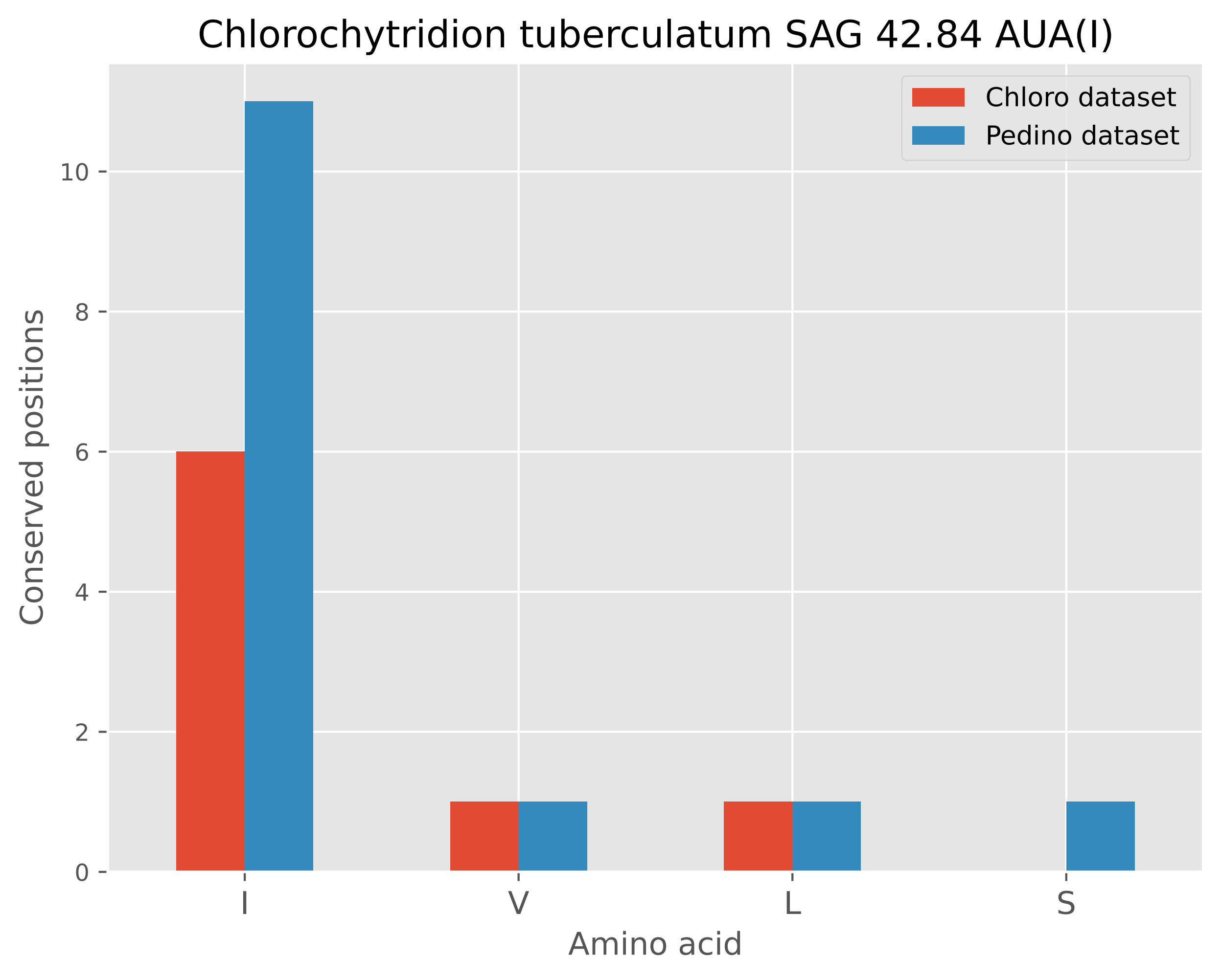

Chlorochytridion tuberculatum SAG 42.84 AUA(I)
Chloro dataset
Pedino dataset
Conserved positions
0
2
4
6
8
10
I
V
L
S
Amino acid

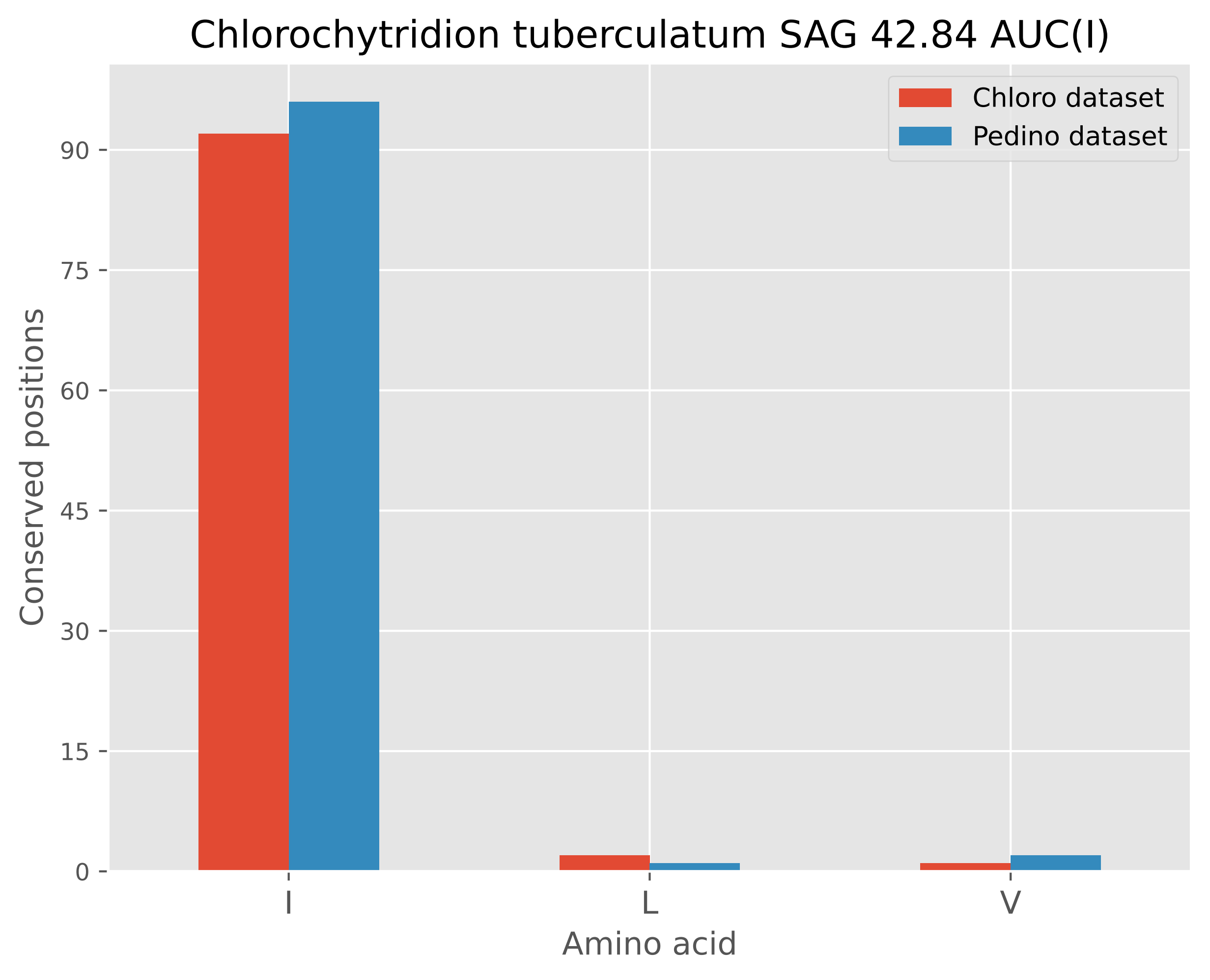
Chlorochytridion tuberculatum SAG 42.84 AUC(I)
Chloro dataset
Pedino dataset
Conserved positions
0
15
30
45
60
75
90
I
L
V
Amino acid

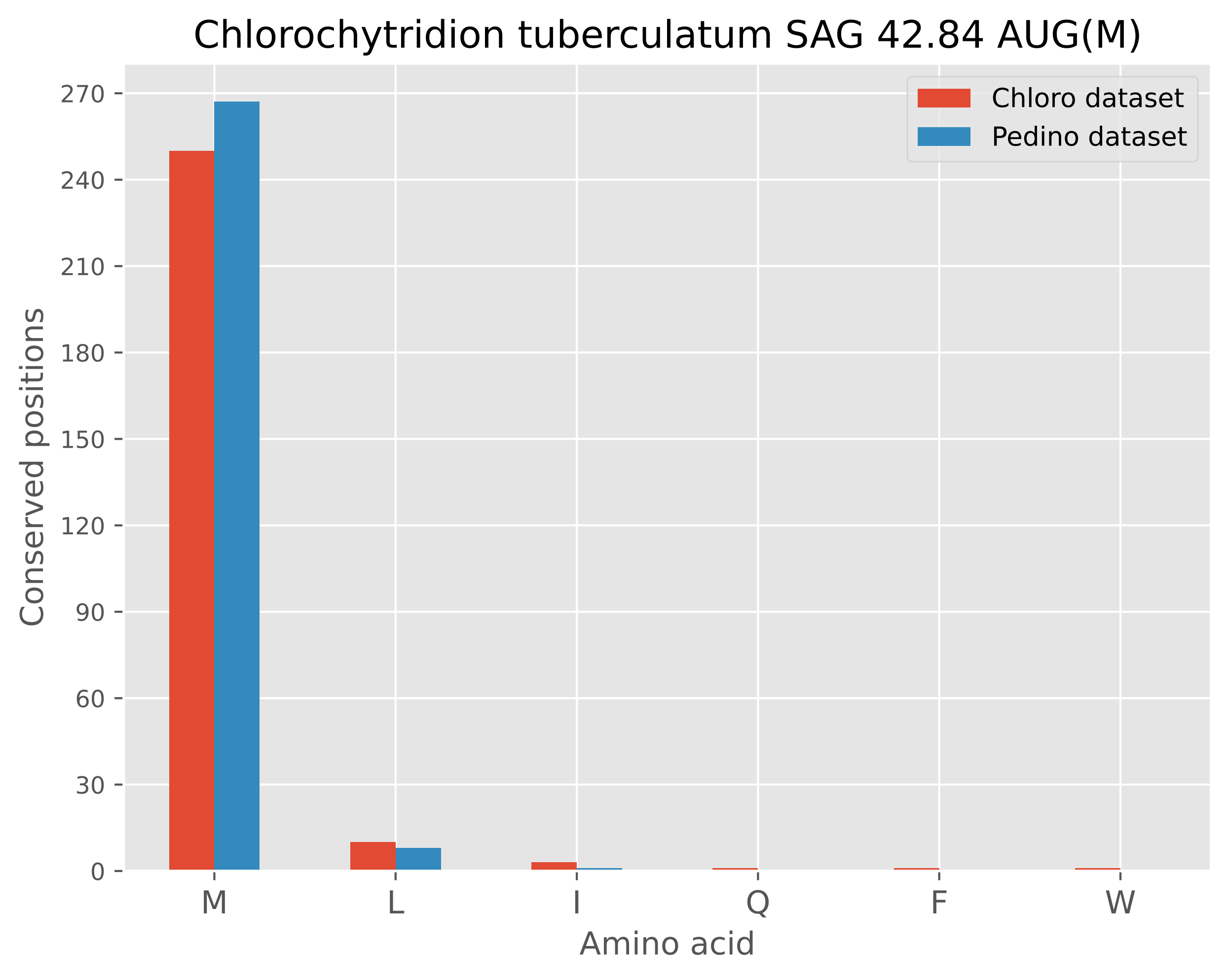
Chlorochytridion tuberculatum SAG 42.84 AUG(M)
Chloro dataset
Pedino dataset
Conserved positions
Amino acid
0
30
60
90
120
150
180
210
240
270
M
L
I
Q
F
W

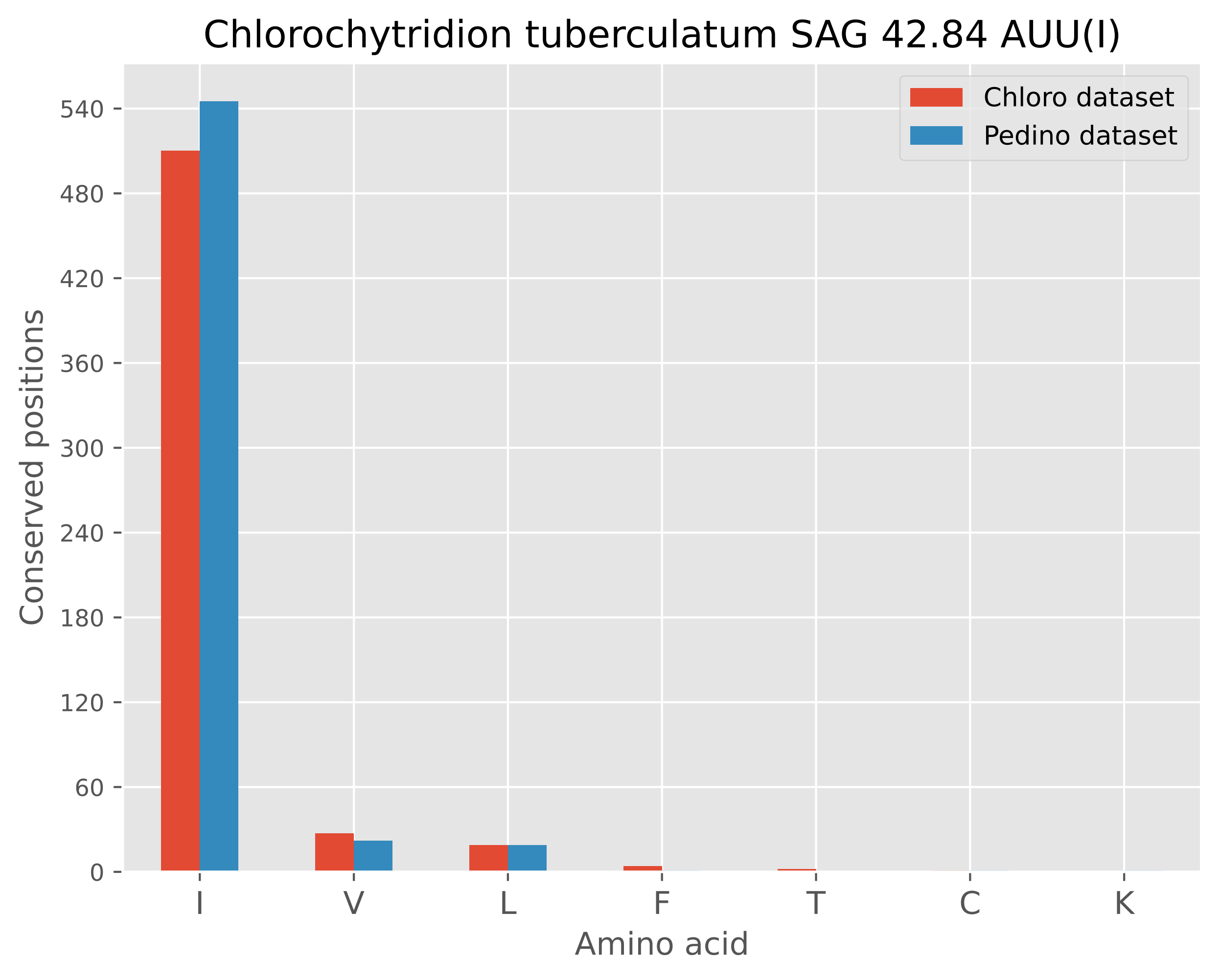
Chlorochytridion tuberculatum SAG 42.84 AUU(I)
Chloro dataset
Pedino dataset
Conserved positions
0
60
120
180
240
300
360
420
480
540
I
V
L
F
T
C
K
Amino acid

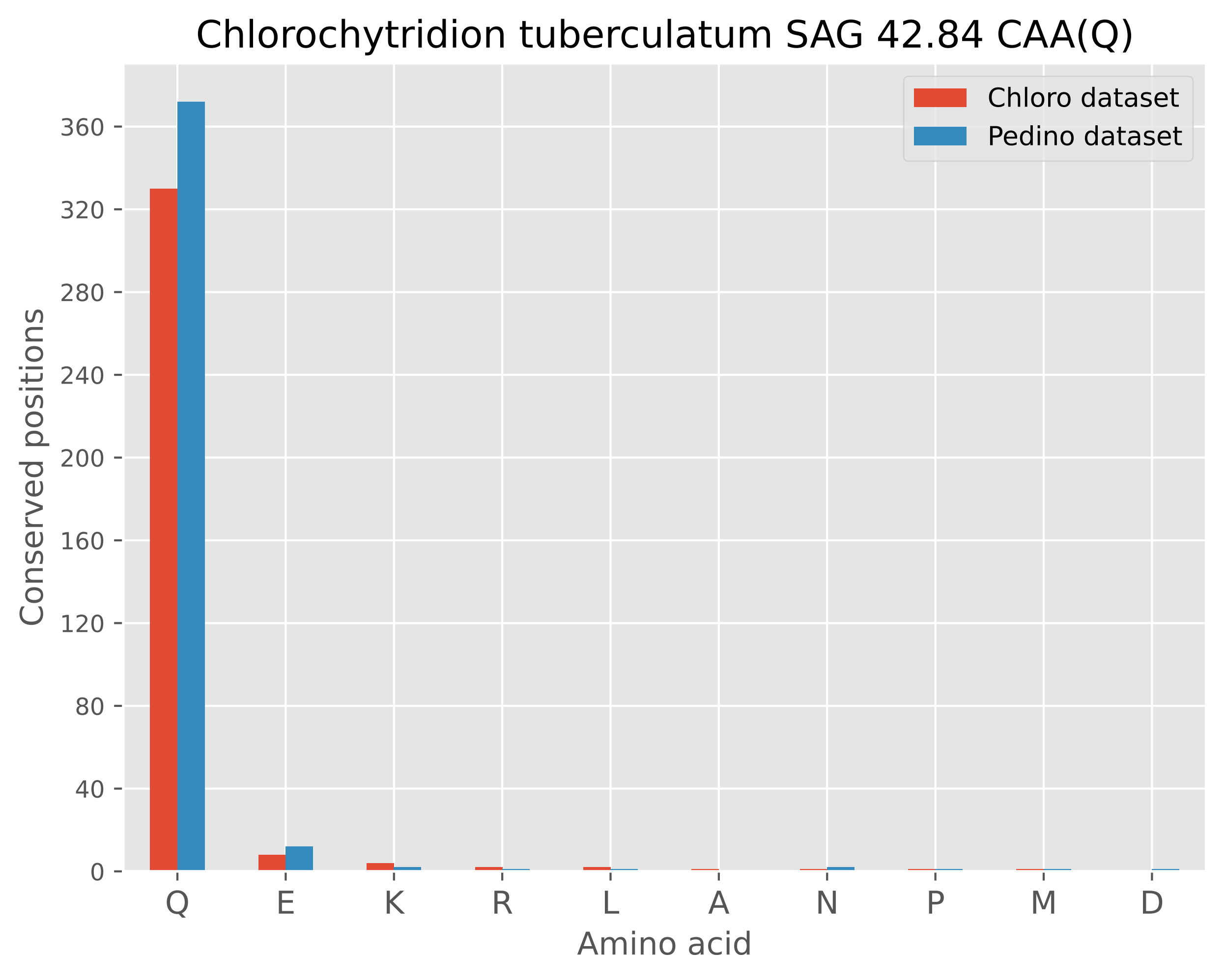
Chlorochytridion tuberculatum SAG 42.84 CAA(Q)
Chloro dataset
Pedino dataset
Conserved positions
0
40
80
120
160
200
240
280
320
360
Q
E
K
R
L
A
N
P
M
D
Amino acid

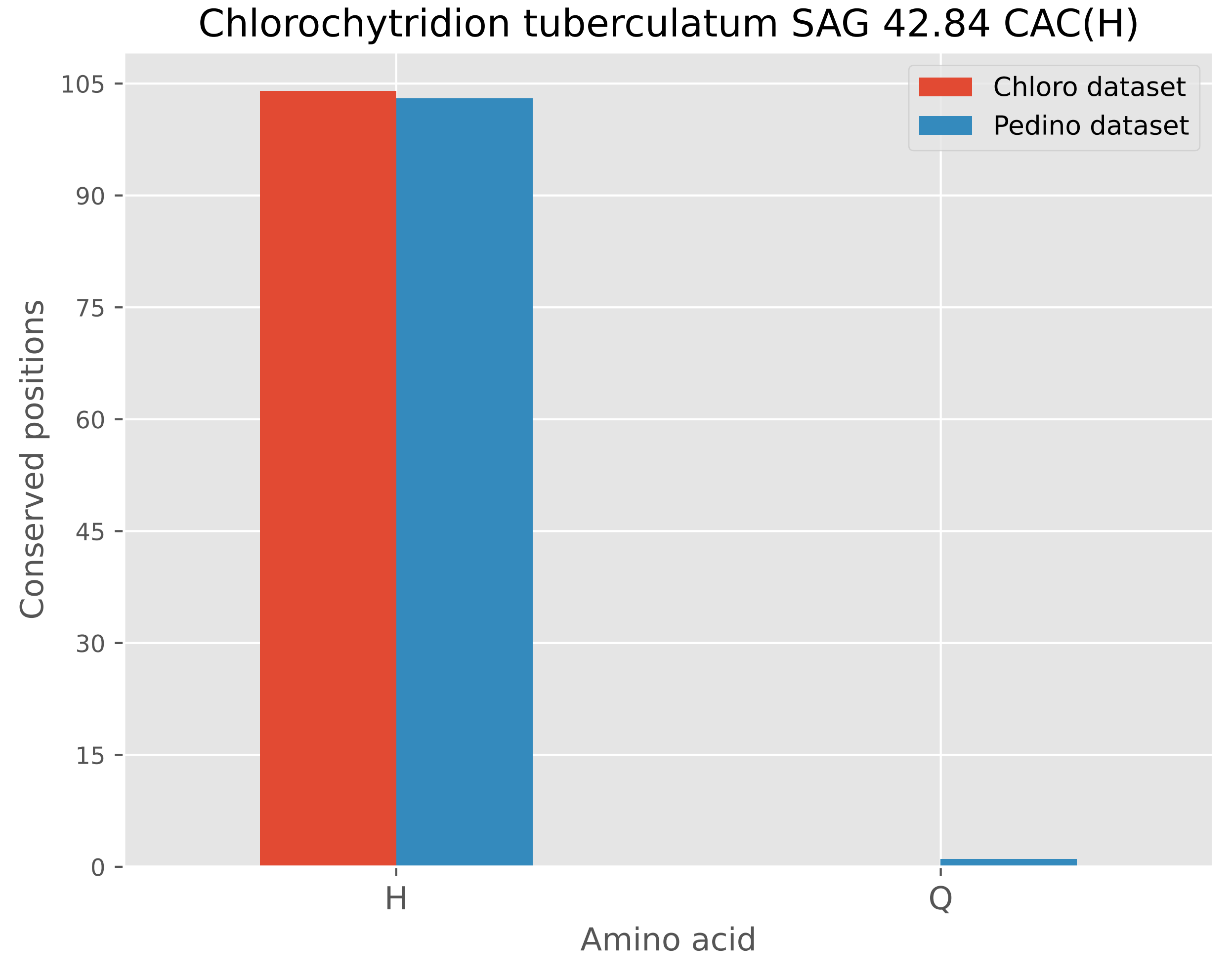

Chlorochytridion tuberculatum SAG 42.84 CAC(H)
Chloro dataset
Pedino dataset
Conserved positions
105
90
75
60
45
30
15
0
H
Q
Amino acid

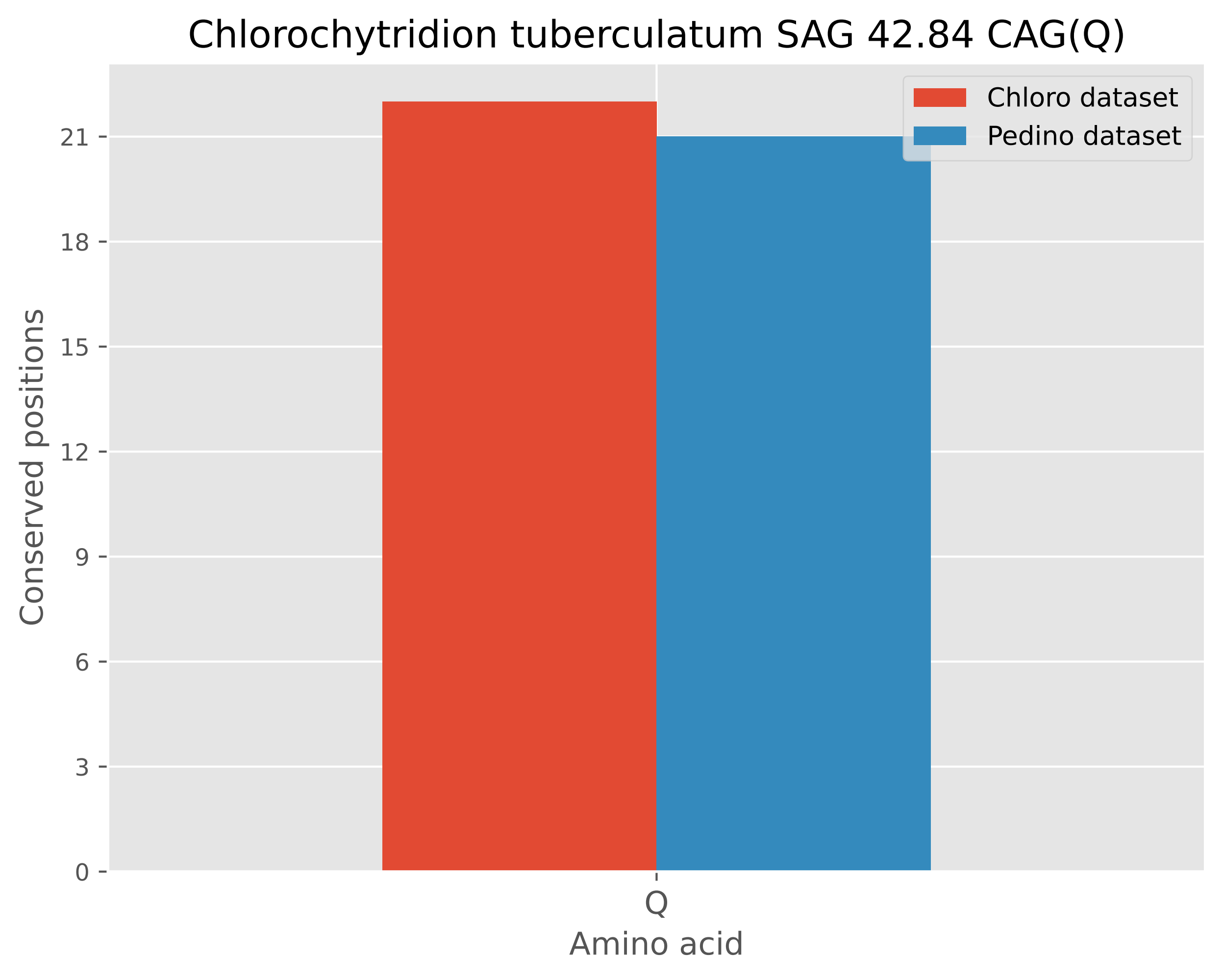
Chlorochytridion tuberculatum SAG 42.84 CAG(Q)
Chloro dataset
Pedino dataset
Conserved positions
0
3
6
9
12
15
18
21
Q
Amino acid

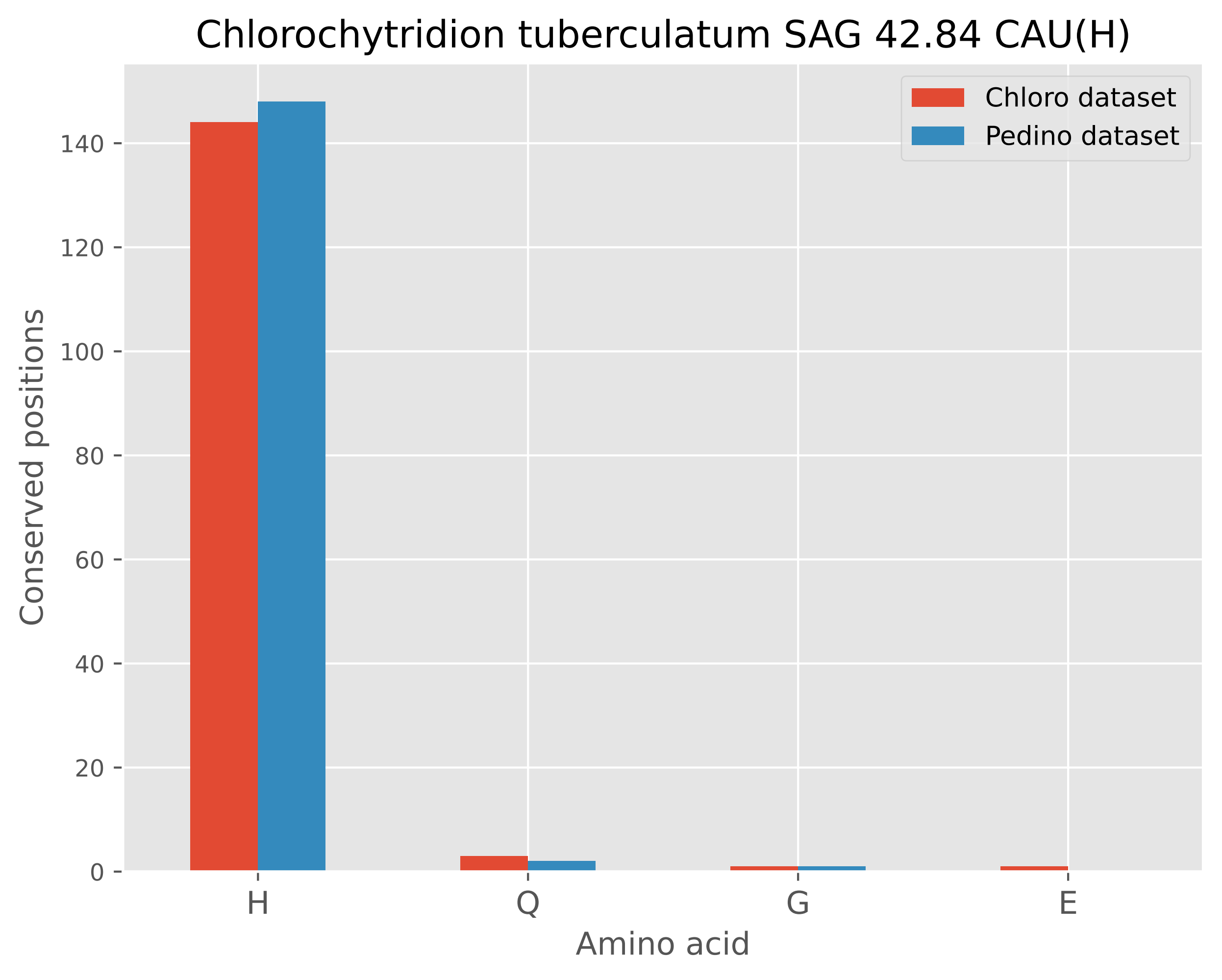

Chlorochytridion tuberculatum SAG 42.84 CAU(H)
Chloro dataset
Pedino dataset
Conserved positions
0
20
40
60
80
100
120
140
H
Q
G
E
Amino acid

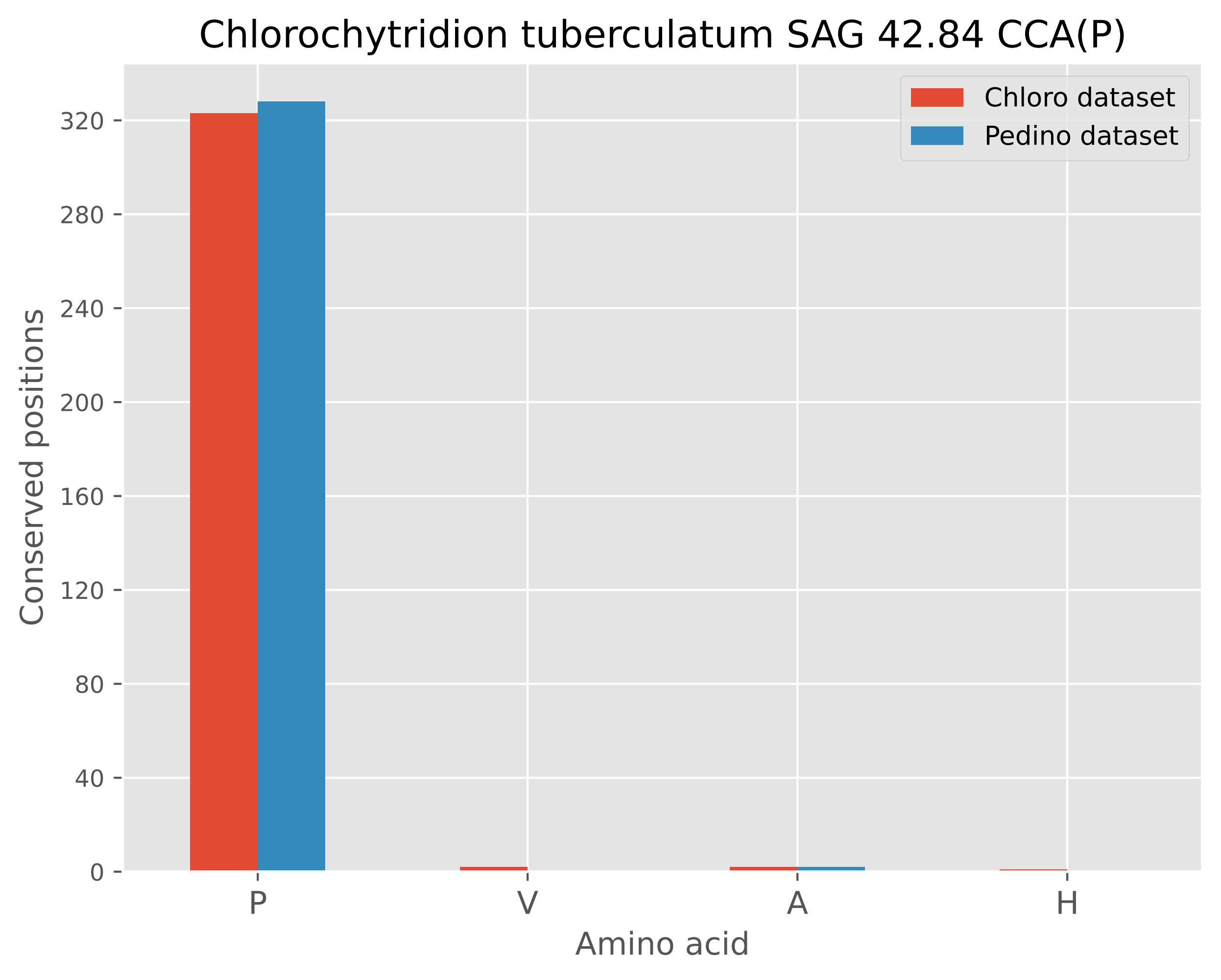
Chlorochytridion tuberculatum SAG 42.84 CCA(P)
Chloro dataset
Pedino dataset
Conserved positions
0
40
80
120
160
200
240
280
320
P
V
A
H
Amino acid

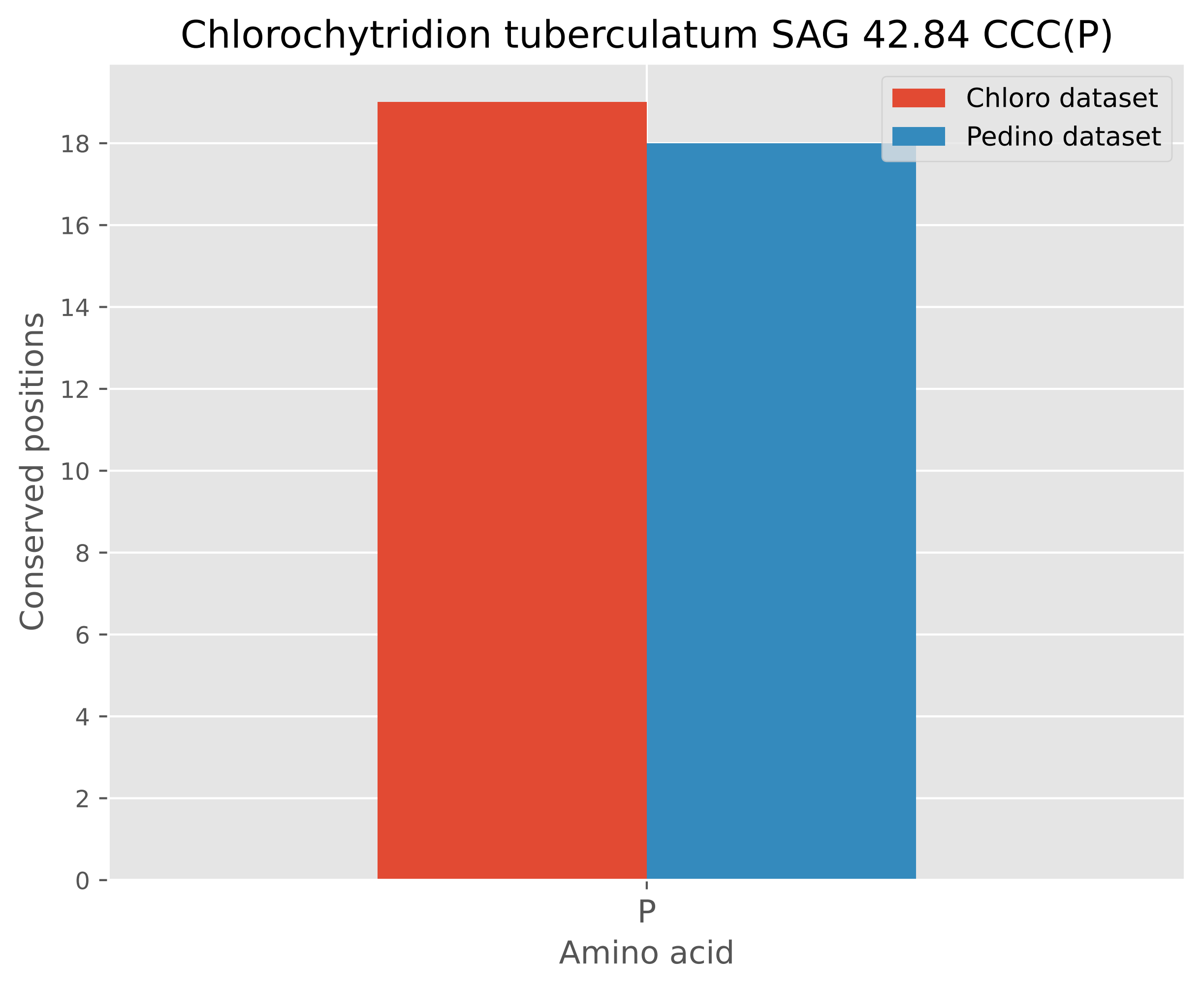
Chlorochytridion tuberculatum SAG 42.84 CCC(P)
Chloro dataset
Pedino dataset
Conserved positions
0
2
4
6
8
10
12
14
16
18
P
Amino acid

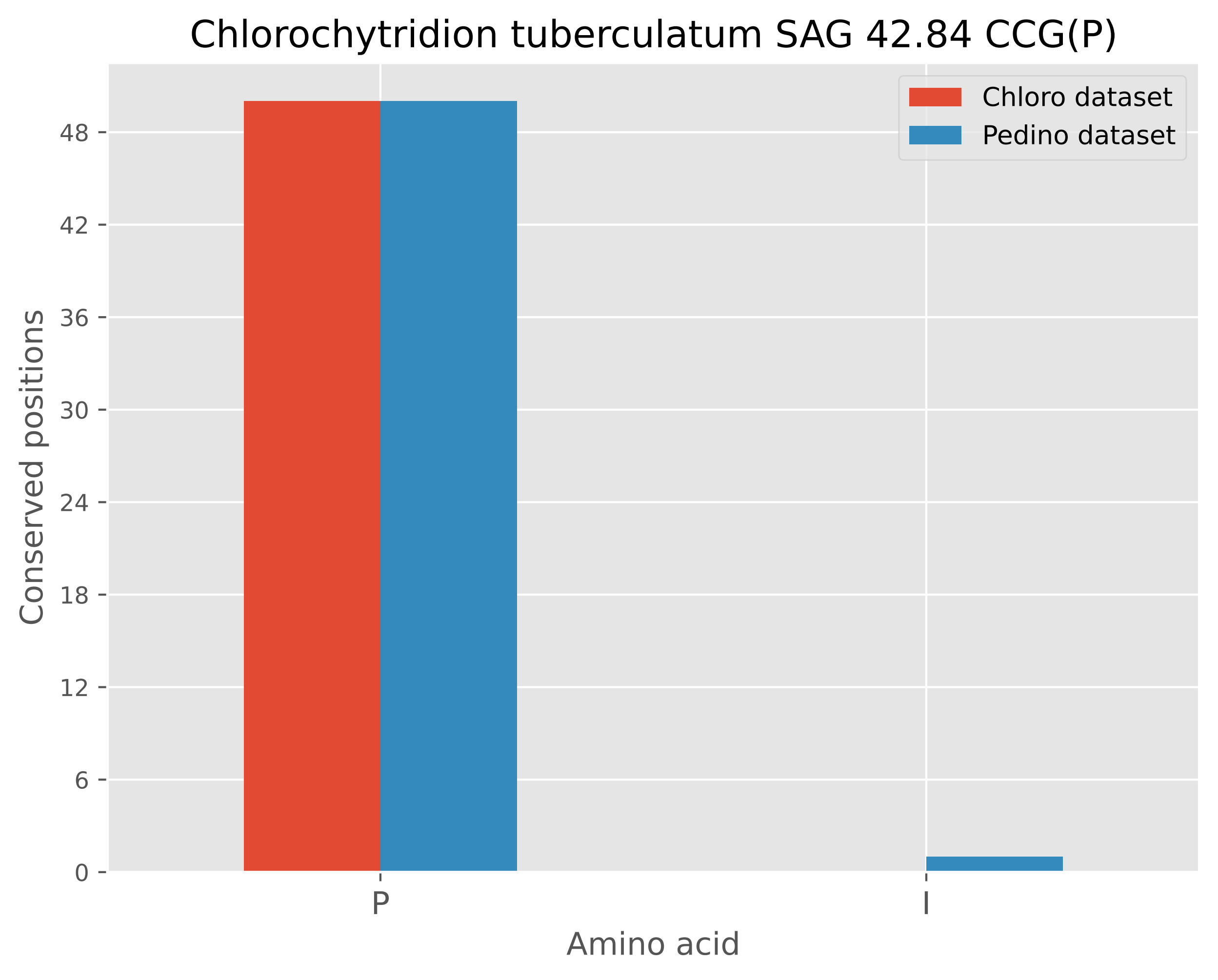
Chlorochytridion tuberculatum SAG 42.84 CCG(P)
Chloro dataset
Pedino dataset
Conserved positions
0
6
12
18
24
30
36
42
48
P
I
Amino acid

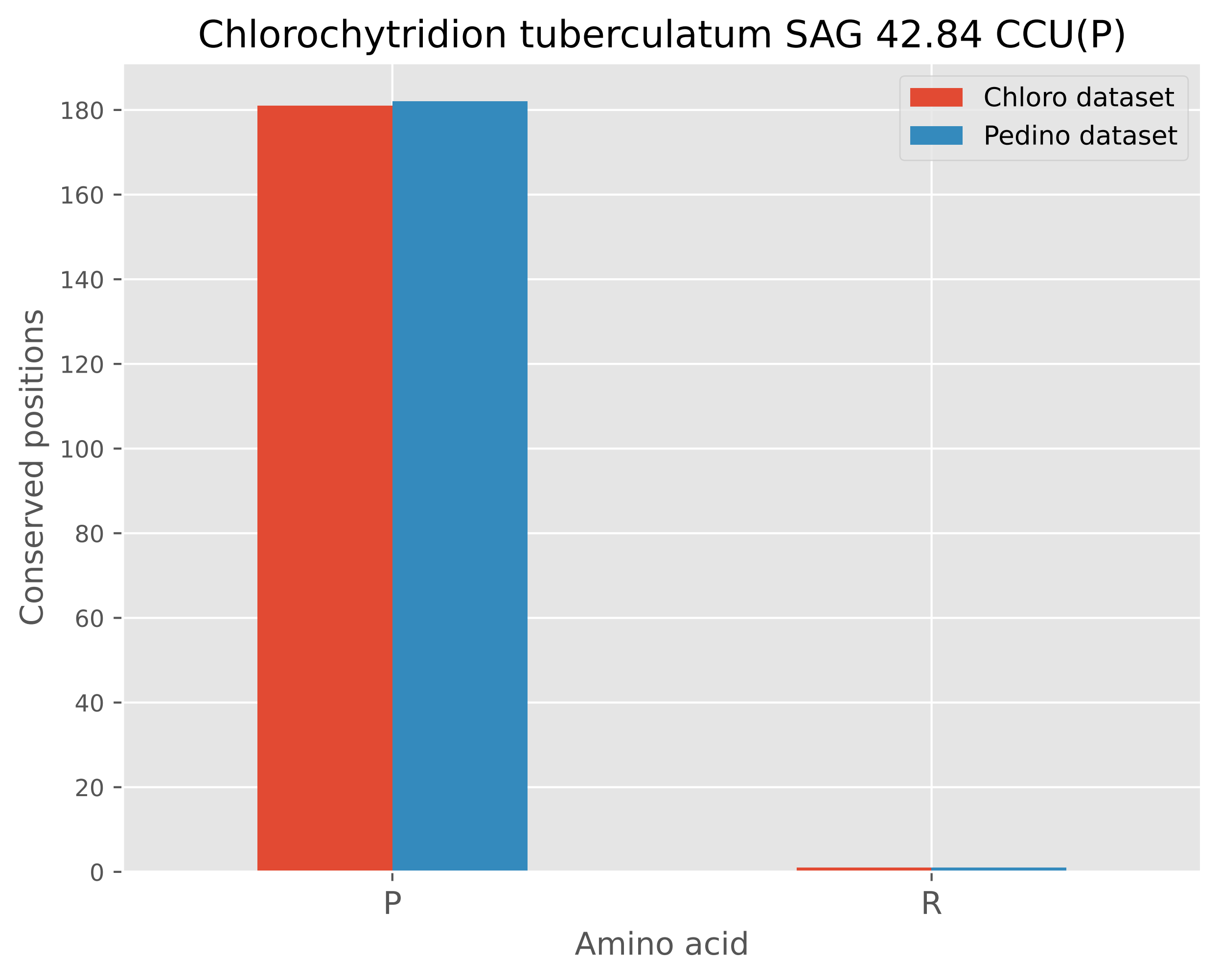
Chlorochytridion tuberculatum SAG 42.84 CCU(P)
Chloro dataset
Pedino dataset
Conserved positions
0
20
40
60
80
100
120
140
160
180
P
R
Amino acid

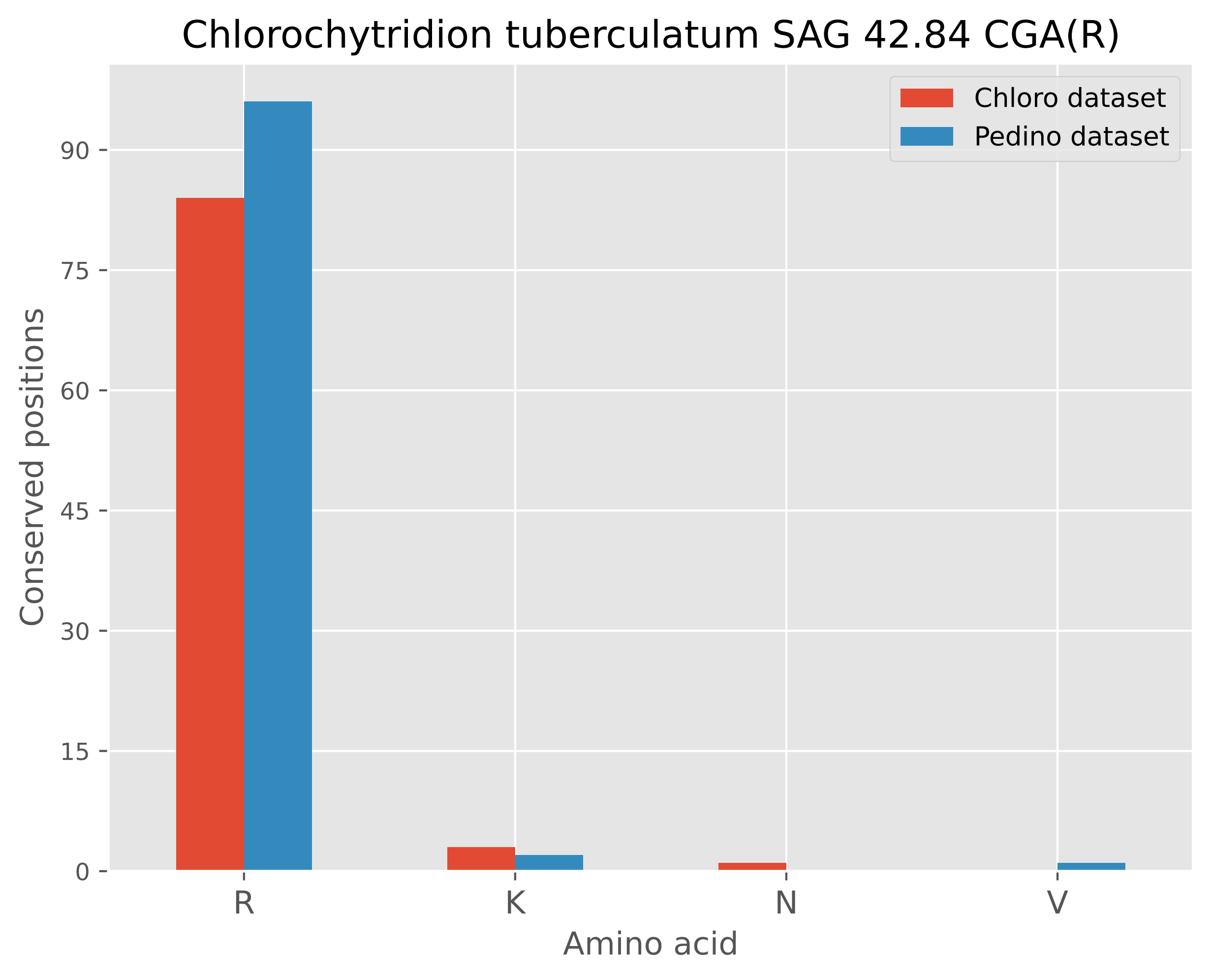
Chlorochytridion tuberculatum SAG 42.84 CGA(R)
Chloro dataset
Pedino dataset
Conserved positions
0
15
30
45
60
75
90
R
K
N
V
Amino acid

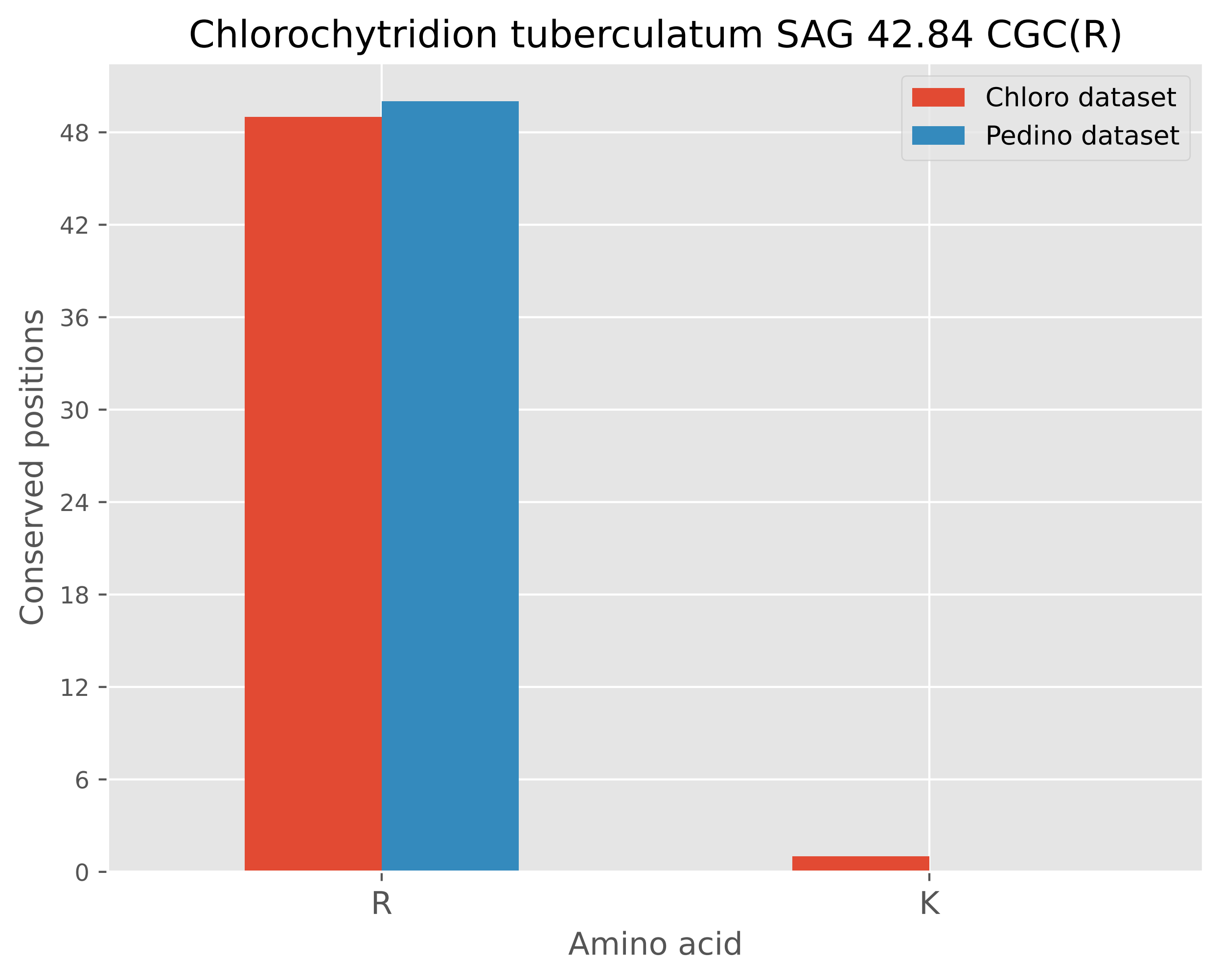

Chlorochytridion tuberculatum SAG 42.84 CGC(R)
Chloro dataset
Pedino dataset
Conserved positions
0
6
12
18
24
30
36
42
48
R
K
Amino acid

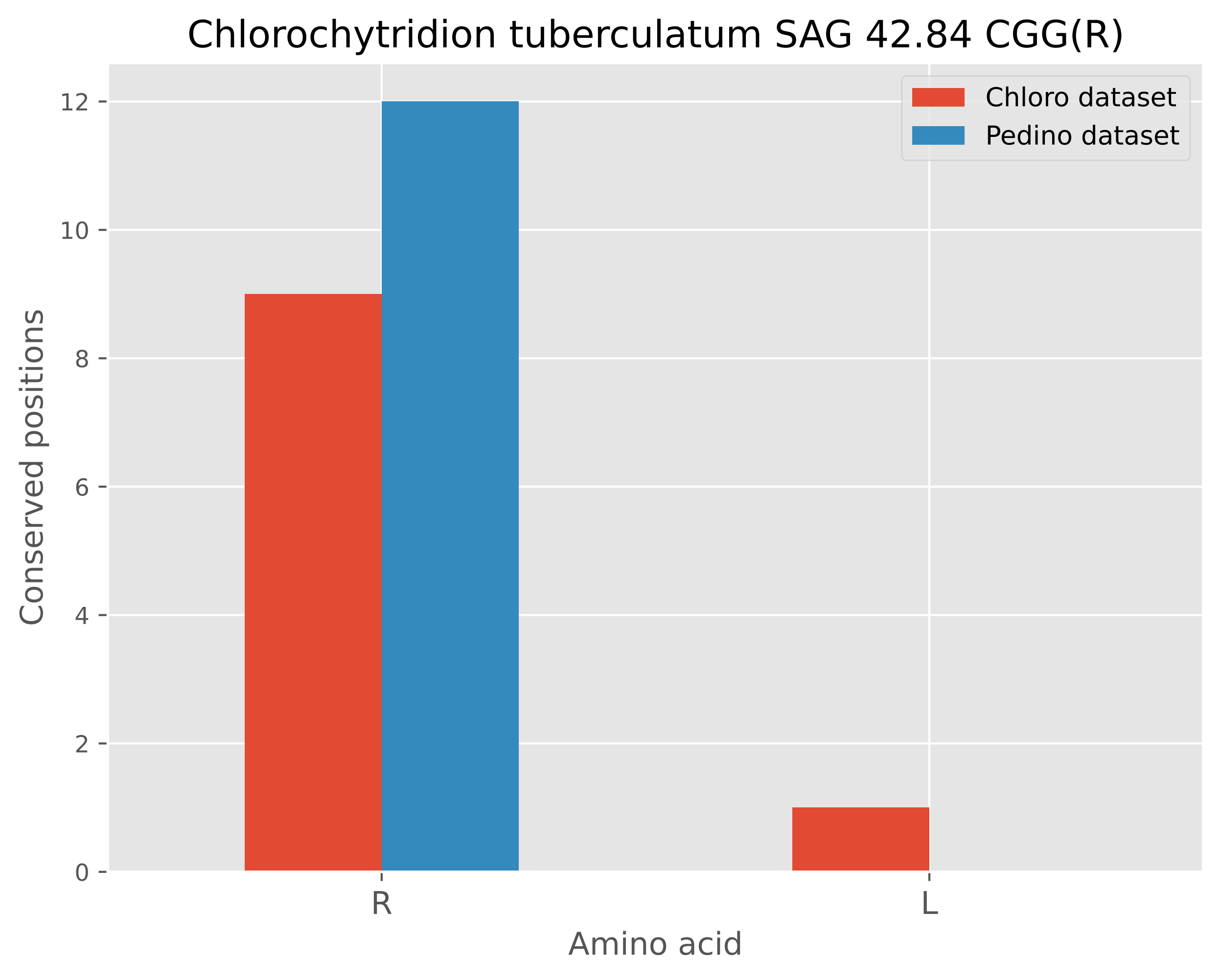

Chlorochytridion tuberculatum SAG 42.84 CGG(R)
Chloro dataset
Pedino dataset
Conserved positions
Amino acid
R
L
0
2
4
6
8
10
12

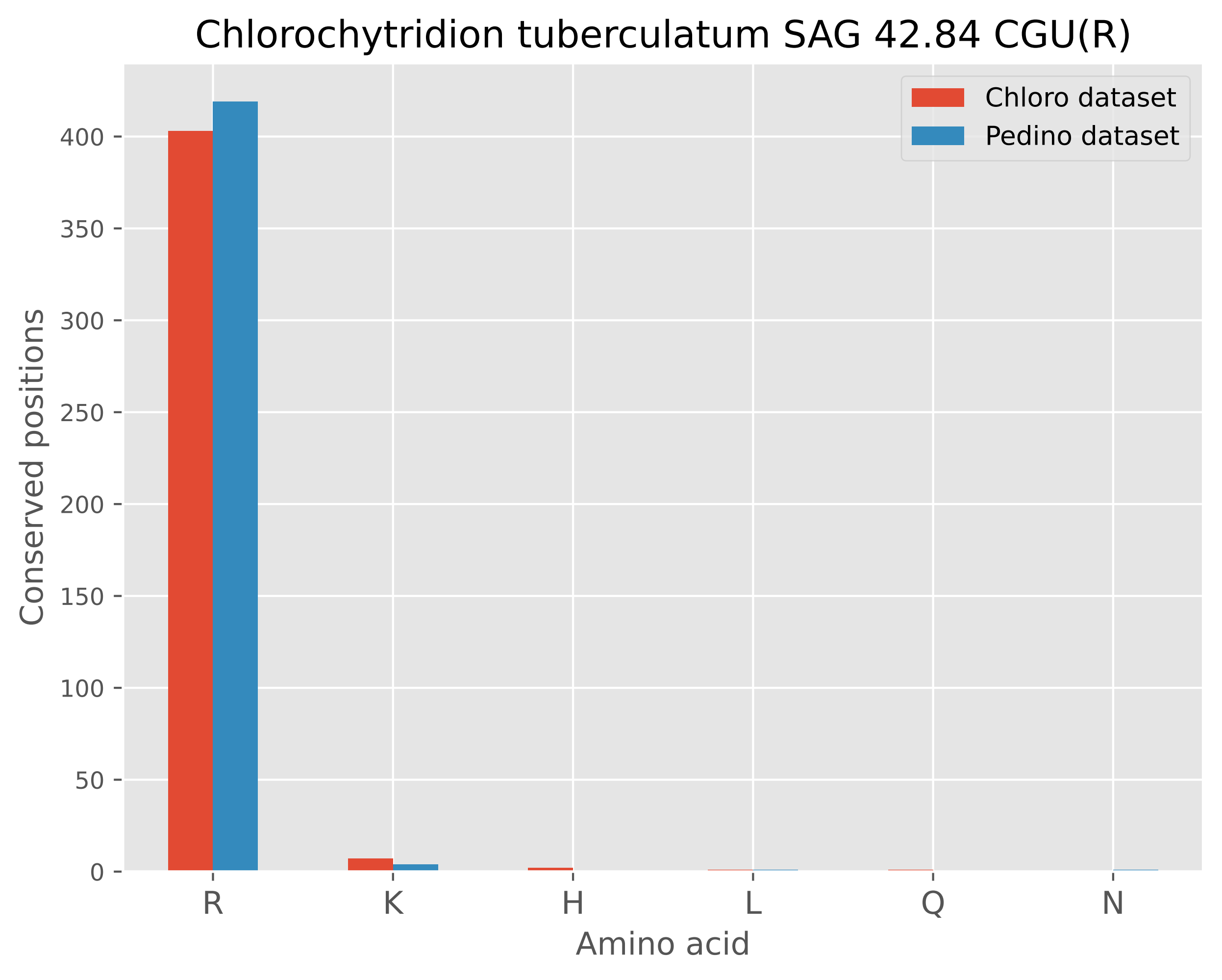

Chlorochytridion tuberculatum SAG 42.84 CGU(R)
Chloro dataset
Pedino dataset
Conserved positions
0
50
100
150
200
250
300
350
400
R
K
H
L
Q
N
Amino acid

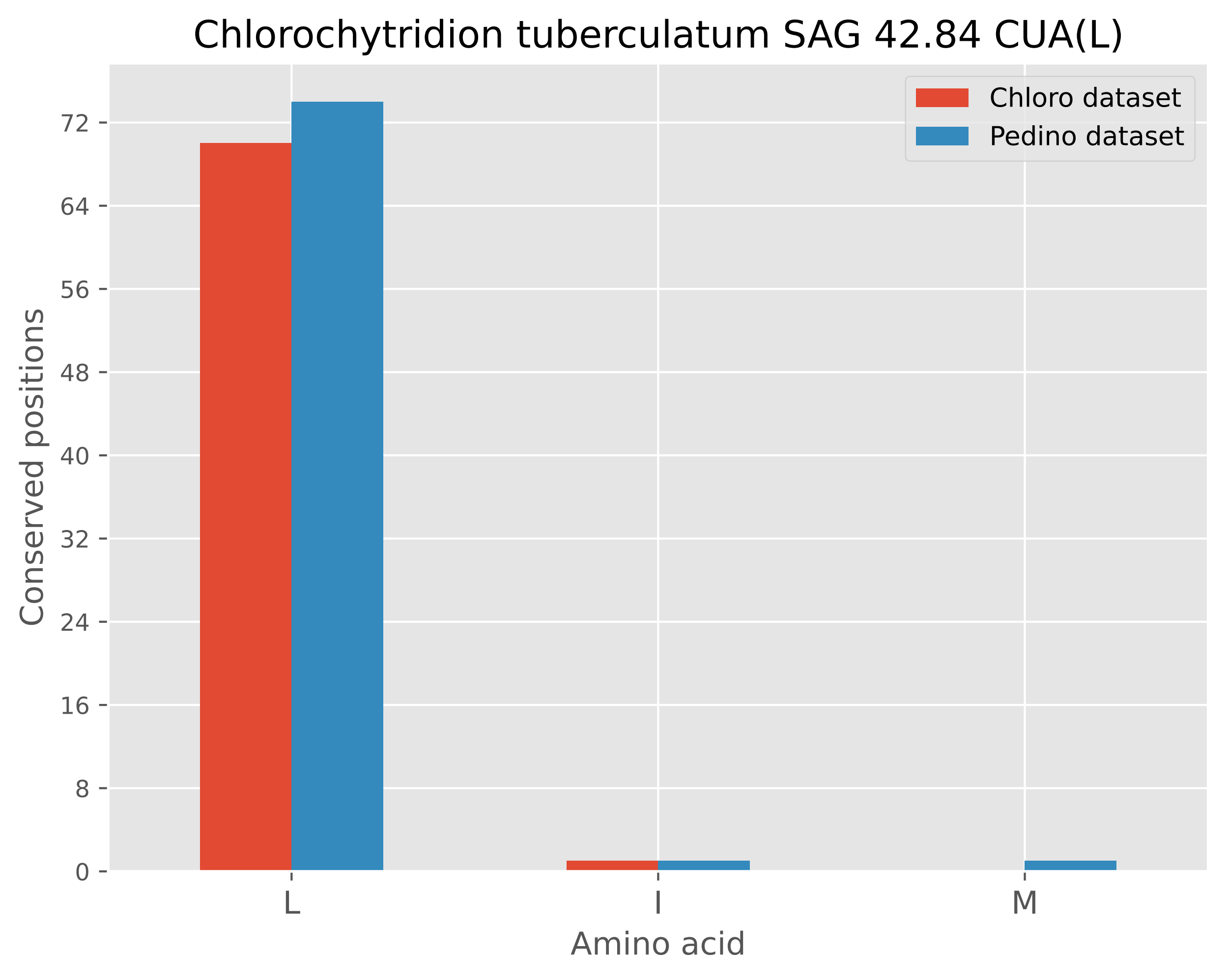

Chlorochytridion tuberculatum SAG 42.84 CUA(L)
Chloro dataset
Pedino dataset
Conserved positions
72
64
56
48
40
32
24
16
8
0
L
I
M
Amino acid

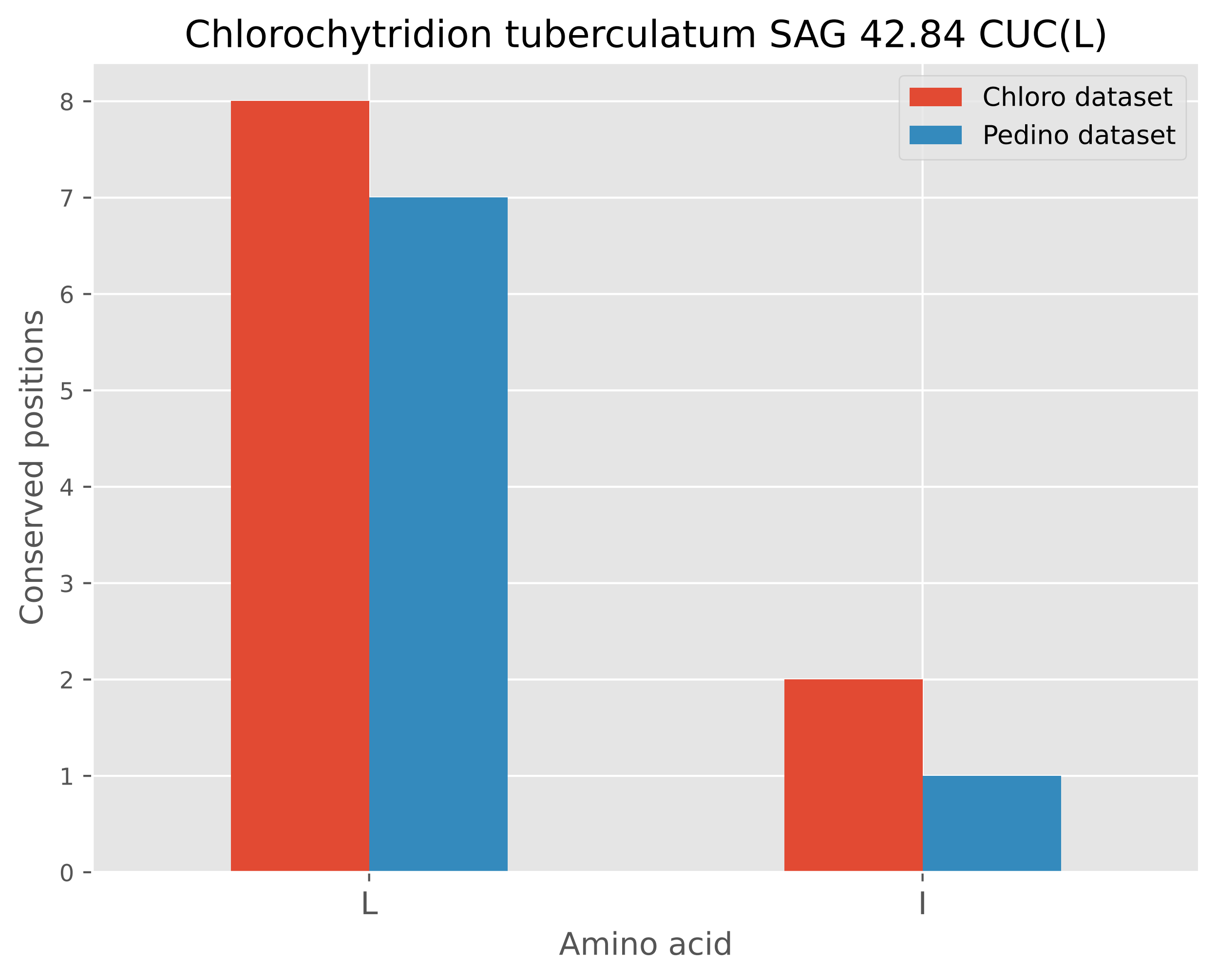
Chlorochytridion tuberculatum SAG 42.84 CUC(L)
Chloro dataset
Pedino dataset
Conserved positions
0
1
2
3
4
5
6
7
8
L
I
Amino acid

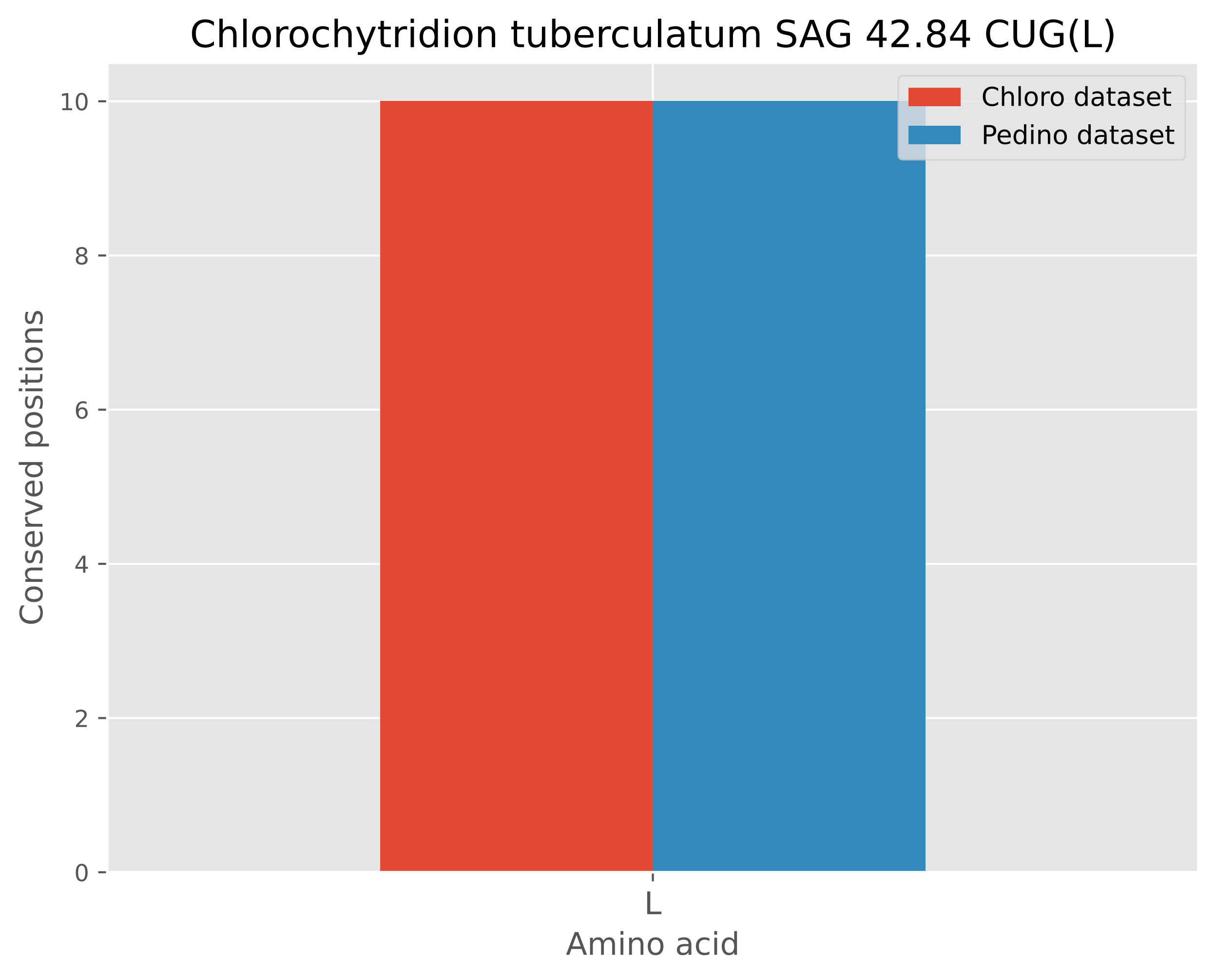
Chlorochytridion tuberculatum SAG 42.84 CUG(L)
Chloro dataset
Pedino dataset
Conserved positions
0
2
4
6
8
10
L
Amino acid

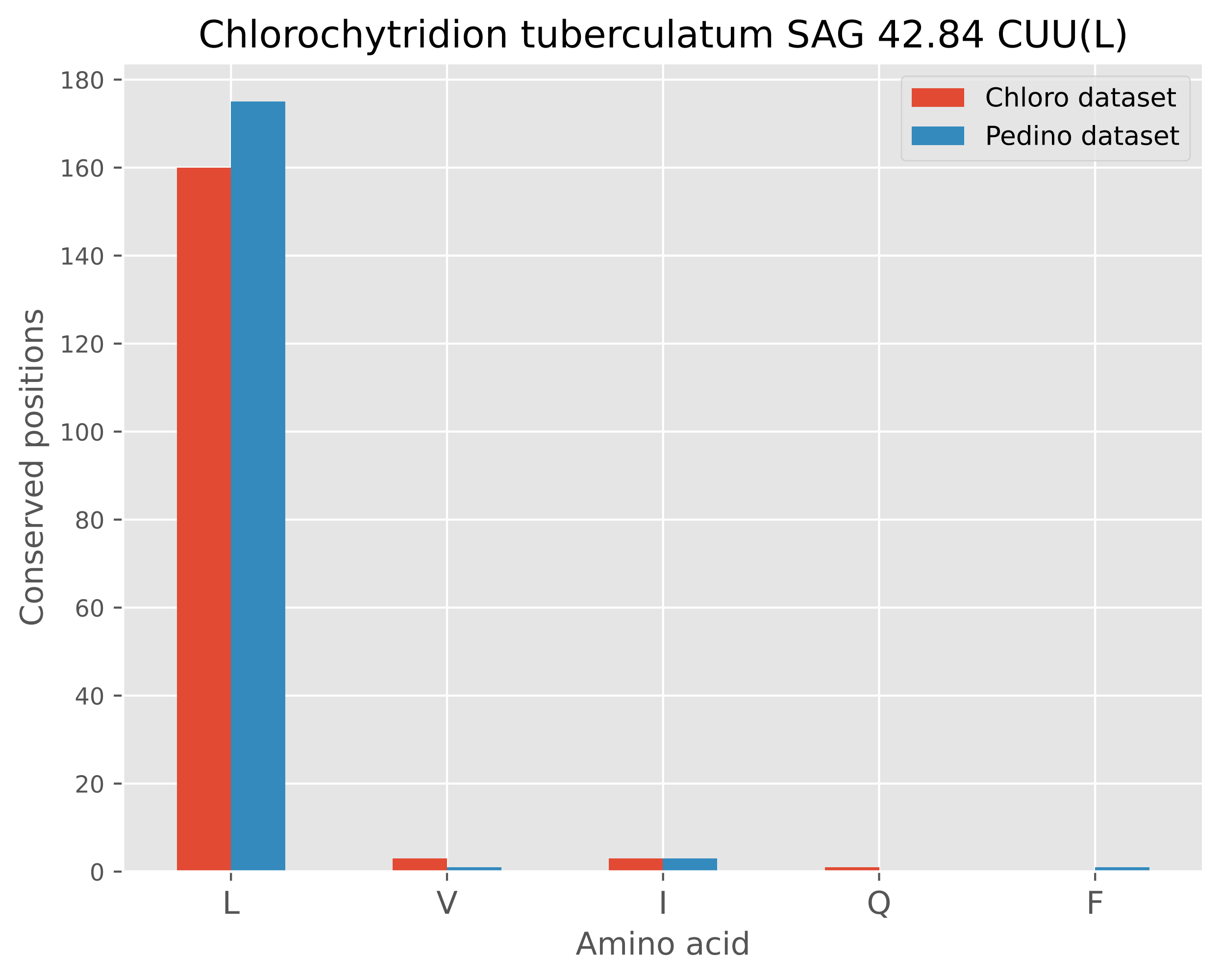
Chlorochytridion tuberculatum SAG 42.84 CUU(L)
Chloro dataset
Pedino dataset
Conserved positions
Amino acid
0
20
40
60
80
100
120
140
160
180
L
V
I
Q
F

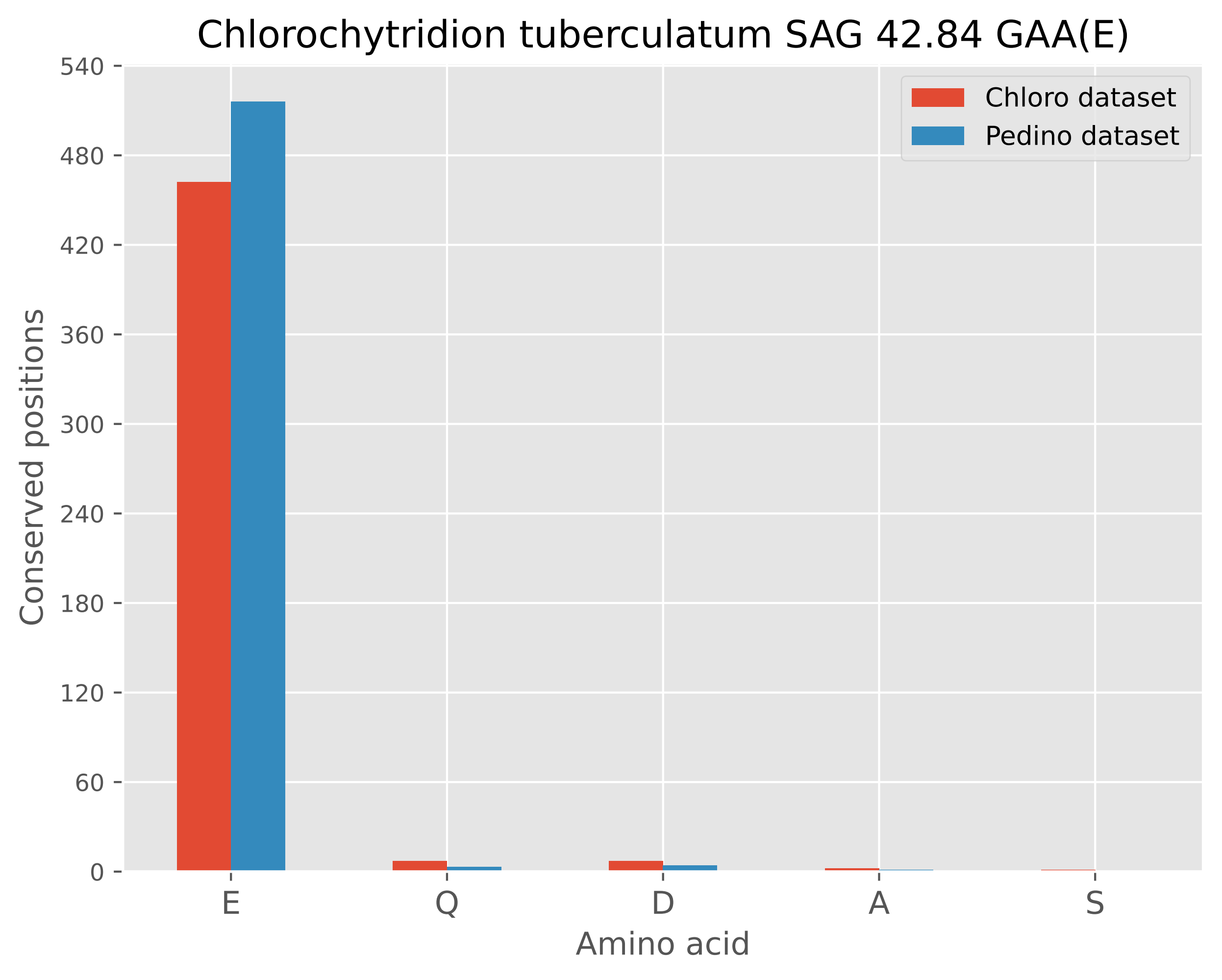

Chlorochytridion tuberculatum SAG 42.84 GAA(E)
Chloro dataset
Pedino dataset
Conserved positions
0
60
120
180
240
300
360
420
480
540
E
Q
D
A
S
Amino acid

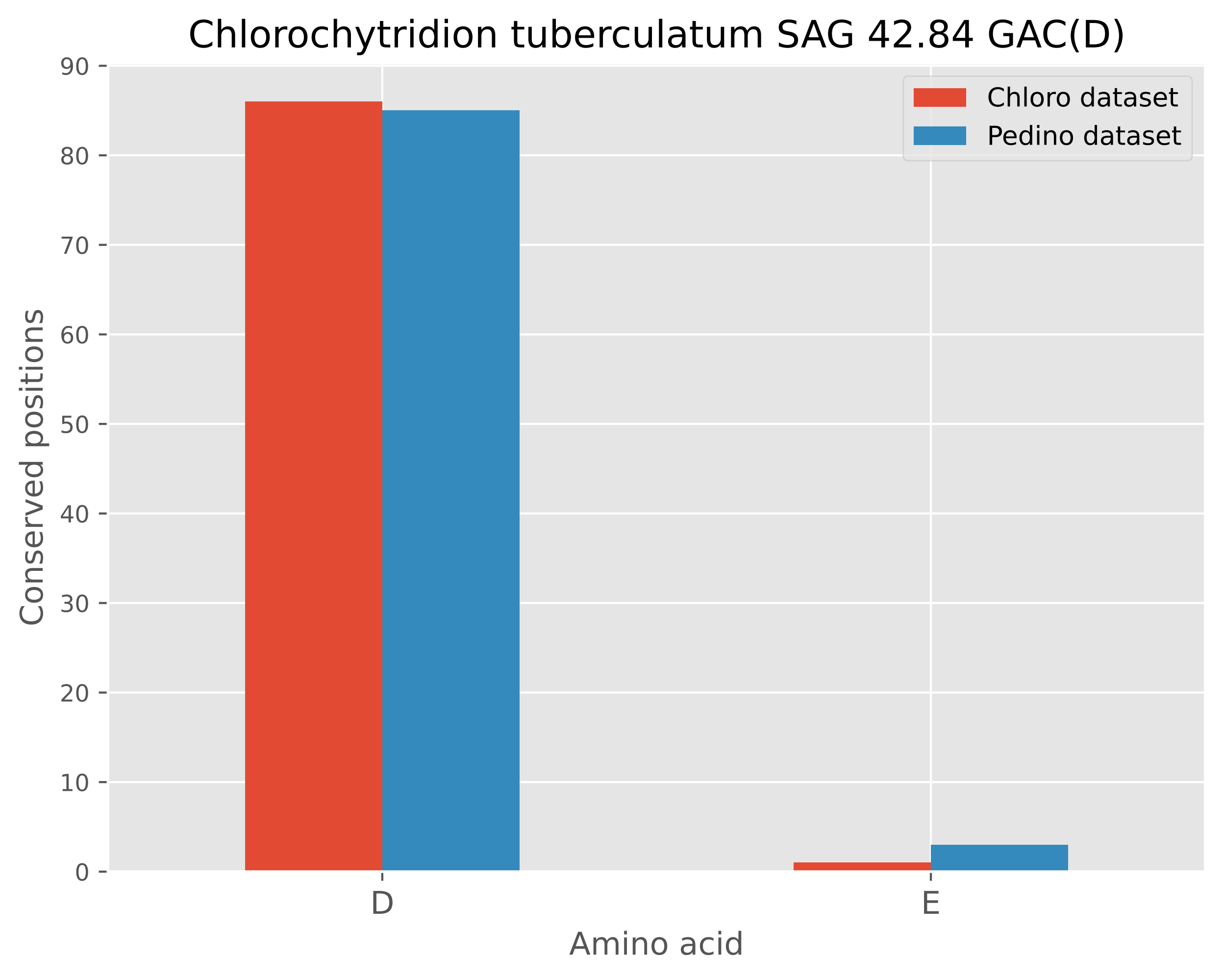
Chlorochytridion tuberculatum SAG 42.84 GAC(D)
Chloro dataset
Pedino dataset
Conserved positions
0
10
20
30
40
50
60
70
80
90
D
E
Amino acid

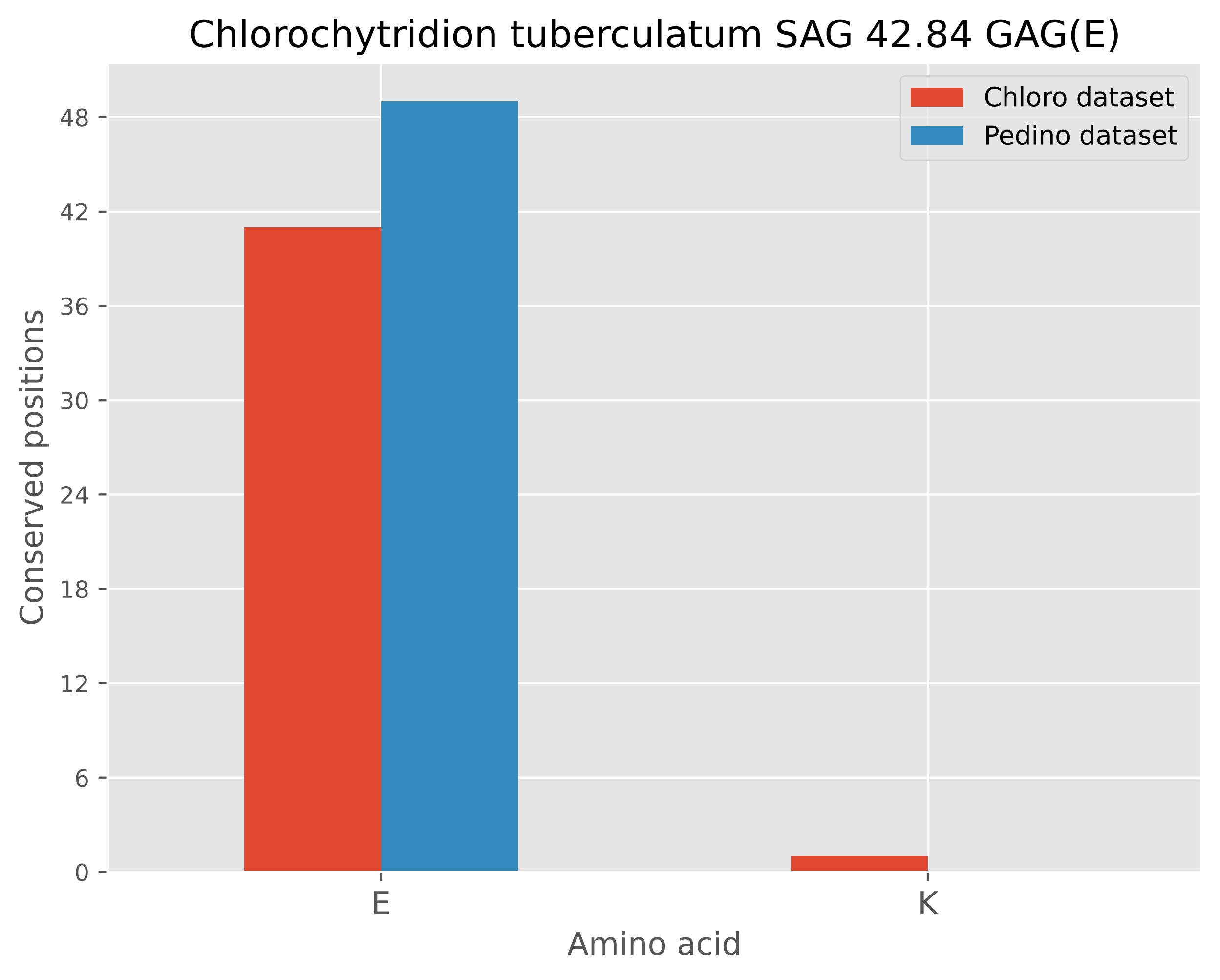

Chlorochytridion tuberculatum SAG 42.84 GAG(E)
Chloro dataset
Pedino dataset
Conserved positions
0
6
12
18
24
30
36
42
48
E
K
Amino acid

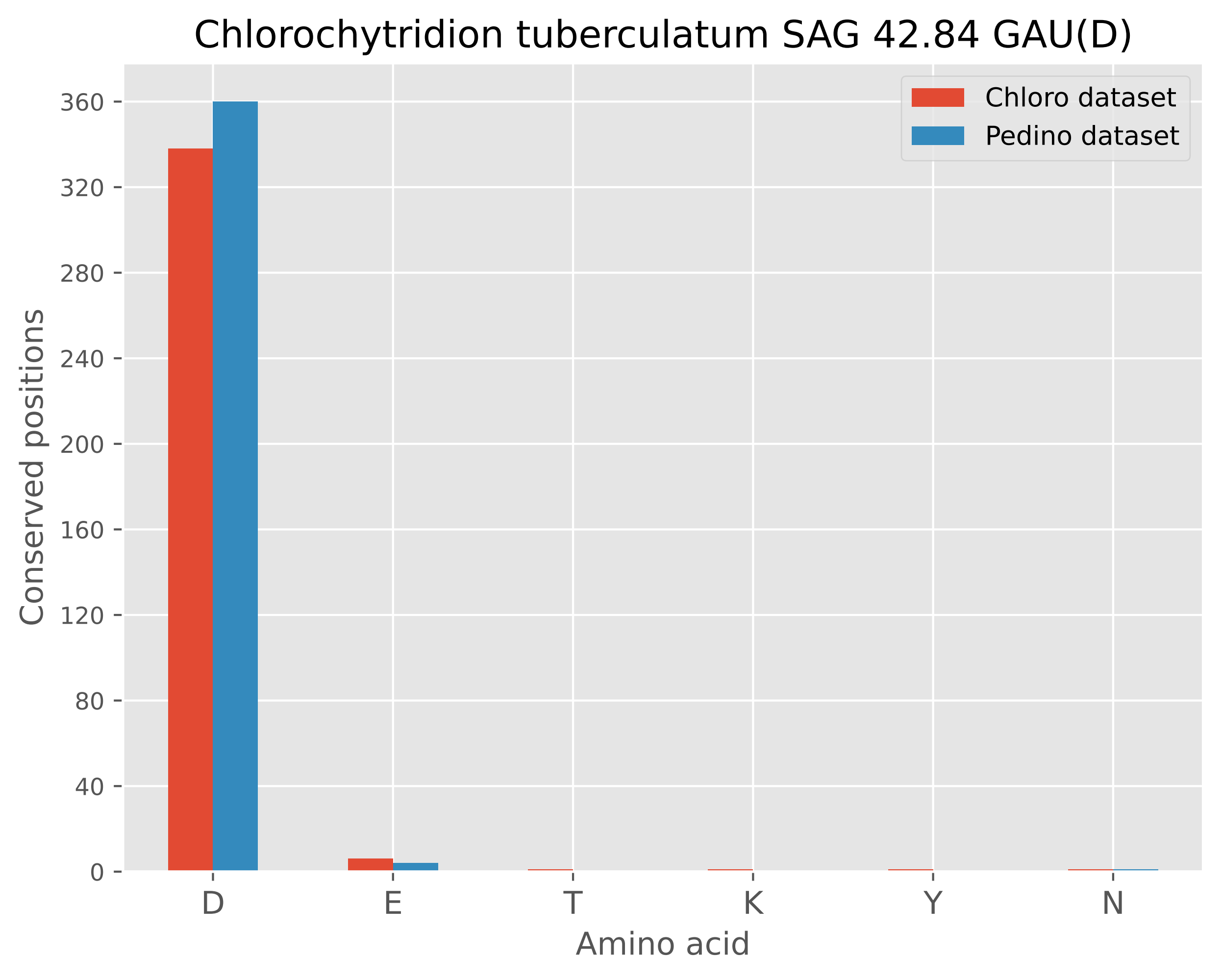

Chlorochytridion tuberculatum SAG 42.84 GAU(D)
Chloro dataset
Pedino dataset
Conserved positions
0
40
80
120
160
200
240
280
320
360
D
E
T
K
Y
N
Amino acid

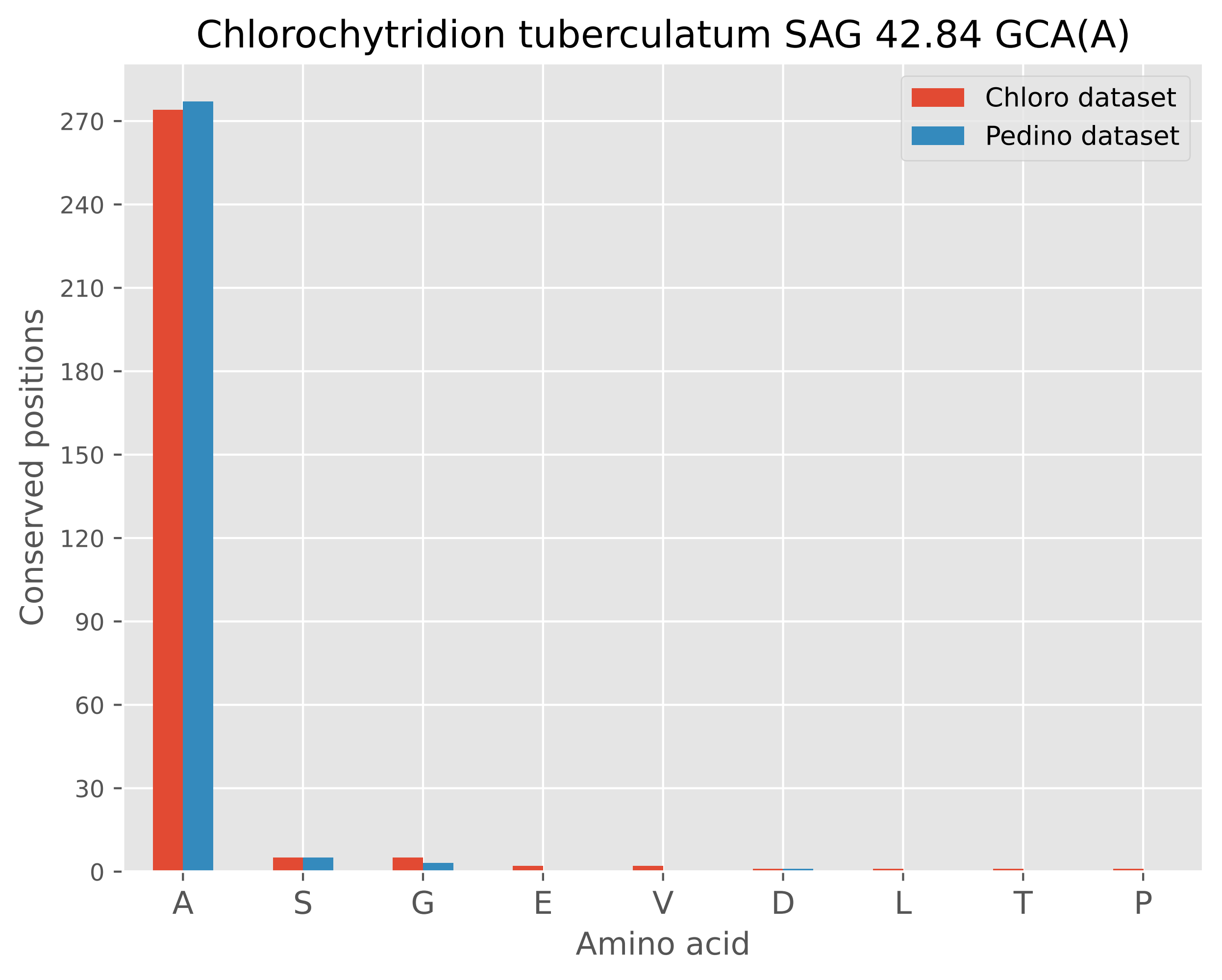

Chlorochytridion tuberculatum SAG 42.84 GCA(A)
Chloro dataset
Pedino dataset
Conserved positions
0
30
60
90
120
150
180
210
240
270
A
S
G
E
V
D
L
T
P
Amino acid

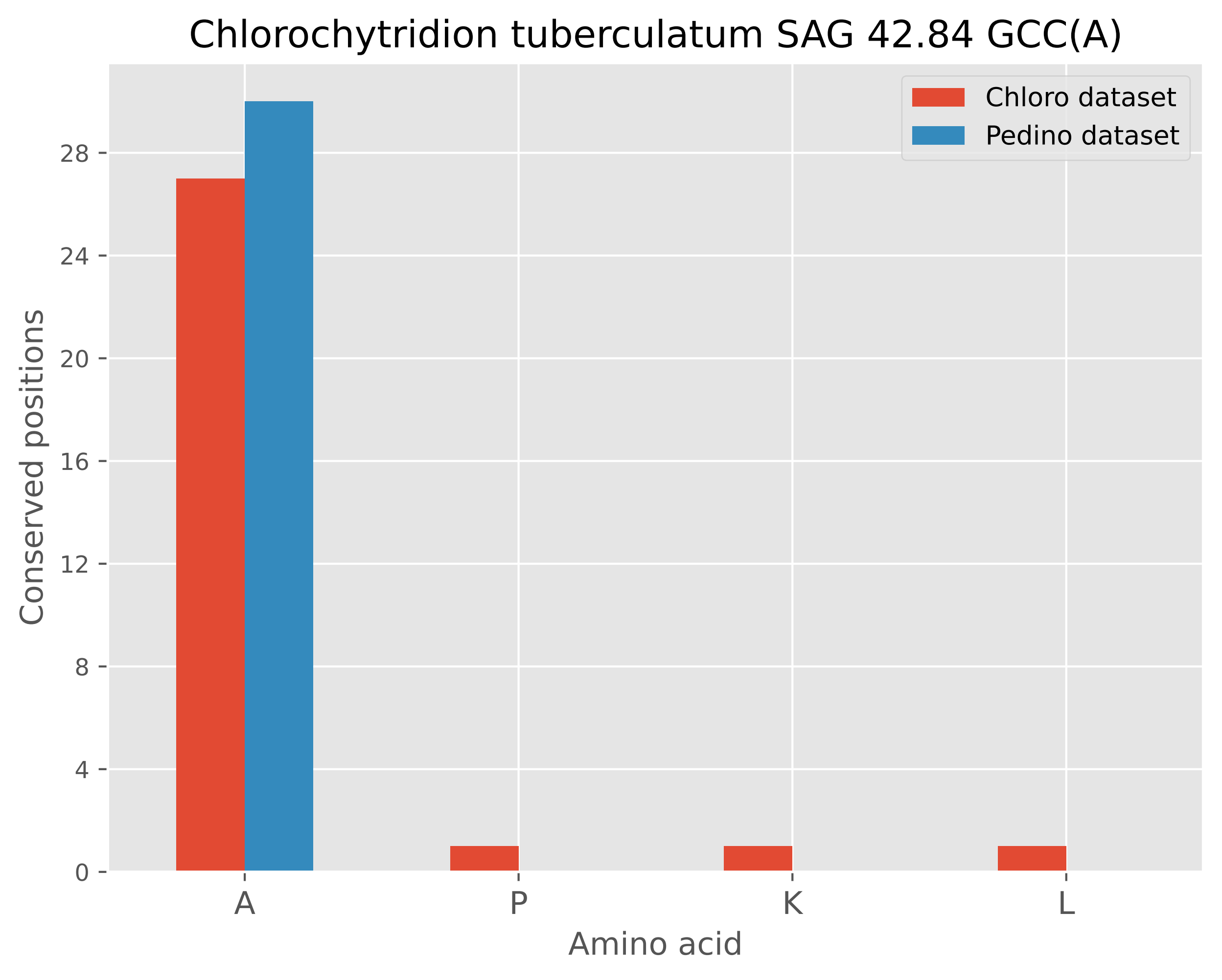

Chlorochytridion tuberculatum SAG 42.84 GCC(A)
Chloro dataset
Pedino dataset
Conserved positions
0
4
8
12
16
20
24
28
A
P
K
L
Amino acid

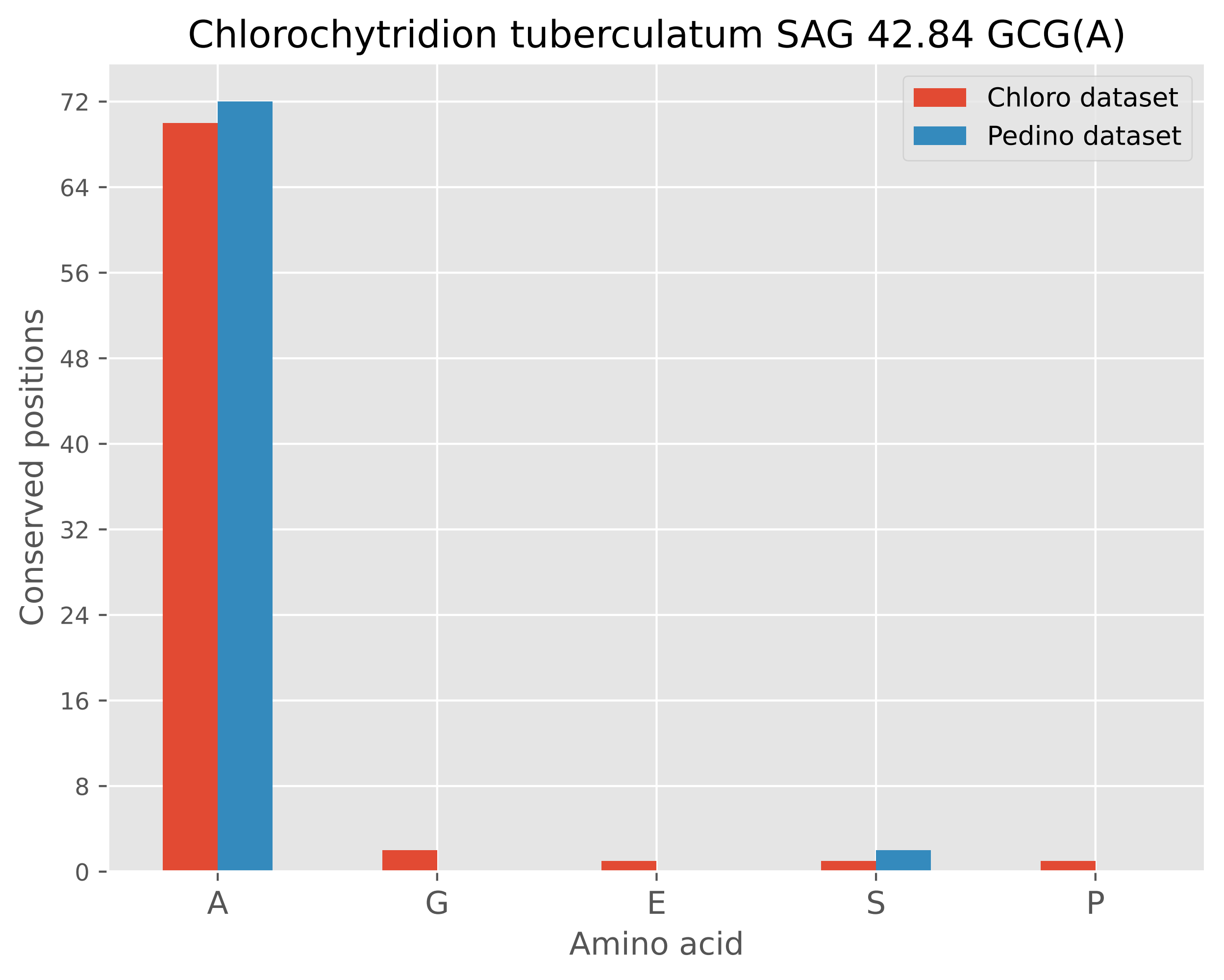

Chlorochytridion tuberculatum SAG 42.84 GCG(A)
Chloro dataset
Pedino dataset
Conserved positions
0
8
16
24
32
40
48
56
64
72
A
G
E
S
P
Amino acid

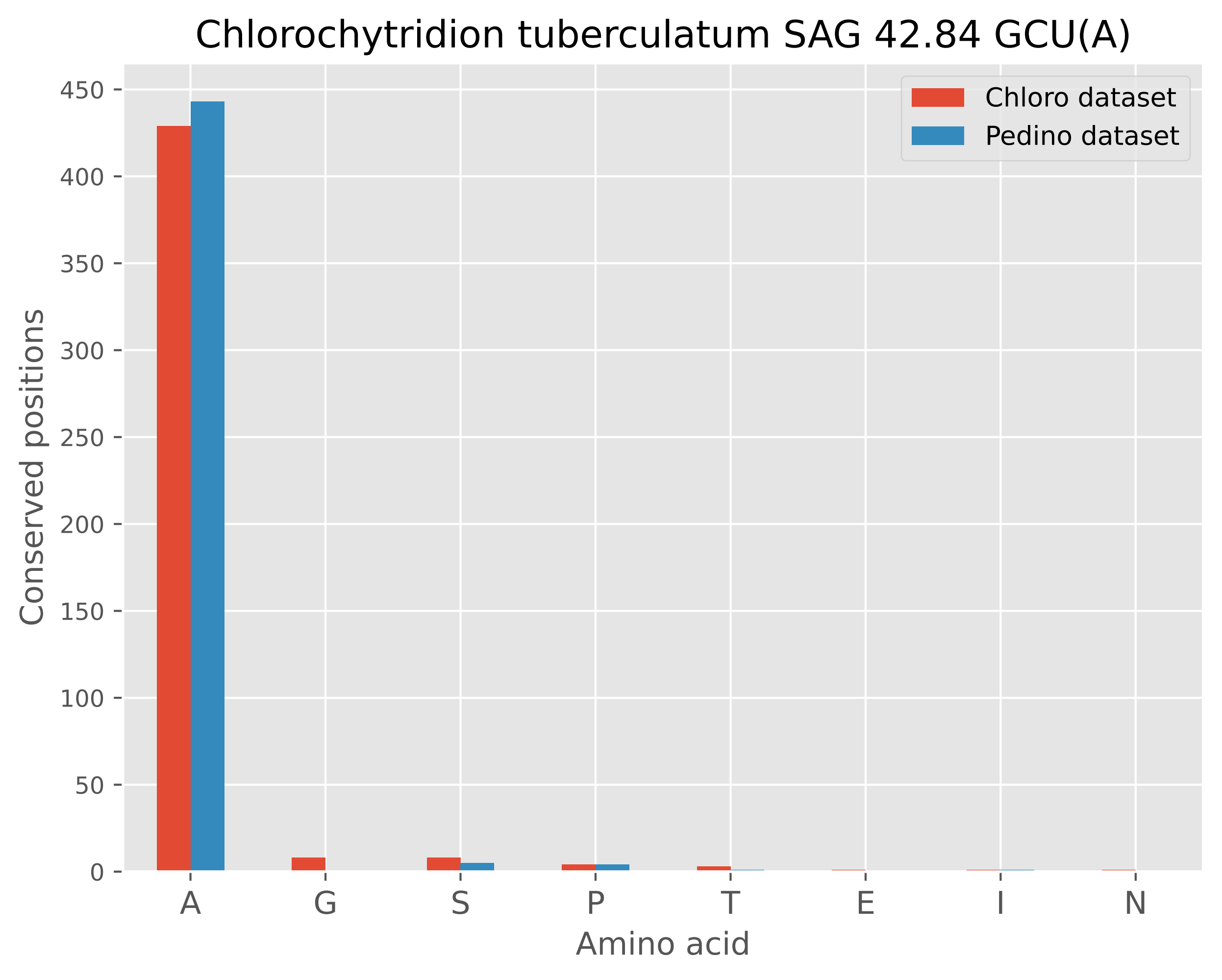

Chlorochytridion tuberculatum SAG 42.84 GCU(A)
Chloro dataset
Pedino dataset
Conserved positions
0
50
100
150
200
250
300
350
400
450
A
G
S
P
T
E
I
N
Amino acid

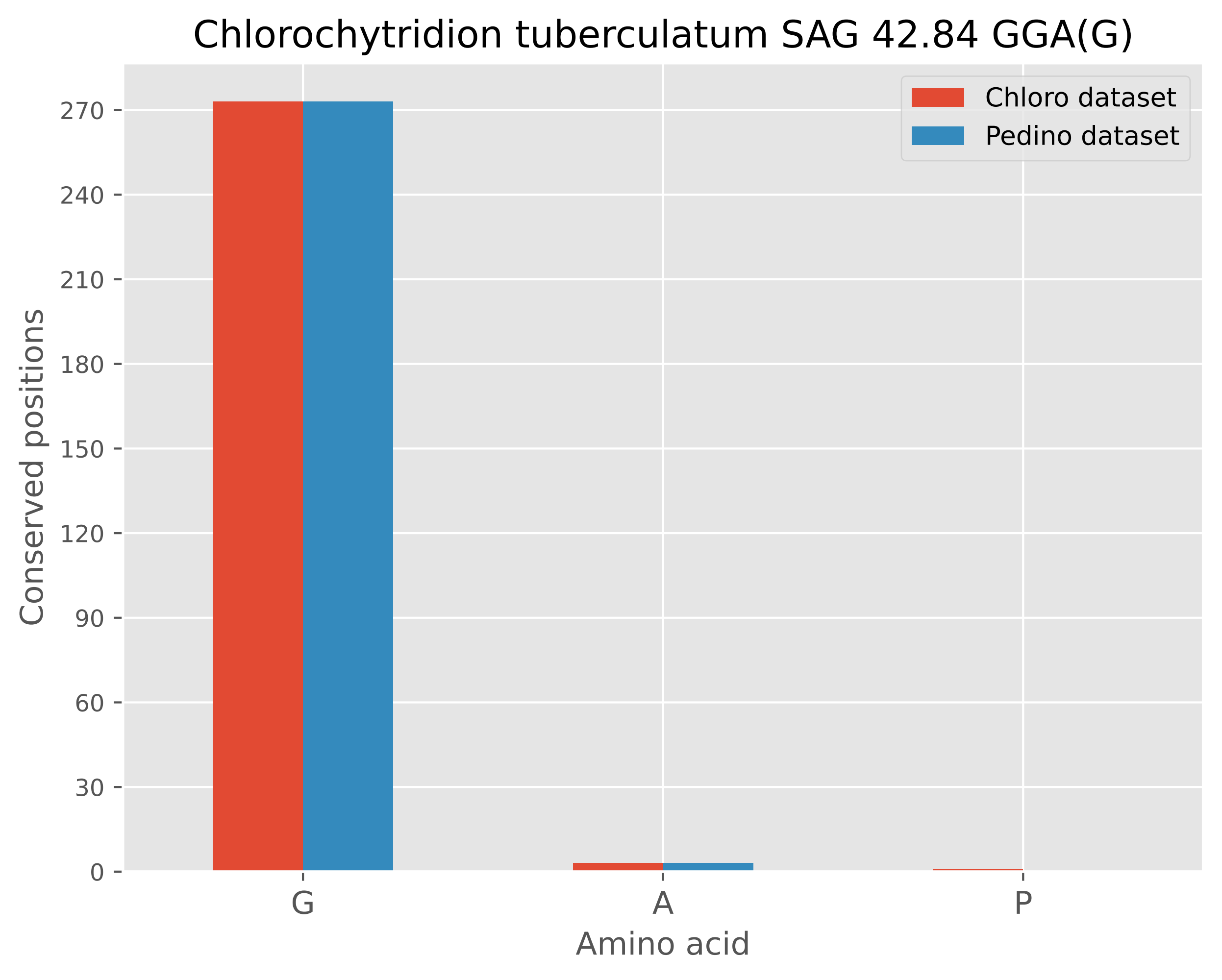
Chlorochytridion tuberculatum SAG 42.84 GGA(G)
Chloro dataset
Pedino dataset
Conserved positions
0
30
60
90
120
150
180
210
240
270
G
A
P
Amino acid

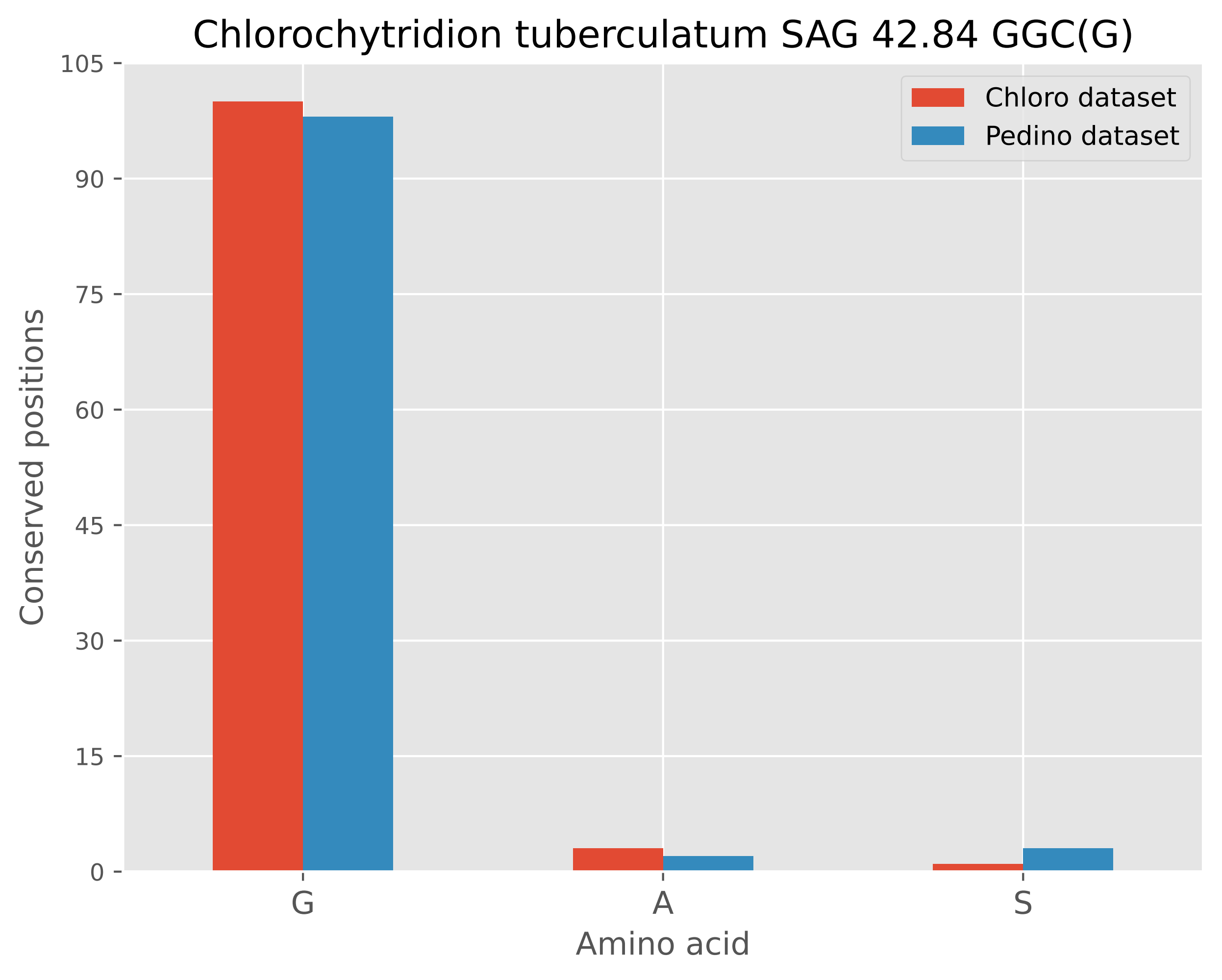
Chlorochytridion tuberculatum SAG 42.84 GGC(G)
Chloro dataset
Pedino dataset
Conserved positions
0
15
30
45
60
75
90
105
G
A
S
Amino acid

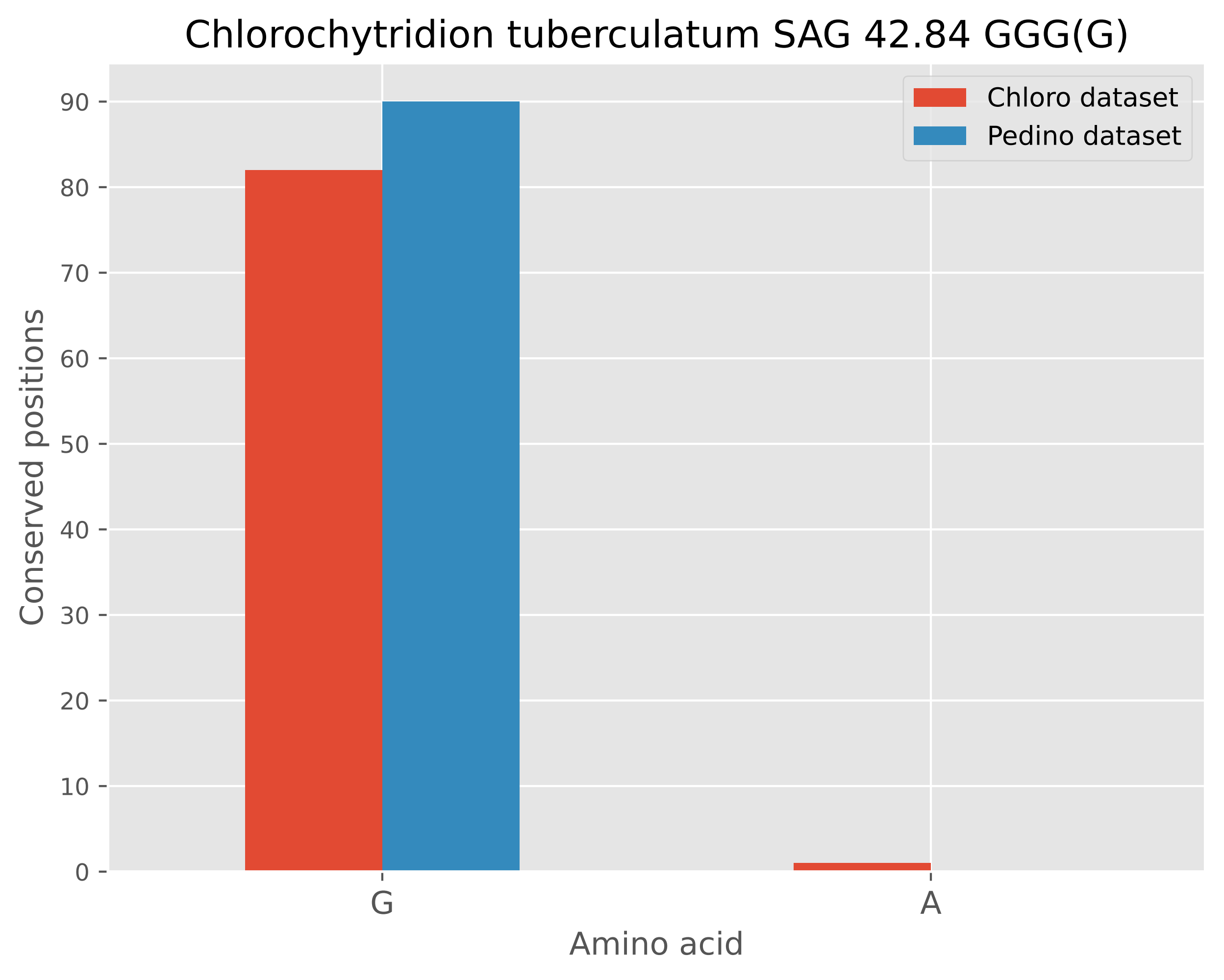

Chlorochytridion tuberculatum SAG 42.84 GGG(G)
Chloro dataset
Pedino dataset
Conserved positions
0
10
20
30
40
50
60
70
80
90
G
A
Amino acid

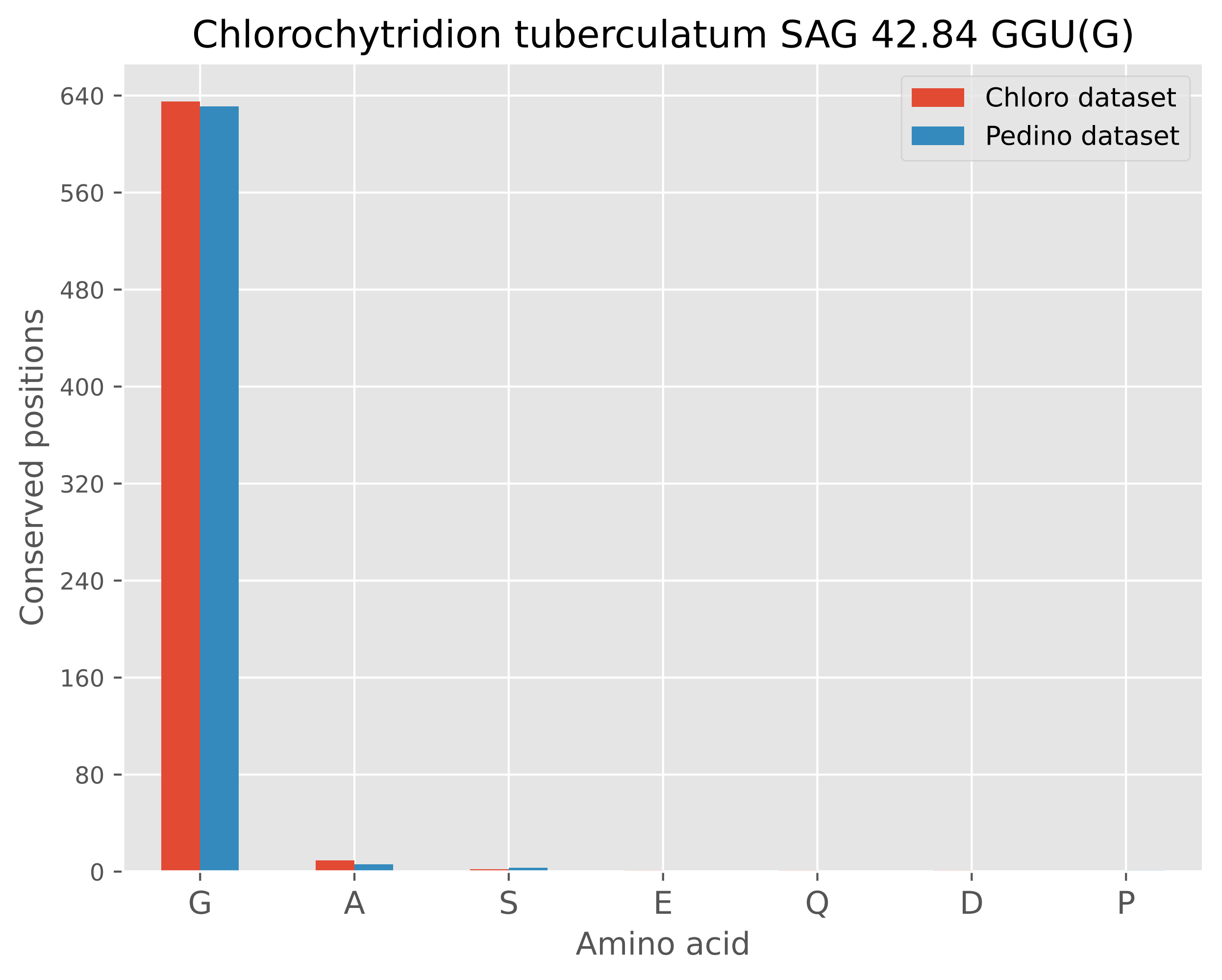

Chlorochytridion tuberculatum SAG 42.84 GGU(G)
Chloro dataset
Pedino dataset
Conserved positions
640
560
480
400
320
240
160
80
0
G
A
S
E
Q
D
P
Amino acid

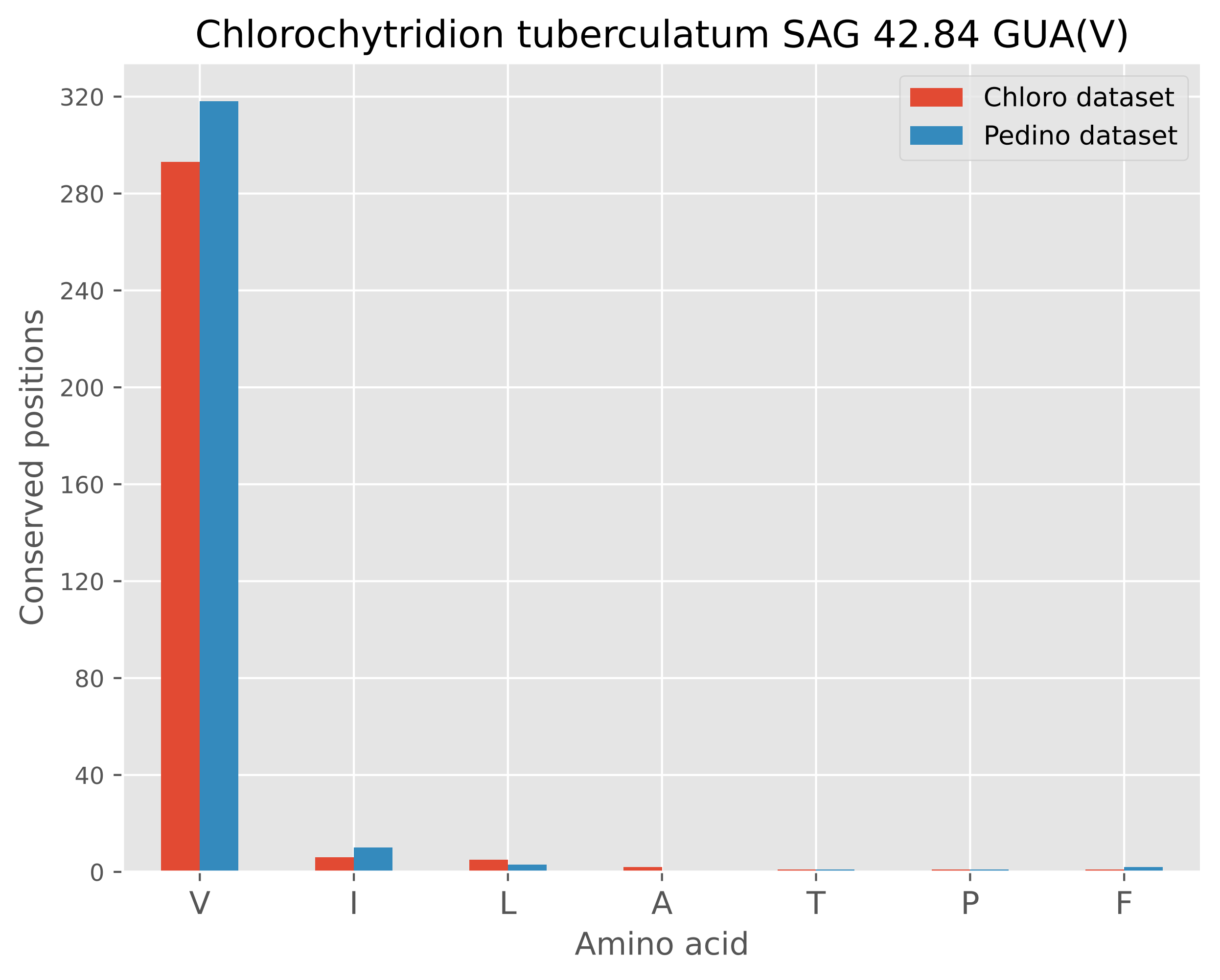

Chlorochytridion tuberculatum SAG 42.84 GUA(V)
Chloro dataset
Pedino dataset
Conserved positions
0
40
80
120
160
200
240
280
320
V
I
L
A
T
P
F
Amino acid

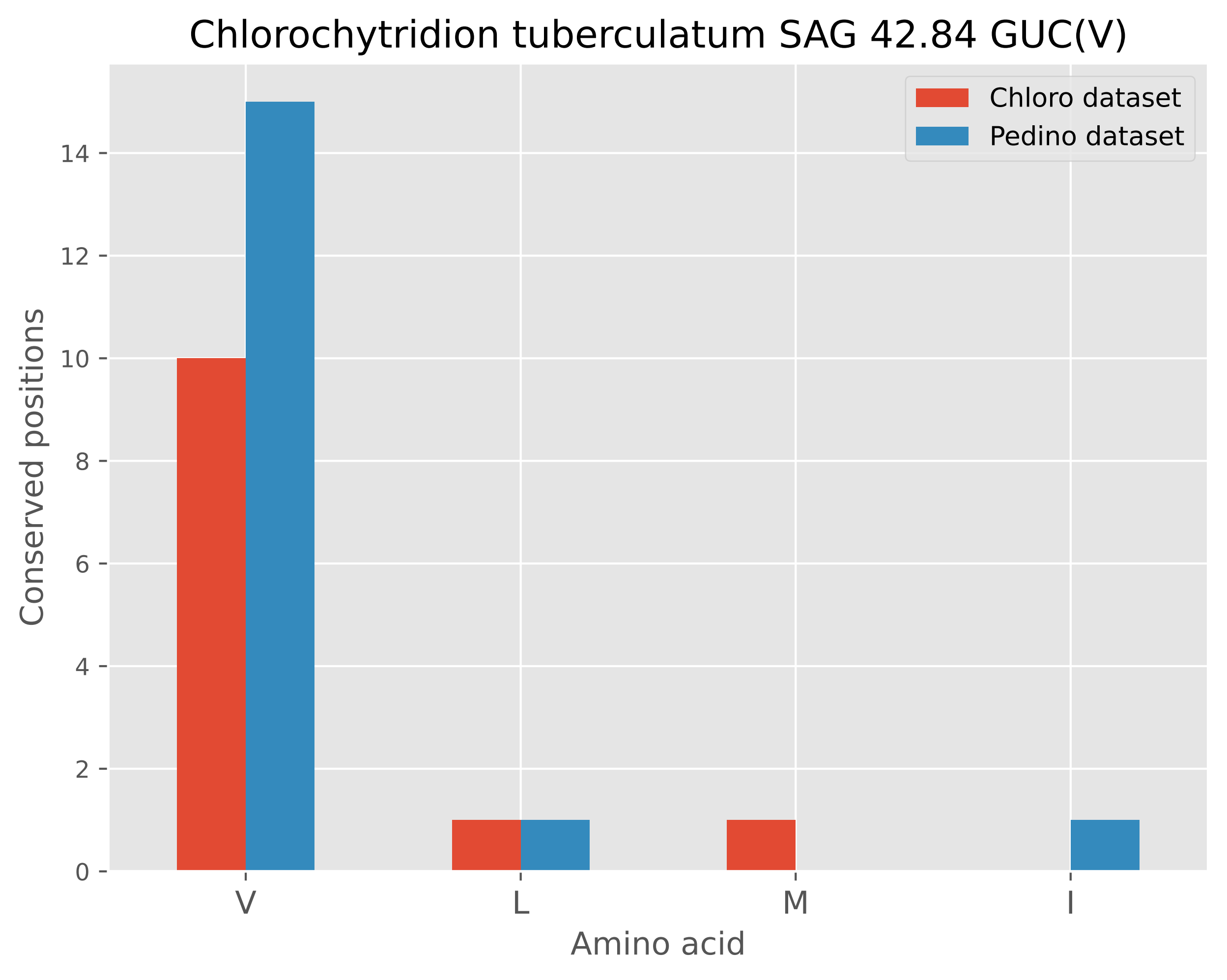

Chlorochytridion tuberculatum SAG 42.84 GUC(V)
Chloro dataset
Pedino dataset
Conserved positions
0
2
4
6
8
10
12
14
V
L
M
I
Amino acid

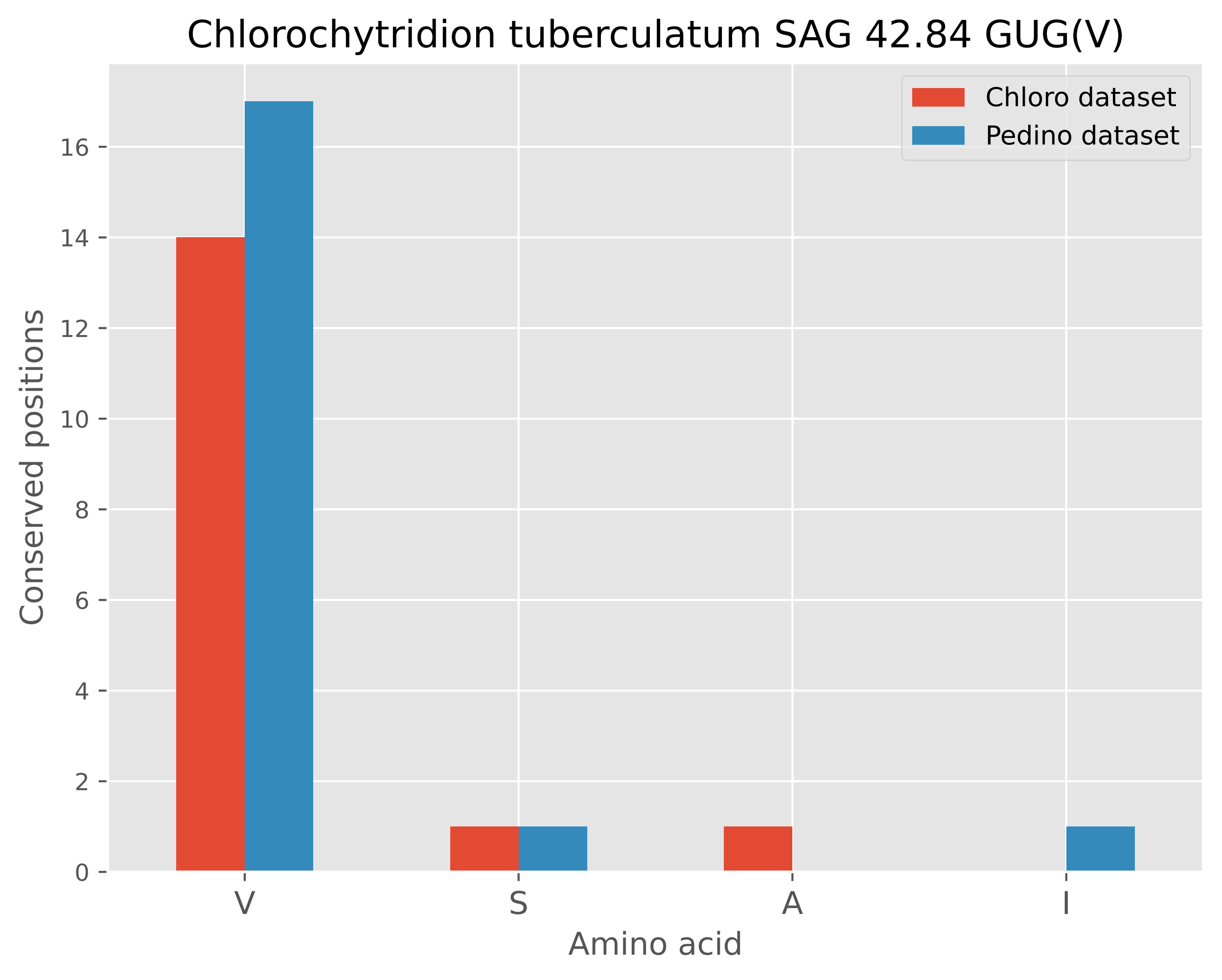

Chlorochytridion tuberculatum SAG 42.84 GUG(V)
Chloro dataset
Pedino dataset
Conserved positions
0
2
4
6
8
10
12
14
16
V
S
A
I
Amino acid

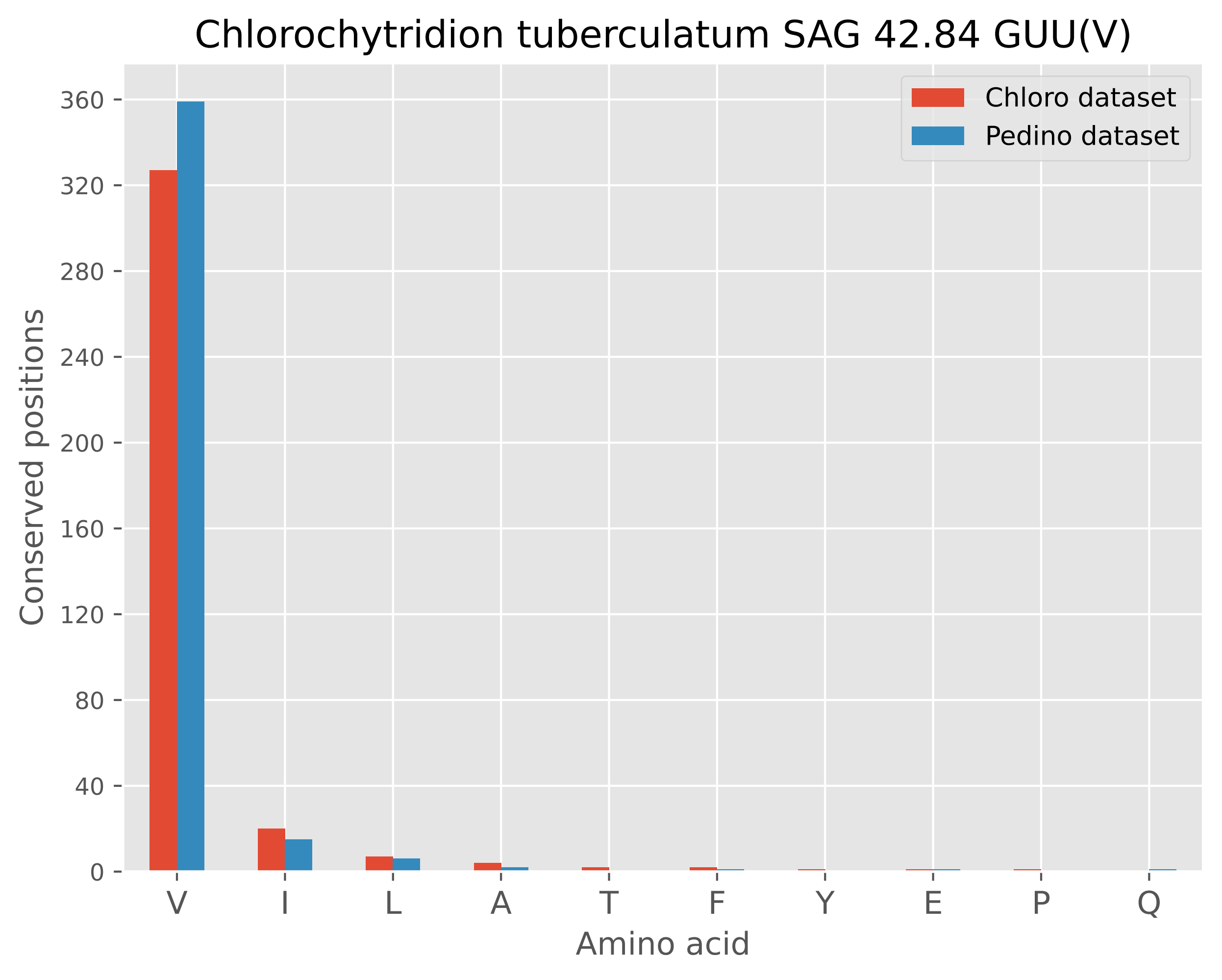
Chlorochytridion tuberculatum SAG 42.84 GUU(V)
Chloro dataset
Pedino dataset
Conserved positions
0
40
80
120
160
200
240
280
320
360
V
I
L
A
T
F
Y
E
P
Q
Amino acid

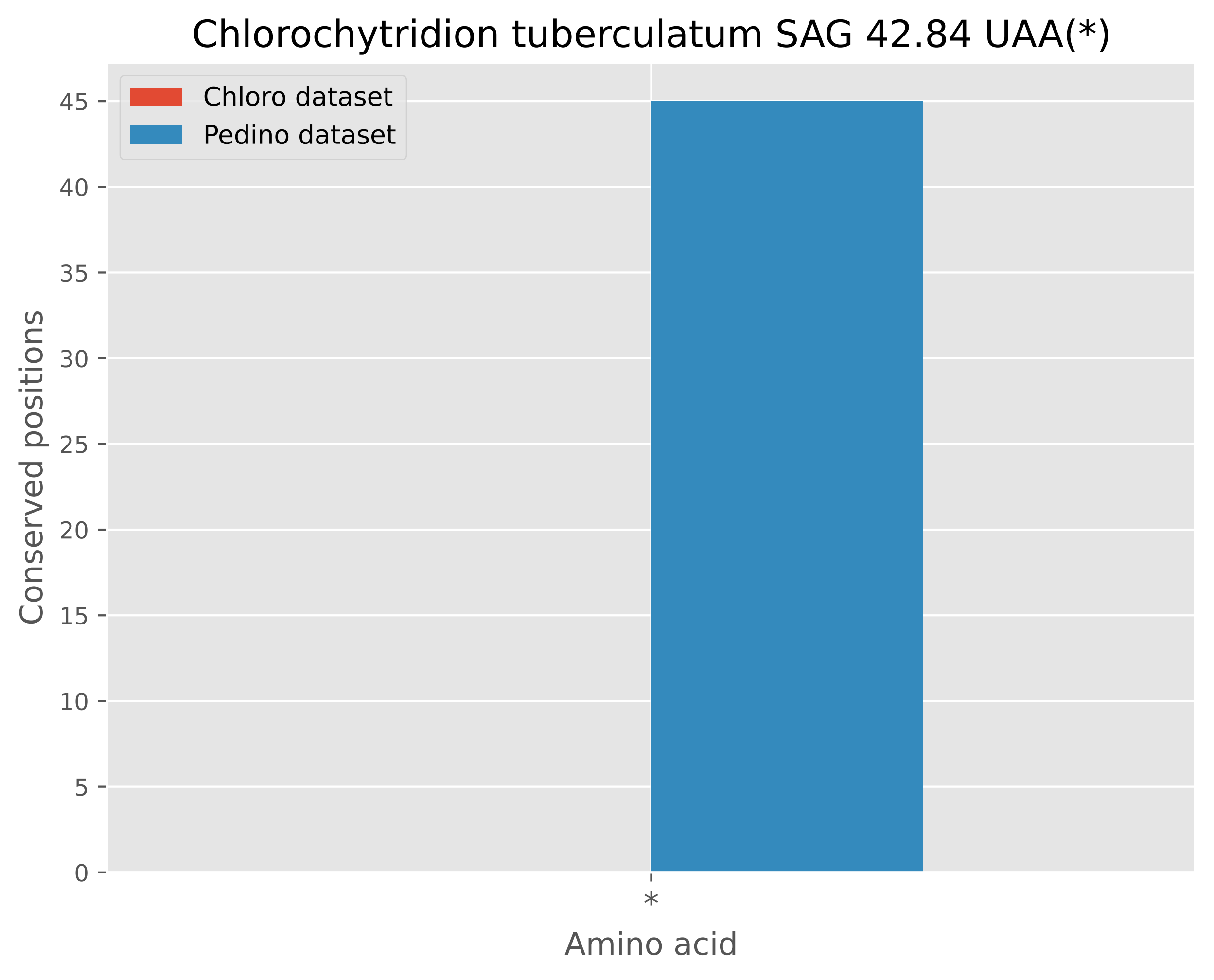
Chlorochytridion tuberculatum SAG 42.84 UAA(*)
Chloro dataset
Pedino dataset
Conserved positions
0
5
10
15
20
25
30
35
40
45
*
Amino acid

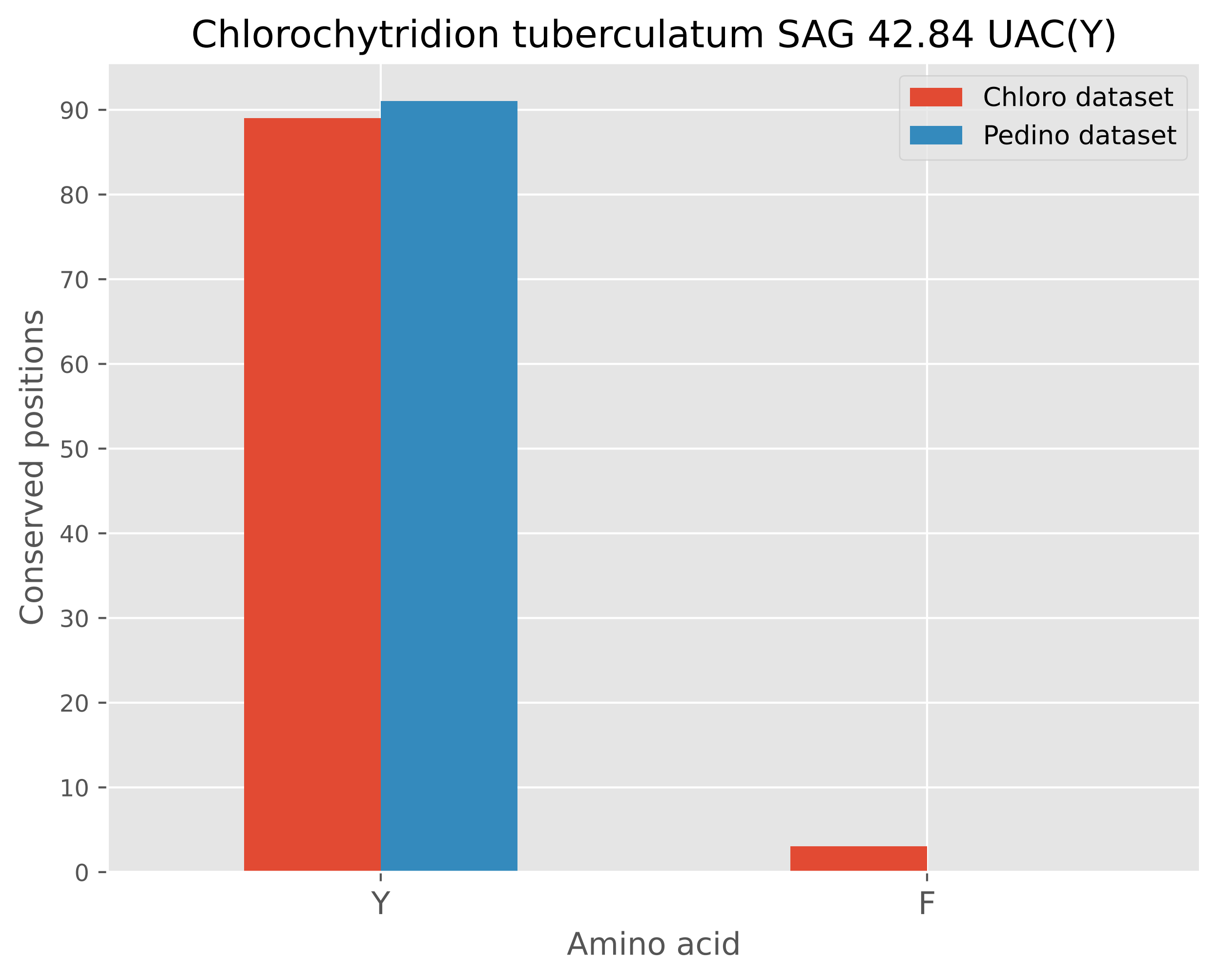

Chlorochytridion tuberculatum SAG 42.84 UAC(Y)
Chloro dataset
Pedino dataset
Conserved positions
0
10
20
30
40
50
60
70
80
90
Y
F
Amino acid

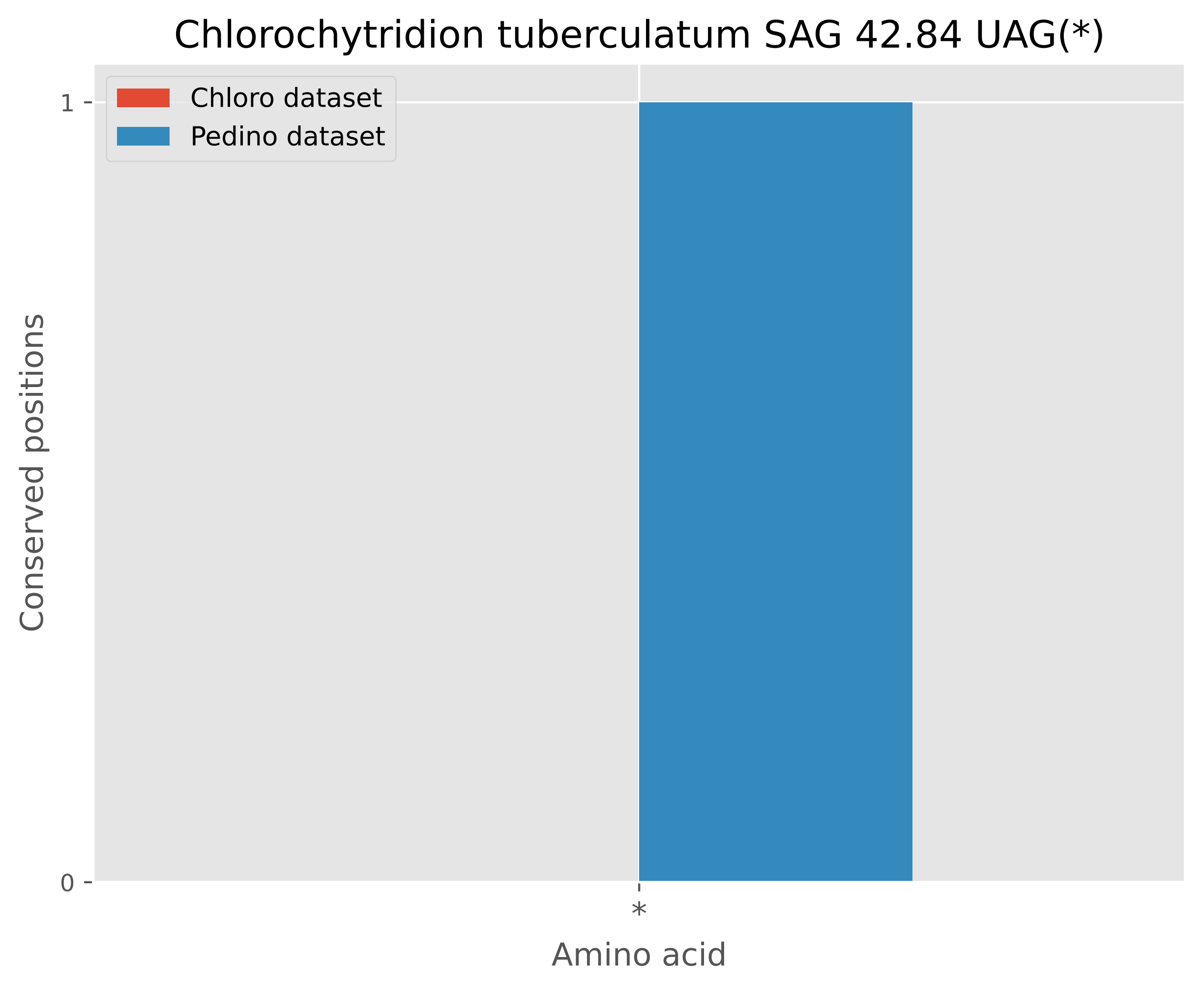

Chlorochytridion tuberculatum SAG 42.84 UAG(*)
Chloro dataset
Pedino dataset
1
0
Conserved positions
*
Amino acid

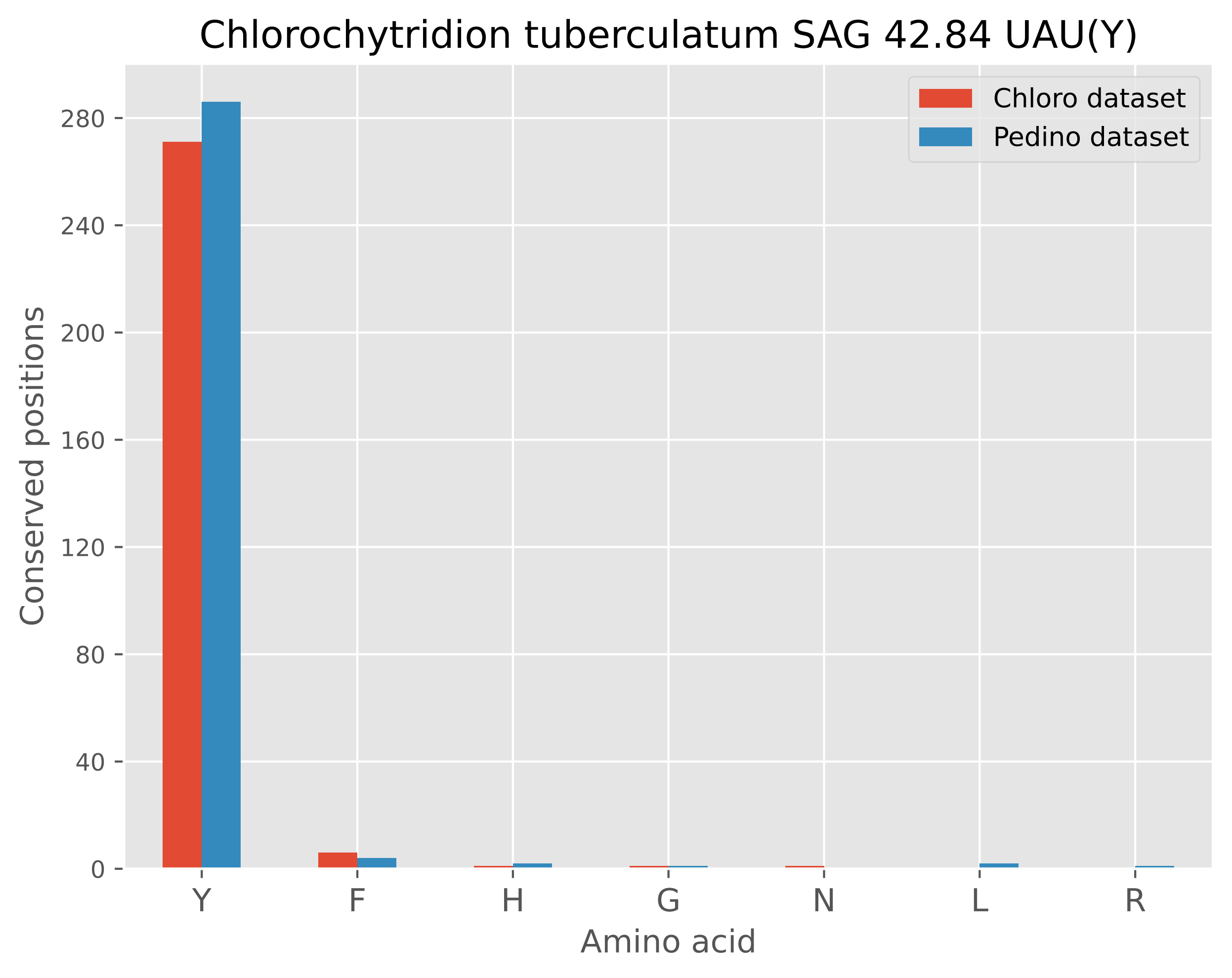
Chlorochytridion tuberculatum SAG 42.84 UAU(Y)
Chloro dataset
Pedino dataset
Conserved positions
0
40
80
120
160
200
240
280
Y
F
H
G
N
L
R
Amino acid

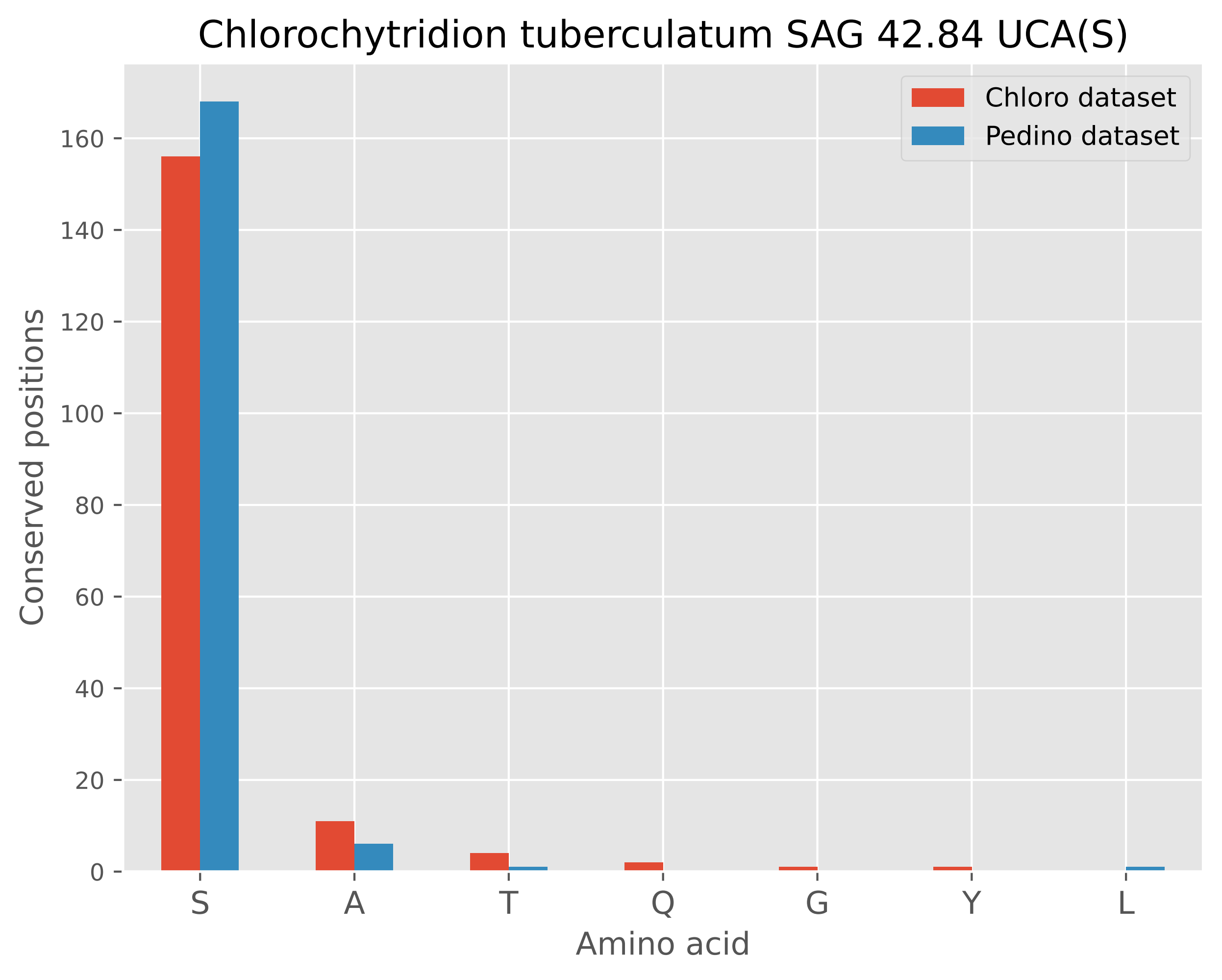

Chlorochytridion tuberculatum SAG 42.84 UCA(S)
Chloro dataset
Pedino dataset
Conserved positions
0
20
40
60
80
100
120
140
160
S
A
T
Q
G
Y
L
Amino acid

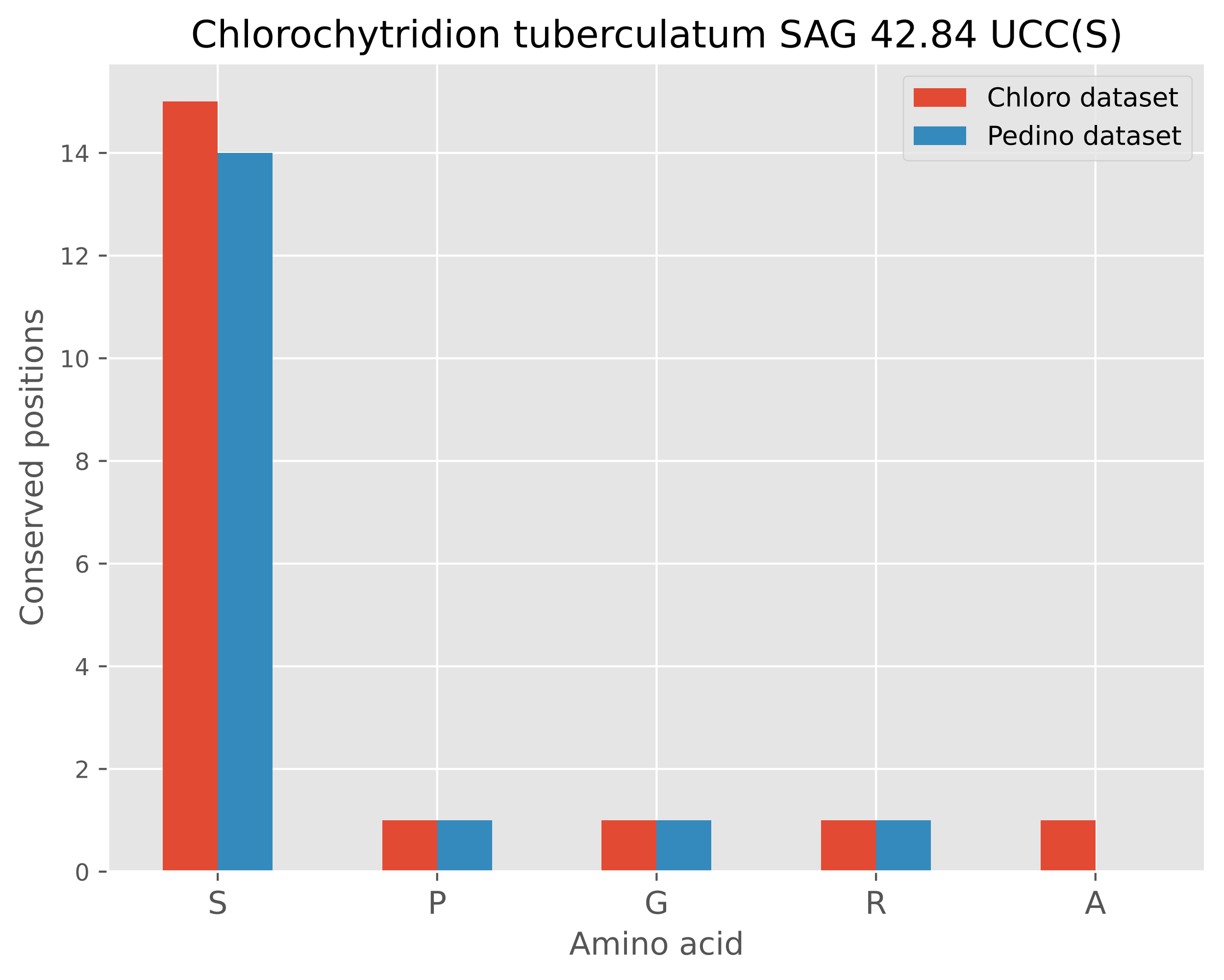
Chlorochytridion tuberculatum SAG 42.84 UCC(S)
Chloro dataset
Pedino dataset
Conserved positions
0
2
4
6
8
10
12
14
S
P
G
R
A
Amino acid

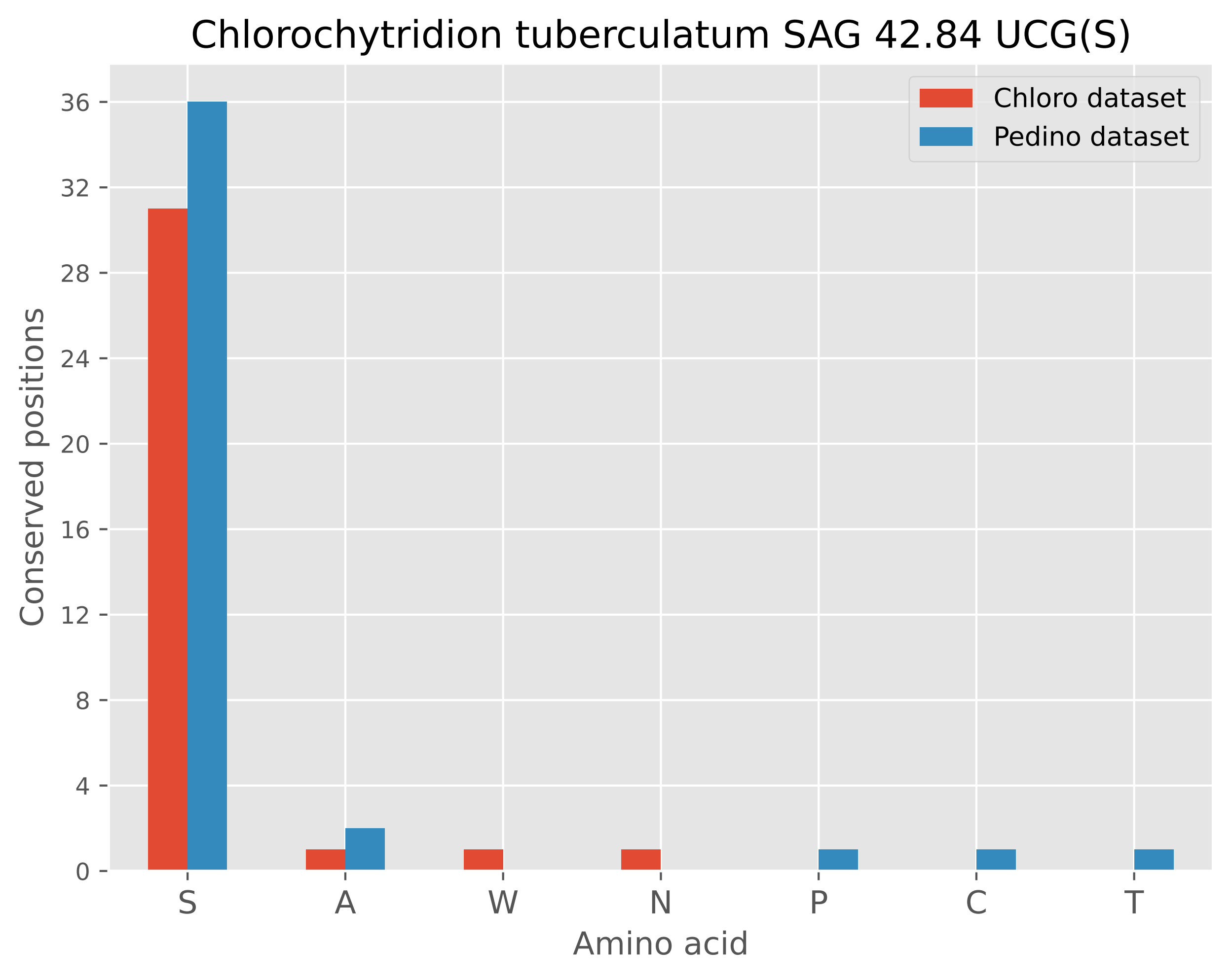
Chlorochytridion tuberculatum SAG 42.84 UCG(S)
Chloro dataset
Pedino dataset
Conserved positions
0
4
8
12
16
20
24
28
32
36
S
A
W
N
P
C
T
Amino acid

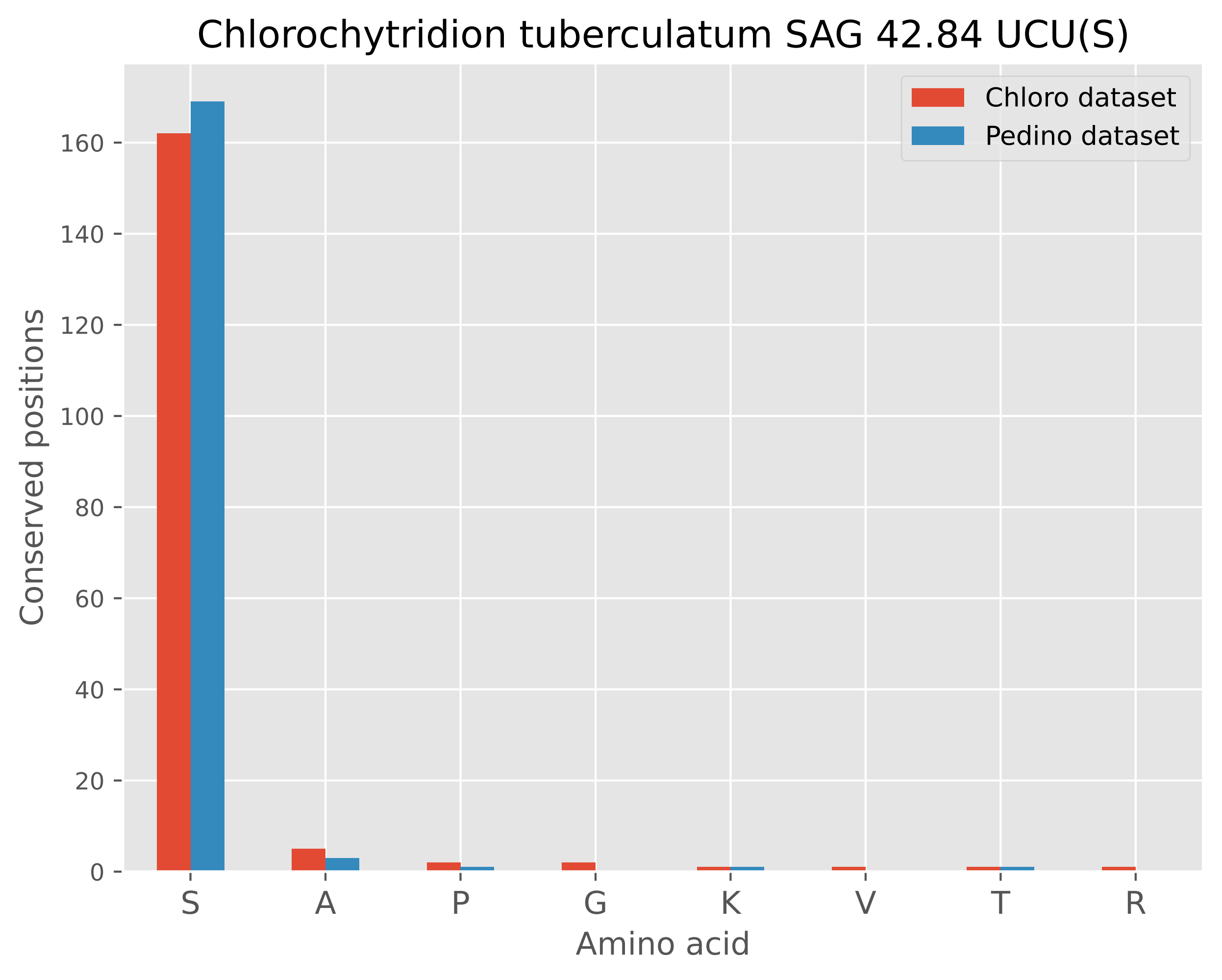
Chlorochytridion tuberculatum SAG 42.84 UCU(S)
Chloro dataset
Pedino dataset
Conserved positions
0
20
40
60
80
100
120
140
160
S
A
P
G
K
V
T
R
Amino acid

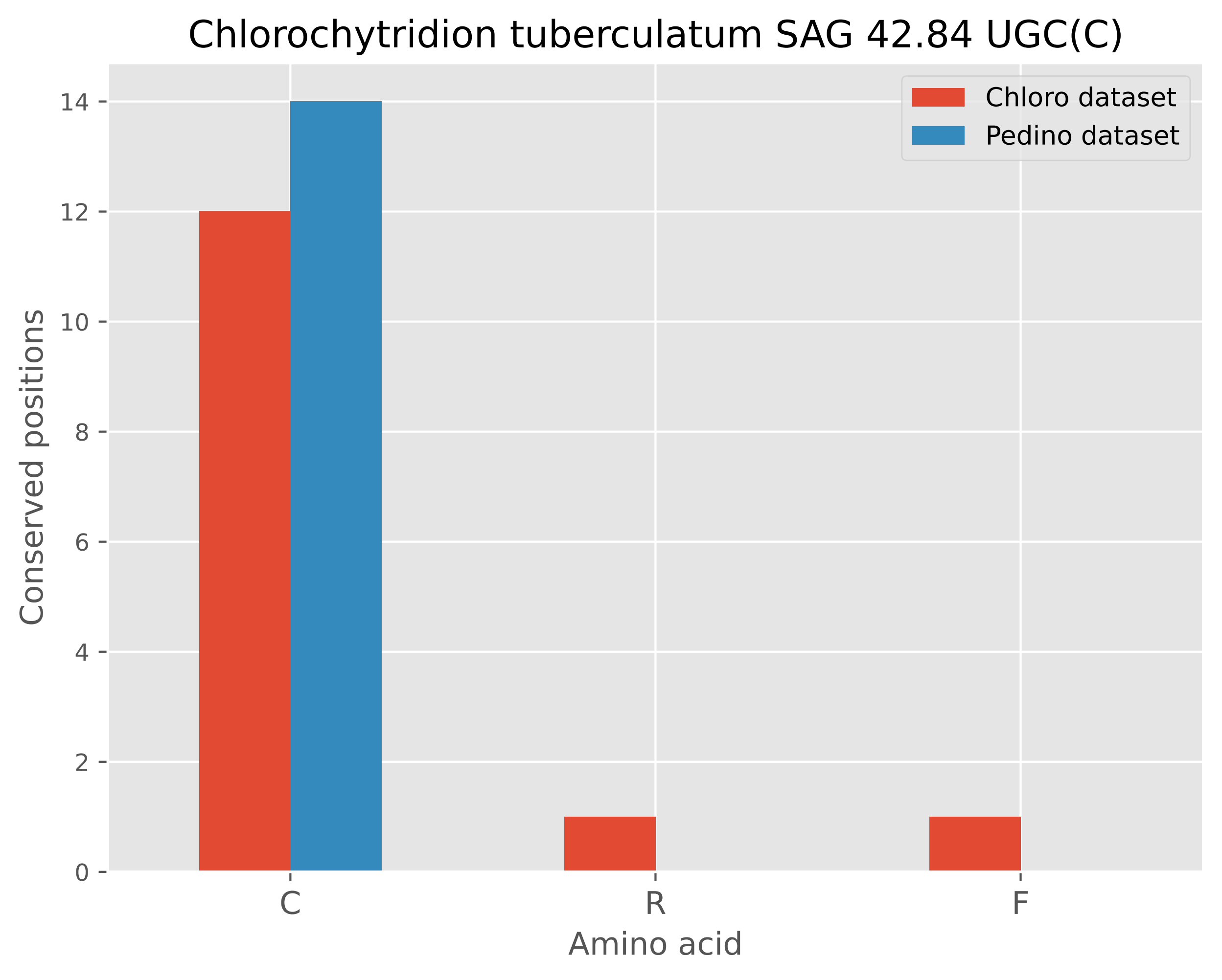

Chlorochytridion tuberculatum SAG 42.84 UGC(C)
Chloro dataset
Pedino dataset
Conserved positions
0
2
4
6
8
10
12
14
C
R
F
Amino acid

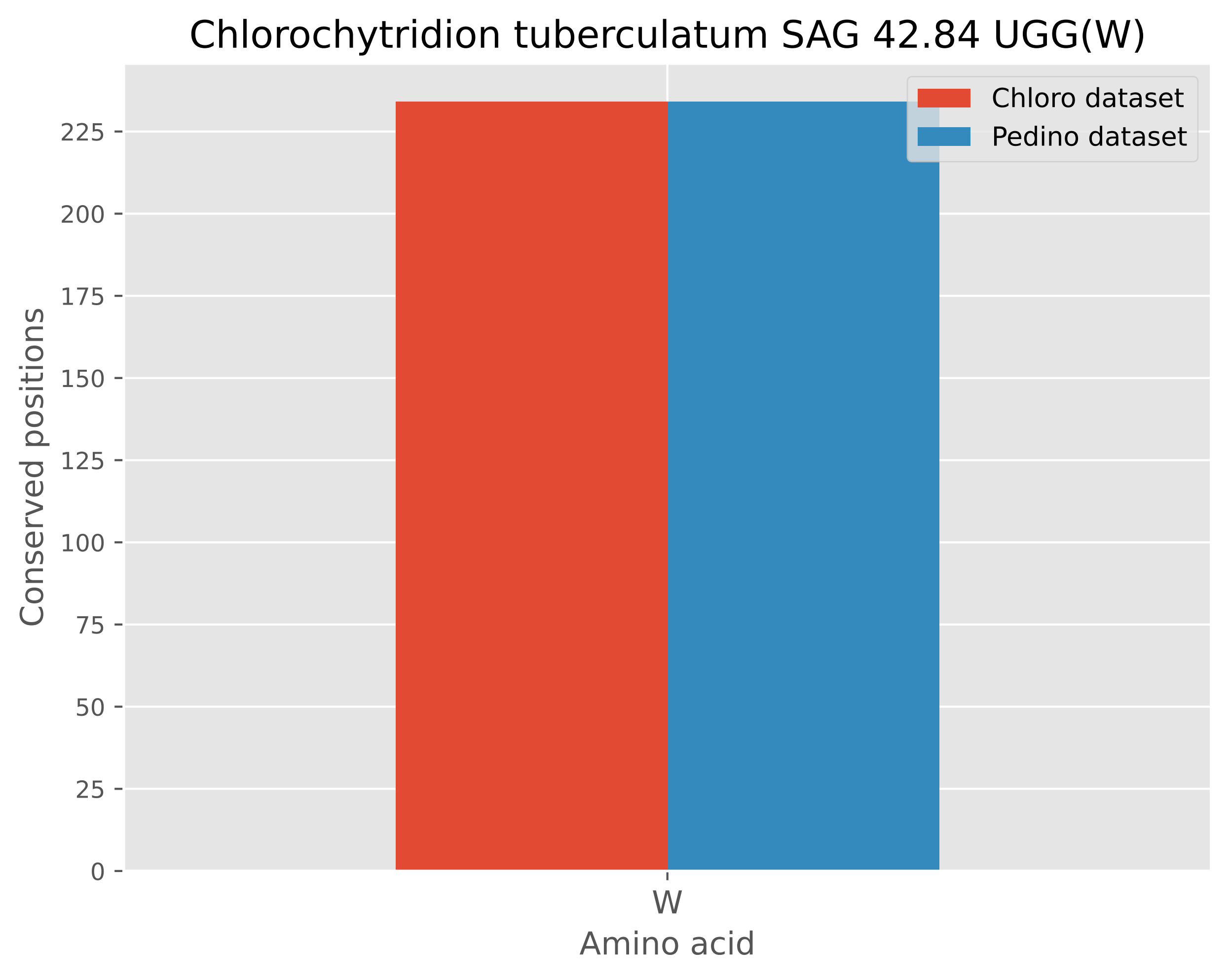

Chlorochytridion tuberculatum SAG 42.84 UGG(W)
Chloro dataset
Pedino dataset
Conserved positions
0
25
50
75
100
125
150
175
200
225
W
Amino acid

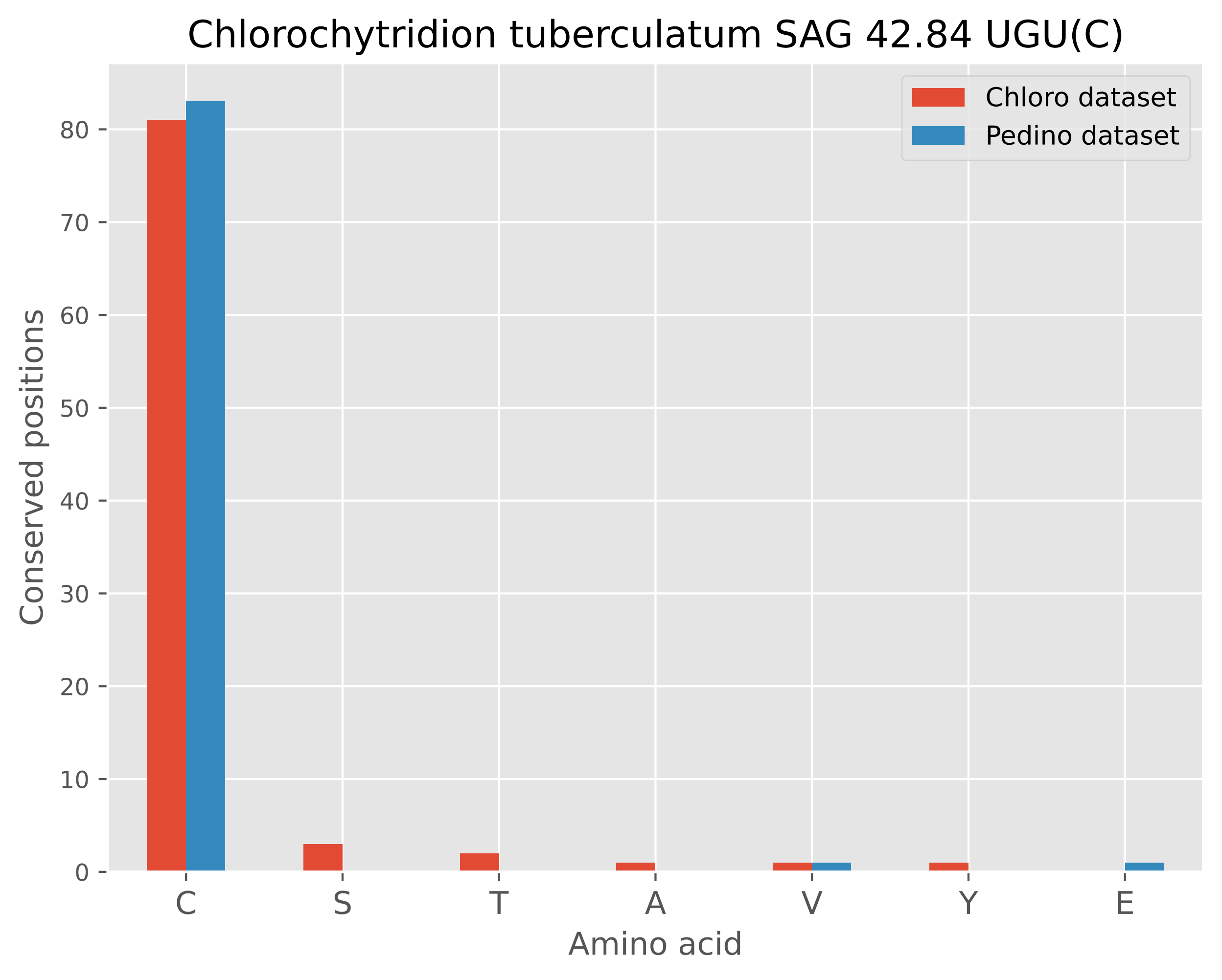
Chlorochytridion tuberculatum SAG 42.84 UGU(C)
Chloro dataset
Pedino dataset
Conserved positions
0
10
20
30
40
50
60
70
80
C
S
T
A
V
Y
E
Amino acid

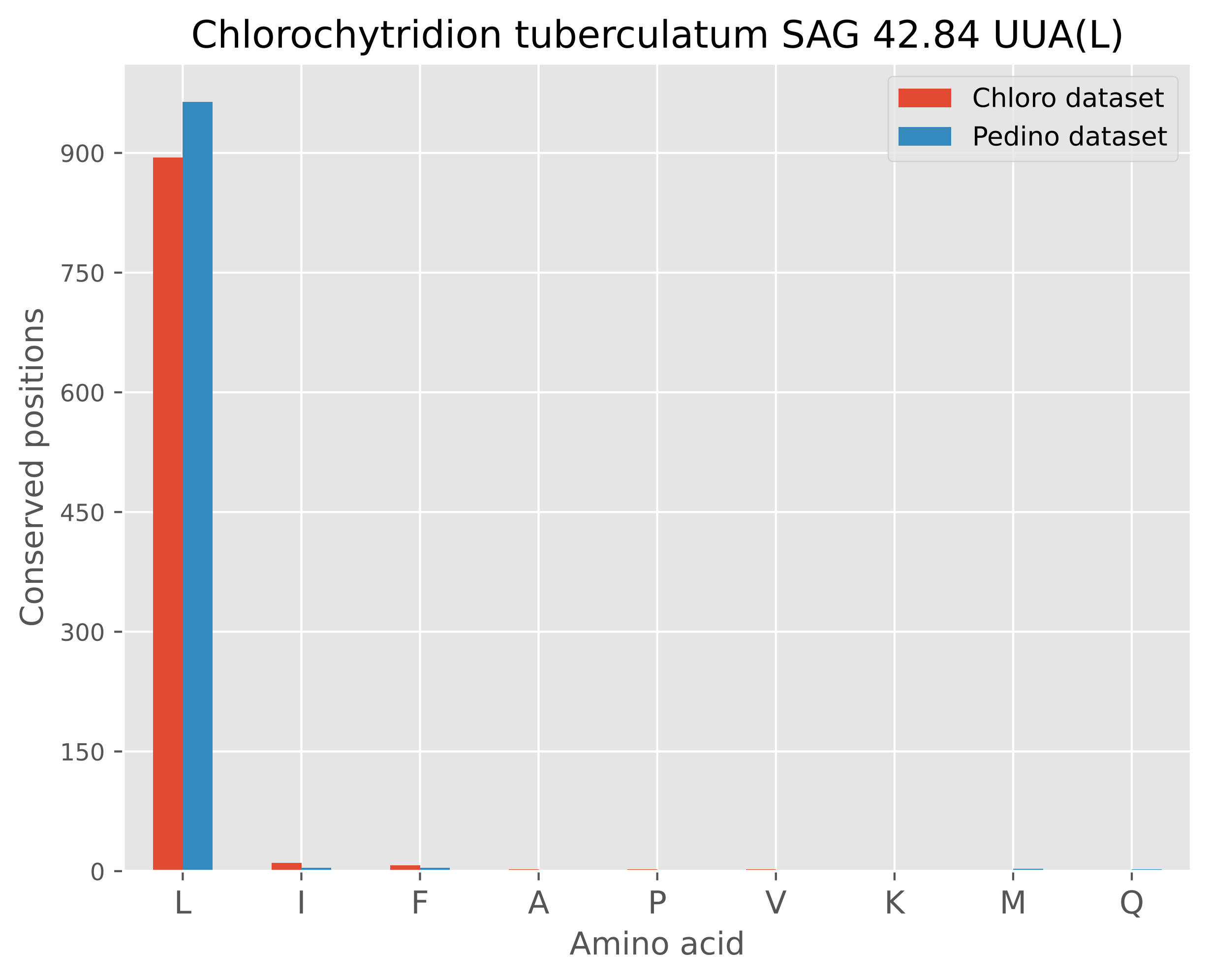
Chlorochytridion tuberculatum SAG 42.84 UUA(L)
Chloro dataset
Pedino dataset
Conserved positions
0
150
300
450
600
750
900
L
I
F
A
P
V
K
M
Q
Amino acid

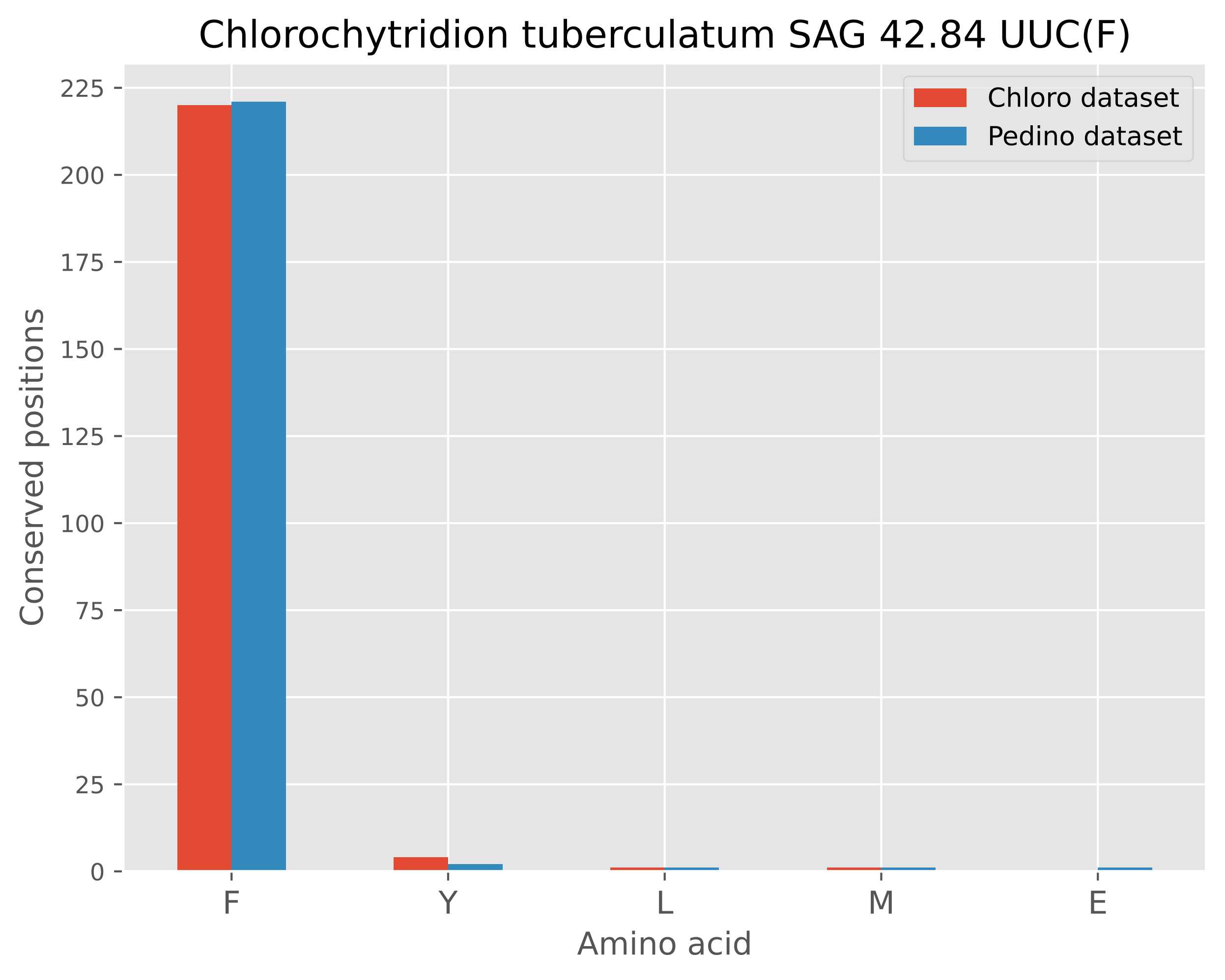

Chlorochytridion tuberculatum SAG 42.84 UUC(F)
Chloro dataset
Pedino dataset
Conserved positions
0
25
50
75
100
125
150
175
200
225
F
Y
L
M
E
Amino acid

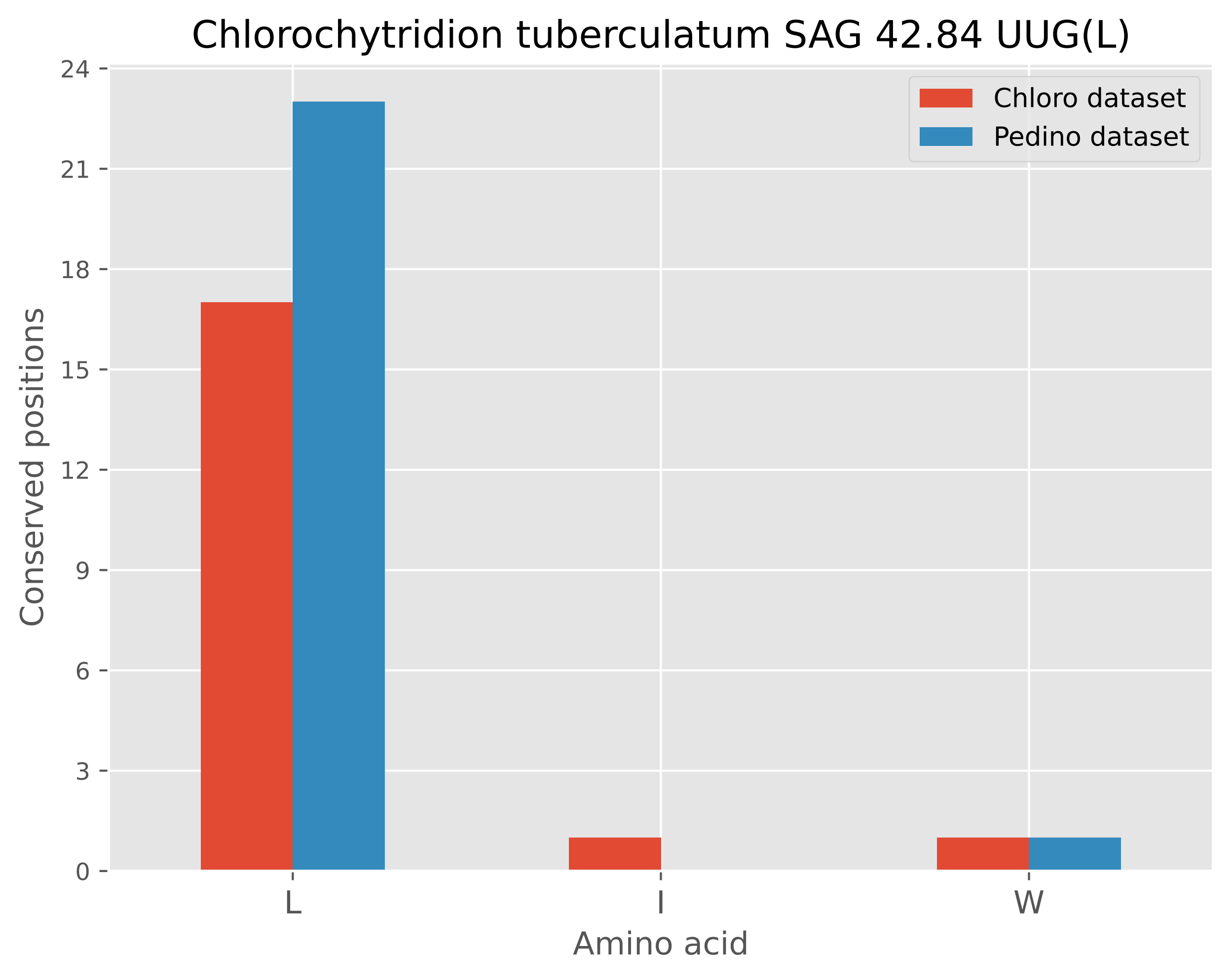

Chlorochytridion tuberculatum SAG 42.84 UUG(L)
Chloro dataset
Pedino dataset
Conserved positions
0
3
6
9
12
15
18
21
24
L
I
W
Amino acid

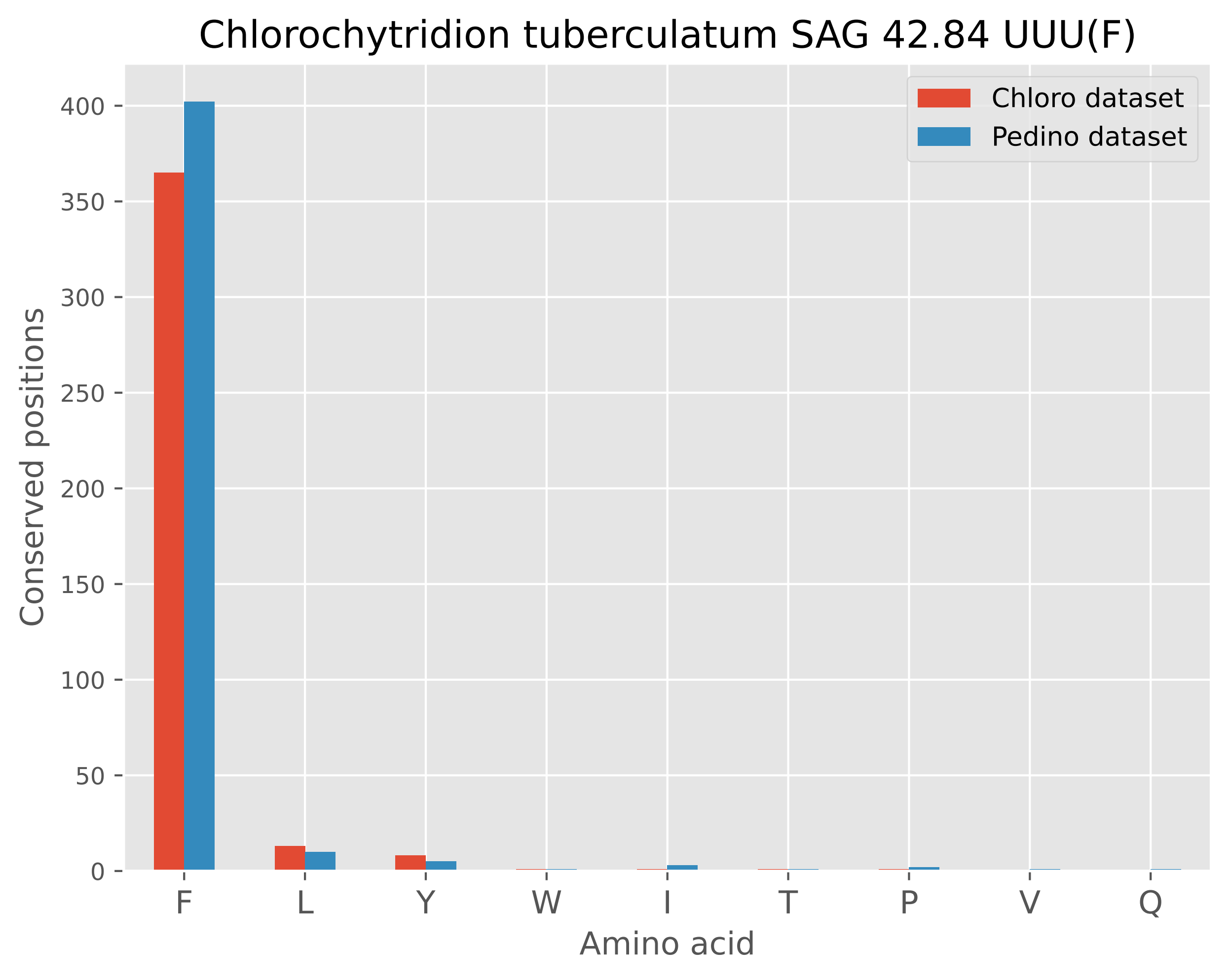

Chlorochytridion tuberculatum SAG 42.84 UUU(F)
Chloro dataset
Pedino dataset
Conserved positions
0
50
100
150
200
250
300
350
400
F
L
Y
W
I
T
P
V
Q
Amino acid

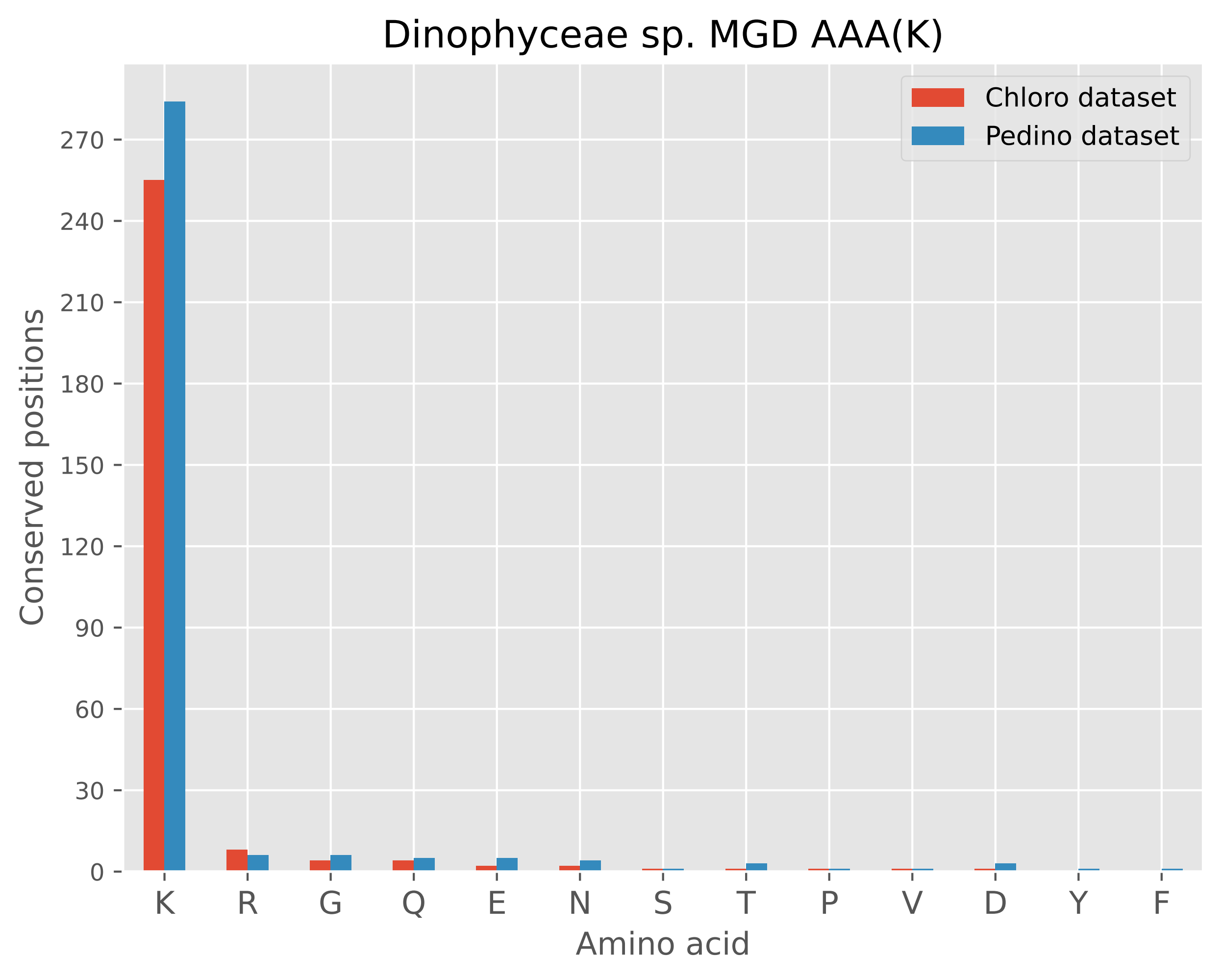
Dinophyceae sp. MGD AAA(K)
Chloro dataset
Pedino dataset
Conserved positions
Amino acid
0
30
60
90
120
150
180
210
240
270
K
R
G
Q
E
N
S
T
P
V
D
Y
F

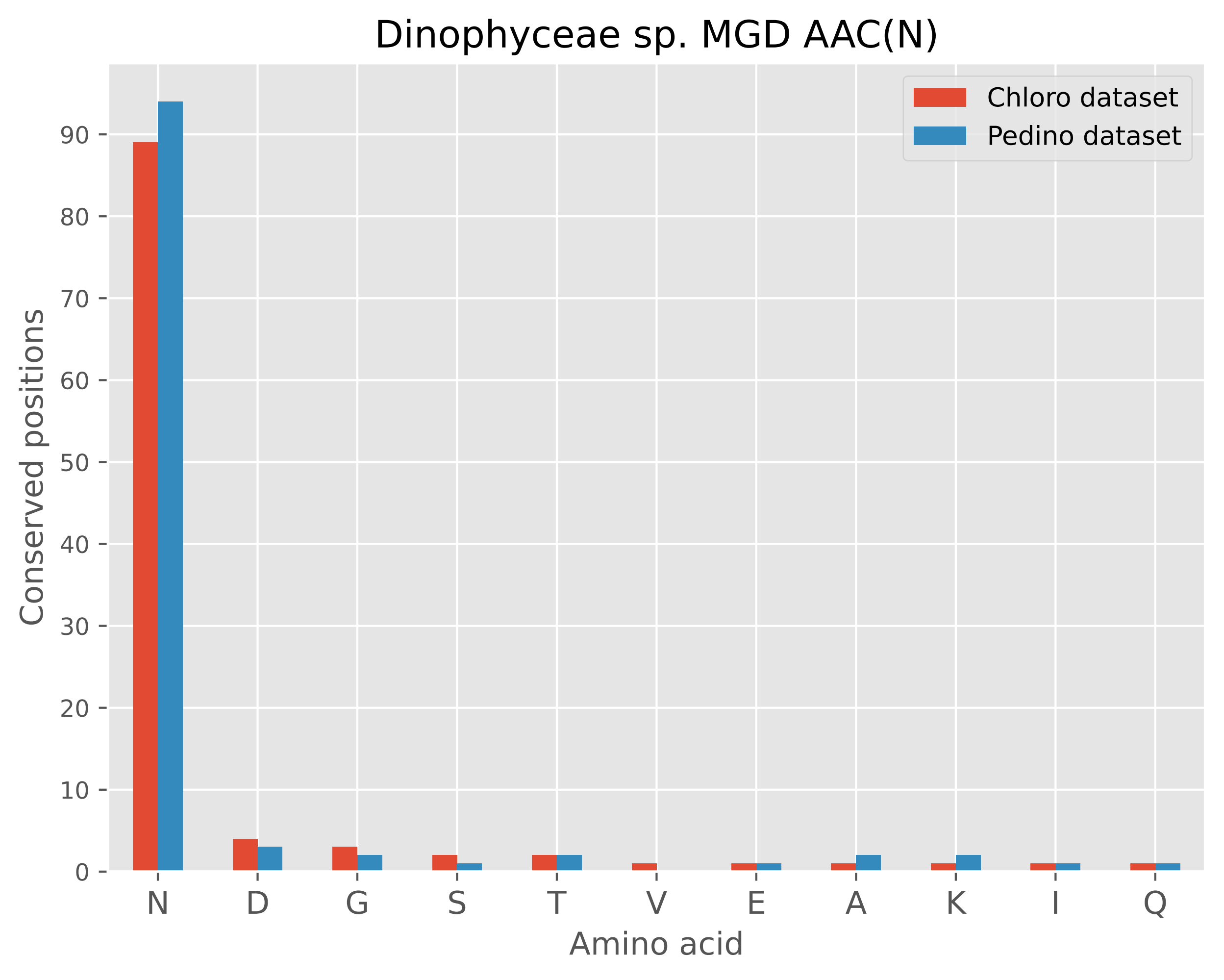
Dinophyceae sp. MGD AAC(N)
Chloro dataset
Pedino dataset
Conserved positions
0
10
20
30
40
50
60
70
80
90
N
D
G
S
T
V
E
A
K
I
Q
Amino acid

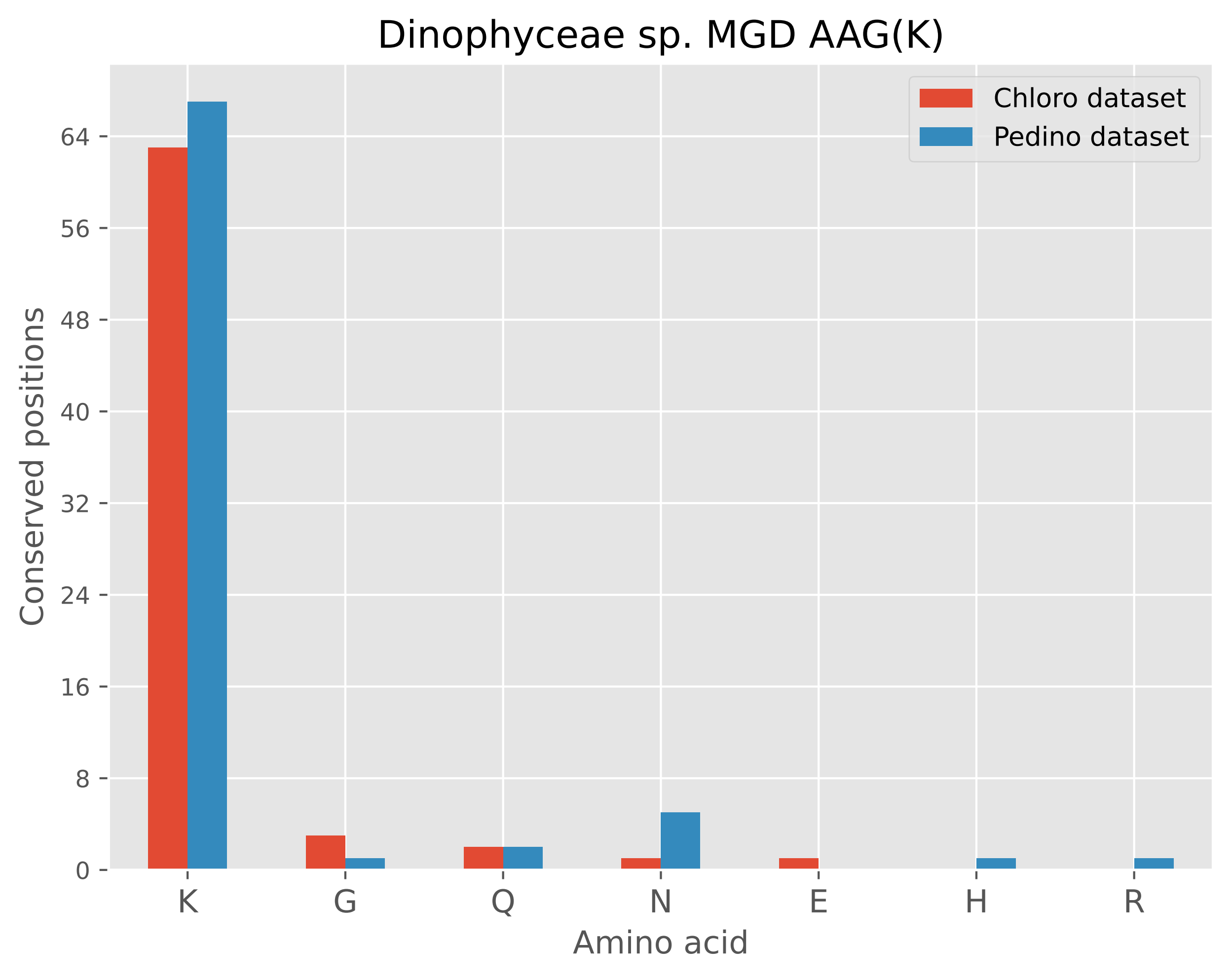

Dinophyceae sp. MGD AAG(K)
Chloro dataset
Pedino dataset
Conserved positions
0
8
16
24
32
40
48
56
64
K
G
Q
N
E
H
R
Amino acid

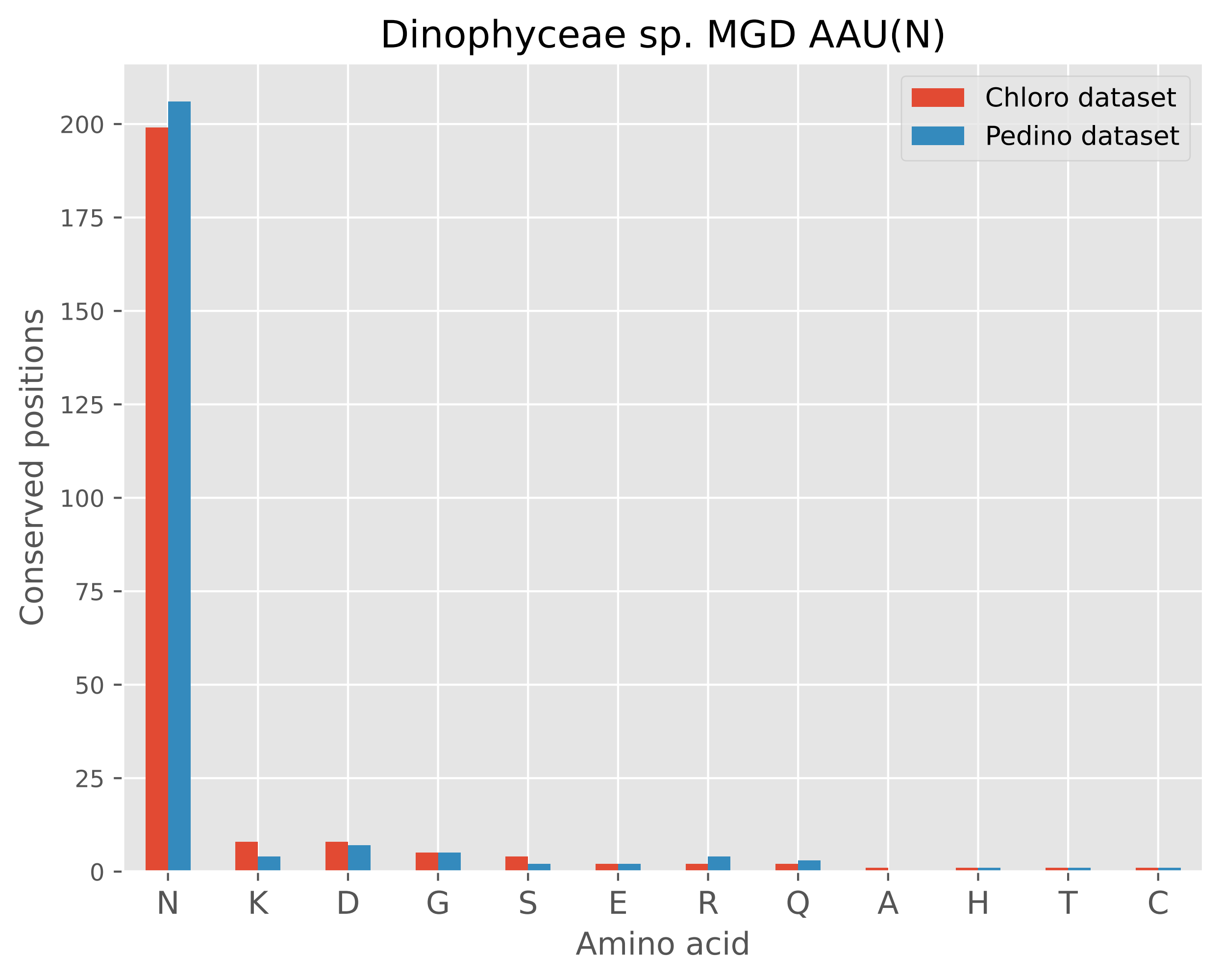

Dinophyceae sp. MGD AAU(N)
Chloro dataset
Pedino dataset
Conserved positions
0
25
50
75
100
125
150
175
200
N
K
D
G
S
E
R
Q
A
H
T
C
Amino acid

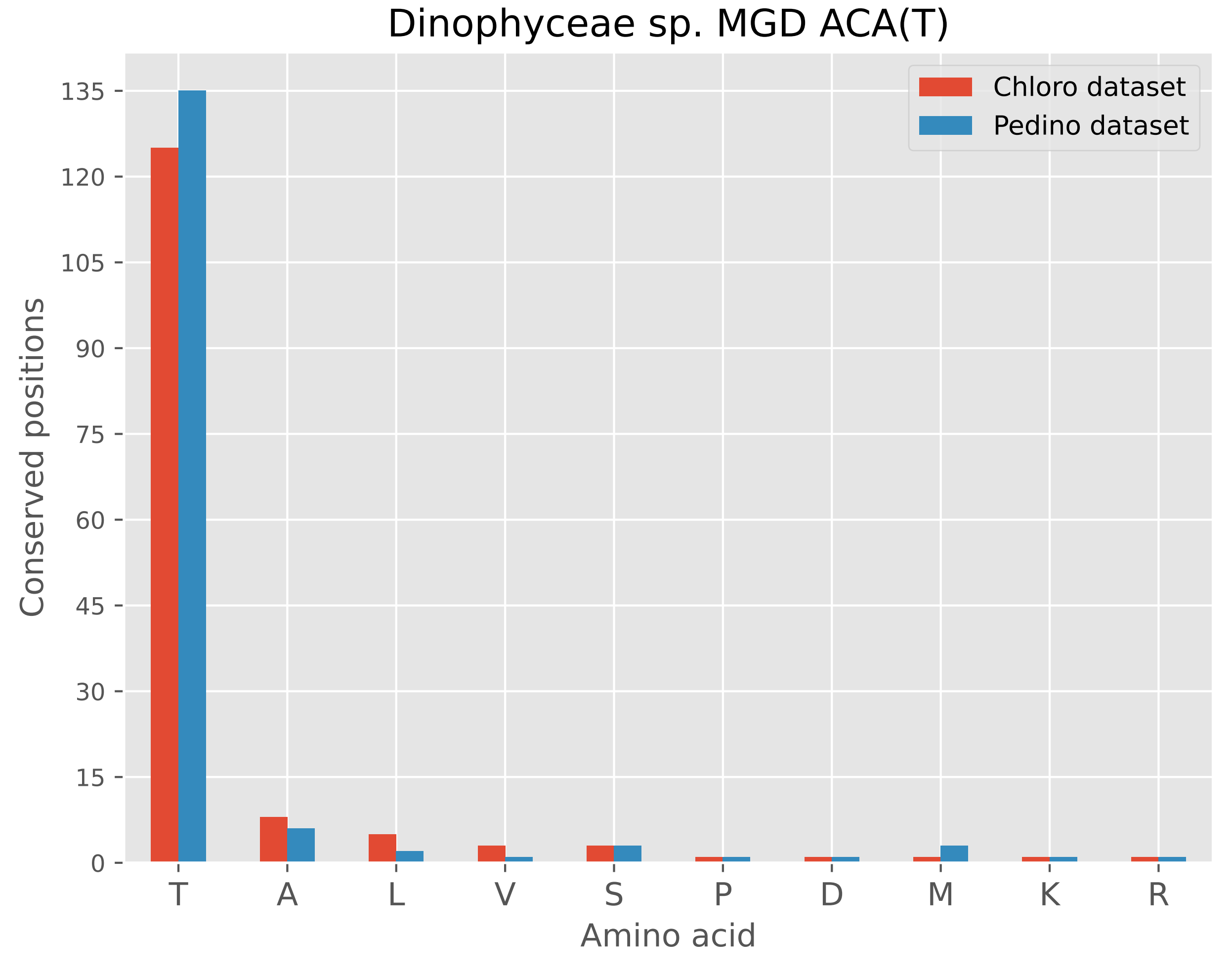

Dinophyceae sp. MGD ACA(T)
Chloro dataset
Pedino dataset
Conserved positions
135
120
105
90
75
60
45
30
15
0
T
A
L
V
S
P
D
M
K
R
Amino acid

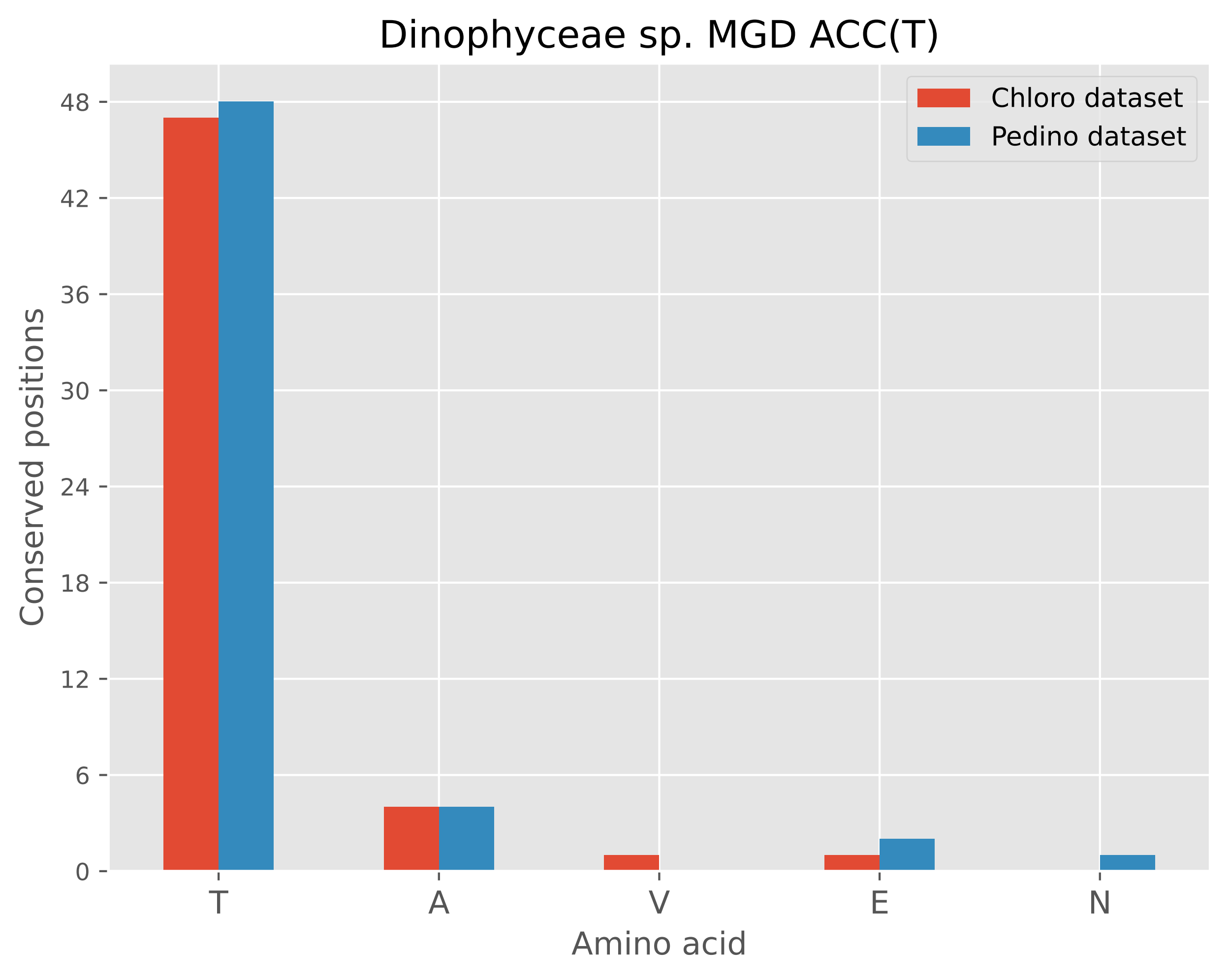

Dinophyceae sp. MGD ACC(T)
Chloro dataset
Pedino dataset
Conserved positions
Amino acid
0
6
12
18
24
30
36
42
48
T
A
V
E
N

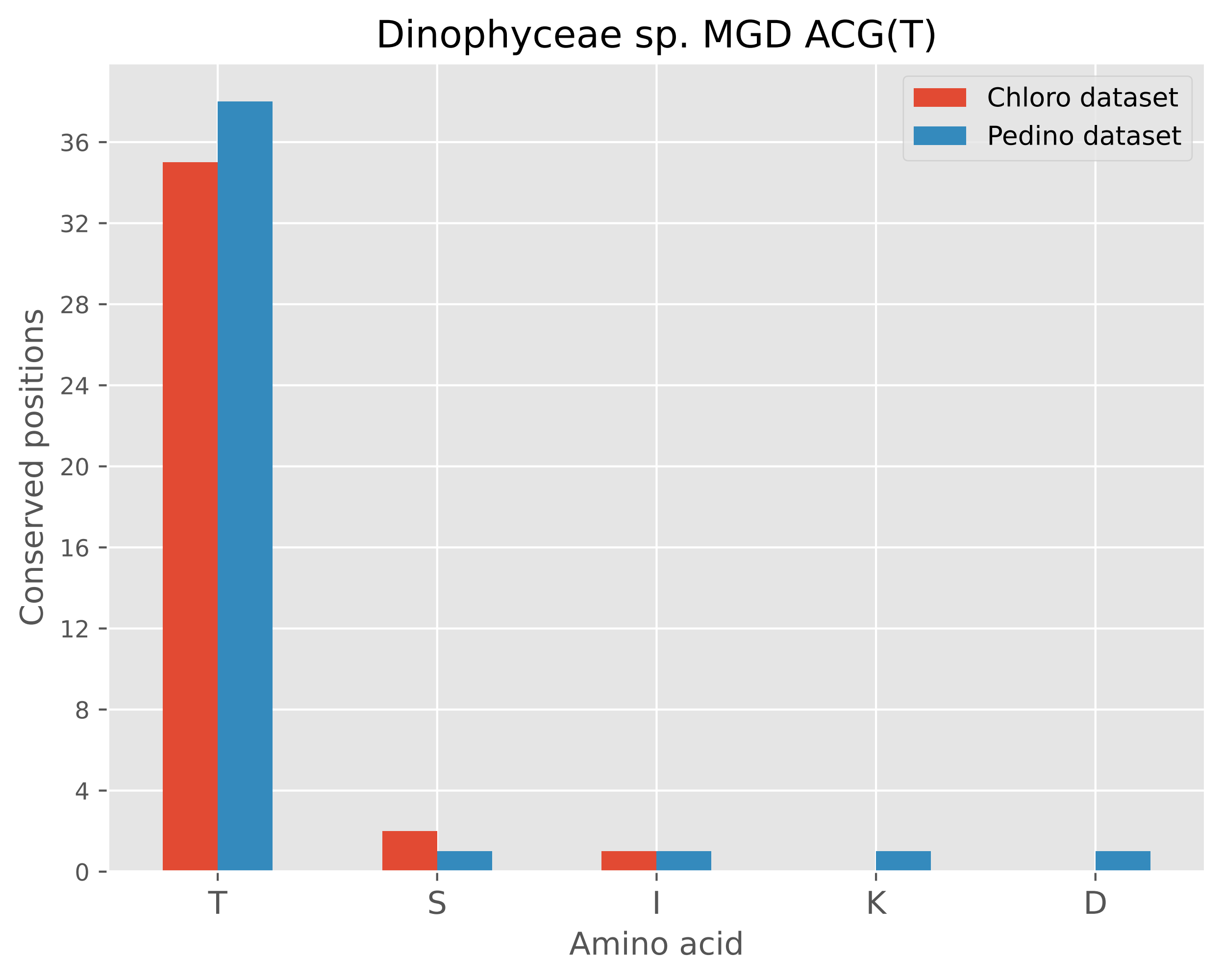

Dinophyceae sp. MGD ACG(T)
Chloro dataset
Pedino dataset
Conserved positions
0
4
8
12
16
20
24
28
32
36
T
S
I
K
D
Amino acid

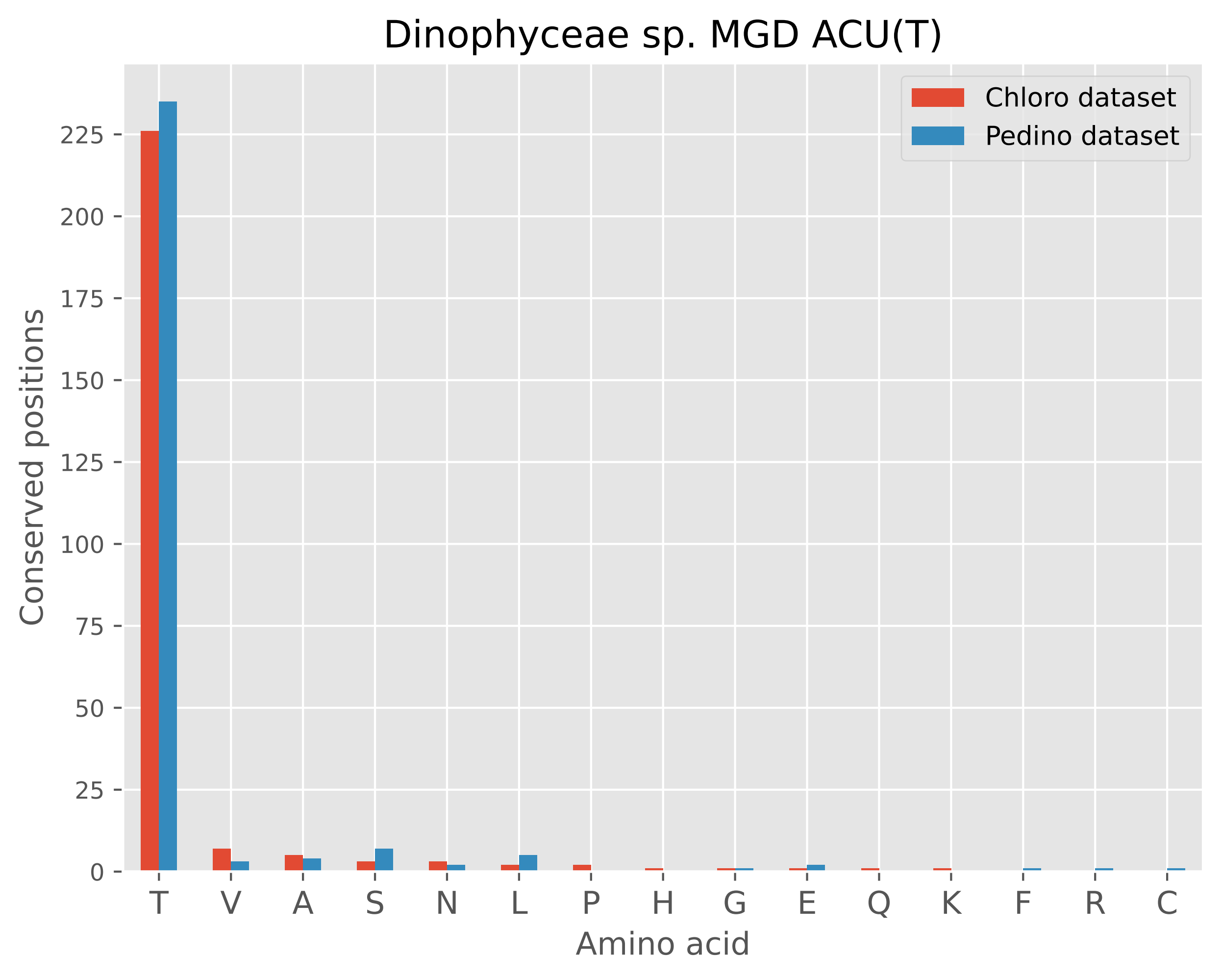

Dinophyceae sp. MGD ACU(T)
Chloro dataset
Pedino dataset
Conserved positions
0
25
50
75
100
125
150
175
200
225
T
V
A
S
N
L
P
H
G
E
Q
K
F
R
C
Amino acid

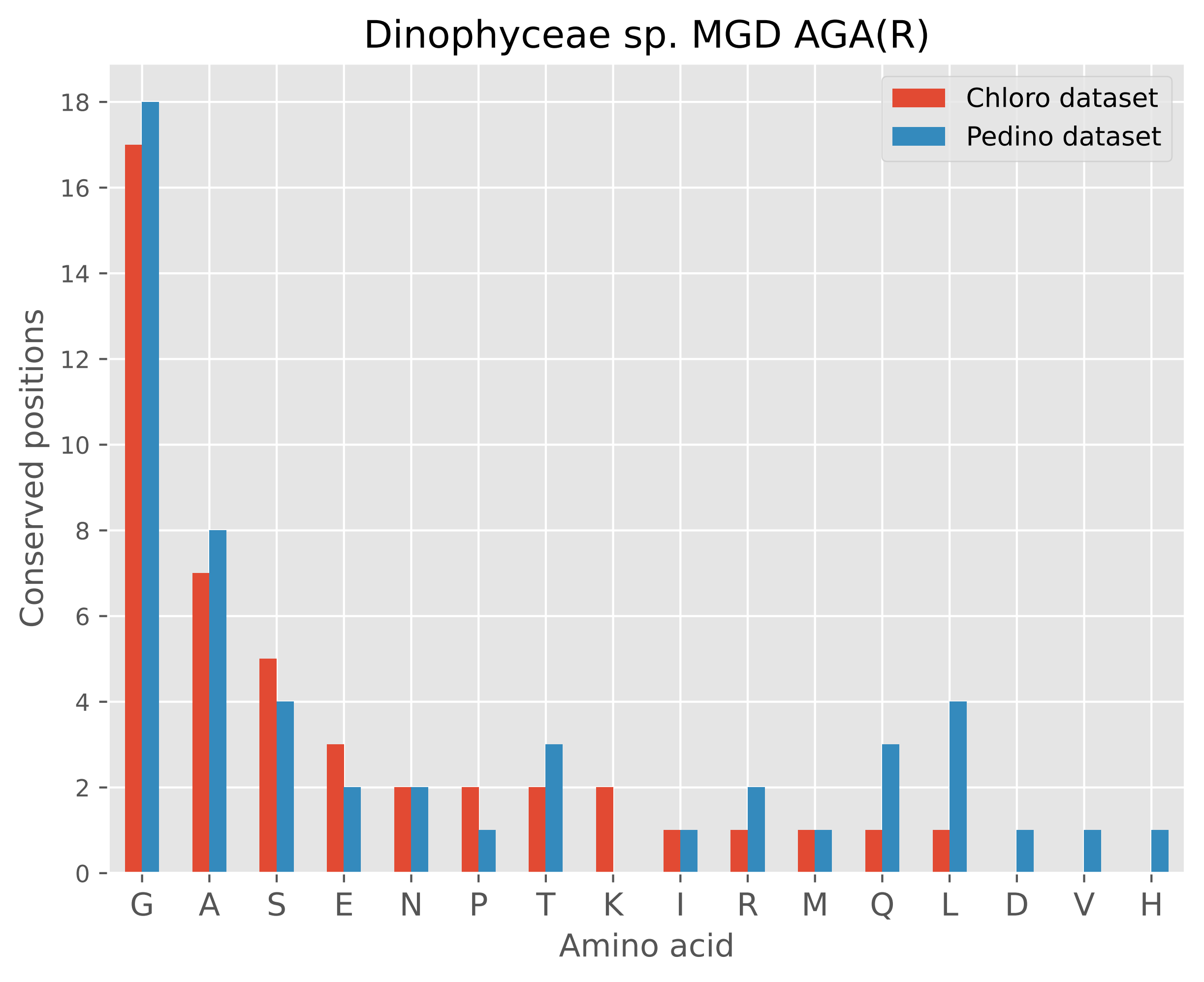

Dinophyceae sp. MGD AGA(R)
Chloro dataset
Pedino dataset
Conserved positions
0
2
4
6
8
10
12
14
16
18
G
A
S
E
N
P
T
K
I
R
M
Q
L
D
V
H
Amino acid

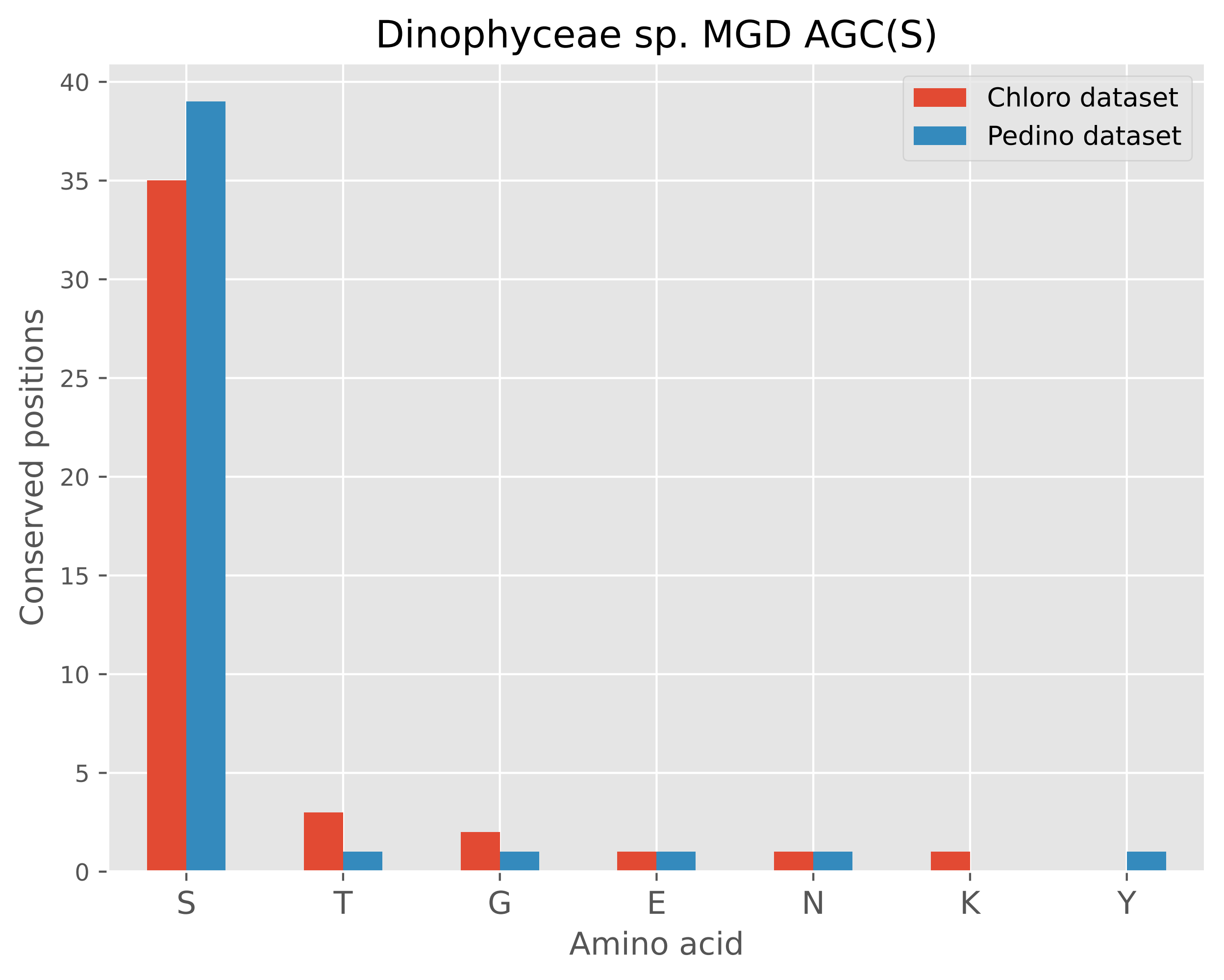

Dinophyceae sp. MGD AGC(S)
Conserved positions
Amino acid
0
5
10
15
20
25
30
35
40
S
T
G
E
N
K
Y
Chloro dataset
Pedino dataset

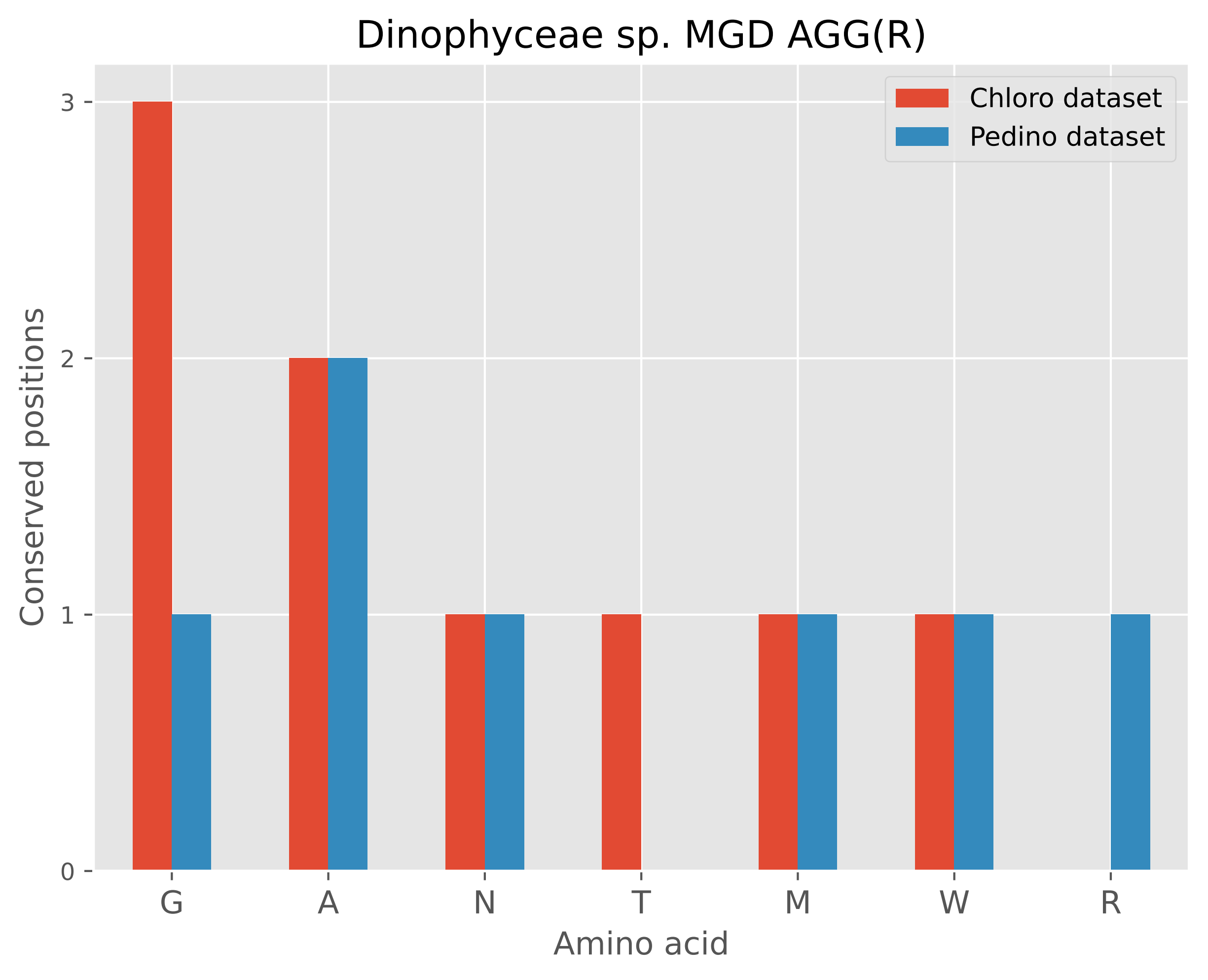
Dinophyceae sp. MGD AGG(R)
Chloro dataset
Pedino dataset
Conserved positions
0
1
2
3
G
A
N
T
M
W
R
Amino acid

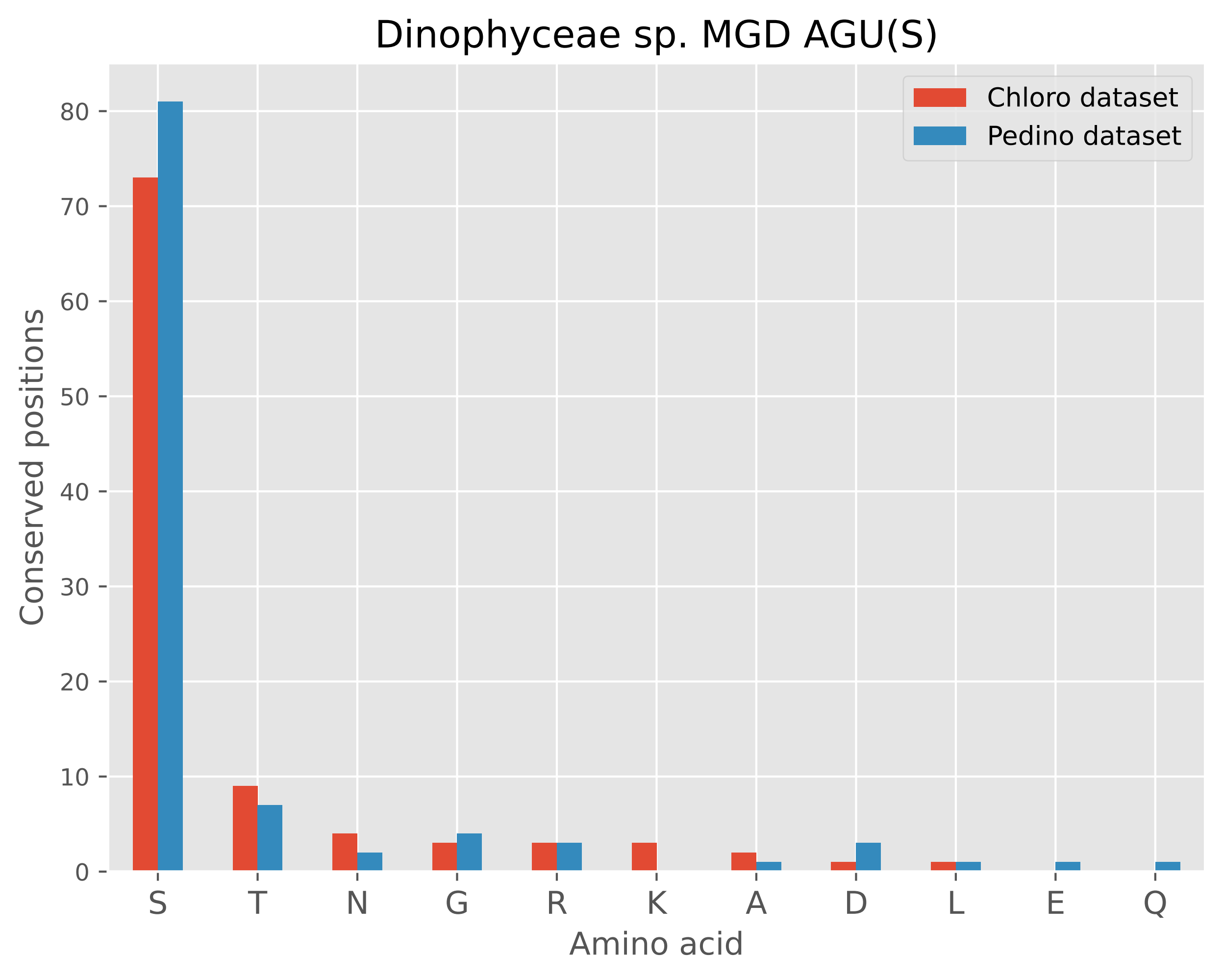
Dinophyceae sp. MGD AGU(S)
Chloro dataset
Pedino dataset
Conserved positions
0
10
20
30
40
50
60
70
80
S
T
N
G
R
K
A
D
L
E
Q
Amino acid

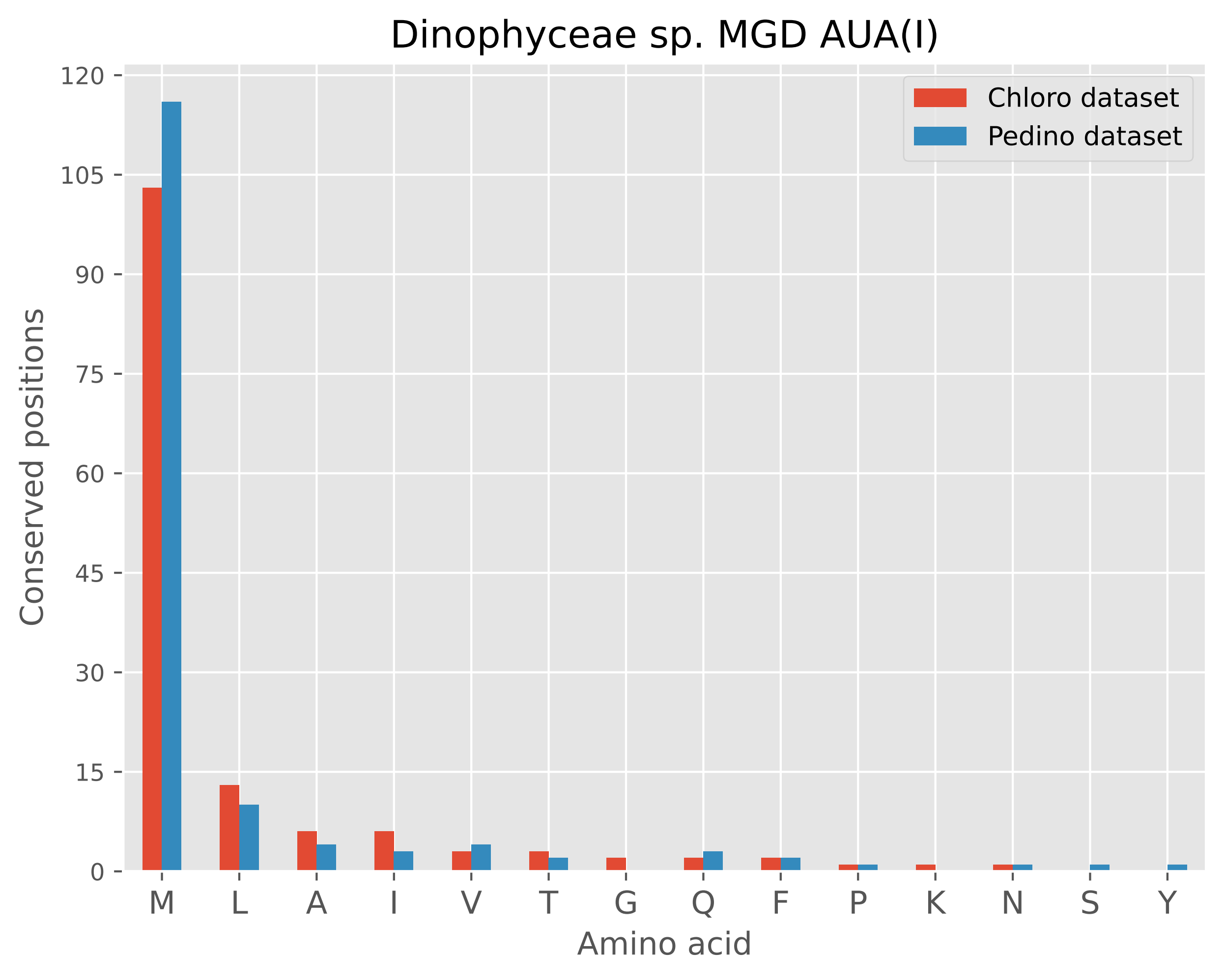
Dinophyceae sp. MGD AUA(I)
Chloro dataset
Pedino dataset
Conserved positions
0
15
30
45
60
75
90
105
120
M
L
A
I
V
T
G
Q
F
P
K
N
S
Y
Amino acid

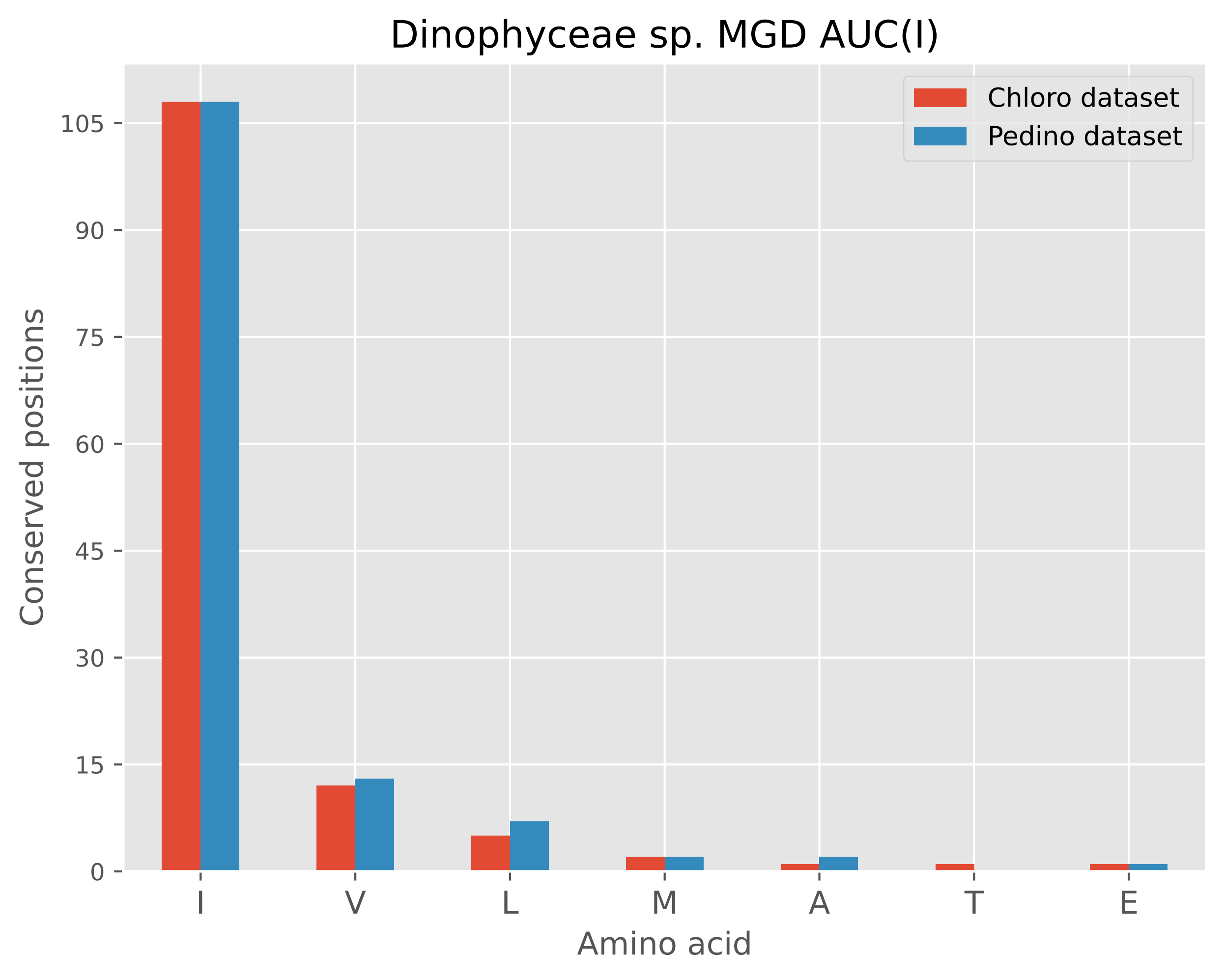

Dinophyceae sp. MGD AUC(I)
Chloro dataset
Pedino dataset
Conserved positions
105
90
75
60
45
30
15
0
I
V
L
M
A
T
E
Amino acid

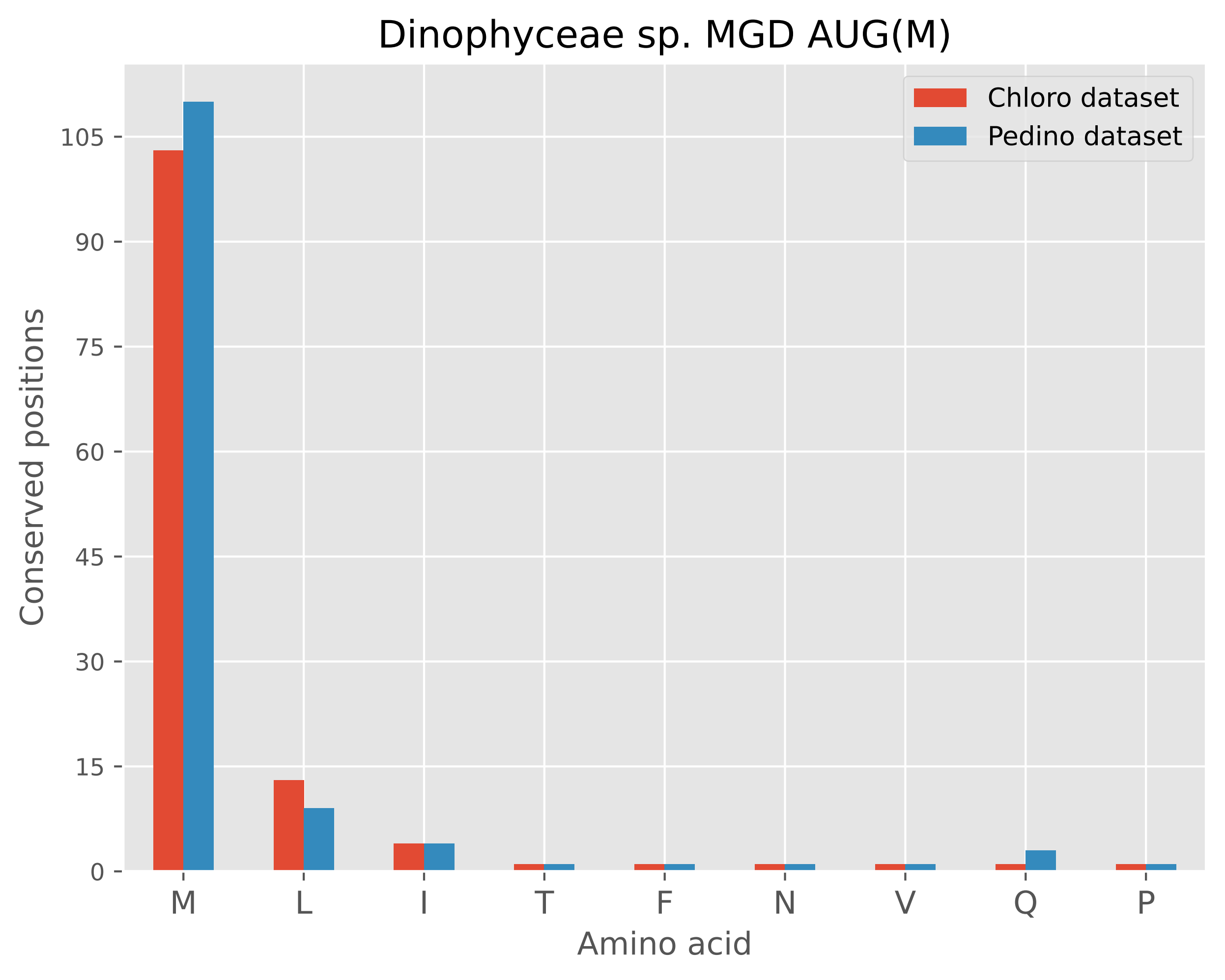

Dinophyceae sp. MGD AUG(M)
Chloro dataset
Pedino dataset
Conserved positions
0
15
30
45
60
75
90
105
M
L
I
T
F
N
V
Q
P
Amino acid

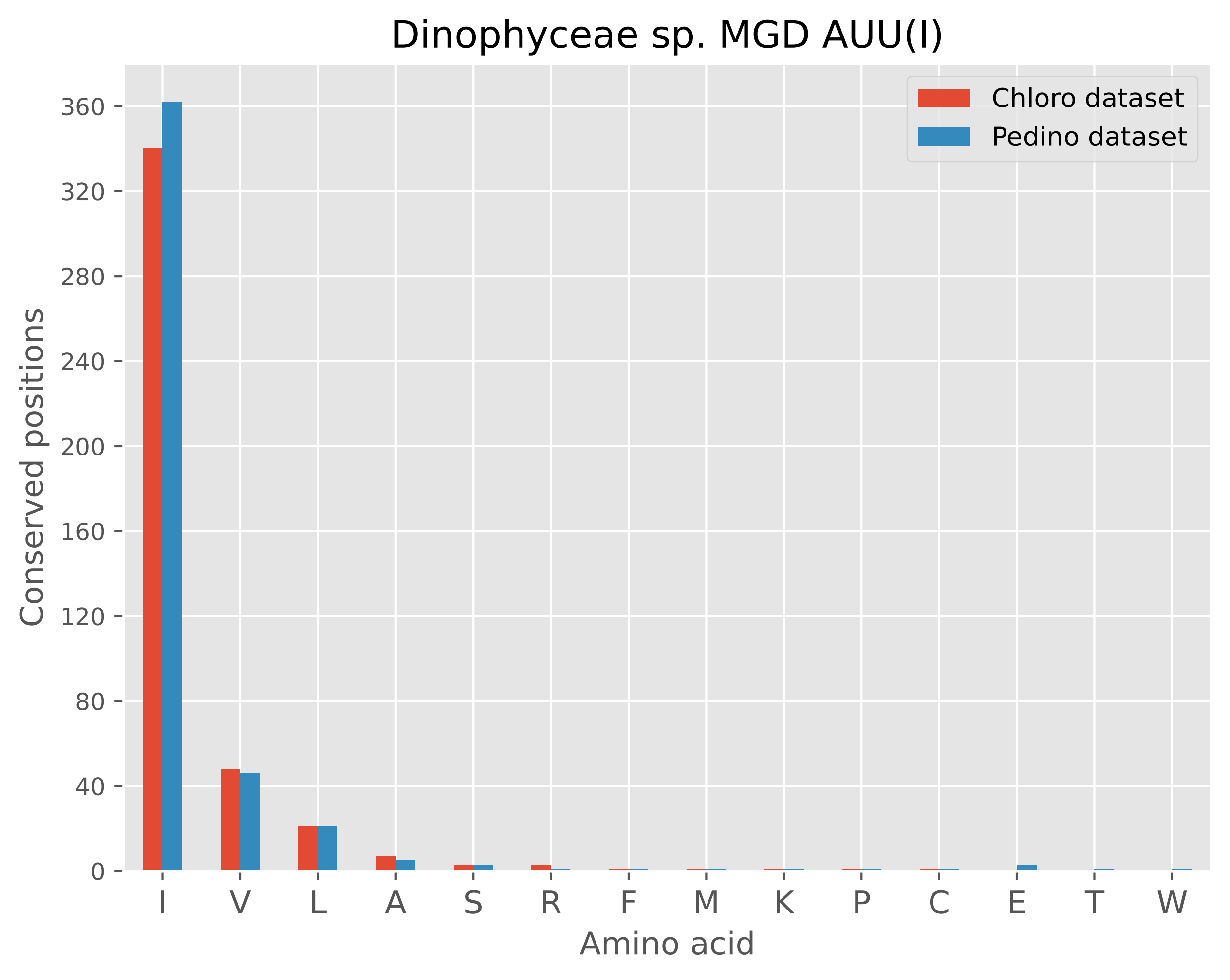

Dinophyceae sp. MGD AUU(I)
Chloro dataset
Pedino dataset
Conserved positions
0
40
80
120
160
200
240
280
320
360
I
V
L
A
S
R
F
M
K
P
C
E
T
W
Amino acid

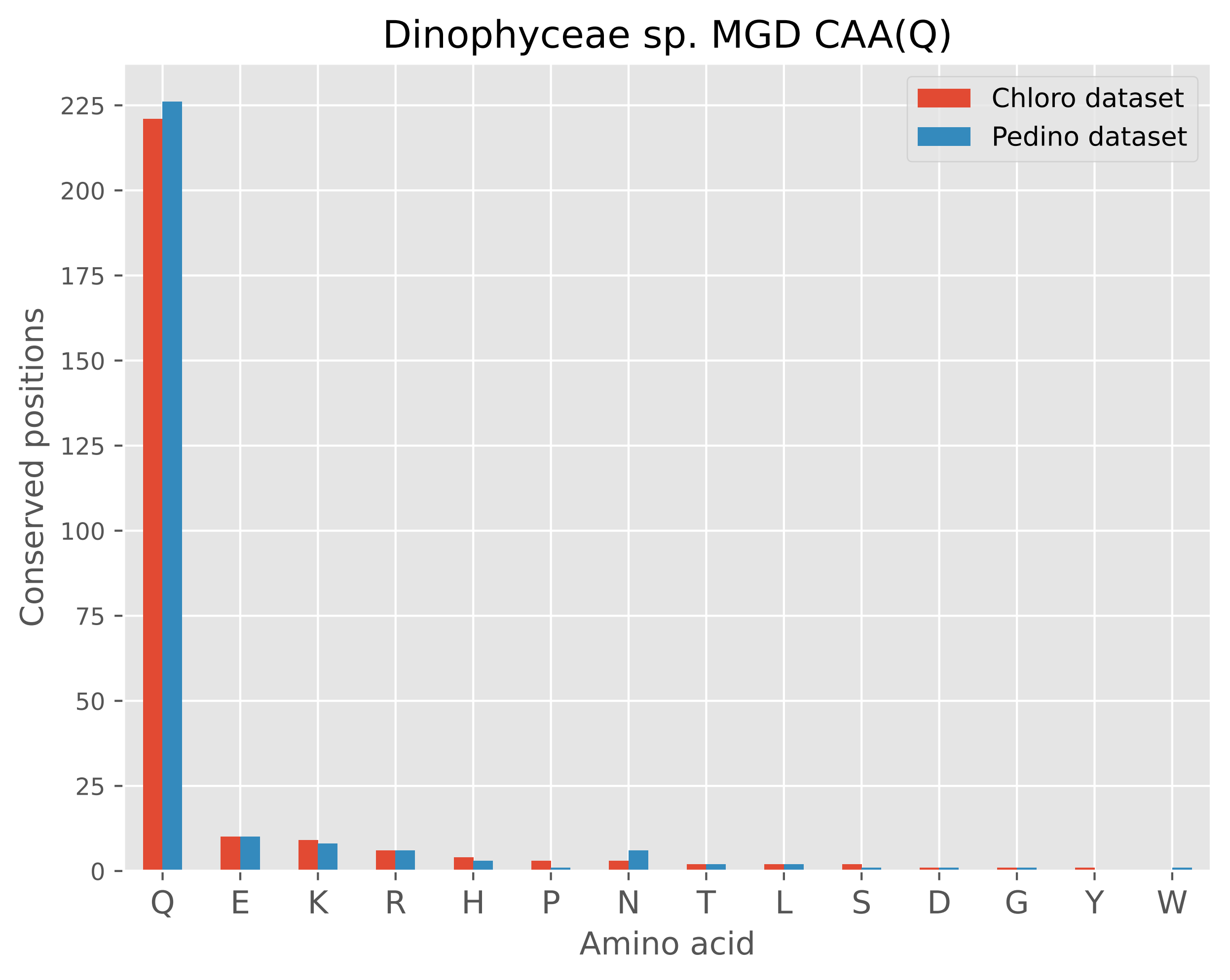

Dinophyceae sp. MGD CAA(Q)
Chloro dataset
Pedino dataset
Conserved positions
0
25
50
75
100
125
150
175
200
225
Q
E
K
R
H
P
N
T
L
S
D
G
Y
W
Amino acid

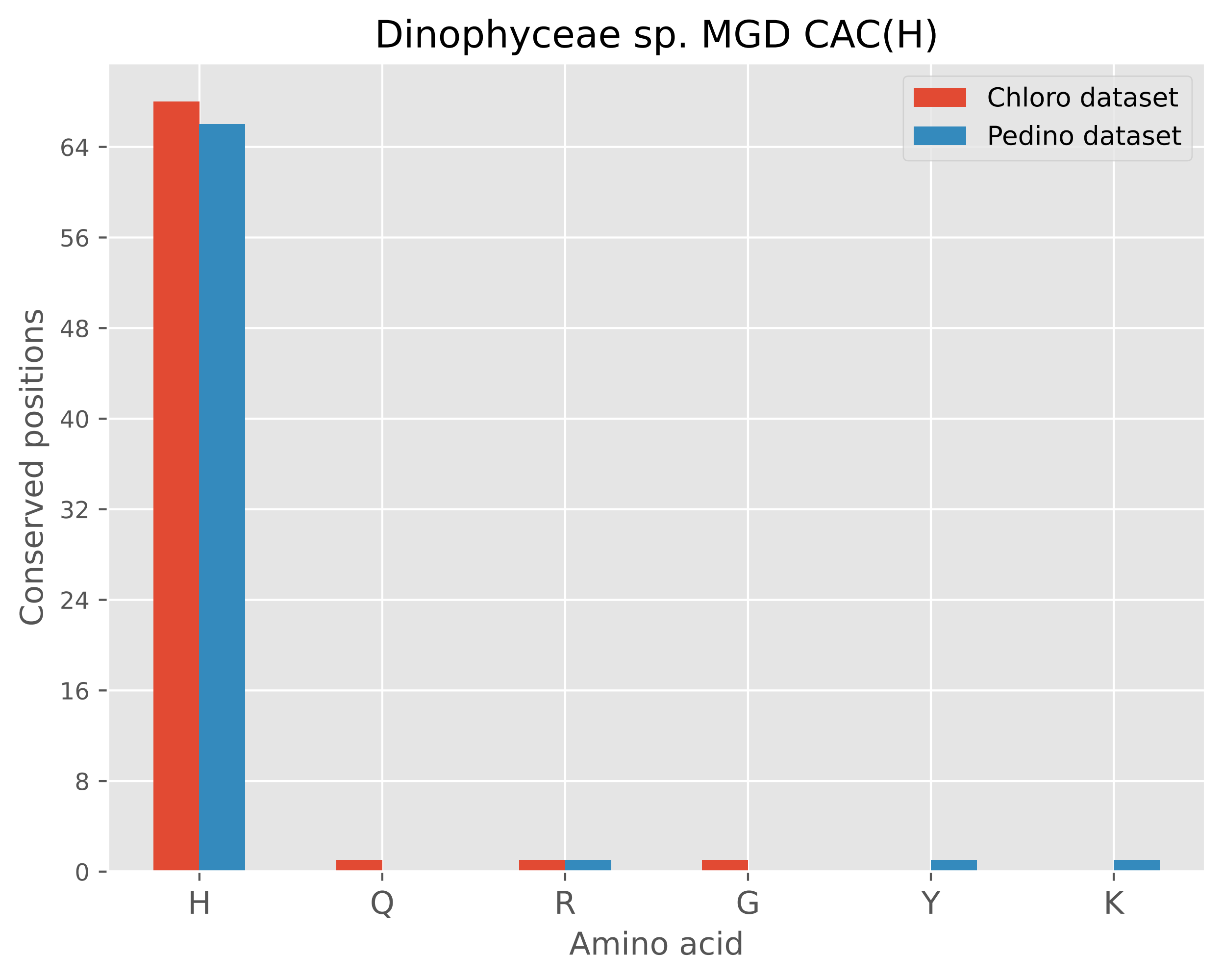

Dinophyceae sp. MGD CAC(H)
Chloro dataset
Pedino dataset
Conserved positions
0
8
16
24
32
40
48
56
64
H
Q
R
G
Y
K
Amino acid

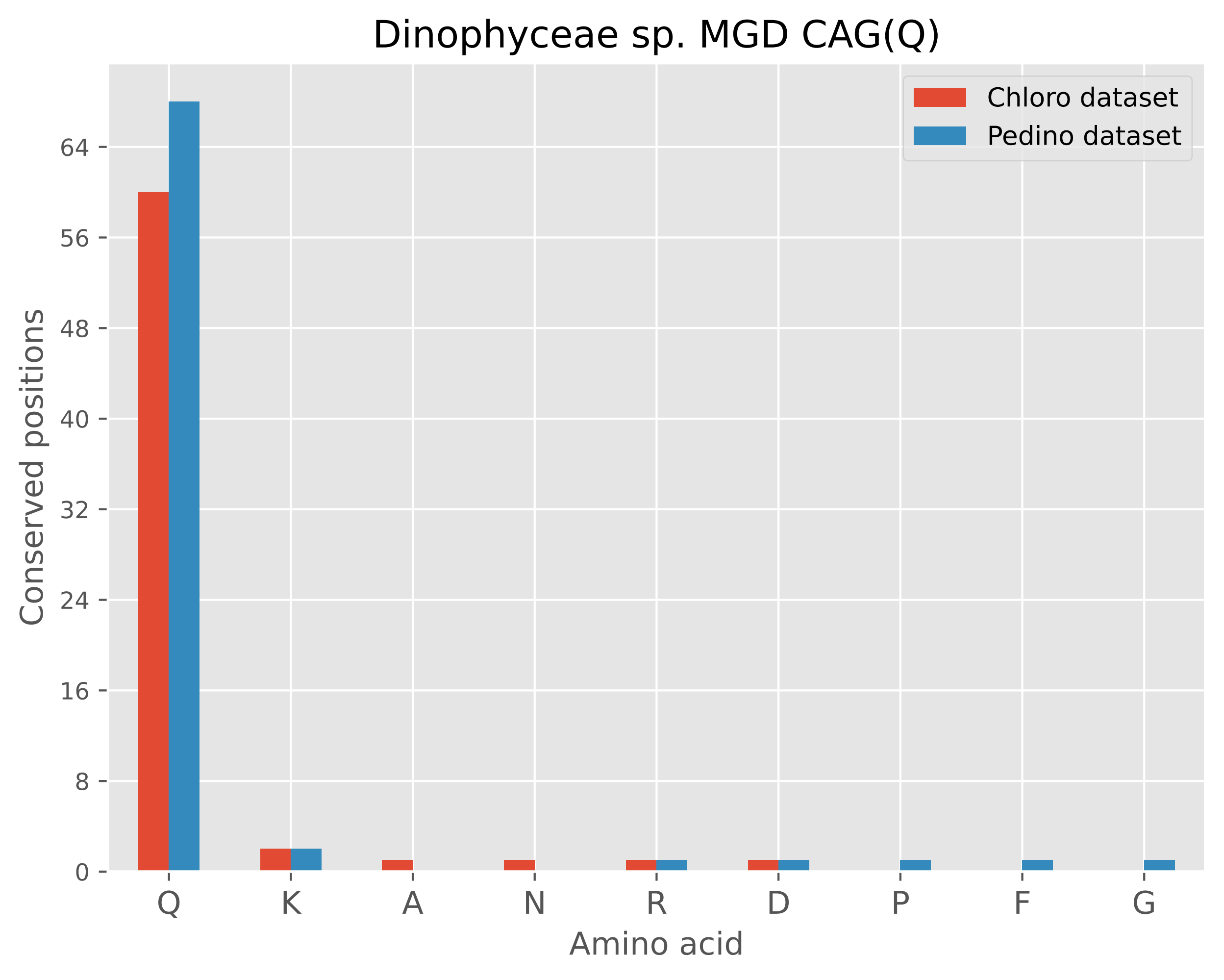
Dinophyceae sp. MGD CAG(Q)
Chloro dataset
Pedino dataset
Conserved positions
0
8
16
24
32
40
48
56
64
Q
K
A
N
R
D
P
F
G
Amino acid

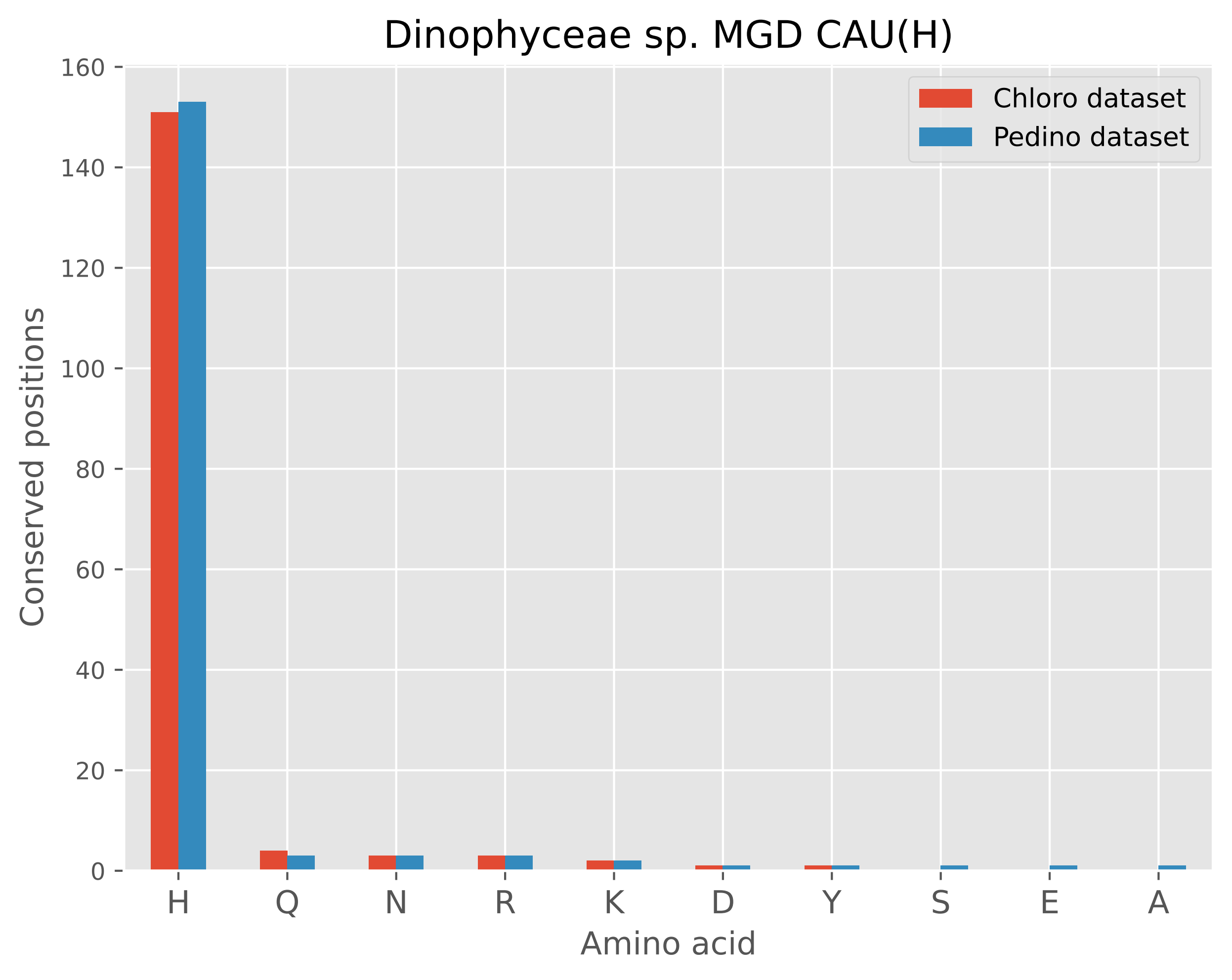

Dinophyceae sp. MGD CAU(H)
Chloro dataset
Pedino dataset
Conserved positions
0
20
40
60
80
100
120
140
160
H
Q
N
R
K
D
Y
S
E
A
Amino acid

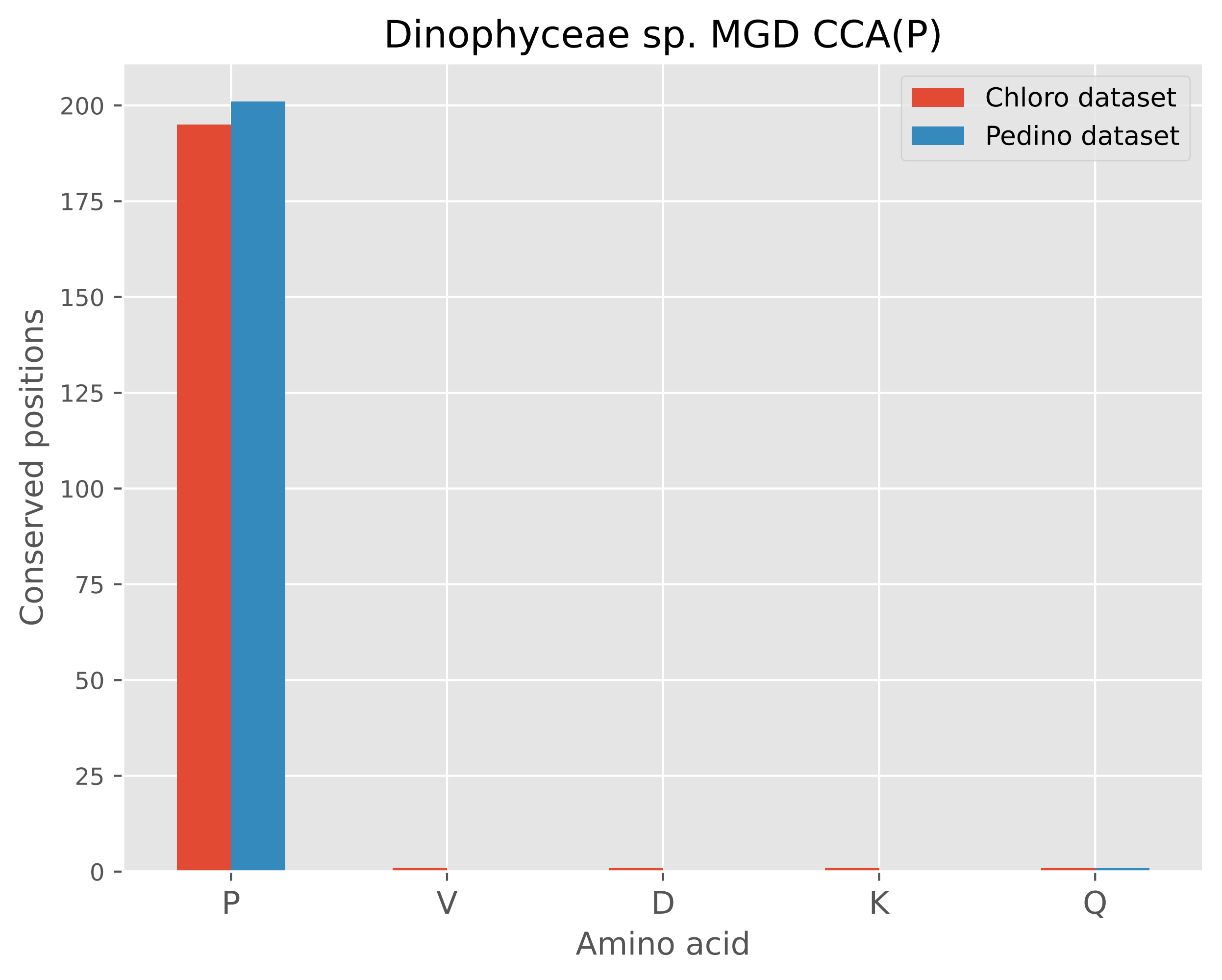

Dinophyceae sp. MGD CCA(P)
Chloro dataset
Pedino dataset
Conserved positions
0
25
50
75
100
125
150
175
200
P
V
D
K
Q
Amino acid

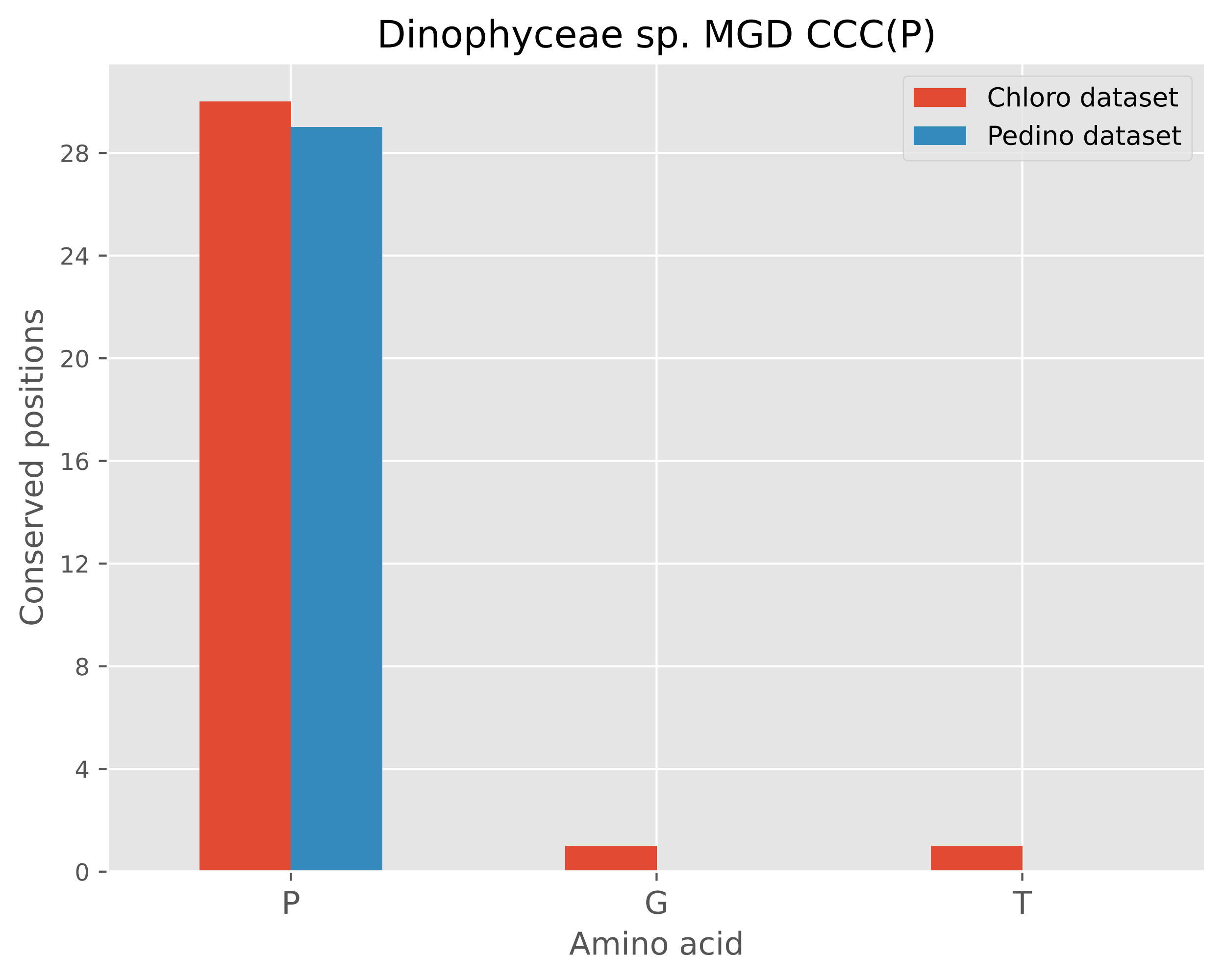

Dinophyceae sp. MGD CCC(P)
Chloro dataset
Pedino dataset
Conserved positions
0
4
8
12
16
20
24
28
P
G
T
Amino acid

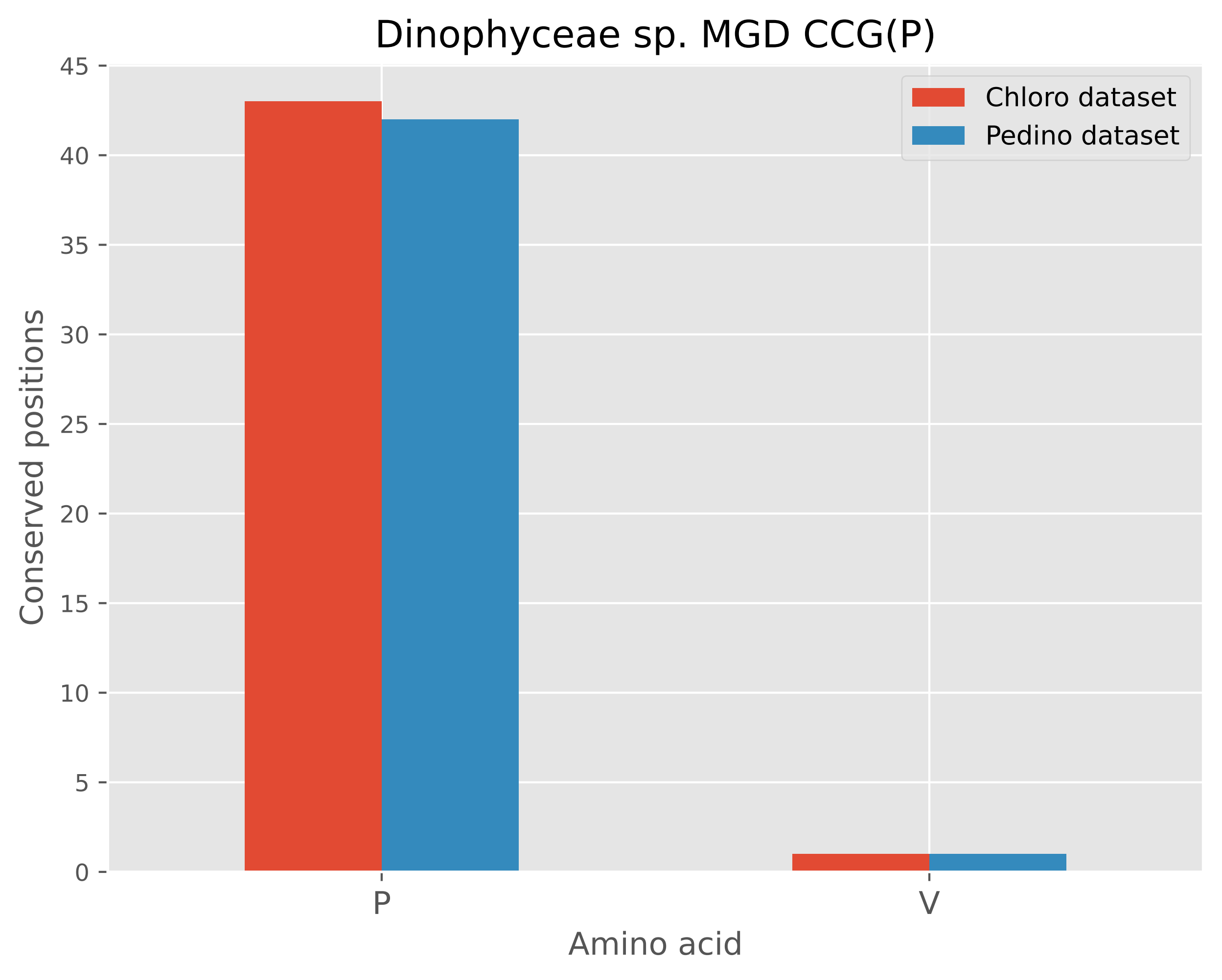
Dinophyceae sp. MGD CCG(P)
Chloro dataset
Pedino dataset
Conserved positions
Amino acid
P
V
0
5
10
15
20
25
30
35
40
45

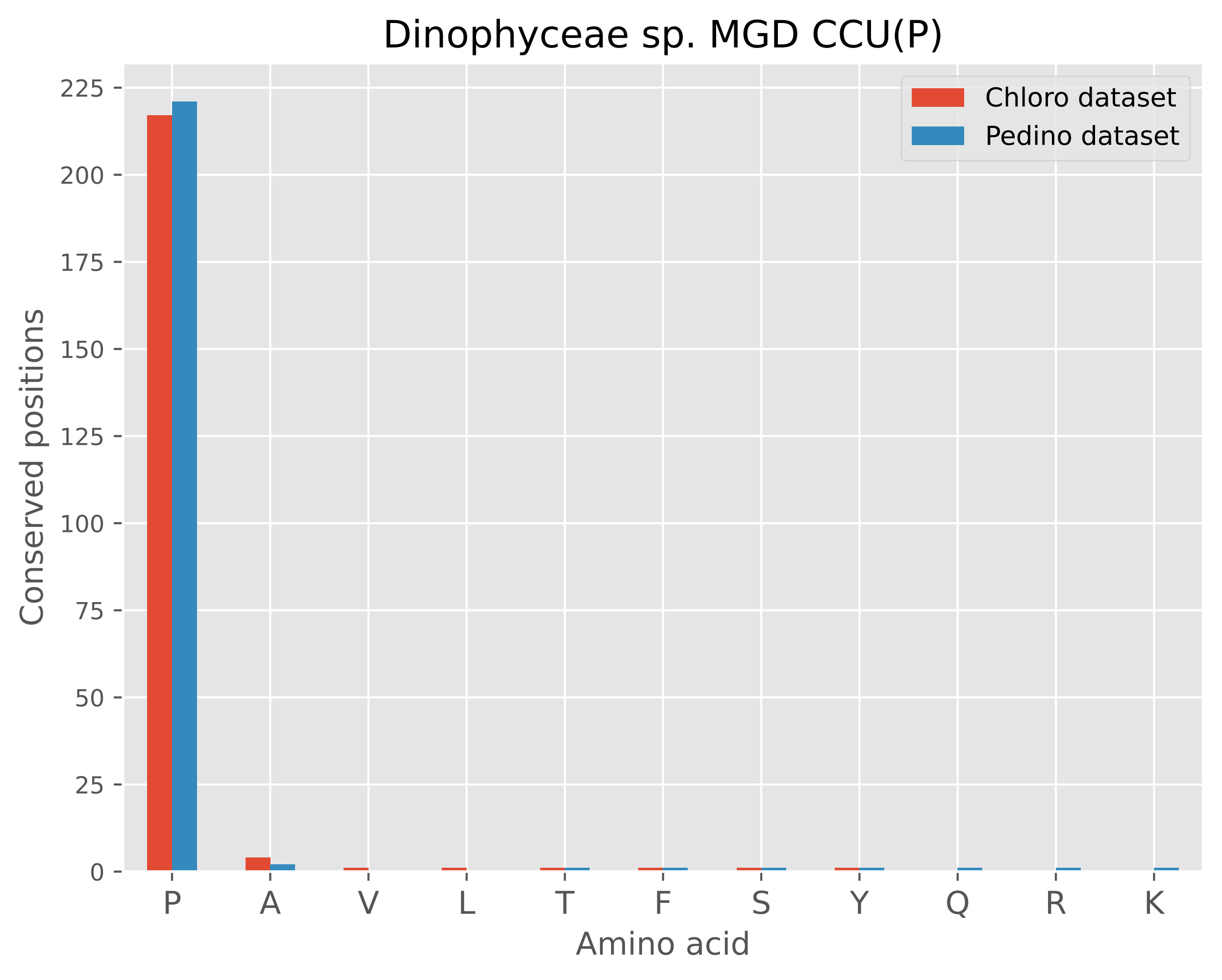

Dinophyceae sp. MGD CCU(P)
Chloro dataset
Pedino dataset
Conserved positions
225
200
175
150
125
100
75
50
25
0
P
A
V
L
T
F
S
Y
Q
R
K
Amino acid

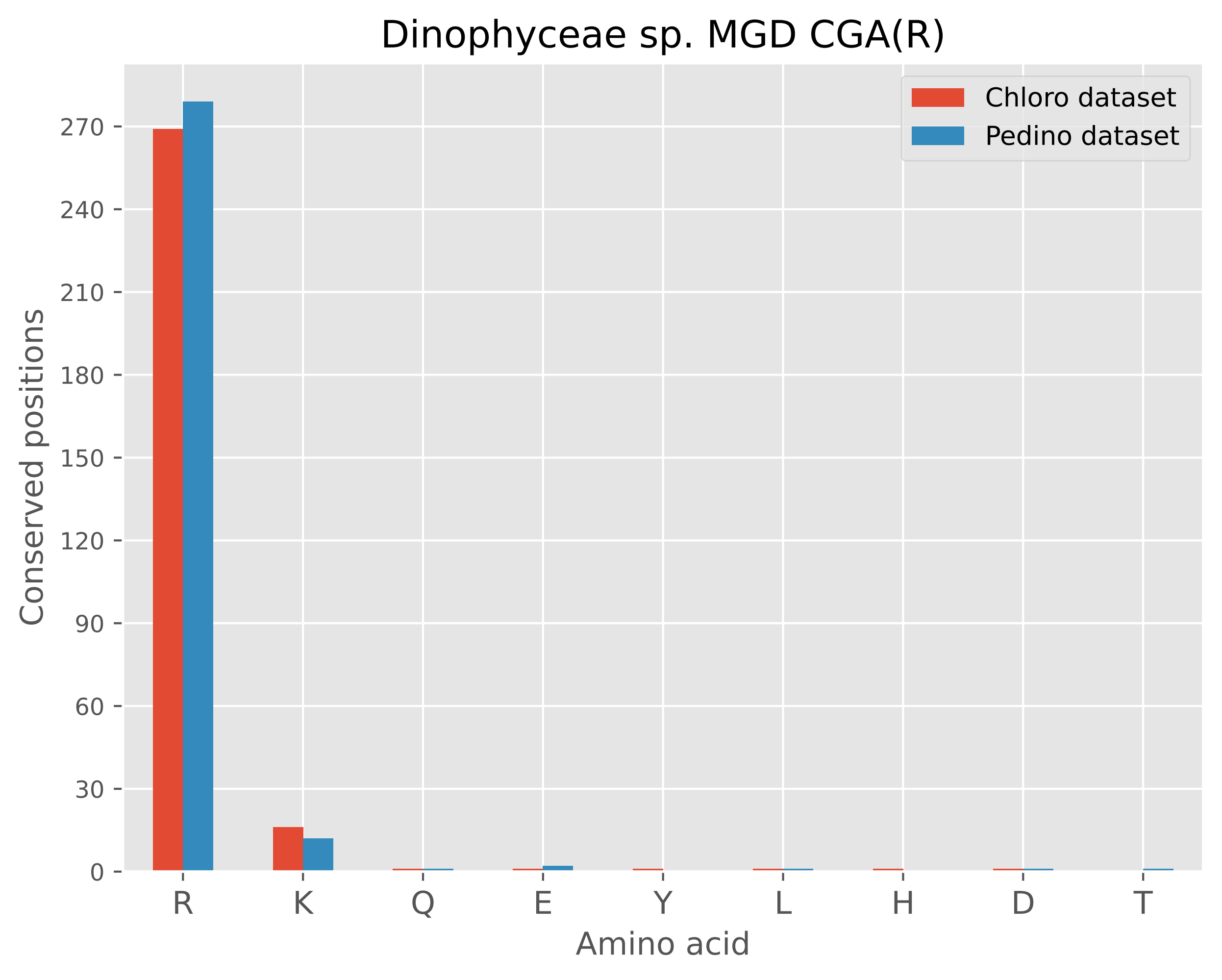

Dinophyceae sp. MGD CGA(R)
Chloro dataset
Pedino dataset
Conserved positions
0
30
60
90
120
150
180
210
240
270
R
K
Q
E
Y
L
H
D
T
Amino acid

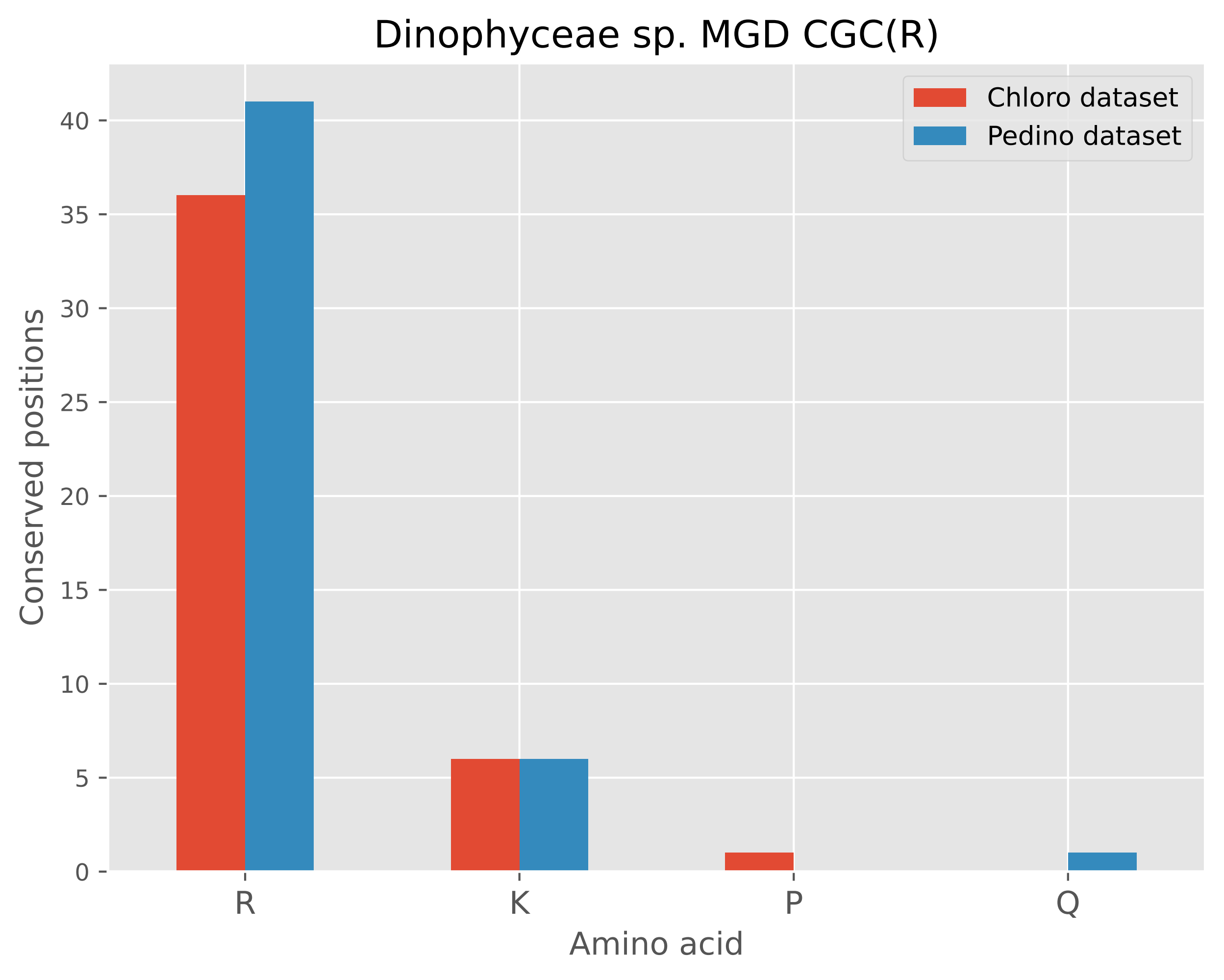

Dinophyceae sp. MGD CGC(R)
Chloro dataset
Pedino dataset
Conserved positions
0
5
10
15
20
25
30
35
40
R
K
P
Q
Amino acid

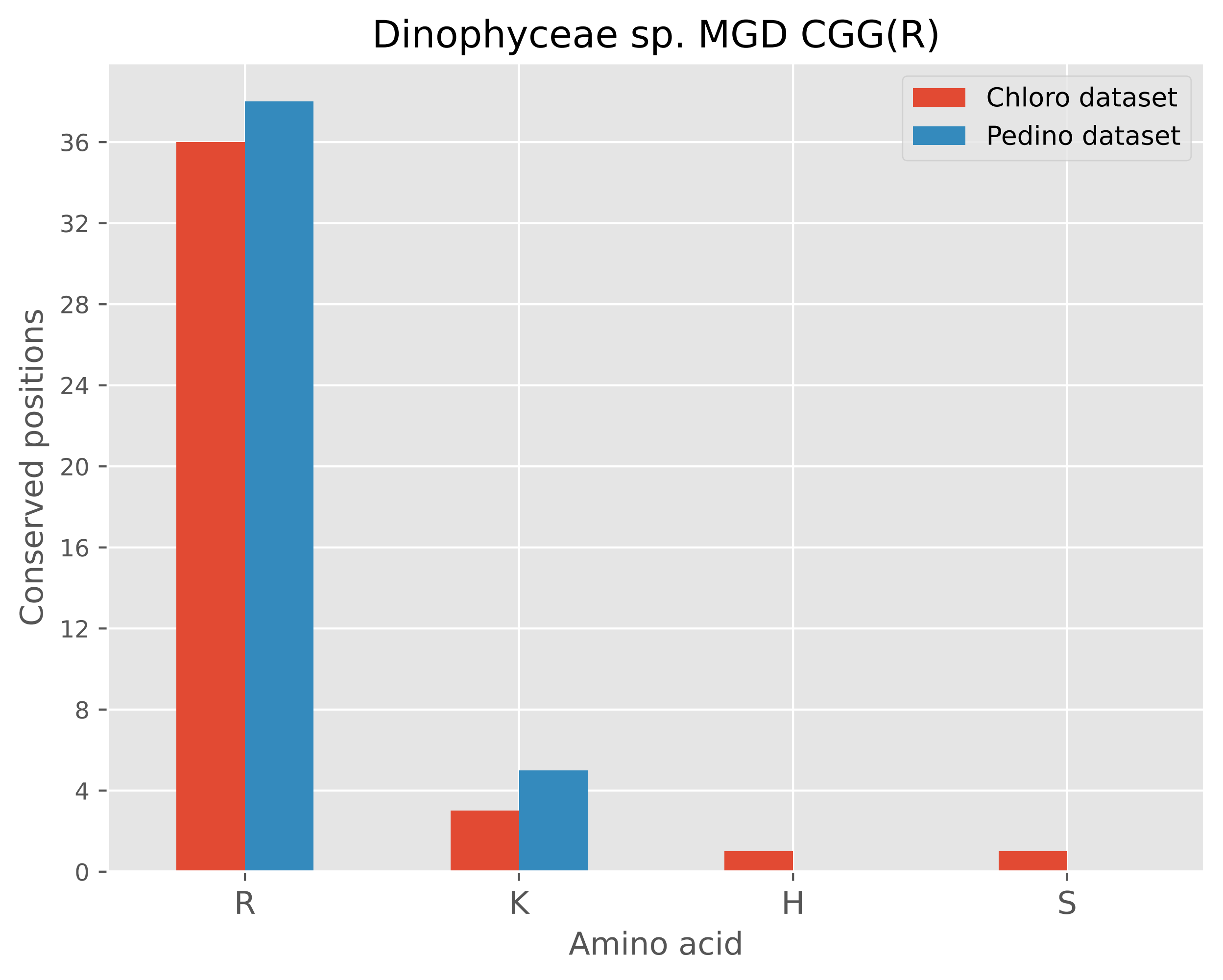

Dinophyceae sp. MGD CGG(R)
Chloro dataset
Pedino dataset
Conserved positions
0
4
8
12
16
20
24
28
32
36
R
K
H
S
Amino acid

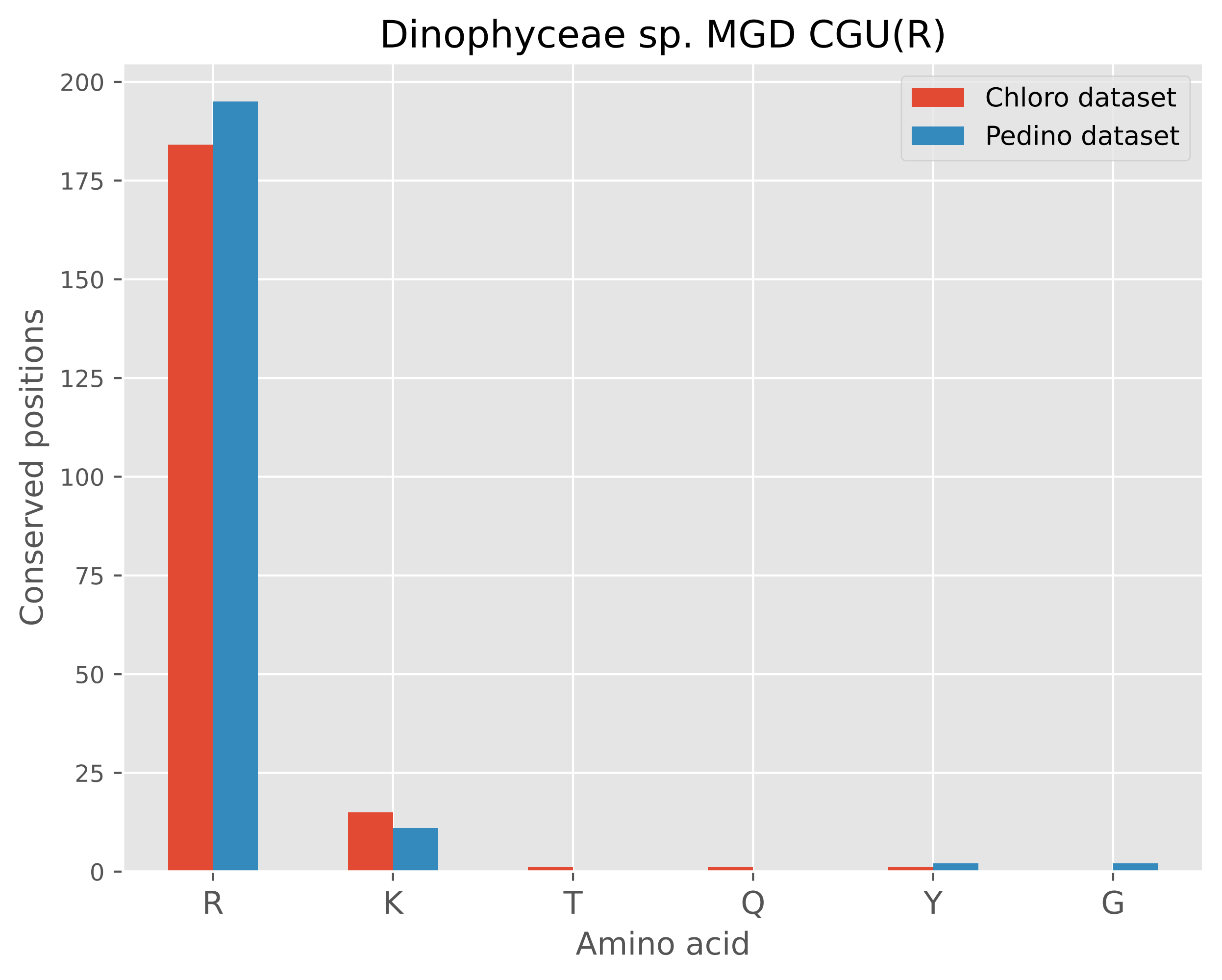
Dinophyceae sp. MGD CGU(R)
Chloro dataset
Pedino dataset
Conserved positions
0
25
50
75
100
125
150
175
200
R
K
T
Q
Y
G
Amino acid

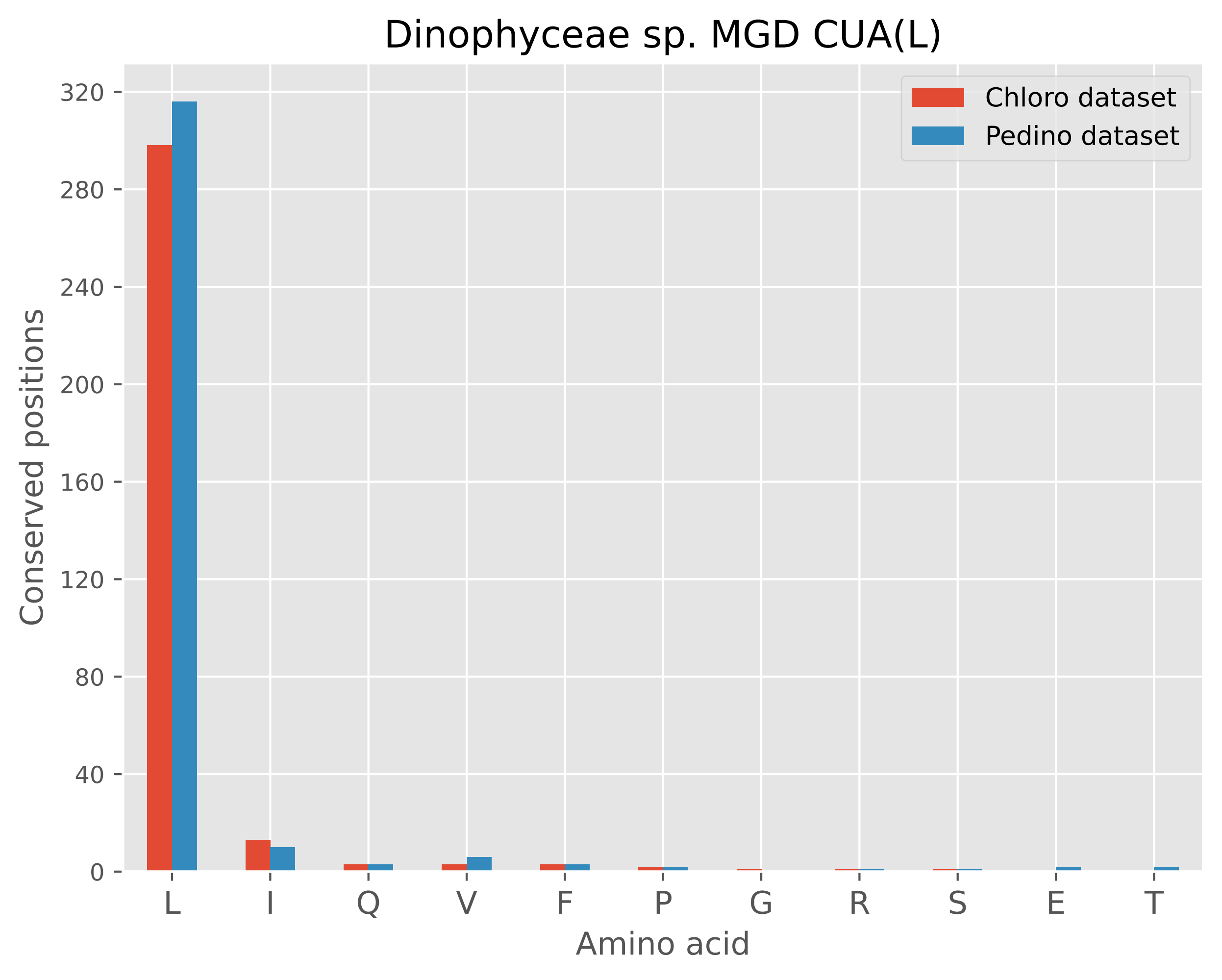

Dinophyceae sp. MGD CUA(L)
Chloro dataset
Pedino dataset
Conserved positions
320
280
240
200
160
120
80
40
0
L
I
Q
V
F
P
G
R
S
E
T
Amino acid

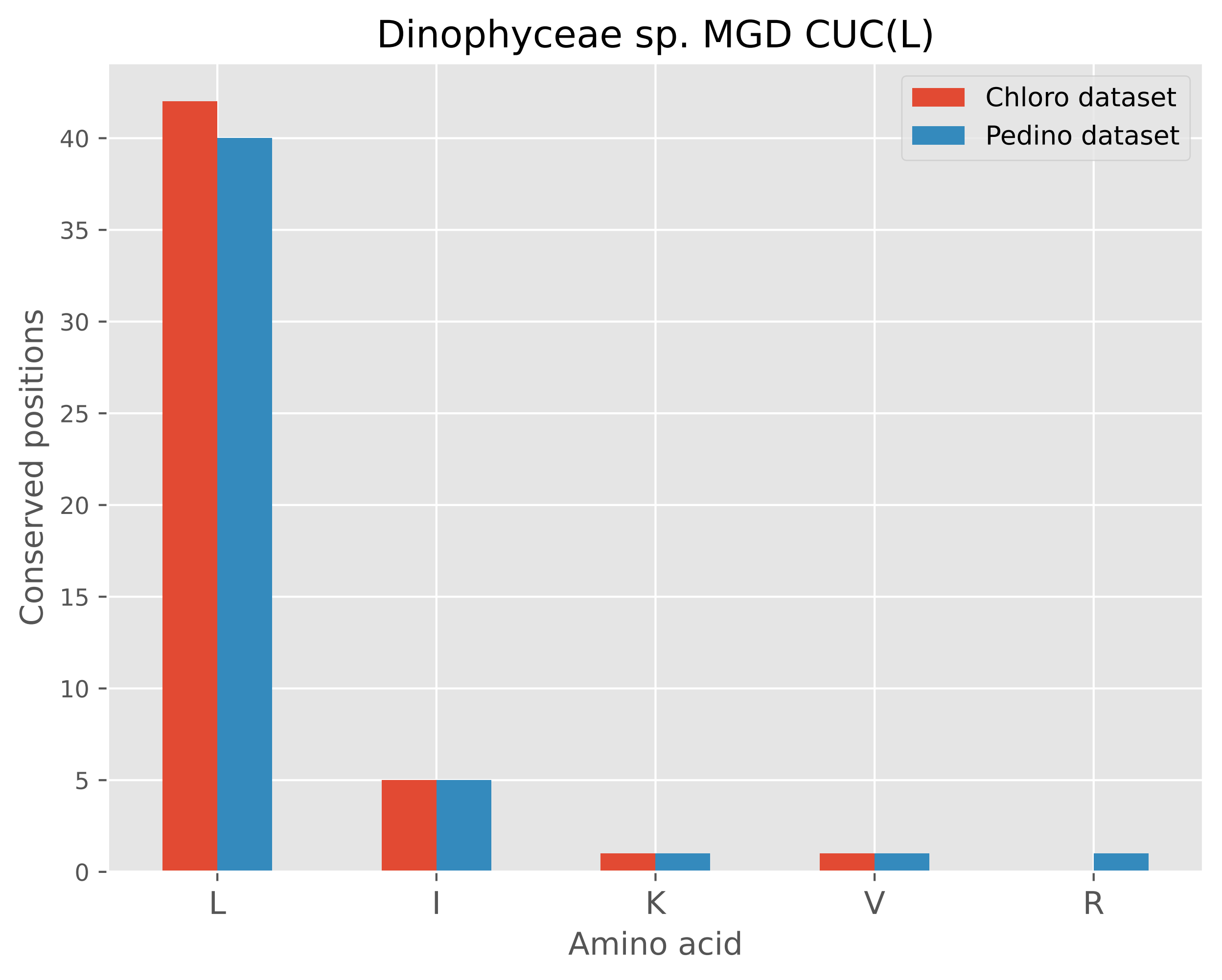

Dinophyceae sp. MGD CUC(L)
Chloro dataset
Pedino dataset
Conserved positions
0
5
10
15
20
25
30
35
40
L
I
K
V
R
Amino acid

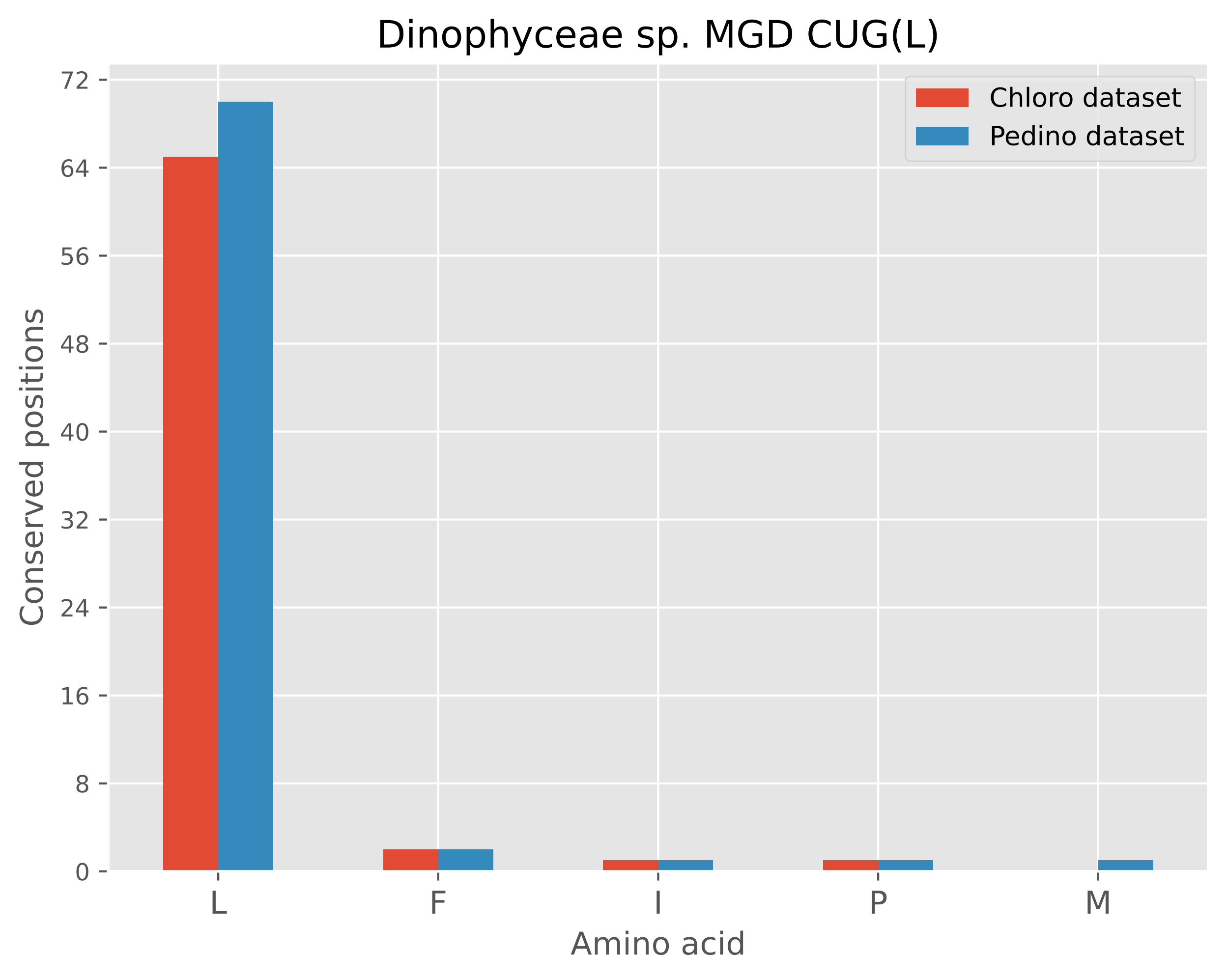

Dinophyceae sp. MGD CUG(L)
Chloro dataset
Pedino dataset
Conserved positions
Amino acid
L
F
I
P
M
0
8
16
24
32
40
48
56
64
72

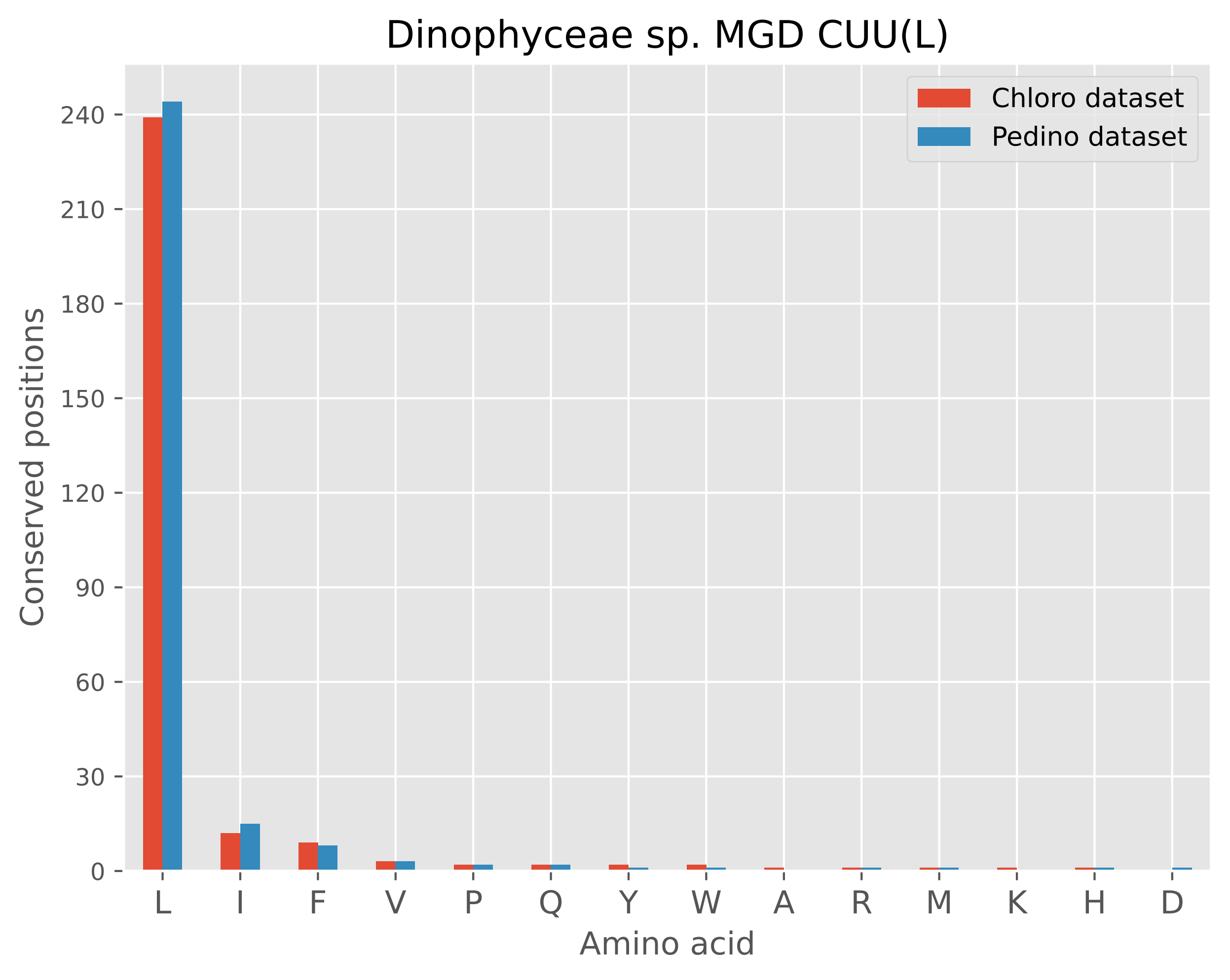

Dinophyceae sp. MGD CUU(L)
Chloro dataset
Pedino dataset
Conserved positions
0
30
60
90
120
150
180
210
240
L
I
F
V
P
Q
Y
W
A
R
M
K
H
D
Amino acid

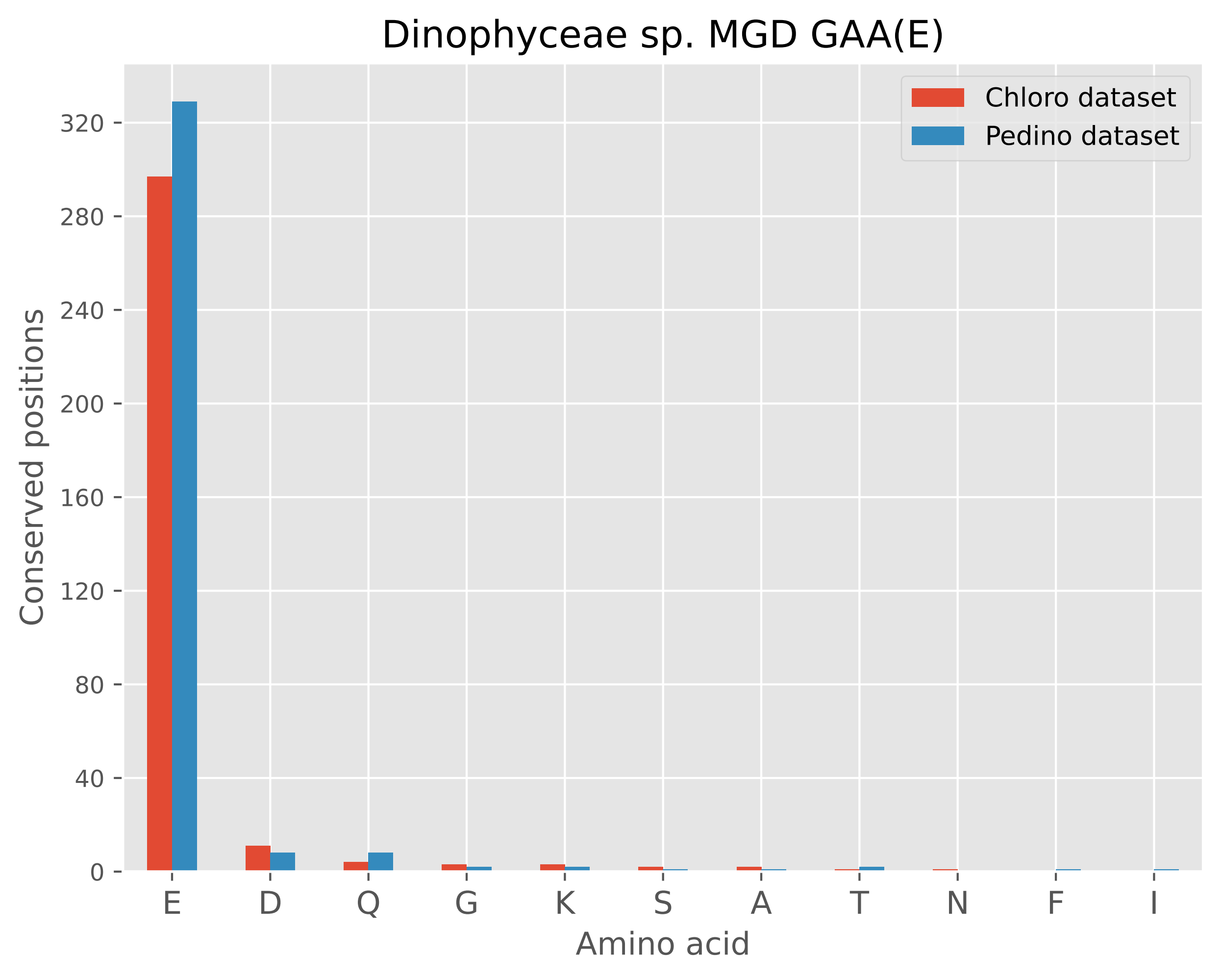

Dinophyceae sp. MGD GAA(E)
Chloro dataset
Pedino dataset
Conserved positions
0
40
80
120
160
200
240
280
320
E
D
Q
G
K
S
A
T
N
F
I
Amino acid

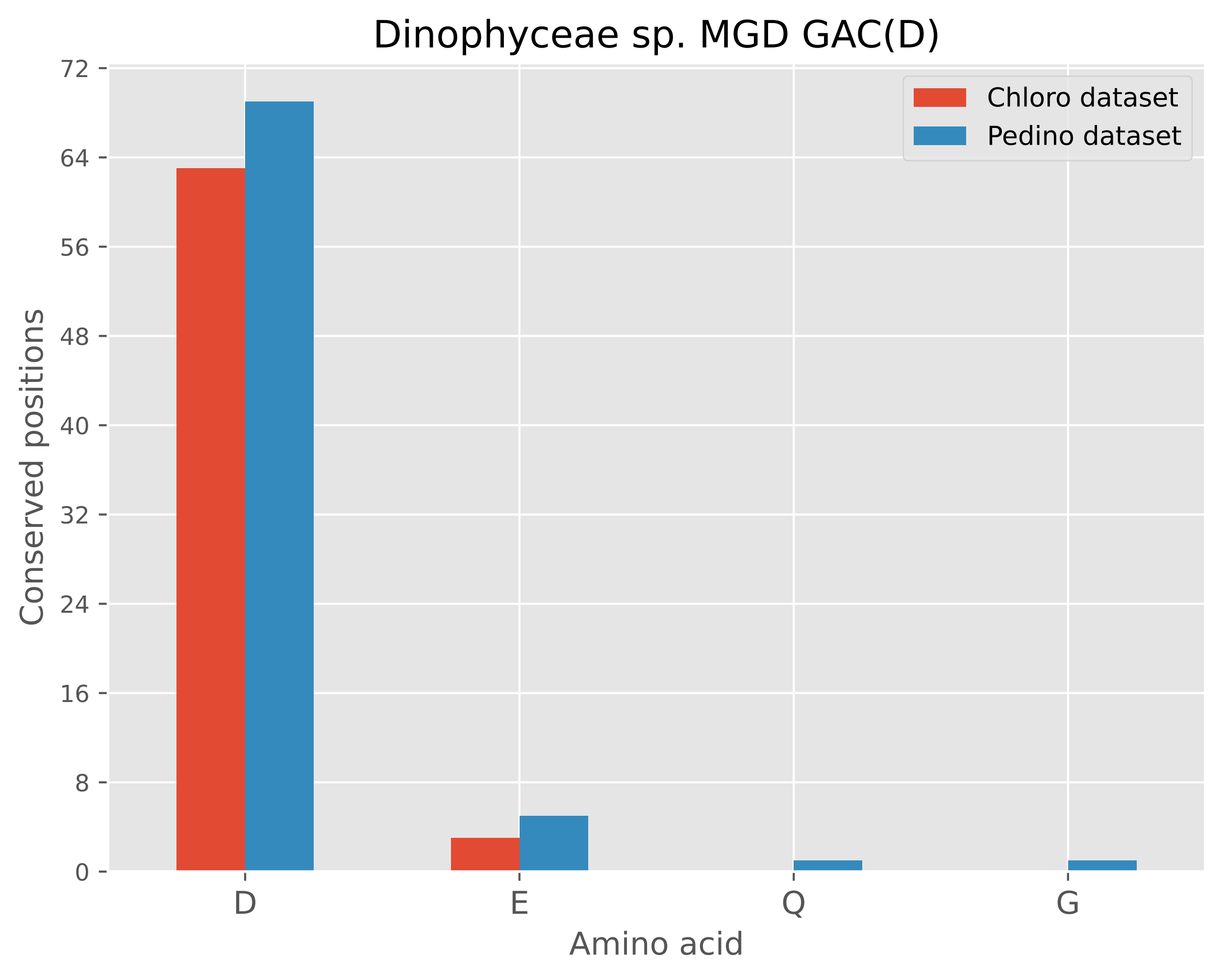

Dinophyceae sp. MGD GAC(D)
Chloro dataset
Pedino dataset
Conserved positions
Amino acid
0
8
16
24
32
40
48
56
64
72
D
E
Q
G

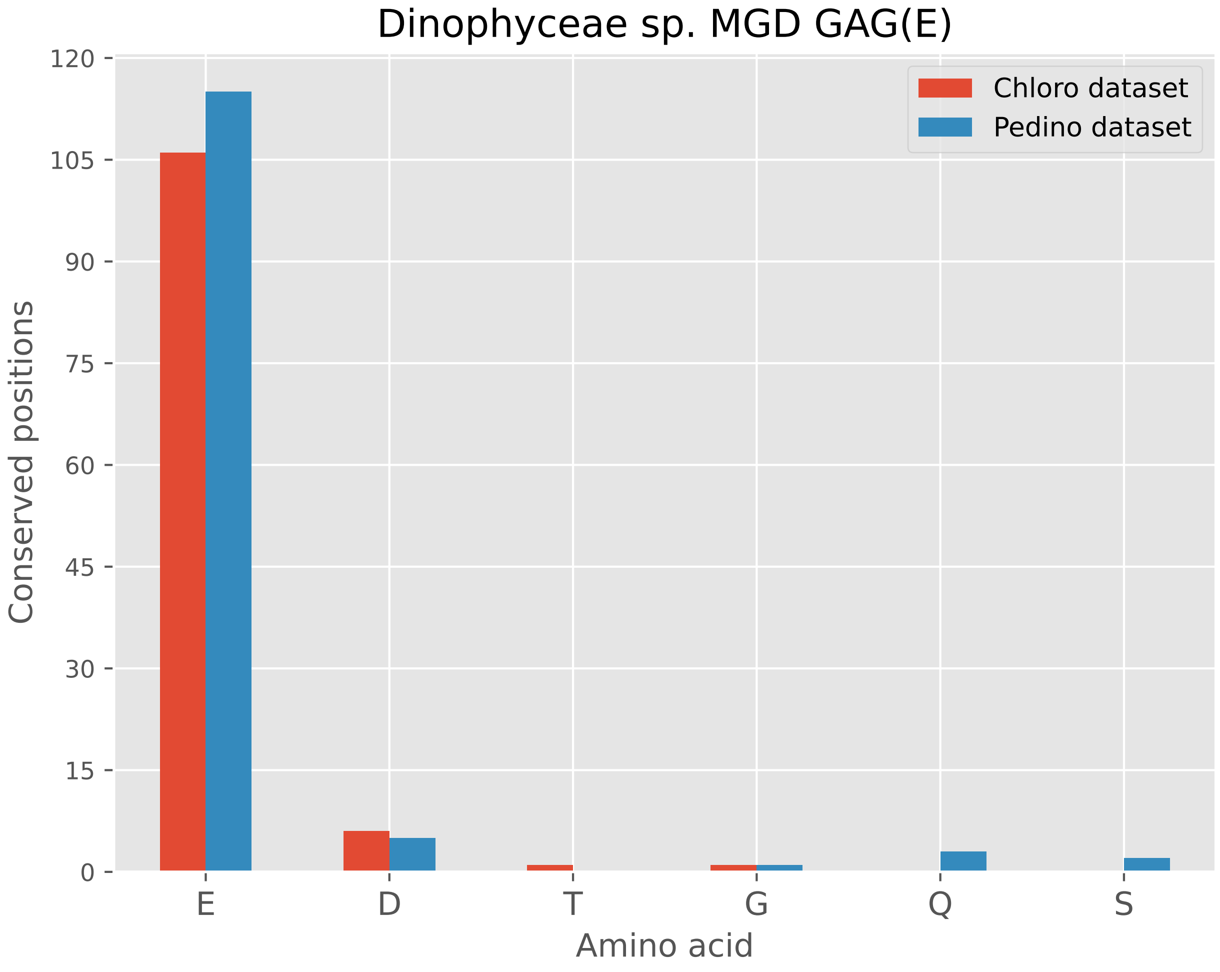

Dinophyceae sp. MGD GAG(E)
Chloro dataset
Pedino dataset
Conserved positions
0
15
30
45
60
75
90
105
120
E
D
T
G
Q
S
Amino acid

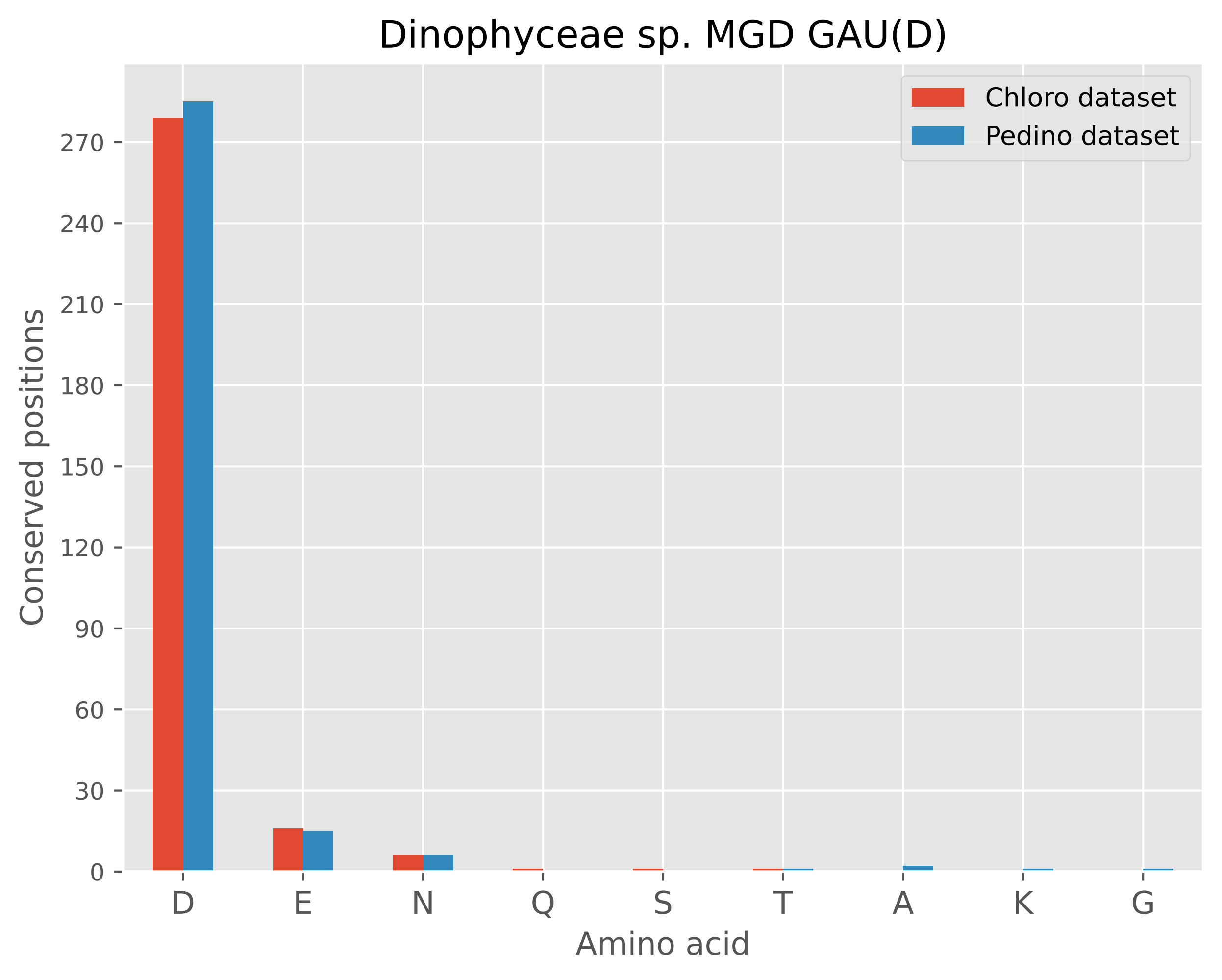

Dinophyceae sp. MGD GAU(D)
Chloro dataset
Pedino dataset
Conserved positions
0
30
60
90
120
150
180
210
240
270
D
E
N
Q
S
T
A
K
G
Amino acid

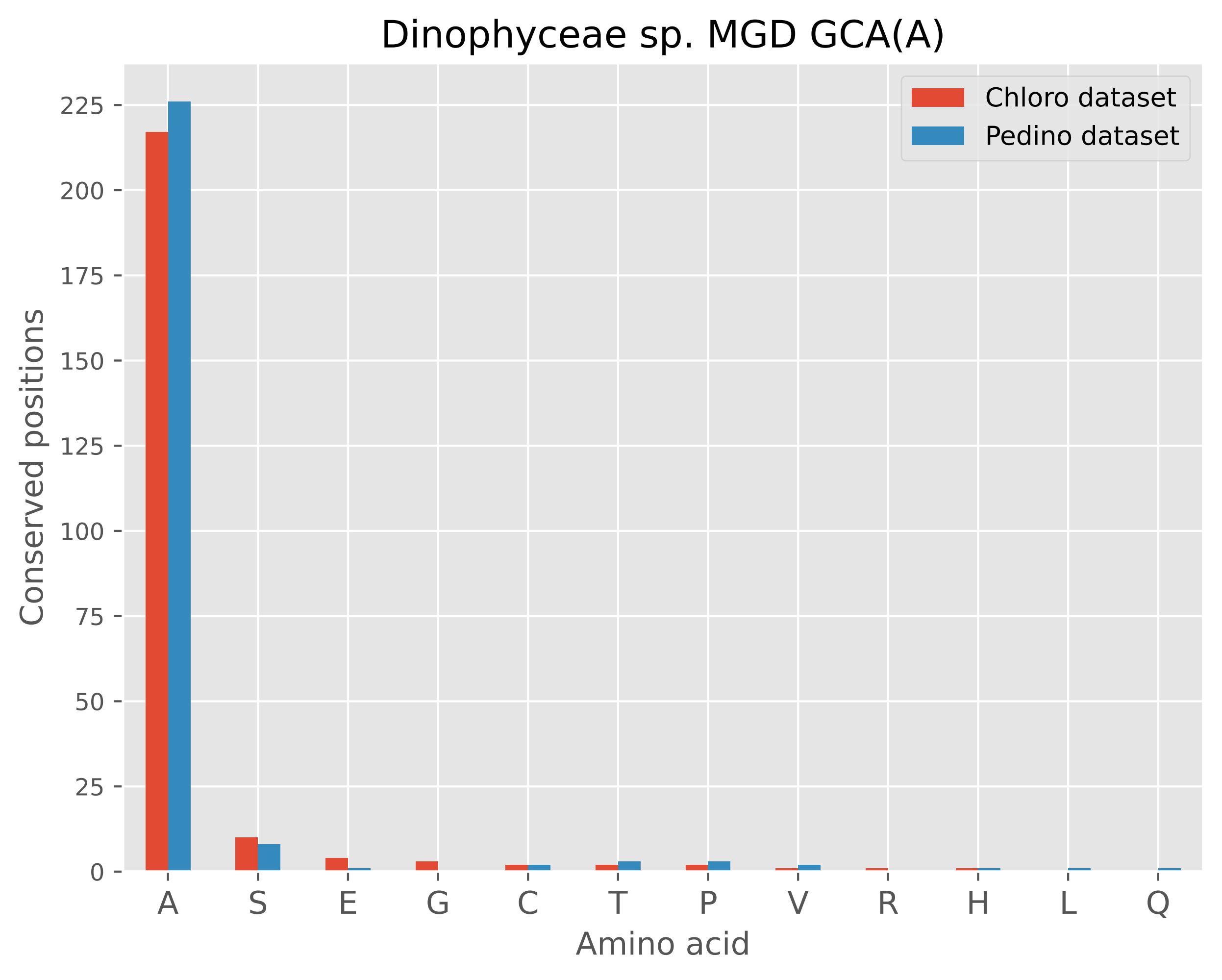

Dinophyceae sp. MGD GCA(A)
Chloro dataset
Pedino dataset
Conserved positions
0
25
50
75
100
125
150
175
200
225
A
S
E
G
C
T
P
V
R
H
L
Q
Amino acid

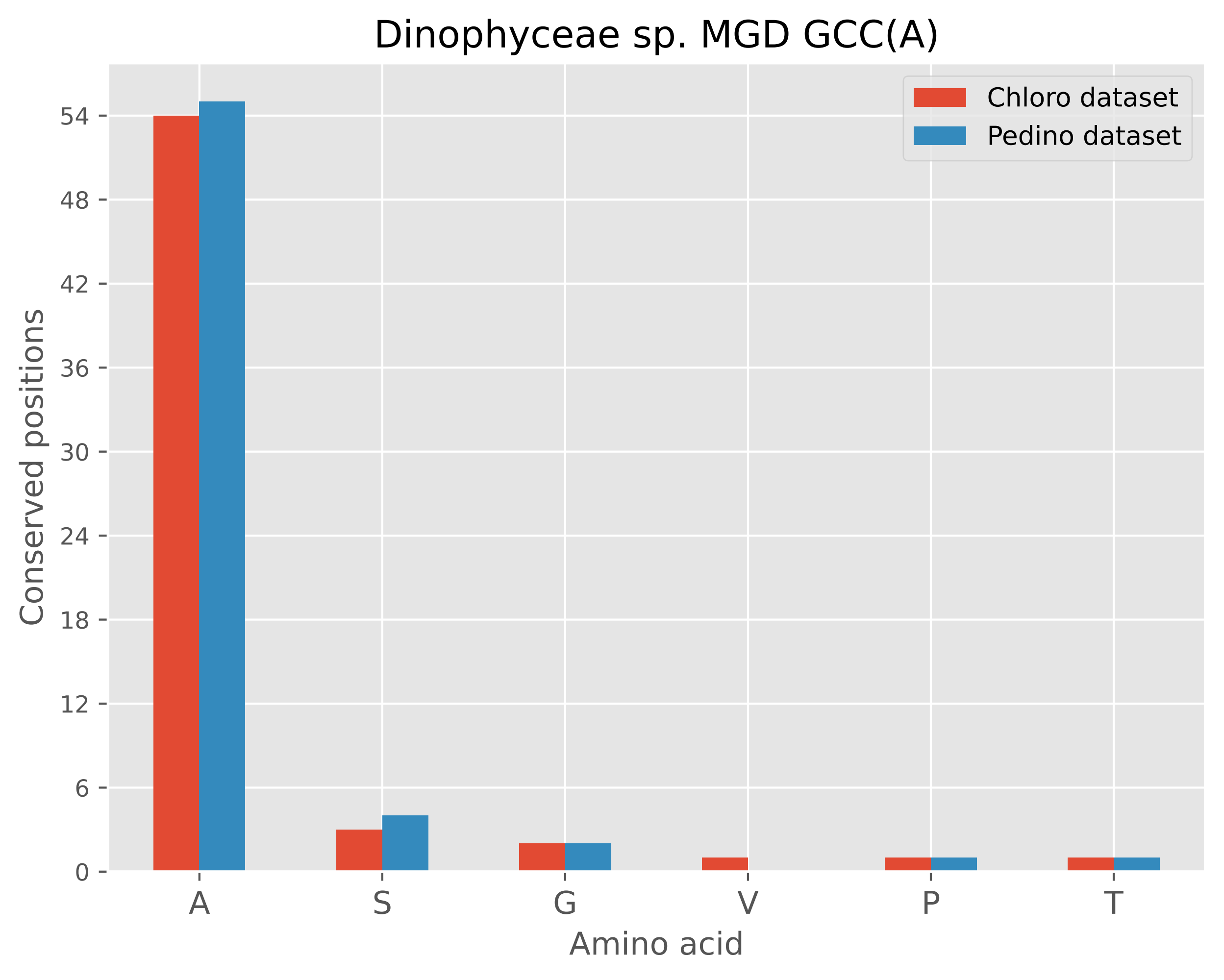
Dinophyceae sp. MGD GCC(A)
Chloro dataset
Pedino dataset
Conserved positions
0
6
12
18
24
30
36
42
48
54
A
S
G
V
P
T
Amino acid

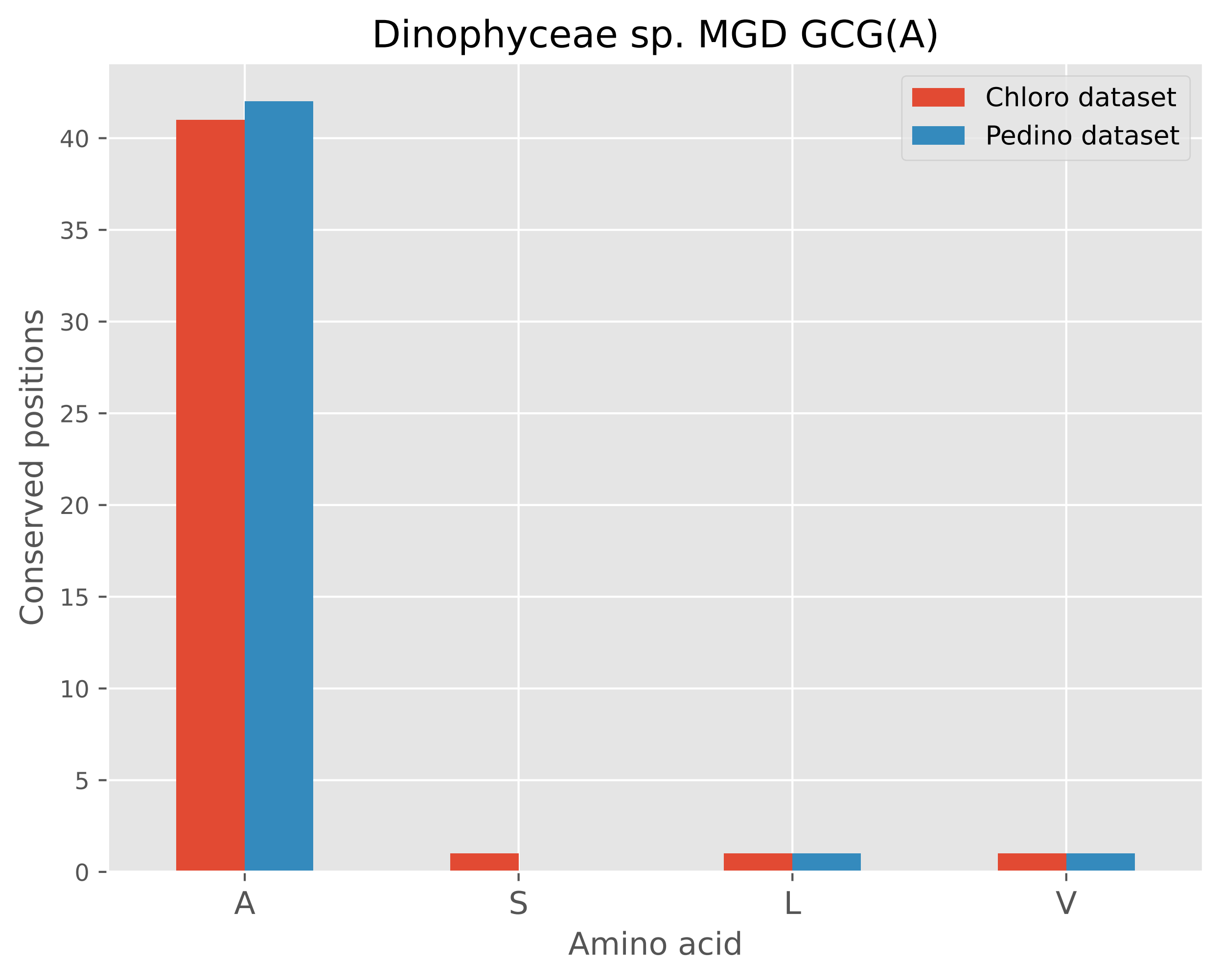
Dinophyceae sp. MGD GCG(A)
Chloro dataset
Pedino dataset
Conserved positions
0
5
10
15
20
25
30
35
40
A
S
L
V
Amino acid

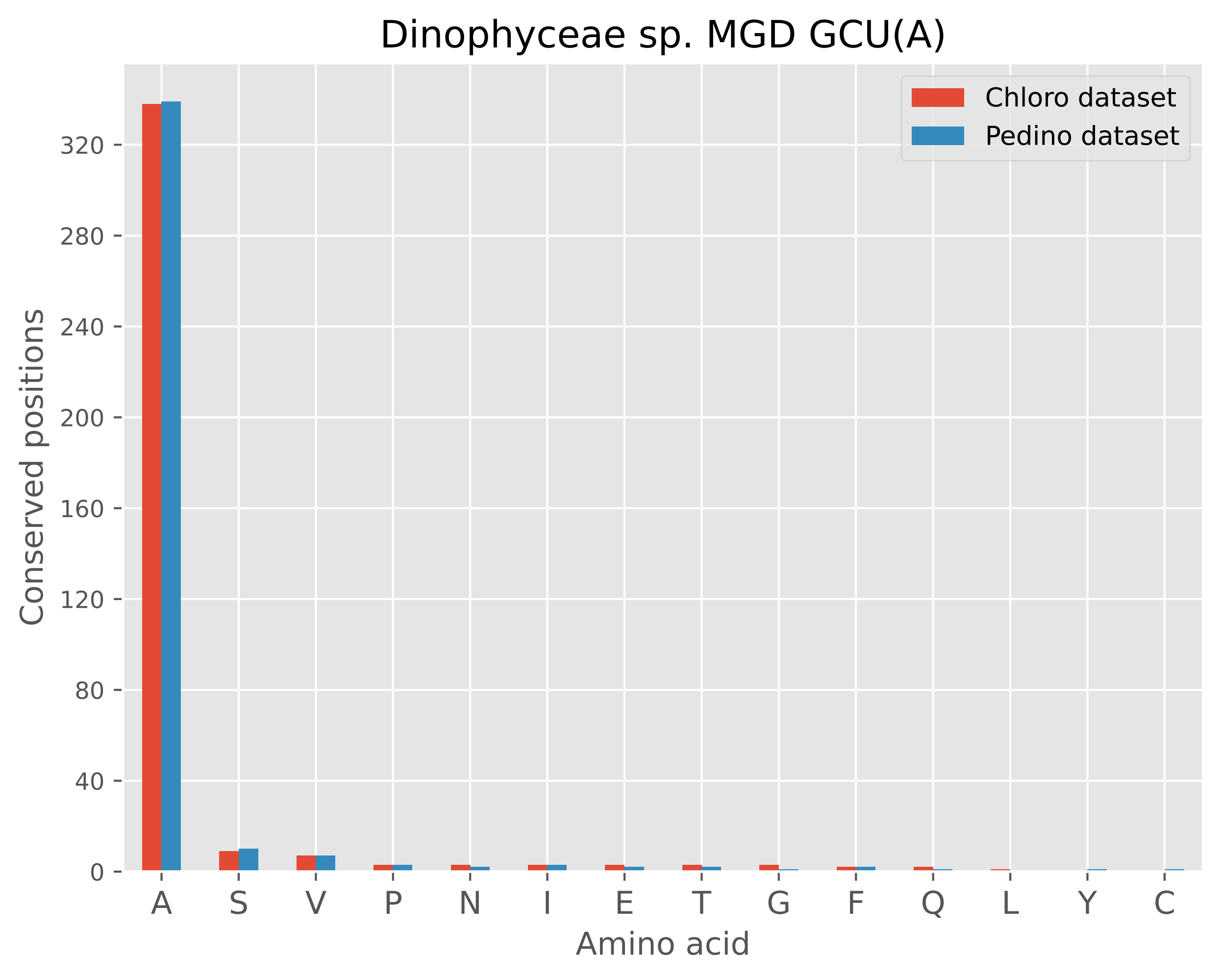
Dinophyceae sp. MGD GCU(A)
Chloro dataset
Pedino dataset
Conserved positions
0
40
80
120
160
200
240
280
320
A
S
V
P
N
I
E
T
G
F
Q
L
Y
C
Amino acid

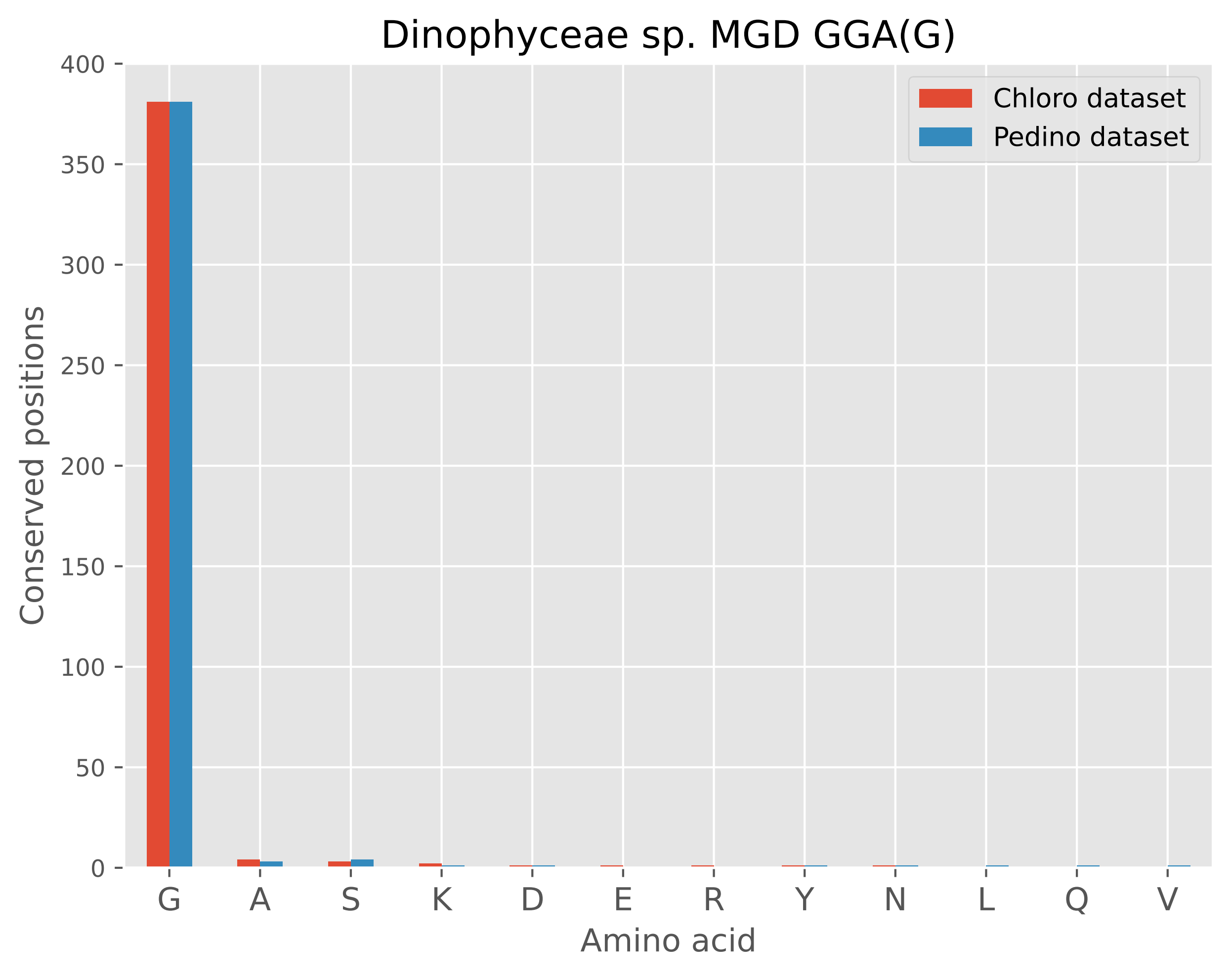

Dinophyceae sp. MGD GGA(G)
Chloro dataset
Pedino dataset
Conserved positions
0
50
100
150
200
250
300
350
400
G
A
S
K
D
E
R
Y
N
L
Q
V
Amino acid

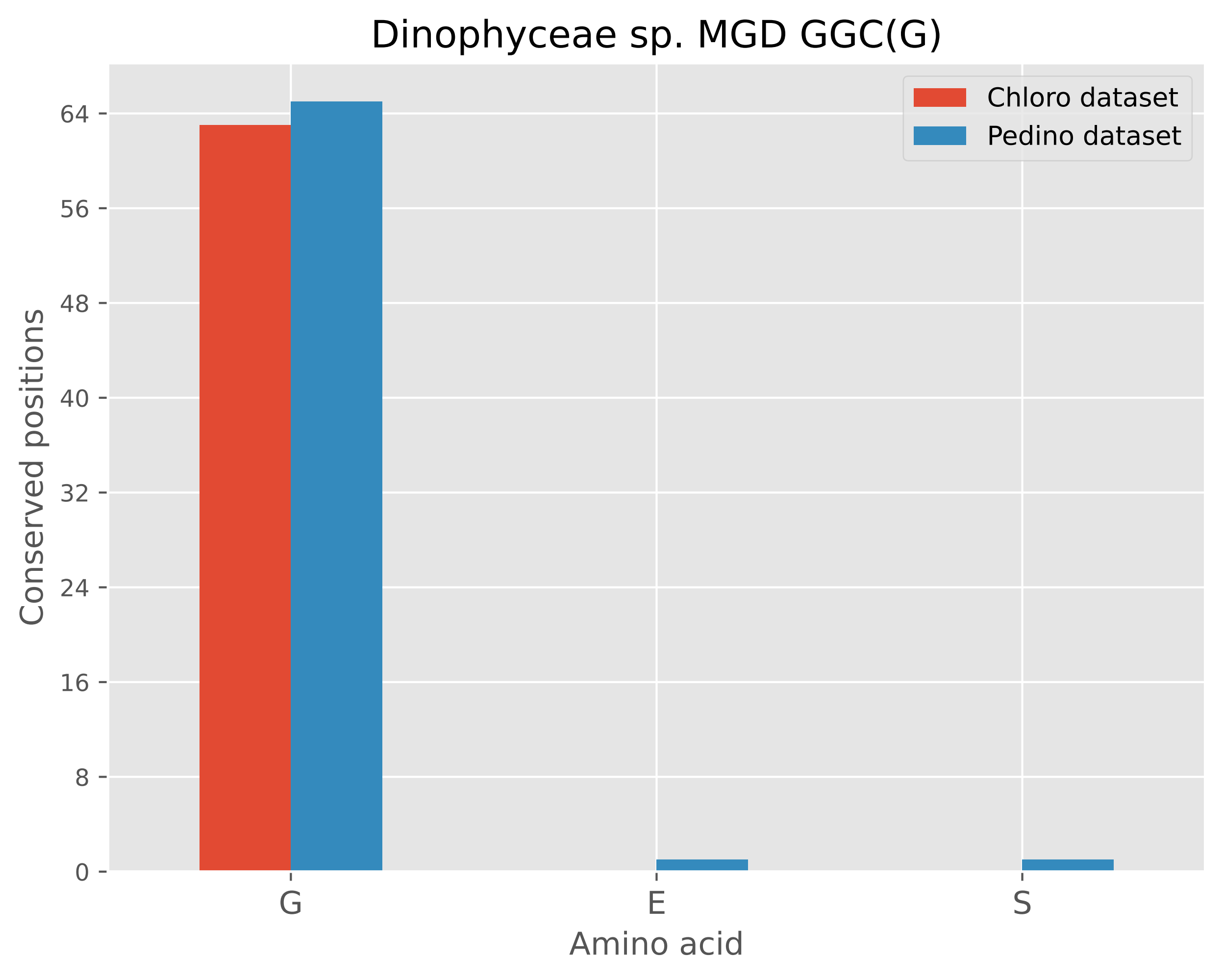

Dinophyceae sp. MGD GGC(G)
Chloro dataset
Pedino dataset
Conserved positions
0
8
16
24
32
40
48
56
64
G
E
S
Amino acid

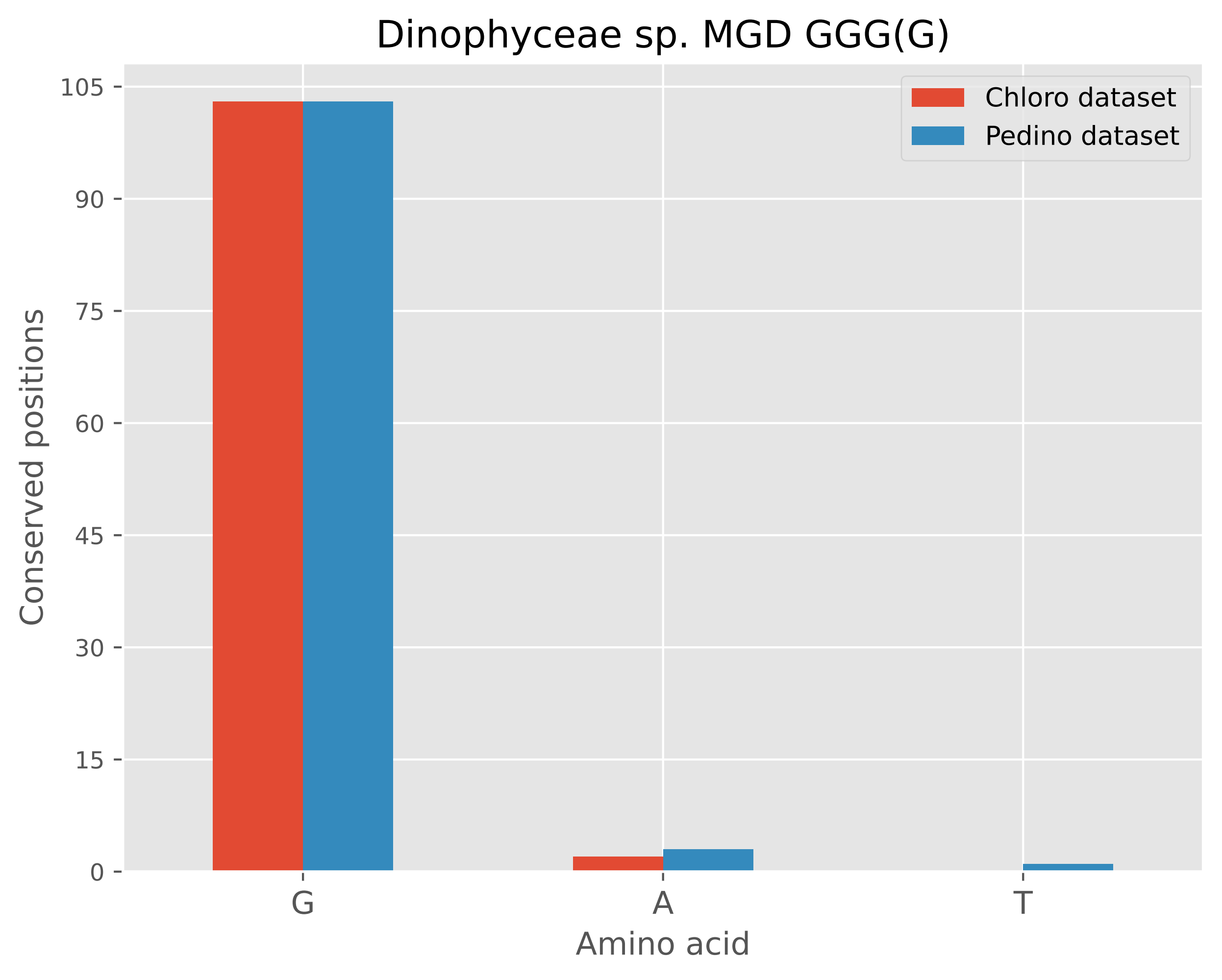
Dinophyceae sp. MGD GGG(G)
Chloro dataset
Pedino dataset
Conserved positions
105
90
75
60
45
30
15
0
G
A
T
Amino acid

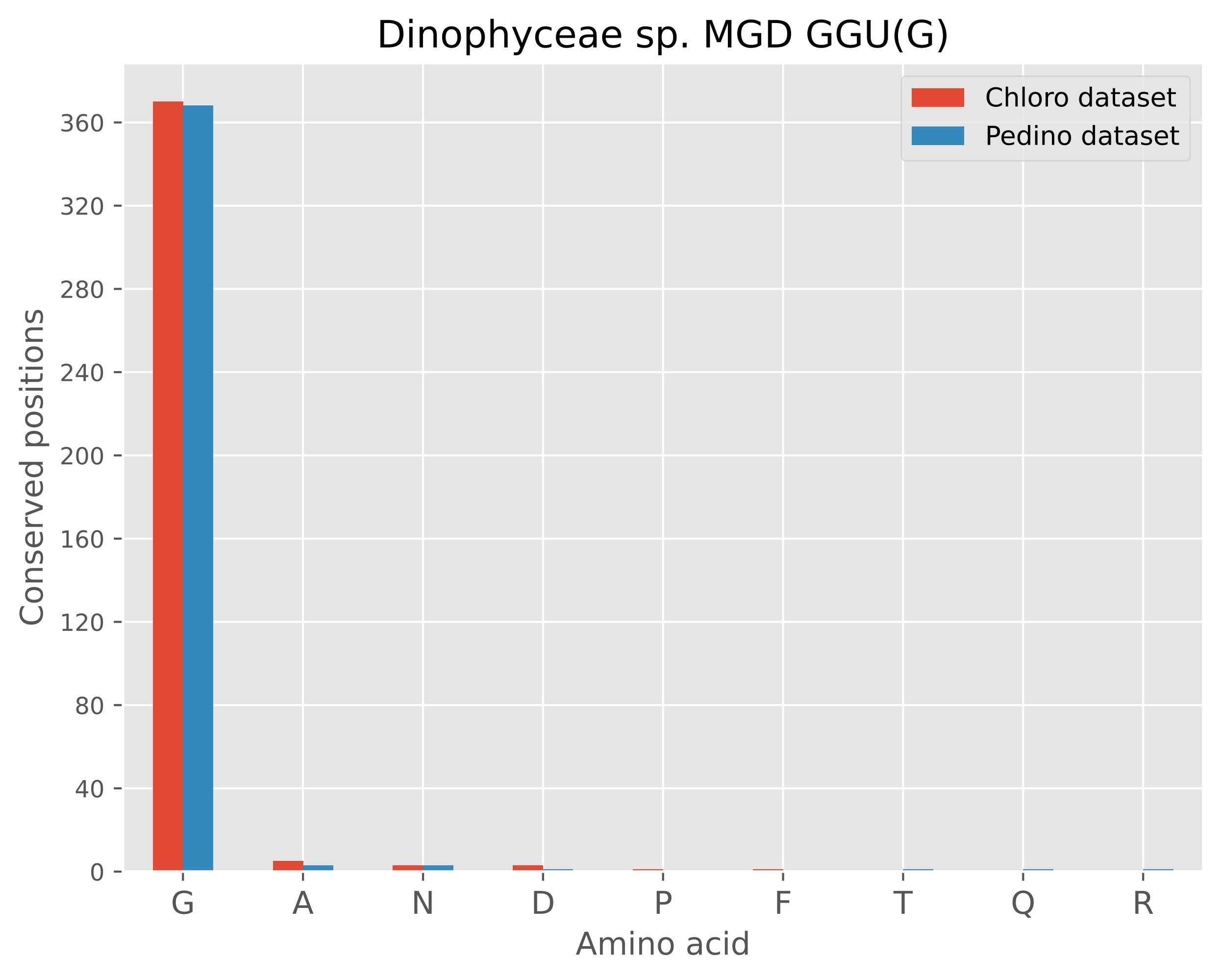

Dinophyceae sp. MGD GGU(G)
Chloro dataset
Pedino dataset
Conserved positions
0
40
80
120
160
200
240
280
320
360
G
A
N
D
P
F
T
Q
R
Amino acid

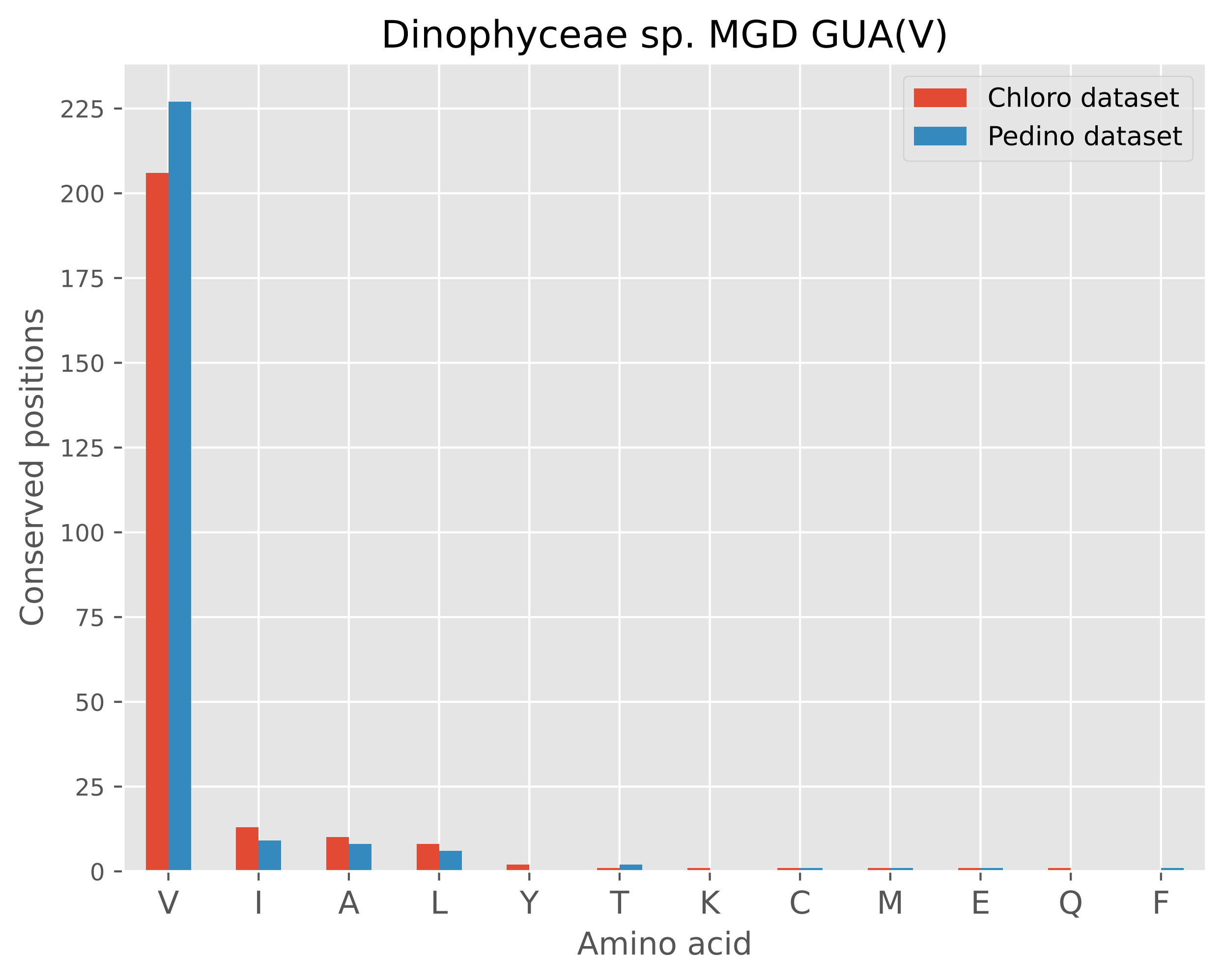

Dinophyceae sp. MGD GUA(V)
Chloro dataset
Pedino dataset
Conserved positions
0
25
50
75
100
125
150
175
200
225
V
I
A
L
Y
T
K
C
M
E
Q
F
Amino acid

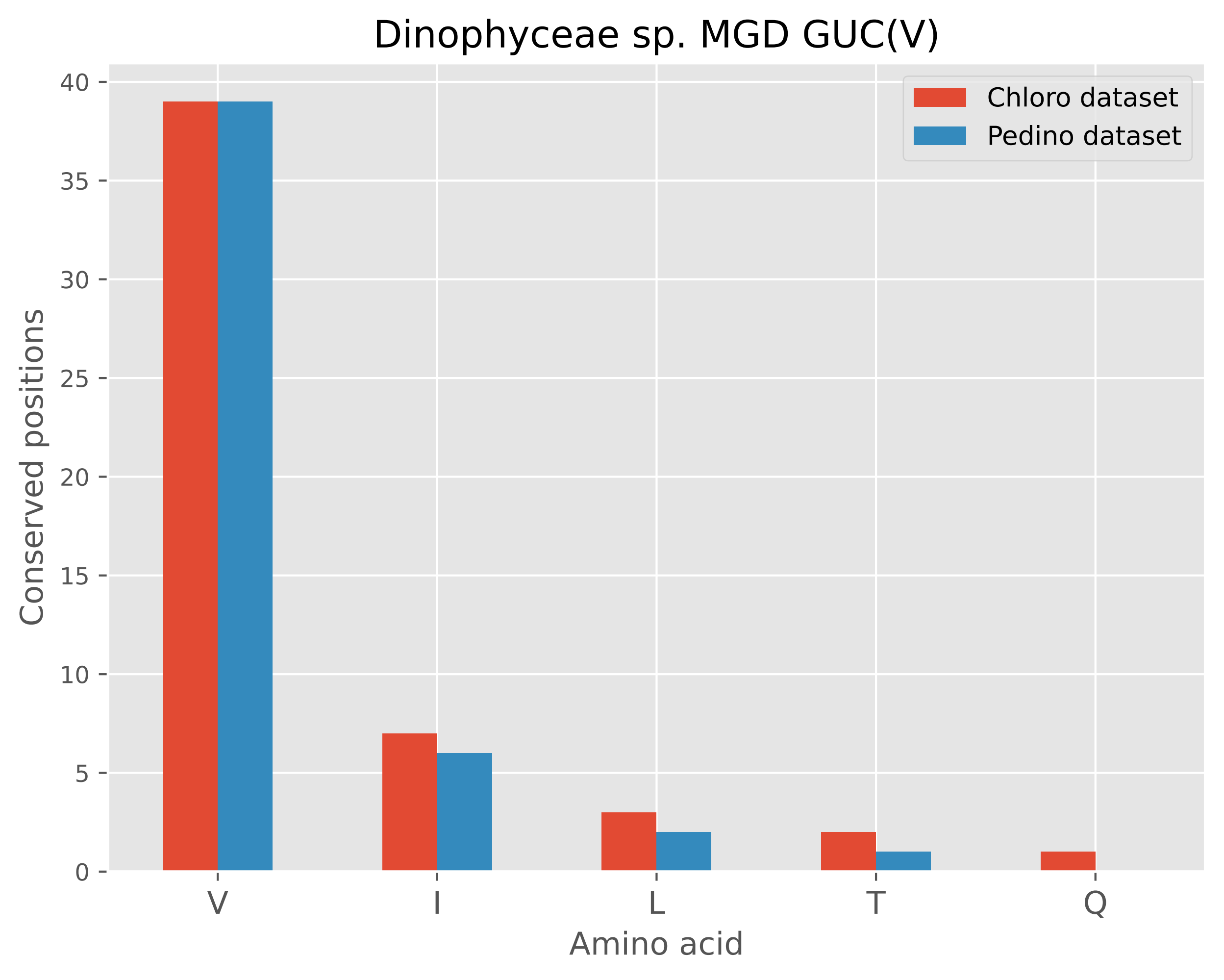

Dinophyceae sp. MGD GUC(V)
Chloro dataset
Pedino dataset
Conserved positions
0
5
10
15
20
25
30
35
40
V
I
L
T
Q
Amino acid

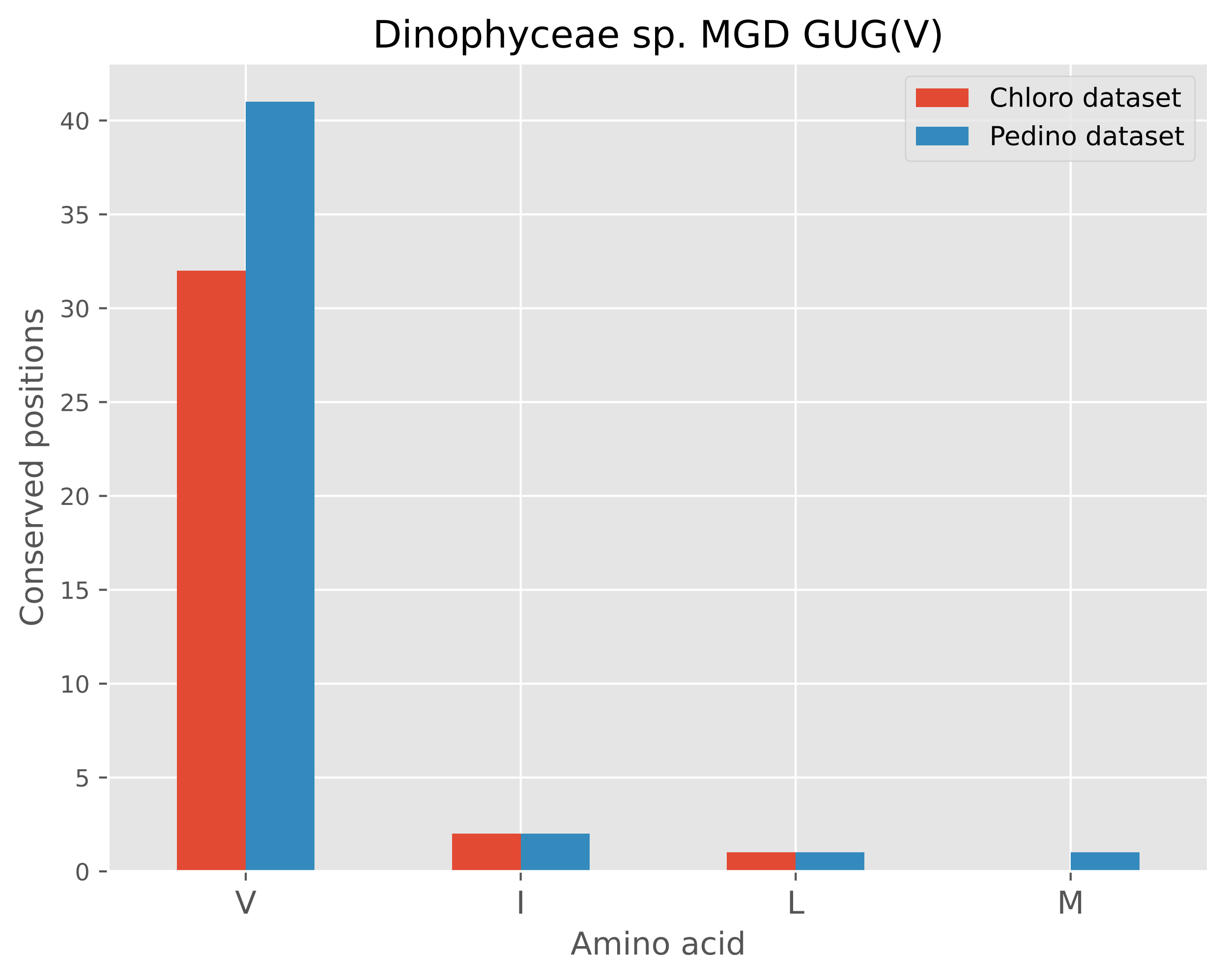

Dinophyceae sp. MGD GUG(V)
Chloro dataset
Pedino dataset
Conserved positions
0
5
10
15
20
25
30
35
40
V
I
L
M
Amino acid

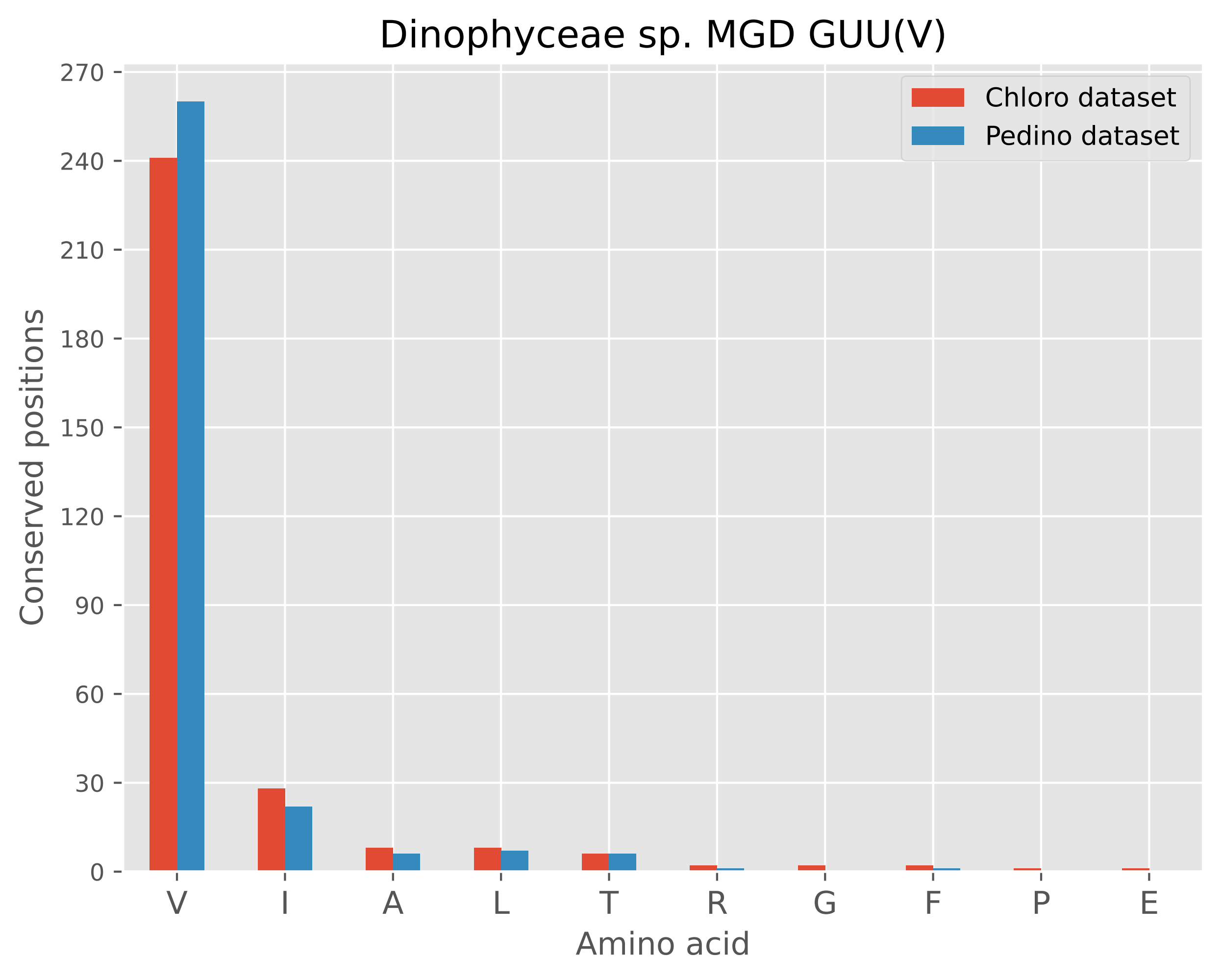

Dinophyceae sp. MGD GUU(V)
Chloro dataset
Pedino dataset
Conserved positions
Amino acid
0
30
60
90
120
150
180
210
240
270
V
I
A
L
T
R
G
F
P
E

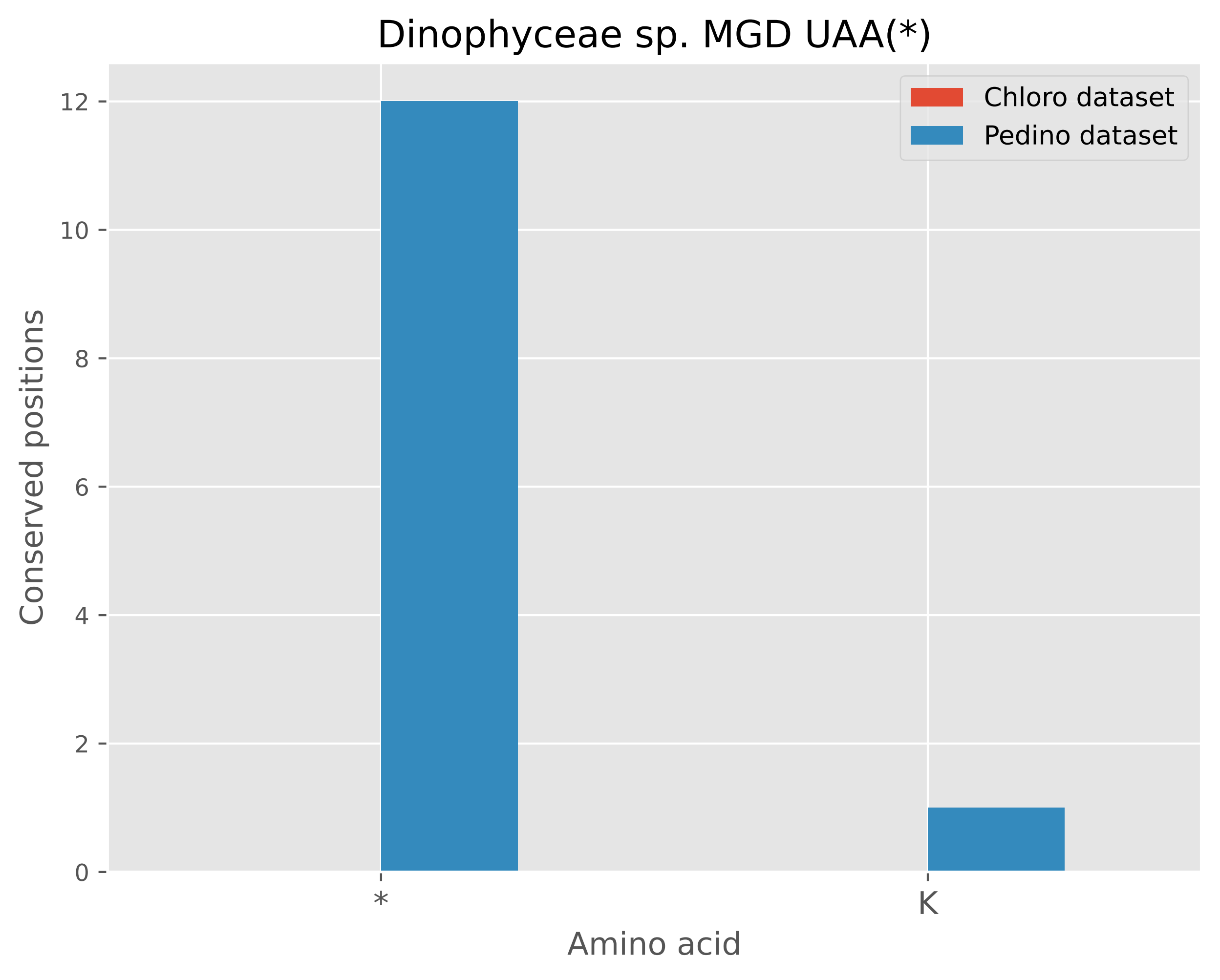

Dinophyceae sp. MGD UAA(*)
Chloro dataset
Pedino dataset
Conserved positions
0
2
4
6
8
10
12
*
K
Amino acid

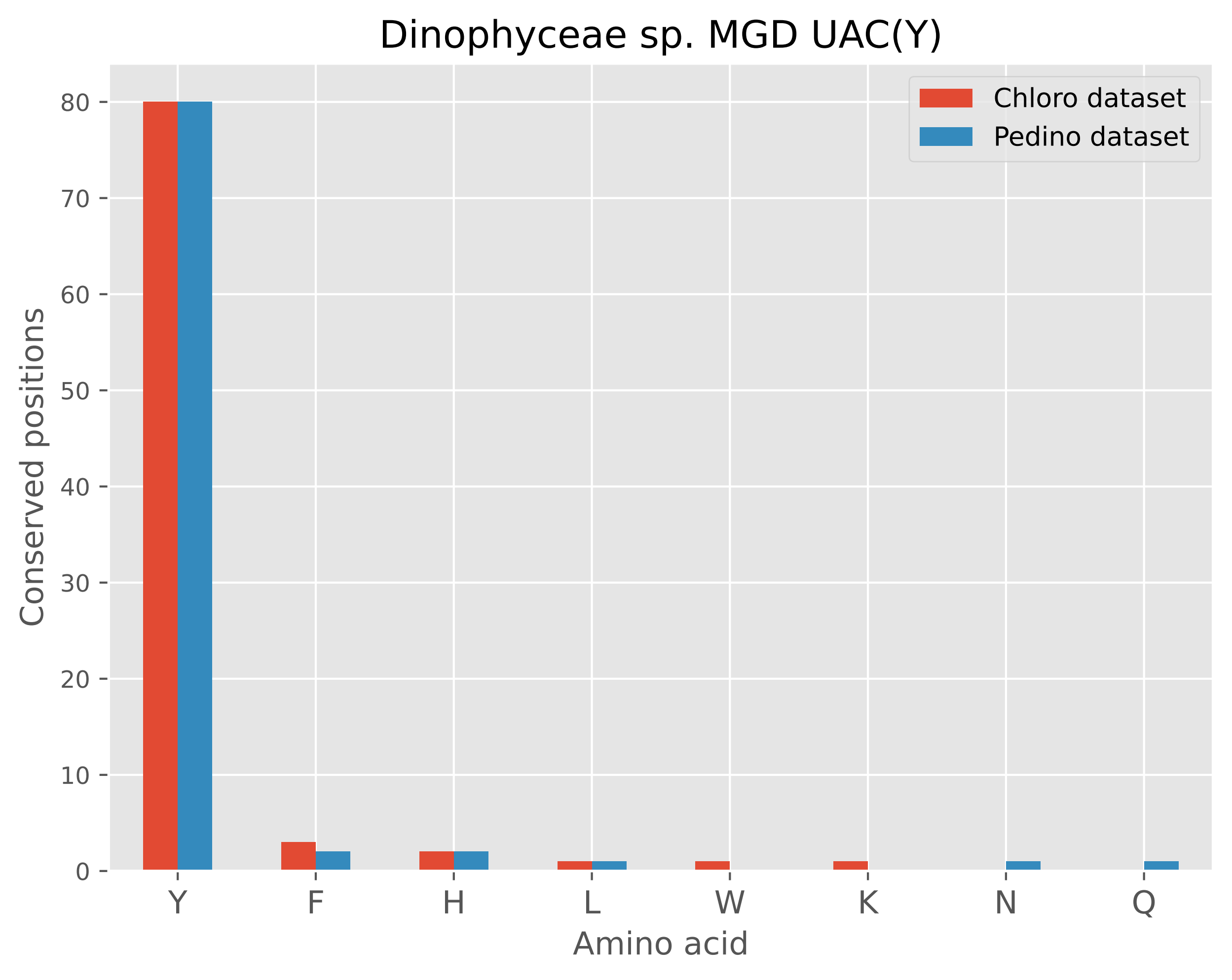
Dinophyceae sp. MGD UAC(Y)
Chloro dataset
Pedino dataset
Conserved positions
0
10
20
30
40
50
60
70
80
Y
F
H
L
W
K
N
Q
Amino acid

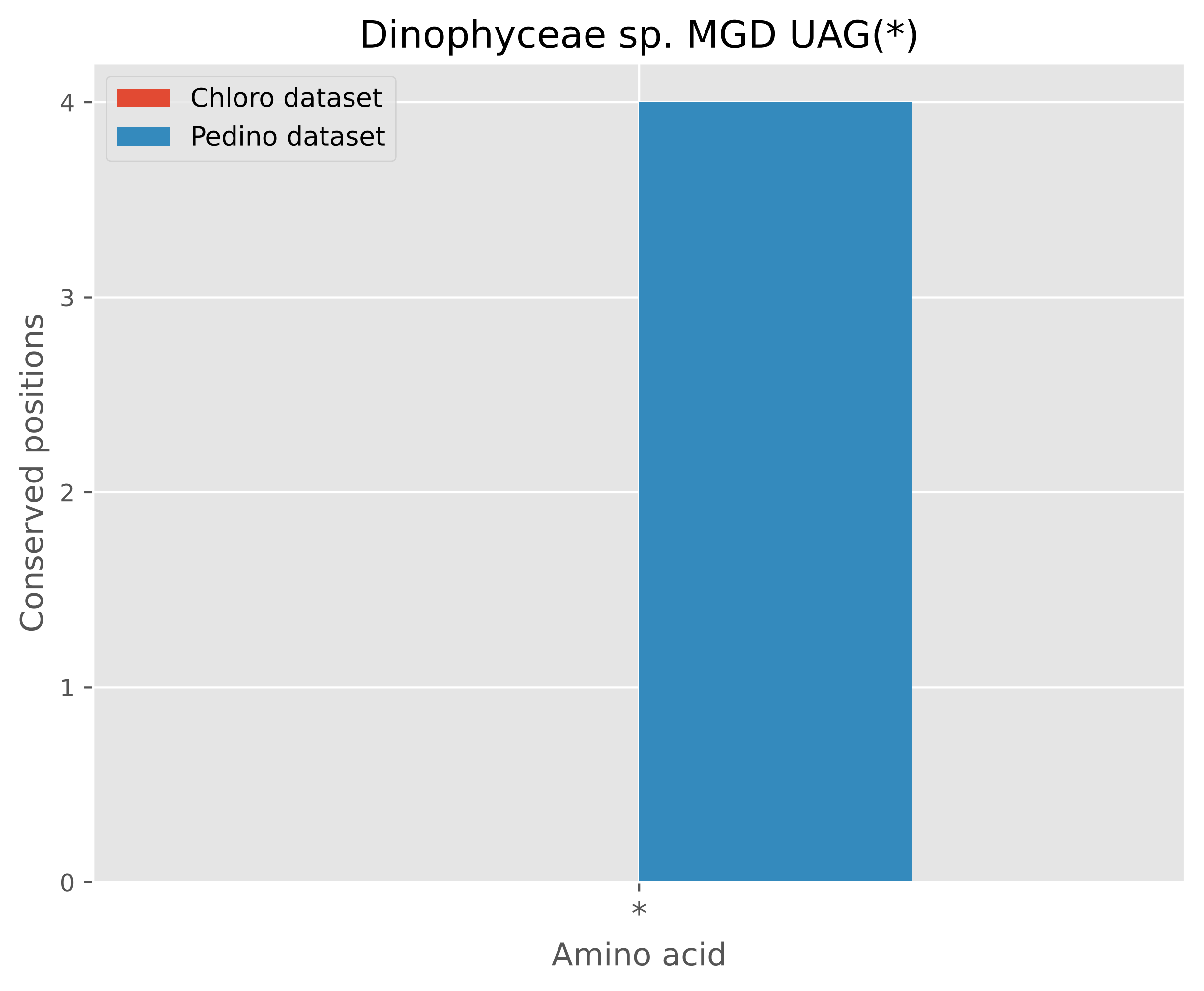

Dinophyceae sp. MGD UAG(*)
Chloro dataset
Pedino dataset
Conserved positions
0
1
2
3
4
*
Amino acid

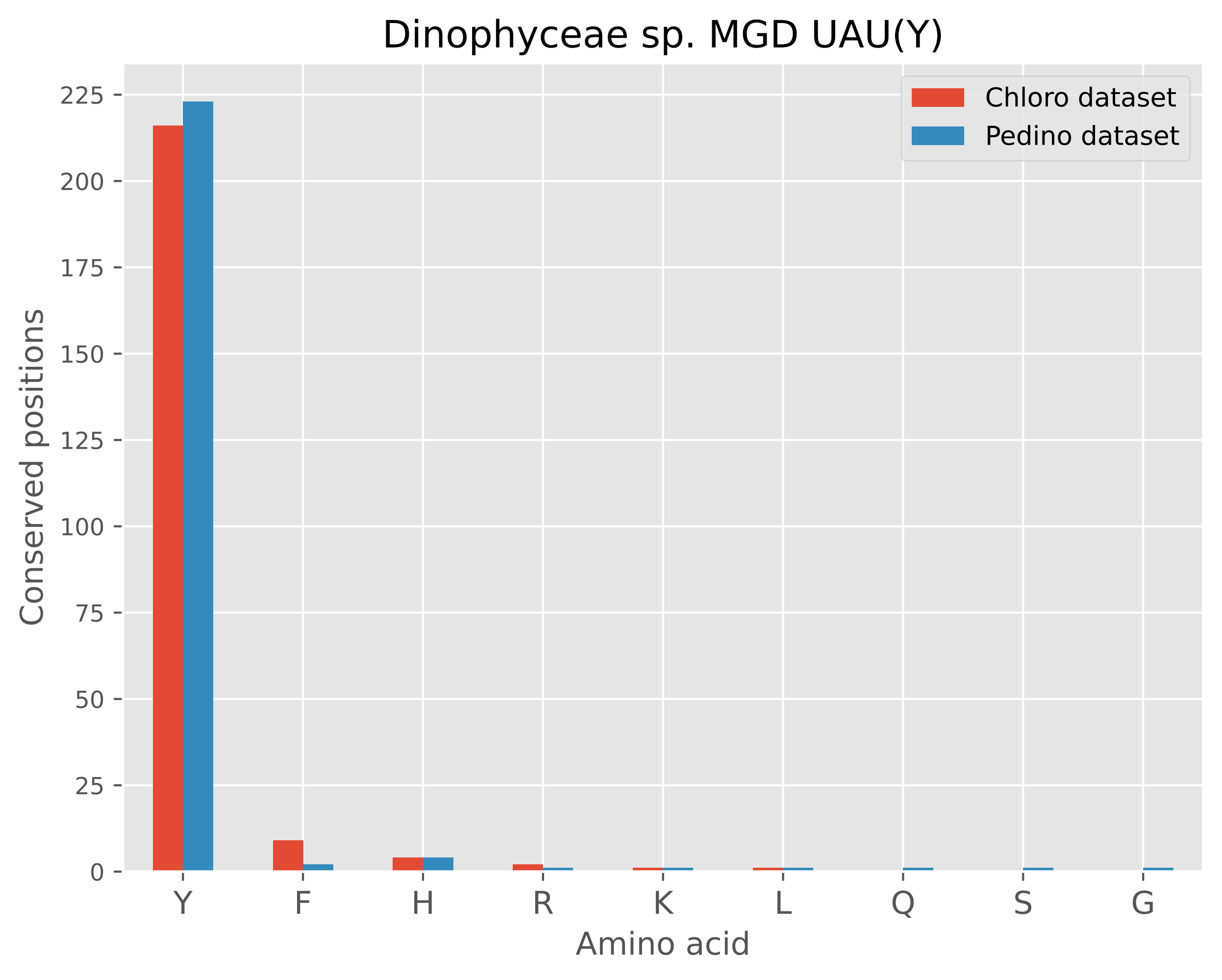
Dinophyceae sp. MGD UAU(Y)
Chloro dataset
Pedino dataset
Conserved positions
0
25
50
75
100
125
150
175
200
225
Y
F
H
R
K
L
Q
S
G
Amino acid

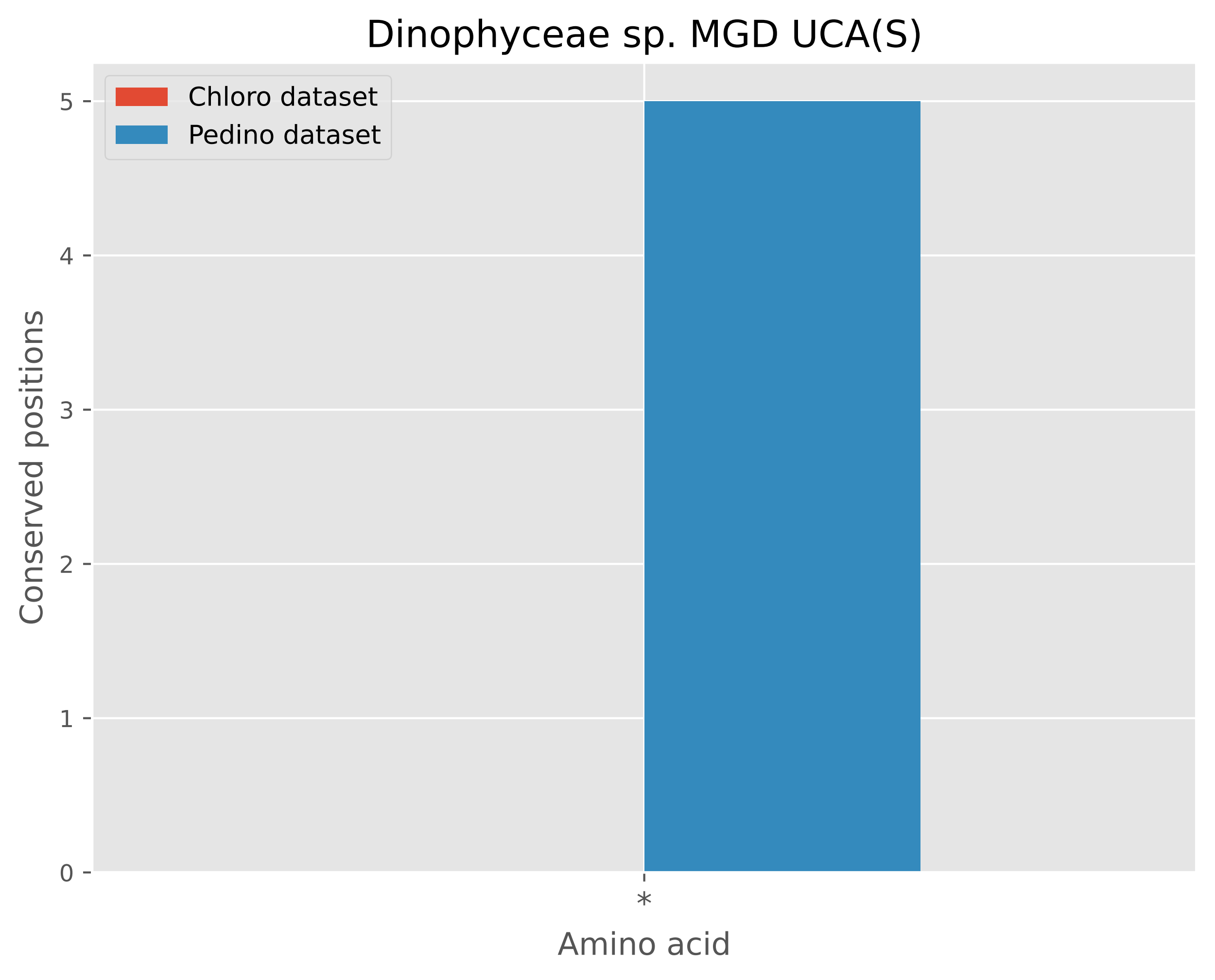

Dinophyceae sp. MGD UCA(S)
Chloro dataset
Pedino dataset
Conserved positions
5
4
3
2
1
0
*
Amino acid

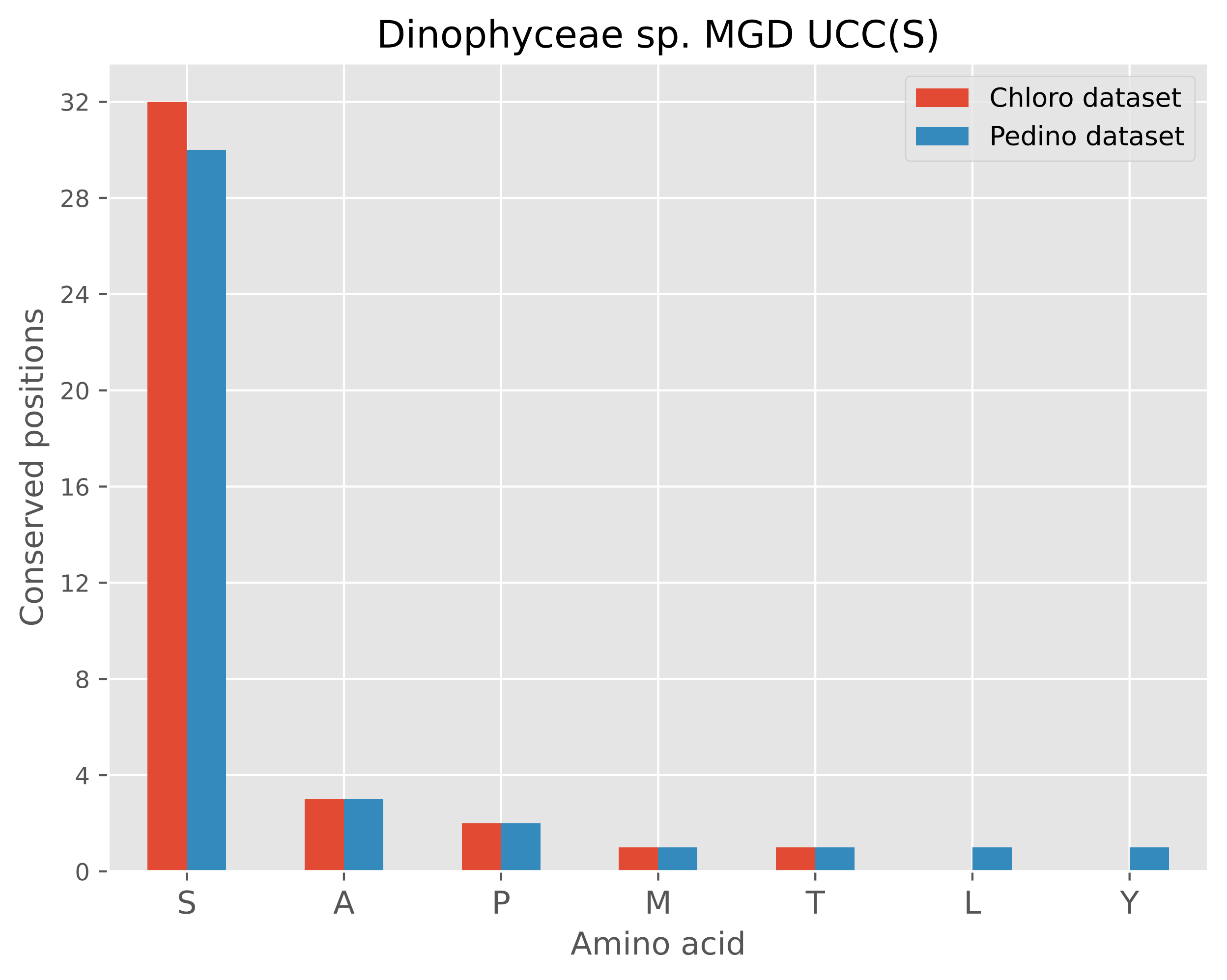
Dinophyceae sp. MGD UCC(S)
Chloro dataset
Pedino dataset
Conserved positions
0
4
8
12
16
20
24
28
32
S
A
P
M
T
L
Y
Amino acid

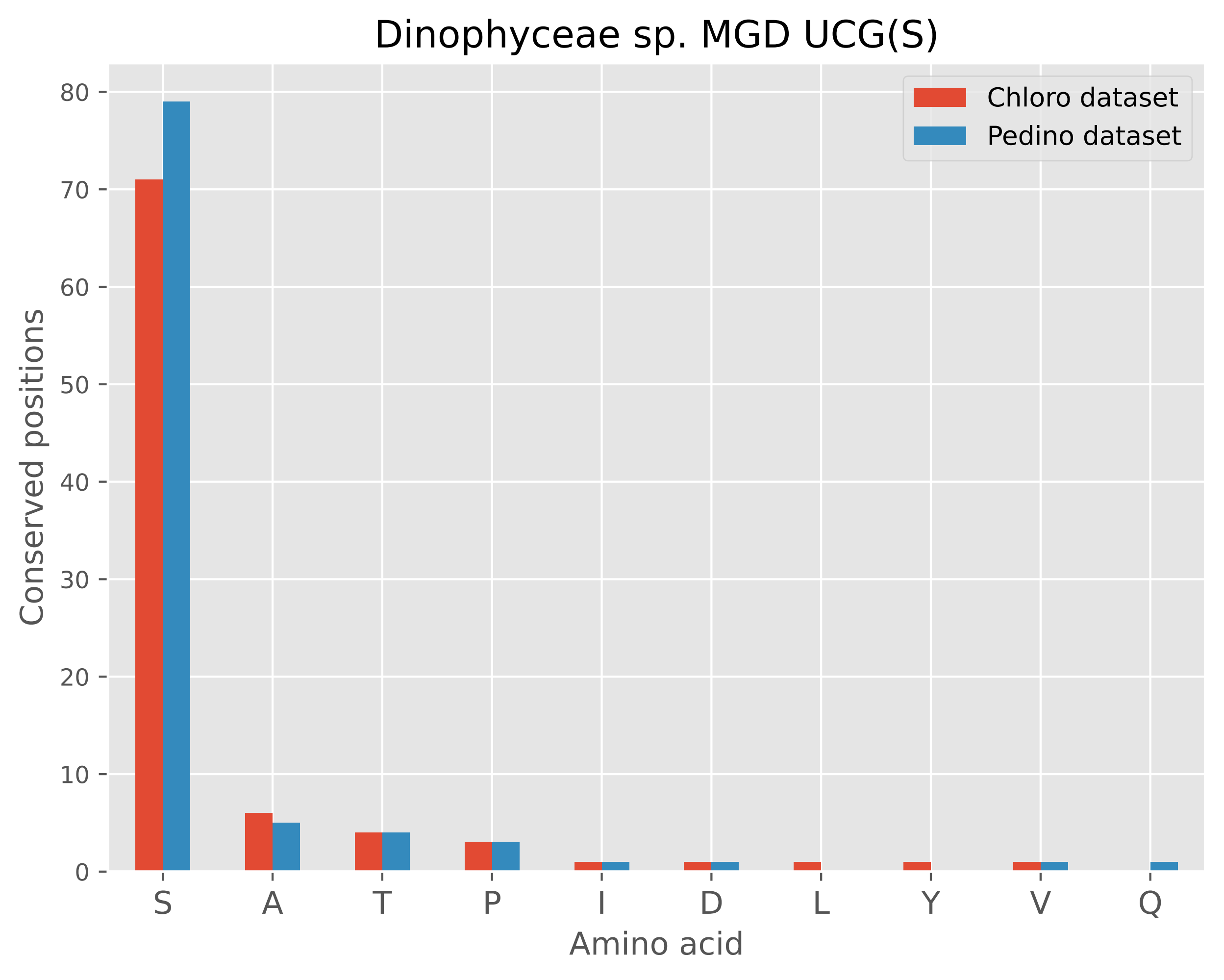

Dinophyceae sp. MGD UCG(S)
Chloro dataset
Pedino dataset
Conserved positions
0
10
20
30
40
50
60
70
80
S
A
T
P
I
D
L
Y
V
Q
Amino acid

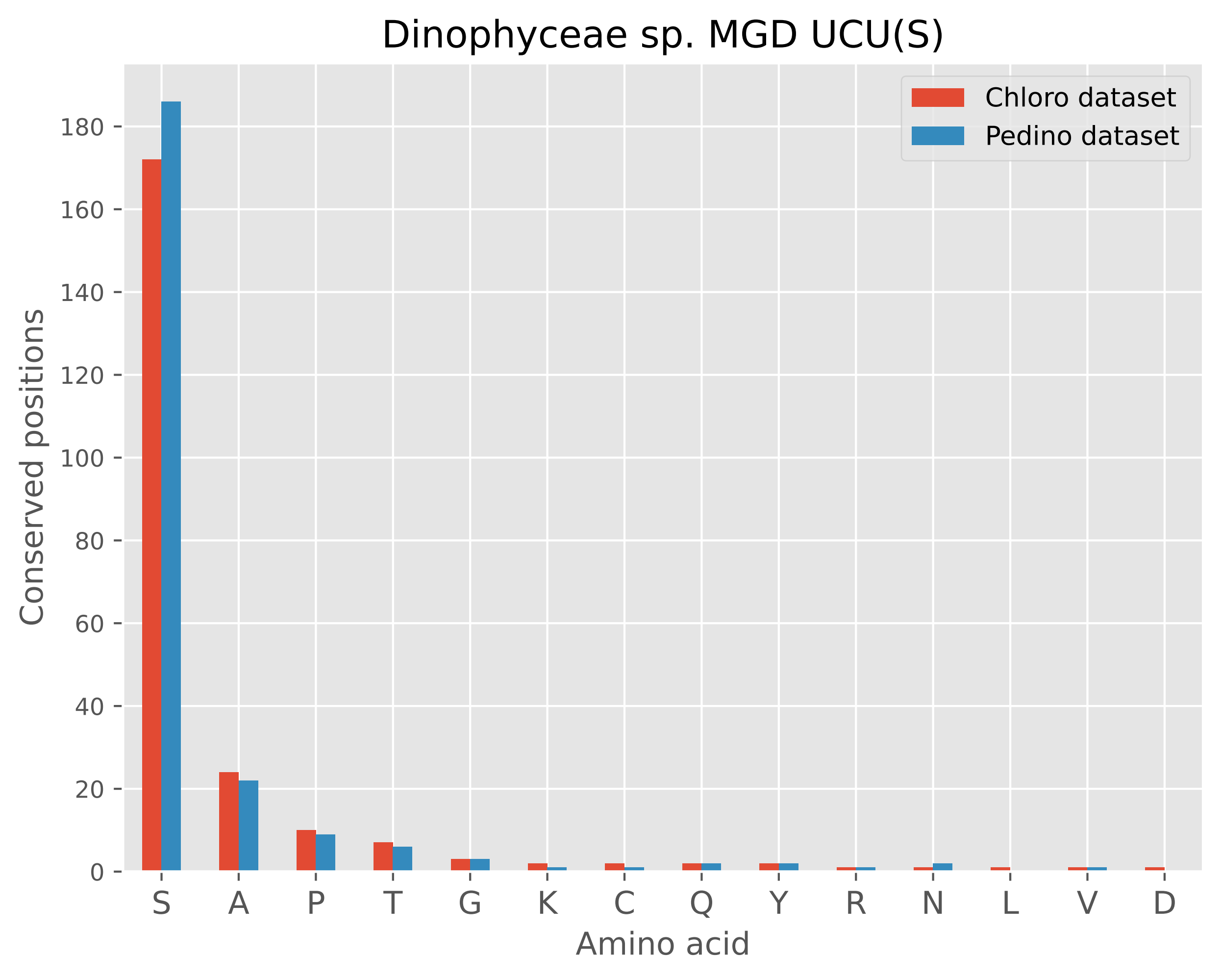

Dinophyceae sp. MGD UCU(S)
Chloro dataset
Pedino dataset
Conserved positions
0
20
40
60
80
100
120
140
160
180
S
A
P
T
G
K
C
Q
Y
R
N
L
V
D
Amino acid

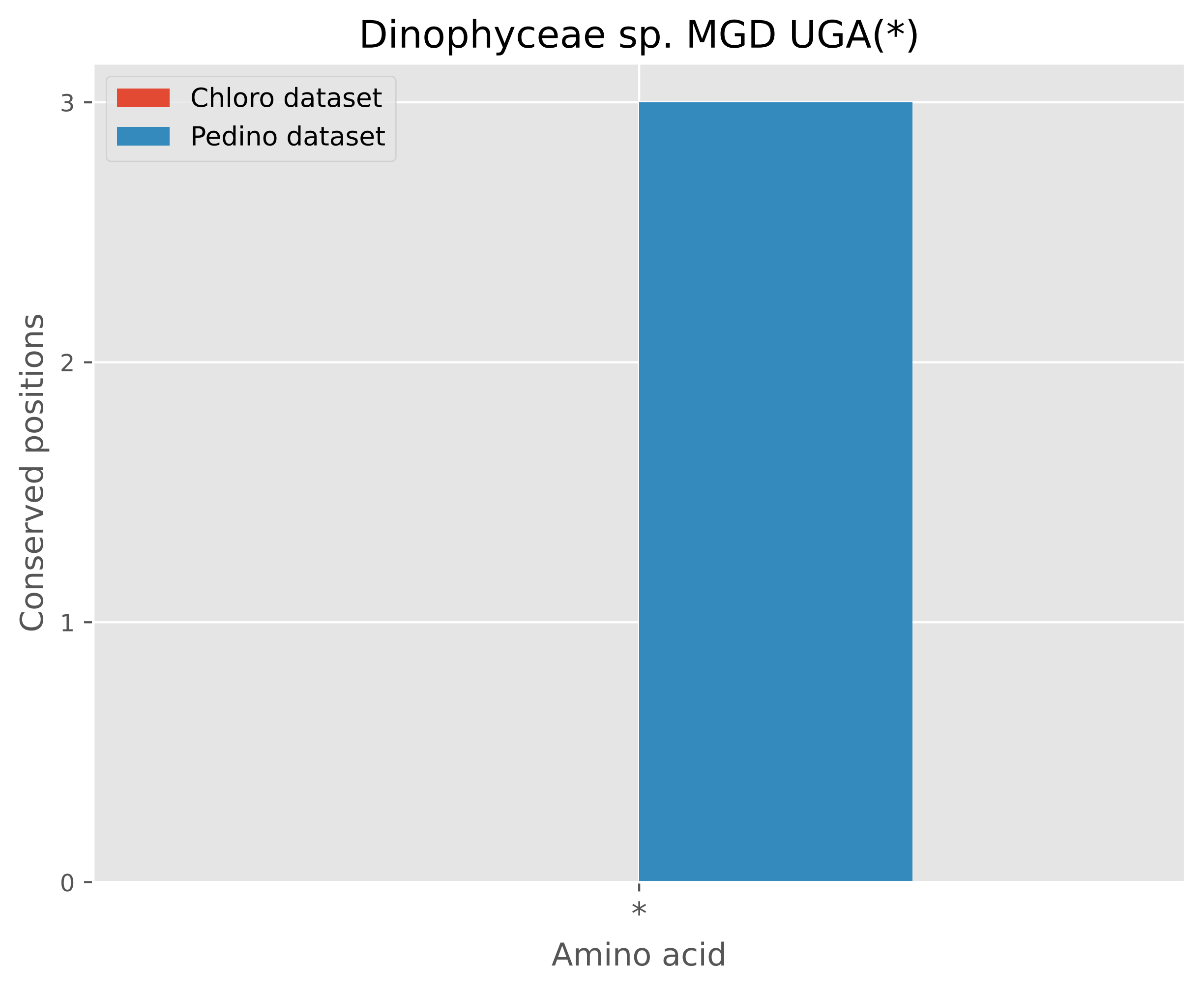
Dinophyceae sp. MGD UGA(*)
Chloro dataset
Pedino dataset
Conserved positions
0
1
2
3
*
Amino acid

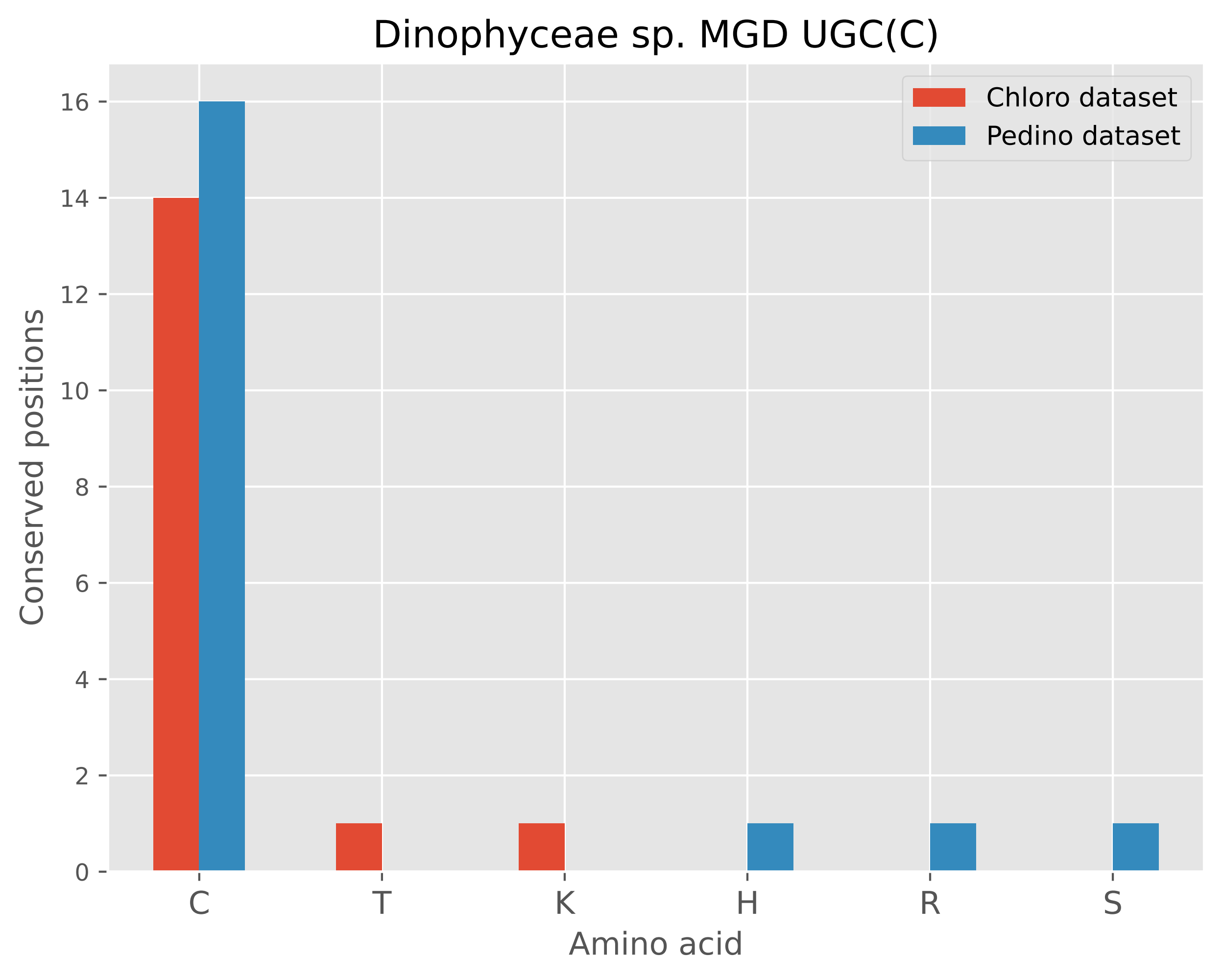
Dinophyceae sp. MGD UGC(C)
Chloro dataset
Pedino dataset
Conserved positions
0
2
4
6
8
10
12
14
16
C
T
K
H
R
S
Amino acid

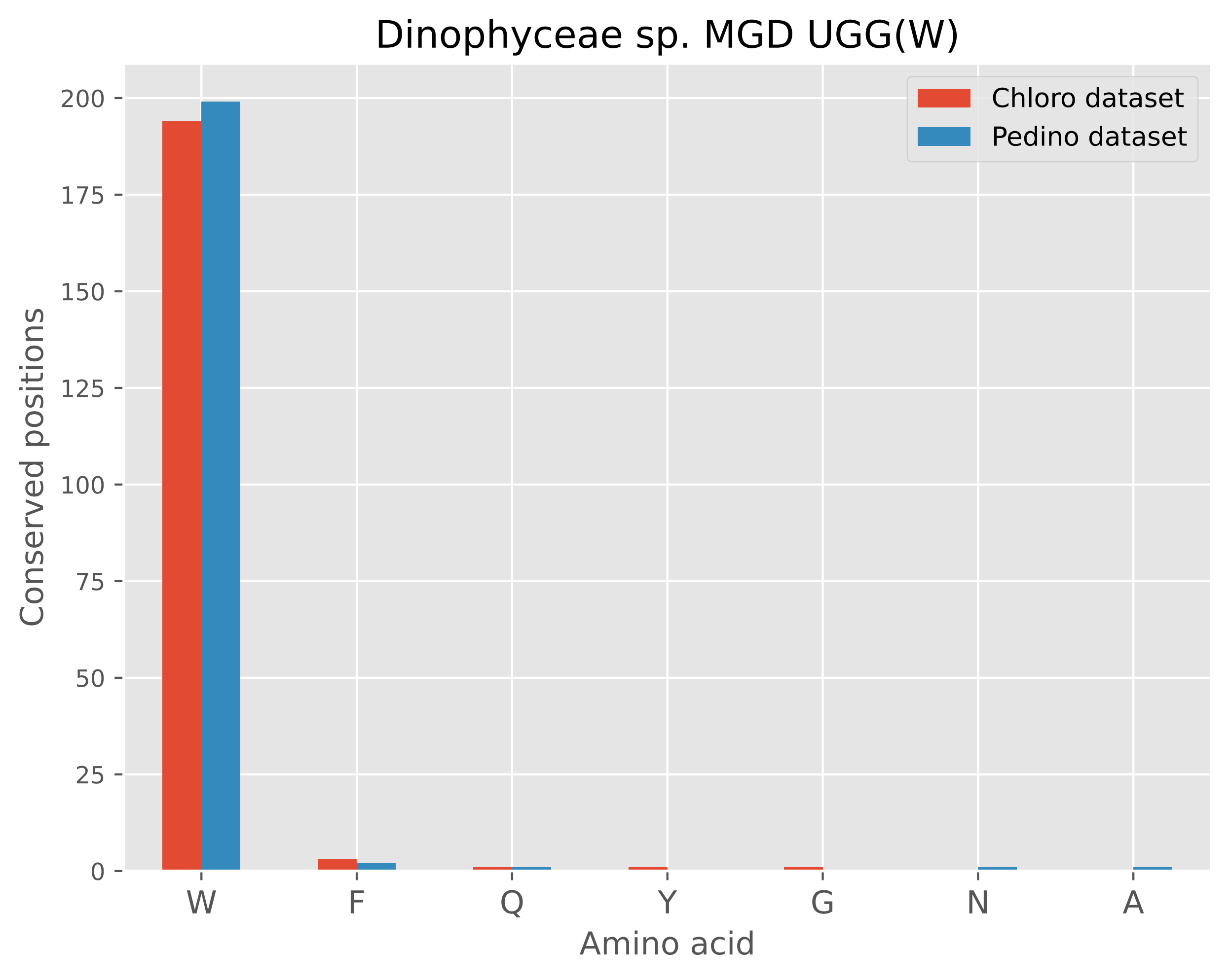

Dinophyceae sp. MGD UGG(W)
Chloro dataset
Pedino dataset
Conserved positions
0
25
50
75
100
125
150
175
200
W
F
Q
Y
G
N
A
Amino acid

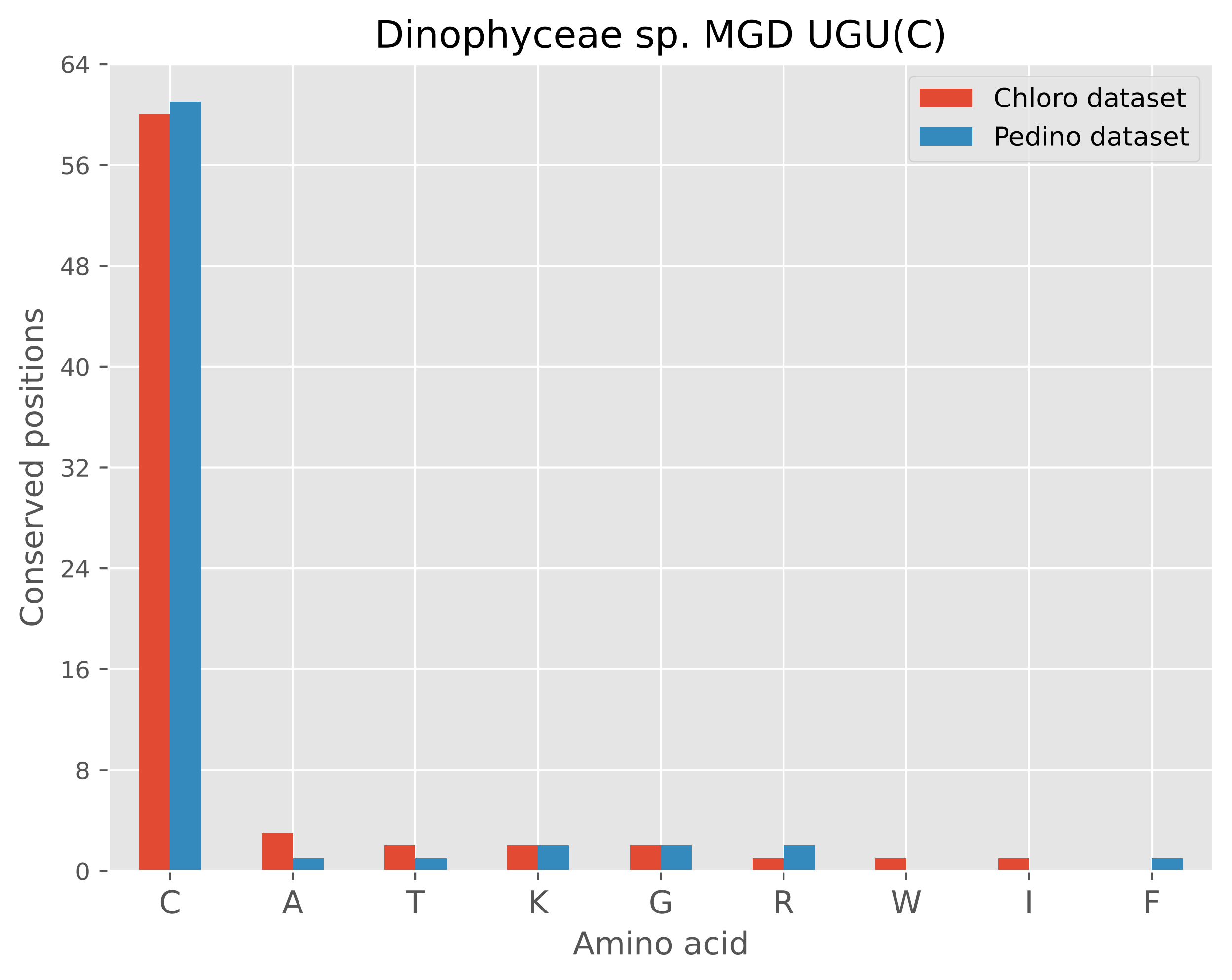

Dinophyceae sp. MGD UGU(C)
Chloro dataset
Pedino dataset
Conserved positions
0
8
16
24
32
40
48
56
64
C
A
T
K
G
R
W
I
F
Amino acid

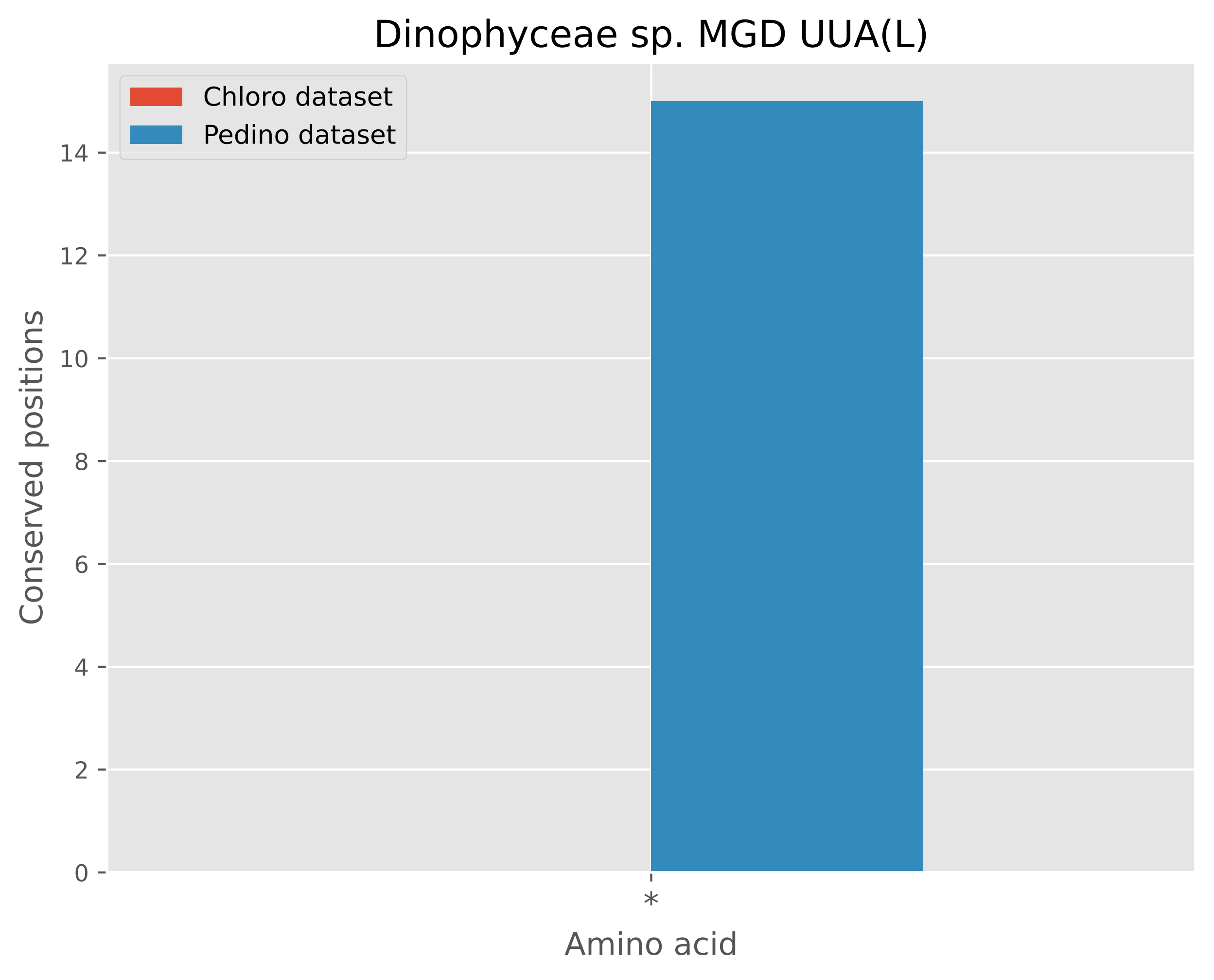

Dinophyceae sp. MGD UUA(L)
Chloro dataset
Pedino dataset
Conserved positions
0
2
4
6
8
10
12
14
*
Amino acid

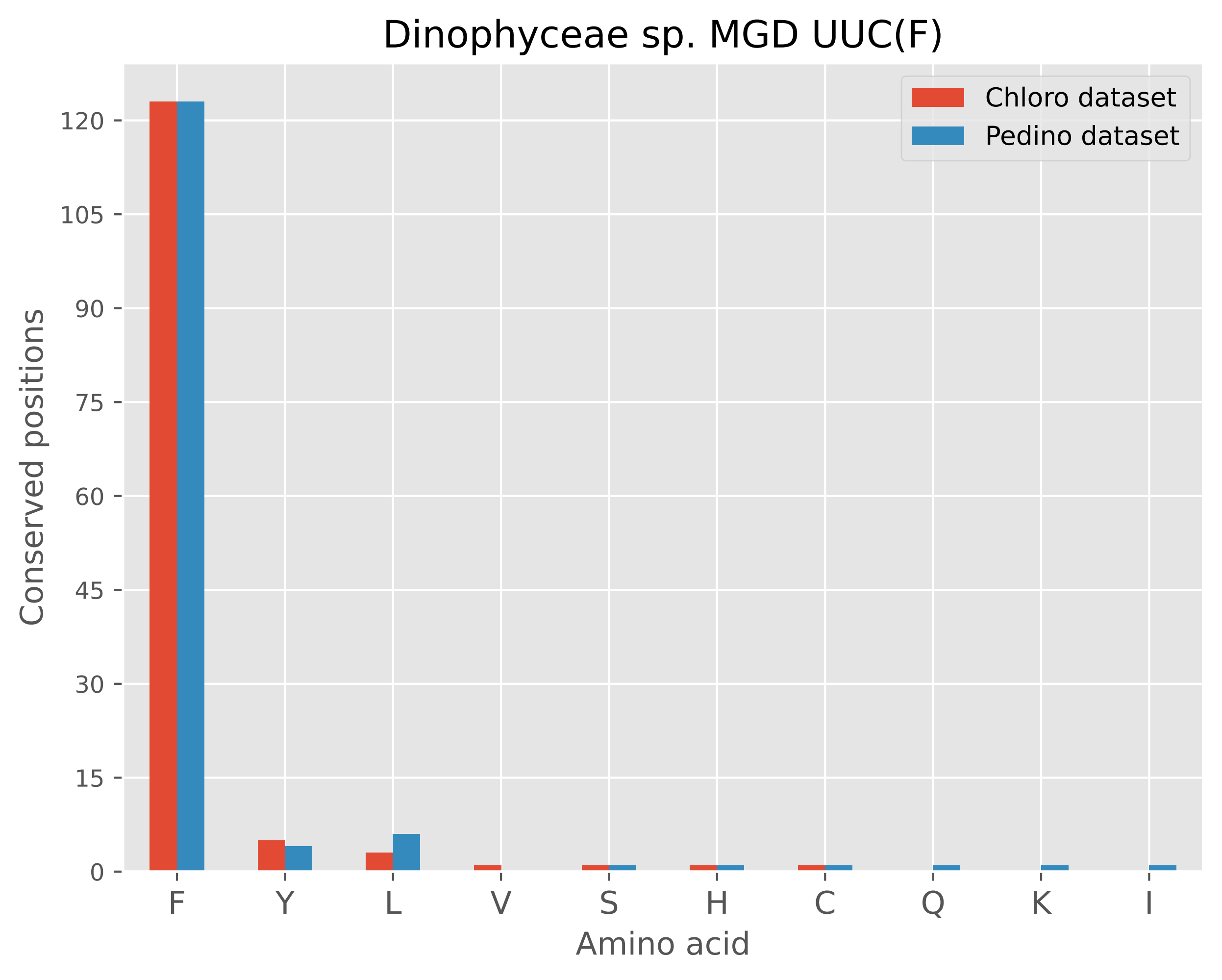

Dinophyceae sp. MGD UUC(F)
Chloro dataset
Pedino dataset
Conserved positions
0
15
30
45
60
75
90
105
120
F
Y
L
V
S
H
C
Q
K
I
Amino acid

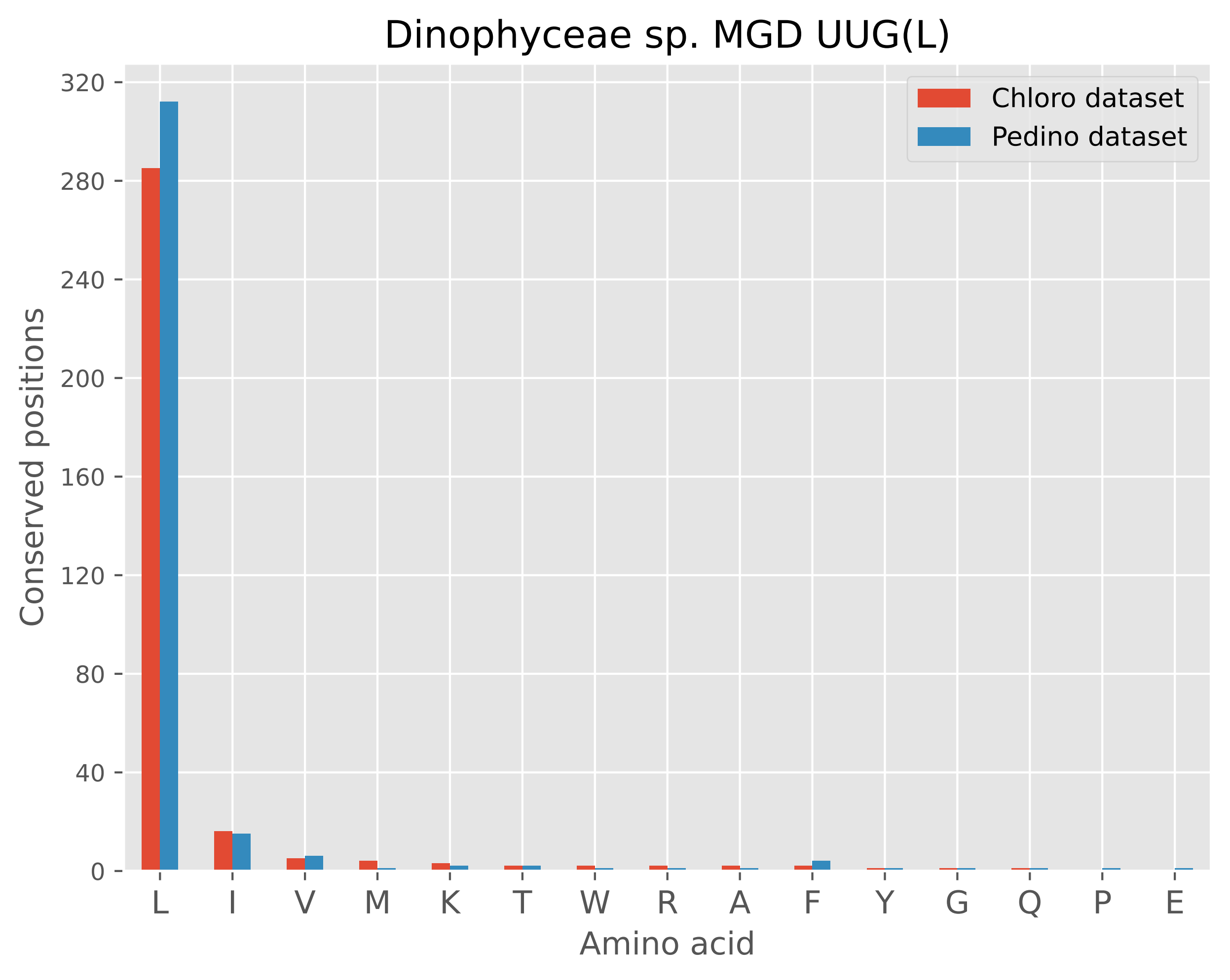

Dinophyceae sp. MGD UUG(L)
Chloro dataset
Pedino dataset
Conserved positions
0
40
80
120
160
200
240
280
320
L
I
V
M
K
T
W
R
A
F
Y
G
Q
P
E
Amino acid

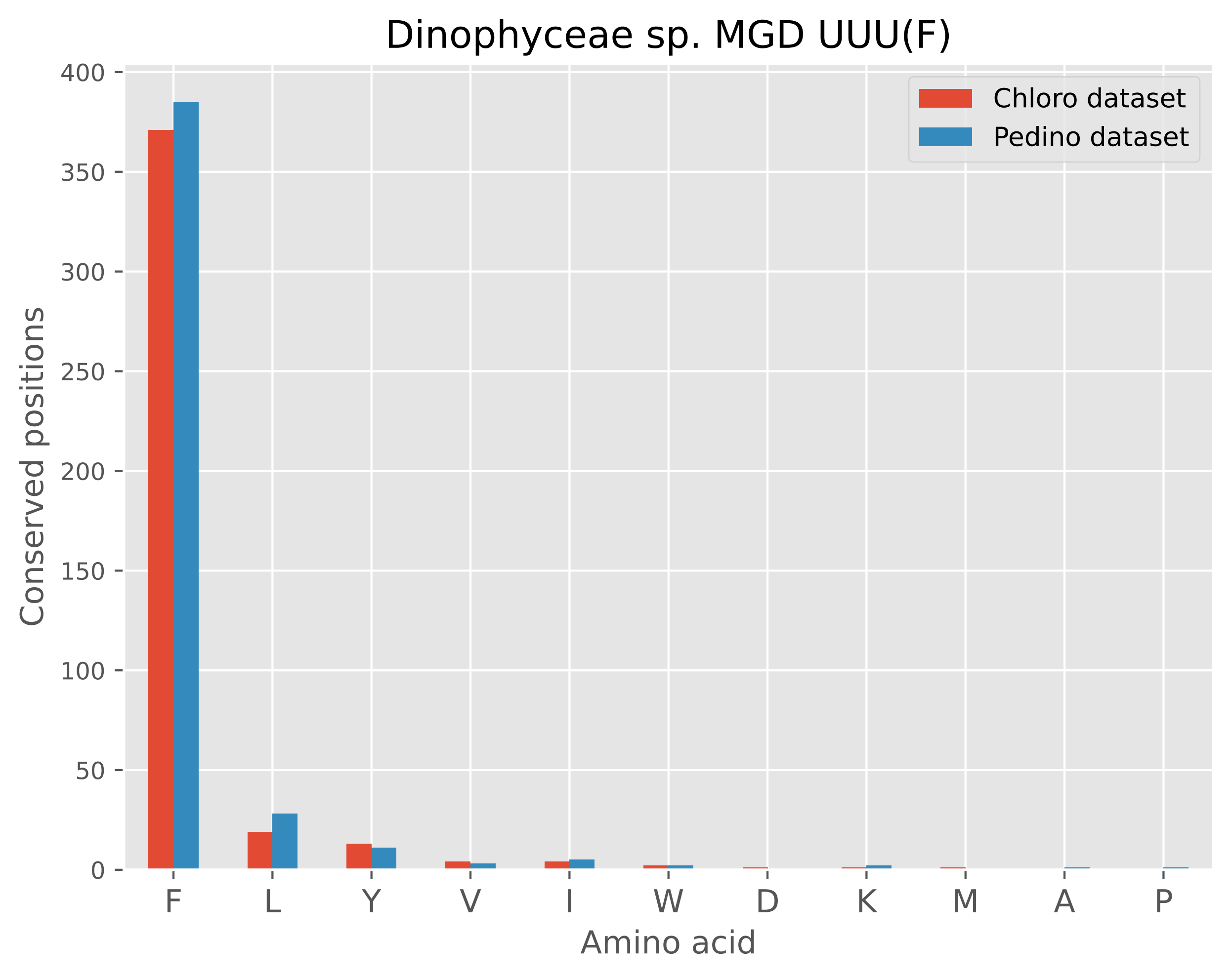
Dinophyceae sp. MGD UUU(F)
Chloro dataset
Pedino dataset
Conserved positions
0
50
100
150
200
250
300
350
400
F
L
Y
V
I
W
D
K
M
A
P
Amino acid

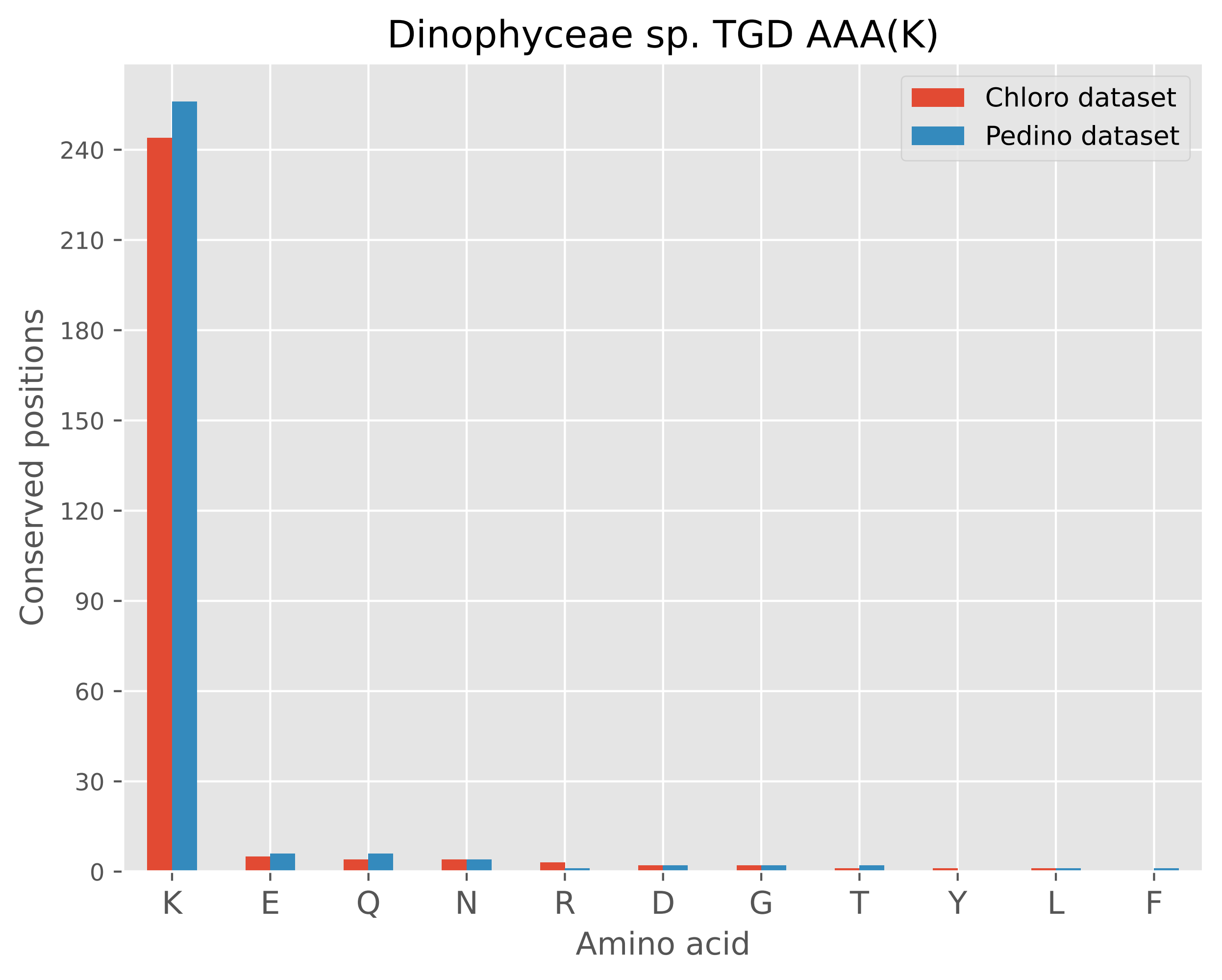

Dinophyceae sp. TGD AAA(K)
Chloro dataset
Pedino dataset
Conserved positions
0
30
60
90
120
150
180
210
240
K
E
Q
N
R
D
G
T
Y
L
F
Amino acid

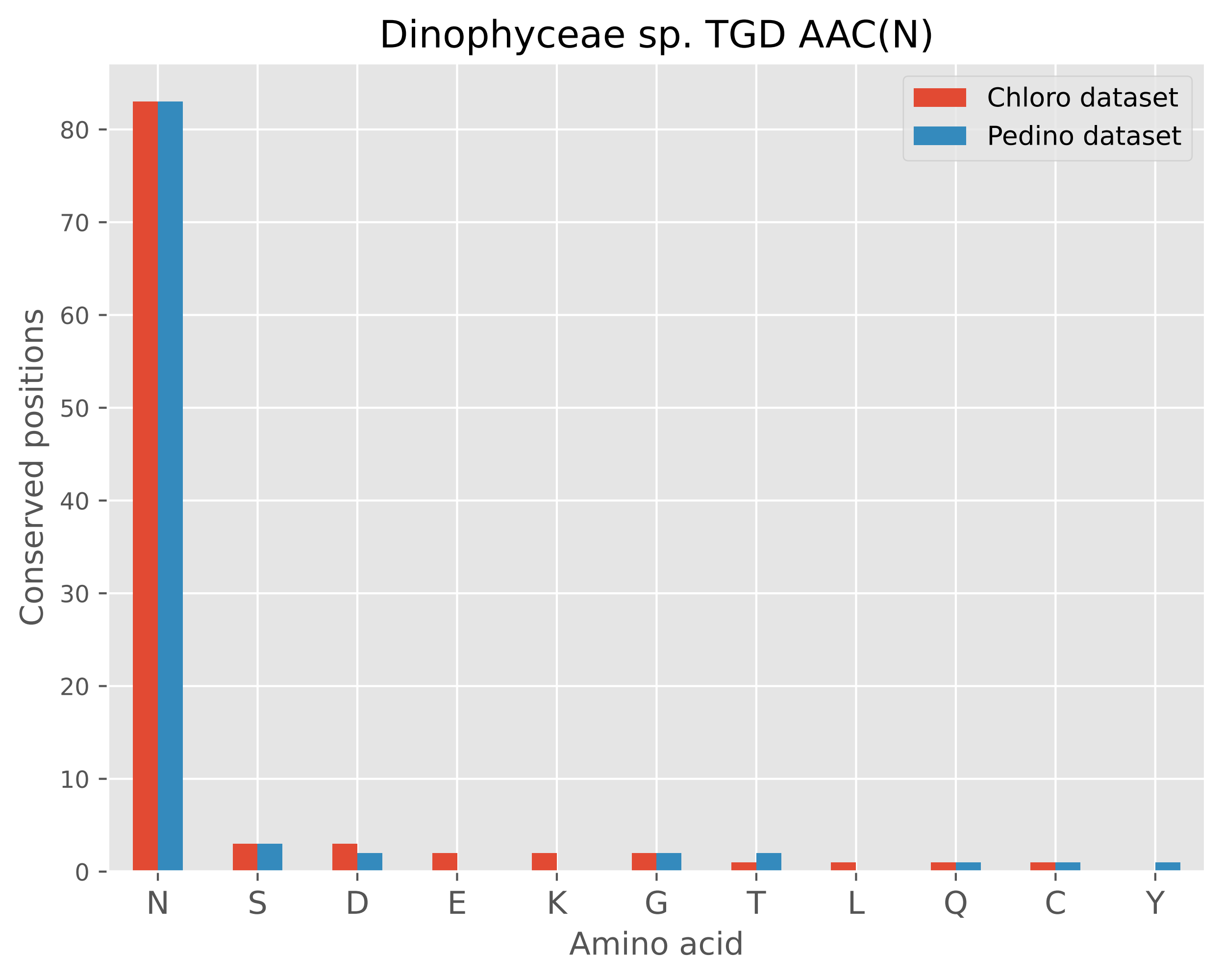
Dinophyceae sp. TGD AAC(N)
Chloro dataset
Pedino dataset
Conserved positions
0
10
20
30
40
50
60
70
80
N
S
D
E
K
G
T
L
Q
C
Y
Amino acid

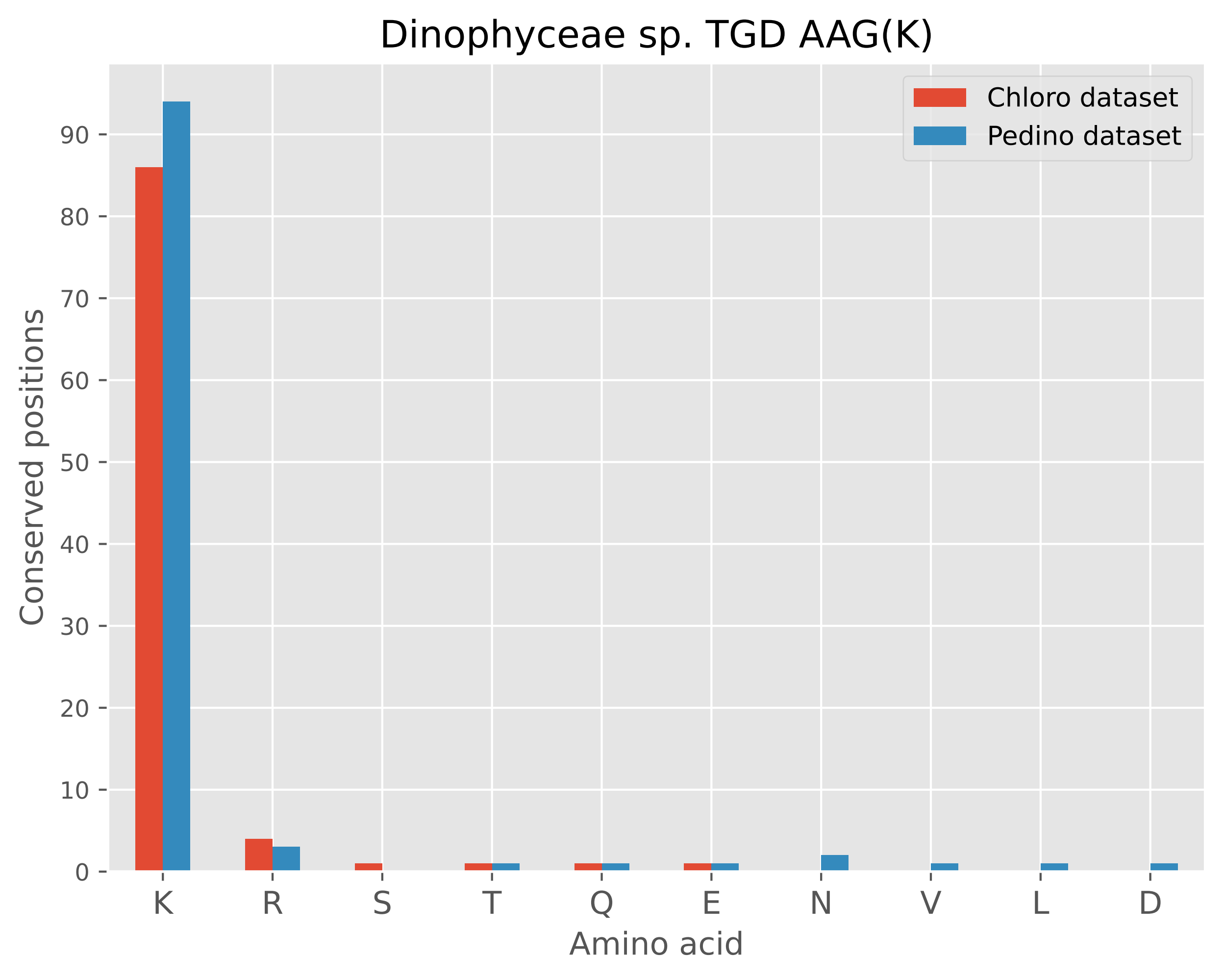

Dinophyceae sp. TGD AAG(K)
Chloro dataset
Pedino dataset
Conserved positions
0
10
20
30
40
50
60
70
80
90
K
R
S
T
Q
E
N
V
L
D
Amino acid

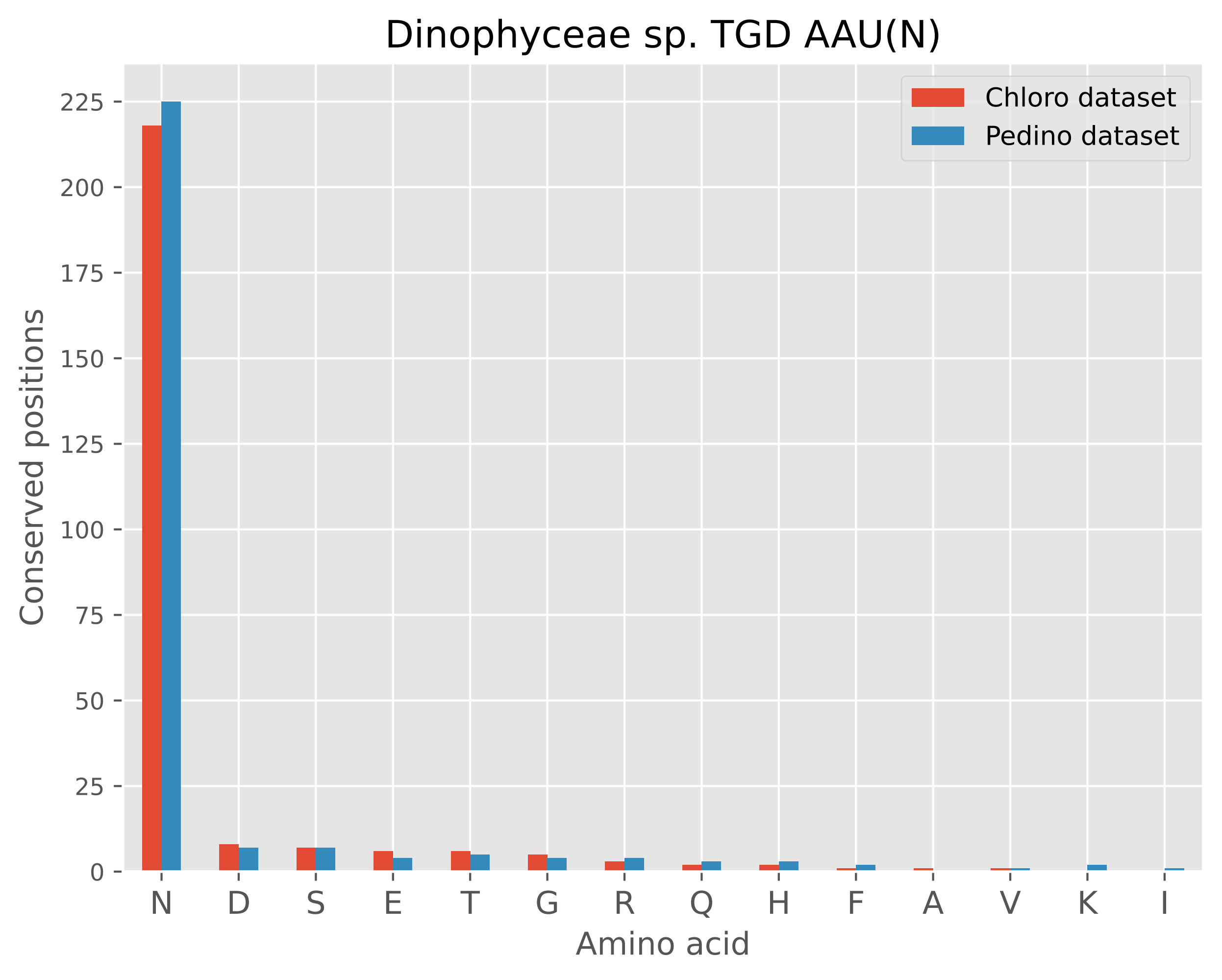

Dinophyceae sp. TGD AAU(N)
Chloro dataset
Pedino dataset
Conserved positions
0
25
50
75
100
125
150
175
200
225
N
D
S
E
T
G
R
Q
H
F
A
V
K
I
Amino acid

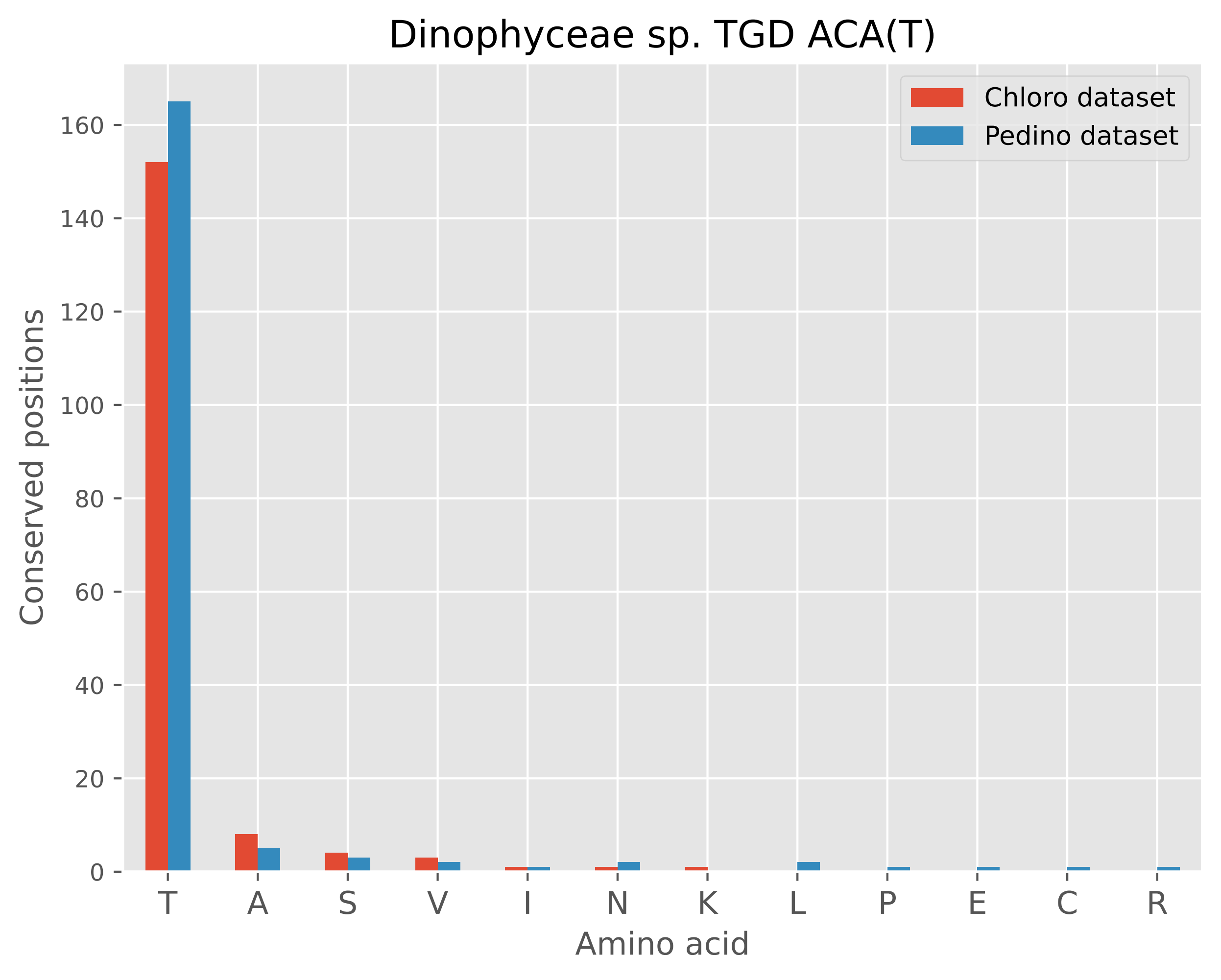
Dinophyceae sp. TGD ACA(T)
Chloro dataset
Pedino dataset
Conserved positions
0
20
40
60
80
100
120
140
160
T
A
S
V
I
N
K
L
P
E
C
R
Amino acid

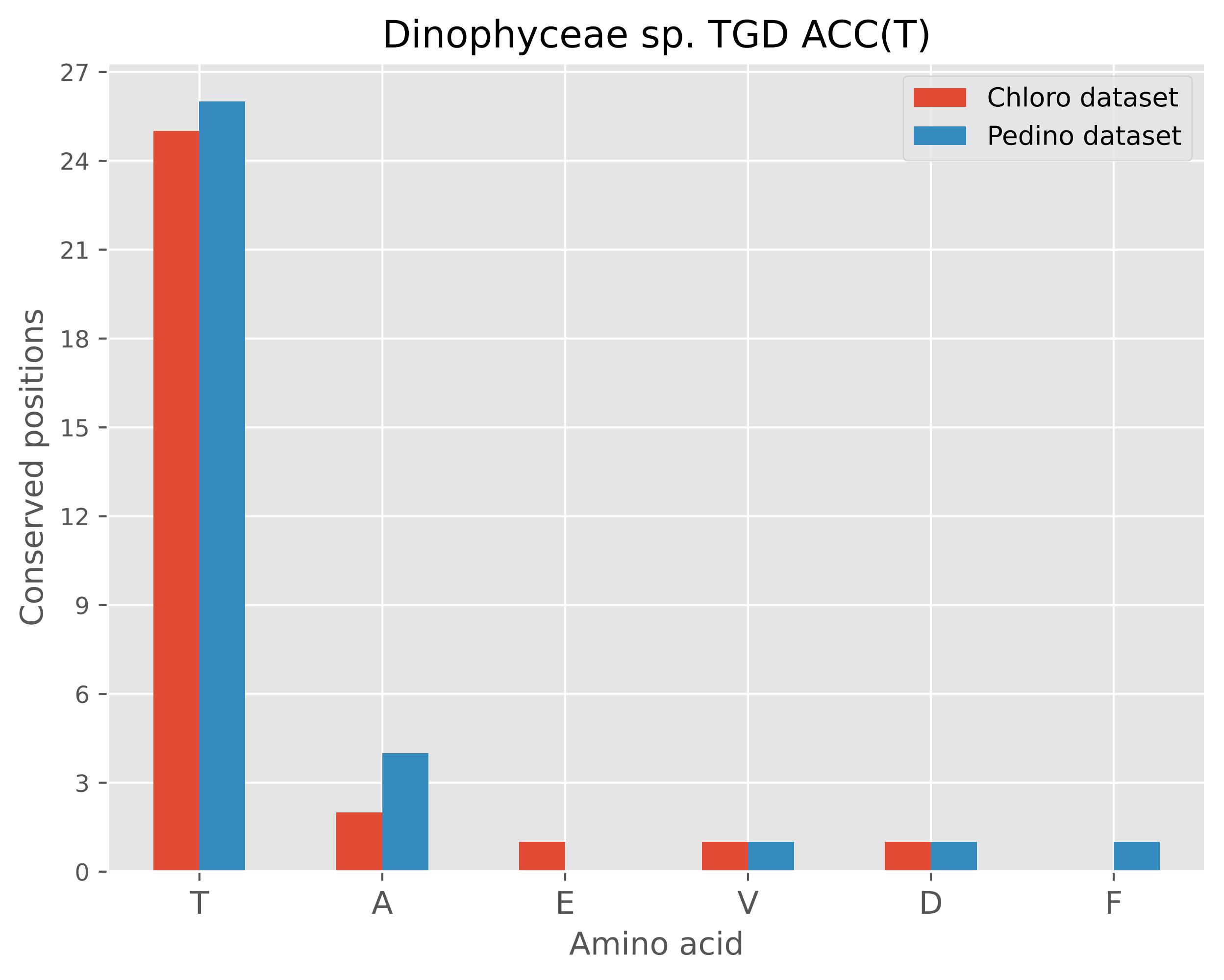
Dinophyceae sp. TGD ACC(T)
Chloro dataset
Pedino dataset
Conserved positions
0
3
6
9
12
15
18
21
24
27
T
A
E
V
D
F
Amino acid

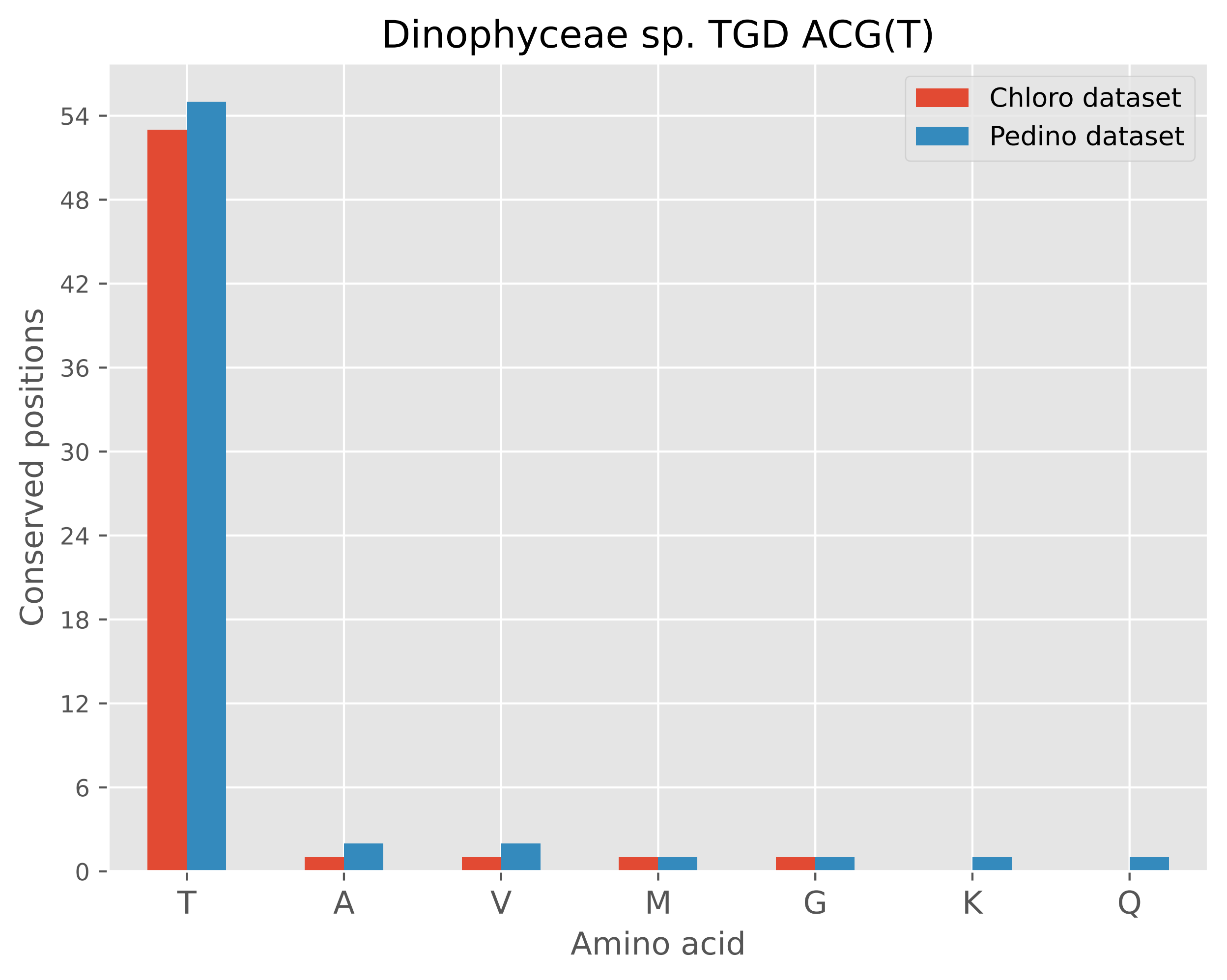

Dinophyceae sp. TGD ACG(T)
Chloro dataset
Pedino dataset
Conserved positions
0
6
12
18
24
30
36
42
48
54
T
A
V
M
G
K
Q
Amino acid

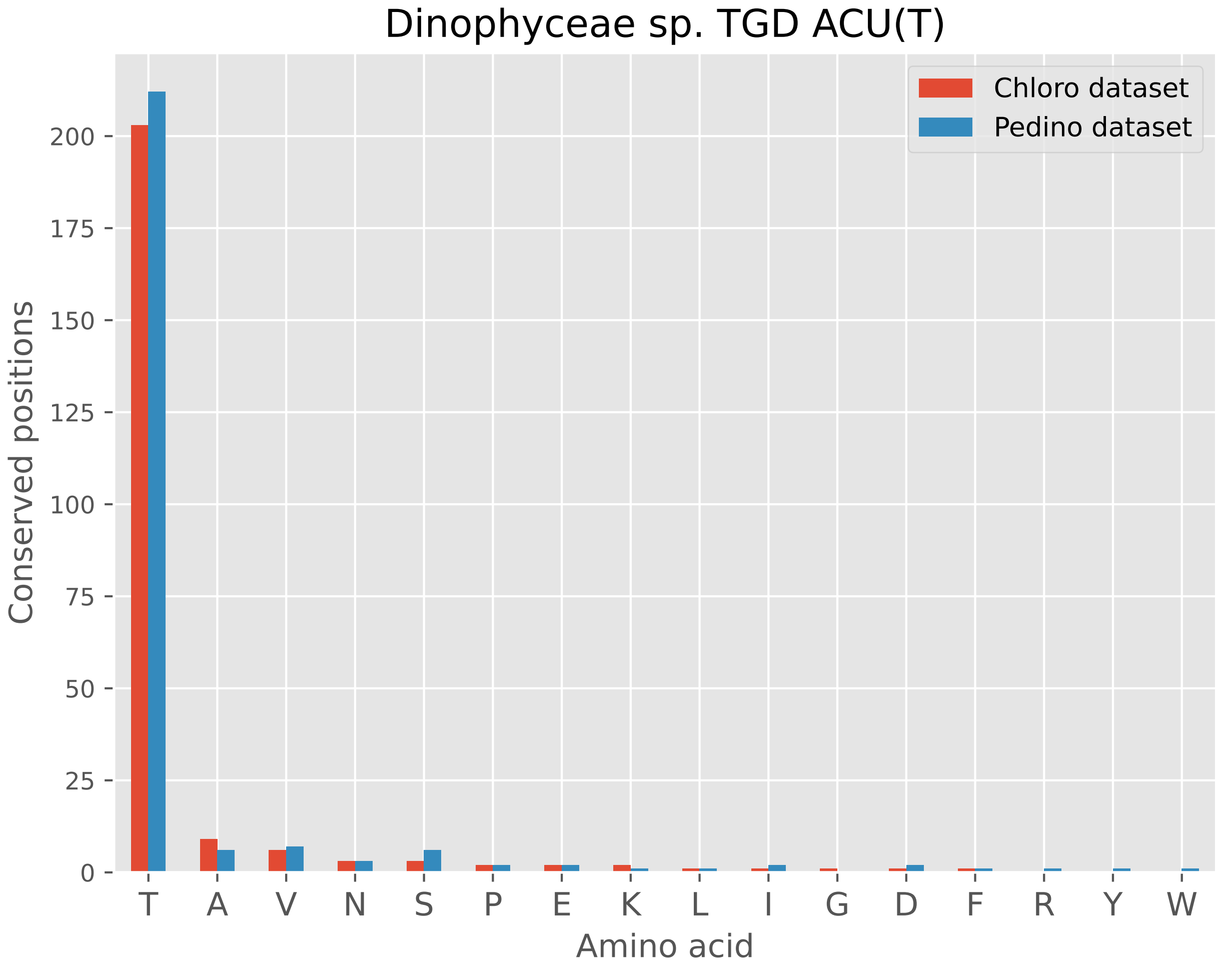

Dinophyceae sp. TGD ACU(T)
Chloro dataset
Pedino dataset
Conserved positions
0
25
50
75
100
125
150
175
200
T
A
V
N
S
P
E
K
L
I
G
D
F
R
Y
W
Amino acid

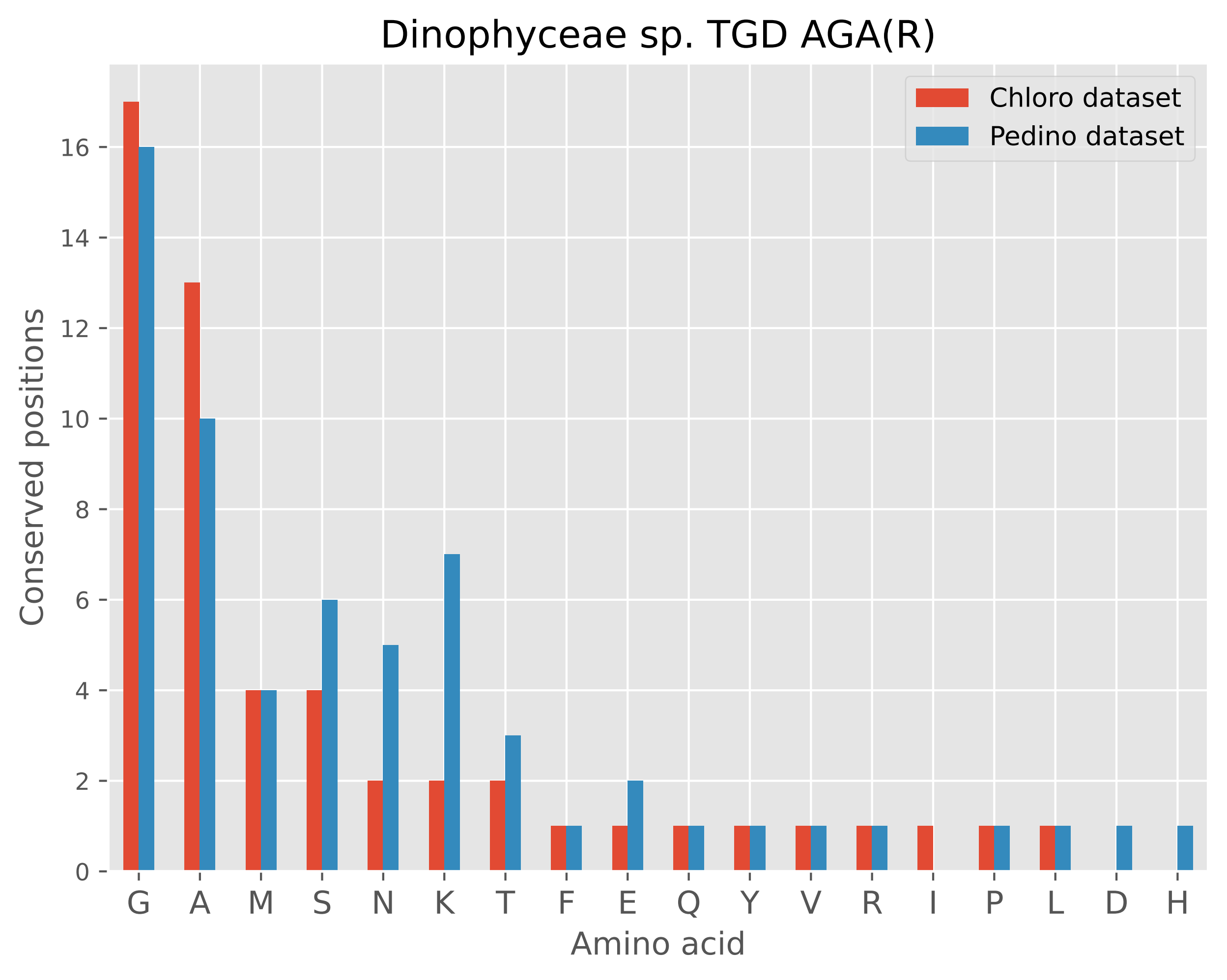
Dinophyceae sp. TGD AGA(R)
Chloro dataset
Pedino dataset
Conserved positions
Amino acid
0
2
4
6
8
10
12
14
16
G
A
M
S
N
K
T
F
E
Q
Y
V
R
I
P
L
D
H

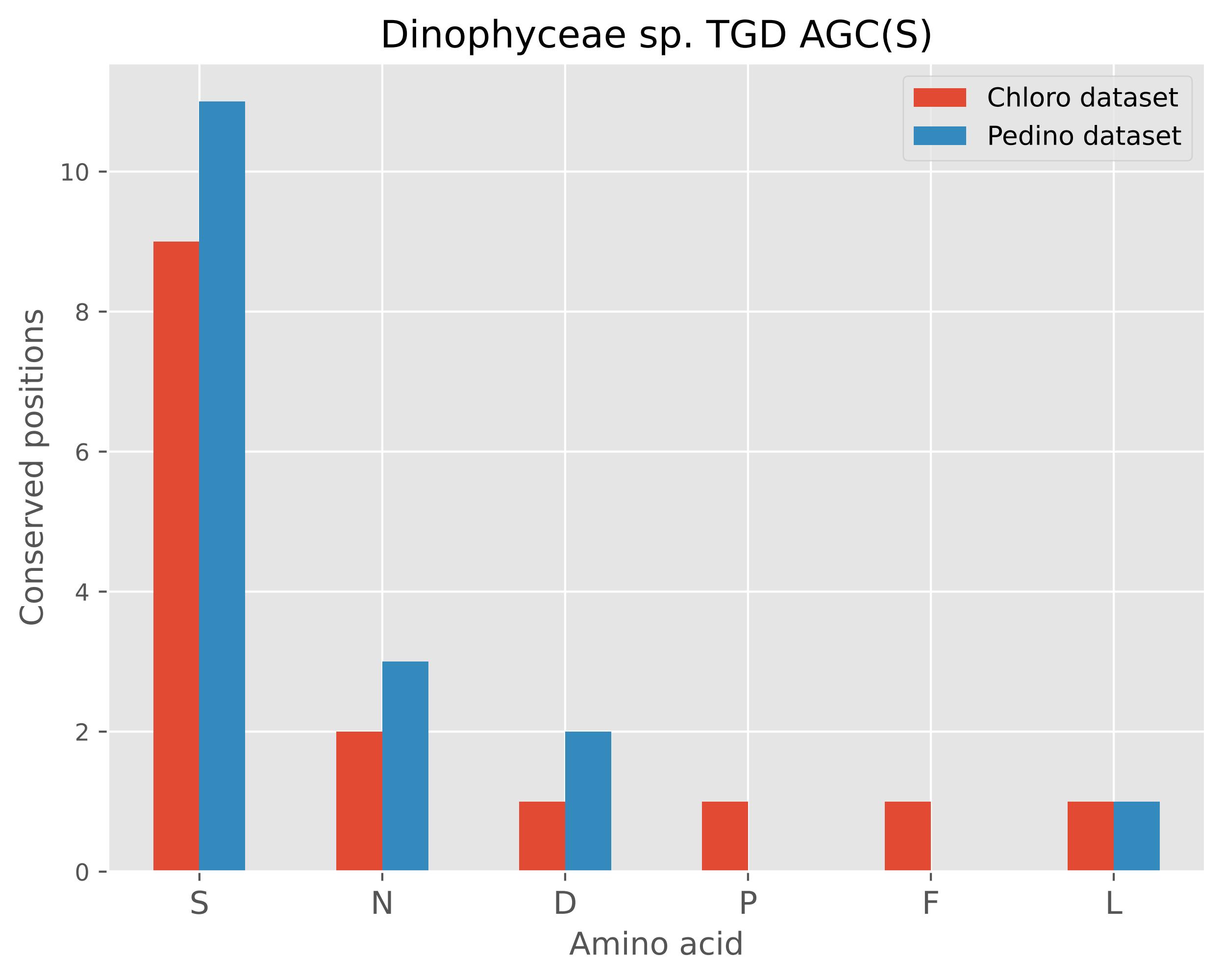
Dinophyceae sp. TGD AGC(S)
Chloro dataset
Pedino dataset
Conserved positions
0
2
4
6
8
10
S
N
D
P
F
L
Amino acid

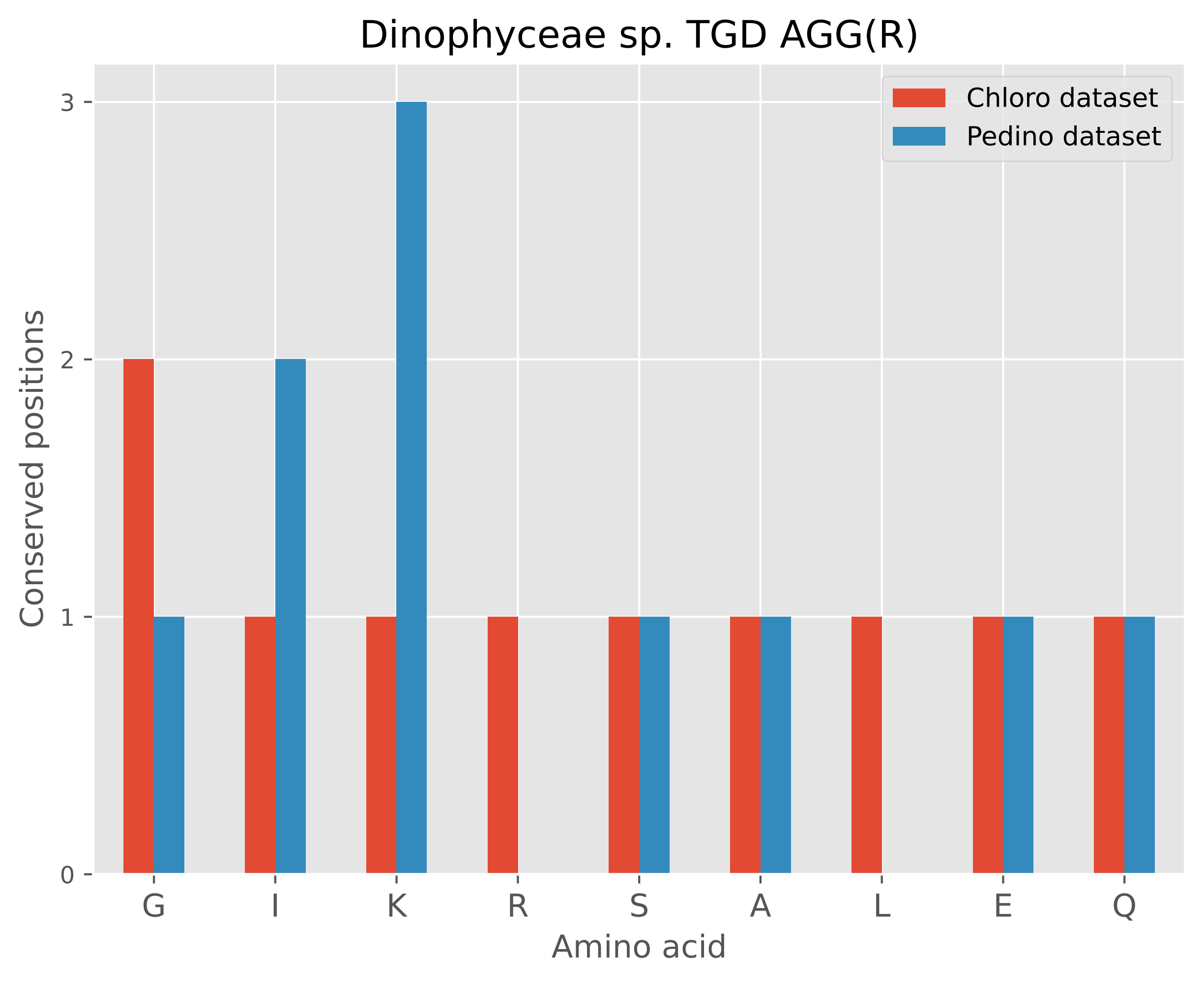
Dinophyceae sp. TGD AGG(R)
Chloro dataset
Pedino dataset
Conserved positions
0
1
2
3
G
I
K
R
S
A
L
E
Q
Amino acid

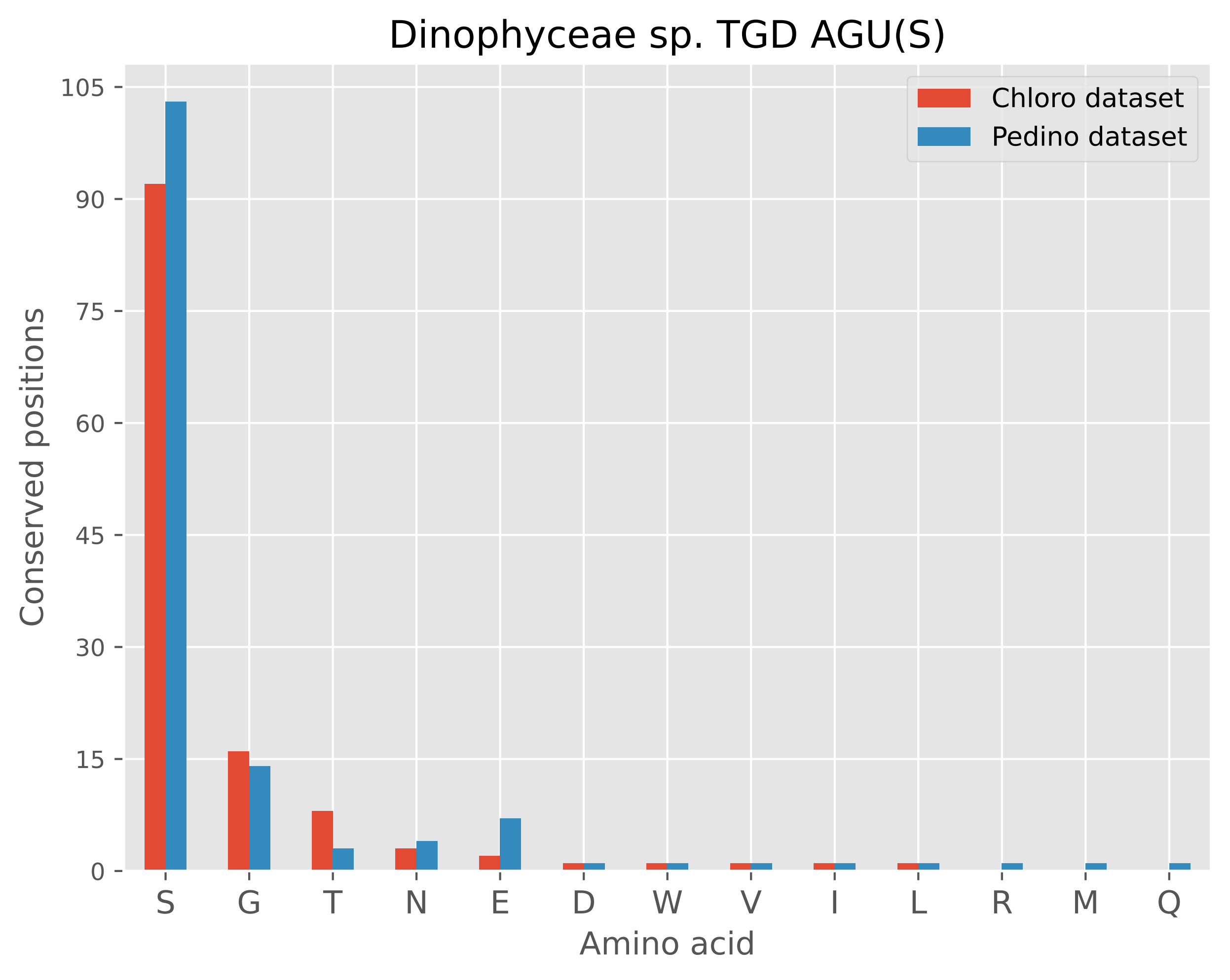
Dinophyceae sp. TGD AGU(S)
Chloro dataset
Pedino dataset
Conserved positions
105
90
75
60
45
30
15
0
S
G
T
N
E
D
W
V
I
L
R
M
Q
Amino acid

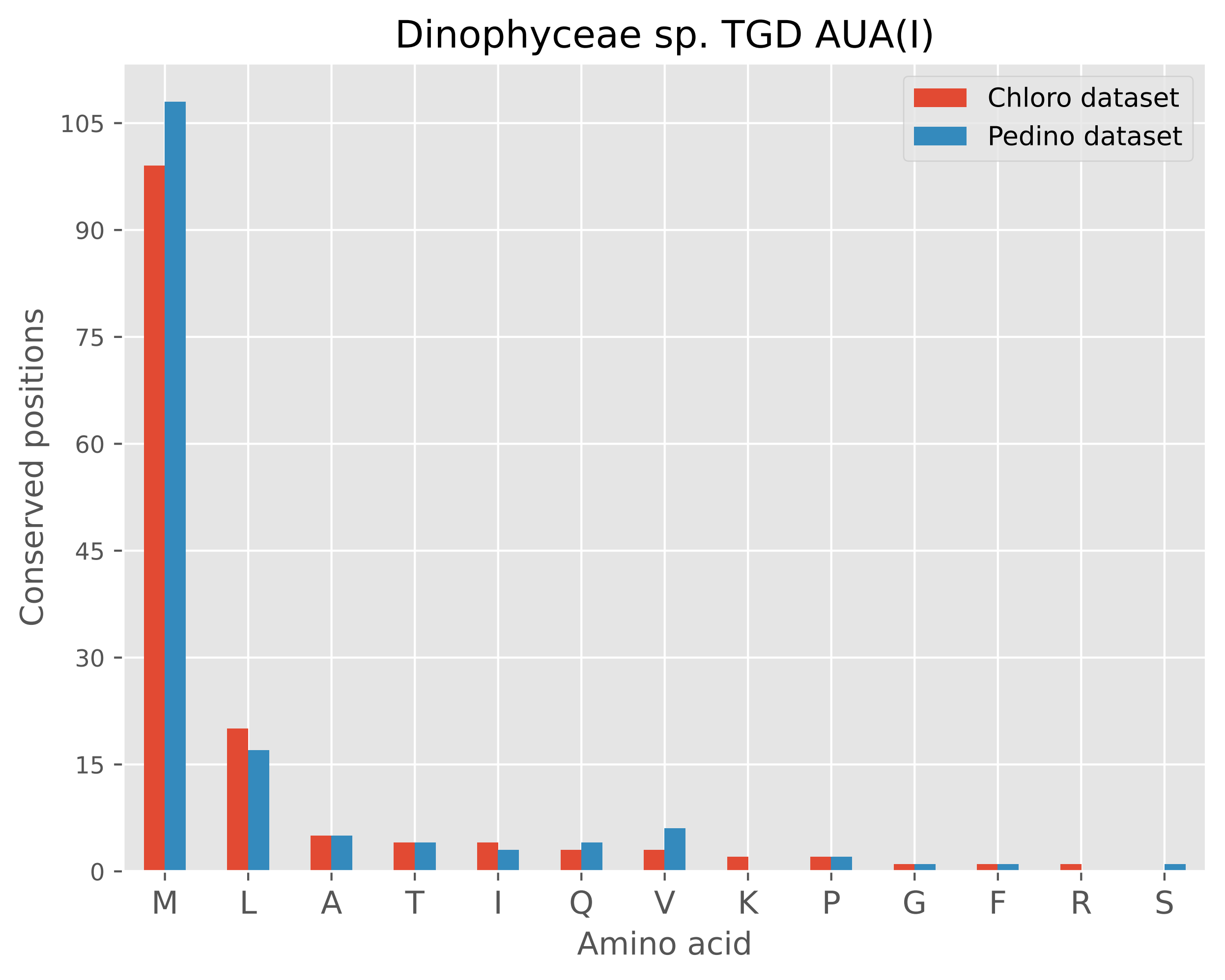
Dinophyceae sp. TGD AUA(I)
Chloro dataset
Pedino dataset
Conserved positions
0
15
30
45
60
75
90
105
M
L
A
T
I
Q
V
K
P
G
F
R
S
Amino acid

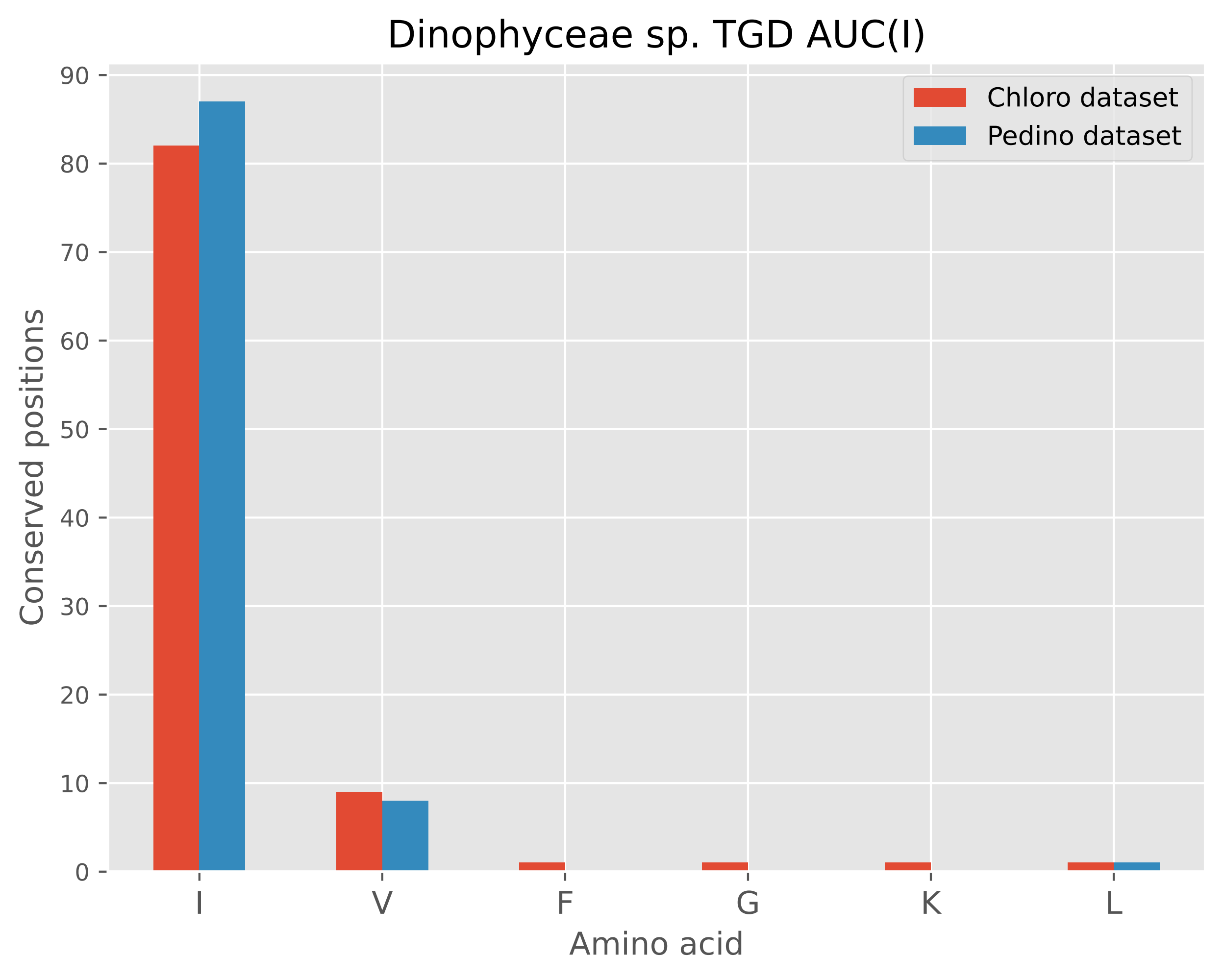

Dinophyceae sp. TGD AUC(I)
Chloro dataset
Pedino dataset
Conserved positions
Amino acid
0
10
20
30
40
50
60
70
80
90
I
V
F
G
K
L

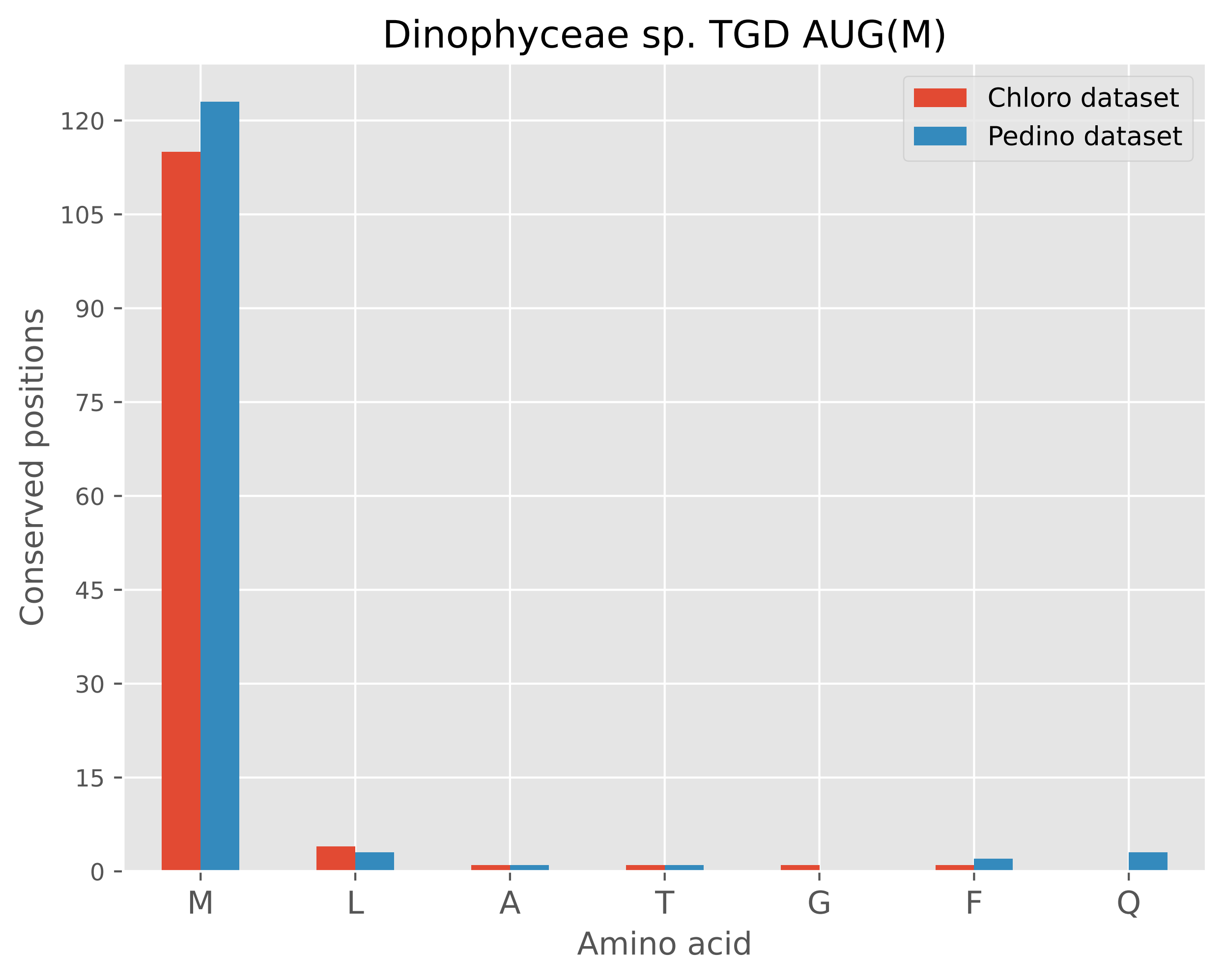
Dinophyceae sp. TGD AUG(M)
Chloro dataset
Pedino dataset
Conserved positions
0
15
30
45
60
75
90
105
120
M
L
A
T
G
F
Q
Amino acid

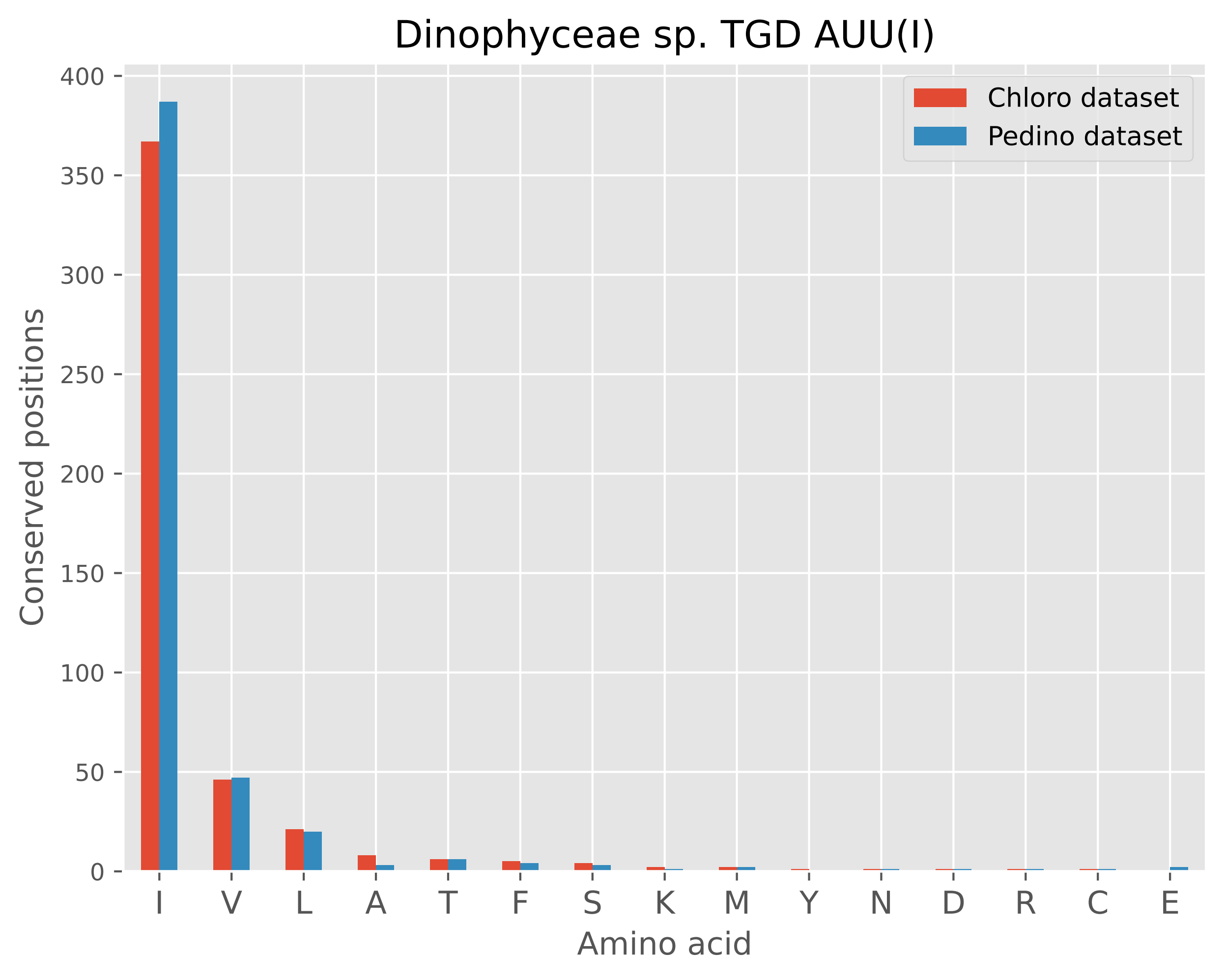
Dinophyceae sp. TGD AUU(I)
Chloro dataset
Pedino dataset
Conserved positions
0
50
100
150
200
250
300
350
400
I
V
L
A
T
F
S
K
M
Y
N
D
R
C
E
Amino acid

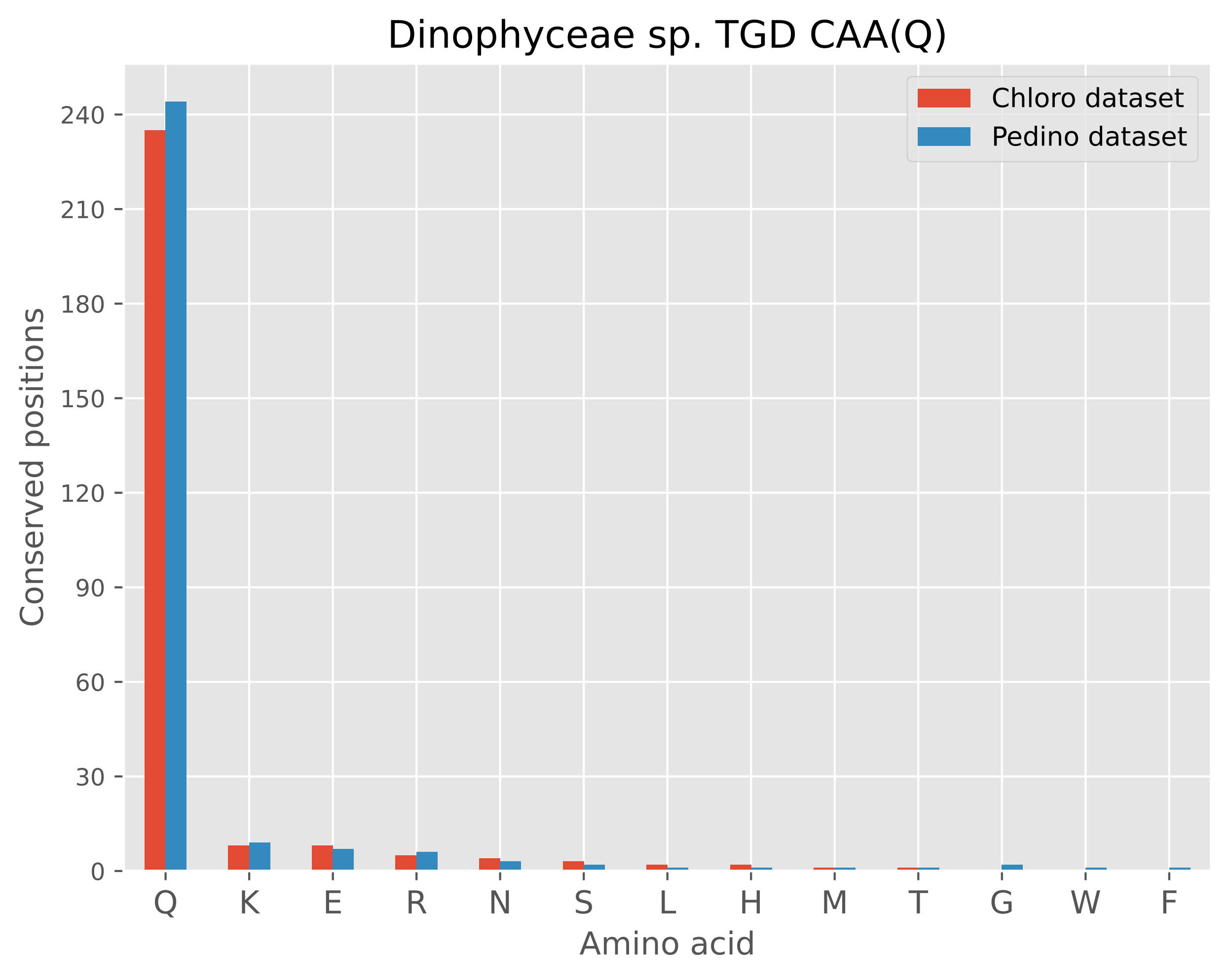

Dinophyceae sp. TGD CAA(Q)
Chloro dataset
Pedino dataset
Conserved positions
0
30
60
90
120
150
180
210
240
Q
K
E
R
N
S
L
H
M
T
G
W
F
Amino acid

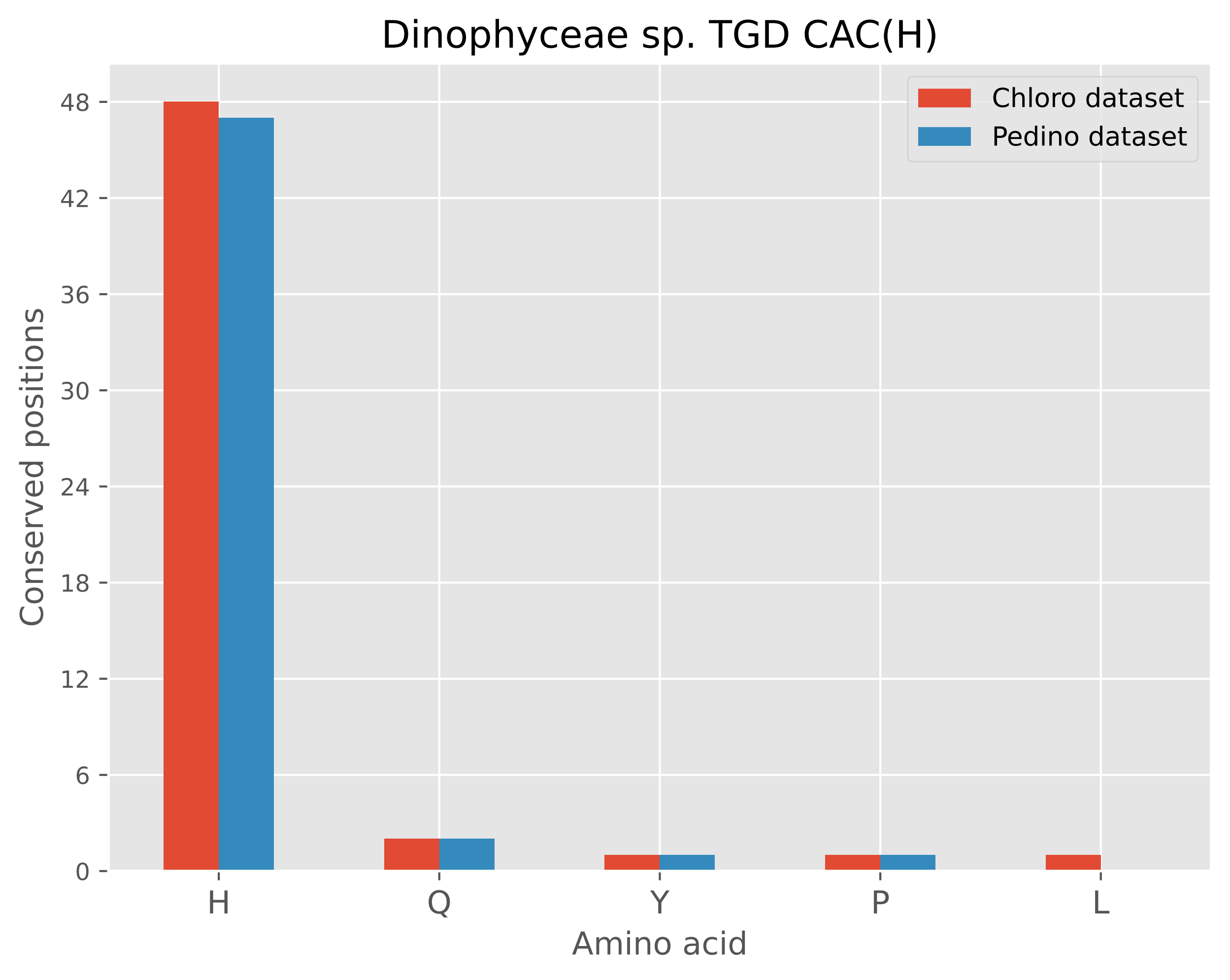

Dinophyceae sp. TGD CAC(H)
Chloro dataset
Pedino dataset
Conserved positions
0
6
12
18
24
30
36
42
48
H
Q
Y
P
L
Amino acid

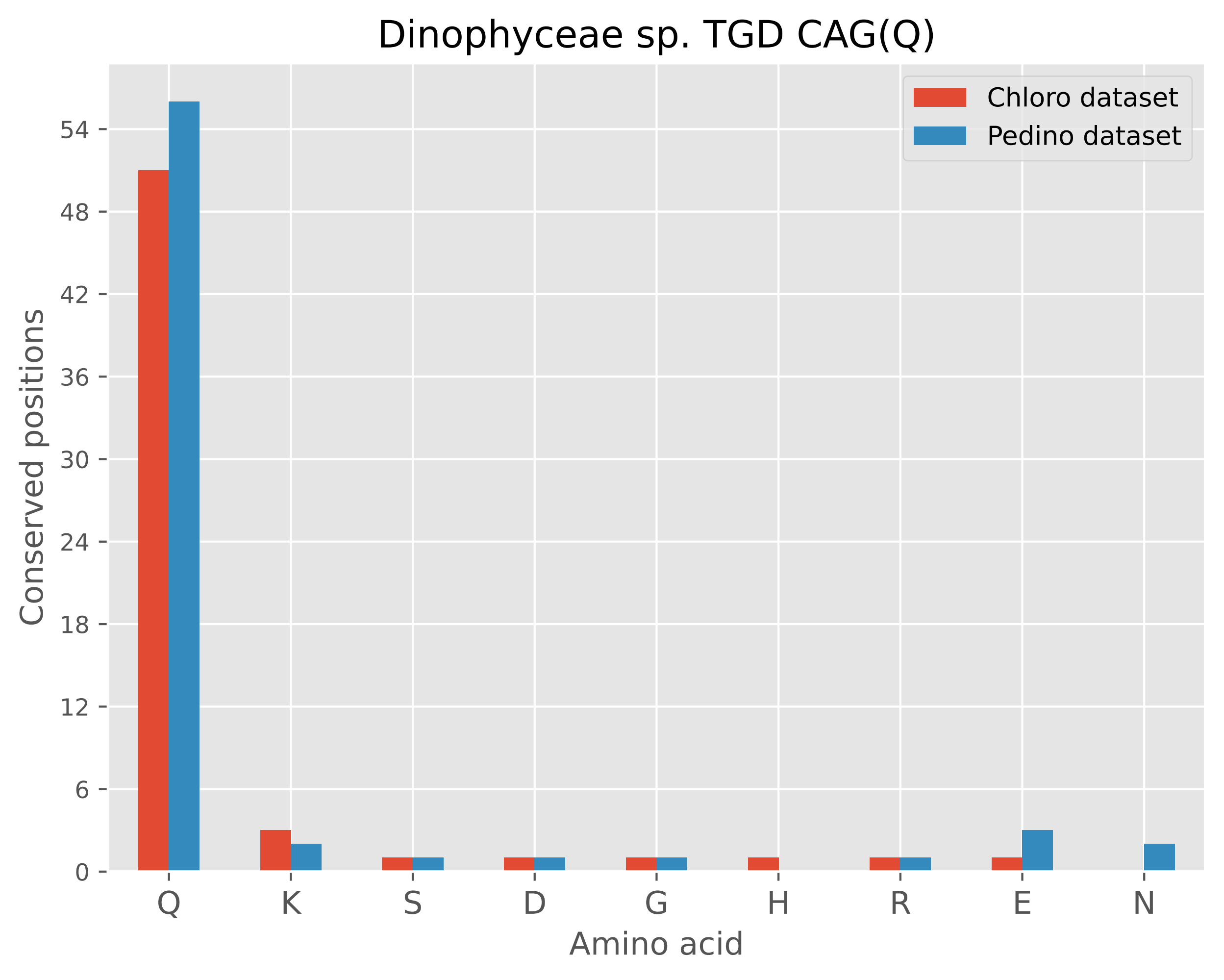

Dinophyceae sp. TGD CAG(Q)
Chloro dataset
Pedino dataset
Conserved positions
0
6
12
18
24
30
36
42
48
54
Q
K
S
D
G
H
R
E
N
Amino acid

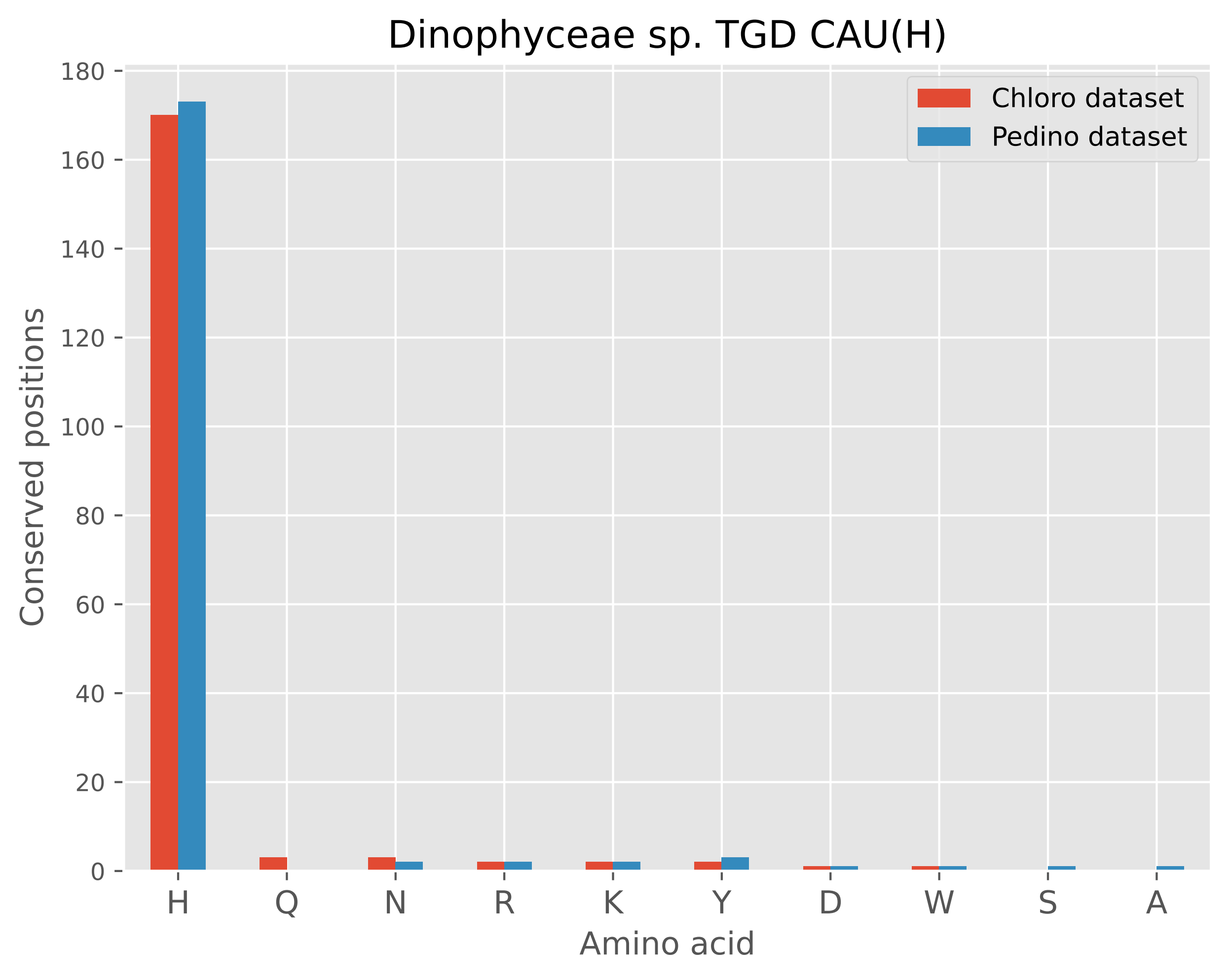

Dinophyceae sp. TGD CAU(H)
Chloro dataset
Pedino dataset
Conserved positions
Amino acid
0
20
40
60
80
100
120
140
160
180
H
Q
N
R
K
Y
D
W
S
A

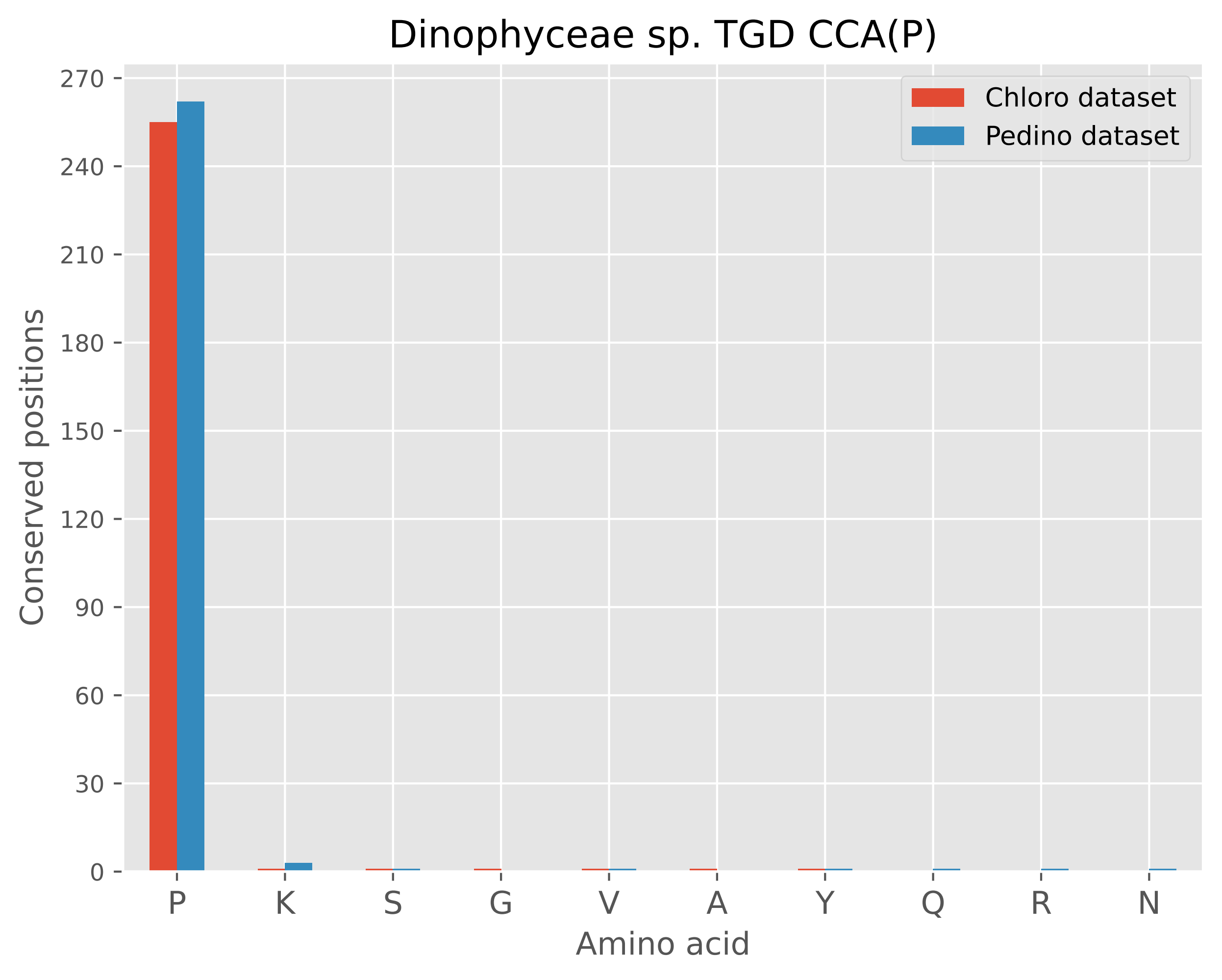

Dinophyceae sp. TGD CCA(P)
Chloro dataset
Pedino dataset
Conserved positions
0
30
60
90
120
150
180
210
240
270
P
K
S
G
V
A
Y
Q
R
N
Amino acid

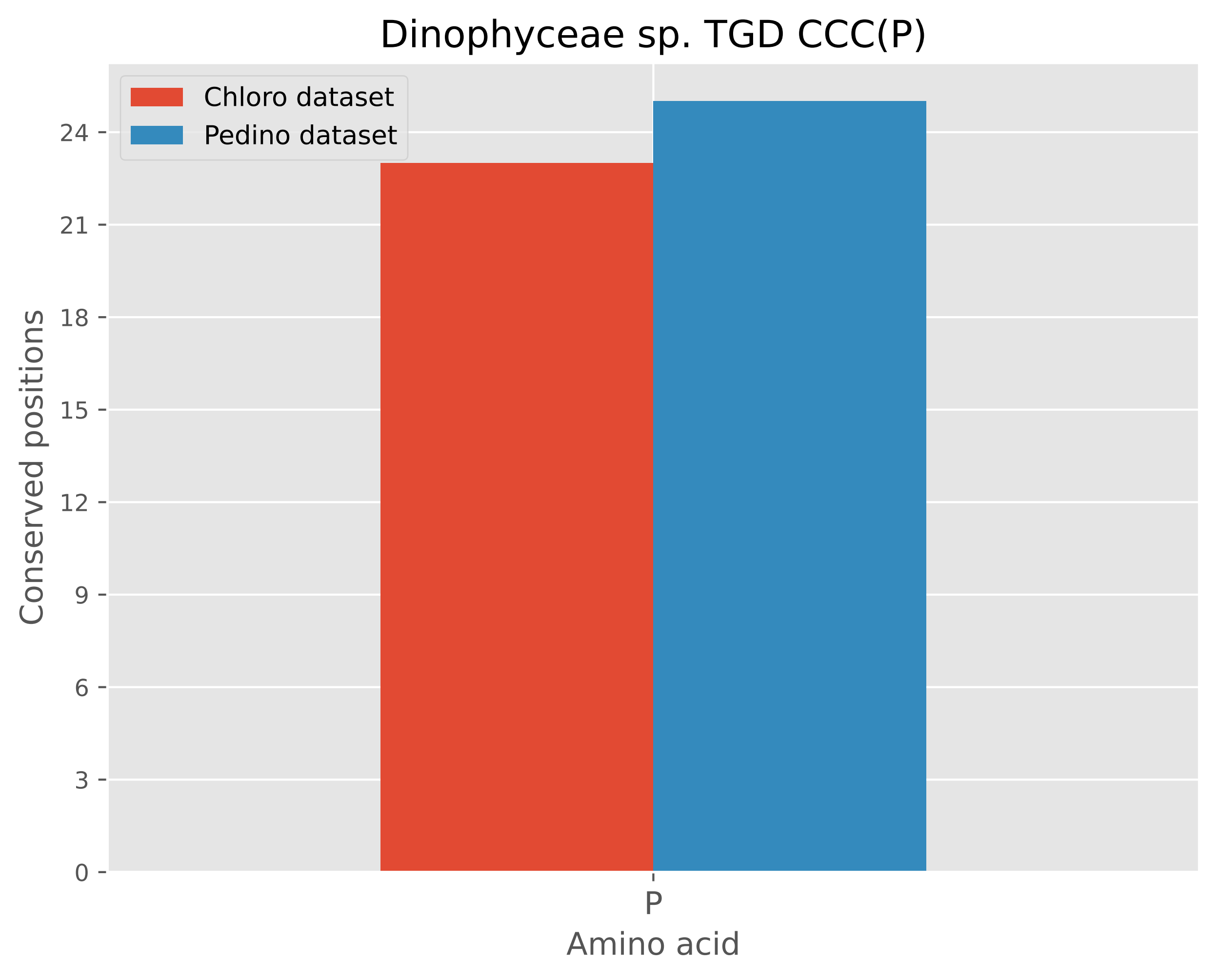
Dinophyceae sp. TGD CCC(P)
Chloro dataset
Pedino dataset
Conserved positions
0
3
6
9
12
15
18
21
24
P
Amino acid

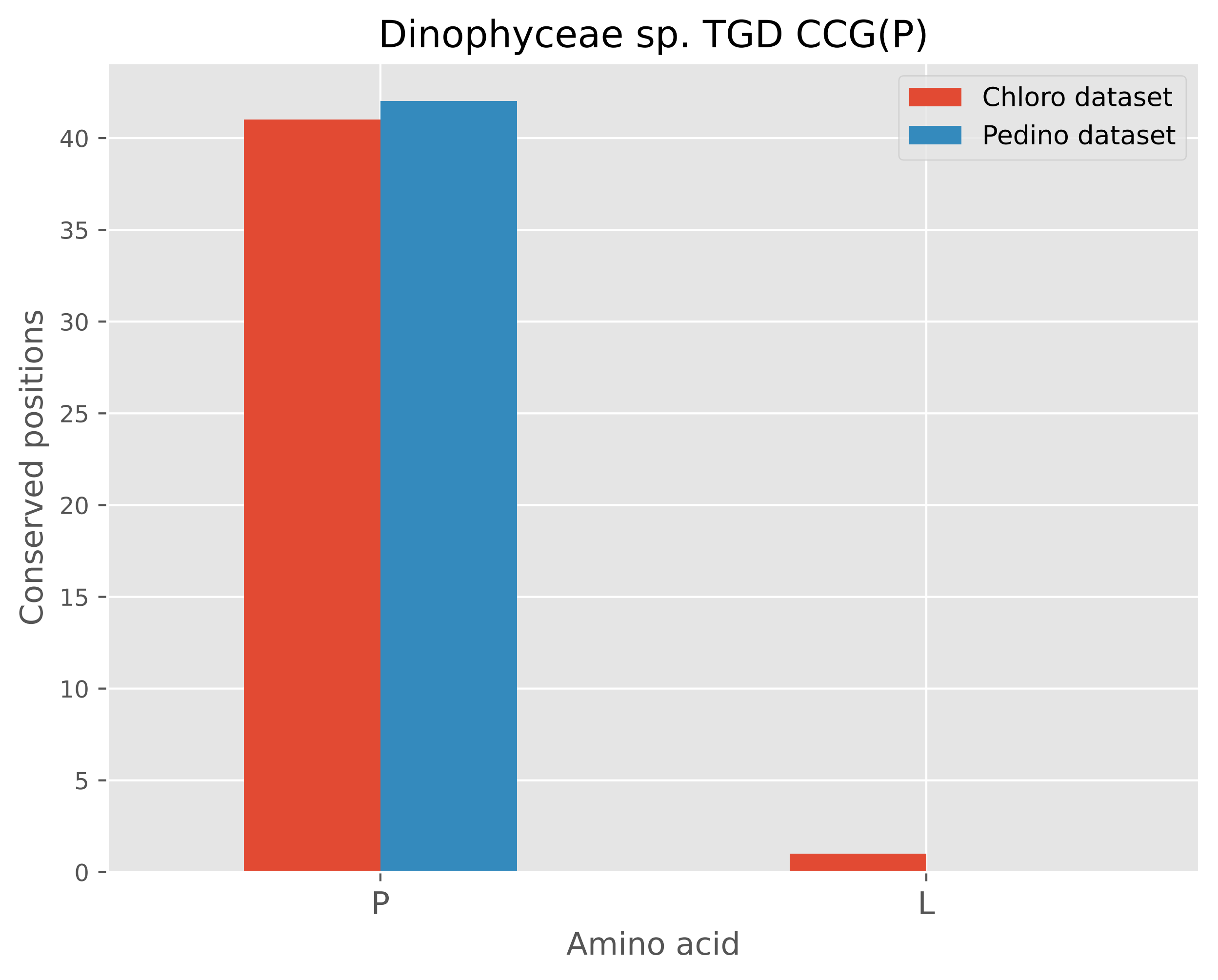

Dinophyceae sp. TGD CCG(P)
Chloro dataset
Pedino dataset
Conserved positions
0
5
10
15
20
25
30
35
40
P
L
Amino acid

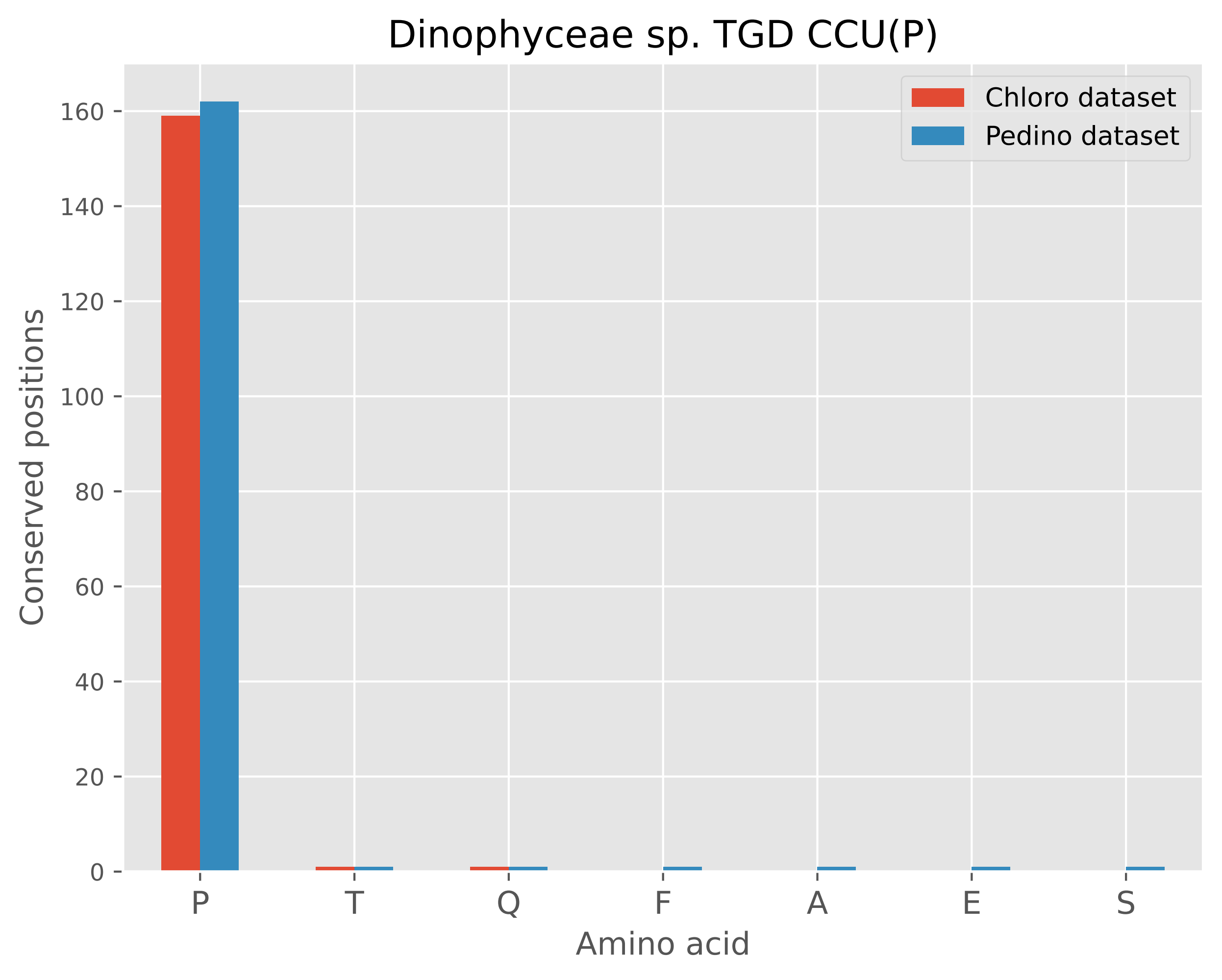
Dinophyceae sp. TGD CCU(P)
Chloro dataset
Pedino dataset
Conserved positions
0
20
40
60
80
100
120
140
160
P
T
Q
F
A
E
S
Amino acid

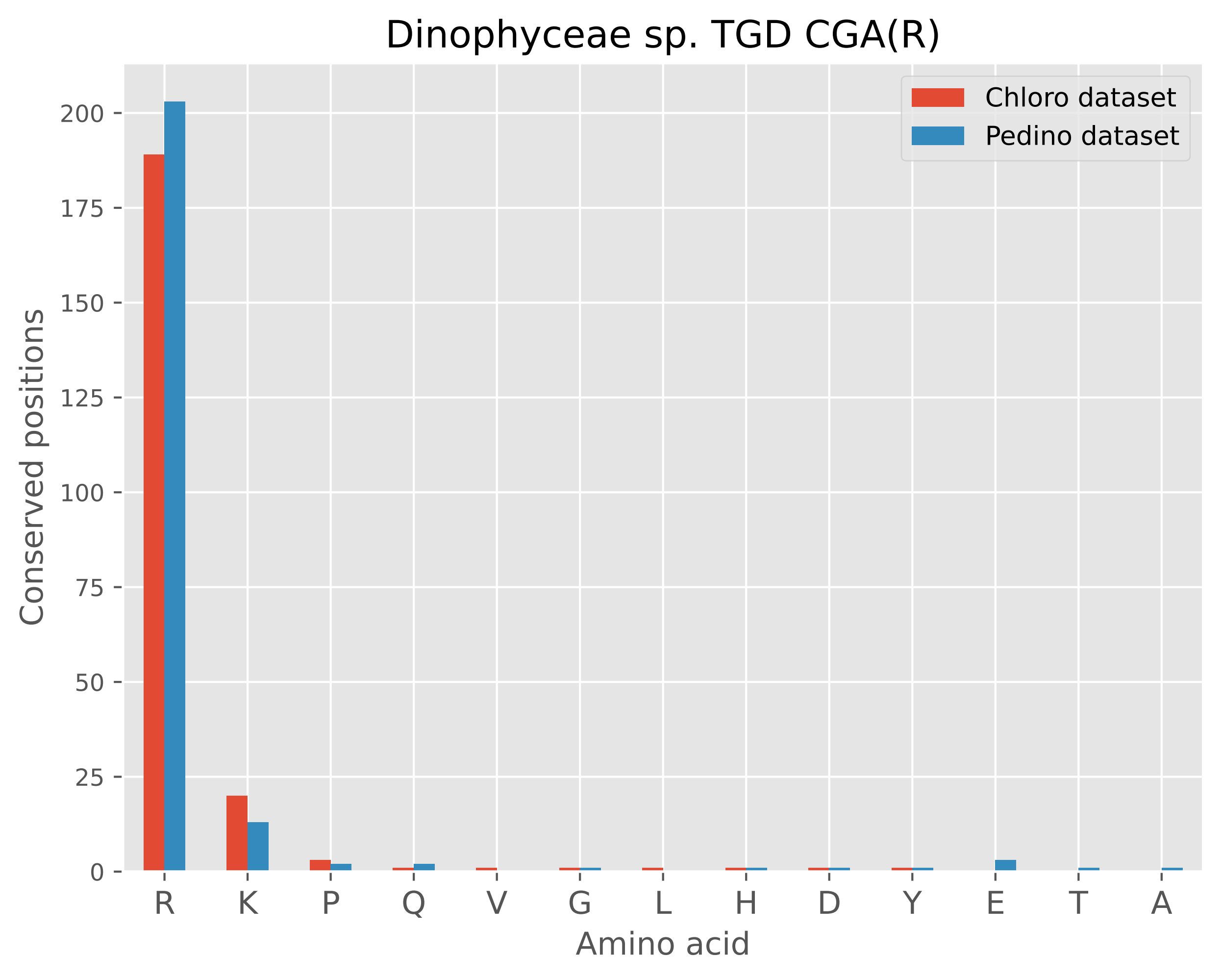

Dinophyceae sp. TGD CGA(R)
Chloro dataset
Pedino dataset
Conserved positions
0
25
50
75
100
125
150
175
200
Amino acid
R
K
P
Q
V
G
L
H
D
Y
E
T
A

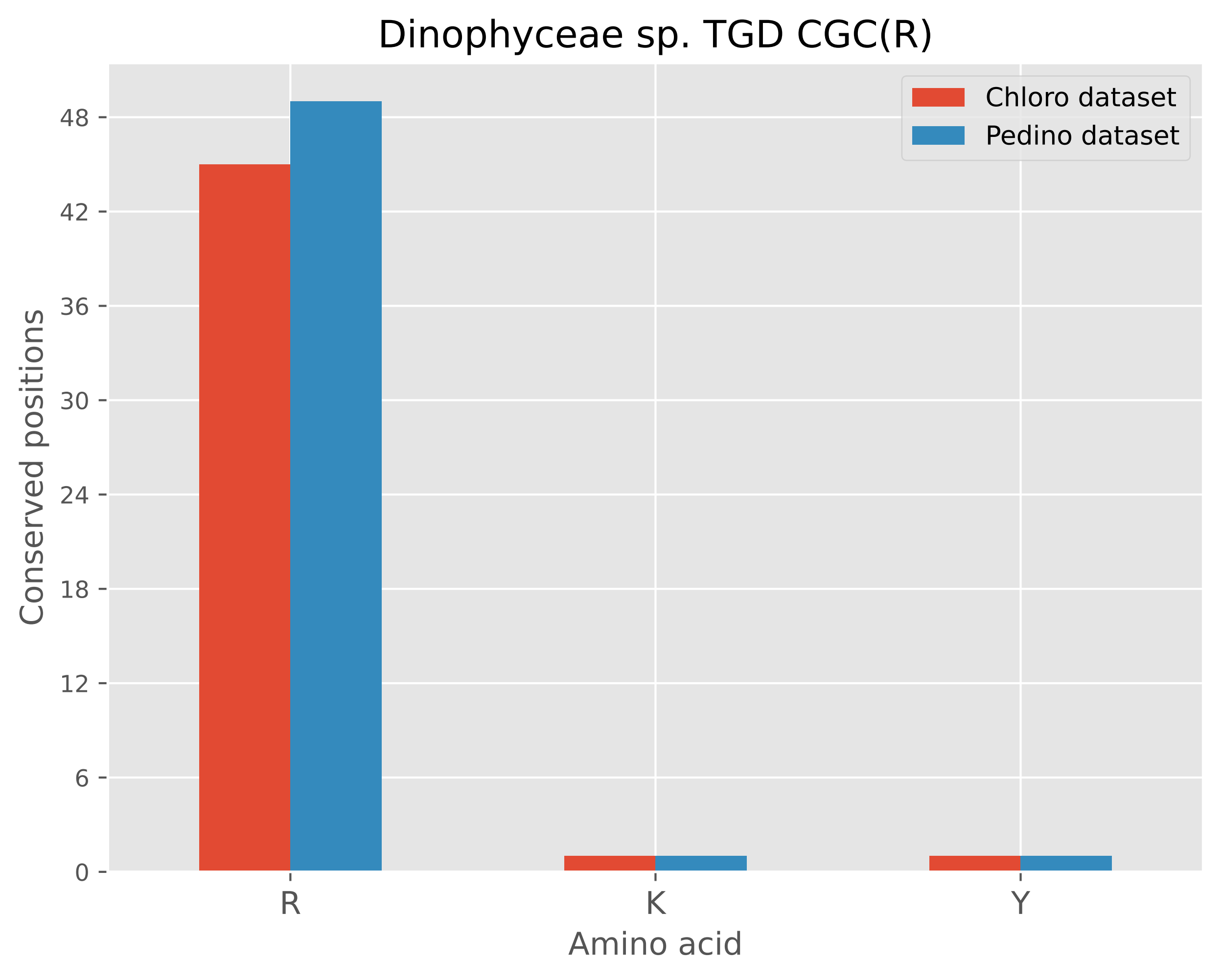

Dinophyceae sp. TGD CGC(R)
Chloro dataset
Pedino dataset
Conserved positions
0
6
12
18
24
30
36
42
48
R
K
Y
Amino acid

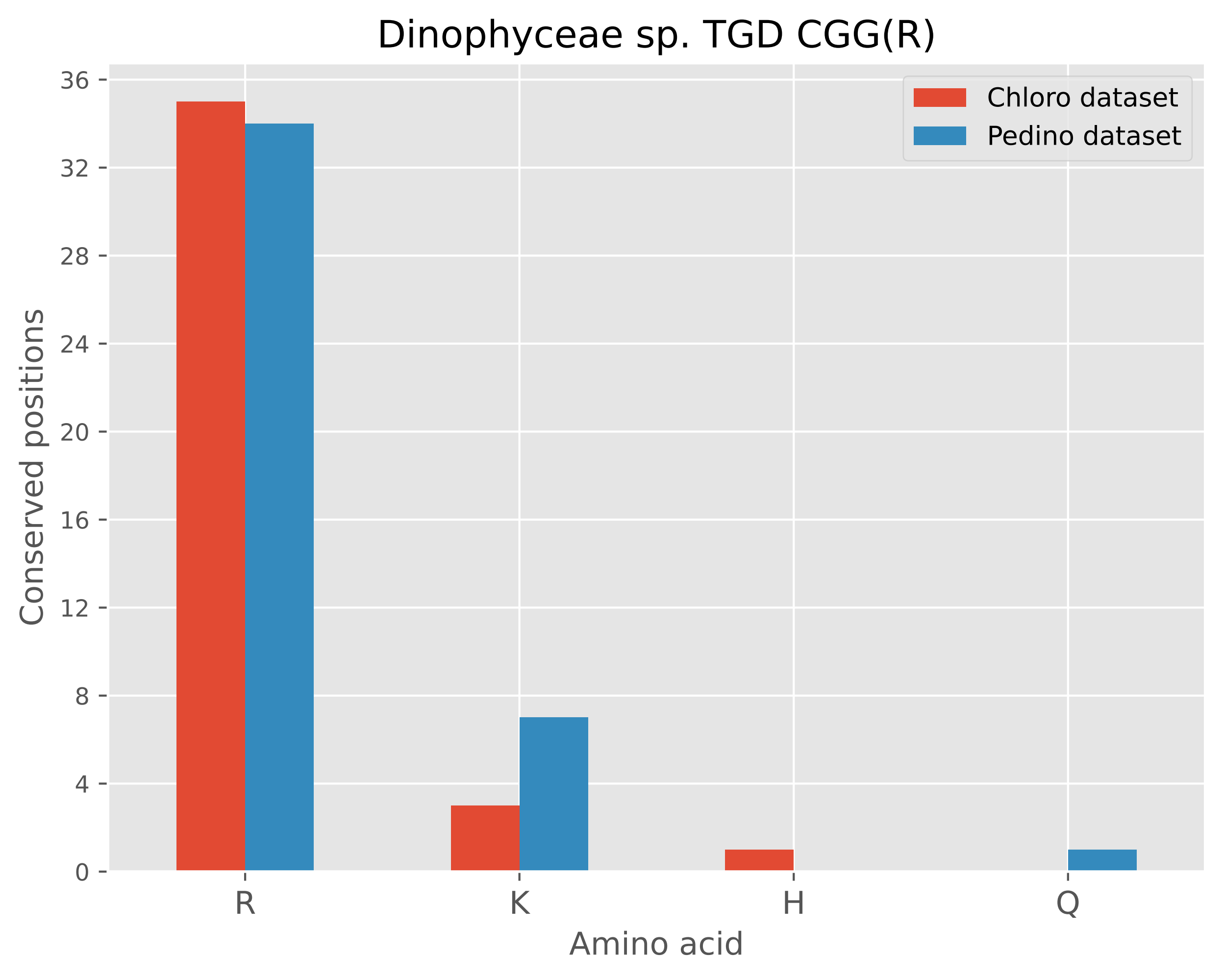

Dinophyceae sp. TGD CGG(R)
Chloro dataset
Pedino dataset
Conserved positions
0
4
8
12
16
20
24
28
32
36
R
K
H
Q
Amino acid

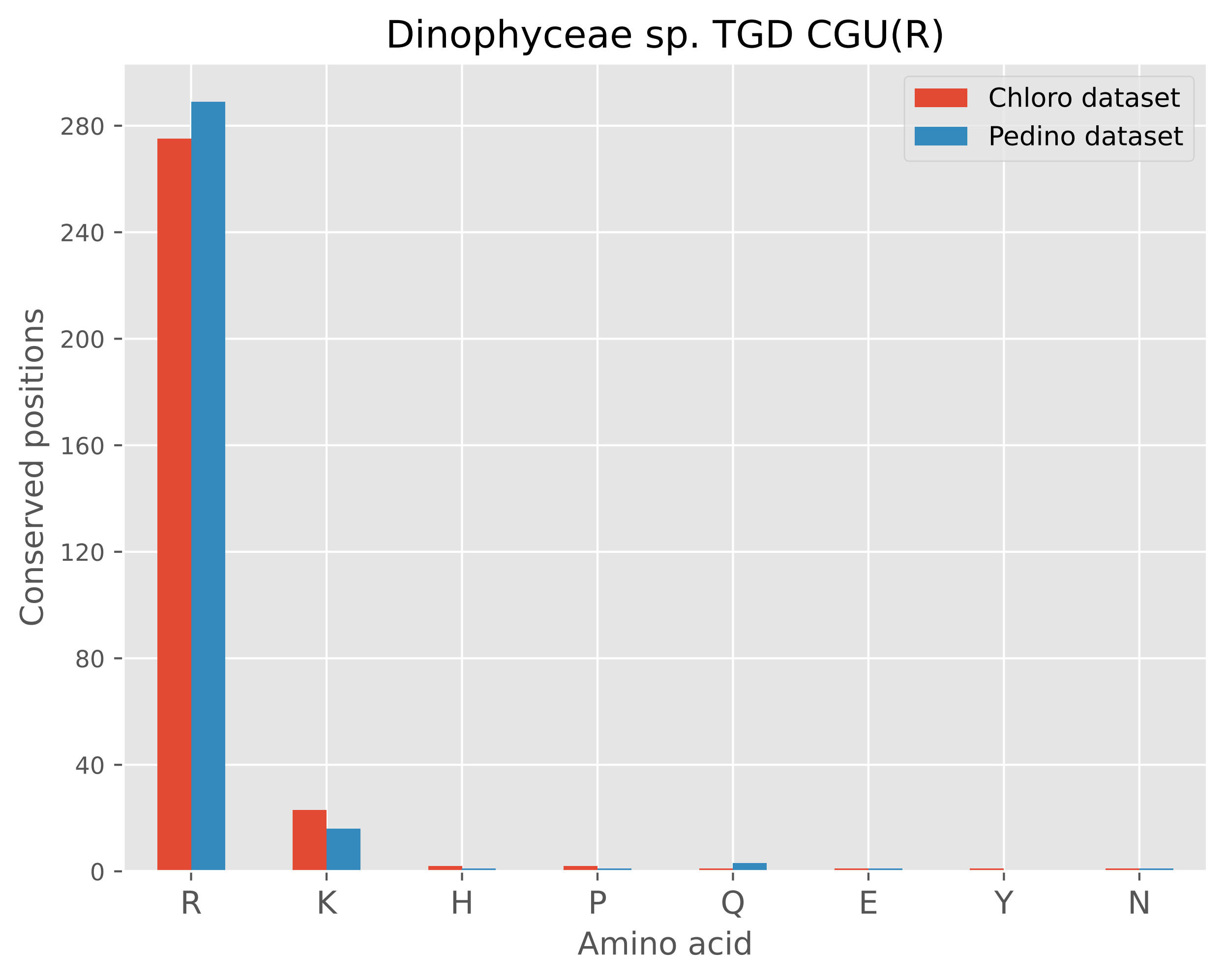
Dinophyceae sp. TGD CGU(R)
Chloro dataset
Pedino dataset
Conserved positions
0
40
80
120
160
200
240
280
R
K
H
P
Q
E
Y
N
Amino acid

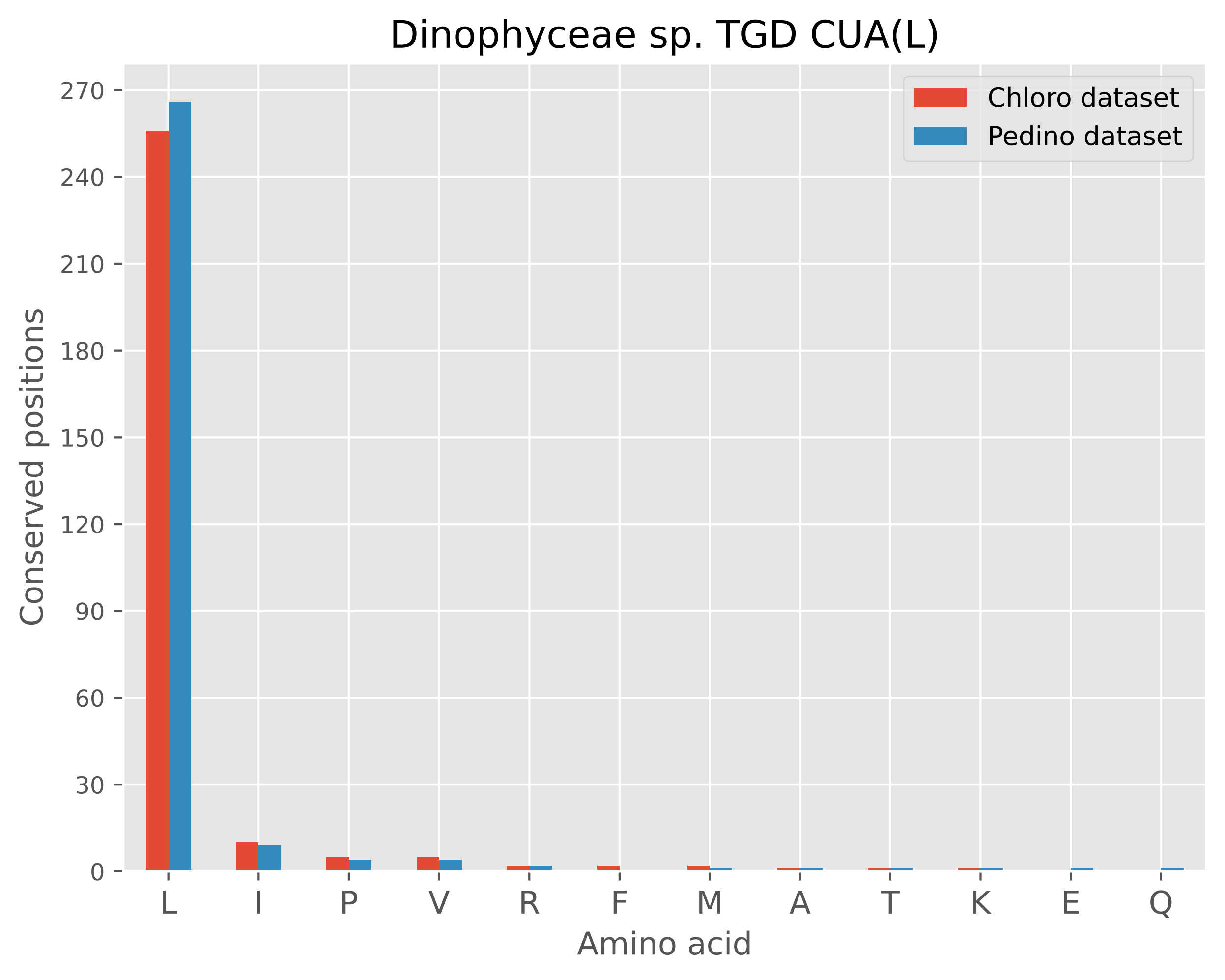

Dinophyceae sp. TGD CUA(L)
Chloro dataset
Pedino dataset
Conserved positions
Amino acid
0
30
60
90
120
150
180
210
240
270
L
I
P
V
R
F
M
A
T
K
E
Q

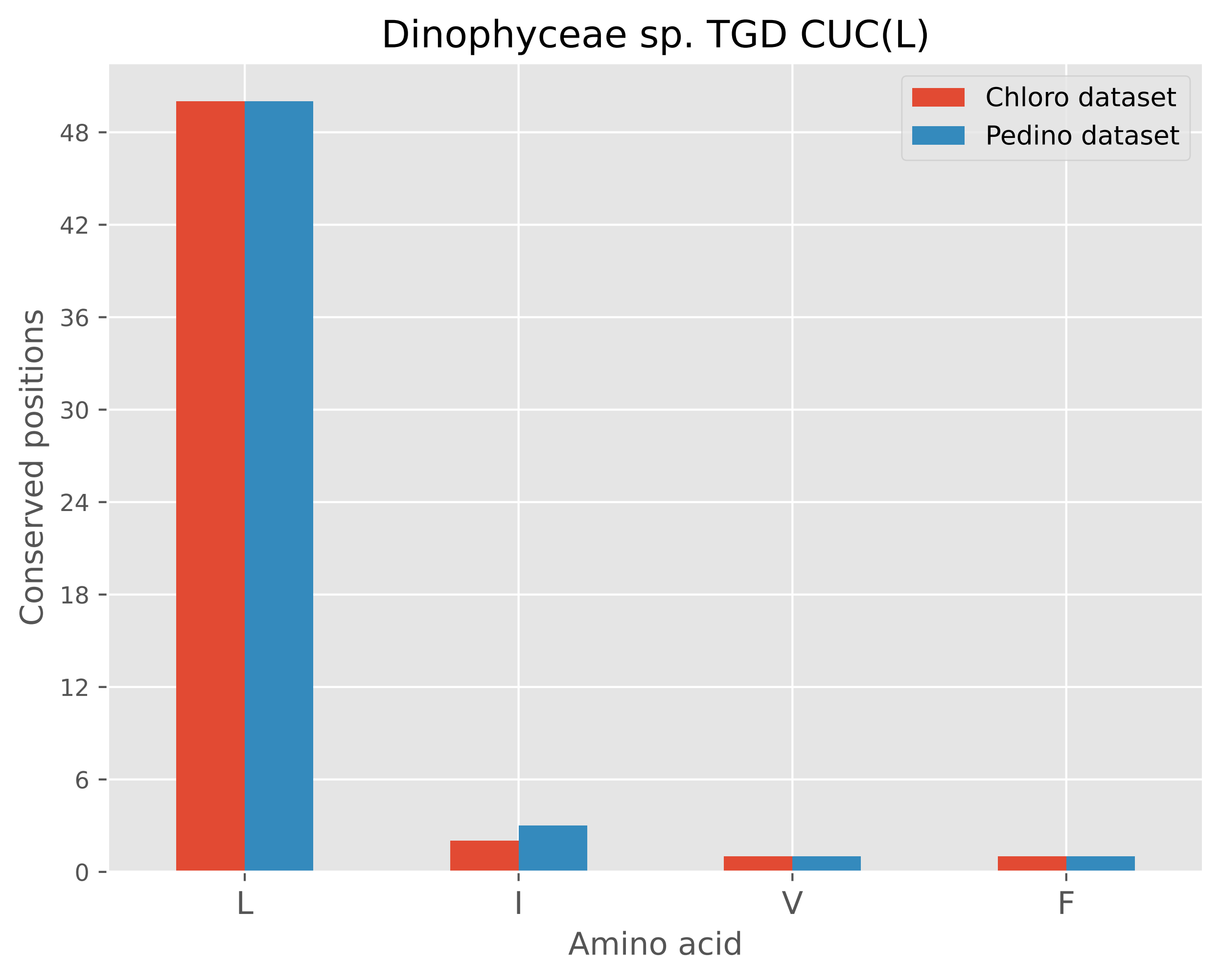

Dinophyceae sp. TGD CUC(L)
Chloro dataset
Pedino dataset
Conserved positions
0
6
12
18
24
30
36
42
48
L
I
V
F
Amino acid

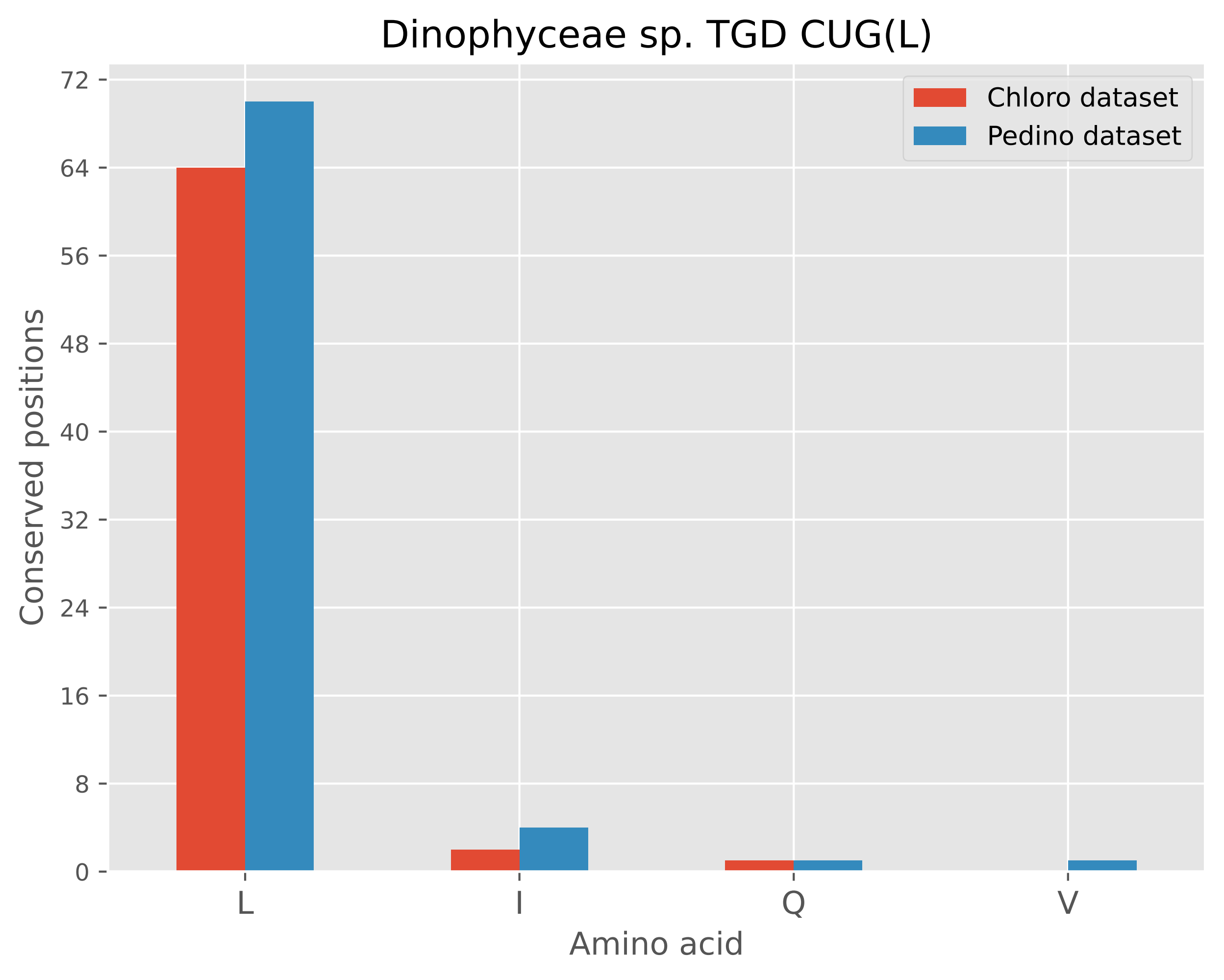

Dinophyceae sp. TGD CUG(L)
Chloro dataset
Pedino dataset
Conserved positions
0
8
16
24
32
40
48
56
64
72
L
I
Q
V
Amino acid

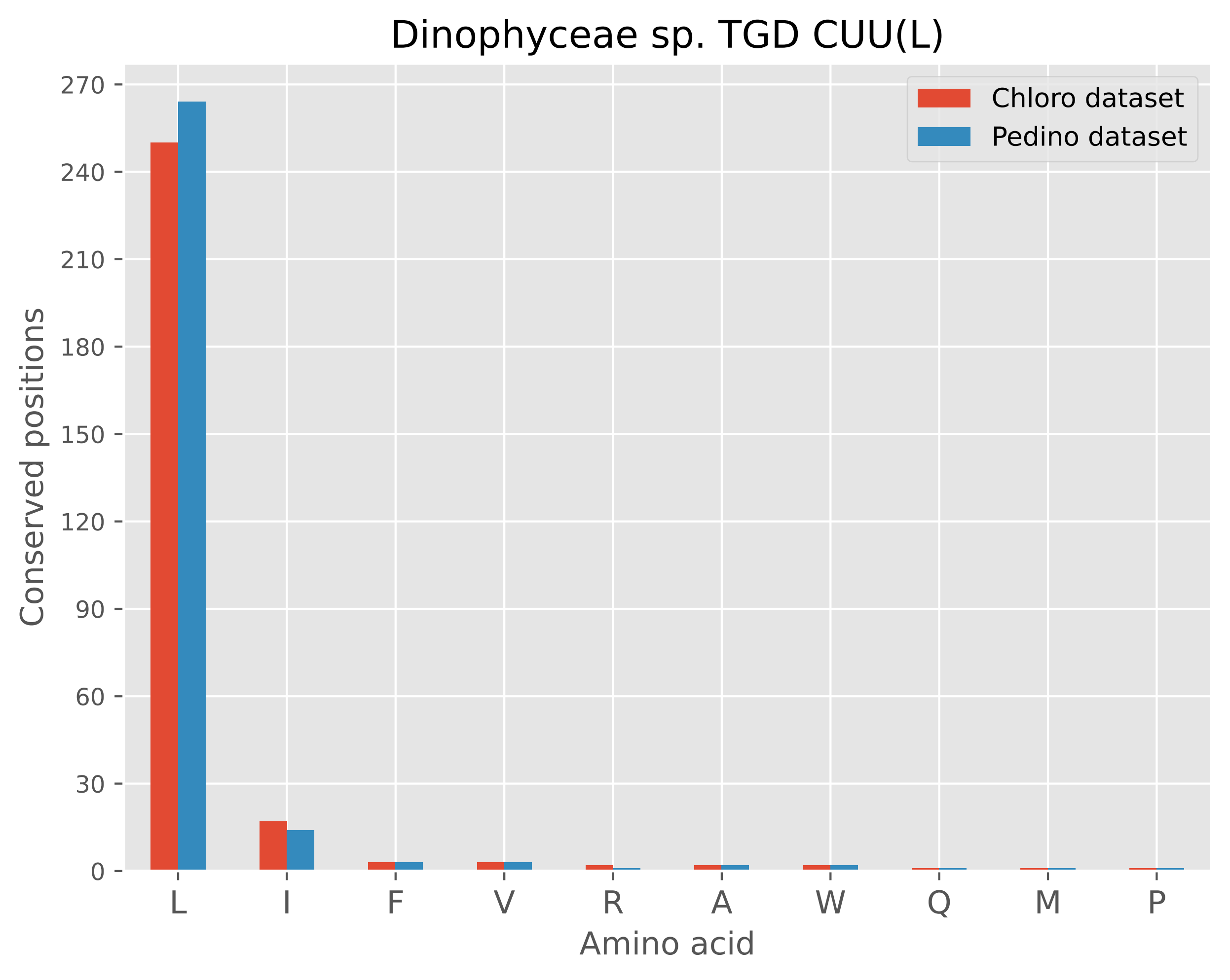
Dinophyceae sp. TGD CUU(L)
Chloro dataset
Pedino dataset
Conserved positions
270
240
210
180
150
120
90
60
30
0
L
I
F
V
R
A
W
Q
M
P
Amino acid

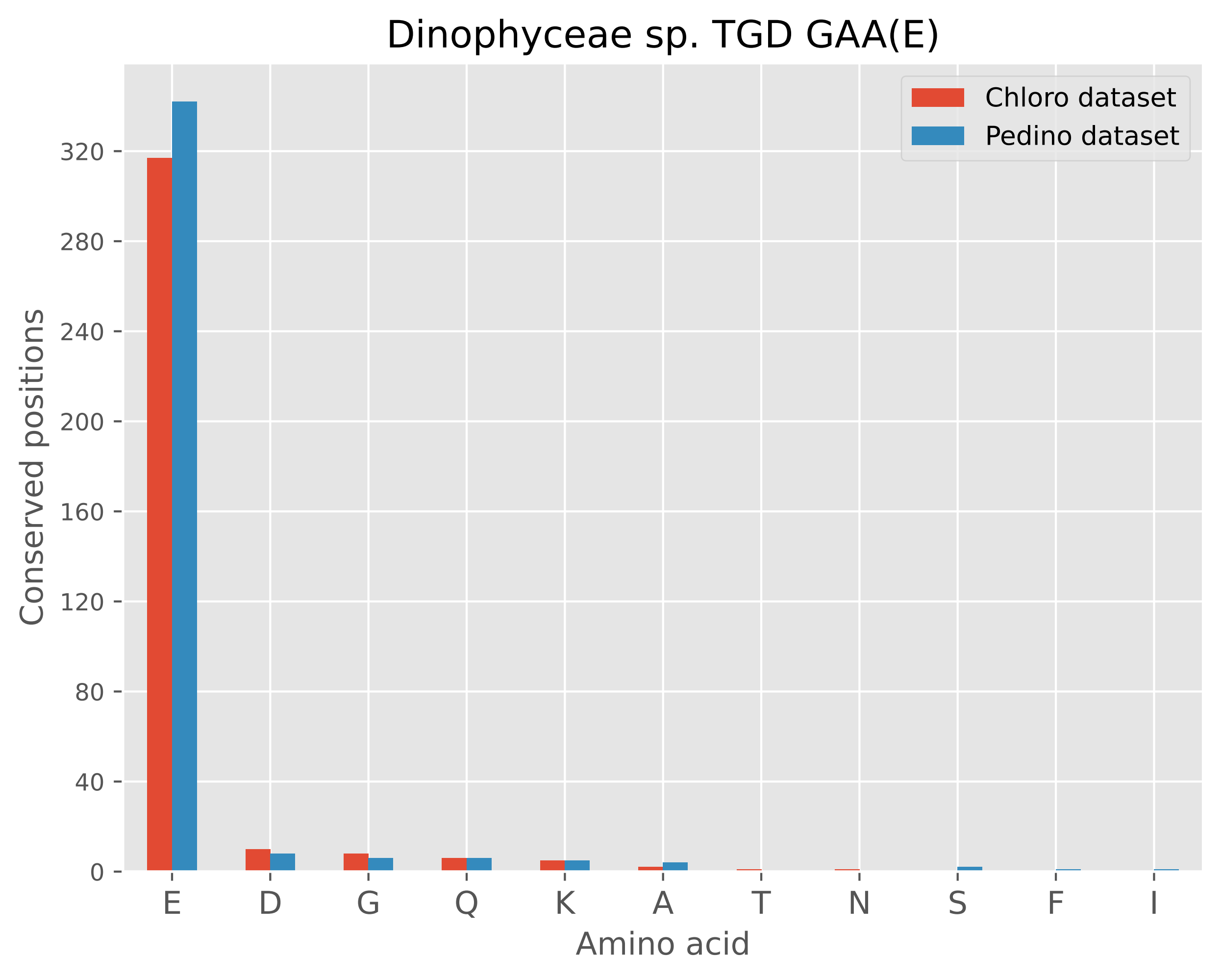
Dinophyceae sp. TGD GAA(E)
Chloro dataset
Pedino dataset
Conserved positions
0
40
80
120
160
200
240
280
320
E
D
G
Q
K
A
T
N
S
F
I
Amino acid

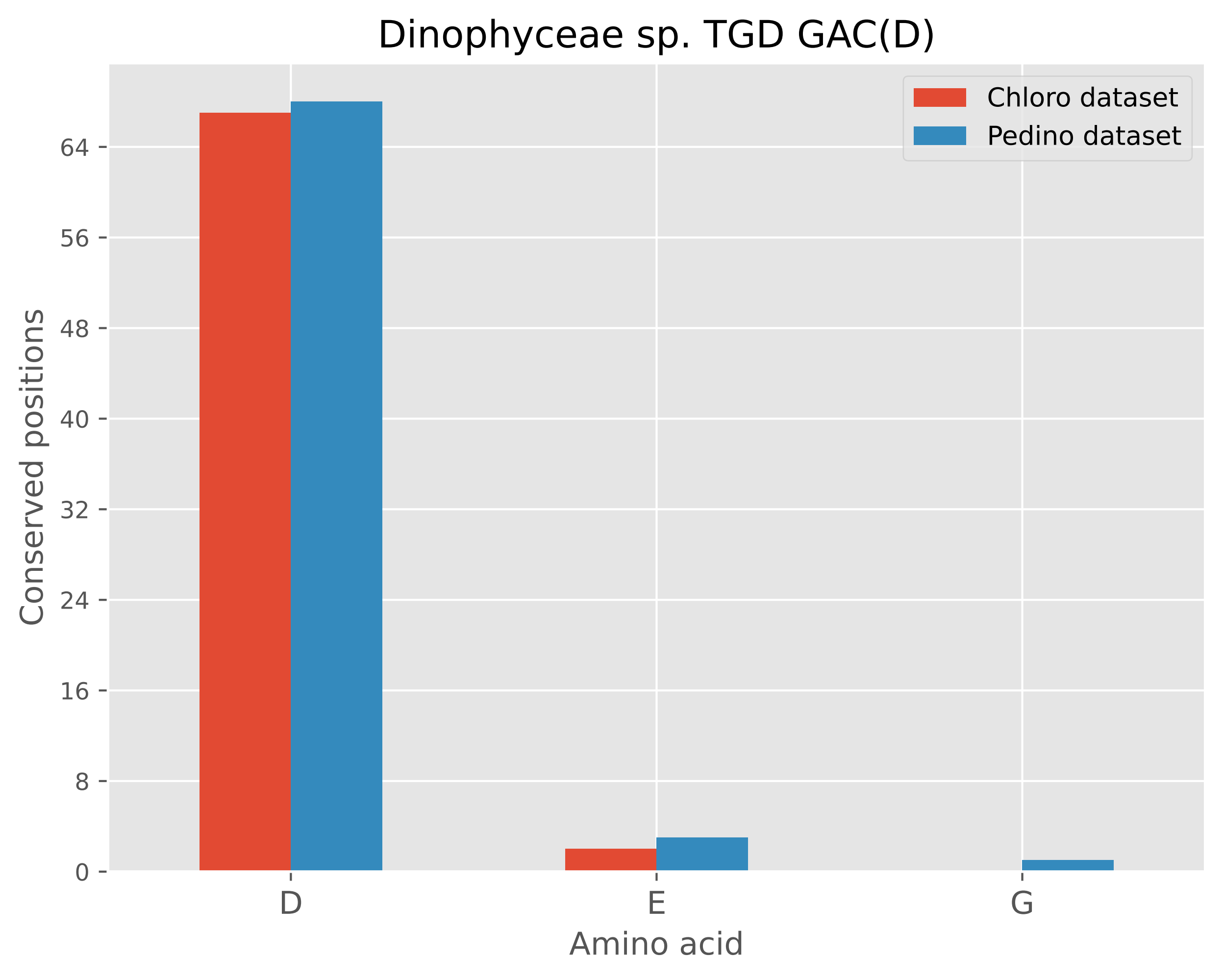

Dinophyceae sp. TGD GAC(D)
Chloro dataset
Pedino dataset
Conserved positions
0
8
16
24
32
40
48
56
64
D
E
G
Amino acid

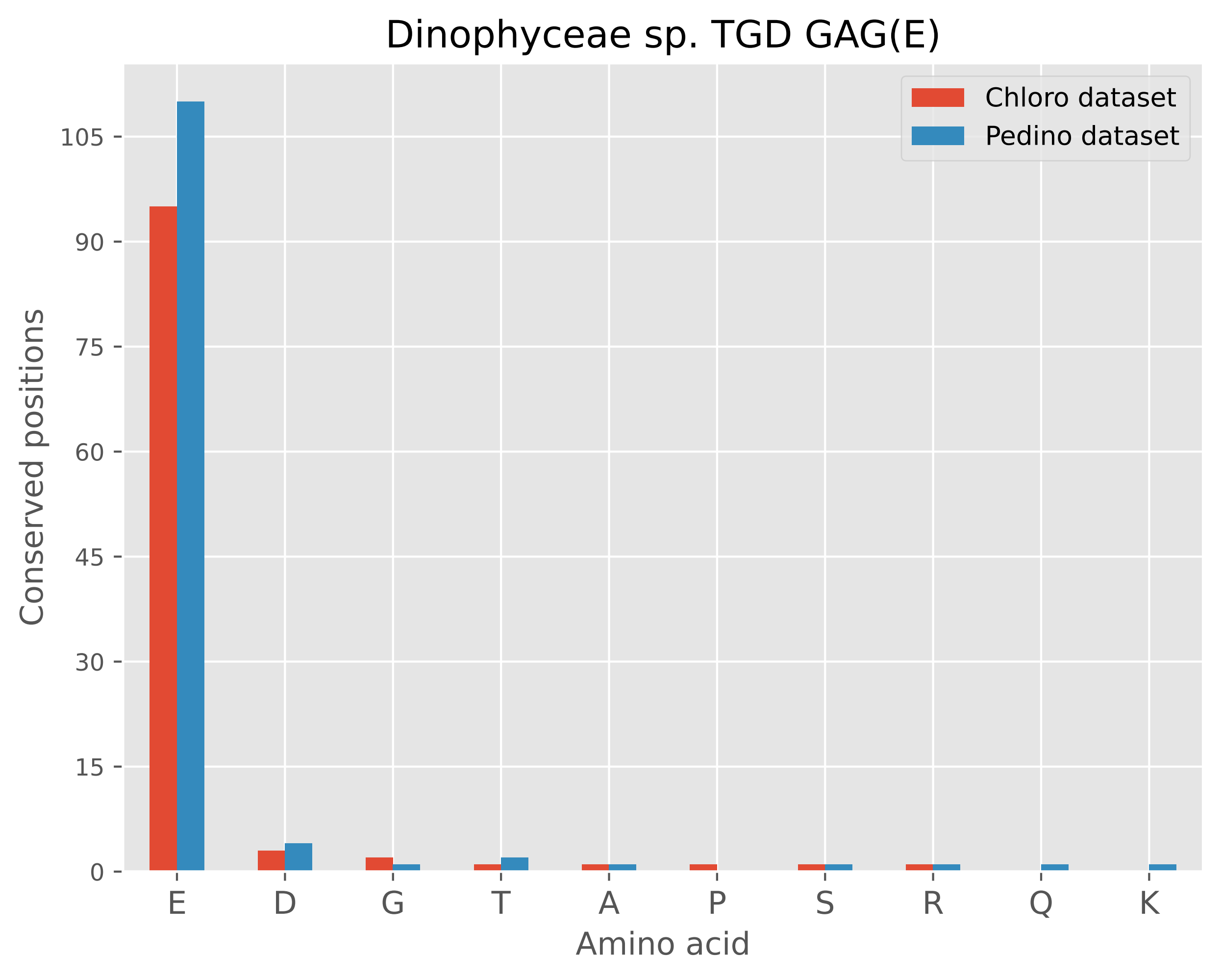

Dinophyceae sp. TGD GAG(E)
Chloro dataset
Pedino dataset
Conserved positions
0
15
30
45
60
75
90
105
E
D
G
T
A
P
S
R
Q
K
Amino acid

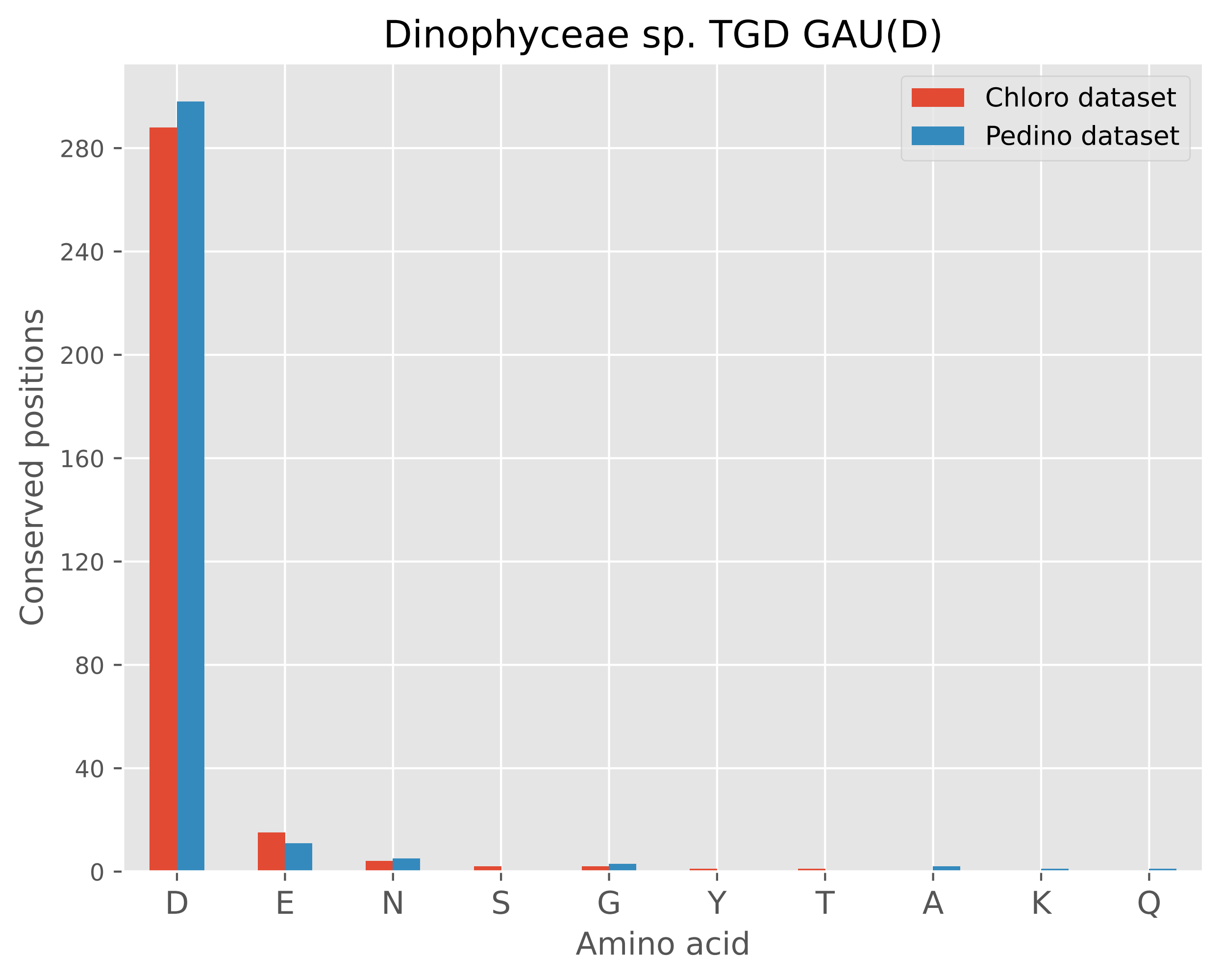

Dinophyceae sp. TGD GAU(D)
Chloro dataset
Pedino dataset
Conserved positions
0
40
80
120
160
200
240
280
D
E
N
S
G
Y
T
A
K
Q
Amino acid

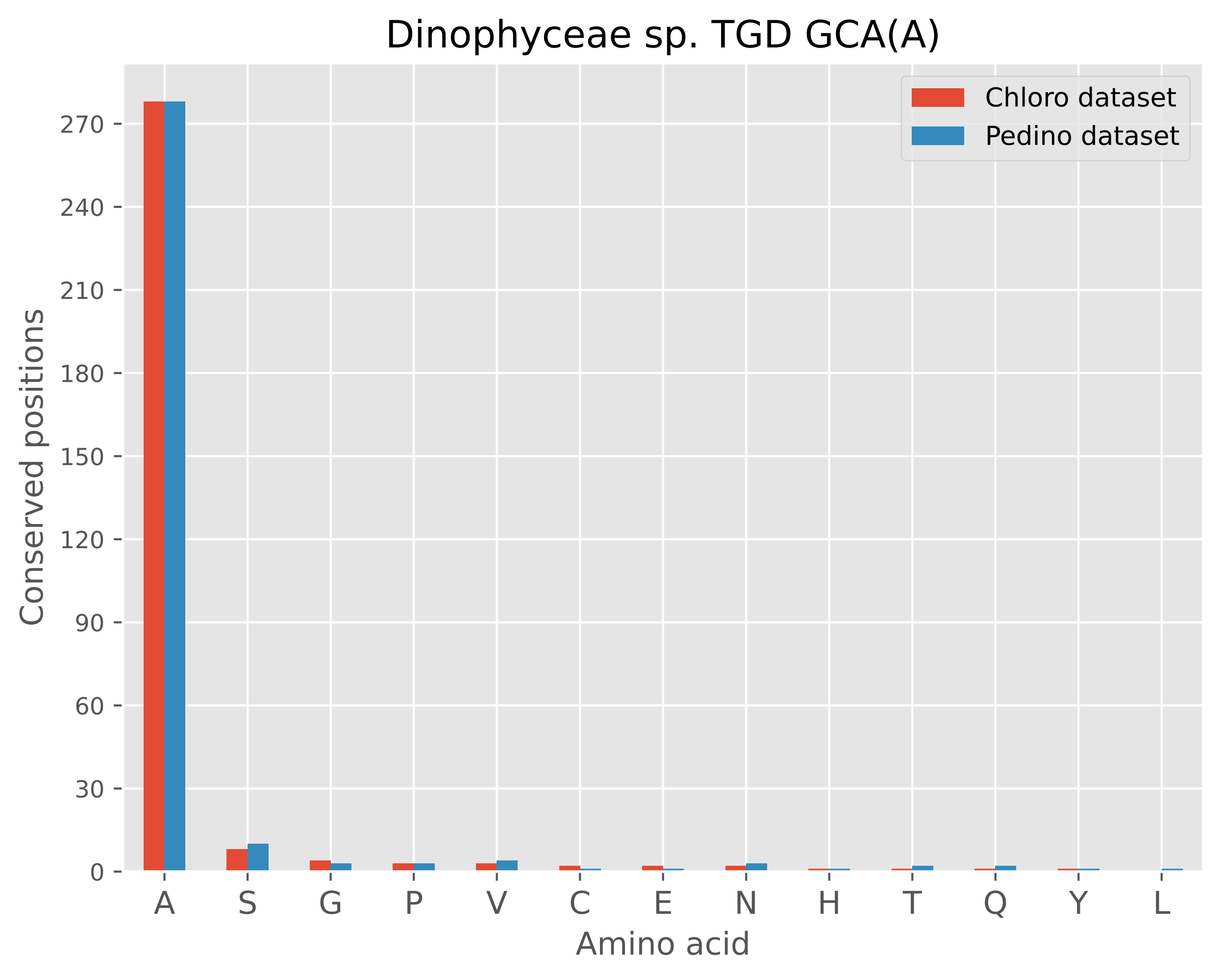
Dinophyceae sp. TGD GCA(A)
Chloro dataset
Pedino dataset
Conserved positions
0
30
60
90
120
150
180
210
240
270
A
S
G
P
V
C
E
N
H
T
Q
Y
L
Amino acid

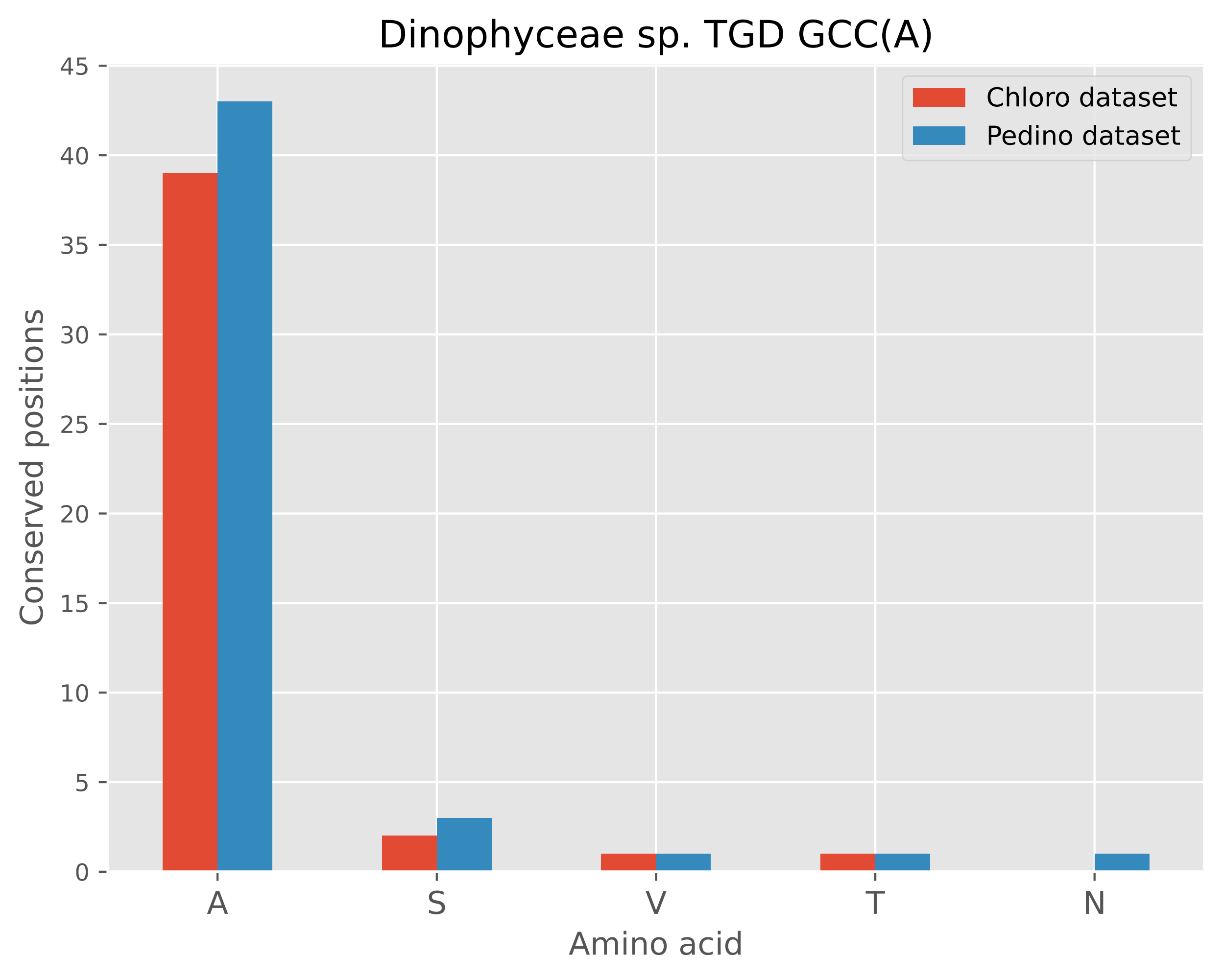
Dinophyceae sp. TGD GCC(A)
Chloro dataset
Pedino dataset
Conserved positions
Amino acid
0
5
10
15
20
25
30
35
40
45
A
S
V
T
N

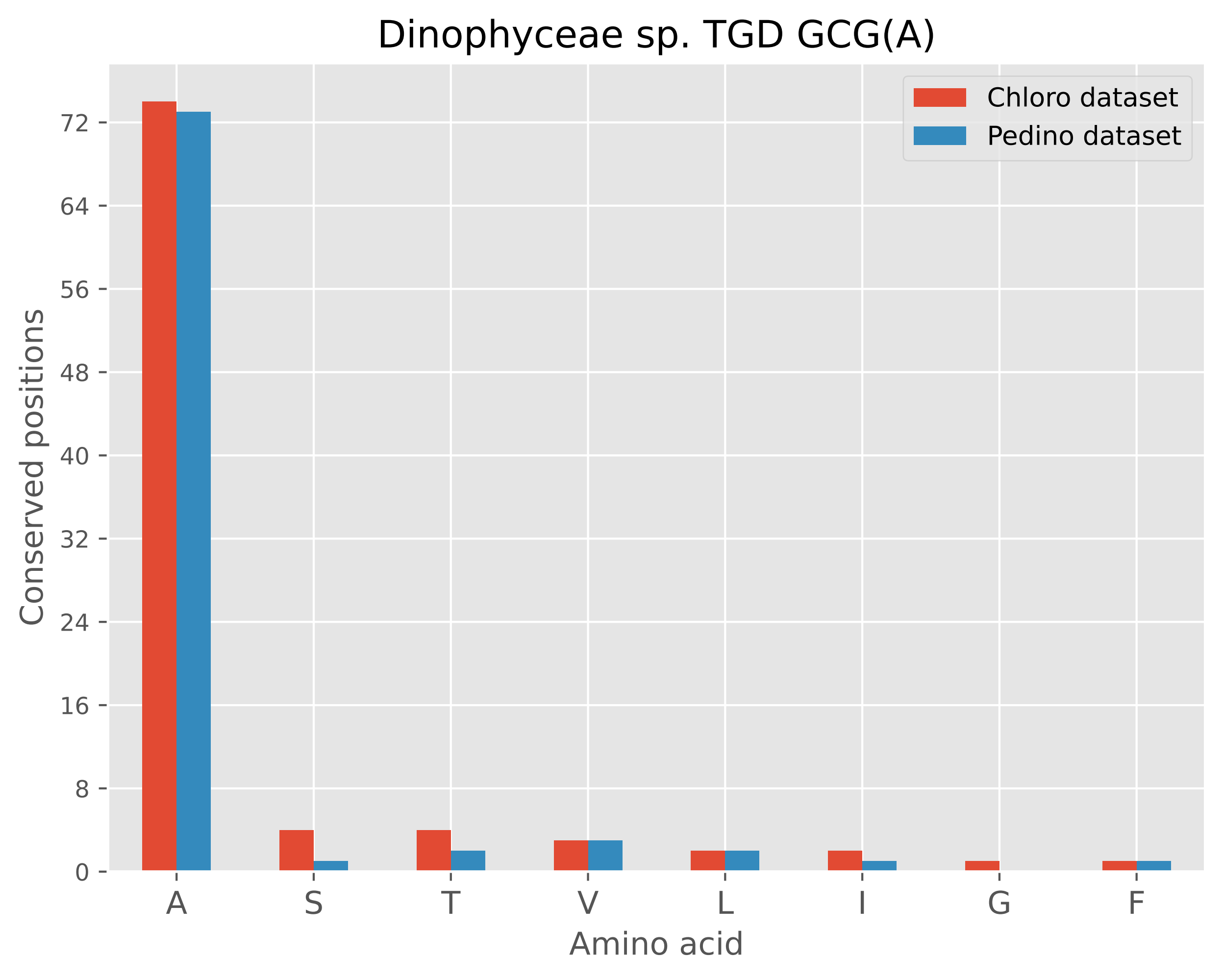
Dinophyceae sp. TGD GCG(A)
Chloro dataset
Pedino dataset
Conserved positions
0
8
16
24
32
40
48
56
64
72
A
S
T
V
L
I
G
F
Amino acid

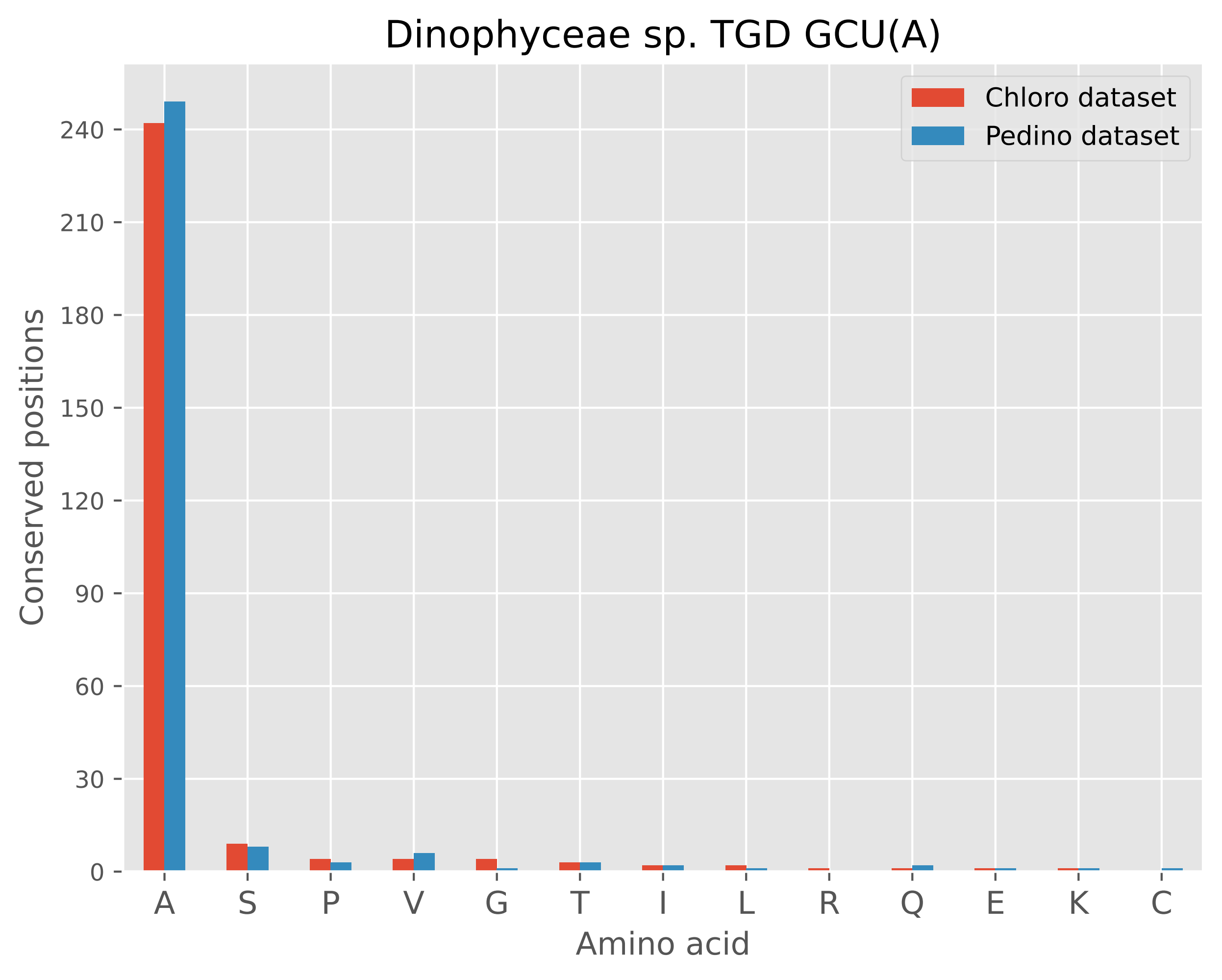

Dinophyceae sp. TGD GCU(A)
Chloro dataset
Pedino dataset
Conserved positions
0
30
60
90
120
150
180
210
240
A
S
P
V
G
T
I
L
R
Q
E
K
C
Amino acid

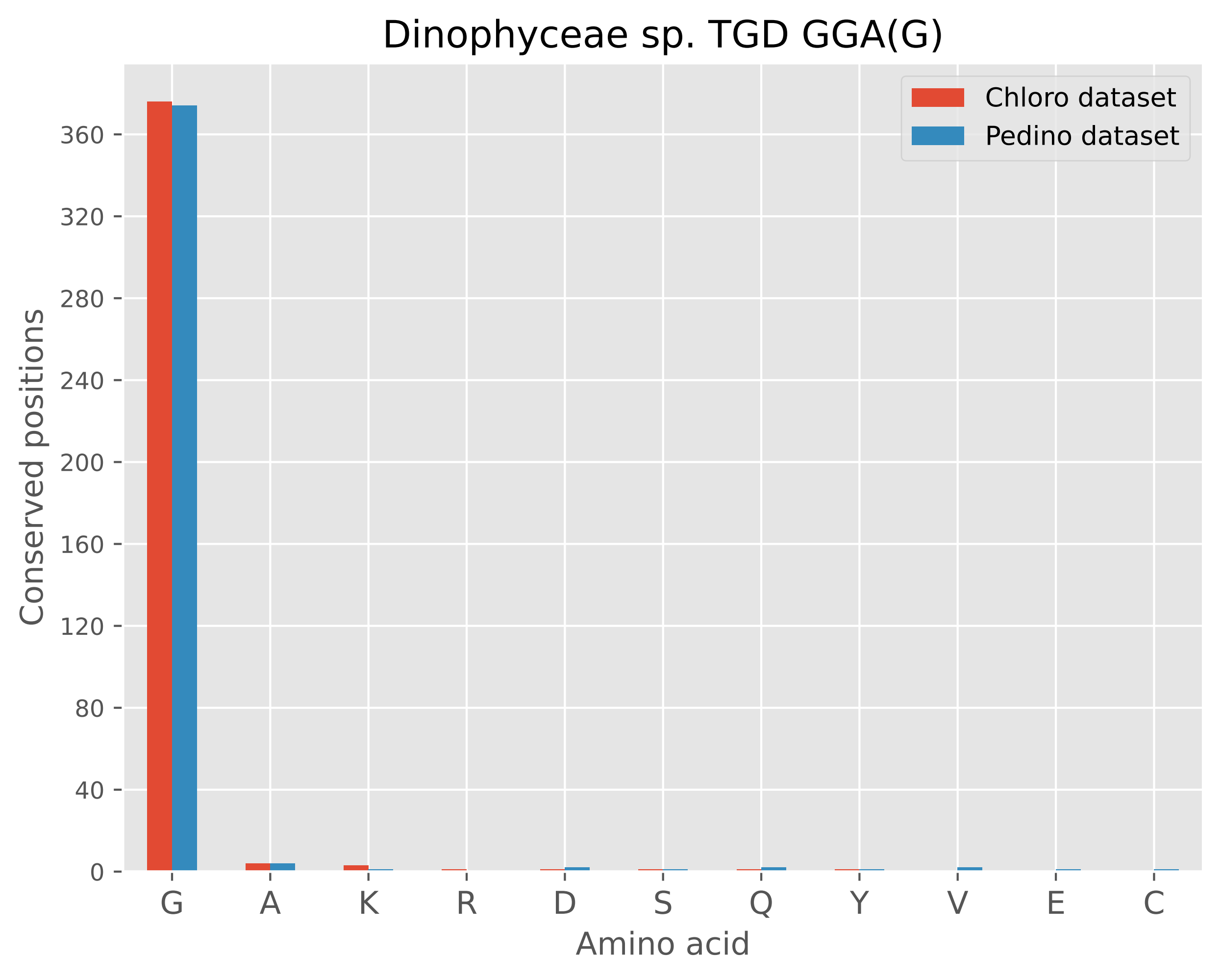

Dinophyceae sp. TGD GGA(G)
Chloro dataset
Pedino dataset
Conserved positions
0
40
80
120
160
200
240
280
320
360
G
A
K
R
D
S
Q
Y
V
E
C
Amino acid

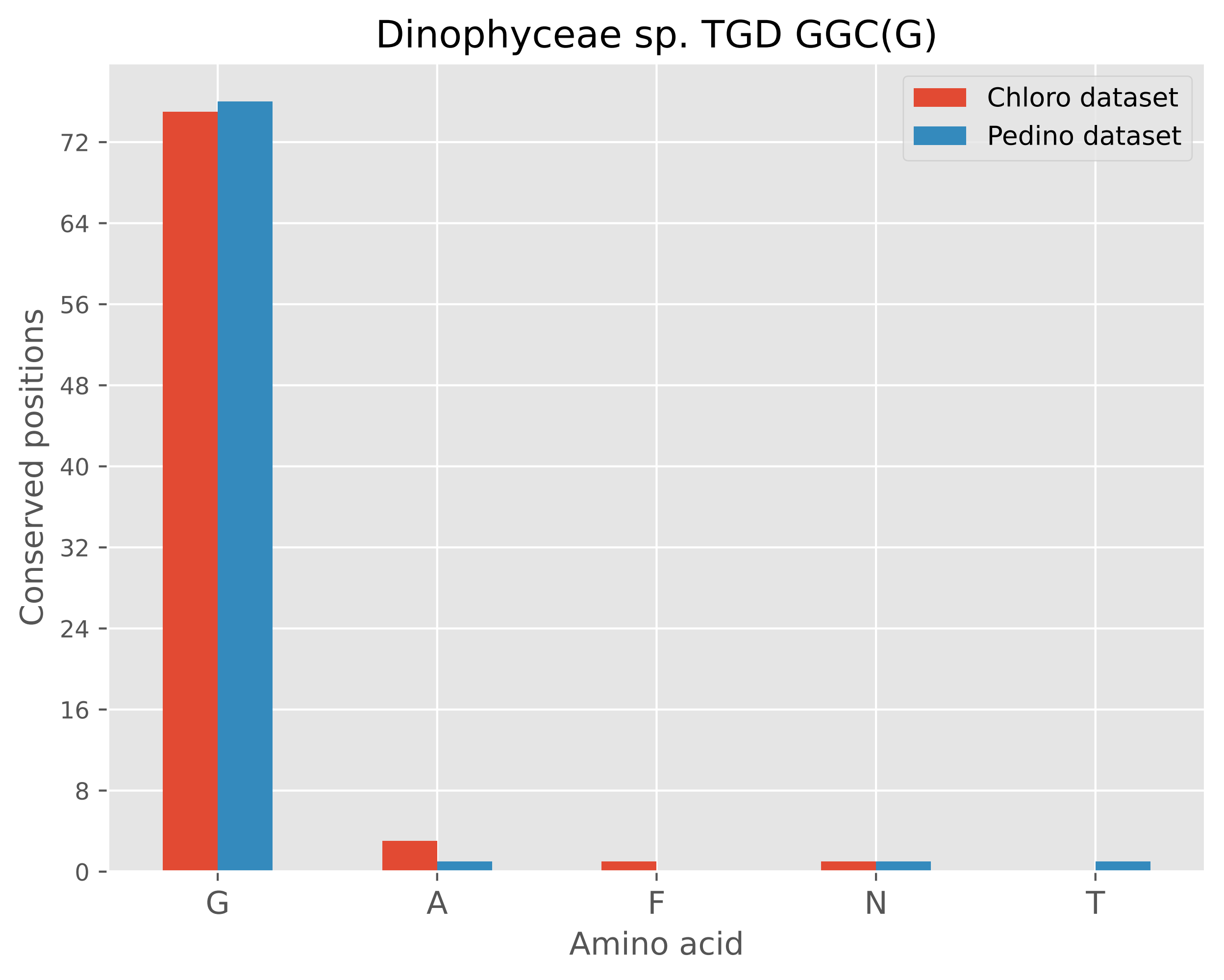
Dinophyceae sp. TGD GGC(G)
Chloro dataset
Pedino dataset
Conserved positions
0
8
16
24
32
40
48
56
64
72
G
A
F
N
T
Amino acid

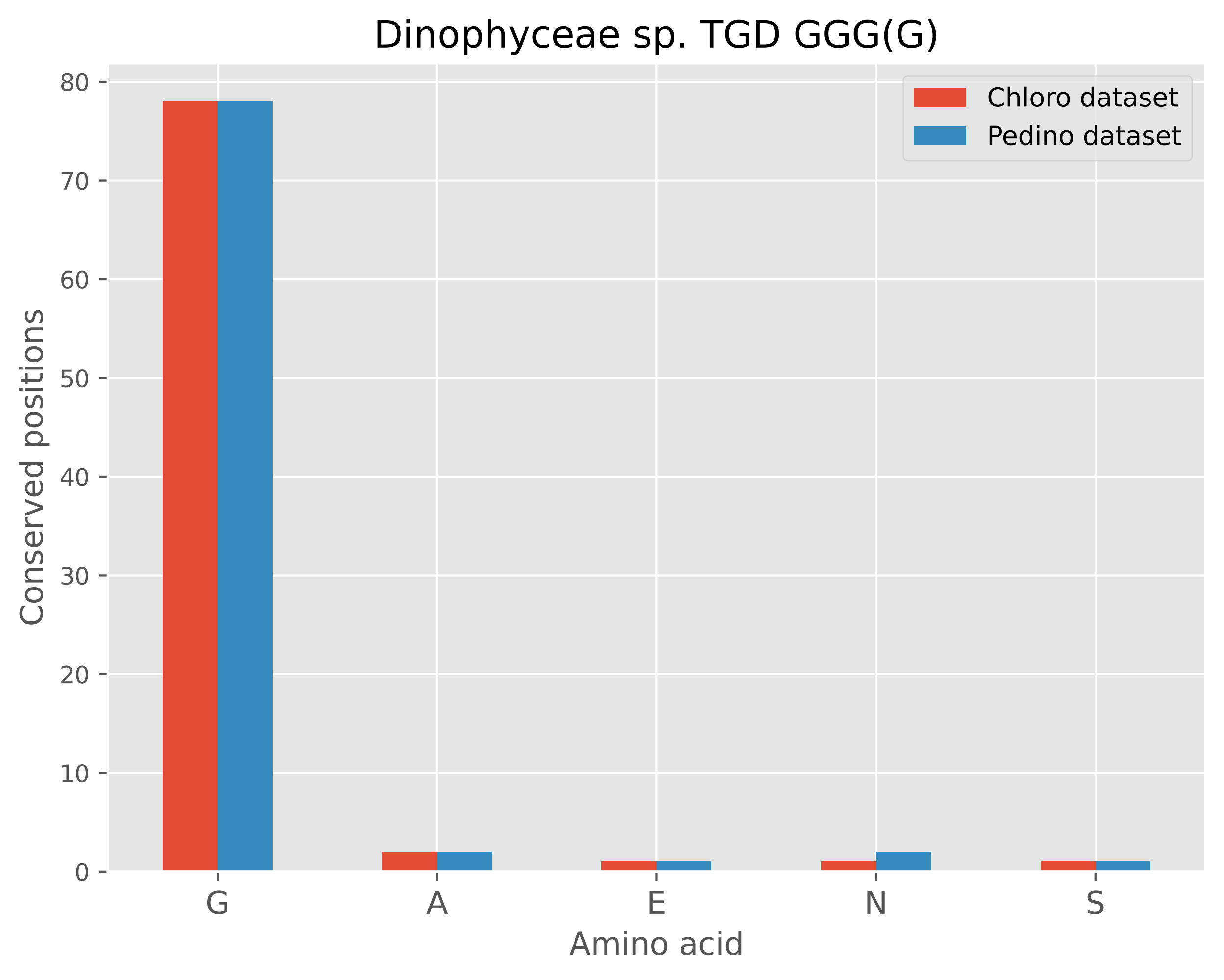

Dinophyceae sp. TGD GGG(G)
Chloro dataset
Pedino dataset
Conserved positions
0
10
20
30
40
50
60
70
80
G
A
E
N
S
Amino acid

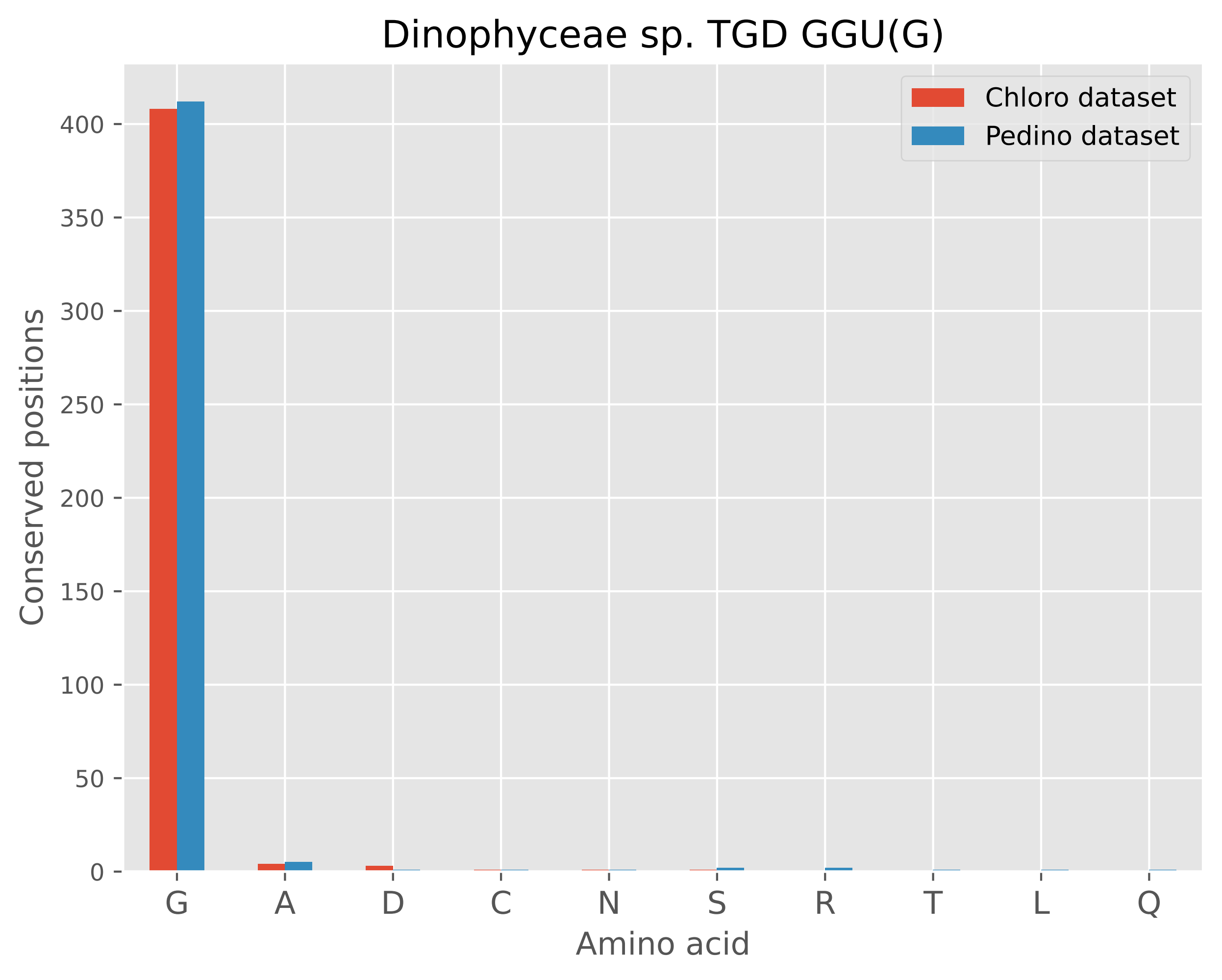
Dinophyceae sp. TGD GGU(G)
Chloro dataset
Pedino dataset
Conserved positions
0
50
100
150
200
250
300
350
400
G
A
D
C
N
S
R
T
L
Q
Amino acid

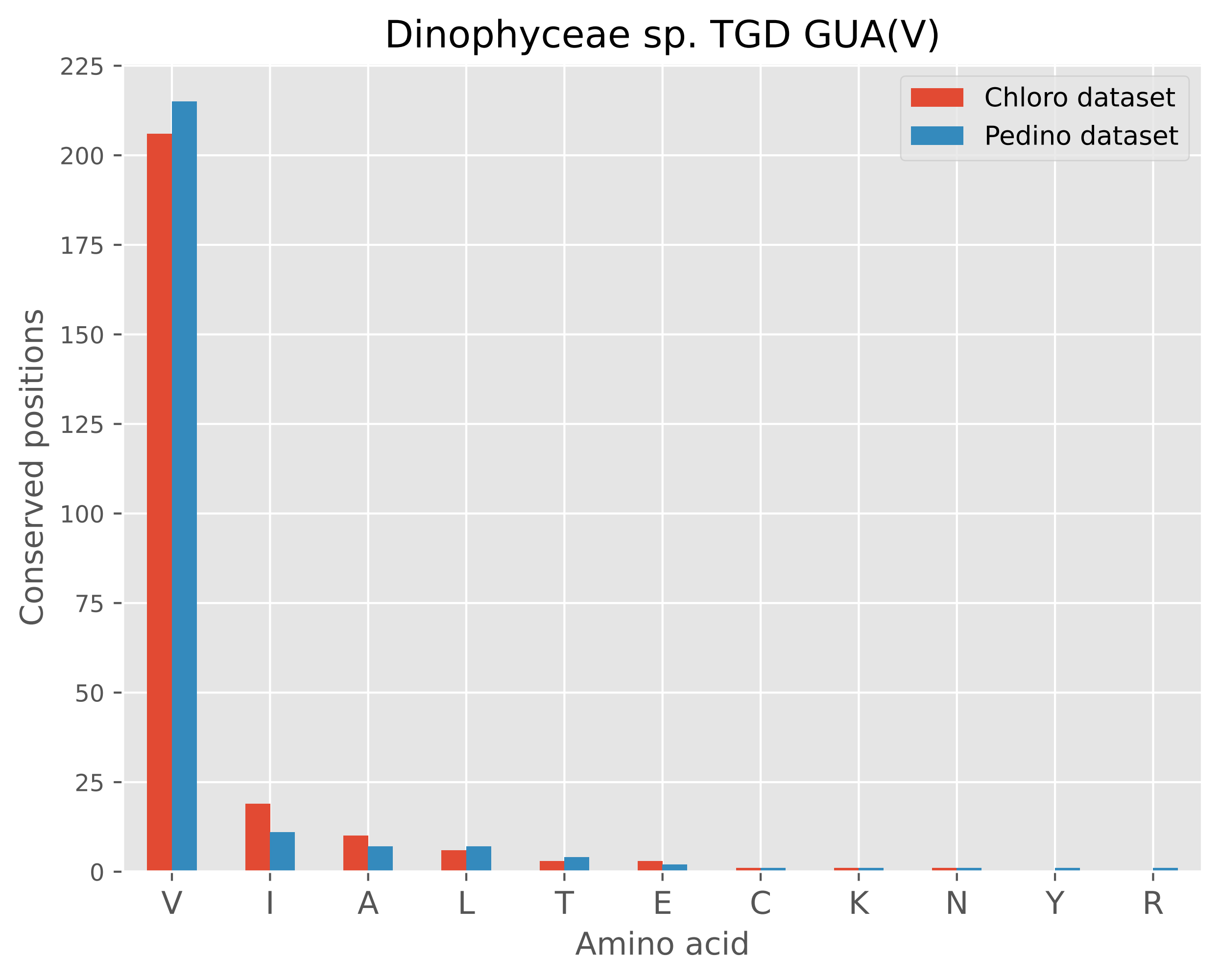

Dinophyceae sp. TGD GUA(V)
Chloro dataset
Pedino dataset
Conserved positions
0
25
50
75
100
125
150
175
200
225
V
I
A
L
T
E
C
K
N
Y
R
Amino acid

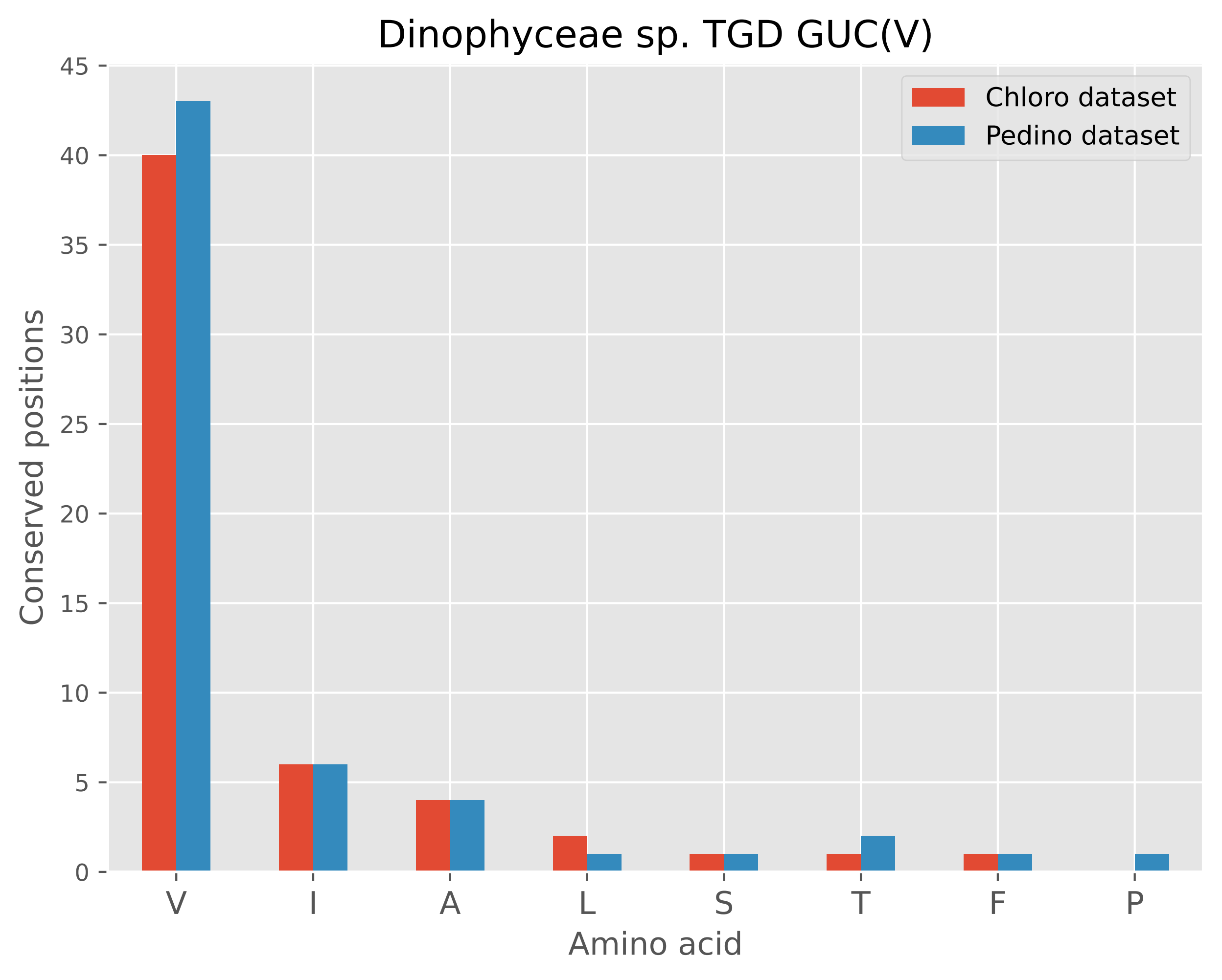

Dinophyceae sp. TGD GUC(V)
Chloro dataset
Pedino dataset
Conserved positions
Amino acid
0
5
10
15
20
25
30
35
40
45
V
I
A
L
S
T
F
P

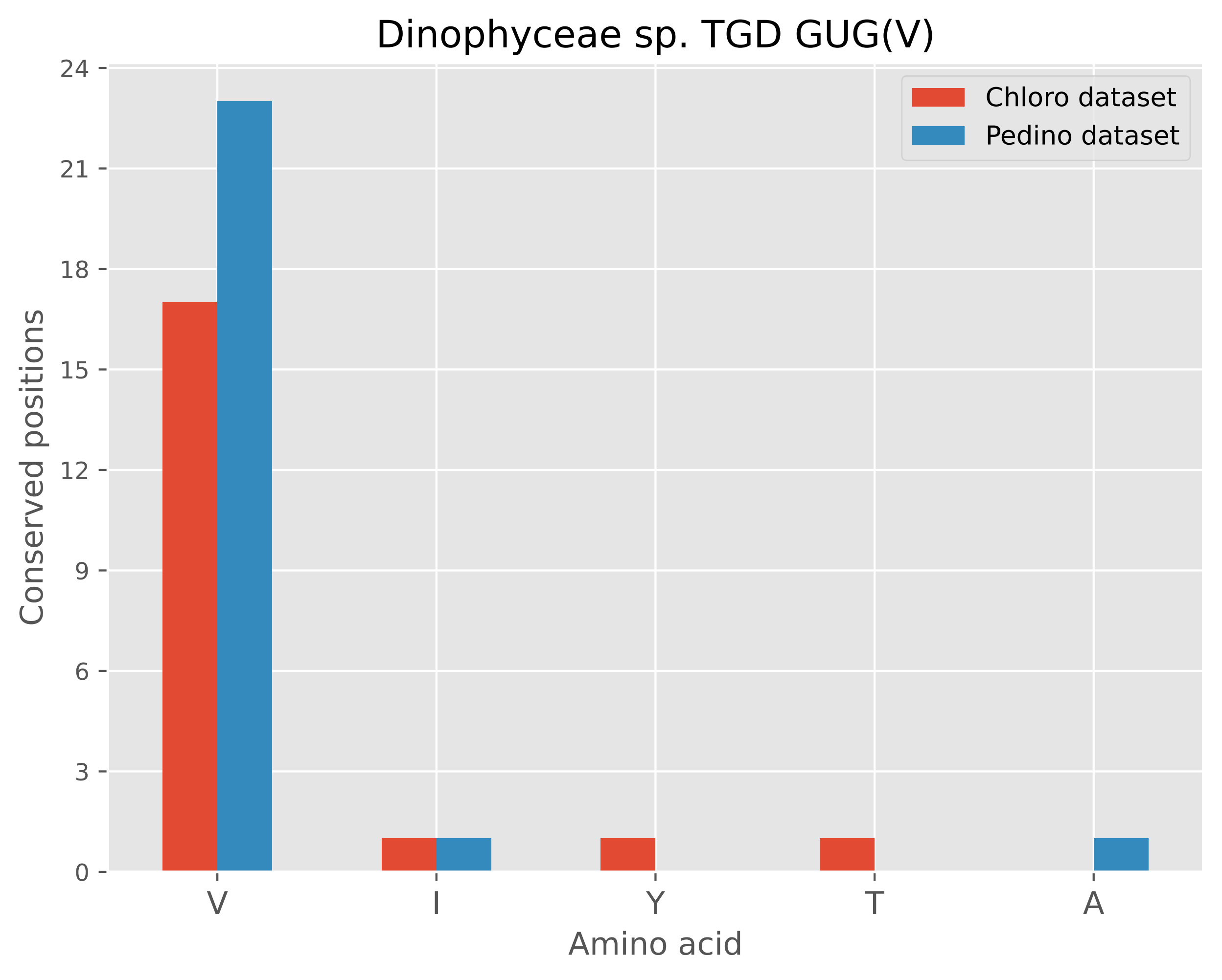

Dinophyceae sp. TGD GUG(V)
Chloro dataset
Pedino dataset
Conserved positions
0
3
6
9
12
15
18
21
24
V
I
Y
T
A
Amino acid

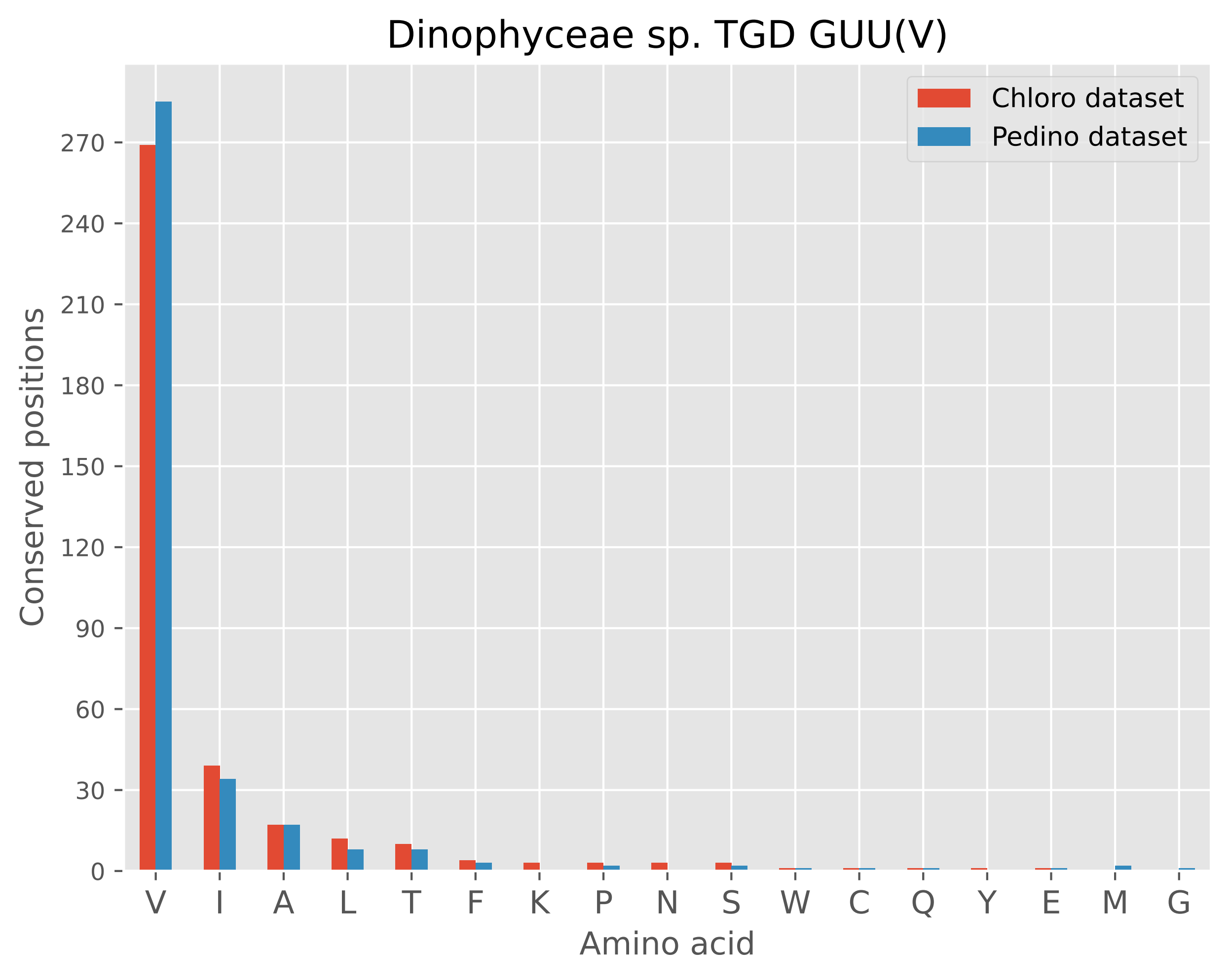

Dinophyceae sp. TGD GUU(V)
Chloro dataset
Pedino dataset
Conserved positions
0
30
60
90
120
150
180
210
240
270
V
I
A
L
T
F
K
P
N
S
W
C
Q
Y
E
M
G
Amino acid

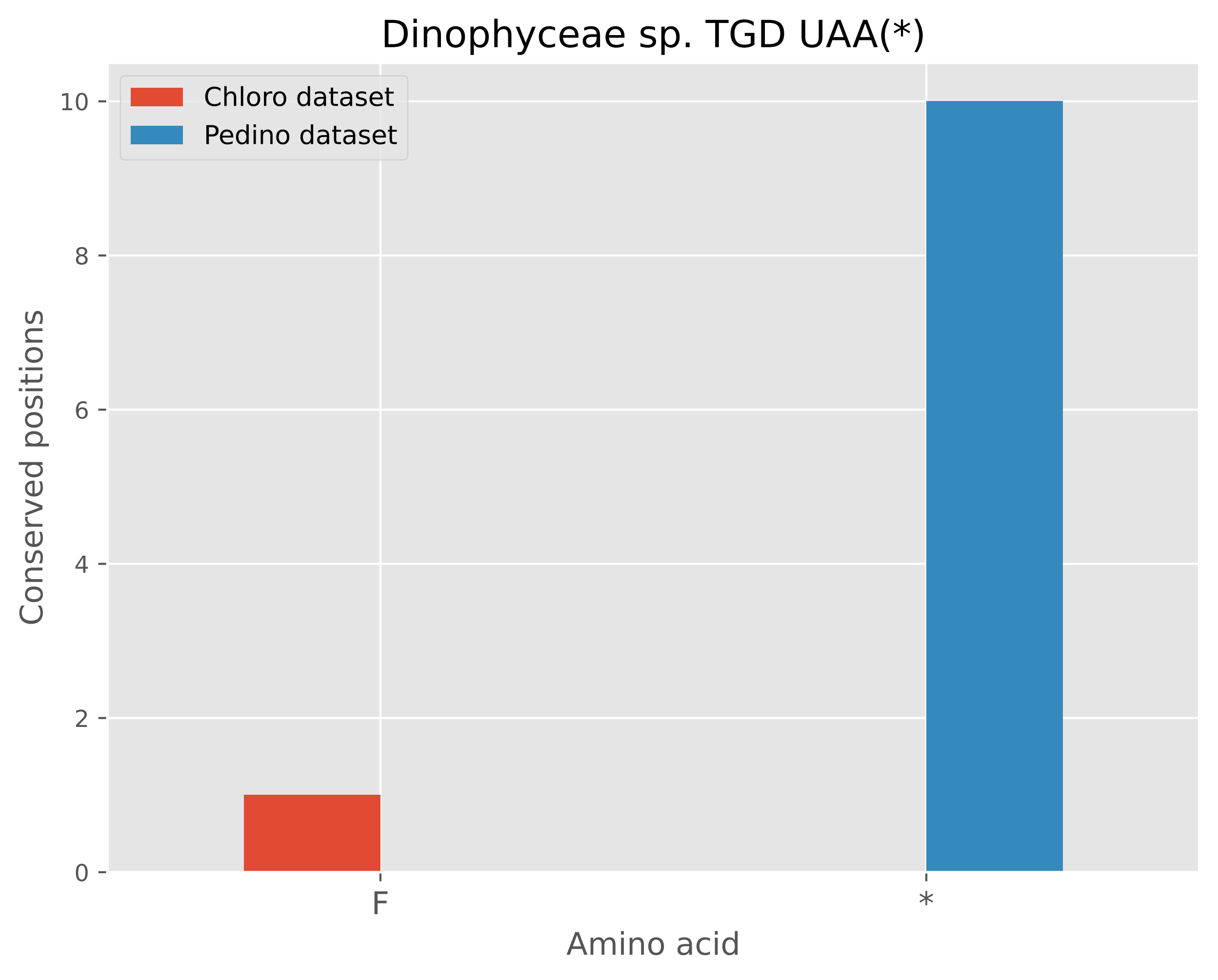
Dinophyceae sp. TGD UAA(*)
Chloro dataset
Pedino dataset
Conserved positions
0
2
4
6
8
10
F
*
Amino acid

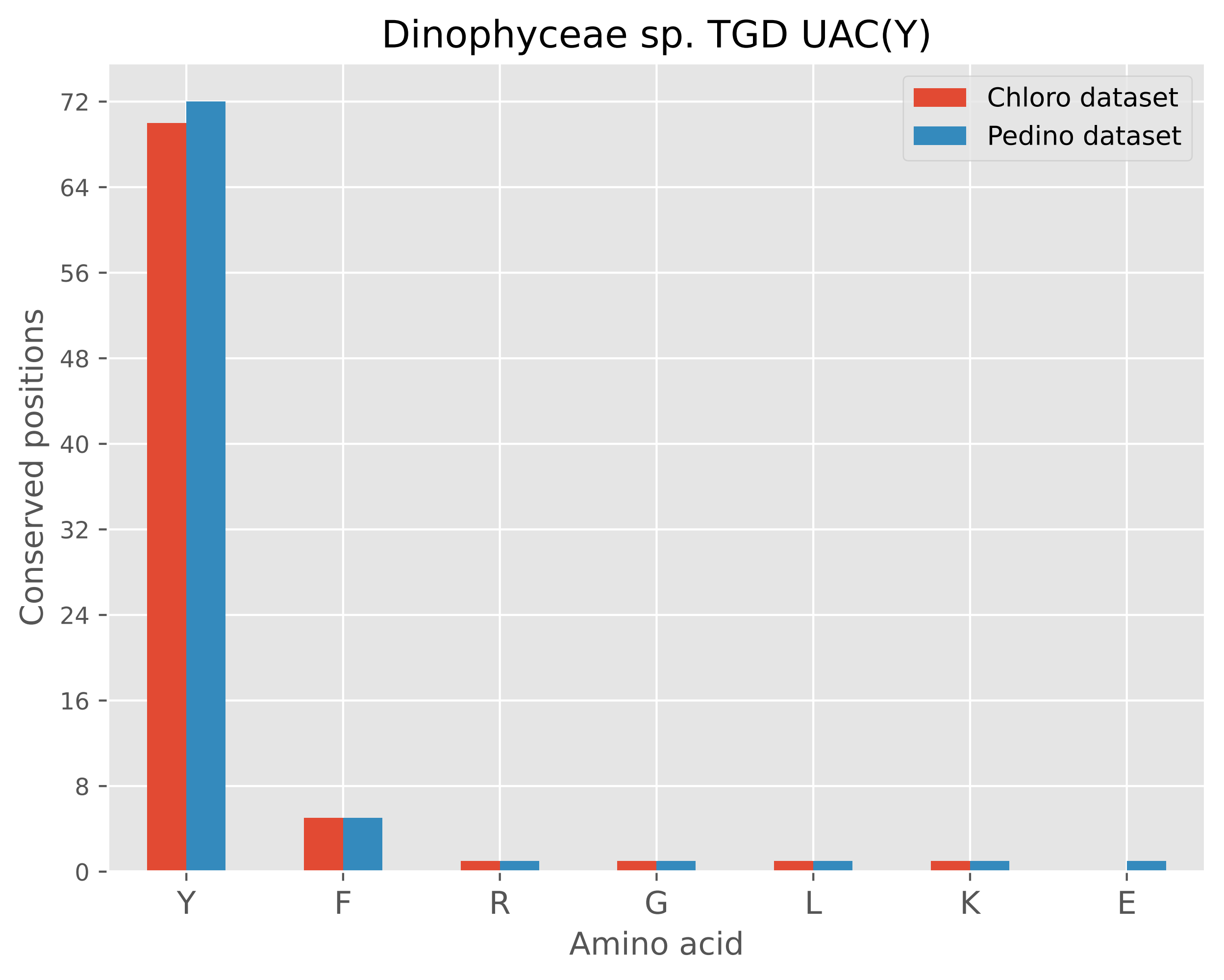

Dinophyceae sp. TGD UAC(Y)
Chloro dataset
Pedino dataset
Conserved positions
0
8
16
24
32
40
48
56
64
72
Y
F
R
G
L
K
E
Amino acid

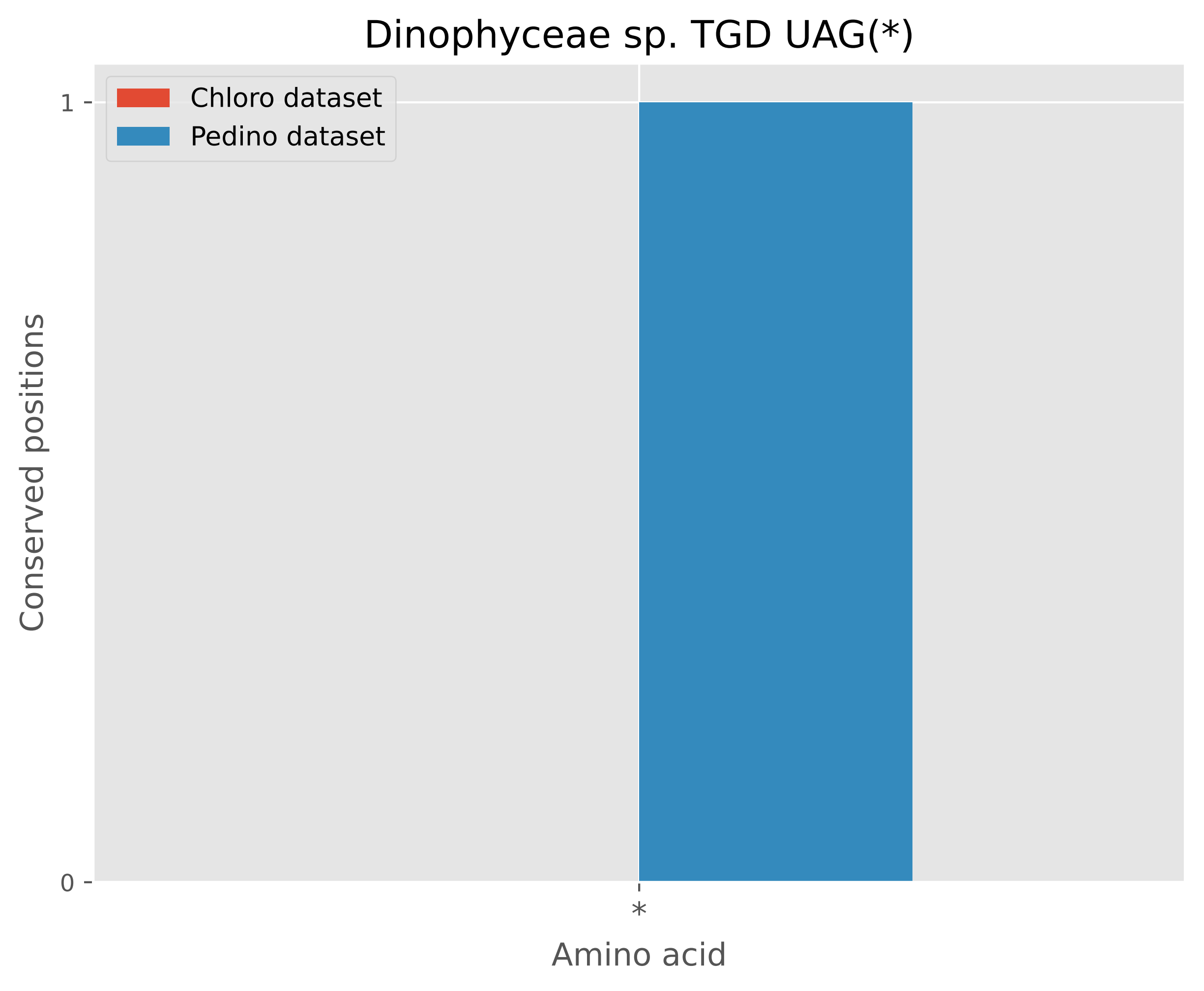
Dinophyceae sp. TGD UAG(*)
Chloro dataset
Pedino dataset
Conserved positions
1
0
*
Amino acid

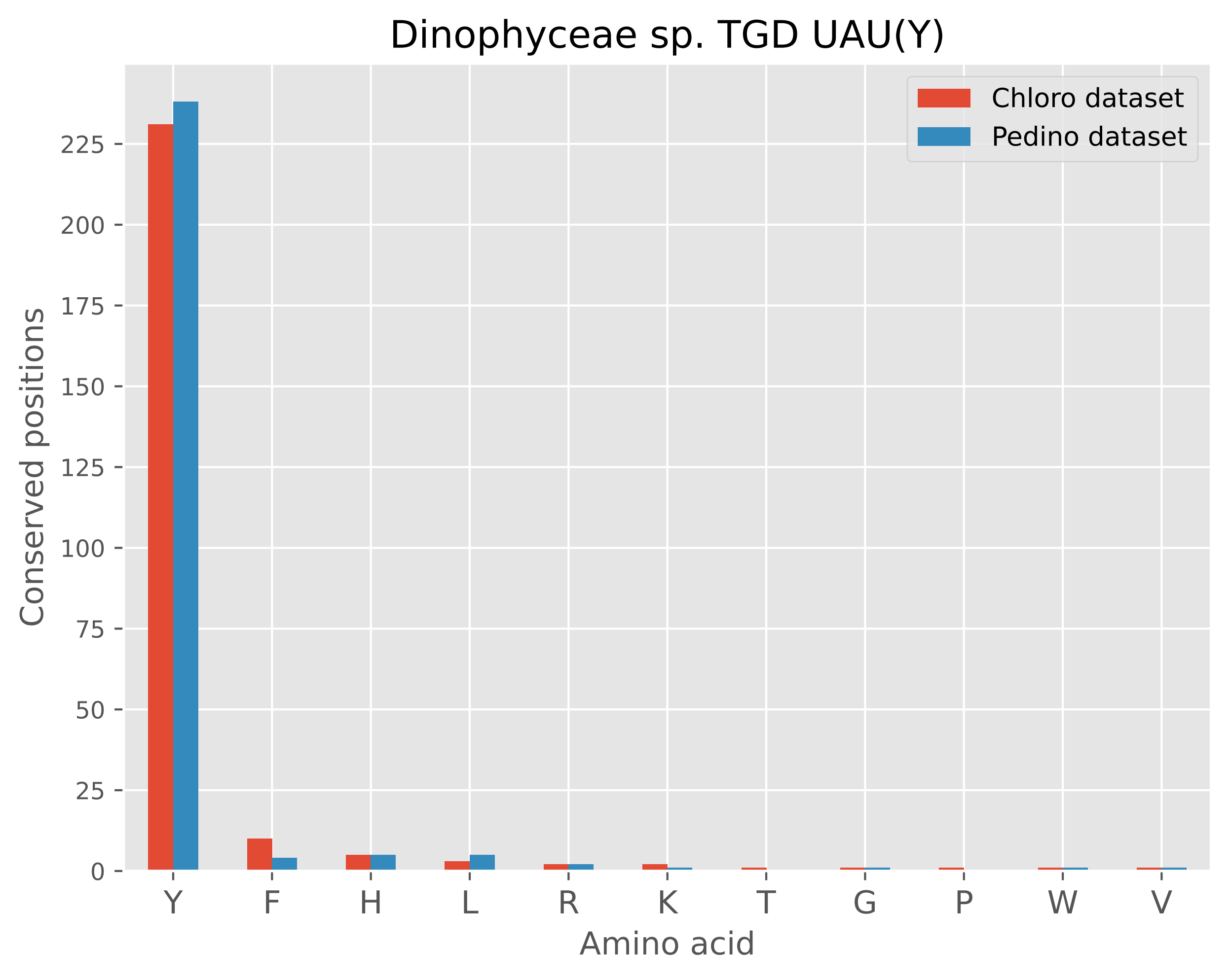
Dinophyceae sp. TGD UAU(Y)
Chloro dataset
Pedino dataset
Conserved positions
0
25
50
75
100
125
150
175
200
225
Y
F
H
L
R
K
T
G
P
W
V
Amino acid

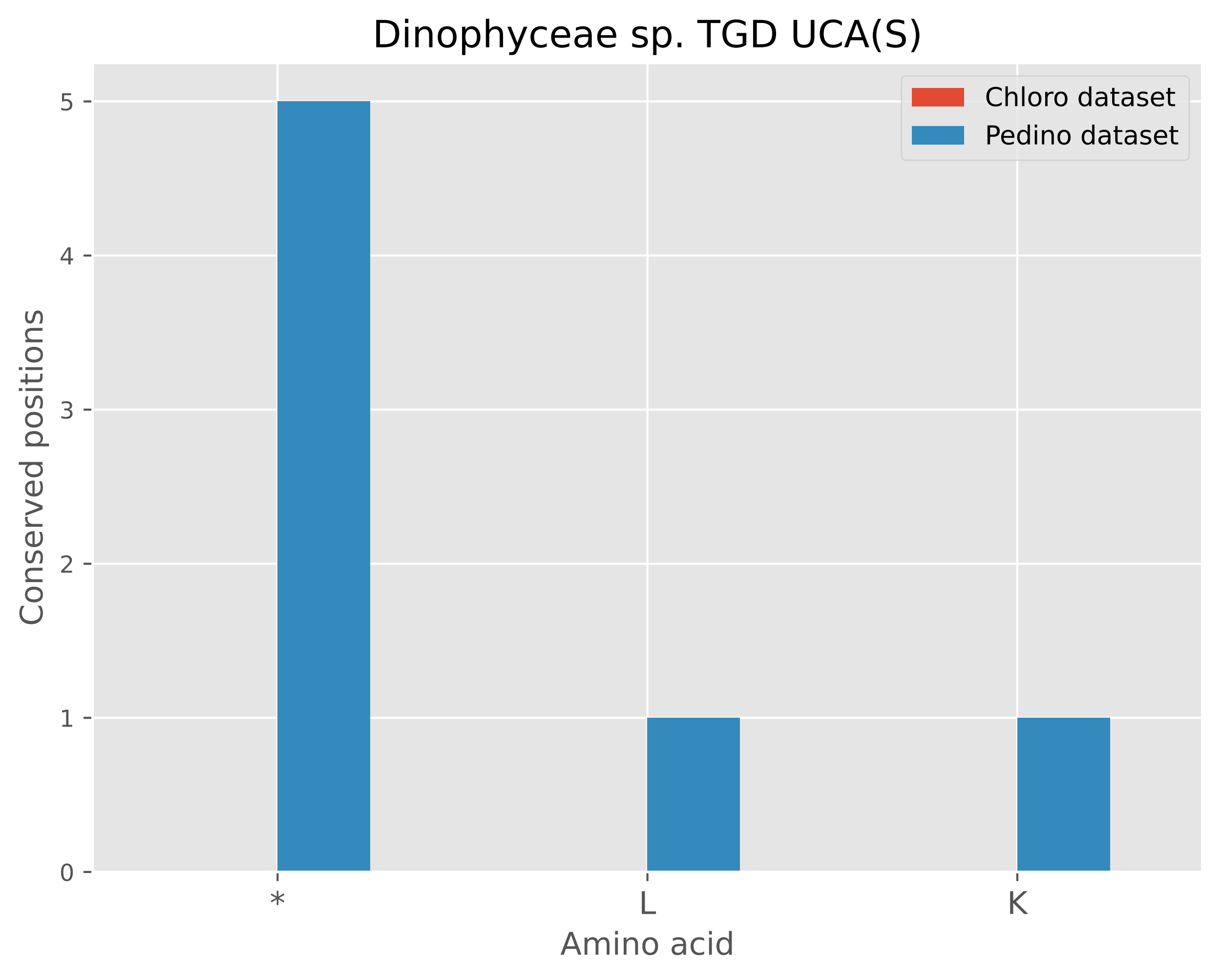
Dinophyceae sp. TGD UCA(S)
Chloro dataset
Pedino dataset
Conserved positions
0
1
2
3
4
5
*
L
K
Amino acid

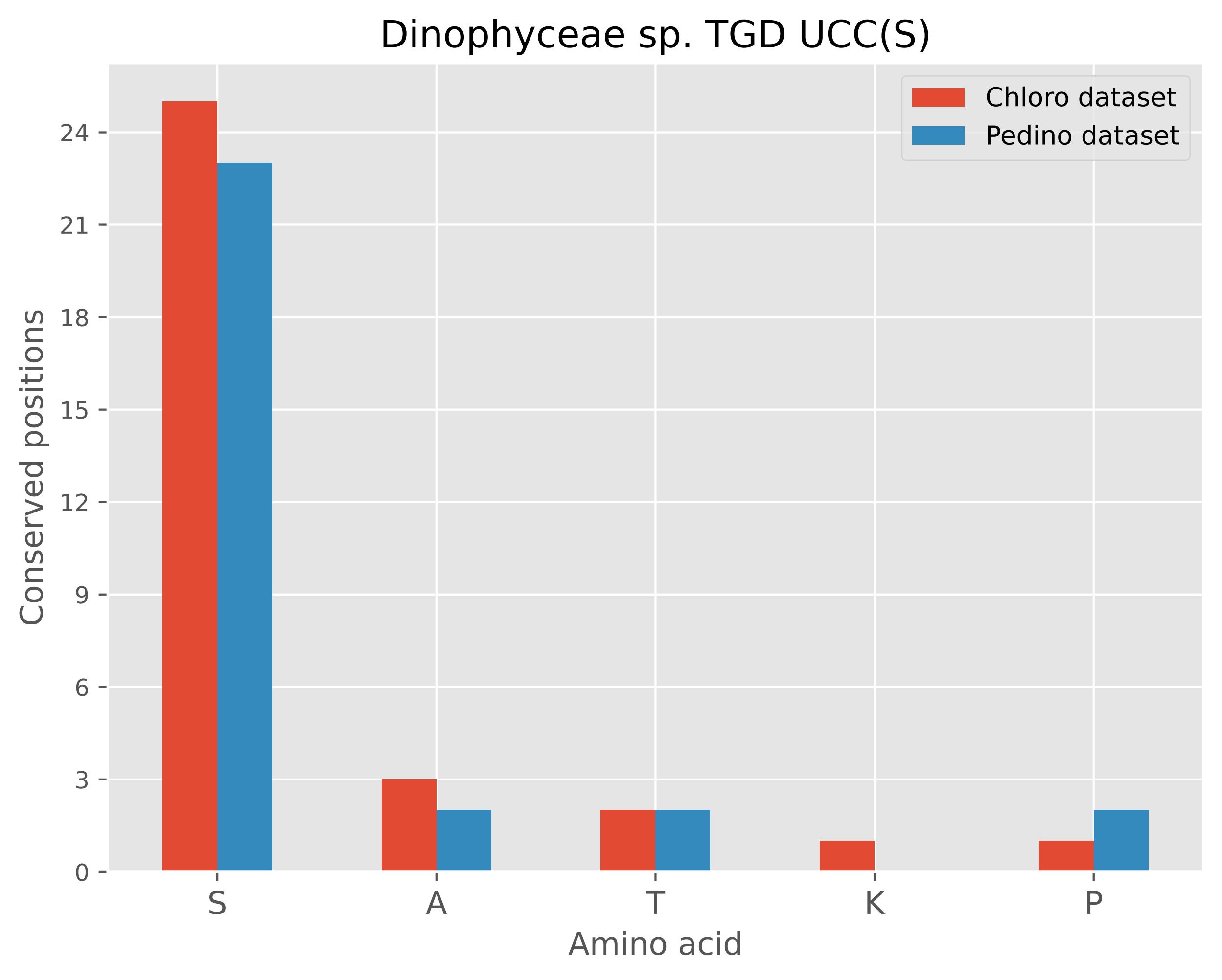

Dinophyceae sp. TGD UCC(S)
Chloro dataset
Pedino dataset
Conserved positions
0
3
6
9
12
15
18
21
24
S
A
T
K
P
Amino acid

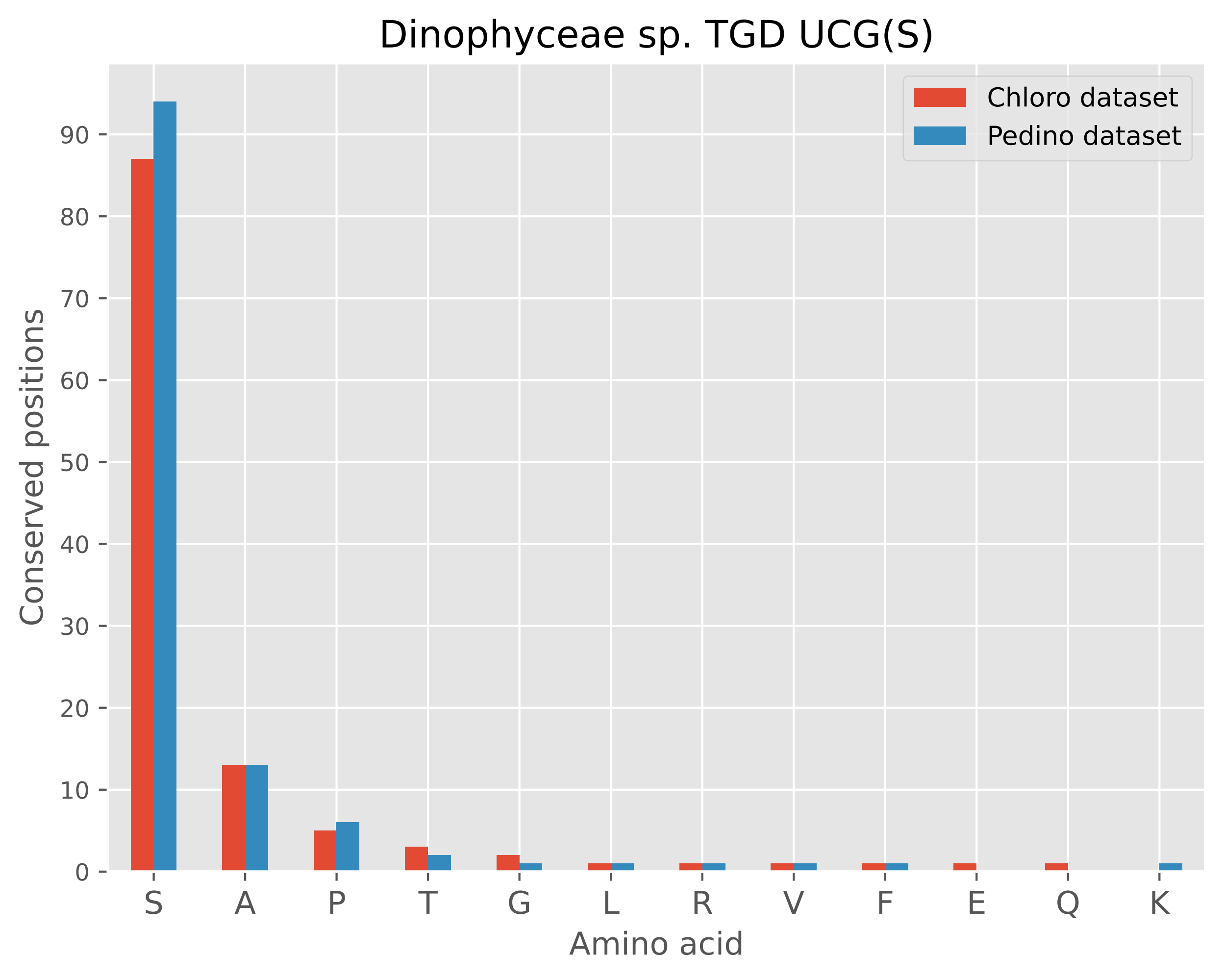

Dinophyceae sp. TGD UCG(S)
Chloro dataset
Pedino dataset
Conserved positions
0
10
20
30
40
50
60
70
80
90
S
A
P
T
G
L
R
V
F
E
Q
K
Amino acid

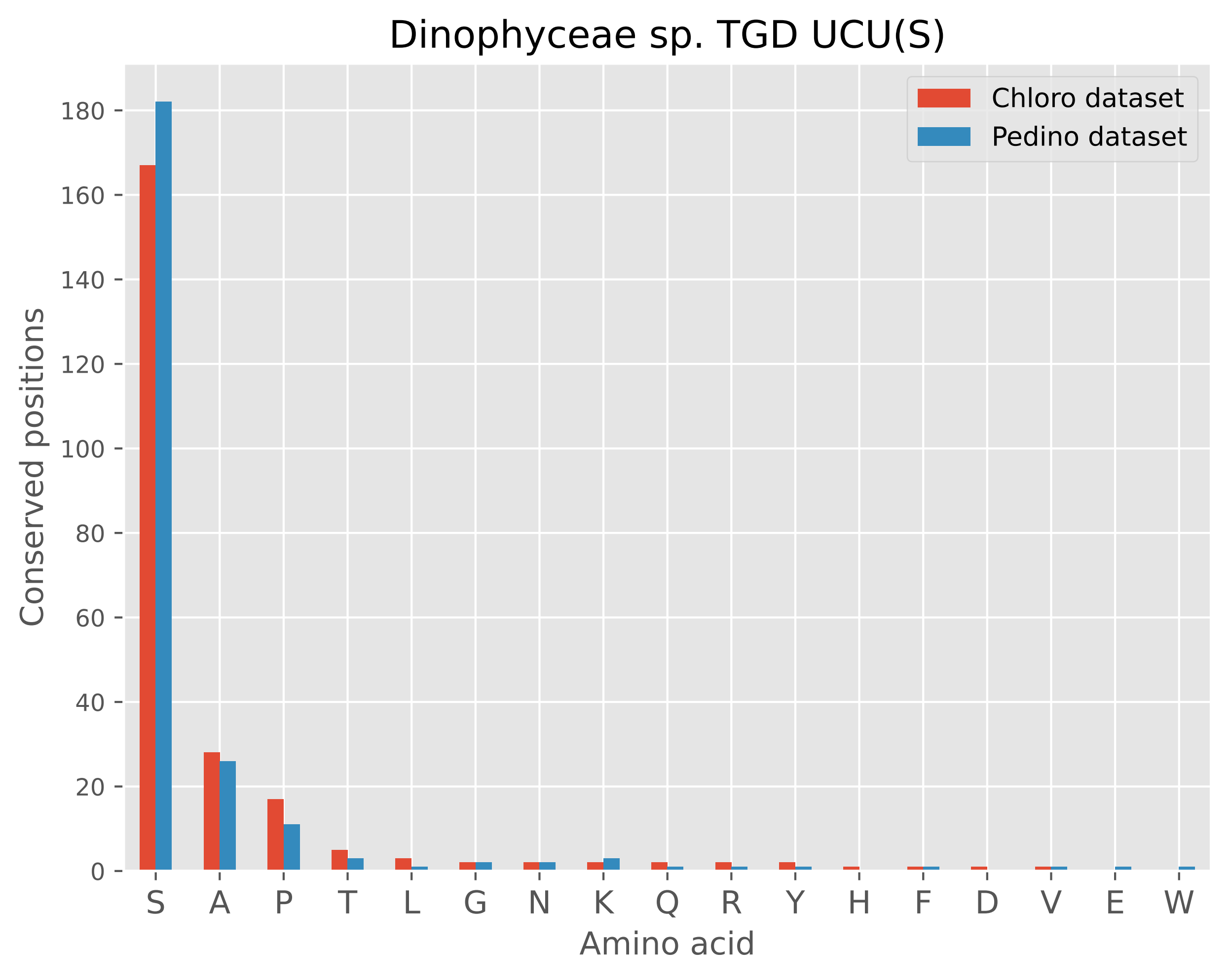

Dinophyceae sp. TGD UCU(S)
Chloro dataset
Pedino dataset
Conserved positions
0
20
40
60
80
100
120
140
160
180
S
A
P
T
L
G
N
K
Q
R
Y
H
F
D
V
E
W
Amino acid

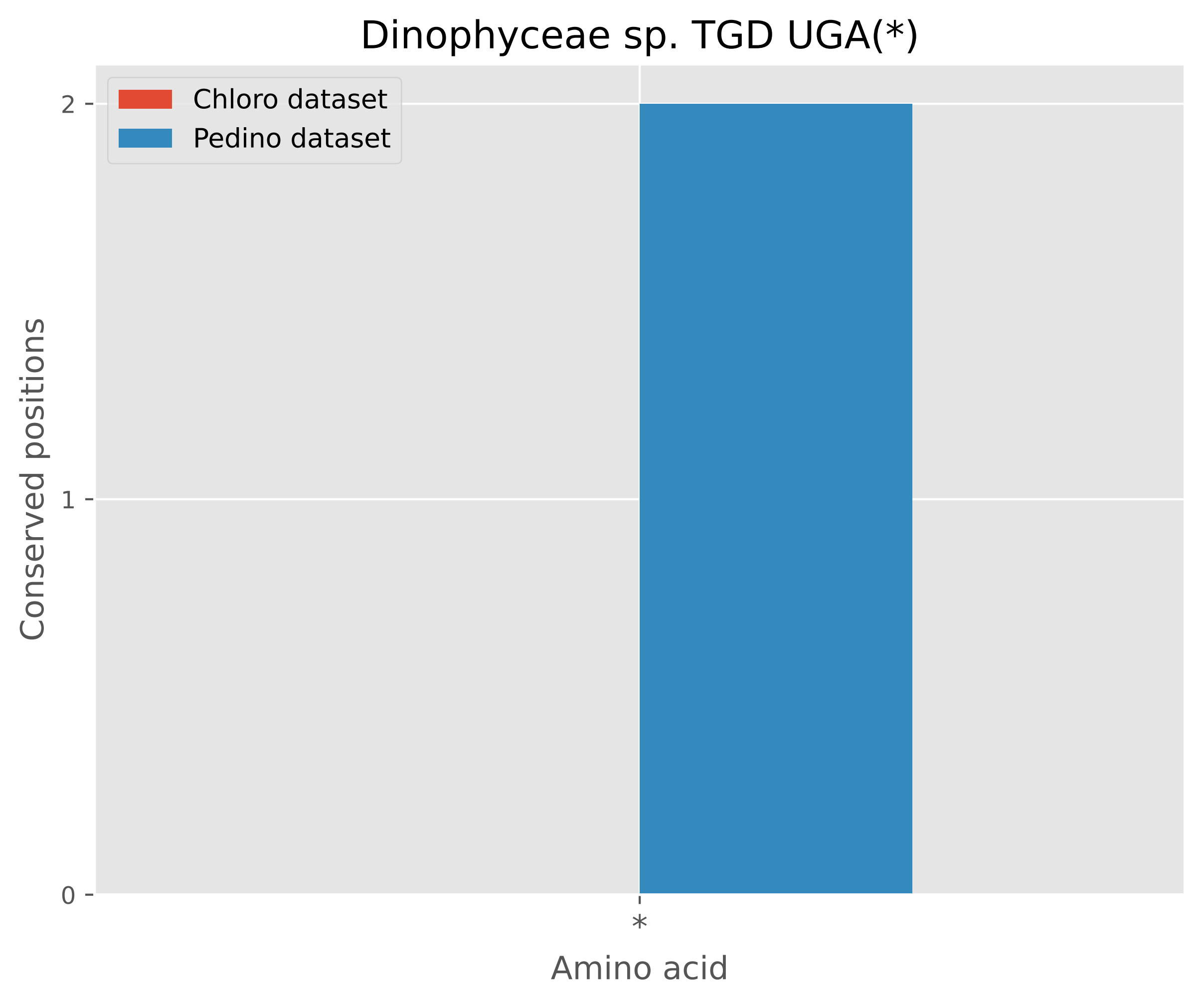

Dinophyceae sp. TGD UGA(*)
Chloro dataset
Pedino dataset
Conserved positions
2
1
0
*
Amino acid

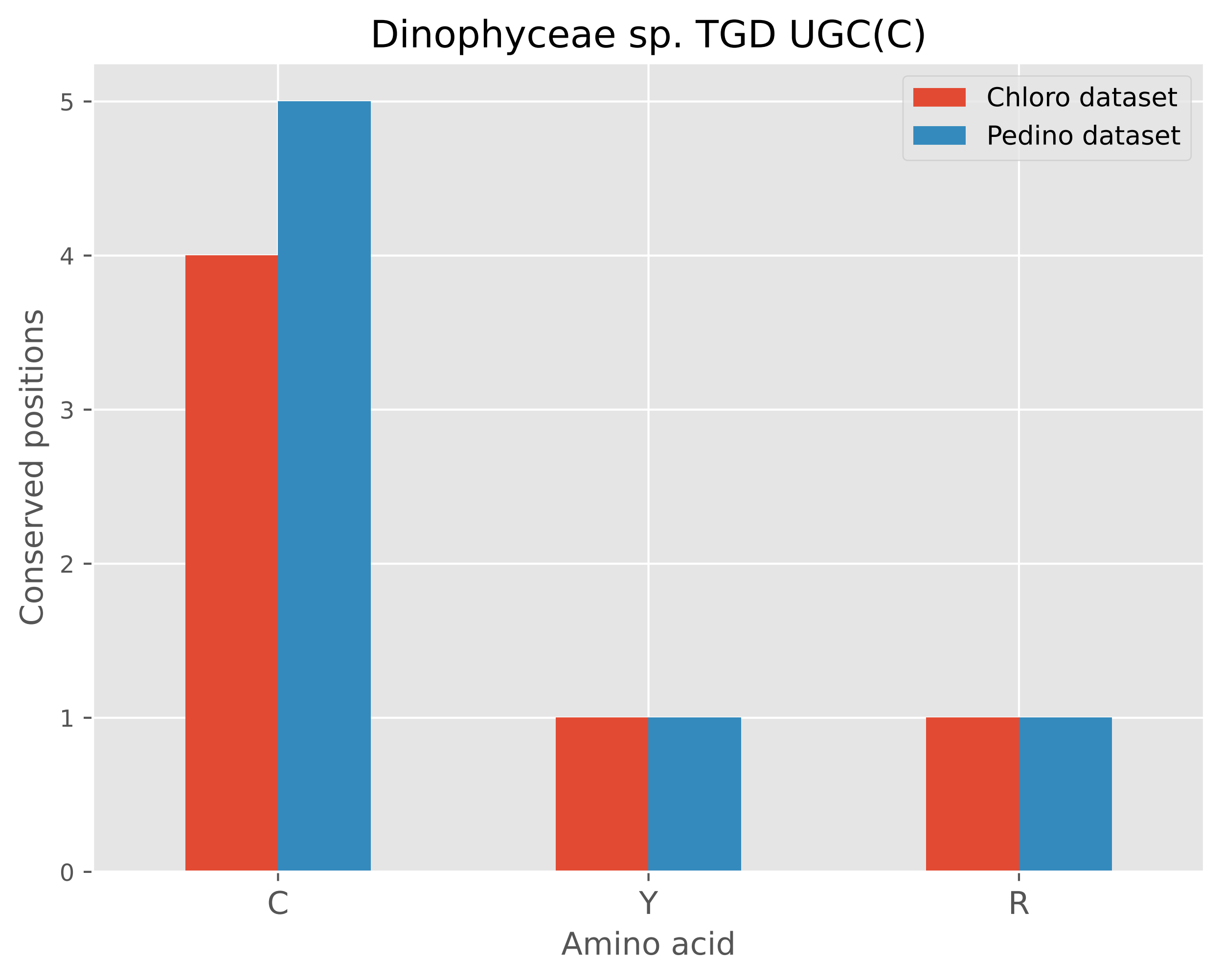

Dinophyceae sp. TGD UGC(C)
Chloro dataset
Pedino dataset
Conserved positions
0
1
2
3
4
5
C
Y
R
Amino acid

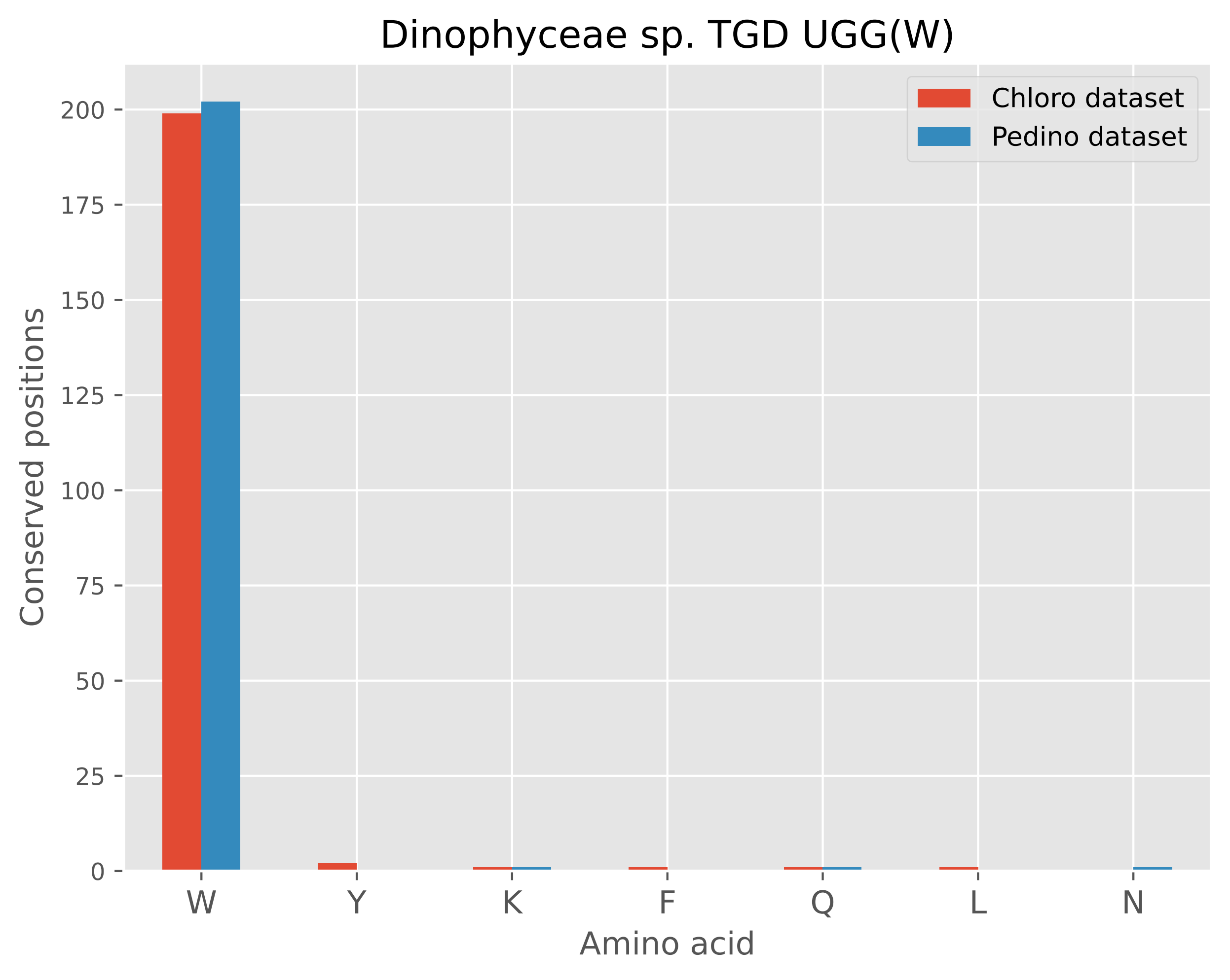

Dinophyceae sp. TGD UGG(W)
Chloro dataset
Pedino dataset
Conserved positions
0
25
50
75
100
125
150
175
200
Amino acid
W
Y
K
F
Q
L
N

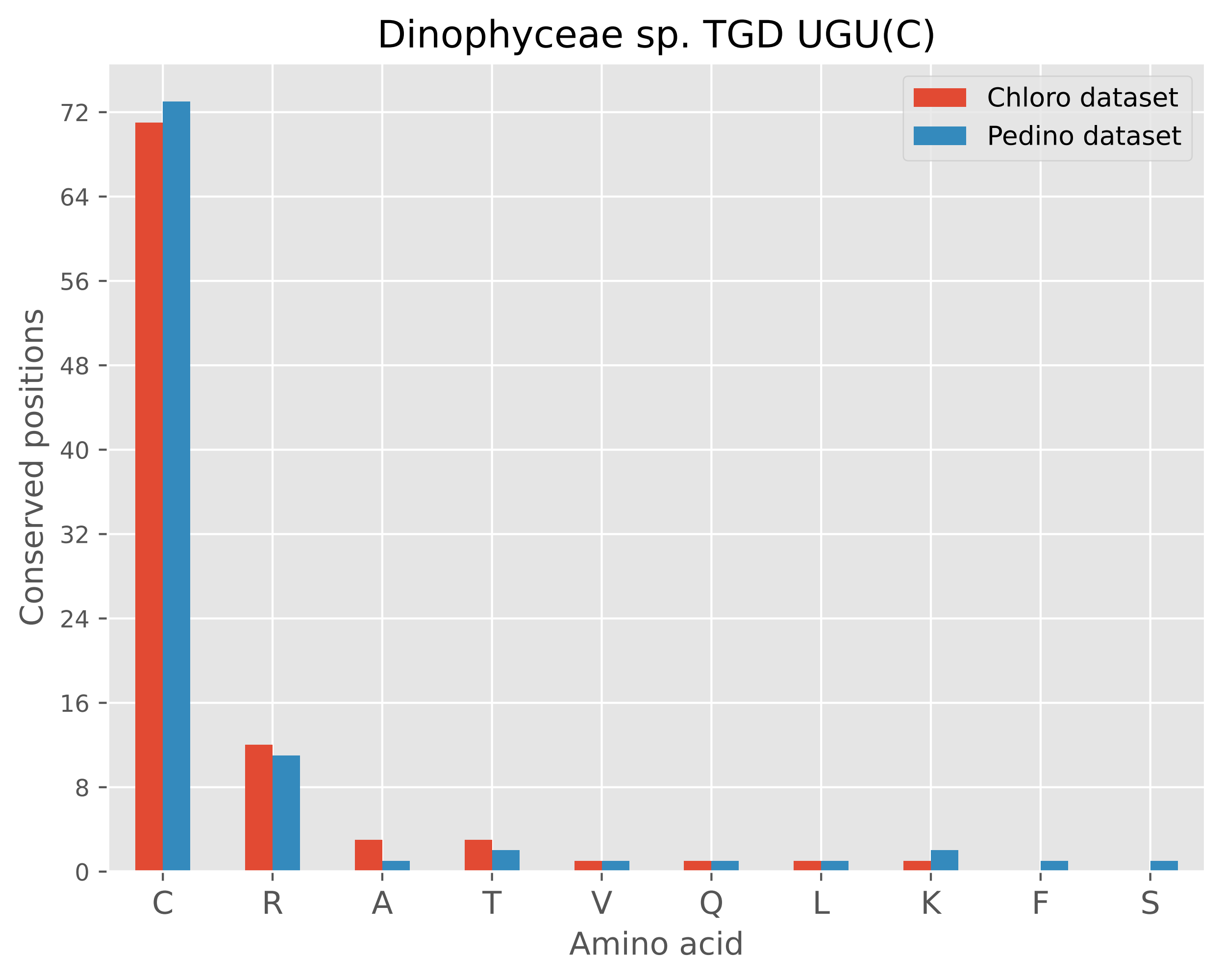

Dinophyceae sp. TGD UGU(C)
Chloro dataset
Pedino dataset
Conserved positions
0
8
16
24
32
40
48
56
64
72
Amino acid
C
R
A
T
V
Q
L
K
F
S

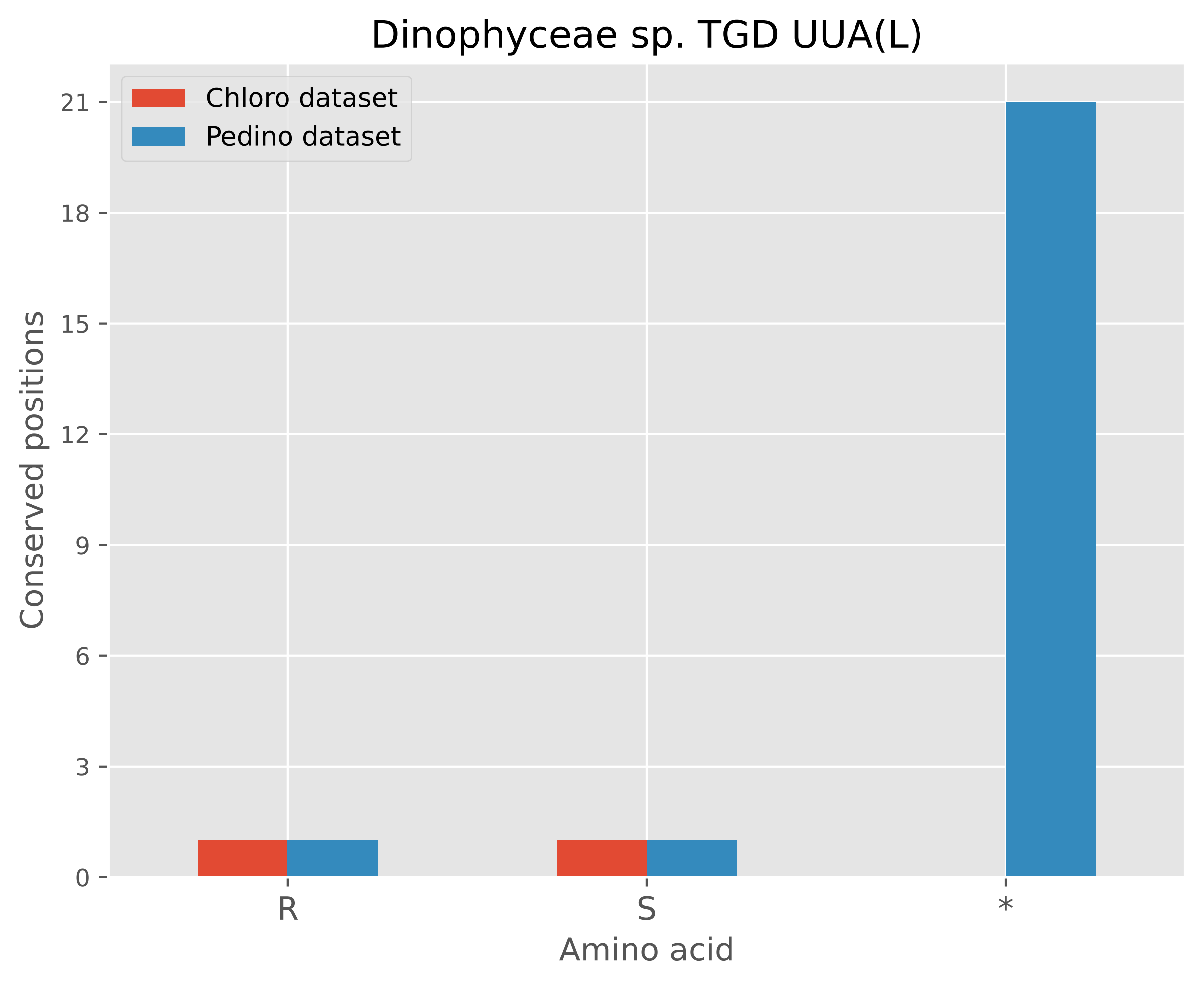
Dinophyceae sp. TGD UUA(L)
Chloro dataset
Pedino dataset
Conserved positions
0
3
6
9
12
15
18
21
R
S
*
Amino acid

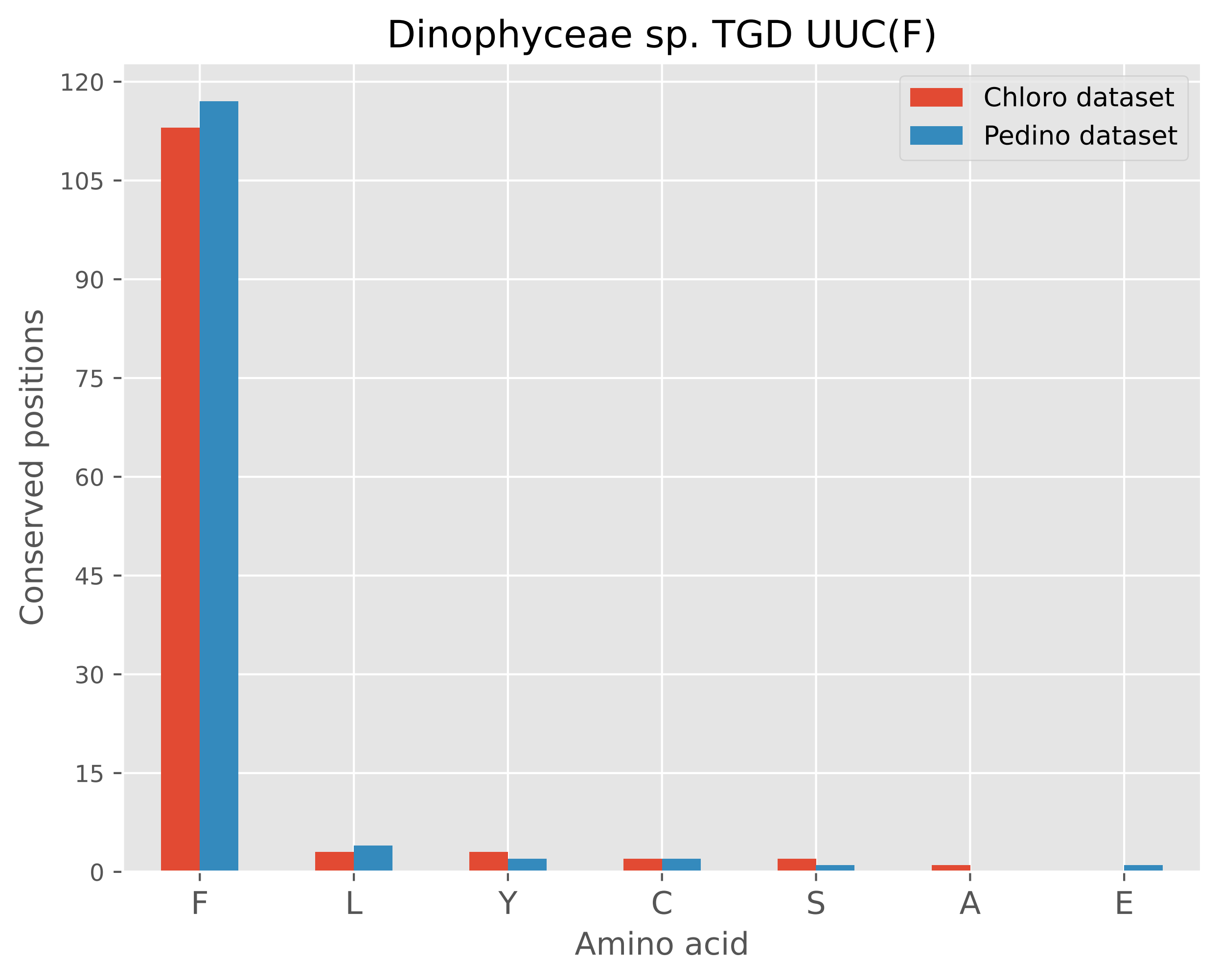

Dinophyceae sp. TGD UUC(F)
Chloro dataset
Pedino dataset
Conserved positions
Amino acid
0
15
30
45
60
75
90
105
120
F
L
Y
C
S
A
E

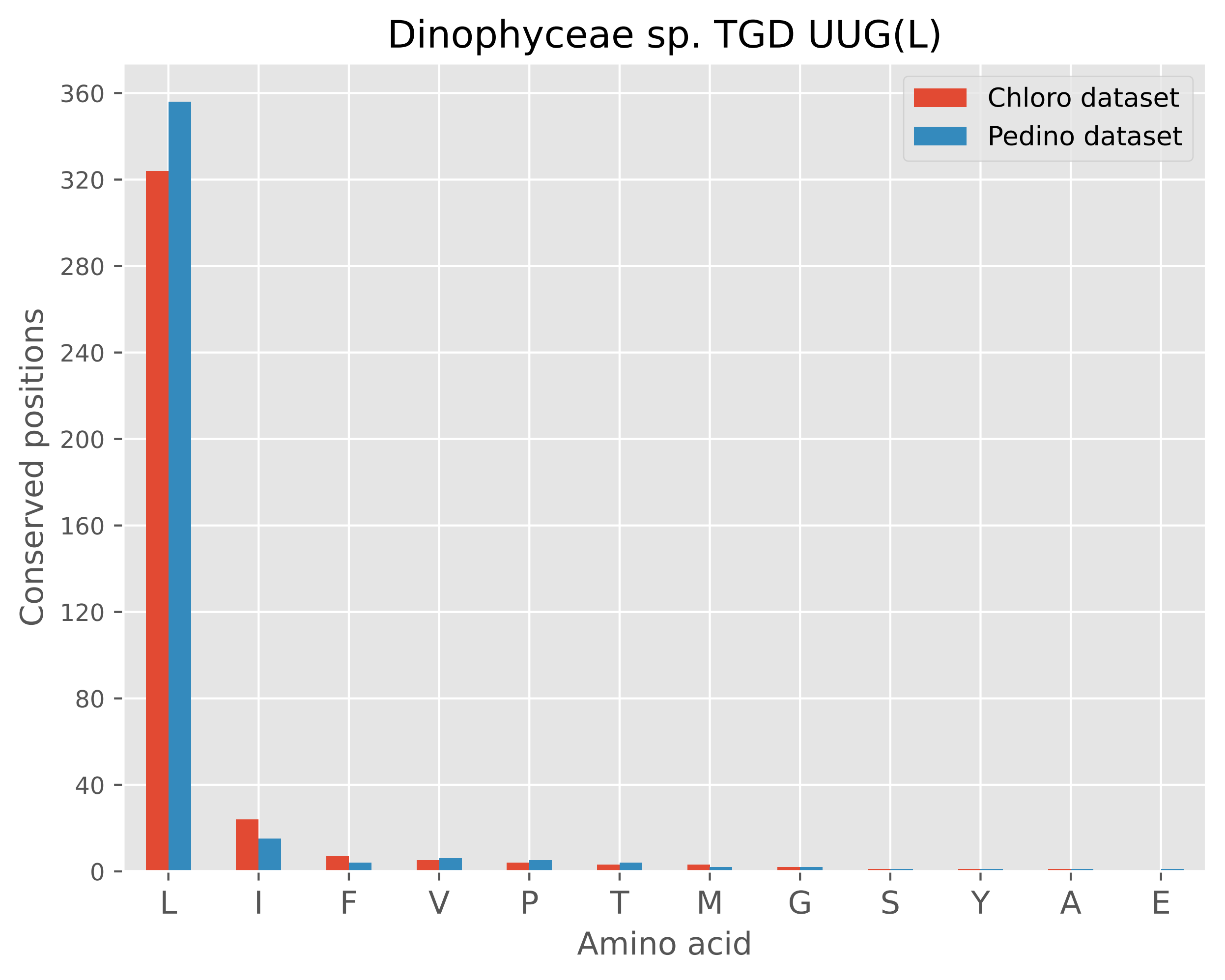
Dinophyceae sp. TGD UUG(L)
Chloro dataset
Pedino dataset
Conserved positions
Amino acid
0
40
80
120
160
200
240
280
320
360
L
I
F
V
P
T
M
G
S
Y
A
E

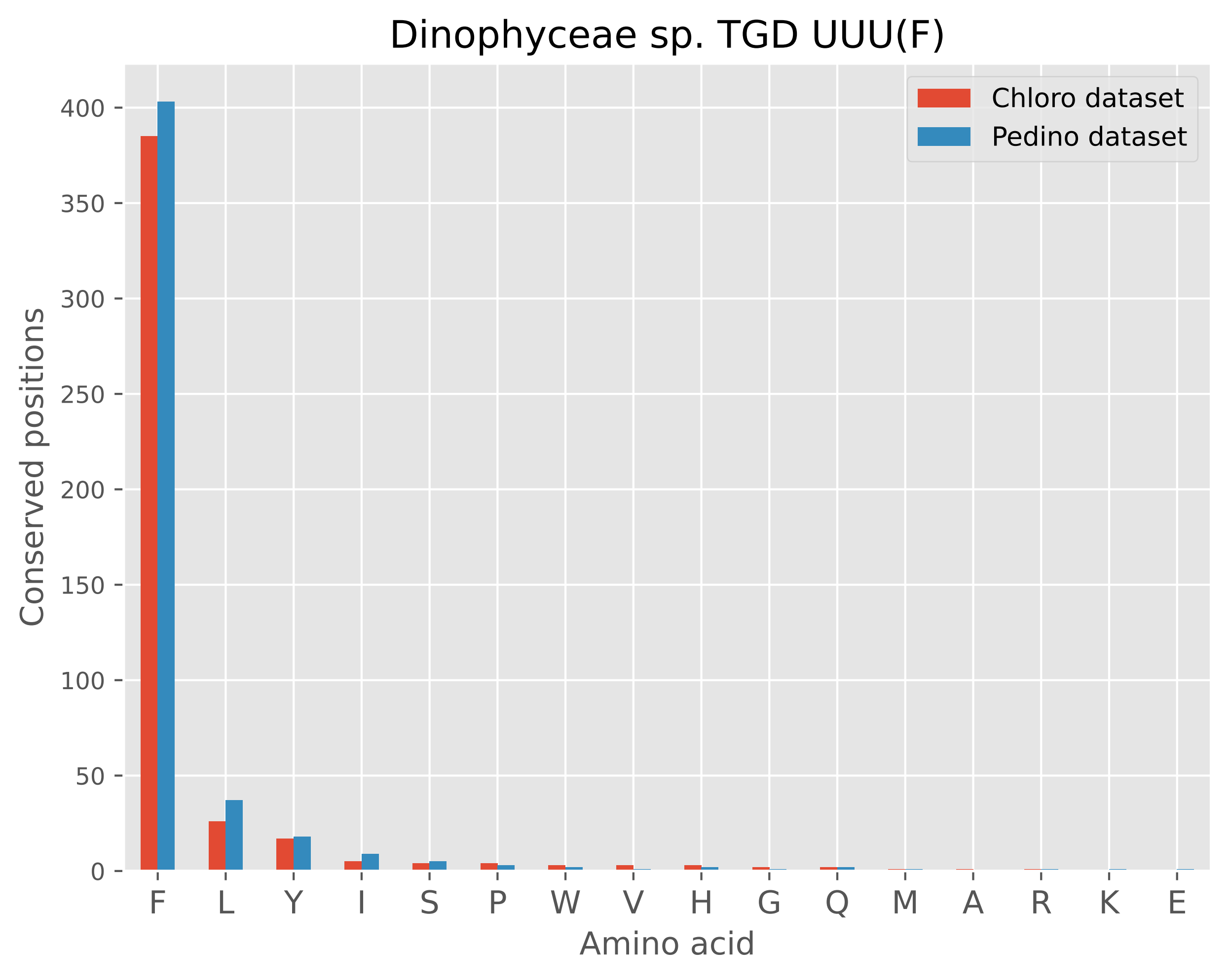
Dinophyceae sp. TGD UUU(F)
Chloro dataset
Pedino dataset
Conserved positions
0
50
100
150
200
250
300
350
400
F
L
Y
I
S
P
W
V
H
G
Q
M
A
R
K
E
Amino acid

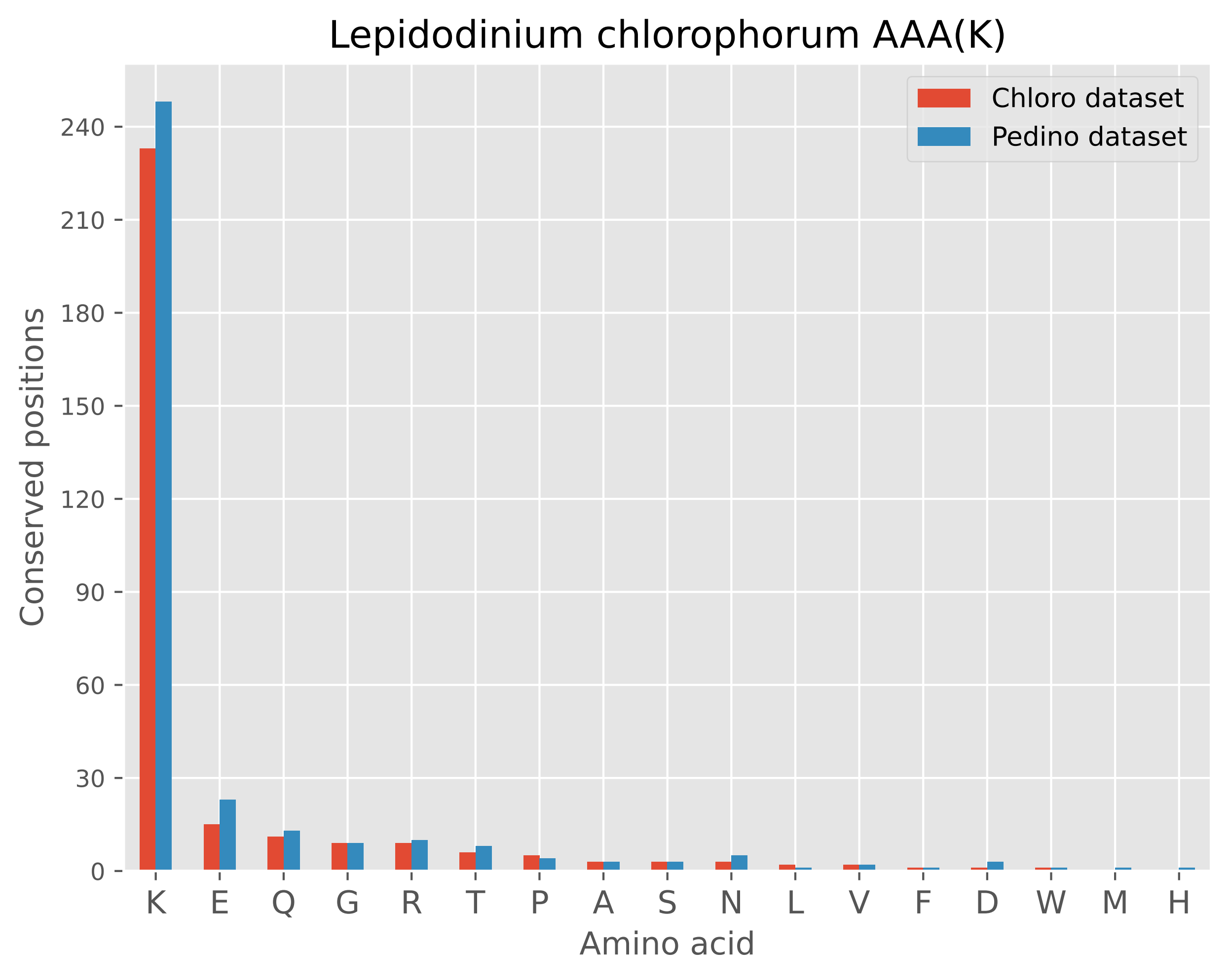
Lepidodinium chlorophorum AAA(K)
Chloro dataset
Pedino dataset
Conserved positions
Amino acid
0
30
60
90
120
150
180
210
240
K
E
Q
G
R
T
P
A
S
N
L
V
F
D
W
M
H

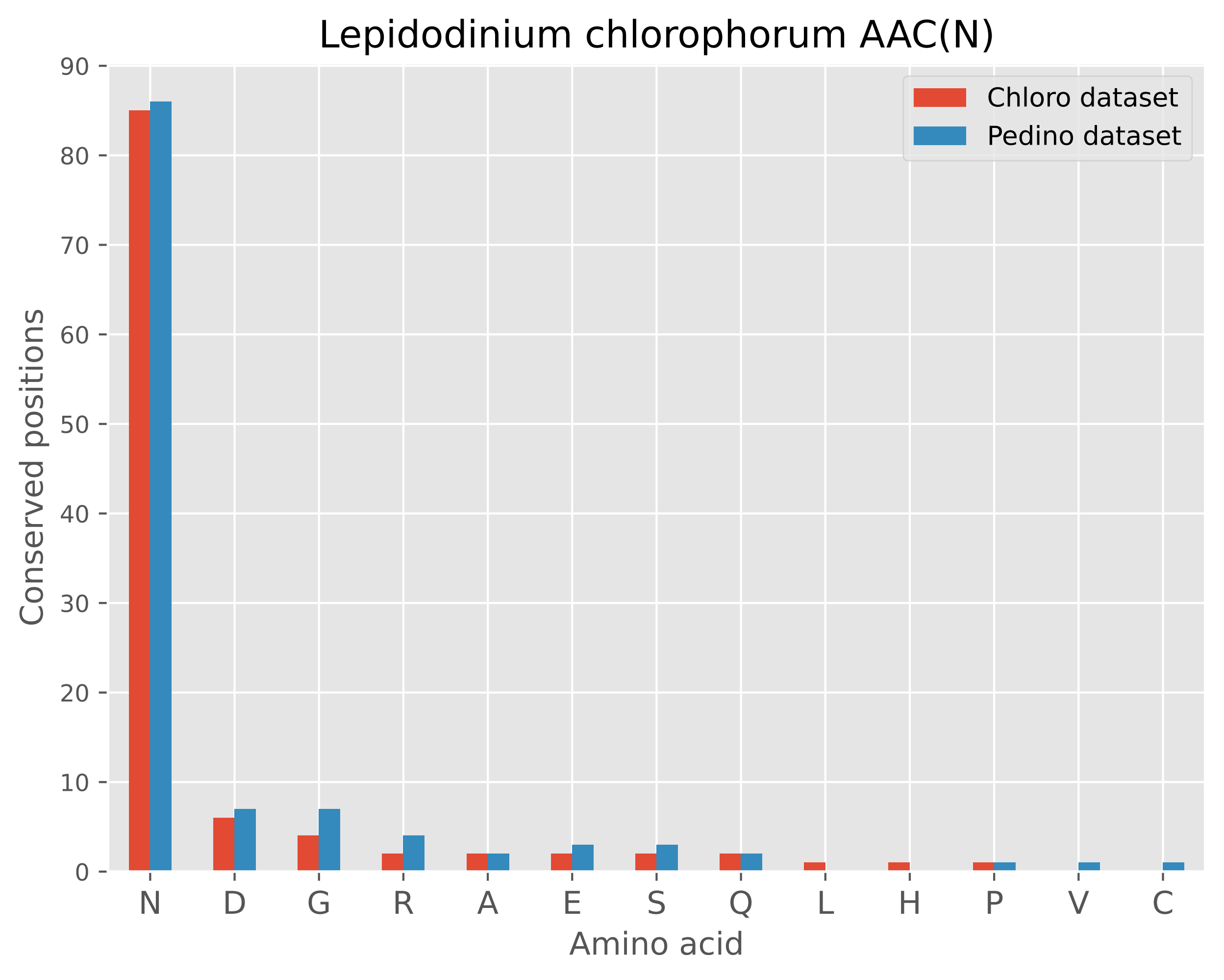

Lepidodinium chlorophorum AAC(N)
Chloro dataset
Pedino dataset
Conserved positions
Amino acid
0
10
20
30
40
50
60
70
80
90
N
D
G
R
A
E
S
Q
L
H
P
V
C

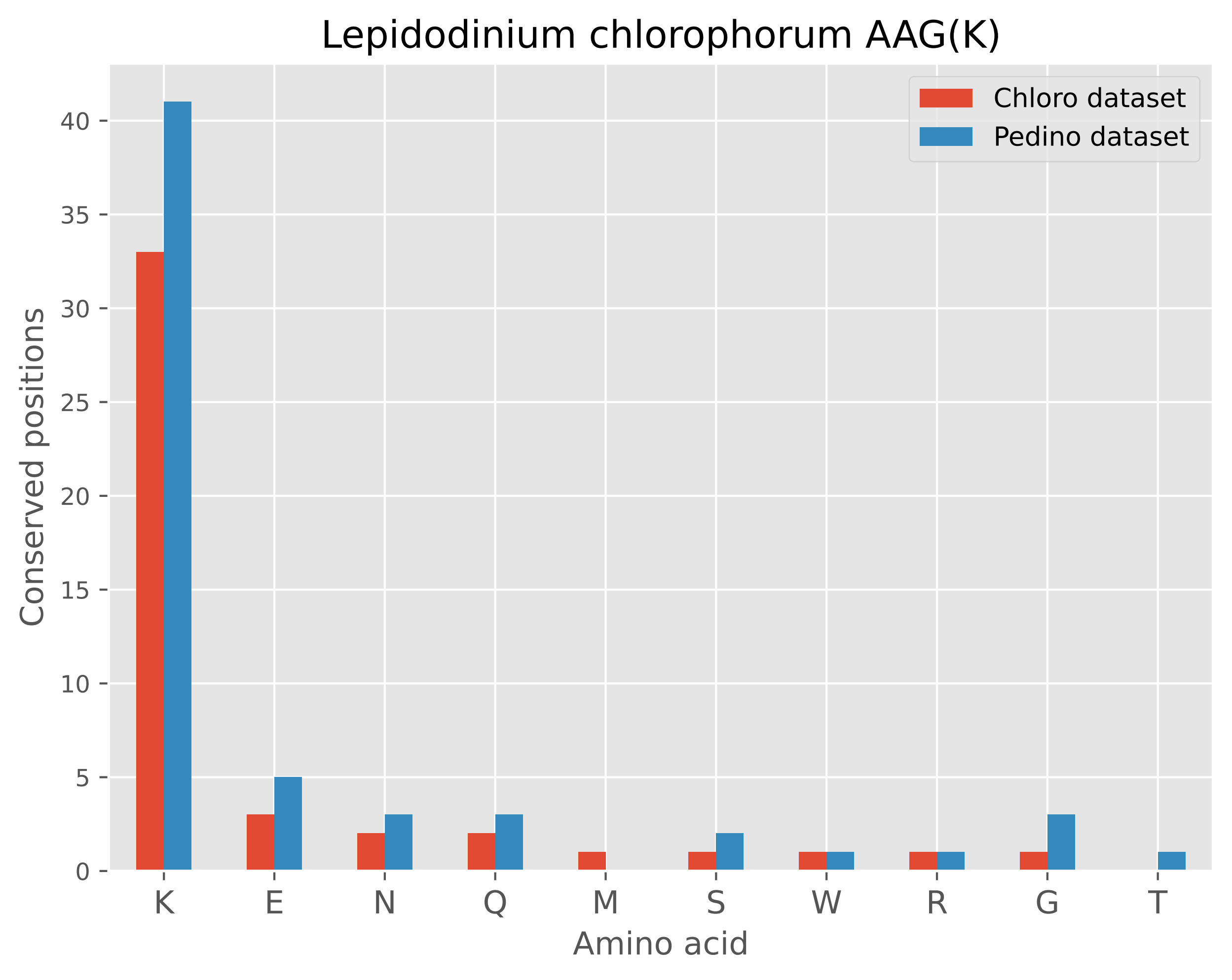

Lepidodinium chlorophorum AAG(K)
Chloro dataset
Pedino dataset
Conserved positions
0
5
10
15
20
25
30
35
40
K
E
N
Q
M
S
W
R
G
T
Amino acid

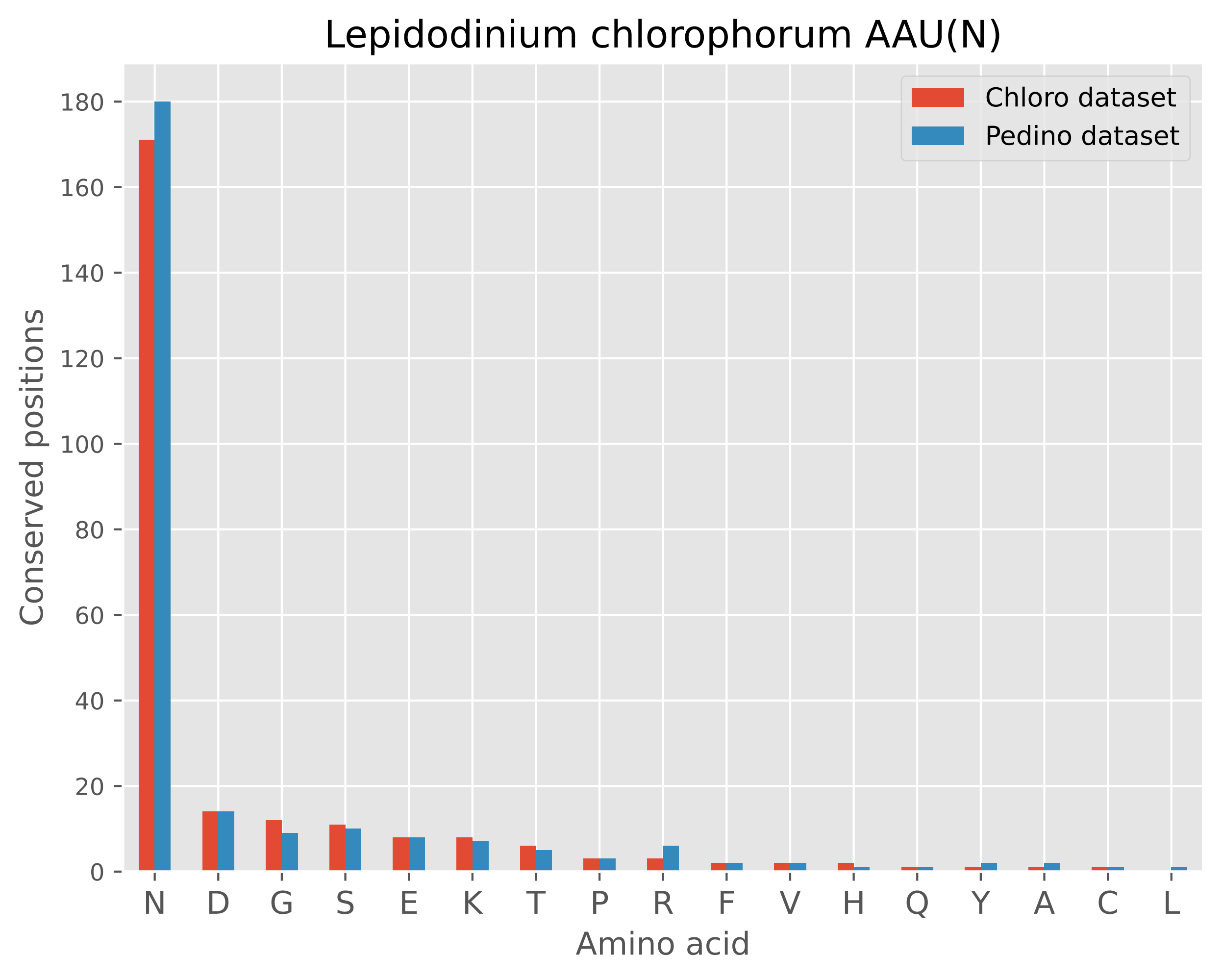

Lepidodinium chlorophorum AAU(N)
Chloro dataset
Pedino dataset
Conserved positions
Amino acid
0
20
40
60
80
100
120
140
160
180
N
D
G
S
E
K
T
P
R
F
V
H
Q
Y
A
C
L

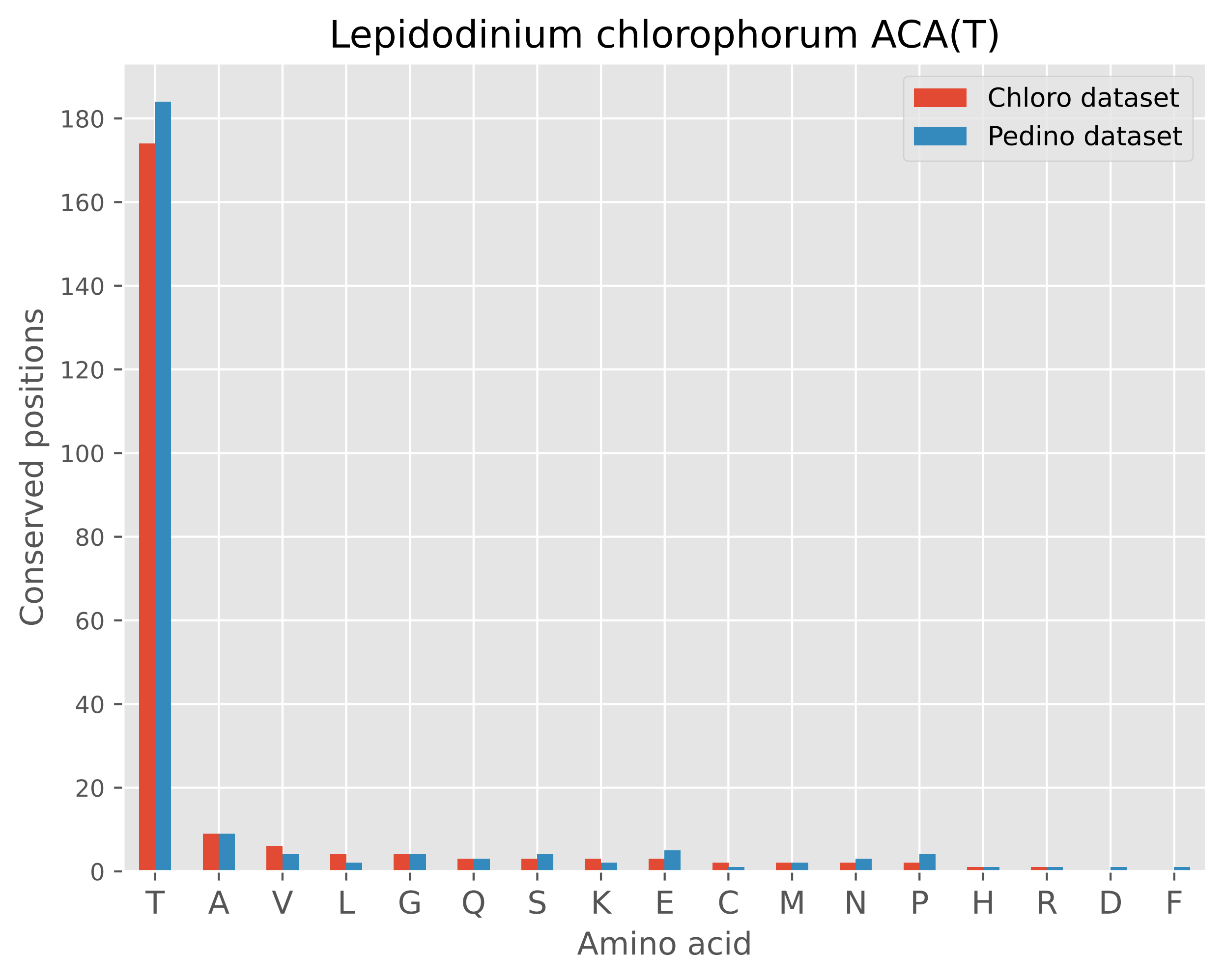

Lepidodinium chlorophorum ACA(T)
Chloro dataset
Pedino dataset
Conserved positions
0
20
40
60
80
100
120
140
160
180
T
A
V
L
G
Q
S
K
E
C
M
N
P
H
R
D
F
Amino acid

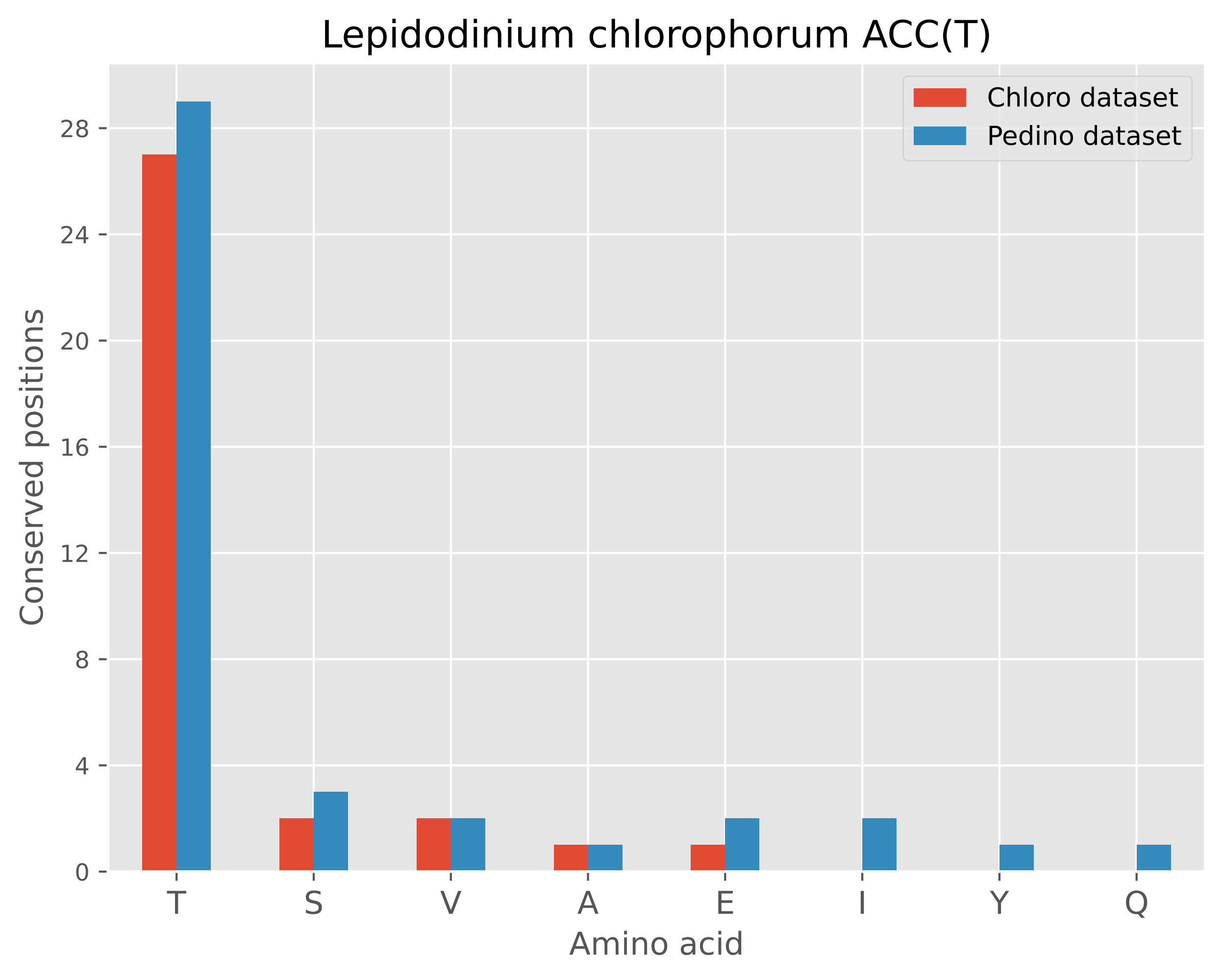

Lepidodinium chlorophorum ACC(T)
Chloro dataset
Pedino dataset
Conserved positions
0
4
8
12
16
20
24
28
T
S
V
A
E
I
Y
Q
Amino acid

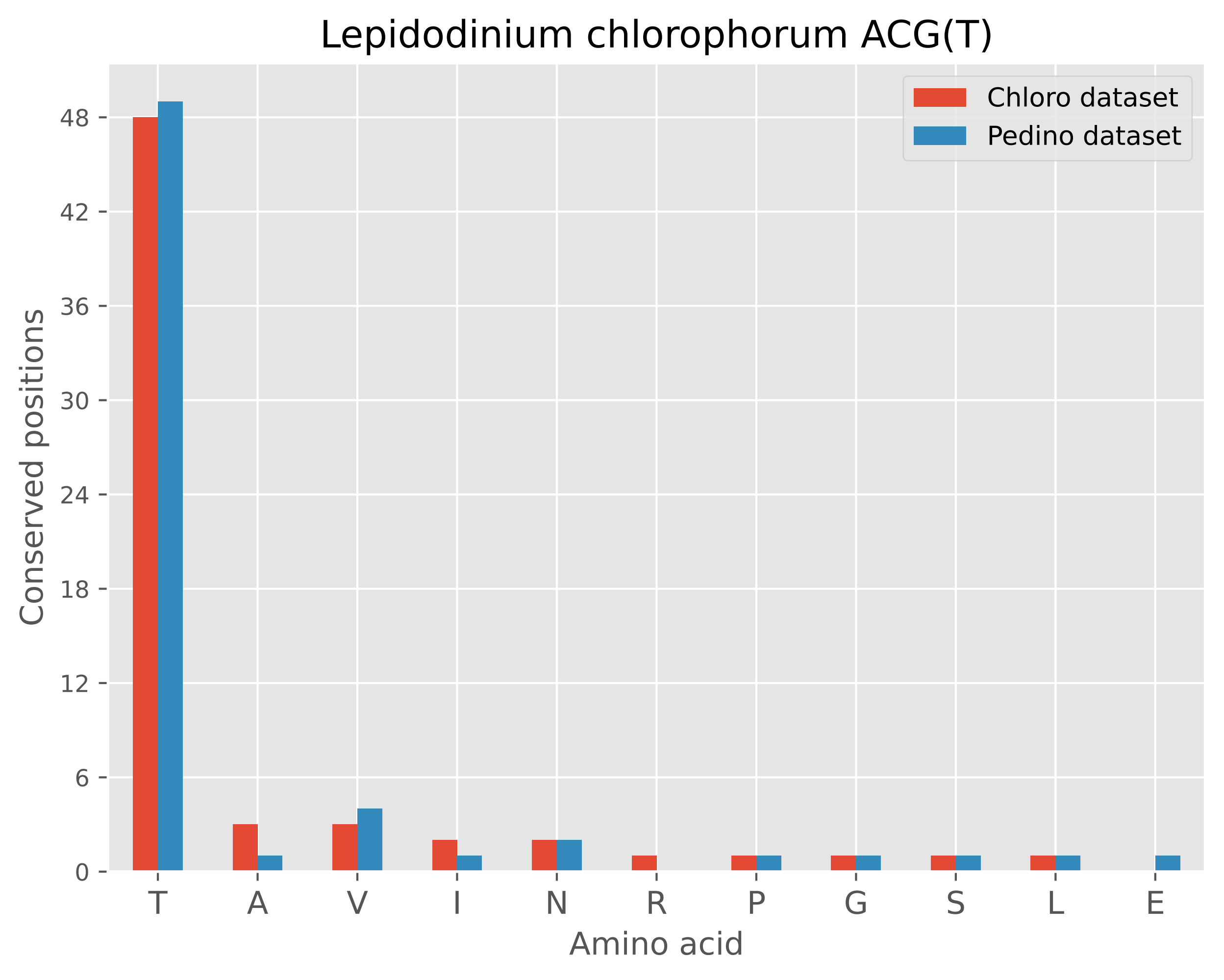

Lepidodinium chlorophorum ACG(T)
Chloro dataset
Pedino dataset
Conserved positions
0
6
12
18
24
30
36
42
48
T
A
V
I
N
R
P
G
S
L
E
Amino acid

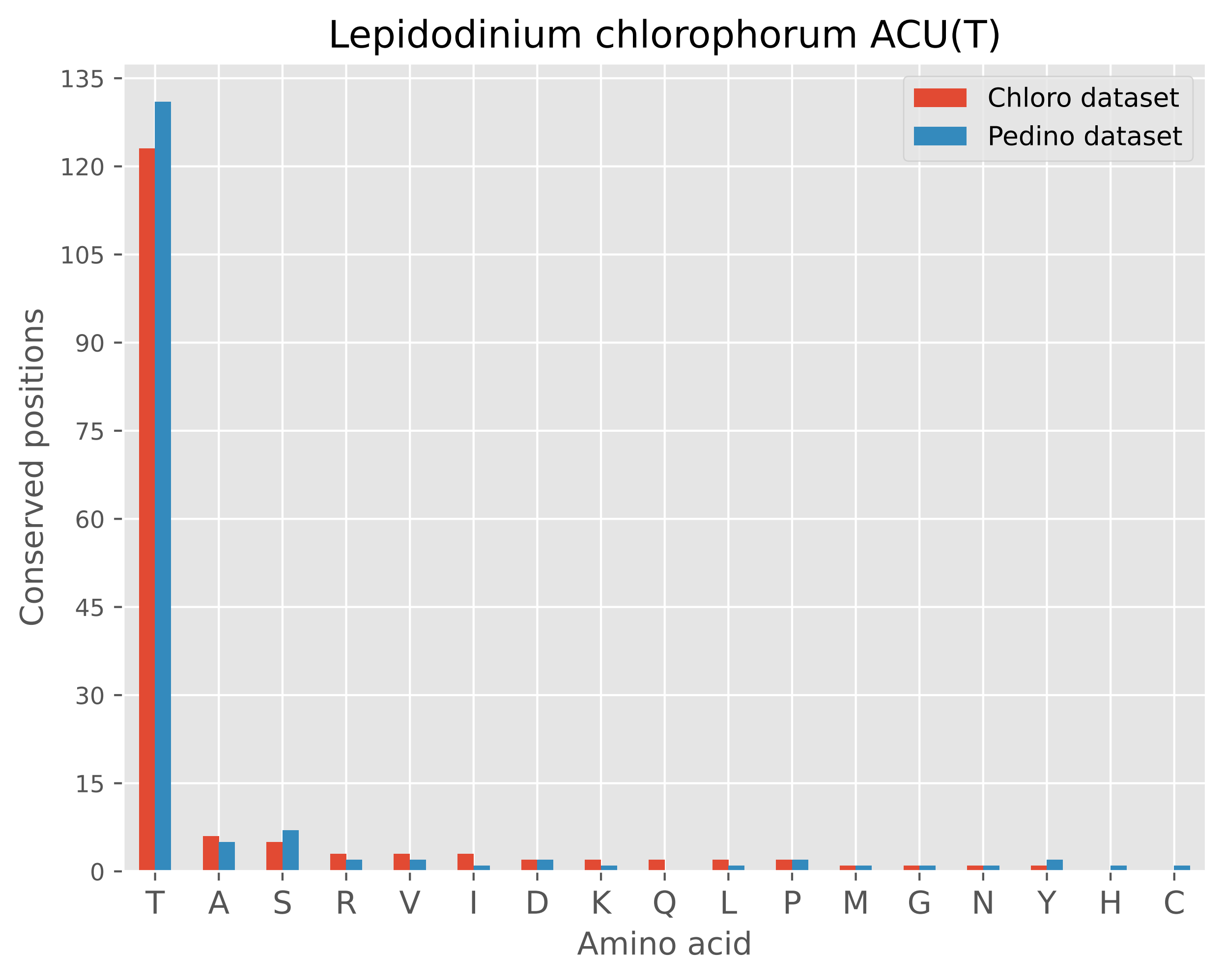

Lepidodinium chlorophorum ACU(T)
Chloro dataset
Pedino dataset
Conserved positions
135
120
105
90
75
60
45
30
15
0
T
A
S
R
V
I
D
K
Q
L
P
M
G
N
Y
H
C
Amino acid

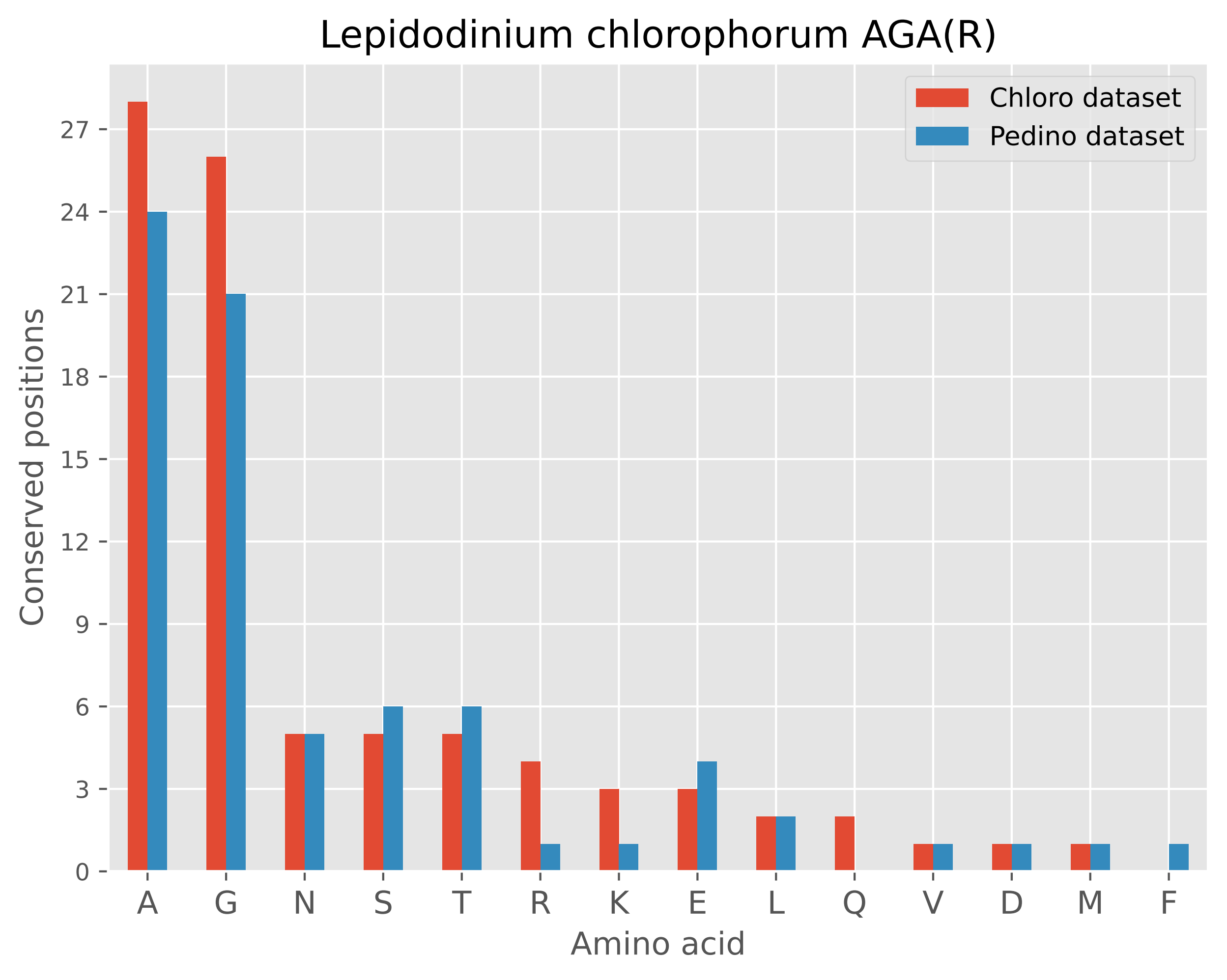

Lepidodinium chlorophorum AGA(R)
Chloro dataset
Pedino dataset
Conserved positions
0
3
6
9
12
15
18
21
24
27
A
G
N
S
T
R
K
E
L
Q
V
D
M
F
Amino acid

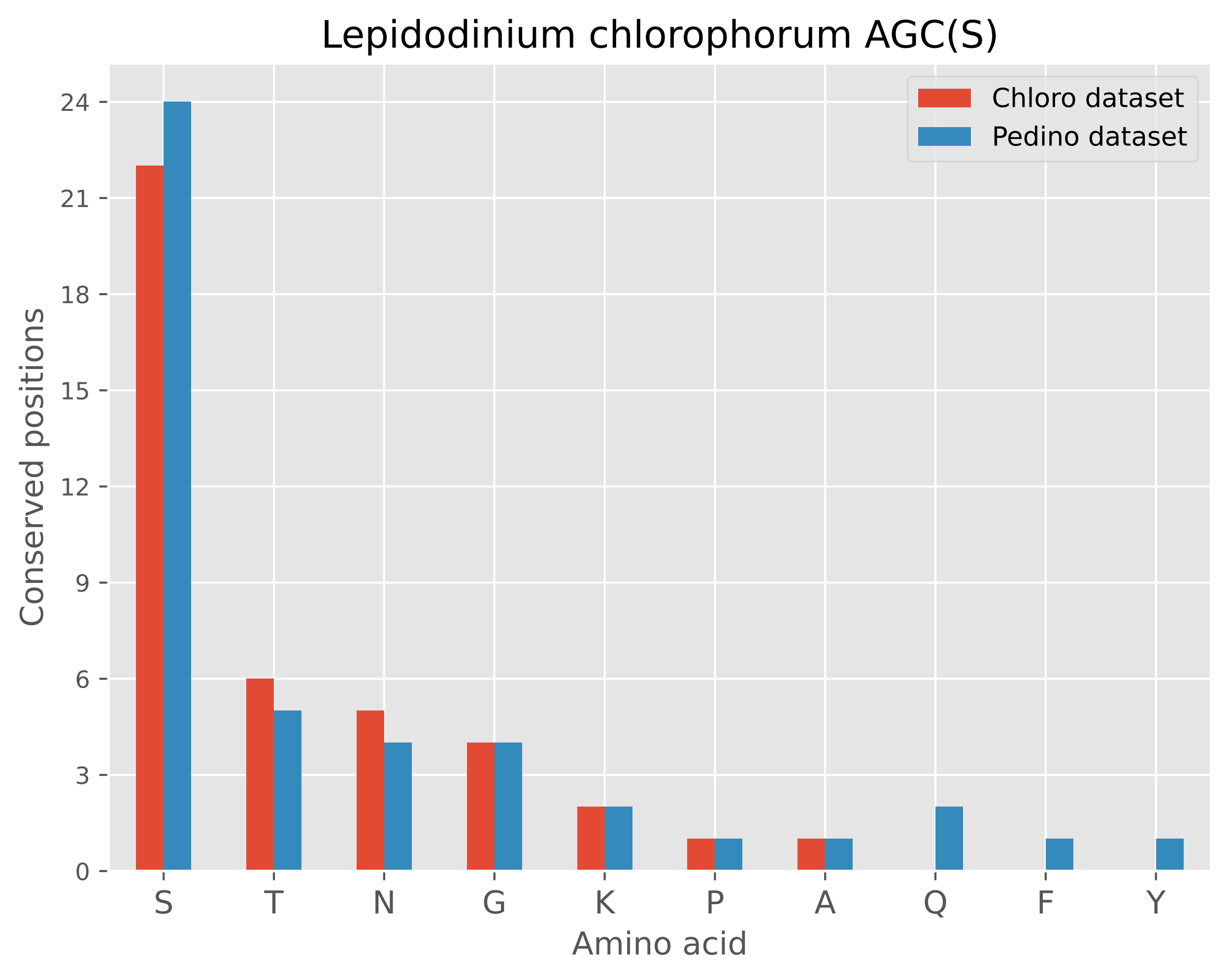
Lepidodinium chlorophorum AGC(S)
Chloro dataset
Pedino dataset
Conserved positions
0
3
6
9
12
15
18
21
24
S
T
N
G
K
P
A
Q
F
Y
Amino acid

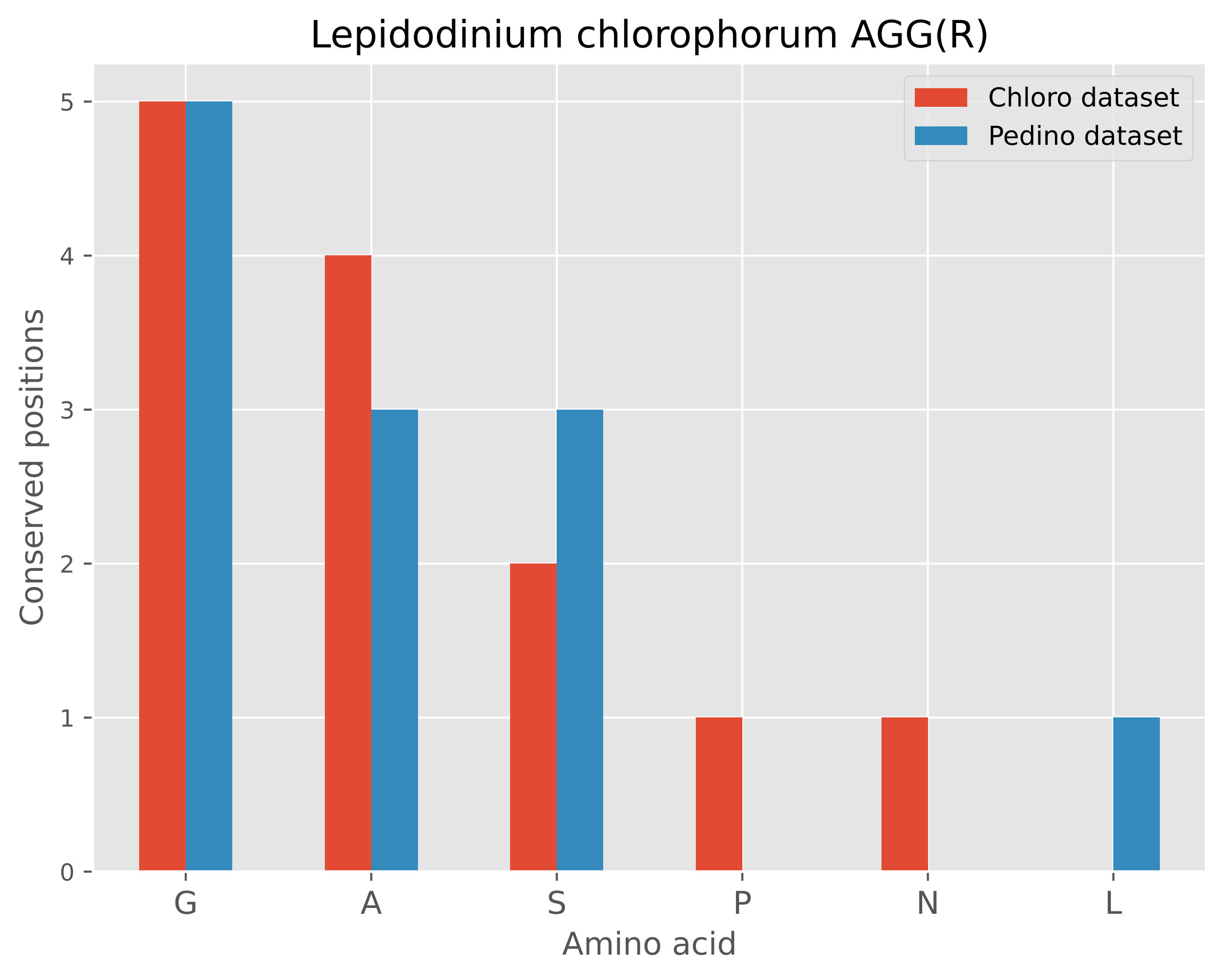
Lepidodinium chlorophorum AGG(R)
Chloro dataset
Pedino dataset
Conserved positions
0
1
2
3
4
5
G
A
S
P
N
L
Amino acid

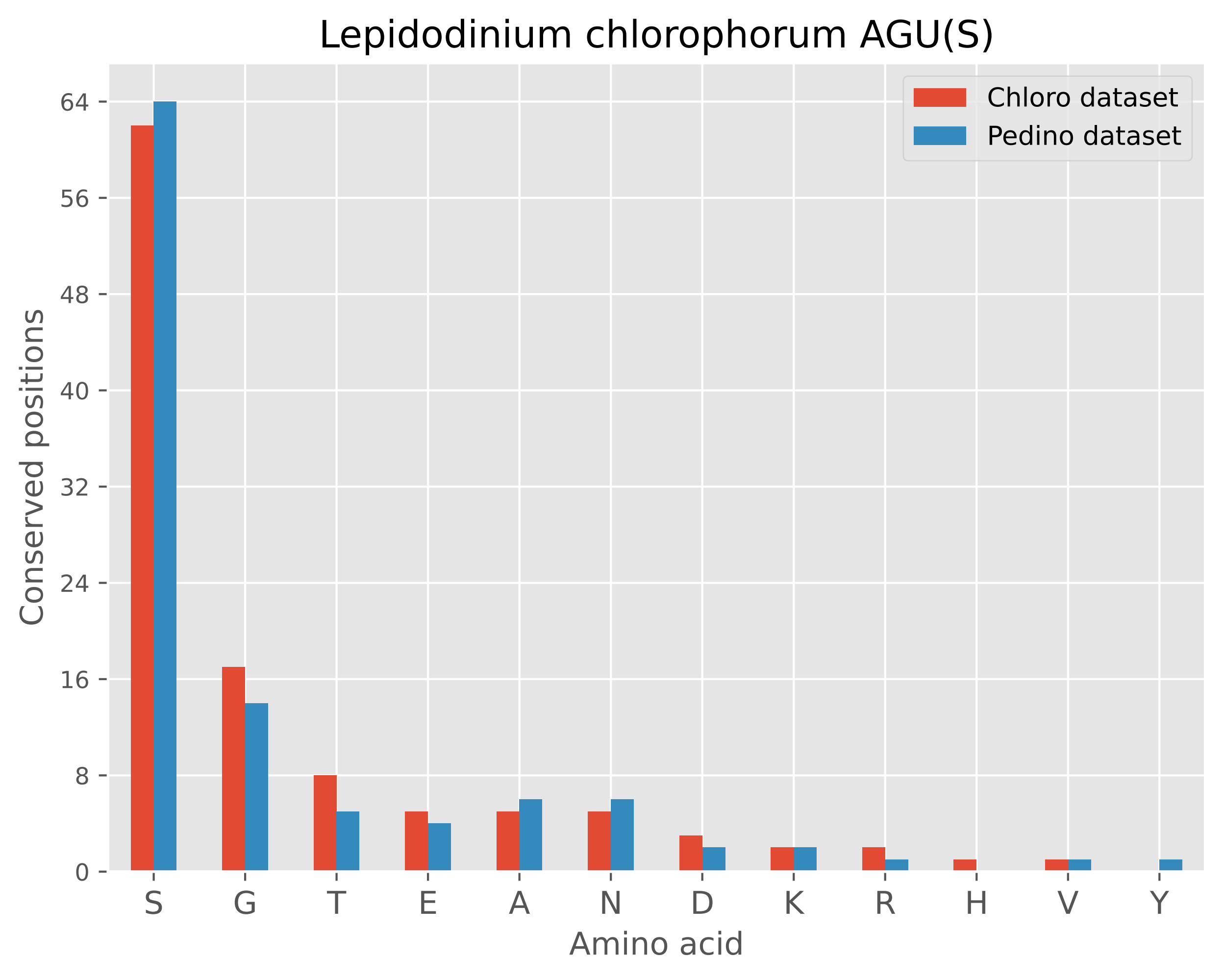
Lepidodinium chlorophorum AGU(S)
Chloro dataset
Pedino dataset
Conserved positions
0
8
16
24
32
40
48
56
64
Amino acid
S
G
T
E
A
N
D
K
R
H
V
Y

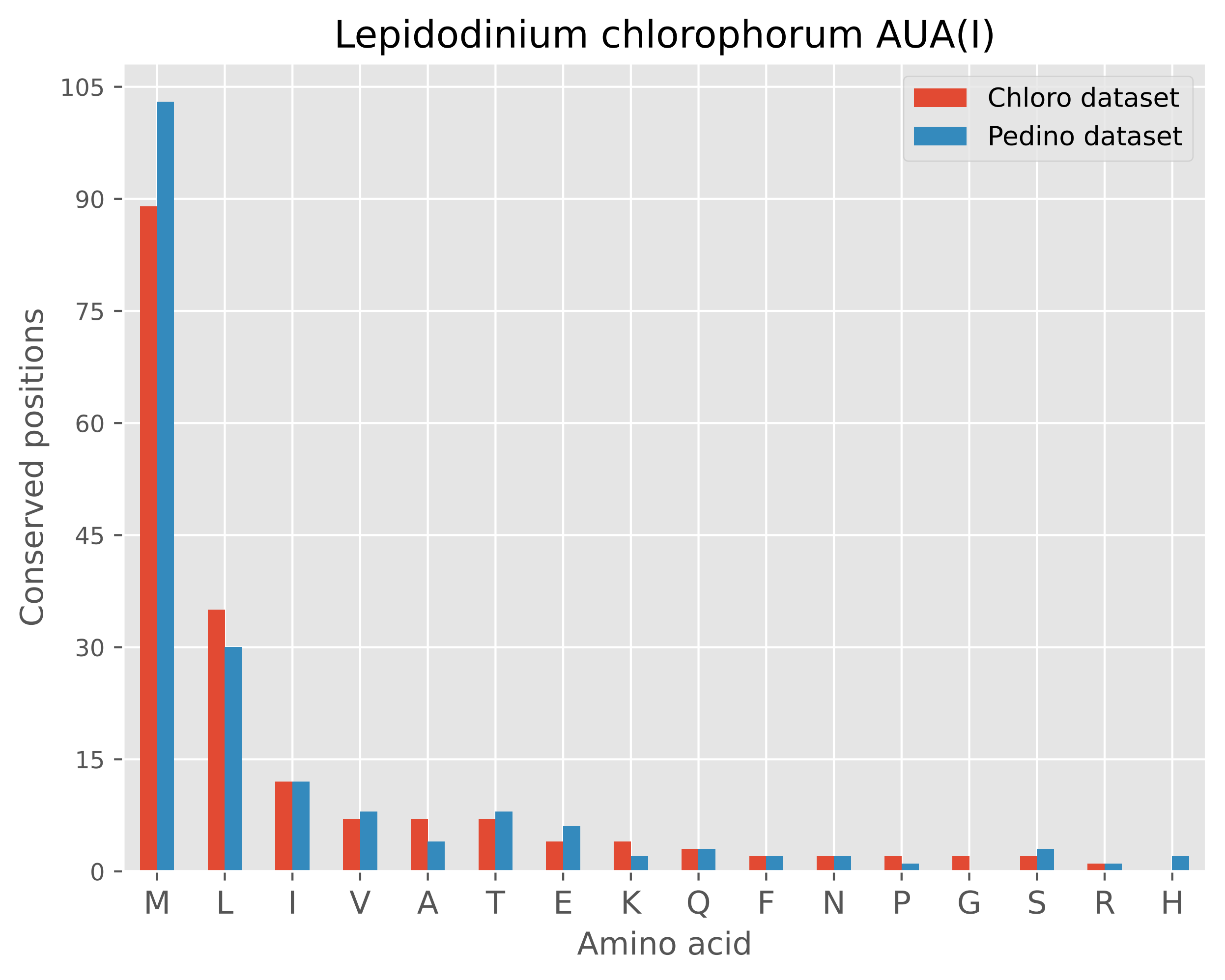

Lepidodinium chlorophorum AUA(I)
Chloro dataset
Pedino dataset
Conserved positions
0
15
30
45
60
75
90
105
M
L
I
V
A
T
E
K
Q
F
N
P
G
S
R
H
Amino acid

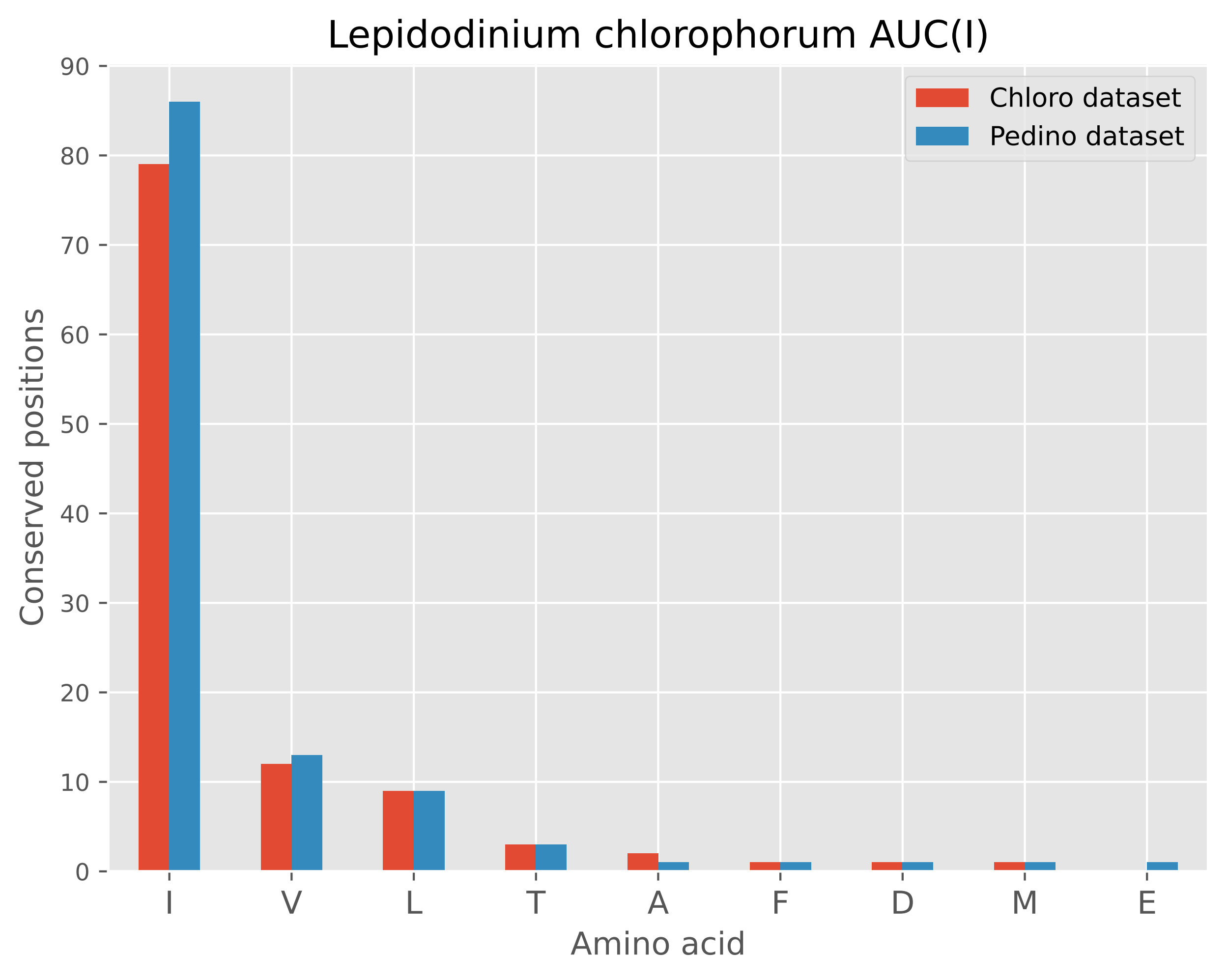

Lepidodinium chlorophorum AUC(I)
Chloro dataset
Pedino dataset
Conserved positions
0
10
20
30
40
50
60
70
80
90
I
V
L
T
A
F
D
M
E
Amino acid

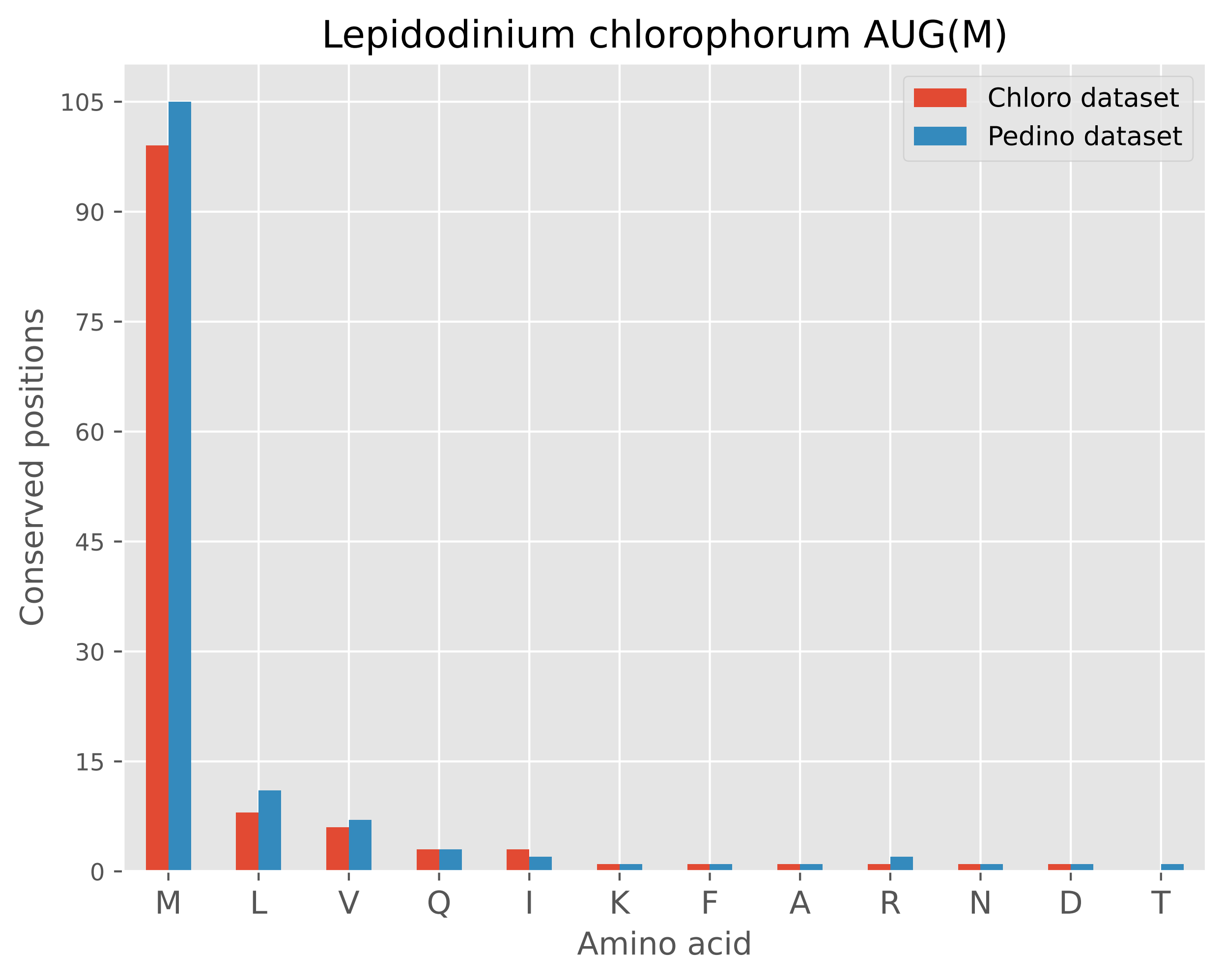

Lepidodinium chlorophorum AUG(M)
Chloro dataset
Pedino dataset
Conserved positions
105
90
75
60
45
30
15
0
M
L
V
Q
I
K
F
A
R
N
D
T
Amino acid

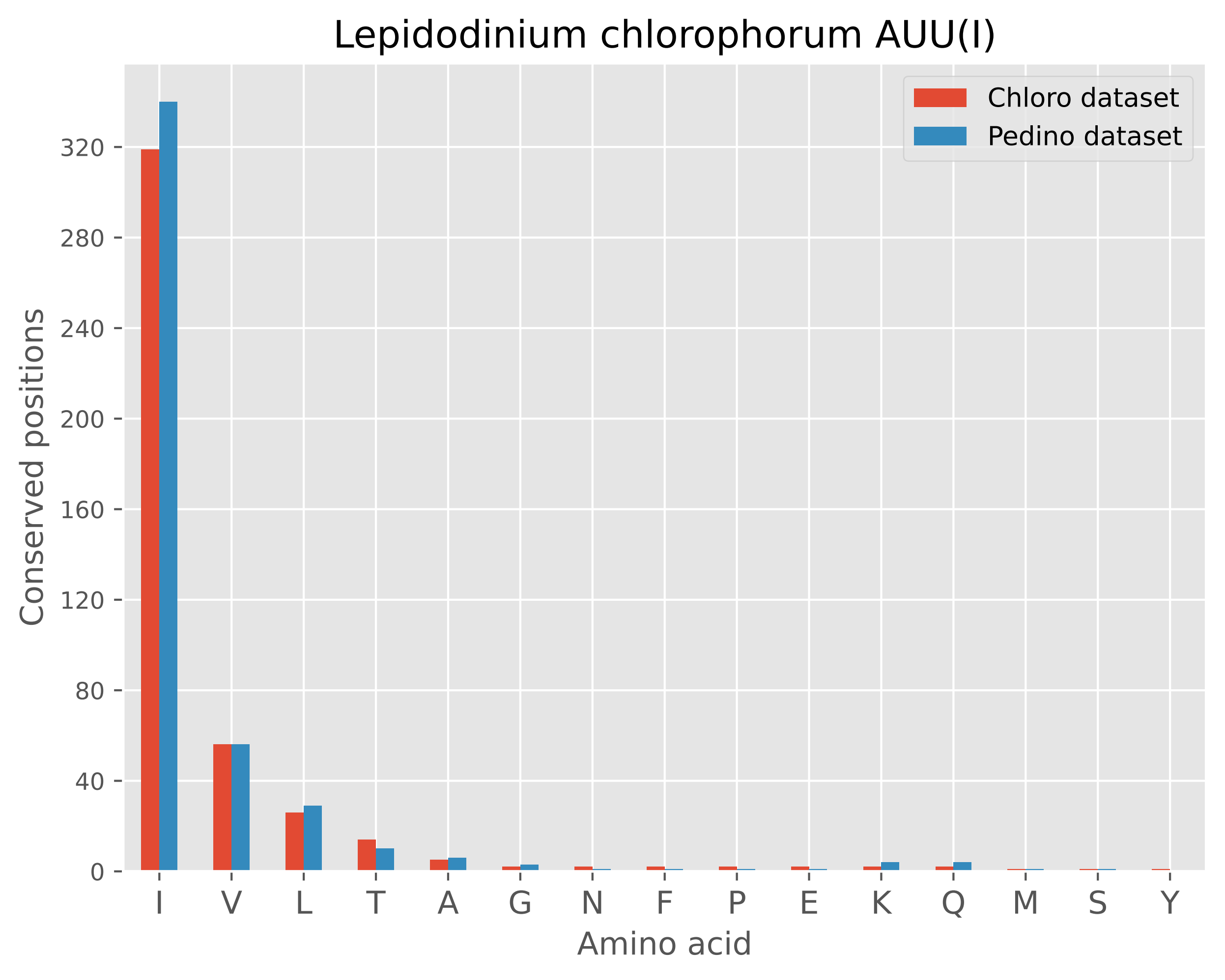

Lepidodinium chlorophorum AUU(I)
Chloro dataset
Pedino dataset
Conserved positions
Amino acid
0
40
80
120
160
200
240
280
320
I
V
L
T
A
G
N
F
P
E
K
Q
M
S
Y

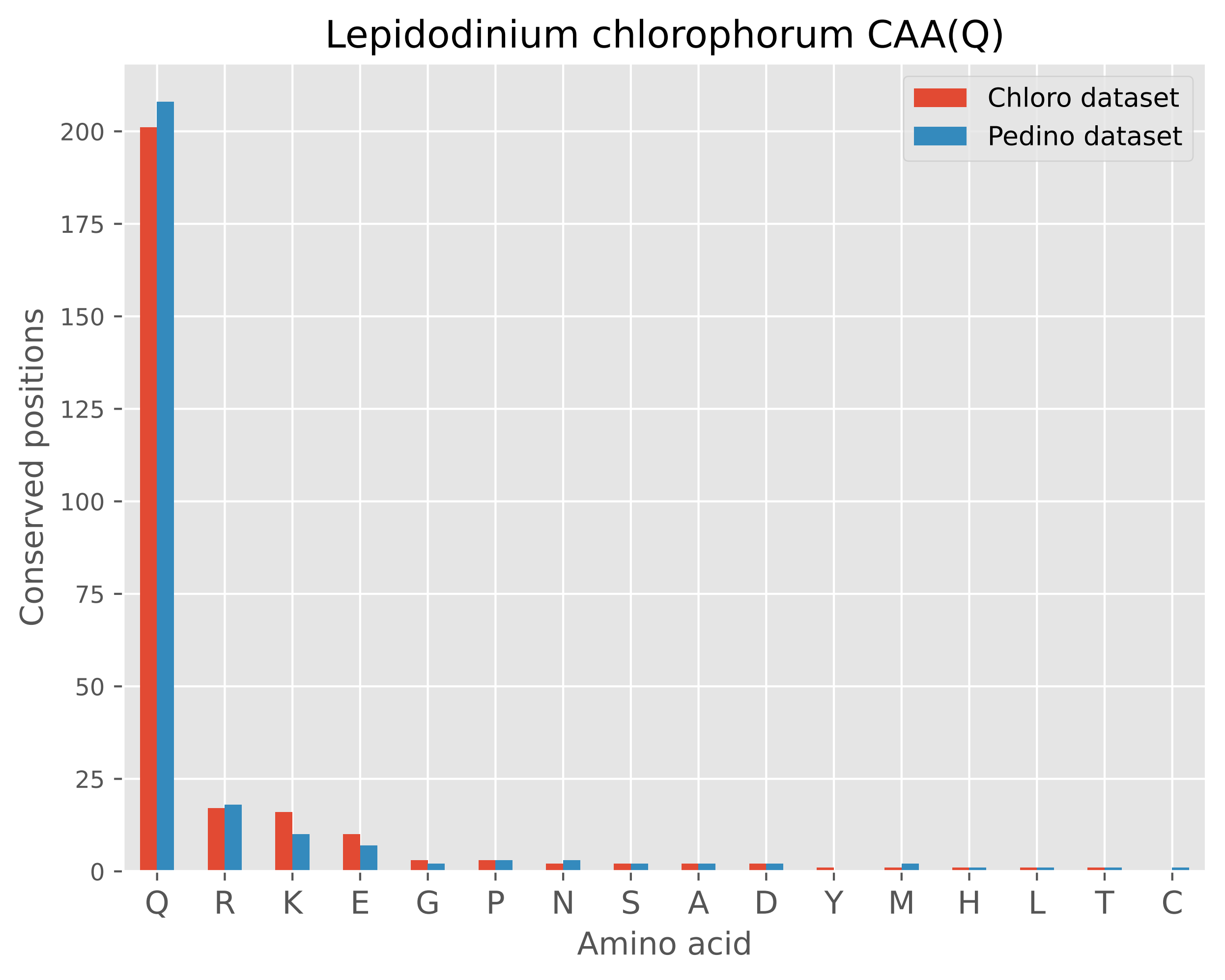

Lepidodinium chlorophorum CAA(Q)
Chloro dataset
Pedino dataset
Conserved positions
0
25
50
75
100
125
150
175
200
Q
R
K
E
G
P
N
S
A
D
Y
M
H
L
T
C
Amino acid

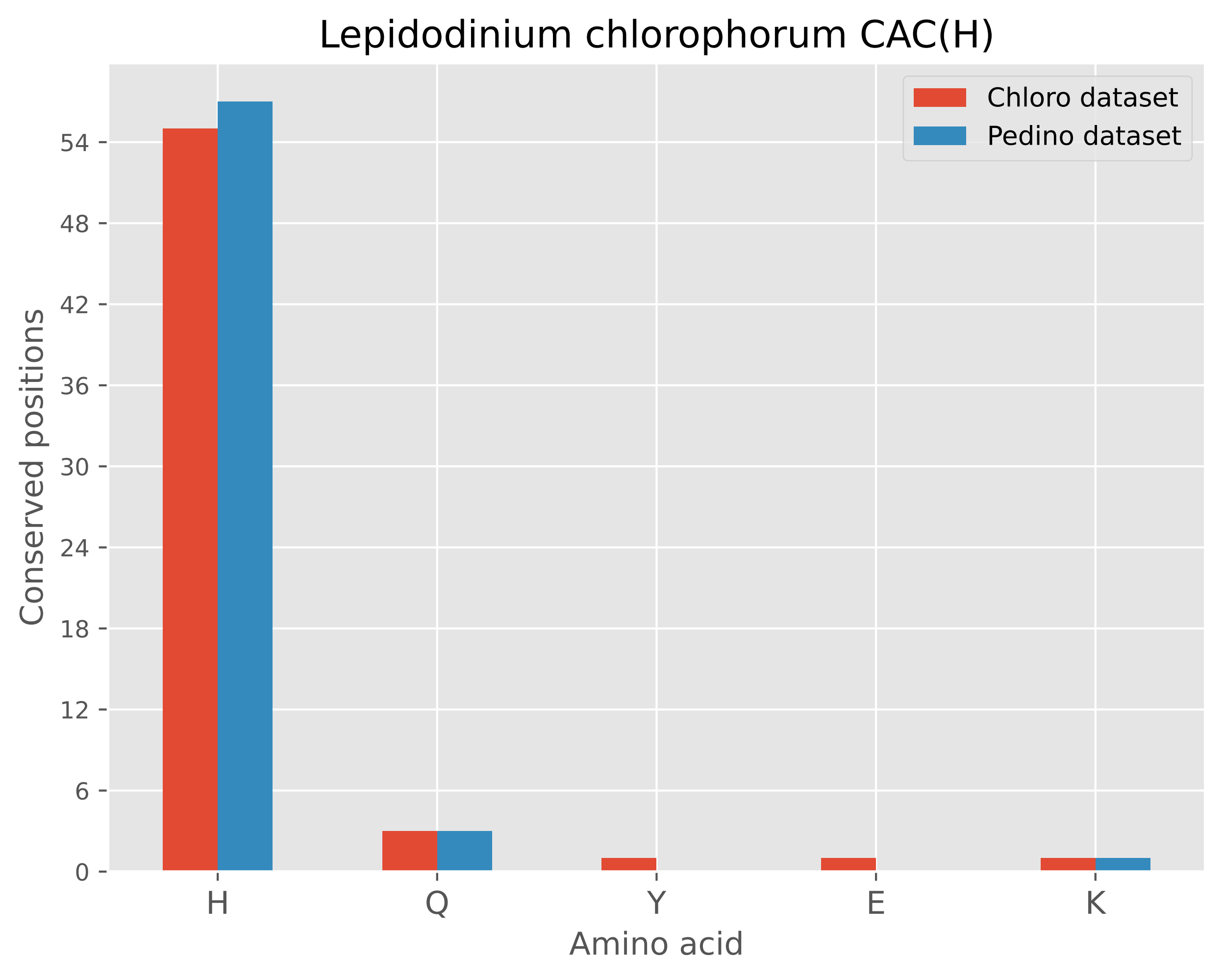

Lepidodinium chlorophorum CAC(H)
Chloro dataset
Pedino dataset
Conserved positions
0
6
12
18
24
30
36
42
48
54
H
Q
Y
E
K
Amino acid

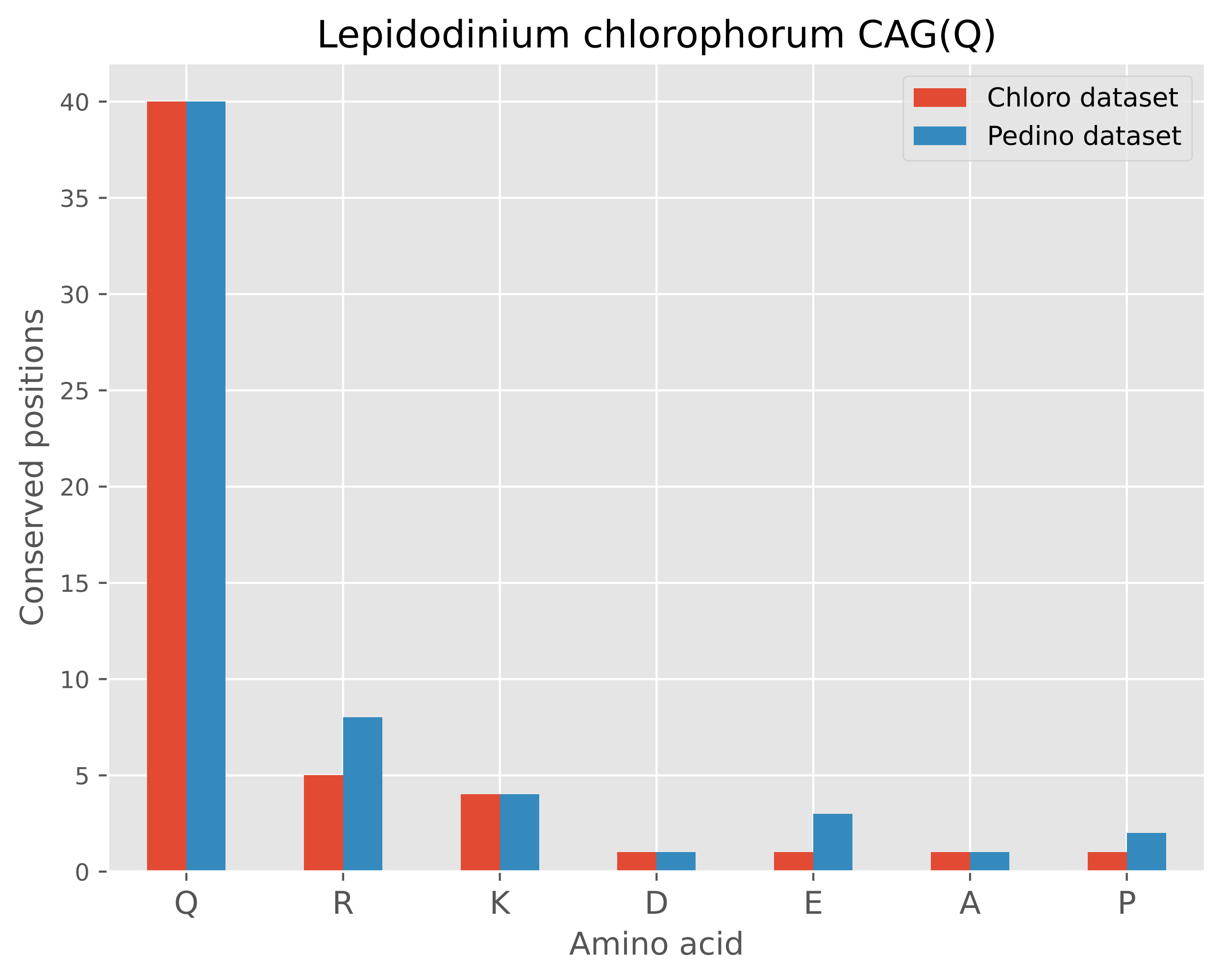

Lepidodinium chlorophorum CAG(Q)
Chloro dataset
Pedino dataset
Conserved positions
0
5
10
15
20
25
30
35
40
Q
R
K
D
E
A
P
Amino acid

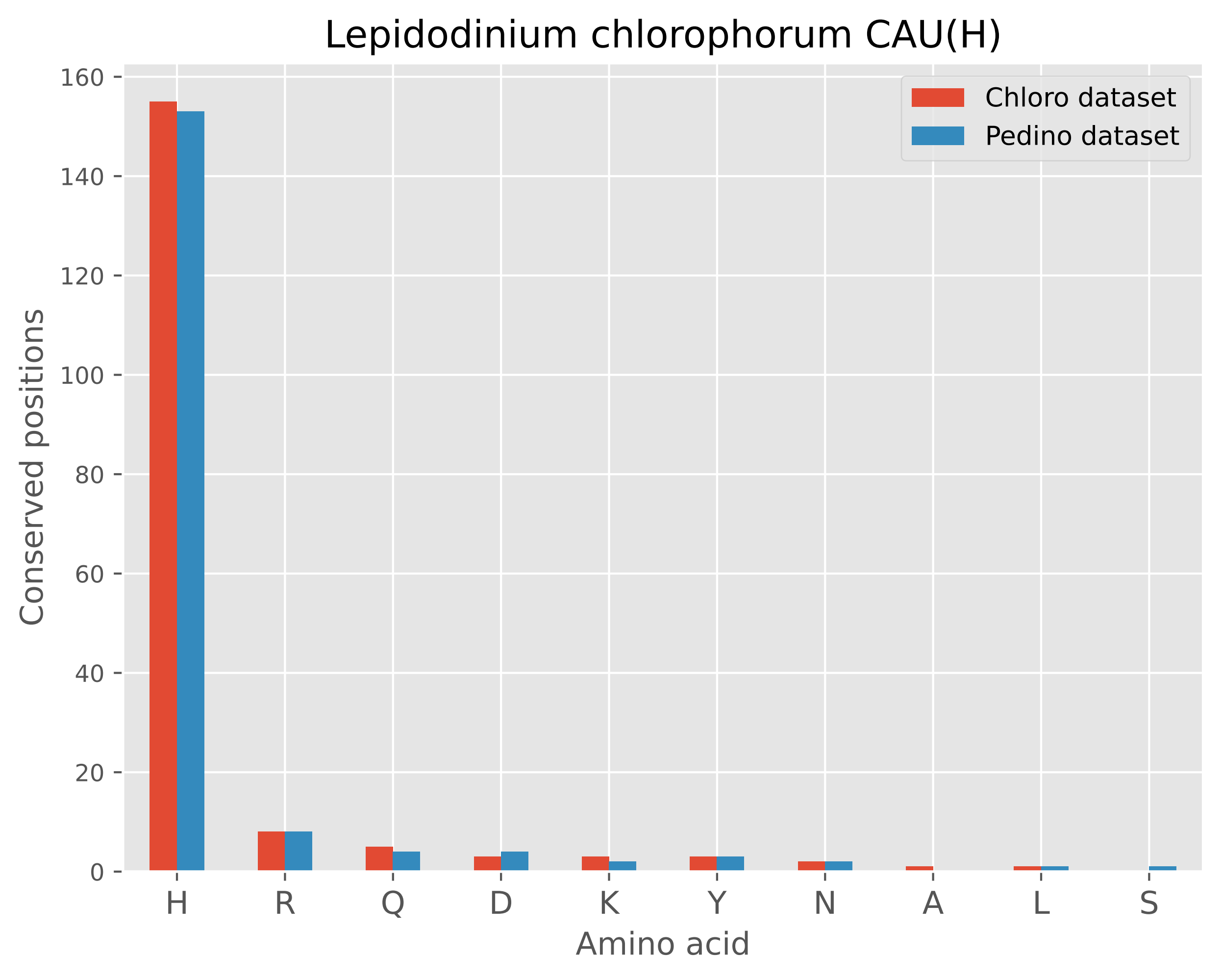

Lepidodinium chlorophorum CAU(H)
Chloro dataset
Pedino dataset
Conserved positions
0
20
40
60
80
100
120
140
160
H
R
Q
D
K
Y
N
A
L
S
Amino acid

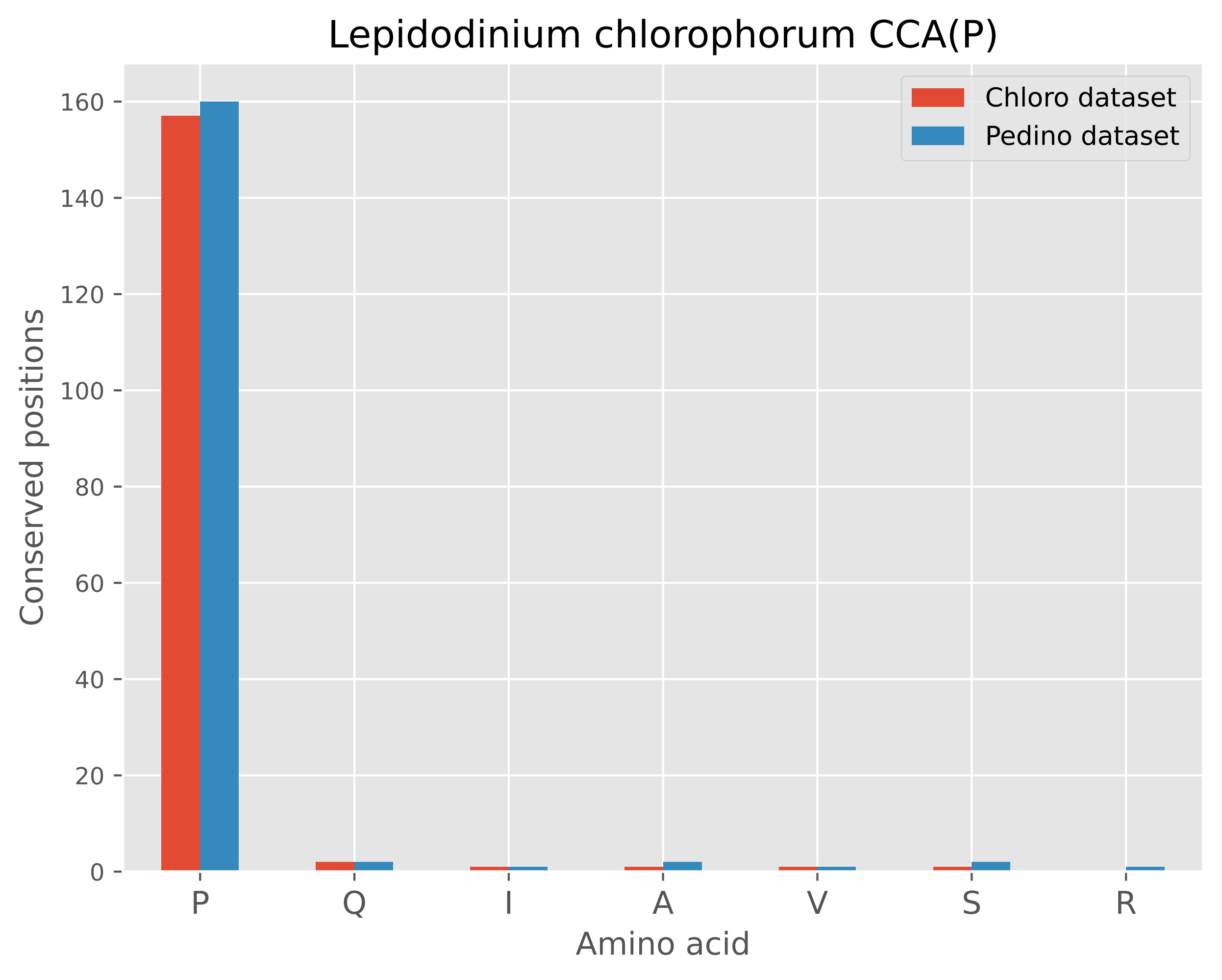
Lepidodinium chlorophorum CCA(P)
Chloro dataset
Pedino dataset
Conserved positions
0
20
40
60
80
100
120
140
160
P
Q
I
A
V
S
R
Amino acid

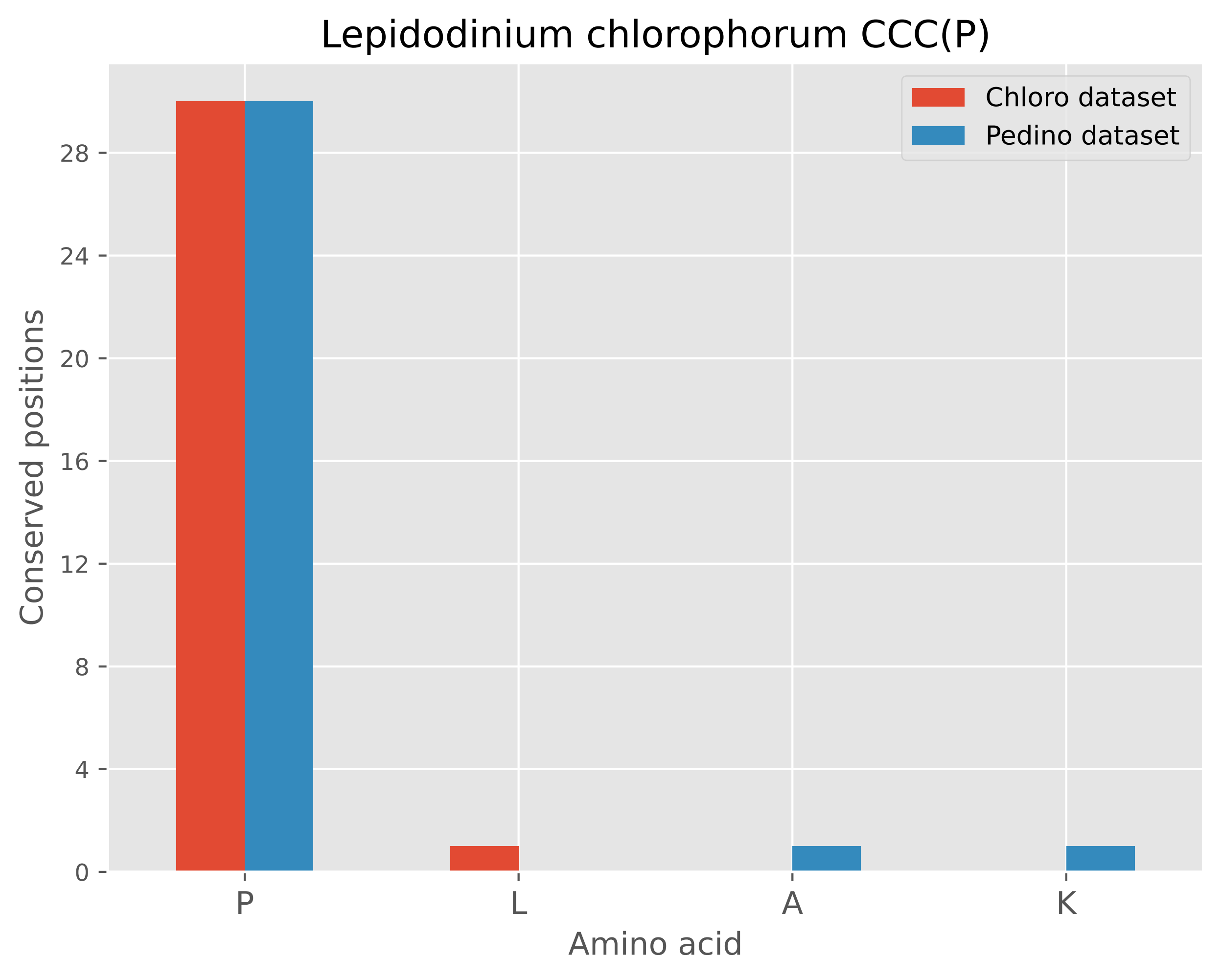

Lepidodinium chlorophorum CCC(P)
Chloro dataset
Pedino dataset
Conserved positions
0
4
8
12
16
20
24
28
P
L
A
K
Amino acid

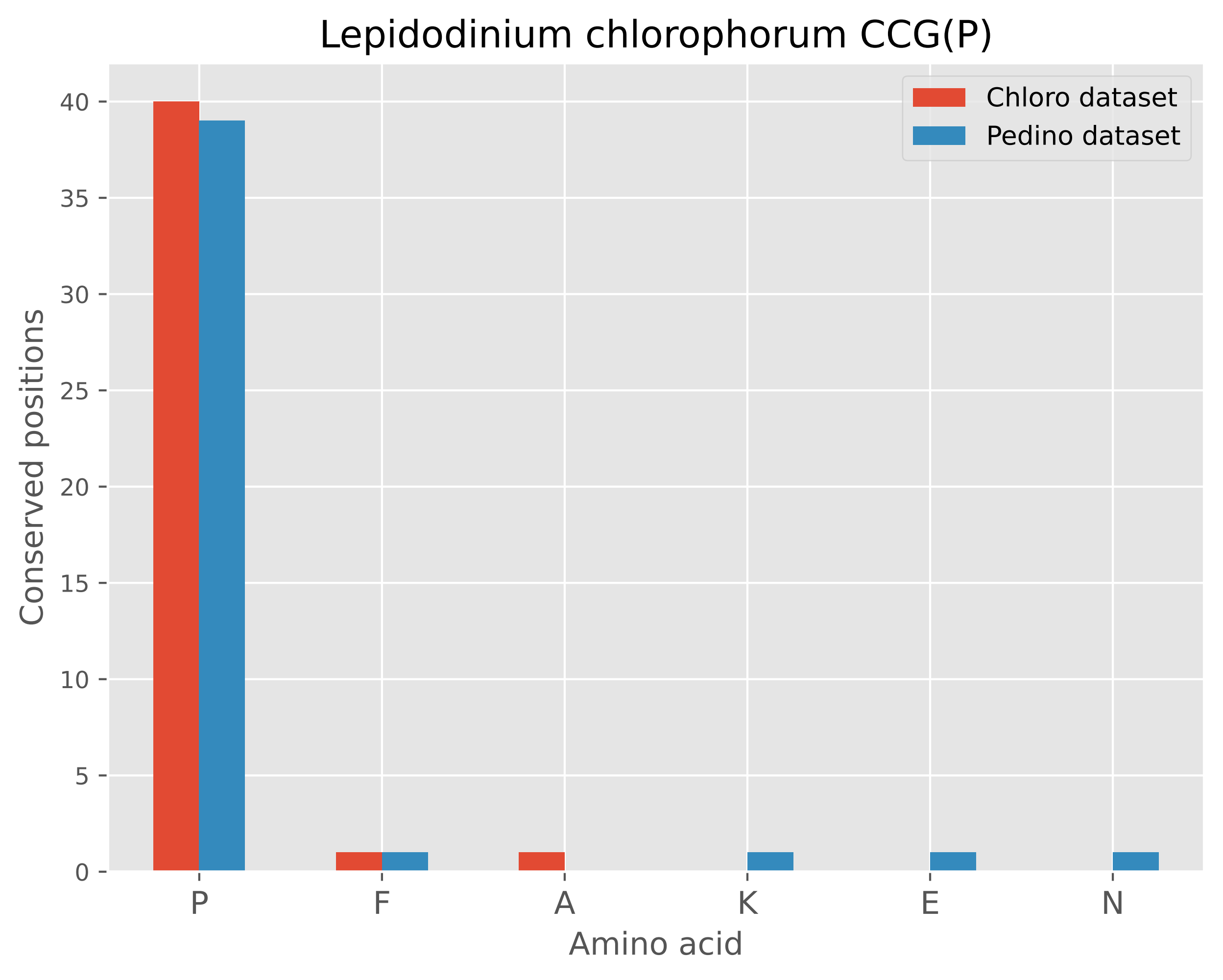

Lepidodinium chlorophorum CCG(P)
Chloro dataset
Pedino dataset
Conserved positions
Amino acid
0
5
10
15
20
25
30
35
40
P
F
A
K
E
N

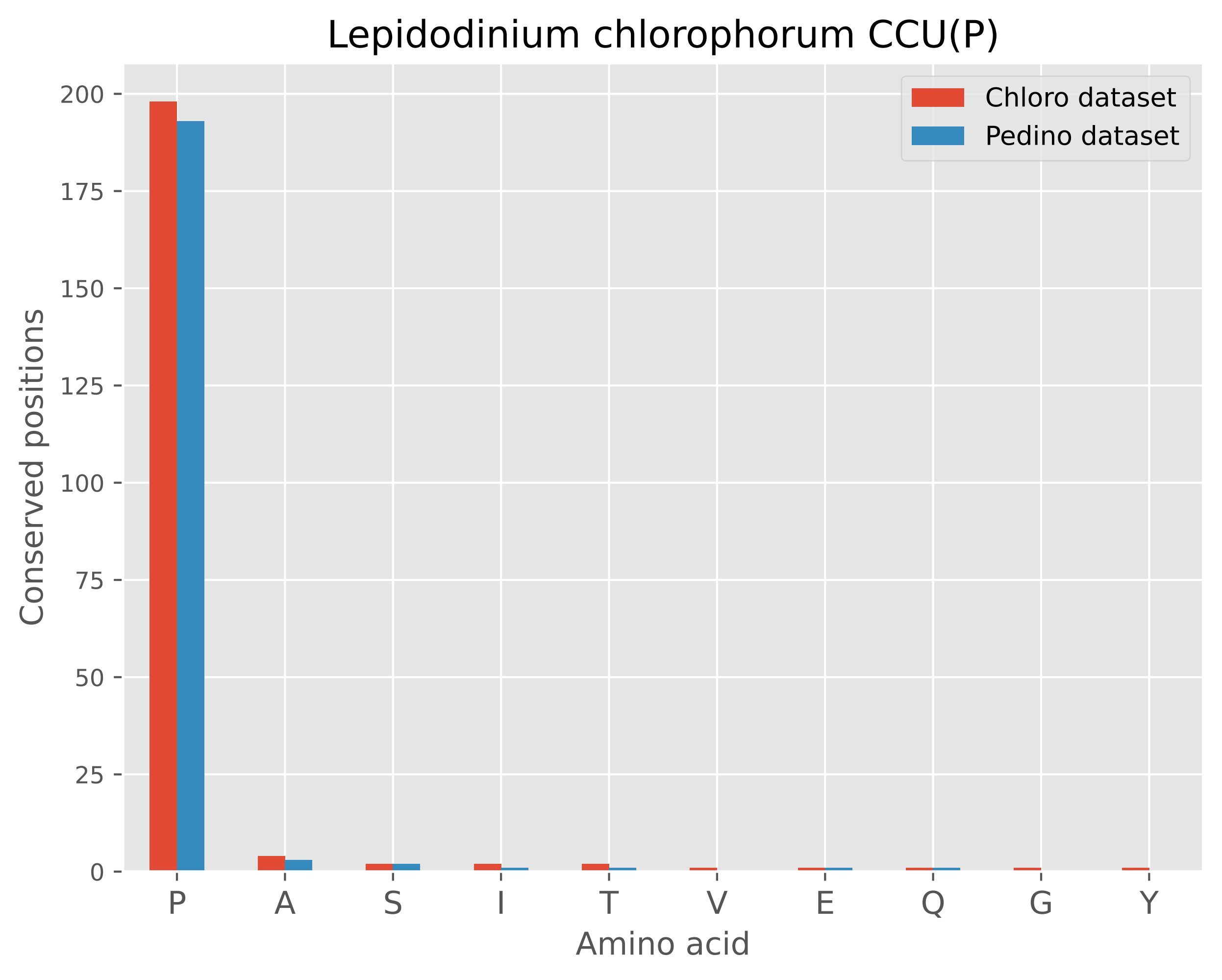

Lepidodinium chlorophorum CCU(P)
Chloro dataset
Pedino dataset
Conserved positions
Amino acid
200
175
150
125
100
75
50
25
0
P
A
S
I
T
V
E
Q
G
Y

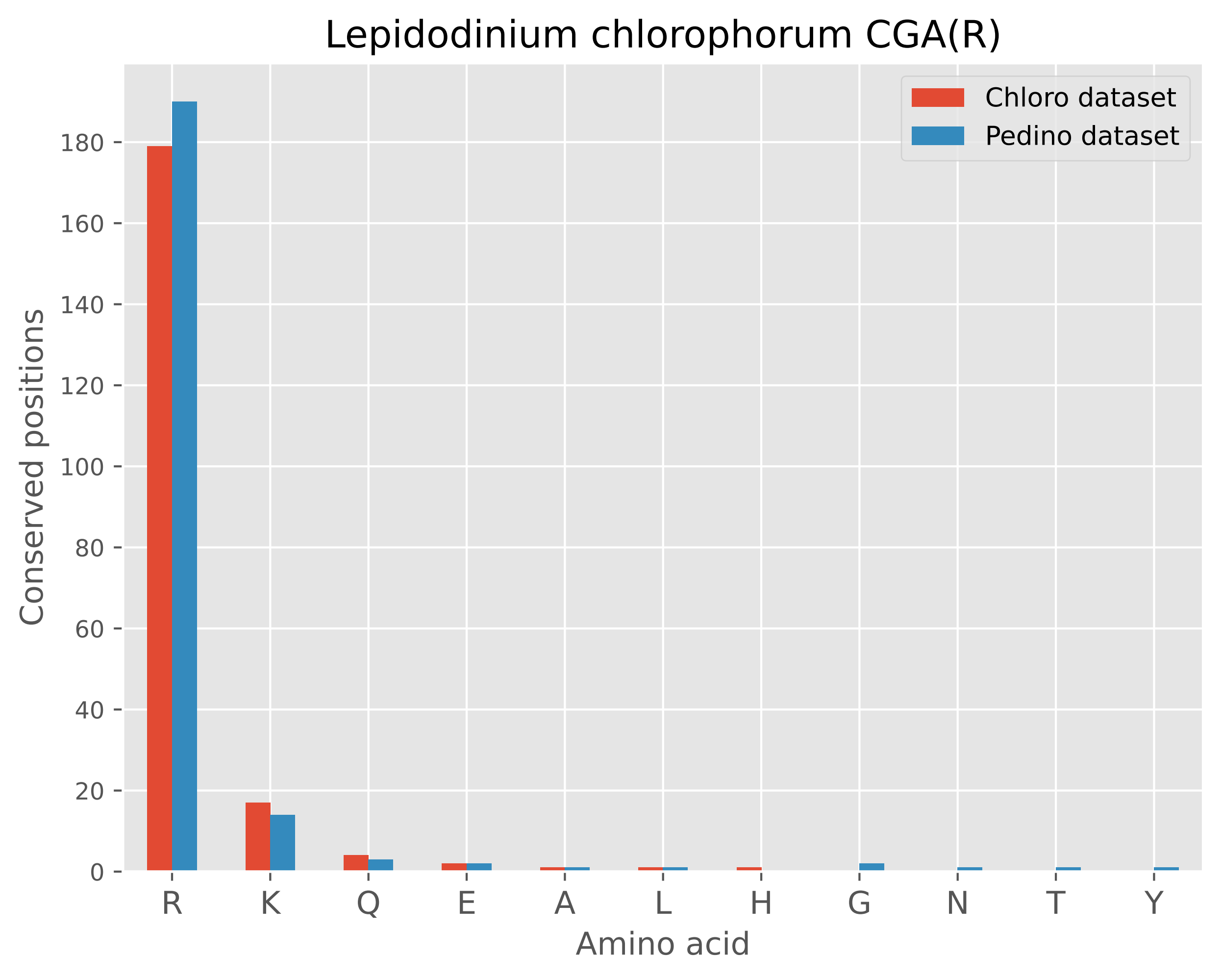

Lepidodinium chlorophorum CGA(R)
Chloro dataset
Pedino dataset
Conserved positions
0
20
40
60
80
100
120
140
160
180
R
K
Q
E
A
L
H
G
N
T
Y
Amino acid

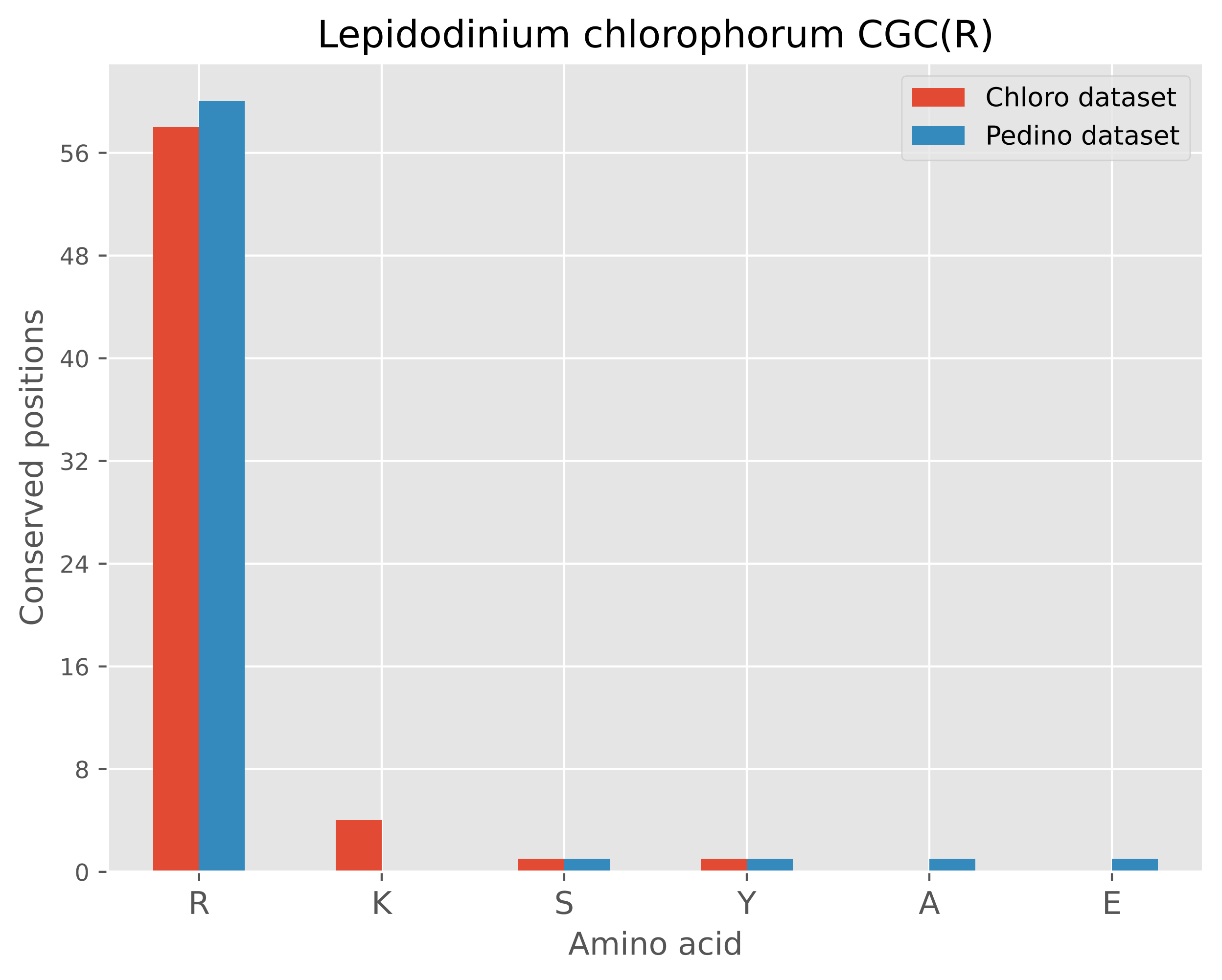

Lepidodinium chlorophorum CGC(R)
Chloro dataset
Pedino dataset
Conserved positions
0
8
16
24
32
40
48
56
R
K
S
Y
A
E
Amino acid

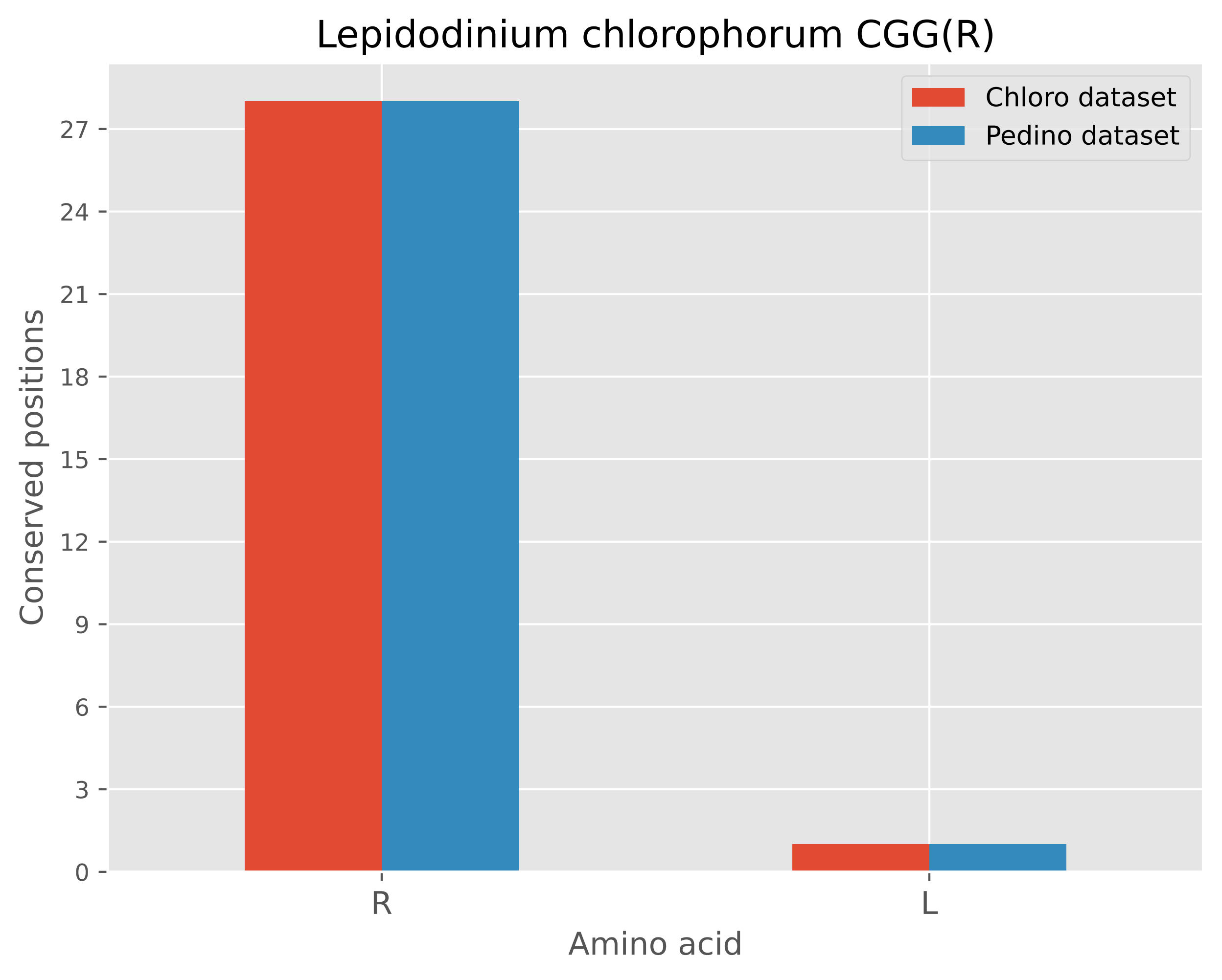

Lepidodinium chlorophorum CGG(R)
Chloro dataset
Pedino dataset
Conserved positions
0
3
6
9
12
15
18
21
24
27
R
L
Amino acid

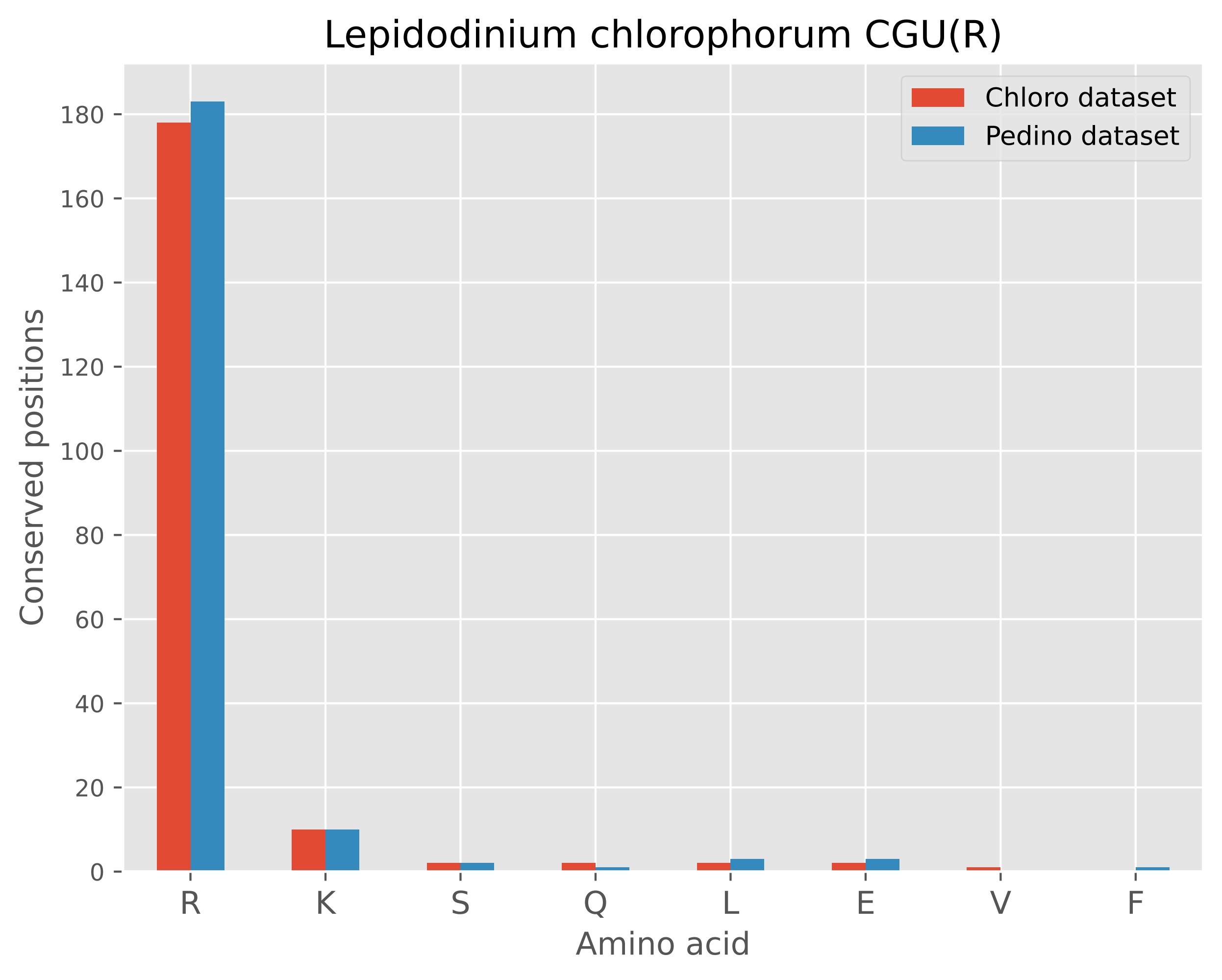

Lepidodinium chlorophorum CGU(R)
Chloro dataset
Pedino dataset
Conserved positions
0
20
40
60
80
100
120
140
160
180
R
K
S
Q
L
E
V
F
Amino acid

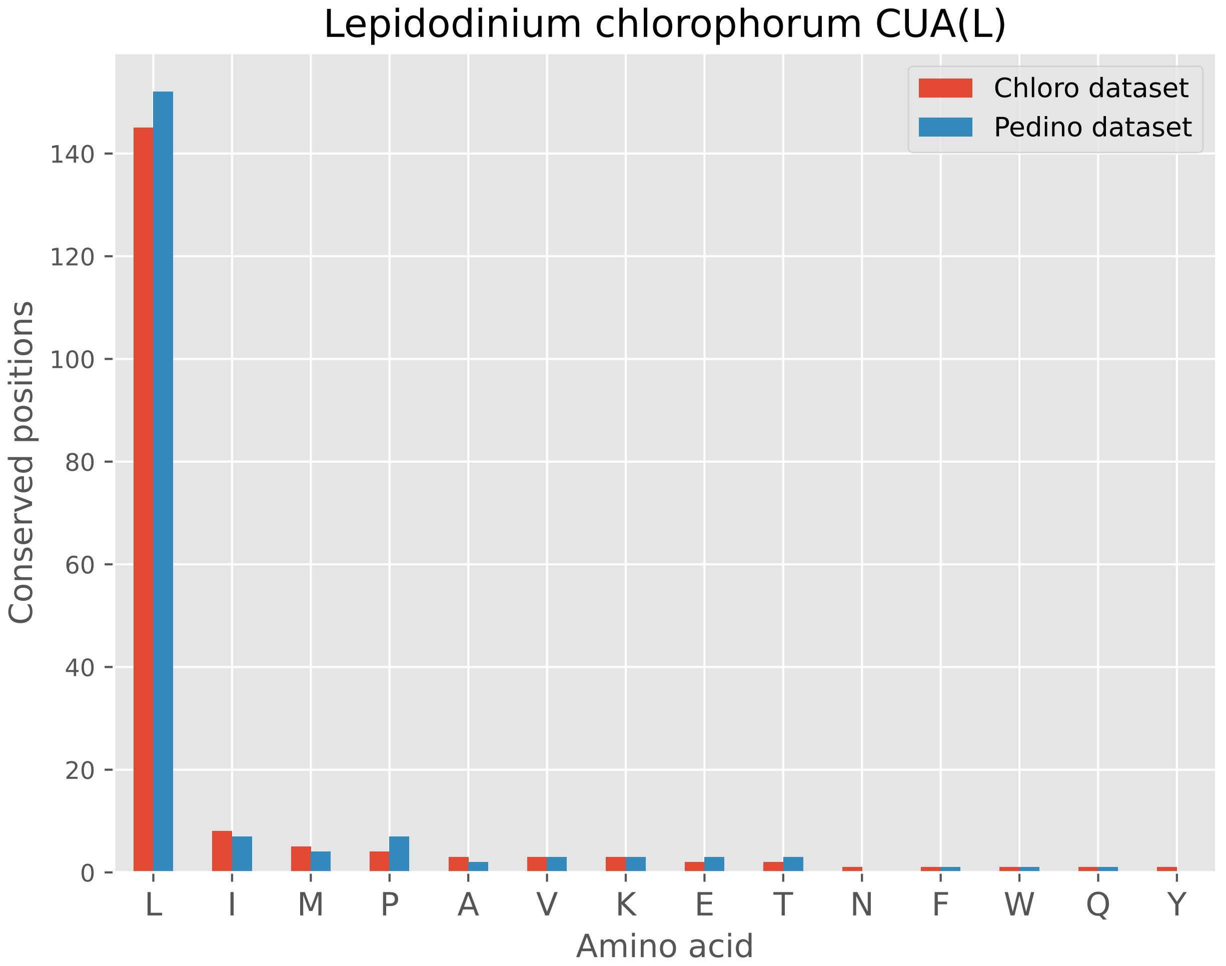

Lepidodinium chlorophorum CUA(L)
Chloro dataset
Pedino dataset
Conserved positions
0
20
40
60
80
100
120
140
L
I
M
P
A
V
K
E
T
N
F
W
Q
Y
Amino acid

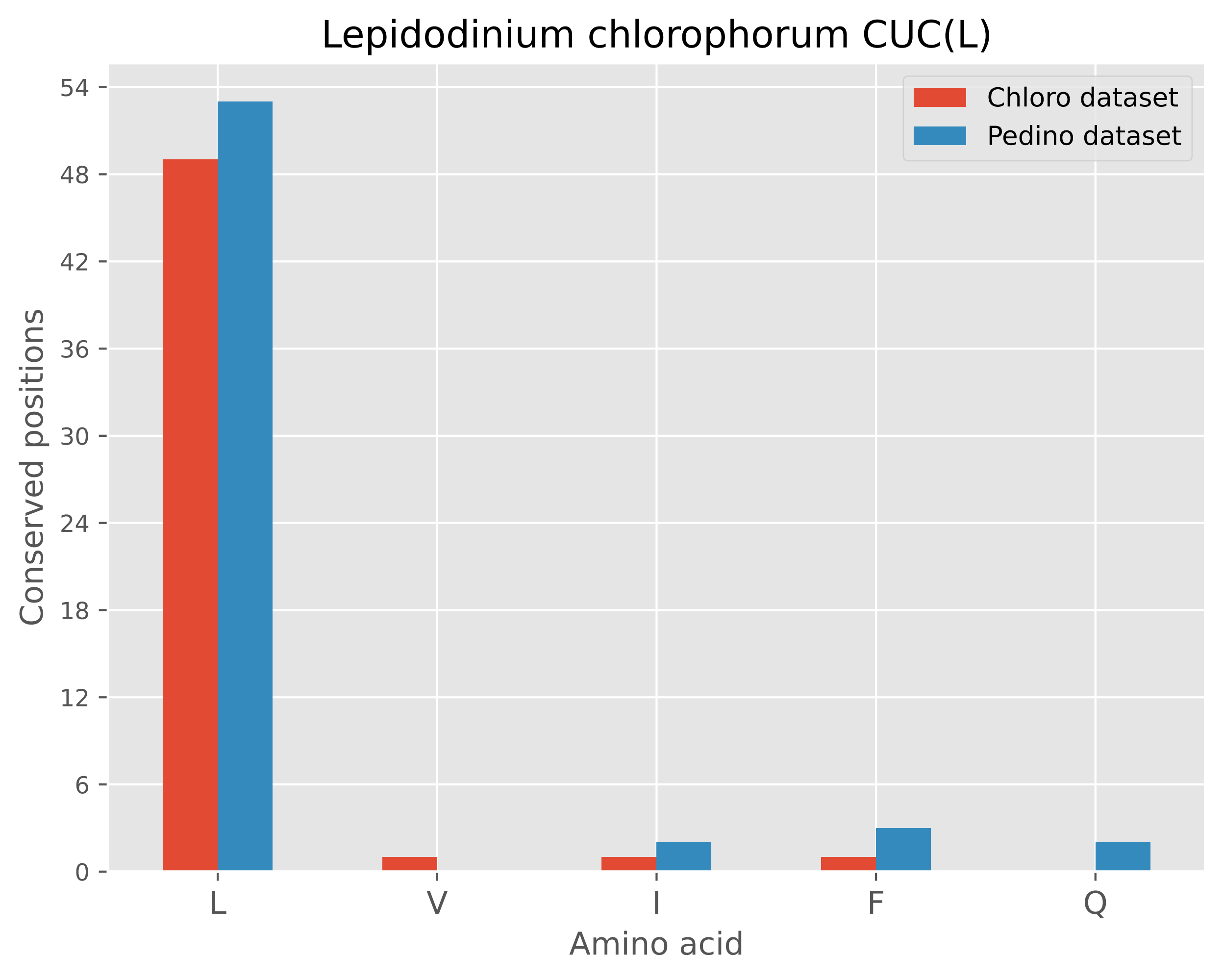

Lepidodinium chlorophorum CUC(L)
Chloro dataset
Pedino dataset
Conserved positions
0
6
12
18
24
30
36
42
48
54
L
V
I
F
Q
Amino acid

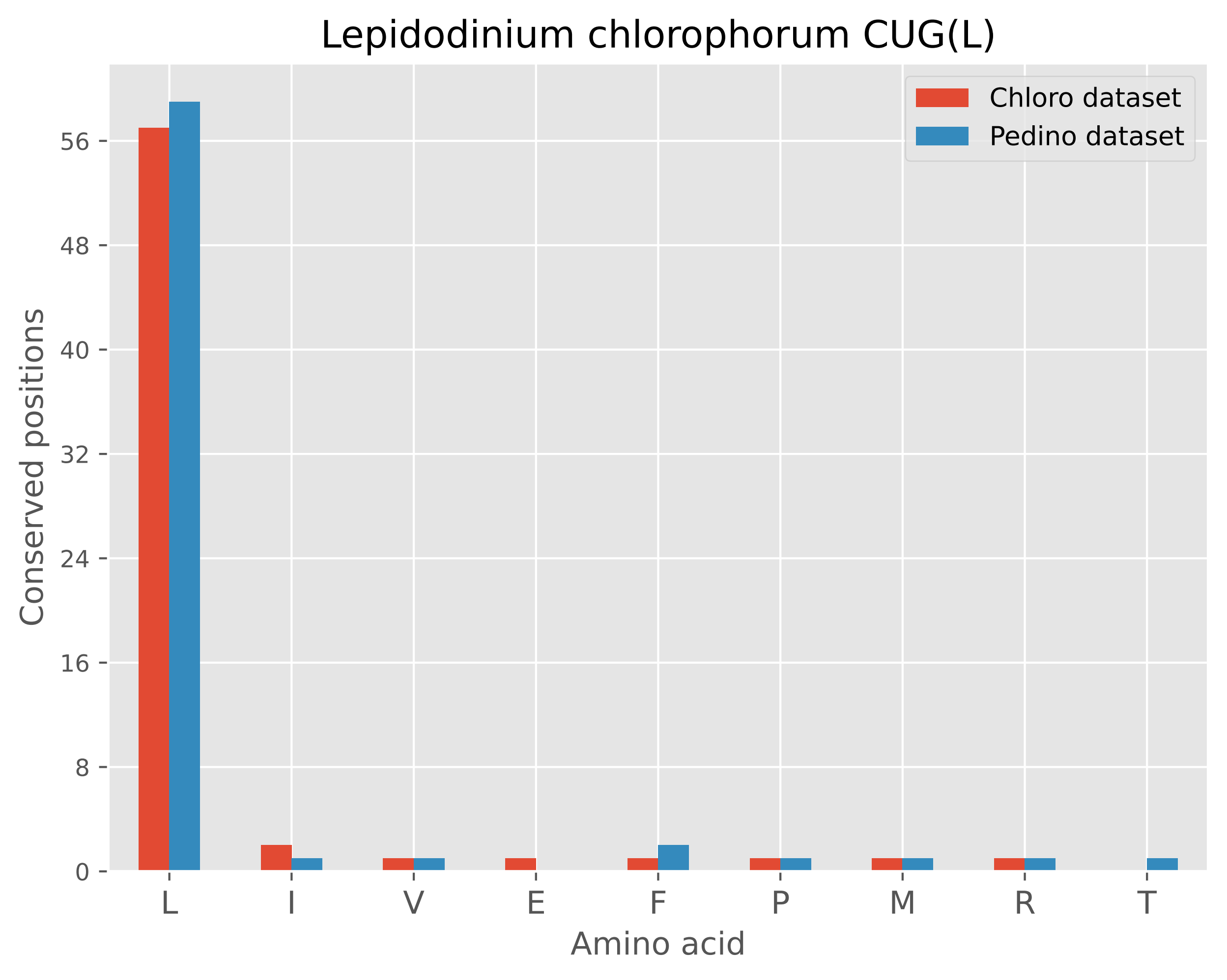

Lepidodinium chlorophorum CUG(L)
Chloro dataset
Pedino dataset
Conserved positions
0
8
16
24
32
40
48
56
L
I
V
E
F
P
M
R
T
Amino acid

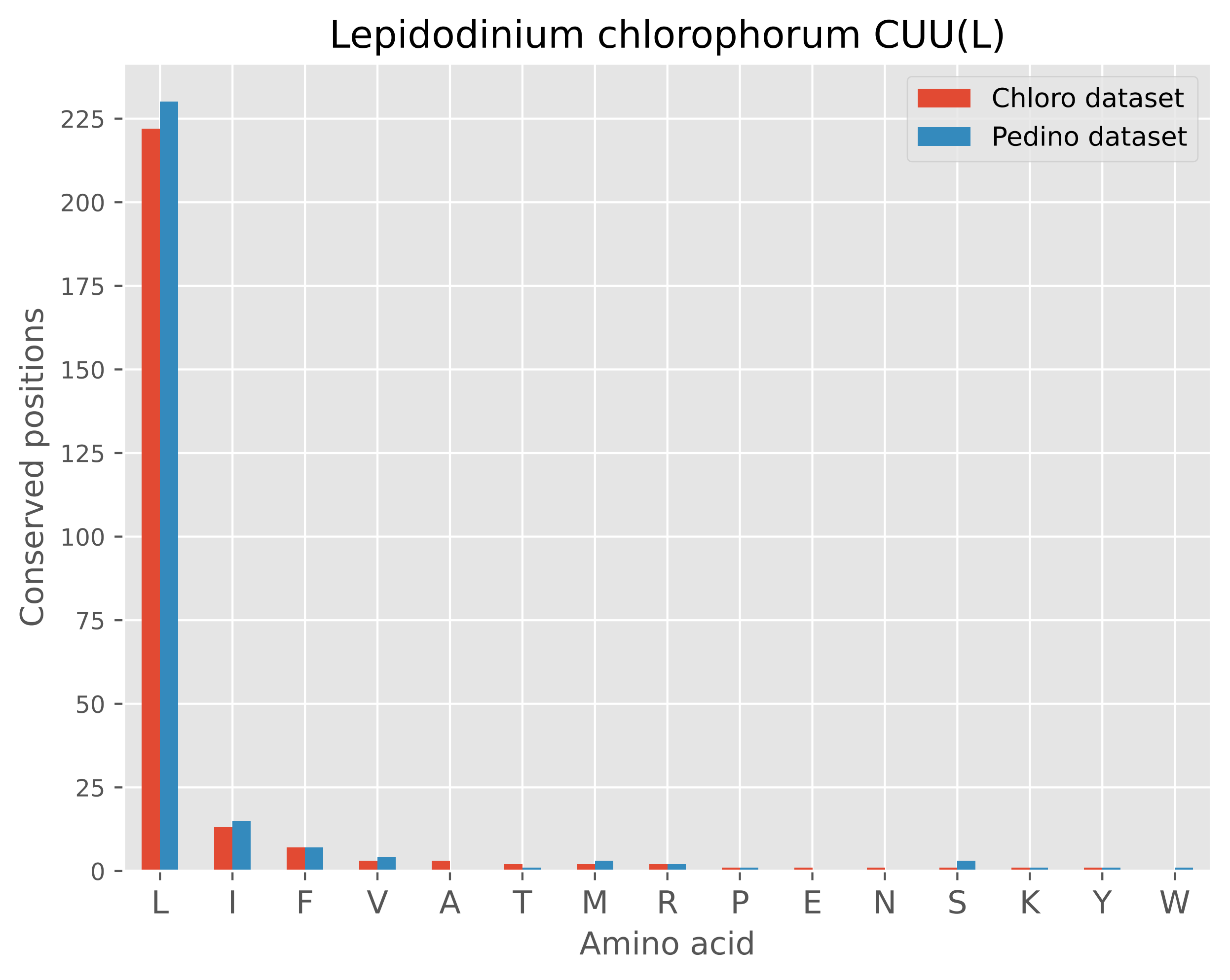

Lepidodinium chlorophorum CUU(L)
Chloro dataset
Pedino dataset
Conserved positions
Amino acid
225
200
175
150
125
100
75
50
25
0
L
I
F
V
A
T
M
R
P
E
N
S
K
Y
W

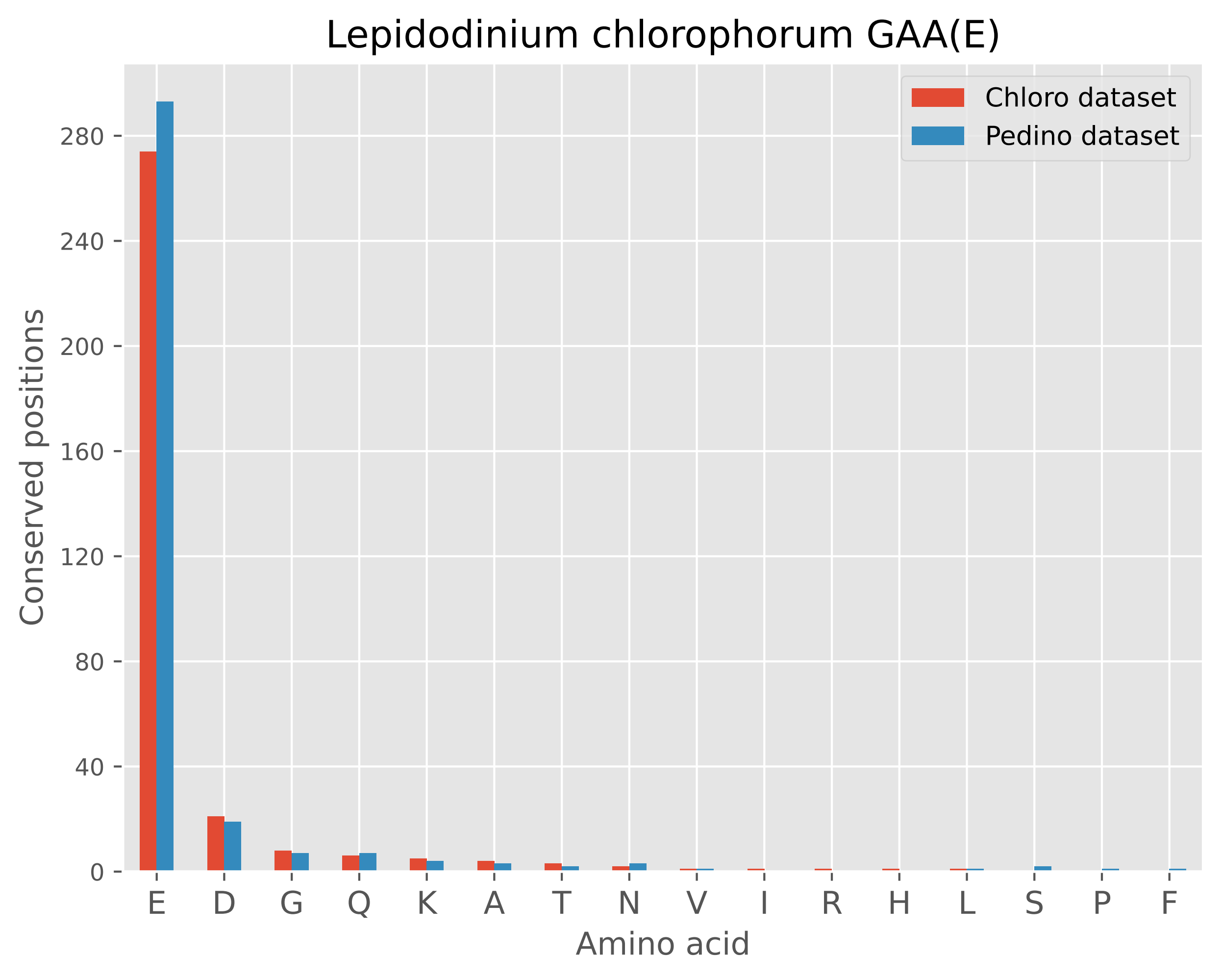

Lepidodinium chlorophorum GAA(E)
Chloro dataset
Pedino dataset
Conserved positions
0
40
80
120
160
200
240
280
E
D
G
Q
K
A
T
N
V
I
R
H
L
S
P
F
Amino acid

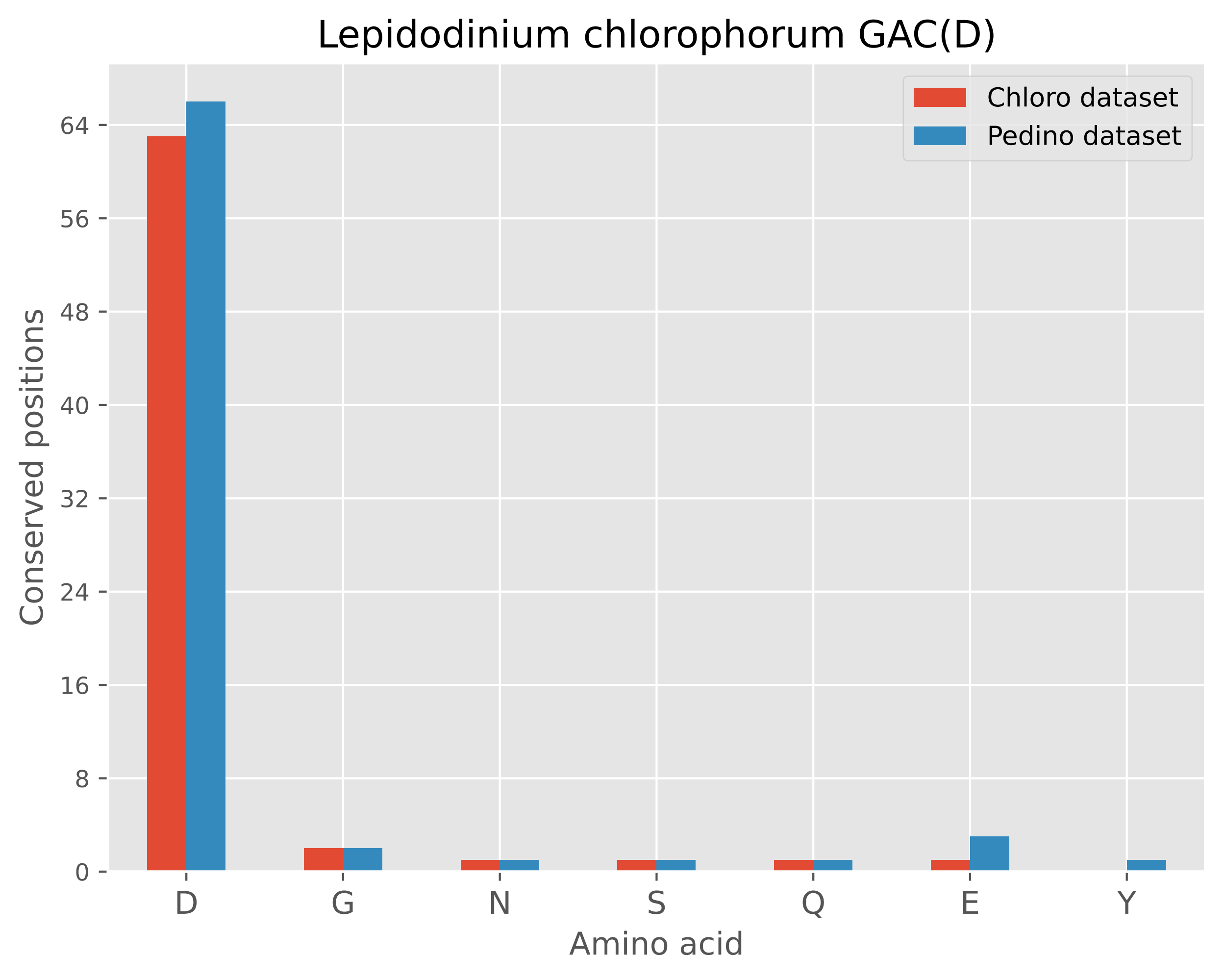
Lepidodinium chlorophorum GAC(D)
Chloro dataset
Pedino dataset
Conserved positions
Amino acid
0
8
16
24
32
40
48
56
64
D
G
N
S
Q
E
Y

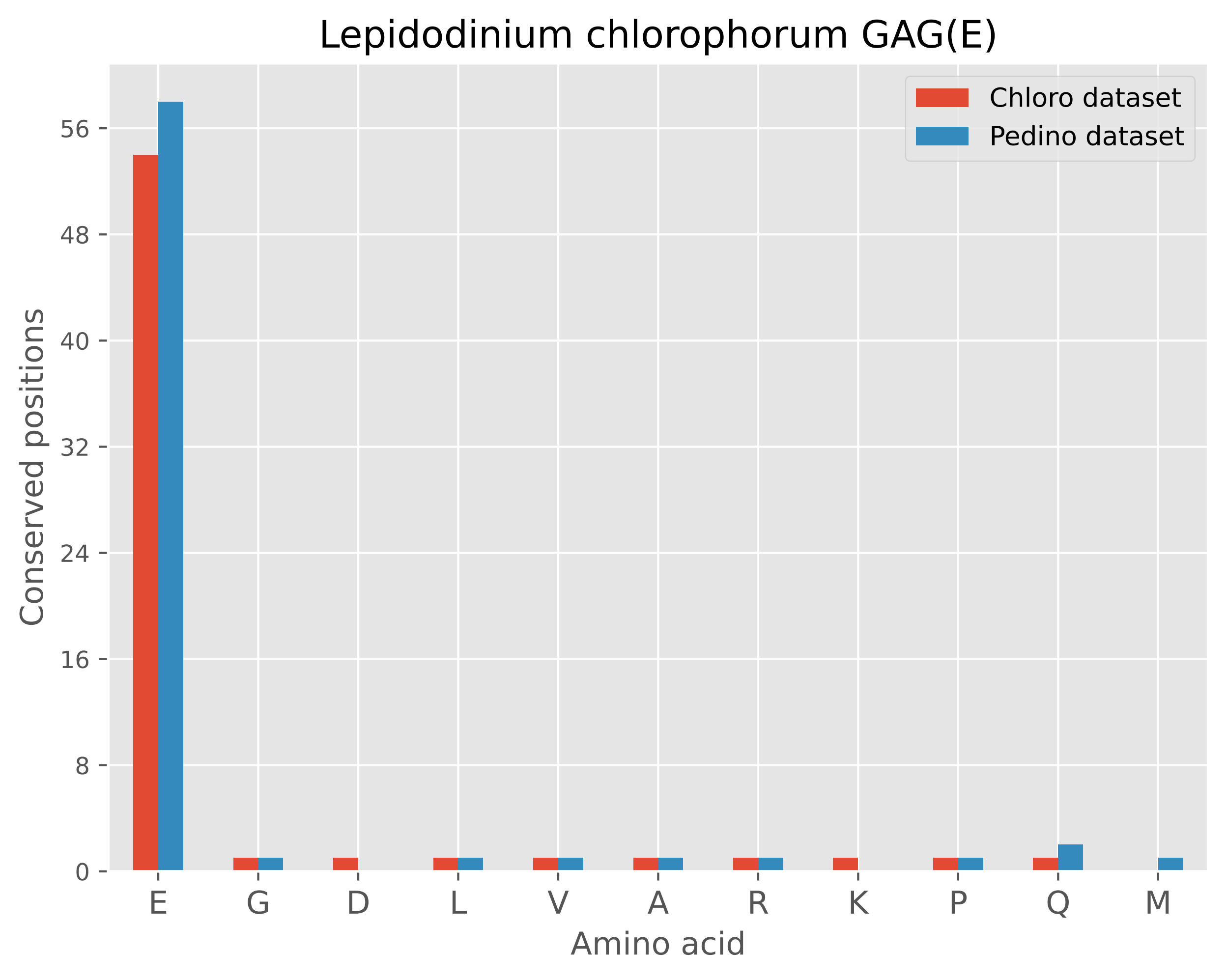

Lepidodinium chlorophorum GAG(E)
Chloro dataset
Pedino dataset
Conserved positions
0
8
16
24
32
40
48
56
E
G
D
L
V
A
R
K
P
Q
M
Amino acid

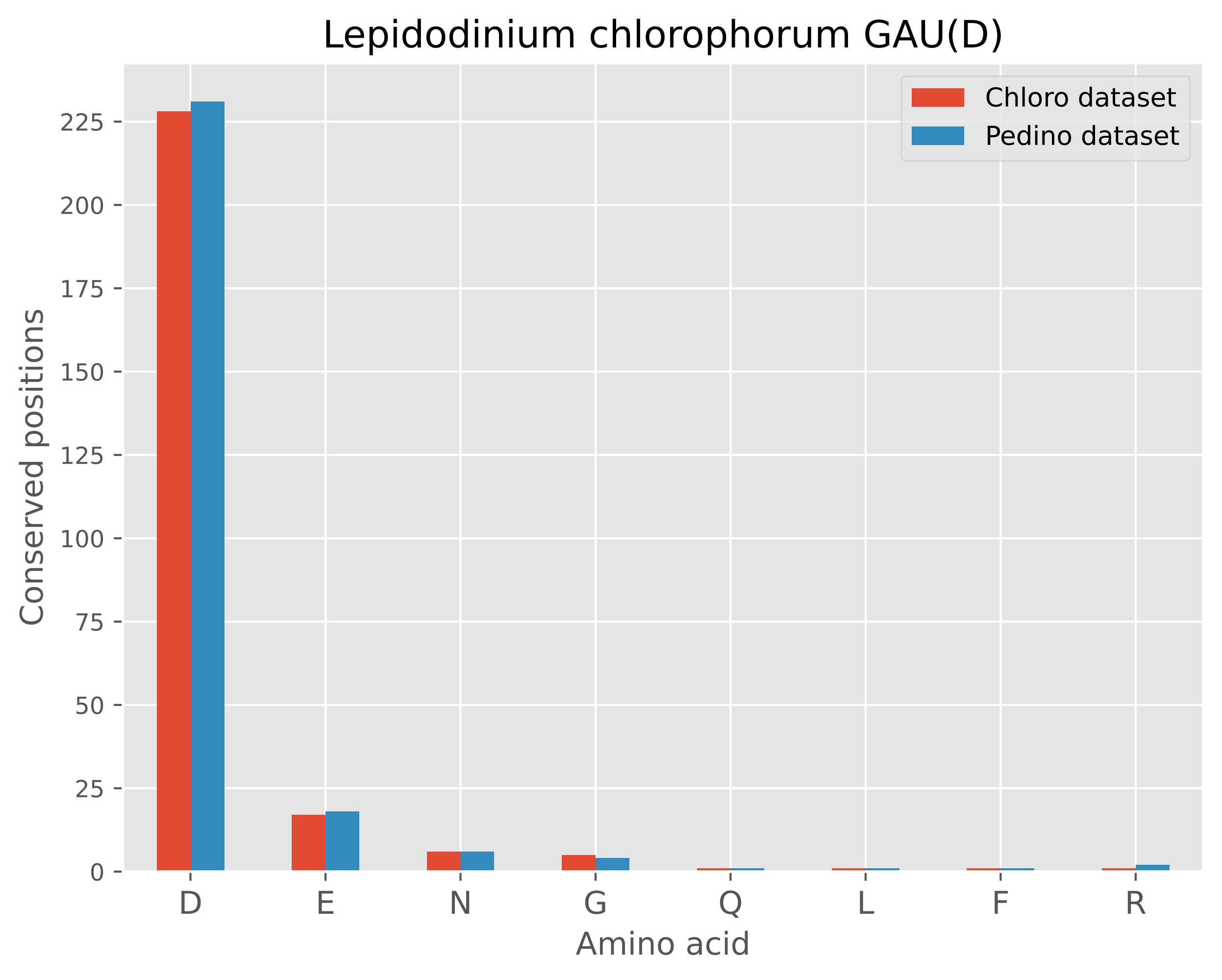

Lepidodinium chlorophorum GAU(D)
Chloro dataset
Pedino dataset
Conserved positions
0
25
50
75
100
125
150
175
200
225
D
E
N
G
Q
L
F
R
Amino acid

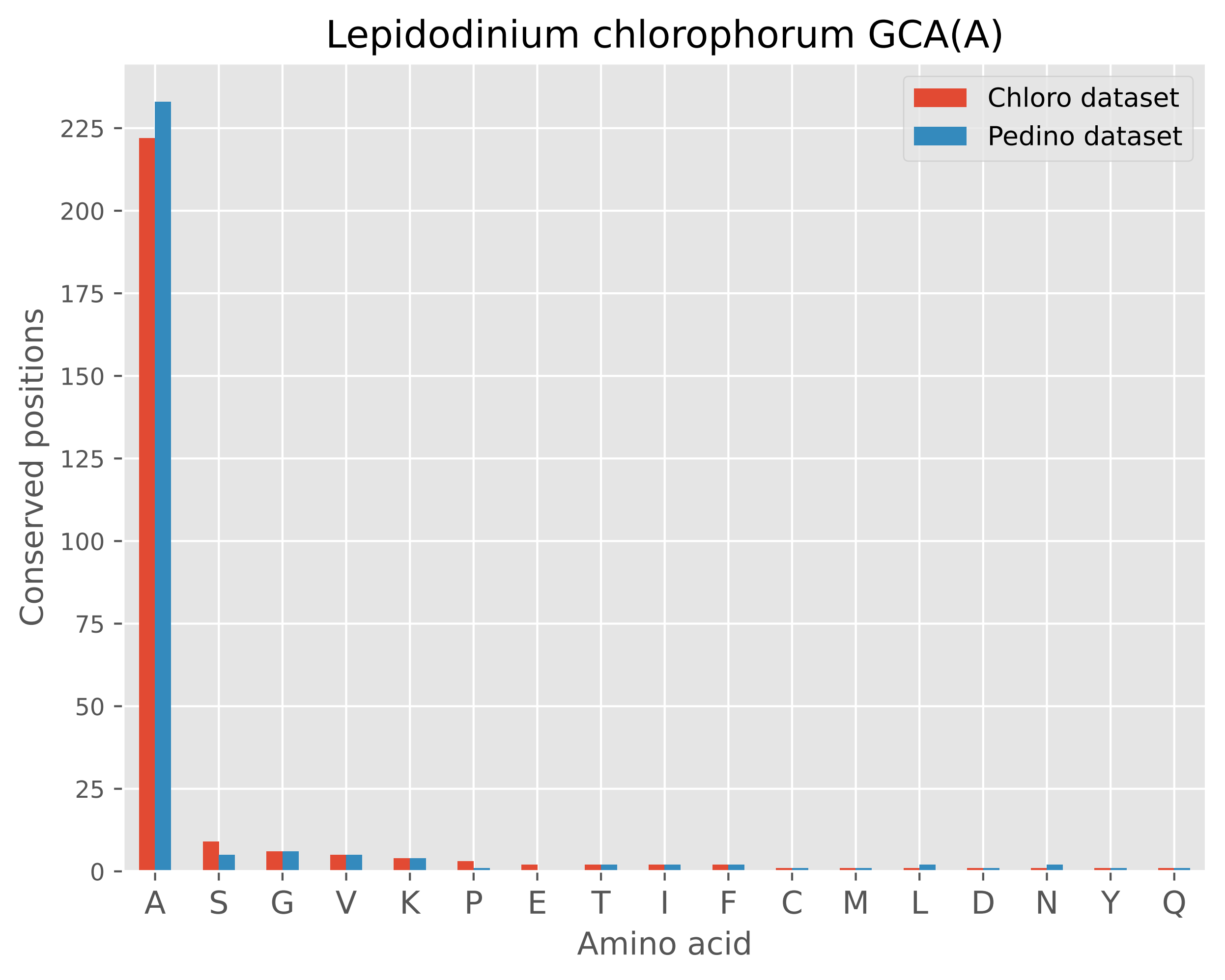
Lepidodinium chlorophorum GCA(A)
Chloro dataset
Pedino dataset
Conserved positions
0
25
50
75
100
125
150
175
200
225
A
S
G
V
K
P
E
T
I
F
C
M
L
D
N
Y
Q
Amino acid

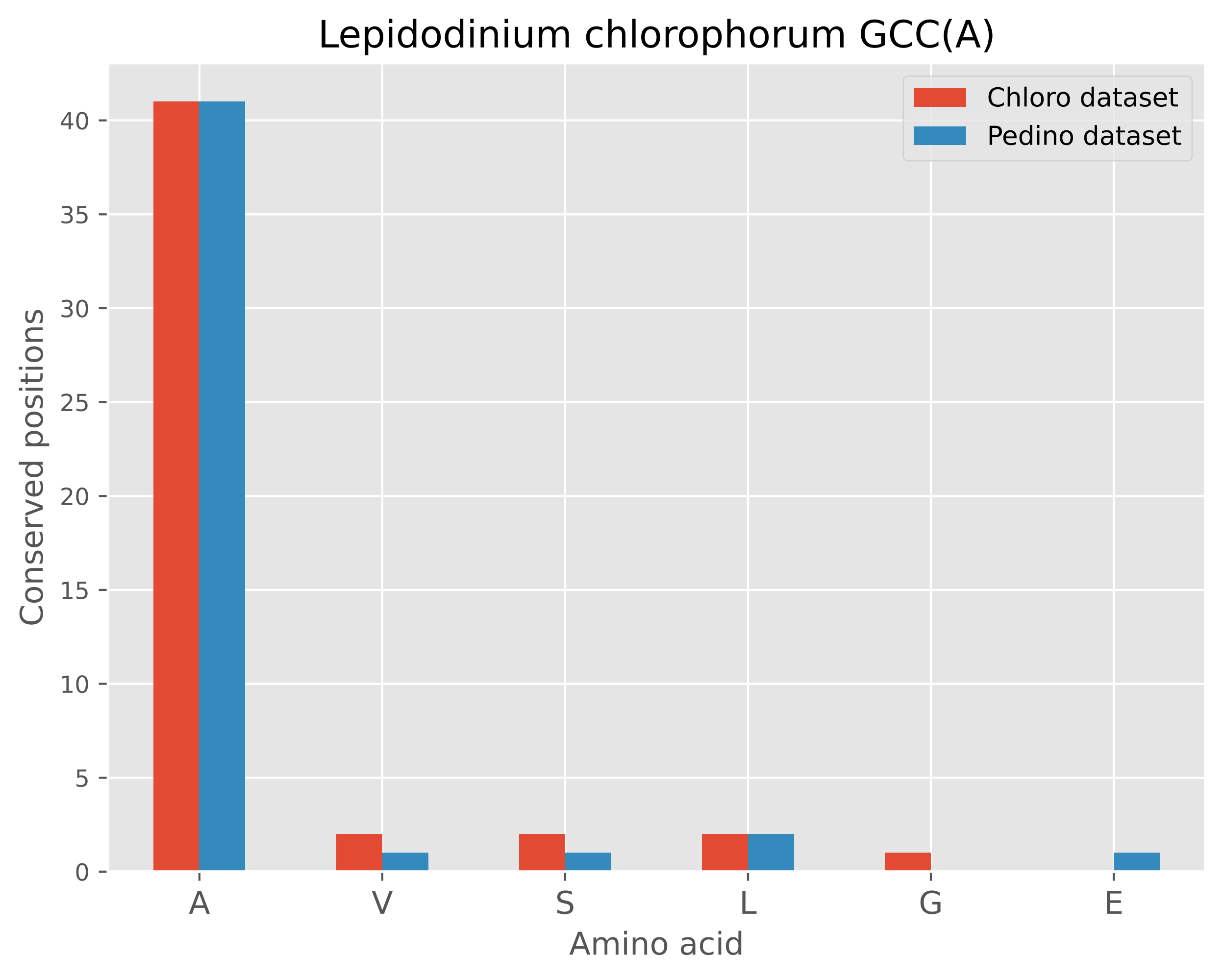

Lepidodinium chlorophorum GCC(A)
Chloro dataset
Pedino dataset
Conserved positions
Amino acid
0
5
10
15
20
25
30
35
40
A
V
S
L
G
E

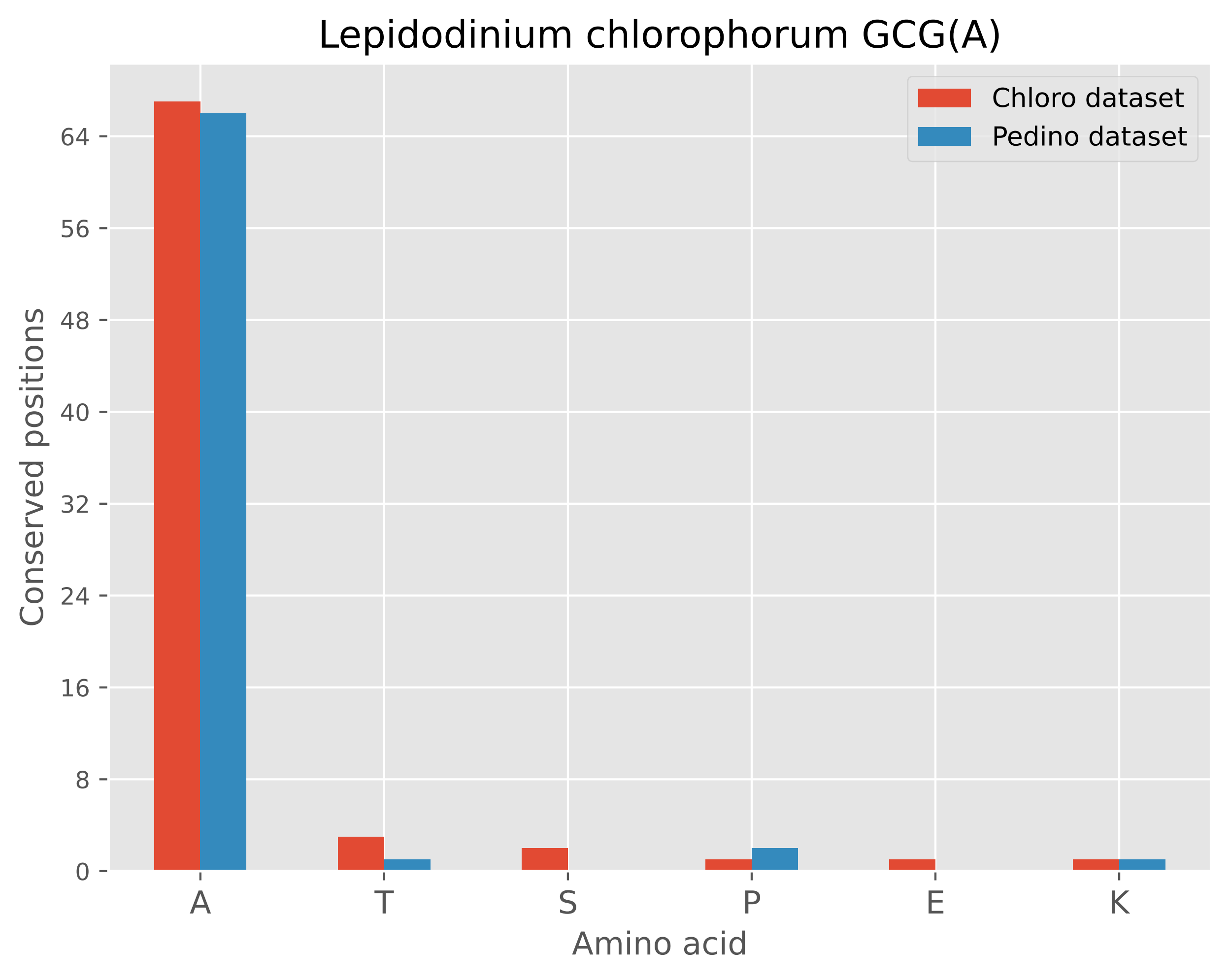

Lepidodinium chlorophorum GCG(A)
Chloro dataset
Pedino dataset
Conserved positions
0
8
16
24
32
40
48
56
64
A
T
S
P
E
K
Amino acid

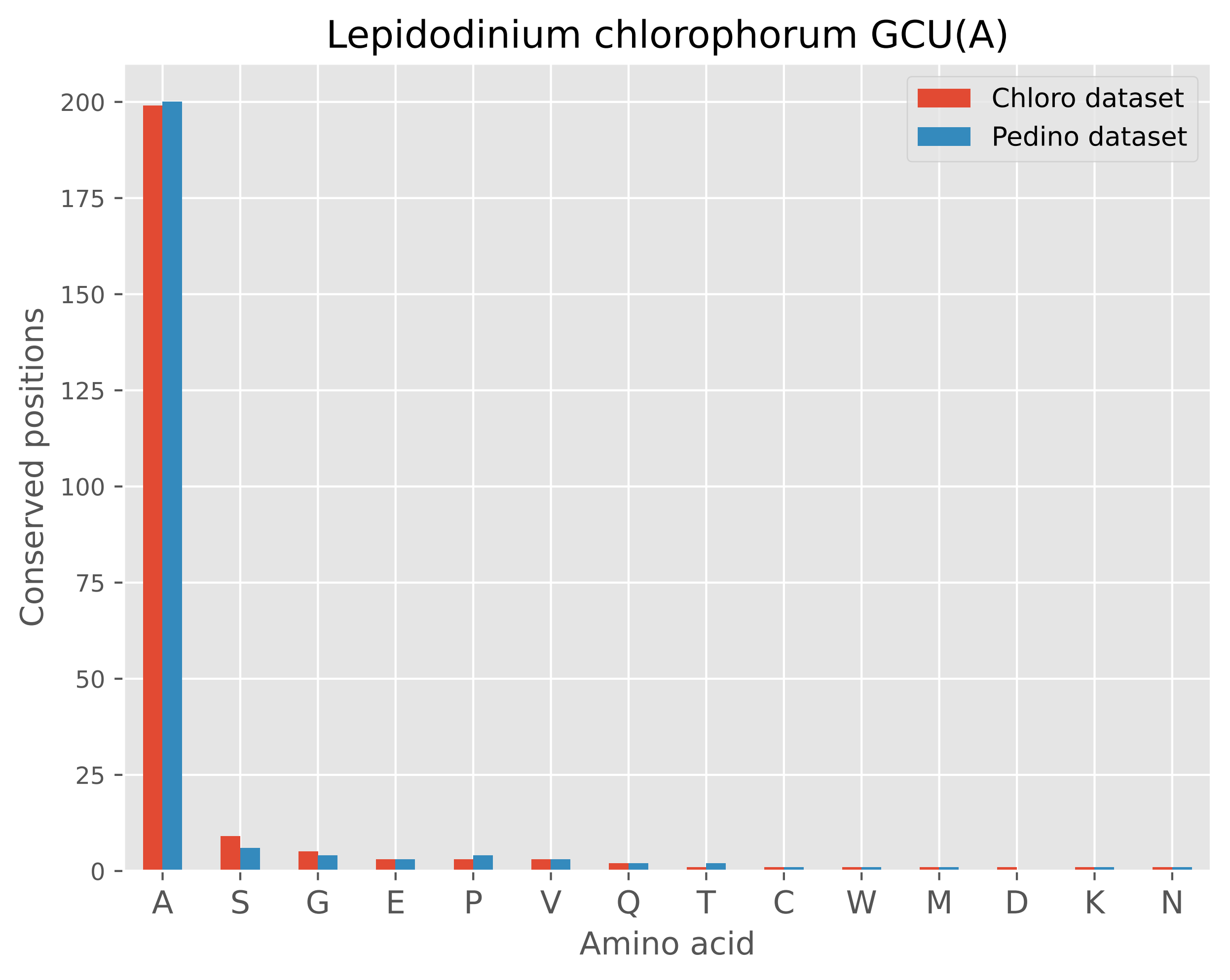

Lepidodinium chlorophorum GCU(A)
Chloro dataset
Pedino dataset
Conserved positions
0
25
50
75
100
125
150
175
200
Amino acid
A
S
G
E
P
V
Q
T
C
W
M
D
K
N

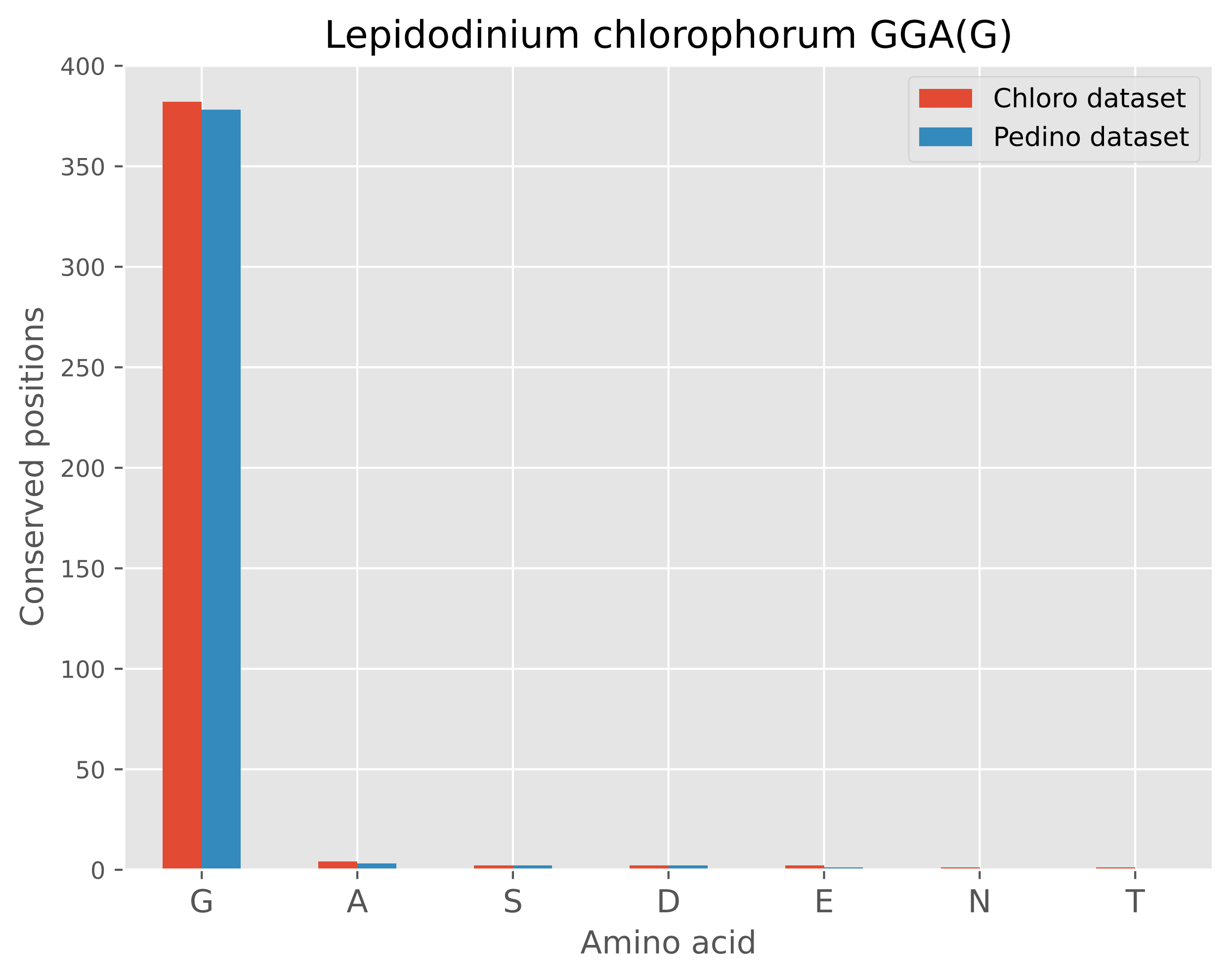

Lepidodinium chlorophorum GGA(G)
Chloro dataset
Pedino dataset
Conserved positions
0
50
100
150
200
250
300
350
400
G
A
S
D
E
N
T
Amino acid

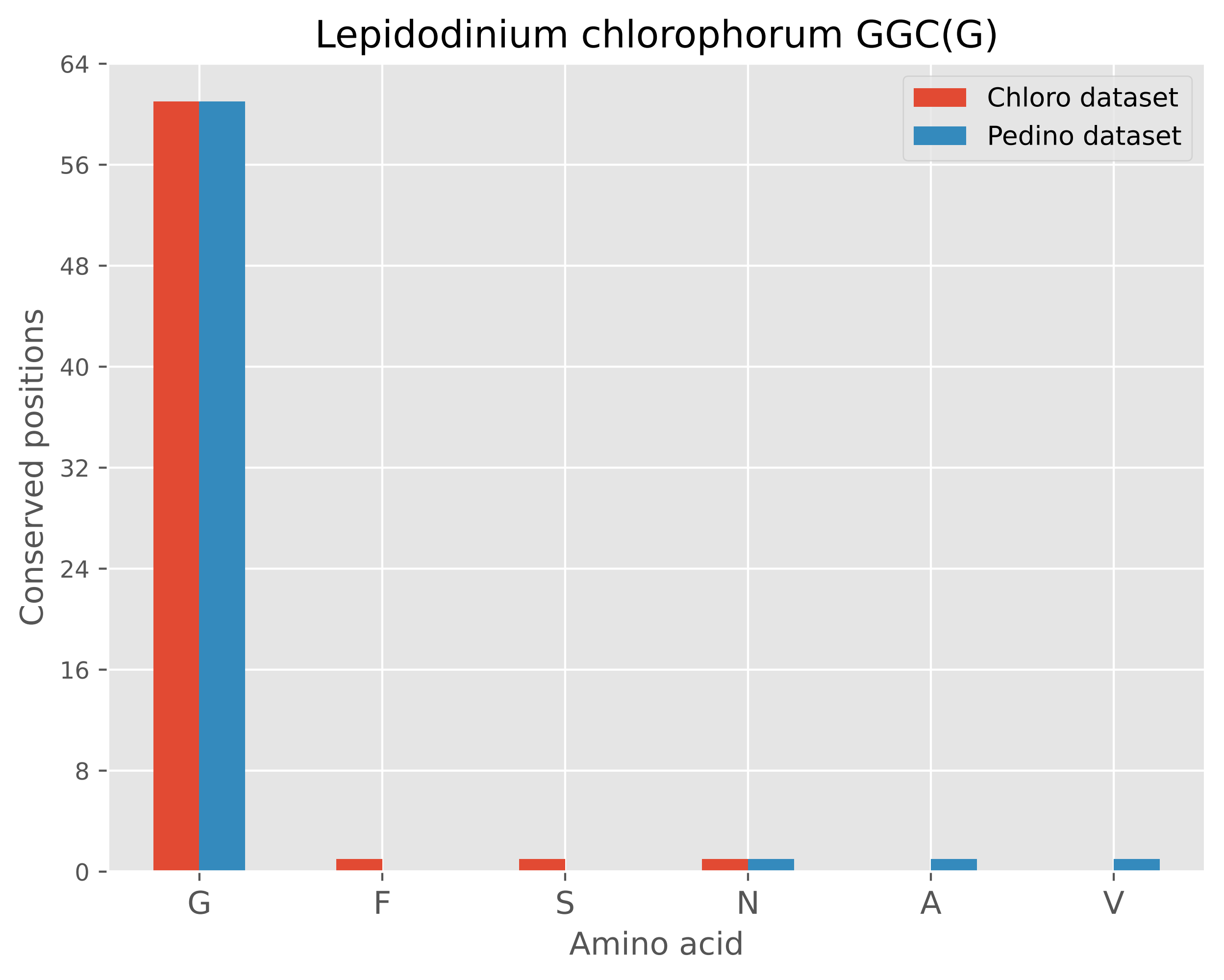
Lepidodinium chlorophorum GGC(G)
Chloro dataset
Pedino dataset
Conserved positions
Amino acid
0
8
16
24
32
40
48
56
64
G
F
S
N
A
V

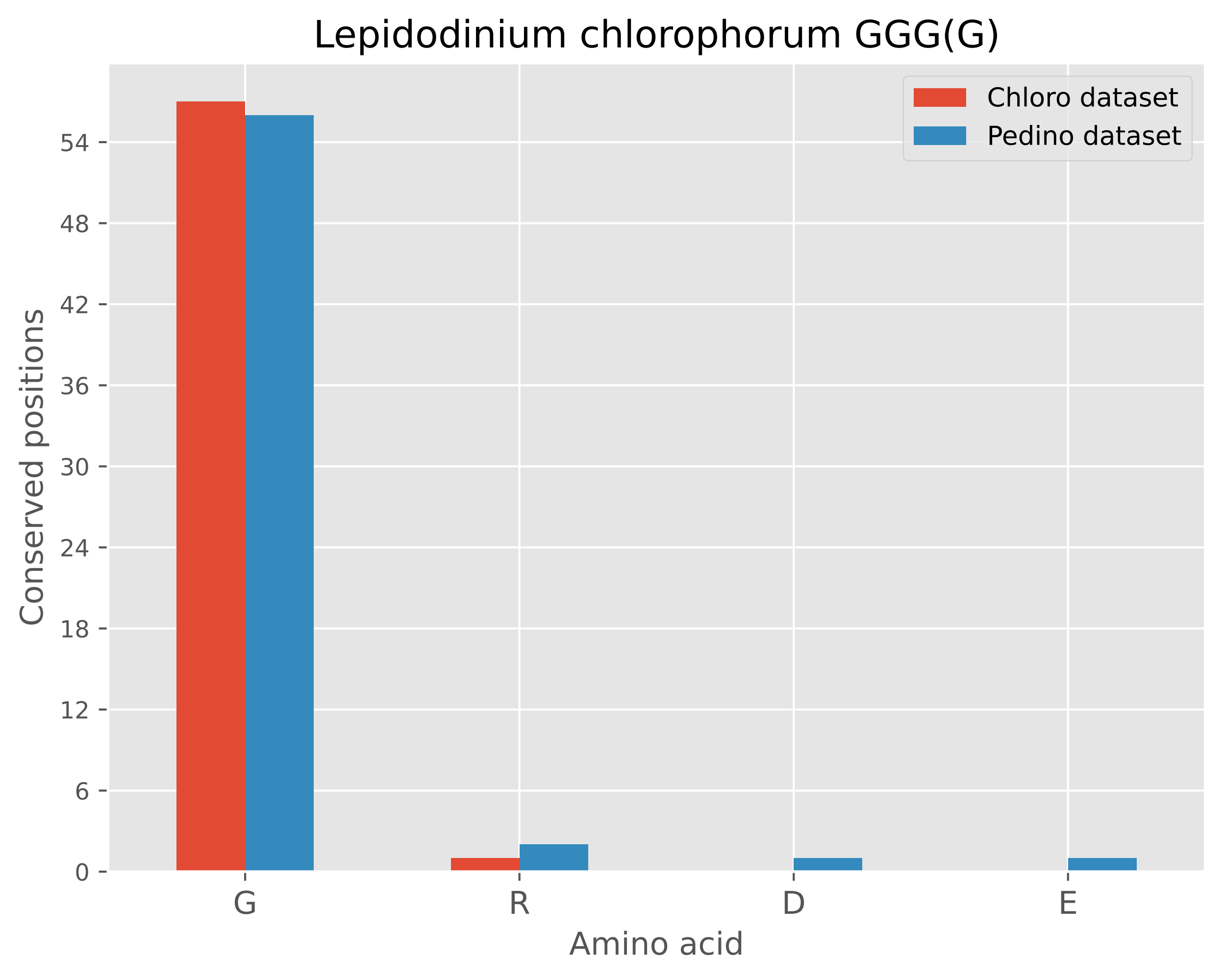

Lepidodinium chlorophorum GGG(G)
Chloro dataset
Pedino dataset
Conserved positions
0
6
12
18
24
30
36
42
48
54
G
R
D
E
Amino acid

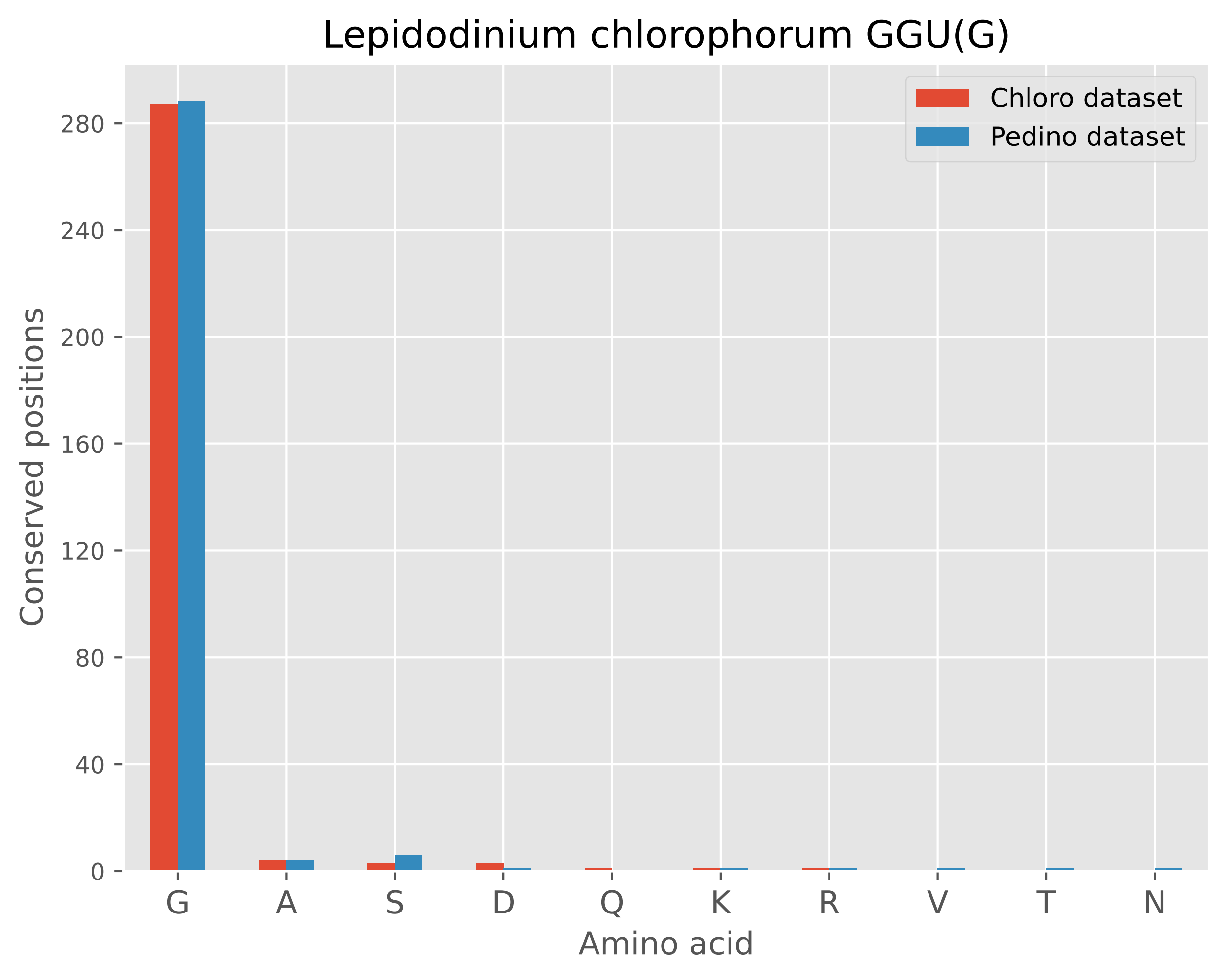
Lepidodinium chlorophorum GGU(G)
Chloro dataset
Pedino dataset
Conserved positions
0
40
80
120
160
200
240
280
G
A
S
D
Q
K
R
V
T
N
Amino acid

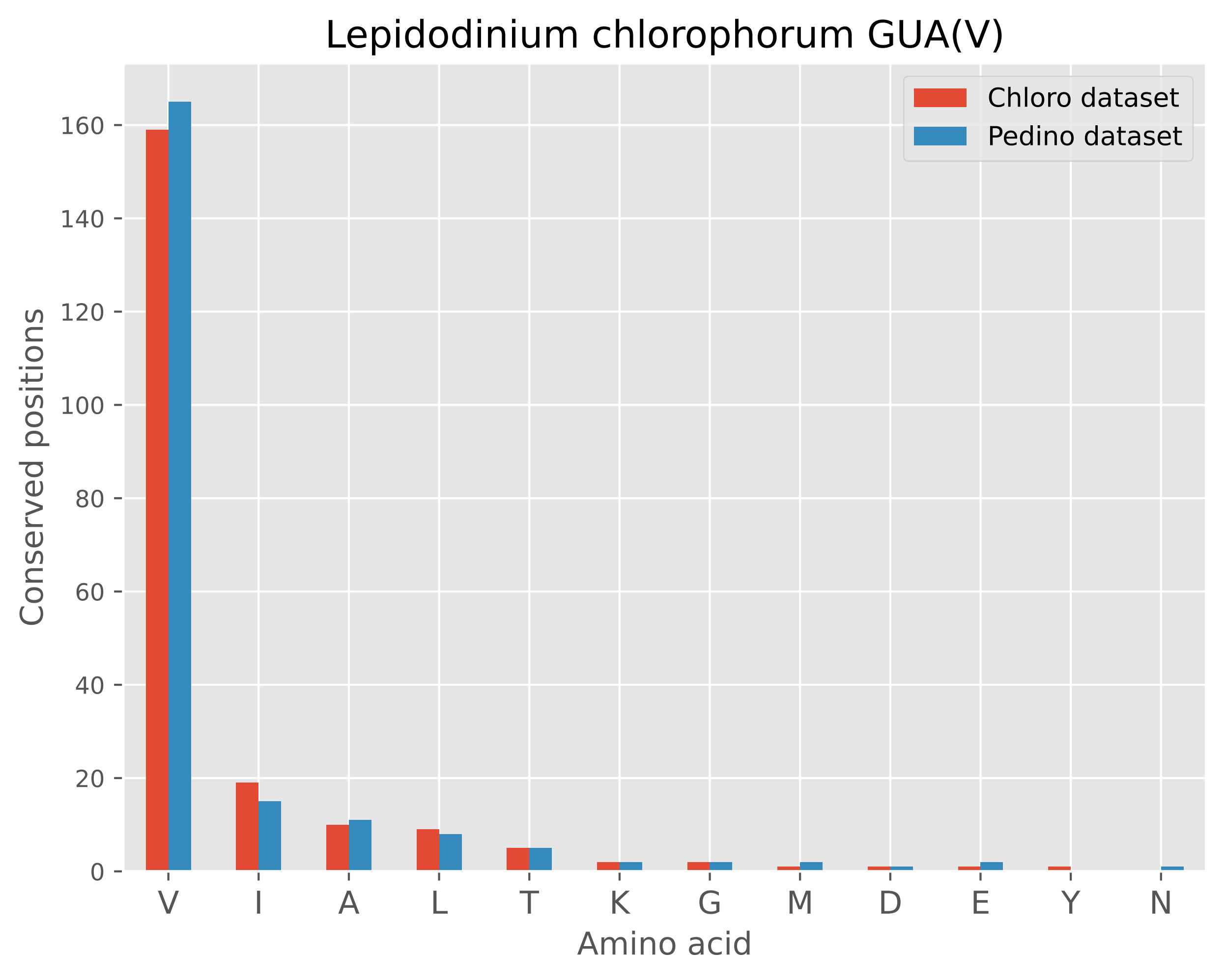

Lepidodinium chlorophorum GUA(V)
Chloro dataset
Pedino dataset
Conserved positions
0
20
40
60
80
100
120
140
160
V
I
A
L
T
K
G
M
D
E
Y
N
Amino acid

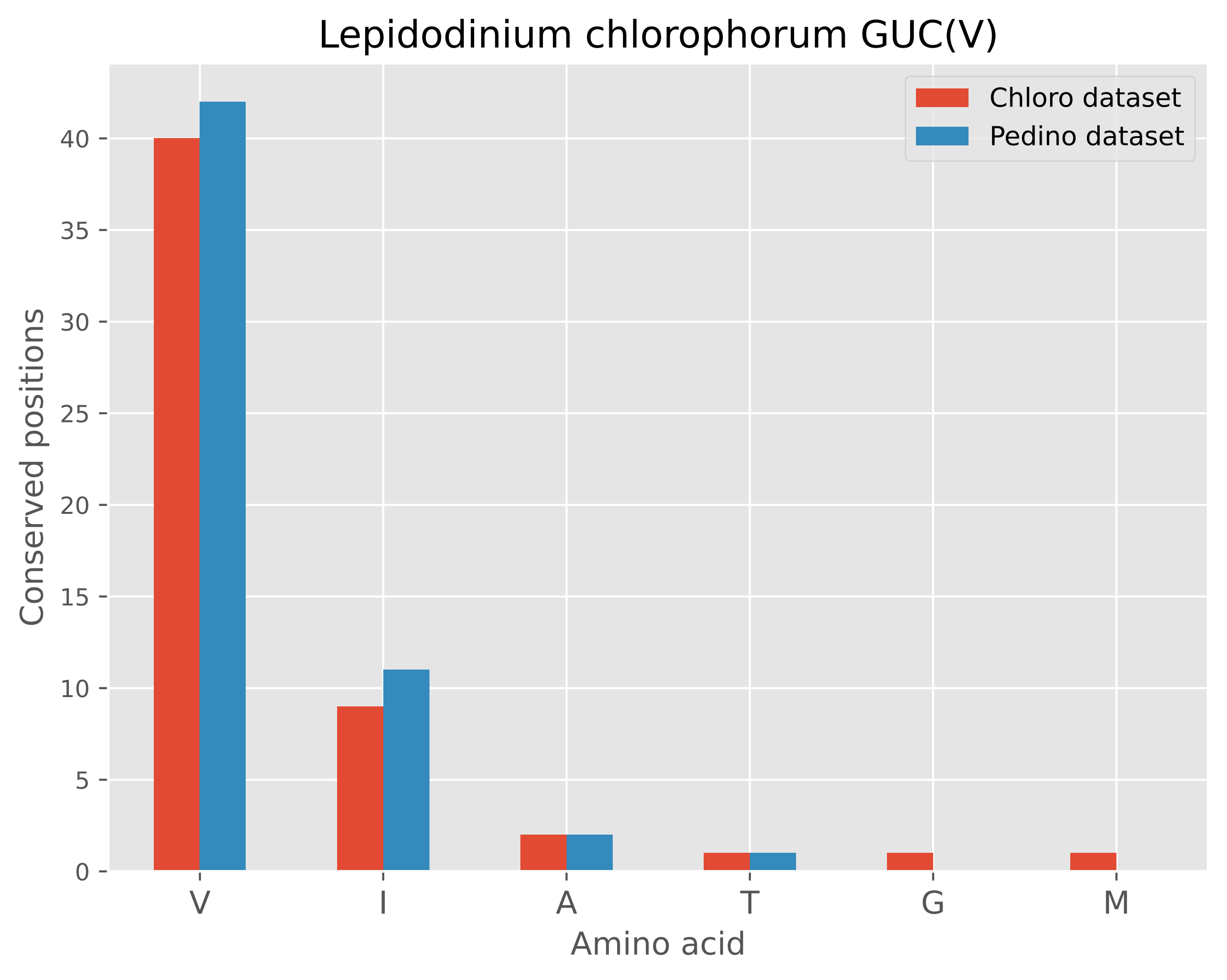
Lepidodinium chlorophorum GUC(V)
Chloro dataset
Pedino dataset
Conserved positions
0
5
10
15
20
25
30
35
40
V
I
A
T
G
M
Amino acid

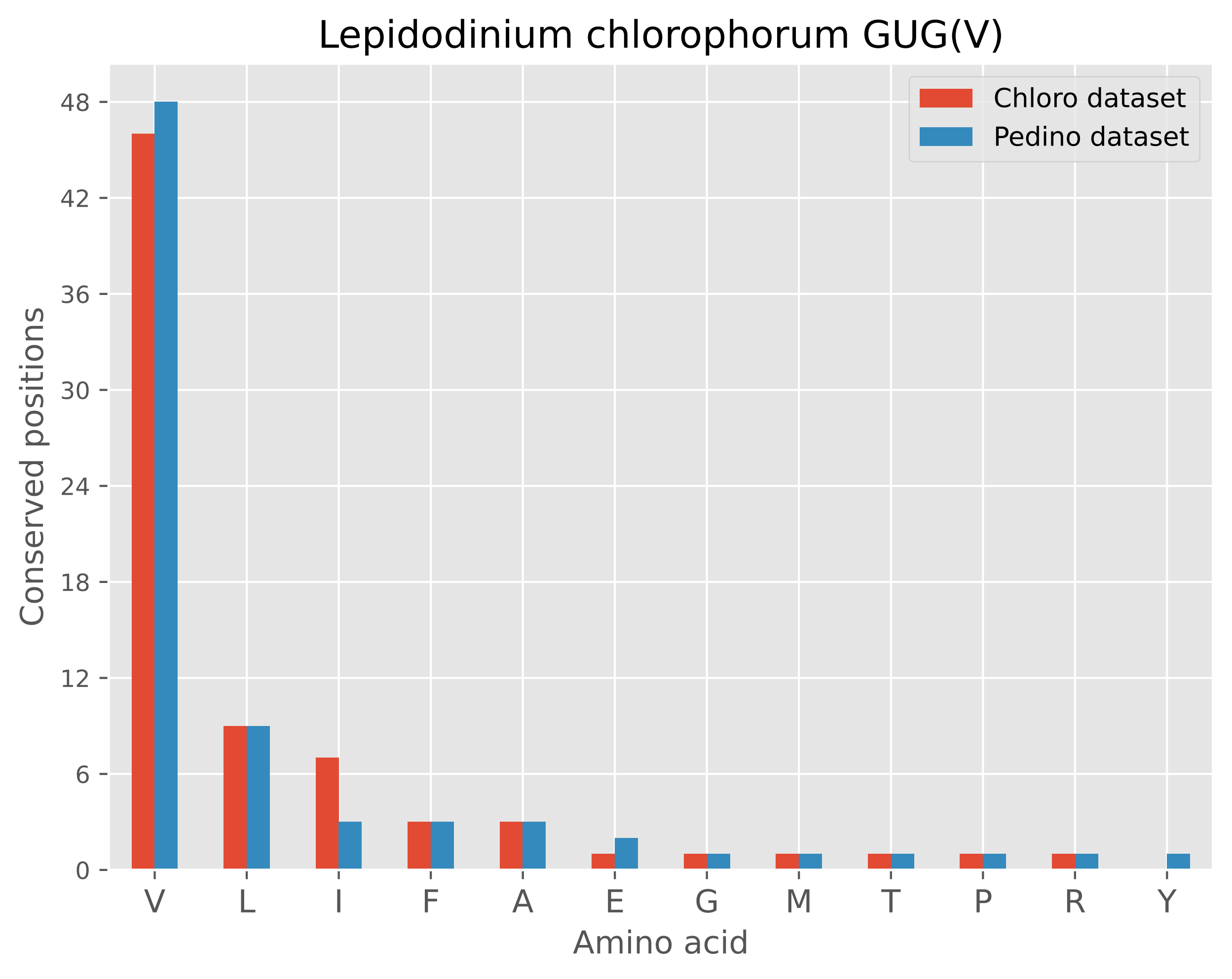
Lepidodinium chlorophorum GUG(V)
Chloro dataset
Pedino dataset
Conserved positions
0
6
12
18
24
30
36
42
48
Amino acid
V
L
I
F
A
E
G
M
T
P
R
Y

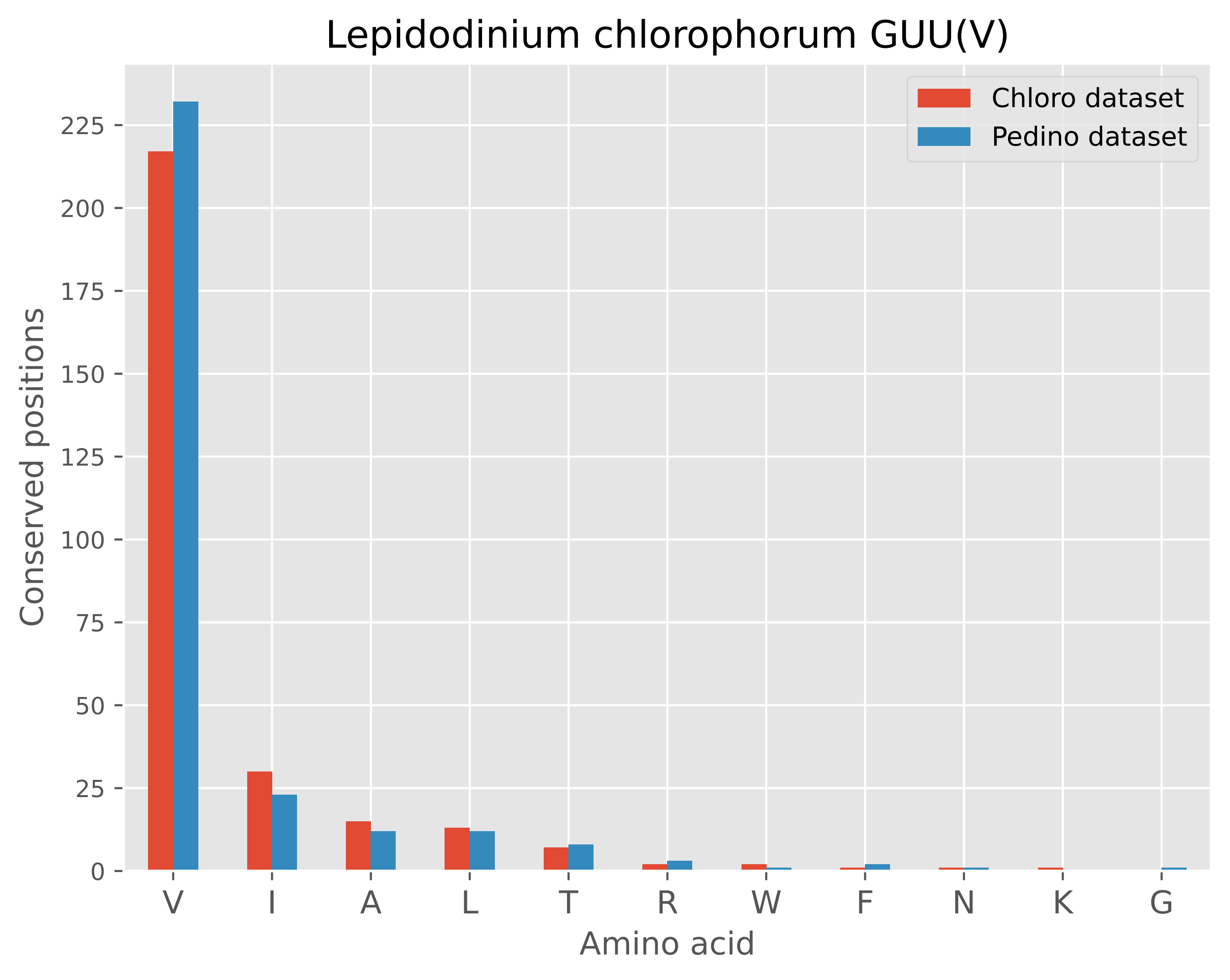
Lepidodinium chlorophorum GUU(V)
Chloro dataset
Pedino dataset
Conserved positions
225
200
175
150
125
100
75
50
25
0
V
I
A
L
T
R
W
F
N
K
G
Amino acid

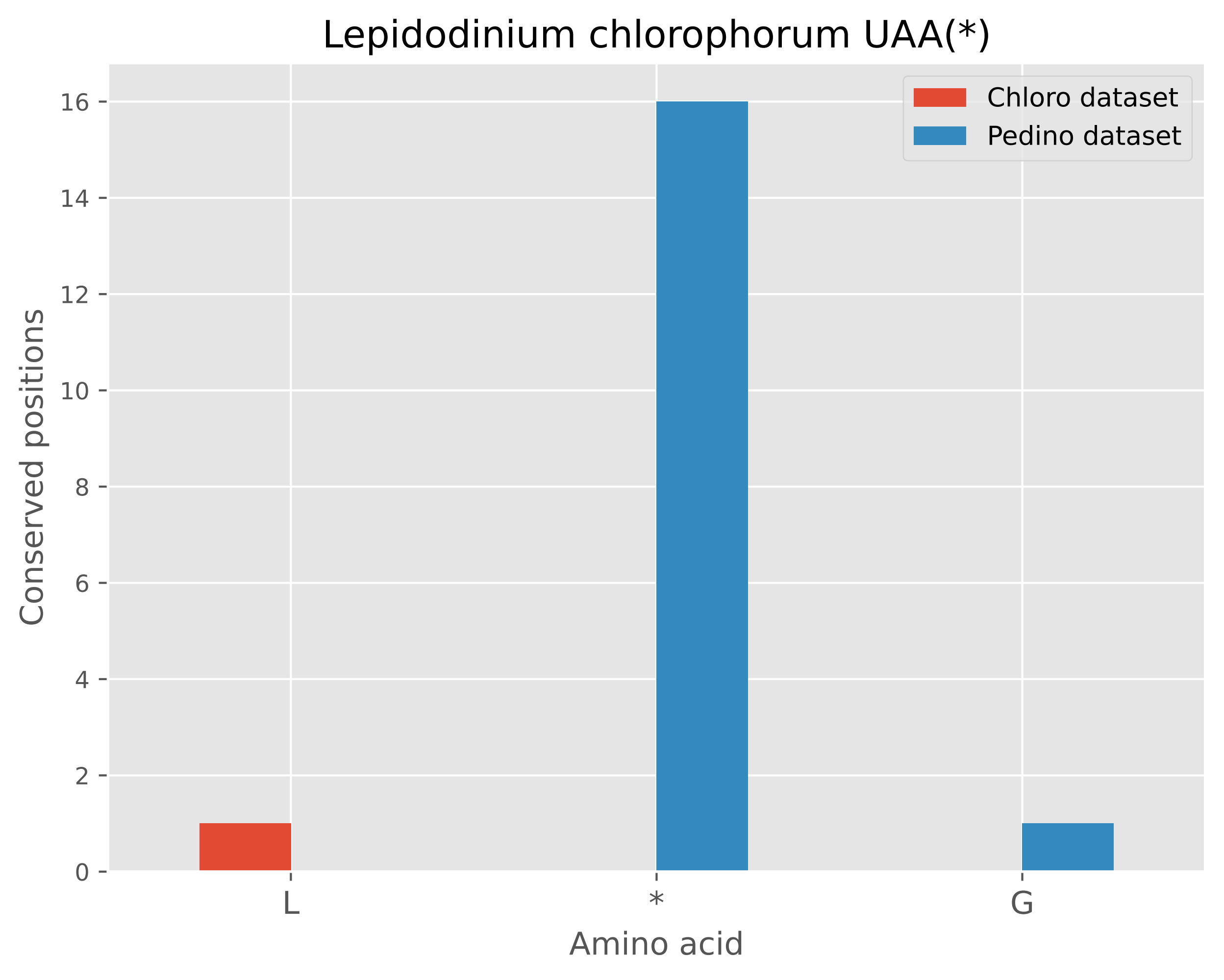
Lepidodinium chlorophorum UAA(*)
Chloro dataset
Pedino dataset
Conserved positions
0
2
4
6
8
10
12
14
16
L
*
G
Amino acid

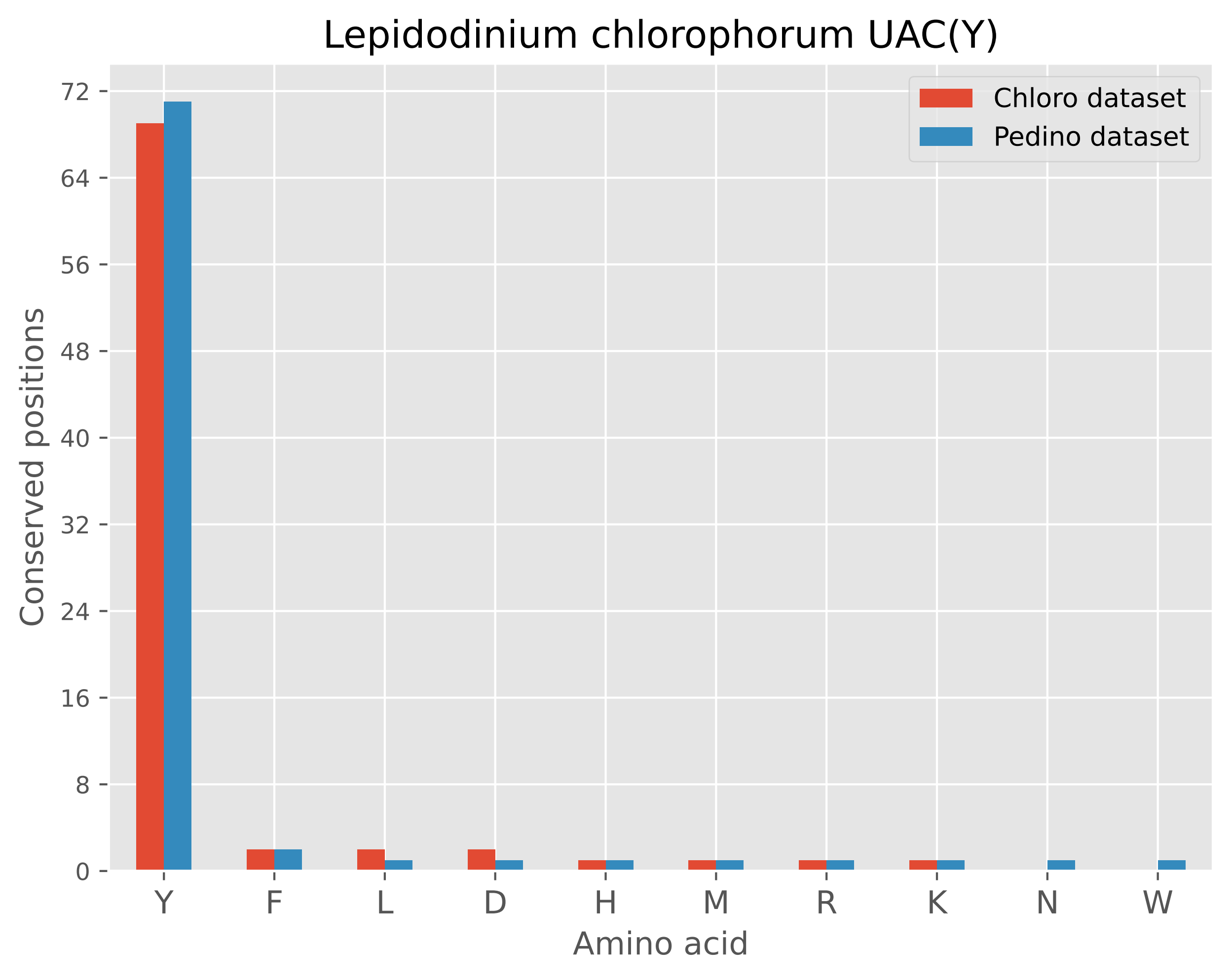

Lepidodinium chlorophorum UAC(Y)
Chloro dataset
Pedino dataset
Conserved positions
0
8
16
24
32
40
48
56
64
72
Y
F
L
D
H
M
R
K
N
W
Amino acid

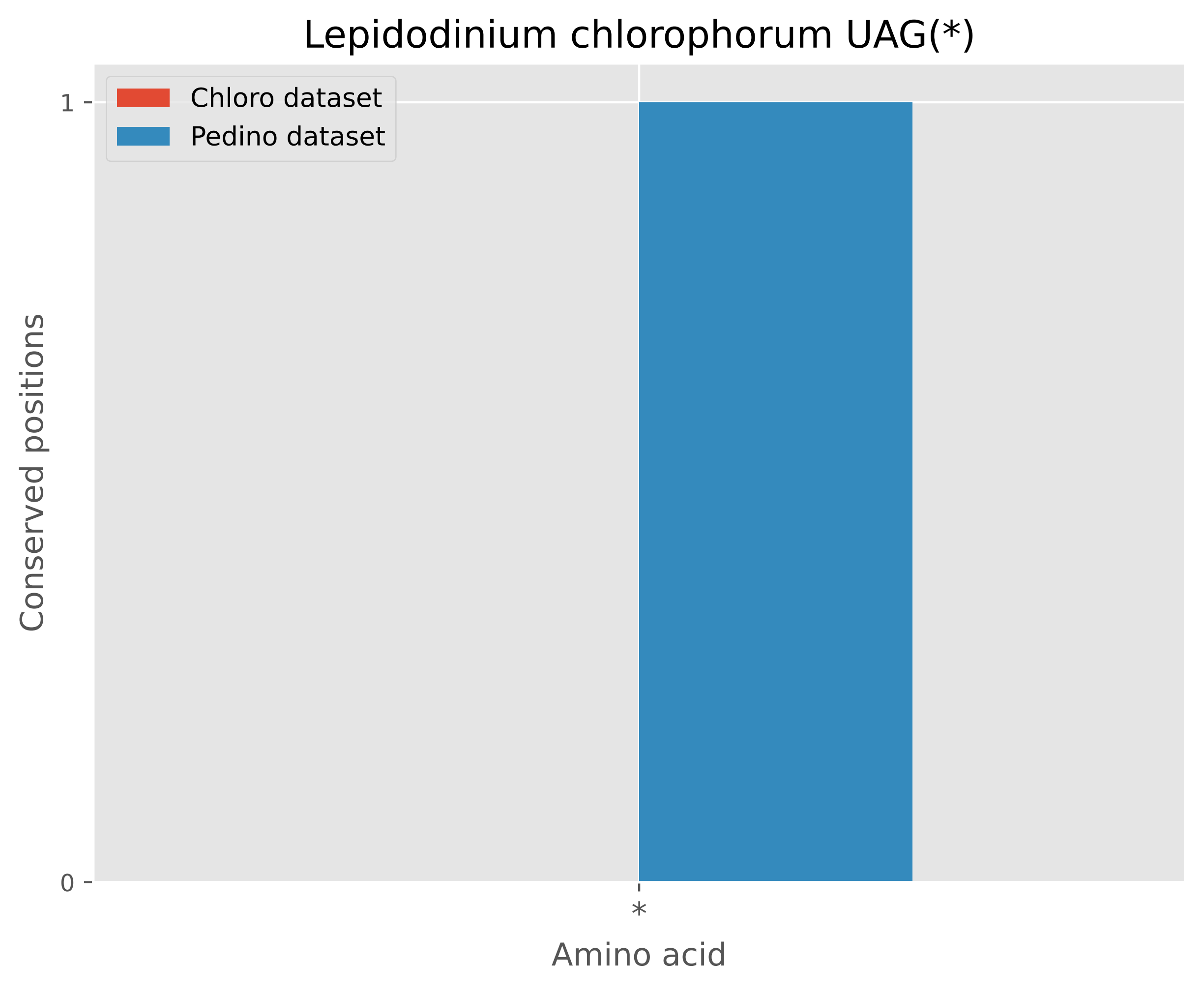
Lepidodinium chlorophorum UAG(*)
Chloro dataset
Pedino dataset
1
0
Conserved positions
*
Amino acid

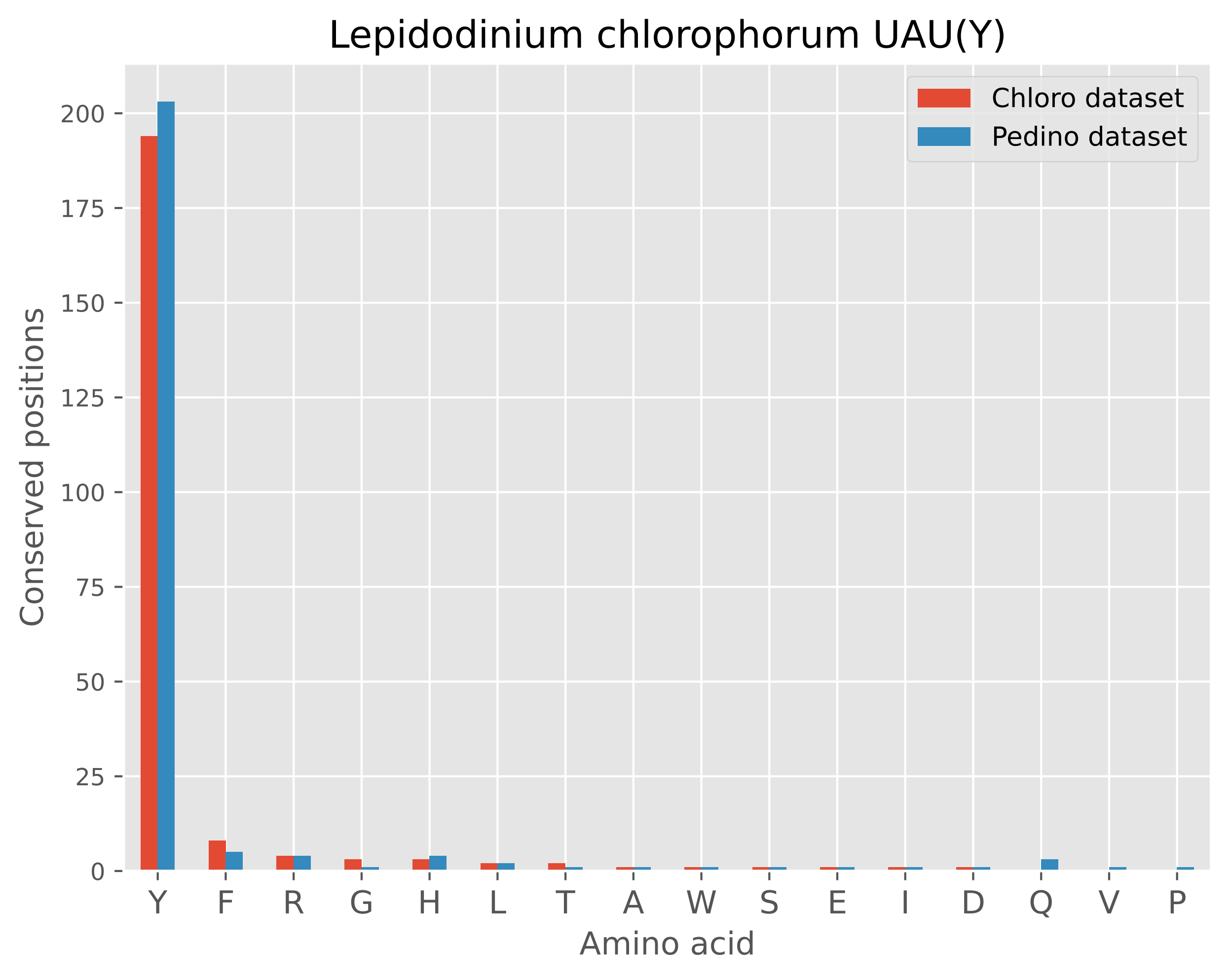

Lepidodinium chlorophorum UAU(Y)
Chloro dataset
Pedino dataset
Conserved positions
0
25
50
75
100
125
150
175
200
Y
F
R
G
H
L
T
A
W
S
E
I
D
Q
V
P
Amino acid

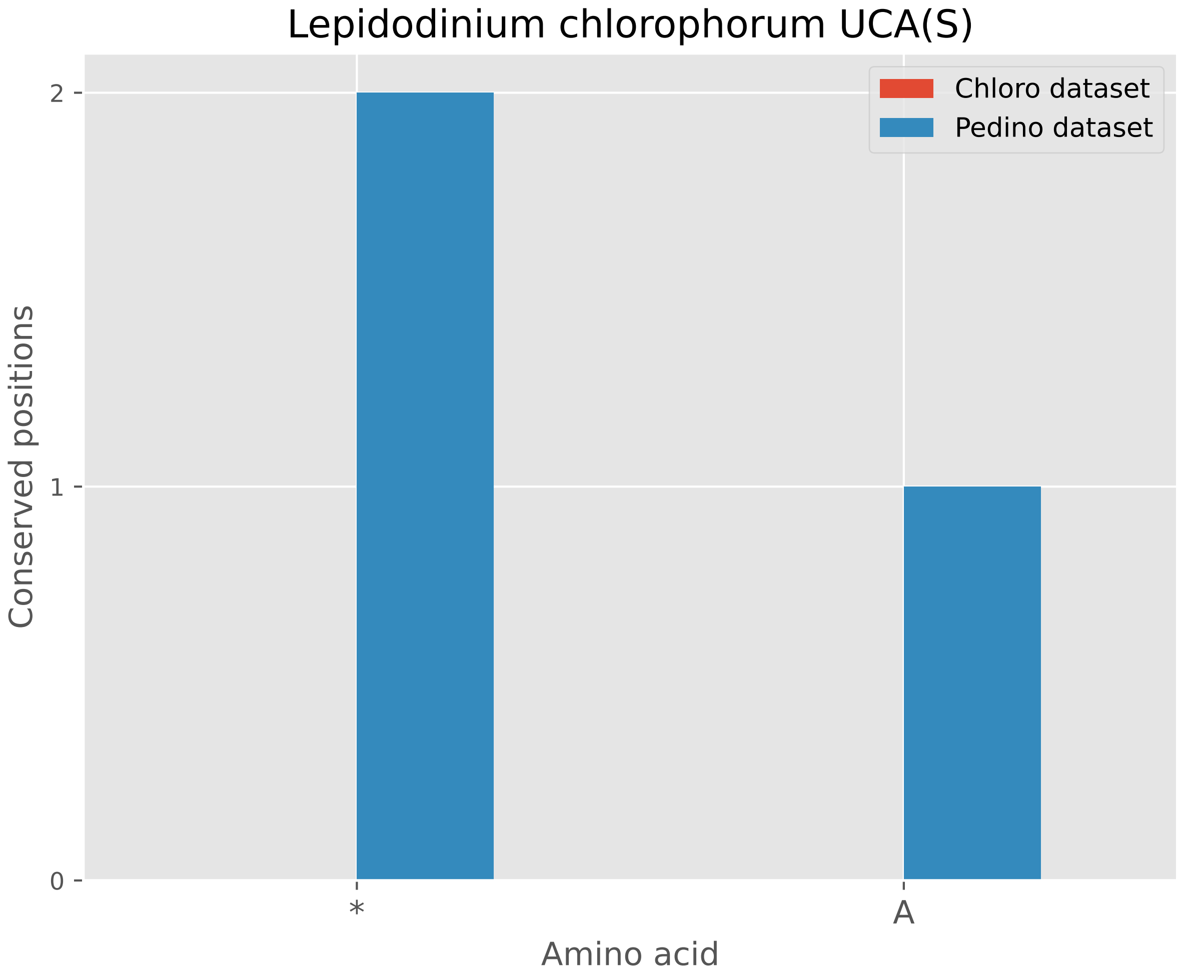

Lepidodinium chlorophorum UCA(S)
Chloro dataset
Pedino dataset
Conserved positions
0
1
2
*
A
Amino acid

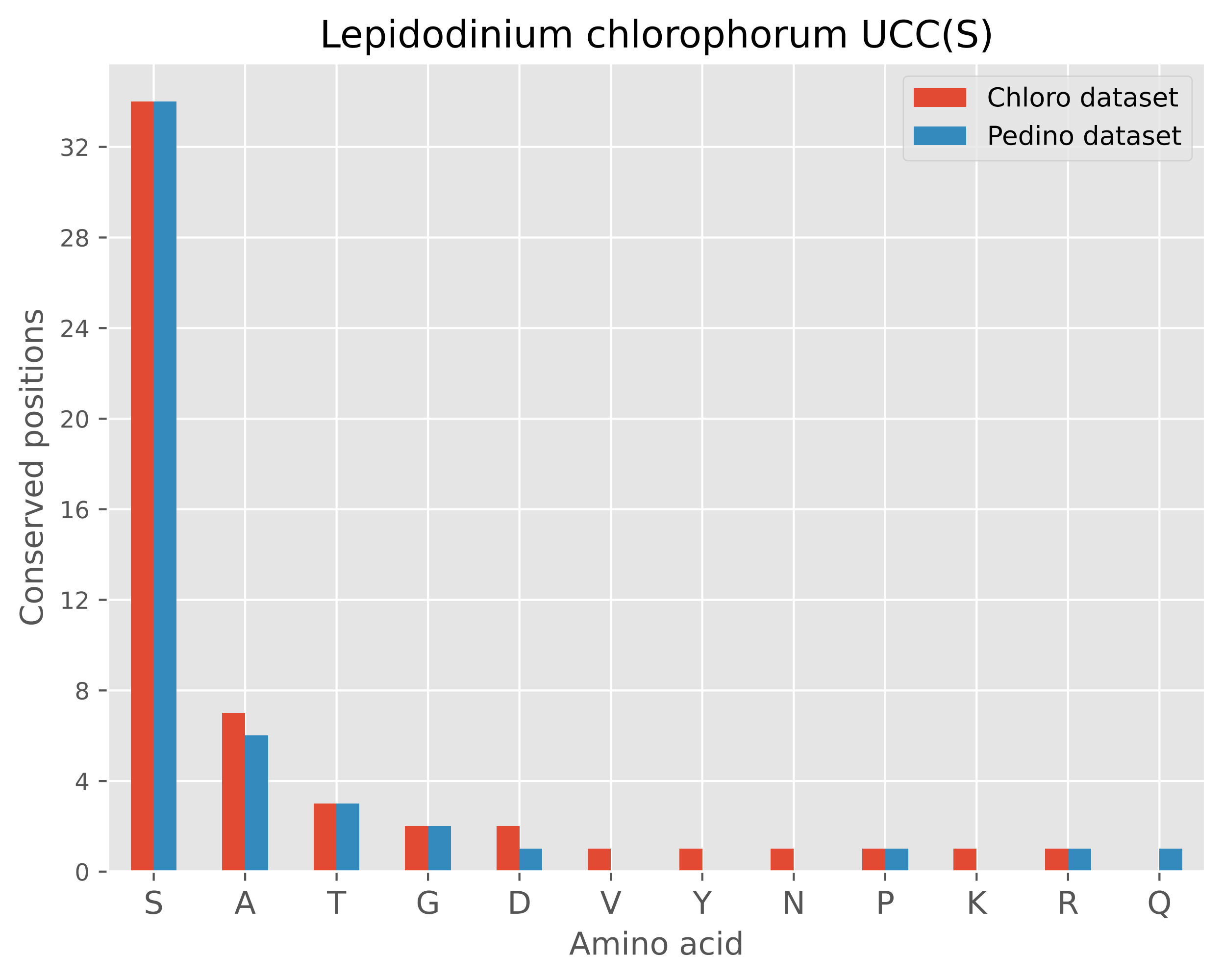

Lepidodinium chlorophorum UCC(S)
Chloro dataset
Pedino dataset
Conserved positions
0
4
8
12
16
20
24
28
32
S
A
T
G
D
V
Y
N
P
K
R
Q
Amino acid

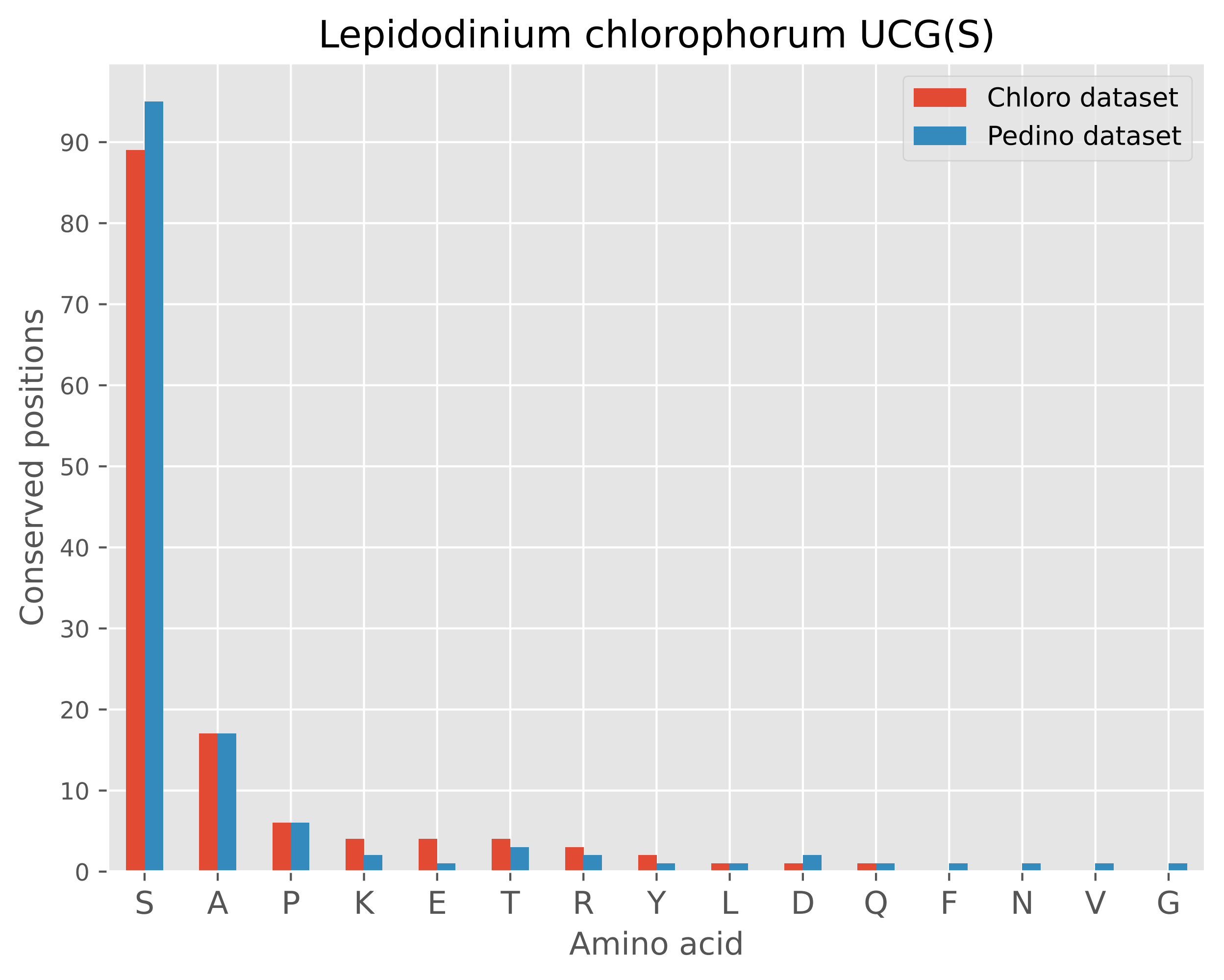

Lepidodinium chlorophorum UCG(S)
Chloro dataset
Pedino dataset
Conserved positions
0
10
20
30
40
50
60
70
80
90
S
A
P
K
E
T
R
Y
L
D
Q
F
N
V
G
Amino acid

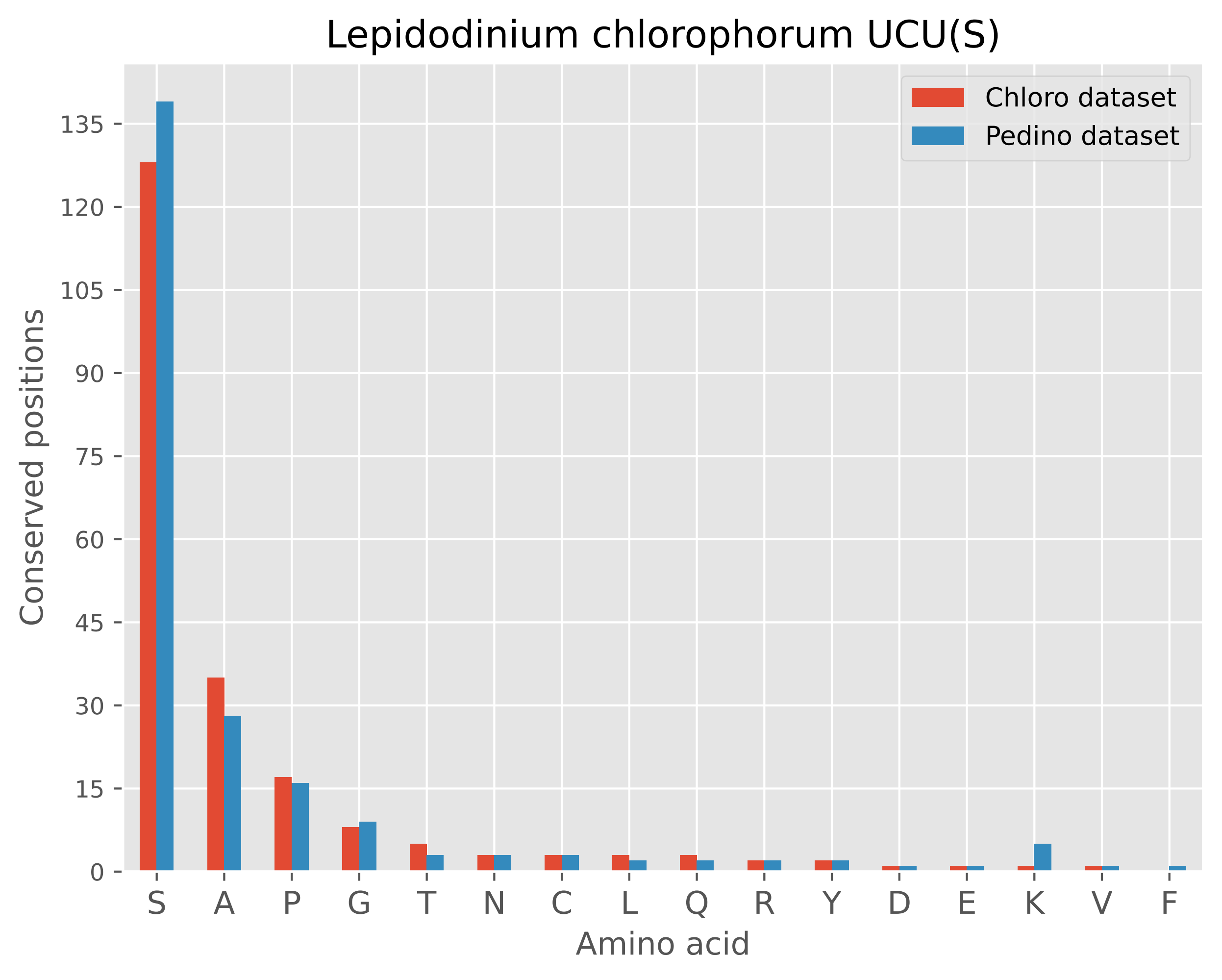

Lepidodinium chlorophorum UCU(S)
Chloro dataset
Pedino dataset
Conserved positions
Amino acid
0
15
30
45
60
75
90
105
120
135
S A P G T N C L Q R Y D E K V F

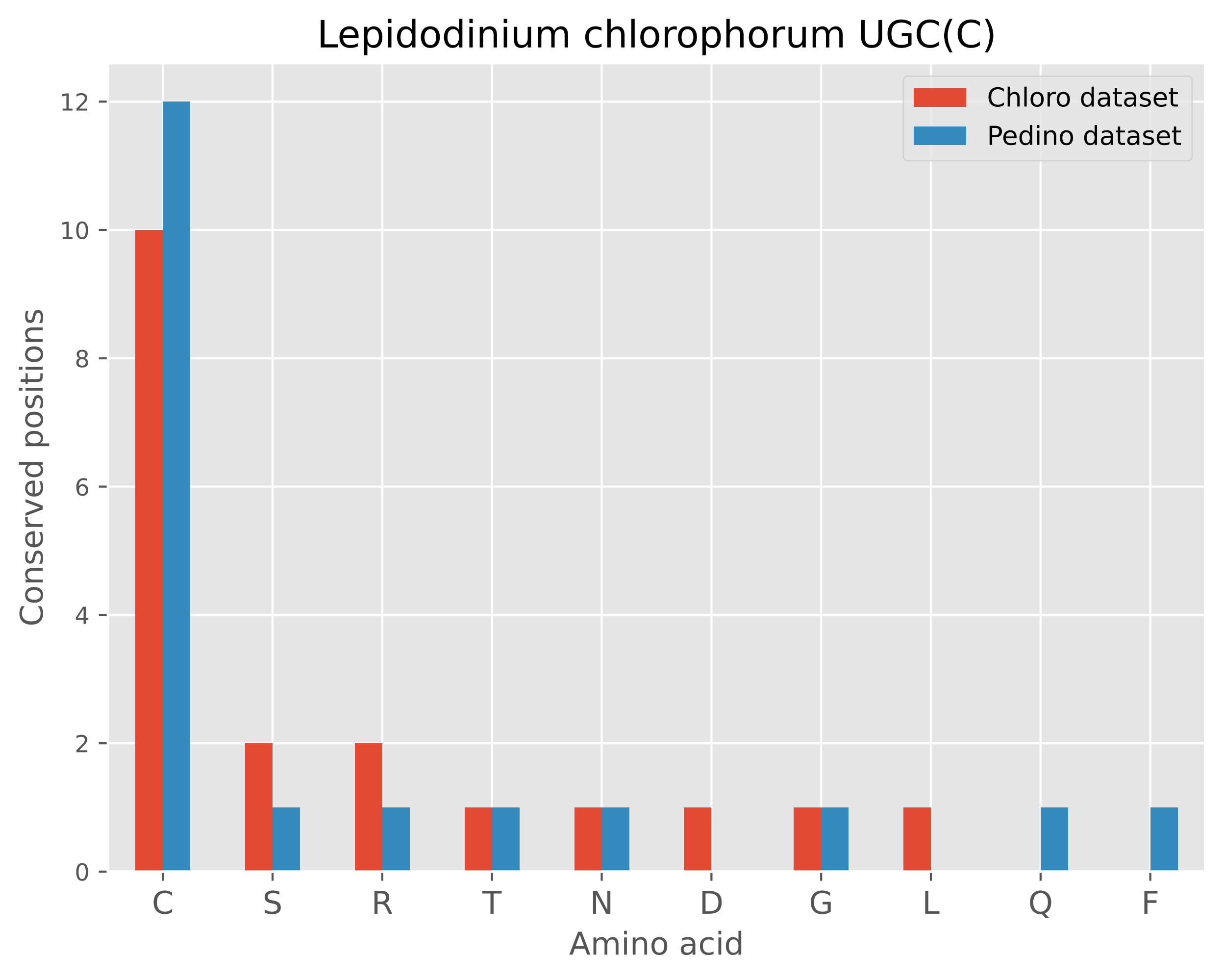

Lepidodinium chlorophorum UGC(C)
Chloro dataset
Pedino dataset
Conserved positions
0
2
4
6
8
10
12
C
S
R
T
N
D
G
L
Q
F
Amino acid

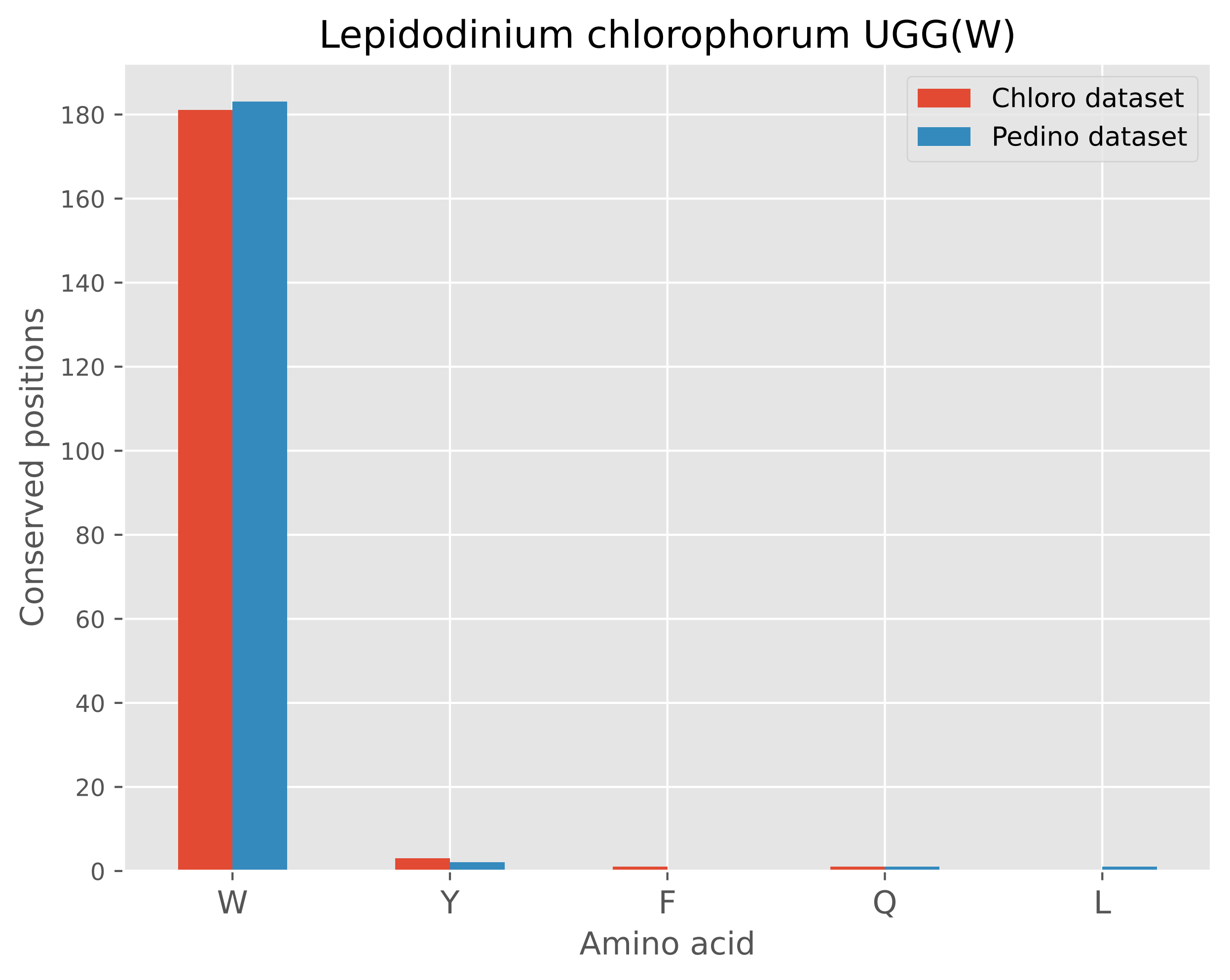

Lepidodinium chlorophorum UGG(W)
Chloro dataset
Pedino dataset
Conserved positions
0
20
40
60
80
100
120
140
160
180
W
Y
F
Q
L
Amino acid

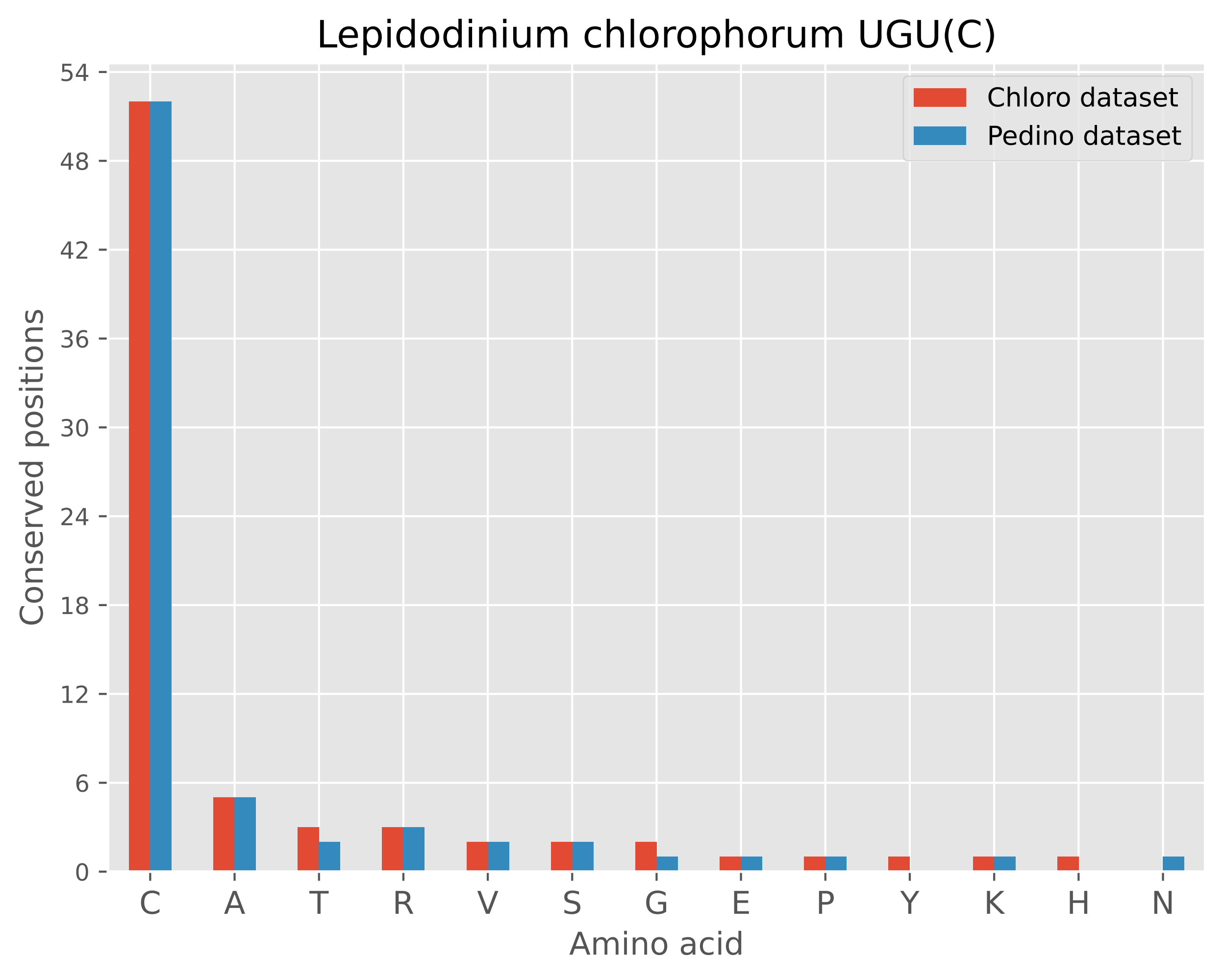

Lepidodinium chlorophorum UGU(C)
Chloro dataset
Pedino dataset
Conserved positions
0
6
12
18
24
30
36
42
48
54
C
A
T
R
V
S
G
E
P
Y
K
H
N
Amino acid

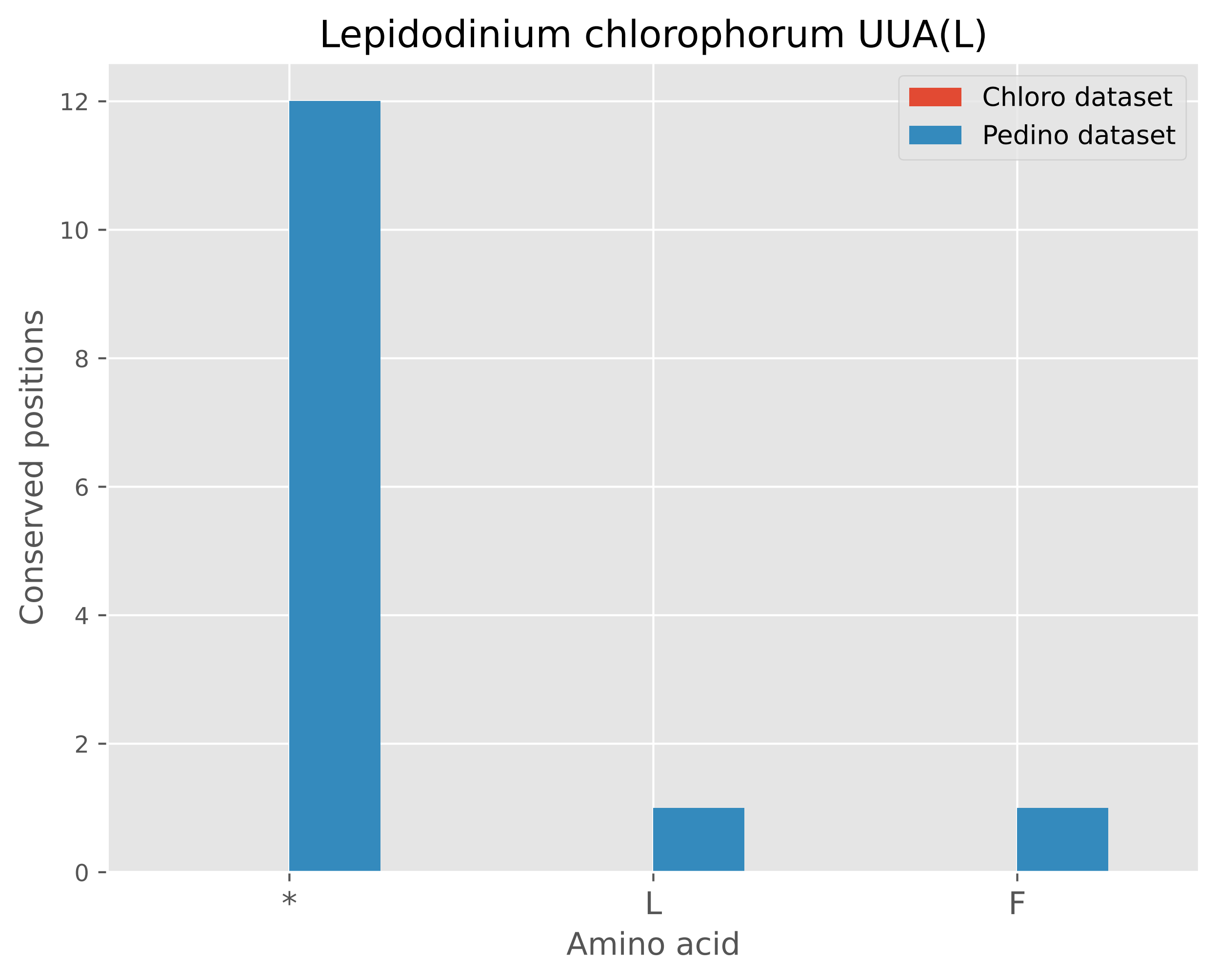

Lepidodinium chlorophorum UUA(L)
Chloro dataset
Pedino dataset
Conserved positions
0
2
4
6
8
10
12
*
L
F
Amino acid

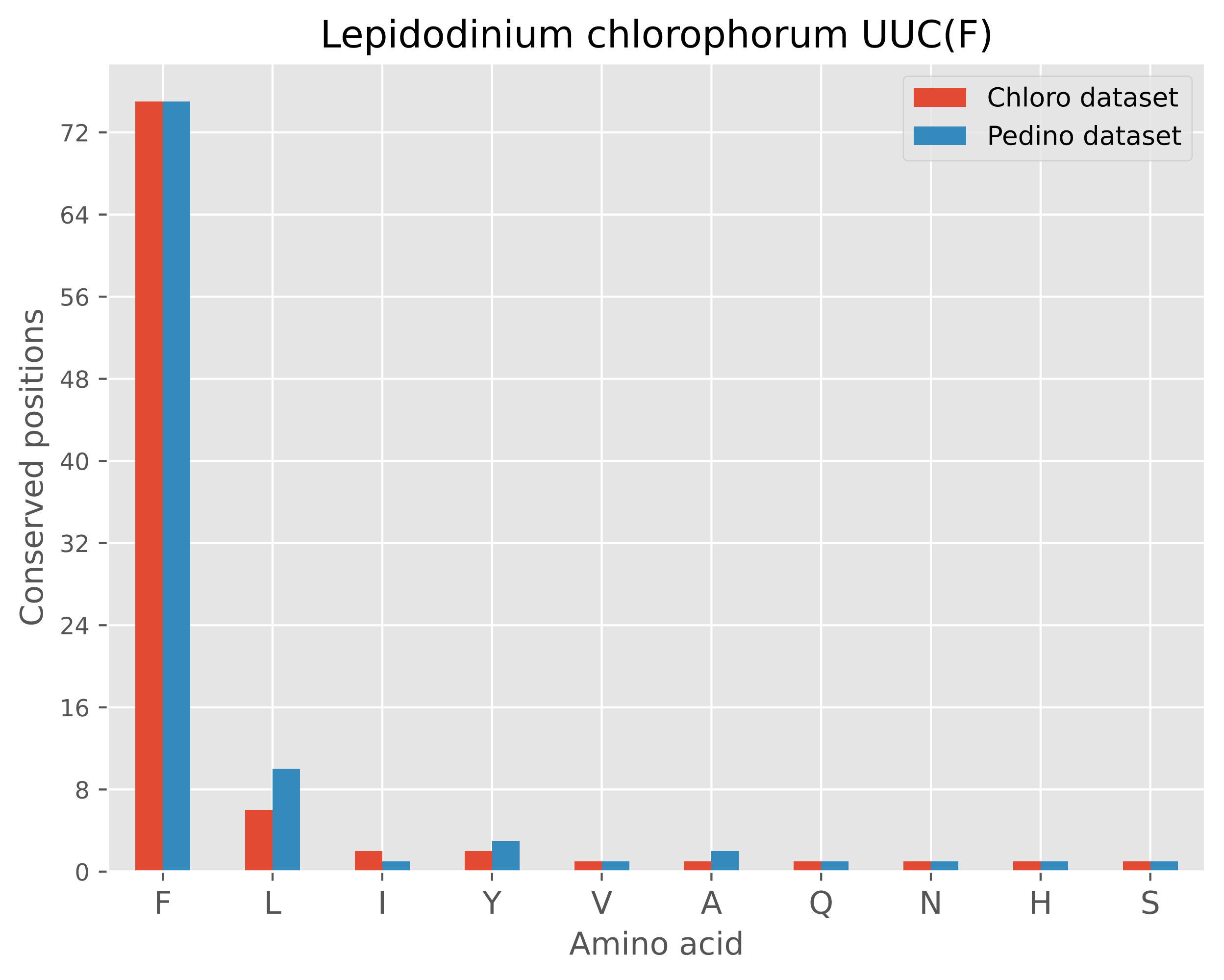

Lepidodinium chlorophorum UUC(F)
Chloro dataset
Pedino dataset
Conserved positions
0
8
16
24
32
40
48
56
64
72
Amino acid
F
L
I
Y
V
A
Q
N
H
S

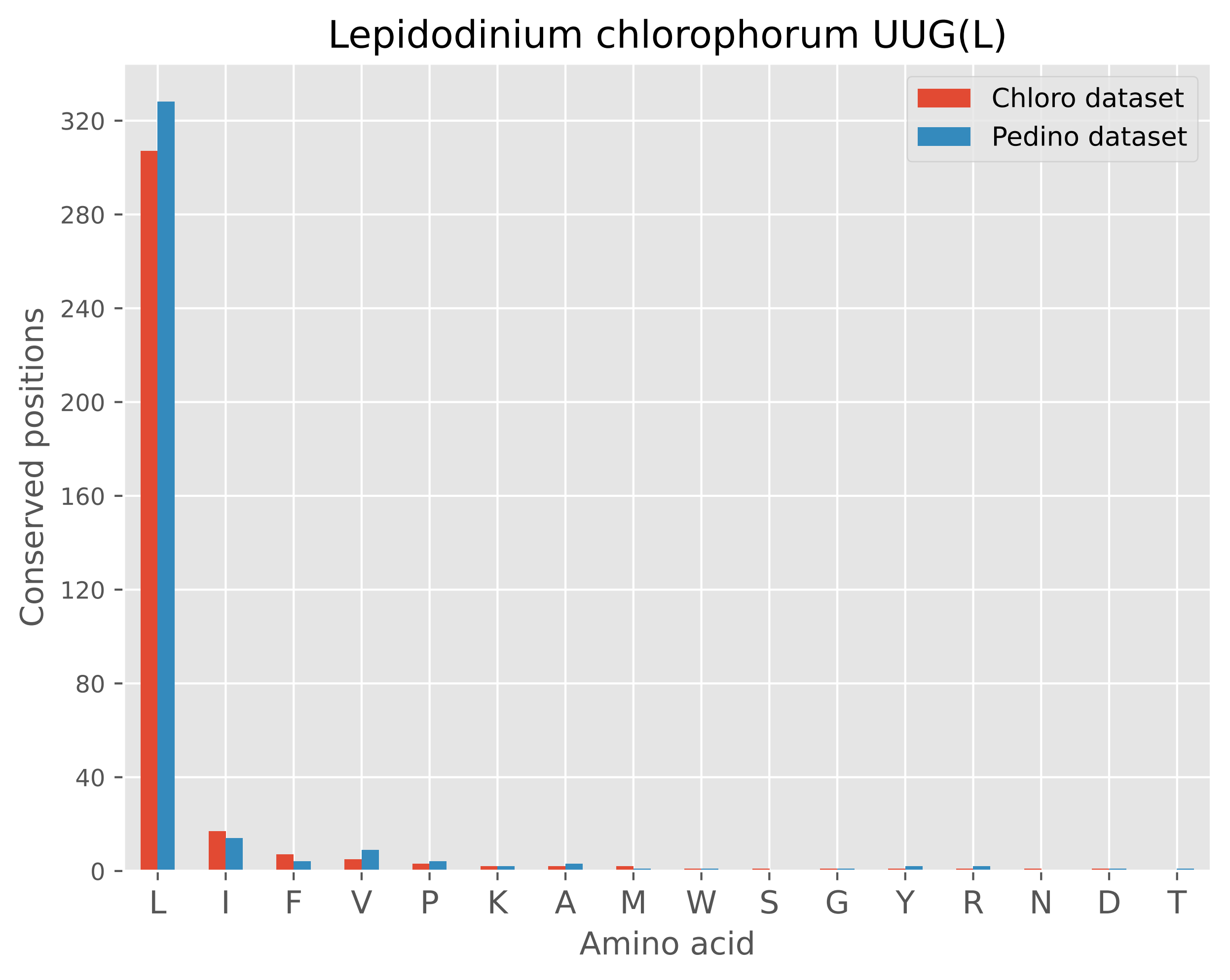

Lepidodinium chlorophorum UUG(L)
Chloro dataset
Pedino dataset
Conserved positions
Amino acid
0
40
80
120
160
200
240
280
320
L
I
F
V
P
K
A
M
W
S
G
Y
R
N
D
T

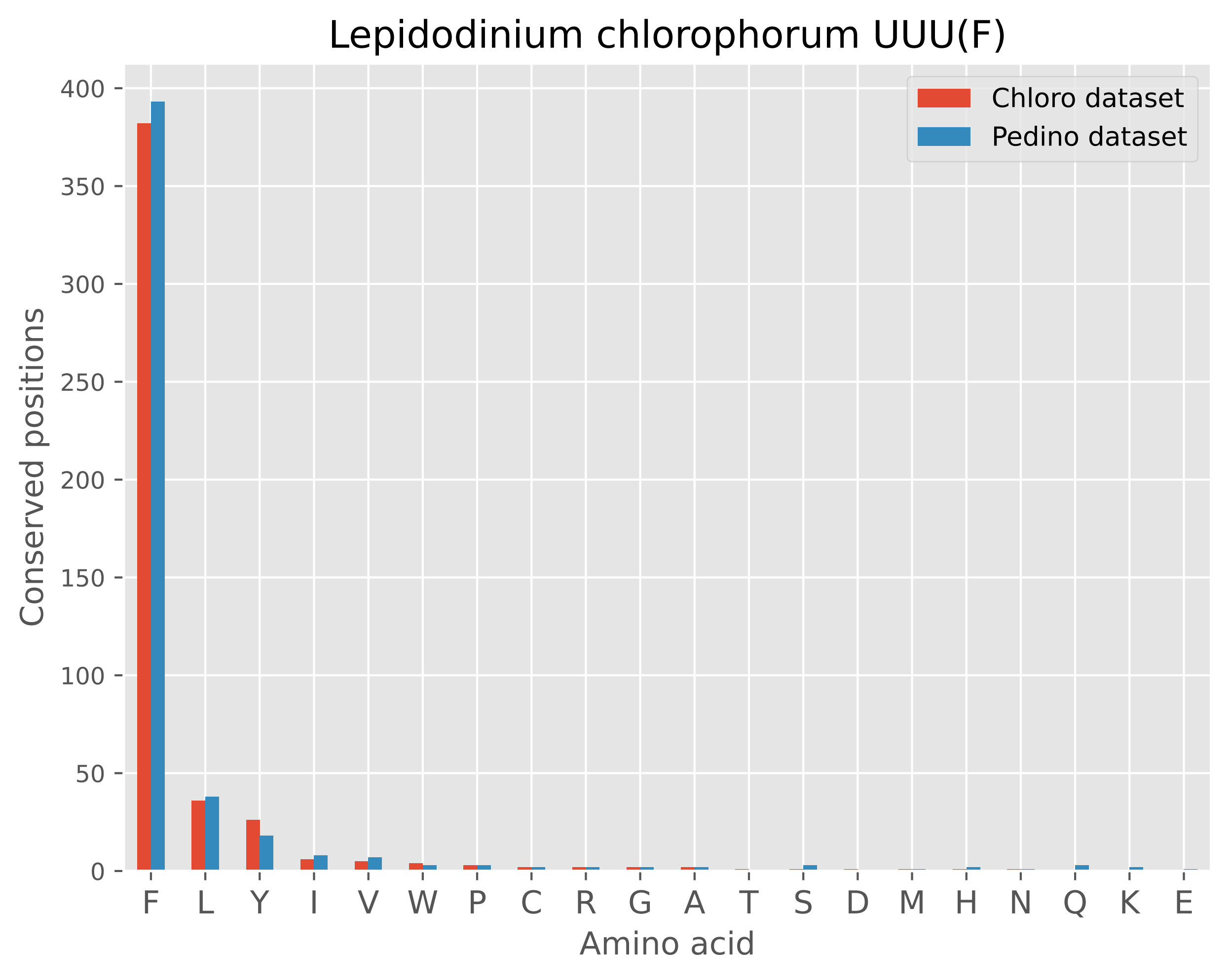
Lepidodinium chlorophorum UUU(F)
Chloro dataset
Pedino dataset
Conserved positions
Amino acid
0
50
100
150
200
250
300
350
400
F
L
Y
I
V
W
P
C
R
G
A
T
S
D
M
H
N
Q
K
E

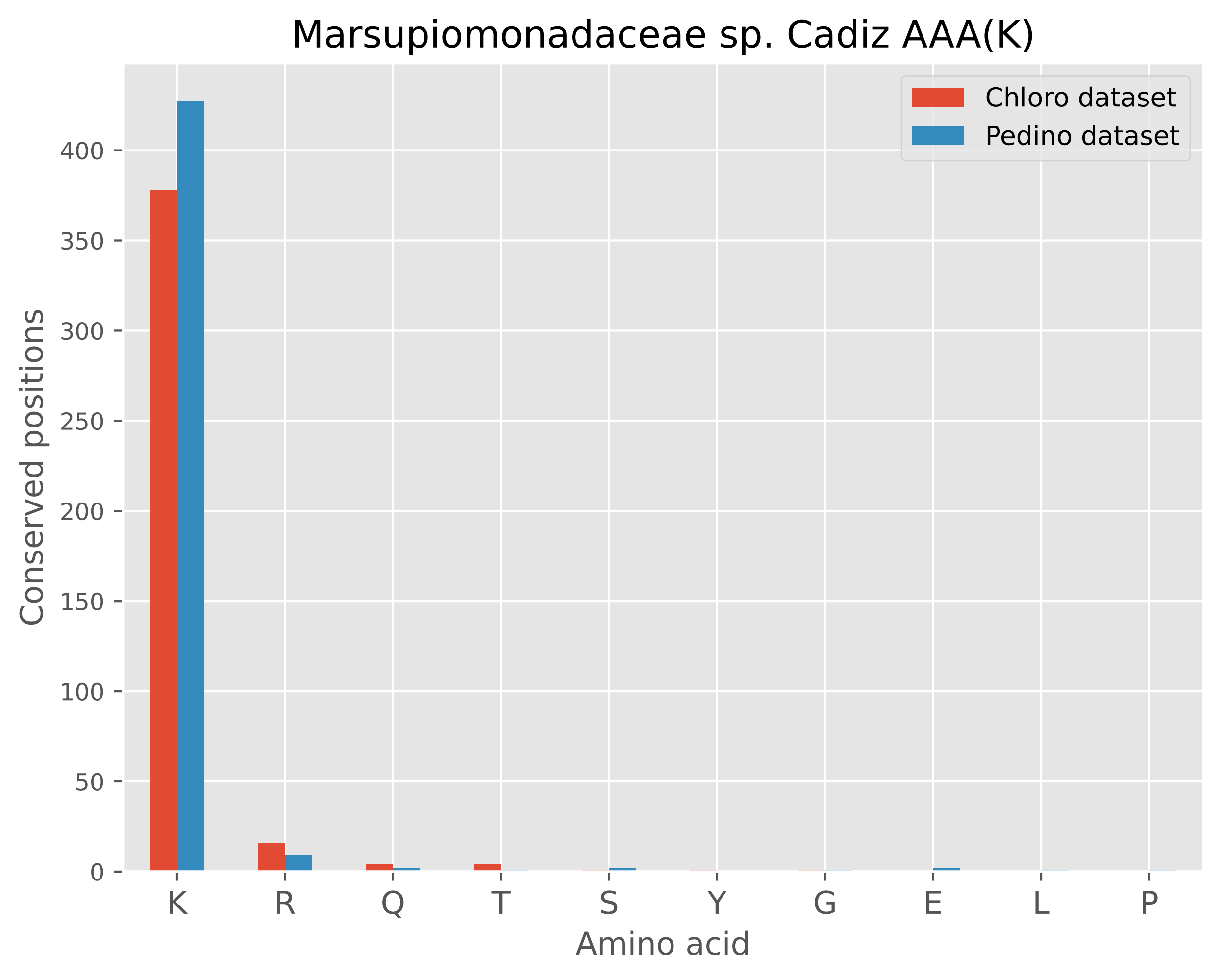

Marsupiomonadaceae sp. Cadiz AAA(K)
Chloro dataset
Pedino dataset
Conserved positions
0
50
100
150
200
250
300
350
400
K
R
Q
T
S
Y
G
E
L
P
Amino acid

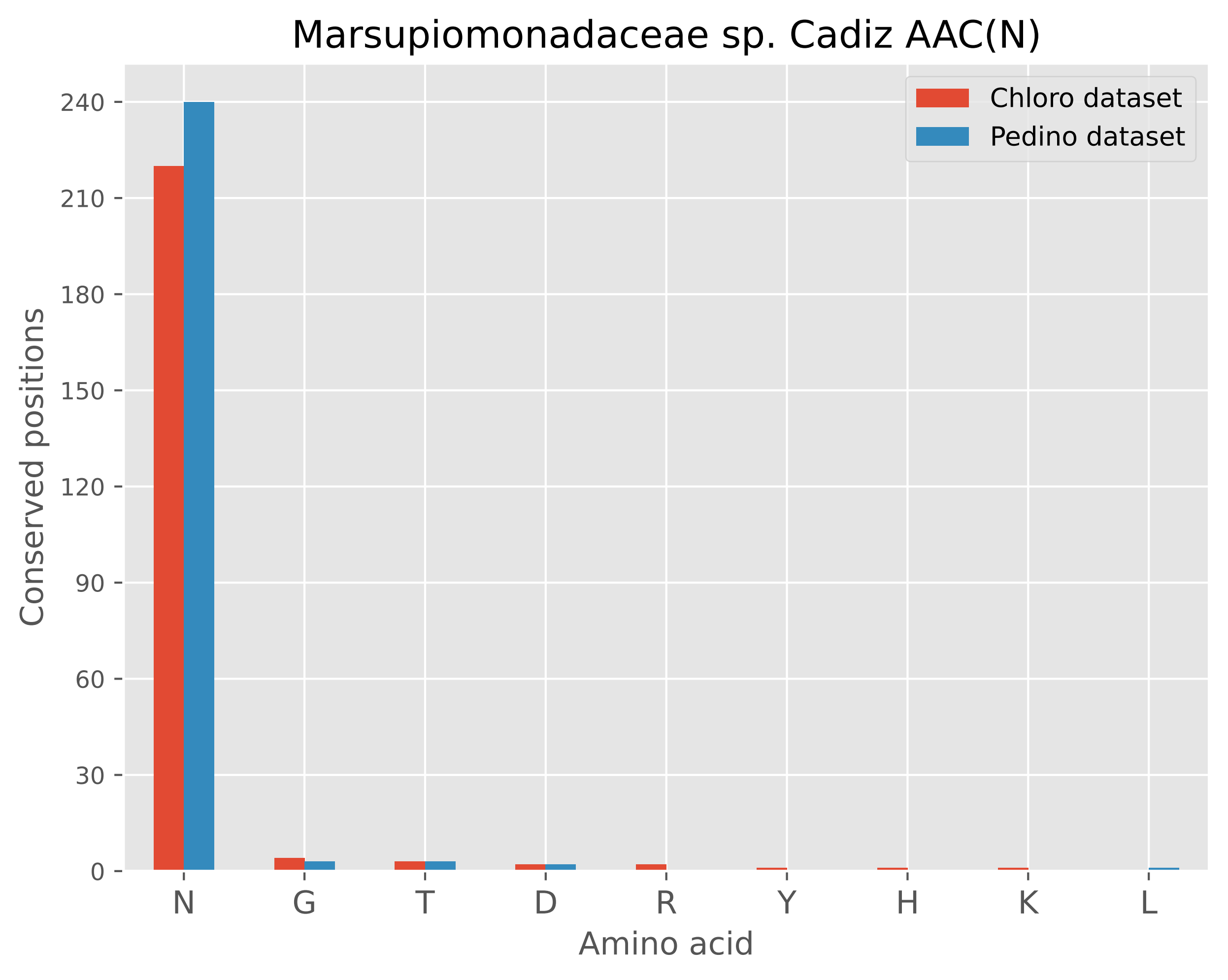

Marsupiomonadaceae sp. Cadiz AAC(N)
Chloro dataset
Pedino dataset
Conserved positions
240
210
180
150
120
90
60
30
0
N
G
T
D
R
Y
H
K
L
Amino acid

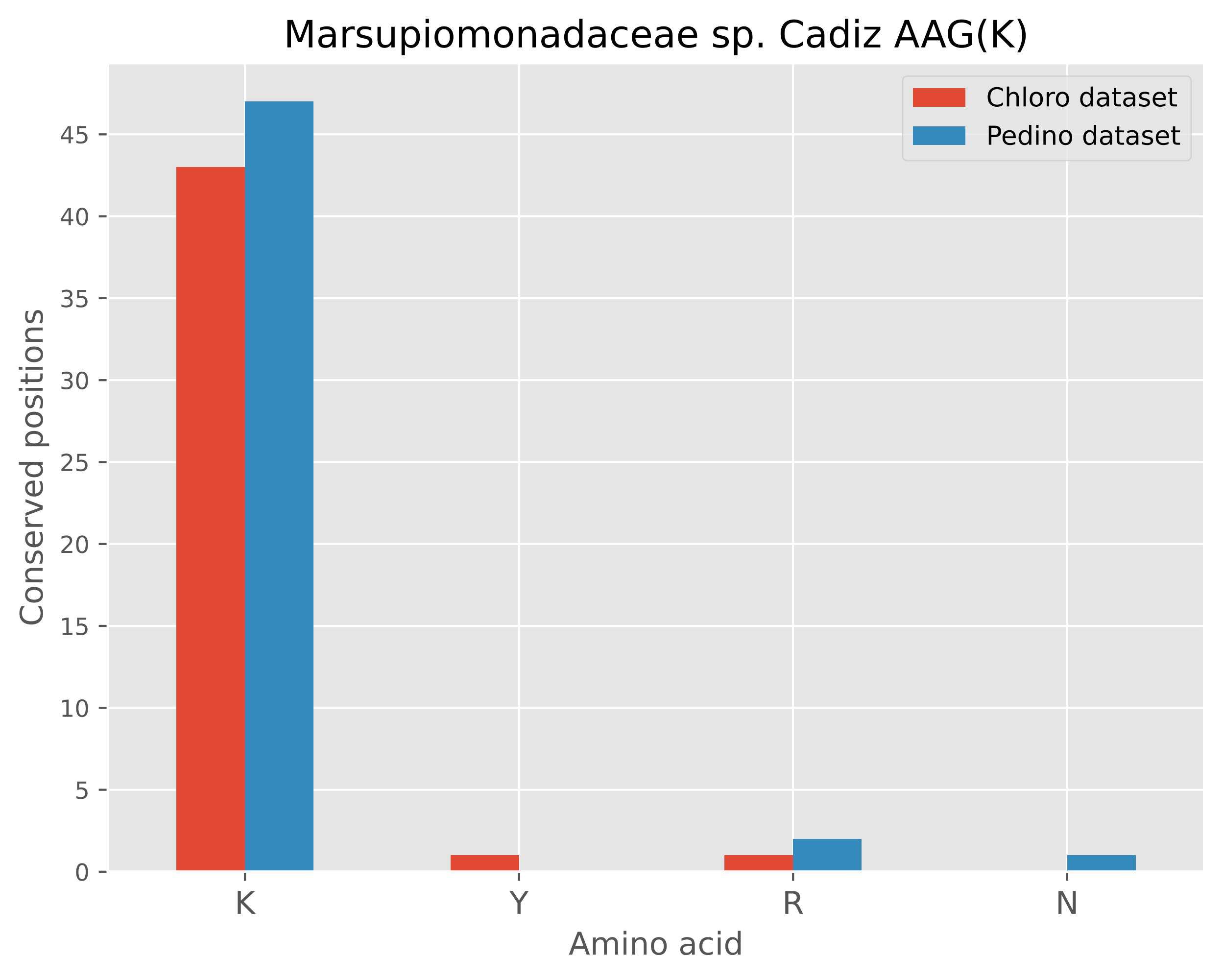

Marsupiomonadaceae sp. Cadiz AAG(K)
Chloro dataset
Pedino dataset
Conserved positions
0
5
10
15
20
25
30
35
40
45
K
Y
R
N
Amino acid

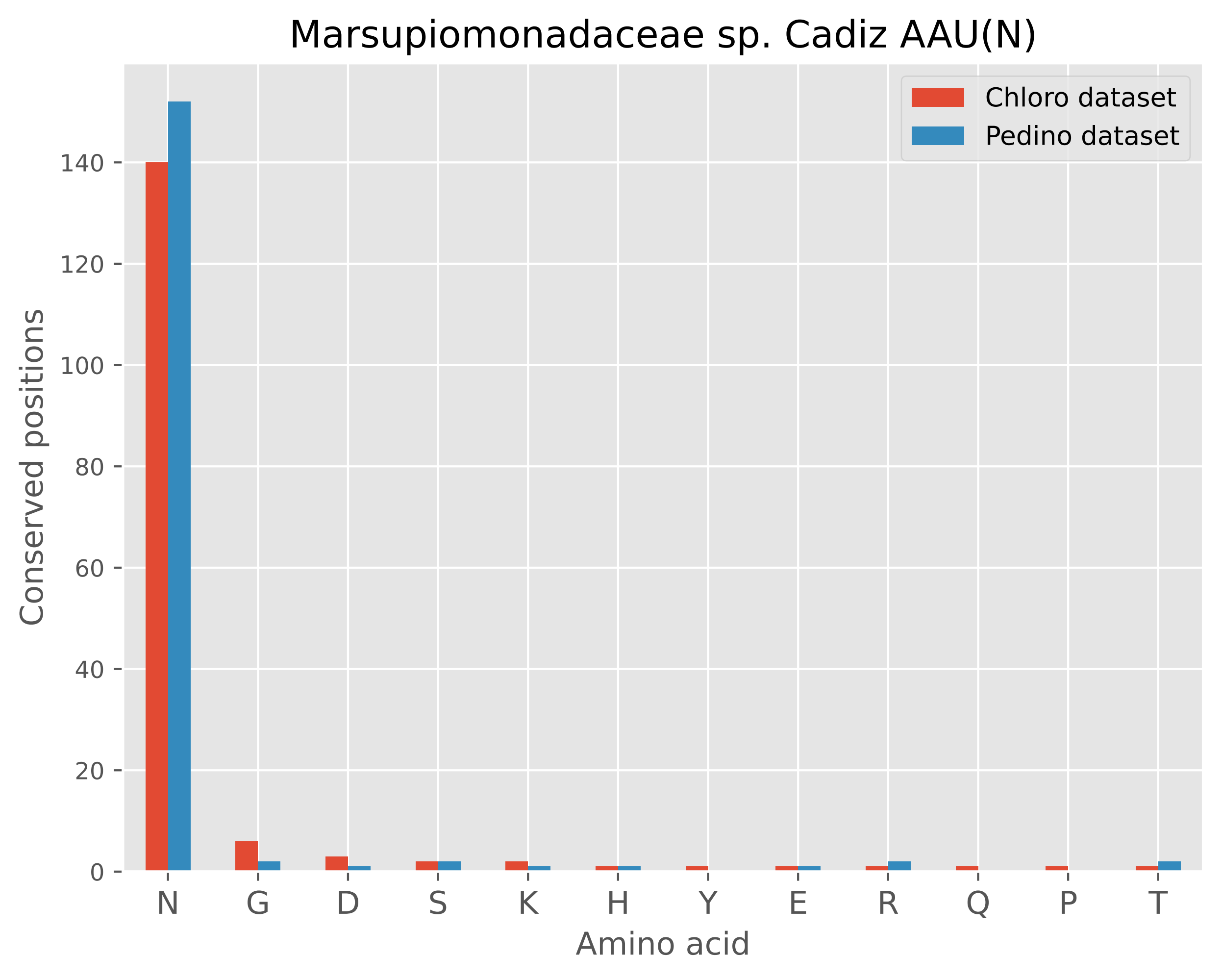
Marsupiomonadaceae sp. Cadiz AAU(N)
Chloro dataset
Pedino dataset
Conserved positions
0
20
40
60
80
100
120
140
N
G
D
S
K
H
Y
E
R
Q
P
T
Amino acid

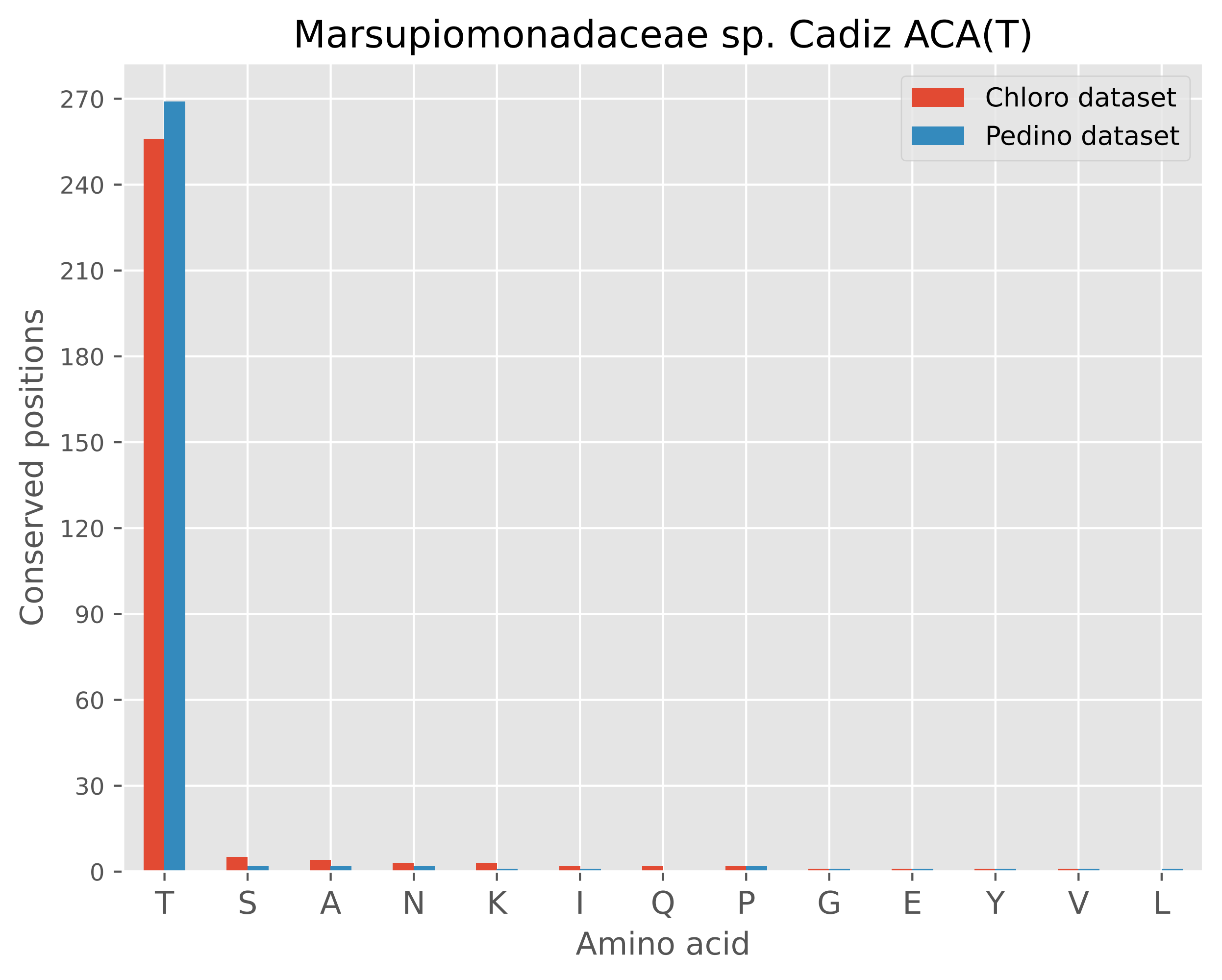

Marsupiomonadaceae sp. Cadiz ACA(T)
Chloro dataset
Pedino dataset
Conserved positions
0
30
60
90
120
150
180
210
240
270
T
S
A
N
K
I
Q
P
G
E
Y
V
L
Amino acid

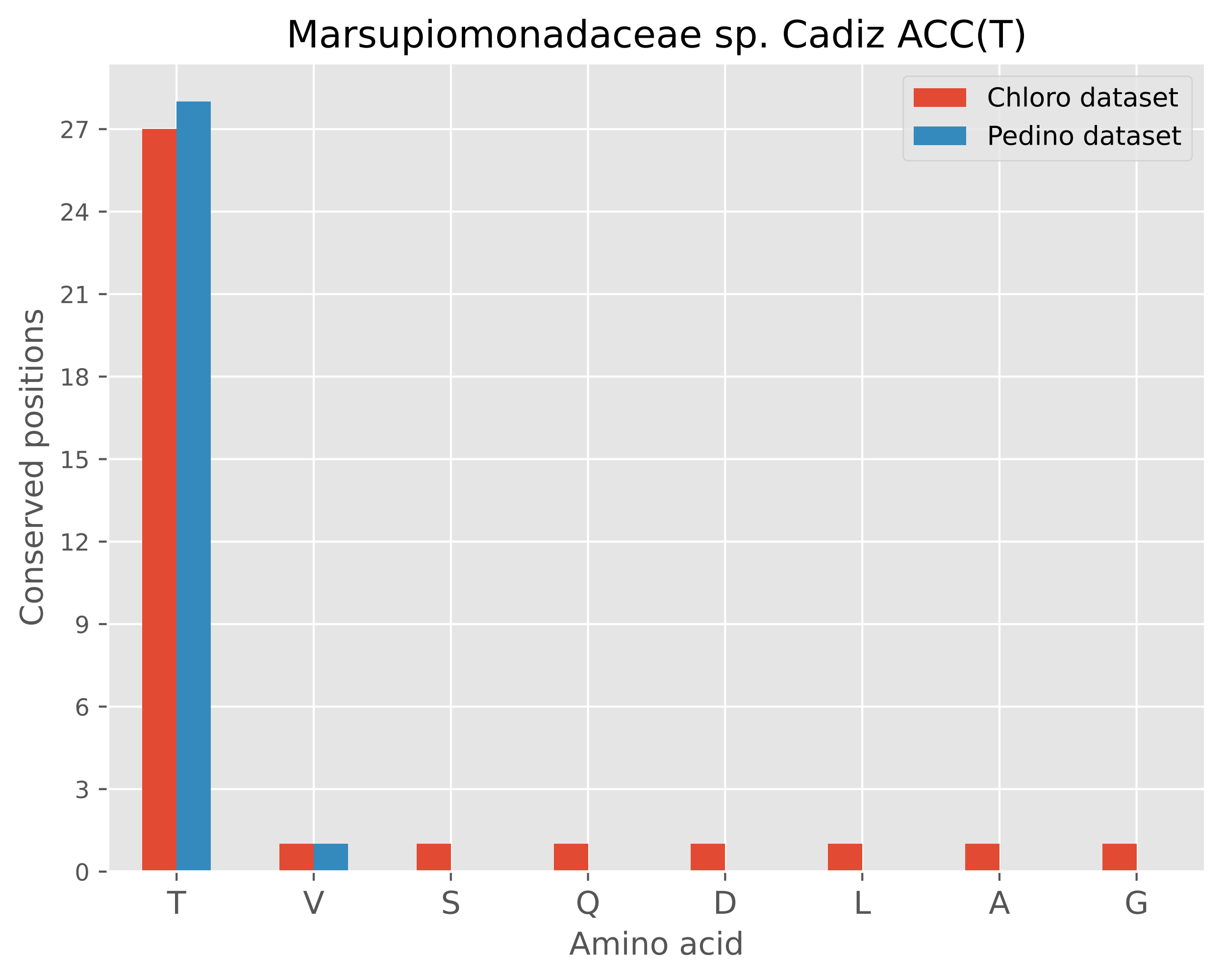
Marsupiomonadaceae sp. Cadiz ACC(T)
Chloro dataset
Pedino dataset
Conserved positions
0
3
6
9
12
15
18
21
24
27
T
V
S
Q
D
L
A
G
Amino acid

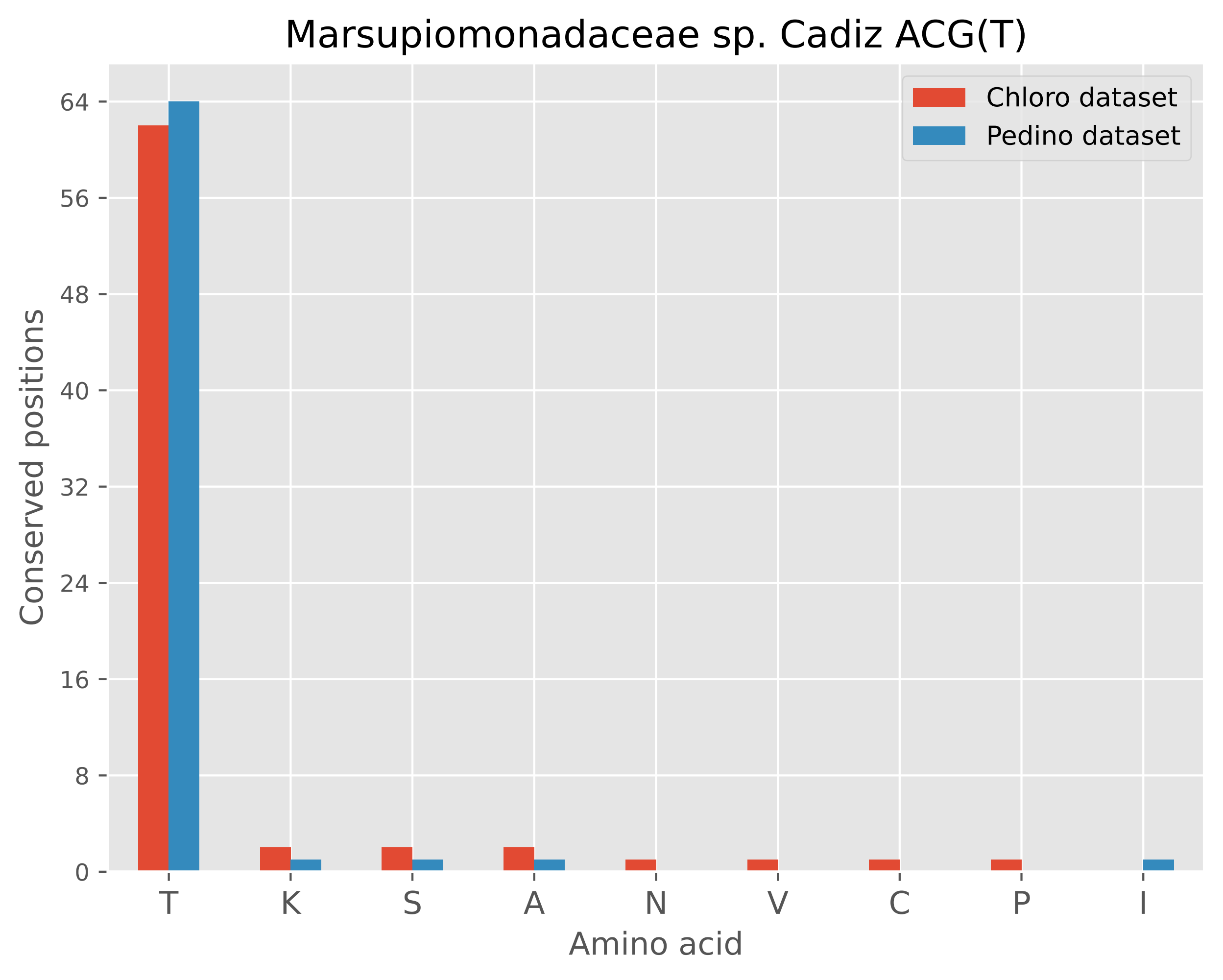
Marsupiomonadaceae sp. Cadiz ACG(T)
Chloro dataset
Pedino dataset
Conserved positions
0
8
16
24
32
40
48
56
64
T
K
S
A
N
V
C
P
I
Amino acid

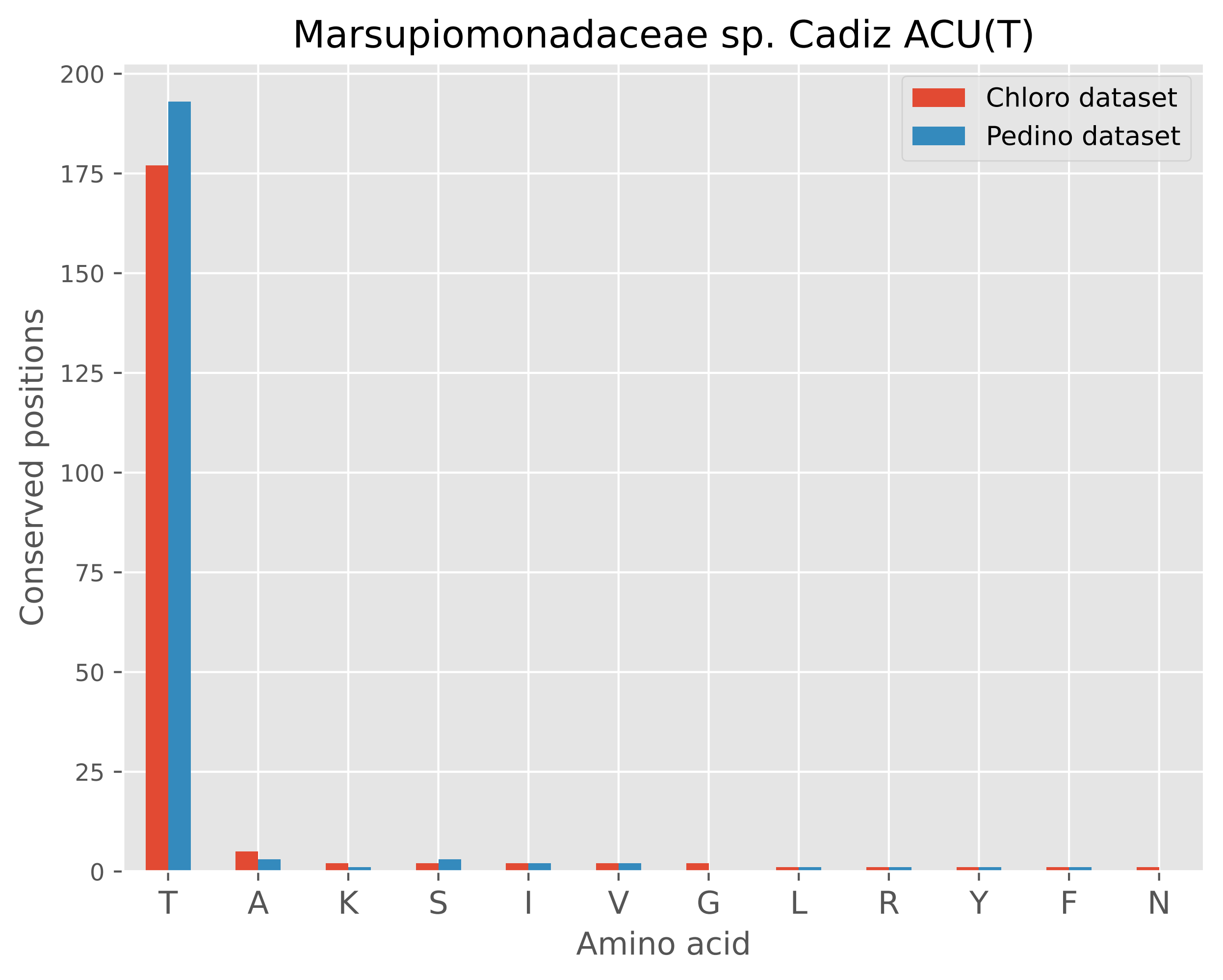

Marsupiomonadaceae sp. Cadiz ACU(T)
Chloro dataset
Pedino dataset
Conserved positions
0
25
50
75
100
125
150
175
200
T
A
K
S
I
V
G
L
R
Y
F
N
Amino acid

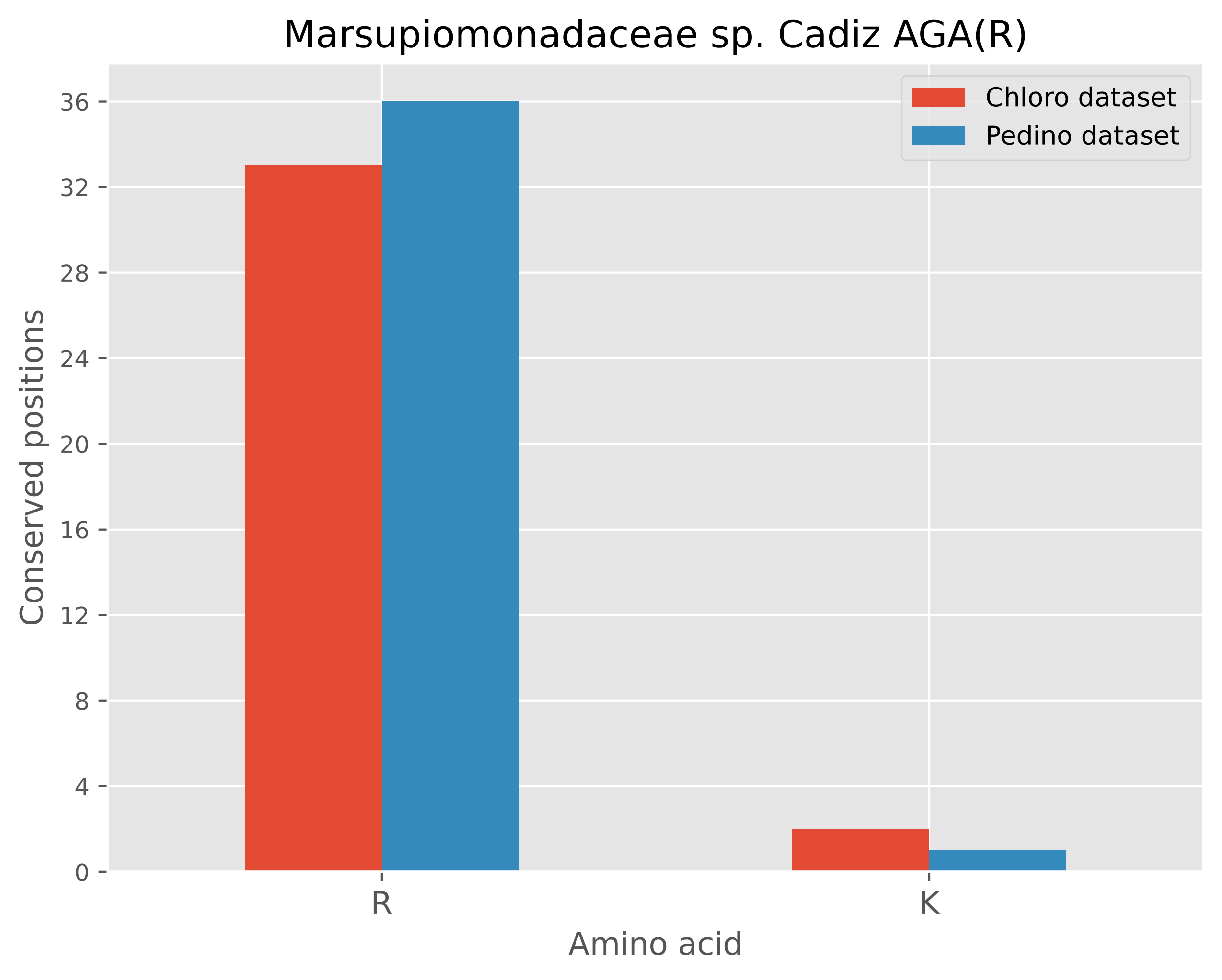

Marsupiomonadaceae sp. Cadiz AGA(R)
Chloro dataset
Pedino dataset
Conserved positions
36
32
28
24
20
16
12
8
4
0
R
K
Amino acid

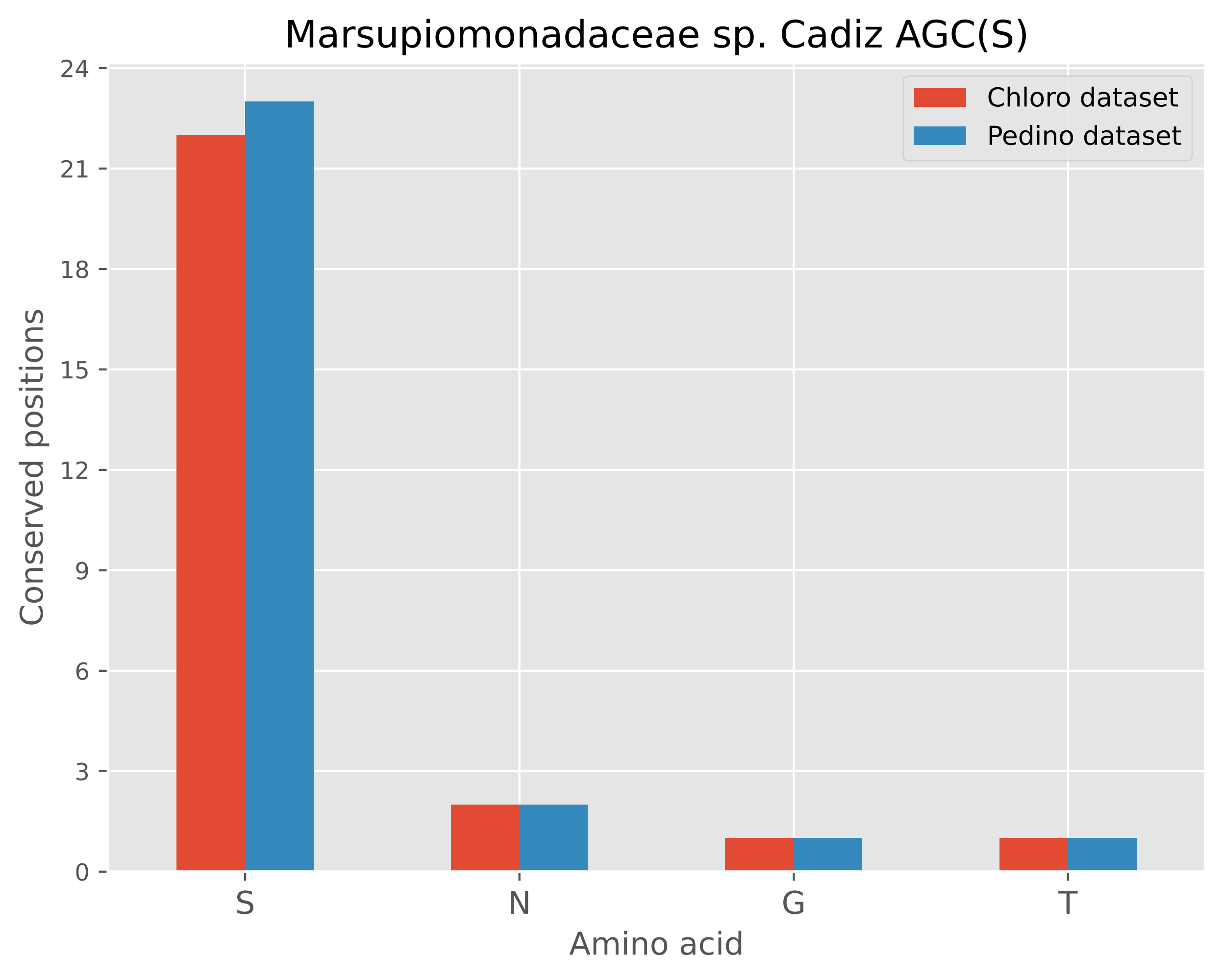

Marsupiomonadaceae sp. Cadiz AGC(S)
Chloro dataset
Pedino dataset
Conserved positions
0
3
6
9
12
15
18
21
24
S
N
G
T
Amino acid

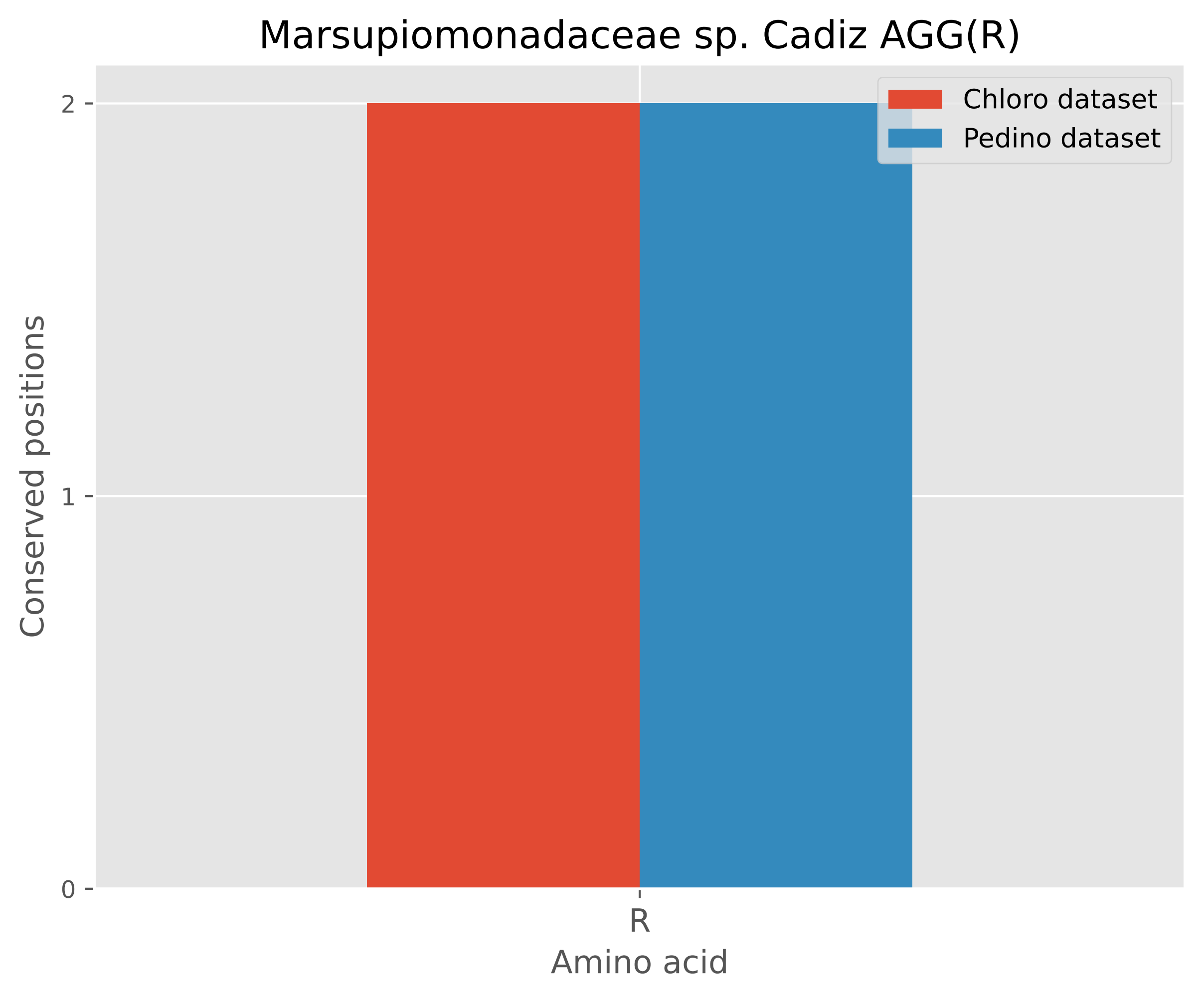
Marsupiomonadaceae sp. Cadiz AGG(R)
Chloro dataset
Pedino dataset
Conserved positions
0
1
2
R
Amino acid

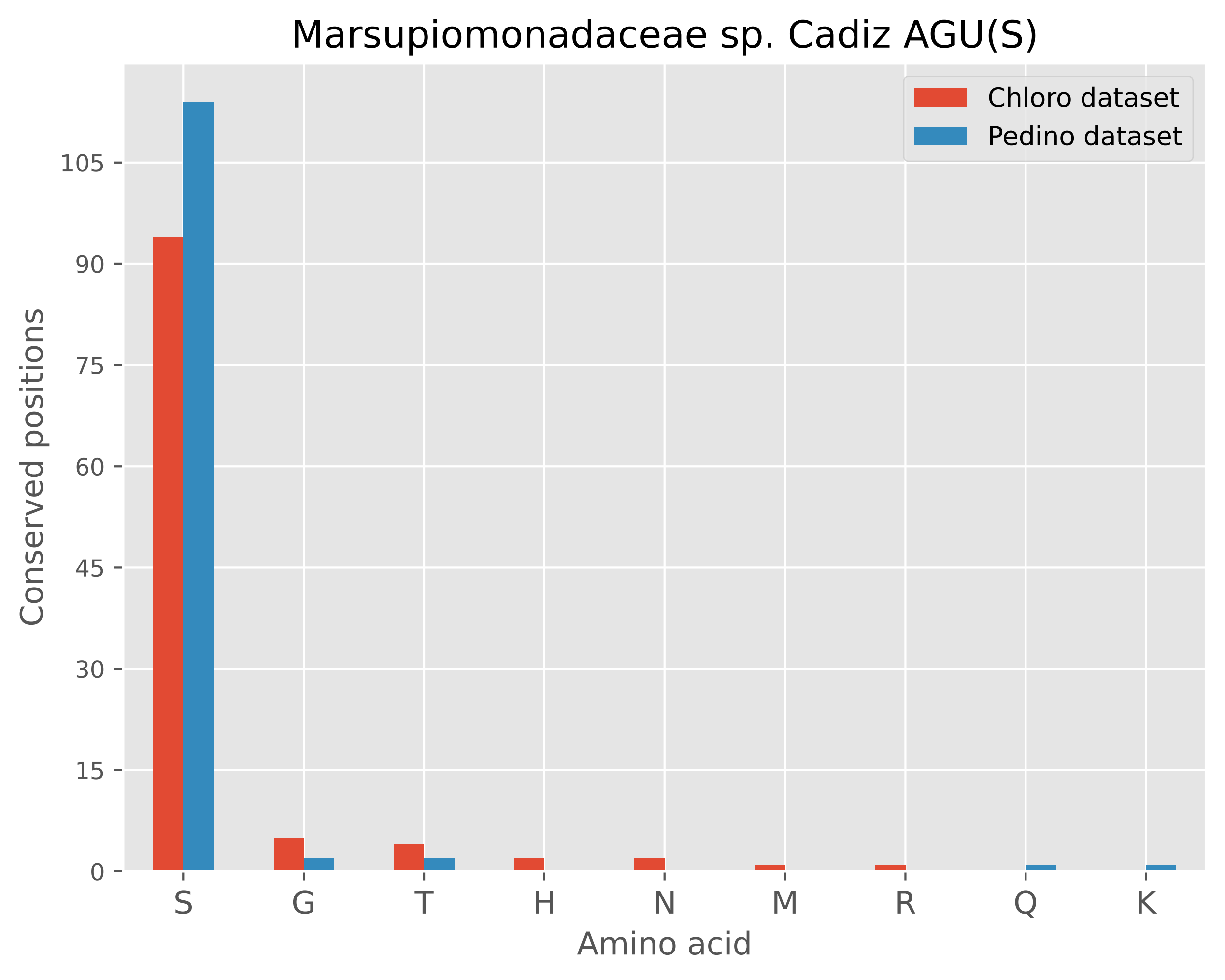

Marsupiomonadaceae sp. Cadiz AGU(S)
Chloro dataset
Pedino dataset
Conserved positions
0
15
30
45
60
75
90
105
S
G
T
H
N
M
R
Q
K
Amino acid

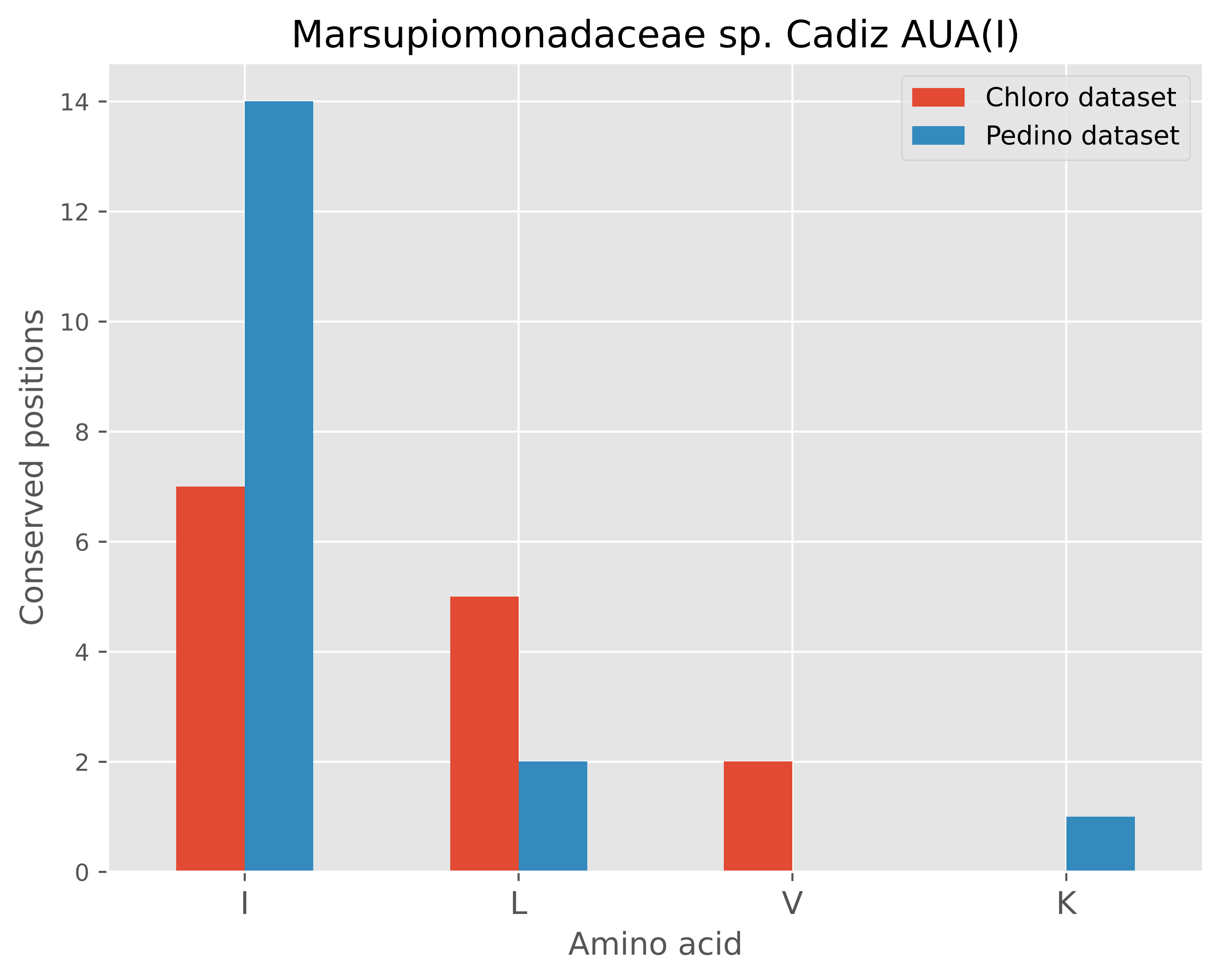

Marsupiomonadaceae sp. Cadiz AUA(I)
Chloro dataset
Pedino dataset
Conserved positions
0
2
4
6
8
10
12
14
I
L
V
K
Amino acid

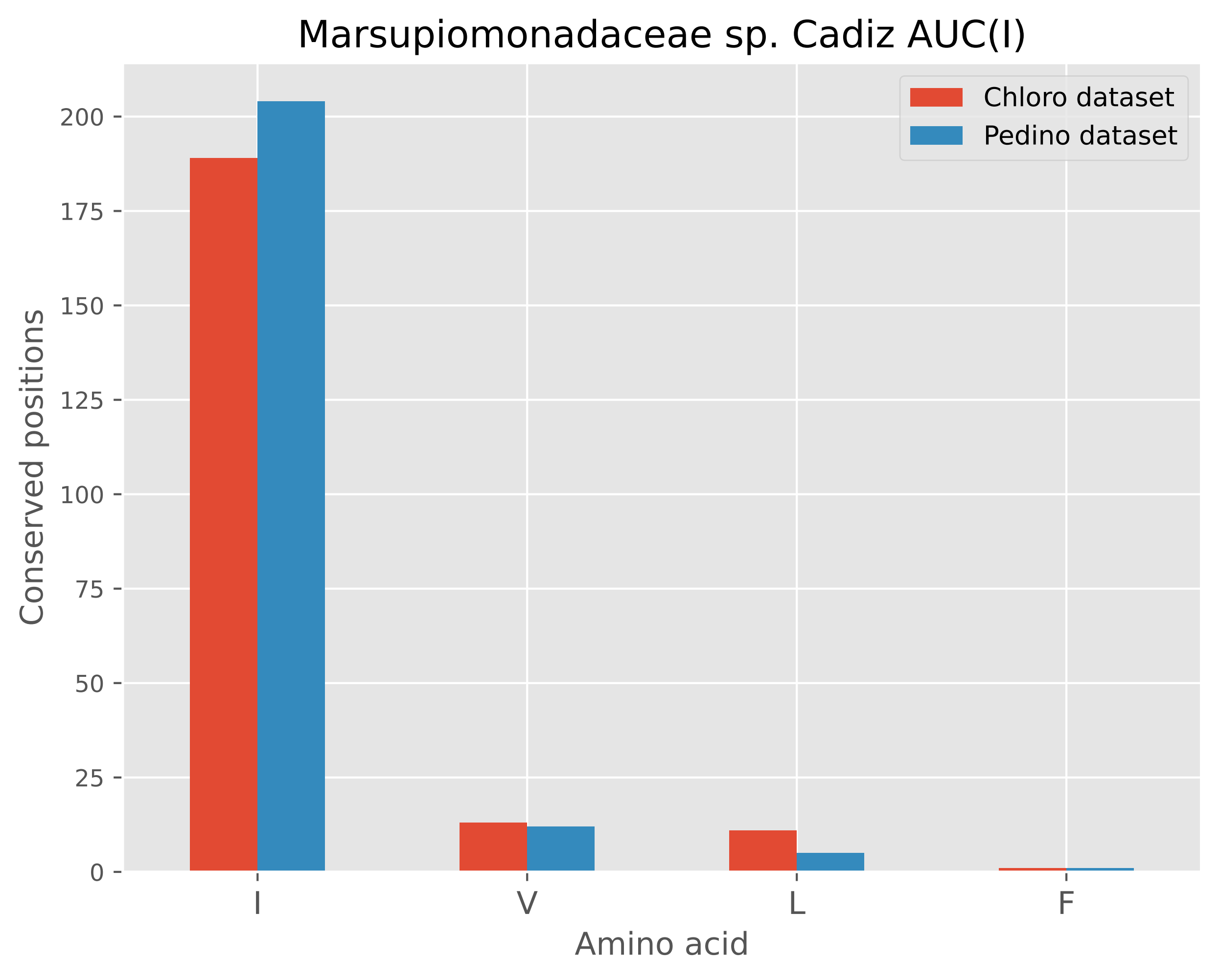

Marsupiomonadaceae sp. Cadiz AUC(I)
Chloro dataset
Pedino dataset
Conserved positions
0
25
50
75
100
125
150
175
200
I
V
L
F
Amino acid

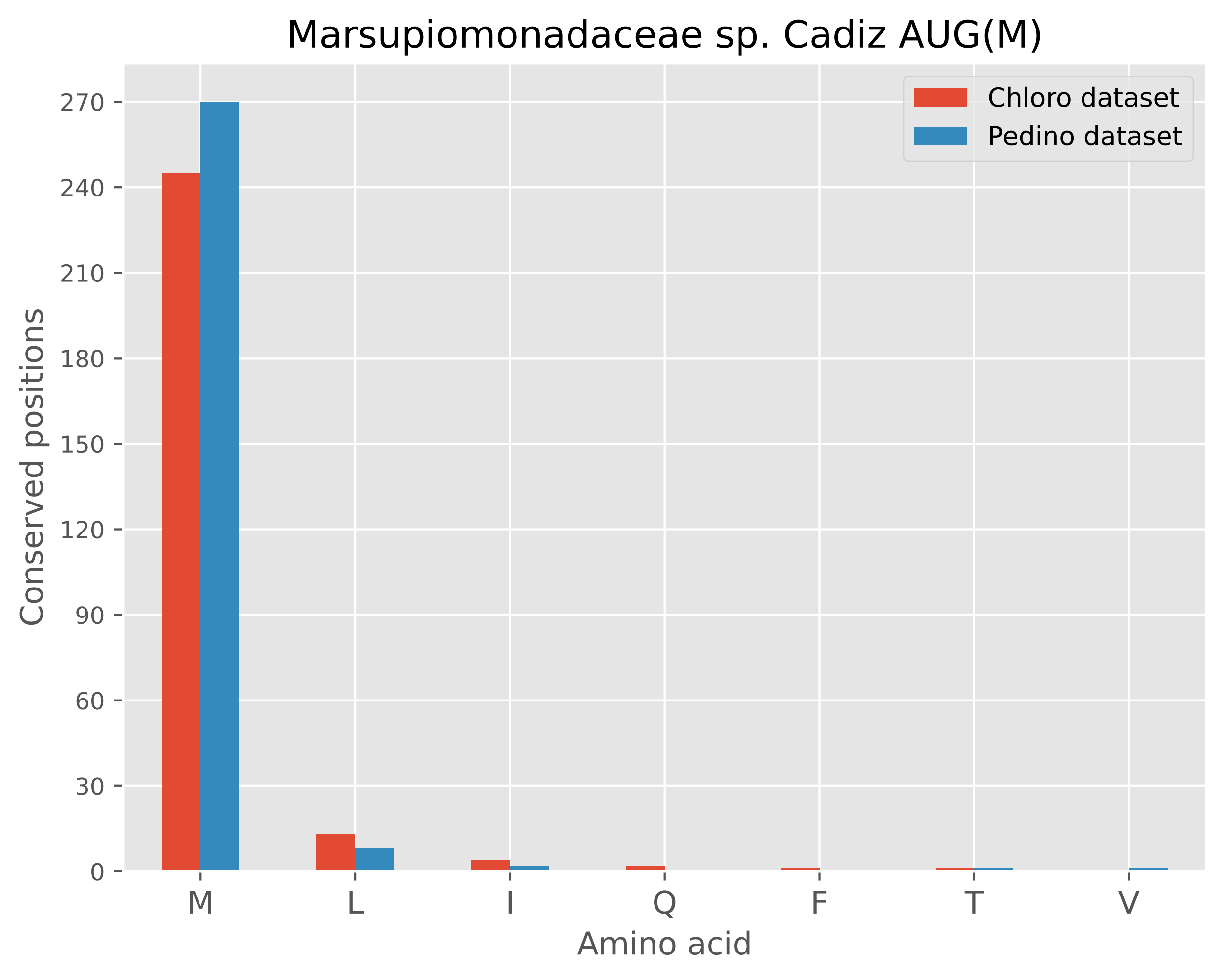
Marsupiomonadaceae sp. Cadiz AUG(M)
Chloro dataset
Pedino dataset
Conserved positions
Amino acid
0
30
60
90
120
150
180
210
240
270
M
L
I
Q
F
T
V

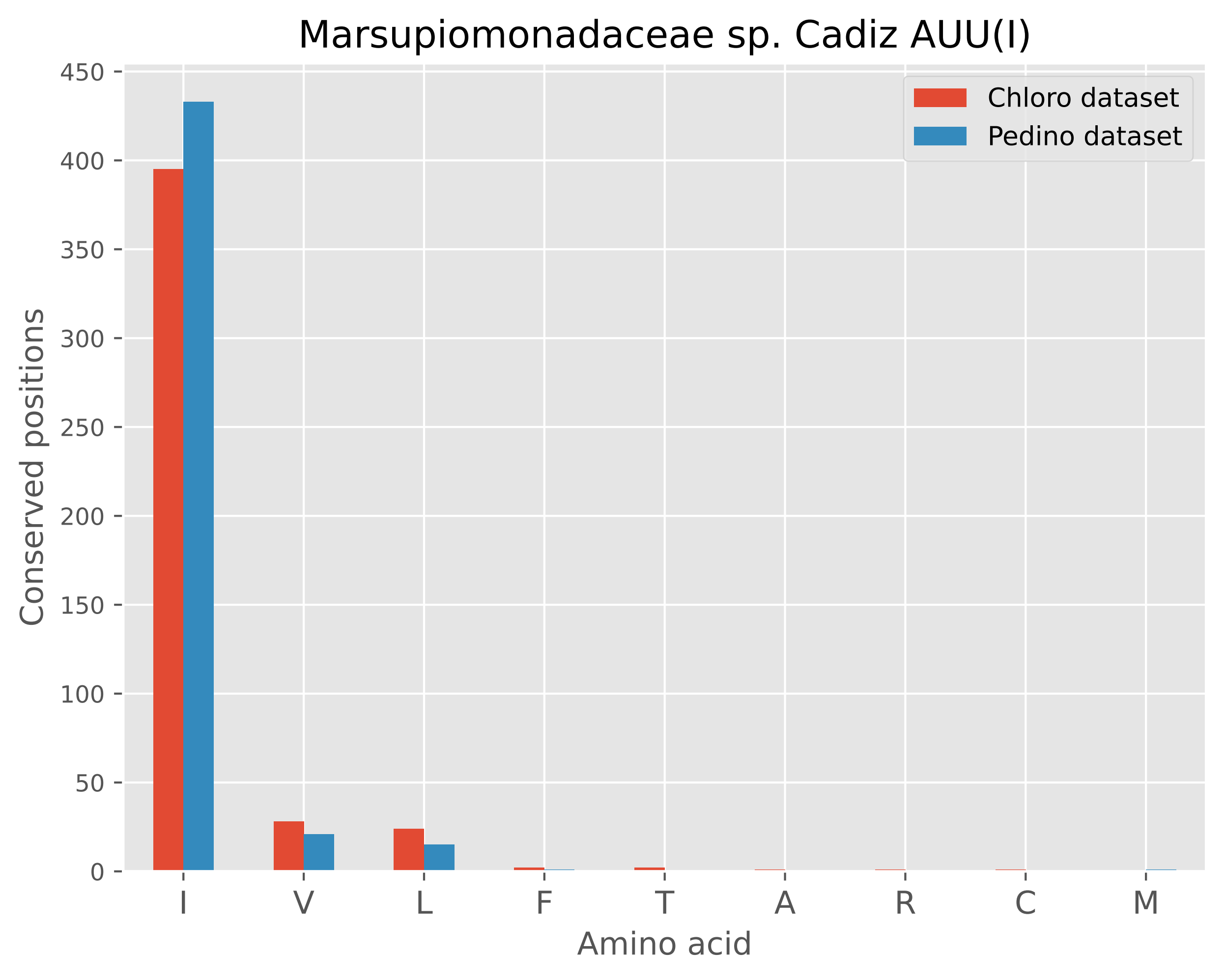
Marsupiomonadaceae sp. Cadiz AUU(I)
Chloro dataset
Pedino dataset
Conserved positions
Amino acid
0
50
100
150
200
250
300
350
400
450
I
V
L
F
T
A
R
C
M

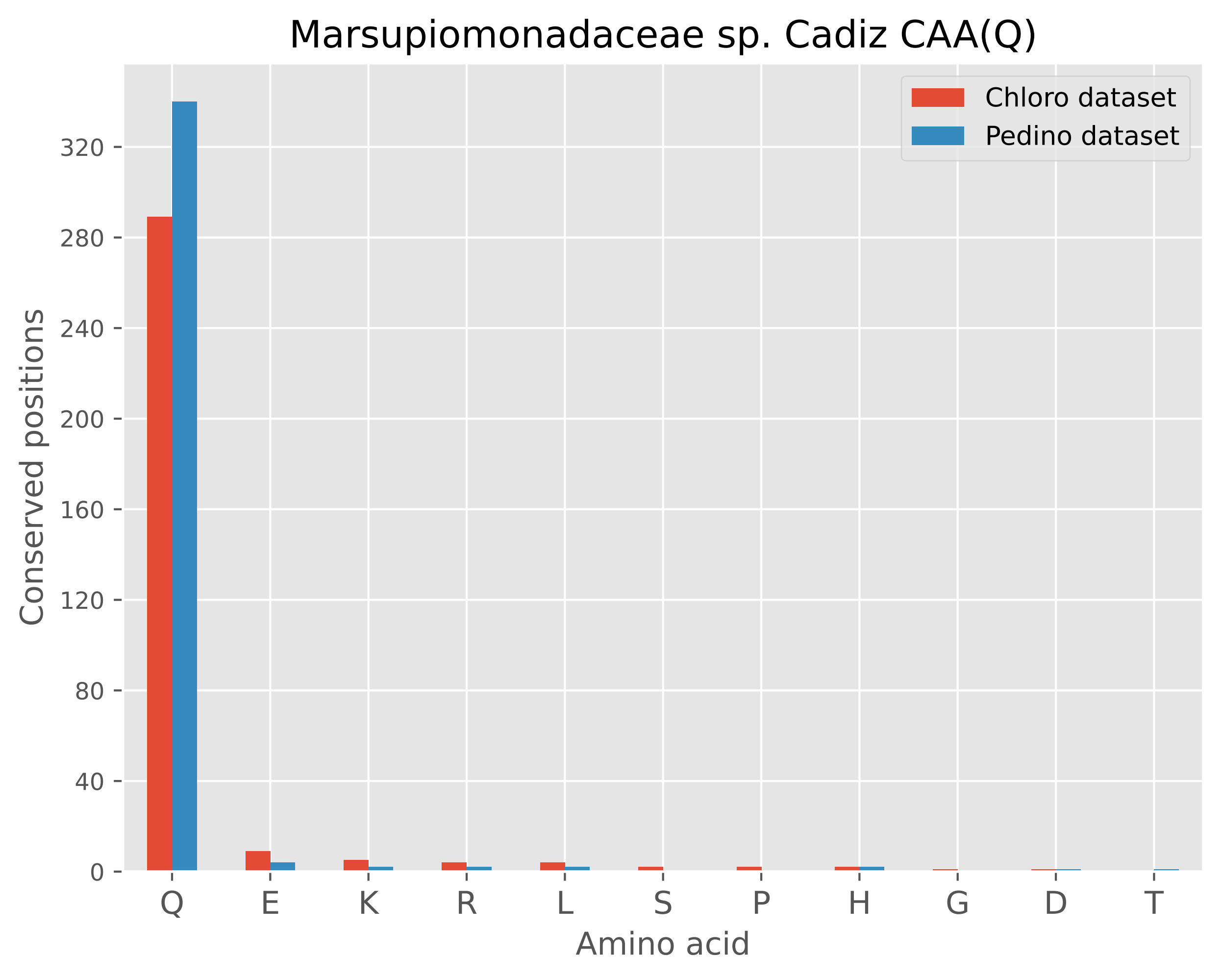

Marsupiomonadaceae sp. Cadiz CAA(Q)
Chloro dataset
Pedino dataset
Conserved positions
320
280
240
200
160
120
80
40
0
Q
E
K
R
L
S
P
H
G
D
T
Amino acid

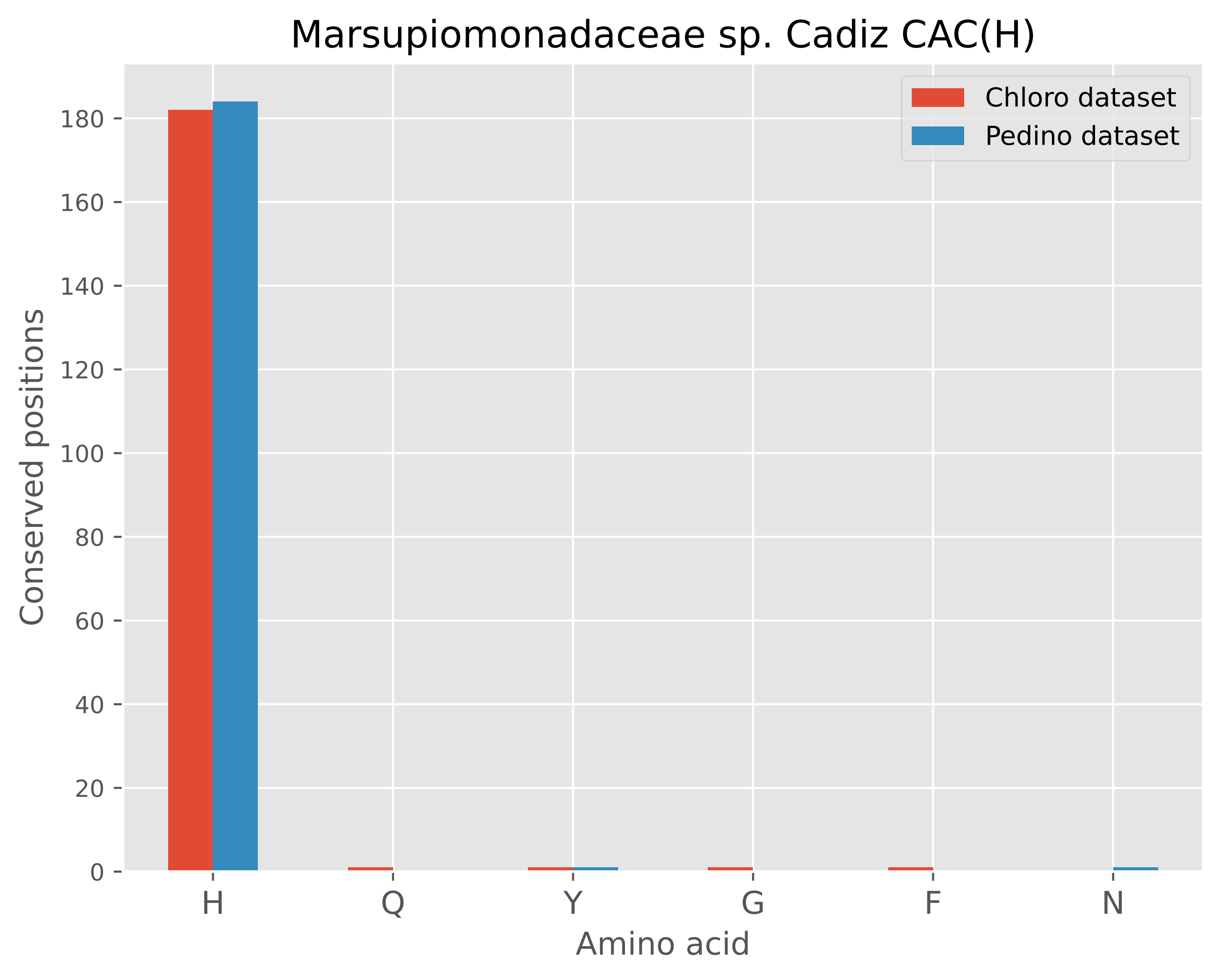

Marsupiomonadaceae sp. Cadiz CAC(H)
Chloro dataset
Pedino dataset
Conserved positions
0
20
40
60
80
100
120
140
160
180
H
Q
Y
G
F
N
Amino acid

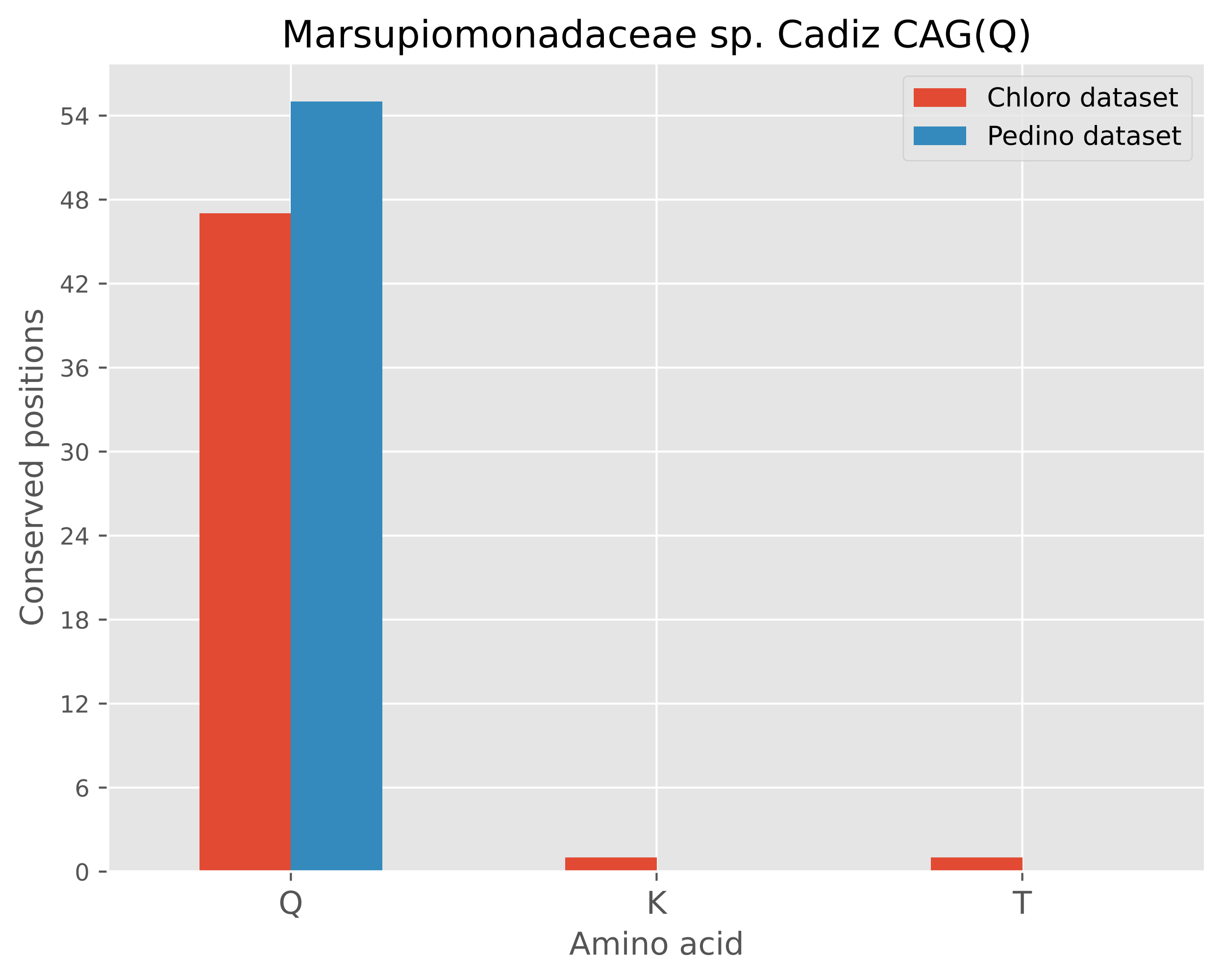

Marsupiomonadaceae sp. Cadiz CAG(Q)
Chloro dataset
Pedino dataset
Conserved positions
0
6
12
18
24
30
36
42
48
54
Q
K
T
Amino acid

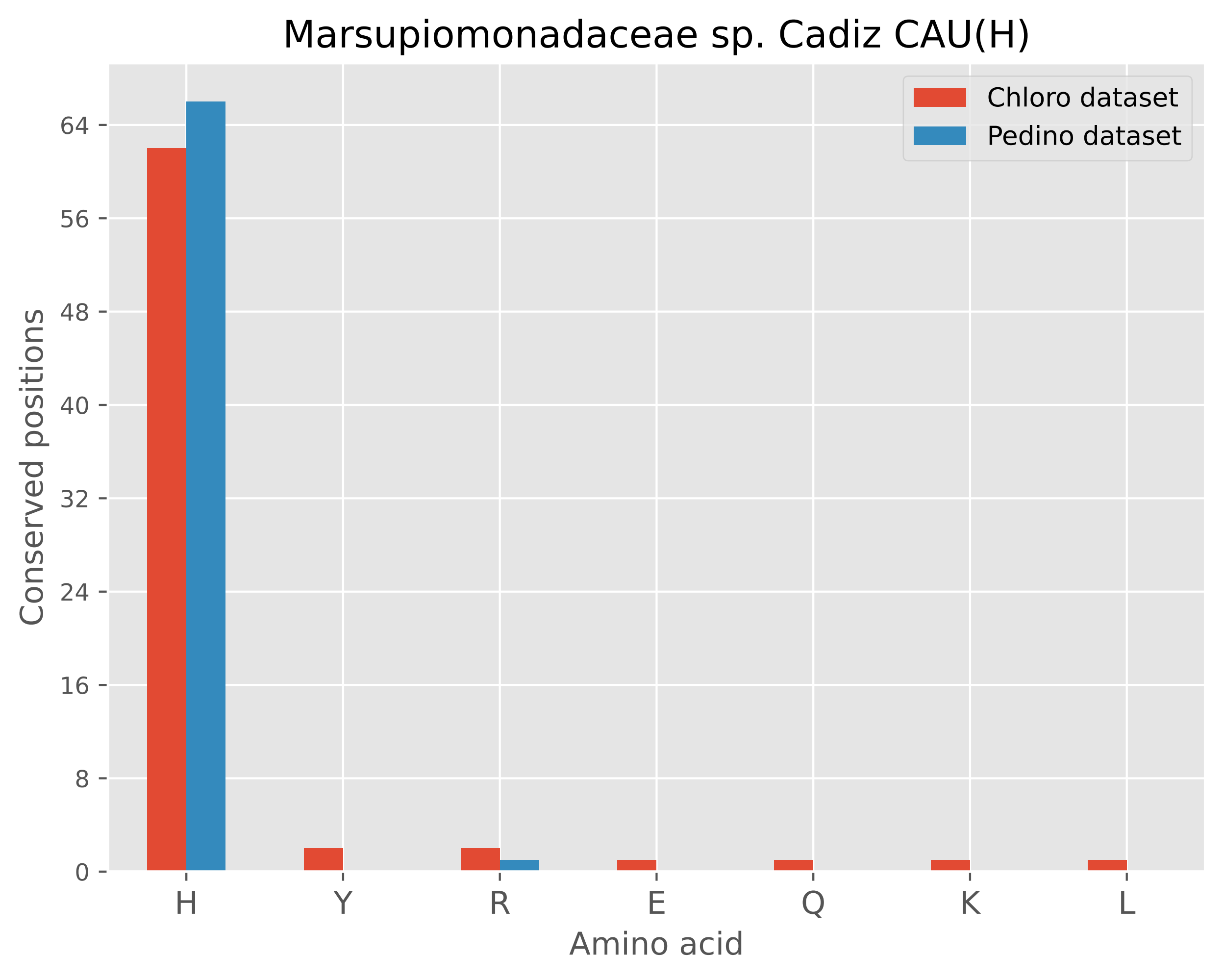
Marsupiomonadaceae sp. Cadiz CAU(H)
Chloro dataset
Pedino dataset
Conserved positions
0
8
16
24
32
40
48
56
64
H
Y
R
E
Q
K
L
Amino acid

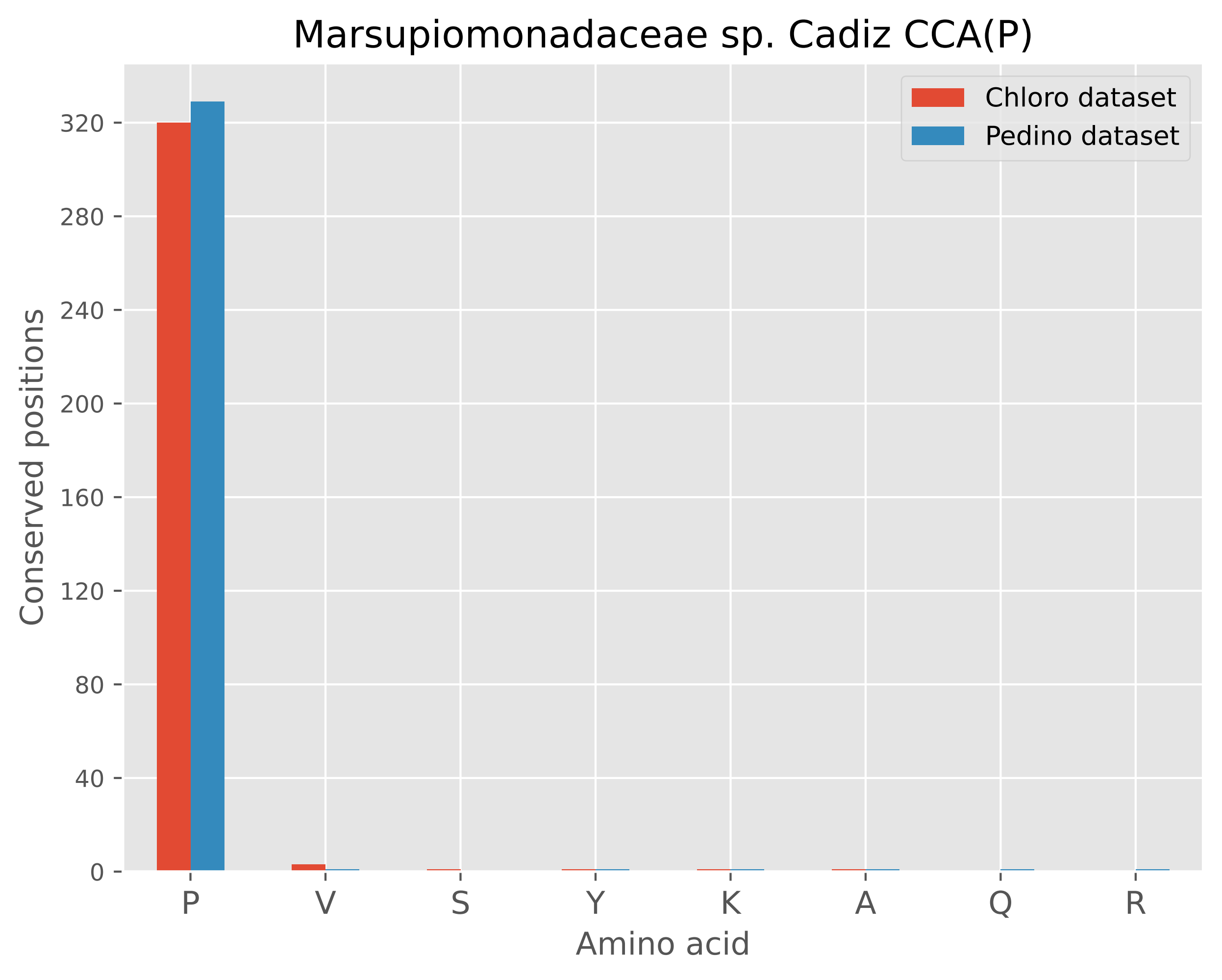

Marsupiomonadaceae sp. Cadiz CCA(P)
Chloro dataset
Pedino dataset
Conserved positions
0
40
80
120
160
200
240
280
320
P
V
S
Y
K
A
Q
R
Amino acid

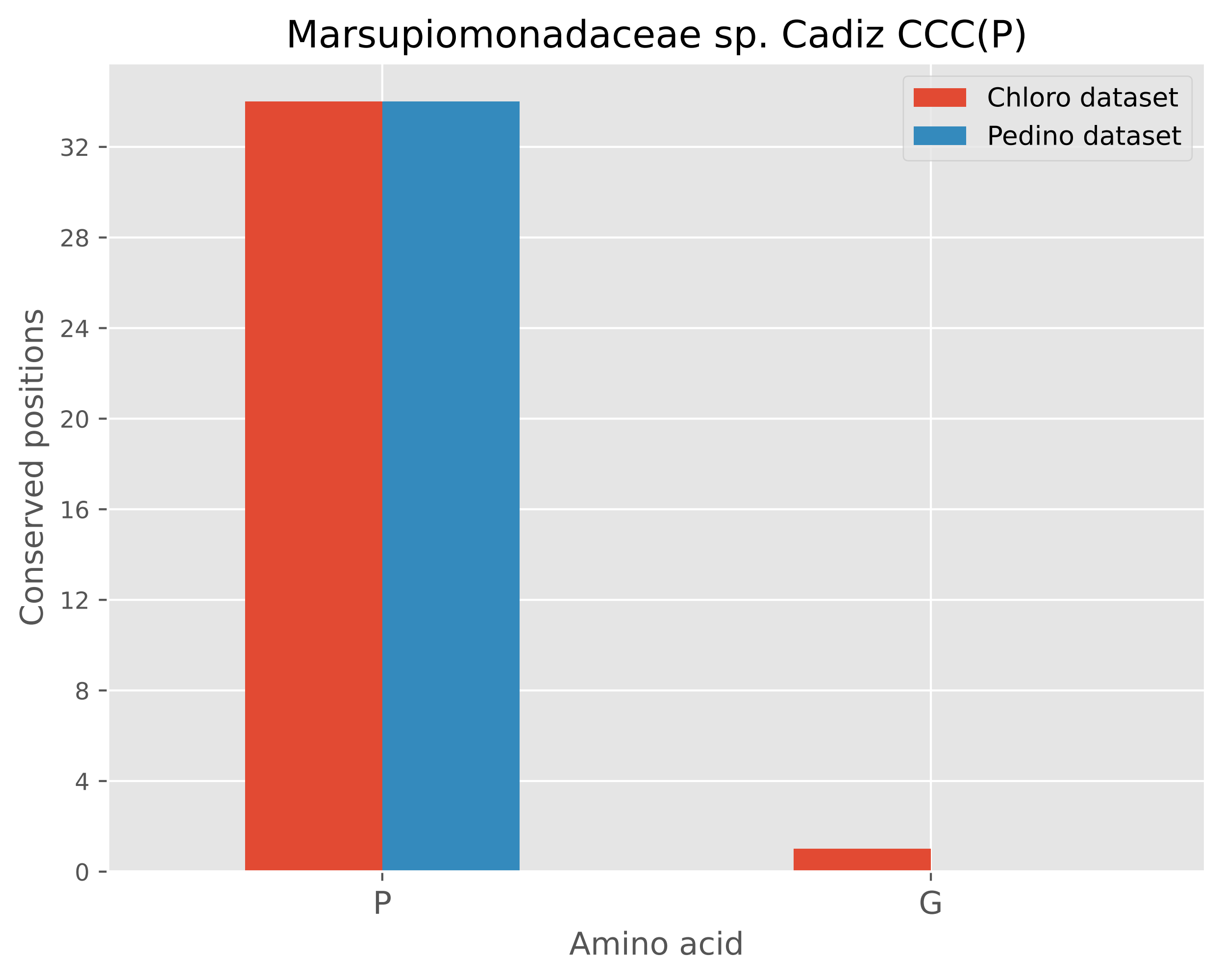
Marsupiomonadaceae sp. Cadiz CCC(P)
Chloro dataset
Pedino dataset
Conserved positions
0
4
8
12
16
20
24
28
32
P
G
Amino acid

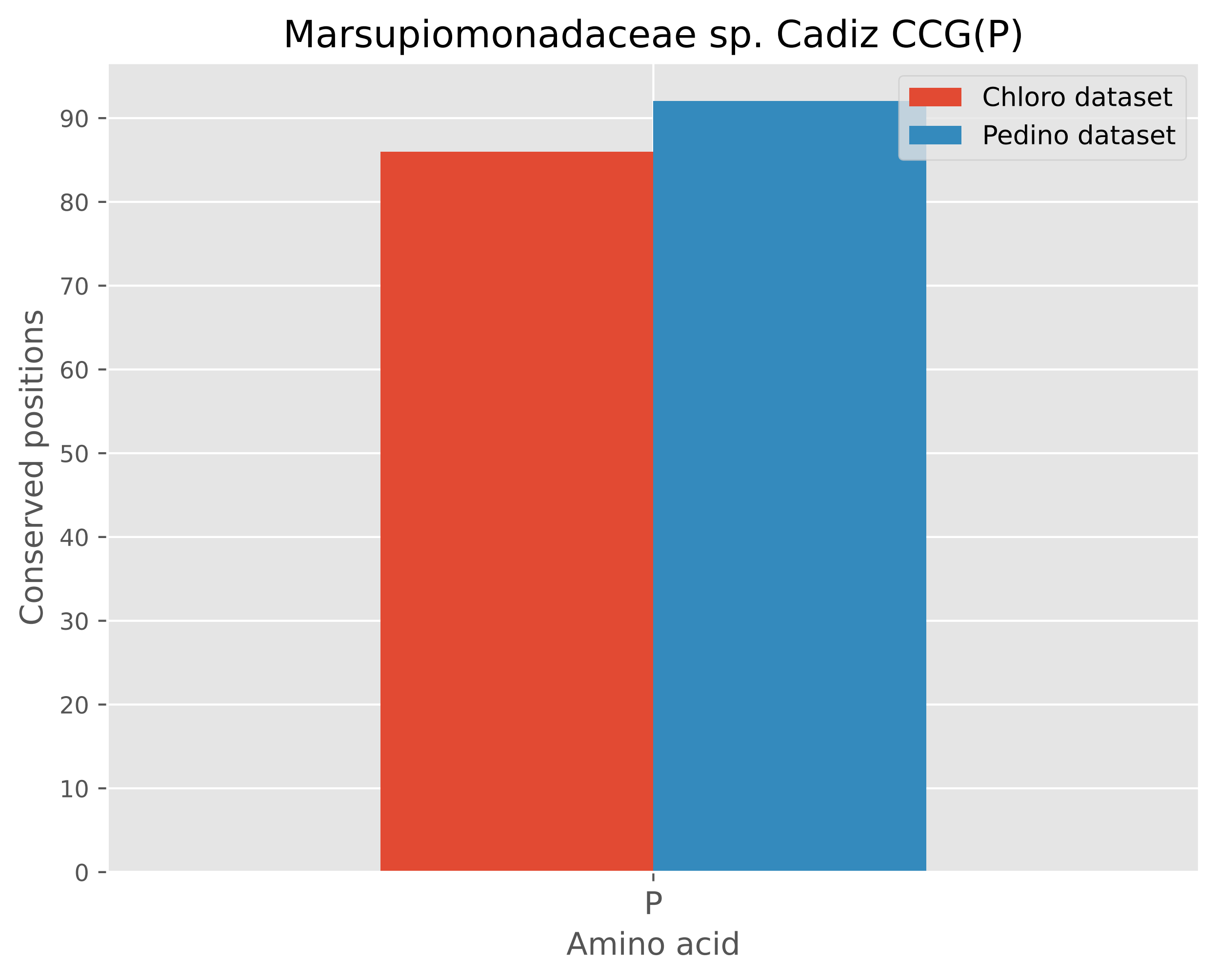

Marsupiomonadaceae sp. Cadiz CCG(P)
Chloro dataset
Pedino dataset
Conserved positions
0
10
20
30
40
50
60
70
80
90
P
Amino acid

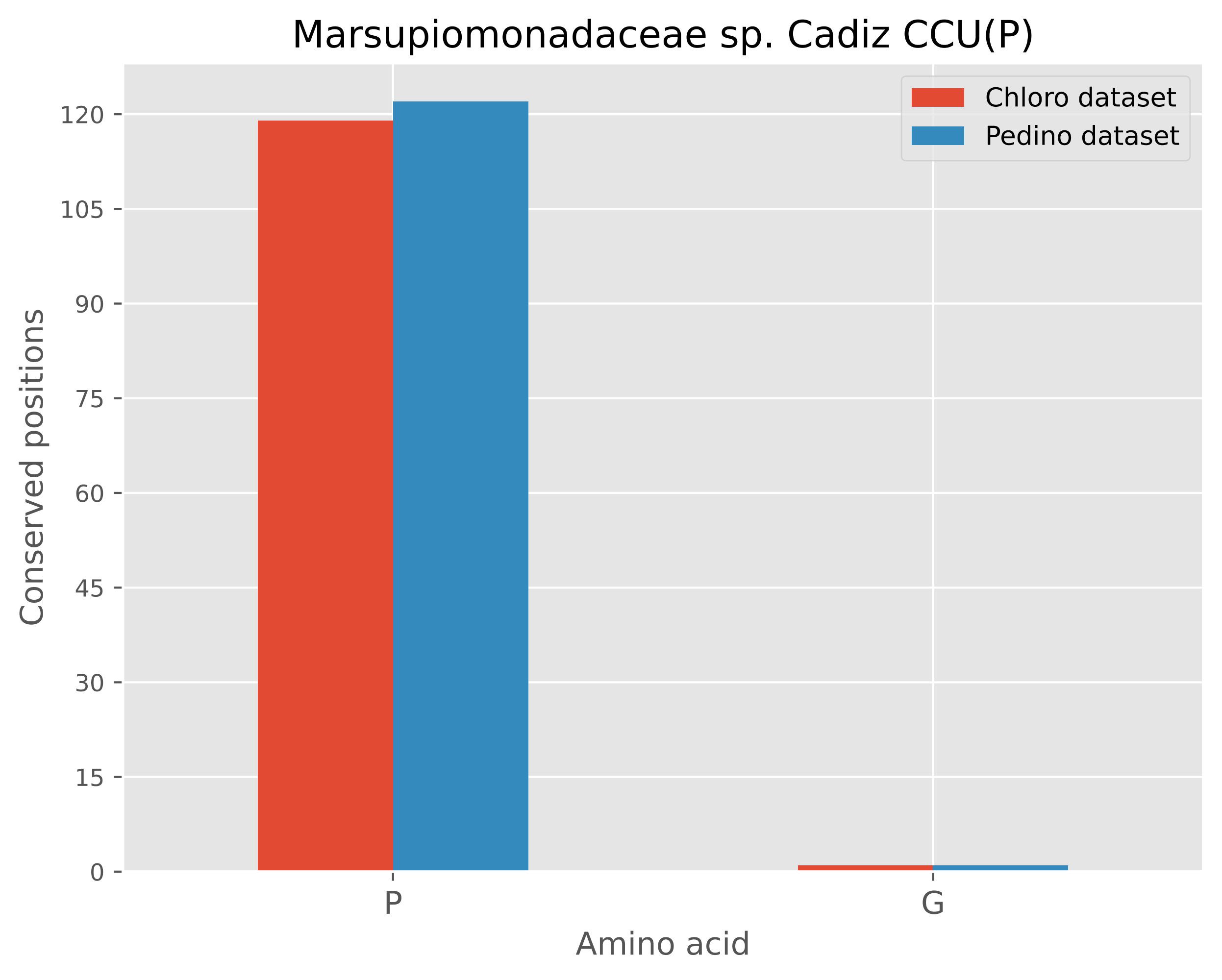

Marsupiomonadaceae sp. Cadiz CCU(P)
Chloro dataset
Pedino dataset
Conserved positions
120
105
90
75
60
45
30
15
0
P
G
Amino acid

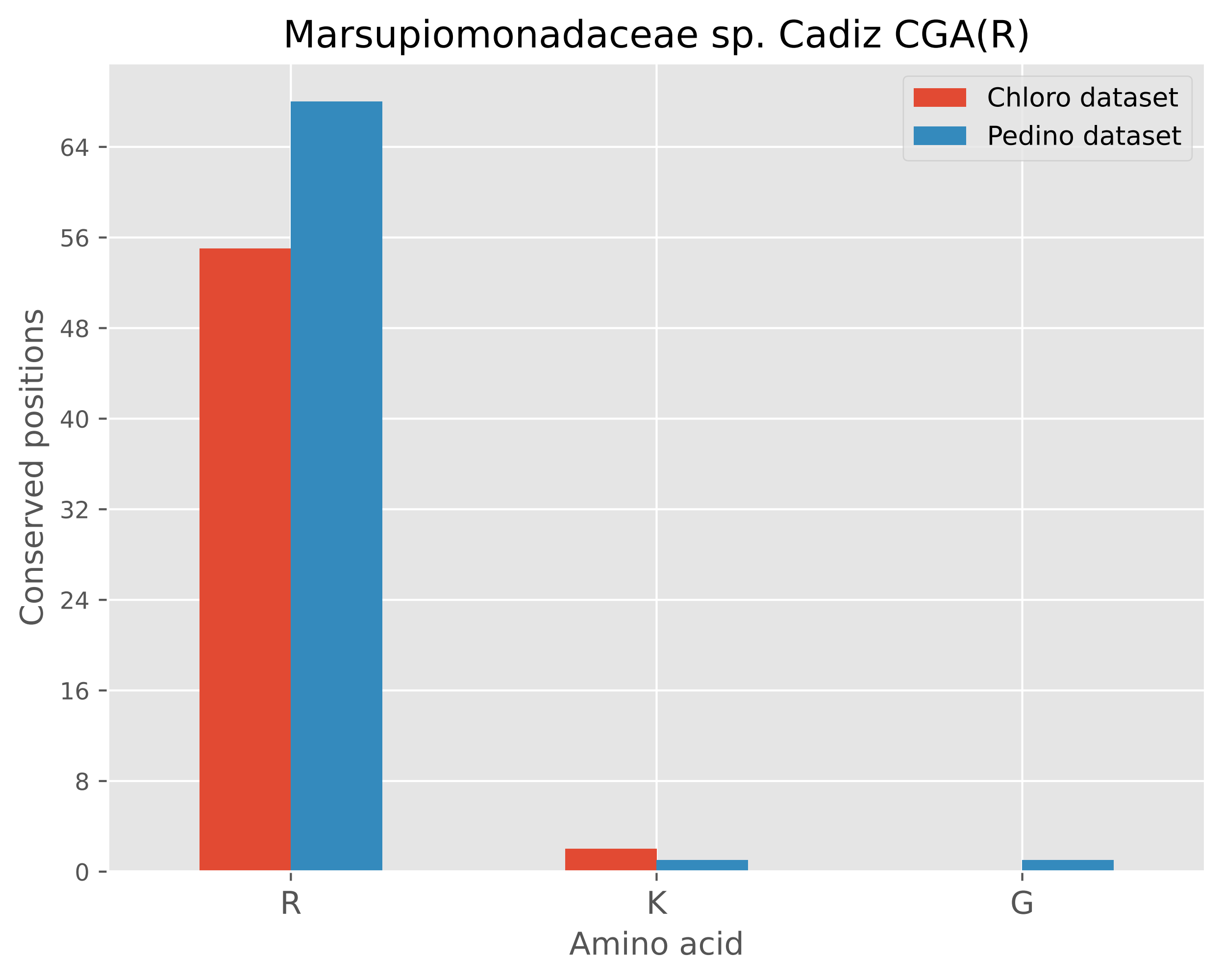

Marsupiomonadaceae sp. Cadiz CGA(R)
Chloro dataset
Pedino dataset
Conserved positions
0
8
16
24
32
40
48
56
64
R
K
G
Amino acid

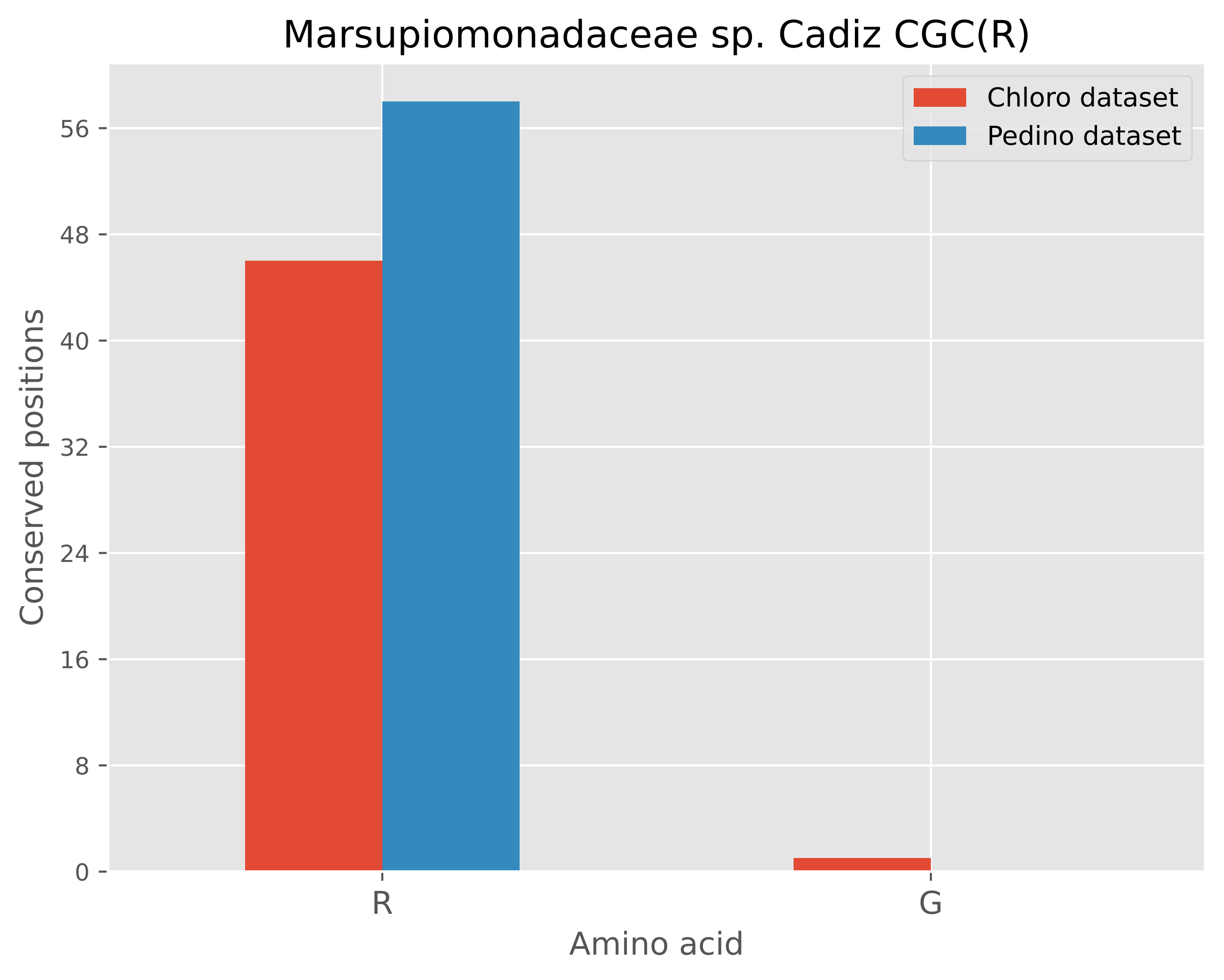

Marsupiomonadaceae sp. Cadiz CGC(R)
Chloro dataset
Pedino dataset
Conserved positions
0
8
16
24
32
40
48
56
R
G
Amino acid

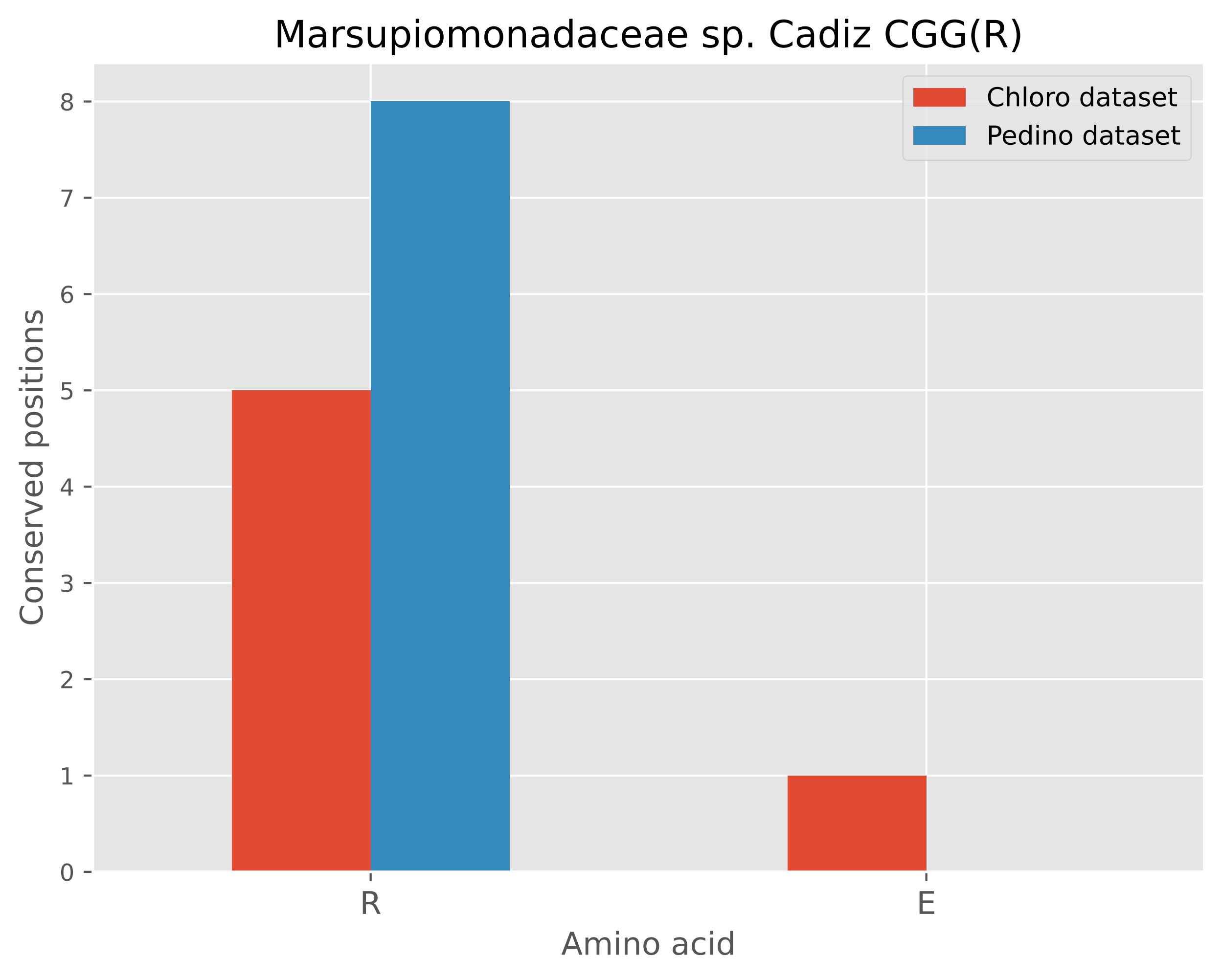

Marsupiomonadaceae sp. Cadiz CGG(R)
Chloro dataset
Pedino dataset
Conserved positions
0
1
2
3
4
5
6
7
8
R
E
Amino acid

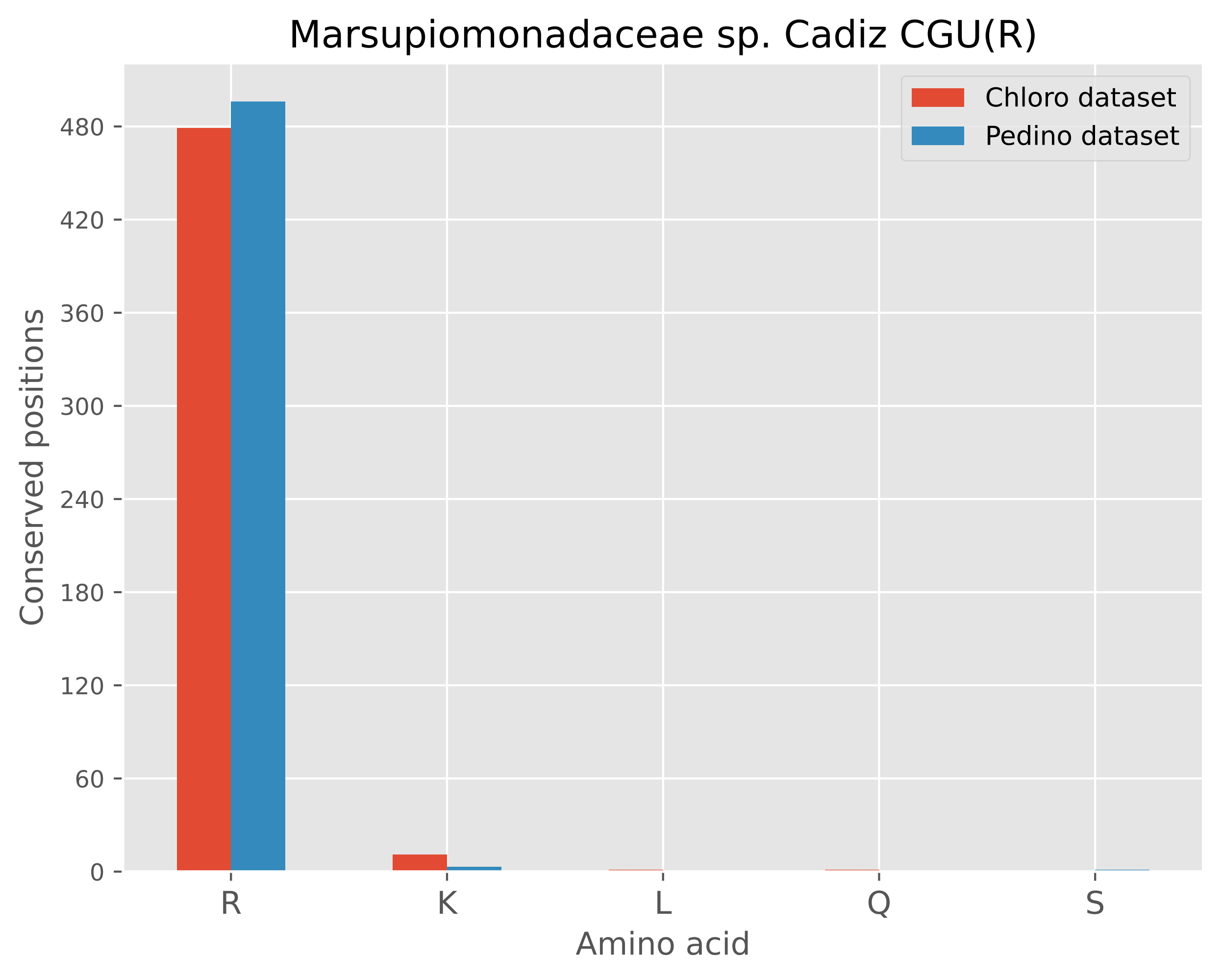
Marsupiomonadaceae sp. Cadiz CGU(R)
Chloro dataset
Pedino dataset
Conserved positions
0
60
120
180
240
300
360
420
480
R
K
L
Q
S
Amino acid

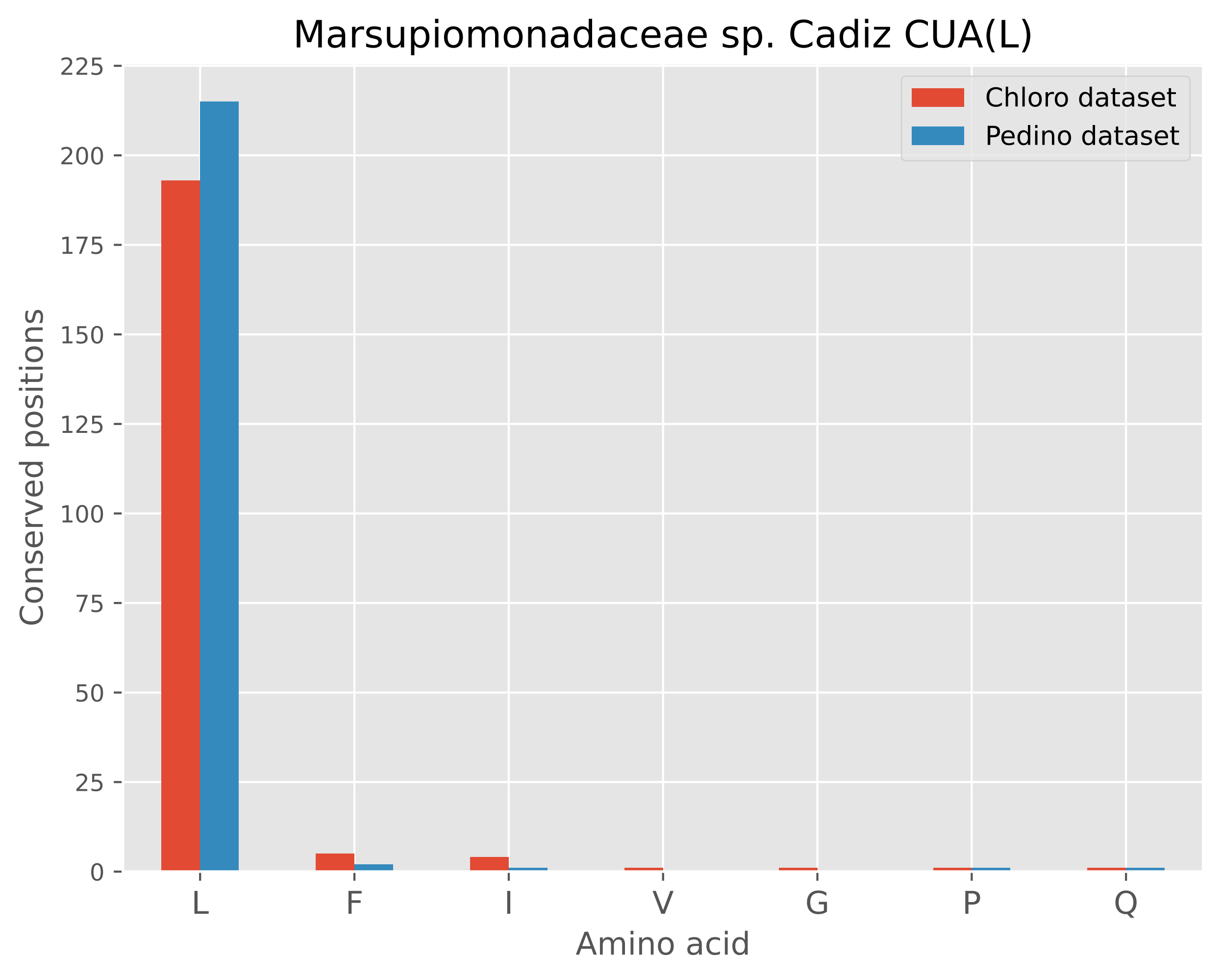

Marsupiomonadaceae sp. Cadiz CUA(L)
Chloro dataset
Pedino dataset
Conserved positions
0
25
50
75
100
125
150
175
200
225
L
F
I
V
G
P
Q
Amino acid

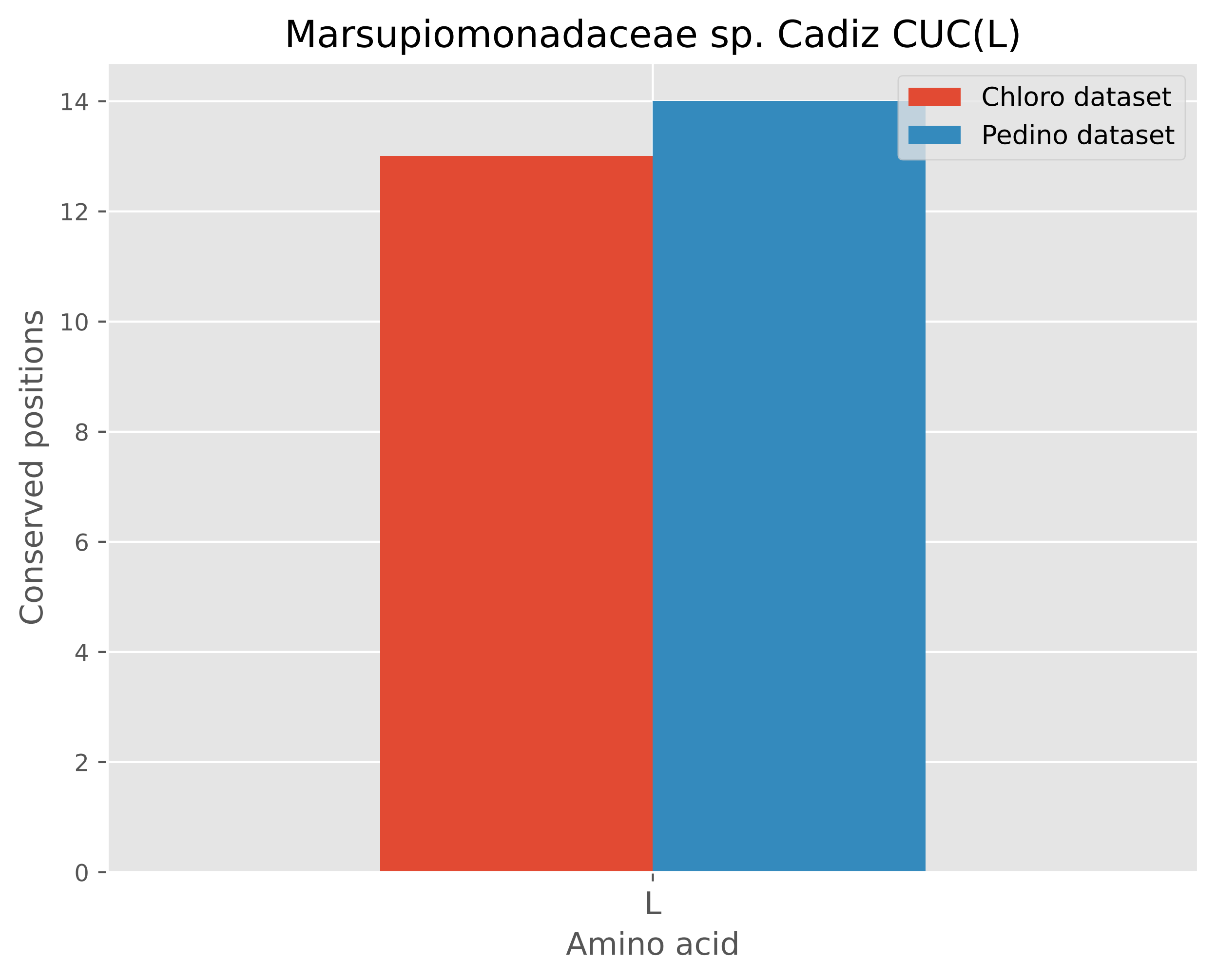
Marsupiomonadaceae sp. Cadiz CUC(L)
Chloro dataset
Pedino dataset
Conserved positions
0
2
4
6
8
10
12
14
L
Amino acid

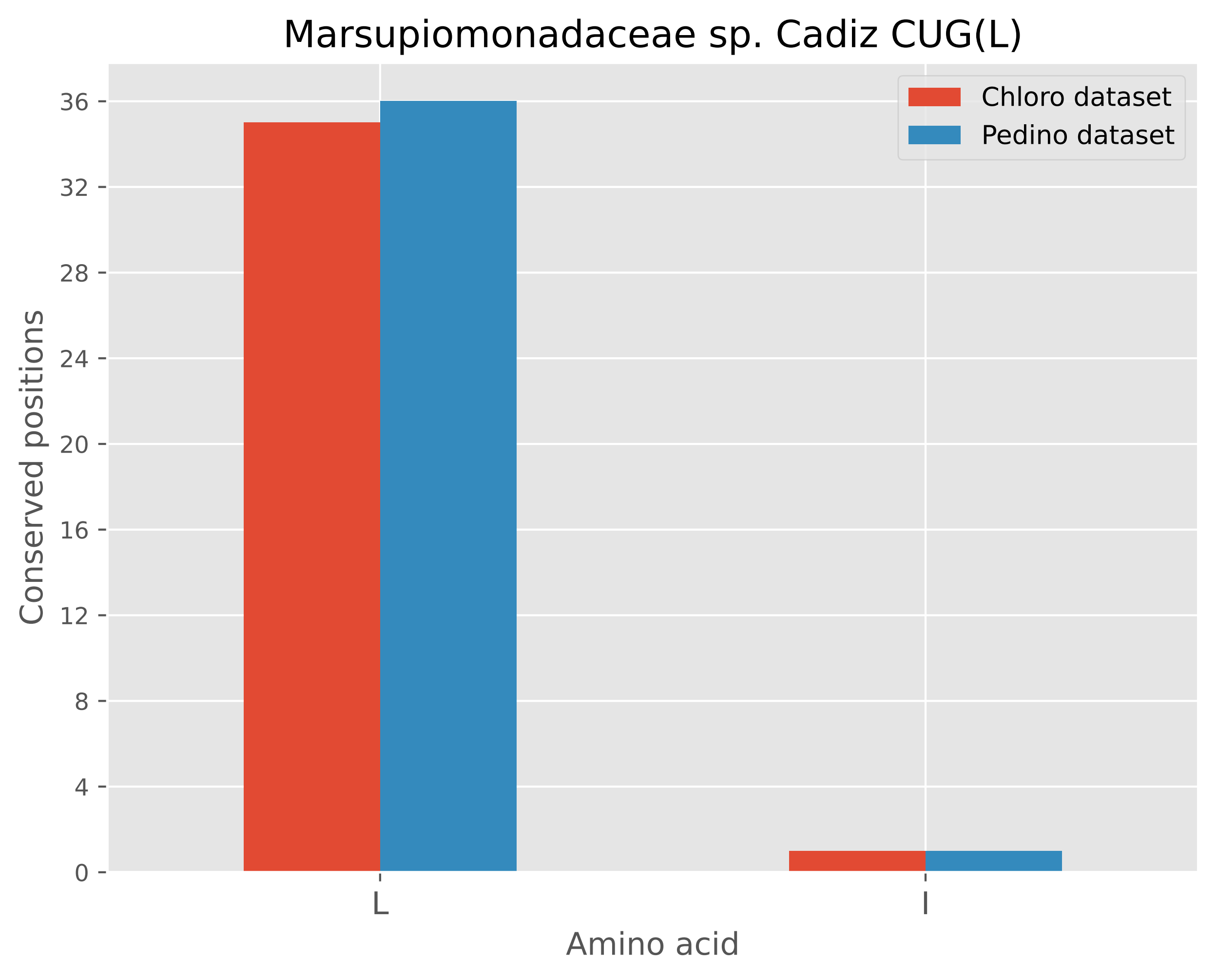

Marsupiomonadaceae sp. Cadiz CUG(L)
Chloro dataset
Pedino dataset
Conserved positions
0
4
8
12
16
20
24
28
32
36
L
I
Amino acid

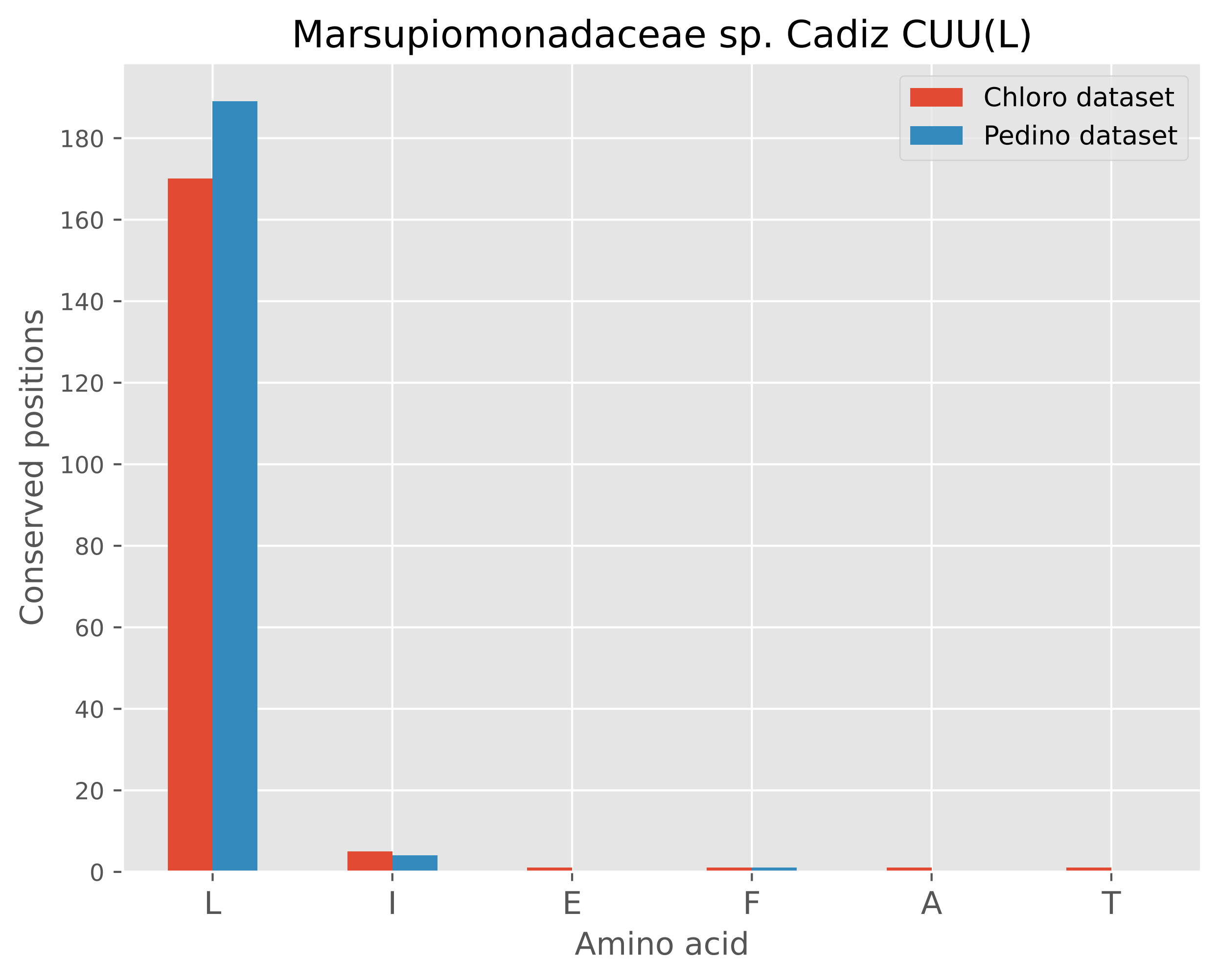
Marsupiomonadaceae sp. Cadiz CUU(L)
Chloro dataset
Pedino dataset
Conserved positions
0
20
40
60
80
100
120
140
160
180
L
I
E
F
A
T
Amino acid

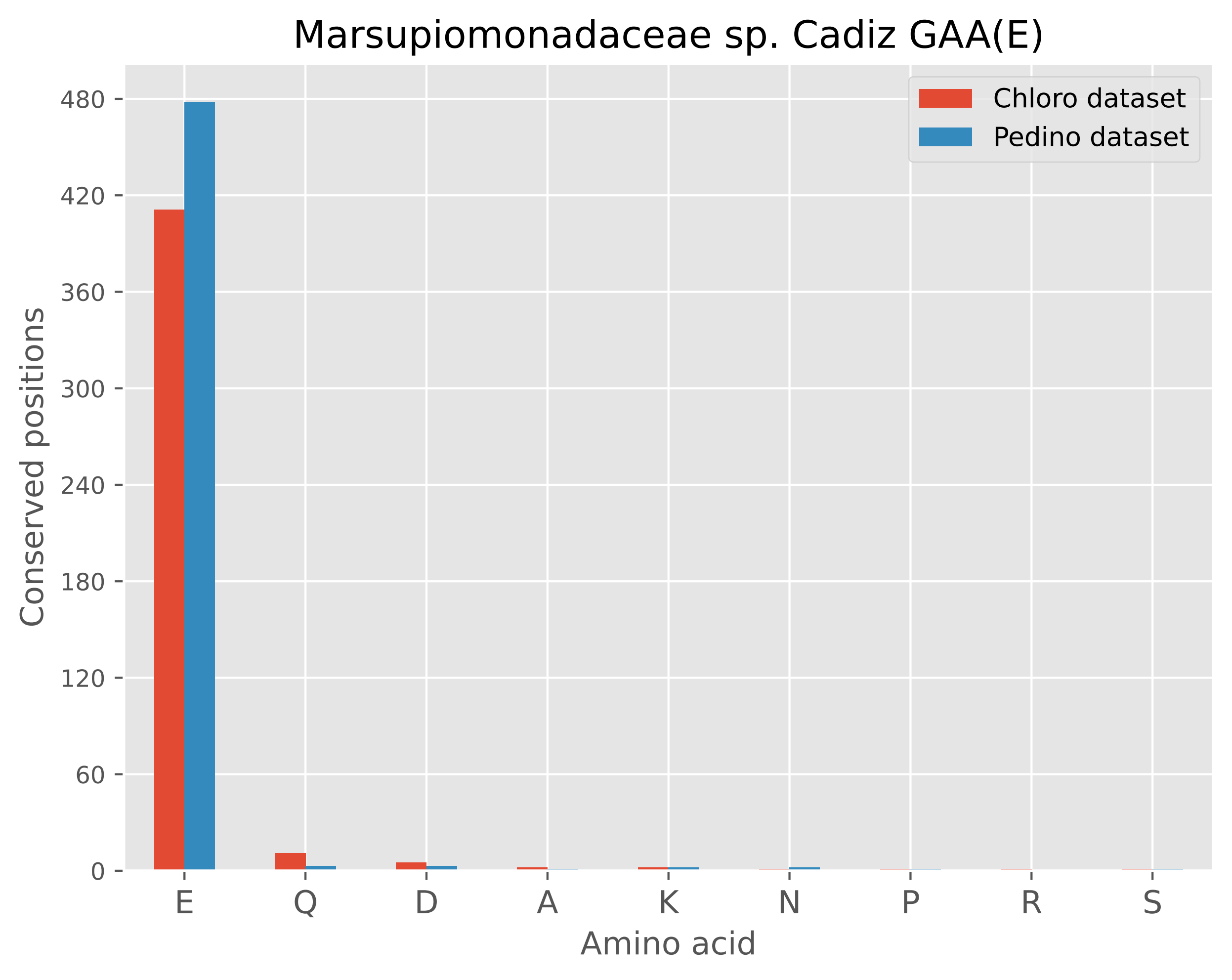

Marsupiomonadaceae sp. Cadiz GAA(E)
Chloro dataset
Pedino dataset
Conserved positions
480
420
360
300
240
180
120
60
0
E
Q
D
A
K
N
P
R
S
Amino acid

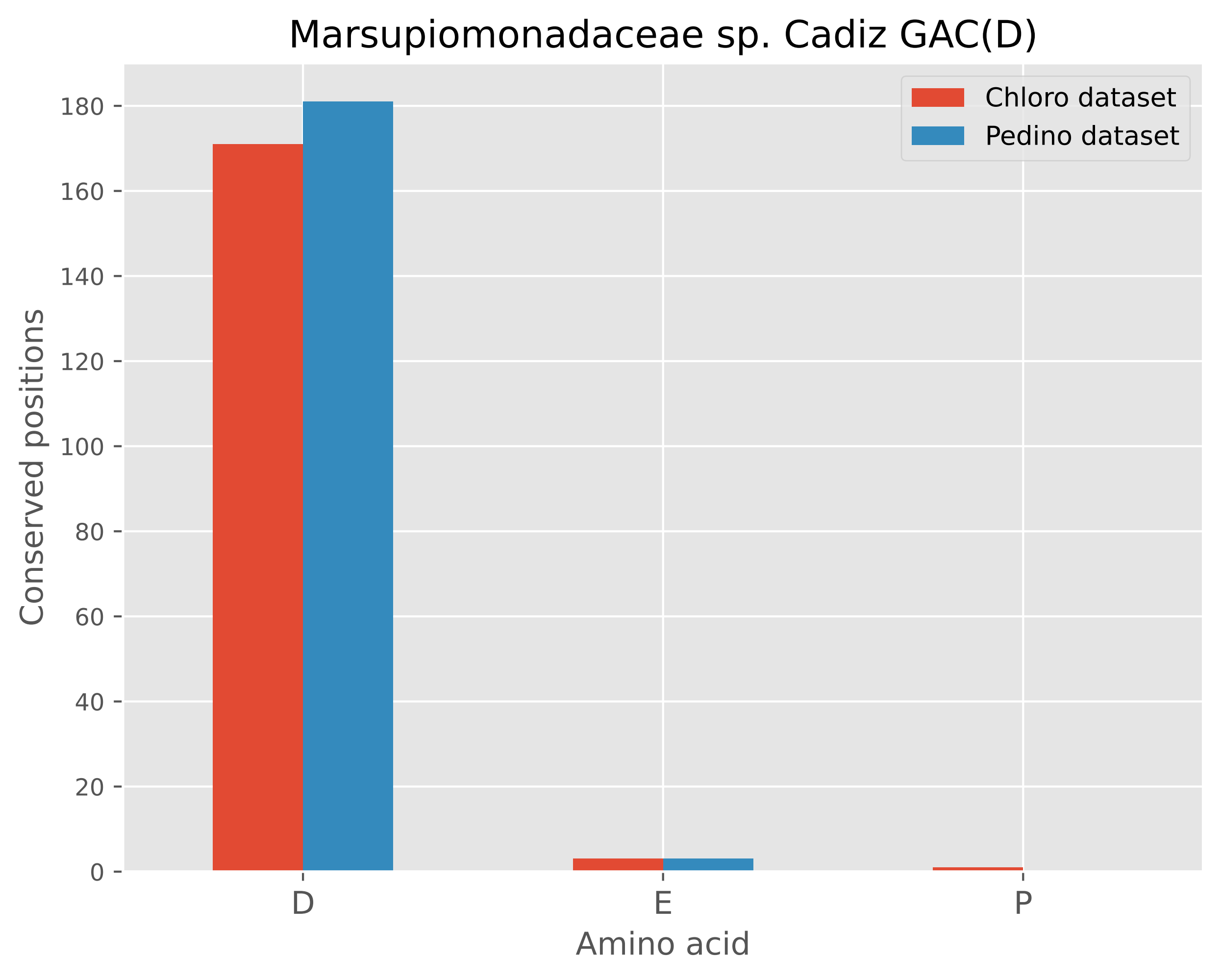

Marsupiomonadaceae sp. Cadiz GAC(D)
Chloro dataset
Pedino dataset
Conserved positions
0
20
40
60
80
100
120
140
160
180
D
E
P
Amino acid

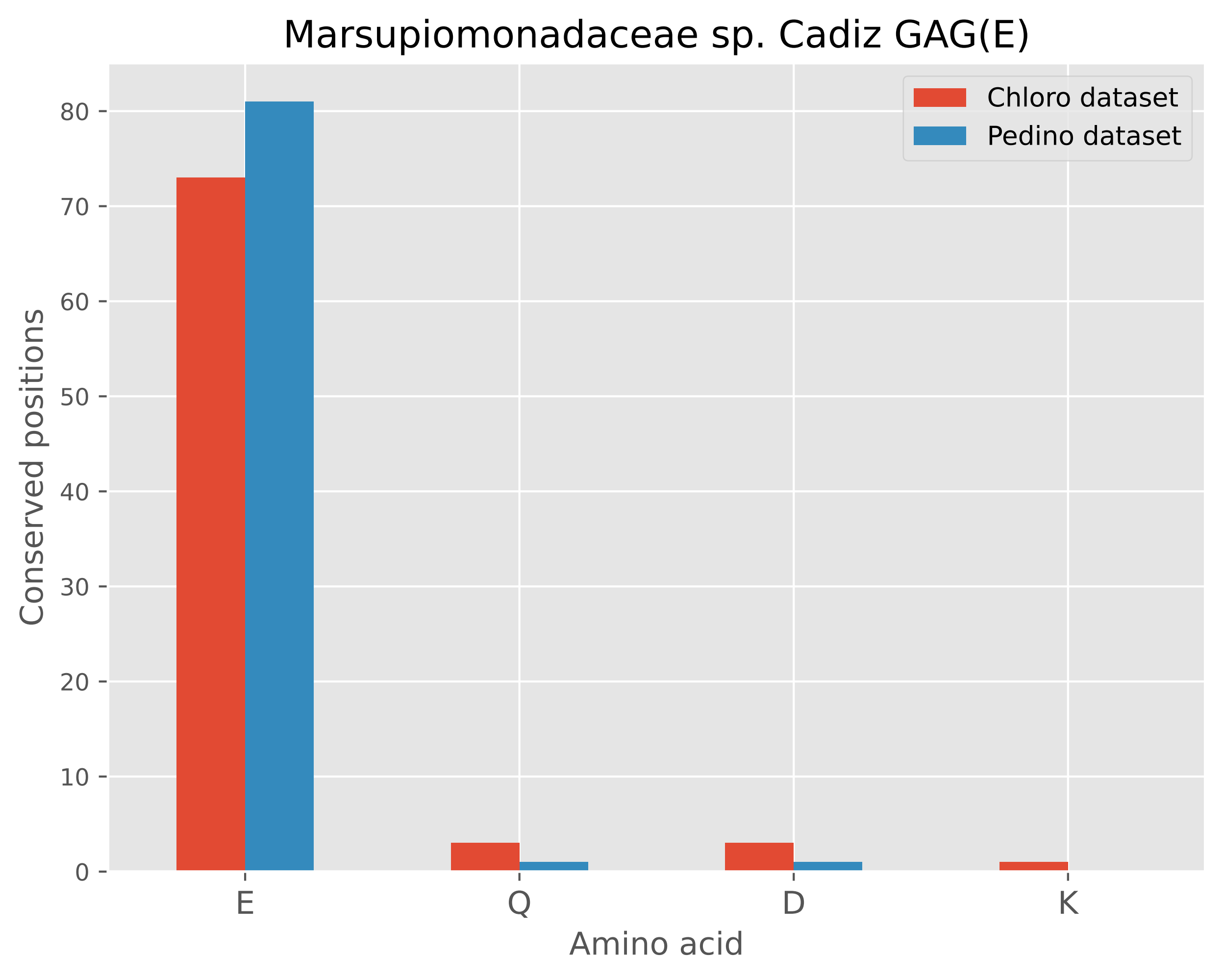

Marsupiomonadaceae sp. Cadiz GAG(E)
Chloro dataset
Pedino dataset
Conserved positions
0
10
20
30
40
50
60
70
80
E
Q
D
K
Amino acid

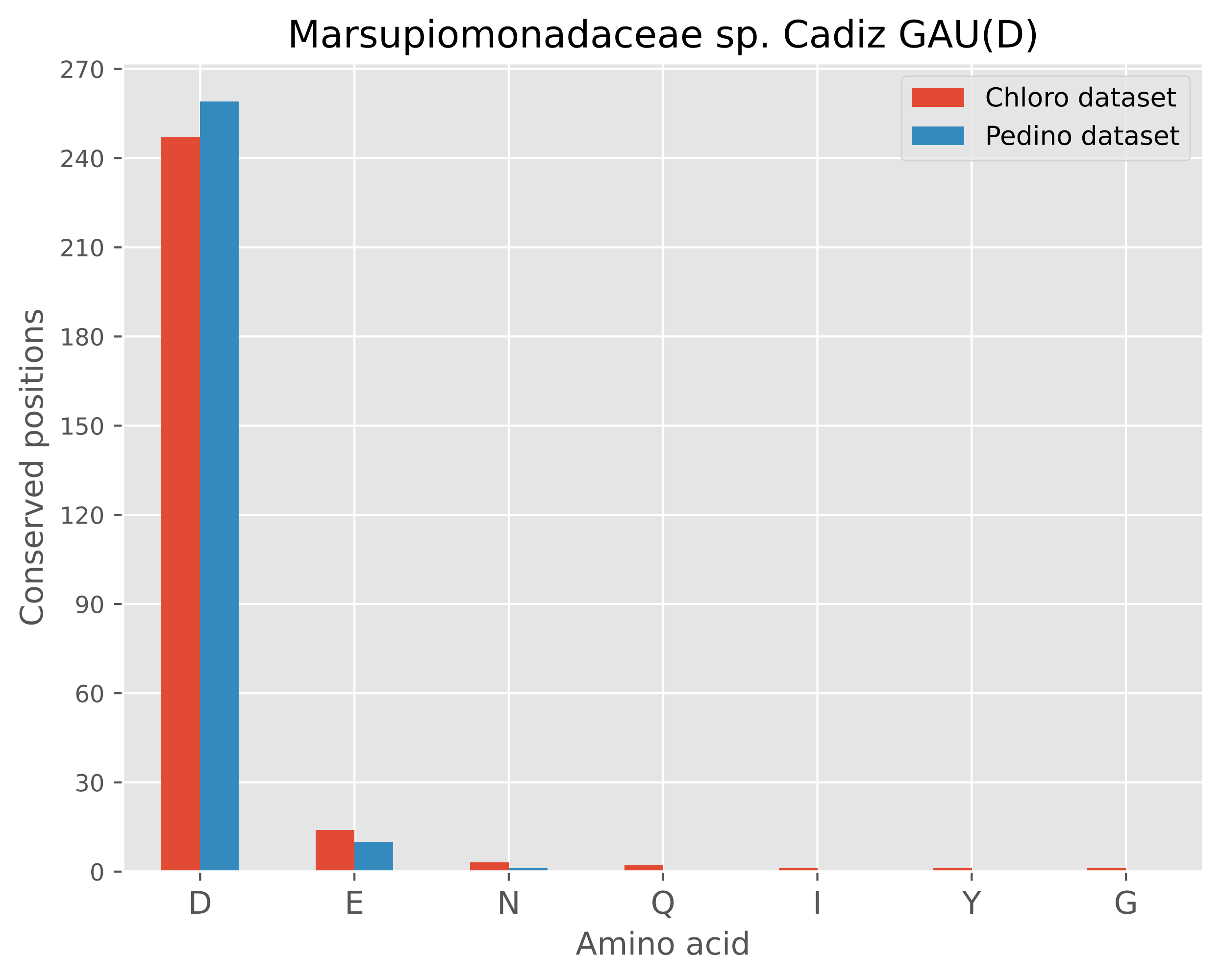
Marsupiomonadaceae sp. Cadiz GAU(D)
Chloro dataset
Pedino dataset
Conserved positions
270
240
210
180
150
120
90
60
30
0
D
E
N
Q
I
Y
G
Amino acid

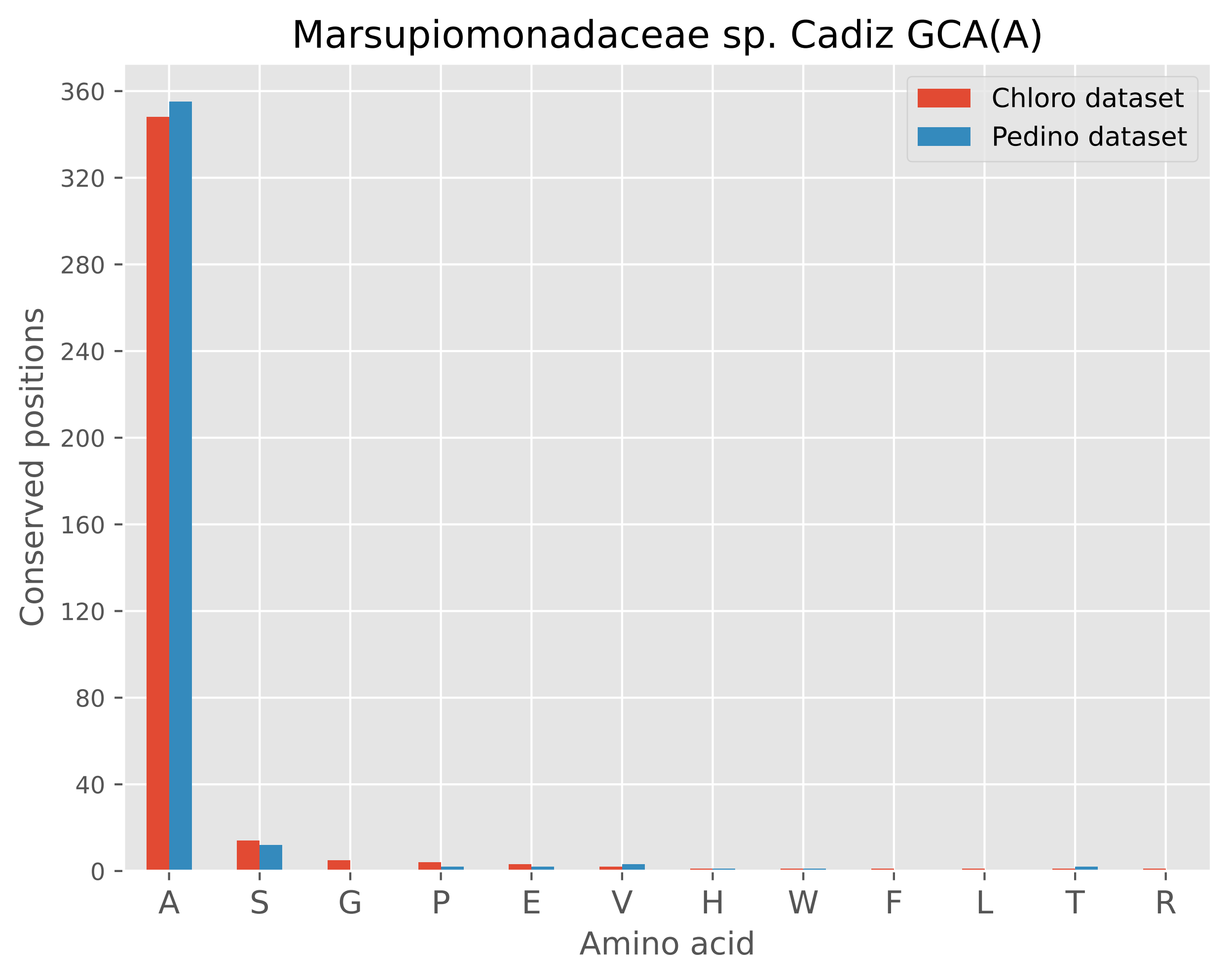

Marsupiomonadaceae sp. Cadiz GCA(A)
Chloro dataset
Pedino dataset
Conserved positions
0
40
80
120
160
200
240
280
320
360
A
S
G
P
E
V
H
W
F
L
T
R
Amino acid

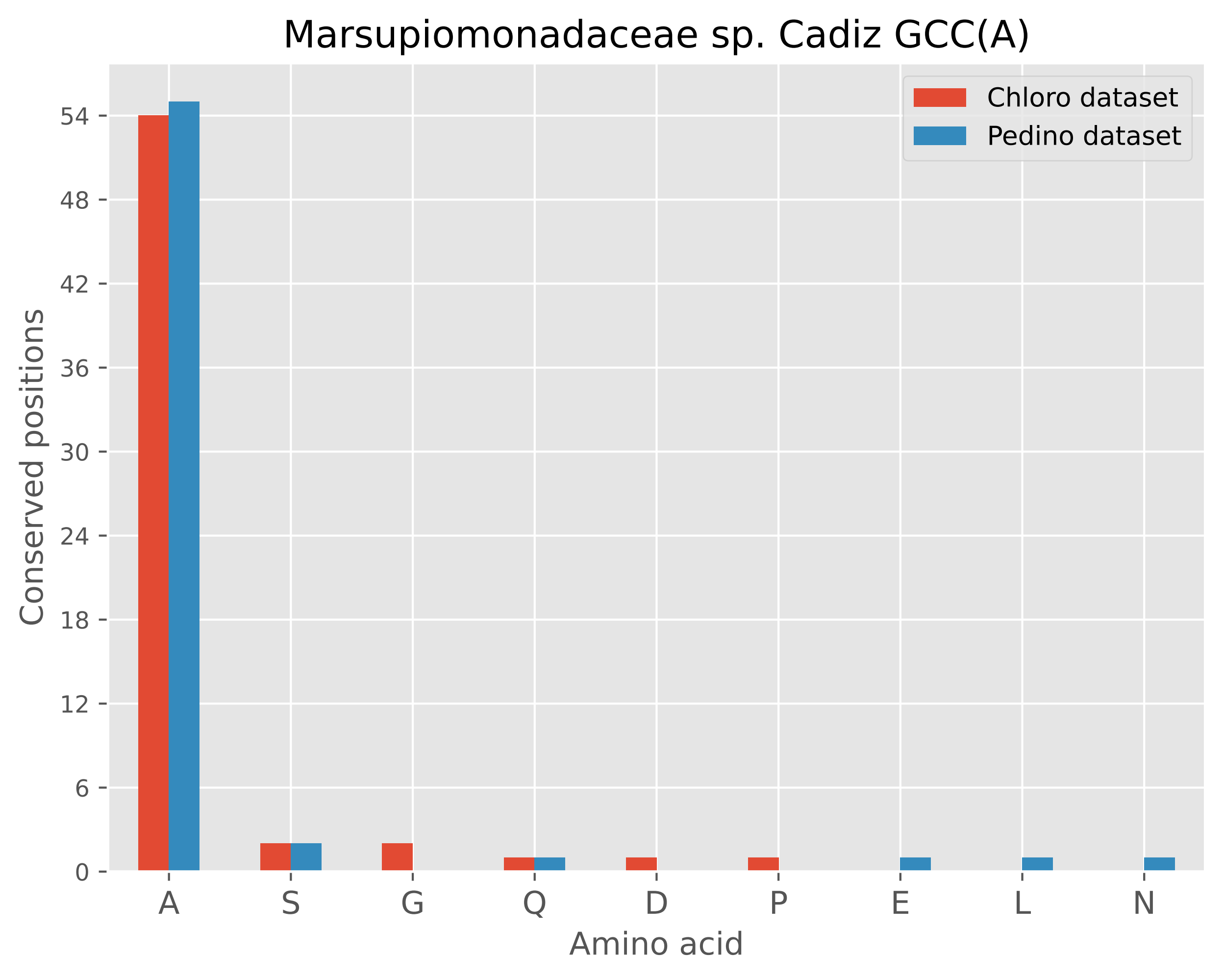

Marsupiomonadaceae sp. Cadiz GCC(A)
Chloro dataset
Pedino dataset
Conserved positions
Amino acid
0
6
12
18
24
30
36
42
48
54
A
S
G
Q
D
P
E
L
N

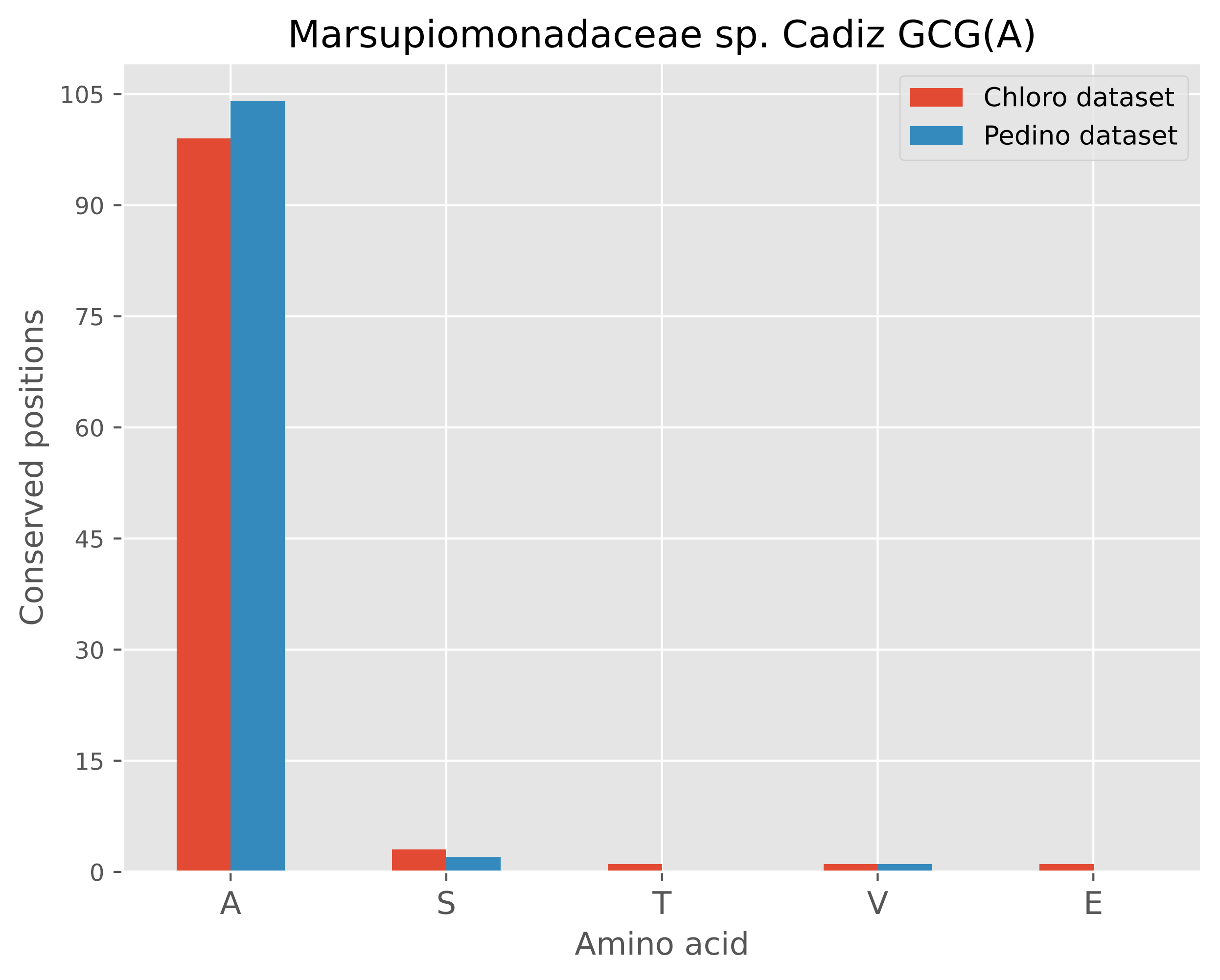

Marsupiomonadaceae sp. Cadiz GCG(A)
Chloro dataset
Pedino dataset
Conserved positions
0
15
30
45
60
75
90
105
A
S
T
V
E
Amino acid

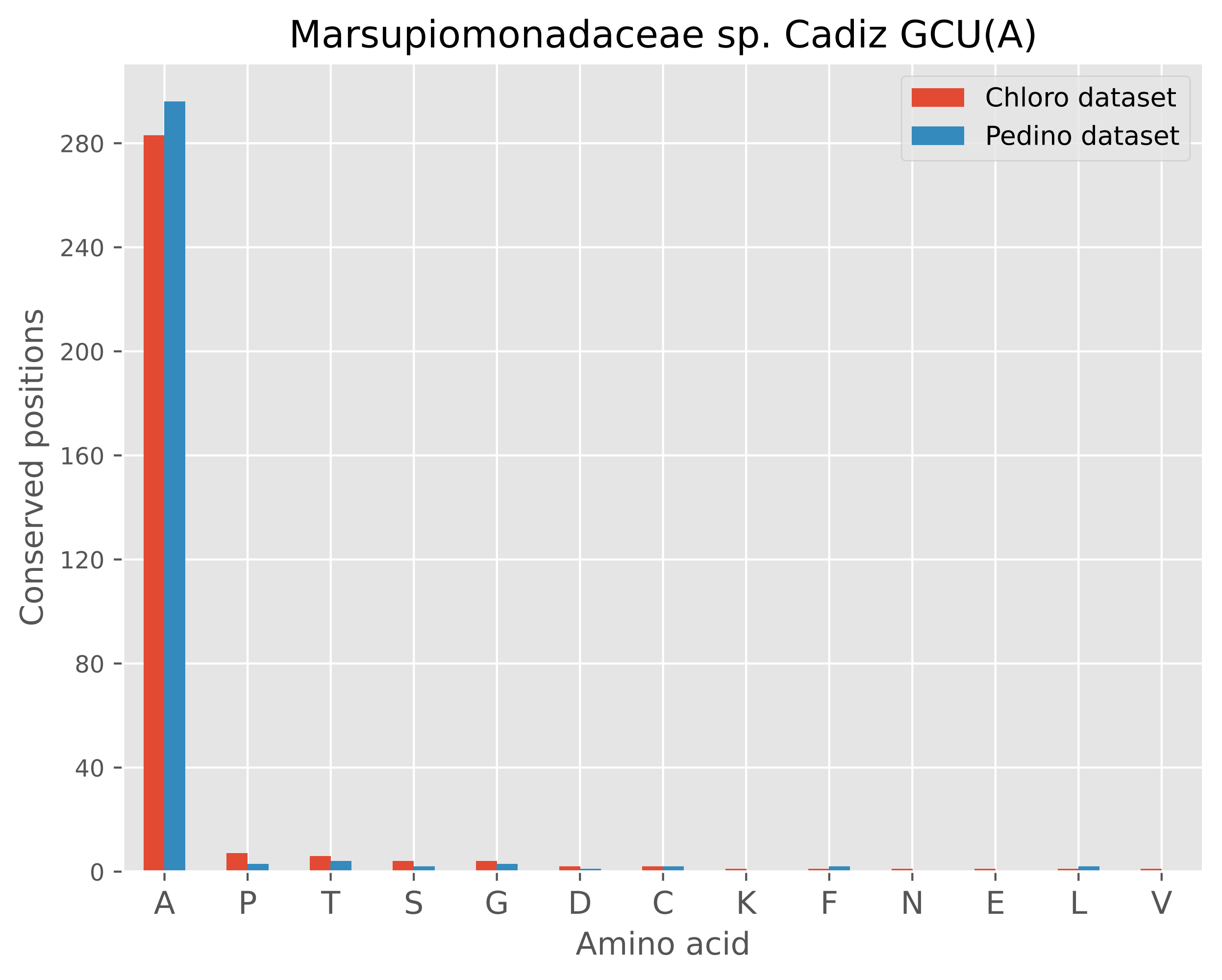
Marsupiomonadaceae sp. Cadiz GCU(A)
Chloro dataset
Pedino dataset
Conserved positions
0
40
80
120
160
200
240
280
A
P
T
S
G
D
C
K
F
N
E
L
V
Amino acid

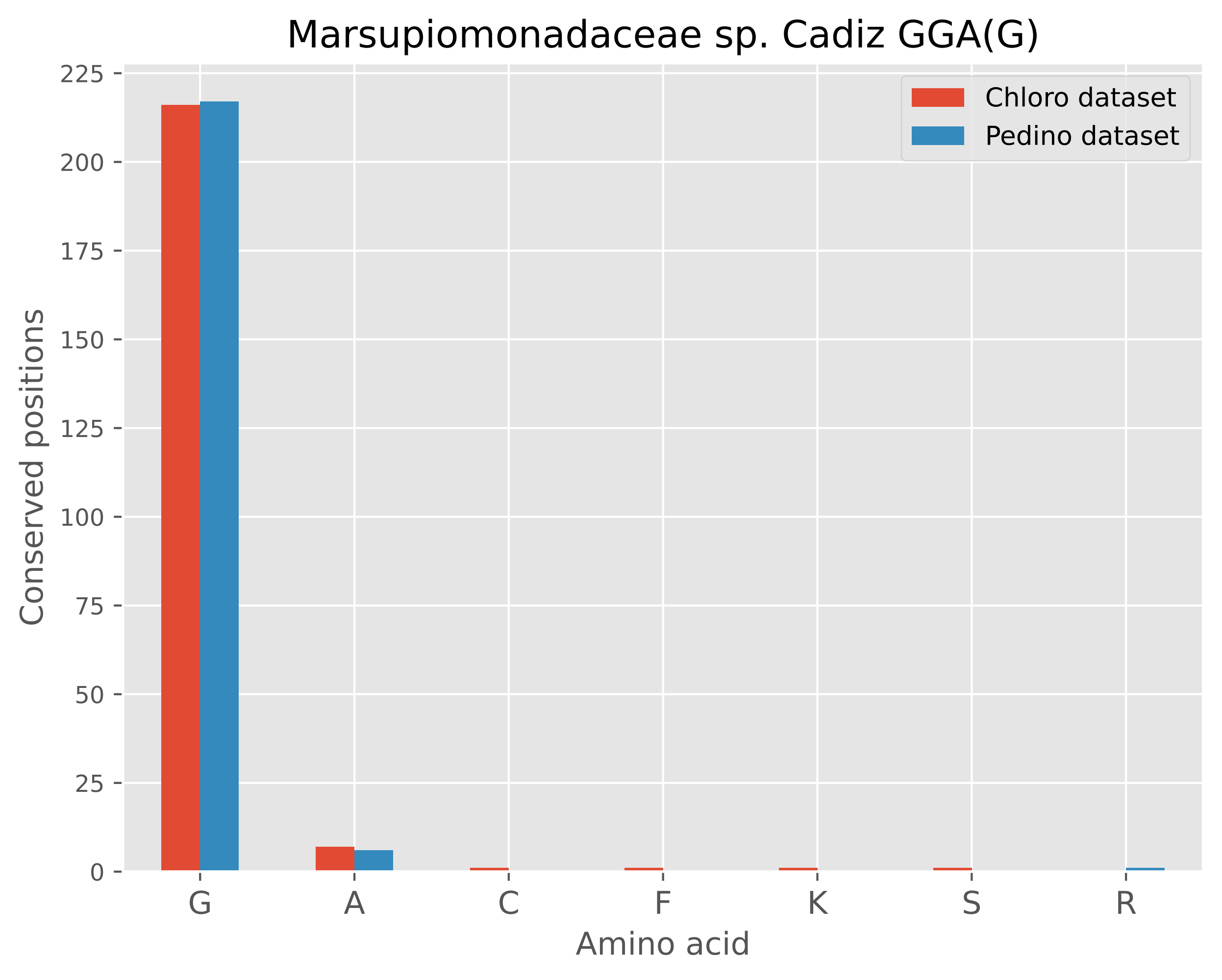
Marsupiomonadaceae sp. Cadiz GGA(G)
Chloro dataset
Pedino dataset
Conserved positions
Amino acid
0
25
50
75
100
125
150
175
200
225
G
A
C
F
K
S
R

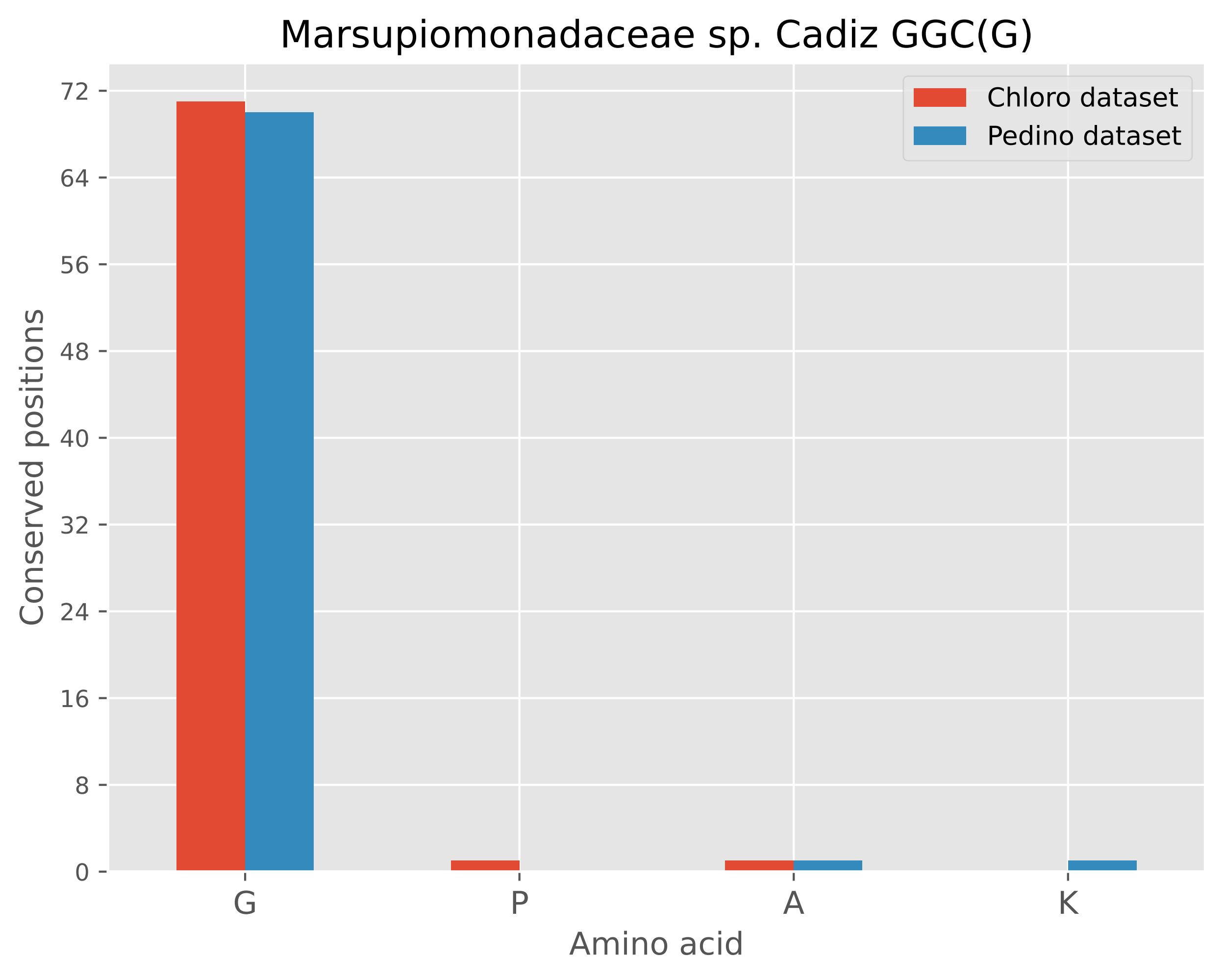

Marsupiomonadaceae sp. Cadiz GGC(G)
Chloro dataset
Pedino dataset
Conserved positions
72
64
56
48
40
32
24
16
8
0
G
P
A
K
Amino acid

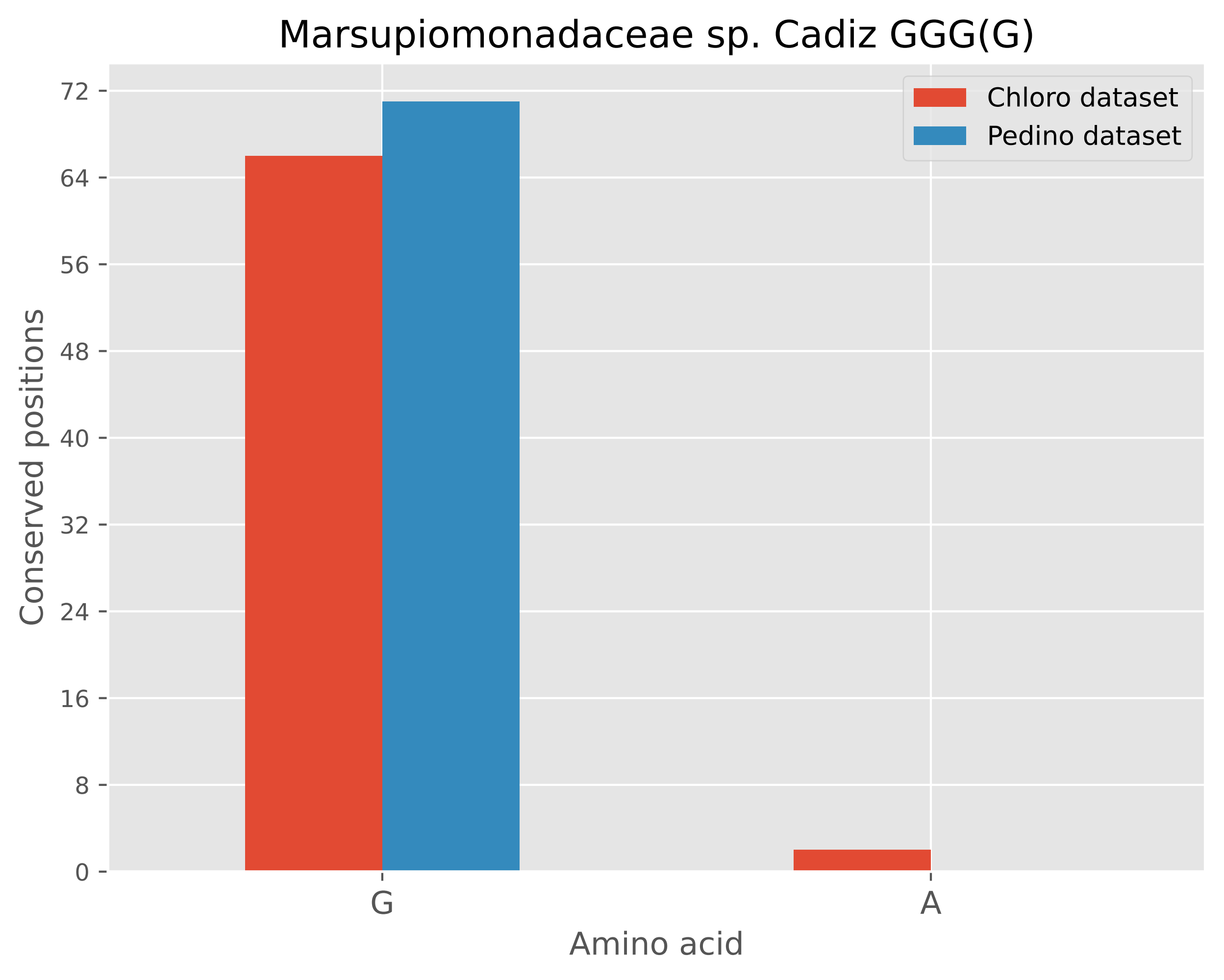
Marsupiomonadaceae sp. Cadiz GGG(G)
Chloro dataset
Pedino dataset
Conserved positions
Amino acid
G
A
0
8
16
24
32
40
48
56
64
72

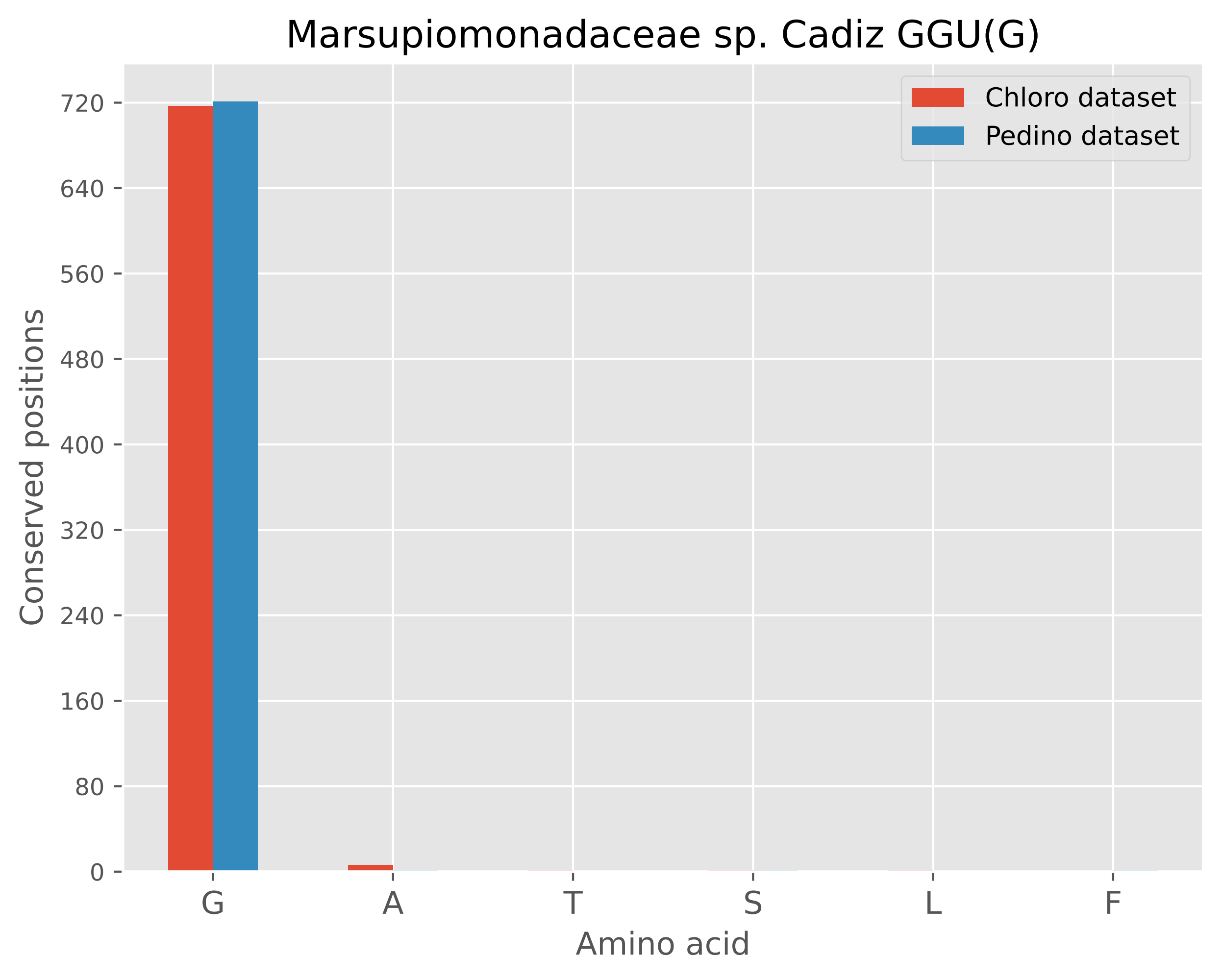

Marsupiomonadaceae sp. Cadiz GGU(G)
Chloro dataset
Pedino dataset
Conserved positions
720
640
560
480
400
320
240
160
80
0
G
A
T
S
L
F
Amino acid

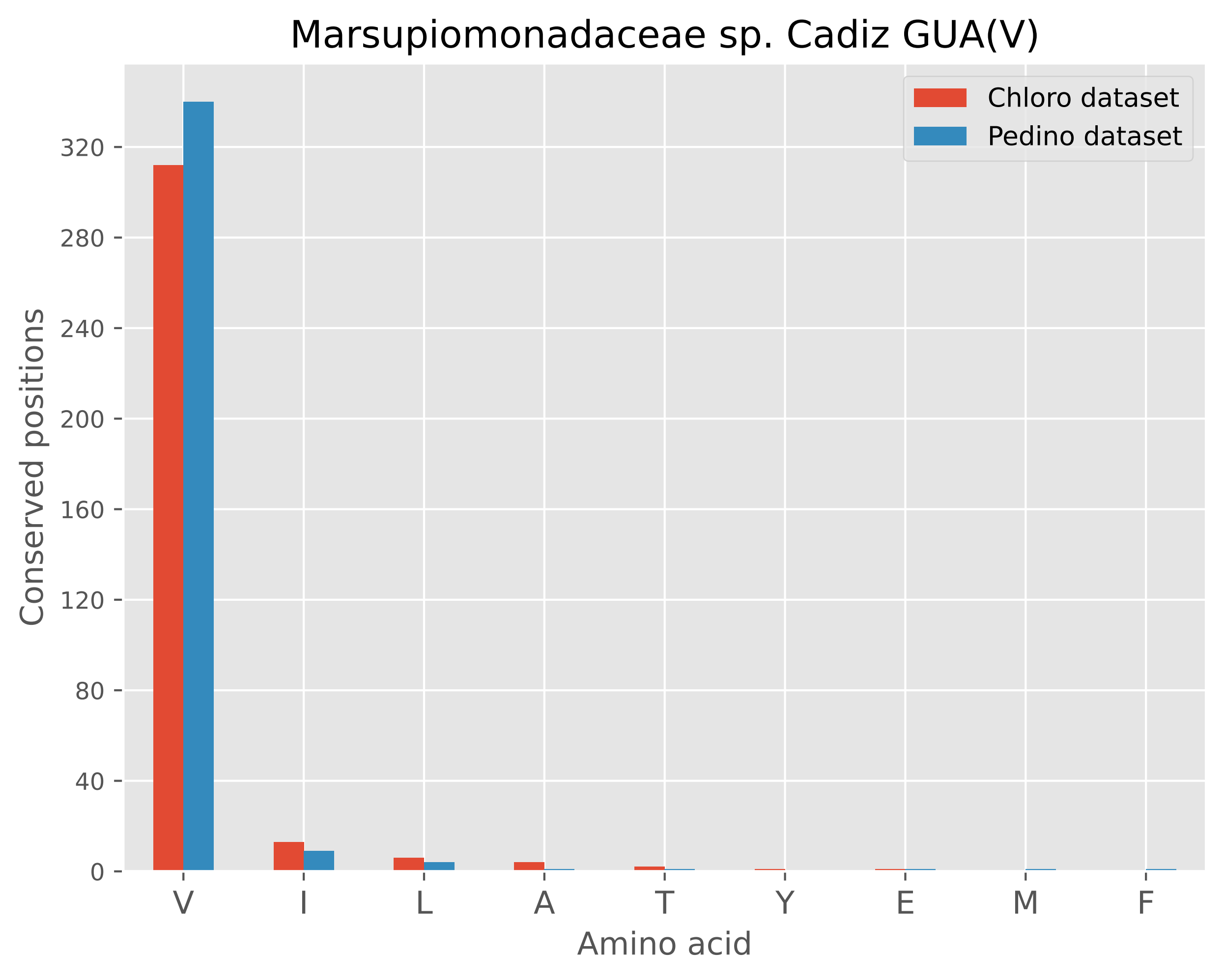

Marsupiomonadaceae sp. Cadiz GUA(V)
Chloro dataset
Pedino dataset
Conserved positions
0
40
80
120
160
200
240
280
320
V
I
L
A
T
Y
E
M
F
Amino acid

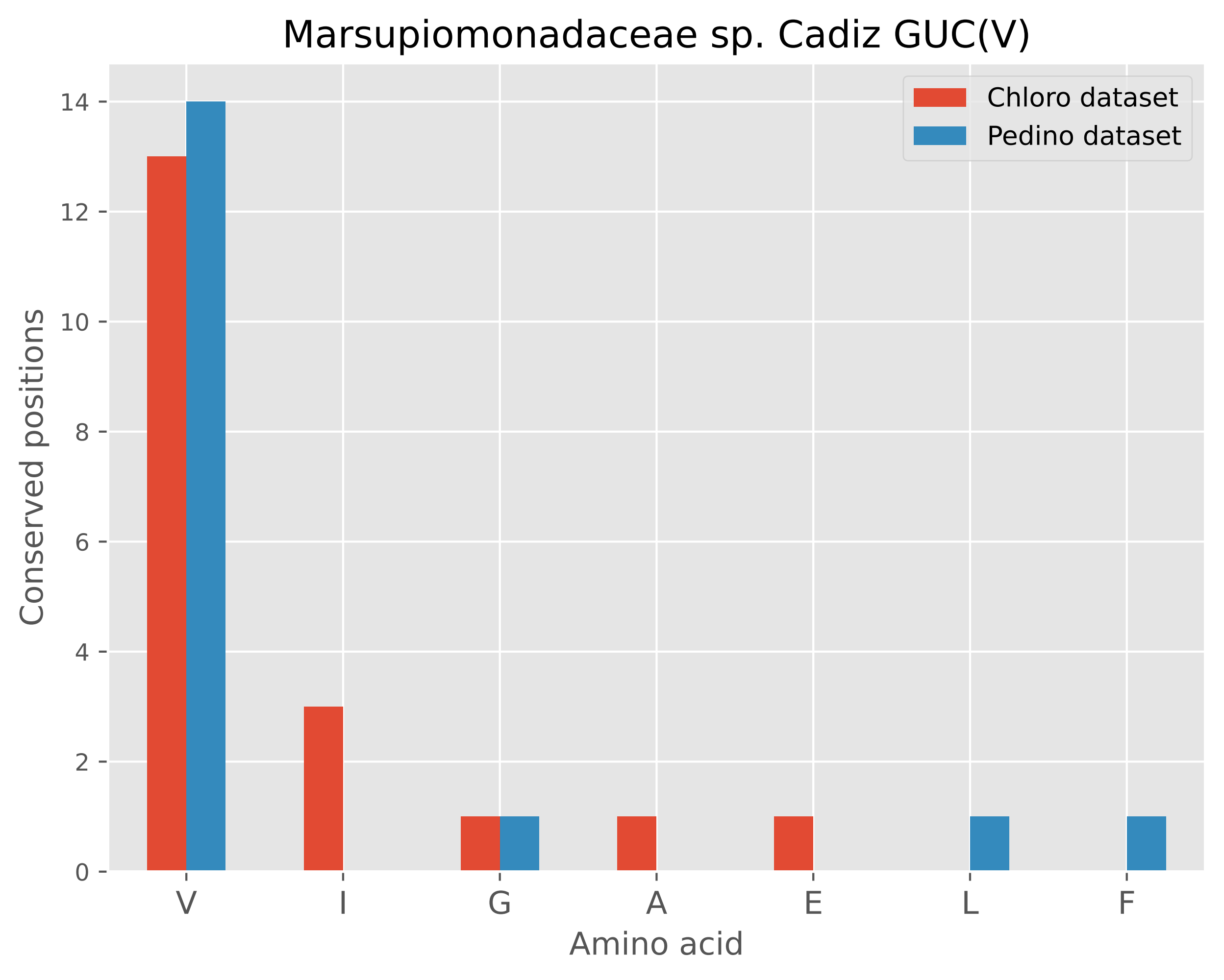

Marsupiomonadaceae sp. Cadiz GUC(V)
Chloro dataset
Pedino dataset
Conserved positions
0
2
4
6
8
10
12
14
V
I
G
A
E
L
F
Amino acid

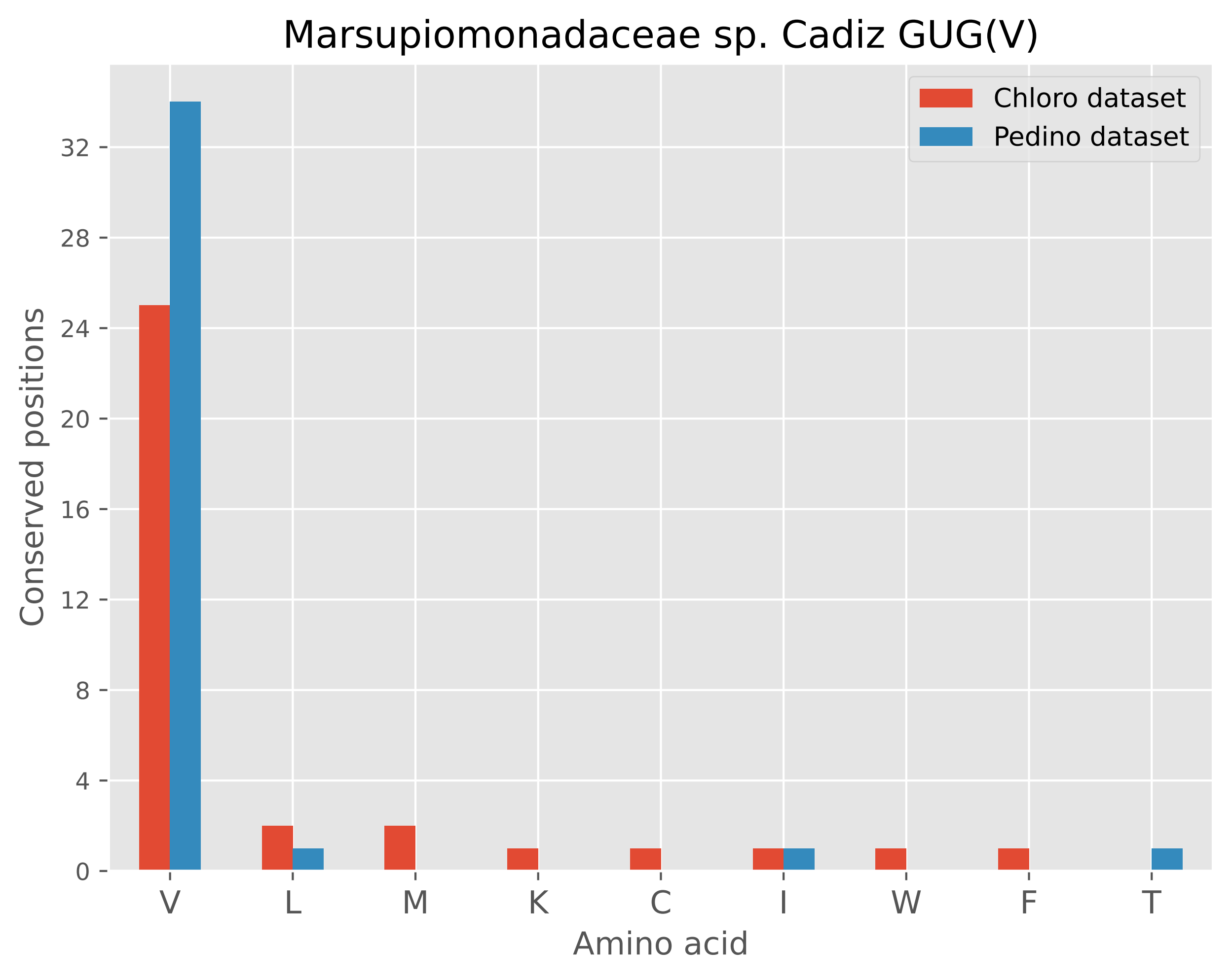
Marsupiomonadaceae sp. Cadiz GUG(V)
Chloro dataset
Pedino dataset
Conserved positions
0
4
8
12
16
20
24
28
32
V
L
M
K
C
I
W
F
T
Amino acid

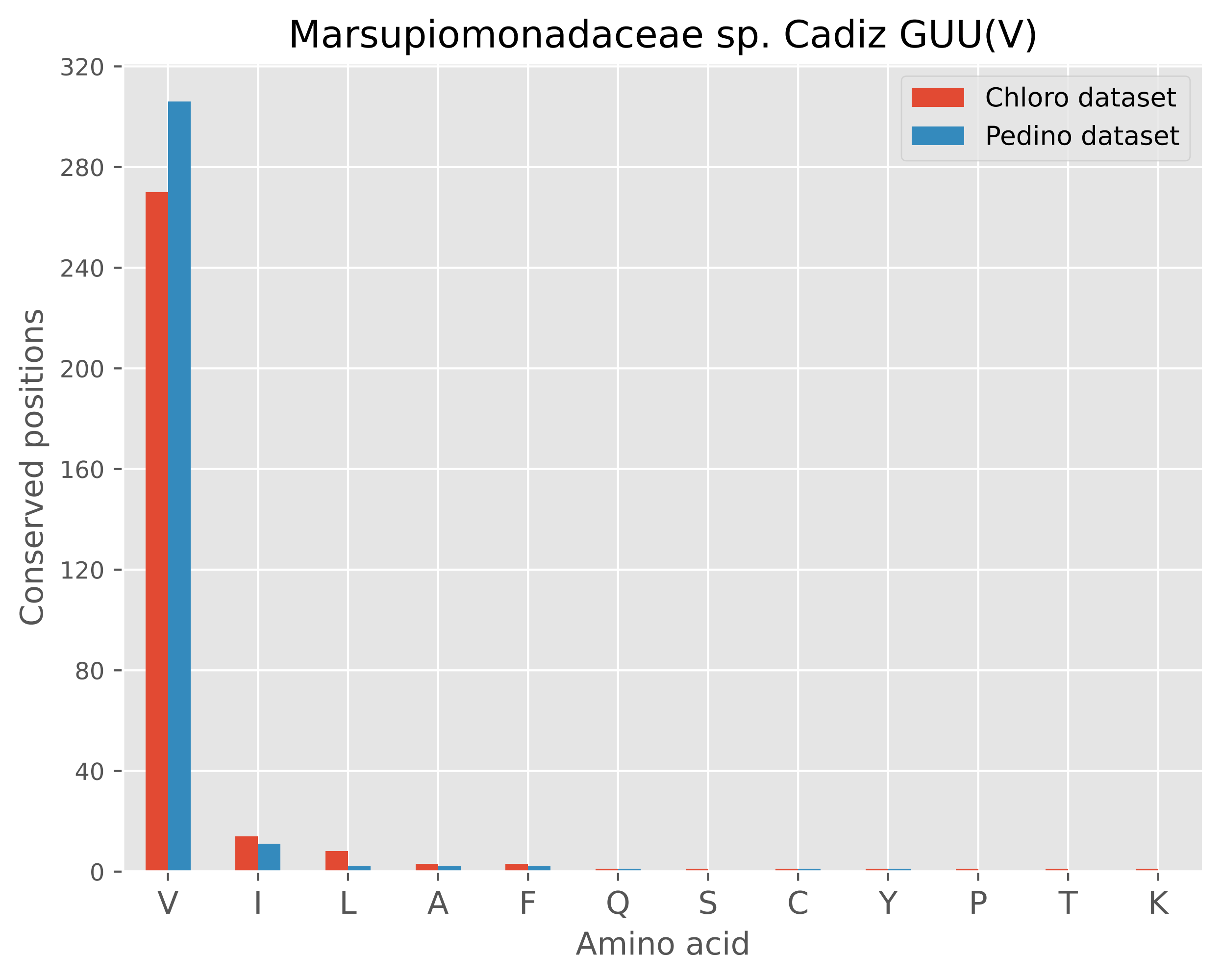

Marsupiomonadaceae sp. Cadiz GUU(V)
Chloro dataset
Pedino dataset
Conserved positions
Amino acid
0
40
80
120
160
200
240
280
320
V
I
L
A
F
Q
S
C
Y
P
T
K

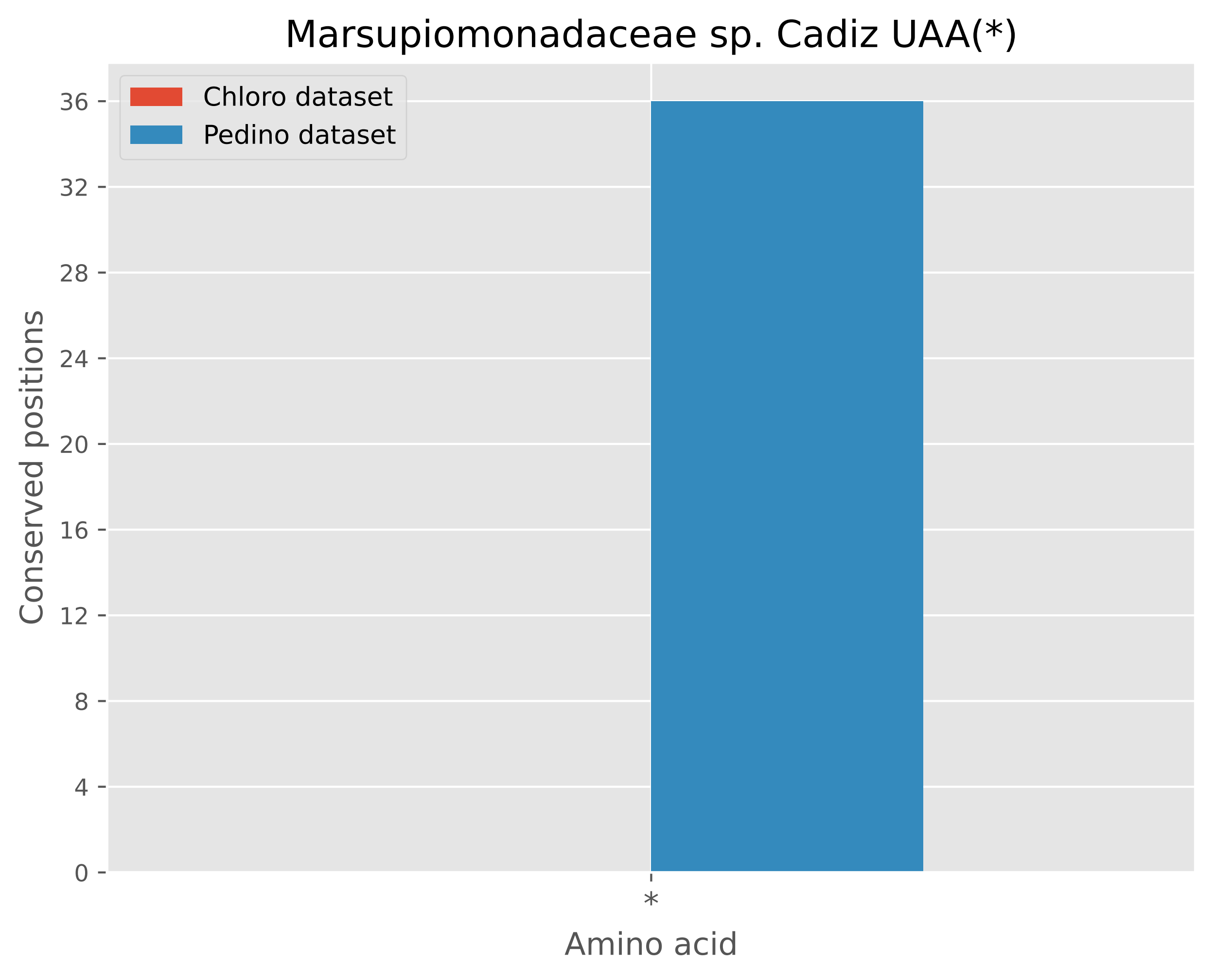

Marsupiomonadaceae sp. Cadiz UAA(*)
Chloro dataset
Pedino dataset
Conserved positions
0
4
8
12
16
20
24
28
32
36
*
Amino acid

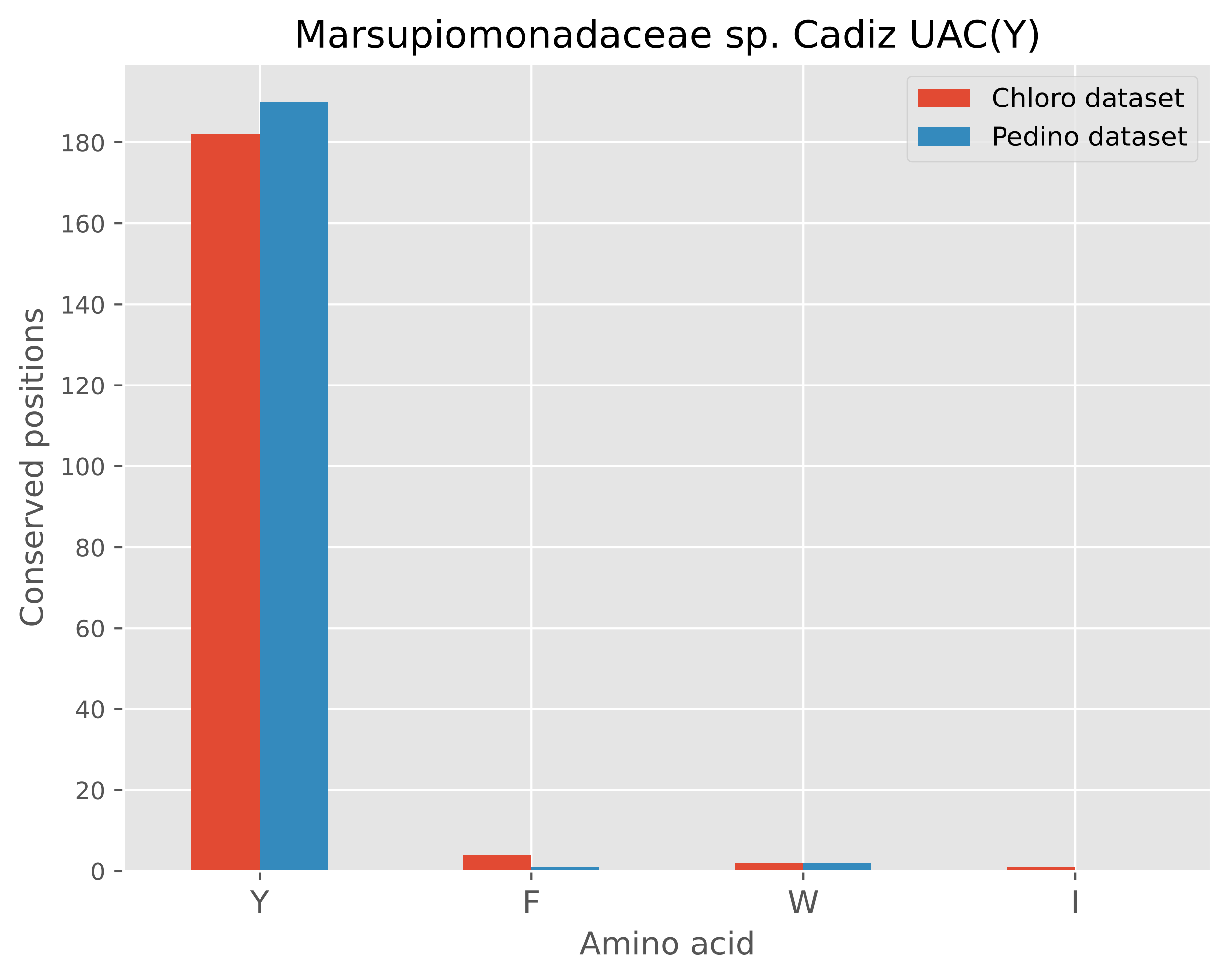
Marsupiomonadaceae sp. Cadiz UAC(Y)
Chloro dataset
Pedino dataset
Conserved positions
0
20
40
60
80
100
120
140
160
180
Y
F
W
I
Amino acid

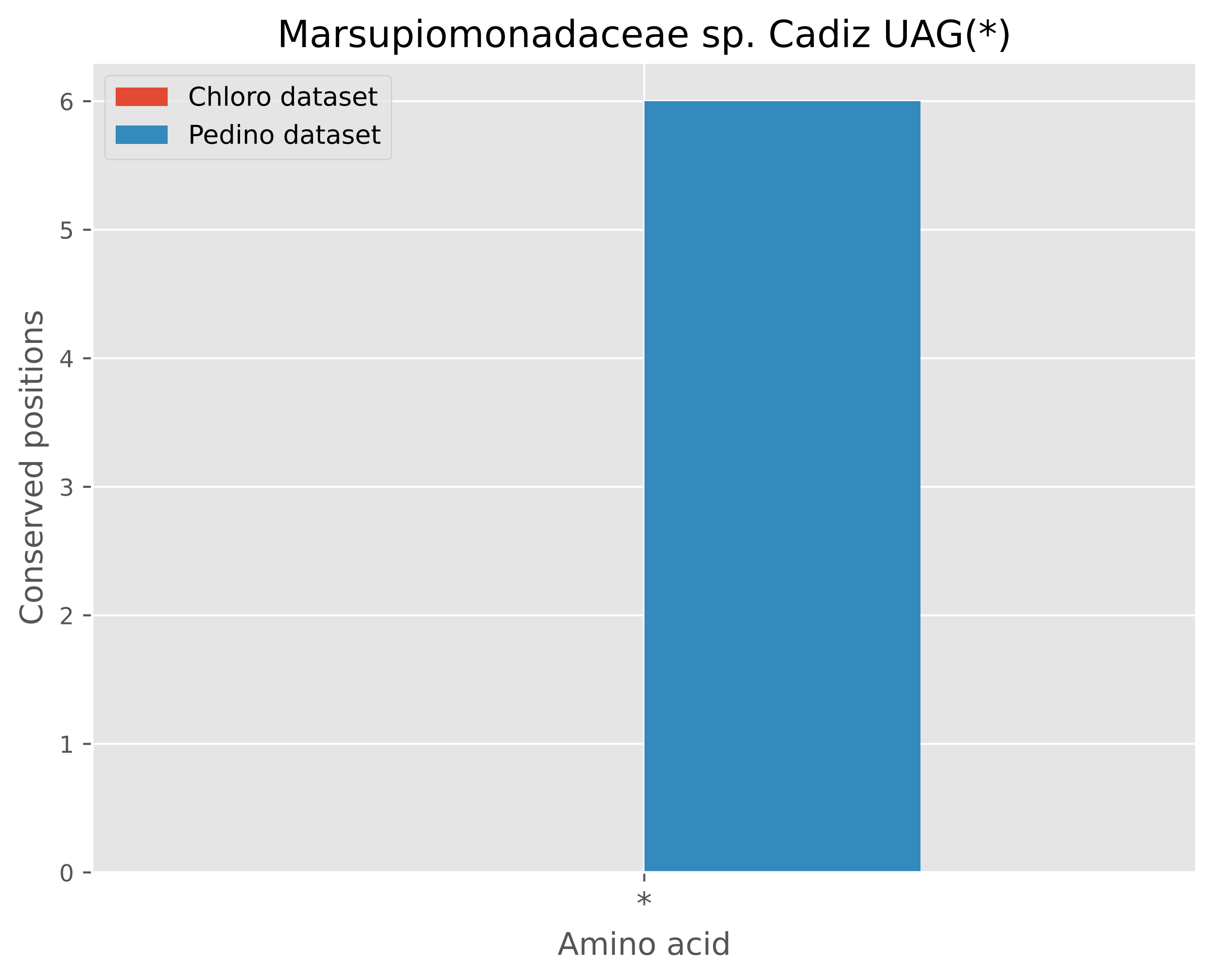
Marsupiomonadaceae sp. Cadiz UAG(*)
Chloro dataset
Pedino dataset
Conserved positions
0
1
2
3
4
5
6
*
Amino acid

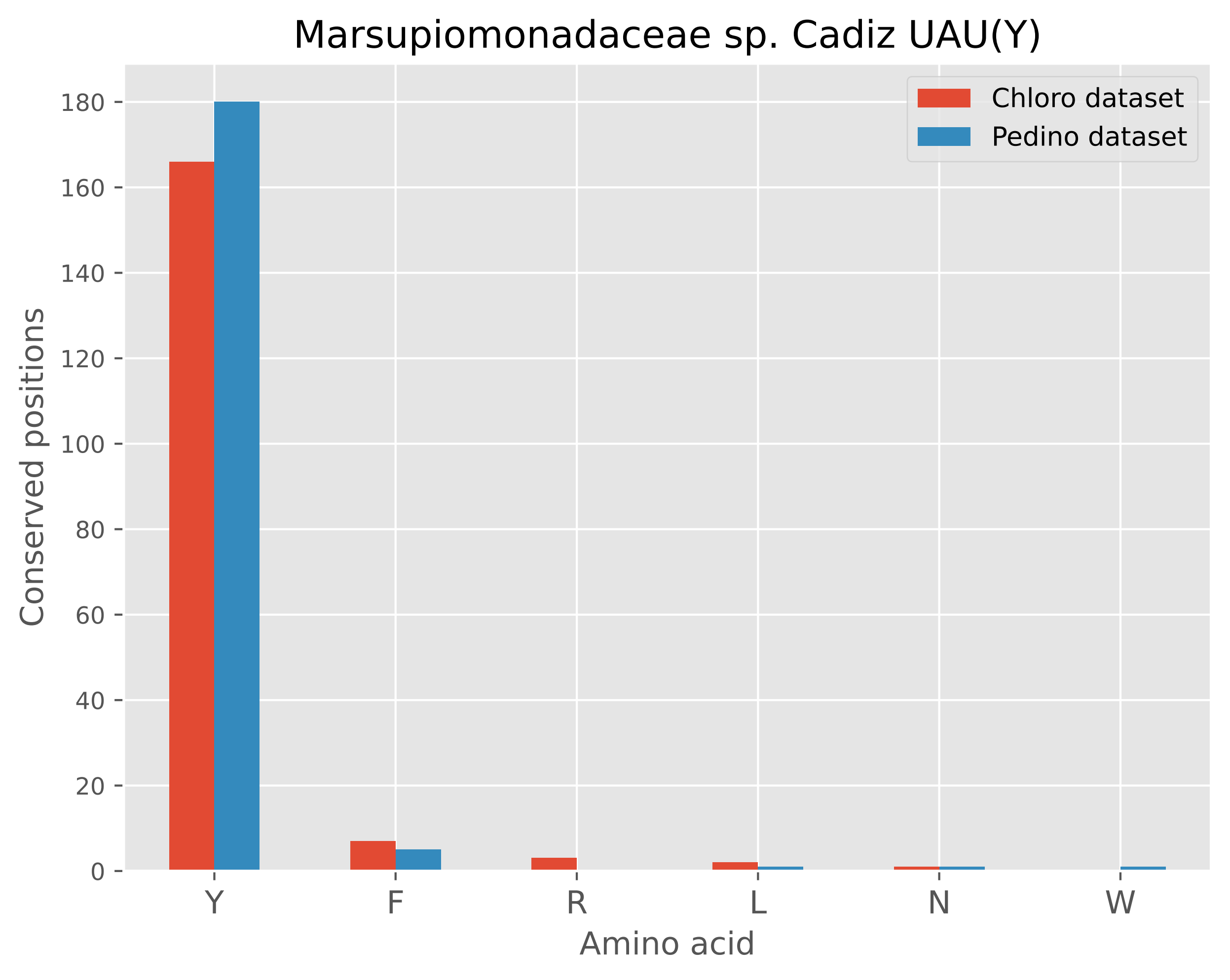

Marsupiomonadaceae sp. Cadiz UAU(Y)
Chloro dataset
Pedino dataset
Conserved positions
0
20
40
60
80
100
120
140
160
180
Y
F
R
L
N
W
Amino acid

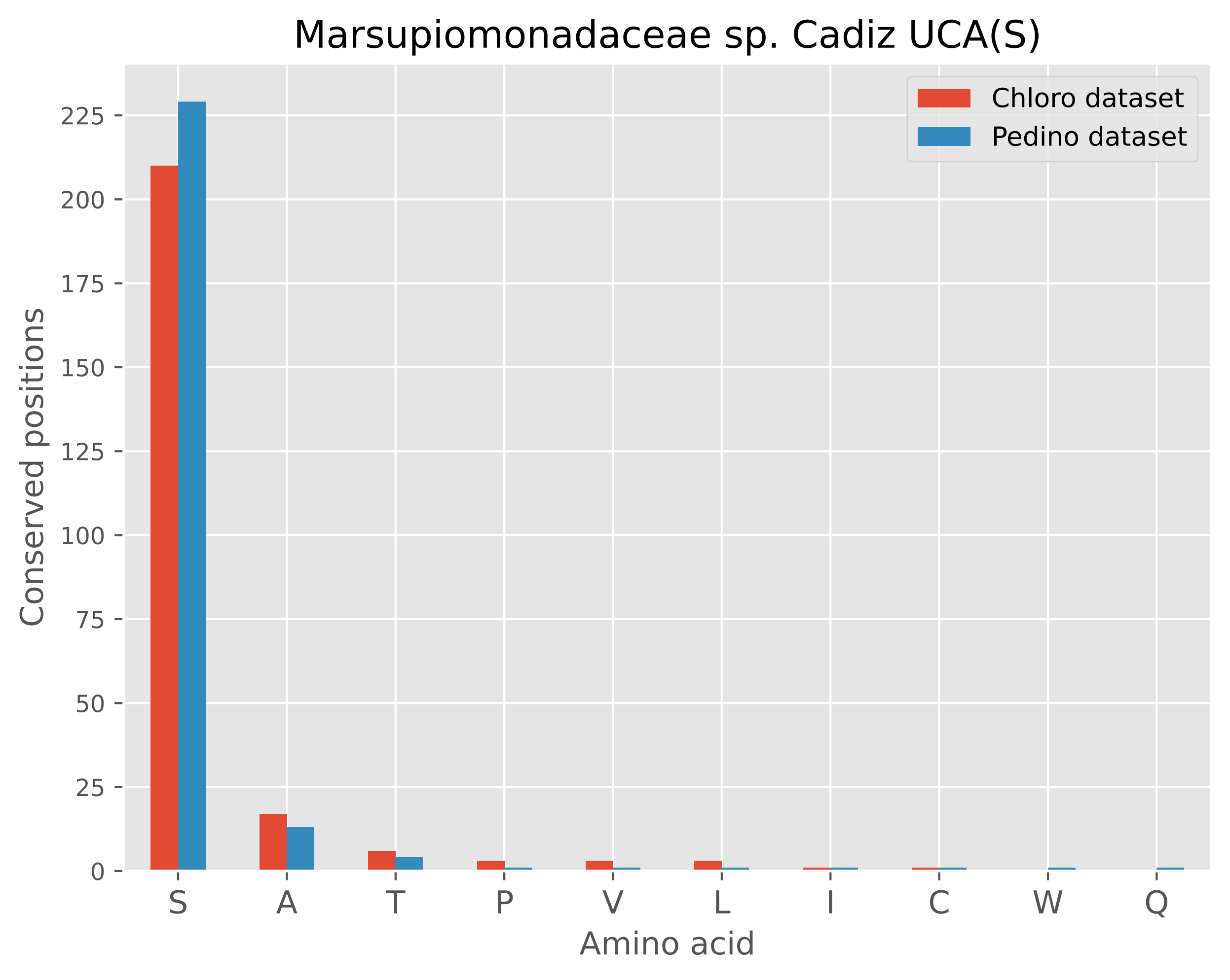

Marsupiomonadaceae sp. Cadiz UCA(S)
Chloro dataset
Pedino dataset
Conserved positions
0
25
50
75
100
125
150
175
200
225
S
A
T
P
V
L
I
C
W
Q
Amino acid

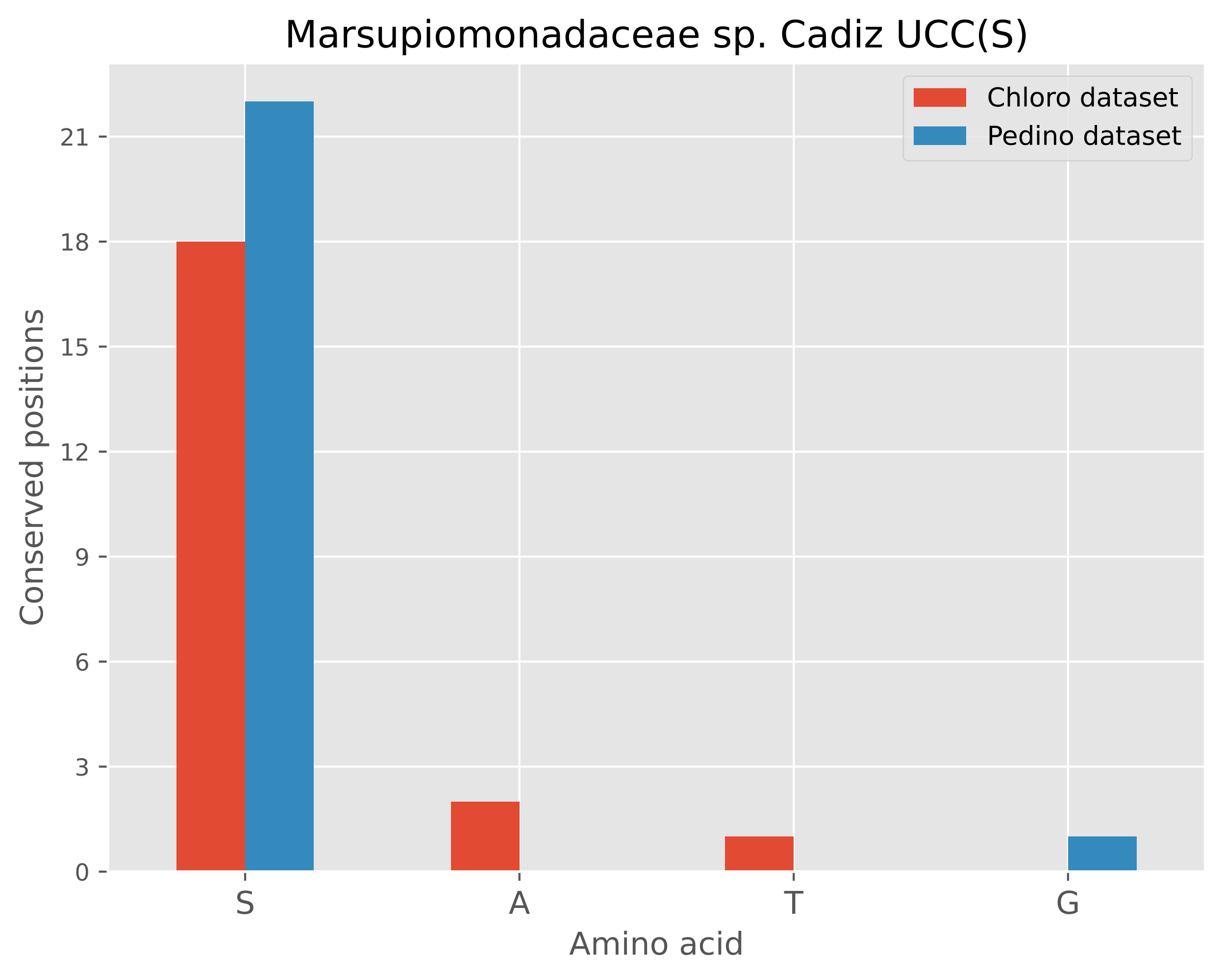
Marsupiomonadaceae sp. Cadiz UCC(S)
Chloro dataset
Pedino dataset
Conserved positions
0
3
6
9
12
15
18
21
S
A
T
G
Amino acid

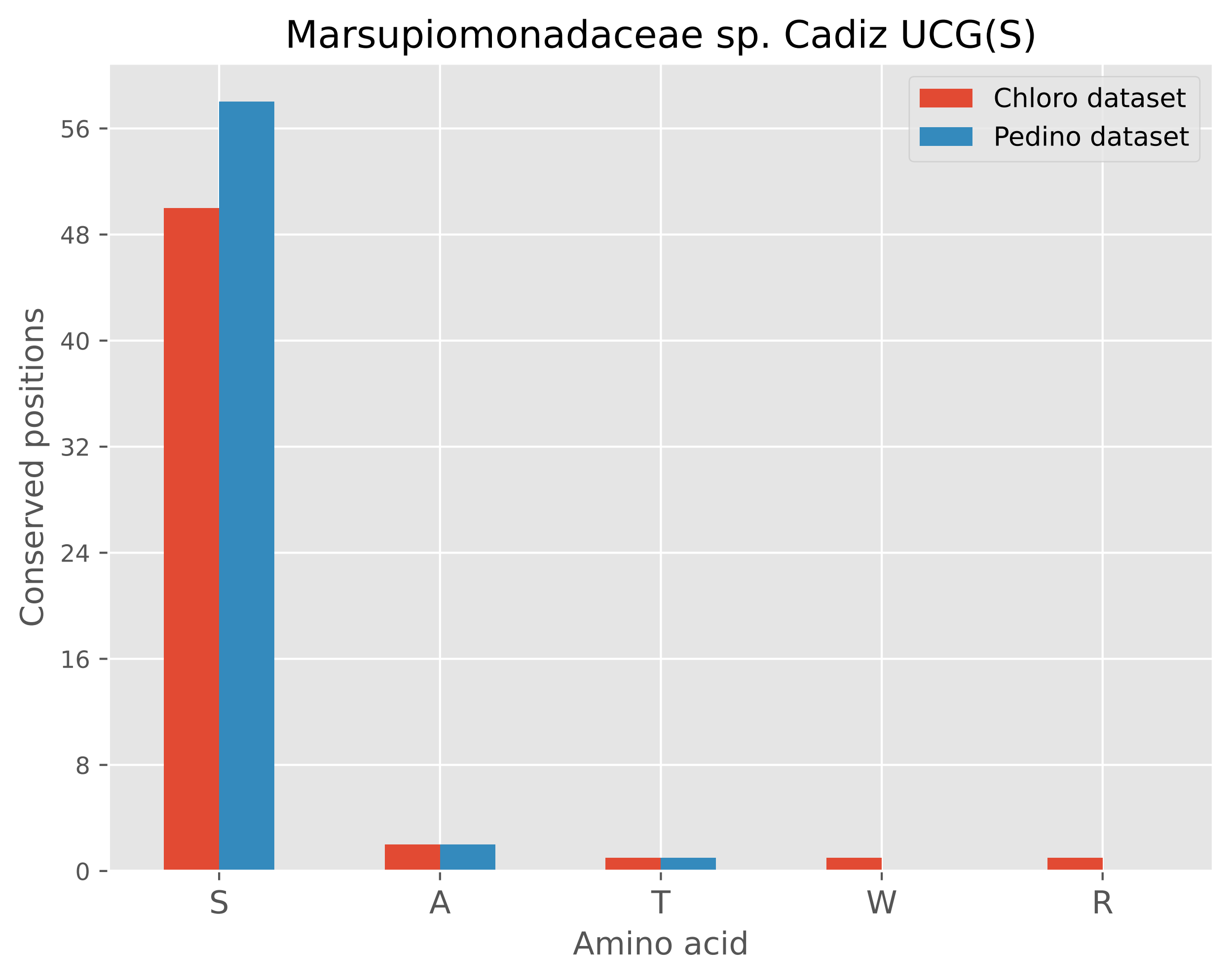

Marsupiomonadaceae sp. Cadiz UCG(S)
Chloro dataset
Pedino dataset
Conserved positions
0
8
16
24
32
40
48
56
S
A
T
W
R
Amino acid

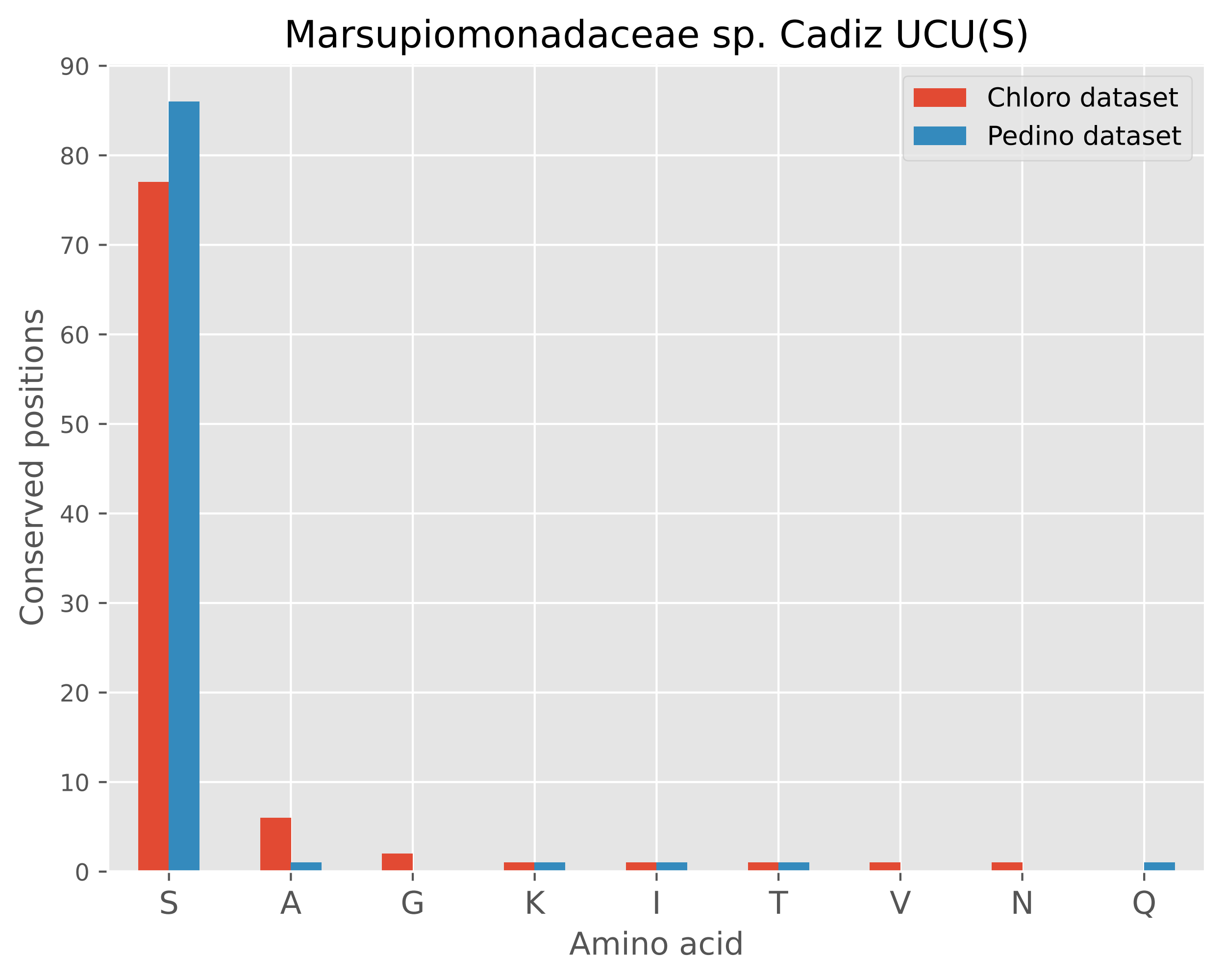
Marsupiomonadaceae sp. Cadiz UCU(S)
Chloro dataset
Pedino dataset
Conserved positions
Amino acid
0
10
20
30
40
50
60
70
80
90
S
A
G
K
I
T
V
N
Q

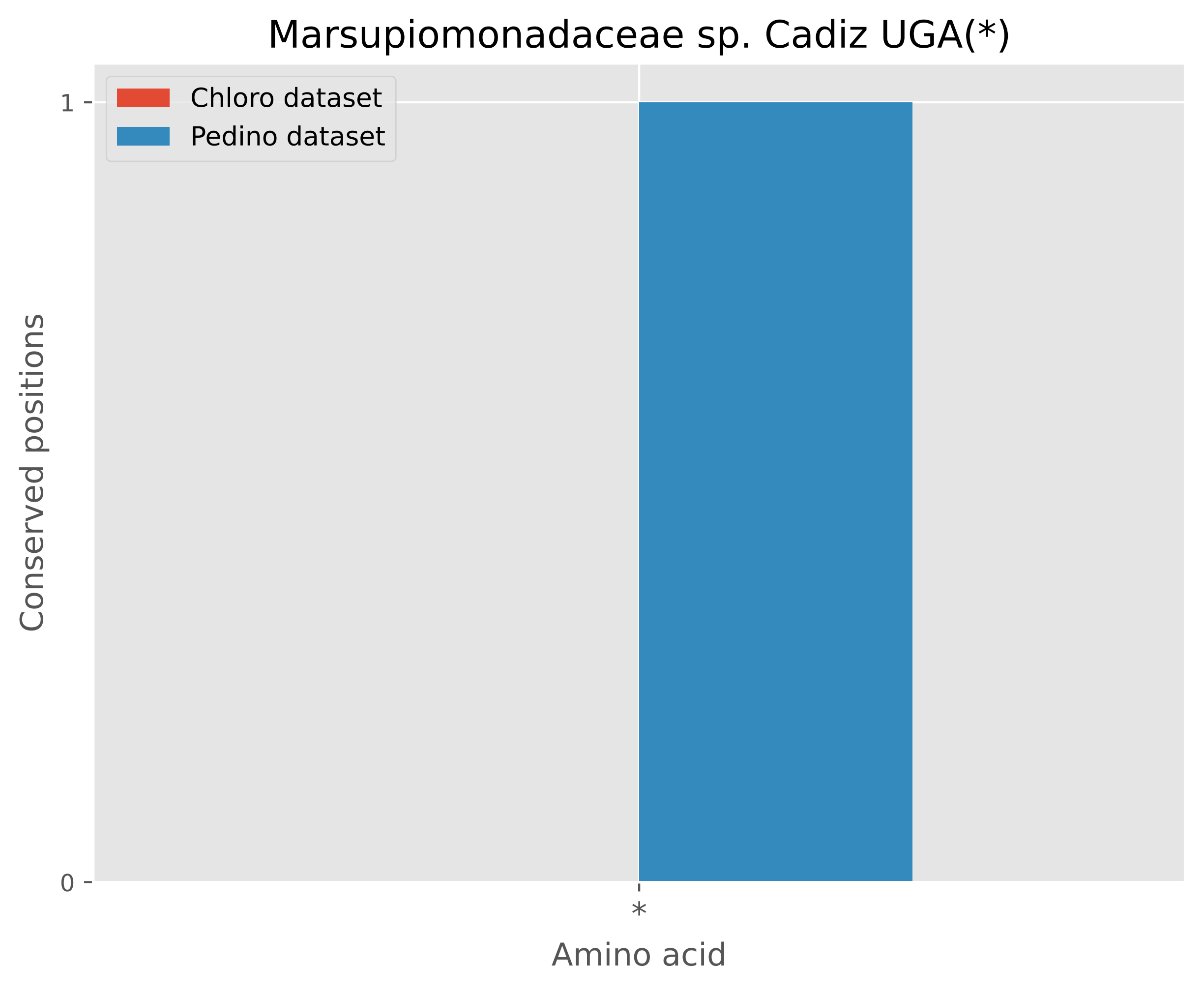
Marsupiomonadaceae sp. Cadiz UGA(*)
Chloro dataset
Pedino dataset
1
0
Conserved positions
*
Amino acid

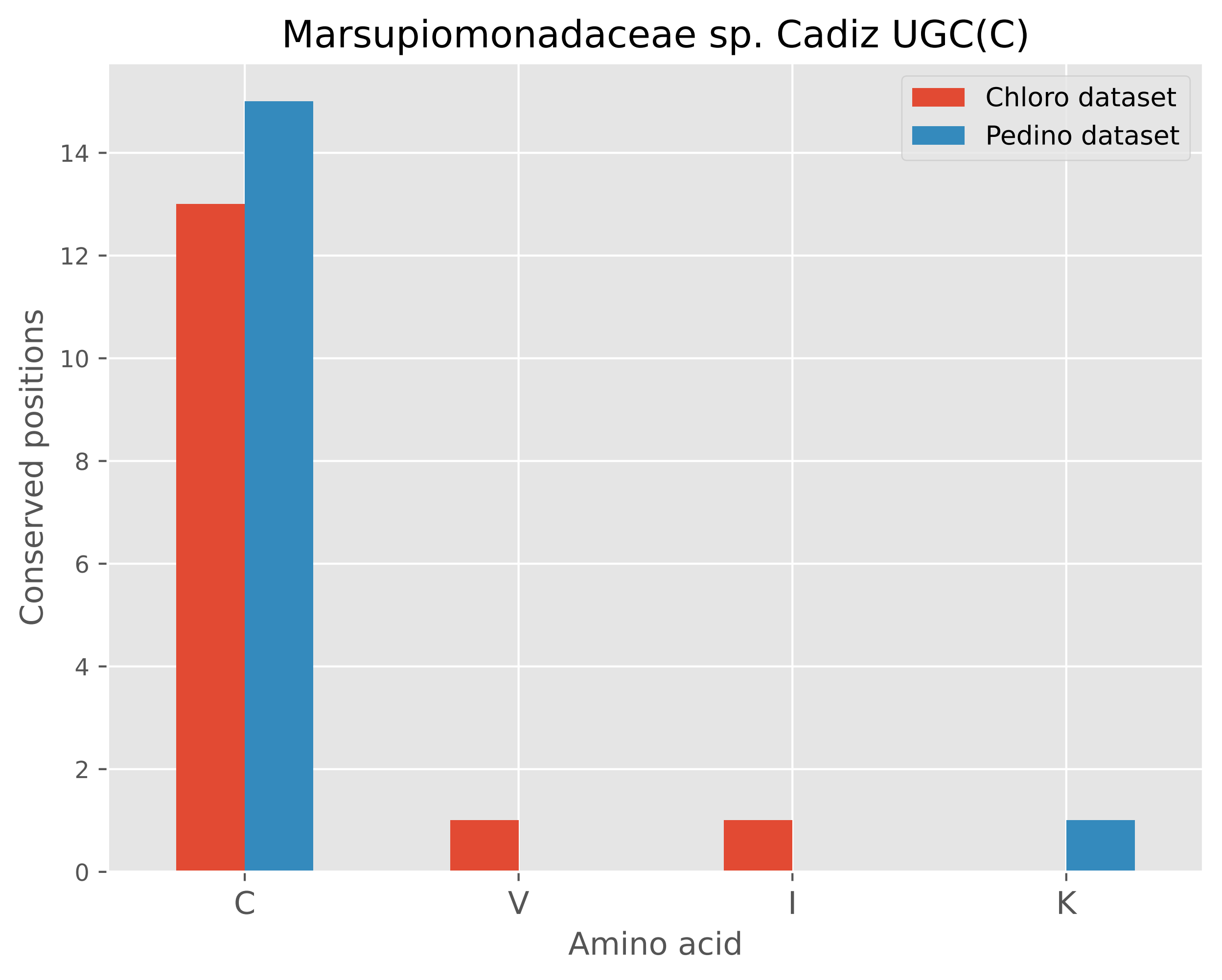

Marsupiomonadaceae sp. Cadiz UGC(C)
Chloro dataset
Pedino dataset
Conserved positions
0
2
4
6
8
10
12
14
C
V
I
K
Amino acid

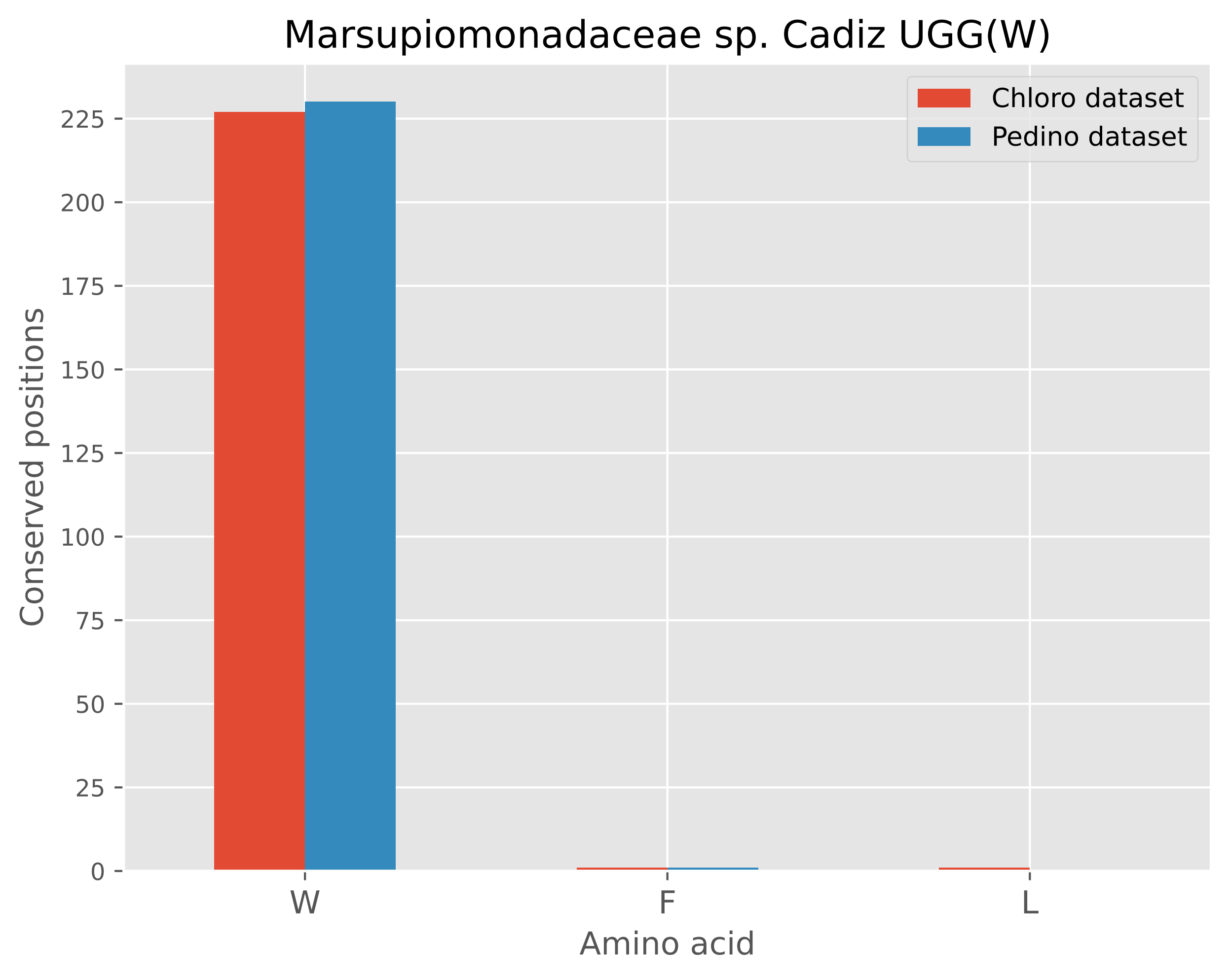

Marsupiomonadaceae sp. Cadiz UGG(W)
Chloro dataset
Pedino dataset
Conserved positions
0
25
50
75
100
125
150
175
200
225
W
F
L
Amino acid

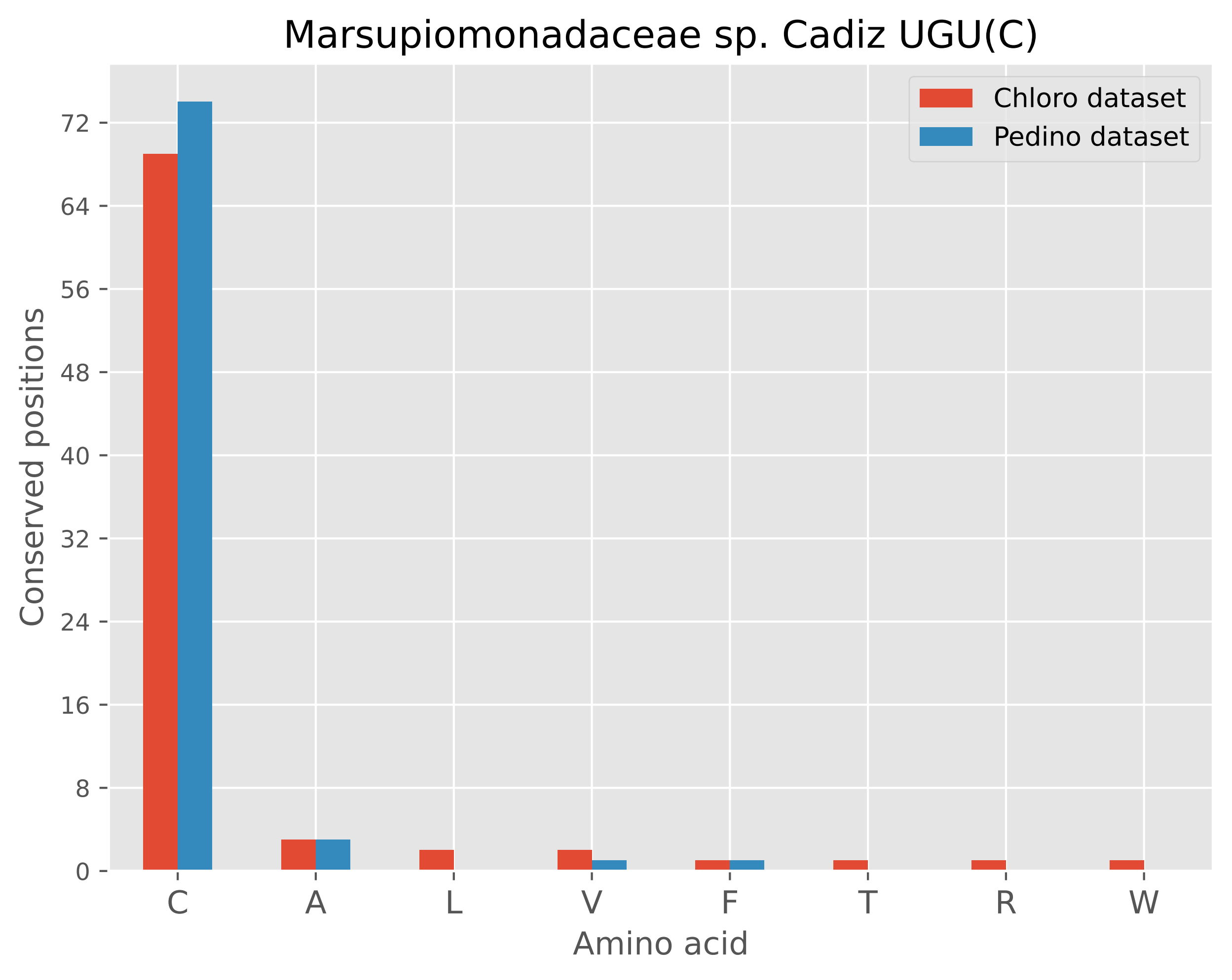
Marsupiomonadaceae sp. Cadiz UGU(C)
Chloro dataset
Pedino dataset
Conserved positions
0
8
16
24
32
40
48
56
64
72
C
A
L
V
F
T
R
W
Amino acid

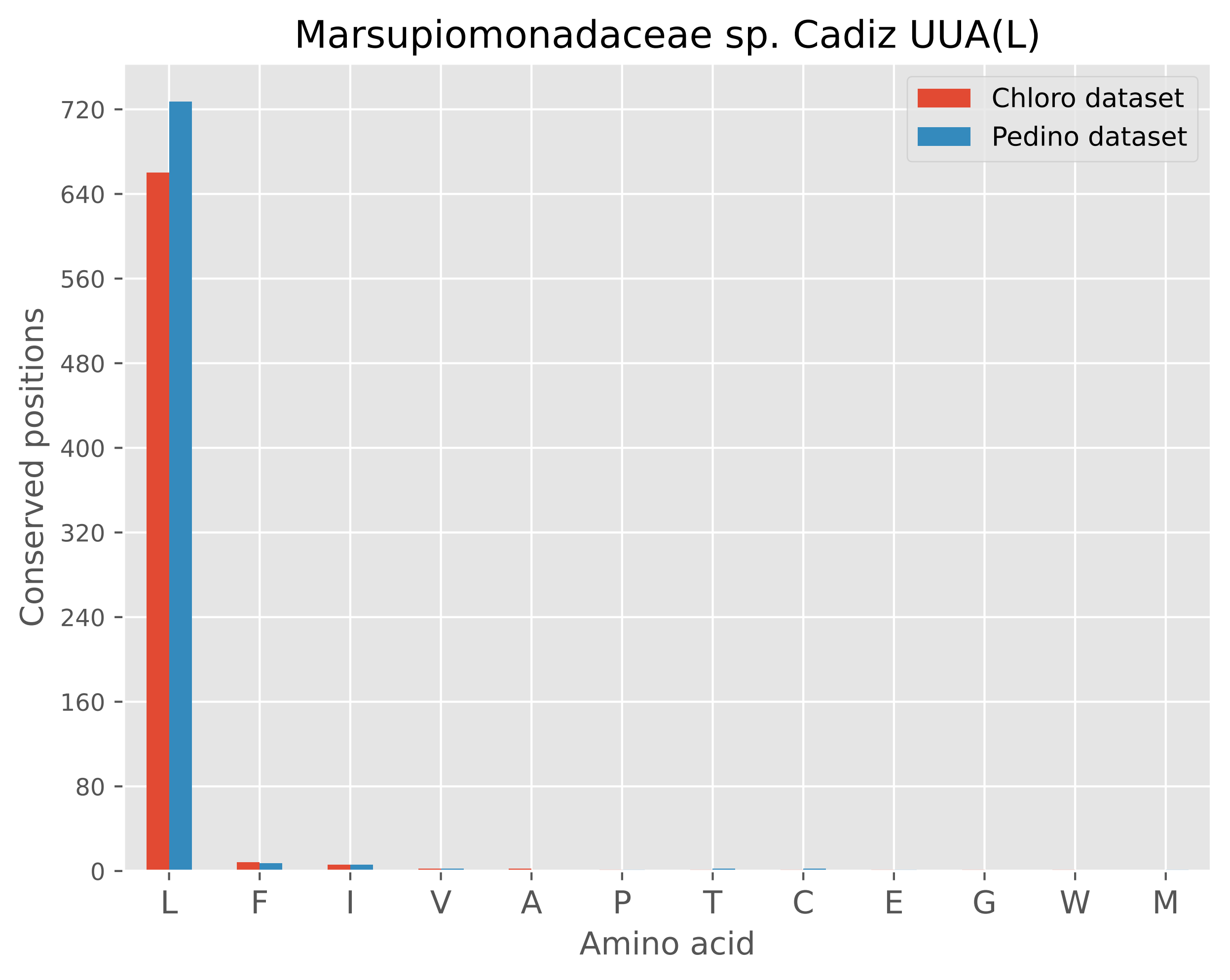
Marsupiomonadaceae sp. Cadiz UUA(L)
Chloro dataset
Pedino dataset
Conserved positions
0
80
160
240
320
400
480
560
640
720
L
F
I
V
A
P
T
C
E
G
W
M
Amino acid

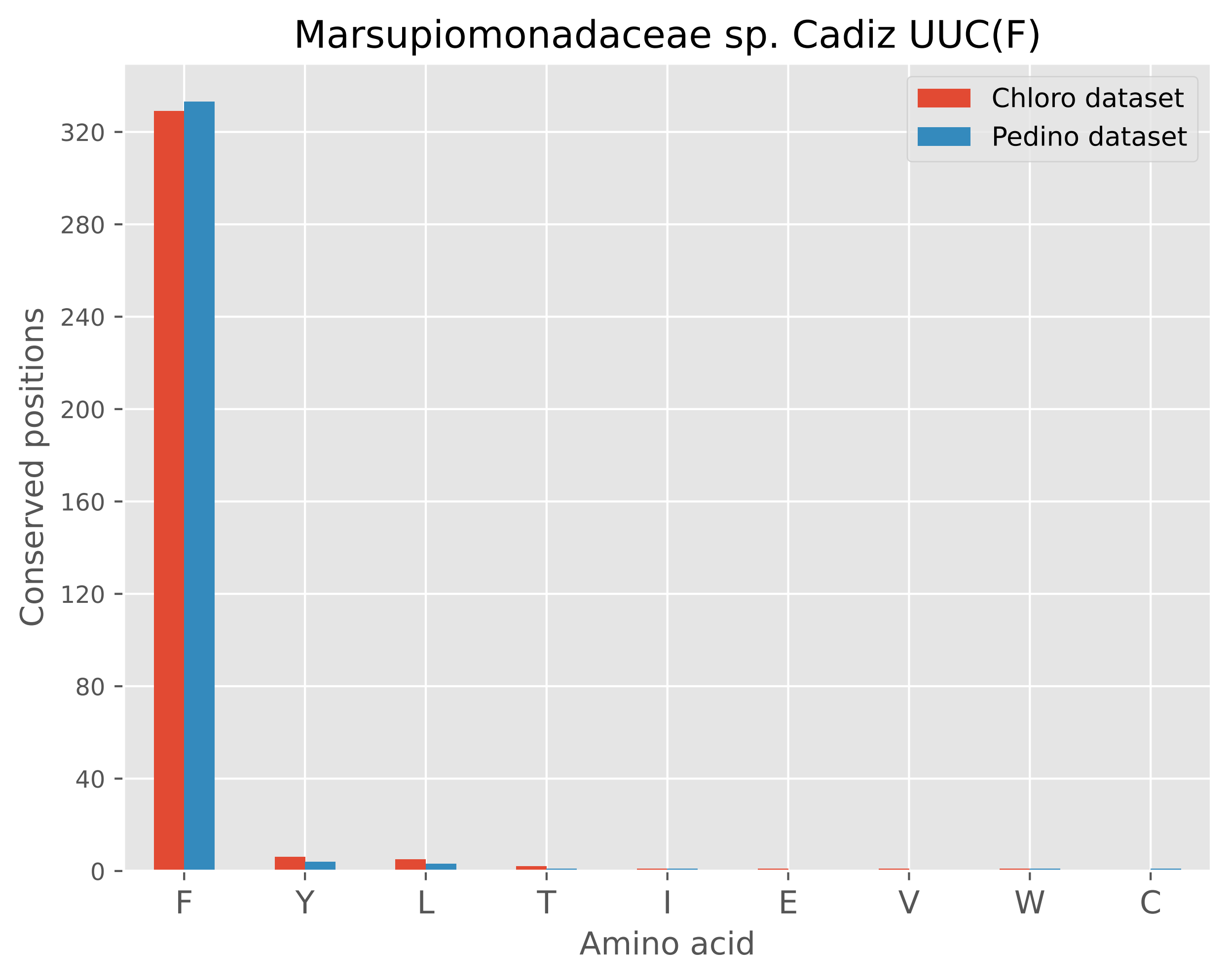
Marsupiomonadaceae sp. Cadiz UUC(F)
Chloro dataset
Pedino dataset
Conserved positions
0
40
80
120
160
200
240
280
320
F
Y
L
T
I
E
V
W
C
Amino acid

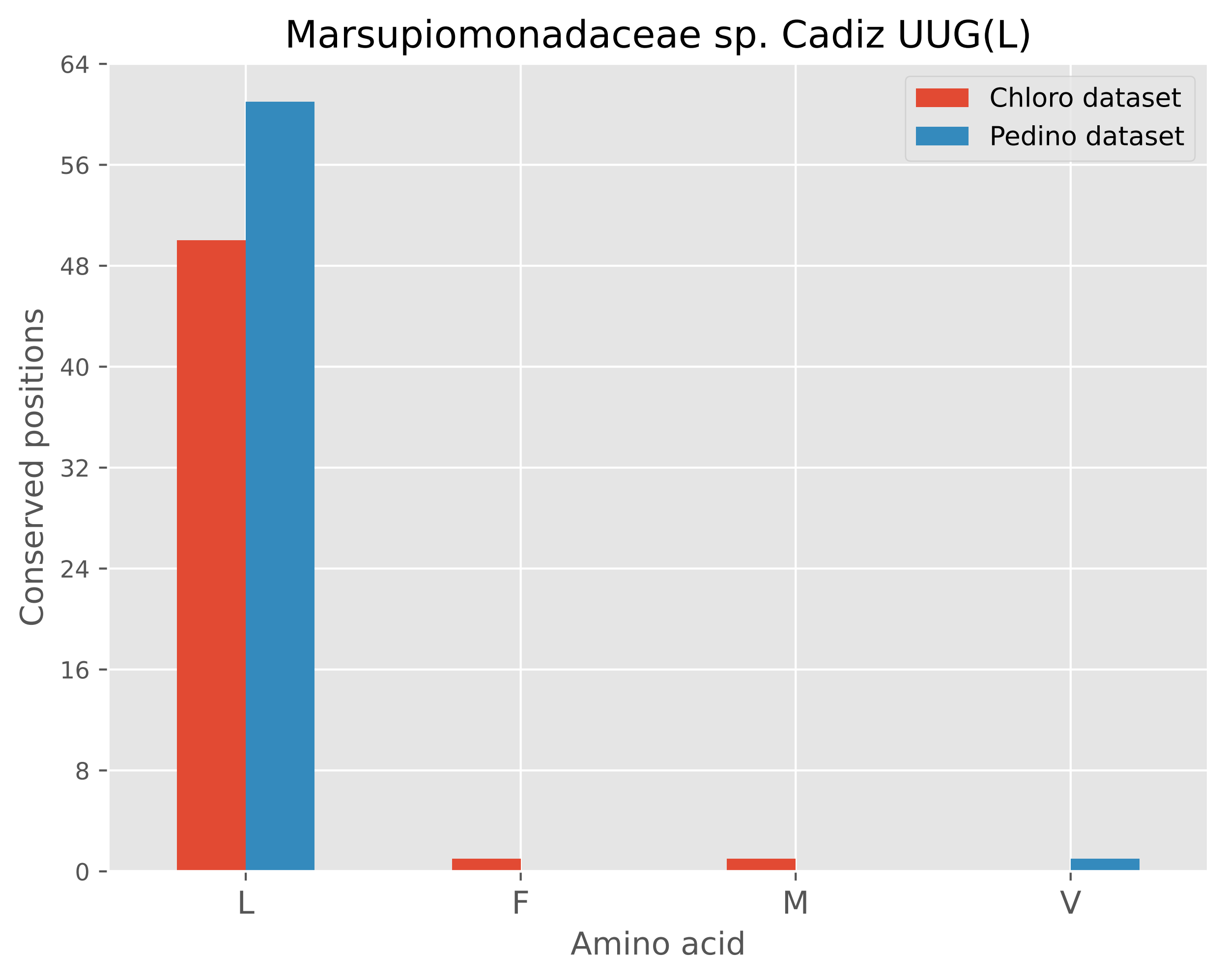

Marsupiomonadaceae sp. Cadiz UUG(L)
Chloro dataset
Pedino dataset
Conserved positions
Amino acid
0
8
16
24
32
40
48
56
64
L
F
M
V

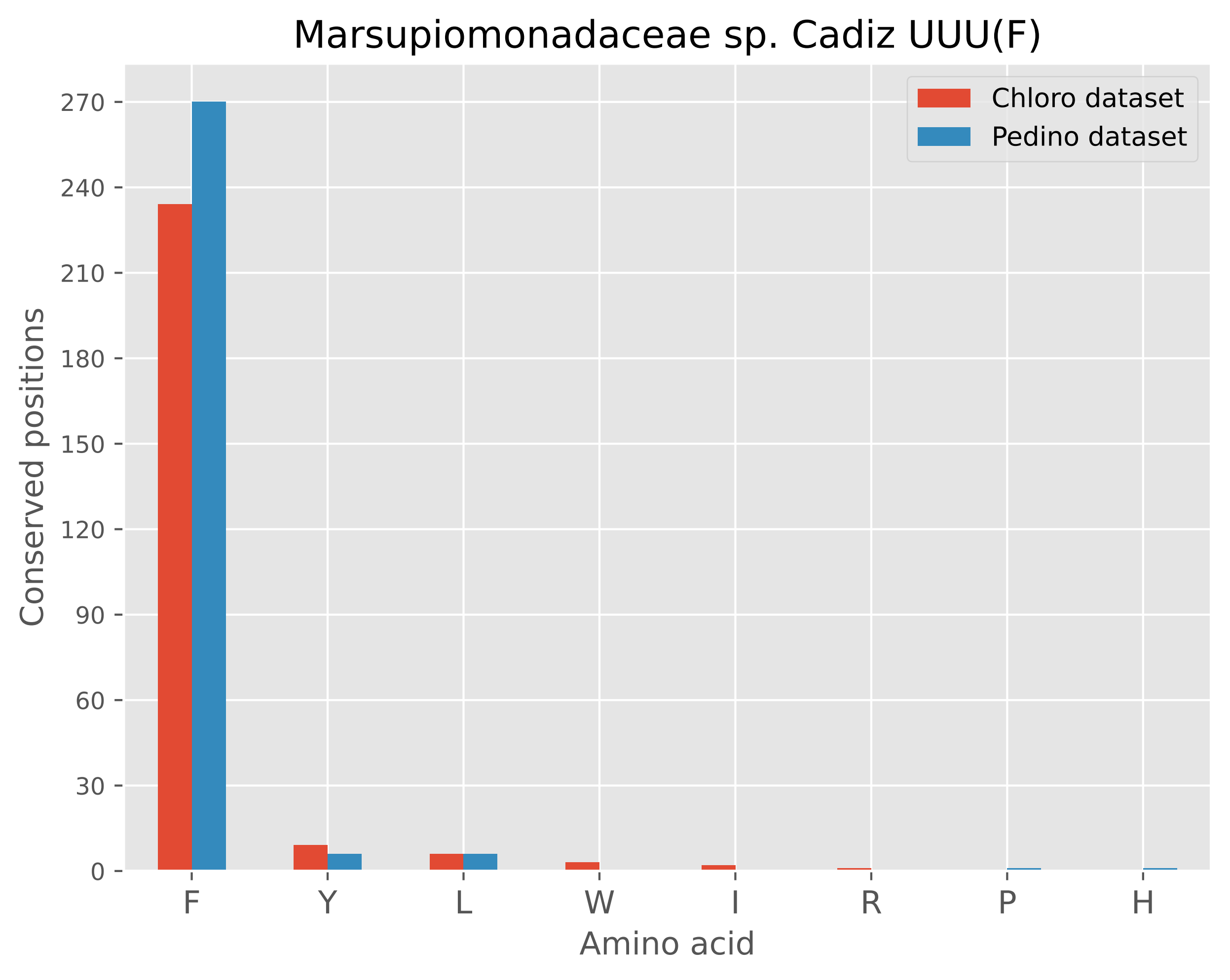

Marsupiomonadaceae sp. Cadiz UUU(F)
Chloro dataset
Pedino dataset
Conserved positions
0
30
60
90
120
150
180
210
240
270
F
Y
L
W
I
R
P
H
Amino acid

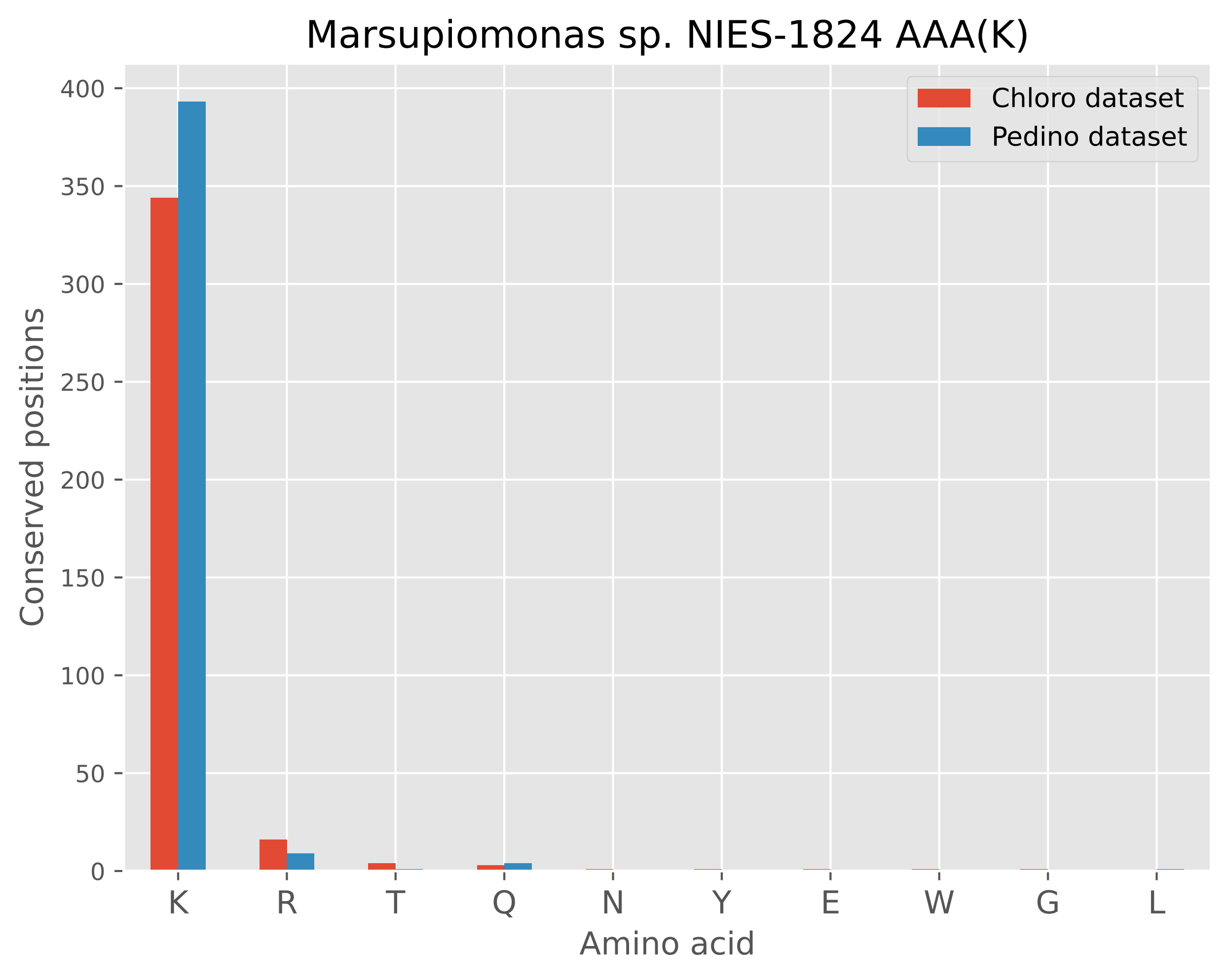
Marsupiomonas sp. NIES-1824 AAA(K)
Chloro dataset
Pedino dataset
Conserved positions
400
350
300
250
200
150
100
50
0
K
R
T
Q
N
Y
E
W
G
L
Amino acid

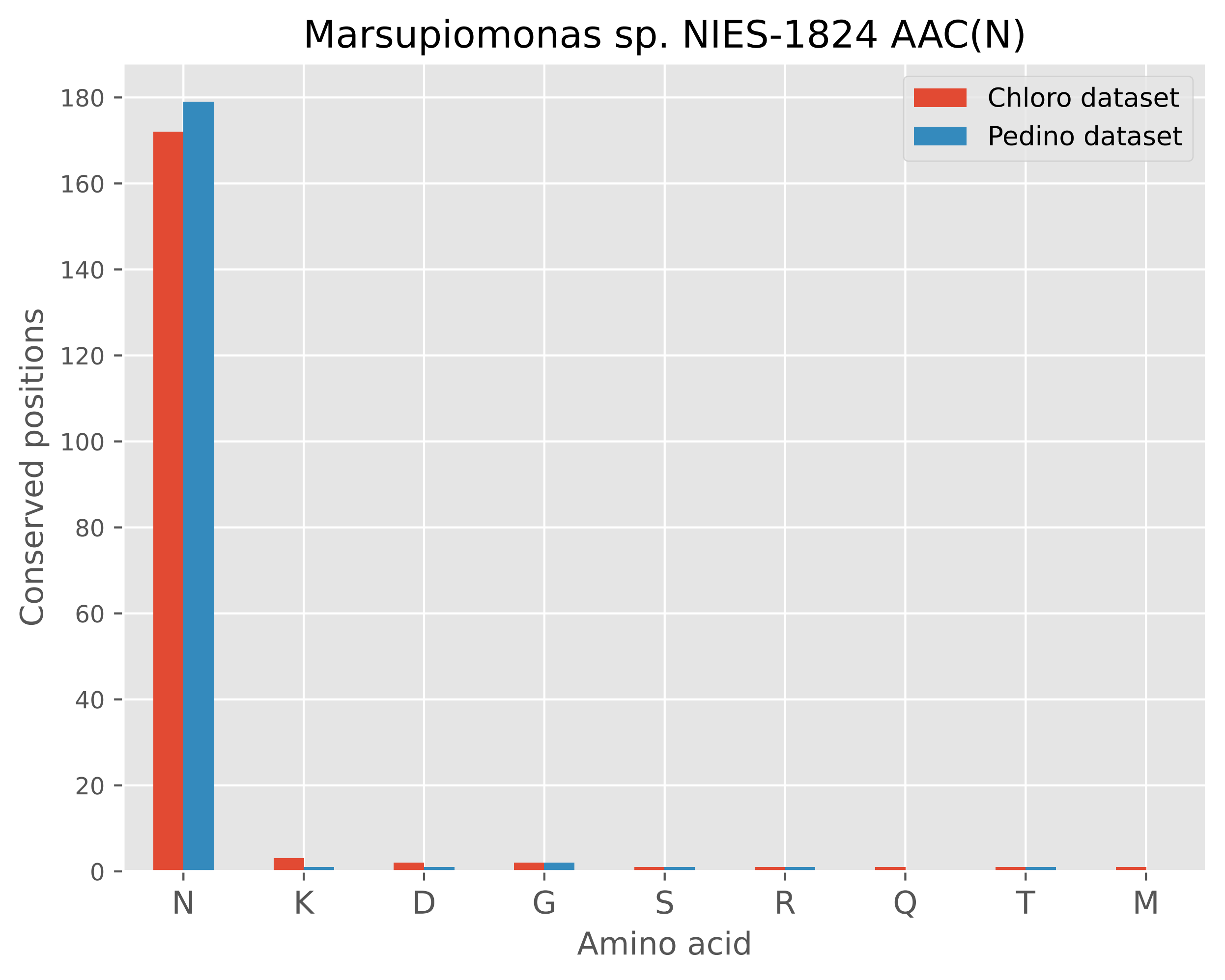

Marsupiomonas sp. NIES-1824 AAC(N)
Chloro dataset
Pedino dataset
Conserved positions
0
20
40
60
80
100
120
140
160
180
N
K
D
G
S
R
Q
T
M
Amino acid

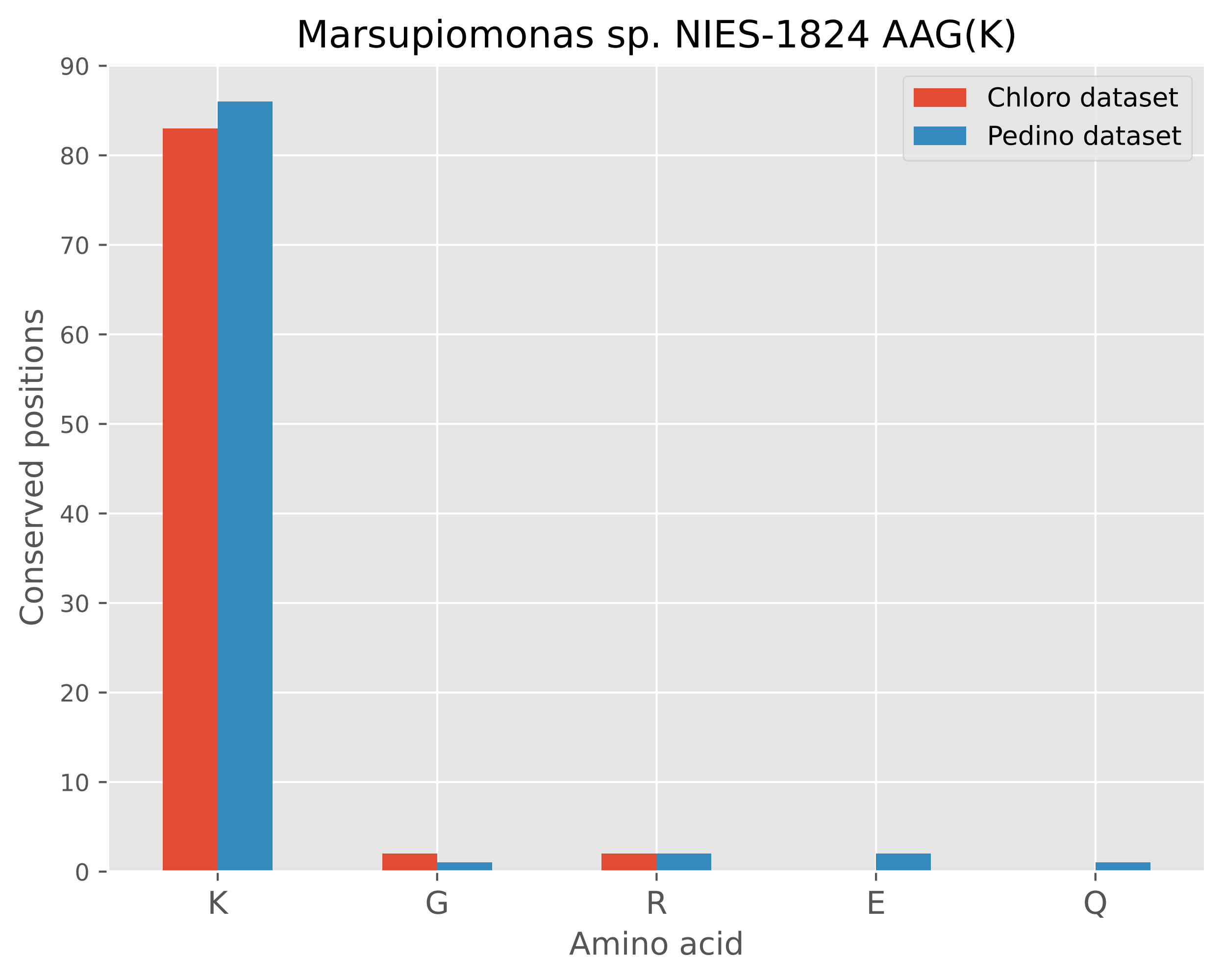

Marsupiomonas sp. NIES-1824 AAG(K)
Chloro dataset
Pedino dataset
Conserved positions
Amino acid
0
10
20
30
40
50
60
70
80
90
K
G
R
E
Q

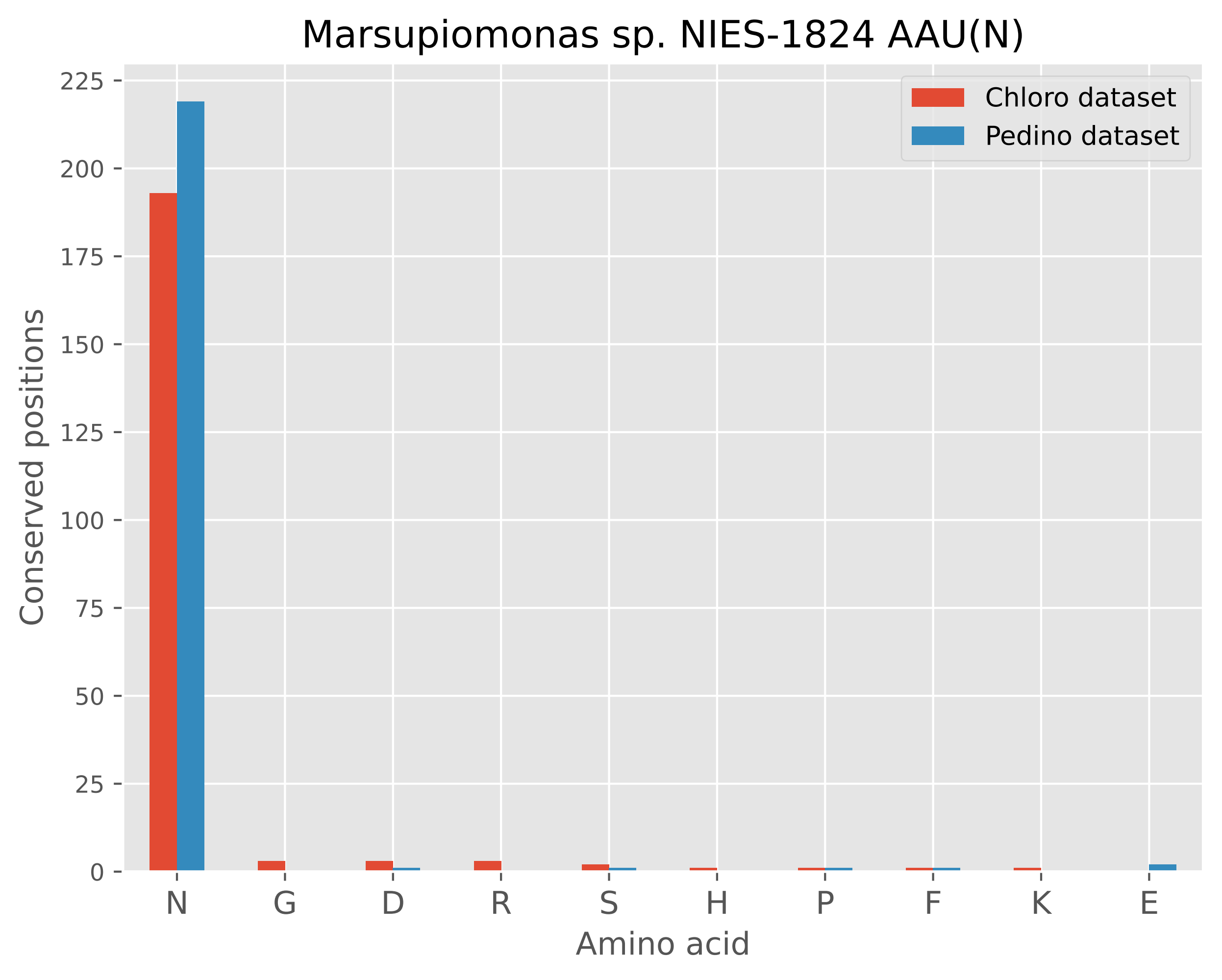

Marsupiomonas sp. NIES-1824 AAU(N)
Chloro dataset
Pedino dataset
Conserved positions
Amino acid
0
25
50
75
100
125
150
175
200
225
N
G
D
R
S
H
P
F
K
E

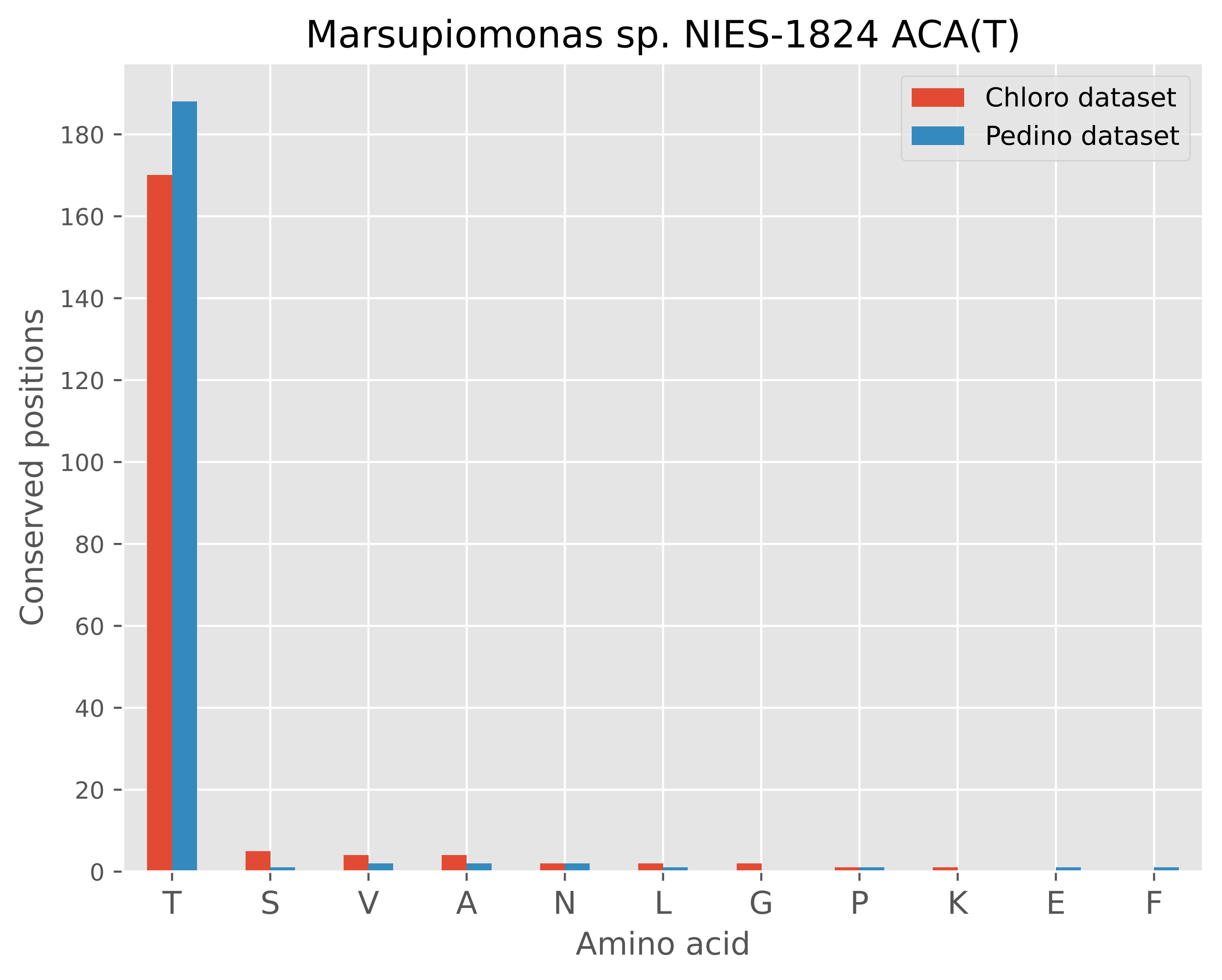

Marsupiomonas sp. NIES-1824 ACA(T)
Chloro dataset
Pedino dataset
Conserved positions
0
20
40
60
80
100
120
140
160
180
T
S
V
A
N
L
G
P
K
E
F
Amino acid

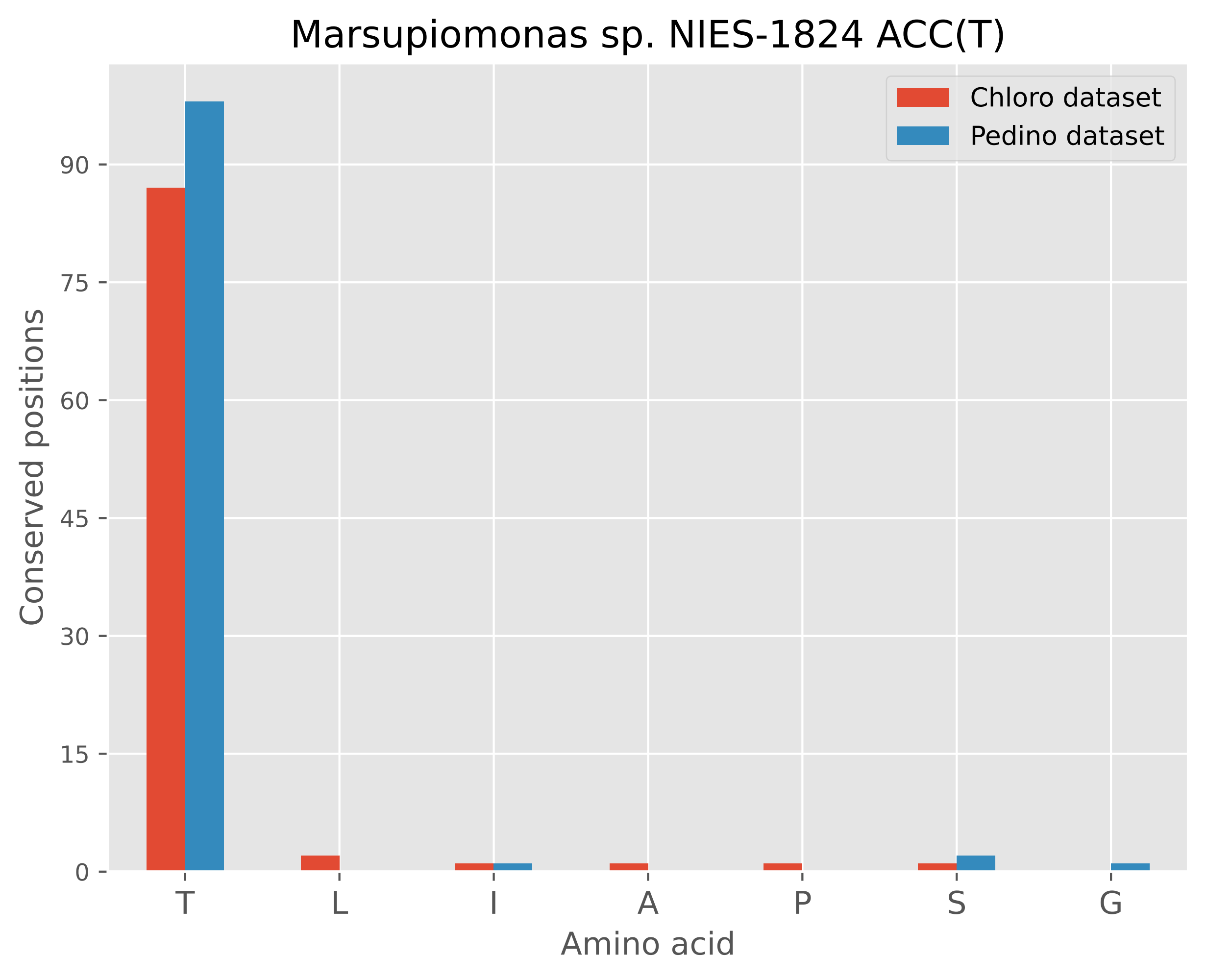
Marsupiomonas sp. NIES-1824 ACC(T)
Chloro dataset
Pedino dataset
Conserved positions
0
15
30
45
60
75
90
T
L
I
A
P
S
G
Amino acid

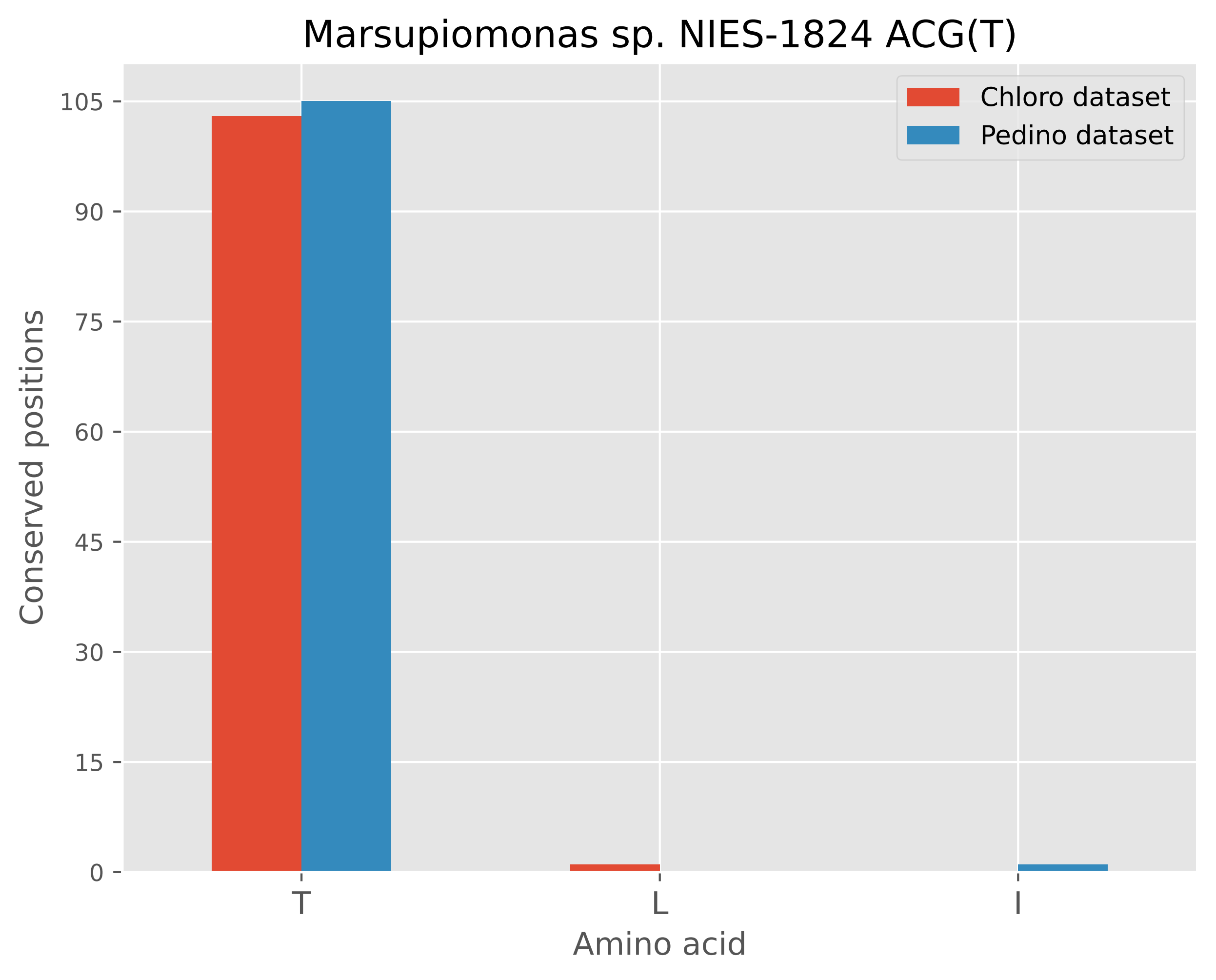

Marsupiomonas sp. NIES-1824 ACG(T)
Chloro dataset
Pedino dataset
Conserved positions
105
90
75
60
45
30
15
0
T
L
I
Amino acid

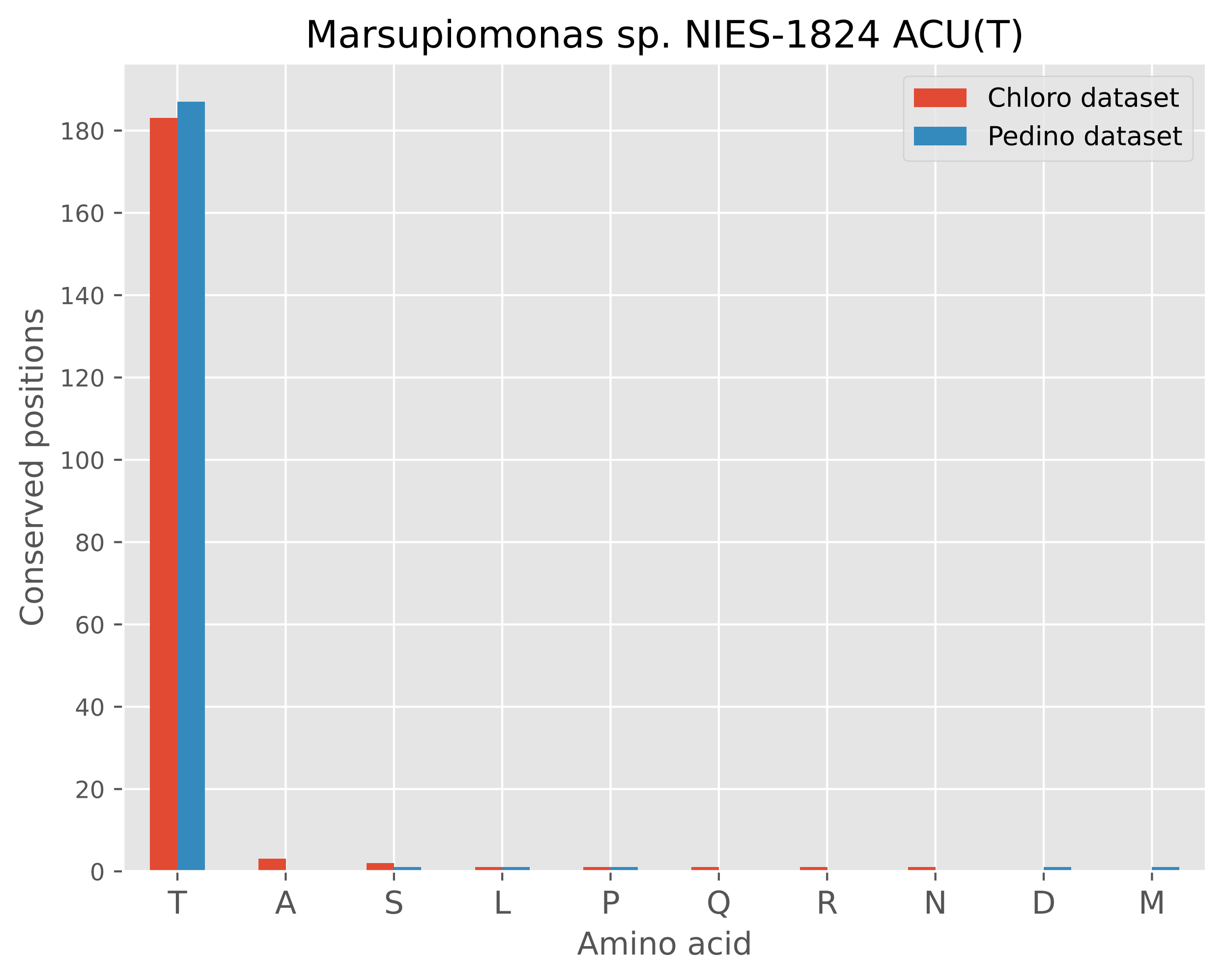

Marsupiomonas sp. NIES-1824 ACU(T)
Chloro dataset
Pedino dataset
Conserved positions
0
20
40
60
80
100
120
140
160
180
T
A
S
L
P
Q
R
N
D
M
Amino acid

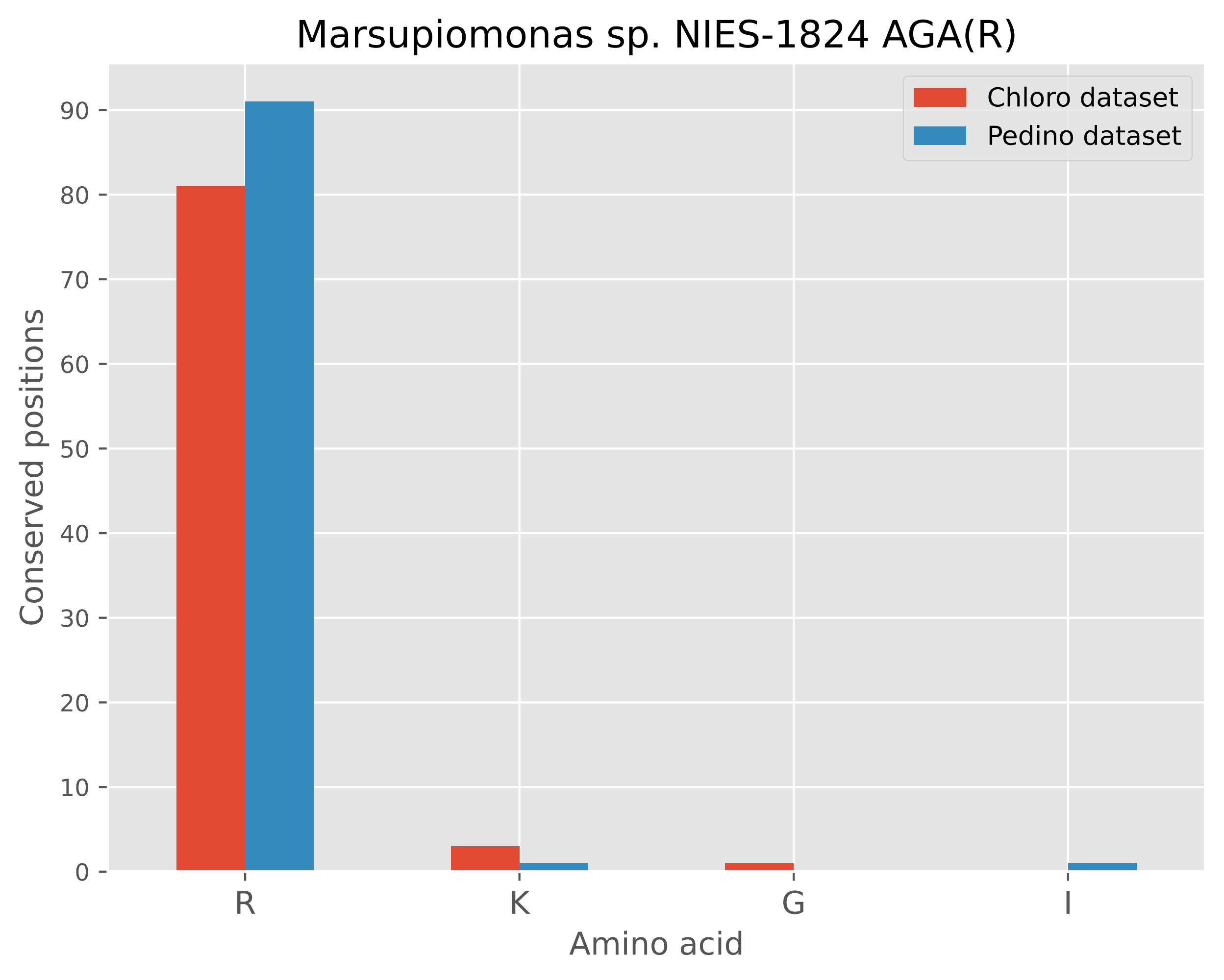

Marsupiomonas sp. NIES-1824 AGA(R)
Chloro dataset
Pedino dataset
Conserved positions
0
10
20
30
40
50
60
70
80
90
R
K
G
I
Amino acid

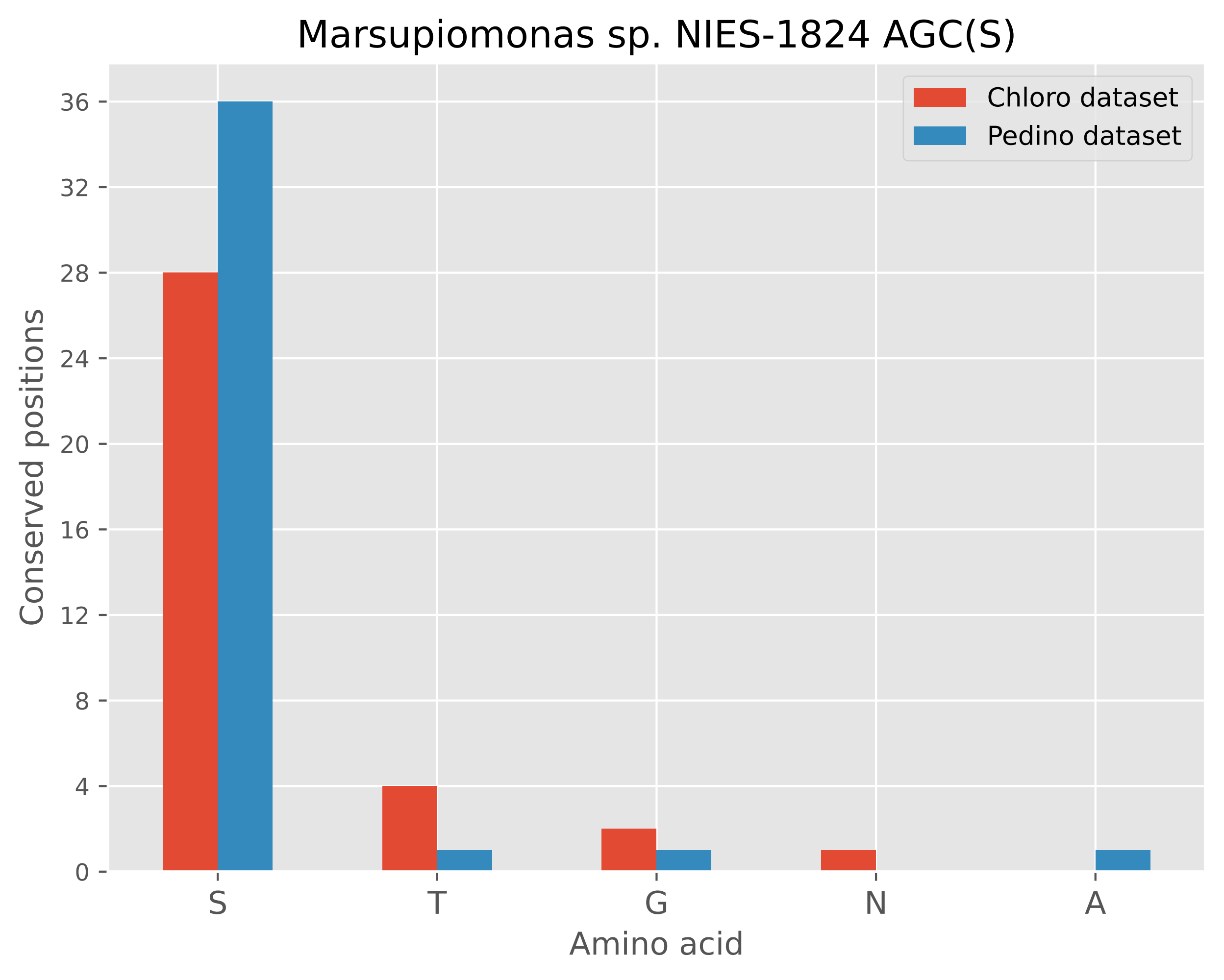

Marsupiomonas sp. NIES-1824 AGC(S)
Chloro dataset
Pedino dataset
Conserved positions
0
4
8
12
16
20
24
28
32
36
S
T
G
N
A
Amino acid

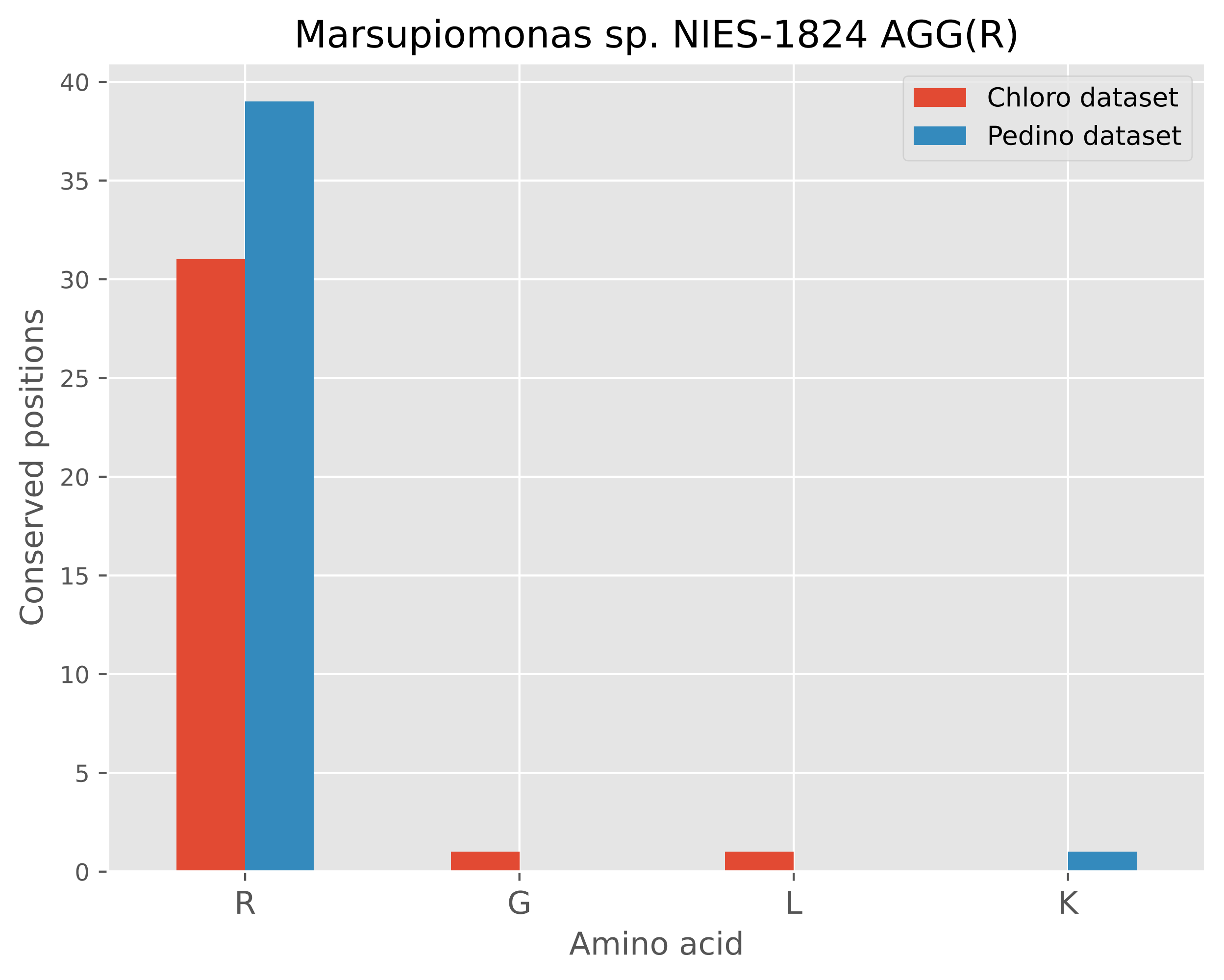

Marsupiomonas sp. NIES-1824 AGG(R)
Chloro dataset
Pedino dataset
Conserved positions
0
5
10
15
20
25
30
35
40
R
G
L
K
Amino acid

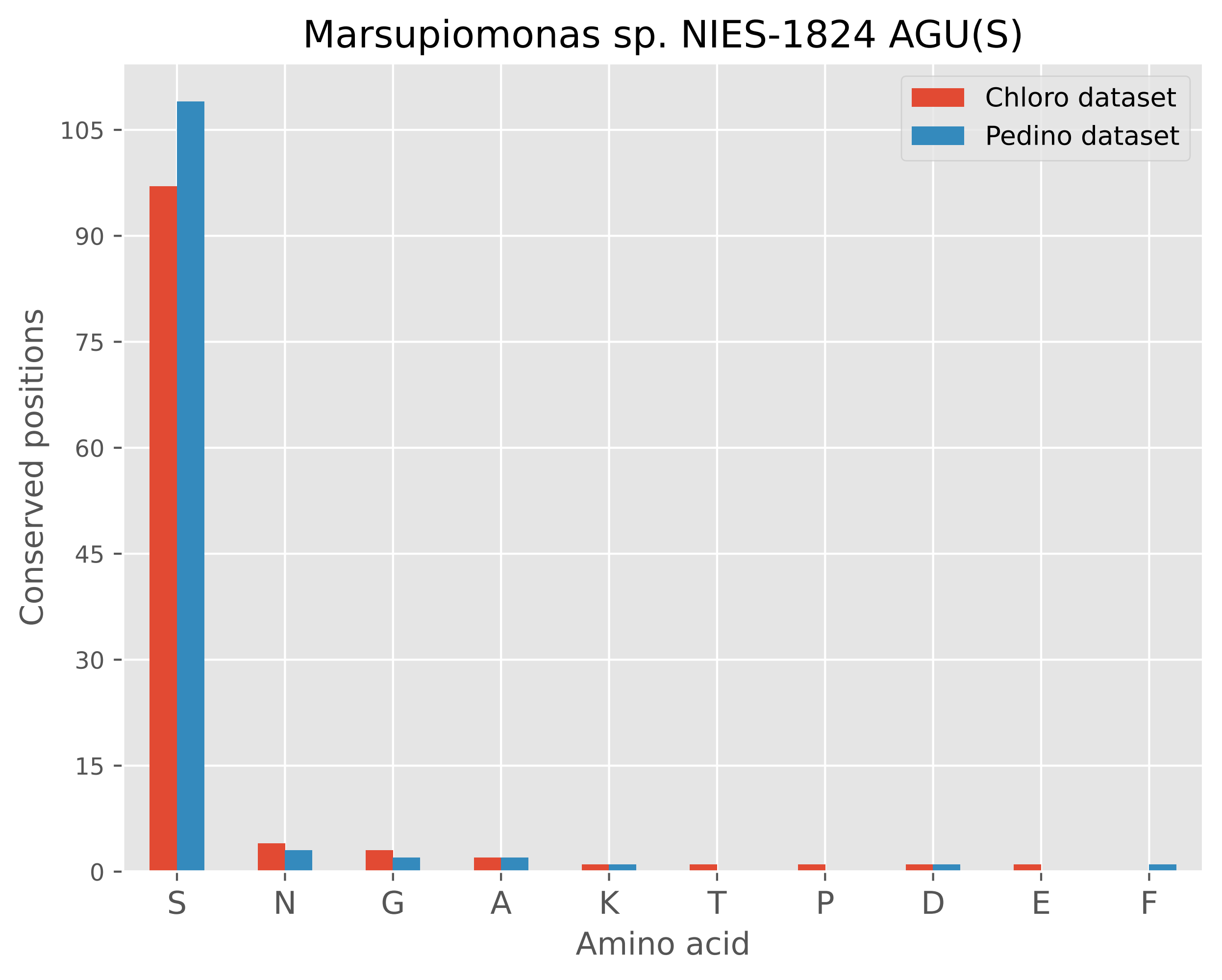
Marsupiomonas sp. NIES-1824 AGU(S)
Chloro dataset
Pedino dataset
Conserved positions
0
15
30
45
60
75
90
105
S
N
G
A
K
T
P
D
E
F
Amino acid

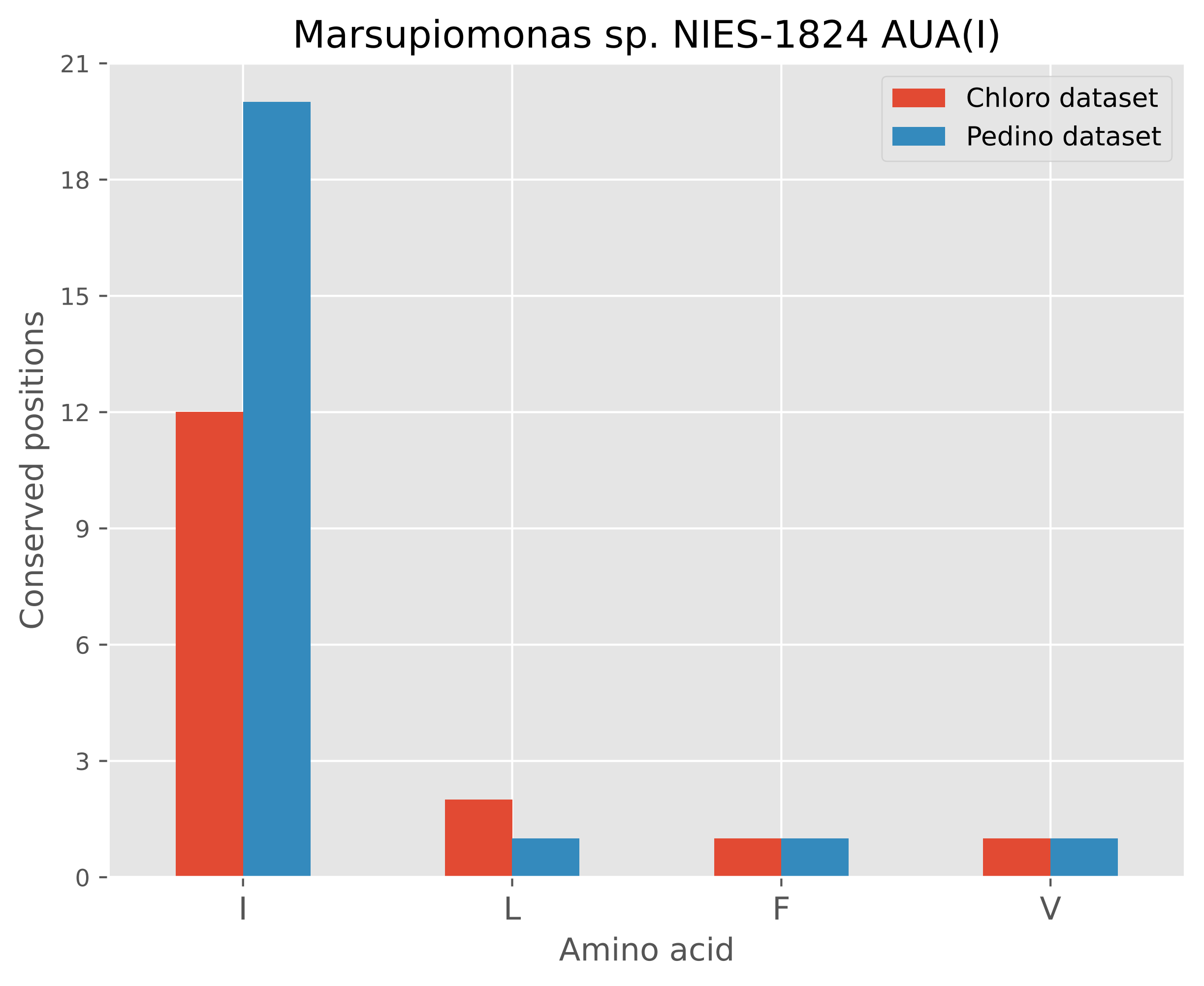
Marsupiomonas sp. NIES-1824 AUA(I)
Chloro dataset
Pedino dataset
Conserved positions
Amino acid
0
3
6
9
12
15
18
21
I
L
F
V

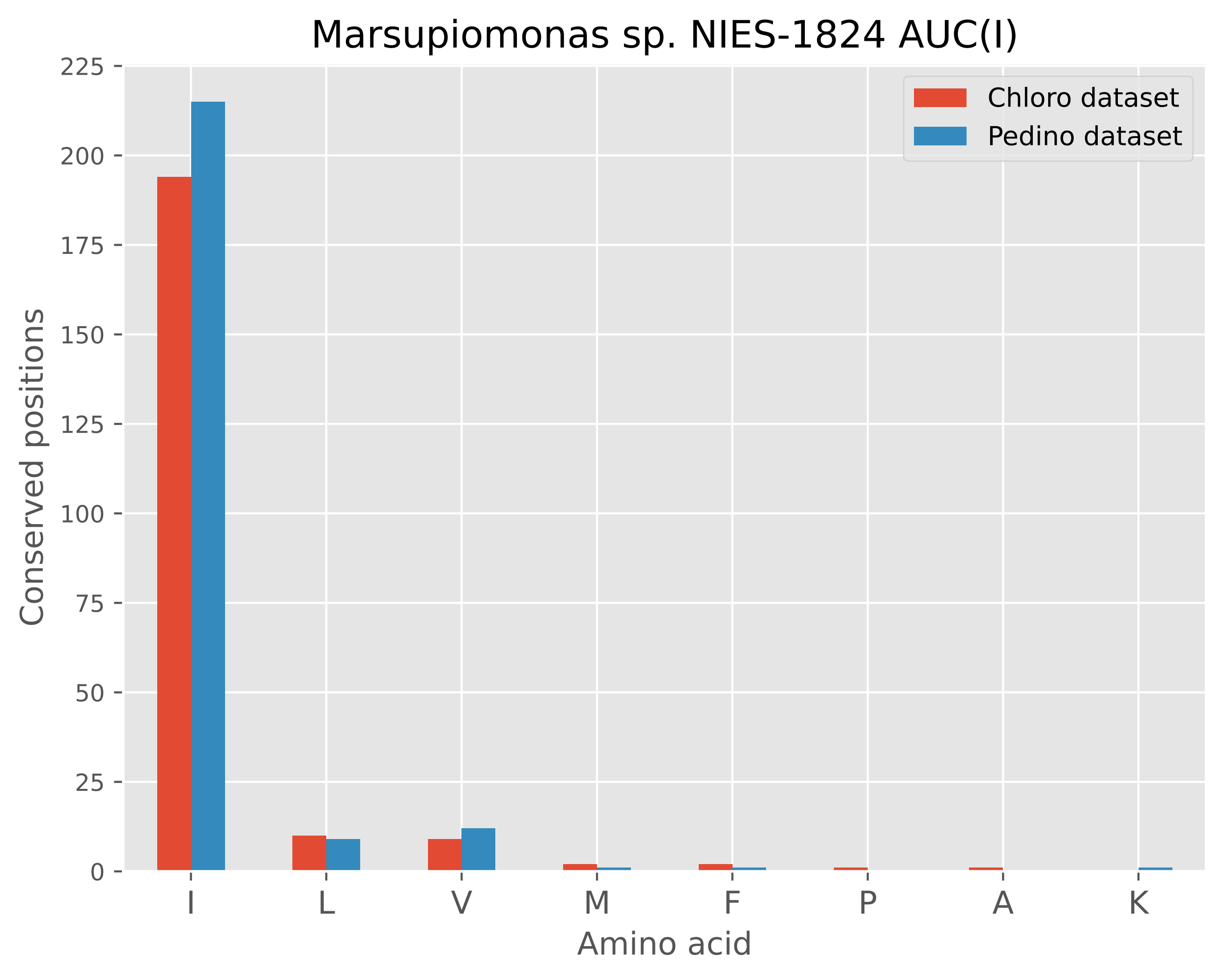
Marsupiomonas sp. NIES-1824 AUC(I)
Chloro dataset
Pedino dataset
Conserved positions
Amino acid
0
25
50
75
100
125
150
175
200
225
I
L
V
M
F
P
A
K

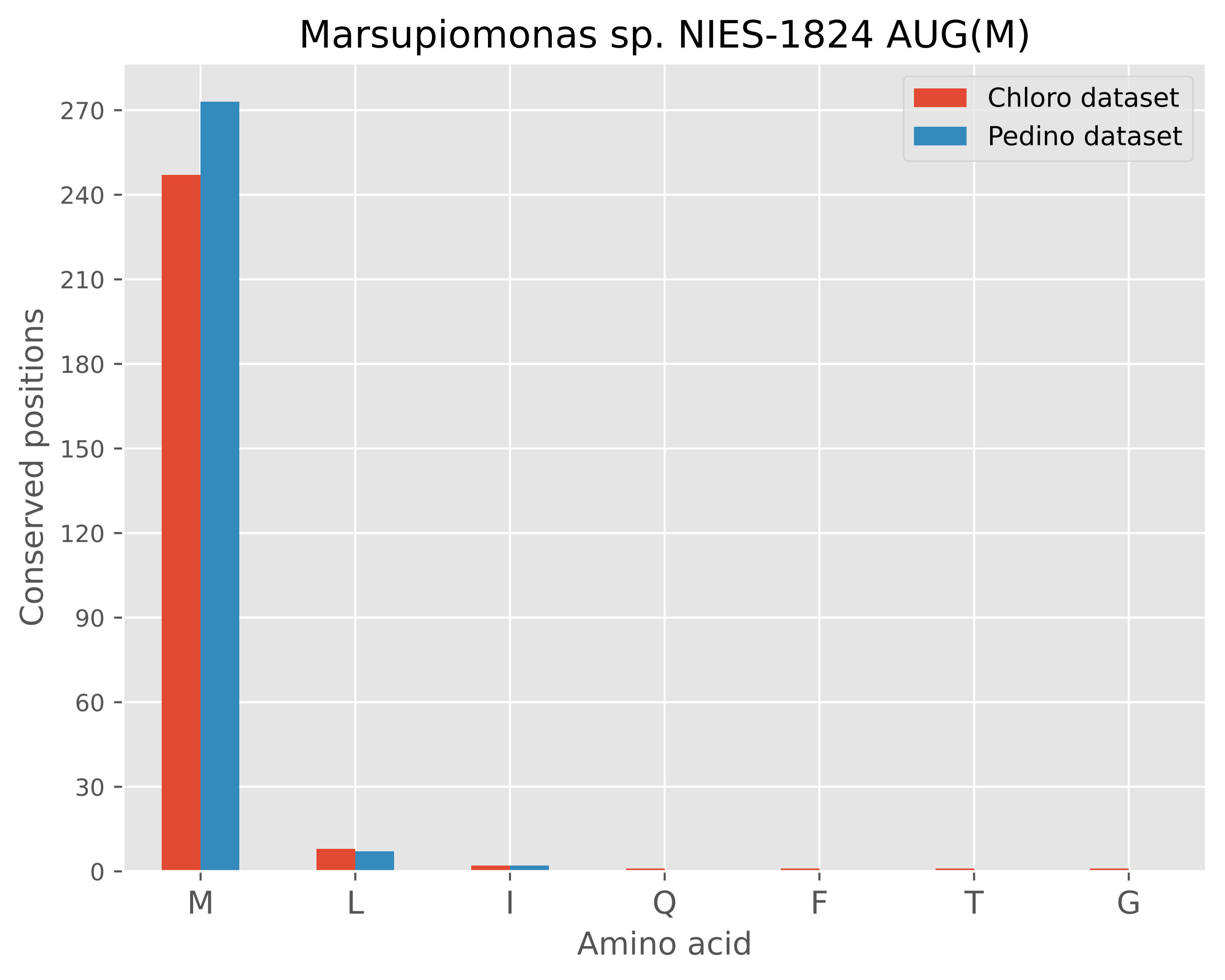

Marsupiomonas sp. NIES-1824 AUG(M)
Chloro dataset
Pedino dataset
Conserved positions
Amino acid
0
30
60
90
120
150
180
210
240
270
M
L
I
Q
F
T
G

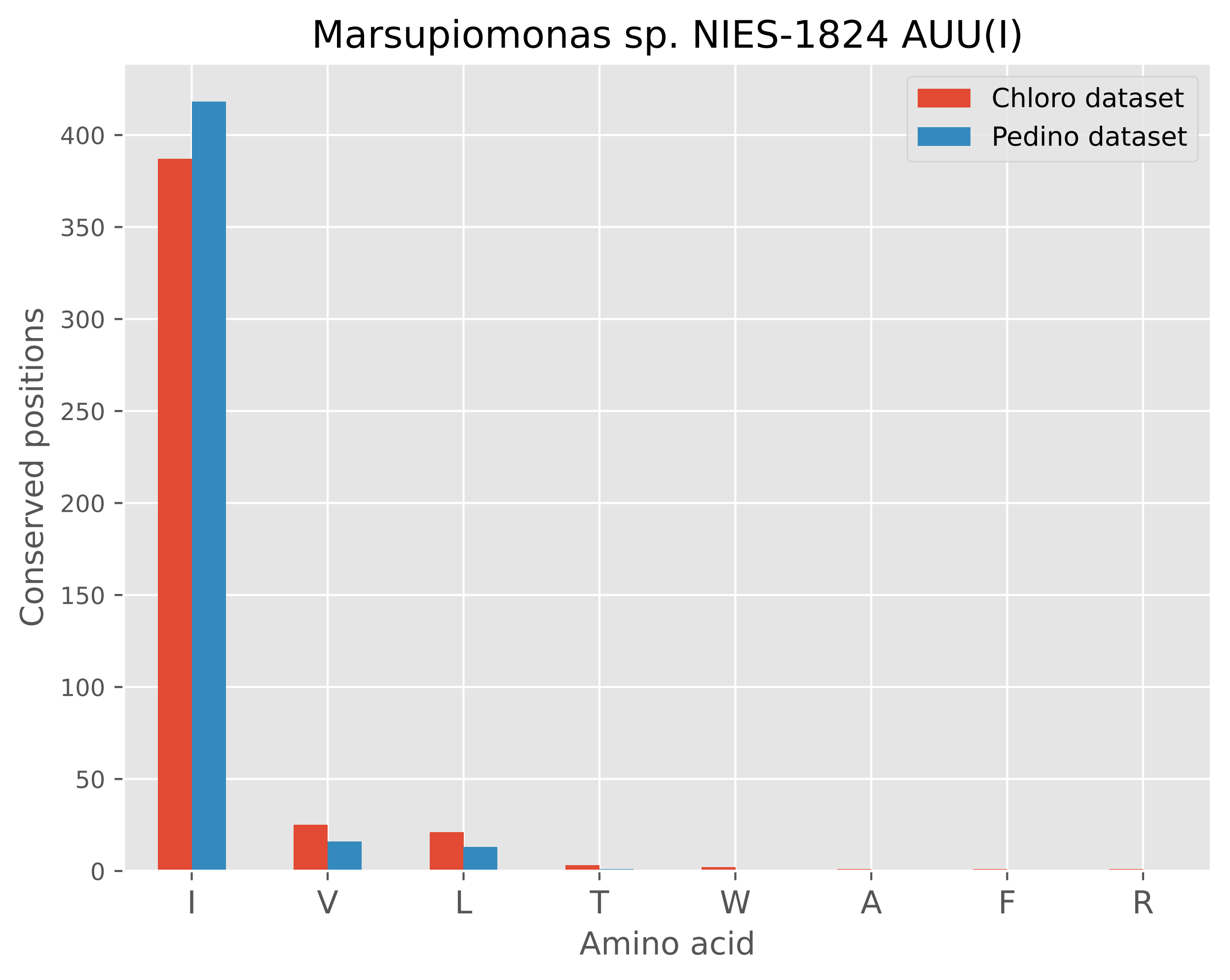
Marsupiomonas sp. NIES-1824 AUU(I)
Chloro dataset
Pedino dataset
Conserved positions
0
50
100
150
200
250
300
350
400
I
V
L
T
W
A
F
R
Amino acid

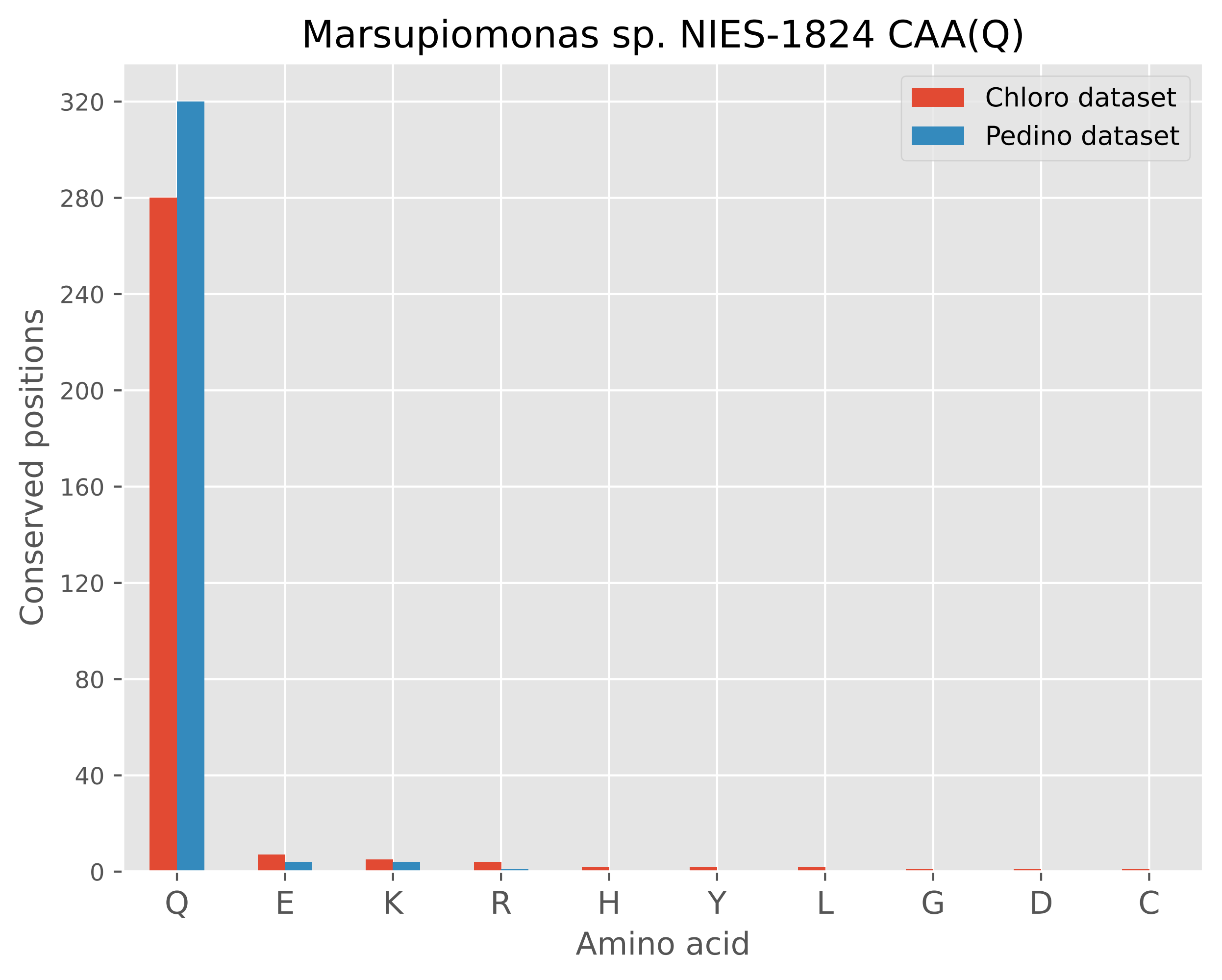

Marsupiomonas sp. NIES-1824 CAA(Q)
Chloro dataset
Pedino dataset
Conserved positions
0
40
80
120
160
200
240
280
320
Q
E
K
R
H
Y
L
G
D
C
Amino acid

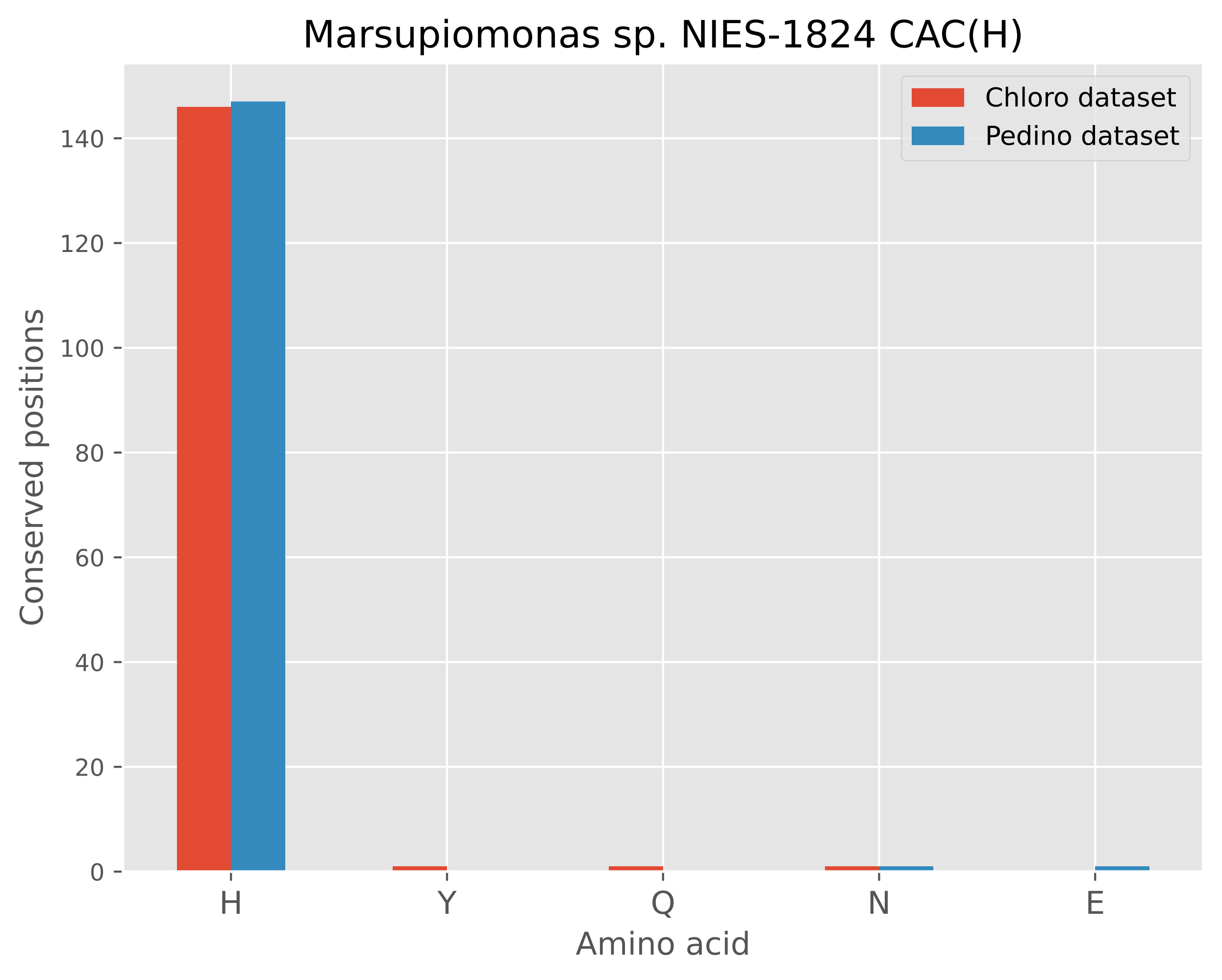
Marsupiomonas sp. NIES-1824 CAC(H)
Chloro dataset
Pedino dataset
Conserved positions
0
20
40
60
80
100
120
140
H
Y
Q
N
E
Amino acid

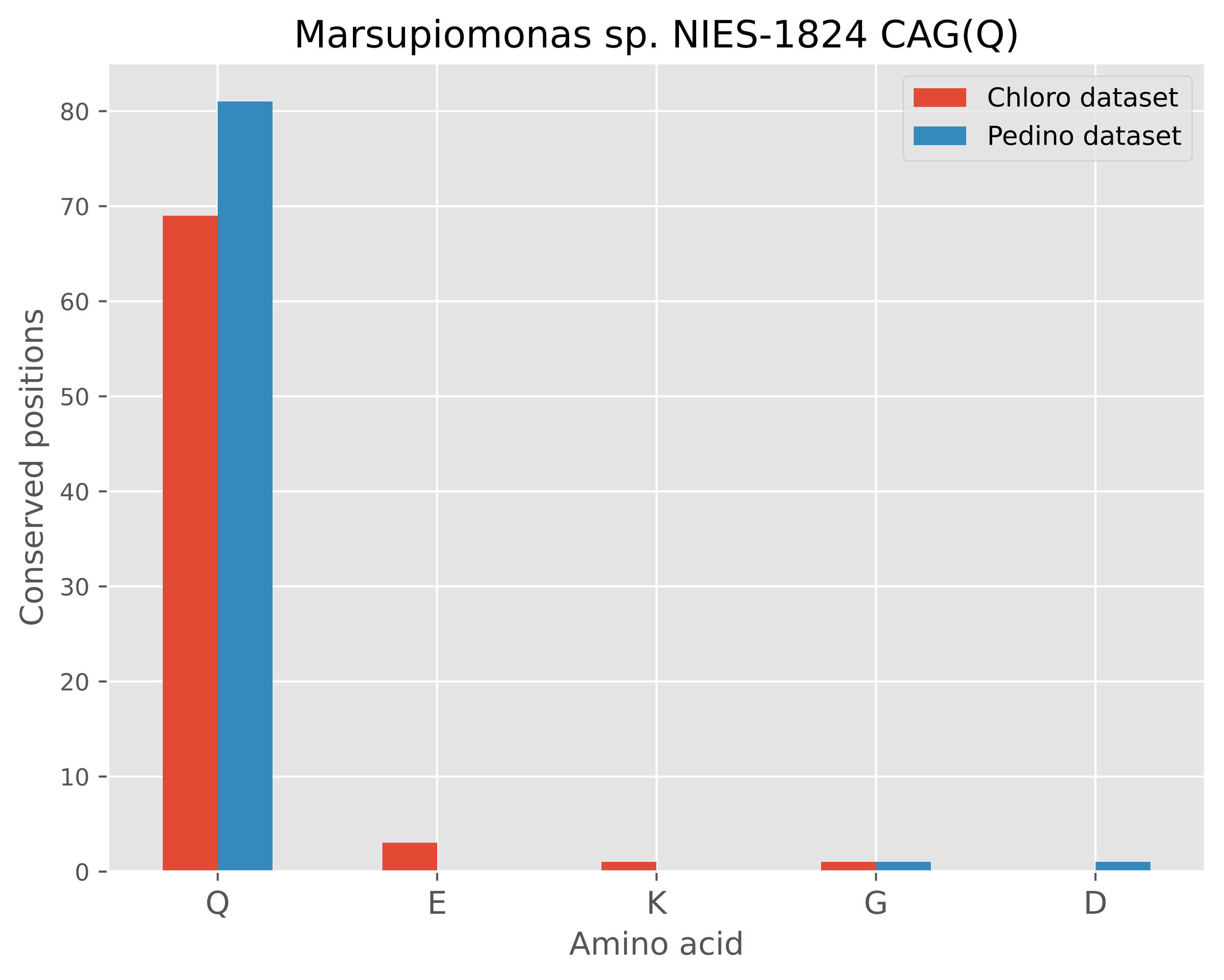

Marsupiomonas sp. NIES-1824 CAG(Q)
Chloro dataset
Pedino dataset
Conserved positions
0
10
20
30
40
50
60
70
80
Q
E
K
G
D
Amino acid

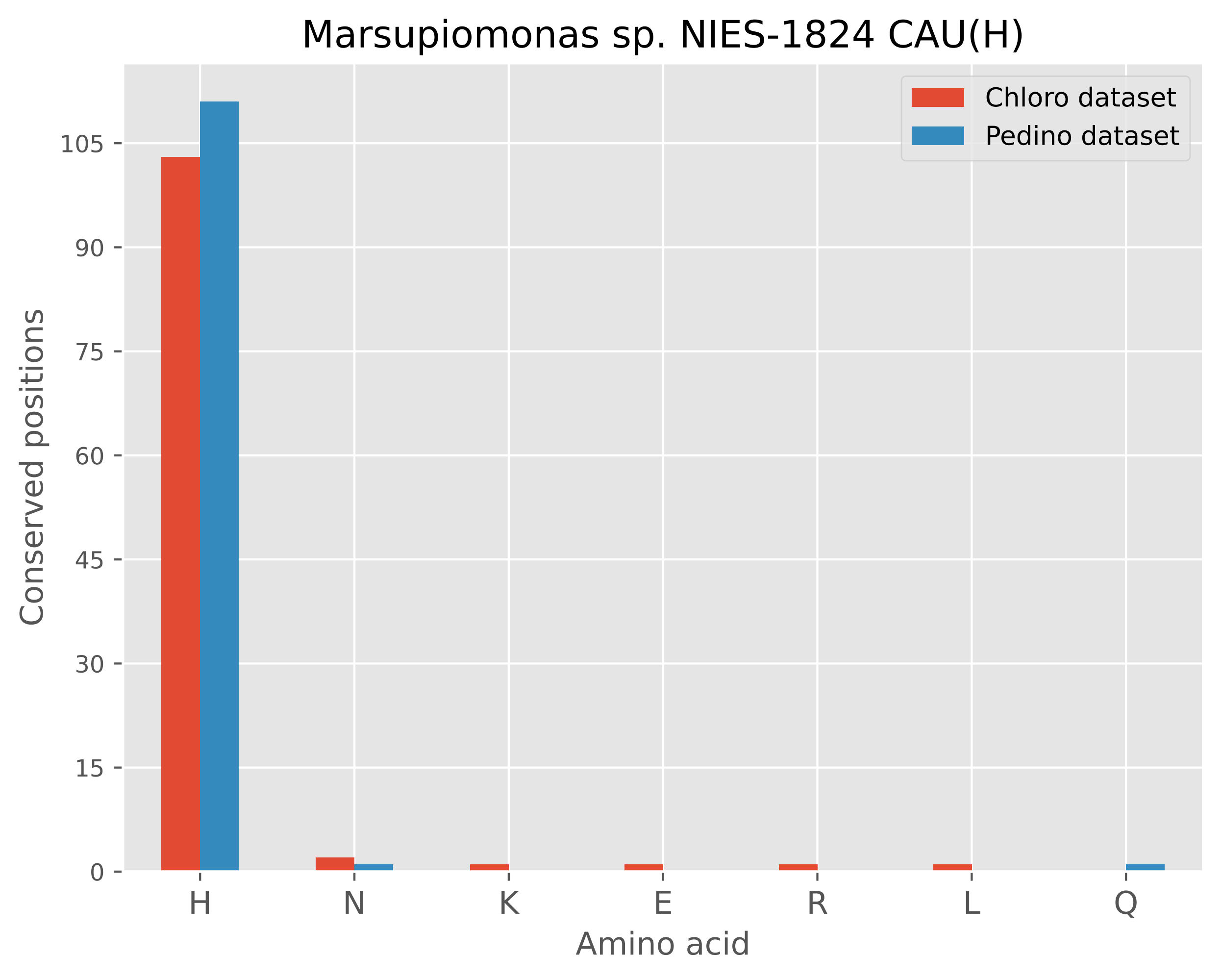

Marsupiomonas sp. NIES-1824 CAU(H)
Chloro dataset
Pedino dataset
Conserved positions
0
15
30
45
60
75
90
105
H
N
K
E
R
L
Q
Amino acid

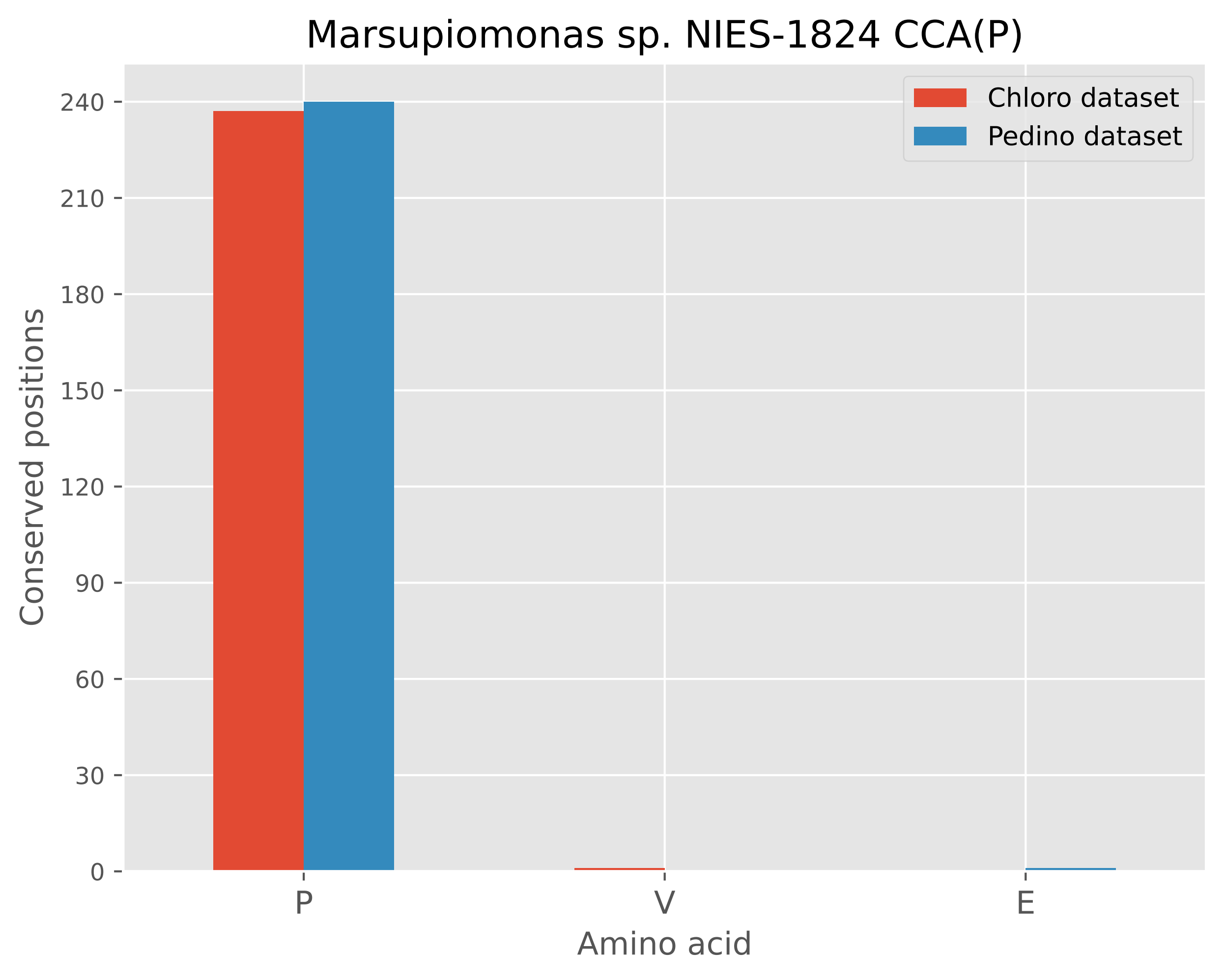
Marsupiomonas sp. NIES-1824 CCA(P)
Chloro dataset
Pedino dataset
Conserved positions
0
30
60
90
120
150
180
210
240
P
V
E
Amino acid

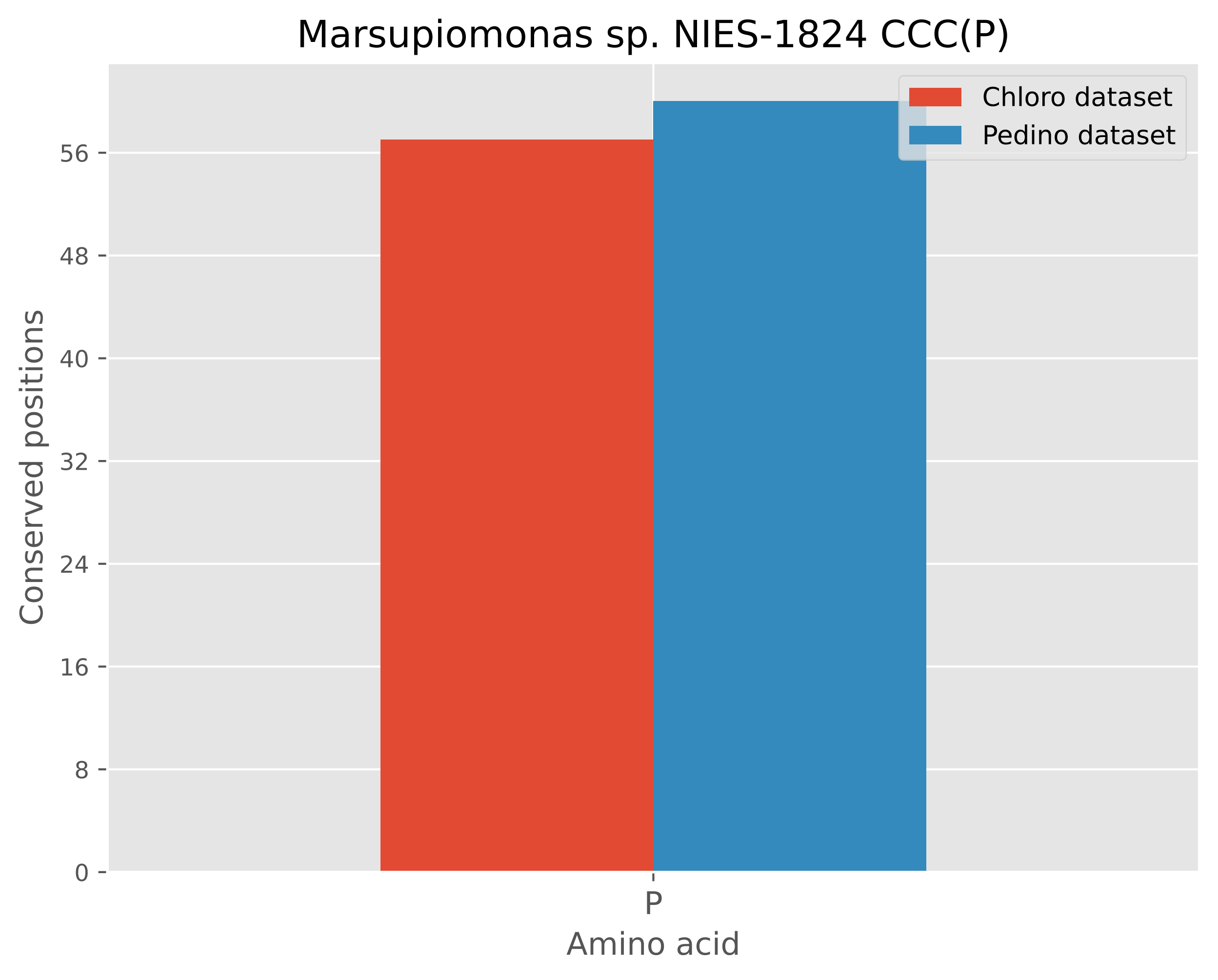

Marsupiomonas sp. NIES-1824 CCC(P)
Chloro dataset
Pedino dataset
Conserved positions
0
8
16
24
32
40
48
56
P
Amino acid

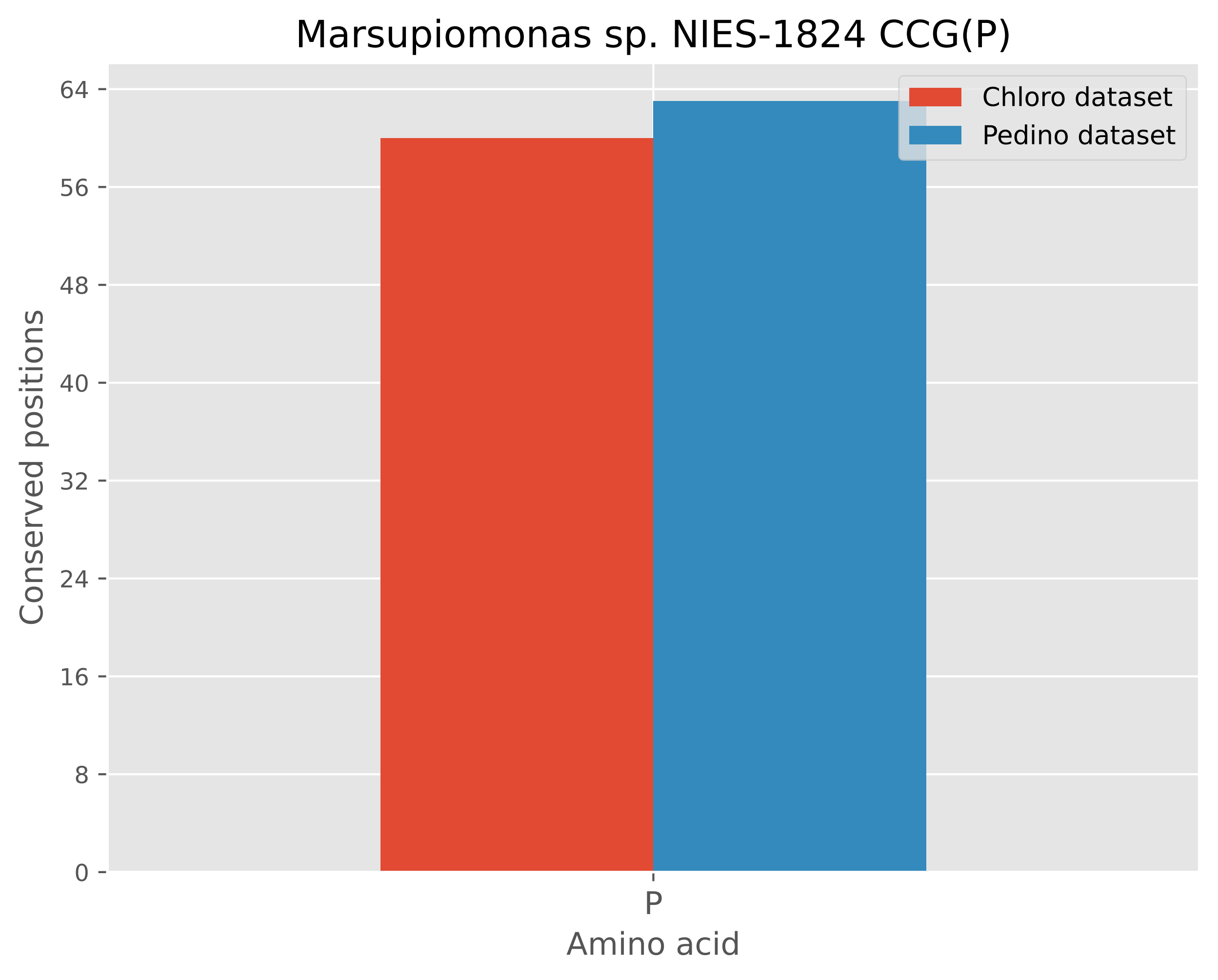
Marsupiomonas sp. NIES-1824 CCG(P)
Chloro dataset
Pedino dataset
Conserved positions
0
8
16
24
32
40
48
56
64
P
Amino acid

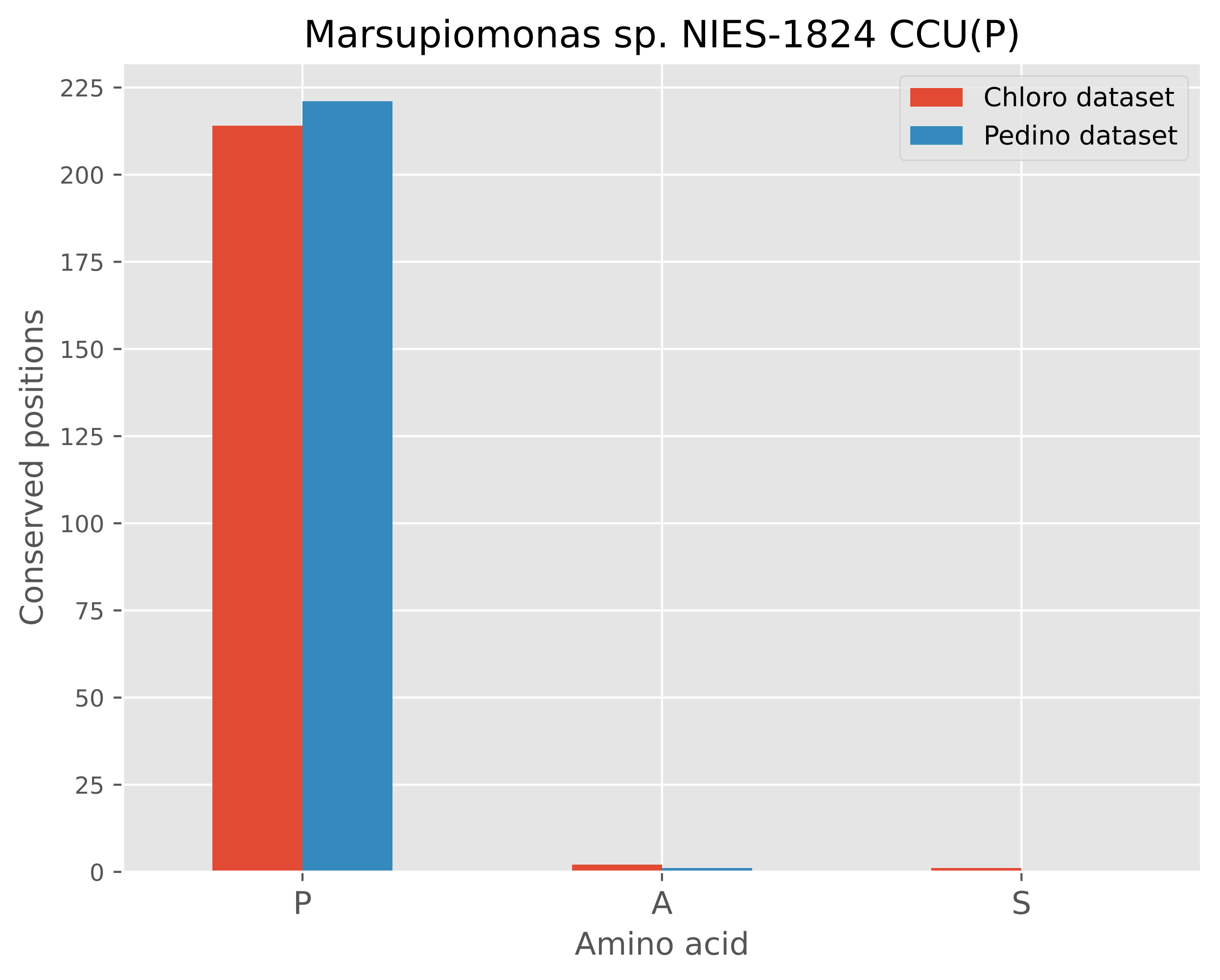
Marsupiomonas sp. NIES-1824 CCU(P)
Chloro dataset
Pedino dataset
Conserved positions
Amino acid
P
A
S
0
25
50
75
100
125
150
175
200
225

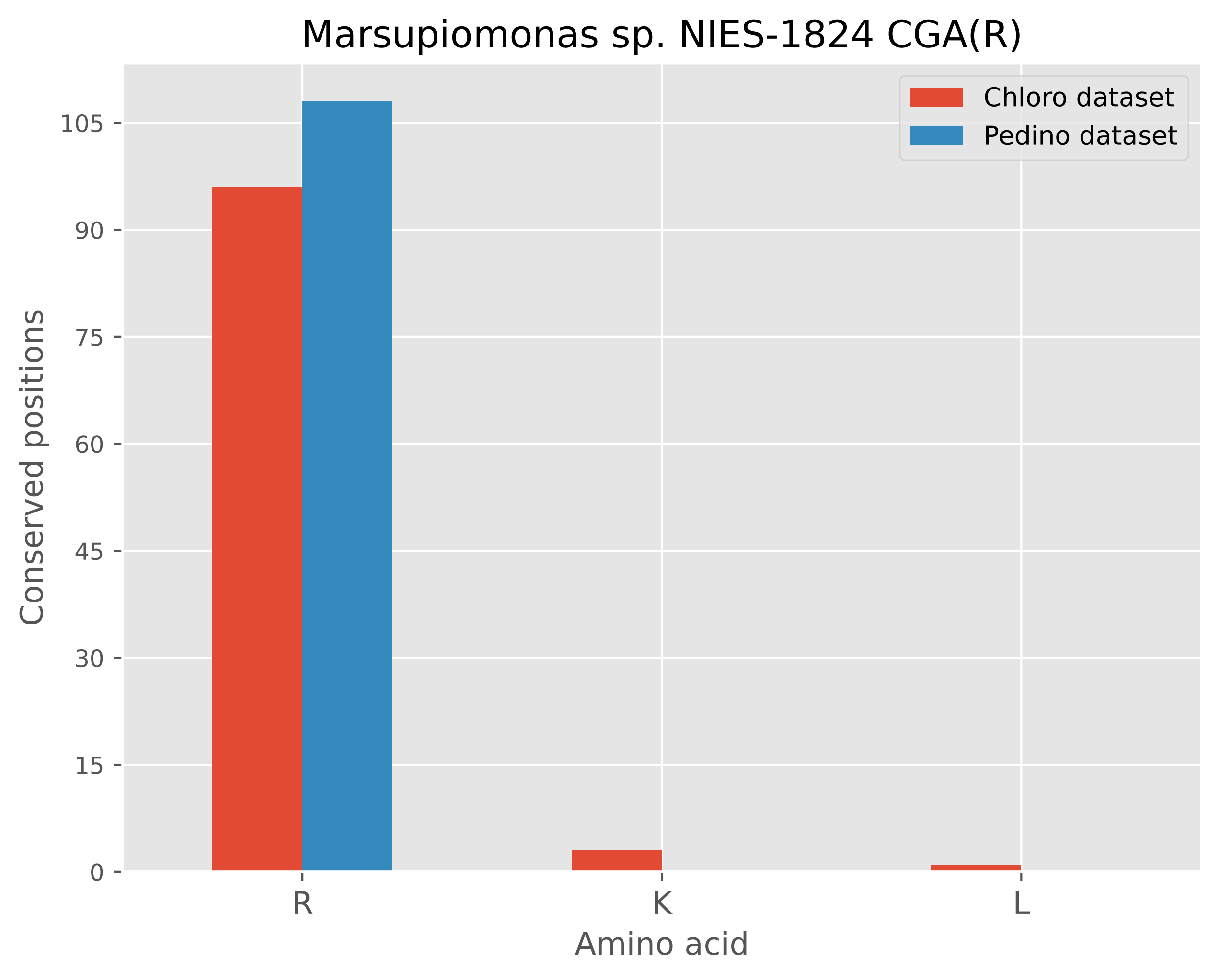
Marsupiomonas sp. NIES-1824 CGA(R)
Chloro dataset
Pedino dataset
Conserved positions
0
15
30
45
60
75
90
105
R
K
L
Amino acid

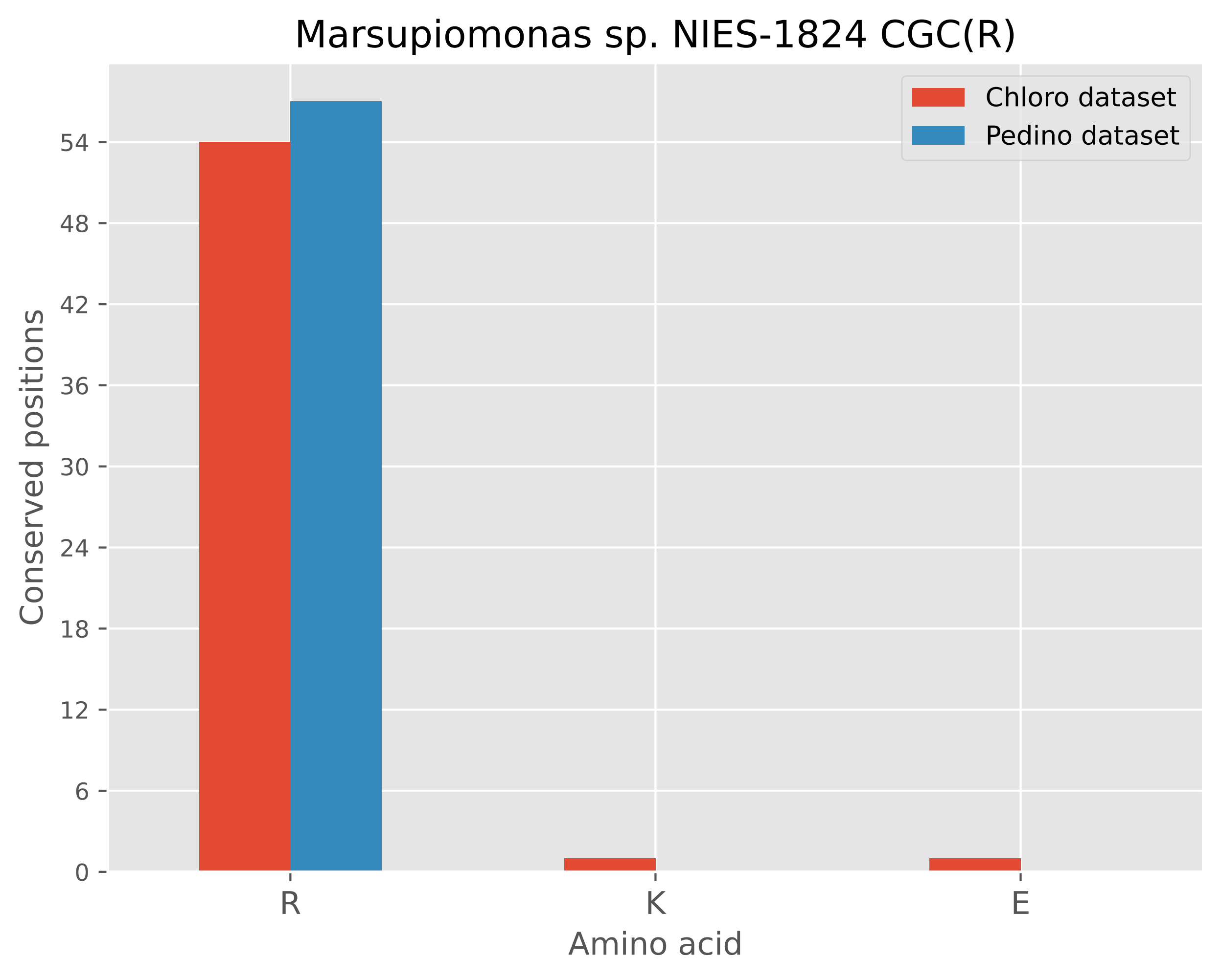

Marsupiomonas sp. NIES-1824 CGC(R)
Chloro dataset
Pedino dataset
Conserved positions
0
6
12
18
24
30
36
42
48
54
R
K
E
Amino acid

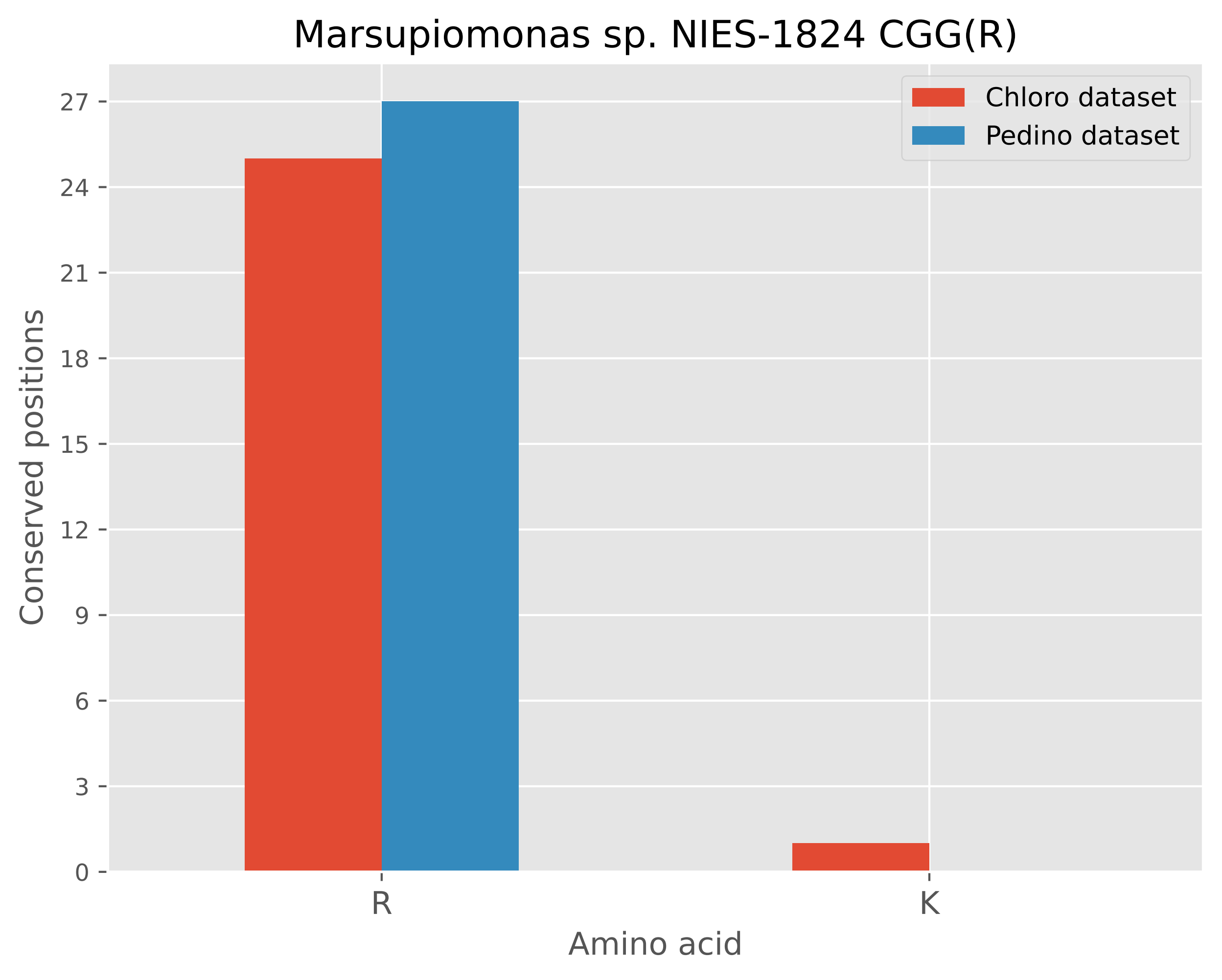

Marsupiomonas sp. NIES-1824 CGG(R)
Chloro dataset
Pedino dataset
Conserved positions
0
3
6
9
12
15
18
21
24
27
R
K
Amino acid

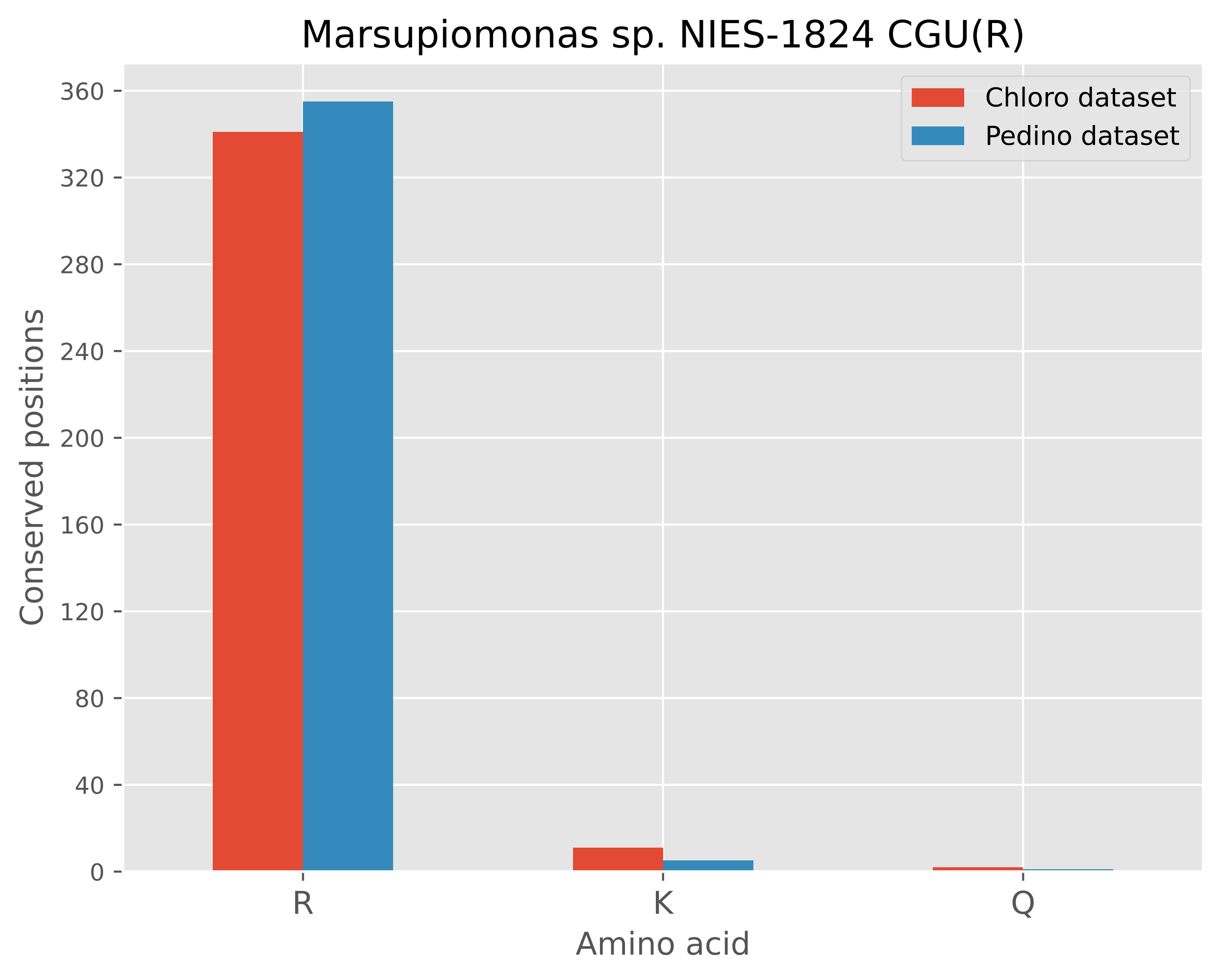
Marsupiomonas sp. NIES-1824 CGU(R)
Chloro dataset
Pedino dataset
Conserved positions
0
40
80
120
160
200
240
280
320
360
R
K
Q
Amino acid

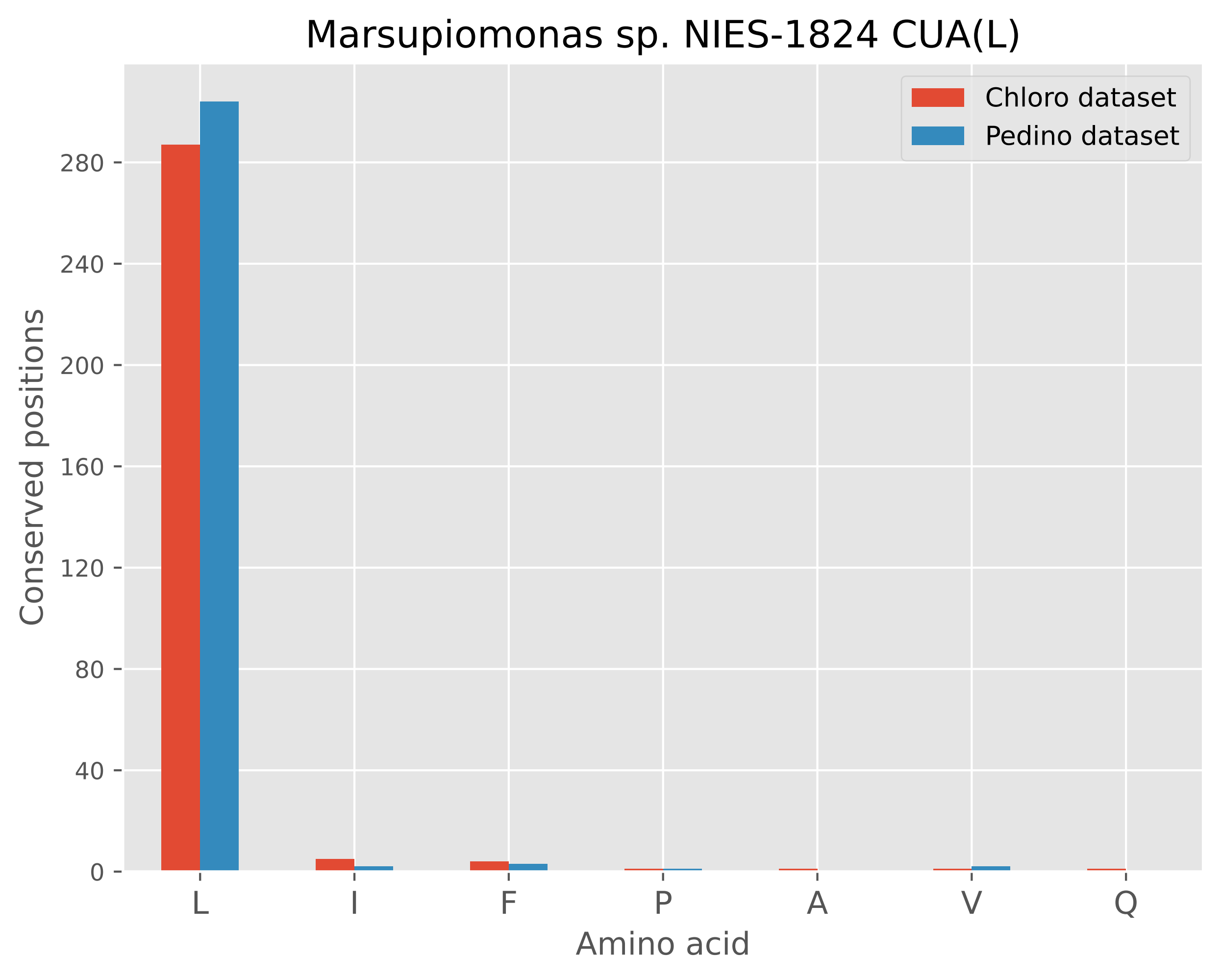

Marsupiomonas sp. NIES-1824 CUA(L)
Chloro dataset
Pedino dataset
Conserved positions
0
40
80
120
160
200
240
280
L
I
F
P
A
V
Q
Amino acid

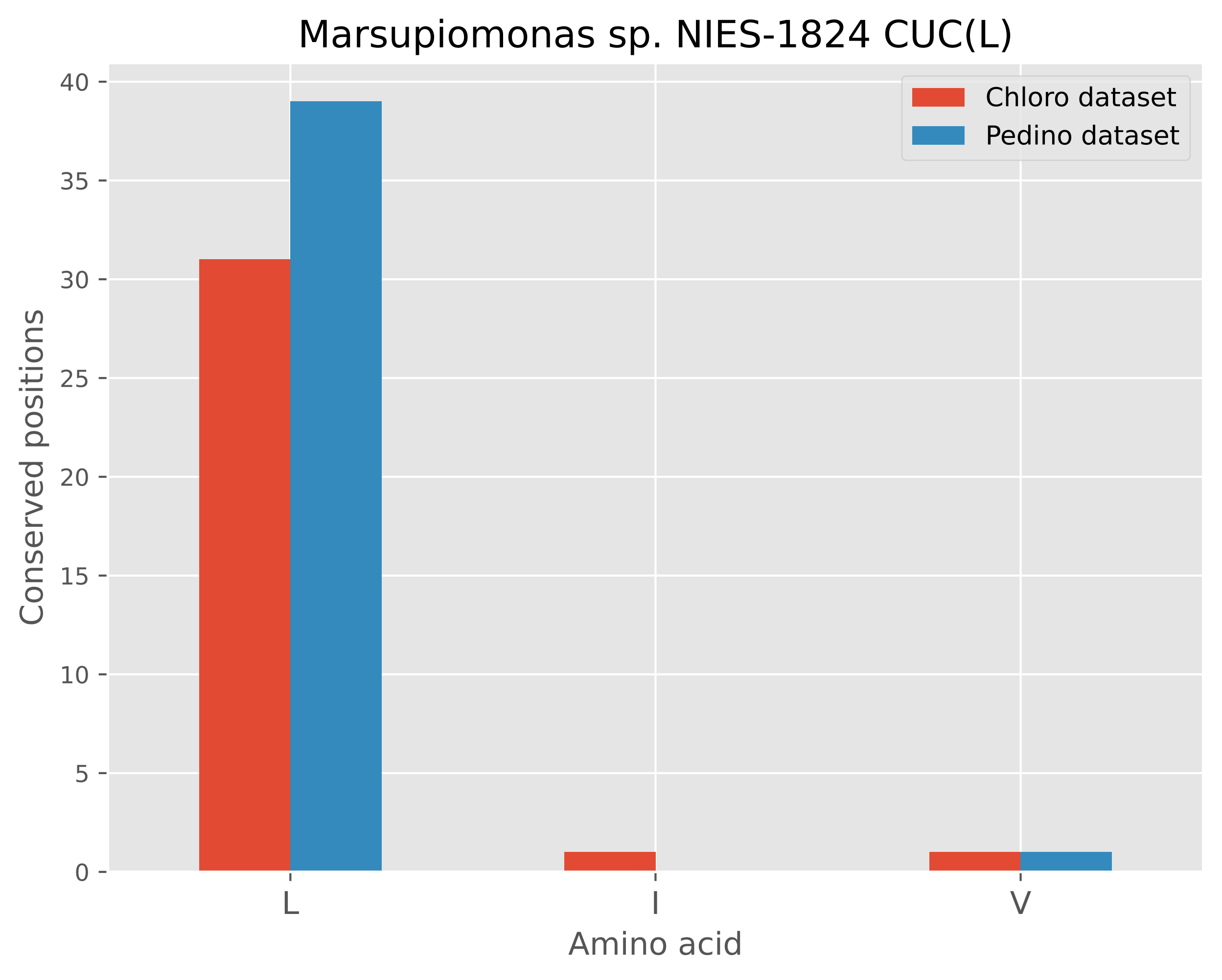
Marsupiomonas sp. NIES-1824 CUC(L)
Chloro dataset
Pedino dataset
Conserved positions
0
5
10
15
20
25
30
35
40
L
I
V
Amino acid

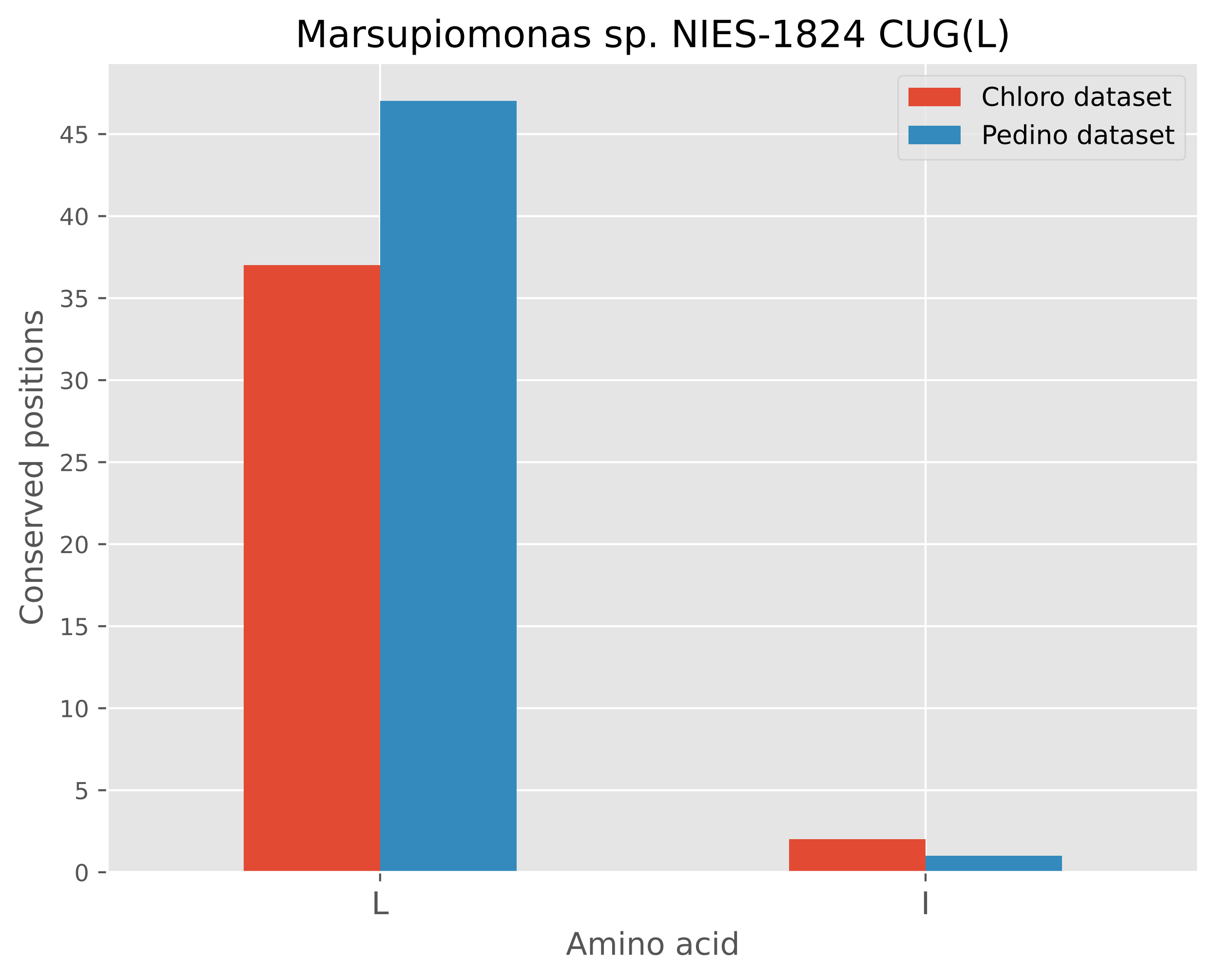
Marsupiomonas sp. NIES-1824 CUG(L)
Chloro dataset
Pedino dataset
Conserved positions
0
5
10
15
20
25
30
35
40
45
L
I
Amino acid

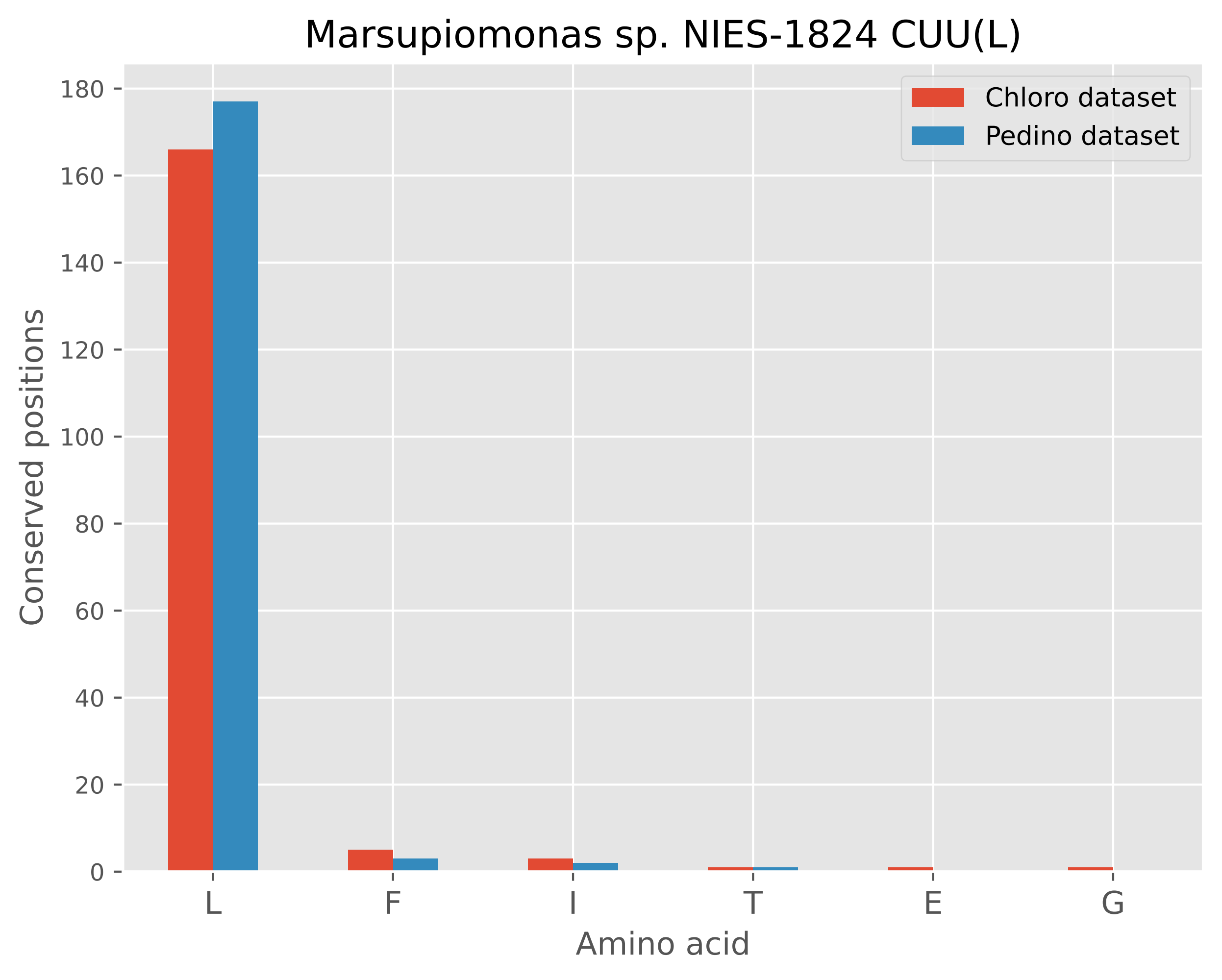
Marsupiomonas sp. NIES-1824 CUU(L)
Chloro dataset
Pedino dataset
Conserved positions
0
20
40
60
80
100
120
140
160
180
L
F
I
T
E
G
Amino acid

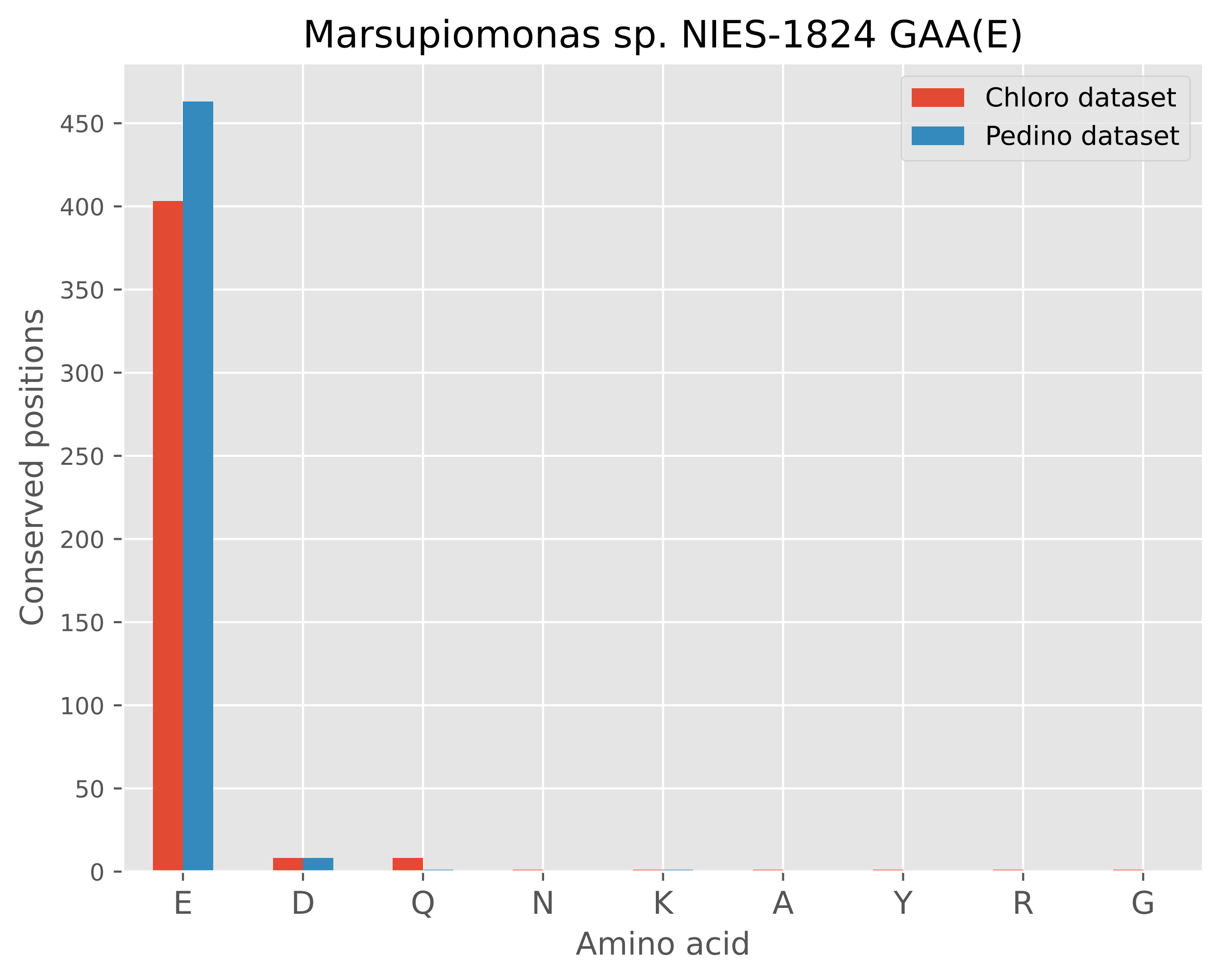

Marsupiomonas sp. NIES-1824 GAA(E)
Chloro dataset
Pedino dataset
Conserved positions
0
50
100
150
200
250
300
350
400
450
E
D
Q
N
K
A
Y
R
G
Amino acid

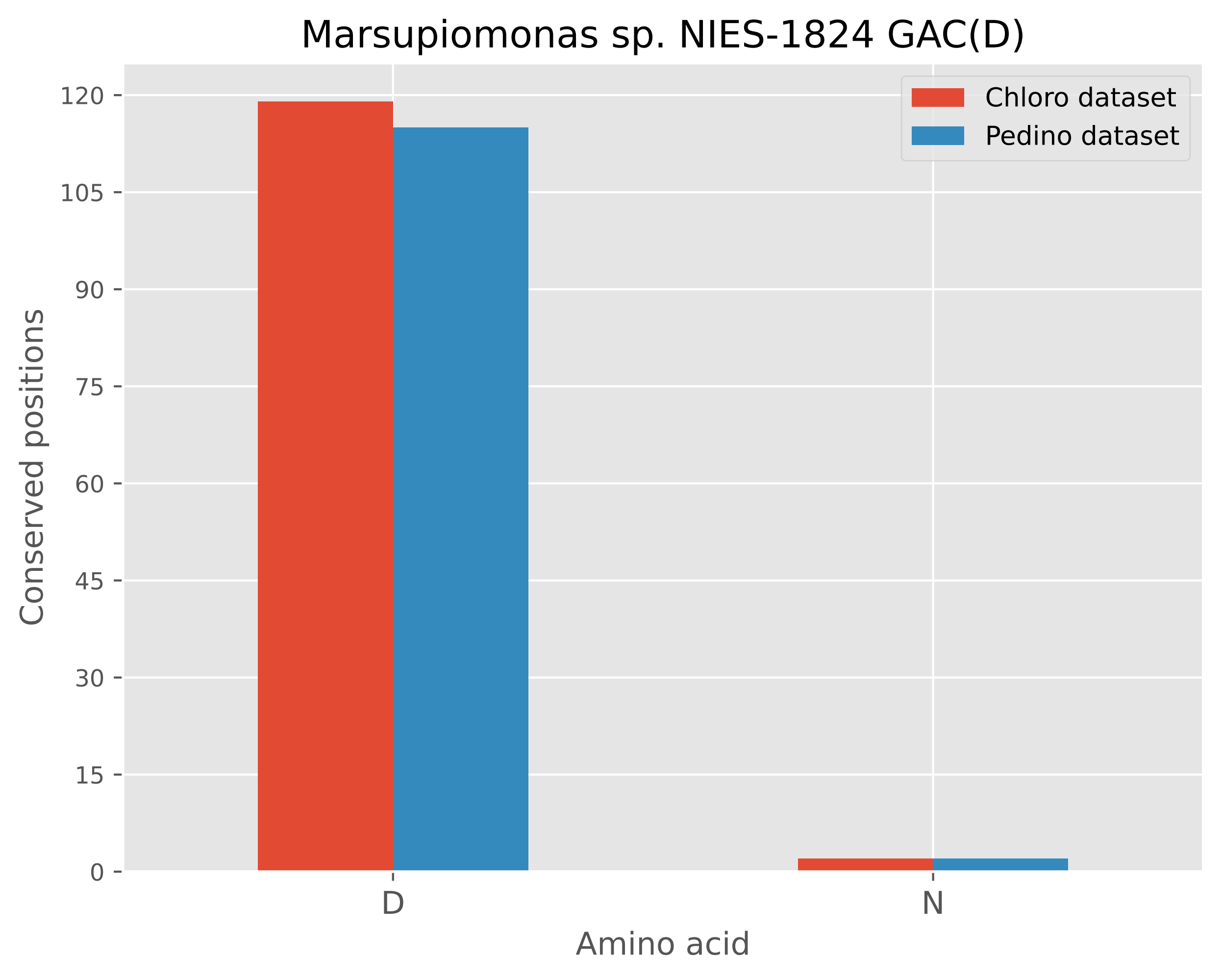
Marsupiomonas sp. NIES-1824 GAC(D)
Chloro dataset
Pedino dataset
Conserved positions
0
15
30
45
60
75
90
105
120
D
N
Amino acid

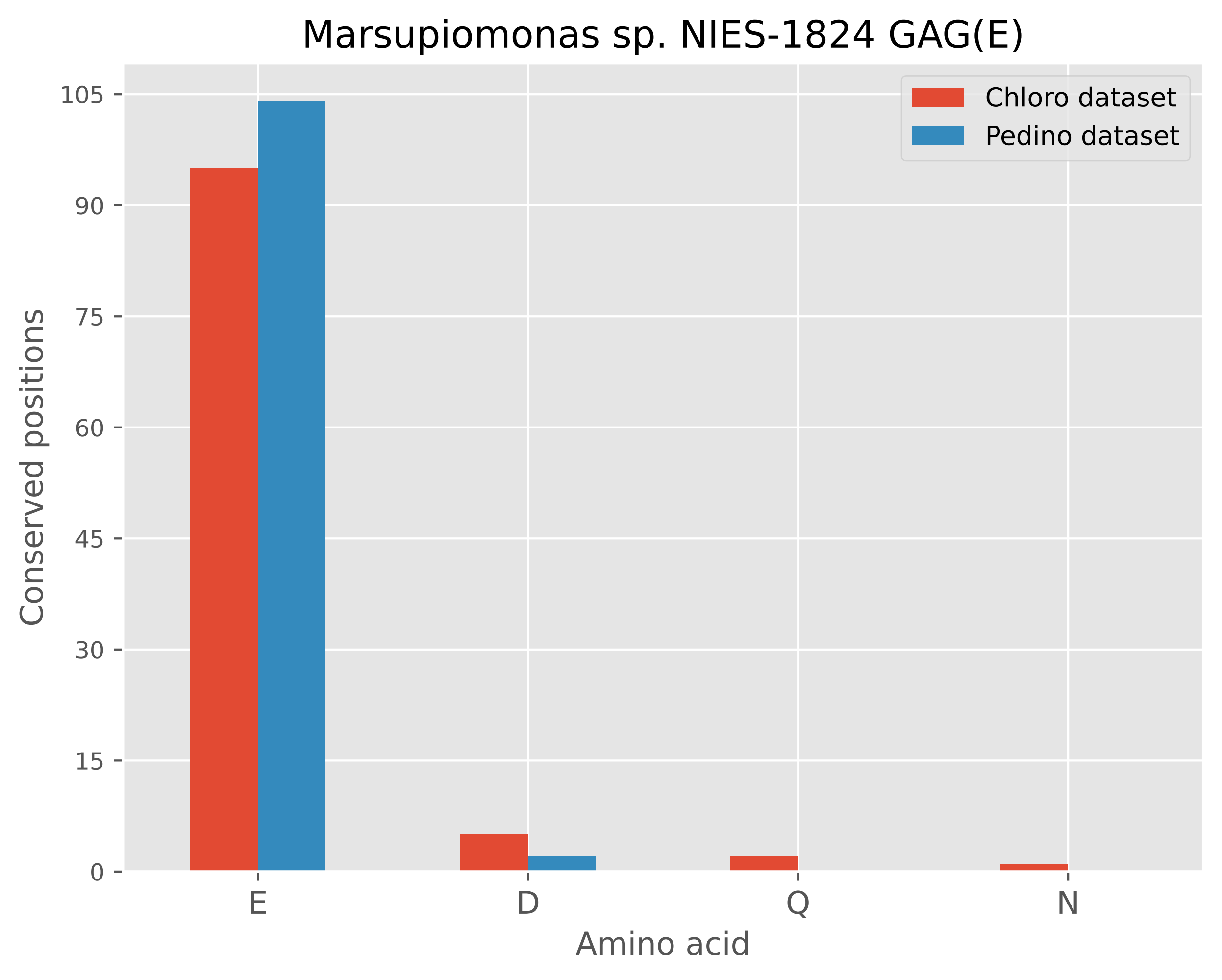

Marsupiomonas sp. NIES-1824 GAG(E)
Chloro dataset
Pedino dataset
Conserved positions
105
90
75
60
45
30
15
0
E
D
Q
N
Amino acid

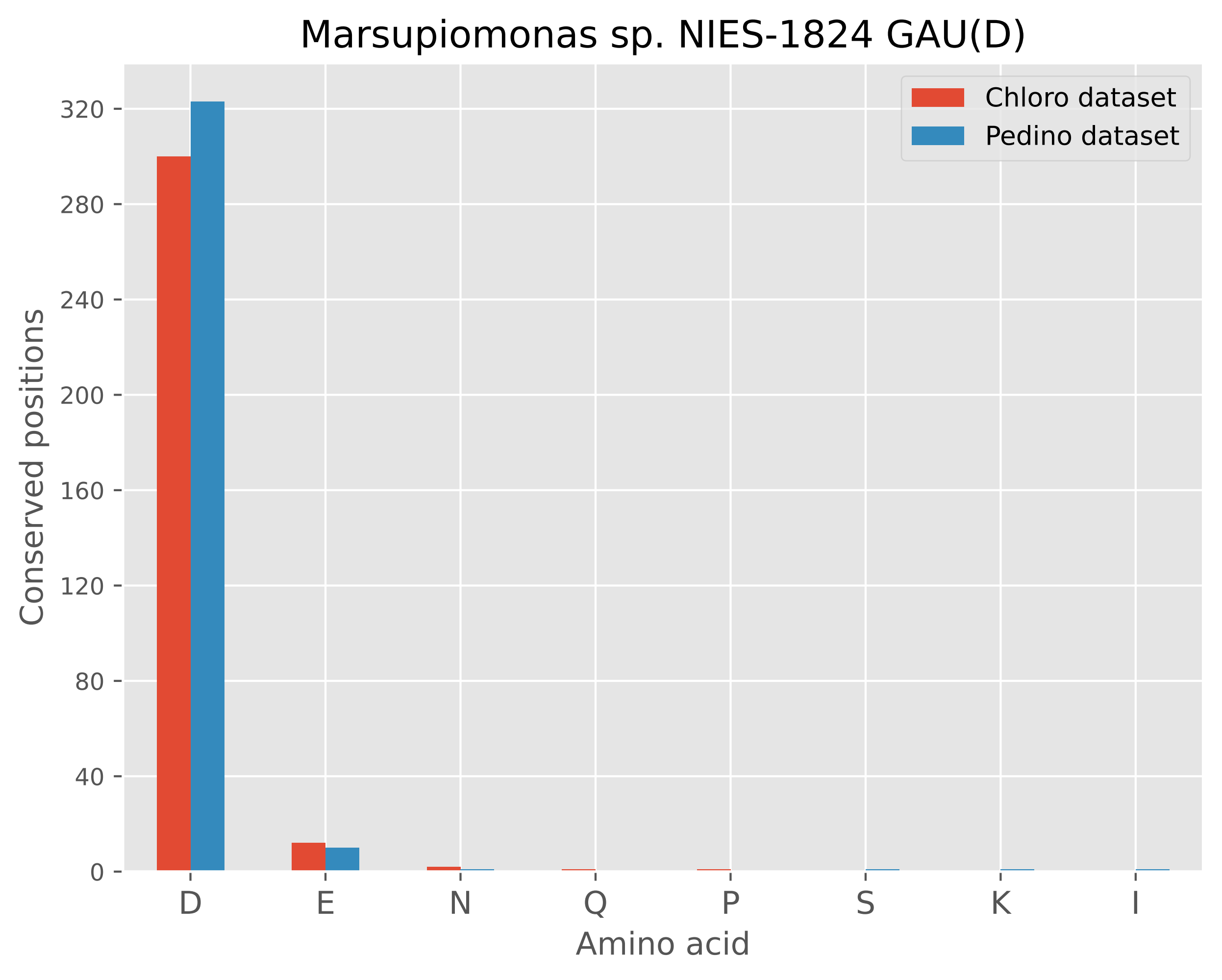

Marsupiomonas sp. NIES-1824 GAU(D)
Chloro dataset
Pedino dataset
Conserved positions
0
40
80
120
160
200
240
280
320
D
E
N
Q
P
S
K
I
Amino acid

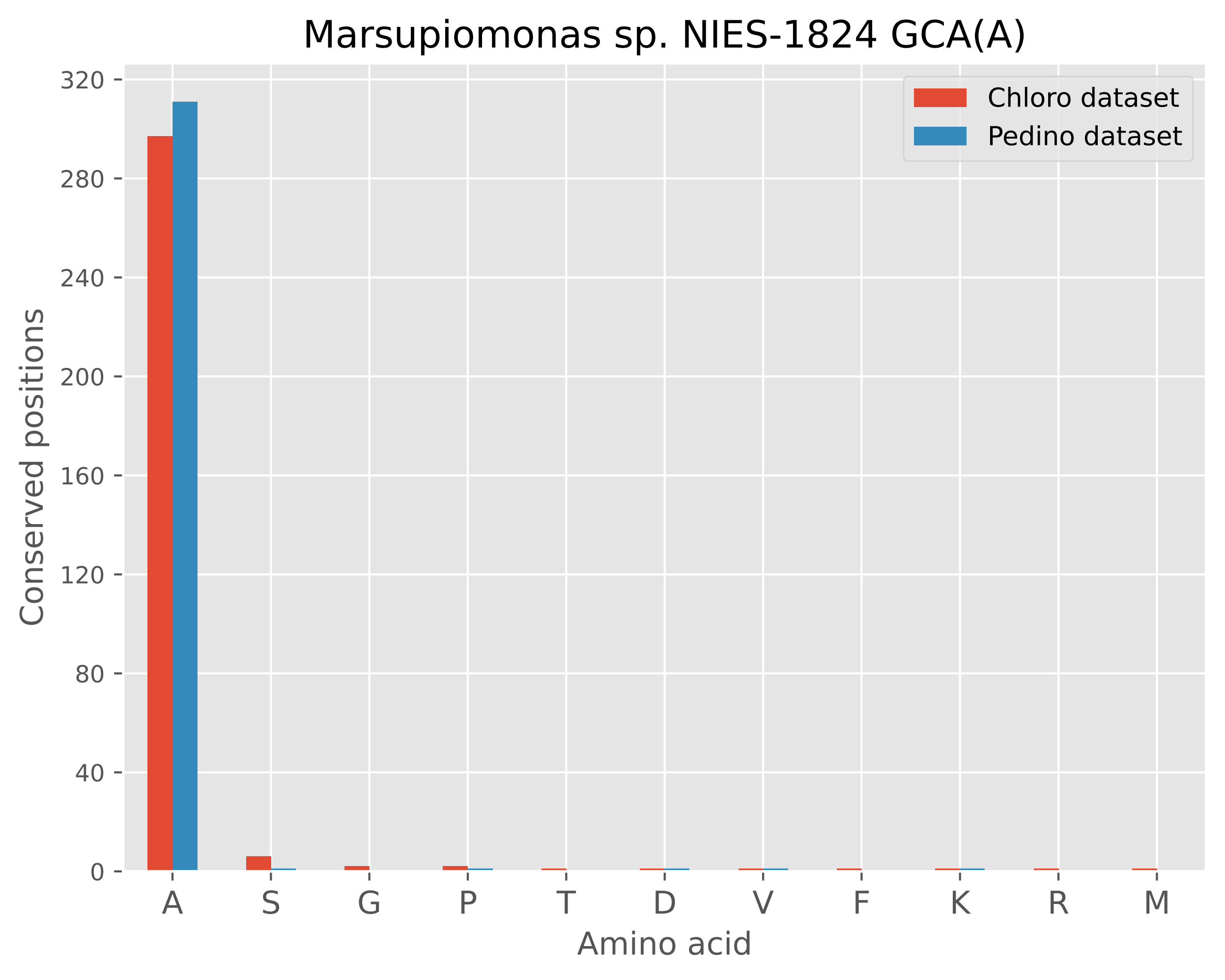

Marsupiomonas sp. NIES-1824 GCA(A)
Chloro dataset
Pedino dataset
Conserved positions
Amino acid
0
40
80
120
160
200
240
280
320
A
S
G
P
T
D
V
F
K
R
M

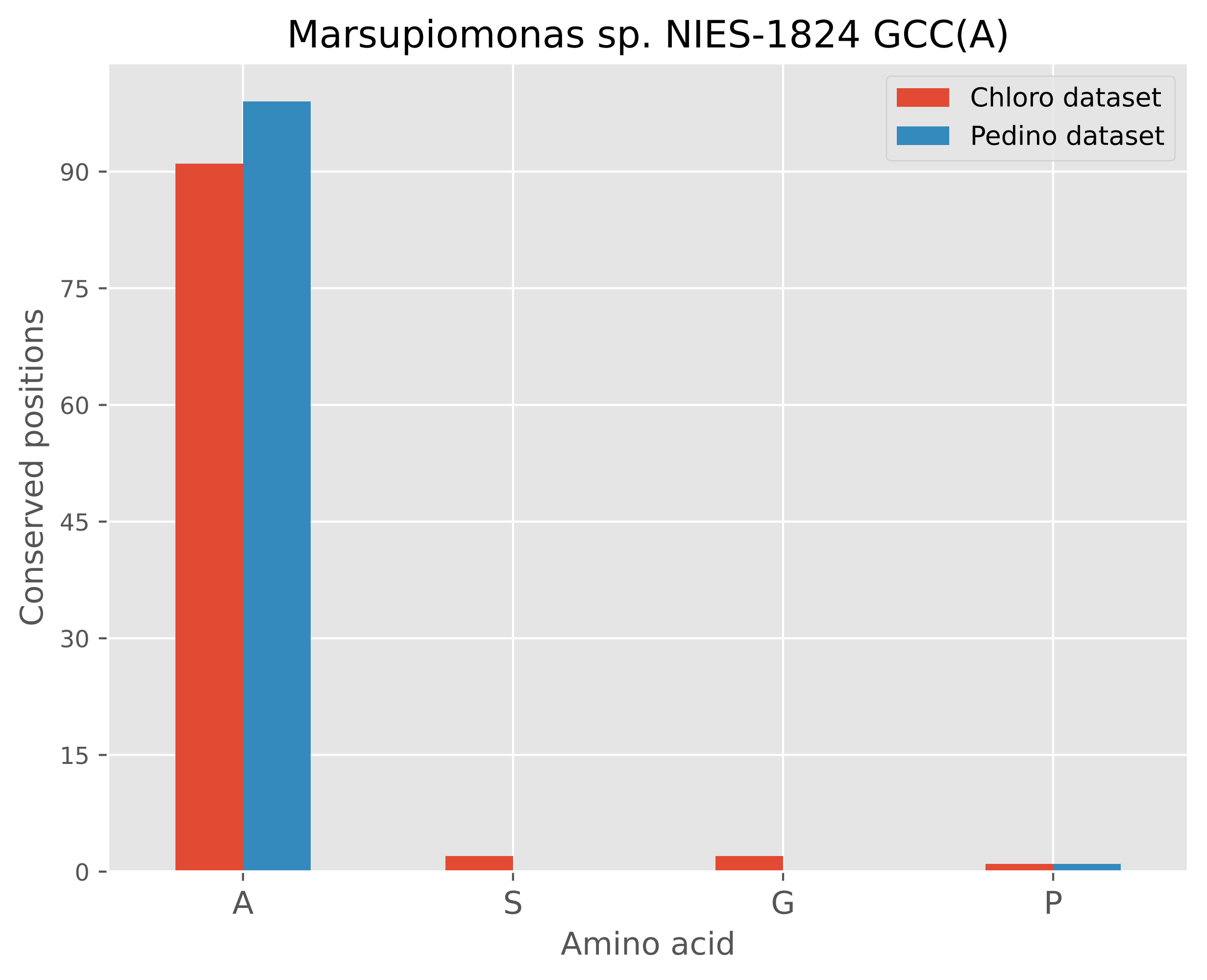
Marsupiomonas sp. NIES-1824 GCC(A)
Chloro dataset
Pedino dataset
Conserved positions
0
15
30
45
60
75
90
A
S
G
P
Amino acid

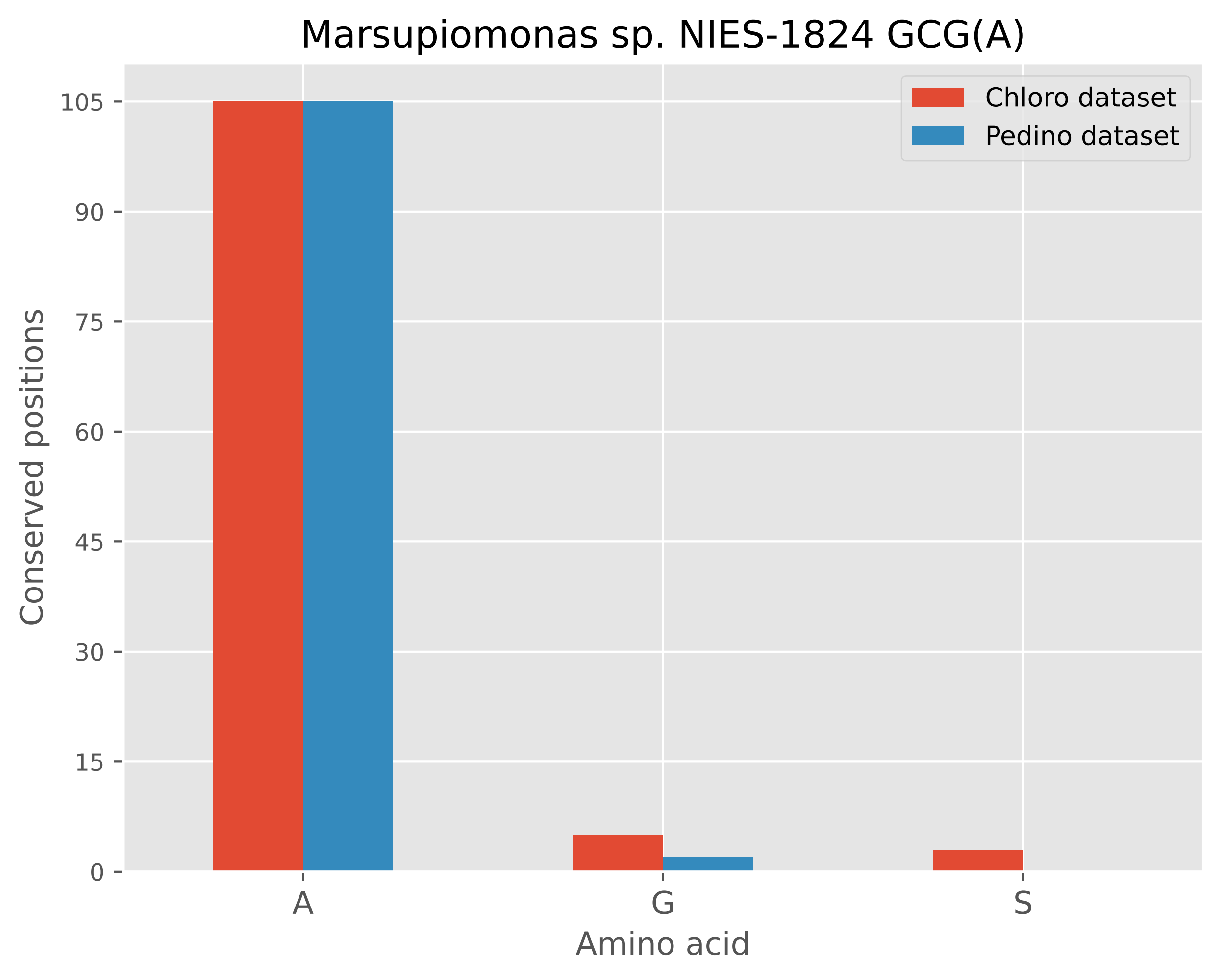
Marsupiomonas sp. NIES-1824 GCG(A)
Chloro dataset
Pedino dataset
Conserved positions
105
90
75
60
45
30
15
0
A
G
S
Amino acid

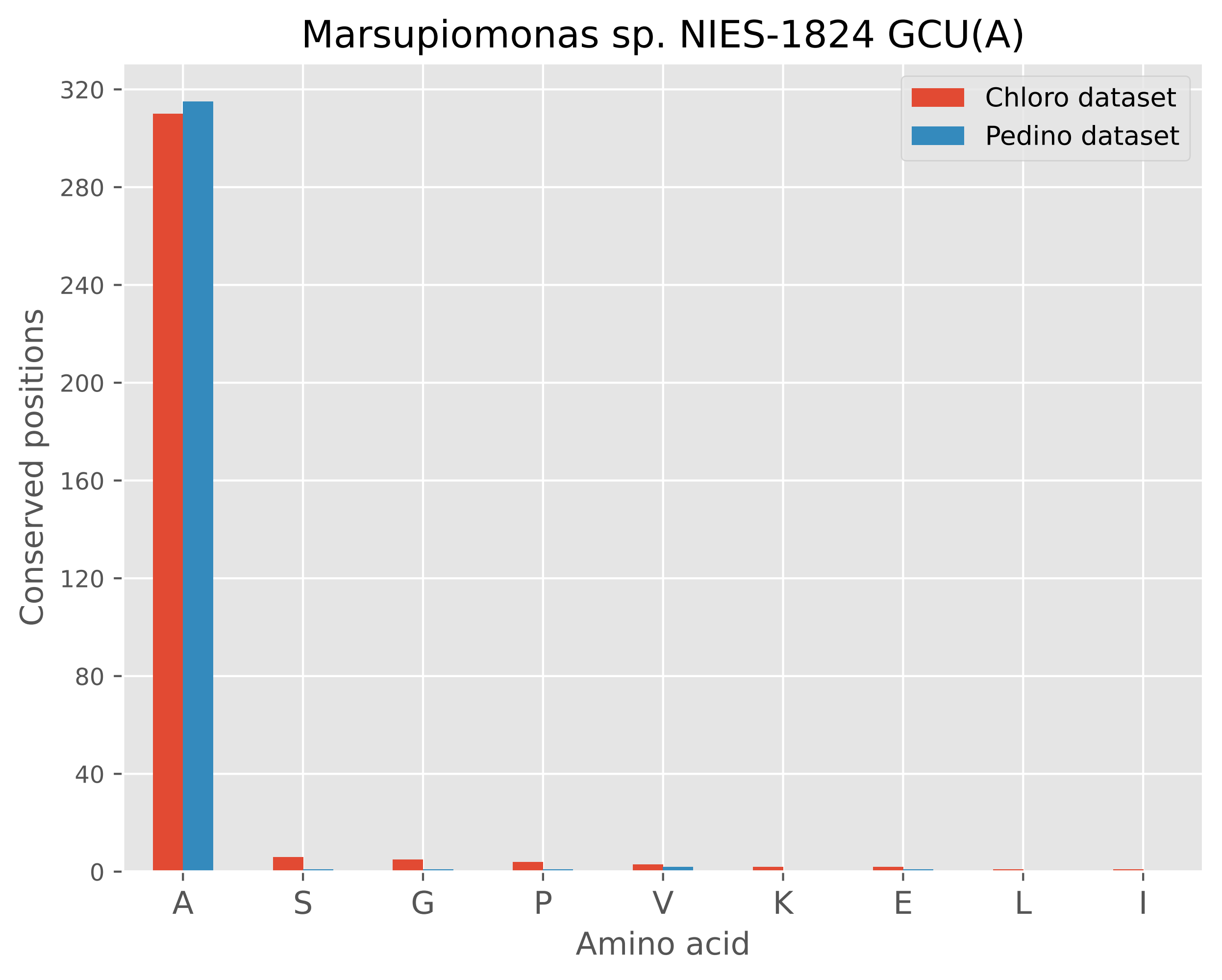

Marsupiomonas sp. NIES-1824 GCU(A)
Chloro dataset
Pedino dataset
Conserved positions
320
280
240
200
160
120
80
40
0
A
S
G
P
V
K
E
L
I
Amino acid

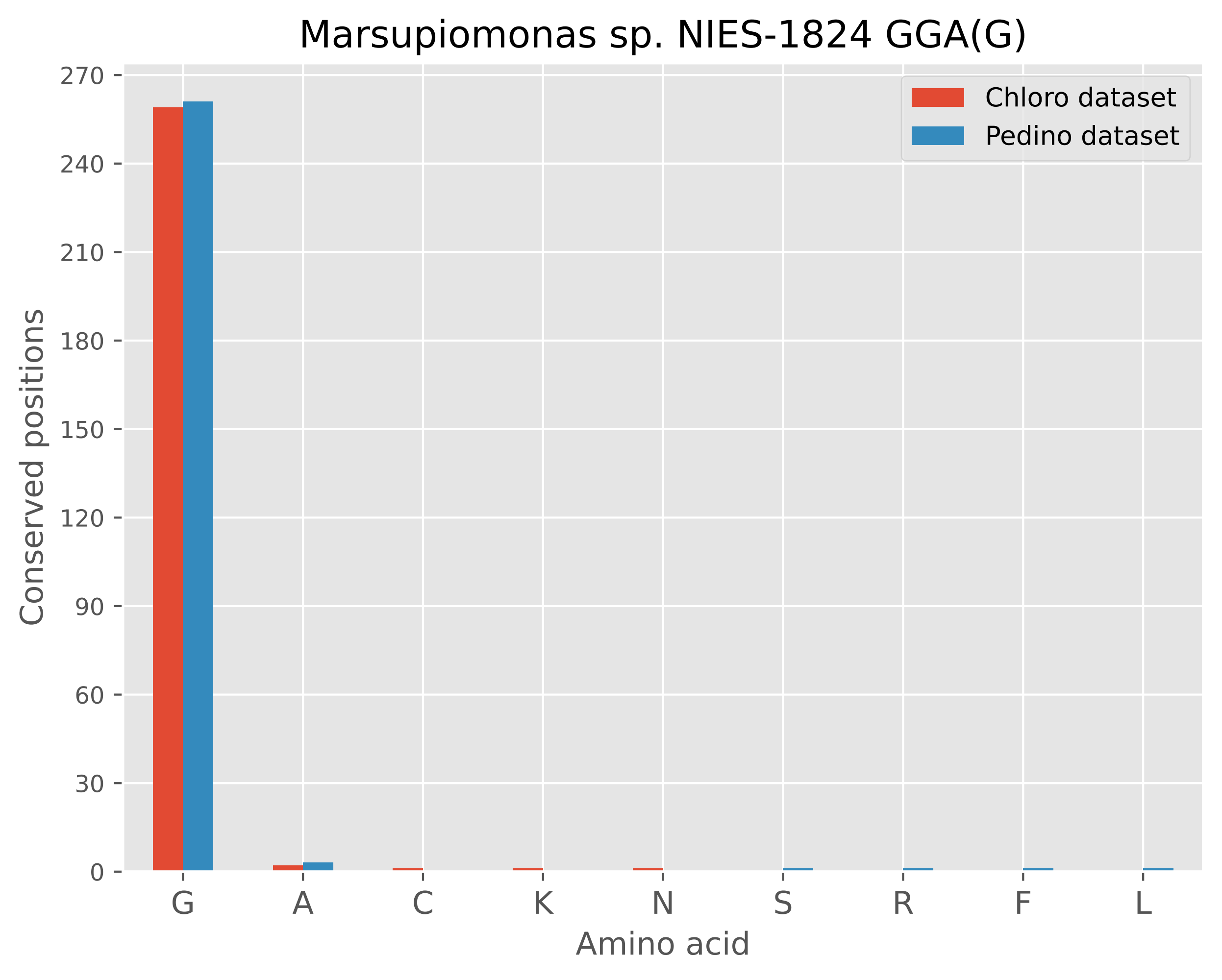

Marsupiomonas sp. NIES-1824 GGA(G)
Chloro dataset
Pedino dataset
Conserved positions
Amino acid
0
30
60
90
120
150
180
210
240
270
G
A
C
K
N
S
R
F
L

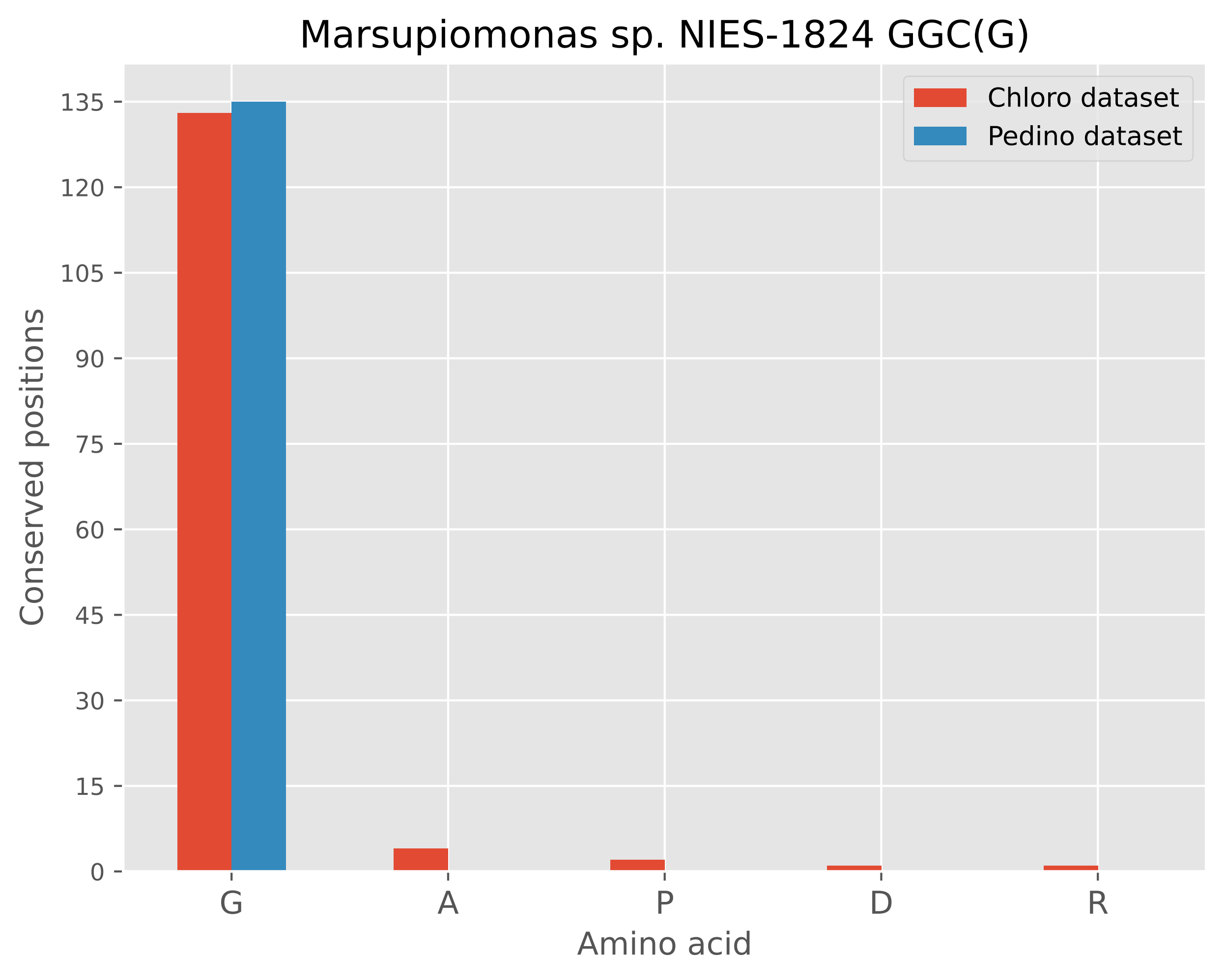

Marsupiomonas sp. NIES-1824 GGC(G)
Chloro dataset
Pedino dataset
Conserved positions
Amino acid
0
15
30
45
60
75
90
105
120
135
G
A
P
D
R

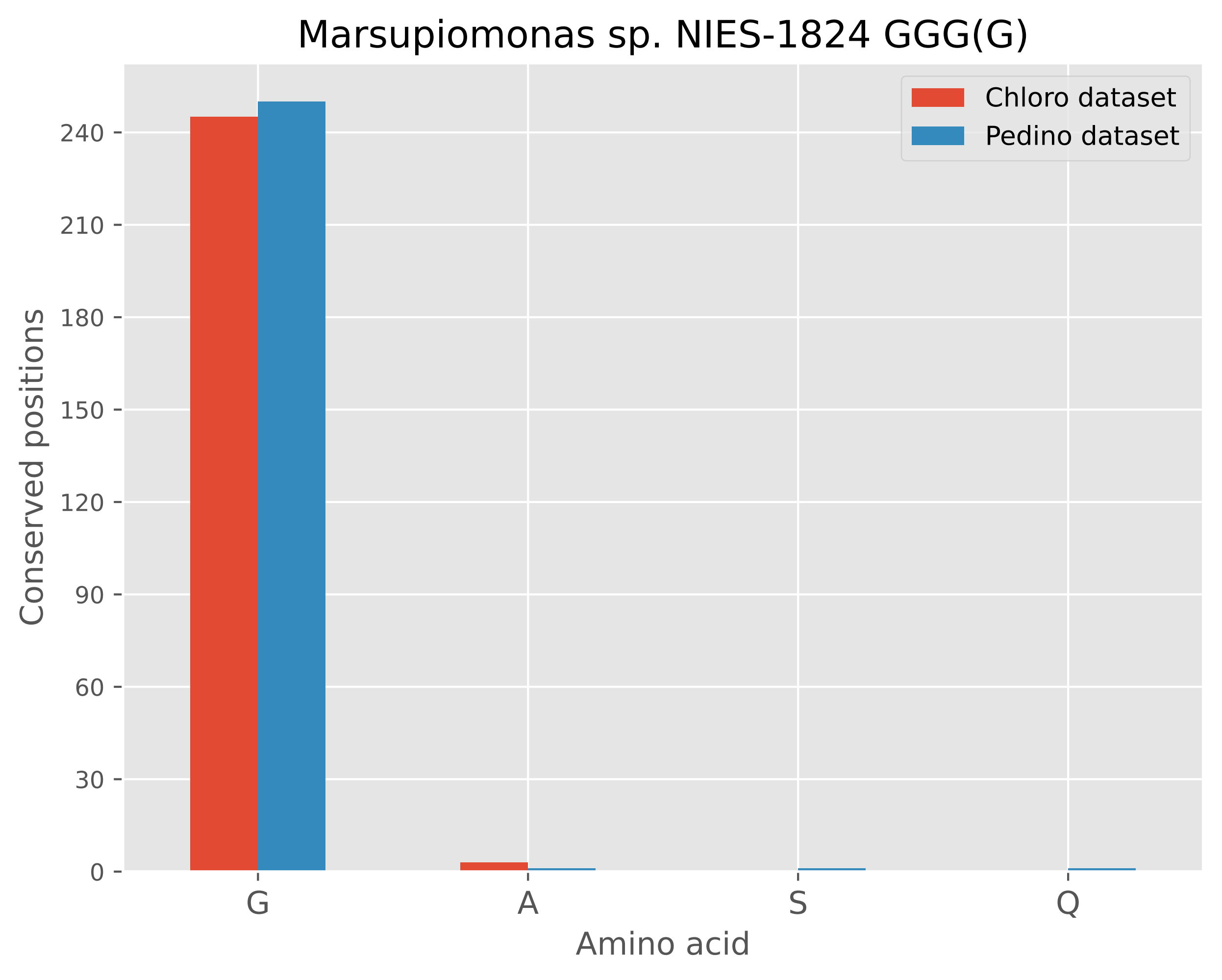

Marsupiomonas sp. NIES-1824 GGG(G)
Chloro dataset
Pedino dataset
Conserved positions
0
30
60
90
120
150
180
210
240
G
A
S
Q
Amino acid

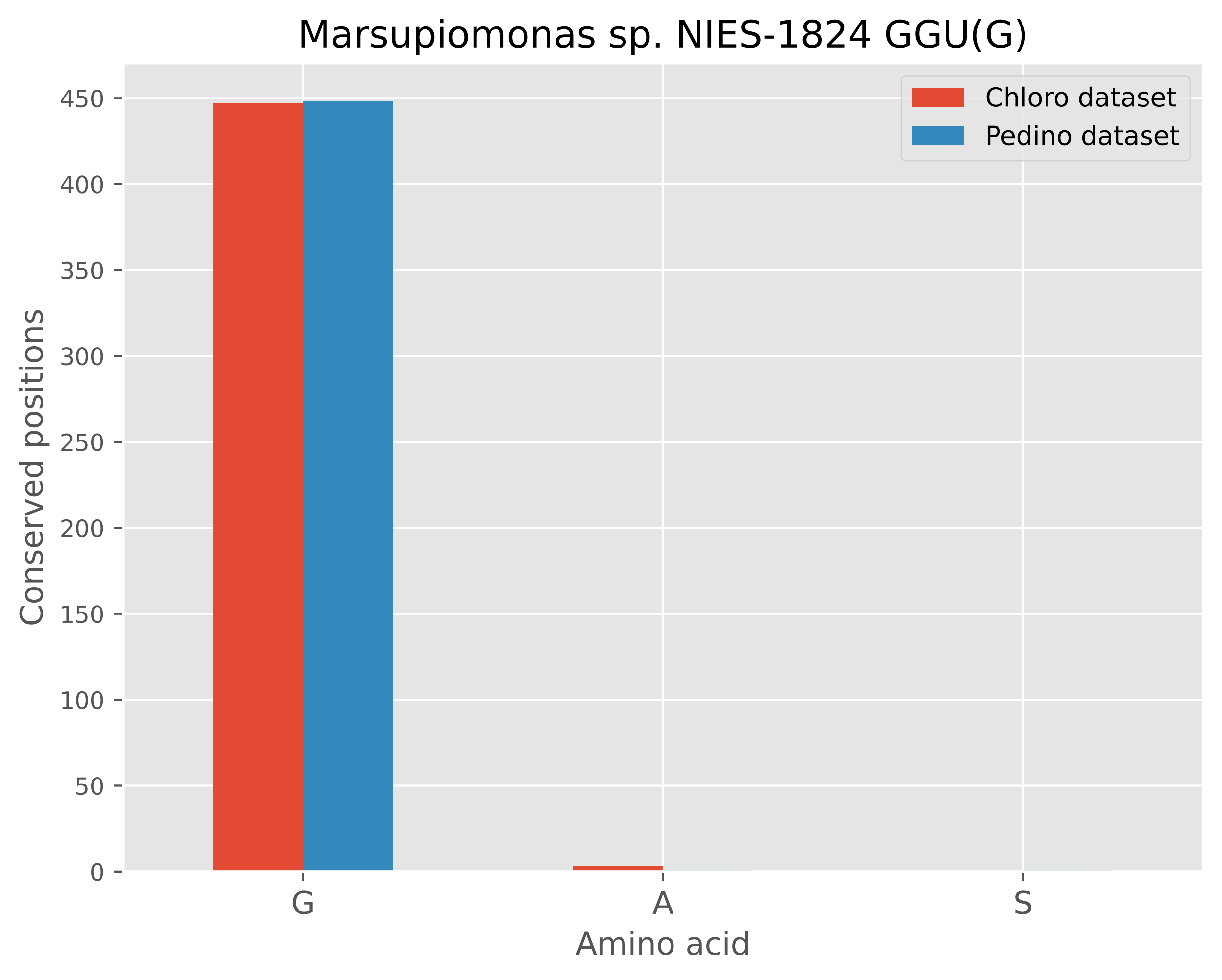
Marsupiomonas sp. NIES-1824 GGU(G)
Chloro dataset
Pedino dataset
Conserved positions
0
50
100
150
200
250
300
350
400
450
G
A
S
Amino acid

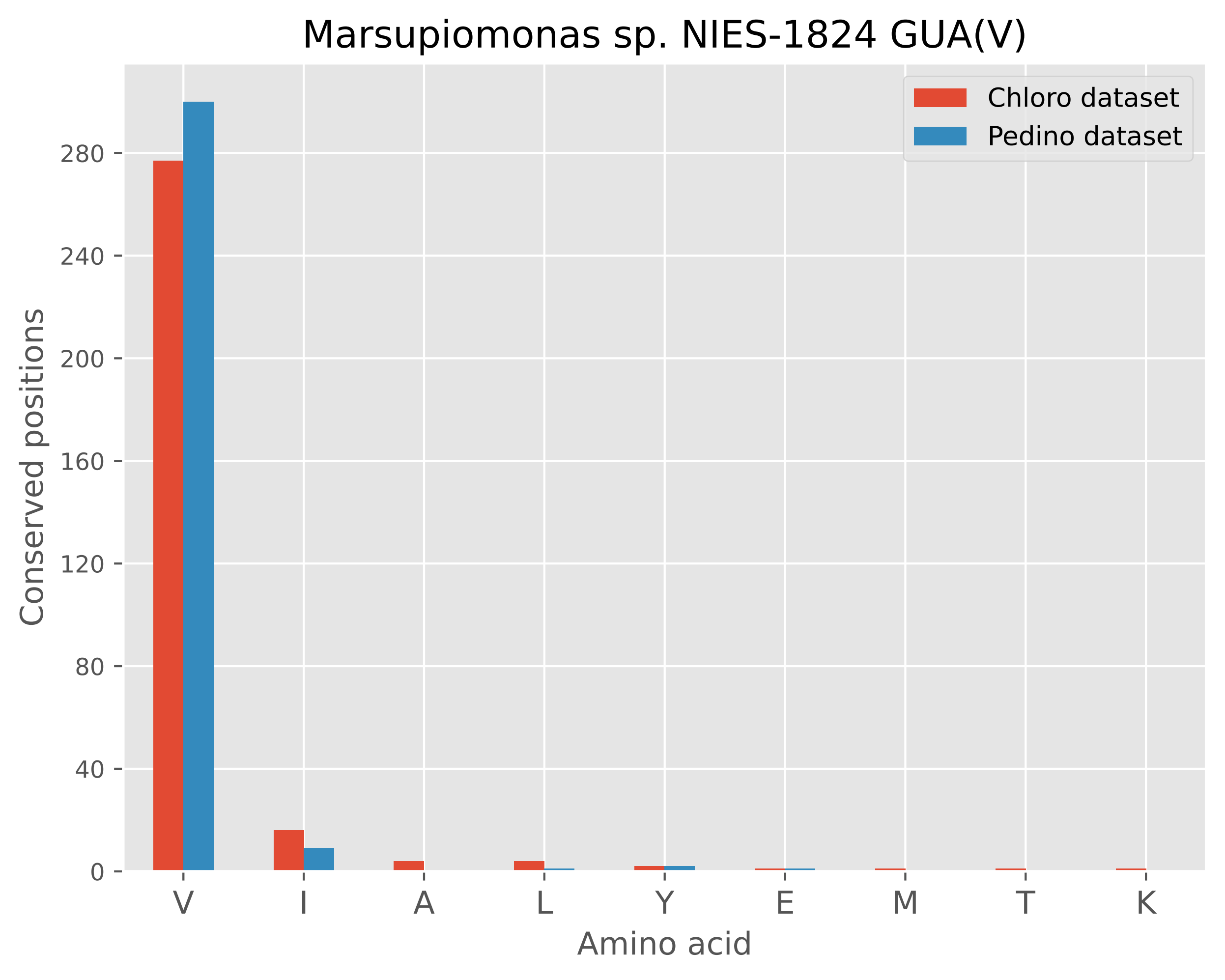

Marsupiomonas sp. NIES-1824 GUA(V)
Chloro dataset
Pedino dataset
Conserved positions
0
40
80
120
160
200
240
280
V
I
A
L
Y
E
M
T
K
Amino acid

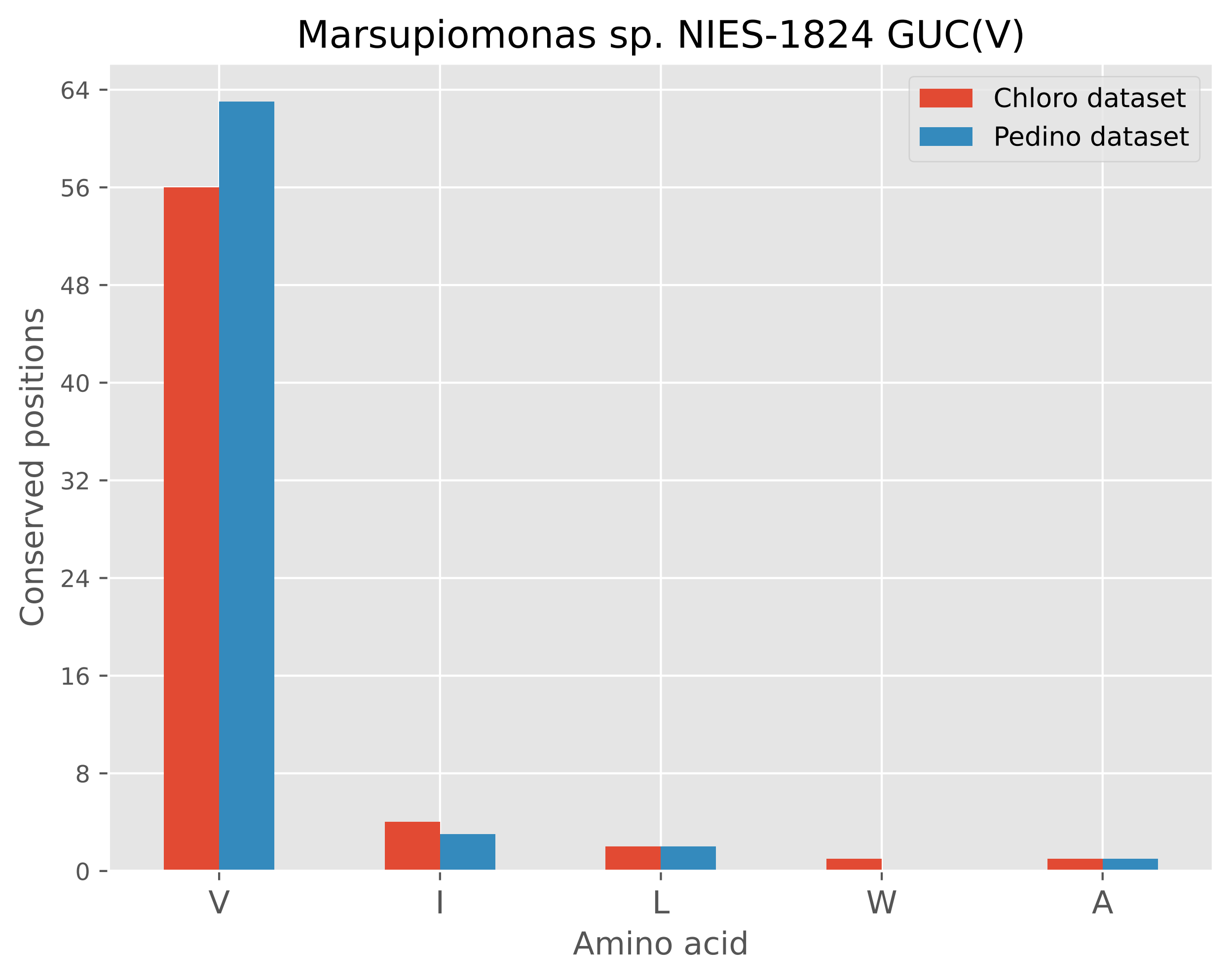
Marsupiomonas sp. NIES-1824 GUC(V)
Chloro dataset
Pedino dataset
Conserved positions
0
8
16
24
32
40
48
56
64
V
I
L
W
A
Amino acid

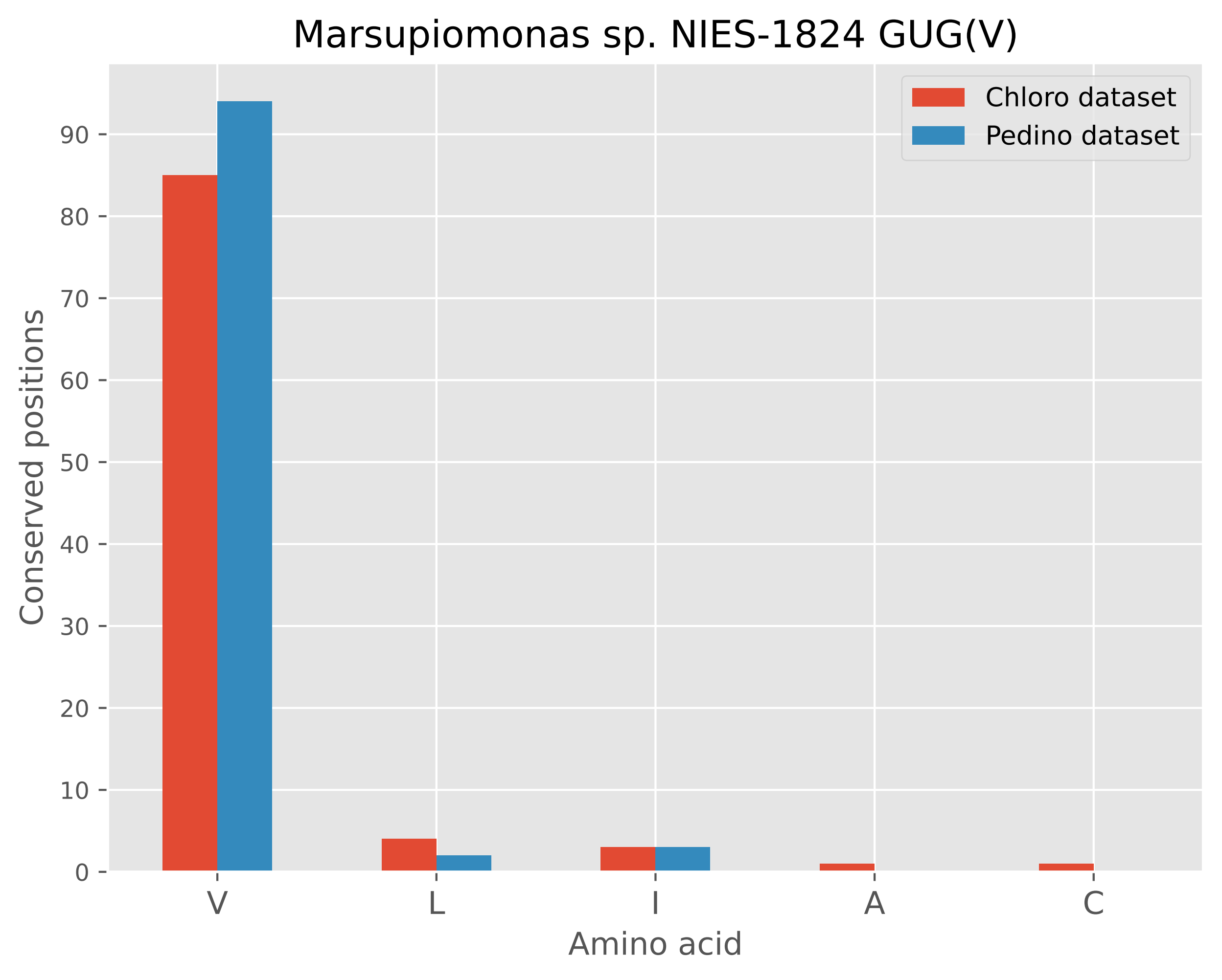

Marsupiomonas sp. NIES-1824 GUG(V)
Chloro dataset
Pedino dataset
Conserved positions
0
10
20
30
40
50
60
70
80
90
V
L
I
A
C
Amino acid

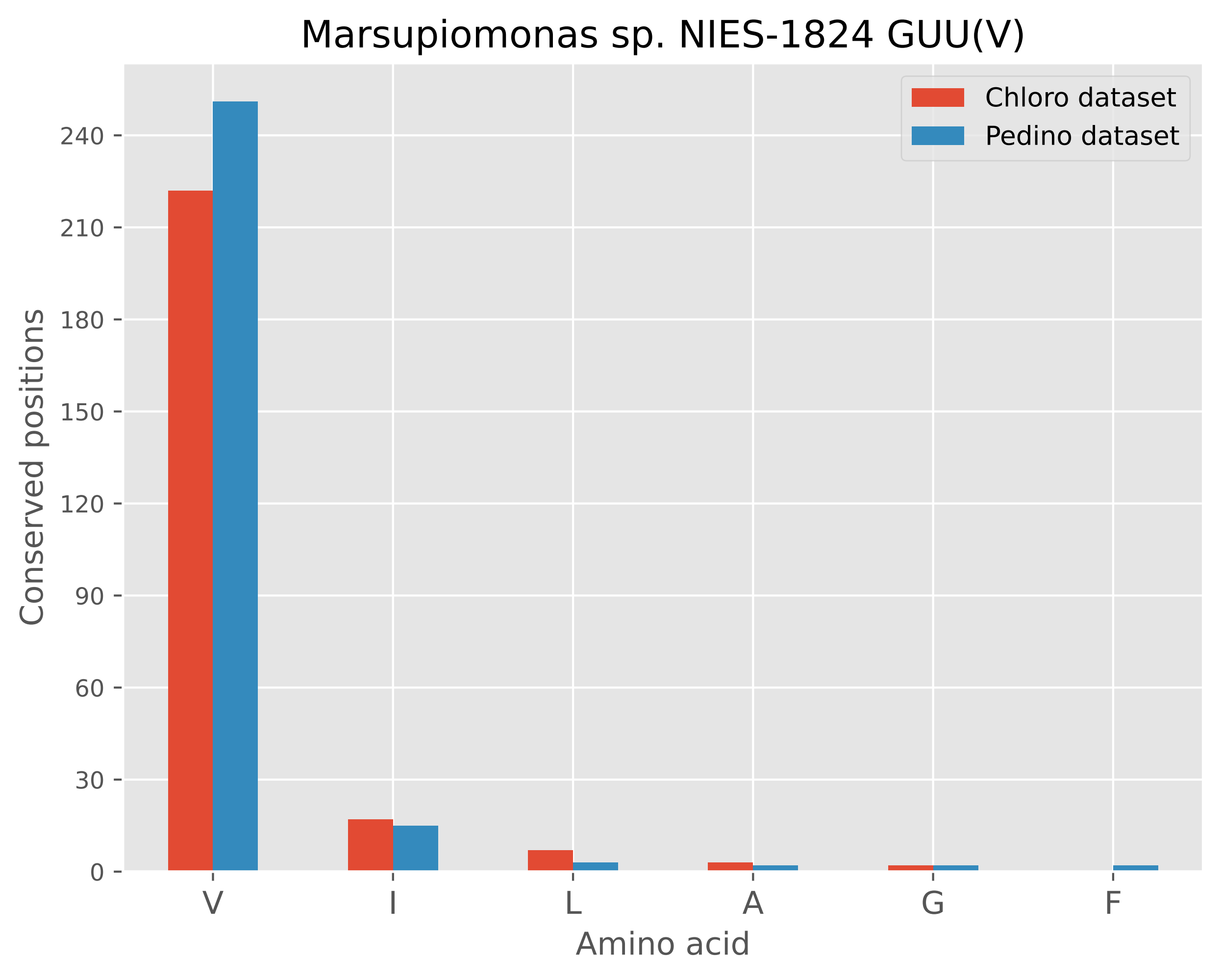

Marsupiomonas sp. NIES-1824 GUU(V)
Chloro dataset
Pedino dataset
Conserved positions
0
30
60
90
120
150
180
210
240
V
I
L
A
G
F
Amino acid

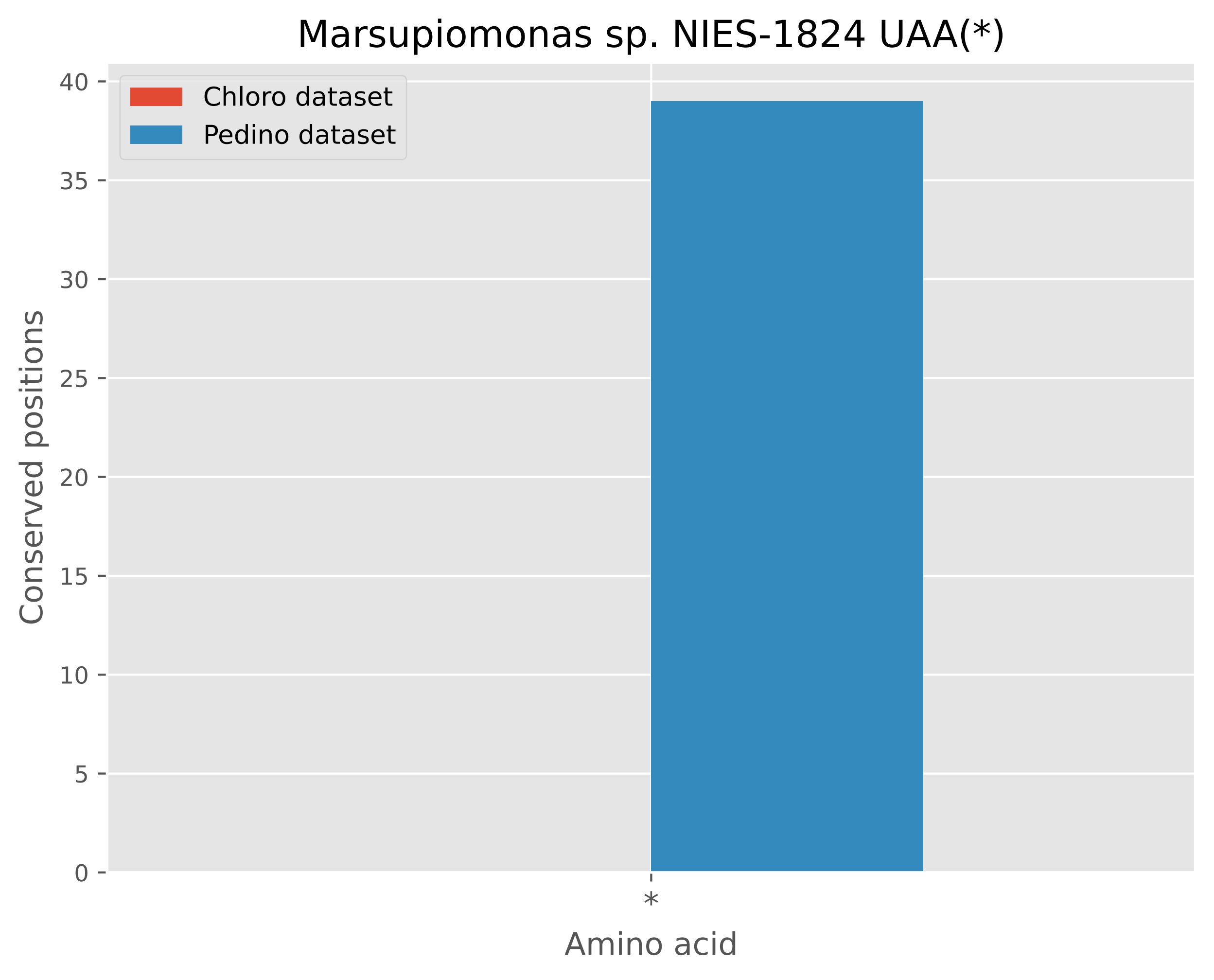

Marsupiomonas sp. NIES-1824 UAA(*)
Chloro dataset
Pedino dataset
Conserved positions
0
5
10
15
20
25
30
35
40
*
Amino acid

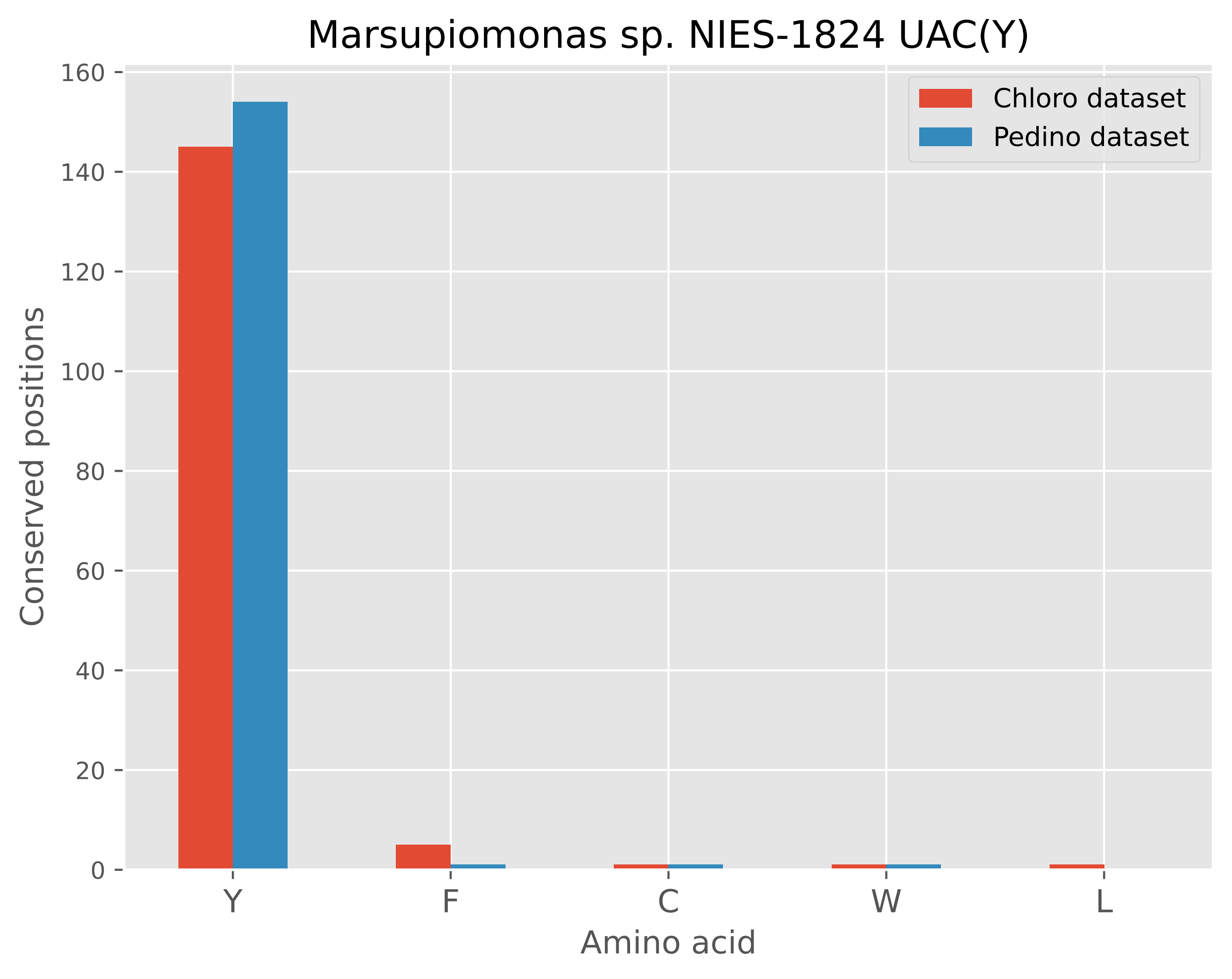

Marsupiomonas sp. NIES-1824 UAC(Y)
Conserved positions
0
20
40
60
80
100
120
140
160
Y
F
C
W
L
Amino acid
Chloro dataset
Pedino dataset

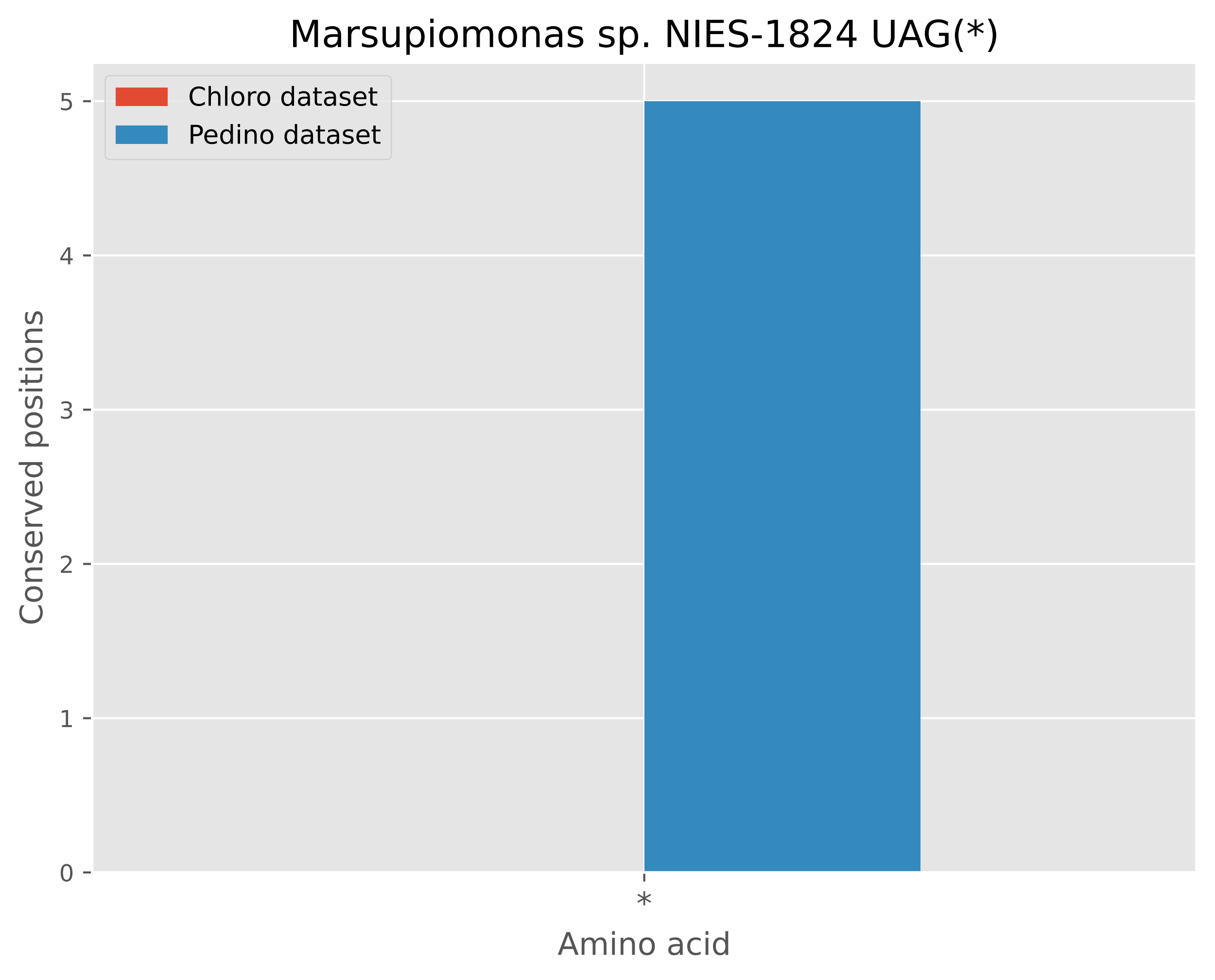

Marsupiomonas sp. NIES-1824 UAG(*)
Chloro dataset
Pedino dataset
Conserved positions
0
1
2
3
4
5
*
Amino acid

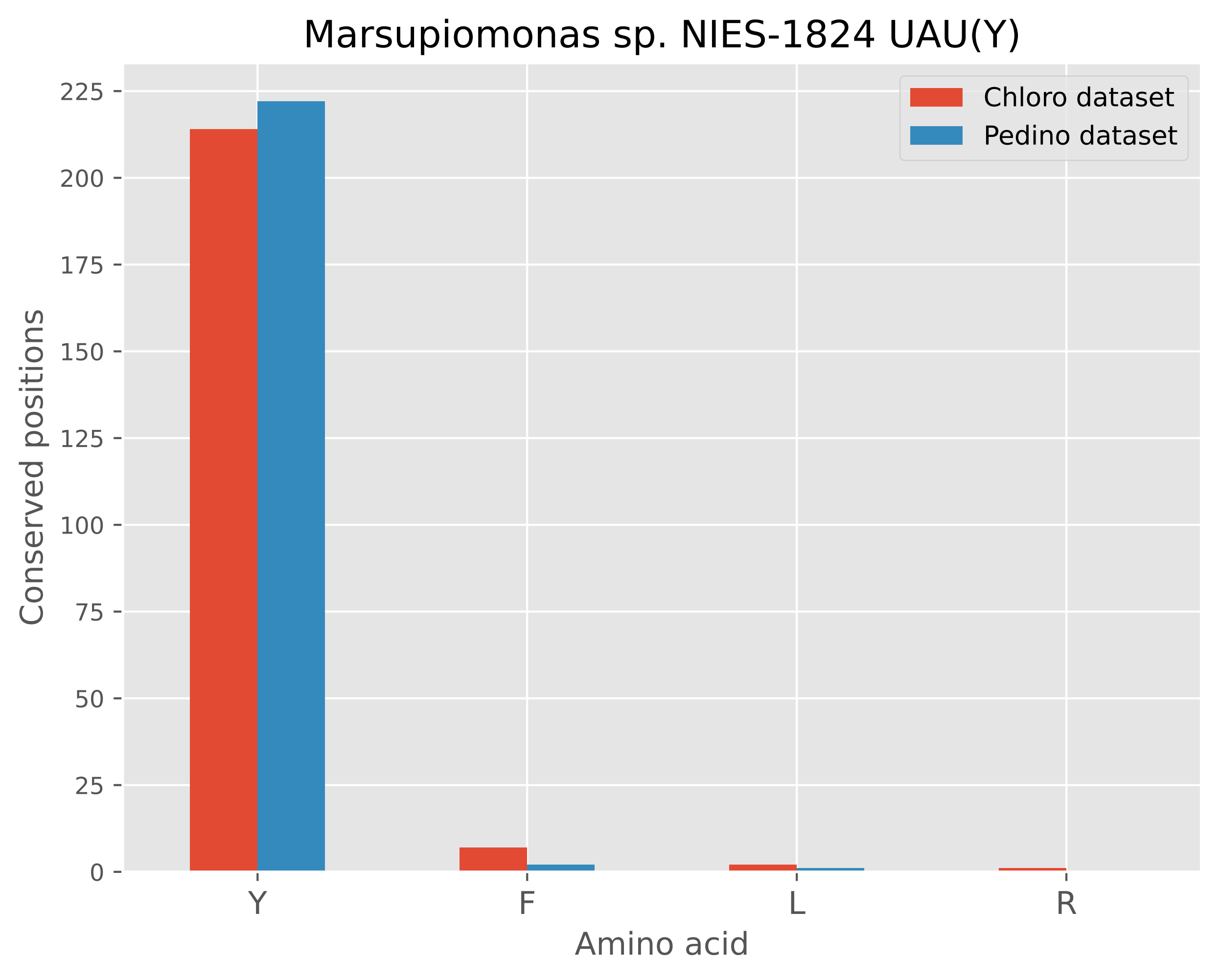

Marsupiomonas sp. NIES-1824 UAU(Y)
Chloro dataset
Pedino dataset
Conserved positions
0
25
50
75
100
125
150
175
200
225
Y
F
L
R
Amino acid

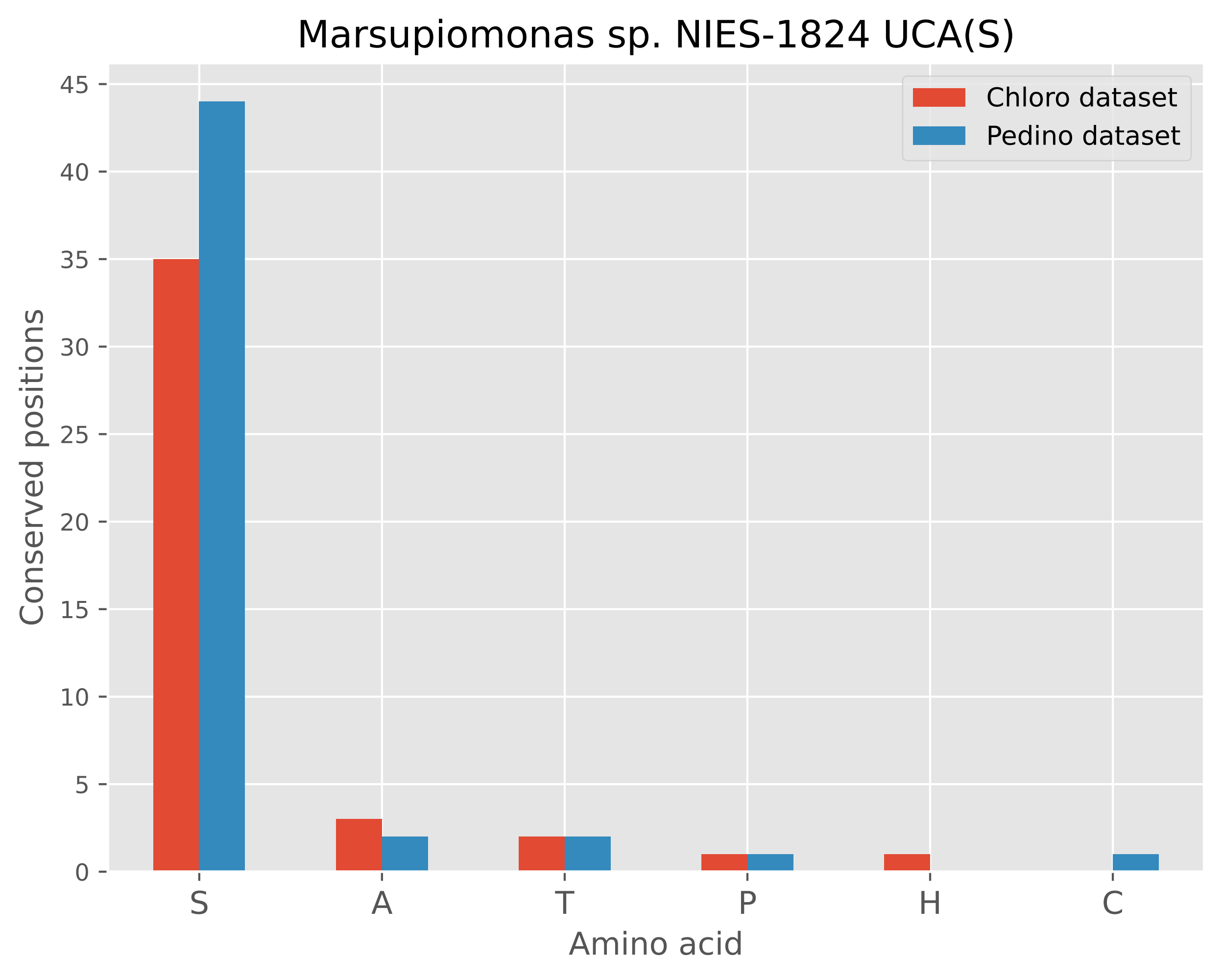

Marsupiomonas sp. NIES-1824 UCA(S)
Chloro dataset
Pedino dataset
Conserved positions
0
5
10
15
20
25
30
35
40
45
S
A
T
P
H
C
Amino acid

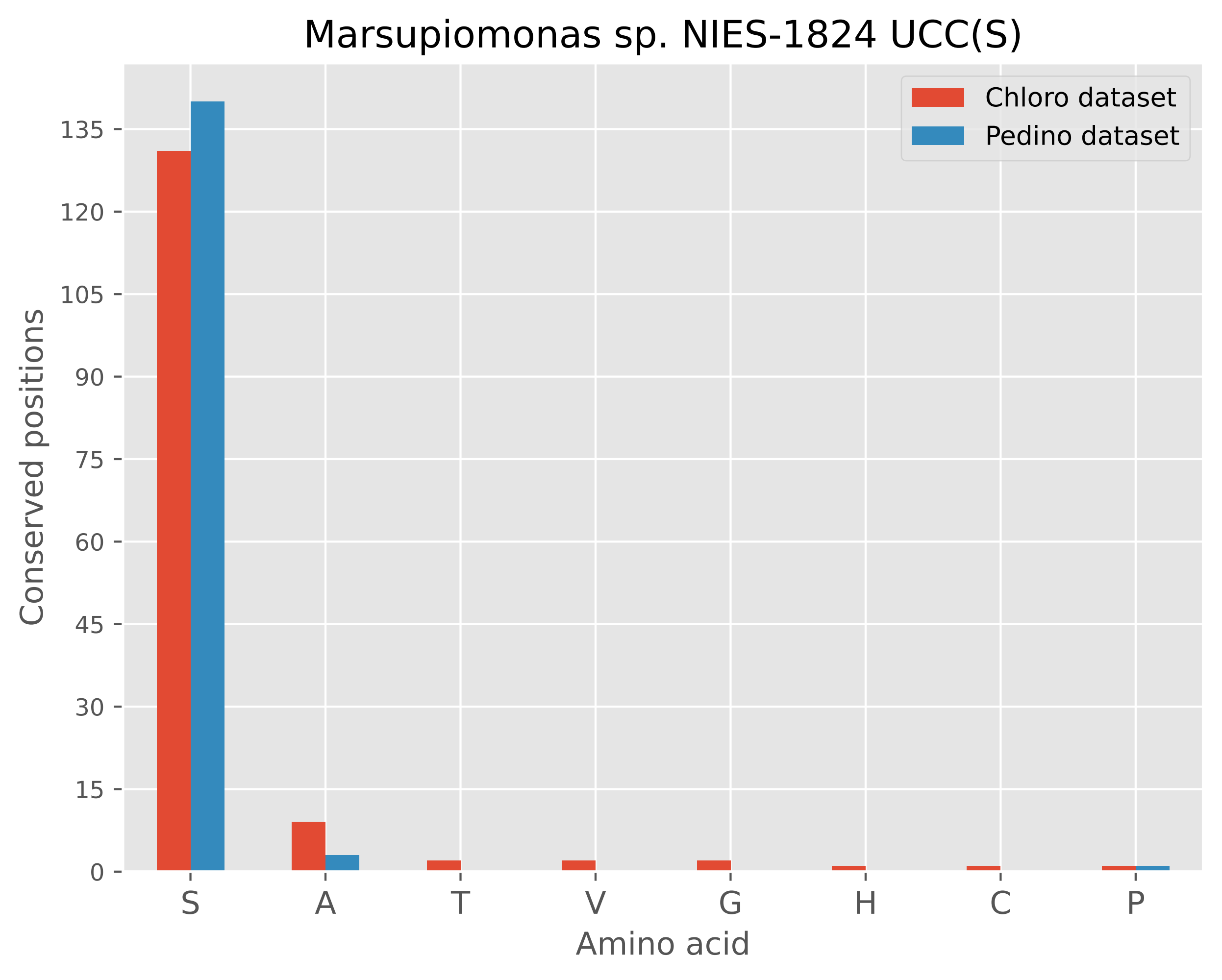

Marsupiomonas sp. NIES-1824 UCC(S)
Chloro dataset
Pedino dataset
Conserved positions
0
15
30
45
60
75
90
105
120
135
S
A
T
V
G
H
C
P
Amino acid

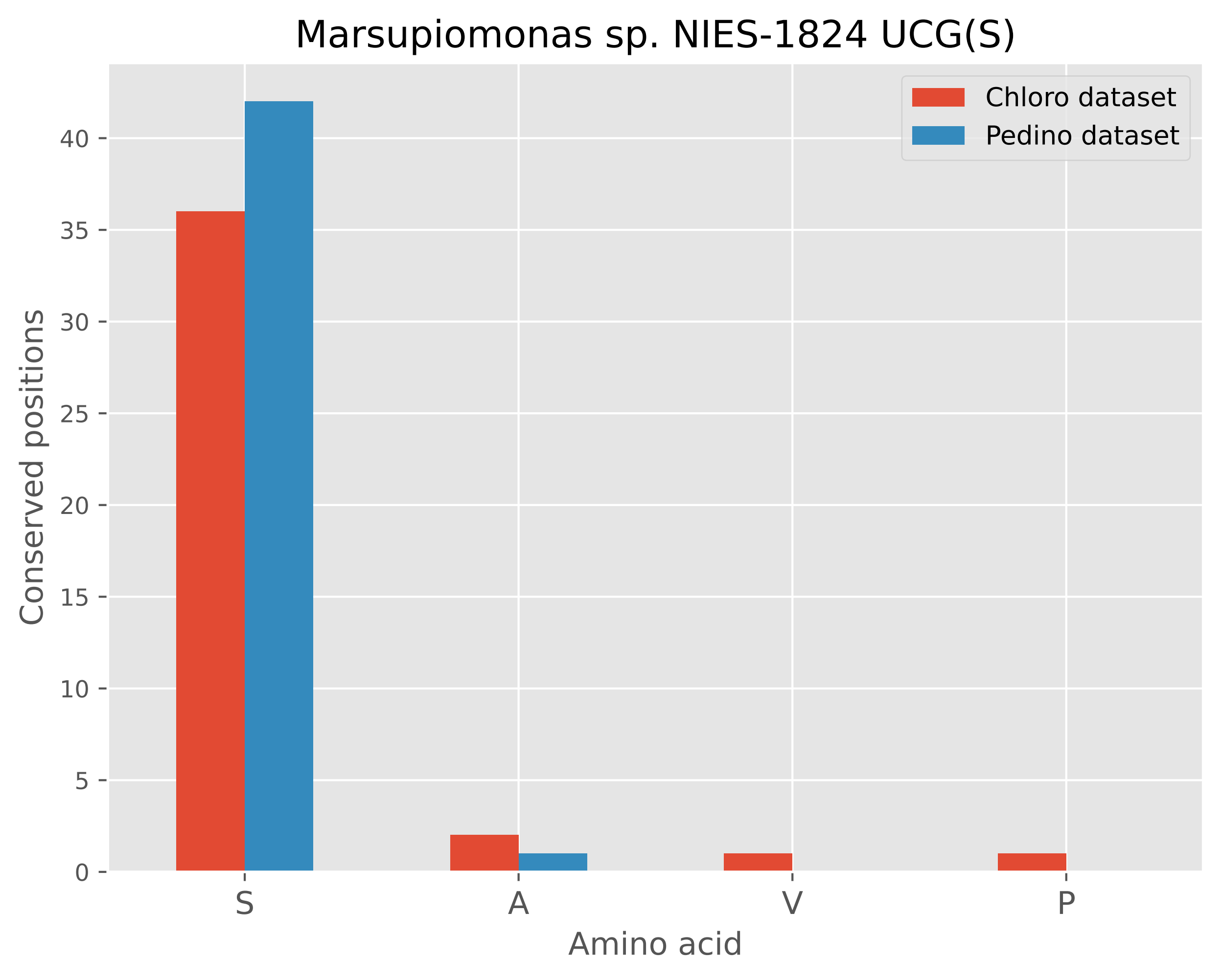

Marsupiomonas sp. NIES-1824 UCG(S)
Chloro dataset
Pedino dataset
Conserved positions
0
5
10
15
20
25
30
35
40
S
A
V
P
Amino acid

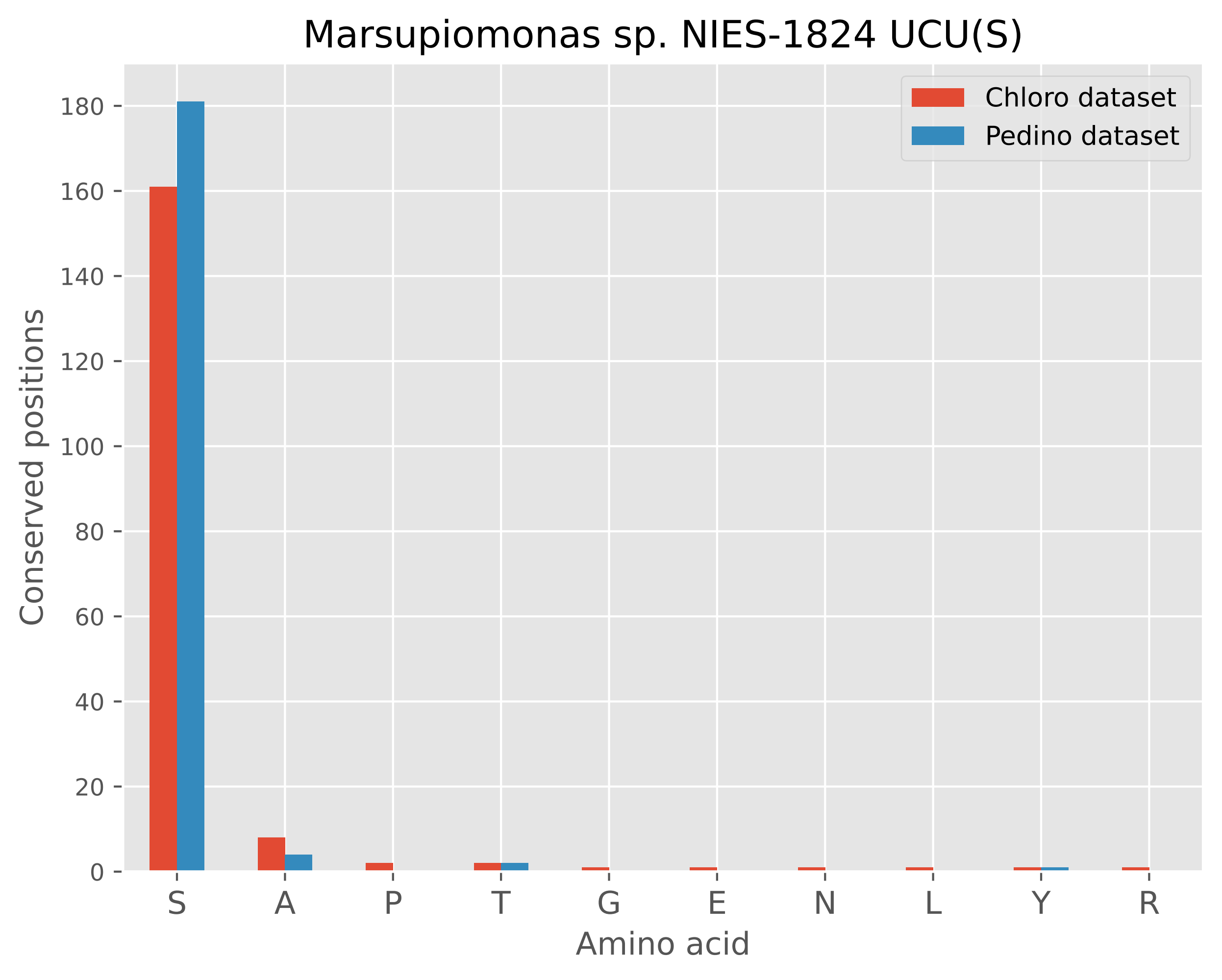

Marsupiomonas sp. NIES-1824 UCU(S)
Chloro dataset
Pedino dataset
Conserved positions
0
20
40
60
80
100
120
140
160
180
S
A
P
T
G
E
N
L
Y
R
Amino acid

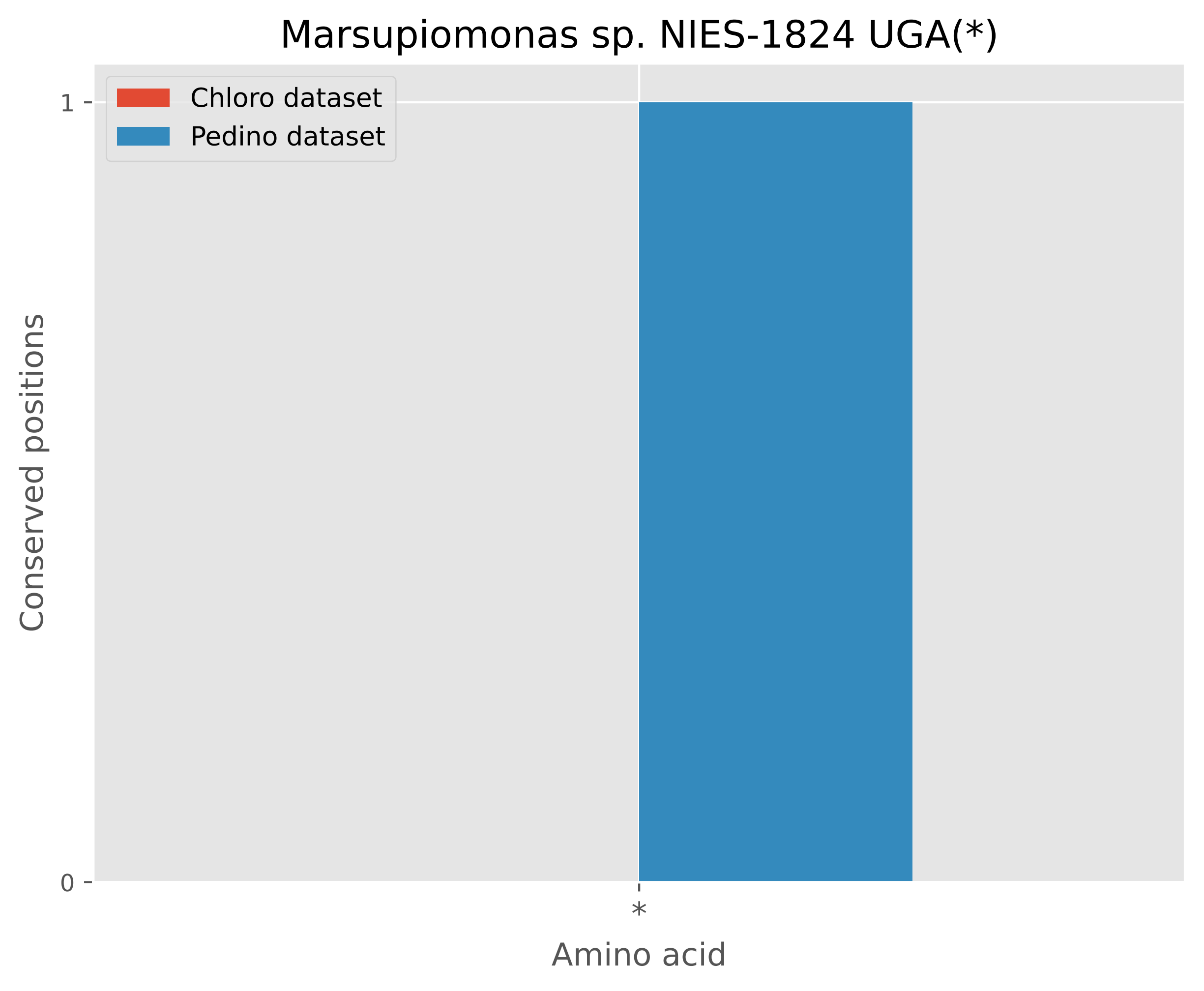
Marsupiomonas sp. NIES-1824 UGA(*)
Chloro dataset
Pedino dataset
1
0
Conserved positions
*
Amino acid

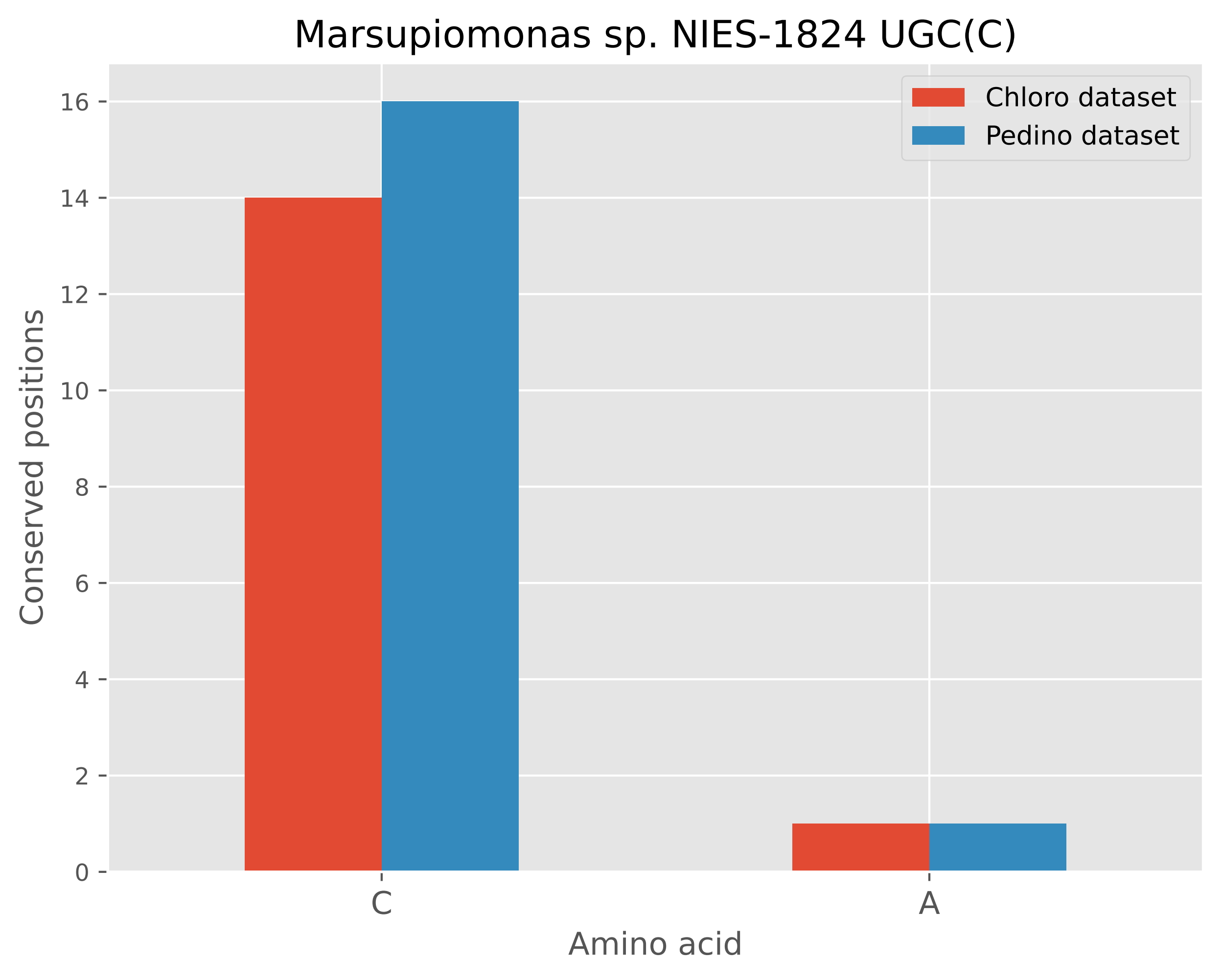
Marsupiomonas sp. NIES-1824 UGC(C)
Chloro dataset
Pedino dataset
Conserved positions
Amino acid
C
A
0
2
4
6
8
10
12
14
16

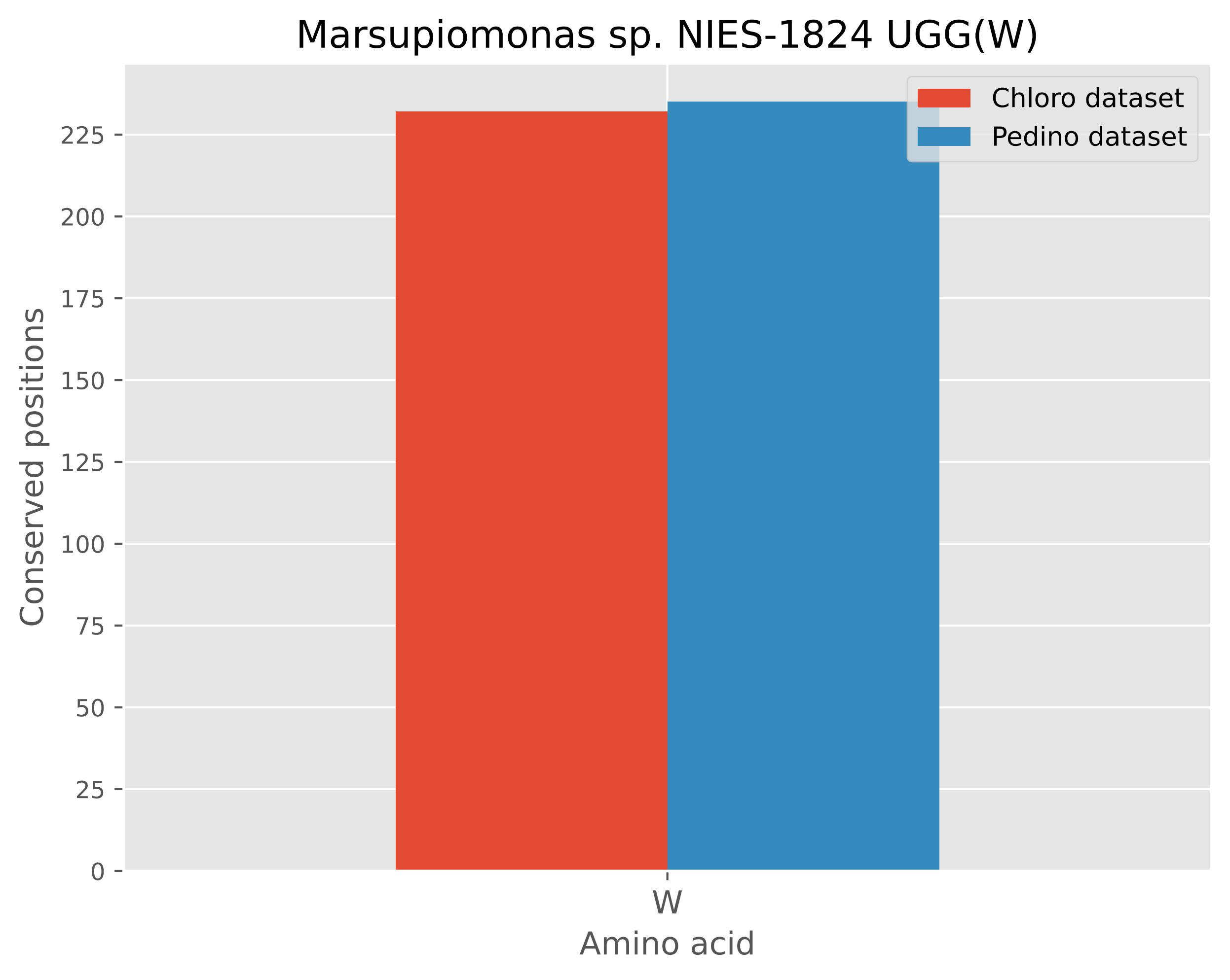
Marsupiomonas sp. NIES-1824 UGG(W)
Chloro dataset
Pedino dataset
Conserved positions
0
25
50
75
100
125
150
175
200
225
W
Amino acid

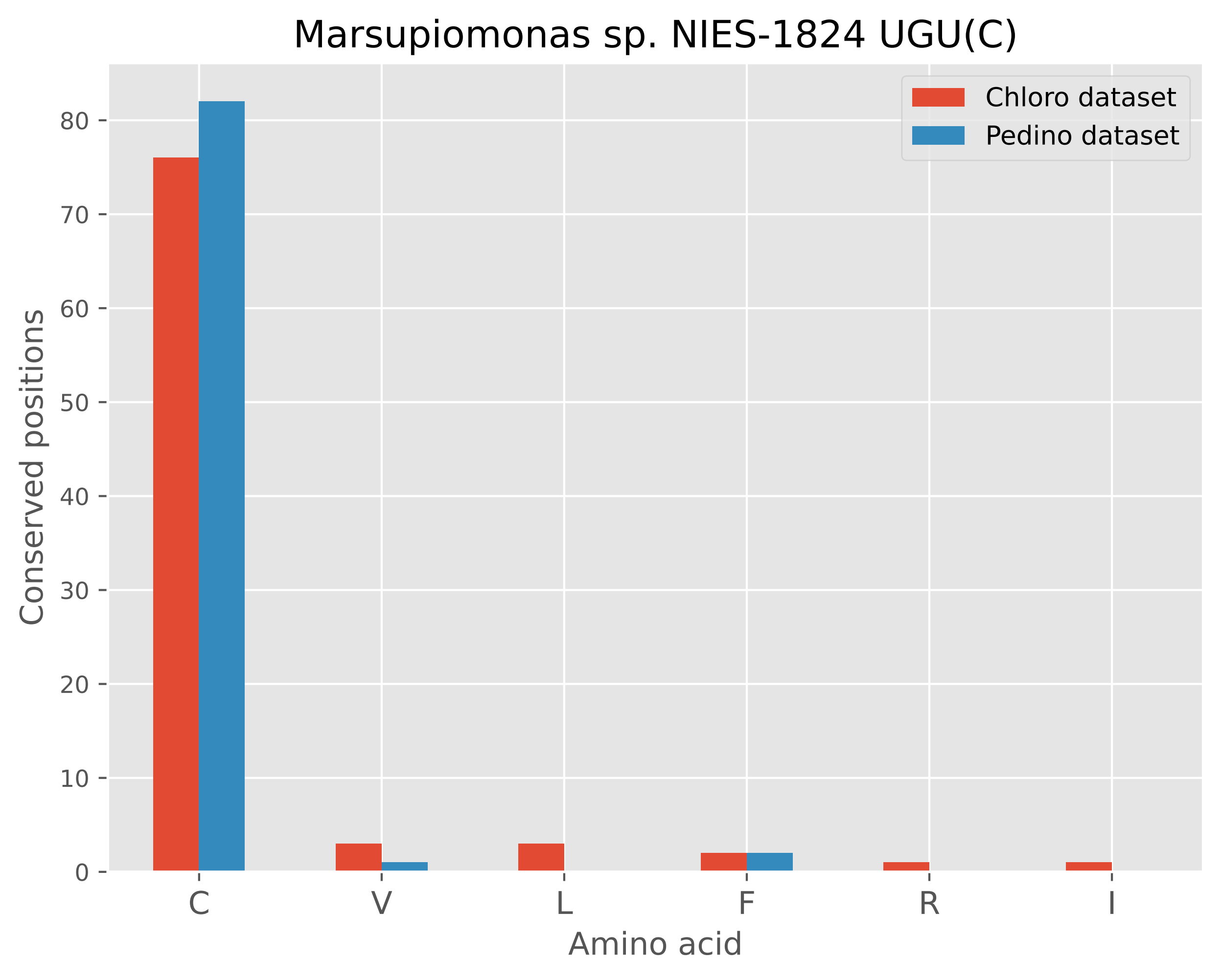

Marsupiomonas sp. NIES-1824 UGU(C)
Chloro dataset
Pedino dataset
Conserved positions
0
10
20
30
40
50
60
70
80
C
V
L
F
R
I
Amino acid

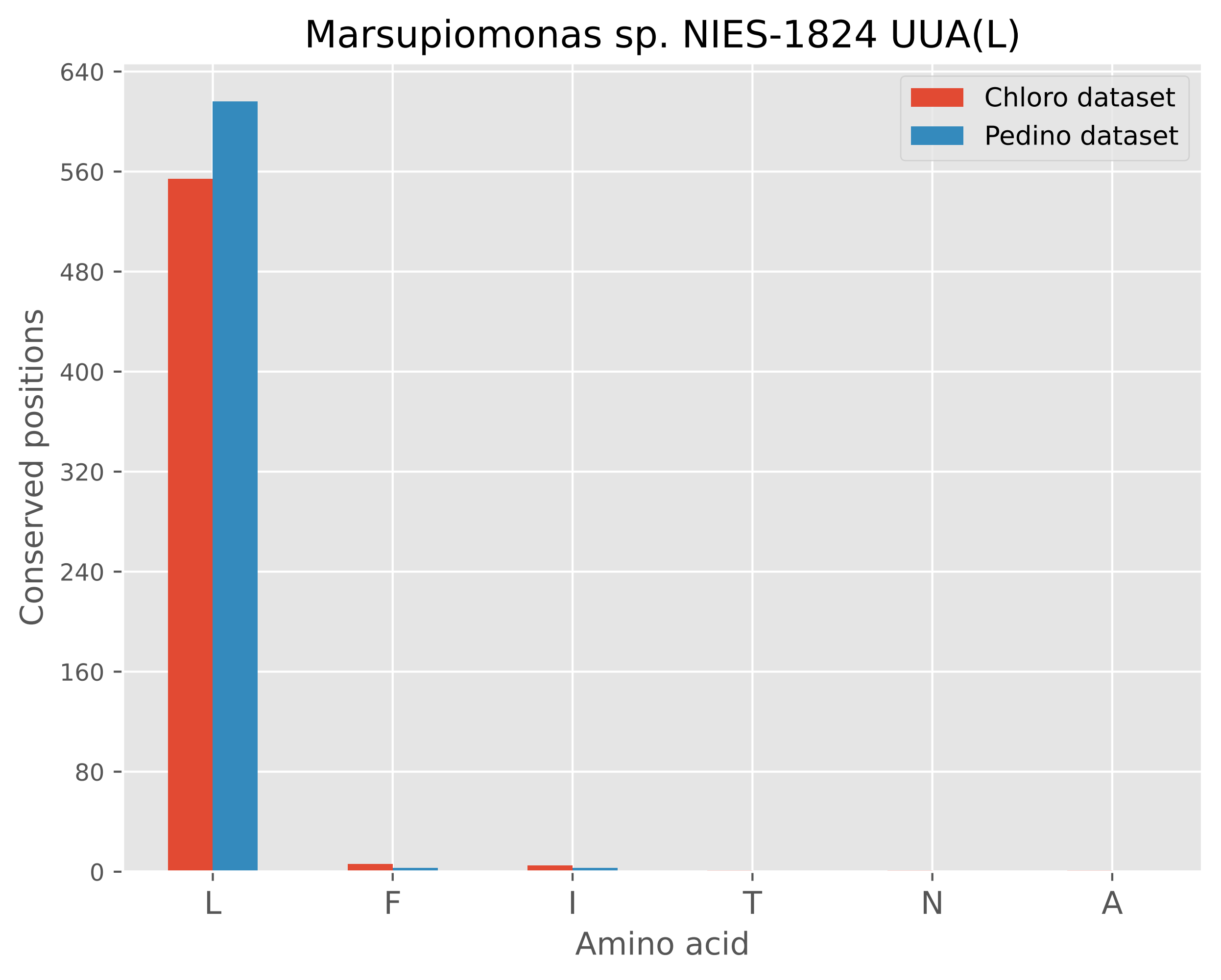

Marsupiomonas sp. NIES-1824 UUA(L)
Chloro dataset
Pedino dataset
Conserved positions
640
560
480
400
320
240
160
80
0
L
F
I
T
N
A
Amino acid

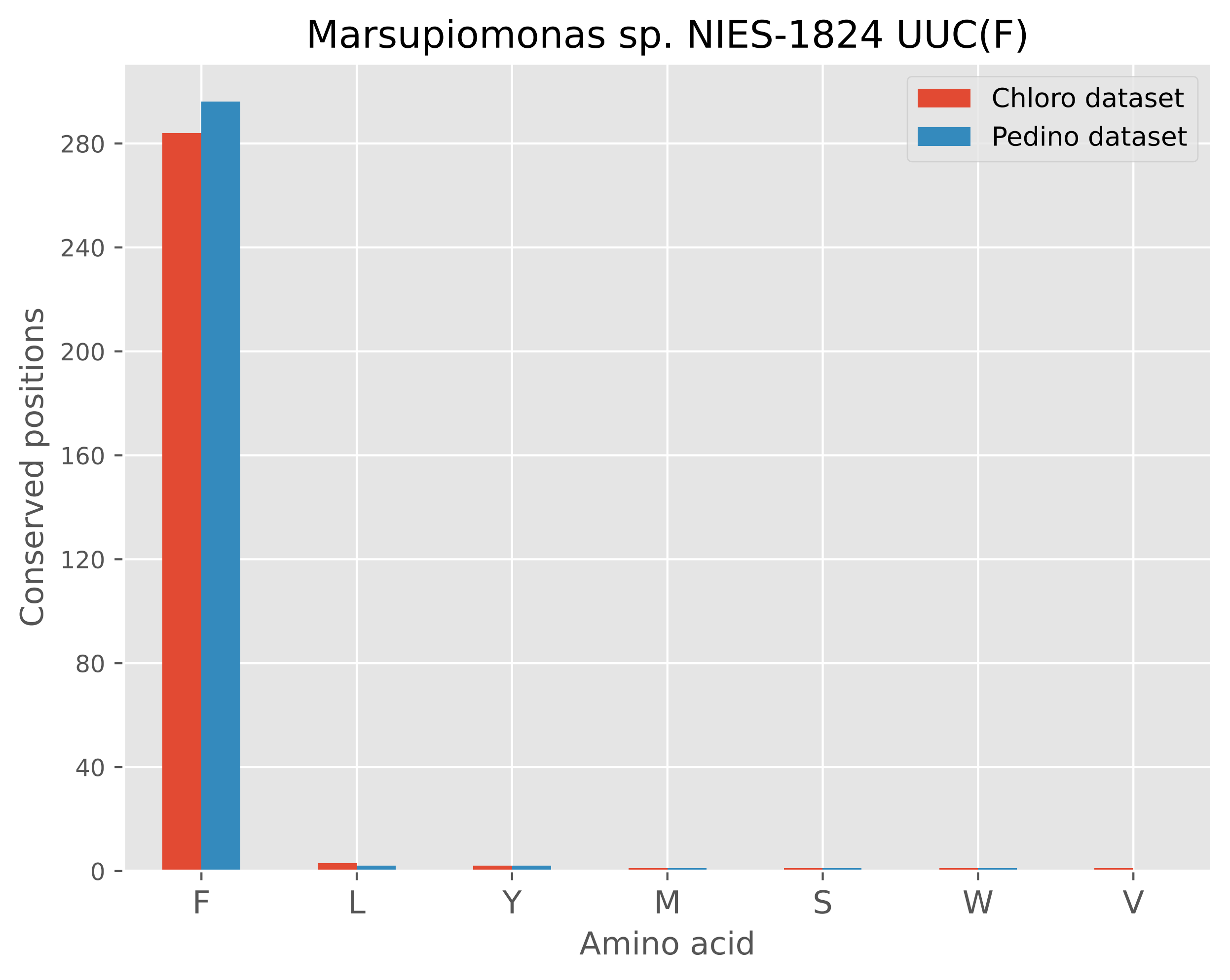

Marsupiomonas sp. NIES-1824 UUC(F)
Chloro dataset
Pedino dataset
Conserved positions
0
40
80
120
160
200
240
280
F
L
Y
M
S
W
V
Amino acid

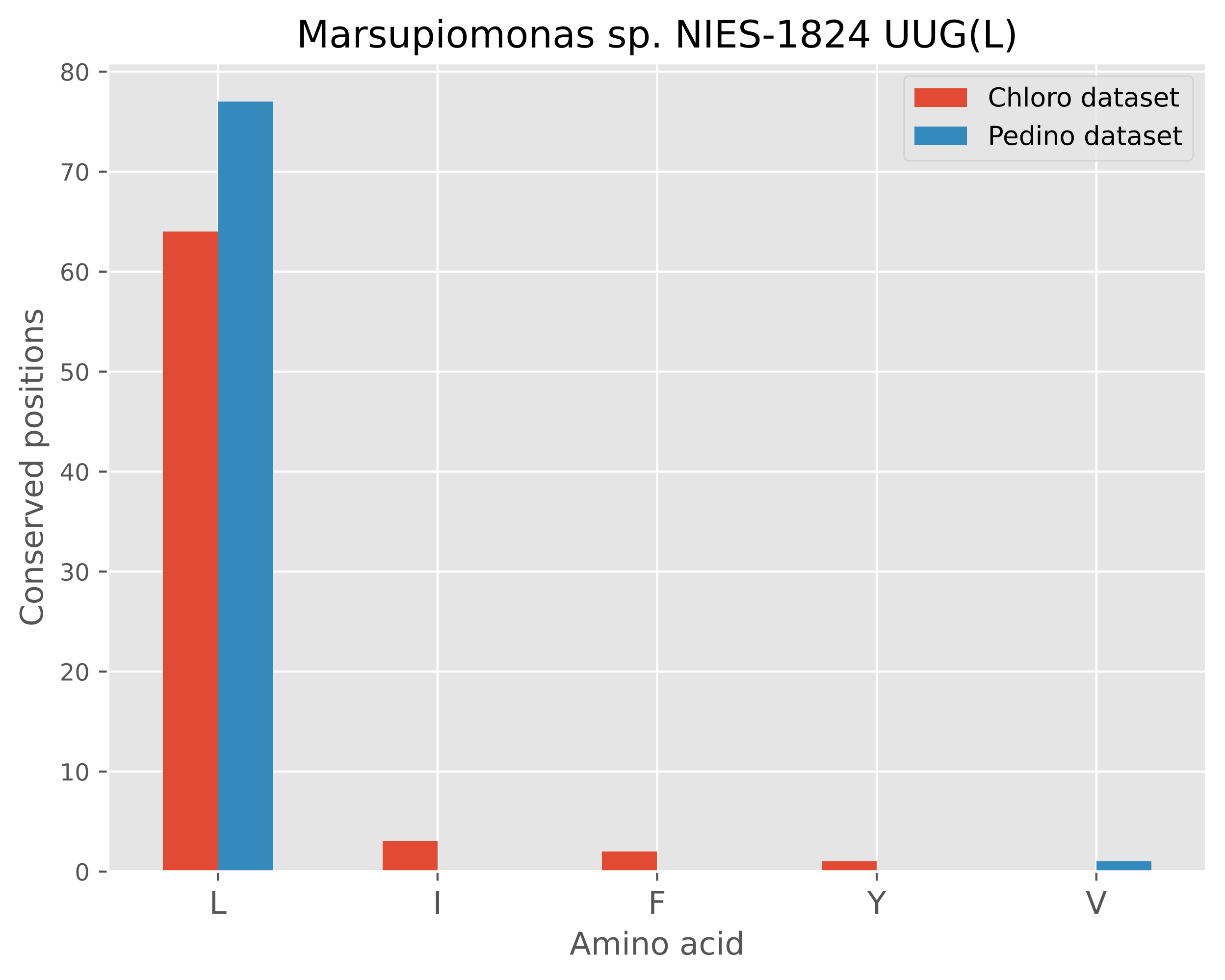

Marsupiomonas sp. NIES-1824 UUG(L)
Chloro dataset
Pedino dataset
Conserved positions
Amino acid
L
I
F
Y
V
0
10
20
30
40
50
60
70
80

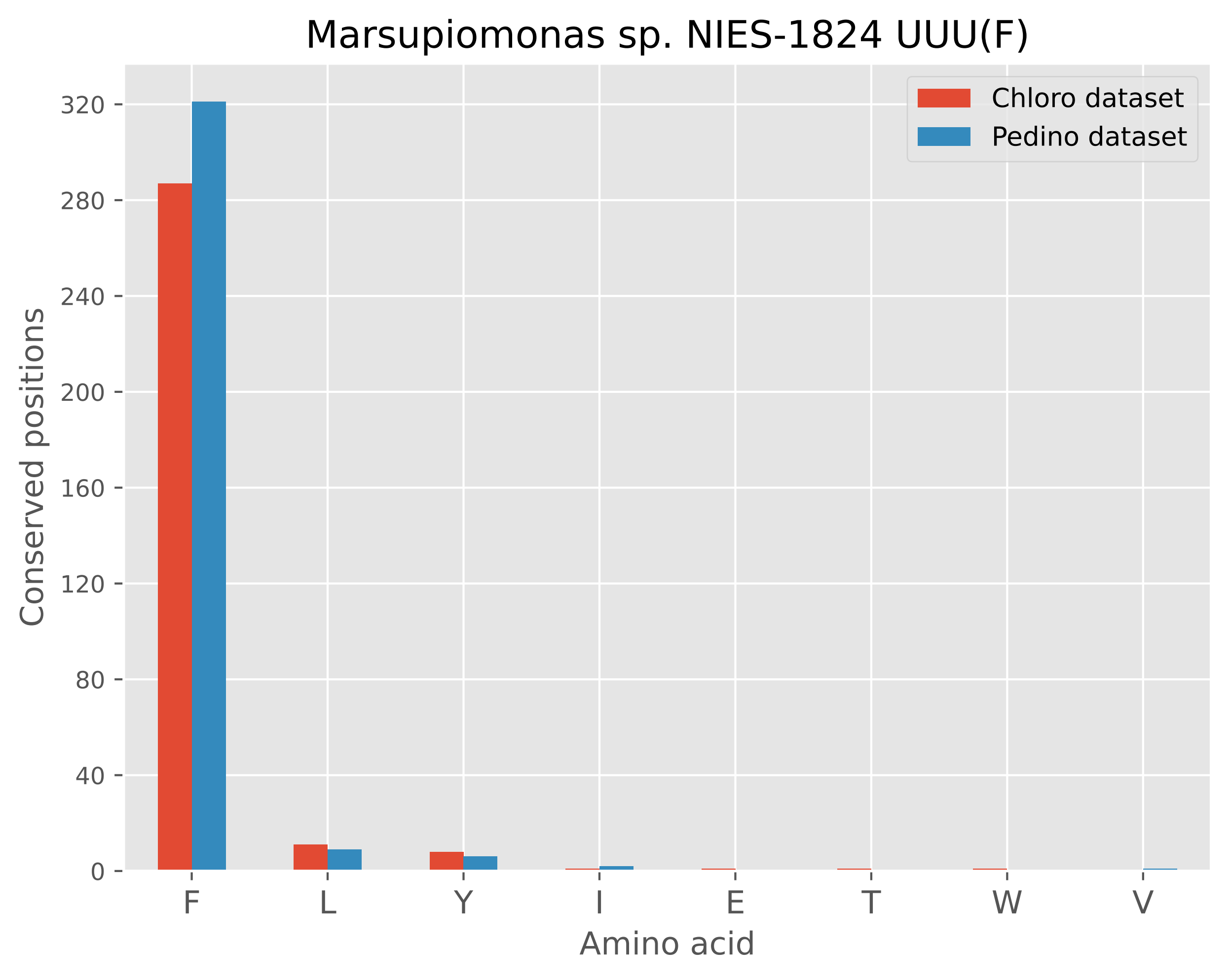

Marsupiomonas sp. NIES-1824 UUU(F)
Chloro dataset
Pedino dataset
Conserved positions
0
40
80
120
160
200
240
280
320
F
L
Y
I
E
T
W
V
Amino acid

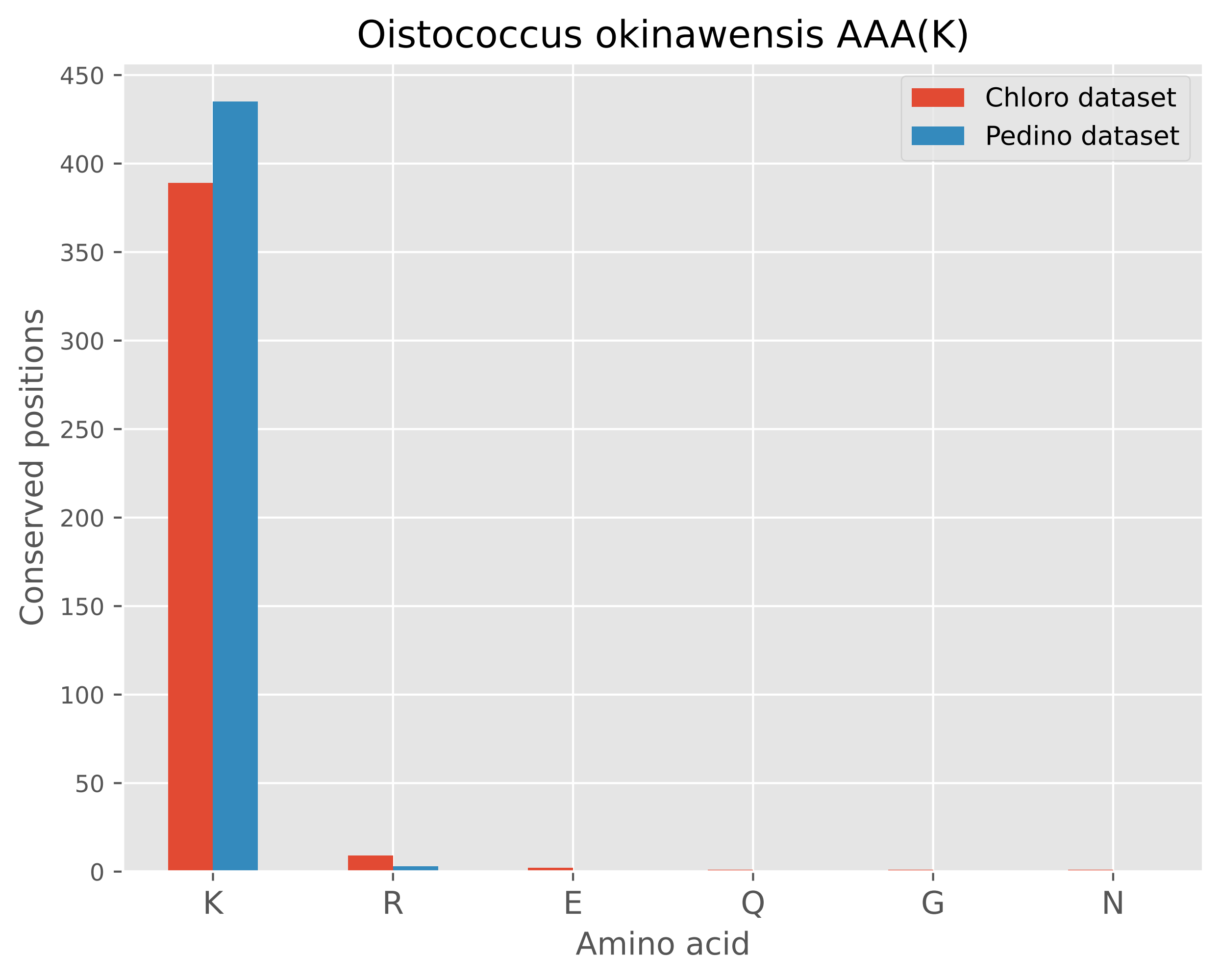

Oistococcus okinawensis AAA(K)
Chloro dataset
Pedino dataset
Conserved positions
0
50
100
150
200
250
300
350
400
450
K
R
E
Q
G
N
Amino acid

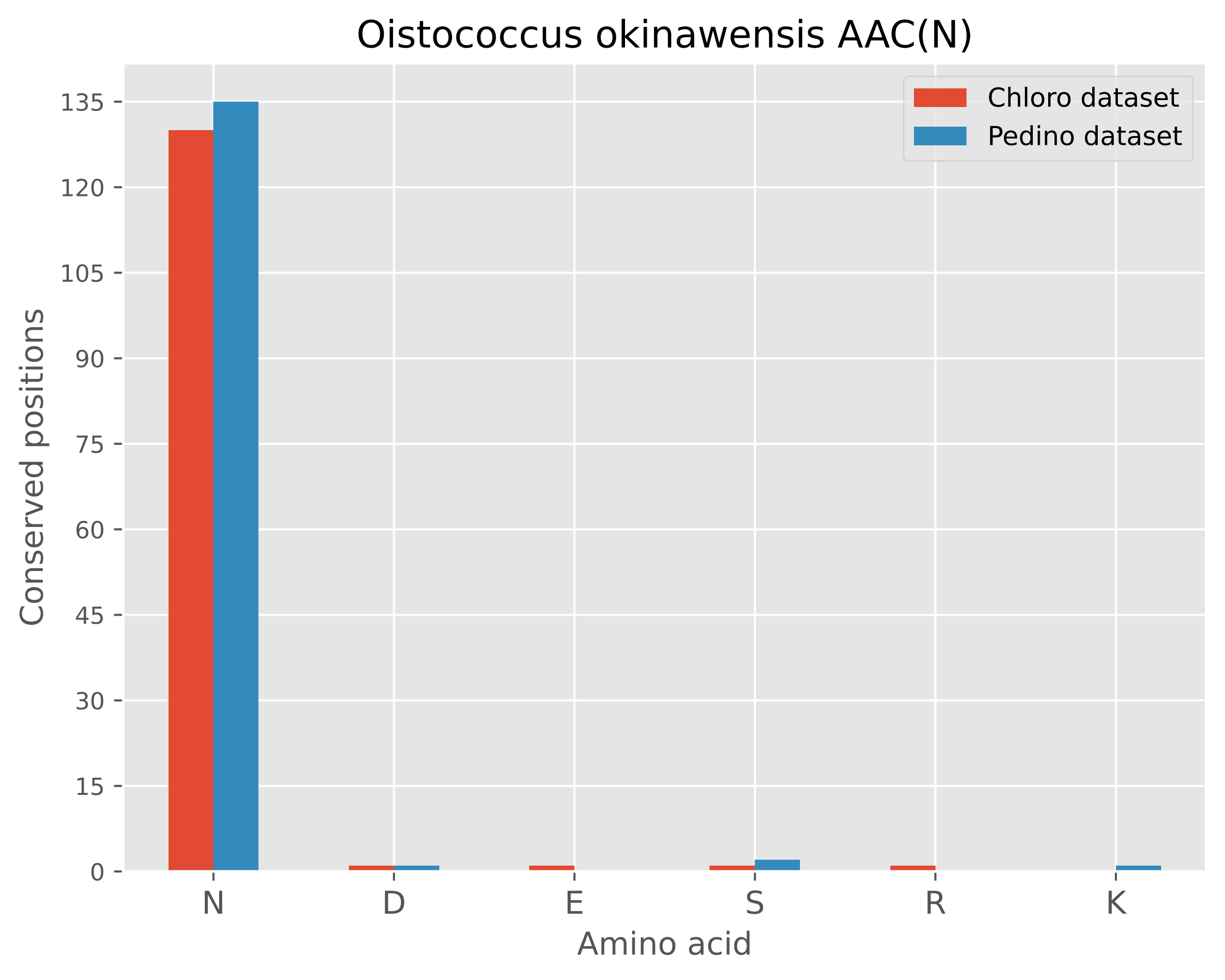
Oistococcus okinawensis AAC(N)
Chloro dataset
Pedino dataset
Conserved positions
135
120
105
90
75
60
45
30
15
0
N
D
E
S
R
K
Amino acid

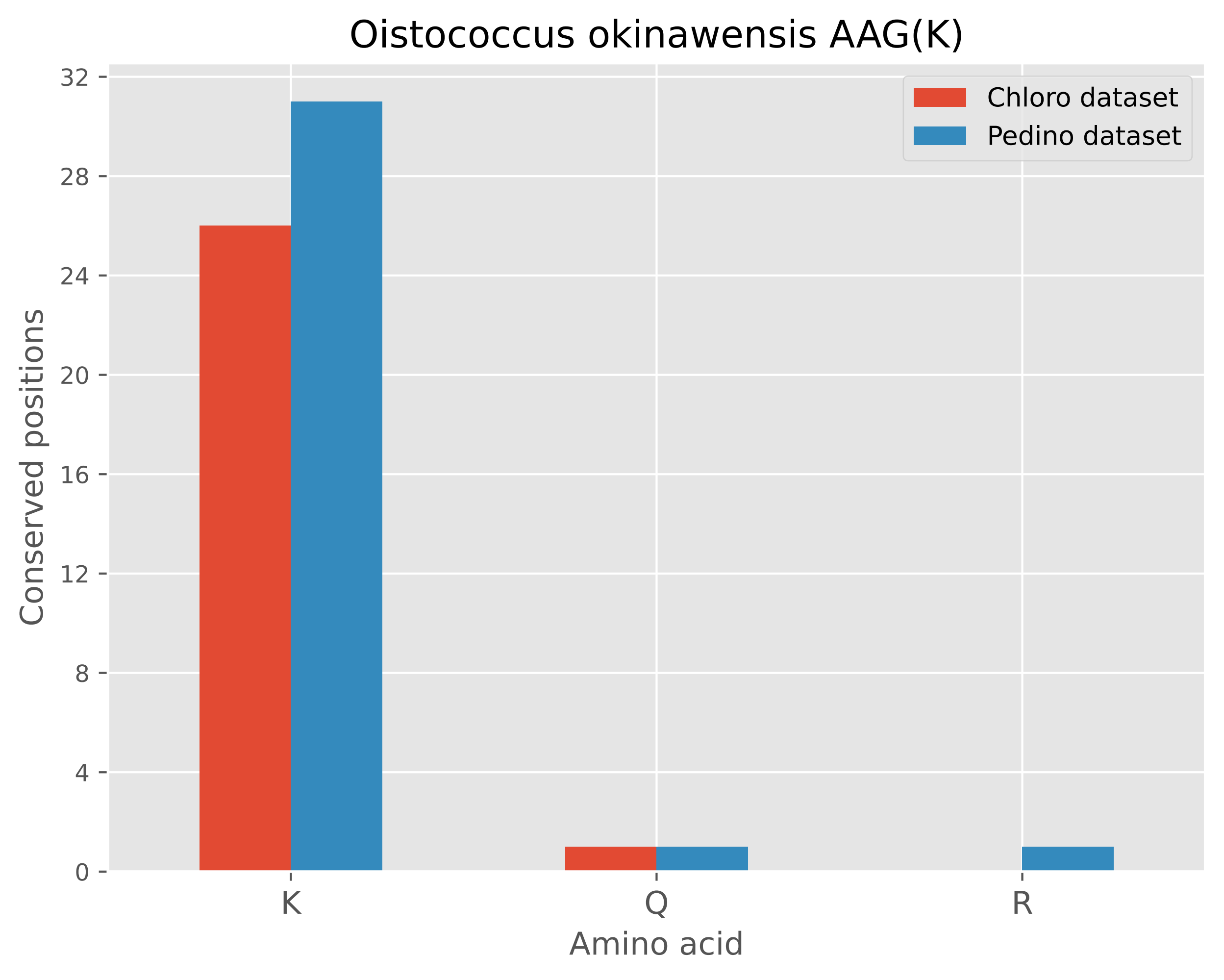
Oistococcus okinawensis AAG(K)
Chloro dataset
Pedino dataset
Conserved positions
0
4
8
12
16
20
24
28
32
K
Q
R
Amino acid

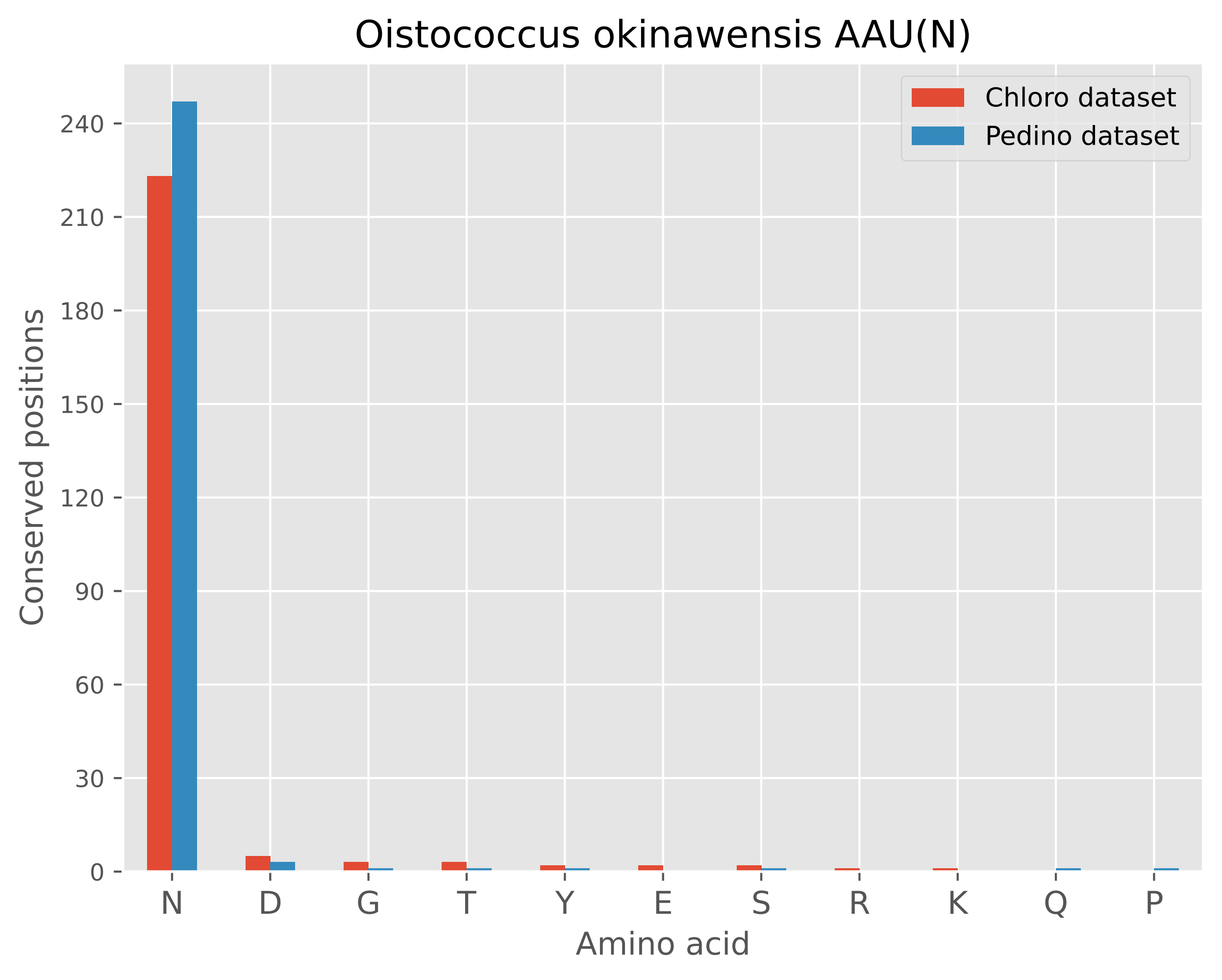

Oistococcus okinawensis AAU(N)
Chloro dataset
Pedino dataset
Conserved positions
0
30
60
90
120
150
180
210
240
N
D
G
T
Y
E
S
R
K
Q
P
Amino acid

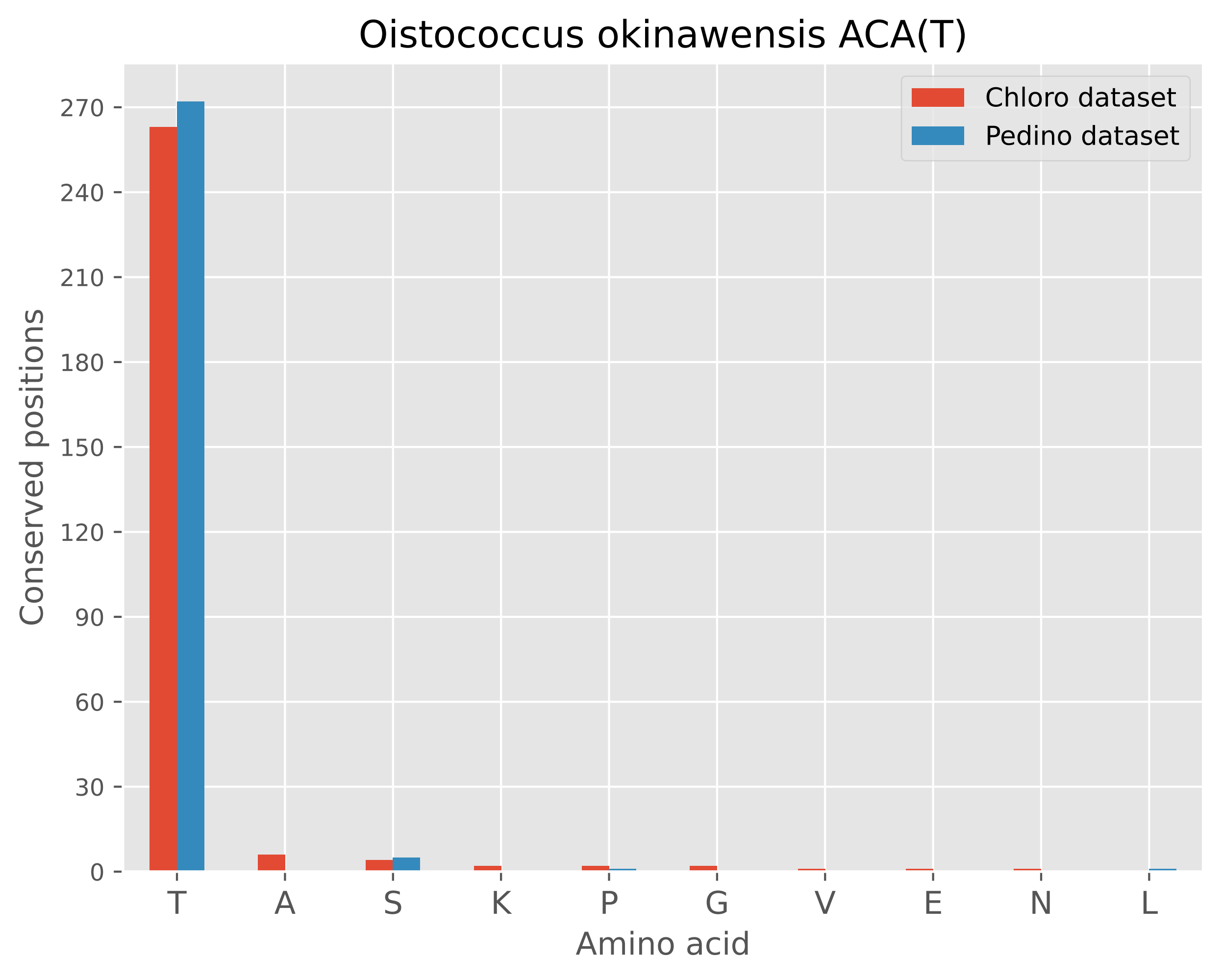

Oistococcus okinawensis ACA(T)
Chloro dataset
Pedino dataset
Conserved positions
270
240
210
180
150
120
90
60
30
0
T
A
S
K
P
G
V
E
N
L
Amino acid

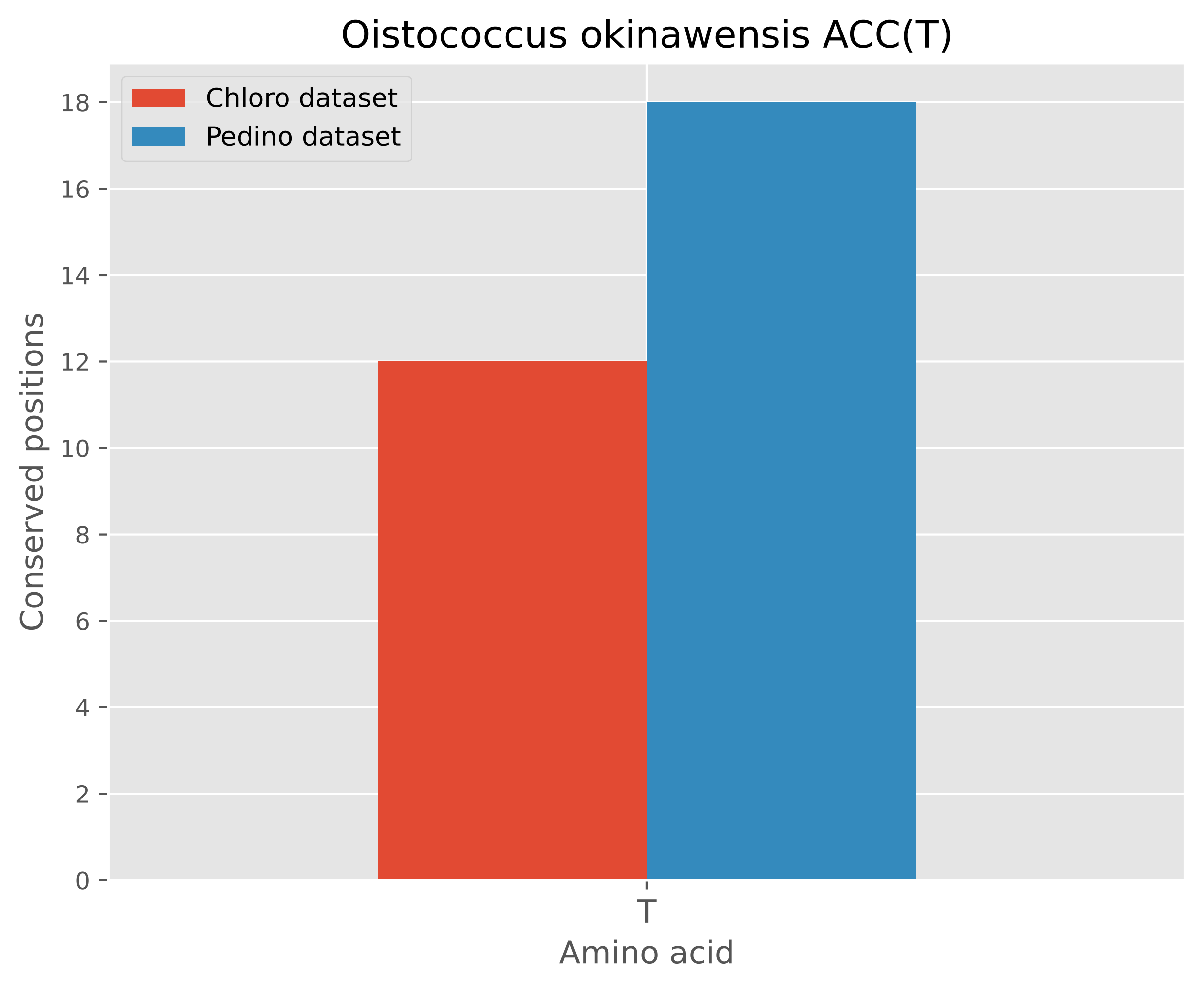

Oistococcus okinawensis ACC(T)
Chloro dataset
Pedino dataset
Conserved positions
0
2
4
6
8
10
12
14
16
18
T
Amino acid

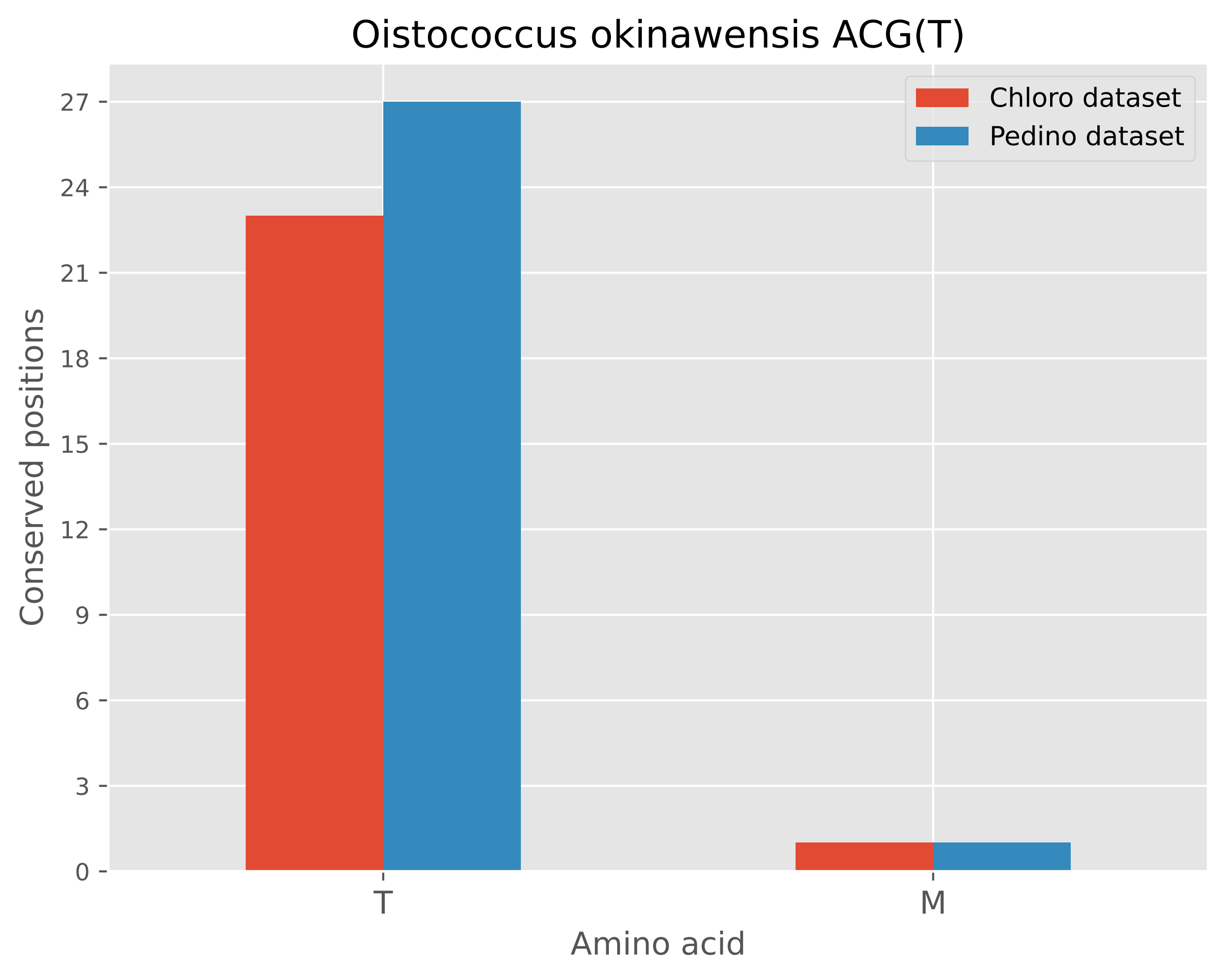

Oistococcus okinawensis ACG(T)
Chloro dataset
Pedino dataset
Conserved positions
0
3
6
9
12
15
18
21
24
27
T
M
Amino acid

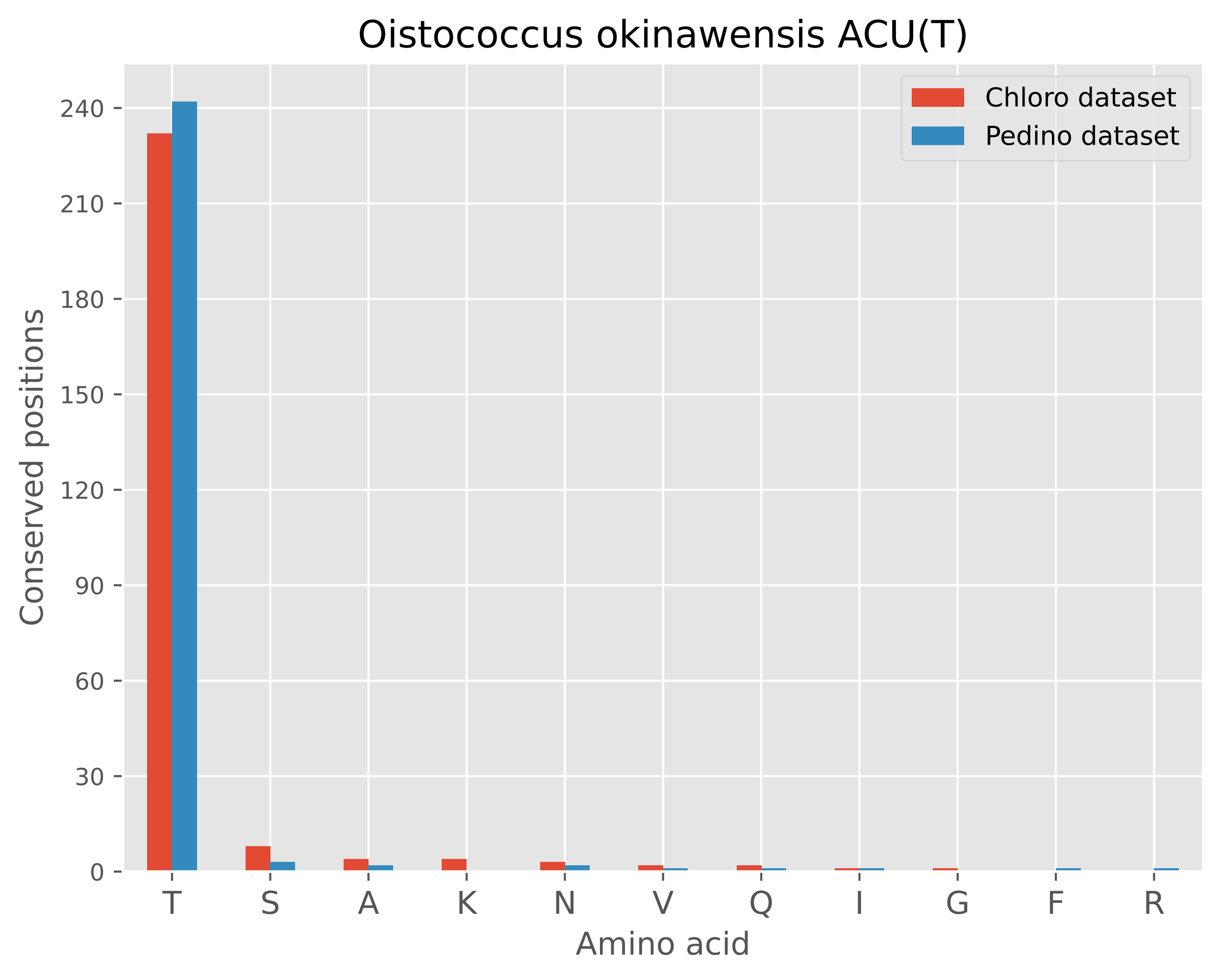
Oistococcus okinawensis ACU(T)
Chloro dataset
Pedino dataset
Conserved positions
0
30
60
90
120
150
180
210
240
T
S
A
K
N
V
Q
I
G
F
R
Amino acid

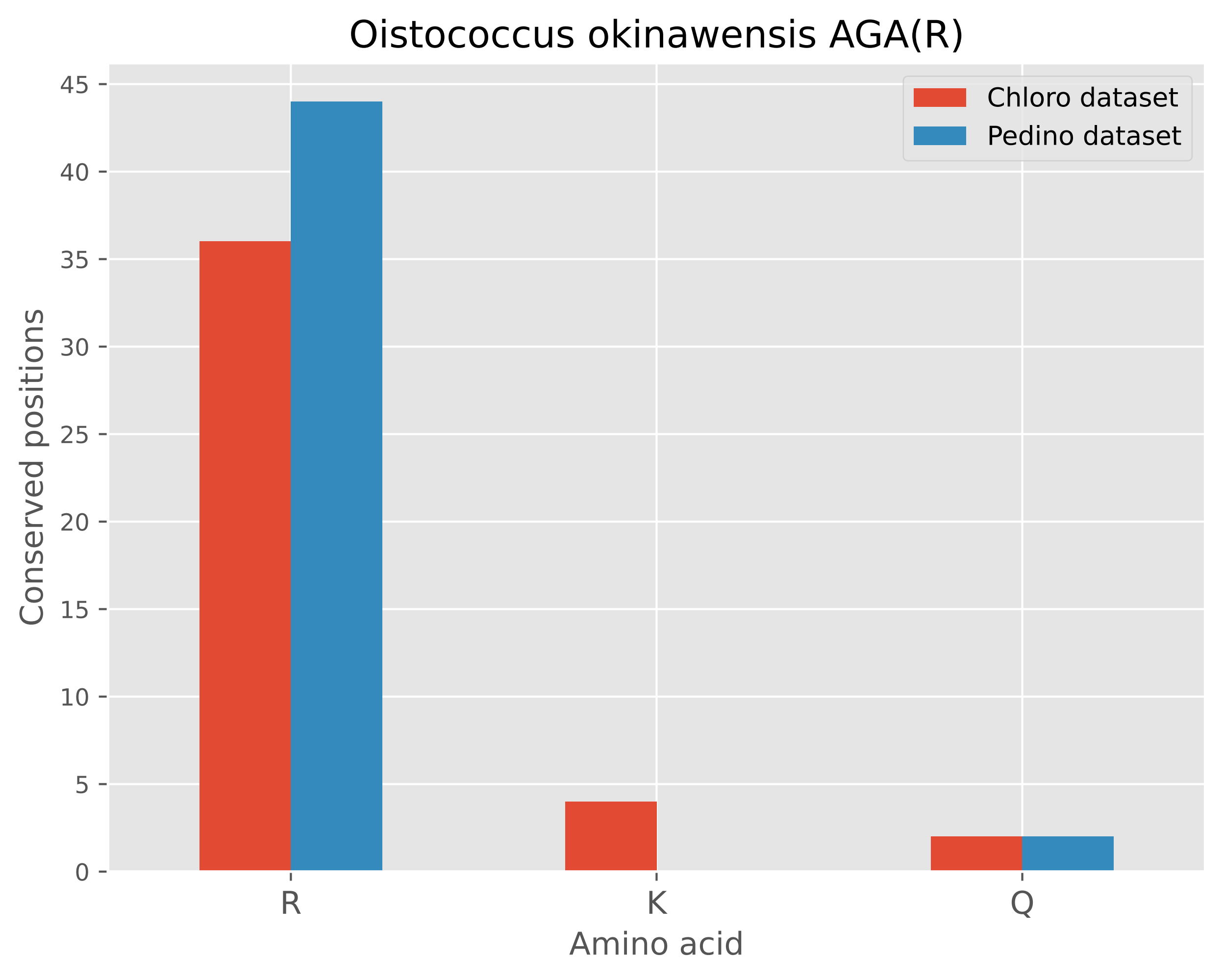
Oistococcus okinawensis AGA(R)
Chloro dataset
Pedino dataset
Conserved positions
0
5
10
15
20
25
30
35
40
45
R
K
Q
Amino acid

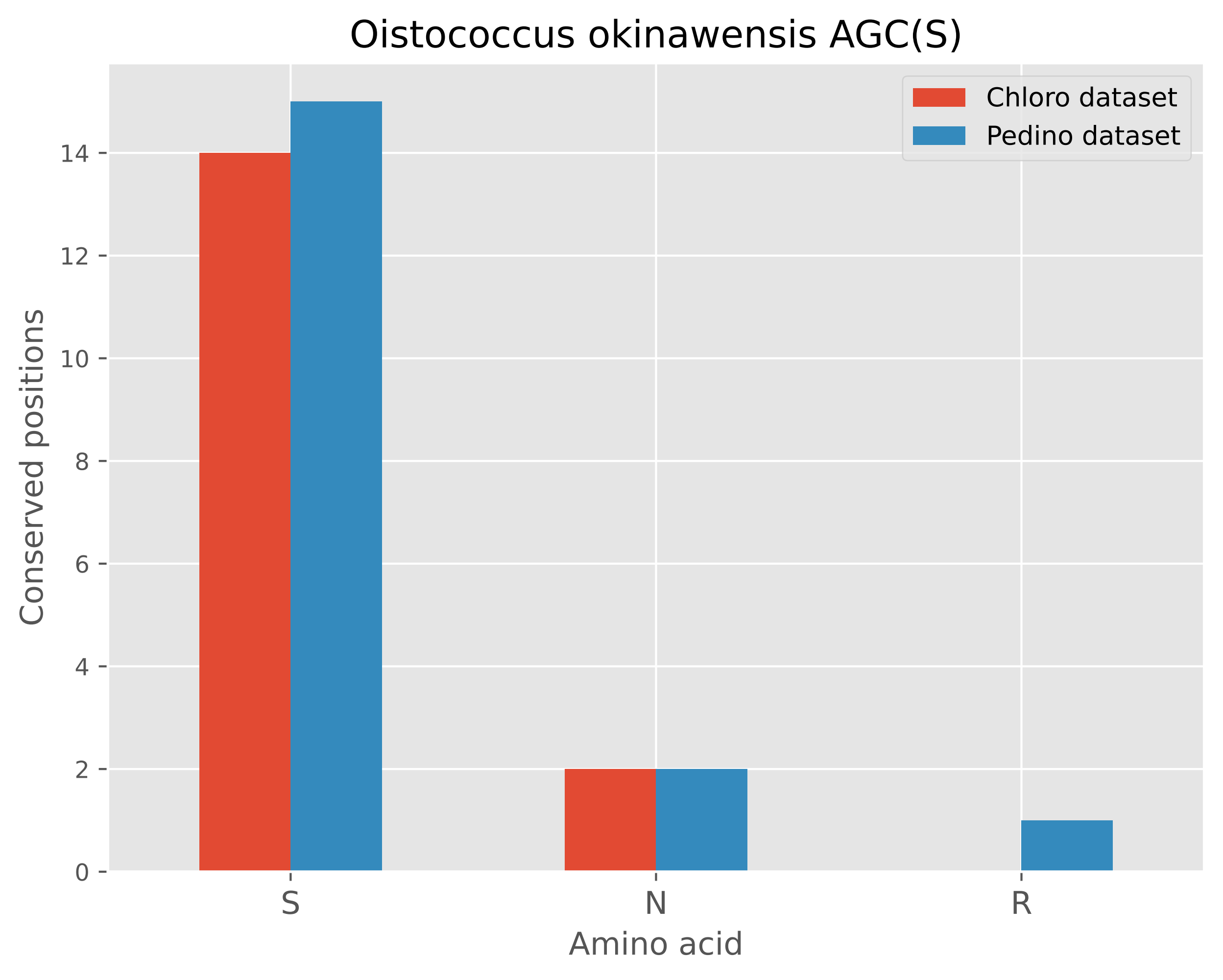

Oistococcus okinawensis AGC(S)
Chloro dataset
Pedino dataset
Conserved positions
0
2
4
6
8
10
12
14
S
N
R
Amino acid

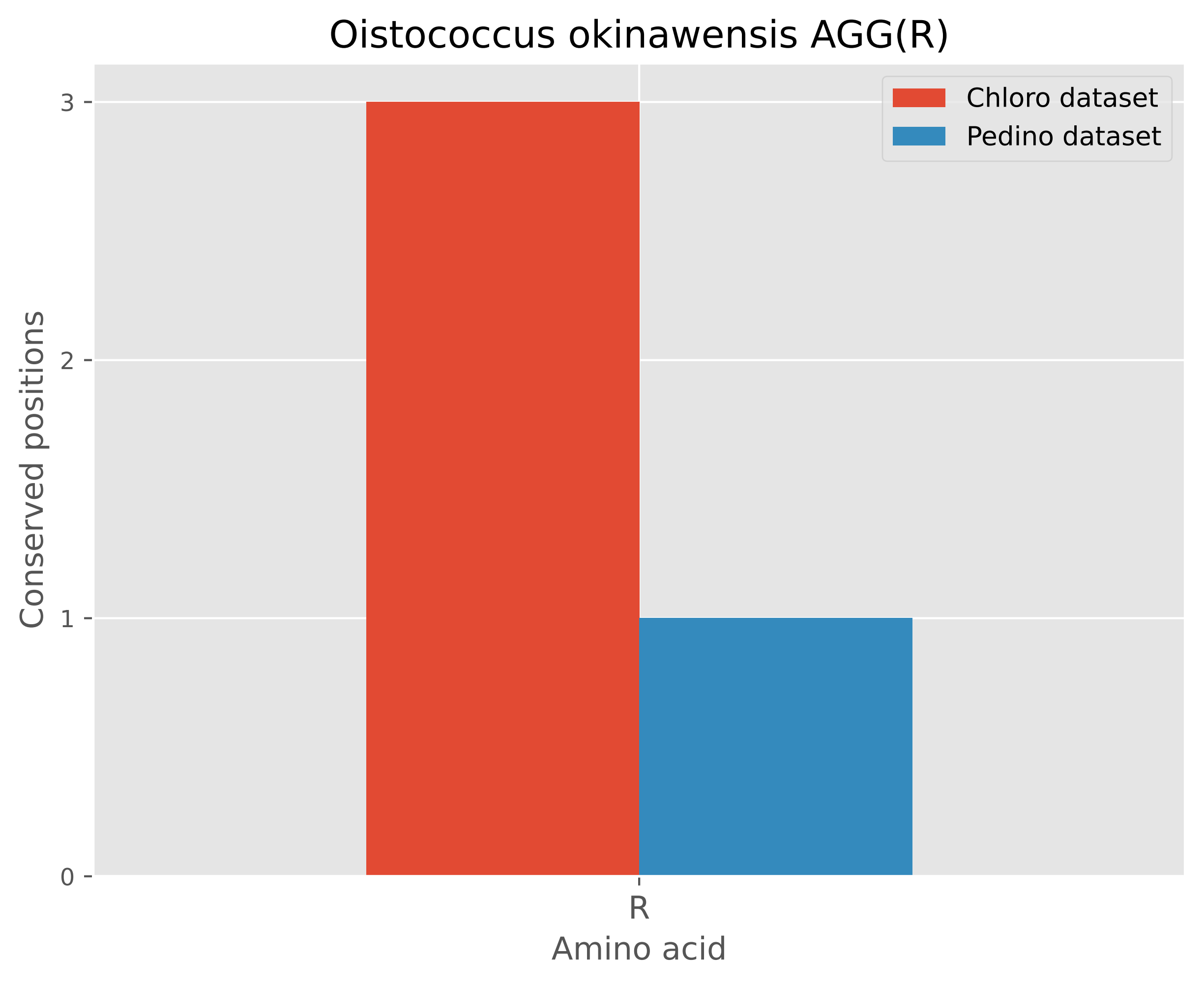

Oistococcus okinawensis AGG(R)
Chloro dataset
Pedino dataset
Conserved positions
0
1
2
3
R
Amino acid

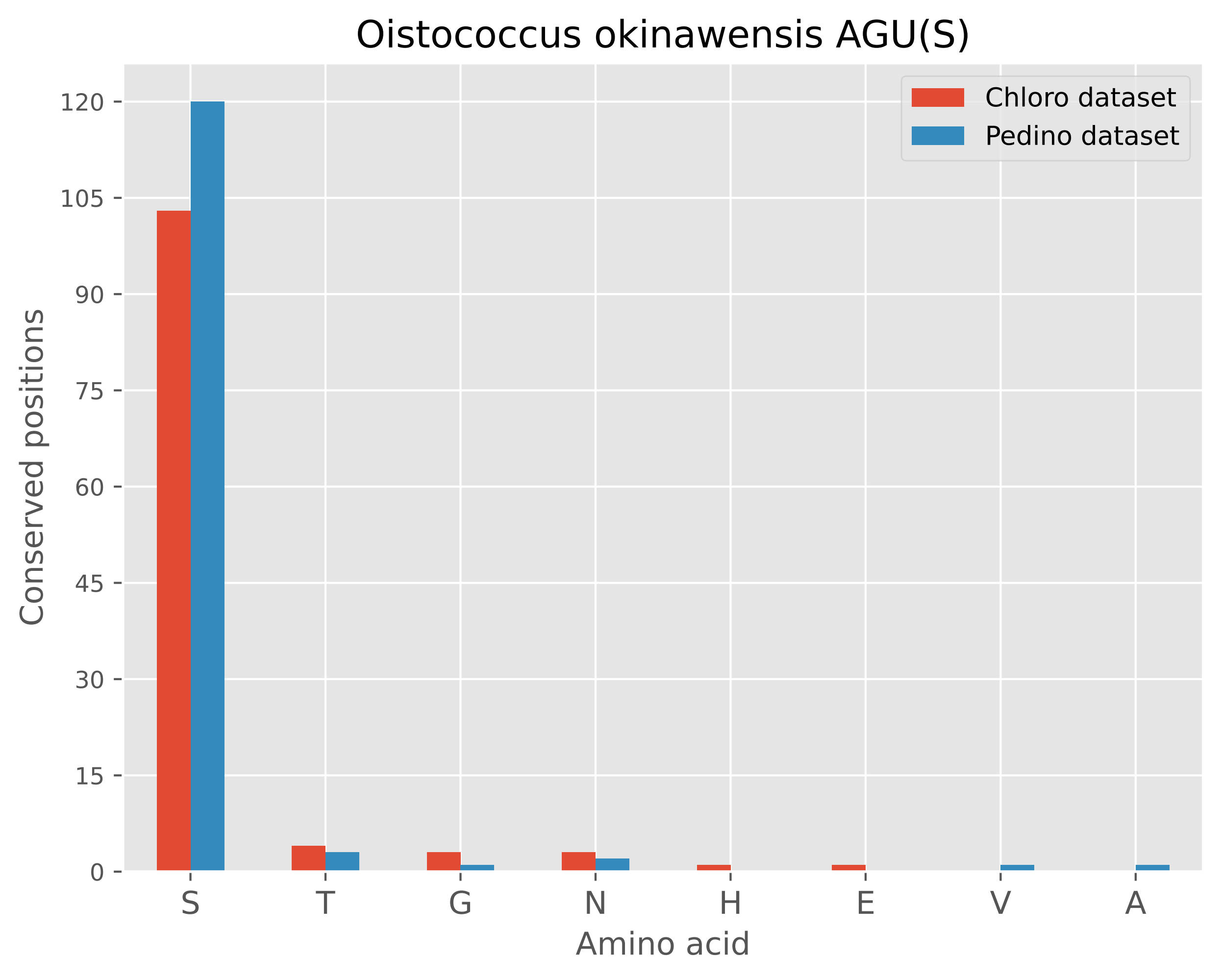

Oistococcus okinawensis AGU(S)
Chloro dataset
Pedino dataset
Conserved positions
0
15
30
45
60
75
90
105
120
S
T
G
N
H
E
V
A
Amino acid

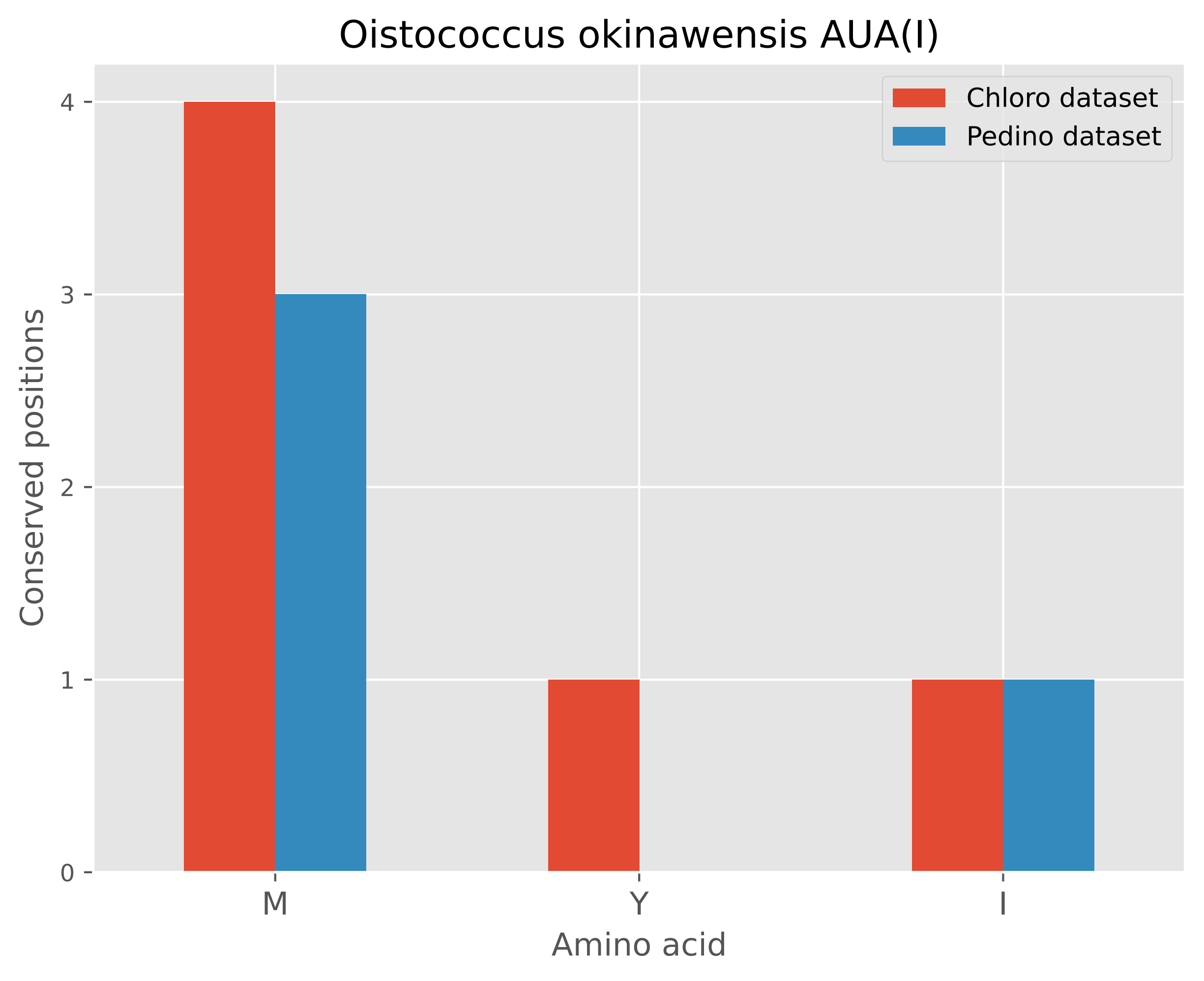

Oistococcus okinawensis AUA(I)
Chloro dataset
Pedino dataset
Conserved positions
0
1
2
3
4
M
Y
I
Amino acid

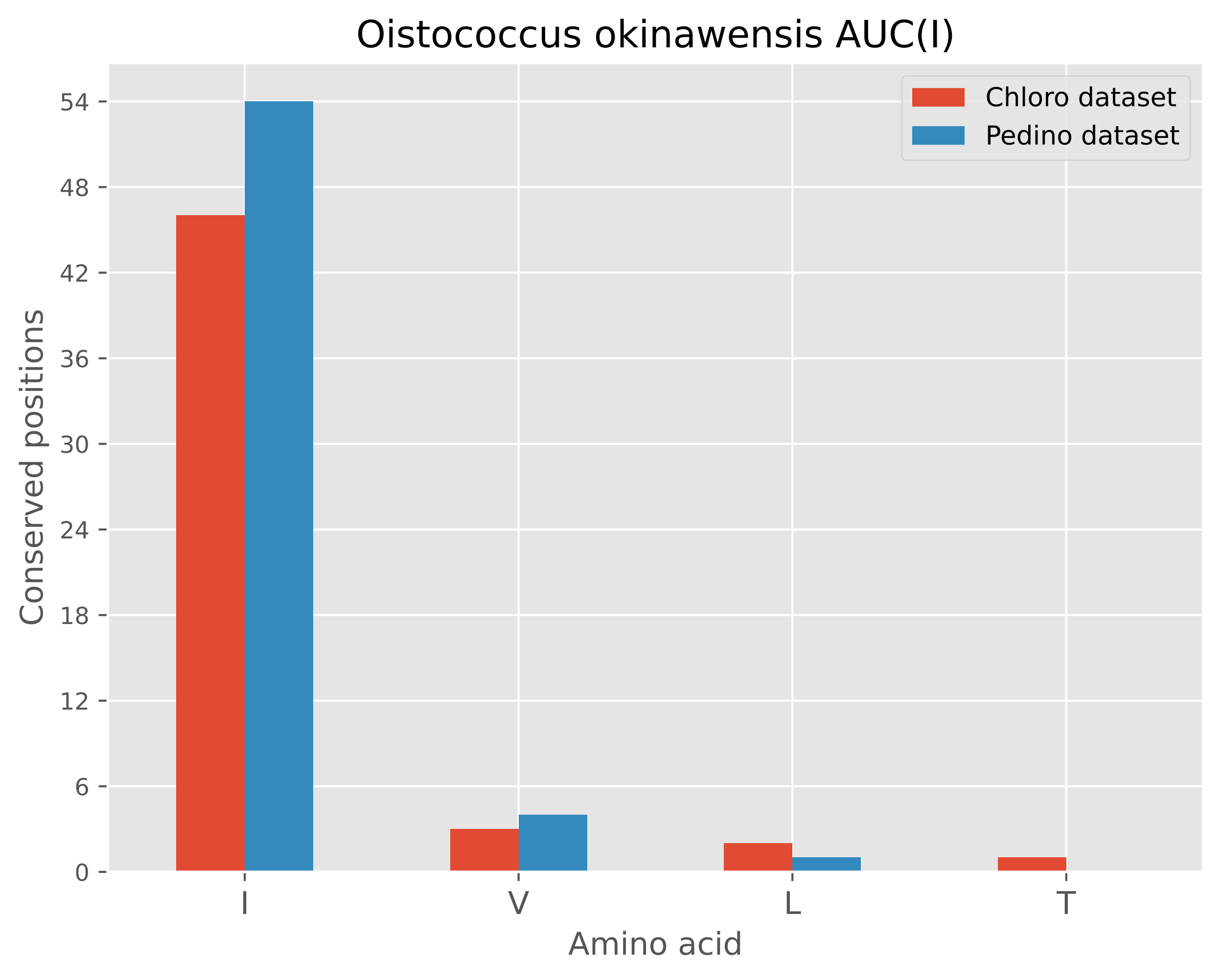
Oistococcus okinawensis AUC(I)
Chloro dataset
Pedino dataset
Conserved positions
0
6
12
18
24
30
36
42
48
54
I
V
L
T
Amino acid

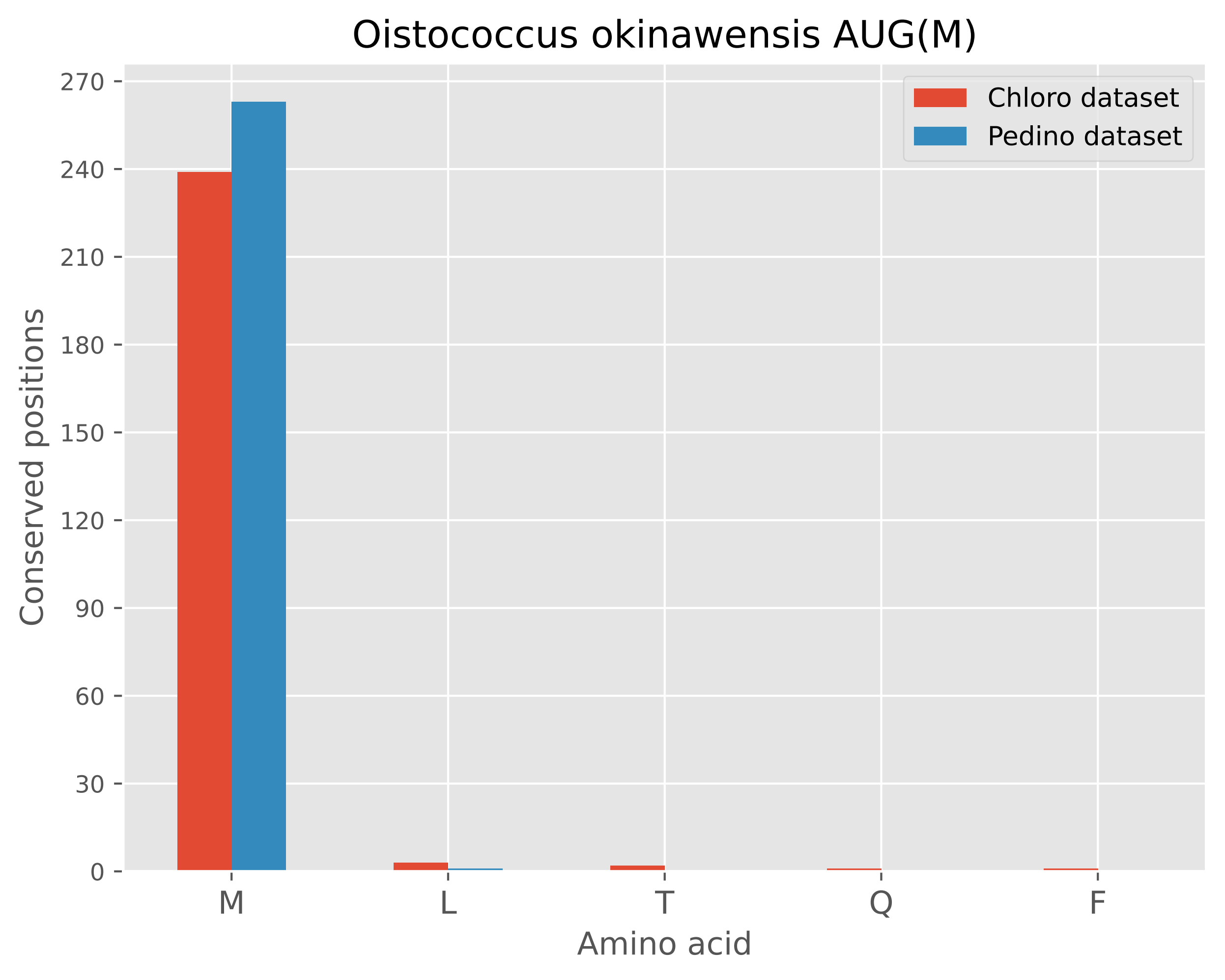

Oistococcus okinawensis AUG(M)
Chloro dataset
Pedino dataset
Conserved positions
270
240
210
180
150
120
90
60
30
0
M
L
T
Q
F
Amino acid

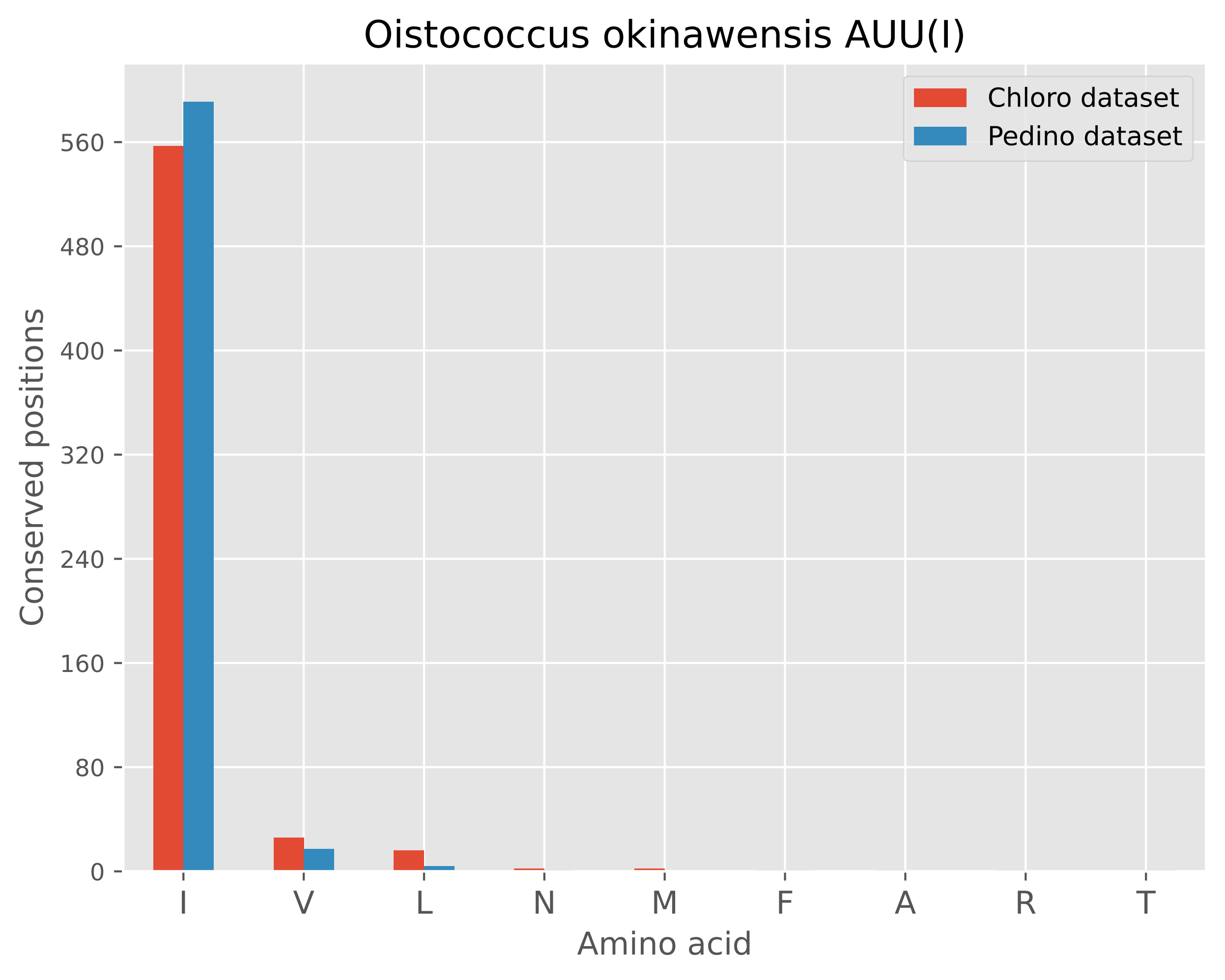
Oistococcus okinawensis AUU(I)
Chloro dataset
Pedino dataset
Conserved positions
0
80
160
240
320
400
480
560
I
V
L
N
M
F
A
R
T
Amino acid

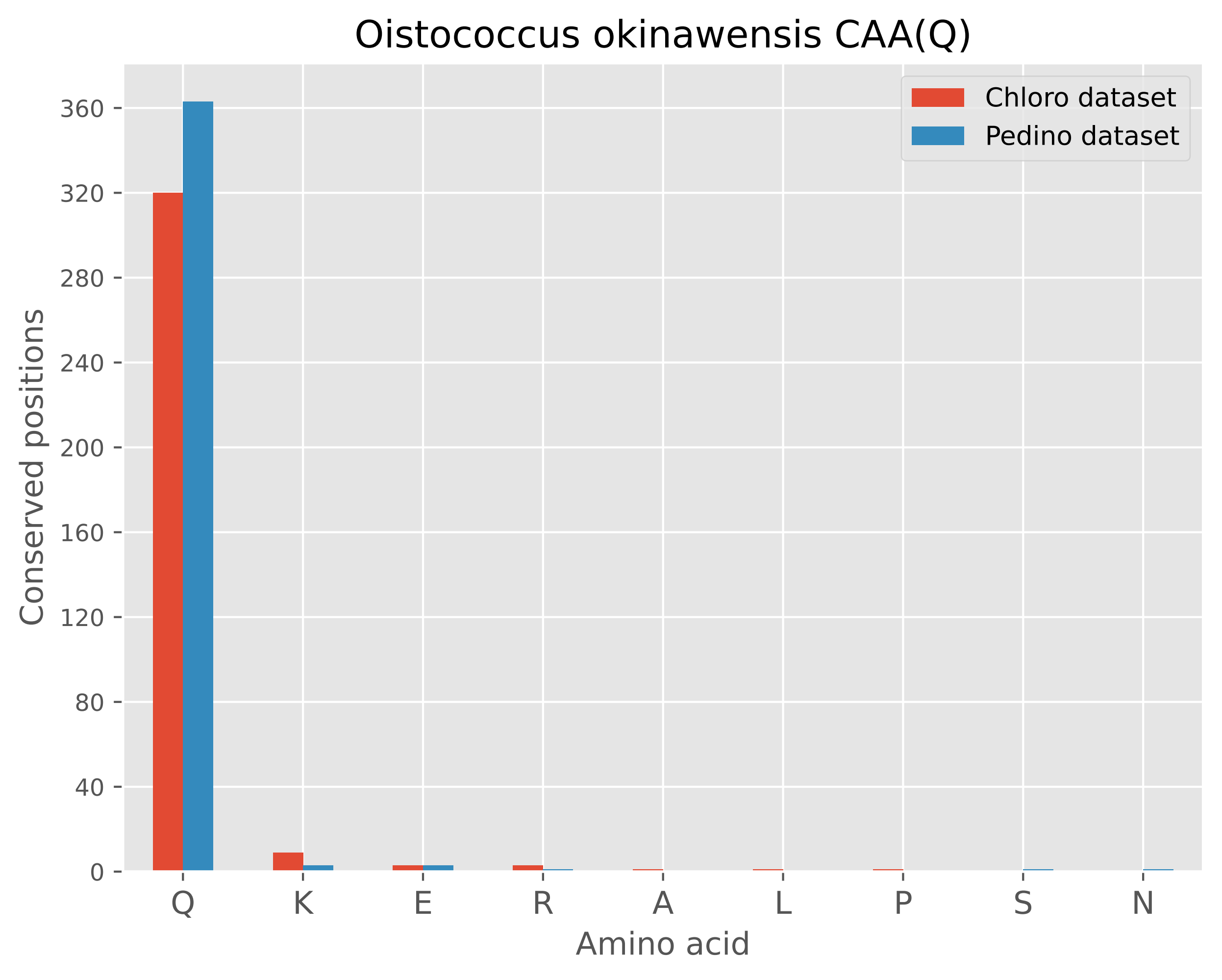

Oistococcus okinawensis CAA(Q)
Chloro dataset
Pedino dataset
Conserved positions
0
40
80
120
160
200
240
280
320
360
Q
K
E
R
A
L
P
S
N
Amino acid

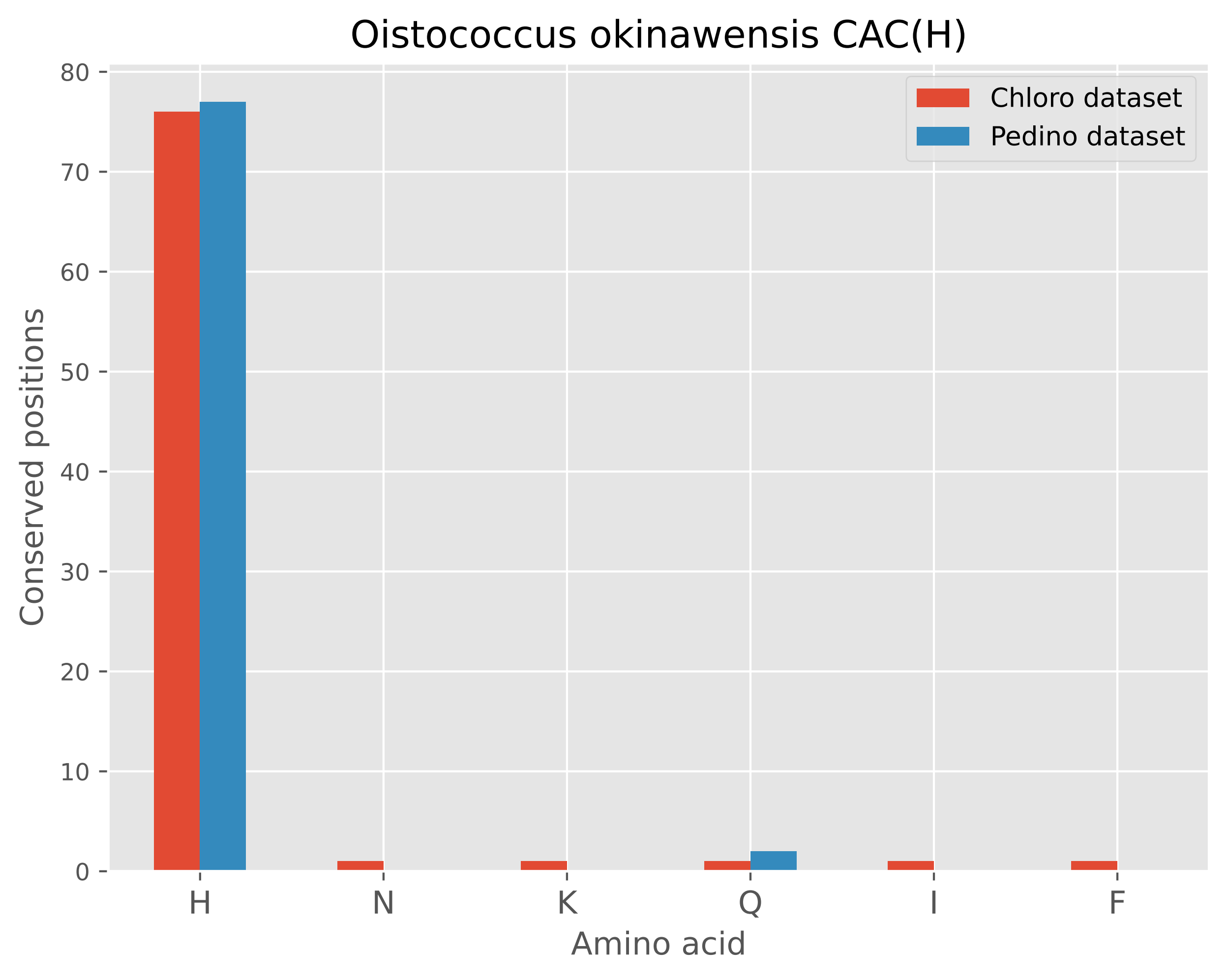
Oistococcus okinawensis CAC(H)
Chloro dataset
Pedino dataset
Conserved positions
Amino acid
0
10
20
30
40
50
60
70
80
H
N
K
Q
I
F

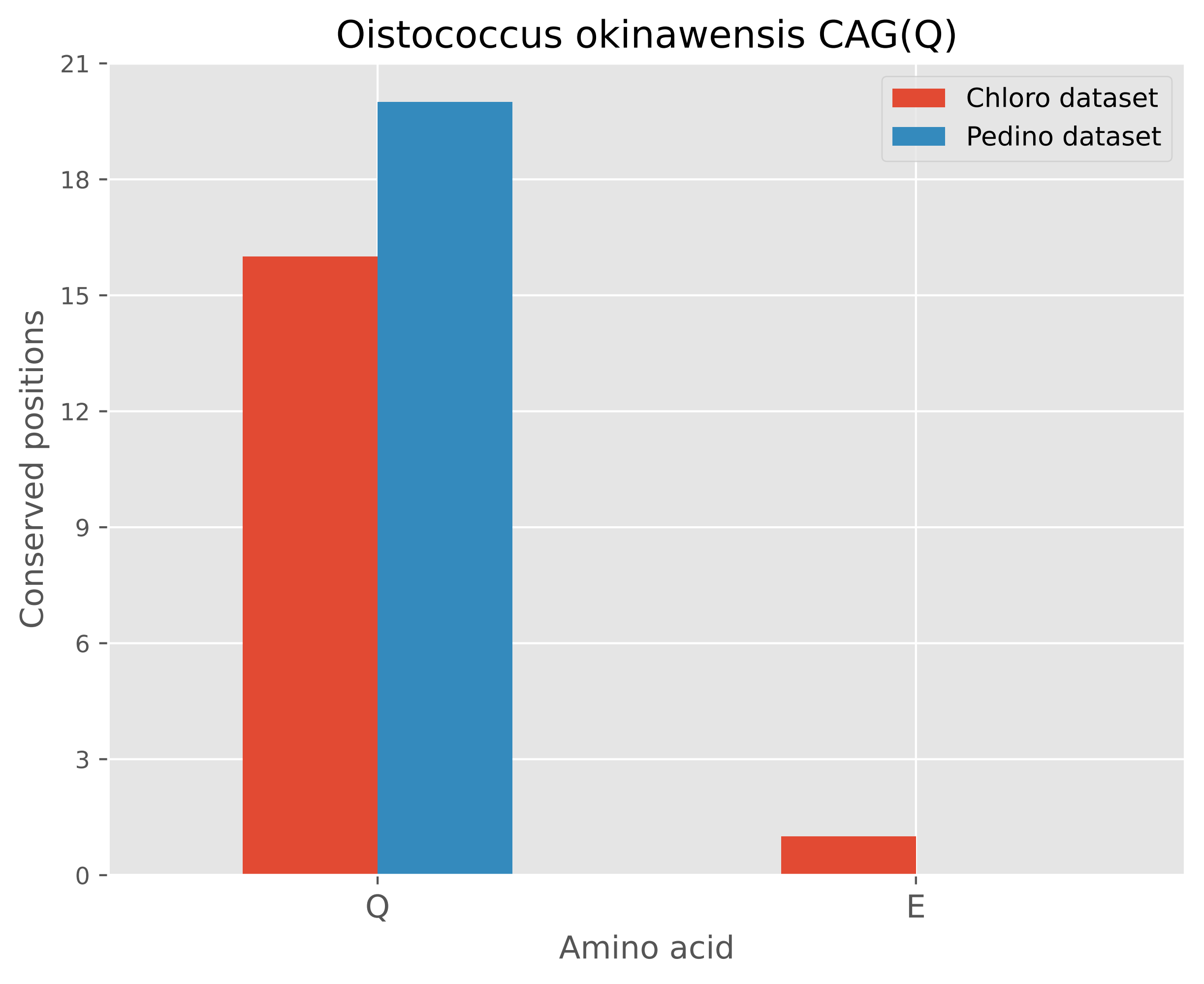

Oistococcus okinawensis CAG(Q)
Chloro dataset
Pedino dataset
Conserved positions
0
3
6
9
12
15
18
21
Q
E
Amino acid

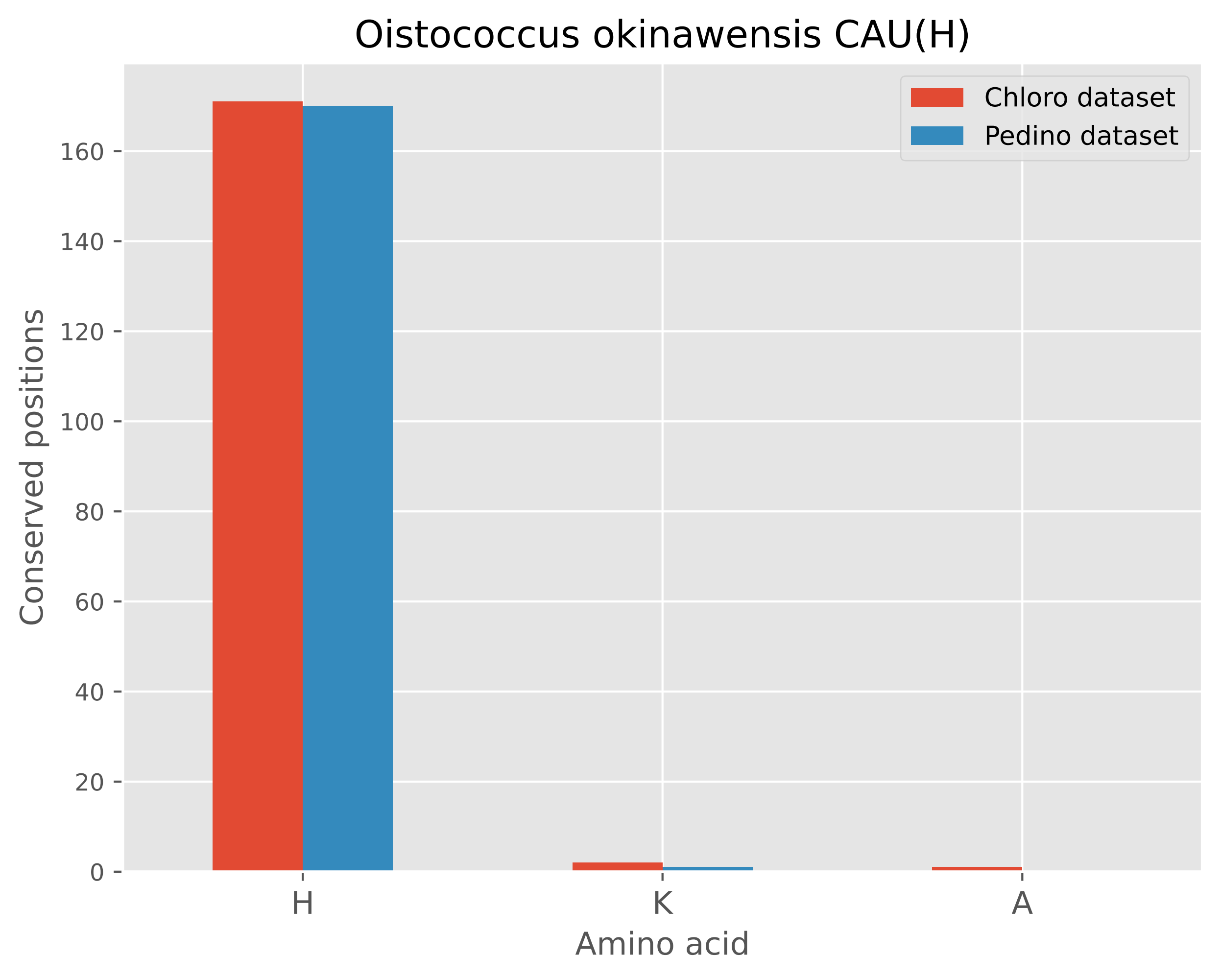
Oistococcus okinawensis CAU(H)
Chloro dataset
Pedino dataset
Conserved positions
0
20
40
60
80
100
120
140
160
H
K
A
Amino acid

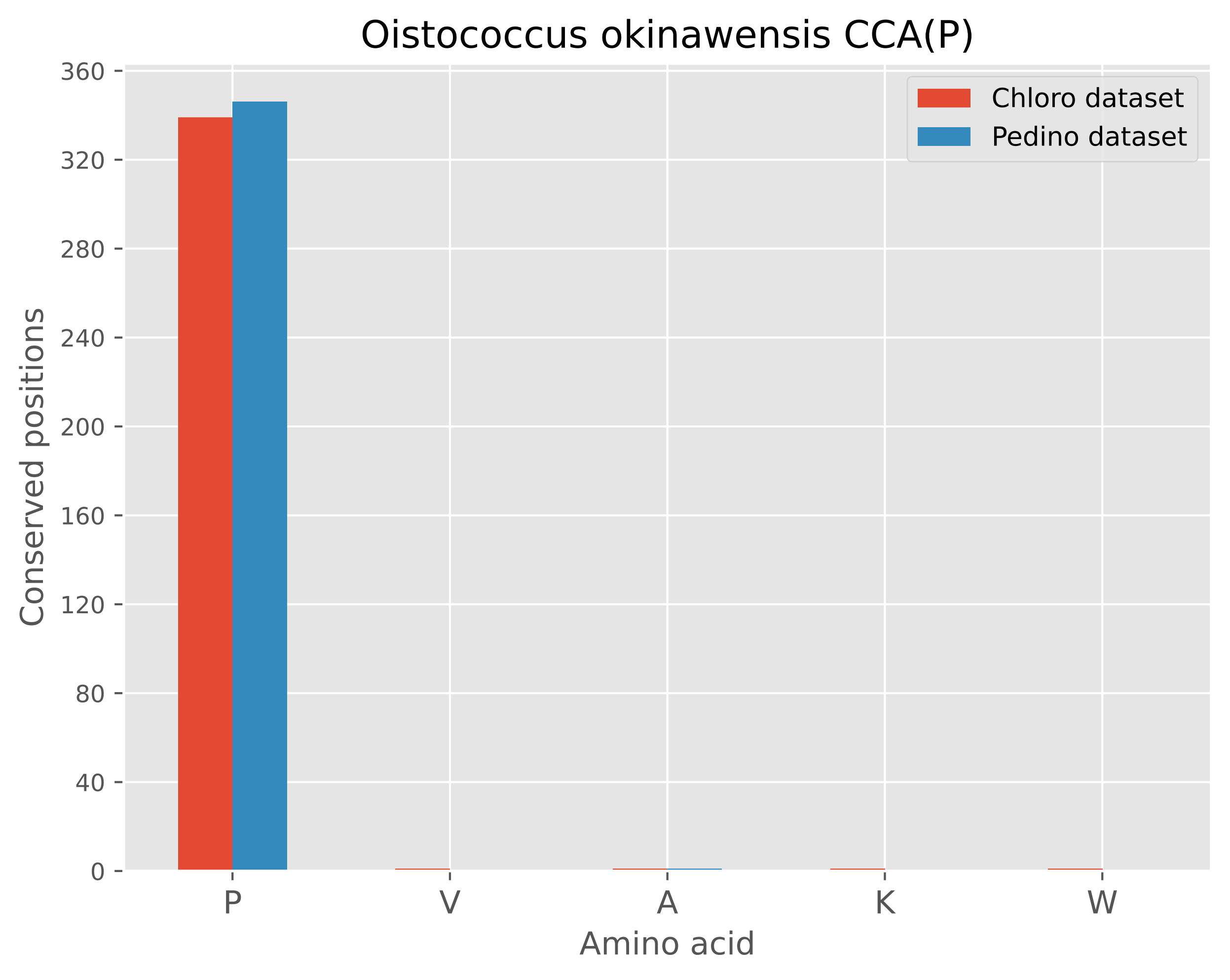

Oistococcus okinawensis CCA(P)
Chloro dataset
Pedino dataset
Conserved positions
0
40
80
120
160
200
240
280
320
360
P
V
A
K
W
Amino acid

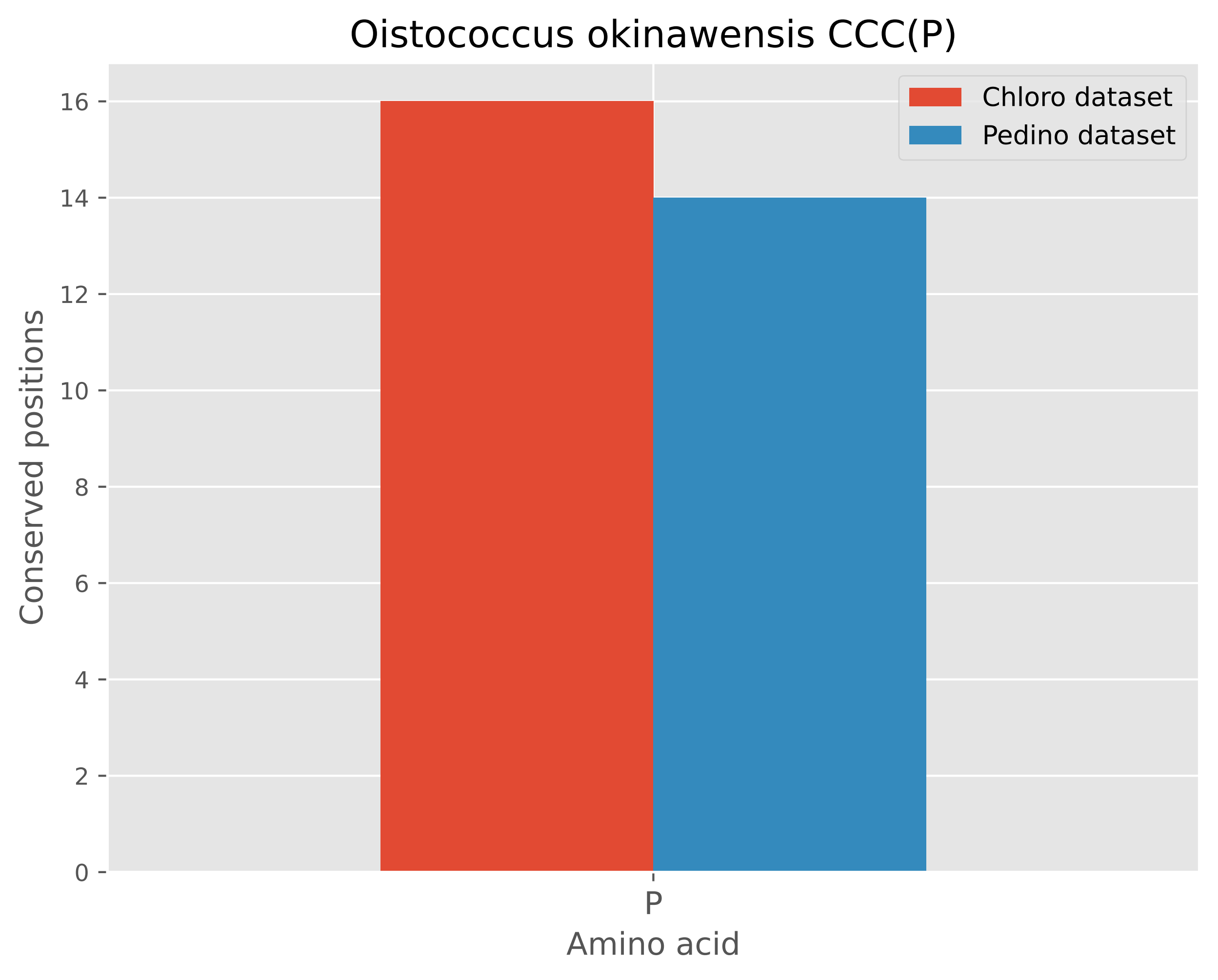

Oistococcus okinawensis CCC(P)
Chloro dataset
Pedino dataset
Conserved positions
0
2
4
6
8
10
12
14
16
P
Amino acid

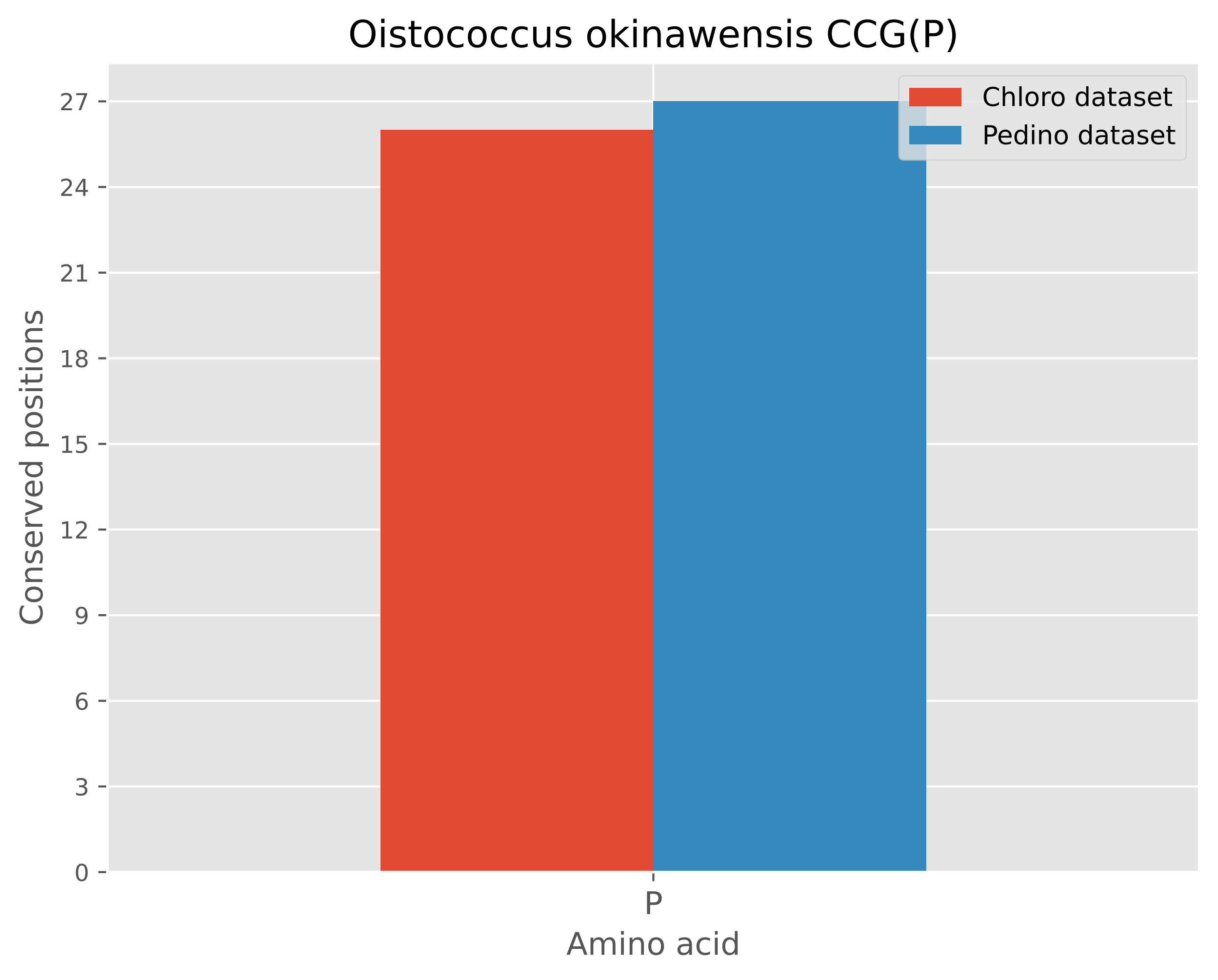
Oistococcus okinawensis CCG(P)
Chloro dataset
Pedino dataset
Conserved positions
0
3
6
9
12
15
18
21
24
27
P
Amino acid

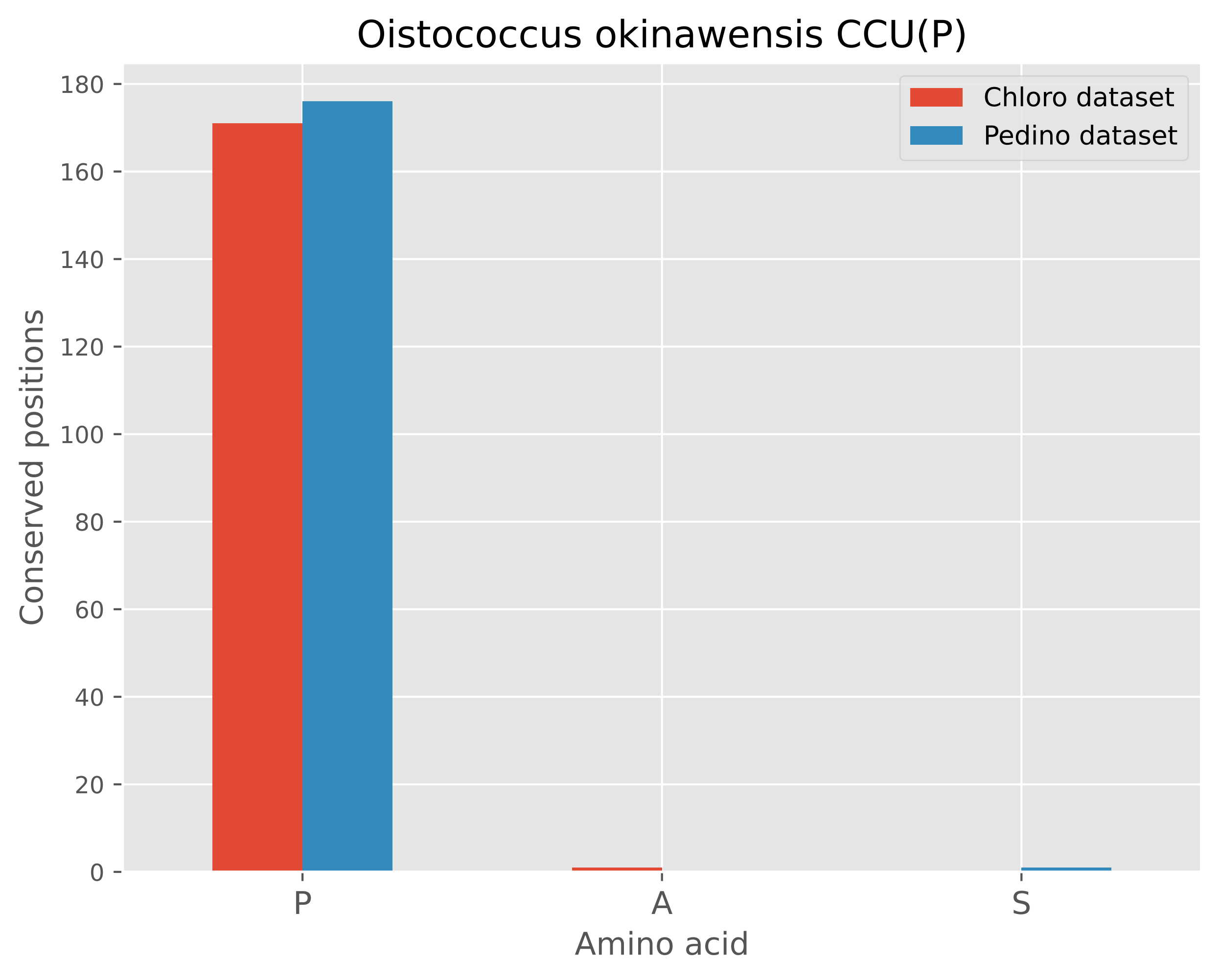
Oistococcus okinawensis CCU(P)
Chloro dataset
Pedino dataset
Conserved positions
0
20
40
60
80
100
120
140
160
180
P
A
S
Amino acid

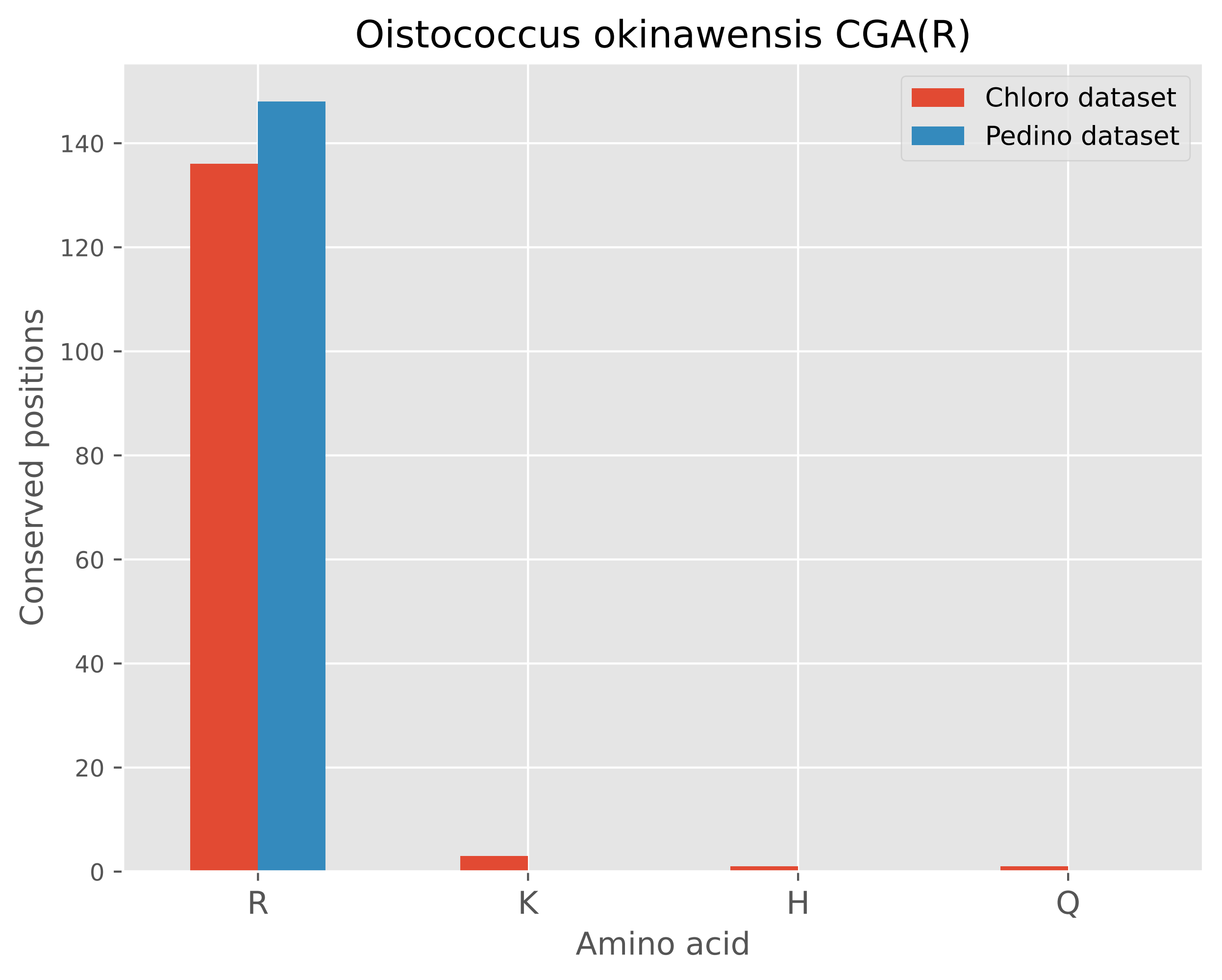
Oistococcus okinawensis CGA(R)
Chloro dataset
Pedino dataset
Conserved positions
0
20
40
60
80
100
120
140
R
K
H
Q
Amino acid

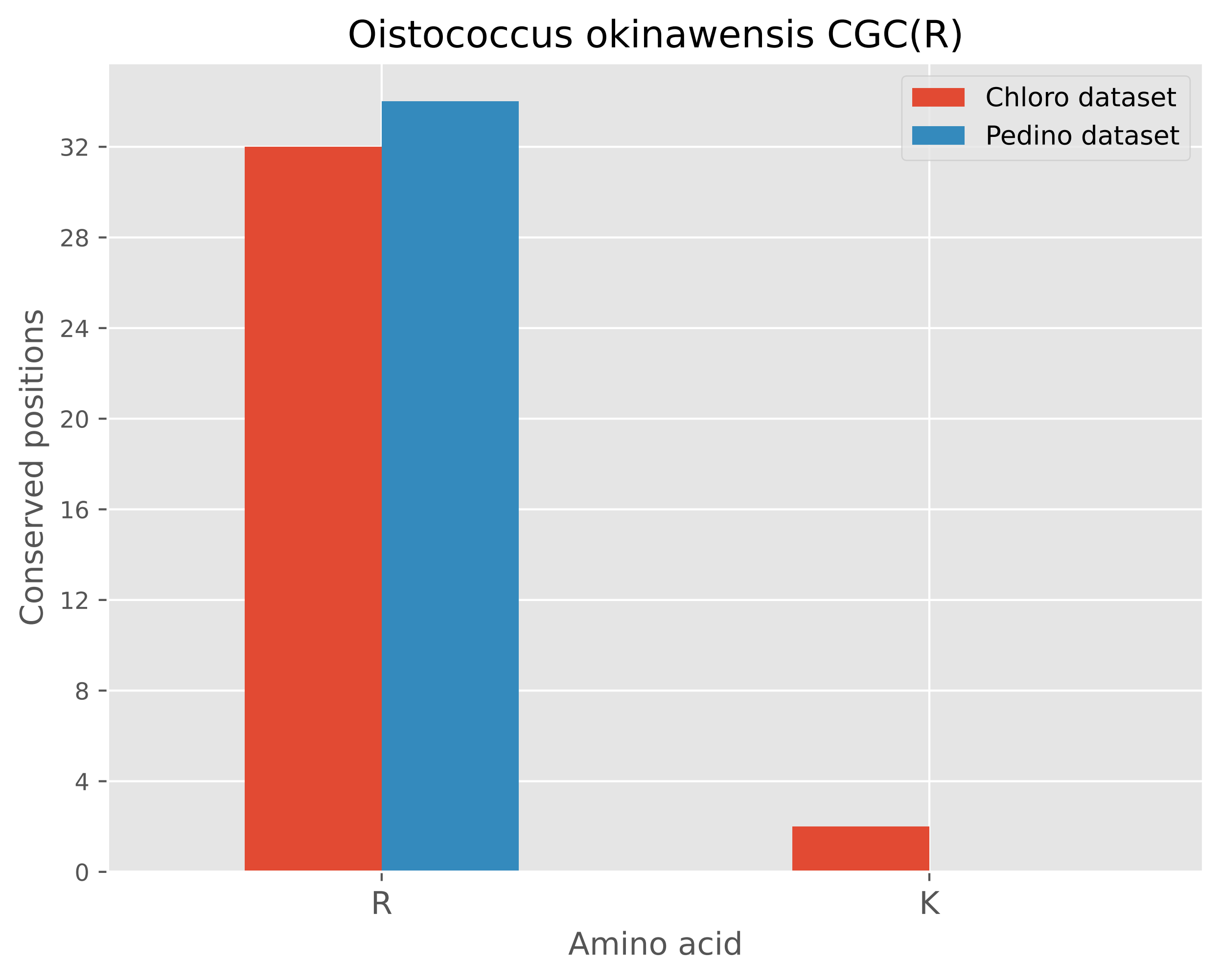

Oistococcus okinawensis CGC(R)
Chloro dataset
Pedino dataset
Conserved positions
0
4
8
12
16
20
24
28
32
R
K
Amino acid

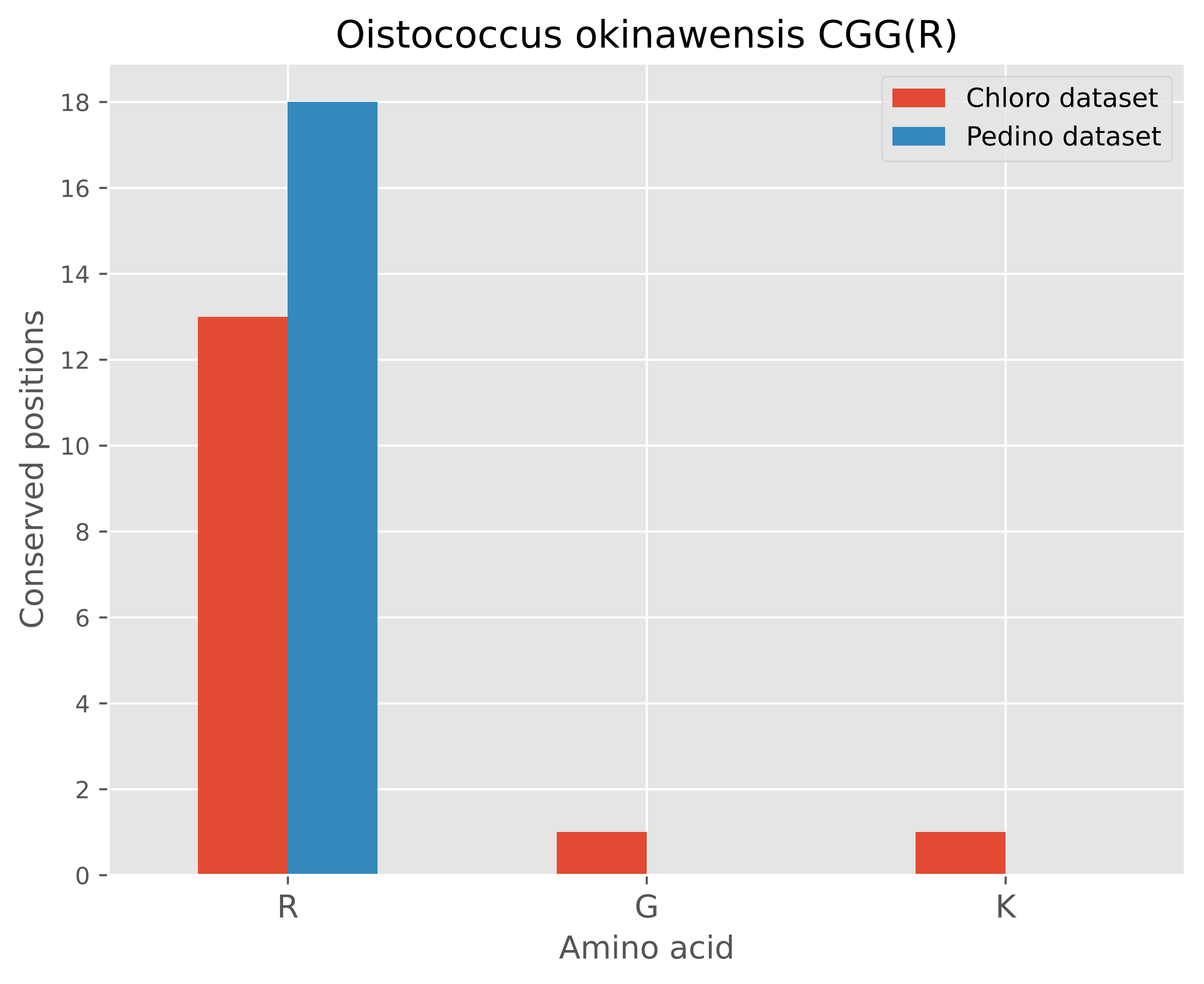
Oistococcus okinawensis CGG(R)
Chloro dataset
Pedino dataset
Conserved positions
0
2
4
6
8
10
12
14
16
18
R
G
K
Amino acid

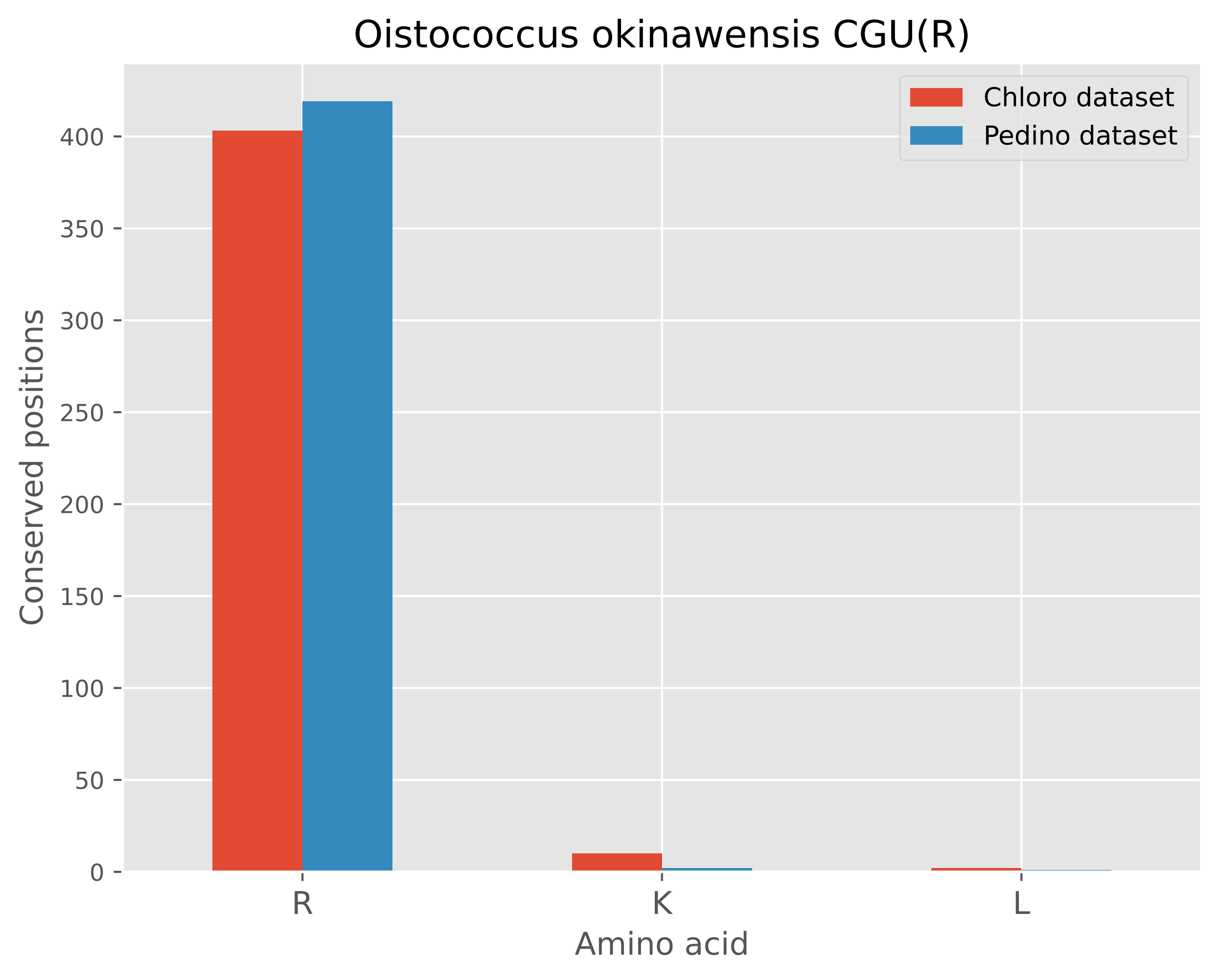

Oistococcus okinawensis CGU(R)
Chloro dataset
Pedino dataset
Conserved positions
0
50
100
150
200
250
300
350
400
R
K
L
Amino acid

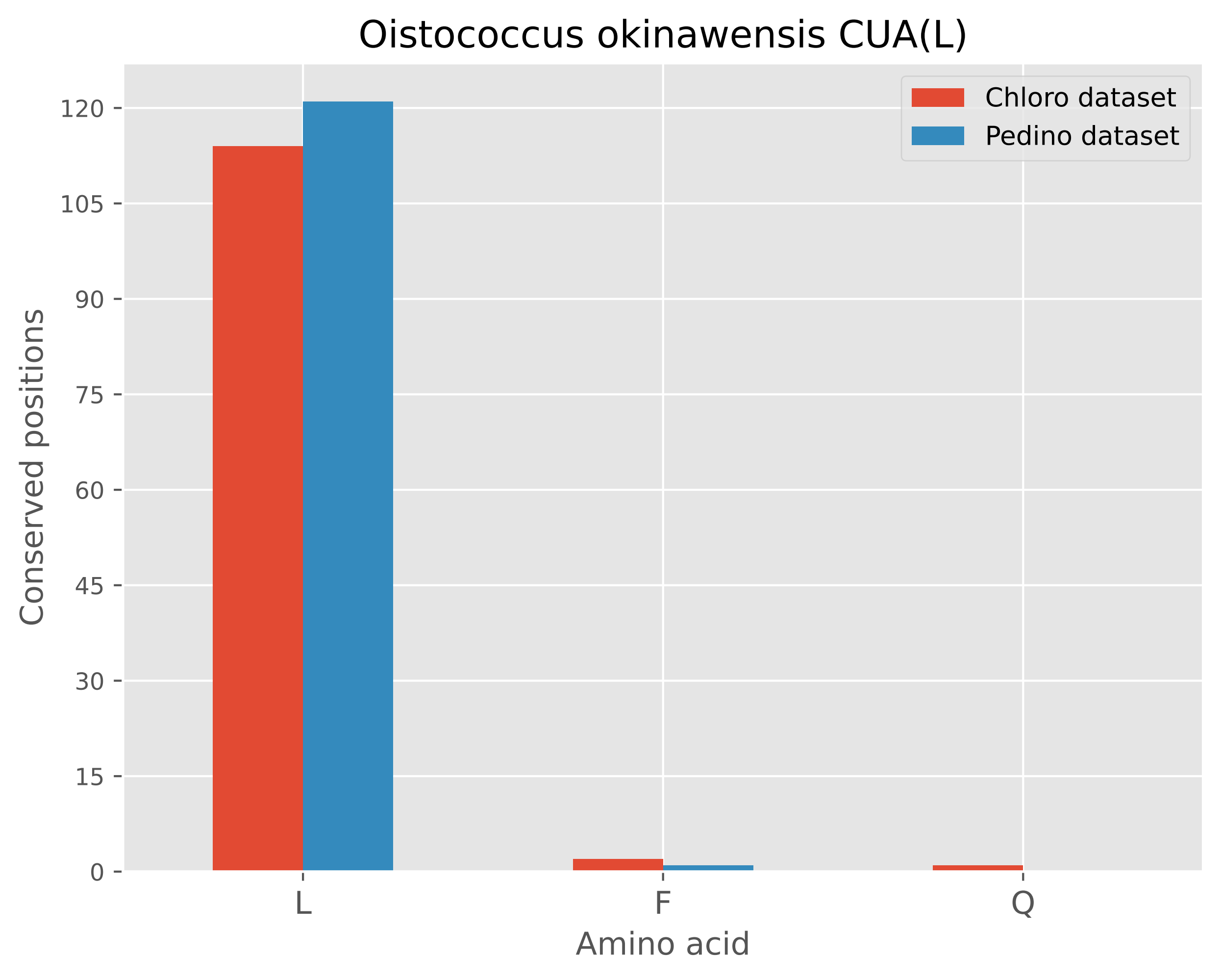
Oistococcus okinawensis CUA(L)
Chloro dataset
Pedino dataset
Conserved positions
0
15
30
45
60
75
90
105
120
L
F
Q
Amino acid

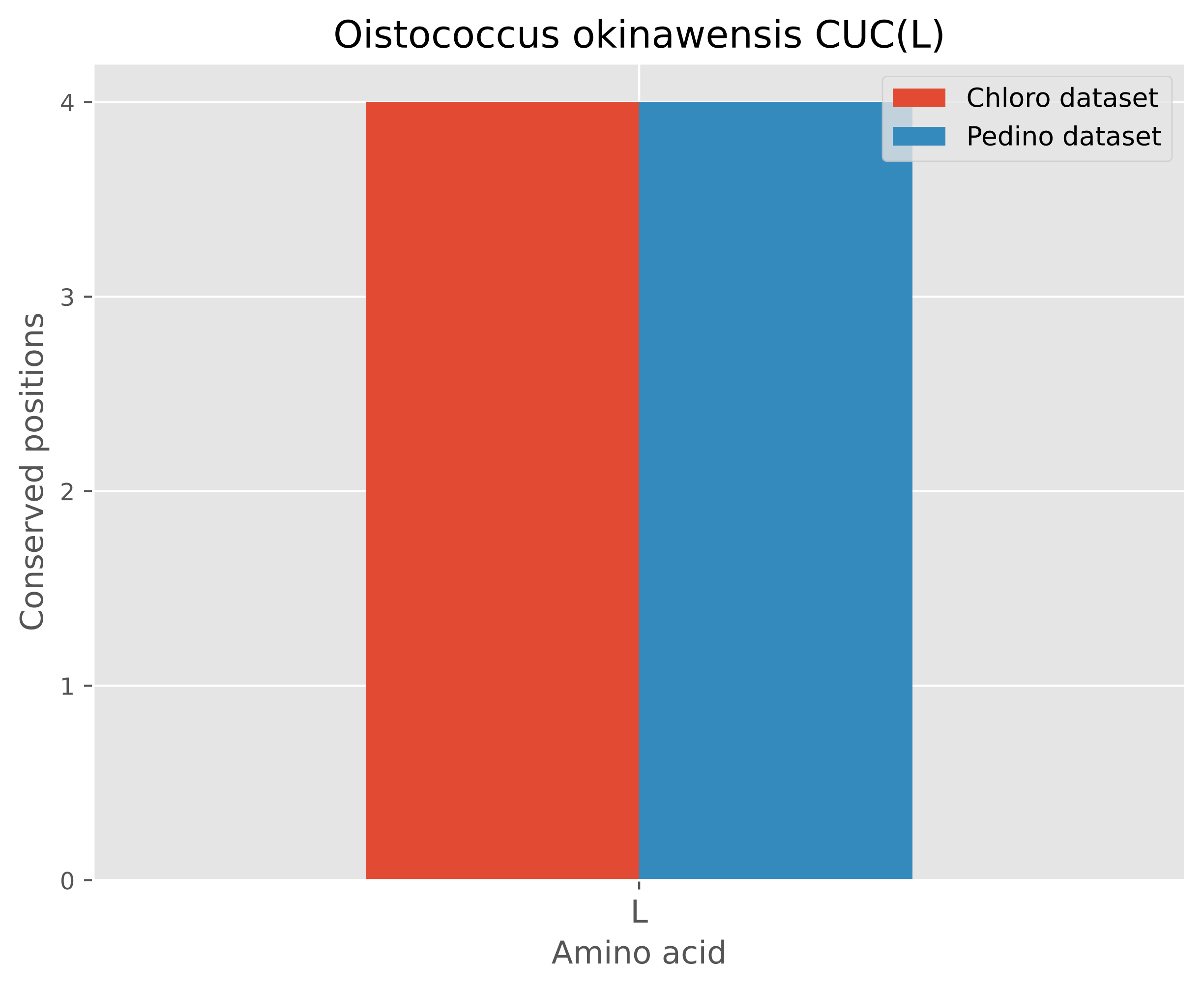

Oistococcus okinawensis CUC(L)
Chloro dataset
Pedino dataset
Conserved positions
0
1
2
3
4
L
Amino acid

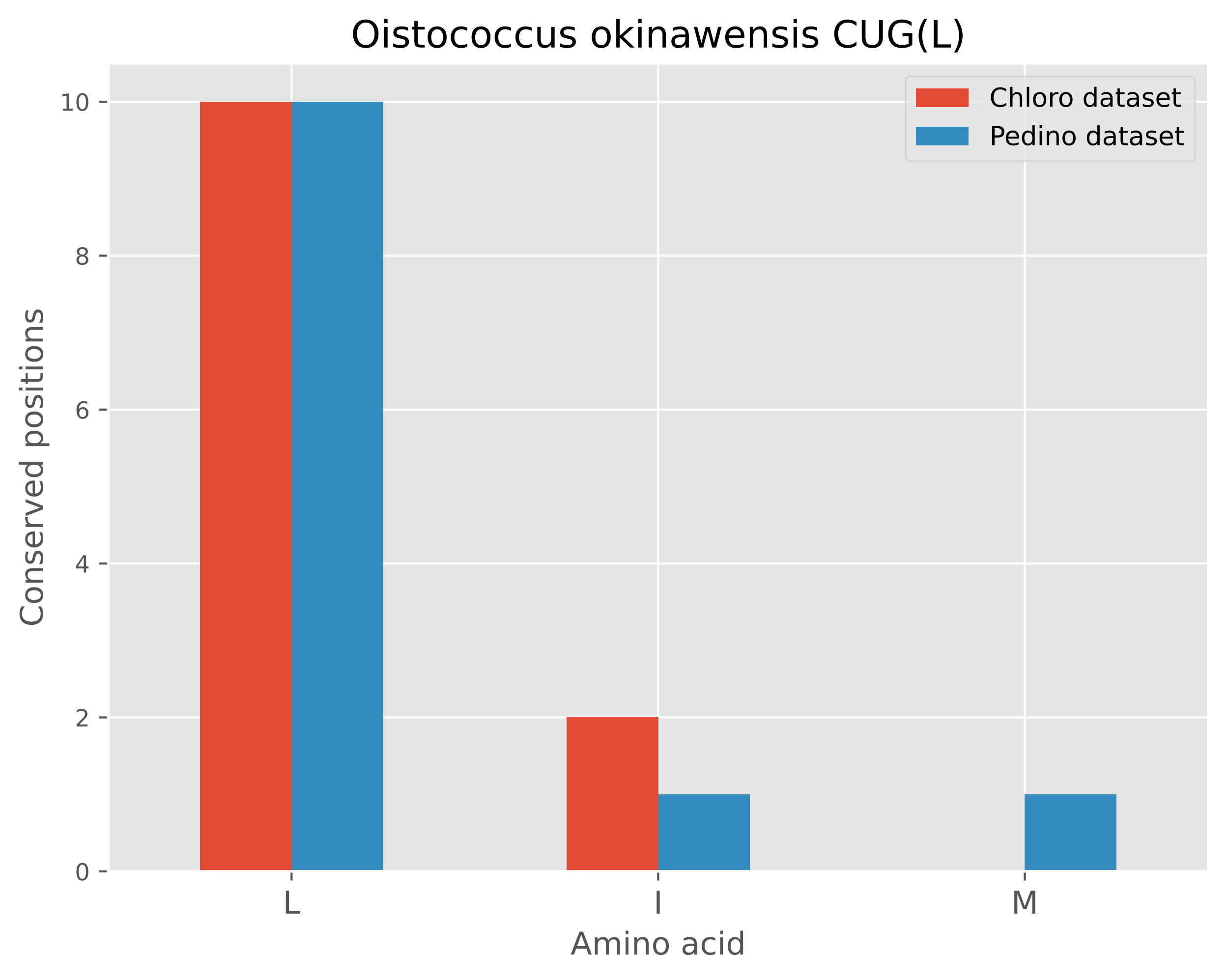

Oistococcus okinawensis CUG(L)
Chloro dataset
Pedino dataset
Conserved positions
0
2
4
6
8
10
L
I
M
Amino acid

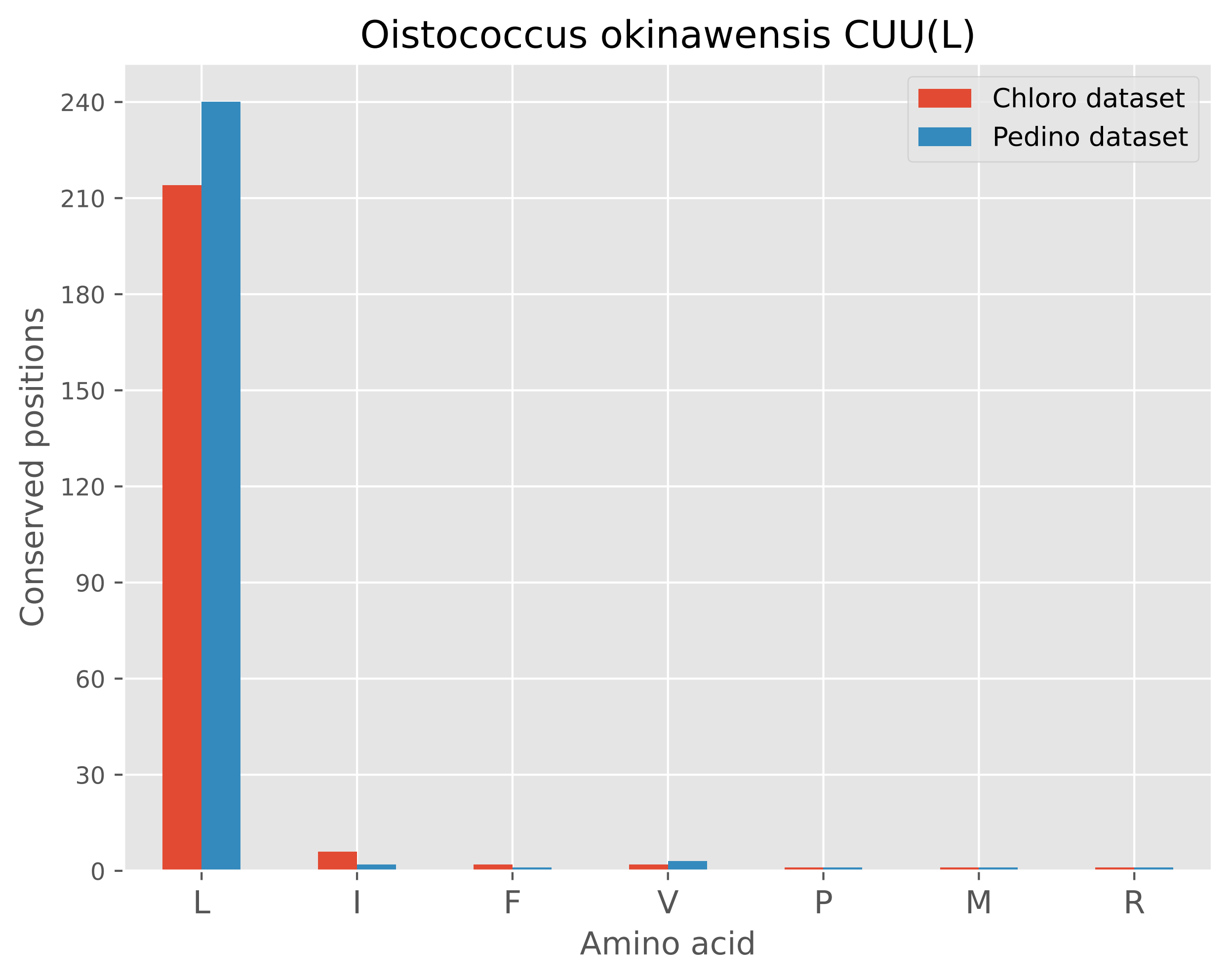

Oistococcus okinawensis CUU(L)
Chloro dataset
Pedino dataset
Conserved positions
240
210
180
150
120
90
60
30
0
L
I
F
V
P
M
R
Amino acid

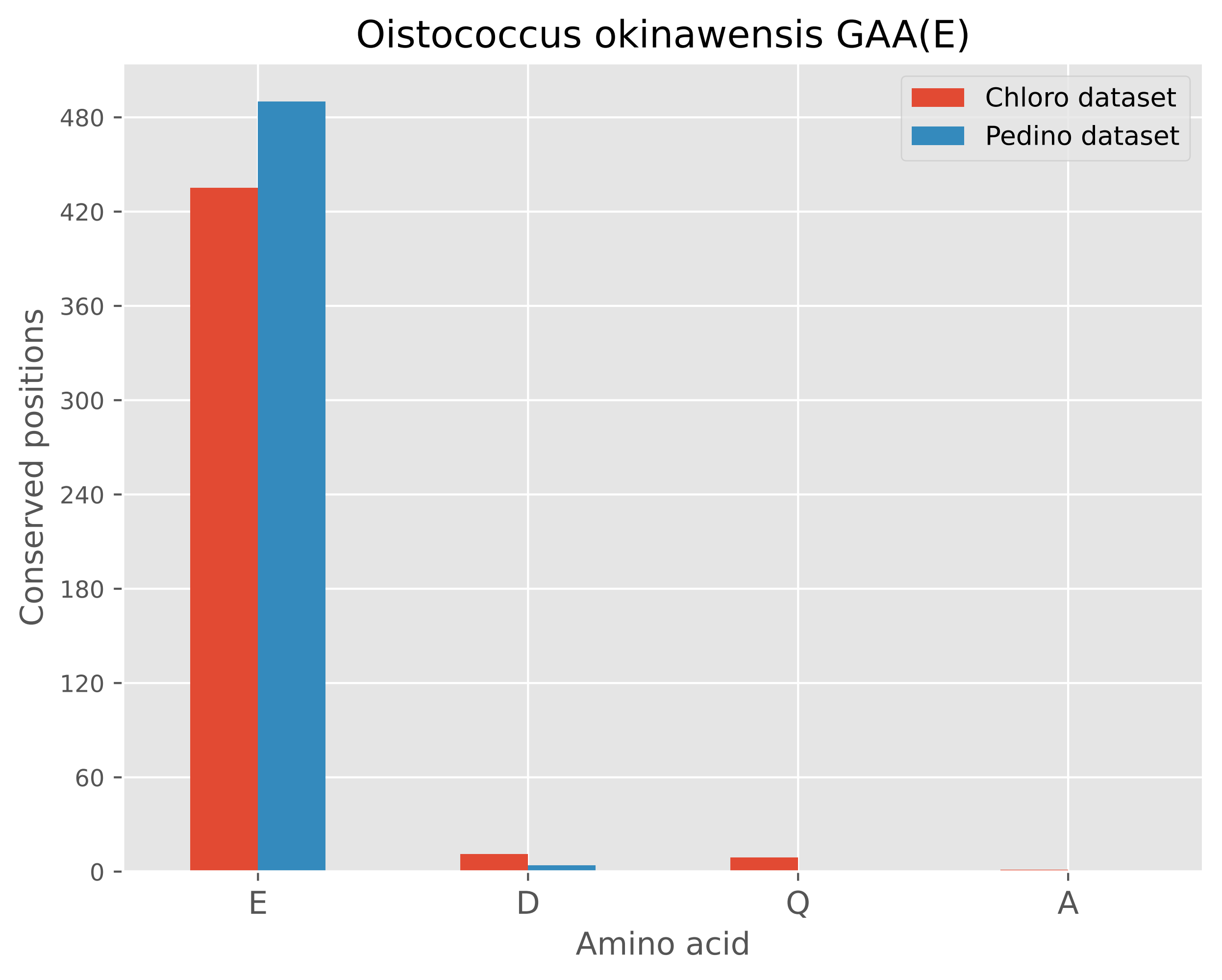
Oistococcus okinawensis GAA(E)
Chloro dataset
Pedino dataset
Conserved positions
0
60
120
180
240
300
360
420
480
E
D
Q
A
Amino acid

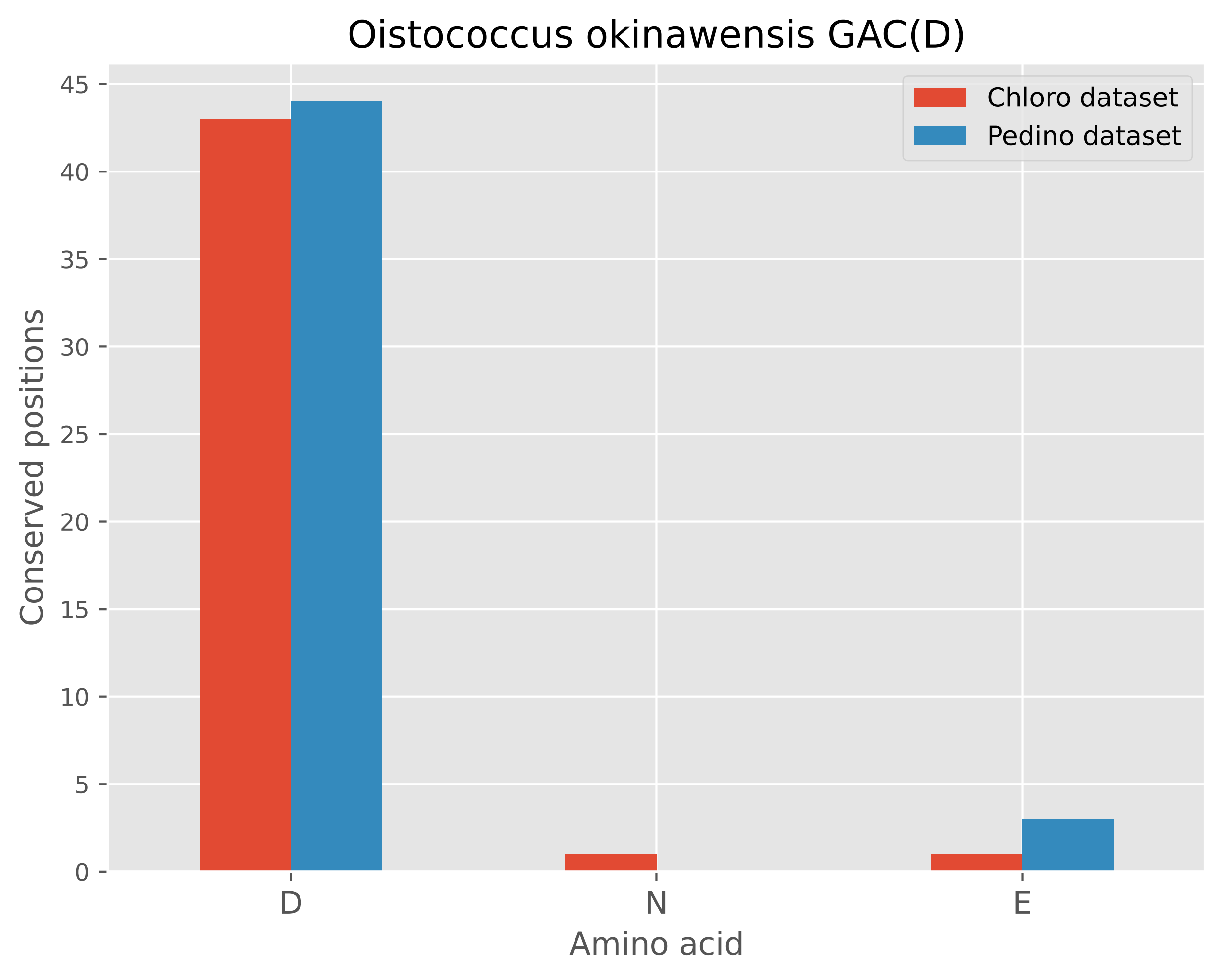

Oistococcus okinawensis GAC(D)
Chloro dataset
Pedino dataset
Conserved positions
0
5
10
15
20
25
30
35
40
45
D
N
E
Amino acid

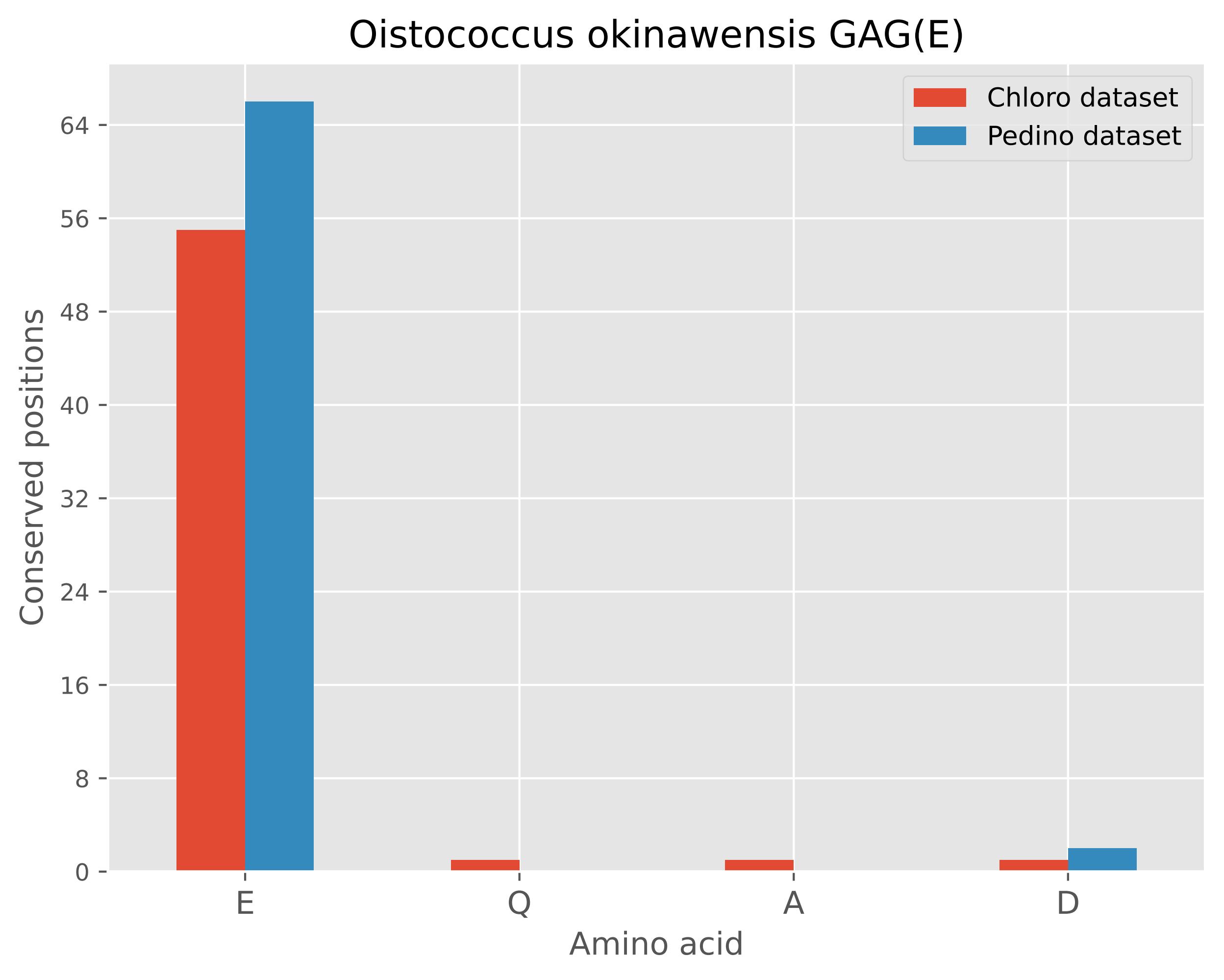
Oistococcus okinawensis GAG(E)
Chloro dataset
Pedino dataset
Conserved positions
0
8
16
24
32
40
48
56
64
E
Q
A
D
Amino acid

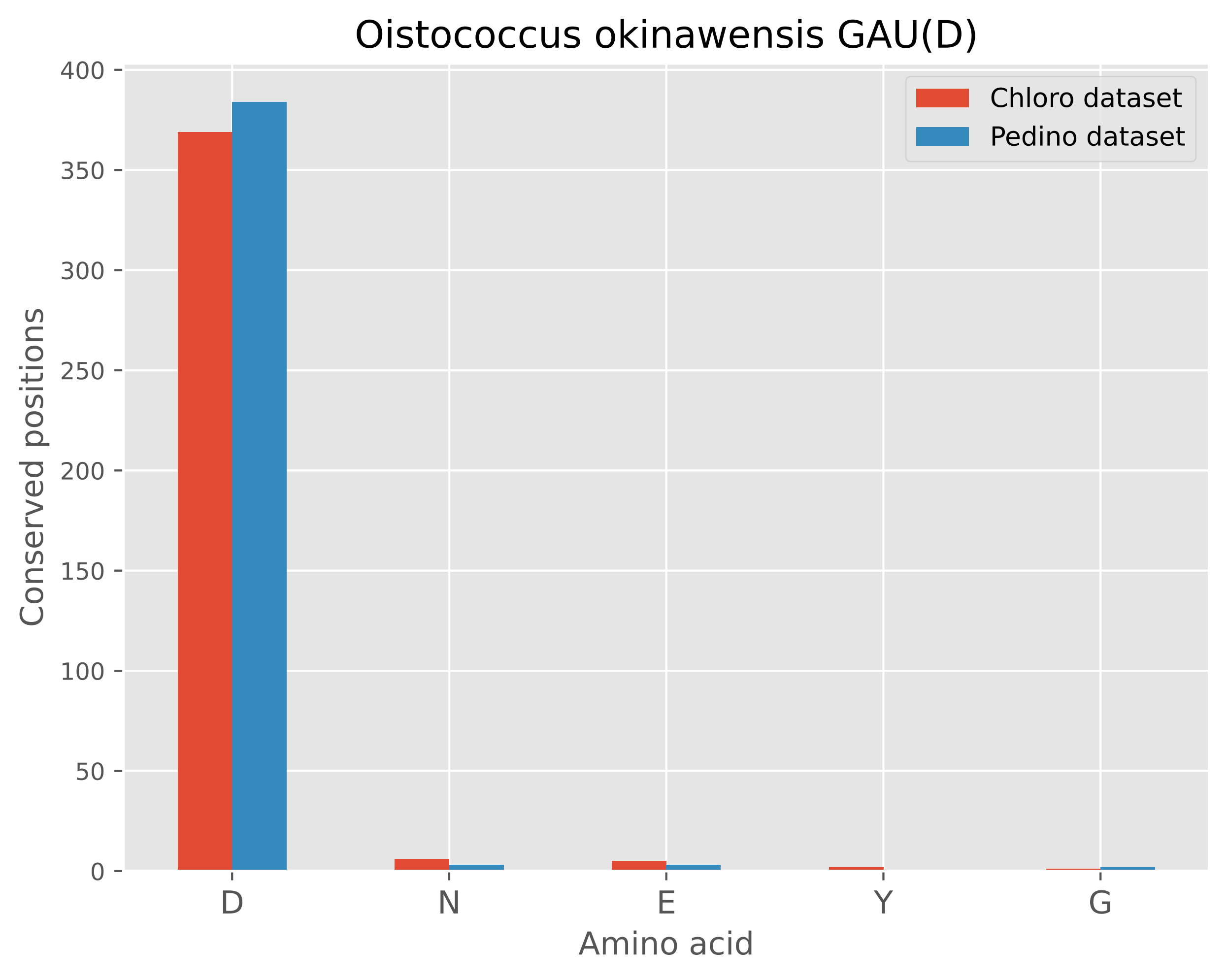
Oistococcus okinawensis GAU(D)
Chloro dataset
Pedino dataset
Conserved positions
0
50
100
150
200
250
300
350
400
D
N
E
Y
G
Amino acid

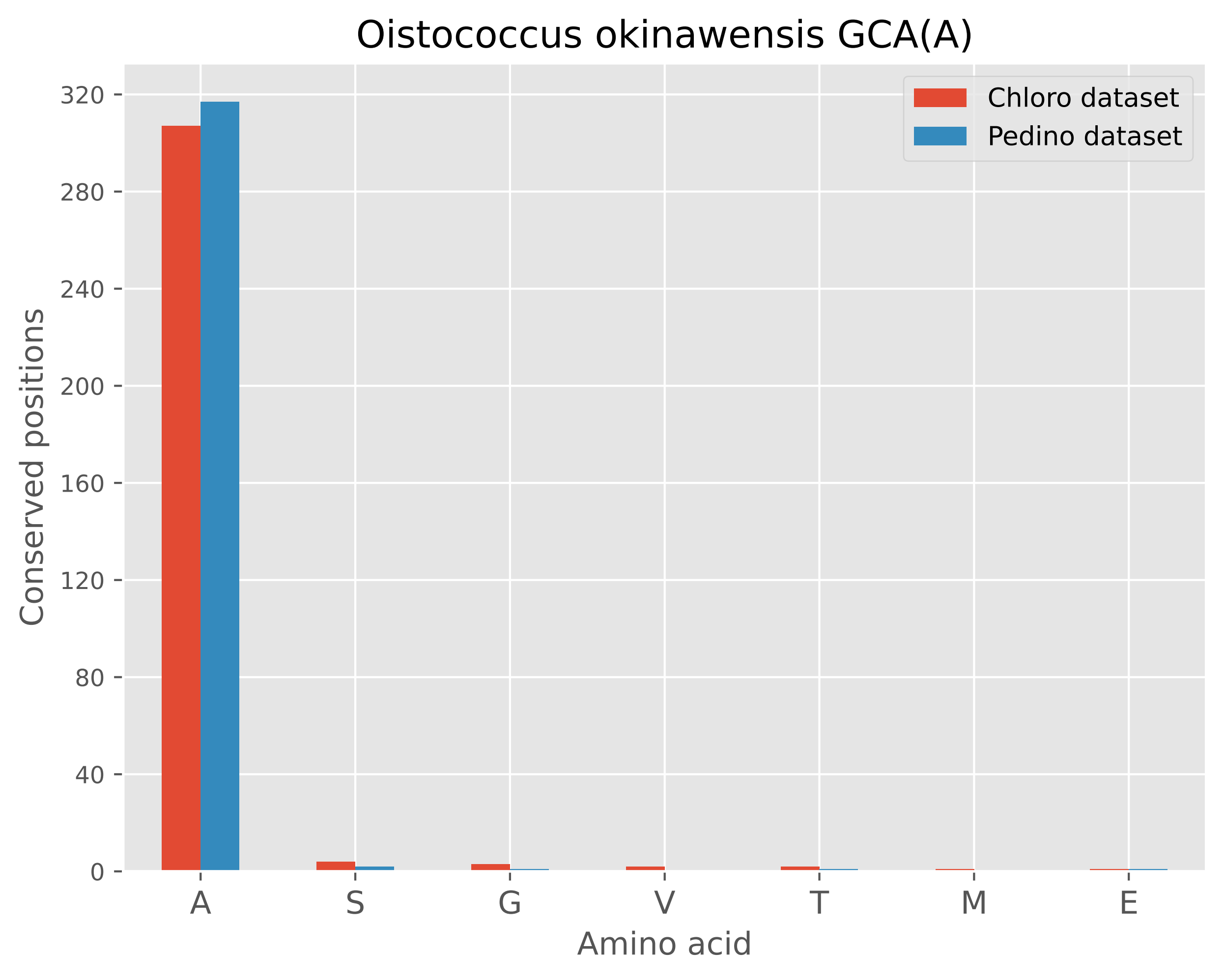
Oistococcus okinawensis GCA(A)
Chloro dataset
Pedino dataset
Conserved positions
0
40
80
120
160
200
240
280
320
A
S
G
V
T
M
E
Amino acid

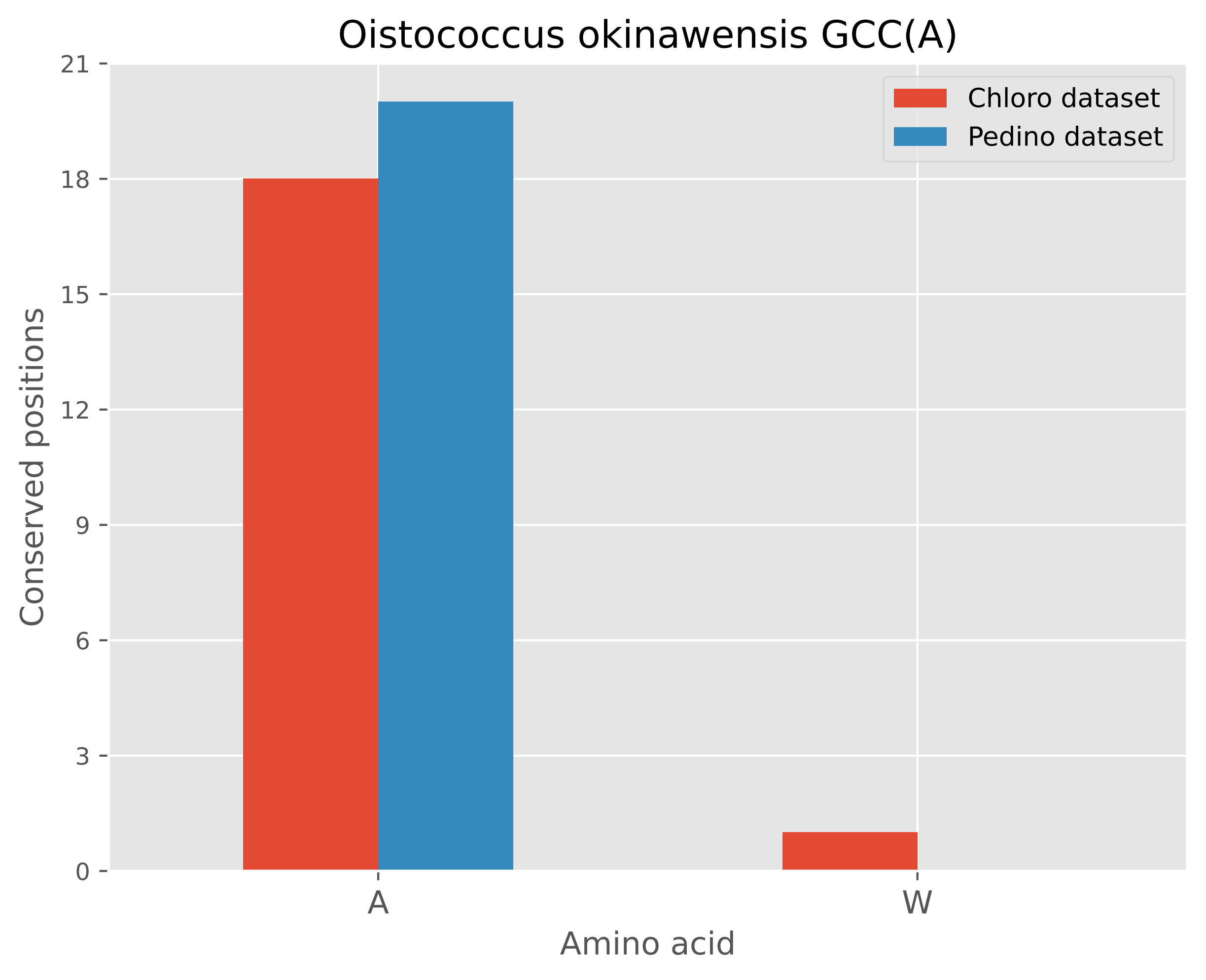

Oistococcus okinawensis GCC(A)
Chloro dataset
Pedino dataset
Conserved positions
Amino acid
A
W
0
3
6
9
12
15
18
21

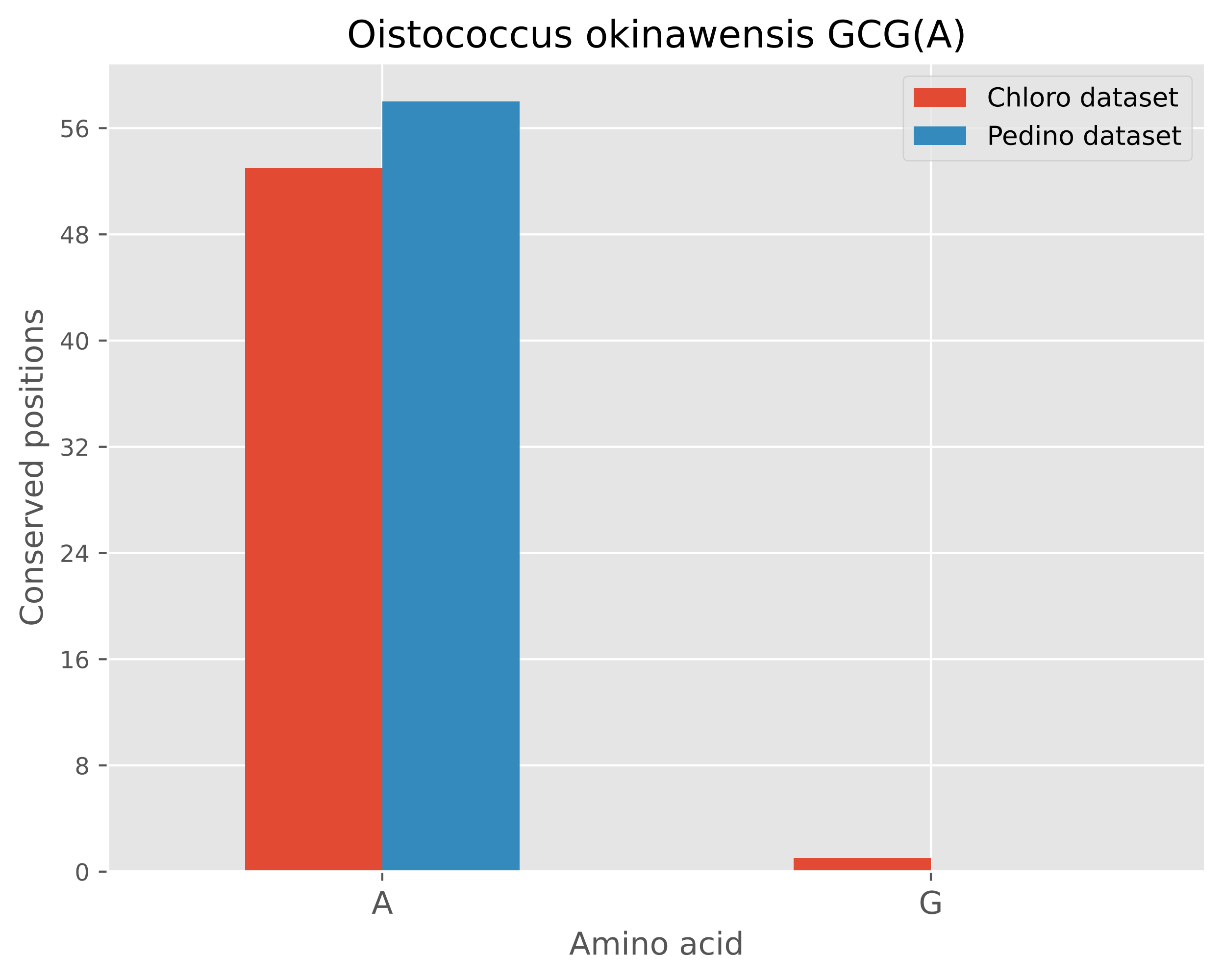

Oistococcus okinawensis GCG(A)
Chloro dataset
Pedino dataset
Conserved positions
0
8
16
24
32
40
48
56
A
G
Amino acid

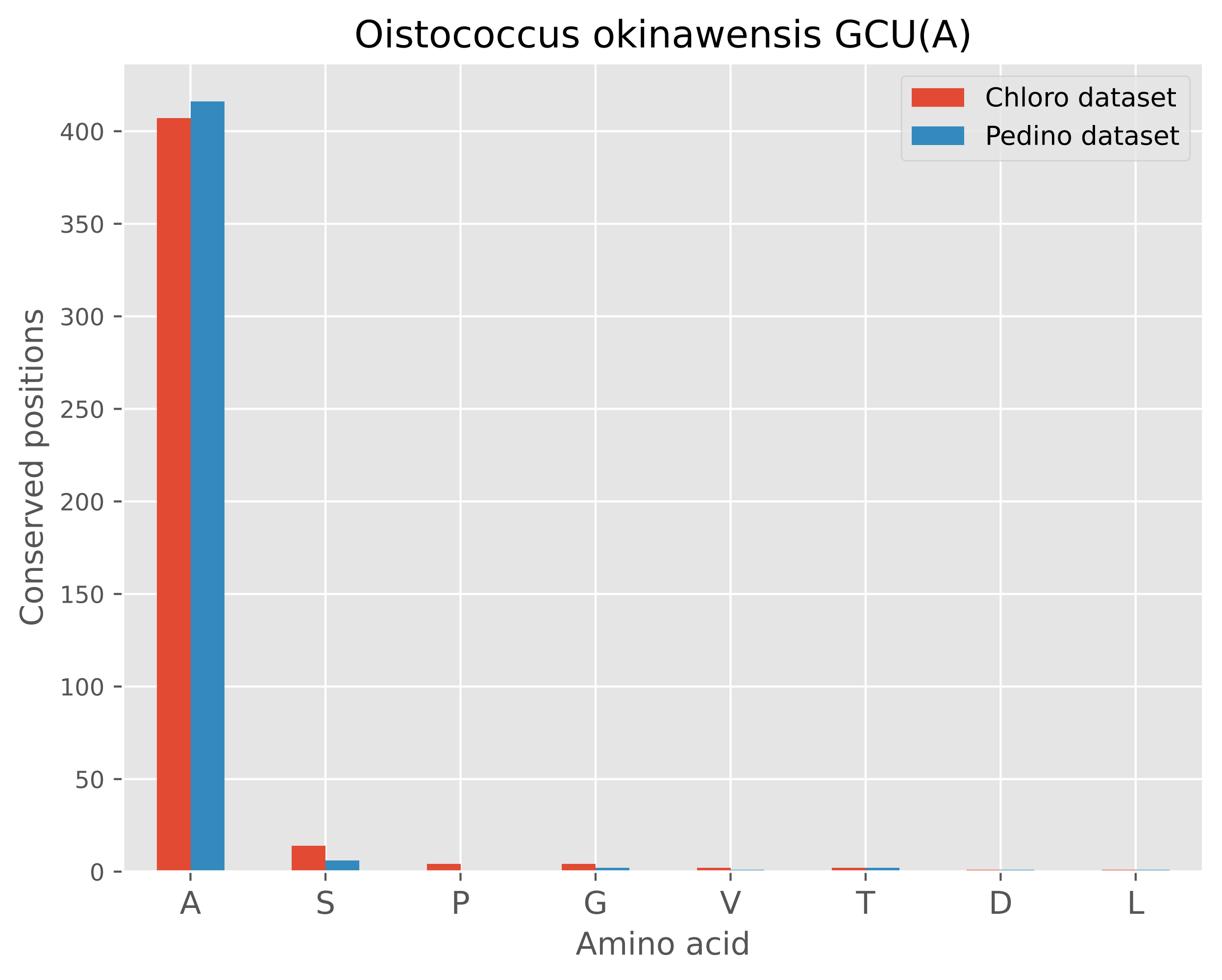

Oistococcus okinawensis GCU(A)
Chloro dataset
Pedino dataset
Conserved positions
0
50
100
150
200
250
300
350
400
A
S
P
G
V
T
D
L
Amino acid

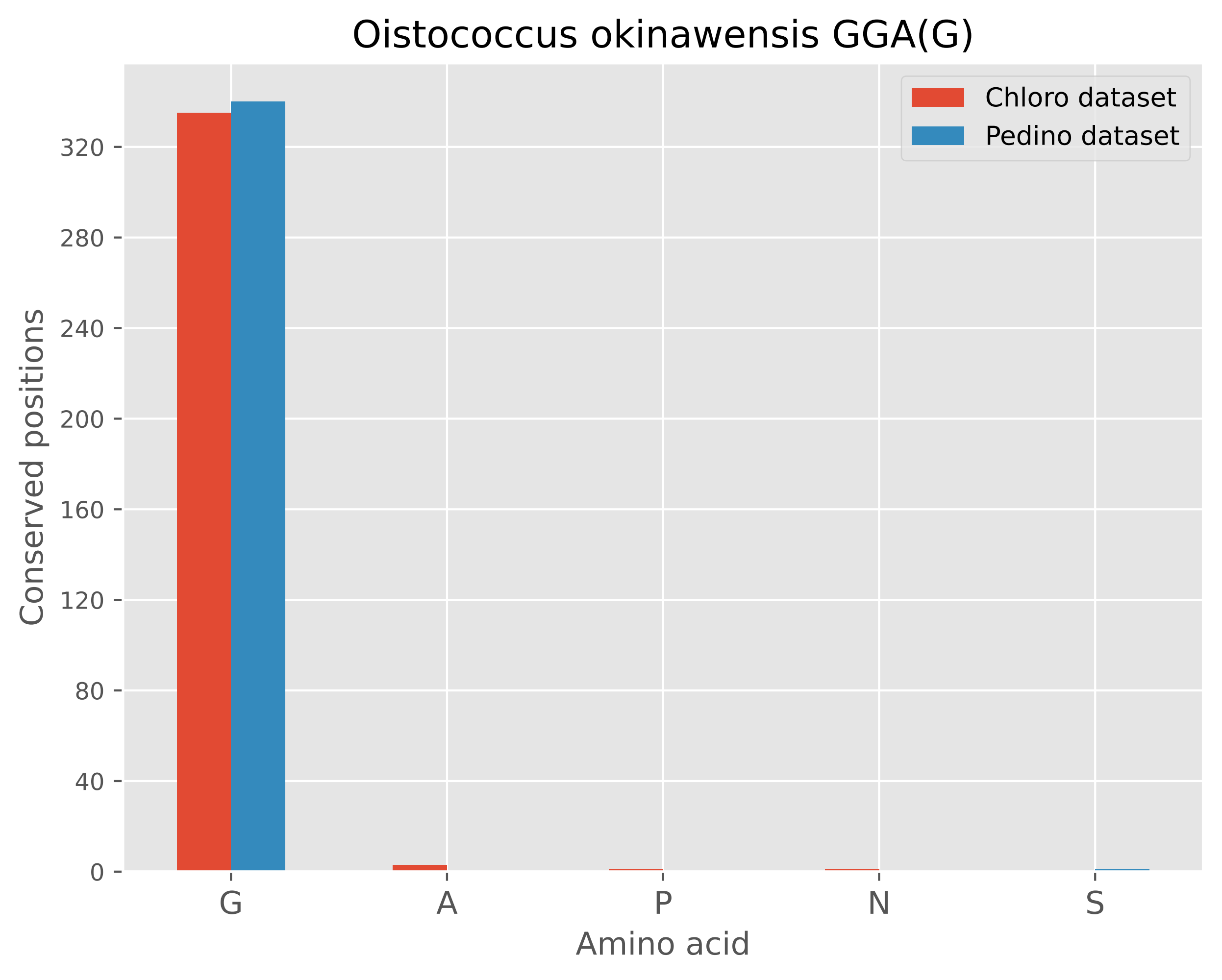

Oistococcus okinawensis GGA(G)
Chloro dataset
Pedino dataset
Conserved positions
0
40
80
120
160
200
240
280
320
G
A
P
N
S
Amino acid

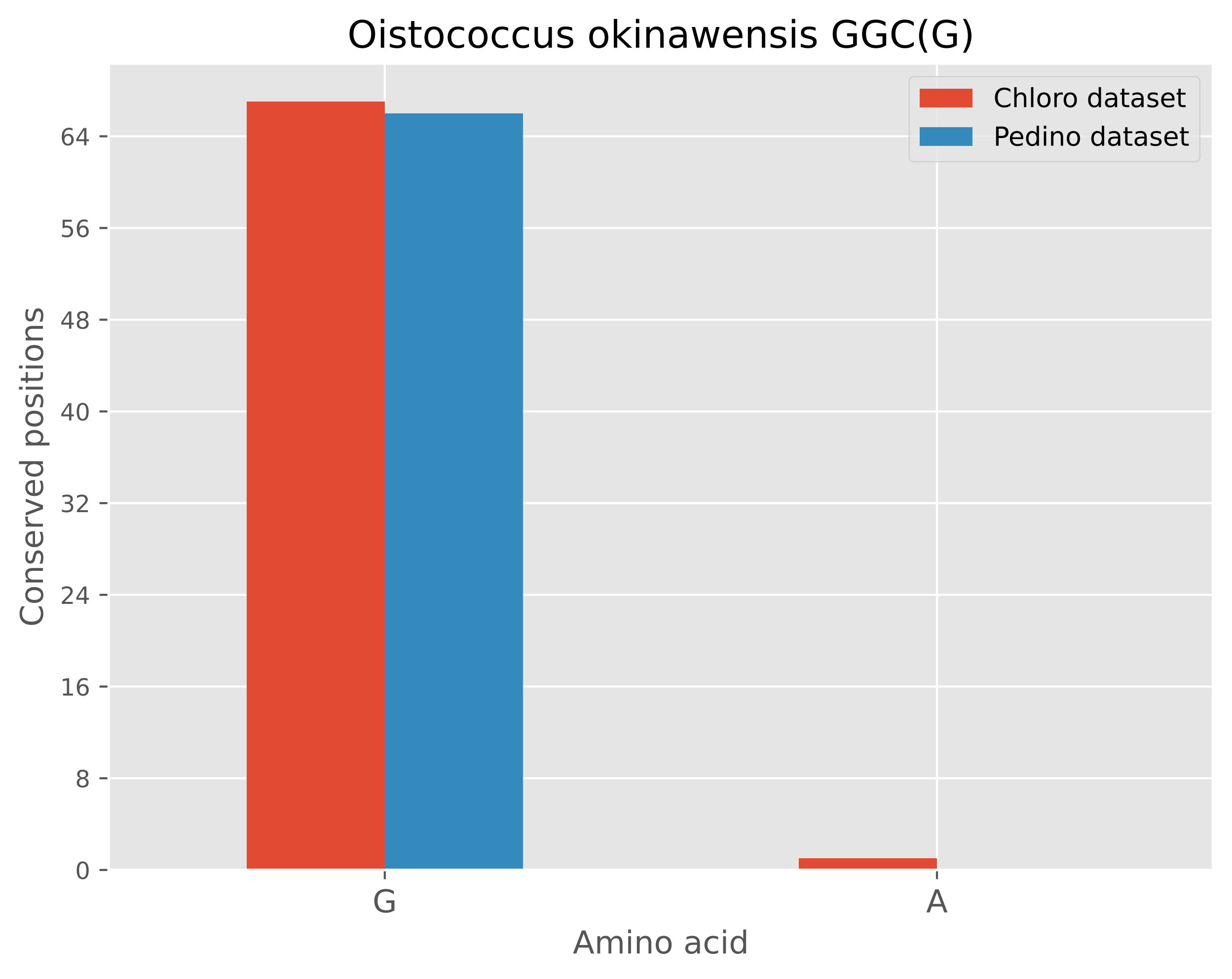

Oistococcus okinawensis GGC(G)
Chloro dataset
Pedino dataset
Conserved positions
0
8
16
24
32
40
48
56
64
G
A
Amino acid

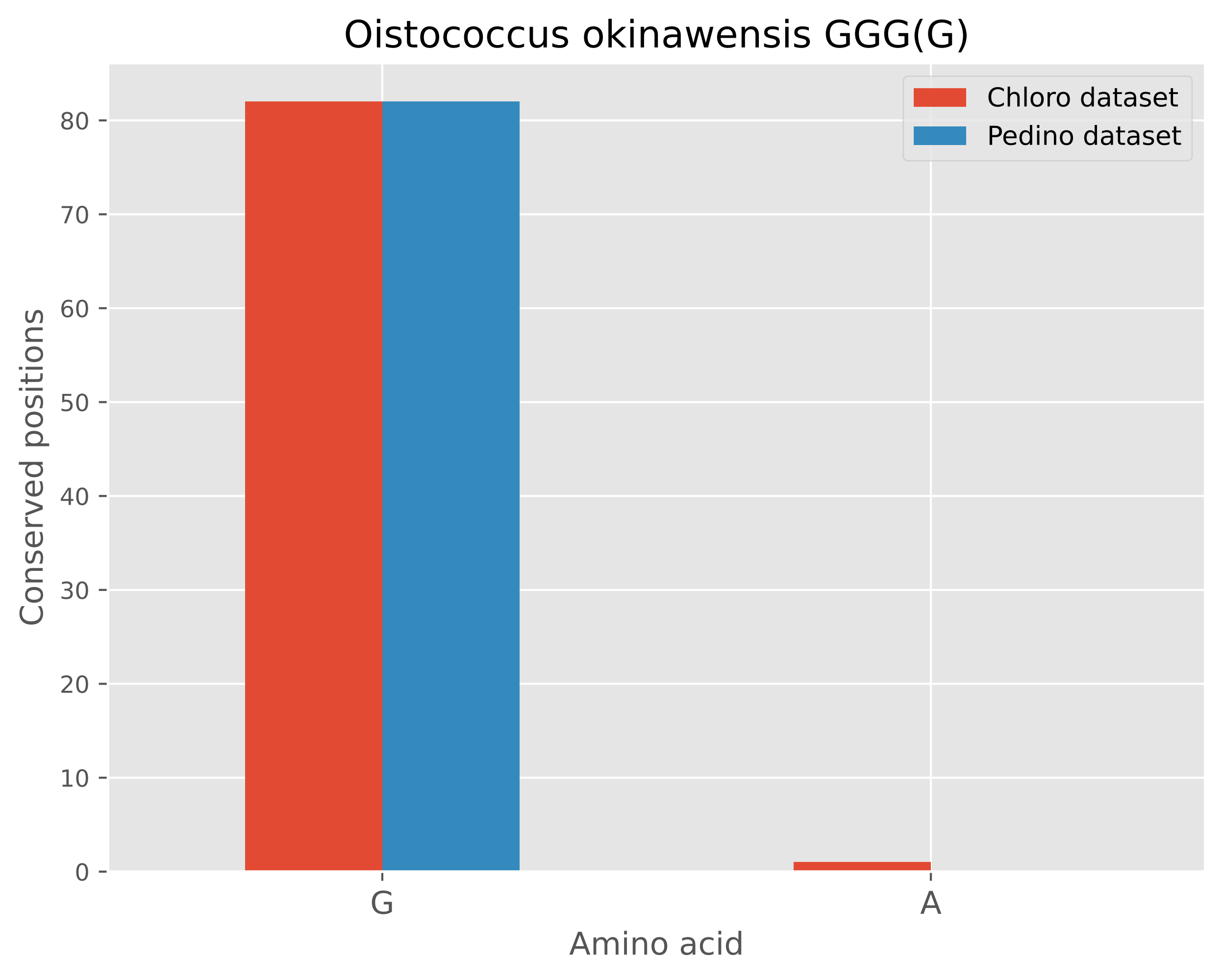

Oistococcus okinawensis GGG(G)
Chloro dataset
Pedino dataset
Conserved positions
0
10
20
30
40
50
60
70
80
G
A
Amino acid

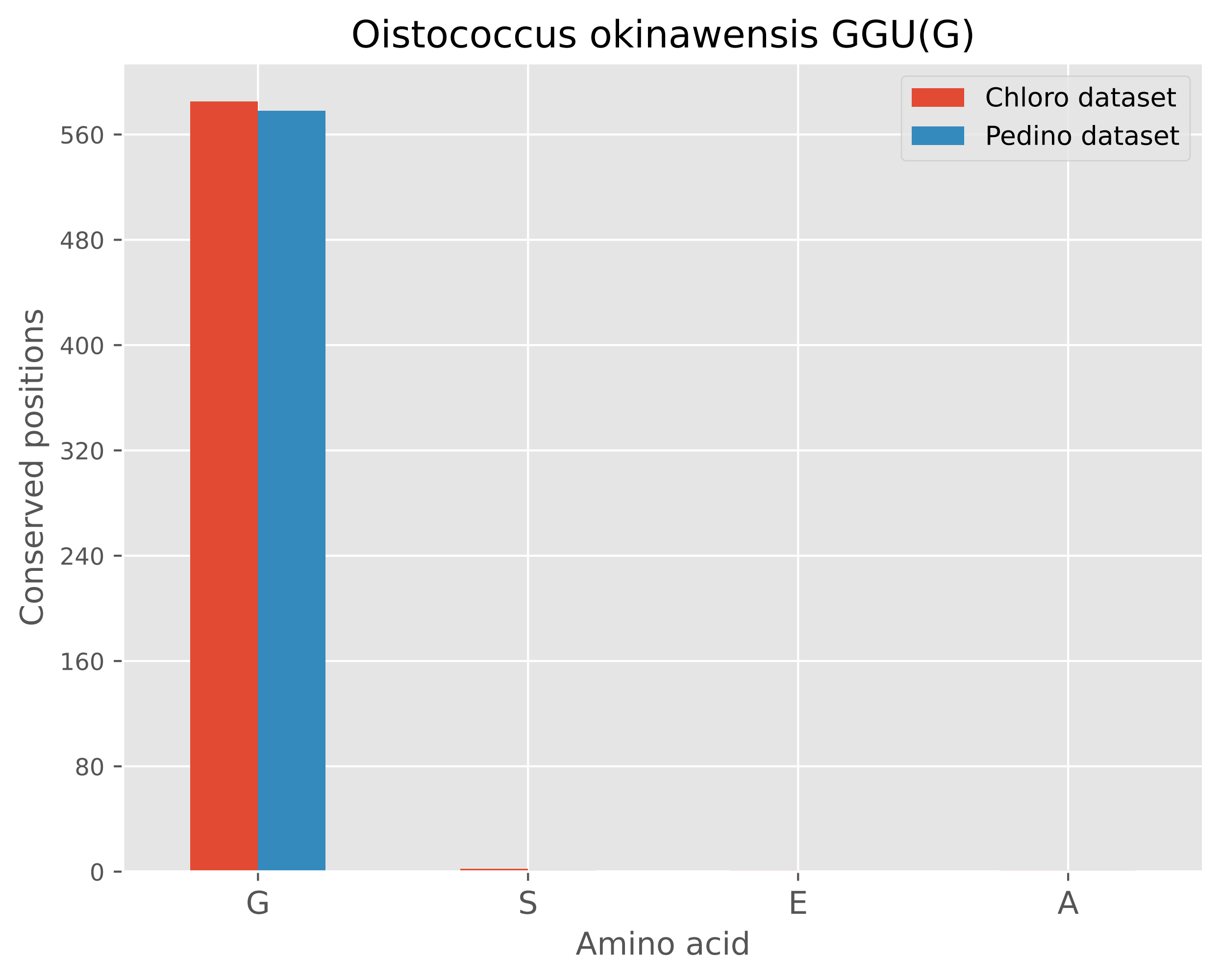

Oistococcus okinawensis GGU(G)
Chloro dataset
Pedino dataset
Conserved positions
0
80
160
240
320
400
480
560
G
S
E
A
Amino acid

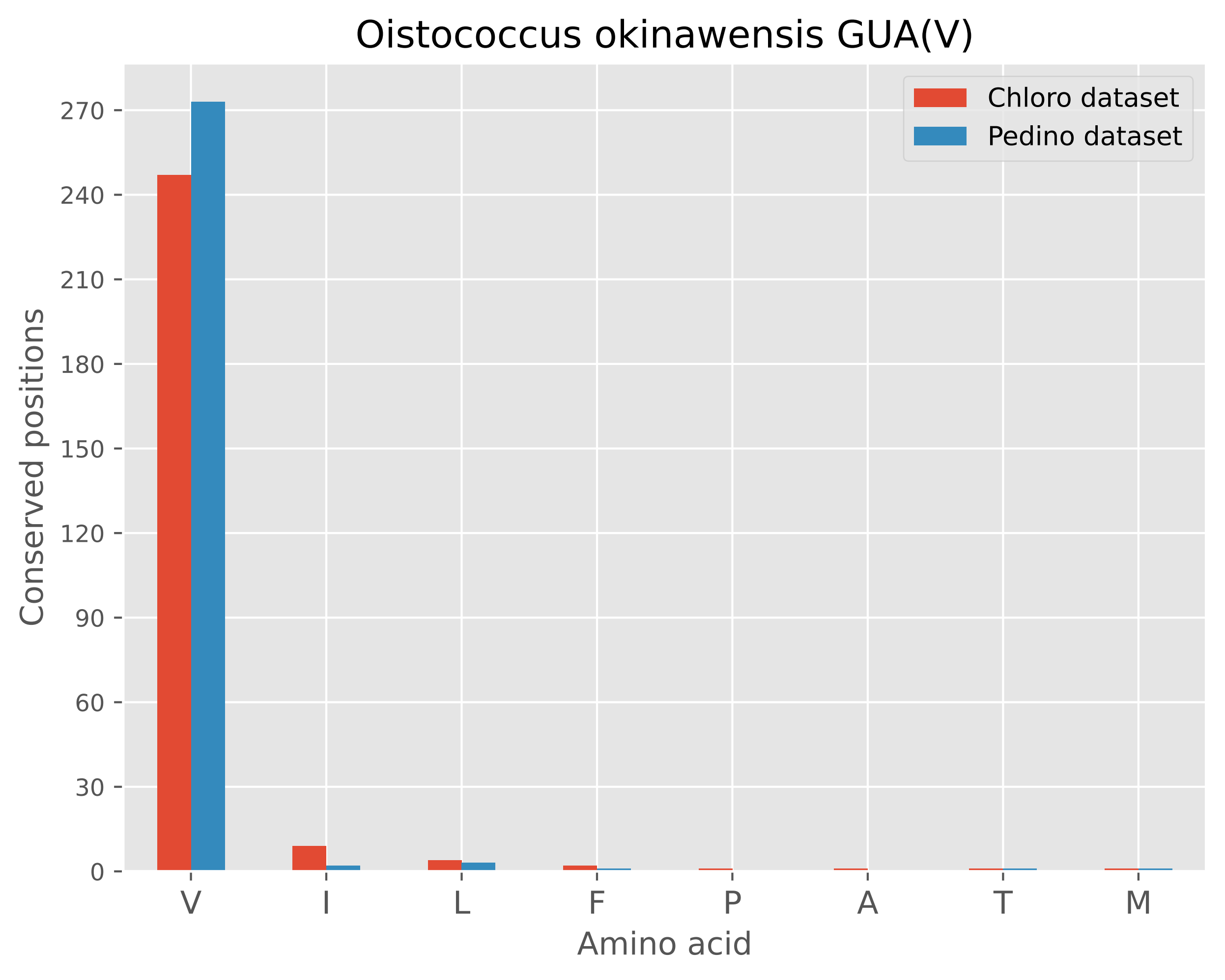

Oistococcus okinawensis GUA(V)
Chloro dataset
Pedino dataset
Conserved positions
0
30
60
90
120
150
180
210
240
270
V
I
L
F
P
A
T
M
Amino acid

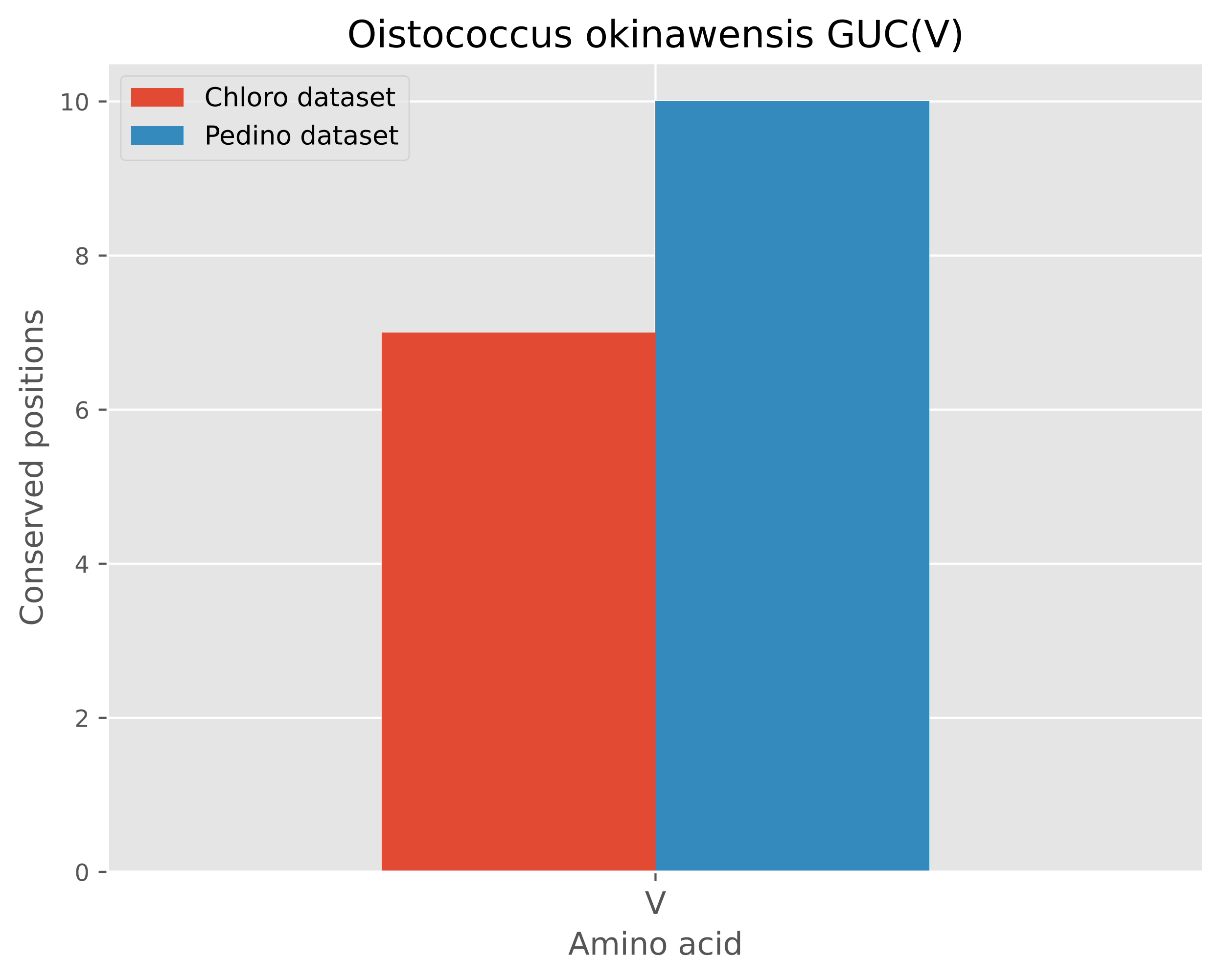

Oistococcus okinawensis GUC(V)
Chloro dataset
Pedino dataset
Conserved positions
0
2
4
6
8
10
V
Amino acid

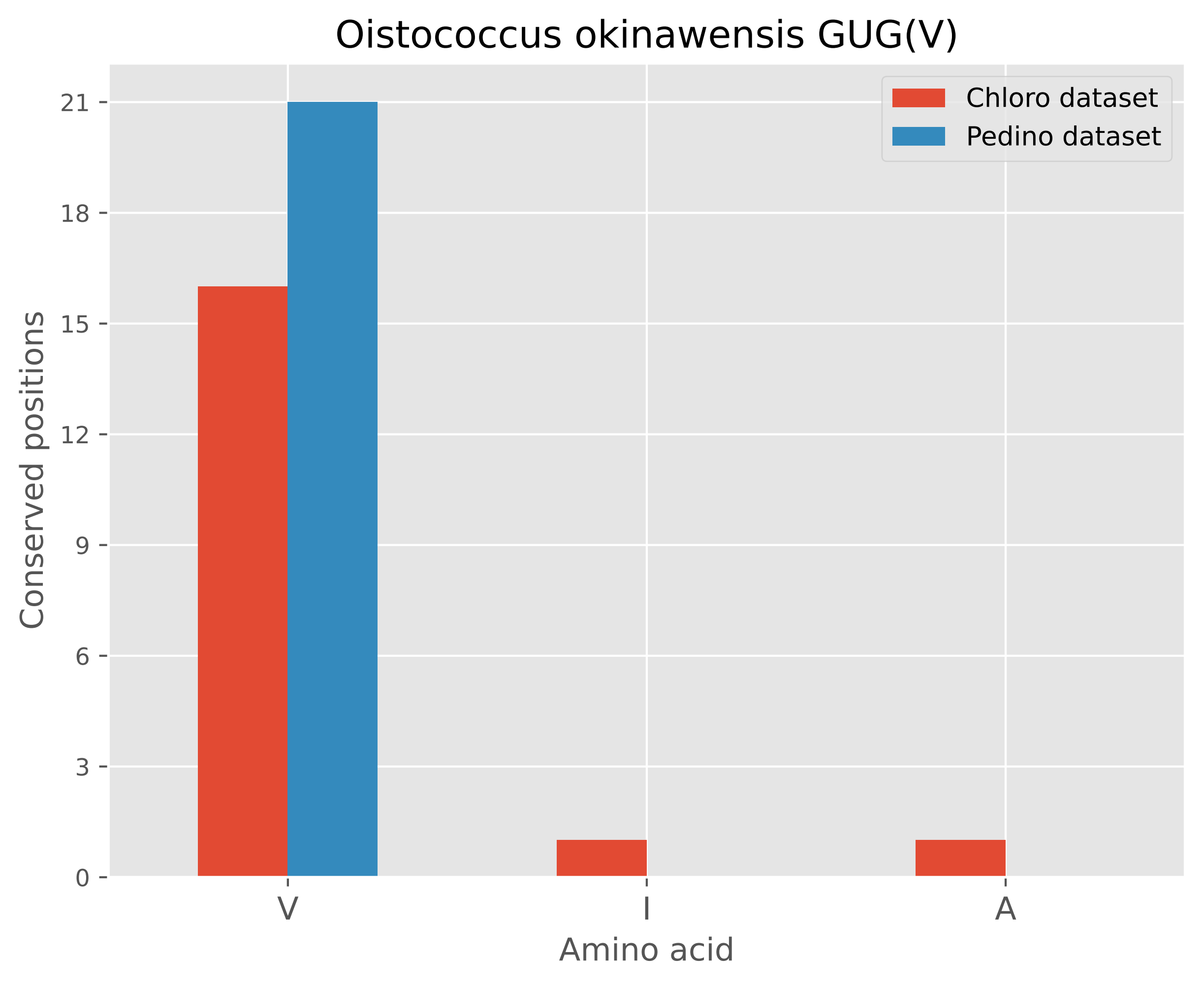
Oistococcus okinawensis GUG(V)
Chloro dataset
Pedino dataset
Conserved positions
0
3
6
9
12
15
18
21
V
I
A
Amino acid

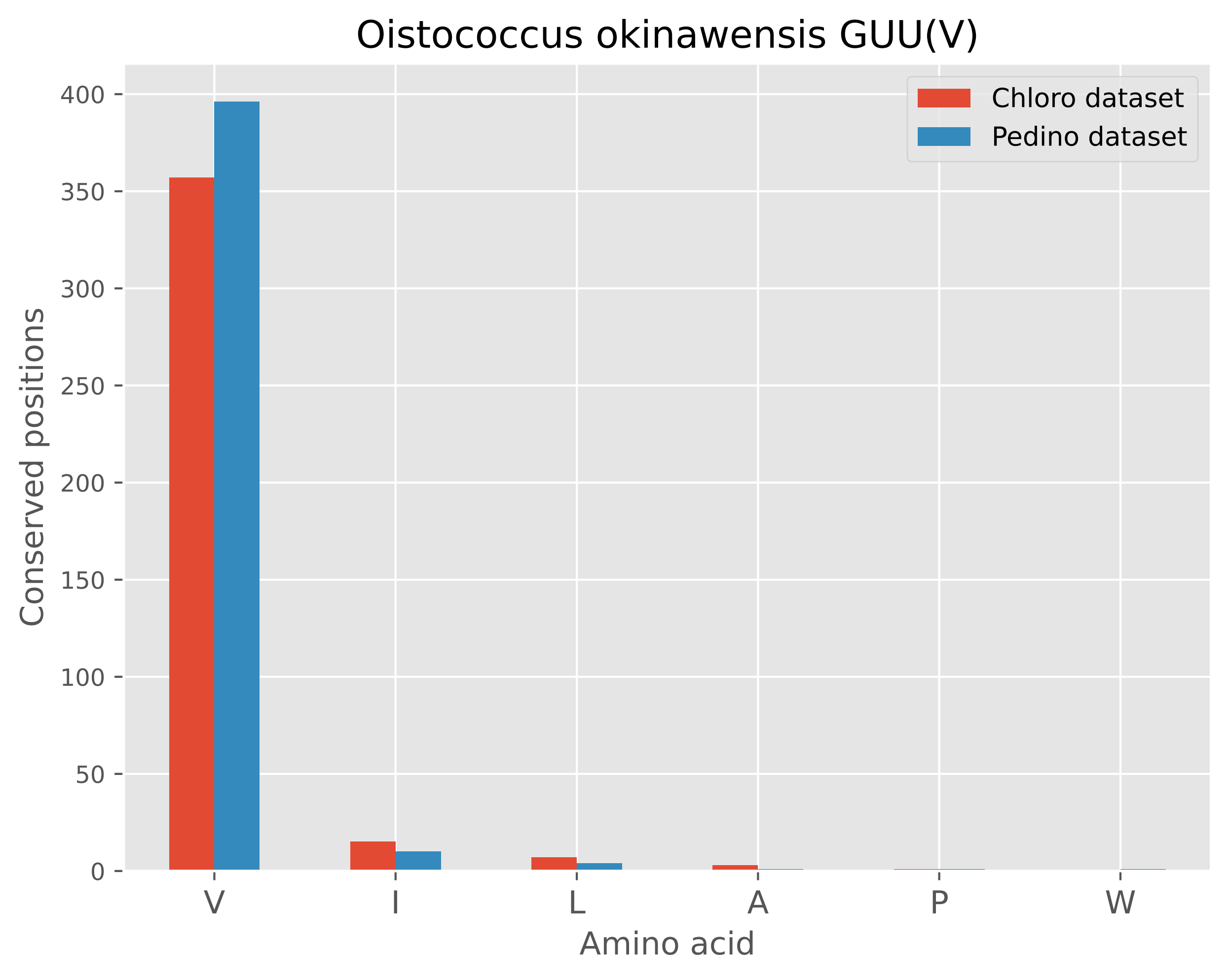
Oistococcus okinawensis GUU(V)
Chloro dataset
Pedino dataset
Conserved positions
0
50
100
150
200
250
300
350
400
V
I
L
A
P
W
Amino acid

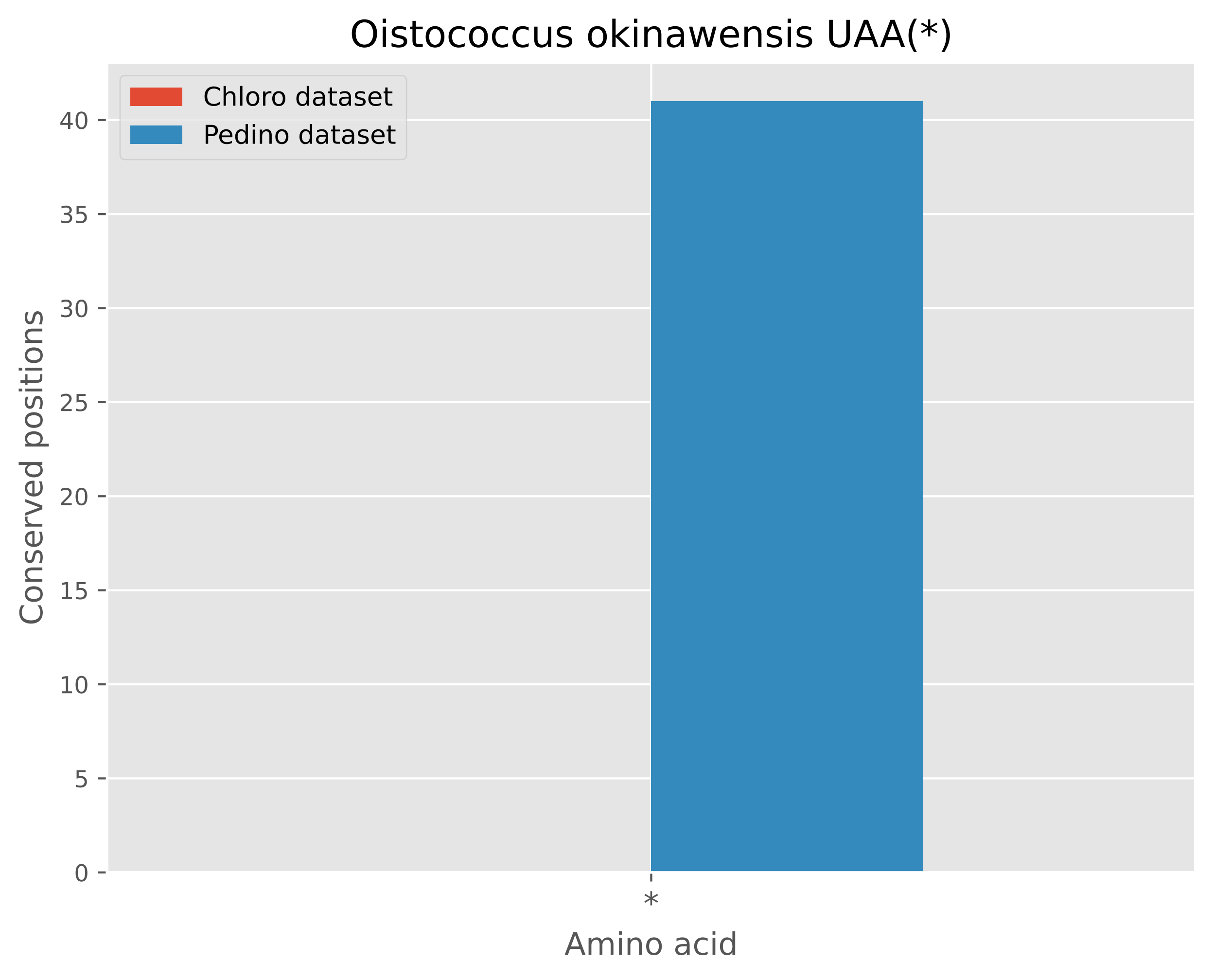
Oistococcus okinawensis UAA(*)
Chloro dataset
Pedino dataset
Conserved positions
0
5
10
15
20
25
30
35
40
*
Amino acid

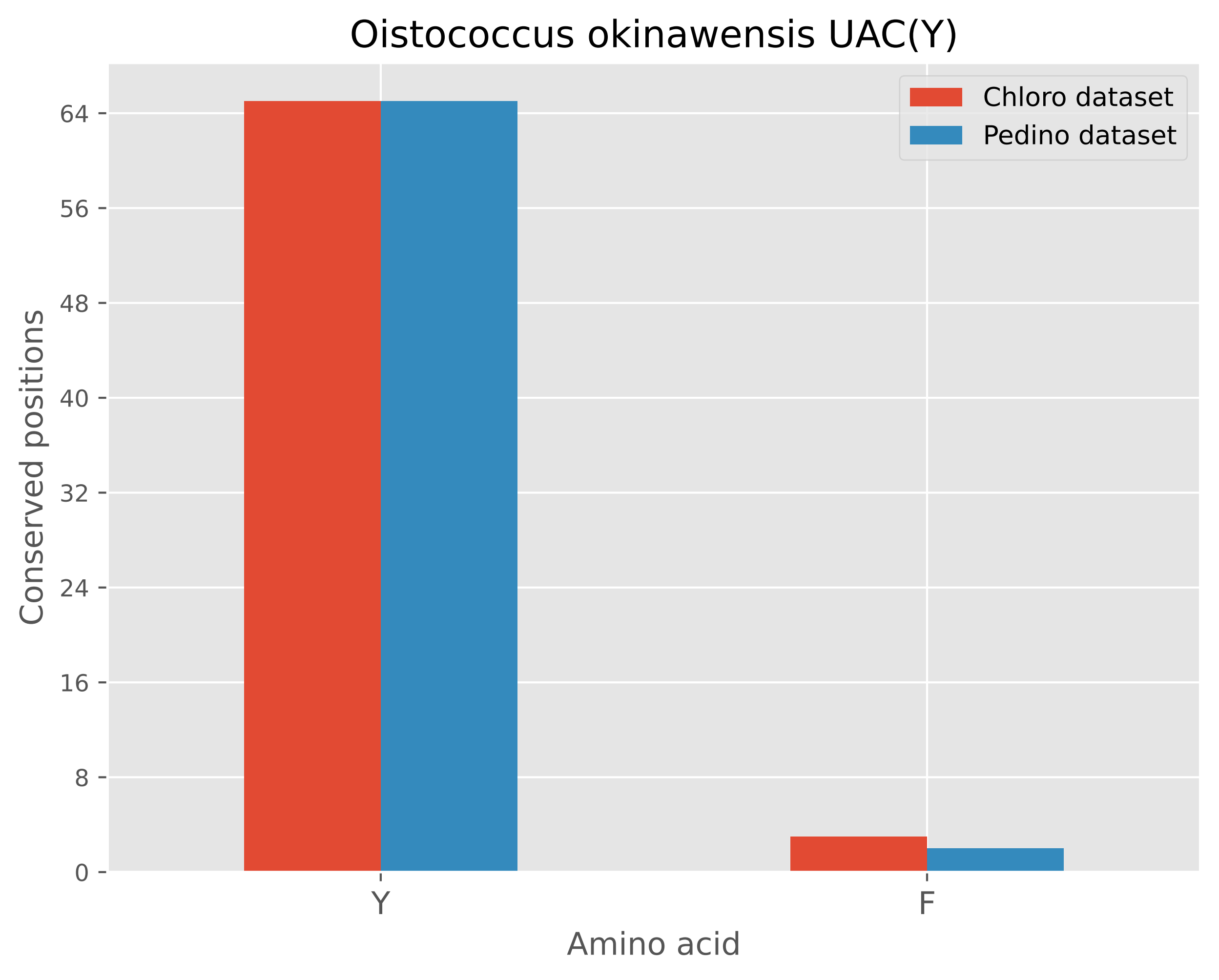

Oistococcus okinawensis UAC(Y)
Chloro dataset
Pedino dataset
Conserved positions
0
8
16
24
32
40
48
56
64
Y
F
Amino acid

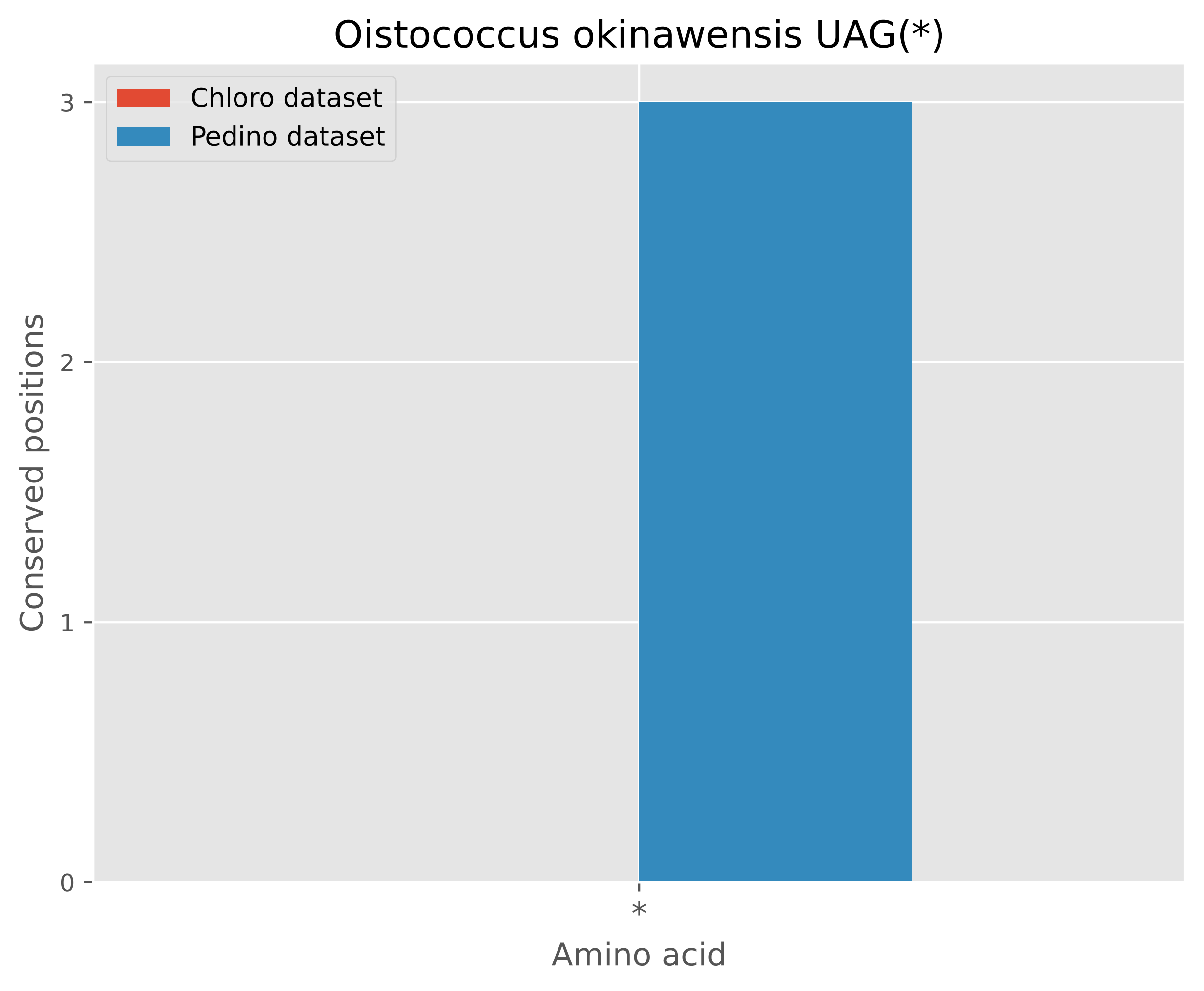

Oistococcus okinawensis UAG(*)
Chloro dataset
Pedino dataset
Conserved positions
3
2
1
0
*
Amino acid

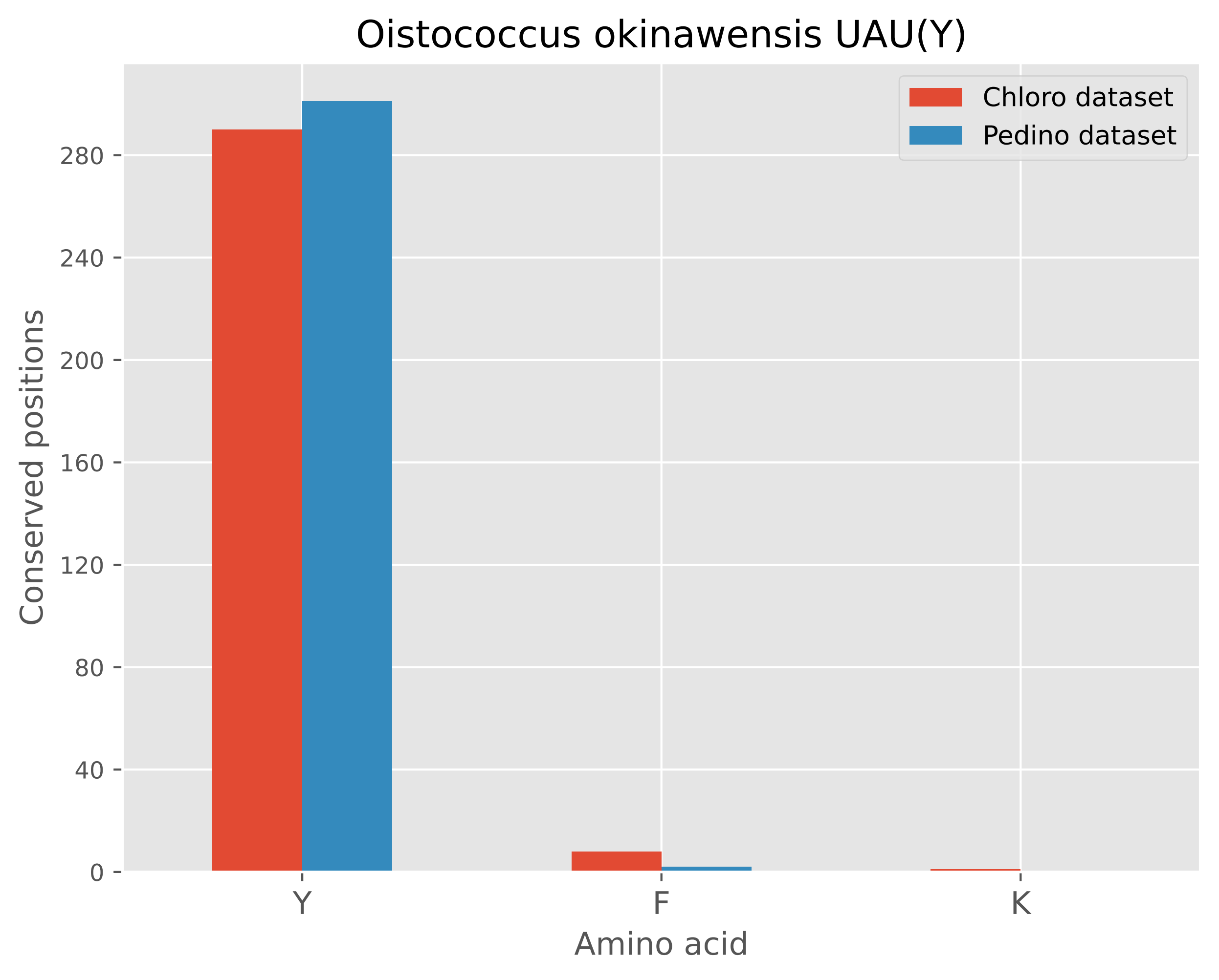
Oistococcus okinawensis UAU(Y)
Chloro dataset
Pedino dataset
Conserved positions
0
40
80
120
160
200
240
280
Y
F
K
Amino acid

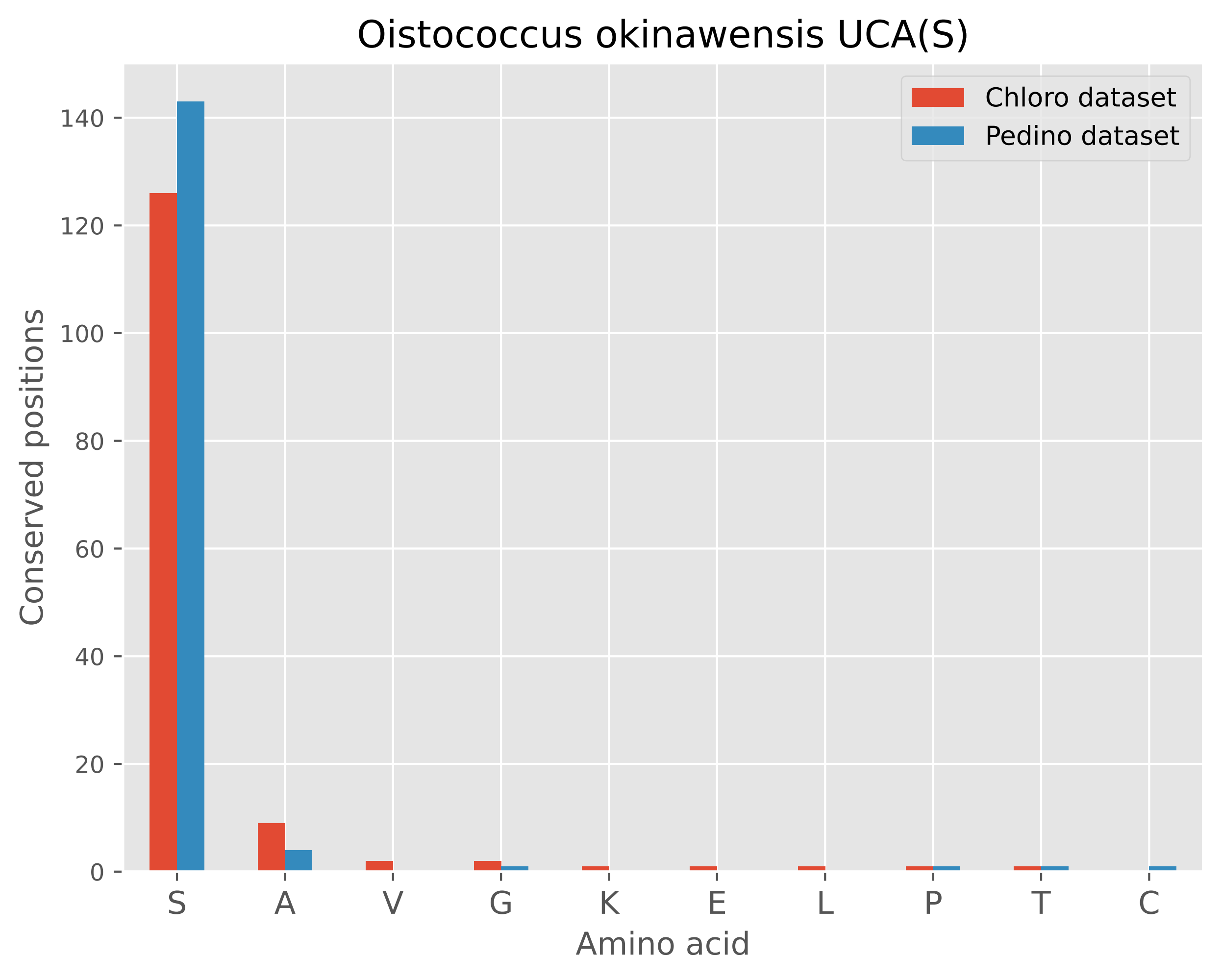

Oistococcus okinawensis UCA(S)
Chloro dataset
Pedino dataset
Conserved positions
0
20
40
60
80
100
120
140
S
A
V
G
K
E
L
P
T
C
Amino acid

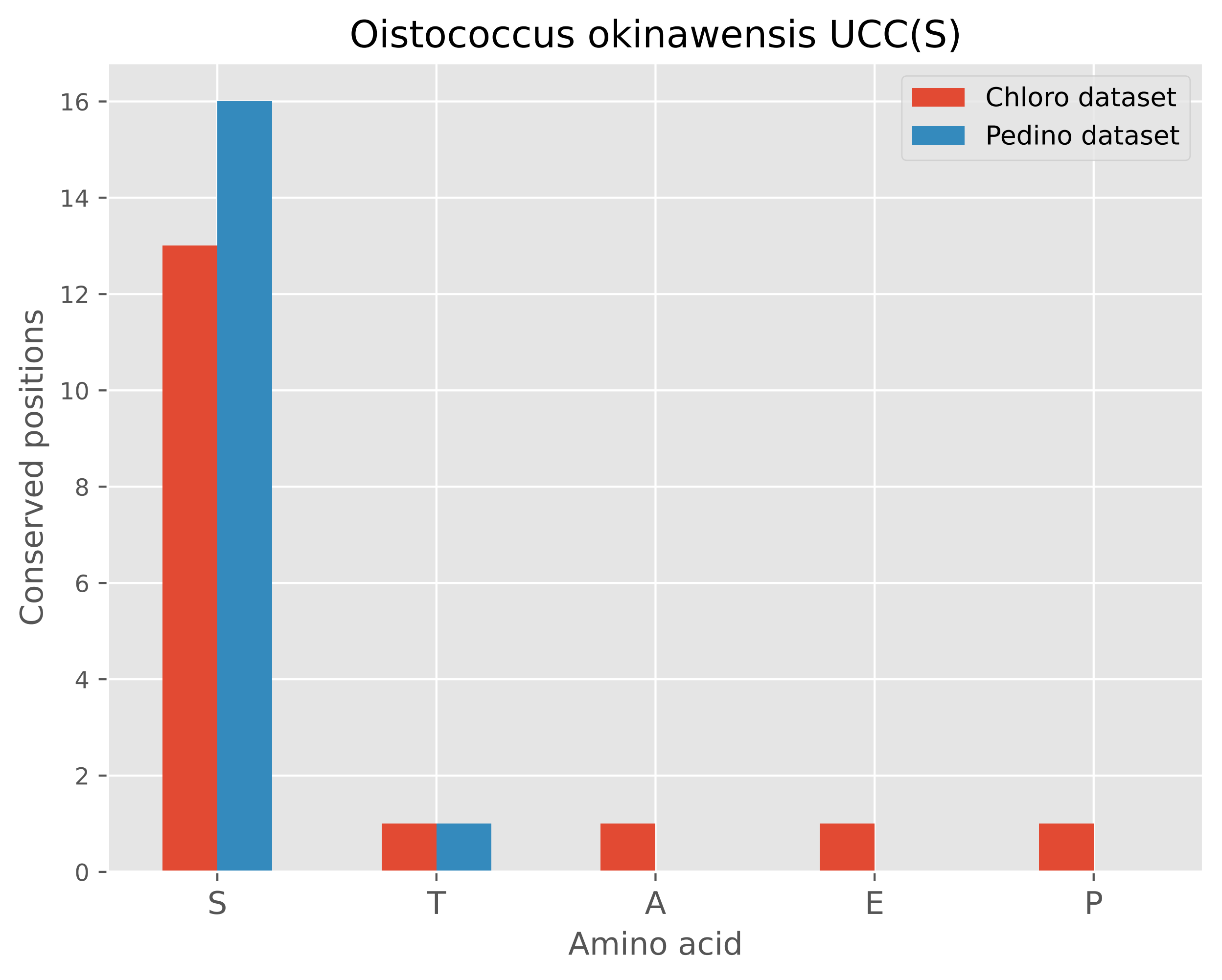

Oistococcus okinawensis UCC(S)
Chloro dataset
Pedino dataset
Conserved positions
0
2
4
6
8
10
12
14
16
S
T
A
E
P
Amino acid

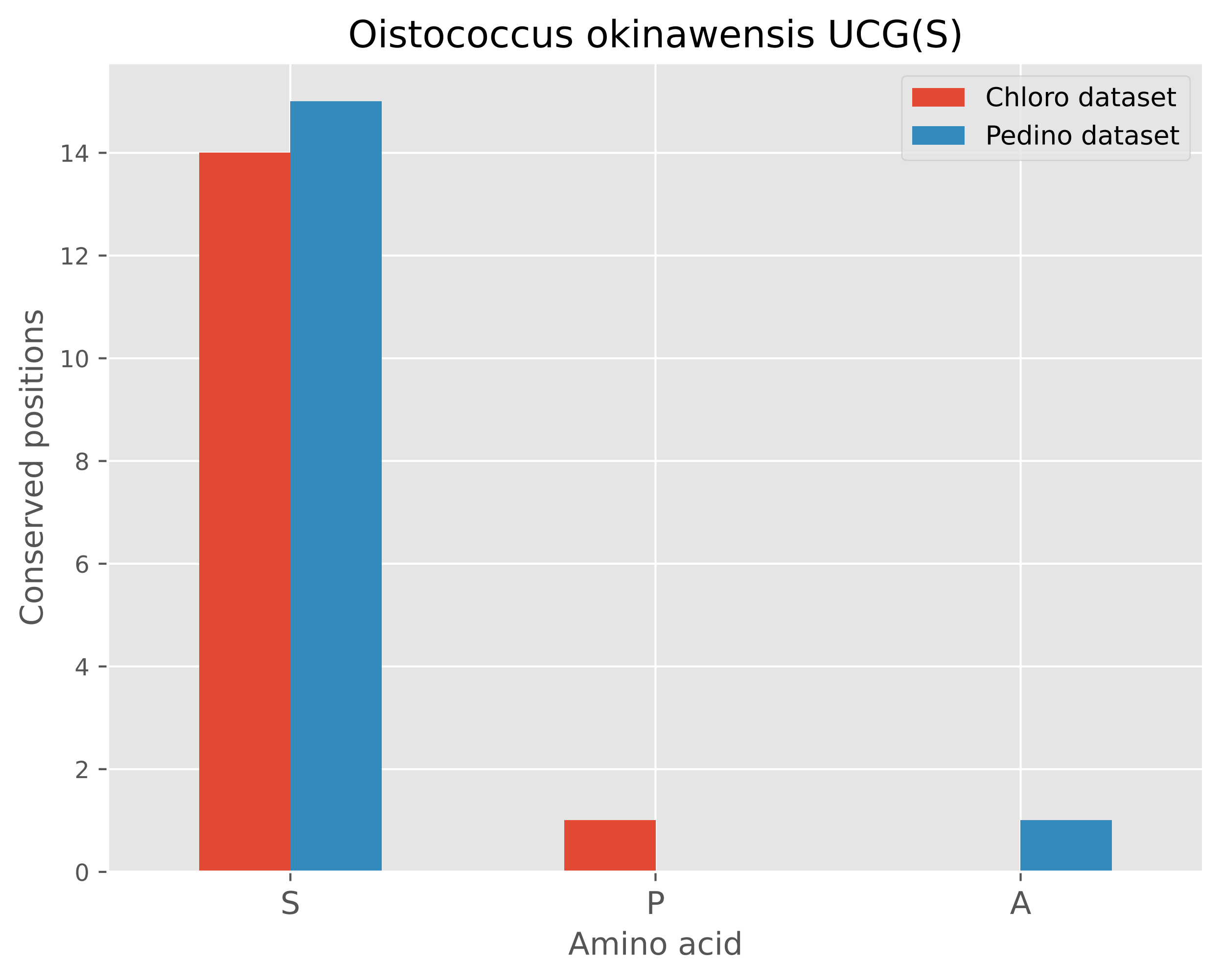
Oistococcus okinawensis UCG(S)
Chloro dataset
Pedino dataset
Conserved positions
0
2
4
6
8
10
12
14
S
P
A
Amino acid

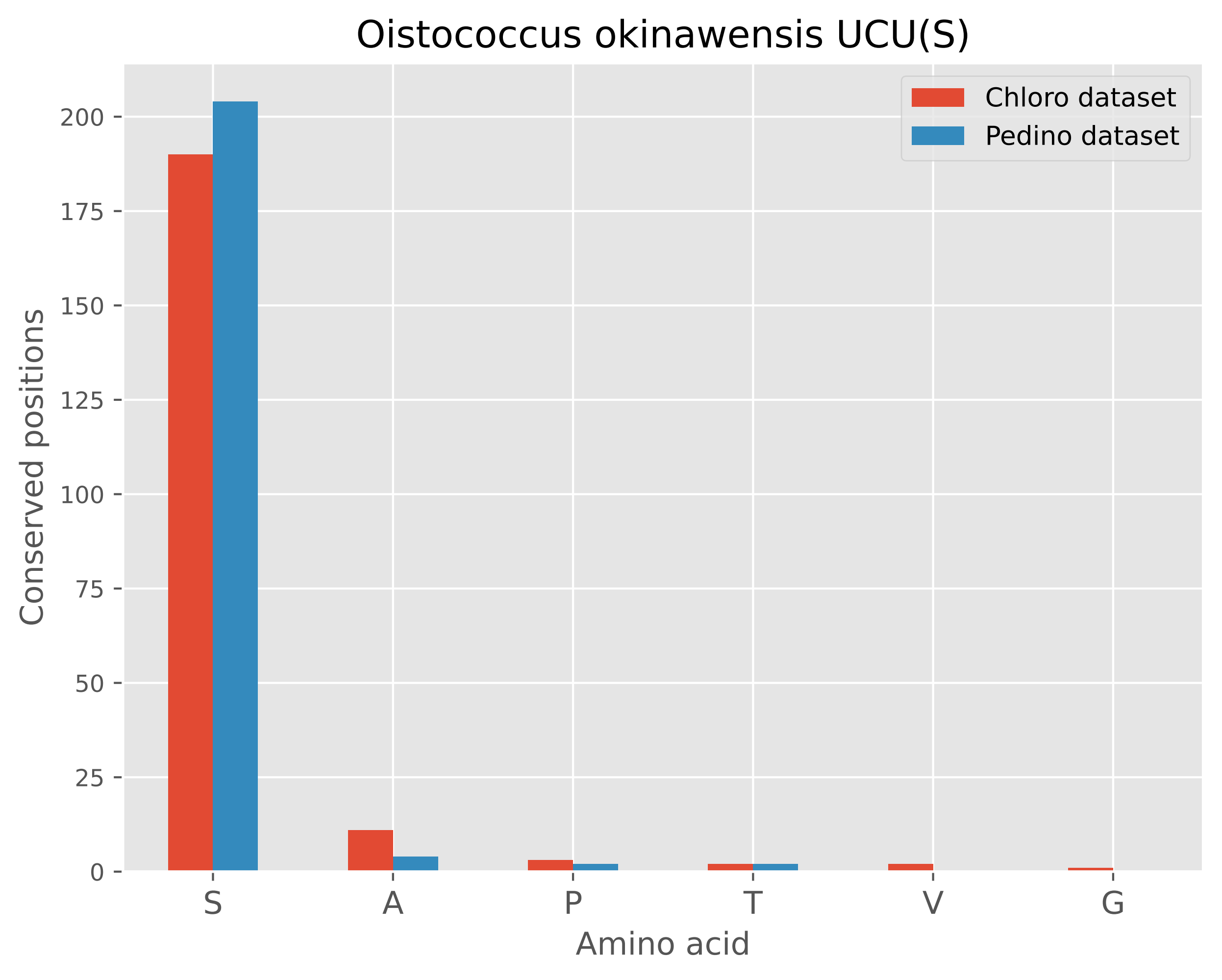

Oistococcus okinawensis UCU(S)
Chloro dataset
Pedino dataset
Conserved positions
0
25
50
75
100
125
150
175
200
S
A
P
T
V
G
Amino acid

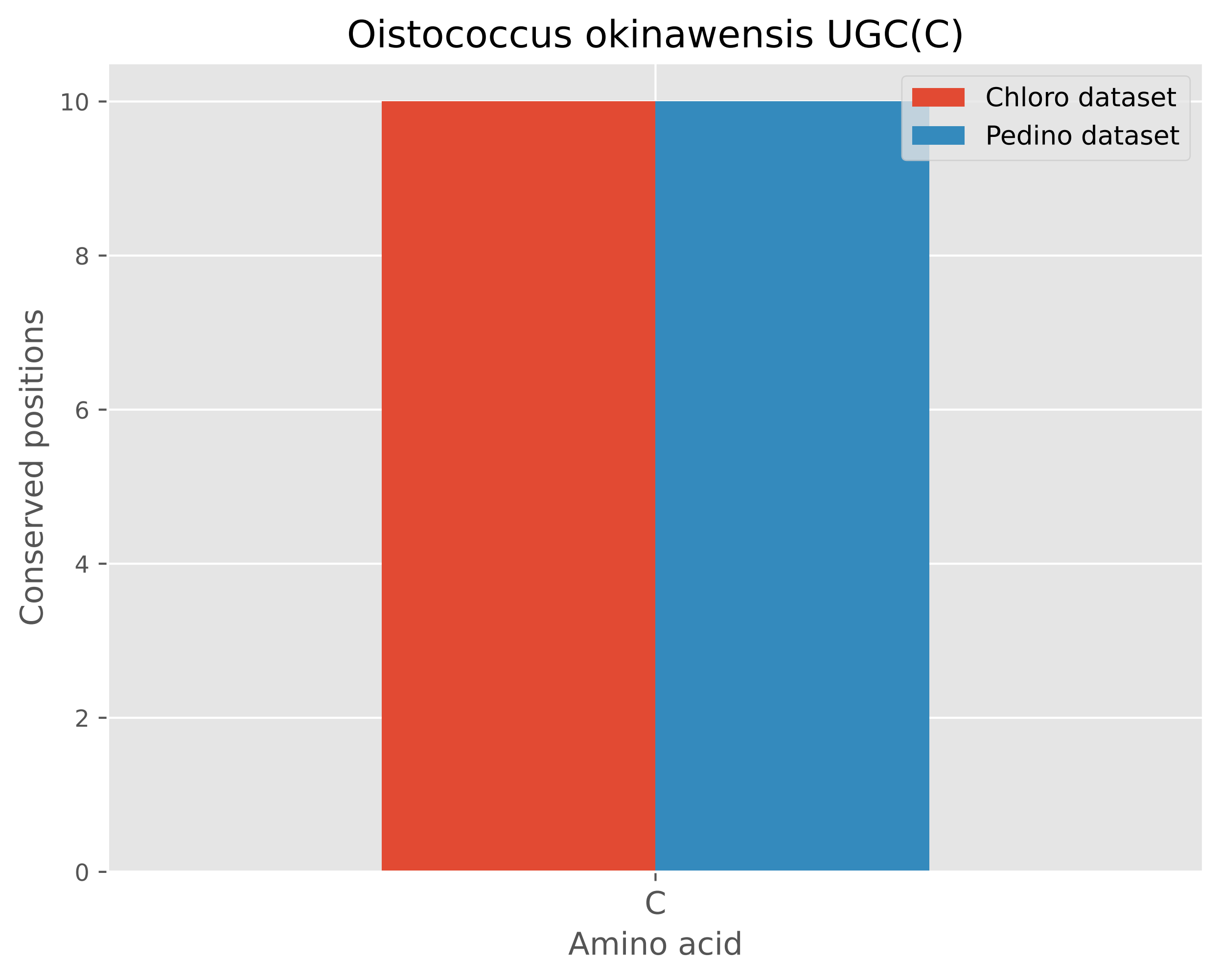

Oistococcus okinawensis UGC(C)
Chloro dataset
Pedino dataset
Conserved positions
0
2
4
6
8
10
C
Amino acid

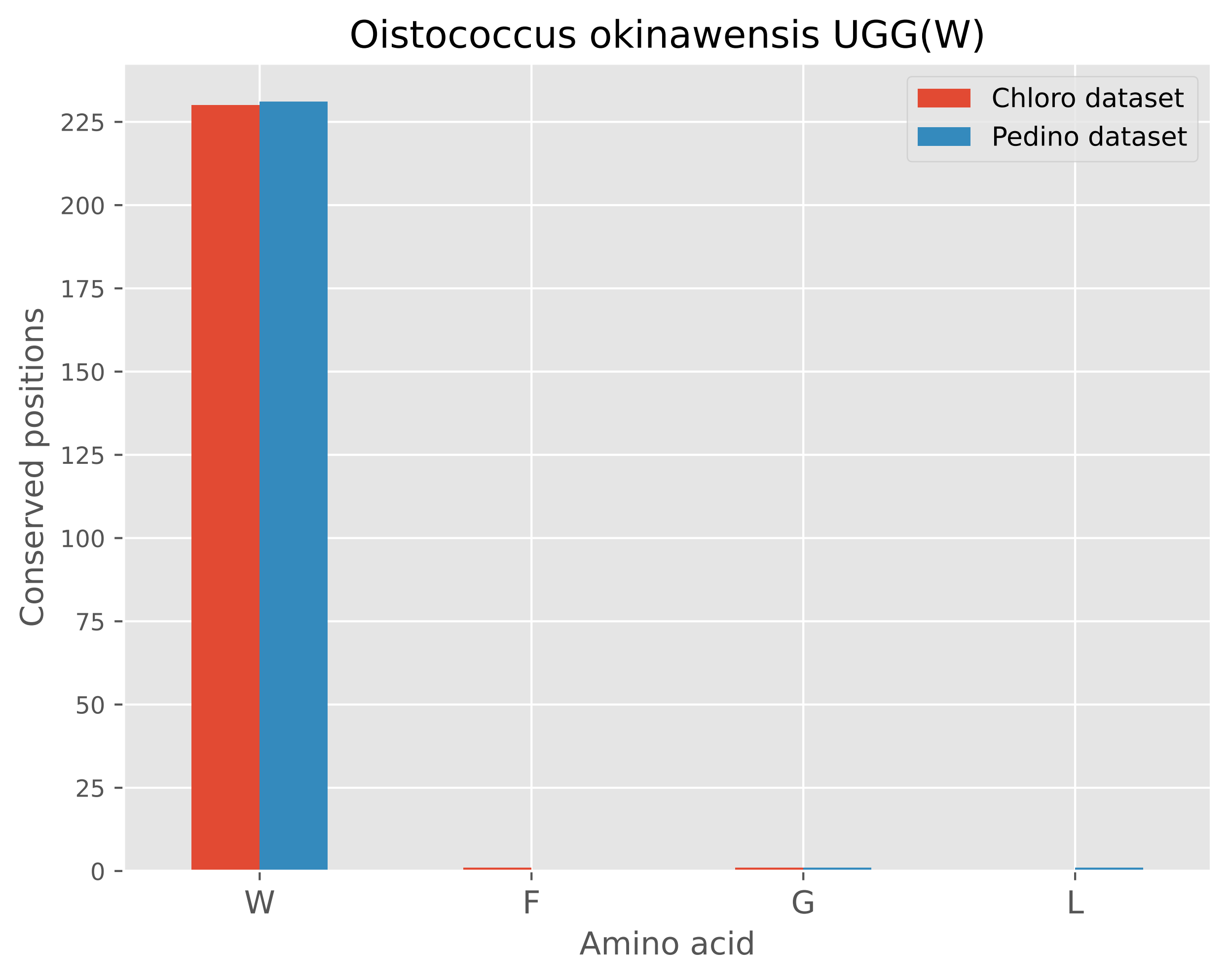
Oistococcus okinawensis UGG(W)
Chloro dataset
Pedino dataset
Conserved positions
0
25
50
75
100
125
150
175
200
225
W
F
G
L
Amino acid

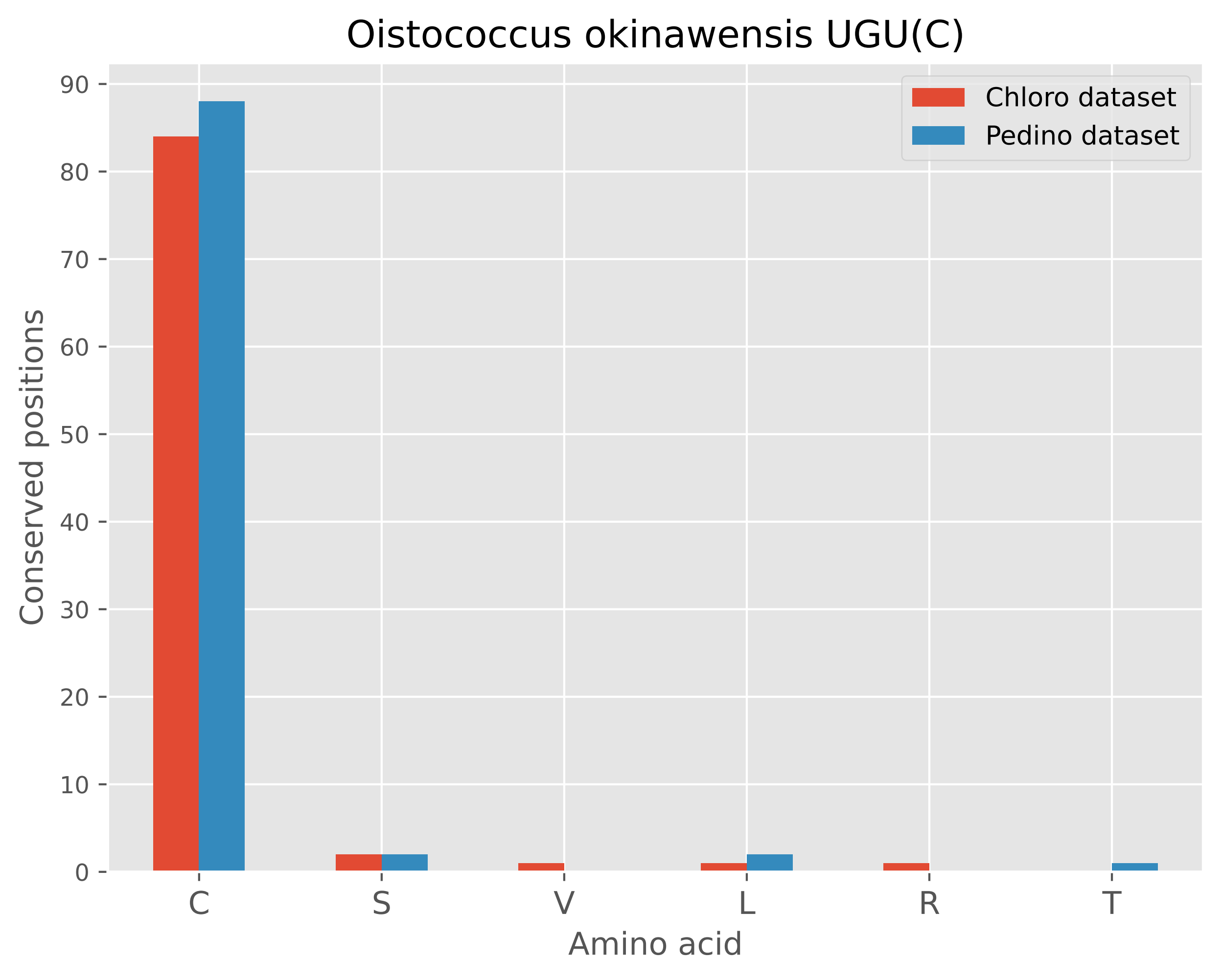

Oistococcus okinawensis UGU(C)
Chloro dataset
Pedino dataset
Conserved positions
0
10
20
30
40
50
60
70
80
90
C
S
V
L
R
T
Amino acid

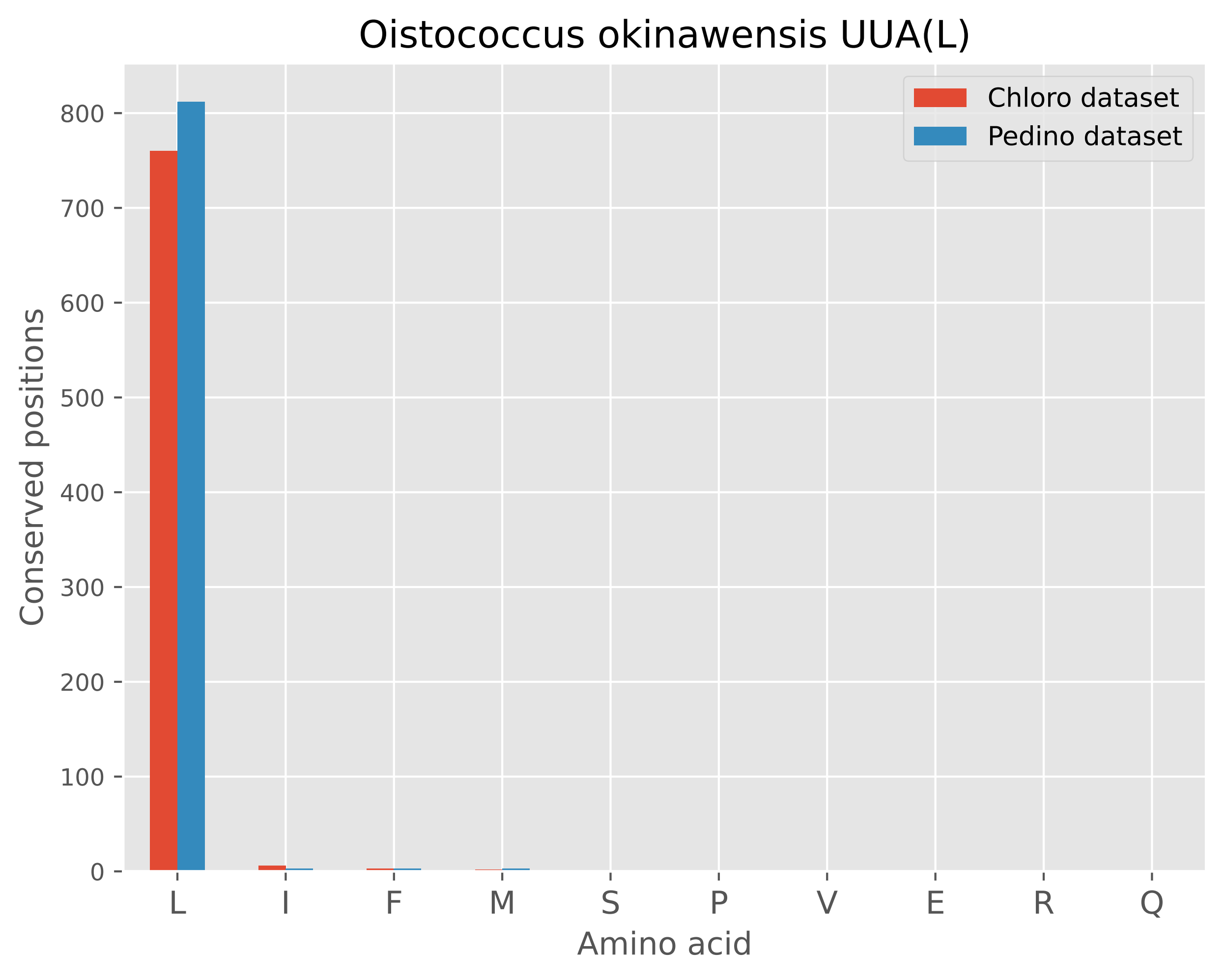

Oistococcus okinawensis UUA(L)
Chloro dataset
Pedino dataset
Conserved positions
0
100
200
300
400
500
600
700
800
L
I
F
M
S
P
V
E
R
Q
Amino acid

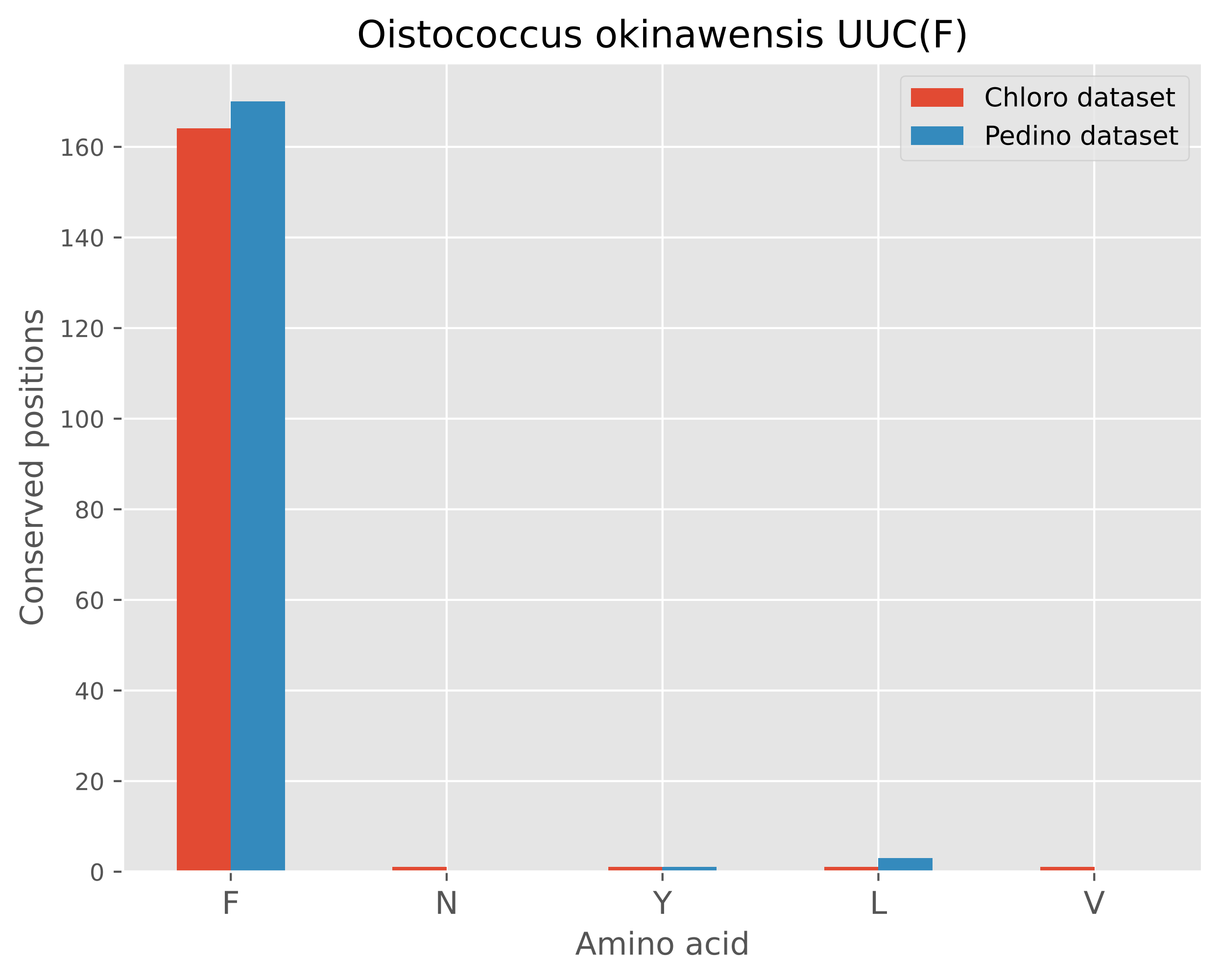
Oistococcus okinawensis UUC(F)
Chloro dataset
Pedino dataset
Conserved positions
0
20
40
60
80
100
120
140
160
F
N
Y
L
V
Amino acid

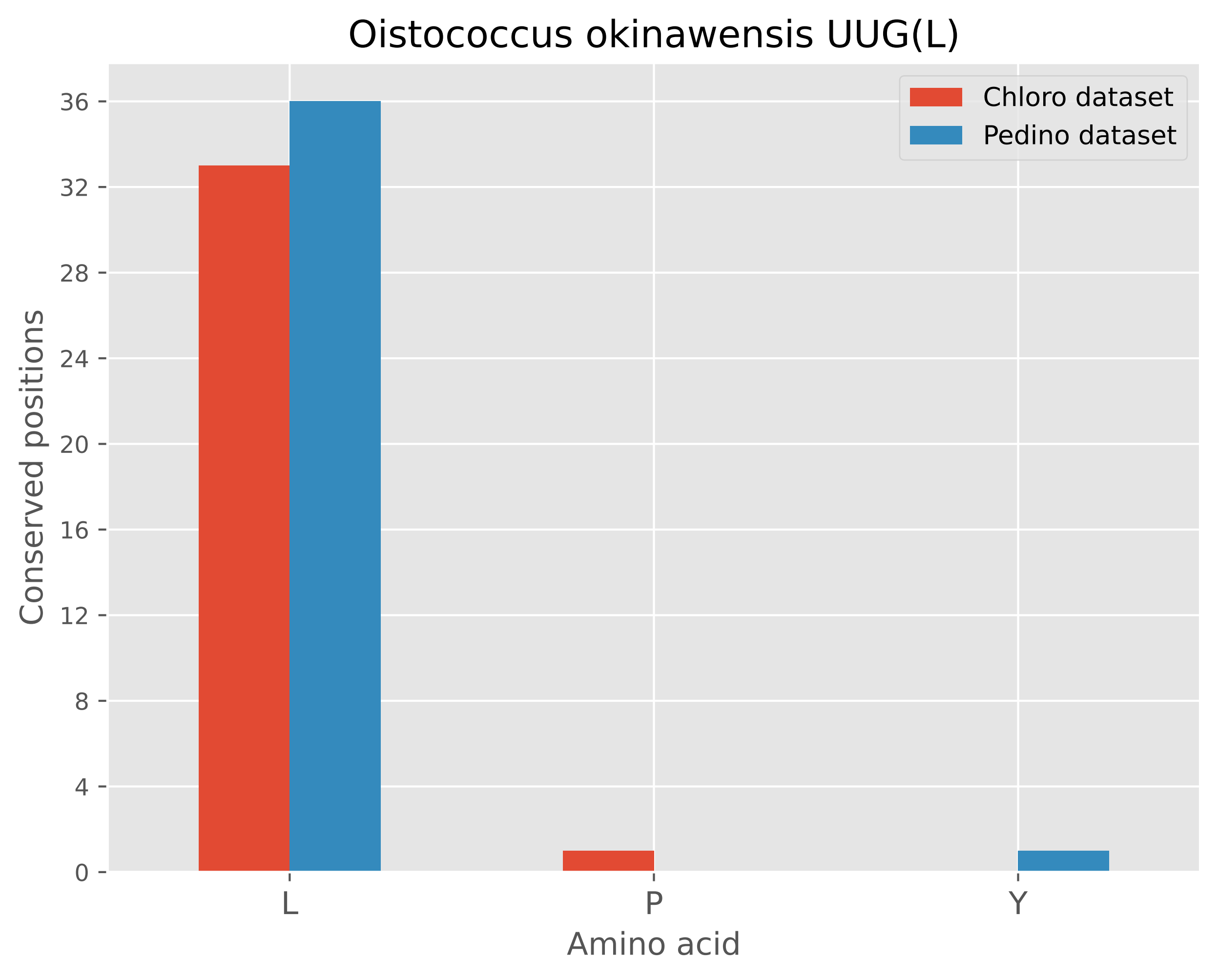
Oistococcus okinawensis UUG(L)
Chloro dataset
Pedino dataset
Conserved positions
0
4
8
12
16
20
24
28
32
36
L
P
Y
Amino acid

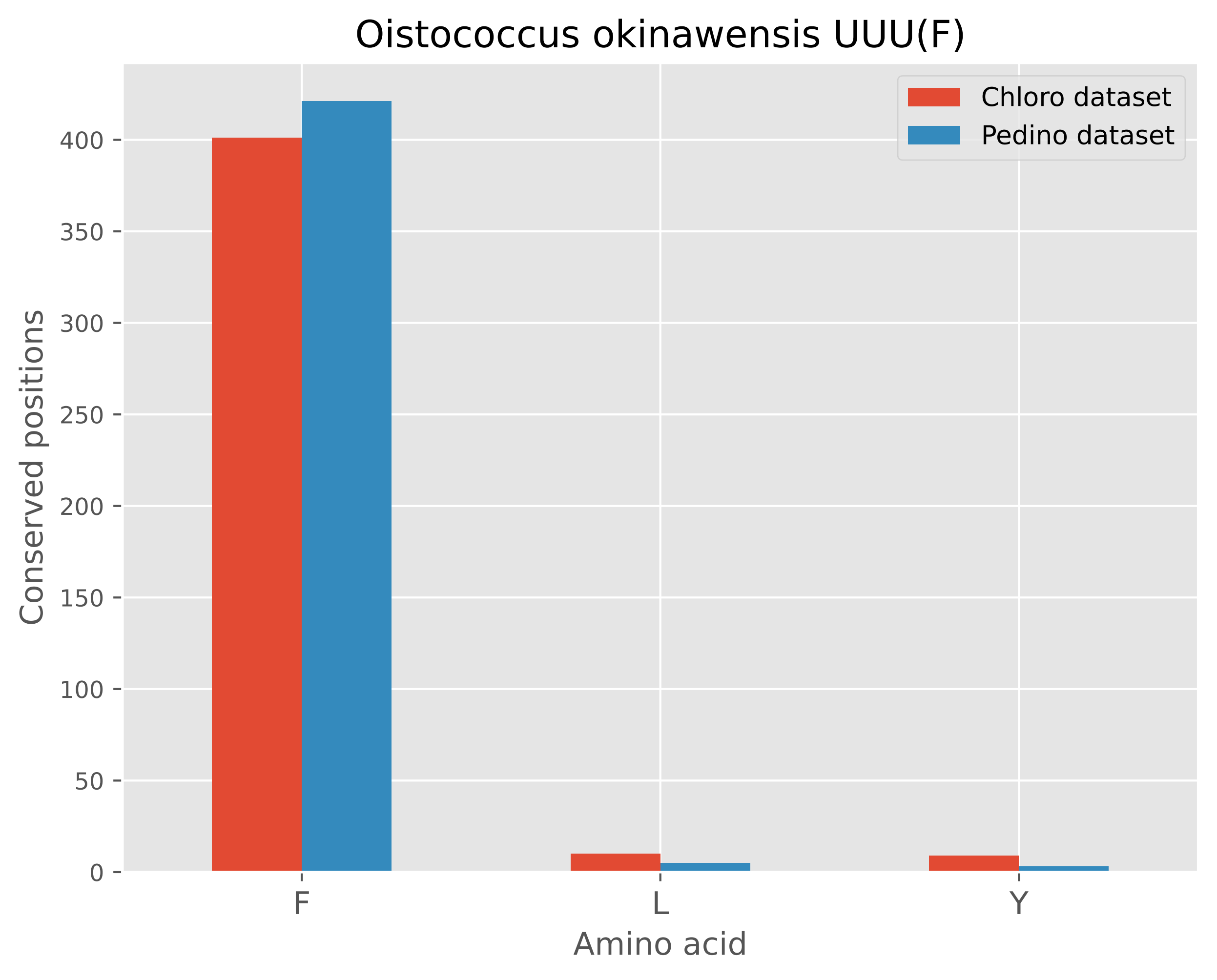

Oistococcus okinawensis UUU(F)
Chloro dataset
Pedino dataset
Conserved positions
0
50
100
150
200
250
300
350
400
F
L
Y
Amino acid

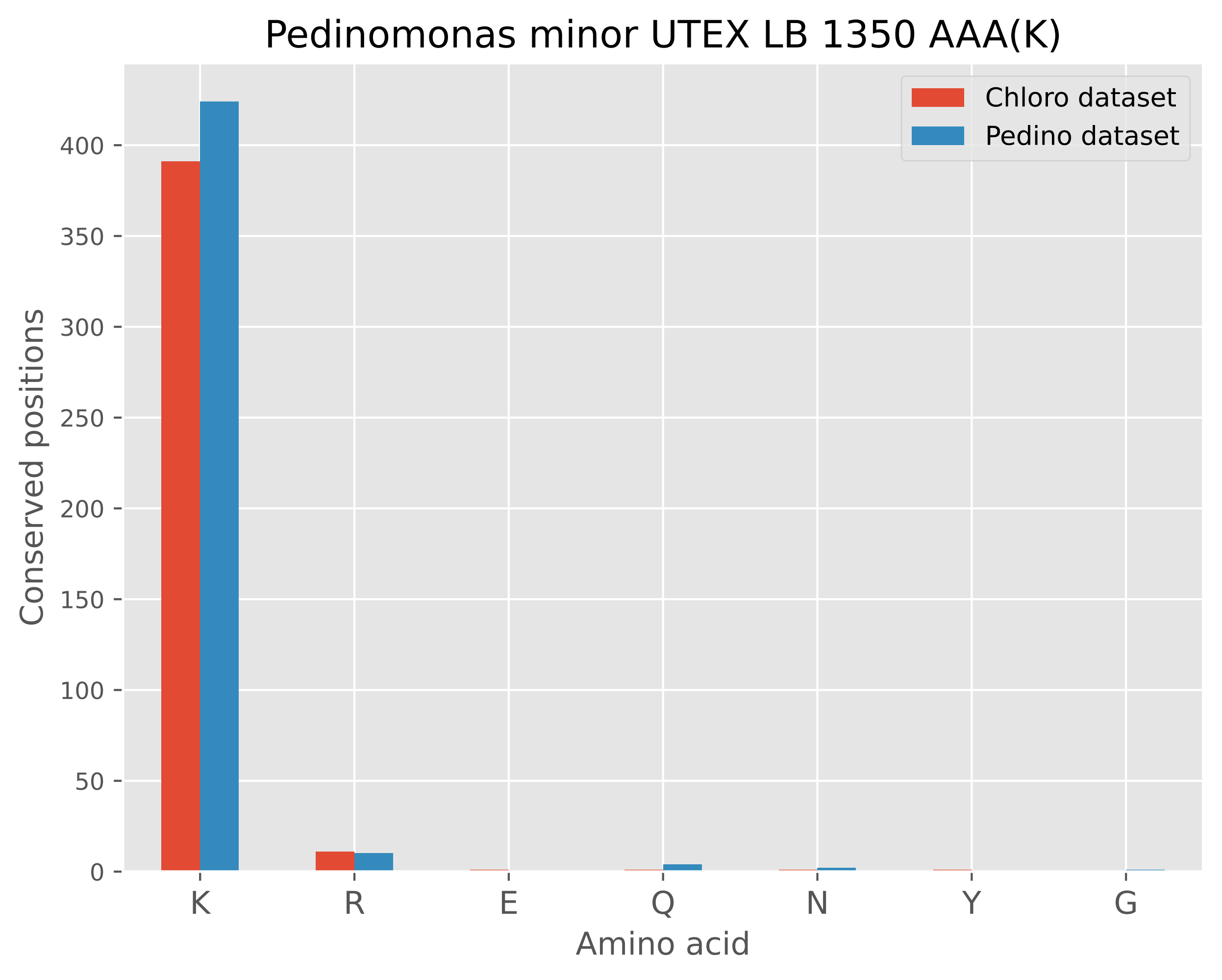
Pedinomonas minor UTEX LB 1350 AAA(K)
Chloro dataset
Pedino dataset
Conserved positions
0
50
100
150
200
250
300
350
400
K
R
E
Q
N
Y
G
Amino acid

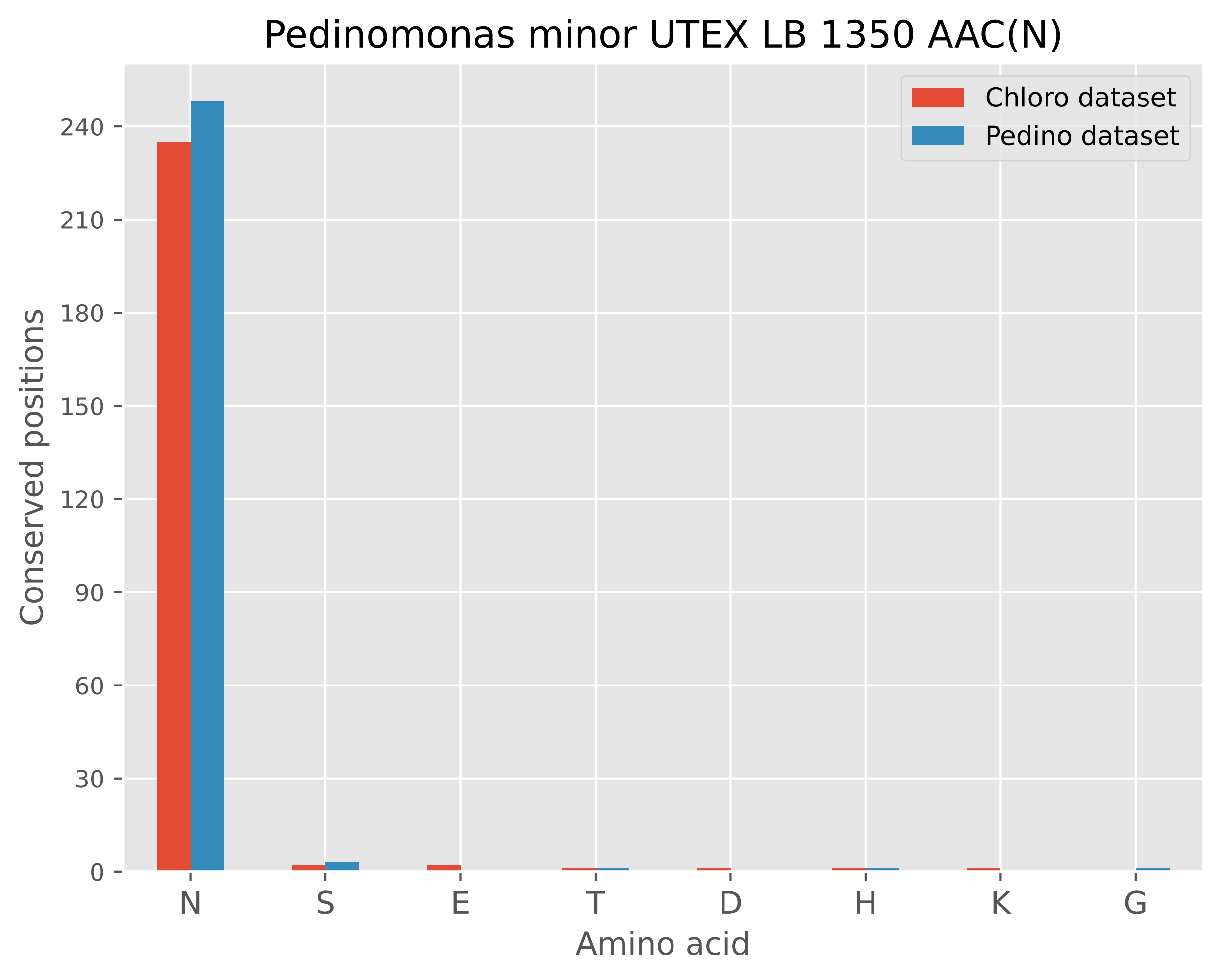

Pedinomonas minor UTEX LB 1350 AAC(N)
Chloro dataset
Pedino dataset
Conserved positions
0
30
60
90
120
150
180
210
240
N
S
E
T
D
H
K
G
Amino acid

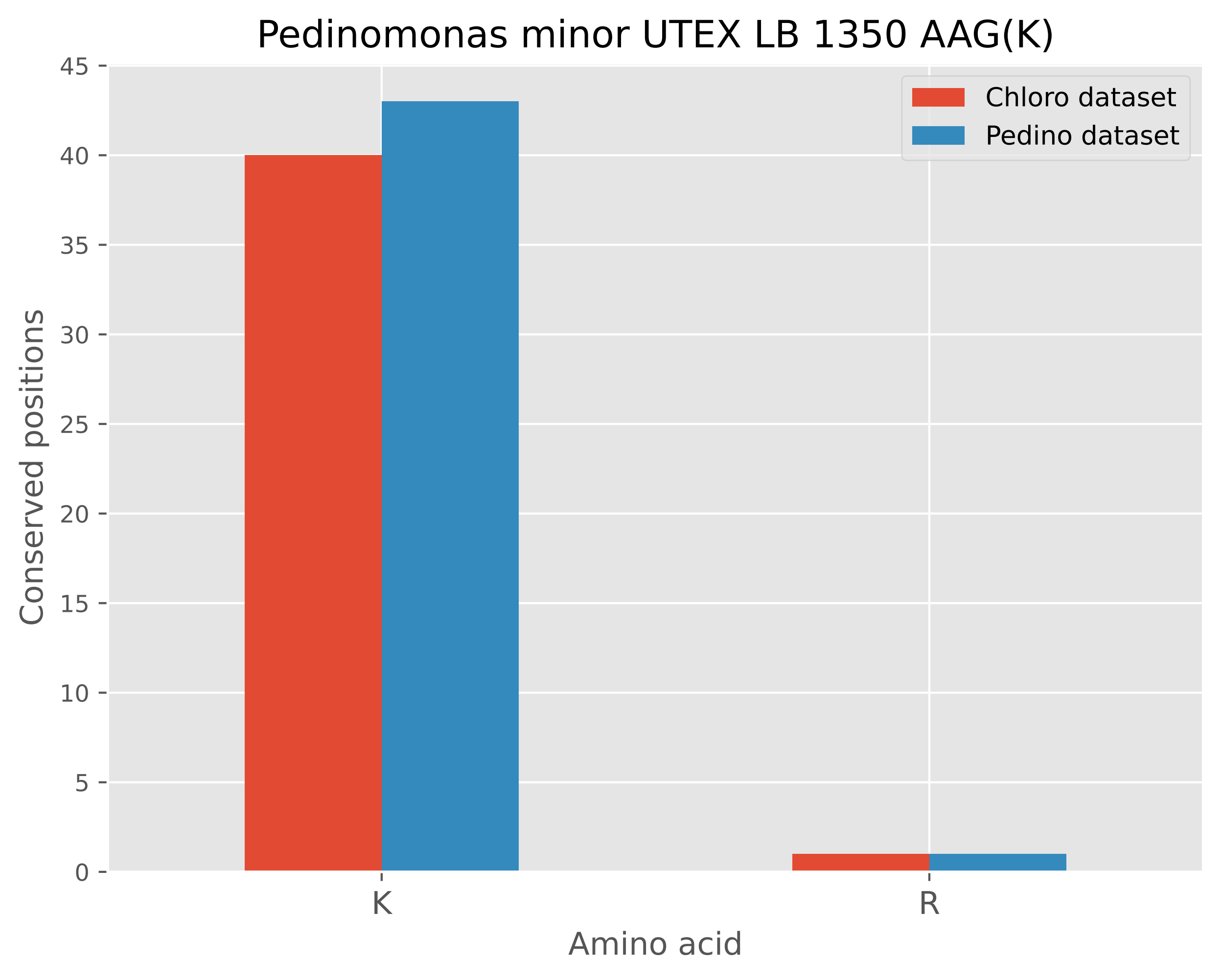

Pedinomonas minor UTEX LB 1350 AAG(K)
Chloro dataset
Pedino dataset
Conserved positions
0
5
10
15
20
25
30
35
40
45
K
R
Amino acid

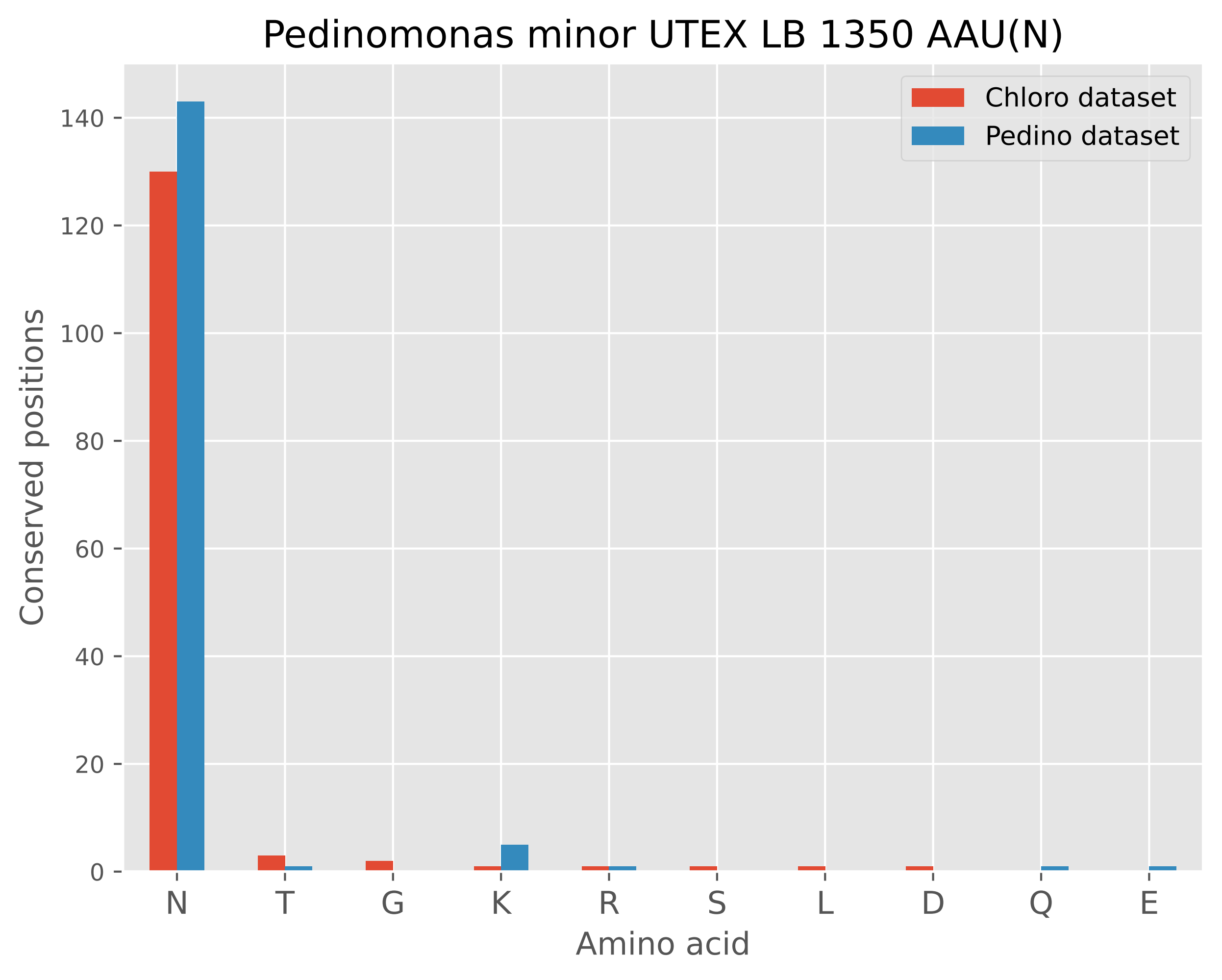

Pedinomonas minor UTEX LB 1350 AAU(N)
Chloro dataset
Pedino dataset
Conserved positions
0
20
40
60
80
100
120
140
N
T
G
K
R
S
L
D
Q
E
Amino acid

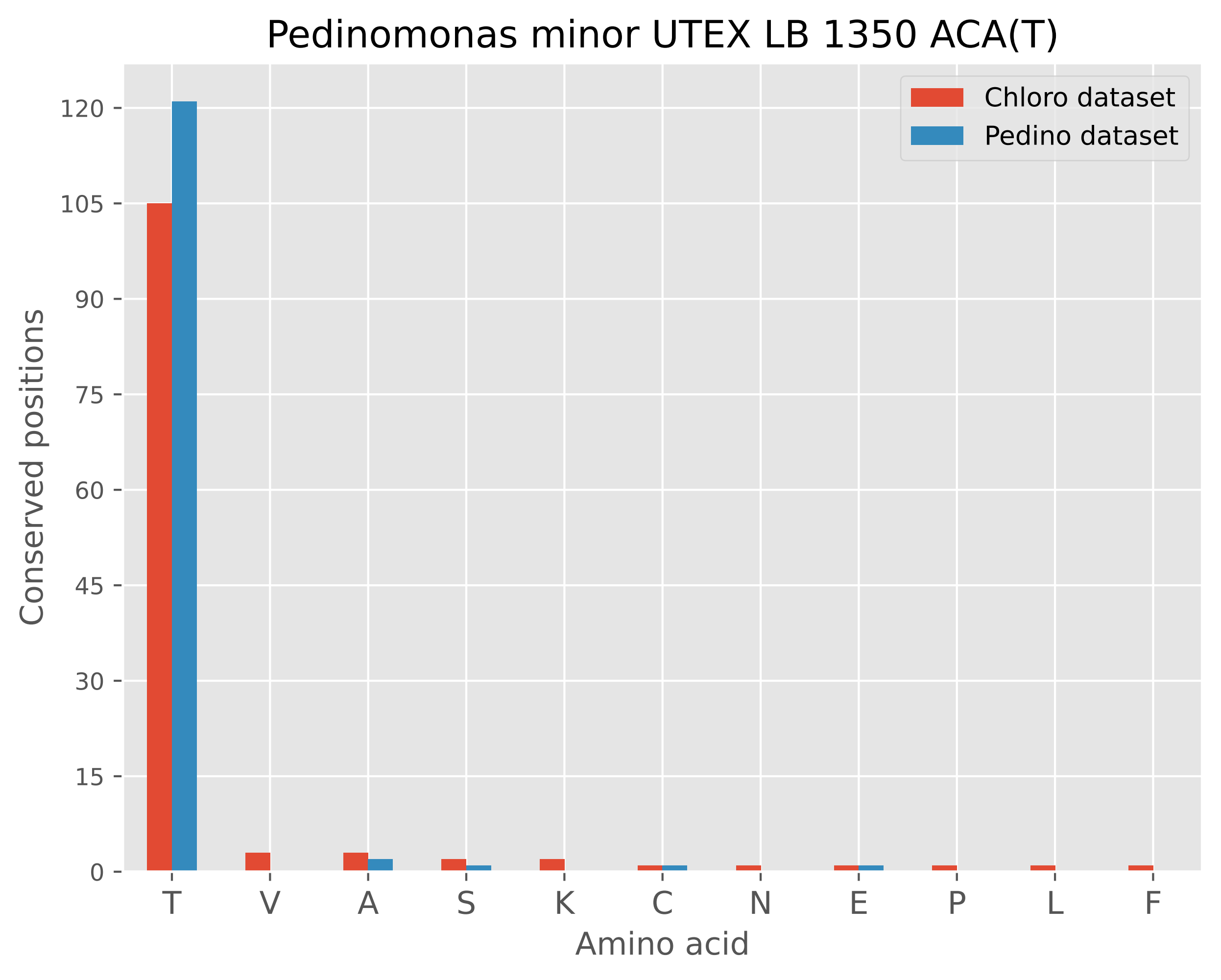
Pedinomonas minor UTEX LB 1350 ACA(T)
Chloro dataset
Pedino dataset
Conserved positions
0
15
30
45
60
75
90
105
120
T
V
A
S
K
C
N
E
P
L
F
Amino acid

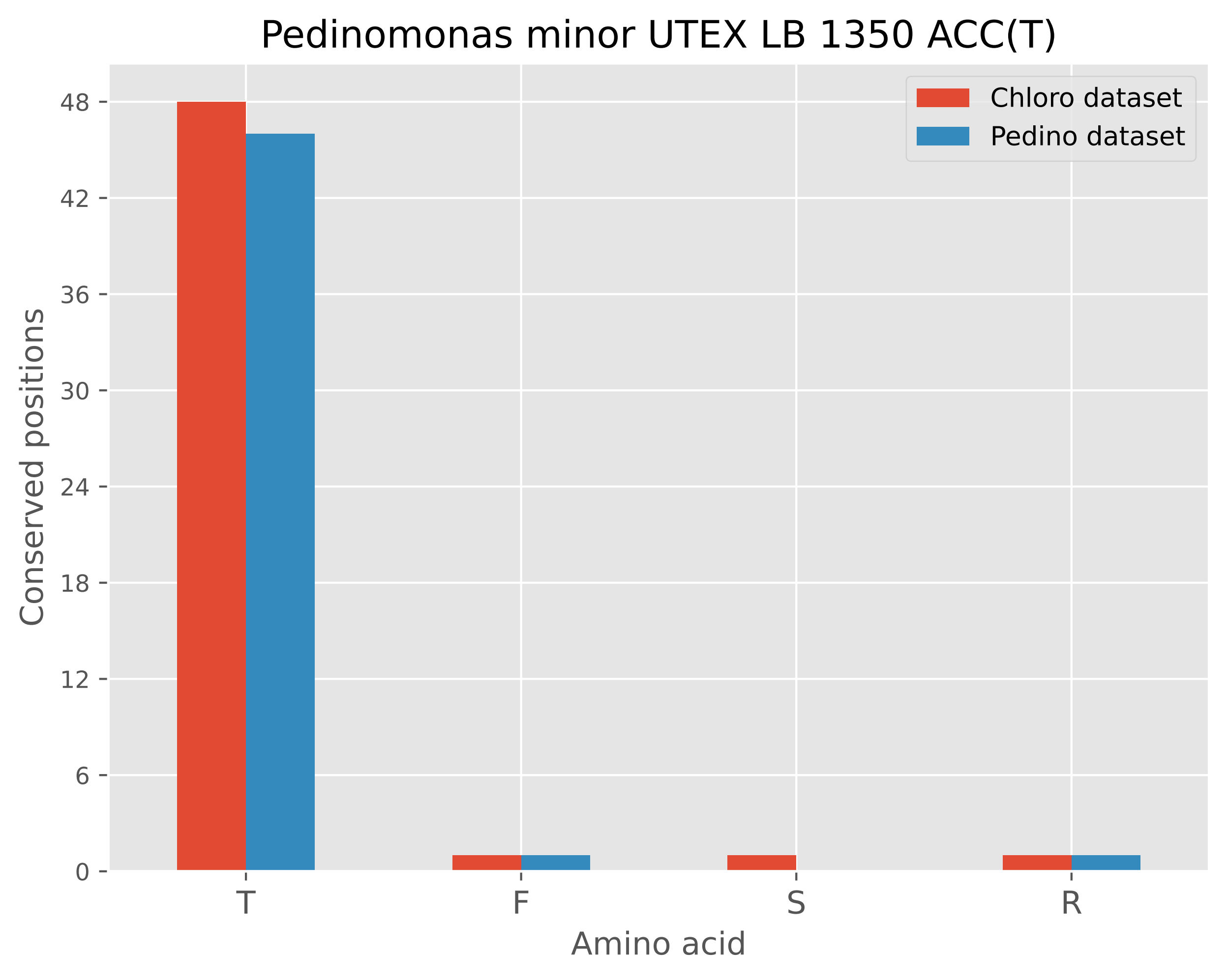

Pedinomonas minor UTEX LB 1350 ACC(T)
Chloro dataset
Pedino dataset
Conserved positions
48
42
36
30
24
18
12
6
0
T
F
S
R
Amino acid

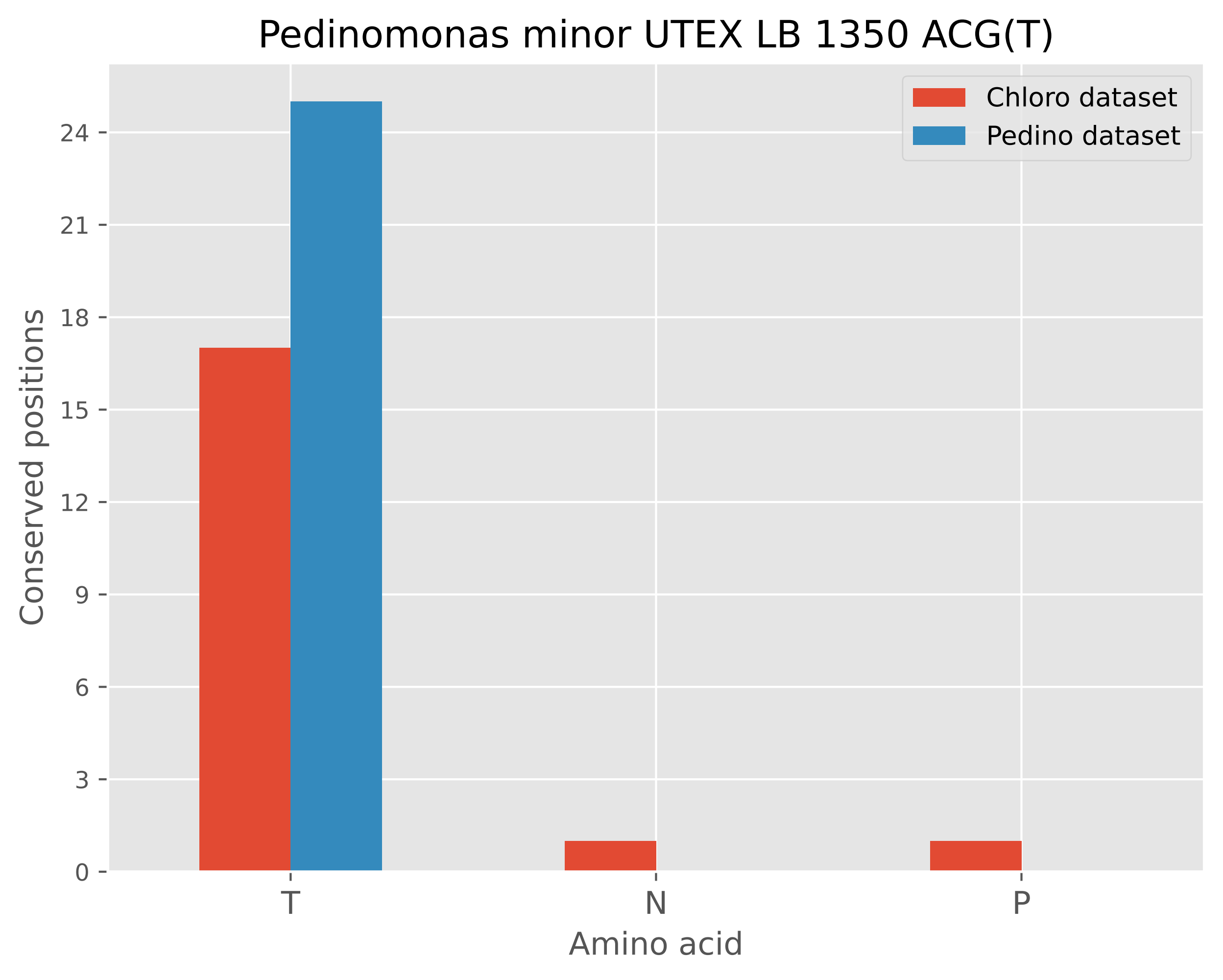
Pedinomonas minor UTEX LB 1350 ACG(T)
Chloro dataset
Pedino dataset
Conserved positions
0
3
6
9
12
15
18
21
24
T
N
P
Amino acid

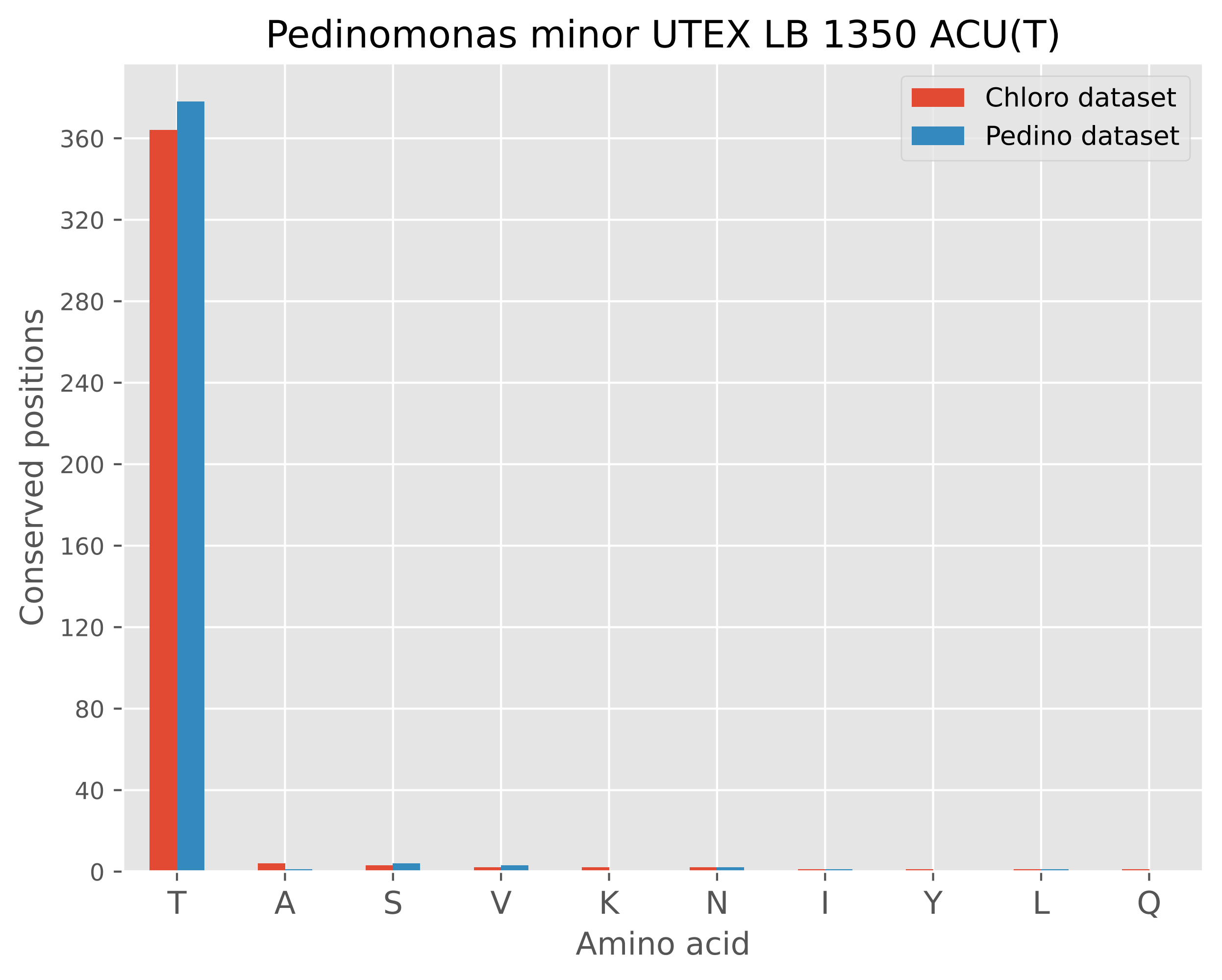

Pedinomonas minor UTEX LB 1350 ACU(T)
Chloro dataset
Pedino dataset
Conserved positions
0
40
80
120
160
200
240
280
320
360
T
A
S
V
K
N
I
Y
L
Q
Amino acid

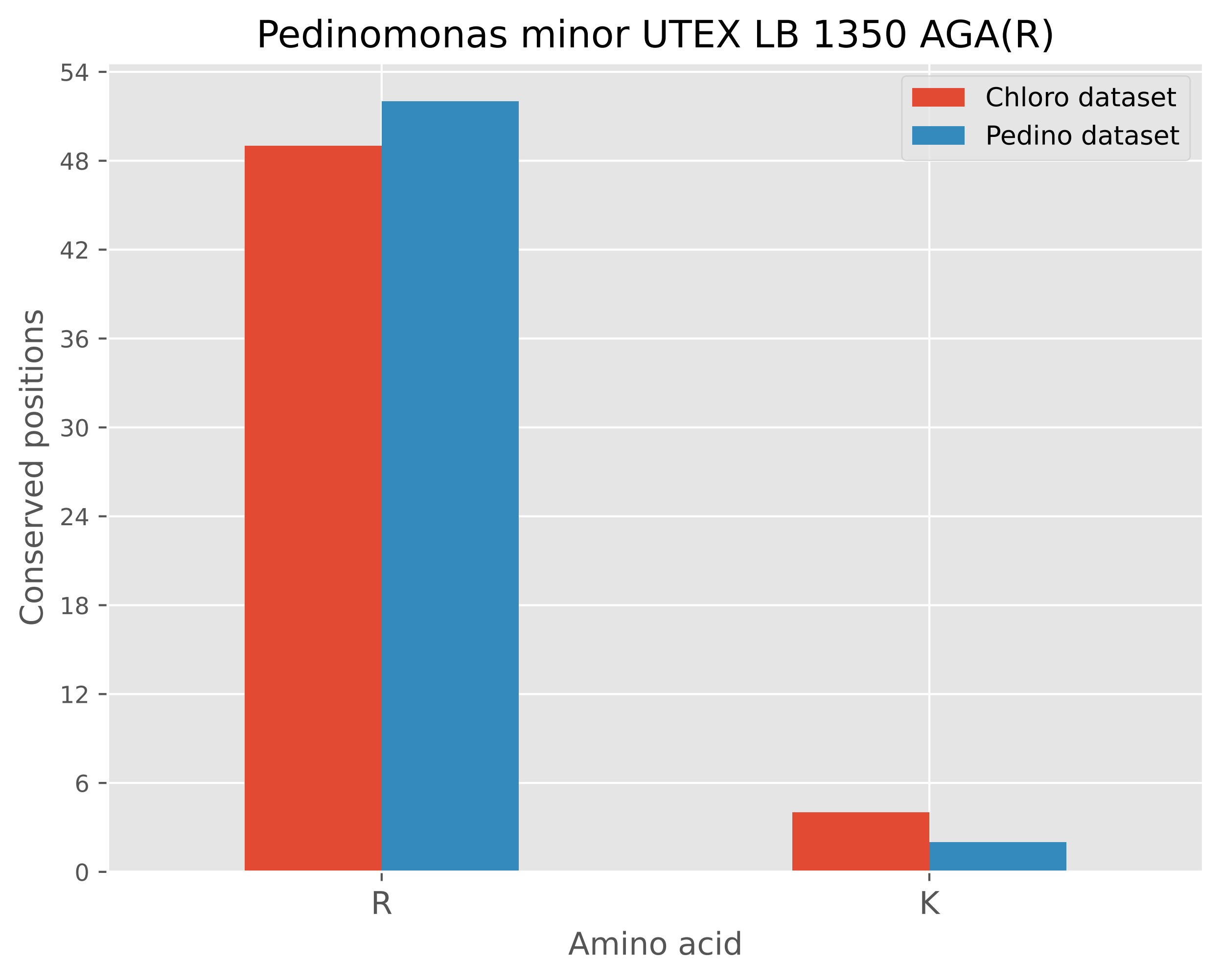

Pedinomonas minor UTEX LB 1350 AGA(R)
Chloro dataset
Pedino dataset
Conserved positions
0
6
12
18
24
30
36
42
48
54
R
K
Amino acid

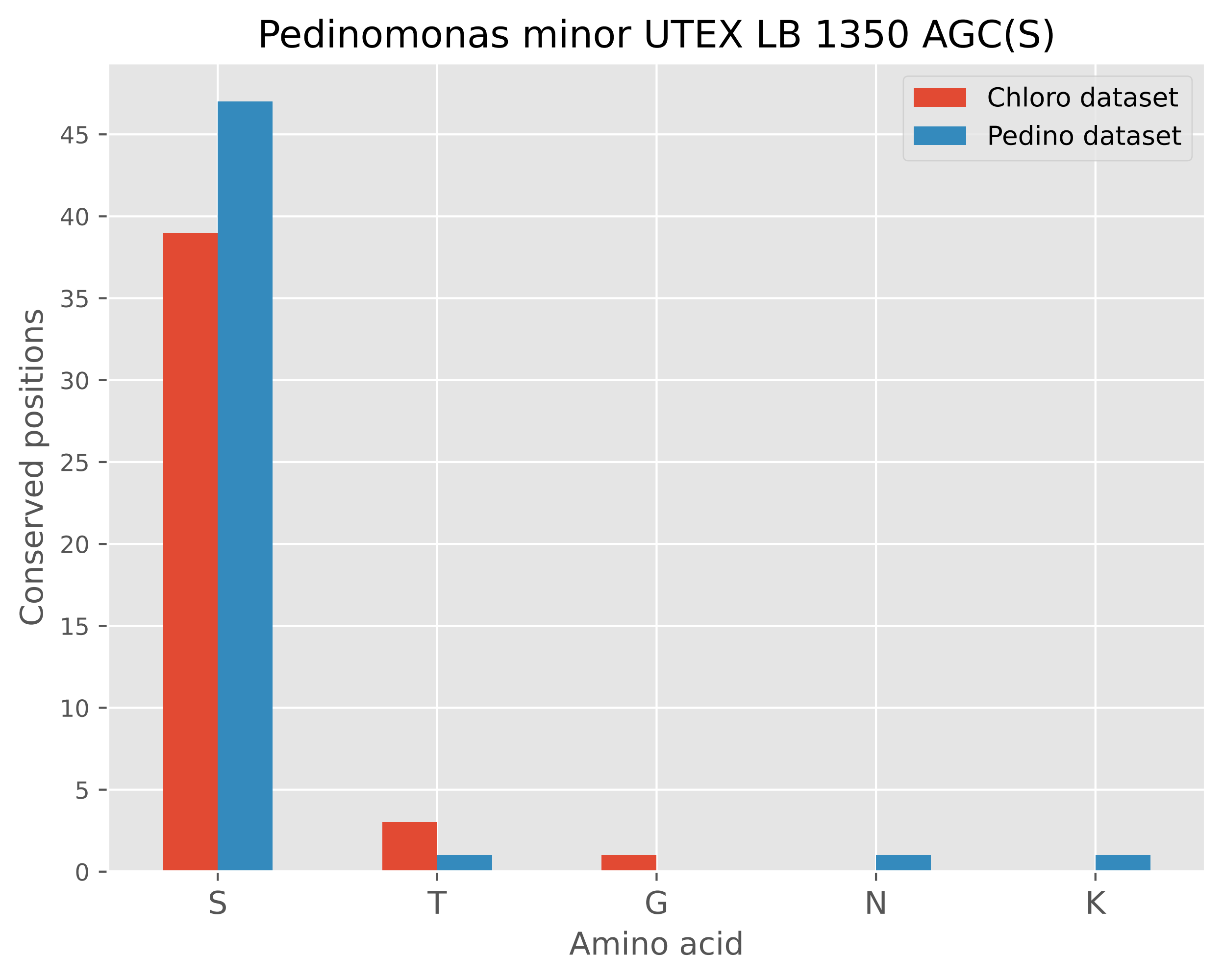

Pedinomonas minor UTEX LB 1350 AGC(S)
Chloro dataset
Pedino dataset
Conserved positions
0
5
10
15
20
25
30
35
40
45
S
T
G
N
K
Amino acid

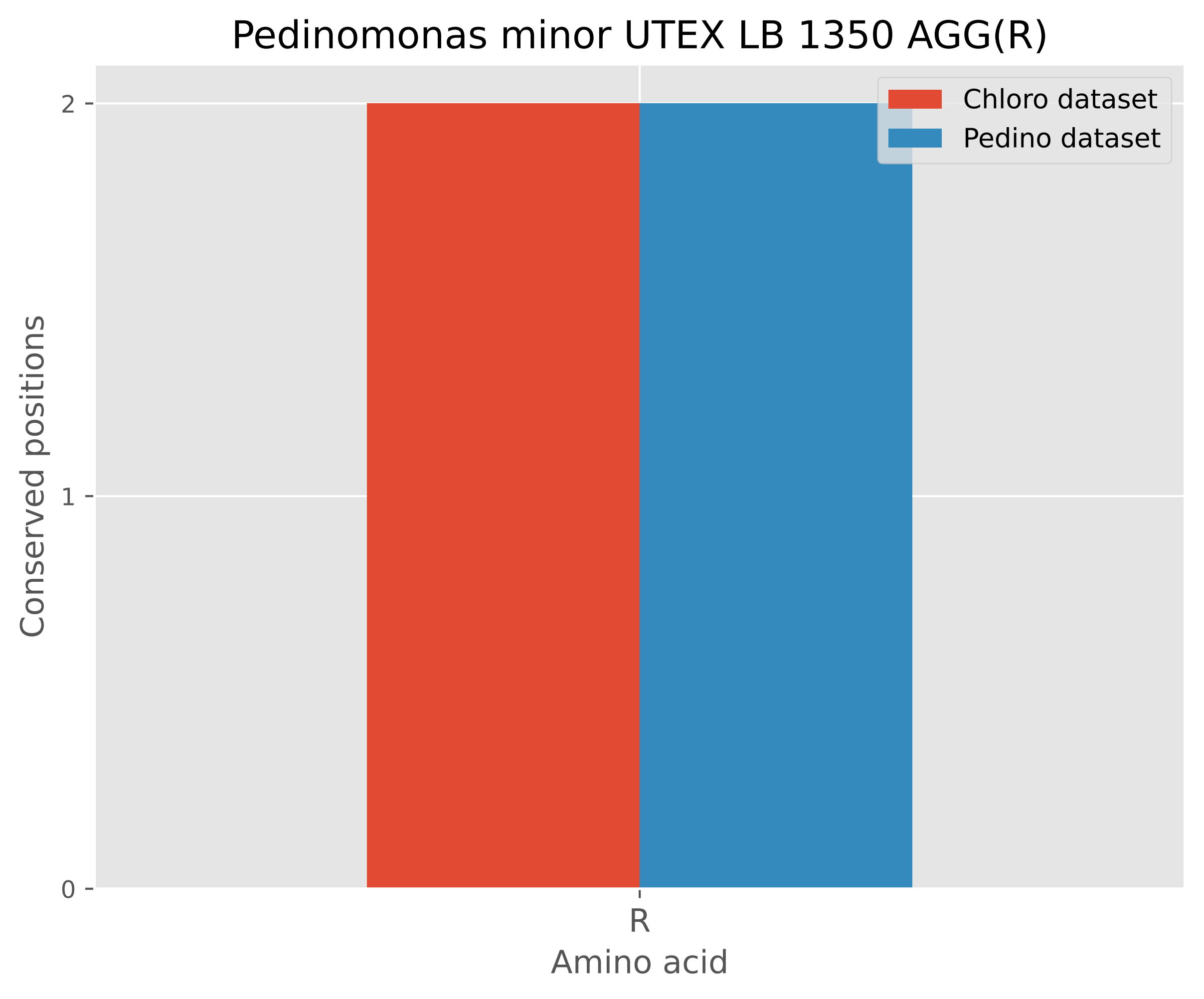
Pedinomonas minor UTEX LB 1350 AGG(R)
Chloro dataset
Pedino dataset
Conserved positions
0
1
2
R
Amino acid

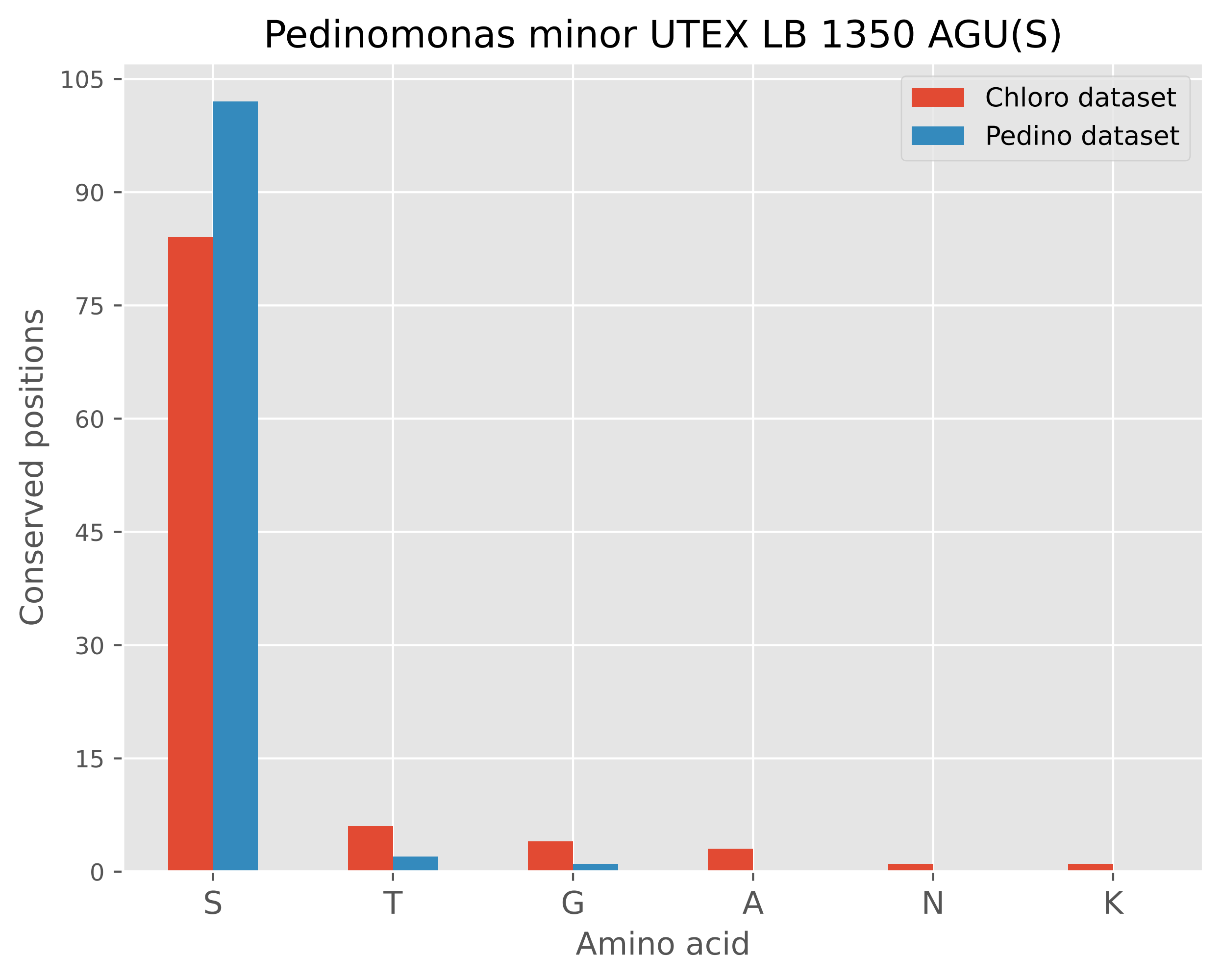
Pedinomonas minor UTEX LB 1350 AGU(S)
Chloro dataset
Pedino dataset
Conserved positions
105
90
75
60
45
30
15
0
S
T
G
A
N
K
Amino acid

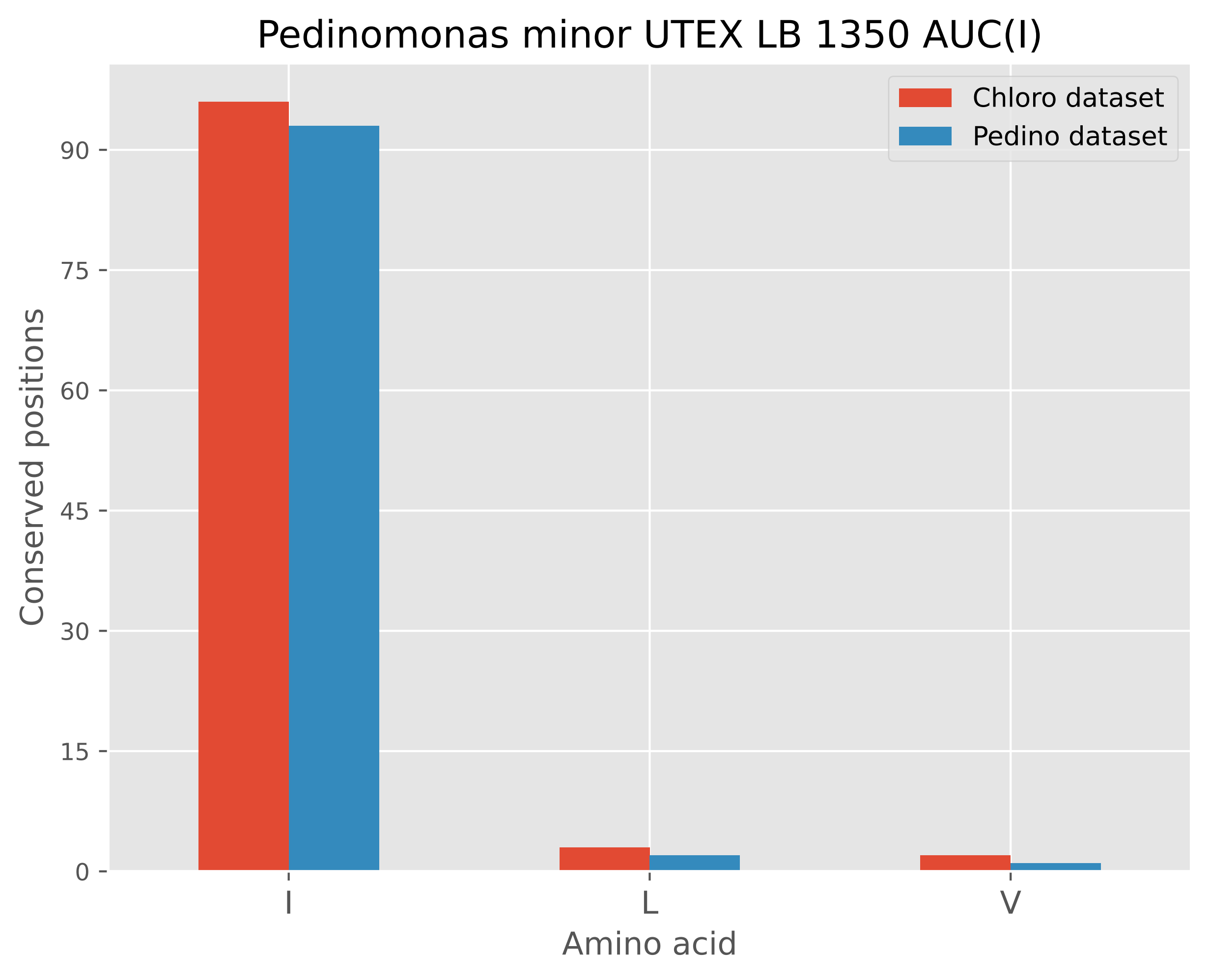

Pedinomonas minor UTEX LB 1350 AUC(I)
Chloro dataset
Pedino dataset
Conserved positions
0
15
30
45
60
75
90
I
L
V
Amino acid

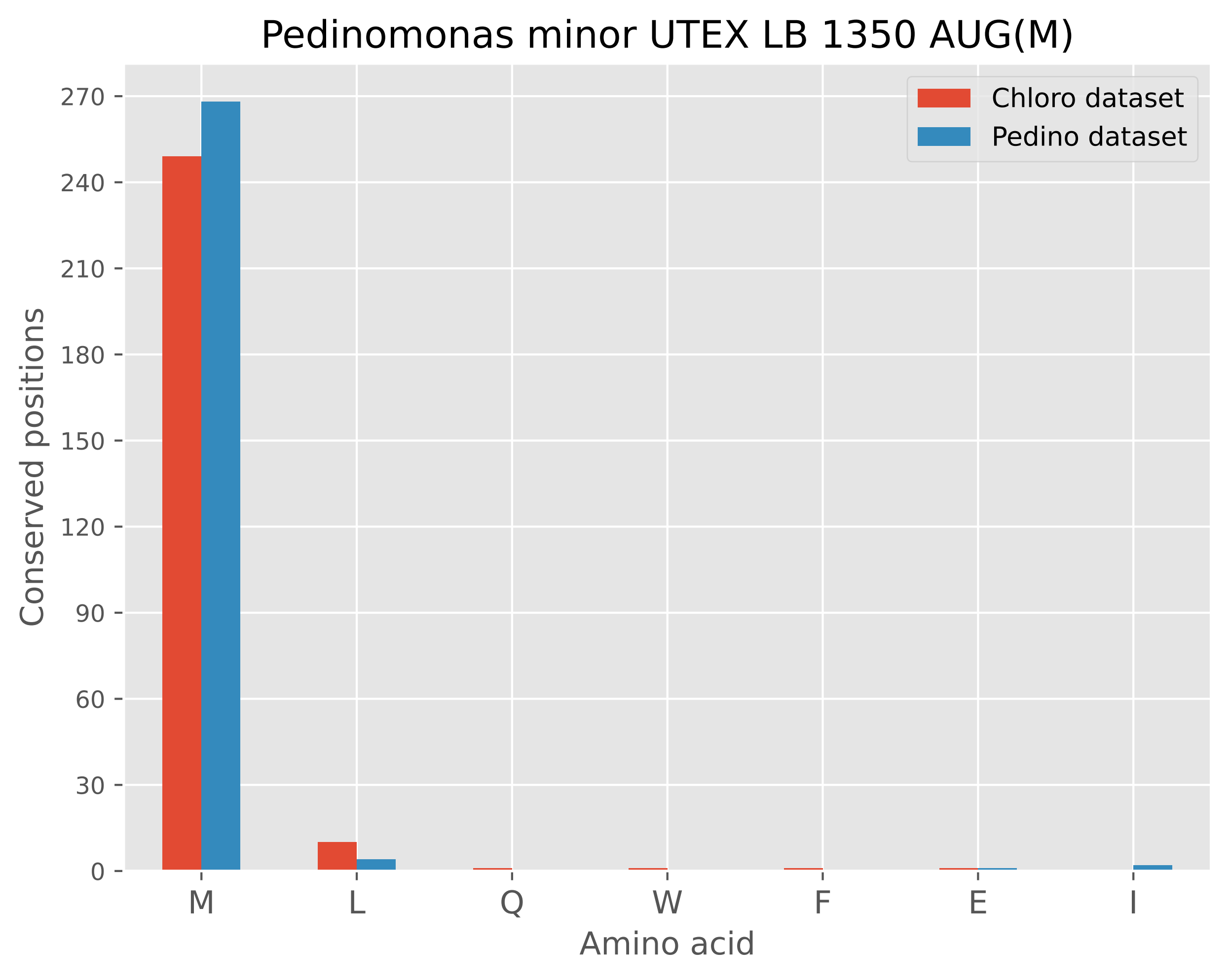
Pedinomonas minor UTEX LB 1350 AUG(M)
Chloro dataset
Pedino dataset
Conserved positions
0
30
60
90
120
150
180
210
240
270
M
L
Q
W
F
E
I
Amino acid

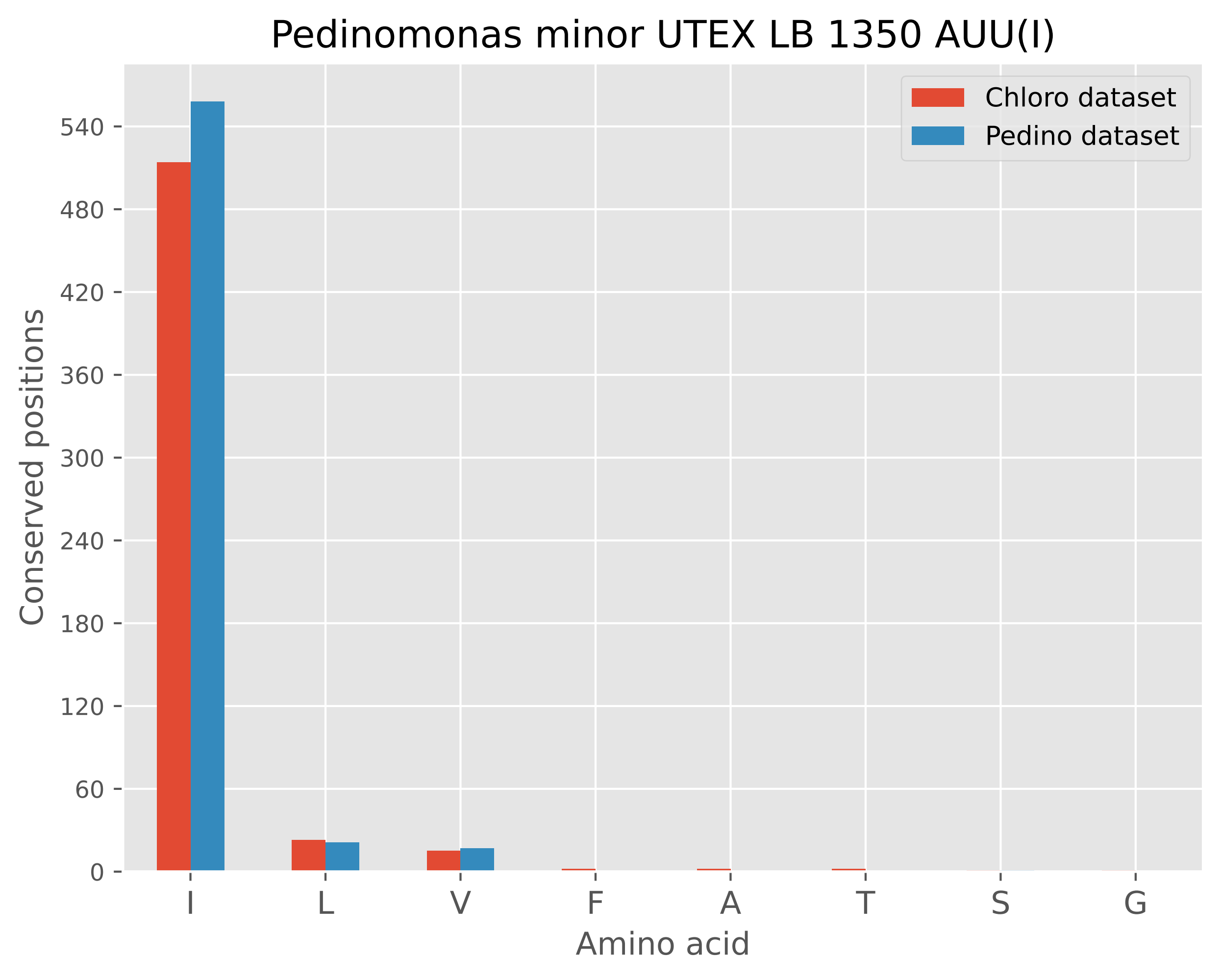

Pedinomonas minor UTEX LB 1350 AUU(I)
Chloro dataset
Pedino dataset
Conserved positions
540
480
420
360
300
240
180
120
60
0
I
L
V
F
A
T
S
G
Amino acid

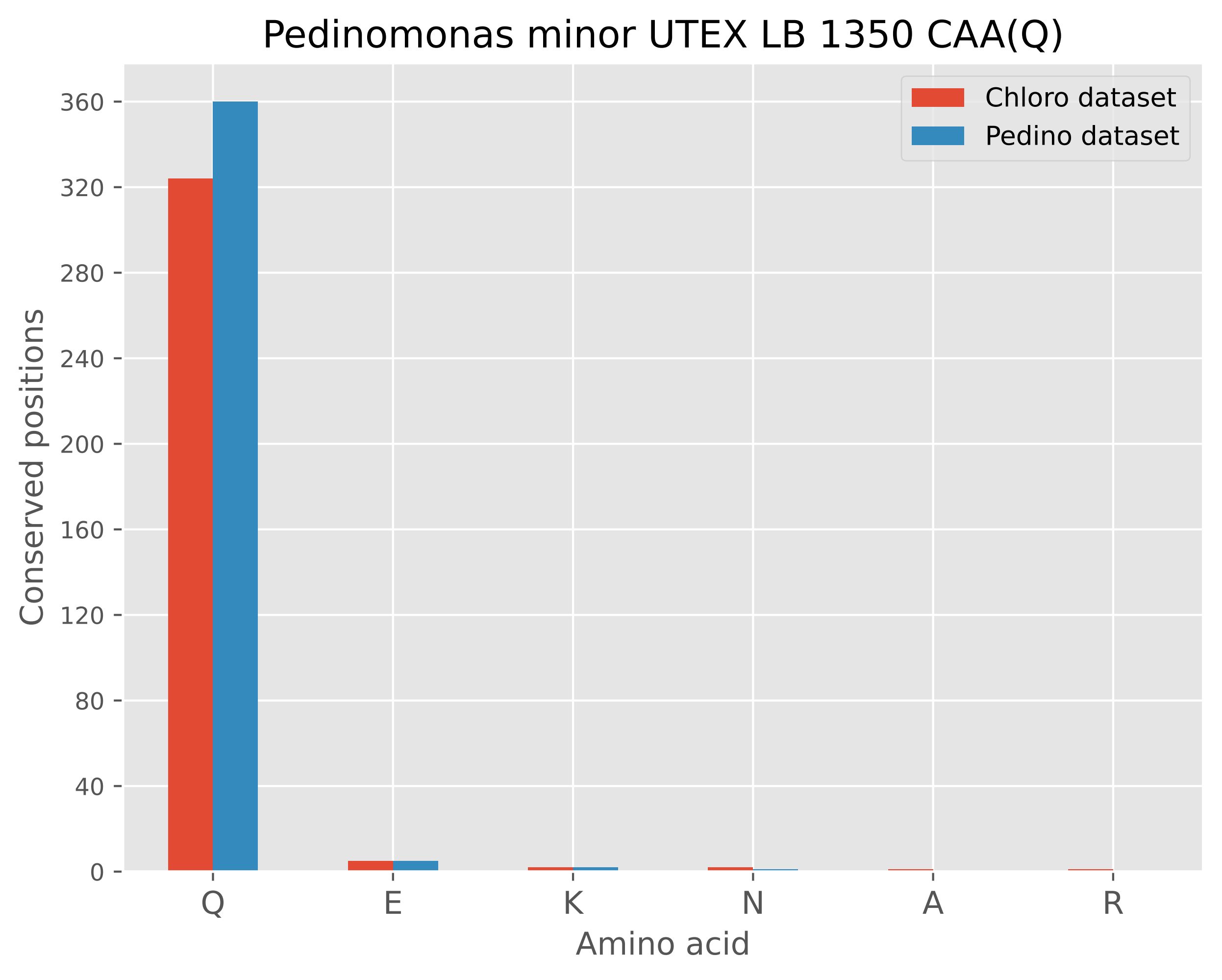

Pedinomonas minor UTEX LB 1350 CAA(Q)
Chloro dataset
Pedino dataset
Conserved positions
0
40
80
120
160
200
240
280
320
360
Q
E
K
N
A
R
Amino acid

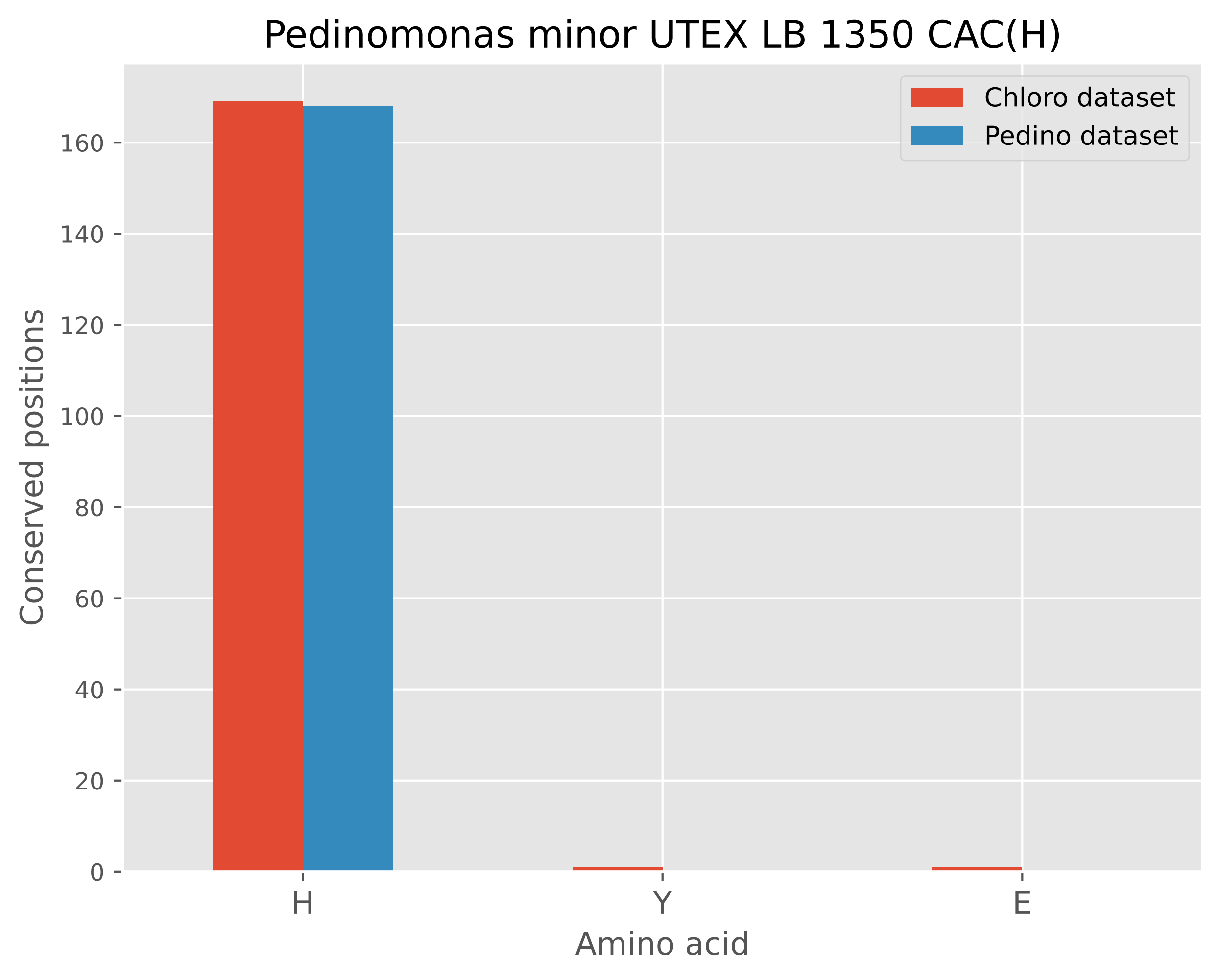

Pedinomonas minor UTEX LB 1350 CAC(H)
Chloro dataset
Pedino dataset
Conserved positions
0
20
40
60
80
100
120
140
160
H
Y
E
Amino acid

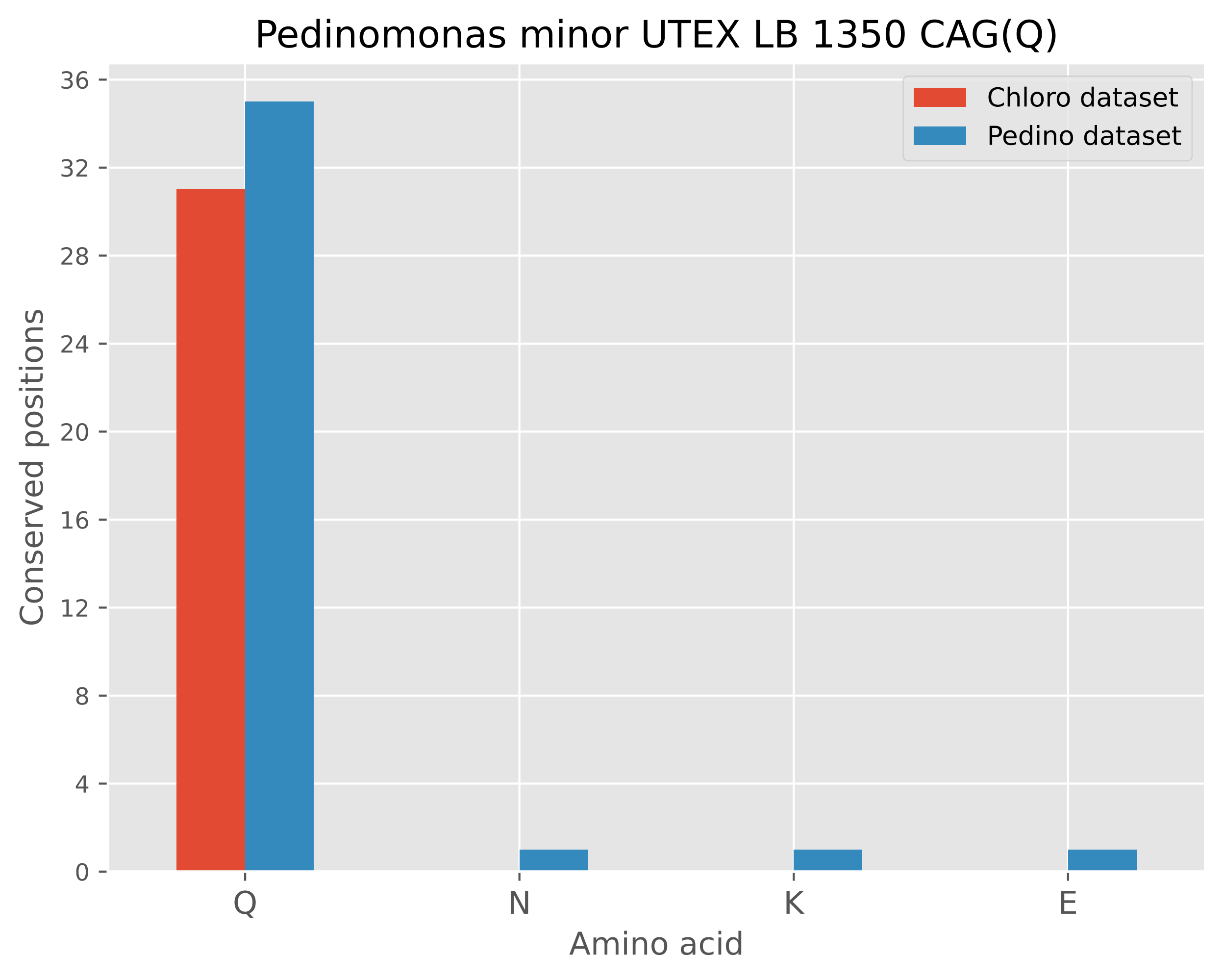

Pedinomonas minor UTEX LB 1350 CAG(Q)
Chloro dataset
Pedino dataset
Conserved positions
Amino acid
0
4
8
12
16
20
24
28
32
36
Q
N
K
E

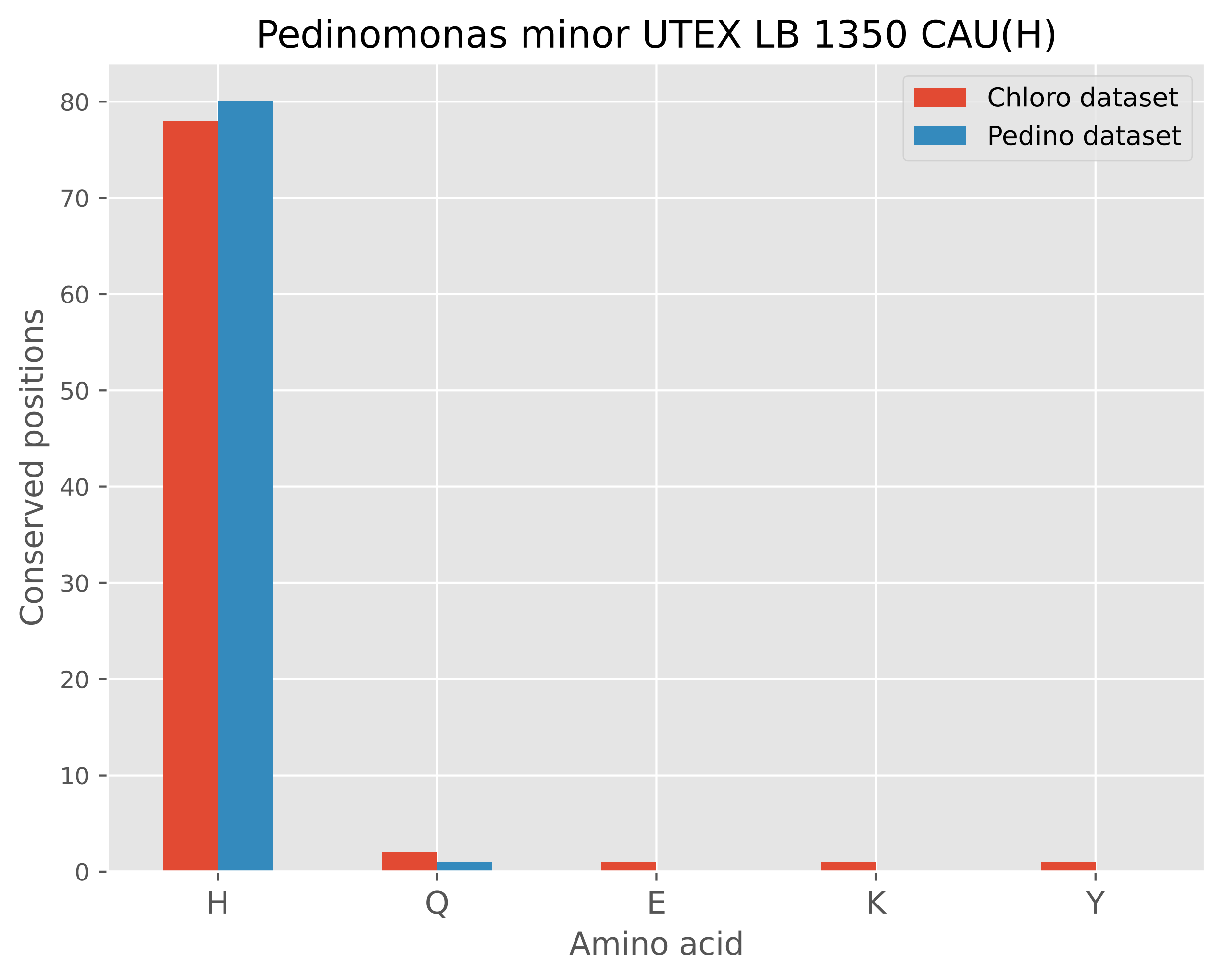
Pedinomonas minor UTEX LB 1350 CAU(H)
Chloro dataset
Pedino dataset
Conserved positions
0
10
20
30
40
50
60
70
80
H
Q
E
K
Y
Amino acid

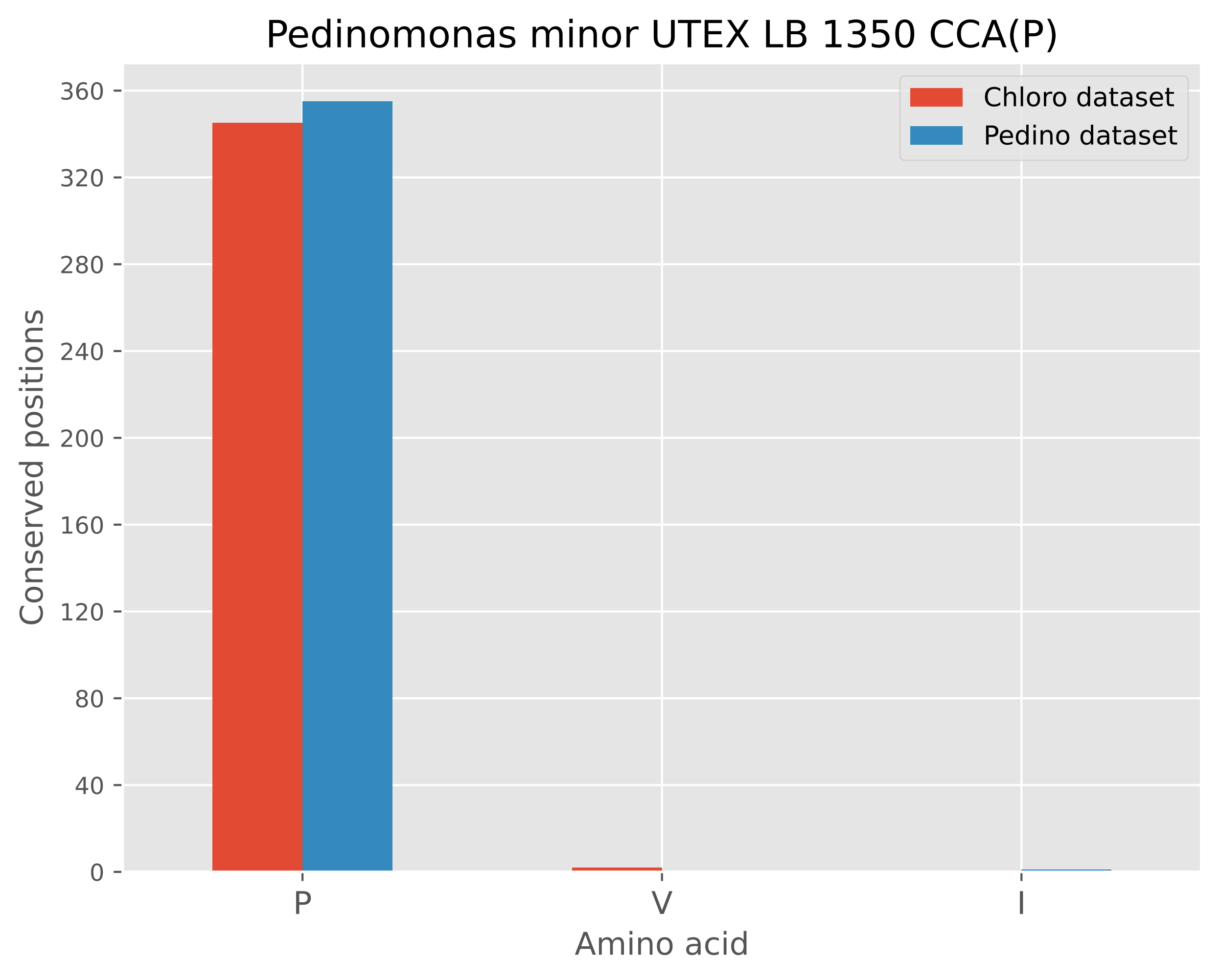

Pedinomonas minor UTEX LB 1350 CCA(P)
Chloro dataset
Pedino dataset
Conserved positions
0
40
80
120
160
200
240
280
320
360
P
V
I
Amino acid

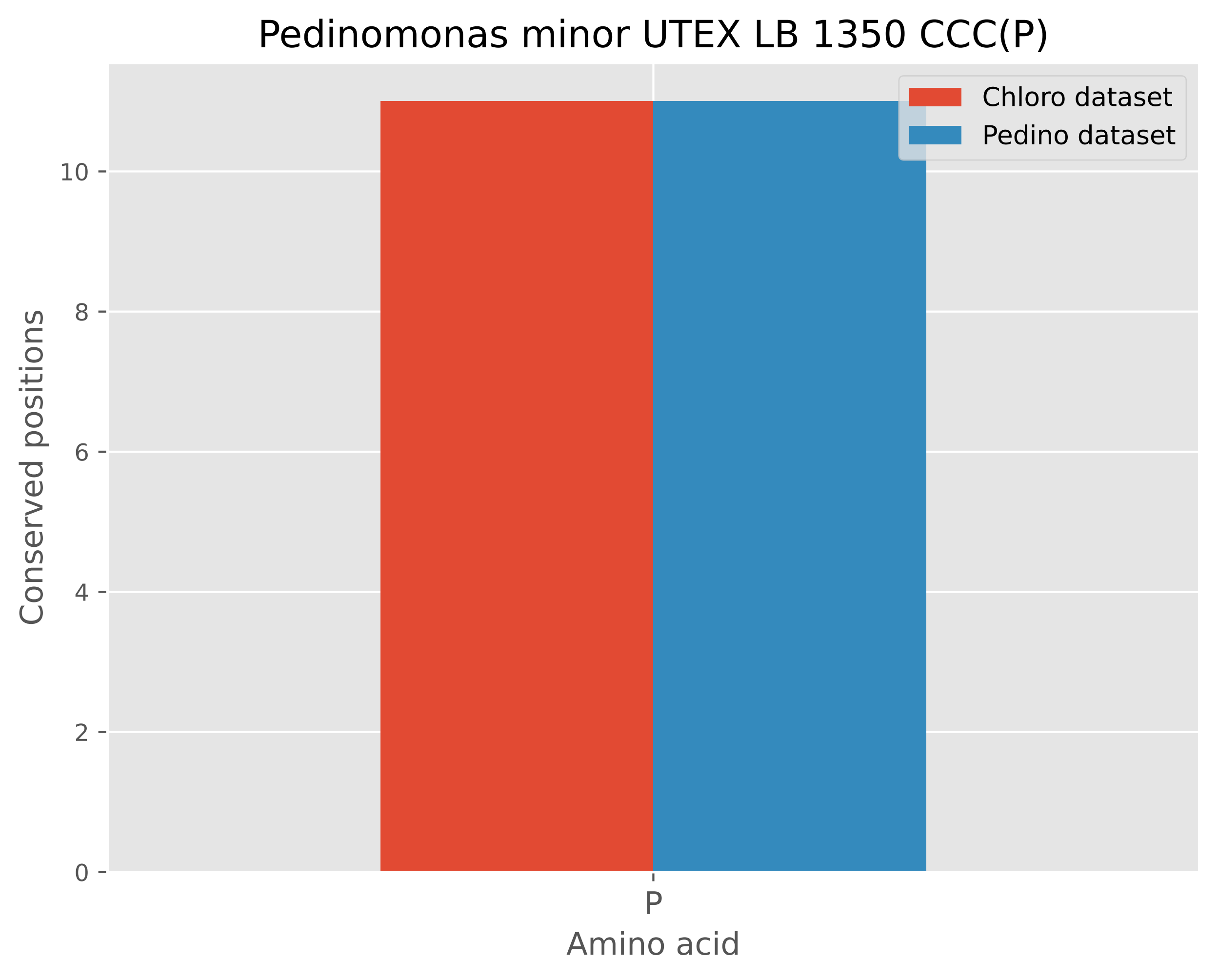

Pedinomonas minor UTEX LB 1350 CCC(P)
Chloro dataset
Pedino dataset
Conserved positions
0
2
4
6
8
10
P
Amino acid

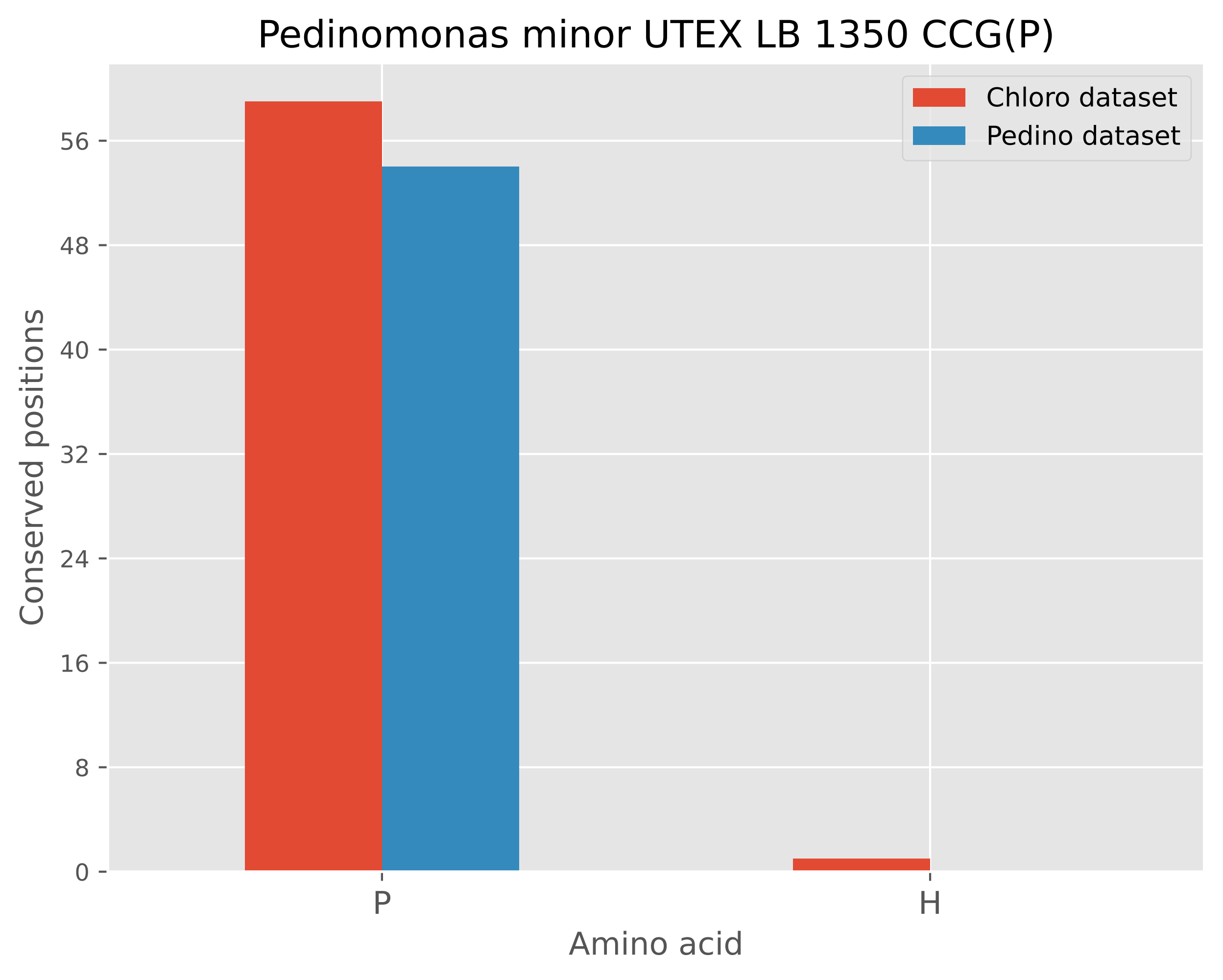

Pedinomonas minor UTEX LB 1350 CCG(P)
Chloro dataset
Pedino dataset
Conserved positions
0
8
16
24
32
40
48
56
P
H
Amino acid

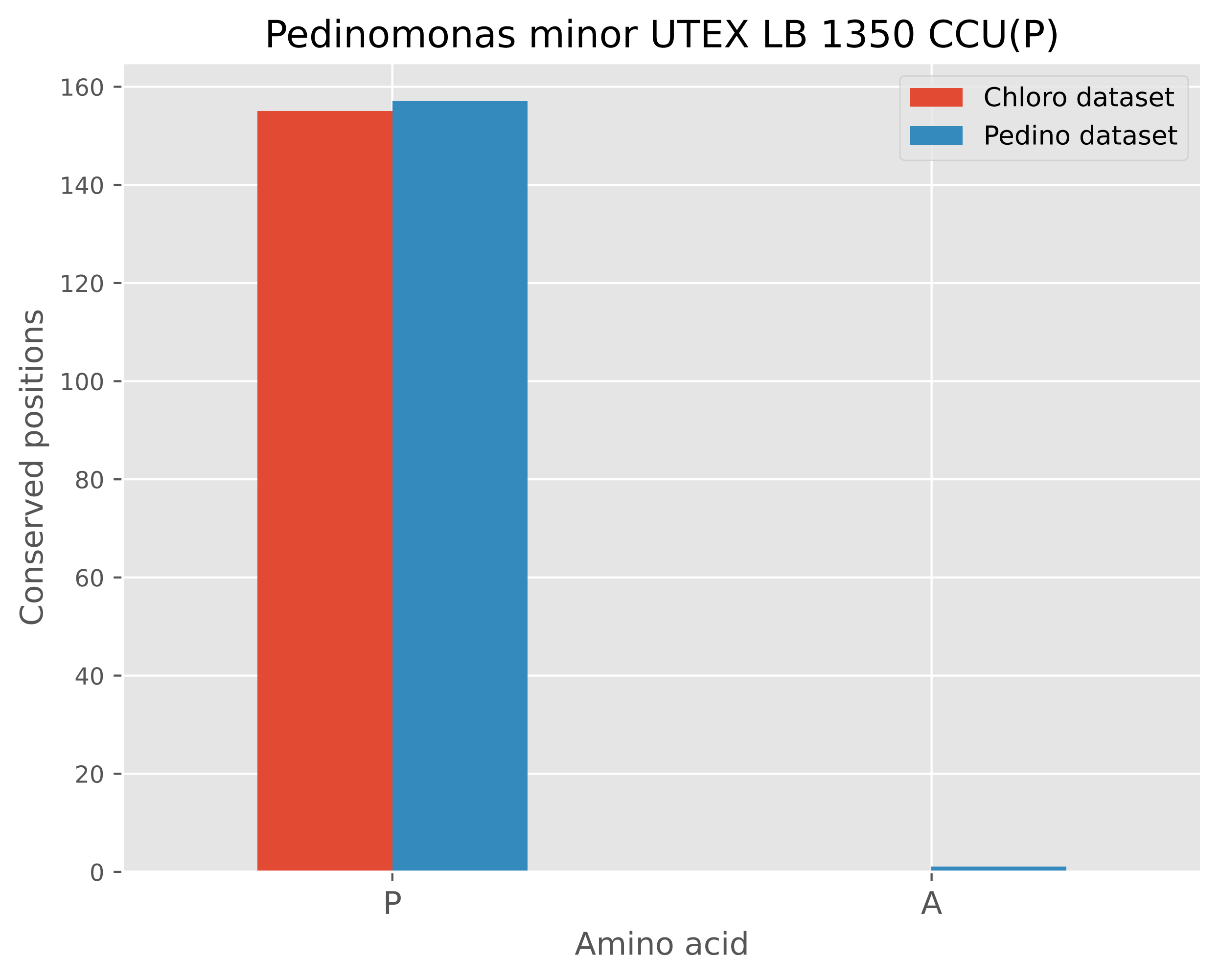
Pedinomonas minor UTEX LB 1350 CCU(P)
Chloro dataset
Pedino dataset
Conserved positions
160
140
120
100
80
60
40
20
0
P
A
Amino acid

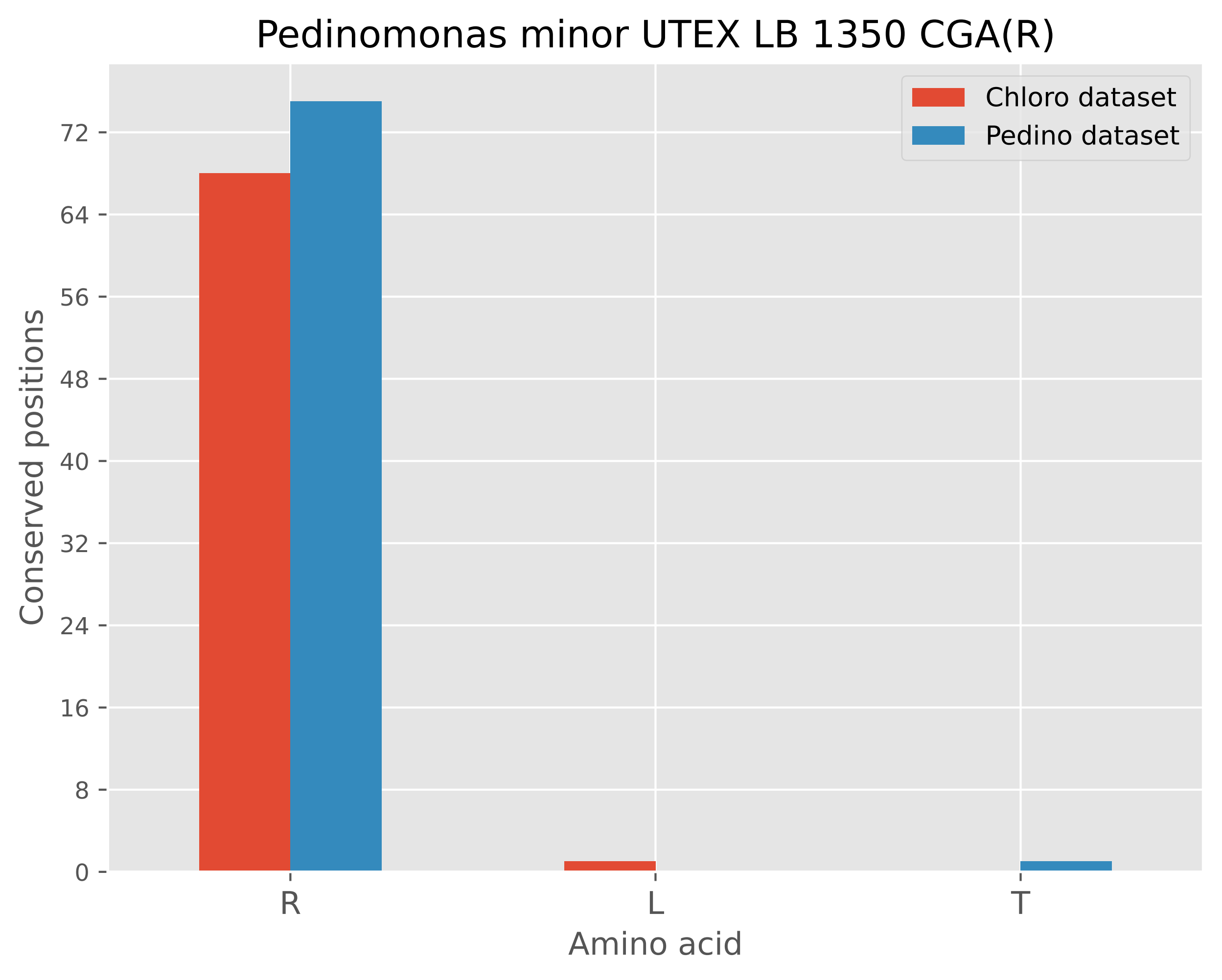

Pedinomonas minor UTEX LB 1350 CGA(R)
Chloro dataset
Pedino dataset
Conserved positions
0
8
16
24
32
40
48
56
64
72
R
L
T
Amino acid

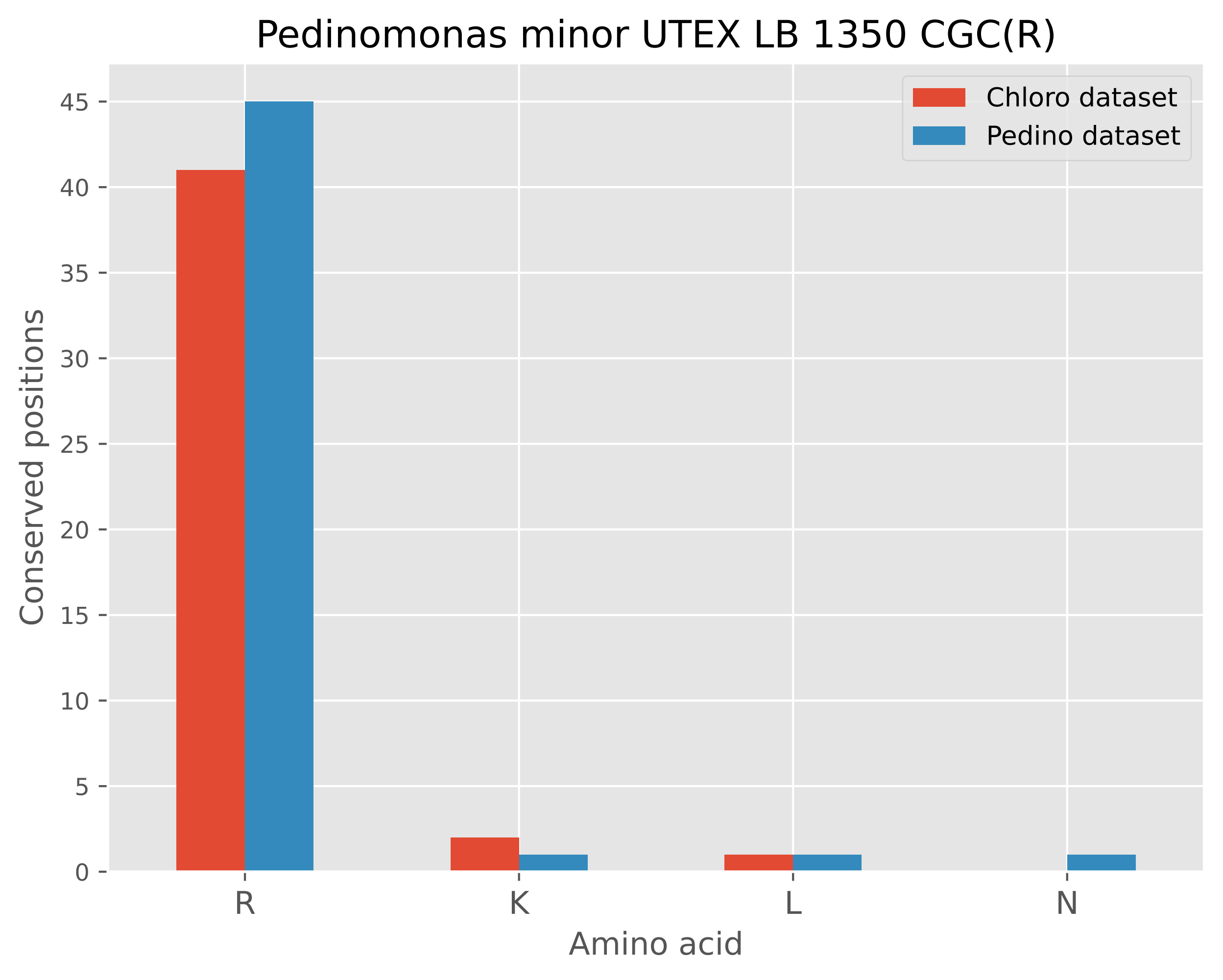

Pedinomonas minor UTEX LB 1350 CGC(R)
Chloro dataset
Pedino dataset
Conserved positions
Amino acid
0
5
10
15
20
25
30
35
40
45
R
K
L
N

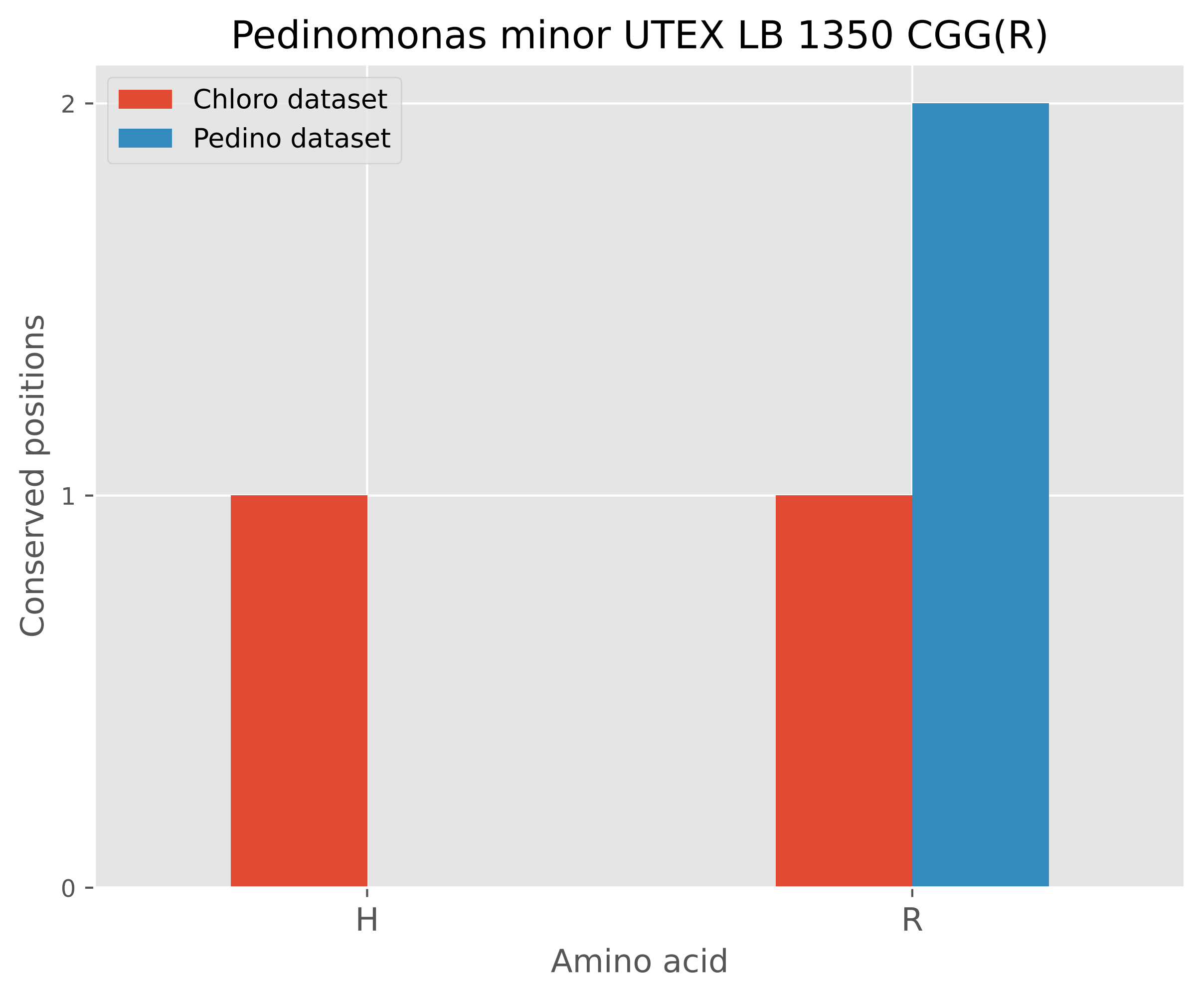

Pedinomonas minor UTEX LB 1350 CGG(R)
Chloro dataset
Pedino dataset
Conserved positions
0
1
2
H
R
Amino acid

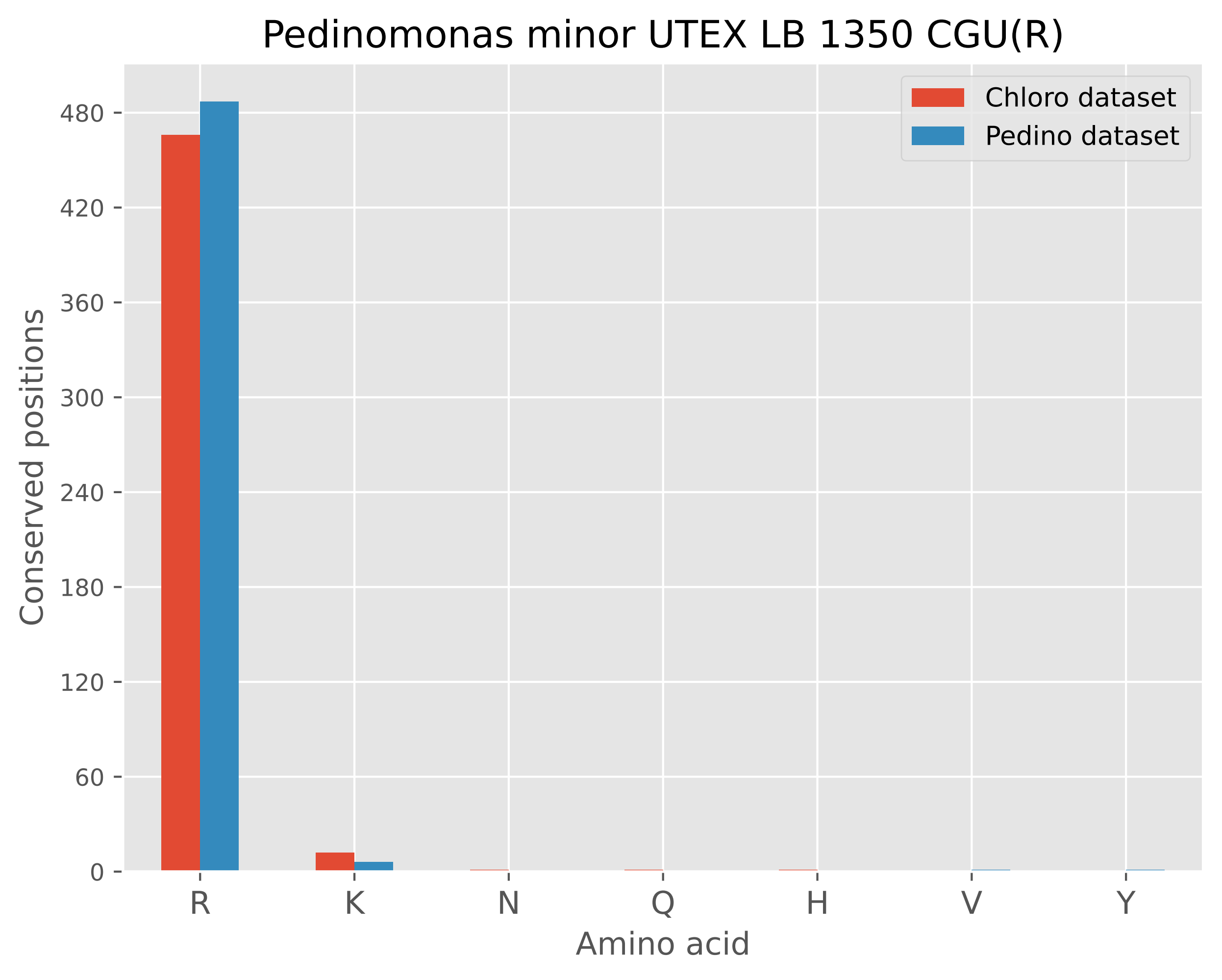
Pedinomonas minor UTEX LB 1350 CGU(R)
Chloro dataset
Pedino dataset
Conserved positions
0
60
120
180
240
300
360
420
480
R
K
N
Q
H
V
Y
Amino acid

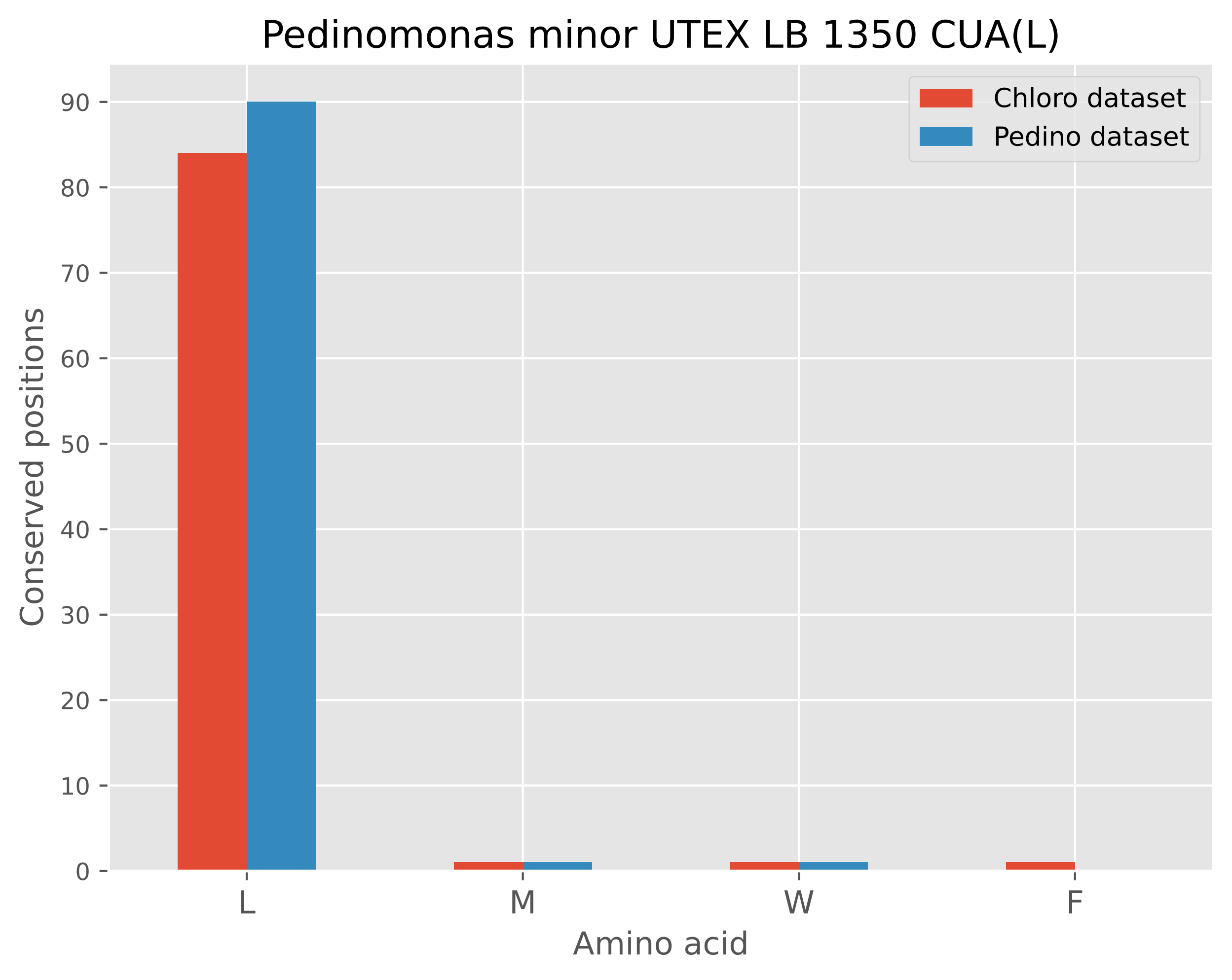
Pedinomonas minor UTEX LB 1350 CUA(L)
Chloro dataset
Pedino dataset
Conserved positions
0
10
20
30
40
50
60
70
80
90
L
M
W
F
Amino acid

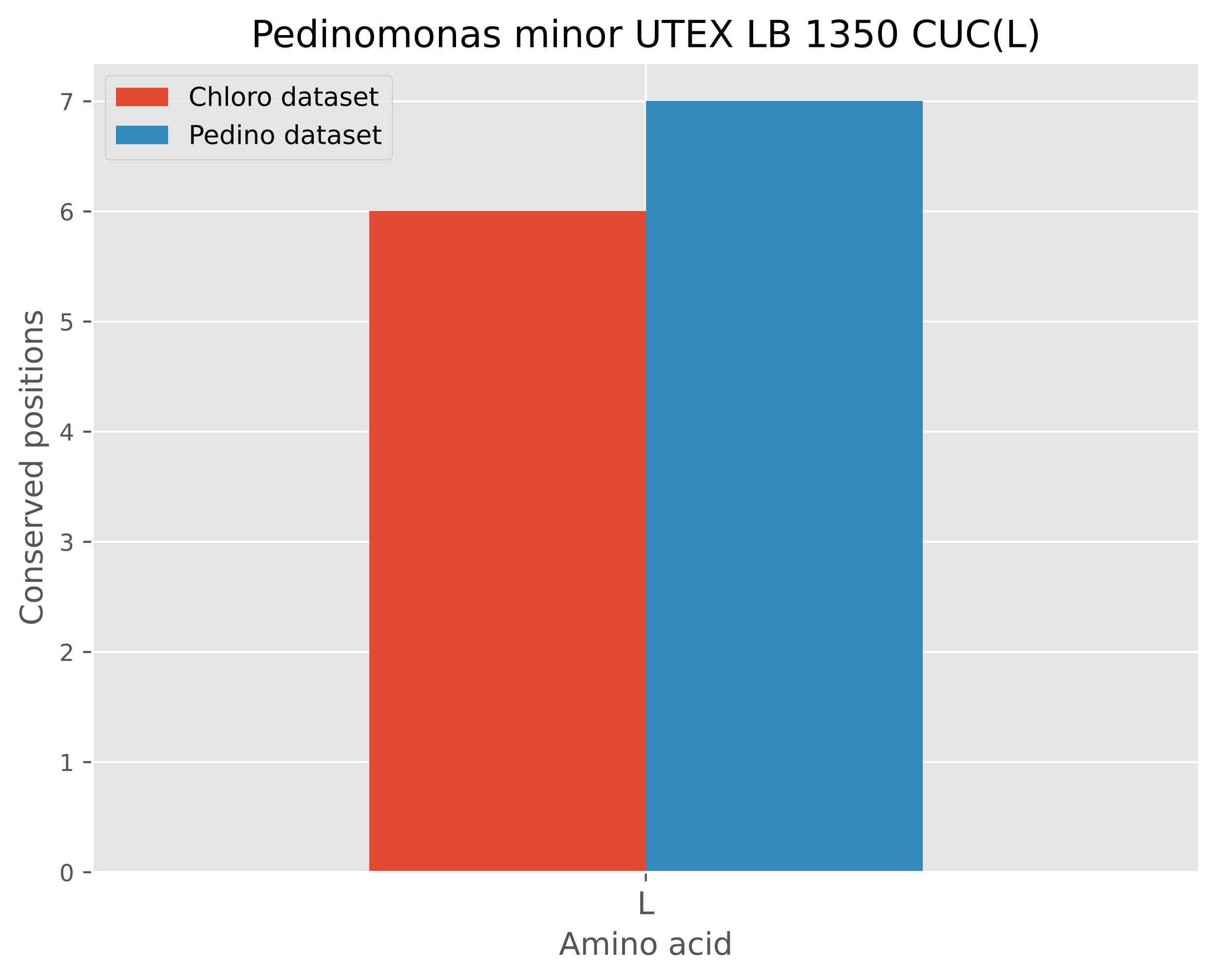

Pedinomonas minor UTEX LB 1350 CUC(L)
Chloro dataset
Pedino dataset
Conserved positions
0
1
2
3
4
5
6
7
L
Amino acid

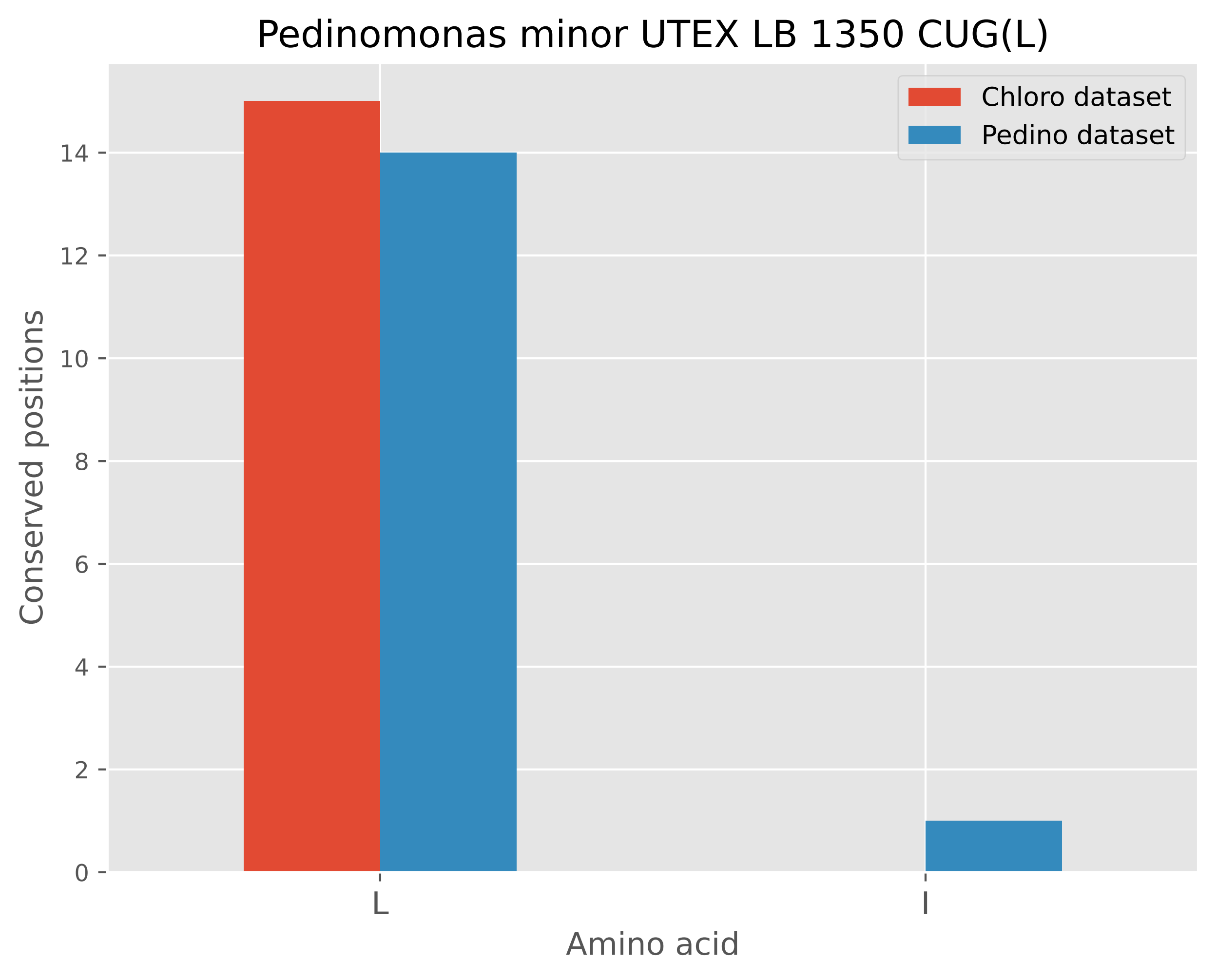
Pedinomonas minor UTEX LB 1350 CUG(L)
Chloro dataset
Pedino dataset
Conserved positions
0
2
4
6
8
10
12
14
L
I
Amino acid

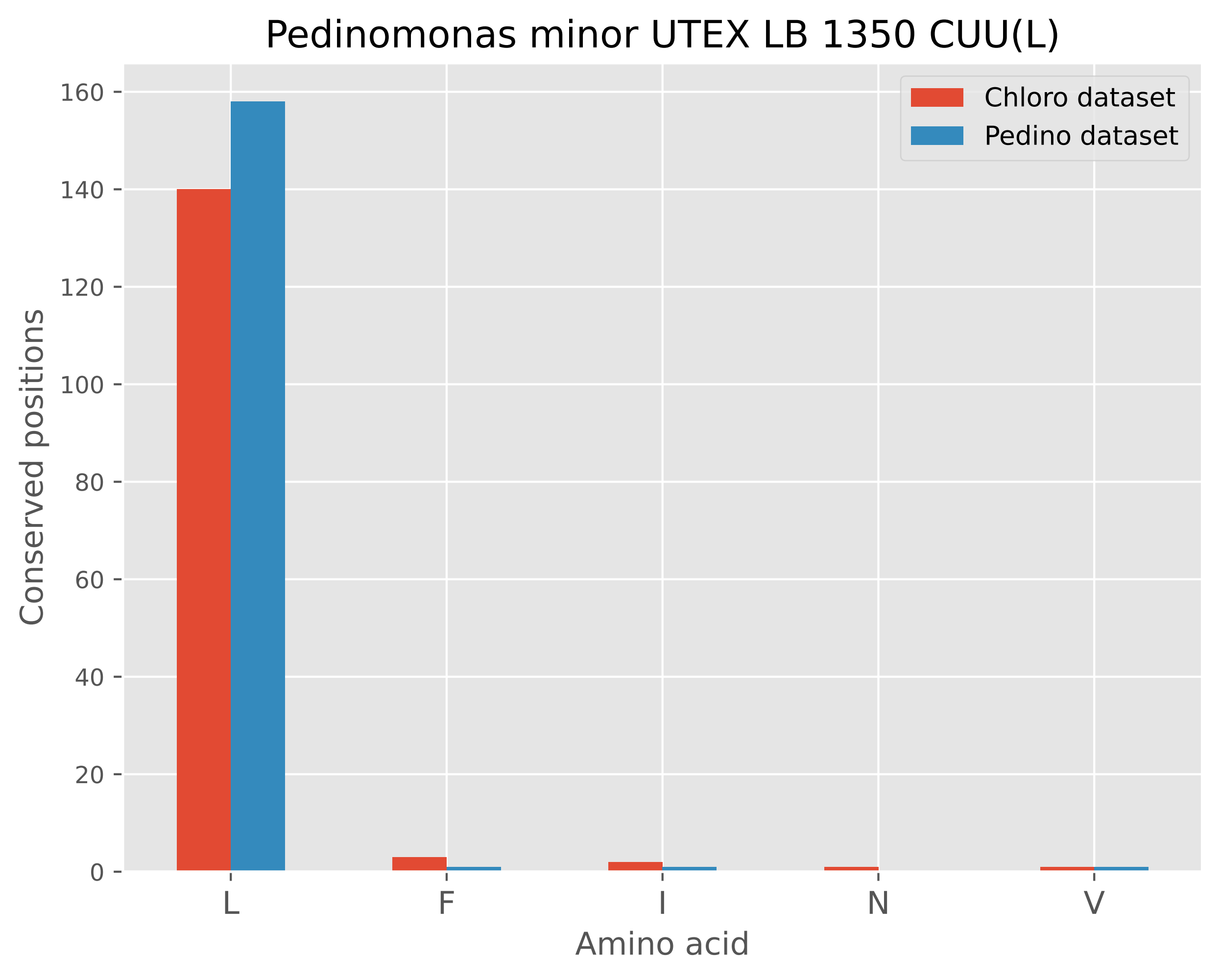

Pedinomonas minor UTEX LB 1350 CUU(L)
Chloro dataset
Pedino dataset
Conserved positions
0
20
40
60
80
100
120
140
160
L
F
I
N
V
Amino acid

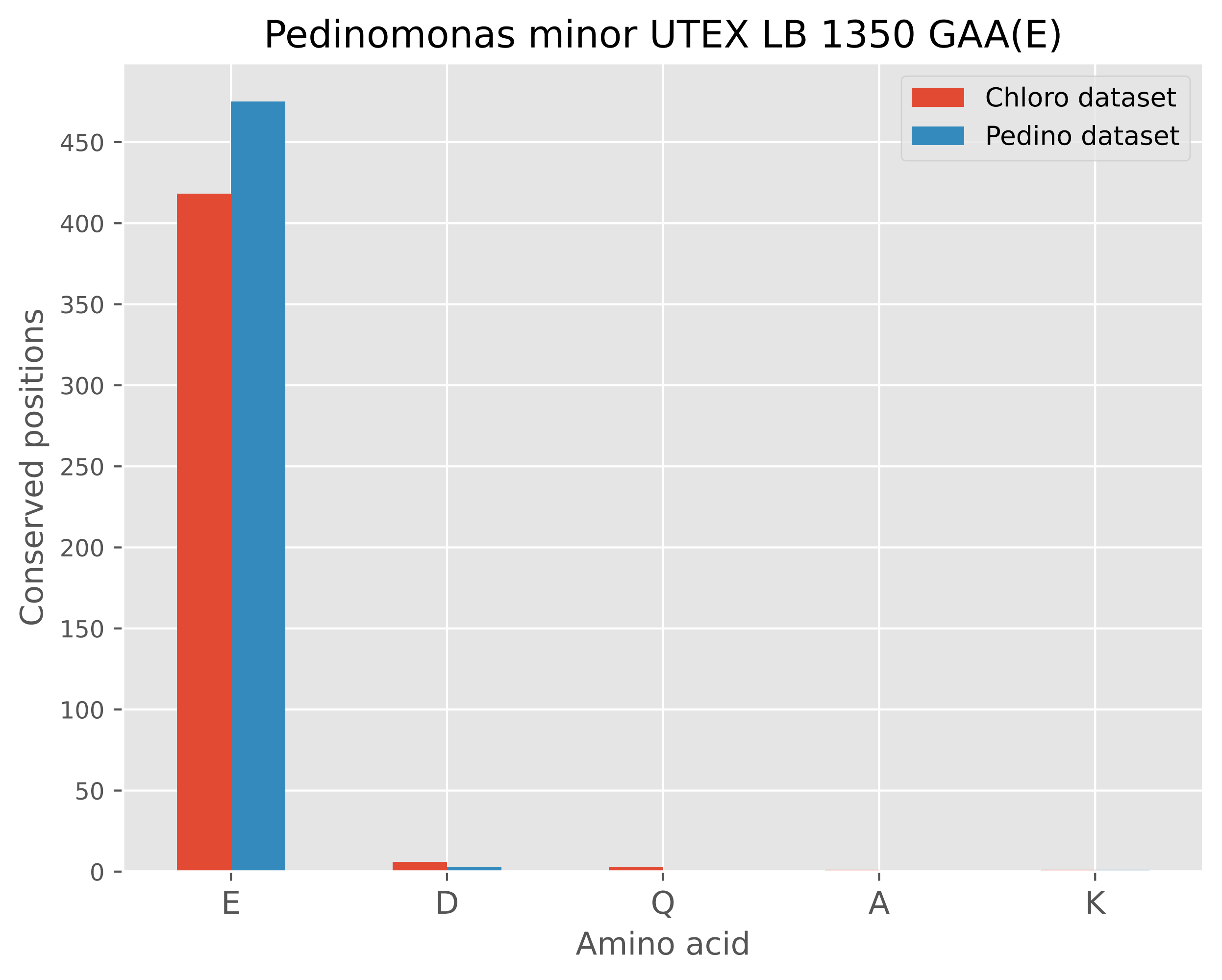
Pedinomonas minor UTEX LB 1350 GAA(E)
Chloro dataset
Pedino dataset
Conserved positions
0
50
100
150
200
250
300
350
400
450
E
D
Q
A
K
Amino acid

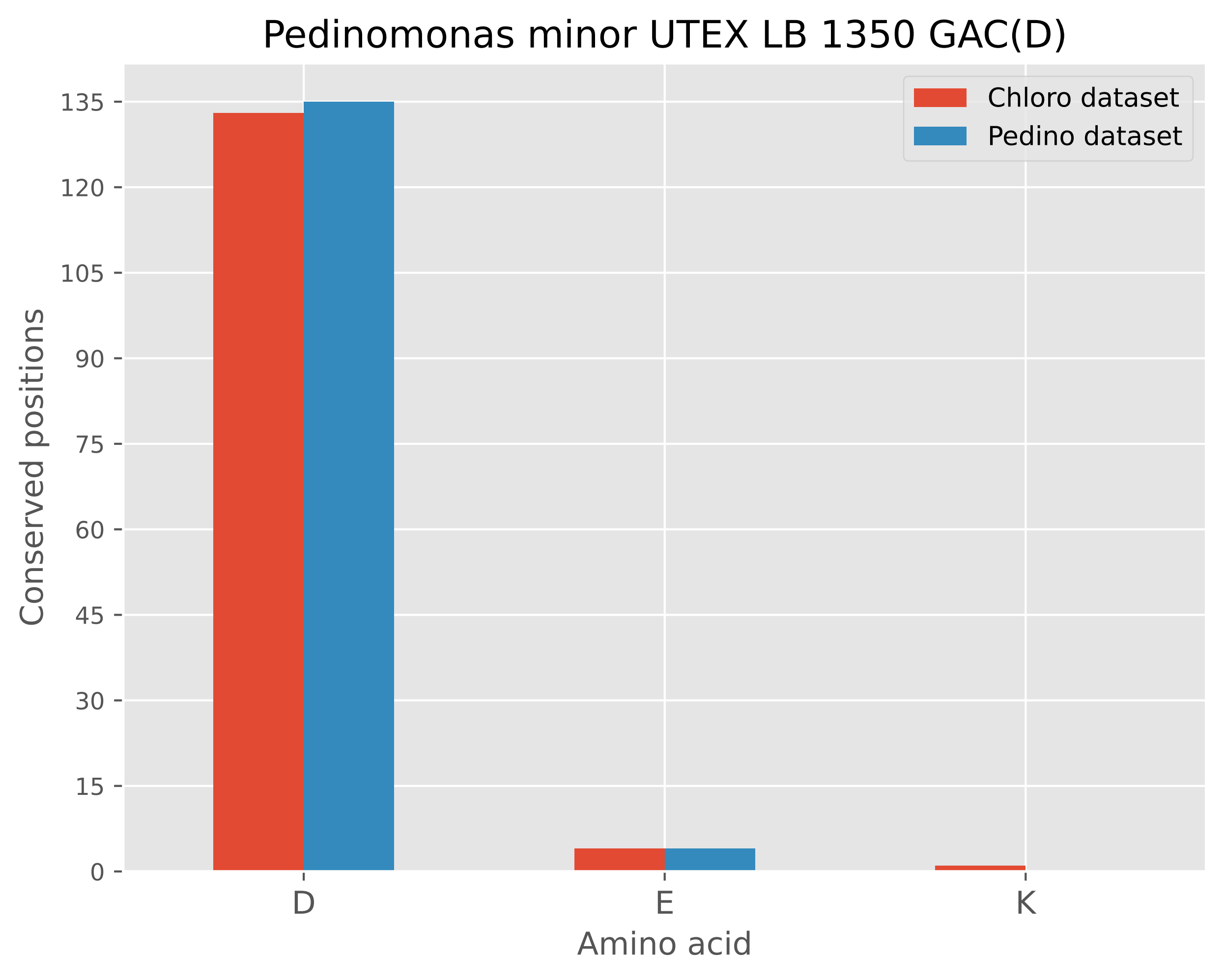

Pedinomonas minor UTEX LB 1350 GAC(D)
Chloro dataset
Pedino dataset
Conserved positions
0
15
30
45
60
75
90
105
120
135
D
E
K
Amino acid

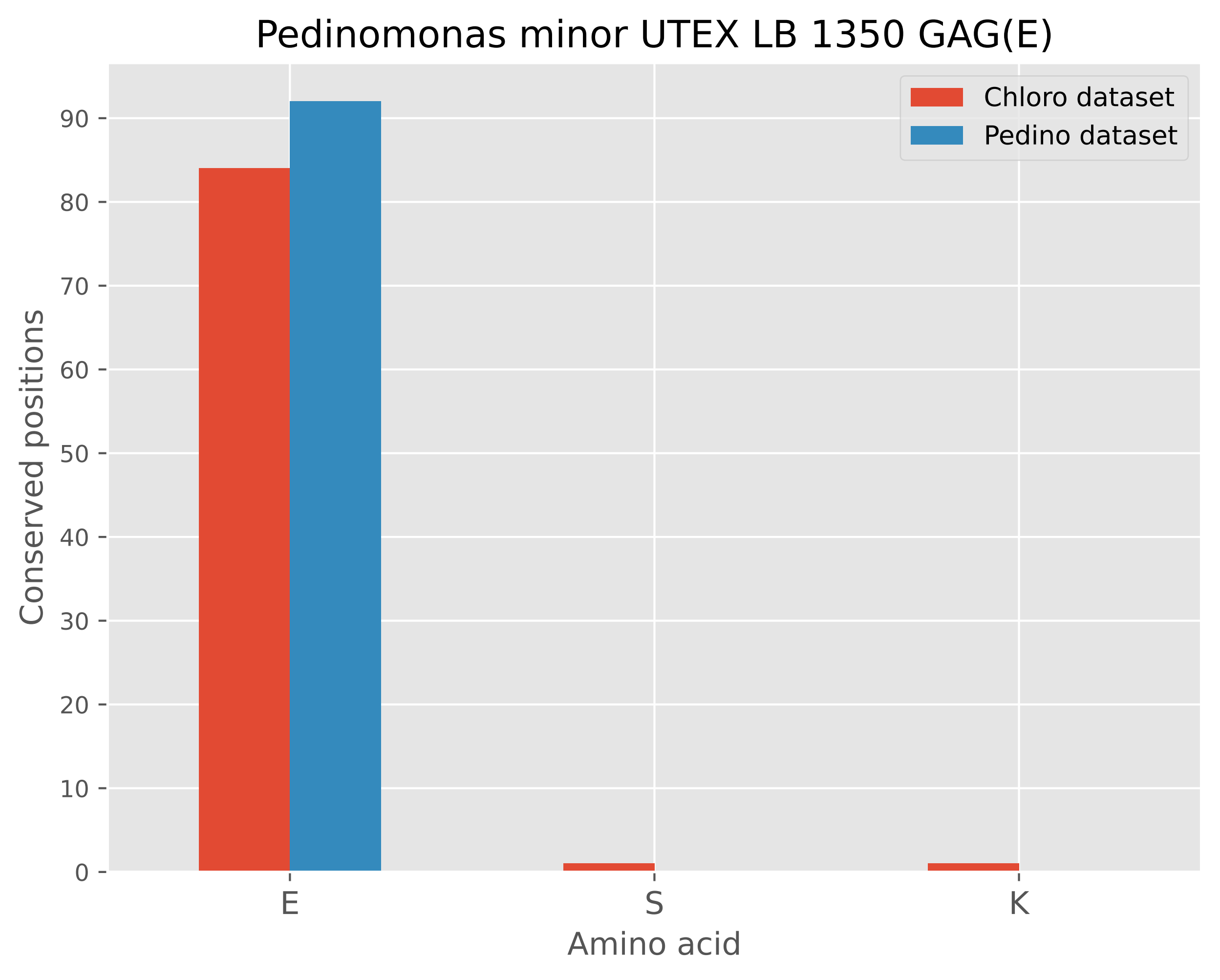
Pedinomonas minor UTEX LB 1350 GAG(E)
Chloro dataset
Pedino dataset
Conserved positions
0
10
20
30
40
50
60
70
80
90
E
S
K
Amino acid

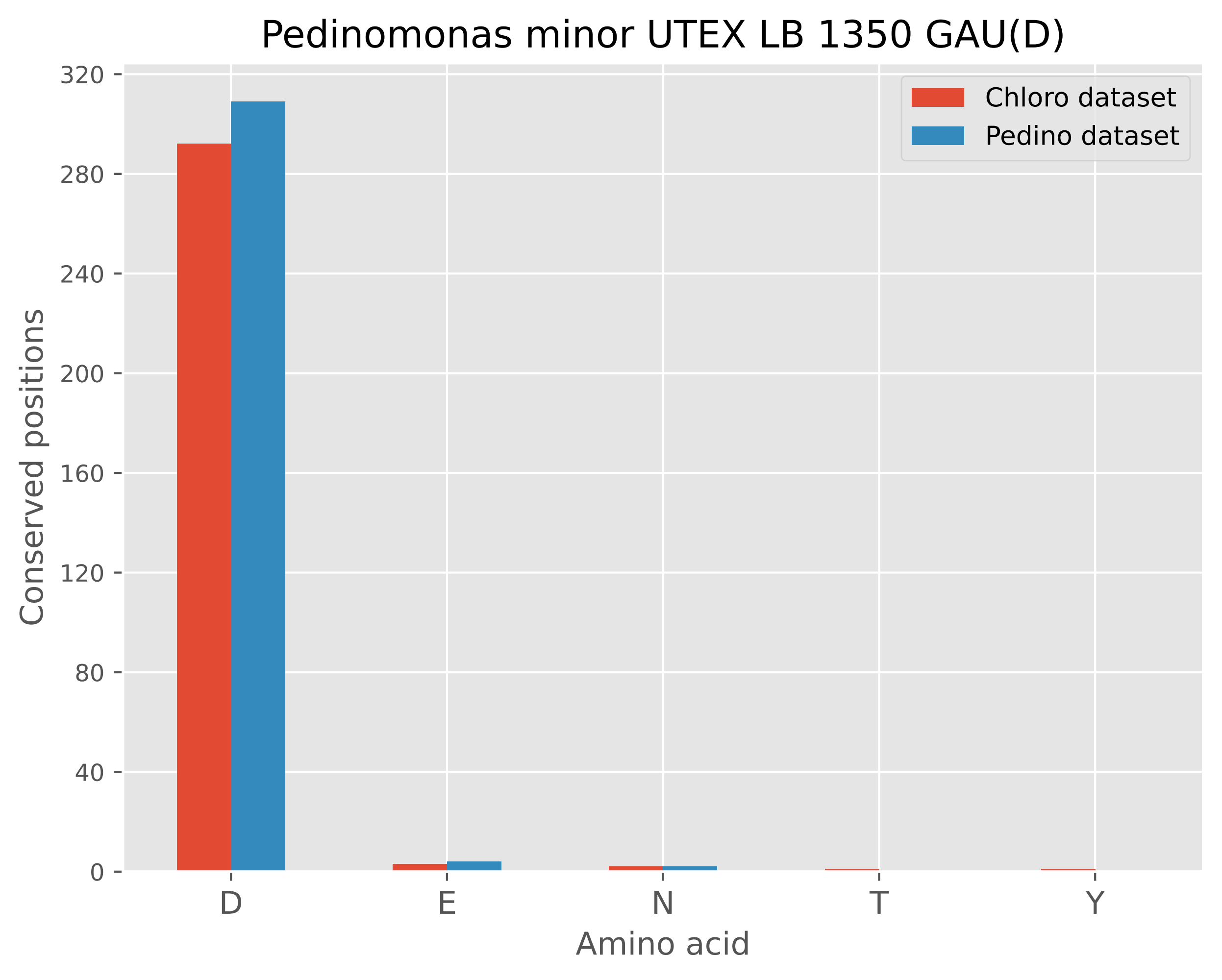

Pedinomonas minor UTEX LB 1350 GAU(D)
Chloro dataset
Pedino dataset
Conserved positions
320
280
240
200
160
120
80
40
0
D
E
N
T
Y
Amino acid

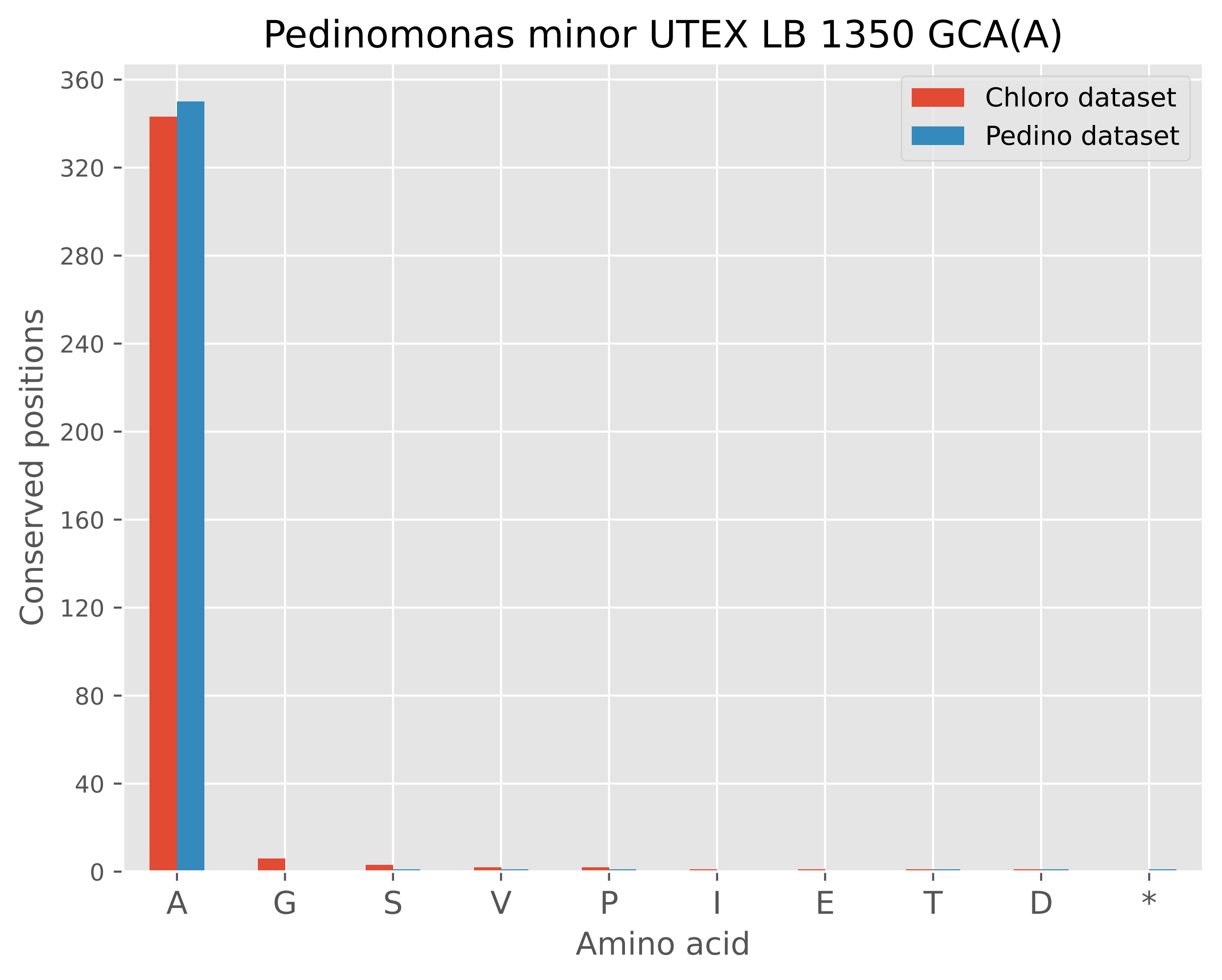
Pedinomonas minor UTEX LB 1350 GCA(A)
Chloro dataset
Pedino dataset
Conserved positions
0
40
80
120
160
200
240
280
320
360
A
G
S
V
P
I
E
T
D
*
Amino acid

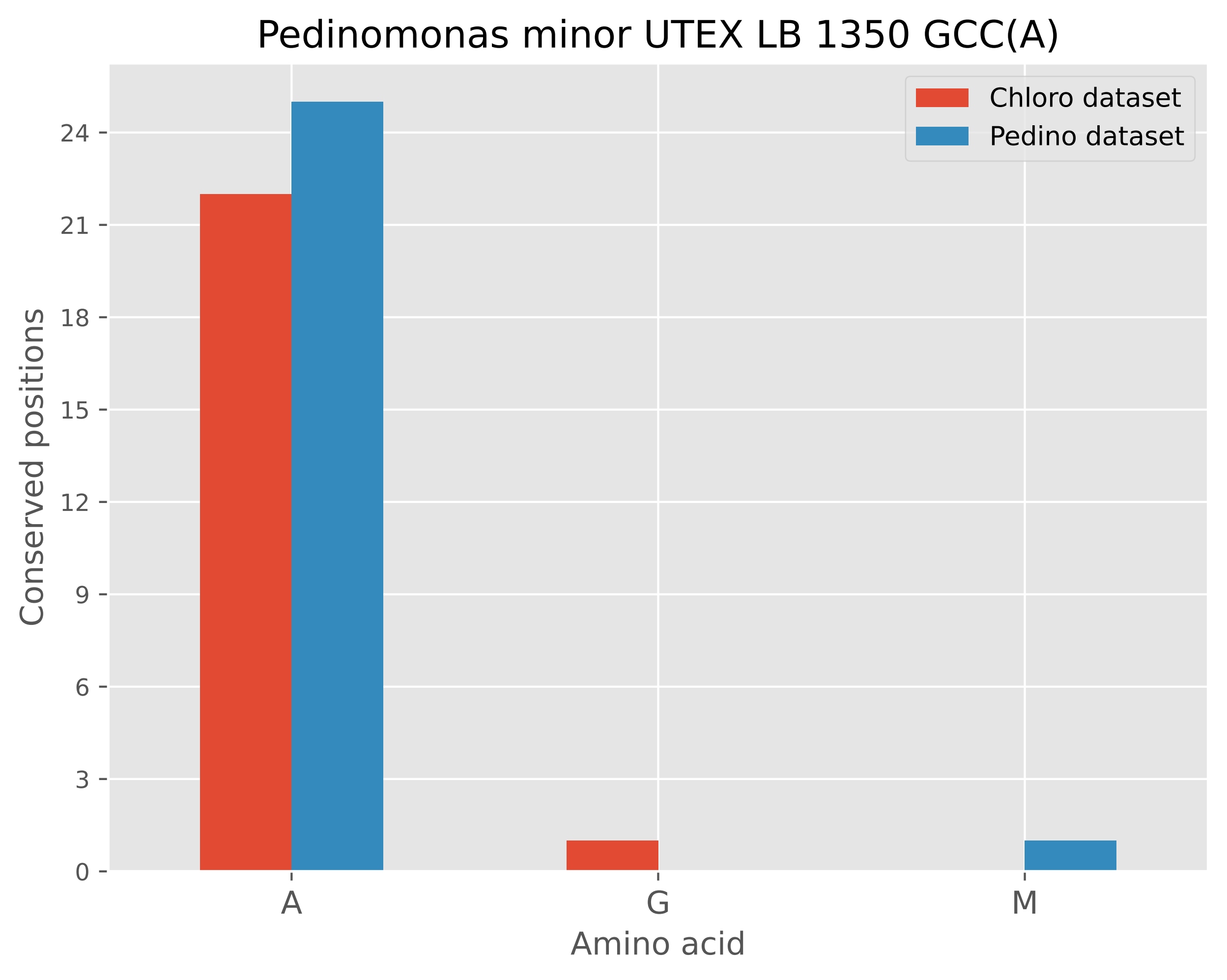
Pedinomonas minor UTEX LB 1350 GCC(A)
Chloro dataset
Pedino dataset
Conserved positions
0
3
6
9
12
15
18
21
24
A
G
M
Amino acid

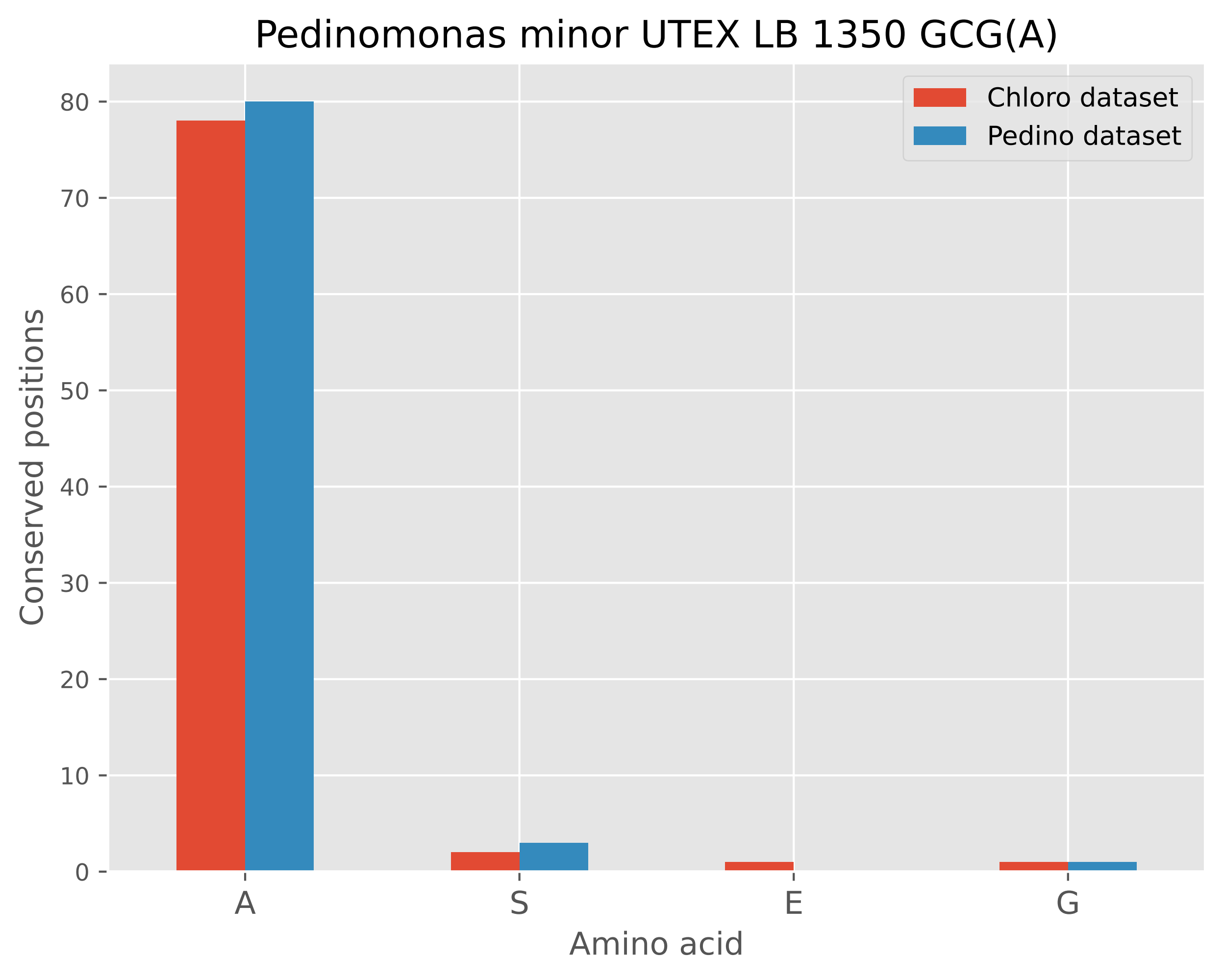
Pedinomonas minor UTEX LB 1350 GCG(A)
Chloro dataset
Pedino dataset
Conserved positions
Amino acid
A
S
E
G
0
10
20
30
40
50
60
70
80

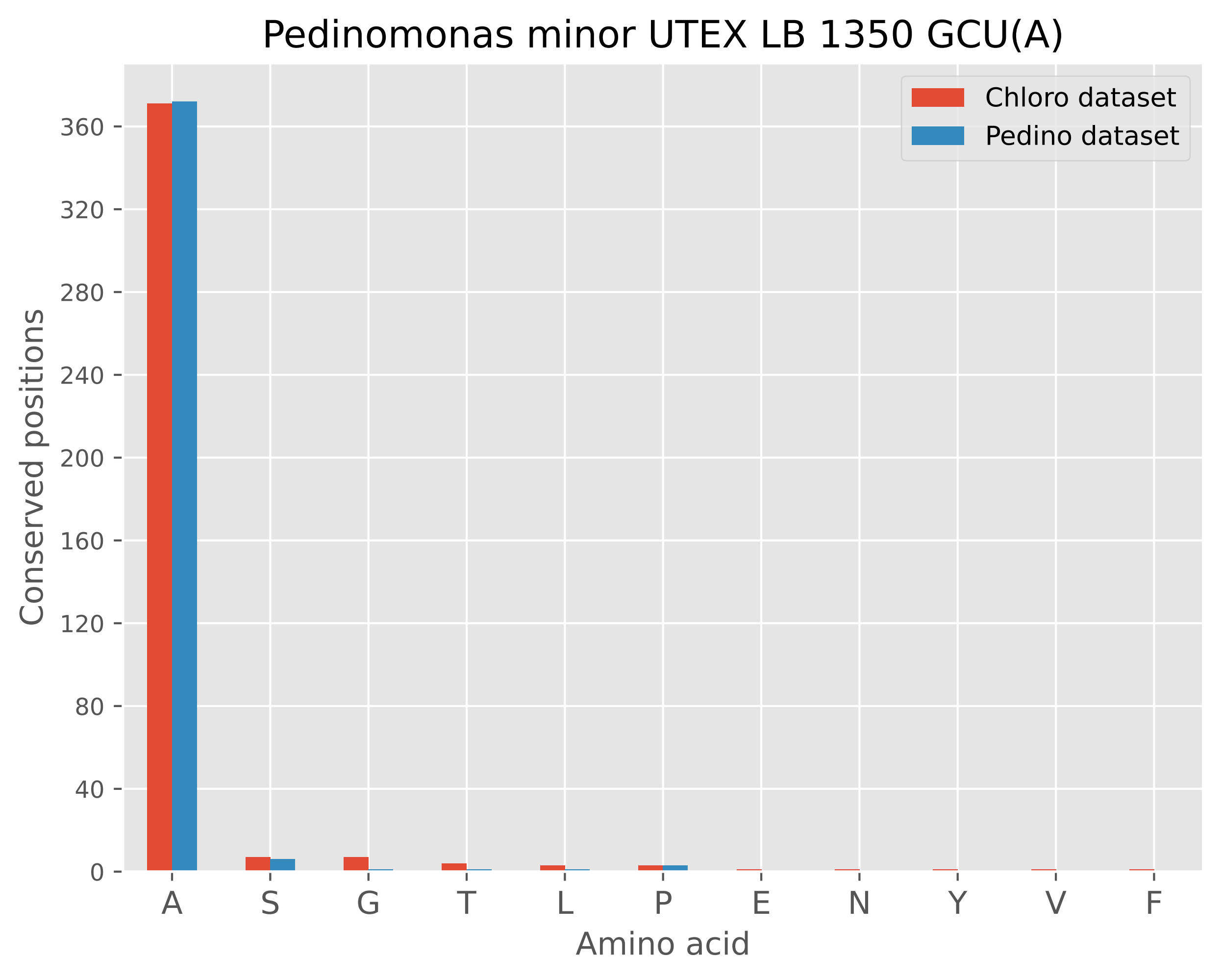

Pedinomonas minor UTEX LB 1350 GCU(A)
Chloro dataset
Pedino dataset
Conserved positions
0
40
80
120
160
200
240
280
320
360
A
S
G
T
L
P
E
N
Y
V
F
Amino acid

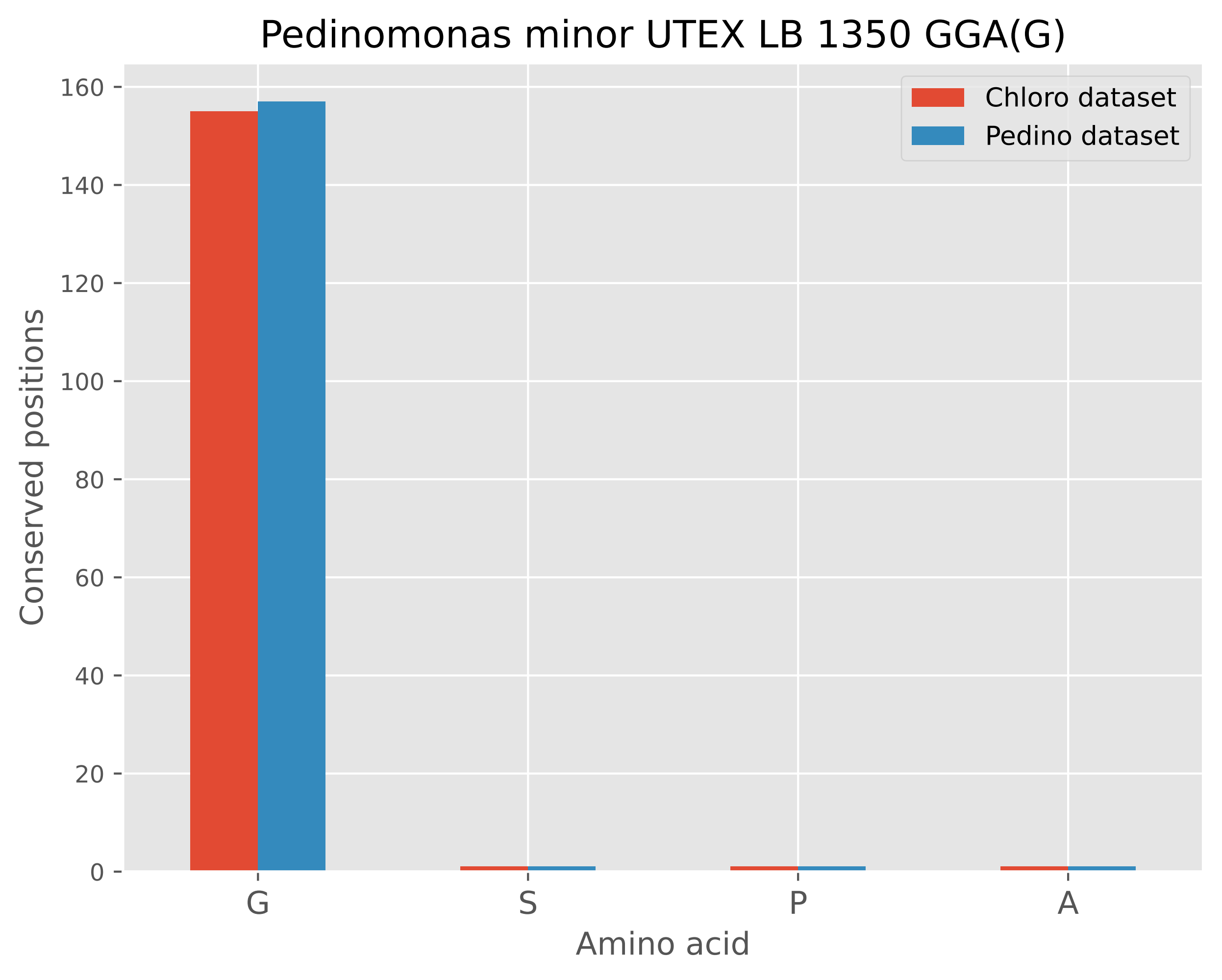

Pedinomonas minor UTEX LB 1350 GGA(G)
Chloro dataset
Pedino dataset
Conserved positions
0
20
40
60
80
100
120
140
160
G
S
P
A
Amino acid

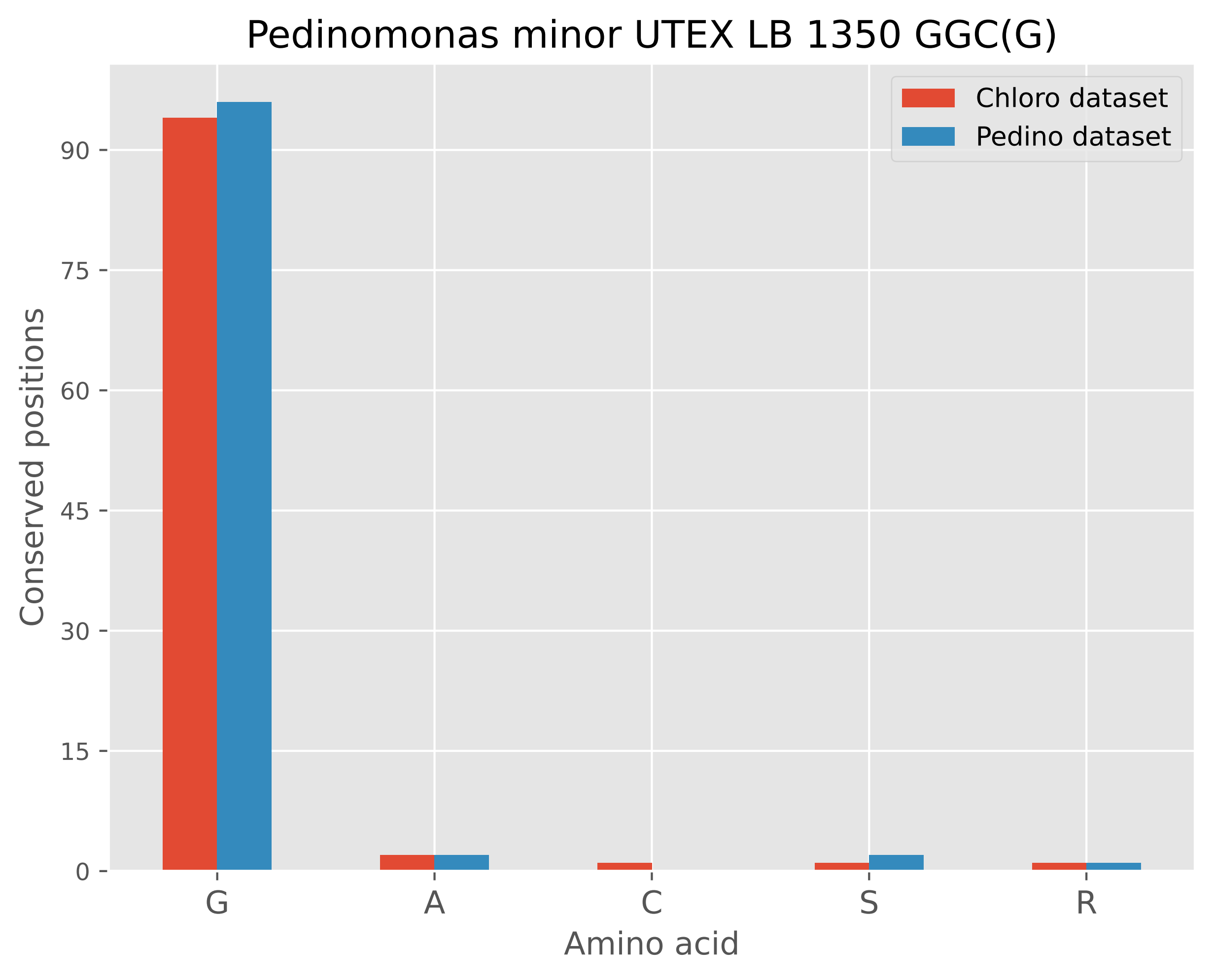

Pedinomonas minor UTEX LB 1350 GGC(G)
Chloro dataset
Pedino dataset
Conserved positions
0
15
30
45
60
75
90
G
A
C
S
R
Amino acid

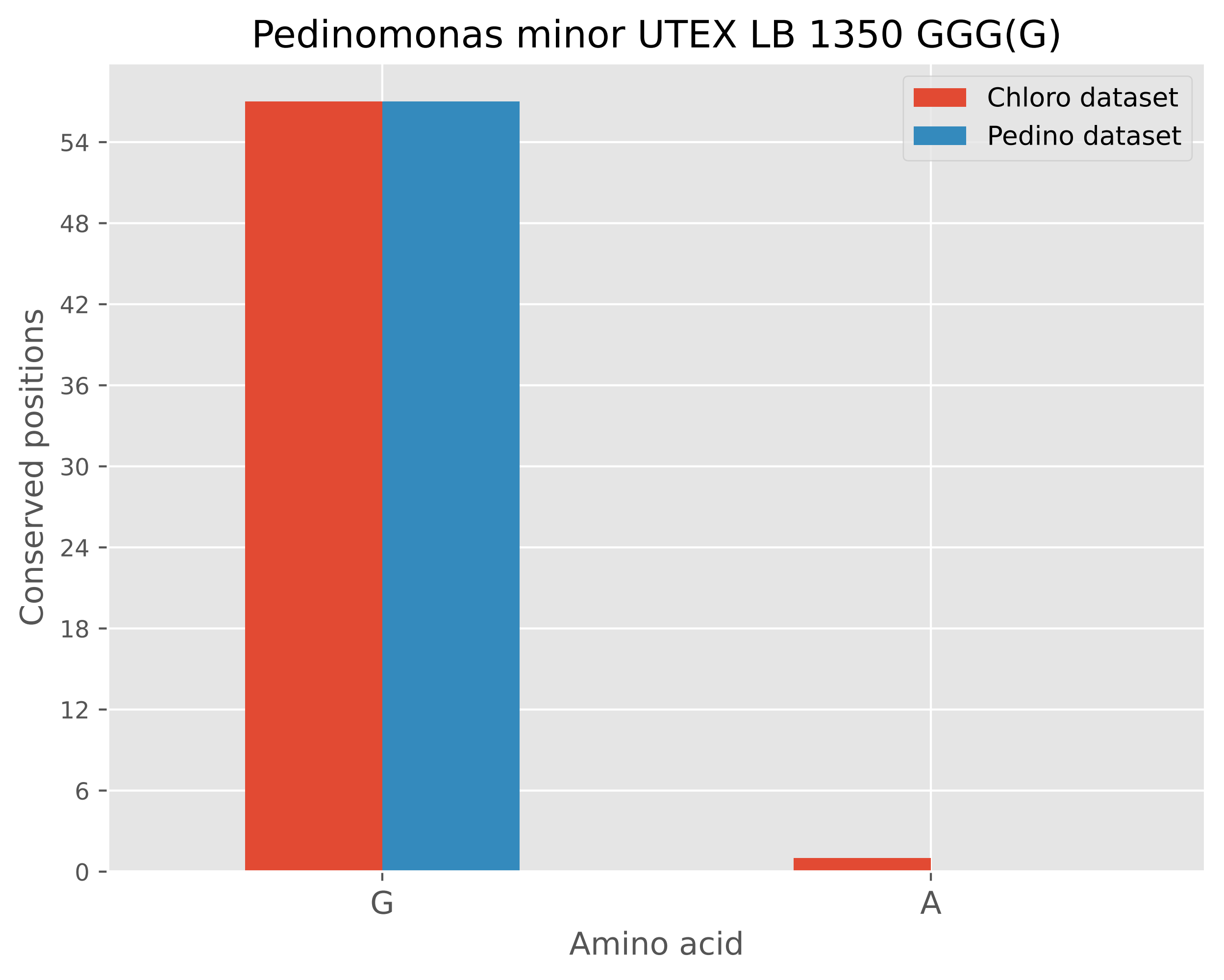

Pedinomonas minor UTEX LB 1350 GGG(G)
Chloro dataset
Pedino dataset
Conserved positions
0
6
12
18
24
30
36
42
48
54
G
A
Amino acid

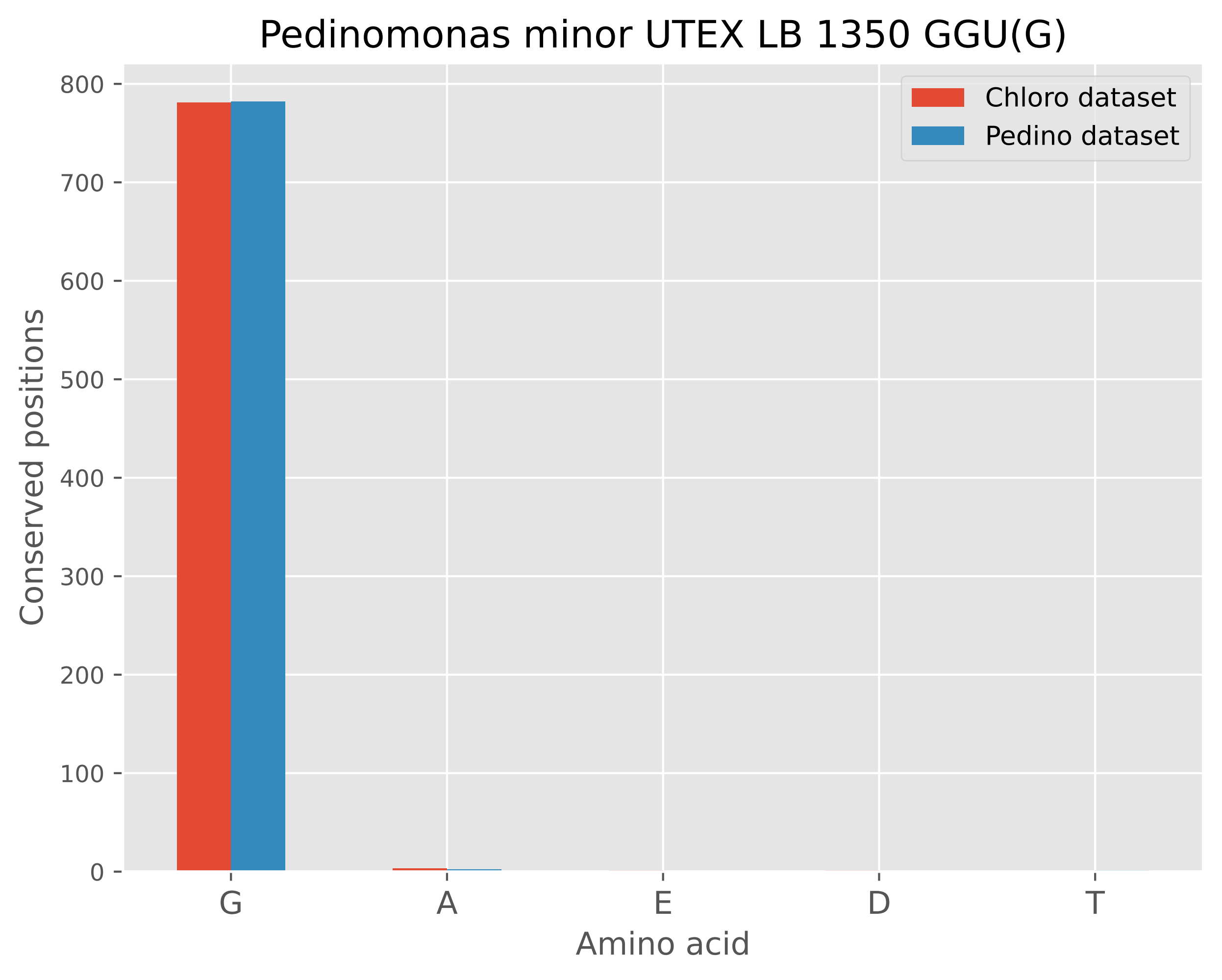

Pedinomonas minor UTEX LB 1350 GGU(G)
Chloro dataset
Pedino dataset
Conserved positions
800
700
600
500
400
300
200
100
0
G
A
E
D
T
Amino acid

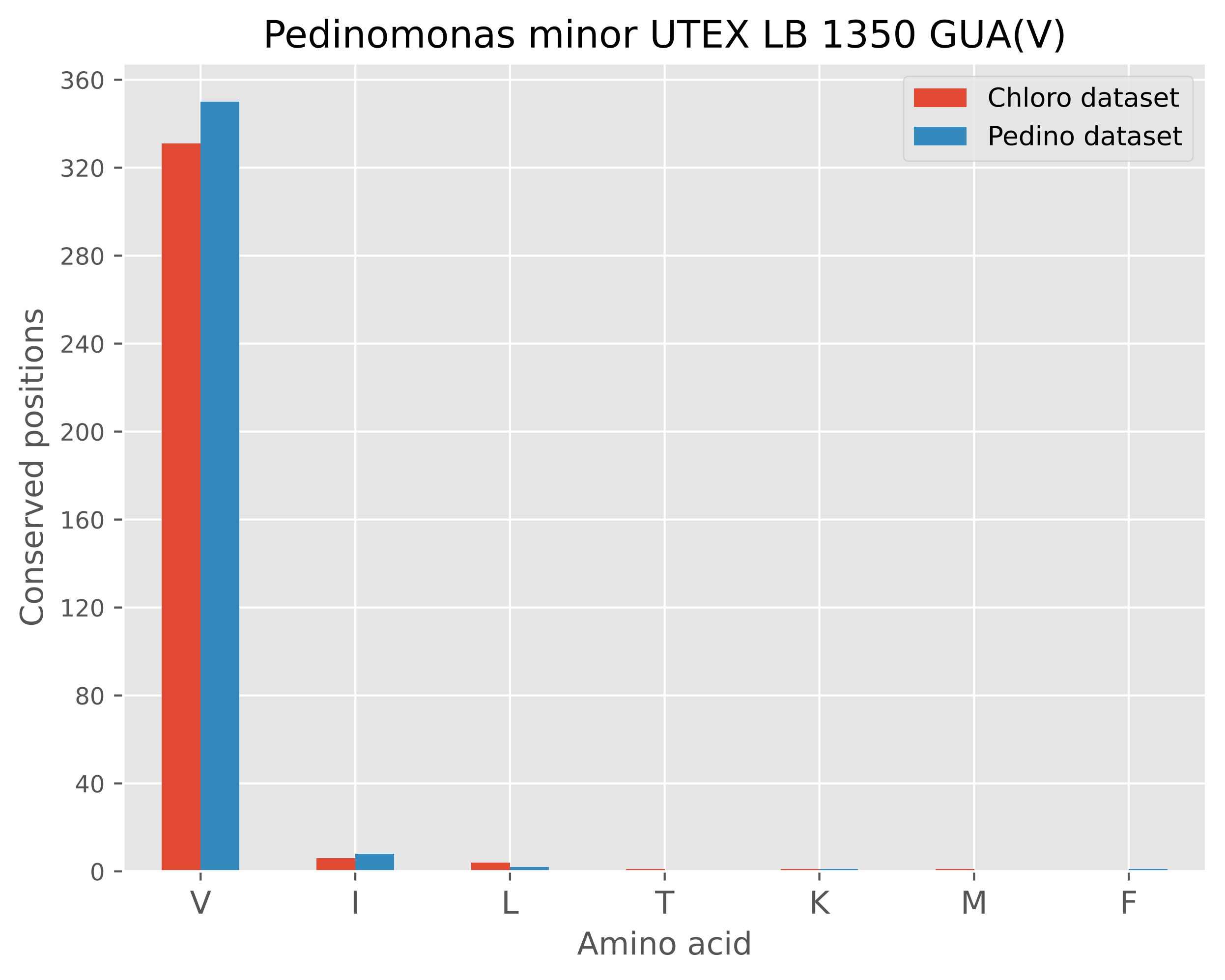

Pedinomonas minor UTEX LB 1350 GUA(V)
Chloro dataset
Pedino dataset
Conserved positions
0
40
80
120
160
200
240
280
320
360
V
I
L
T
K
M
F
Amino acid

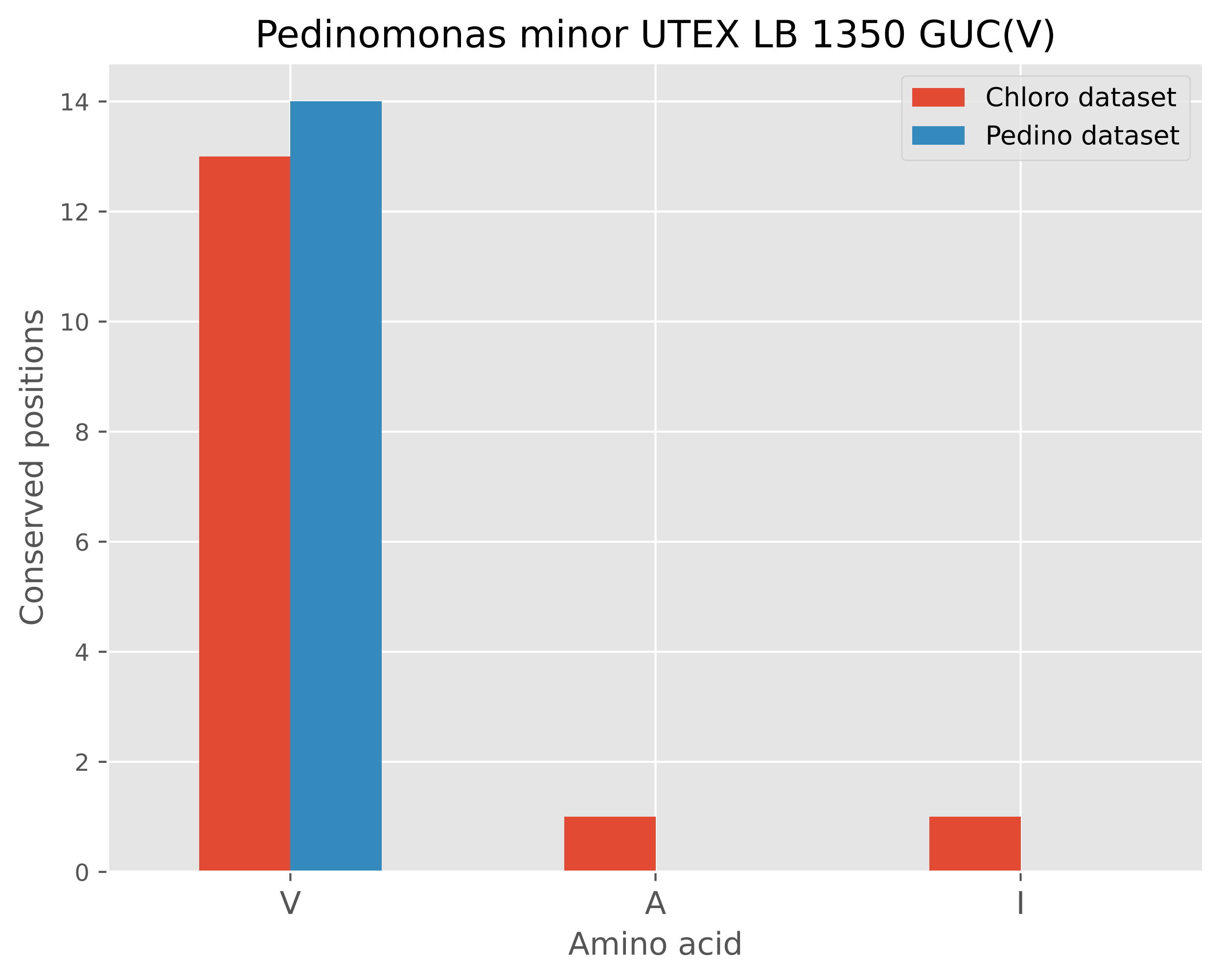

Pedinomonas minor UTEX LB 1350 GUC(V)
Chloro dataset
Pedino dataset
Conserved positions
0
2
4
6
8
10
12
14
V
A
I
Amino acid

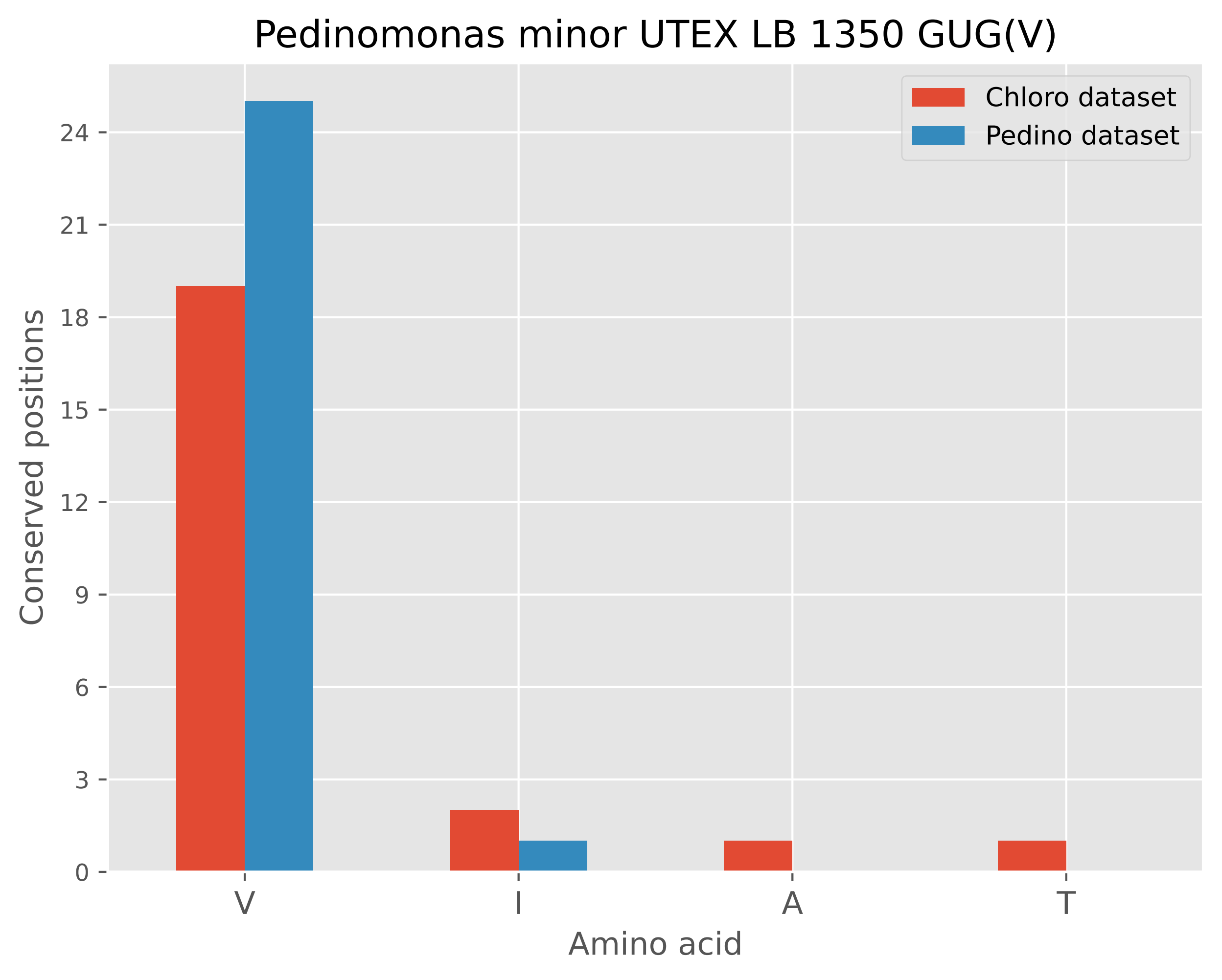
Pedinomonas minor UTEX LB 1350 GUG(V)
Chloro dataset
Pedino dataset
Conserved positions
0
3
6
9
12
15
18
21
24
V
I
A
T
Amino acid

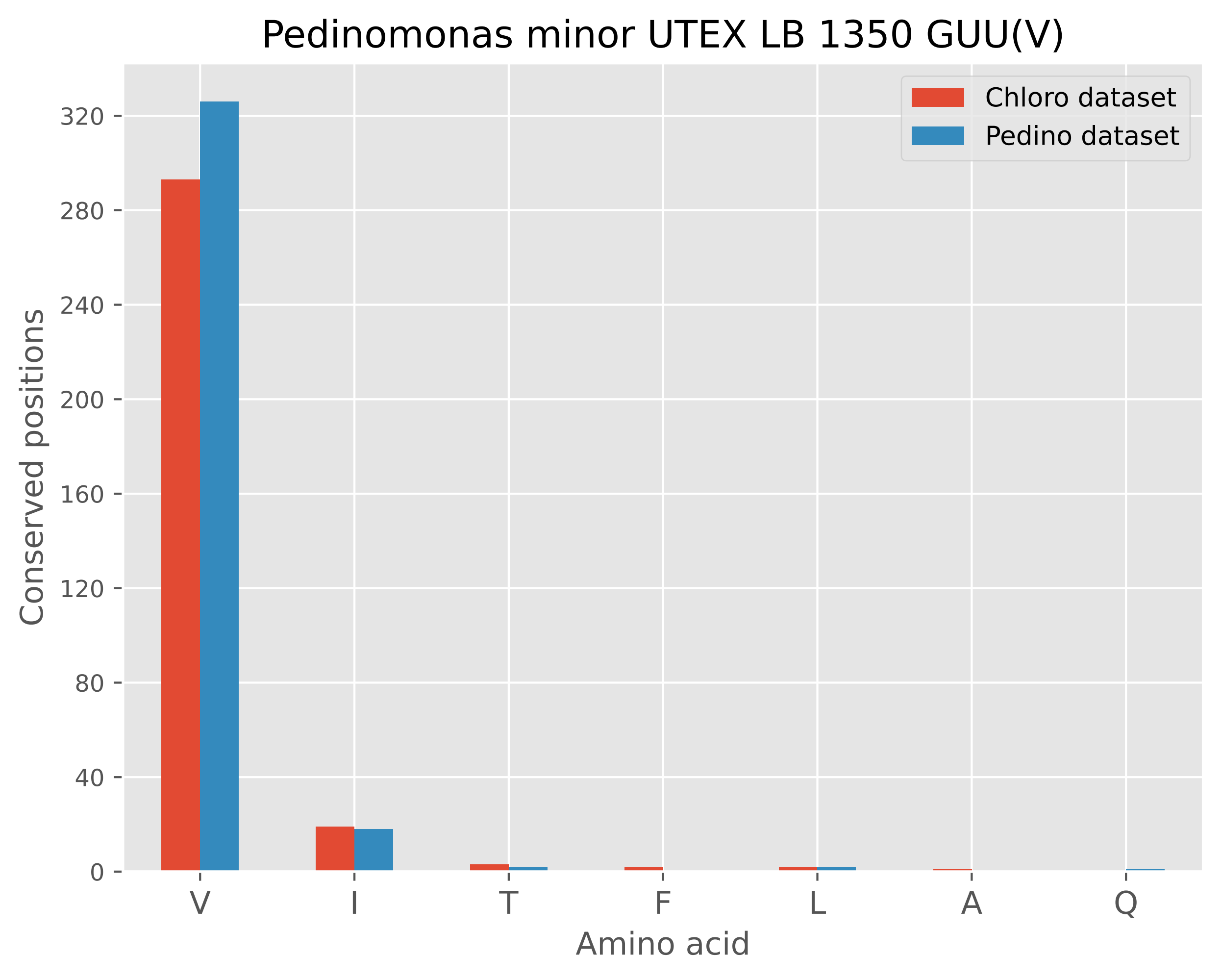
Pedinomonas minor UTEX LB 1350 GUU(V)
Chloro dataset
Pedino dataset
Conserved positions
0
40
80
120
160
200
240
280
320
V
I
T
F
L
A
Q
Amino acid

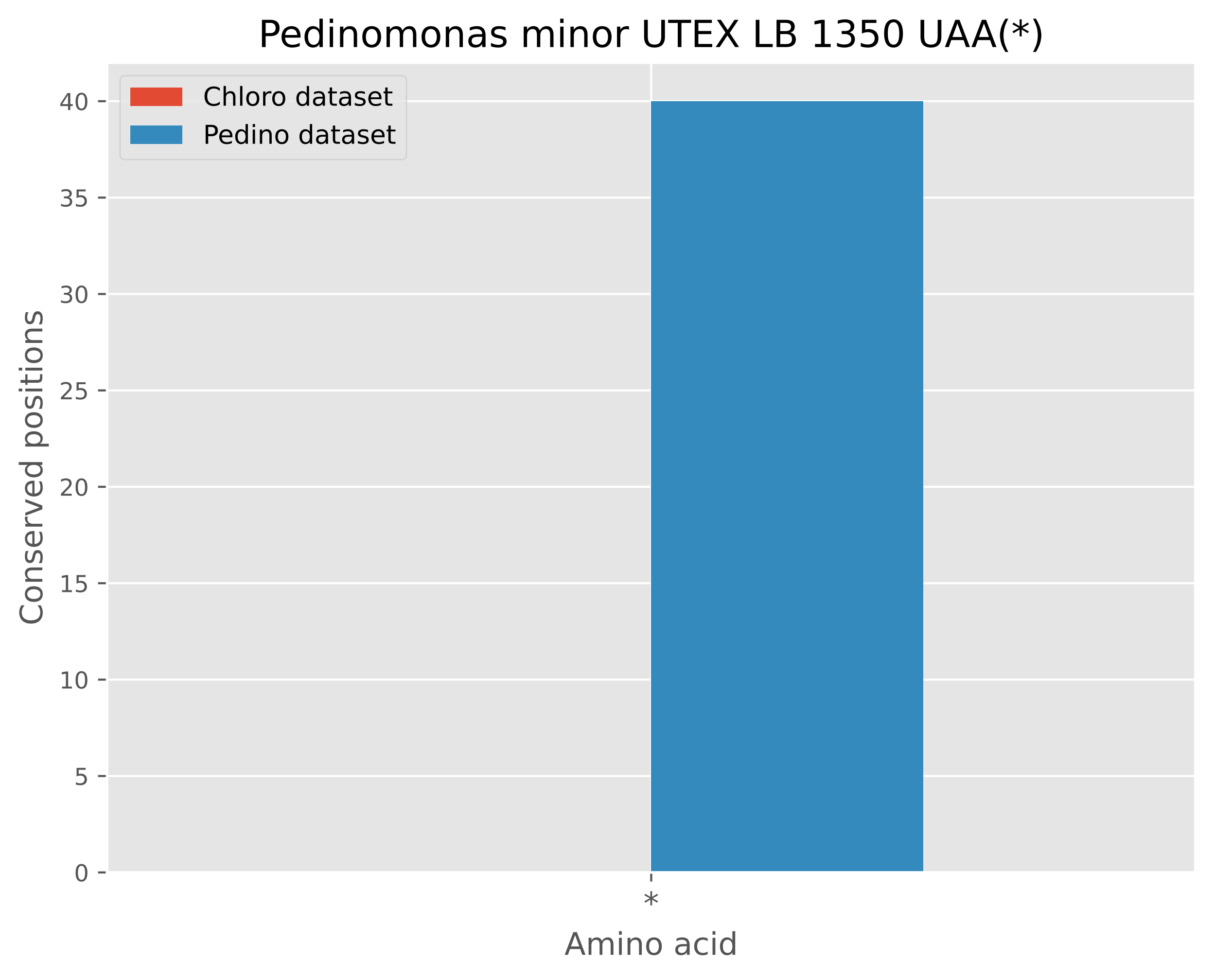

Pedinomonas minor UTEX LB 1350 UAA(*)
Chloro dataset
Pedino dataset
Conserved positions
40
35
30
25
20
15
10
5
0
*
Amino acid

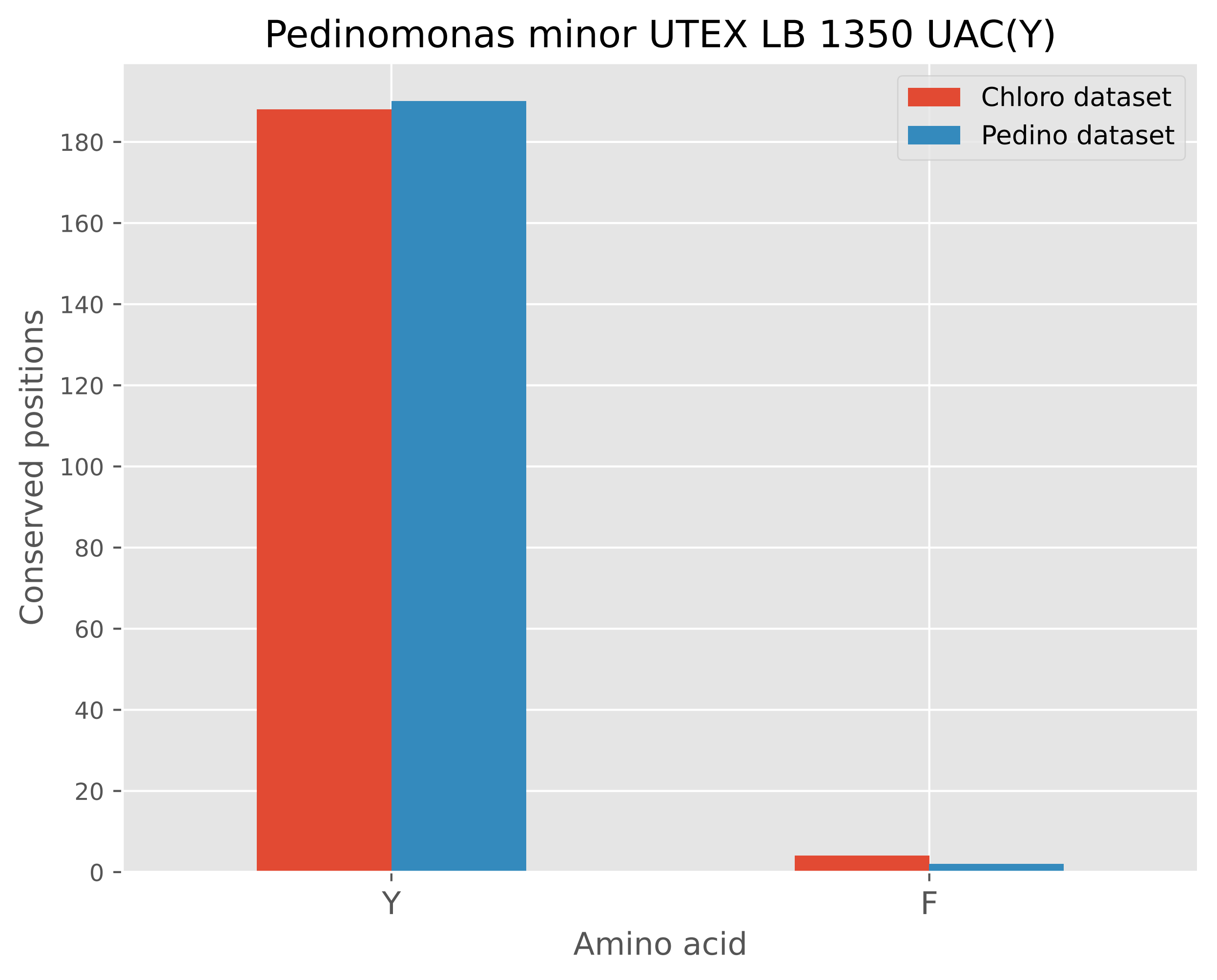
Pedinomonas minor UTEX LB 1350 UAC(Y)
Chloro dataset
Pedino dataset
Conserved positions
0
20
40
60
80
100
120
140
160
180
Y
F
Amino acid

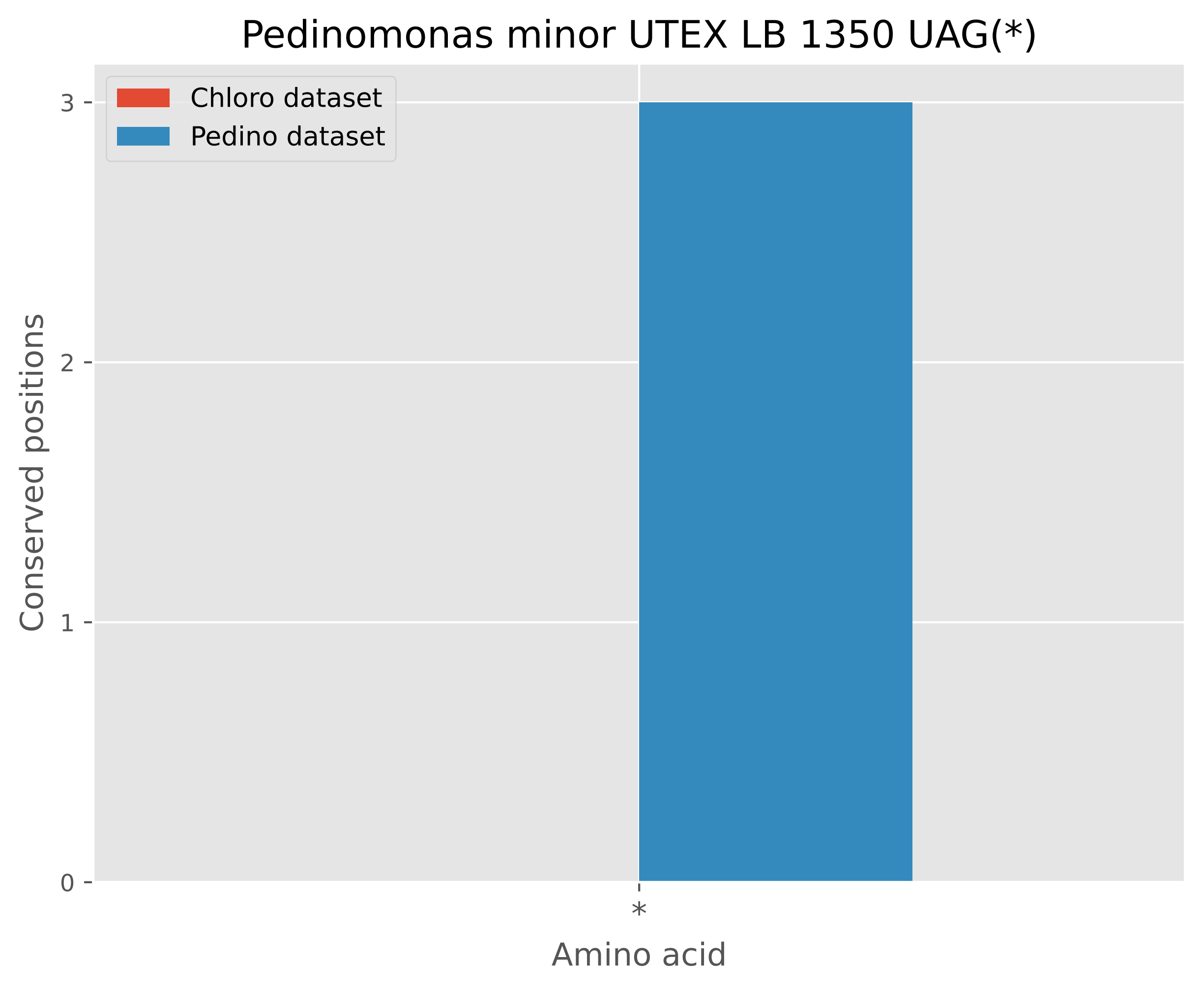
Pedinomonas minor UTEX LB 1350 UAG(*)
Chloro dataset
Pedino dataset
Conserved positions
0
1
2
3
*
Amino acid

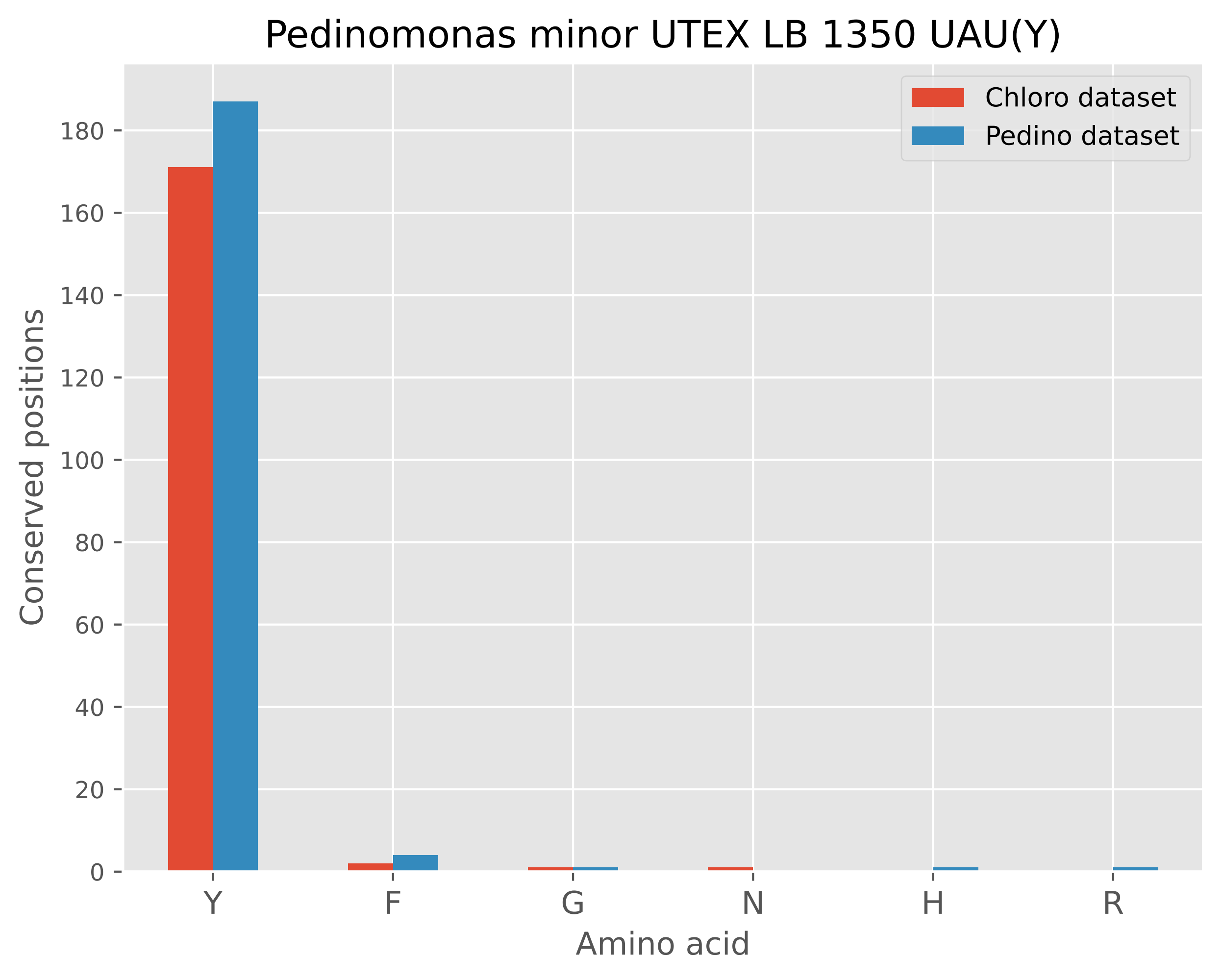
Pedinomonas minor UTEX LB 1350 UAU(Y)
Chloro dataset
Pedino dataset
Conserved positions
0
20
40
60
80
100
120
140
160
180
Y
F
G
N
H
R
Amino acid

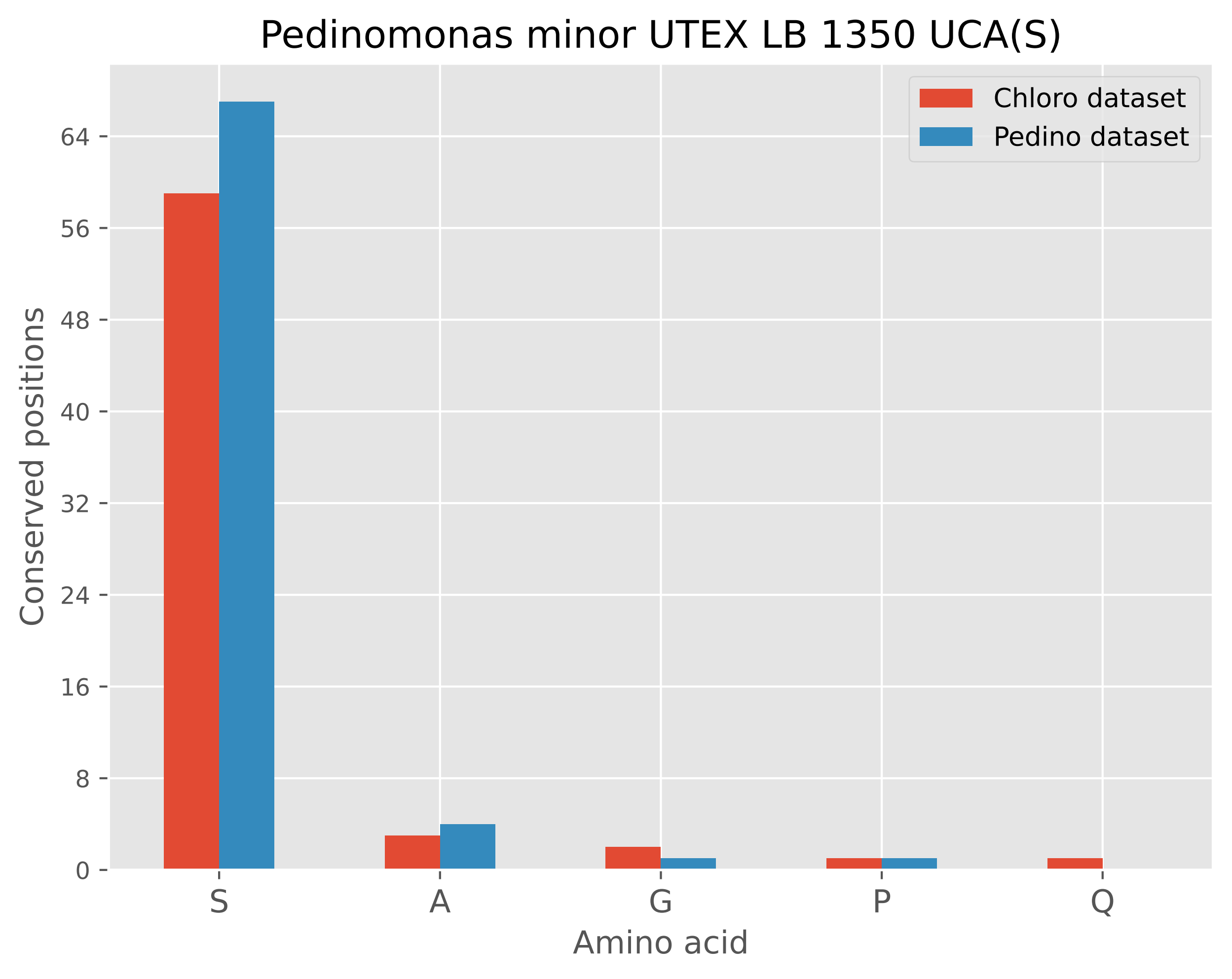

Pedinomonas minor UTEX LB 1350 UCA(S)
Chloro dataset
Pedino dataset
Conserved positions
0
8
16
24
32
40
48
56
64
S
A
G
P
Q
Amino acid

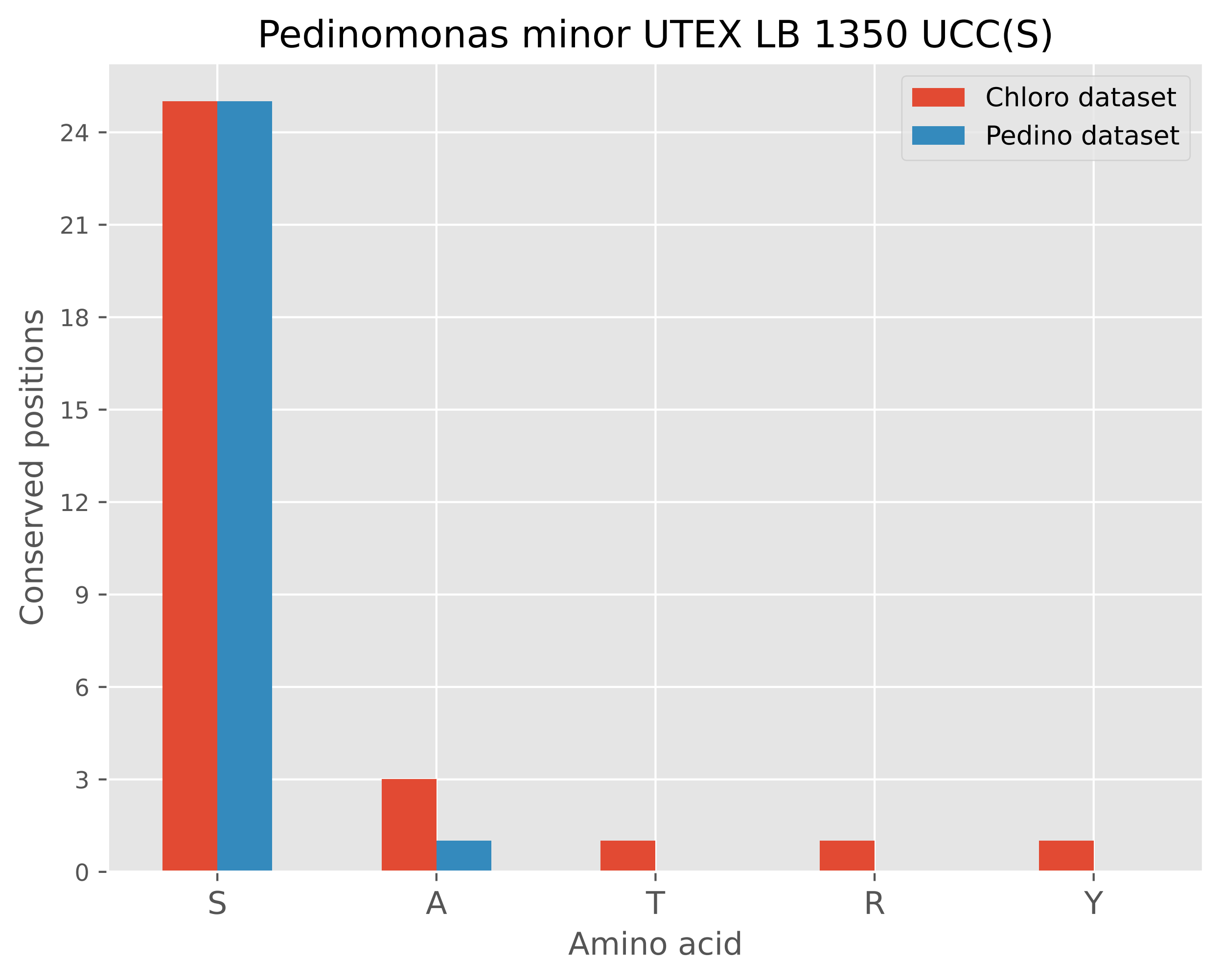
Pedinomonas minor UTEX LB 1350 UCC(S)
Chloro dataset
Pedino dataset
Conserved positions
0
3
6
9
12
15
18
21
24
S
A
T
R
Y
Amino acid

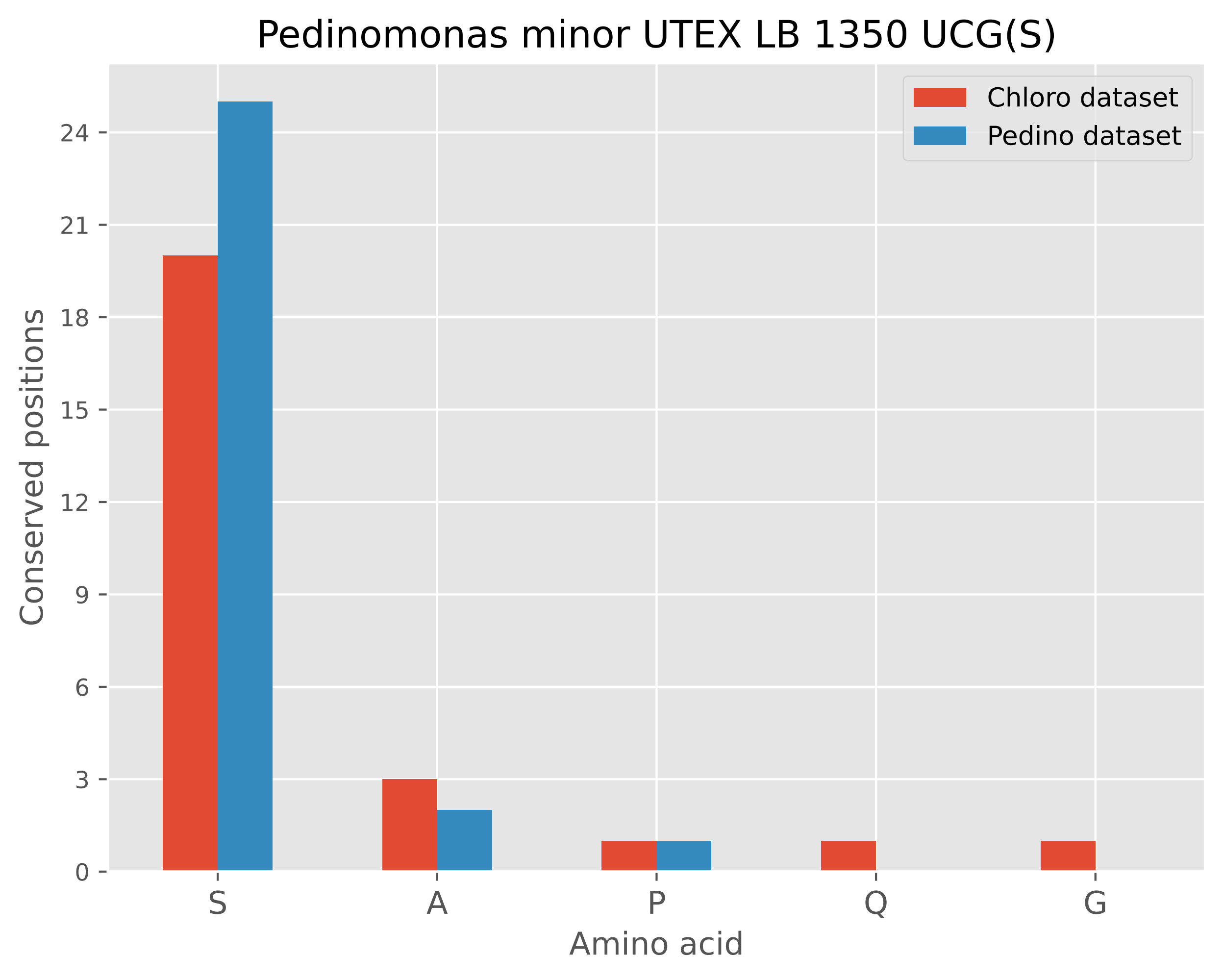

Pedinomonas minor UTEX LB 1350 UCG(S)
Chloro dataset
Pedino dataset
Conserved positions
0
3
6
9
12
15
18
21
24
S
A
P
Q
G
Amino acid

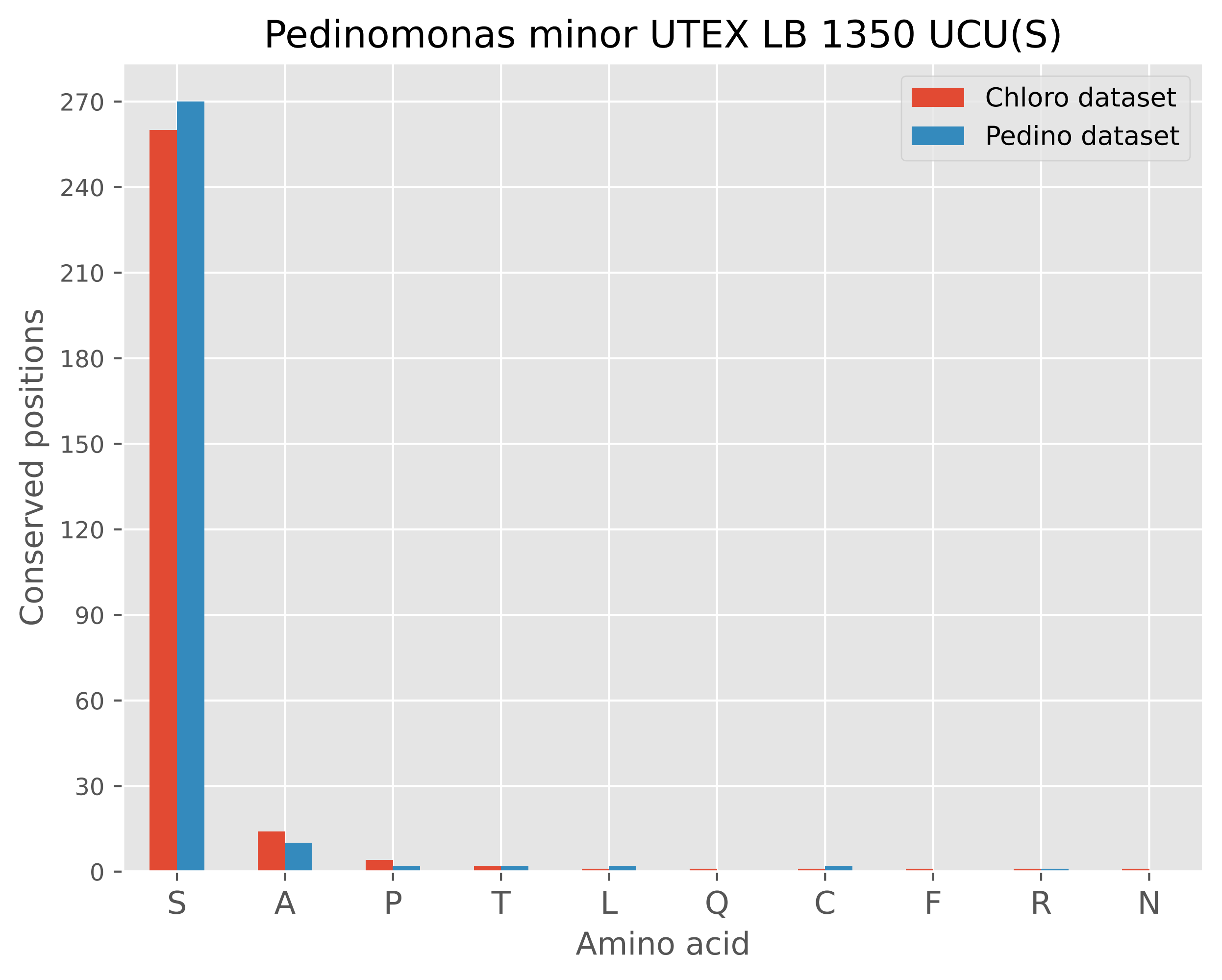

Pedinomonas minor UTEX LB 1350 UCU(S)
Chloro dataset
Pedino dataset
Conserved positions
0
30
60
90
120
150
180
210
240
270
S
A
P
T
L
Q
C
F
R
N
Amino acid

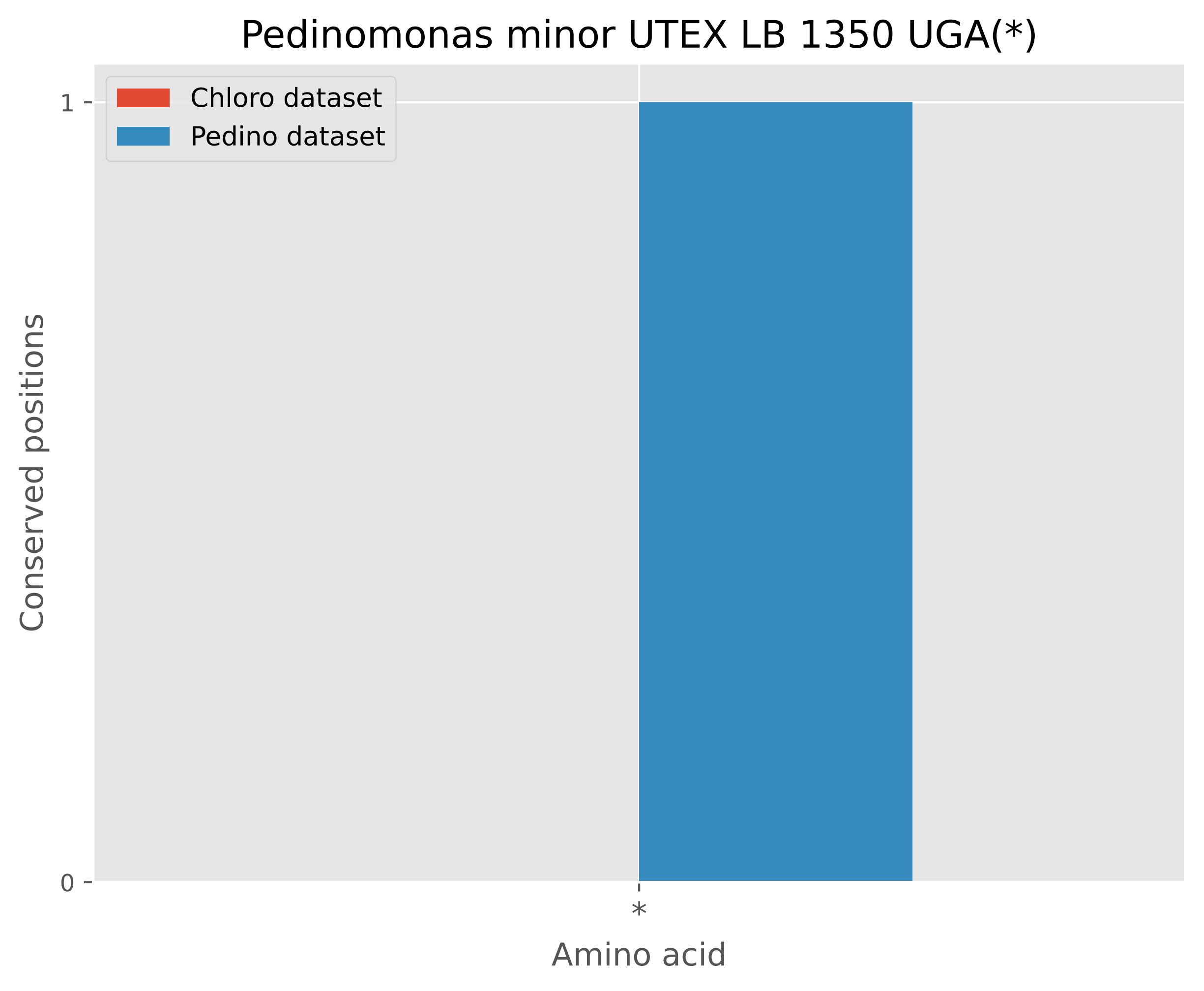

Pedinomonas minor UTEX LB 1350 UGA(*)
Chloro dataset
Pedino dataset
1
0
Conserved positions
*
Amino acid

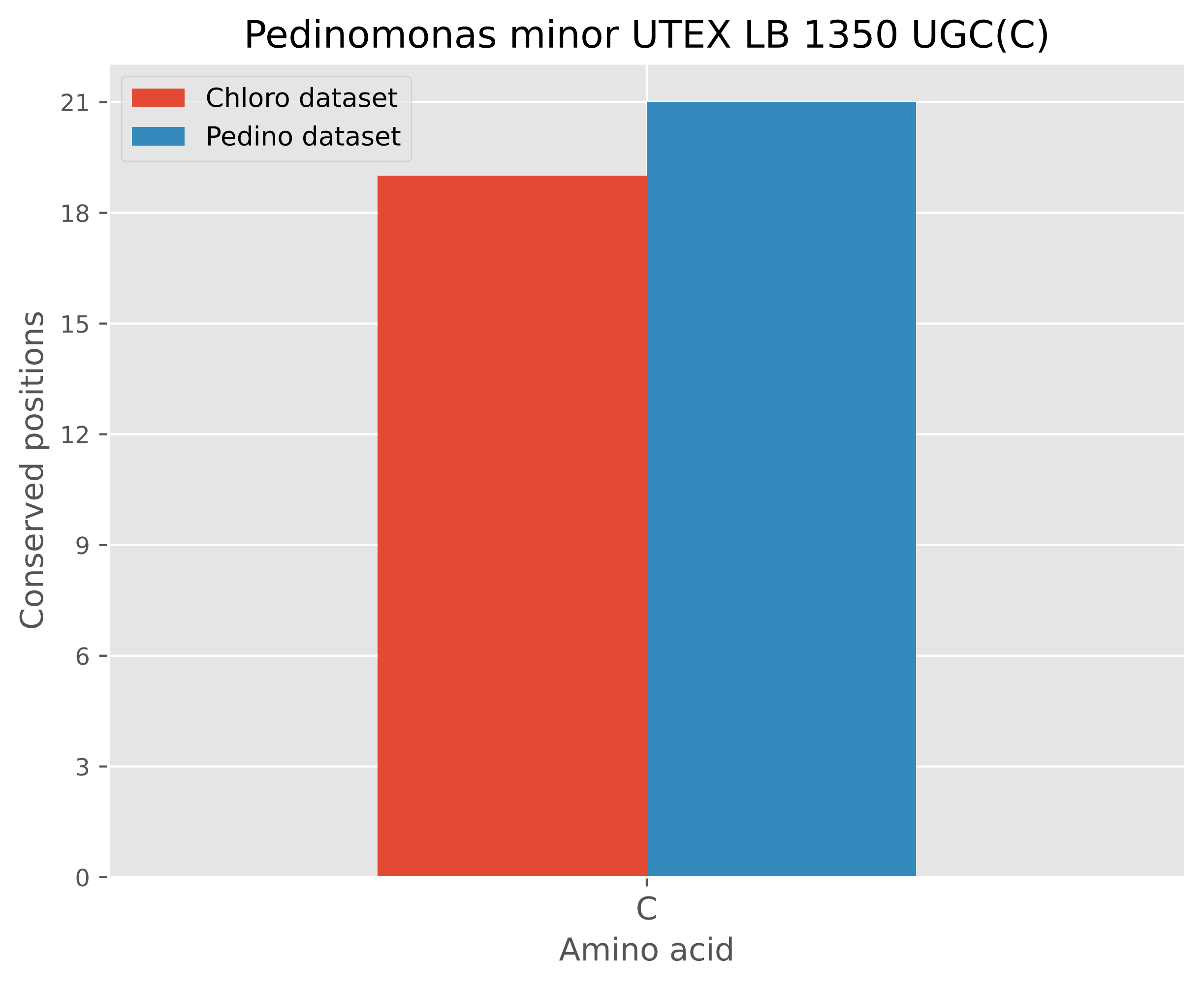

Pedinomonas minor UTEX LB 1350 UGC(C)
Chloro dataset
Pedino dataset
Conserved positions
0
3
6
9
12
15
18
21
C
Amino acid

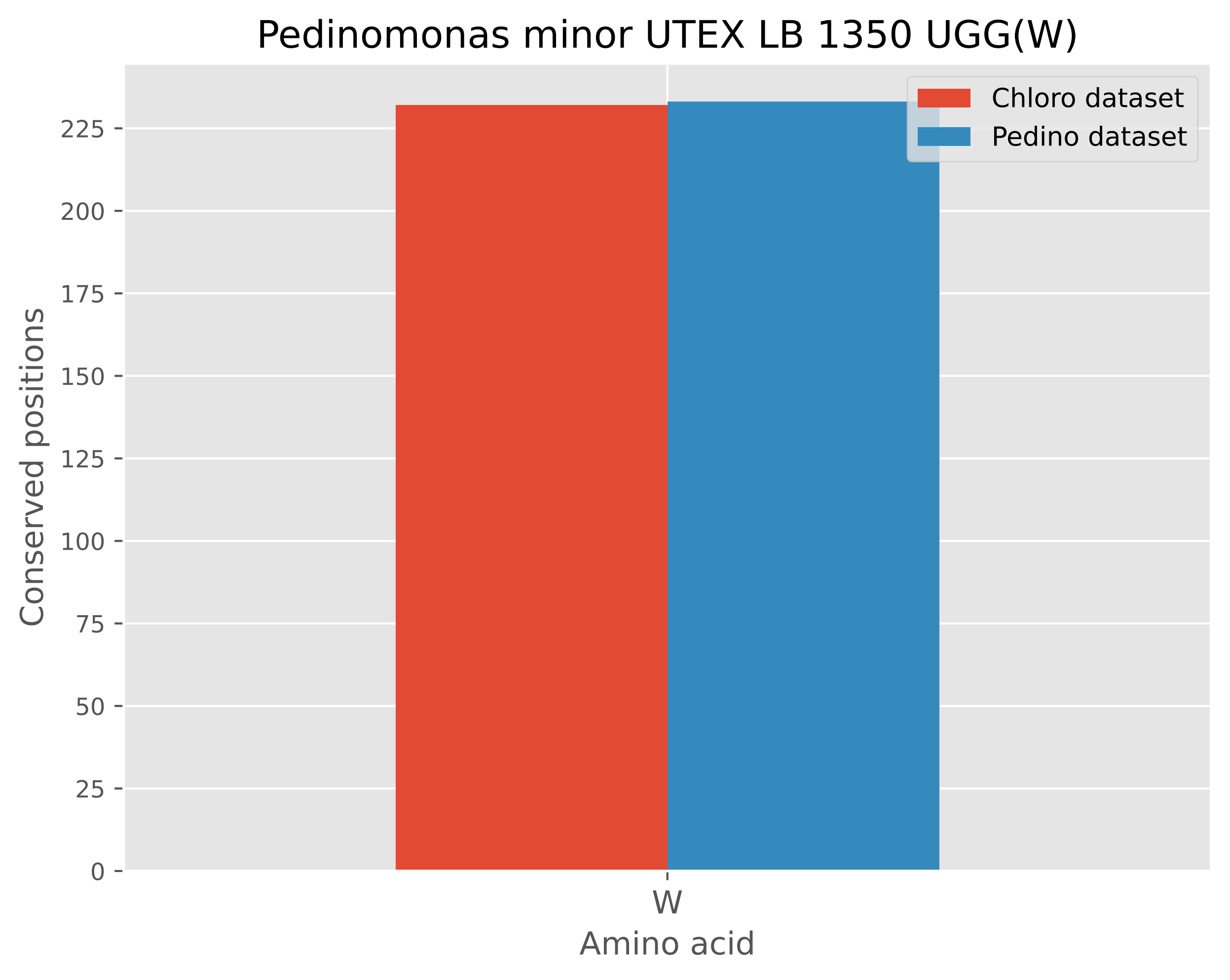

Pedinomonas minor UTEX LB 1350 UGG(W)
Conserved positions
0
25
50
75
100
125
150
175
200
225
W
Amino acid
Chloro dataset
Pedino dataset

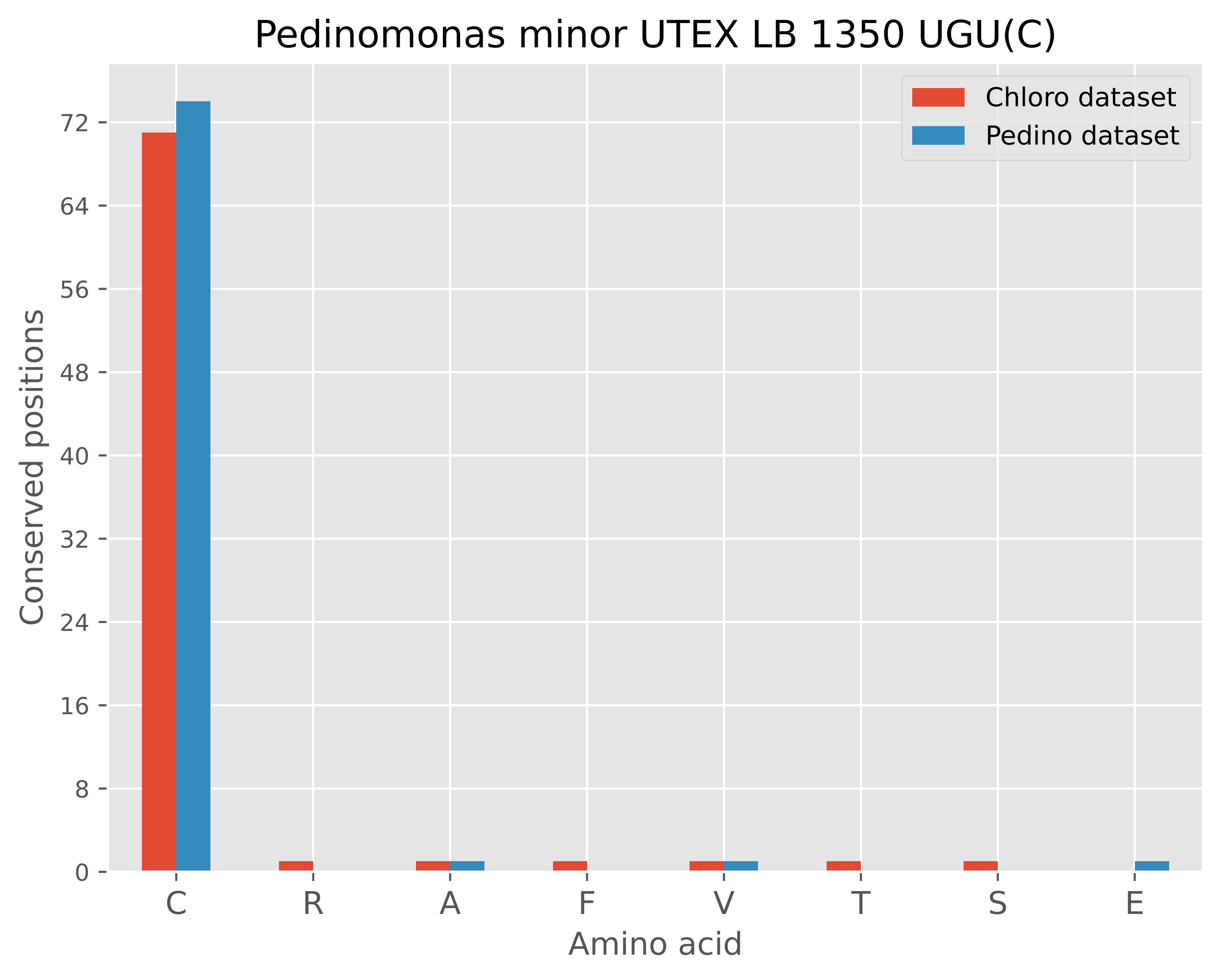

Pedinomonas minor UTEX LB 1350 UGU(C)
Chloro dataset
Pedino dataset
Conserved positions
Amino acid
0
8
16
24
32
40
48
56
64
72
C
R
A
F
V
T
S
E

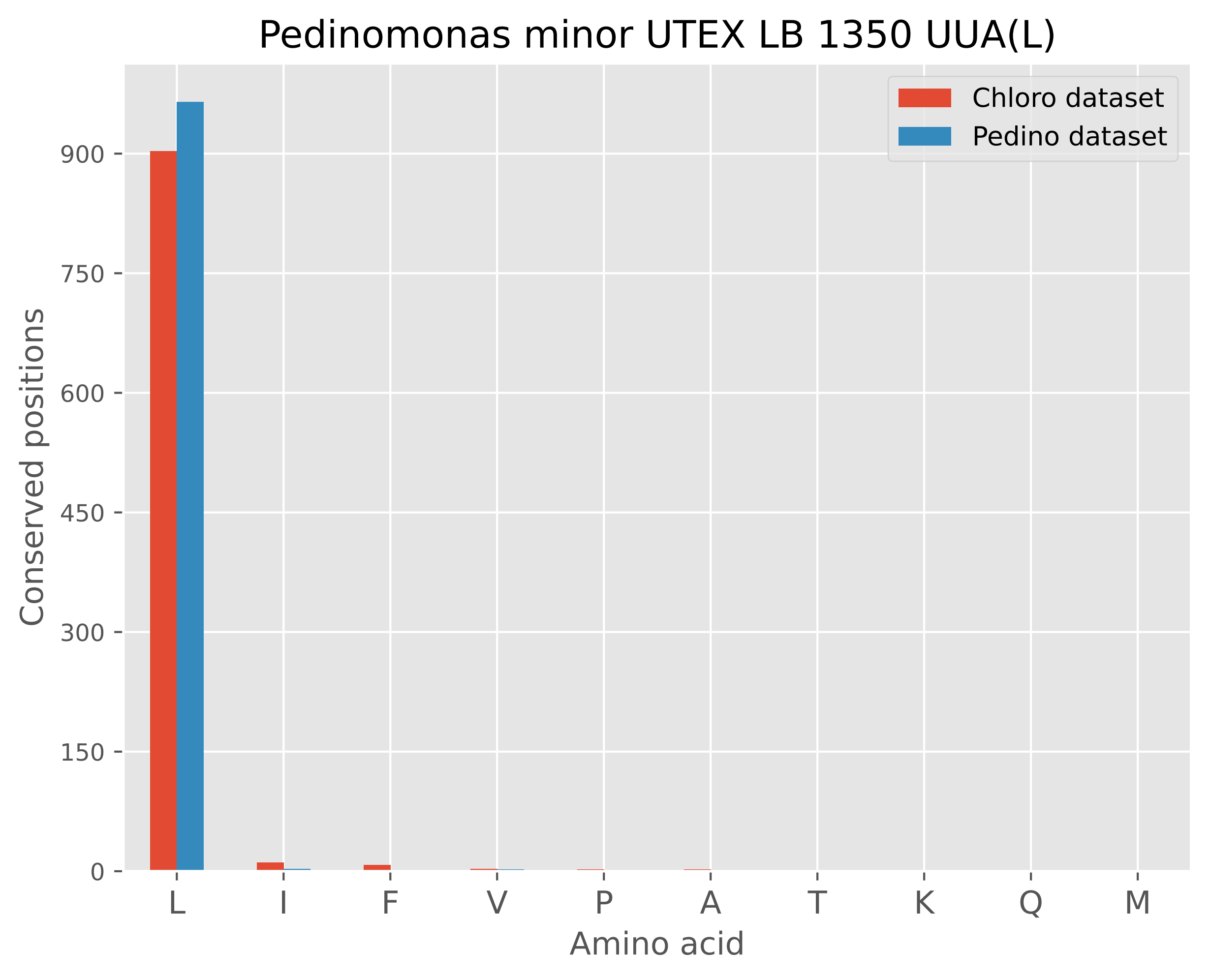

Pedinomonas minor UTEX LB 1350 UUA(L)
Chloro dataset
Pedino dataset
Conserved positions
0
150
300
450
600
750
900
L
I
F
V
P
A
T
K
Q
M
Amino acid

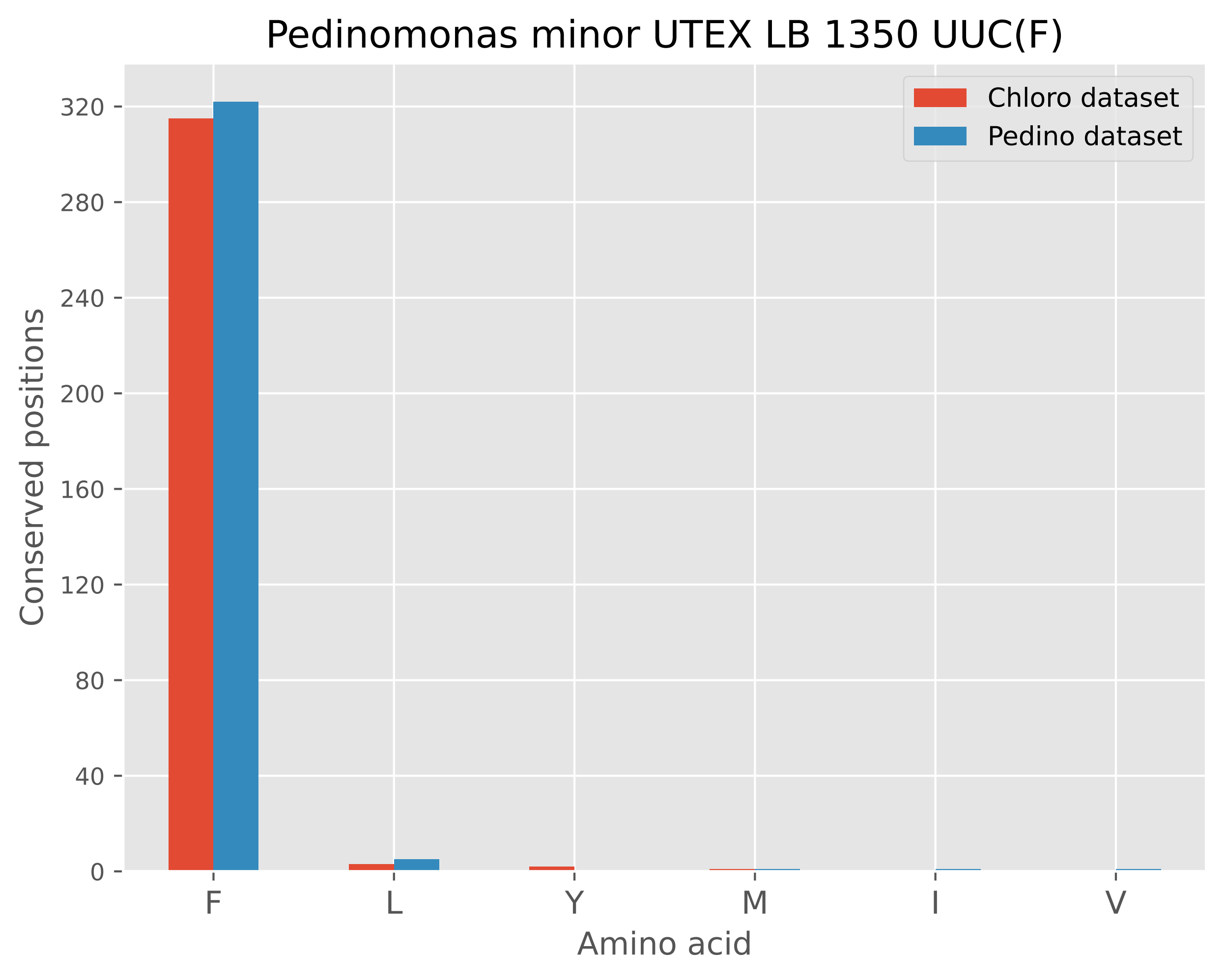

Pedinomonas minor UTEX LB 1350 UUC(F)
Chloro dataset
Pedino dataset
Conserved positions
0
40
80
120
160
200
240
280
320
F
L
Y
M
I
V
Amino acid

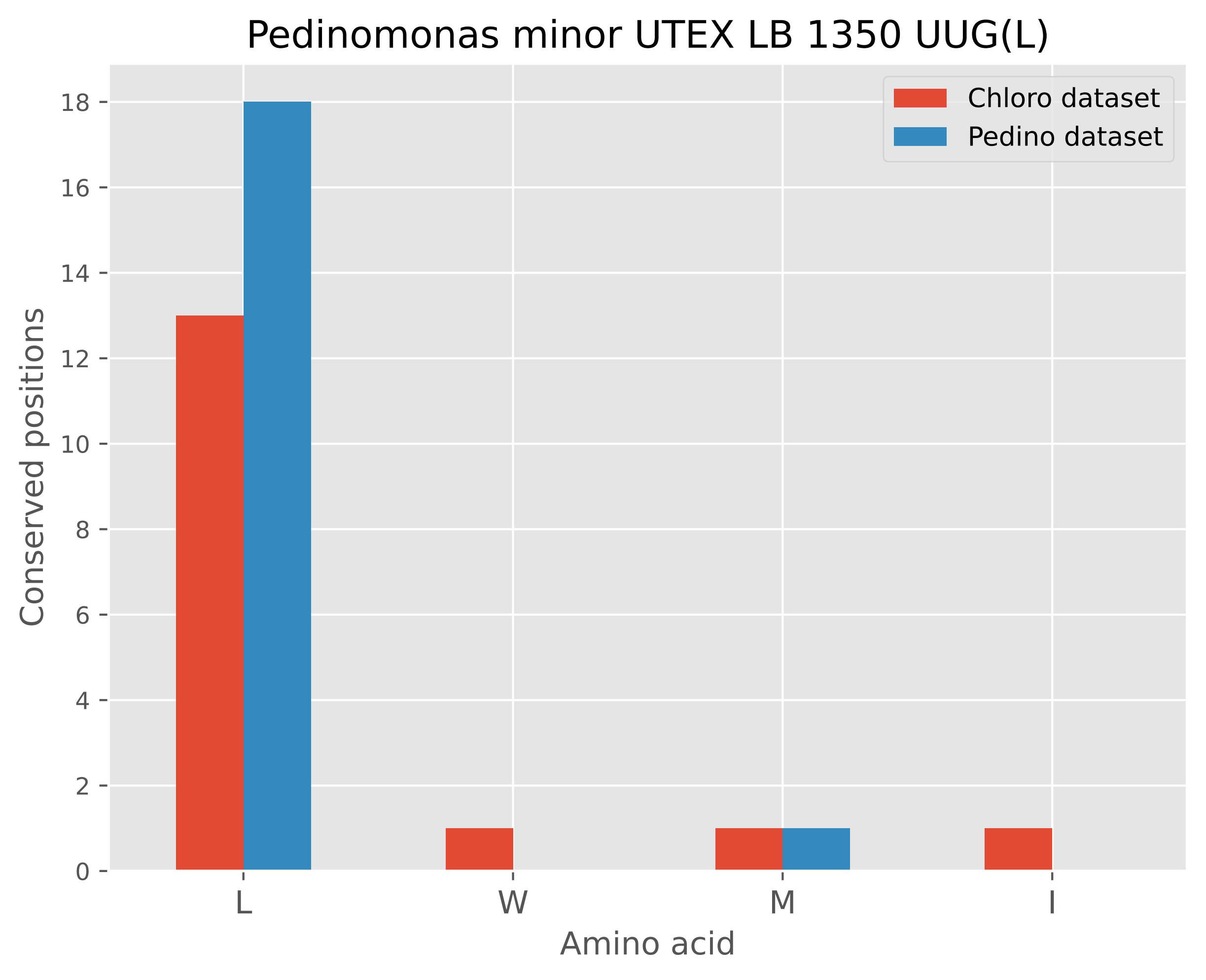
Pedinomonas minor UTEX LB 1350 UUG(L)
Chloro dataset
Pedino dataset
Conserved positions
0
2
4
6
8
10
12
14
16
18
L
W
M
I
Amino acid

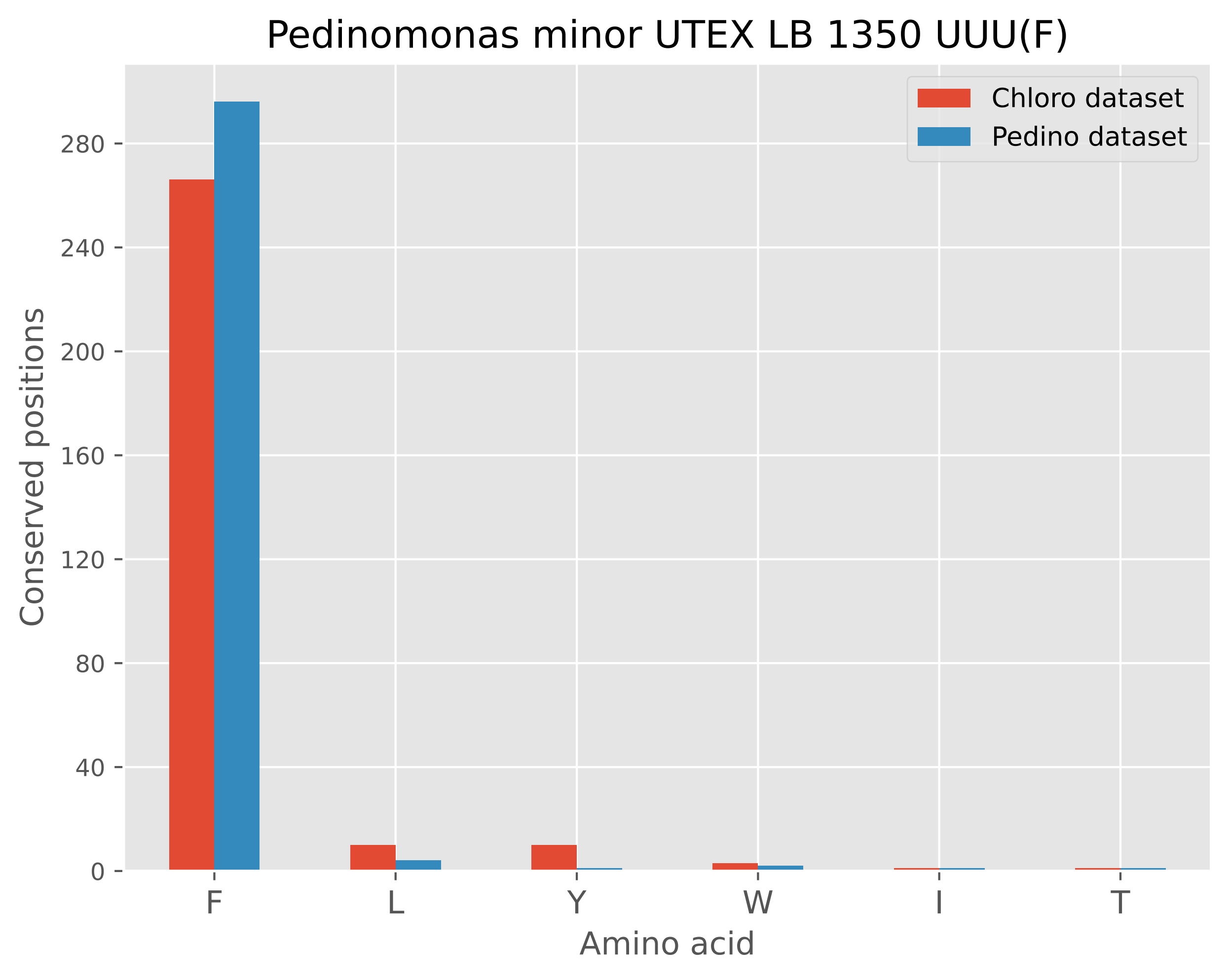
Pedinomonas minor UTEX LB 1350 UUU(F)
Chloro dataset
Pedino dataset
Conserved positions
0
40
80
120
160
200
240
280
F
L
Y
W
I
T
Amino acid

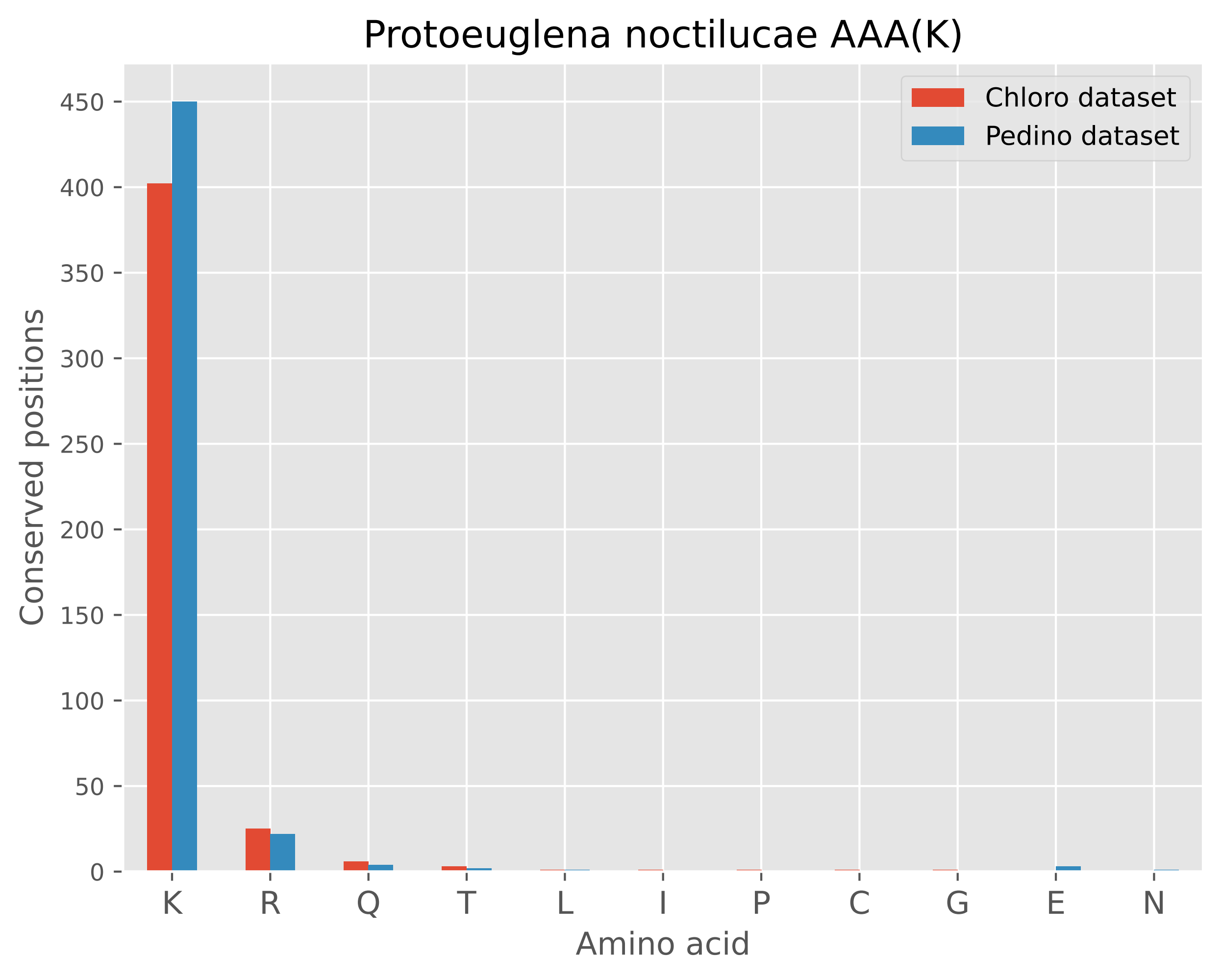

Protoeuglena noctilucae AAA(K)
Chloro dataset
Pedino dataset
Conserved positions
0
50
100
150
200
250
300
350
400
450
K
R
Q
T
L
I
P
C
G
E
N
Amino acid

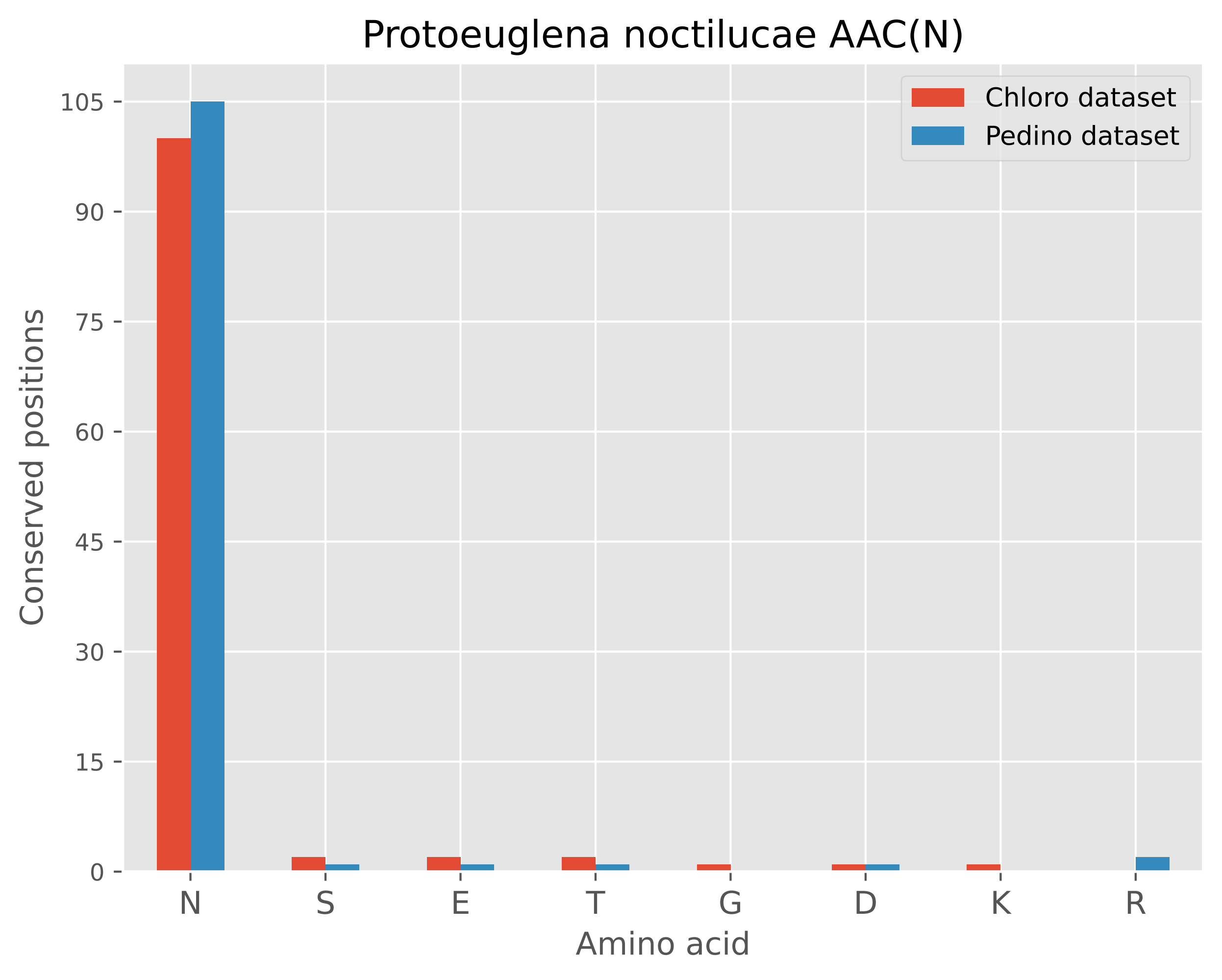

Protoeuglena noctilucae AAC(N)
Chloro dataset
Pedino dataset
Conserved positions
105
90
75
60
45
30
15
0
N
S
E
T
G
D
K
R
Amino acid

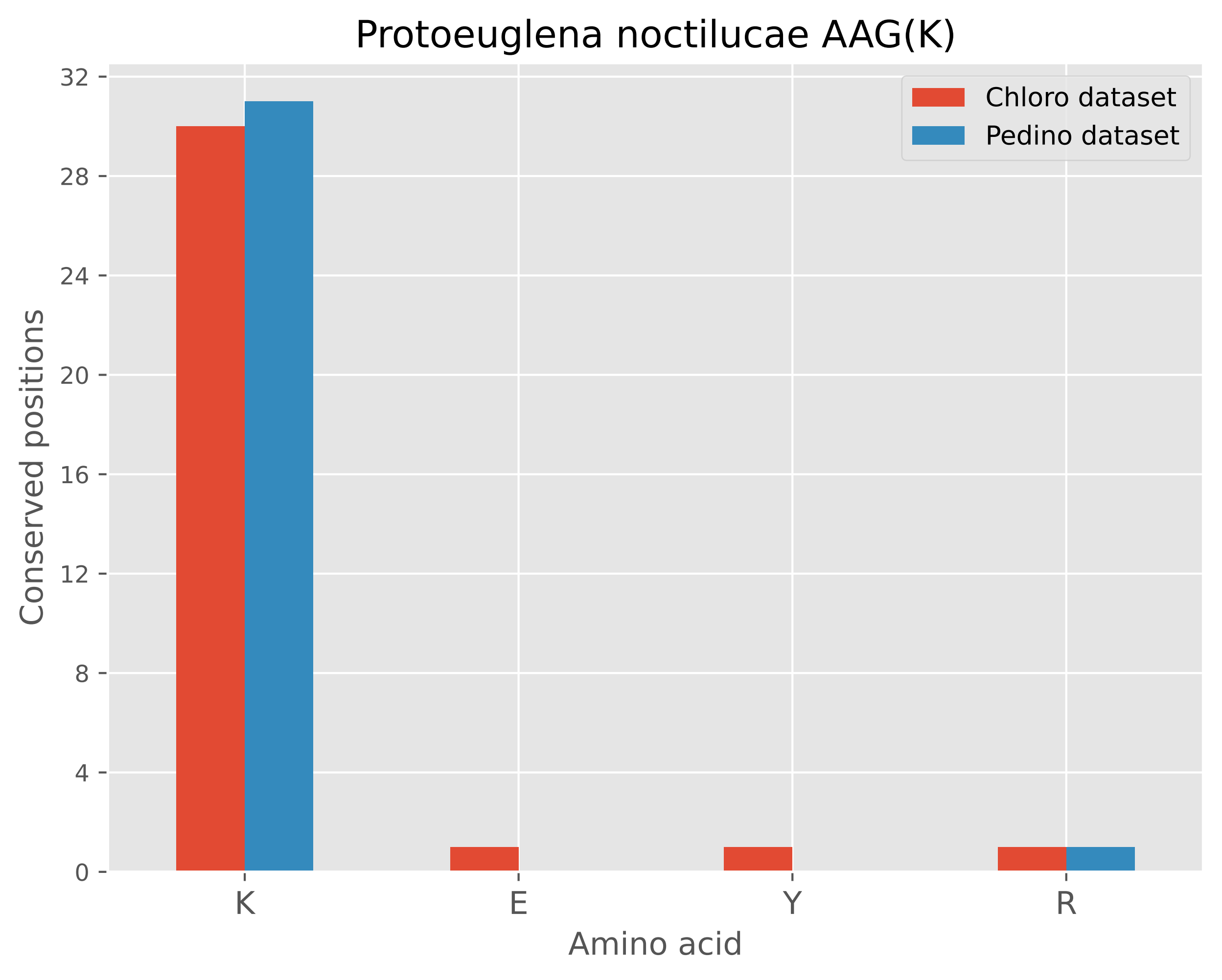

Protoeuglena noctilucae AAG(K)
Chloro dataset
Pedino dataset
Conserved positions
0
4
8
12
16
20
24
28
32
K
E
Y
R
Amino acid

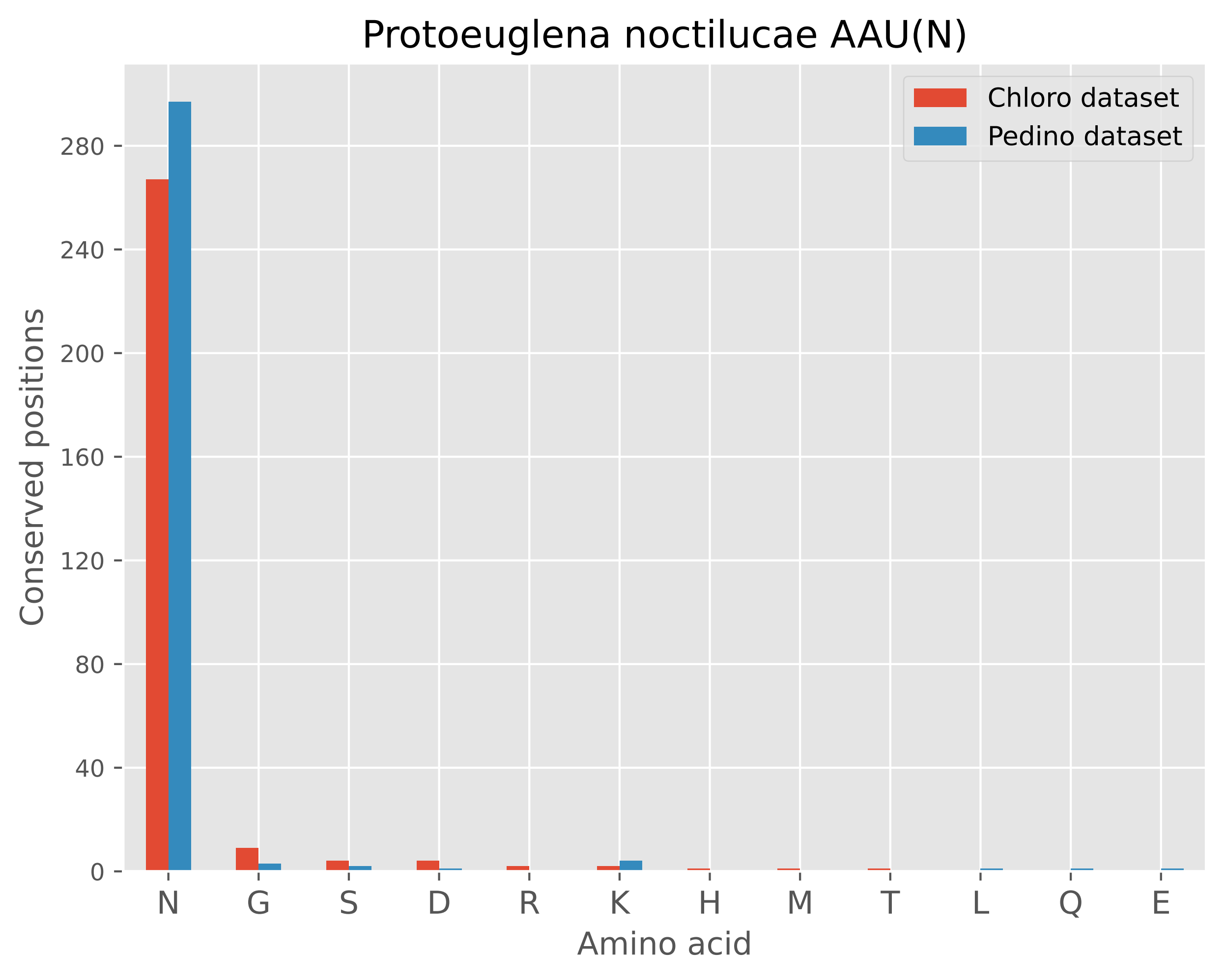

Protoeuglena noctilucae AAU(N)
Chloro dataset
Pedino dataset
Conserved positions
0
40
80
120
160
200
240
280
N
G
S
D
R
K
H
M
T
L
Q
E
Amino acid

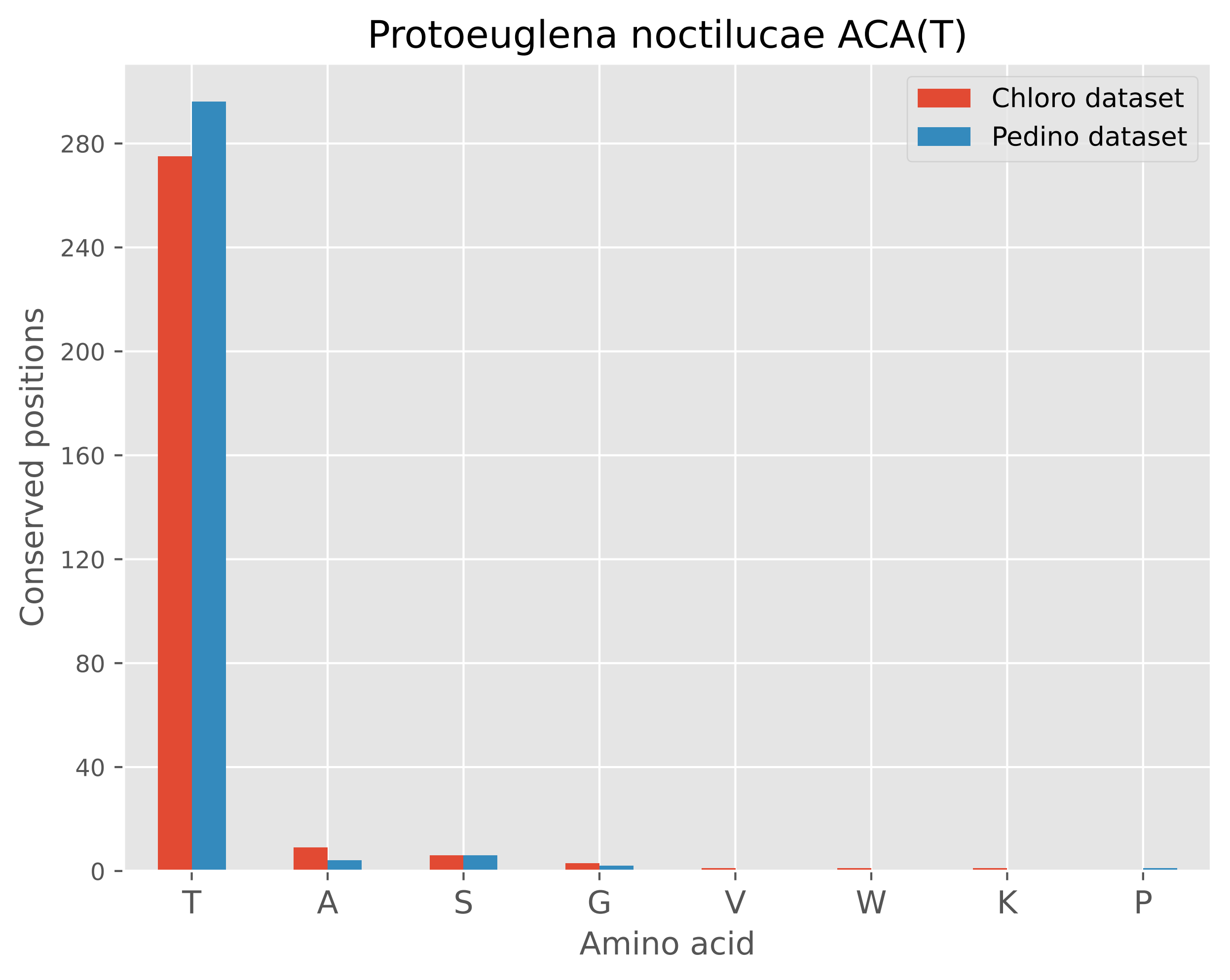

Protoeuglena noctilucae ACA(T)
Chloro dataset
Pedino dataset
Conserved positions
0
40
80
120
160
200
240
280
T
A
S
G
V
W
K
P
Amino acid

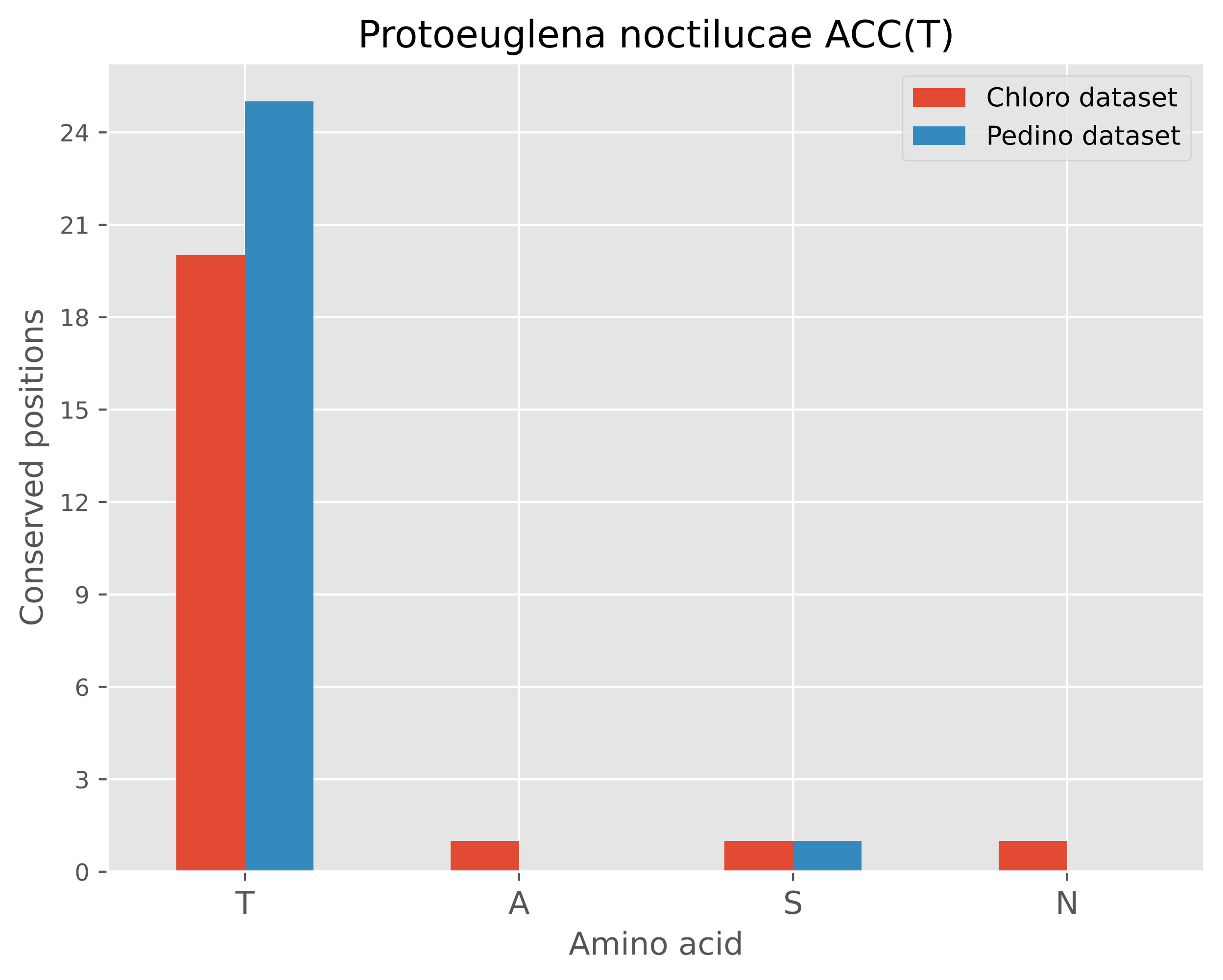

Protoeuglena noctilucae ACC(T)
Chloro dataset
Pedino dataset
Conserved positions
0
3
6
9
12
15
18
21
24
T
A
S
N
Amino acid

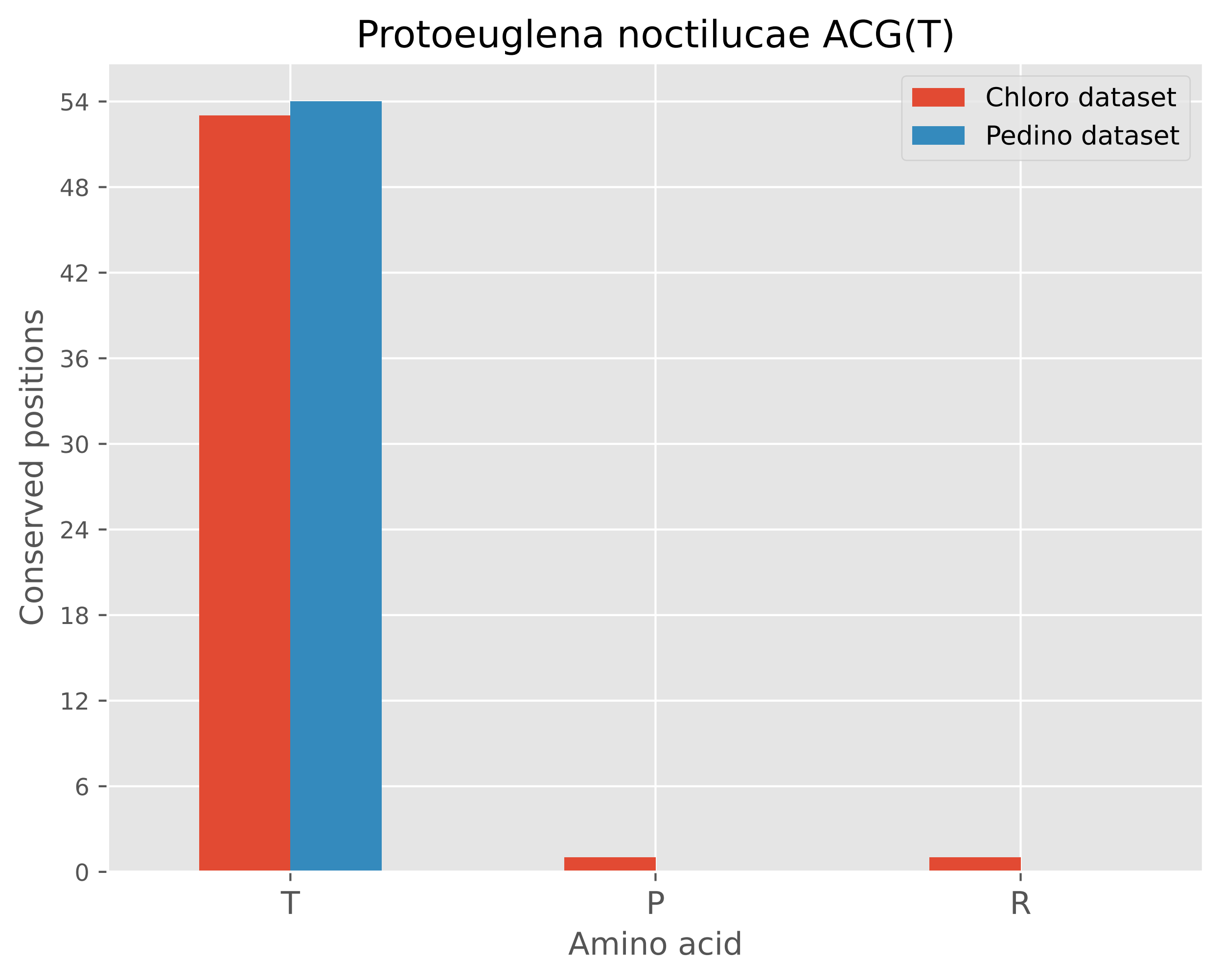

Protoeuglena noctilucae ACG(T)
Chloro dataset
Pedino dataset
Conserved positions
0
6
12
18
24
30
36
42
48
54
T
P
R
Amino acid

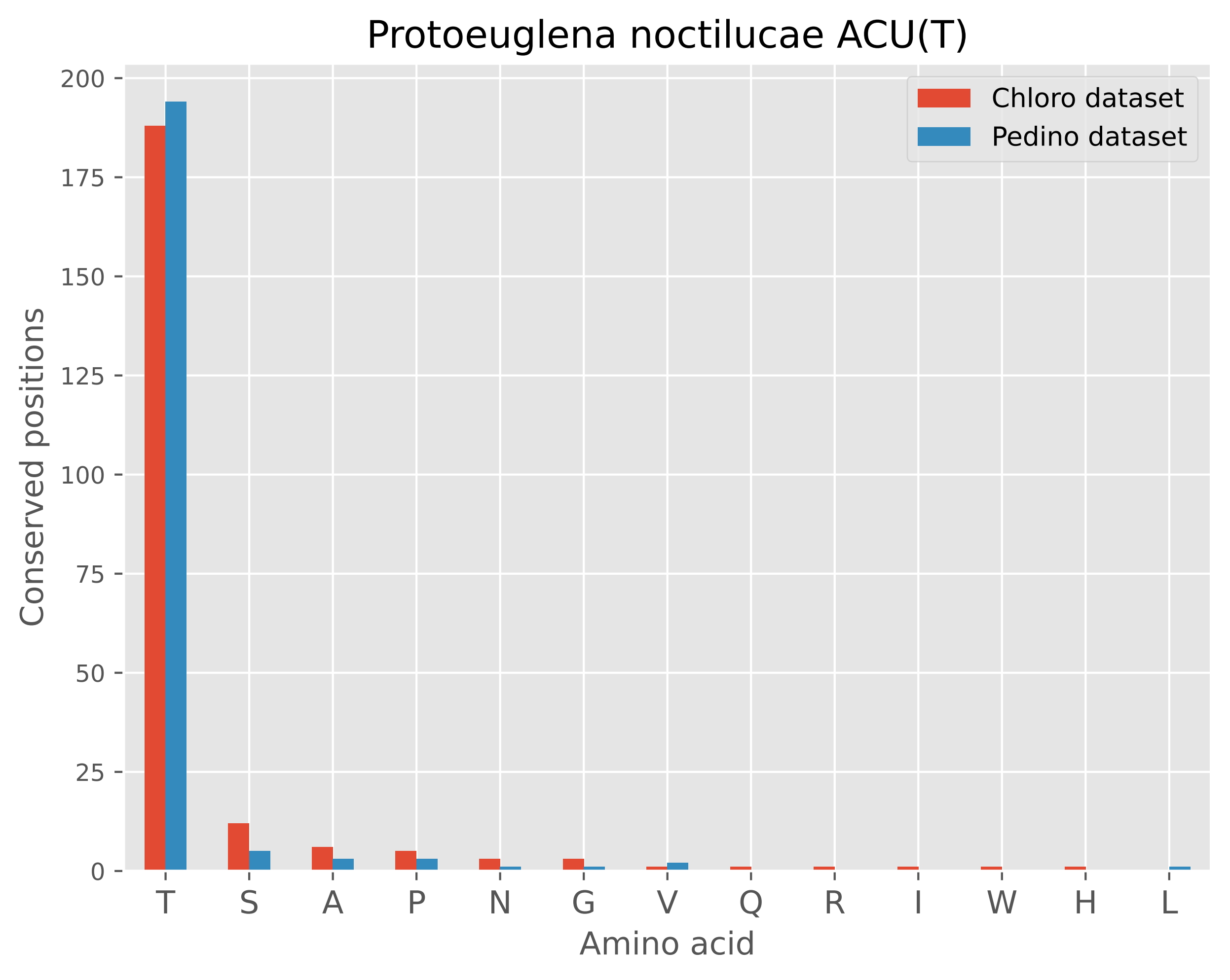

Protoeuglena noctilucae ACU(T)
Chloro dataset
Pedino dataset
Conserved positions
0
25
50
75
100
125
150
175
200
T
S
A
P
N
G
V
Q
R
I
W
H
L
Amino acid

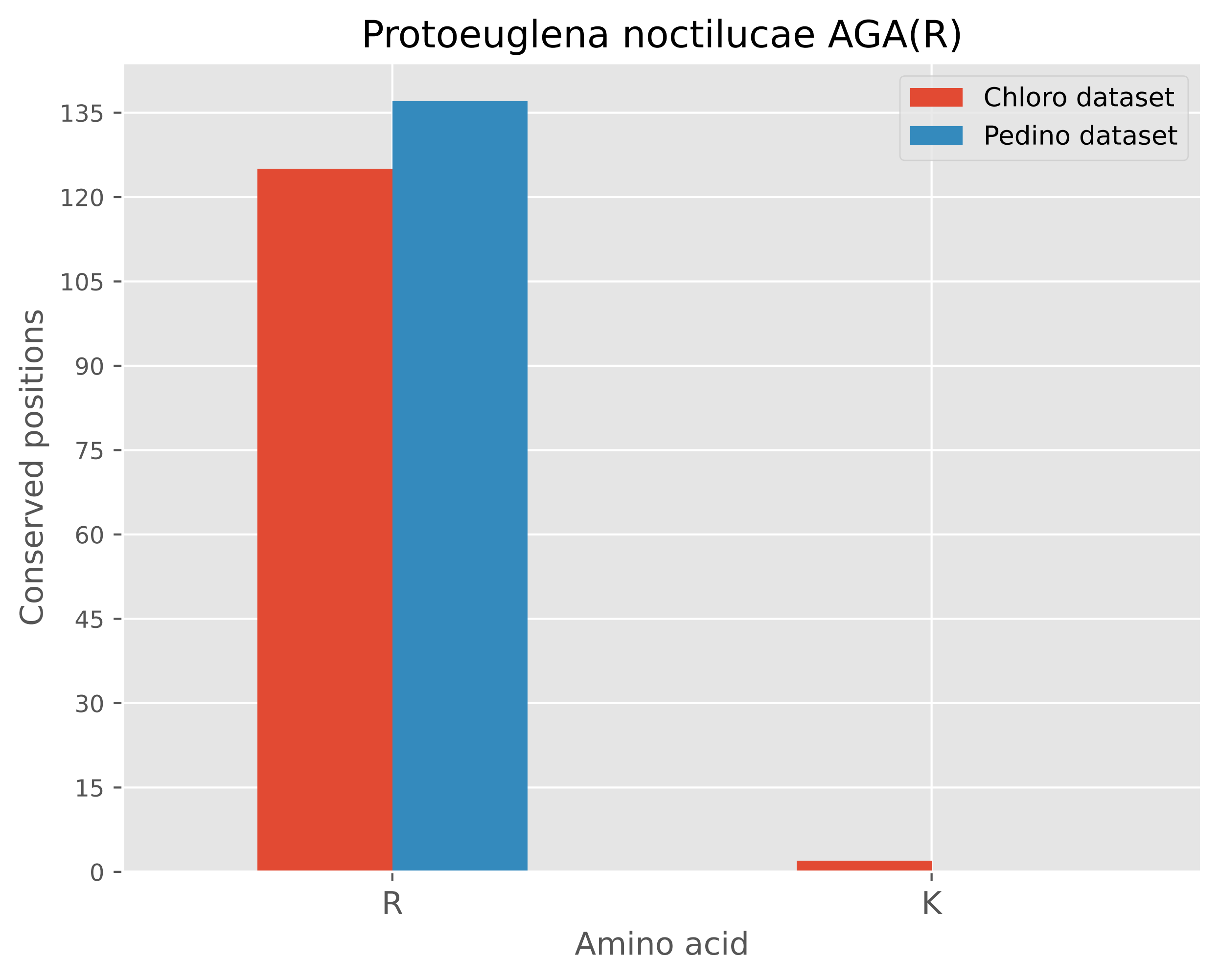

Protoeuglena noctilucae AGA(R)
Chloro dataset
Pedino dataset
Conserved positions
0
15
30
45
60
75
90
105
120
135
R
K
Amino acid

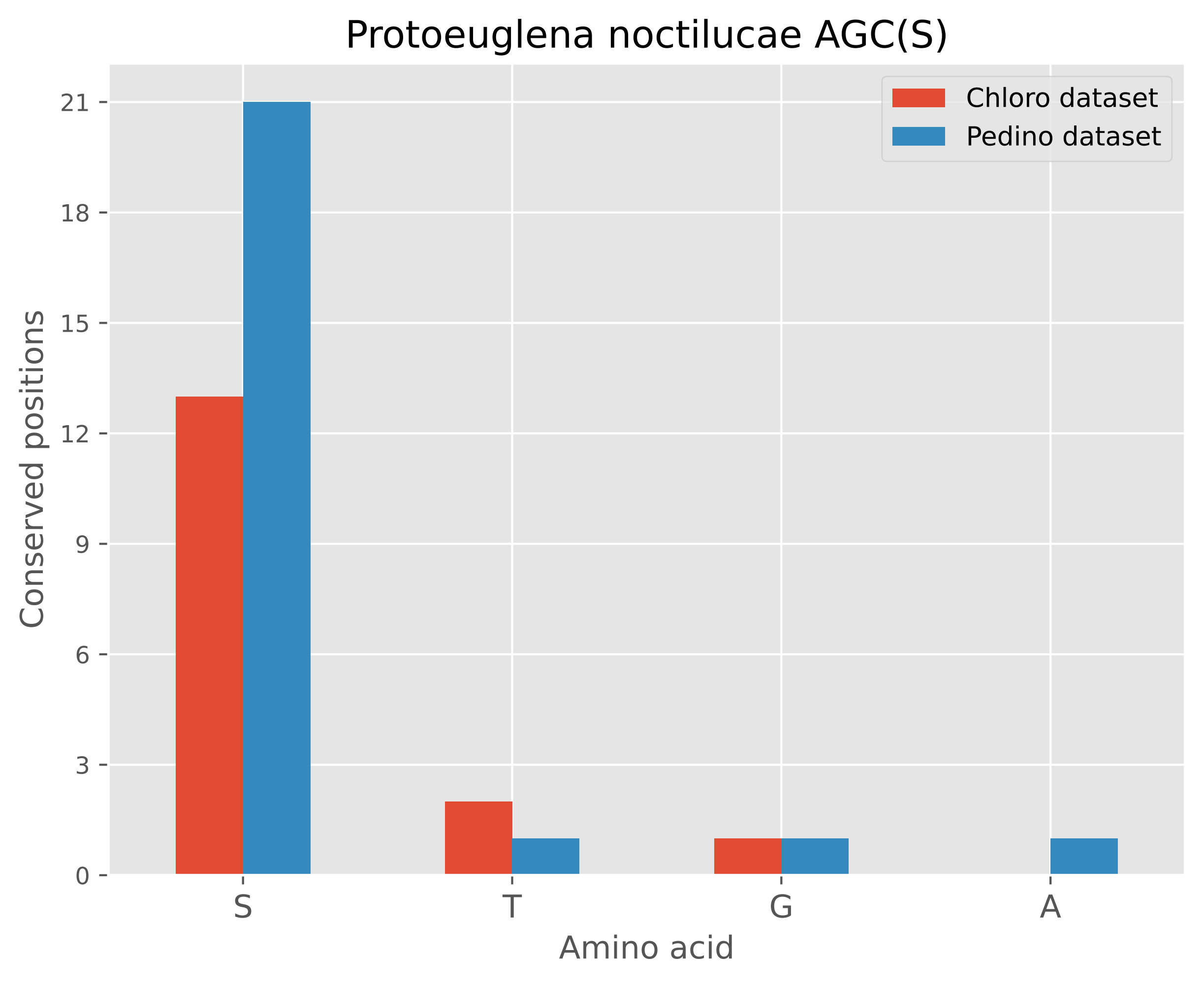
Protoeuglena noctilucae AGC(S)
Chloro dataset
Pedino dataset
Conserved positions
0
3
6
9
12
15
18
21
S
T
G
A
Amino acid

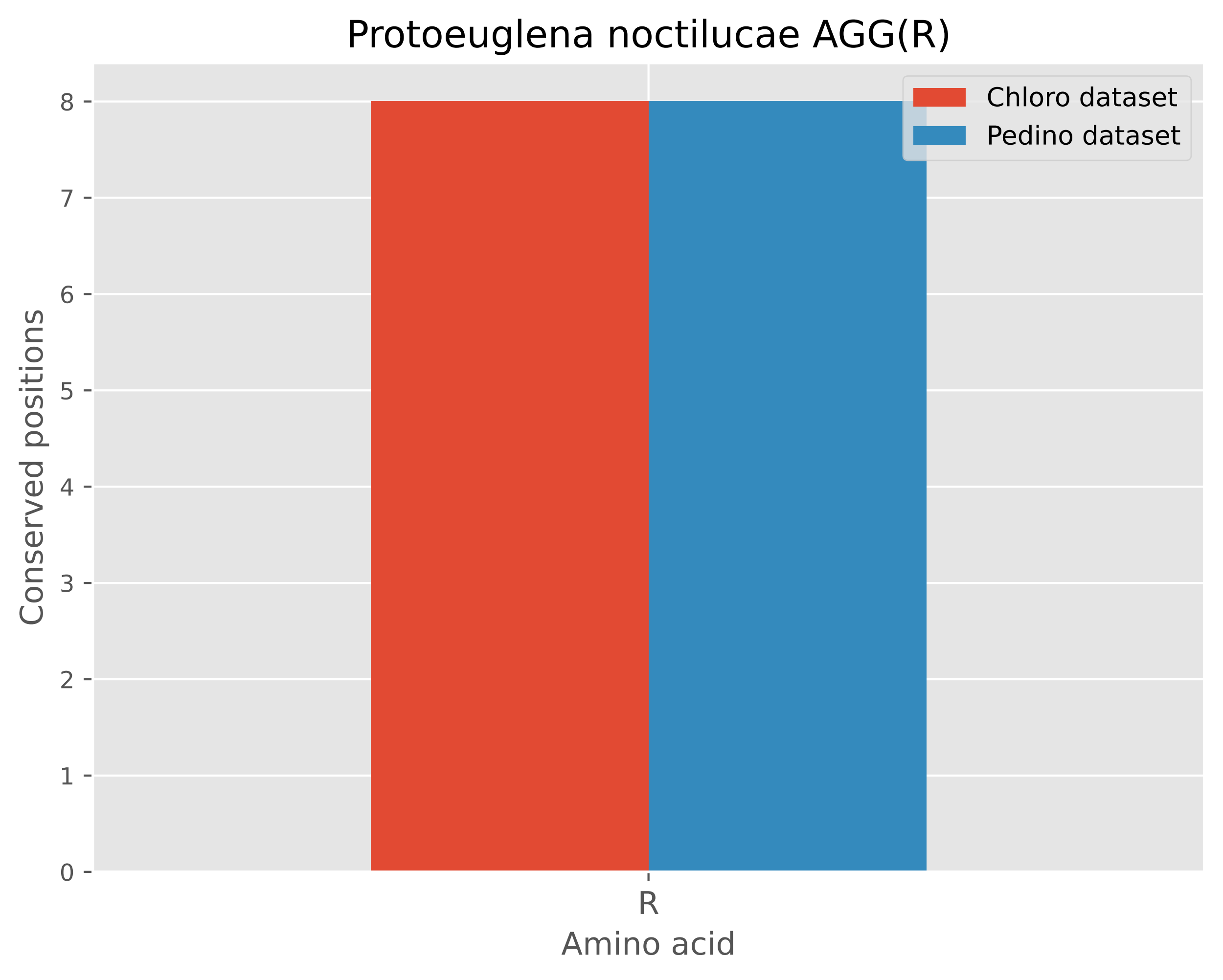

Protoeuglena noctilucae AGG(R)
Chloro dataset
Pedino dataset
Conserved positions
0
1
2
3
4
5
6
7
8
R
Amino acid

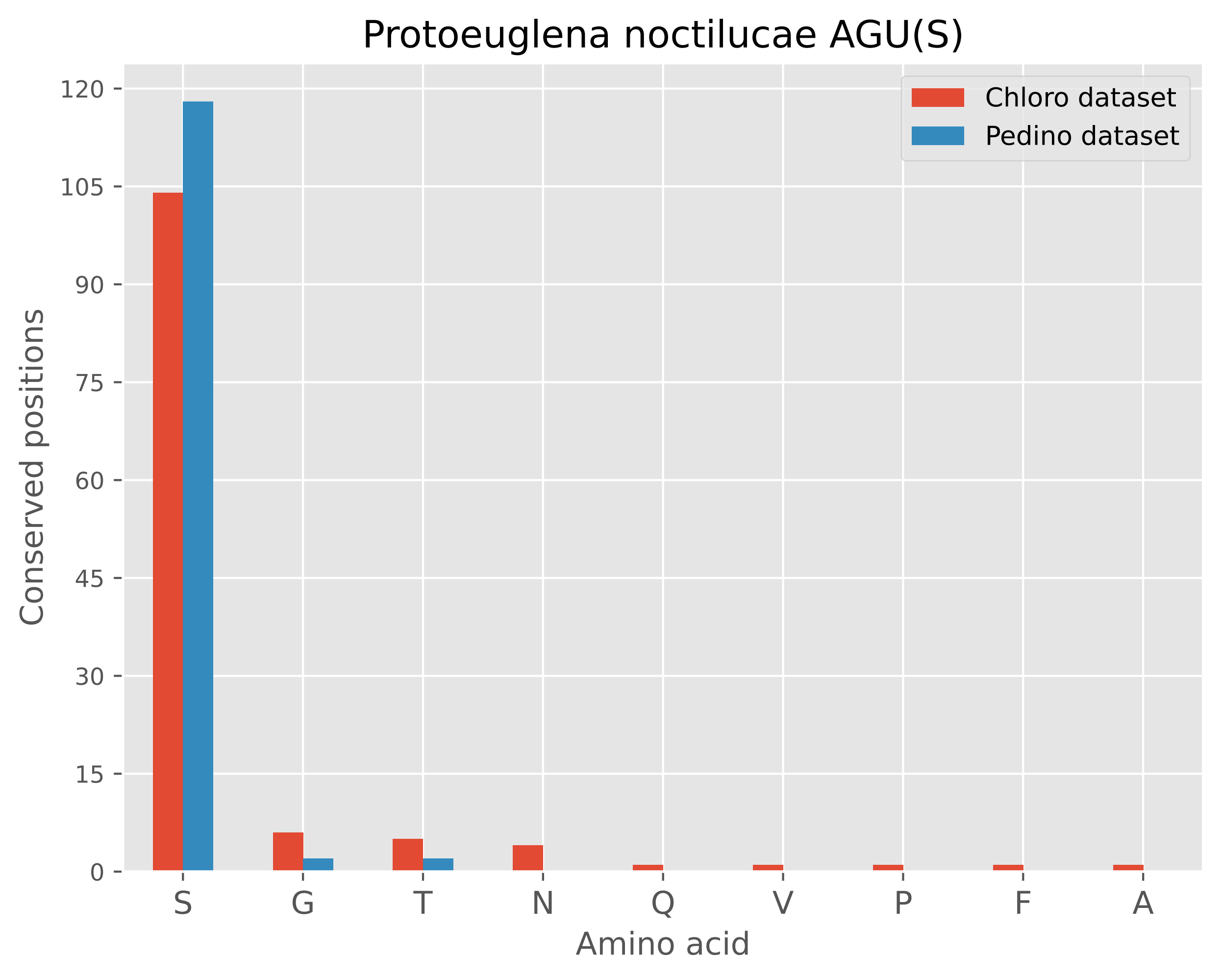

Protoeuglena noctilucae AGU(S)
Chloro dataset
Pedino dataset
Conserved positions
0
15
30
45
60
75
90
105
120
S
G
T
N
Q
V
P
F
A
Amino acid

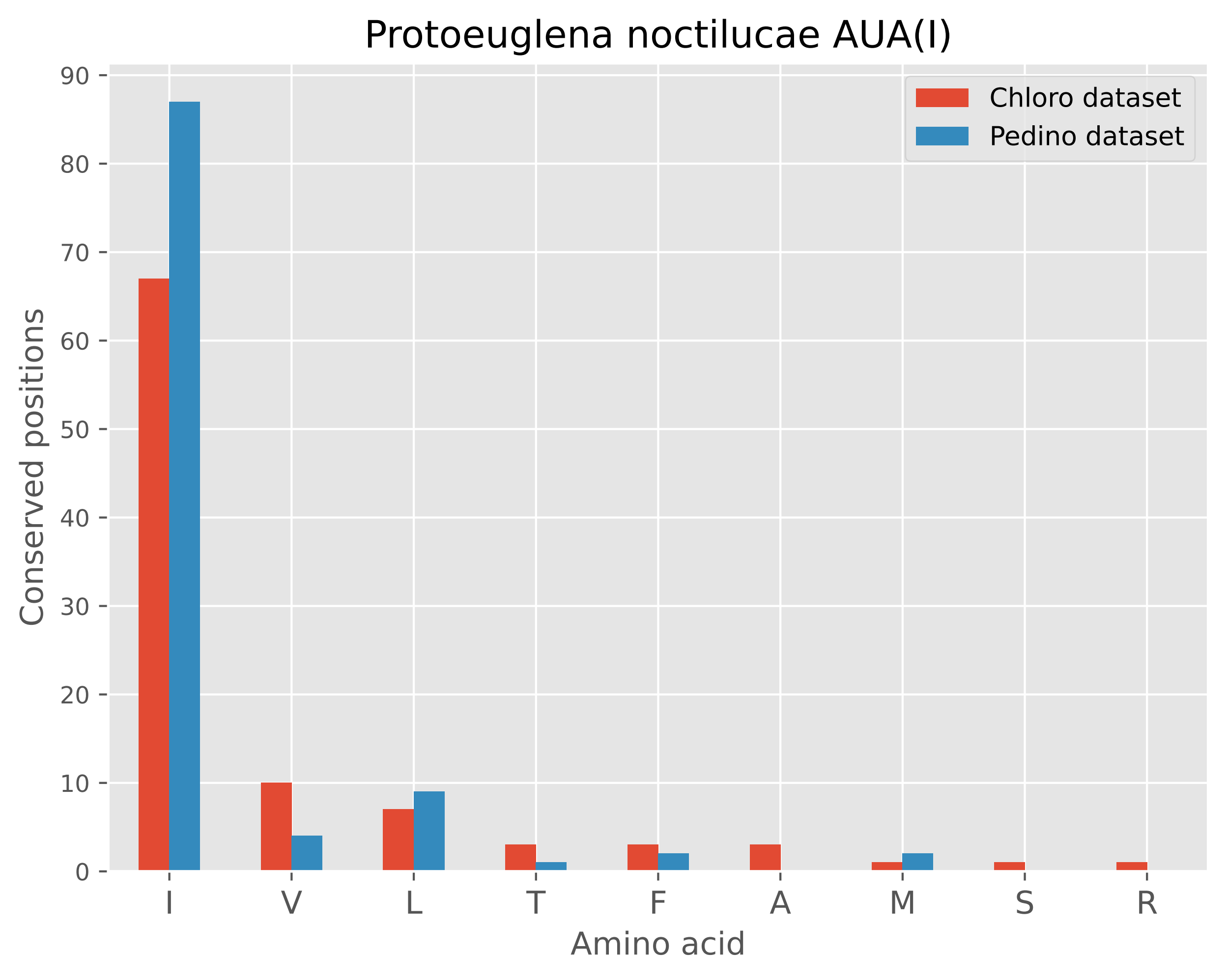

Protoeuglena noctilucae AUA(I)
Chloro dataset
Pedino dataset
Conserved positions
Amino acid
0
10
20
30
40
50
60
70
80
90
I
V
L
T
F
A
M
S
R

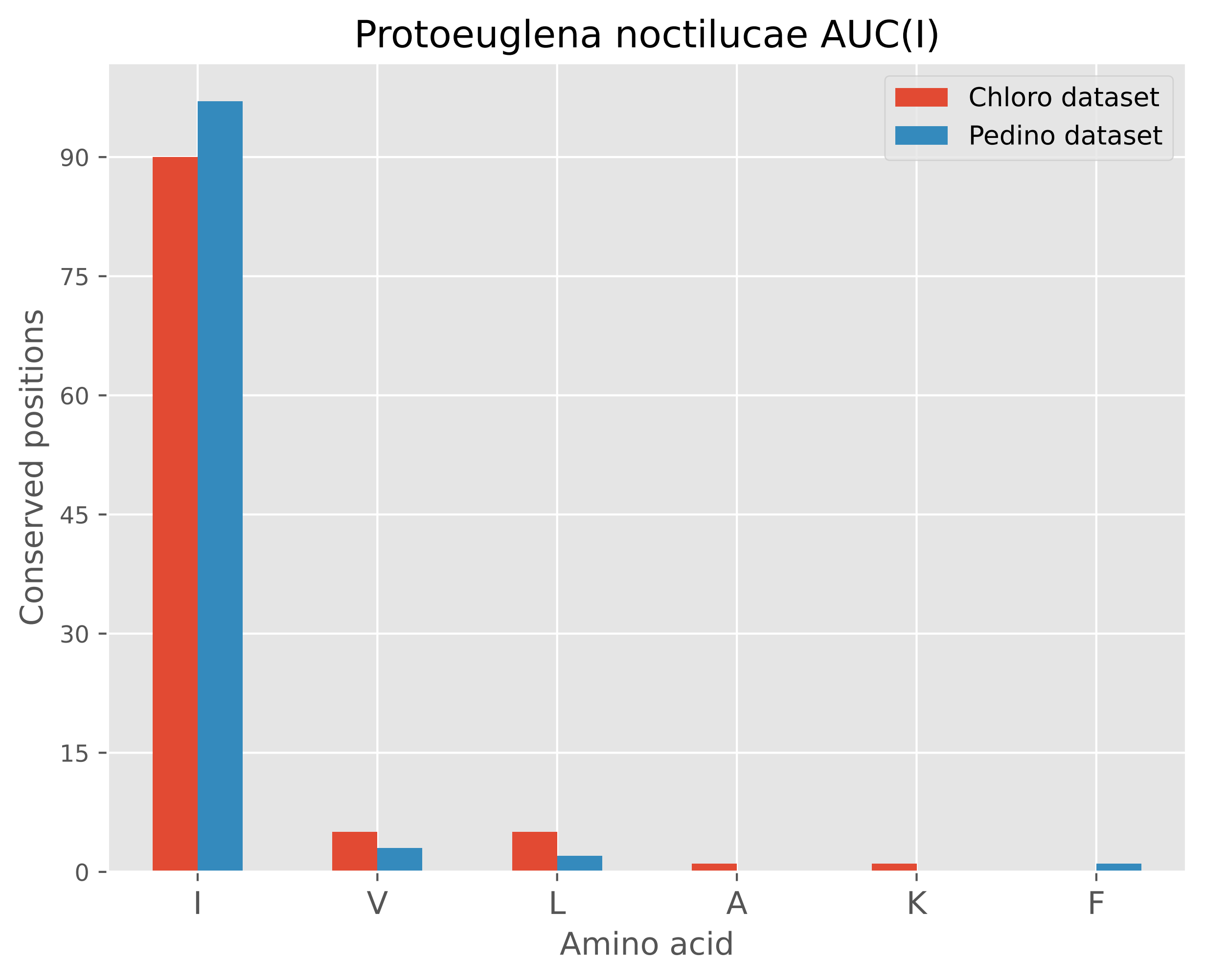
Protoeuglena noctilucae AUC(I)
Chloro dataset
Pedino dataset
Conserved positions
0
15
30
45
60
75
90
I
V
L
A
K
F
Amino acid

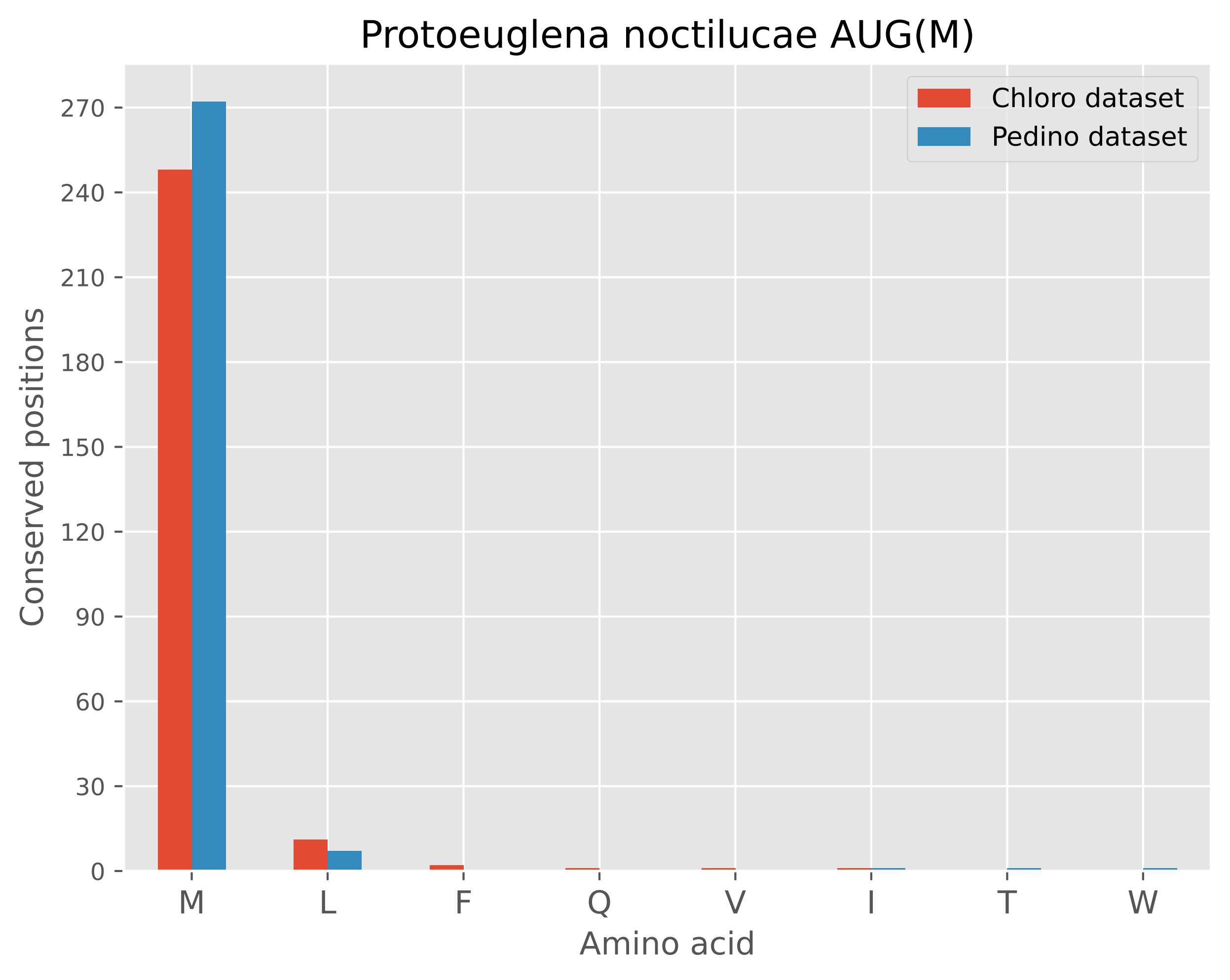

Protoeuglena noctilucae AUG(M)
Chloro dataset
Pedino dataset
Conserved positions
0
30
60
90
120
150
180
210
240
270
M
L
F
Q
V
I
T
W
Amino acid

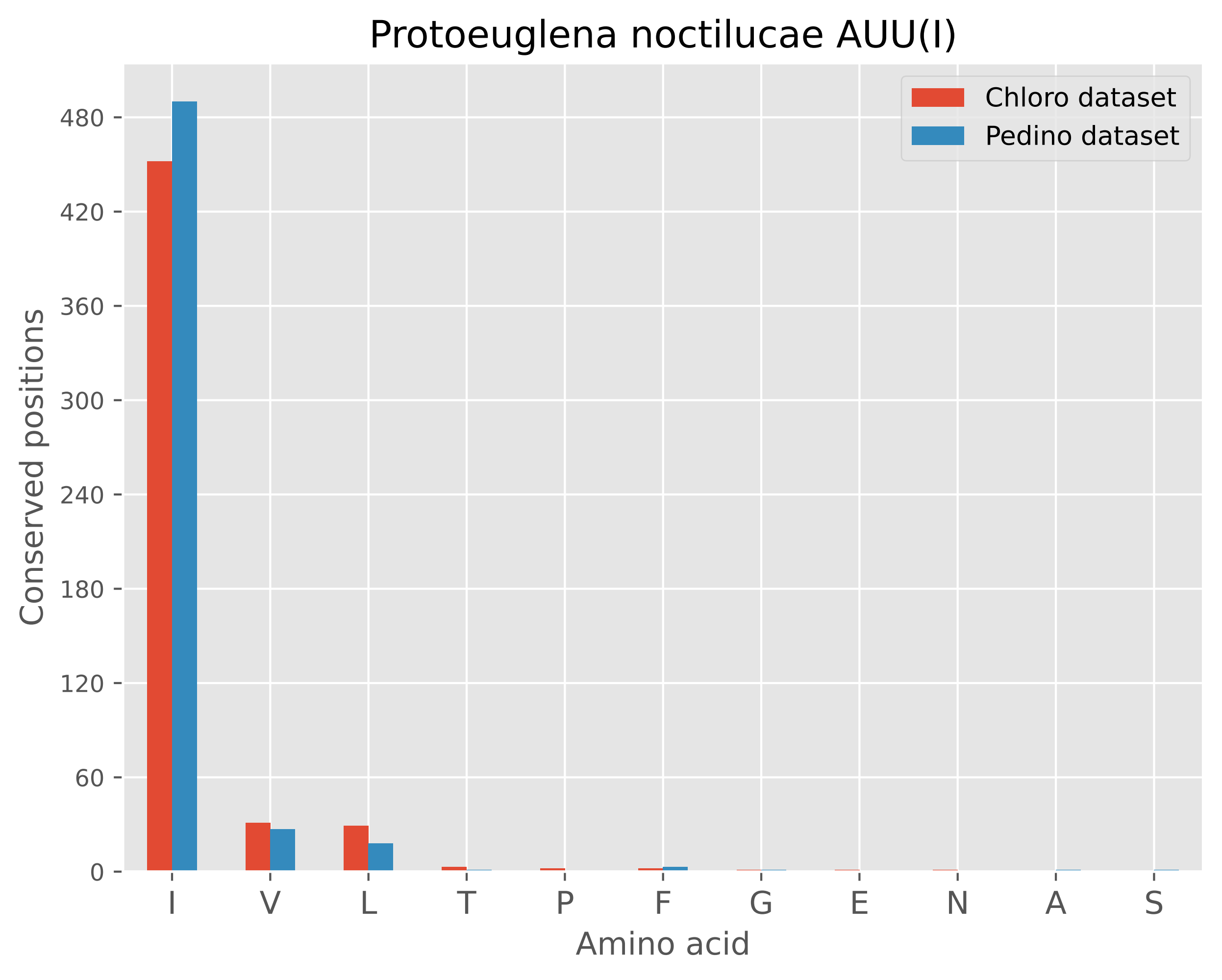

Protoeuglena noctilucae AUU(I)
Chloro dataset
Pedino dataset
Conserved positions
0
60
120
180
240
300
360
420
480
I
V
L
T
P
F
G
E
N
A
S
Amino acid

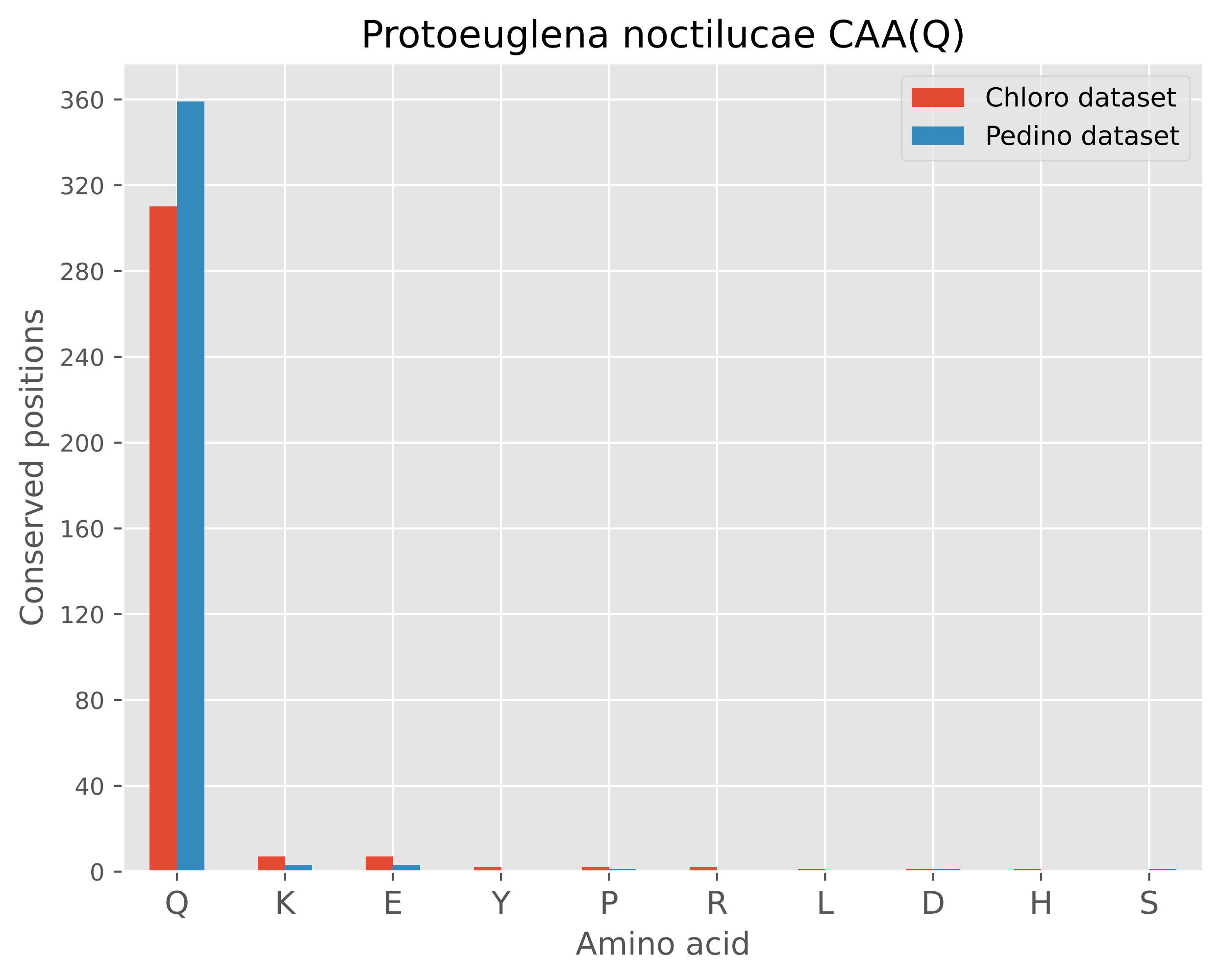
Protoeuglena noctilucae CAA(Q)
Chloro dataset
Pedino dataset
Conserved positions
360
320
280
240
200
160
120
80
40
0
Q
K
E
Y
P
R
L
D
H
S
Amino acid

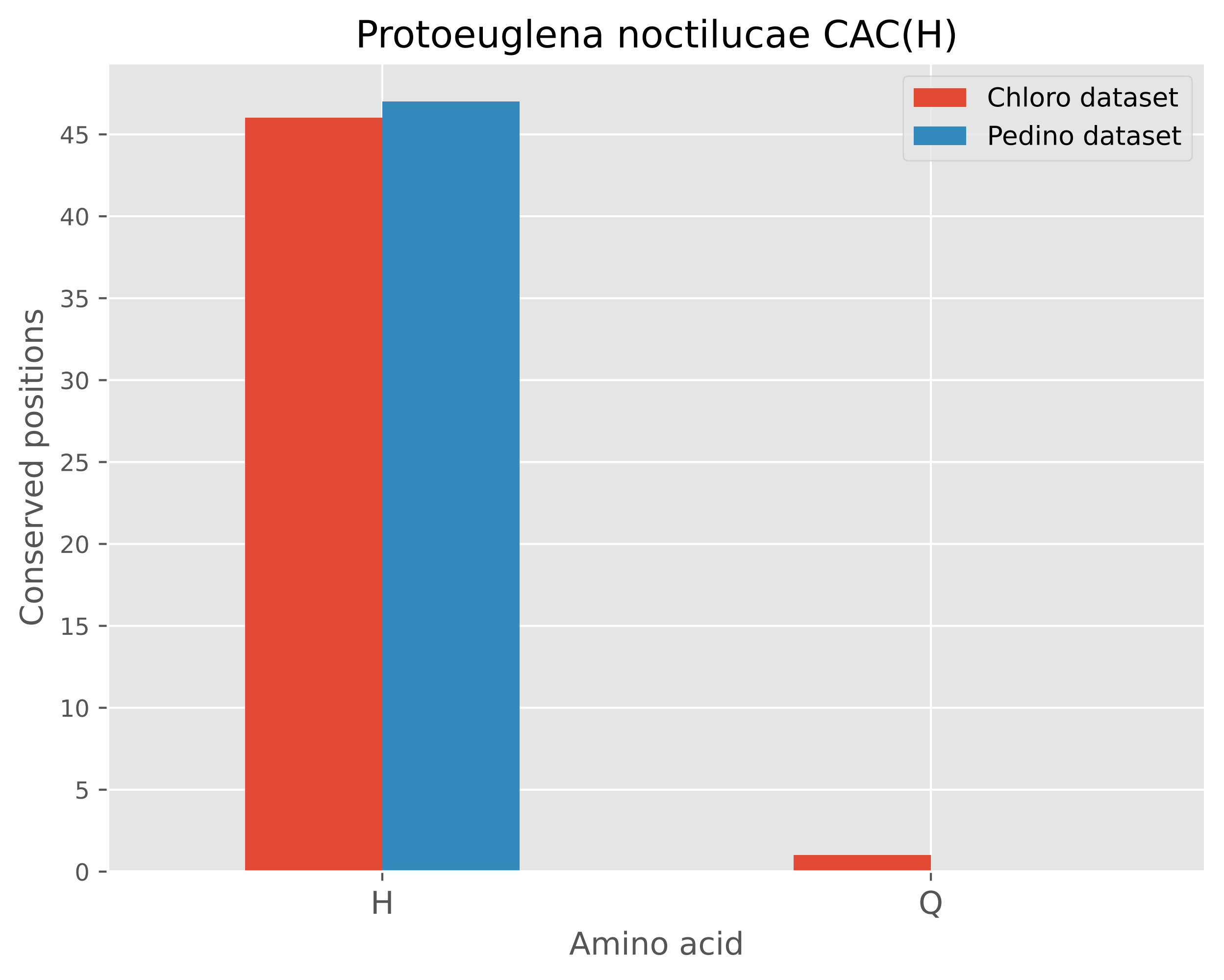

Protoeuglena noctilucae CAC(H)
Chloro dataset
Pedino dataset
Conserved positions
0
5
10
15
20
25
30
35
40
45
H
Q
Amino acid

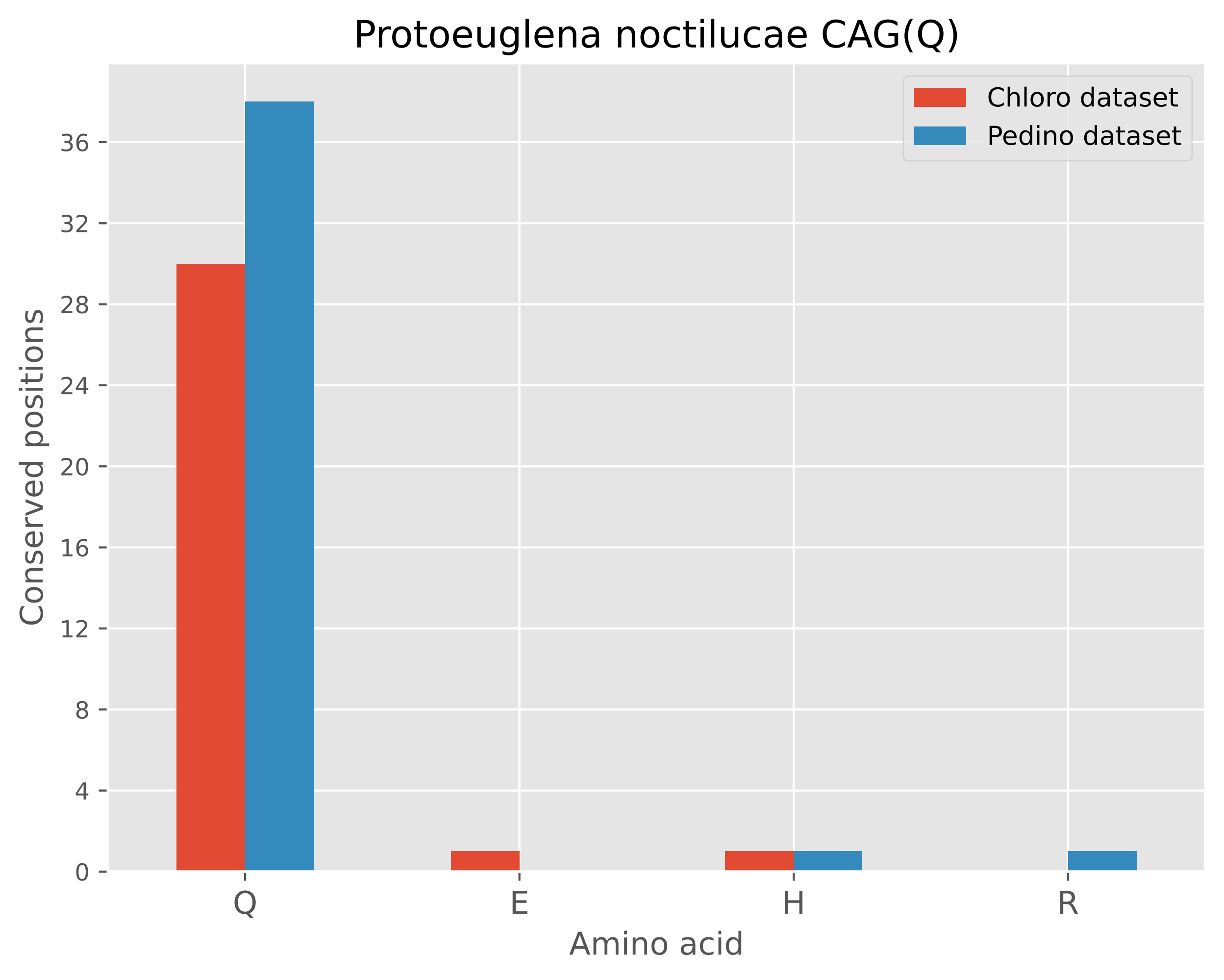

Protoeuglena noctilucae CAG(Q)
Chloro dataset
Pedino dataset
Conserved positions
0
4
8
12
16
20
24
28
32
36
Q
E
H
R
Amino acid

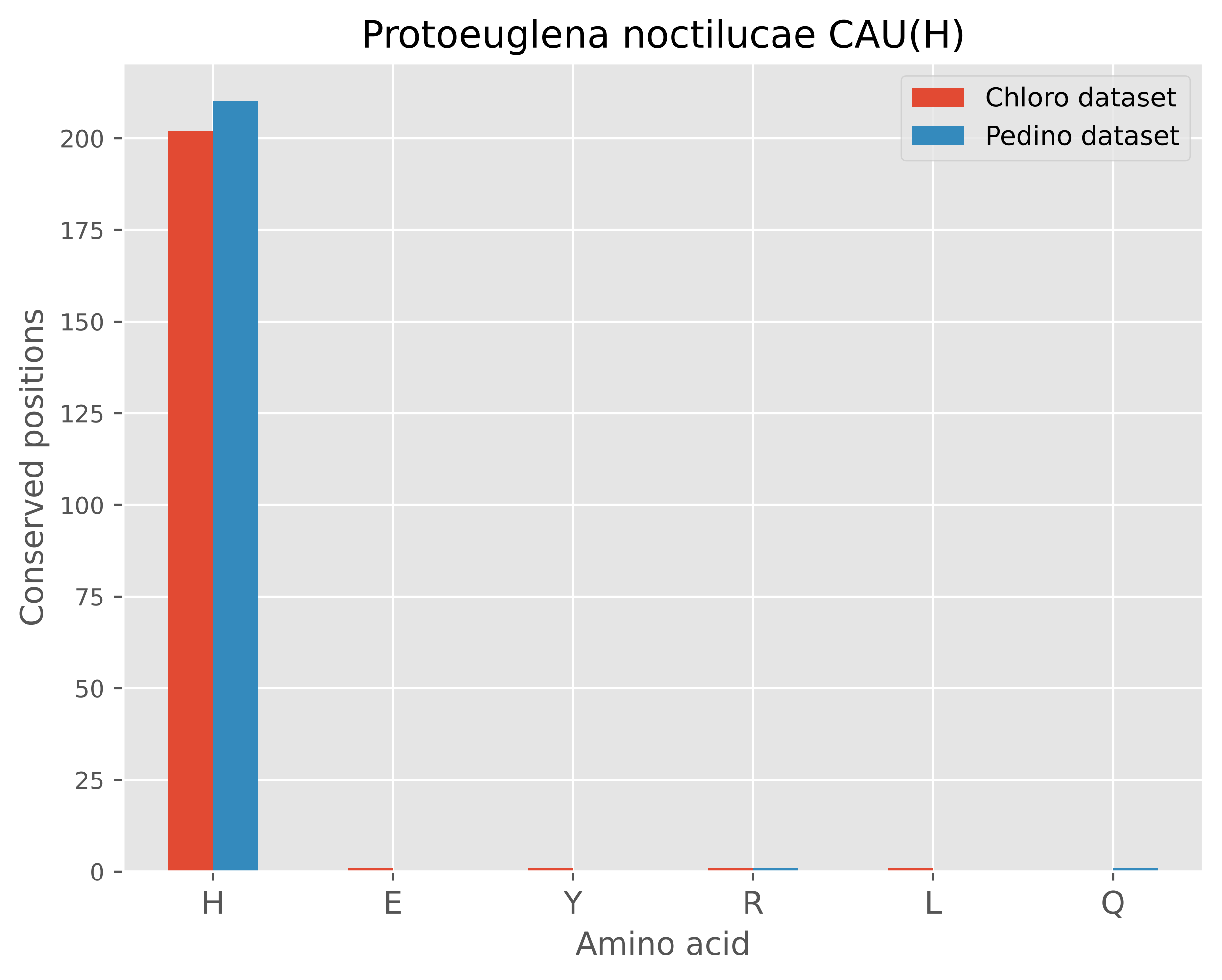

Protoeuglena noctilucae CAU(H)
Chloro dataset
Pedino dataset
Conserved positions
0
25
50
75
100
125
150
175
200
H
E
Y
R
L
Q
Amino acid

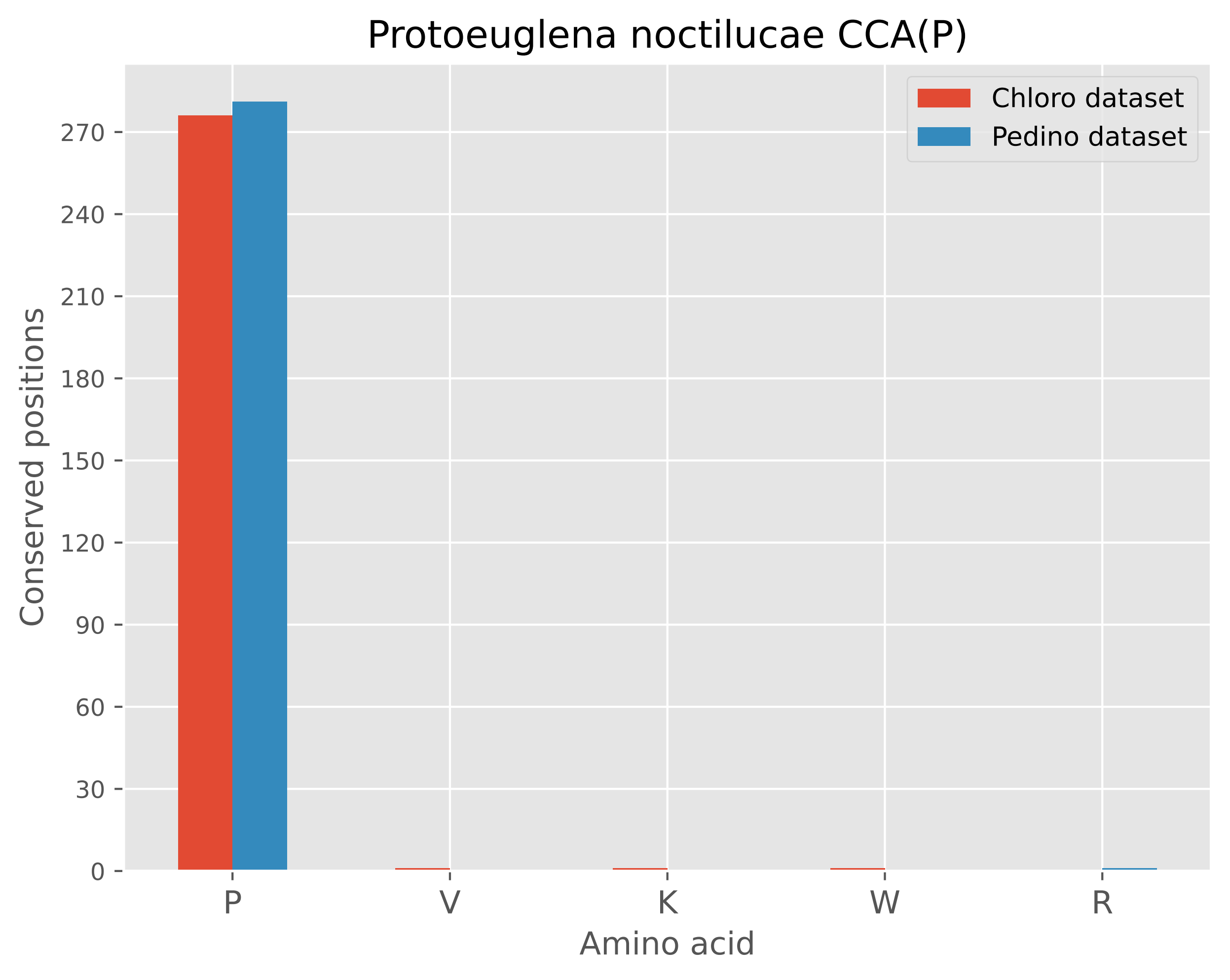

Protoeuglena noctilucae CCA(P)
Chloro dataset
Pedino dataset
Conserved positions
0
30
60
90
120
150
180
210
240
270
P
V
K
W
R
Amino acid

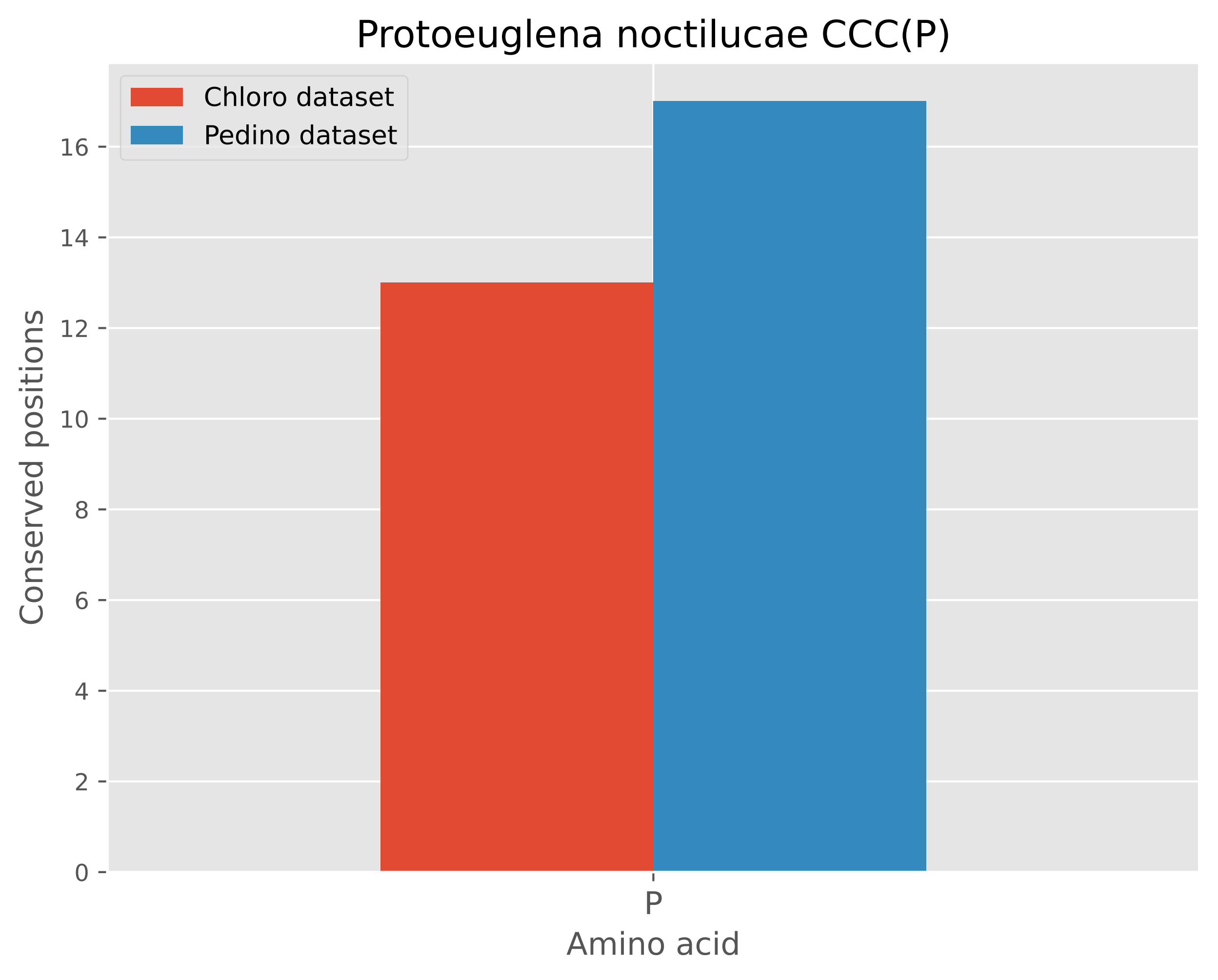

Protoeuglena noctilucae CCC(P)
Chloro dataset
Pedino dataset
Conserved positions
0
2
4
6
8
10
12
14
16
P
Amino acid

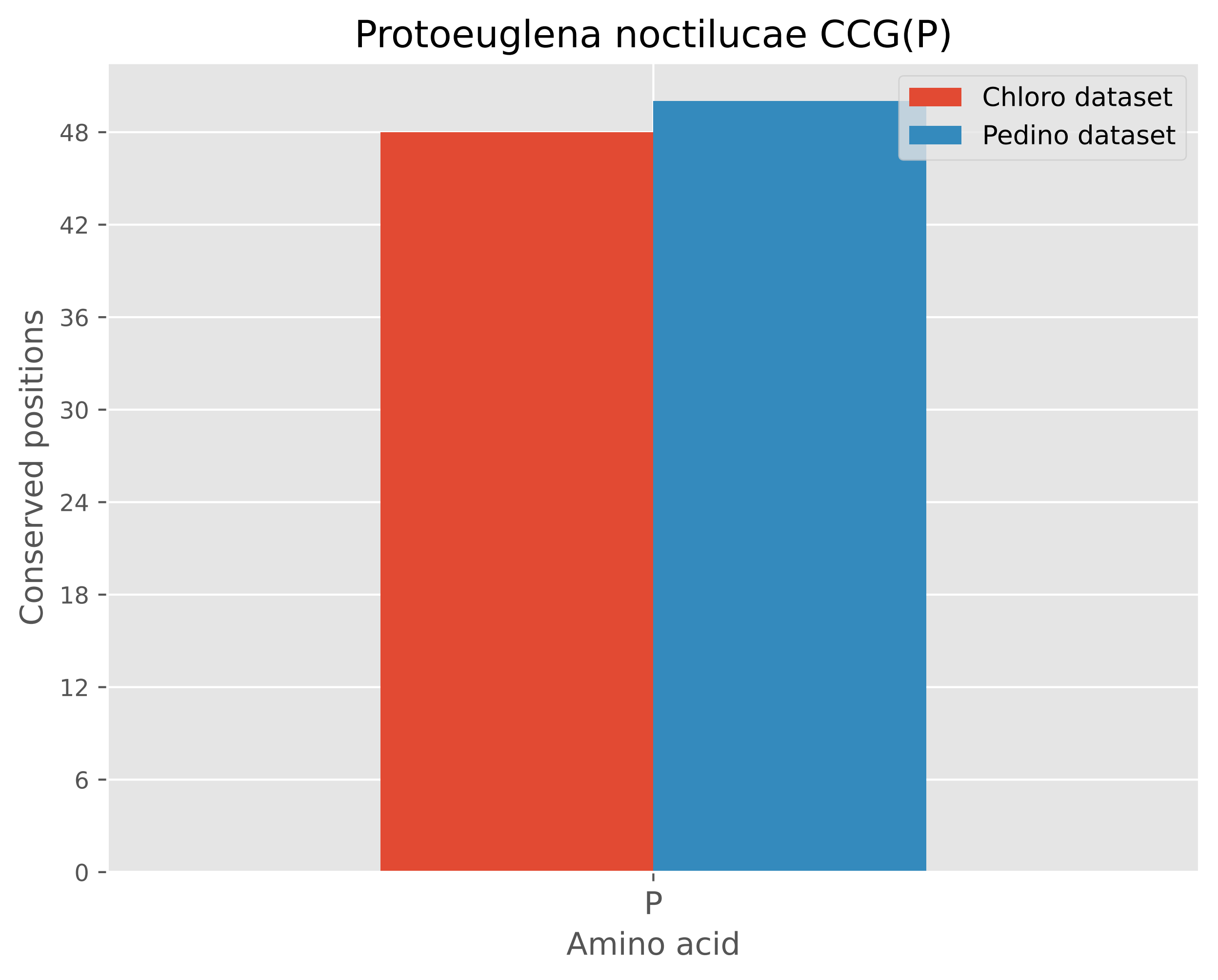

Protoeuglena noctilucae CCG(P)
Chloro dataset
Pedino dataset
Conserved positions
0
6
12
18
24
30
36
42
48
P
Amino acid

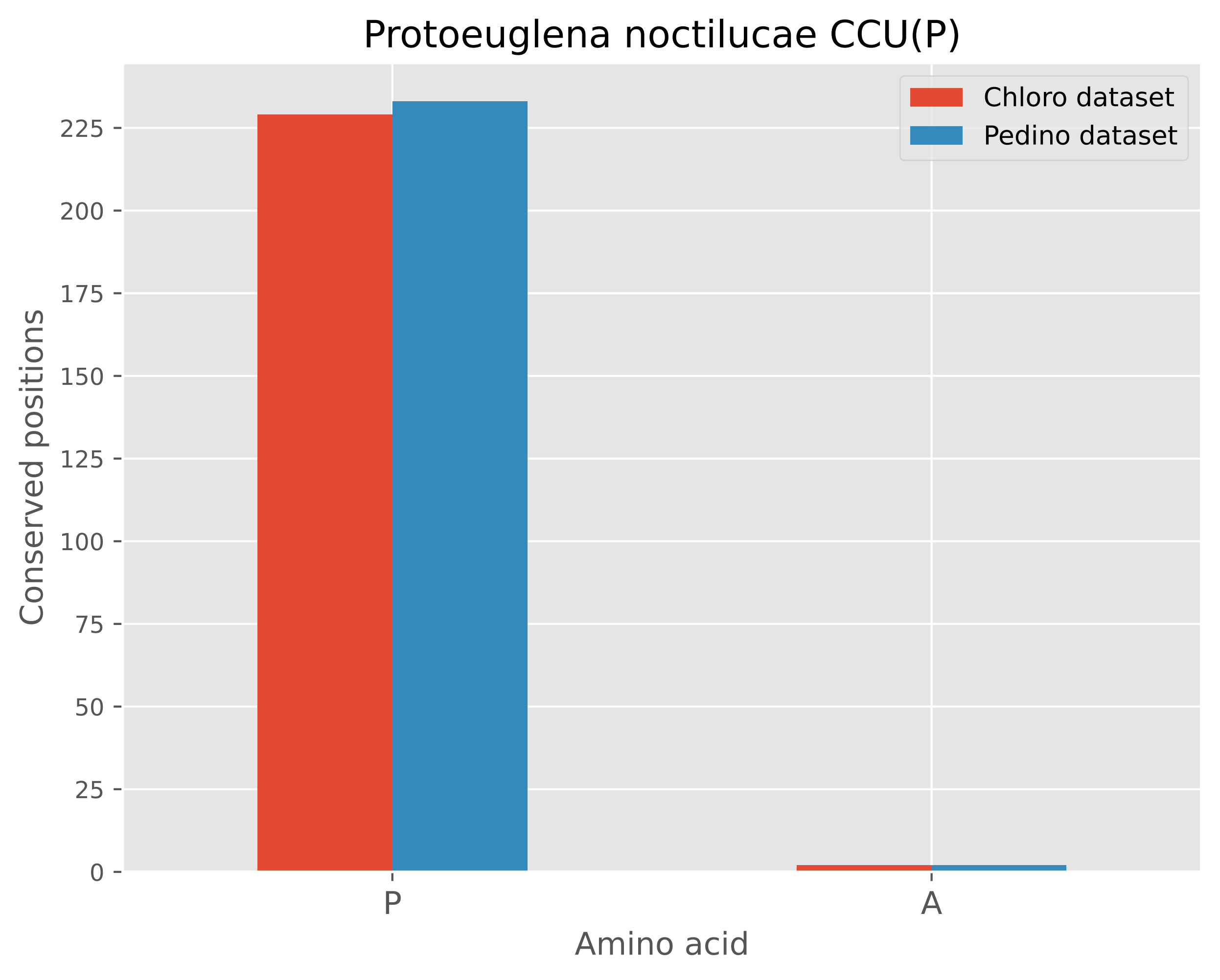
Protoeuglena noctilucae CCU(P)
Chloro dataset
Pedino dataset
Conserved positions
0
25
50
75
100
125
150
175
200
225
P
A
Amino acid

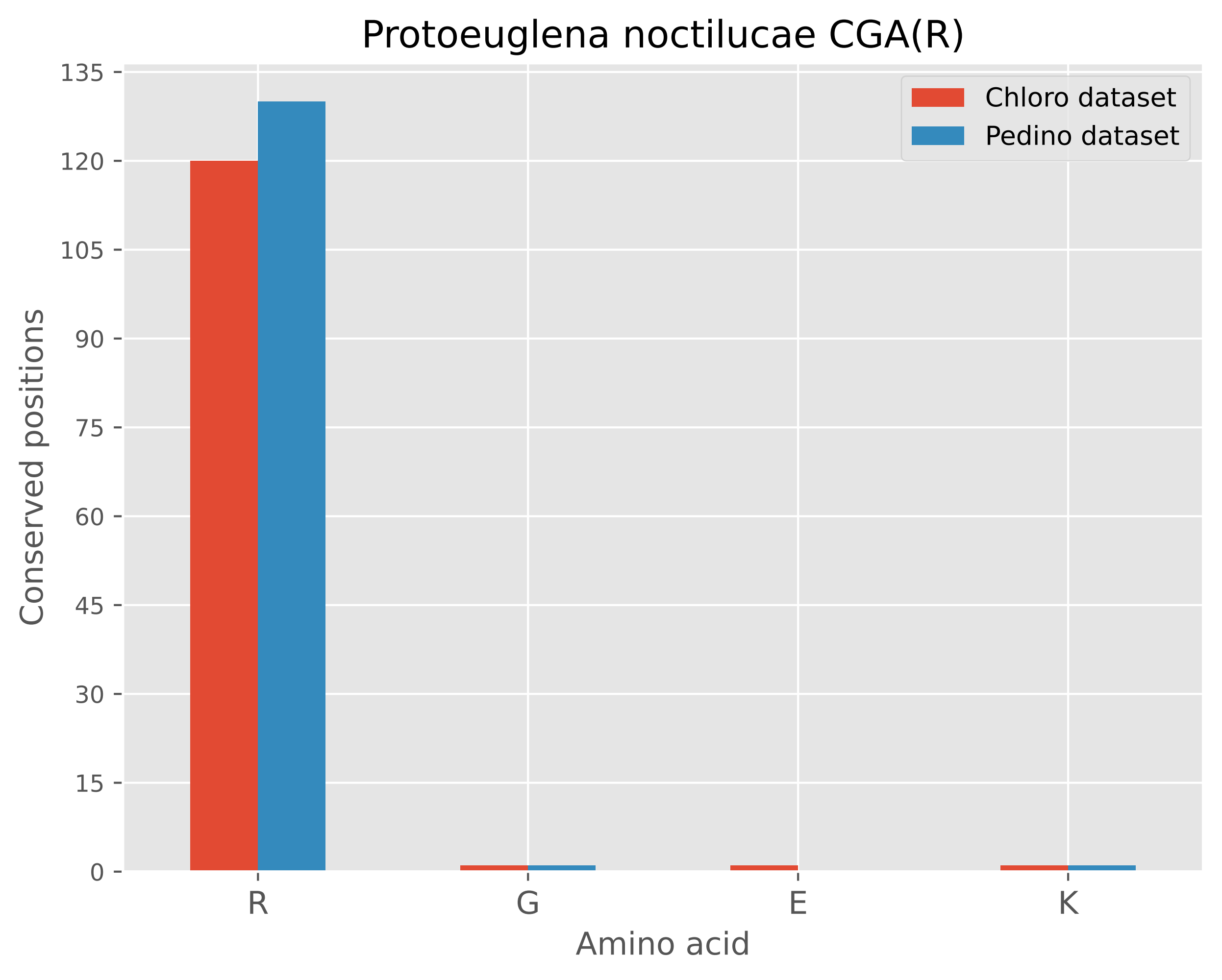

Protoeuglena noctilucae CGA(R)
Chloro dataset
Pedino dataset
Conserved positions
0
15
30
45
60
75
90
105
120
135
R
G
E
K
Amino acid

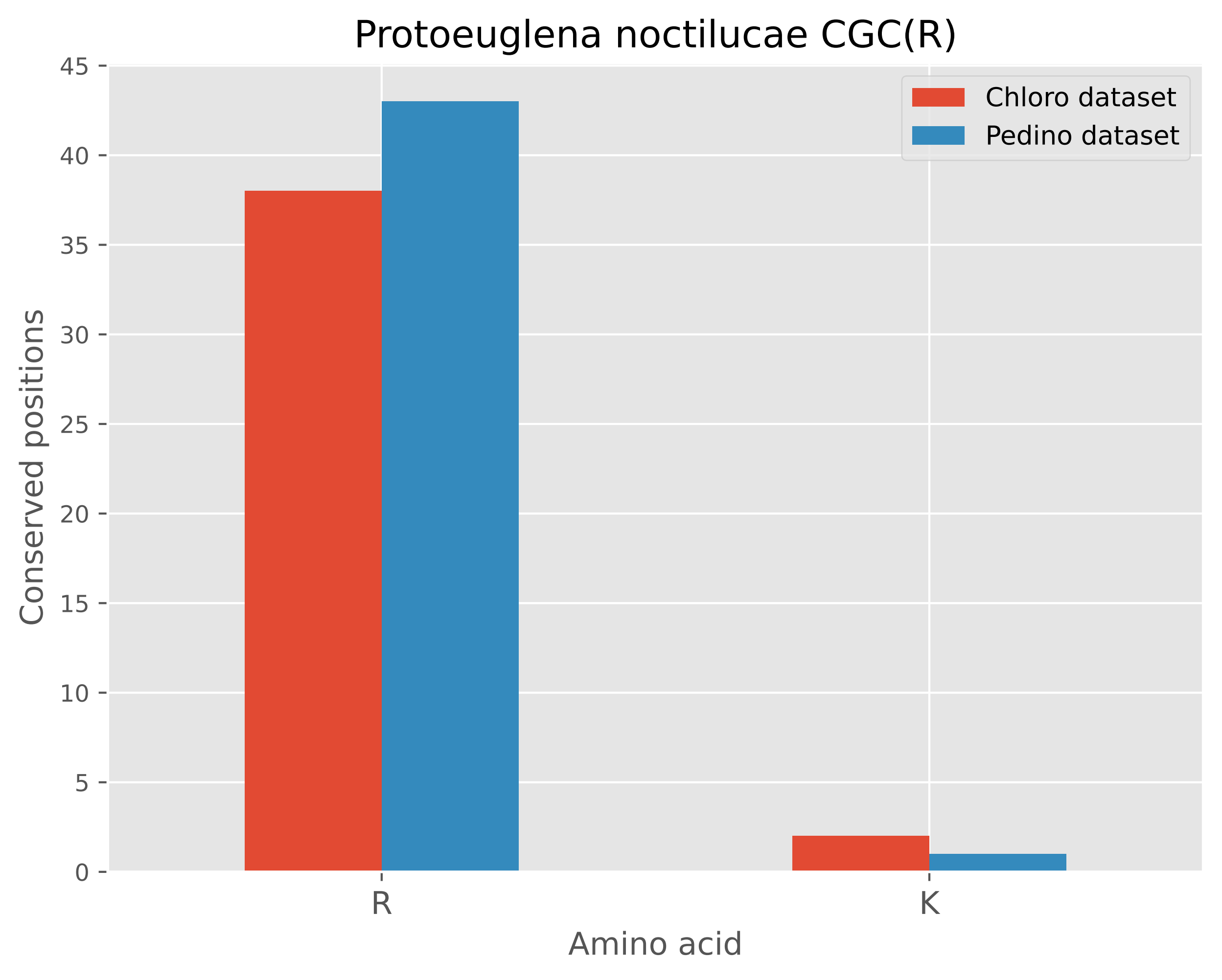
Protoeuglena noctilucae CGC(R)
Chloro dataset
Pedino dataset
Conserved positions
0
5
10
15
20
25
30
35
40
45
R
K
Amino acid

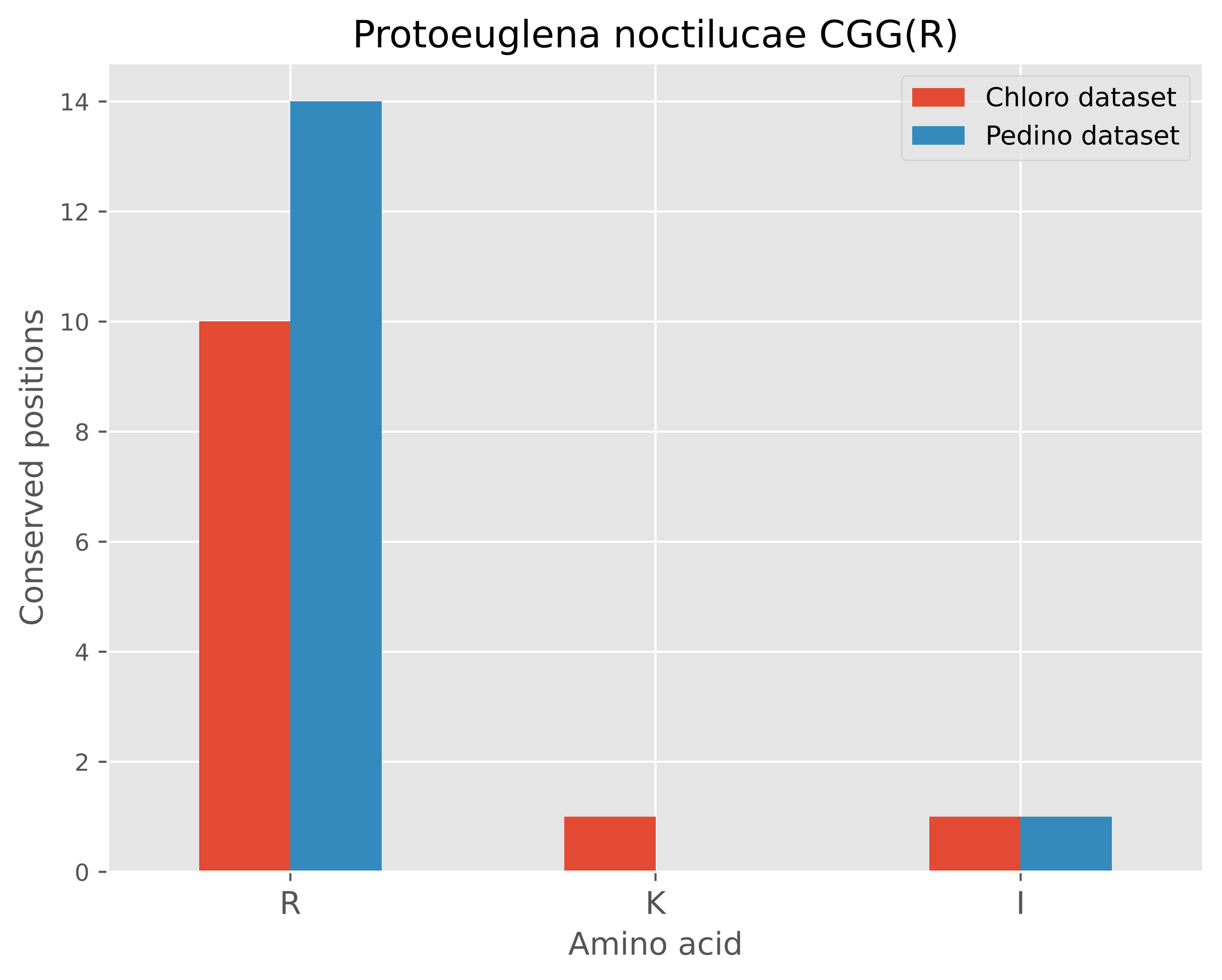

Protoeuglena noctilucae CGG(R)
Chloro dataset
Pedino dataset
Conserved positions
0
2
4
6
8
10
12
14
R
K
I
Amino acid

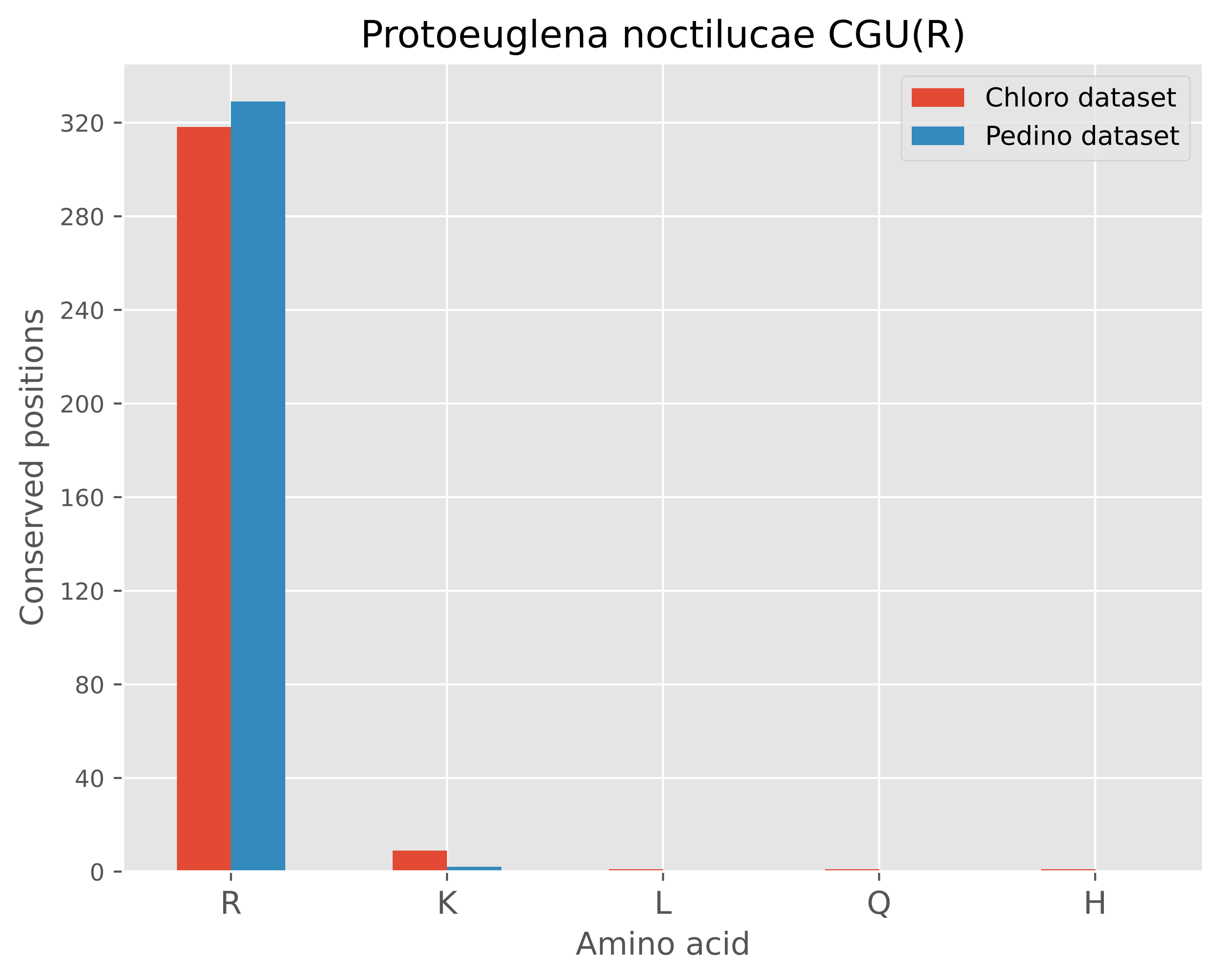

Protoeuglena noctilucae CGU(R)
Chloro dataset
Pedino dataset
Conserved positions
0
40
80
120
160
200
240
280
320
R
K
L
Q
H
Amino acid

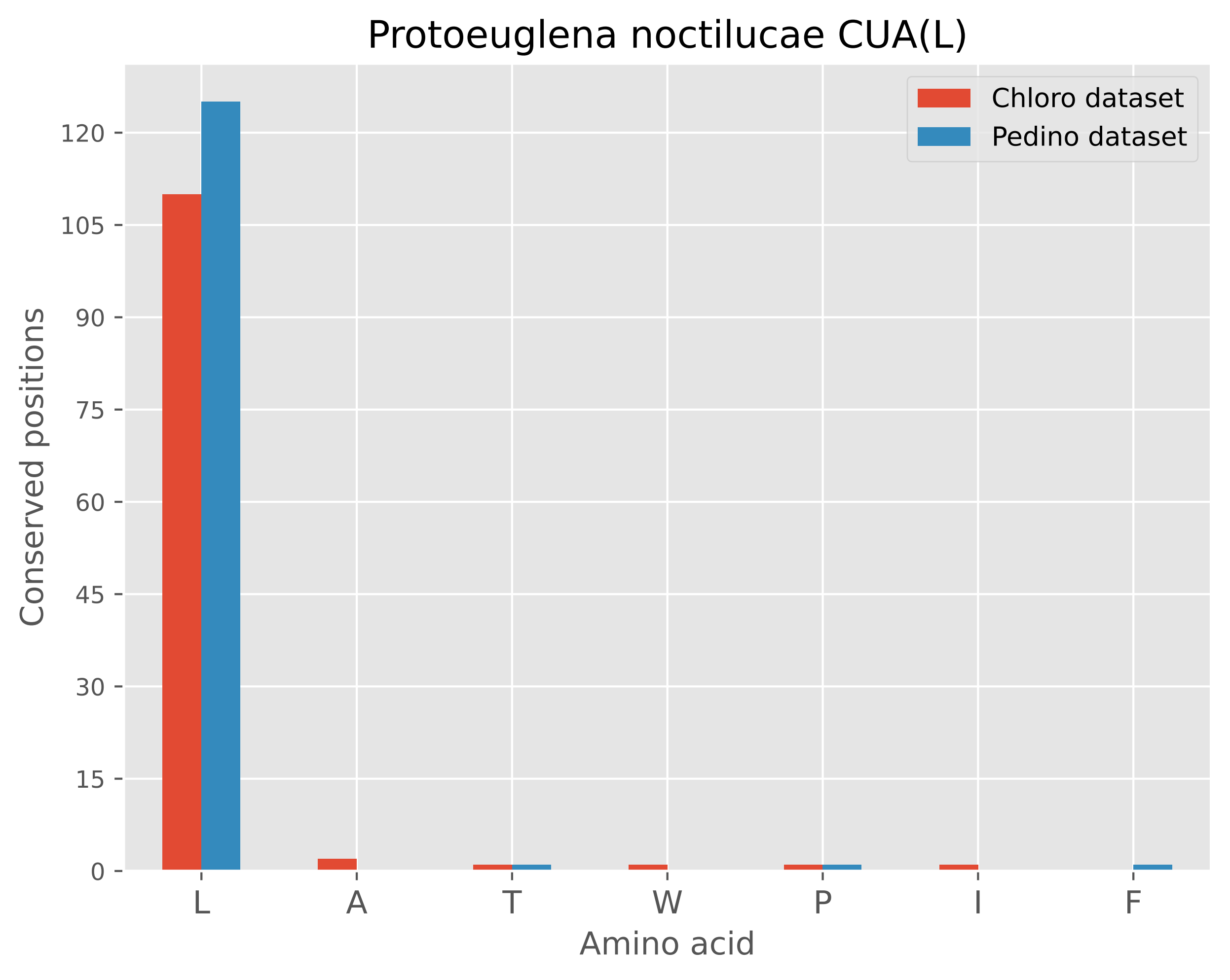

Protoeuglena noctilucae CUA(L)
Chloro dataset
Pedino dataset
Conserved positions
0
15
30
45
60
75
90
105
120
L
A
T
W
P
I
F
Amino acid

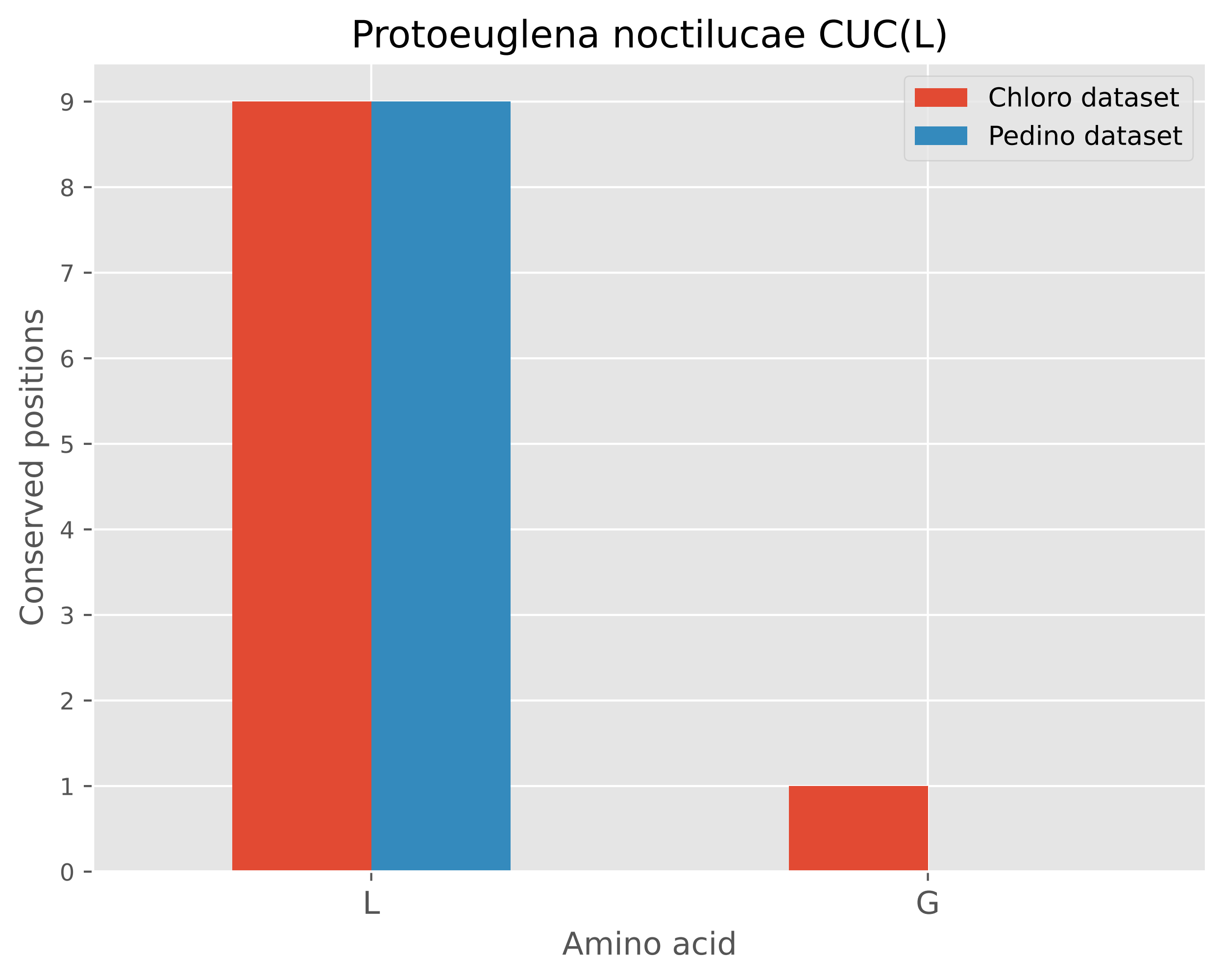
Protoeuglena noctilucae CUC(L)
Chloro dataset
Pedino dataset
Conserved positions
0
1
2
3
4
5
6
7
8
9
L
G
Amino acid

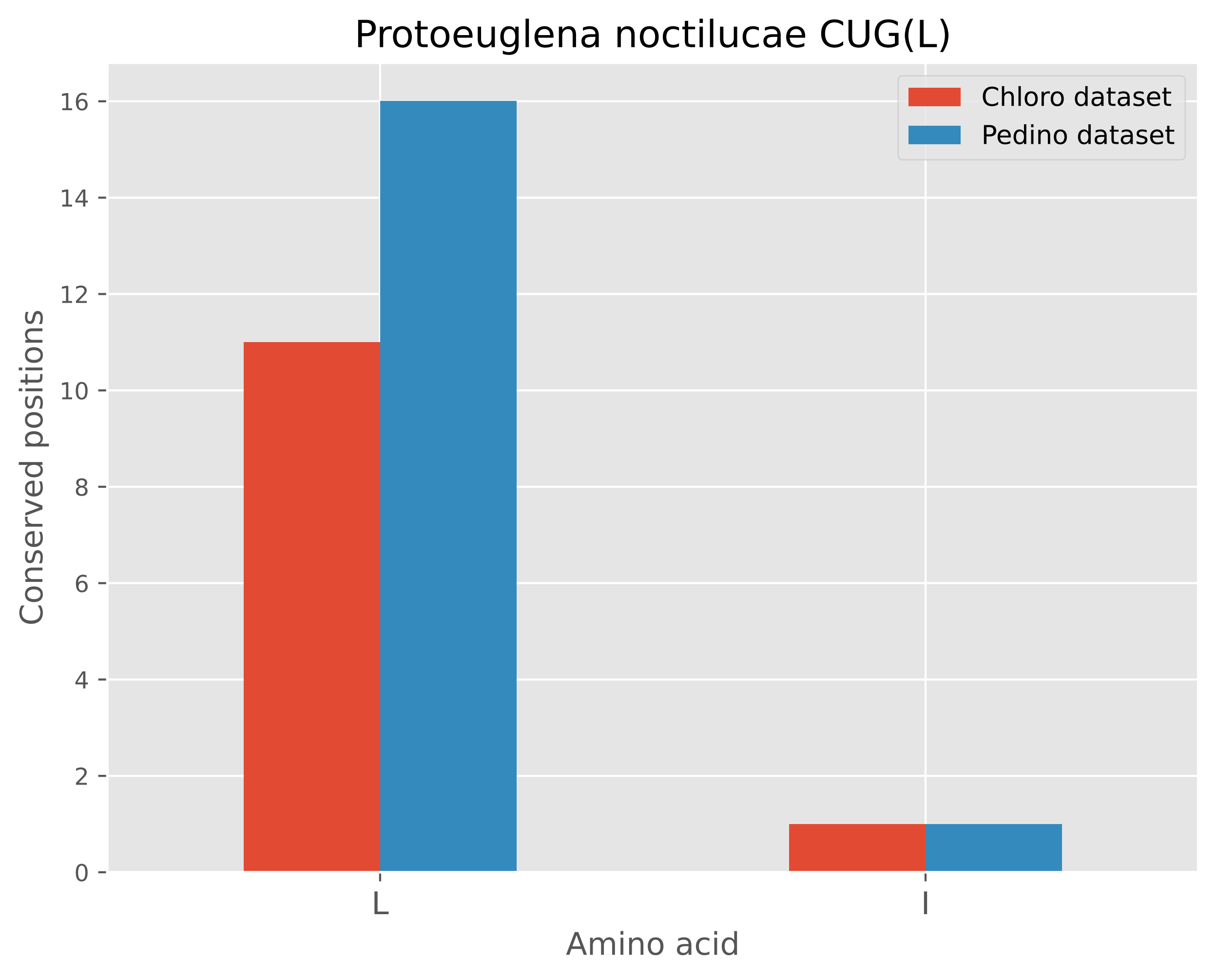
Protoeuglena noctilucae CUG(L)
Chloro dataset
Pedino dataset
Conserved positions
0
2
4
6
8
10
12
14
16
L
I
Amino acid

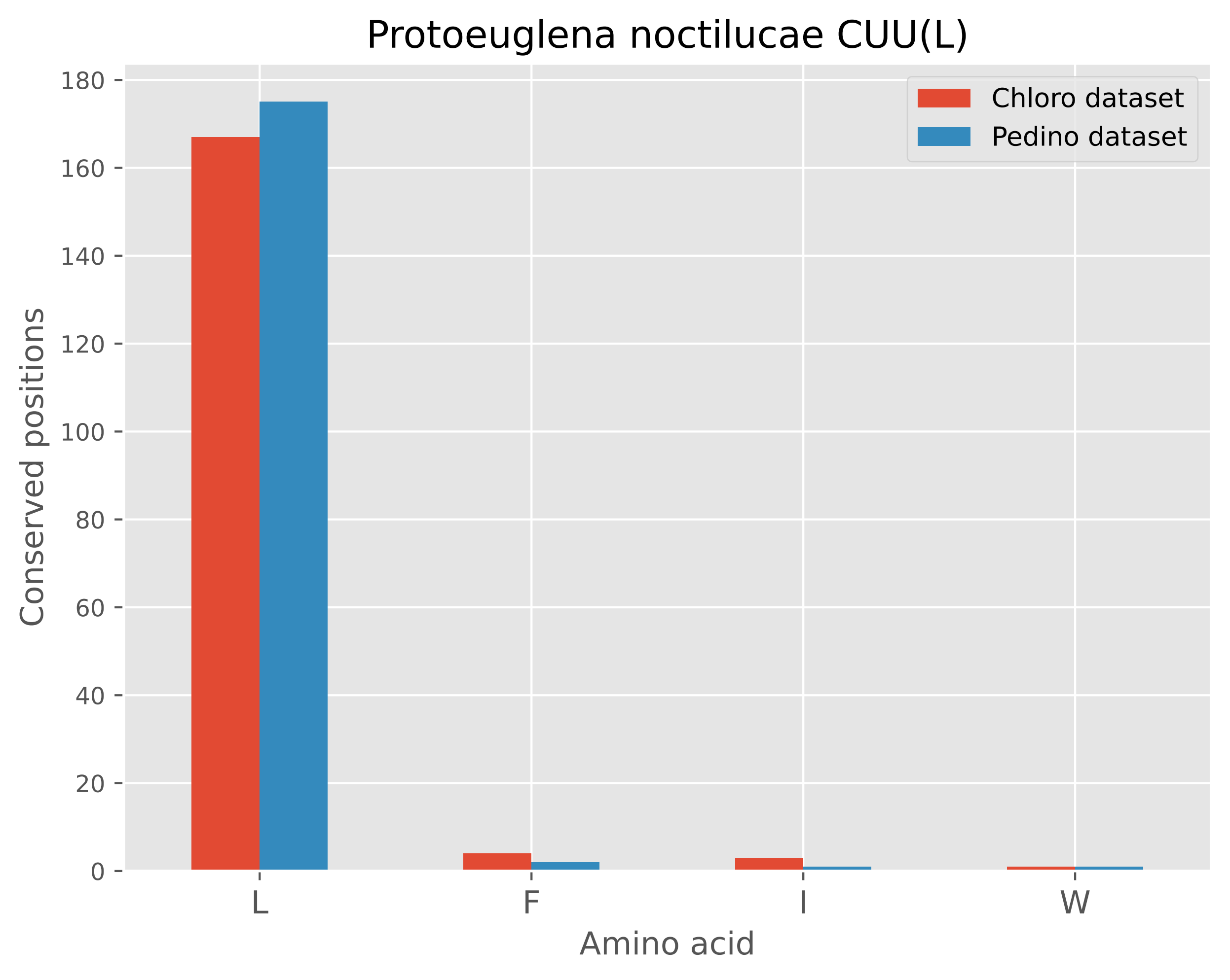

Protoeuglena noctilucae CUU(L)
Chloro dataset
Pedino dataset
Conserved positions
0
20
40
60
80
100
120
140
160
180
L
F
I
W
Amino acid

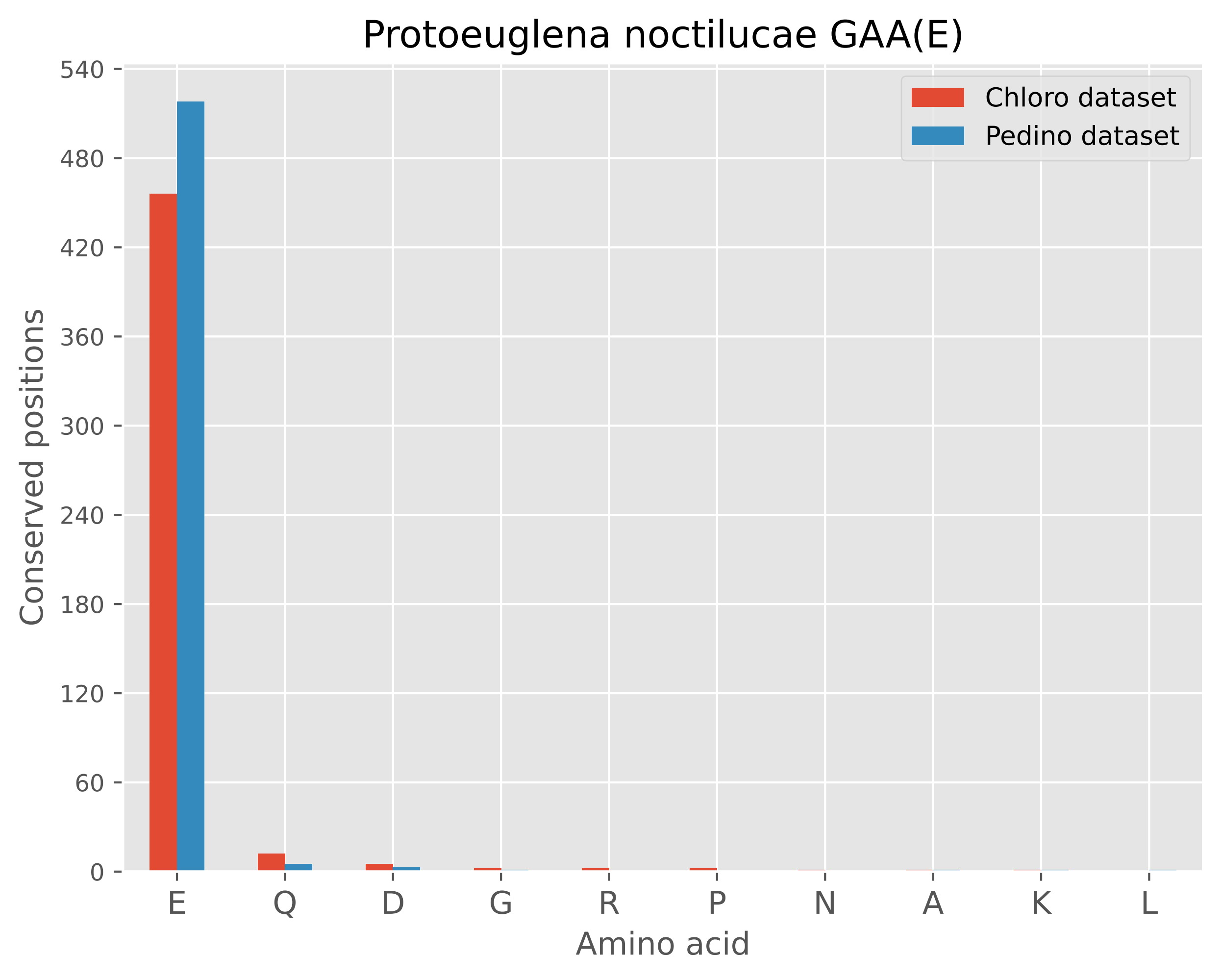

Protoeuglena noctilucae GAA(E)
Chloro dataset
Pedino dataset
Conserved positions
540
480
420
360
300
240
180
120
60
0
E
Q
D
G
R
P
N
A
K
L
Amino acid

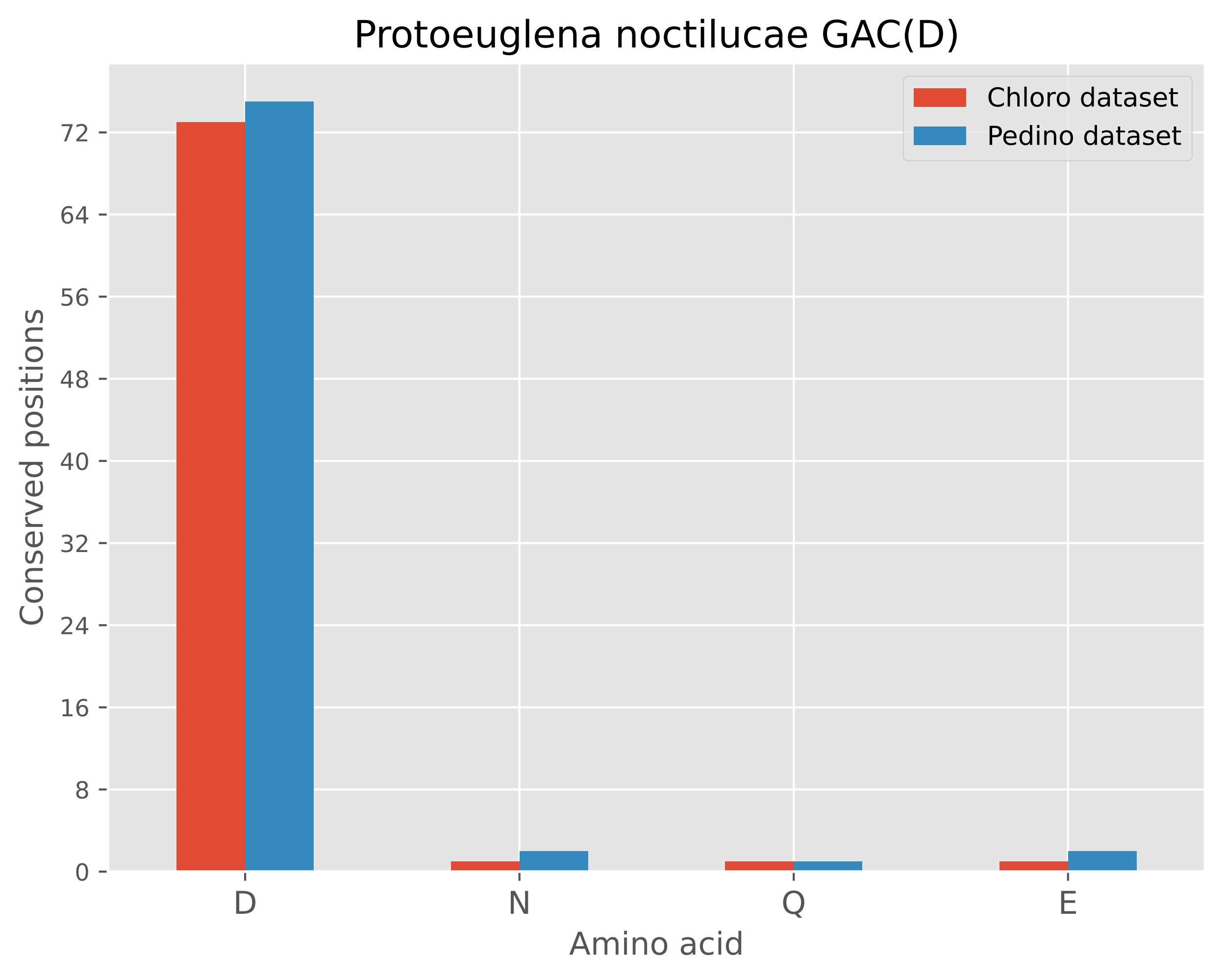
Protoeuglena noctilucae GAC(D)
Chloro dataset
Pedino dataset
Conserved positions
0
8
16
24
32
40
48
56
64
72
D
N
Q
E
Amino acid

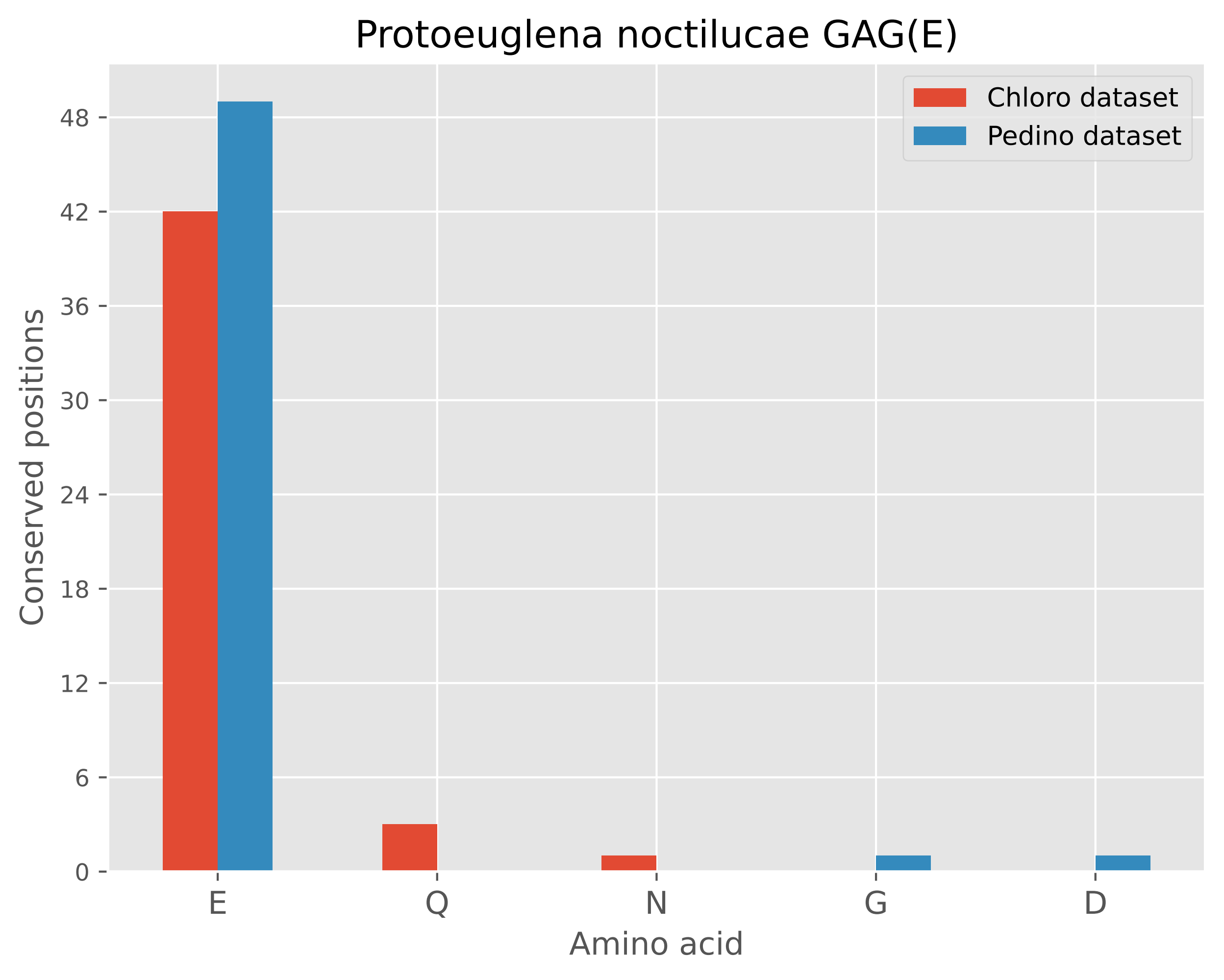
Protoeuglena noctilucae GAG(E)
Chloro dataset
Pedino dataset
Conserved positions
0
6
12
18
24
30
36
42
48
E
Q
N
G
D
Amino acid

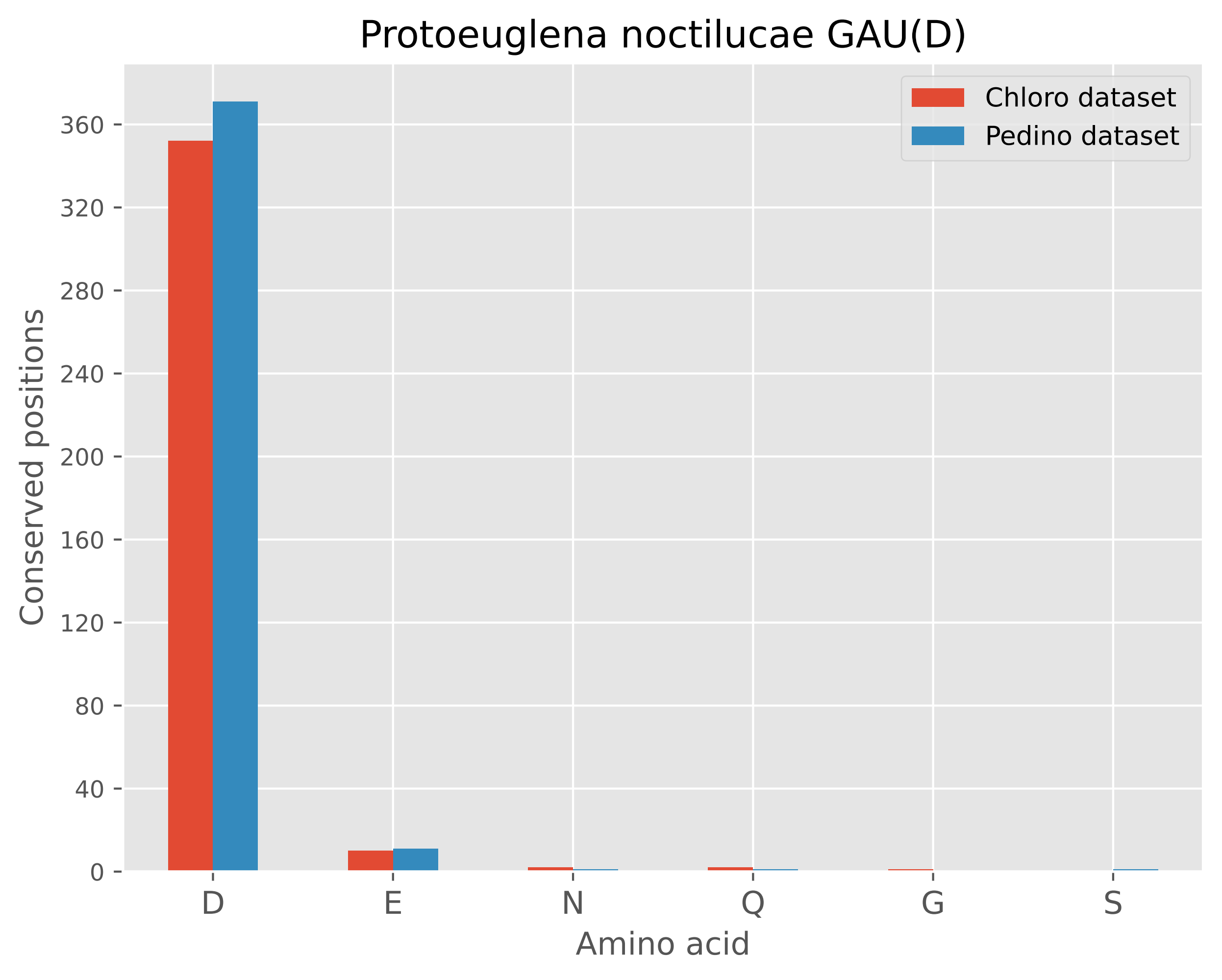

Protoeuglena noctilucae GAU(D)
Chloro dataset
Pedino dataset
Conserved positions
0
40
80
120
160
200
240
280
320
360
D
E
N
Q
G
S
Amino acid

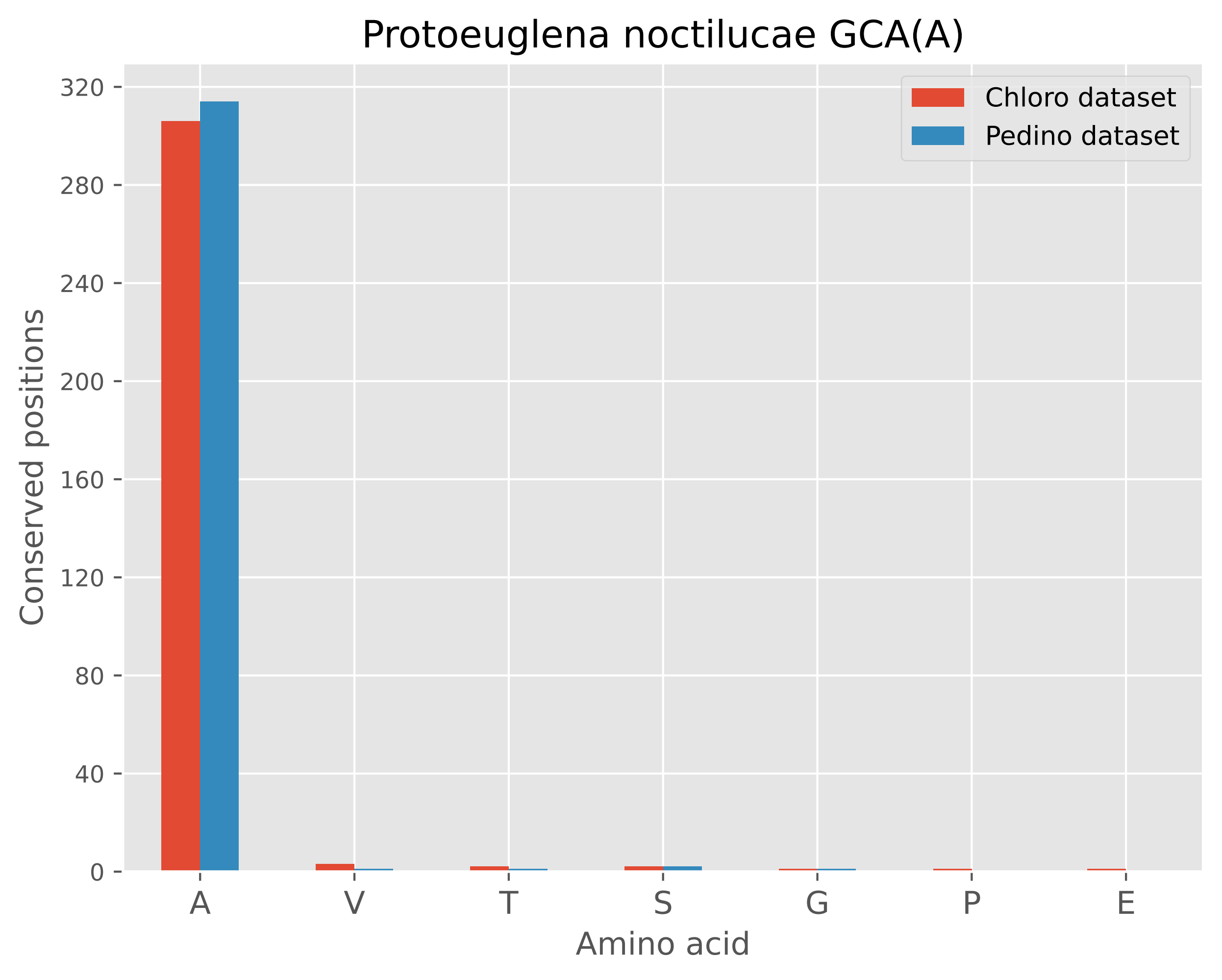
Protoeuglena noctilucae GCA(A)
Chloro dataset
Pedino dataset
Conserved positions
Amino acid
0
40
80
120
160
200
240
280
320
A
V
T
S
G
P
E

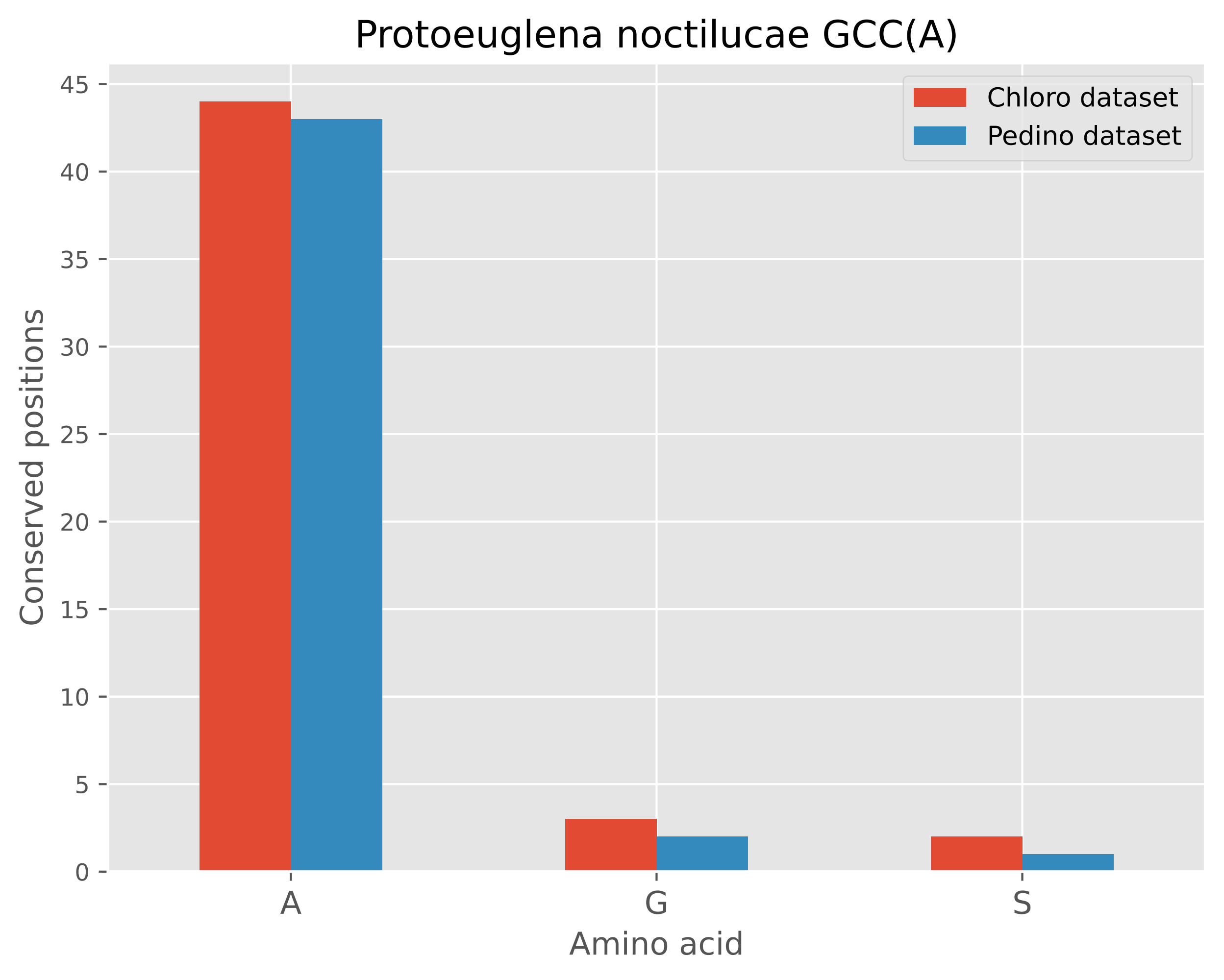

Protoeuglena noctilucae GCC(A)
Chloro dataset
Pedino dataset
Conserved positions
45
40
35
30
25
20
15
10
5
0
A
G
S
Amino acid

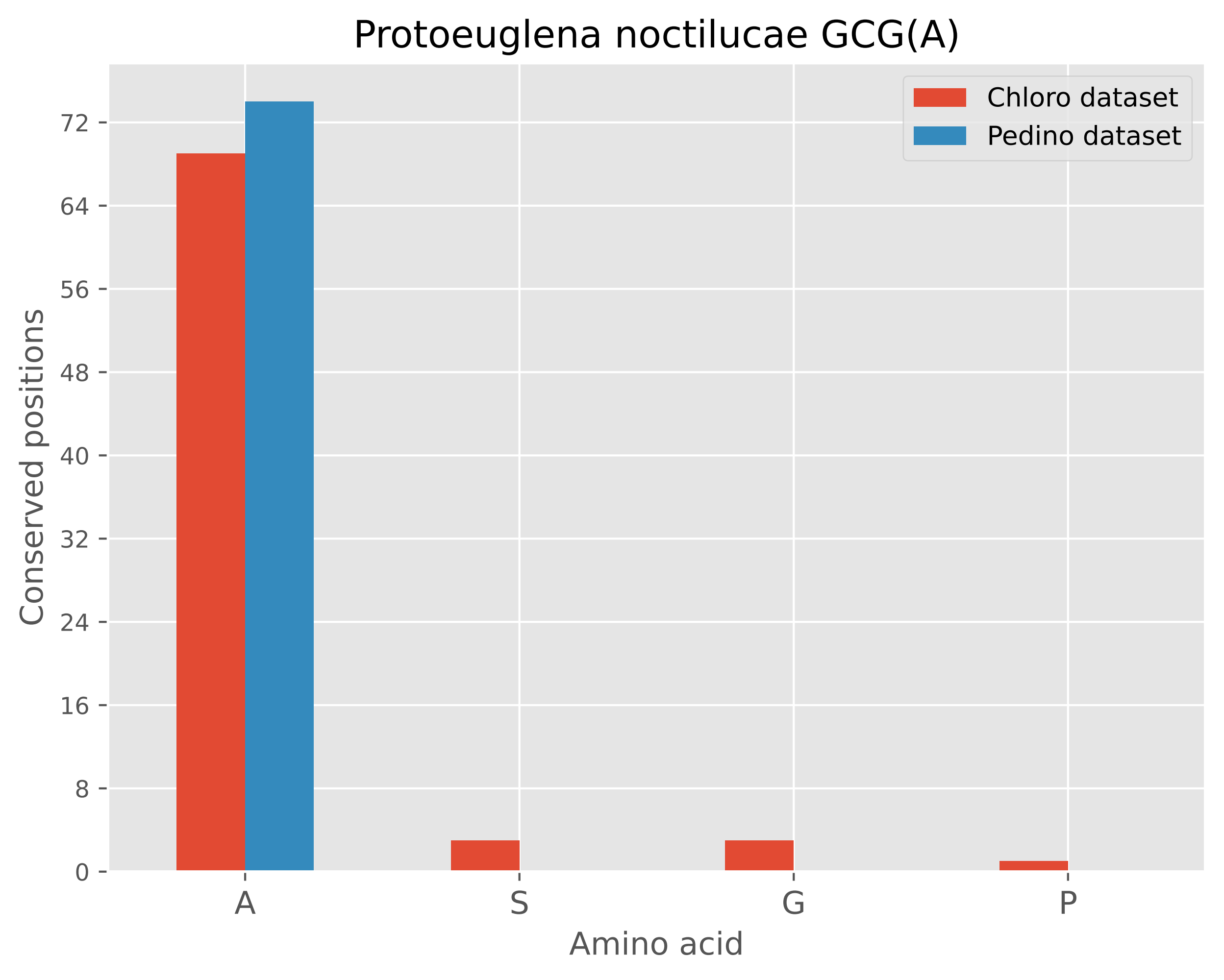

Protoeuglena noctilucae GCG(A)
Chloro dataset
Pedino dataset
Conserved positions
0
8
16
24
32
40
48
56
64
72
A
S
G
P
Amino acid

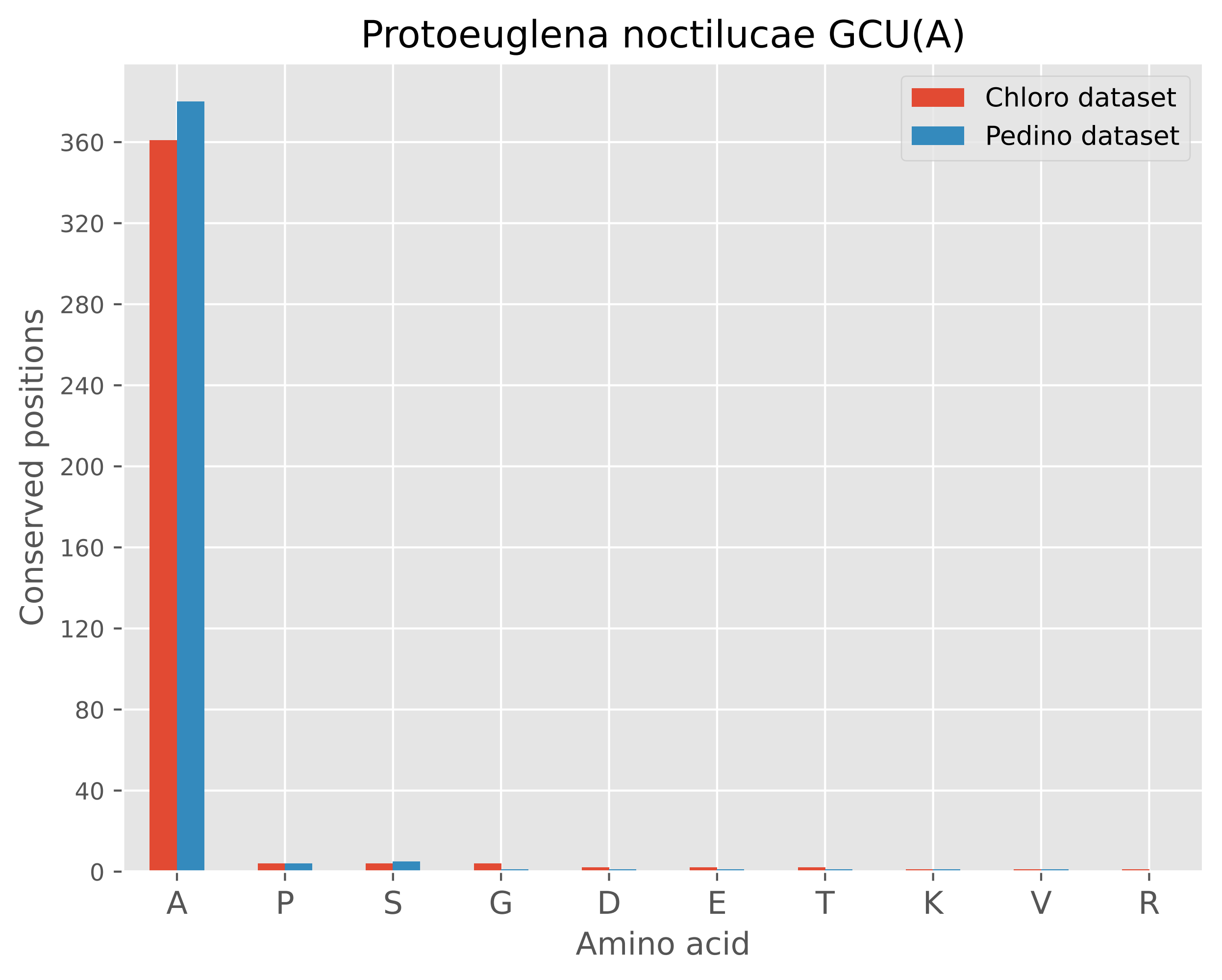
Protoeuglena noctilucae GCU(A)
Chloro dataset
Pedino dataset
Conserved positions
0
40
80
120
160
200
240
280
320
360
A
P
S
G
D
E
T
K
V
R
Amino acid

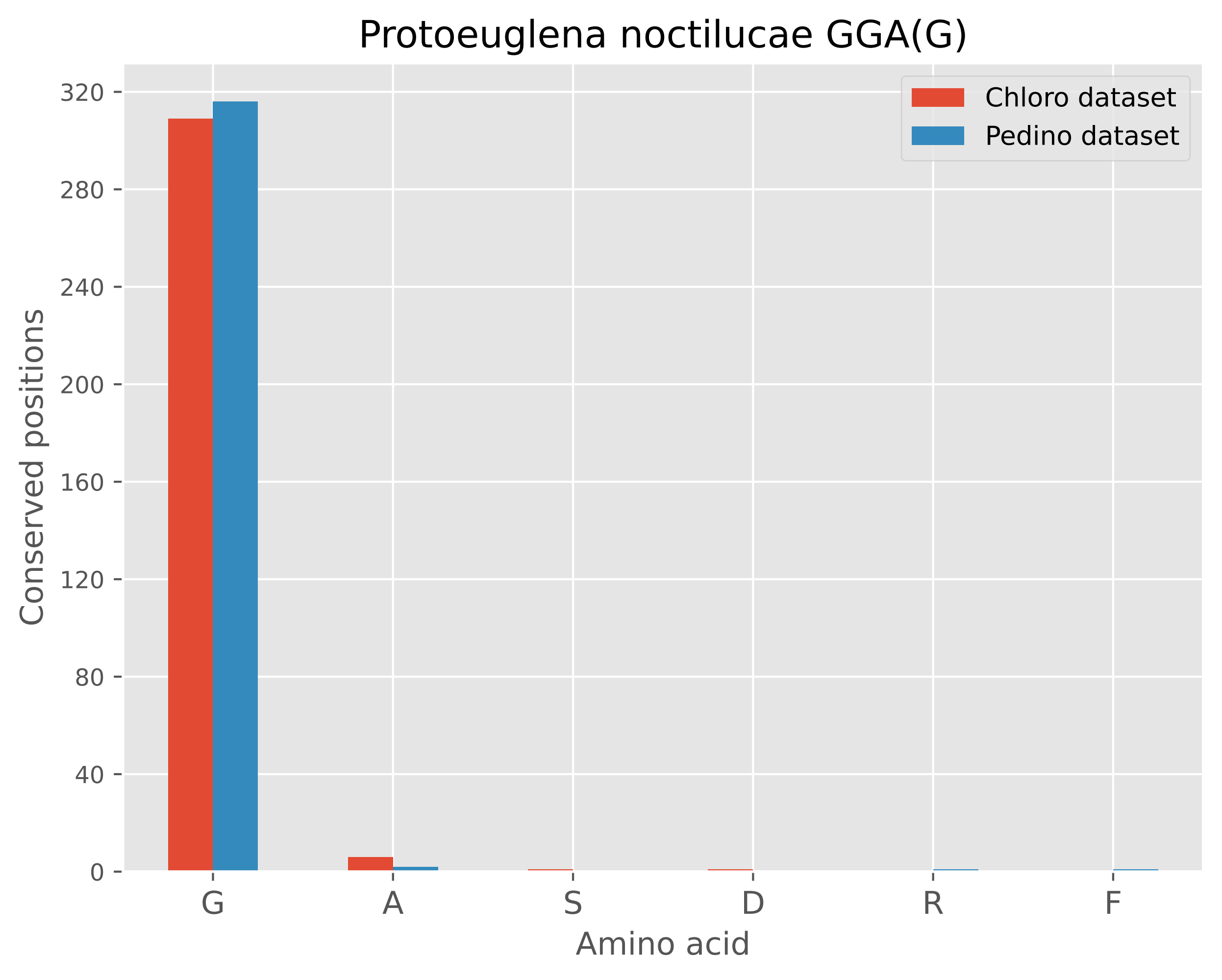

Protoeuglena noctilucae GGA(G)
Chloro dataset
Pedino dataset
Conserved positions
0
40
80
120
160
200
240
280
320
G
A
S
D
R
F
Amino acid

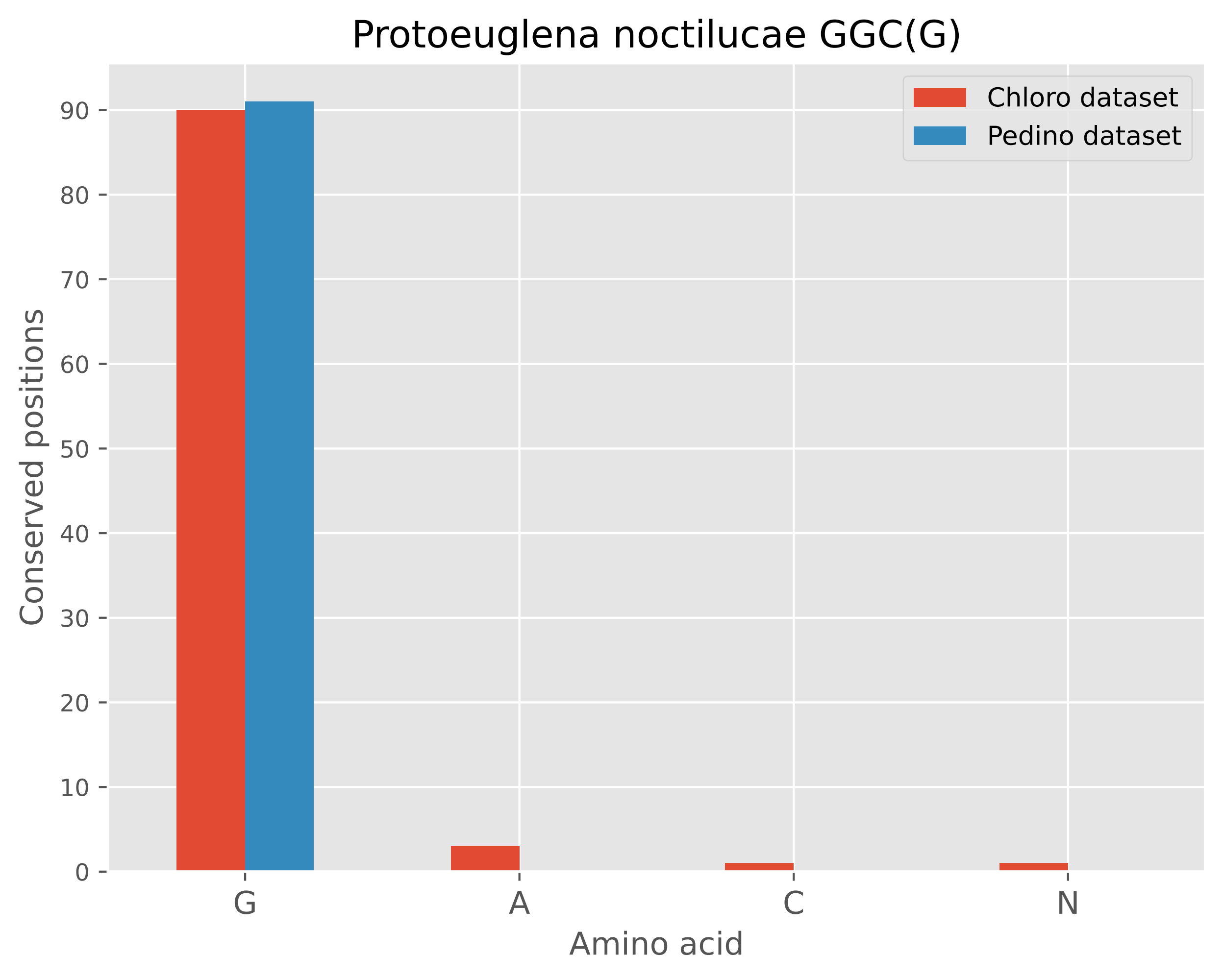

Protoeuglena noctilucae GGC(G)
Chloro dataset
Pedino dataset
Conserved positions
0
10
20
30
40
50
60
70
80
90
G
A
C
N
Amino acid

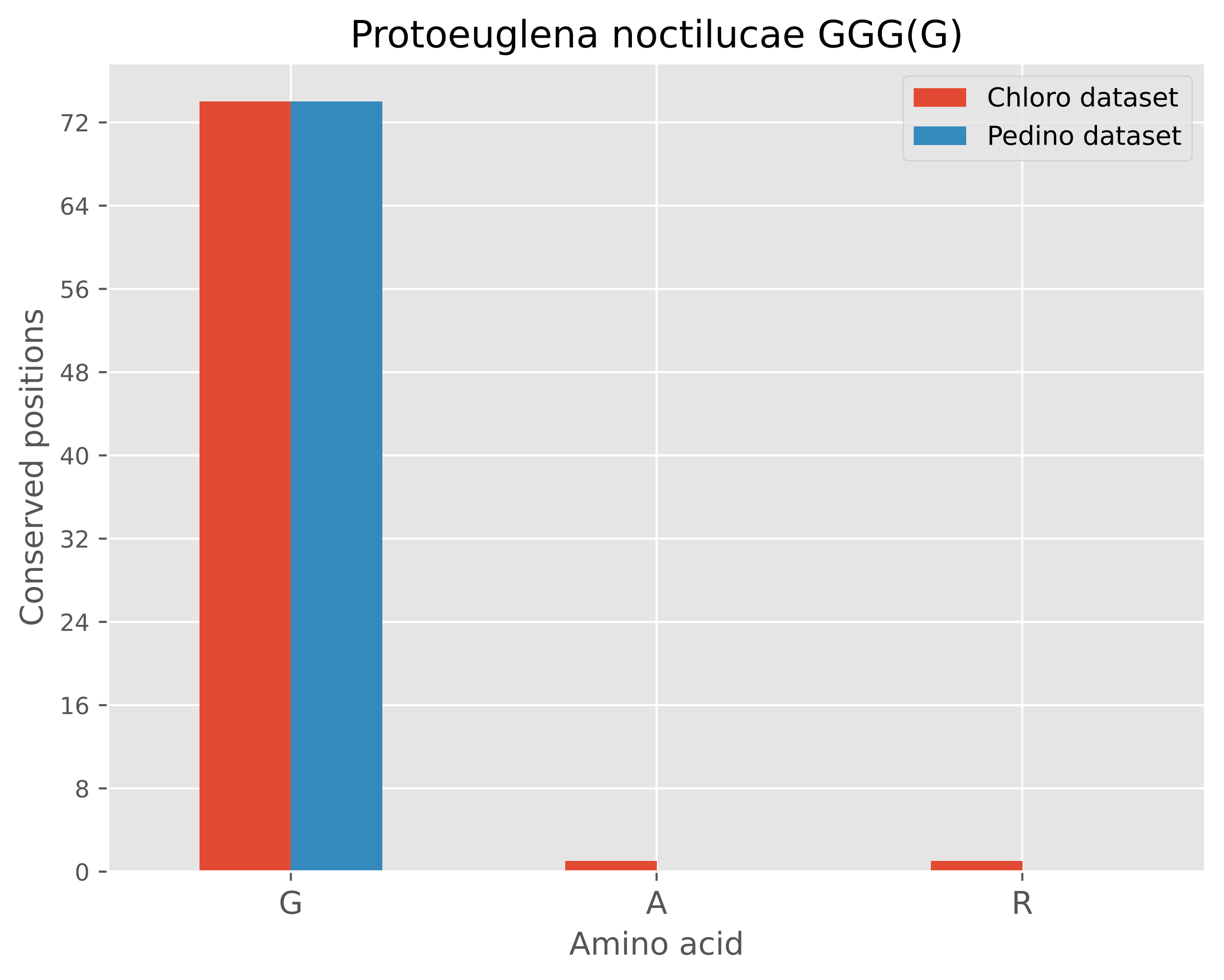

Protoeuglena noctilucae GGG(G)
Chloro dataset
Pedino dataset
Conserved positions
0
8
16
24
32
40
48
56
64
72
G
A
R
Amino acid

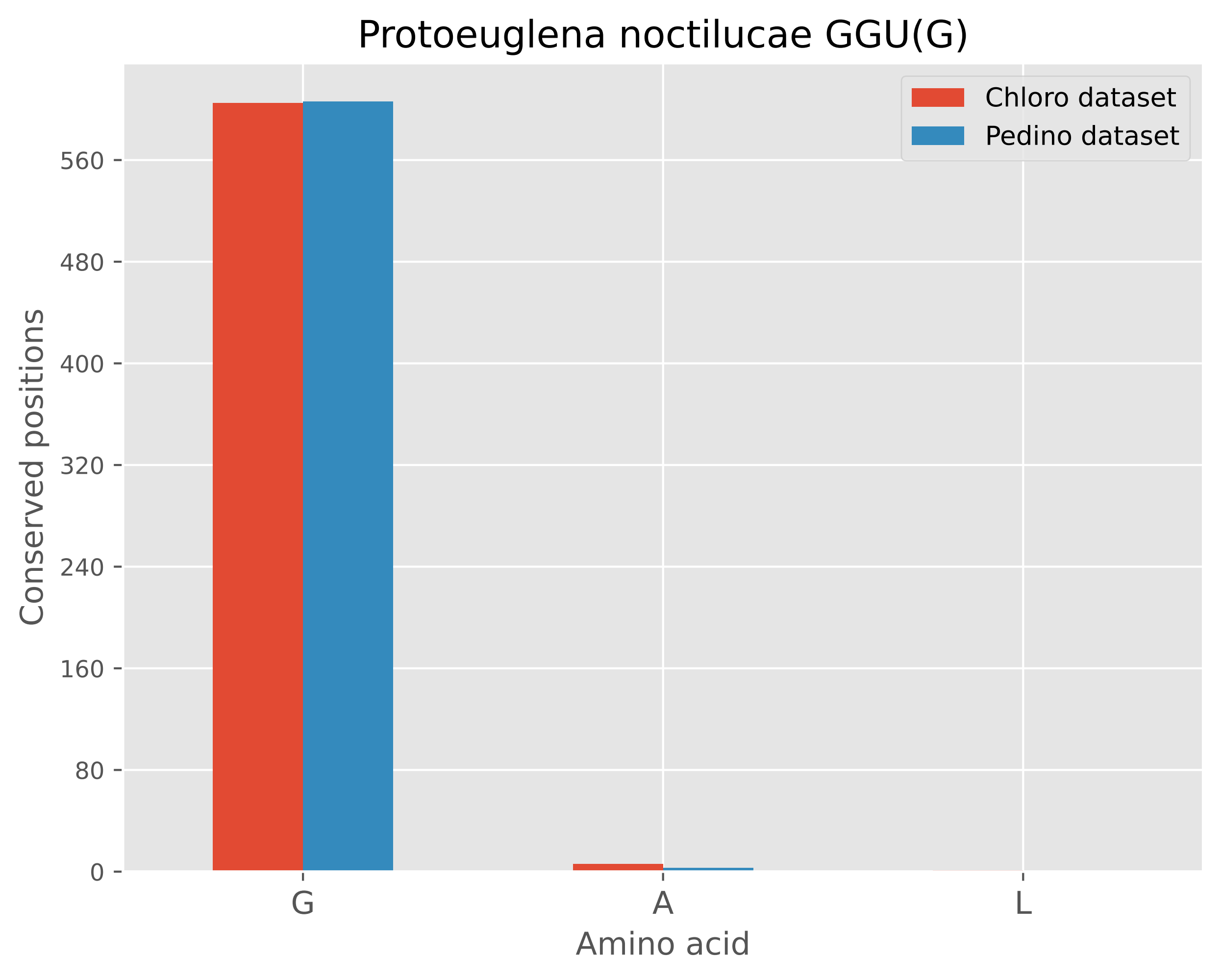

Protoeuglena noctilucae GGU(G)
Chloro dataset
Pedino dataset
Conserved positions
0
80
160
240
320
400
480
560
G
A
L
Amino acid

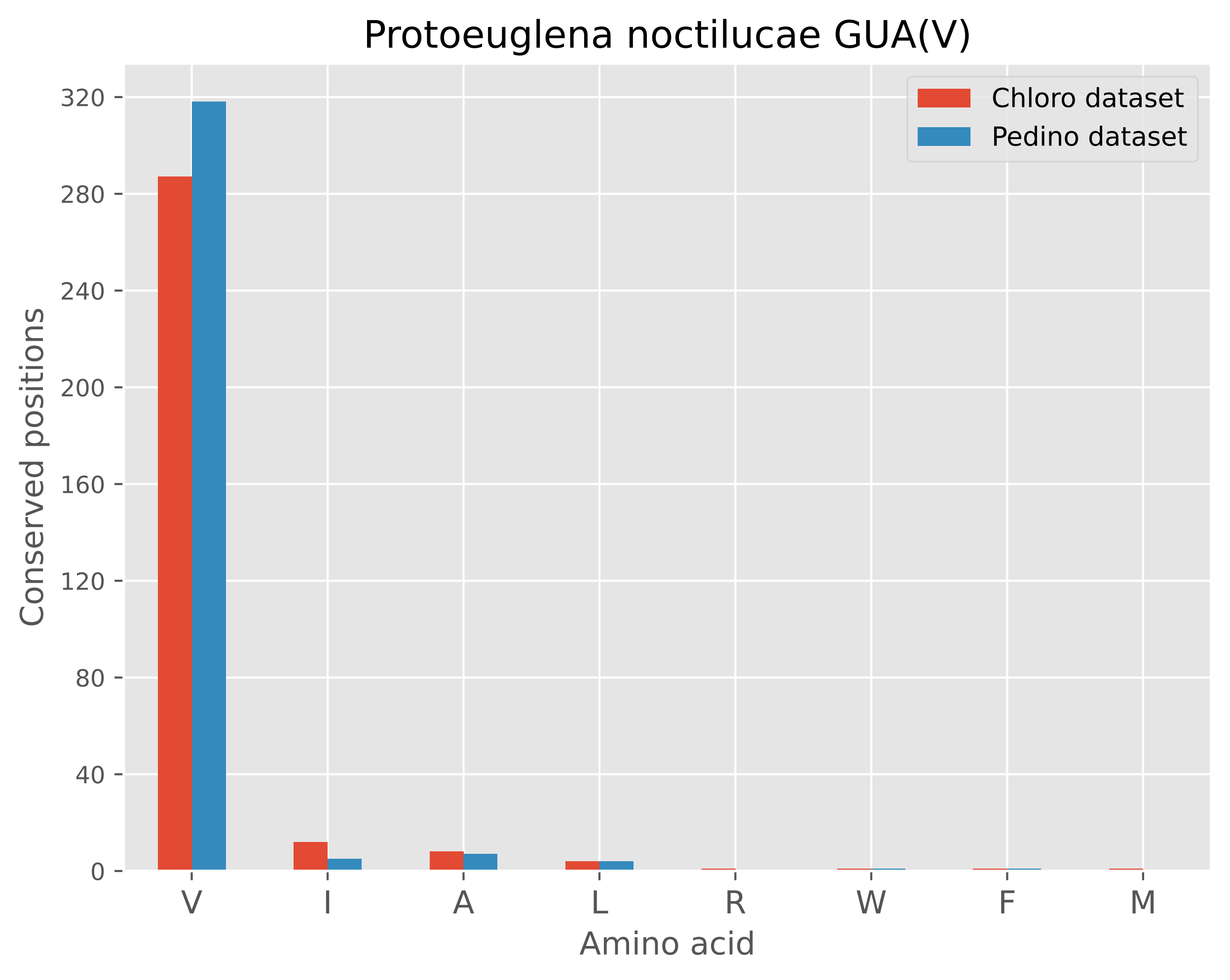
Protoeuglena noctilucae GUA(V)
Chloro dataset
Pedino dataset
Conserved positions
0
40
80
120
160
200
240
280
320
V
I
A
L
R
W
F
M
Amino acid

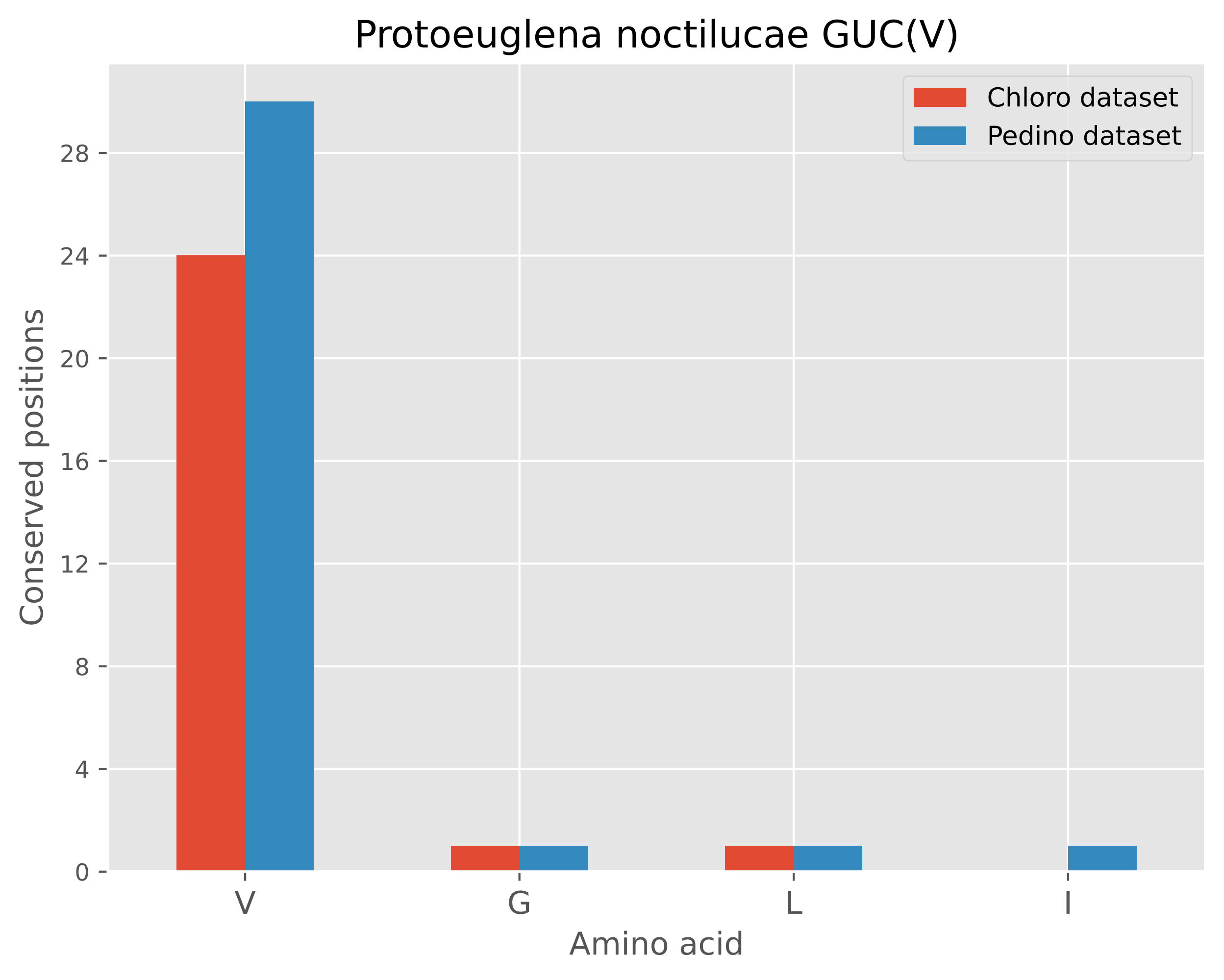
Protoeuglena noctilucae GUC(V)
Chloro dataset
Pedino dataset
Conserved positions
0
4
8
12
16
20
24
28
V
G
L
I
Amino acid

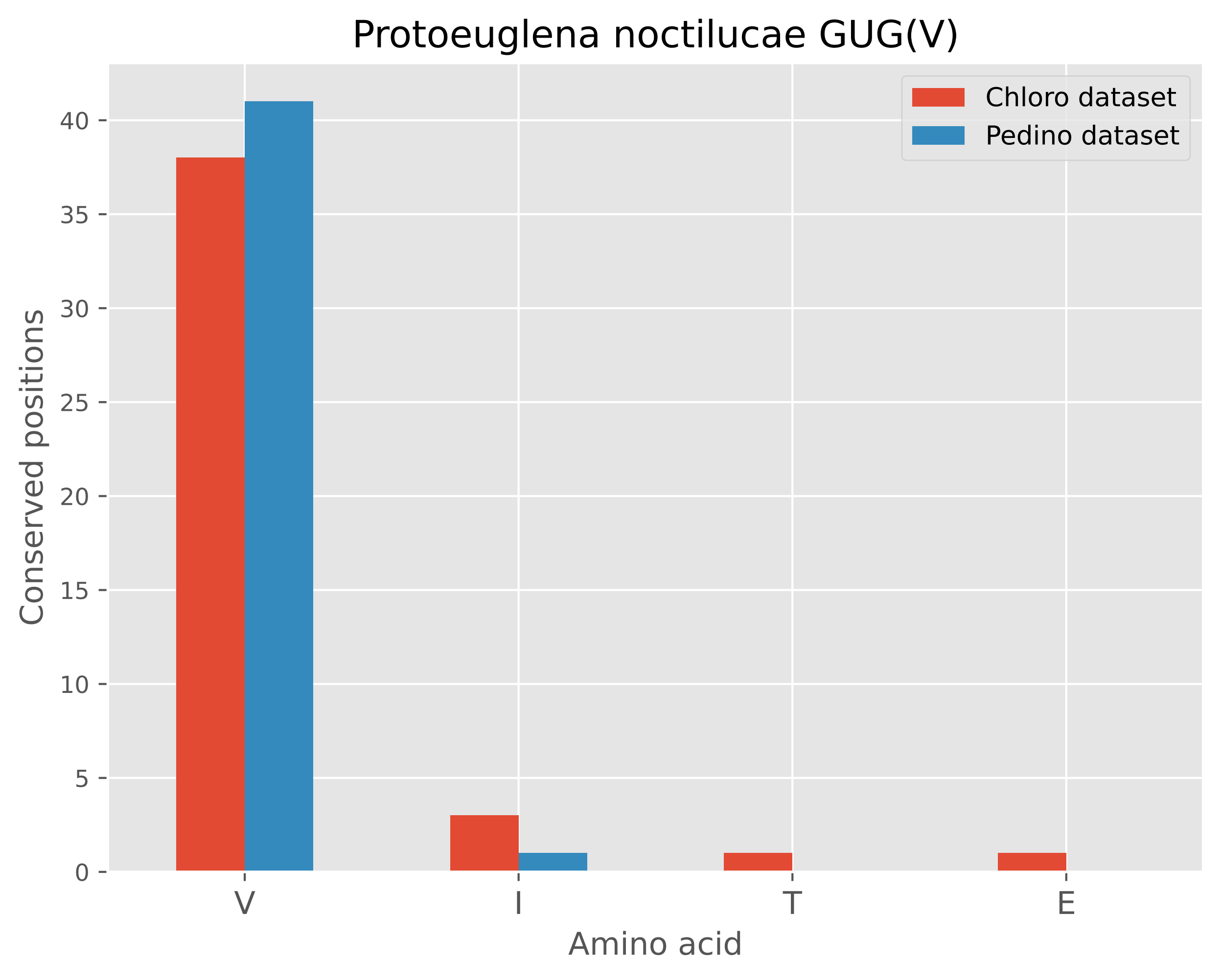

Protoeuglena noctilucae GUG(V)
Chloro dataset
Pedino dataset
Conserved positions
0
5
10
15
20
25
30
35
40
V
I
T
E
Amino acid

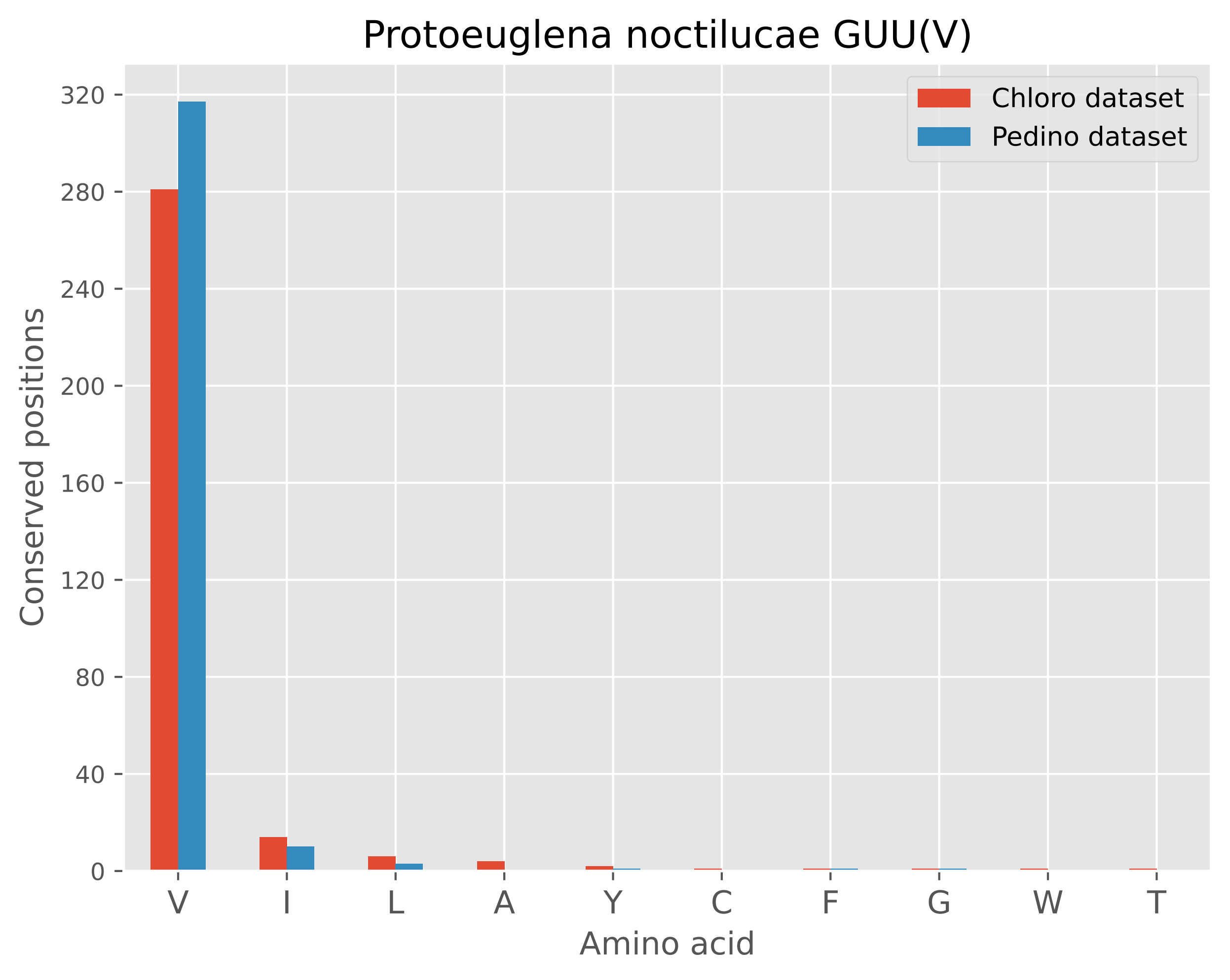

Protoeuglena noctilucae GUU(V)
Chloro dataset
Pedino dataset
Conserved positions
0
40
80
120
160
200
240
280
320
V
I
L
A
Y
C
F
G
W
T
Amino acid

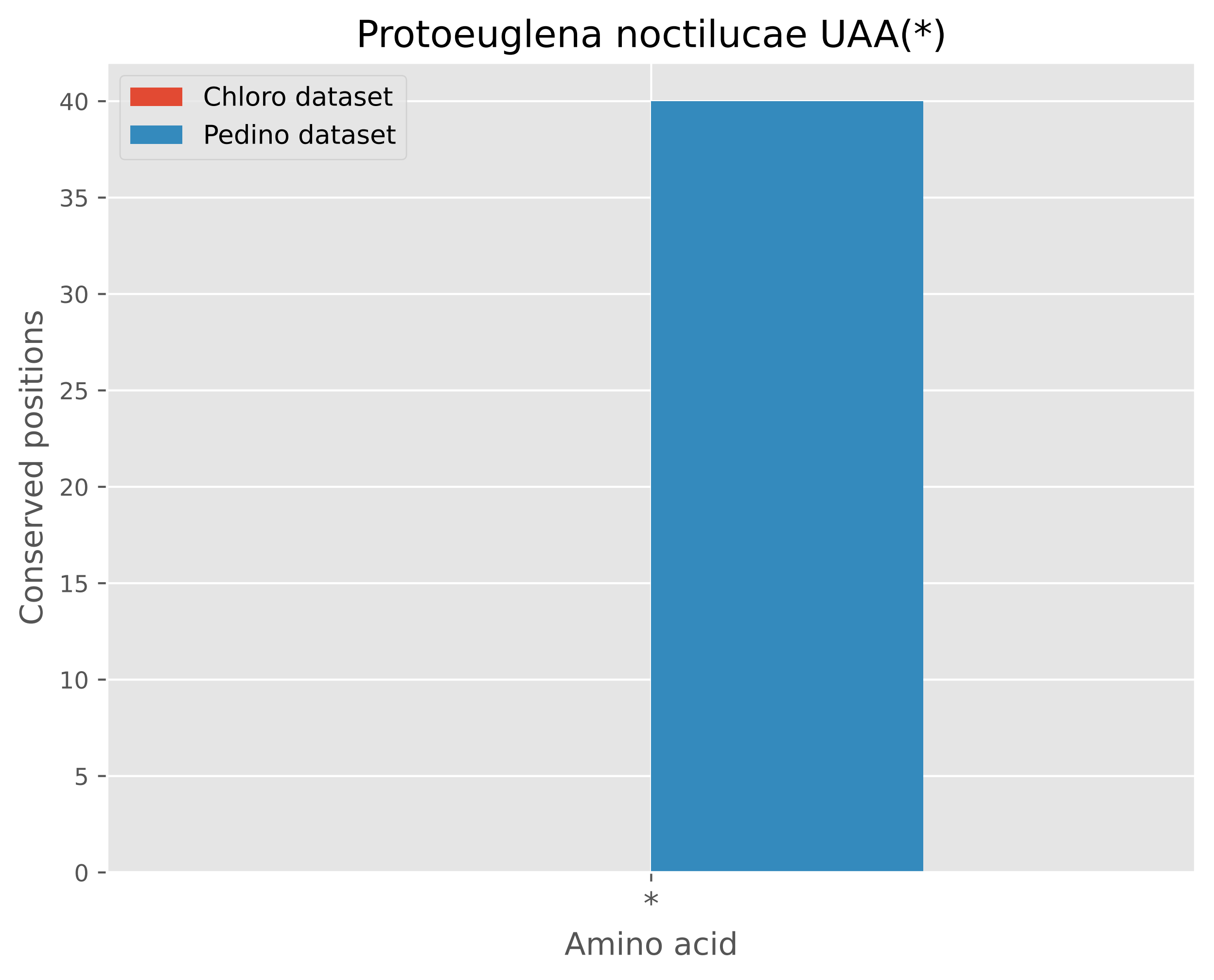
Protoeuglena noctilucae UAA(*)
Chloro dataset
Pedino dataset
Conserved positions
0
5
10
15
20
25
30
35
40
*
Amino acid

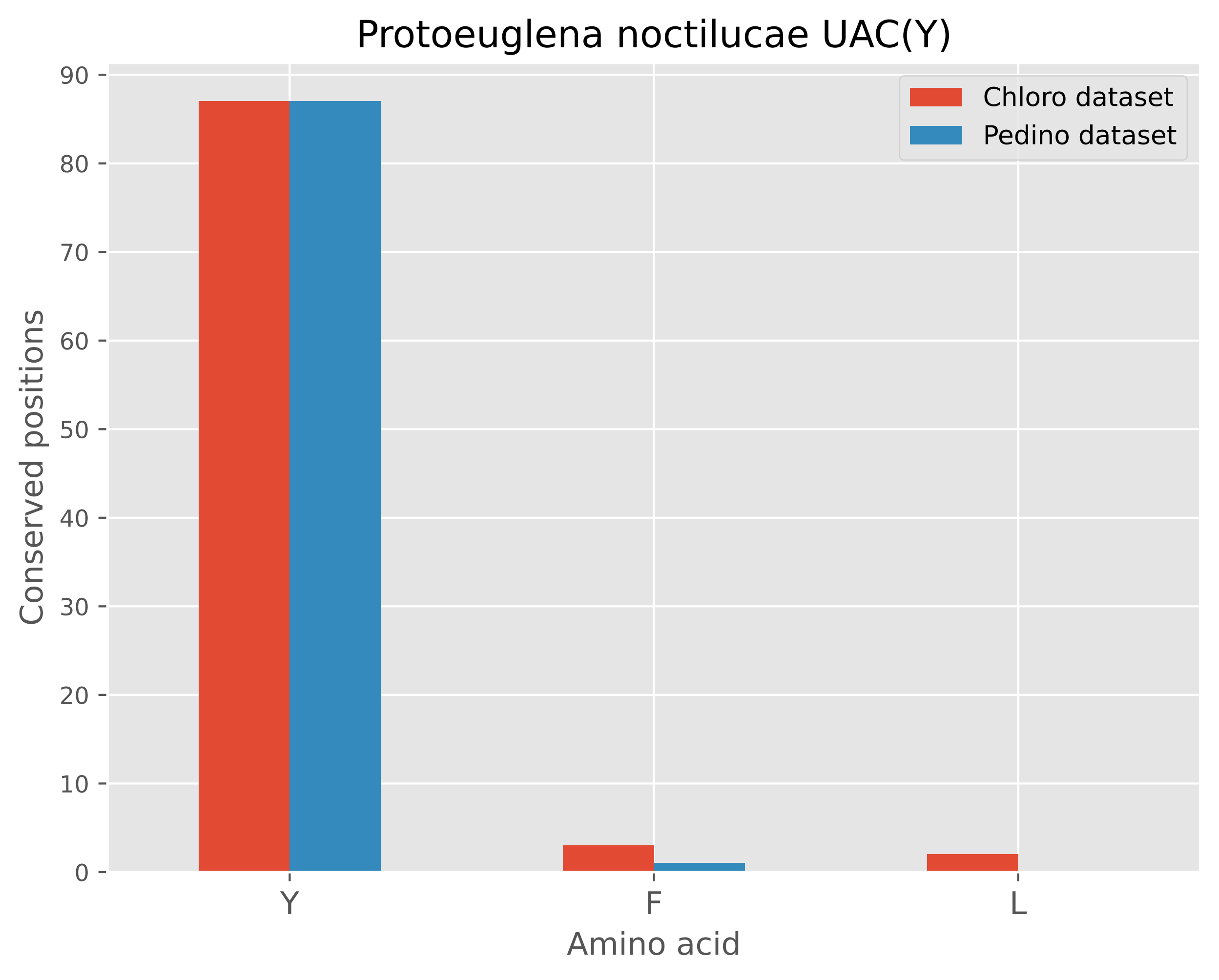

Protoeuglena noctilucae UAC(Y)
Chloro dataset
Pedino dataset
Conserved positions
0
10
20
30
40
50
60
70
80
90
Y
F
L
Amino acid

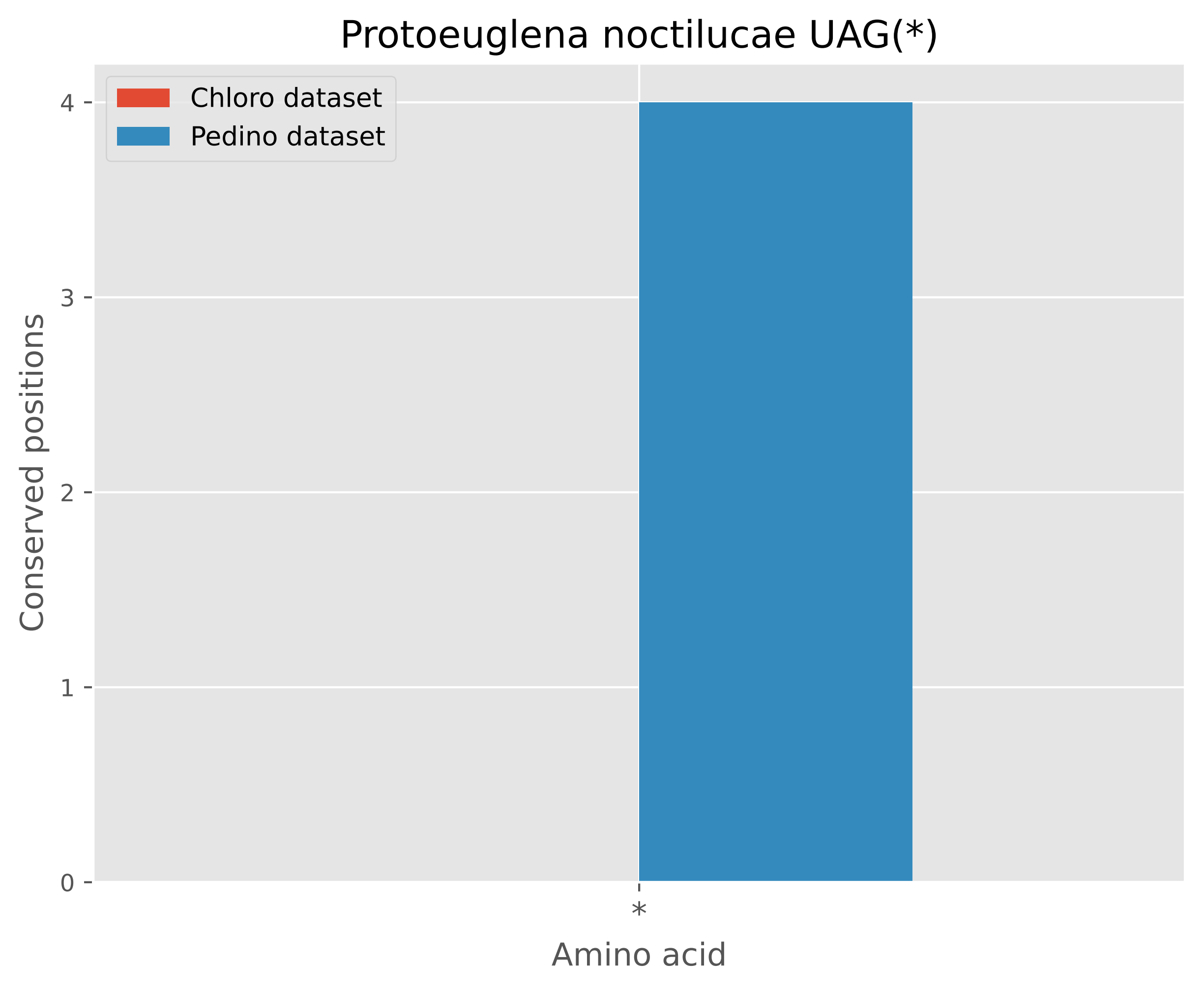

Protoeuglena noctilucae UAG(*)
Chloro dataset
Pedino dataset
Conserved positions
0
1
2
3
4
*
Amino acid

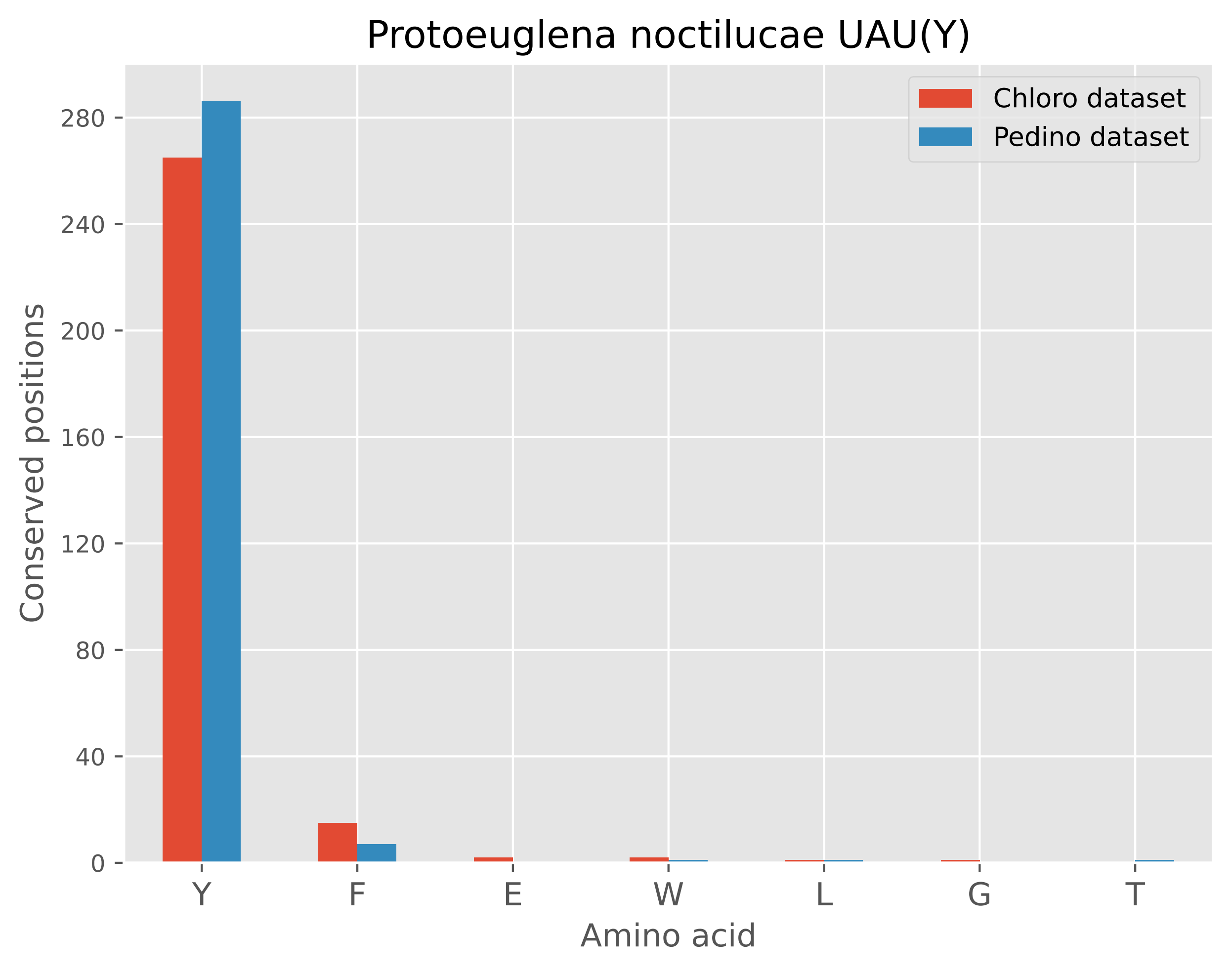

Protoeuglena noctilucae UAU(Y)
Chloro dataset
Pedino dataset
Conserved positions
0
40
80
120
160
200
240
280
Y
F
E
W
L
G
T
Amino acid

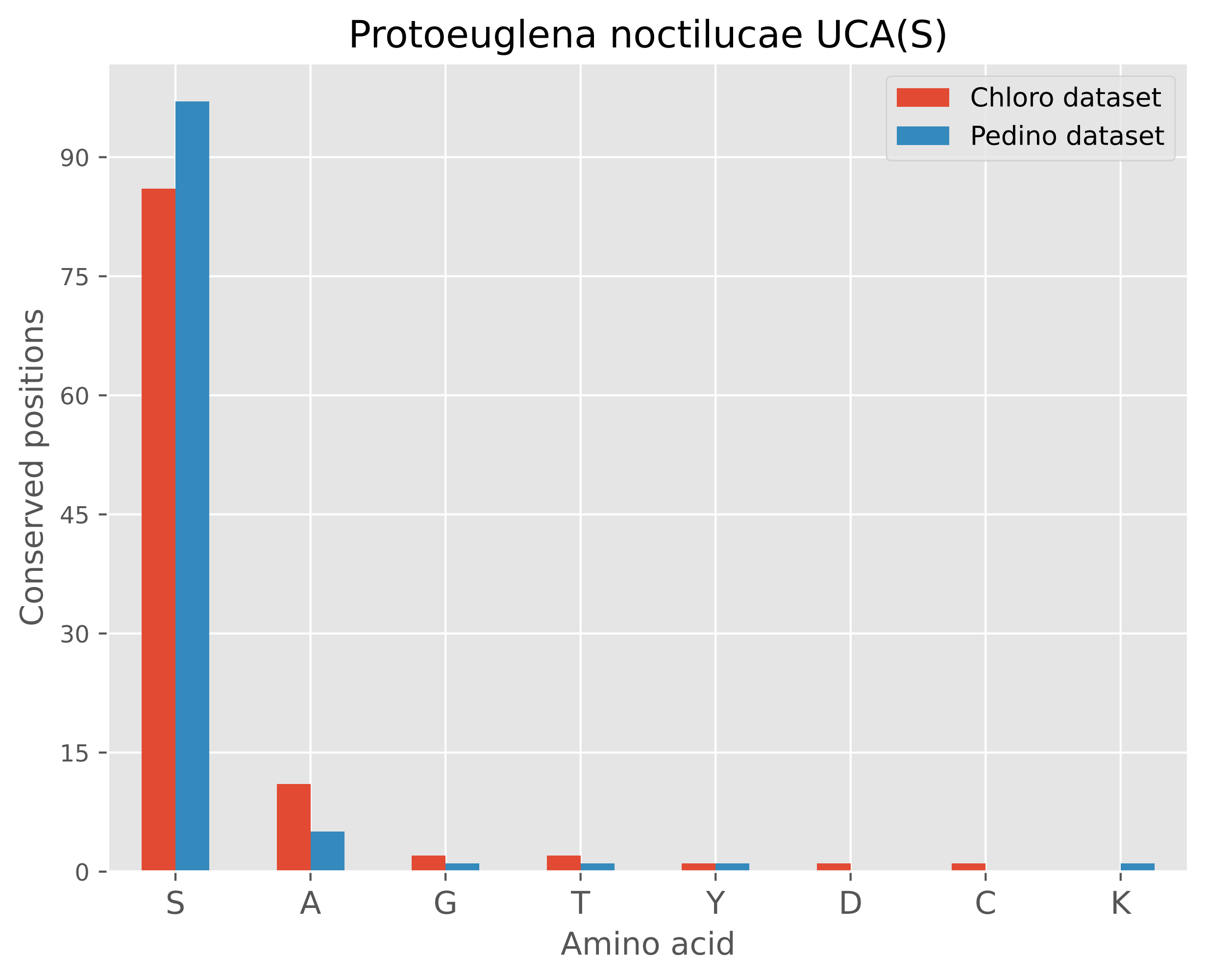
Protoeuglena noctilucae UCA(S)
Chloro dataset
Pedino dataset
Conserved positions
0
15
30
45
60
75
90
S
A
G
T
Y
D
C
K
Amino acid

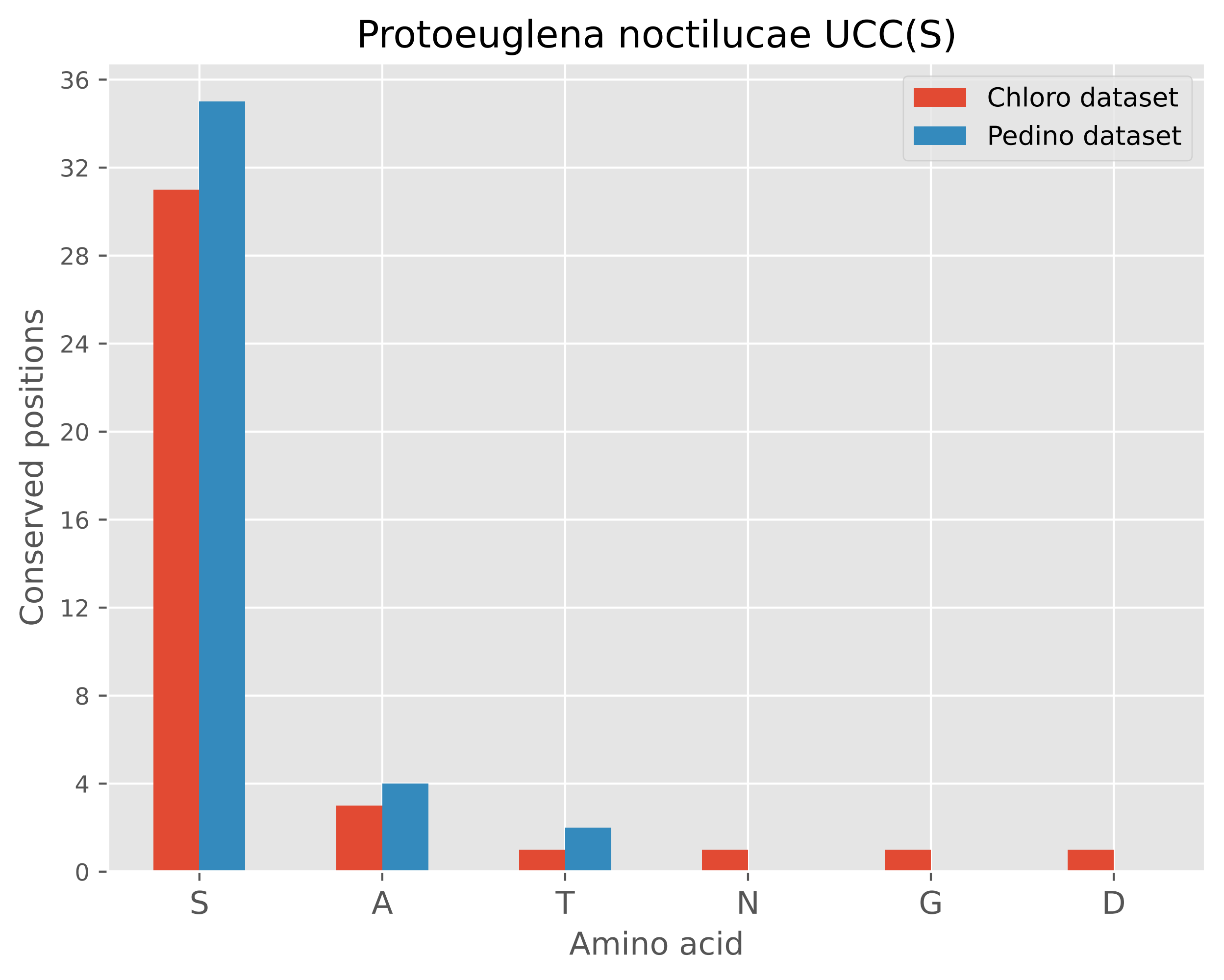

Protoeuglena noctilucae UCC(S)
Chloro dataset
Pedino dataset
Conserved positions
0
4
8
12
16
20
24
28
32
36
S
A
T
N
G
D
Amino acid

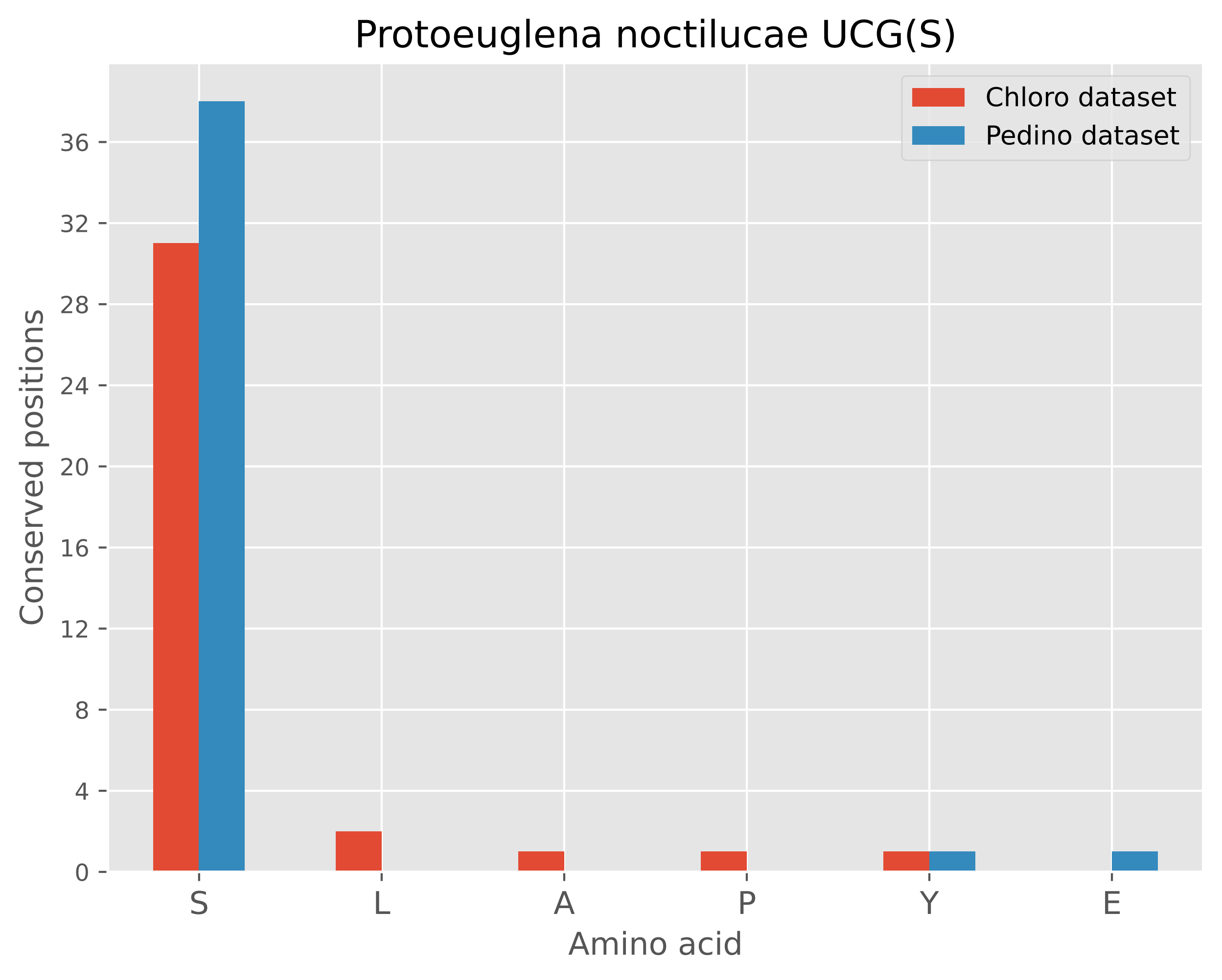

Protoeuglena noctilucae UCG(S)
Chloro dataset
Pedino dataset
Conserved positions
0
4
8
12
16
20
24
28
32
36
S
L
A
P
Y
E
Amino acid

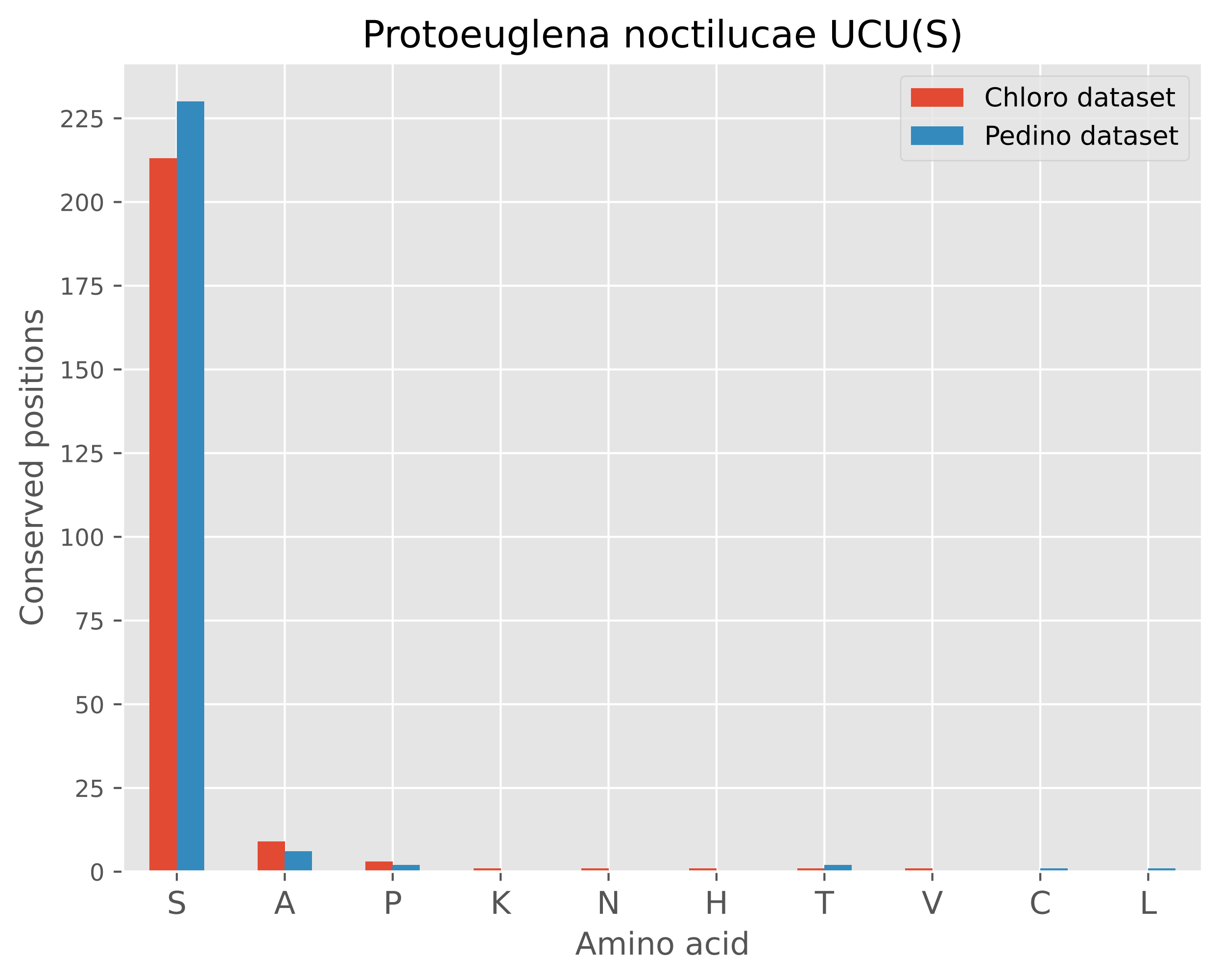

Protoeuglena noctilucae UCU(S)
Chloro dataset
Pedino dataset
Conserved positions
0
25
50
75
100
125
150
175
200
225
S
A
P
K
N
H
T
V
C
L
Amino acid

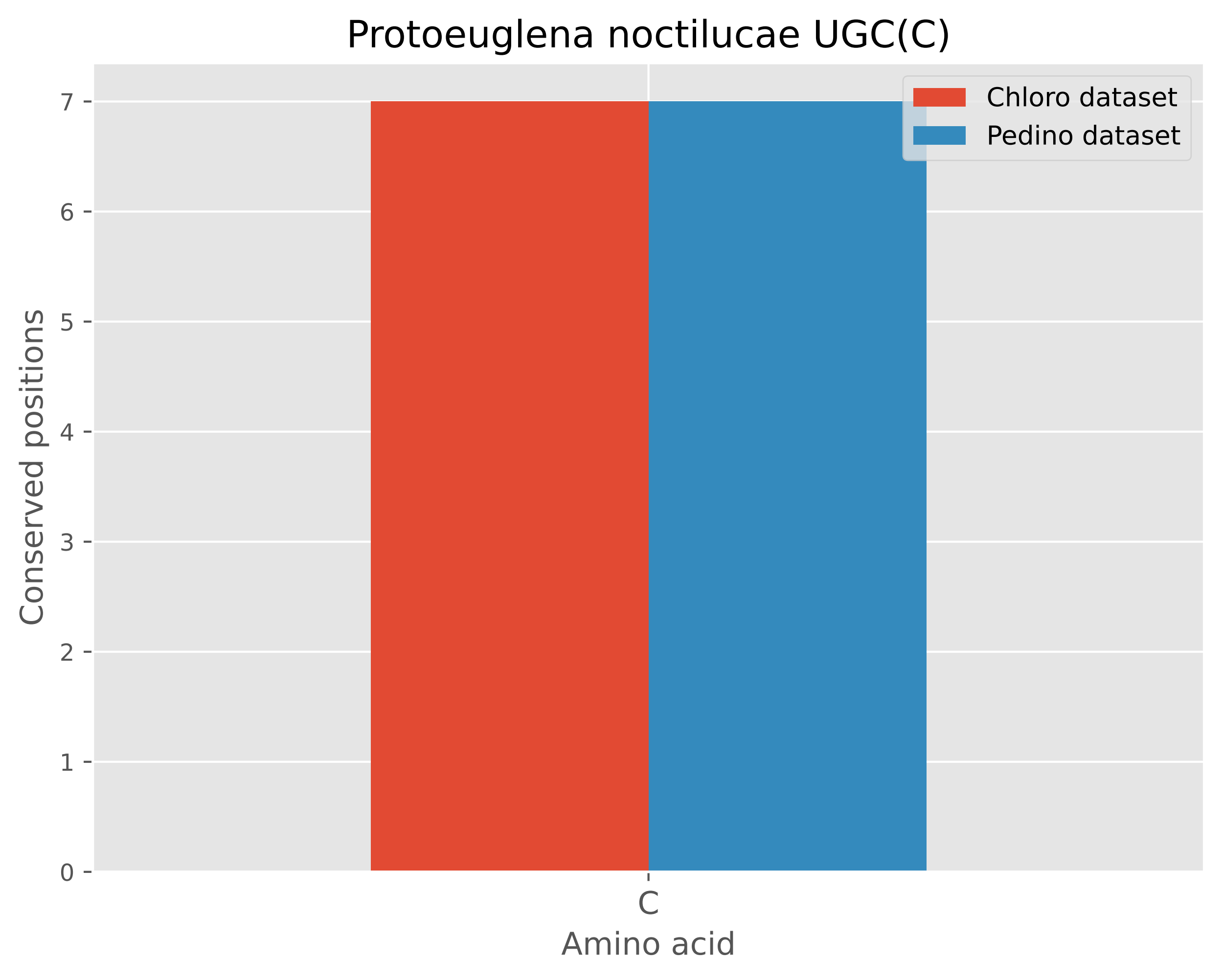
Protoeuglena noctilucae UGC(C)
Chloro dataset
Pedino dataset
Conserved positions
0
1
2
3
4
5
6
7
C
Amino acid

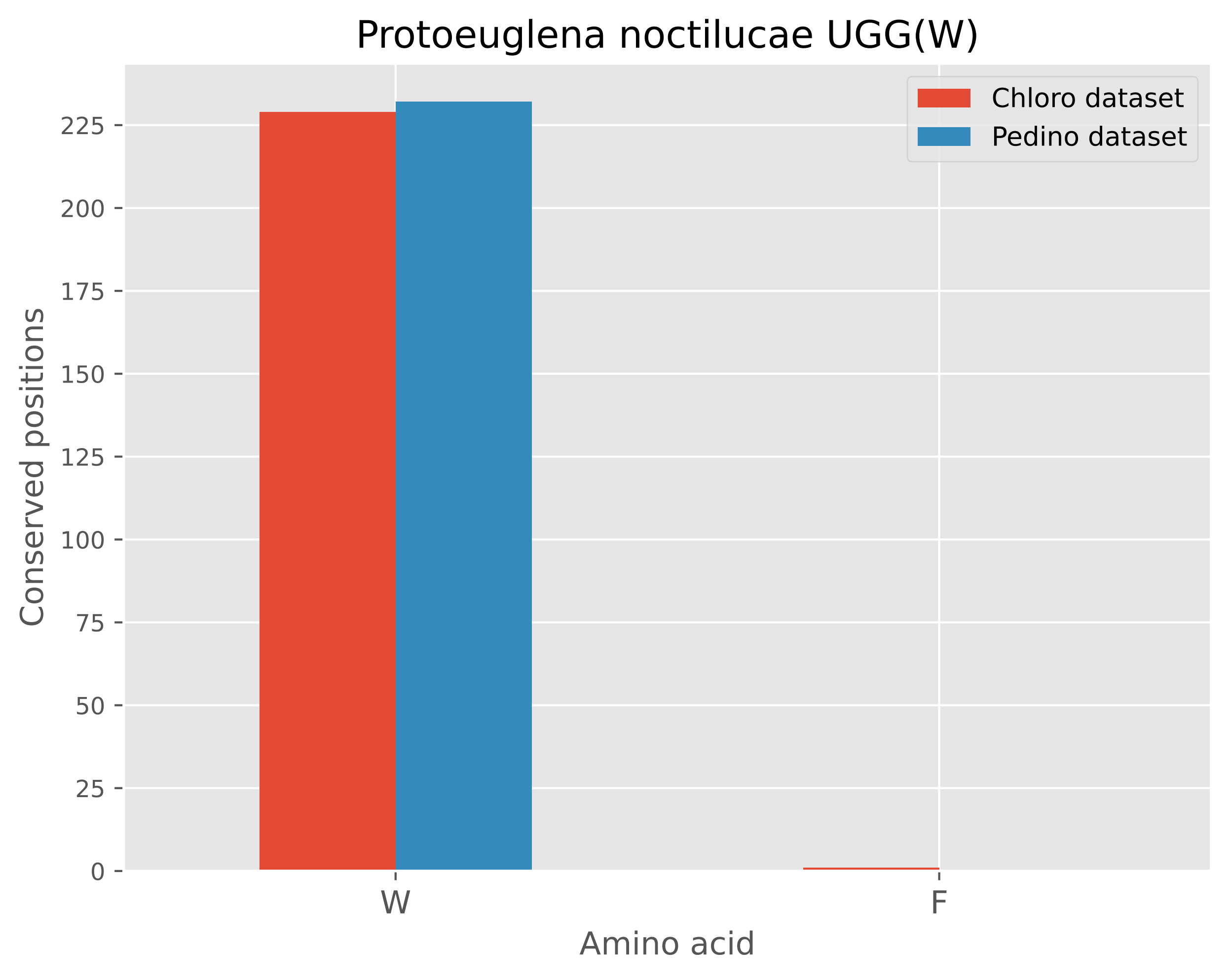

Protoeuglena noctilucae UGG(W)
Chloro dataset
Pedino dataset
Conserved positions
0
25
50
75
100
125
150
175
200
225
W
F
Amino acid

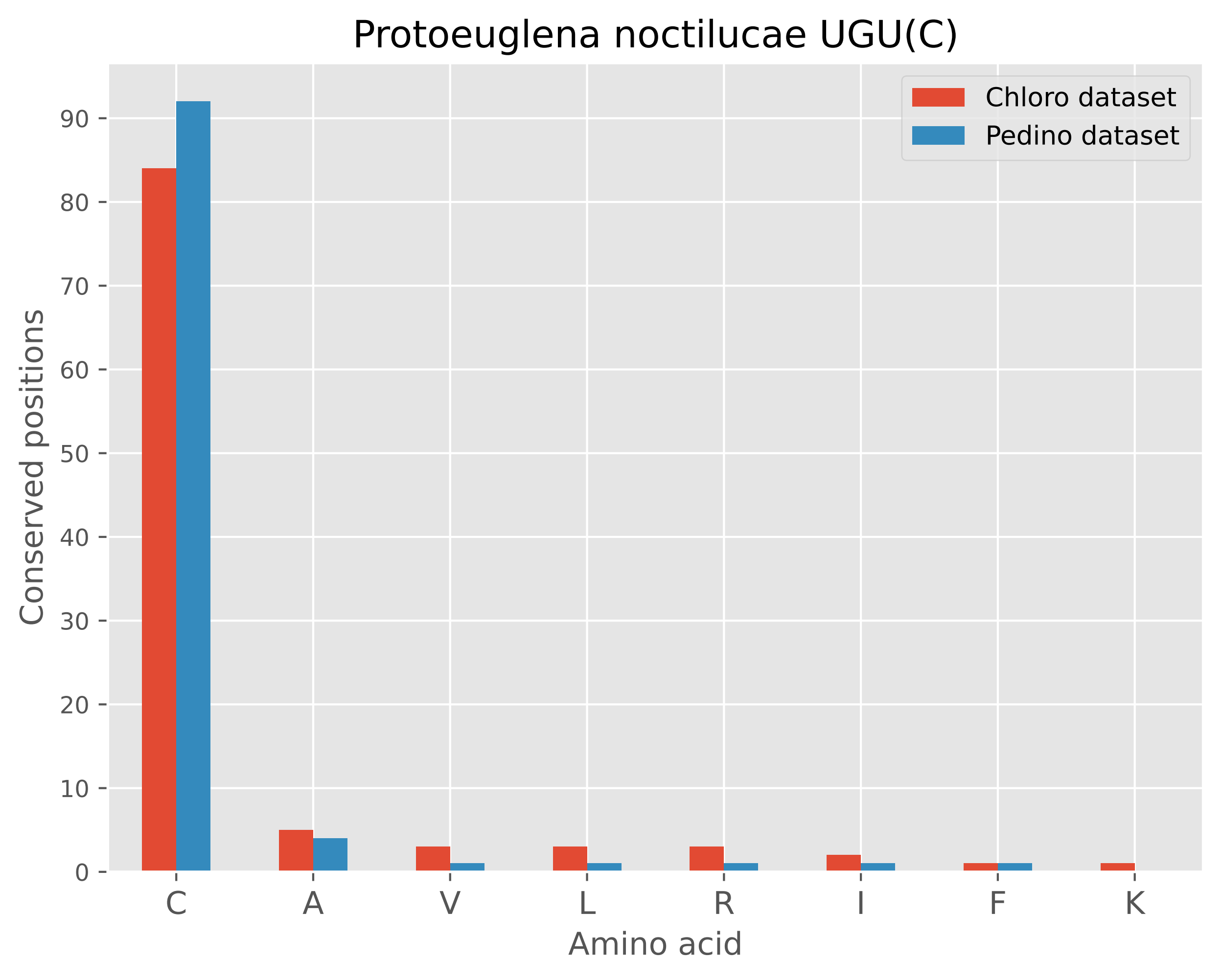
Protoeuglena noctilucae UGU(C)
Chloro dataset
Pedino dataset
Conserved positions
0
10
20
30
40
50
60
70
80
90
C
A
V
L
R
I
F
K
Amino acid

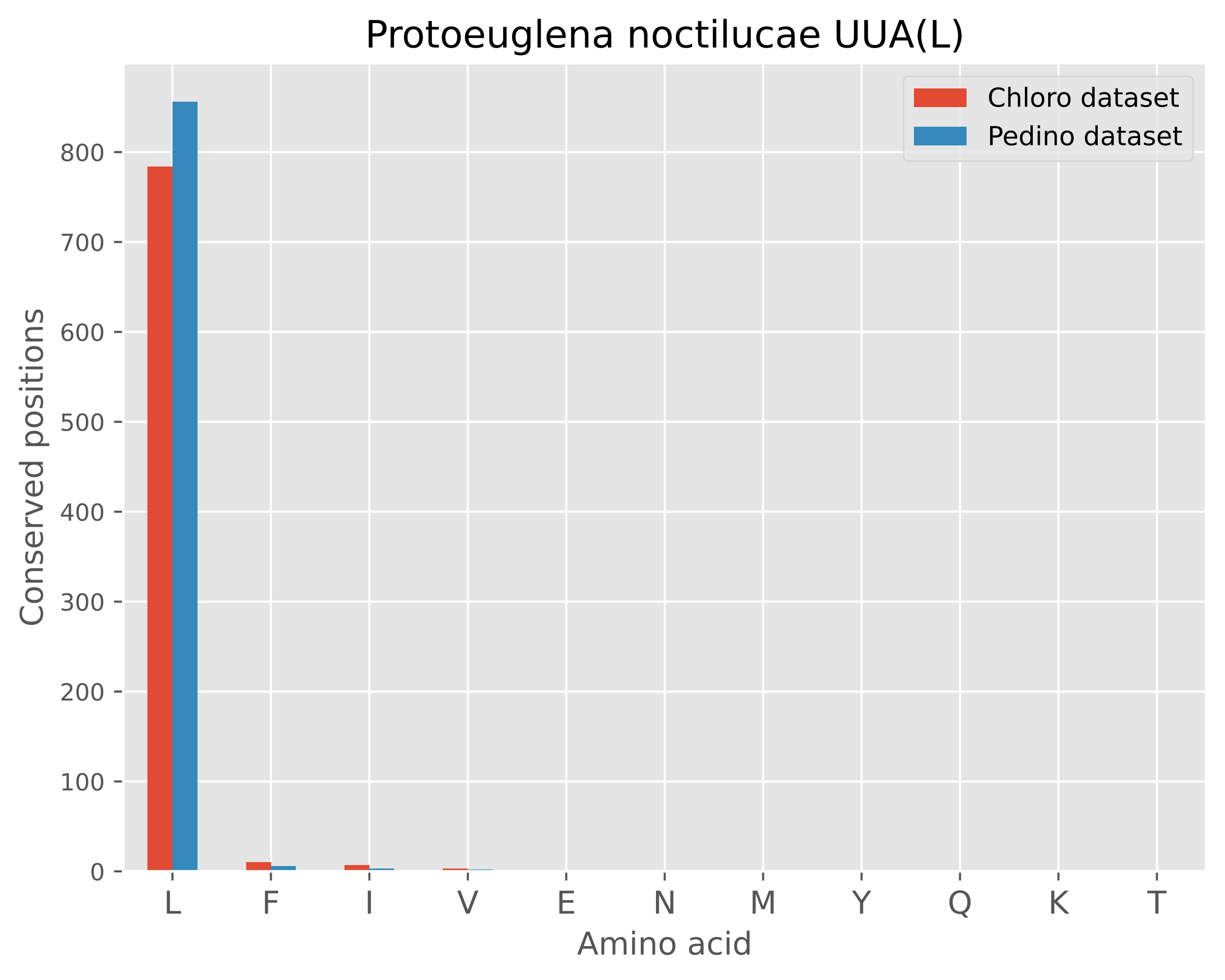

Protoeuglena noctilucae UUA(L)
Chloro dataset
Pedino dataset
Conserved positions
800
700
600
500
400
300
200
100
0
L
F
I
V
E
N
M
Y
Q
K
T
Amino acid

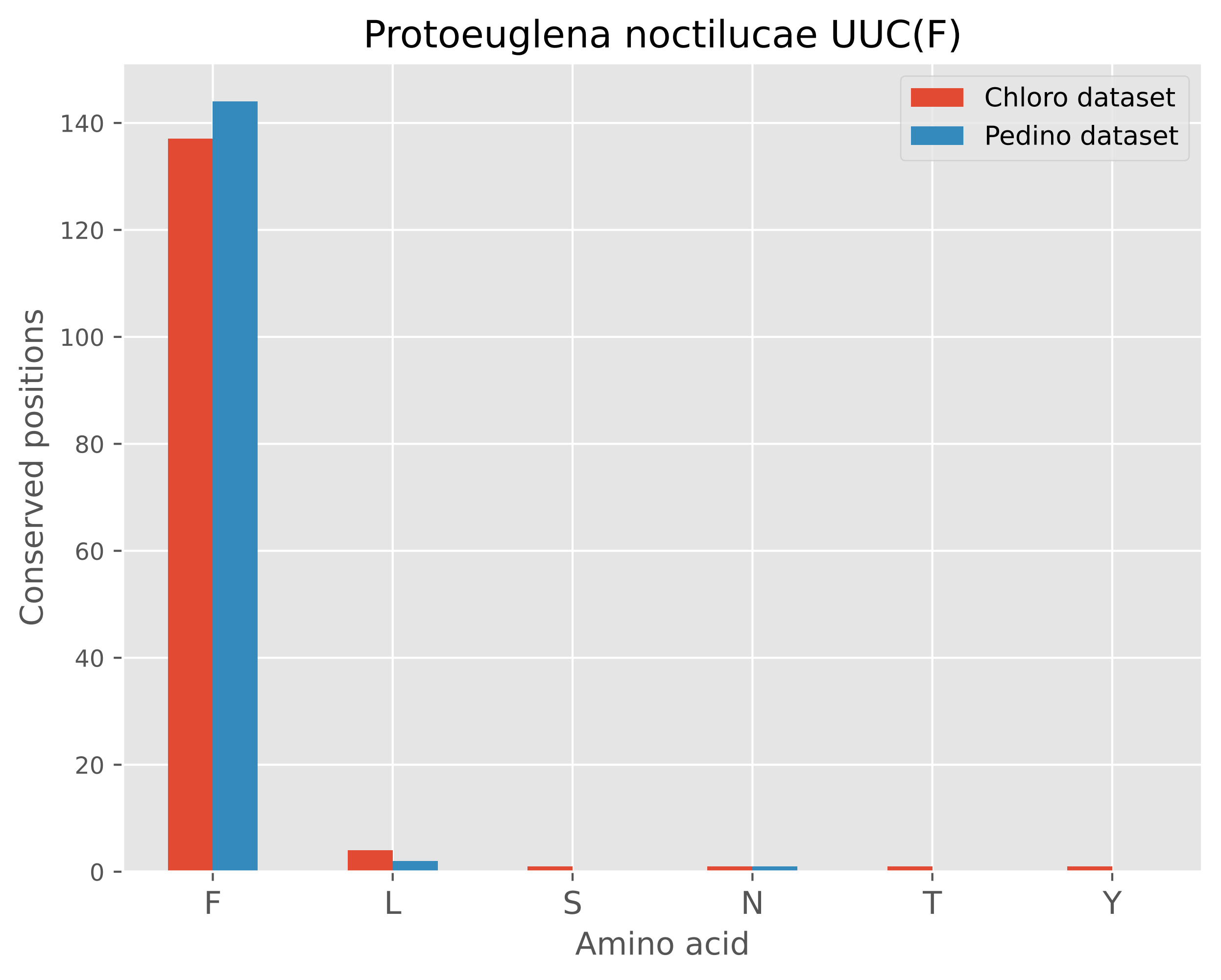
Protoeuglena noctilucae UUC(F)
Chloro dataset
Pedino dataset
Conserved positions
0
20
40
60
80
100
120
140
F
L
S
N
T
Y
Amino acid

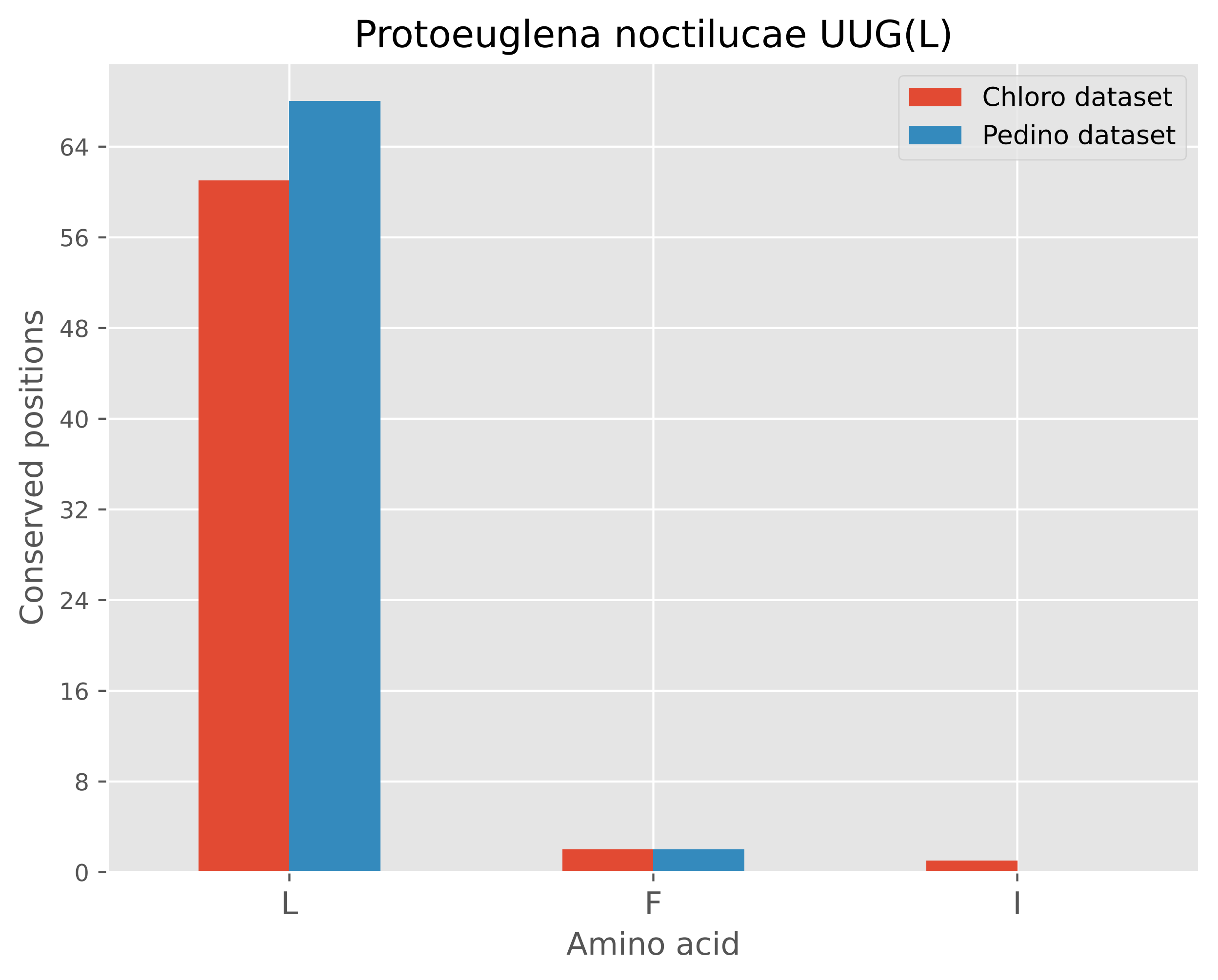

Protoeuglena noctilucae UUG(L)
Chloro dataset
Pedino dataset
Conserved positions
0
8
16
24
32
40
48
56
64
L
F
I
Amino acid

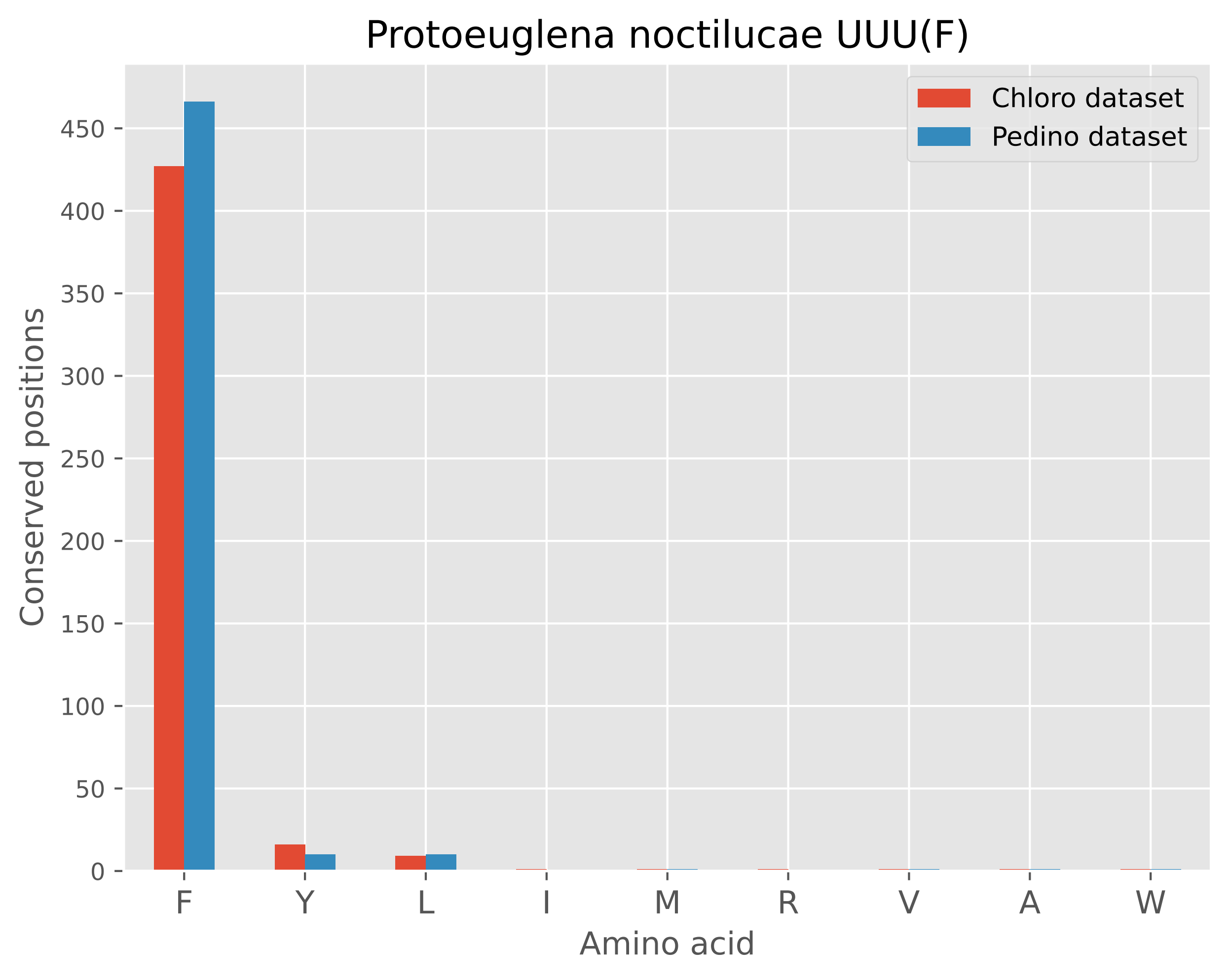
Protoeuglena noctilucae UUU(F)
Chloro dataset
Pedino dataset
Conserved positions
0
50
100
150
200
250
300
350
400
450
F
Y
L
I
M
R
V
A
W
Amino acid

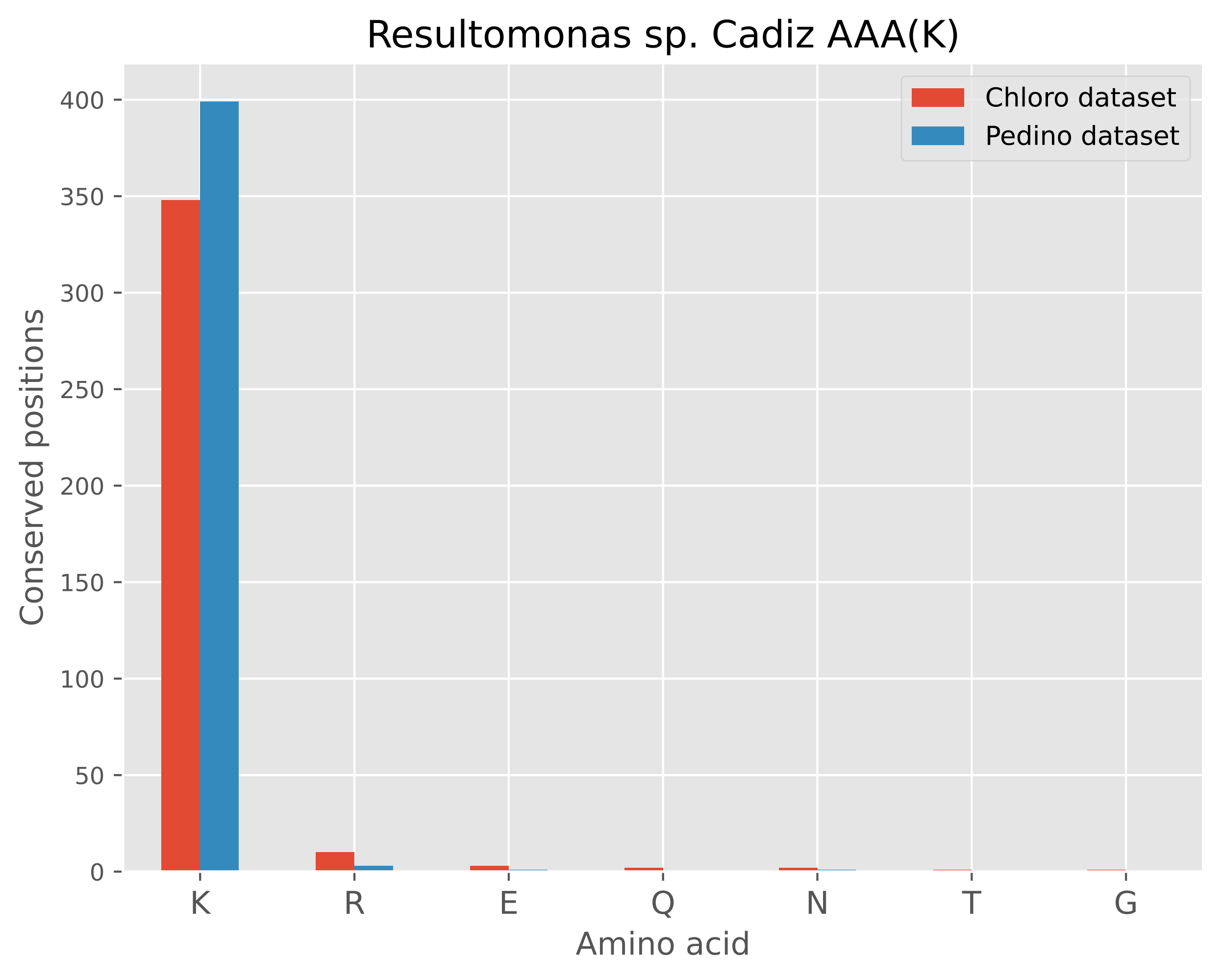

Resultomonas sp. Cadiz AAA(K)
Chloro dataset
Pedino dataset
Conserved positions
400
350
300
250
200
150
100
50
0
K
R
E
Q
N
T
G
Amino acid

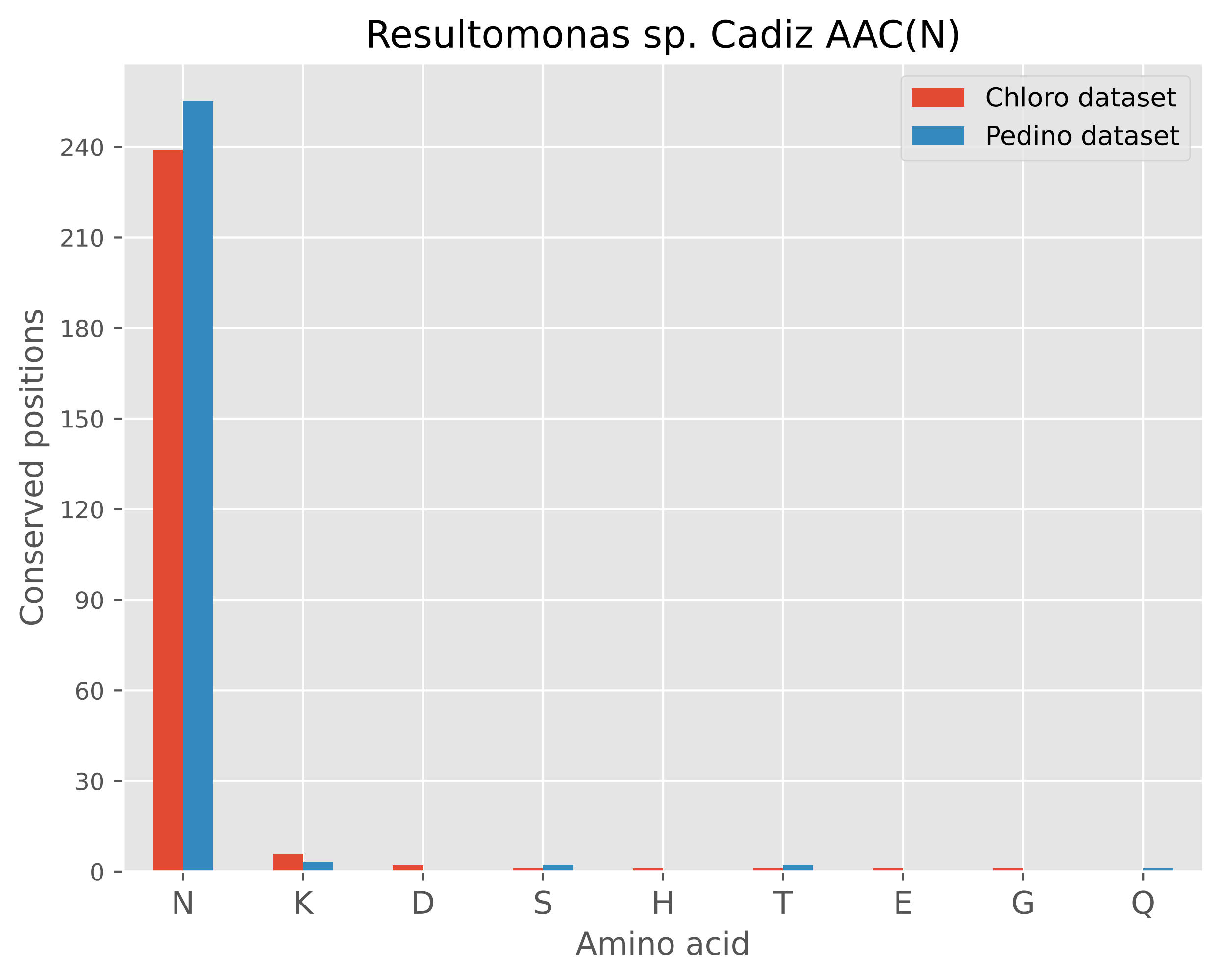

Resultomonas sp. Cadiz AAC(N)
Chloro dataset
Pedino dataset
Conserved positions
0
30
60
90
120
150
180
210
240
N
K
D
S
H
T
E
G
Q
Amino acid

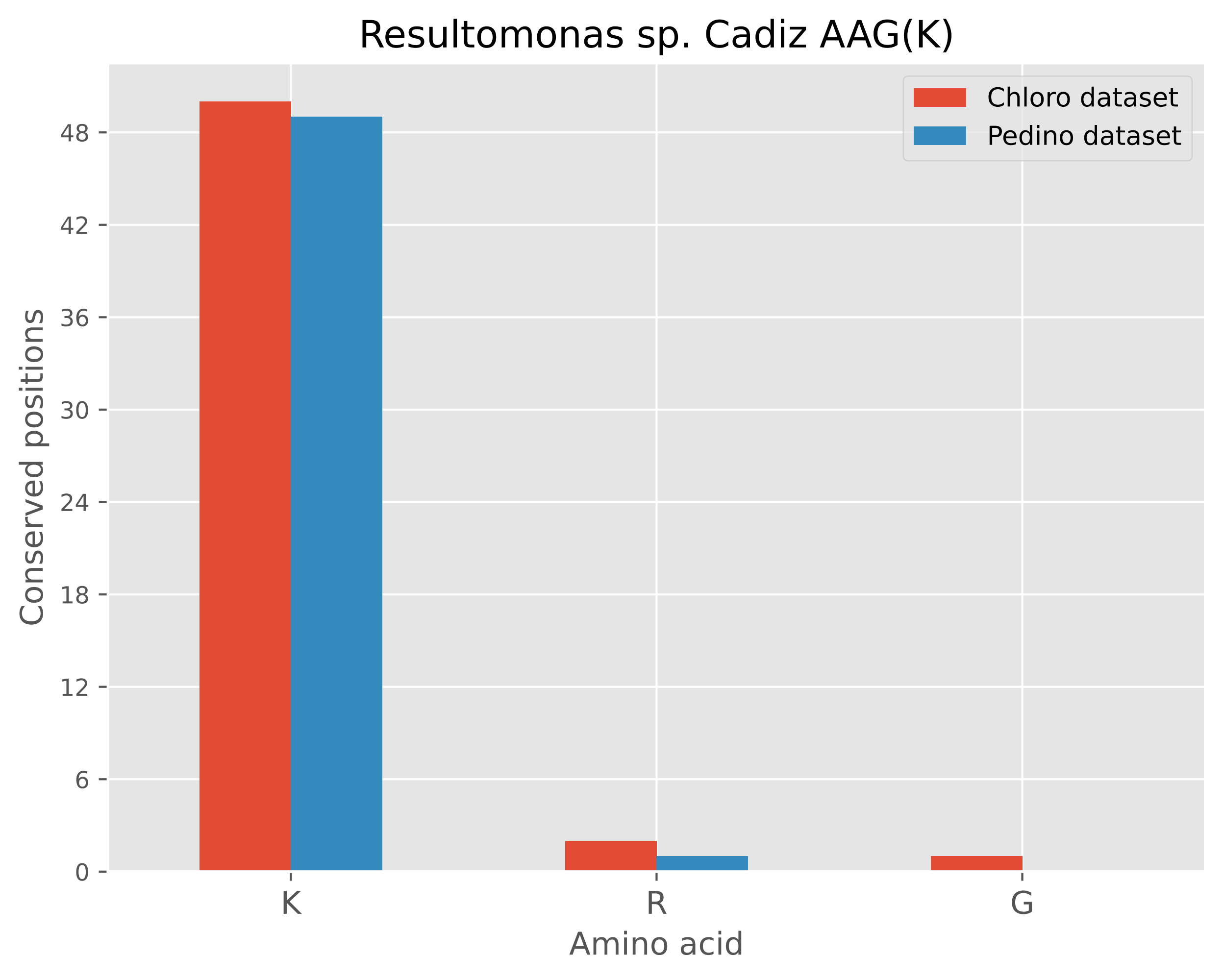

Resultomonas sp. Cadiz AAG(K)
Chloro dataset
Pedino dataset
Conserved positions
0
6
12
18
24
30
36
42
48
K
R
G
Amino acid

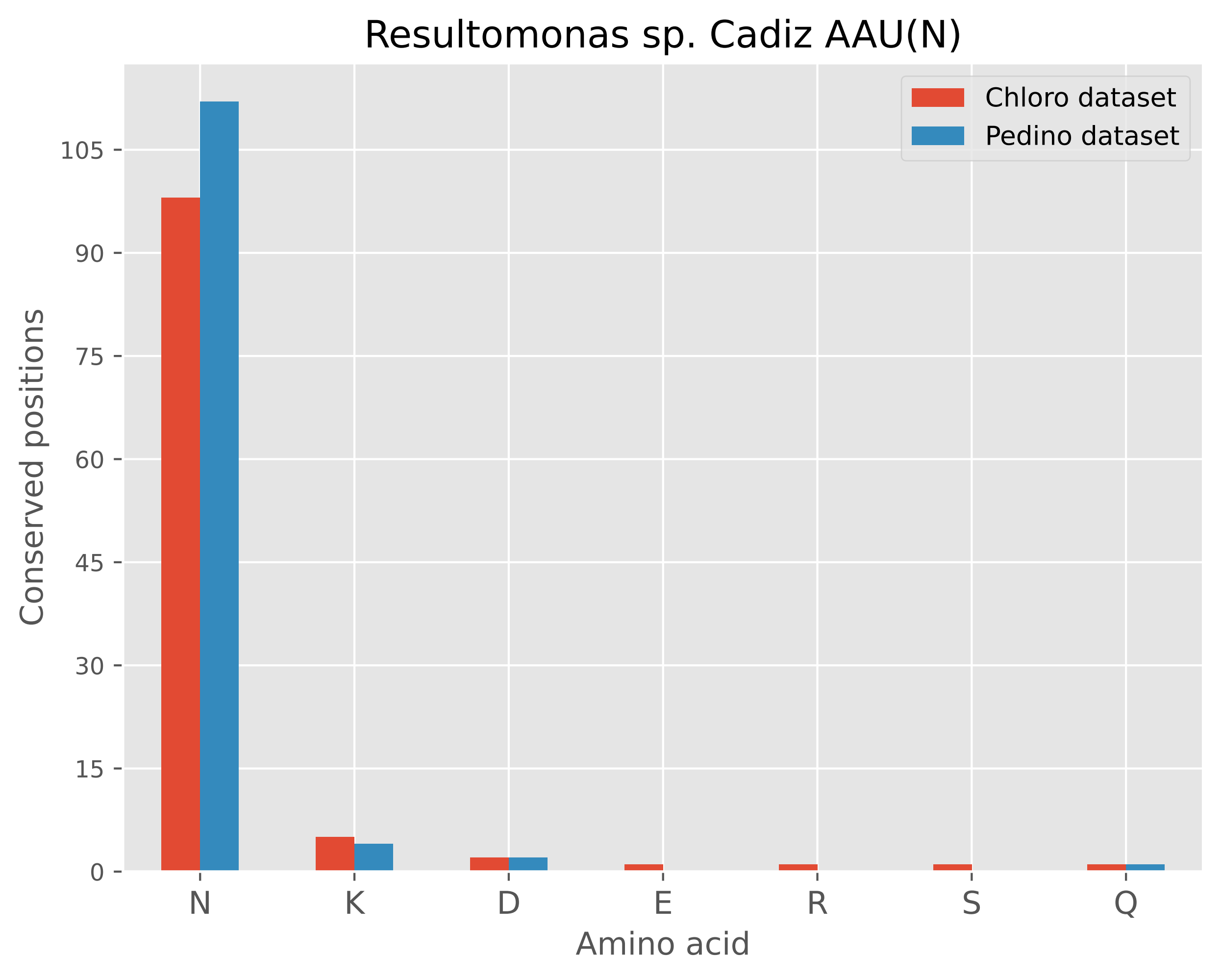

Resultomonas sp. Cadiz AAU(N)
Chloro dataset
Pedino dataset
Conserved positions
105
90
75
60
45
30
15
0
N
K
D
E
R
S
Q
Amino acid

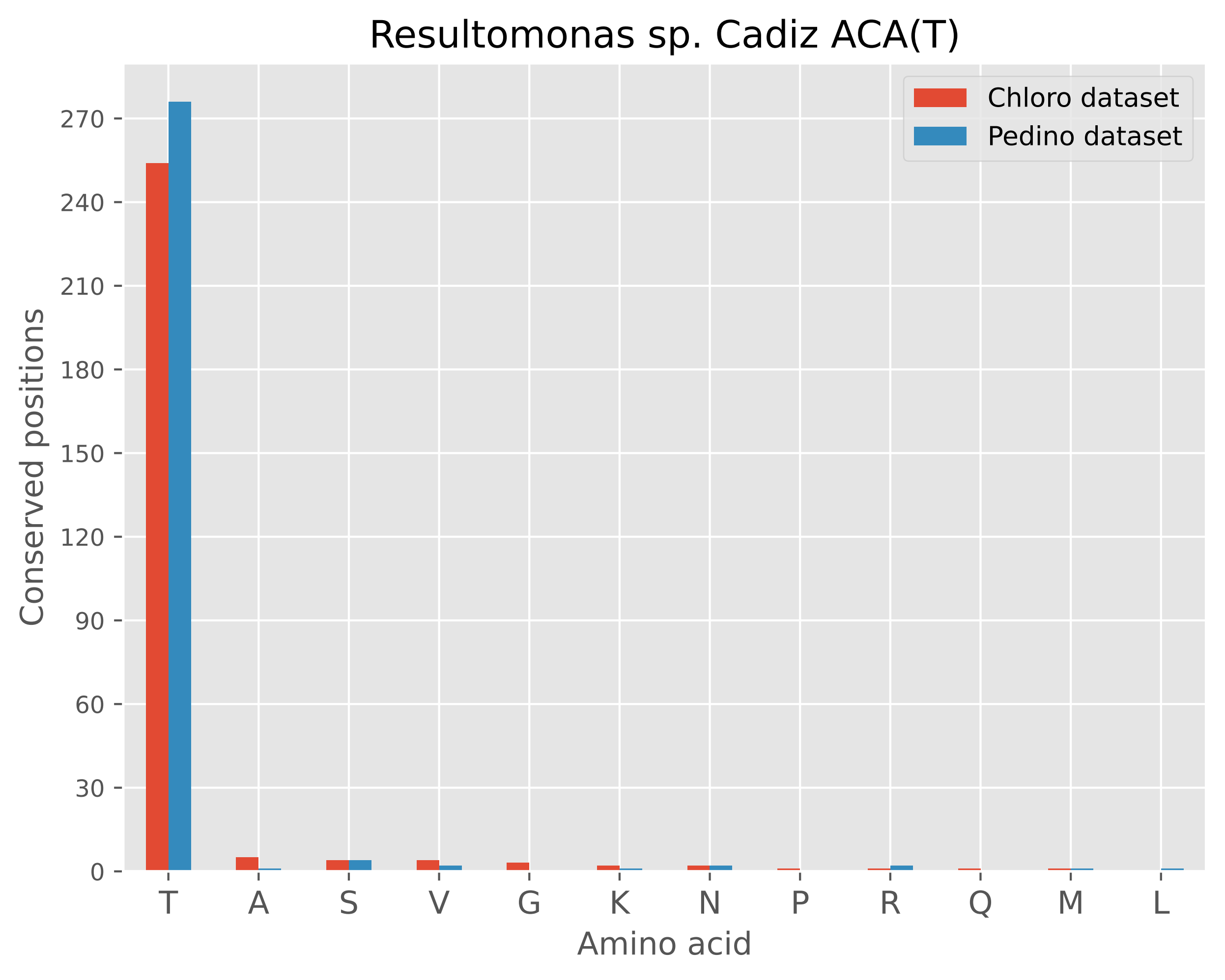
Resultomonas sp. Cadiz ACA(T)
Chloro dataset
Pedino dataset
Conserved positions
0
30
60
90
120
150
180
210
240
270
T
A
S
V
G
K
N
P
R
Q
M
L
Amino acid

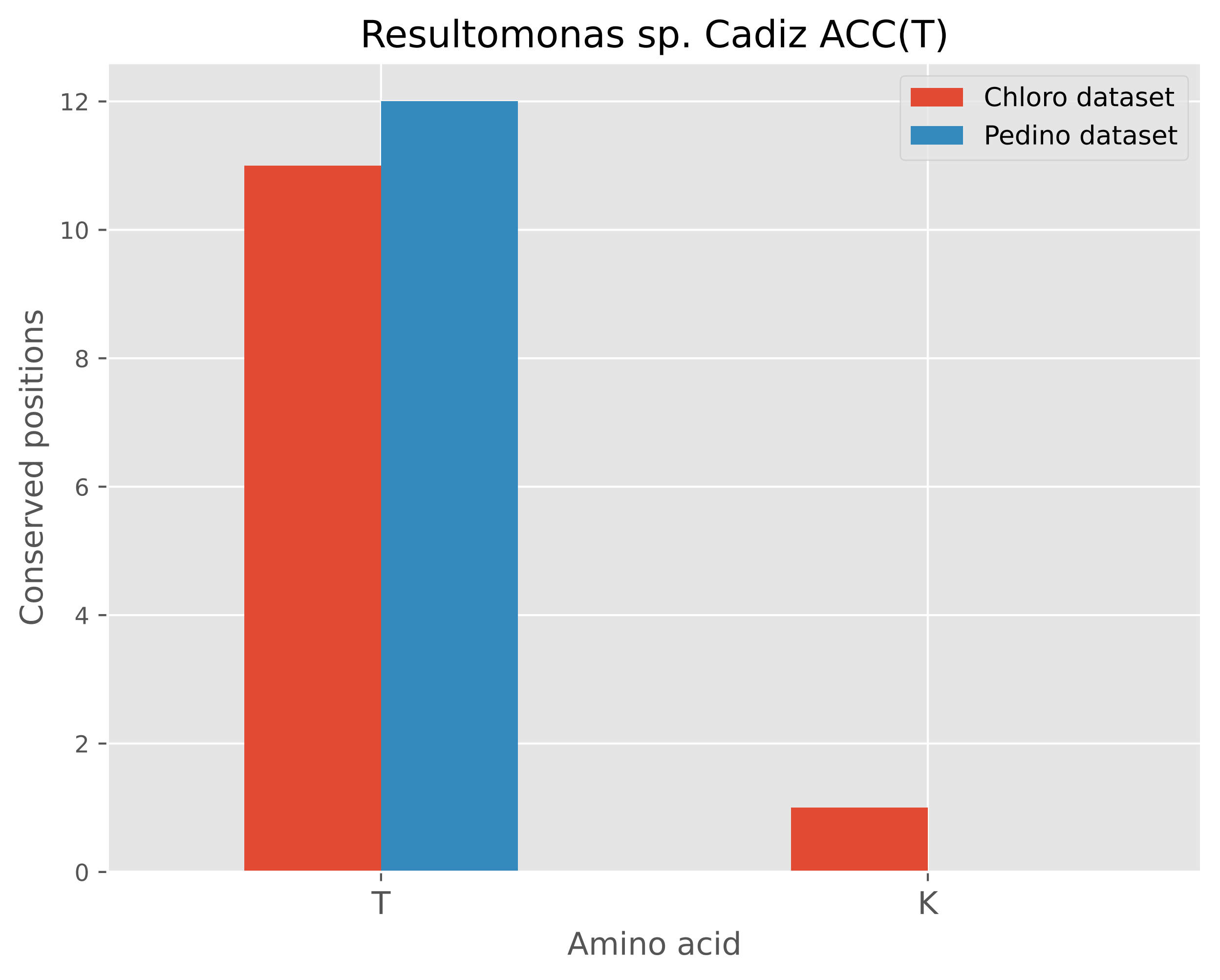

Resultomonas sp. Cadiz ACC(T)
Chloro dataset
Pedino dataset
Conserved positions
Amino acid
0
2
4
6
8
10
12
T
K

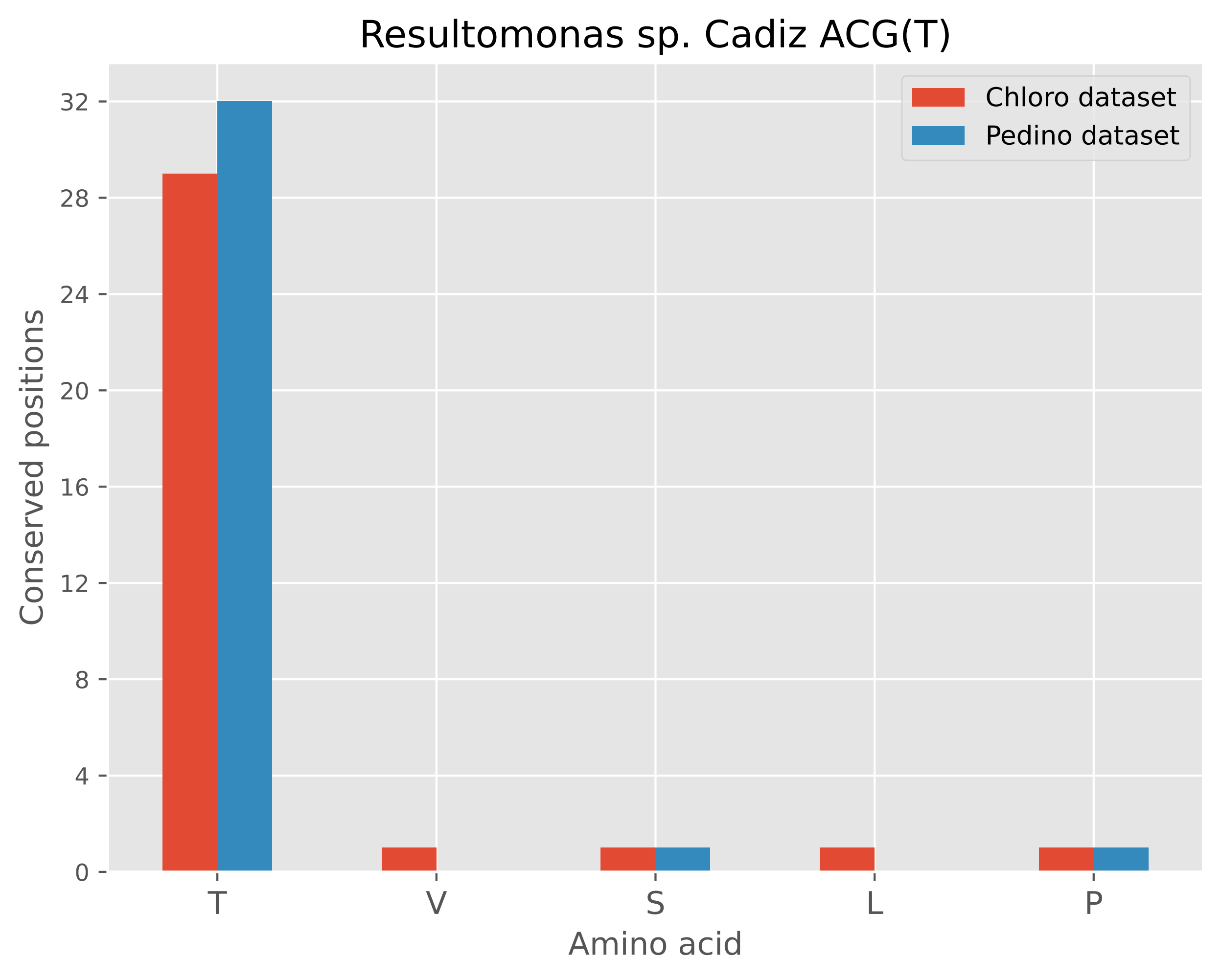

Resultomonas sp. Cadiz ACG(T)
Chloro dataset
Pedino dataset
Conserved positions
0
4
8
12
16
20
24
28
32
T
V
S
L
P
Amino acid

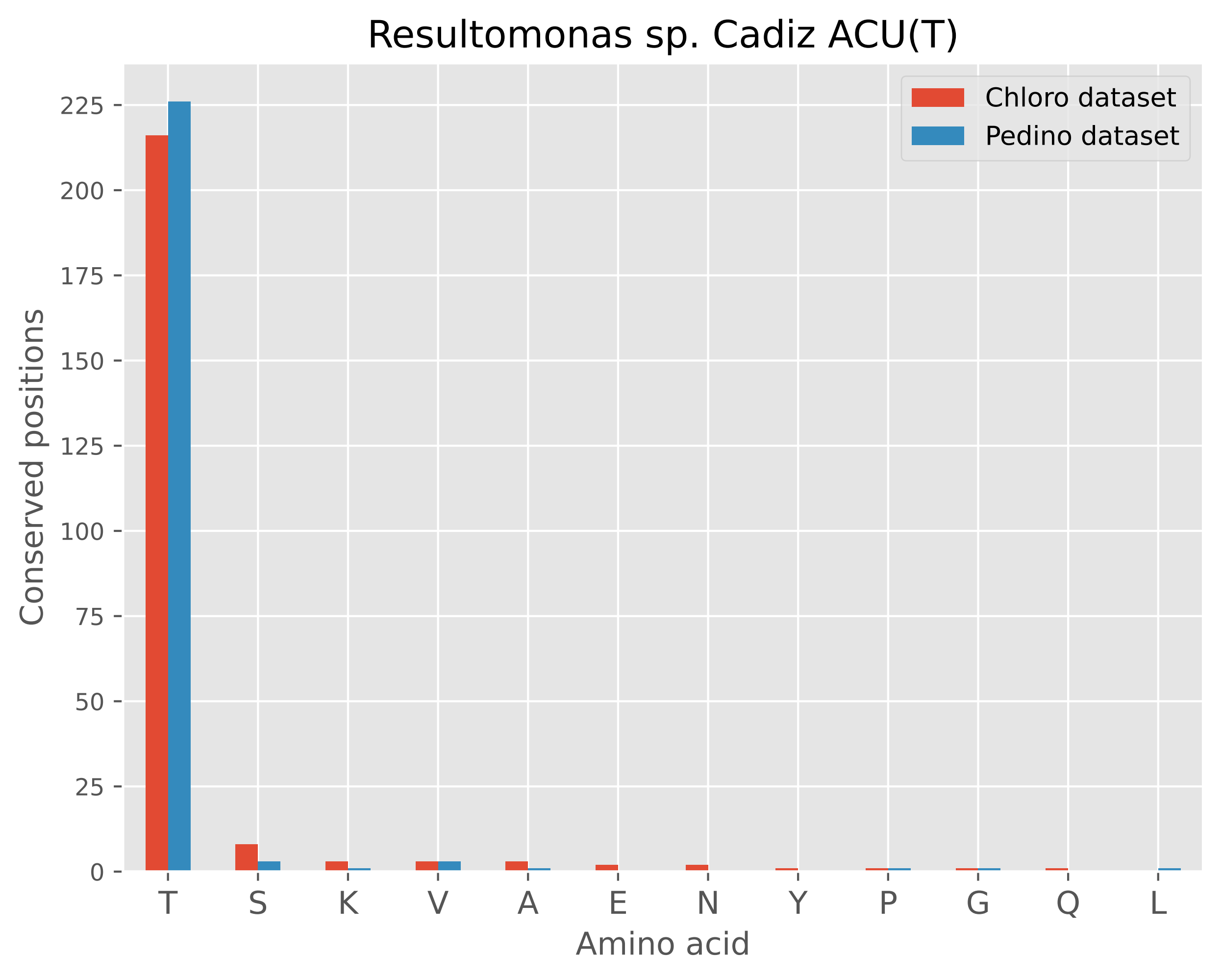
Resultomonas sp. Cadiz ACU(T)
Chloro dataset
Pedino dataset
Conserved positions
0
25
50
75
100
125
150
175
200
225
T
S
K
V
A
E
N
Y
P
G
Q
L
Amino acid

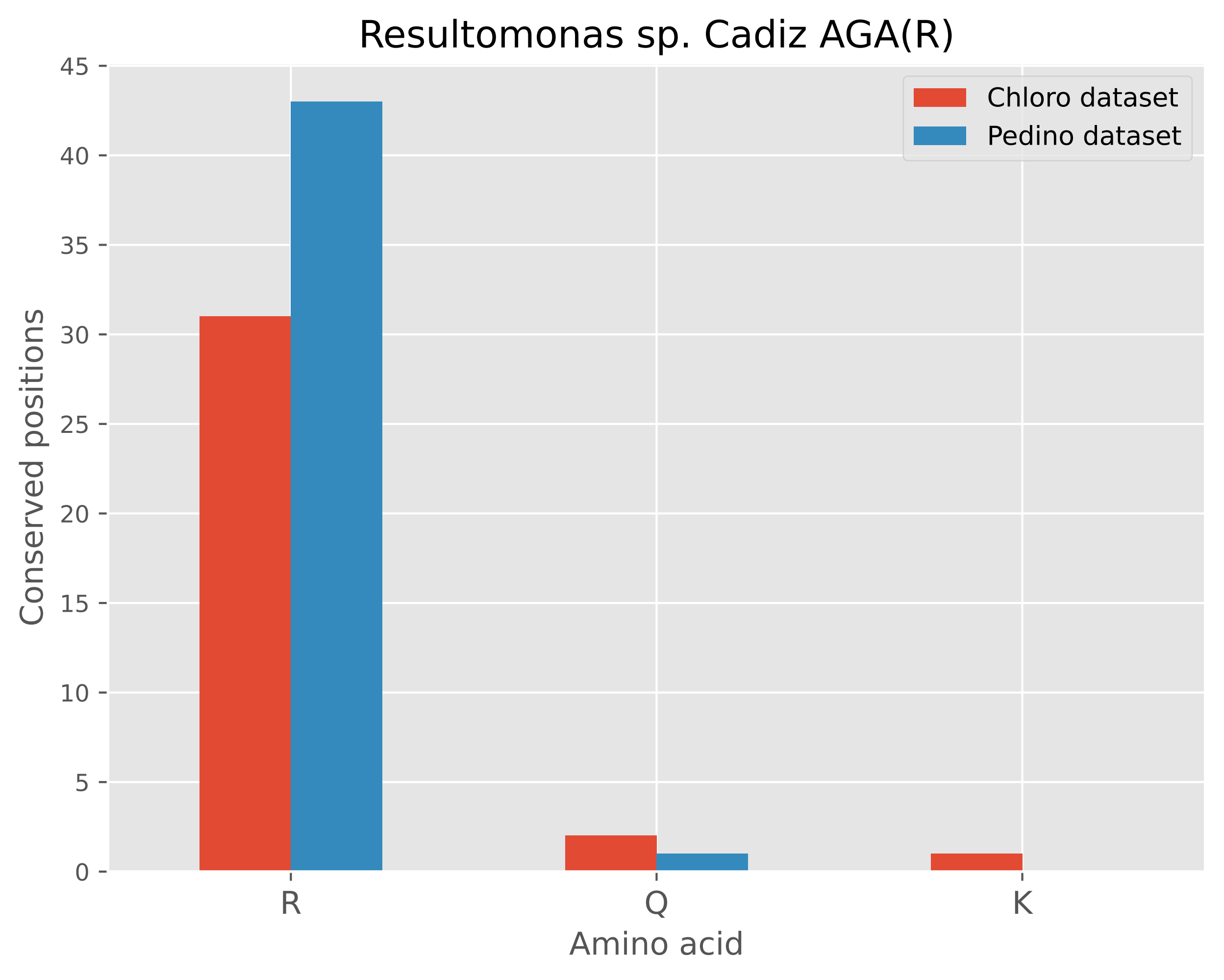
Resultomonas sp. Cadiz AGA(R)
Chloro dataset
Pedino dataset
Conserved positions
0
5
10
15
20
25
30
35
40
45
R
Q
K
Amino acid

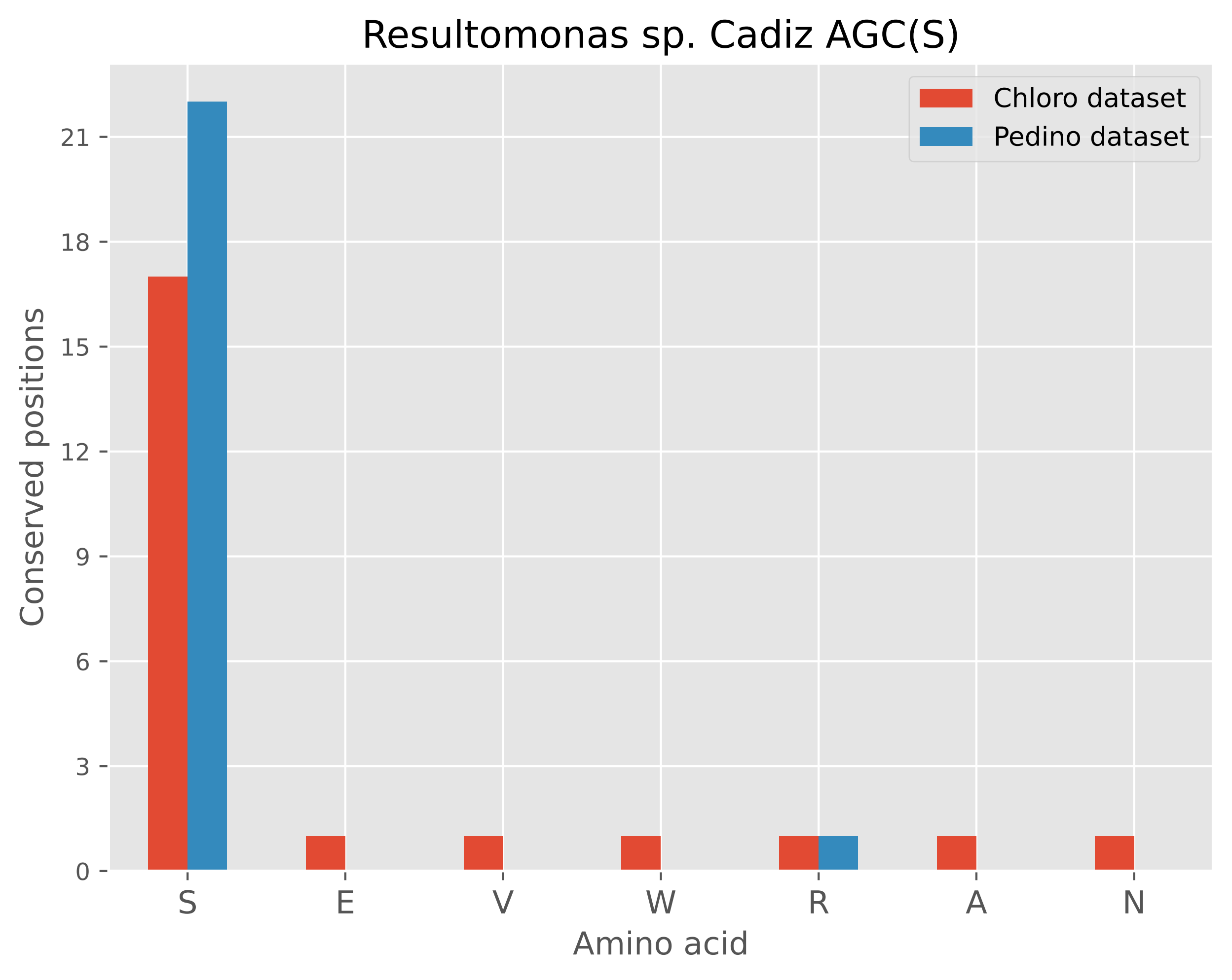
Resultomonas sp. Cadiz AGC(S)
Chloro dataset
Pedino dataset
Conserved positions
Amino acid
0
3
6
9
12
15
18
21
S
E
V
W
R
A
N

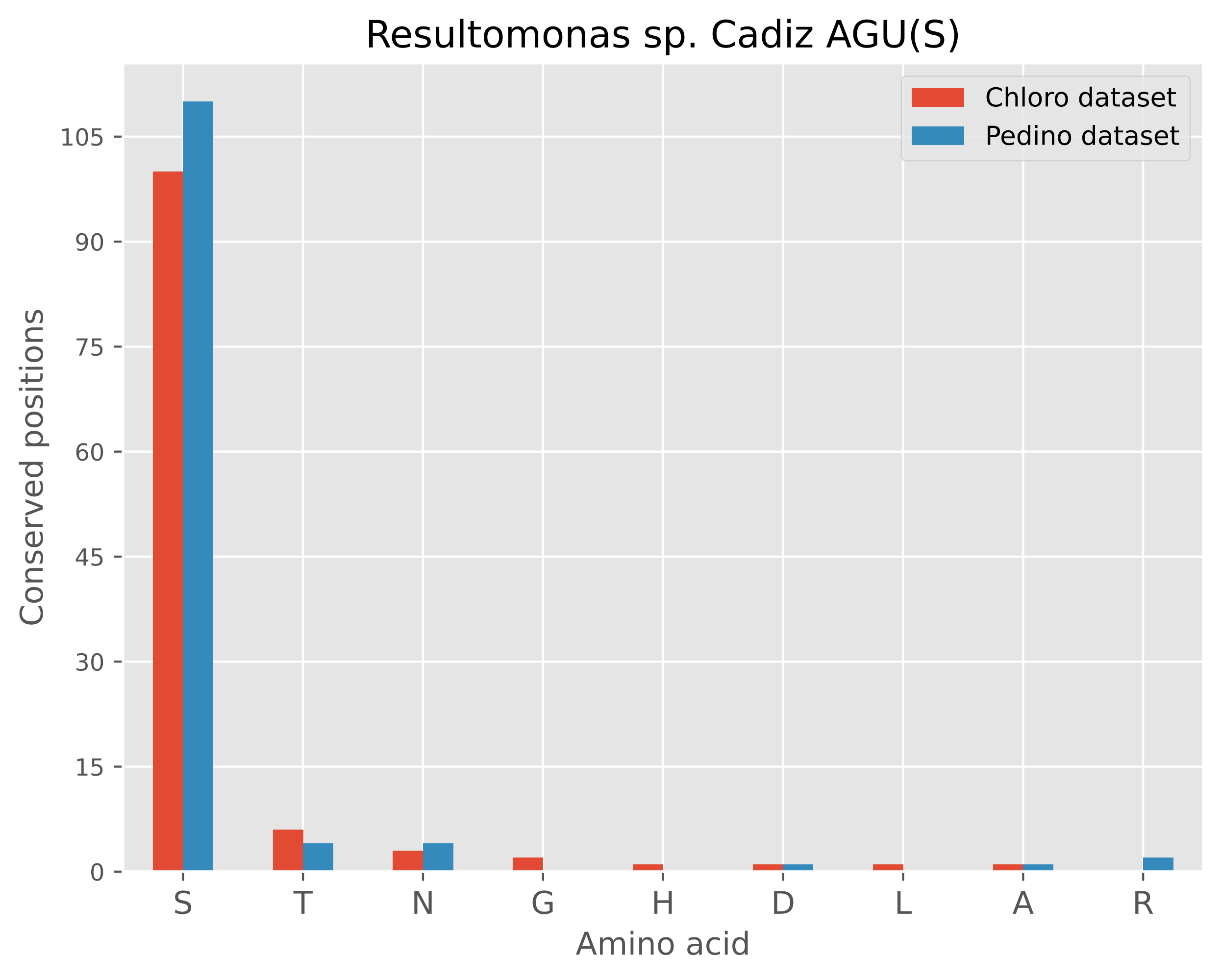
Resultomonas sp. Cadiz AGU(S)
Chloro dataset
Pedino dataset
Conserved positions
0
15
30
45
60
75
90
105
S
T
N
G
H
D
L
A
R
Amino acid

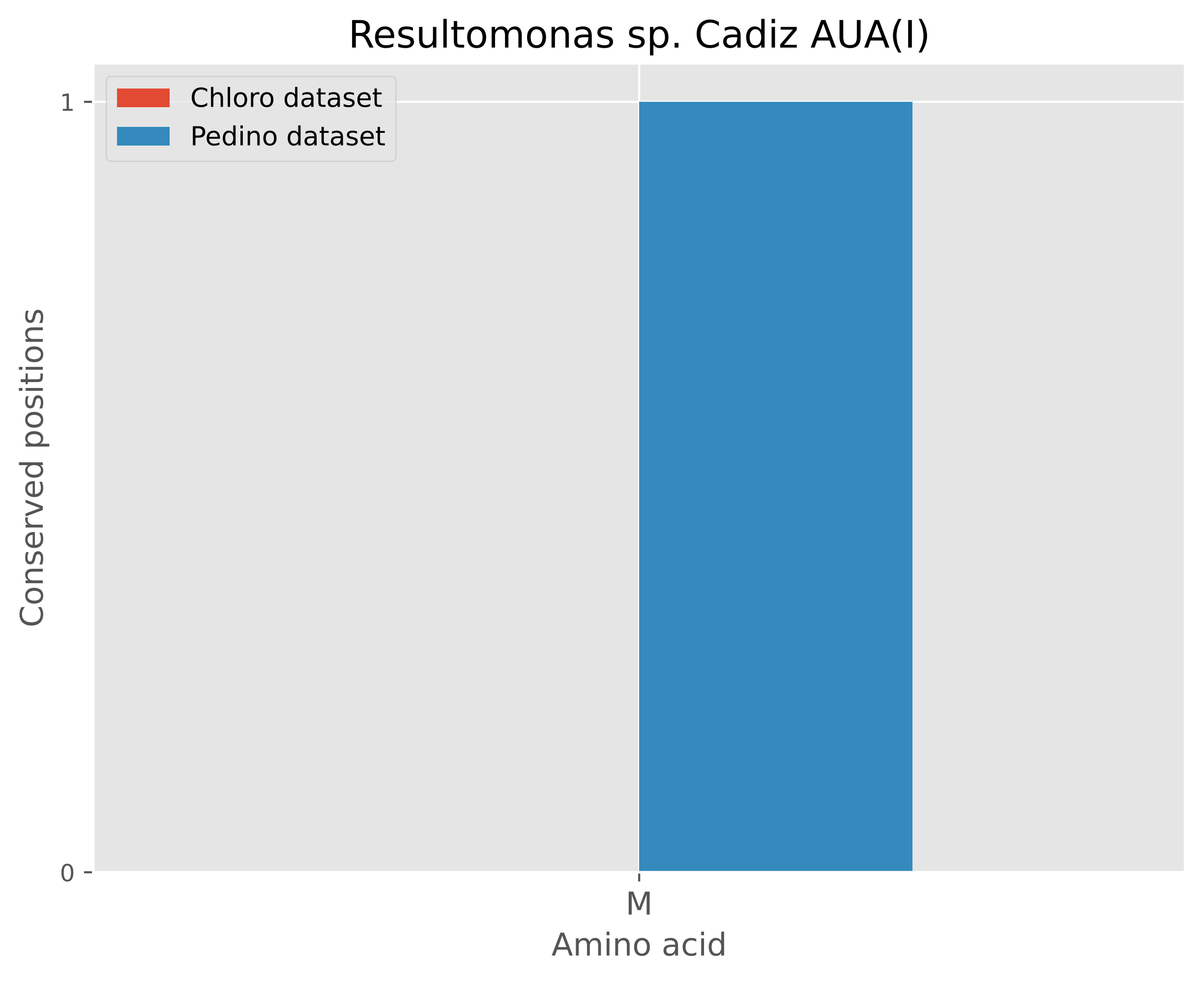
Resultomonas sp. Cadiz AUA(I)
Chloro dataset
Pedino dataset
Conserved positions
1
0
M
Amino acid

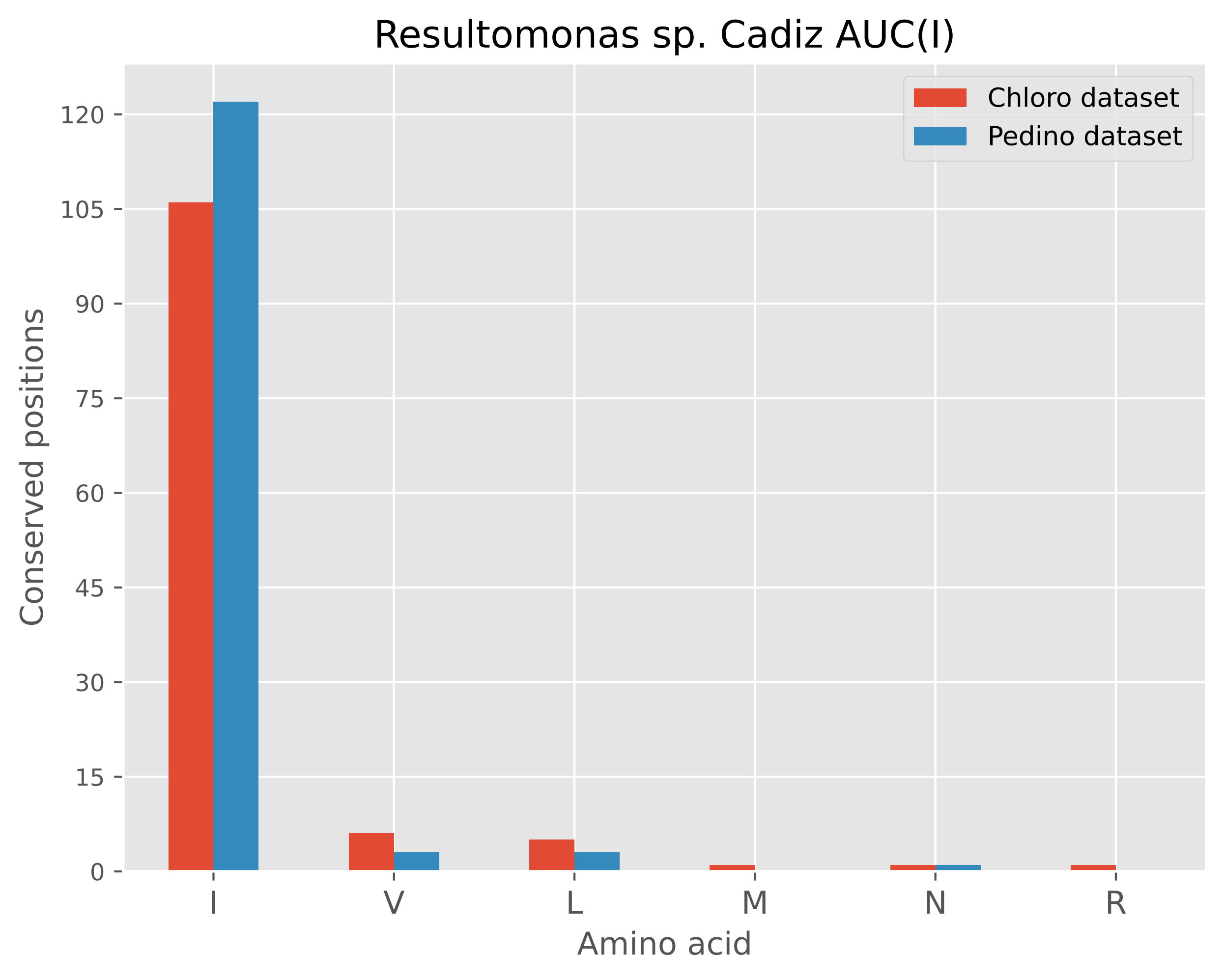

Resultomonas sp. Cadiz AUC(I)
Chloro dataset
Pedino dataset
Conserved positions
0
15
30
45
60
75
90
105
120
I
V
L
M
N
R
Amino acid

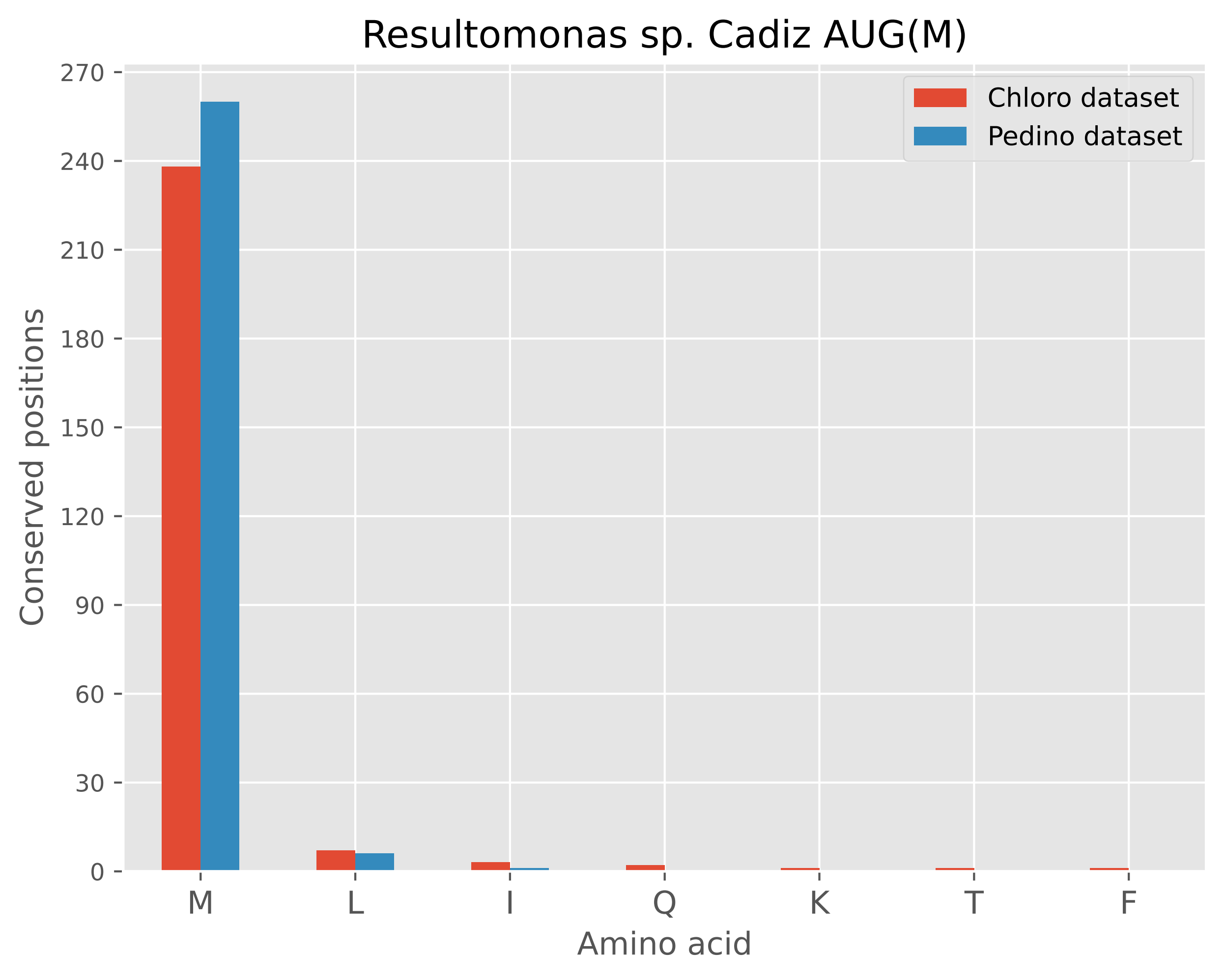
Resultomonas sp. Cadiz AUG(M)
Chloro dataset
Pedino dataset
Conserved positions
Amino acid
0
30
60
90
120
150
180
210
240
270
M
L
I
Q
K
T
F

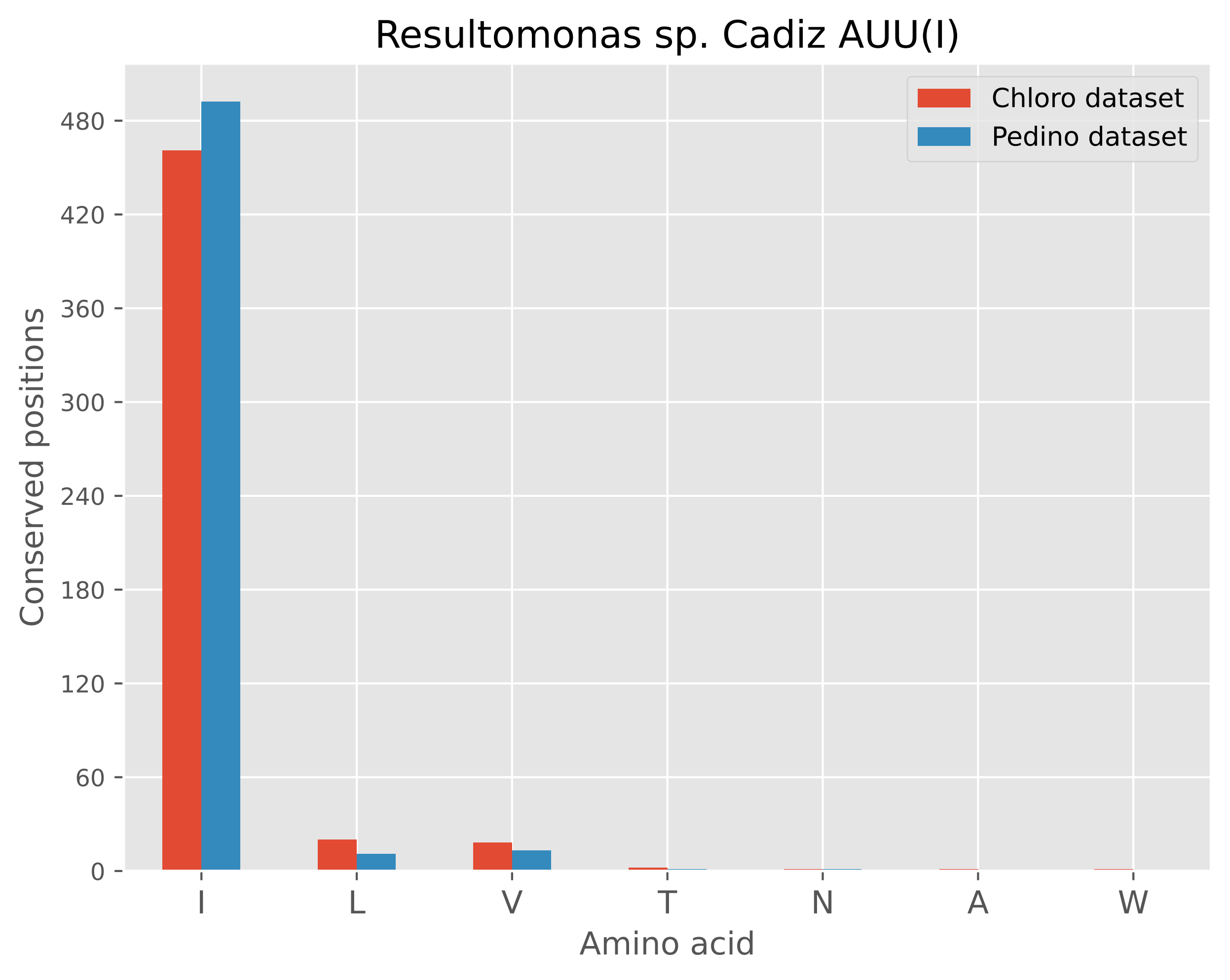

Resultomonas sp. Cadiz AUU(I)
Chloro dataset
Pedino dataset
Conserved positions
0
60
120
180
240
300
360
420
480
I
L
V
T
N
A
W
Amino acid

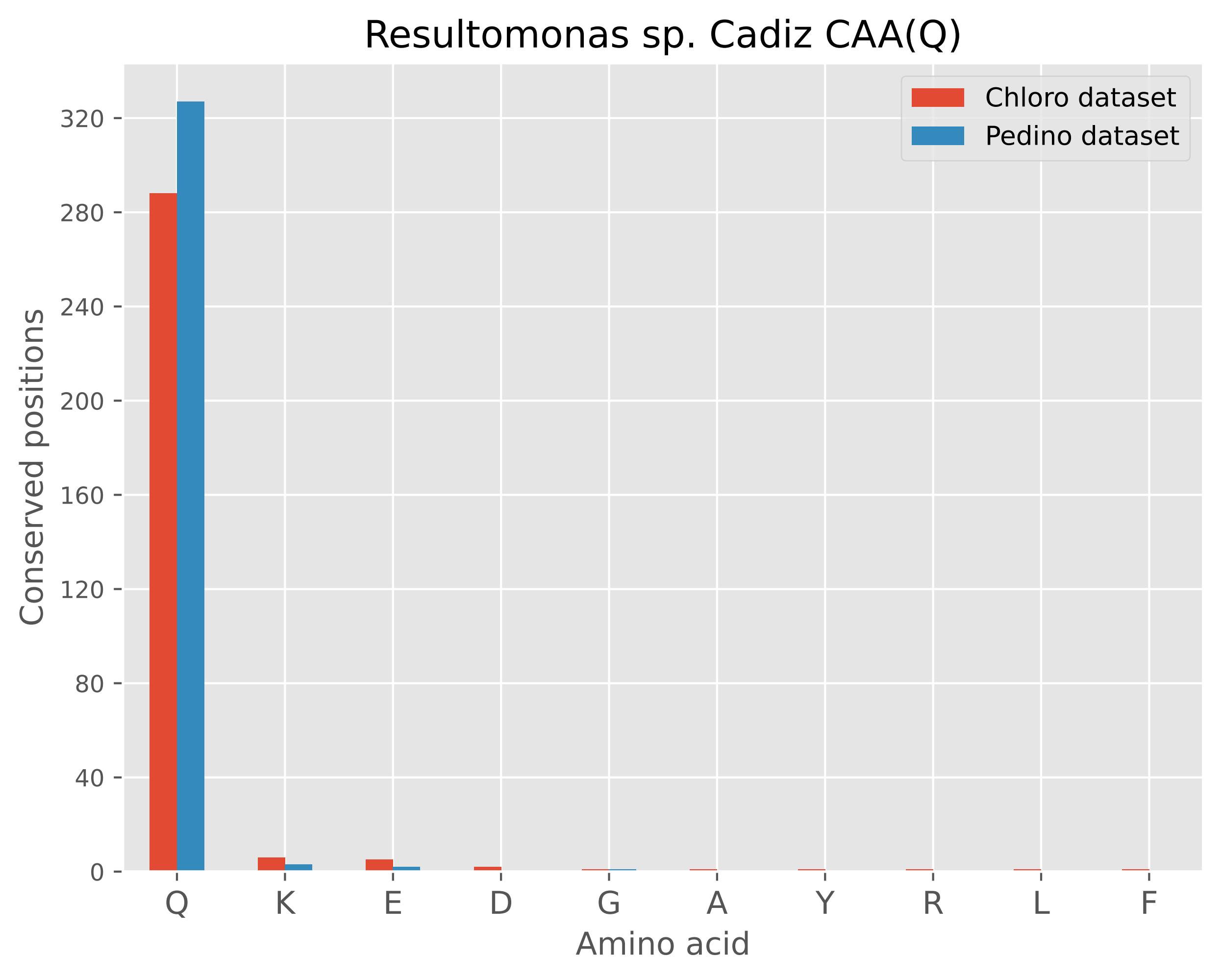
Resultomonas sp. Cadiz CAA(Q)
Chloro dataset
Pedino dataset
Conserved positions
0
40
80
120
160
200
240
280
320
Q
K
E
D
G
A
Y
R
L
F
Amino acid

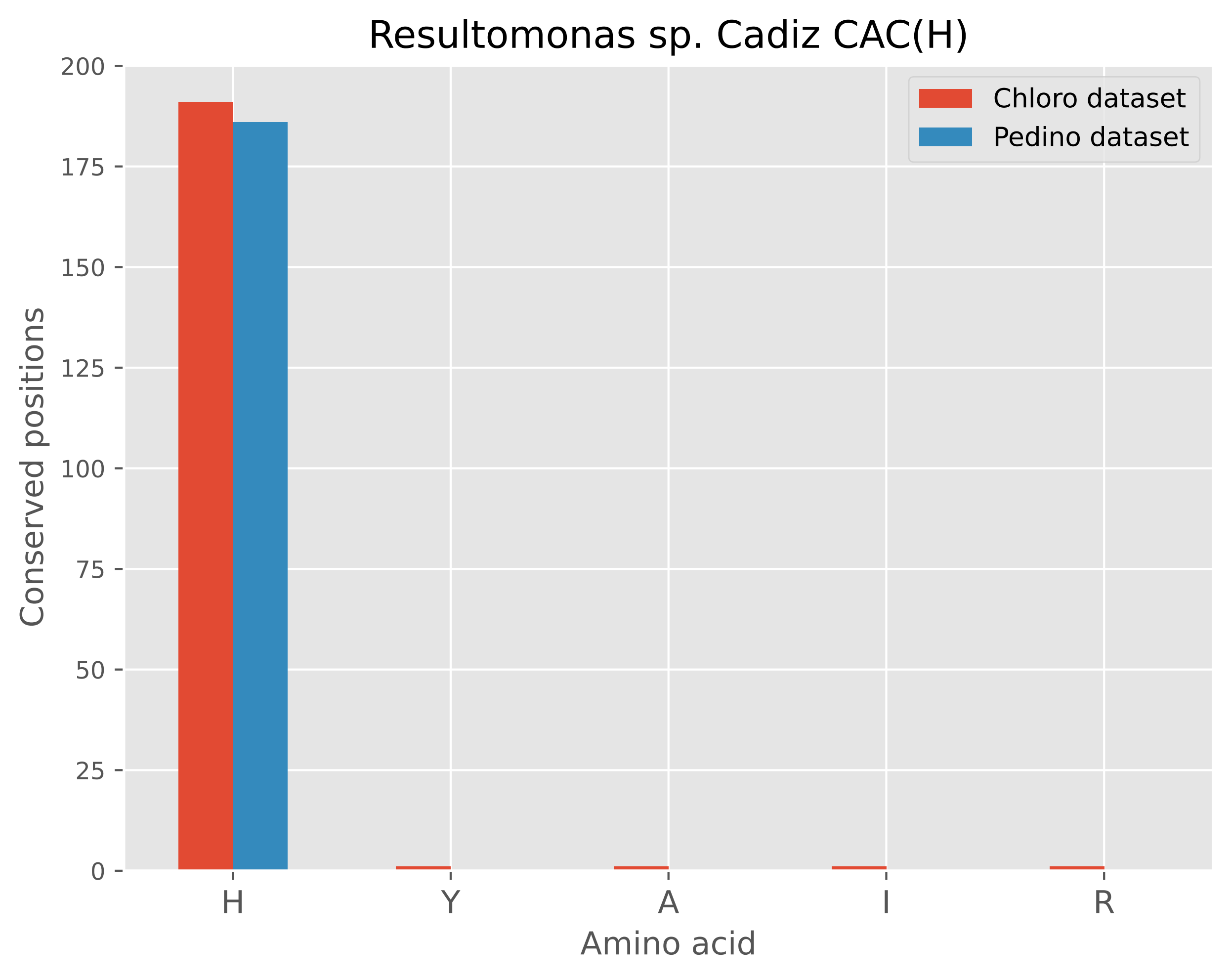

Resultomonas sp. Cadiz CAC(H)
Chloro dataset
Pedino dataset
Conserved positions
200
175
150
125
100
75
50
25
0
H
Y
A
I
R
Amino acid

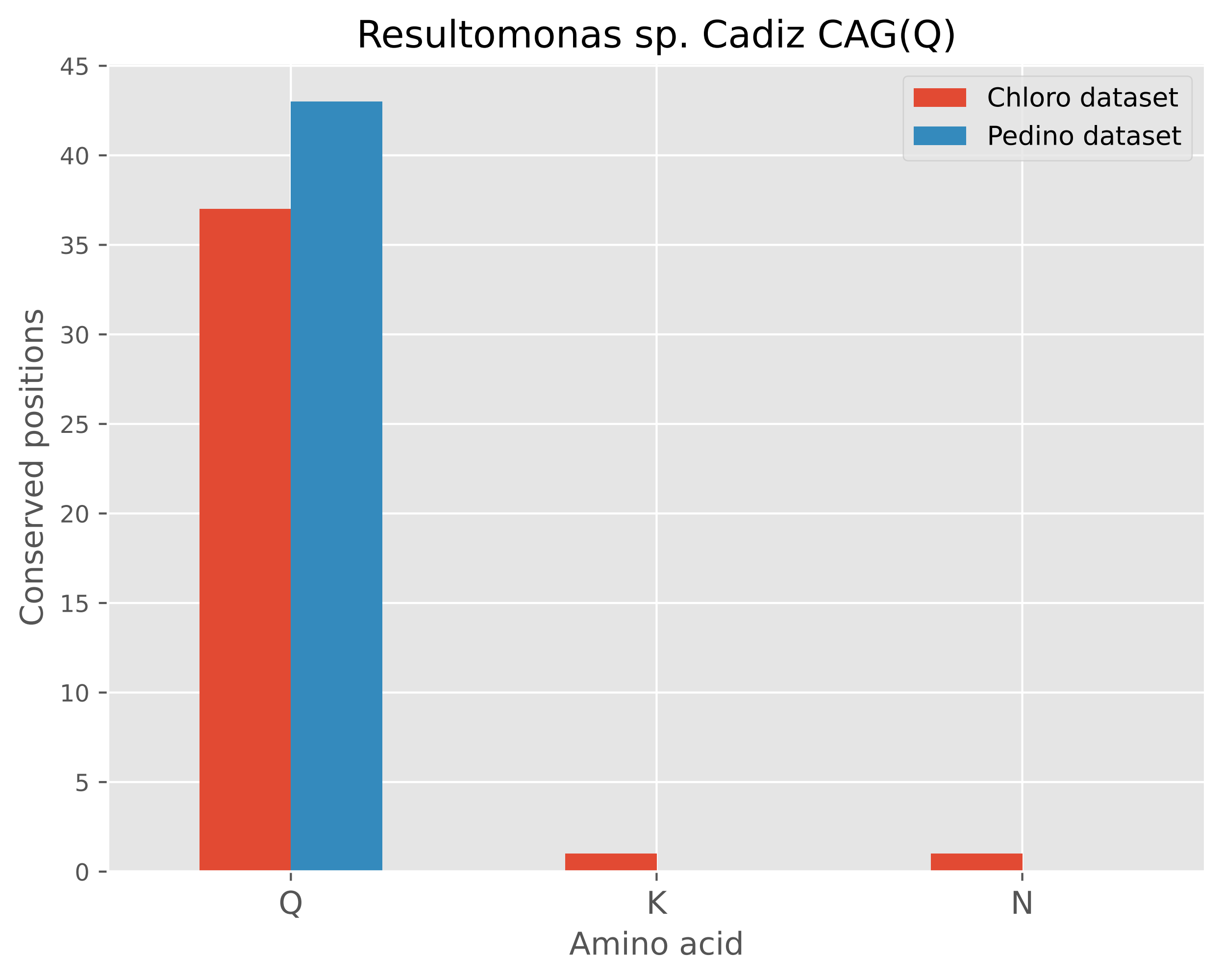

Resultomonas sp. Cadiz CAG(Q)
Chloro dataset
Pedino dataset
Conserved positions
Amino acid
0
5
10
15
20
25
30
35
40
45
Q
K
N

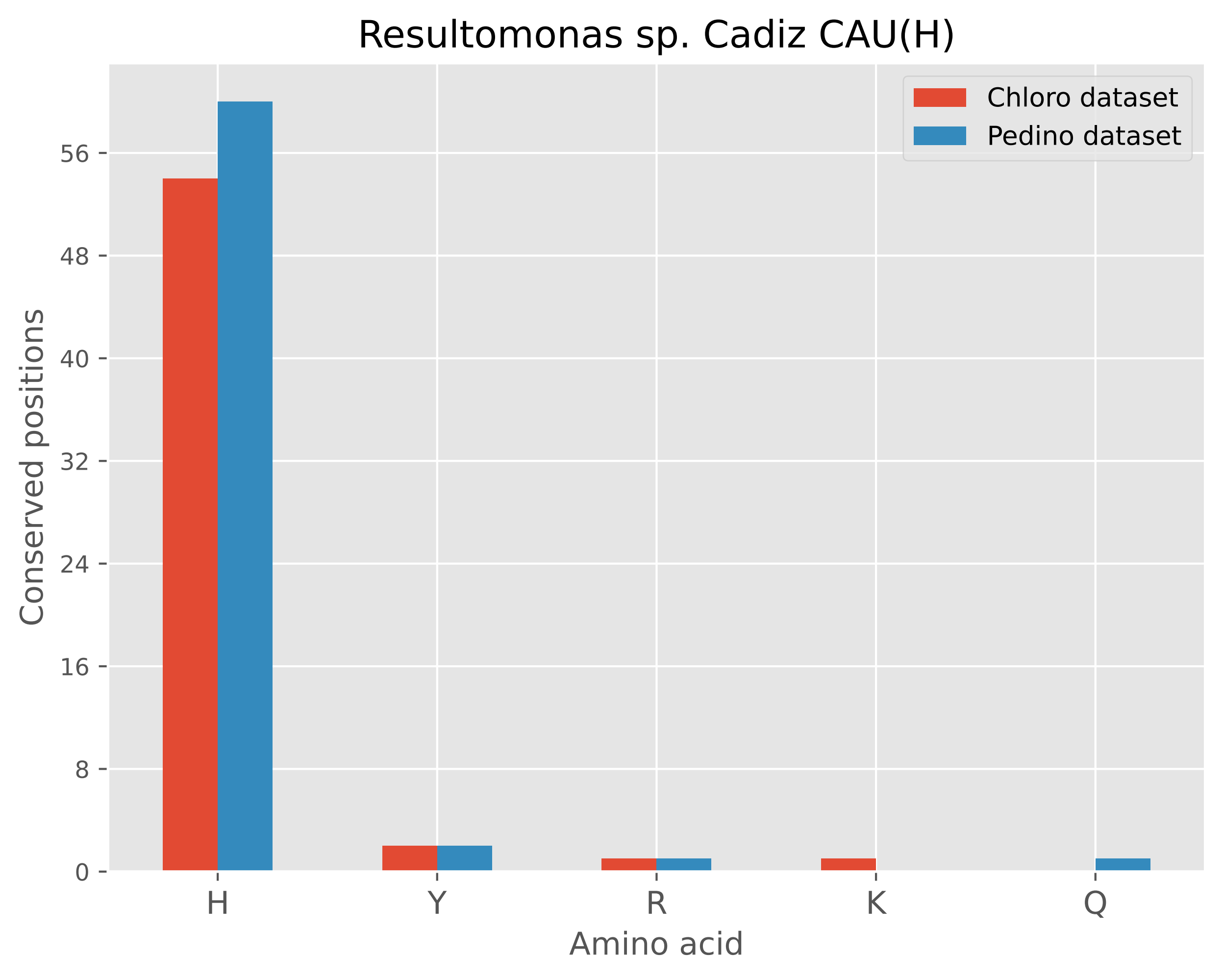

Resultomonas sp. Cadiz CAU(H)
Chloro dataset
Pedino dataset
Conserved positions
0
8
16
24
32
40
48
56
H
Y
R
K
Q
Amino acid

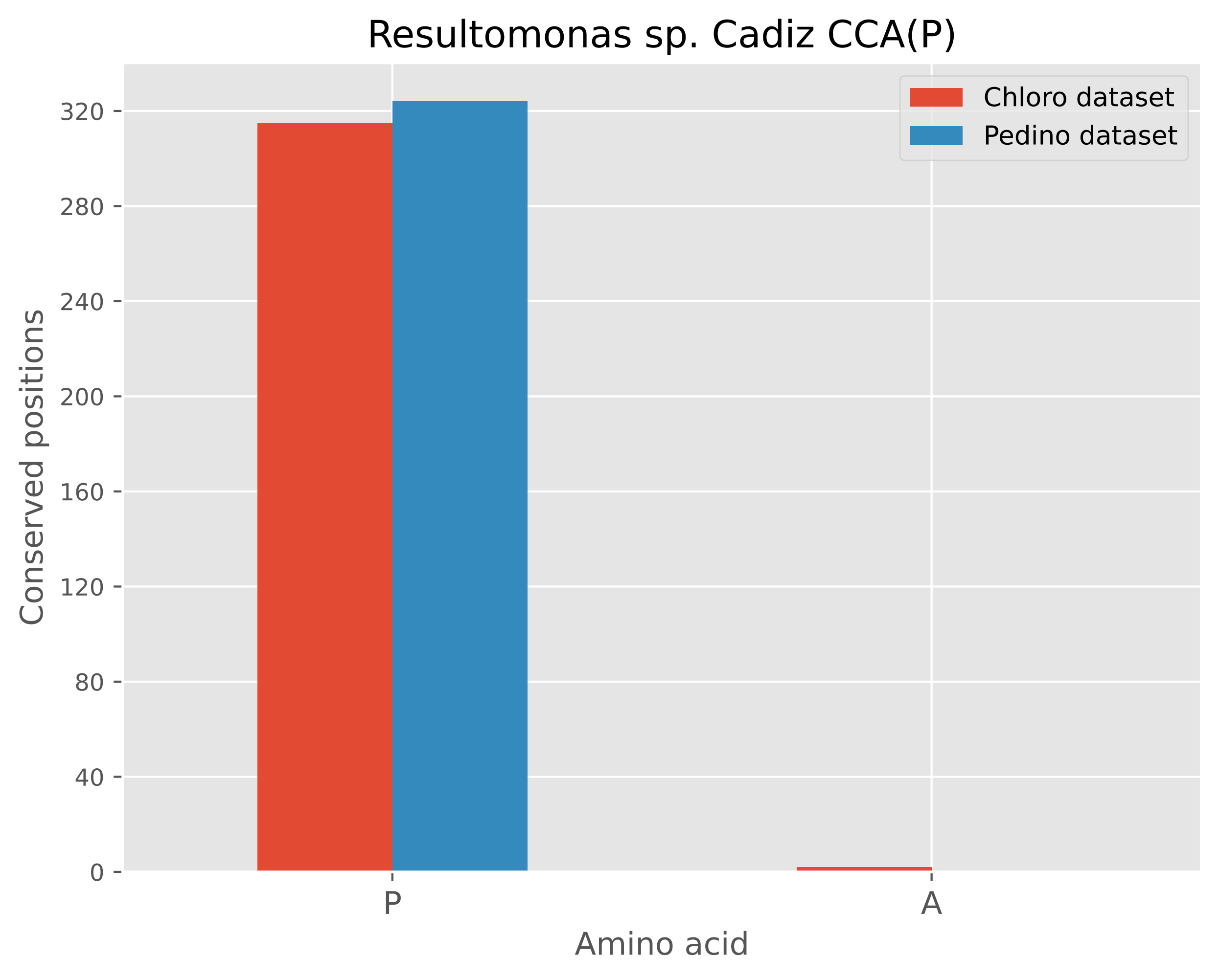

Resultomonas sp. Cadiz CCA(P)
Chloro dataset
Pedino dataset
Conserved positions
0
40
80
120
160
200
240
280
320
P
A
Amino acid

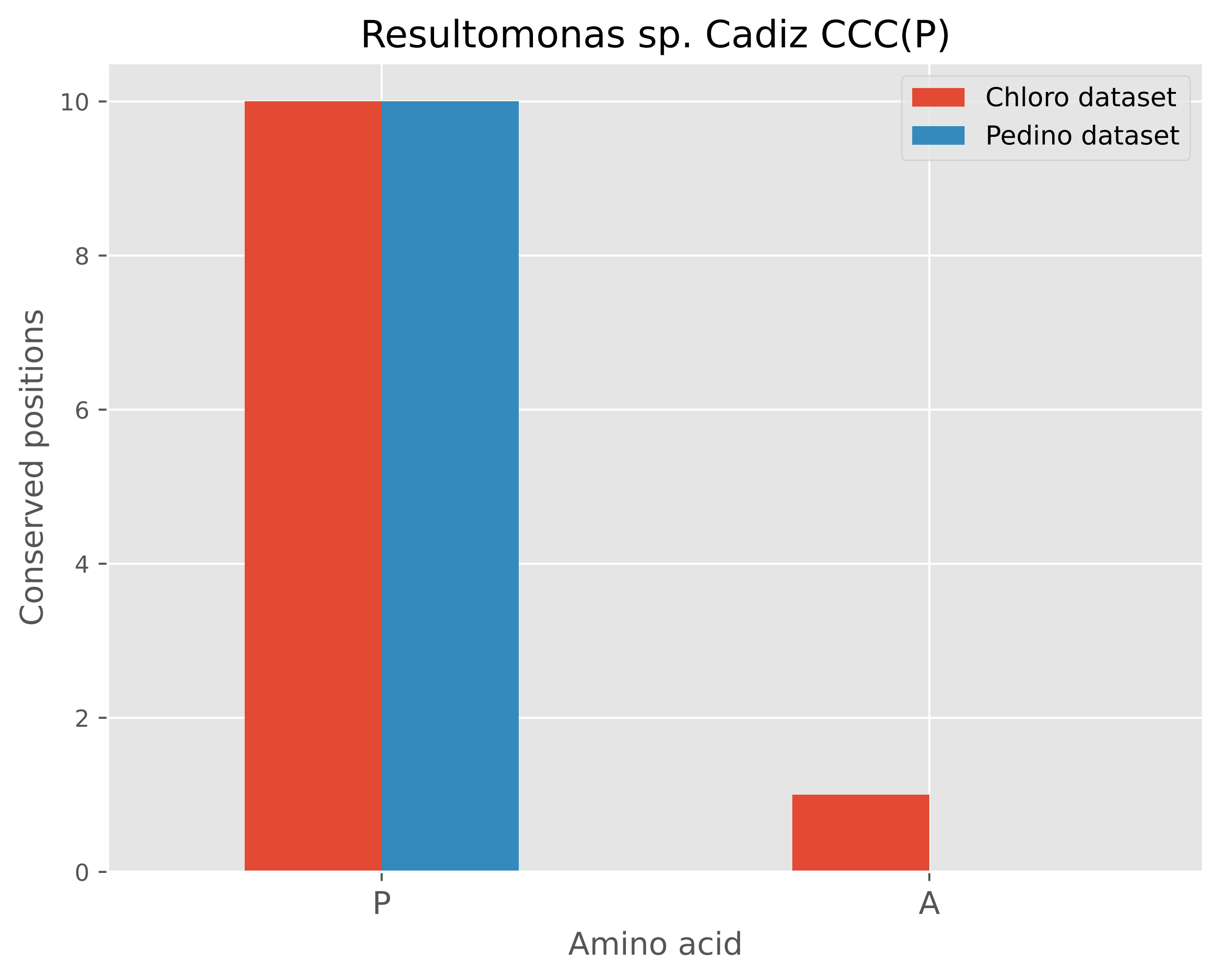
Resultomonas sp. Cadiz CCC(P)
Chloro dataset
Pedino dataset
Conserved positions
0
2
4
6
8
10
P
A
Amino acid

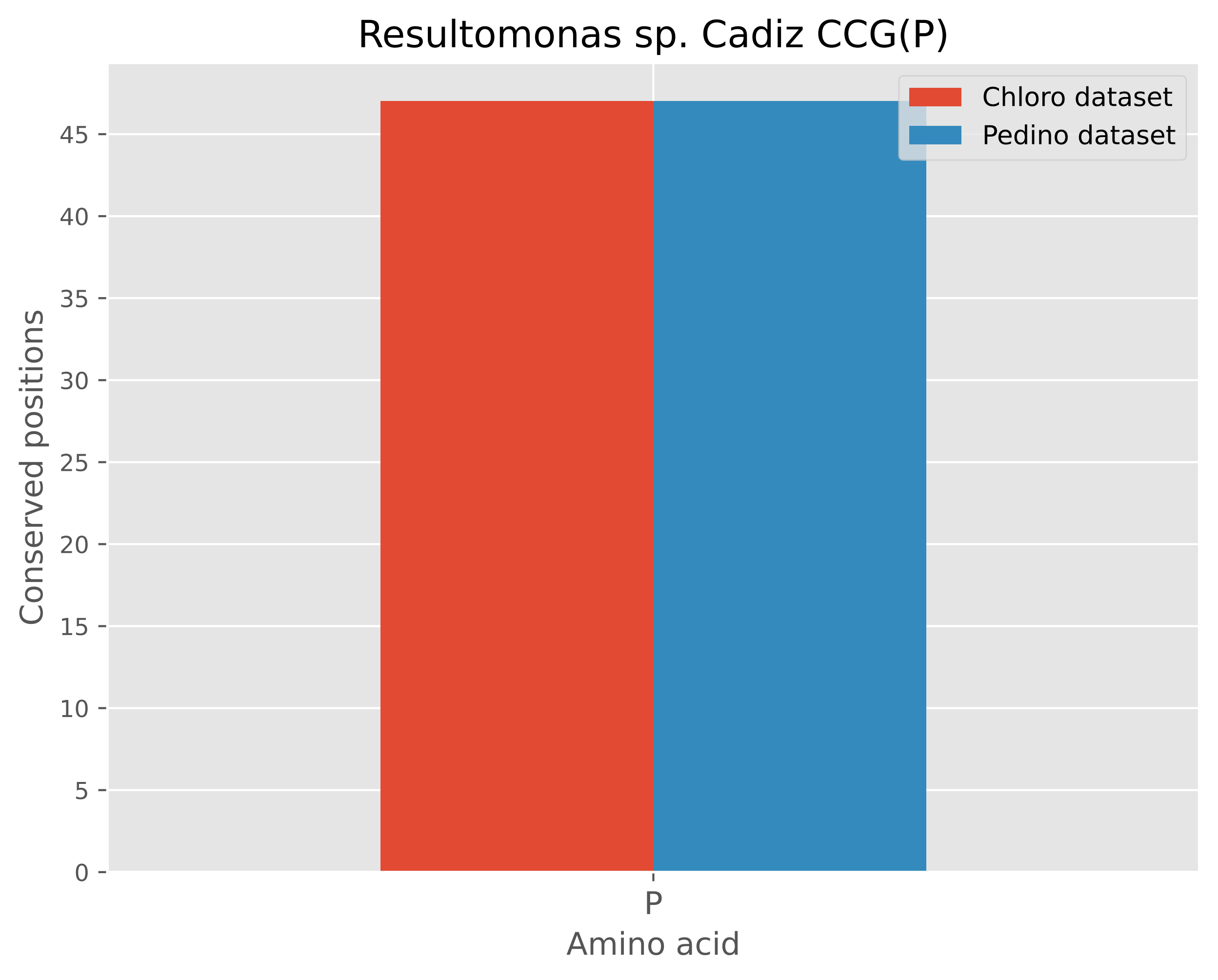

Resultomonas sp. Cadiz CCG(P)
Chloro dataset
Pedino dataset
Conserved positions
0
5
10
15
20
25
30
35
40
45
P
Amino acid

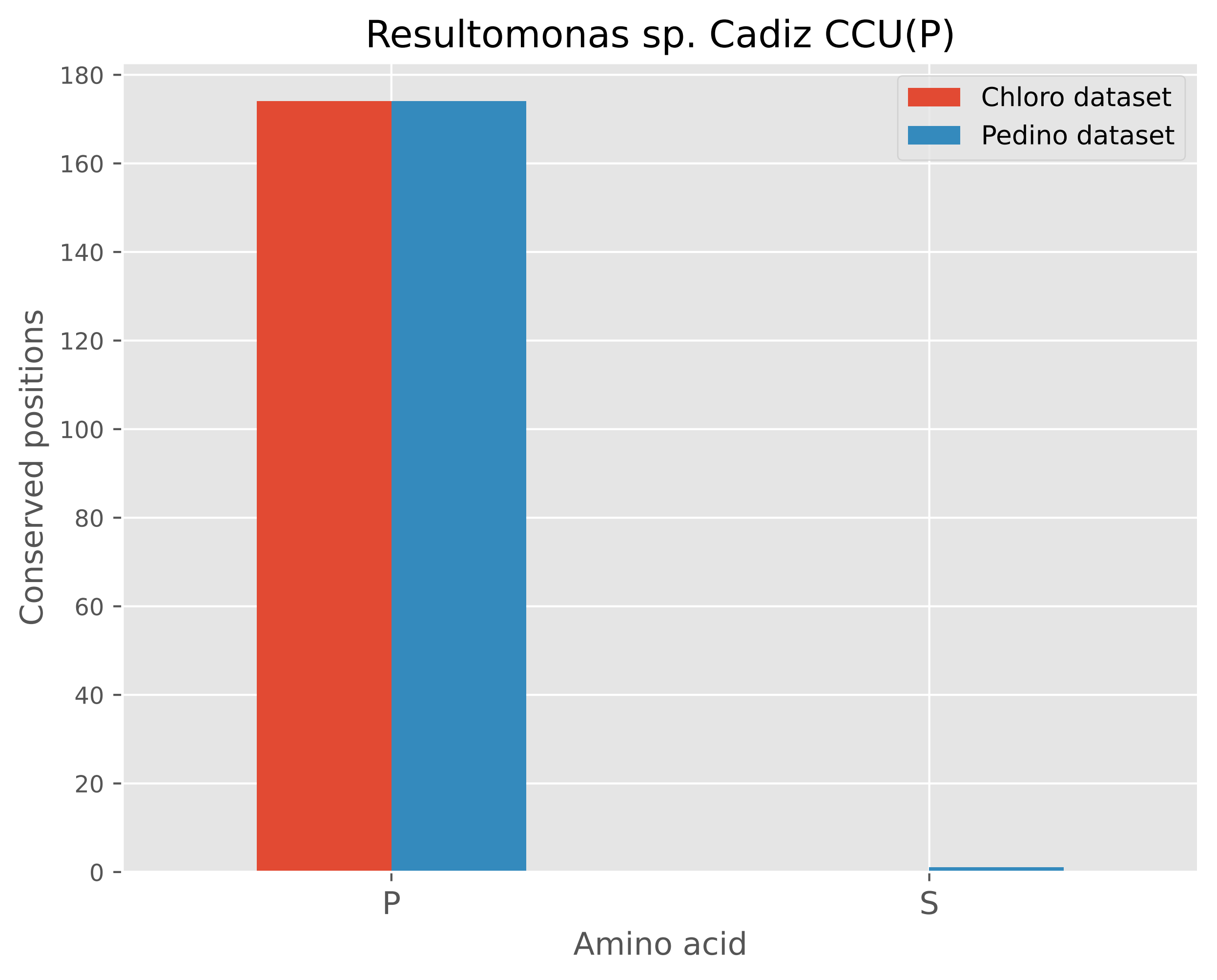

Resultomonas sp. Cadiz CCU(P)
Chloro dataset
Pedino dataset
Conserved positions
Amino acid
0
20
40
60
80
100
120
140
160
180
P
S

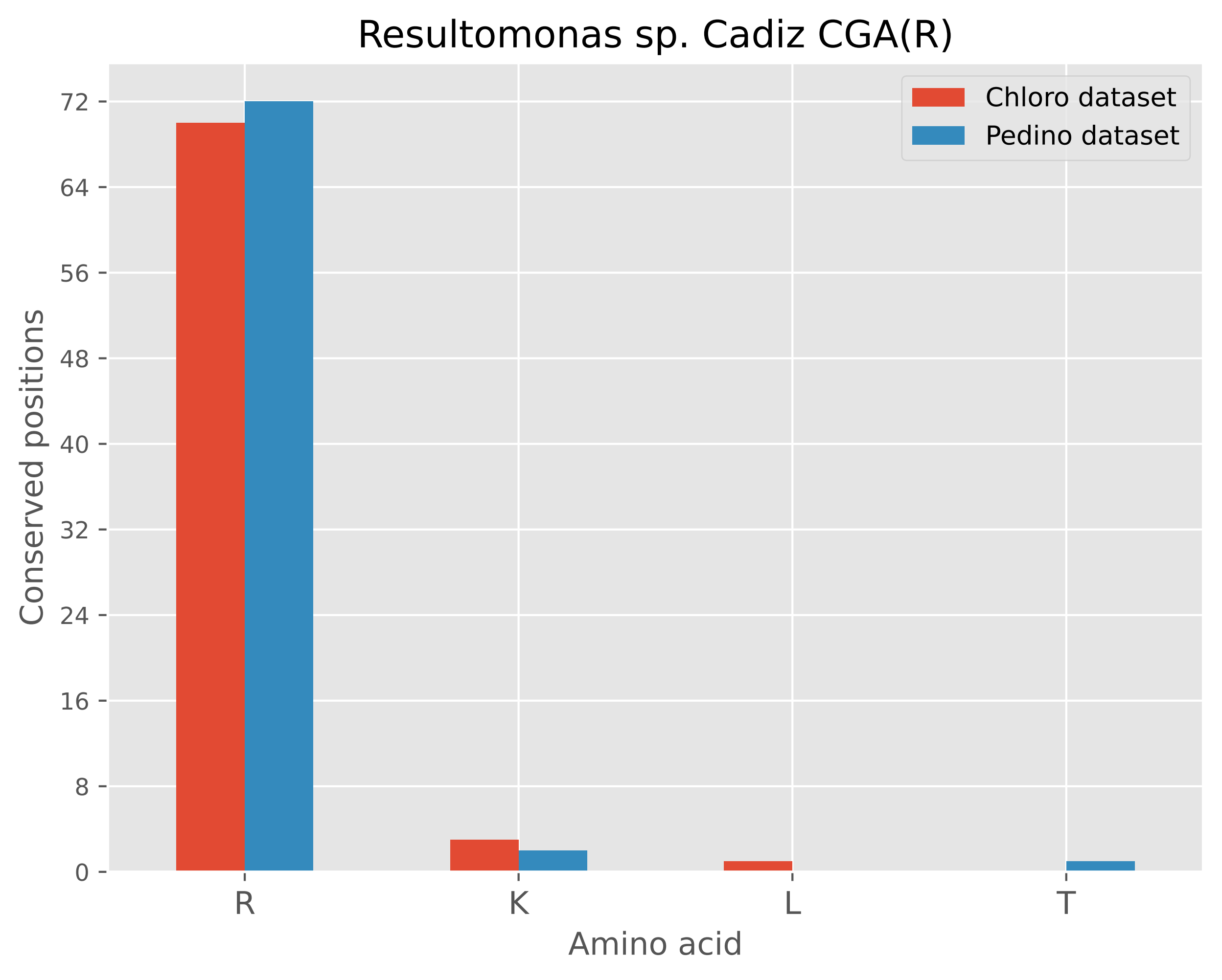

Resultomonas sp. Cadiz CGA(R)
Chloro dataset
Pedino dataset
Conserved positions
72
64
56
48
40
32
24
16
8
0
R
K
L
T
Amino acid

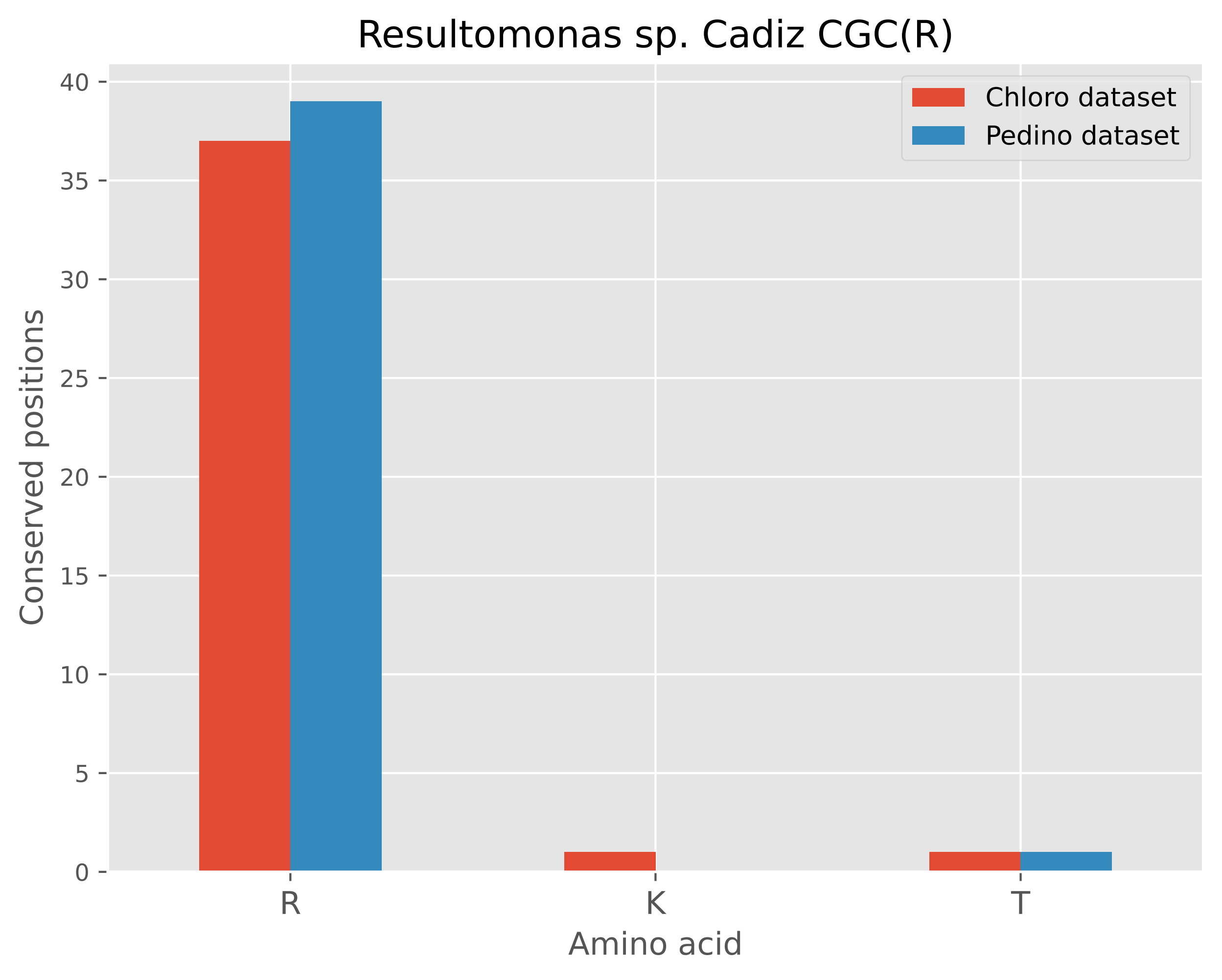
Resultomonas sp. Cadiz CGC(R)
Chloro dataset
Pedino dataset
Conserved positions
0
5
10
15
20
25
30
35
40
R
K
T
Amino acid

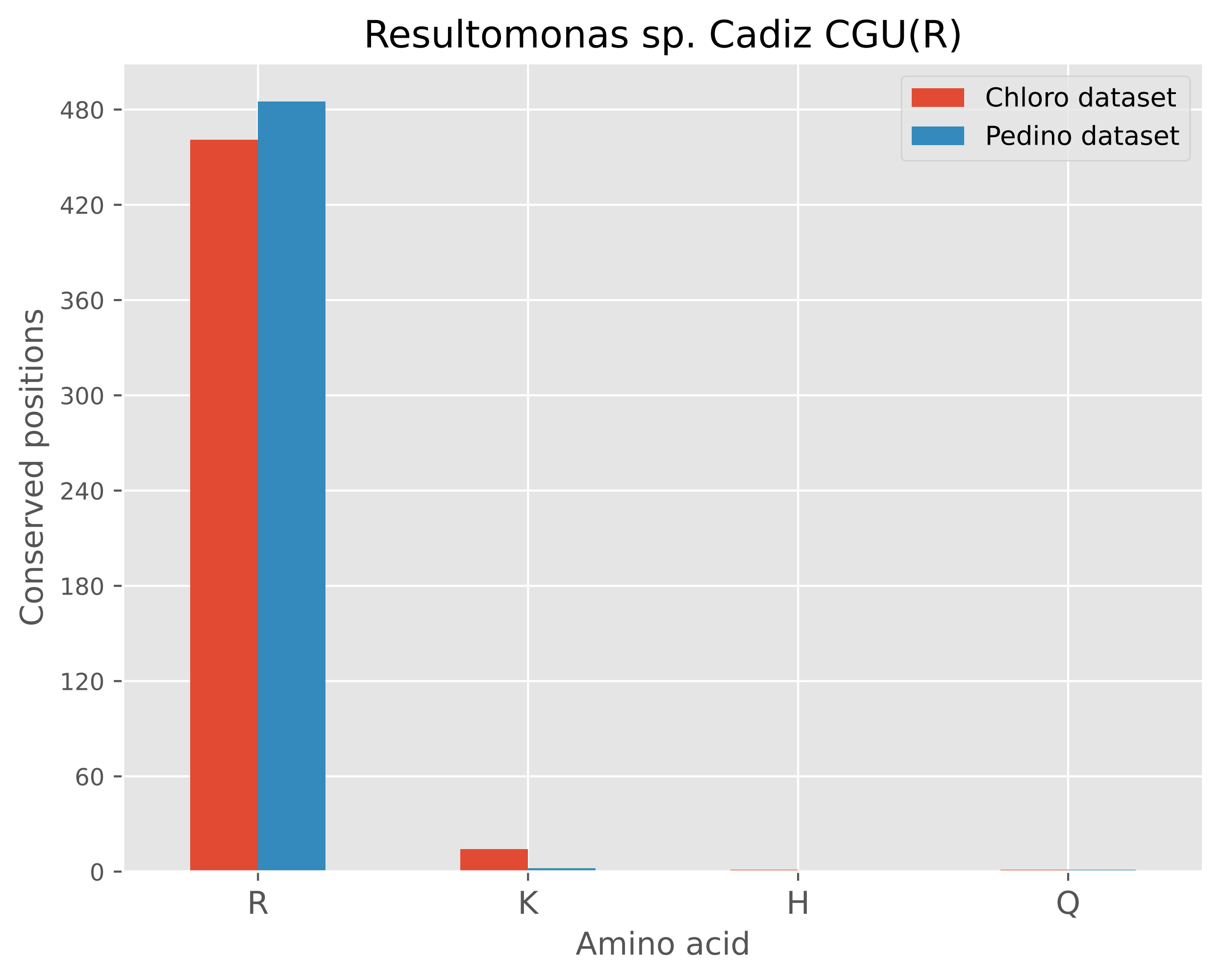

Resultomonas sp. Cadiz CGU(R)
Chloro dataset
Pedino dataset
Conserved positions
0
60
120
180
240
300
360
420
480
R
K
H
Q
Amino acid

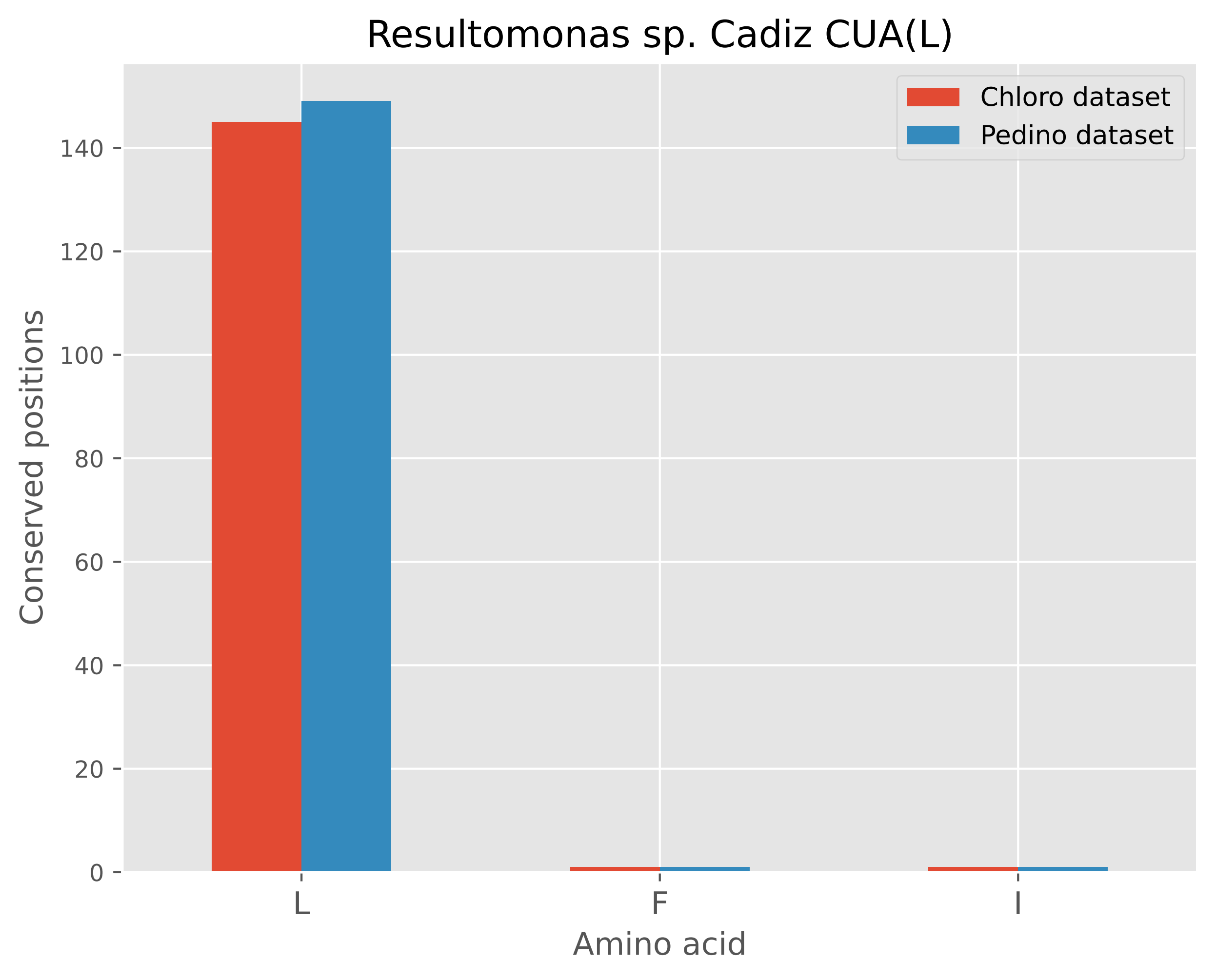

Resultomonas sp. Cadiz CUA(L)
Chloro dataset
Pedino dataset
Conserved positions
0
20
40
60
80
100
120
140
L
F
I
Amino acid

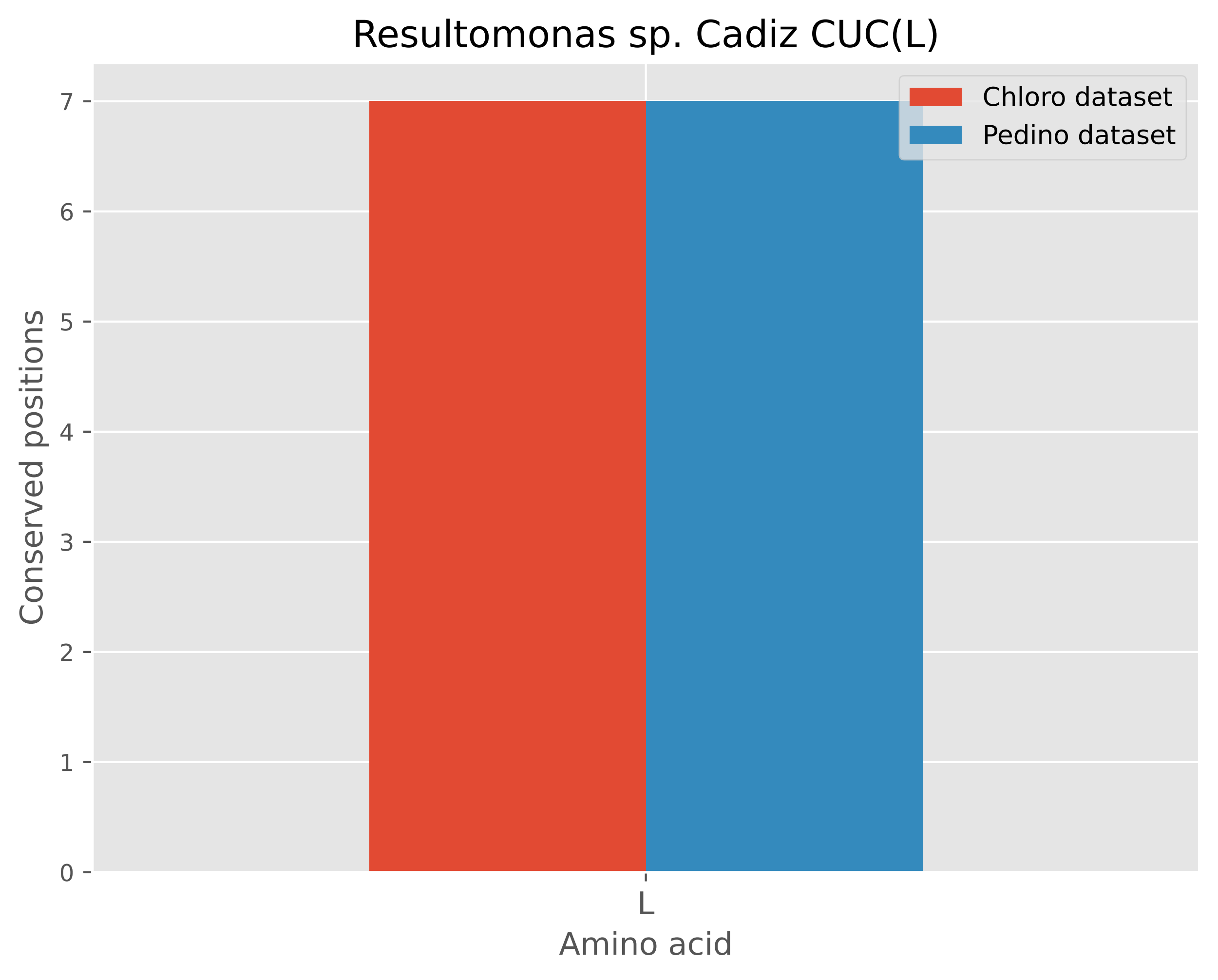
Resultomonas sp. Cadiz CUC(L)
Chloro dataset
Pedino dataset
Conserved positions
0
1
2
3
4
5
6
7
L
Amino acid

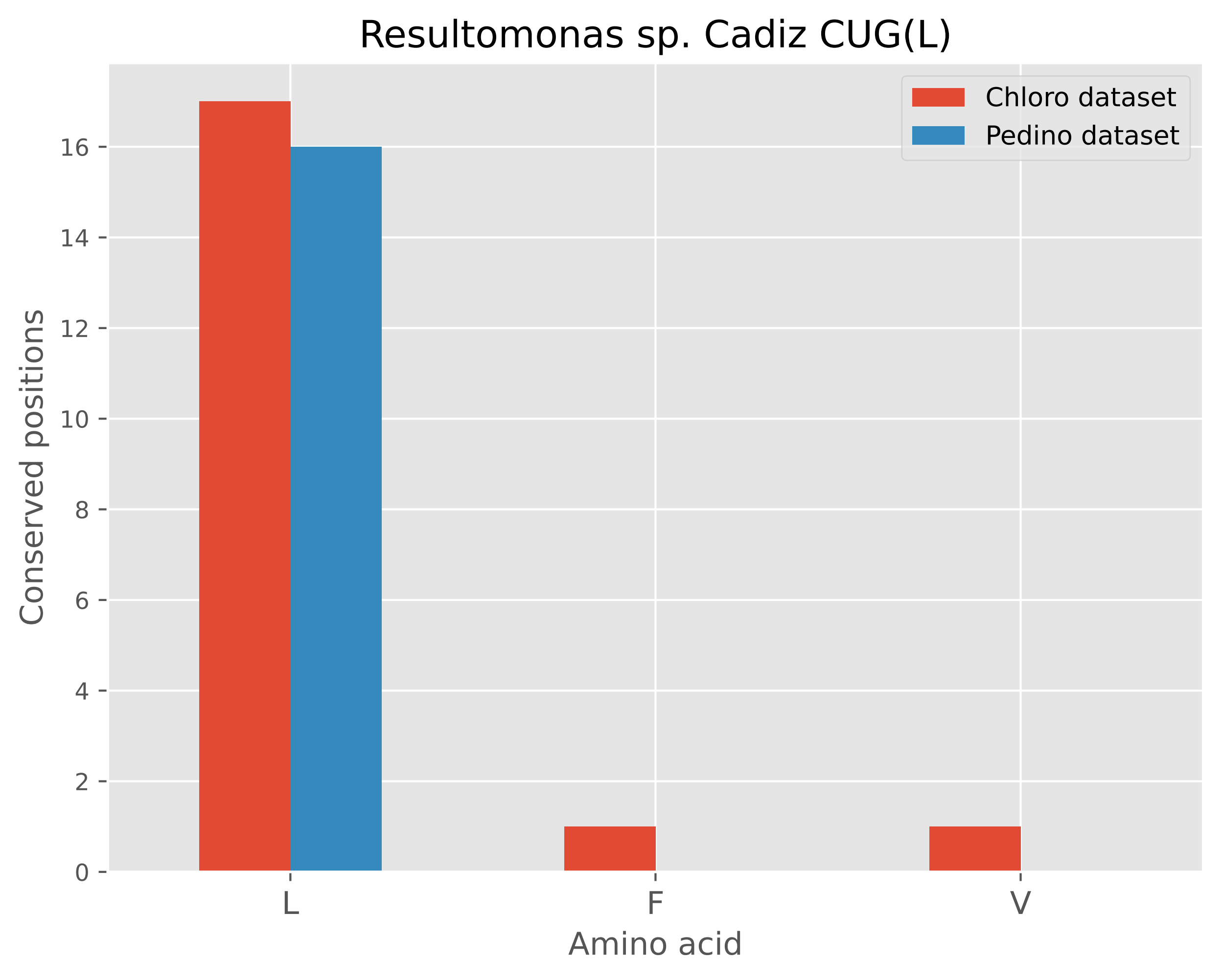

Resultomonas sp. Cadiz CUG(L)
Chloro dataset
Pedino dataset
Conserved positions
0
2
4
6
8
10
12
14
16
L
F
V
Amino acid

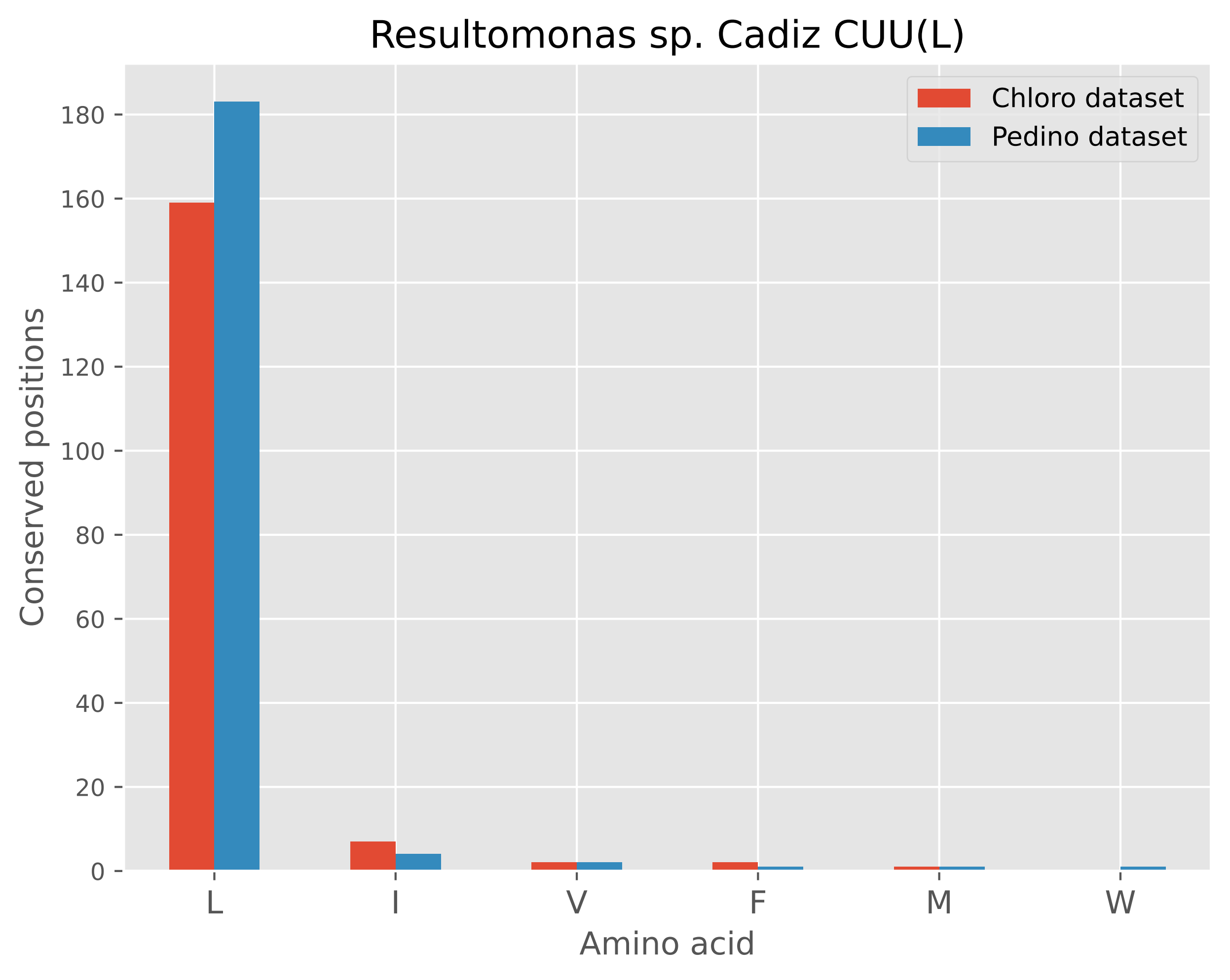

Resultomonas sp. Cadiz CUU(L)
Chloro dataset
Pedino dataset
Conserved positions
0
20
40
60
80
100
120
140
160
180
L
I
V
F
M
W
Amino acid

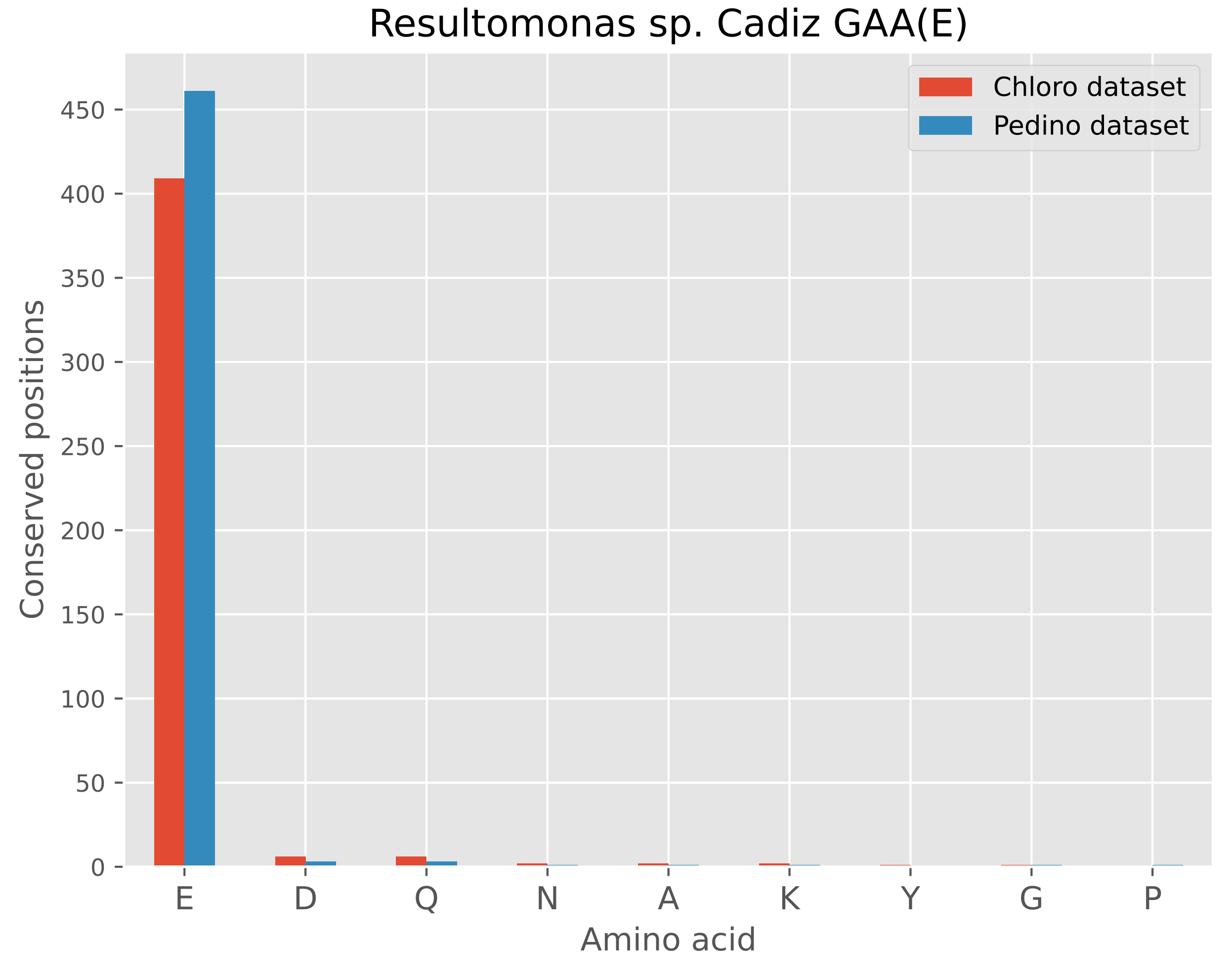

Resultomonas sp. Cadiz GAA(E)
Chloro dataset
Pedino dataset
Conserved positions
0
50
100
150
200
250
300
350
400
450
E
D
Q
N
A
K
Y
G
P
Amino acid

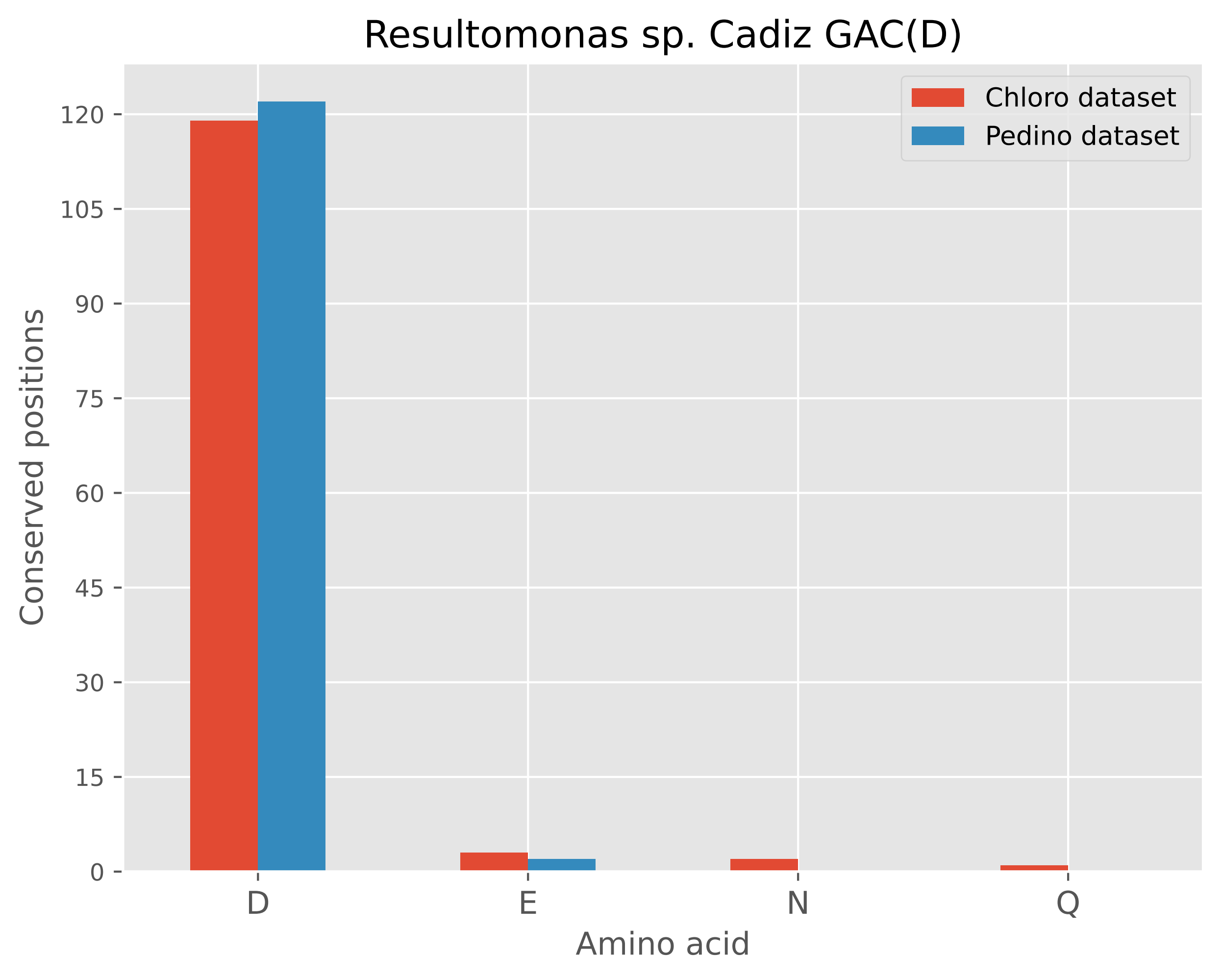
Resultomonas sp. Cadiz GAC(D)
Chloro dataset
Pedino dataset
Conserved positions
0
15
30
45
60
75
90
105
120
D
E
N
Q
Amino acid

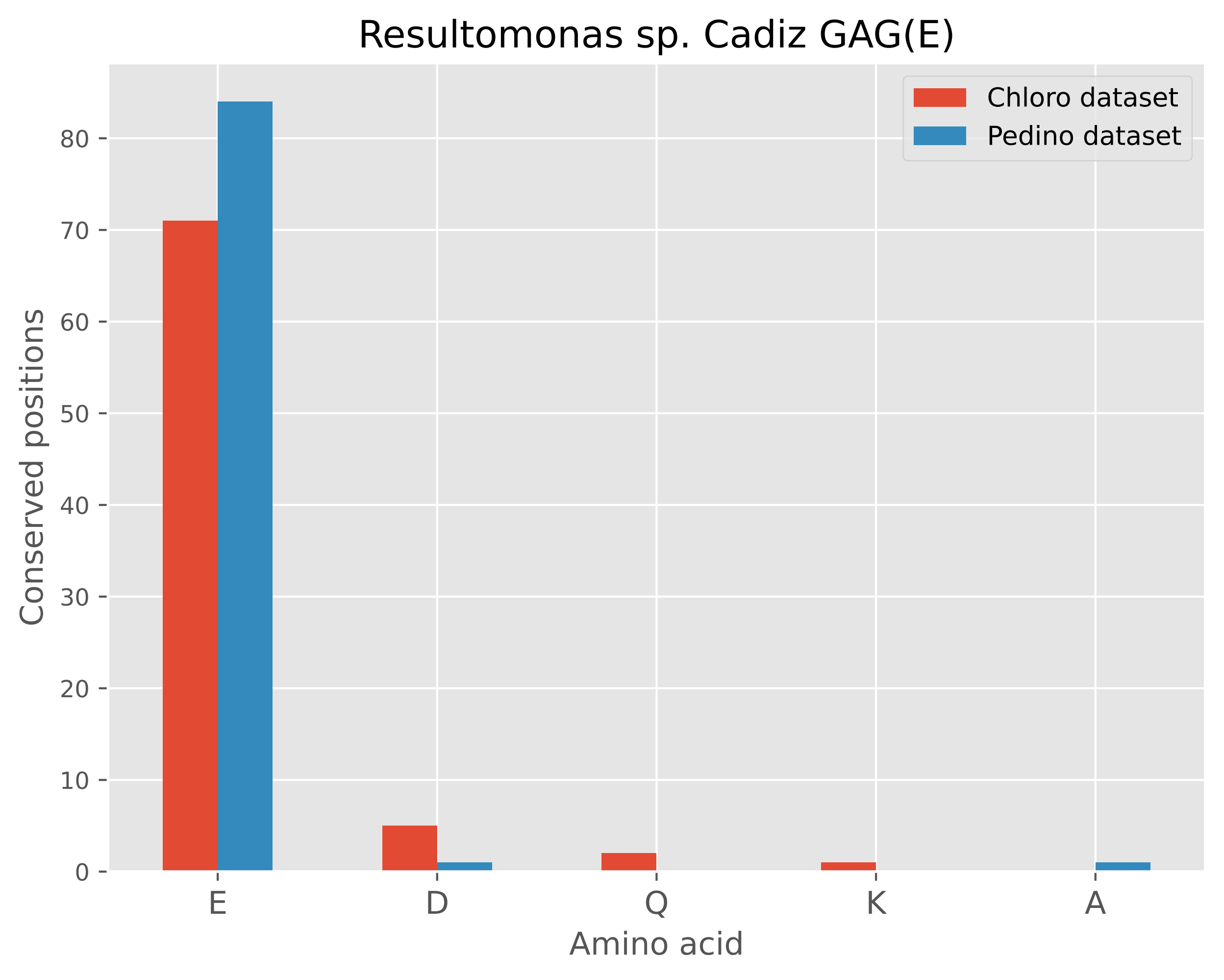

Resultomonas sp. Cadiz GAG(E)
Chloro dataset
Pedino dataset
Conserved positions
0
10
20
30
40
50
60
70
80
E
D
Q
K
A
Amino acid

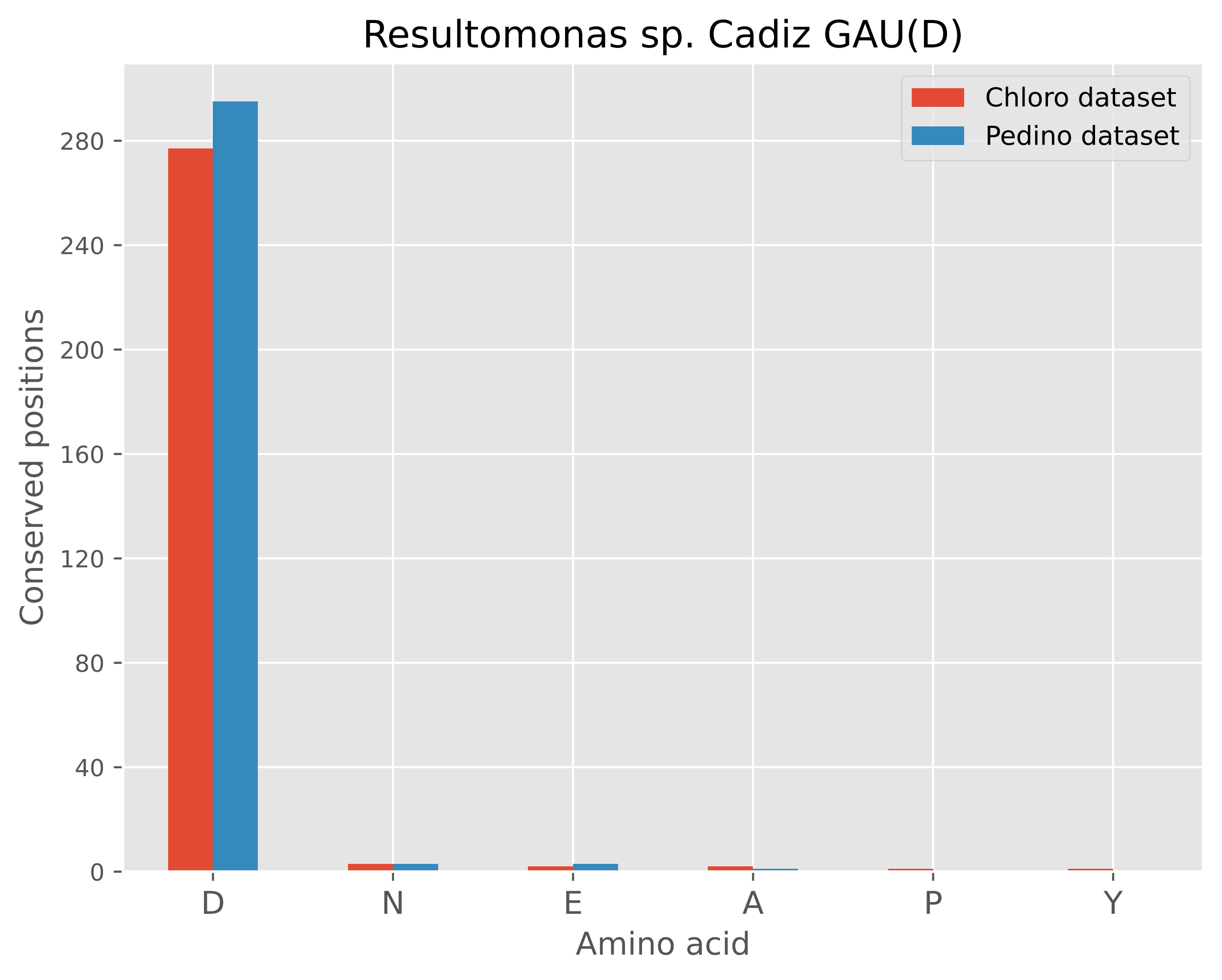
Resultomonas sp. Cadiz GAU(D)
Chloro dataset
Pedino dataset
Conserved positions
0
40
80
120
160
200
240
280
D
N
E
A
P
Y
Amino acid

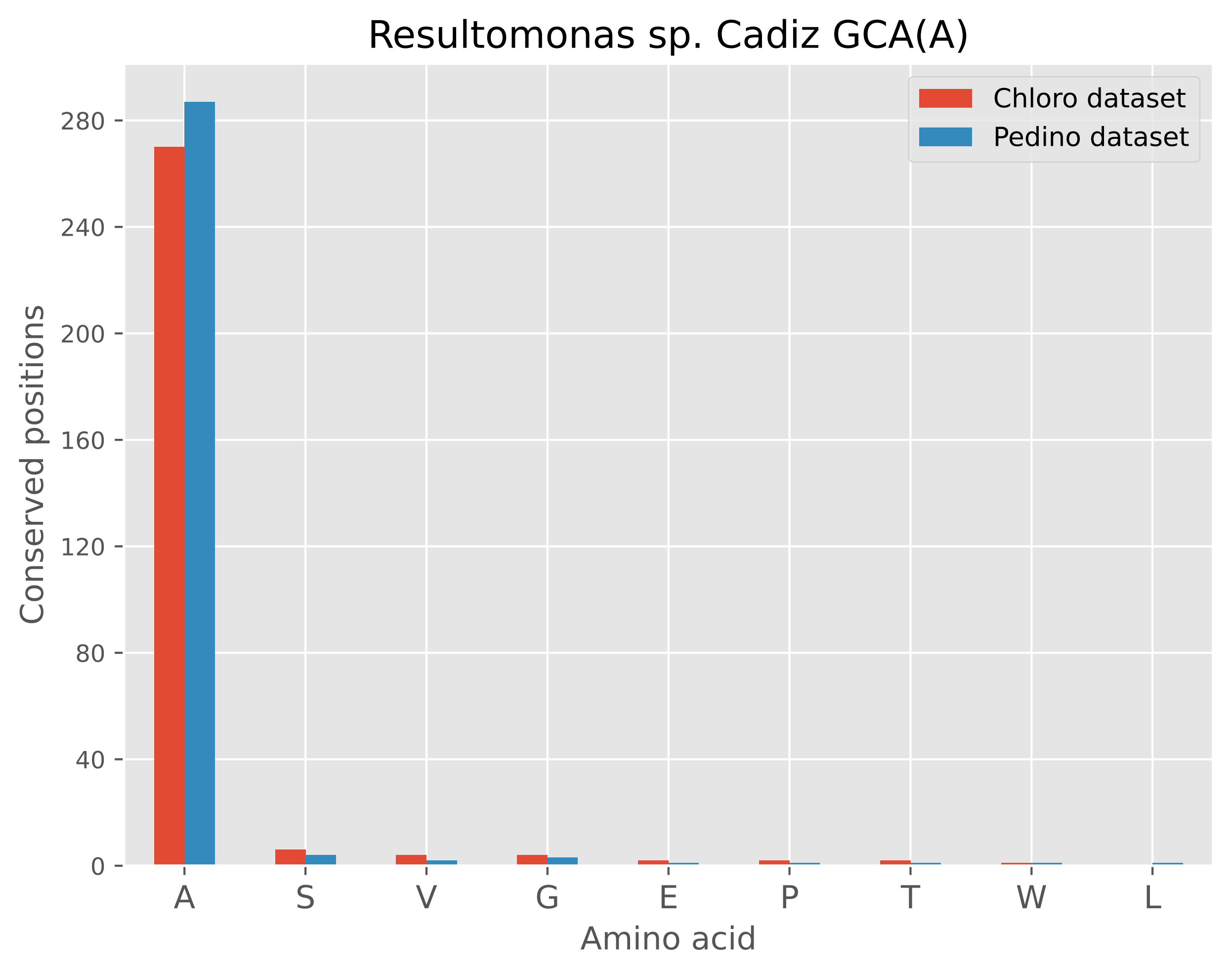
Resultomonas sp. Cadiz GCA(A)
Chloro dataset
Pedino dataset
Conserved positions
0
40
80
120
160
200
240
280
A
S
V
G
E
P
T
W
L
Amino acid

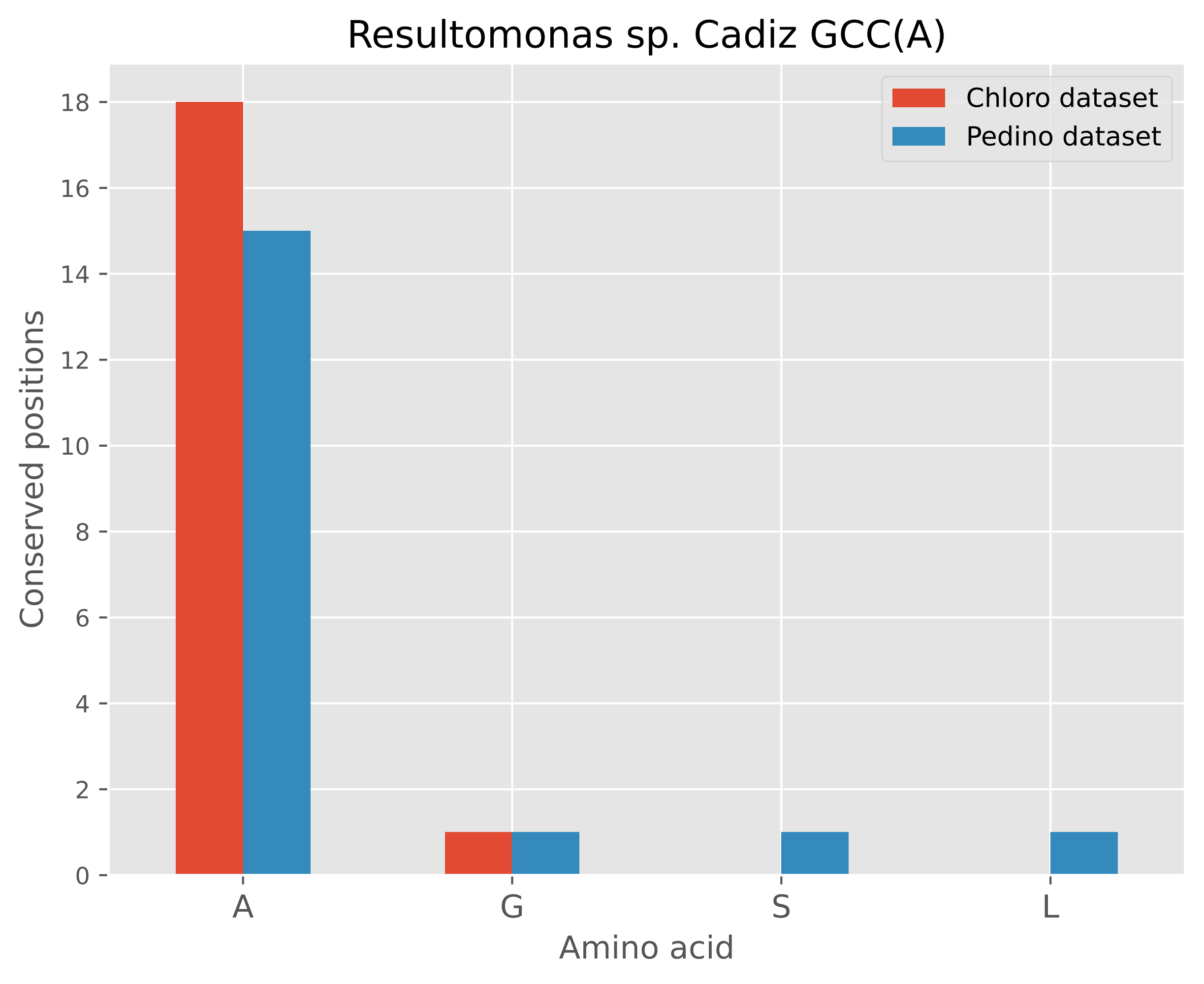
Resultomonas sp. Cadiz GCC(A)
Conserved positions
Amino acid
0
2
4
6
8
10
12
14
16
18
A
G
S
L
Chloro dataset
Pedino dataset

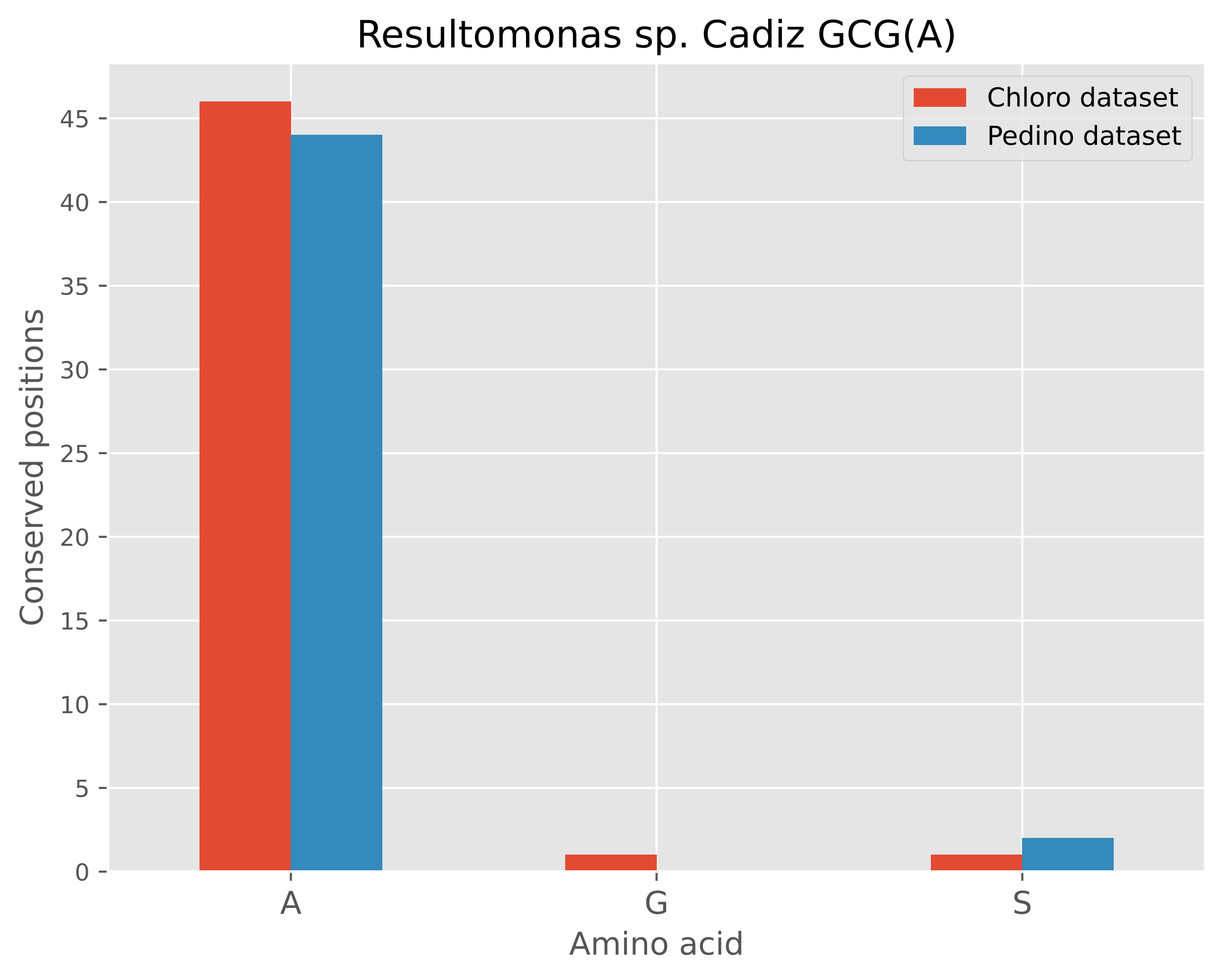
Resultomonas sp. Cadiz GCG(A)
Chloro dataset
Pedino dataset
Conserved positions
0
5
10
15
20
25
30
35
40
45
A
G
S
Amino acid

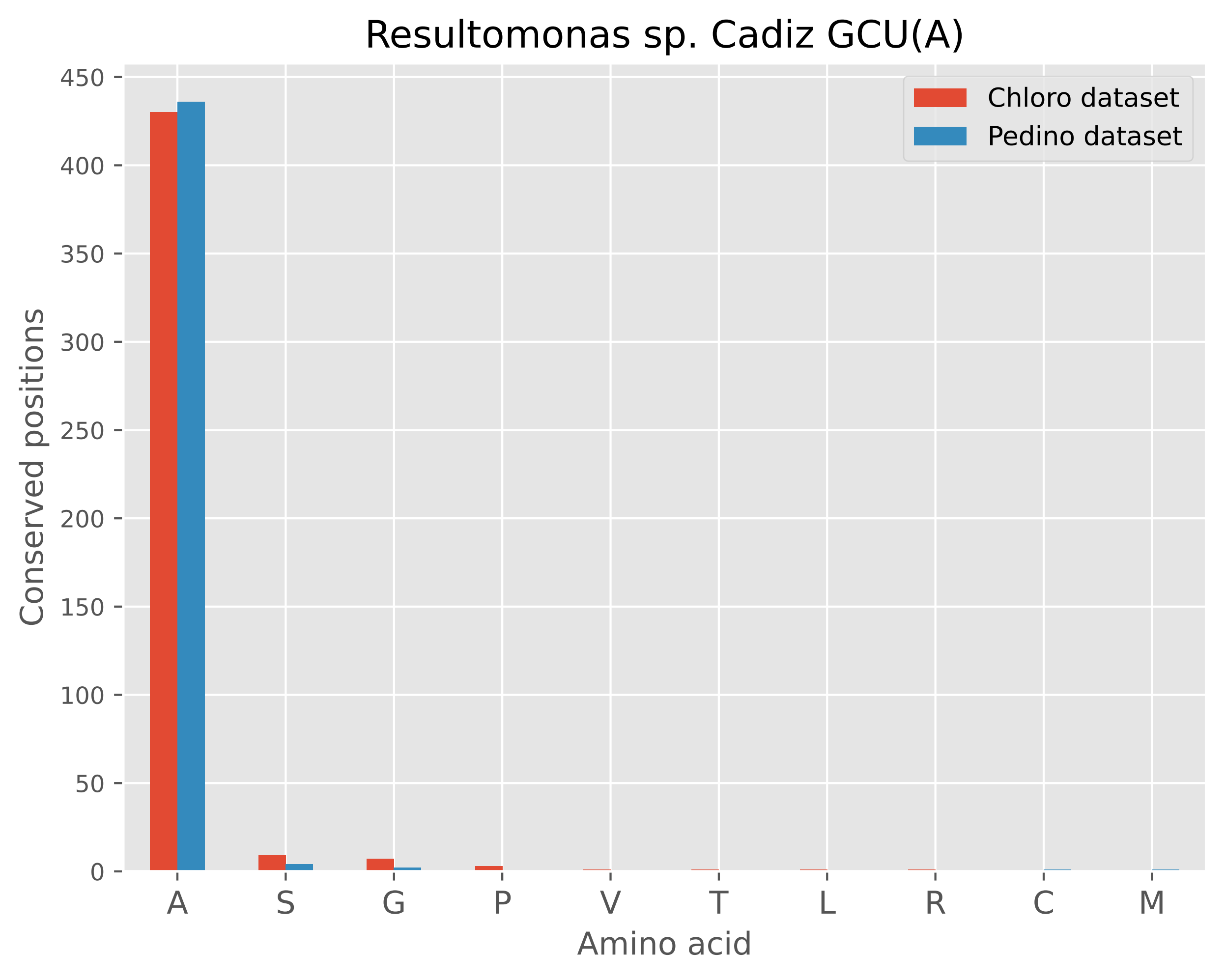
Resultomonas sp. Cadiz GCU(A)
Chloro dataset
Pedino dataset
Conserved positions
450
400
350
300
250
200
150
100
50
0
A
S
G
P
V
T
L
R
C
M
Amino acid

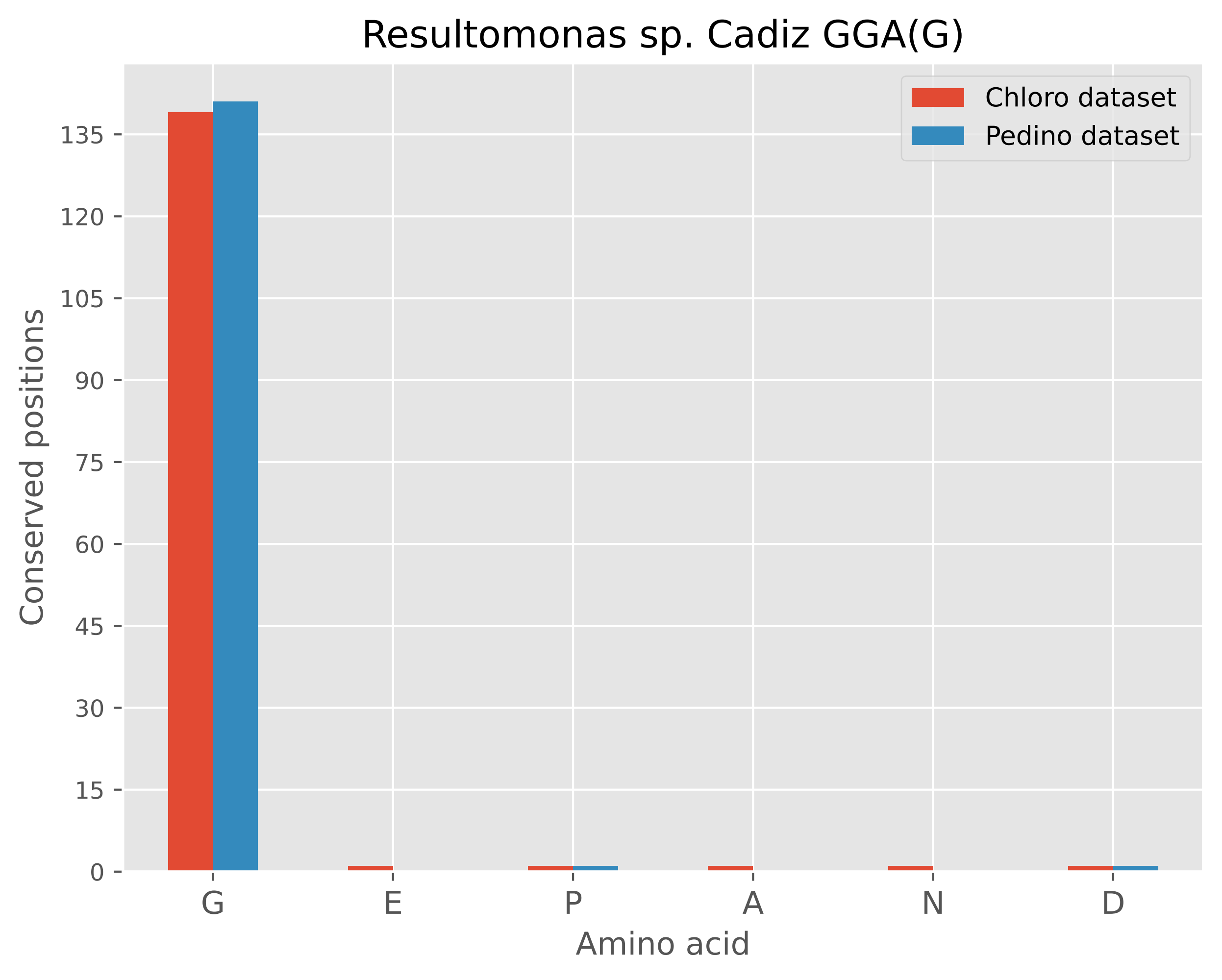

Resultomonas sp. Cadiz GGA(G)
Chloro dataset
Pedino dataset
Conserved positions
0
15
30
45
60
75
90
105
120
135
G
E
P
A
N
D
Amino acid

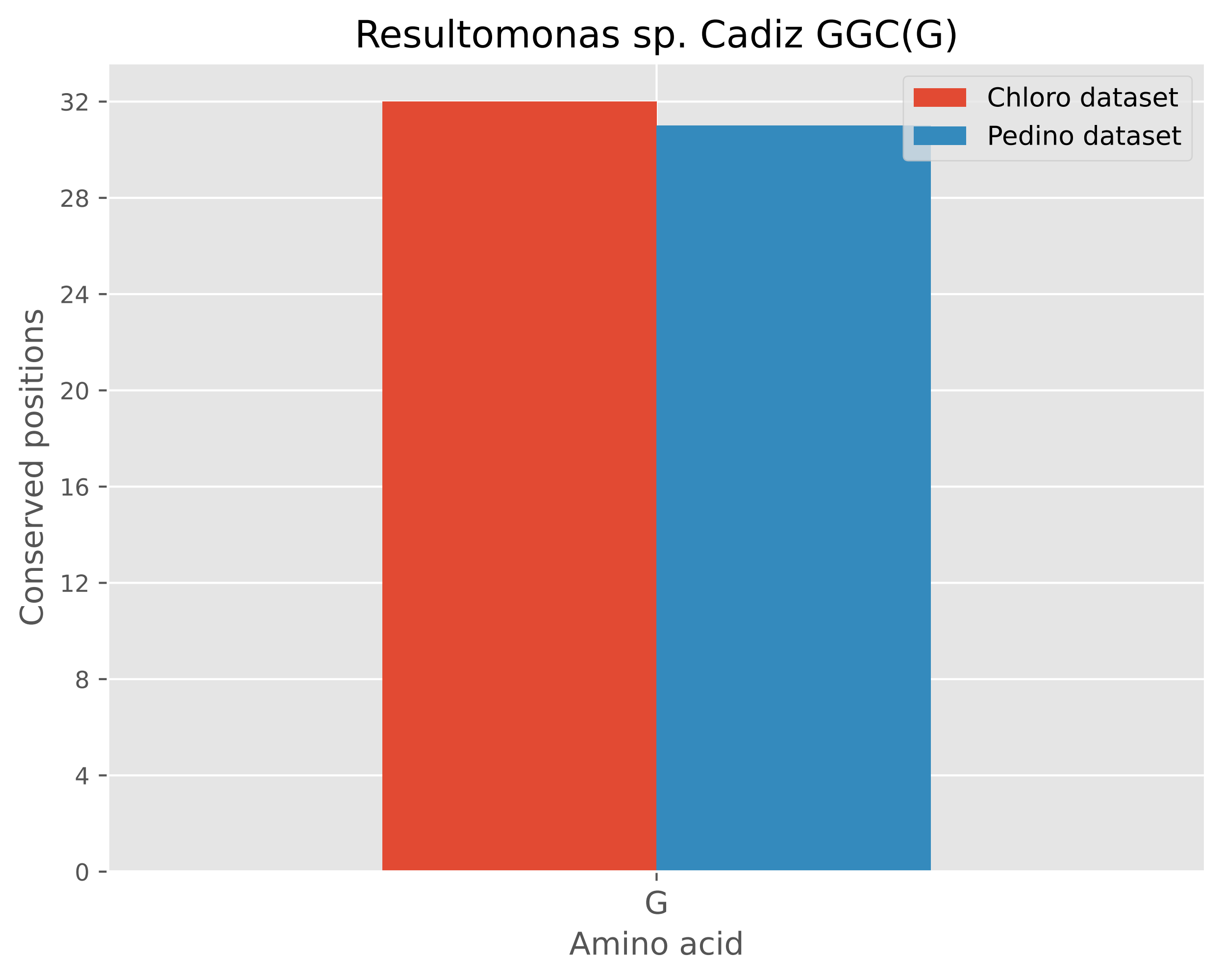

Resultomonas sp. Cadiz GGC(G)
Chloro dataset
Pedino dataset
Conserved positions
0
4
8
12
16
20
24
28
32
G
Amino acid

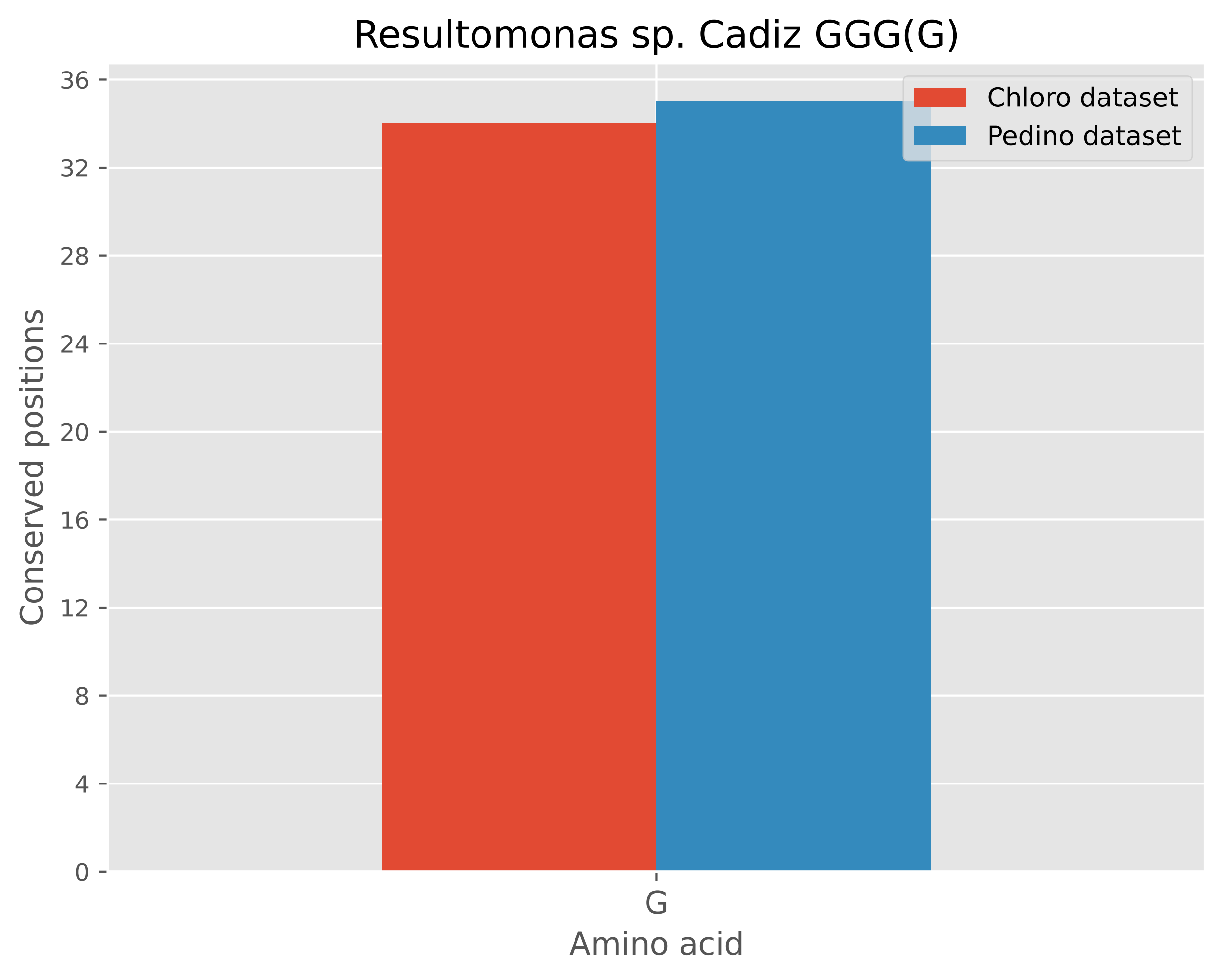
Resultomonas sp. Cadiz GGG(G)
Chloro dataset
Pedino dataset
Conserved positions
0
4
8
12
16
20
24
28
32
36
G
Amino acid

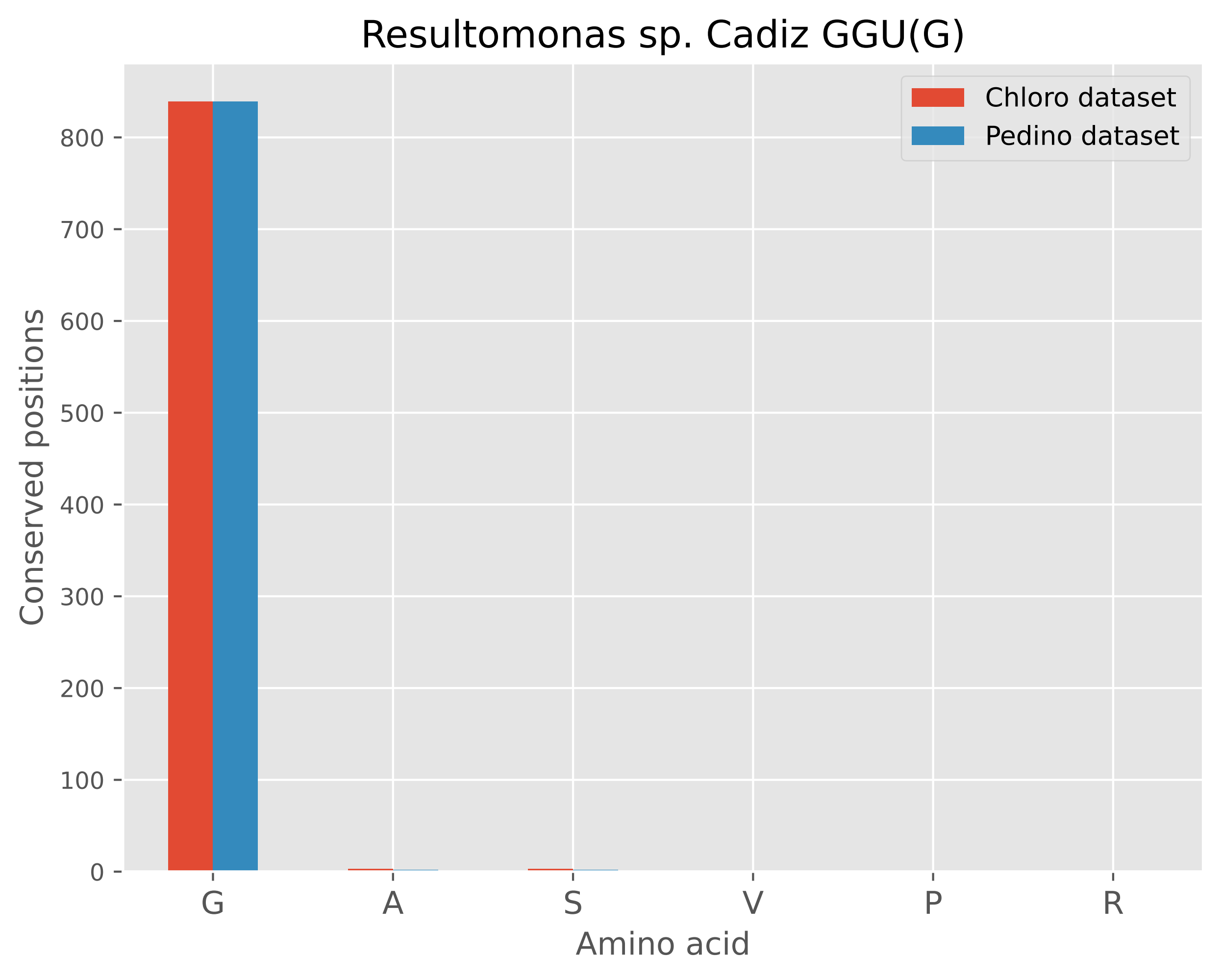

Resultomonas sp. Cadiz GGU(G)
Chloro dataset
Pedino dataset
Conserved positions
800
700
600
500
400
300
200
100
0
G
A
S
V
P
R
Amino acid

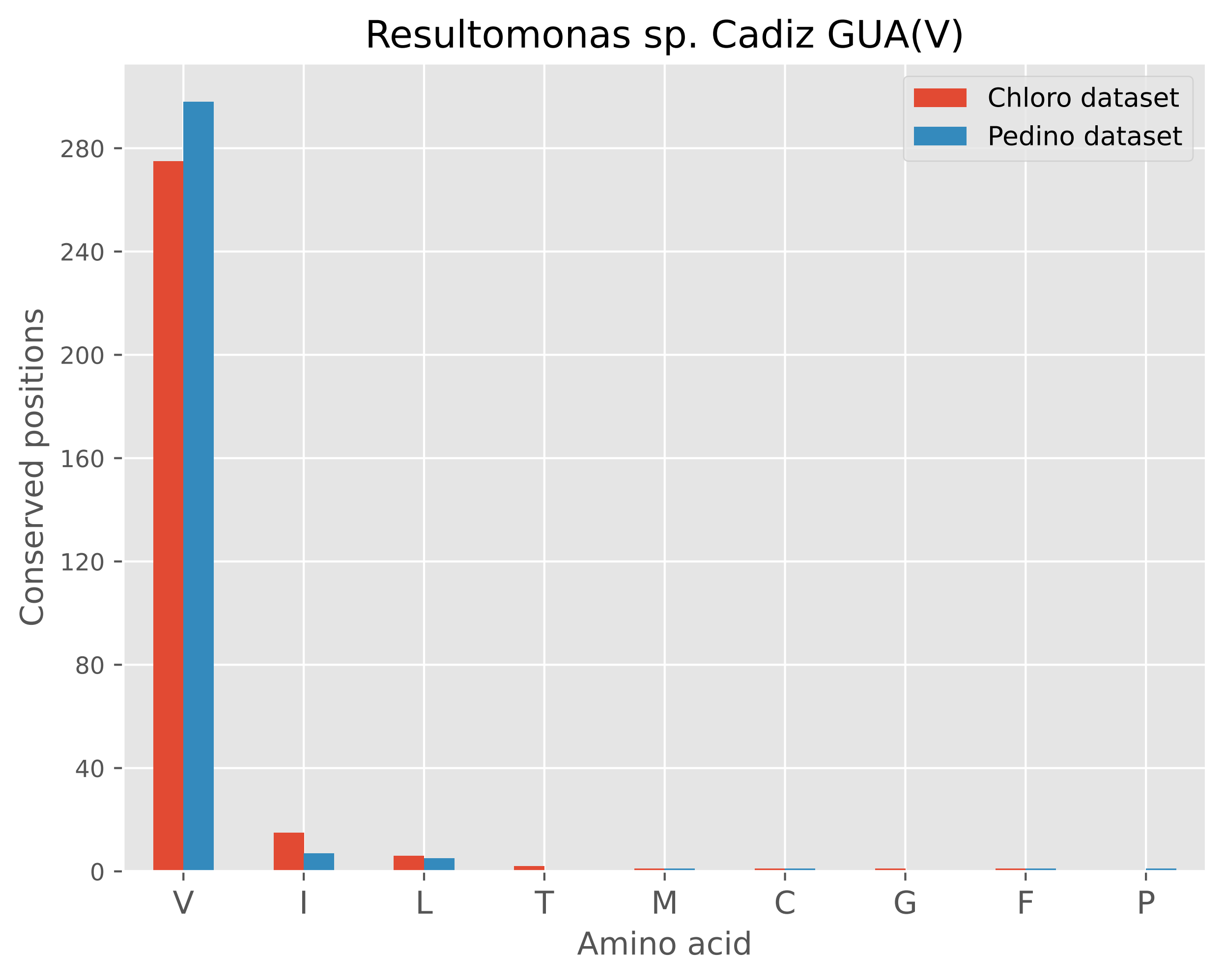

Resultomonas sp. Cadiz GUA(V)
Chloro dataset
Pedino dataset
Conserved positions
0
40
80
120
160
200
240
280
V
I
L
T
M
C
G
F
P
Amino acid

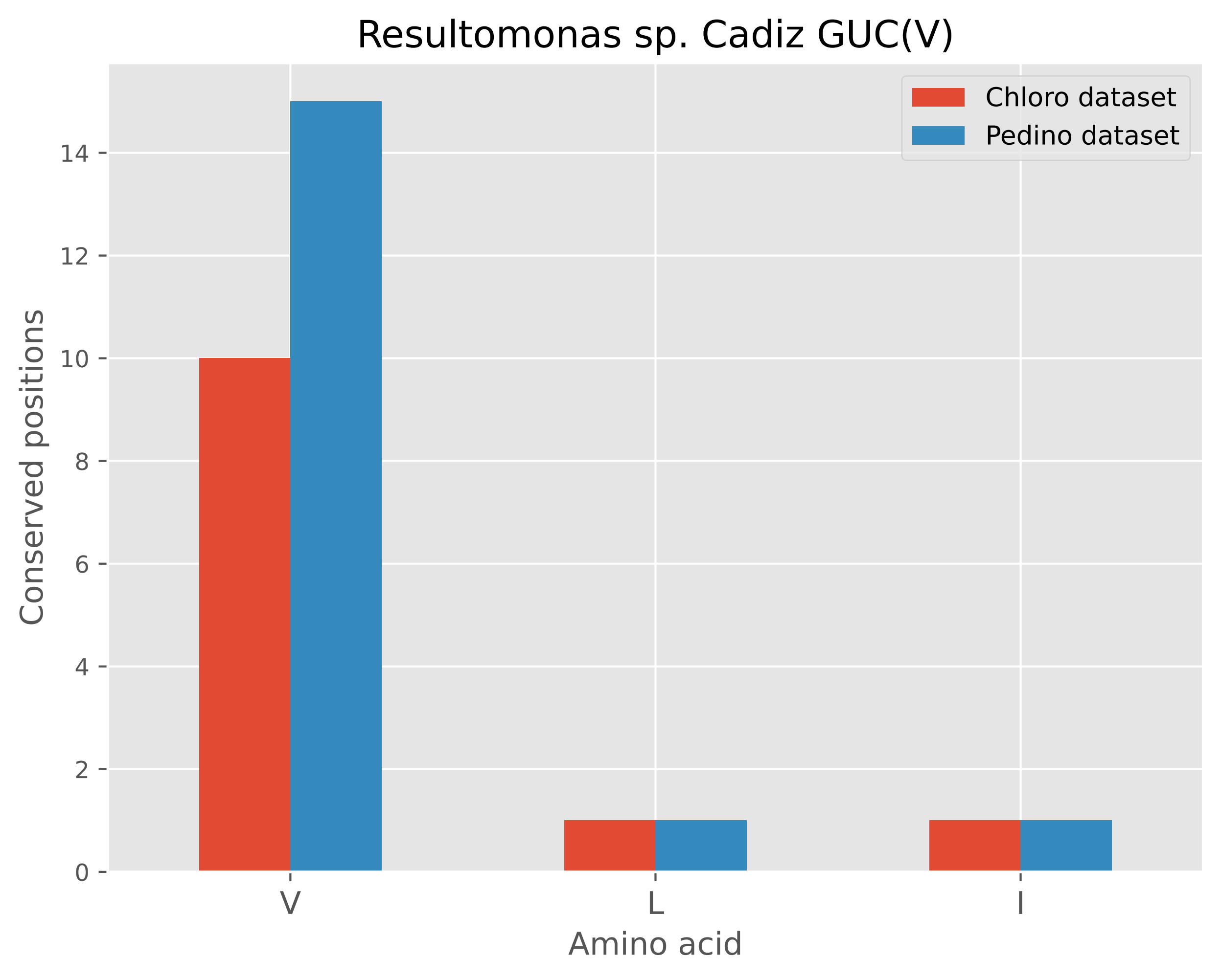
Resultomonas sp. Cadiz GUC(V)
Chloro dataset
Pedino dataset
Conserved positions
0
2
4
6
8
10
12
14
V
L
I
Amino acid

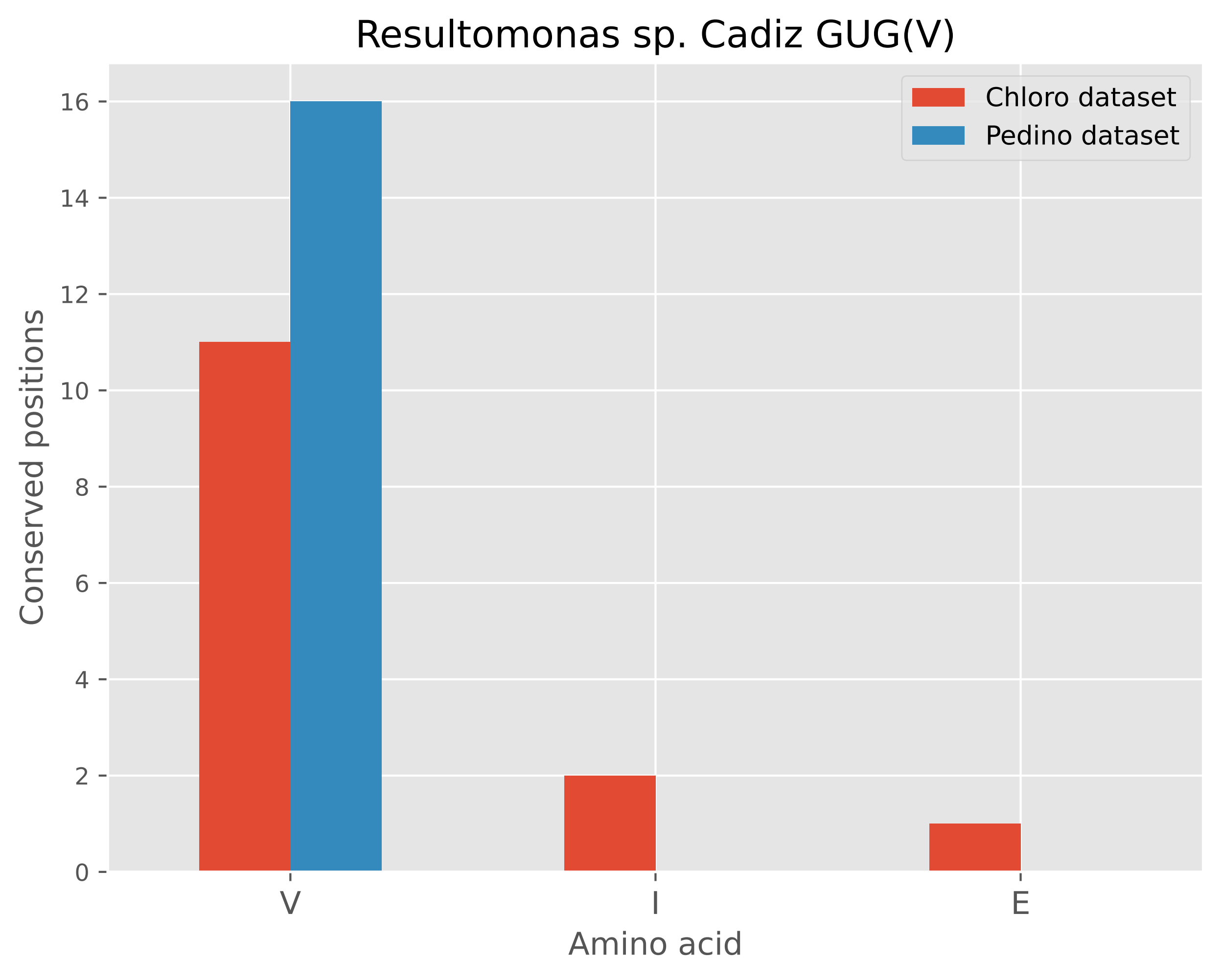

Resultomonas sp. Cadiz GUG(V)
Chloro dataset
Pedino dataset
Conserved positions
0
2
4
6
8
10
12
14
16
V
I
E
Amino acid

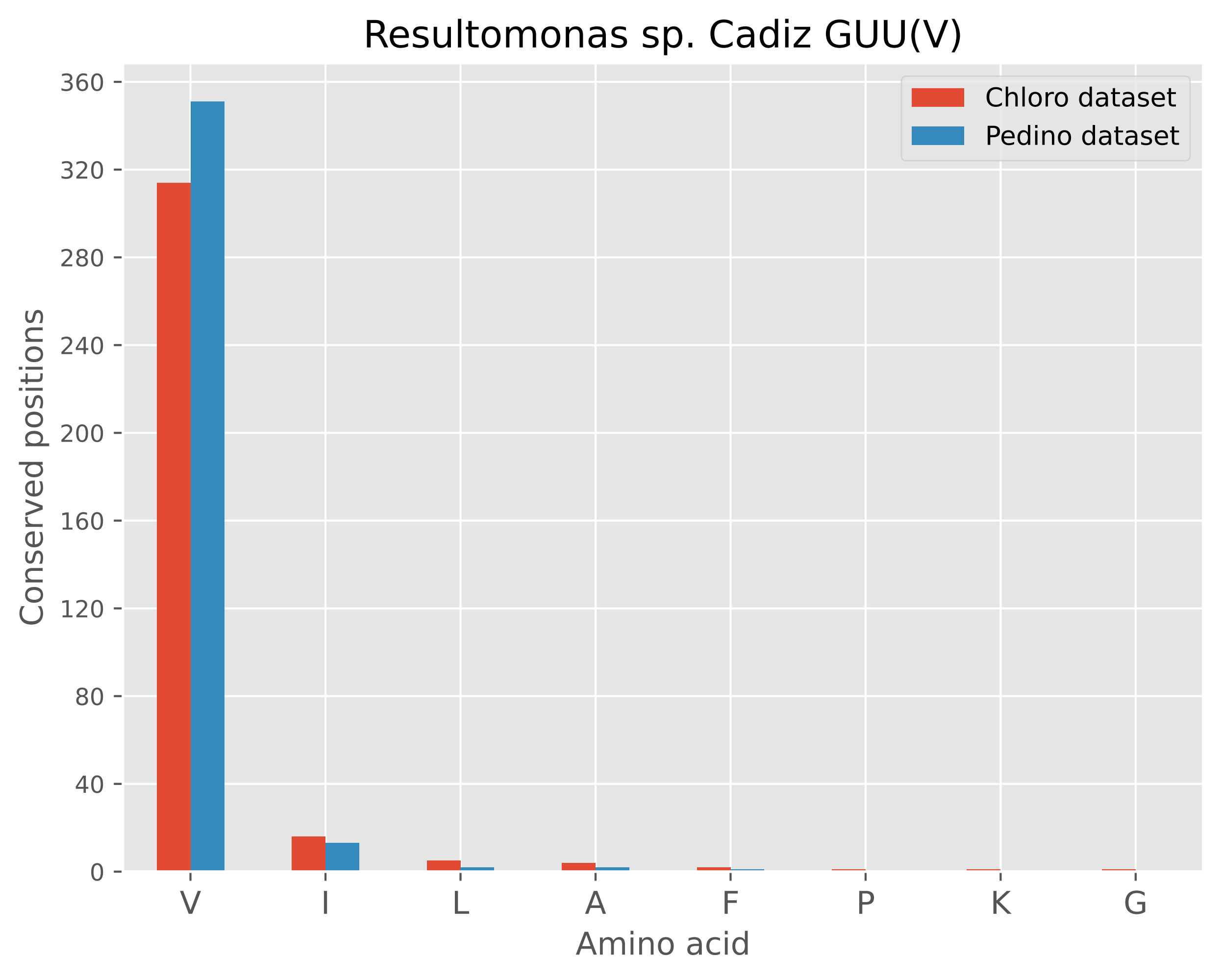
Resultomonas sp. Cadiz GUU(V)
Chloro dataset
Pedino dataset
Conserved positions
0
40
80
120
160
200
240
280
320
360
V
I
L
A
F
P
K
G
Amino acid

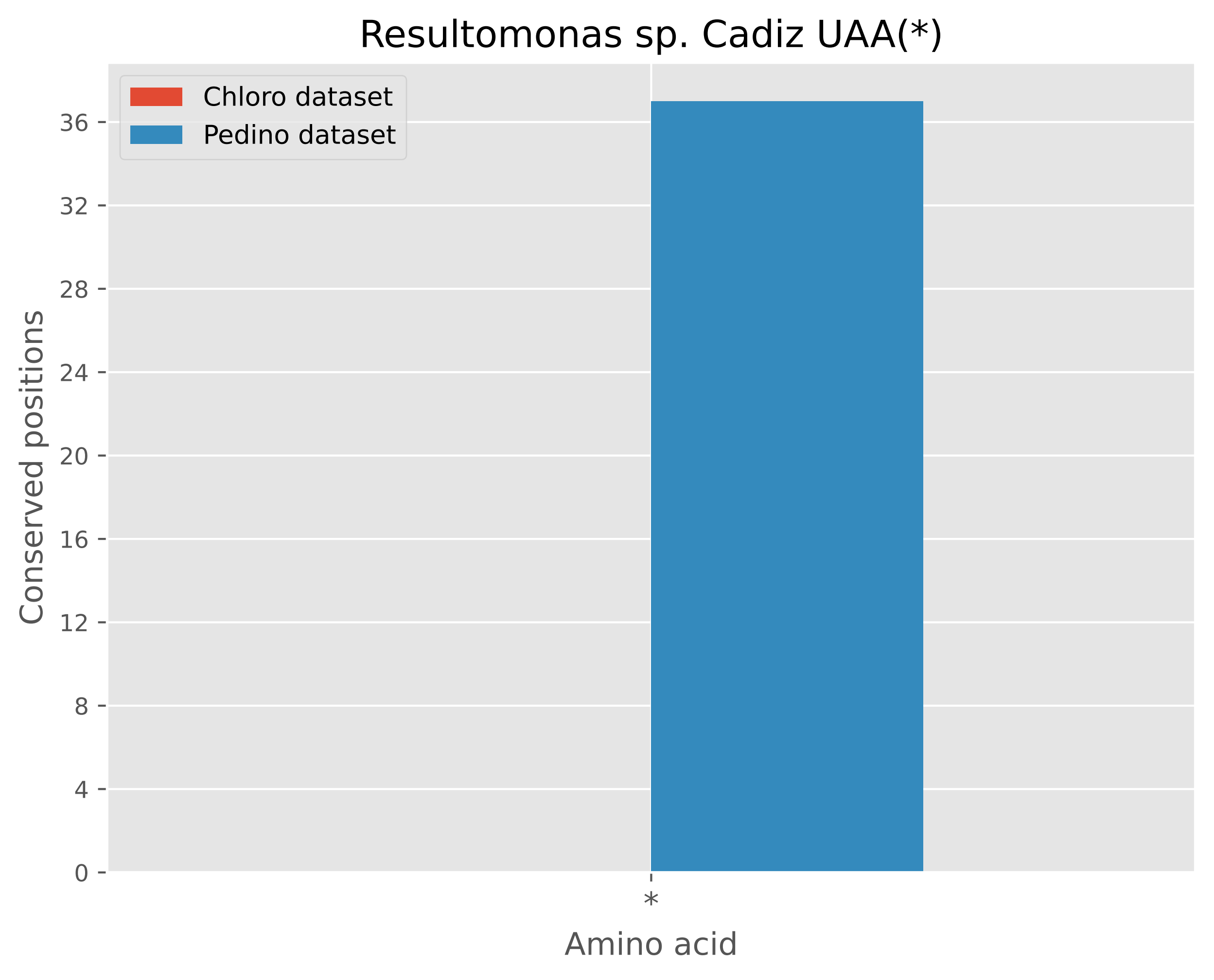
Resultomonas sp. Cadiz UAA(*)
Chloro dataset
Pedino dataset
Conserved positions
0
4
8
12
16
20
24
28
32
36
*
Amino acid

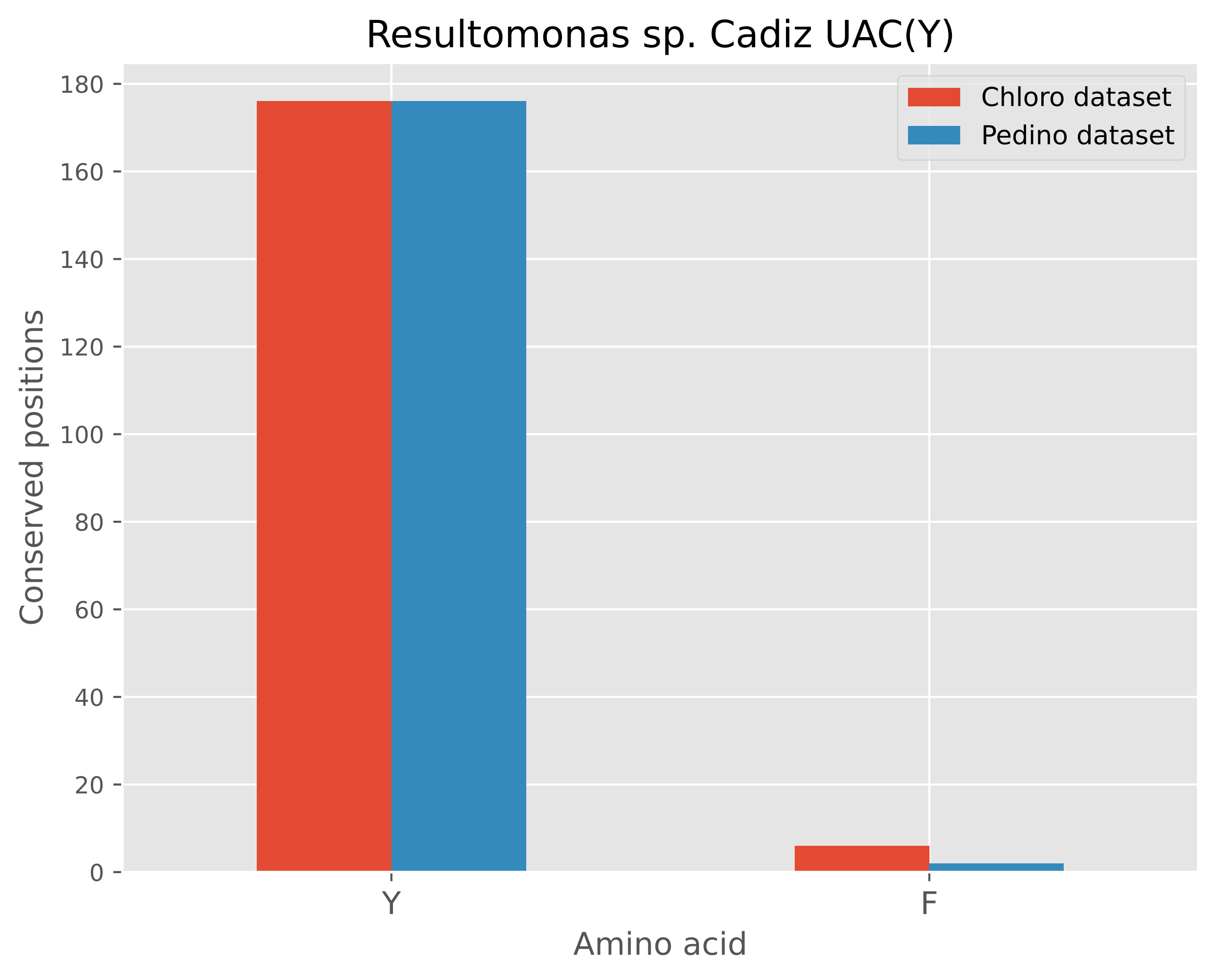

Resultomonas sp. Cadiz UAC(Y)
Chloro dataset
Pedino dataset
Conserved positions
Amino acid
Y
F
0
20
40
60
80
100
120
140
160
180

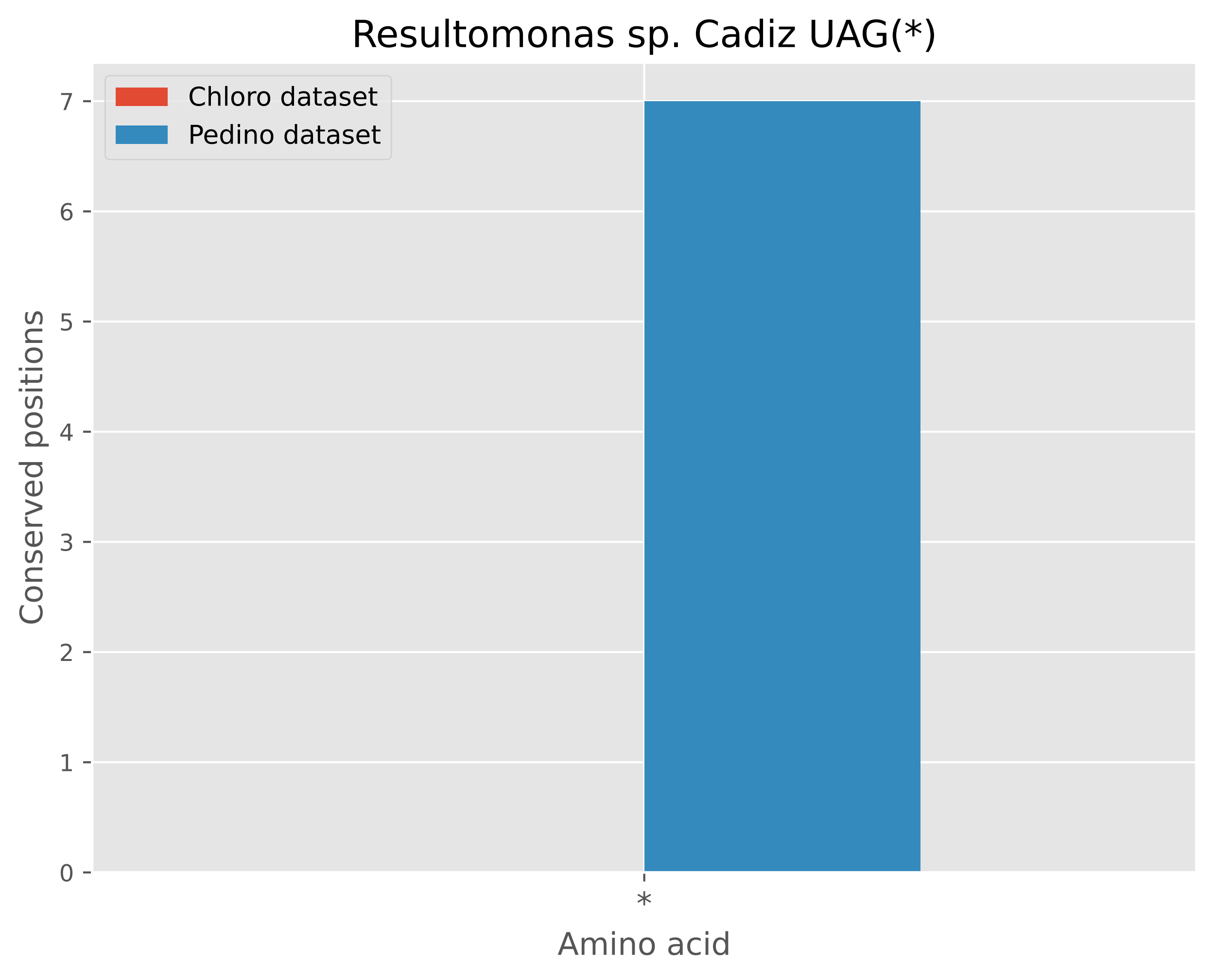

Resultomonas sp. Cadiz UAG(*)
Chloro dataset
Pedino dataset
Conserved positions
0
1
2
3
4
5
6
7
*
Amino acid

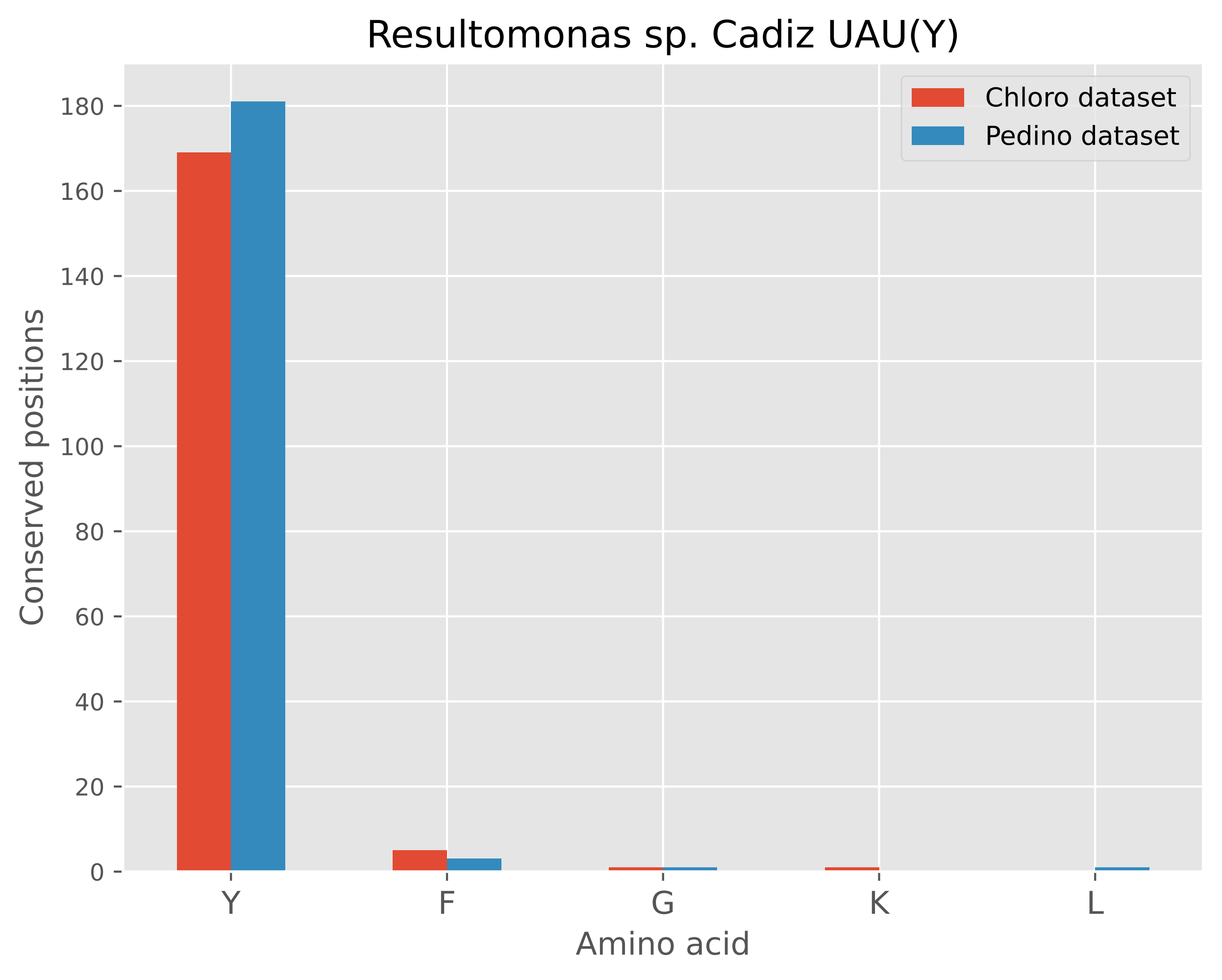

Resultomonas sp. Cadiz UAU(Y)
Chloro dataset
Pedino dataset
Conserved positions
0
20
40
60
80
100
120
140
160
180
Y
F
G
K
L
Amino acid

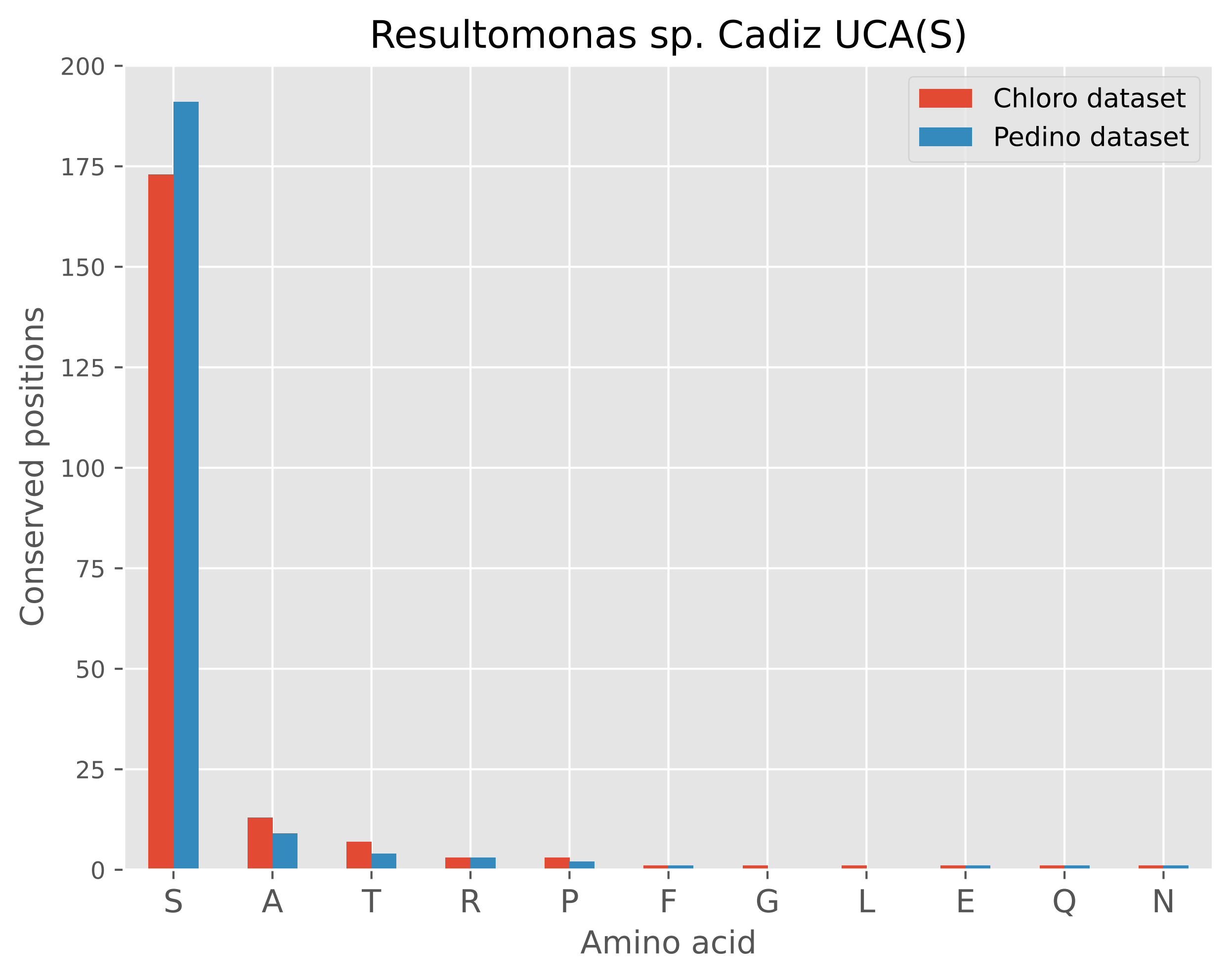
Resultomonas sp. Cadiz UCA(S)
Chloro dataset
Pedino dataset
Conserved positions
Amino acid
0
25
50
75
100
125
150
175
200
S
A
T
R
P
F
G
L
E
Q
N

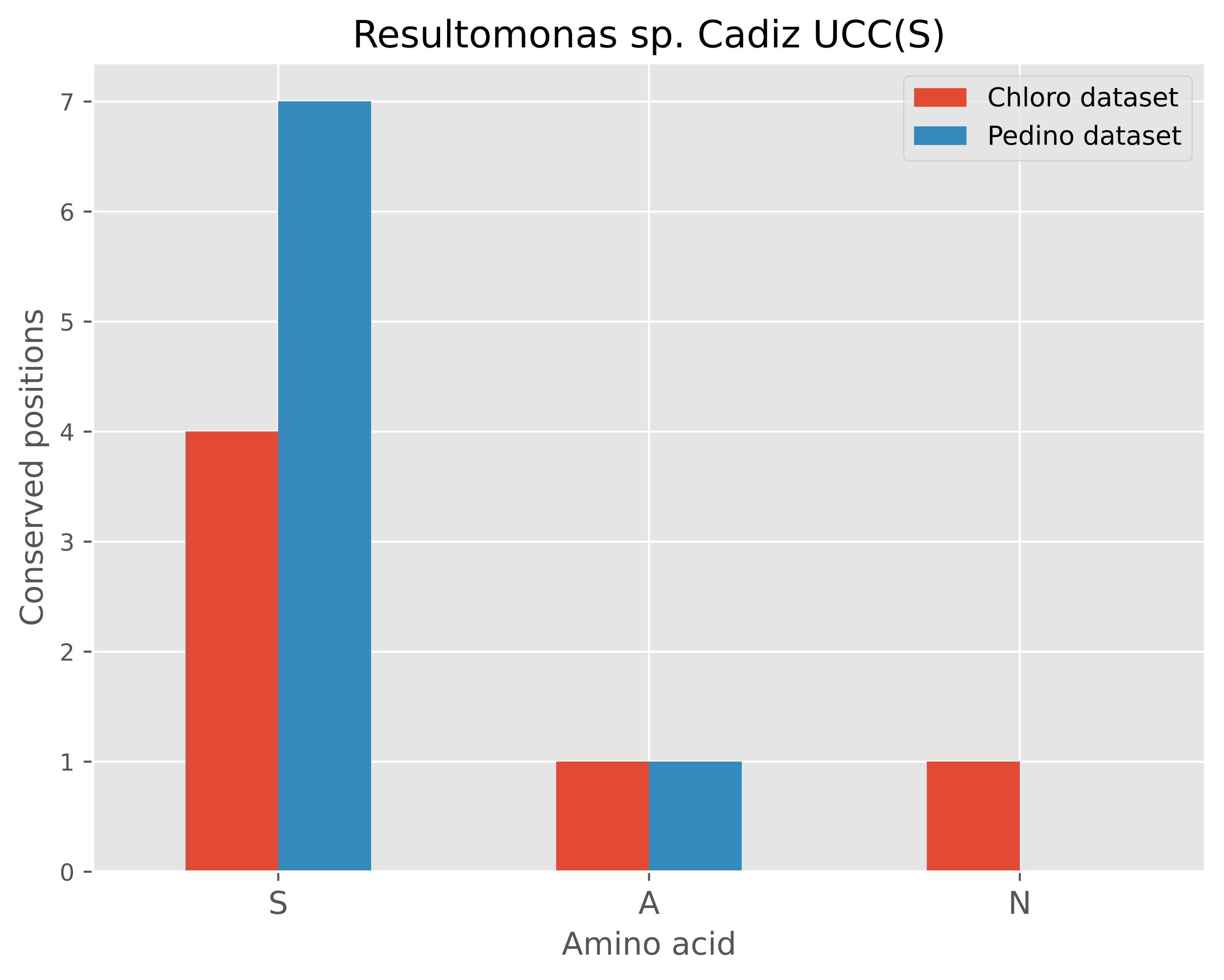

Resultomonas sp. Cadiz UCC(S)
Chloro dataset
Pedino dataset
Conserved positions
0
1
2
3
4
5
6
7
S
A
N
Amino acid

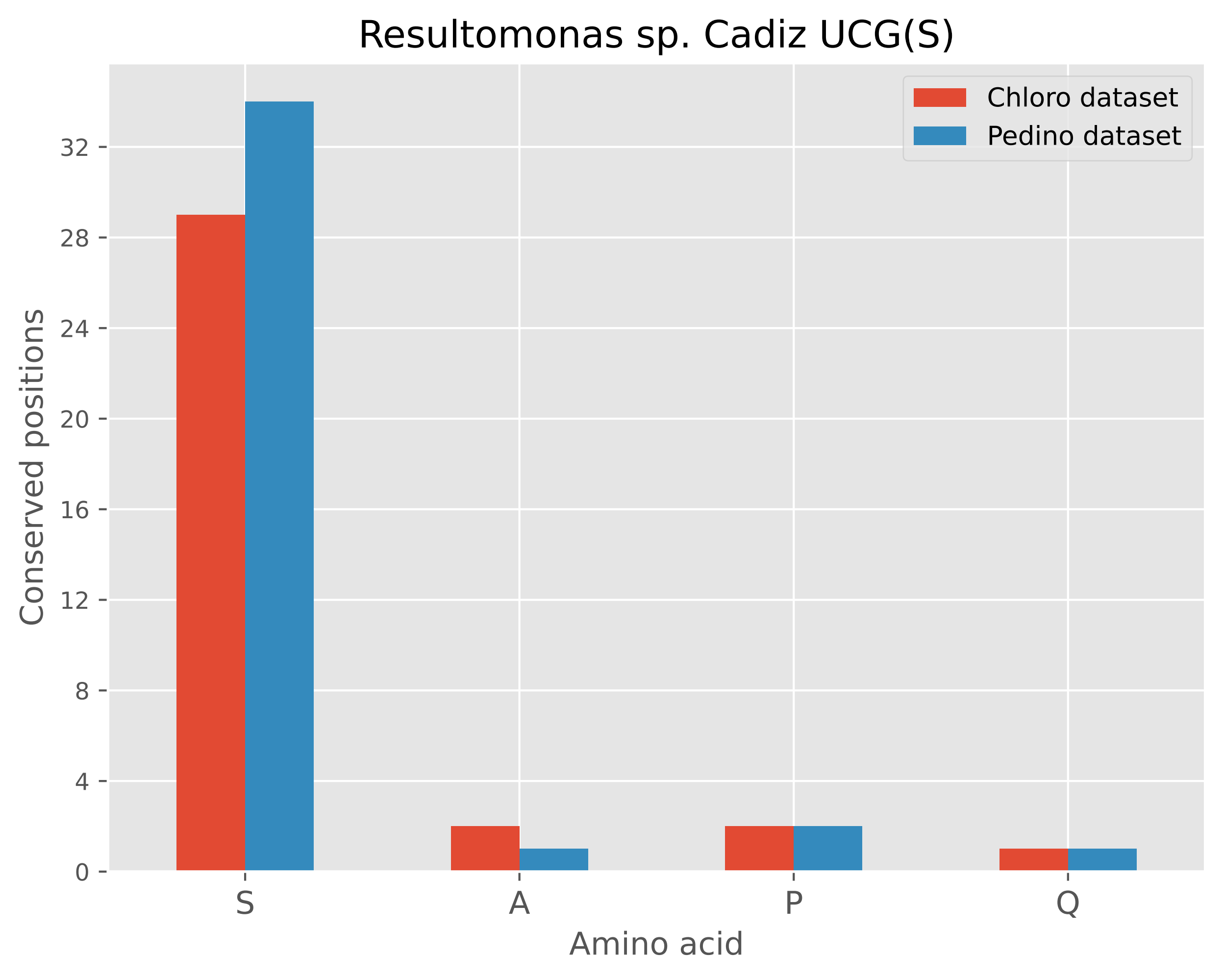

Resultomonas sp. Cadiz UCG(S)
Chloro dataset
Pedino dataset
Conserved positions
0
4
8
12
16
20
24
28
32
S
A
P
Q
Amino acid

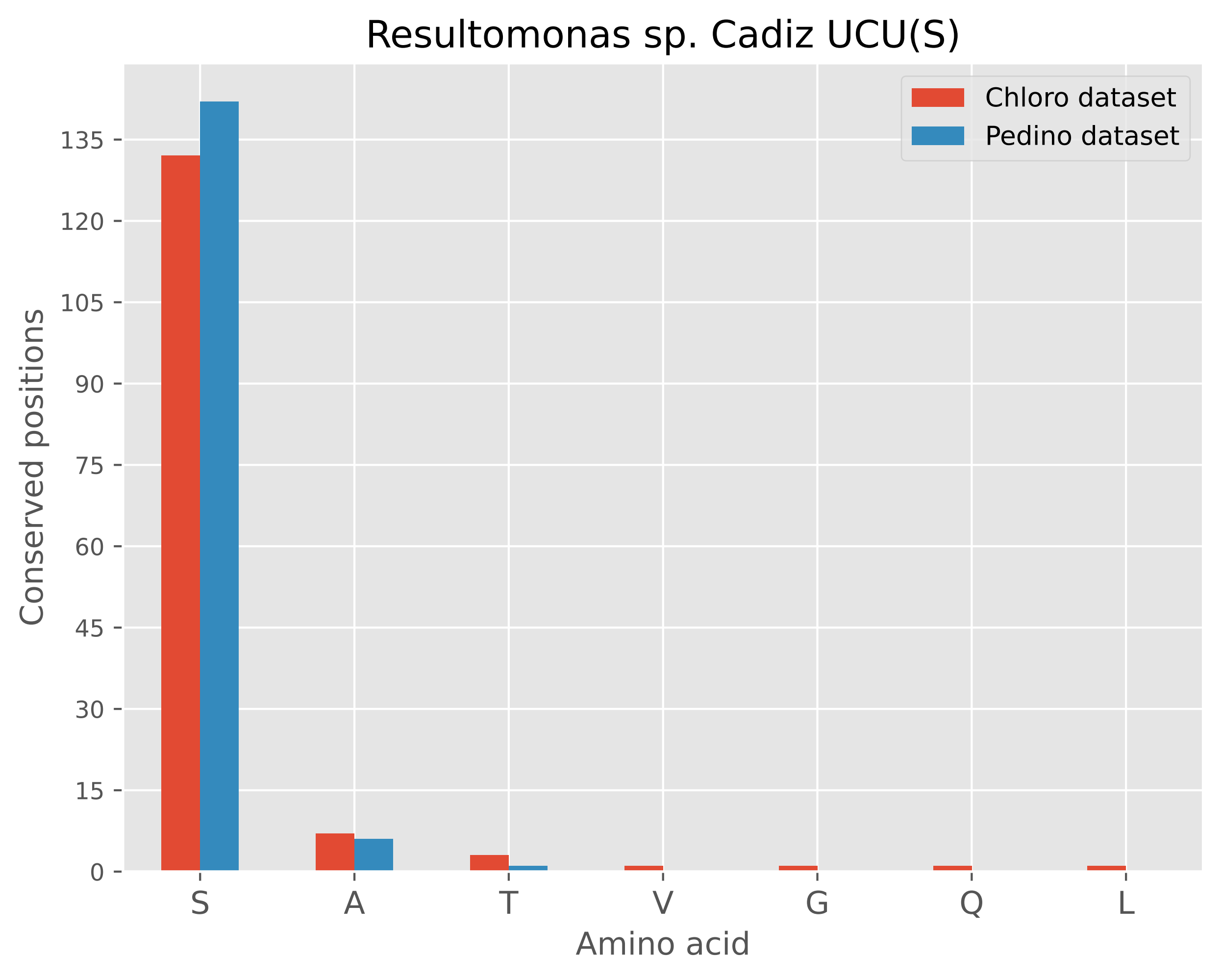

Resultomonas sp. Cadiz UCU(S)
Chloro dataset
Pedino dataset
Conserved positions
0
15
30
45
60
75
90
105
120
135
S
A
T
V
G
Q
L
Amino acid

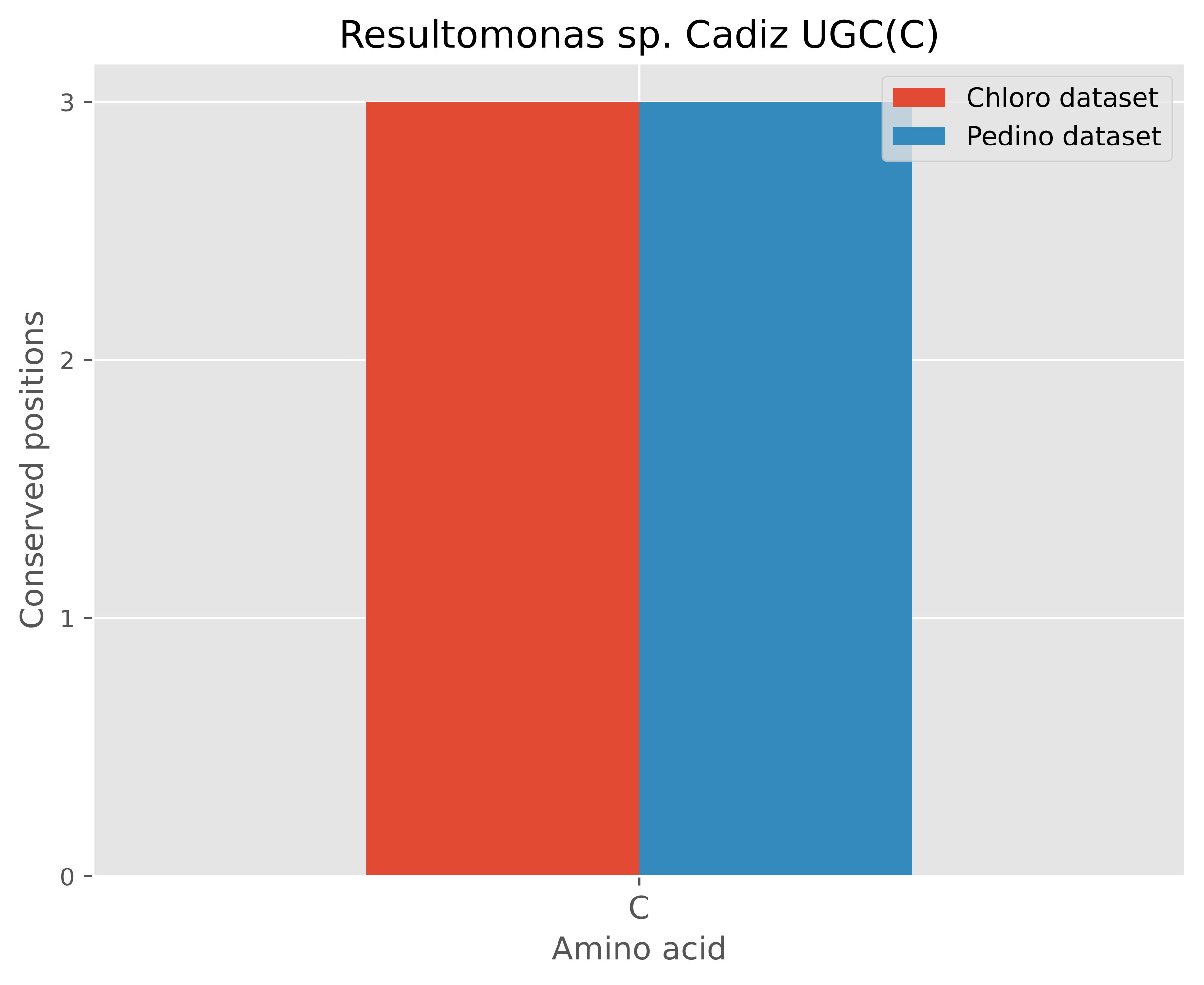
Resultomonas sp. Cadiz UGC(C)
Chloro dataset
Pedino dataset
Conserved positions
0
1
2
3
C
Amino acid

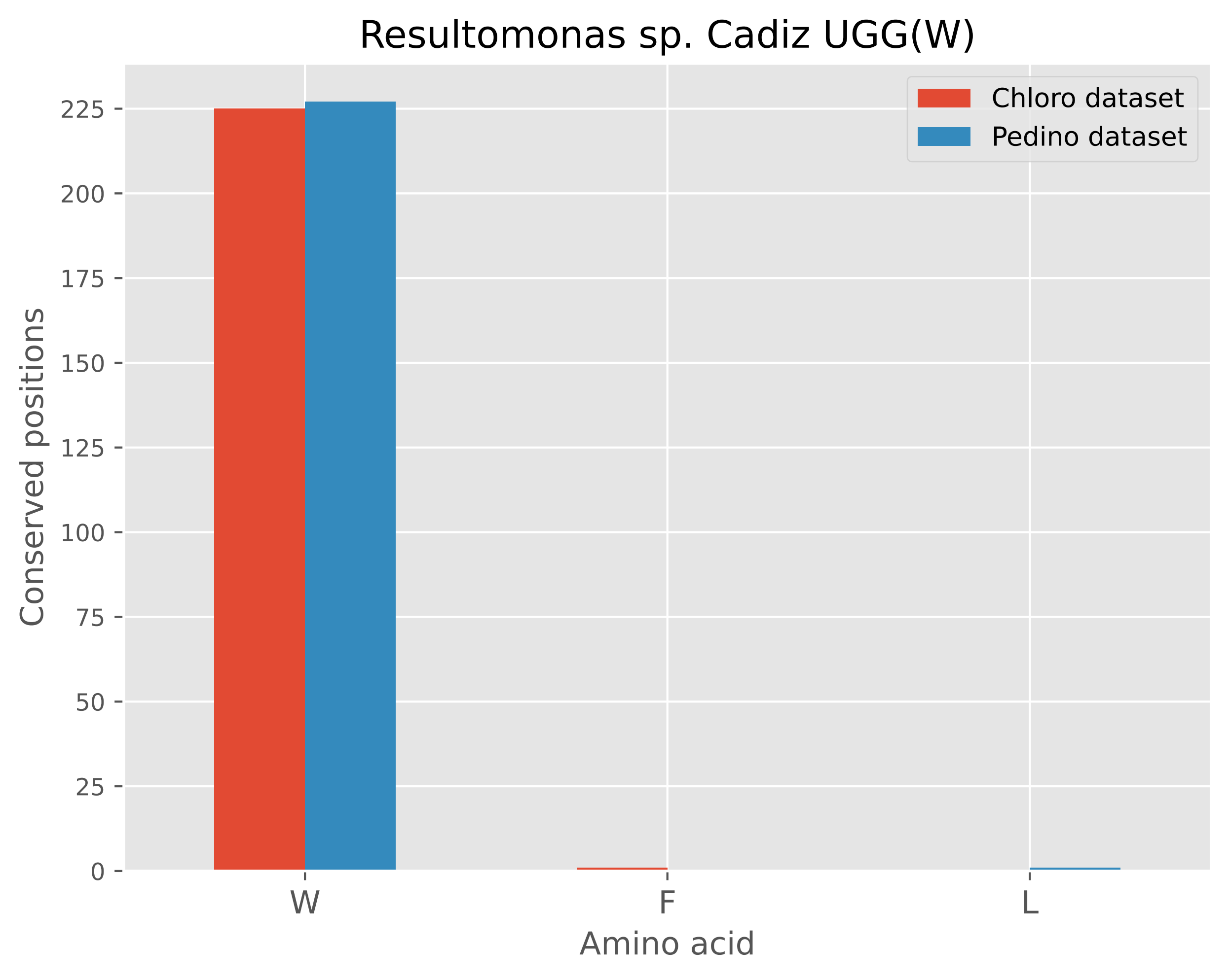

Resultomonas sp. Cadiz UGG(W)
Chloro dataset
Pedino dataset
Conserved positions
0
25
50
75
100
125
150
175
200
225
W
F
L
Amino acid

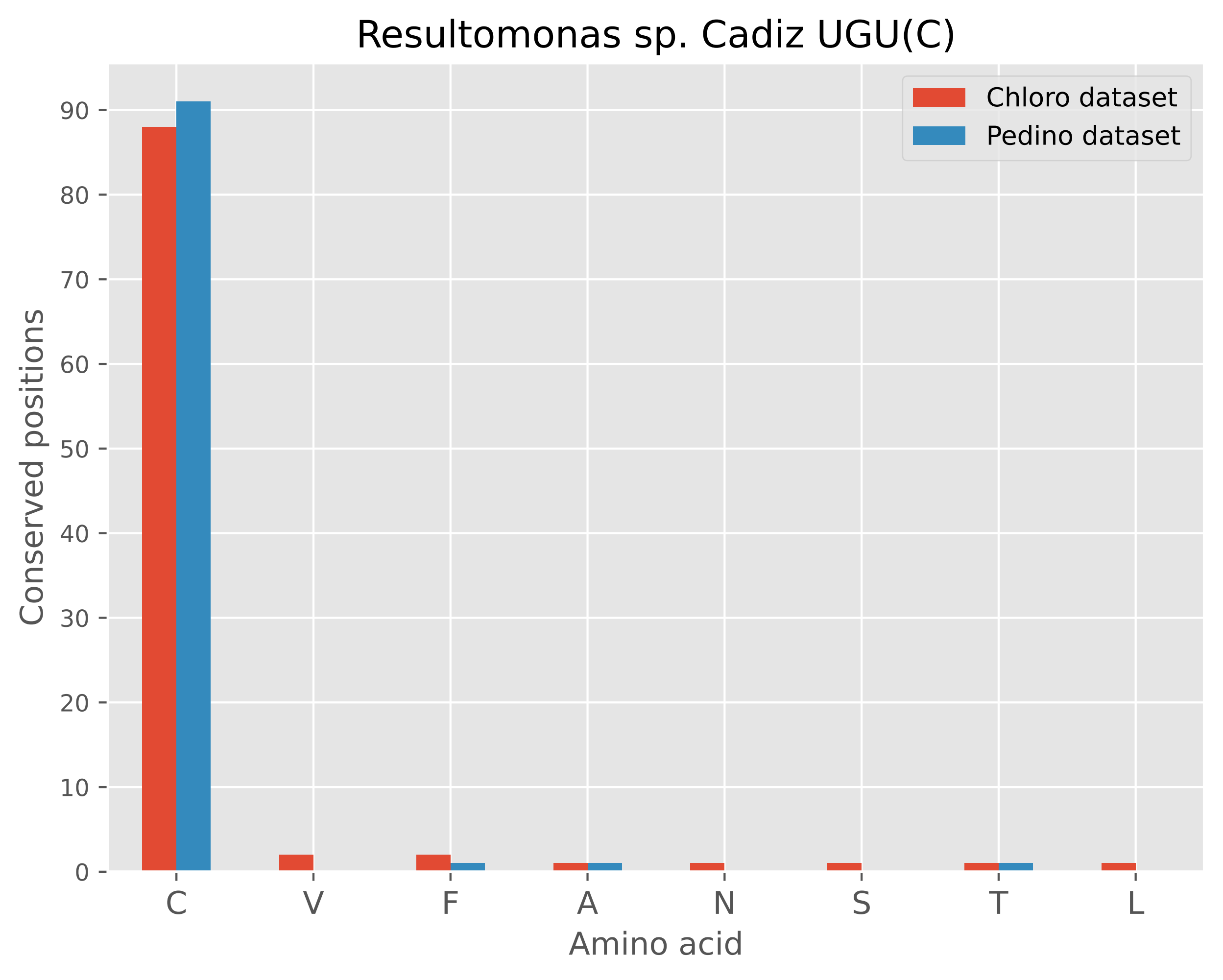
Resultomonas sp. Cadiz UGU(C)
Chloro dataset
Pedino dataset
Conserved positions
0
10
20
30
40
50
60
70
80
90
C
V
F
A
N
S
T
L
Amino acid

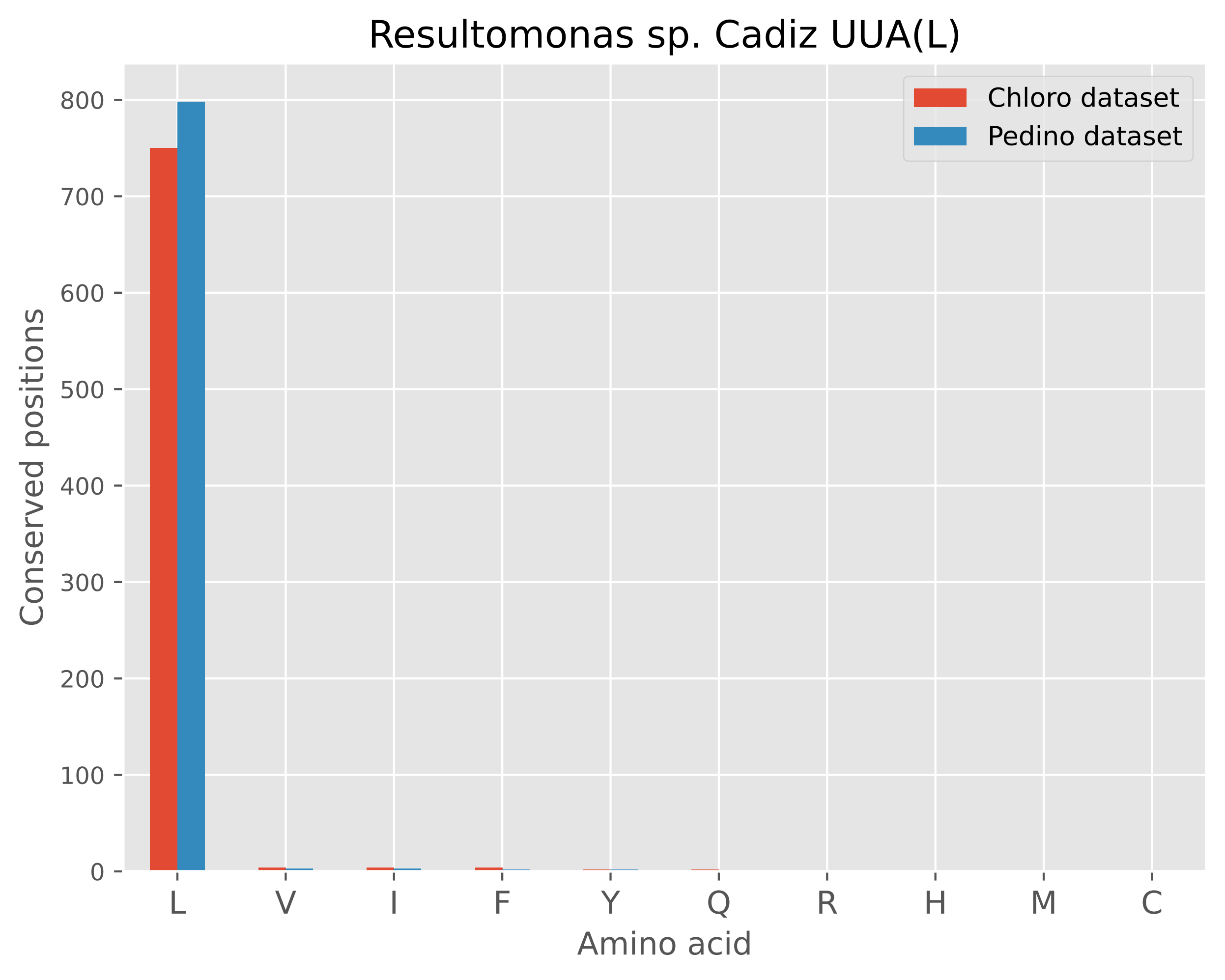

Resultomonas sp. Cadiz UUA(L)
Chloro dataset
Pedino dataset
Conserved positions
0
100
200
300
400
500
600
700
800
L
V
I
F
Y
Q
R
H
M
C
Amino acid

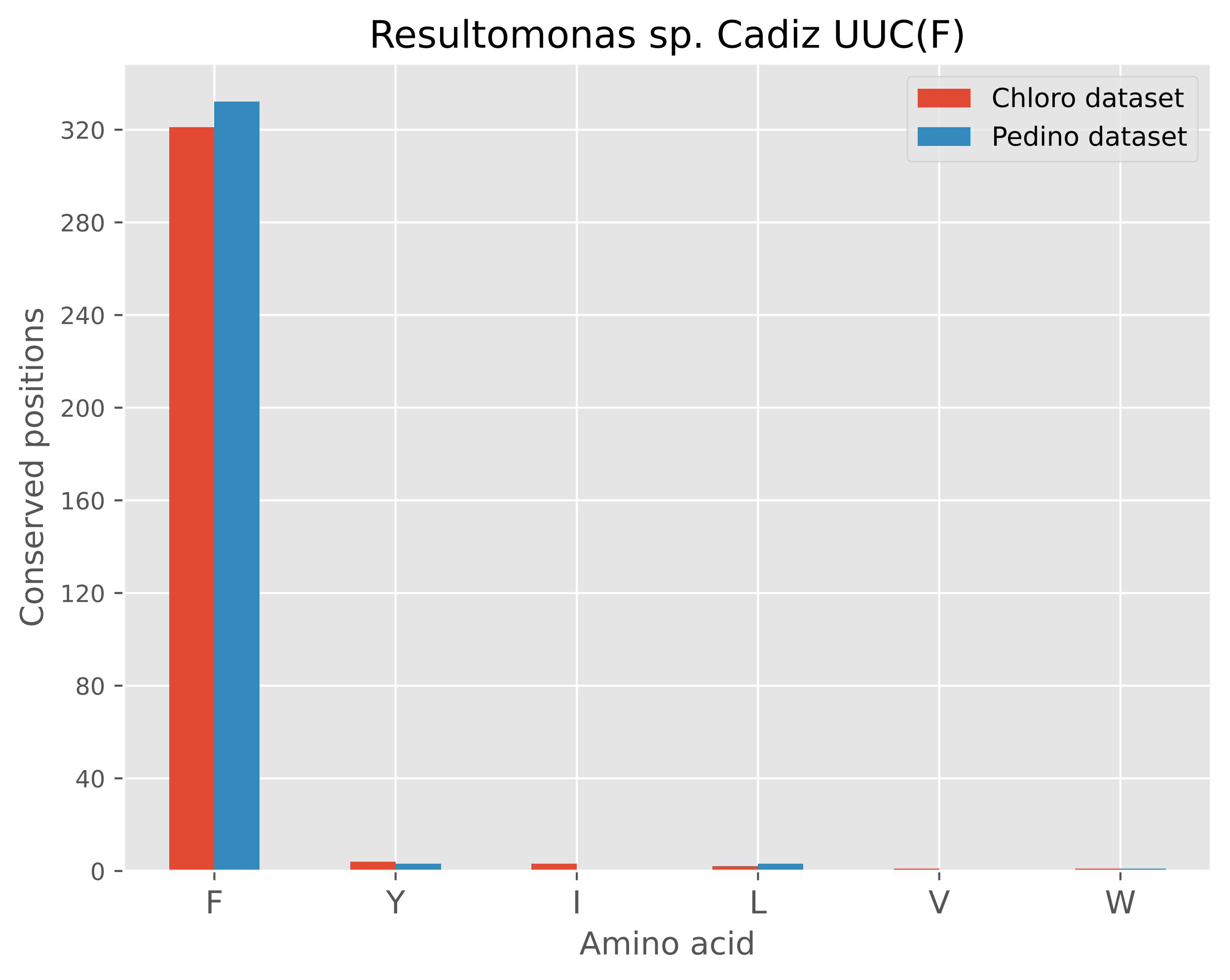

Resultomonas sp. Cadiz UUC(F)
Chloro dataset
Pedino dataset
Conserved positions
0
40
80
120
160
200
240
280
320
F
Y
I
L
V
W
Amino acid

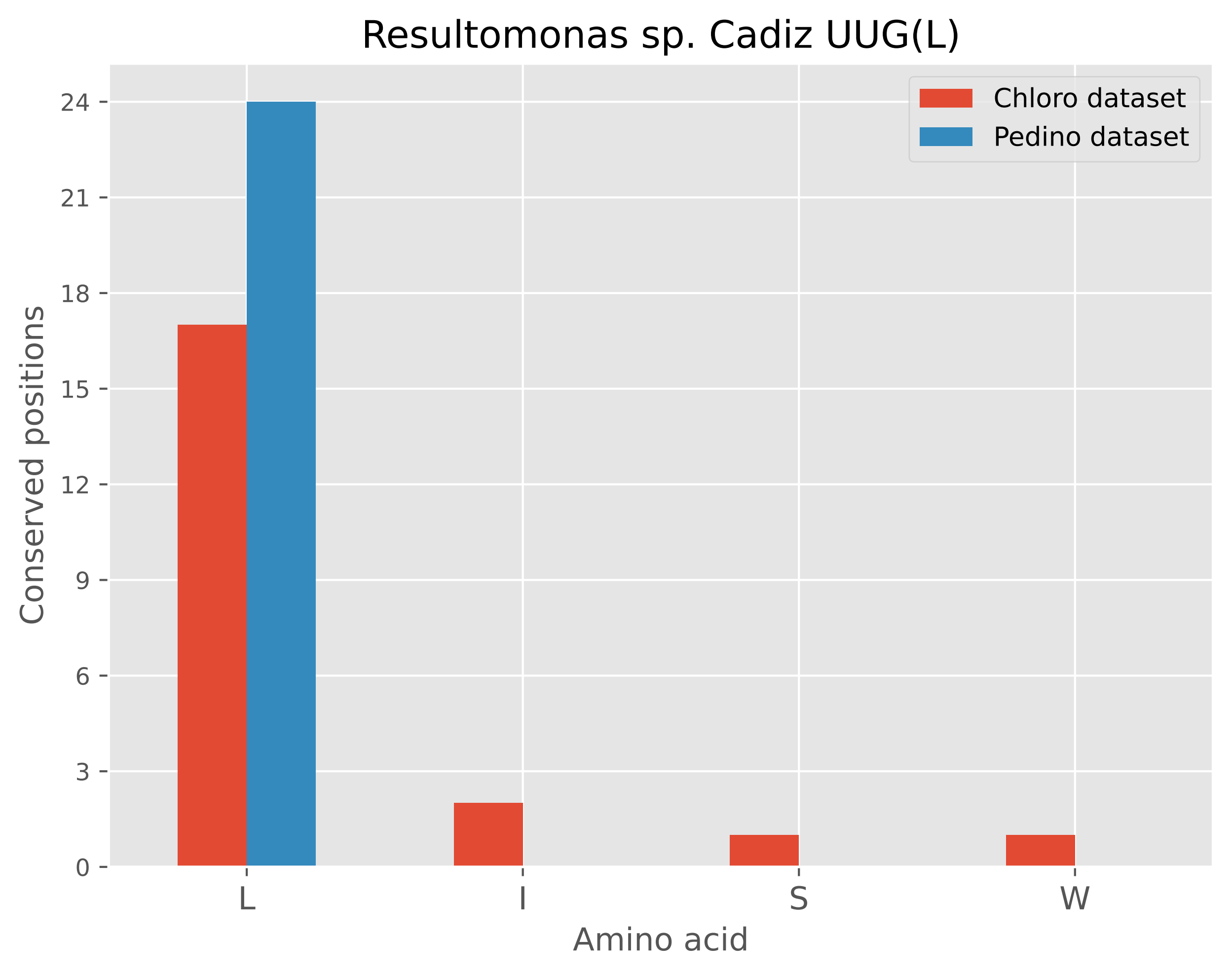

Resultomonas sp. Cadiz UUG(L)
Chloro dataset
Pedino dataset
Conserved positions
0
3
6
9
12
15
18
21
24
L
I
S
W
Amino acid

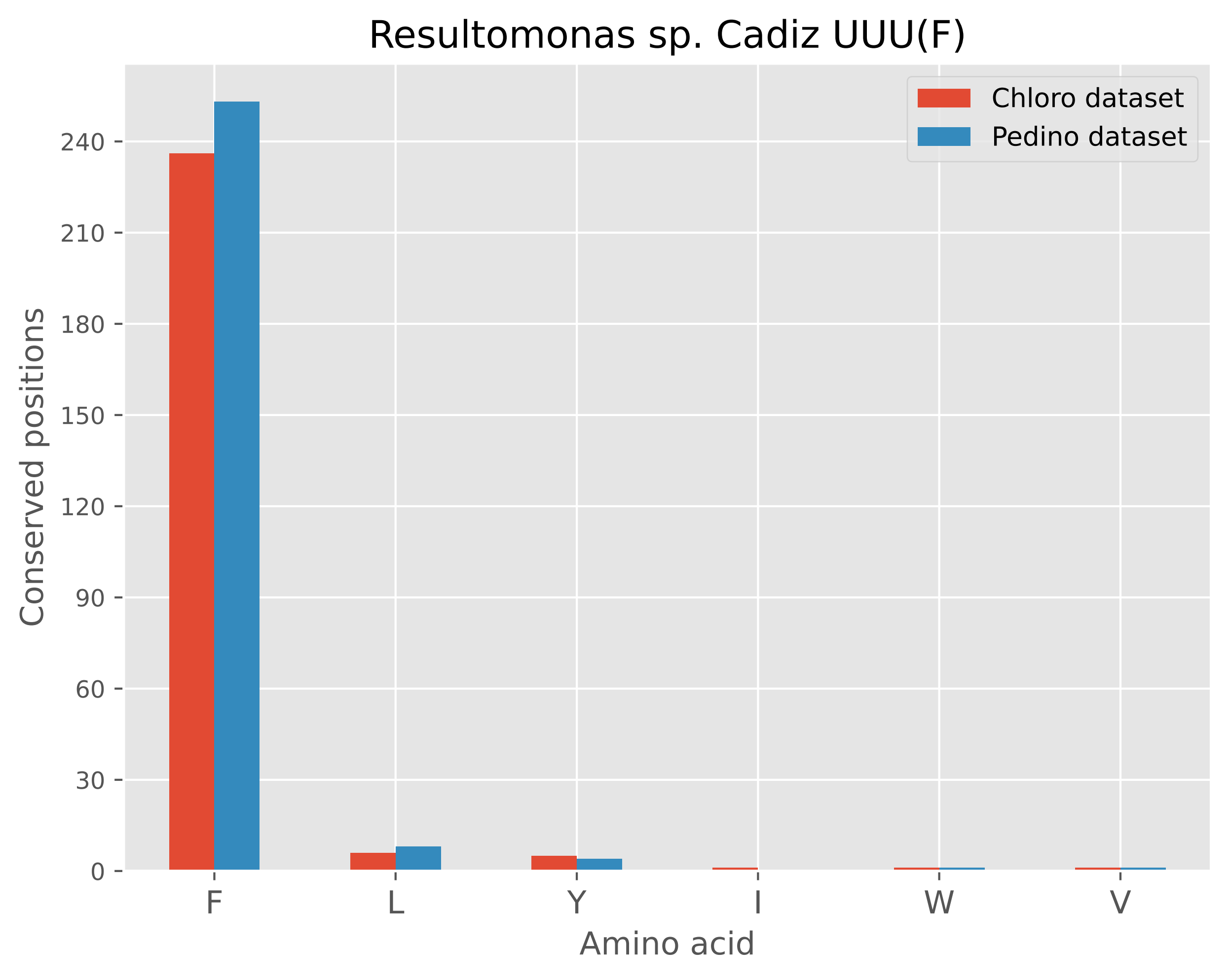

Resultomonas sp. Cadiz UUU(F)
Chloro dataset
Pedino dataset
Conserved positions
0
30
60
90
120
150
180
210
240
F
L
Y
I
W
V
Amino acid

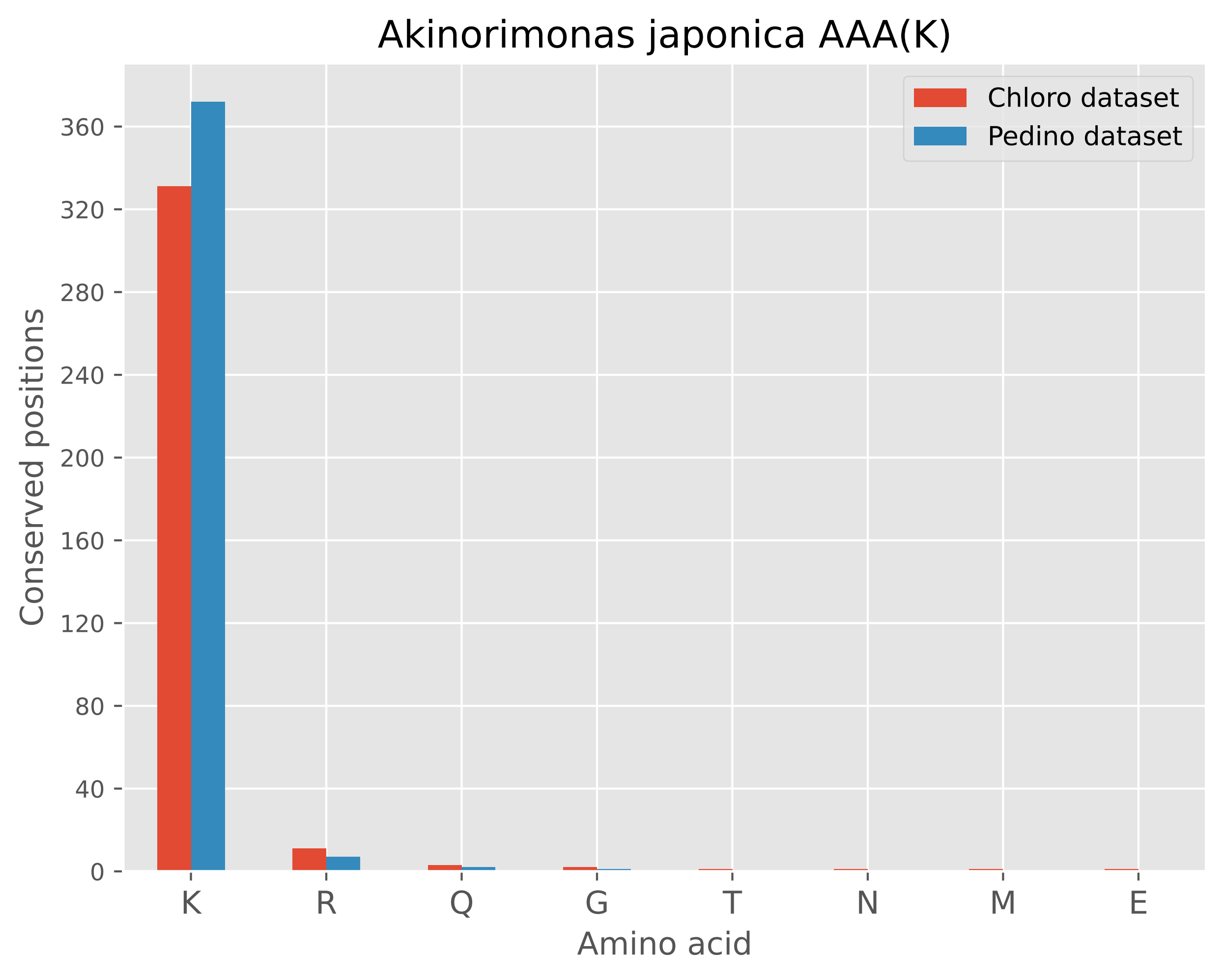

Akinorimonas japonica AAA(K)
Chloro dataset
Pedino dataset
Conserved positions
0
40
80
120
160
200
240
280
320
360
K
R
Q
G
T
N
M
E
Amino acid

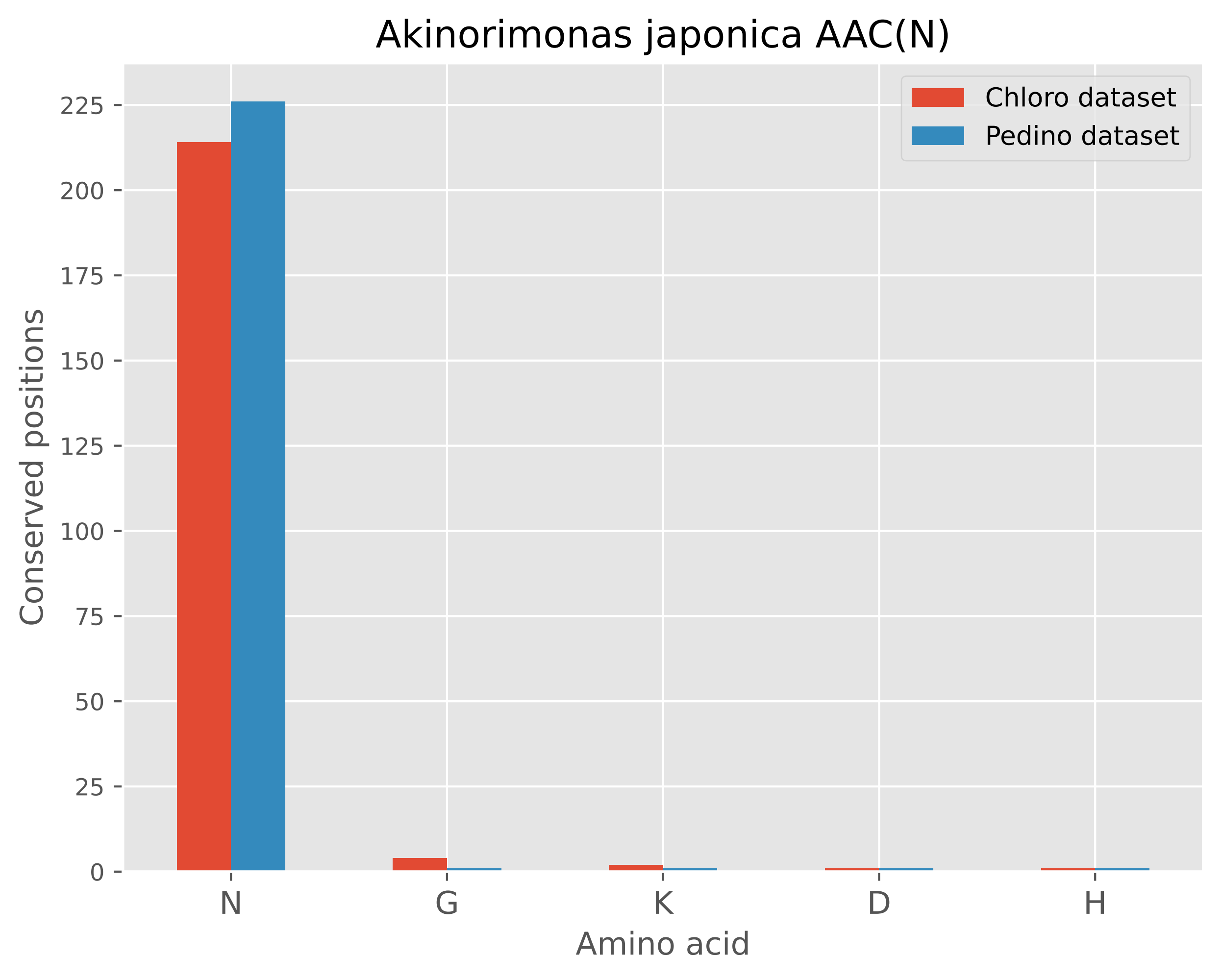
Akinorimonas japonica AAC(N)
Chloro dataset
Pedino dataset
Conserved positions
0
25
50
75
100
125
150
175
200
225
N
G
K
D
H
Amino acid

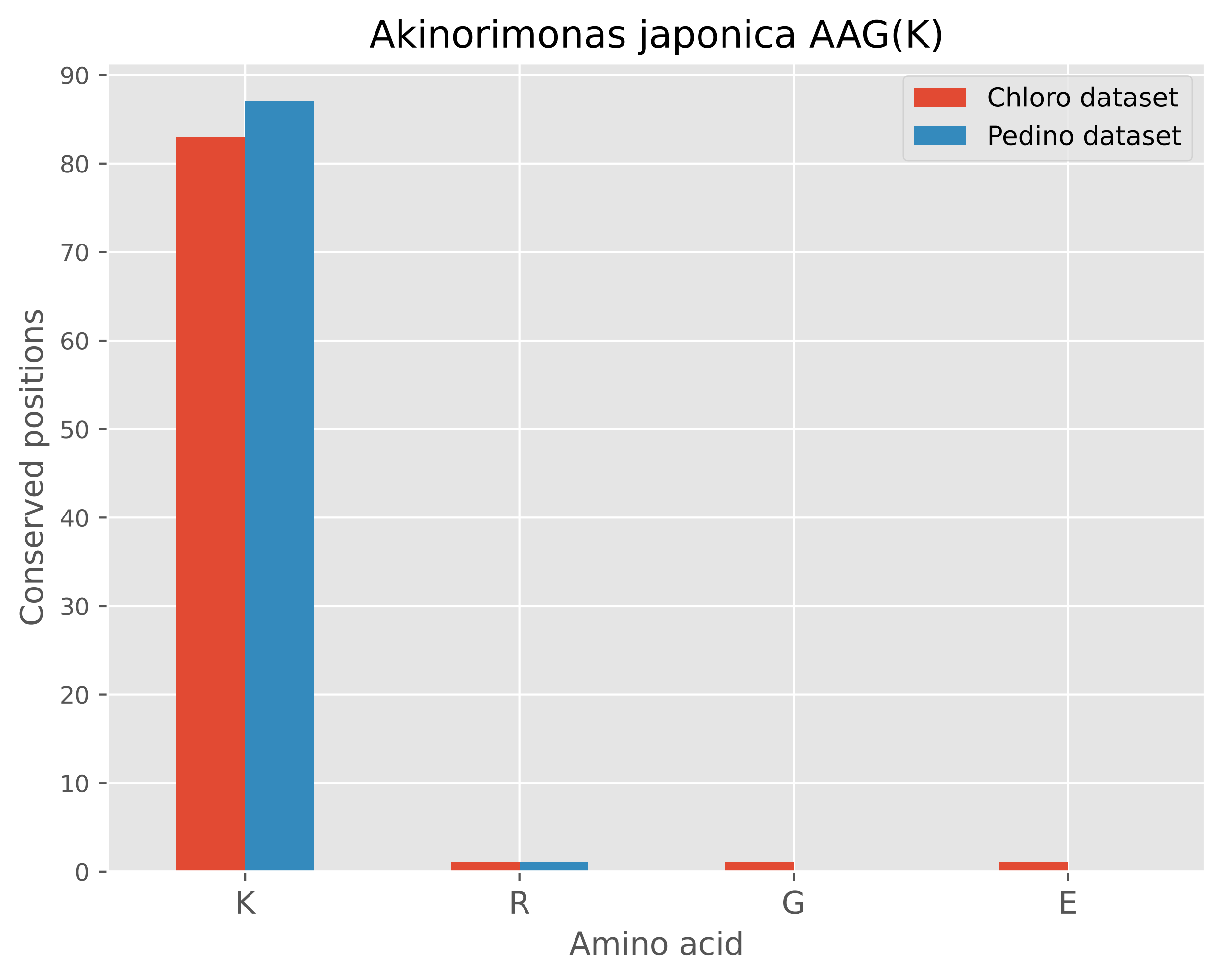
Akinorimonas japonica AAG(K)
Chloro dataset
Pedino dataset
Conserved positions
0
10
20
30
40
50
60
70
80
90
K
R
G
E
Amino acid

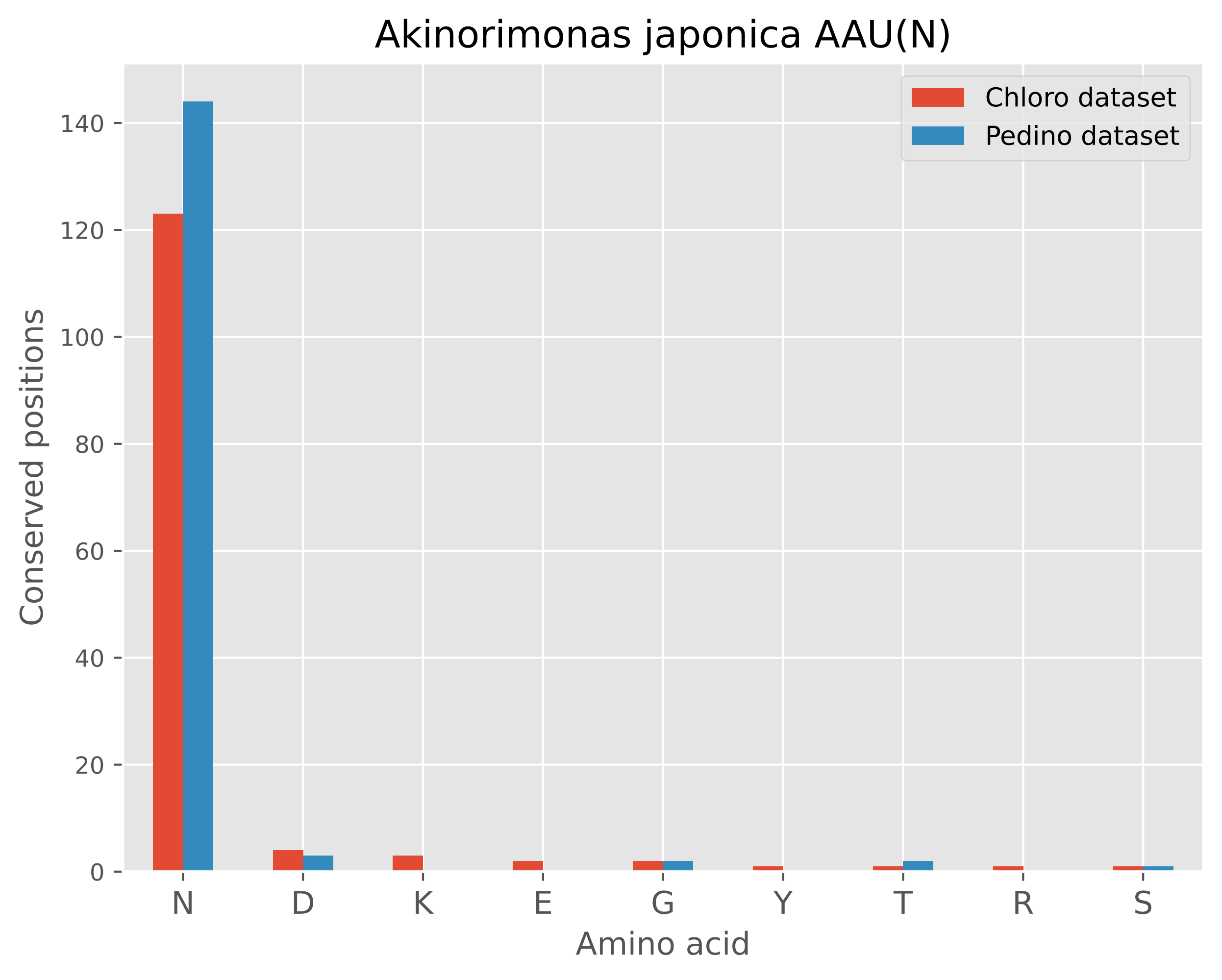
Akinorimonas japonica AAU(N)
Chloro dataset
Pedino dataset
Conserved positions
0
20
40
60
80
100
120
140
N
D
K
E
G
Y
T
R
S
Amino acid

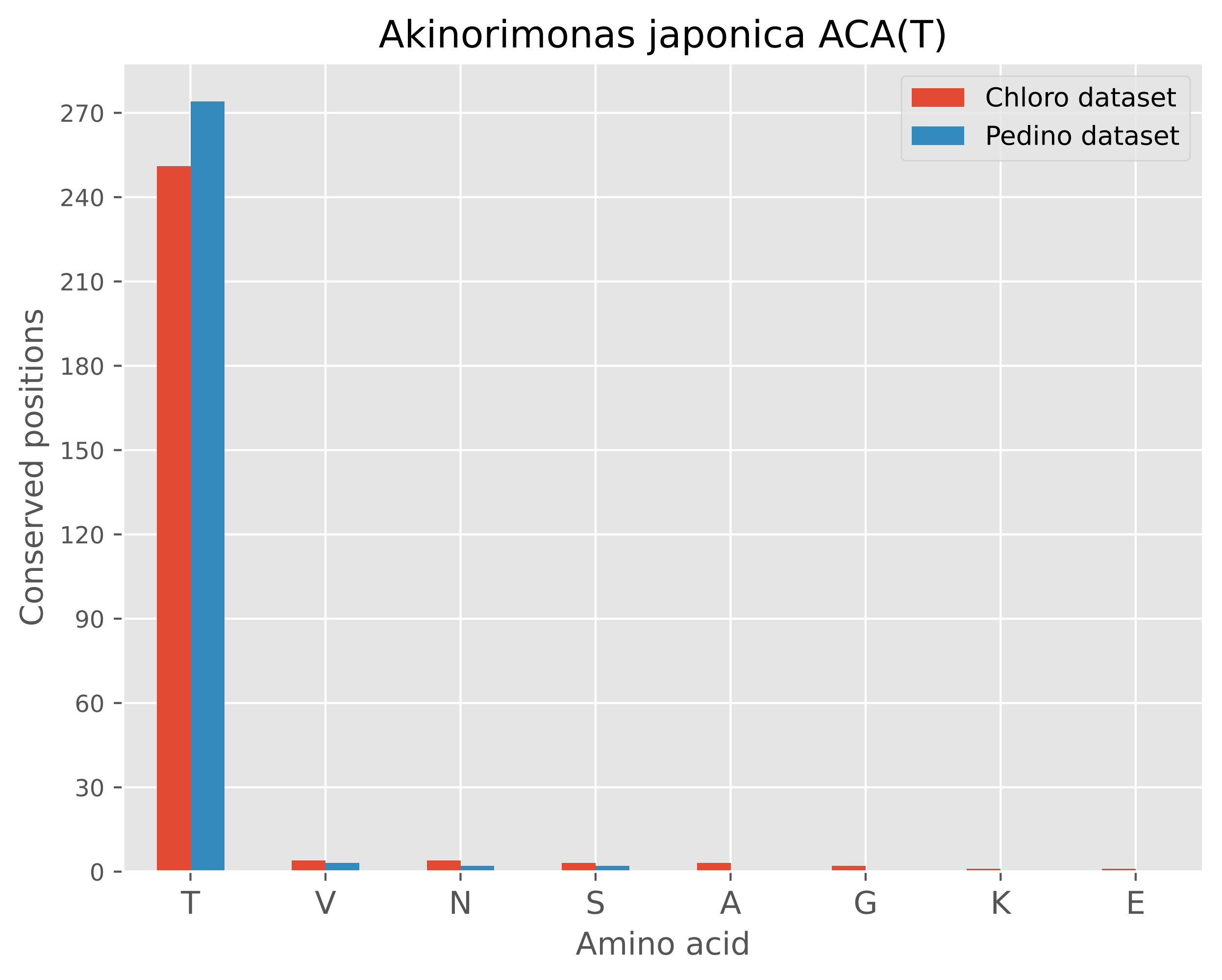
Akinorimonas japonica ACA(T)
Chloro dataset
Pedino dataset
Conserved positions
0
30
60
90
120
150
180
210
240
270
T
V
N
S
A
G
K
E
Amino acid

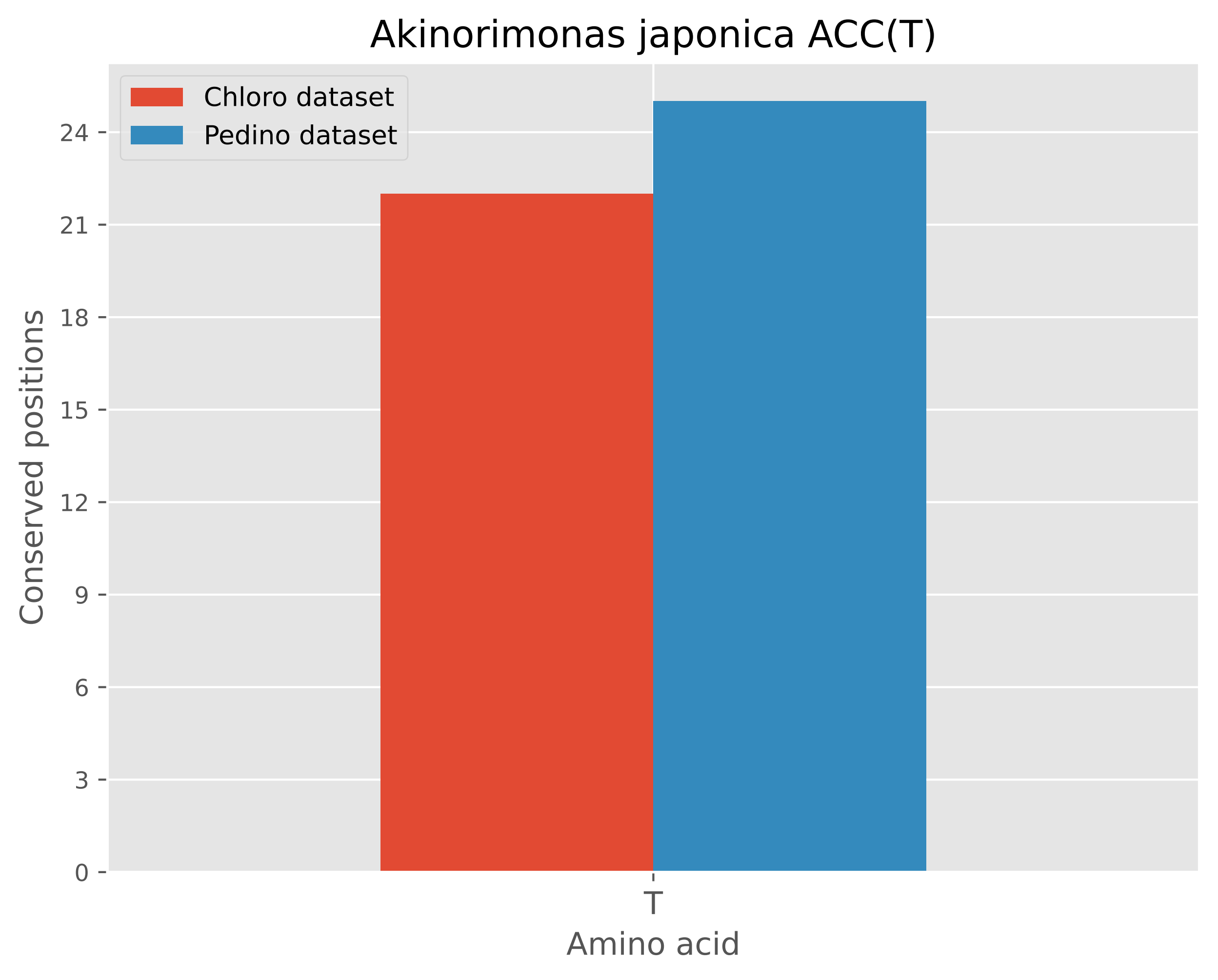
Akinorimonas japonica ACC(T)
Chloro dataset
Pedino dataset
Conserved positions
0
3
6
9
12
15
18
21
24
T
Amino acid

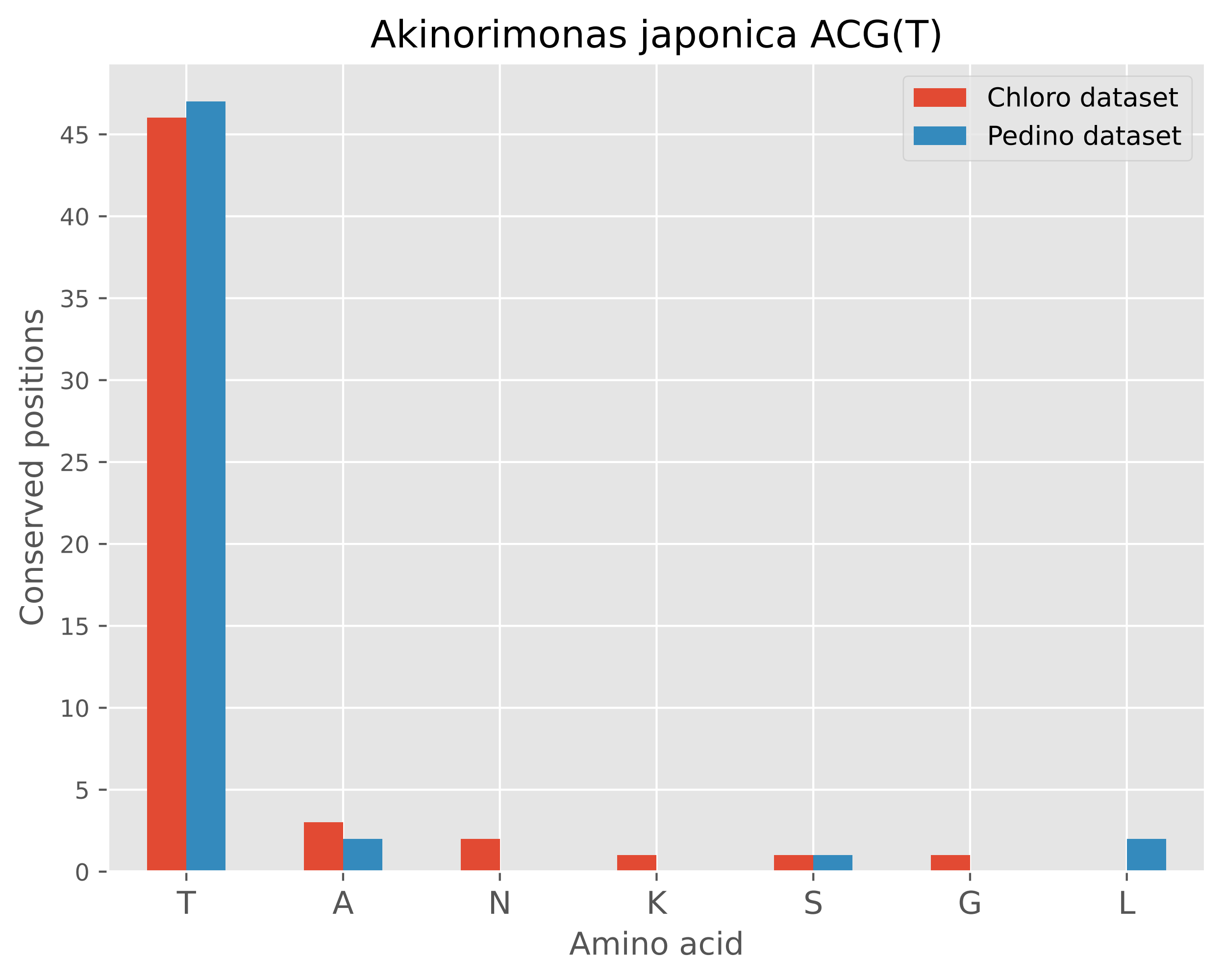

Akinorimonas japonica ACG(T)
Chloro dataset
Pedino dataset
Conserved positions
0
5
10
15
20
25
30
35
40
45
T
A
N
K
S
G
L
Amino acid

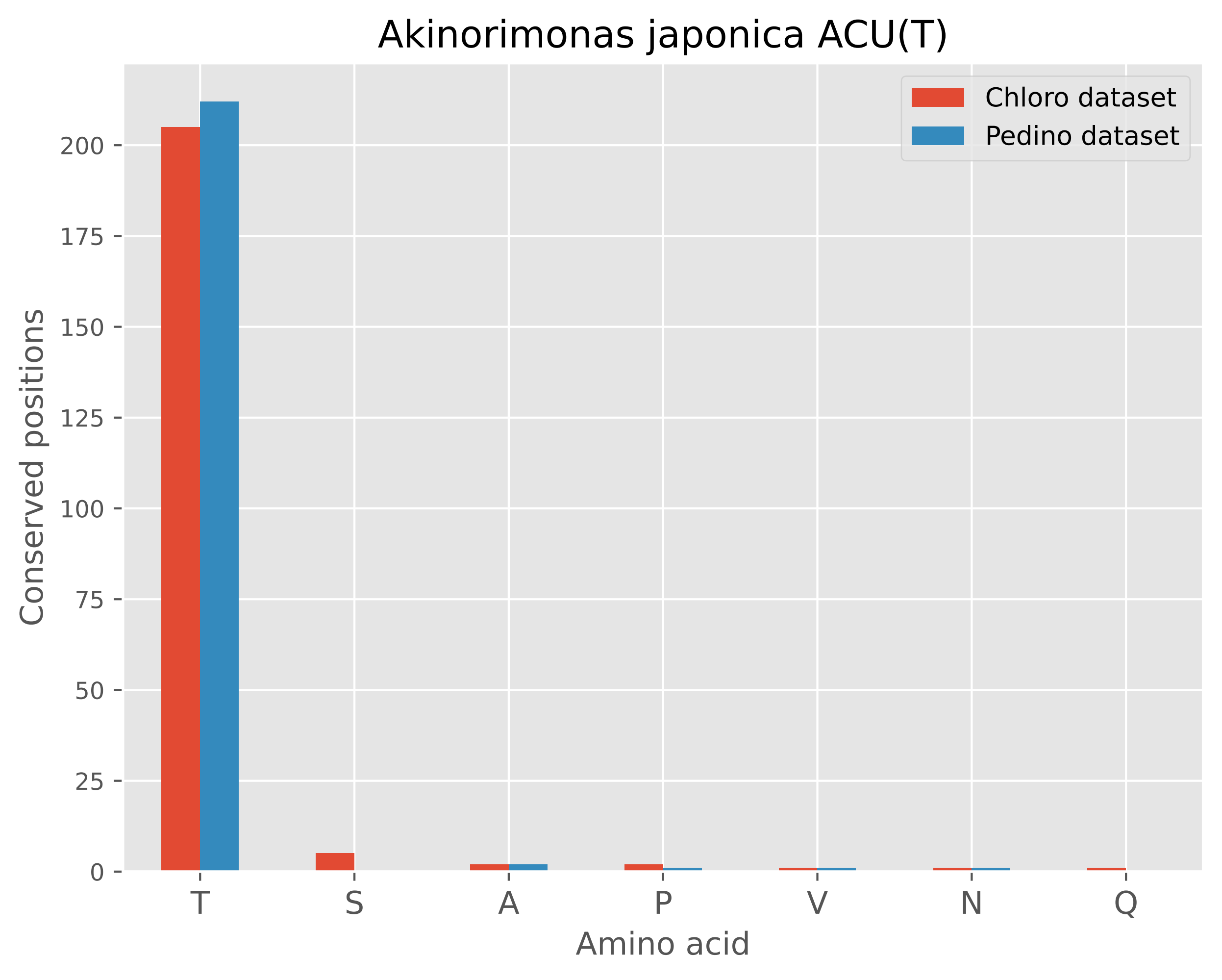

Akinorimonas japonica ACU(T)
Chloro dataset
Pedino dataset
Conserved positions
0
25
50
75
100
125
150
175
200
T
S
A
P
V
N
Q
Amino acid

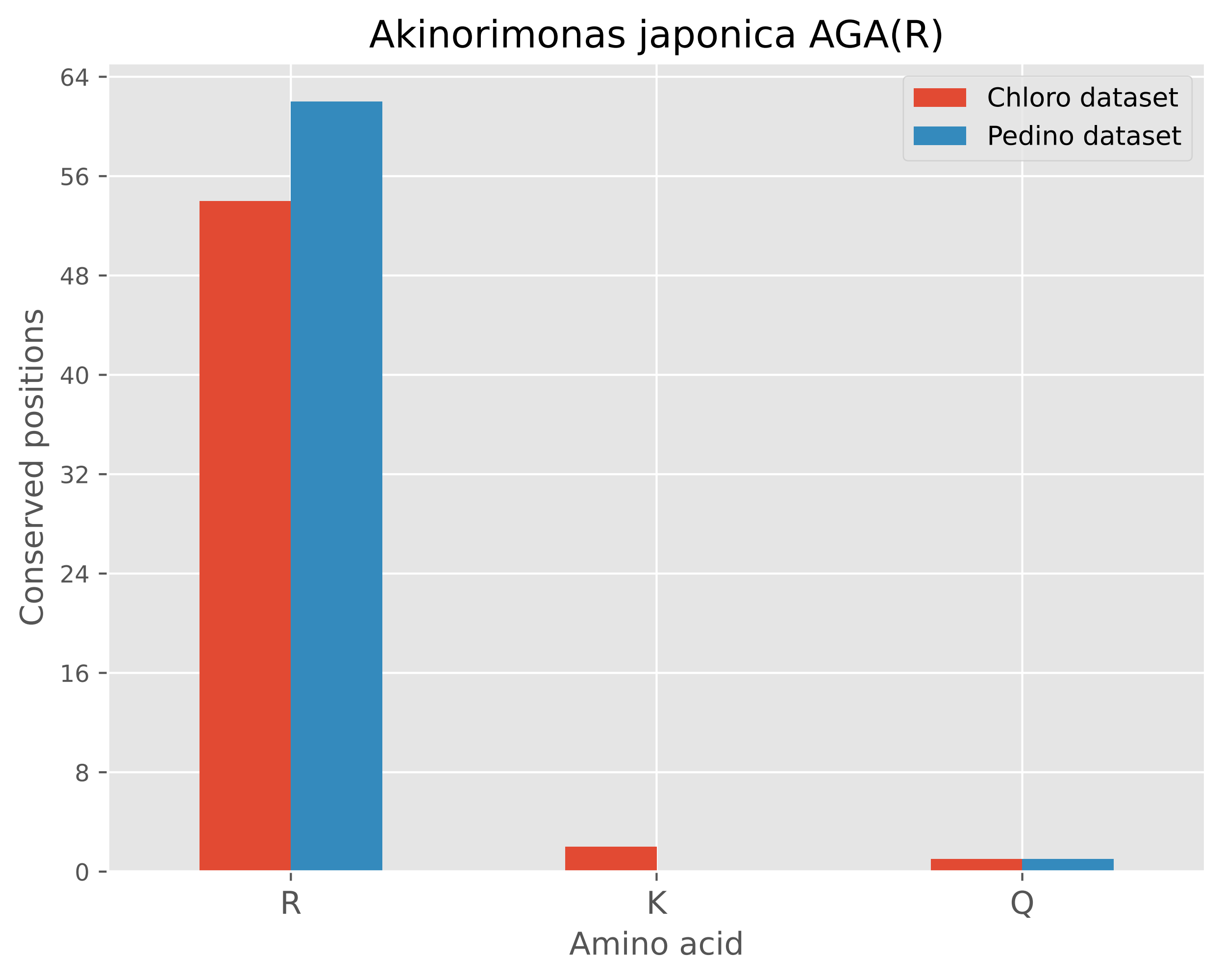
Akinorimonas japonica AGA(R)
Chloro dataset
Pedino dataset
Conserved positions
0
8
16
24
32
40
48
56
64
R
K
Q
Amino acid

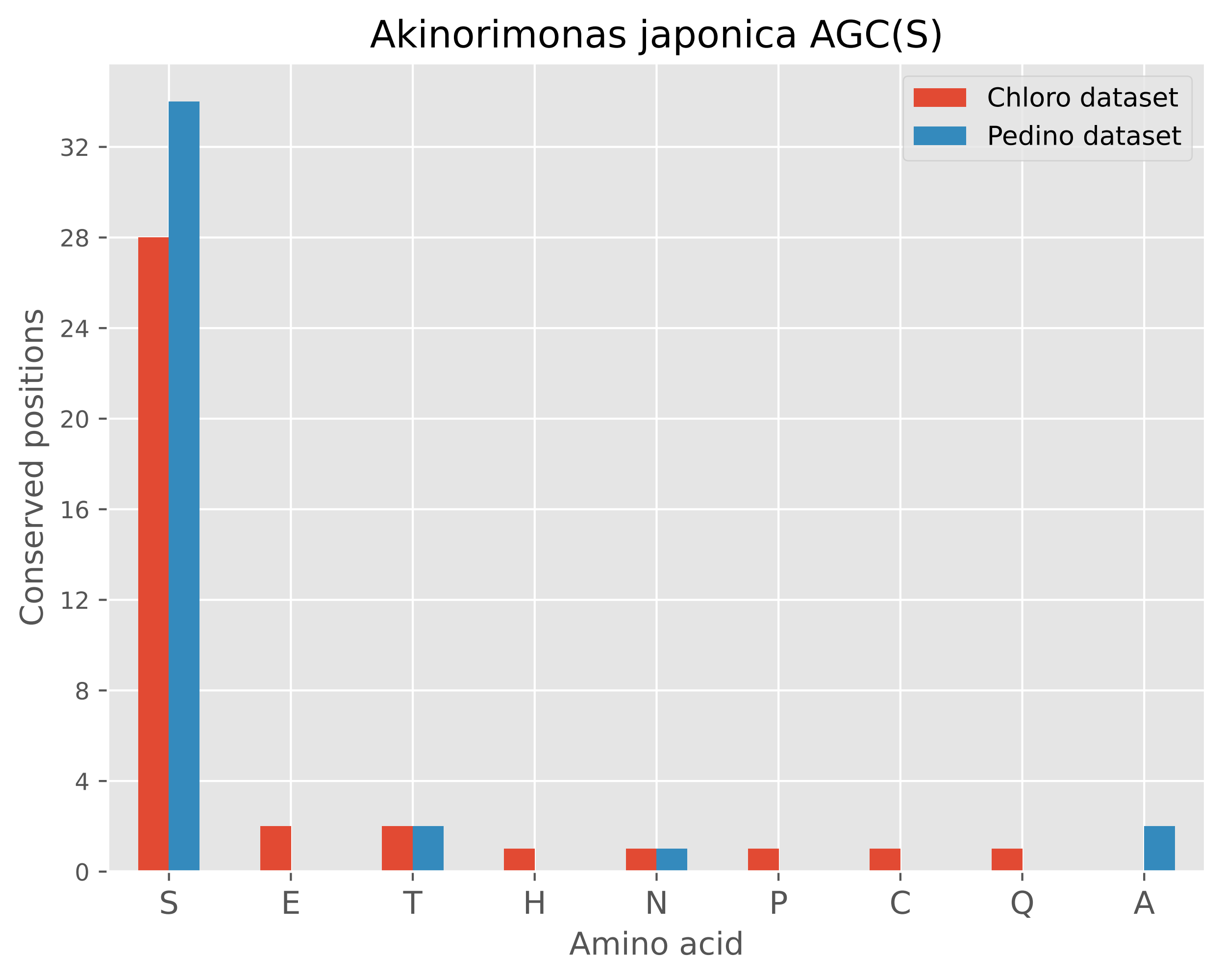

Akinorimonas japonica AGC(S)
Chloro dataset
Pedino dataset
Conserved positions
0
4
8
12
16
20
24
28
32
S
E
T
H
N
P
C
Q
A
Amino acid

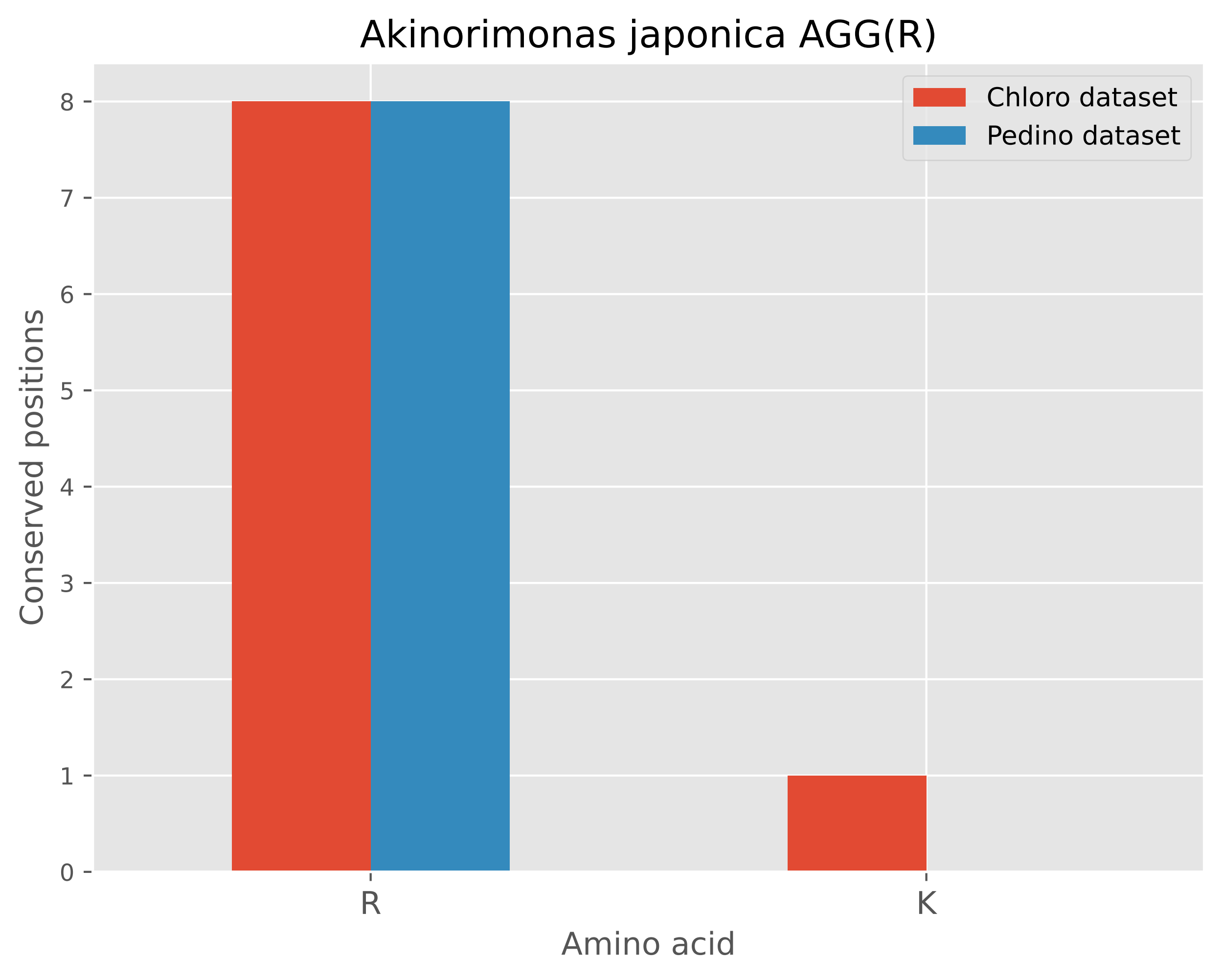

Akinorimonas japonica AGG(R)
Chloro dataset
Pedino dataset
Conserved positions
0
1
2
3
4
5
6
7
8
R
K
Amino acid

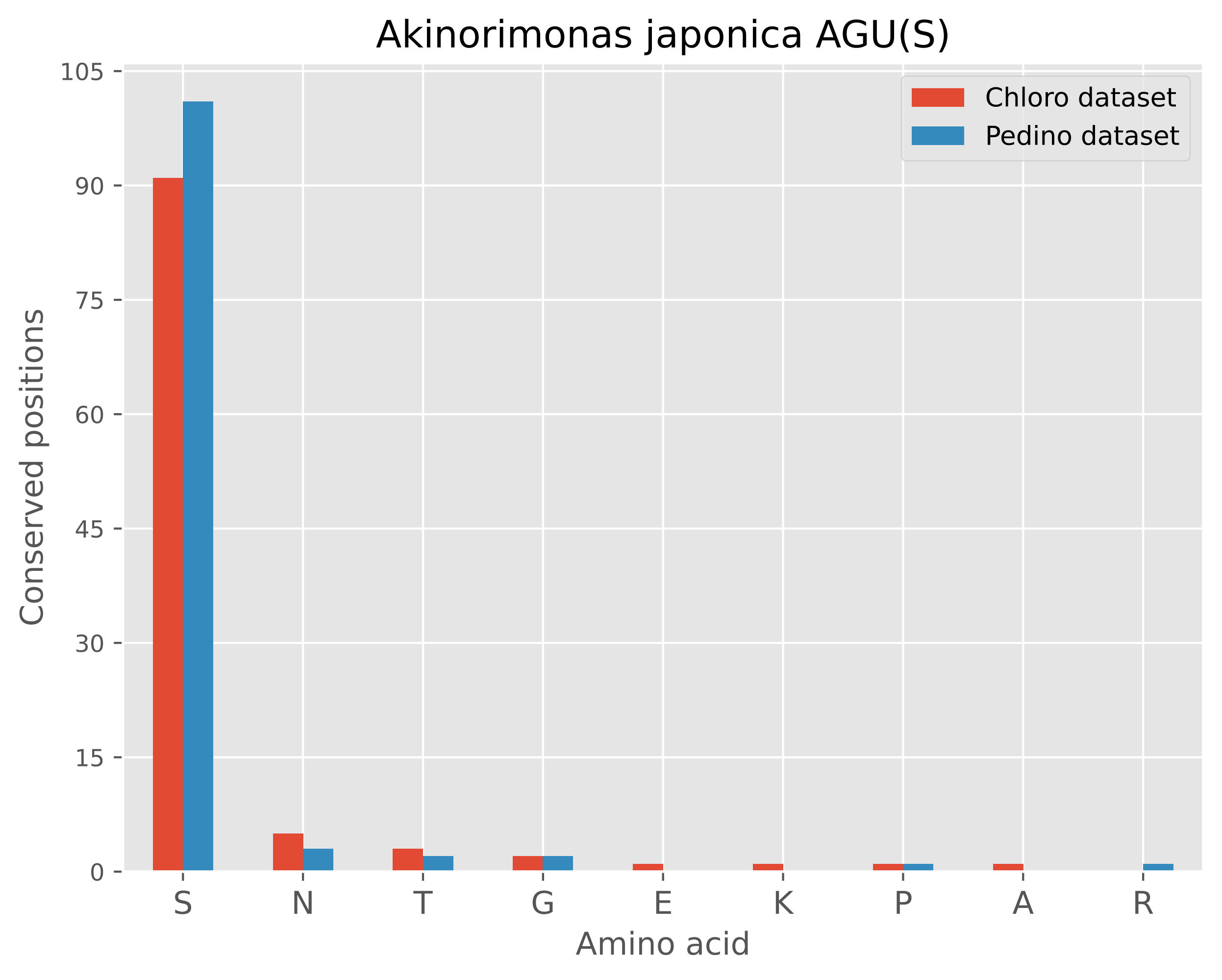
Akinorimonas japonica AGU(S)
Chloro dataset
Pedino dataset
Conserved positions
0
15
30
45
60
75
90
105
S
N
T
G
E
K
P
A
R
Amino acid

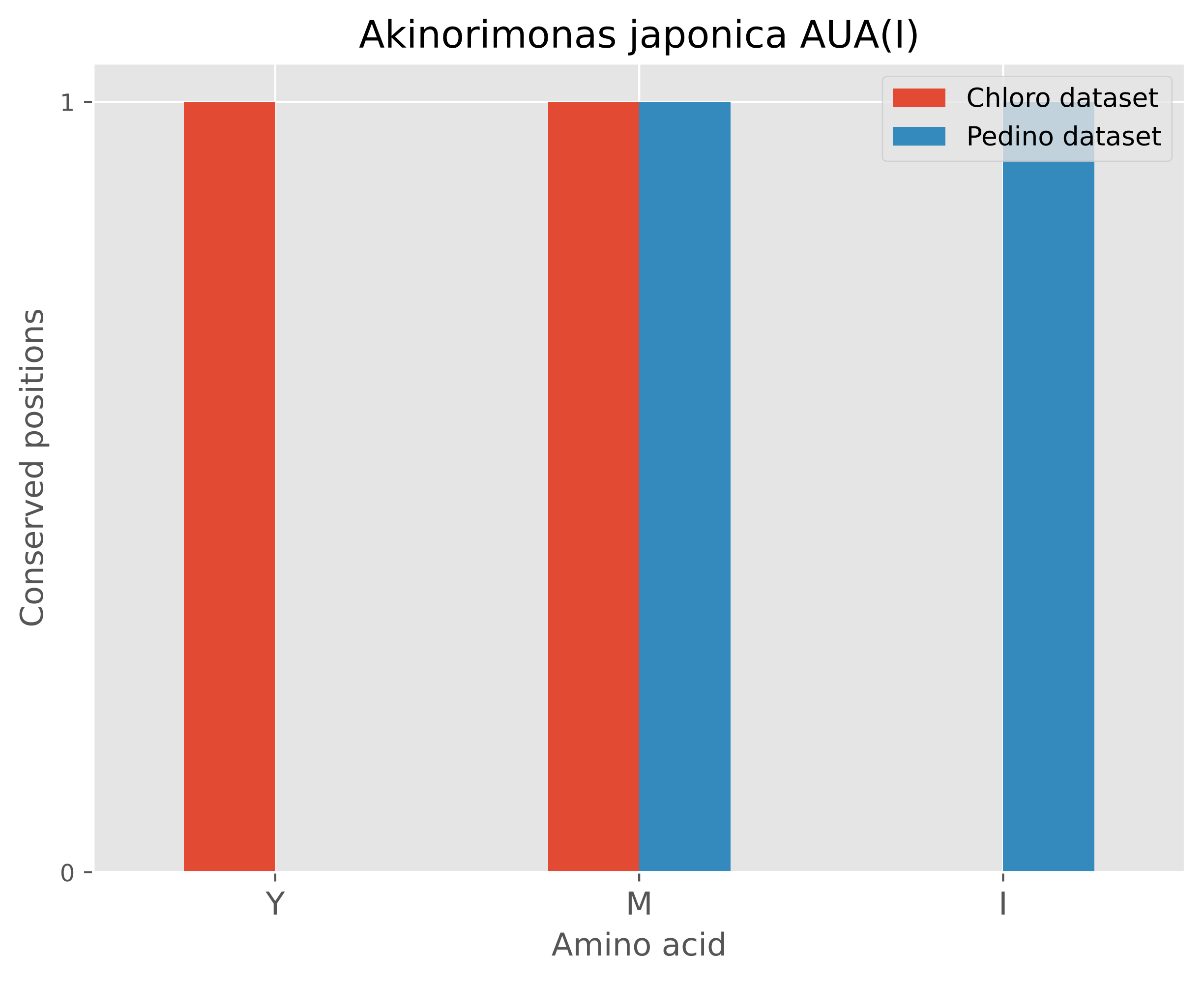

Akinorimonas japonica AUA(I)
Chloro dataset
Pedino dataset
Conserved positions
1
0
Y
M
I
Amino acid

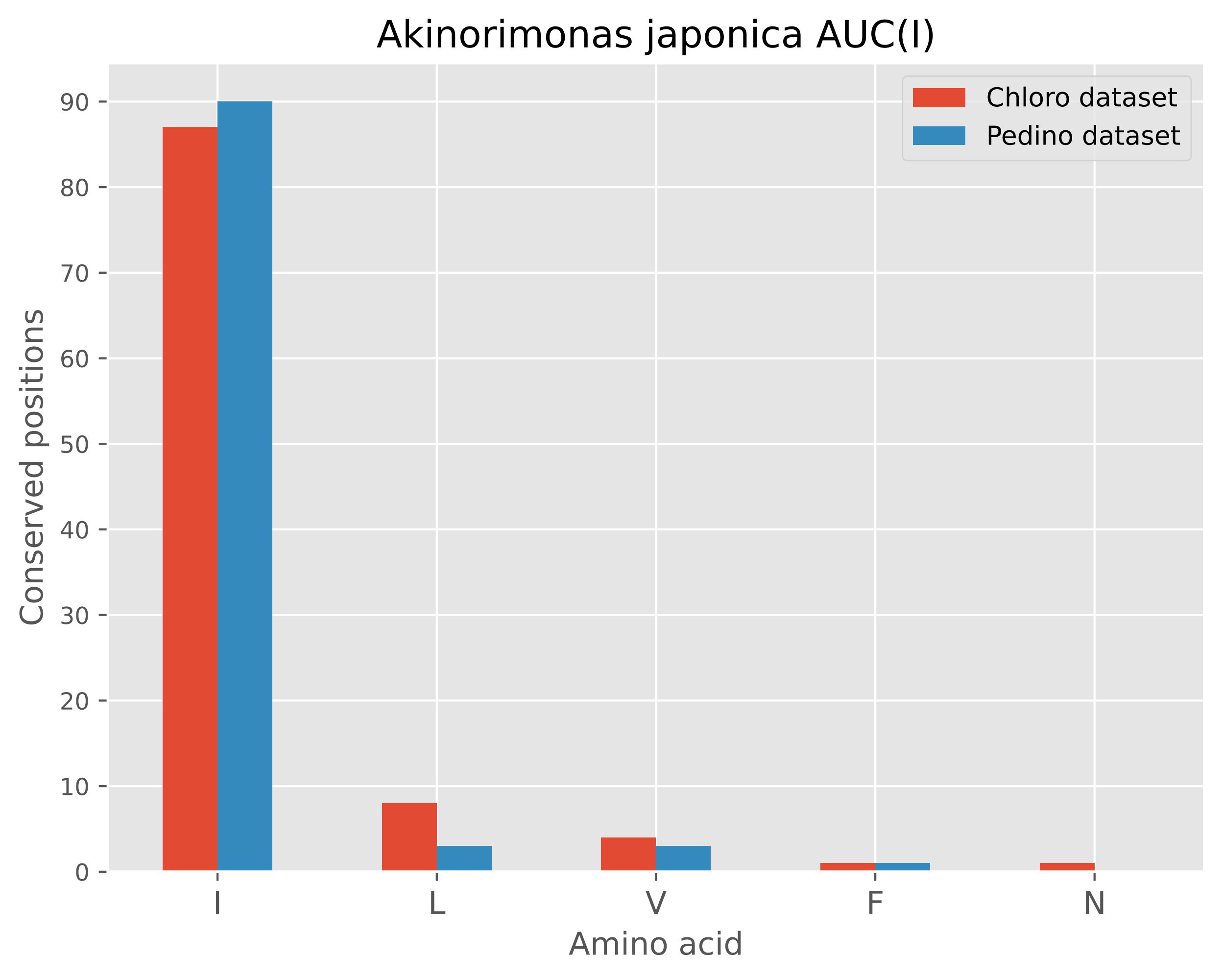
Akinorimonas japonica AUC(I)
Chloro dataset
Pedino dataset
Conserved positions
Amino acid
0
10
20
30
40
50
60
70
80
90
I
L
V
F
N

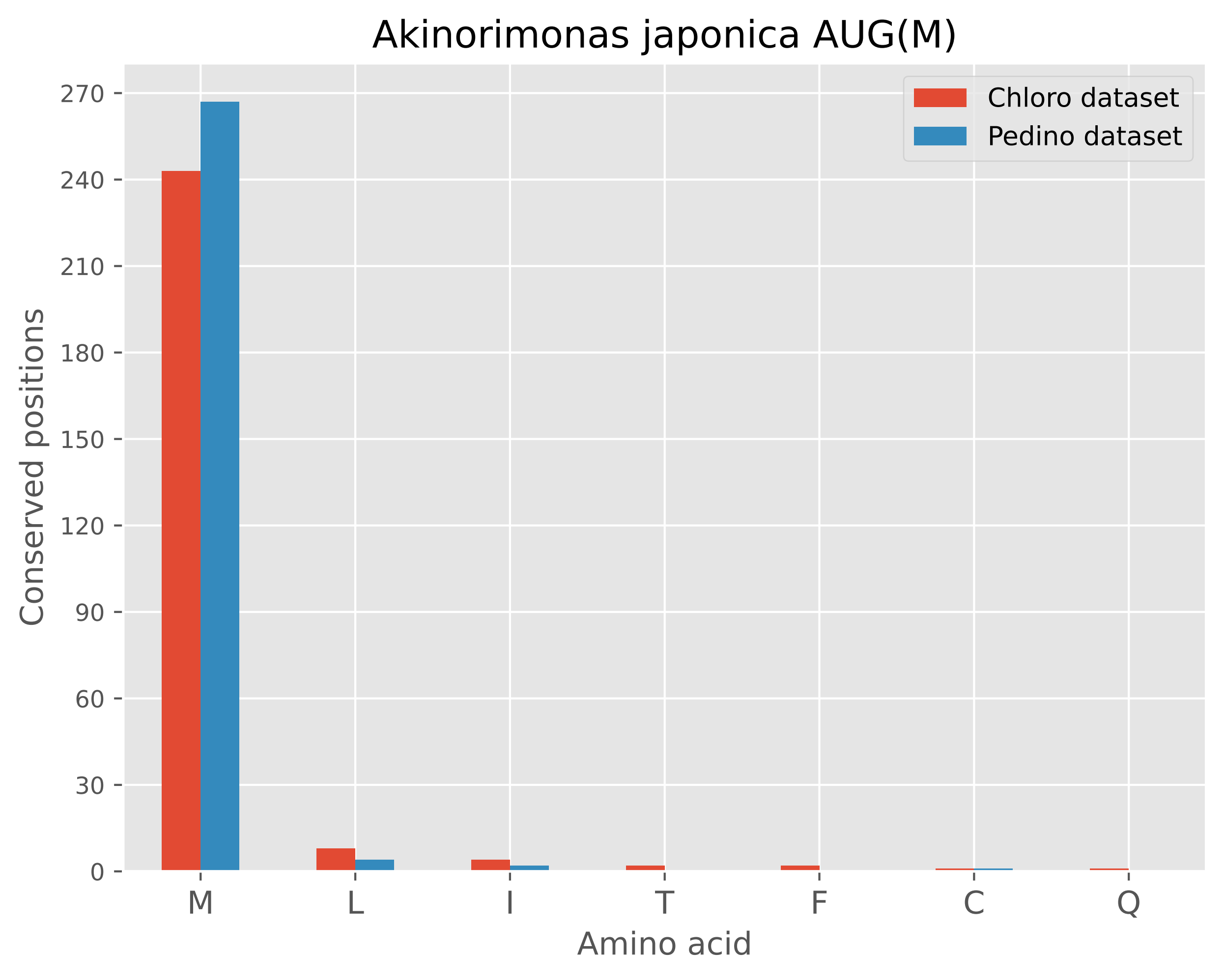

Akinorimonas japonica AUG(M)
Chloro dataset
Pedino dataset
Conserved positions
0
30
60
90
120
150
180
210
240
270
M
L
I
T
F
C
Q
Amino acid

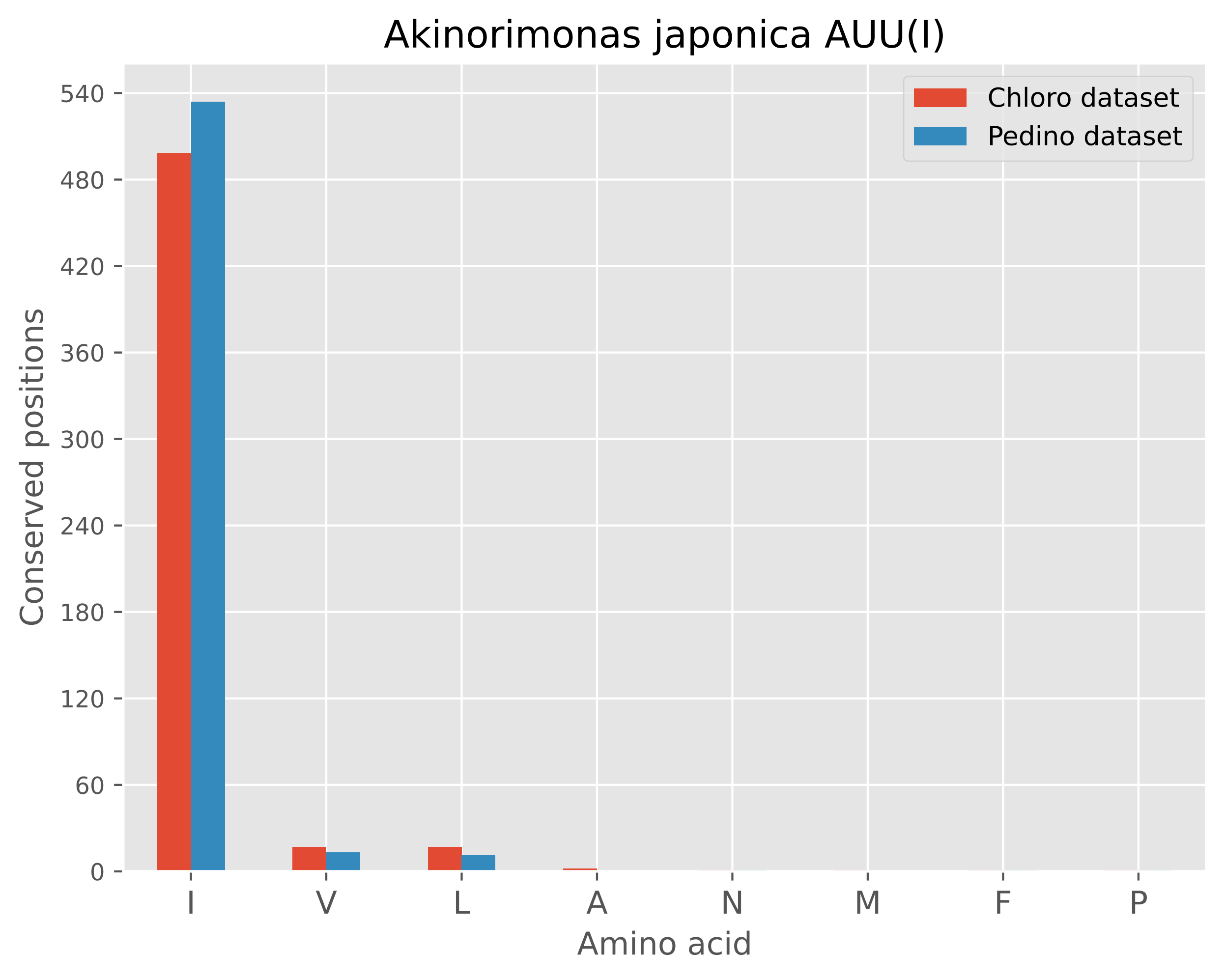
Akinorimonas japonica AUU(I)
Chloro dataset
Pedino dataset
Conserved positions
540
480
420
360
300
240
180
120
60
0
I
V
L
A
N
M
F
P
Amino acid

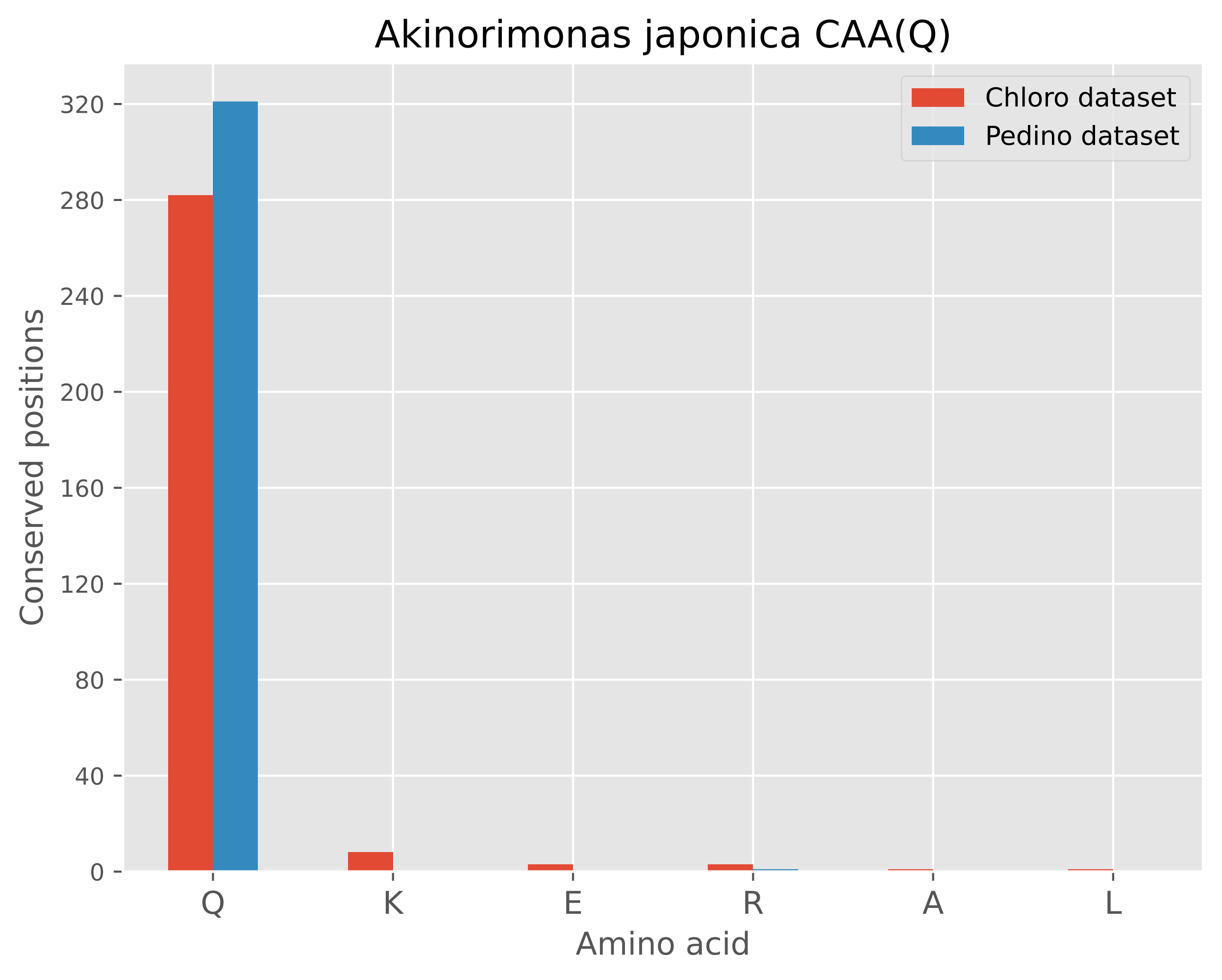
Akinorimonas japonica CAA(Q)
Chloro dataset
Pedino dataset
Conserved positions
0
40
80
120
160
200
240
280
320
Q
K
E
R
A
L
Amino acid

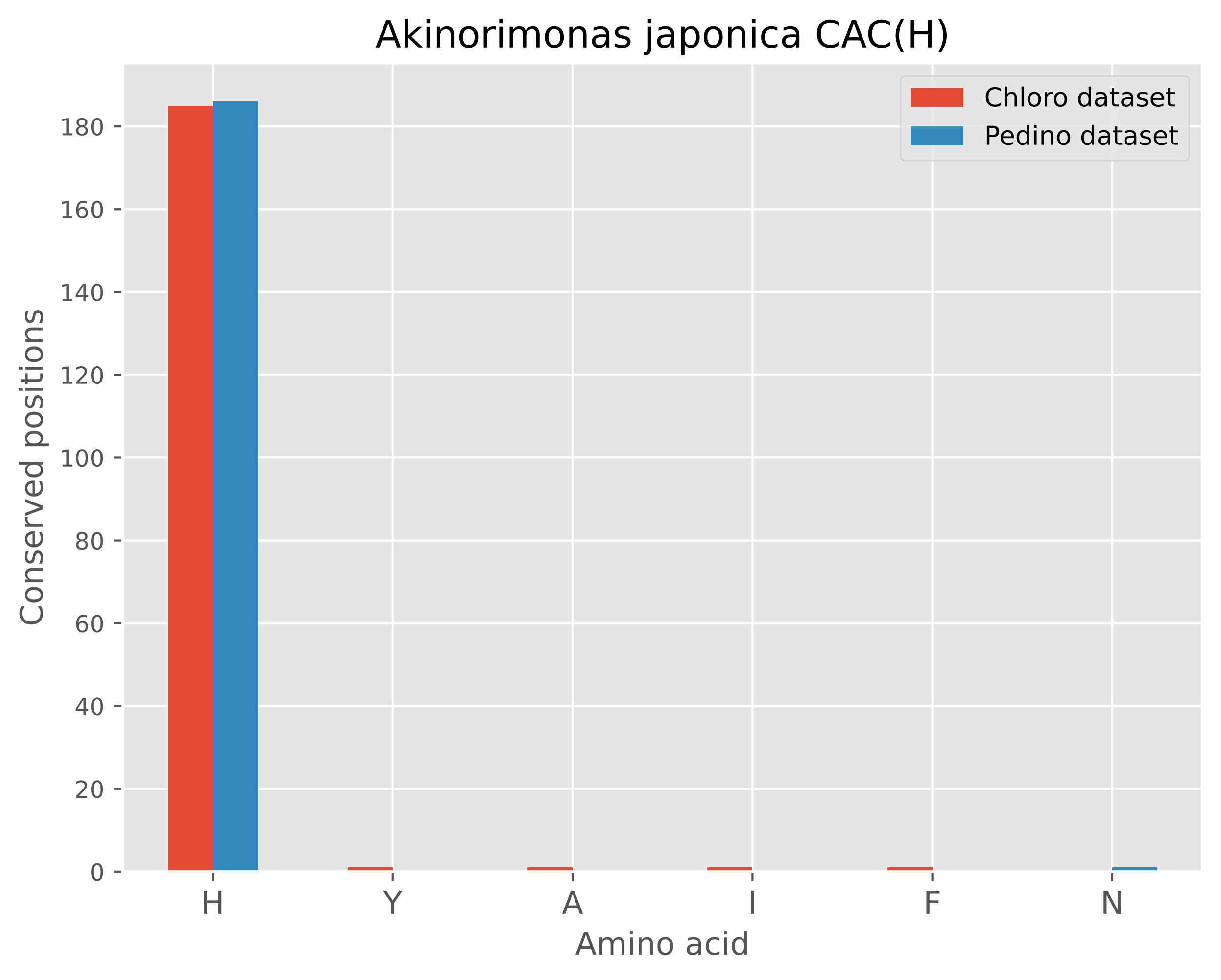

Akinorimonas japonica CAC(H)
Chloro dataset
Pedino dataset
Conserved positions
0
20
40
60
80
100
120
140
160
180
H
Y
A
I
F
N
Amino acid

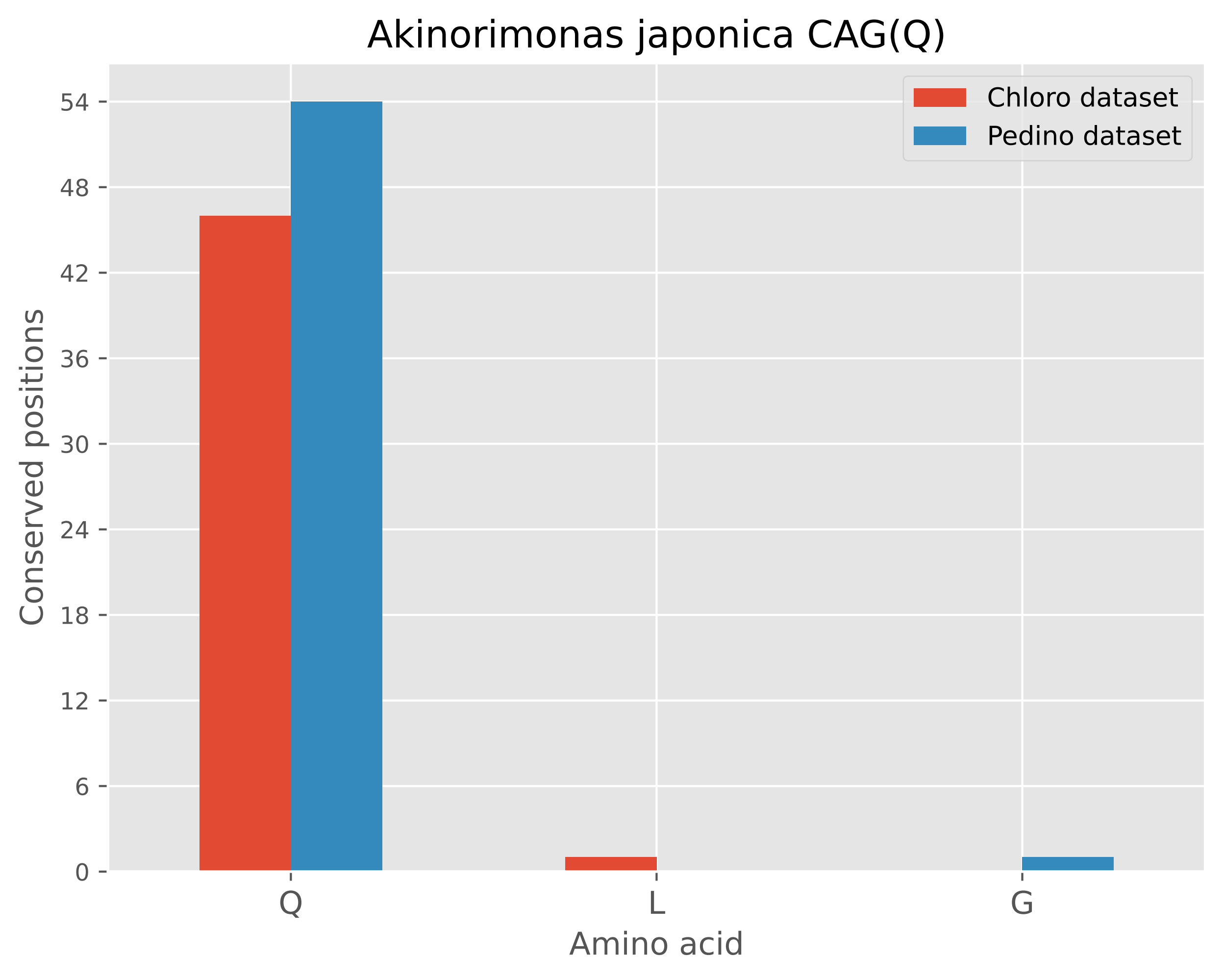
Akinorimonas japonica CAG(Q)
Chloro dataset
Pedino dataset
Conserved positions
0
6
12
18
24
30
36
42
48
54
Q
L
G
Amino acid

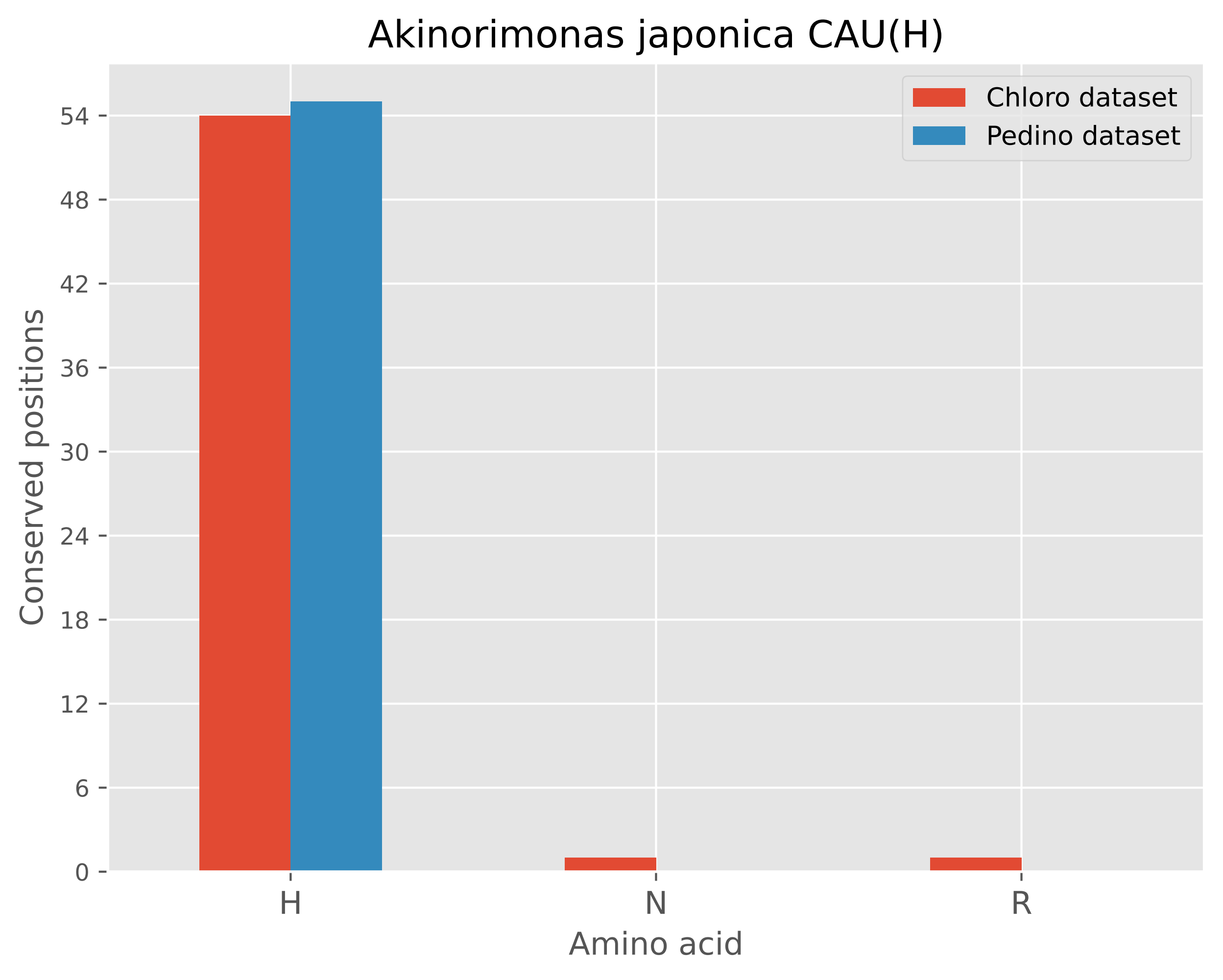
Akinorimonas japonica CAU(H)
Chloro dataset
Pedino dataset
Conserved positions
0
6
12
18
24
30
36
42
48
54
H
N
R
Amino acid

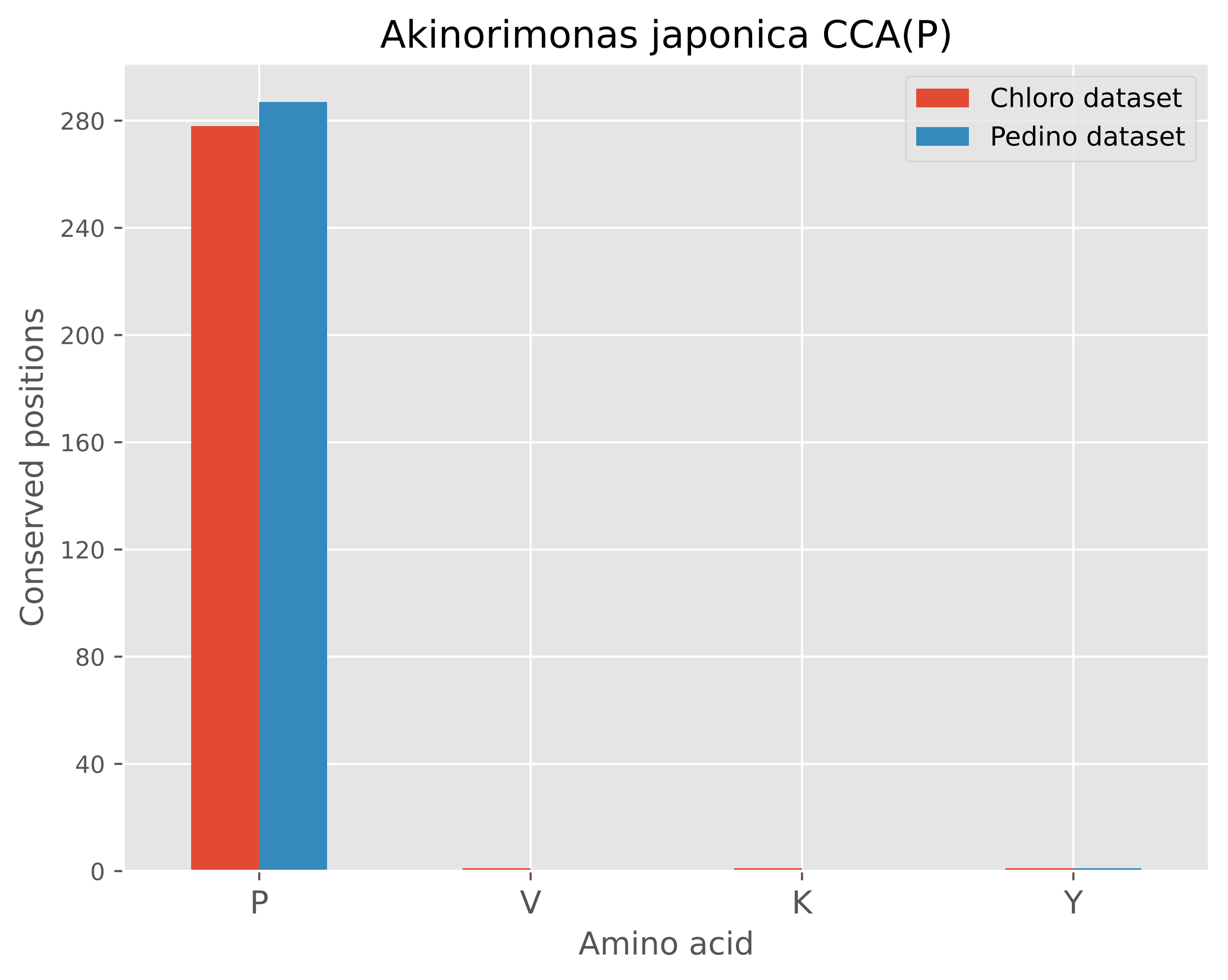

Akinorimonas japonica CCA(P)
Chloro dataset
Pedino dataset
Conserved positions
0
40
80
120
160
200
240
280
P
V
K
Y
Amino acid

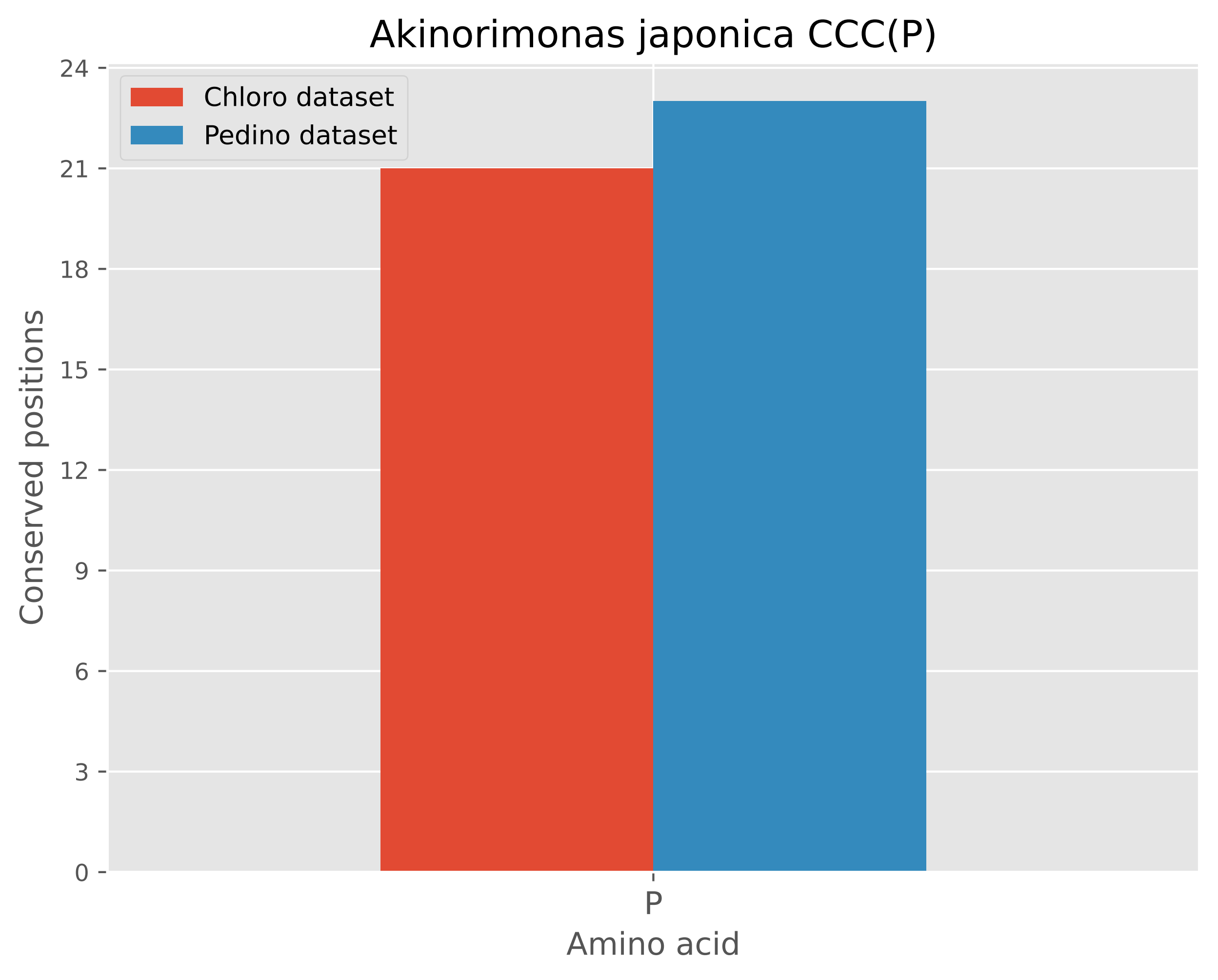

Akinorimonas japonica CCC(P)
Chloro dataset
Pedino dataset
Conserved positions
0
3
6
9
12
15
18
21
24
P
Amino acid

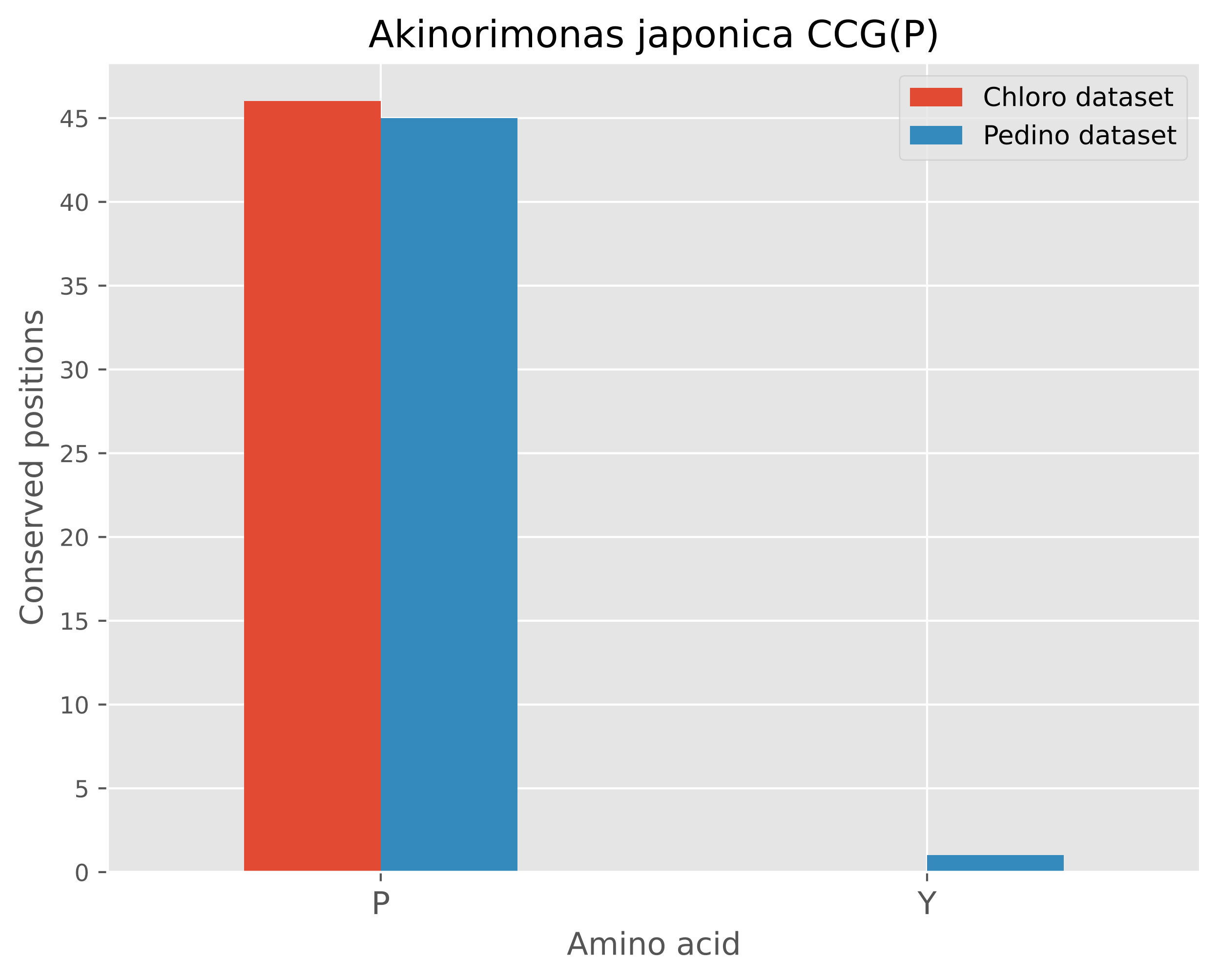

Akinorimonas japonica CCG(P)
Chloro dataset
Pedino dataset
Conserved positions
0
5
10
15
20
25
30
35
40
45
P
Y
Amino acid

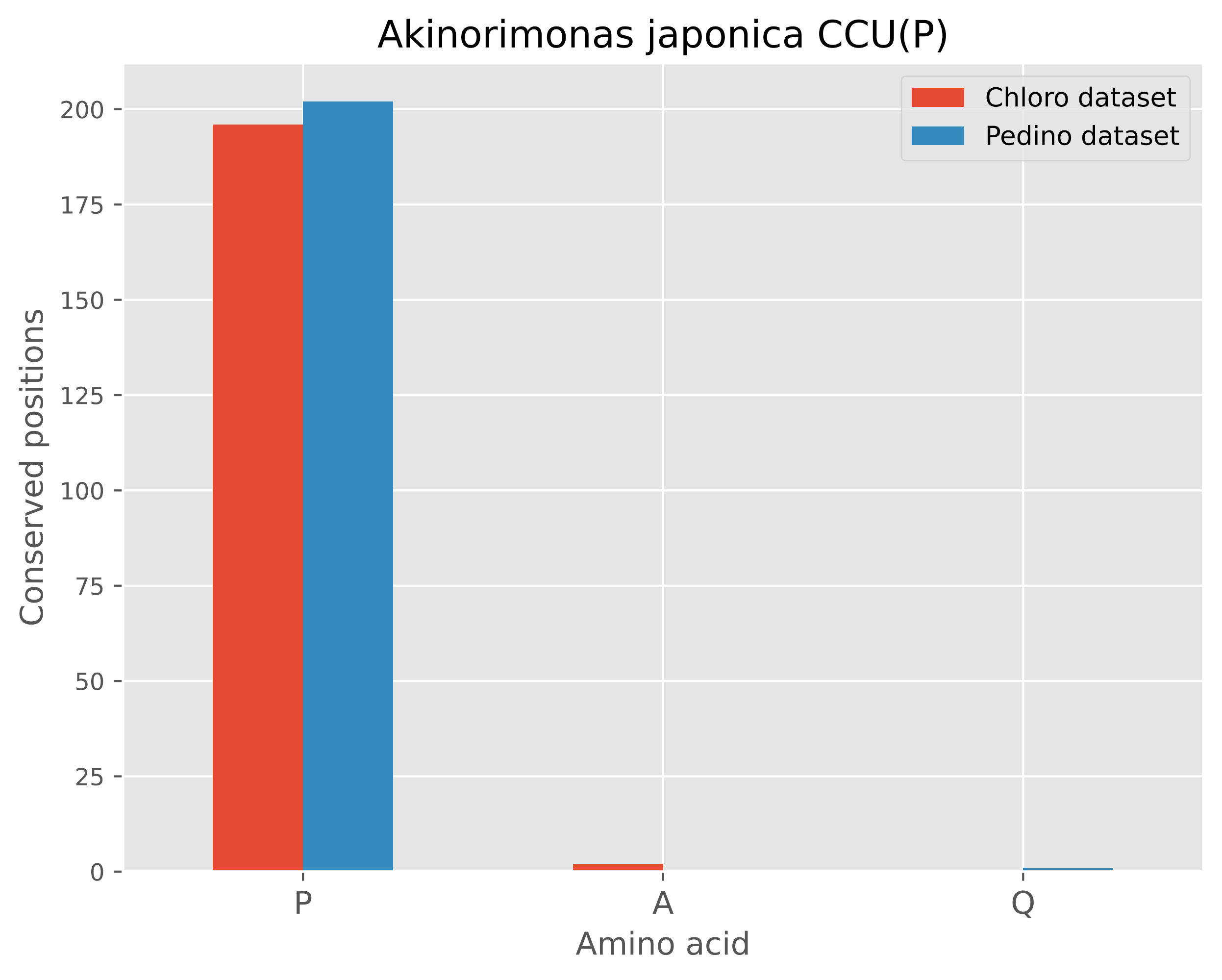
Akinorimonas japonica CCU(P)
Chloro dataset
Pedino dataset
Conserved positions
0
25
50
75
100
125
150
175
200
P
A
Q
Amino acid

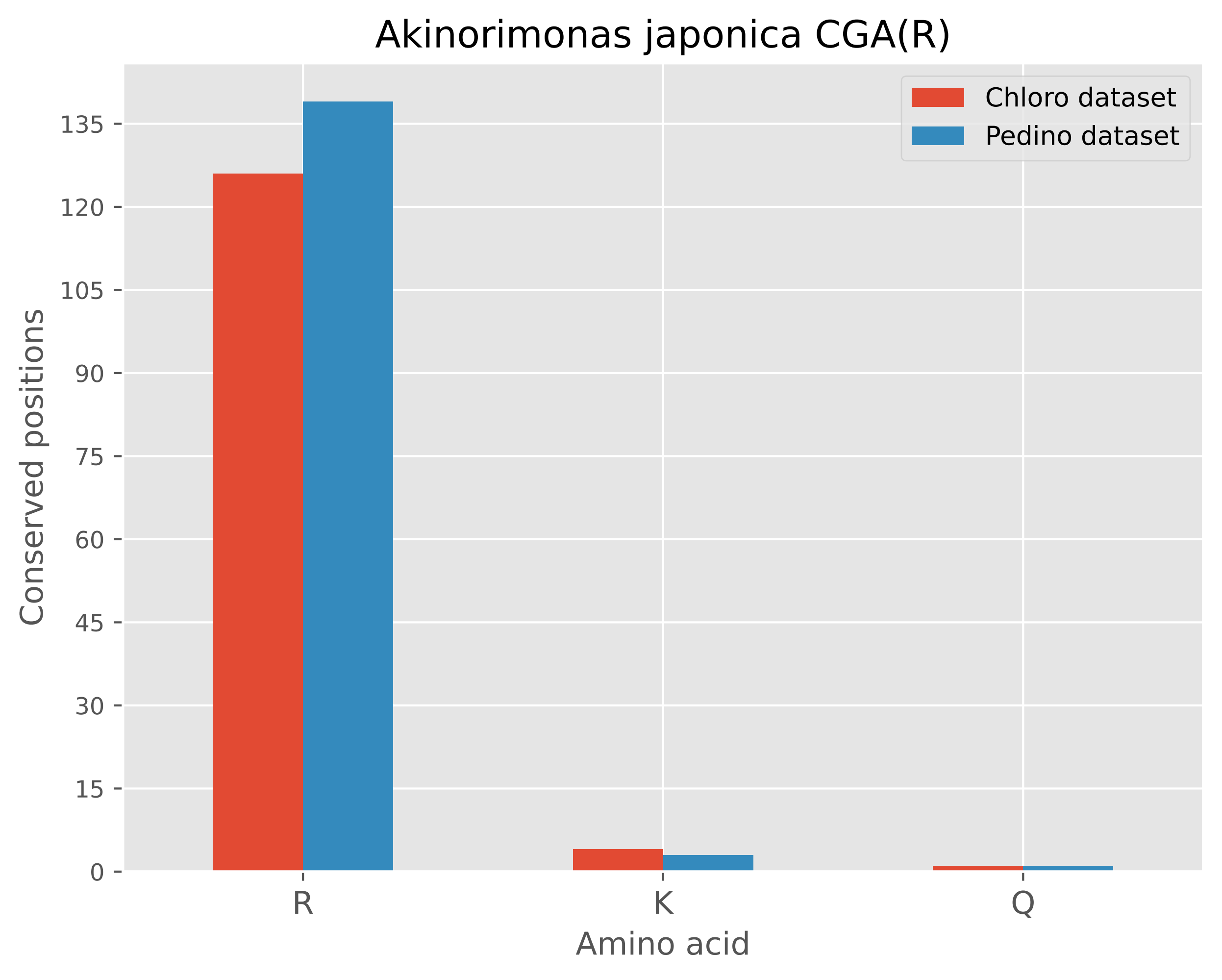
Akinorimonas japonica CGA(R)
Chloro dataset
Pedino dataset
Conserved positions
0
15
30
45
60
75
90
105
120
135
R
K
Q
Amino acid

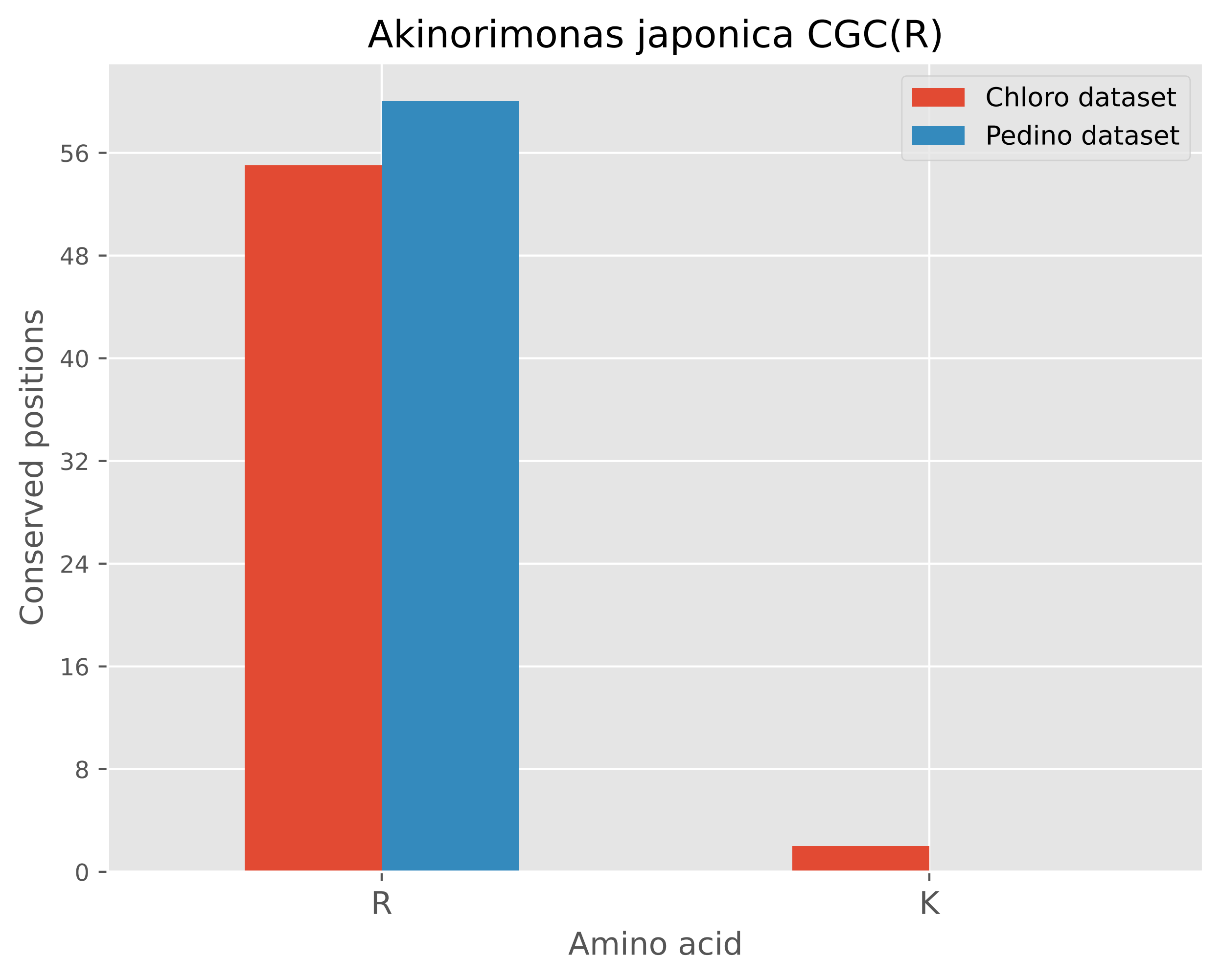

Akinorimonas japonica CGC(R)
Chloro dataset
Pedino dataset
Conserved positions
0
8
16
24
32
40
48
56
R
K
Amino acid

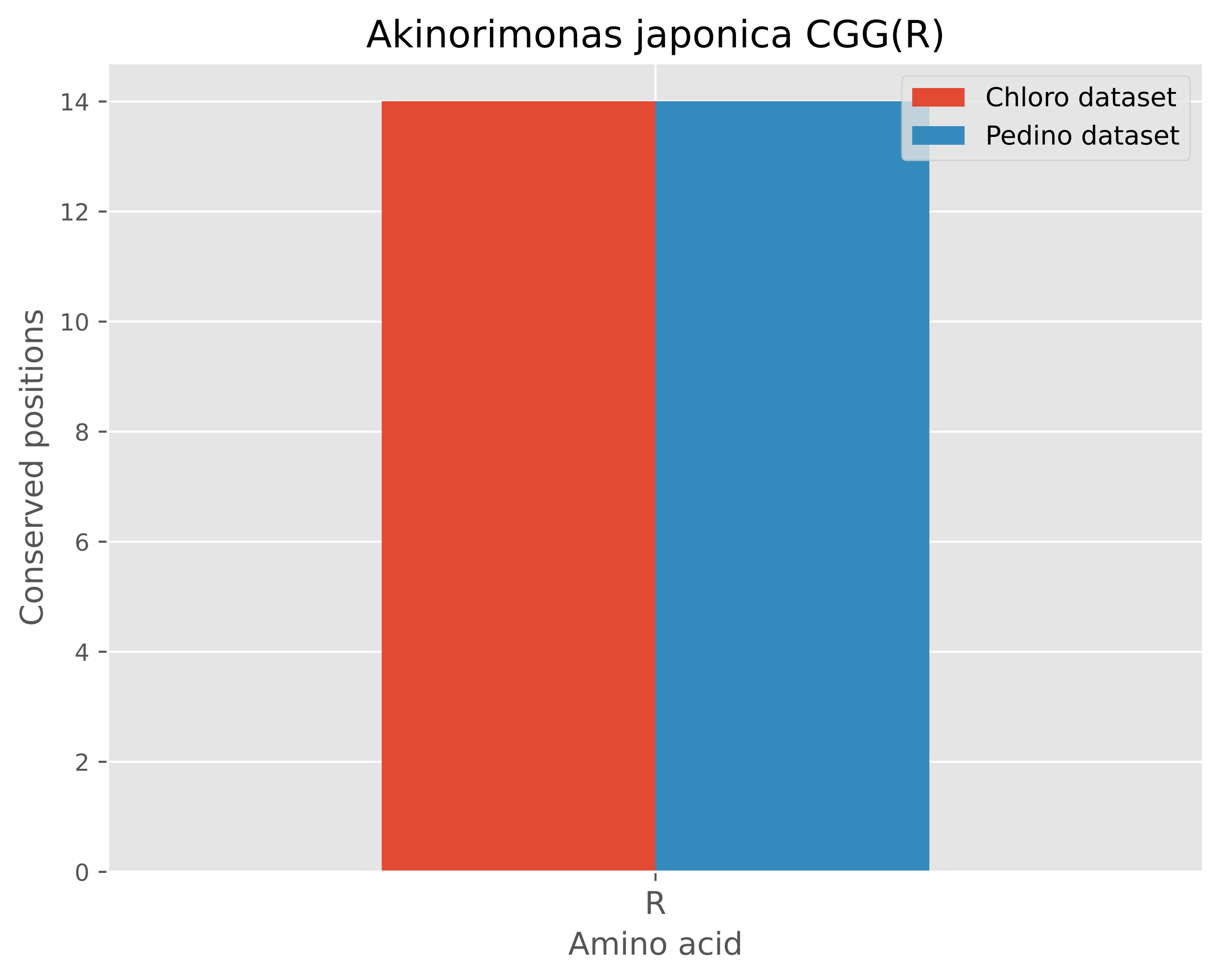

Akinorimonas japonica CGG(R)
Chloro dataset
Pedino dataset
Conserved positions
0
2
4
6
8
10
12
14
R
Amino acid

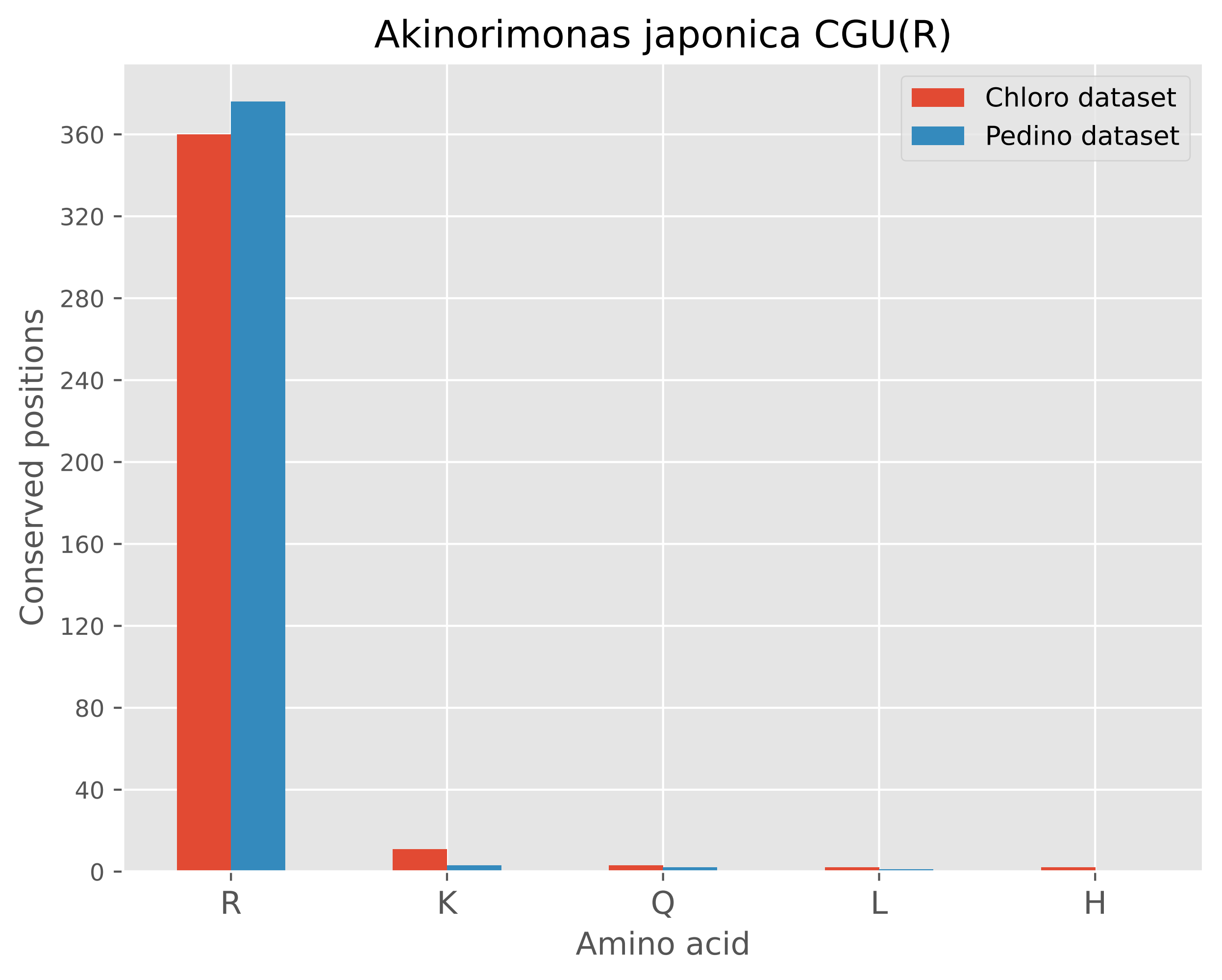

Akinorimonas japonica CGU(R)
Chloro dataset
Pedino dataset
Conserved positions
0
40
80
120
160
200
240
280
320
360
R
K
Q
L
H
Amino acid

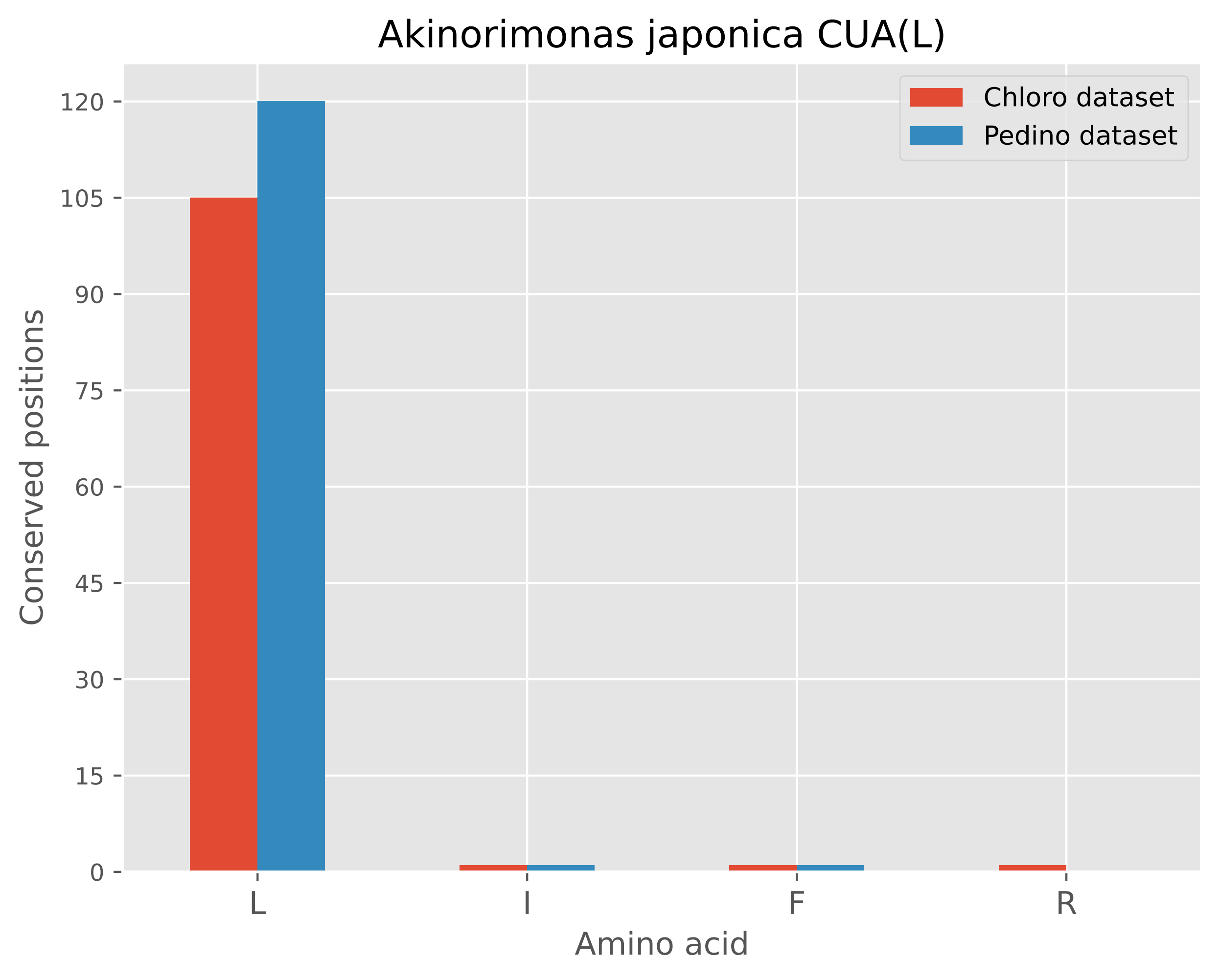
Akinorimonas japonica CUA(L)
Chloro dataset
Pedino dataset
Conserved positions
120
105
90
75
60
45
30
15
0
L
I
F
R
Amino acid

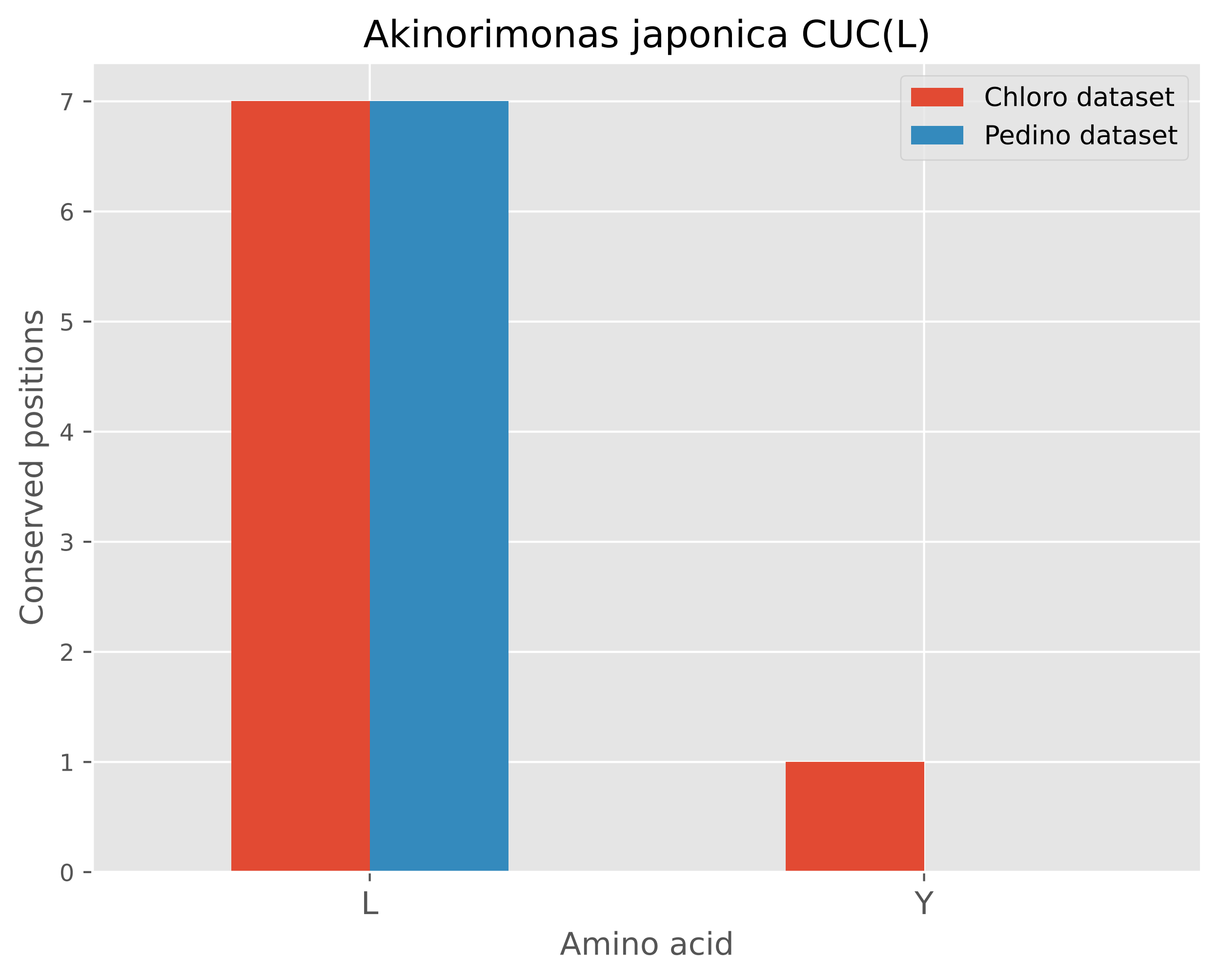
Akinorimonas japonica CUC(L)
Chloro dataset
Pedino dataset
Conserved positions
0
1
2
3
4
5
6
7
L
Y
Amino acid

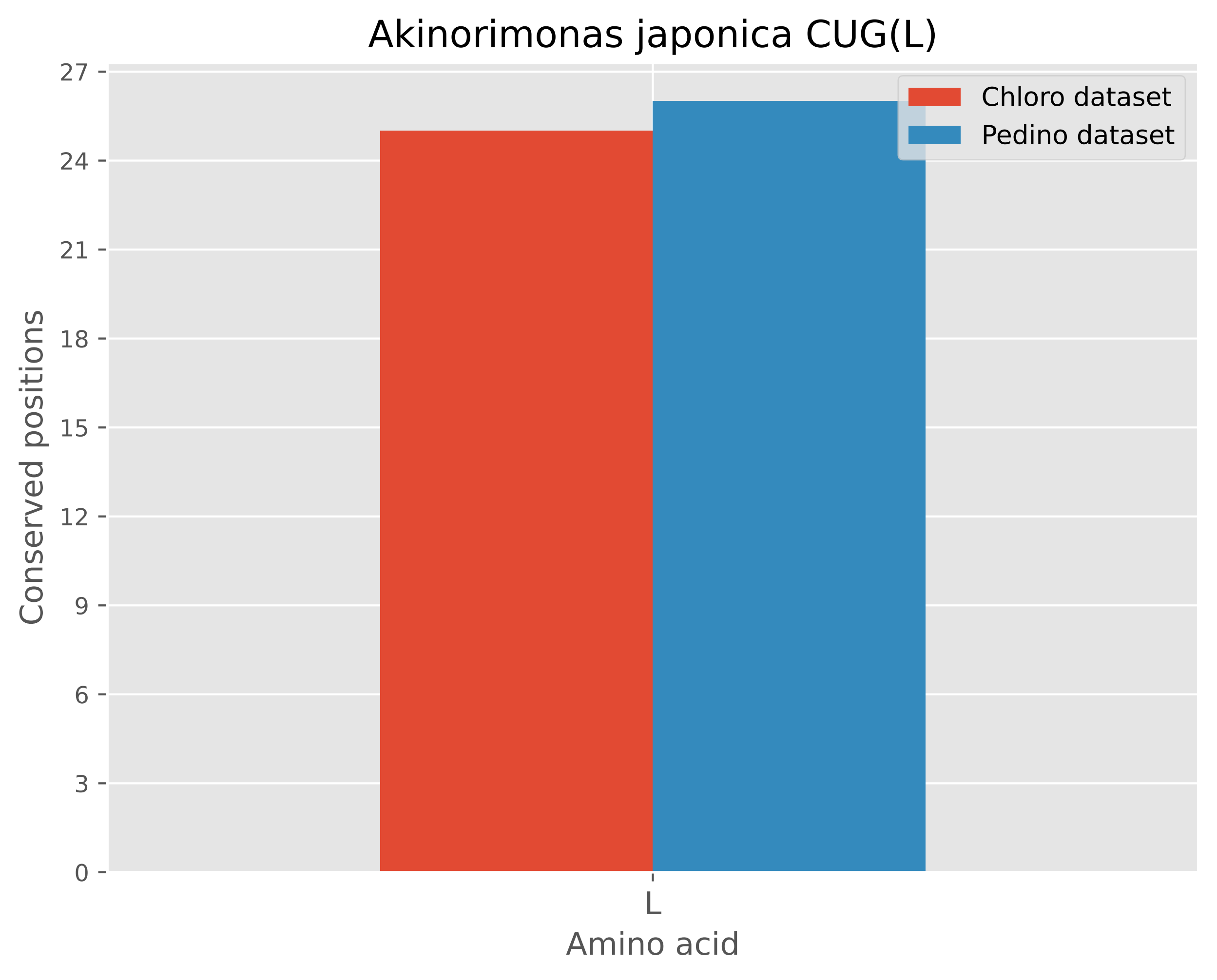
Akinorimonas japonica CUG(L)
Chloro dataset
Pedino dataset
Conserved positions
0
3
6
9
12
15
18
21
24
27
L
Amino acid

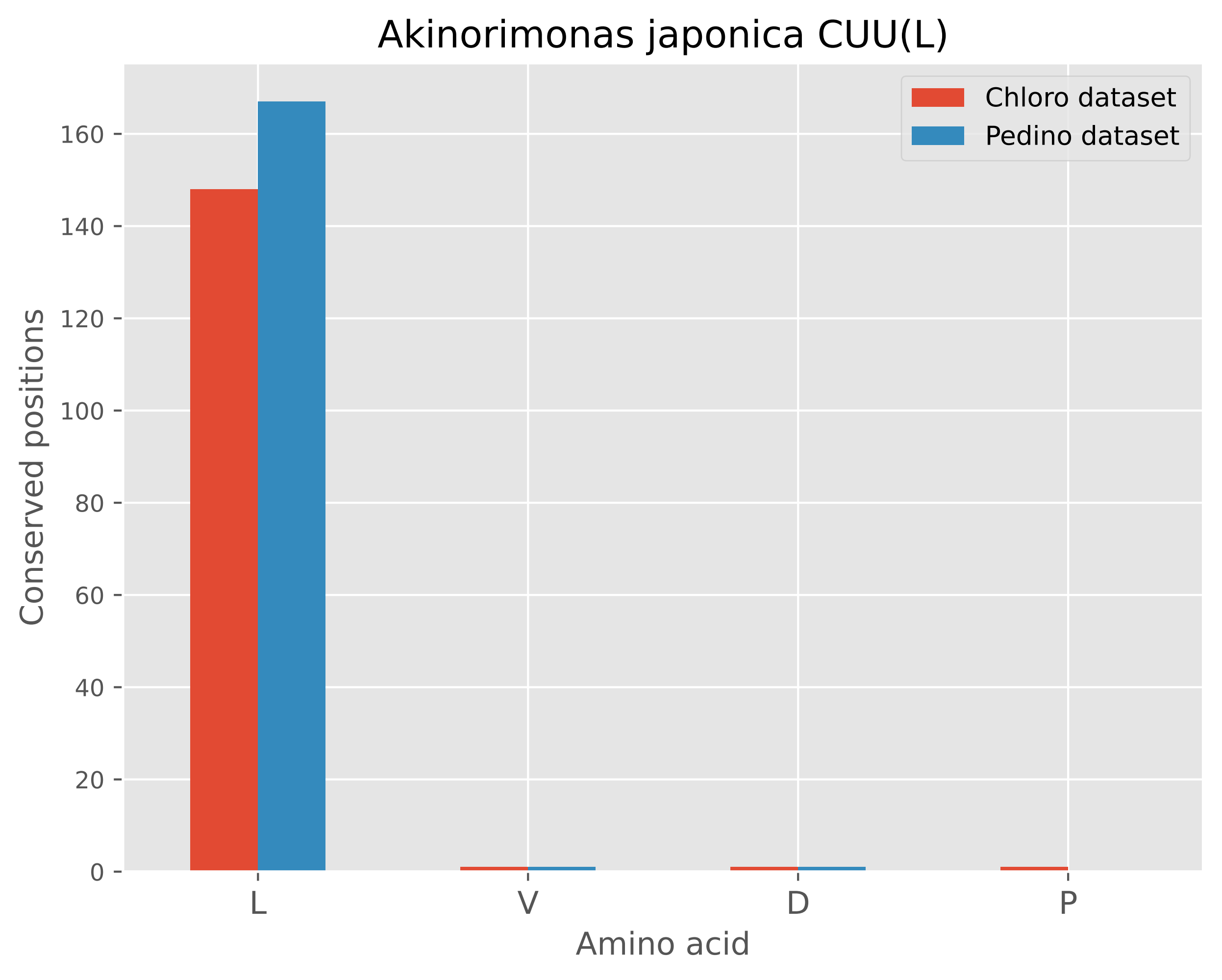
Akinorimonas japonica CUU(L)
Chloro dataset
Pedino dataset
Conserved positions
0
20
40
60
80
100
120
140
160
L
V
D
P
Amino acid

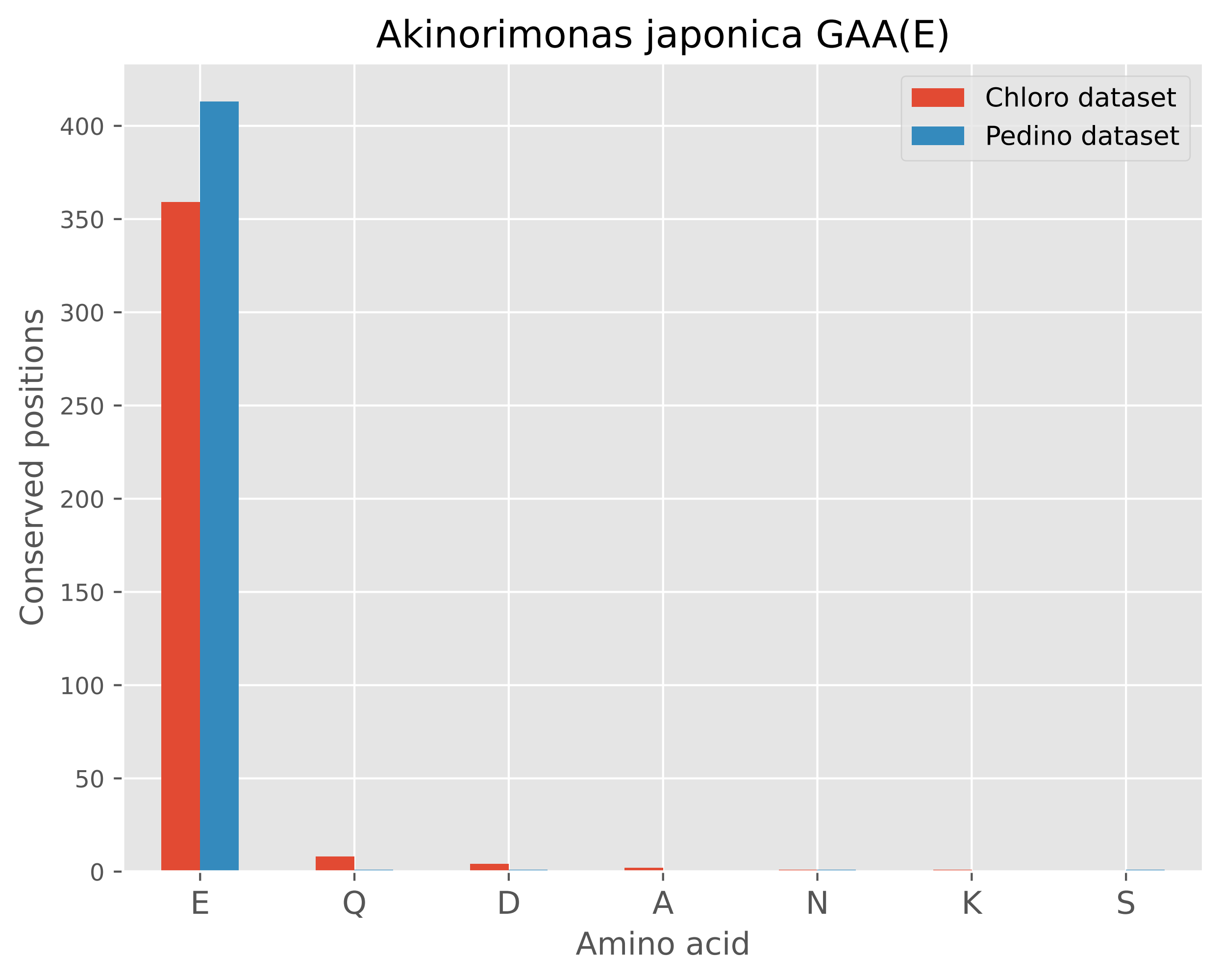
Akinorimonas japonica GAA(E)
Chloro dataset
Pedino dataset
Conserved positions
0
50
100
150
200
250
300
350
400
E
Q
D
A
N
K
S
Amino acid

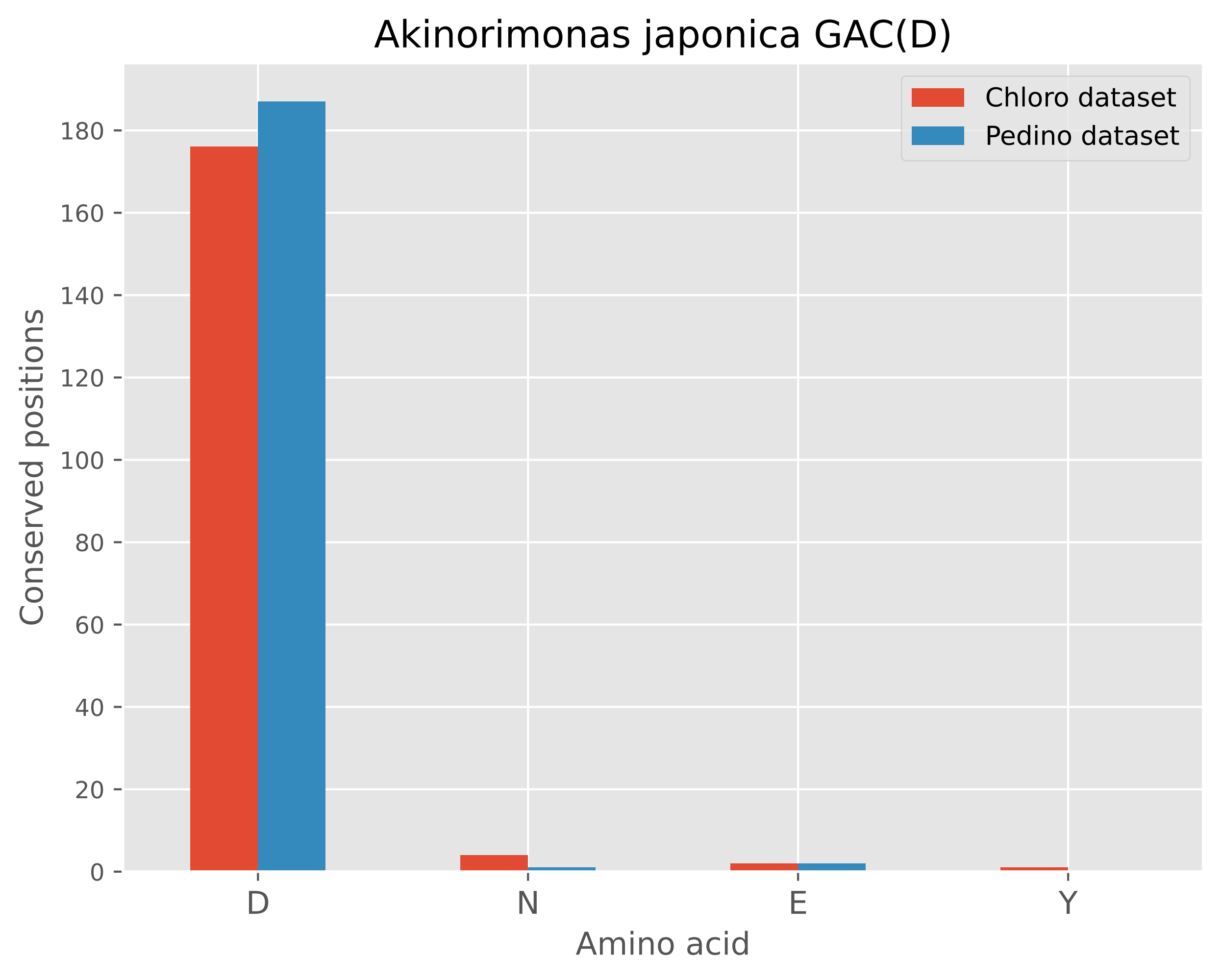

Akinorimonas japonica GAC(D)
Chloro dataset
Pedino dataset
Conserved positions
0
20
40
60
80
100
120
140
160
180
D
N
E
Y
Amino acid

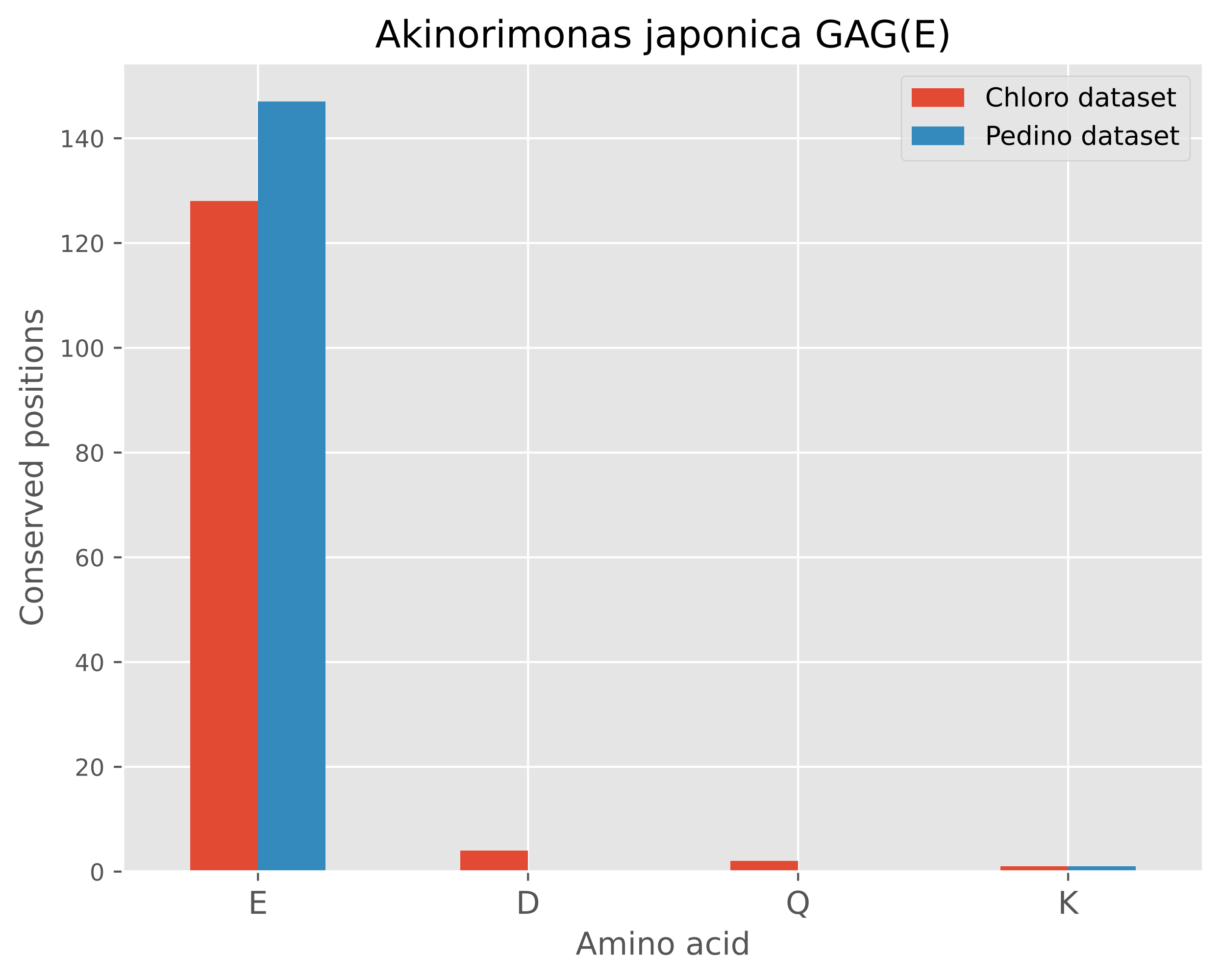

Akinorimonas japonica GAG(E)
Chloro dataset
Pedino dataset
Conserved positions
0
20
40
60
80
100
120
140
E
D
Q
K
Amino acid

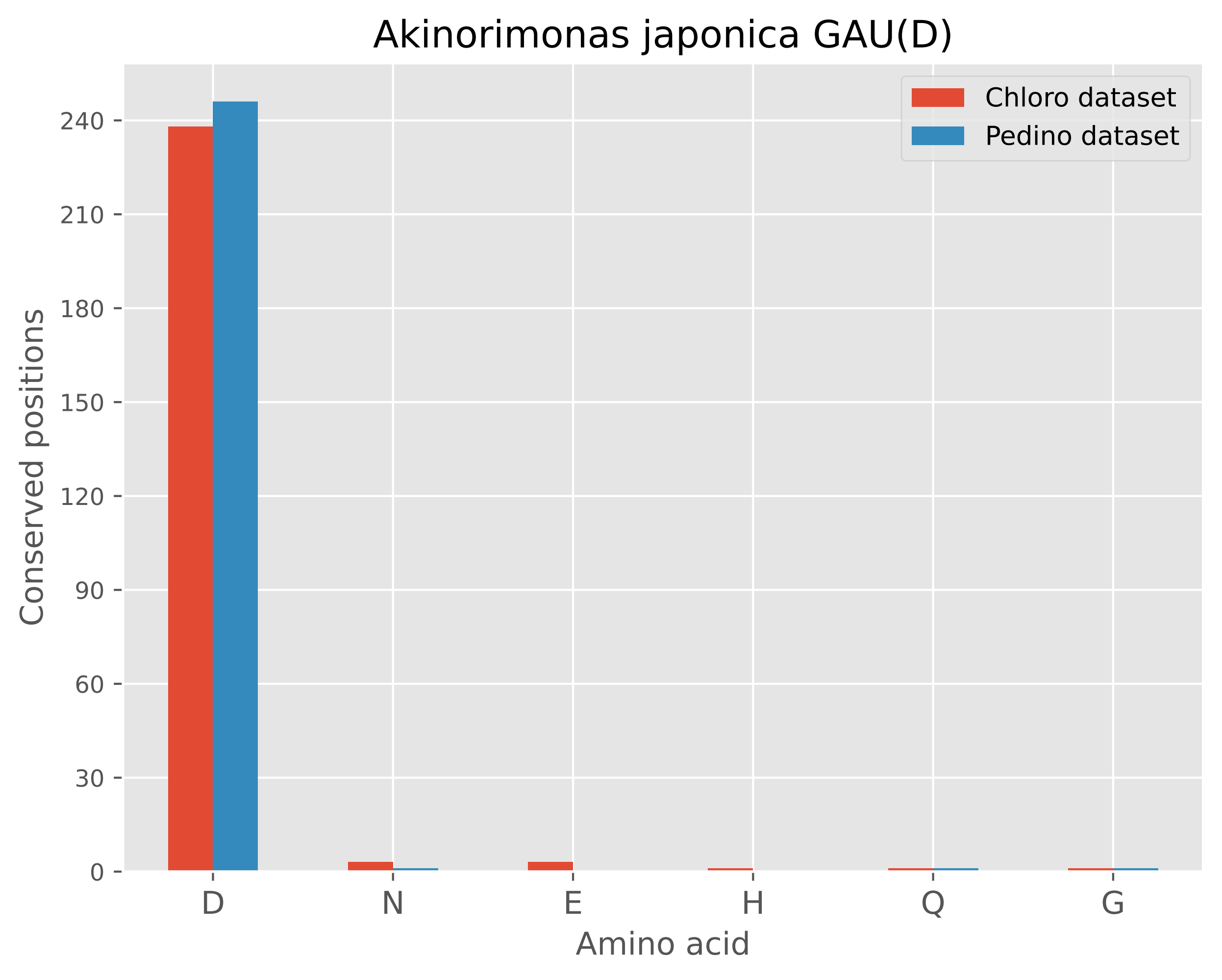
Akinorimonas japonica GAU(D)
Chloro dataset
Pedino dataset
Conserved positions
0
30
60
90
120
150
180
210
240
D
N
E
H
Q
G
Amino acid

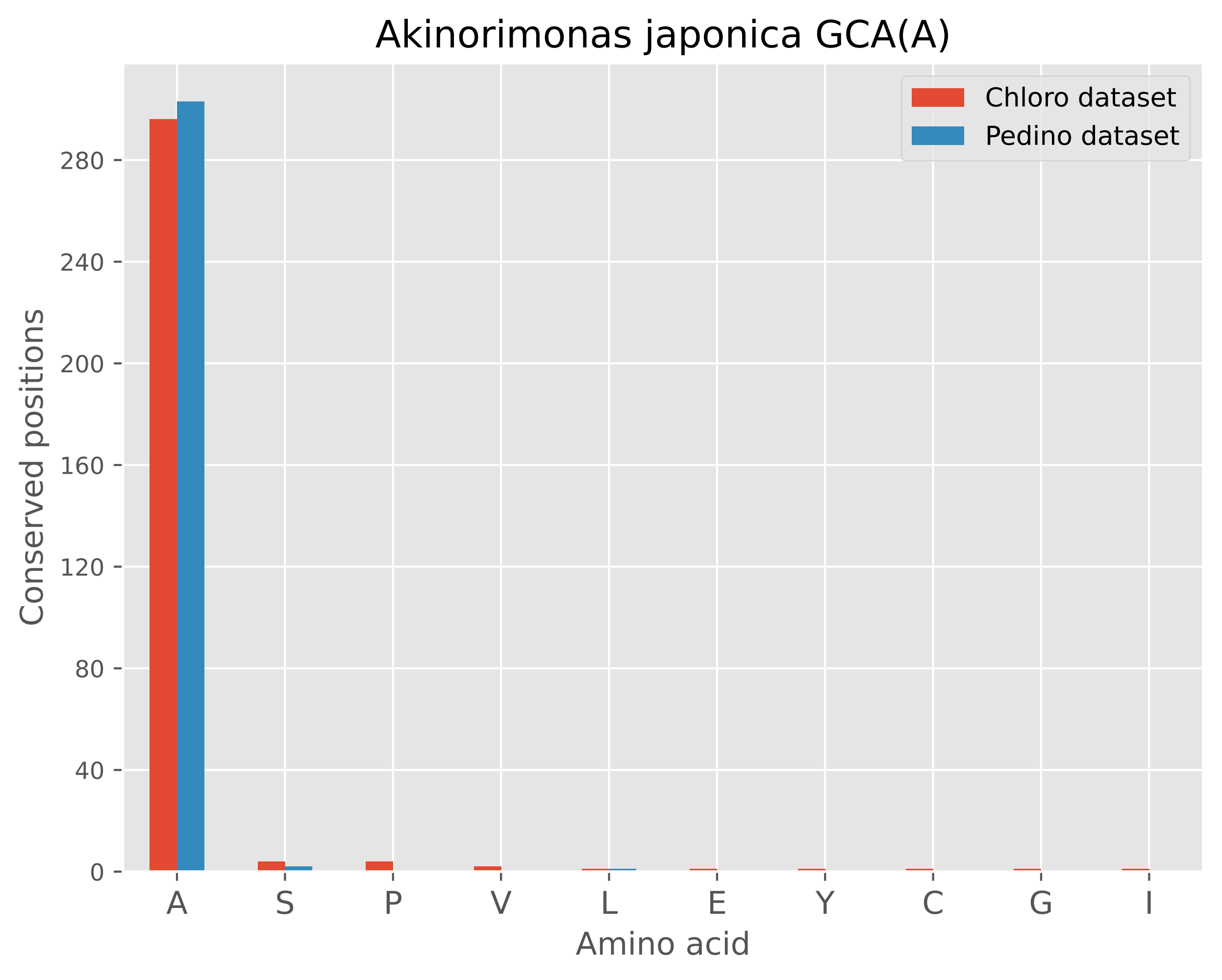
Akinorimonas japonica GCA(A)
Chloro dataset
Pedino dataset
Conserved positions
0
40
80
120
160
200
240
280
A
S
P
V
L
E
Y
C
G
I
Amino acid

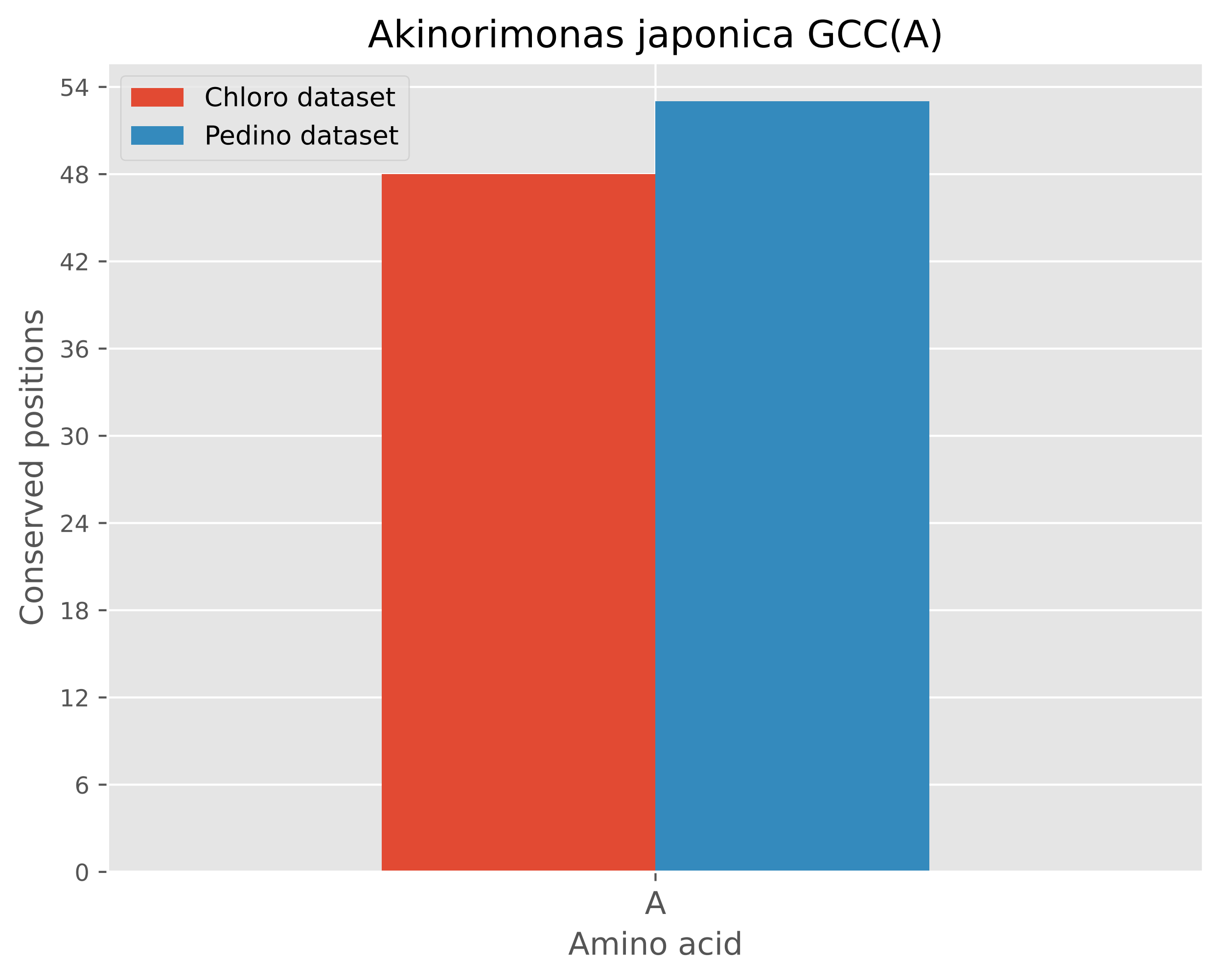

Akinorimonas japonica GCC(A)
Chloro dataset
Pedino dataset
Conserved positions
0
6
12
18
24
30
36
42
48
54
A
Amino acid

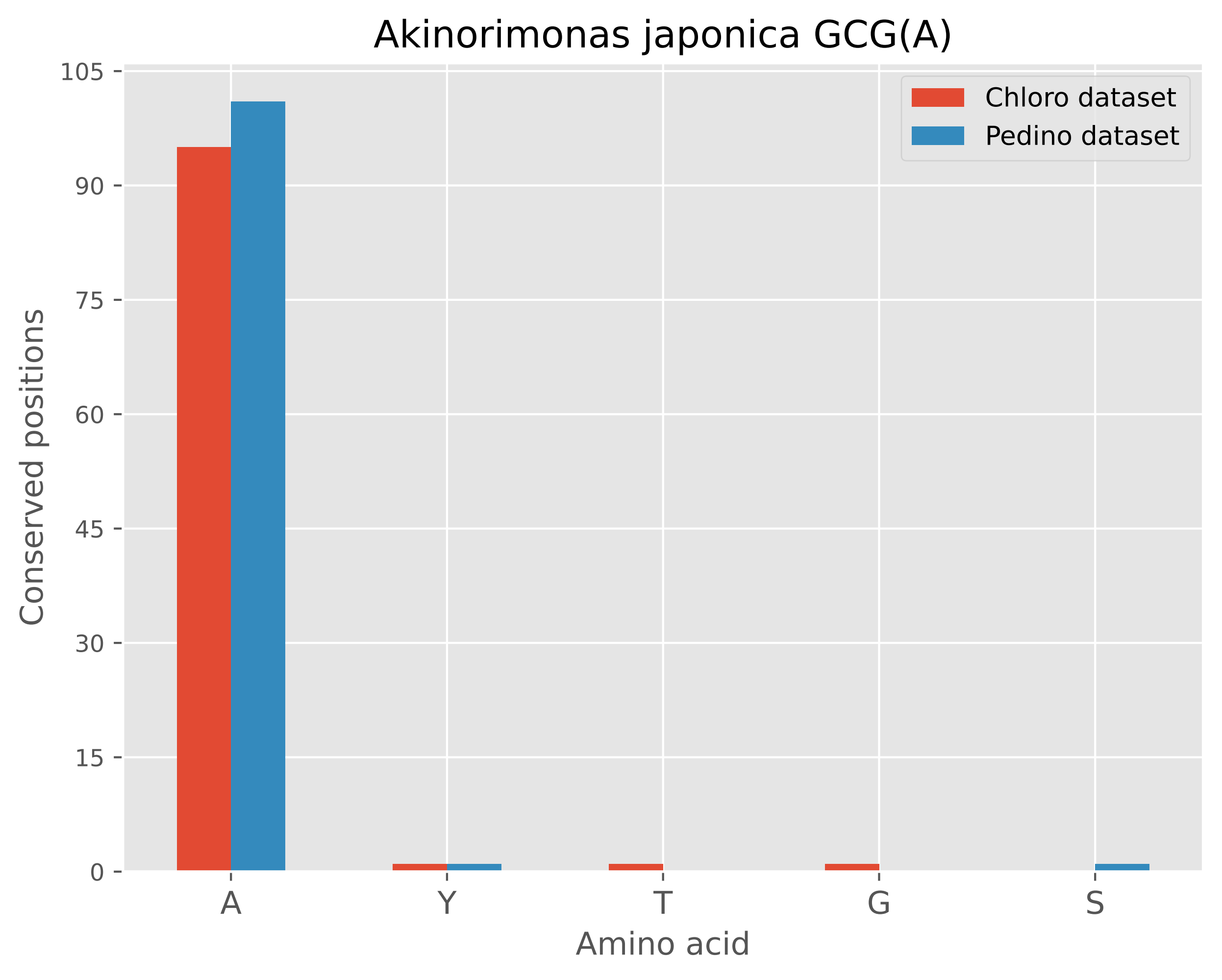

Akinorimonas japonica GCG(A)
Chloro dataset
Pedino dataset
Conserved positions
0
15
30
45
60
75
90
105
A
Y
T
G
S
Amino acid

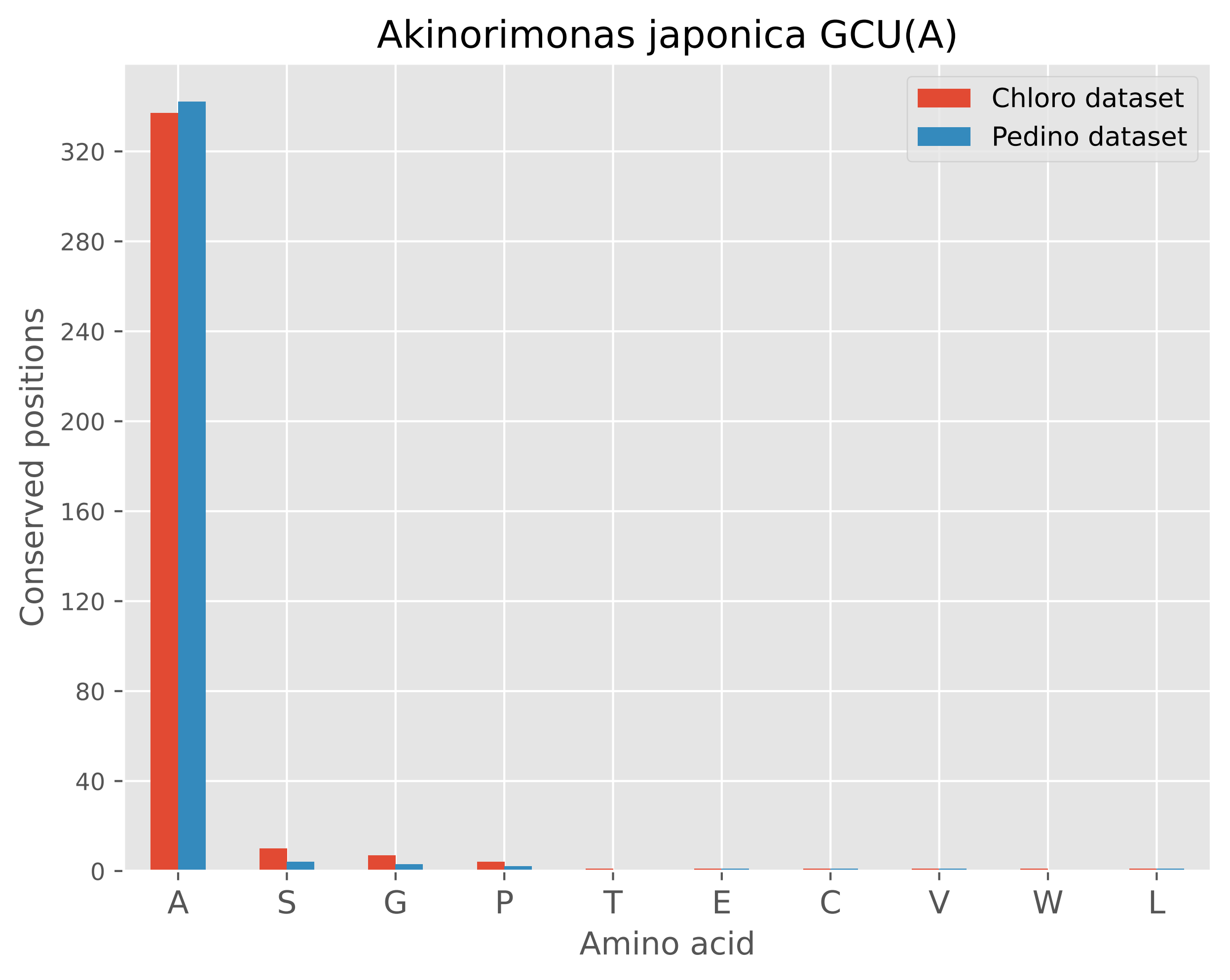

Akinorimonas japonica GCU(A)
Chloro dataset
Pedino dataset
Conserved positions
0
40
80
120
160
200
240
280
320
A
S
G
P
T
E
C
V
W
L
Amino acid

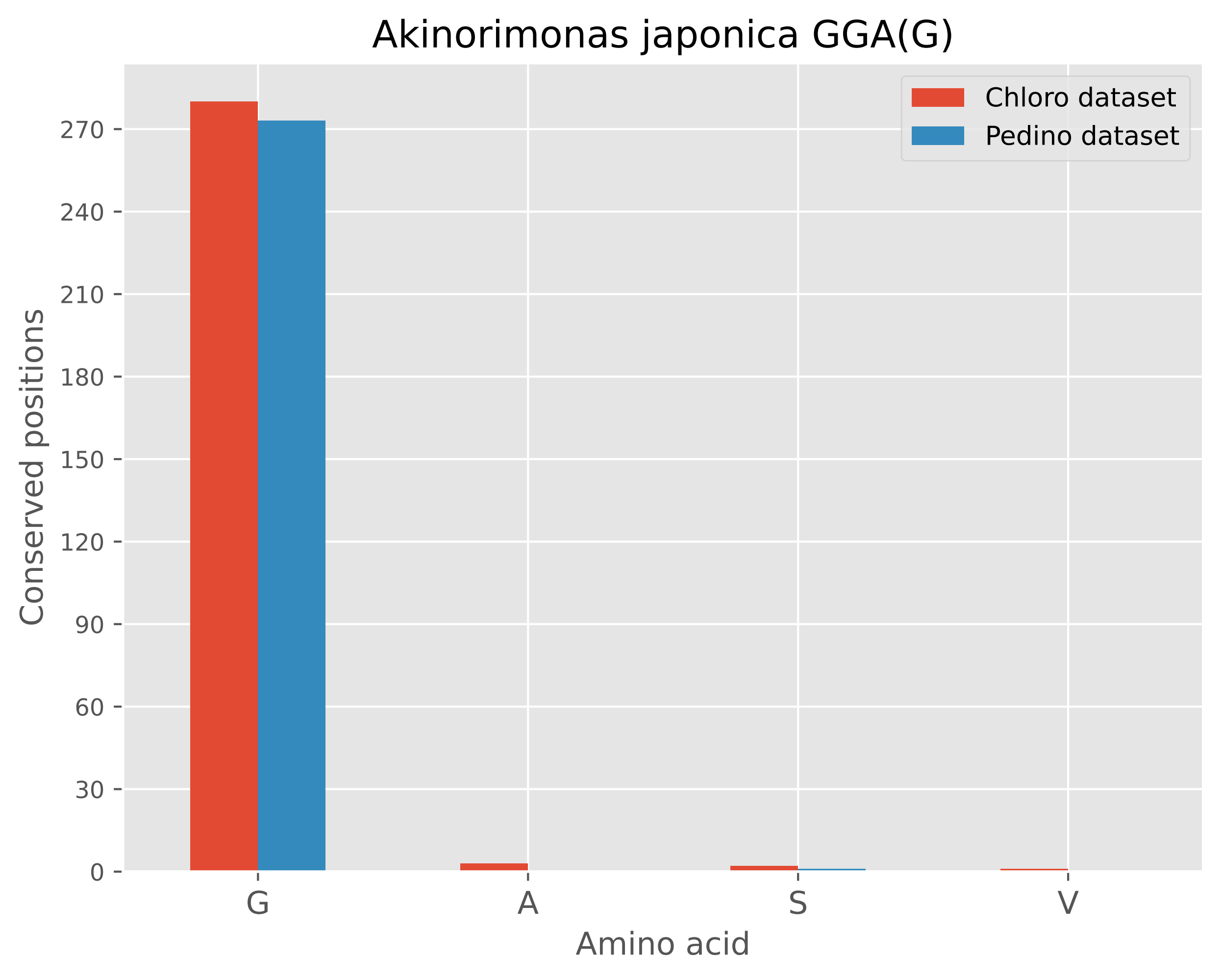
Akinorimonas japonica GGA(G)
Chloro dataset
Pedino dataset
Conserved positions
0
30
60
90
120
150
180
210
240
270
G
A
S
V
Amino acid

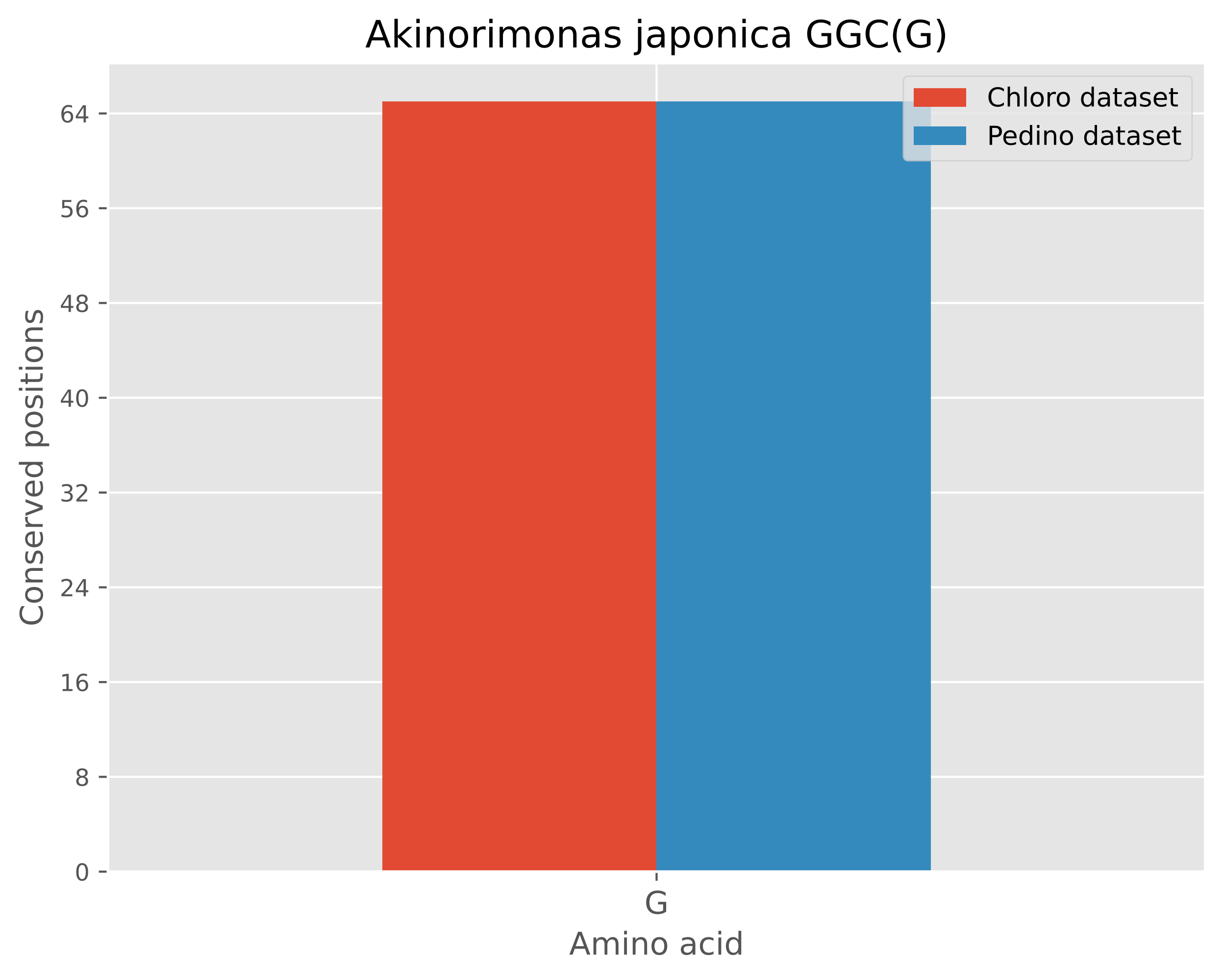
Akinorimonas japonica GGC(G)
Chloro dataset
Pedino dataset
Conserved positions
0
8
16
24
32
40
48
56
64
G
Amino acid

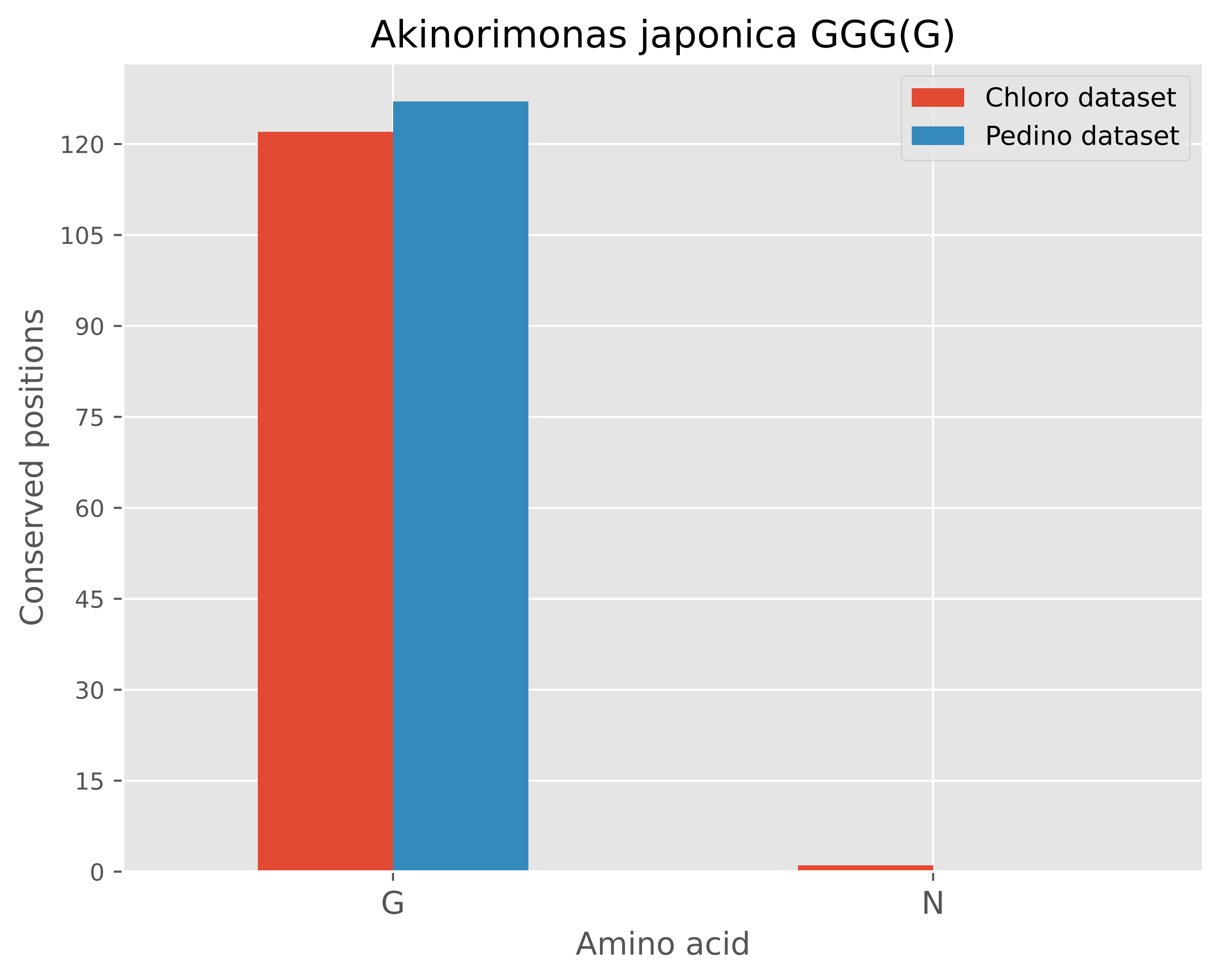

Akinorimonas japonica GGG(G)
Chloro dataset
Pedino dataset
Conserved positions
0
15
30
45
60
75
90
105
120
G
N
Amino acid

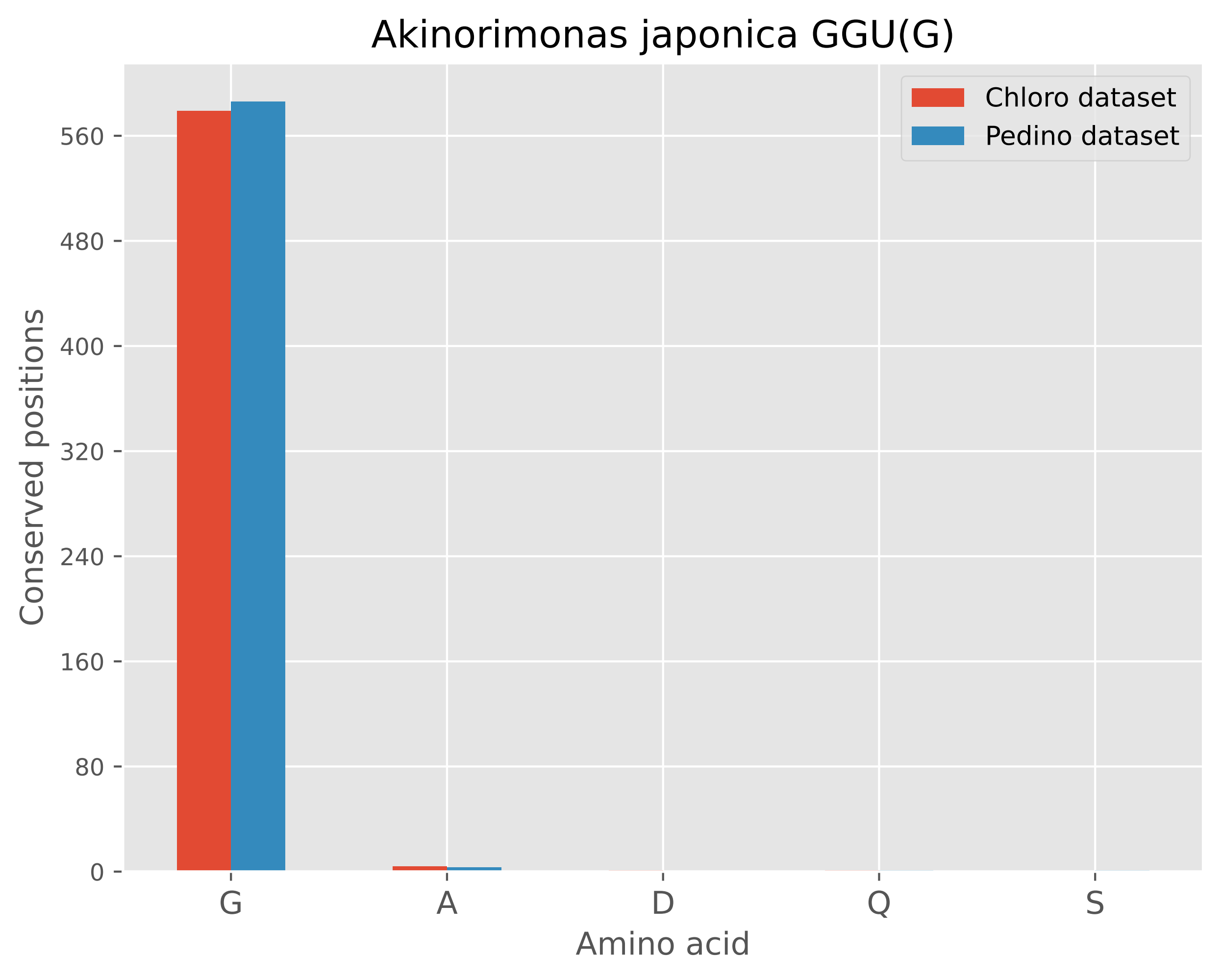

Akinorimonas japonica GGU(G)
Chloro dataset
Pedino dataset
Conserved positions
560
480
400
320
240
160
80
0
G
A
D
Q
S
Amino acid

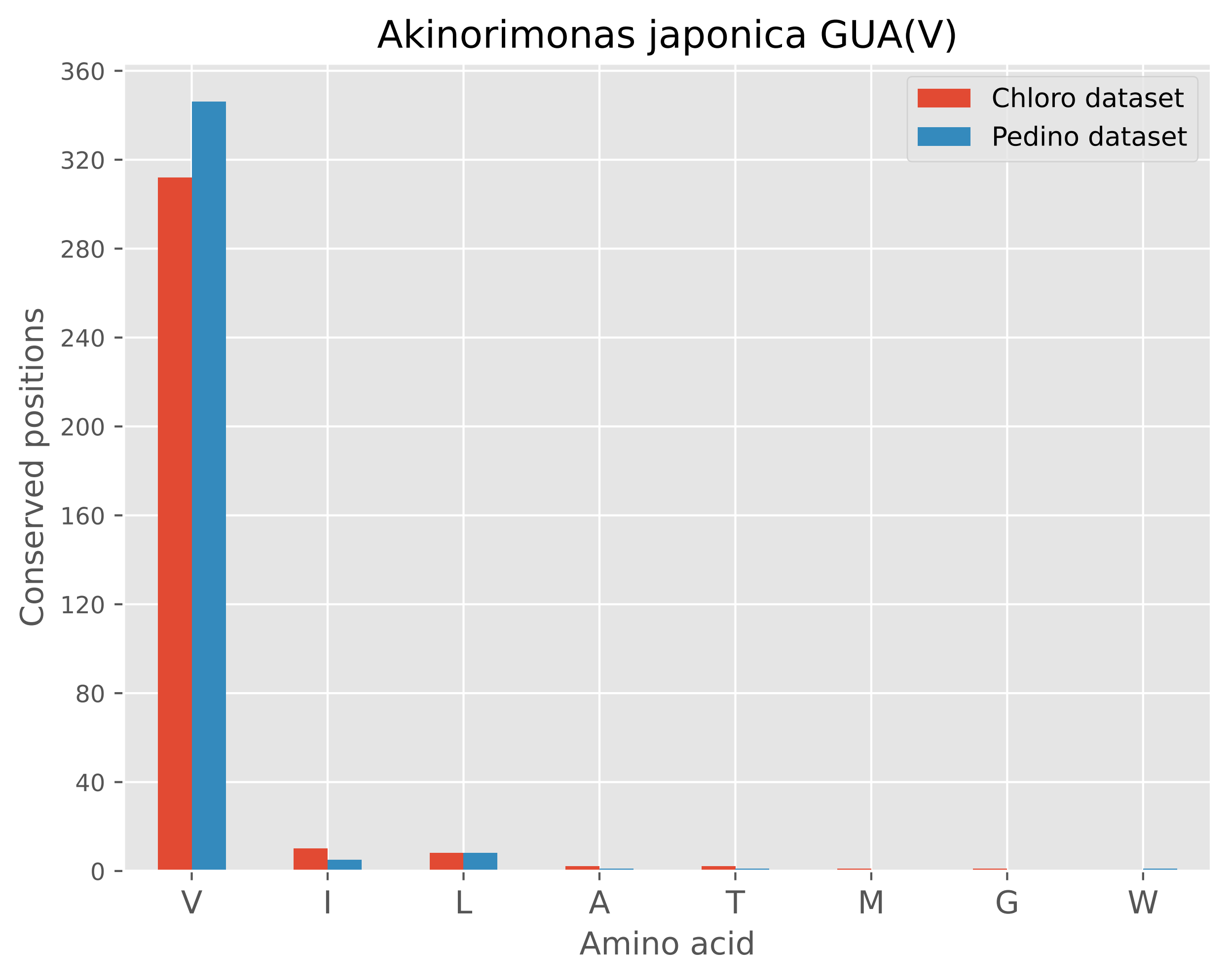

Akinorimonas japonica GUA(V)
Chloro dataset
Pedino dataset
Conserved positions
0
40
80
120
160
200
240
280
320
360
V
I
L
A
T
M
G
W
Amino acid

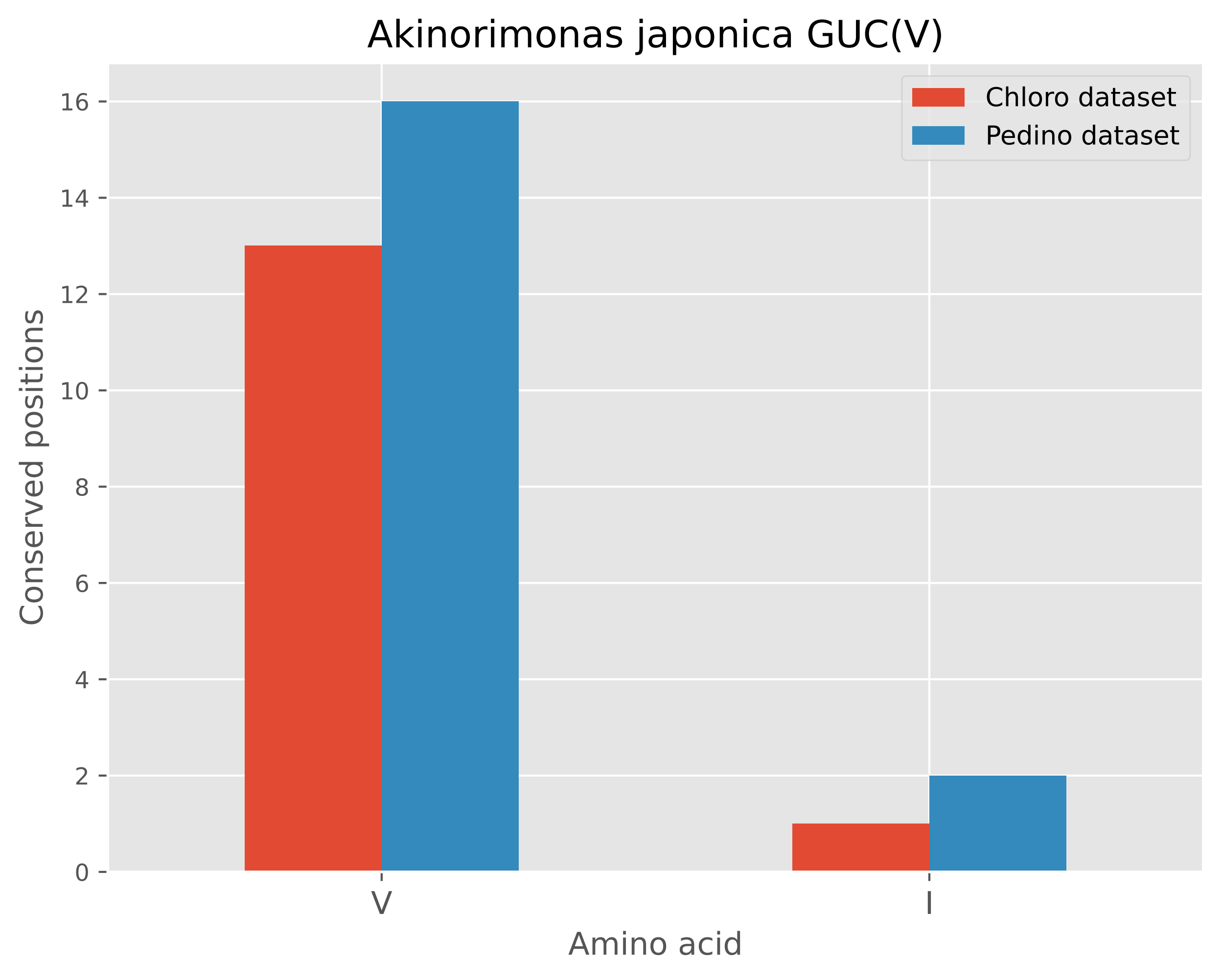

Akinorimonas japonica GUC(V)
Chloro dataset
Pedino dataset
Conserved positions
0
2
4
6
8
10
12
14
16
V
I
Amino acid

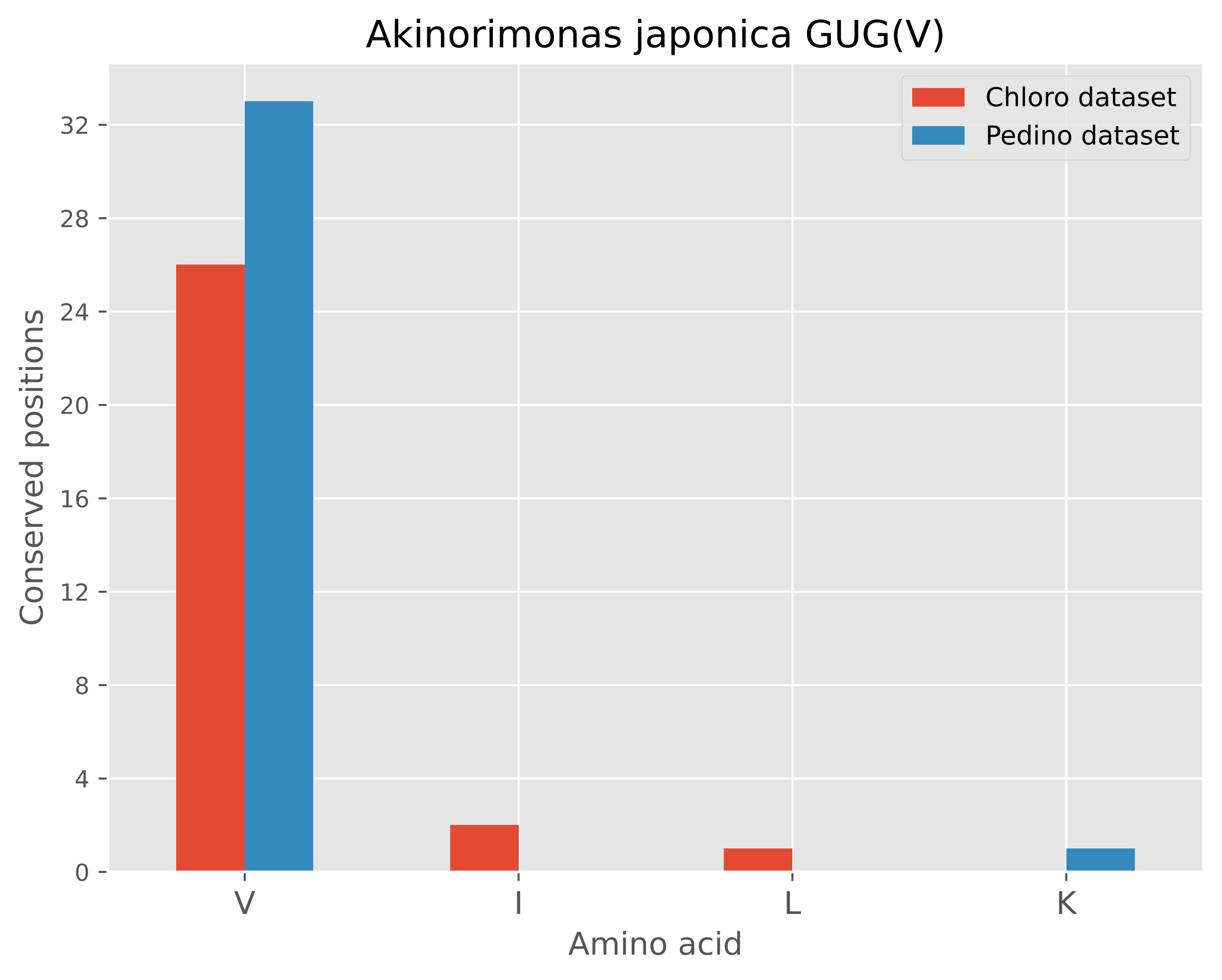
Akinorimonas japonica GUG(V)
Chloro dataset
Pedino dataset
Conserved positions
0
4
8
12
16
20
24
28
32
V
I
L
K
Amino acid

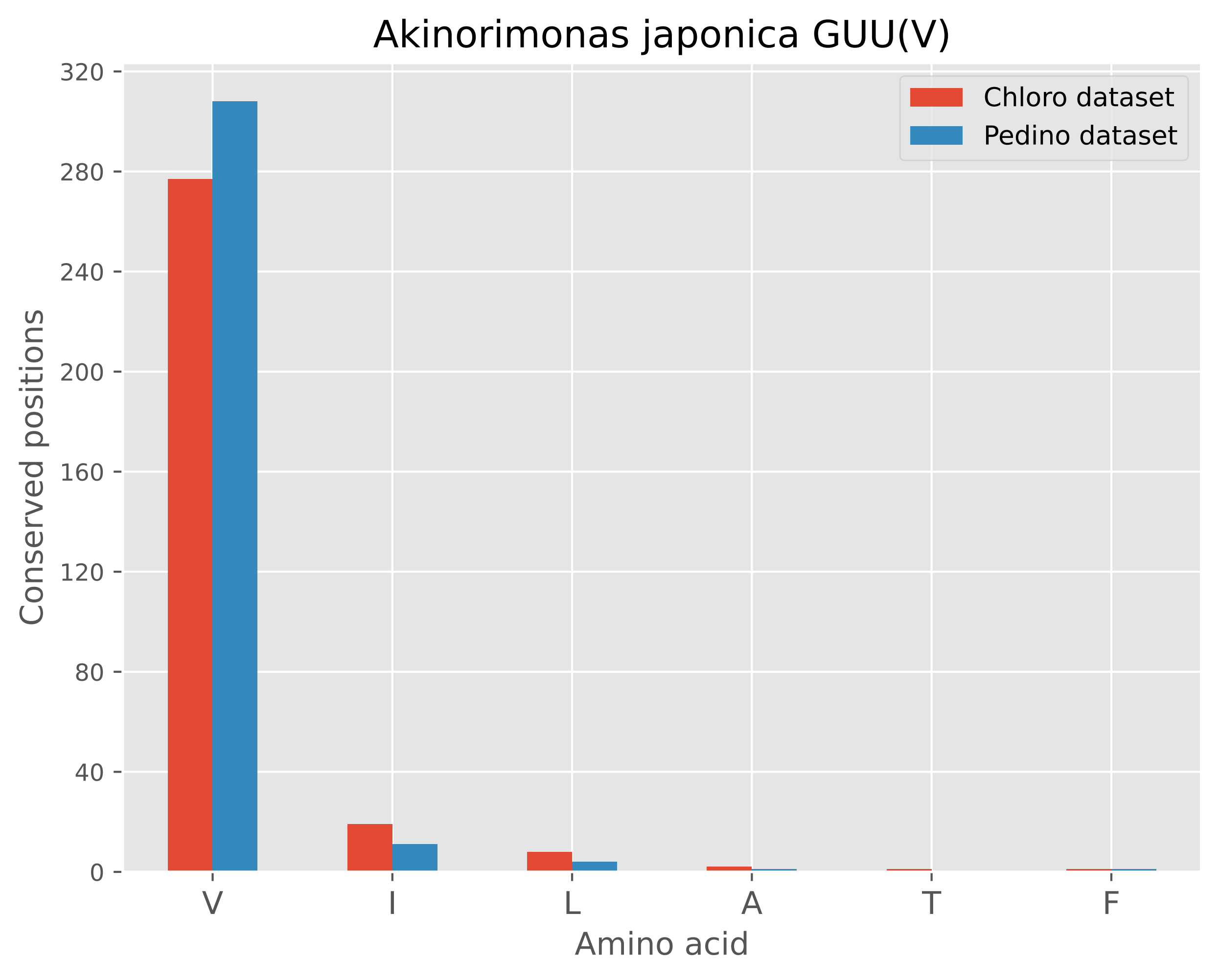

Akinorimonas japonica GUU(V)
Chloro dataset
Pedino dataset
Conserved positions
0
40
80
120
160
200
240
280
320
V
I
L
A
T
F
Amino acid

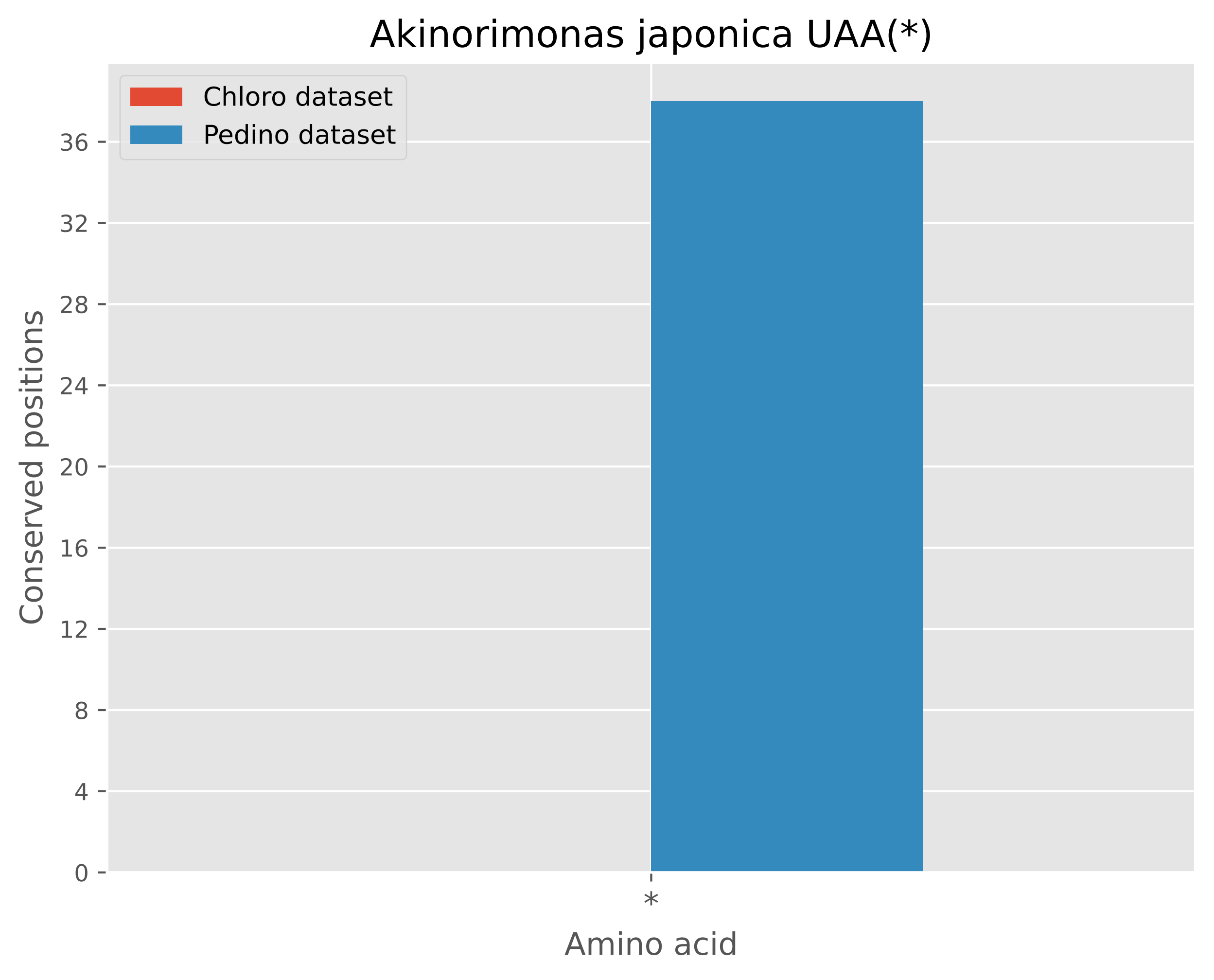

Akinorimonas japonica UAA(*)
Chloro dataset
Pedino dataset
Conserved positions
0
4
8
12
16
20
24
28
32
36
*
Amino acid

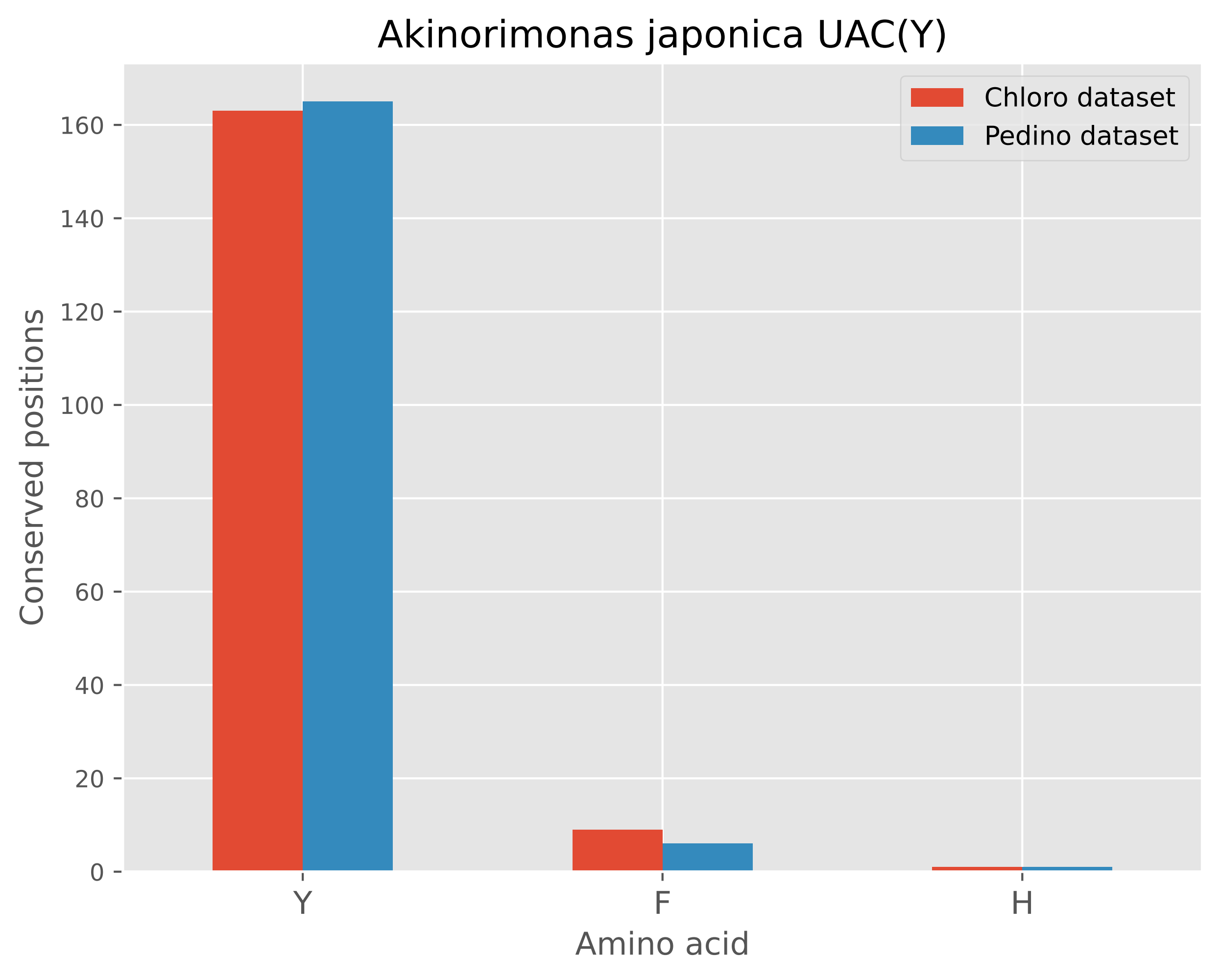

Akinorimonas japonica UAC(Y)
Chloro dataset
Pedino dataset
Conserved positions
0
20
40
60
80
100
120
140
160
Y
F
H
Amino acid

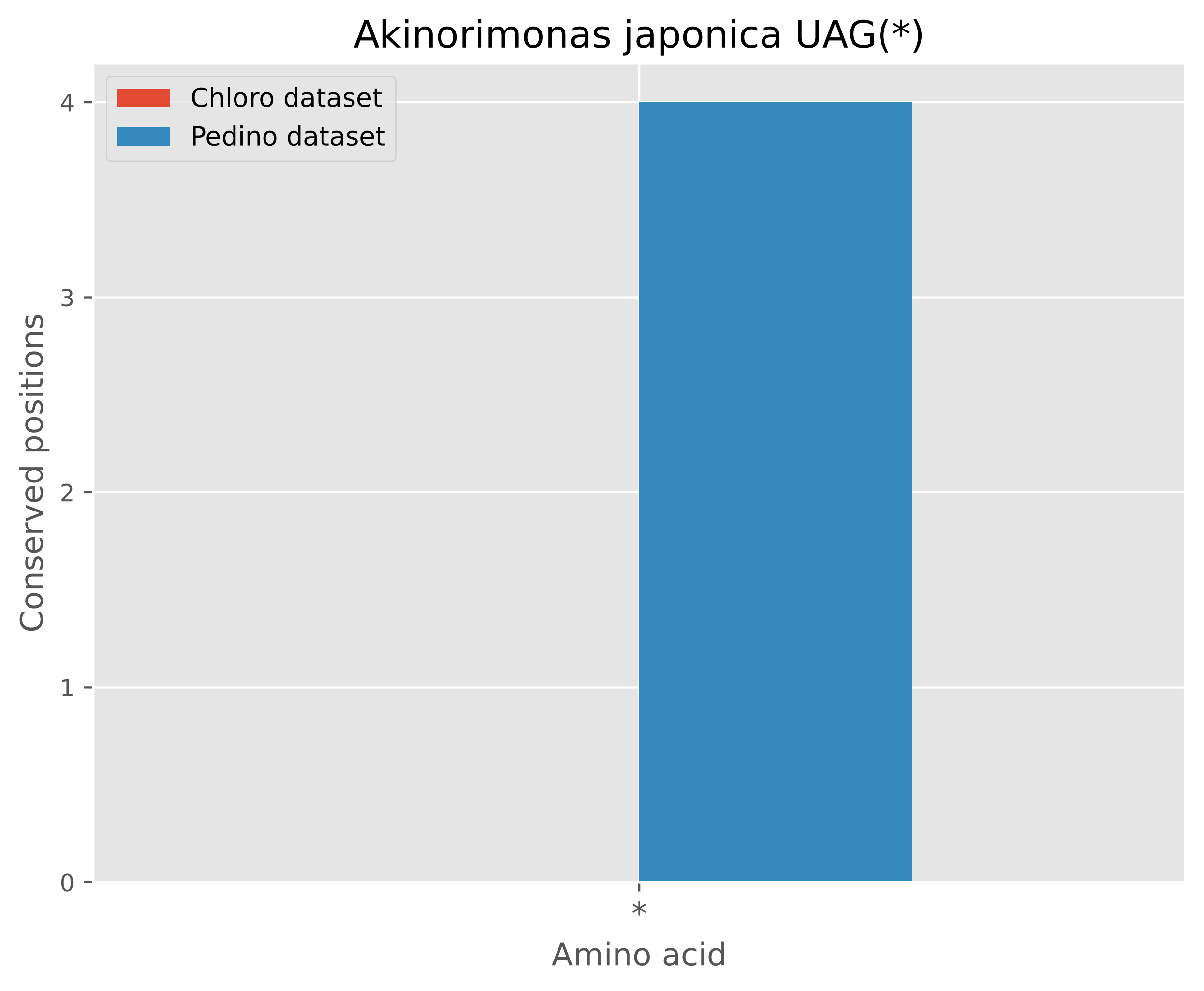
Akinorimonas japonica UAG(*)
Chloro dataset
Pedino dataset
Conserved positions
0
1
2
3
4
*
Amino acid

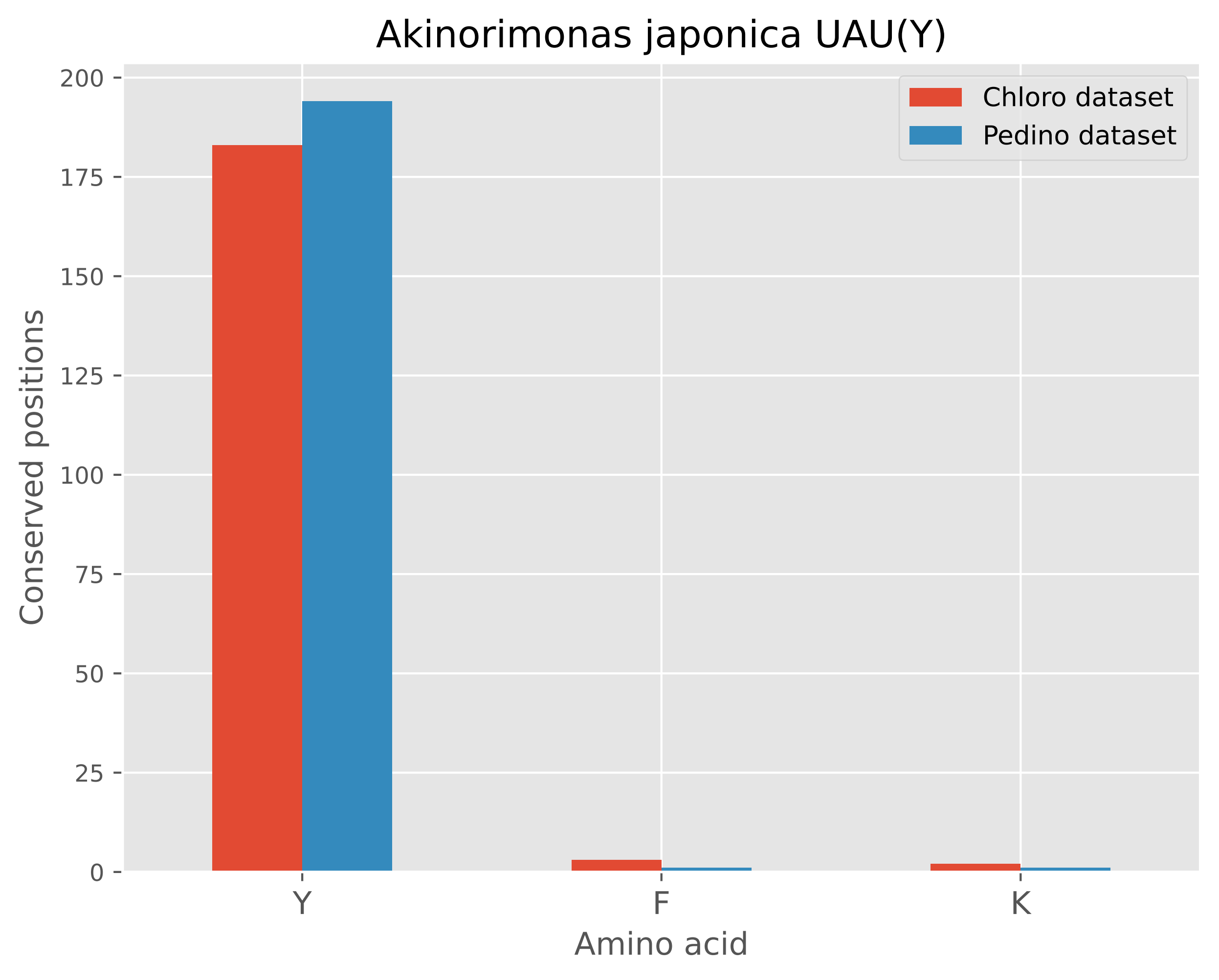

Akinorimonas japonica UAU(Y)
Chloro dataset
Pedino dataset
Conserved positions
0
25
50
75
100
125
150
175
200
Y
F
K
Amino acid

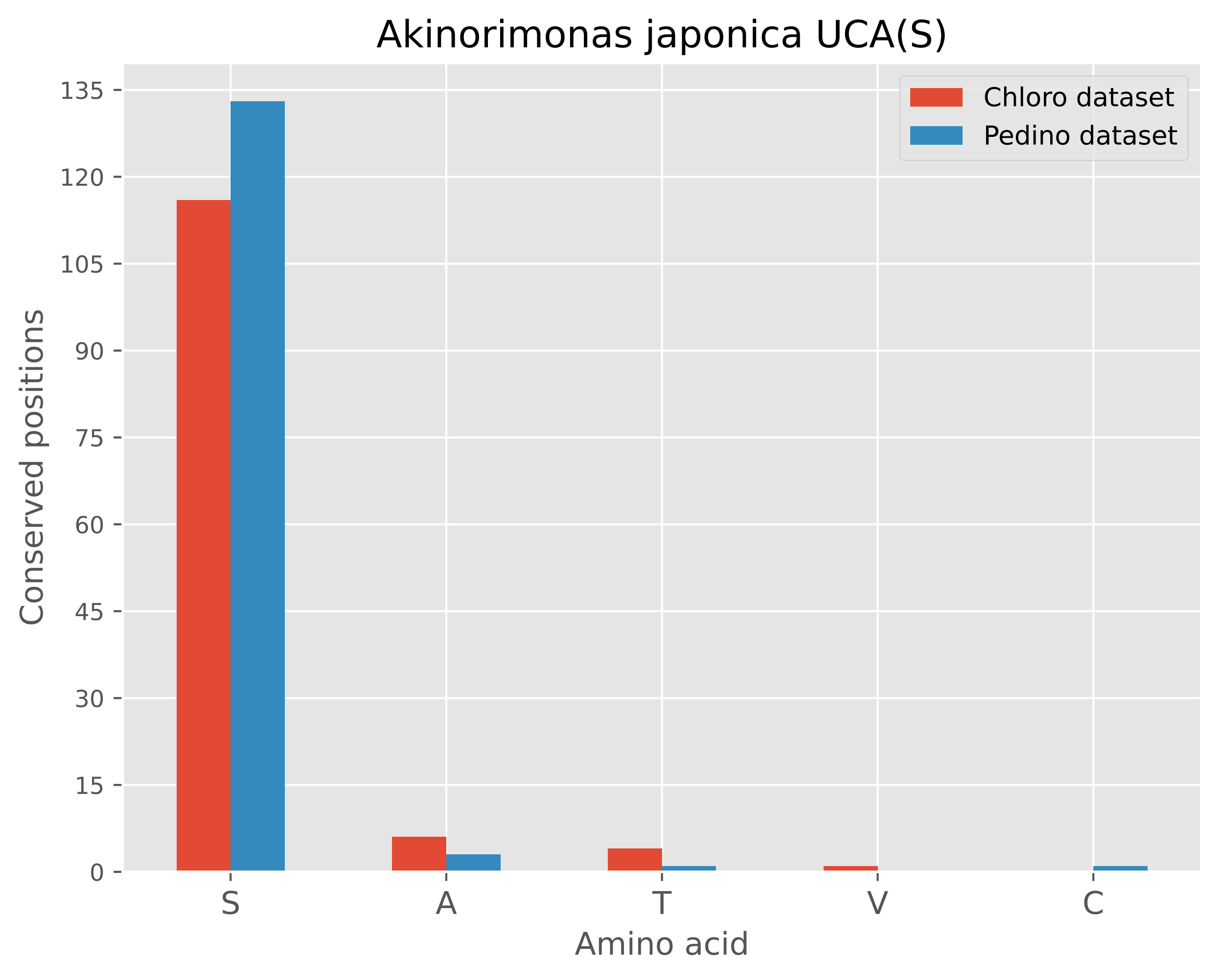

Akinorimonas japonica UCA(S)
Chloro dataset
Pedino dataset
Conserved positions
0
15
30
45
60
75
90
105
120
135
S
A
T
V
C
Amino acid

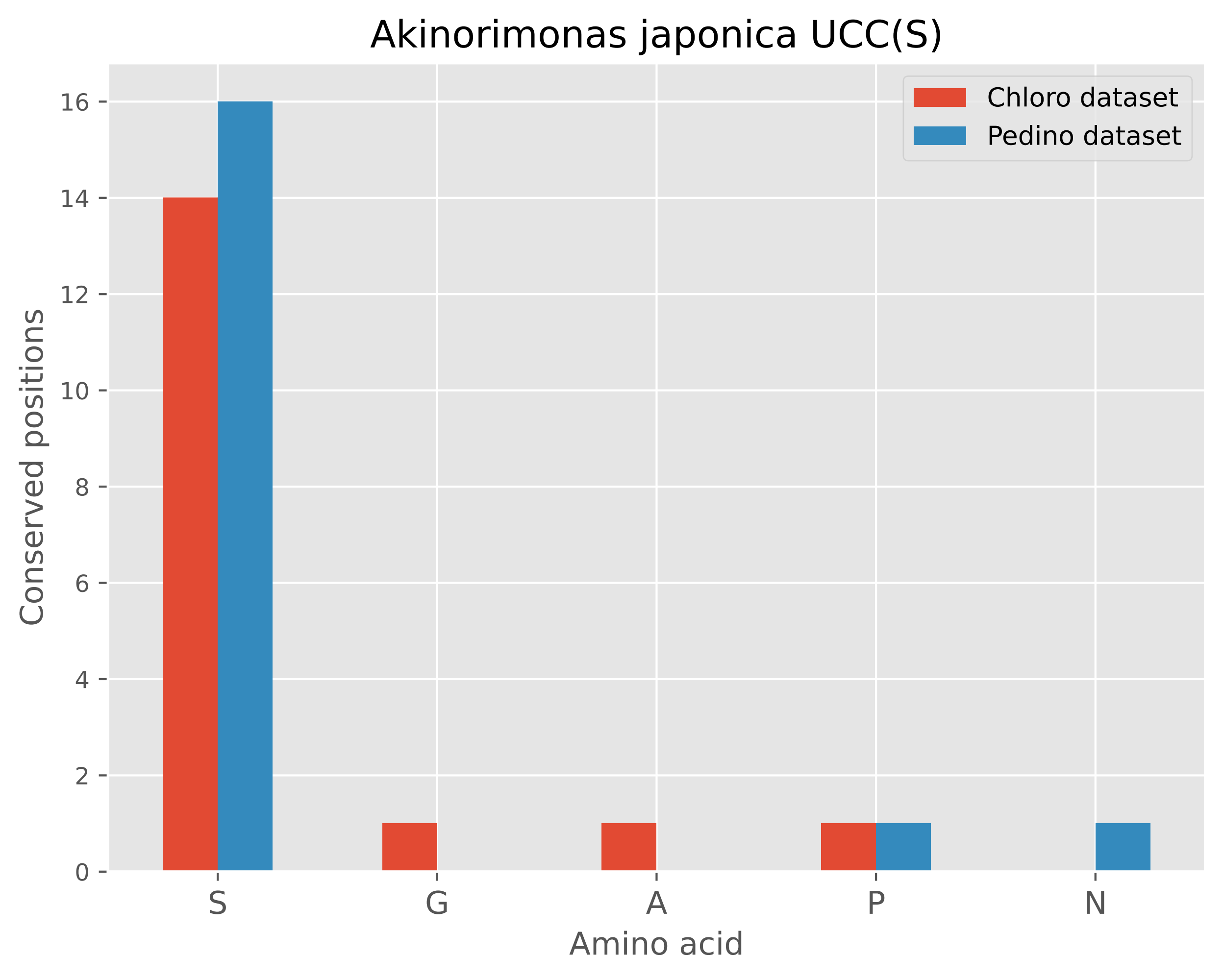

Akinorimonas japonica UCC(S)
Chloro dataset
Pedino dataset
Conserved positions
0
2
4
6
8
10
12
14
16
S
G
A
P
N
Amino acid

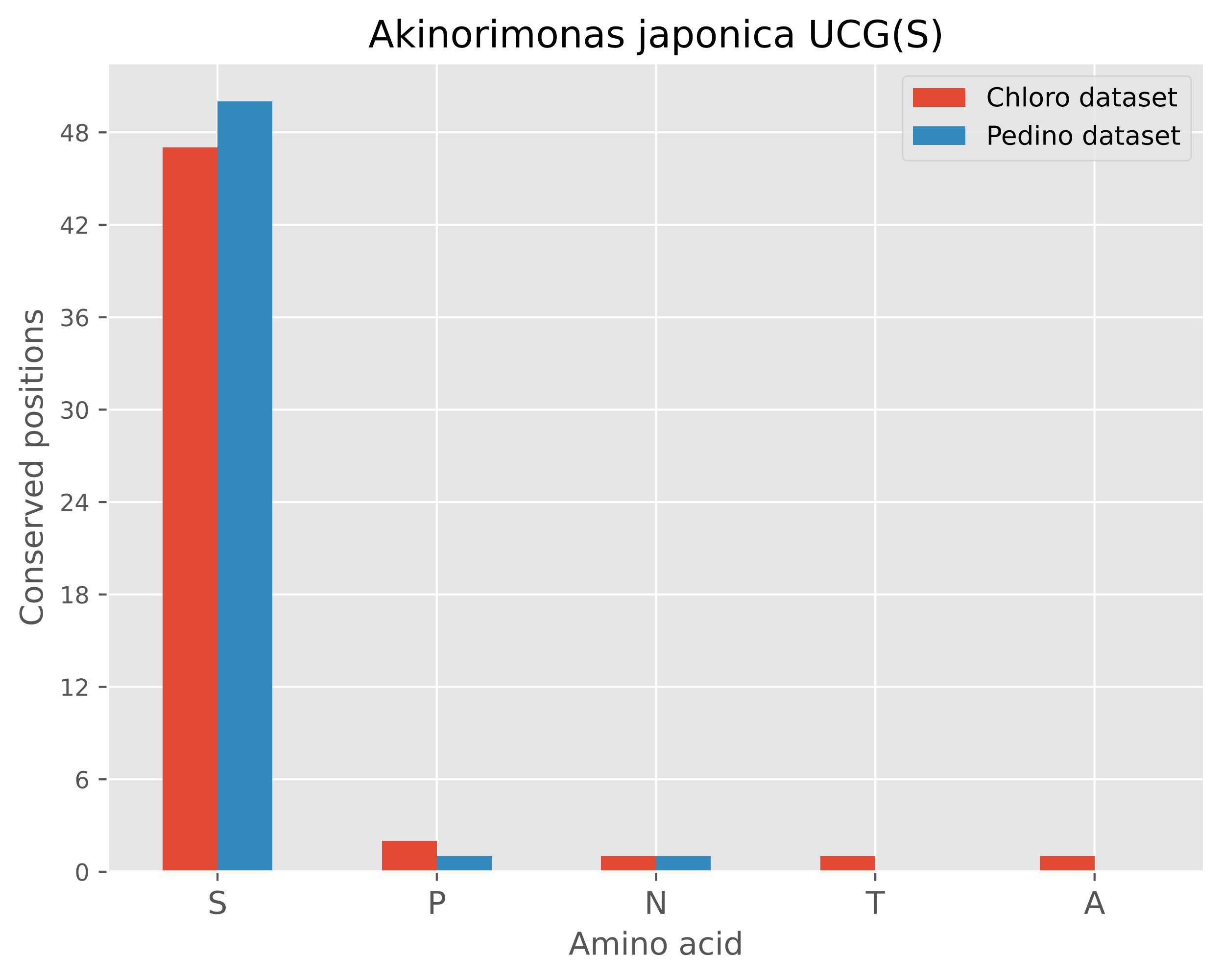

Akinorimonas japonica UCG(S)
Chloro dataset
Pedino dataset
Conserved positions
0
6
12
18
24
30
36
42
48
S
P
N
T
A
Amino acid

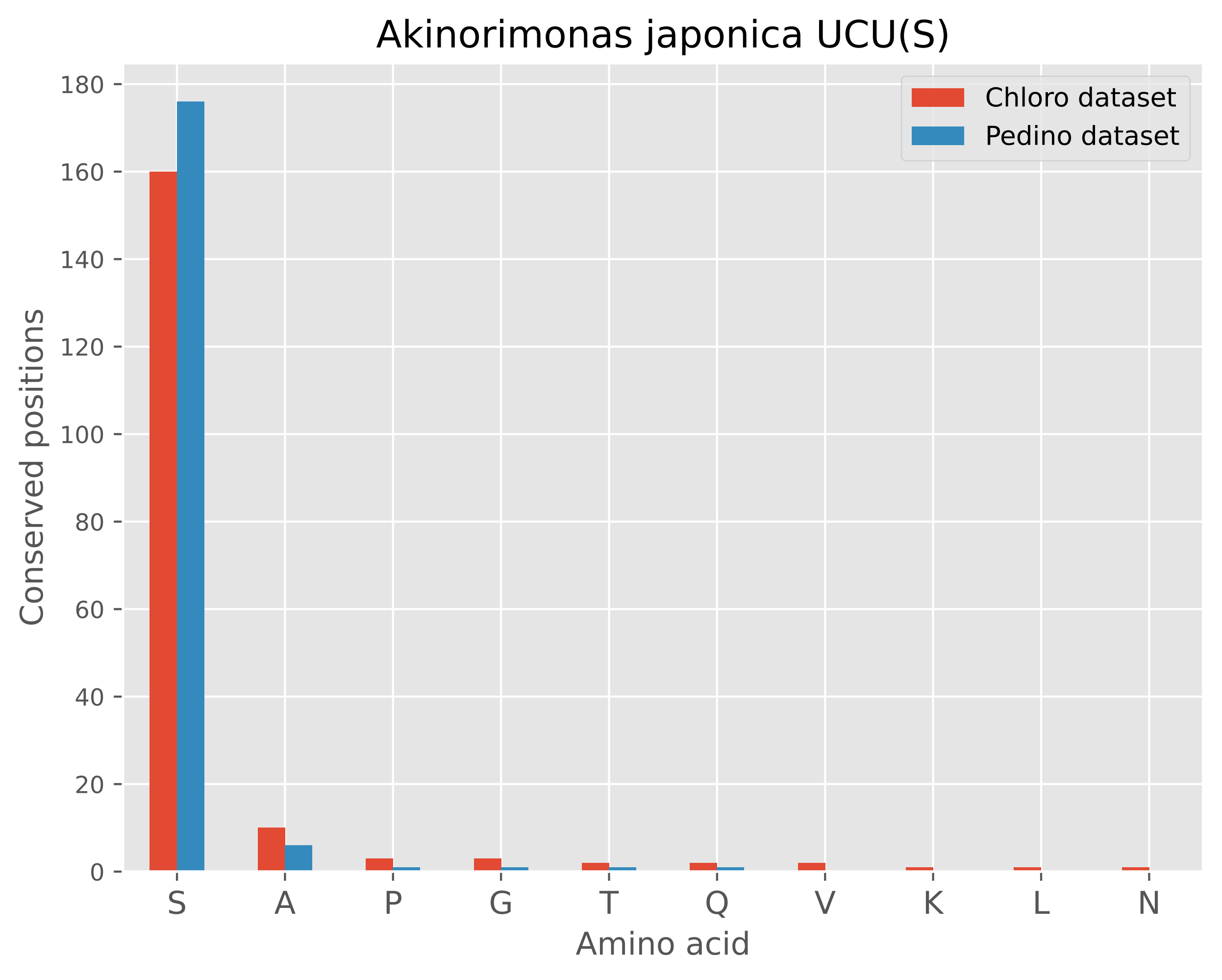

Akinorimonas japonica UCU(S)
Chloro dataset
Pedino dataset
Conserved positions
0
20
40
60
80
100
120
140
160
180
S
A
P
G
T
Q
V
K
L
N
Amino acid

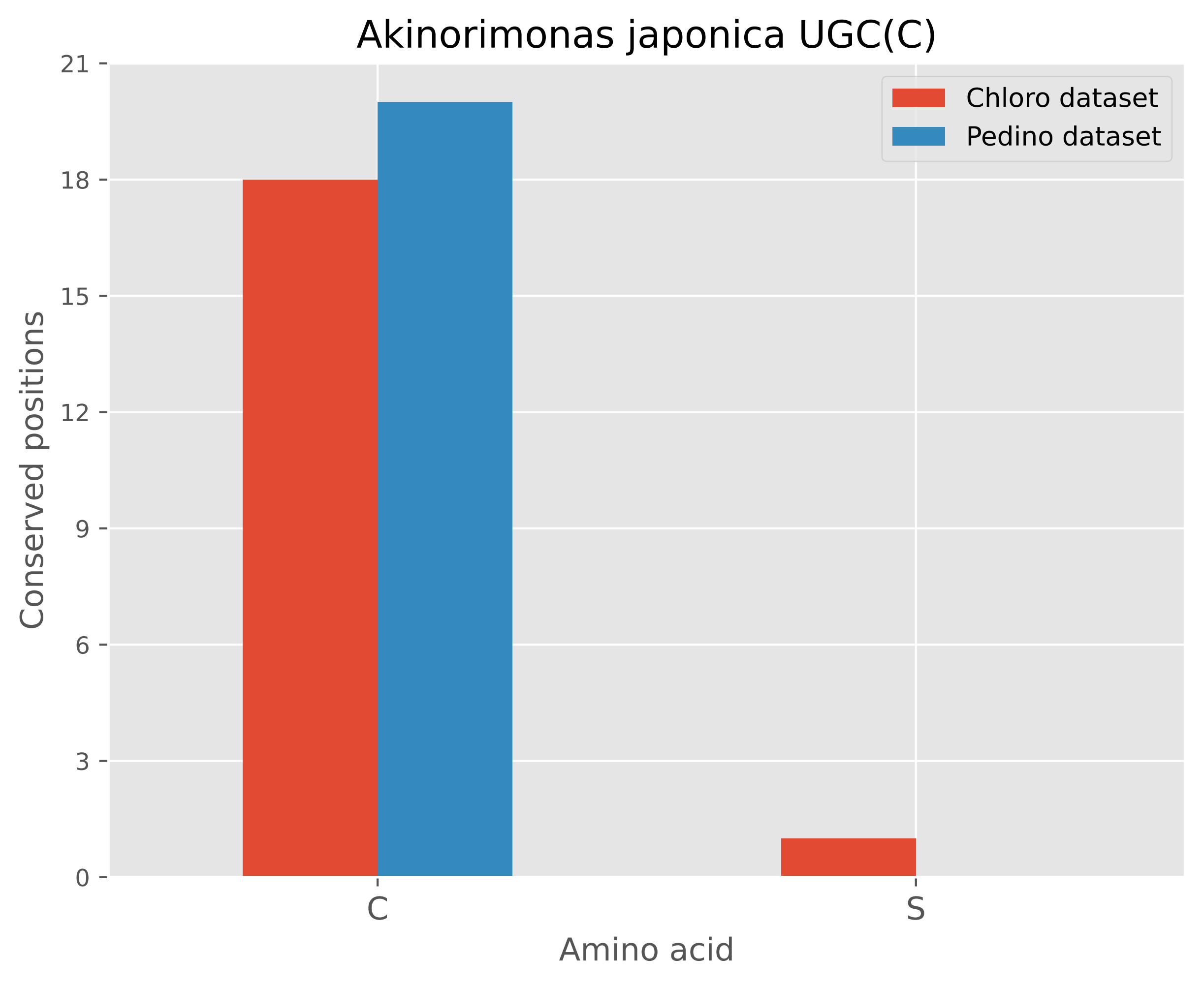

Akinorimonas japonica UGC(C)
Chloro dataset
Pedino dataset
Conserved positions
0
3
6
9
12
15
18
21
C
S
Amino acid

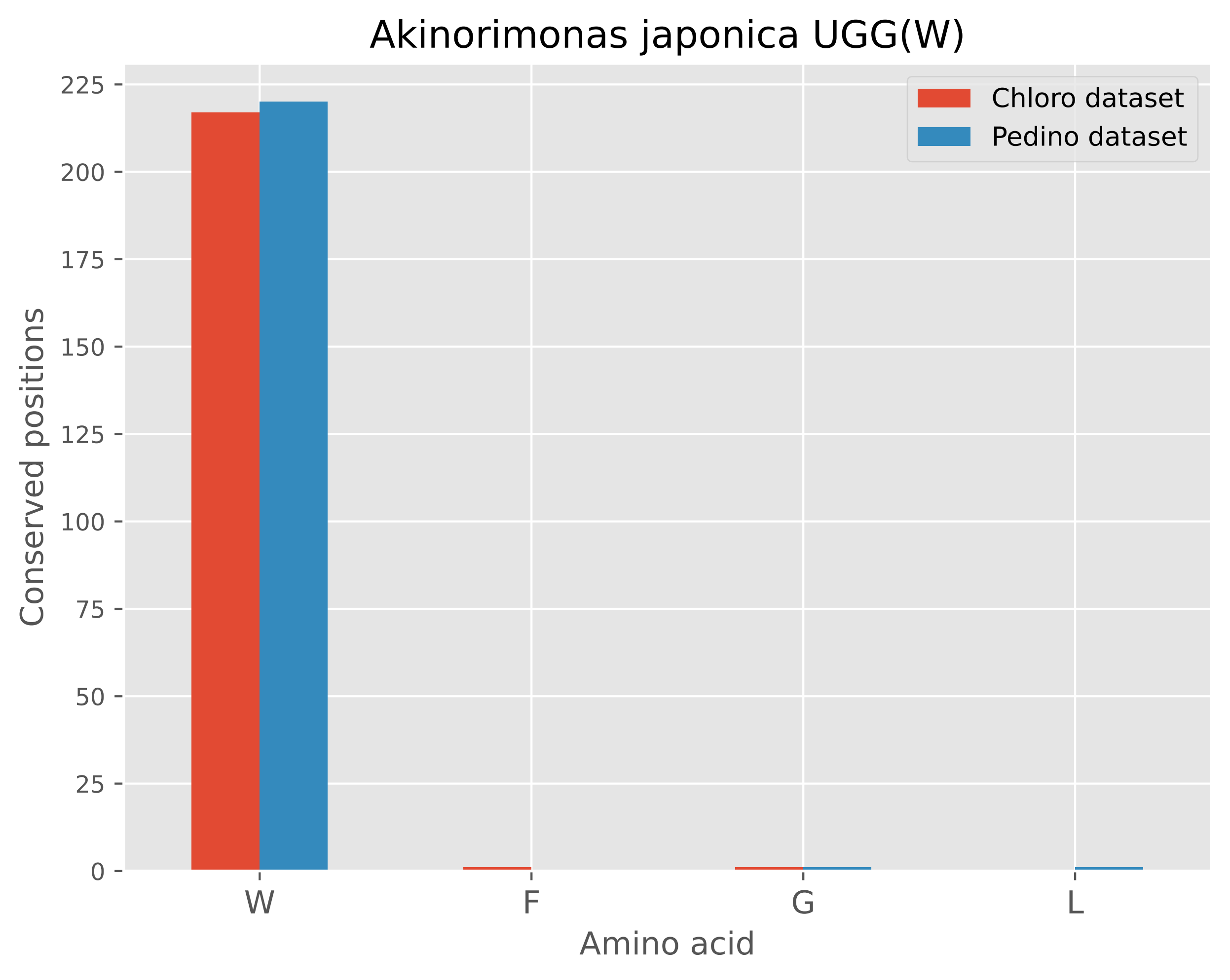

Akinorimonas japonica UGG(W)
Chloro dataset
Pedino dataset
Conserved positions
0
25
50
75
100
125
150
175
200
225
W
F
G
L
Amino acid

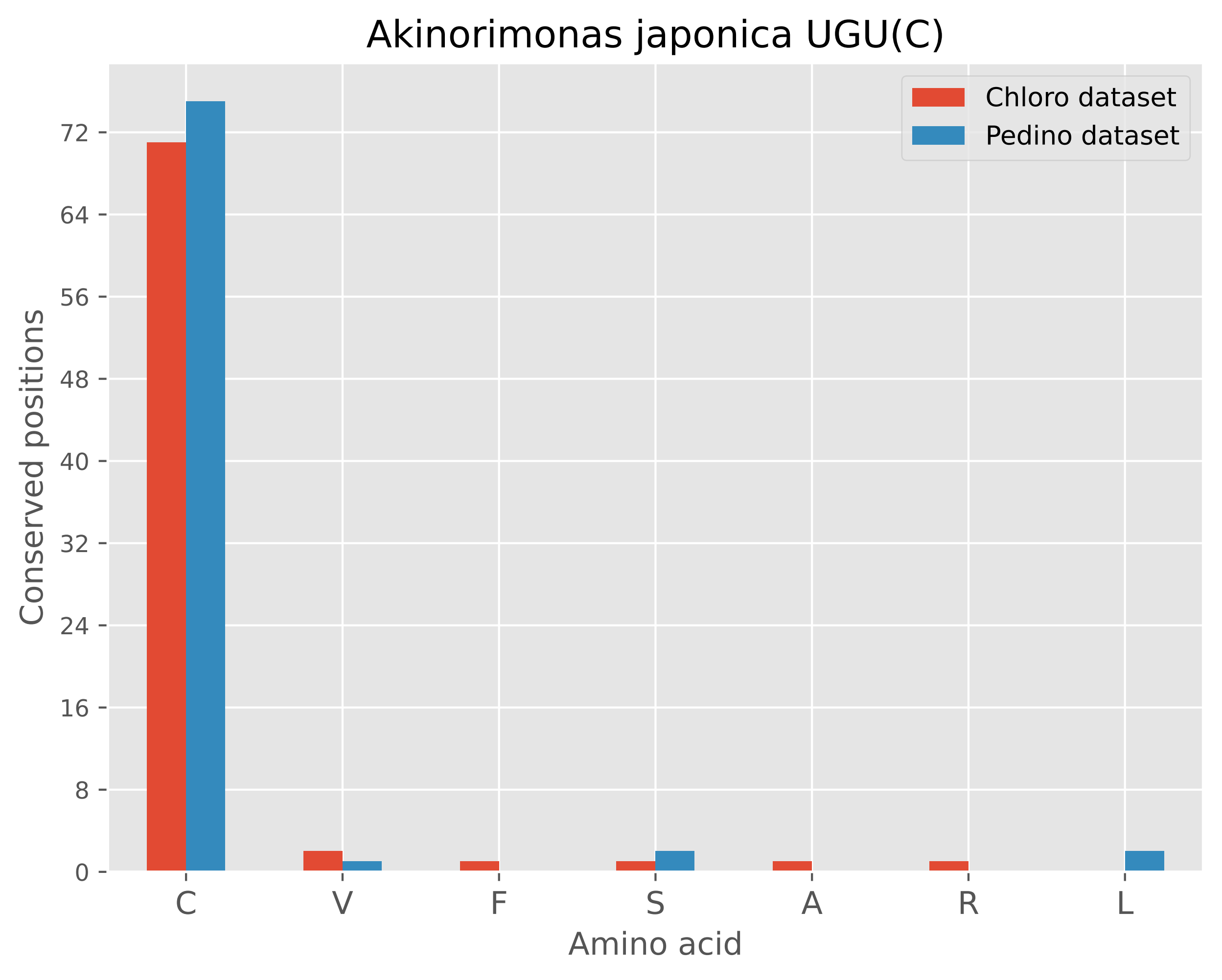
Akinorimonas japonica UGU(C)
Chloro dataset
Pedino dataset
Conserved positions
0
8
16
24
32
40
48
56
64
72
C
V
F
S
A
R
L
Amino acid

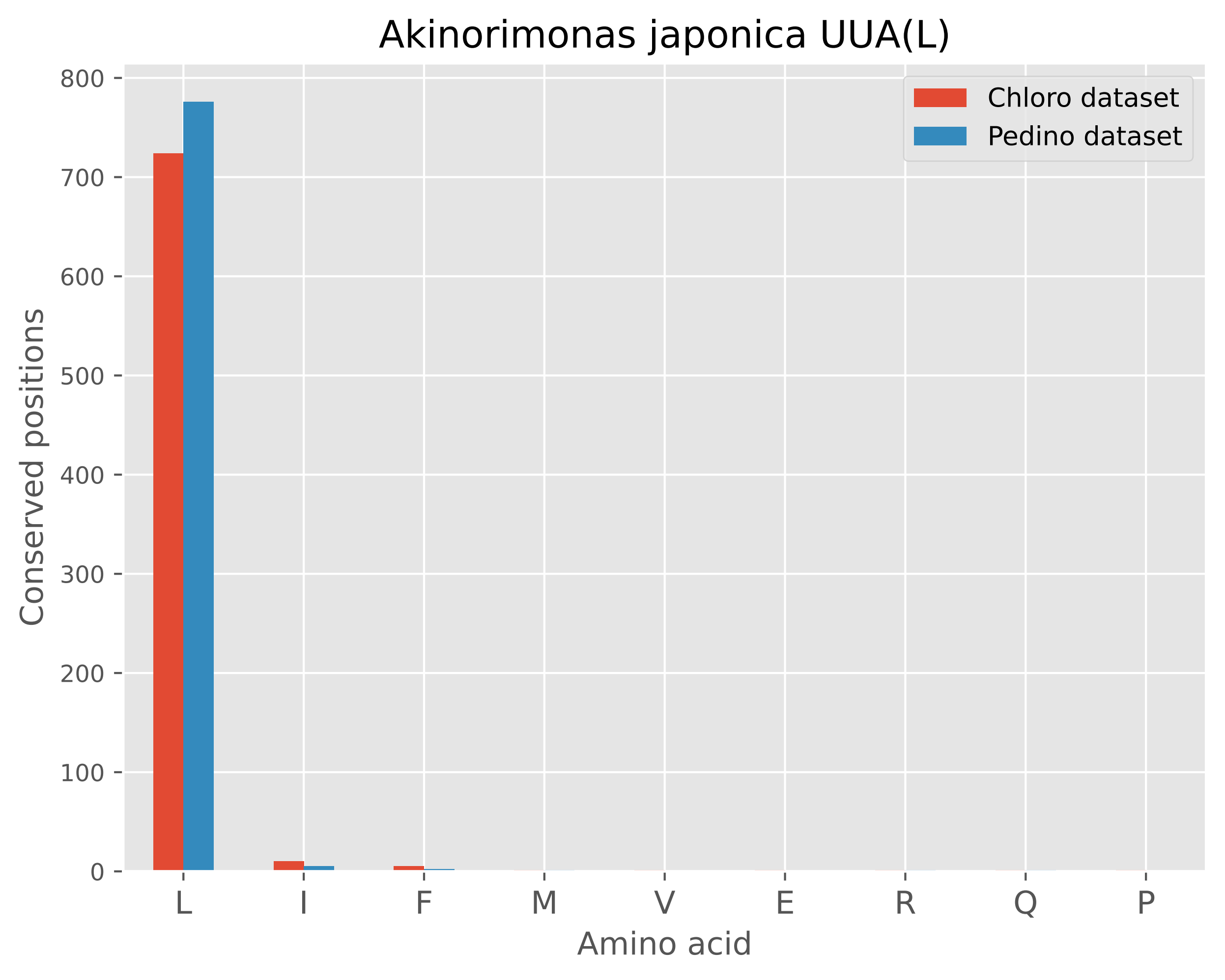

Akinorimonas japonica UUA(L)
Chloro dataset
Pedino dataset
Conserved positions
0
100
200
300
400
500
600
700
800
L
I
F
M
V
E
R
Q
P
Amino acid

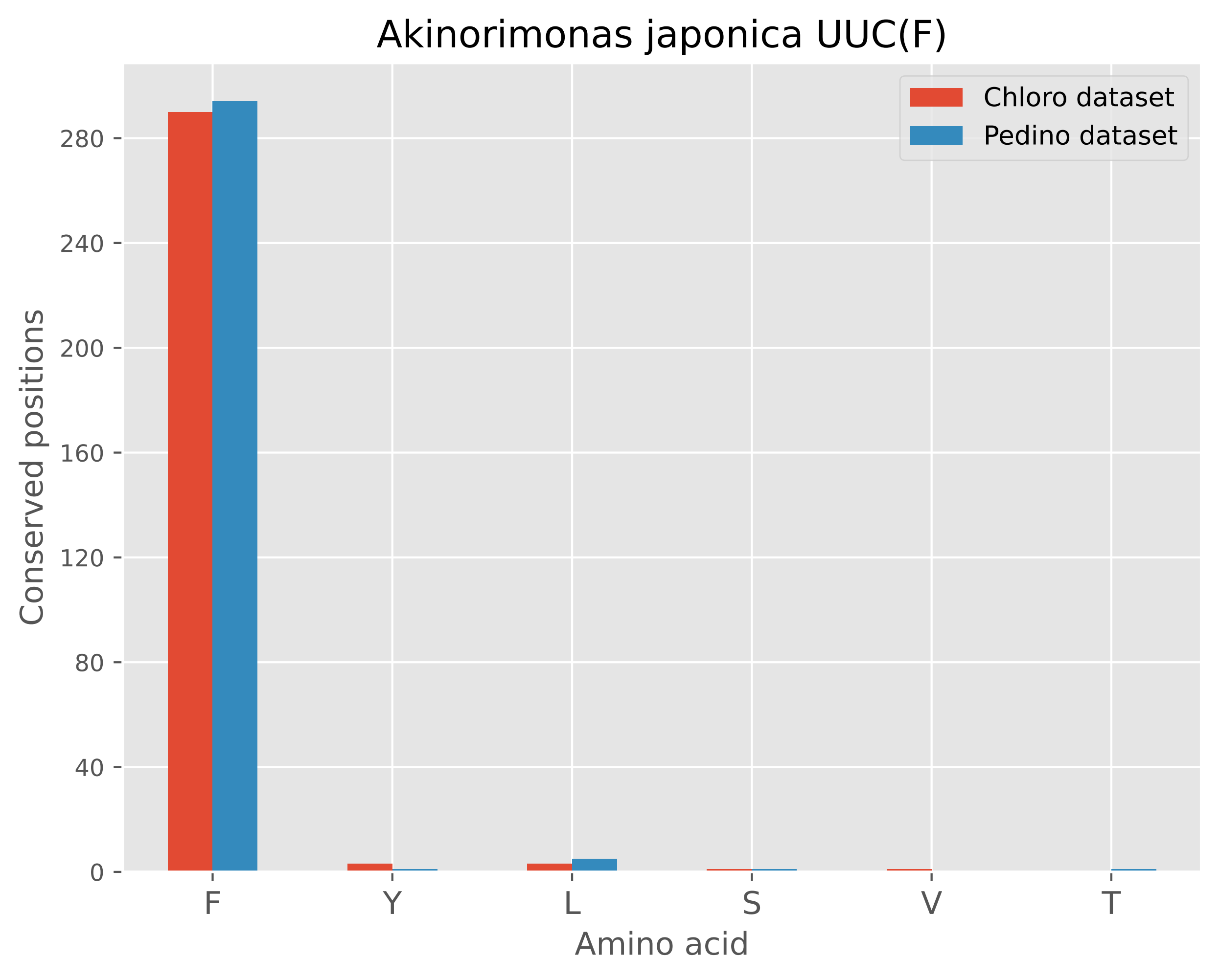

Akinorimonas japonica UUC(F)
Chloro dataset
Pedino dataset
Conserved positions
0
40
80
120
160
200
240
280
F
Y
L
S
V
T
Amino acid

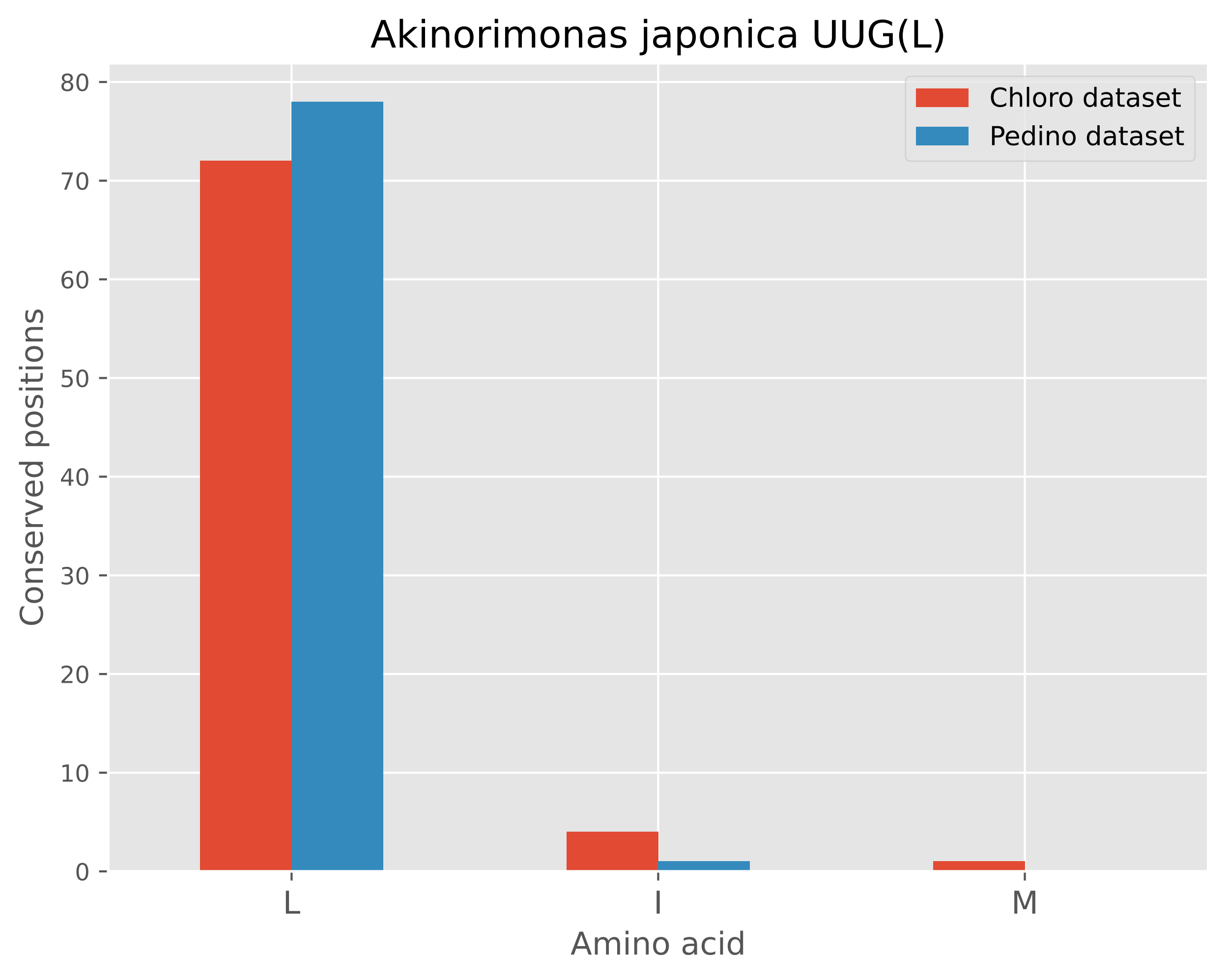
Akinorimonas japonica UUG(L)
Chloro dataset
Pedino dataset
Conserved positions
0
10
20
30
40
50
60
70
80
L
I
M
Amino acid

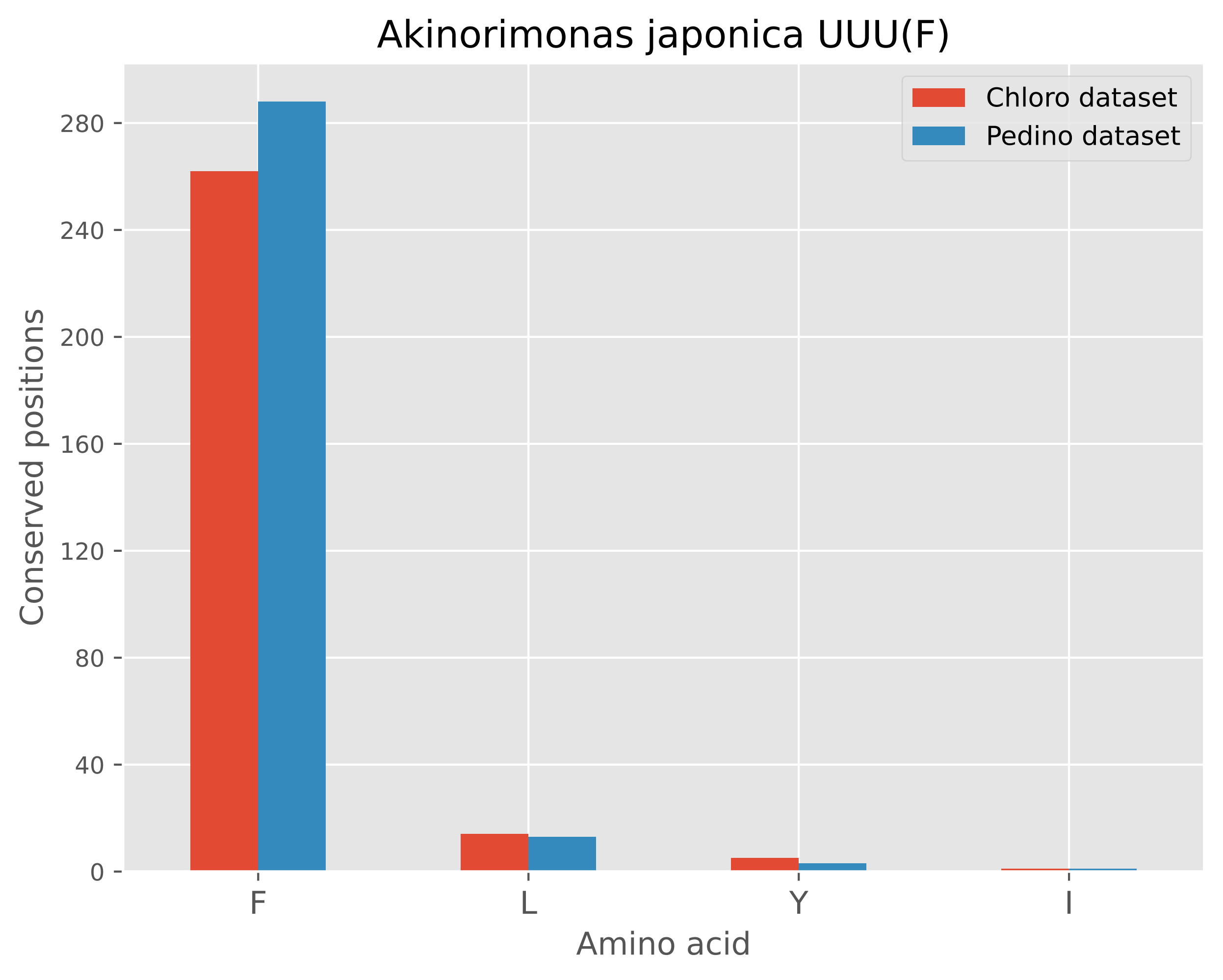
Akinorimonas japonica UUU(F)
Chloro dataset
Pedino dataset
Conserved positions
0
40
80
120
160
200
240
280
F
L
Y
I
Amino acid
