# Supplementary material for: Expanded diversity of pedinophytes provides a window into the evolution of the genetic code in organelles
Source: PLoS Genet. 2025 Oct 22;21(10):e1011901. doi: 10.1371/journal.pgen.1011901 (PMC12574857; doi:10.1371/journal.pgen.1011901)
Supplement: S2 Dataset — Absence of a plot for a given codon in a given taxon means the codon was not present at any position deemed conserved. (PDF) [file pgen.1011901.s032.pdf]

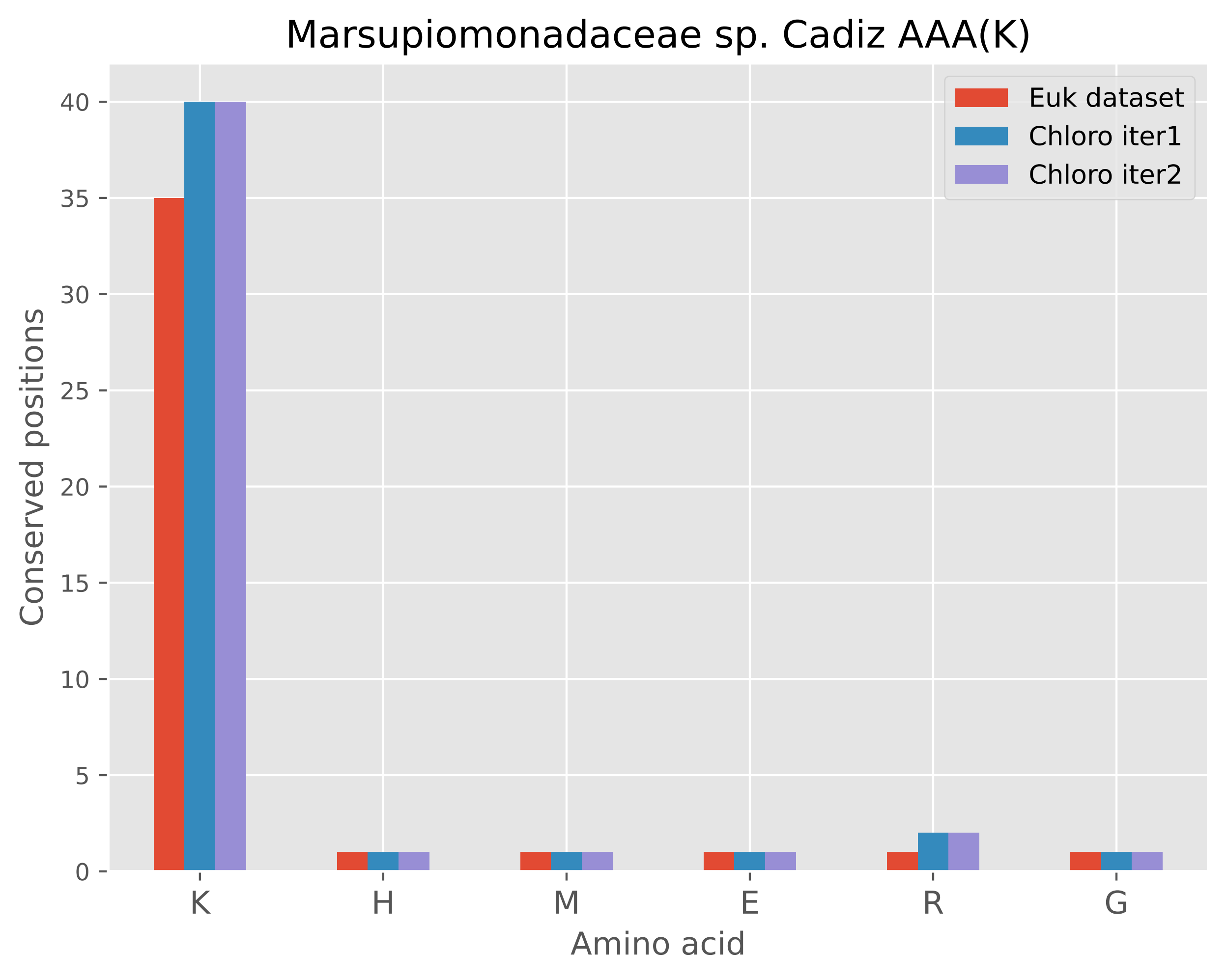
Marsupiomonadaceae sp. Cadiz AAA(K)
Euk dataset
Chloro iter1
Chloro iter2
Conserved positions
0
5
10
15
20
25
30
35
40
K
H
M
E
R
G
Amino acid

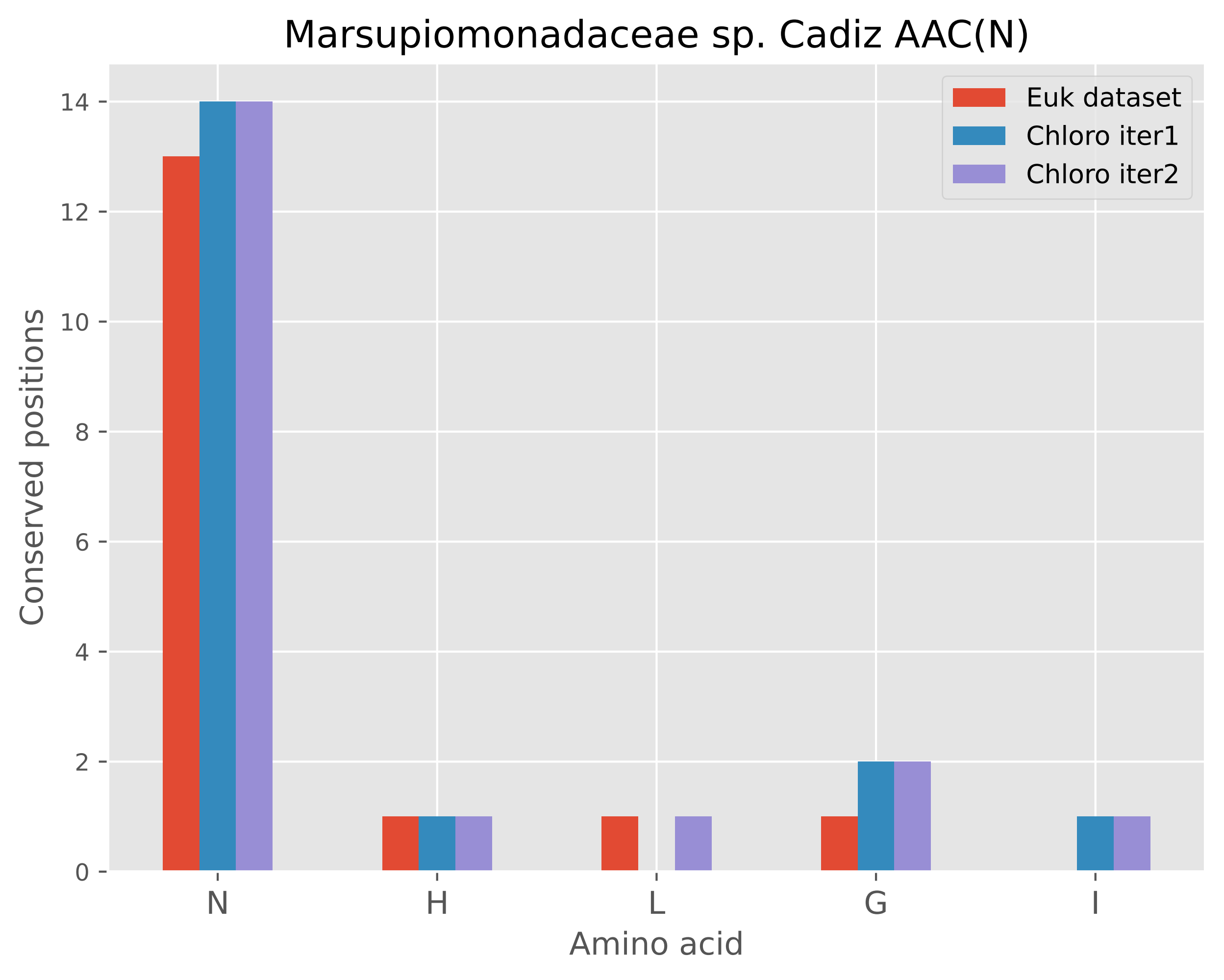
Marsupiomonadaceae sp. Cadiz AAC(N)
Euk dataset
Chloro iter1
Chloro iter2
Conserved positions
0
2
4
6
8
10
12
14
N
H
L
G
I
Amino acid

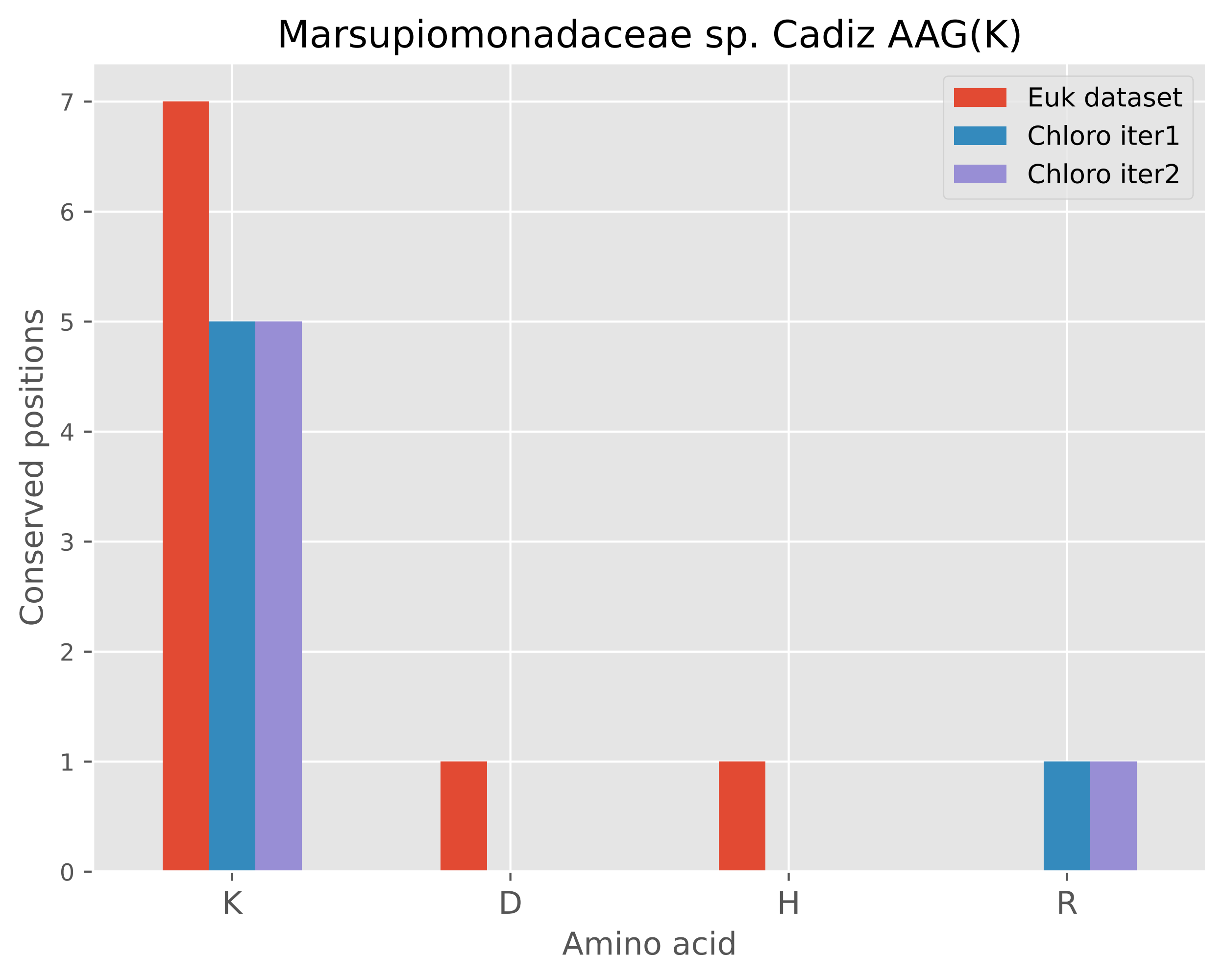

Marsupiomonadaceae sp. Cadiz AAG(K)
Euk dataset
Chloro iter1
Chloro iter2
Conserved positions
0
1
2
3
4
5
6
7
K
D
H
R
Amino acid

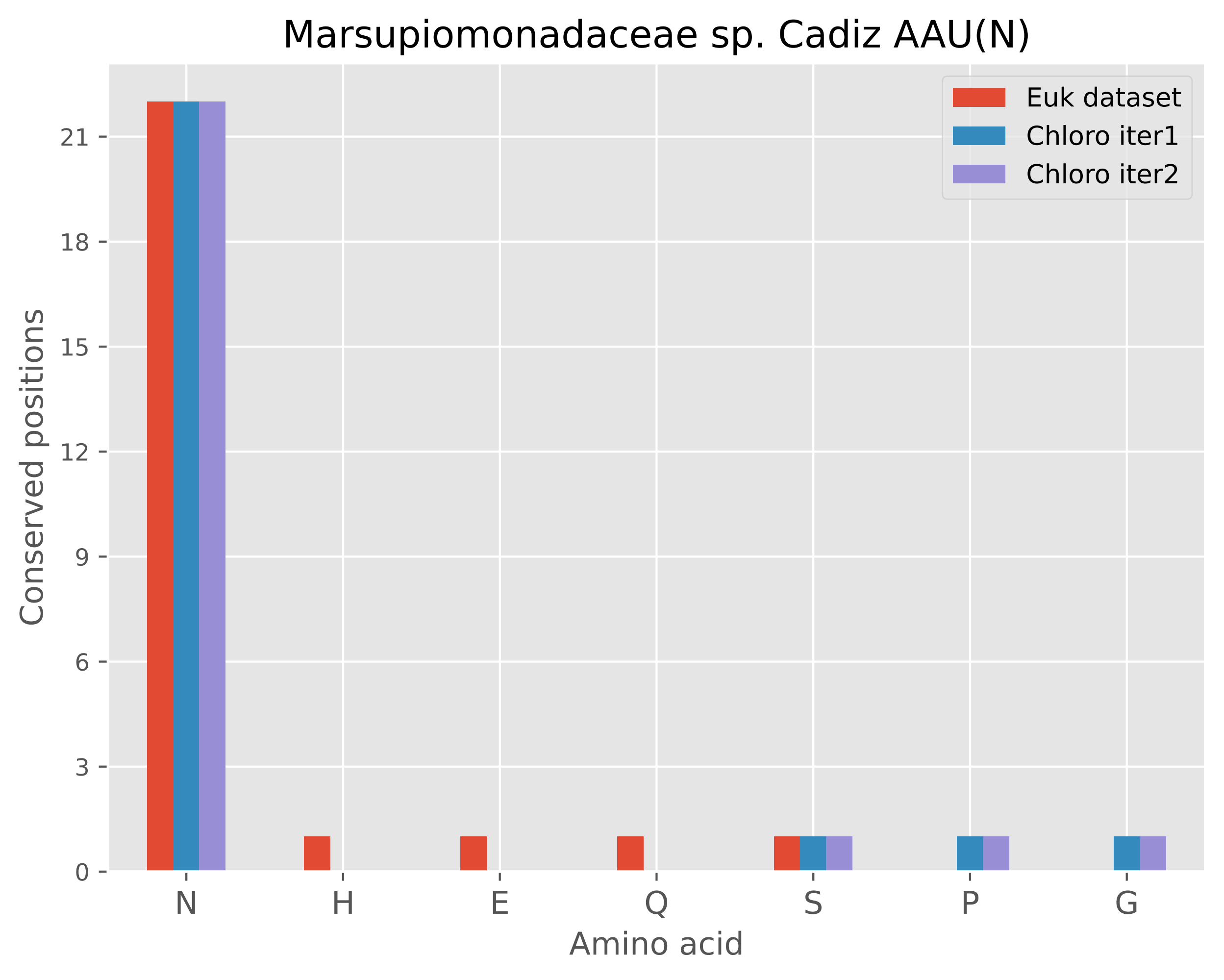

Marsupiomonadaceae sp. Cadiz AAU(N)
Euk dataset
Chloro iter1
Chloro iter2
Conserved positions
0
3
6
9
12
15
18
21
N
H
E
Q
S
P
G
Amino acid

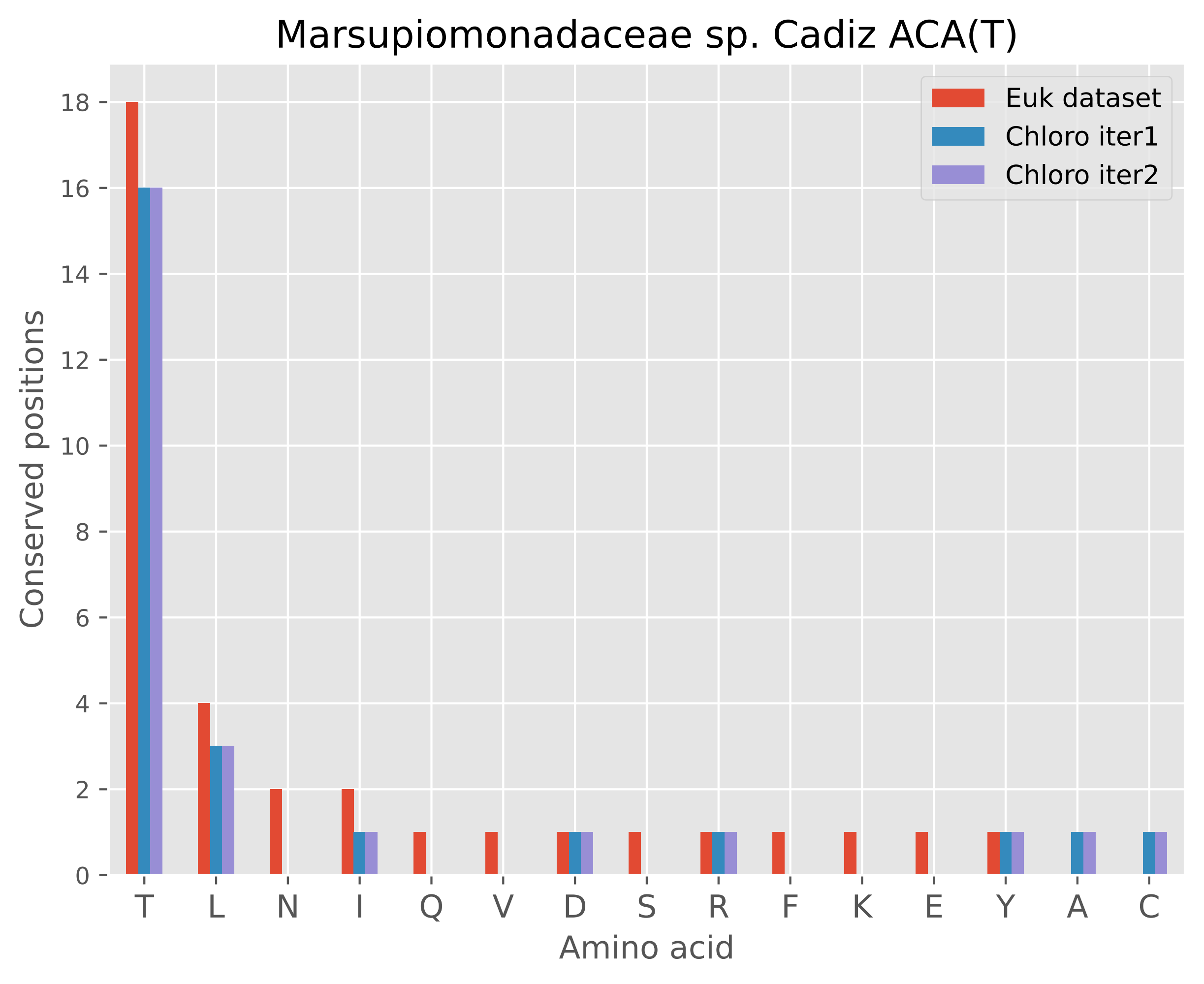
Marsupiomonadaceae sp. Cadiz ACA(T)
Euk dataset
Chloro iter1
Chloro iter2
Conserved positions
Amino acid
0
2
4
6
8
10
12
14
16
18
T
L
N
I
Q
V
D
S
R
F
K
E
Y
A
C

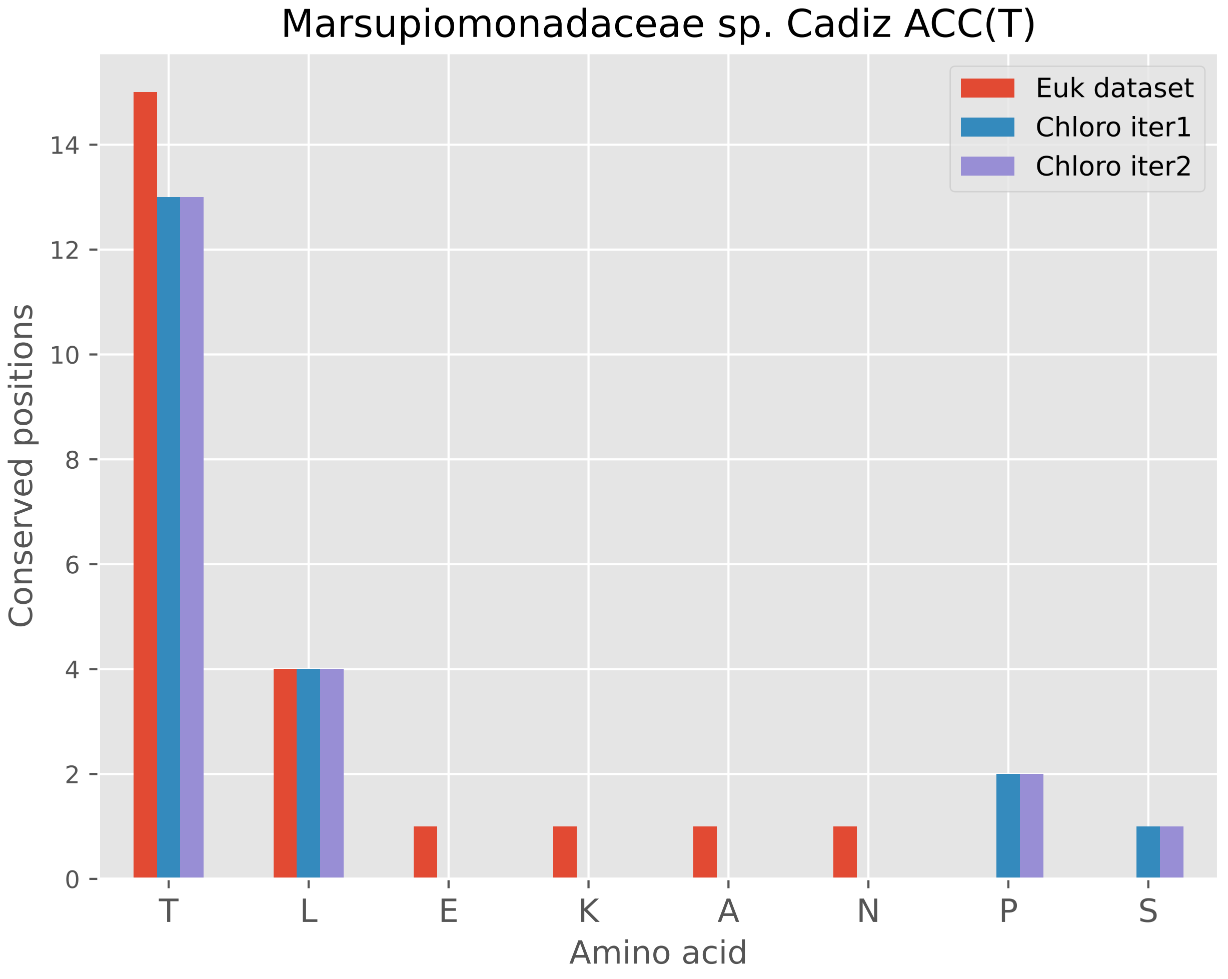
Marsupiomonadaceae sp. Cadiz ACC(T)
Euk dataset
Chloro iter1
Chloro iter2
Conserved positions
0
2
4
6
8
10
12
14
T
L
E
K
A
N
P
S
Amino acid

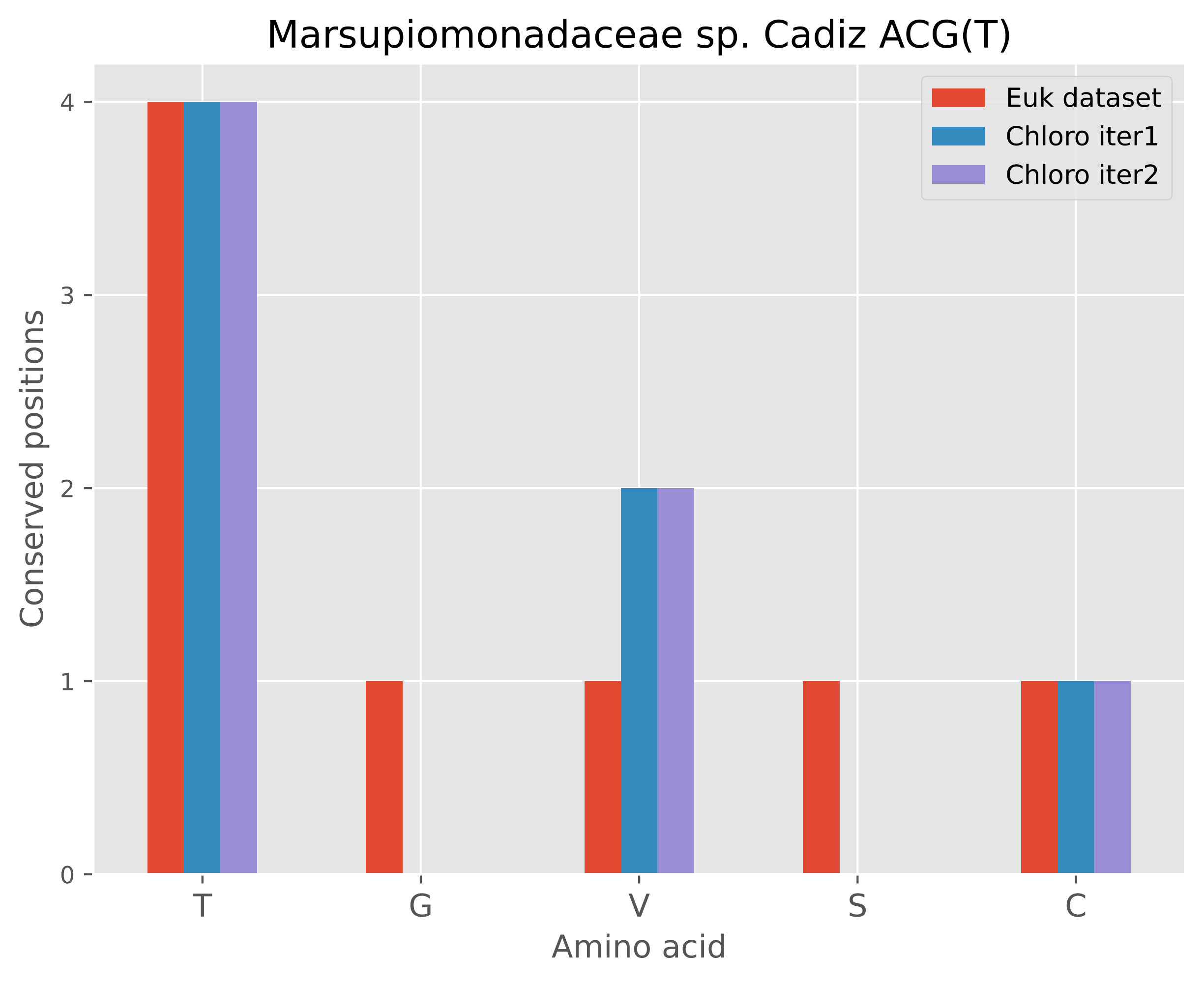
Marsupiomonadaceae sp. Cadiz ACG(T)
Euk dataset
Chloro iter1
Chloro iter2
Conserved positions
0
1
2
3
4
T
G
V
S
C
Amino acid

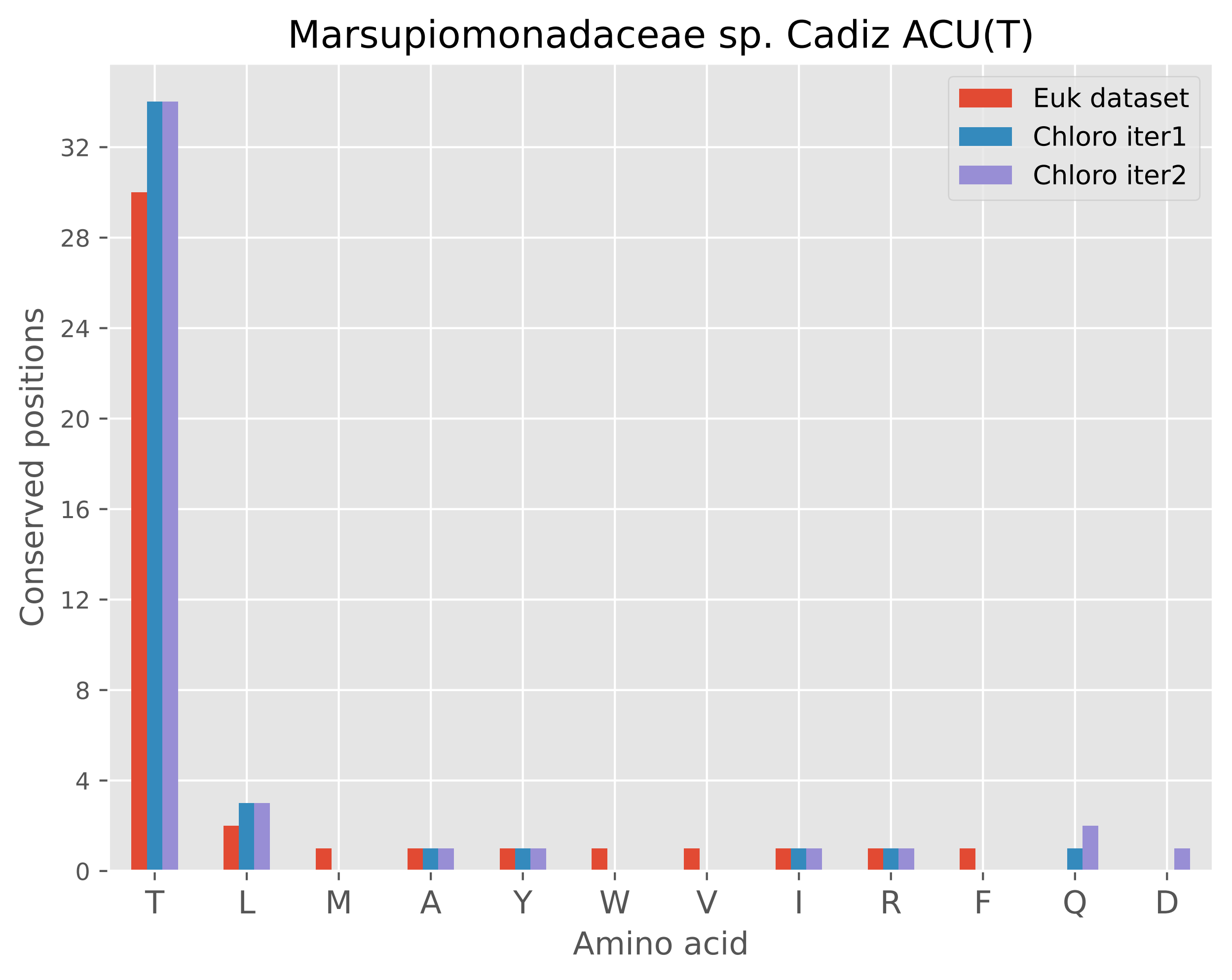
Marsupiomonadaceae sp. Cadiz ACU(T)
Euk dataset
Chloro iter1
Chloro iter2
Conserved positions
0
4
8
12
16
20
24
28
32
T
L
M
A
Y
W
V
I
R
F
Q
D
Amino acid

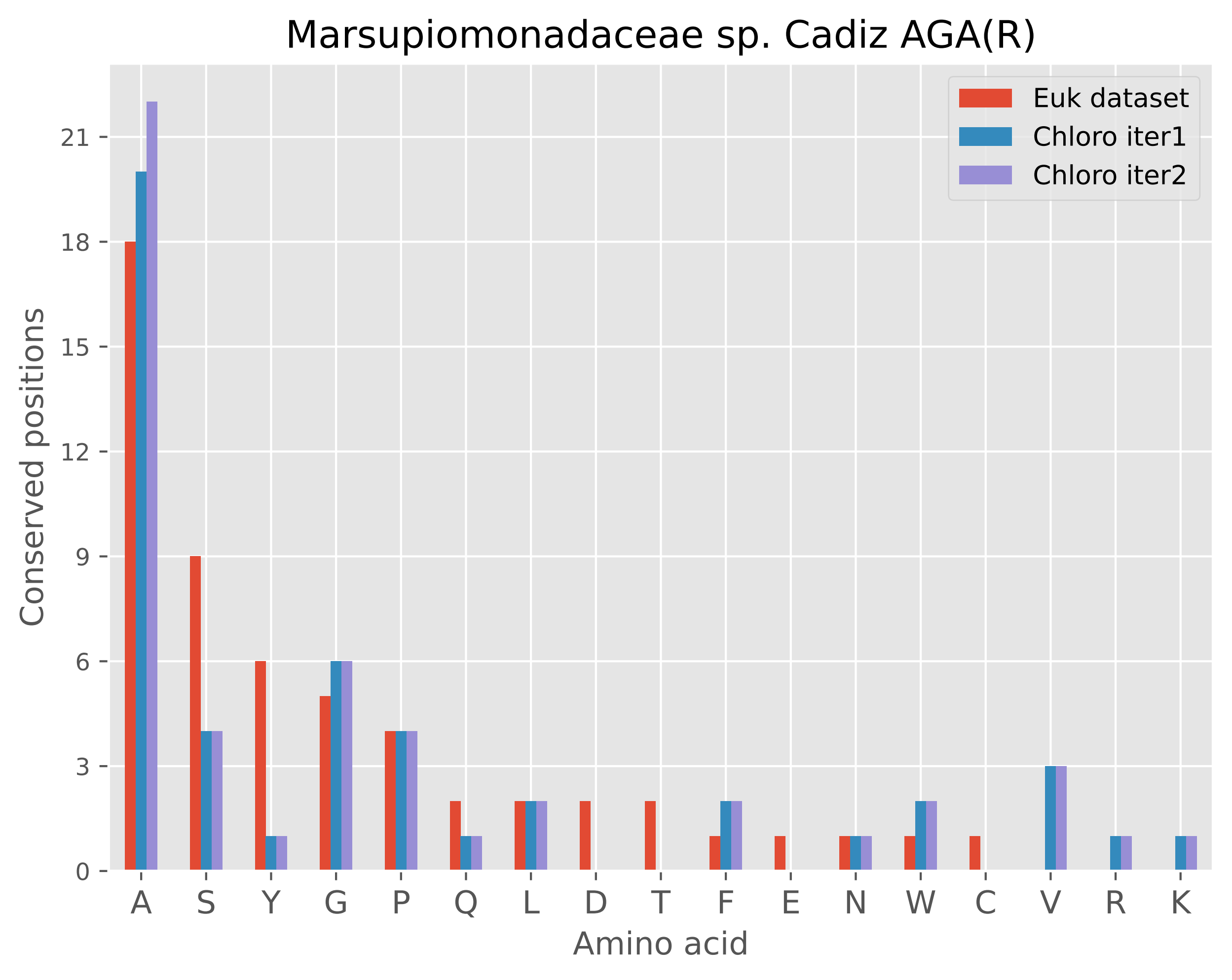
Marsupiomonadaceae sp. Cadiz AGA(R)
Euk dataset
Chloro iter1
Chloro iter2
Conserved positions
0
3
6
9
12
15
18
21
A
S
Y
G
P
Q
L
D
T
F
E
N
W
C
V
R
K
Amino acid

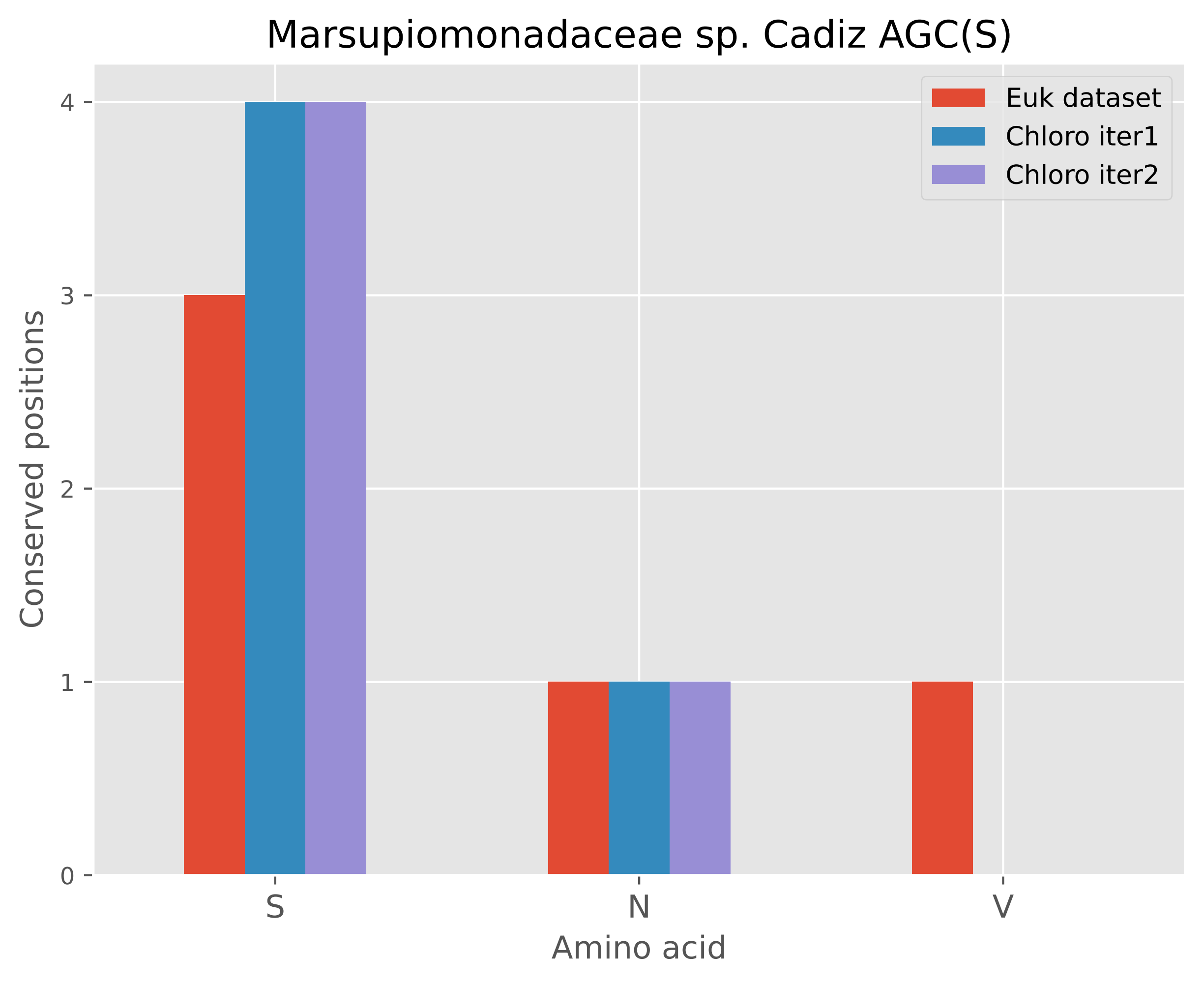
Marsupiomonadaceae sp. Cadiz AGC(S)
Euk dataset
Chloro iter1
Chloro iter2
Conserved positions
0
1
2
3
4
S
N
V
Amino acid

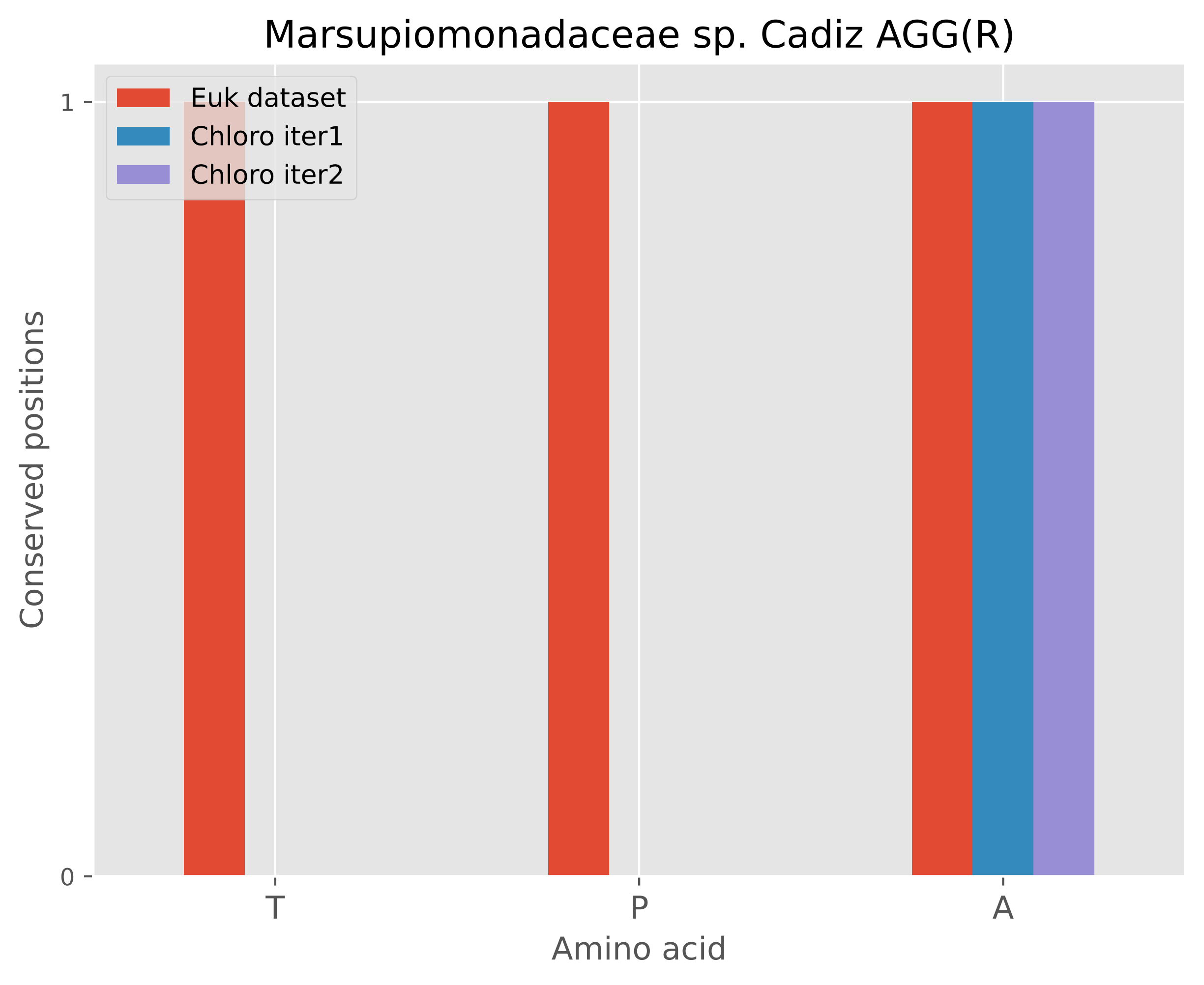

Marsupiomonadaceae sp. Cadiz AGG(R)
Euk dataset
Chloro iter1
Chloro iter2
1
0
Conserved positions
T
P
A
Amino acid

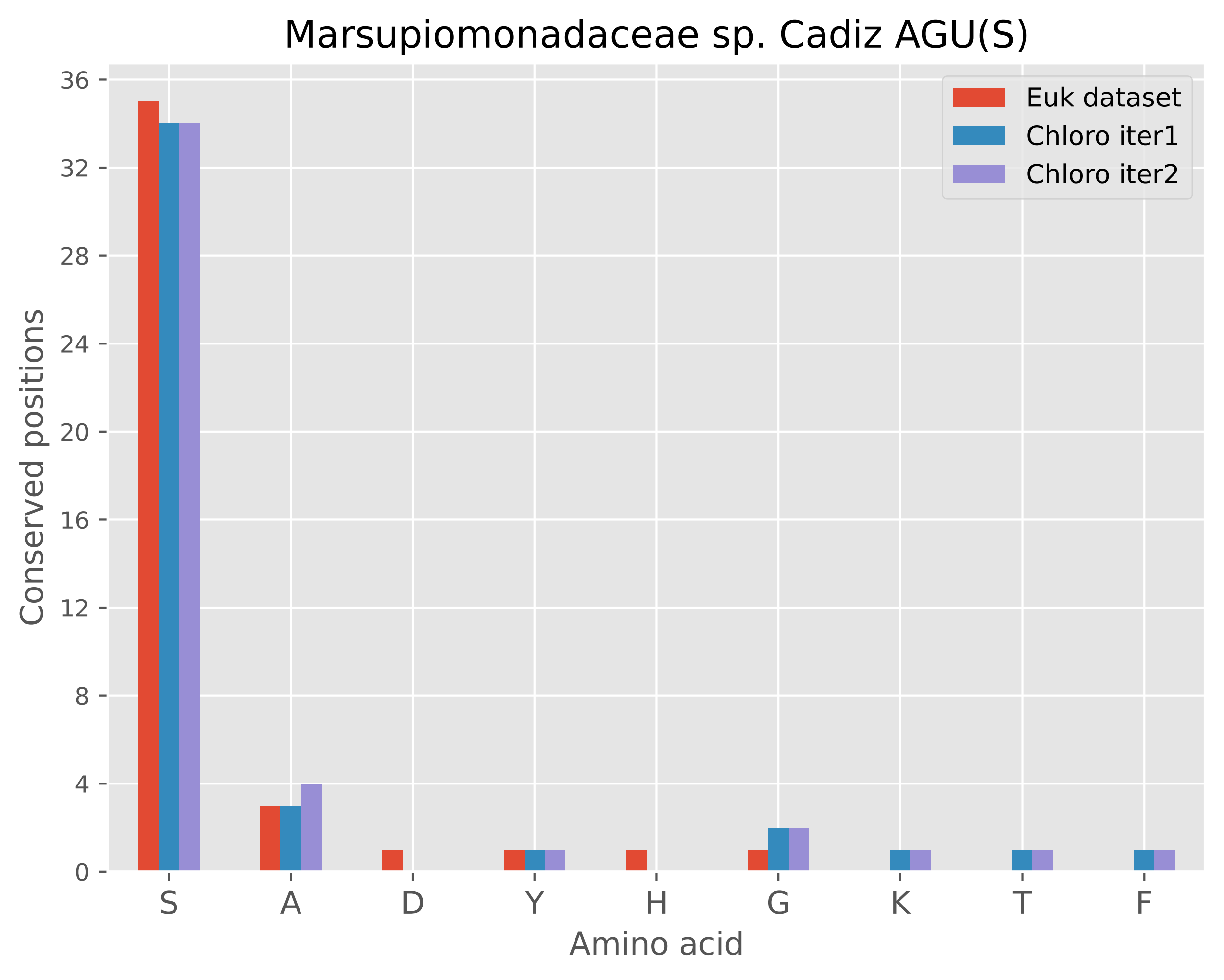

Marsupiomonadaceae sp. Cadiz AGU(S)
Euk dataset
Chloro iter1
Chloro iter2
Conserved positions
Amino acid
0
4
8
12
16
20
24
28
32
36
S
A
D
Y
H
G
K
T
F

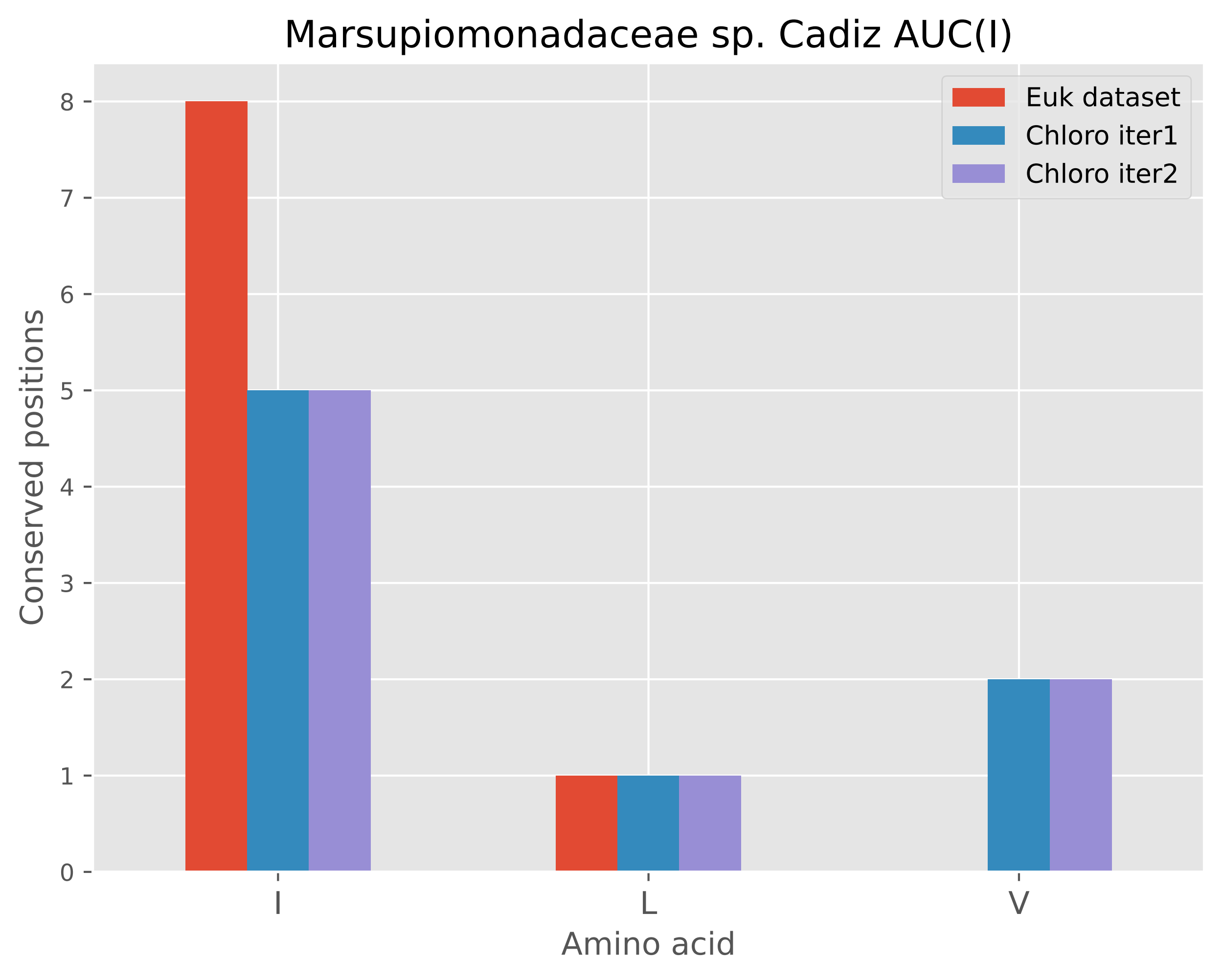

Marsupiomonadaceae sp. Cadiz AUC(I)
Euk dataset
Chloro iter1
Chloro iter2
Conserved positions
0
1
2
3
4
5
6
7
8
I
L
V
Amino acid

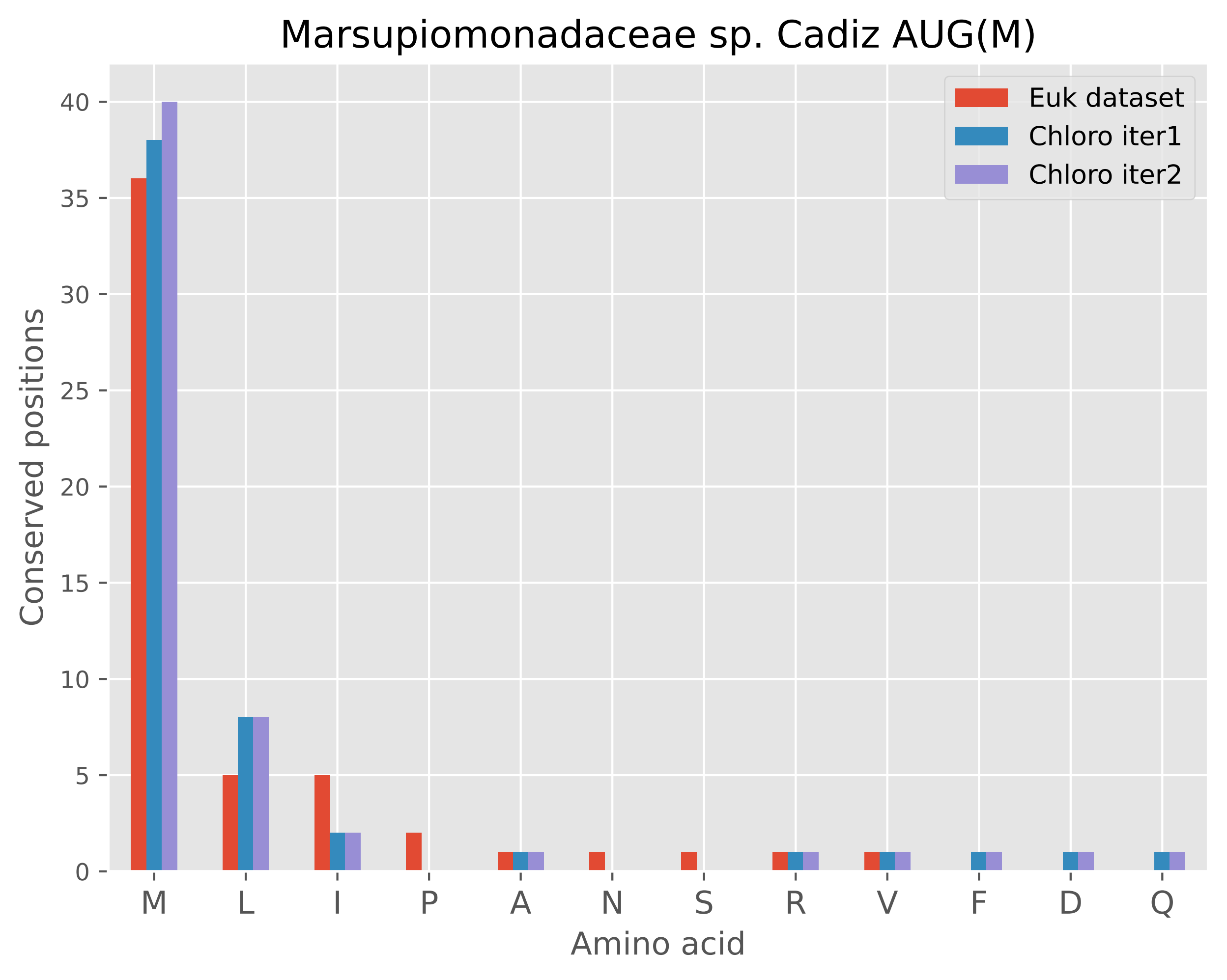

Marsupiomonadaceae sp. Cadiz AUG(M)
Euk dataset
Chloro iter1
Chloro iter2
Conserved positions
0
5
10
15
20
25
30
35
40
M
L
I
P
A
N
S
R
V
F
D
Q
Amino acid

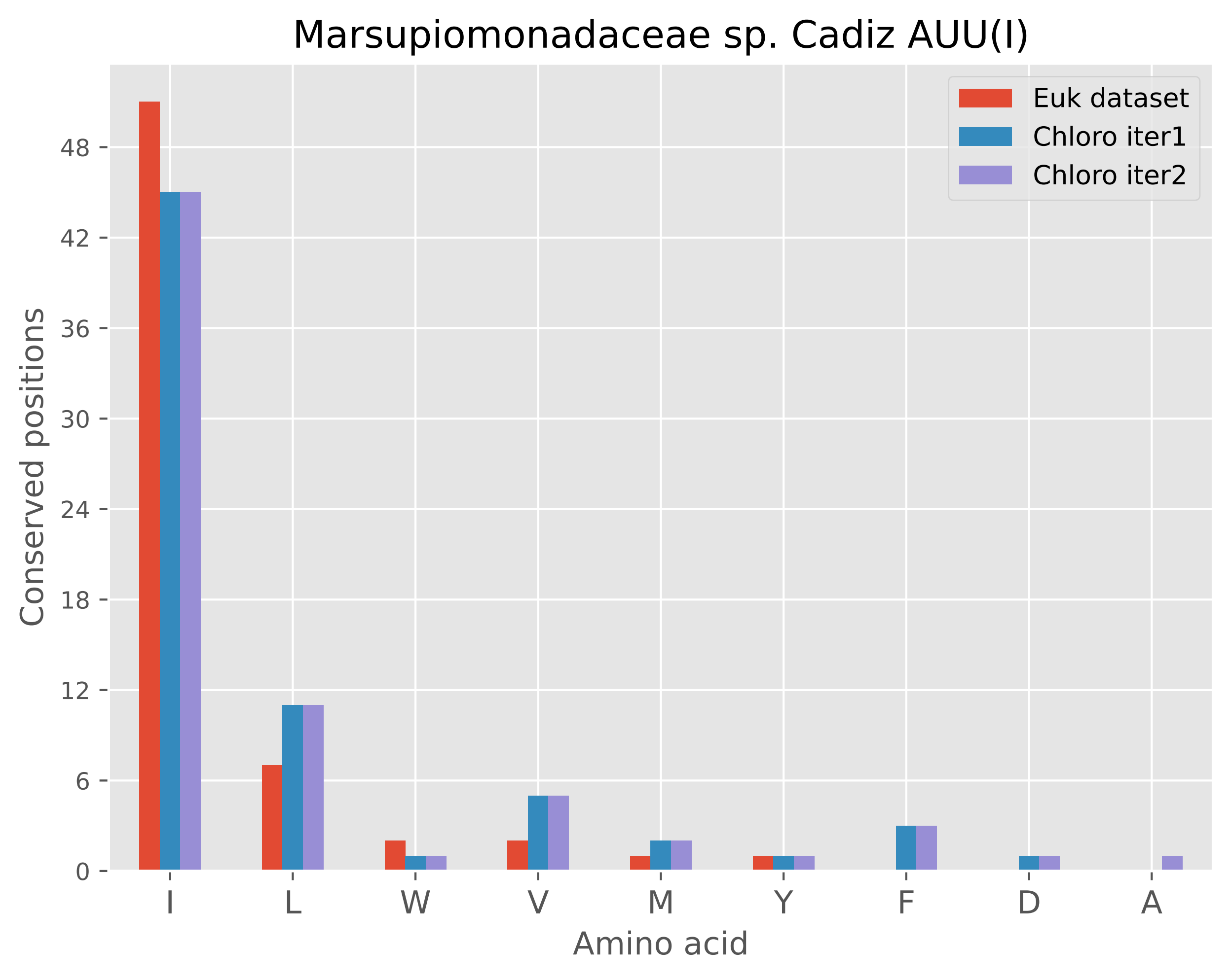
Marsupiomonadaceae sp. Cadiz AUU(I)
Euk dataset
Chloro iter1
Chloro iter2
Conserved positions
0
6
12
18
24
30
36
42
48
I
L
W
V
M
Y
F
D
A
Amino acid

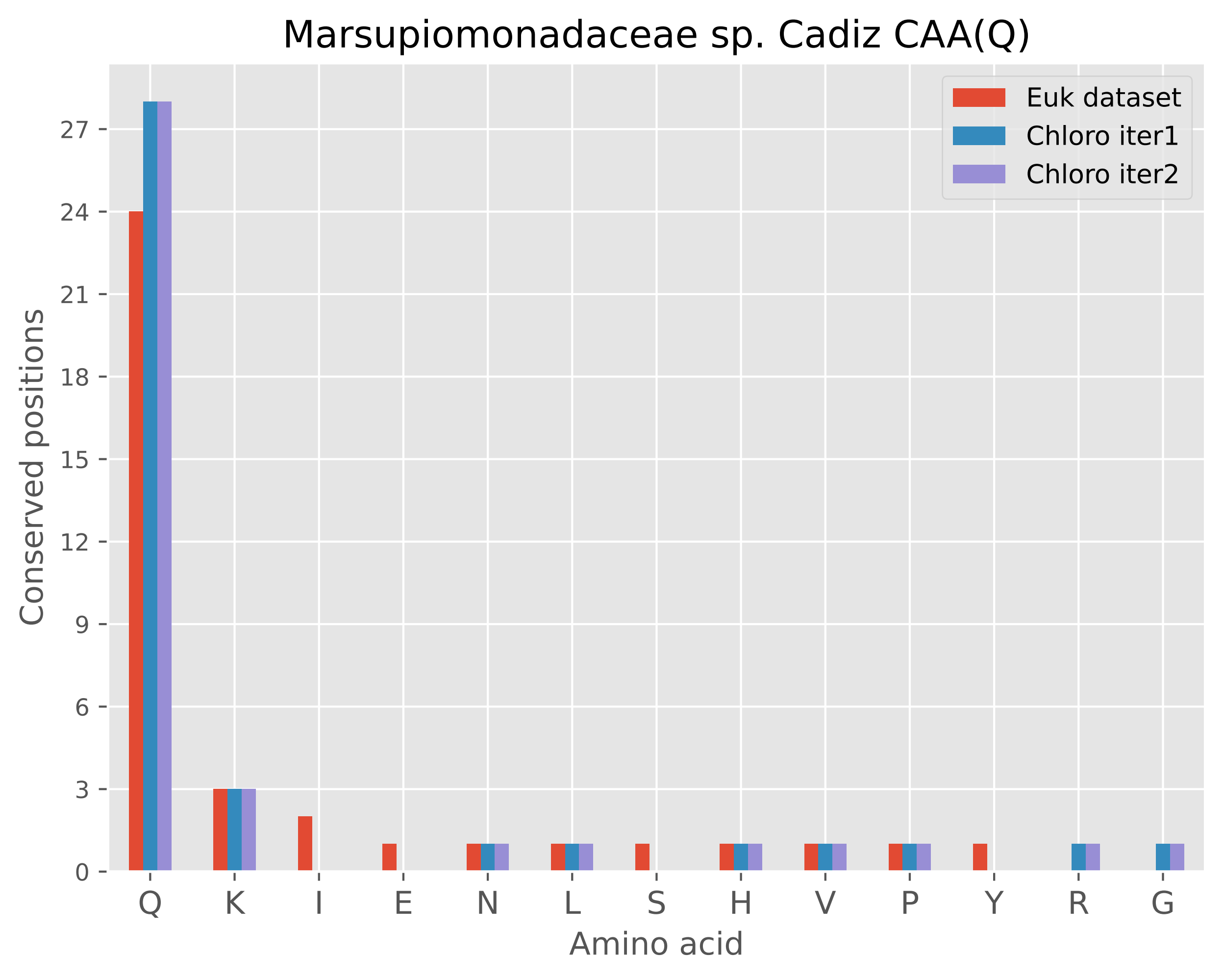

Marsupiomonadaceae sp. Cadiz CAA(Q)
Euk dataset
Chloro iter1
Chloro iter2
Conserved positions
0
3
6
9
12
15
18
21
24
27
Q
K
I
E
N
L
S
H
V
P
Y
R
G
Amino acid

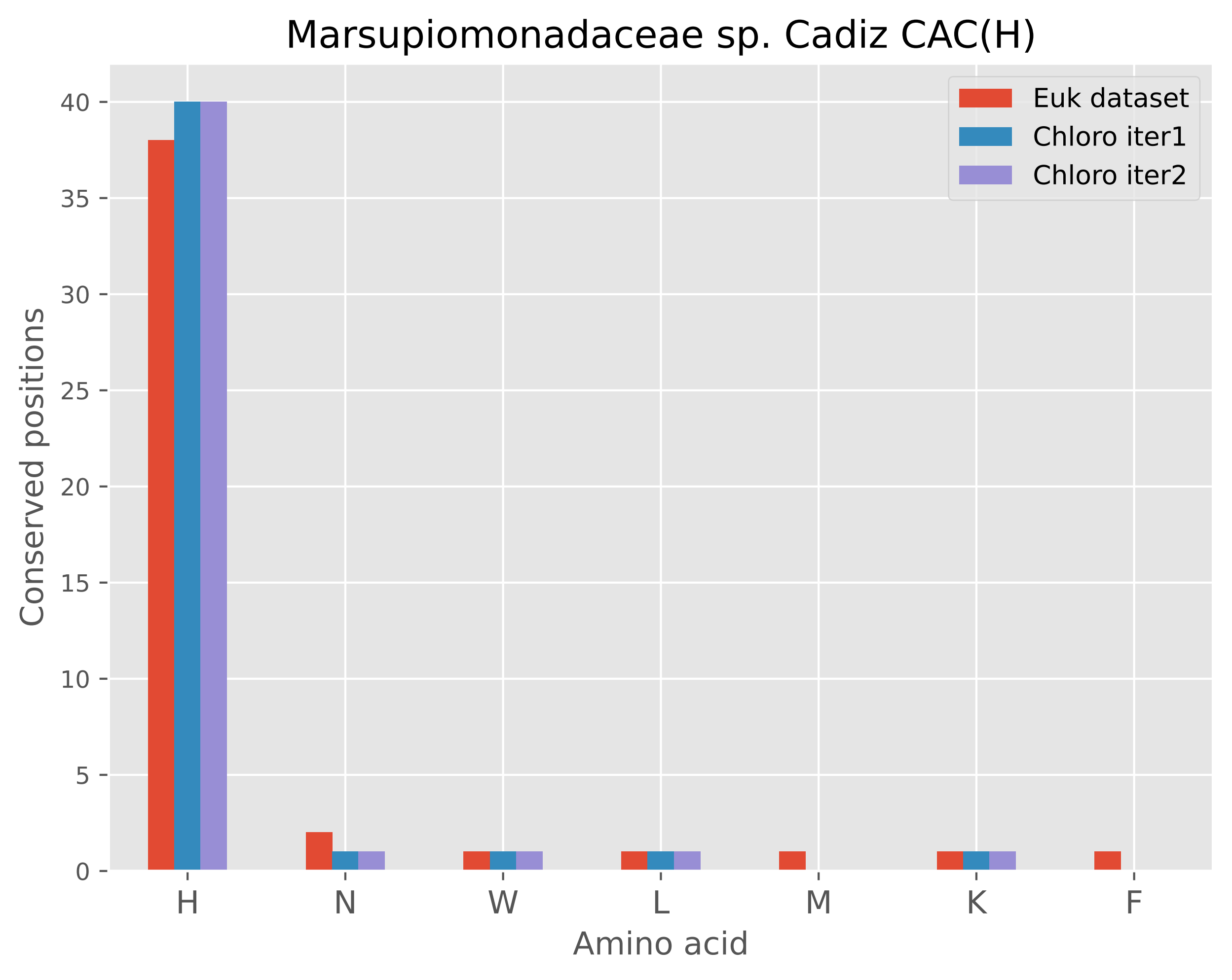

Marsupiomonadaceae sp. Cadiz CAC(H)
Euk dataset
Chloro iter1
Chloro iter2
Conserved positions
Amino acid
0
5
10
15
20
25
30
35
40
H
N
W
L
M
K
F

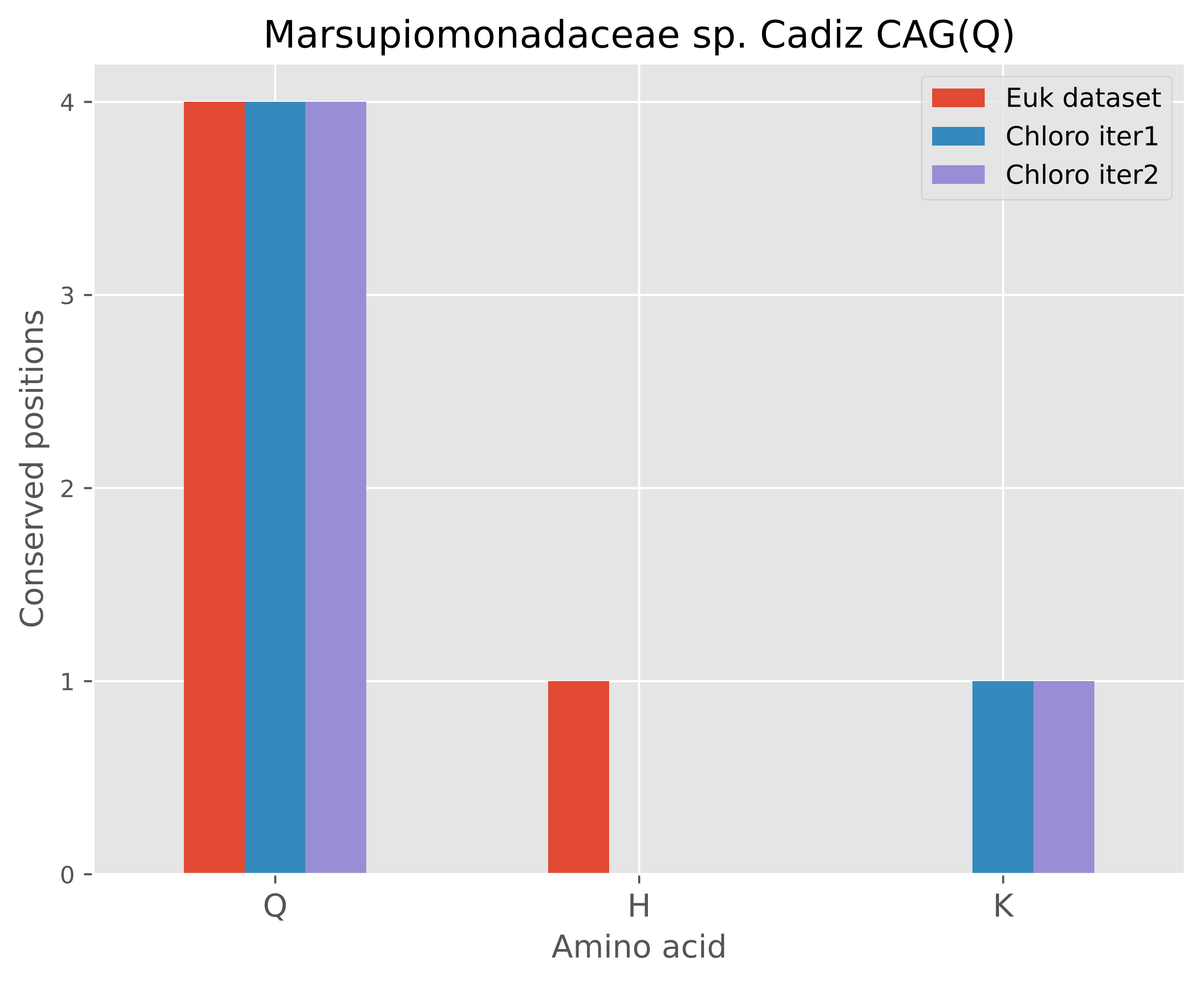
Marsupiomonadaceae sp. Cadiz CAG(Q)
Euk dataset
Chloro iter1
Chloro iter2
Conserved positions
0
1
2
3
4
Q
H
K
Amino acid

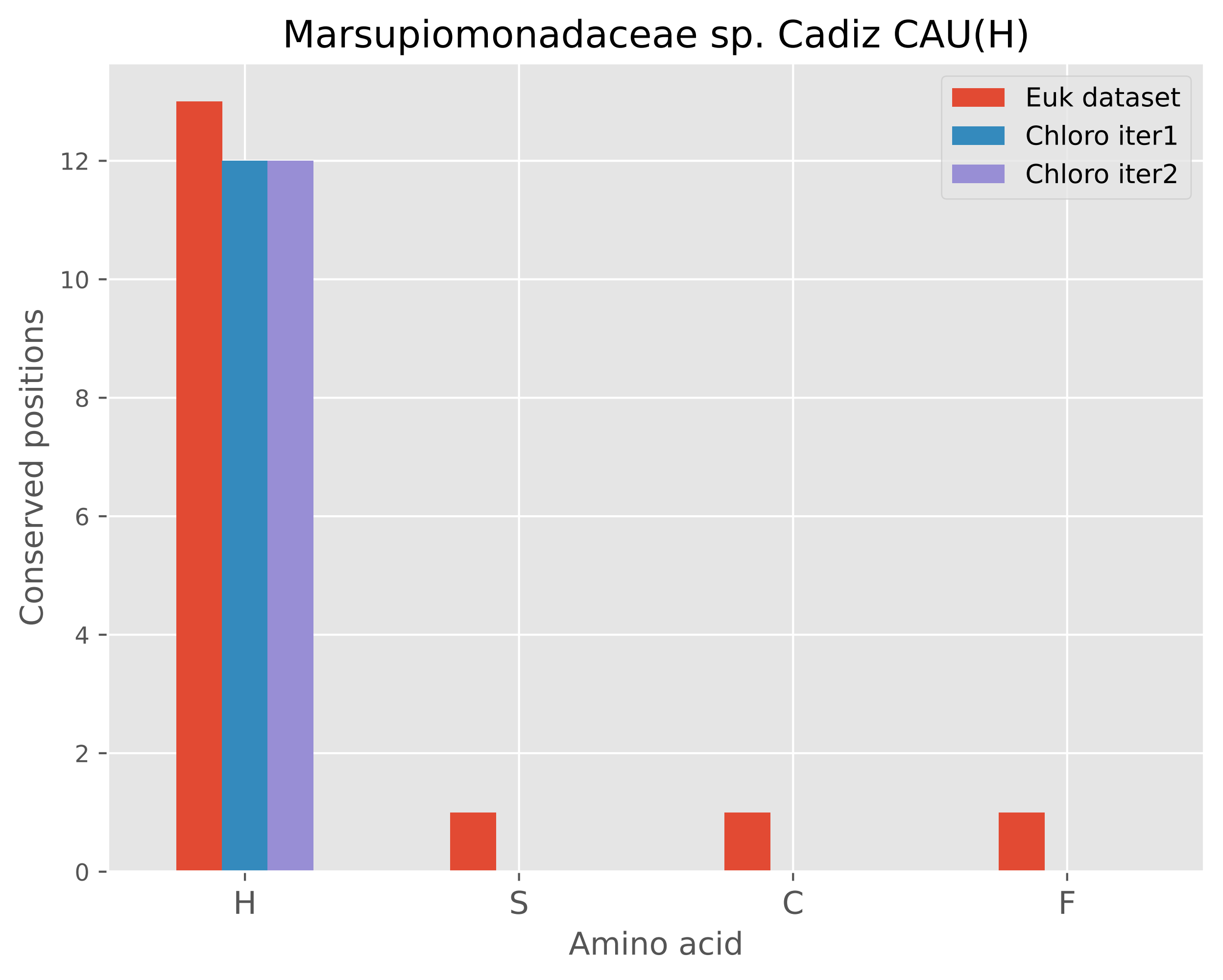

Marsupiomonadaceae sp. Cadiz CAU(H)
Euk dataset
Chloro iter1
Chloro iter2
Conserved positions
0
2
4
6
8
10
12
H
S
C
F
Amino acid

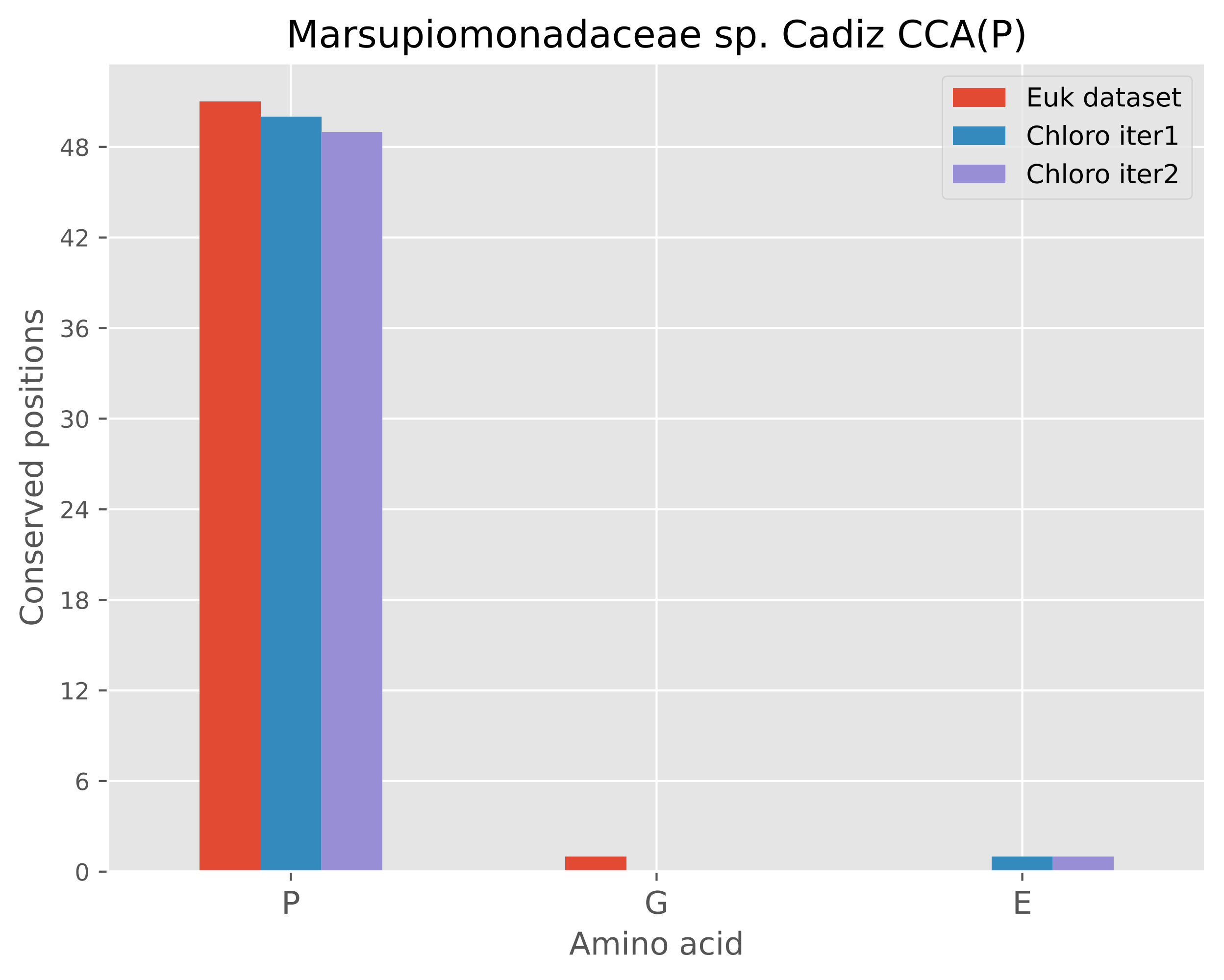

Marsupiomonadaceae sp. Cadiz CCA(P)
Euk dataset
Chloro iter1
Chloro iter2
Conserved positions
0
6
12
18
24
30
36
42
48
P
G
E
Amino acid

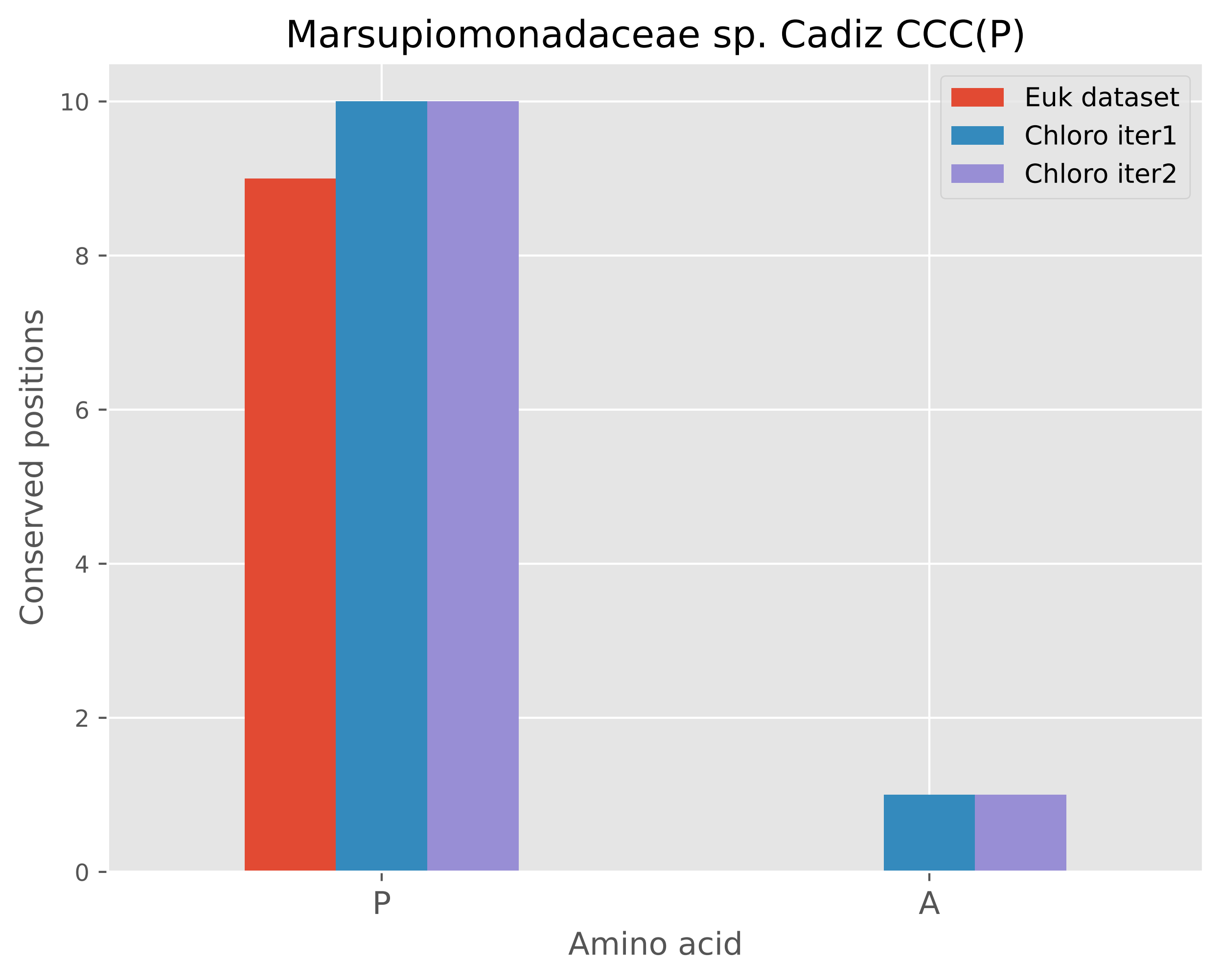

Marsupiomonadaceae sp. Cadiz CCC(P)
Euk dataset
Chloro iter1
Chloro iter2
Conserved positions
0
2
4
6
8
10
P
A
Amino acid

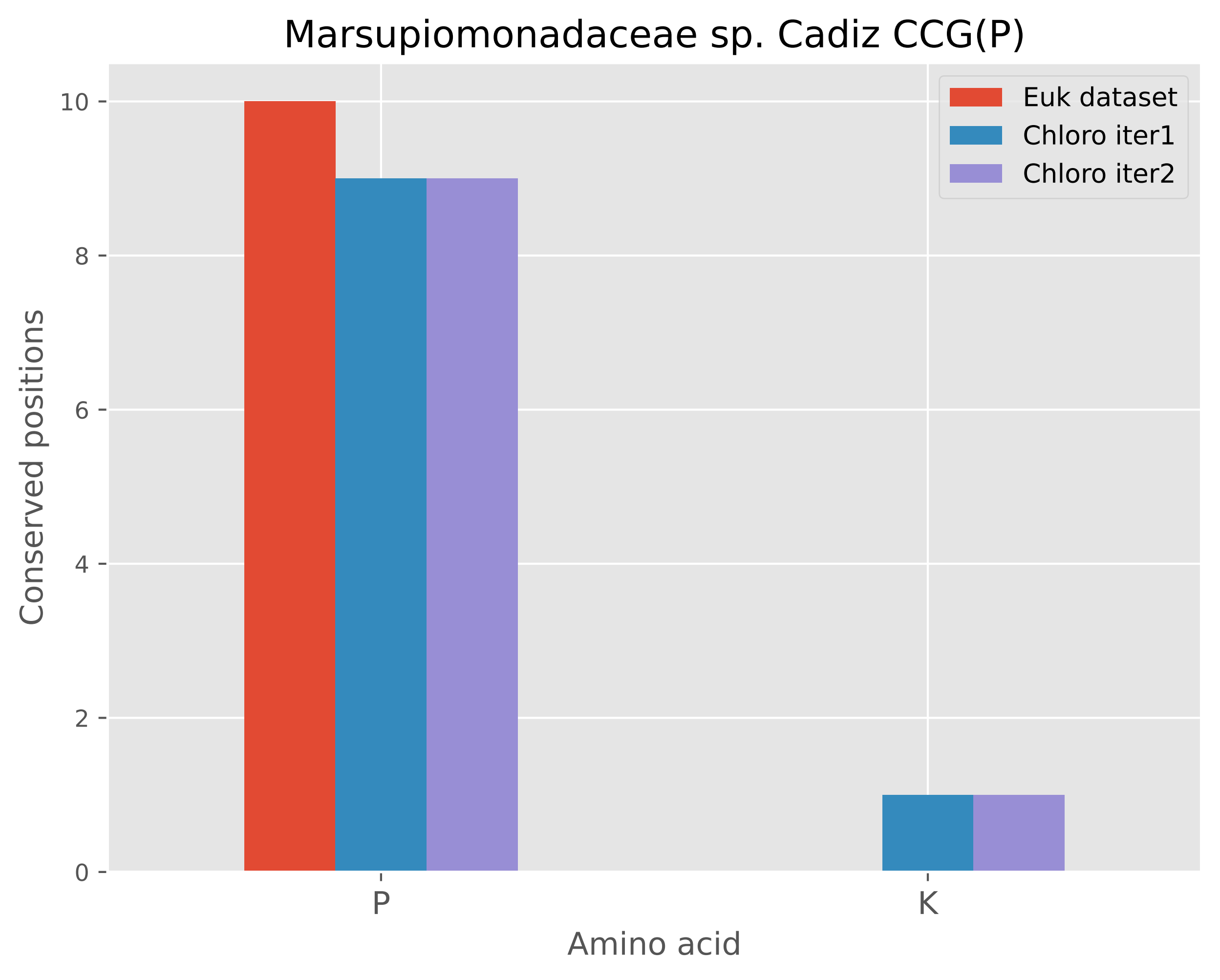

Marsupiomonadaceae sp. Cadiz CCG(P)
Euk dataset
Chloro iter1
Chloro iter2
Conserved positions
0
2
4
6
8
10
P
K
Amino acid

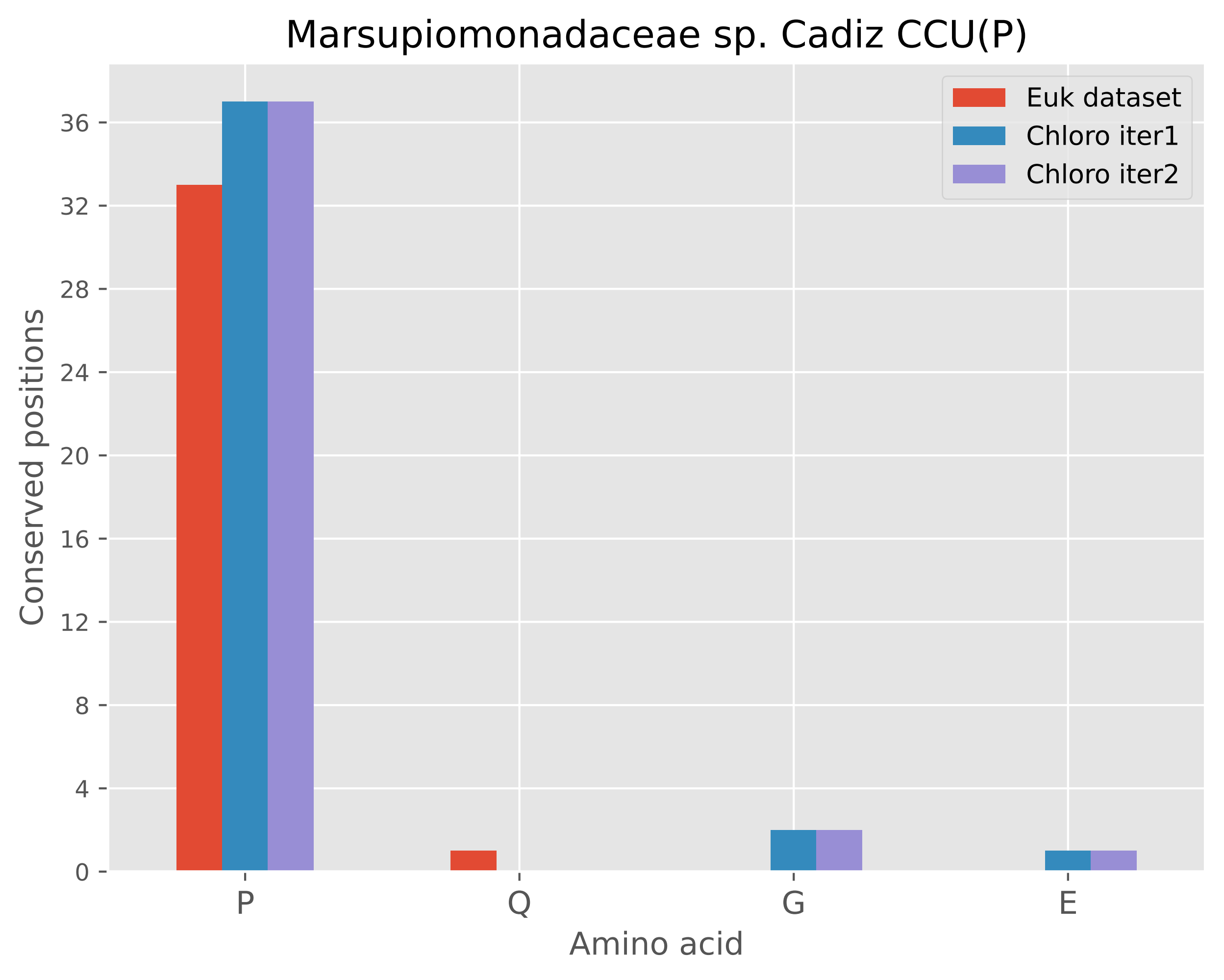

Marsupiomonadaceae sp. Cadiz CCU(P)
Euk dataset
Chloro iter1
Chloro iter2
Conserved positions
0
4
8
12
16
20
24
28
32
36
P
Q
G
E
Amino acid

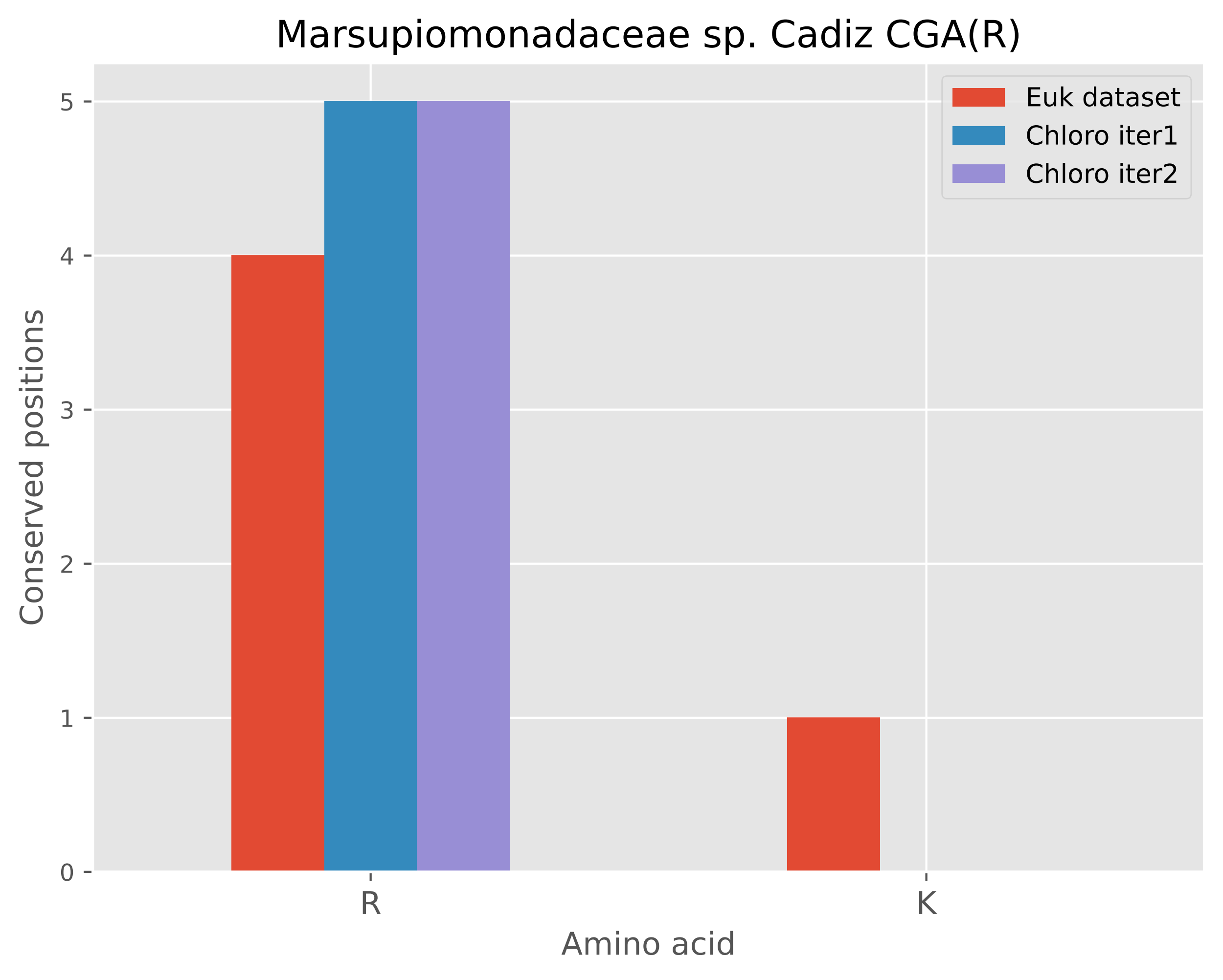

Marsupiomonadaceae sp. Cadiz CGA(R)
Euk dataset
Chloro iter1
Chloro iter2
Conserved positions
Amino acid
R
K
0
1
2
3
4
5

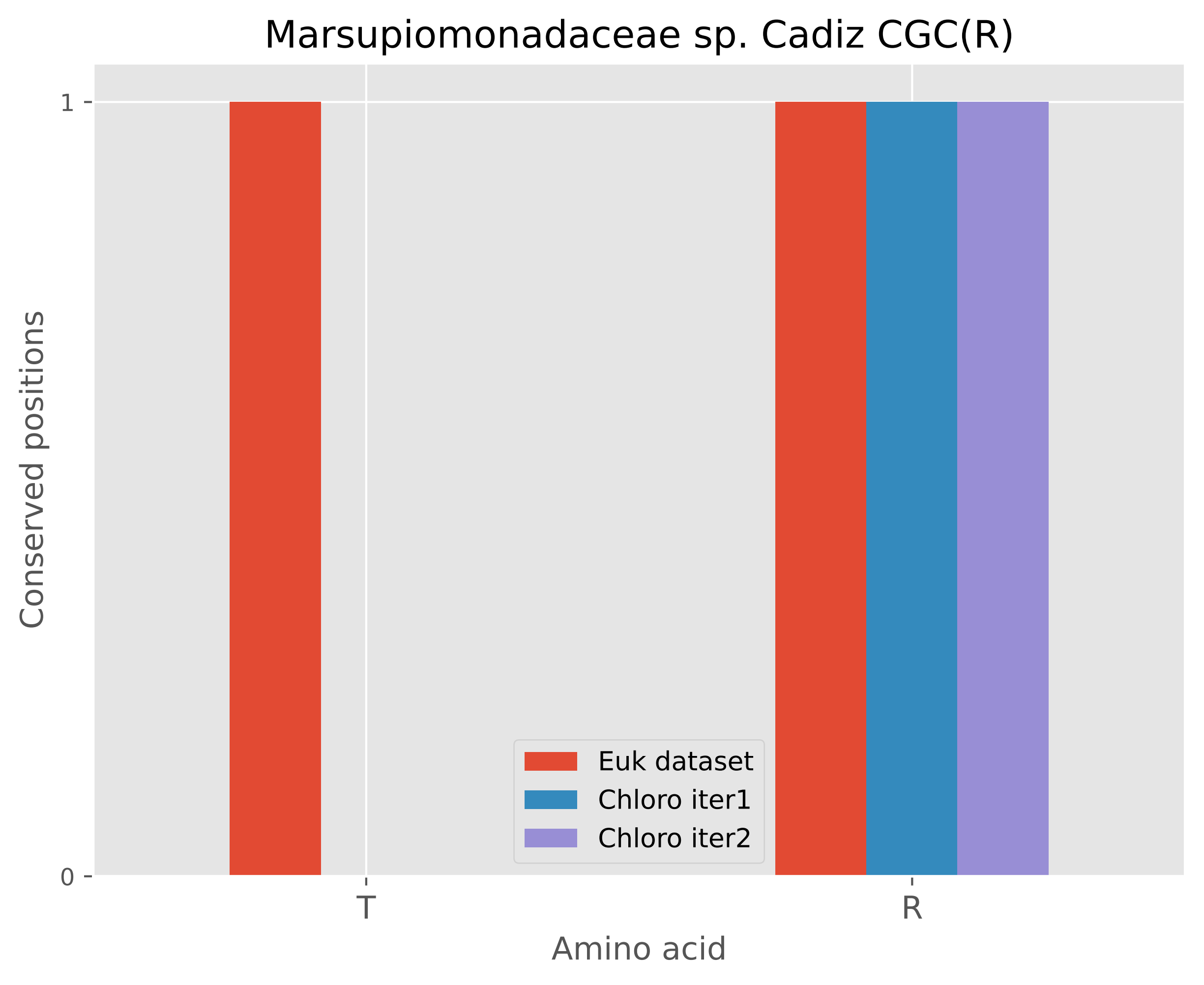
Marsupiomonadaceae sp. Cadiz CGC(R)
Conserved positions
1
0
T
R
Amino acid
Euk dataset
Chloro iter1
Chloro iter2

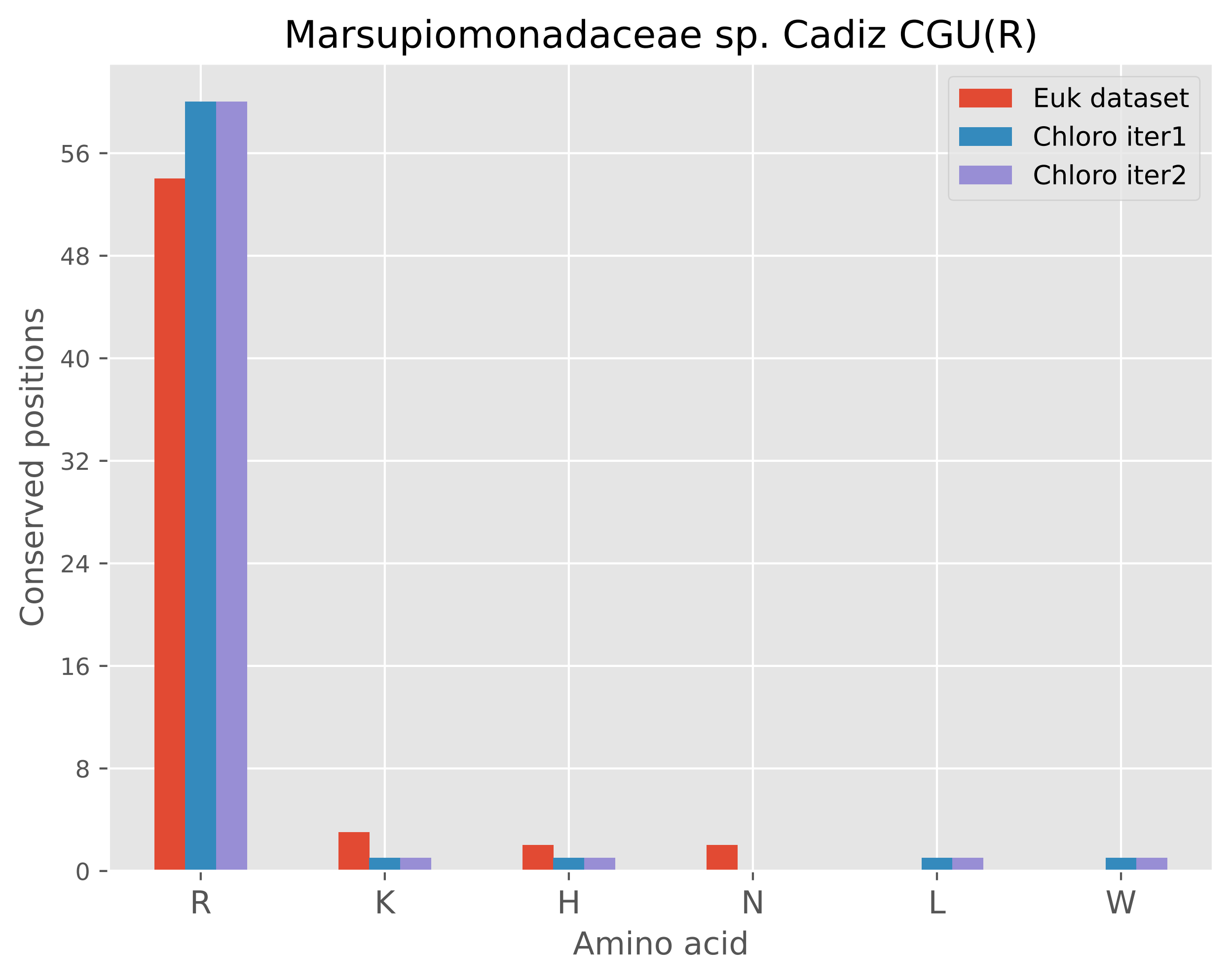

Marsupiomonadaceae sp. Cadiz CGU(R)
Euk dataset
Chloro iter1
Chloro iter2
Conserved positions
0
8
16
24
32
40
48
56
R
K
H
N
L
W
Amino acid

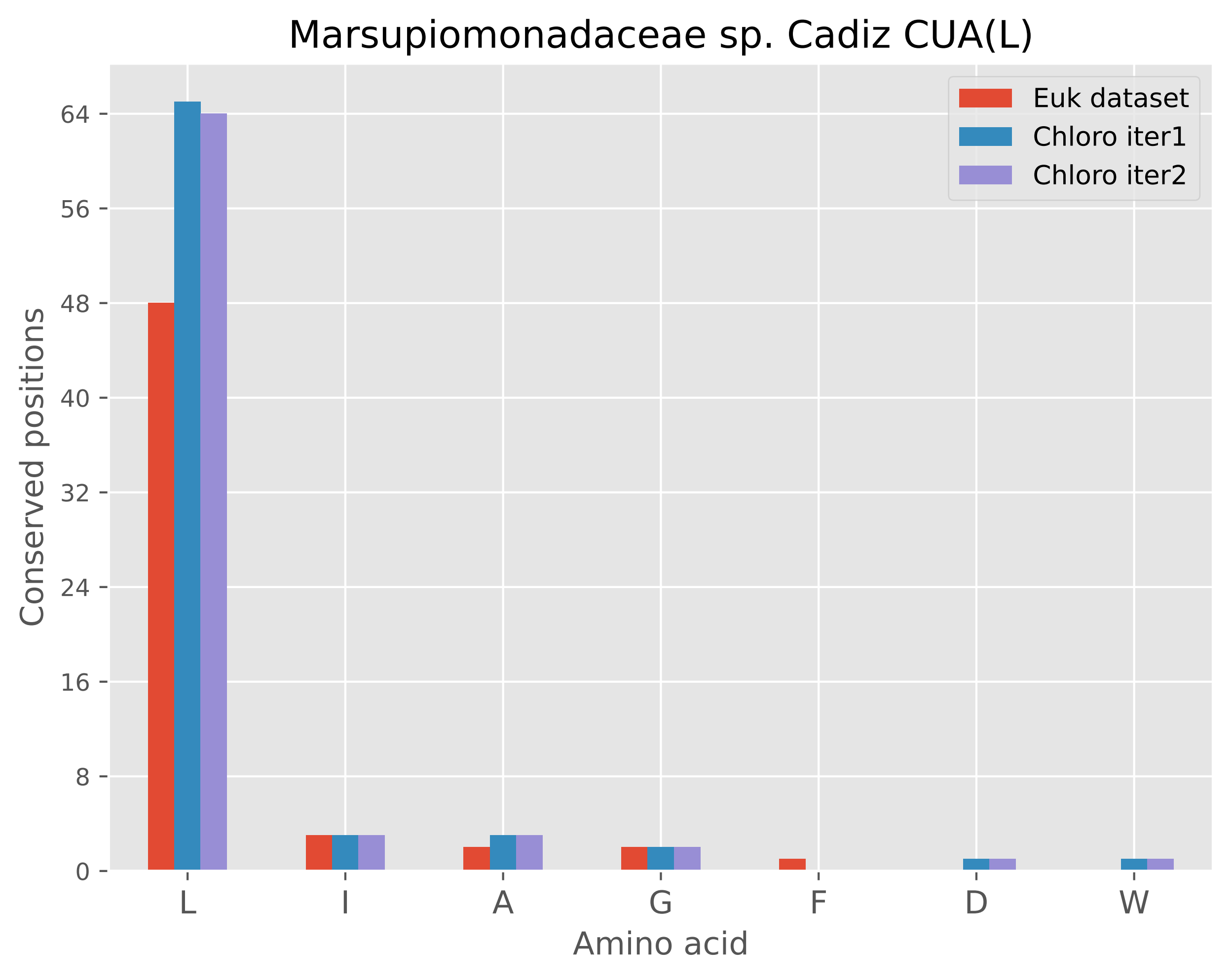

Marsupiomonadaceae sp. Cadiz CUA(L)
Euk dataset
Chloro iter1
Chloro iter2
Conserved positions
0
8
16
24
32
40
48
56
64
L
I
A
G
F
D
W
Amino acid

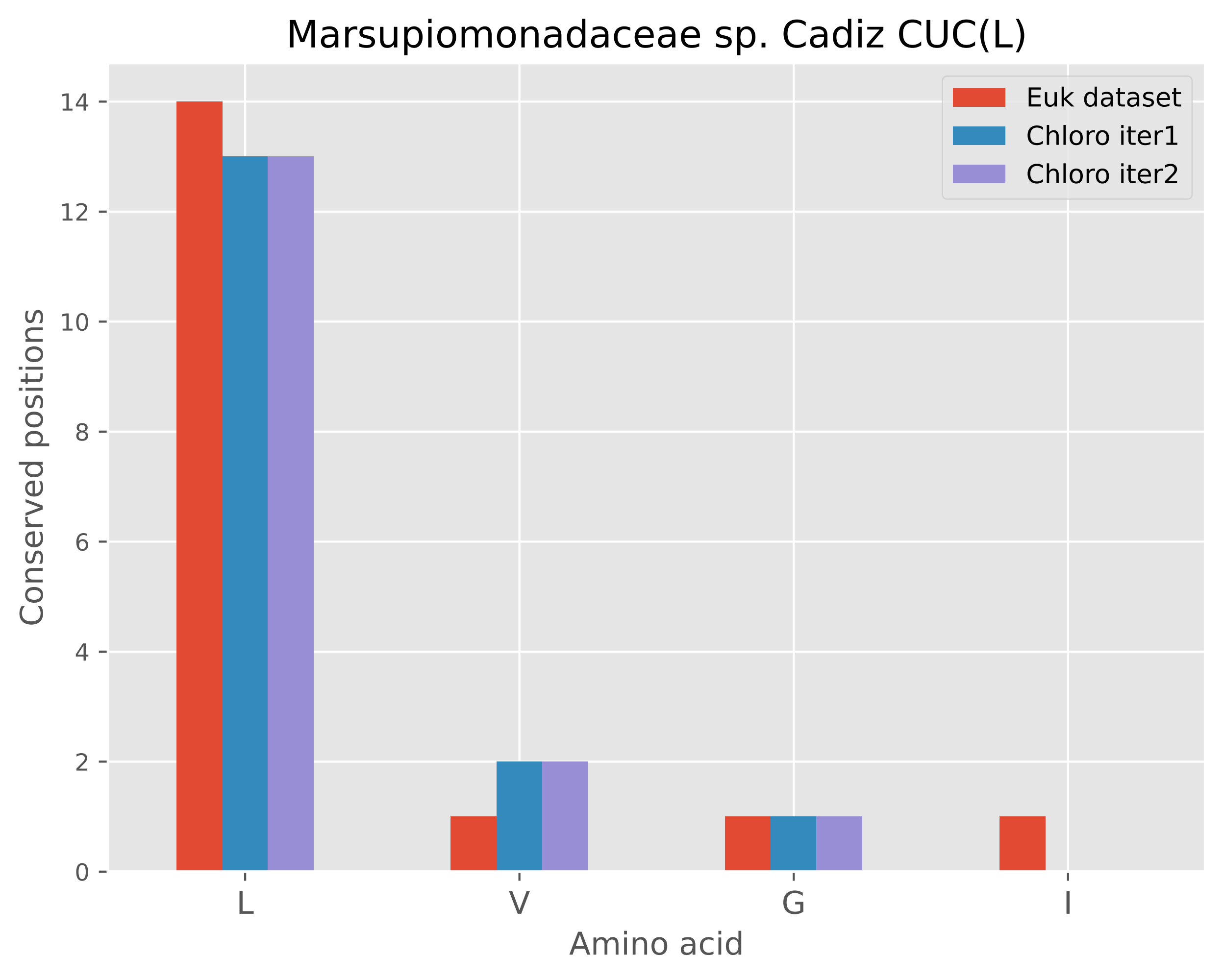
Marsupiomonadaceae sp. Cadiz CUC(L)
Euk dataset
Chloro iter1
Chloro iter2
Conserved positions
0
2
4
6
8
10
12
14
L
V
G
I
Amino acid

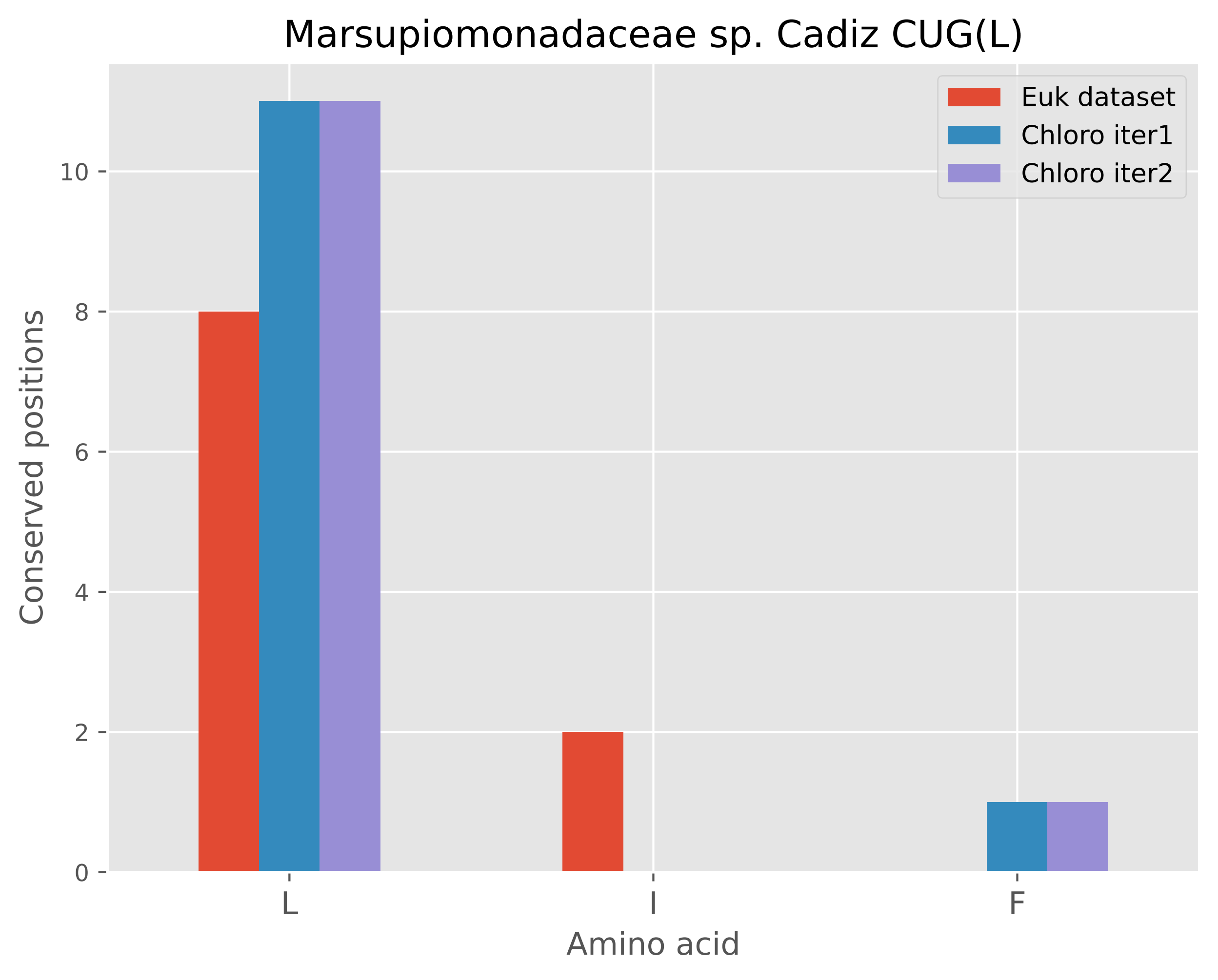
Marsupiomonadaceae sp. Cadiz CUG(L)
Euk dataset
Chloro iter1
Chloro iter2
Conserved positions
0
2
4
6
8
10
L
I
F
Amino acid

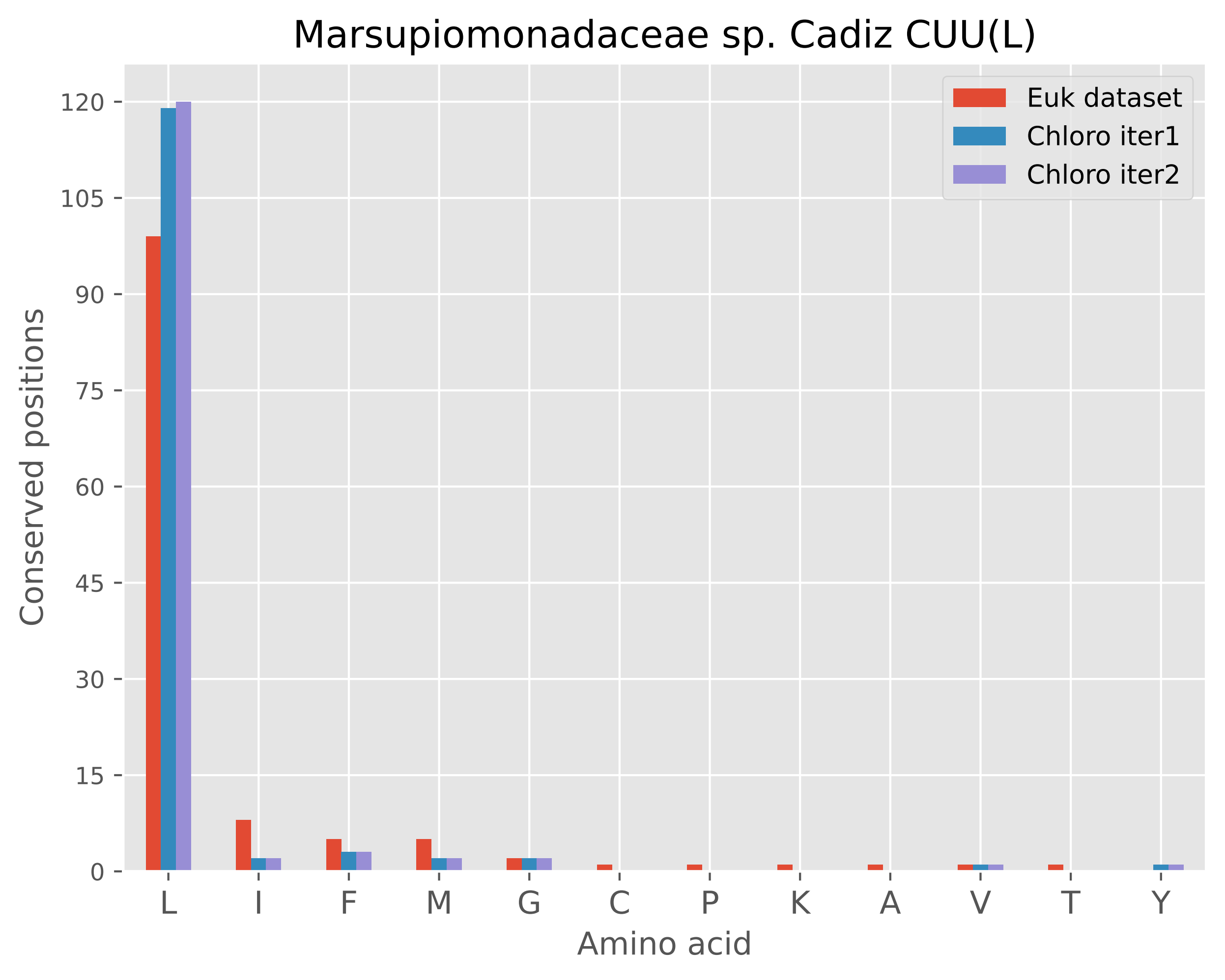

Marsupiomonadaceae sp. Cadiz CUU(L)
Euk dataset
Chloro iter1
Chloro iter2
Conserved positions
Amino acid
0
15
30
45
60
75
90
105
120
L
I
F
M
G
C
P
K
A
V
T
Y

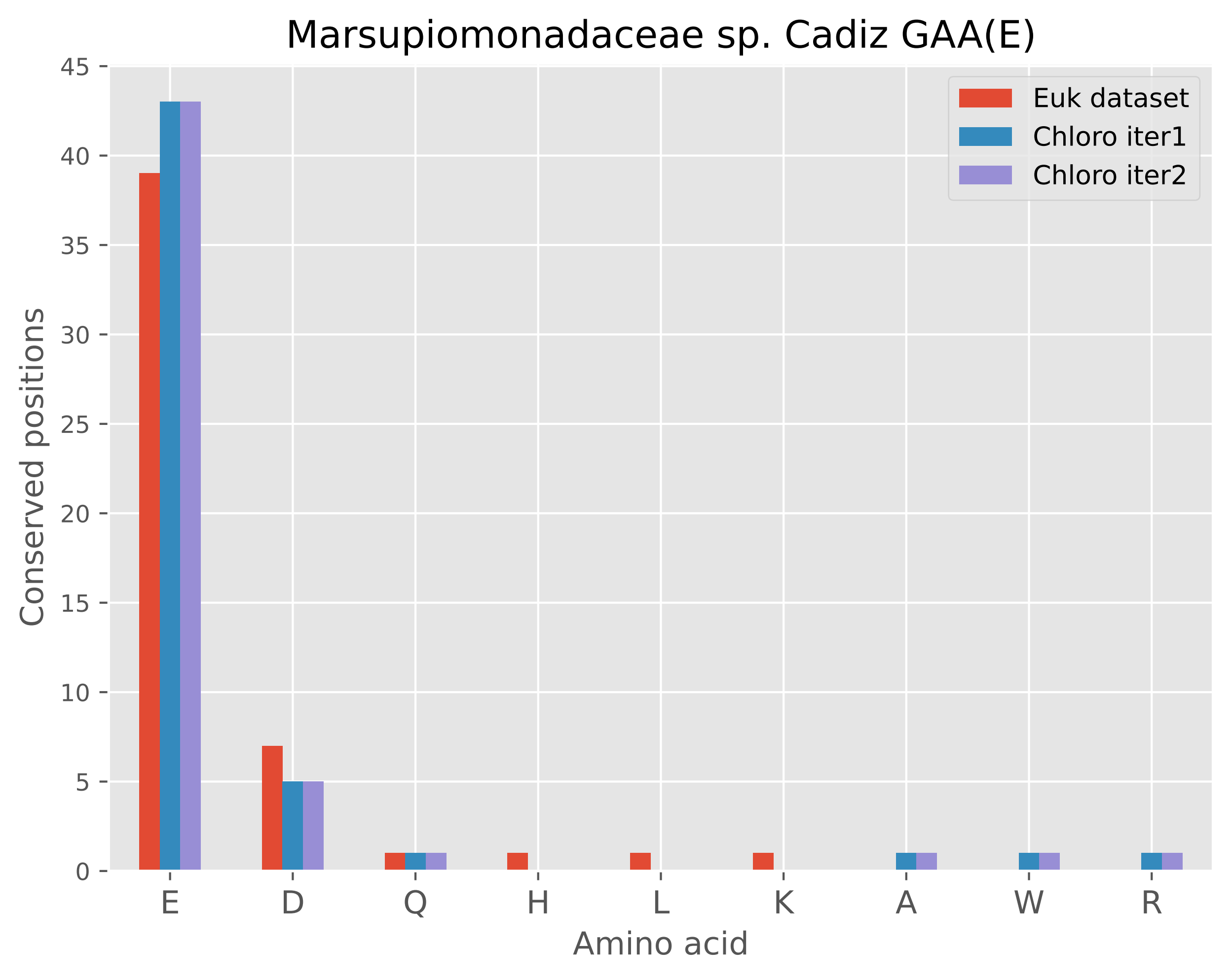

Marsupiomonadaceae sp. Cadiz GAA(E)
Euk dataset
Chloro iter1
Chloro iter2
Conserved positions
Amino acid
0
5
10
15
20
25
30
35
40
45
E
D
Q
H
L
K
A
W
R

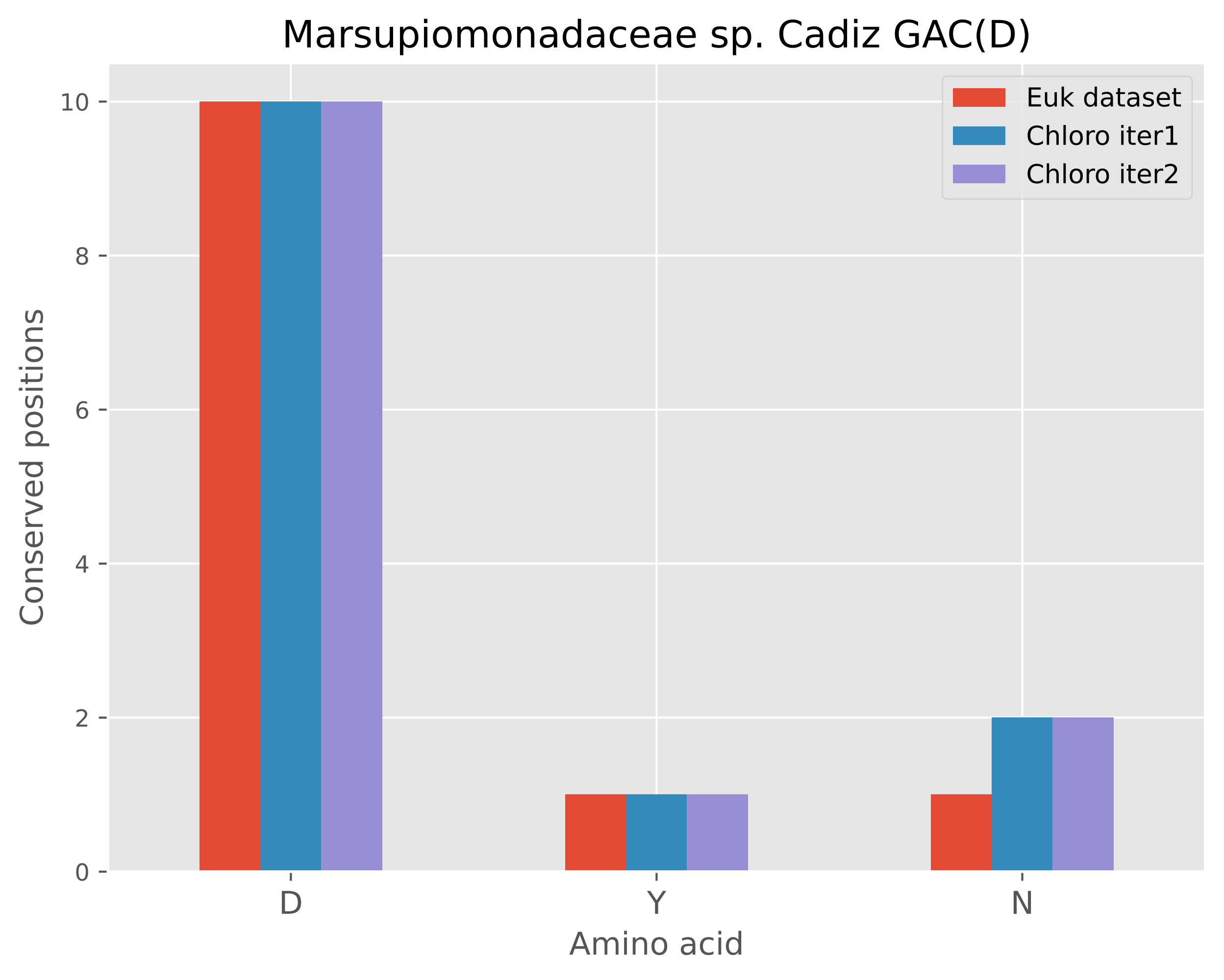
Marsupiomonadaceae sp. Cadiz GAC(D)
Euk dataset
Chloro iter1
Chloro iter2
Conserved positions
0
2
4
6
8
10
D
Y
N
Amino acid

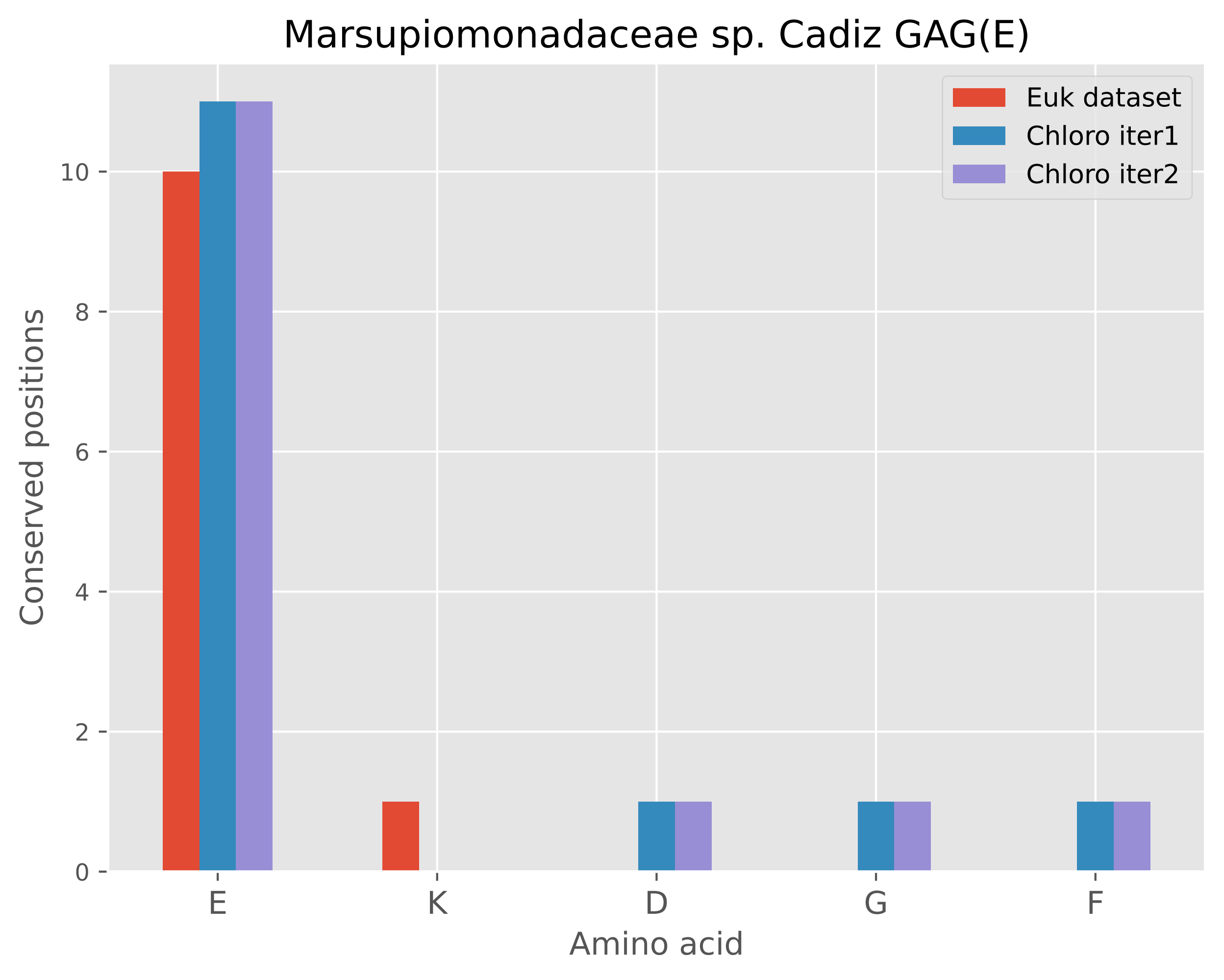
Marsupiomonadaceae sp. Cadiz GAG(E)
Euk dataset
Chloro iter1
Chloro iter2
Conserved positions
0
2
4
6
8
10
E
K
D
G
F
Amino acid

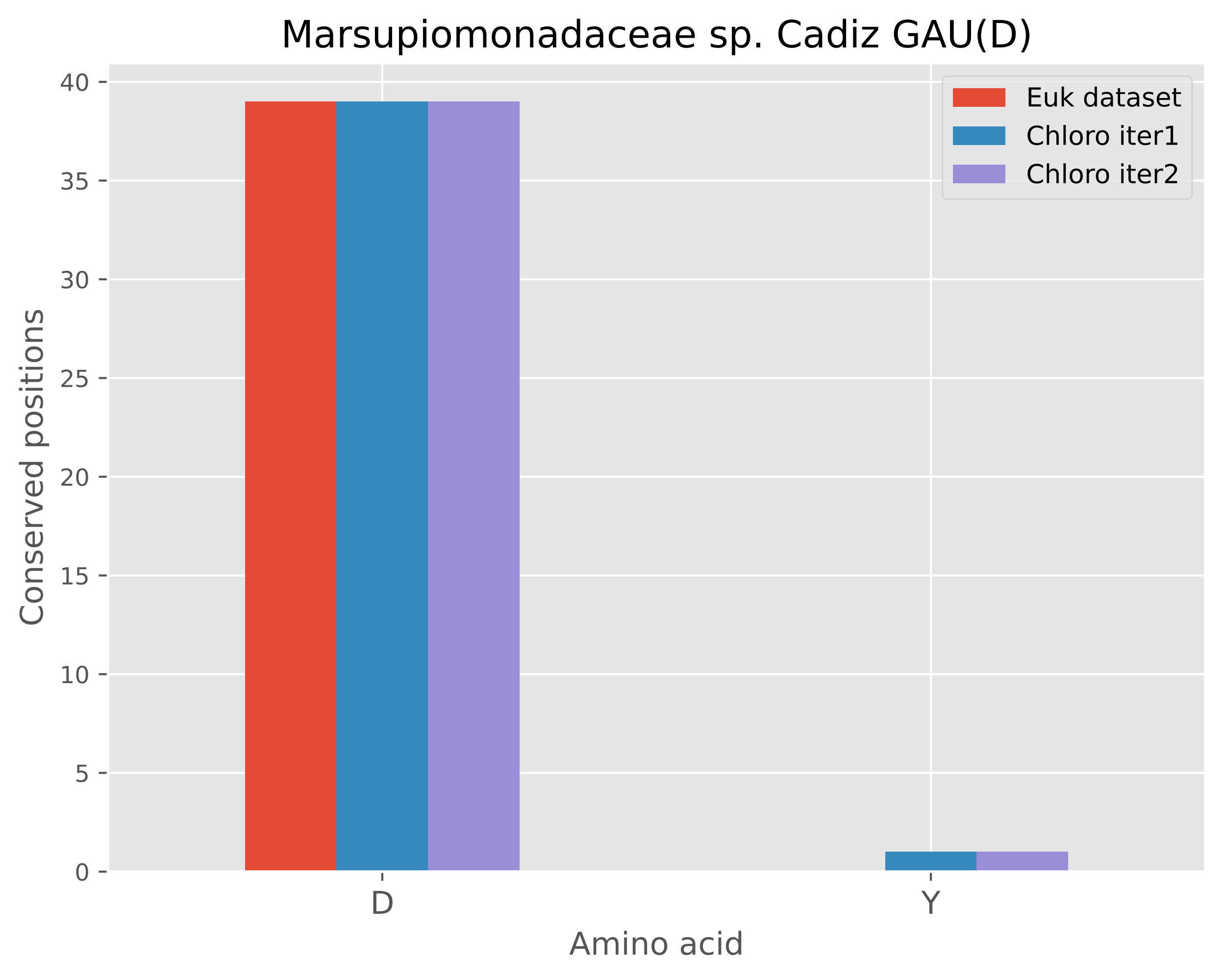

Marsupiomonadaceae sp. Cadiz GAU(D)
Euk dataset
Chloro iter1
Chloro iter2
Conserved positions
0
5
10
15
20
25
30
35
40
D
Y
Amino acid

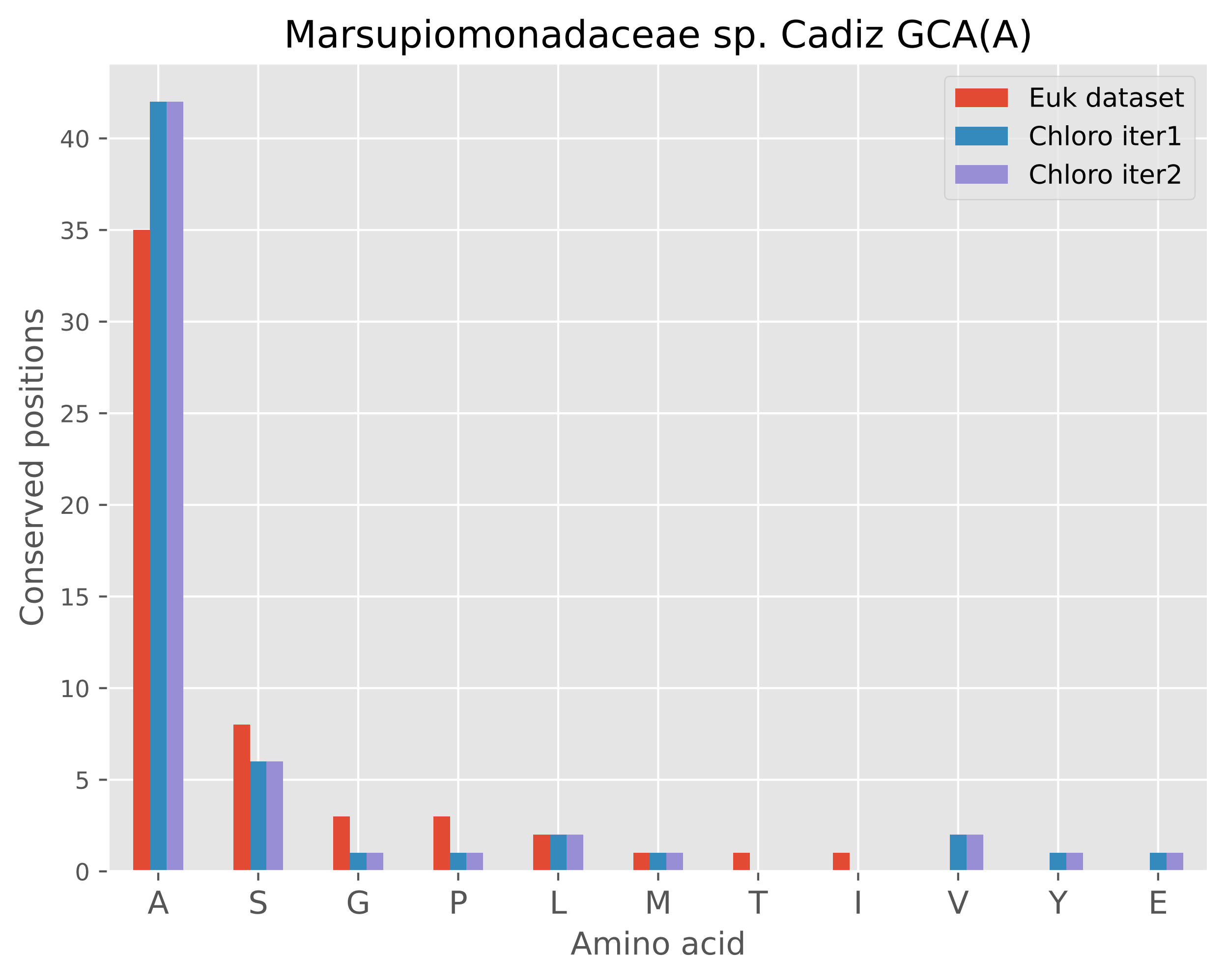

Marsupiomonadaceae sp. Cadiz GCA(A)
Euk dataset
Chloro iter1
Chloro iter2
Conserved positions
0
5
10
15
20
25
30
35
40
A
S
G
P
L
M
T
I
V
Y
E
Amino acid

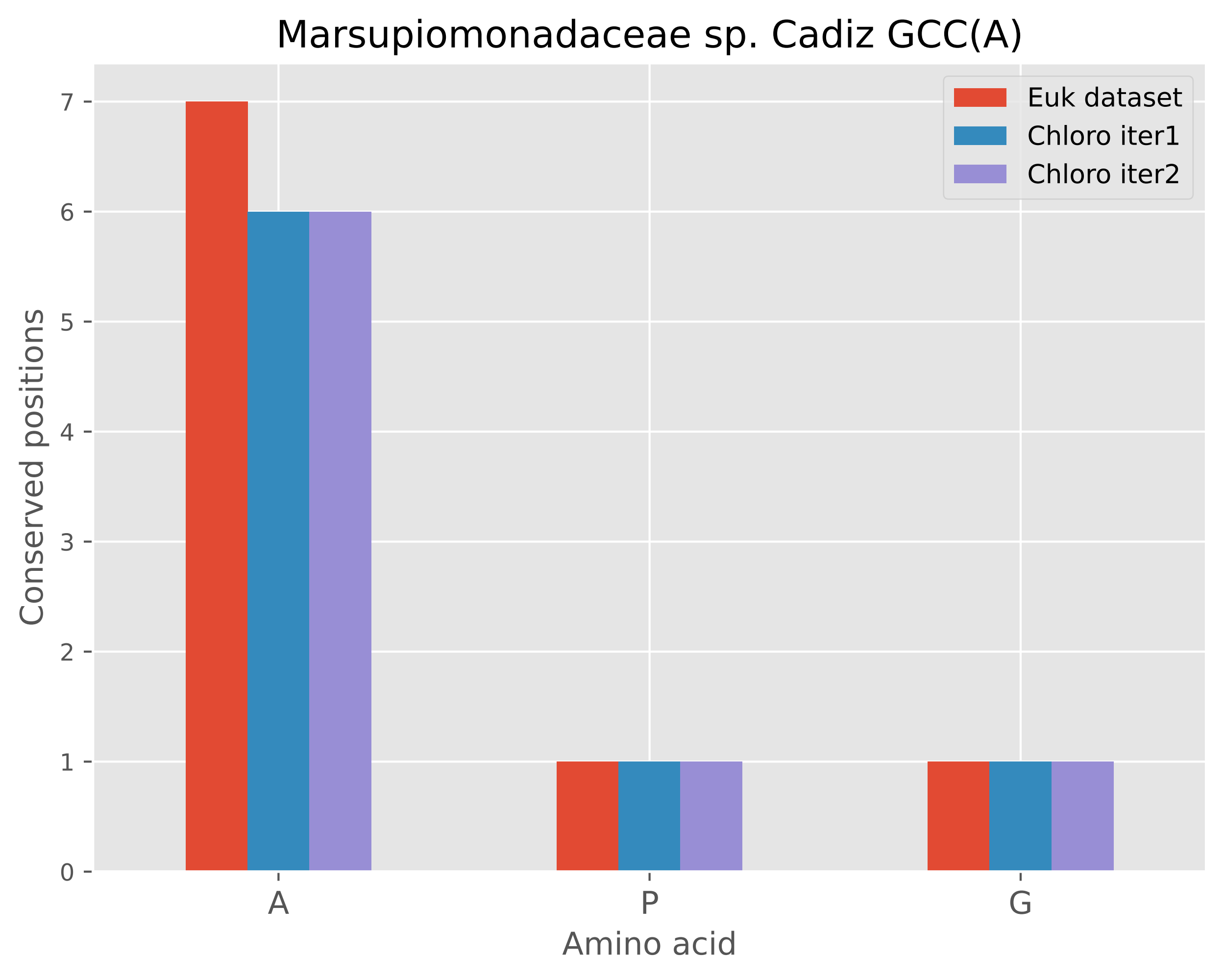

Marsupiomonadaceae sp. Cadiz GCC(A)
Euk dataset
Chloro iter1
Chloro iter2
Conserved positions
0
1
2
3
4
5
6
7
A
P
G
Amino acid

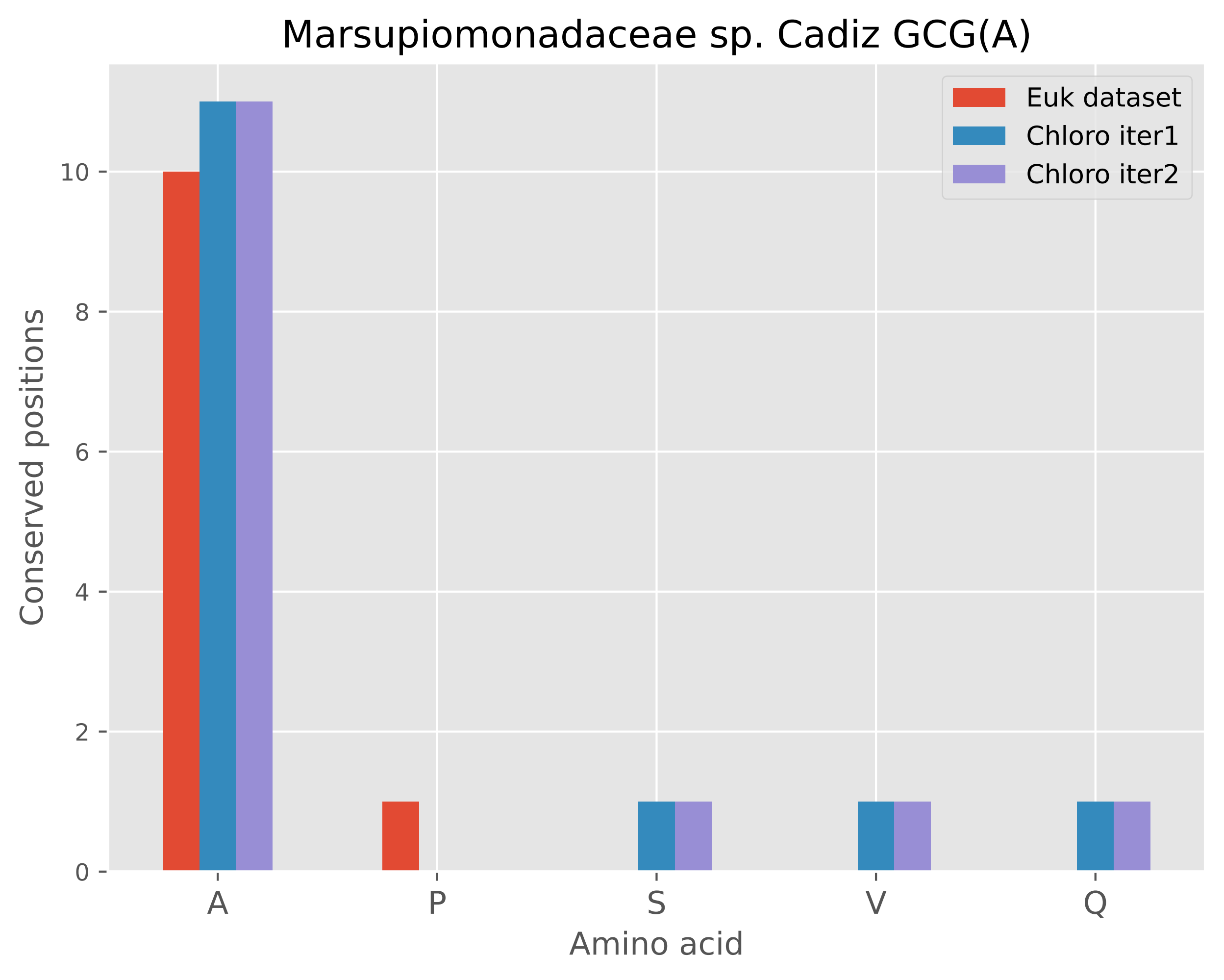

Marsupiomonadaceae sp. Cadiz GCG(A)
Euk dataset
Chloro iter1
Chloro iter2
Conserved positions
0
2
4
6
8
10
A
P
S
V
Q
Amino acid

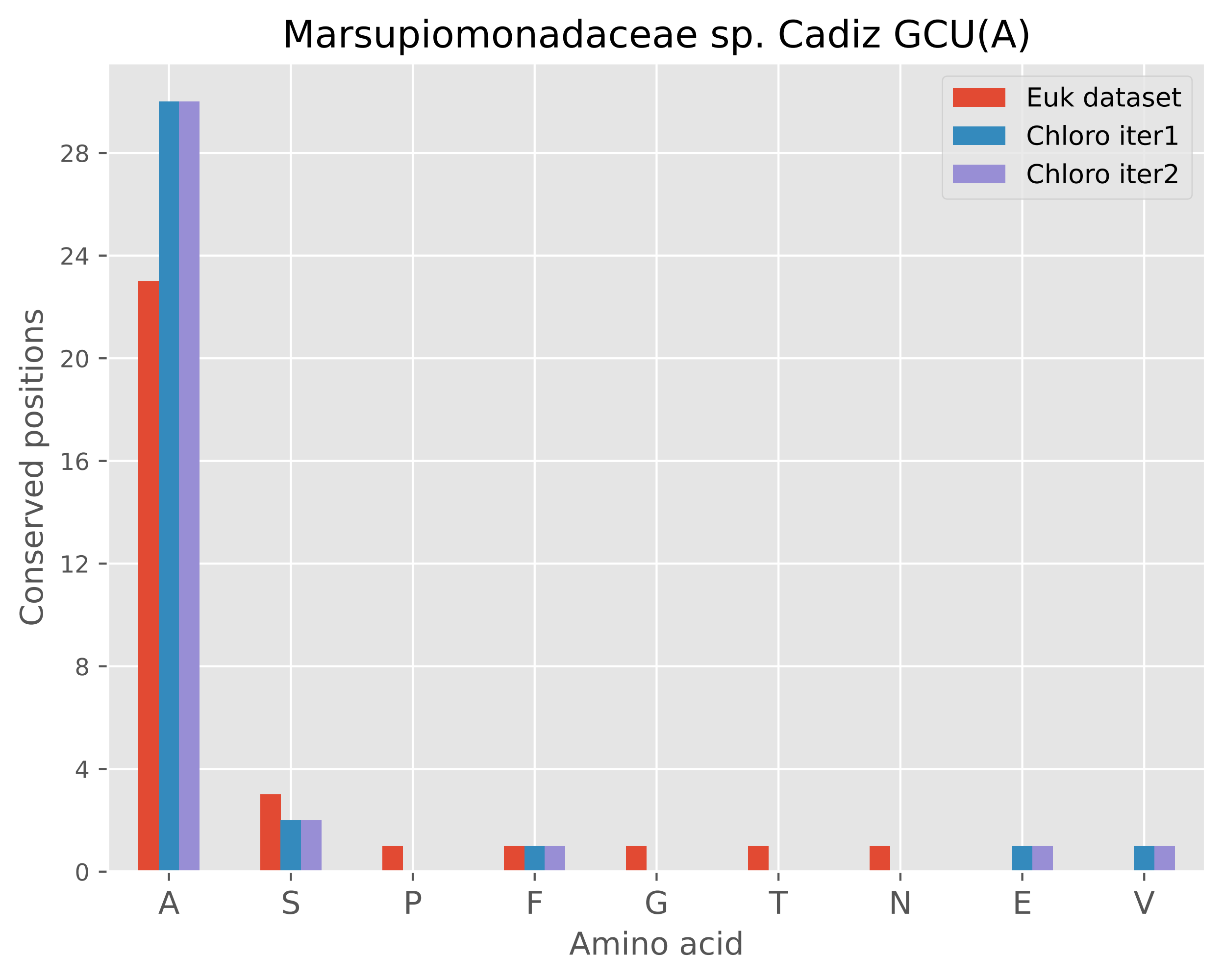

Marsupiomonadaceae sp. Cadiz GCU(A)
Euk dataset
Chloro iter1
Chloro iter2
Conserved positions
0
4
8
12
16
20
24
28
A
S
P
F
G
T
N
E
V
Amino acid

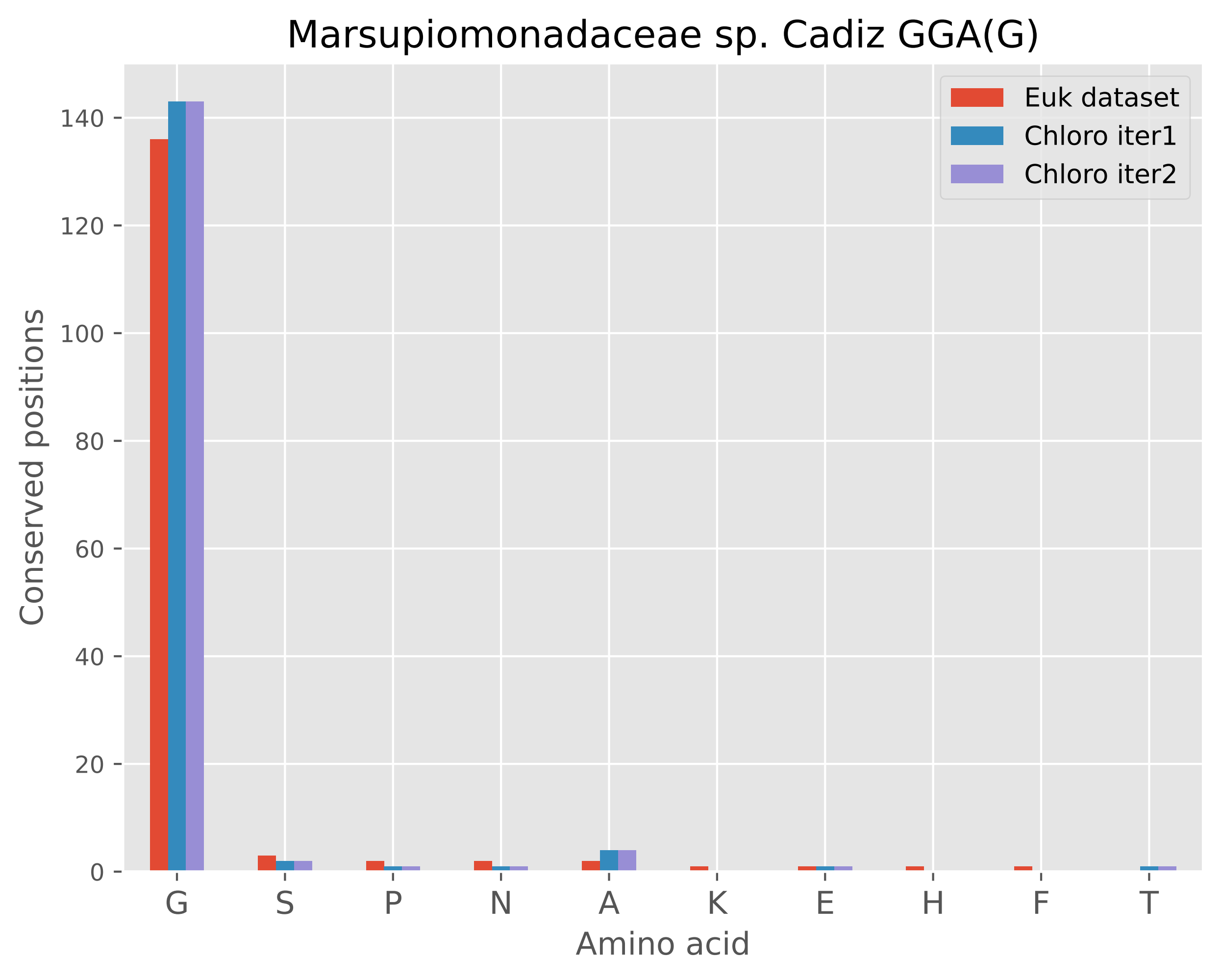

Marsupiomonadaceae sp. Cadiz GGA(G)
Euk dataset
Chloro iter1
Chloro iter2
Conserved positions
0
20
40
60
80
100
120
140
G
S
P
N
A
K
E
H
F
T
Amino acid

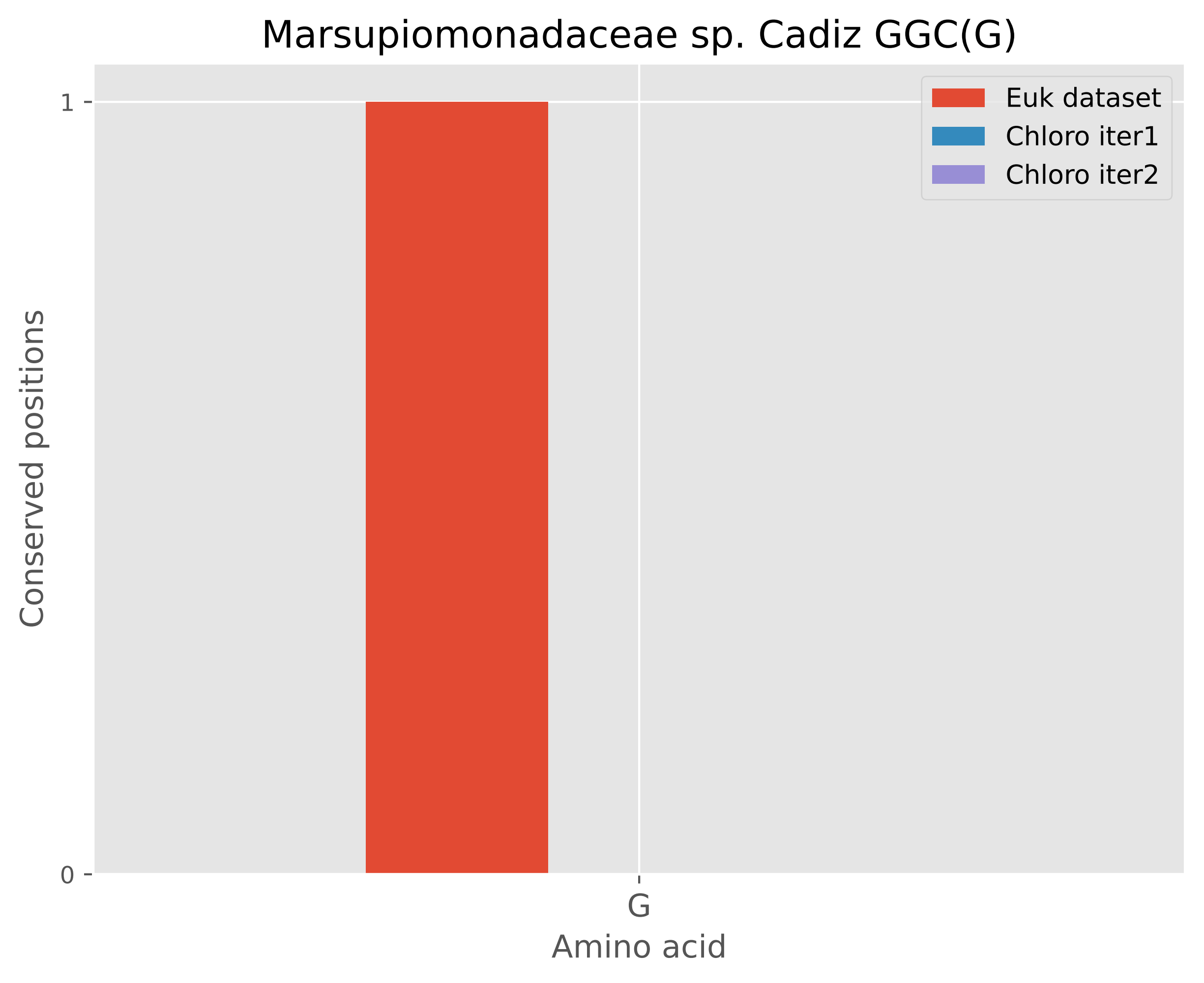

Marsupiomonadaceae sp. Cadiz GGC(G)
Euk dataset
Chloro iter1
Chloro iter2
Conserved positions
1
0
G
Amino acid

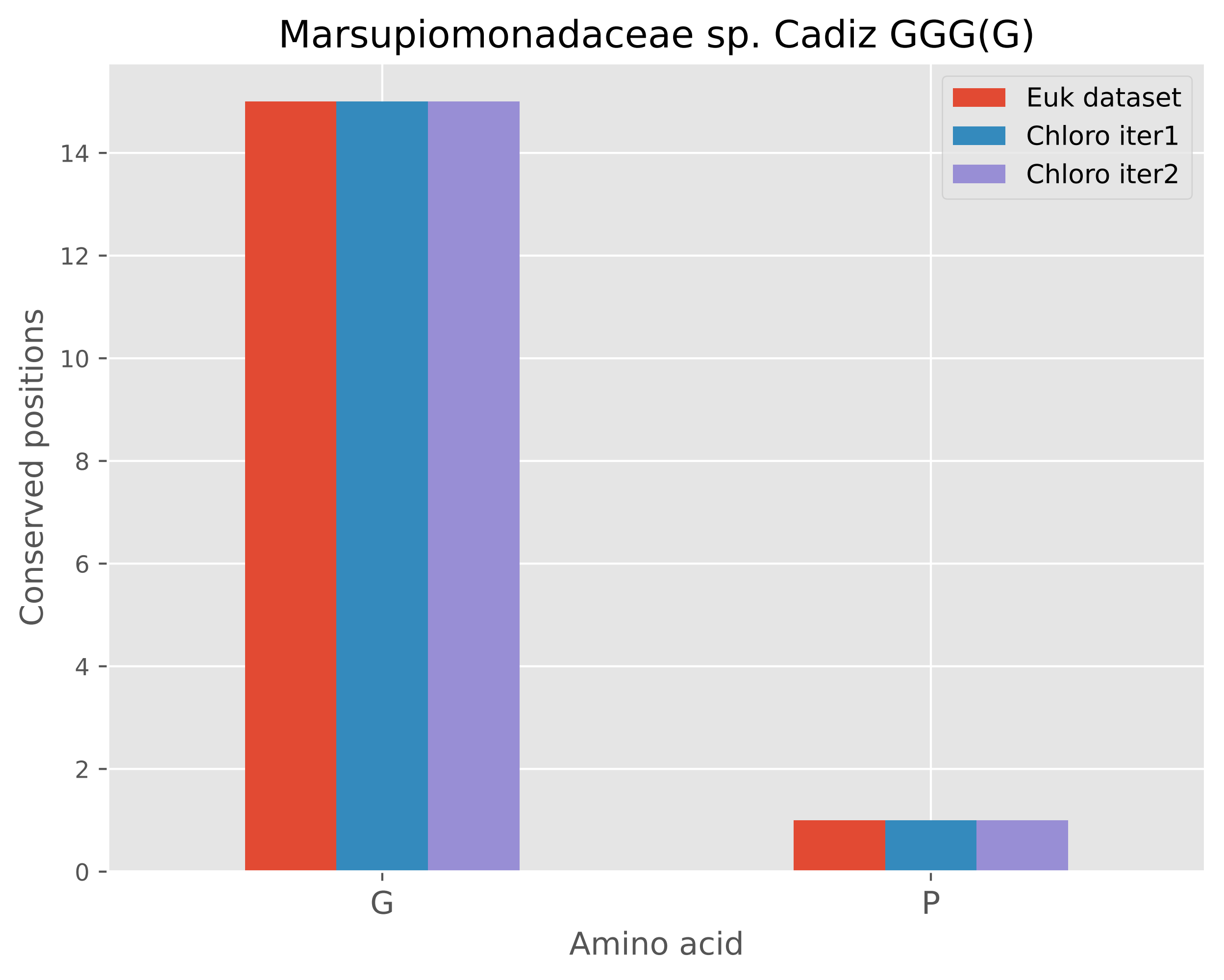

Marsupiomonadaceae sp. Cadiz GGG(G)
Euk dataset
Chloro iter1
Chloro iter2
Conserved positions
0
2
4
6
8
10
12
14
G
P
Amino acid

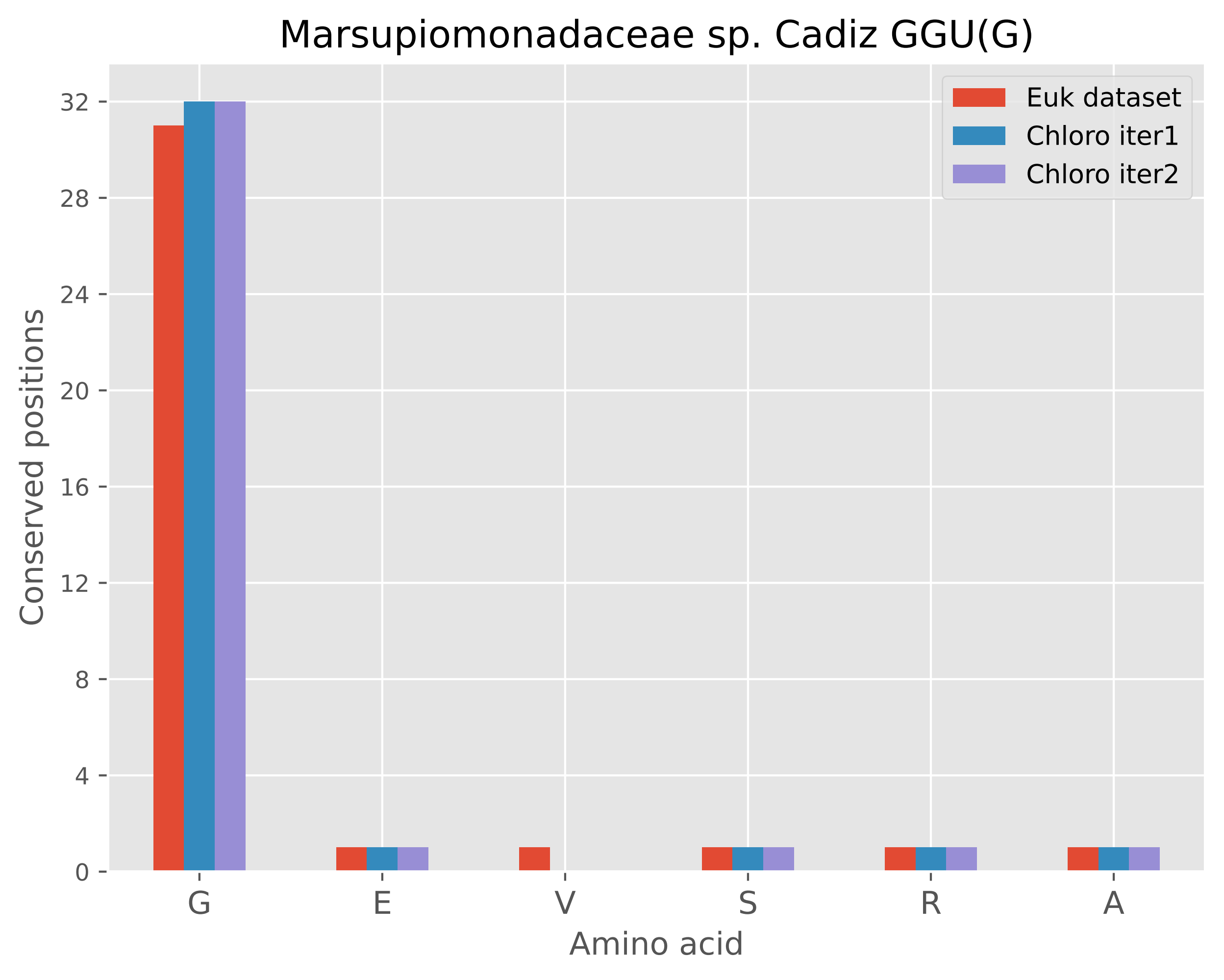

Marsupiomonadaceae sp. Cadiz GGU(G)
Euk dataset
Chloro iter1
Chloro iter2
Conserved positions
0
4
8
12
16
20
24
28
32
G
E
V
S
R
A
Amino acid

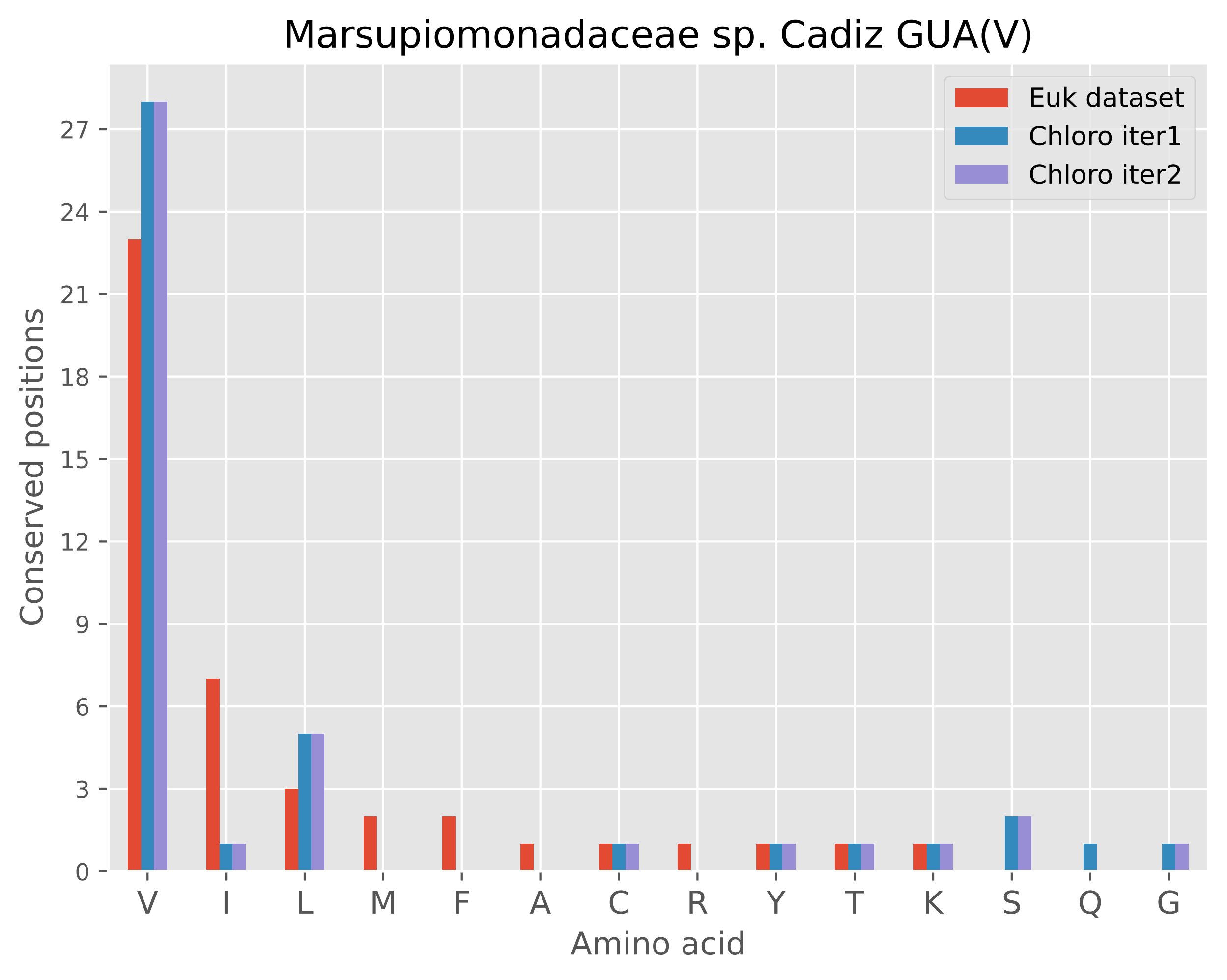

Marsupiomonadaceae sp. Cadiz GUA(V)
Euk dataset
Chloro iter1
Chloro iter2
Conserved positions
Amino acid
0
3
6
9
12
15
18
21
24
27
V
I
L
M
F
A
C
R
Y
T
K
S
Q
G

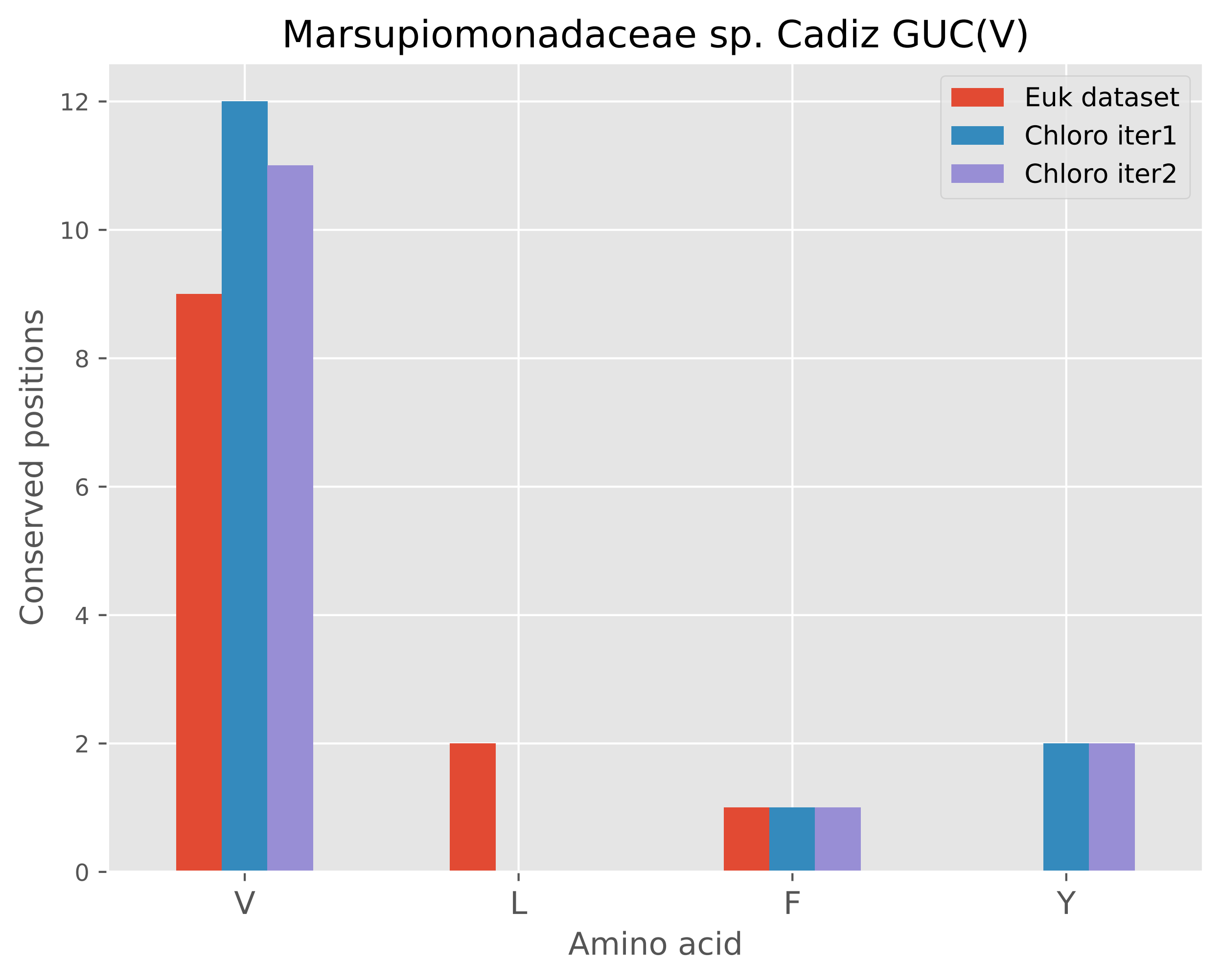

Marsupiomonadaceae sp. Cadiz GUC(V)
Euk dataset
Chloro iter1
Chloro iter2
Conserved positions
0
2
4
6
8
10
12
V
L
F
Y
Amino acid

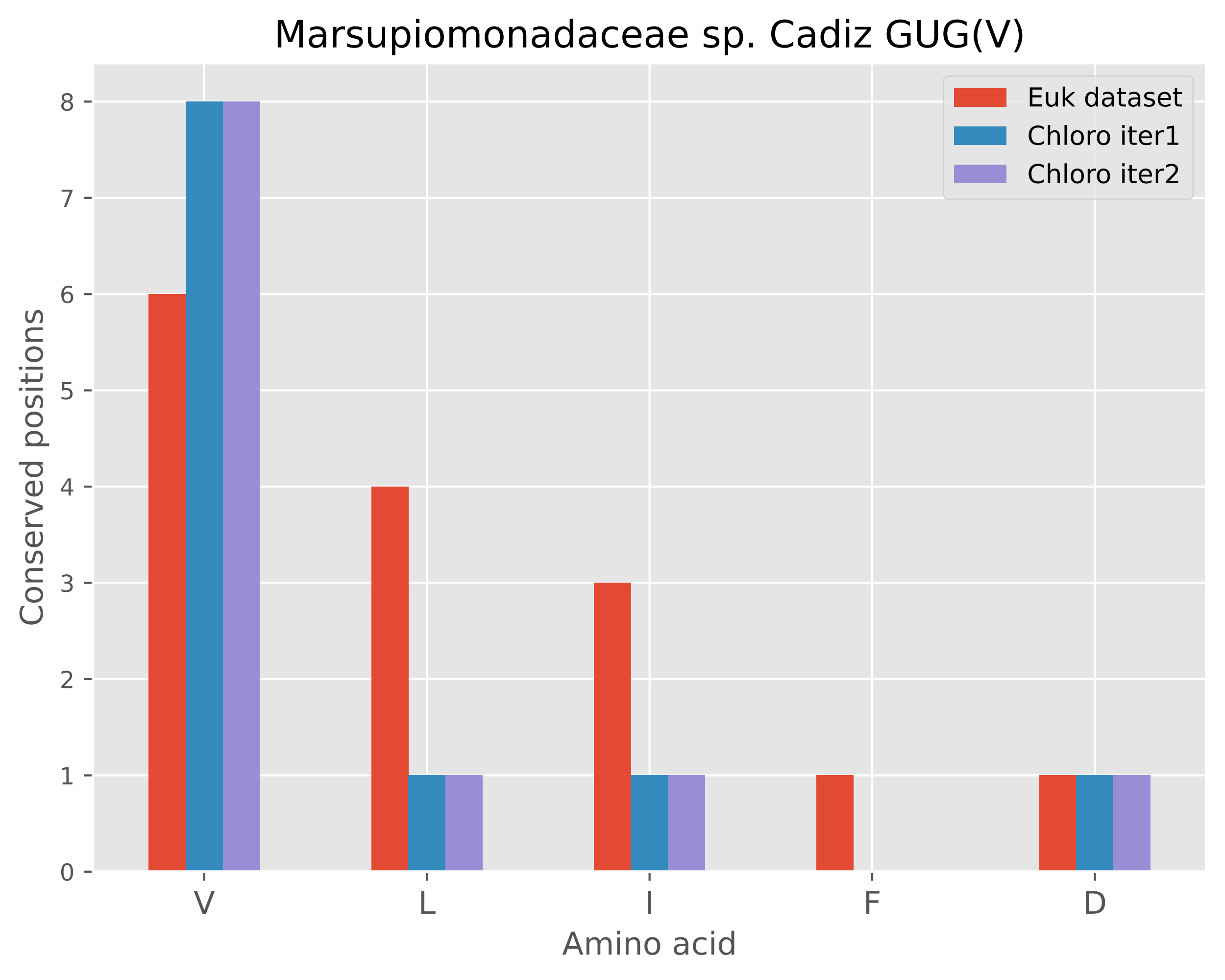

Marsupiomonadaceae sp. Cadiz GUG(V)
Euk dataset
Chloro iter1
Chloro iter2
Conserved positions
Amino acid
0
1
2
3
4
5
6
7
8
V
L
I
F
D

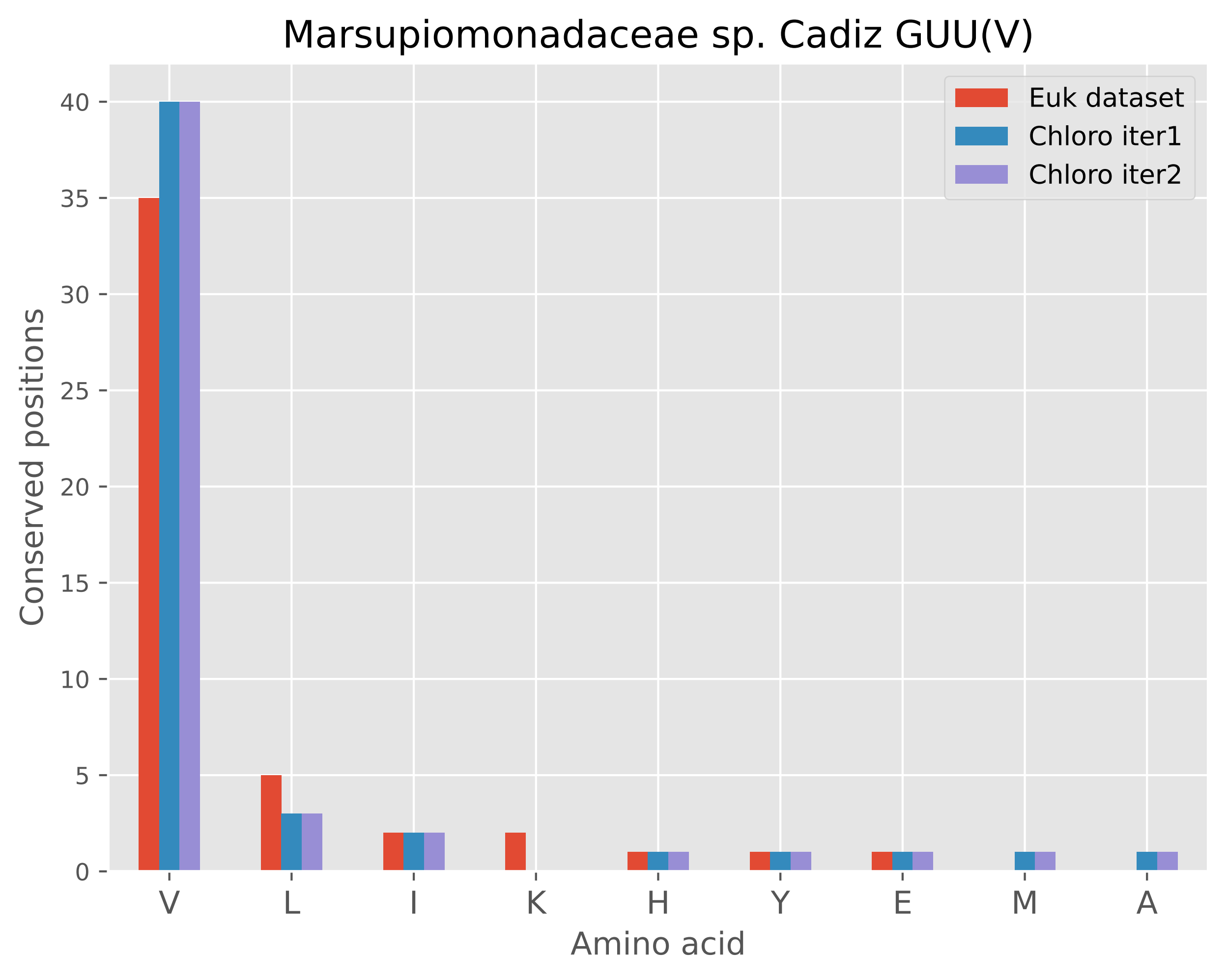
Marsupiomonadaceae sp. Cadiz GUU(V)
Euk dataset
Chloro iter1
Chloro iter2
Conserved positions
0
5
10
15
20
25
30
35
40
V
L
I
K
H
Y
E
M
A
Amino acid

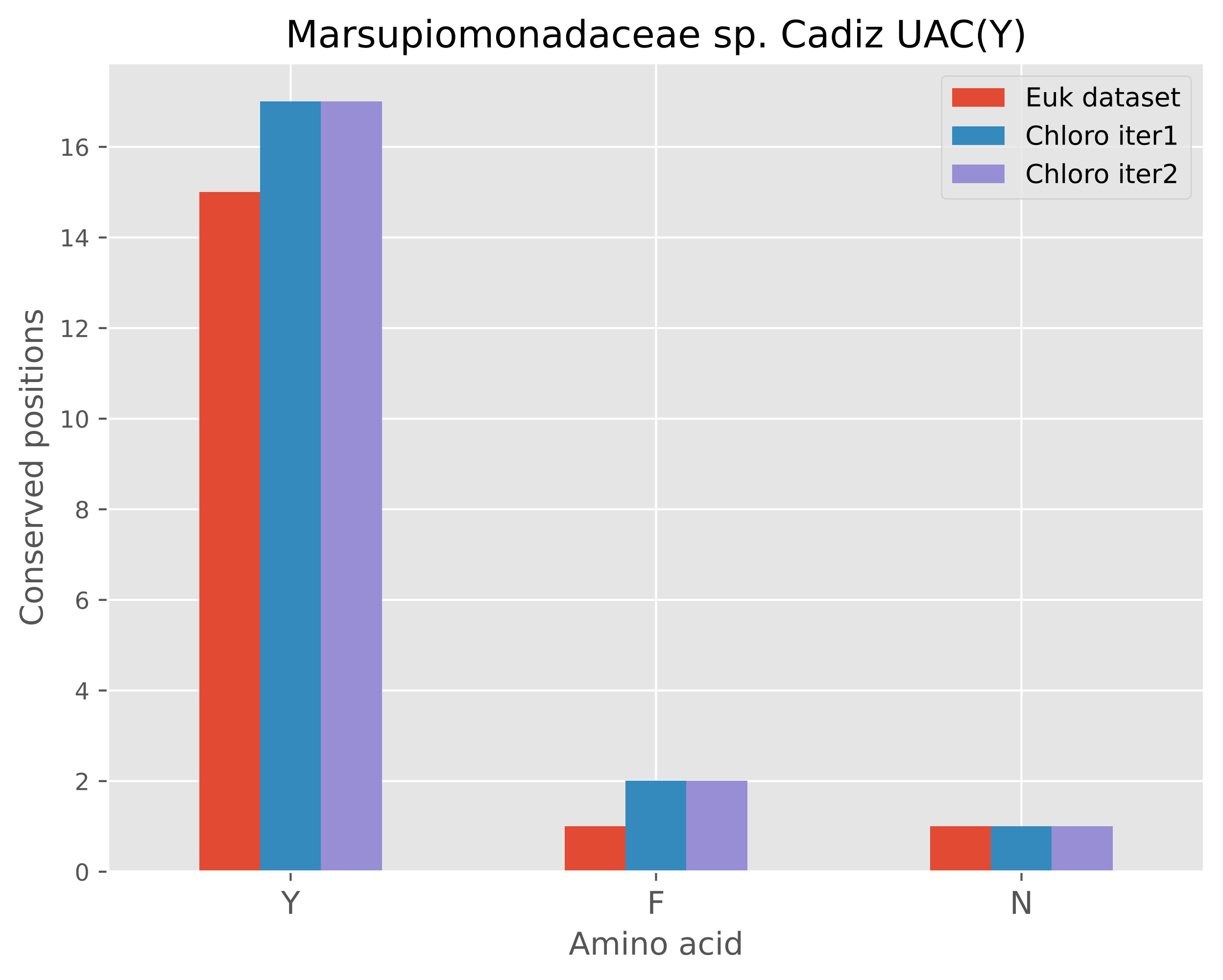

Marsupiomonadaceae sp. Cadiz UAC(Y)
Euk dataset
Chloro iter1
Chloro iter2
Conserved positions
0
2
4
6
8
10
12
14
16
Y
F
N
Amino acid

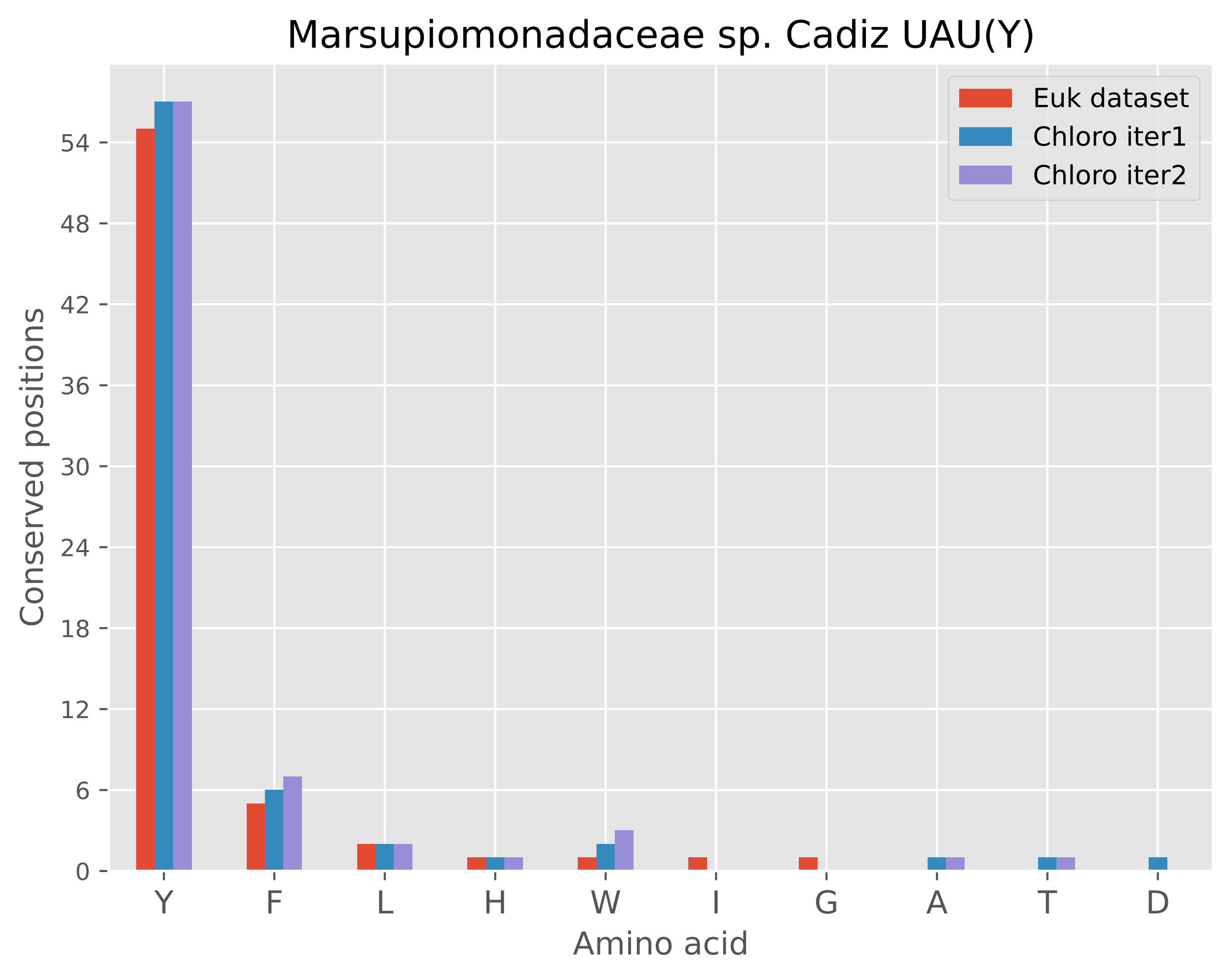

Marsupiomonadaceae sp. Cadiz UAU(Y)
Euk dataset
Chloro iter1
Chloro iter2
Conserved positions
0
6
12
18
24
30
36
42
48
54
Y
F
L
H
W
I
G
A
T
D
Amino acid

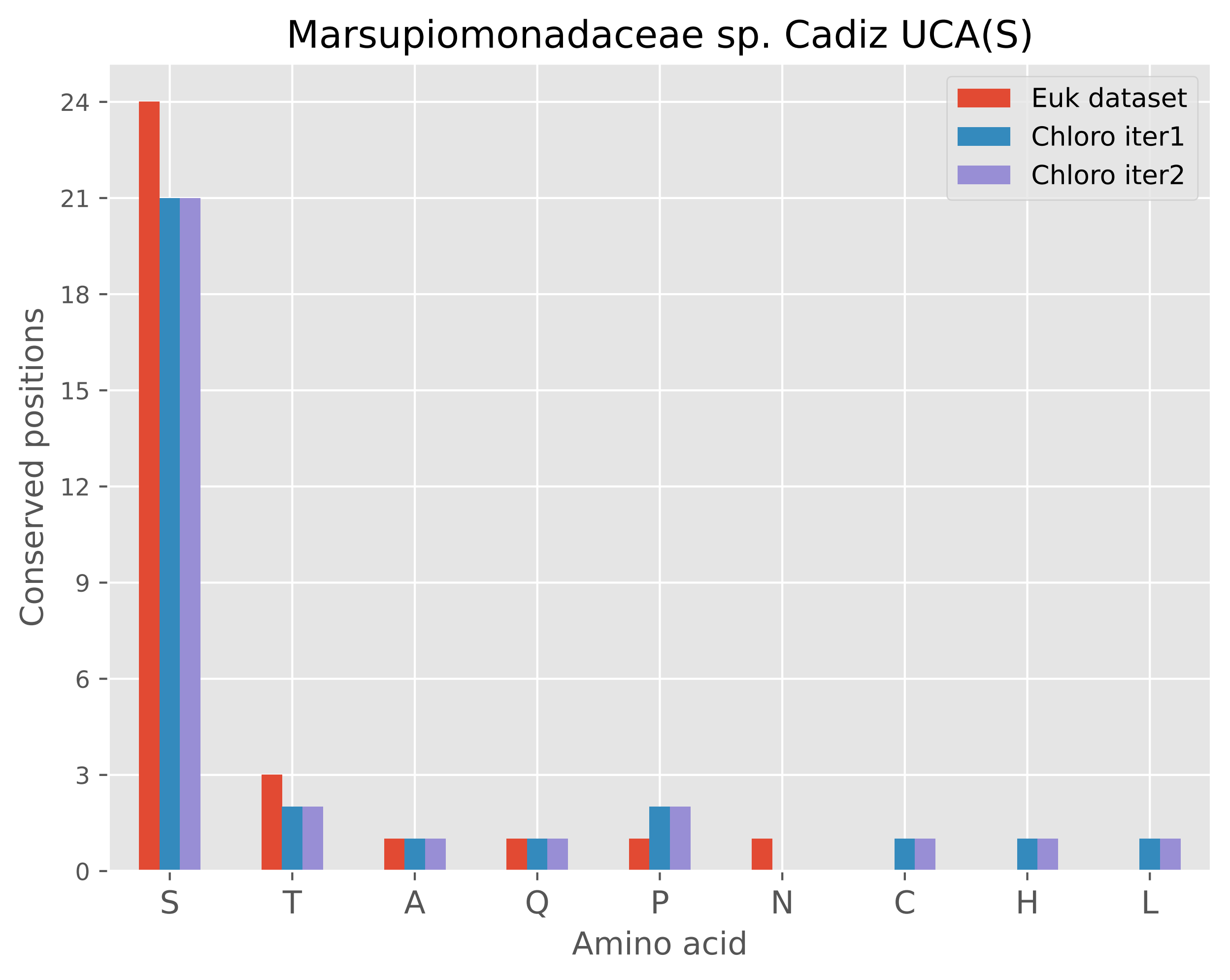
Marsupiomonadaceae sp. Cadiz UCA(S)
Euk dataset
Chloro iter1
Chloro iter2
Conserved positions
0
3
6
9
12
15
18
21
24
S
T
A
Q
P
N
C
H
L
Amino acid

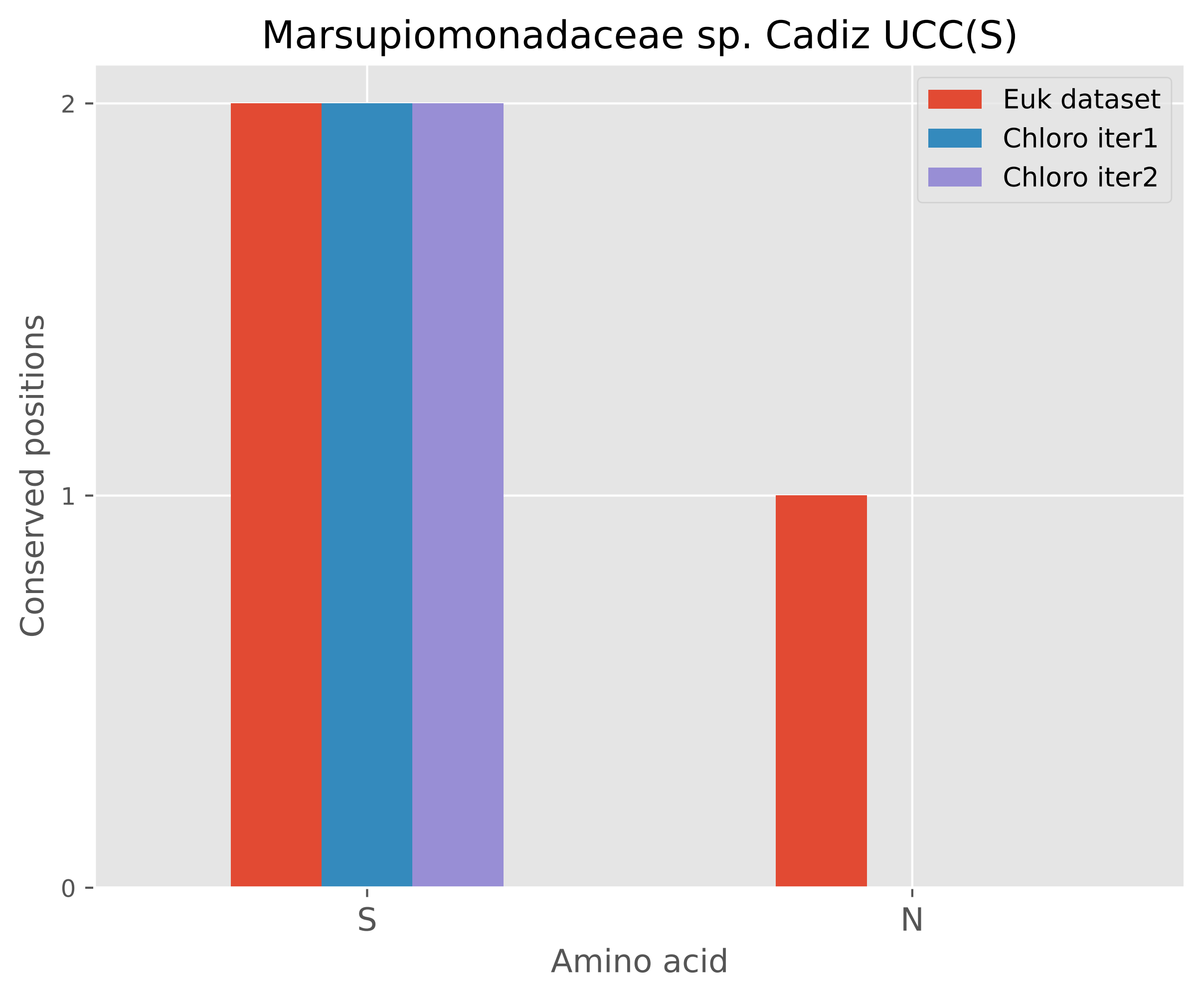
Marsupiomonadaceae sp. Cadiz UCC(S)
Euk dataset
Chloro iter1
Chloro iter2
Conserved positions
2
1
0
S
N
Amino acid

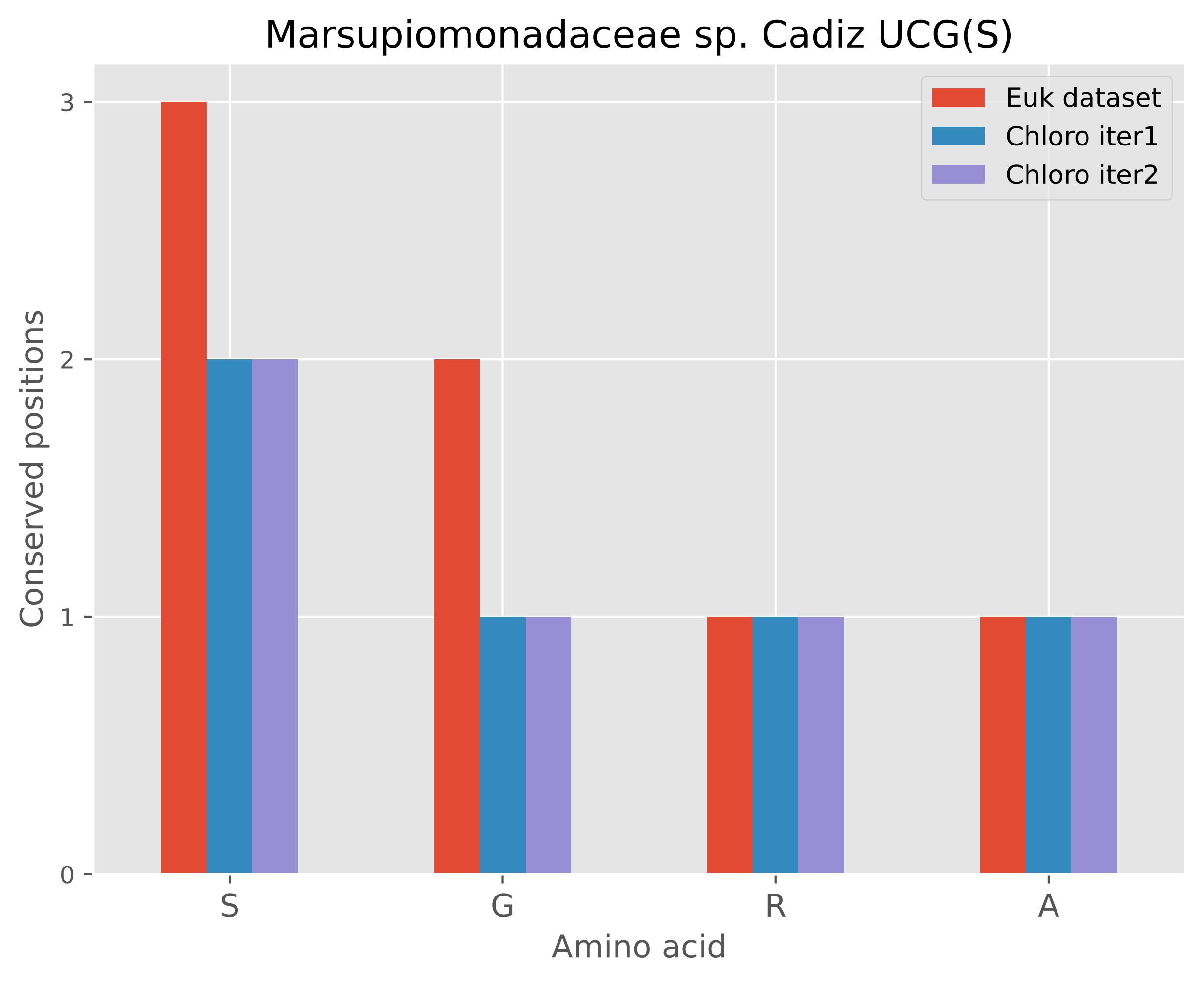
Marsupiomonadaceae sp. Cadiz UCG(S)
Euk dataset
Chloro iter1
Chloro iter2
Conserved positions
0
1
2
3
S
G
R
A
Amino acid

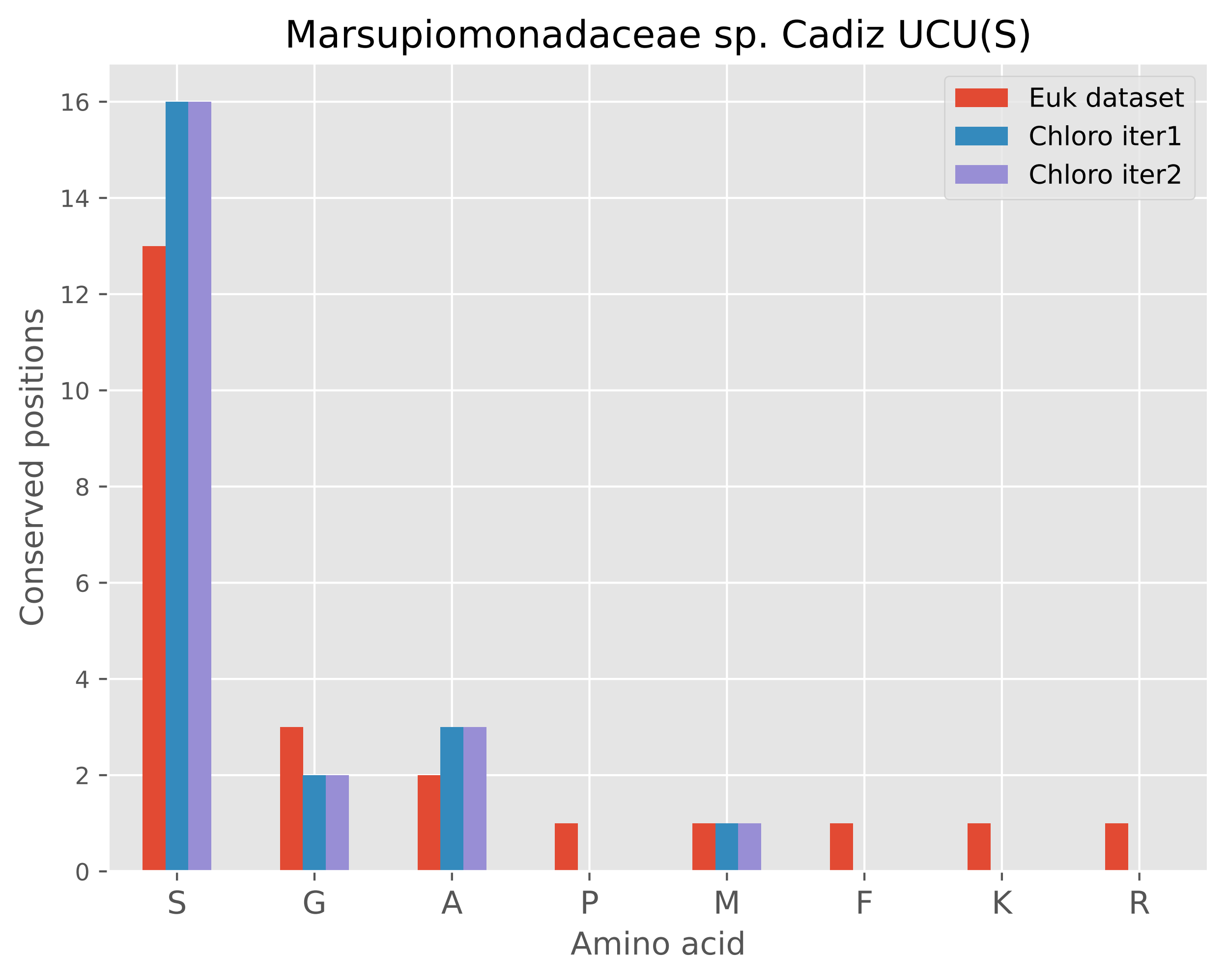

Marsupiomonadaceae sp. Cadiz UCU(S)
Euk dataset
Chloro iter1
Chloro iter2
Conserved positions
0
2
4
6
8
10
12
14
16
S
G
A
P
M
F
K
R
Amino acid

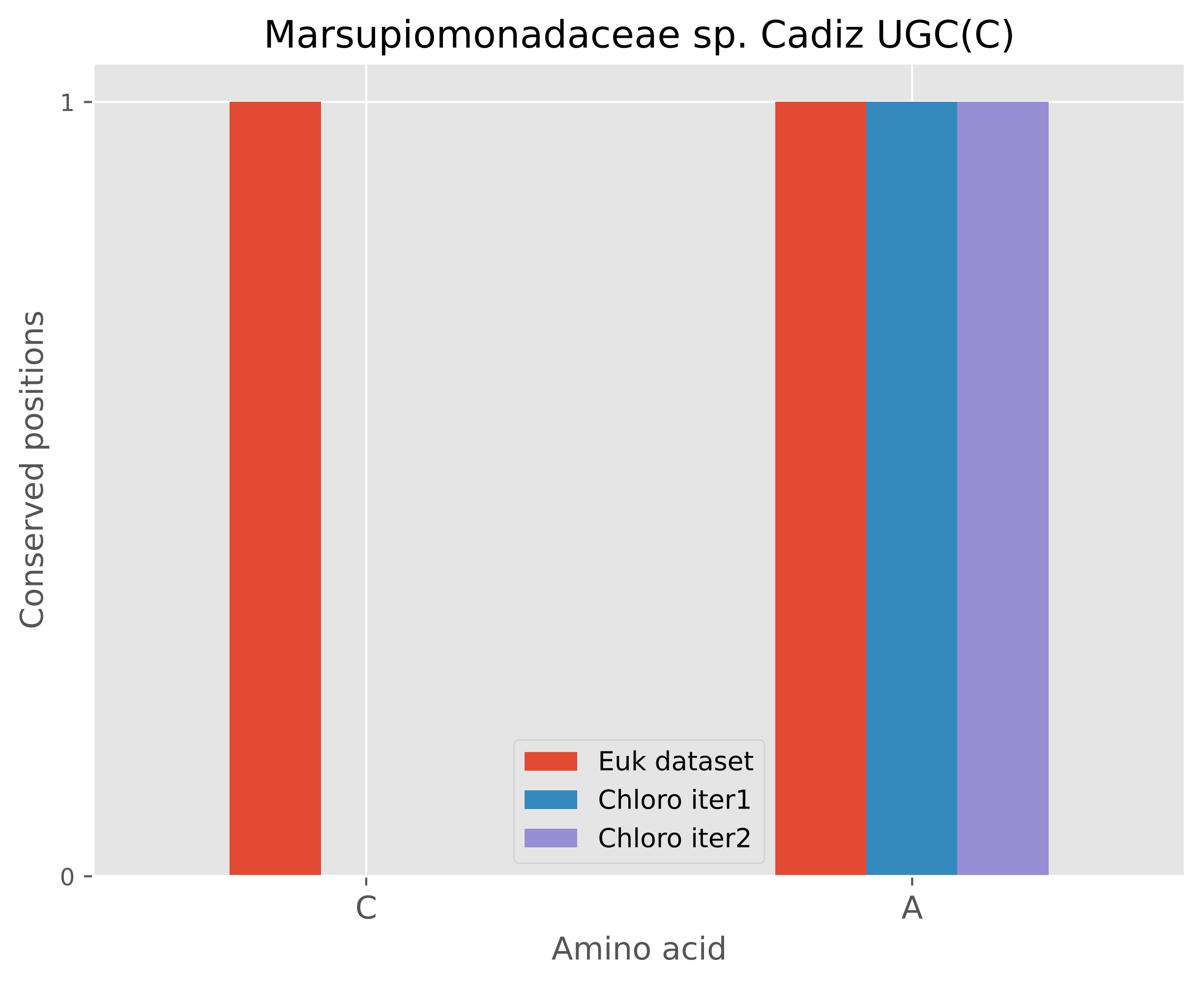
Marsupiomonadaceae sp. Cadiz UGC(C)
1
0
Conserved positions
C
A
Amino acid
Euk dataset
Chloro iter1
Chloro iter2

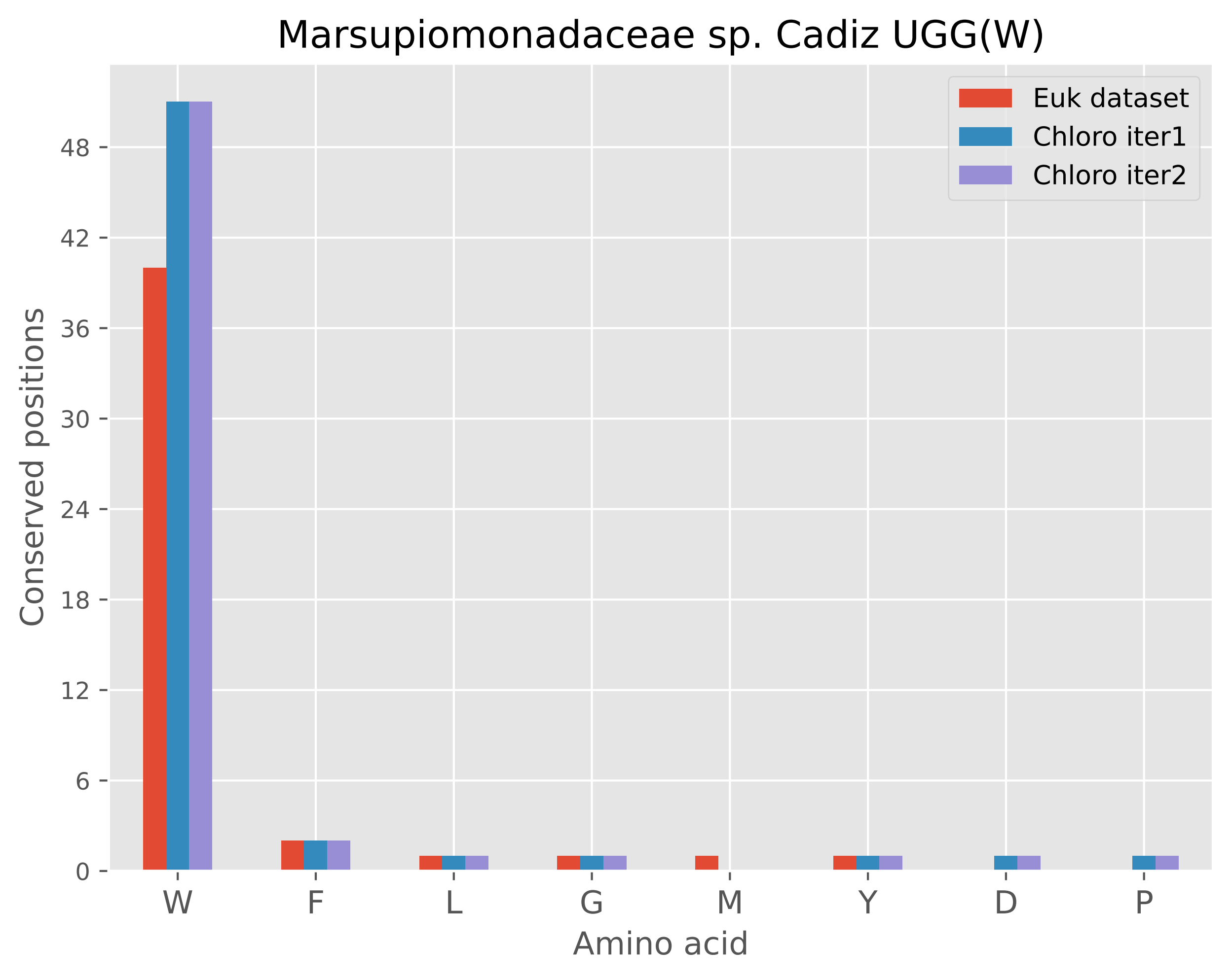

Marsupiomonadaceae sp. Cadiz UGG(W)
Euk dataset
Chloro iter1
Chloro iter2
Conserved positions
0
6
12
18
24
30
36
42
48
W
F
L
G
M
Y
D
P
Amino acid

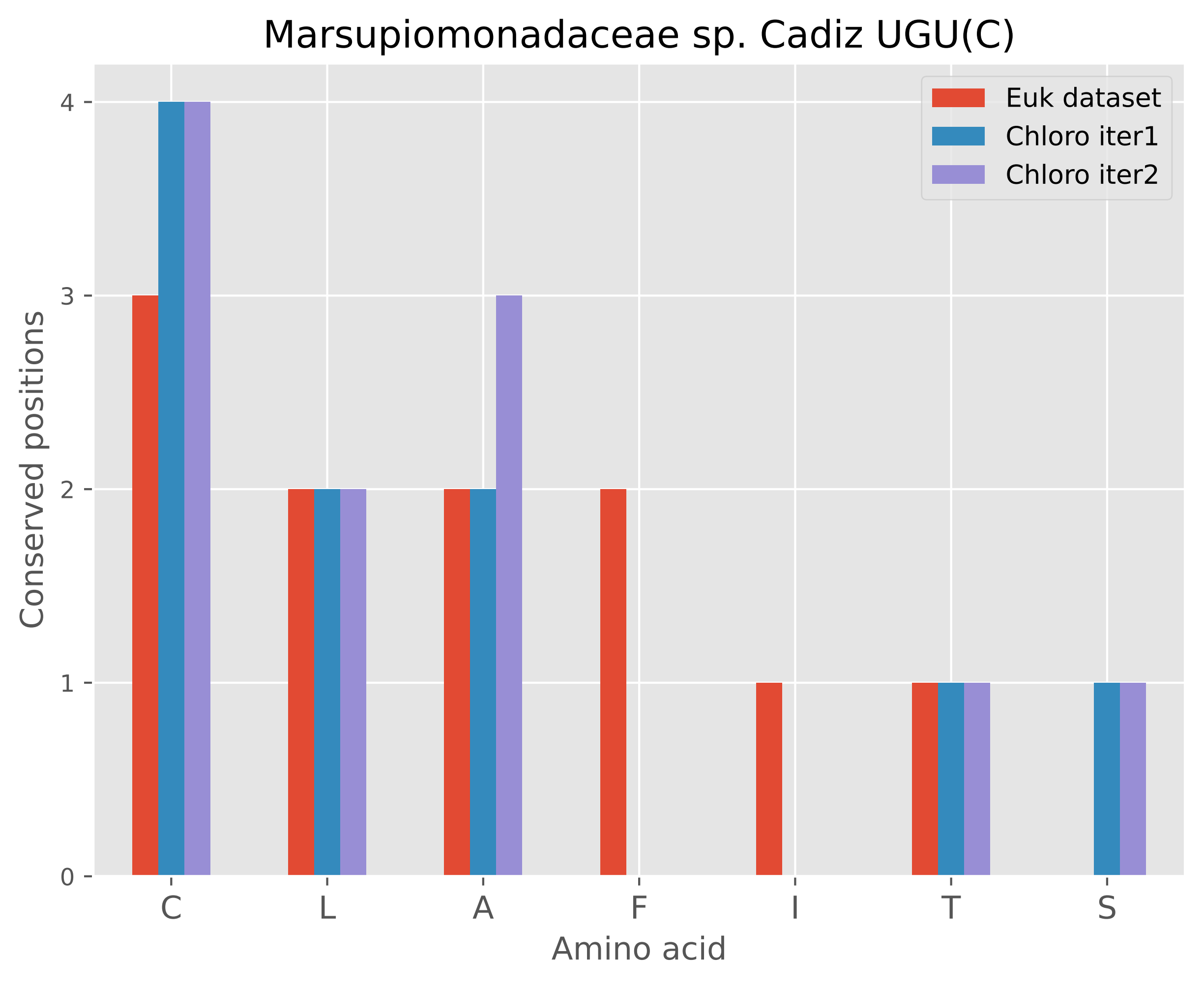

Marsupiomonadaceae sp. Cadiz UGU(C)
Euk dataset
Chloro iter1
Chloro iter2
Conserved positions
0
1
2
3
4
C
L
A
F
I
T
S
Amino acid

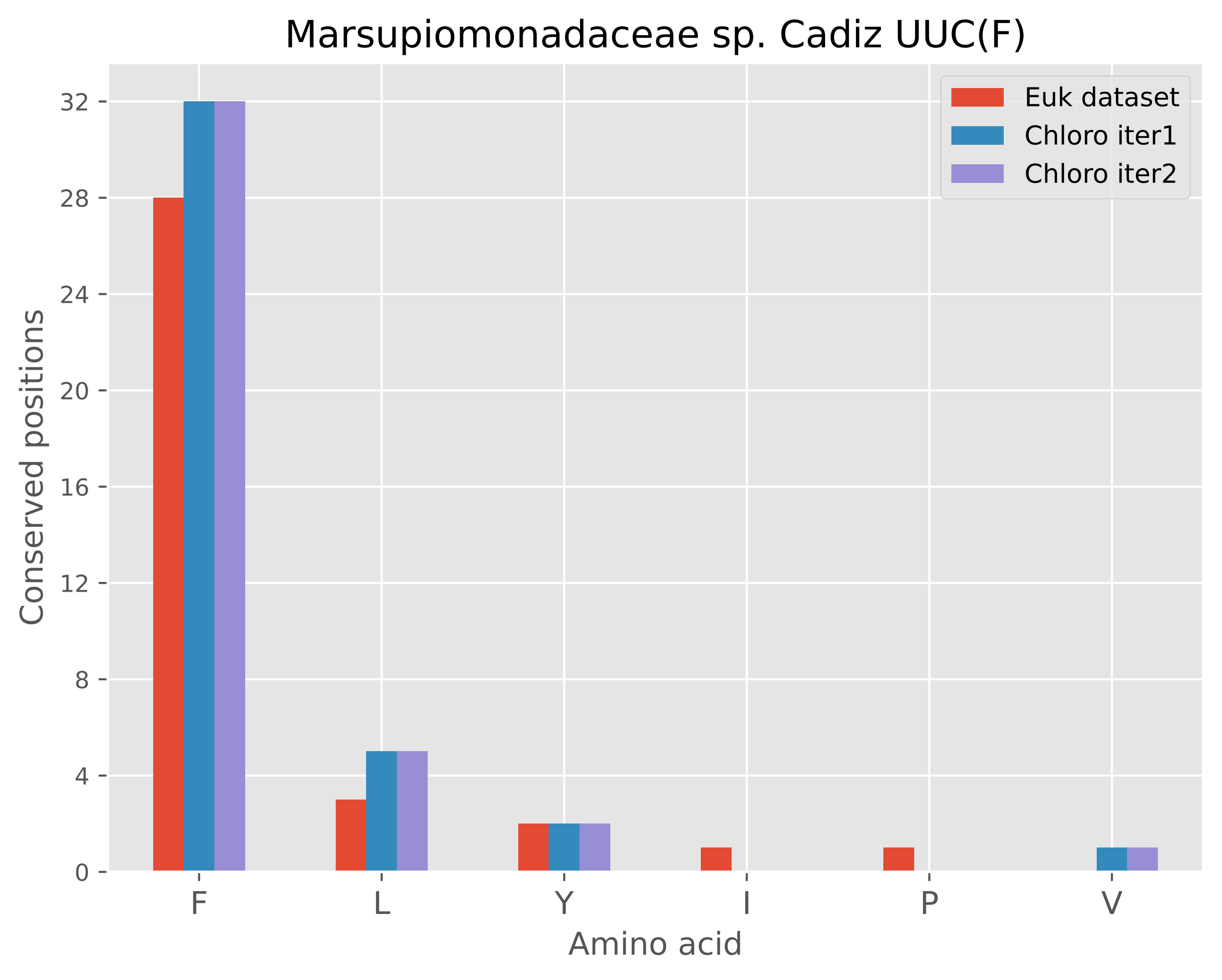
Marsupiomonadaceae sp. Cadiz UUC(F)
Euk dataset
Chloro iter1
Chloro iter2
Conserved positions
0
4
8
12
16
20
24
28
32
F
L
Y
I
P
V
Amino acid

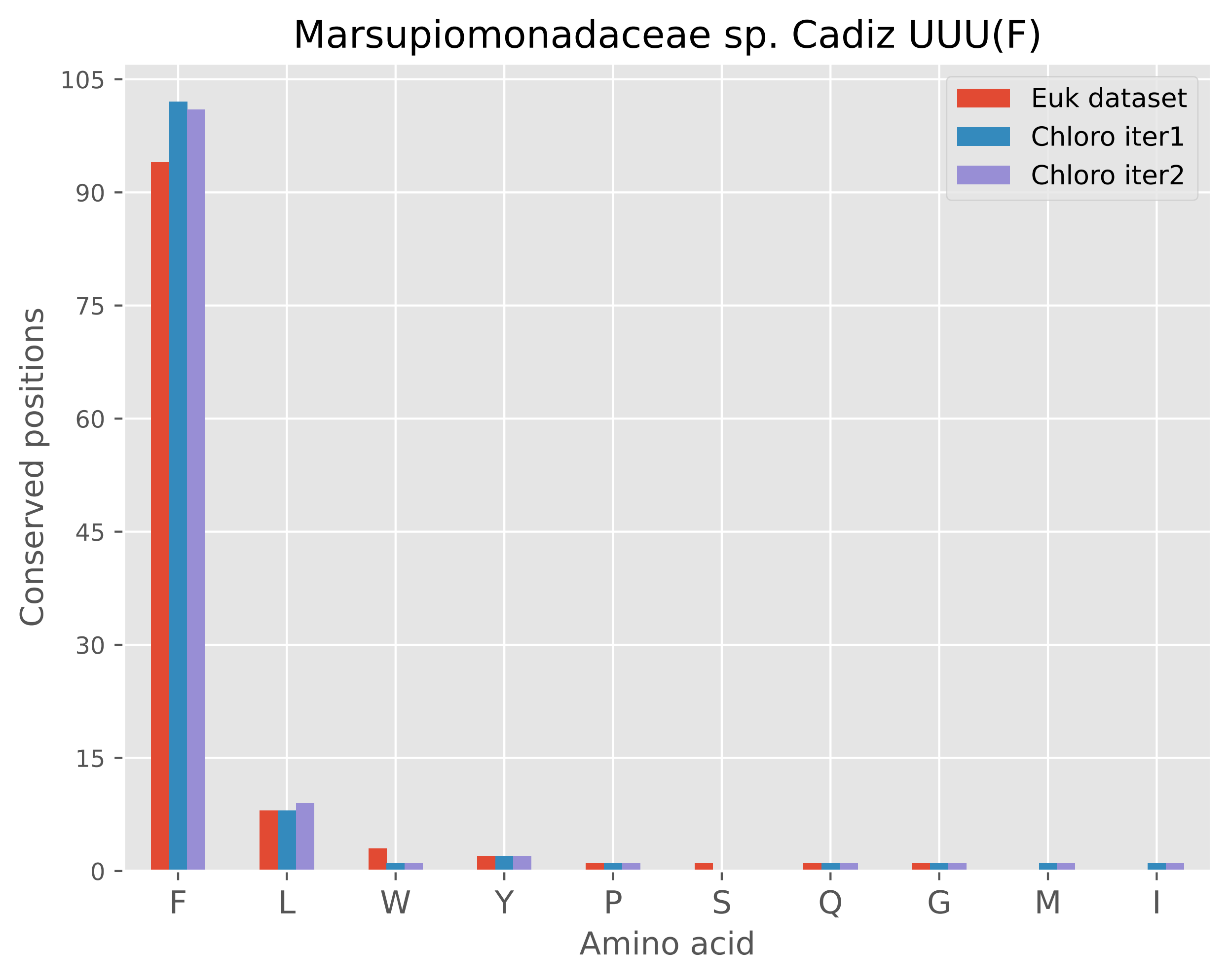
Marsupiomonadaceae sp. Cadiz UUU(F)
Euk dataset
Chloro iter1
Chloro iter2
Conserved positions
0
15
30
45
60
75
90
105
F
L
W
Y
P
S
Q
G
M
I
Amino acid

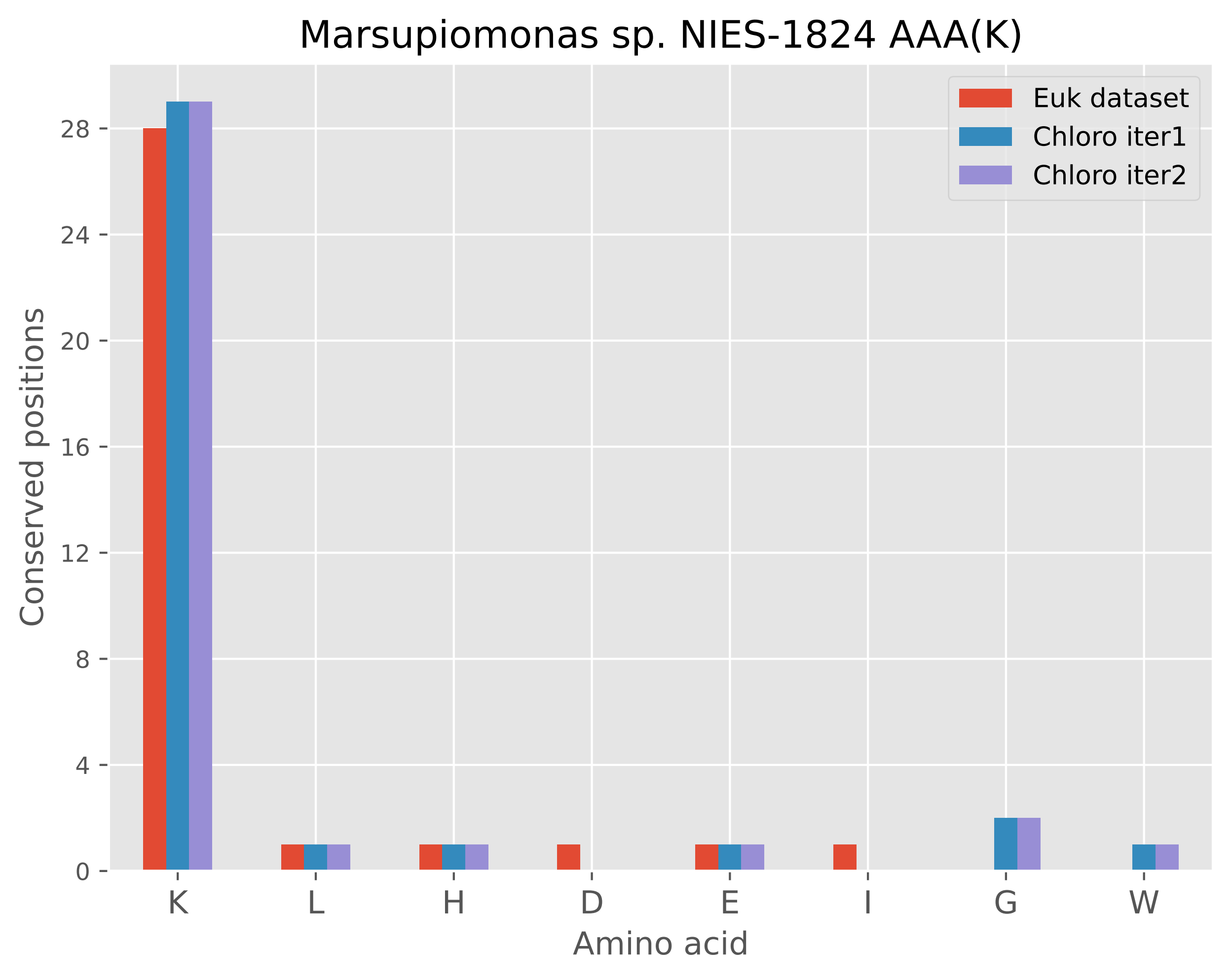

Marsupiomonas sp. NIES-1824 AAA(K)
Euk dataset
Chloro iter1
Chloro iter2
Conserved positions
0
4
8
12
16
20
24
28
K
L
H
D
E
I
G
W
Amino acid

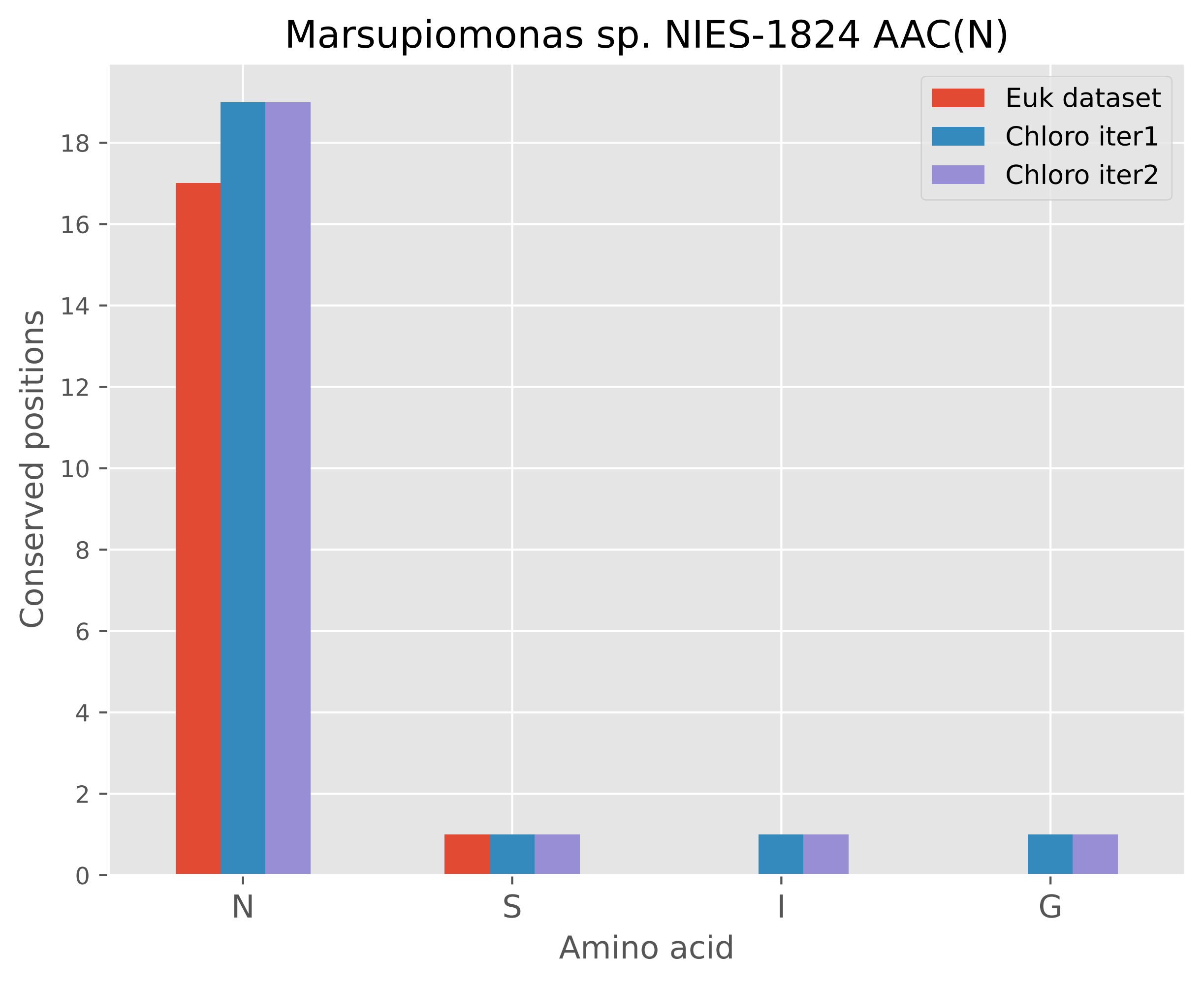

Marsupiomonas sp. NIES-1824 AAC(N)
Euk dataset
Chloro iter1
Chloro iter2
Conserved positions
0
2
4
6
8
10
12
14
16
18
N
S
I
G
Amino acid

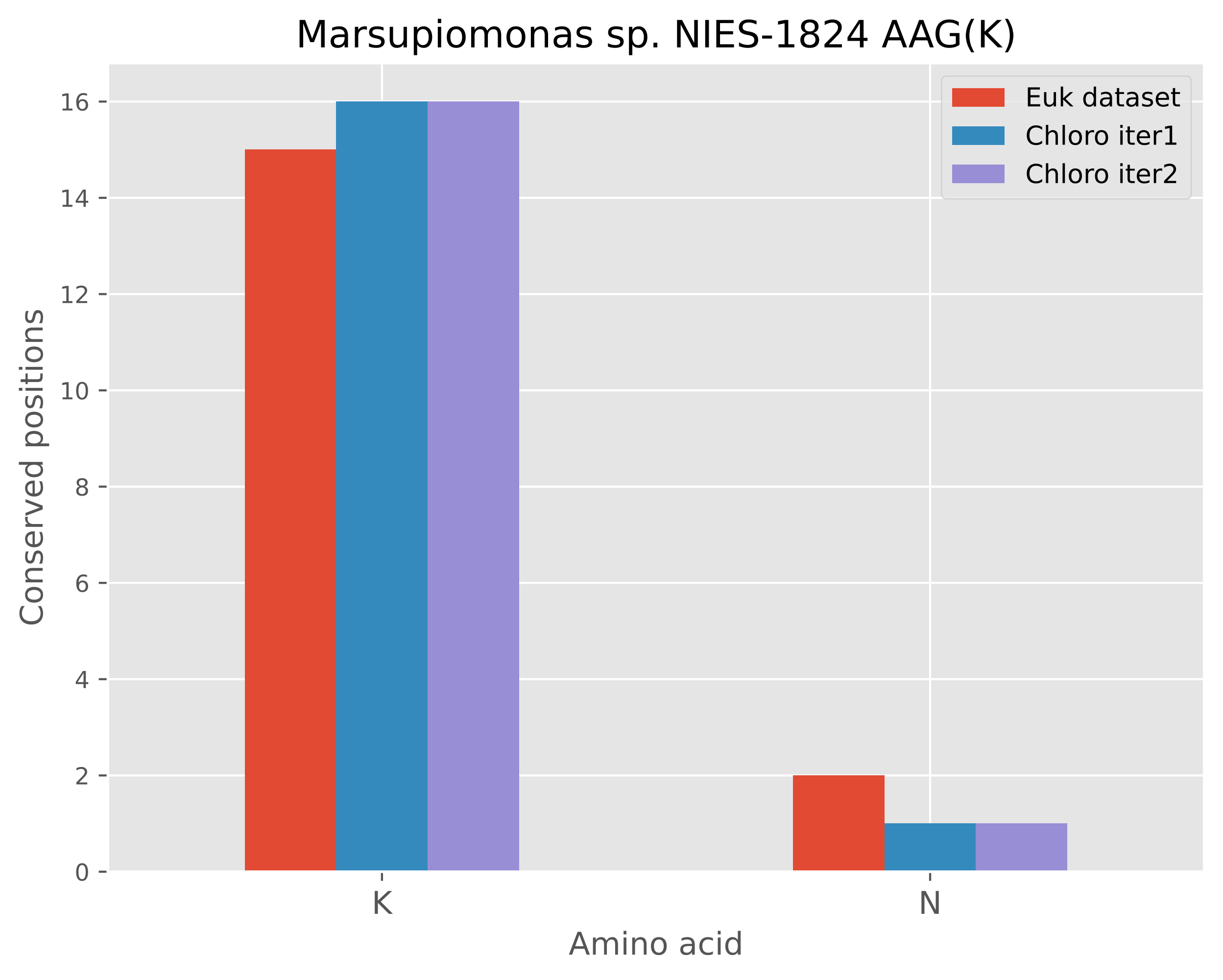

Marsupiomonas sp. NIES-1824 AAG(K)
Euk dataset
Chloro iter1
Chloro iter2
Conserved positions
0
2
4
6
8
10
12
14
16
K
N
Amino acid

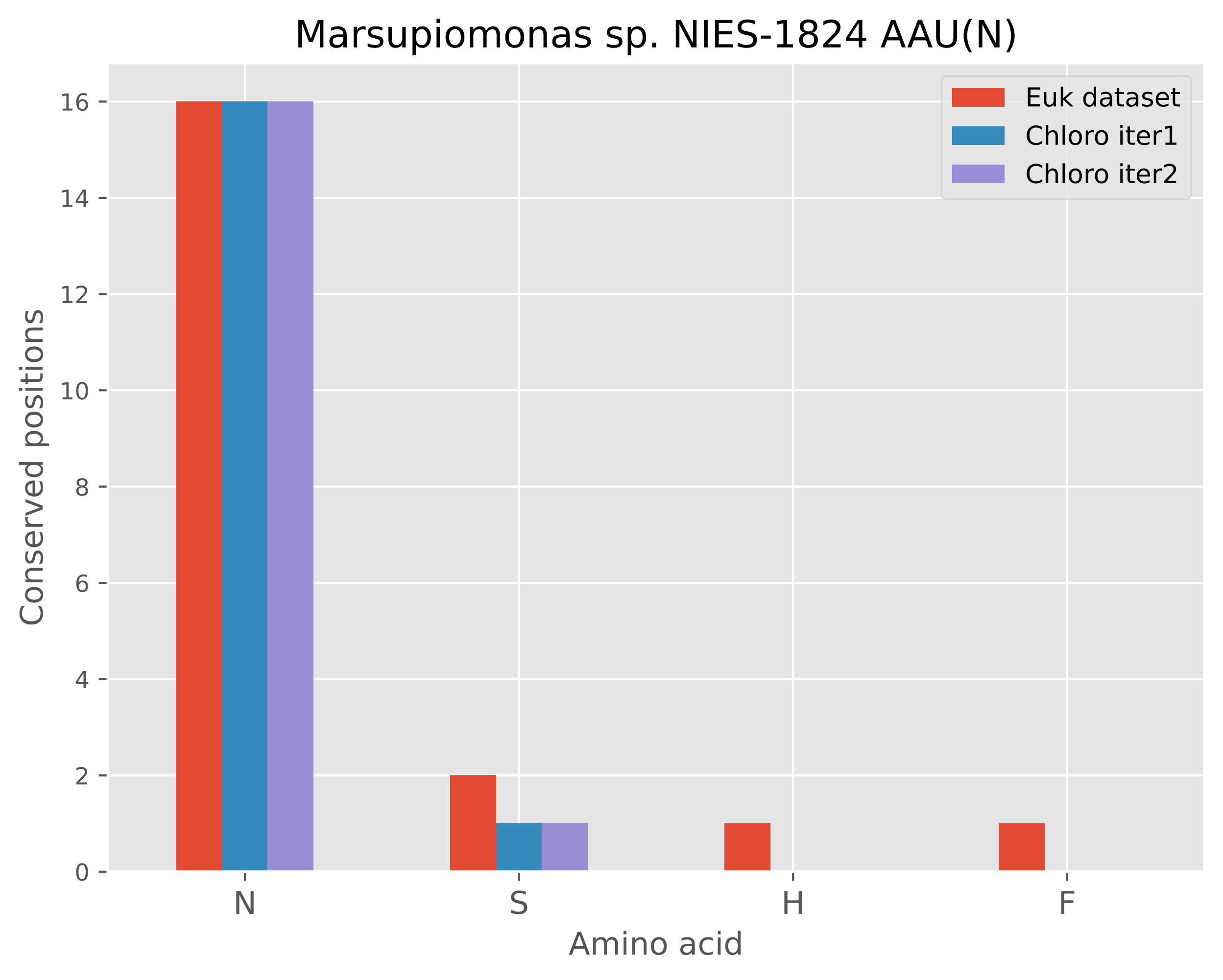
Marsupiomonas sp. NIES-1824 AAU(N)
Euk dataset
Chloro iter1
Chloro iter2
Conserved positions
0
2
4
6
8
10
12
14
16
N
S
H
F
Amino acid

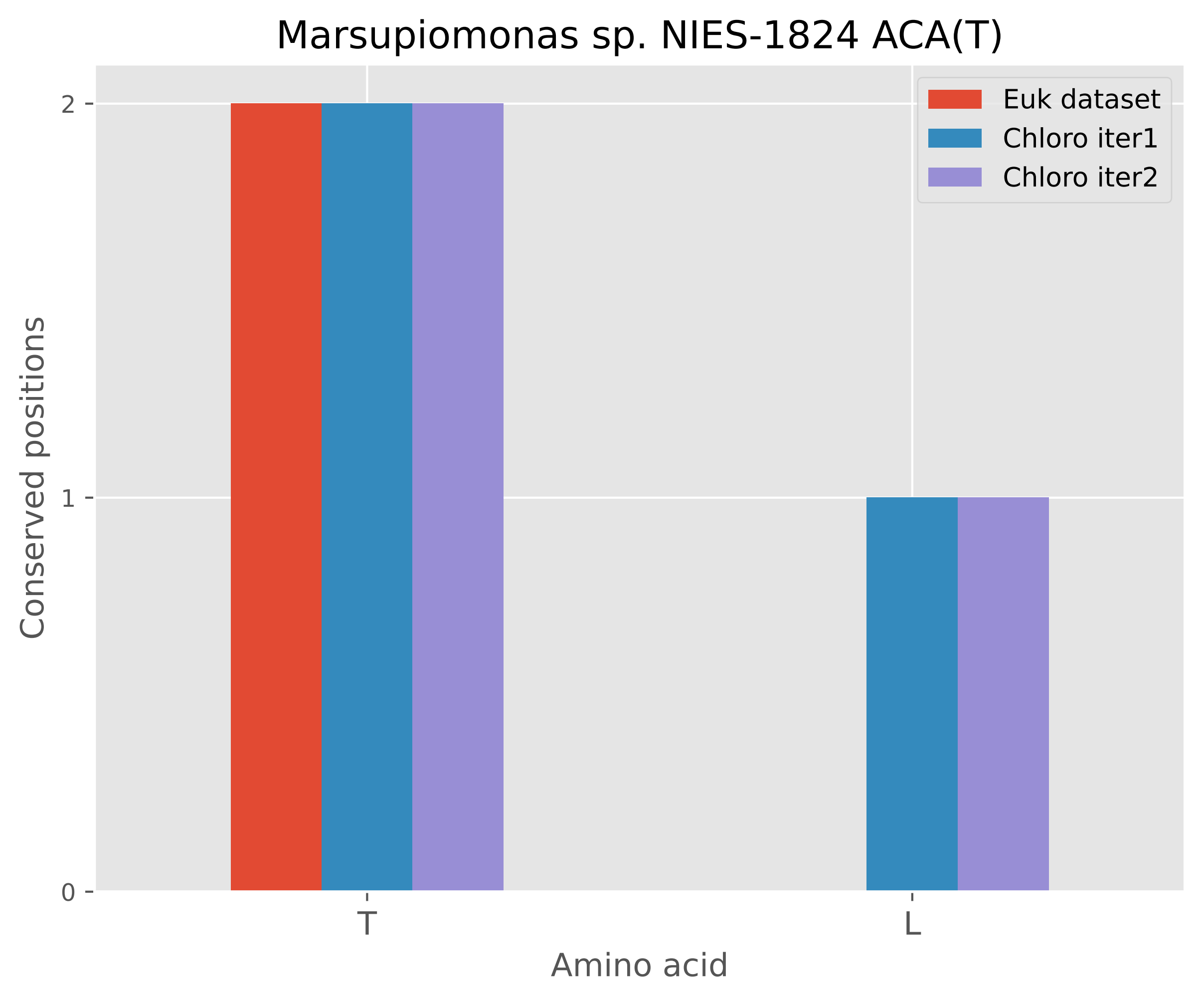

Marsupiomonas sp. NIES-1824 ACA(T)
Euk dataset
Chloro iter1
Chloro iter2
Conserved positions
2
1
0
T
L
Amino acid

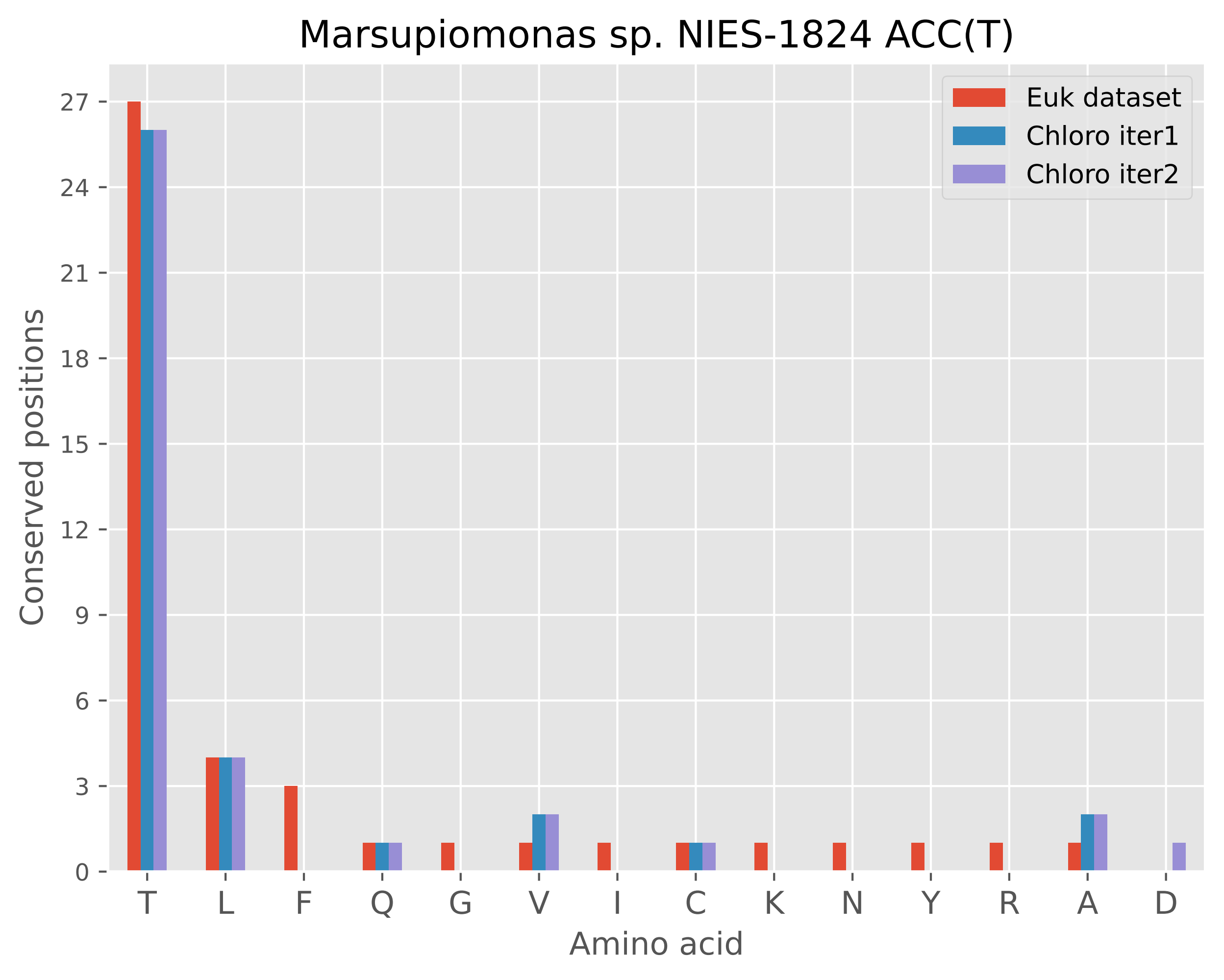
Marsupiomonas sp. NIES-1824 ACC(T)
Conserved positions
0
3
6
9
12
15
18
21
24
27
Amino acid
T
L
F
Q
G
V
I
C
K
N
Y
R
A
D
Euk dataset
Chloro iter1
Chloro iter2

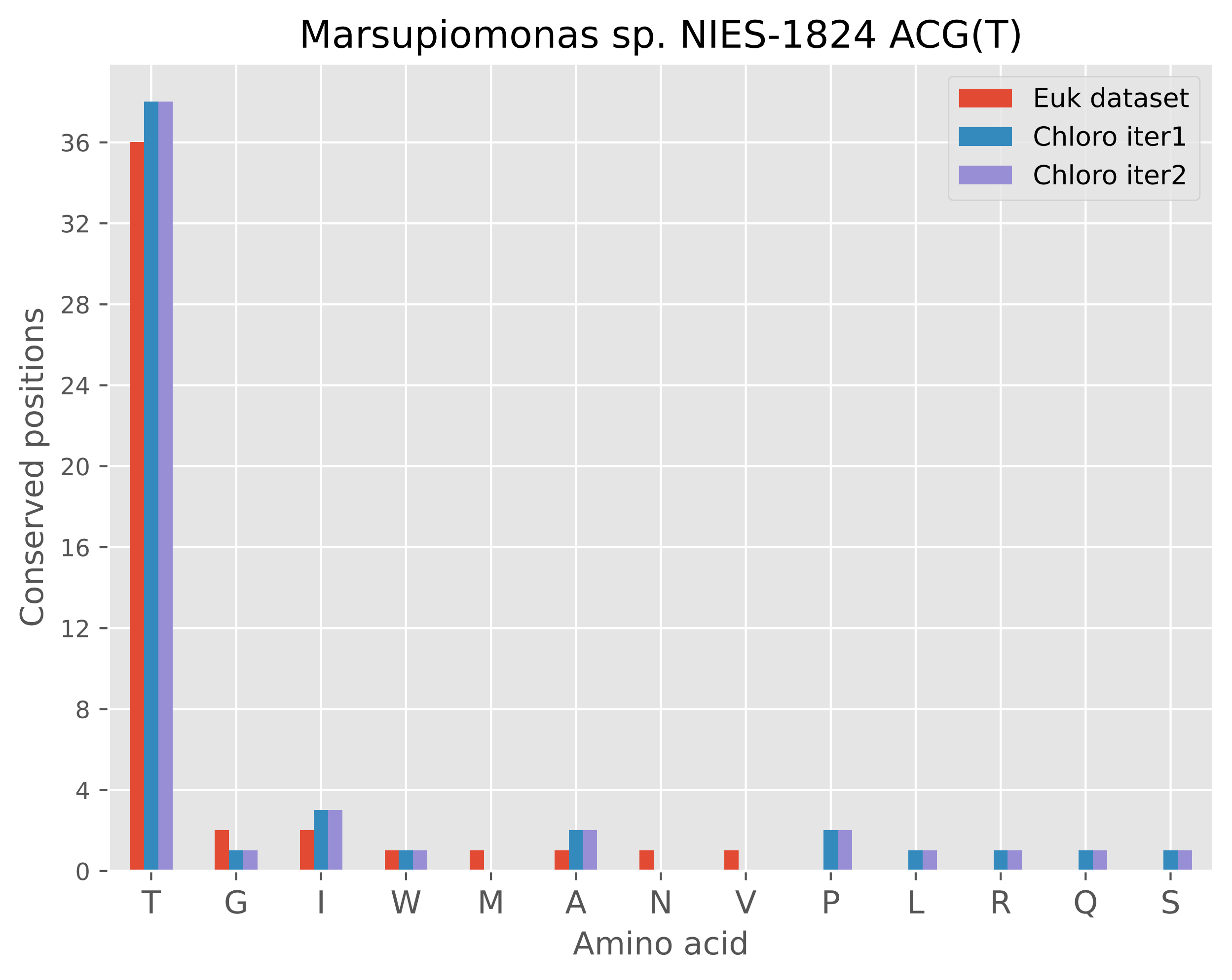

Marsupiomonas sp. NIES-1824 ACG(T)
Euk dataset
Chloro iter1
Chloro iter2
Conserved positions
0
4
8
12
16
20
24
28
32
36
T
G
I
W
M
A
N
V
P
L
R
Q
S
Amino acid

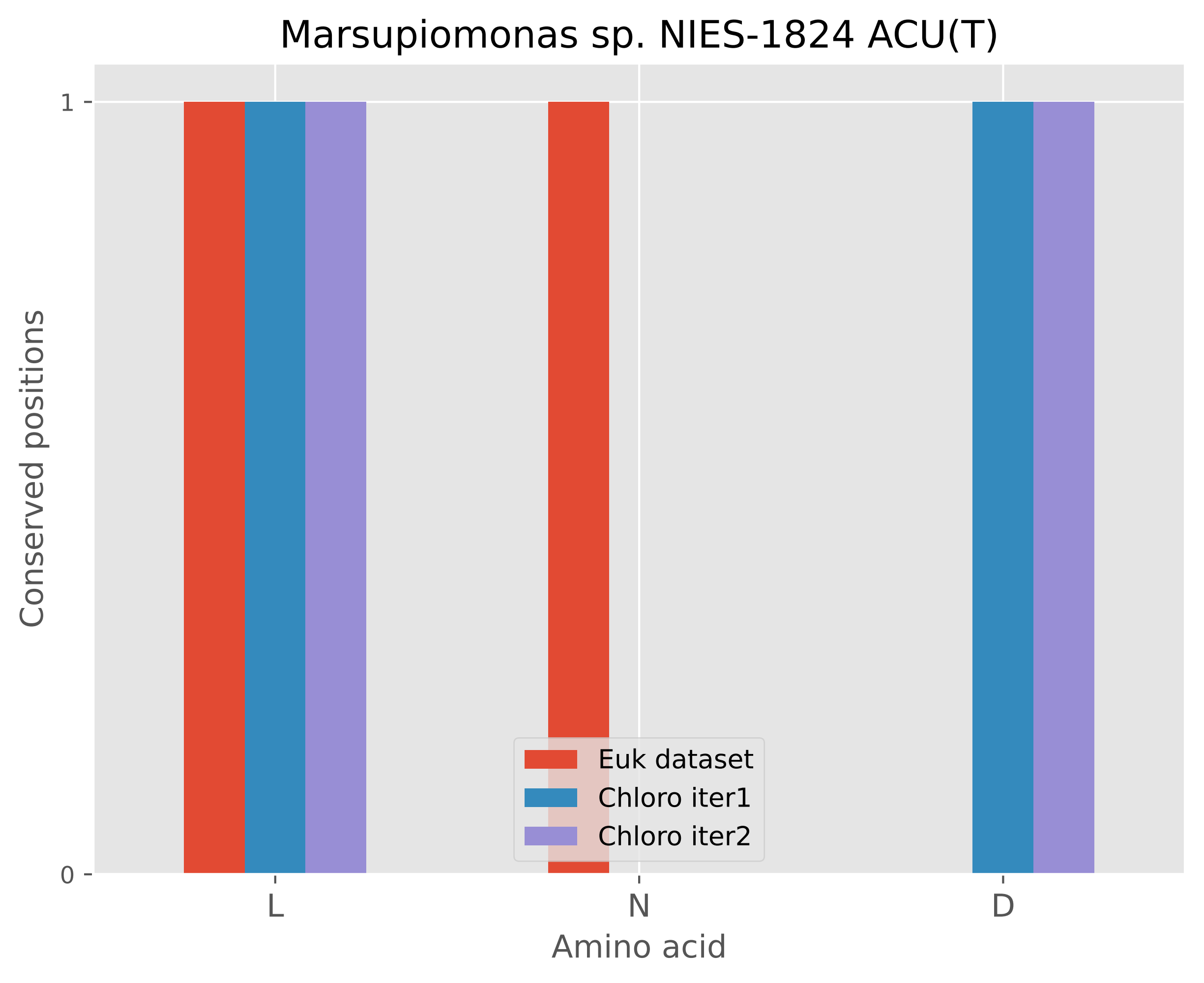
Marsupiomonas sp. NIES-1824 ACU(T)
Conserved positions
1
0
L
N
D
Amino acid
Euk dataset
Chloro iter1
Chloro iter2

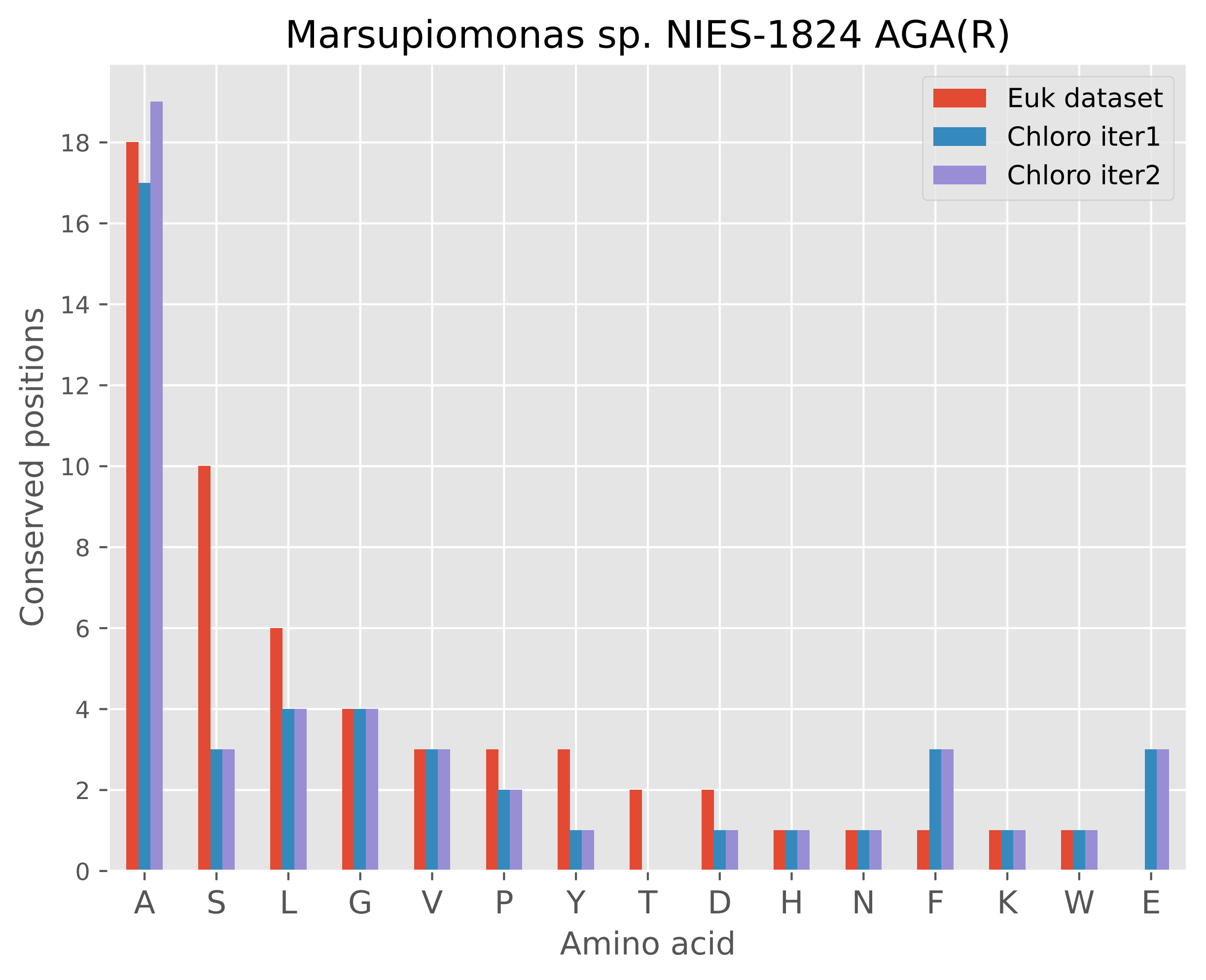

Marsupiomonas sp. NIES-1824 AGA(R)
Euk dataset
Chloro iter1
Chloro iter2
Conserved positions
0
2
4
6
8
10
12
14
16
18
A
S
L
G
V
P
Y
T
D
H
N
F
K
W
E
Amino acid

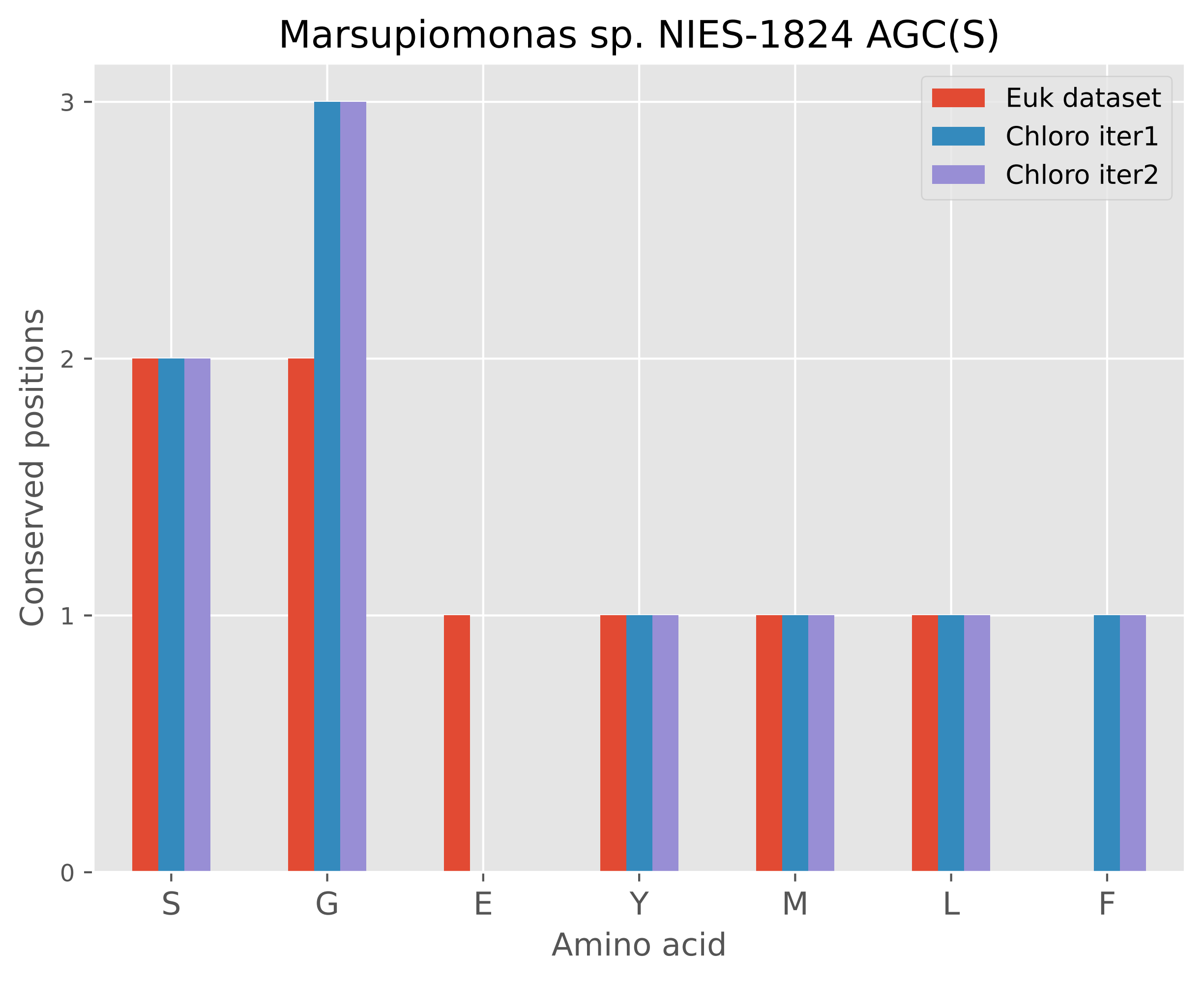
Marsupiomonas sp. NIES-1824 AGC(S)
Euk dataset
Chloro iter1
Chloro iter2
Conserved positions
0
1
2
3
S
G
E
Y
M
L
F
Amino acid

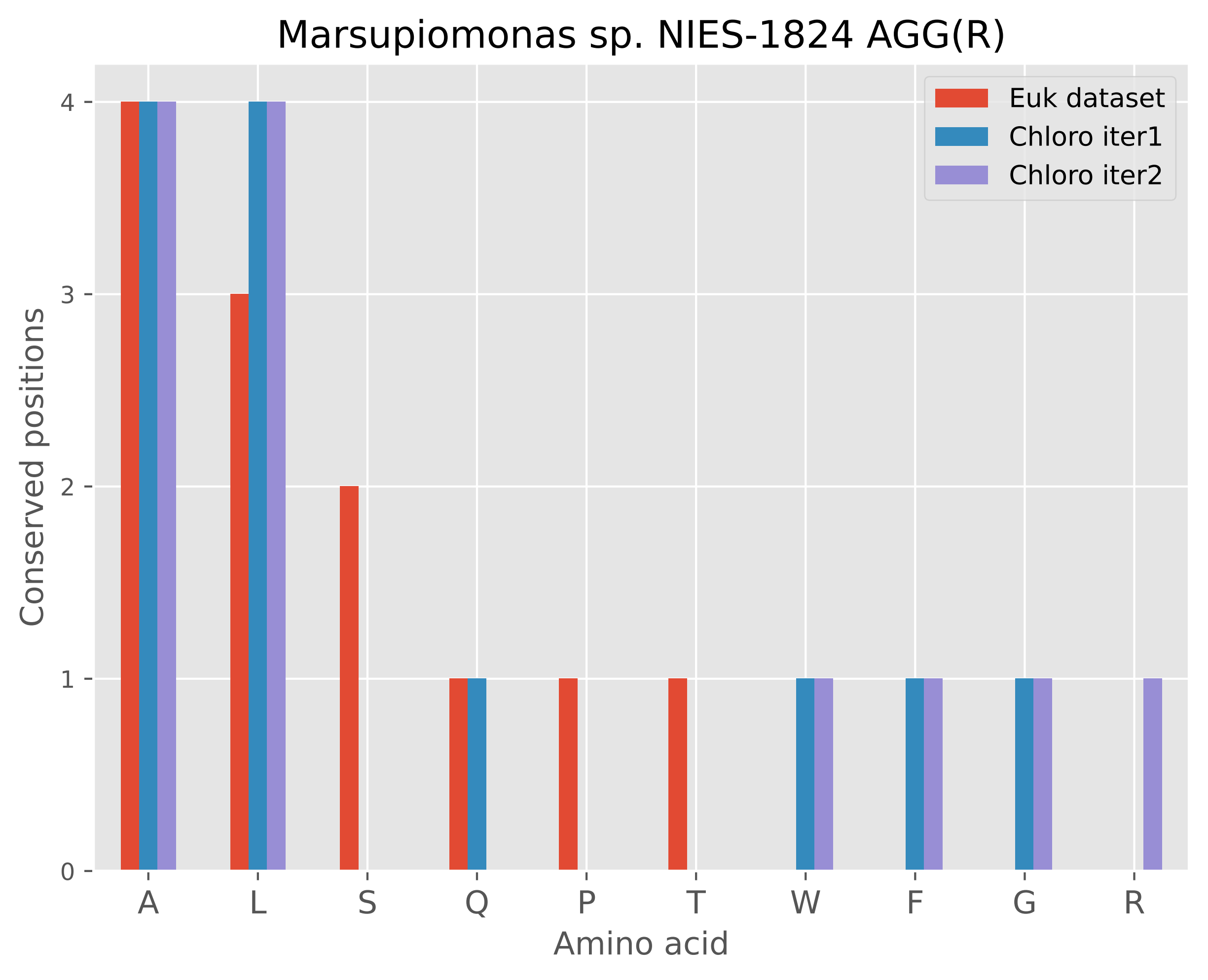
Marsupiomonas sp. NIES-1824 AGG(R)
Euk dataset
Chloro iter1
Chloro iter2
Conserved positions
0
1
2
3
4
A
L
S
Q
P
T
W
F
G
R
Amino acid

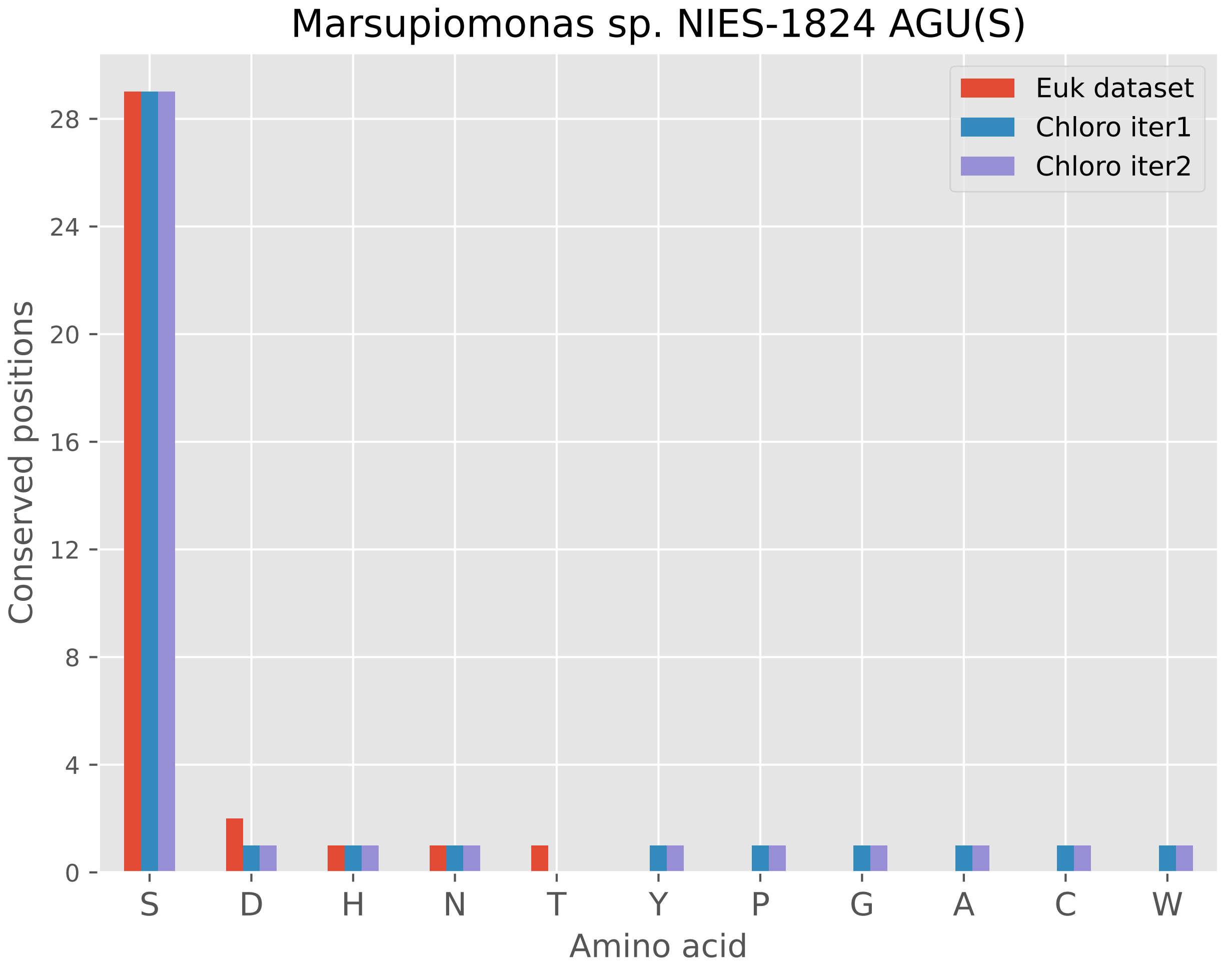

Marsupiomonas sp. NIES-1824 AGU(S)
Euk dataset
Chloro iter1
Chloro iter2
Conserved positions
0
4
8
12
16
20
24
28
S
D
H
N
T
Y
P
G
A
C
W
Amino acid

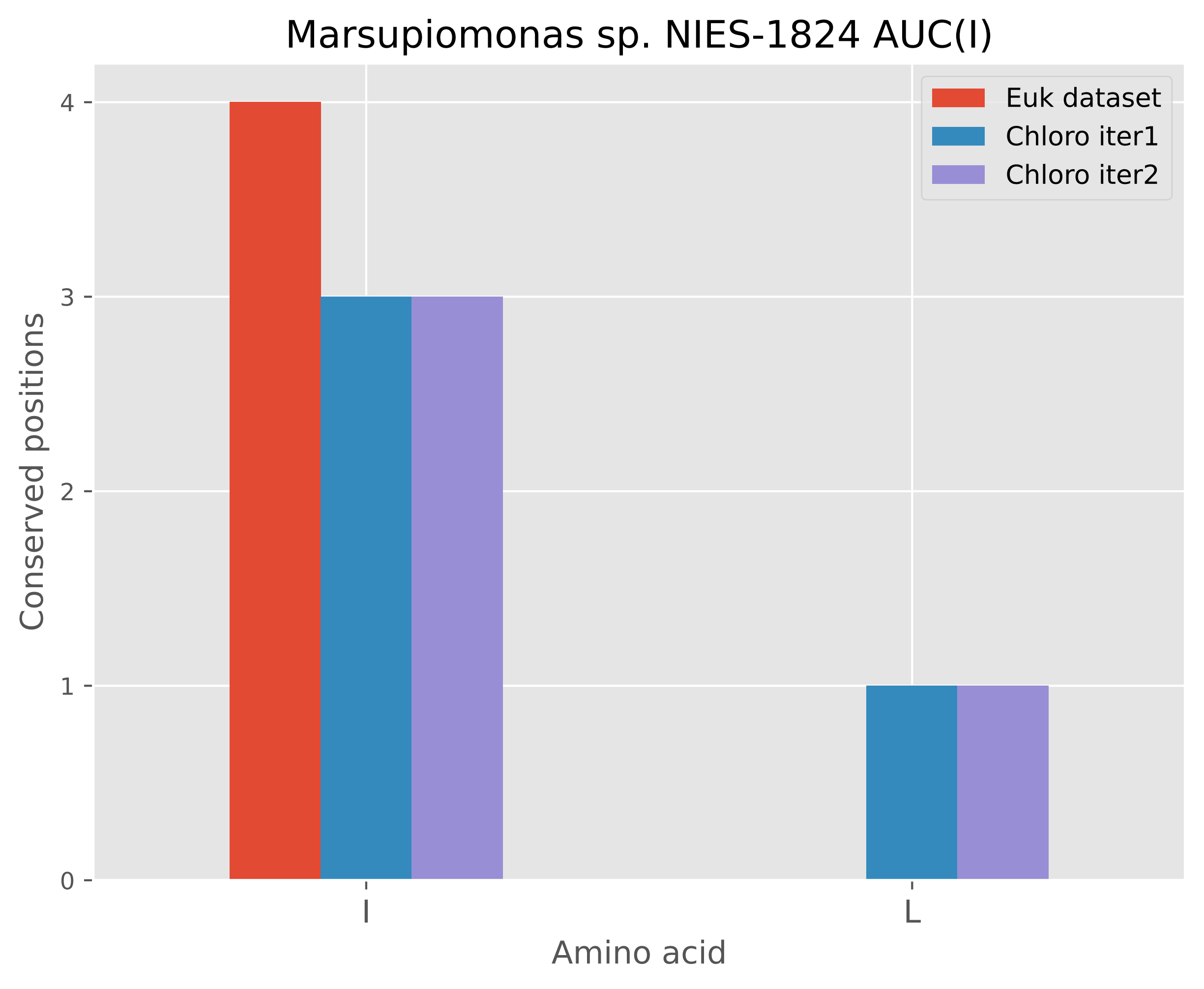
Marsupiomonas sp. NIES-1824 AUC(I)
Euk dataset
Chloro iter1
Chloro iter2
Conserved positions
0
1
2
3
4
I
L
Amino acid

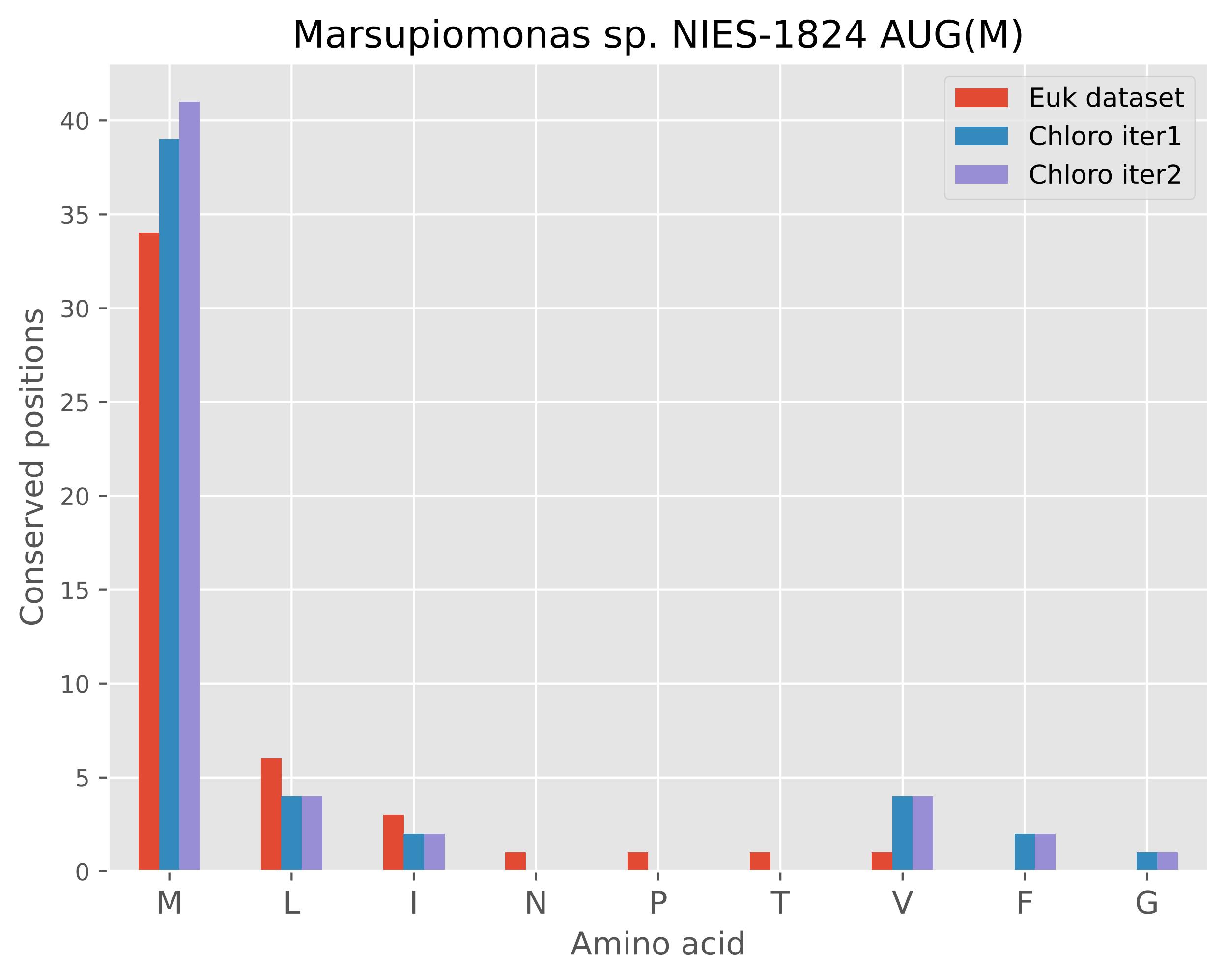
Marsupiomonas sp. NIES-1824 AUG(M)
Euk dataset
Chloro iter1
Chloro iter2
Conserved positions
0
5
10
15
20
25
30
35
40
M
L
I
N
P
T
V
F
G
Amino acid

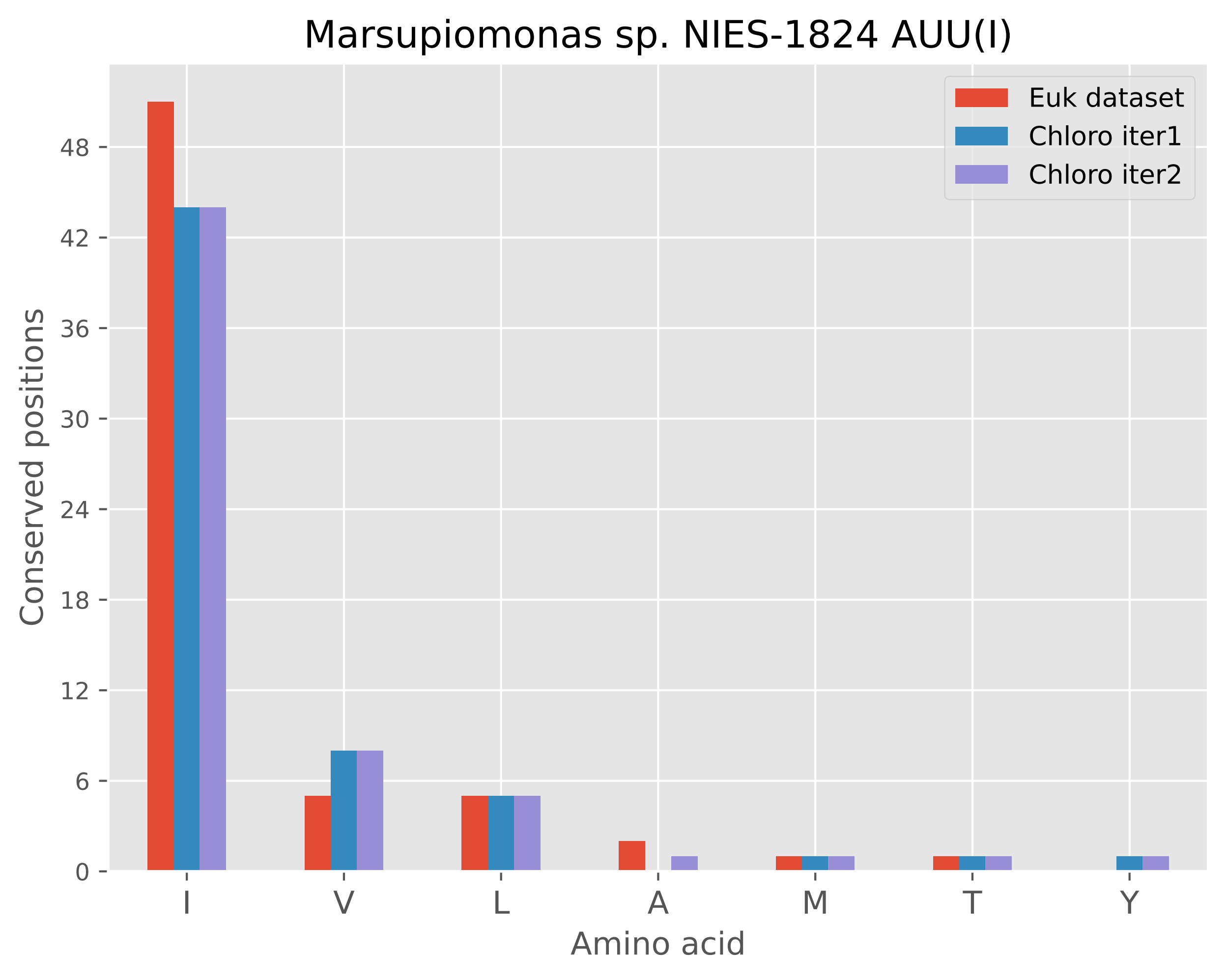
Marsupiomonas sp. NIES-1824 AUU(I)
Euk dataset
Chloro iter1
Chloro iter2
Conserved positions
0
6
12
18
24
30
36
42
48
I
V
L
A
M
T
Y
Amino acid

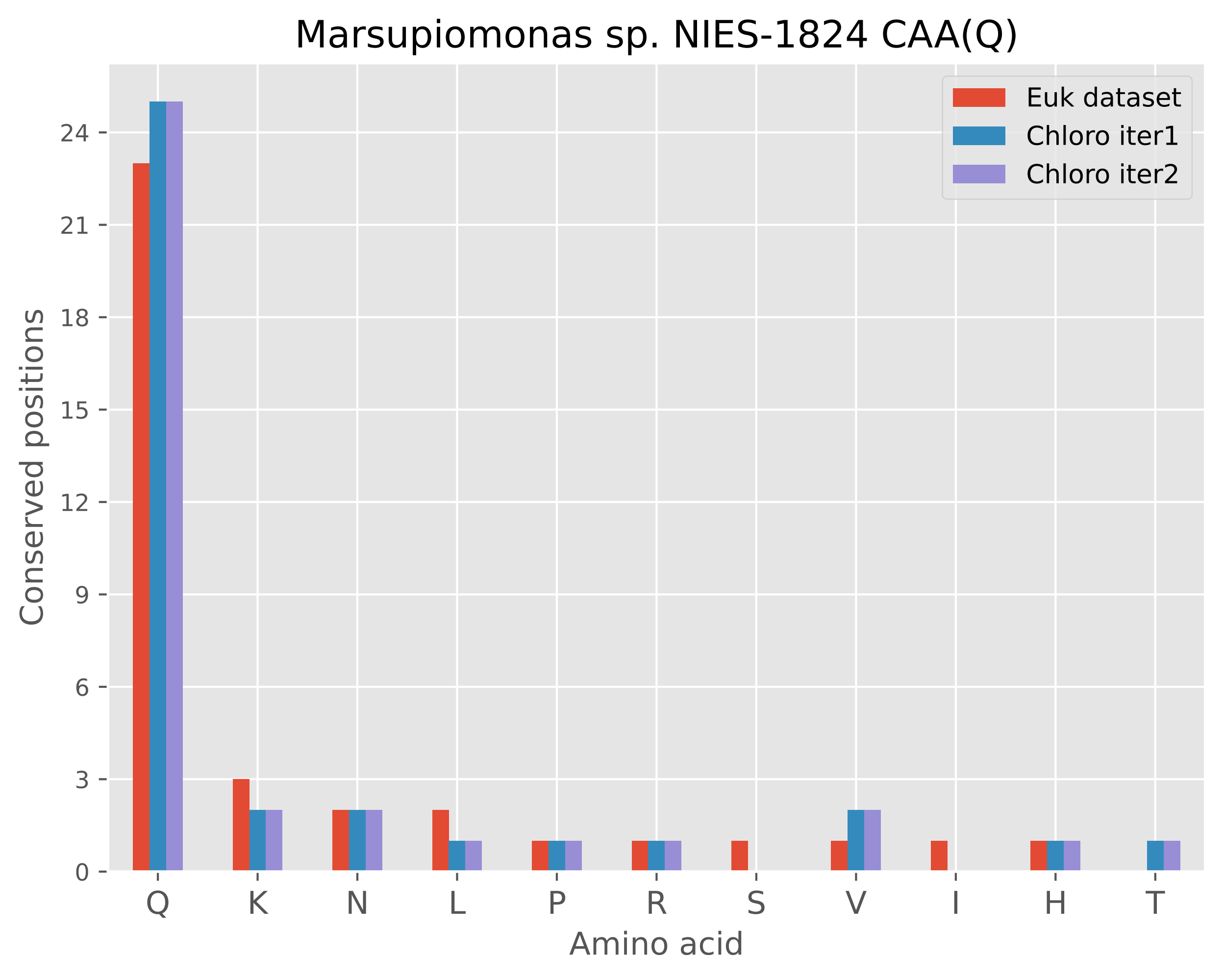
Marsupiomonas sp. NIES-1824 CAA(Q)
Euk dataset
Chloro iter1
Chloro iter2
Conserved positions
0
3
6
9
12
15
18
21
24
Q
K
N
L
P
R
S
V
I
H
T
Amino acid

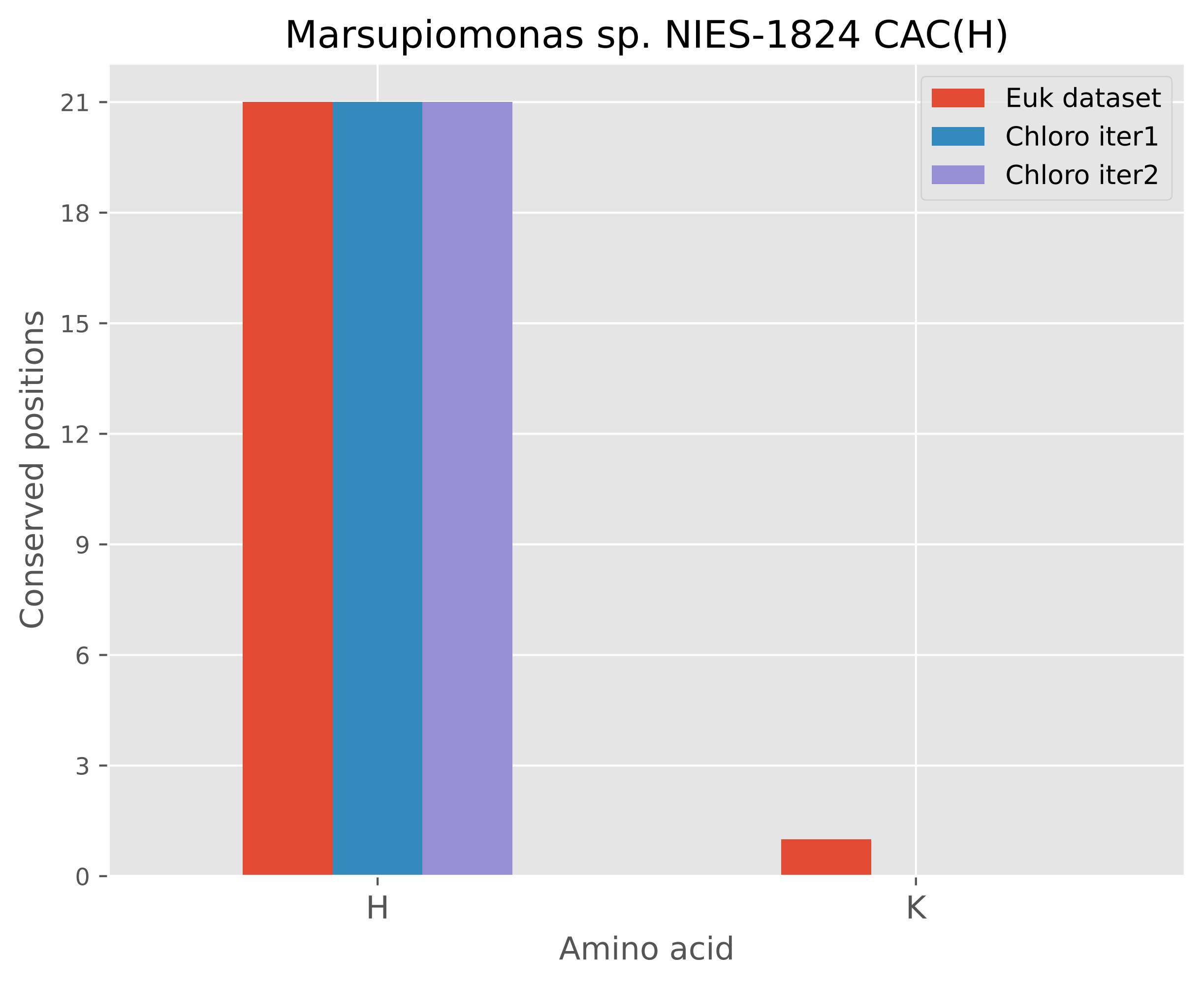

Marsupiomonas sp. NIES-1824 CAC(H)
Euk dataset
Chloro iter1
Chloro iter2
Conserved positions
0
3
6
9
12
15
18
21
H
K
Amino acid

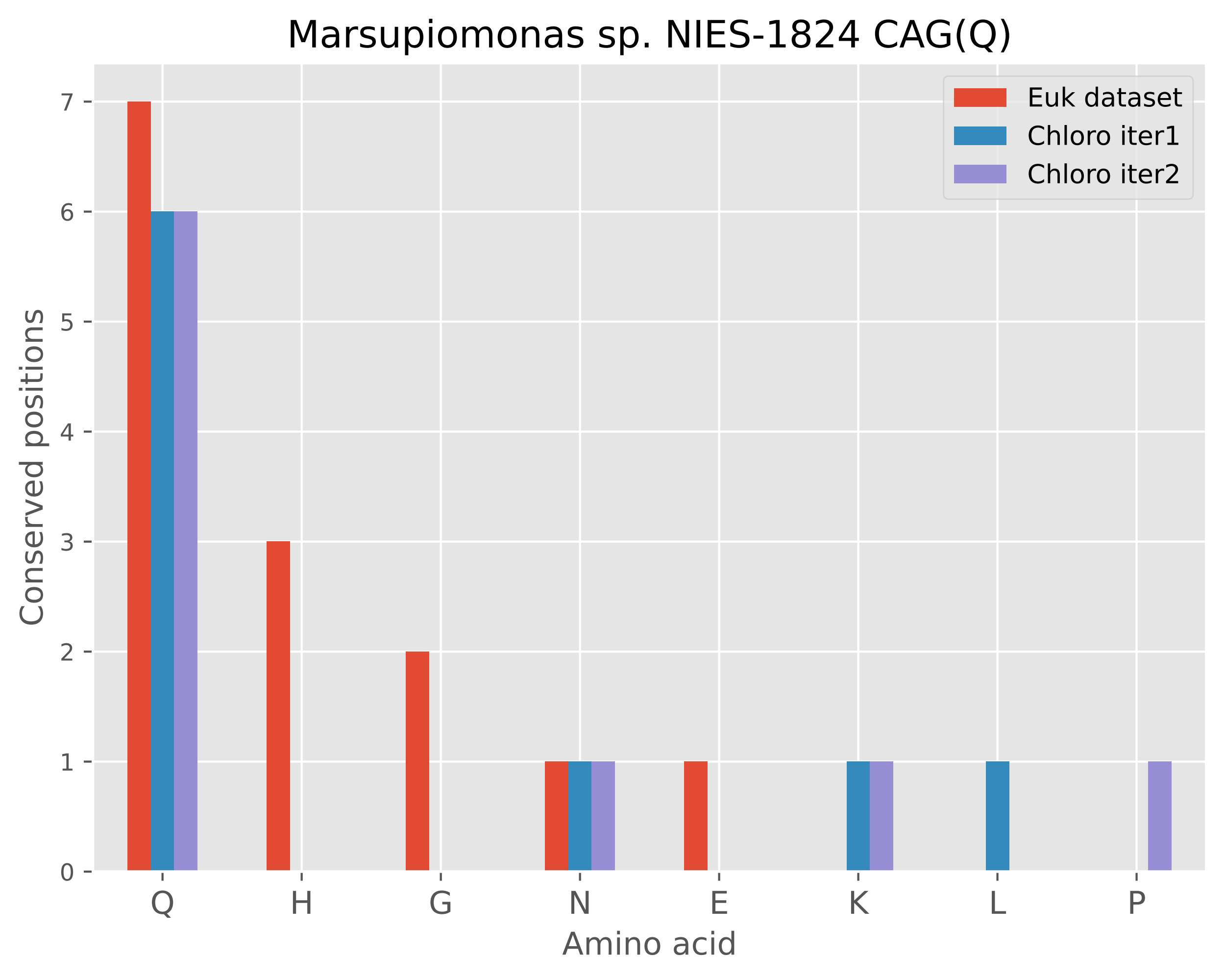

Marsupiomonas sp. NIES-1824 CAG(Q)
Euk dataset
Chloro iter1
Chloro iter2
Conserved positions
0
1
2
3
4
5
6
7
Q
H
G
N
E
K
L
P
Amino acid

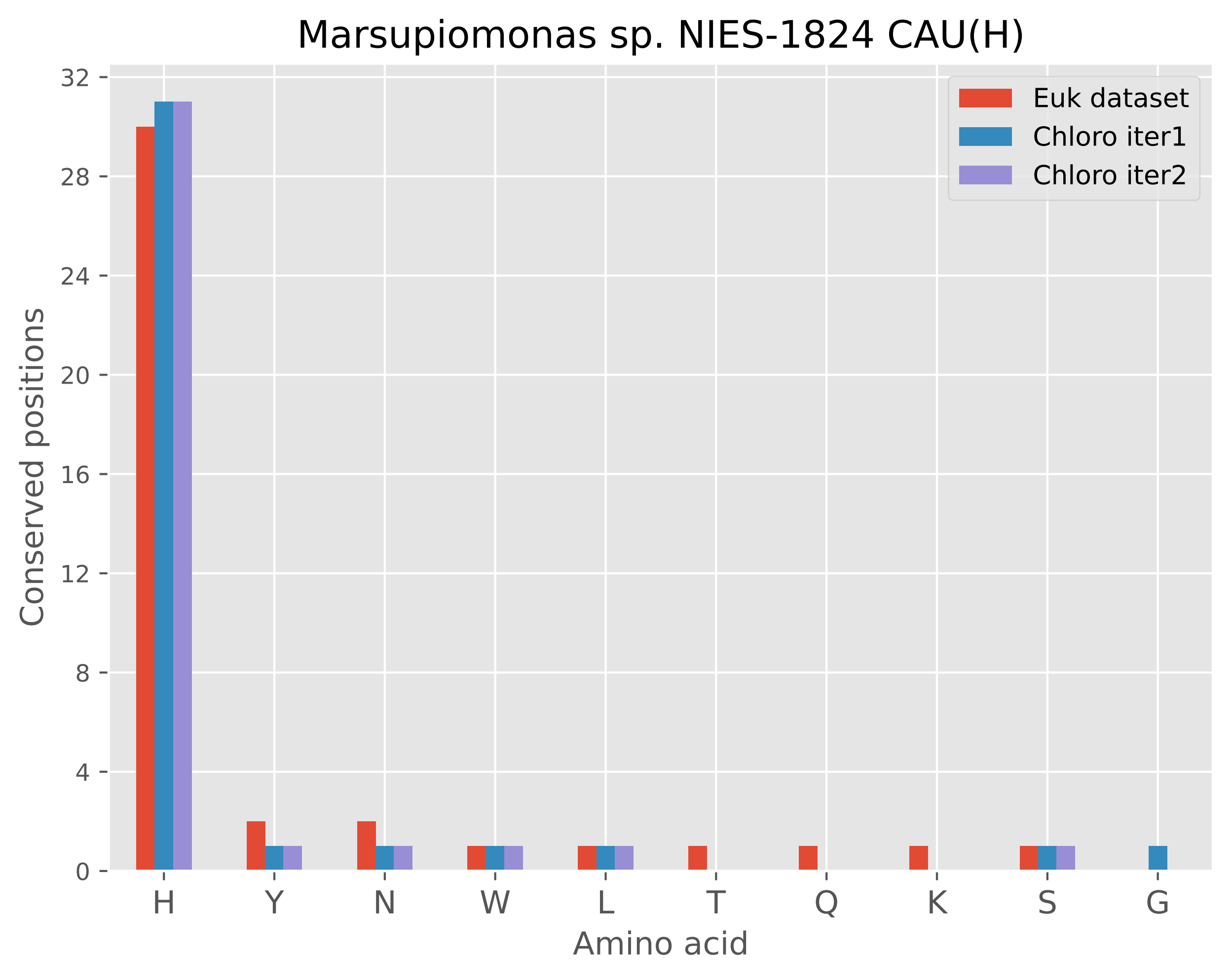
Marsupiomonas sp. NIES-1824 CAU(H)
Euk dataset
Chloro iter1
Chloro iter2
Conserved positions
0
4
8
12
16
20
24
28
32
H
Y
N
W
L
T
Q
K
S
G
Amino acid

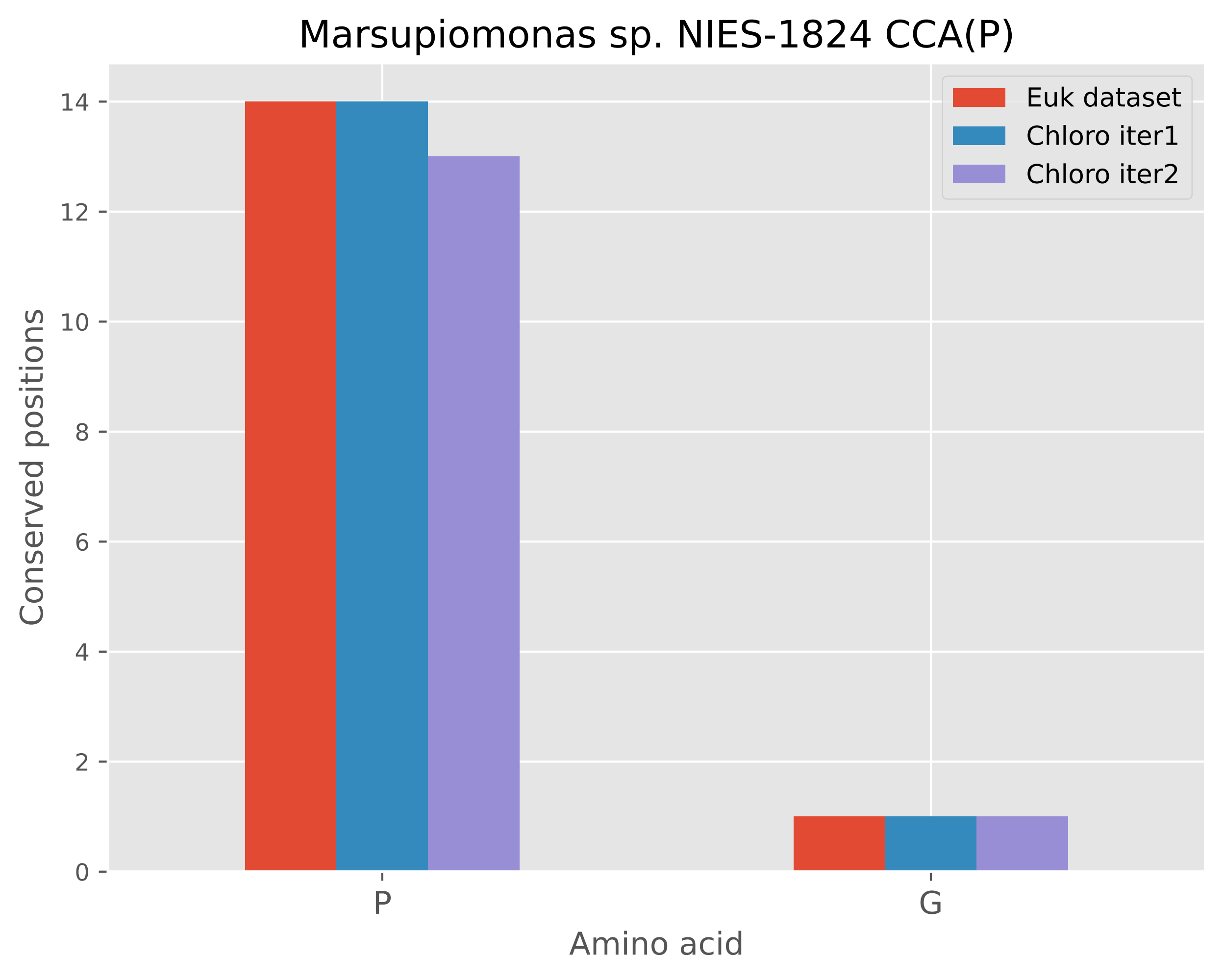
Marsupiomonas sp. NIES-1824 CCA(P)
Euk dataset
Chloro iter1
Chloro iter2
Conserved positions
0
2
4
6
8
10
12
14
P
G
Amino acid

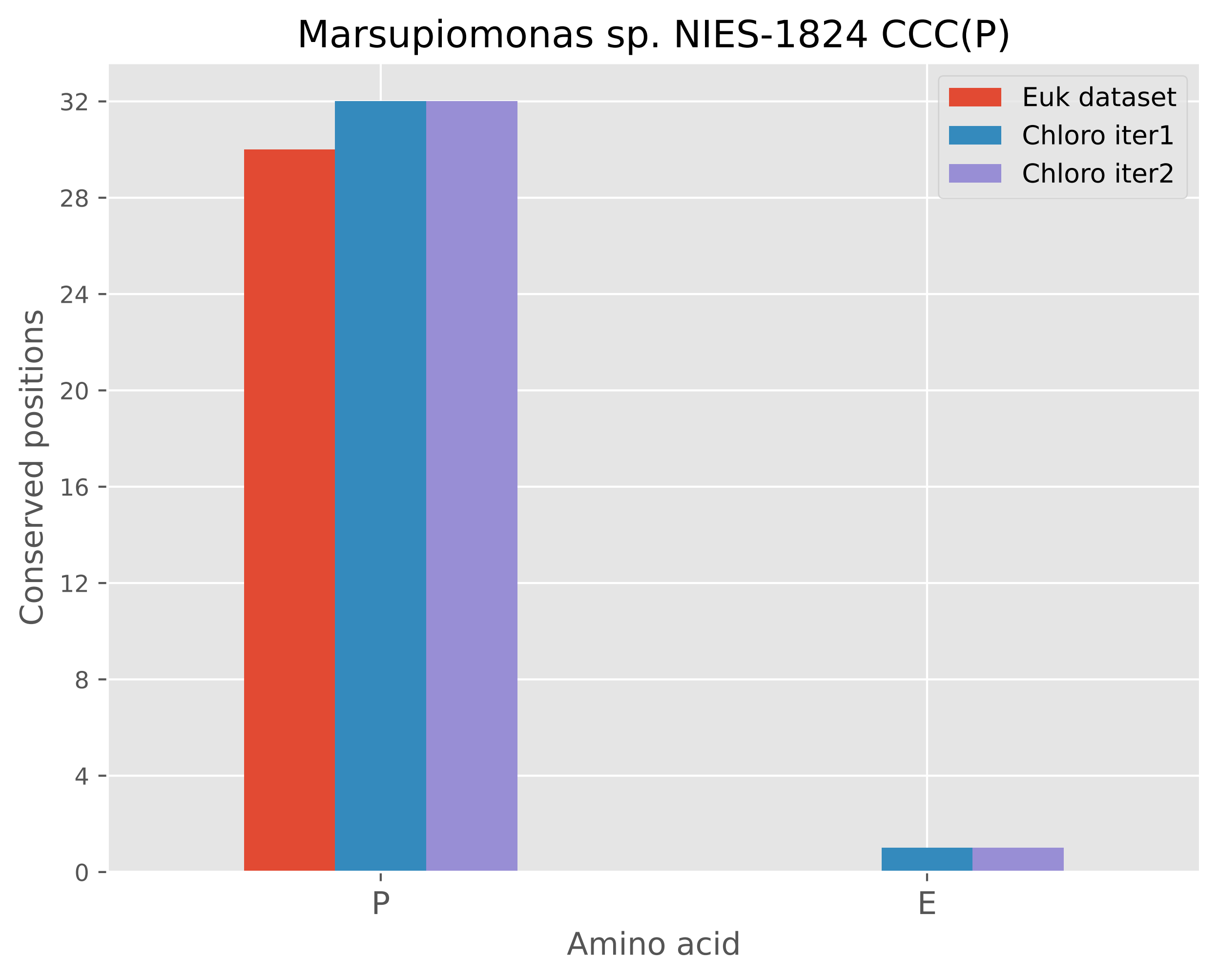

Marsupiomonas sp. NIES-1824 CCC(P)
Euk dataset
Chloro iter1
Chloro iter2
Conserved positions
0
4
8
12
16
20
24
28
32
P
E
Amino acid

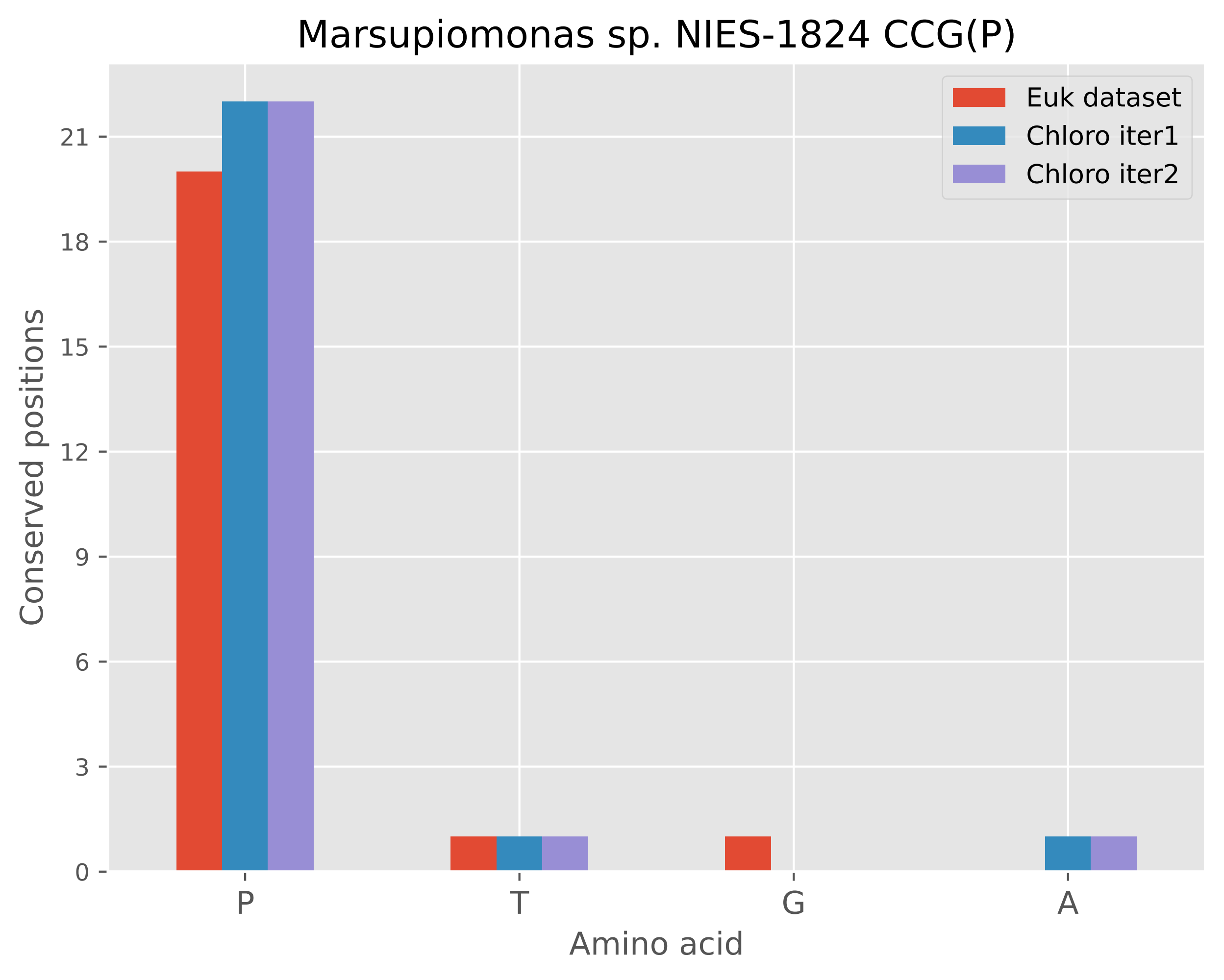
Marsupiomonas sp. NIES-1824 CCG(P)
Euk dataset
Chloro iter1
Chloro iter2
Conserved positions
0
3
6
9
12
15
18
21
P
T
G
A
Amino acid

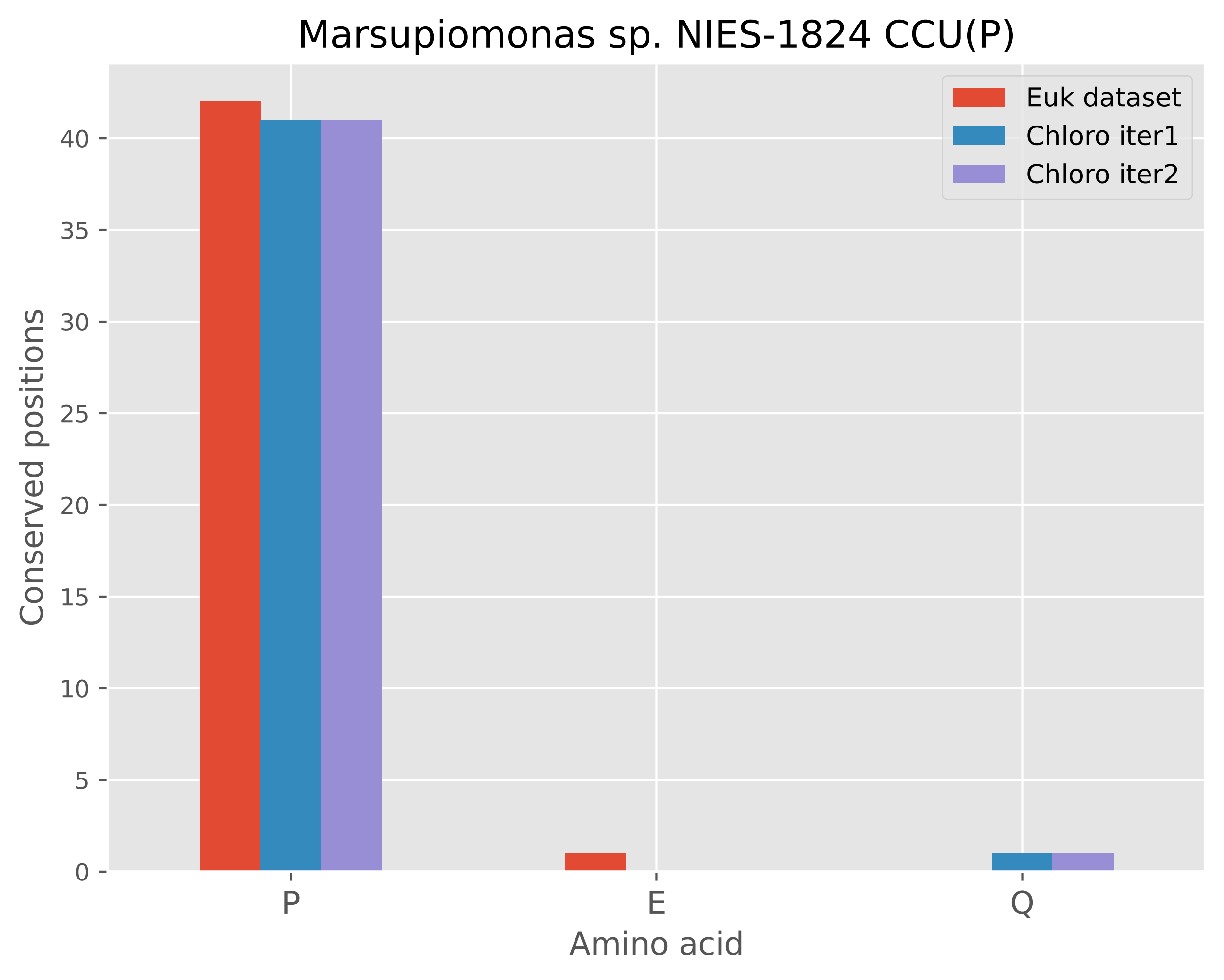

Marsupiomonas sp. NIES-1824 CCU(P)
Euk dataset
Chloro iter1
Chloro iter2
Conserved positions
0
5
10
15
20
25
30
35
40
P
E
Q
Amino acid

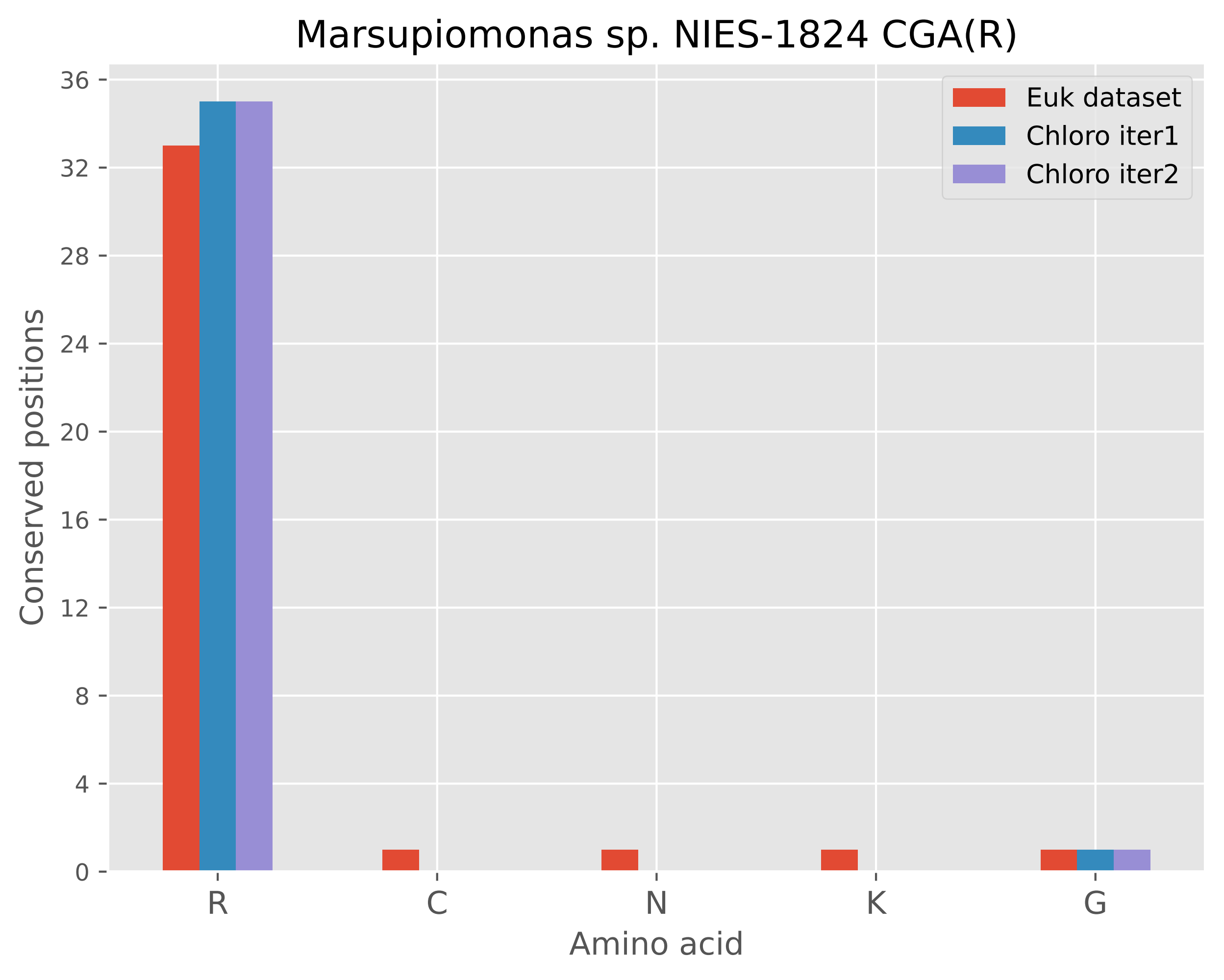
Marsupiomonas sp. NIES-1824 CGA(R)
Euk dataset
Chloro iter1
Chloro iter2
Conserved positions
0
4
8
12
16
20
24
28
32
36
R
C
N
K
G
Amino acid

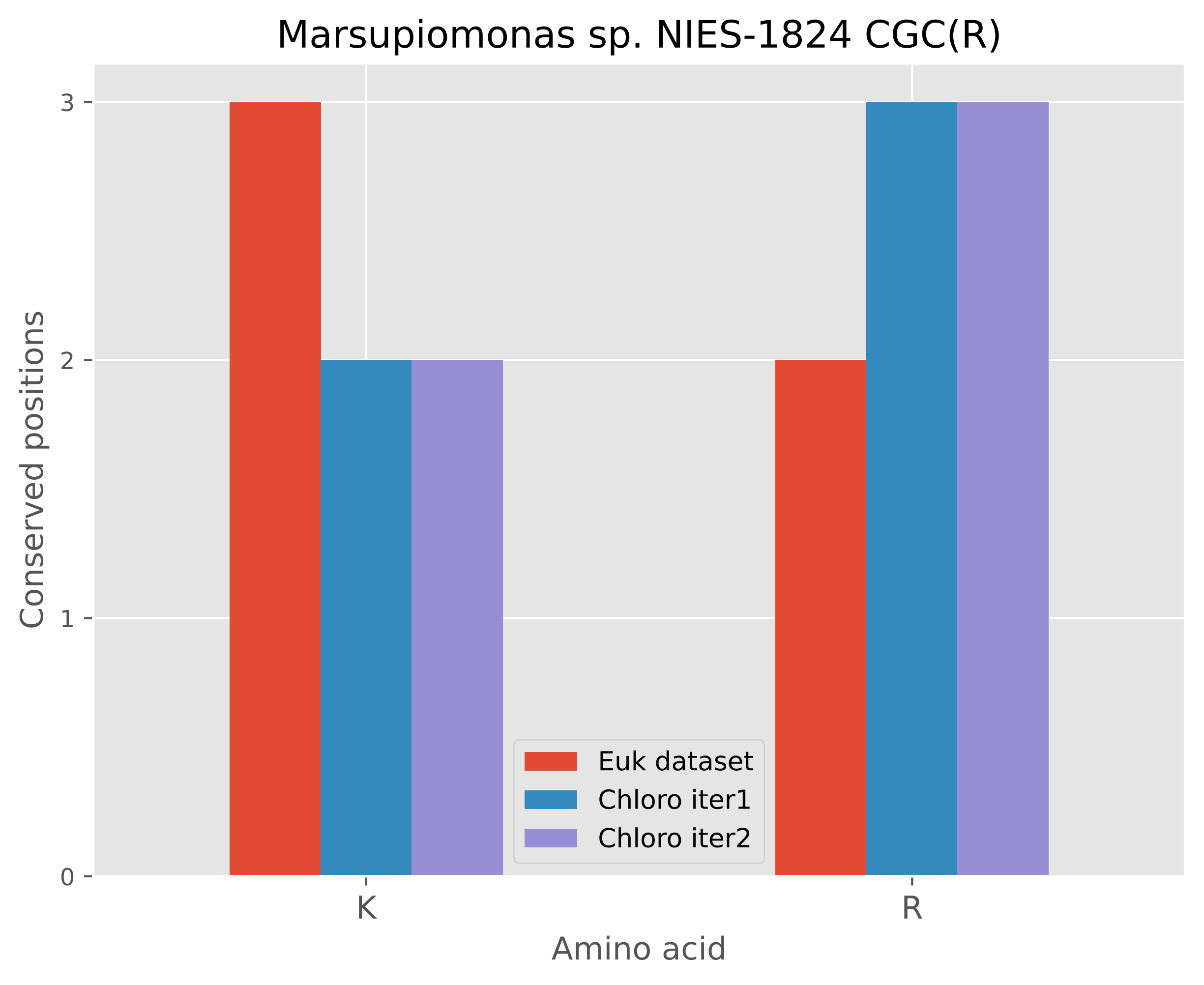
Marsupiomonas sp. NIES-1824 CGC(R)
Conserved positions
0
1
2
3
K
R
Amino acid
Euk dataset
Chloro iter1
Chloro iter2

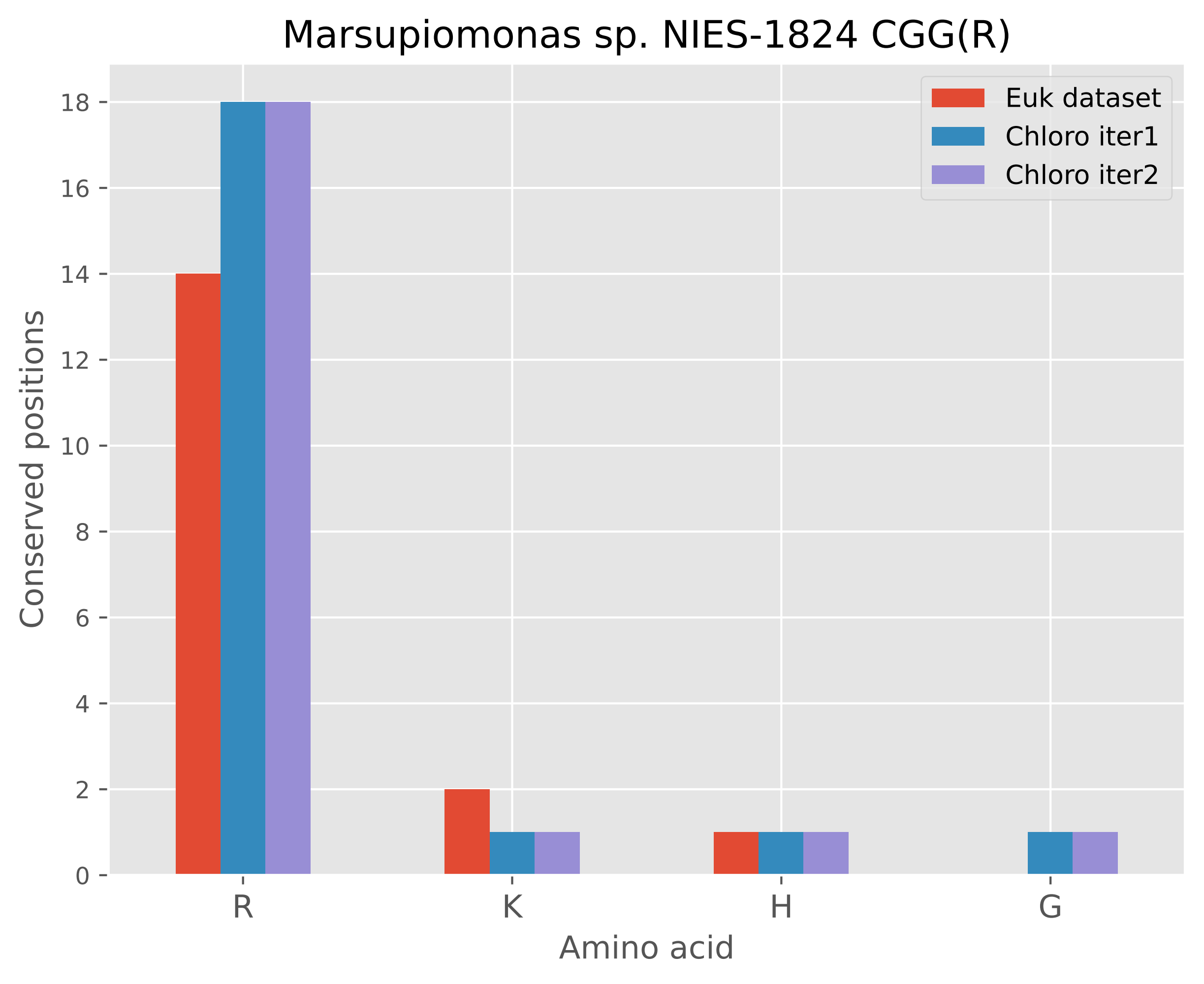

Marsupiomonas sp. NIES-1824 CGG(R)
Conserved positions
Amino acid
Euk dataset
Chloro iter1
Chloro iter2
R
K
H
G
0
2
4
6
8
10
12
14
16
18

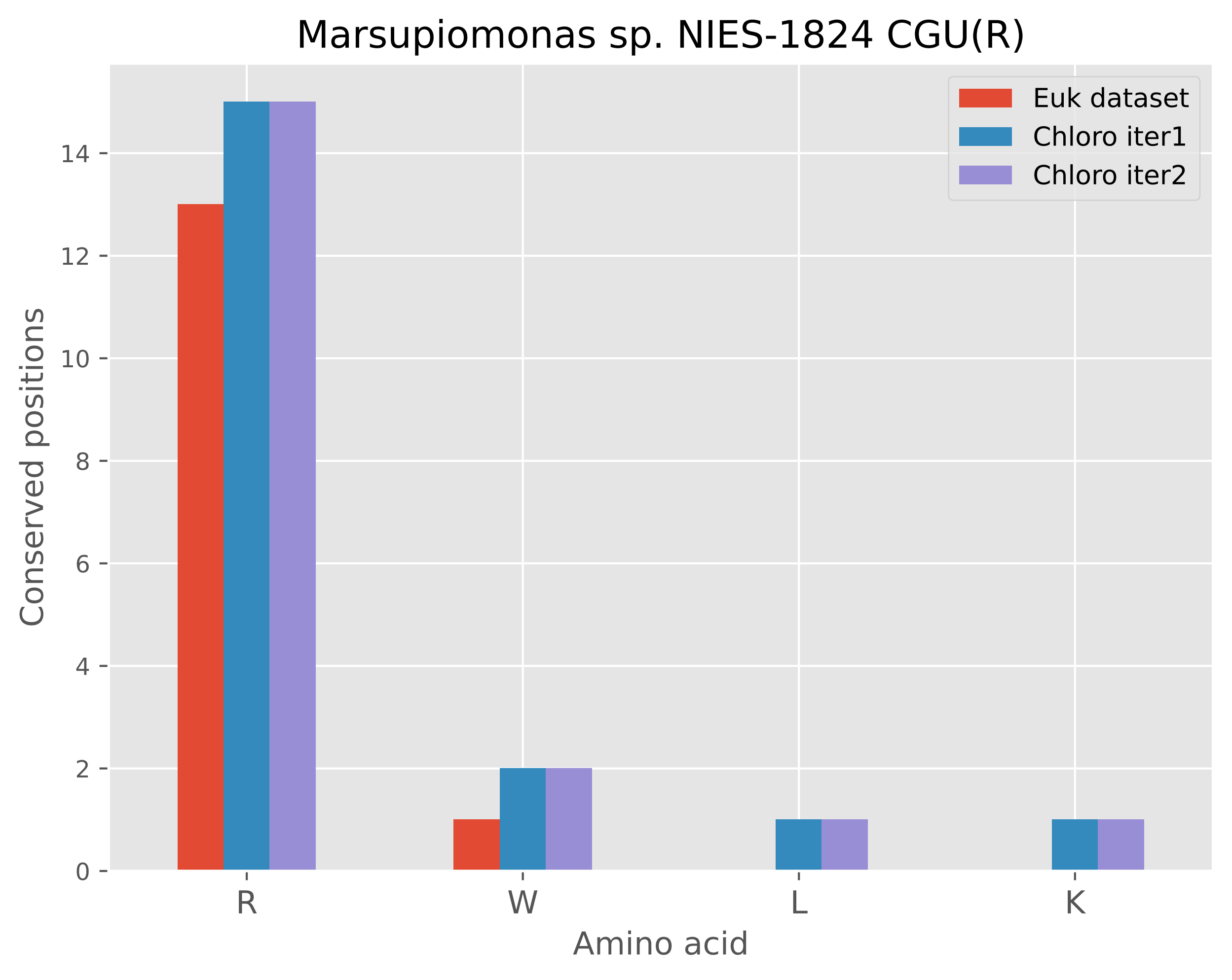

Marsupiomonas sp. NIES-1824 CGU(R)
Euk dataset
Chloro iter1
Chloro iter2
Conserved positions
0
2
4
6
8
10
12
14
R
W
L
K
Amino acid

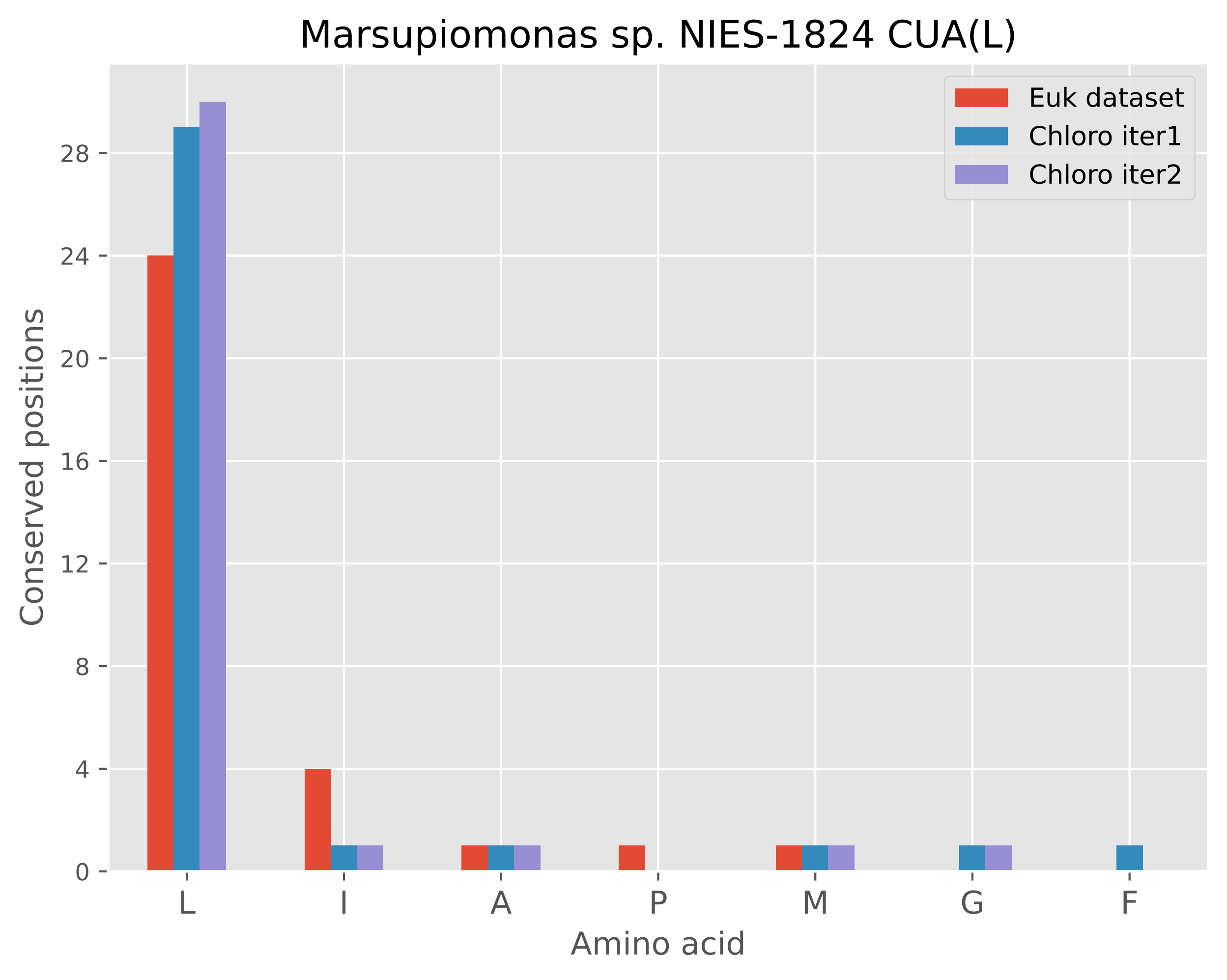
Marsupiomonas sp. NIES-1824 CUA(L)
Euk dataset
Chloro iter1
Chloro iter2
Conserved positions
0
4
8
12
16
20
24
28
L
I
A
P
M
G
F
Amino acid

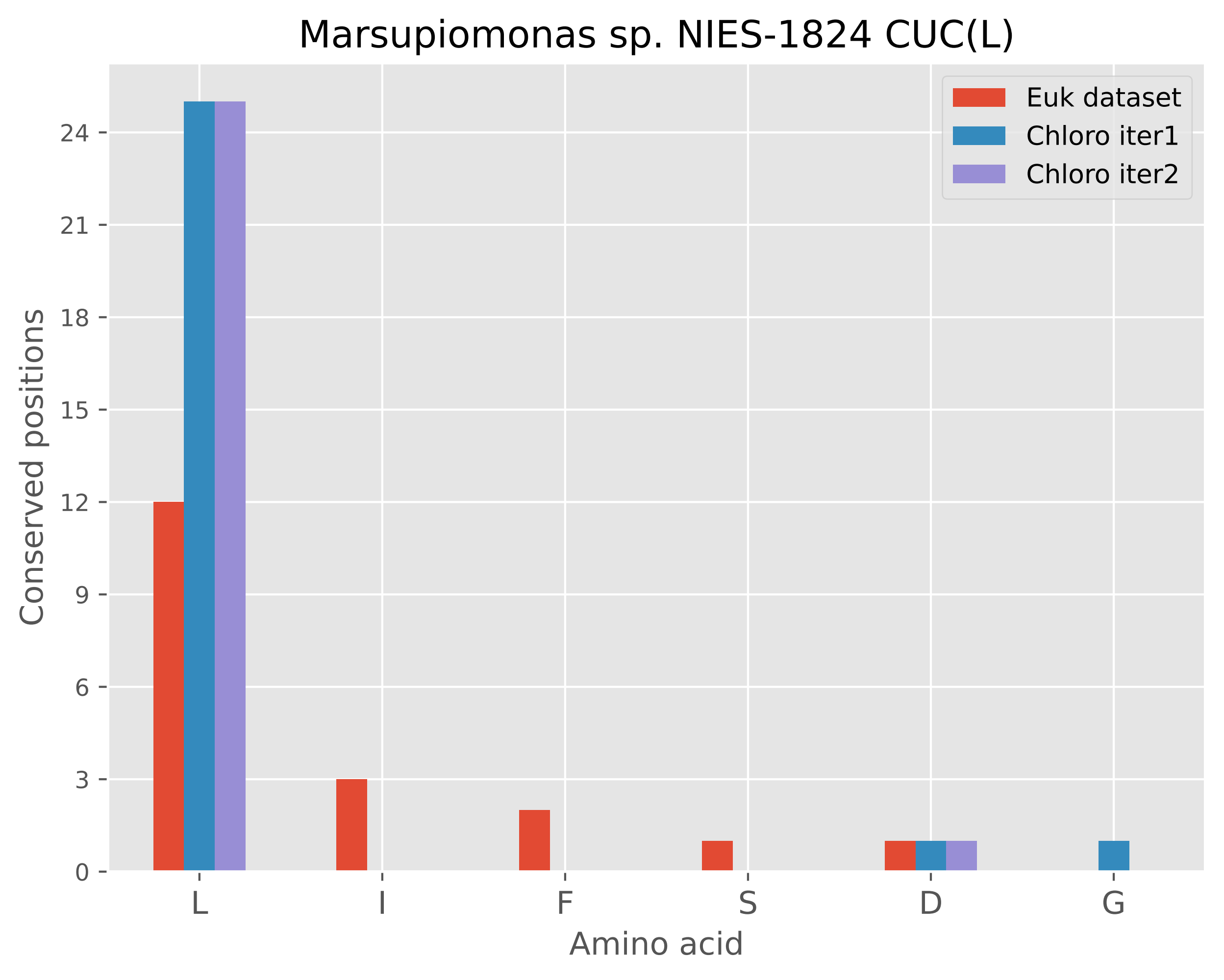
Marsupiomonas sp. NIES-1824 CUC(L)
Euk dataset
Chloro iter1
Chloro iter2
Conserved positions
0
3
6
9
12
15
18
21
24
L
I
F
S
D
G
Amino acid

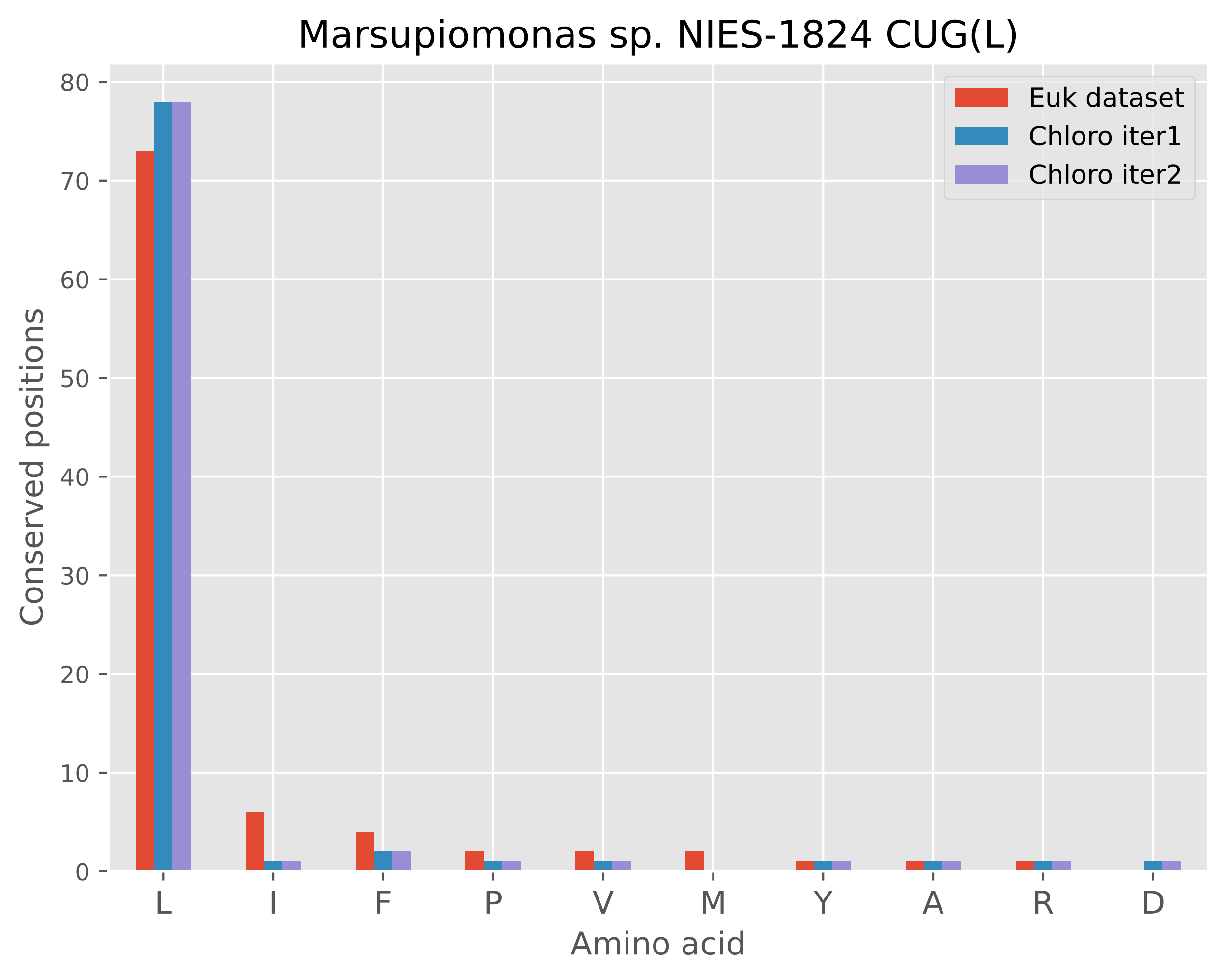

Marsupiomonas sp. NIES-1824 CUG(L)
Euk dataset
Chloro iter1
Chloro iter2
Conserved positions
0
10
20
30
40
50
60
70
80
L
I
F
P
V
M
Y
A
R
D
Amino acid

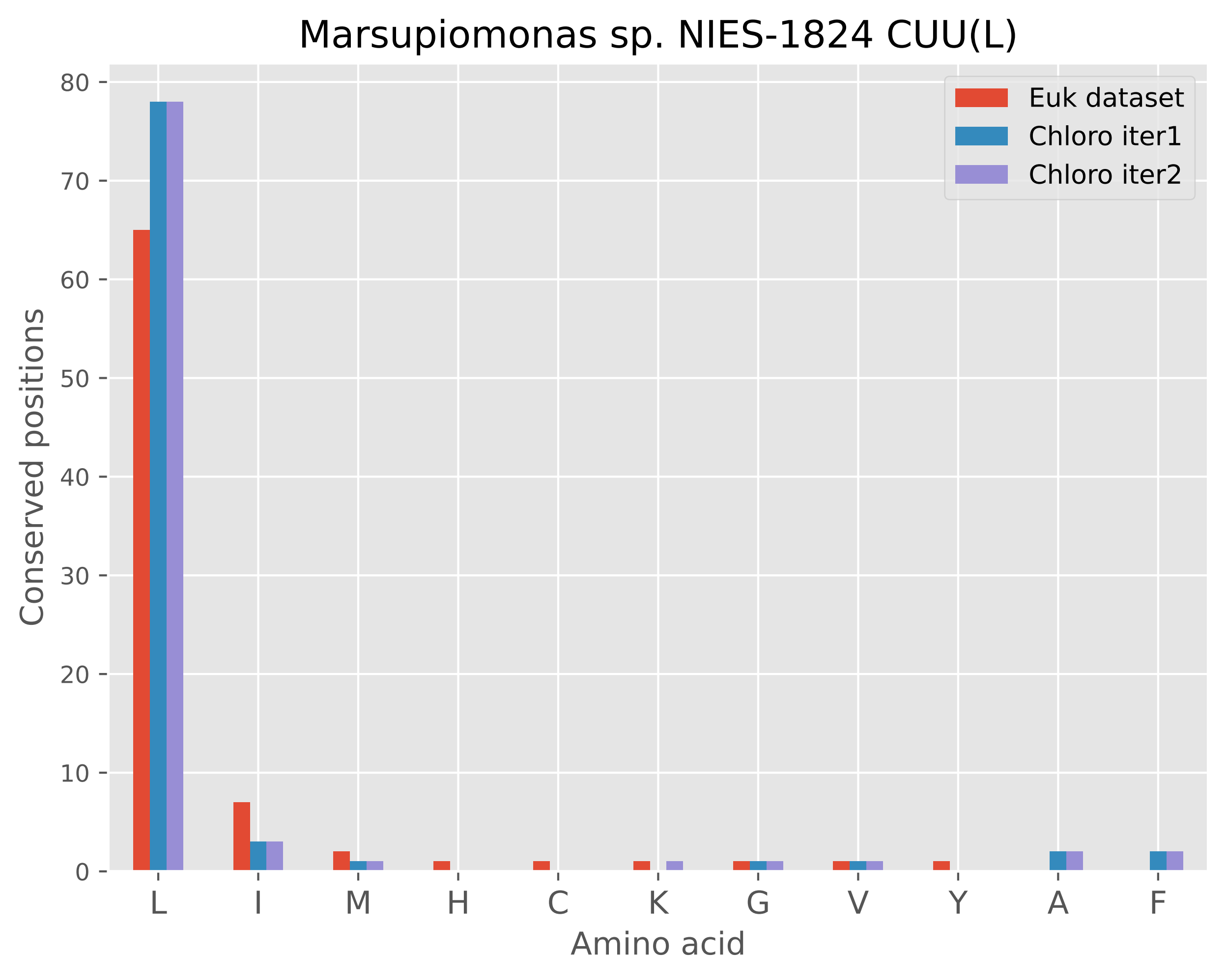
Marsupiomonas sp. NIES-1824 CUU(L)
Euk dataset
Chloro iter1
Chloro iter2
Conserved positions
0
10
20
30
40
50
60
70
80
L
I
M
H
C
K
G
V
Y
A
F
Amino acid

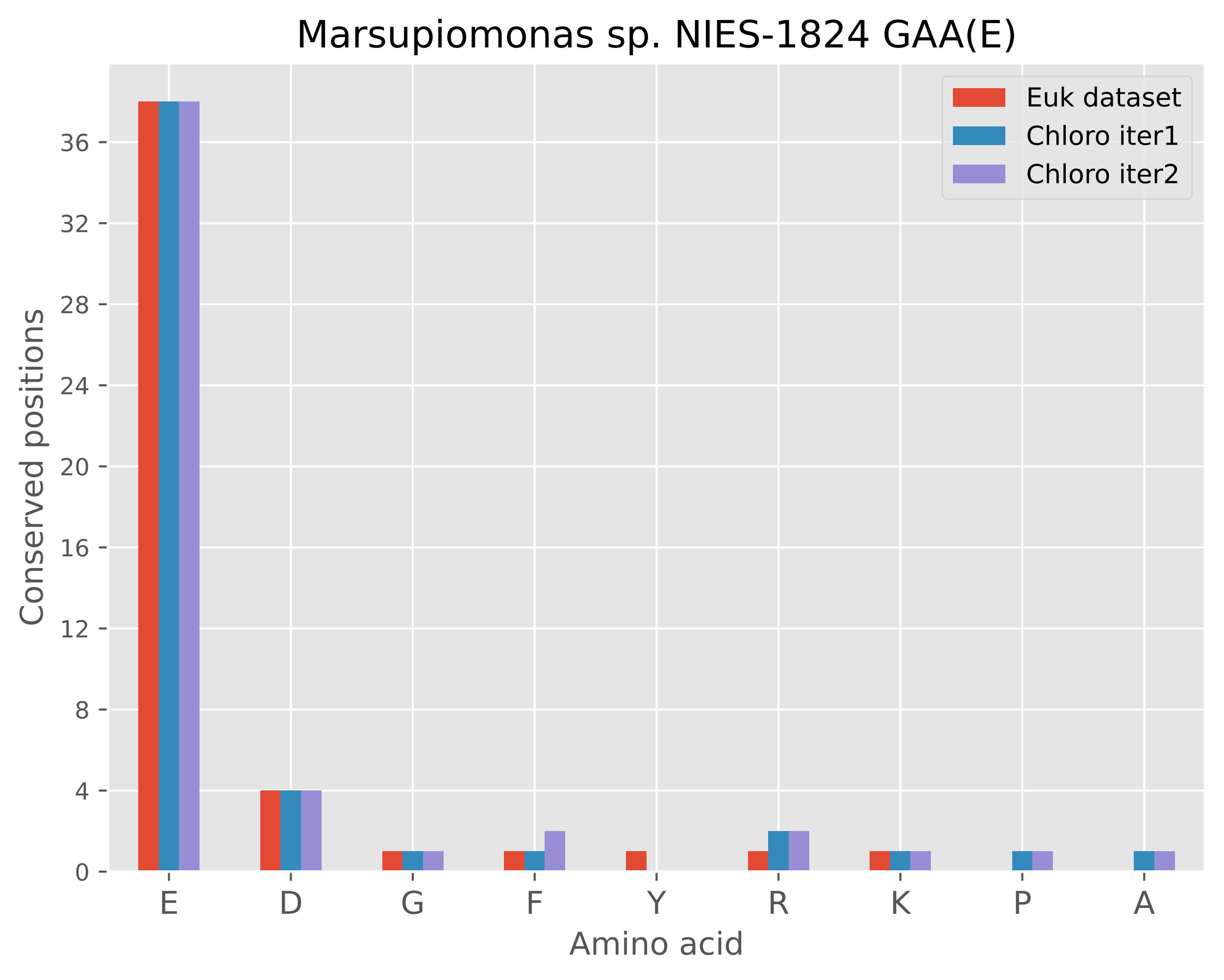

Marsupiomonas sp. NIES-1824 GAA(E)
Euk dataset
Chloro iter1
Chloro iter2
Conserved positions
0
4
8
12
16
20
24
28
32
36
E
D
G
F
Y
R
K
P
A
Amino acid

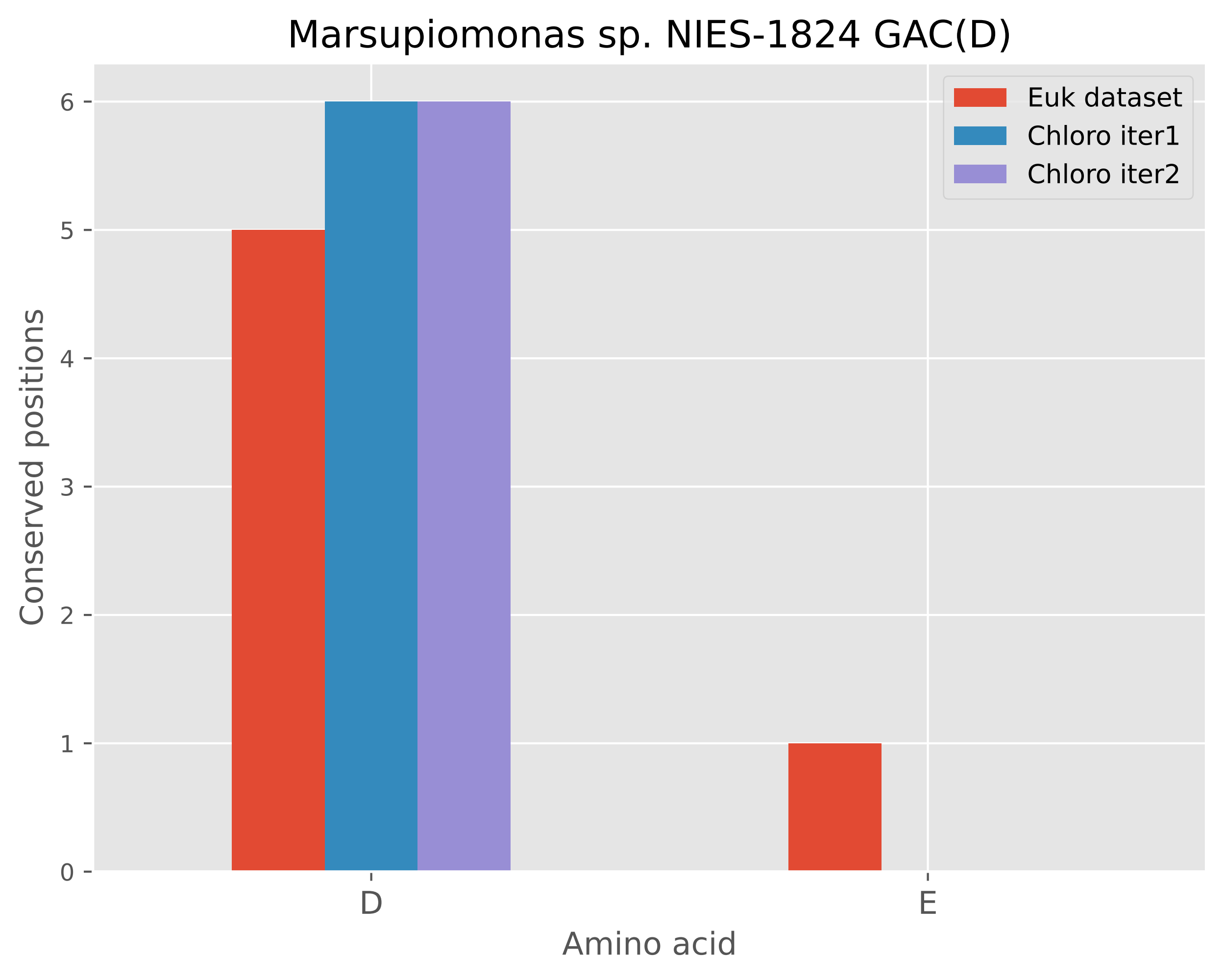
Marsupiomonas sp. NIES-1824 GAC(D)
Euk dataset
Chloro iter1
Chloro iter2
Conserved positions
0
1
2
3
4
5
6
D
E
Amino acid

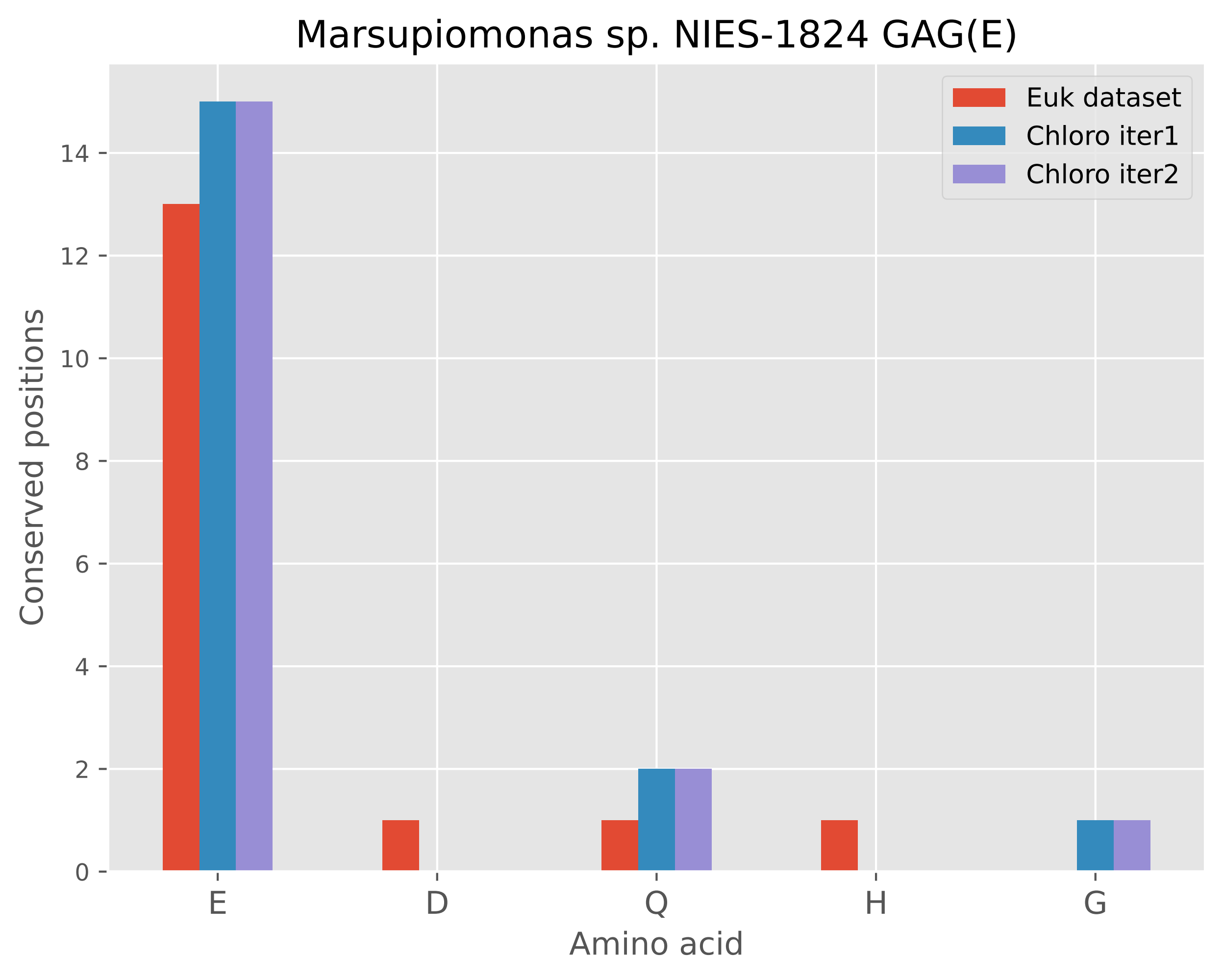
Marsupiomonas sp. NIES-1824 GAG(E)
Euk dataset
Chloro iter1
Chloro iter2
Conserved positions
0
2
4
6
8
10
12
14
E
D
Q
H
G
Amino acid

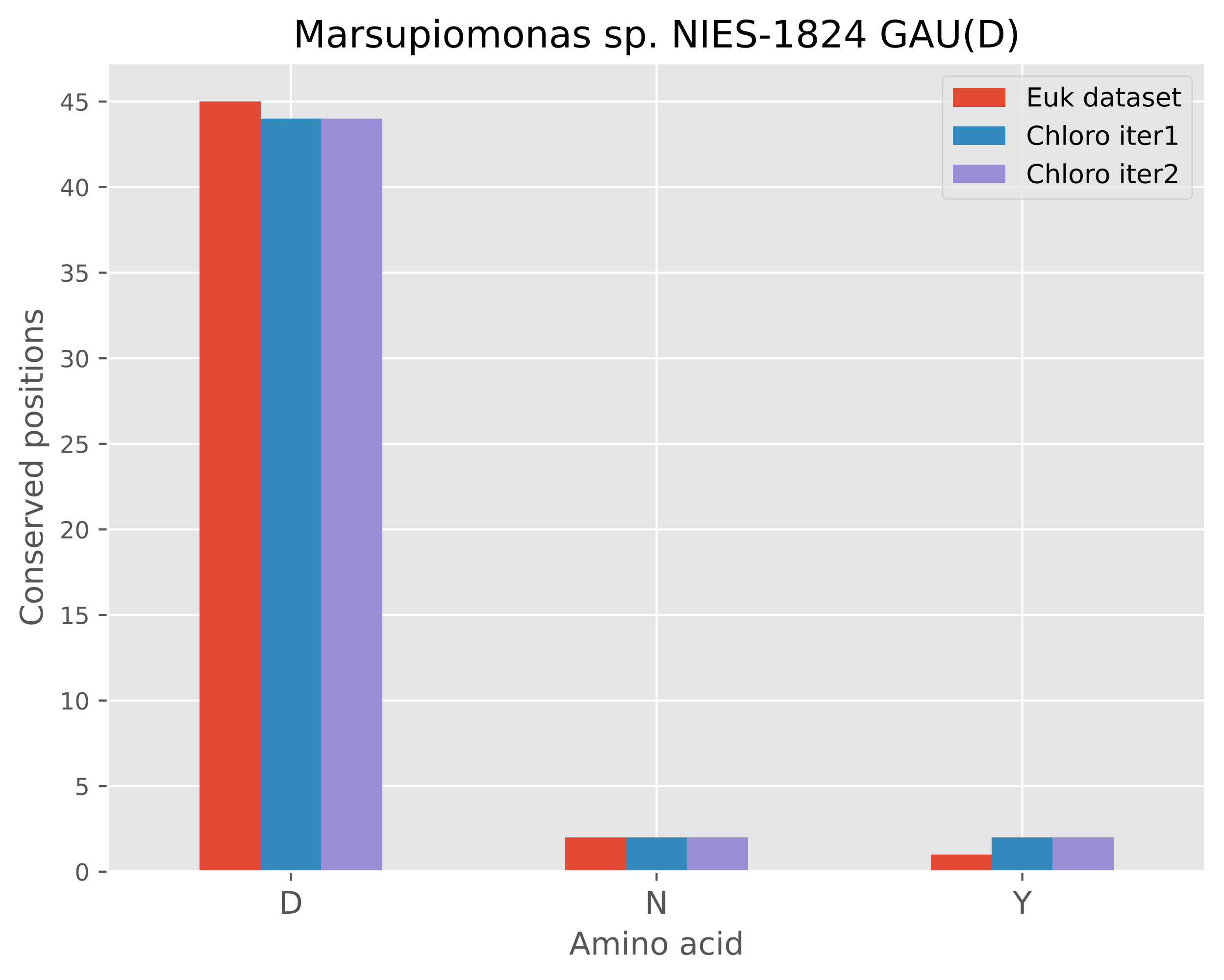

Marsupiomonas sp. NIES-1824 GAU(D)
Euk dataset
Chloro iter1
Chloro iter2
Conserved positions
0
5
10
15
20
25
30
35
40
45
D
N
Y
Amino acid

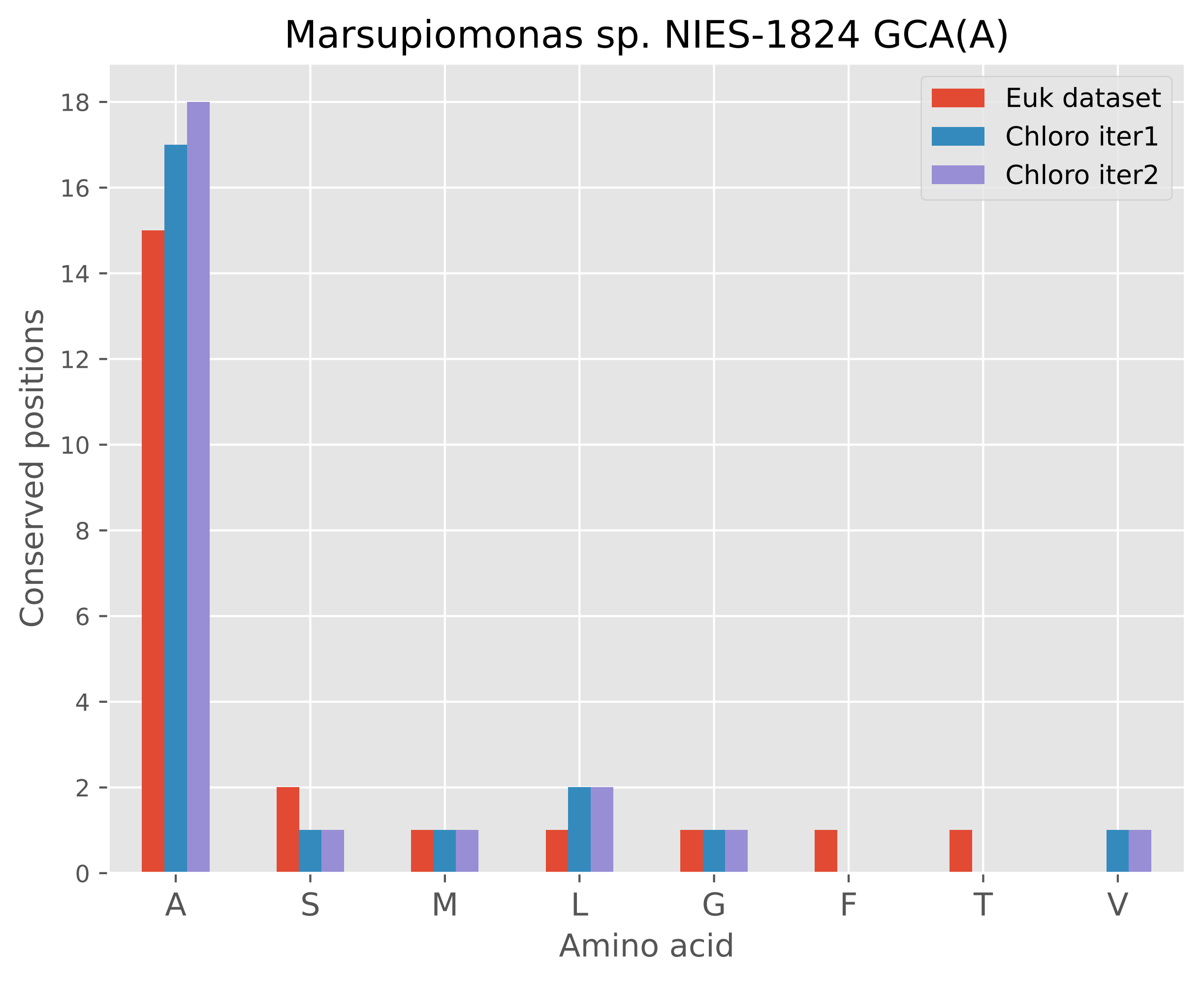
Marsupiomonas sp. NIES-1824 GCA(A)
Euk dataset
Chloro iter1
Chloro iter2
Conserved positions
0
2
4
6
8
10
12
14
16
18
A
S
M
L
G
F
T
V
Amino acid

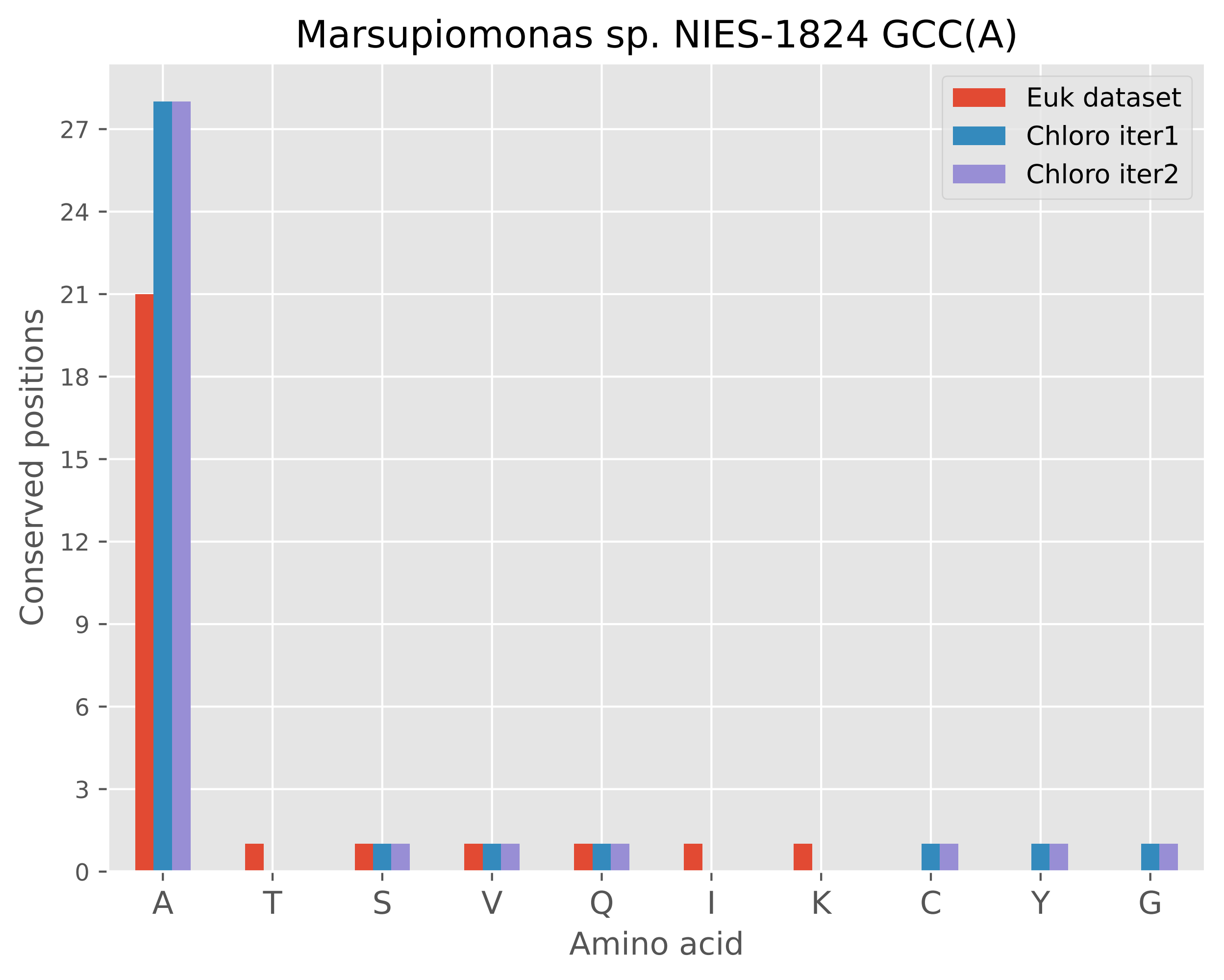

Marsupiomonas sp. NIES-1824 GCC(A)
Euk dataset
Chloro iter1
Chloro iter2
Conserved positions
0
3
6
9
12
15
18
21
24
27
A
T
S
V
Q
I
K
C
Y
G
Amino acid

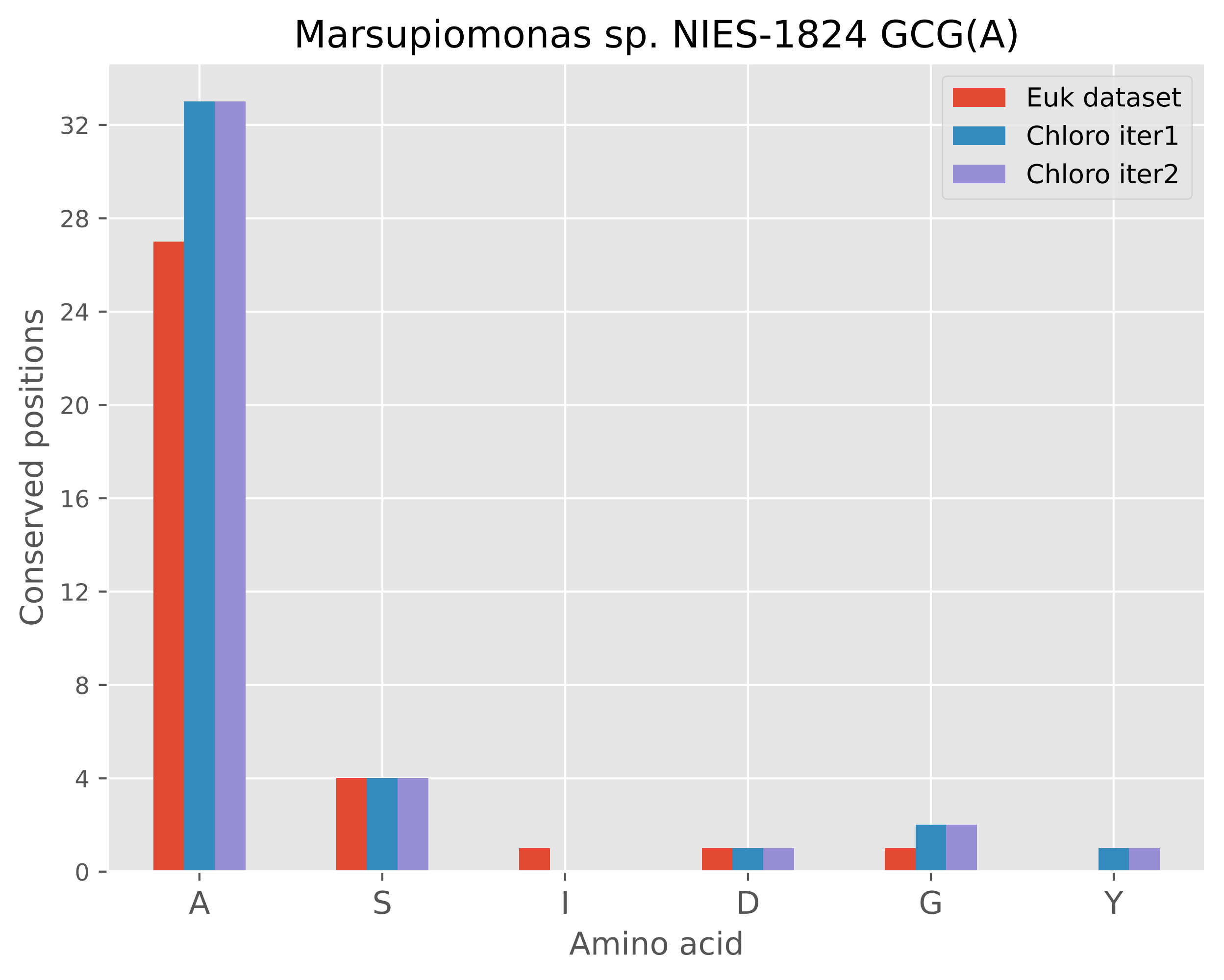

Marsupiomonas sp. NIES-1824 GCG(A)
Euk dataset
Chloro iter1
Chloro iter2
Conserved positions
0
4
8
12
16
20
24
28
32
A
S
I
D
G
Y
Amino acid

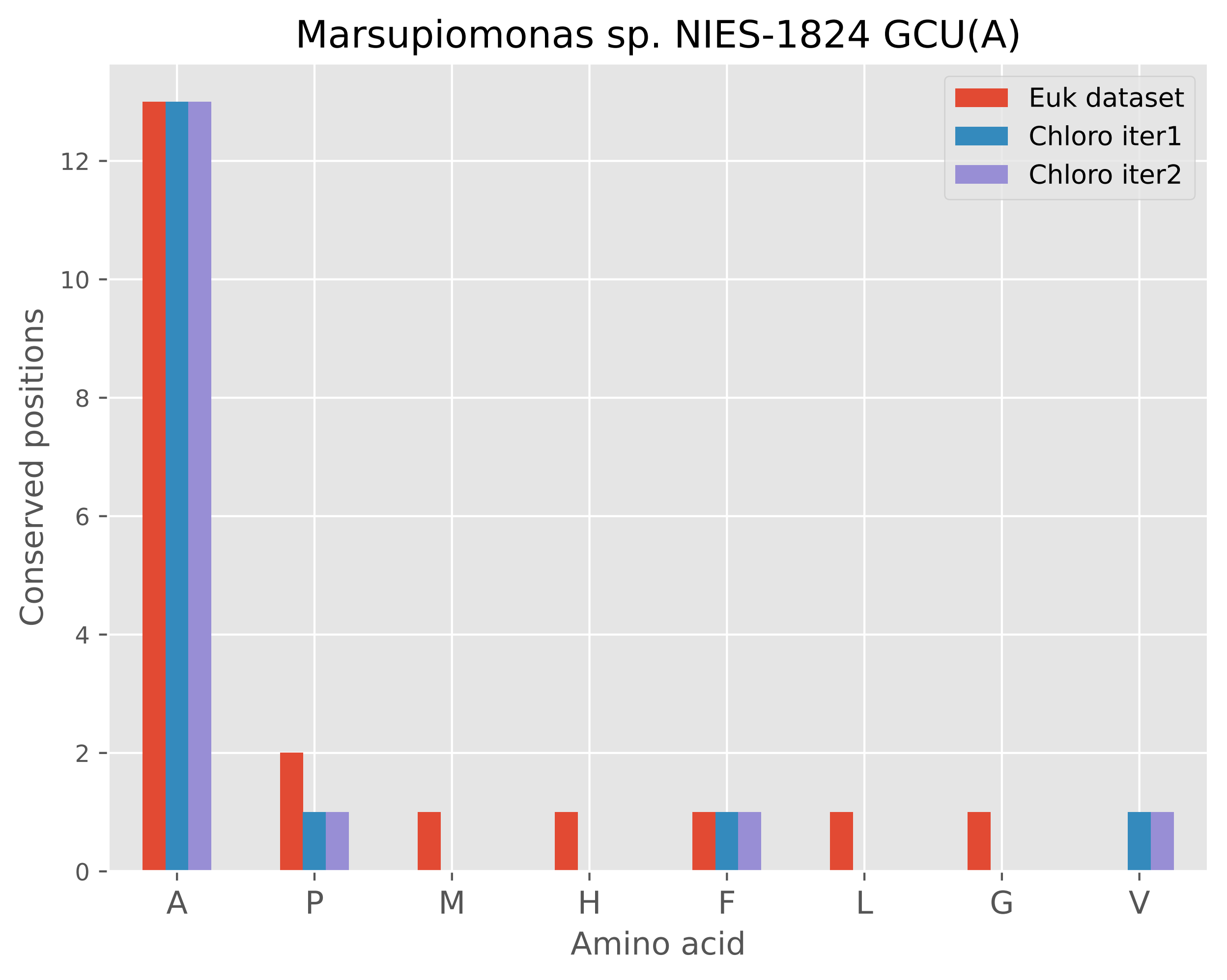
Marsupiomonas sp. NIES-1824 GCU(A)
Euk dataset
Chloro iter1
Chloro iter2
Conserved positions
0
2
4
6
8
10
12
A
P
M
H
F
L
G
V
Amino acid

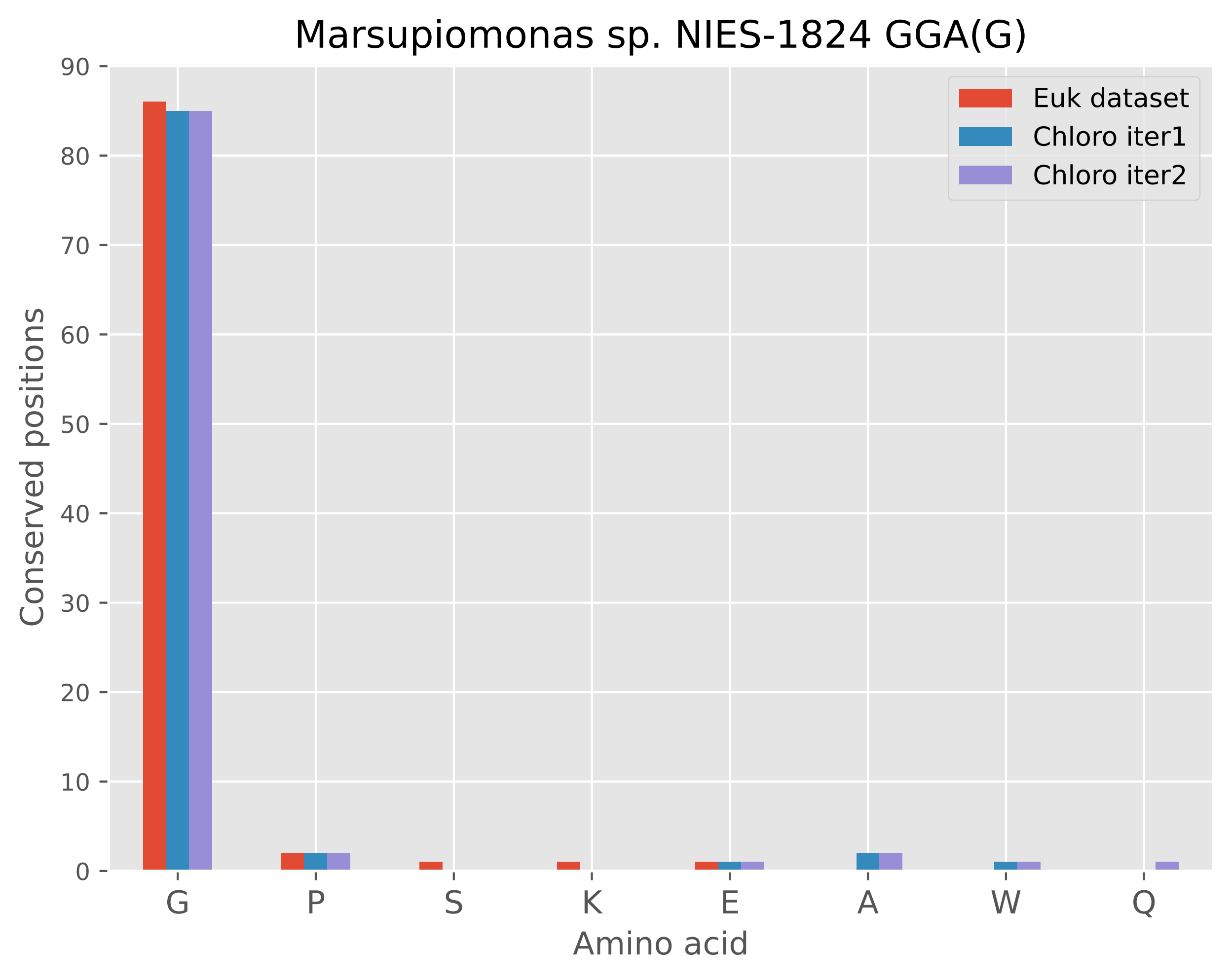
Marsupiomonas sp. NIES-1824 GGA(G)
Euk dataset
Chloro iter1
Chloro iter2
Conserved positions
0
10
20
30
40
50
60
70
80
90
G
P
S
K
E
A
W
Q
Amino acid

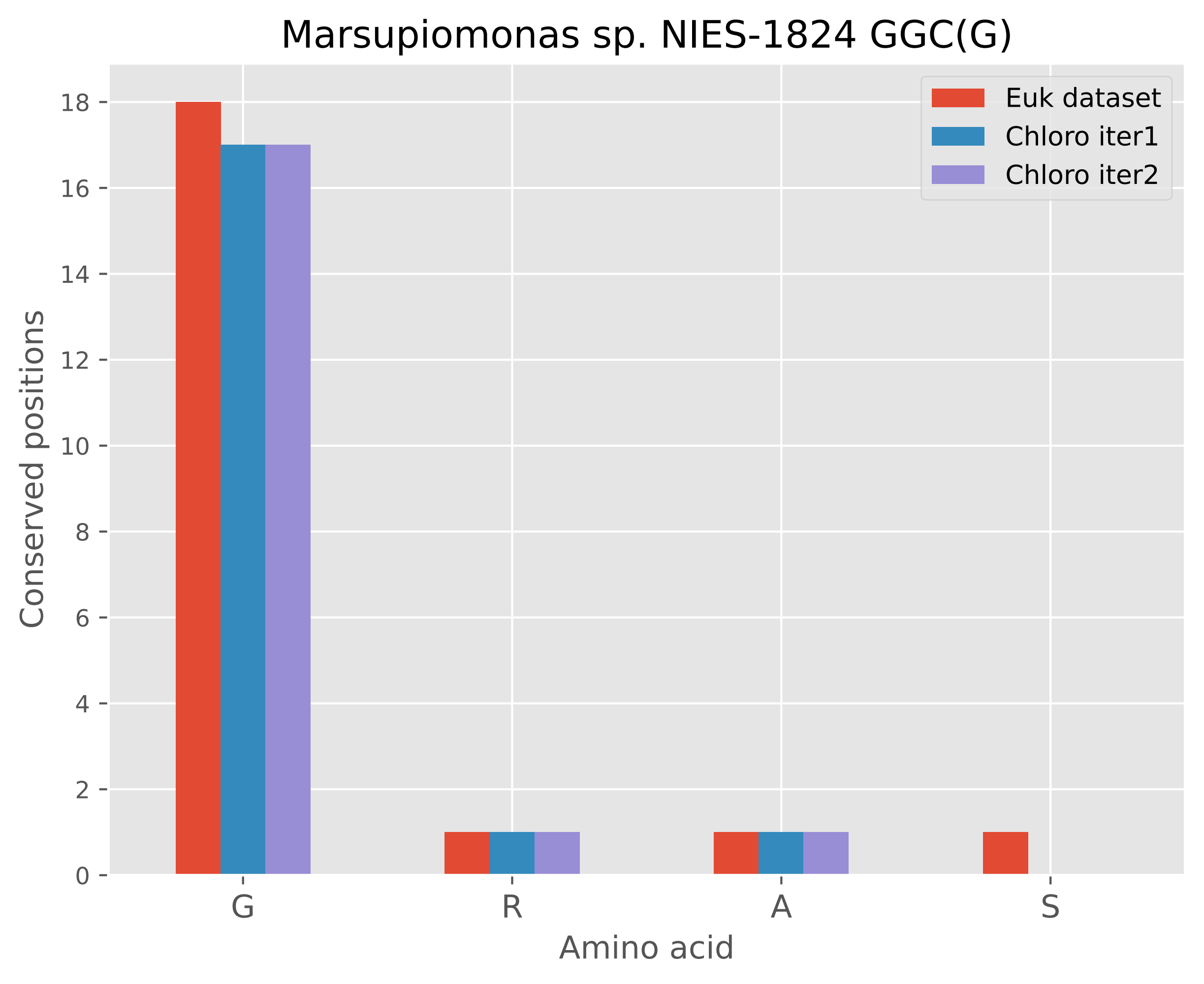

Marsupiomonas sp. NIES-1824 GGC(G)
Euk dataset
Chloro iter1
Chloro iter2
Conserved positions
0
2
4
6
8
10
12
14
16
18
G
R
A
S
Amino acid

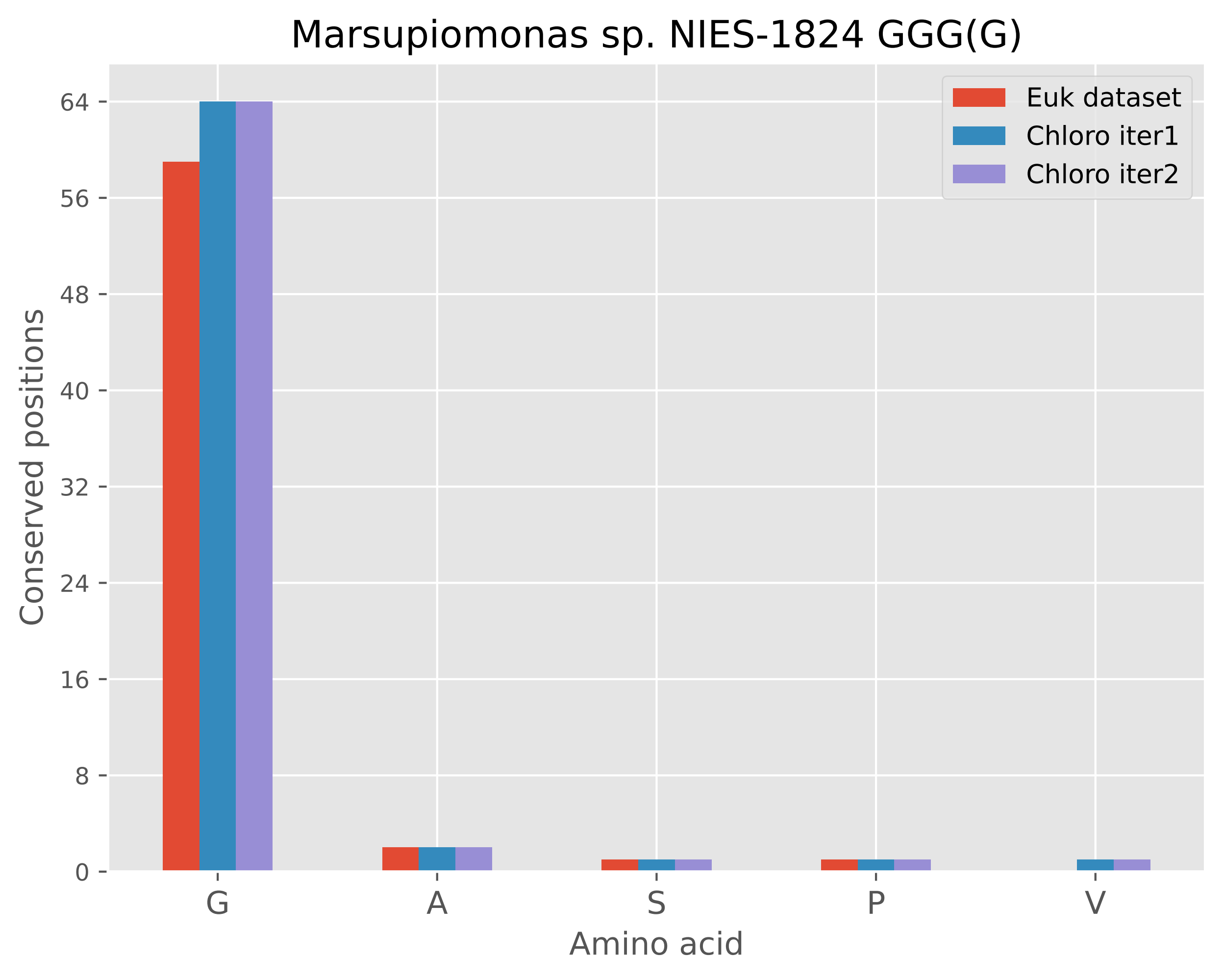
Marsupiomonas sp. NIES-1824 GGG(G)
Euk dataset
Chloro iter1
Chloro iter2
Conserved positions
0
8
16
24
32
40
48
56
64
G
A
S
P
V
Amino acid

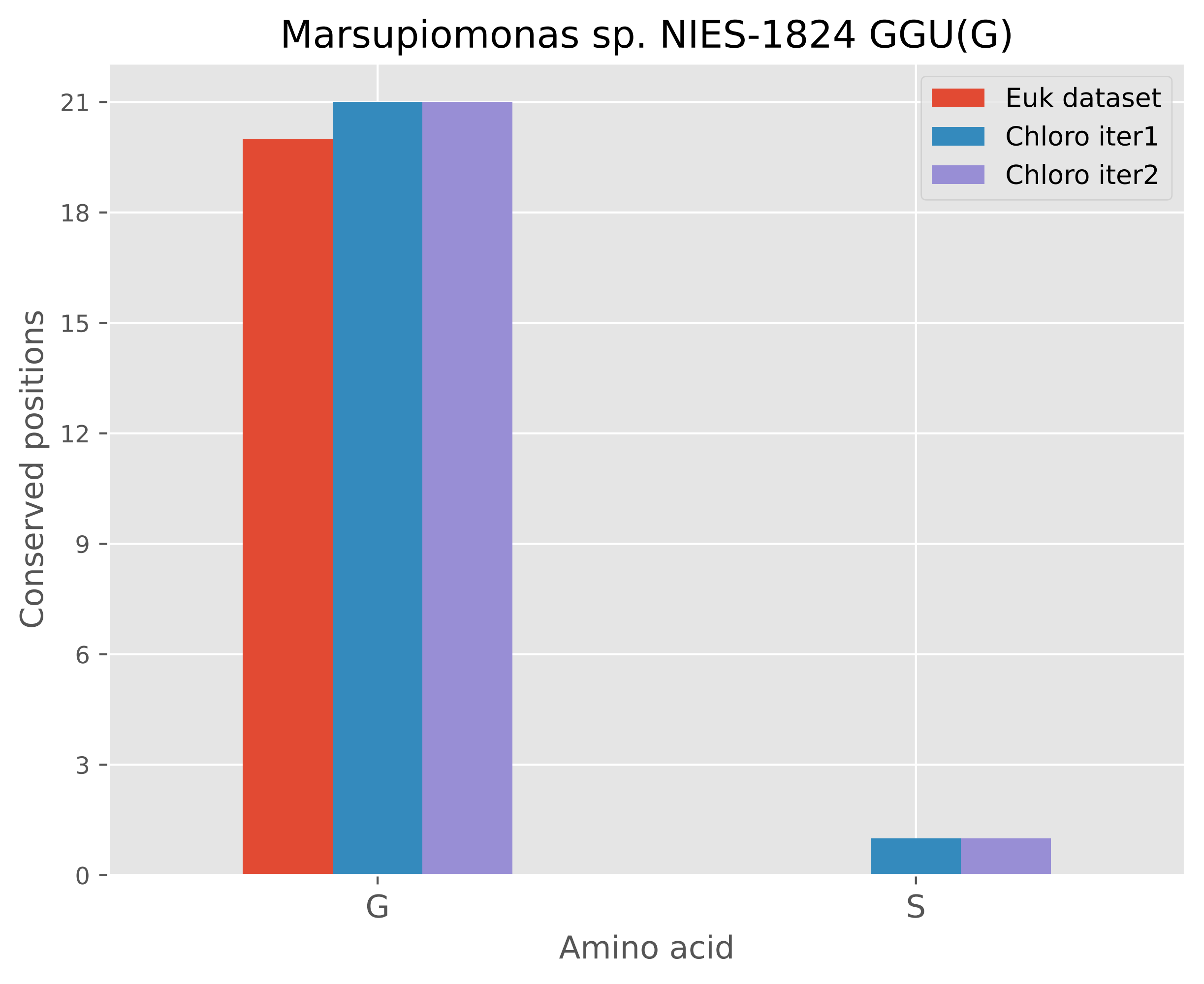

Marsupiomonas sp. NIES-1824 GGU(G)
Euk dataset
Chloro iter1
Chloro iter2
Conserved positions
0
3
6
9
12
15
18
21
G
S
Amino acid

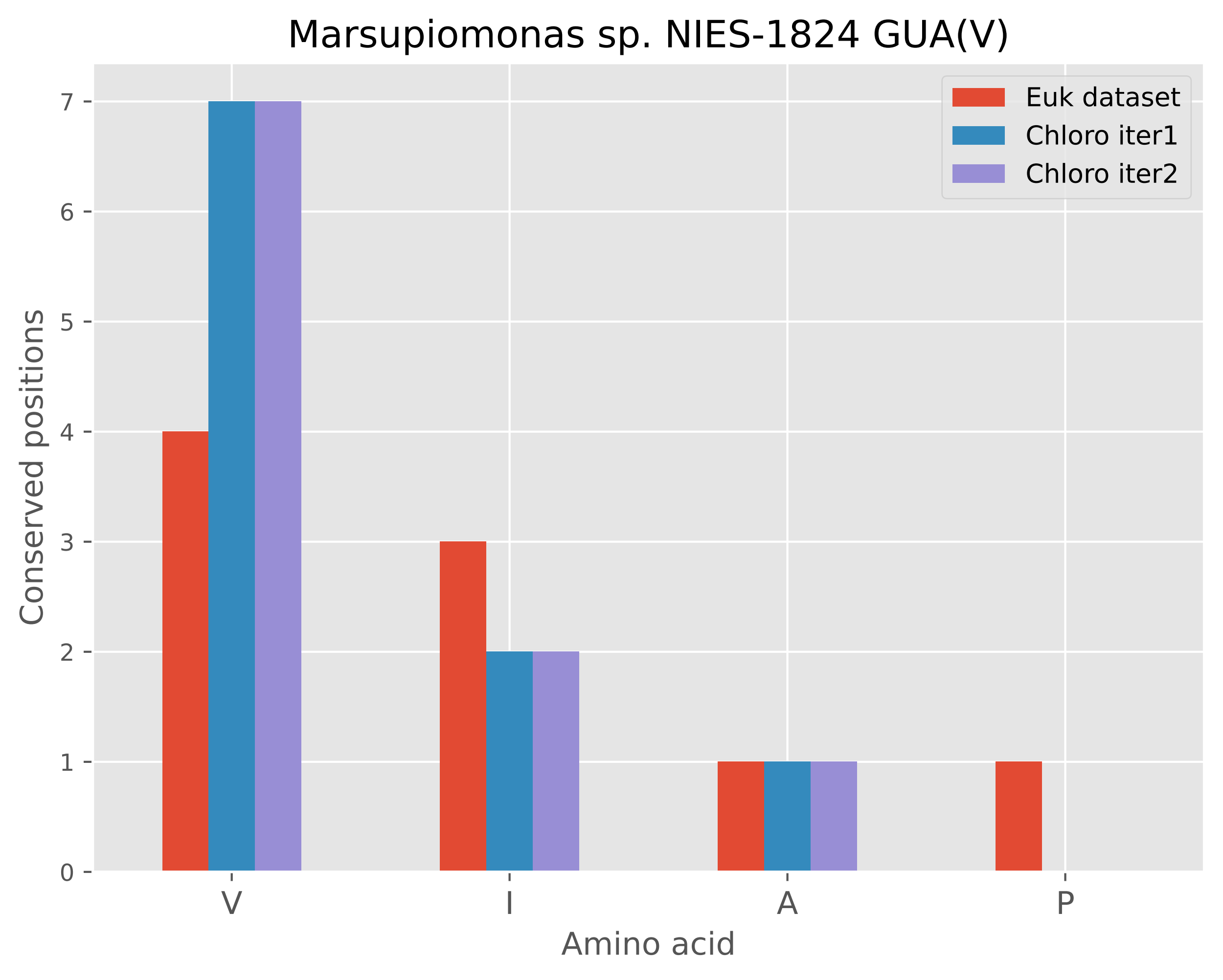
Marsupiomonas sp. NIES-1824 GUA(V)
Euk dataset
Chloro iter1
Chloro iter2
Conserved positions
0
1
2
3
4
5
6
7
V
I
A
P
Amino acid

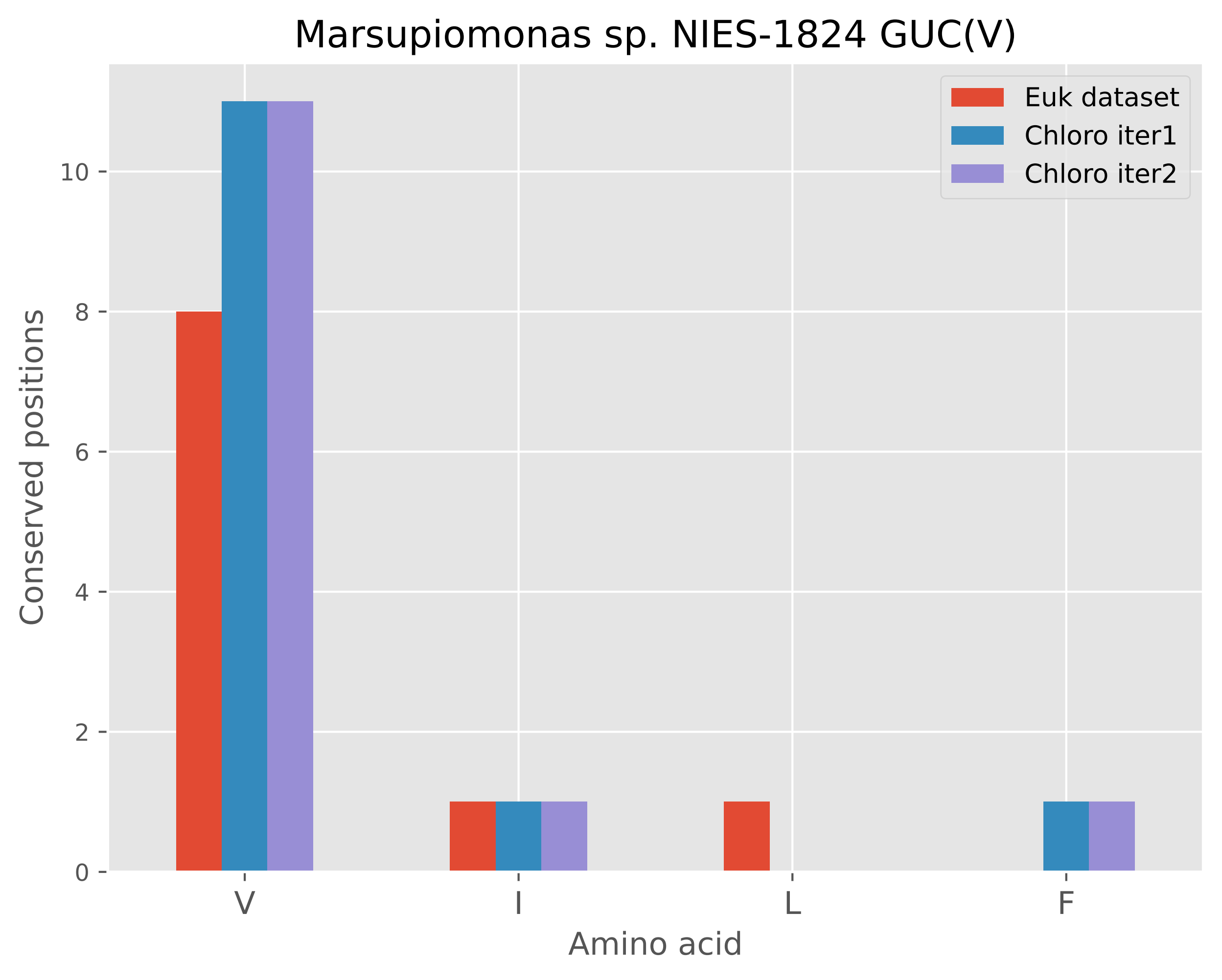
Marsupiomonas sp. NIES-1824 GUC(V)
Euk dataset
Chloro iter1
Chloro iter2
Conserved positions
0
2
4
6
8
10
V
I
L
F
Amino acid

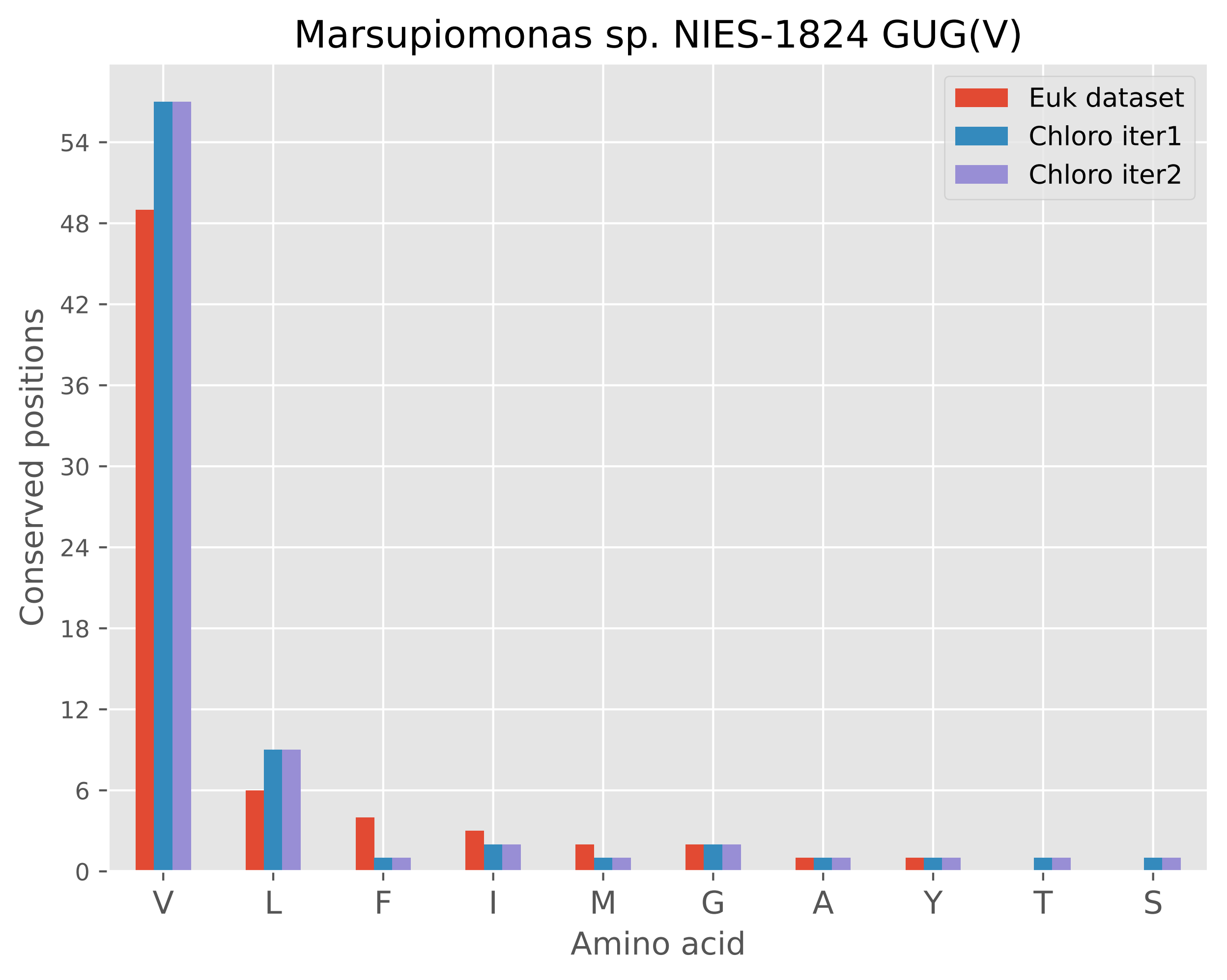

Marsupiomonas sp. NIES-1824 GUG(V)
Euk dataset
Chloro iter1
Chloro iter2
Conserved positions
0
6
12
18
24
30
36
42
48
54
V
L
F
I
M
G
A
Y
T
S
Amino acid

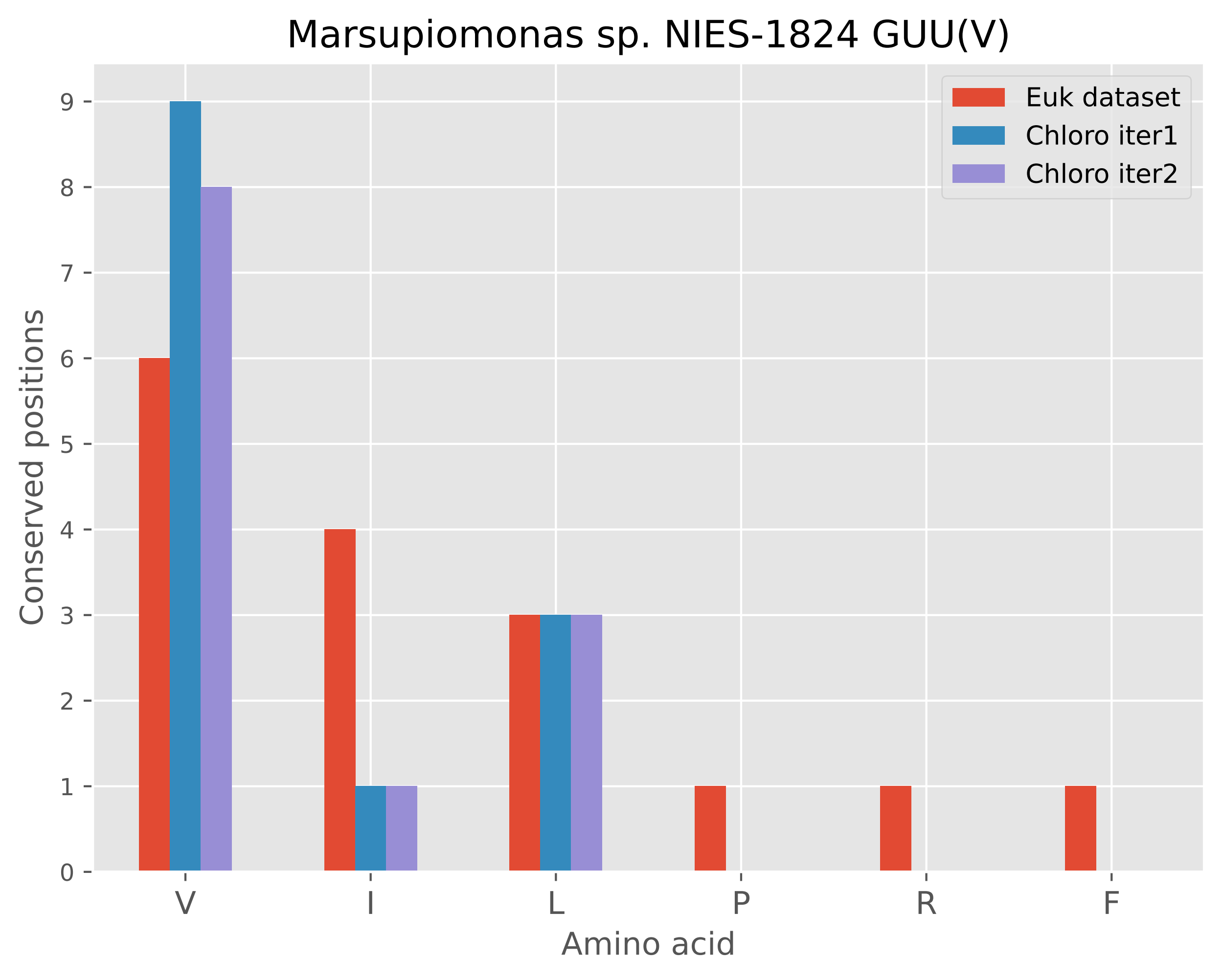

Marsupiomonas sp. NIES-1824 GUU(V)
Euk dataset
Chloro iter1
Chloro iter2
Conserved positions
0
1
2
3
4
5
6
7
8
9
V
I
L
P
R
F
Amino acid

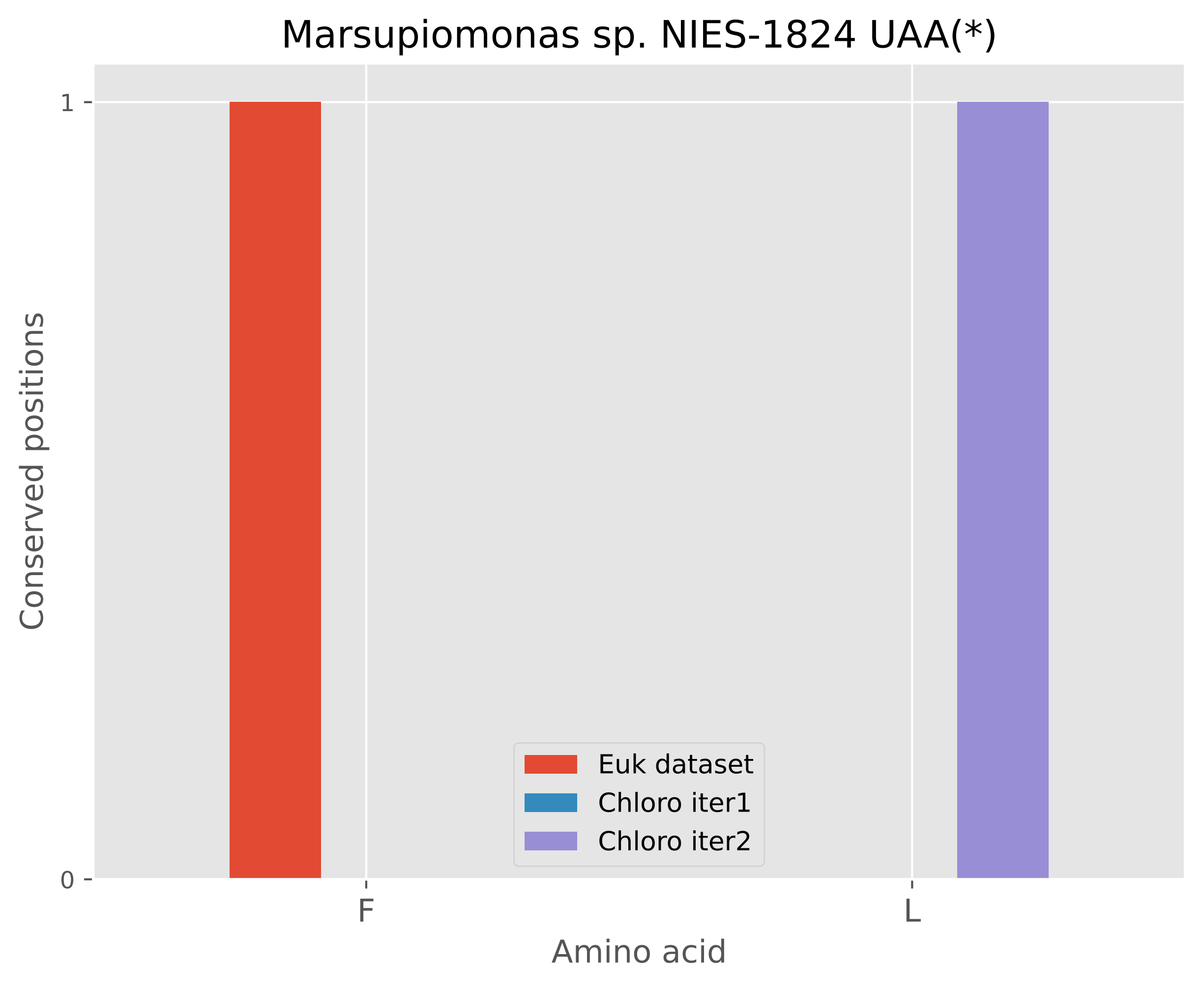
Marsupiomonas sp. NIES-1824 UAA(*)
Conserved positions
1
0
F
L
Amino acid
Euk dataset
Chloro iter1
Chloro iter2

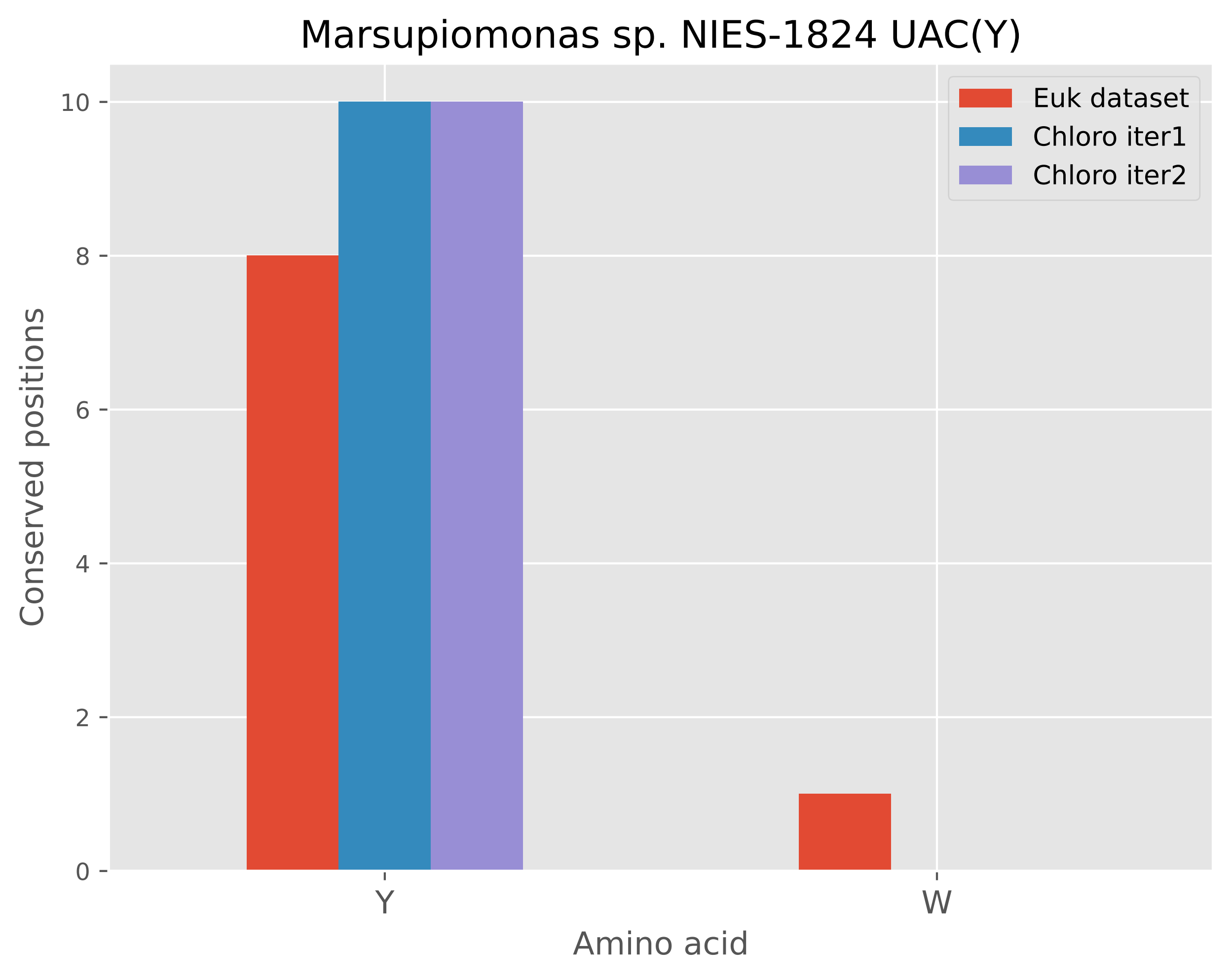

Marsupiomonas sp. NIES-1824 UAC(Y)
Euk dataset
Chloro iter1
Chloro iter2
Conserved positions
0
2
4
6
8
10
Y
W
Amino acid

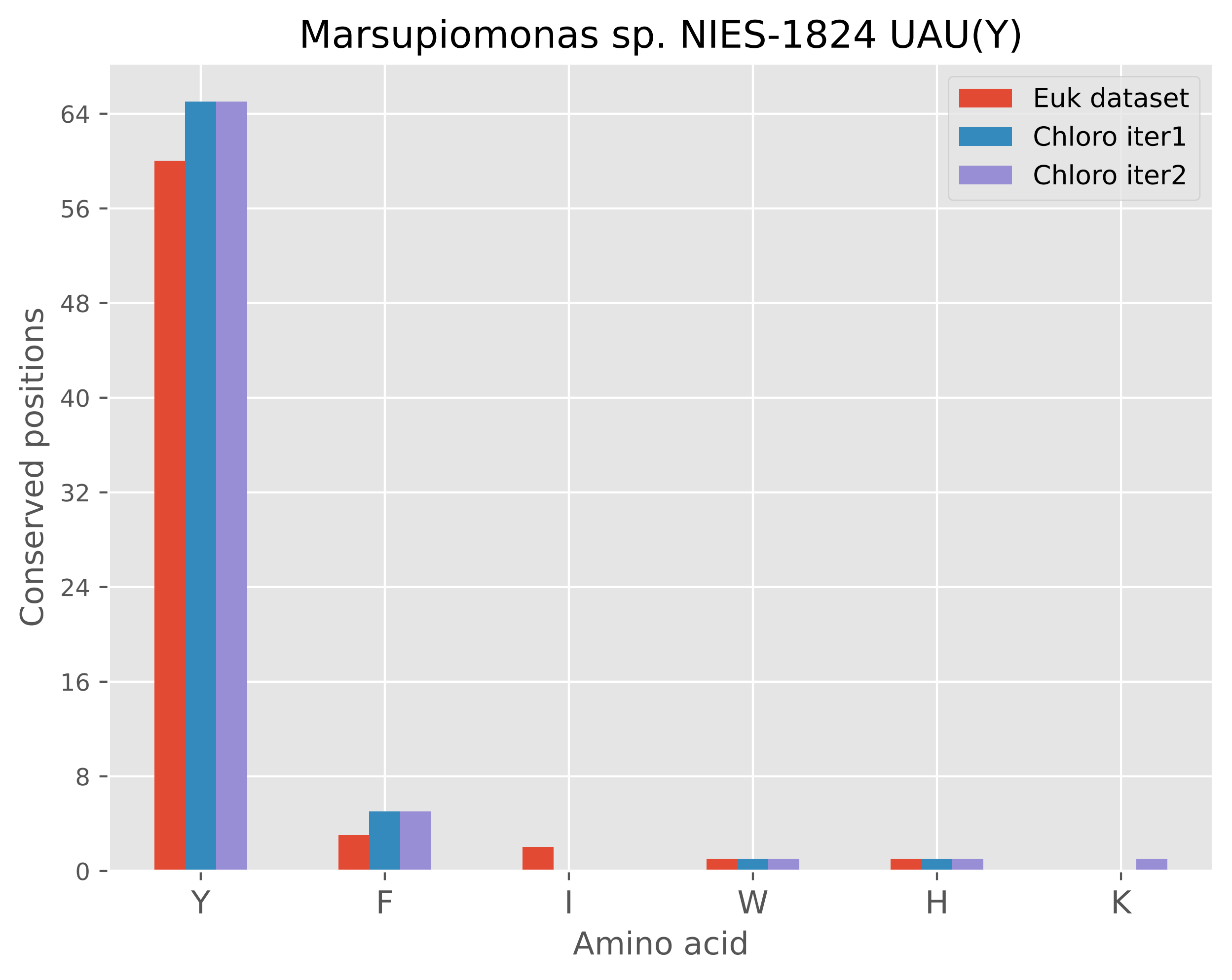
Marsupiomonas sp. NIES-1824 UAU(Y)
Euk dataset
Chloro iter1
Chloro iter2
Conserved positions
0
8
16
24
32
40
48
56
64
Y
F
I
W
H
K
Amino acid

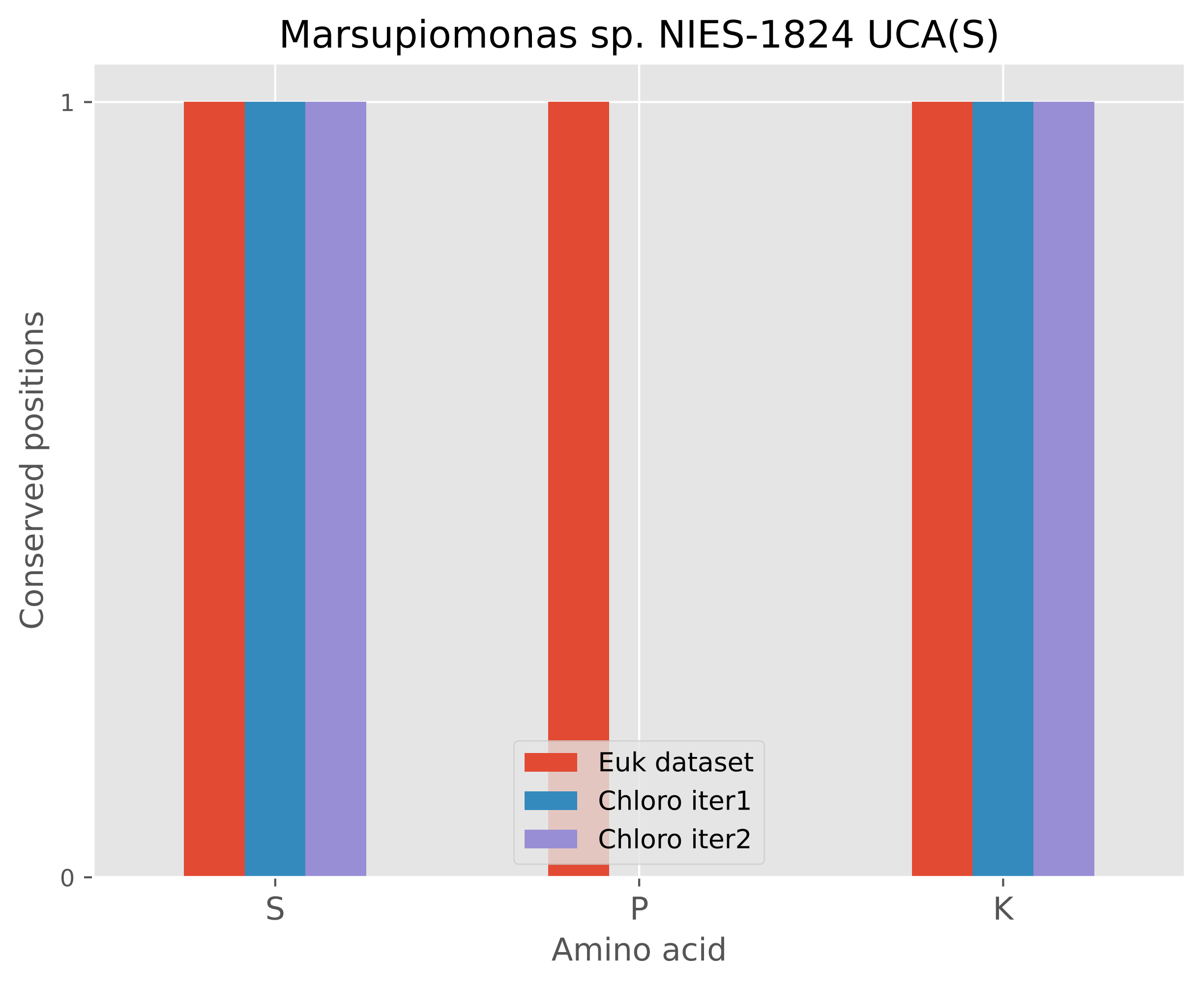
Marsupiomonas sp. NIES-1824 UCA(S)
Conserved positions
1
0
S
P
K
Amino acid
Euk dataset
Chloro iter1
Chloro iter2

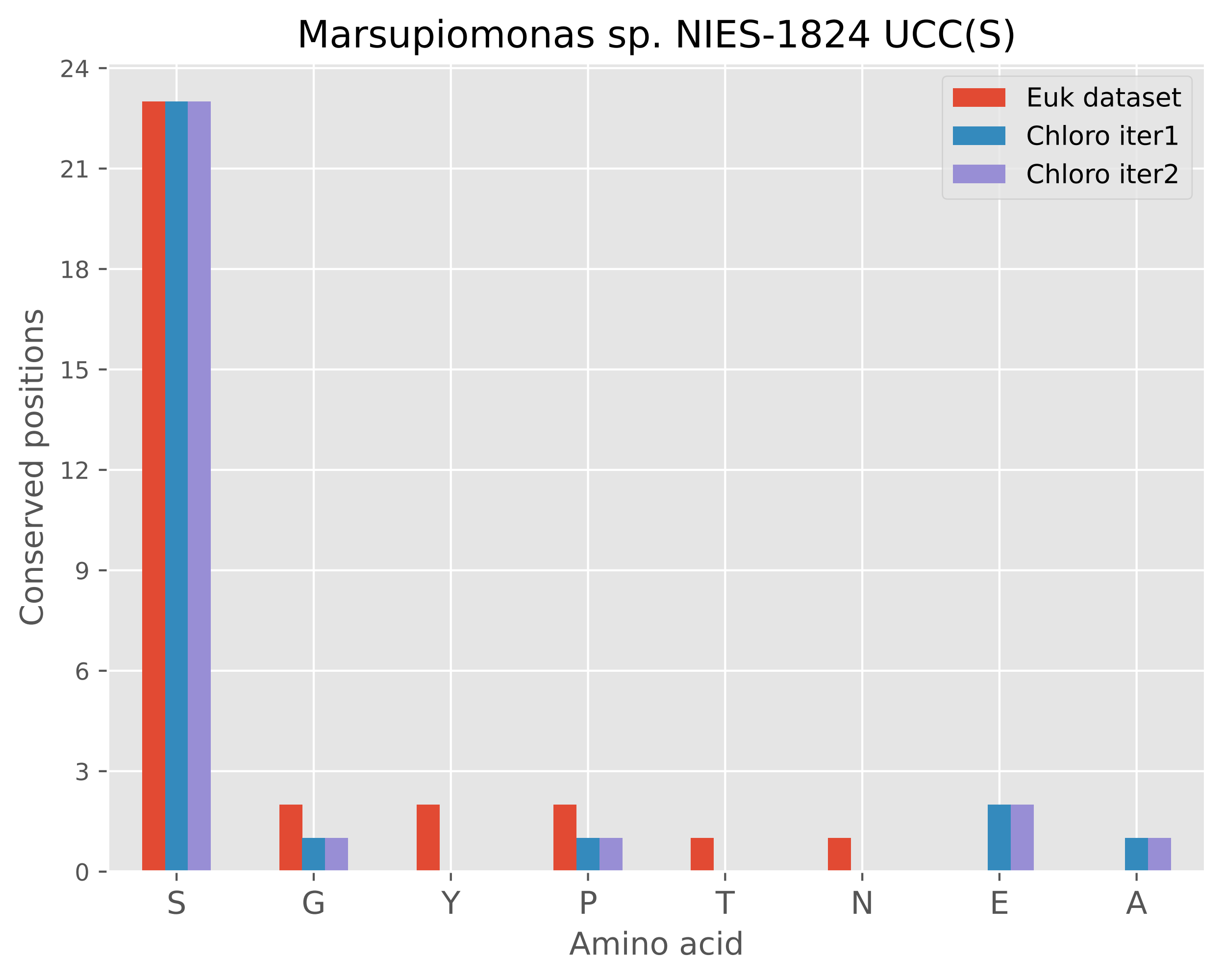

Marsupiomonas sp. NIES-1824 UCC(S)
Conserved positions
Amino acid
Euk dataset
Chloro iter1
Chloro iter2
0
3
6
9
12
15
18
21
24
S
G
Y
P
T
N
E
A

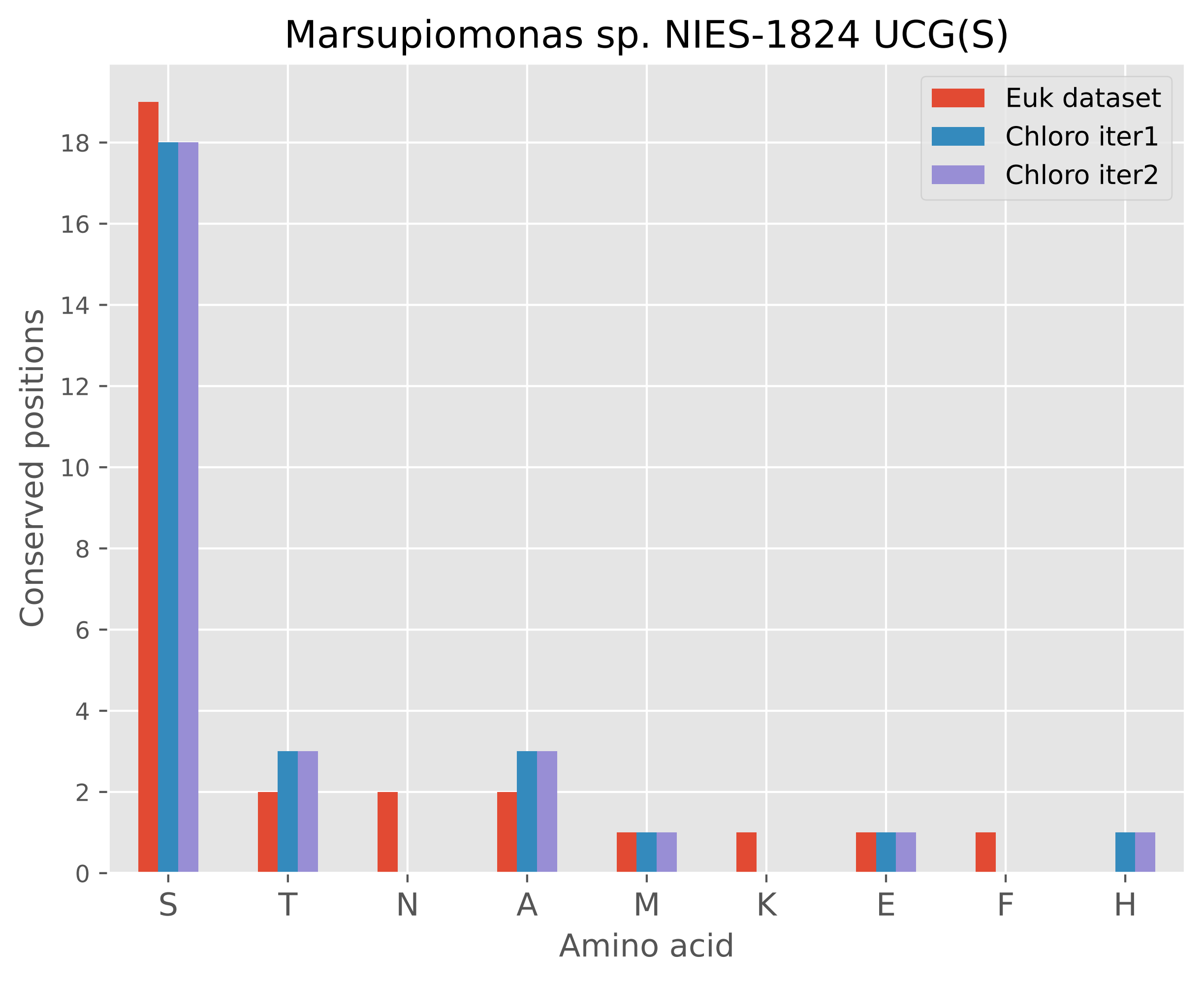

Marsupiomonas sp. NIES-1824 UCG(S)
Euk dataset
Chloro iter1
Chloro iter2
Conserved positions
0
2
4
6
8
10
12
14
16
18
S
T
N
A
M
K
E
F
H
Amino acid

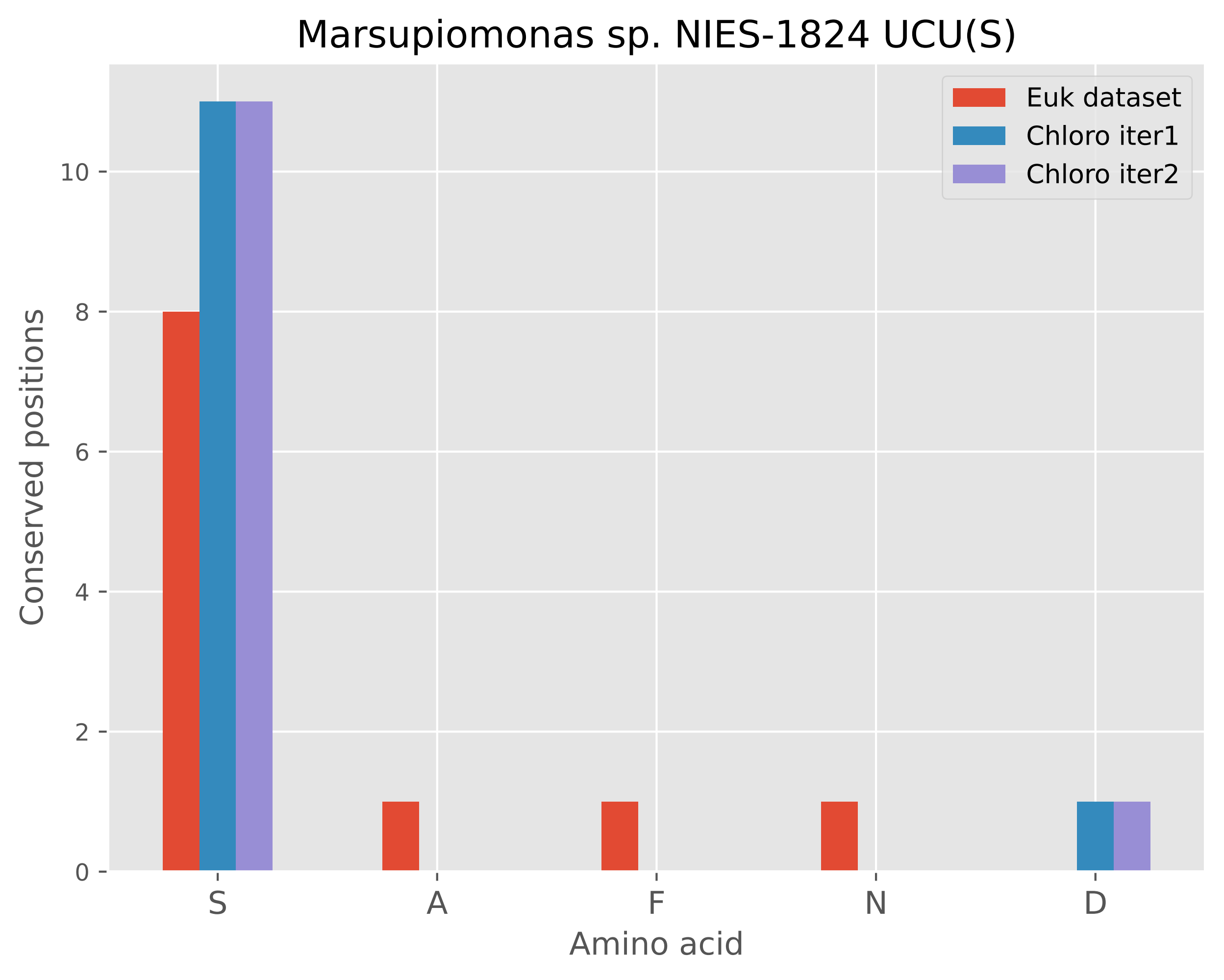

Marsupiomonas sp. NIES-1824 UCU(S)
Euk dataset
Chloro iter1
Chloro iter2
Conserved positions
0
2
4
6
8
10
S
A
F
N
D
Amino acid

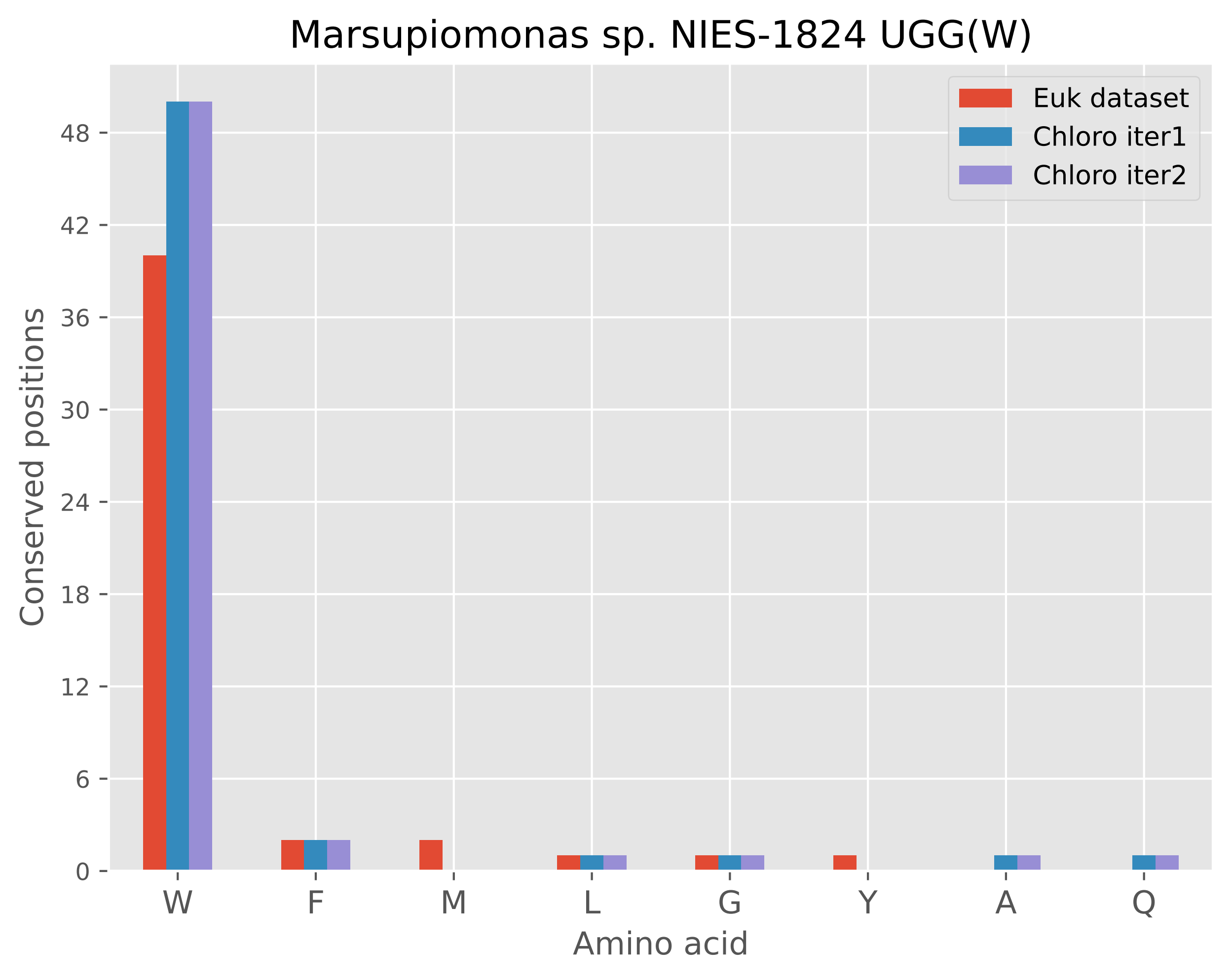
Marsupiomonas sp. NIES-1824 UGG(W)
Euk dataset
Chloro iter1
Chloro iter2
Conserved positions
0
6
12
18
24
30
36
42
48
W
F
M
L
G
Y
A
Q
Amino acid

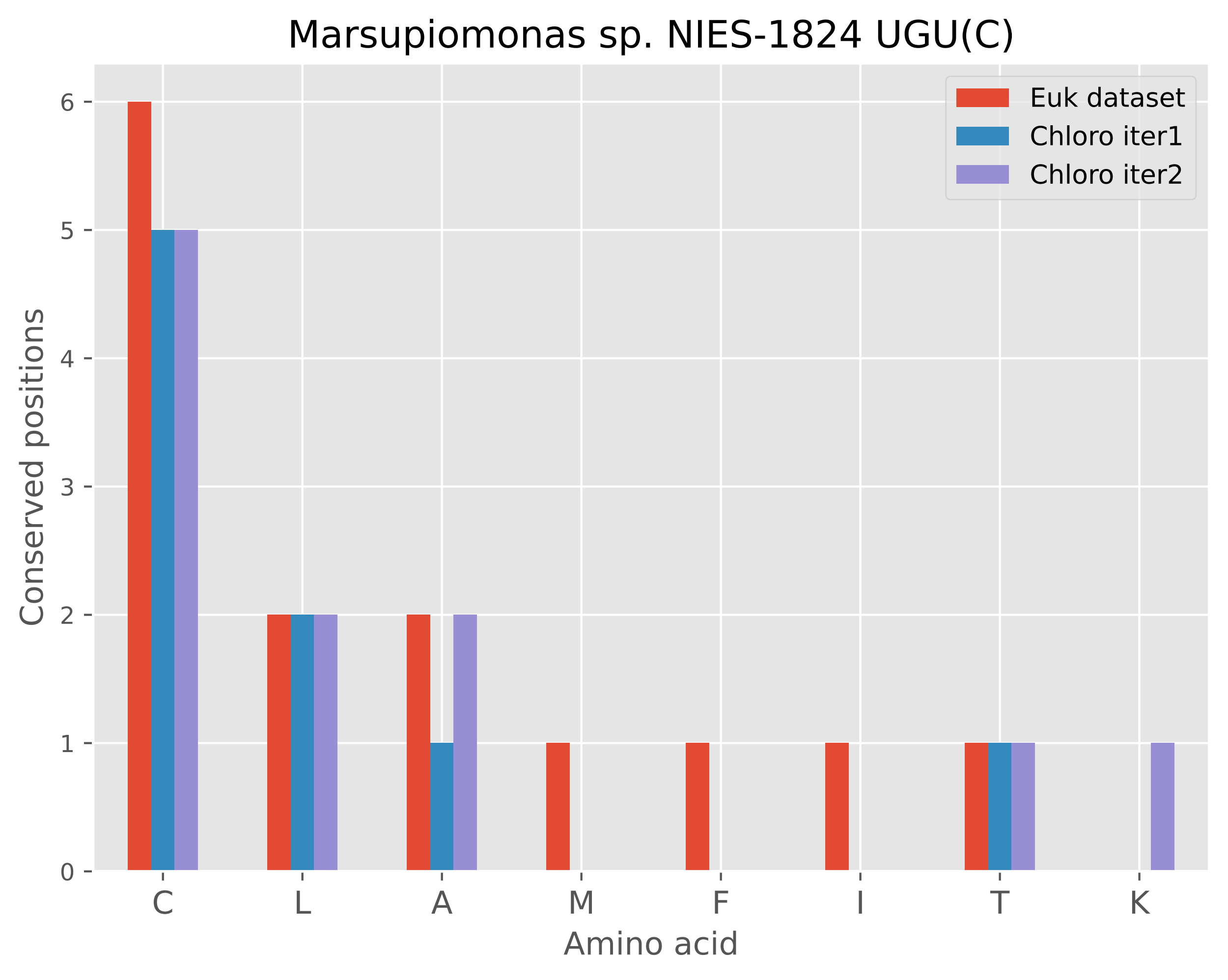

Marsupiomonas sp. NIES-1824 UGU(C)
Euk dataset
Chloro iter1
Chloro iter2
Conserved positions
0
1
2
3
4
5
6
C
L
A
M
F
I
T
K
Amino acid

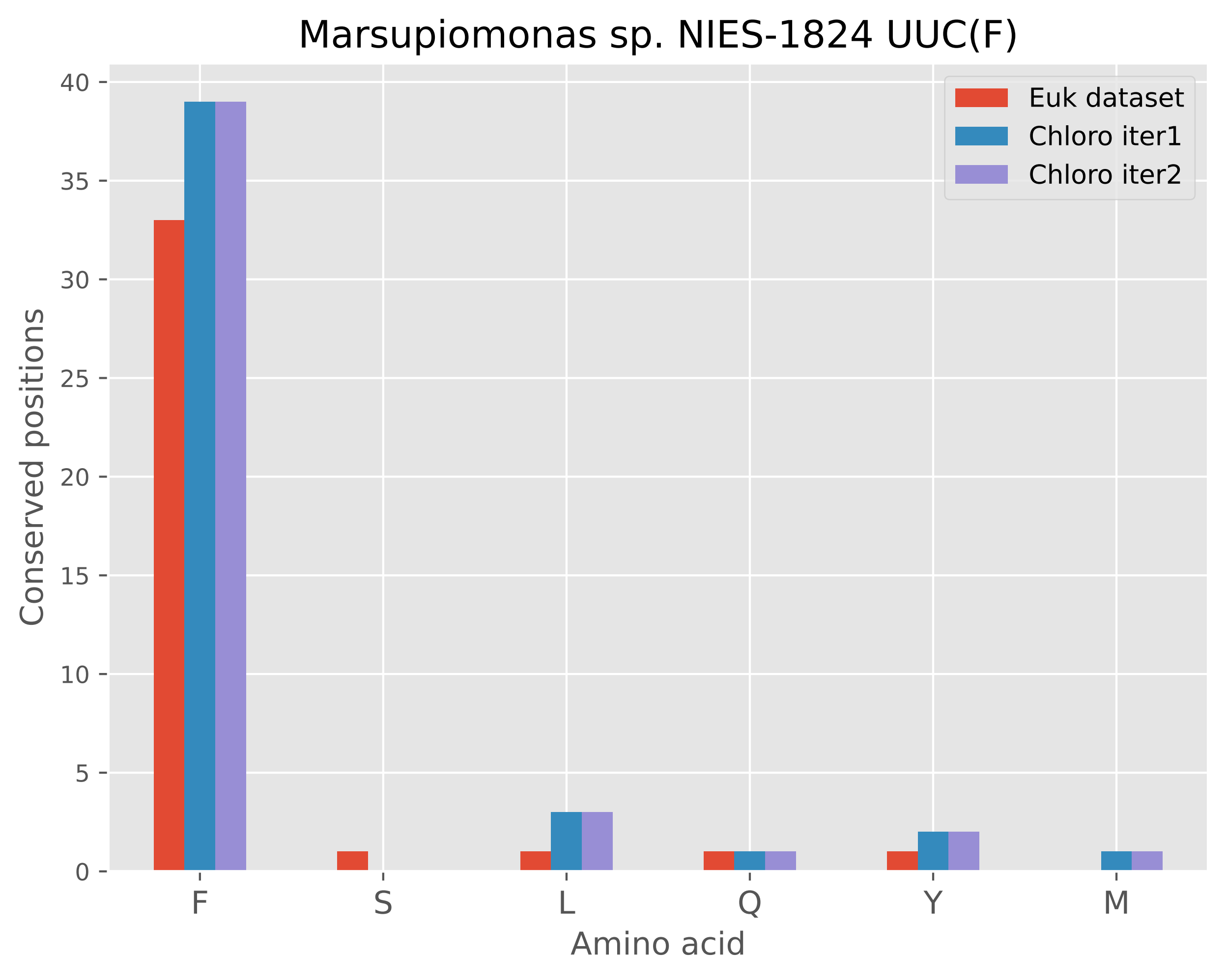
Marsupiomonas sp. NIES-1824 UUC(F)
Euk dataset
Chloro iter1
Chloro iter2
Conserved positions
0
5
10
15
20
25
30
35
40
F
S
L
Q
Y
M
Amino acid

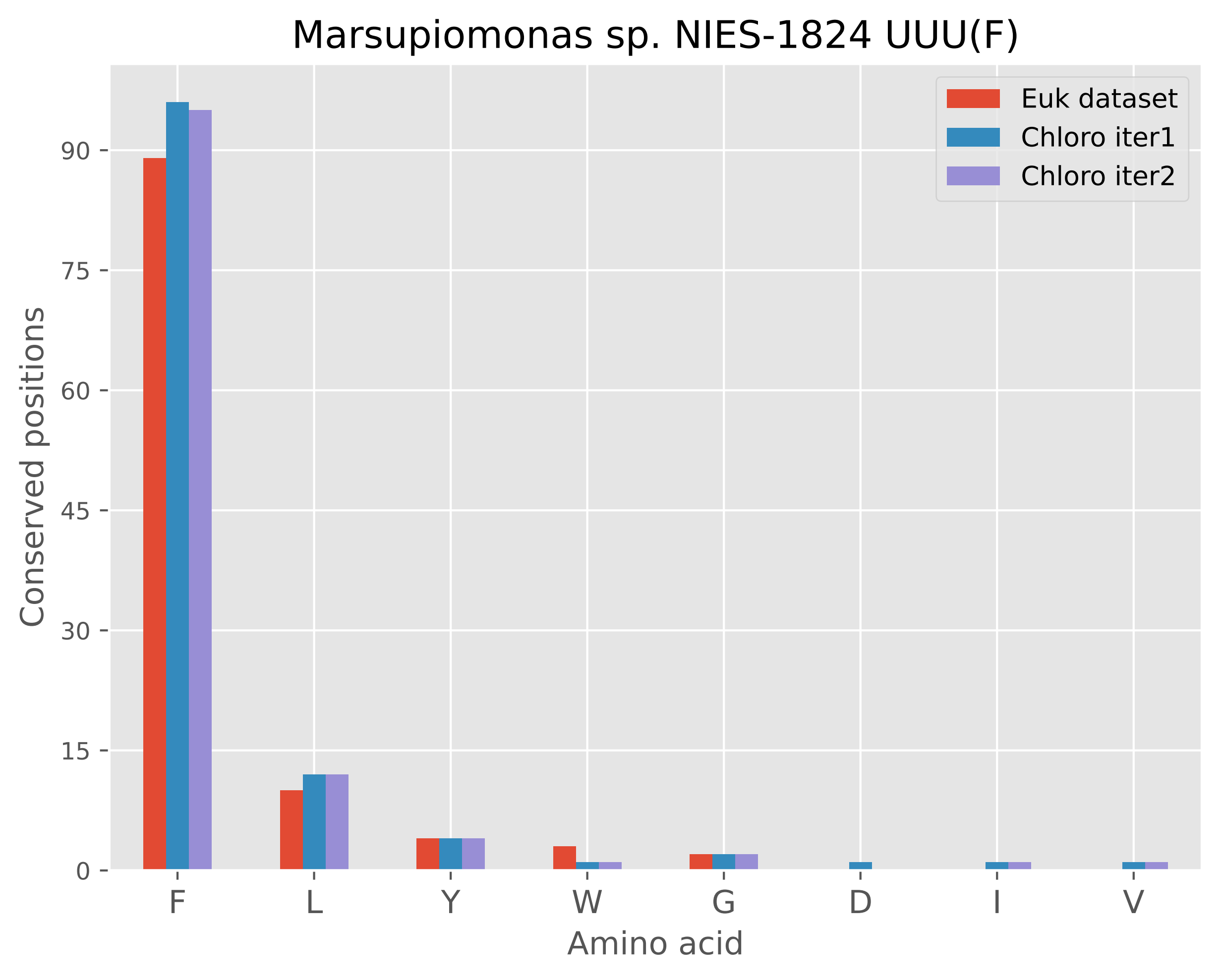

Marsupiomonas sp. NIES-1824 UUU(F)
Euk dataset
Chloro iter1
Chloro iter2
Conserved positions
0
15
30
45
60
75
90
F
L
Y
W
G
D
I
V
Amino acid

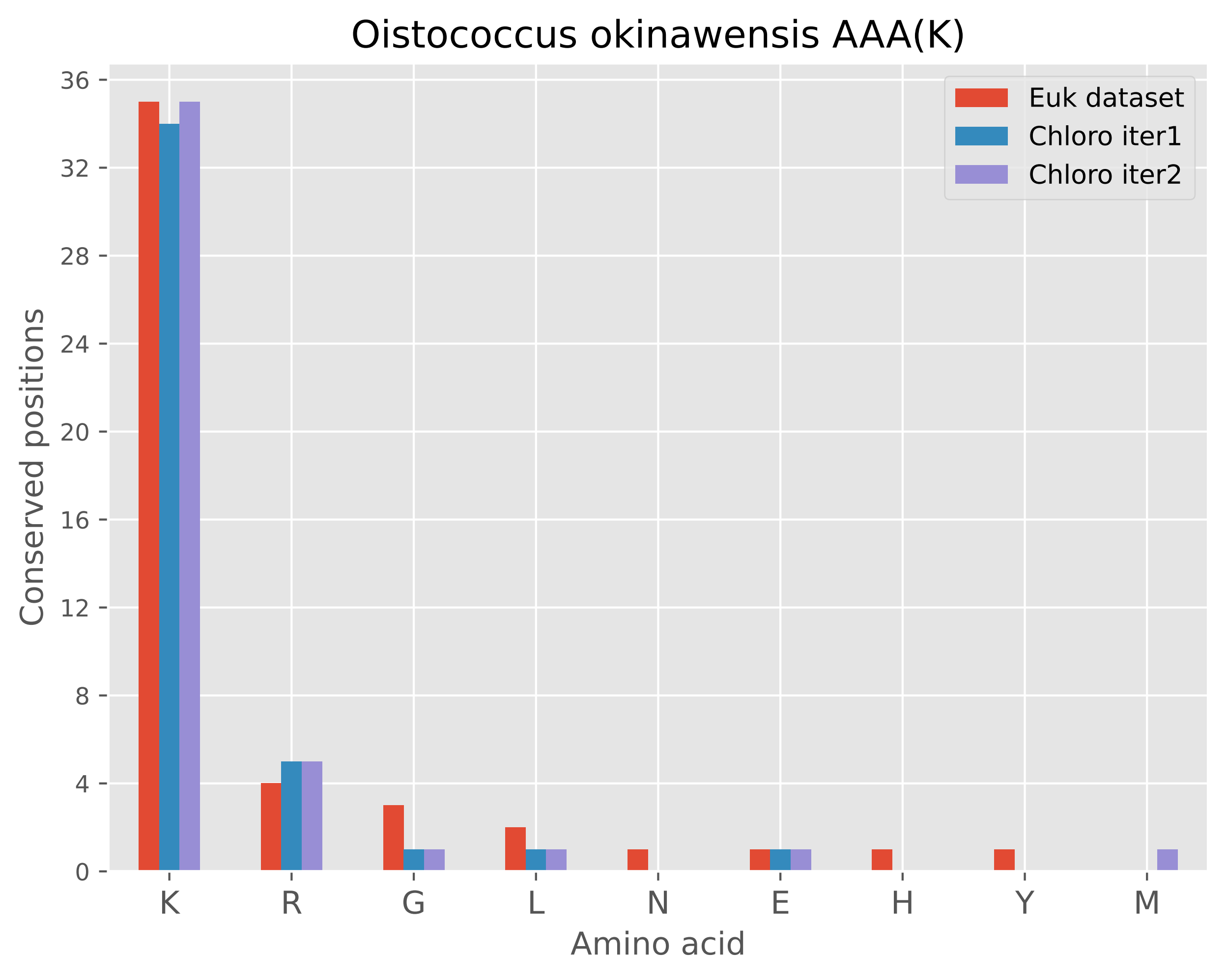
Oistococcus okinawensis AAA(K)
Euk dataset
Chloro iter1
Chloro iter2
Conserved positions
0
4
8
12
16
20
24
28
32
36
K
R
G
L
N
E
H
Y
M
Amino acid

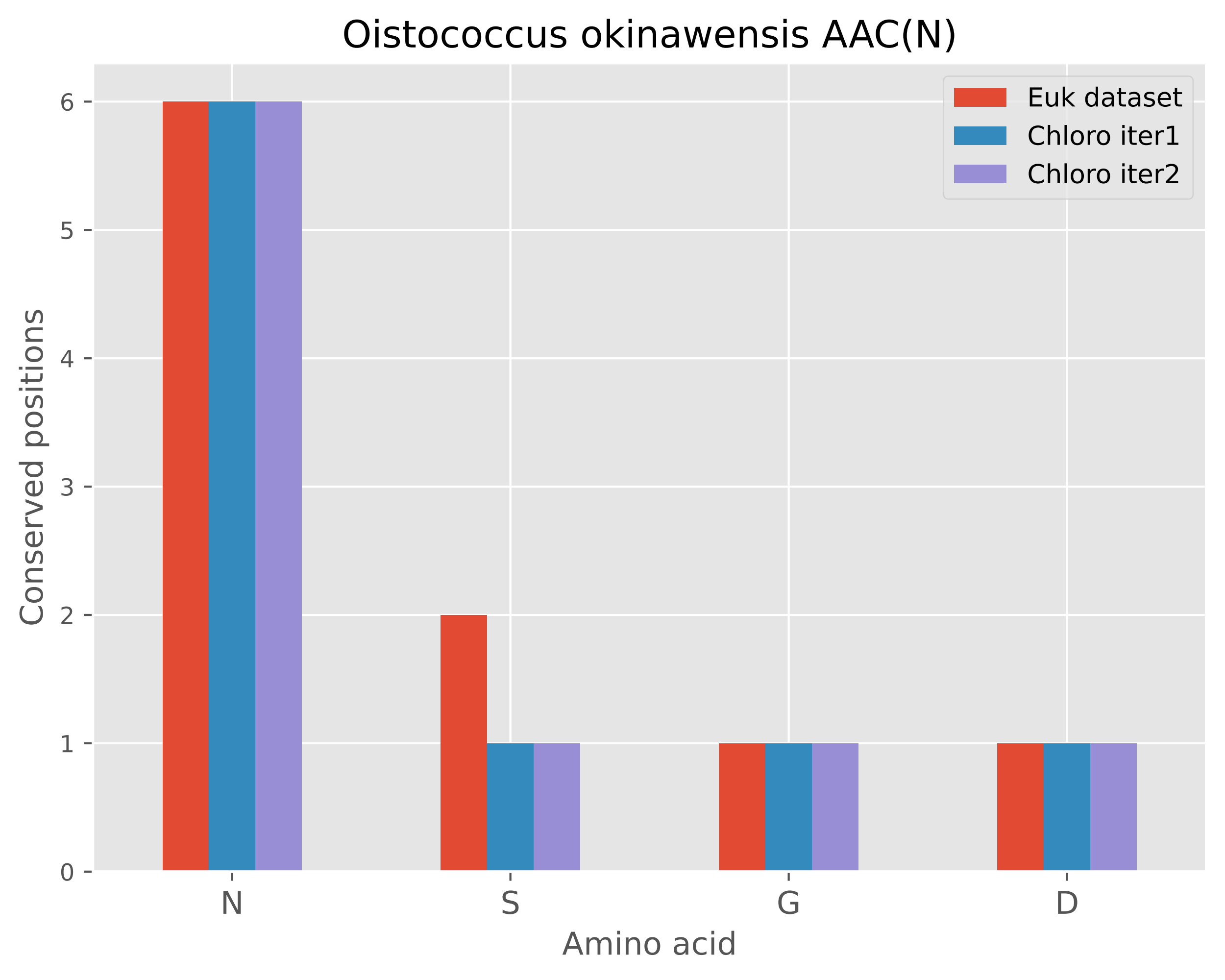
Oistococcus okinawensis AAC(N)
Euk dataset
Chloro iter1
Chloro iter2
Conserved positions
0
1
2
3
4
5
6
N
S
G
D
Amino acid

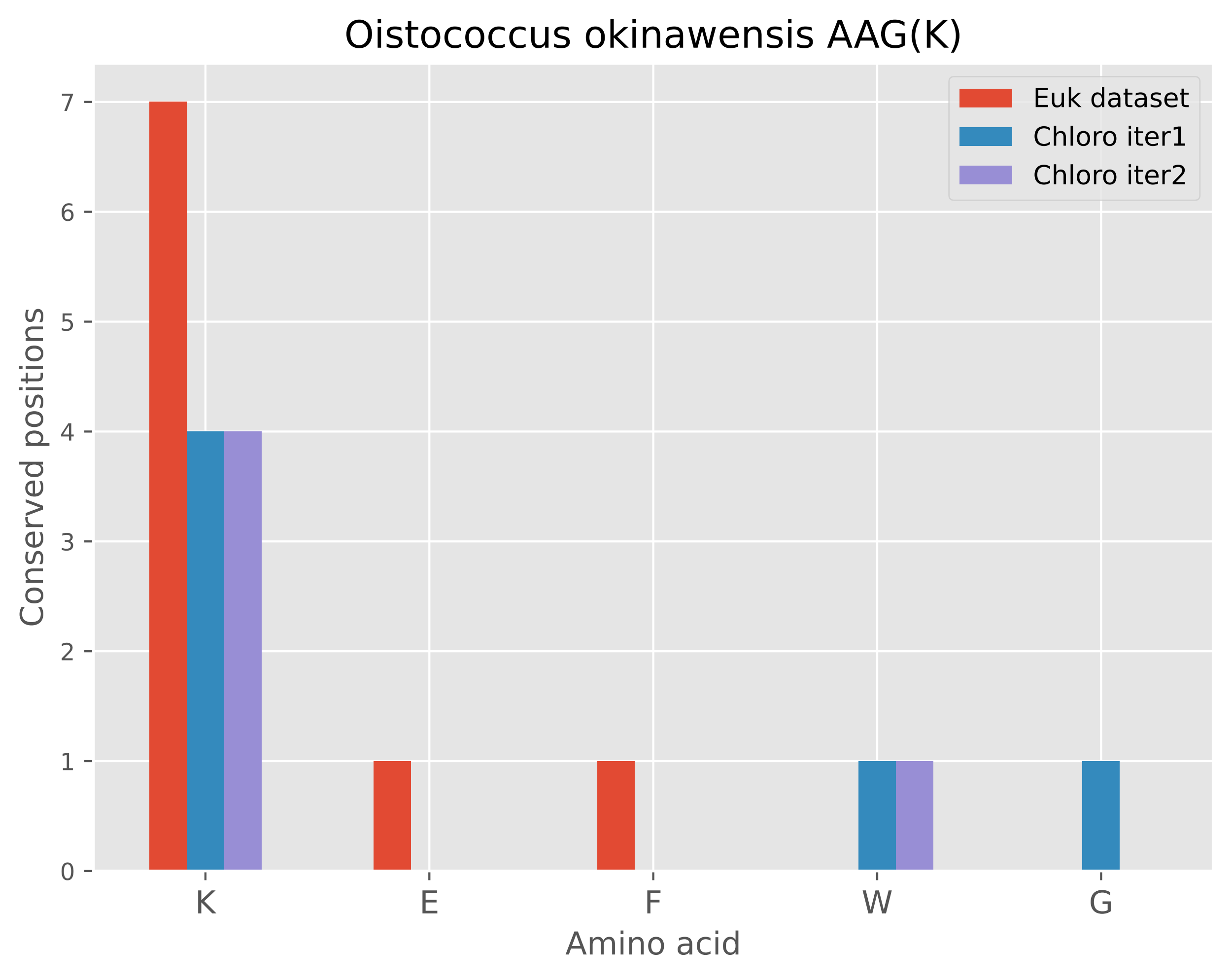
Oistococcus okinawensis AAG(K)
Euk dataset
Chloro iter1
Chloro iter2
Conserved positions
0
1
2
3
4
5
6
7
K
E
F
W
G
Amino acid

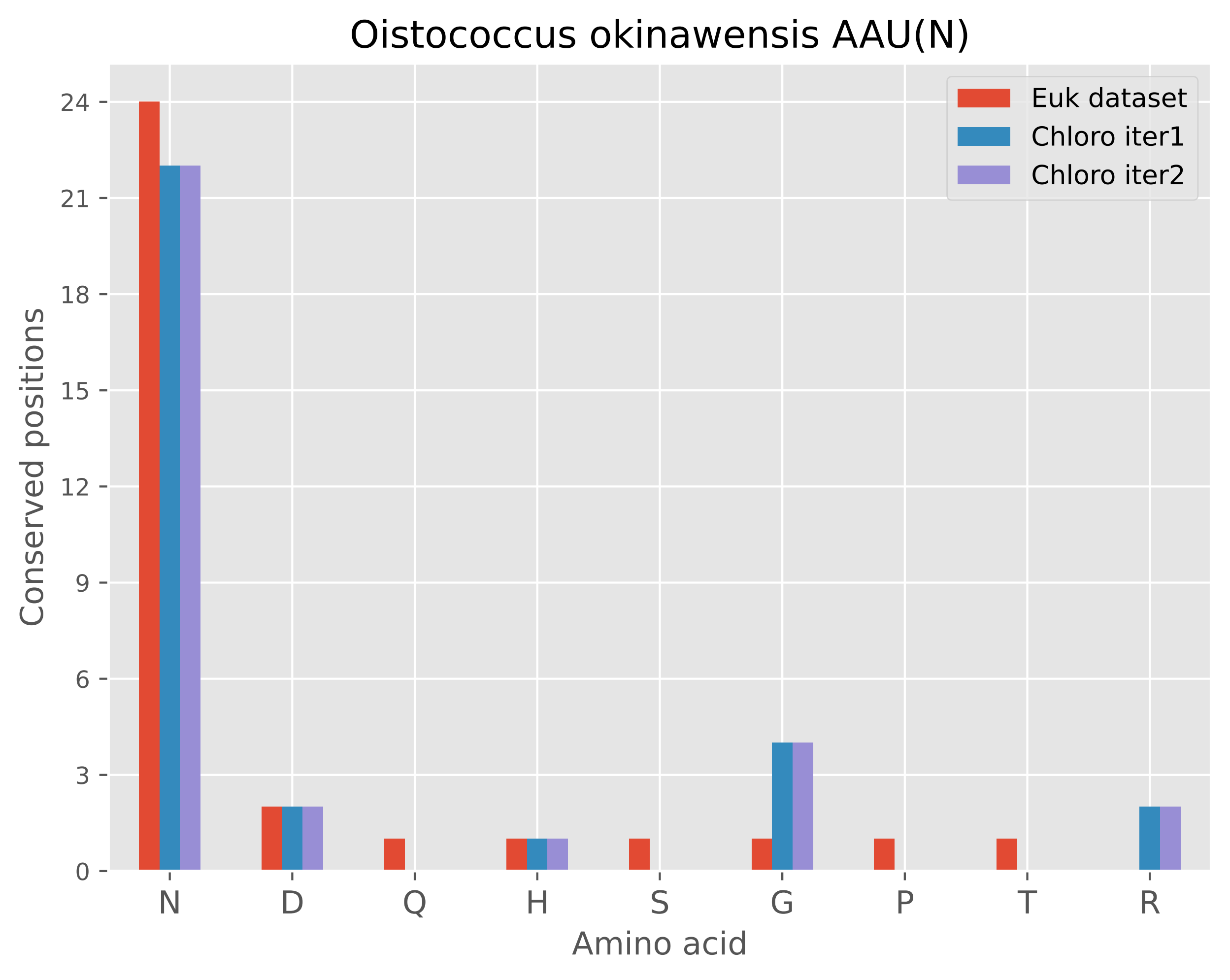

Oistococcus okinawensis AAU(N)
Euk dataset
Chloro iter1
Chloro iter2
Conserved positions
0
3
6
9
12
15
18
21
24
N
D
Q
H
S
G
P
T
R
Amino acid

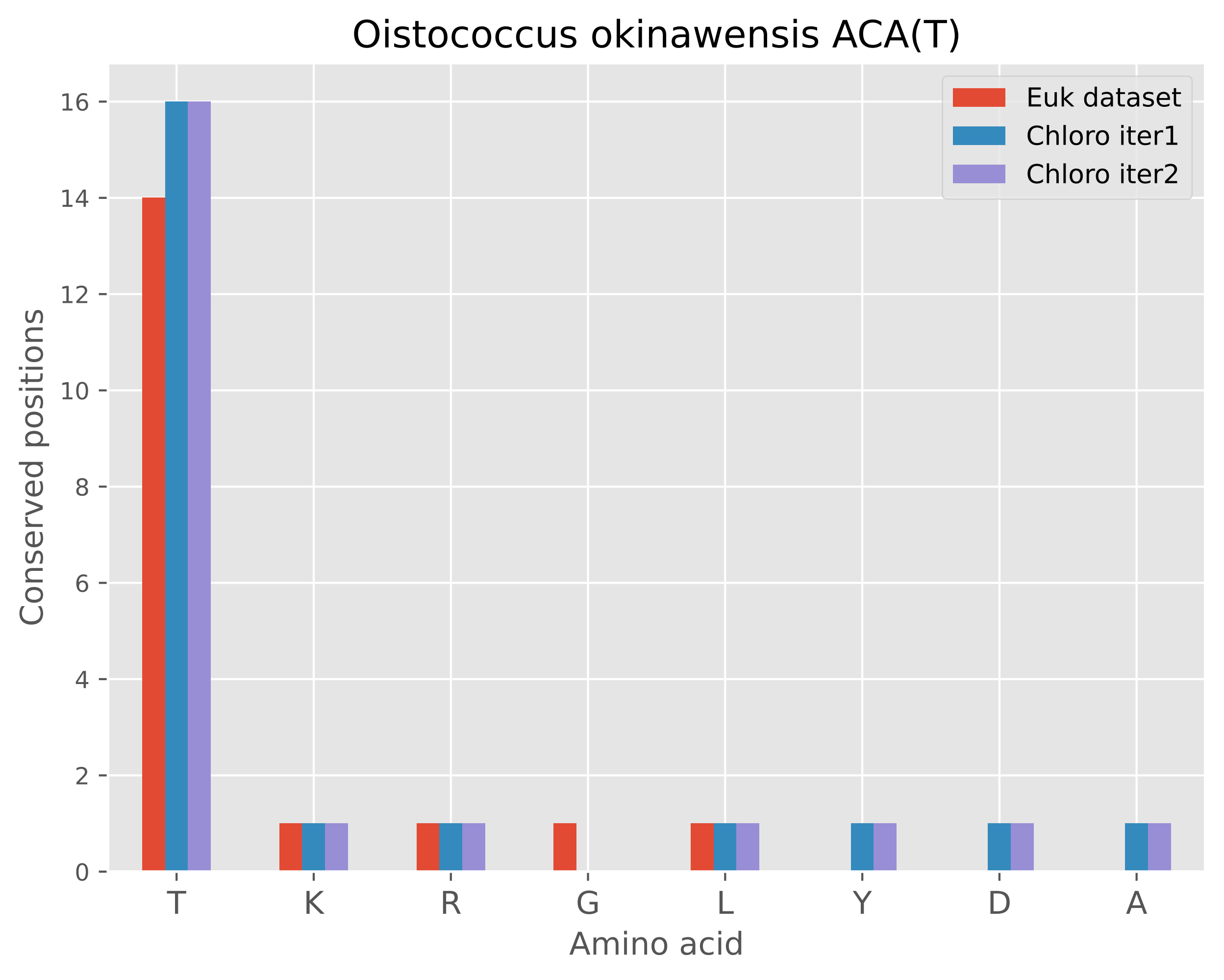
Oistococcus okinawensis ACA(T)
Euk dataset
Chloro iter1
Chloro iter2
Conserved positions
Amino acid
0
2
4
6
8
10
12
14
16
T
K
R
G
L
Y
D
A

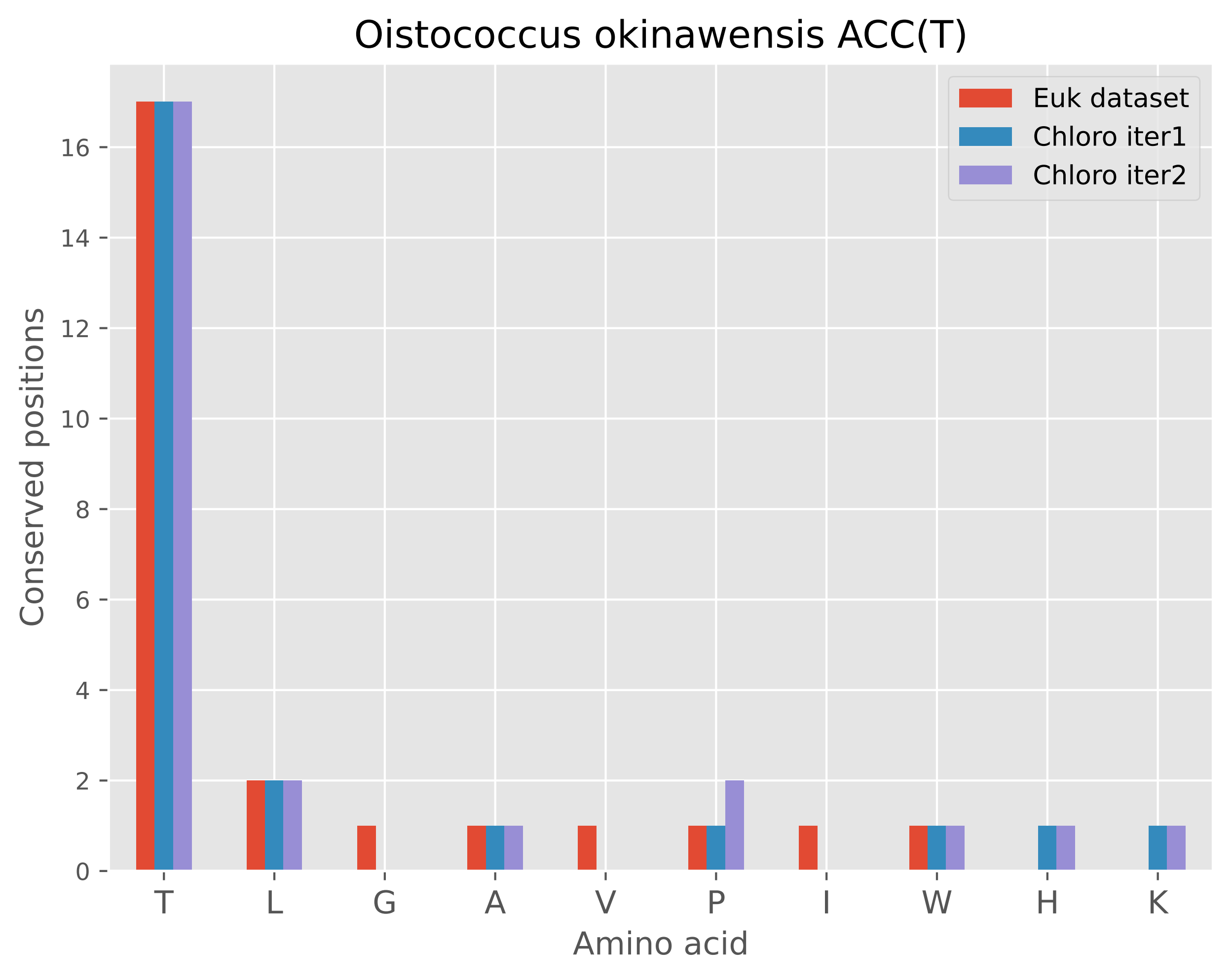

Oistococcus okinawensis ACC(T)
Euk dataset
Chloro iter1
Chloro iter2
Conserved positions
0
2
4
6
8
10
12
14
16
T
L
G
A
V
P
I
W
H
K
Amino acid

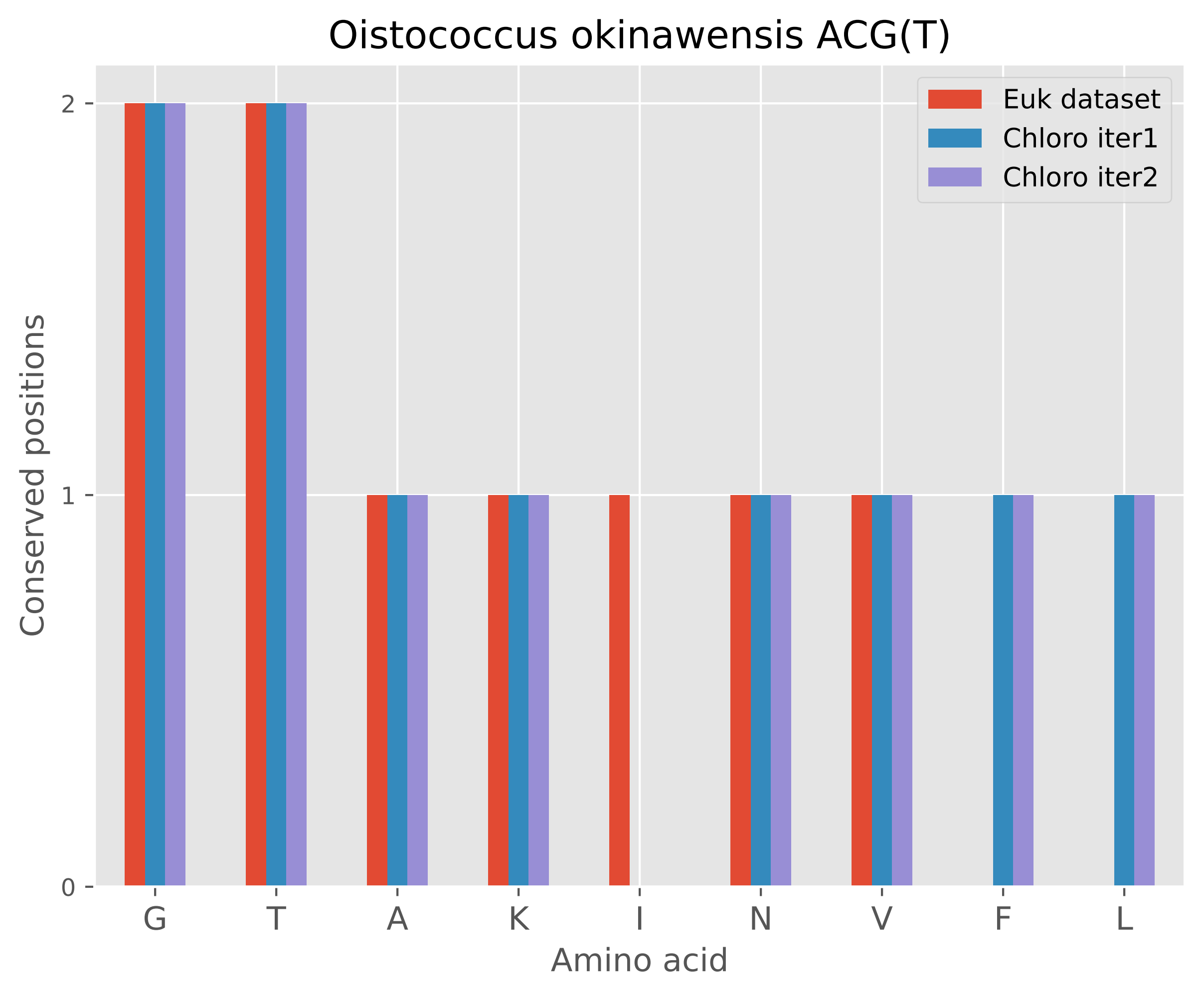
Oistococcus okinawensis ACG(T)
Euk dataset
Chloro iter1
Chloro iter2
Conserved positions
0
1
2
G
T
A
K
I
N
V
F
L
Amino acid

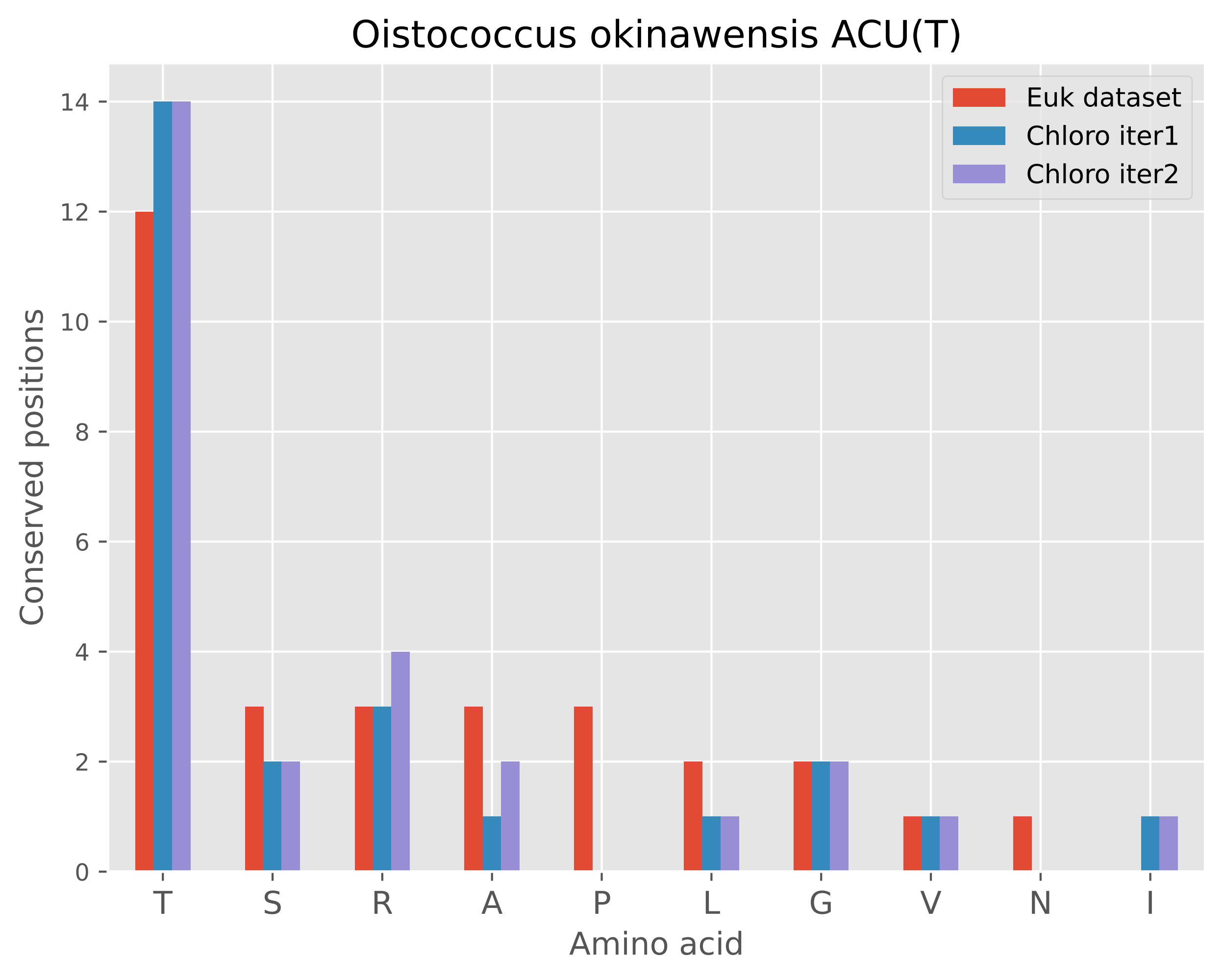
Oistococcus okinawensis ACU(T)
Euk dataset
Chloro iter1
Chloro iter2
Conserved positions
0
2
4
6
8
10
12
14
T
S
R
A
P
L
G
V
N
I
Amino acid

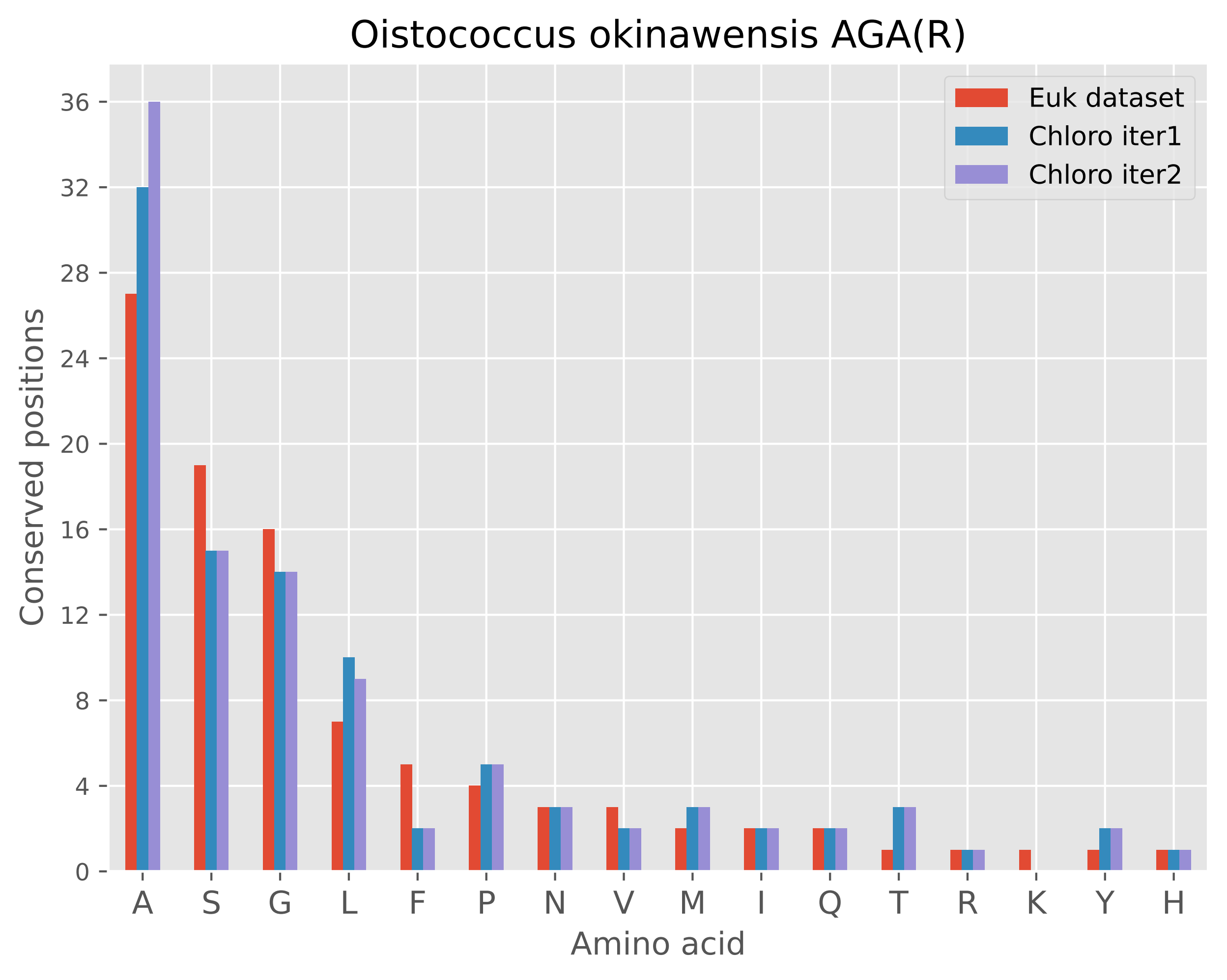

Oistococcus okinawensis AGA(R)
Euk dataset
Chloro iter1
Chloro iter2
Conserved positions
Amino acid
0
4
8
12
16
20
24
28
32
36
A
S
G
L
F
P
N
V
M
I
Q
T
R
K
Y
H

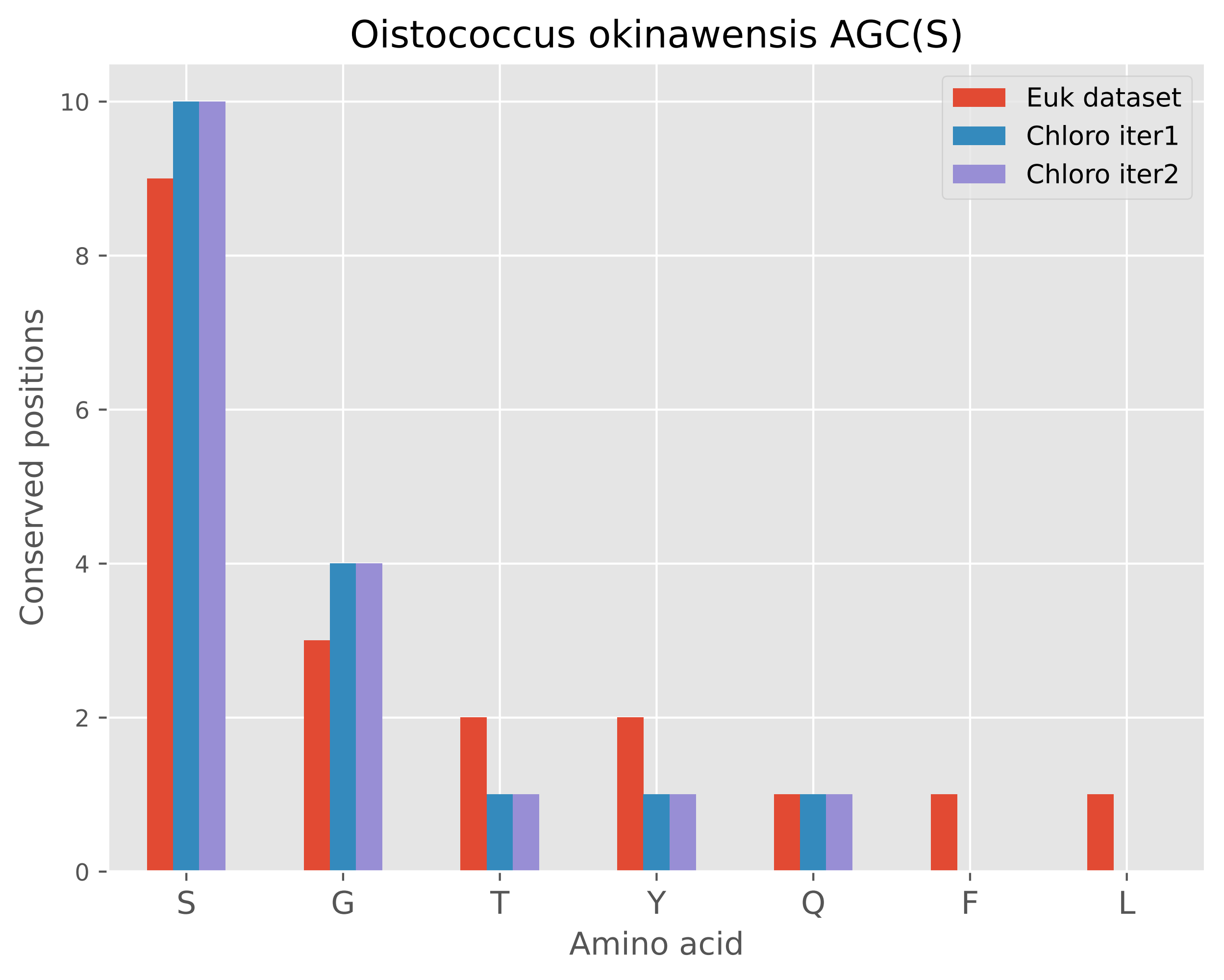
Oistococcus okinawensis AGC(S)
Euk dataset
Chloro iter1
Chloro iter2
Conserved positions
0
2
4
6
8
10
S
G
T
Y
Q
F
L
Amino acid

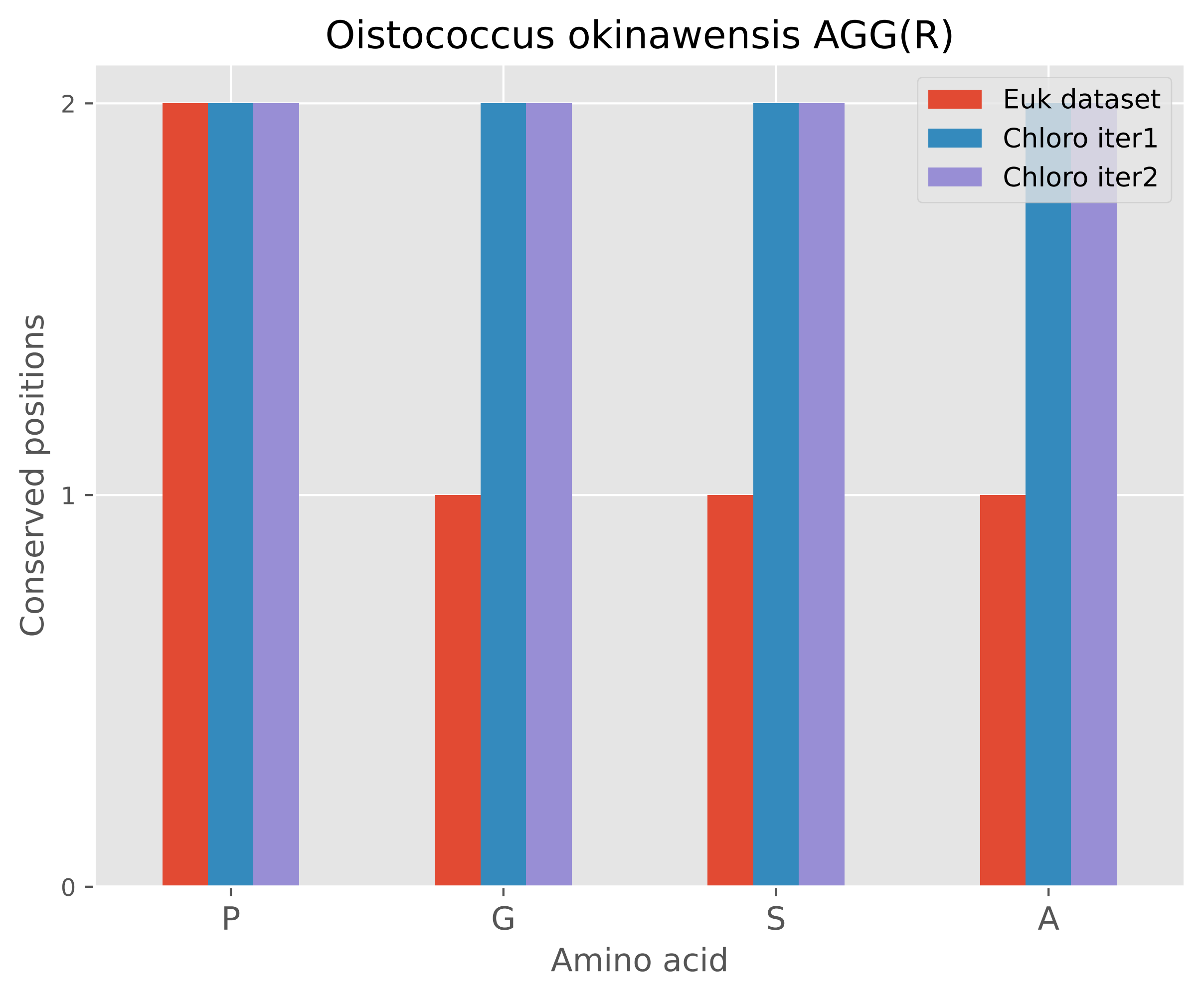

Oistococcus okinawensis AGG(R)
Euk dataset
Chloro iter1
Chloro iter2
Conserved positions
0
1
2
P
G
S
A
Amino acid

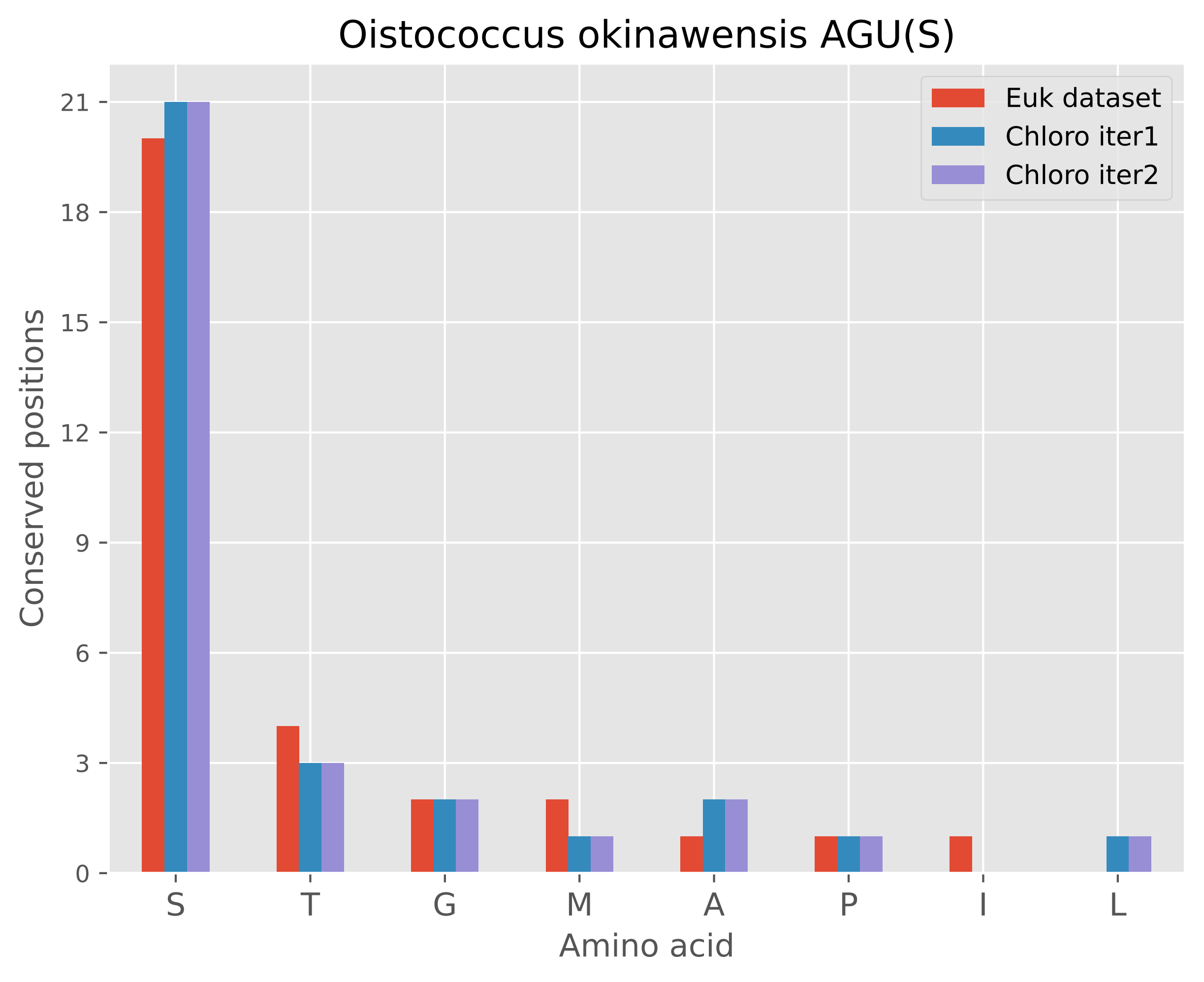

Oistococcus okinawensis AGU(S)
Euk dataset
Chloro iter1
Chloro iter2
Conserved positions
0
3
6
9
12
15
18
21
S
T
G
M
A
P
I
L
Amino acid

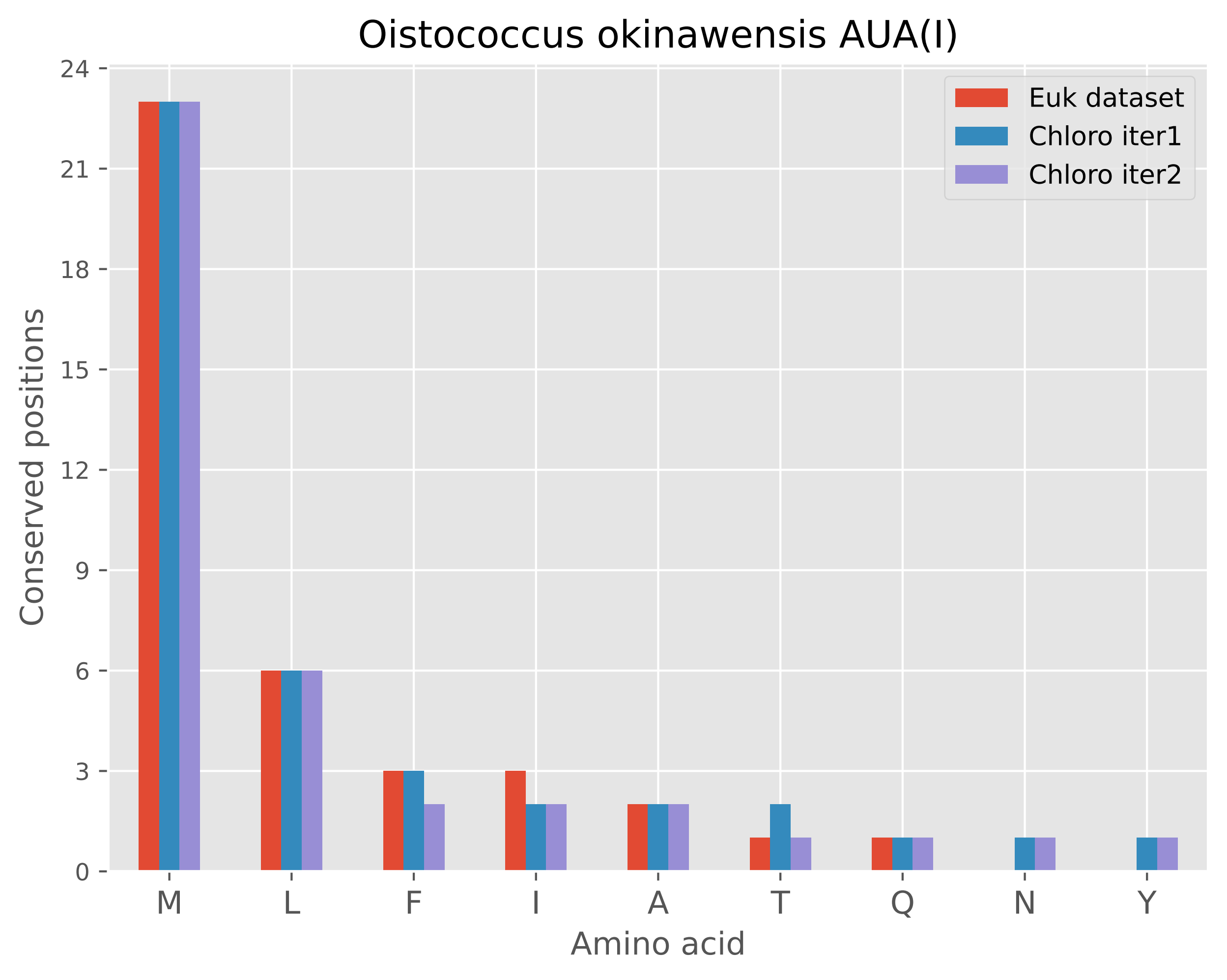
Oistococcus okinawensis AUA(I)
Euk dataset
Chloro iter1
Chloro iter2
Conserved positions
0
3
6
9
12
15
18
21
24
M
L
F
I
A
T
Q
N
Y
Amino acid

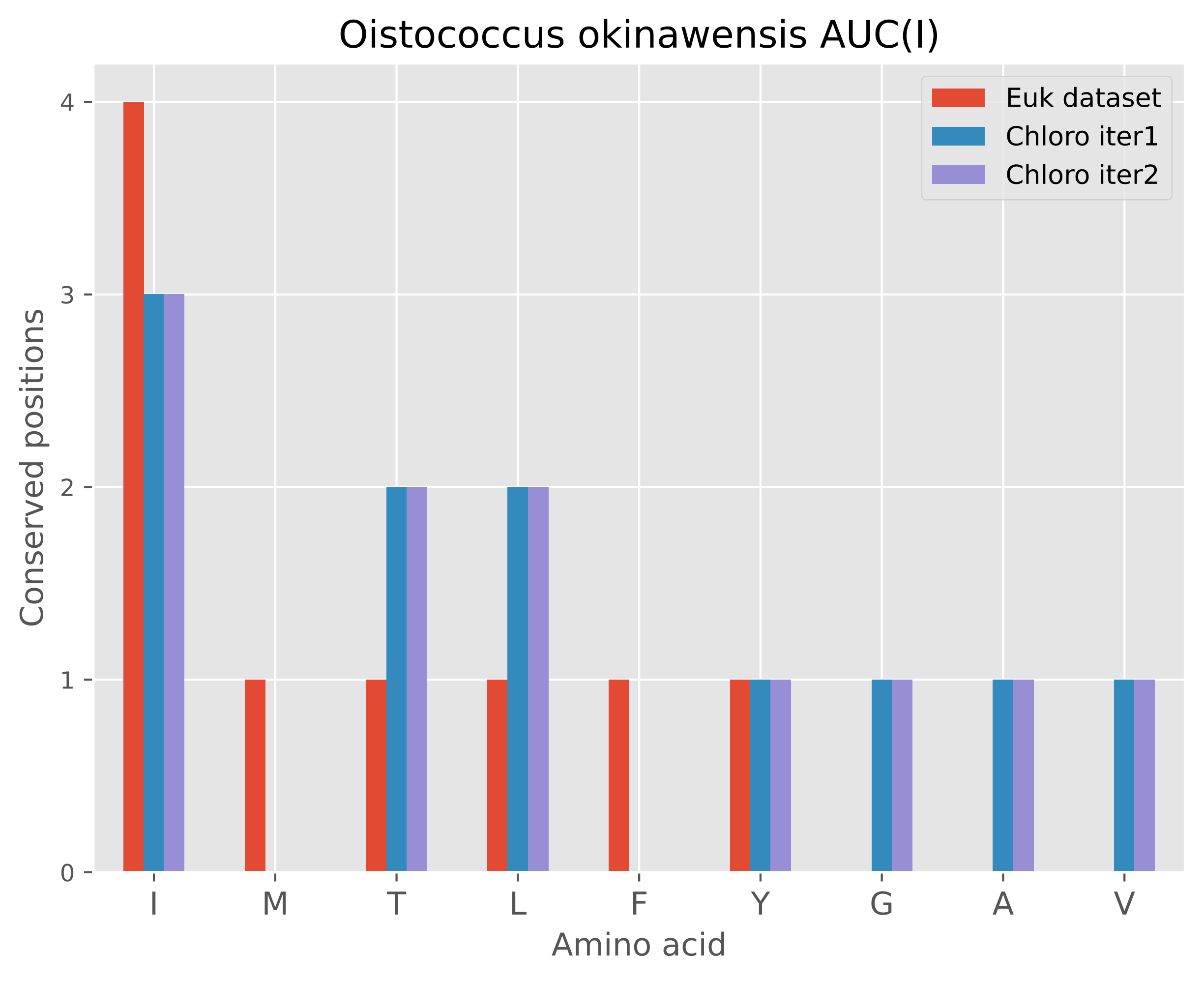
Oistococcus okinawensis AUC(I)
Conserved positions
Amino acid
Euk dataset
Chloro iter1
Chloro iter2
0
1
2
3
4
I
M
T
L
F
Y
G
A
V

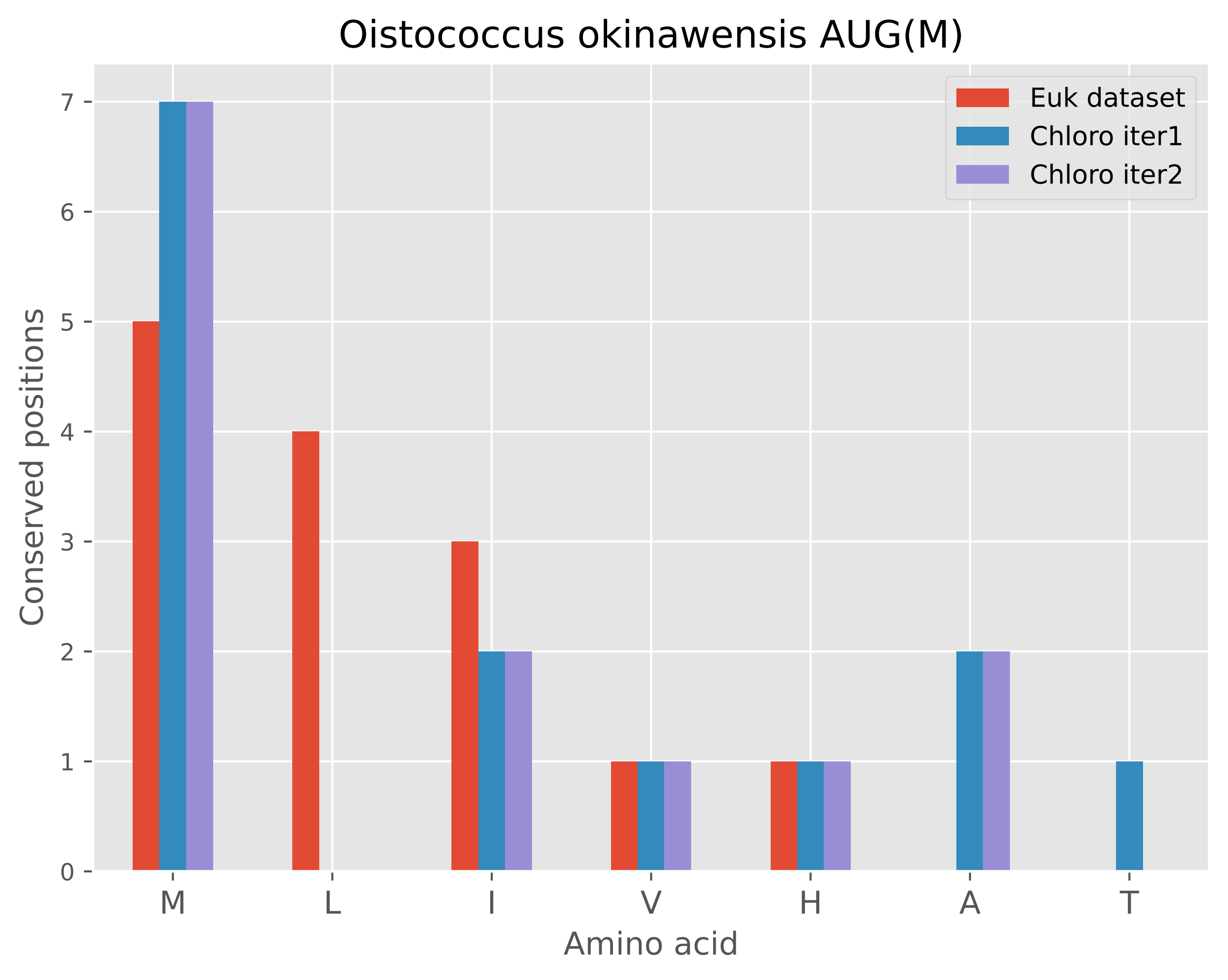
Oistococcus okinawensis AUG(M)
Euk dataset
Chloro iter1
Chloro iter2
Conserved positions
Amino acid
0
1
2
3
4
5
6
7
M
L
I
V
H
A
T

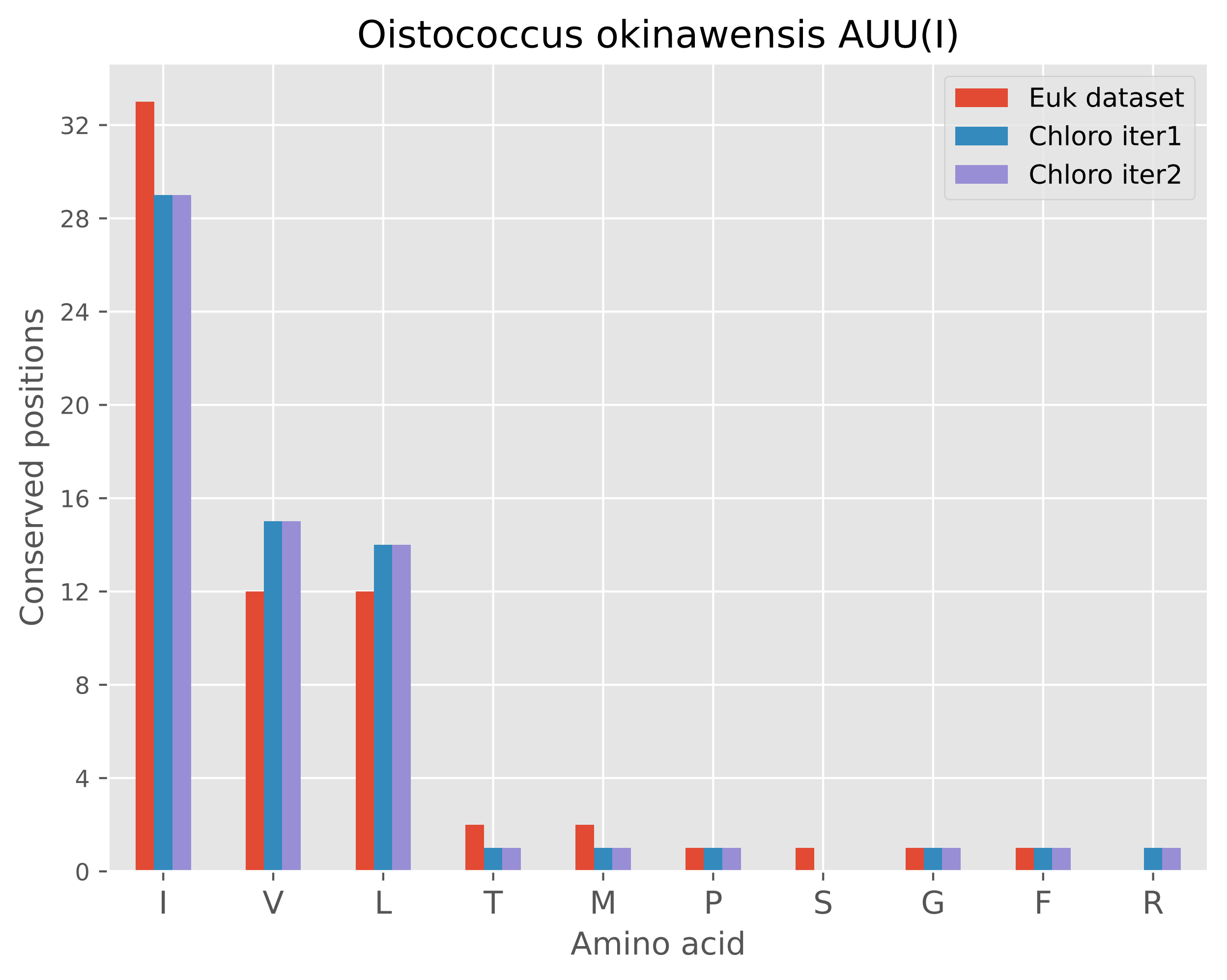
Oistococcus okinawensis AUU(I)
Conserved positions
0
4
8
12
16
20
24
28
32
Euk dataset
Chloro iter1
Chloro iter2
I
V
L
T
M
P
S
G
F
R
Amino acid

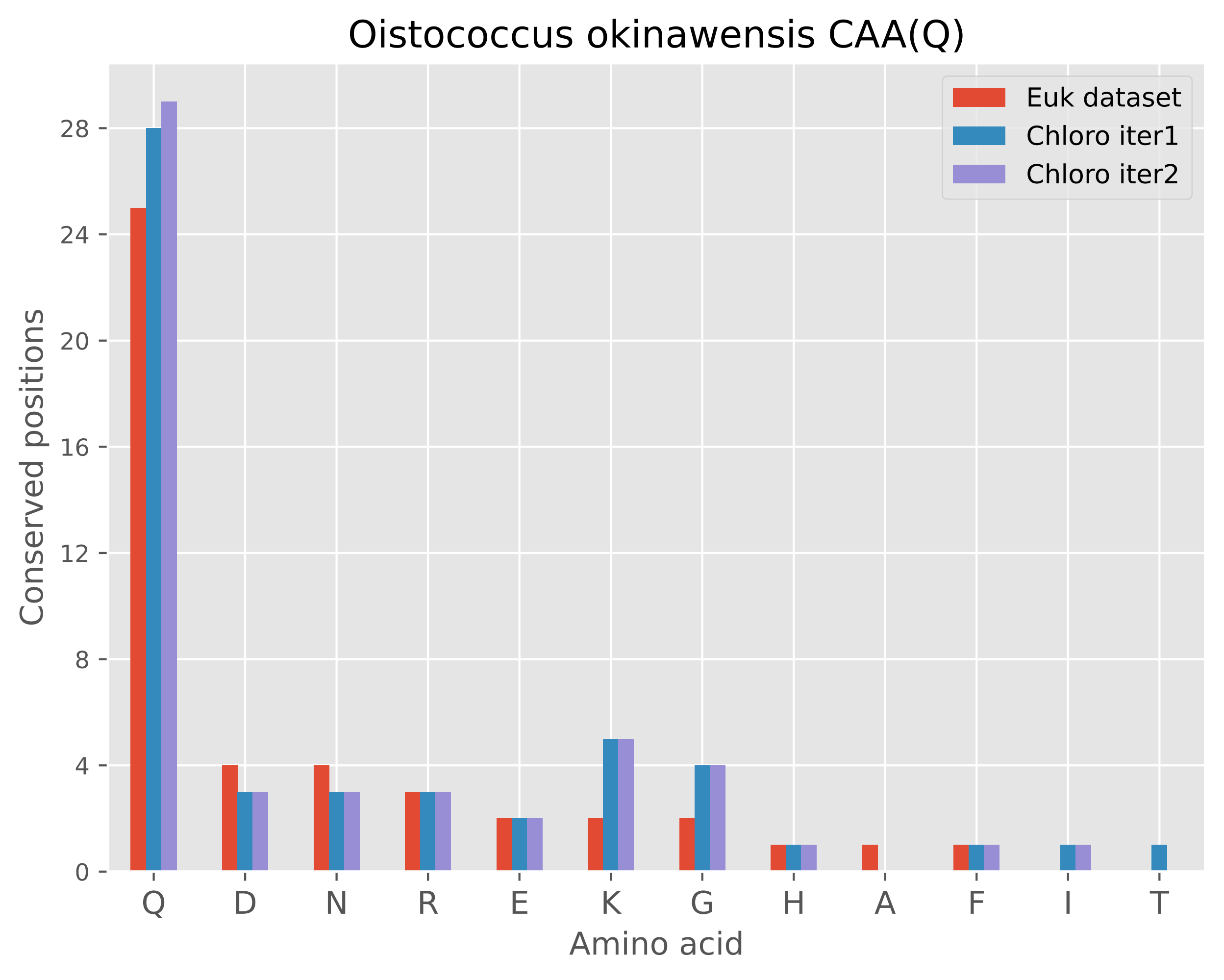

Oistococcus okinawensis CAA(Q)
Euk dataset
Chloro iter1
Chloro iter2
Conserved positions
0
4
8
12
16
20
24
28
Q
D
N
R
E
K
G
H
A
F
I
T
Amino acid

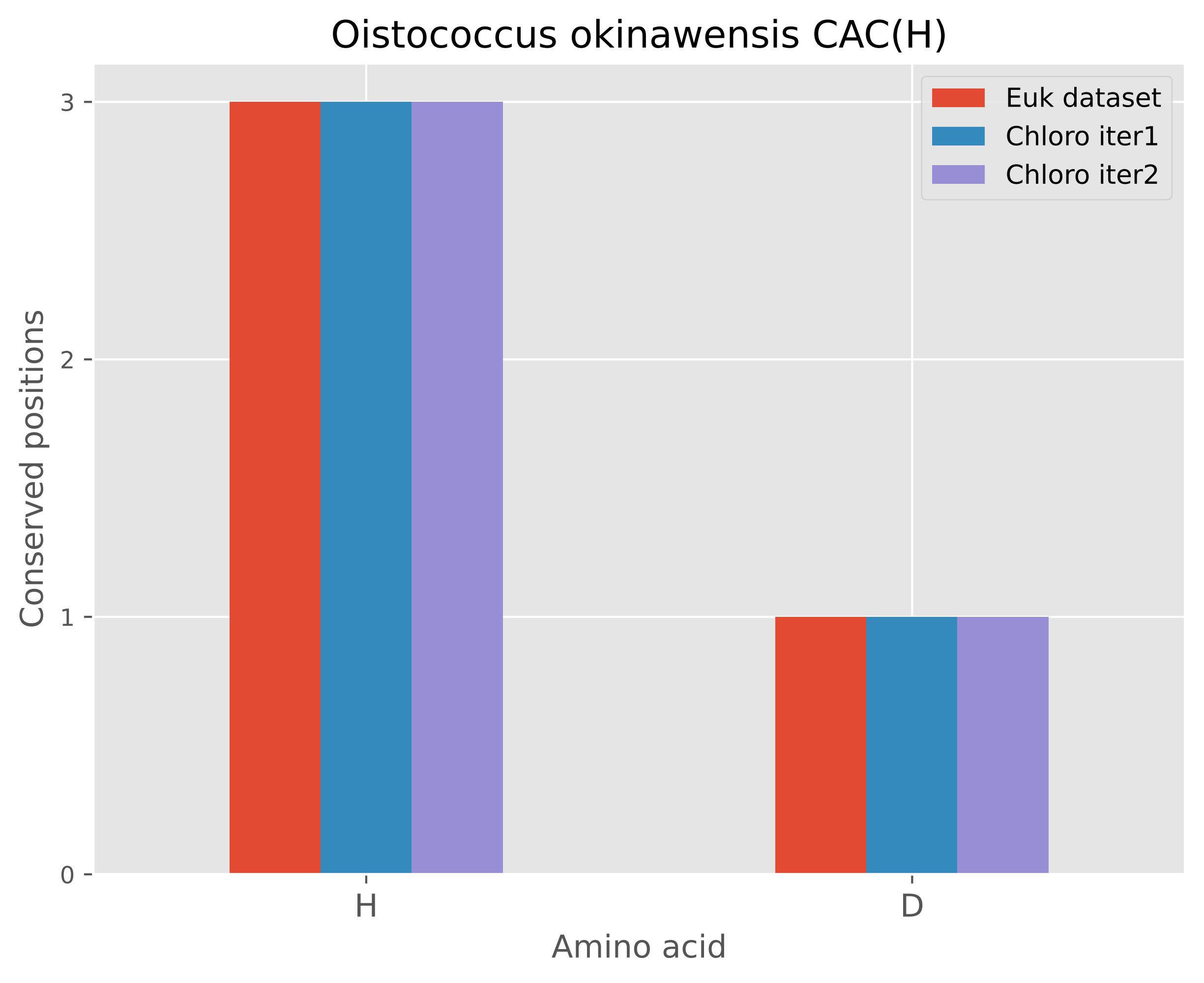
Oistococcus okinawensis CAC(H)
Euk dataset
Chloro iter1
Chloro iter2
Conserved positions
0
1
2
3
H
D
Amino acid

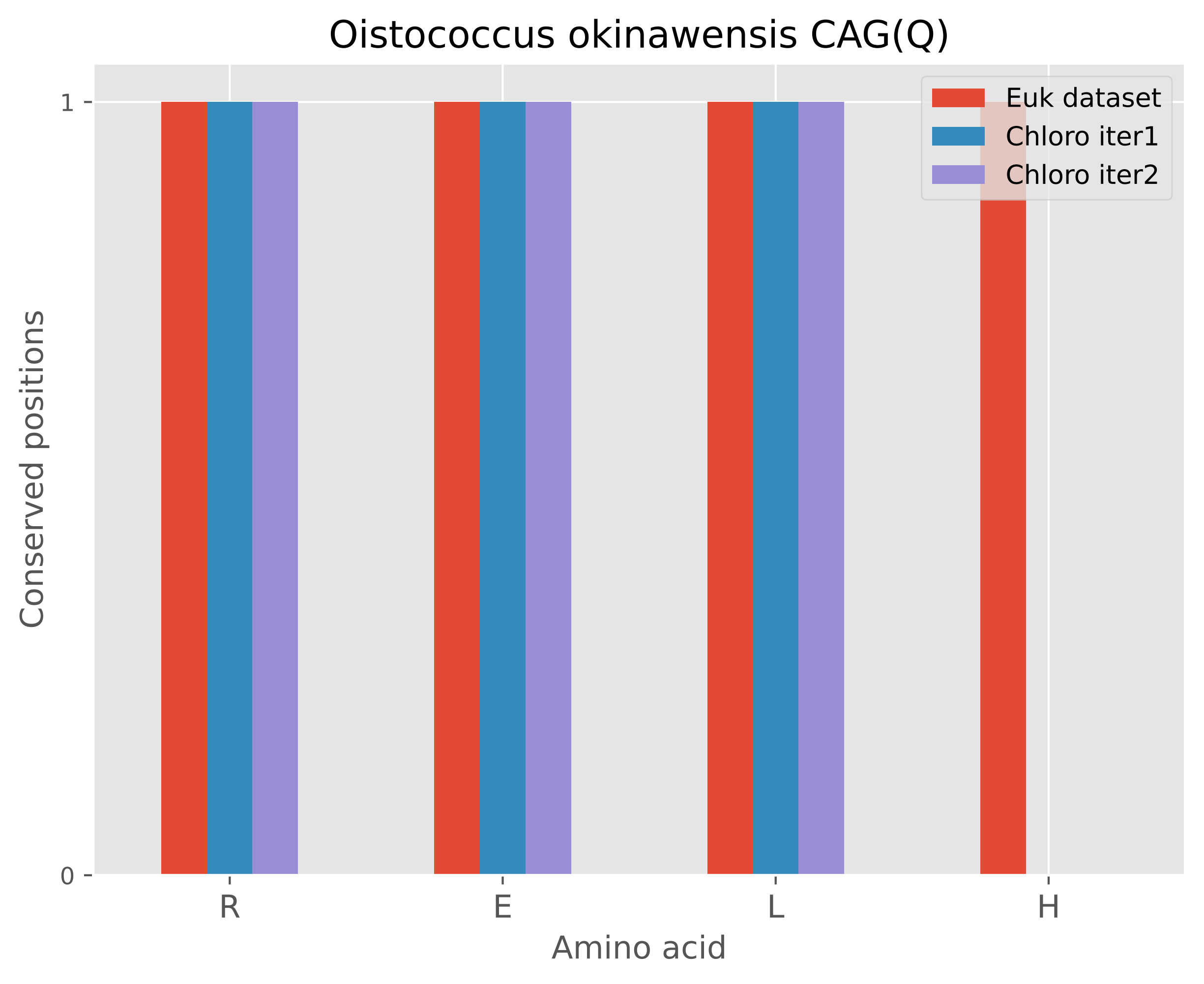
Oistococcus okinawensis CAG(Q)
Euk dataset
Chloro iter1
Chloro iter2
Conserved positions
1
0
R
E
L
H
Amino acid

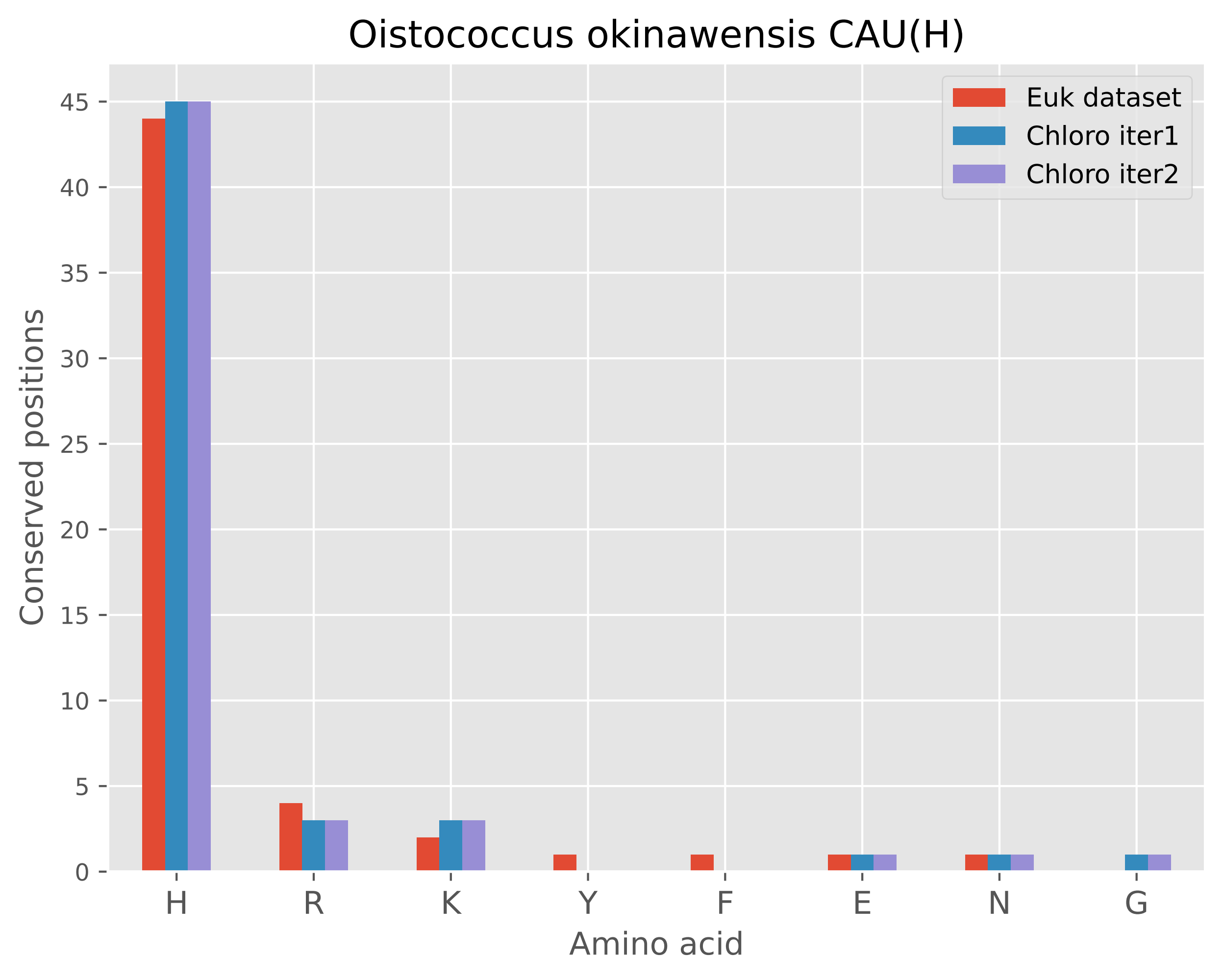
Oistococcus okinawensis CAU(H)
Euk dataset
Chloro iter1
Chloro iter2
Conserved positions
0
5
10
15
20
25
30
35
40
45
H
R
K
Y
F
E
N
G
Amino acid

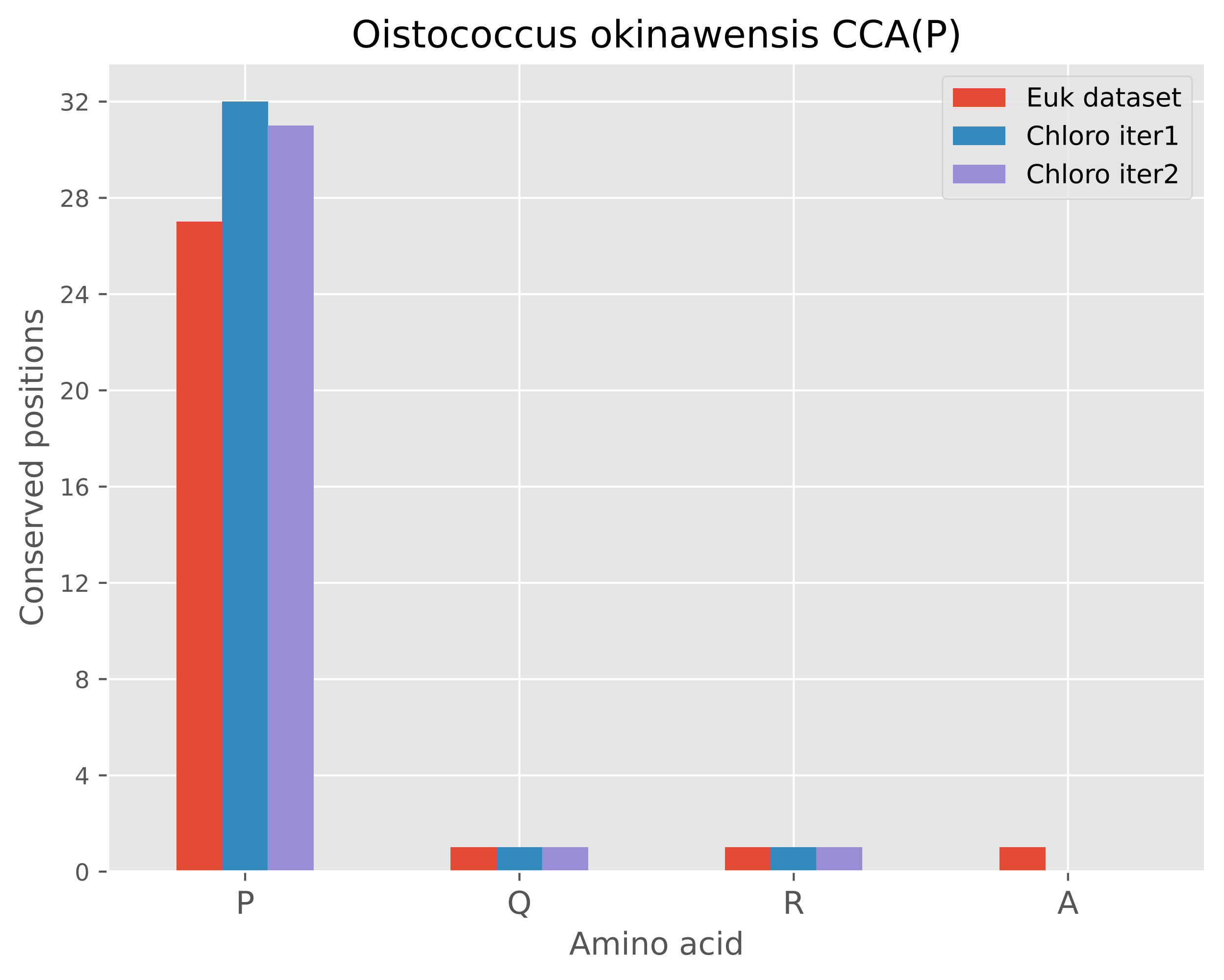

Oistococcus okinawensis CCA(P)
Euk dataset
Chloro iter1
Chloro iter2
Conserved positions
0
4
8
12
16
20
24
28
32
P
Q
R
A
Amino acid

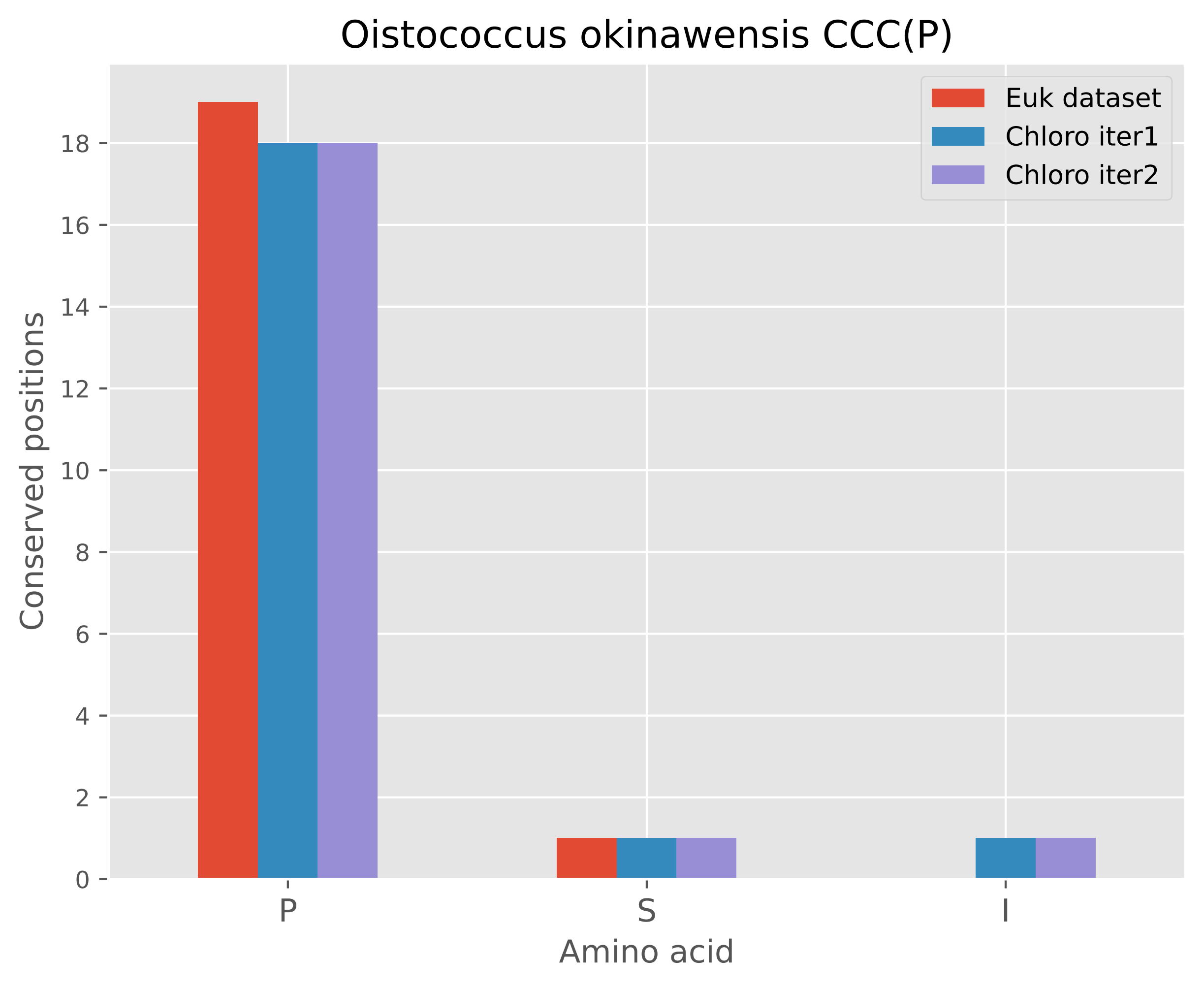

Oistococcus okinawensis CCC(P)
Euk dataset
Chloro iter1
Chloro iter2
Conserved positions
0
2
4
6
8
10
12
14
16
18
P
S
I
Amino acid

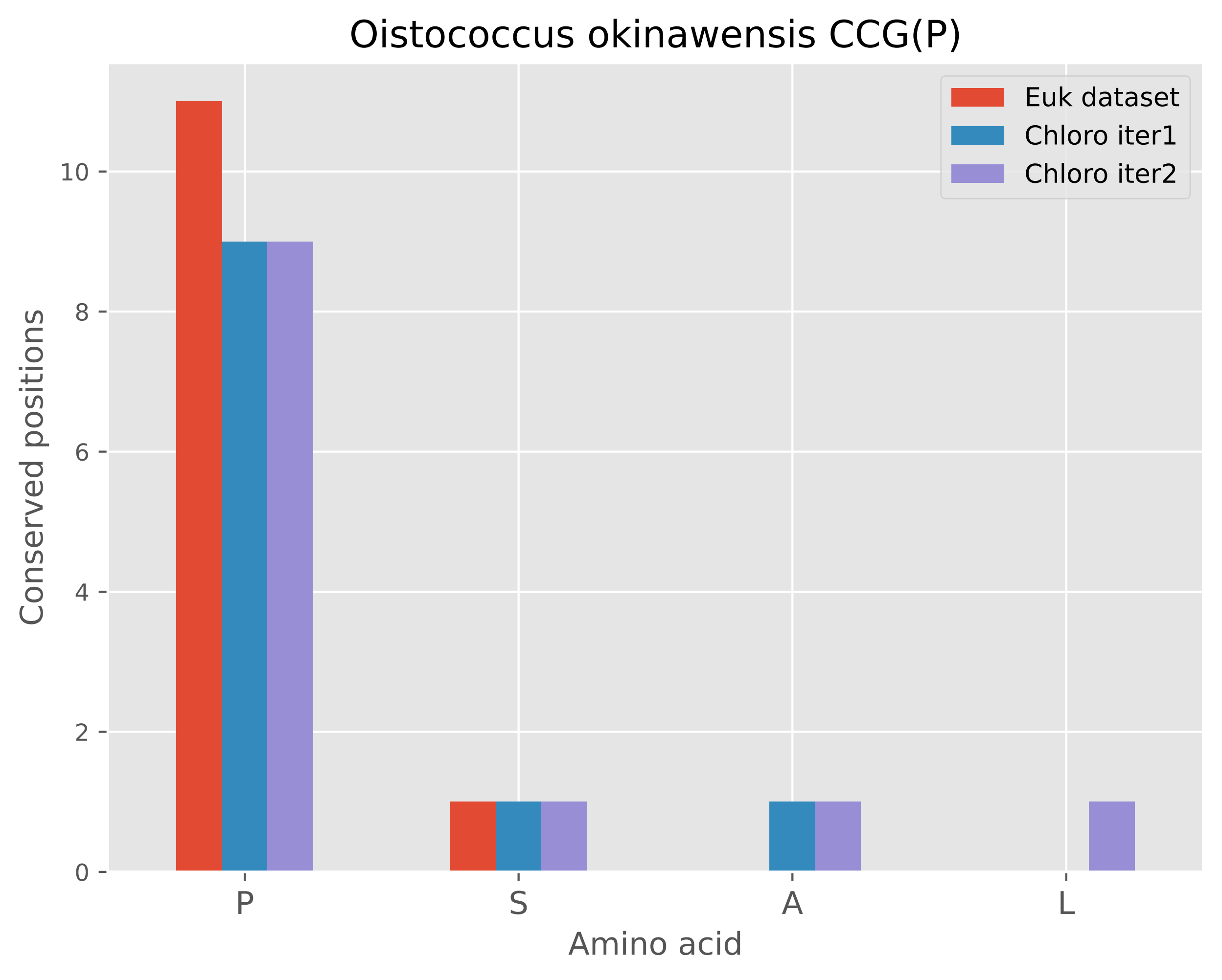
Oistococcus okinawensis CCG(P)
Euk dataset
Chloro iter1
Chloro iter2
Conserved positions
0
2
4
6
8
10
P
S
A
L
Amino acid

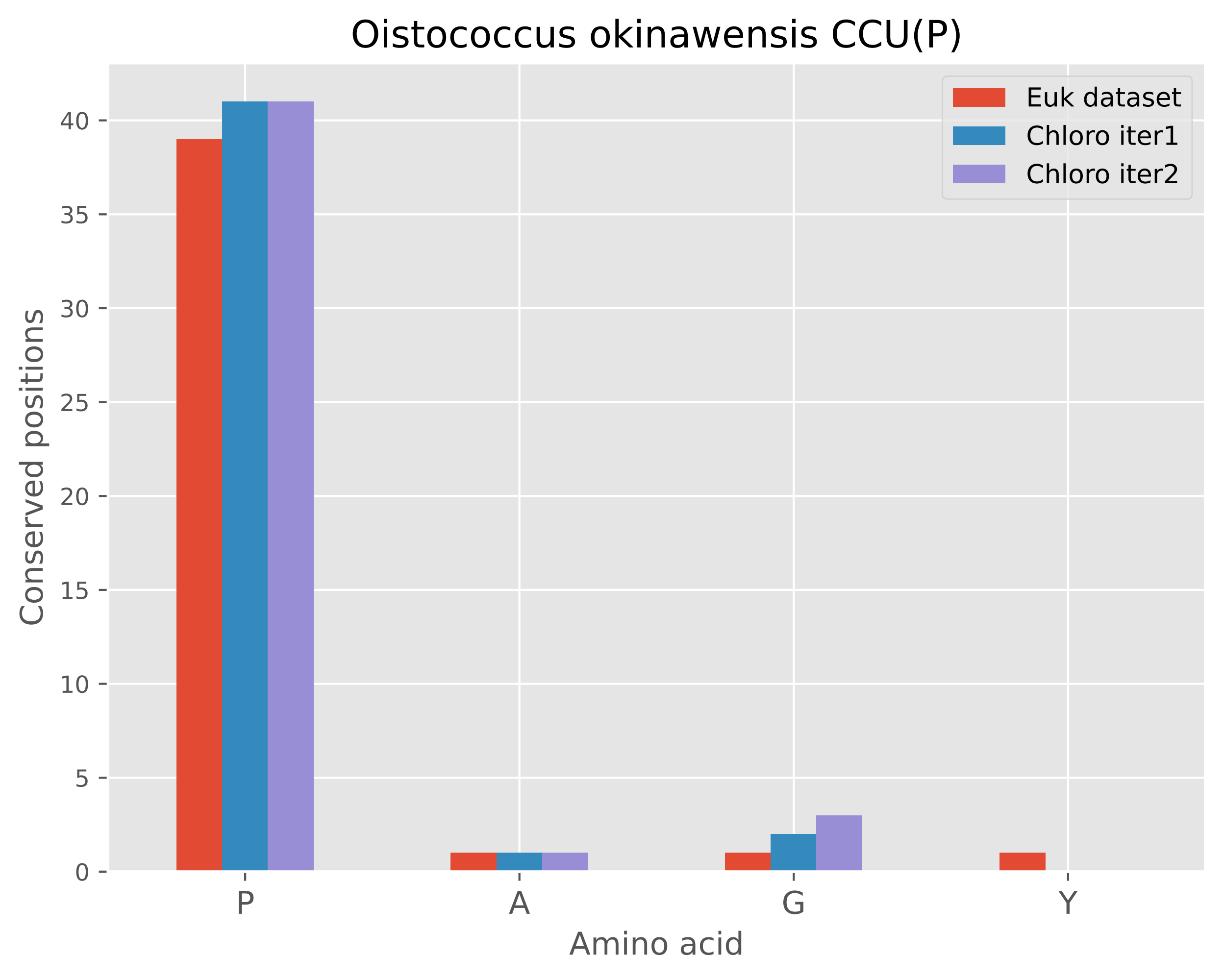
Oistococcus okinawensis CCU(P)
Euk dataset
Chloro iter1
Chloro iter2
Conserved positions
0
5
10
15
20
25
30
35
40
P
A
G
Y
Amino acid

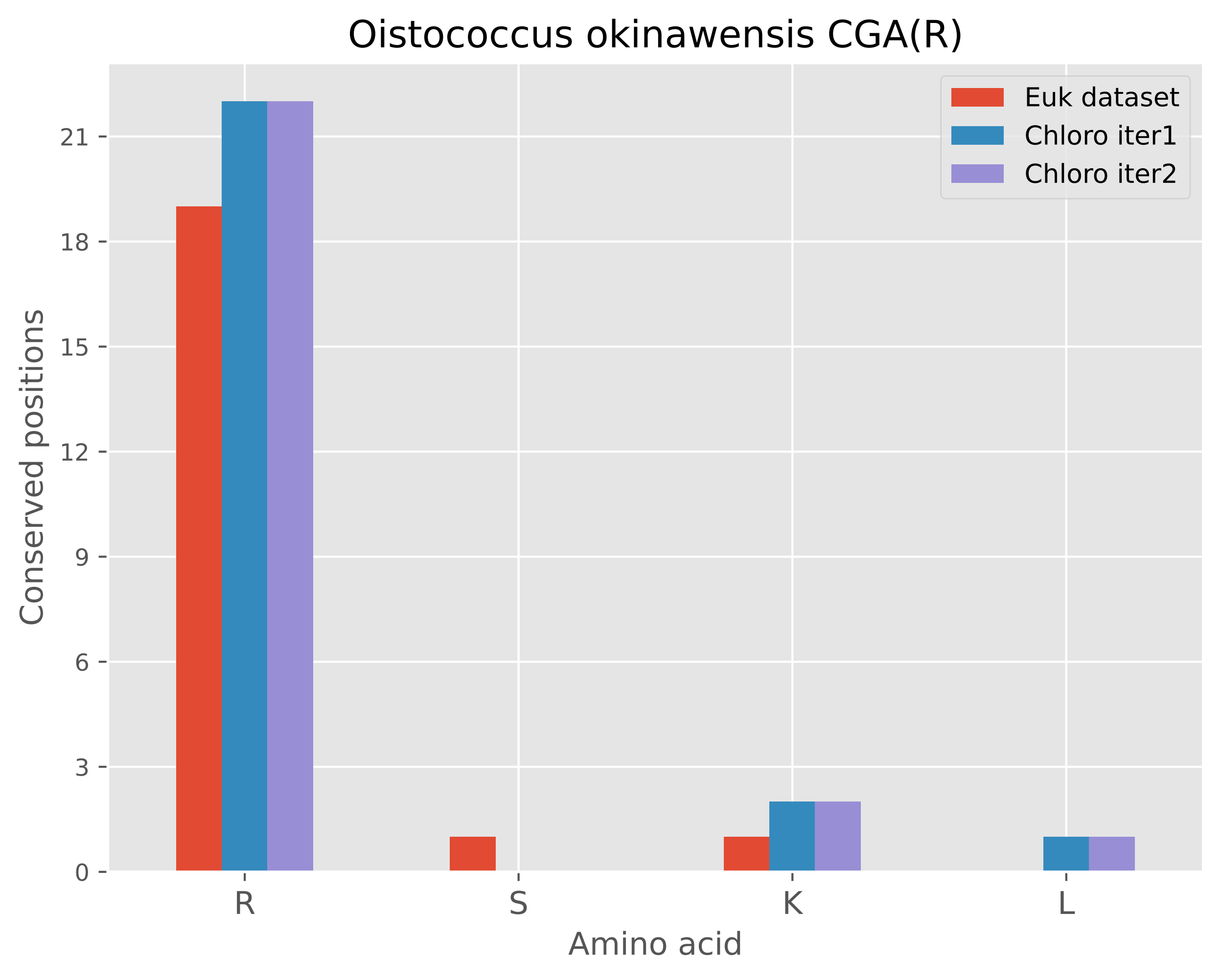

Oistococcus okinawensis CGA(R)
Euk dataset
Chloro iter1
Chloro iter2
Conserved positions
0
3
6
9
12
15
18
21
R
S
K
L
Amino acid

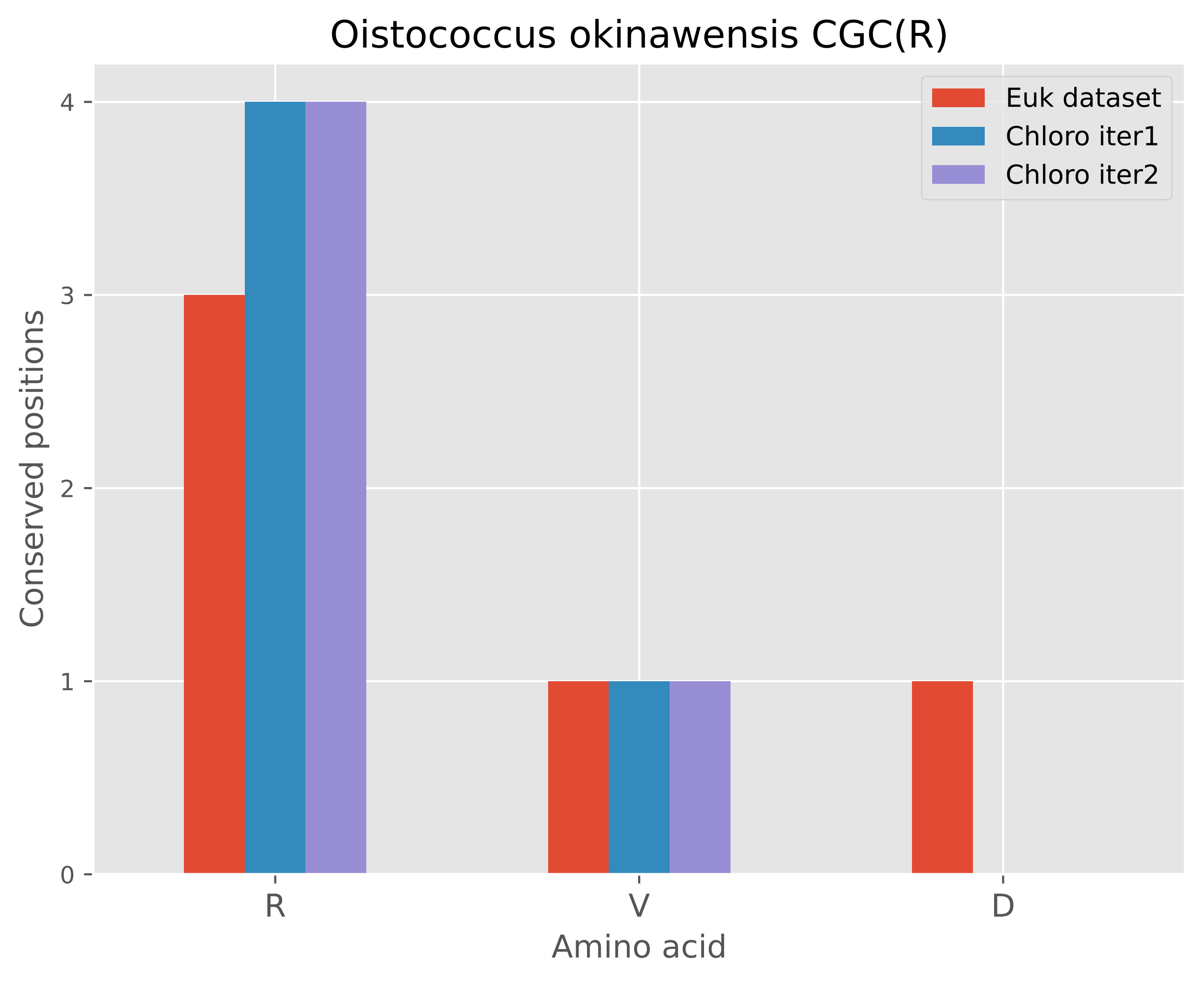
Oistococcus okinawensis CGC(R)
Conserved positions
Amino acid
0
1
2
3
4
R
V
D
Euk dataset
Chloro iter1
Chloro iter2

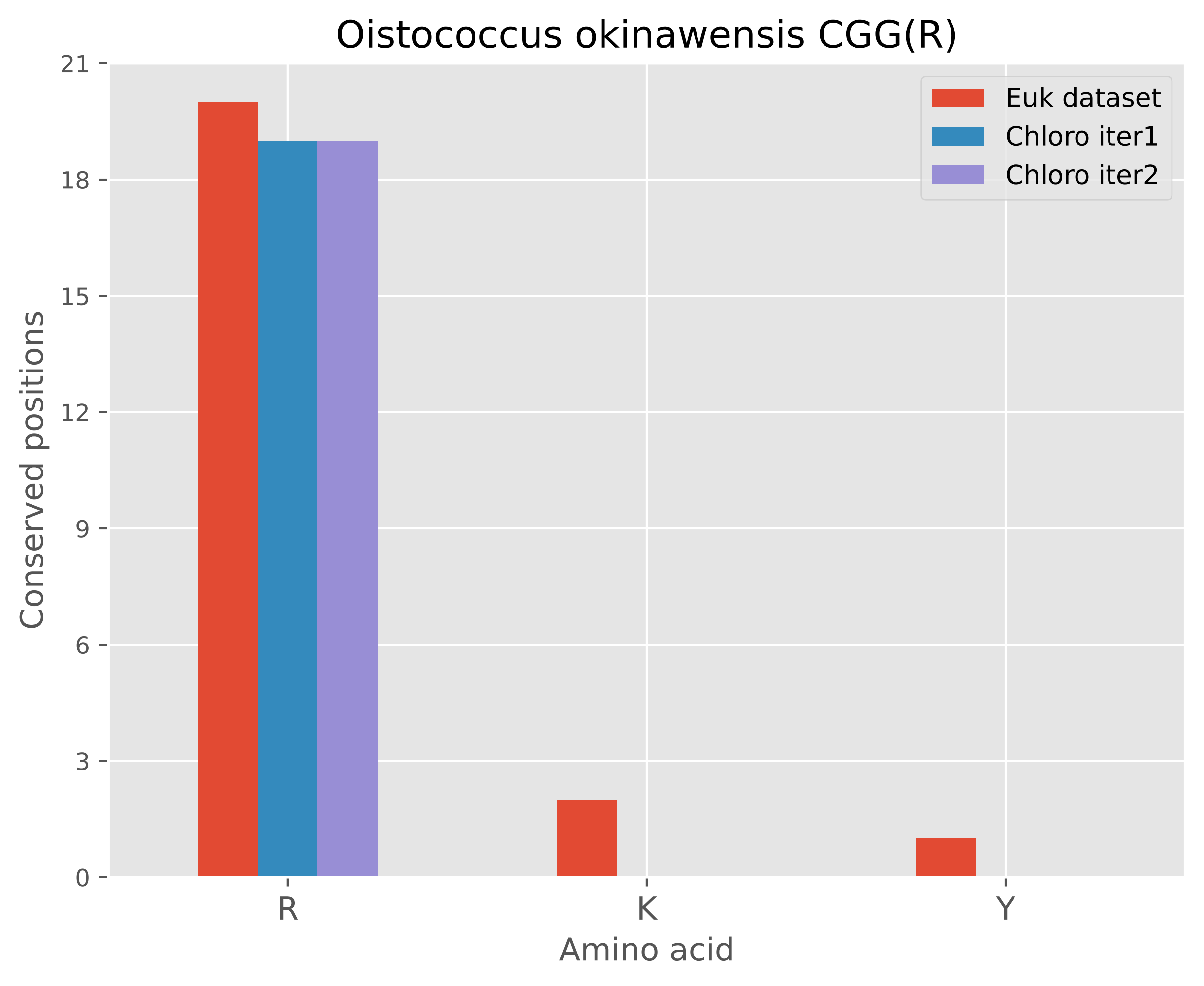
Oistococcus okinawensis CGG(R)
Conserved positions
Amino acid
0
3
6
9
12
15
18
21
R
K
Y
Euk dataset
Chloro iter1
Chloro iter2

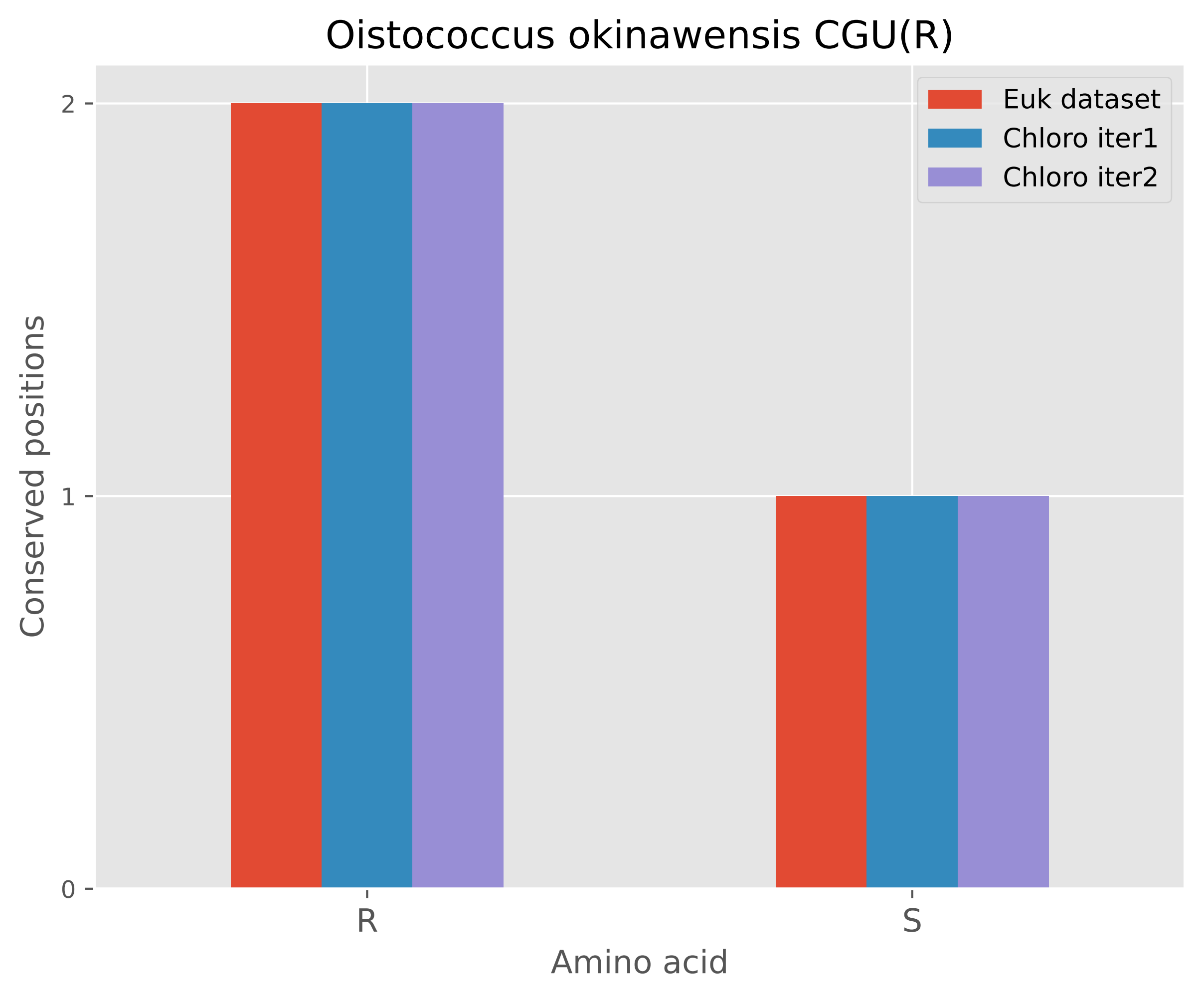
Oistococcus okinawensis CGU(R)
Euk dataset
Chloro iter1
Chloro iter2
Conserved positions
Amino acid
0
1
2
R
S

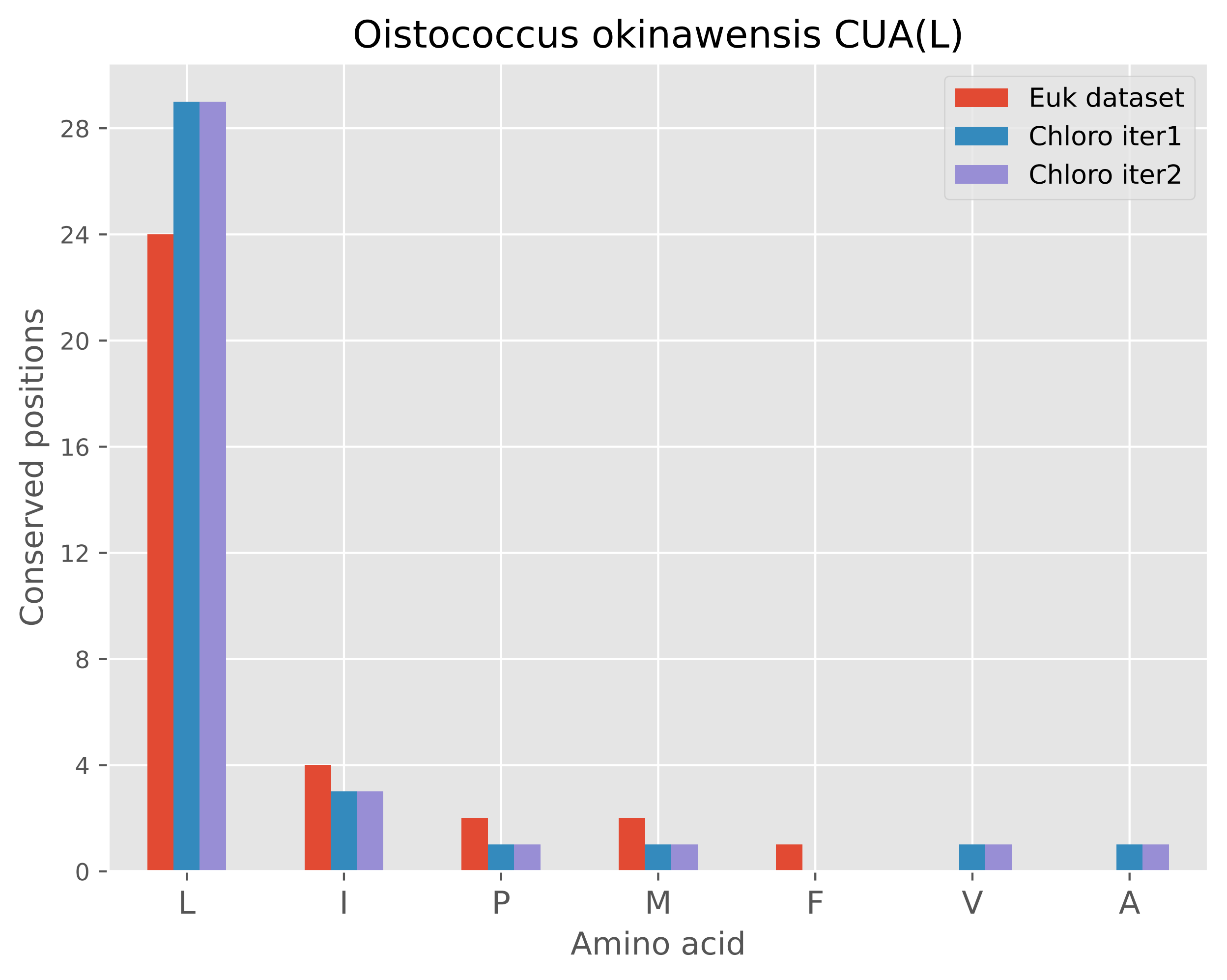

Oistococcus okinawensis CUA(L)
Euk dataset
Chloro iter1
Chloro iter2
Conserved positions
0
4
8
12
16
20
24
28
L
I
P
M
F
V
A
Amino acid

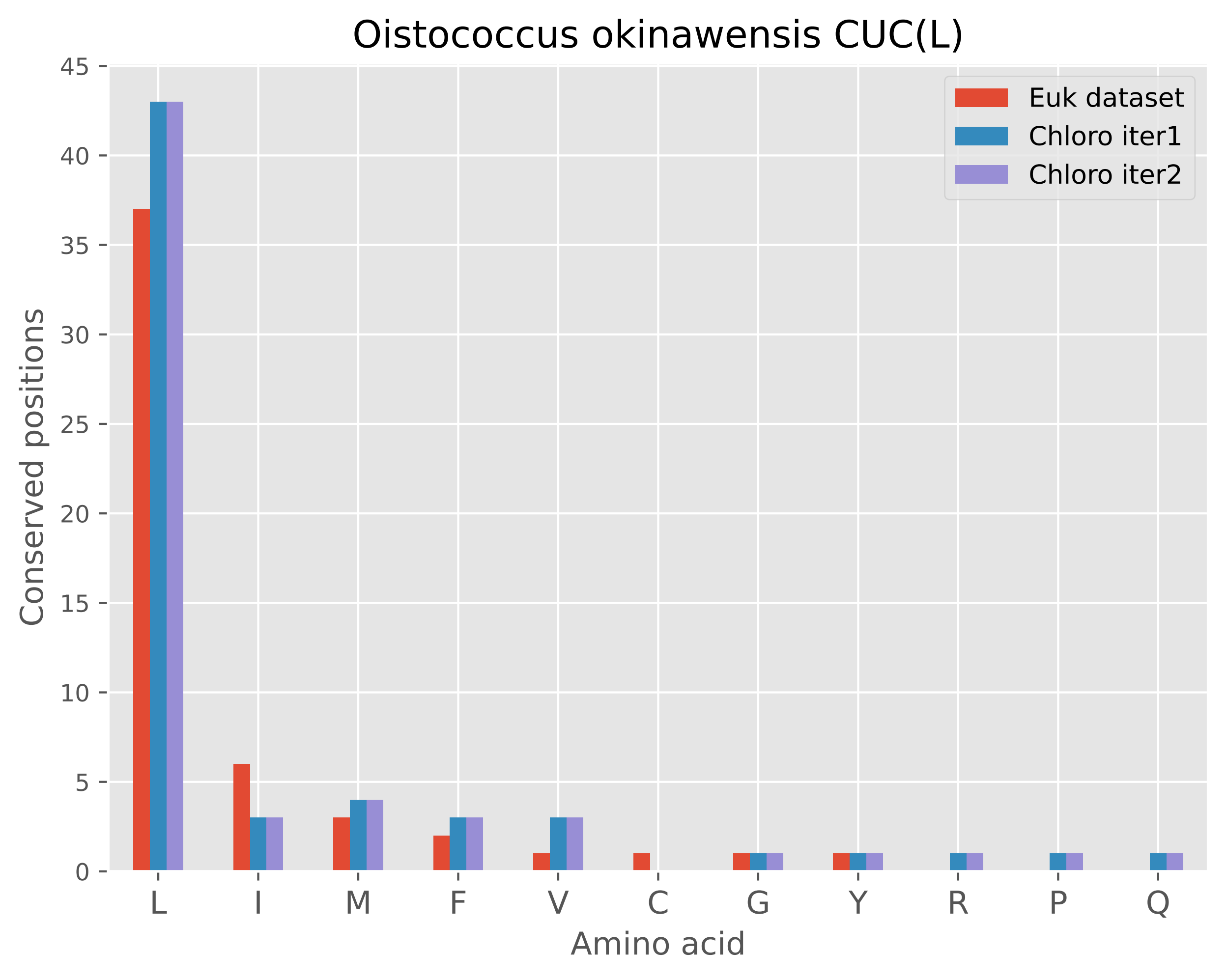

Oistococcus okinawensis CUC(L)
Conserved positions
Amino acid
Euk dataset
Chloro iter1
Chloro iter2
0
5
10
15
20
25
30
35
40
45
L
I
M
F
V
C
G
Y
R
P
Q

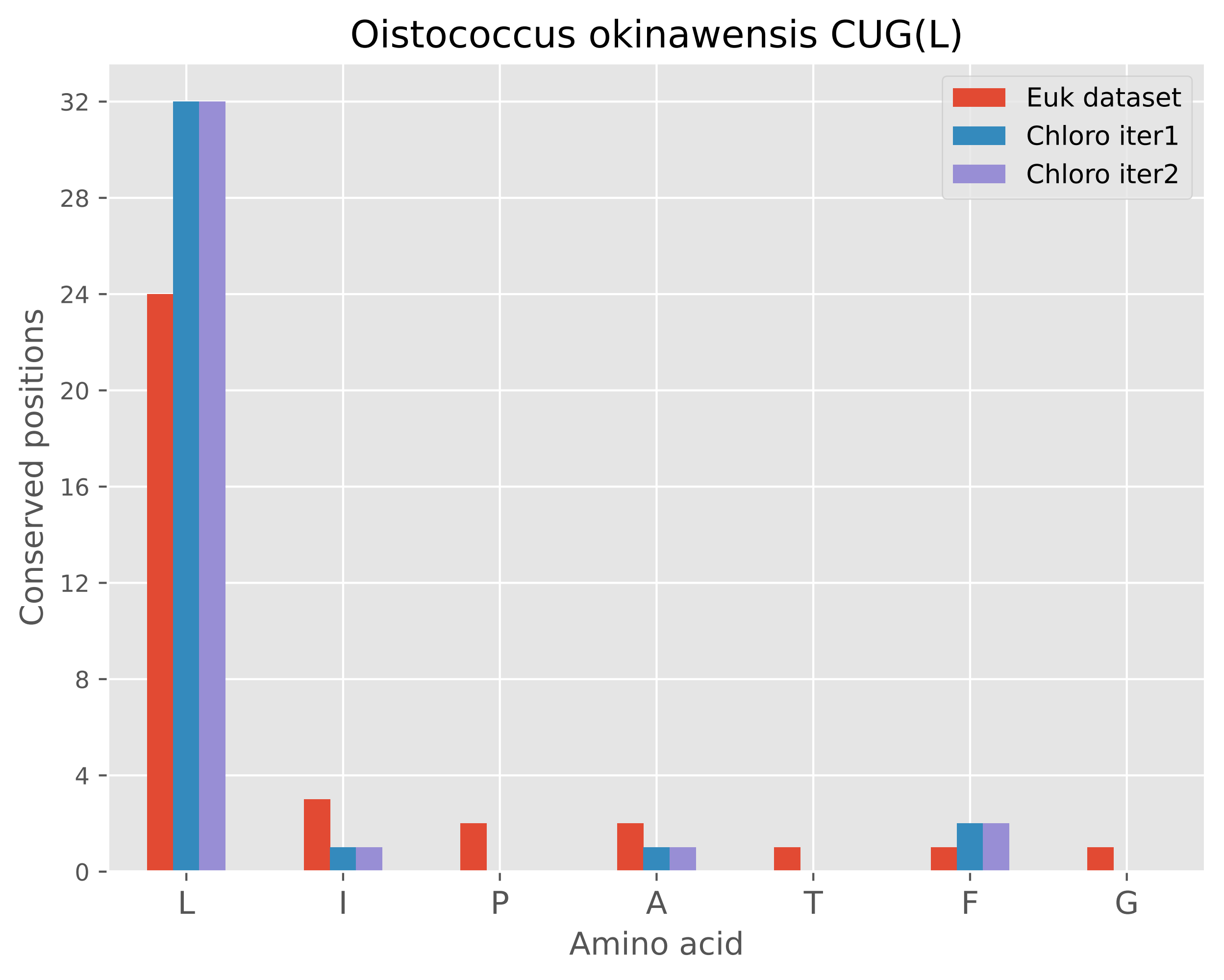
Oistococcus okinawensis CUG(L)
Euk dataset
Chloro iter1
Chloro iter2
Conserved positions
0
4
8
12
16
20
24
28
32
L
I
P
A
T
F
G
Amino acid

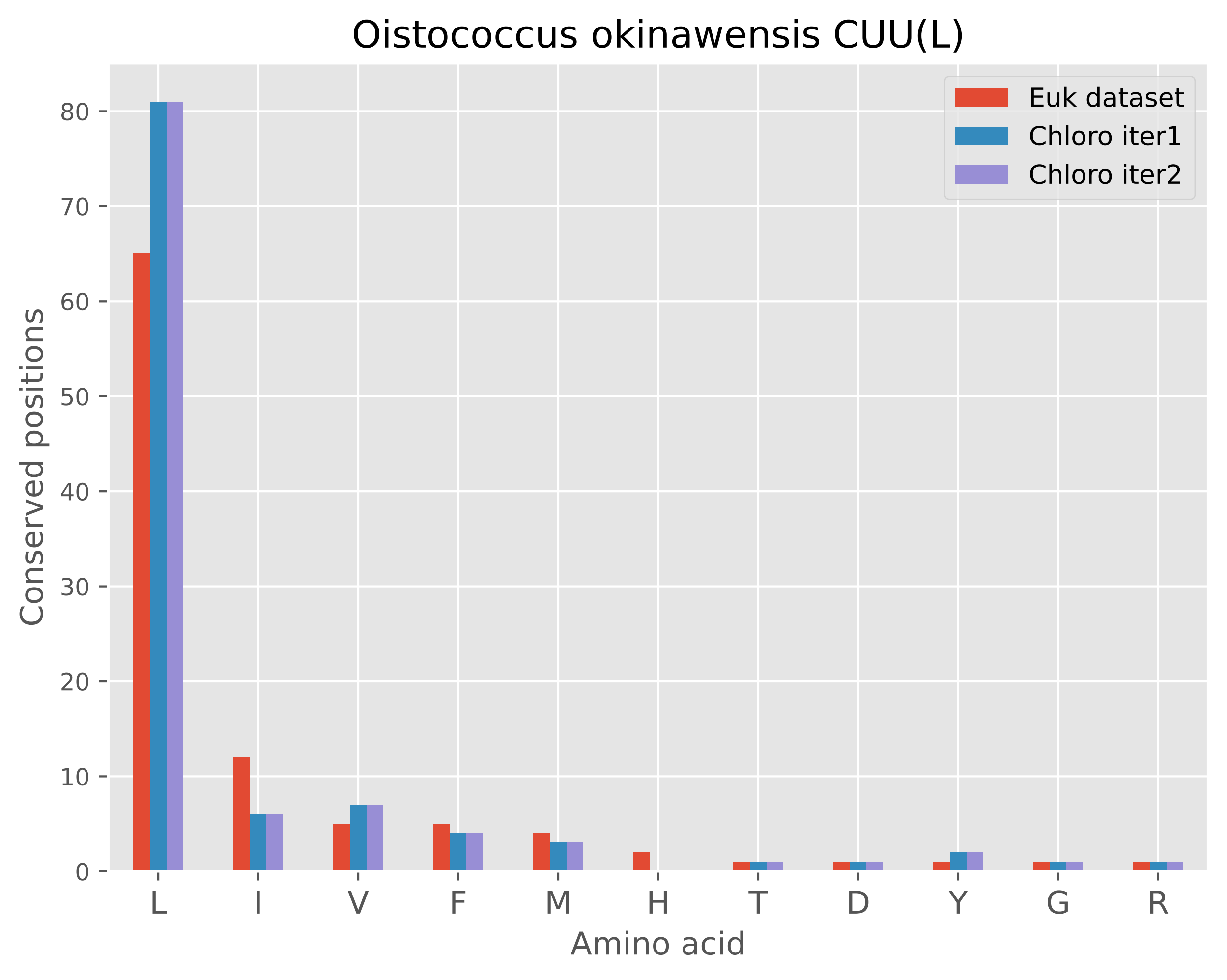

Oistococcus okinawensis CUU(L)
Euk dataset
Chloro iter1
Chloro iter2
Conserved positions
0
10
20
30
40
50
60
70
80
L
I
V
F
M
H
T
D
Y
G
R
Amino acid

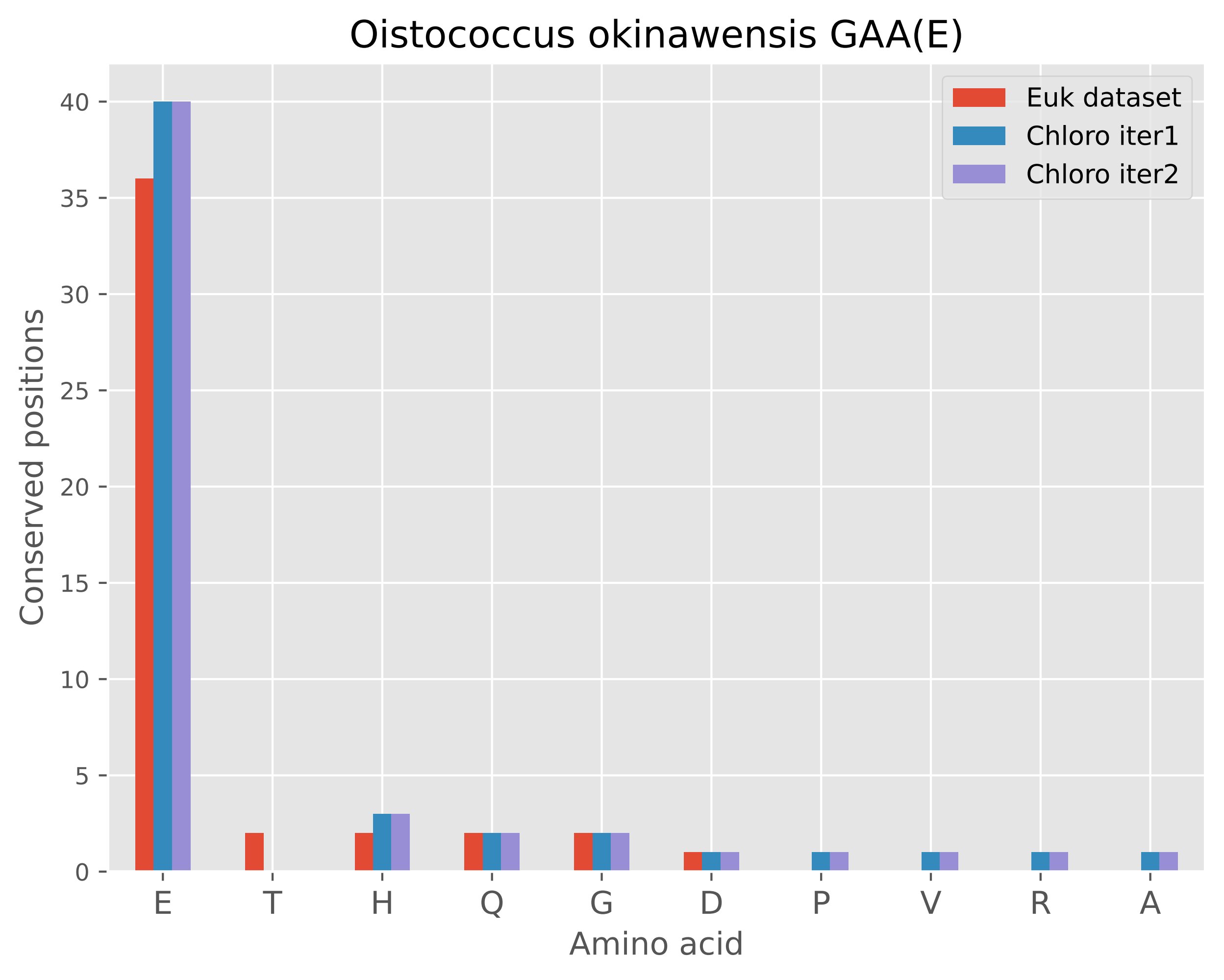

Oistococcus okinawensis GAA(E)
Euk dataset
Chloro iter1
Chloro iter2
Conserved positions
0
5
10
15
20
25
30
35
40
E
T
H
Q
G
D
P
V
R
A
Amino acid

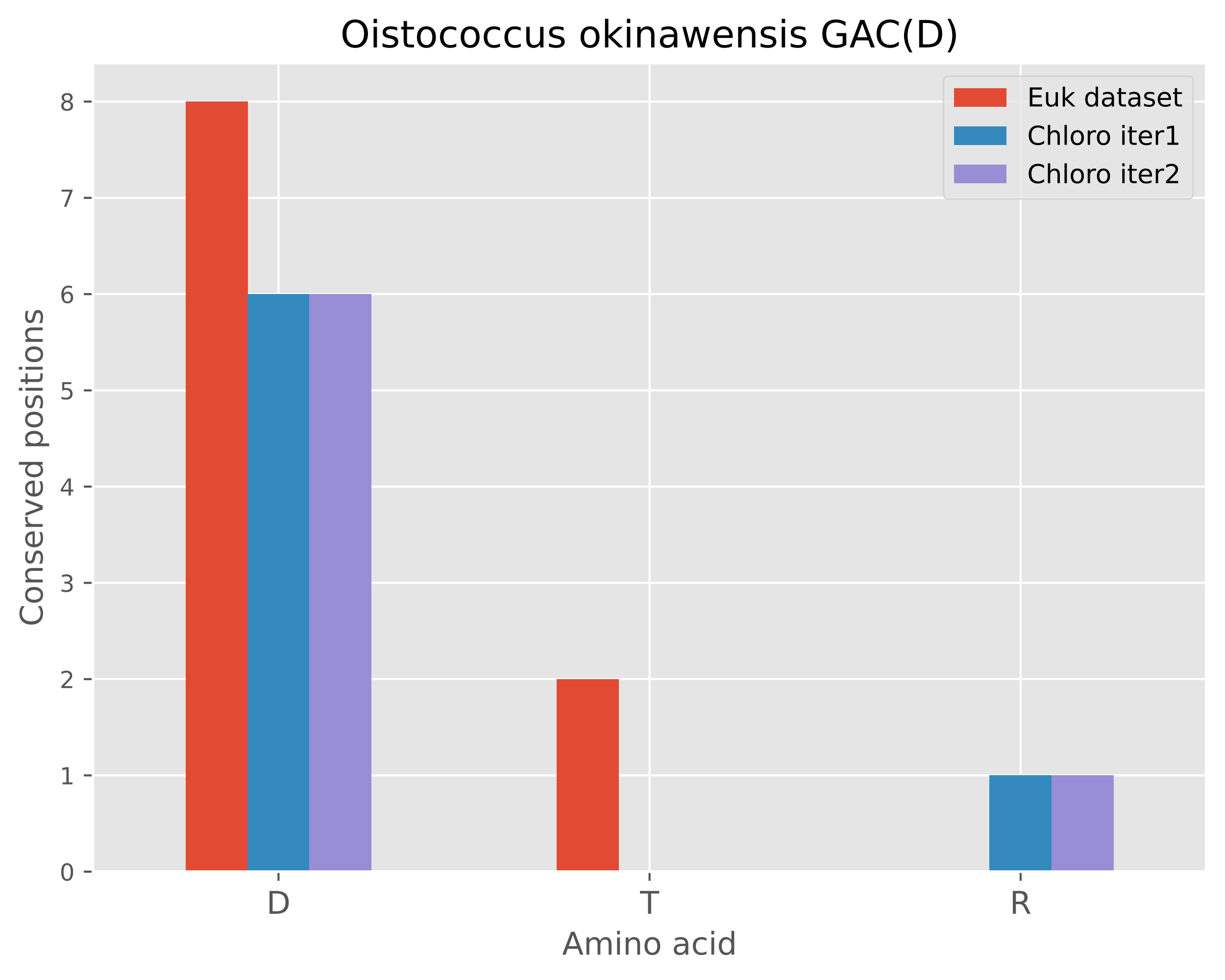
Oistococcus okinawensis GAC(D)
Euk dataset
Chloro iter1
Chloro iter2
Conserved positions
0
1
2
3
4
5
6
7
8
D
T
R
Amino acid

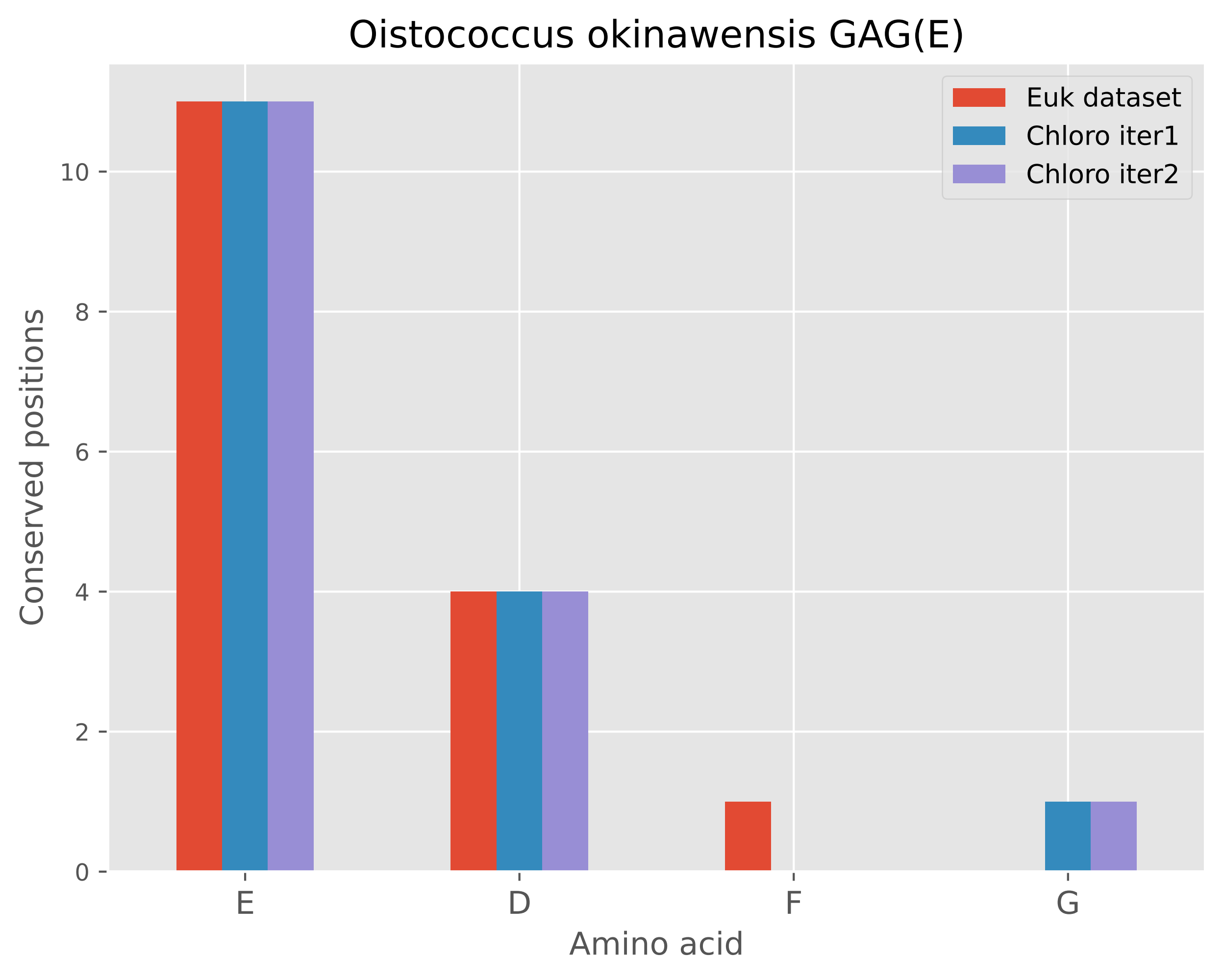

Oistococcus okinawensis GAG(E)
Euk dataset
Chloro iter1
Chloro iter2
Conserved positions
0
2
4
6
8
10
E
D
F
G
Amino acid

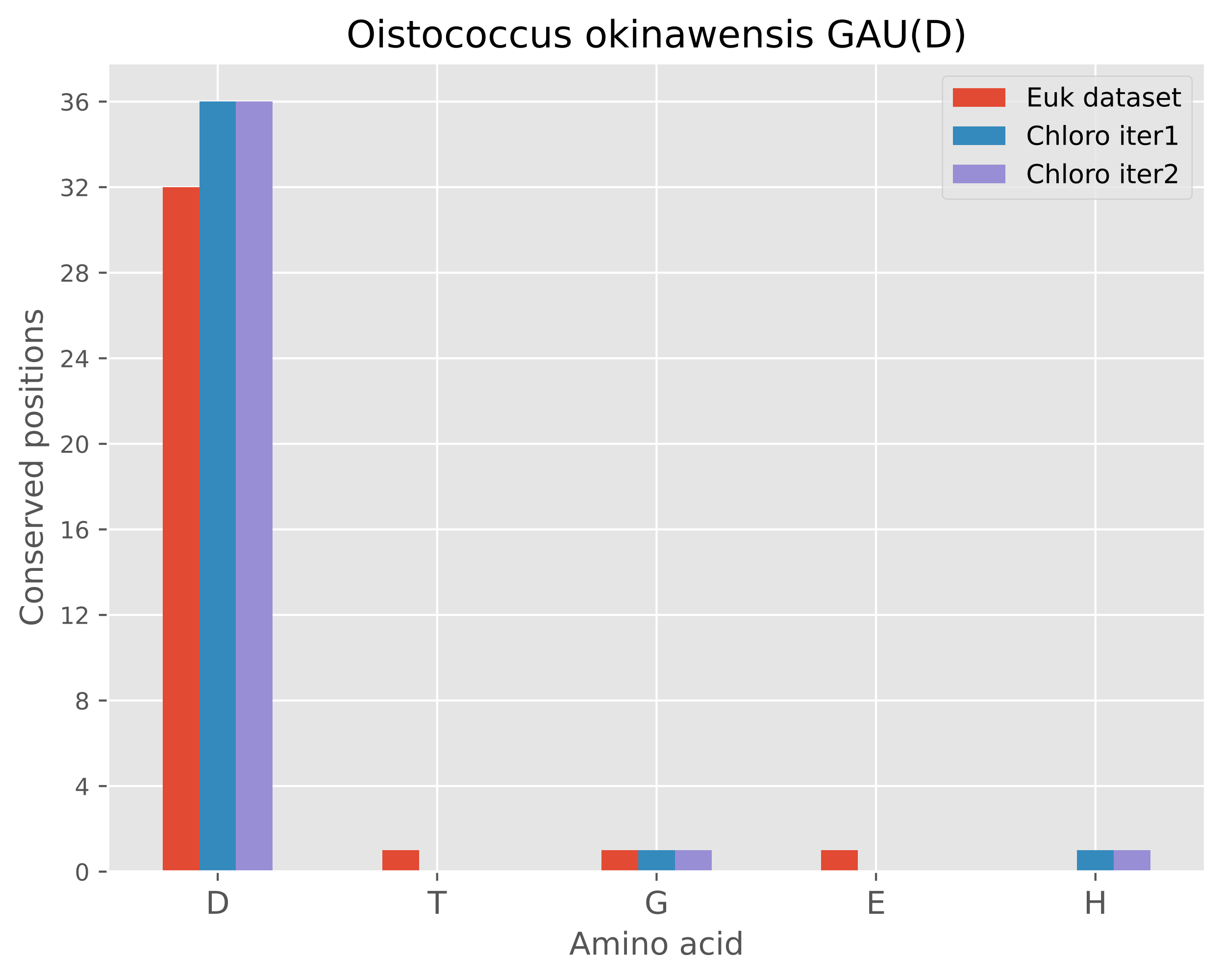
Oistococcus okinawensis GAU(D)
Euk dataset
Chloro iter1
Chloro iter2
Conserved positions
0
4
8
12
16
20
24
28
32
36
D
T
G
E
H
Amino acid

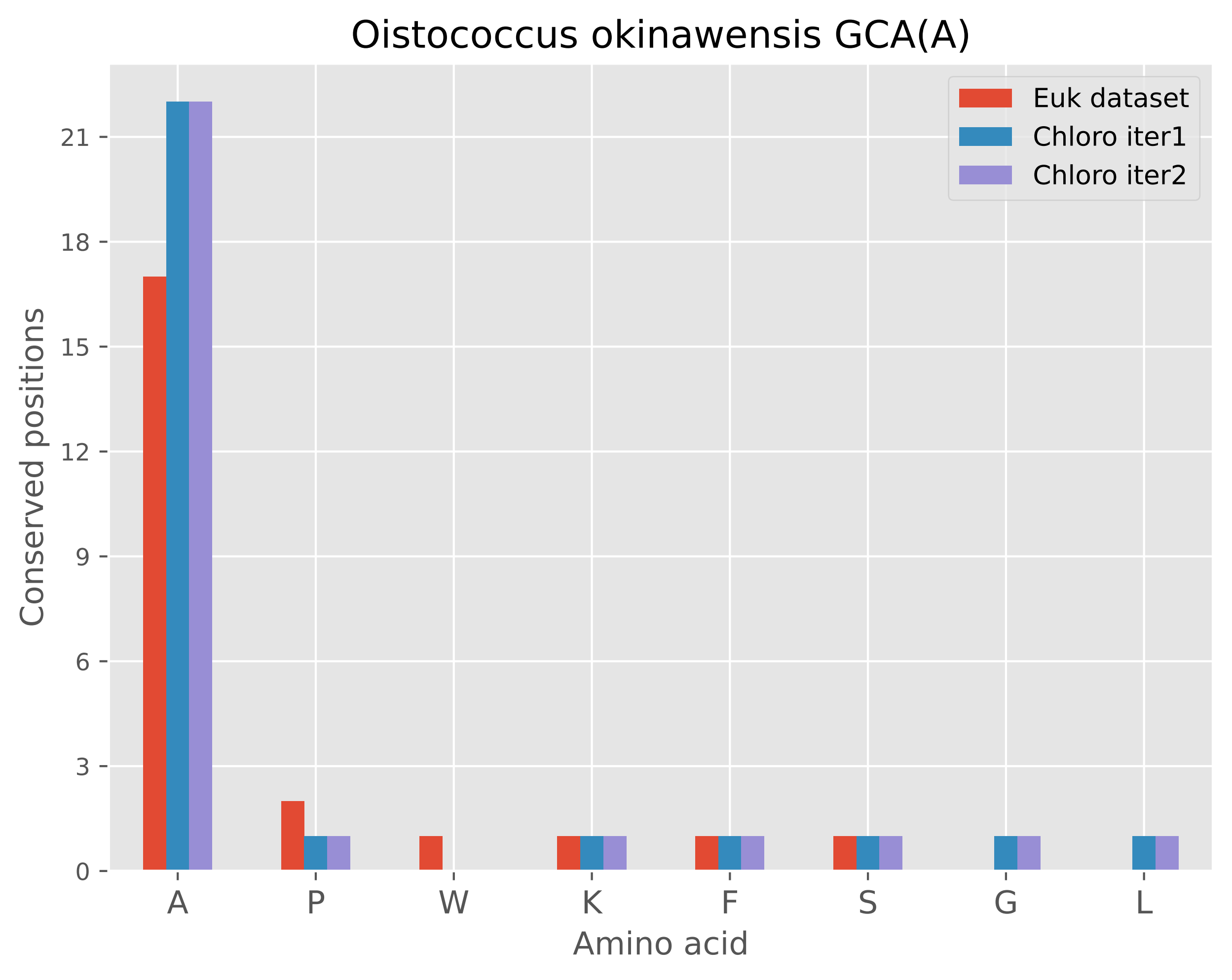
Oistococcus okinawensis GCA(A)
Euk dataset
Chloro iter1
Chloro iter2
Conserved positions
0
3
6
9
12
15
18
21
A
P
W
K
F
S
G
L
Amino acid

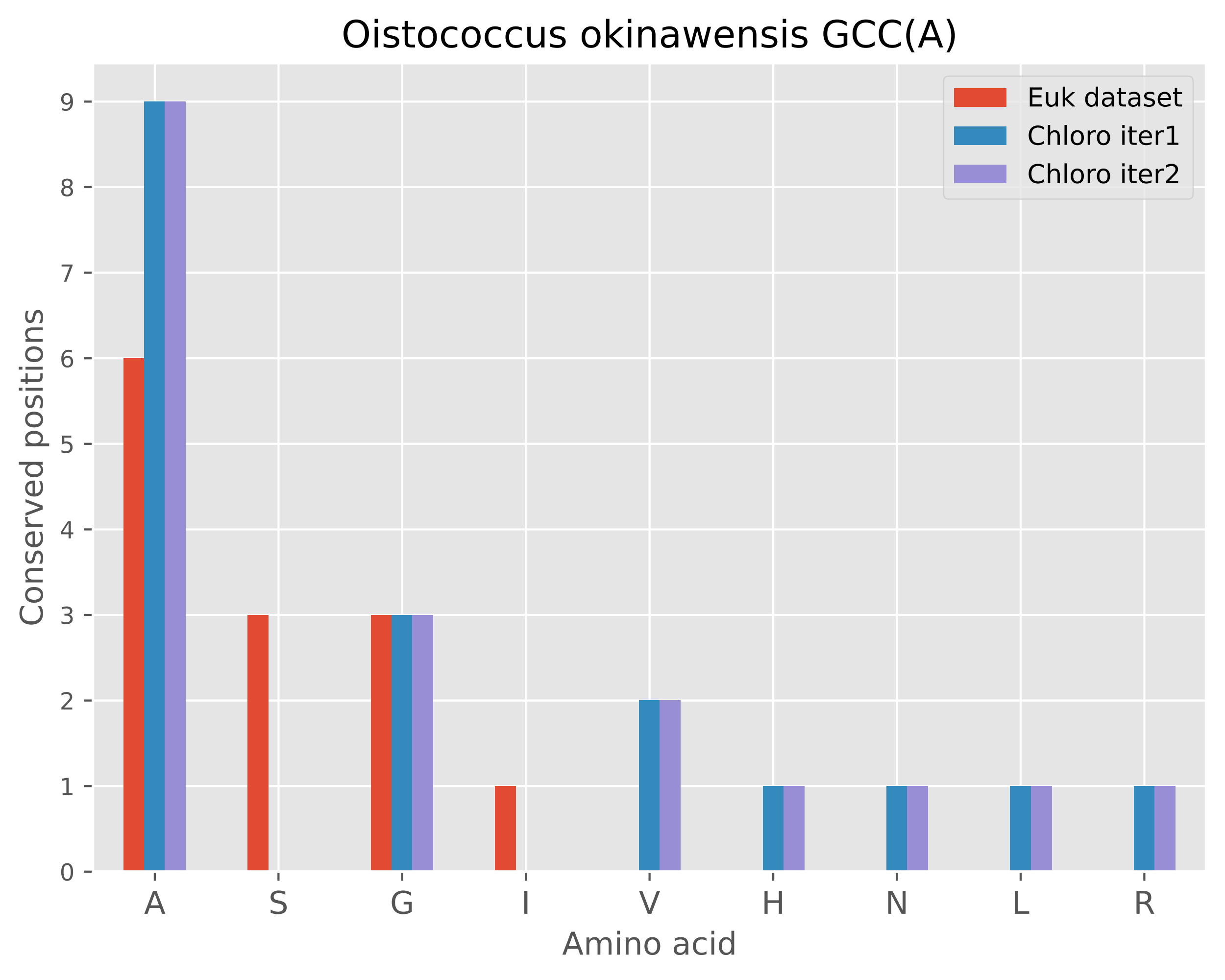

Oistococcus okinawensis GCC(A)
Euk dataset
Chloro iter1
Chloro iter2
Conserved positions
0
1
2
3
4
5
6
7
8
9
A
S
G
I
V
H
N
L
R
Amino acid

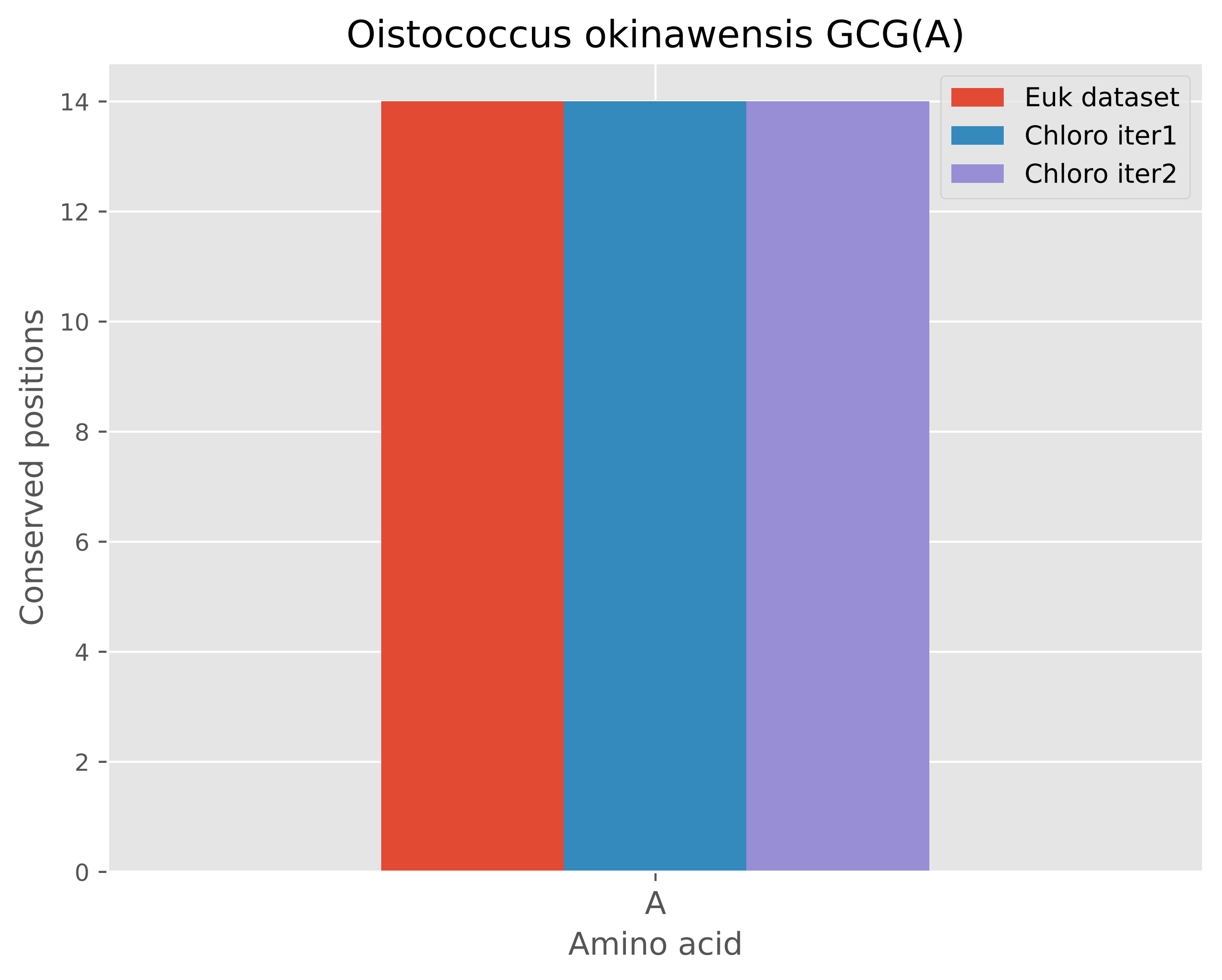
Oistococcus okinawensis GCG(A)
Euk dataset
Chloro iter1
Chloro iter2
Conserved positions
0
2
4
6
8
10
12
14
A
Amino acid

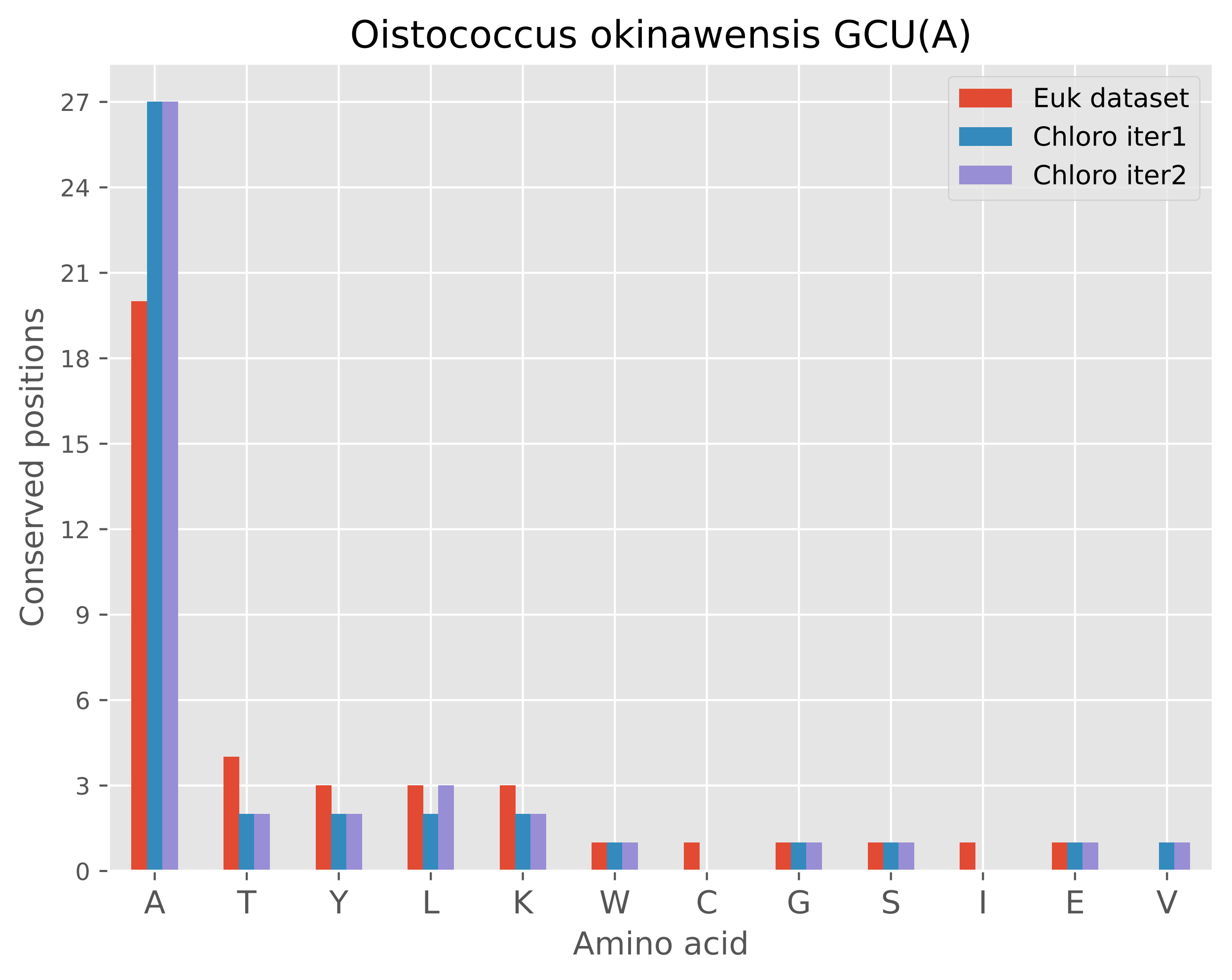

Oistococcus okinawensis GCU(A)
Euk dataset
Chloro iter1
Chloro iter2
Conserved positions
0
3
6
9
12
15
18
21
24
27
A
T
Y
L
K
W
C
G
S
I
E
V
Amino acid

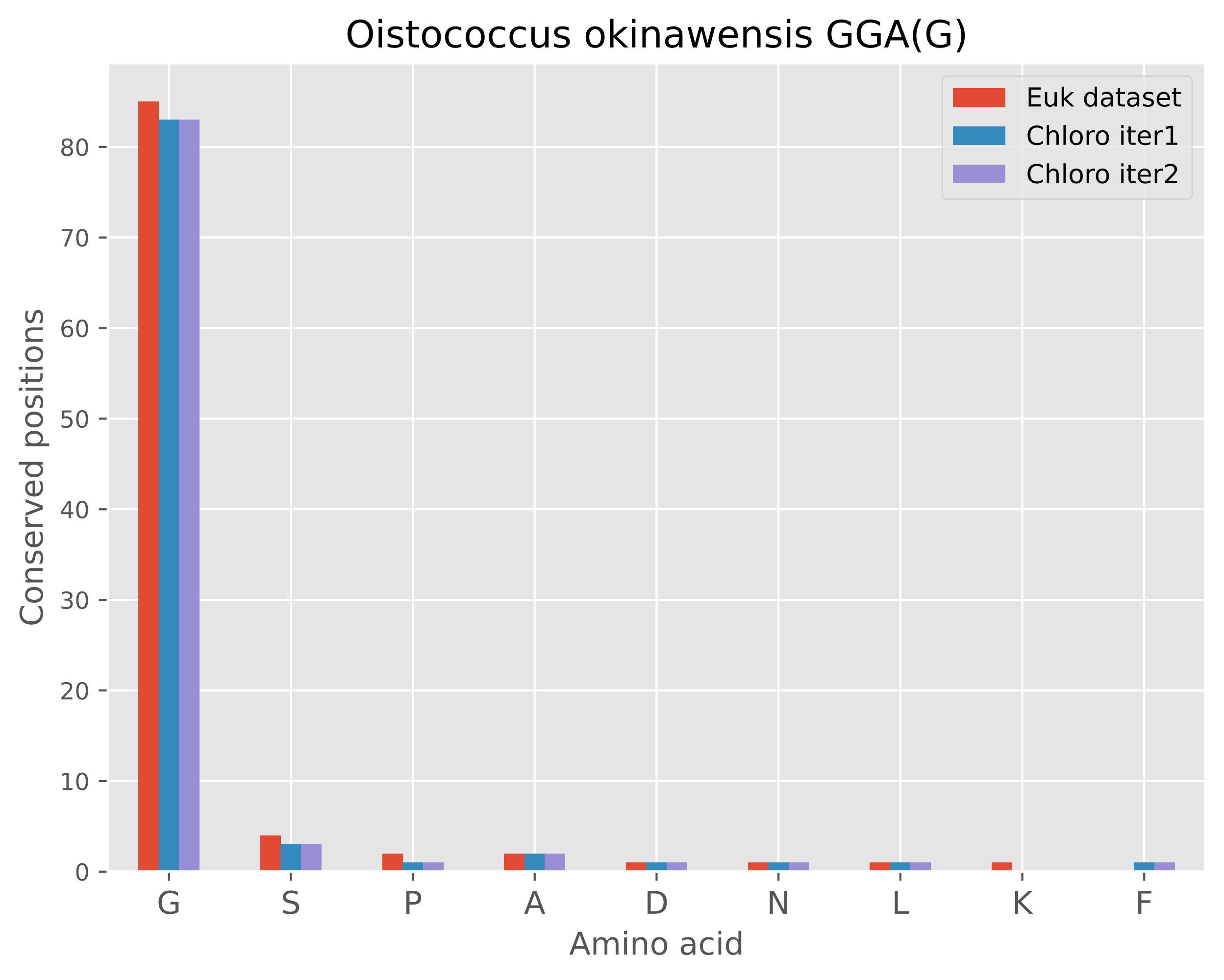

Oistococcus okinawensis GGA(G)
Euk dataset
Chloro iter1
Chloro iter2
Conserved positions
0
10
20
30
40
50
60
70
80
G
S
P
A
D
N
L
K
F
Amino acid

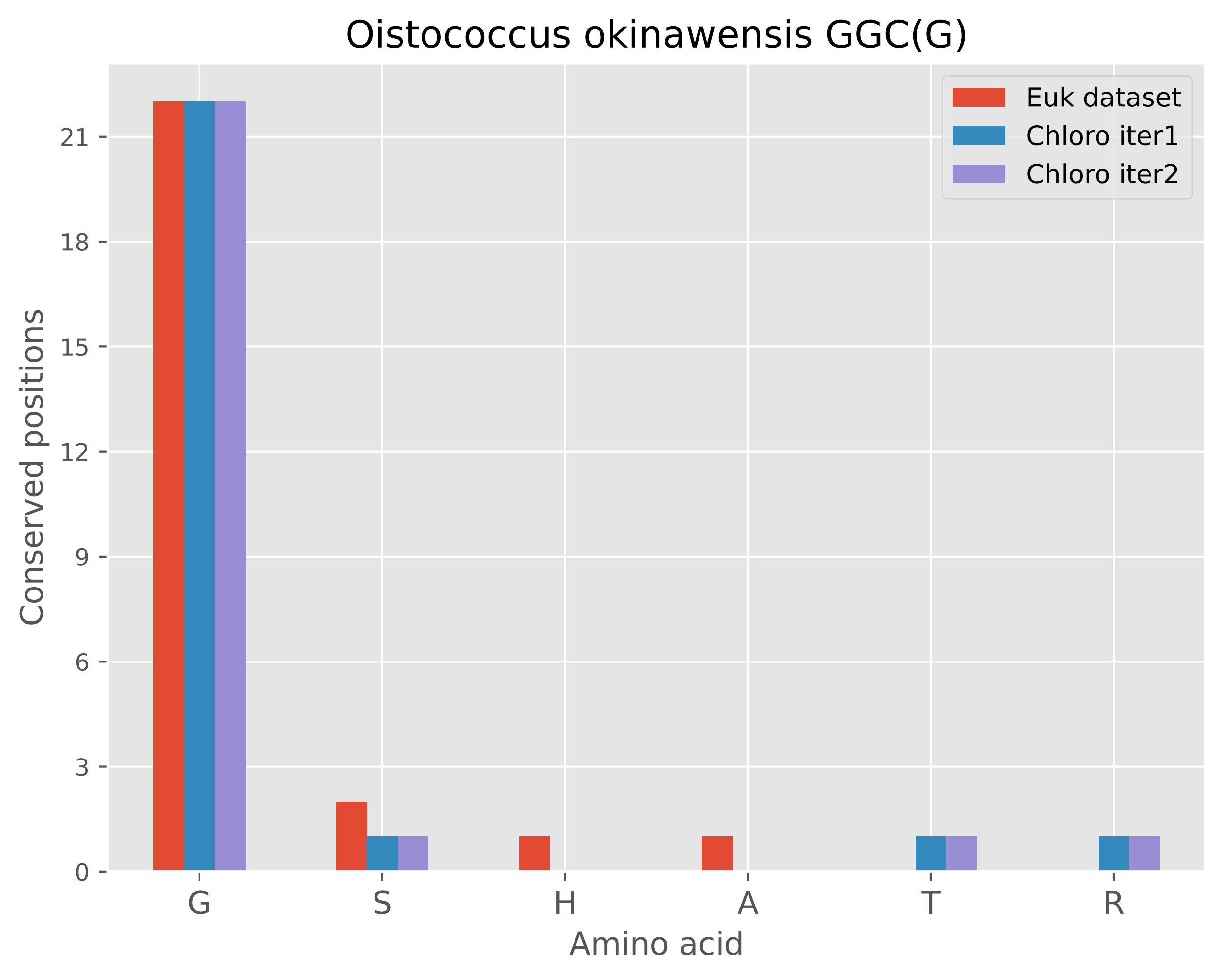

Oistococcus okinawensis GGC(G)
Euk dataset
Chloro iter1
Chloro iter2
Conserved positions
0
3
6
9
12
15
18
21
G
S
H
A
T
R
Amino acid

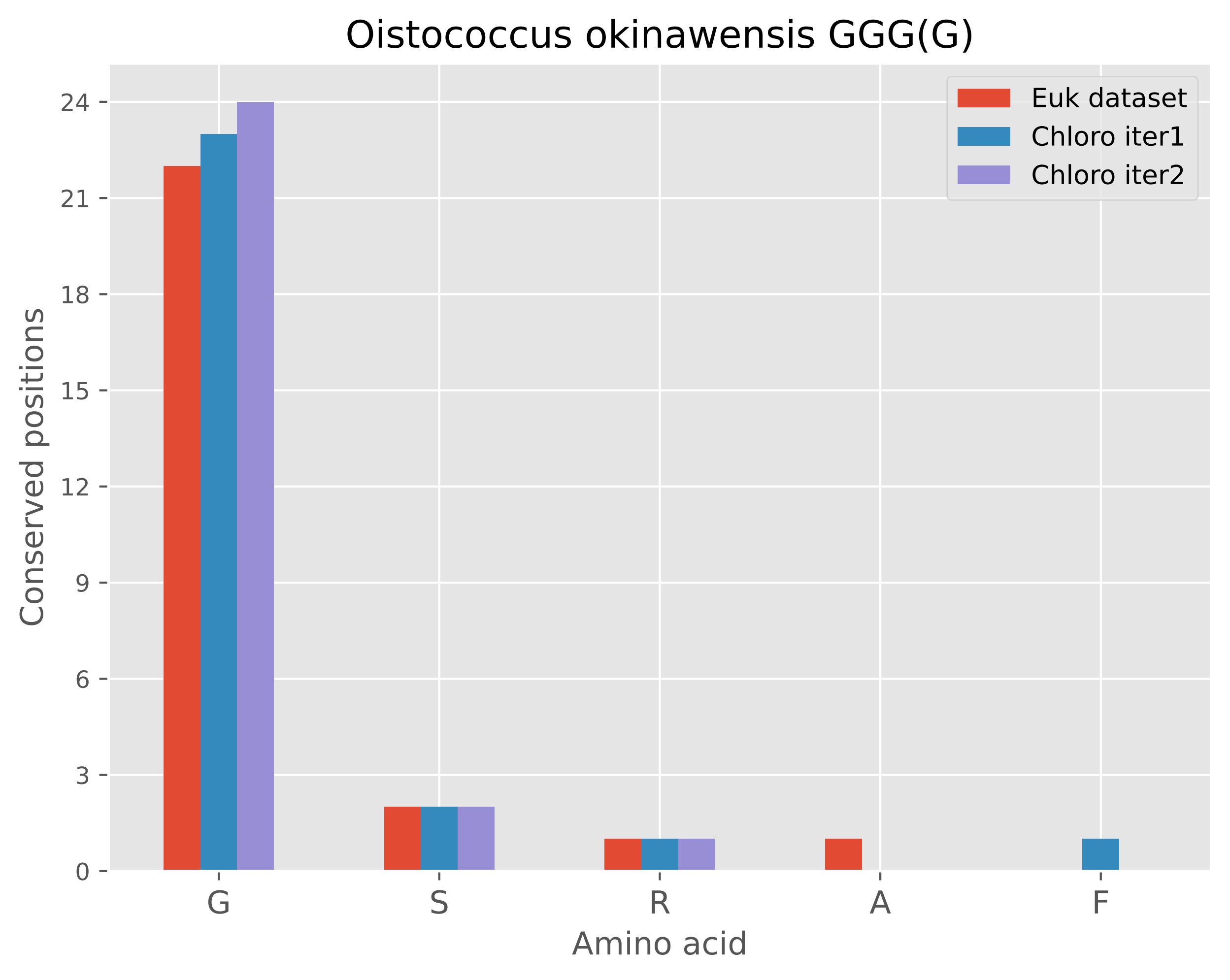
Oistococcus okinawensis GGG(G)
Euk dataset
Chloro iter1
Chloro iter2
Conserved positions
0
3
6
9
12
15
18
21
24
G
S
R
A
F
Amino acid

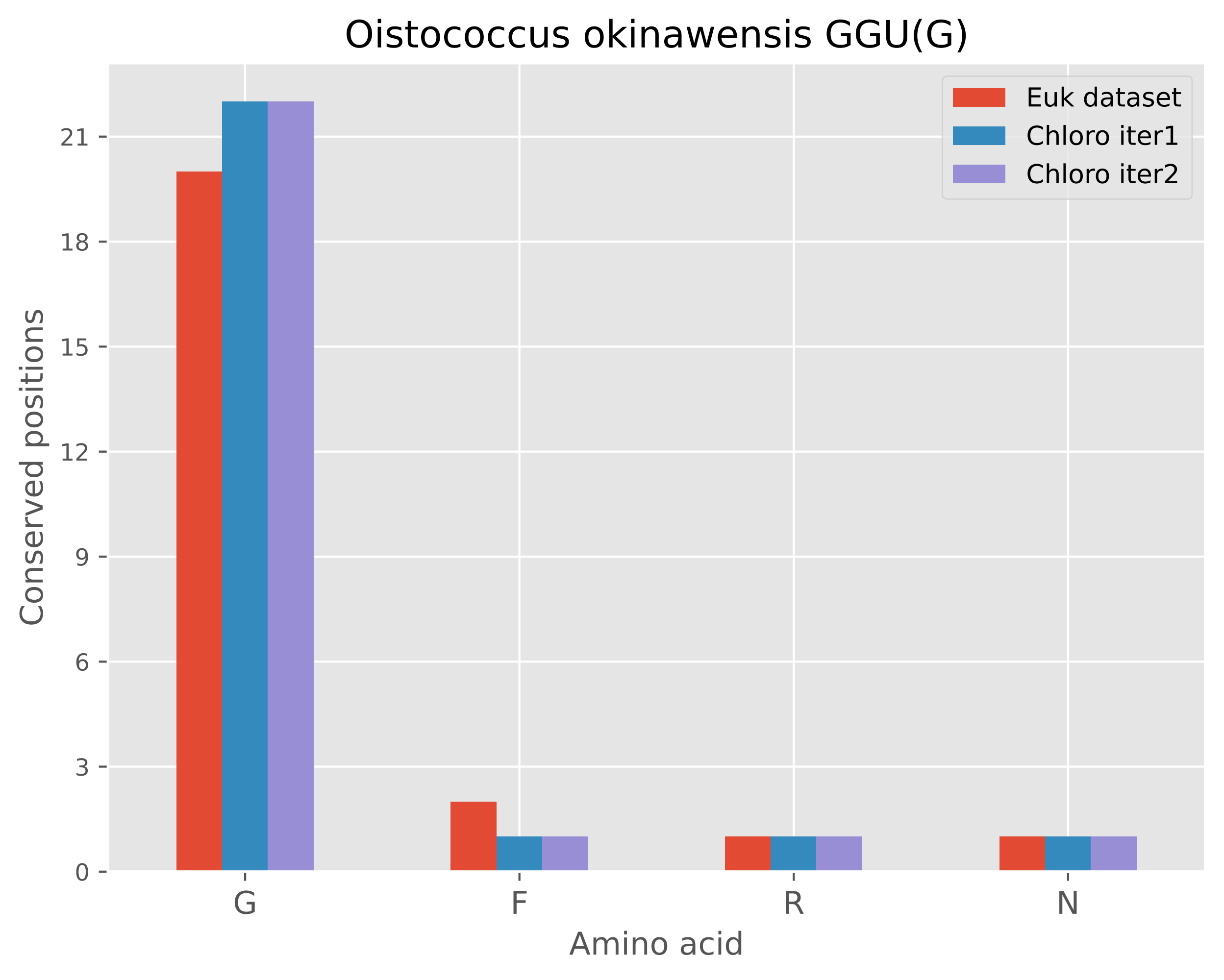

Oistococcus okinawensis GGU(G)
Euk dataset
Chloro iter1
Chloro iter2
Conserved positions
0
3
6
9
12
15
18
21
G
F
R
N
Amino acid

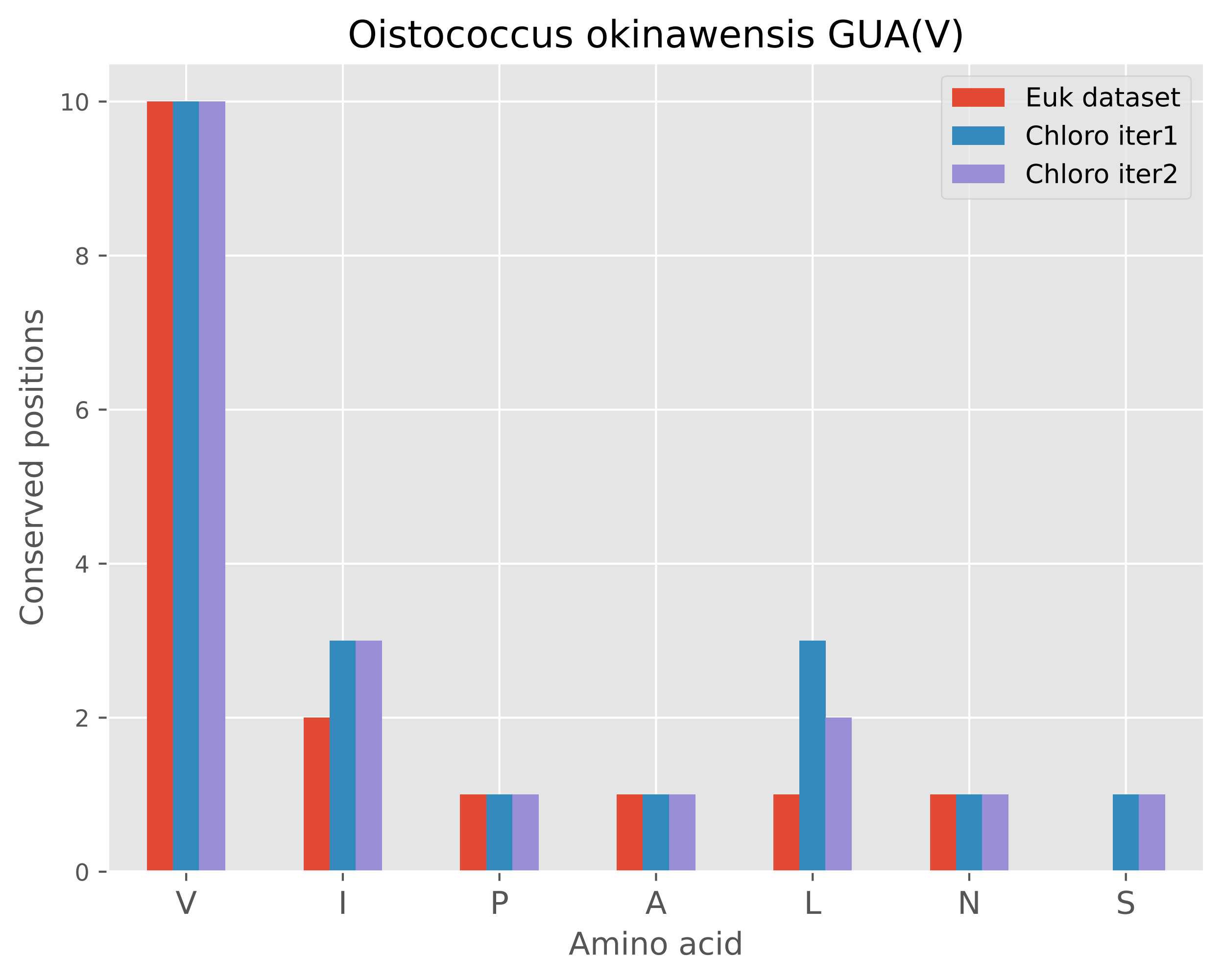
Oistococcus okinawensis GUA(V)
Euk dataset
Chloro iter1
Chloro iter2
Conserved positions
0
2
4
6
8
10
V
I
P
A
L
N
S
Amino acid

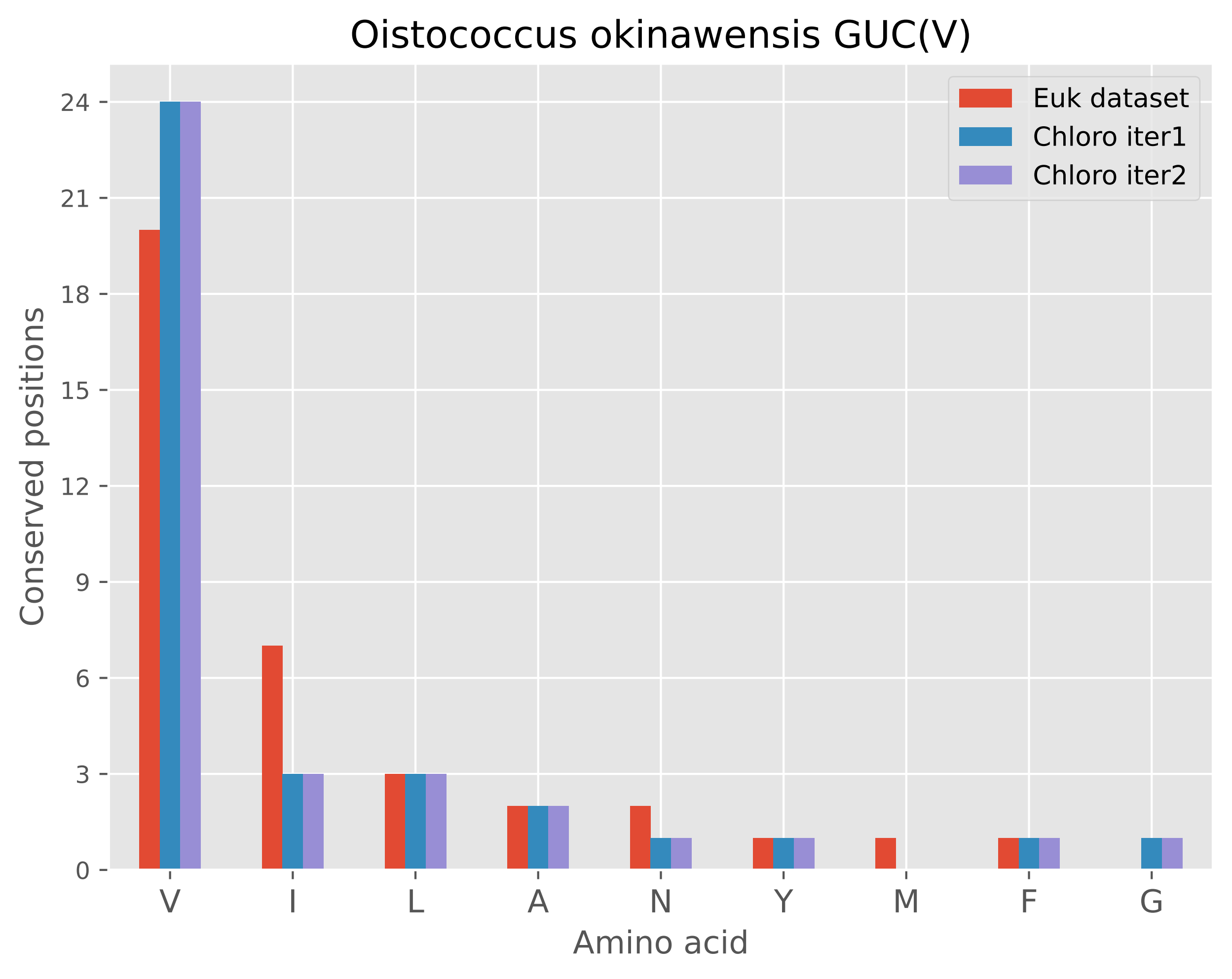
Oistococcus okinawensis GUC(V)
Euk dataset
Chloro iter1
Chloro iter2
Conserved positions
0
3
6
9
12
15
18
21
24
V
I
L
A
N
Y
M
F
G
Amino acid

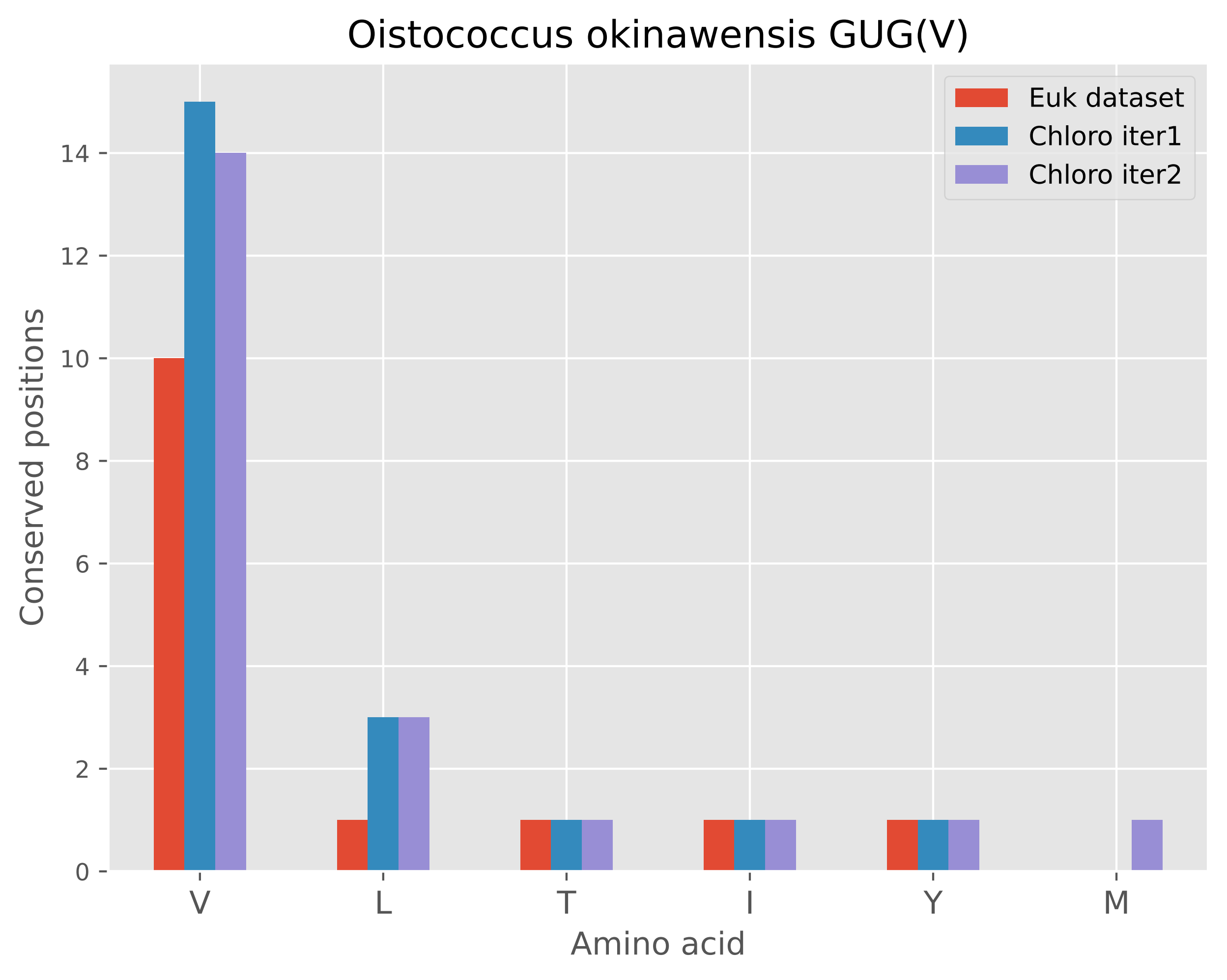
Oistococcus okinawensis GUG(V)
Euk dataset
Chloro iter1
Chloro iter2
Conserved positions
0
2
4
6
8
10
12
14
V
L
T
I
Y
M
Amino acid

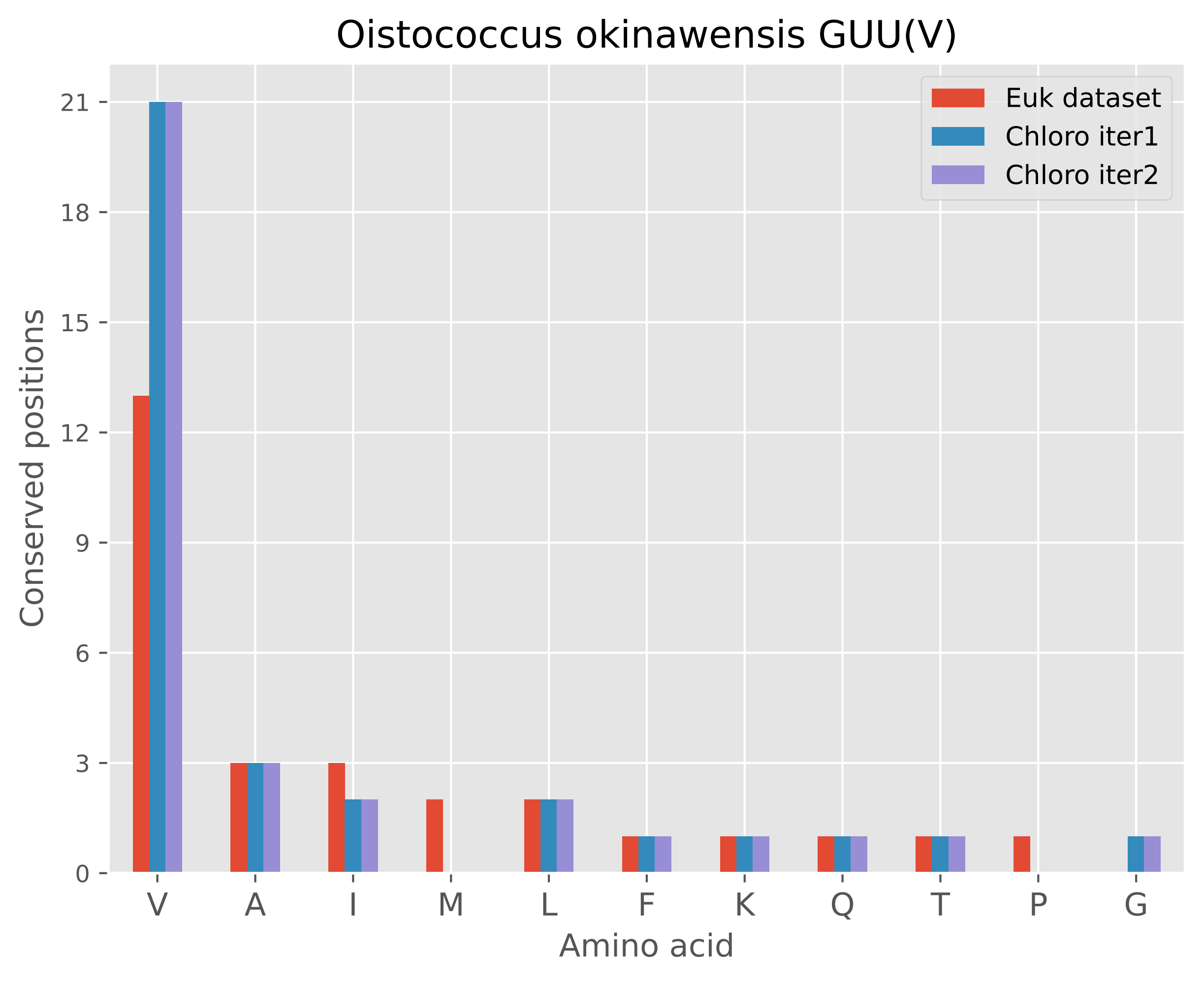

Oistococcus okinawensis GUU(V)
Euk dataset
Chloro iter1
Chloro iter2
Conserved positions
0
3
6
9
12
15
18
21
V
A
I
M
L
F
K
Q
T
P
G
Amino acid

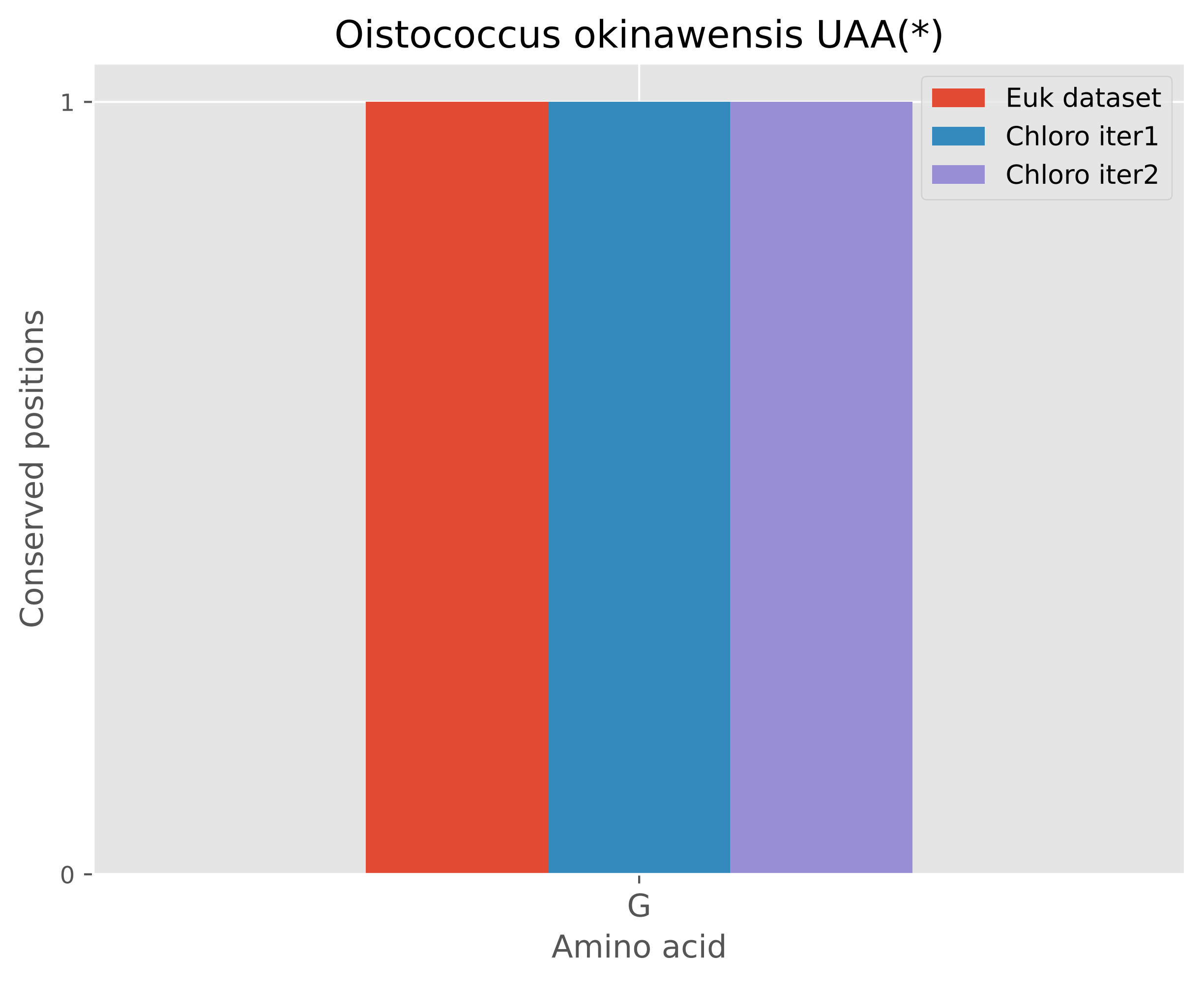
Oistococcus okinawensis UAA(*)
Euk dataset
Chloro iter1
Chloro iter2
Conserved positions
1
0
G
Amino acid

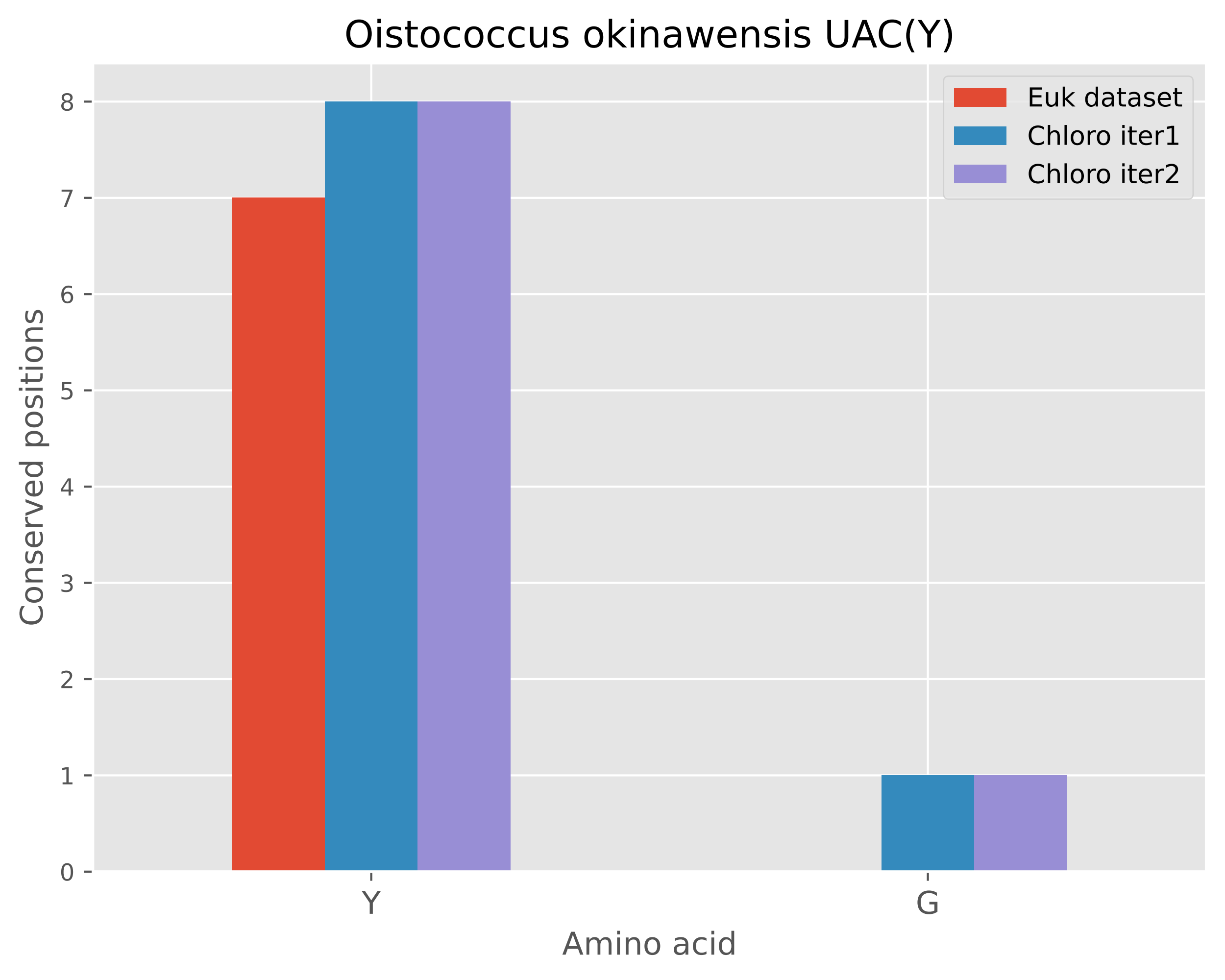

Oistococcus okinawensis UAC(Y)
Euk dataset
Chloro iter1
Chloro iter2
Conserved positions
0
1
2
3
4
5
6
7
8
Y
G
Amino acid

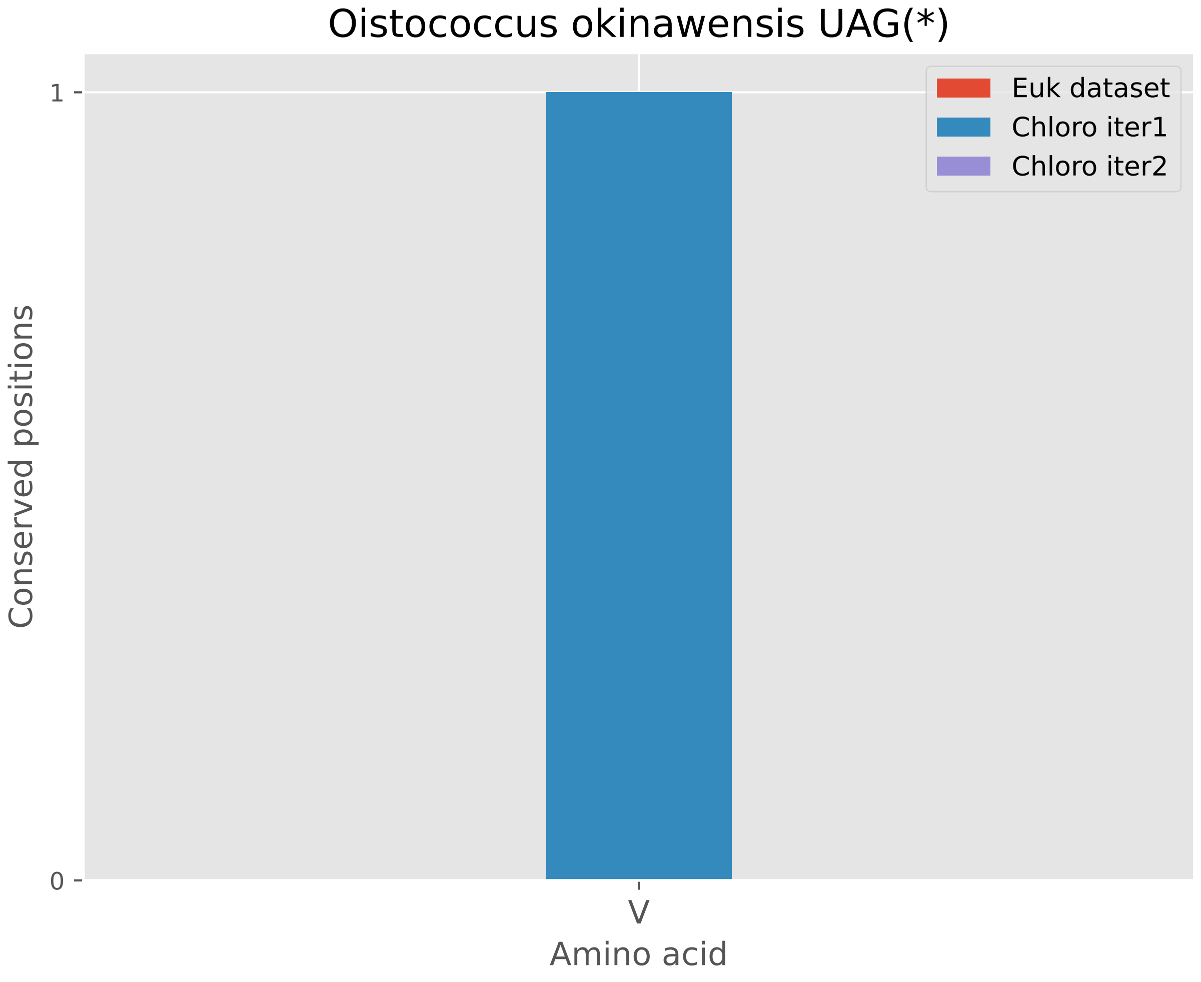

Oistococcus okinawensis UAG(*)
Euk dataset
Chloro iter1
Chloro iter2
Conserved positions
1
0
V
Amino acid

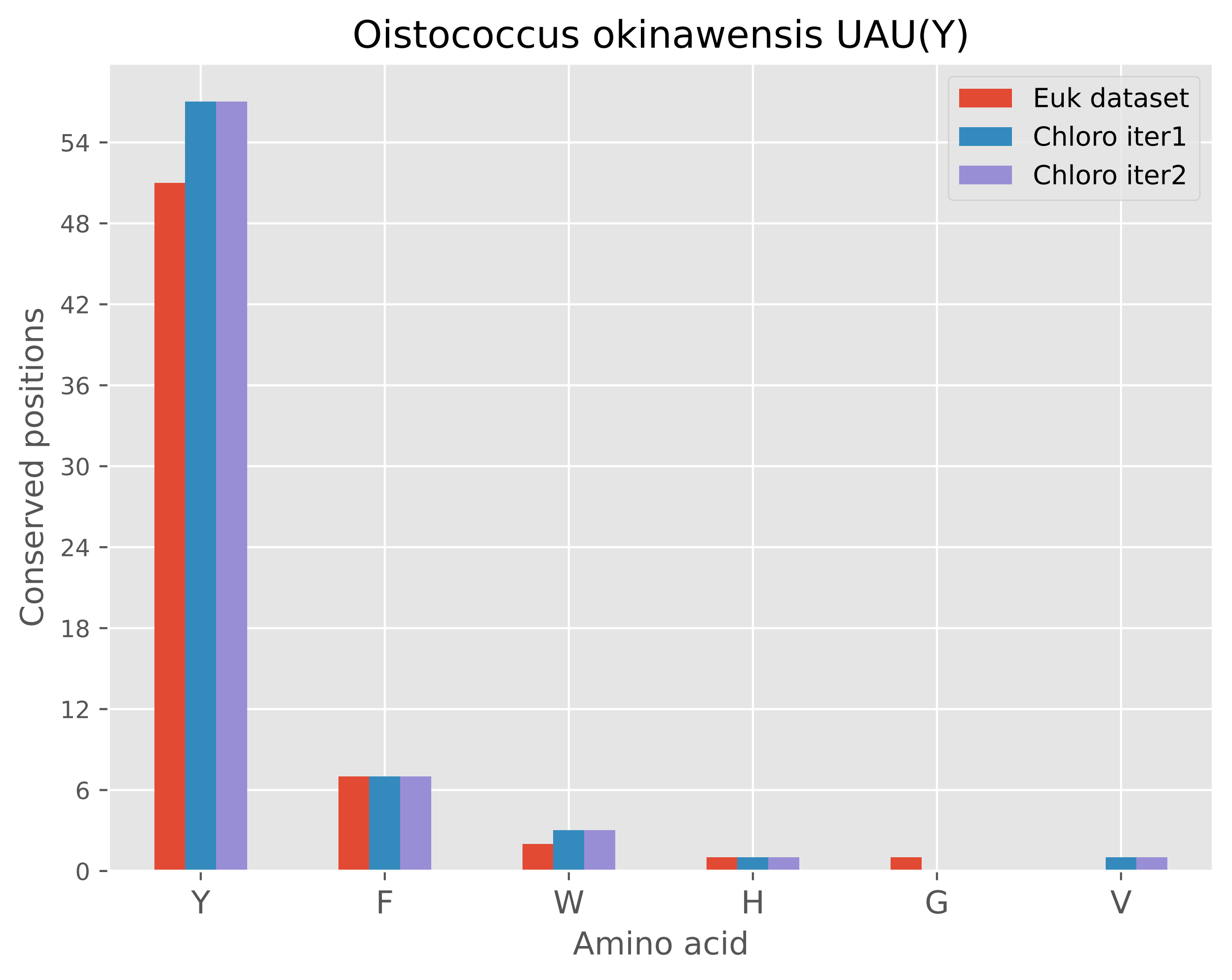
Oistococcus okinawensis UAU(Y)
Euk dataset
Chloro iter1
Chloro iter2
Conserved positions
0
6
12
18
24
30
36
42
48
54
Y
F
W
H
G
V
Amino acid

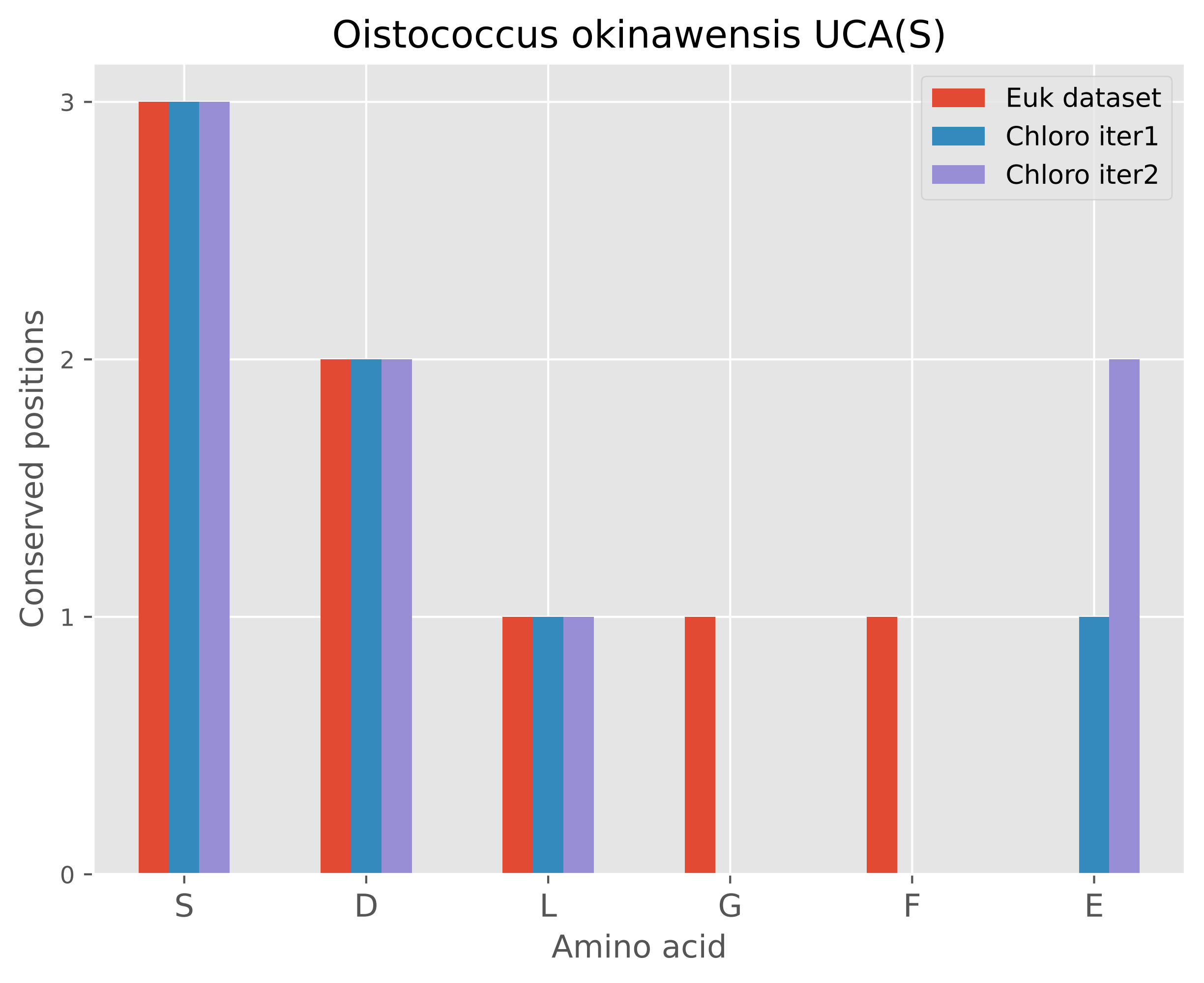

Oistococcus okinawensis UCA(S)
Euk dataset
Chloro iter1
Chloro iter2
Conserved positions
0
1
2
3
S
D
L
G
F
E
Amino acid

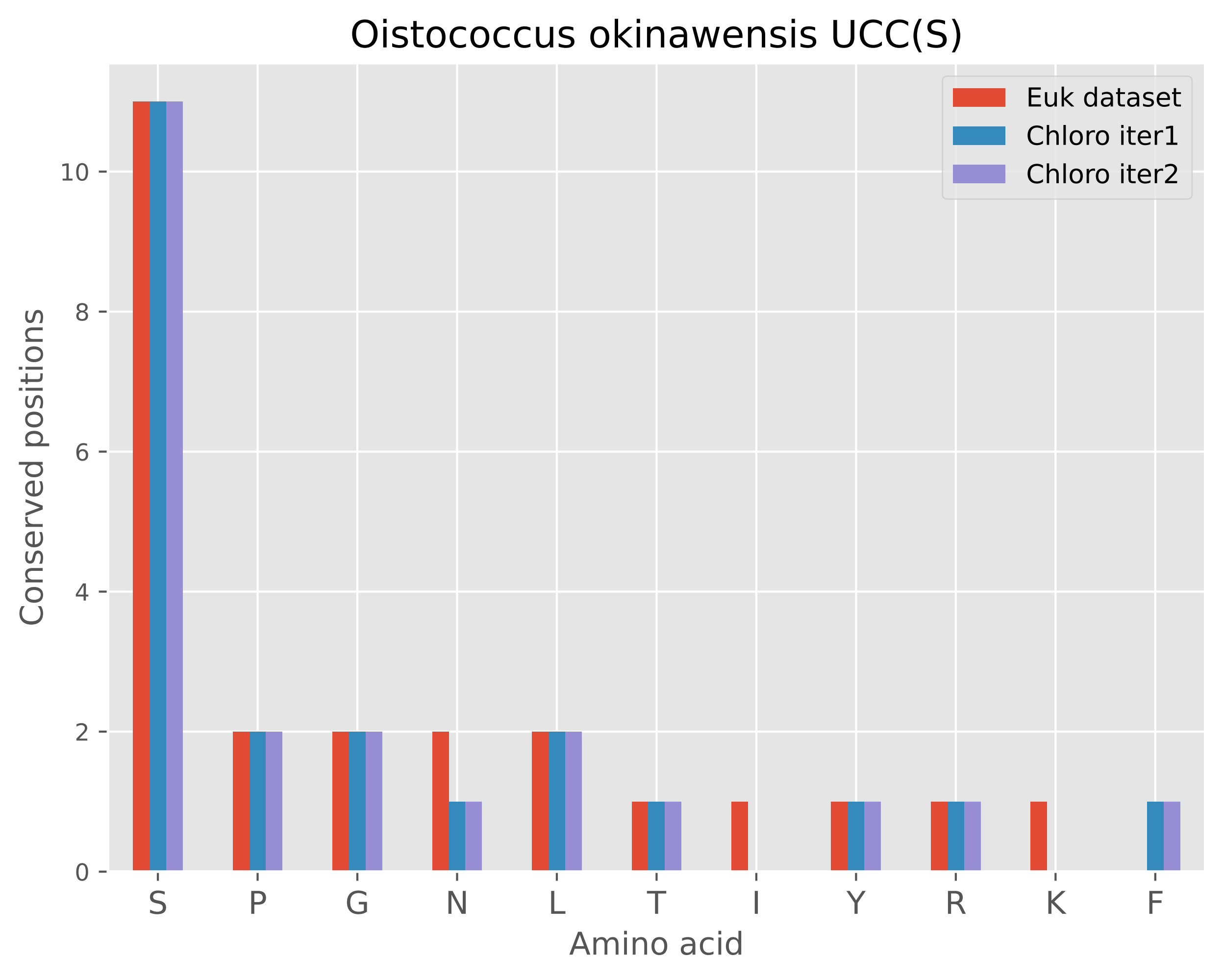

Oistococcus okinawensis UCC(S)
Euk dataset
Chloro iter1
Chloro iter2
Conserved positions
0
2
4
6
8
10
S
P
G
N
L
T
I
Y
R
K
F
Amino acid

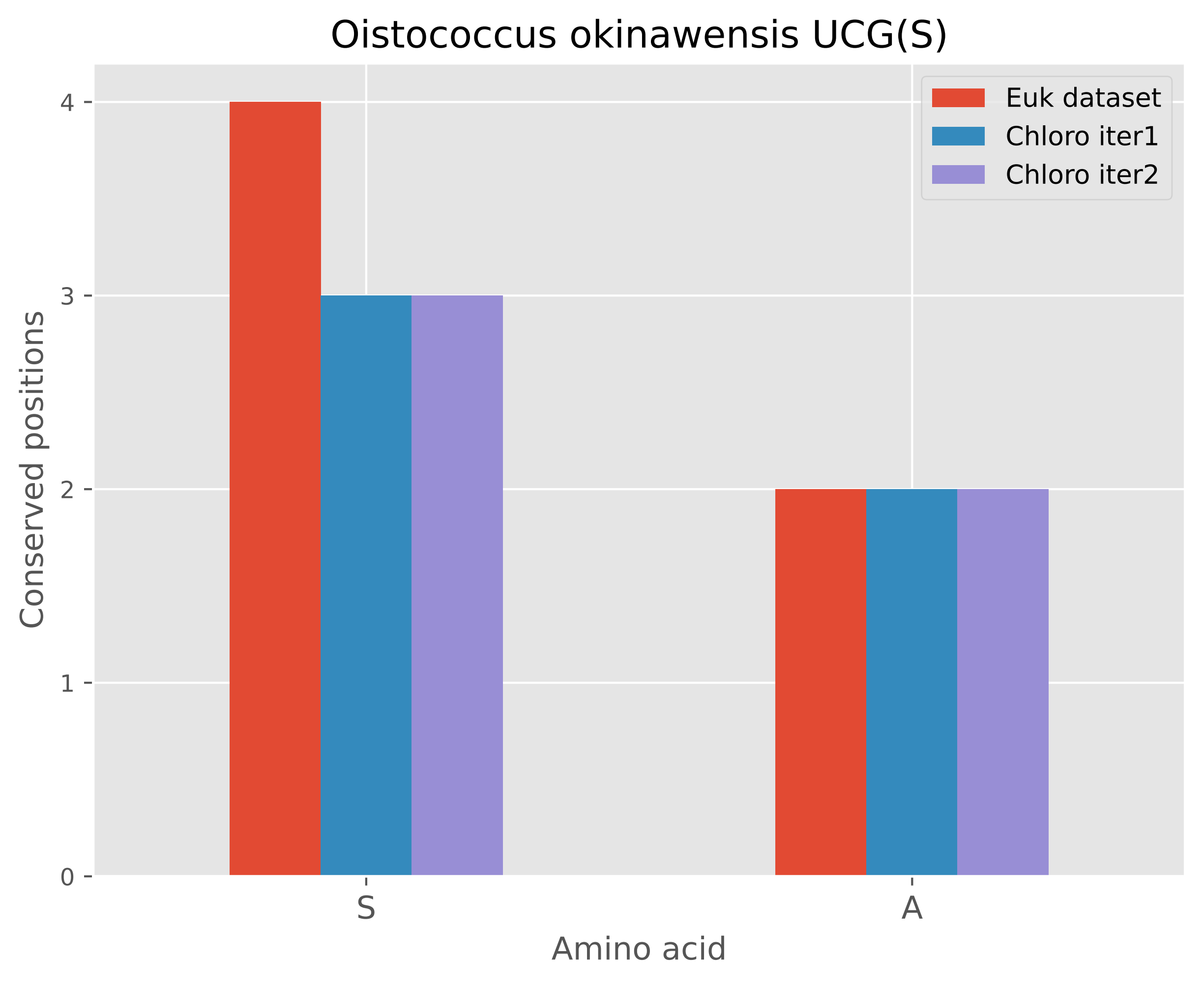
Oistococcus okinawensis UCG(S)
Euk dataset
Chloro iter1
Chloro iter2
Conserved positions
0
1
2
3
4
S
A
Amino acid

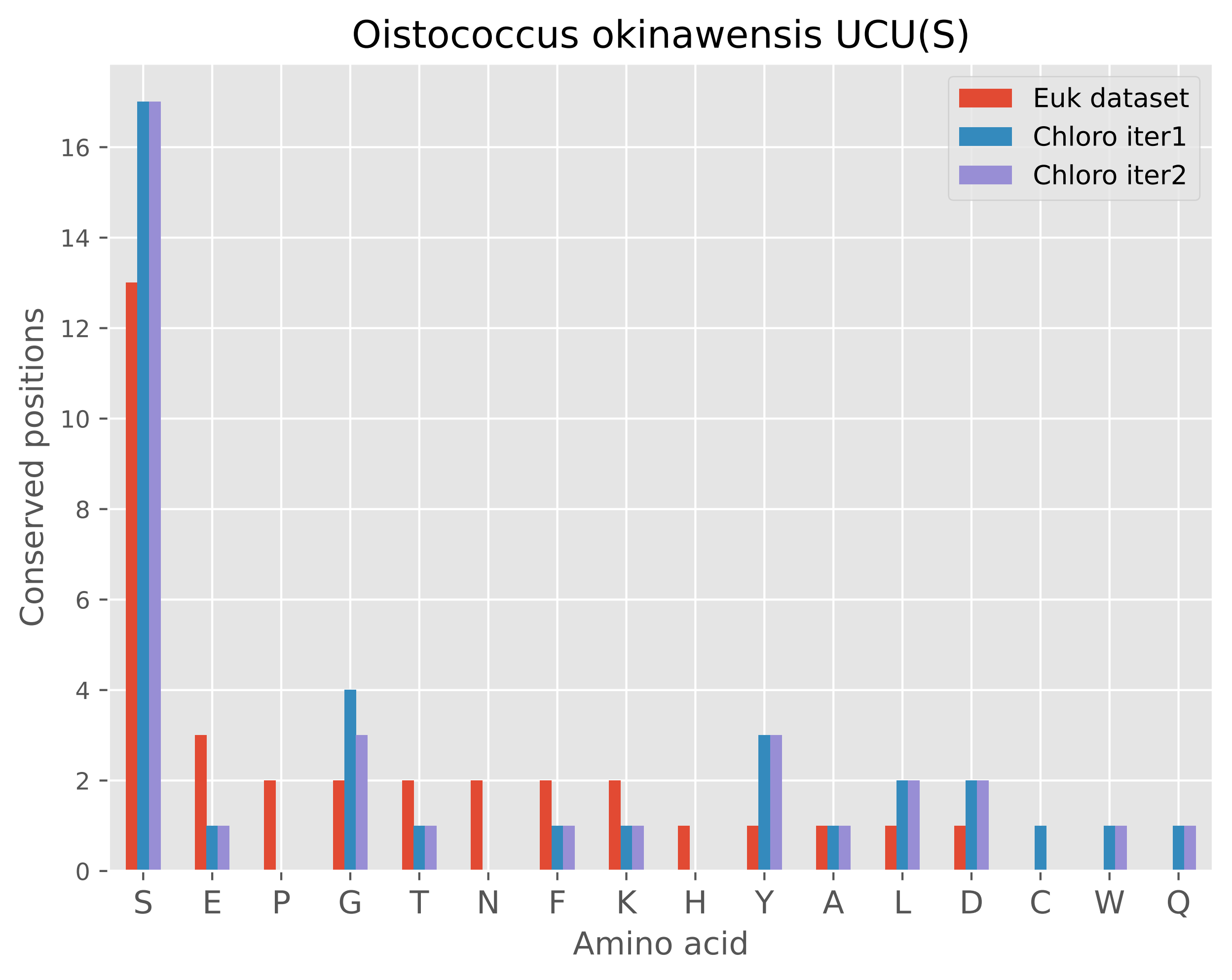

Oistococcus okinawensis UCU(S)
Euk dataset
Chloro iter1
Chloro iter2
Conserved positions
0
2
4
6
8
10
12
14
16
S
E
P
G
T
N
F
K
H
Y
A
L
D
C
W
Q
Amino acid

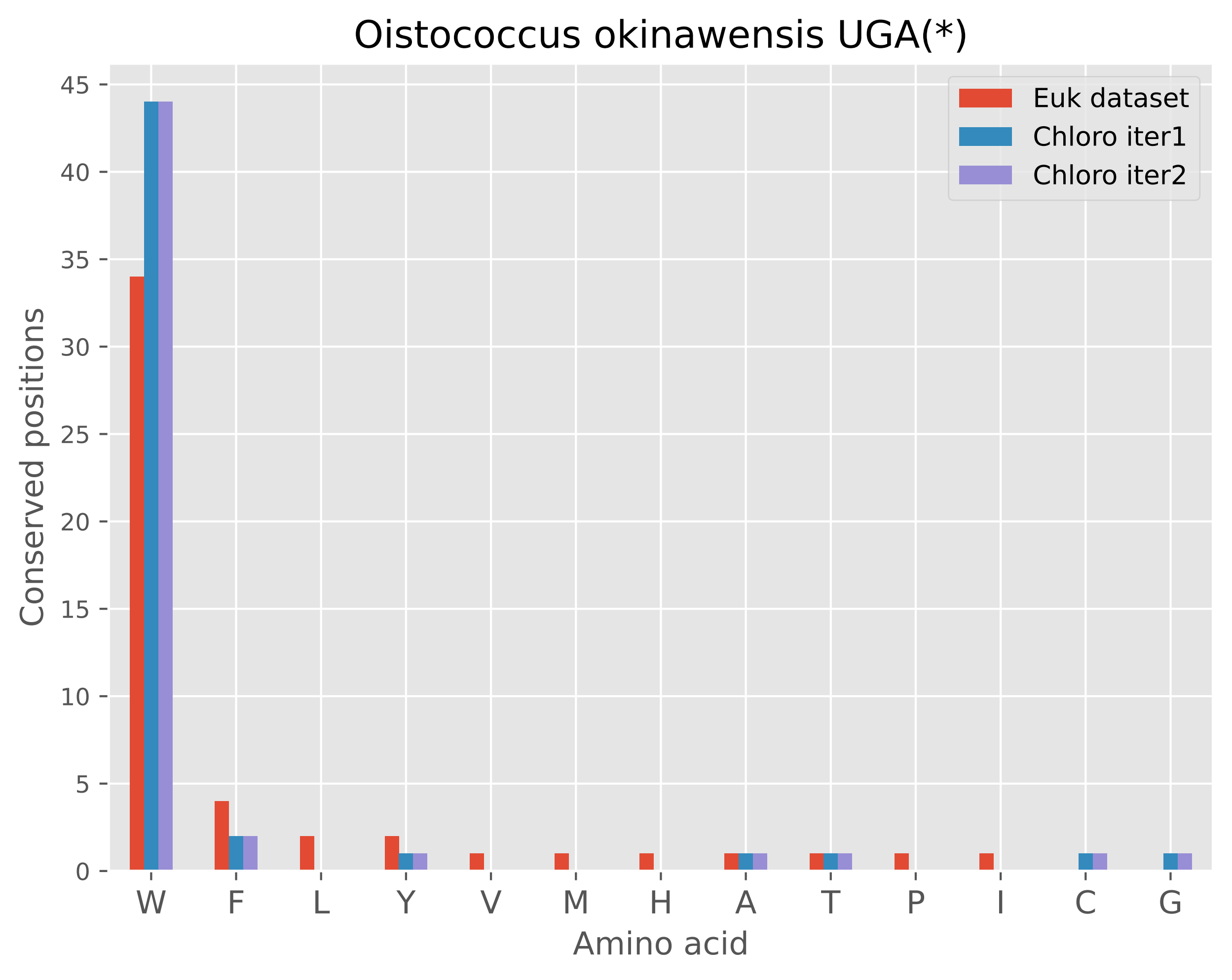

Oistococcus okinawensis UGA(*)
Euk dataset
Chloro iter1
Chloro iter2
Conserved positions
0
5
10
15
20
25
30
35
40
45
W
F
L
Y
V
M
H
A
T
P
I
C
G
Amino acid

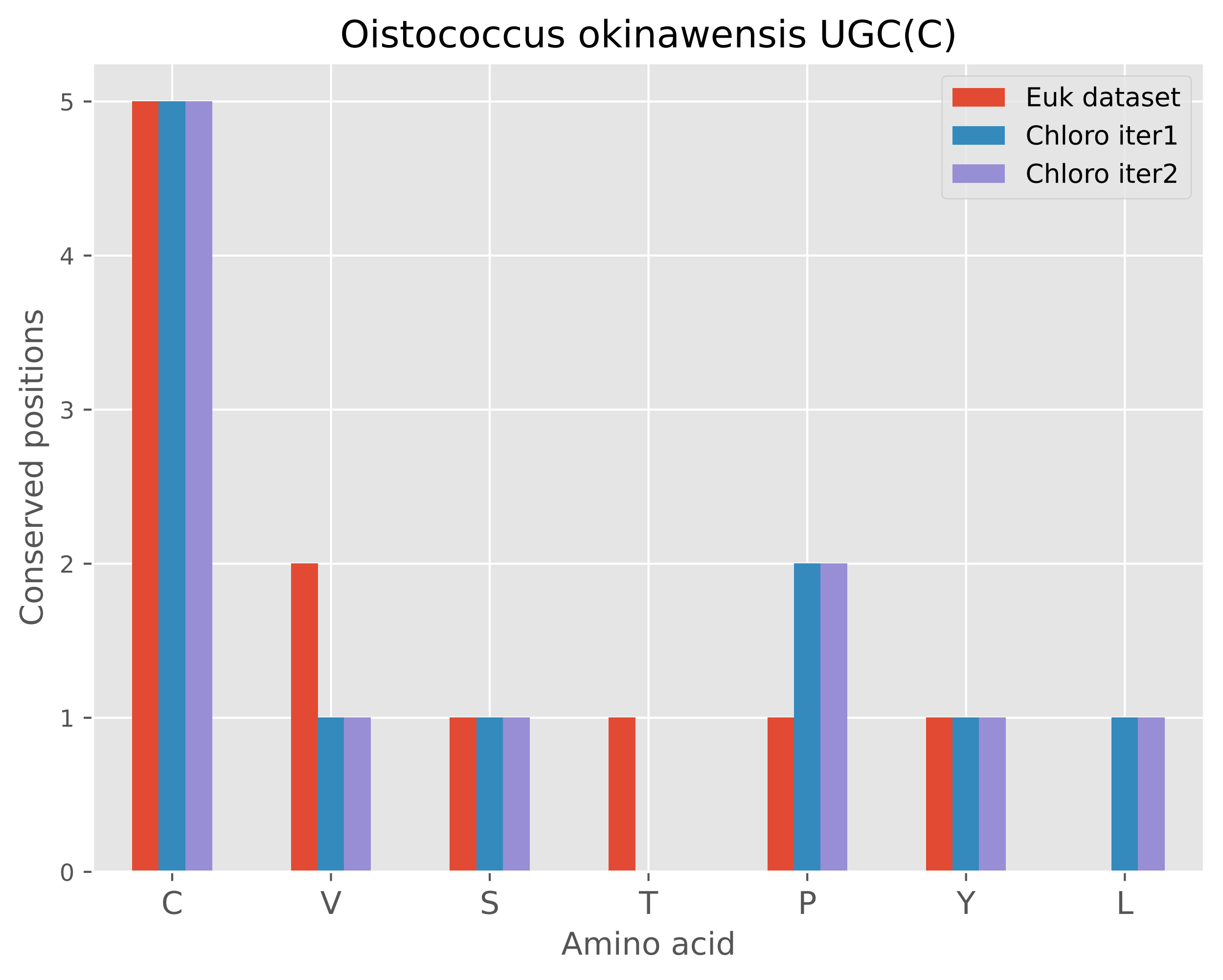

Oistococcus okinawensis UGC(C)
Euk dataset
Chloro iter1
Chloro iter2
Conserved positions
0
1
2
3
4
5
C
V
S
T
P
Y
L
Amino acid

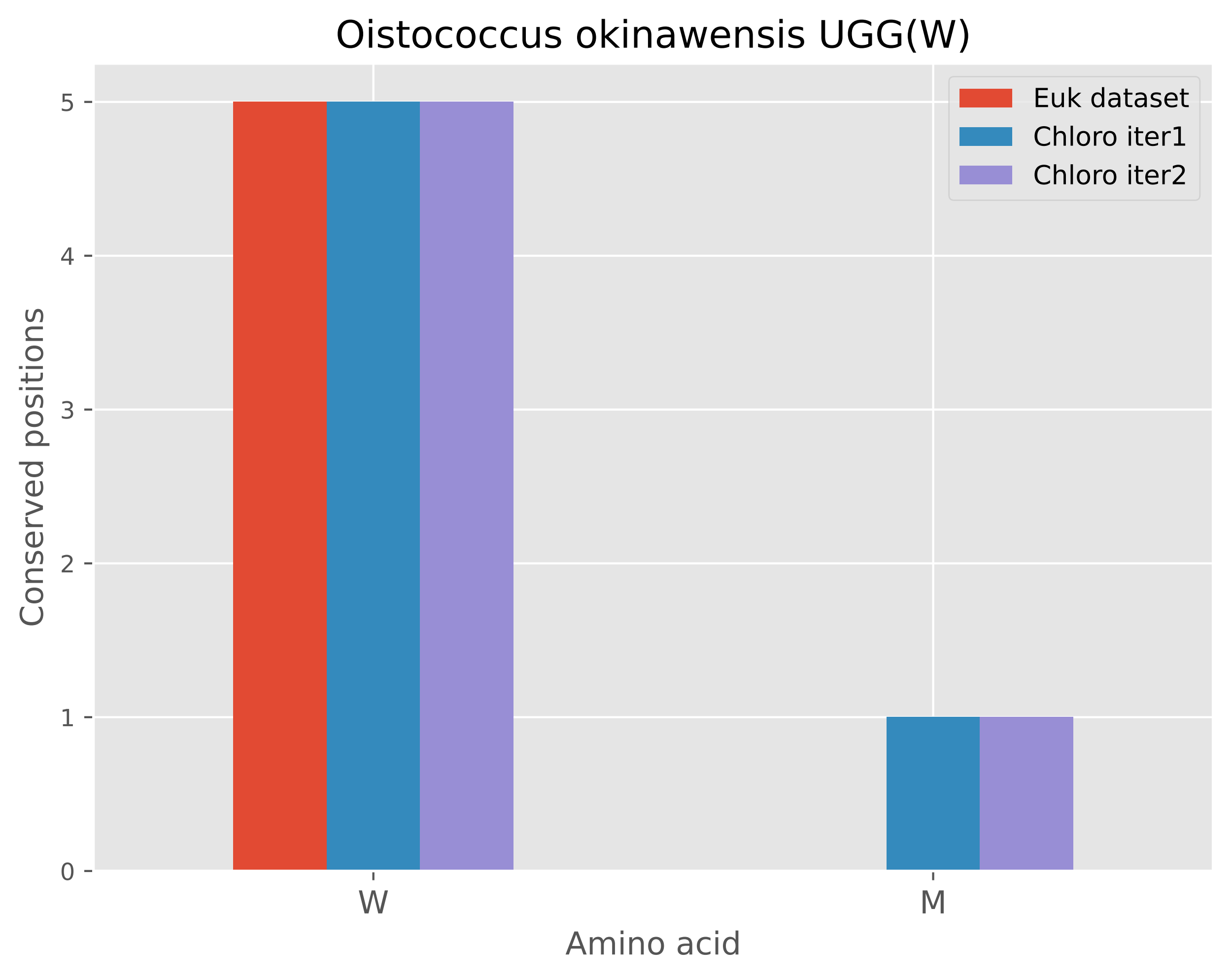

Oistococcus okinawensis UGG(W)
Euk dataset
Chloro iter1
Chloro iter2
Conserved positions
0
1
2
3
4
5
W
M
Amino acid

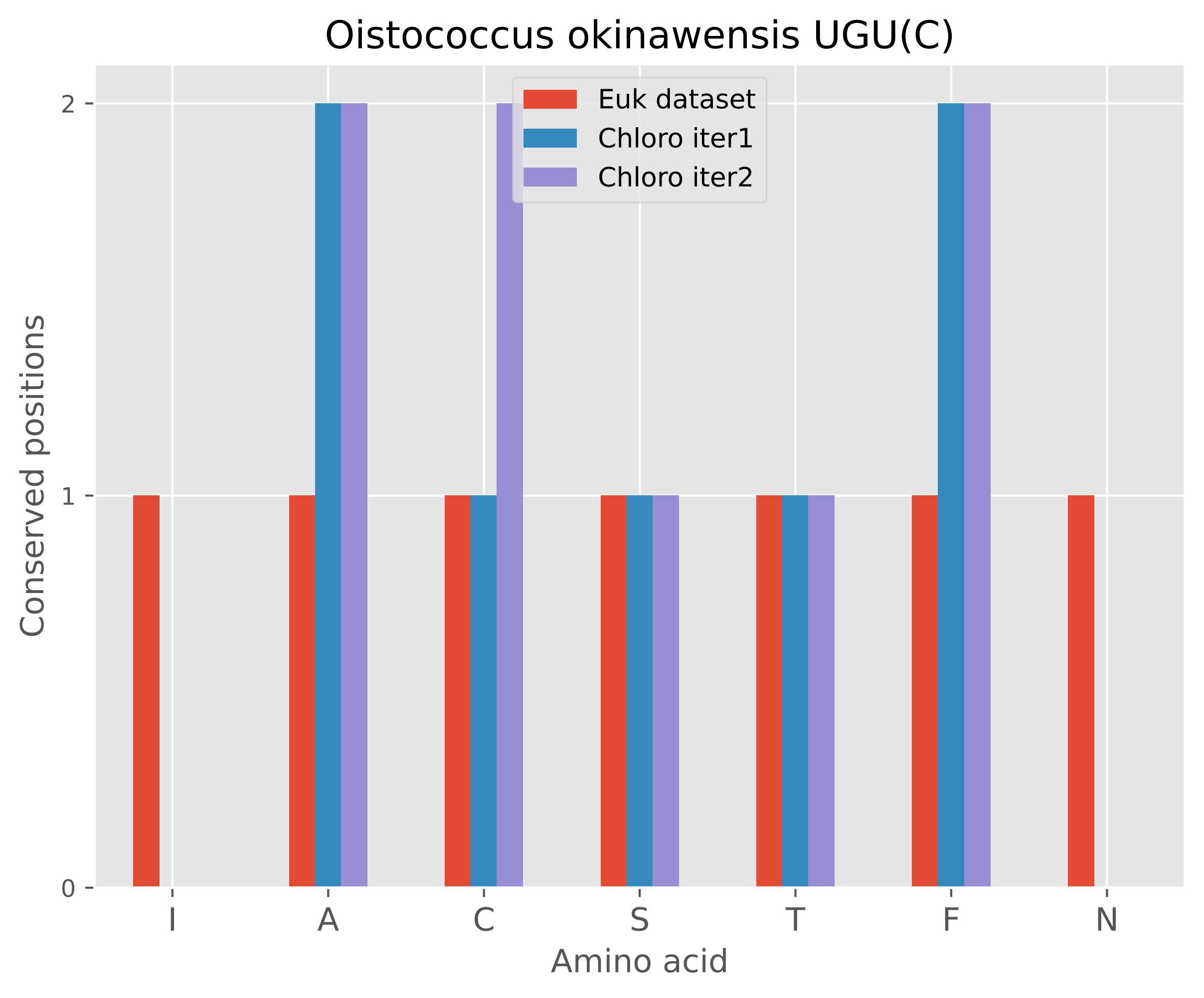

Oistococcus okinawensis UGU(C)
Euk dataset
Chloro iter1
Chloro iter2
Conserved positions
0
1
2
I
A
C
S
T
F
N
Amino acid

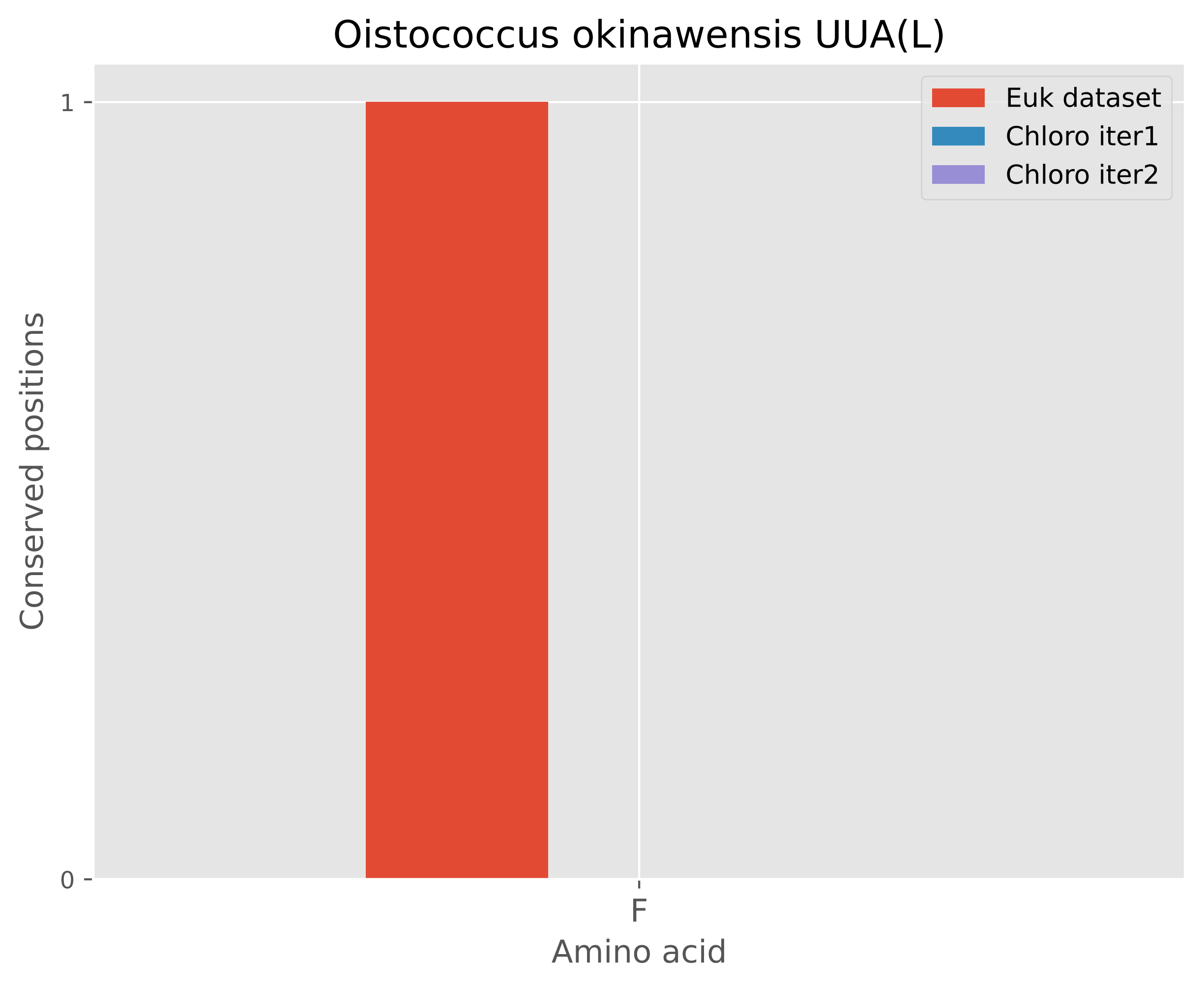

Oistococcus okinawensis UUA(L)
Euk dataset
Chloro iter1
Chloro iter2
Conserved positions
1
0
F
Amino acid

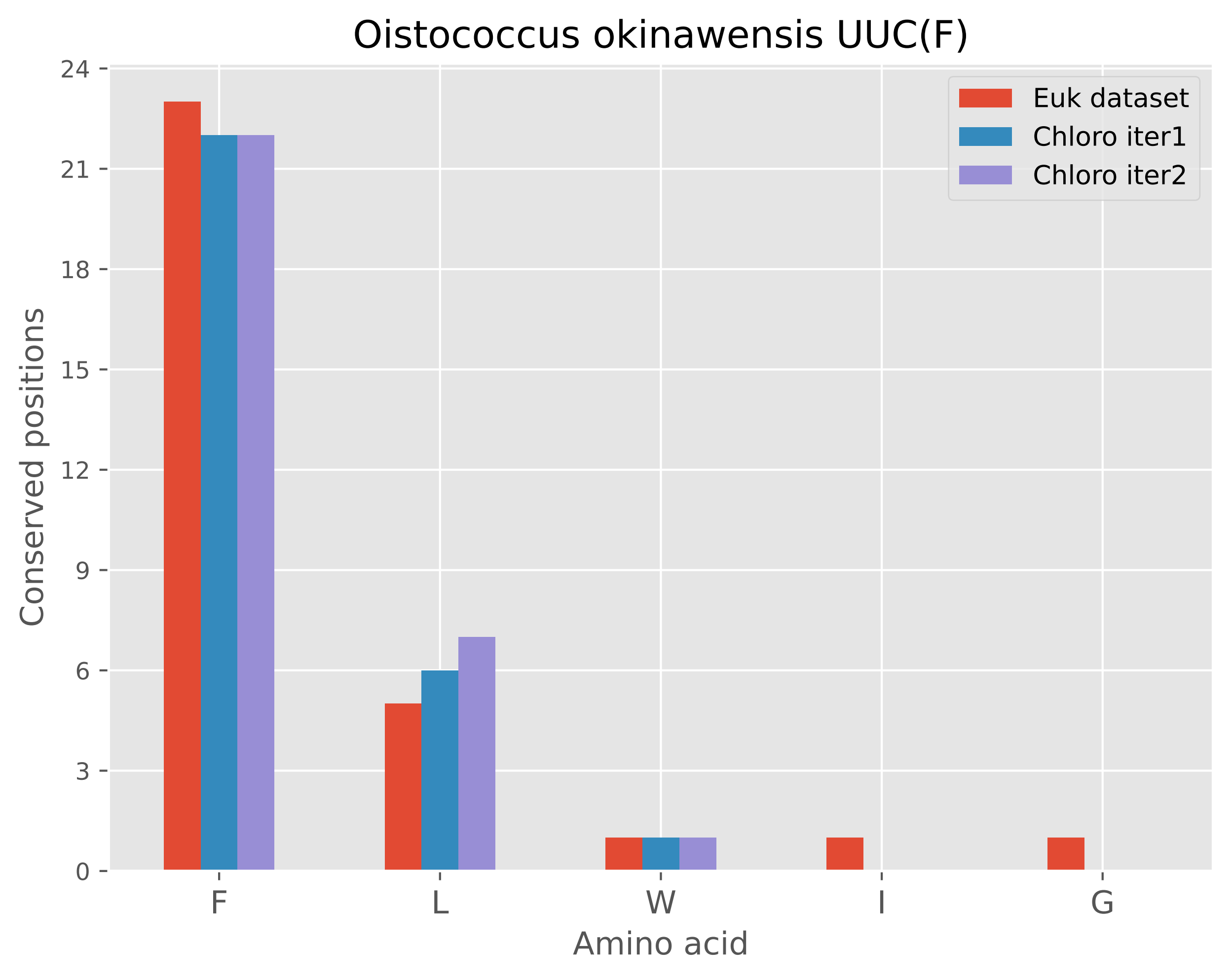

Oistococcus okinawensis UUC(F)
Euk dataset
Chloro iter1
Chloro iter2
Conserved positions
0
3
6
9
12
15
18
21
24
F
L
W
I
G
Amino acid

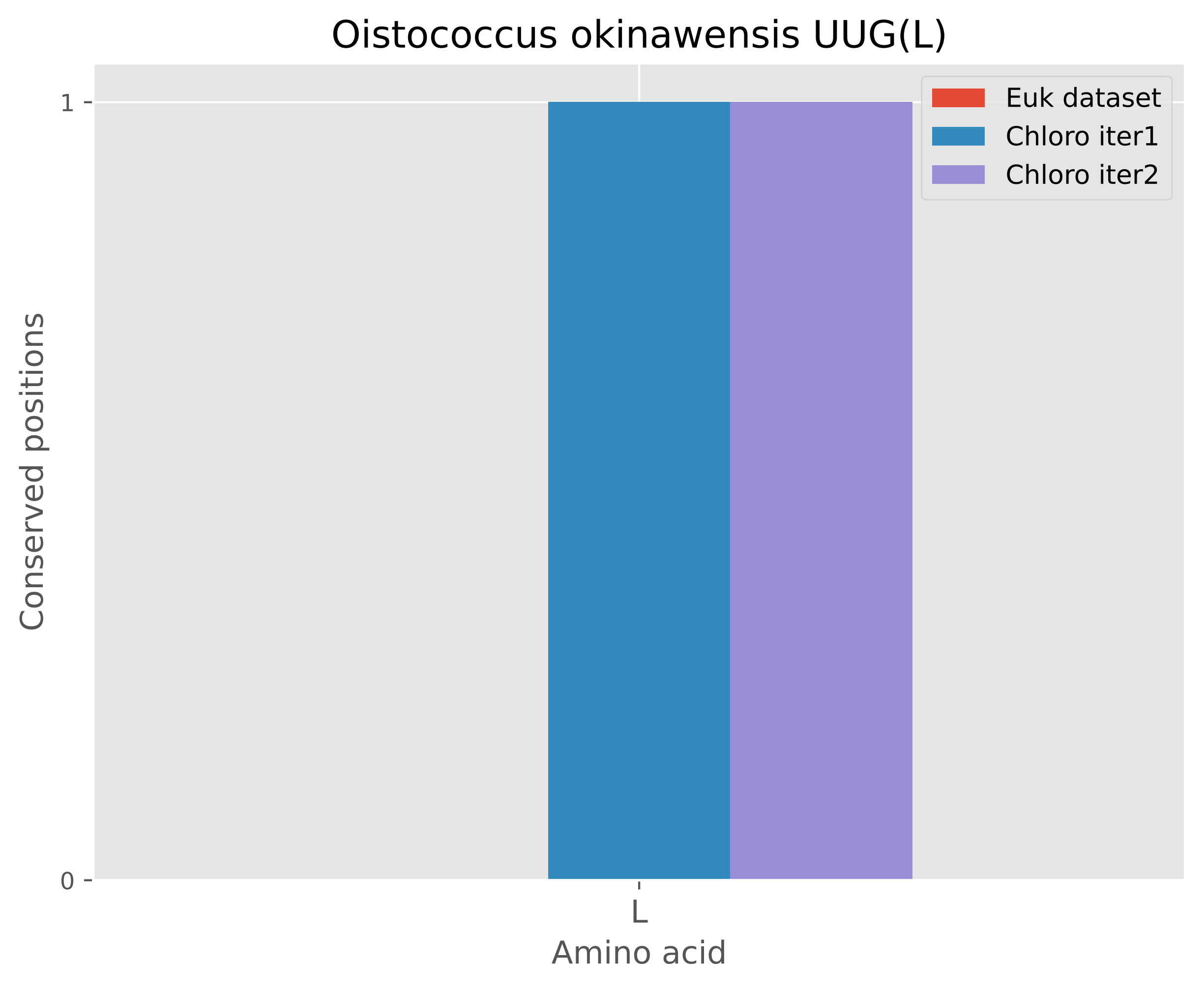

Oistococcus okinawensis UUG(L)
Euk dataset
Chloro iter1
Chloro iter2
Conserved positions
1
0
L
Amino acid

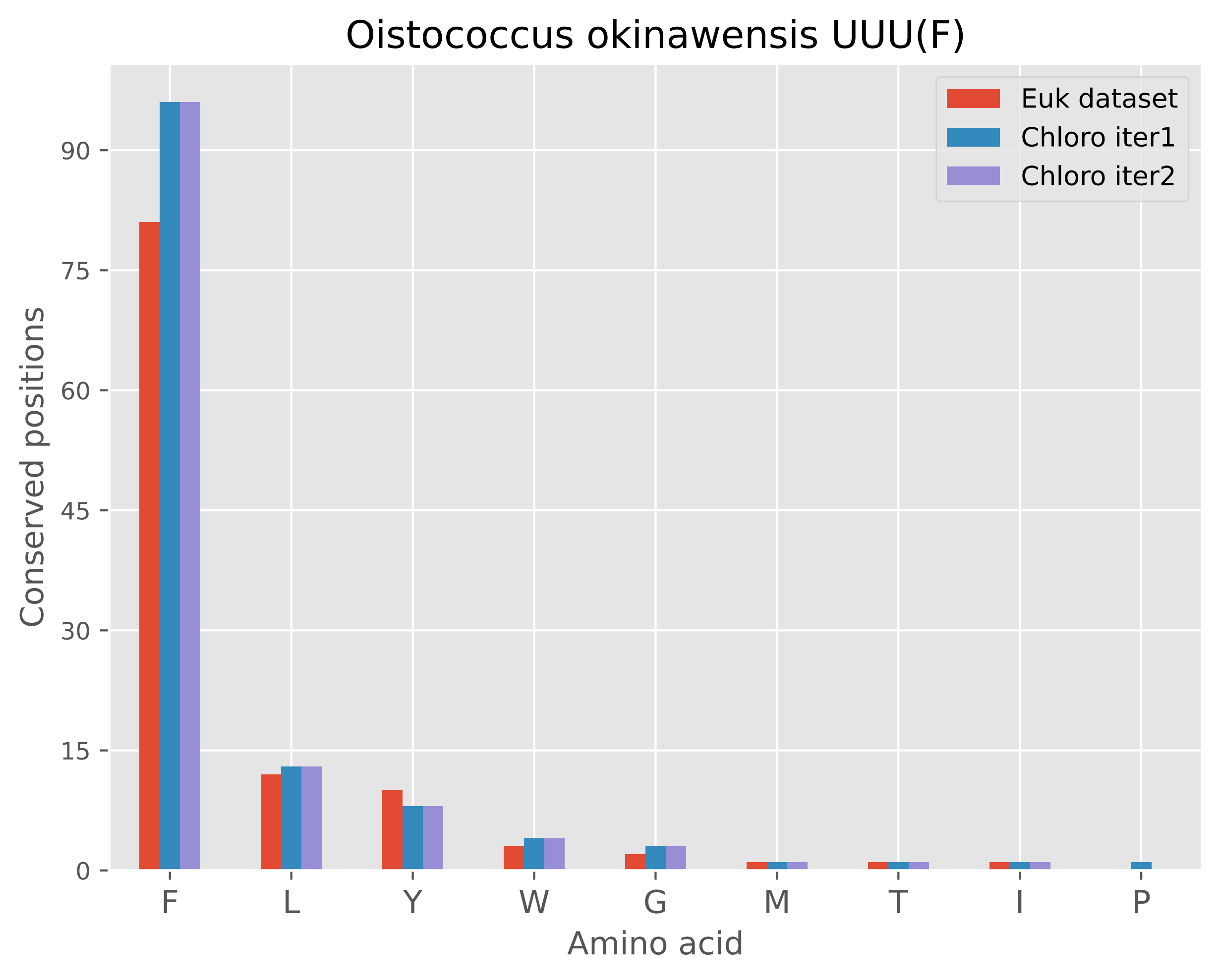
Oistococcus okinawensis UUU(F)
Conserved positions
Amino acid
0
15
30
45
60
75
90
F
L
Y
W
G
M
T
I
P
Euk dataset
Chloro iter1
Chloro iter2

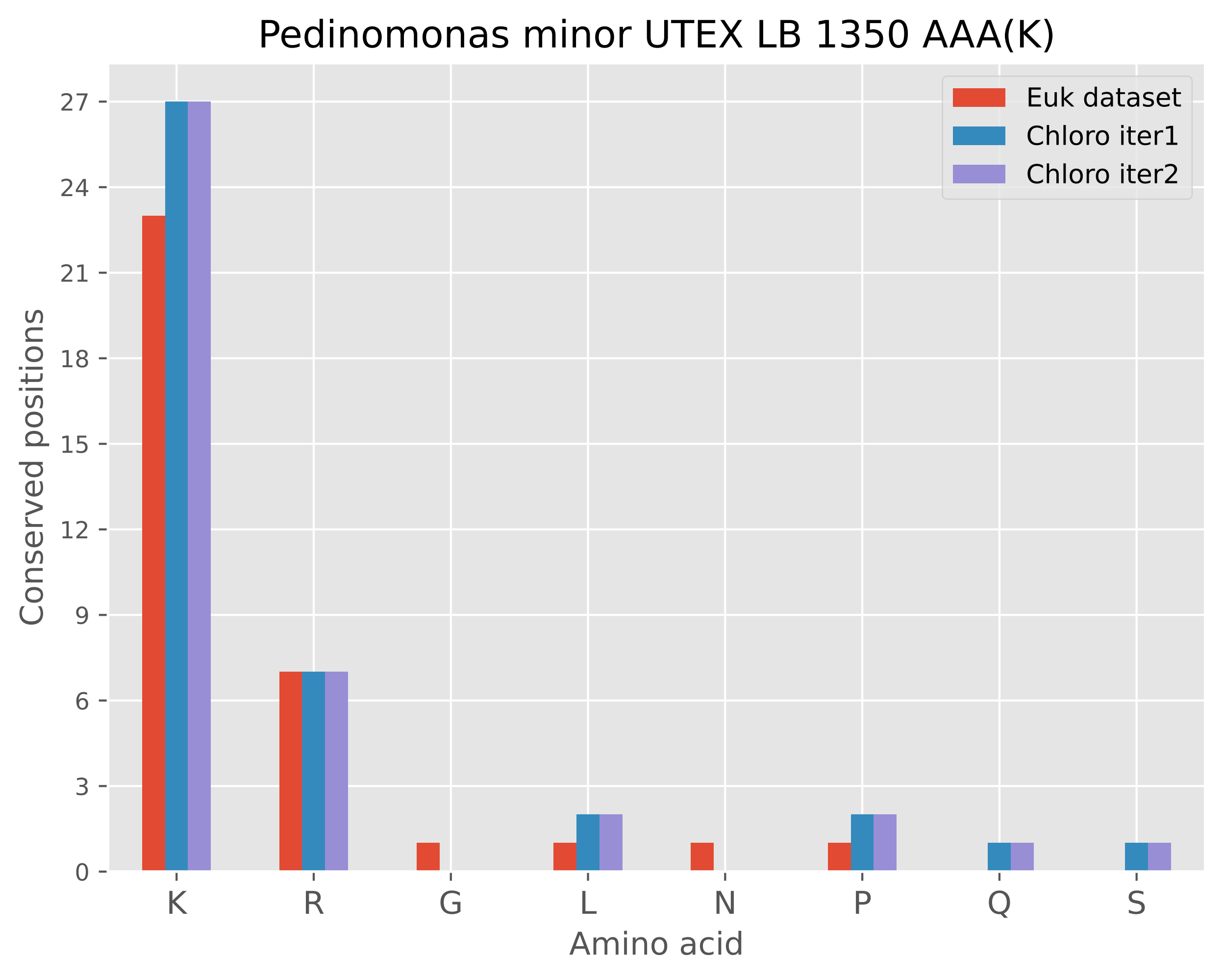

Pedinomonas minor UTEX LB 1350 AAA(K)
Conserved positions
Amino acid
Euk dataset
Chloro iter1
Chloro iter2
0
3
6
9
12
15
18
21
24
27
K
R
G
L
N
P
Q
S

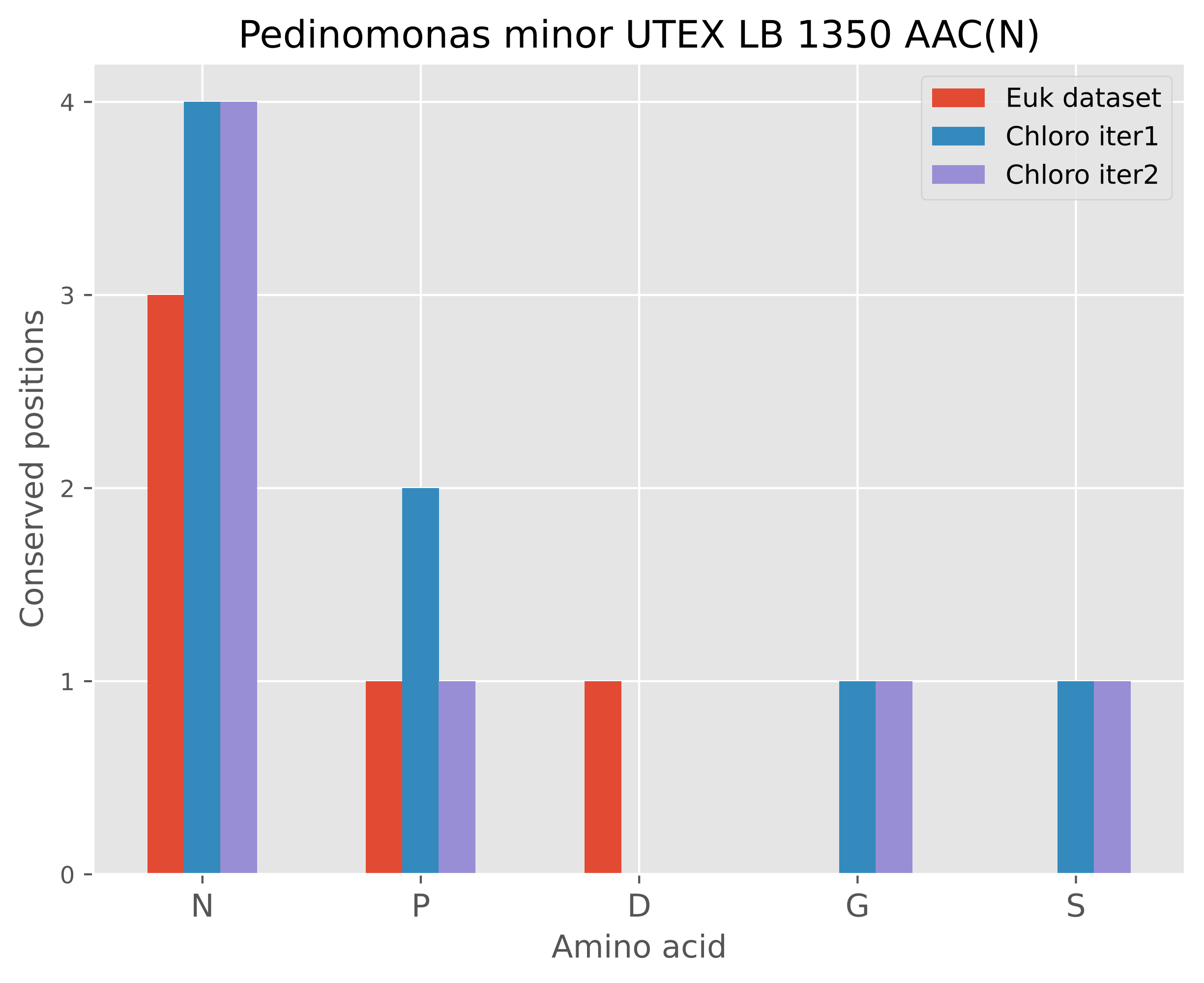

Pedinomonas minor UTEX LB 1350 AAC(N)
Euk dataset
Chloro iter1
Chloro iter2
Conserved positions
0
1
2
3
4
N
P
D
G
S
Amino acid

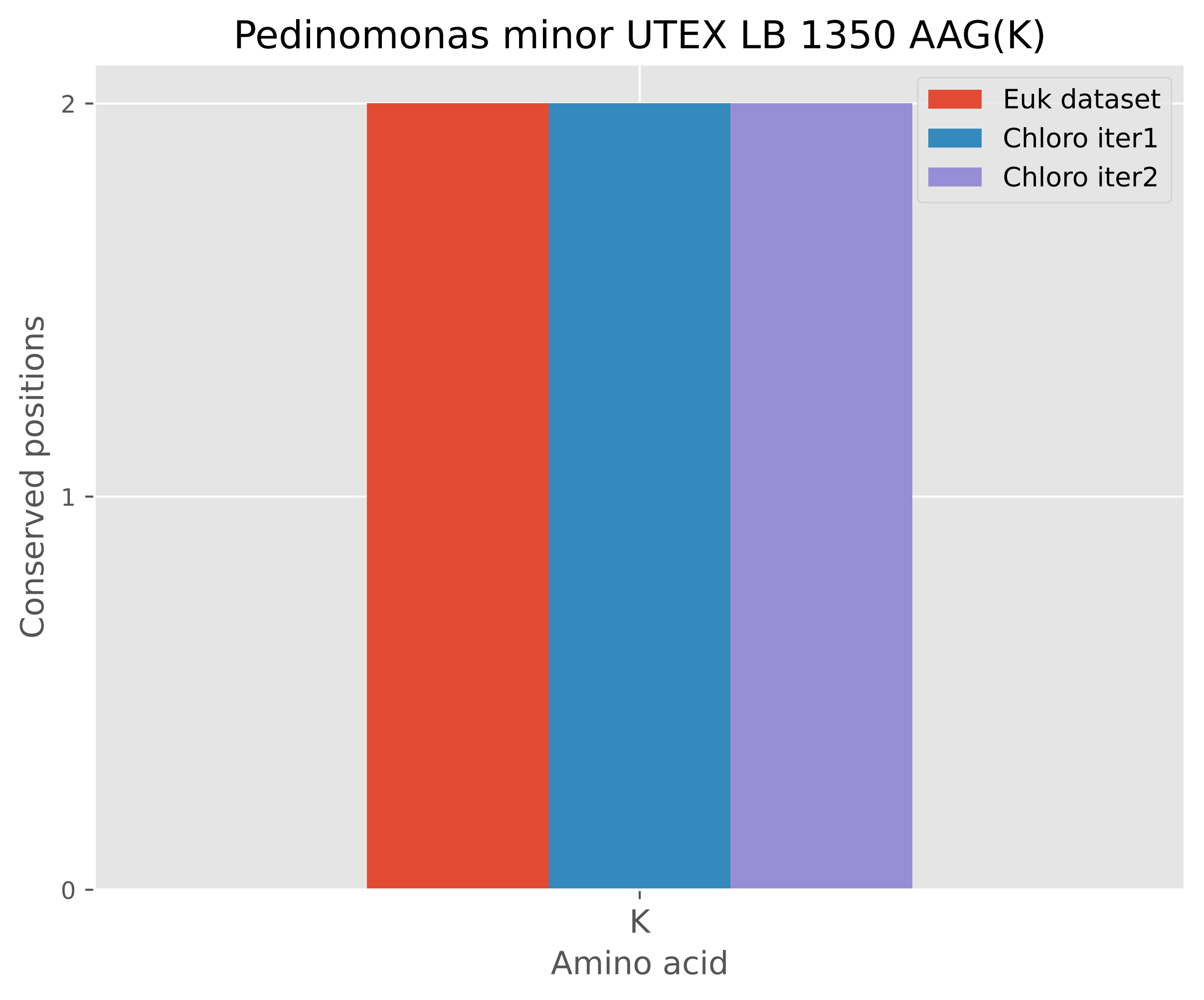
Pedinomonas minor UTEX LB 1350 AAG(K)
Conserved positions
2
1
0
K
Amino acid
Euk dataset
Chloro iter1
Chloro iter2

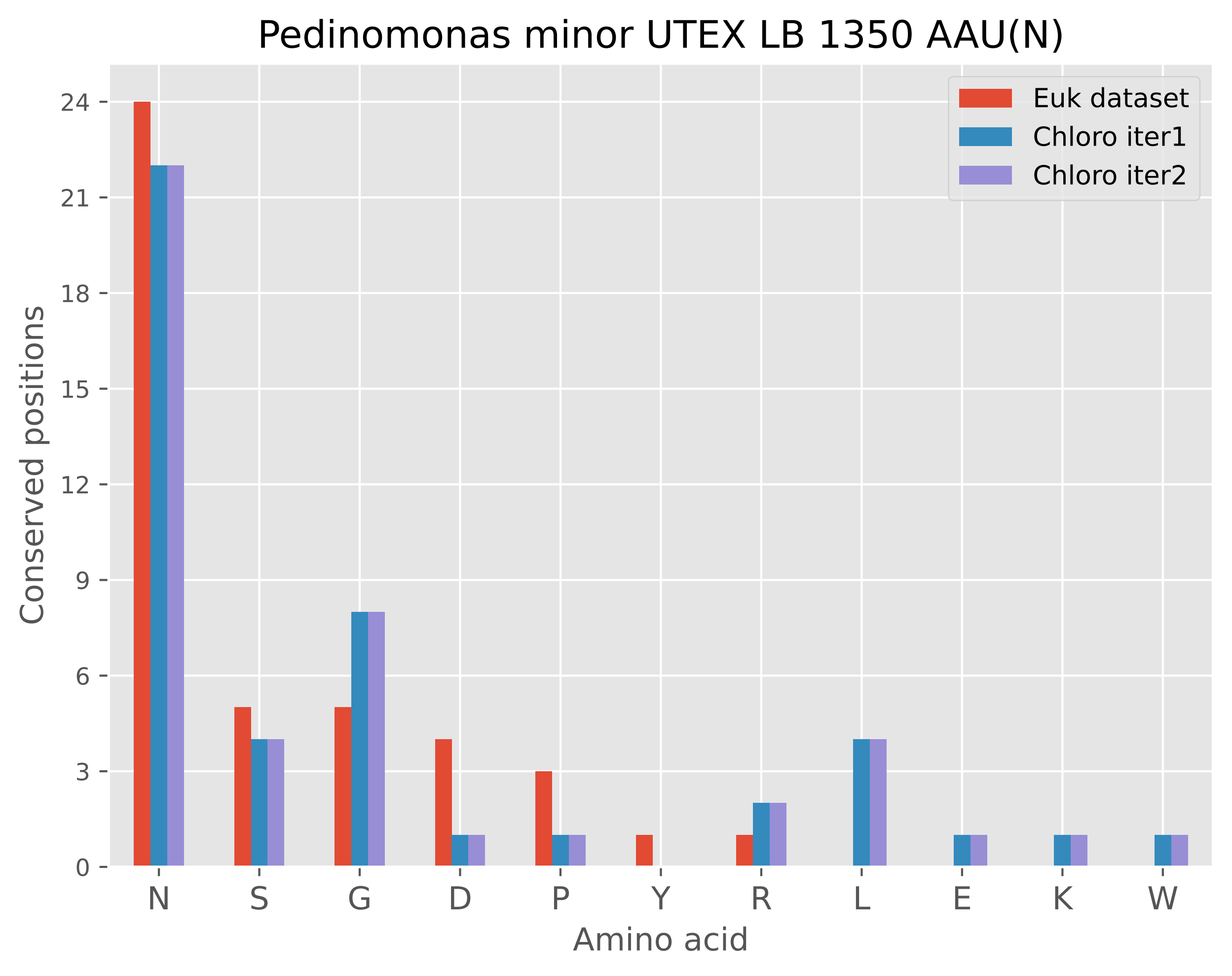
Pedinomonas minor UTEX LB 1350 AAU(N)
Euk dataset
Chloro iter1
Chloro iter2
Conserved positions
0
3
6
9
12
15
18
21
24
N
S
G
D
P
Y
R
L
E
K
W
Amino acid

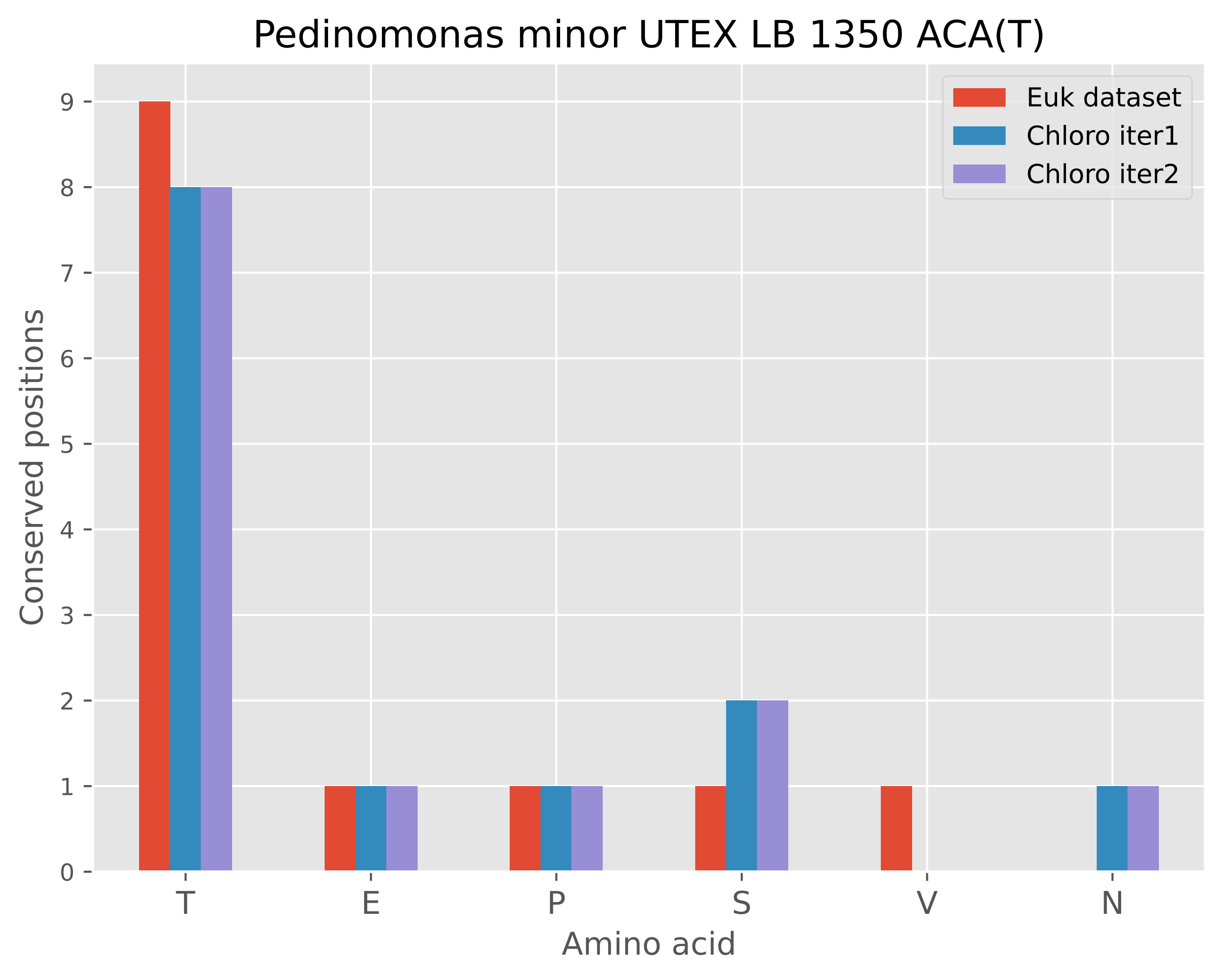

Pedinomonas minor UTEX LB 1350 ACA(T)
Euk dataset
Chloro iter1
Chloro iter2
Conserved positions
0
1
2
3
4
5
6
7
8
9
T
E
P
S
V
N
Amino acid

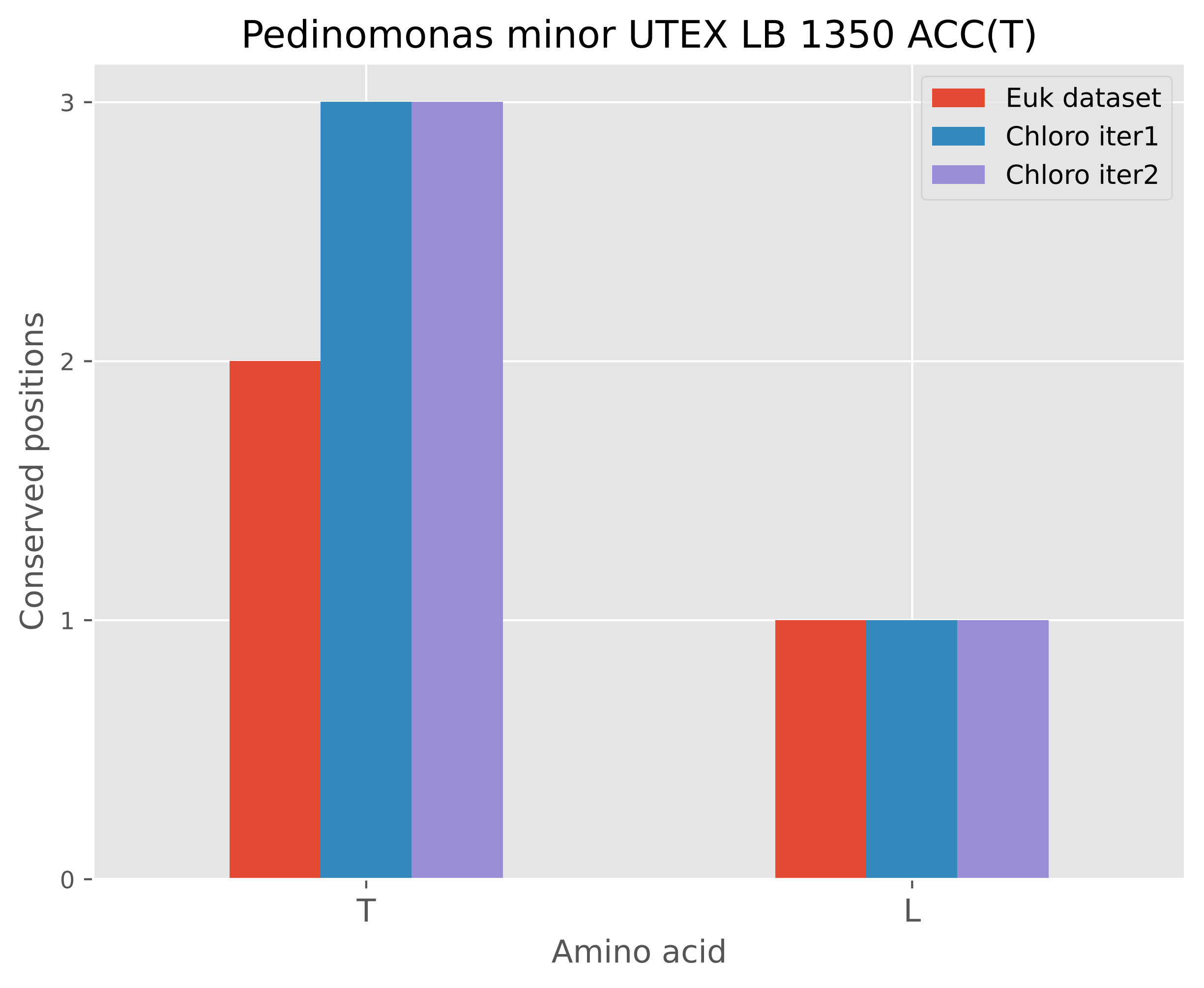
Pedinomonas minor UTEX LB 1350 ACC(T)
Euk dataset
Chloro iter1
Chloro iter2
Conserved positions
0
1
2
3
T
L
Amino acid

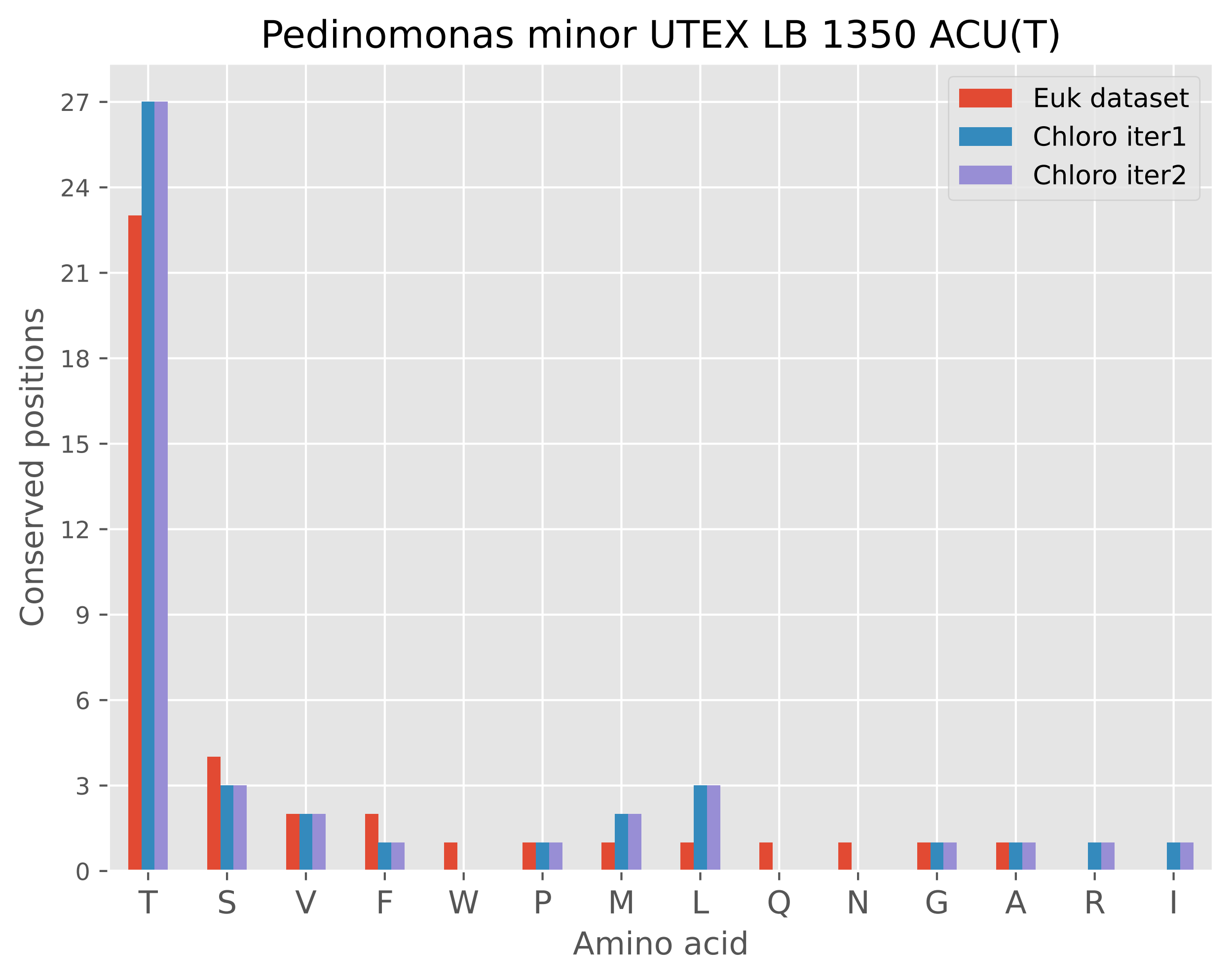

Pedinomonas minor UTEX LB 1350 ACU(T)
Euk dataset
Chloro iter1
Chloro iter2
Conserved positions
0
3
6
9
12
15
18
21
24
27
T
S
V
F
W
P
M
L
Q
N
G
A
R
I
Amino acid

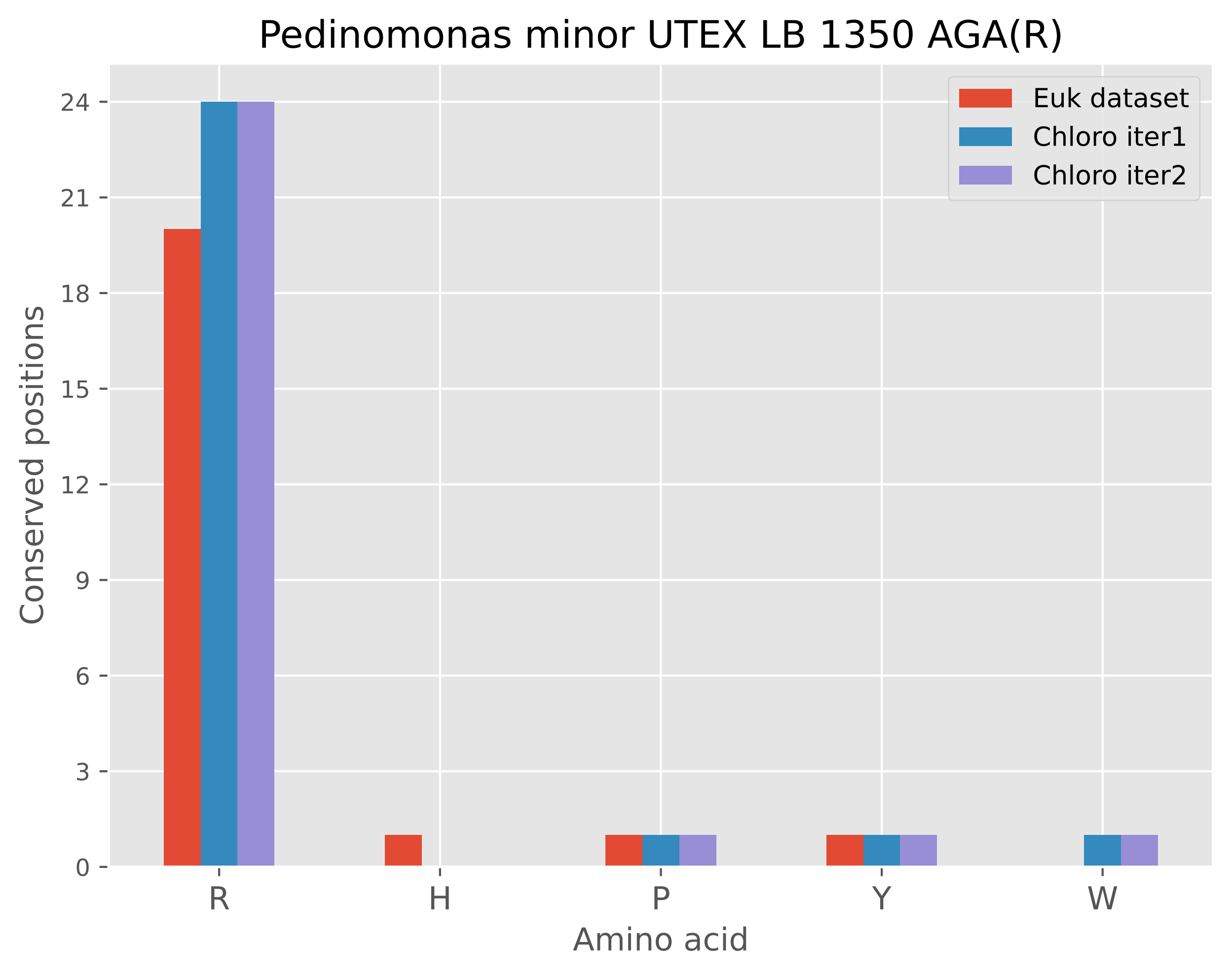
Pedinomonas minor UTEX LB 1350 AGA(R)
Euk dataset
Chloro iter1
Chloro iter2
Conserved positions
0
3
6
9
12
15
18
21
24
R
H
P
Y
W
Amino acid

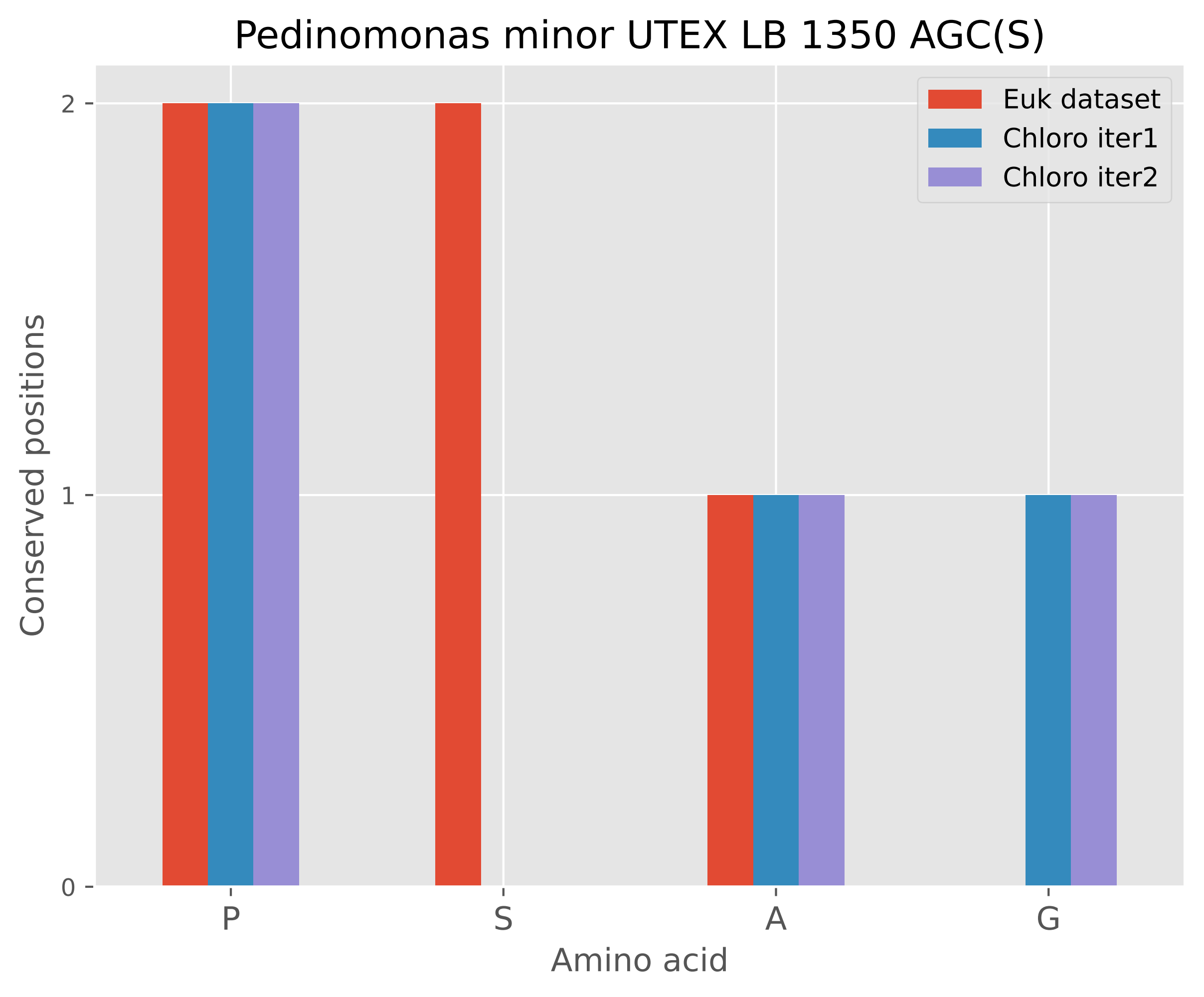
Pedinomonas minor UTEX LB 1350 AGC(S)
Euk dataset
Chloro iter1
Chloro iter2
Conserved positions
2
1
0
P
S
A
G
Amino acid

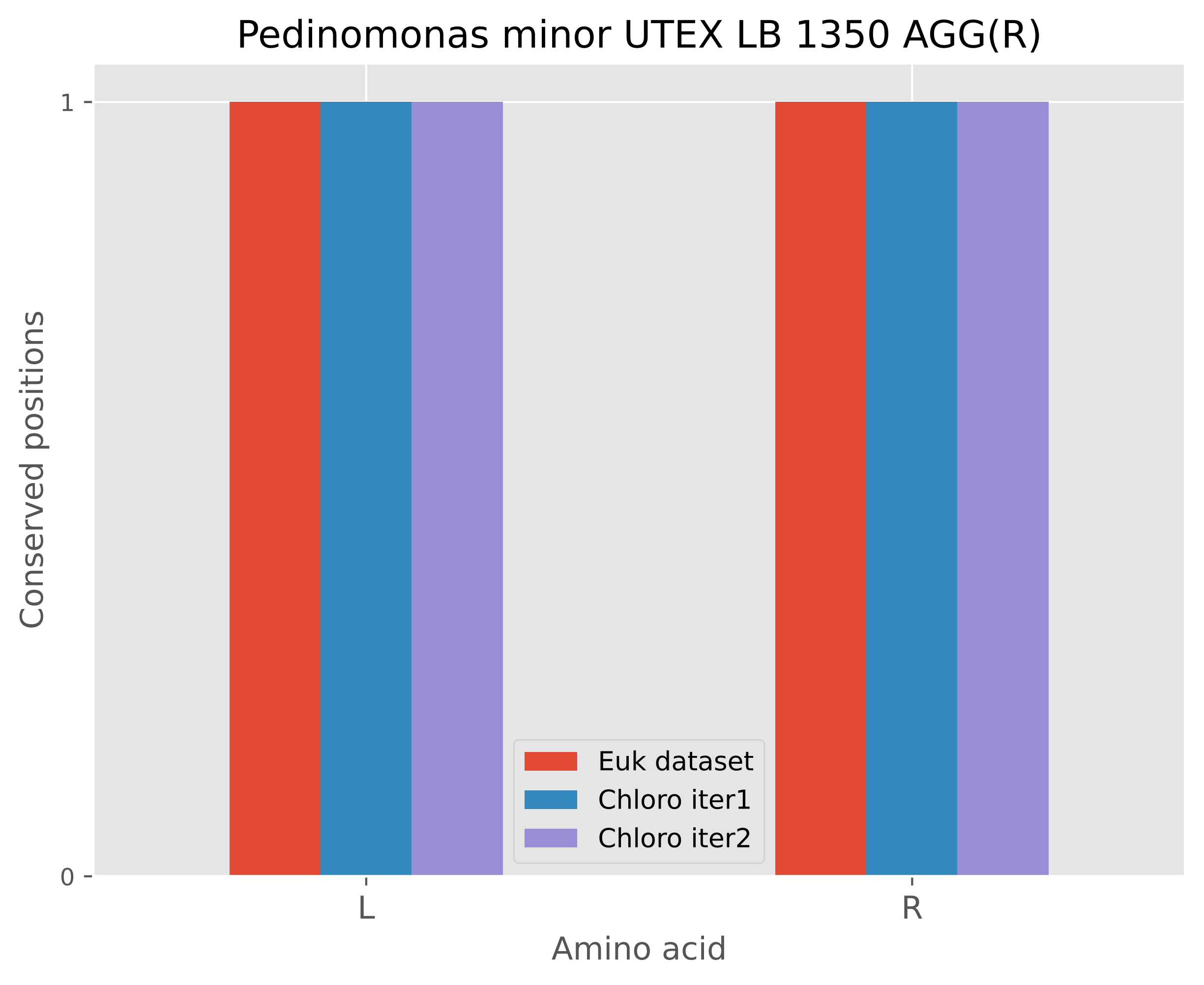
Pedinomonas minor UTEX LB 1350 AGG(R)
Conserved positions
1
0
L
R
Amino acid
Euk dataset
Chloro iter1
Chloro iter2

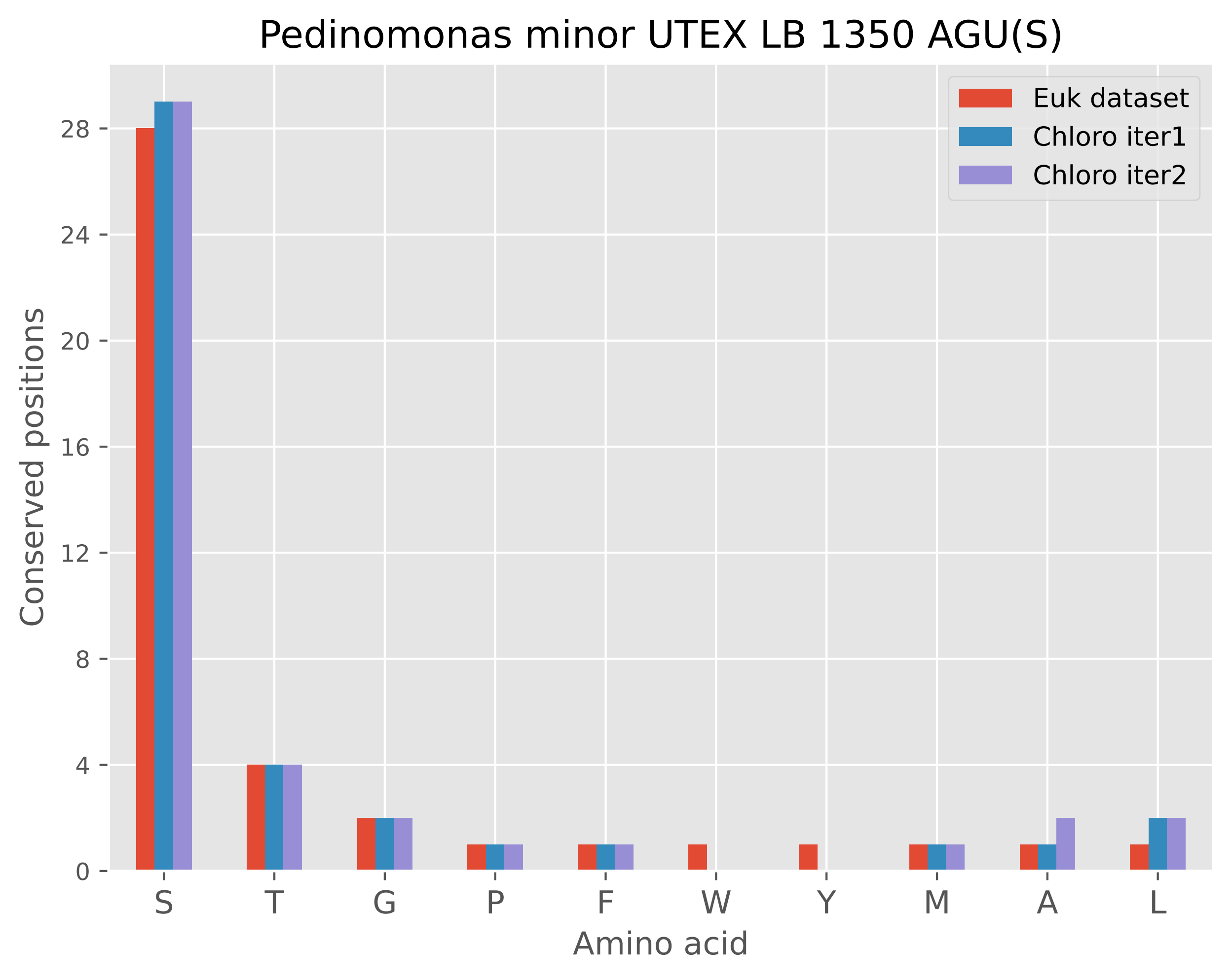

Pedinomonas minor UTEX LB 1350 AGU(S)
Euk dataset
Chloro iter1
Chloro iter2
Conserved positions
0
4
8
12
16
20
24
28
S
T
G
P
F
W
Y
M
A
L
Amino acid

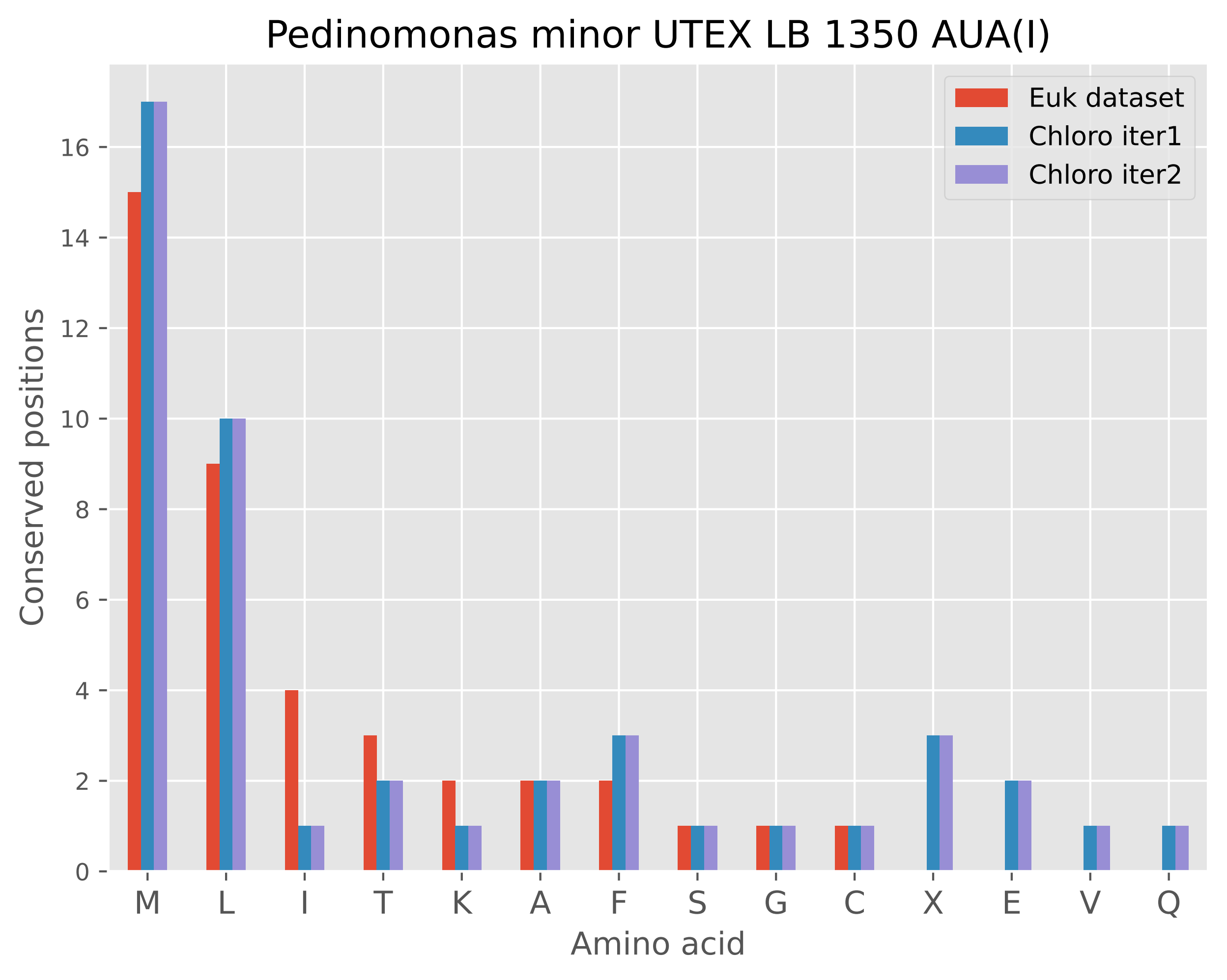
Pedinomonas minor UTEX LB 1350 AUA(I)
Euk dataset
Chloro iter1
Chloro iter2
Conserved positions
0
2
4
6
8
10
12
14
16
M
L
I
T
K
A
F
S
G
C
X
E
V
Q
Amino acid

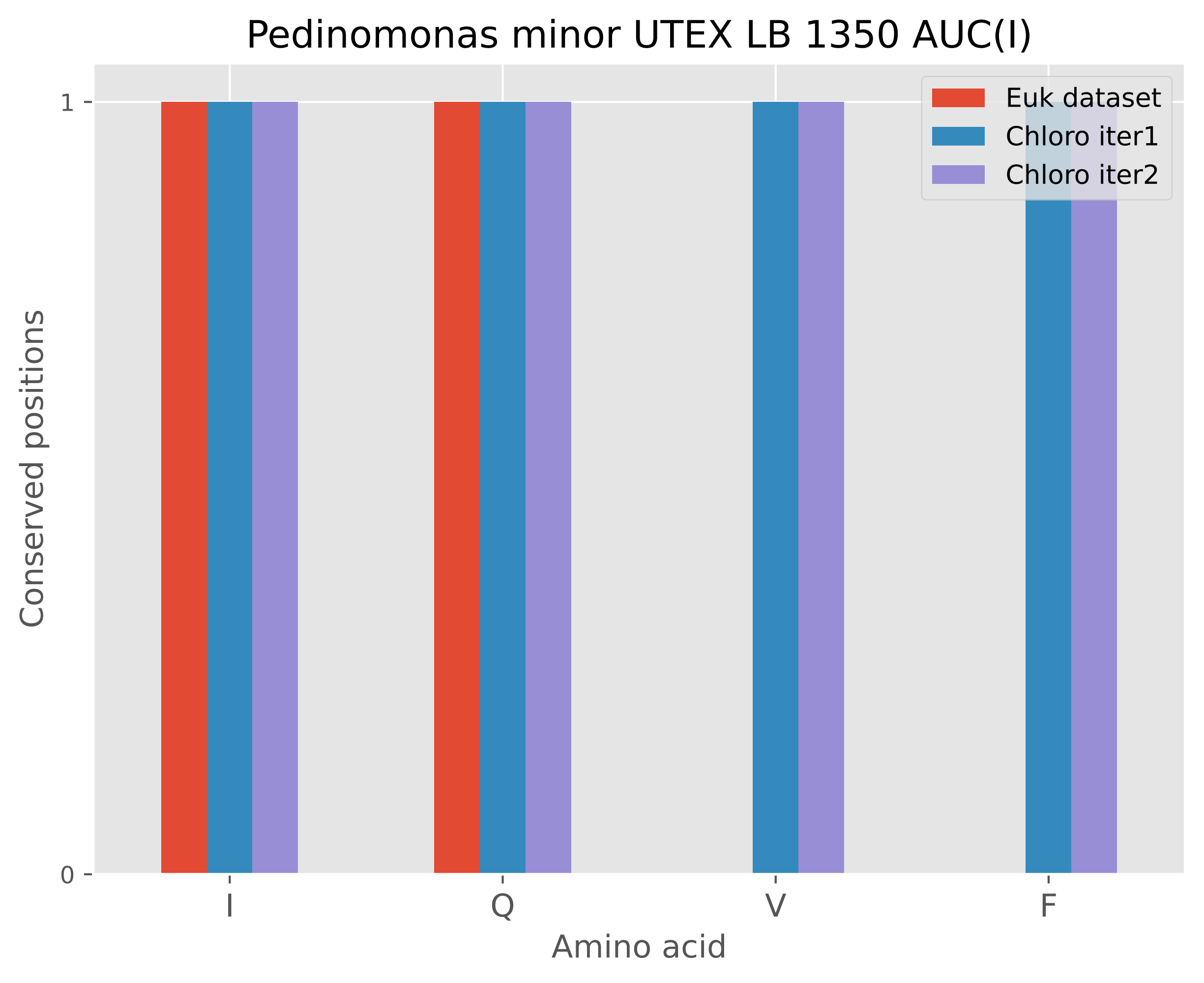

Pedinomonas minor UTEX LB 1350 AUC(I)
Euk dataset
Chloro iter1
Chloro iter2
Conserved positions
1
0
I
Q
V
F
Amino acid

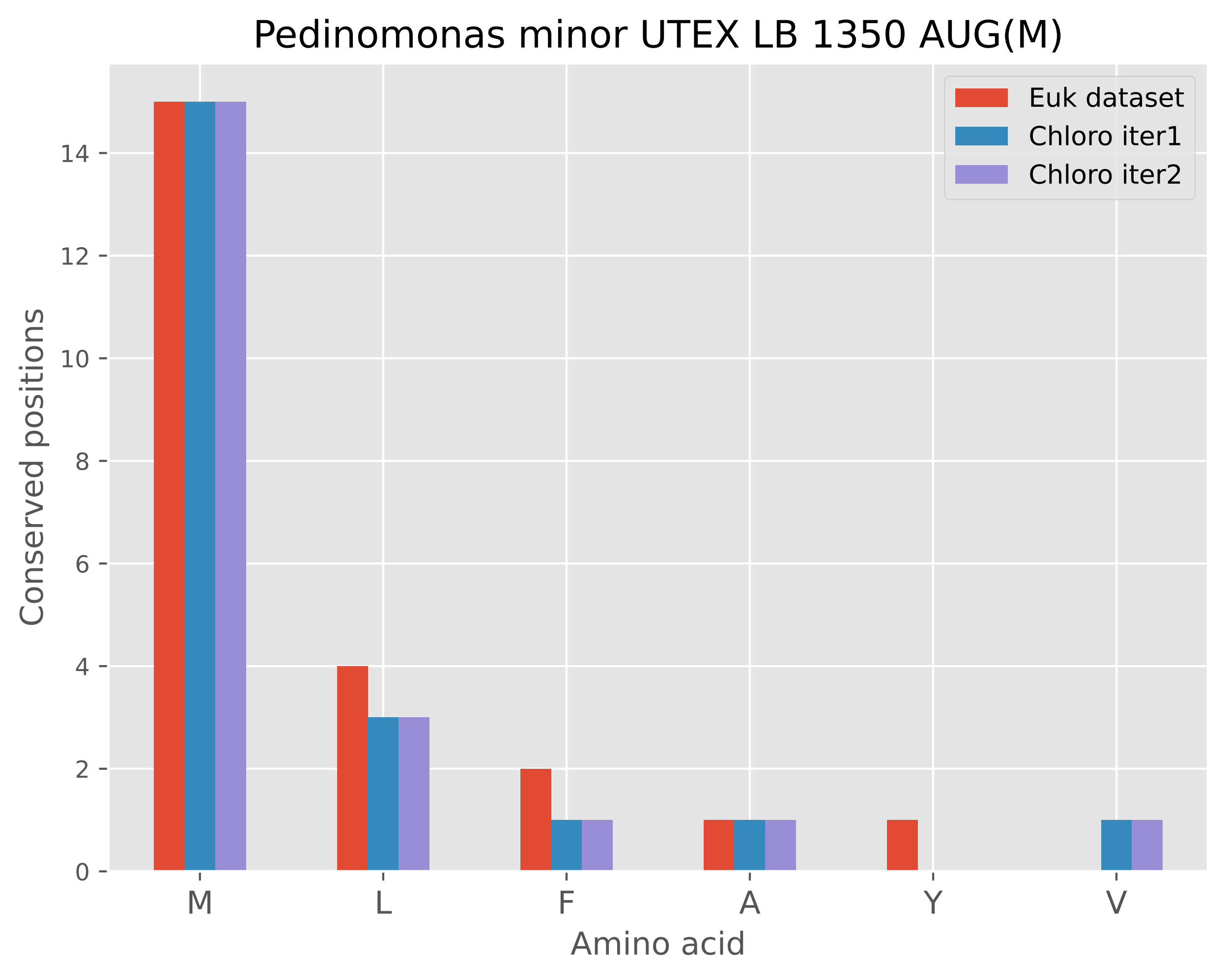

Pedinomonas minor UTEX LB 1350 AUG(M)
Euk dataset
Chloro iter1
Chloro iter2
Conserved positions
0
2
4
6
8
10
12
14
M
L
F
A
Y
V
Amino acid

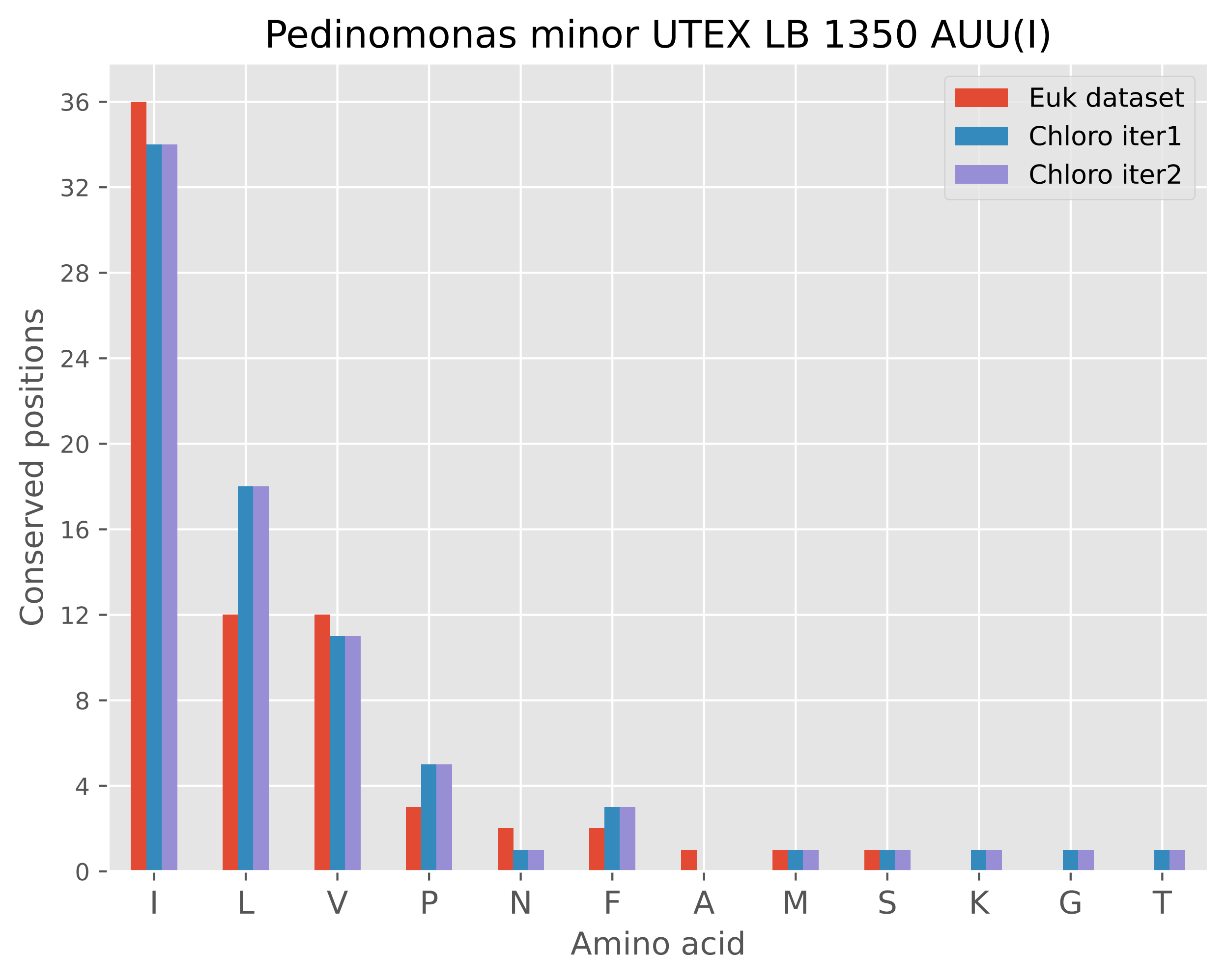

Pedinomonas minor UTEX LB 1350 AUU(I)
Euk dataset
Chloro iter1
Chloro iter2
Conserved positions
0
4
8
12
16
20
24
28
32
36
I
L
V
P
N
F
A
M
S
K
G
T
Amino acid

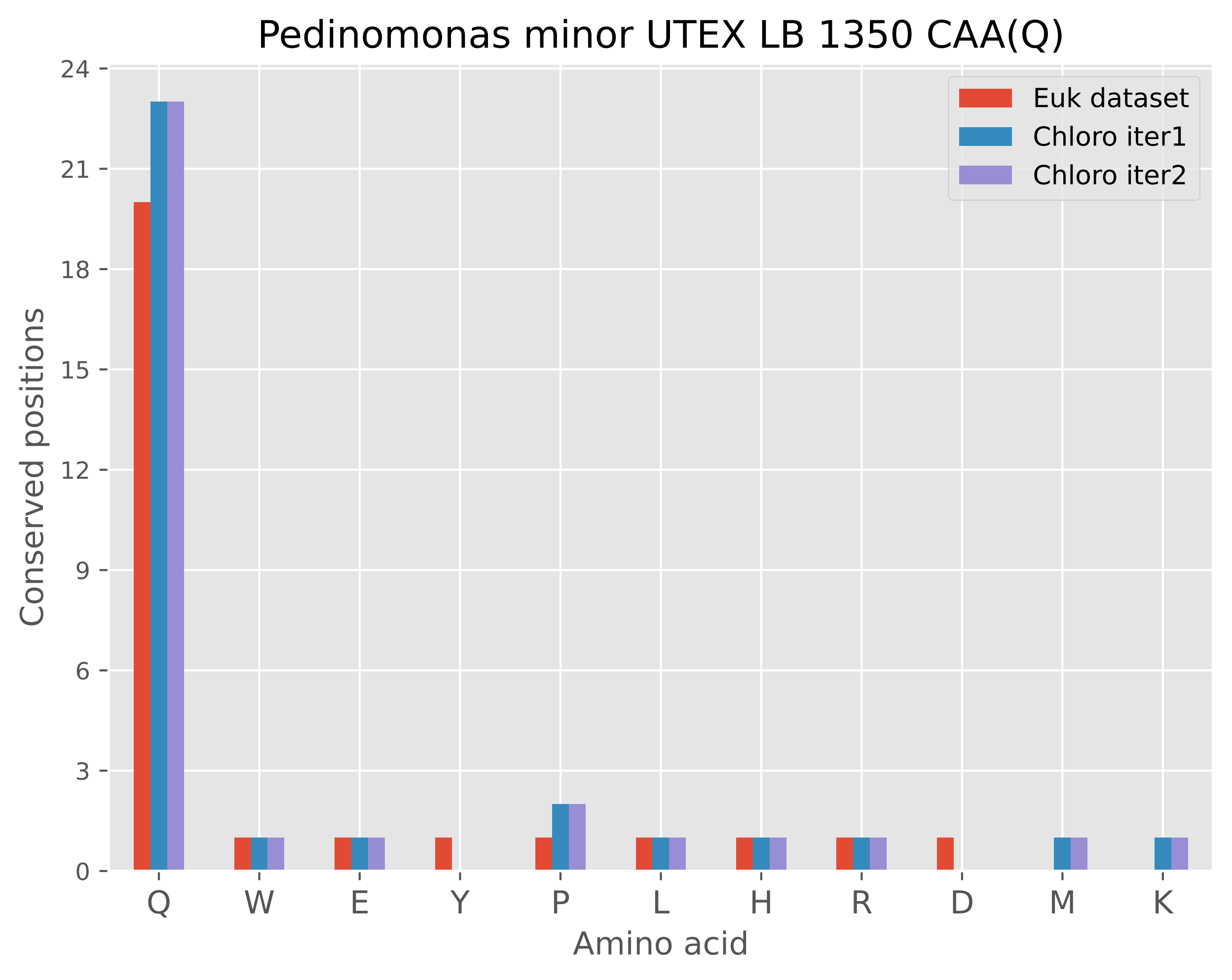

Pedinomonas minor UTEX LB 1350 CAA(Q)
Euk dataset
Chloro iter1
Chloro iter2
Conserved positions
0
3
6
9
12
15
18
21
24
Q
W
E
Y
P
L
H
R
D
M
K
Amino acid

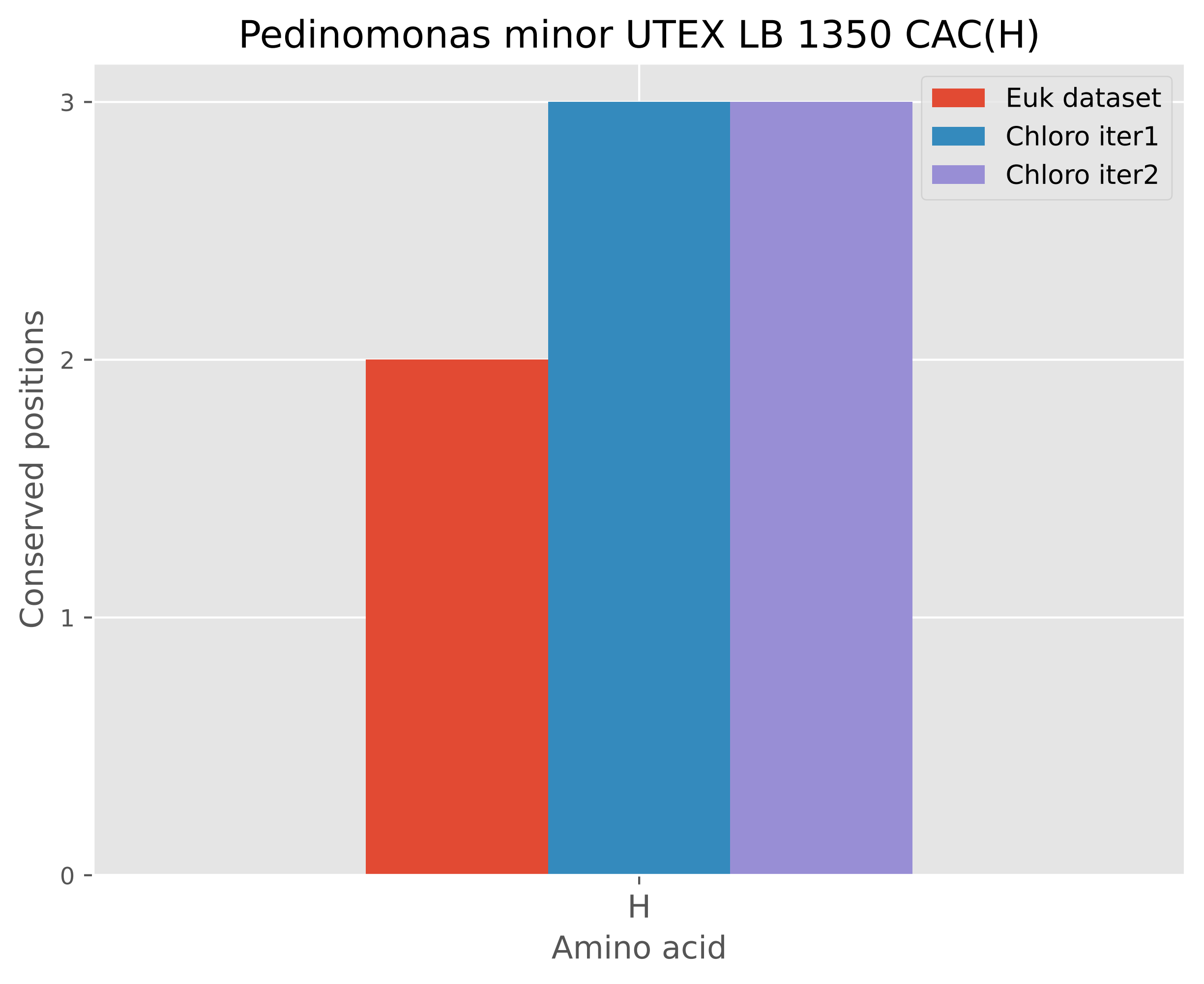
Pedinomonas minor UTEX LB 1350 CAC(H)
Euk dataset
Chloro iter1
Chloro iter2
Conserved positions
0
1
2
3
H
Amino acid

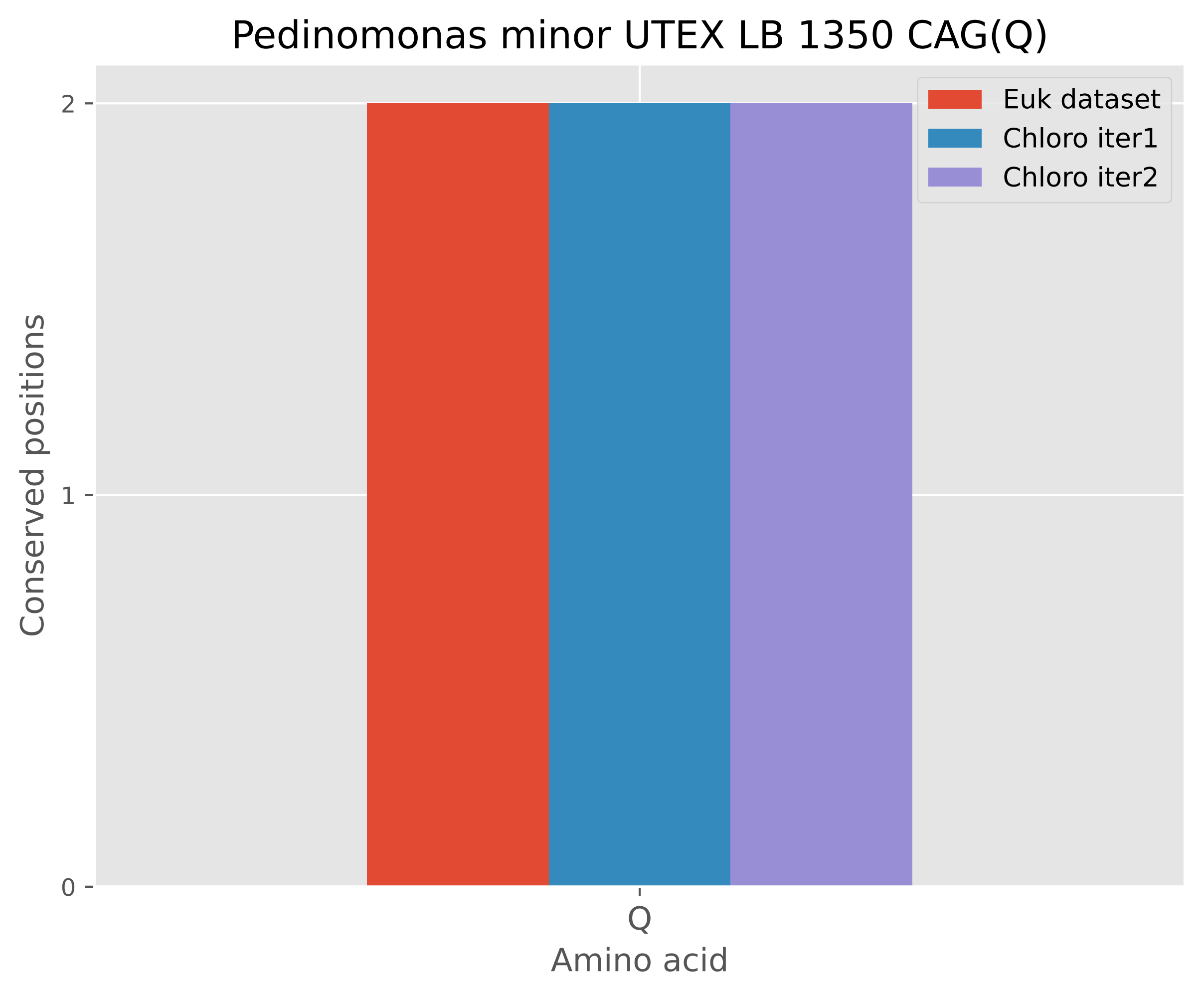
Pedinomonas minor UTEX LB 1350 CAG(Q)
Euk dataset
Chloro iter1
Chloro iter2
Conserved positions
2
1
0
Q
Amino acid

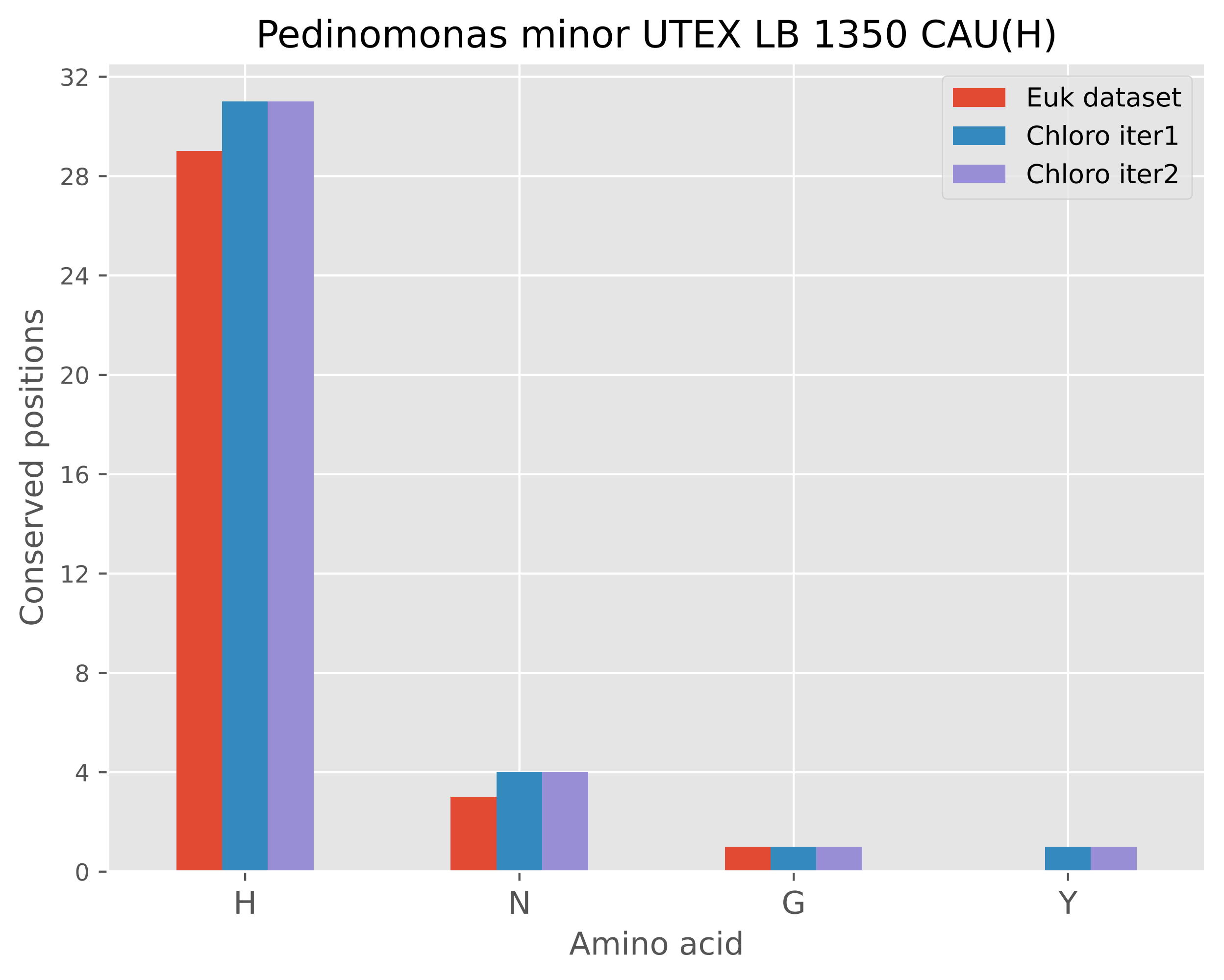

Pedinomonas minor UTEX LB 1350 CAU(H)
Conserved positions
0
4
8
12
16
20
24
28
32
Euk dataset
Chloro iter1
Chloro iter2
H
N
G
Y
Amino acid

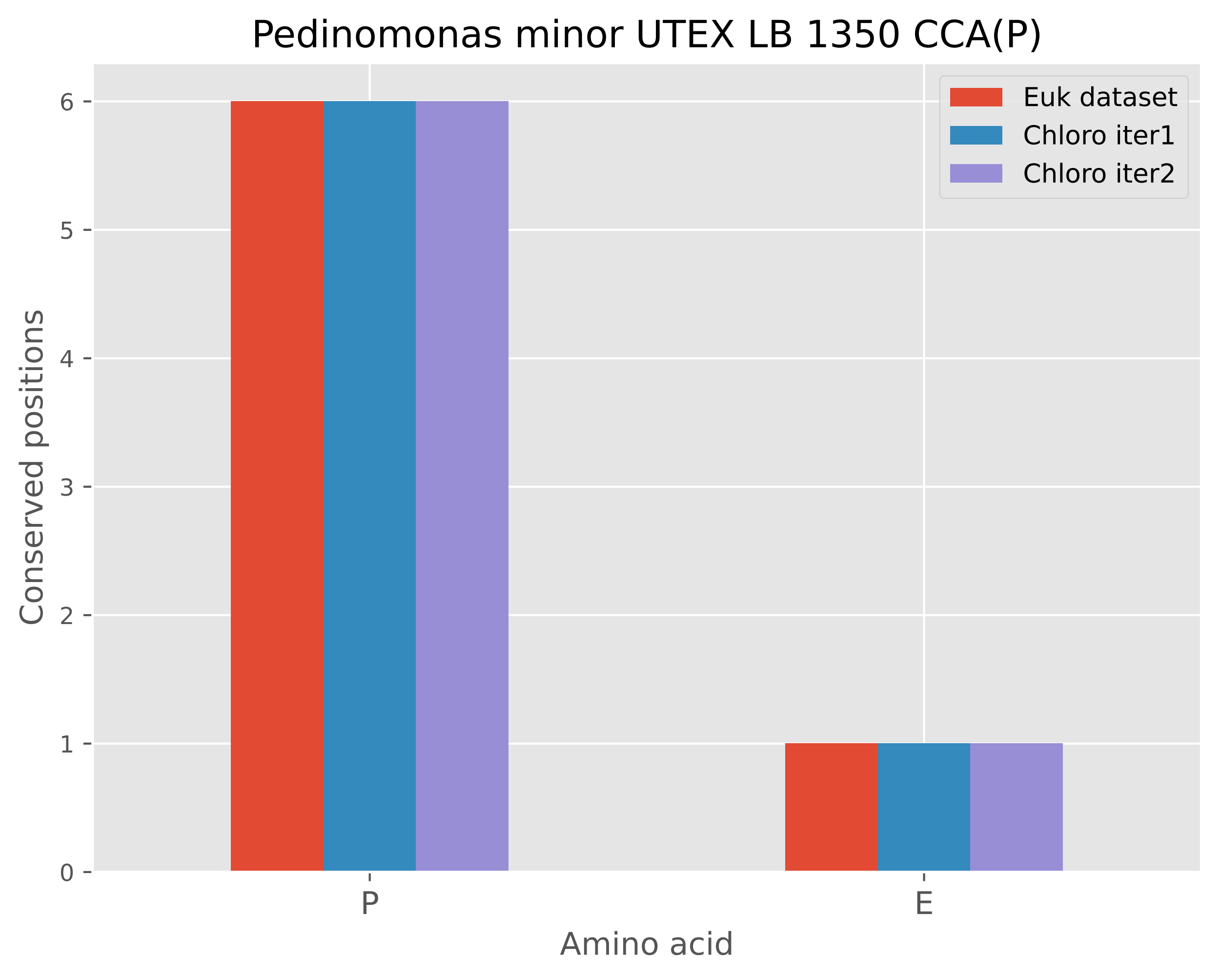
Pedinomonas minor UTEX LB 1350 CCA(P)
Euk dataset
Chloro iter1
Chloro iter2
Conserved positions
0
1
2
3
4
5
6
P
E
Amino acid

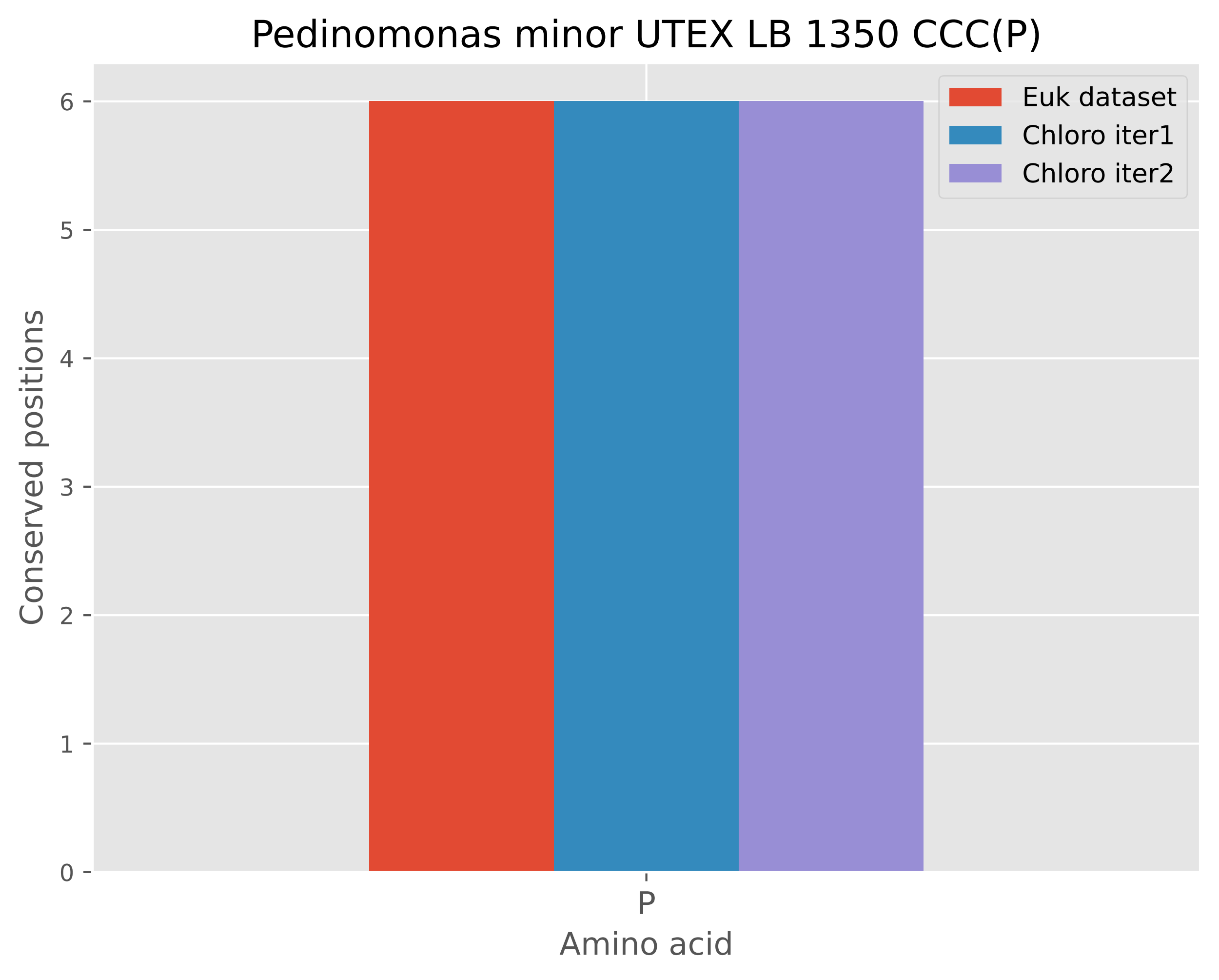

Pedinomonas minor UTEX LB 1350 CCC(P)
Euk dataset
Chloro iter1
Chloro iter2
Conserved positions
0
1
2
3
4
5
6
P
Amino acid

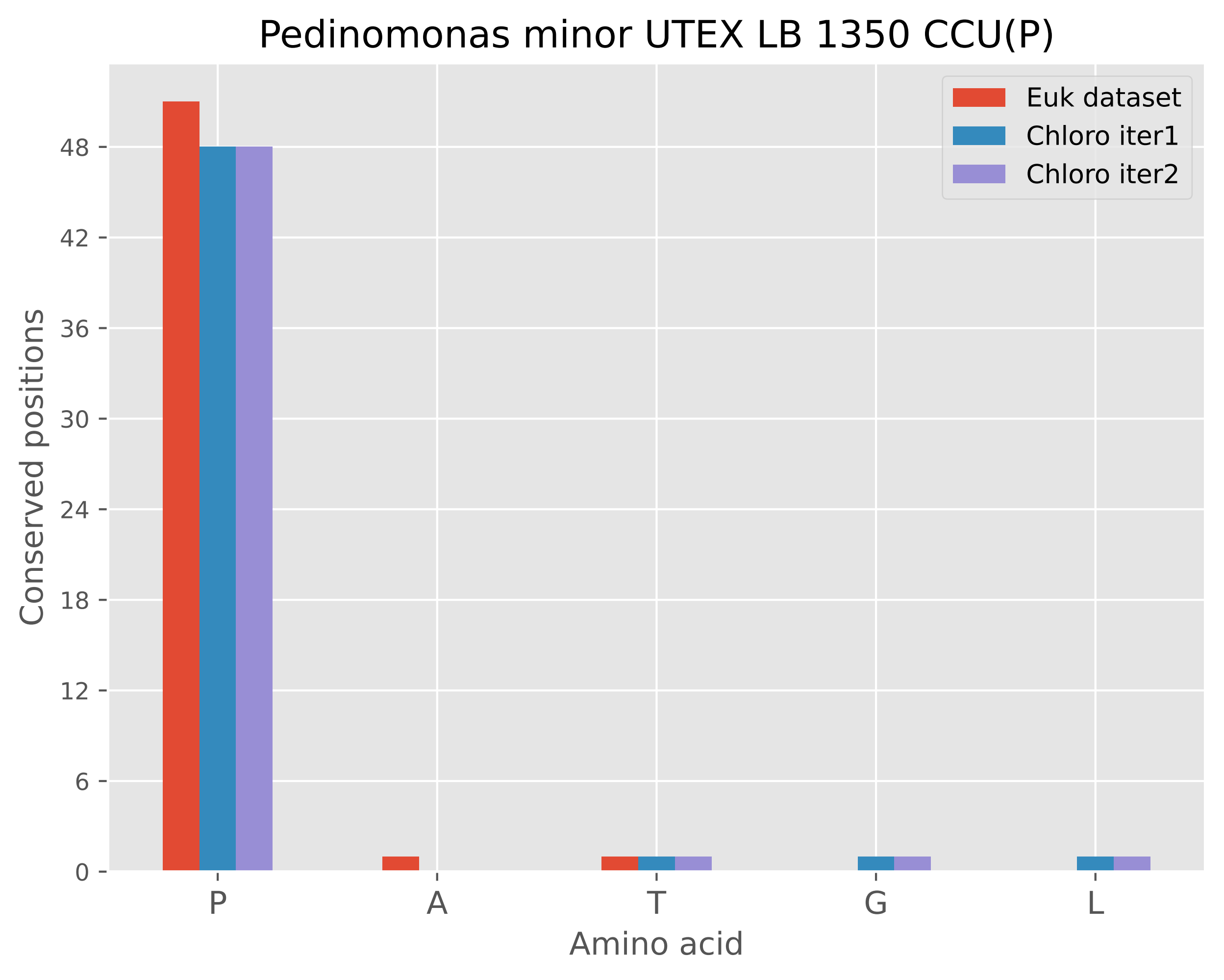
Pedinomonas minor UTEX LB 1350 CCU(P)
Euk dataset
Chloro iter1
Chloro iter2
Conserved positions
0
6
12
18
24
30
36
42
48
P
A
T
G
L
Amino acid

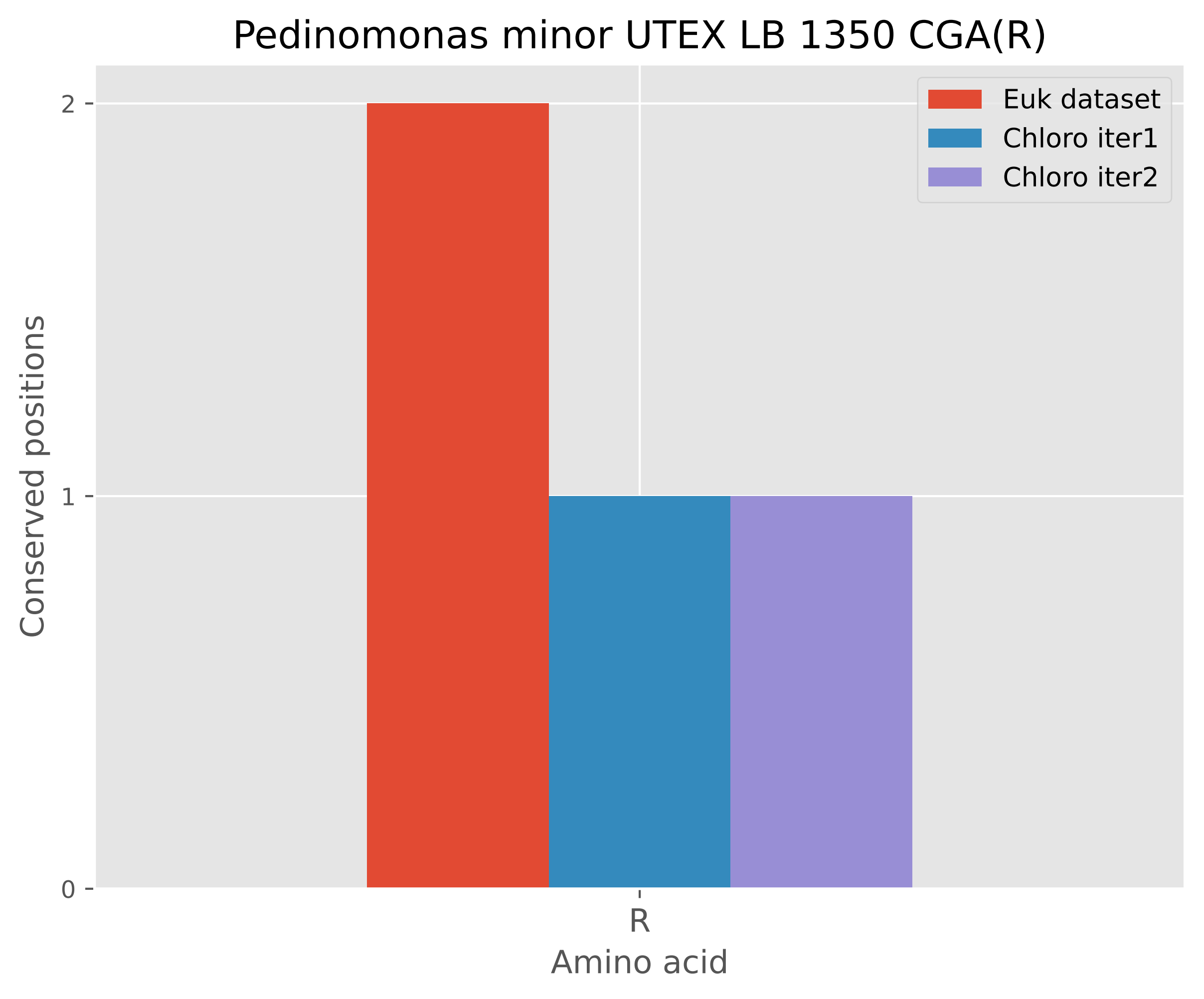
Pedinomonas minor UTEX LB 1350 CGA(R)
Euk dataset
Chloro iter1
Chloro iter2
Conserved positions
2
1
0
R
Amino acid

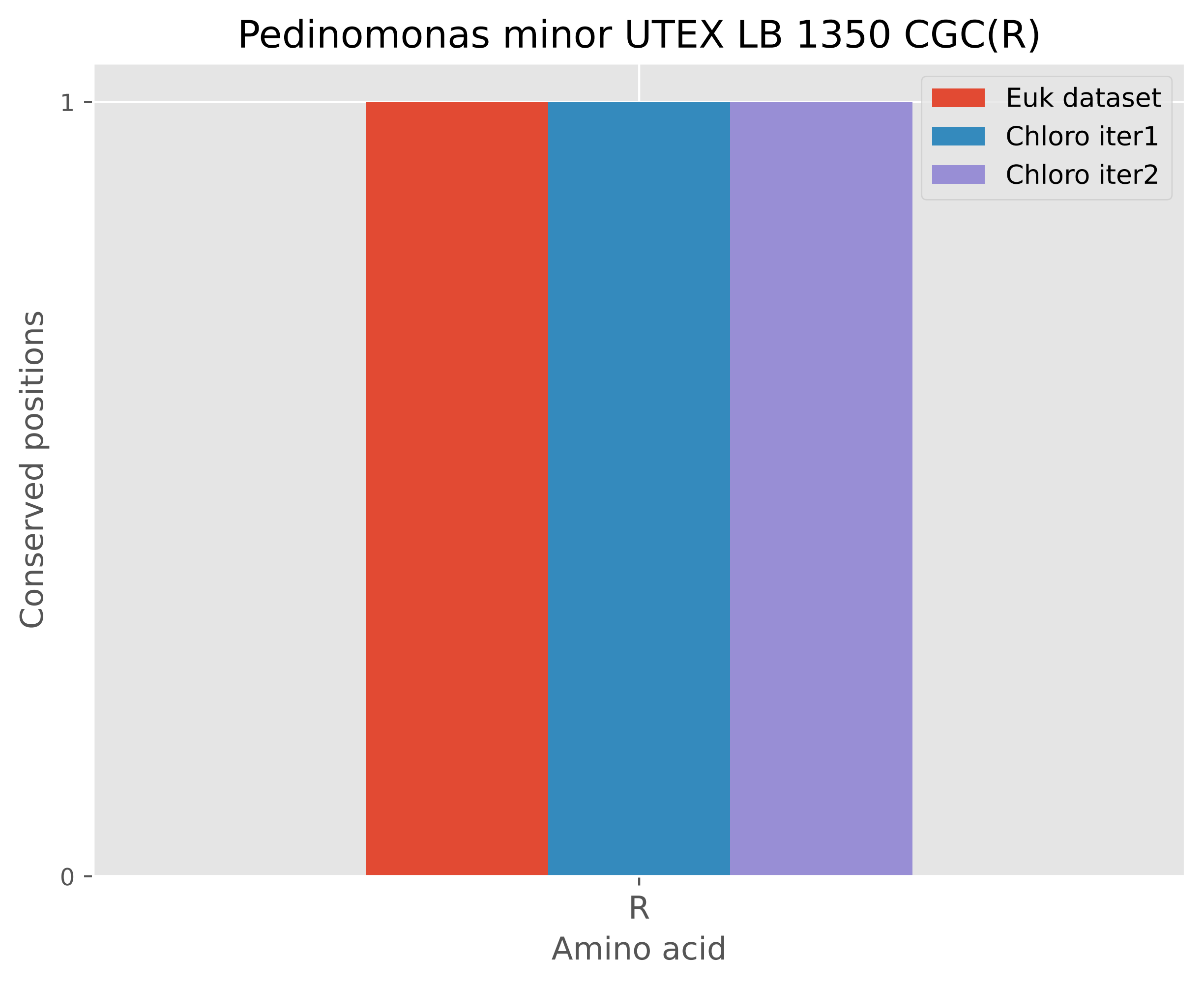

Pedinomonas minor UTEX LB 1350 CGC(R)
Euk dataset
Chloro iter1
Chloro iter2
1
0
Conserved positions
R
Amino acid

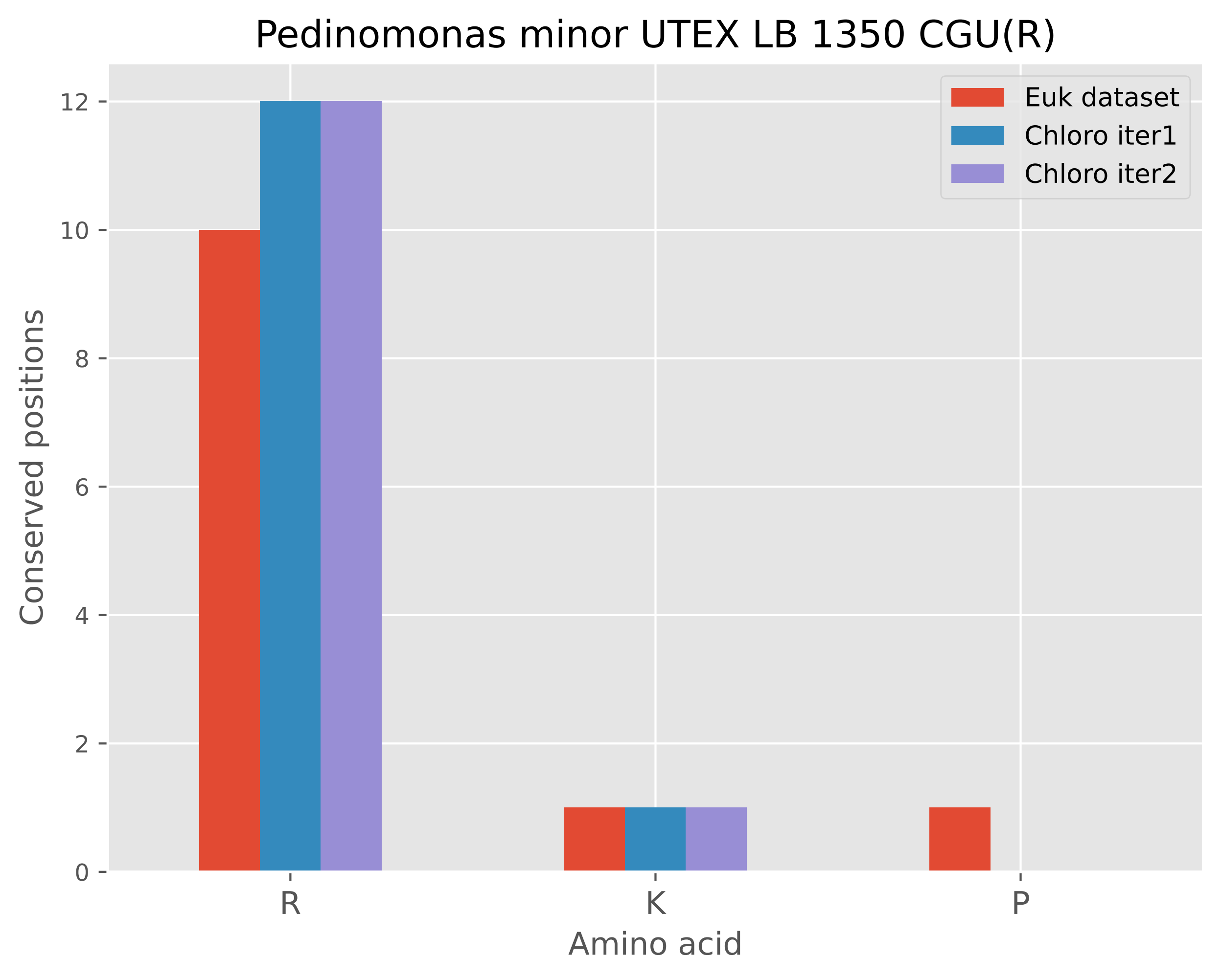
Pedinomonas minor UTEX LB 1350 CGU(R)
Euk dataset
Chloro iter1
Chloro iter2
Conserved positions
0
2
4
6
8
10
12
R
K
P
Amino acid

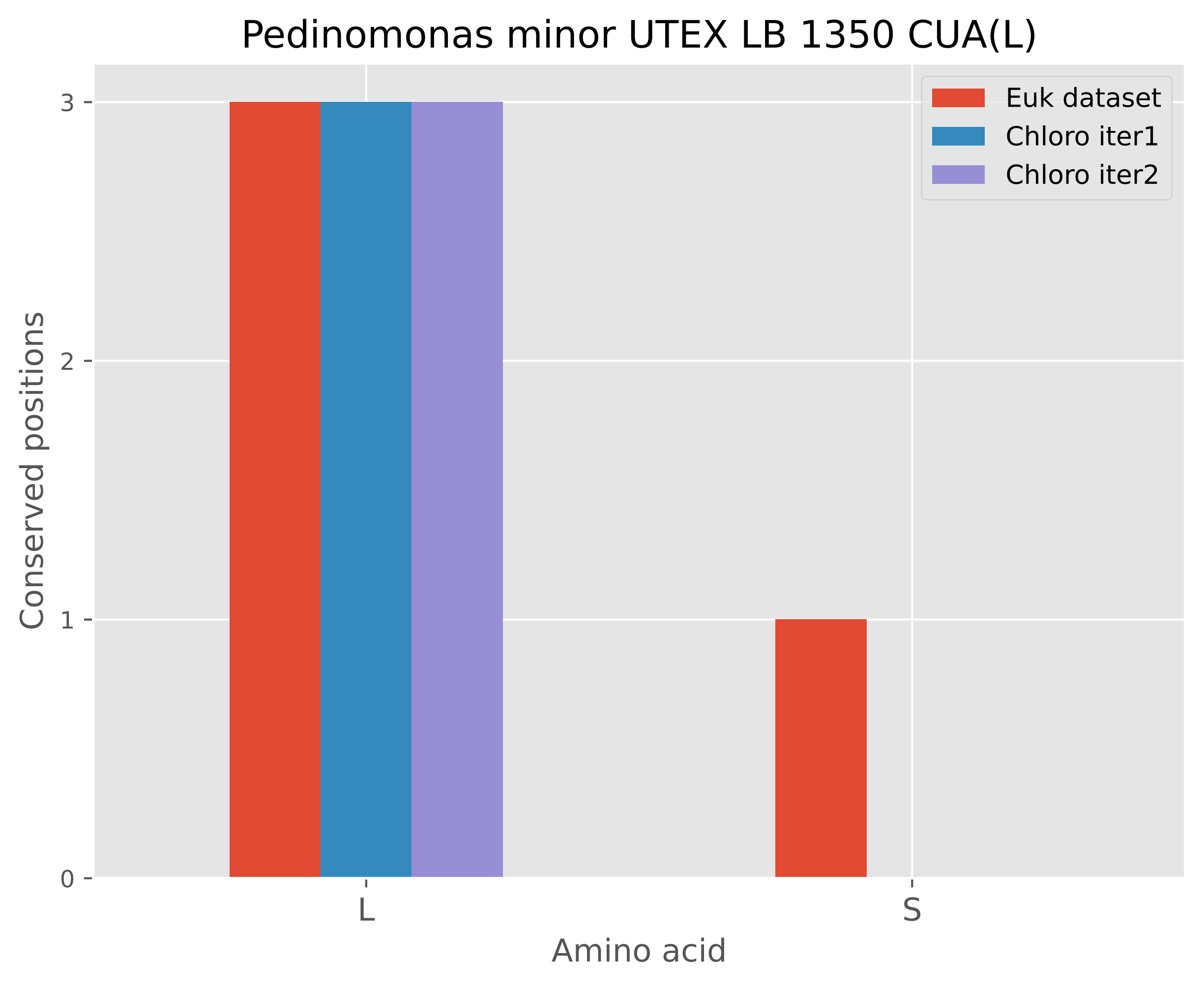

Pedinomonas minor UTEX LB 1350 CUA(L)
Euk dataset
Chloro iter1
Chloro iter2
Conserved positions
0
1
2
3
L
S
Amino acid

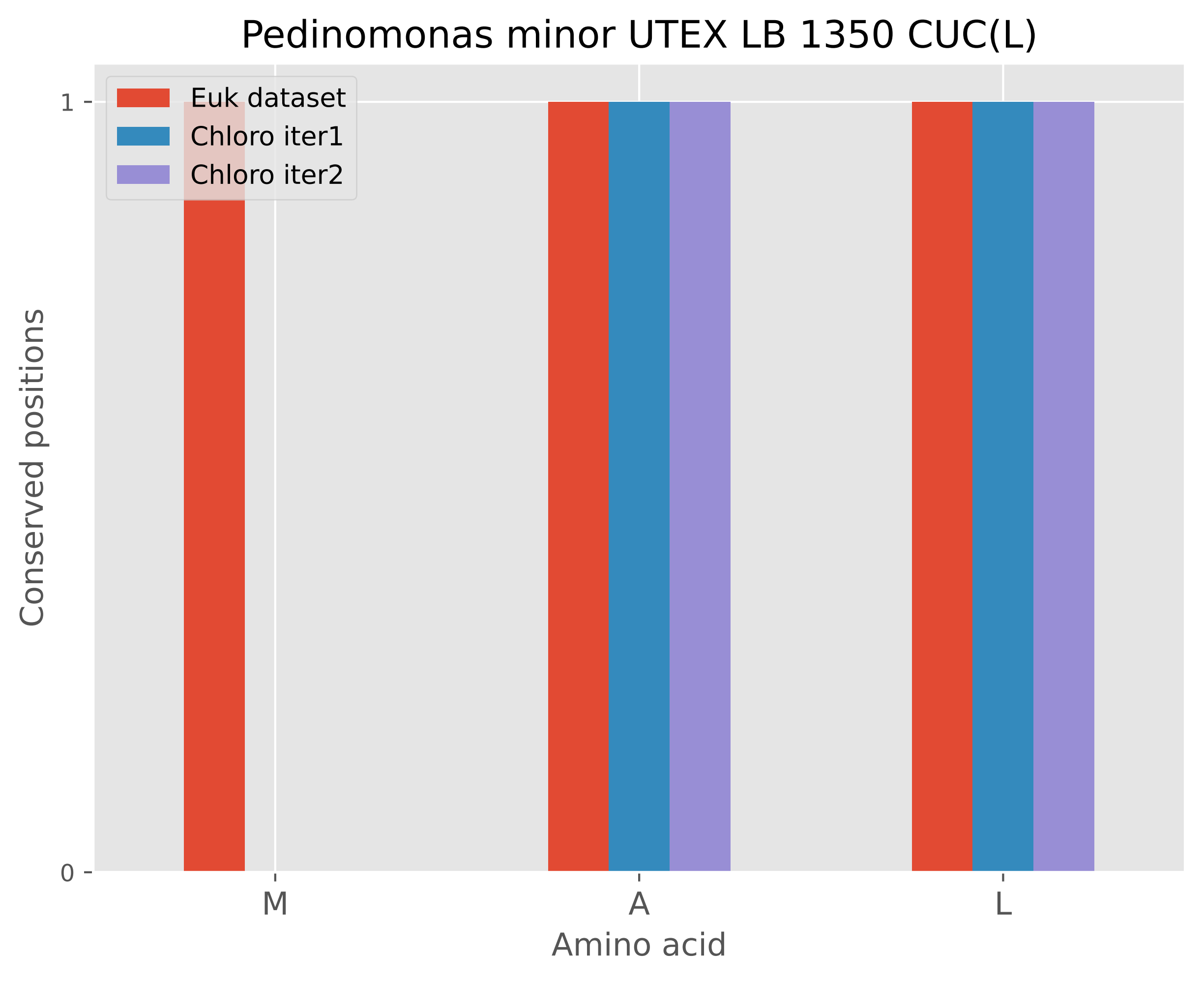
Pedinomonas minor UTEX LB 1350 CUC(L)
Euk dataset
Chloro iter1
Chloro iter2
Conserved positions
1
0
M
A
L
Amino acid

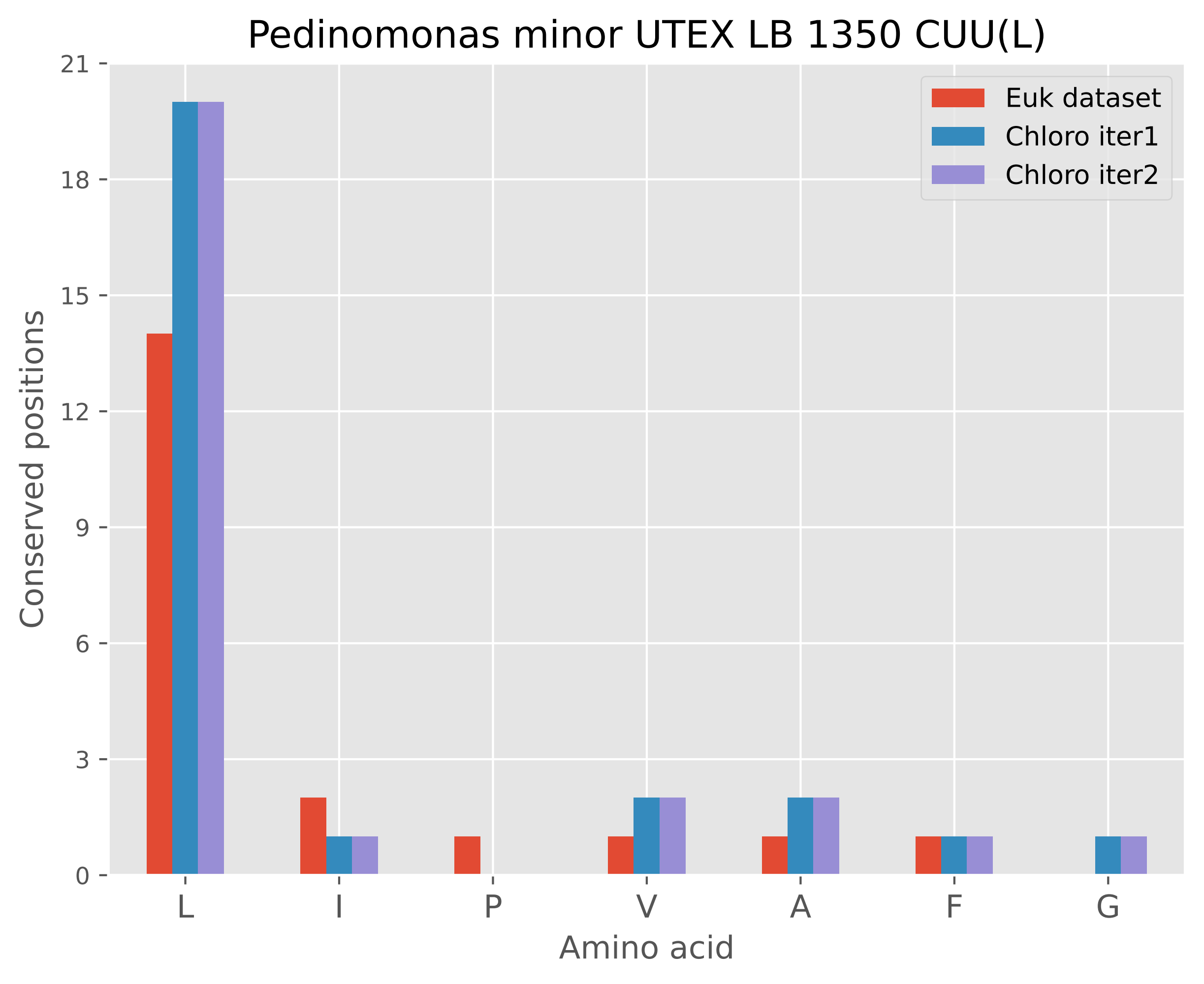

Pedinomonas minor UTEX LB 1350 CUU(L)
Conserved positions
Amino acid
L
I
P
V
A
F
G
0
3
6
9
12
15
18
21
Euk dataset
Chloro iter1
Chloro iter2

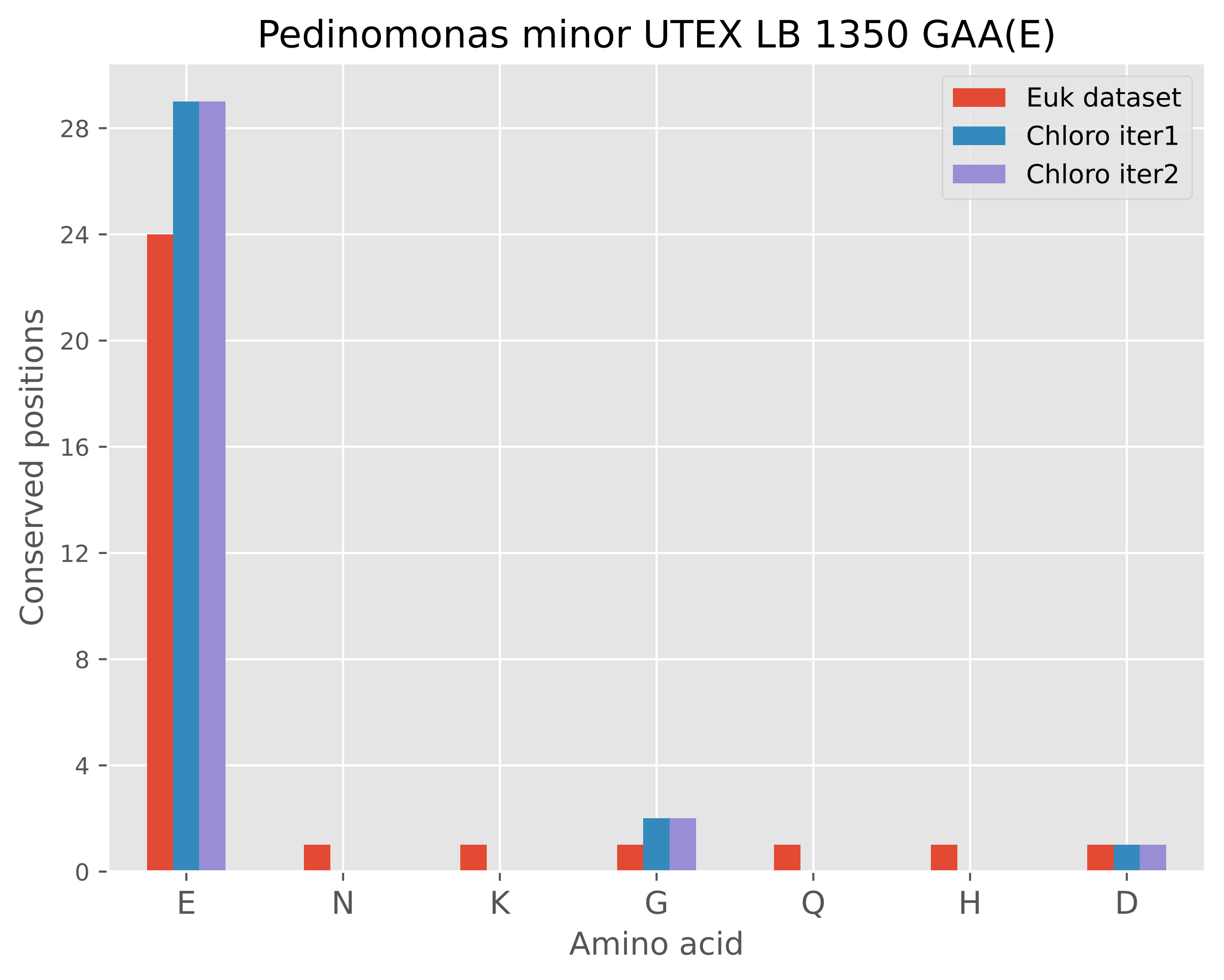

Pedinomonas minor UTEX LB 1350 GAA(E)
Euk dataset
Chloro iter1
Chloro iter2
Conserved positions
0
4
8
12
16
20
24
28
E
N
K
G
Q
H
D
Amino acid

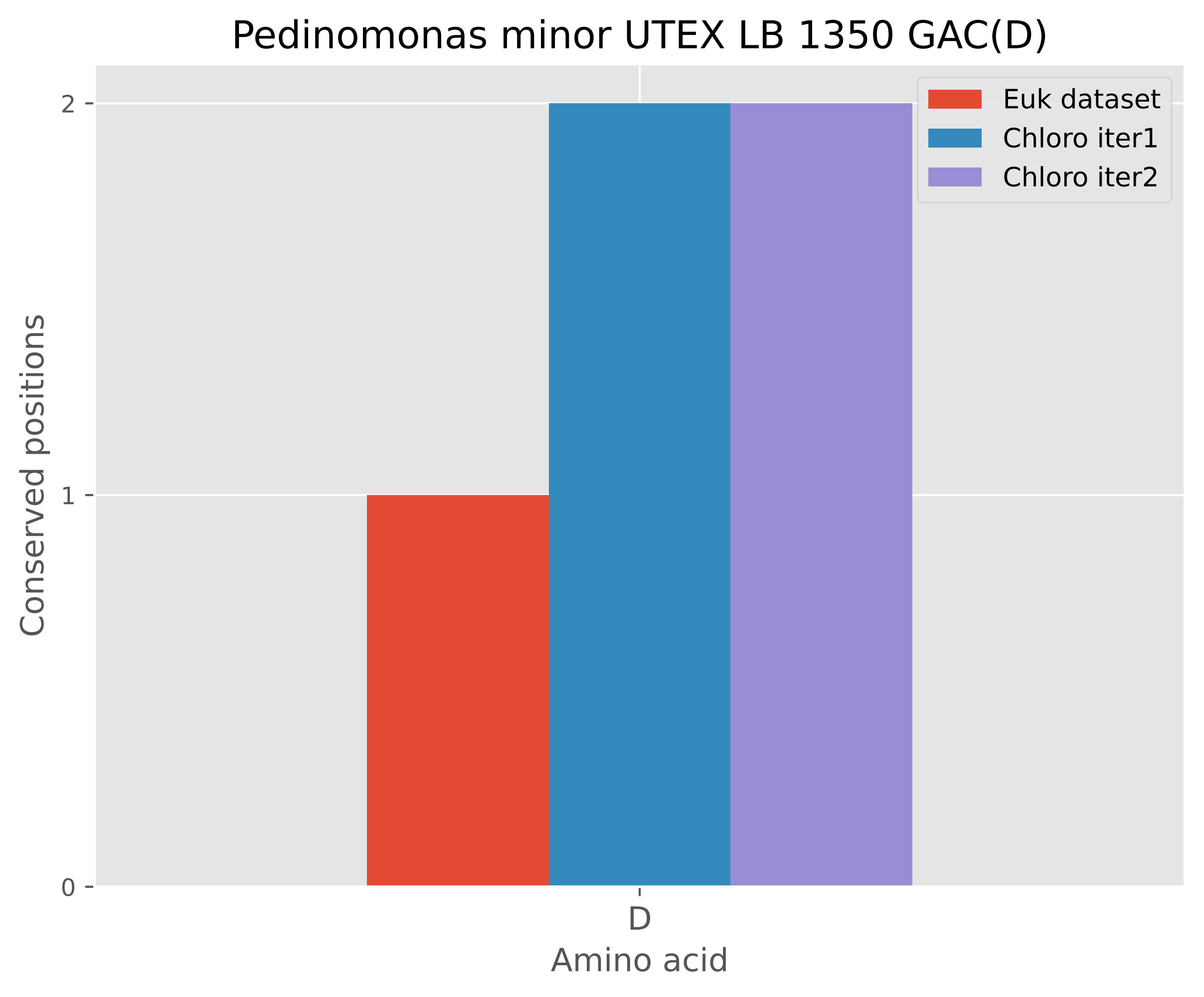

Pedinomonas minor UTEX LB 1350 GAC(D)
Euk dataset
Chloro iter1
Chloro iter2
Conserved positions
0
1
2
D
Amino acid

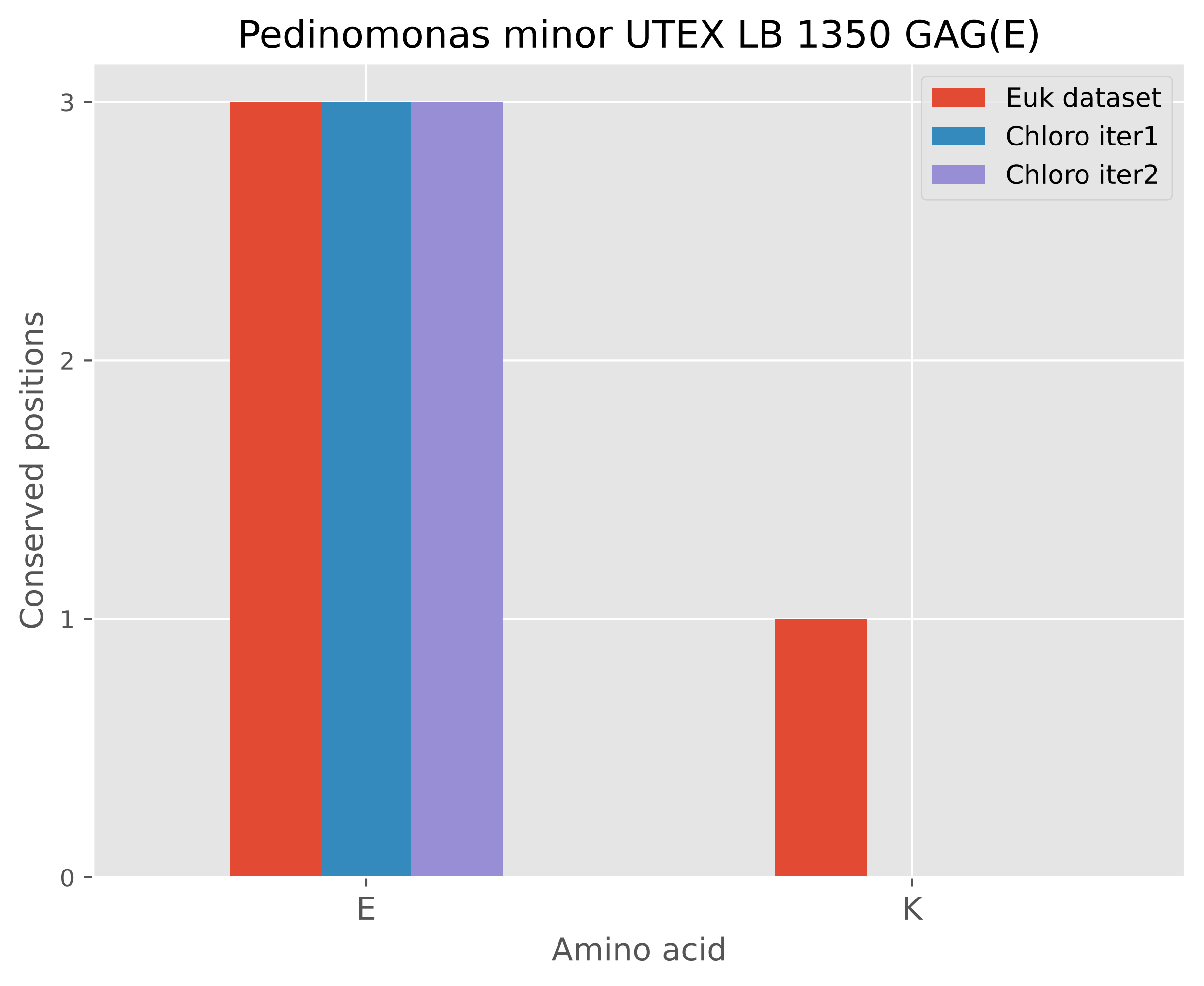
Pedinomonas minor UTEX LB 1350 GAG(E)
Euk dataset
Chloro iter1
Chloro iter2
Conserved positions
0
1
2
3
E
K
Amino acid

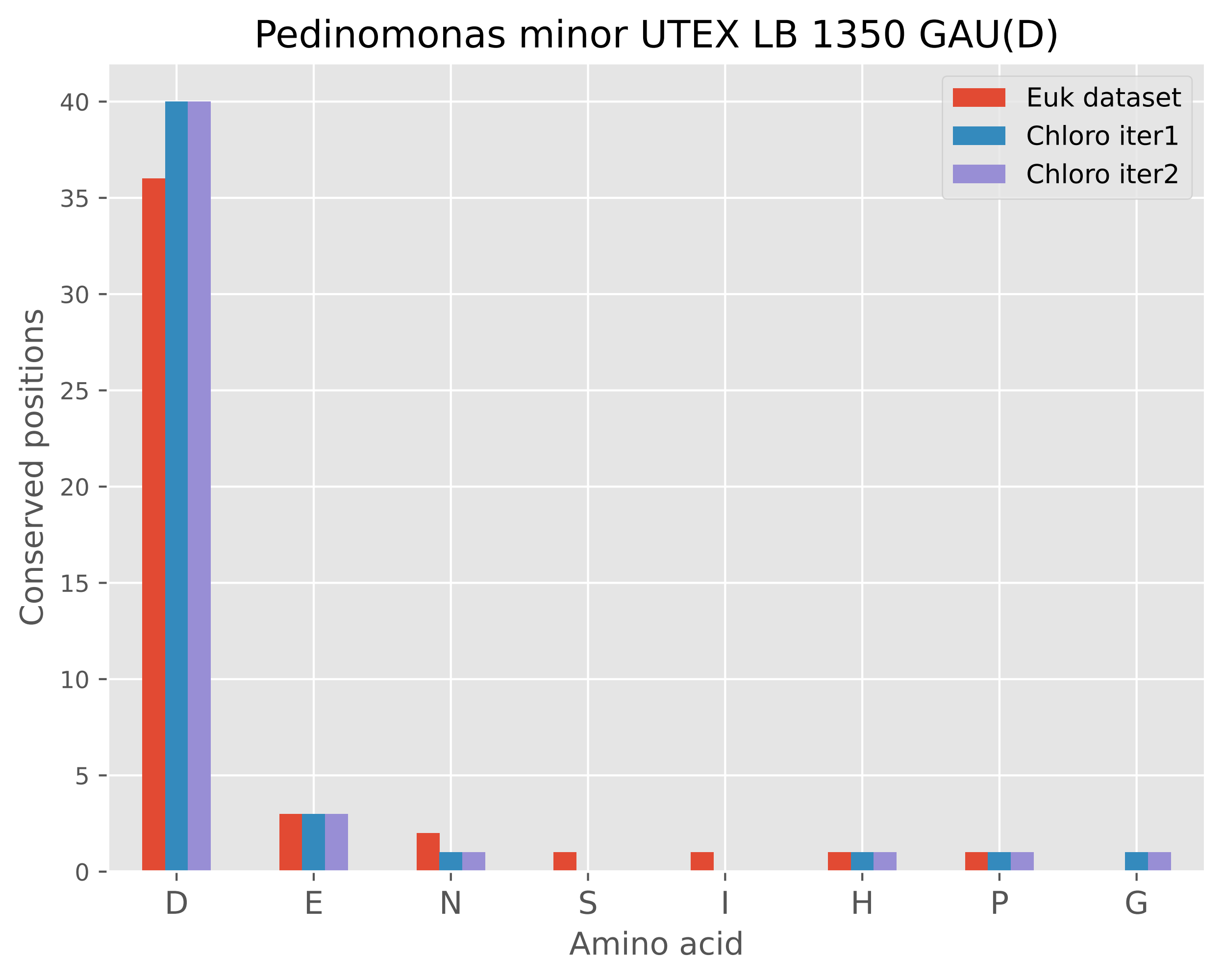

Pedinomonas minor UTEX LB 1350 GAU(D)
Euk dataset
Chloro iter1
Chloro iter2
Conserved positions
0
5
10
15
20
25
30
35
40
D
E
N
S
I
H
P
G
Amino acid

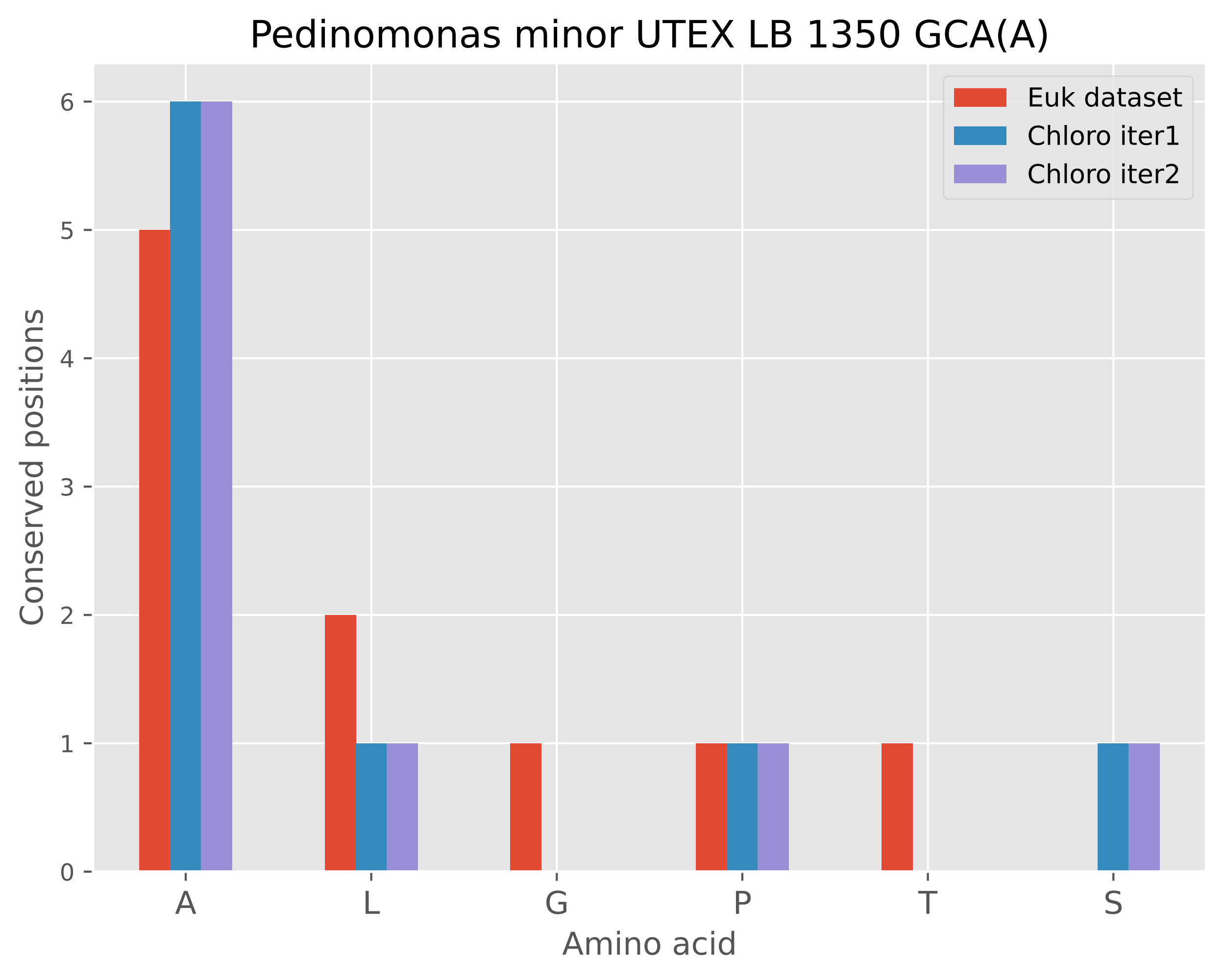
Pedinomonas minor UTEX LB 1350 GCA(A)
Euk dataset
Chloro iter1
Chloro iter2
Conserved positions
0
1
2
3
4
5
6
A
L
G
P
T
S
Amino acid

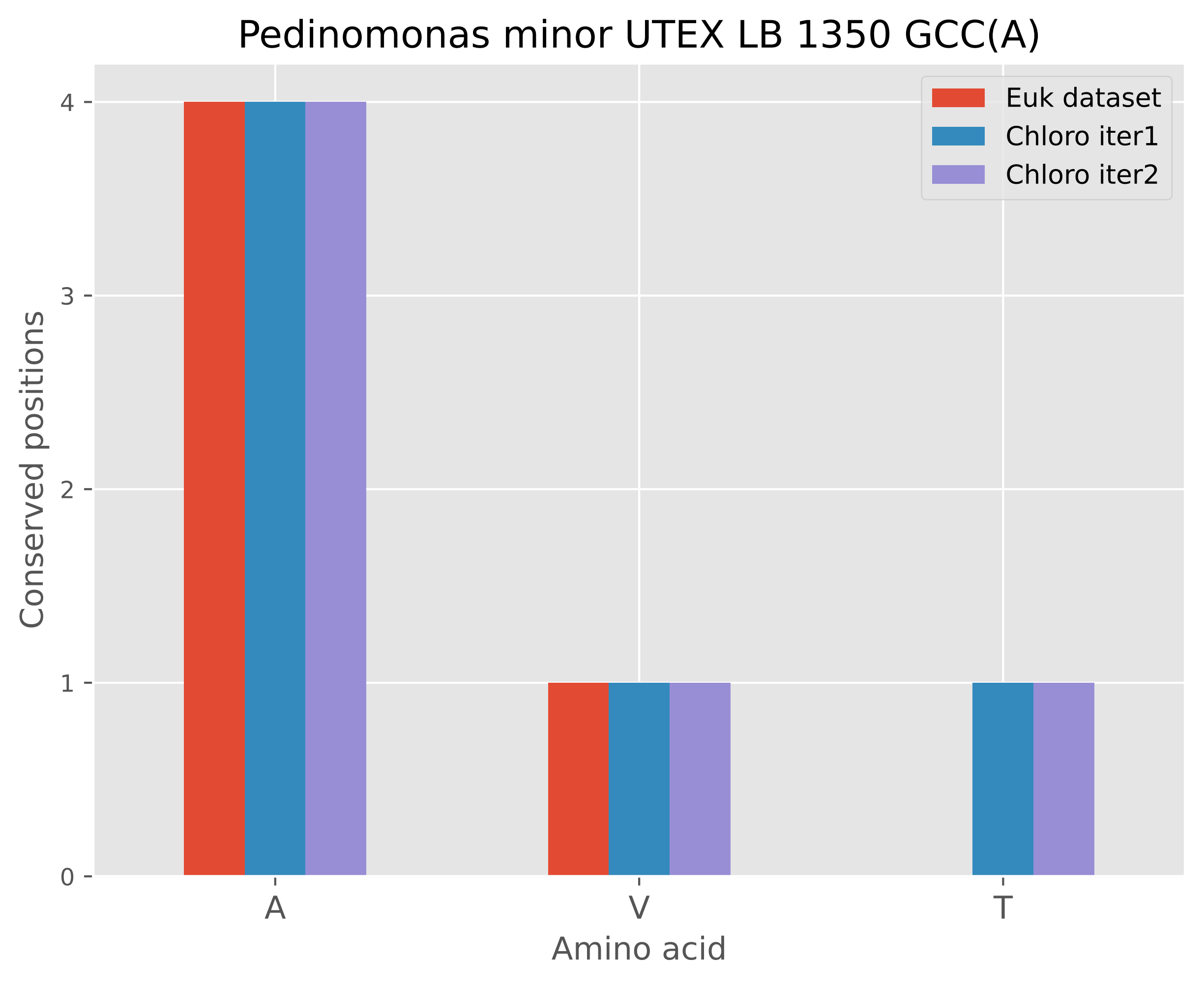
Pedinomonas minor UTEX LB 1350 GCC(A)
Conserved positions
0
1
2
3
4
Euk dataset
Chloro iter1
Chloro iter2
A
V
T
Amino acid

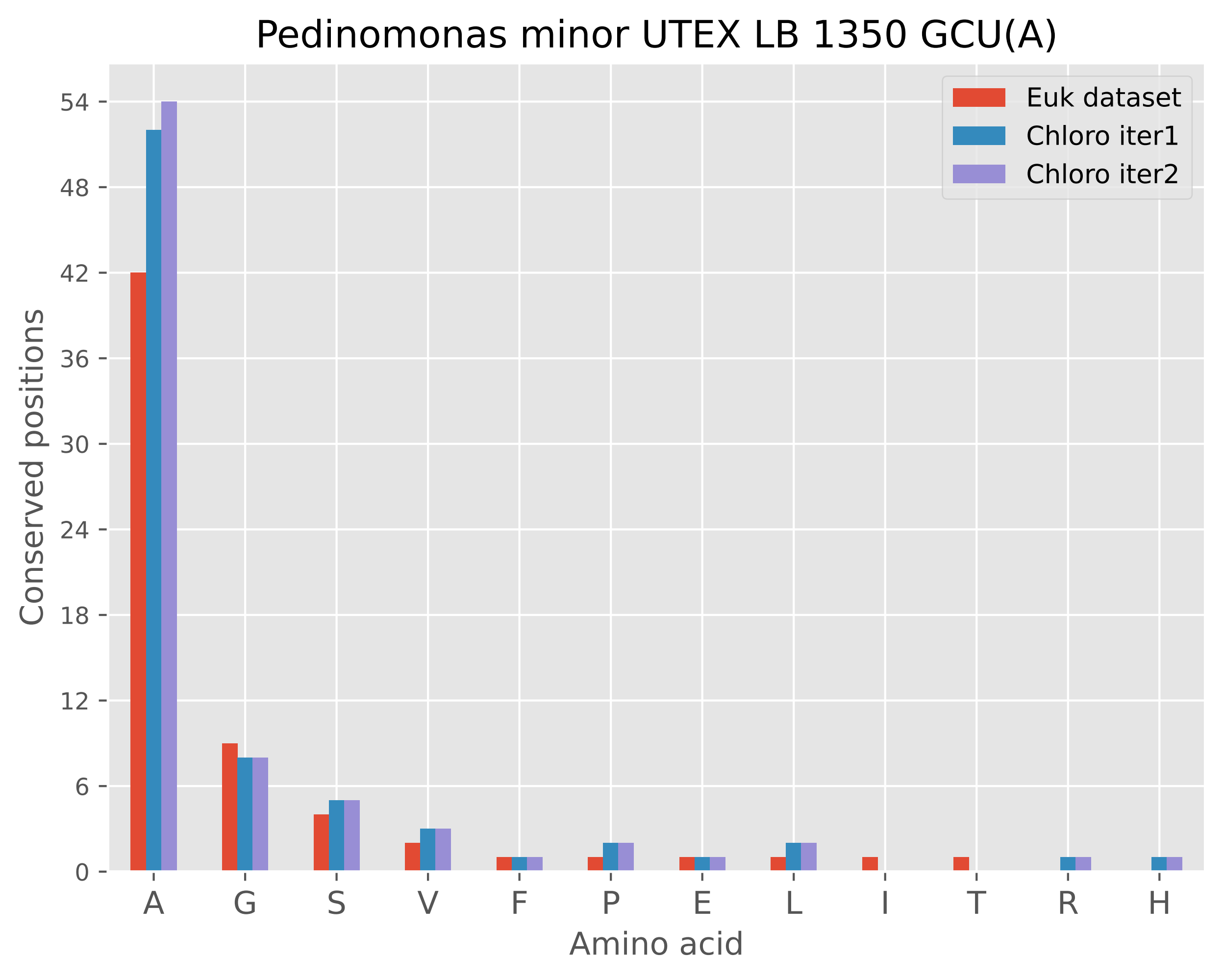

Pedinomonas minor UTEX LB 1350 GCU(A)
Euk dataset
Chloro iter1
Chloro iter2
Conserved positions
0
6
12
18
24
30
36
42
48
54
A
G
S
V
F
P
E
L
I
T
R
H
Amino acid

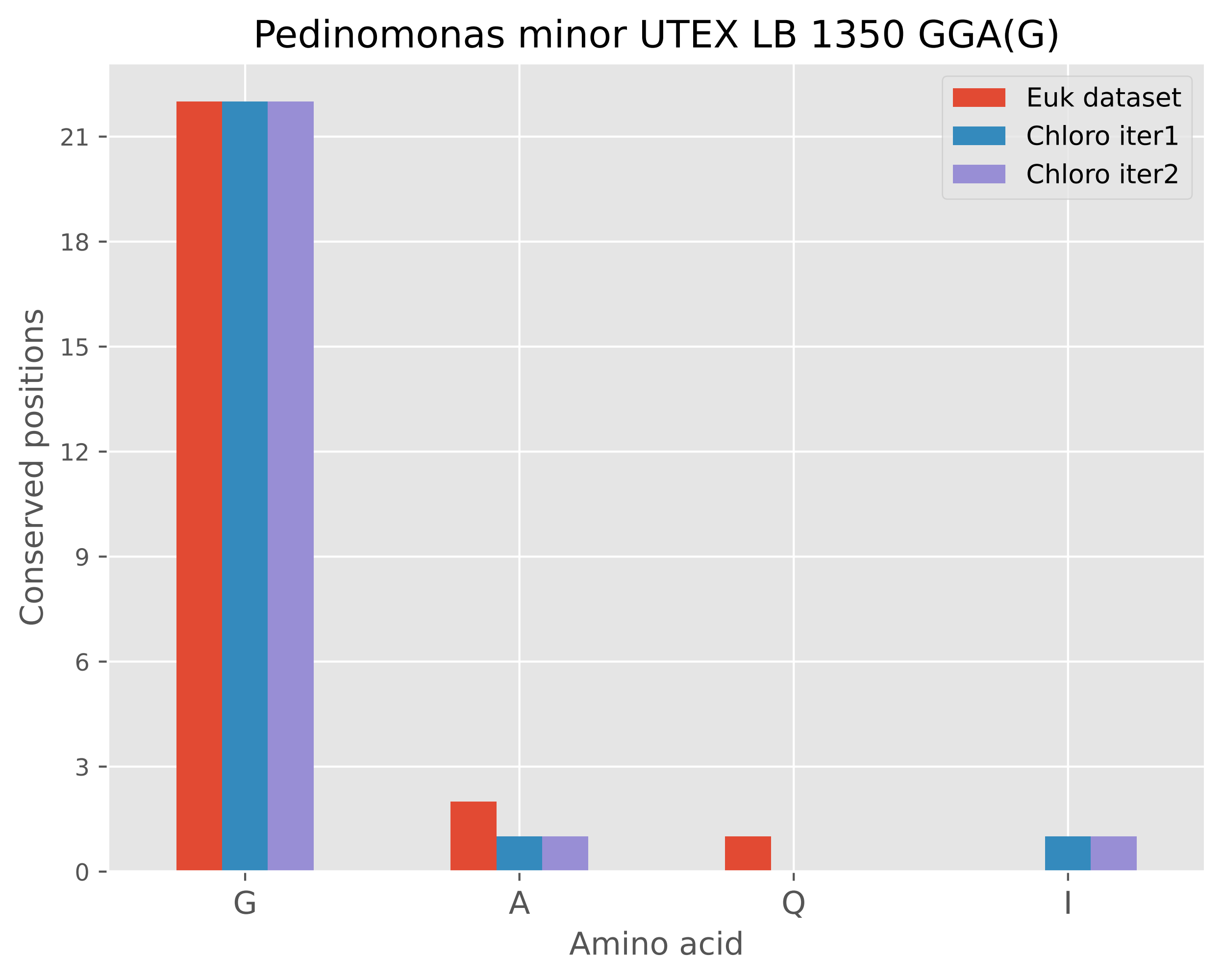
Pedinomonas minor UTEX LB 1350 GGA(G)
Euk dataset
Chloro iter1
Chloro iter2
Conserved positions
0
3
6
9
12
15
18
21
G
A
Q
I
Amino acid

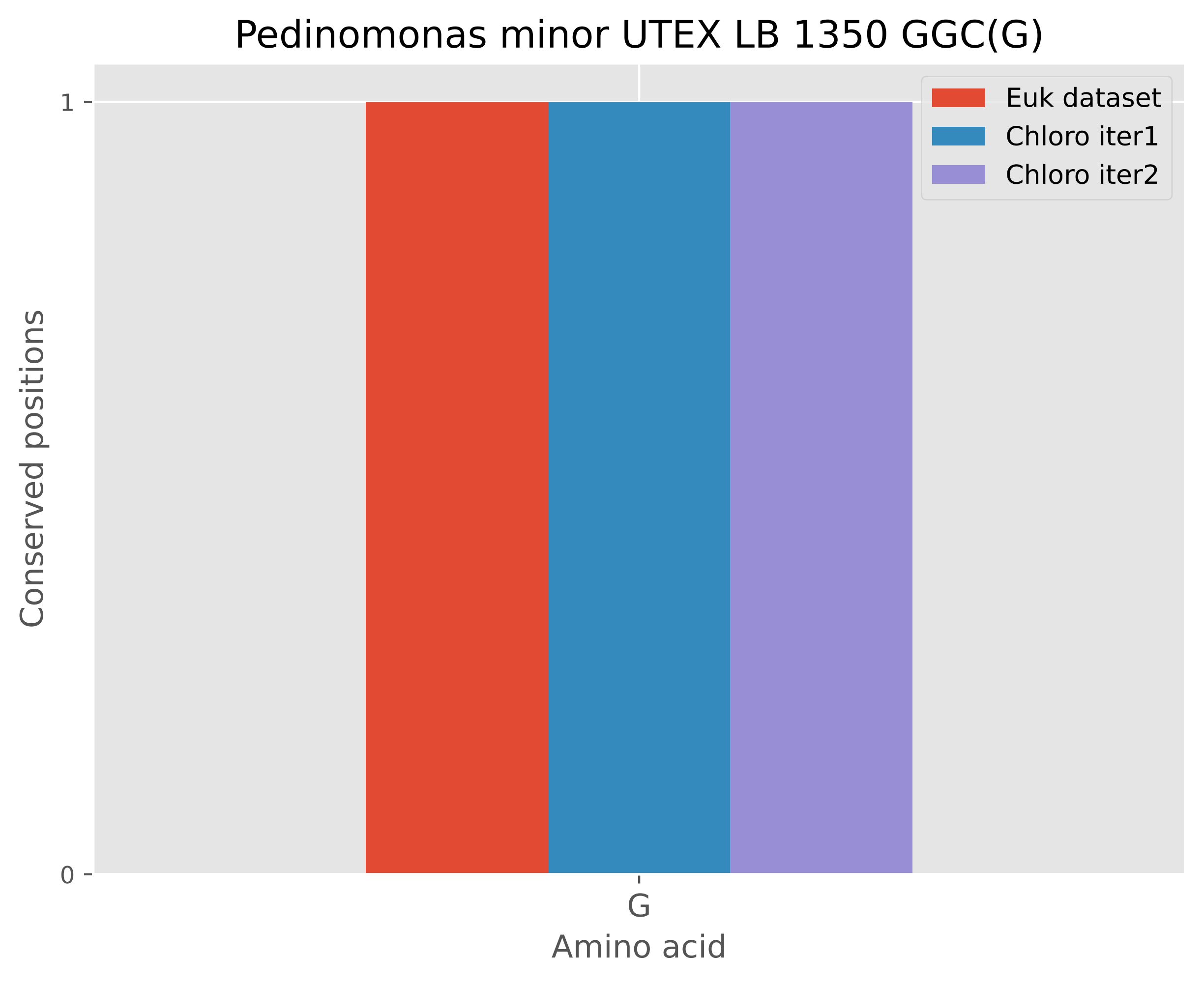
Pedinomonas minor UTEX LB 1350 GGC(G)
Euk dataset
Chloro iter1
Chloro iter2
1
0
Conserved positions
G
Amino acid

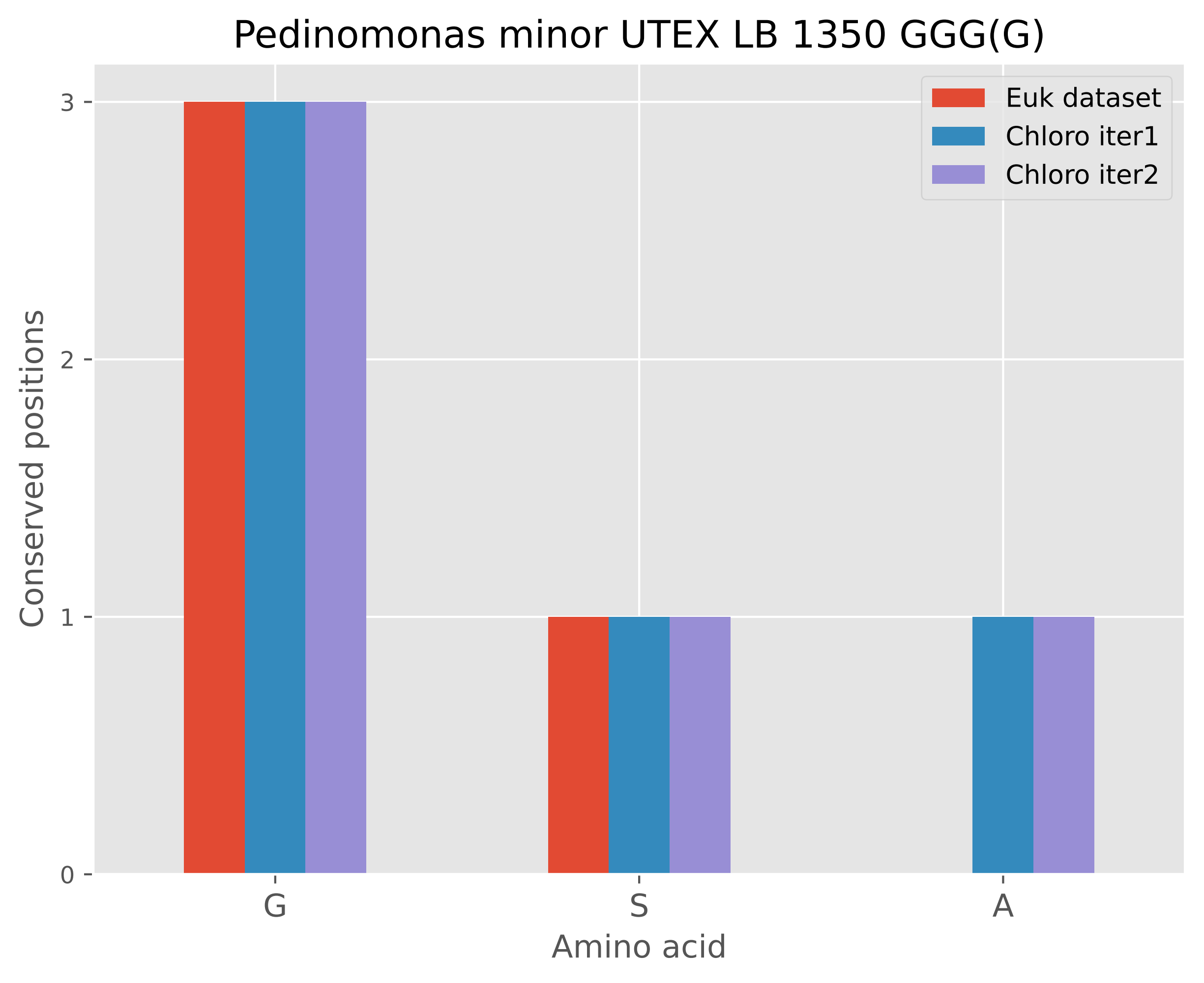
Pedinomonas minor UTEX LB 1350 GGG(G)
Euk dataset
Chloro iter1
Chloro iter2
Conserved positions
0
1
2
3
G
S
A
Amino acid

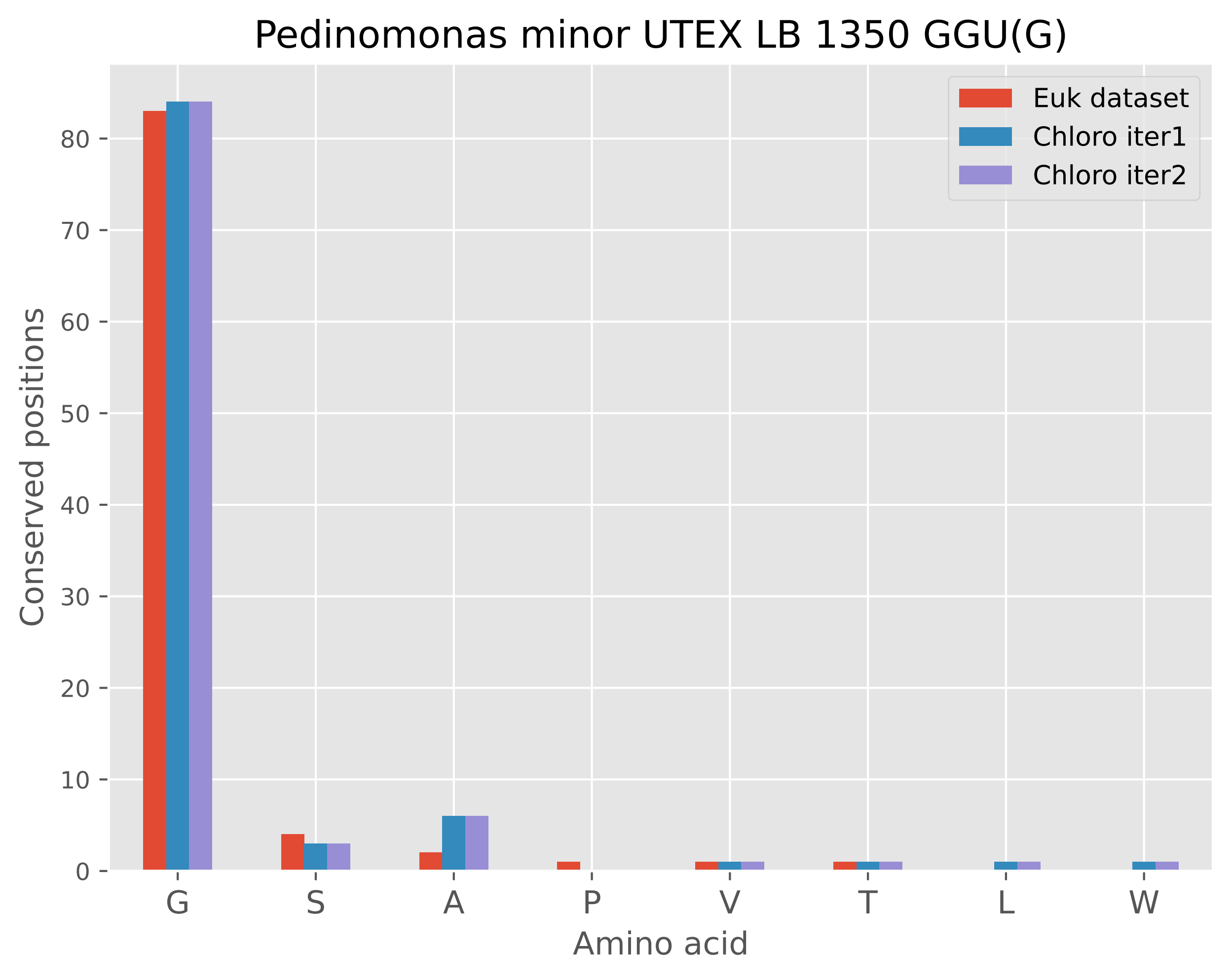
Pedinomonas minor UTEX LB 1350 GGU(G)
Euk dataset
Chloro iter1
Chloro iter2
Conserved positions
0
10
20
30
40
50
60
70
80
G
S
A
P
V
T
L
W
Amino acid

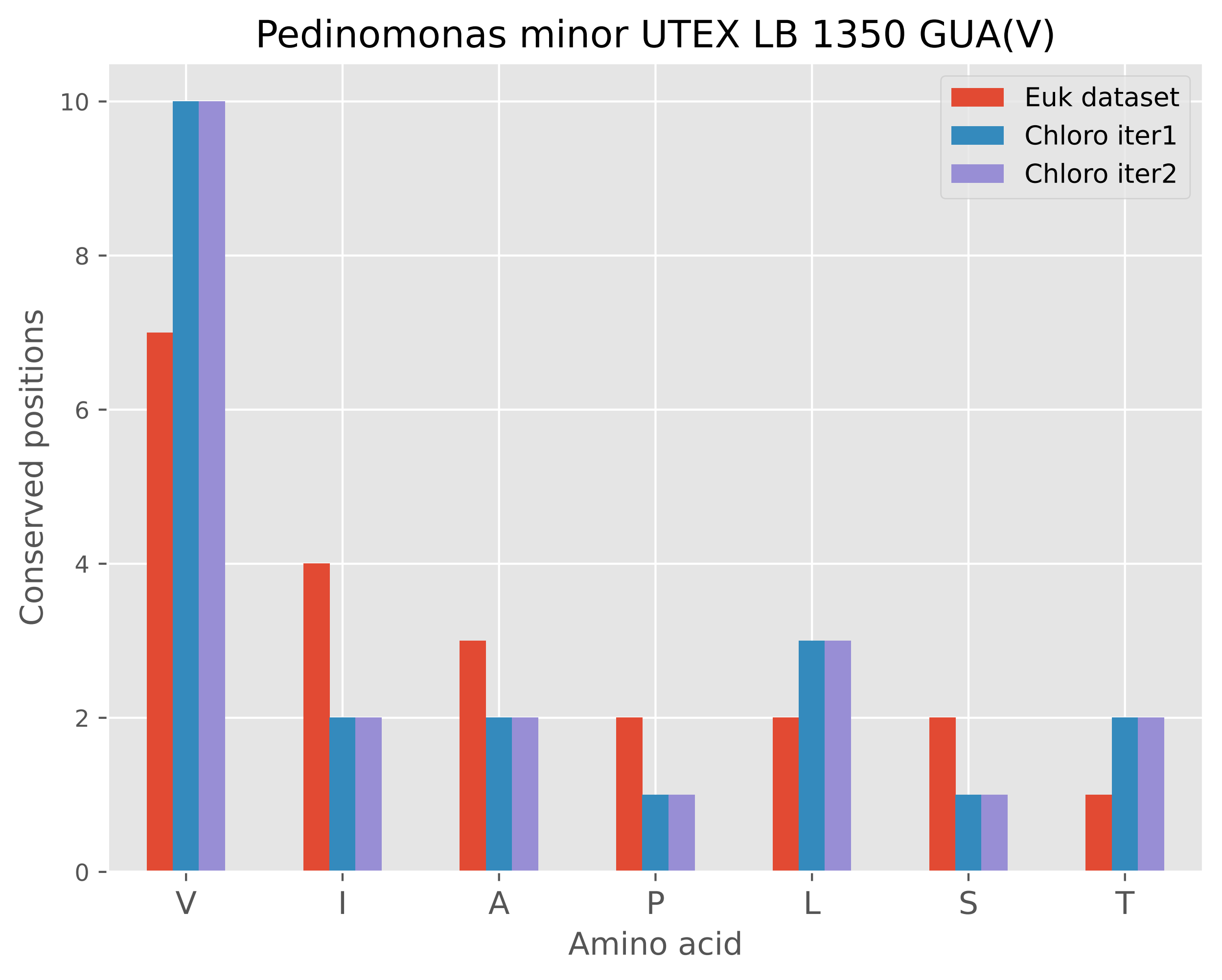

Pedinomonas minor UTEX LB 1350 GUA(V)
Euk dataset
Chloro iter1
Chloro iter2
Conserved positions
0
2
4
6
8
10
V
I
A
P
L
S
T
Amino acid

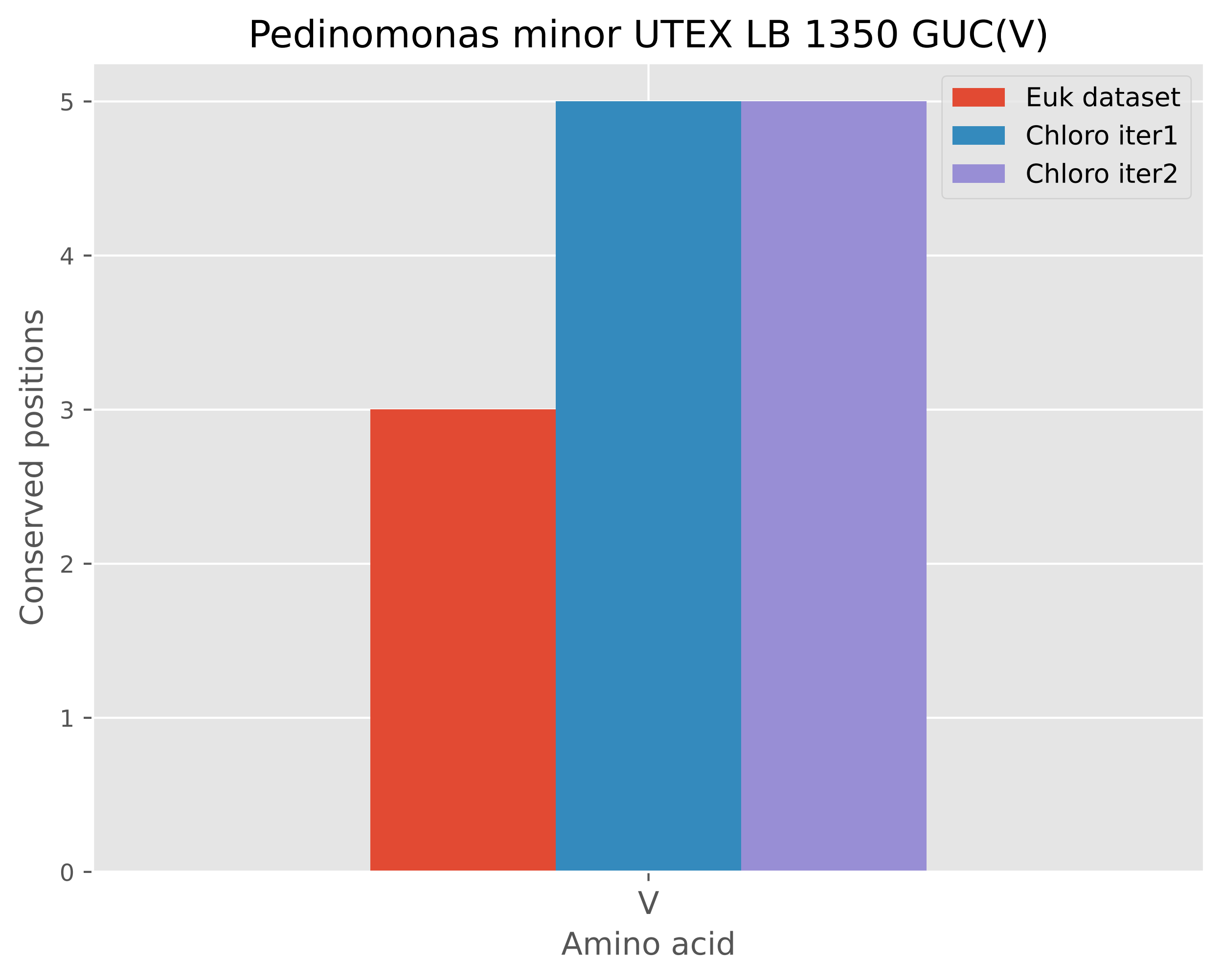

Pedinomonas minor UTEX LB 1350 GUC(V)
Euk dataset
Chloro iter1
Chloro iter2
Conserved positions
0
1
2
3
4
5
V
Amino acid

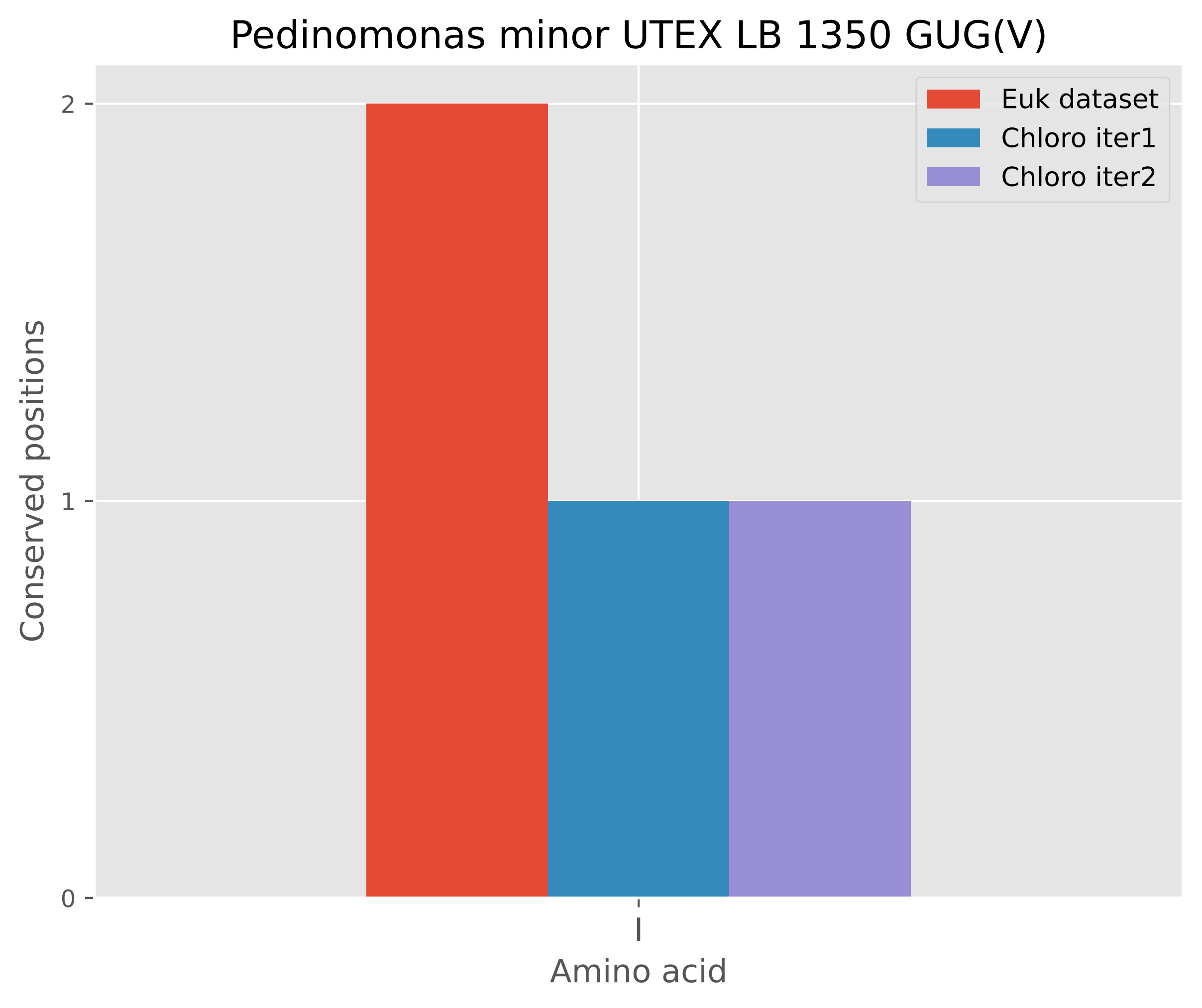
Pedinomonas minor UTEX LB 1350 GUG(V)
Euk dataset
Chloro iter1
Chloro iter2
Conserved positions
2
1
0
I
Amino acid

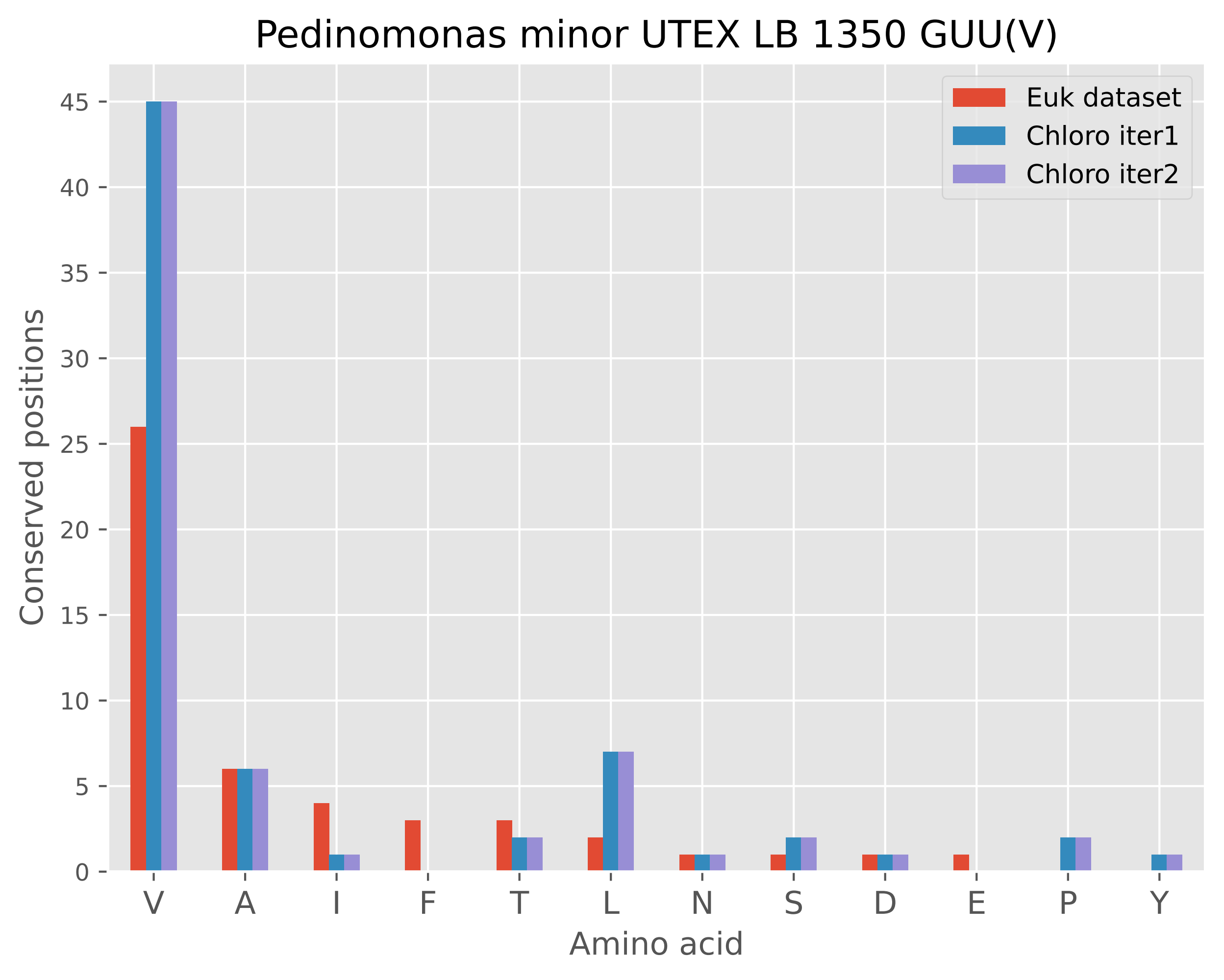

Pedinomonas minor UTEX LB 1350 GUU(V)
Euk dataset
Chloro iter1
Chloro iter2
Conserved positions
0
5
10
15
20
25
30
35
40
45
V
A
I
F
T
L
N
S
D
E
P
Y
Amino acid

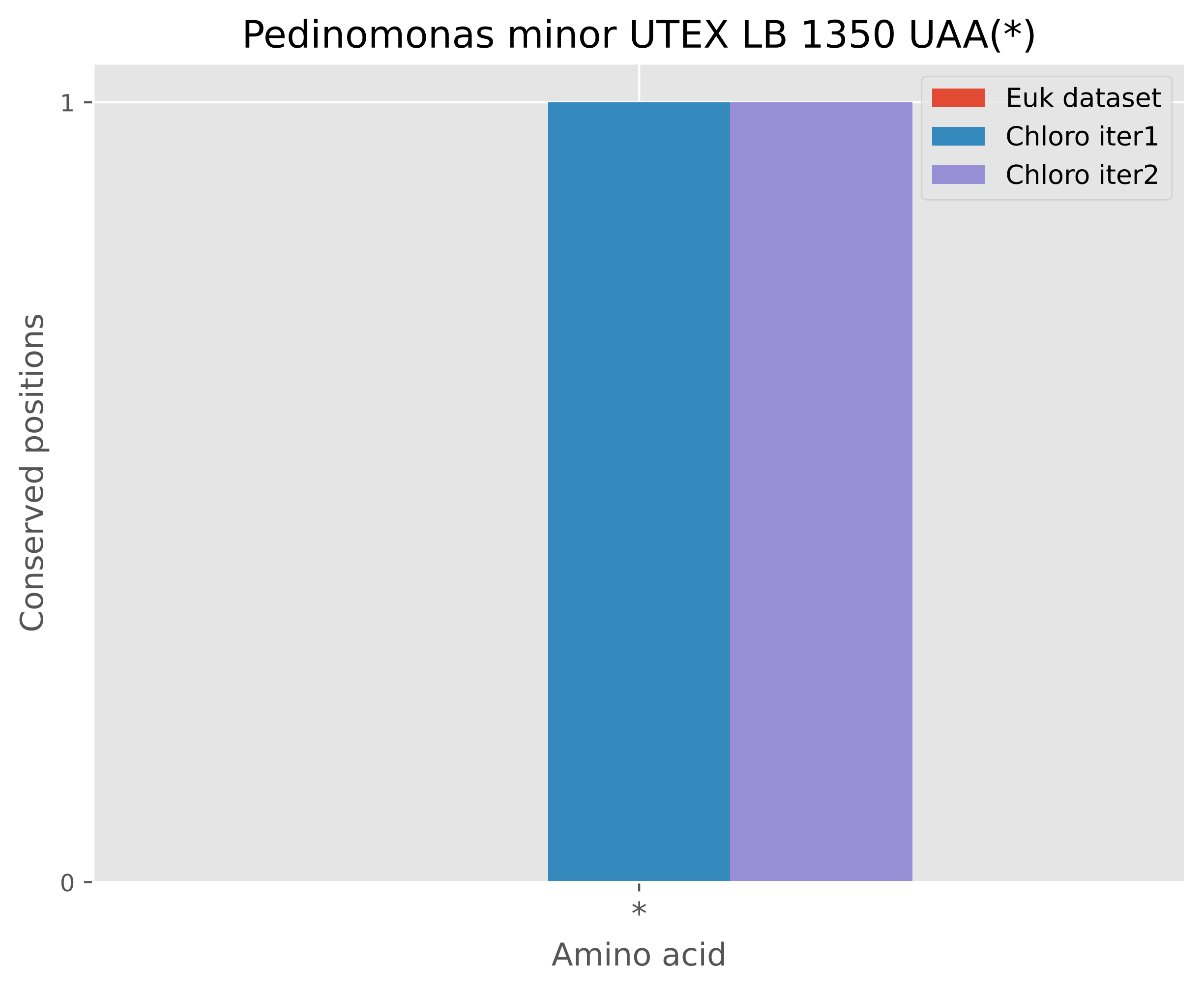
Pedinomonas minor UTEX LB 1350 UAA(*)
Euk dataset
Chloro iter1
Chloro iter2
Conserved positions
1
0
*
Amino acid

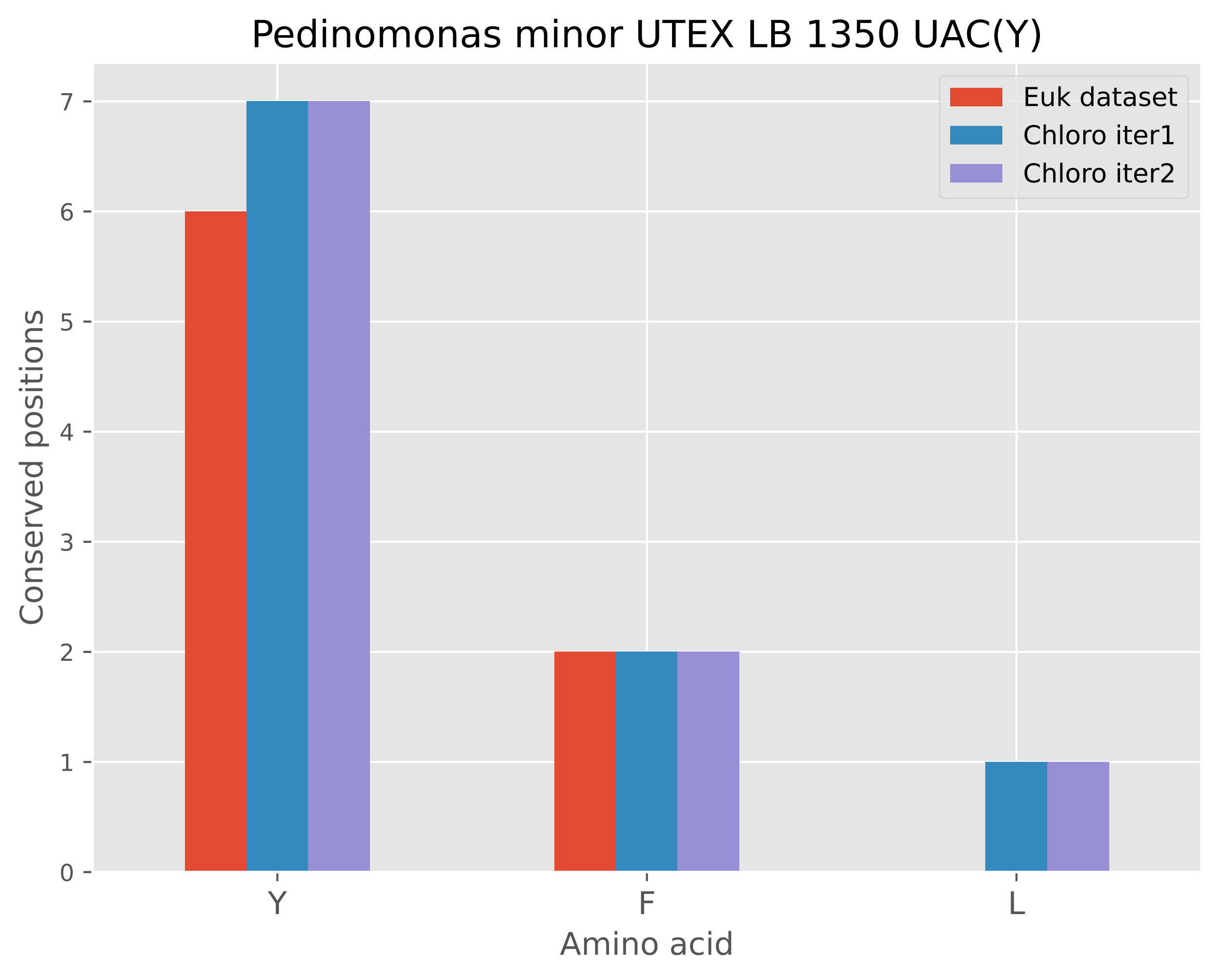
Pedinomonas minor UTEX LB 1350 UAC(Y)
Euk dataset
Chloro iter1
Chloro iter2
Conserved positions
0
1
2
3
4
5
6
7
Y
F
L
Amino acid

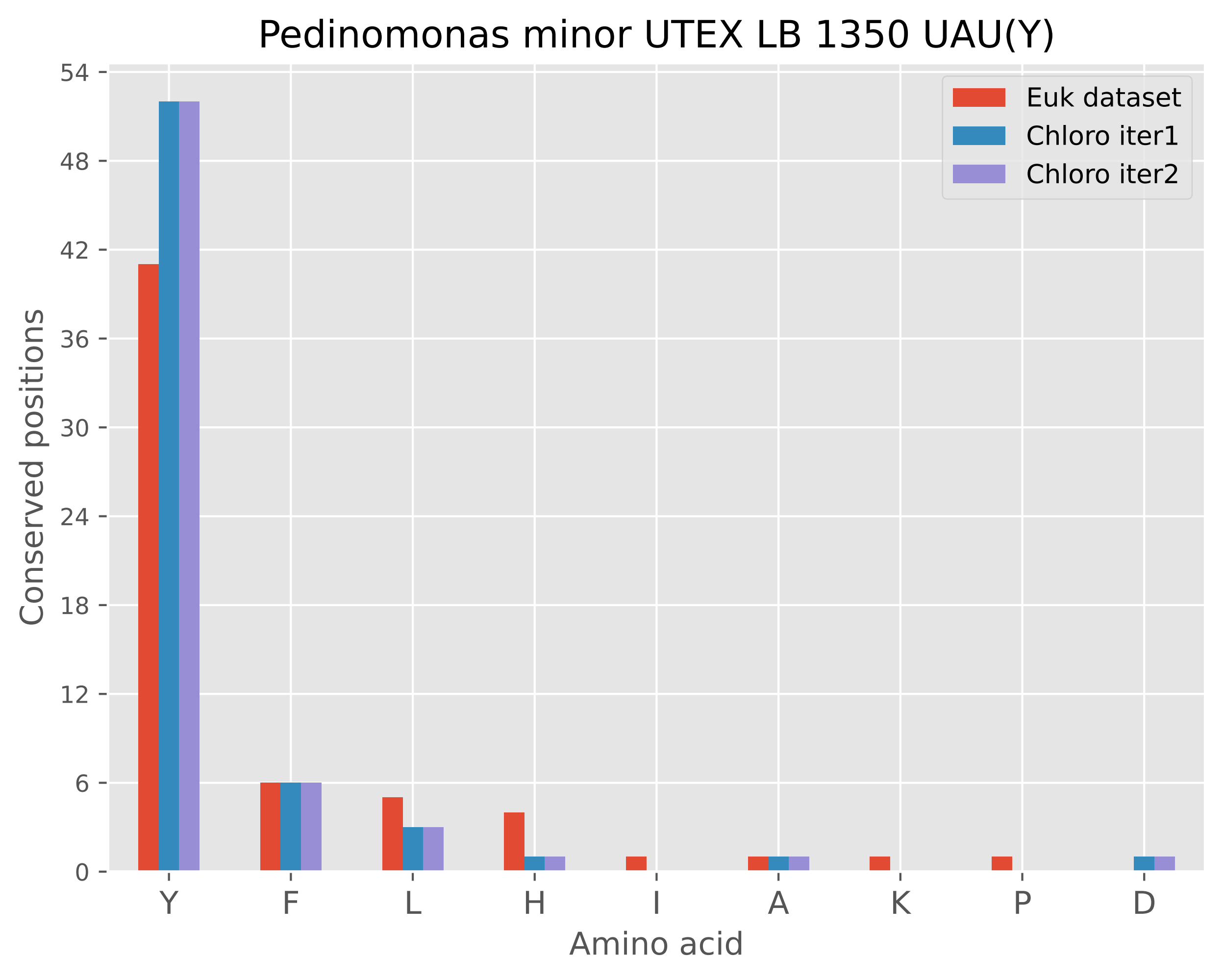

Pedinomonas minor UTEX LB 1350 UAU(Y)
Euk dataset
Chloro iter1
Chloro iter2
Conserved positions
0
6
12
18
24
30
36
42
48
54
Y
F
L
H
I
A
K
P
D
Amino acid

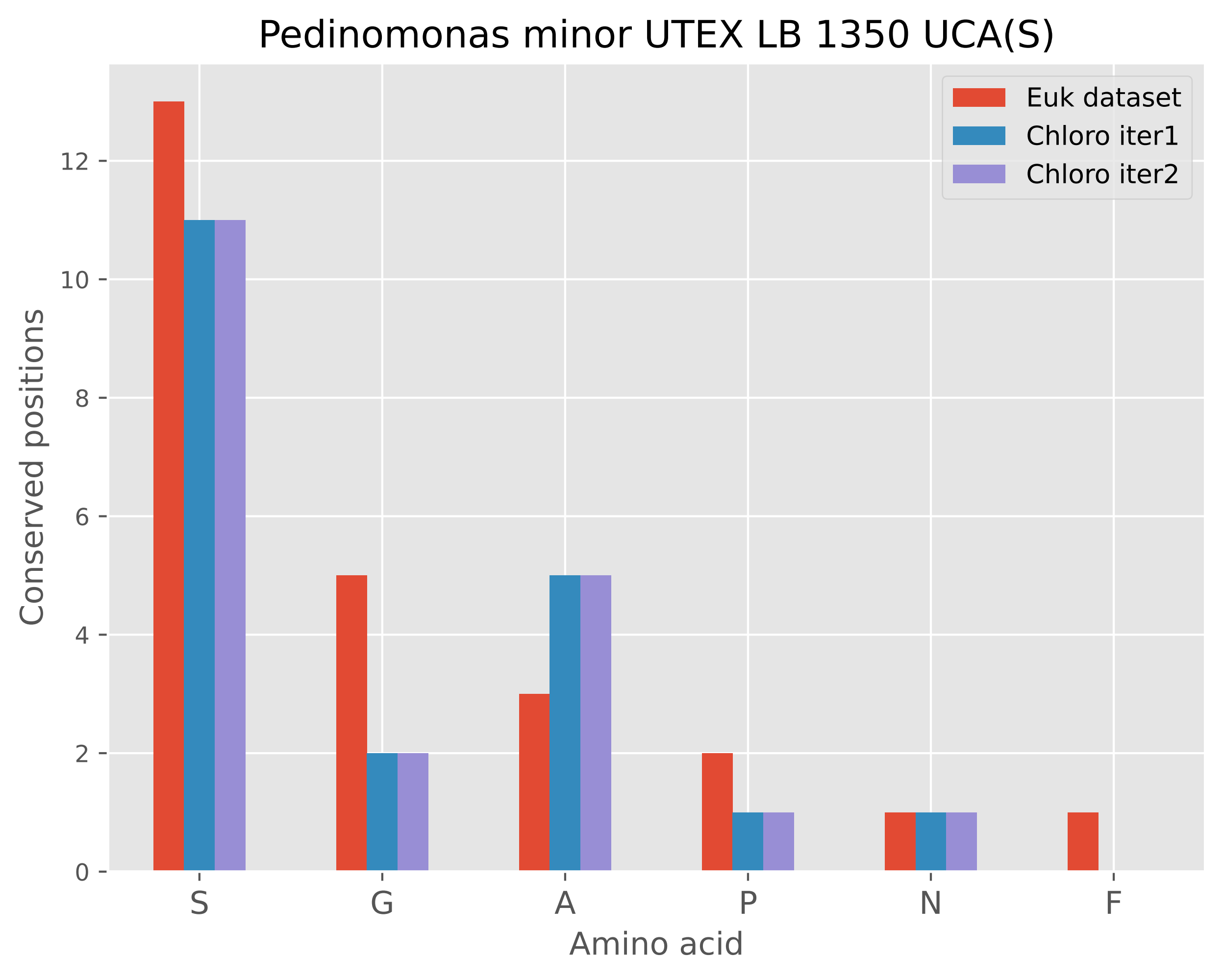

Pedinomonas minor UTEX LB 1350 UCA(S)
Euk dataset
Chloro iter1
Chloro iter2
Conserved positions
0
2
4
6
8
10
12
S
G
A
P
N
F
Amino acid

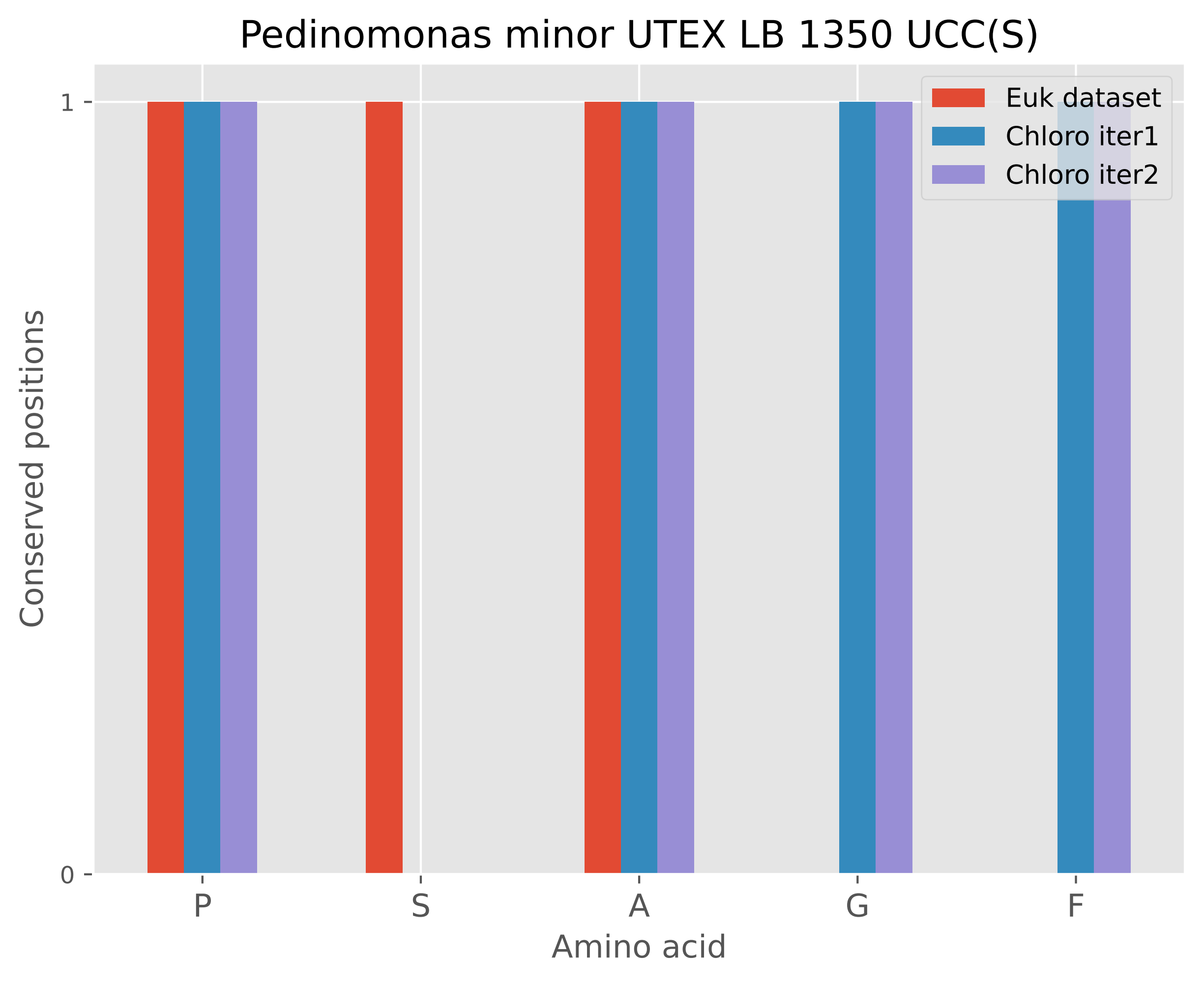
Pedinomonas minor UTEX LB 1350 UCC(S)
Euk dataset
Chloro iter1
Chloro iter2
Conserved positions
1
0
P
S
A
G
F
Amino acid

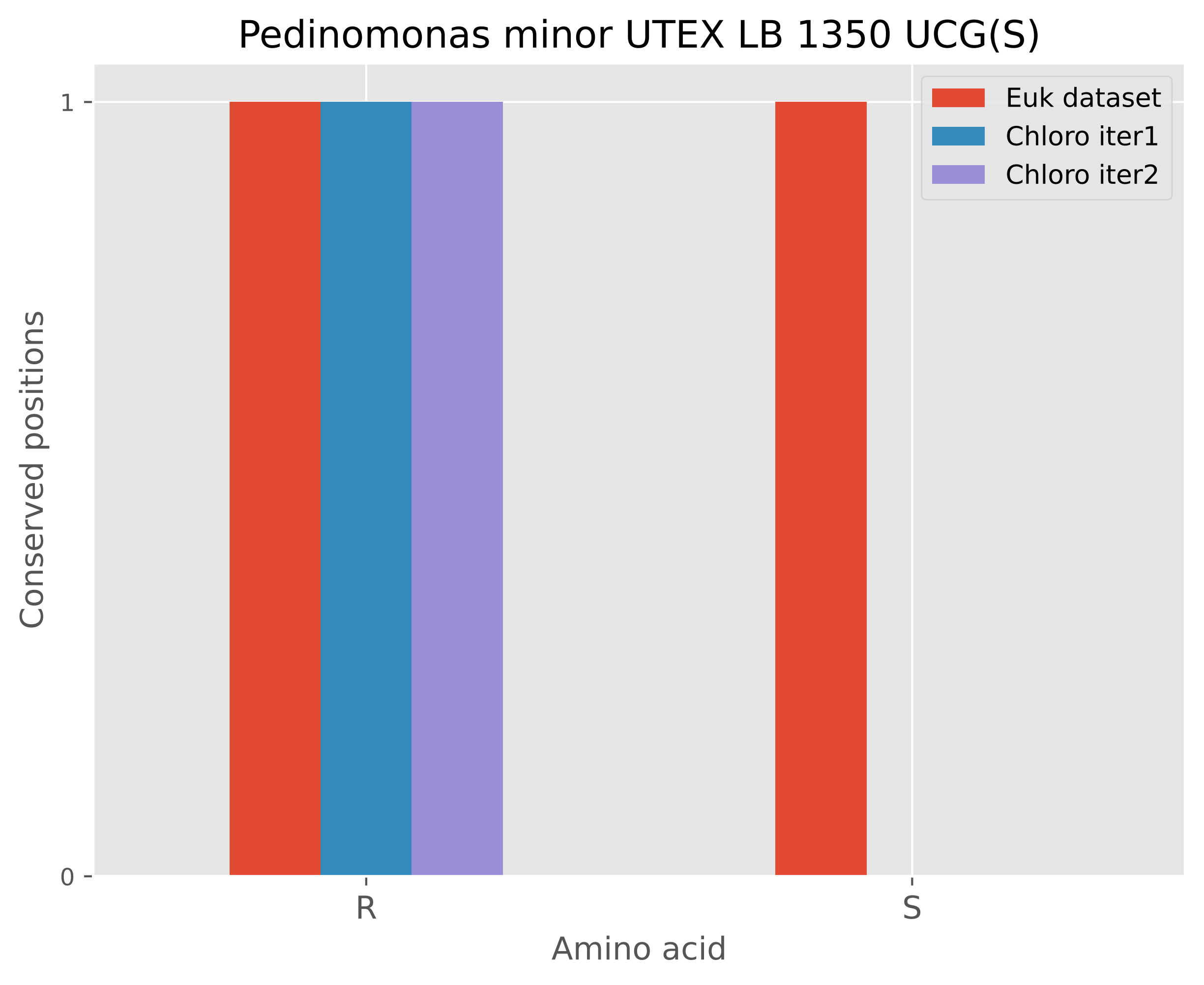
Pedinomonas minor UTEX LB 1350 UCG(S)
Euk dataset
Chloro iter1
Chloro iter2
Conserved positions
1
0
R
S
Amino acid

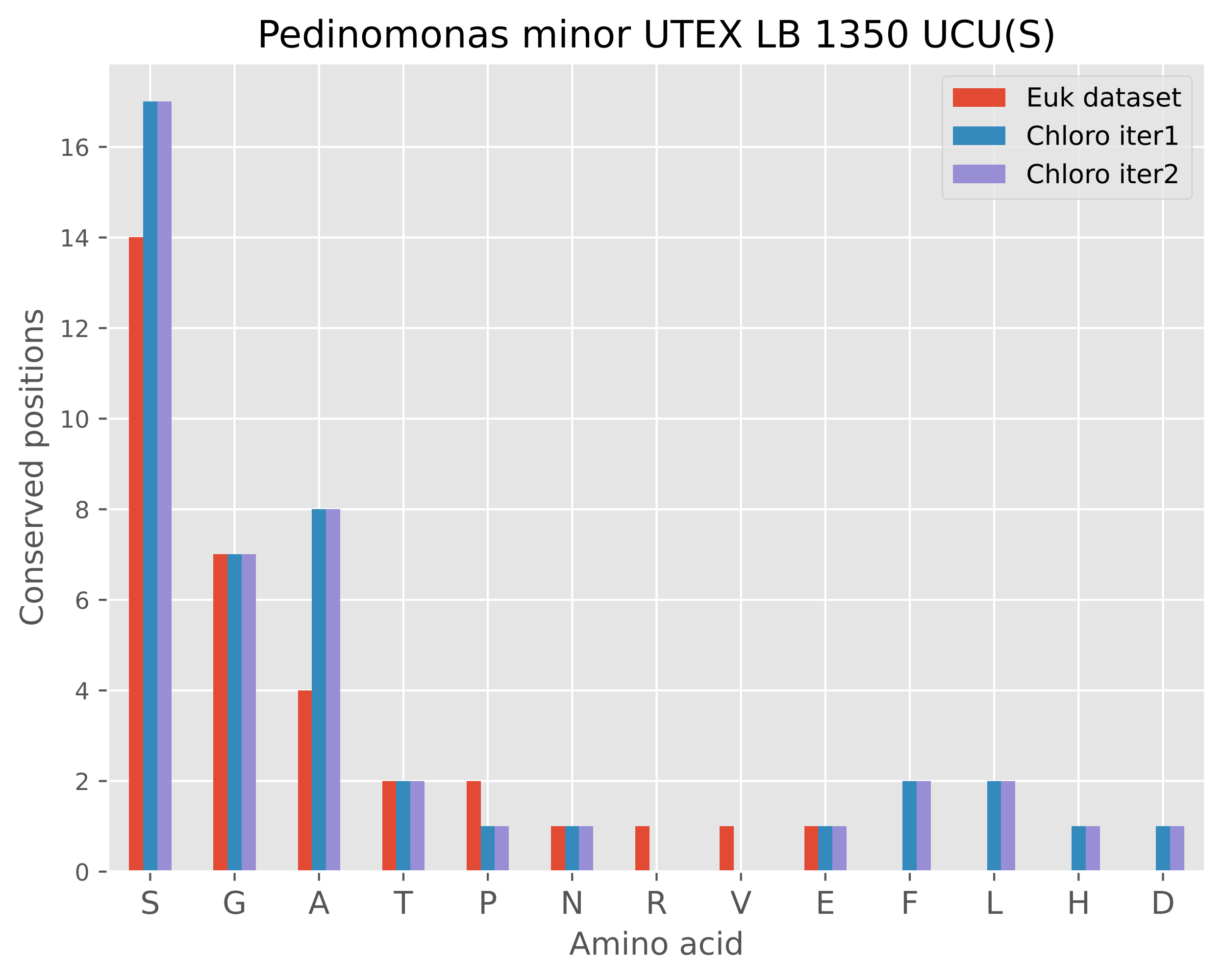
Pedinomonas minor UTEX LB 1350 UCU(S)
Euk dataset
Chloro iter1
Chloro iter2
Conserved positions
0
2
4
6
8
10
12
14
16
S
G
A
T
P
N
R
V
E
F
L
H
D
Amino acid

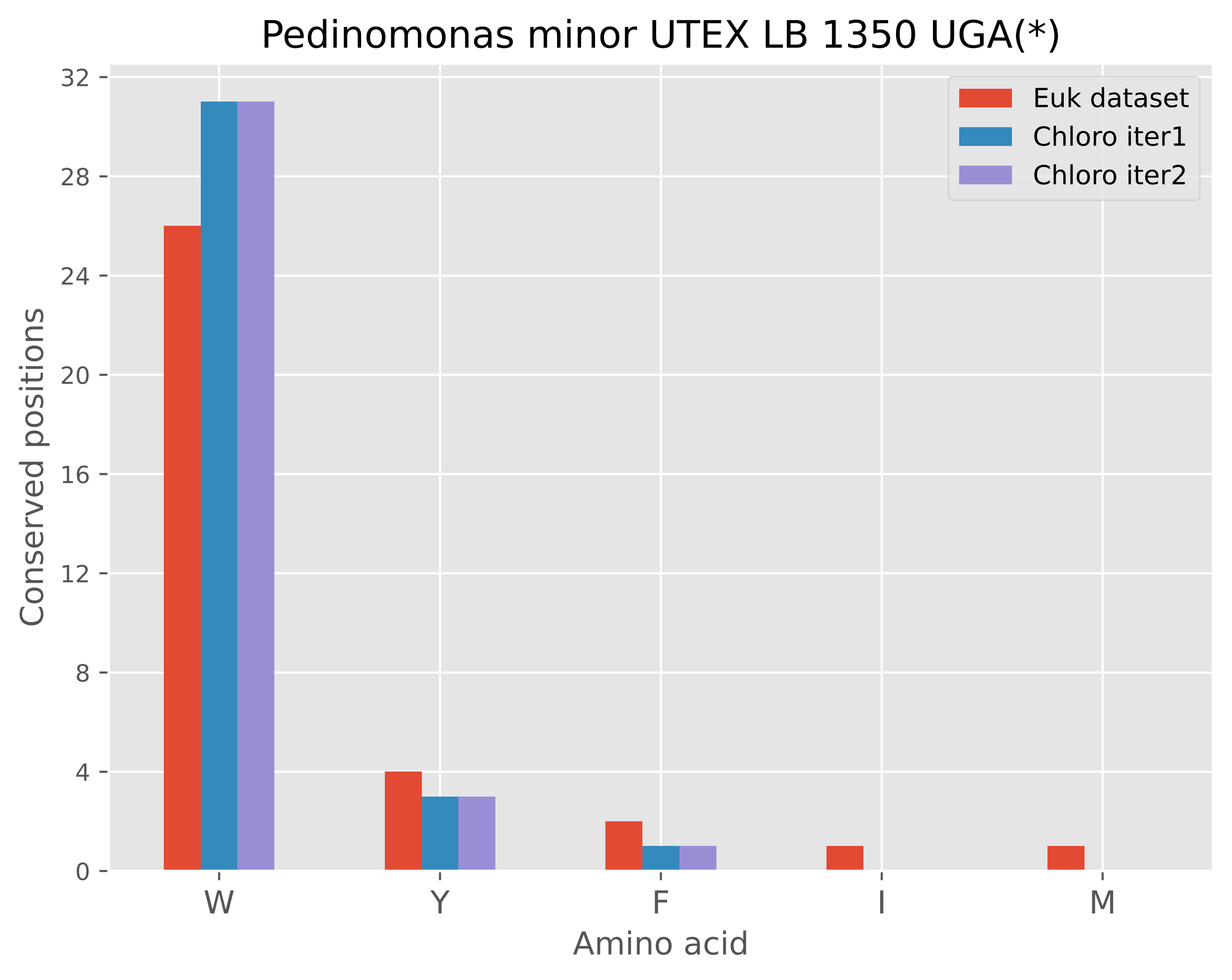
Pedinomonas minor UTEX LB 1350 UGA(*)
Euk dataset
Chloro iter1
Chloro iter2
Conserved positions
0
4
8
12
16
20
24
28
32
W
Y
F
I
M
Amino acid

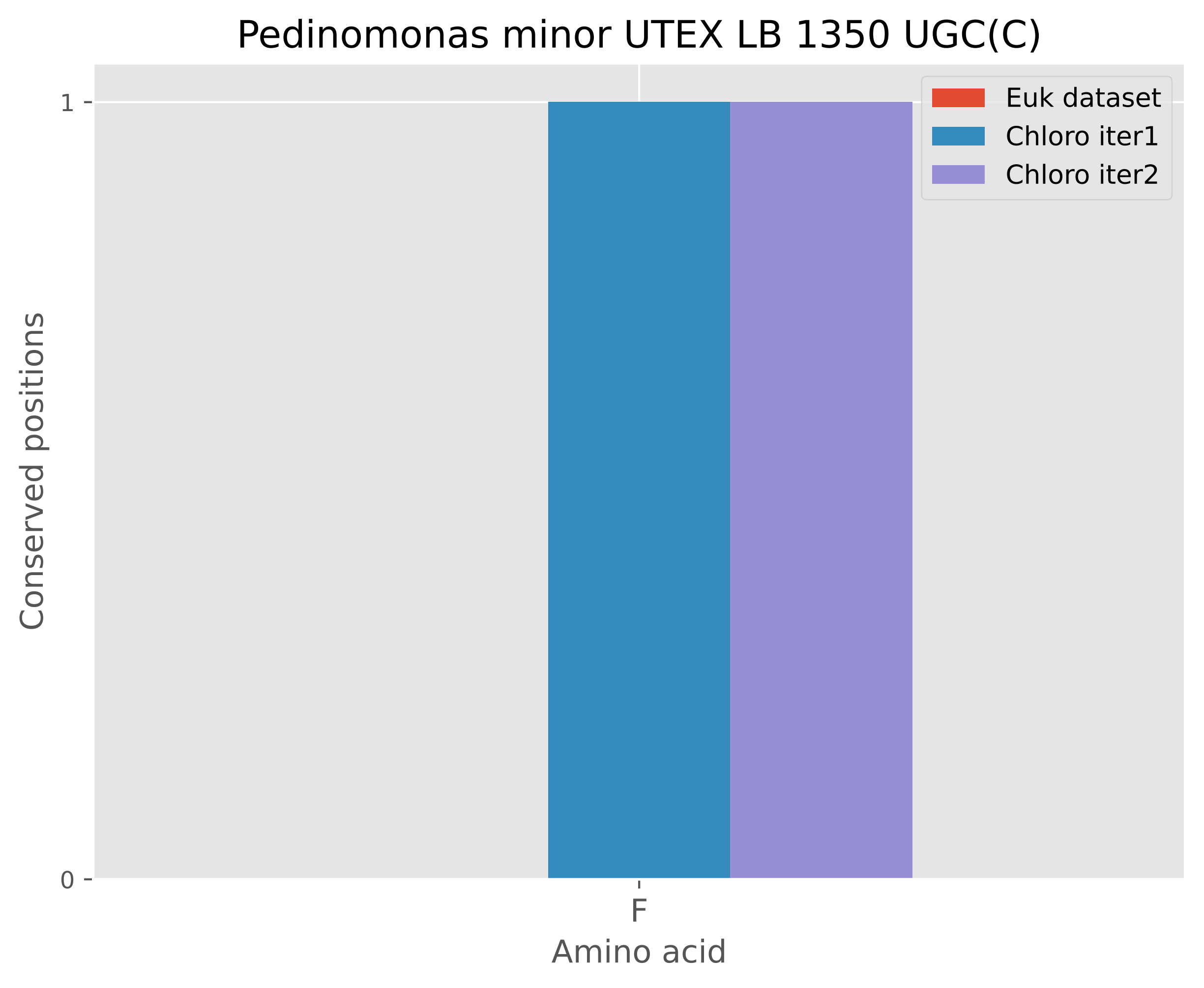
Pedinomonas minor UTEX LB 1350 UGC(C)
Euk dataset
Chloro iter1
Chloro iter2
Conserved positions
1
0
F
Amino acid

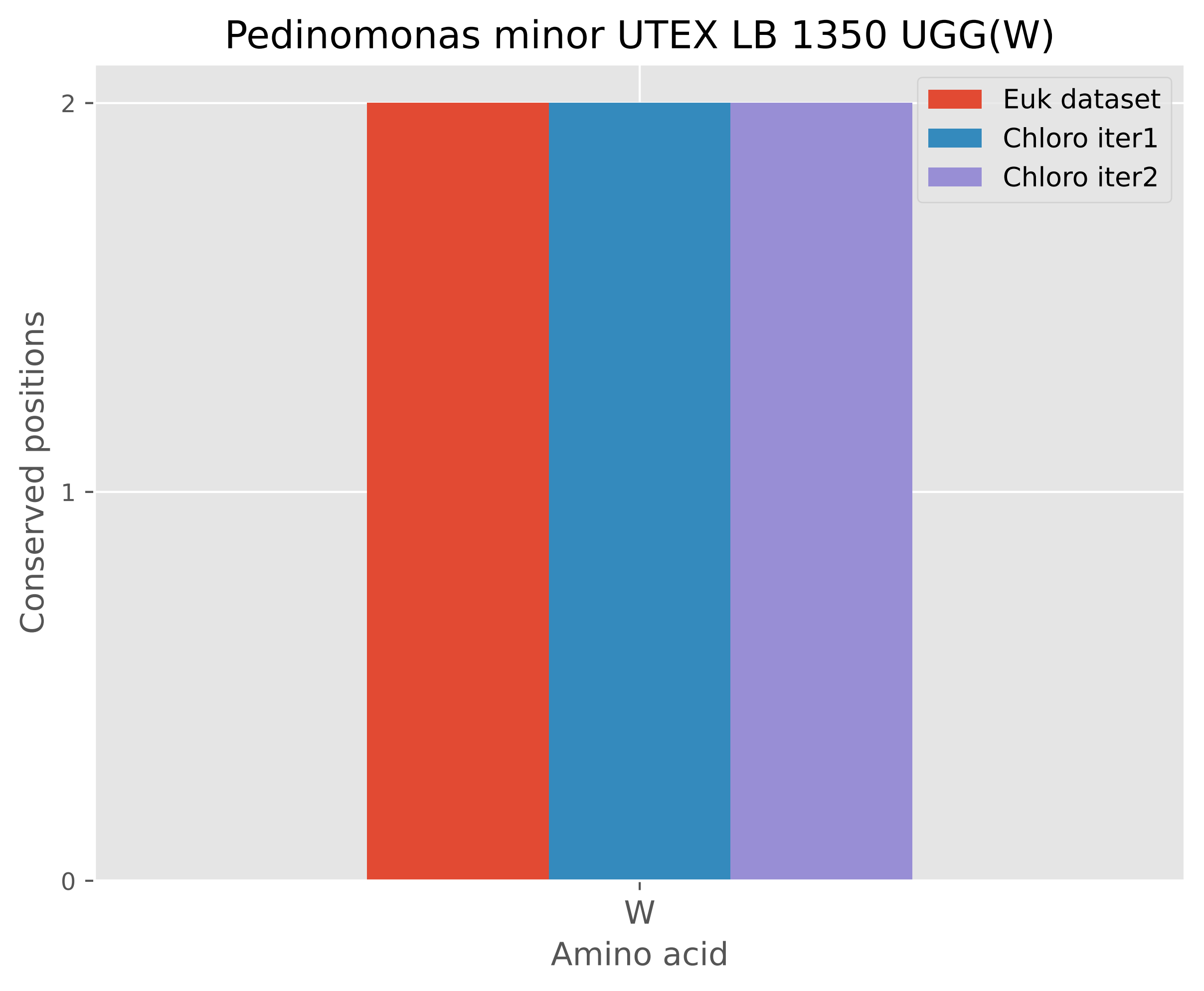
Pedinomonas minor UTEX LB 1350 UGG(W)
Euk dataset
Chloro iter1
Chloro iter2
Conserved positions
0
1
2
W
Amino acid

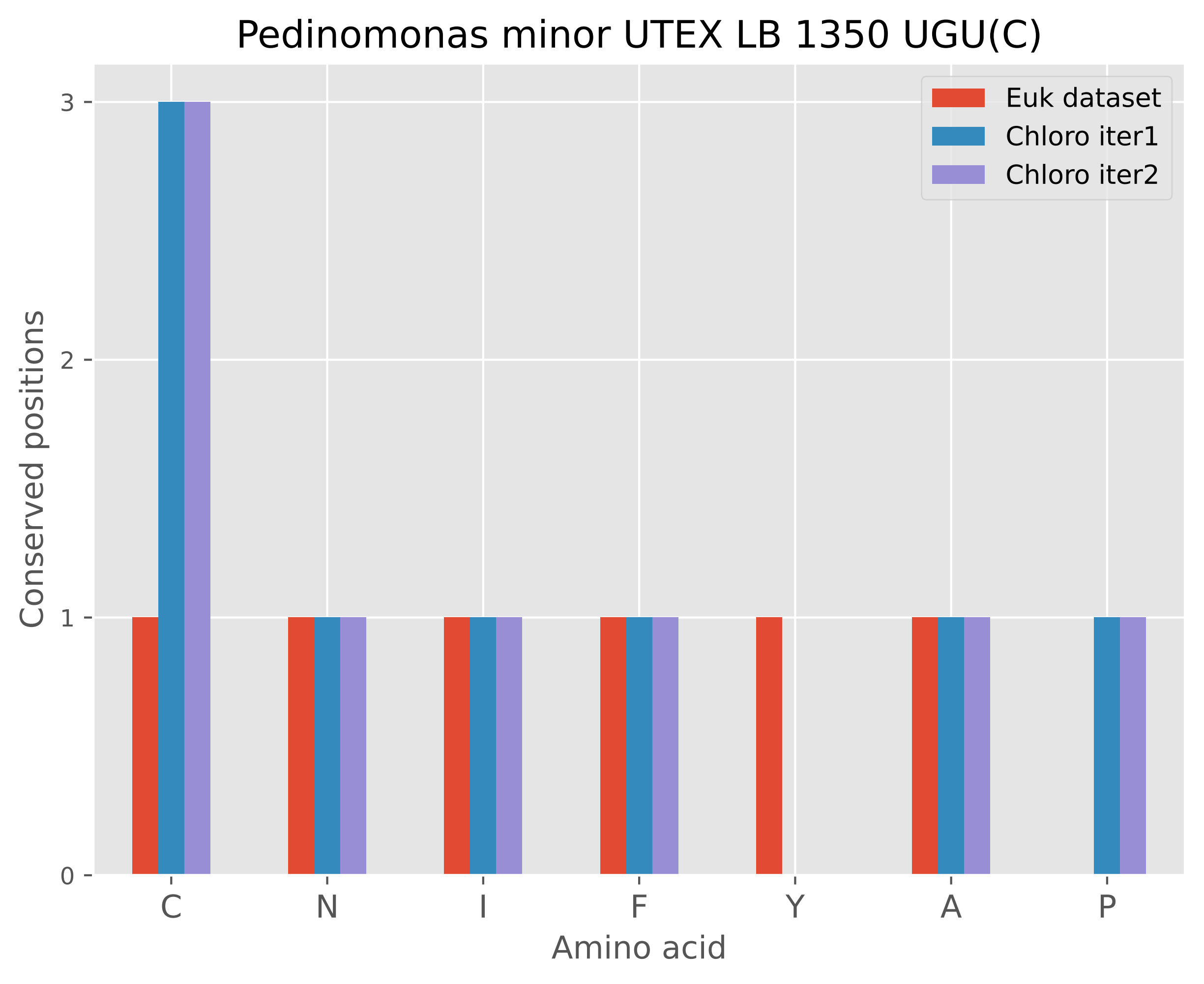

Pedinomonas minor UTEX LB 1350 UGU(C)
Euk dataset
Chloro iter1
Chloro iter2
Conserved positions
Amino acid
0
1
2
3
C
N
I
F
Y
A
P

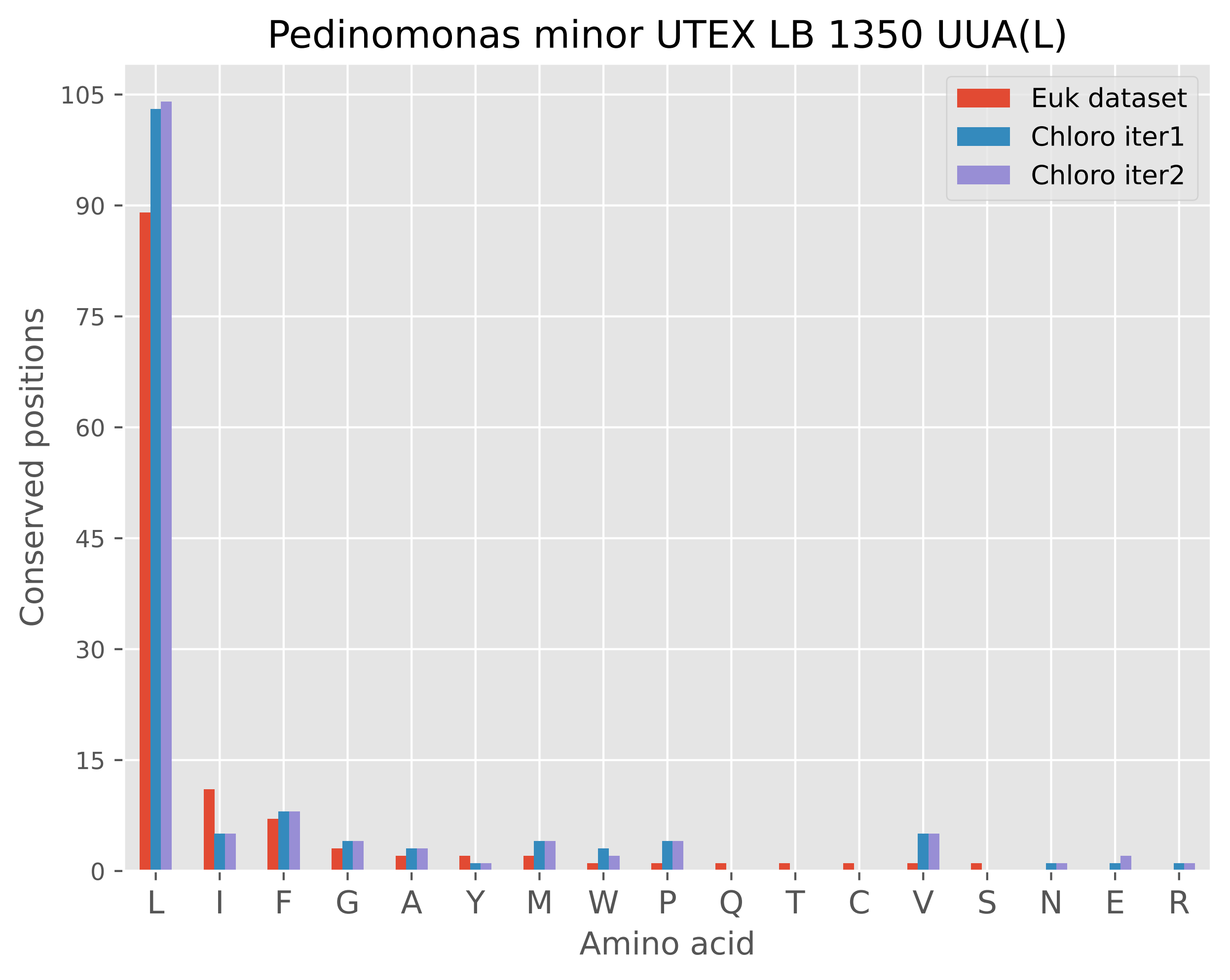

Pedinomonas minor UTEX LB 1350 UUA(L)
Euk dataset
Chloro iter1
Chloro iter2
Conserved positions
0
15
30
45
60
75
90
105
L
I
F
G
A
Y
M
W
P
Q
T
C
V
S
N
E
R
Amino acid

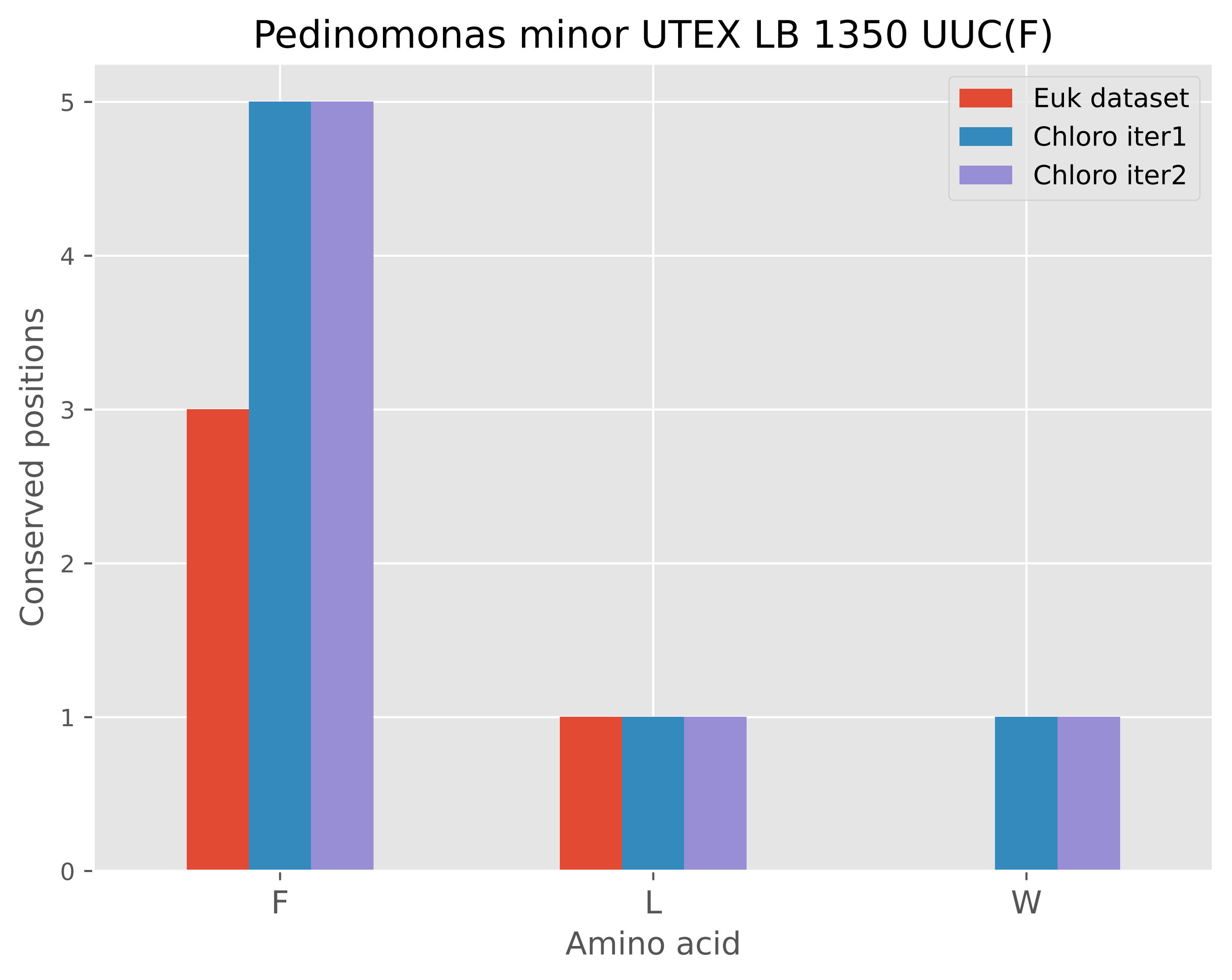
Pedinomonas minor UTEX LB 1350 UUC(F)
Euk dataset
Chloro iter1
Chloro iter2
Conserved positions
0
1
2
3
4
5
F
L
W
Amino acid

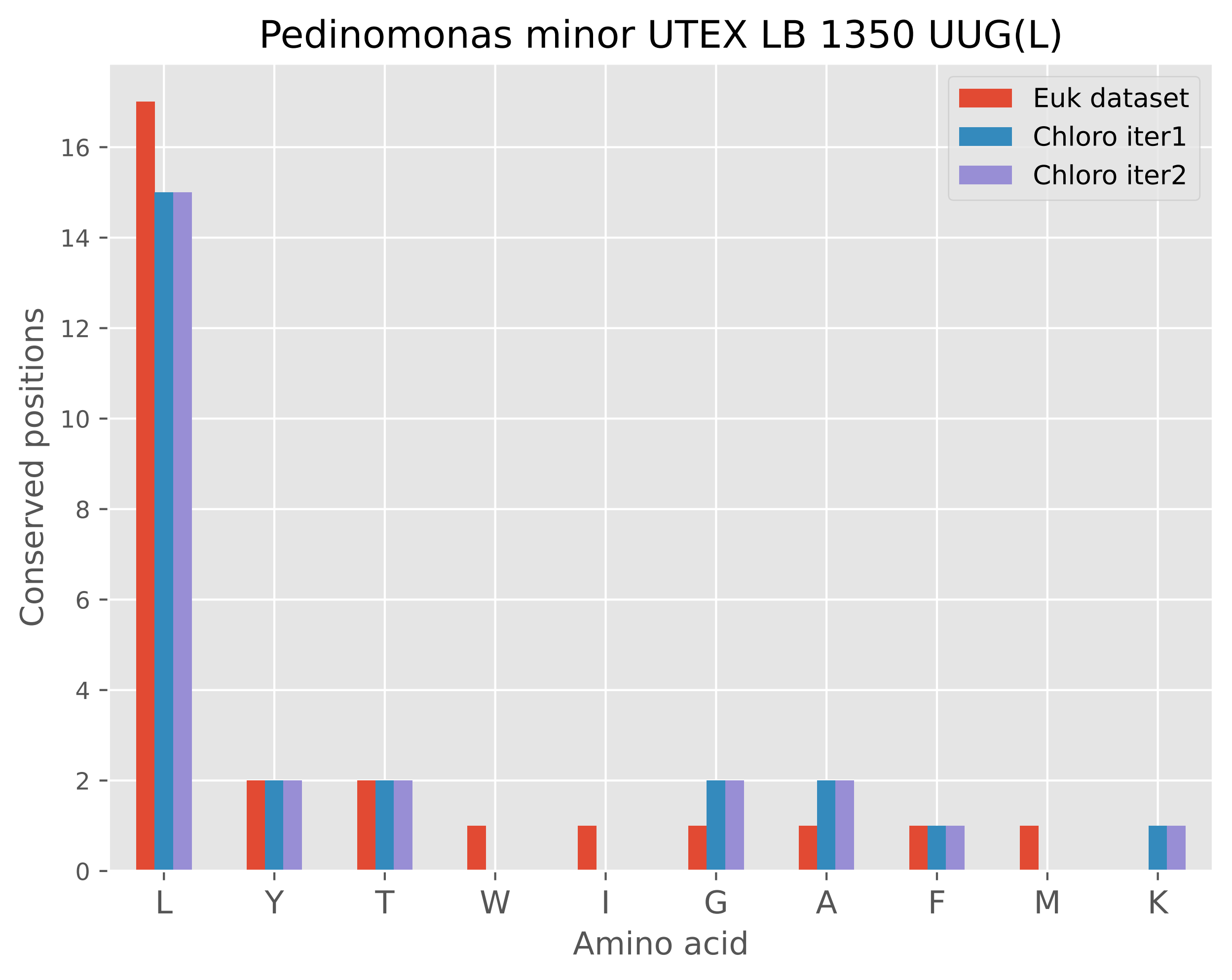

Pedinomonas minor UTEX LB 1350 UUG(L)
Euk dataset
Chloro iter1
Chloro iter2
Conserved positions
0
2
4
6
8
10
12
14
16
L
Y
T
W
I
G
A
F
M
K
Amino acid

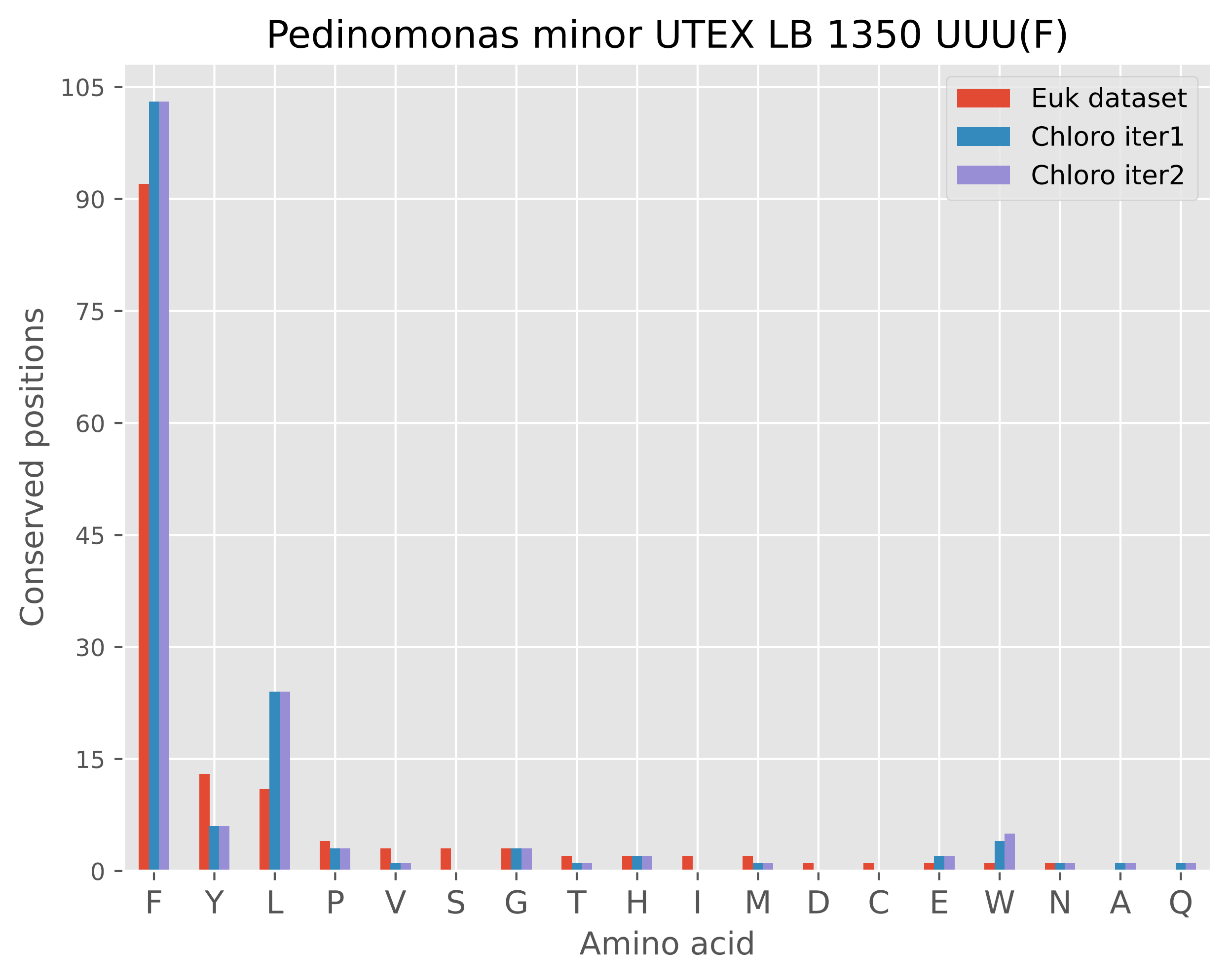

Pedinomonas minor UTEX LB 1350 UUU(F)
Euk dataset
Chloro iter1
Chloro iter2
Conserved positions
0
15
30
45
60
75
90
105
F
Y
L
P
V
S
G
T
H
I
M
D
C
E
W
N
A
Q
Amino acid

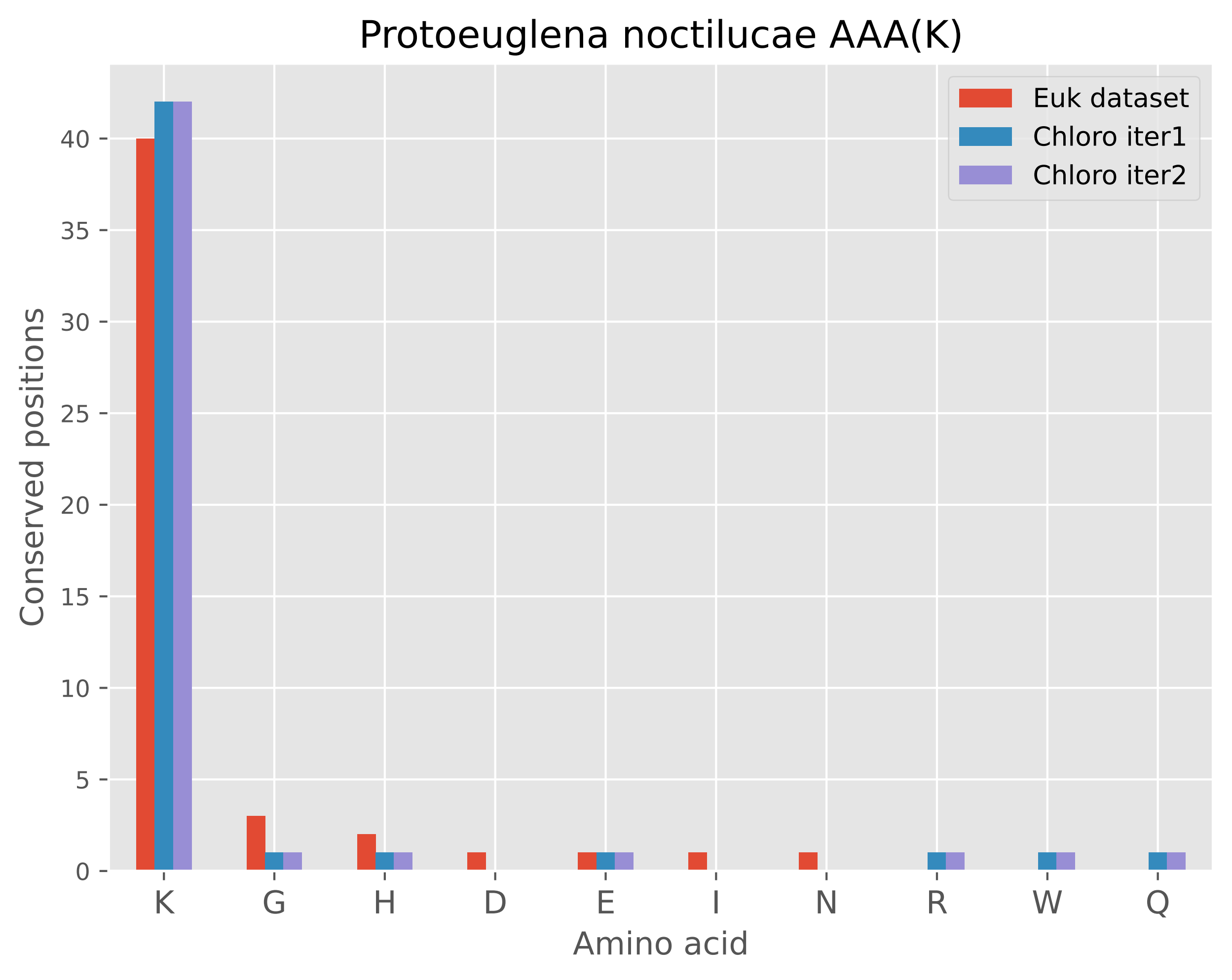
Protoeuglena noctilucae AAA(K)
Euk dataset
Chloro iter1
Chloro iter2
Conserved positions
0
5
10
15
20
25
30
35
40
K
G
H
D
E
I
N
R
W
Q
Amino acid

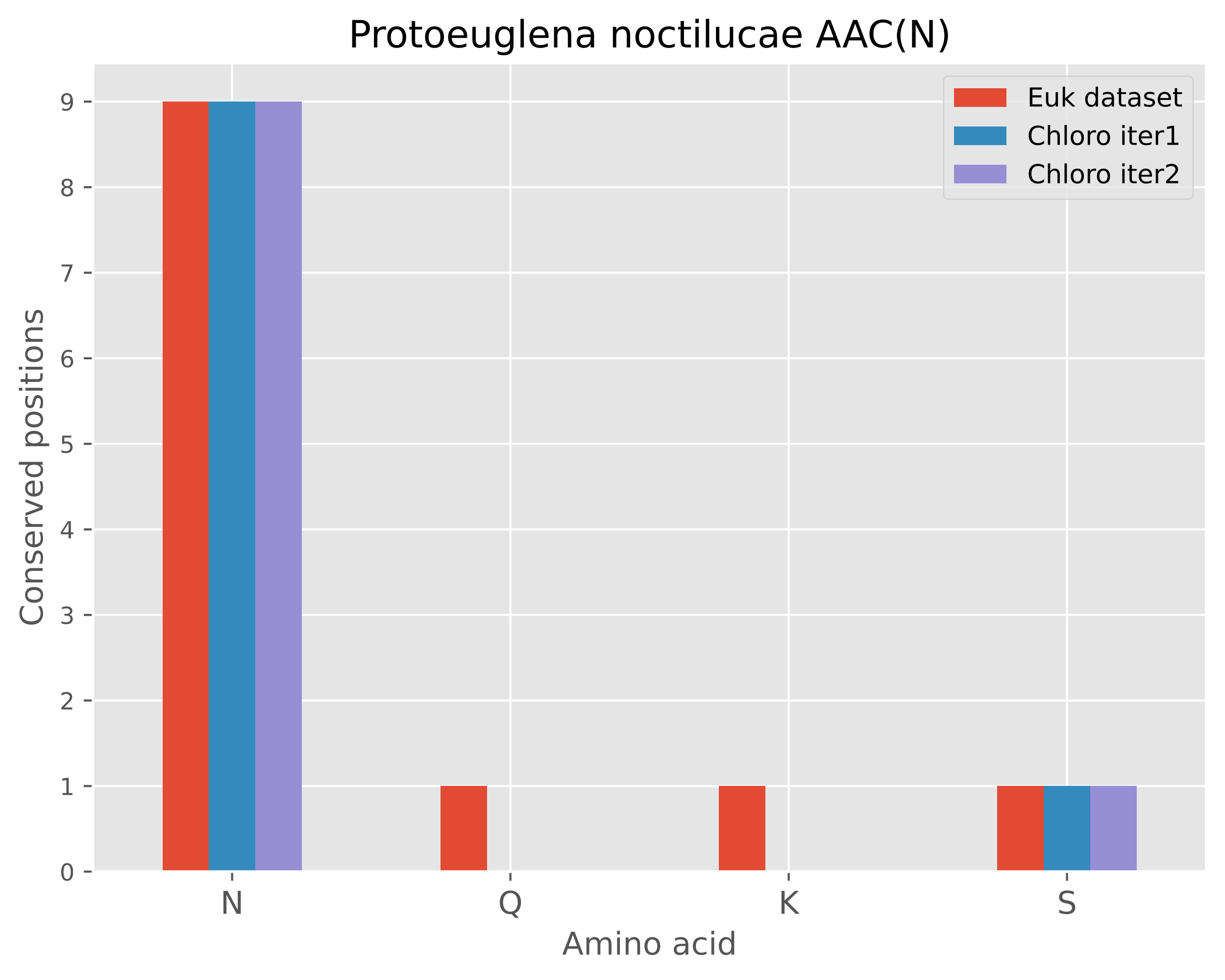
Protoeuglena noctilucae AAC(N)
Euk dataset
Chloro iter1
Chloro iter2
Conserved positions
0
1
2
3
4
5
6
7
8
9
N
Q
K
S
Amino acid

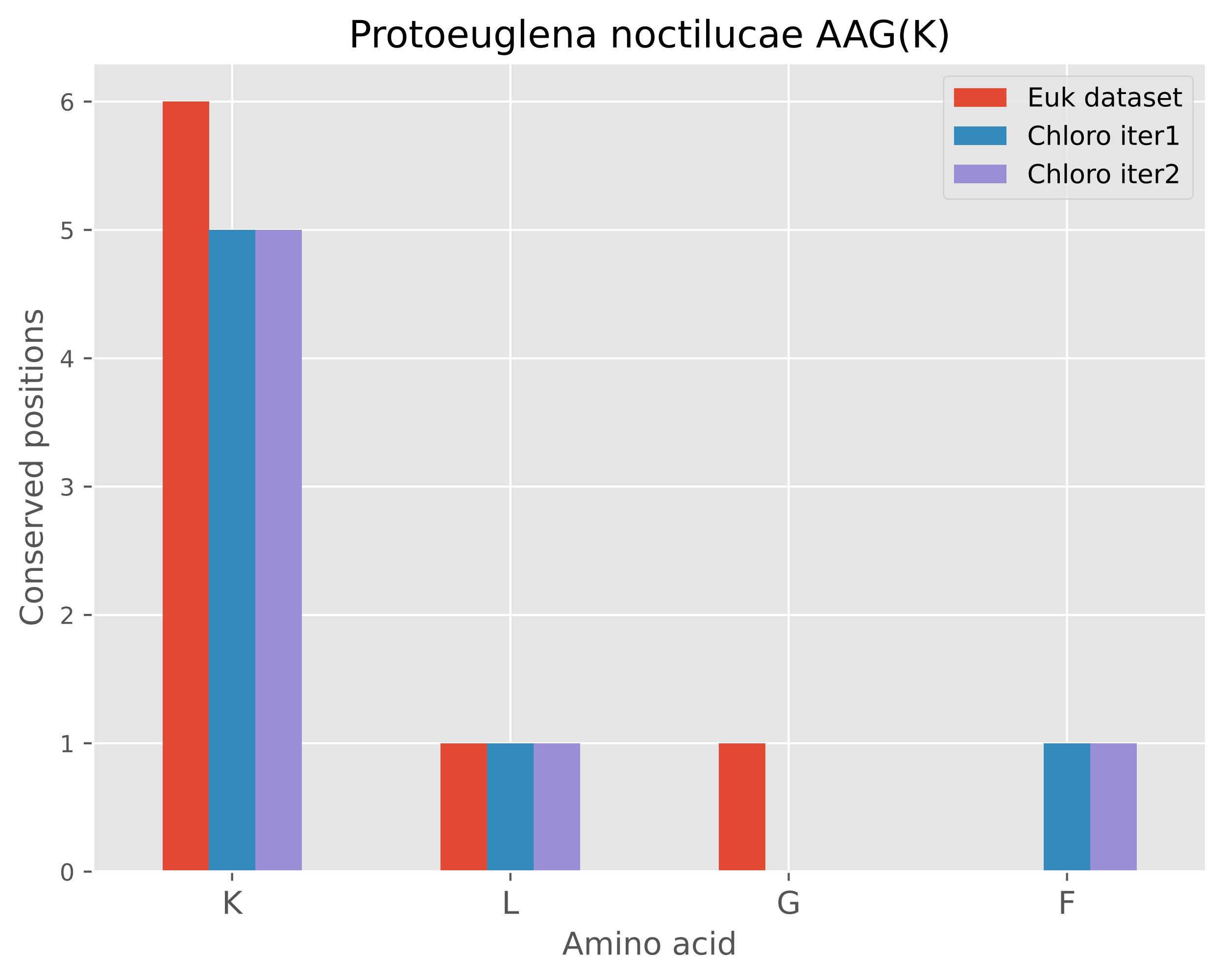

Protoeuglena noctilucae AAG(K)
Euk dataset
Chloro iter1
Chloro iter2
Conserved positions
0
1
2
3
4
5
6
K
L
G
F
Amino acid

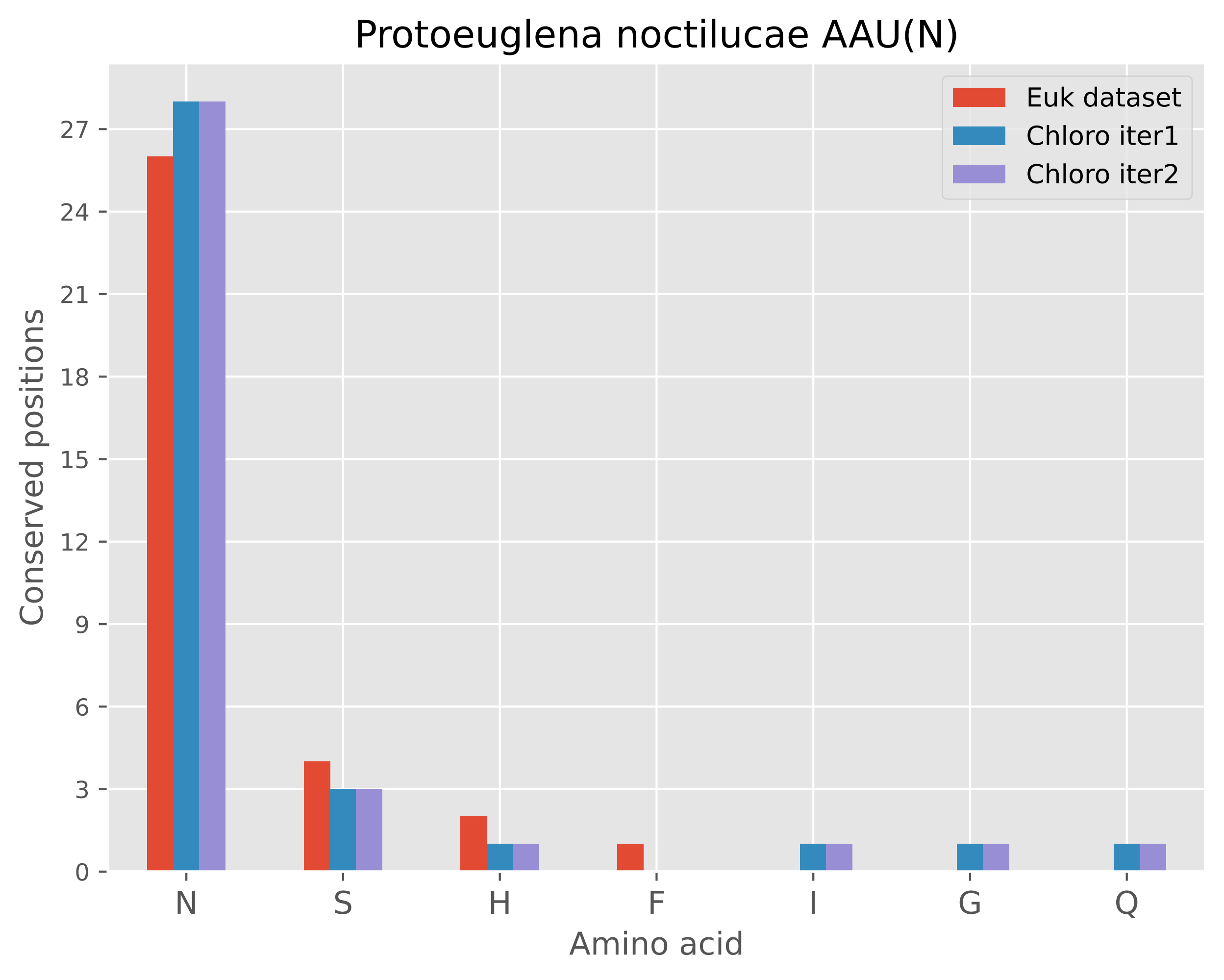

Protoeuglena noctilucae AAU(N)
Euk dataset
Chloro iter1
Chloro iter2
Conserved positions
0
3
6
9
12
15
18
21
24
27
N
S
H
F
I
G
Q
Amino acid

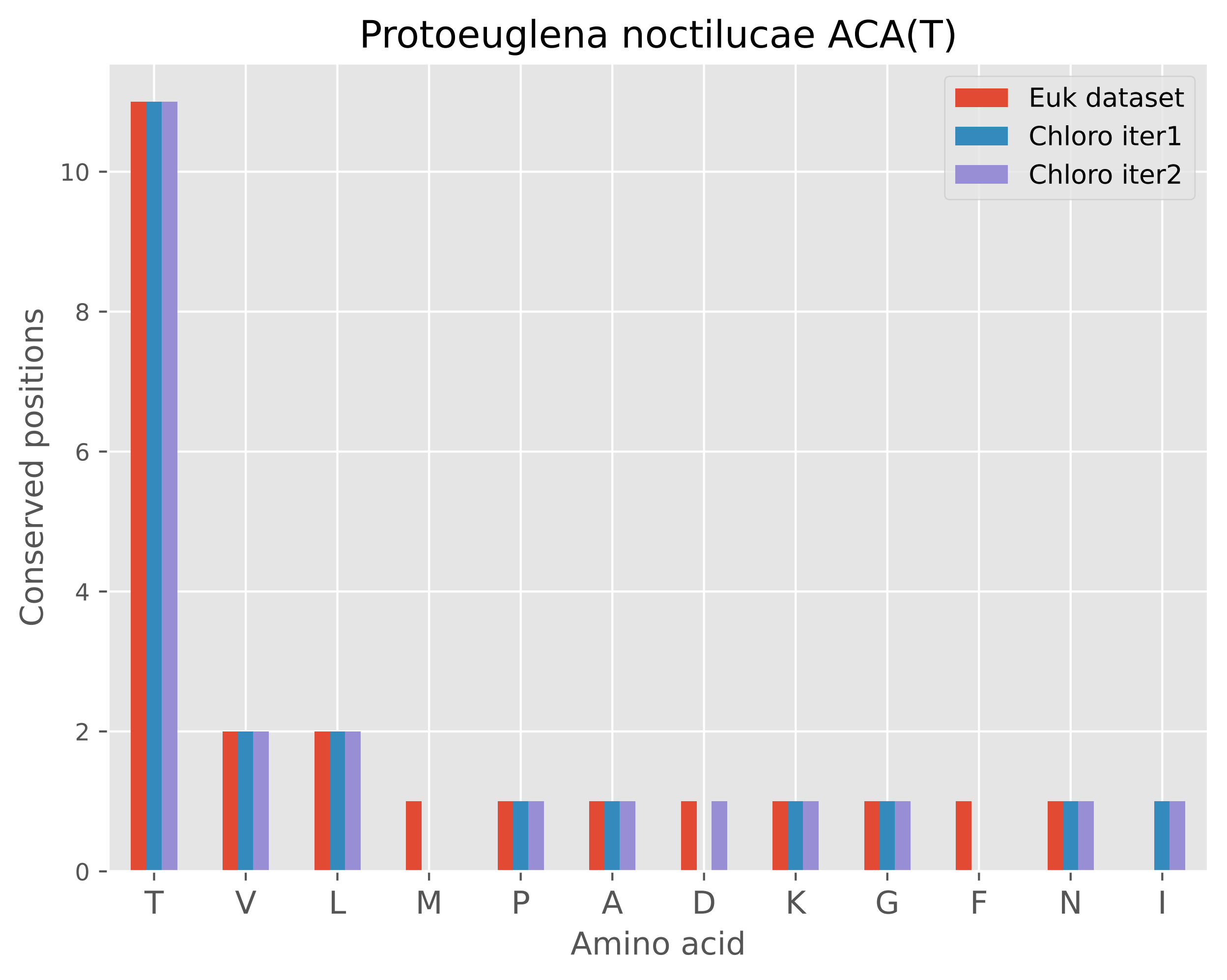

Protoeuglena noctilucae ACA(T)
Euk dataset
Chloro iter1
Chloro iter2
Conserved positions
0
2
4
6
8
10
T
V
L
M
P
A
D
K
G
F
N
I
Amino acid

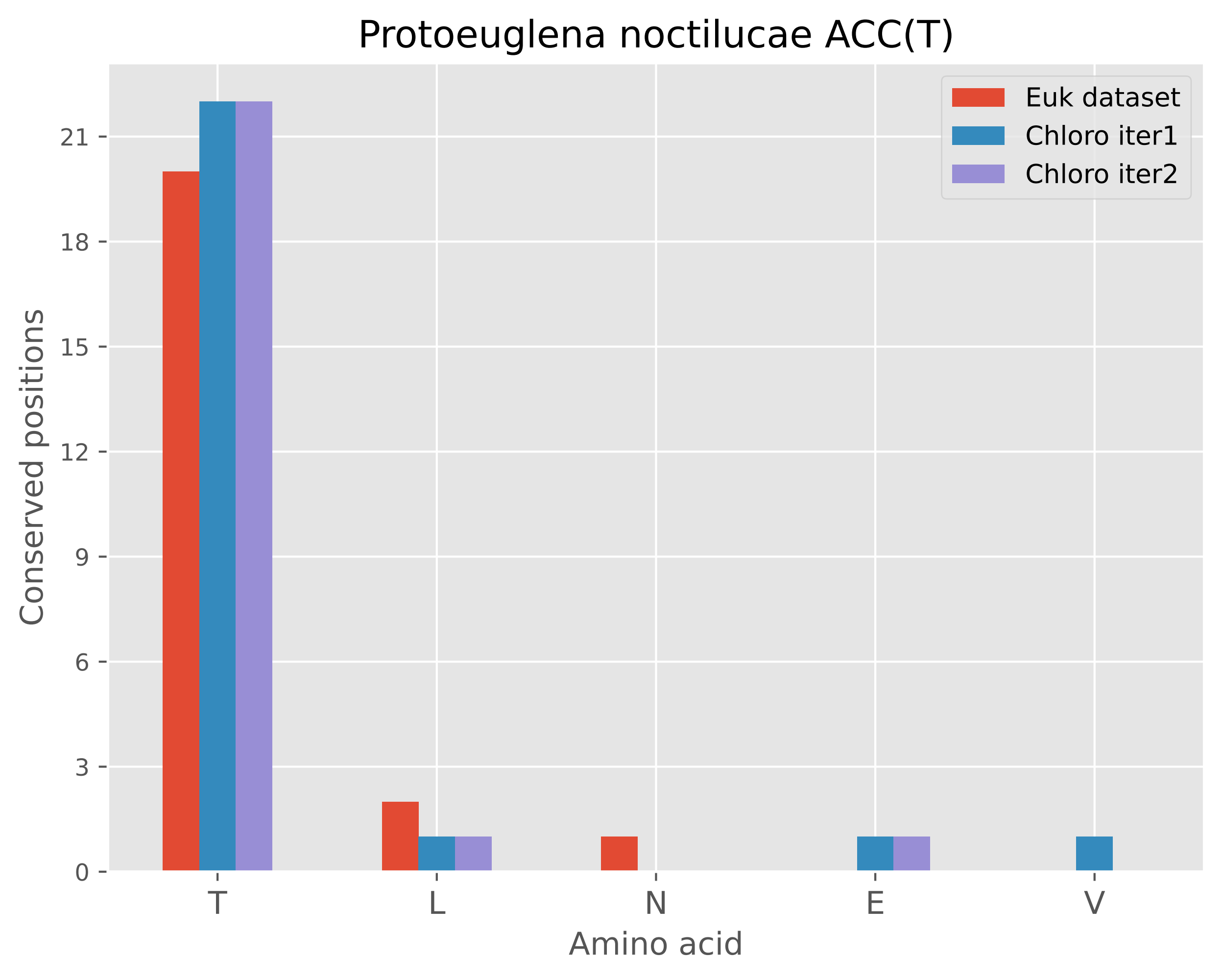
Protoeuglena noctilucae ACC(T)
Euk dataset
Chloro iter1
Chloro iter2
Conserved positions
0
3
6
9
12
15
18
21
T
L
N
E
V
Amino acid

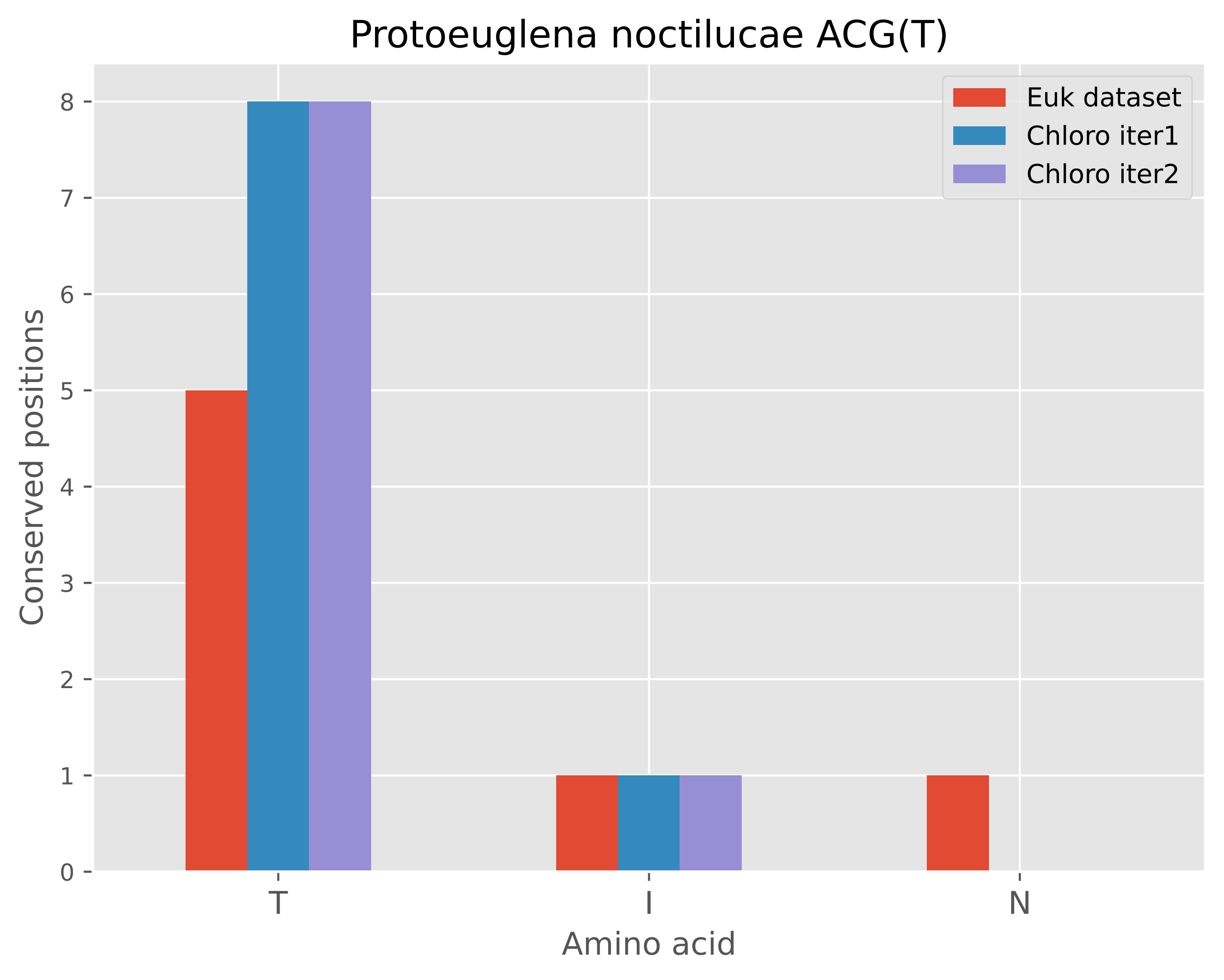

Protoeuglena noctilucae ACG(T)
Euk dataset
Chloro iter1
Chloro iter2
Conserved positions
0
1
2
3
4
5
6
7
8
T
I
N
Amino acid

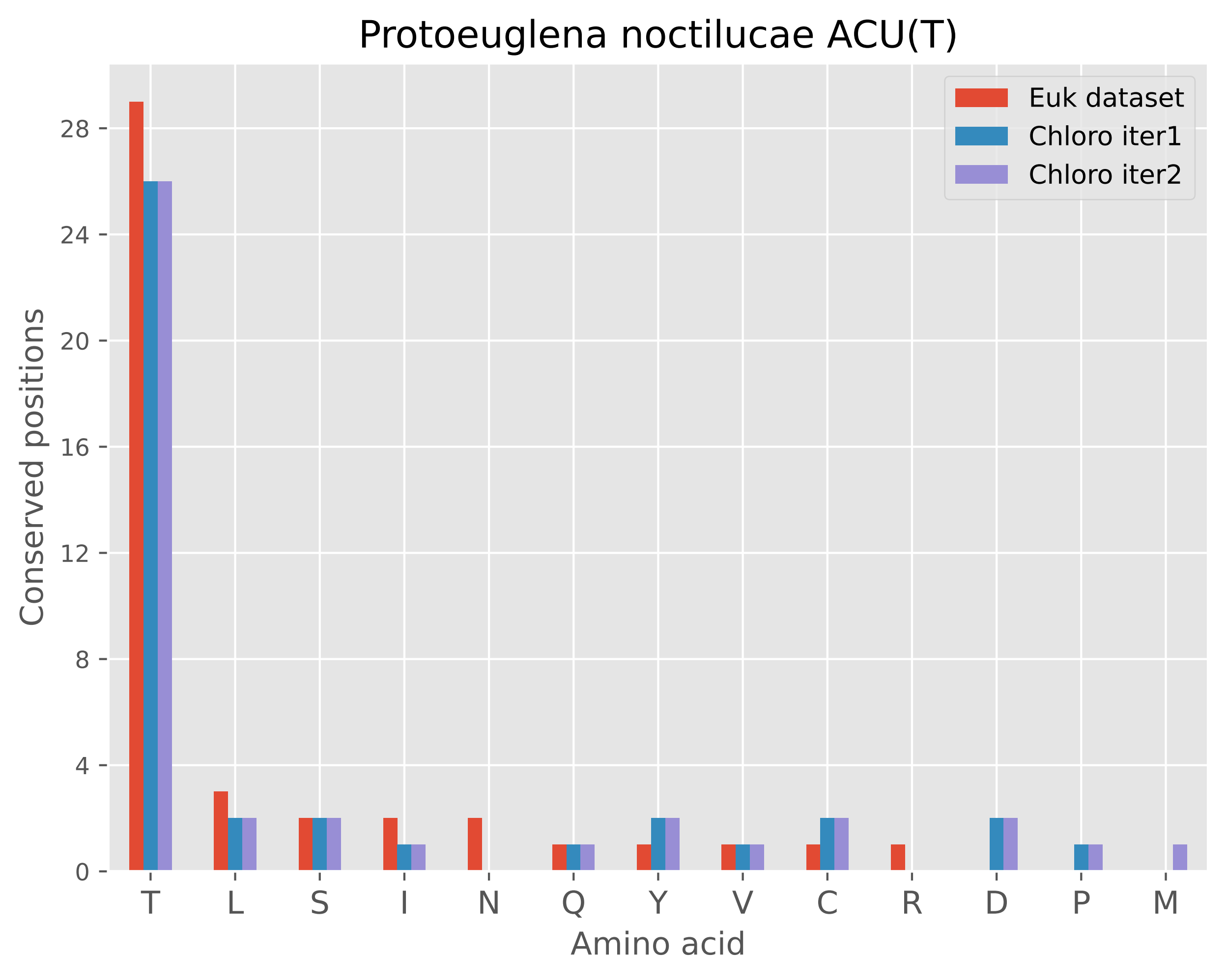
Protoeuglena noctilucae ACU(T)
Euk dataset
Chloro iter1
Chloro iter2
Conserved positions
0
4
8
12
16
20
24
28
T
L
S
I
N
Q
Y
V
C
R
D
P
M
Amino acid

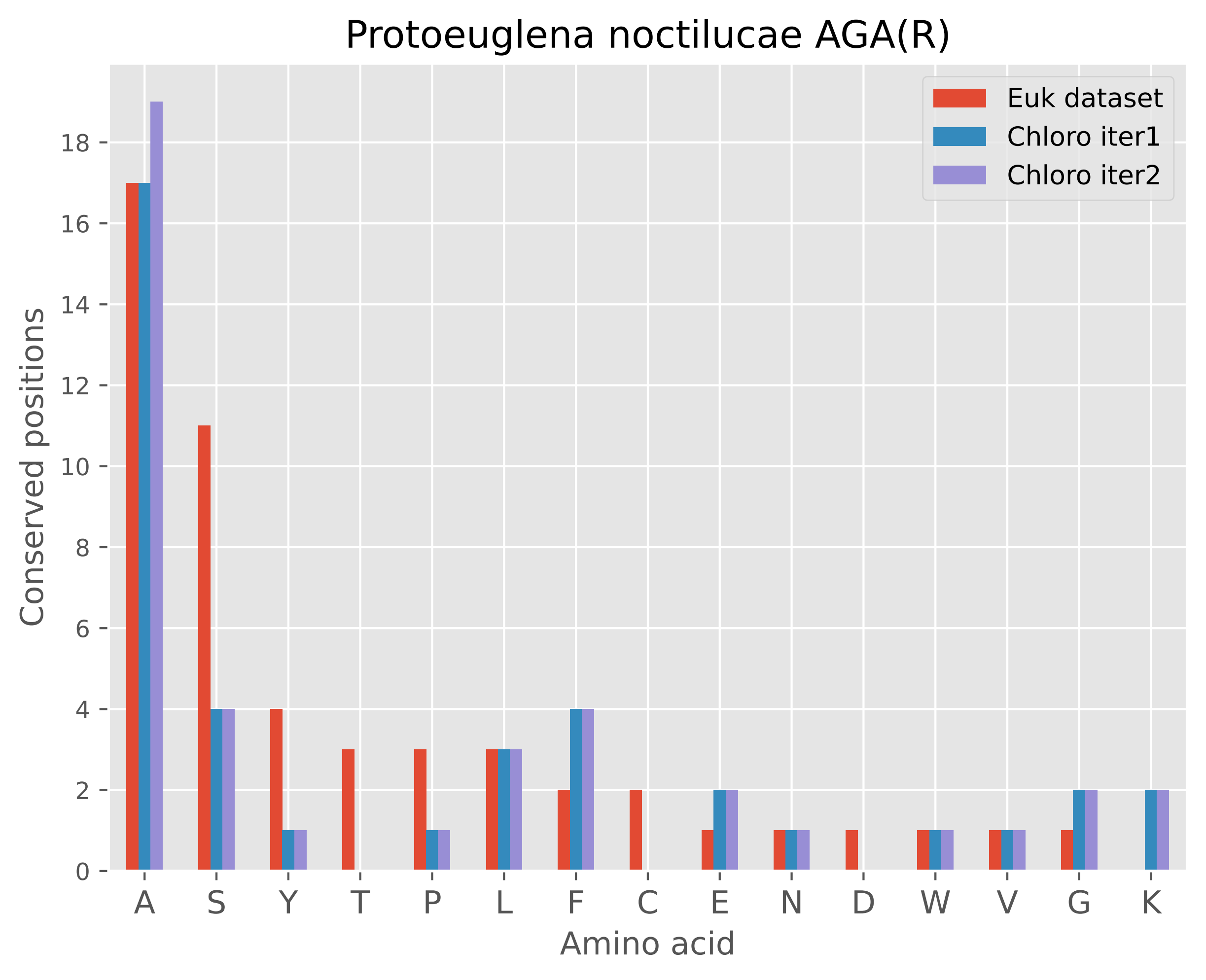

Protoeuglena noctilucae AGA(R)
Euk dataset
Chloro iter1
Chloro iter2
Conserved positions
0
2
4
6
8
10
12
14
16
18
A
S
Y
T
P
L
F
C
E
N
D
W
V
G
K
Amino acid

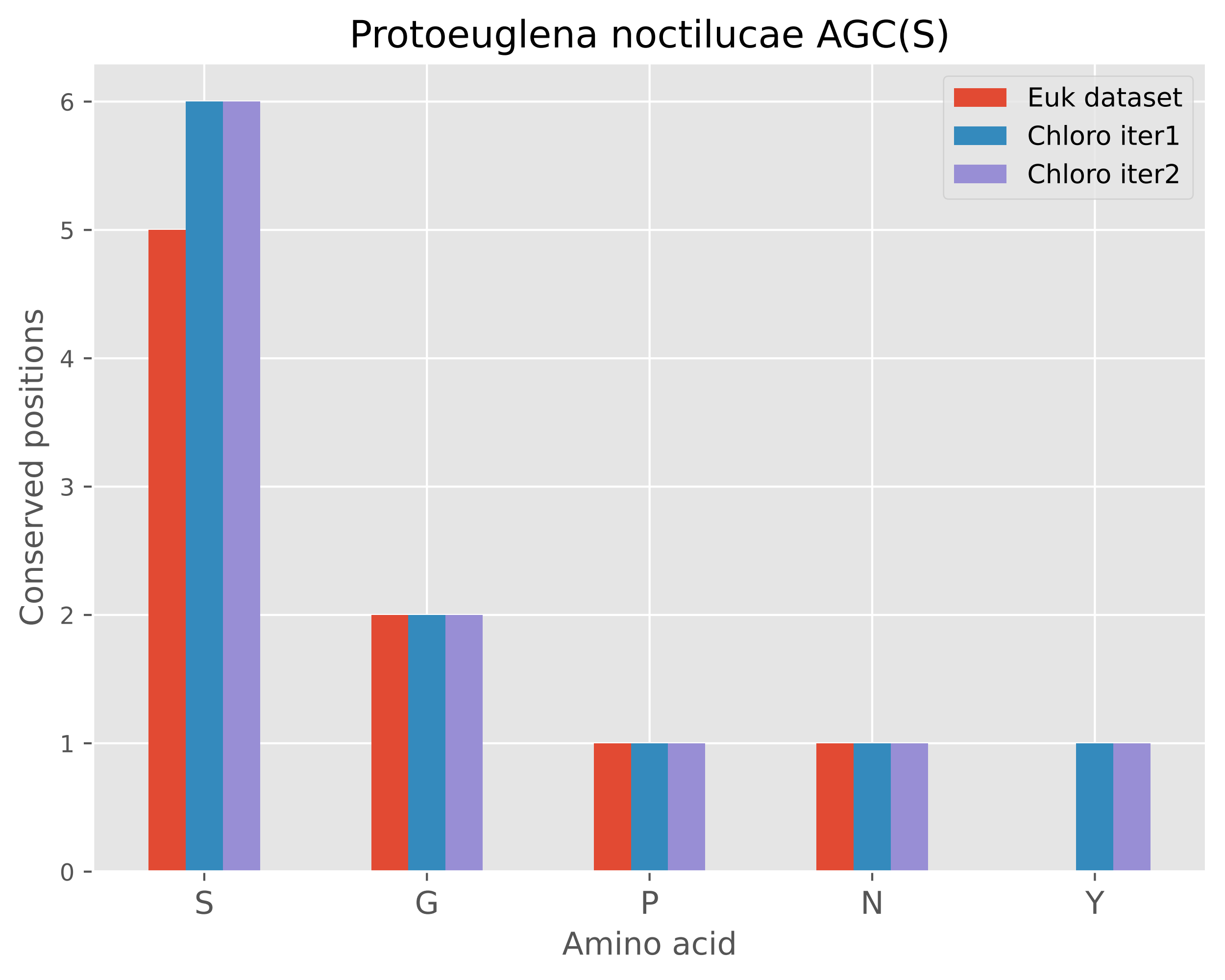
Protoeuglena noctilucae AGC(S)
Conserved positions
0
1
2
3
4
5
6
Euk dataset
Chloro iter1
Chloro iter2
S
G
P
N
Y
Amino acid

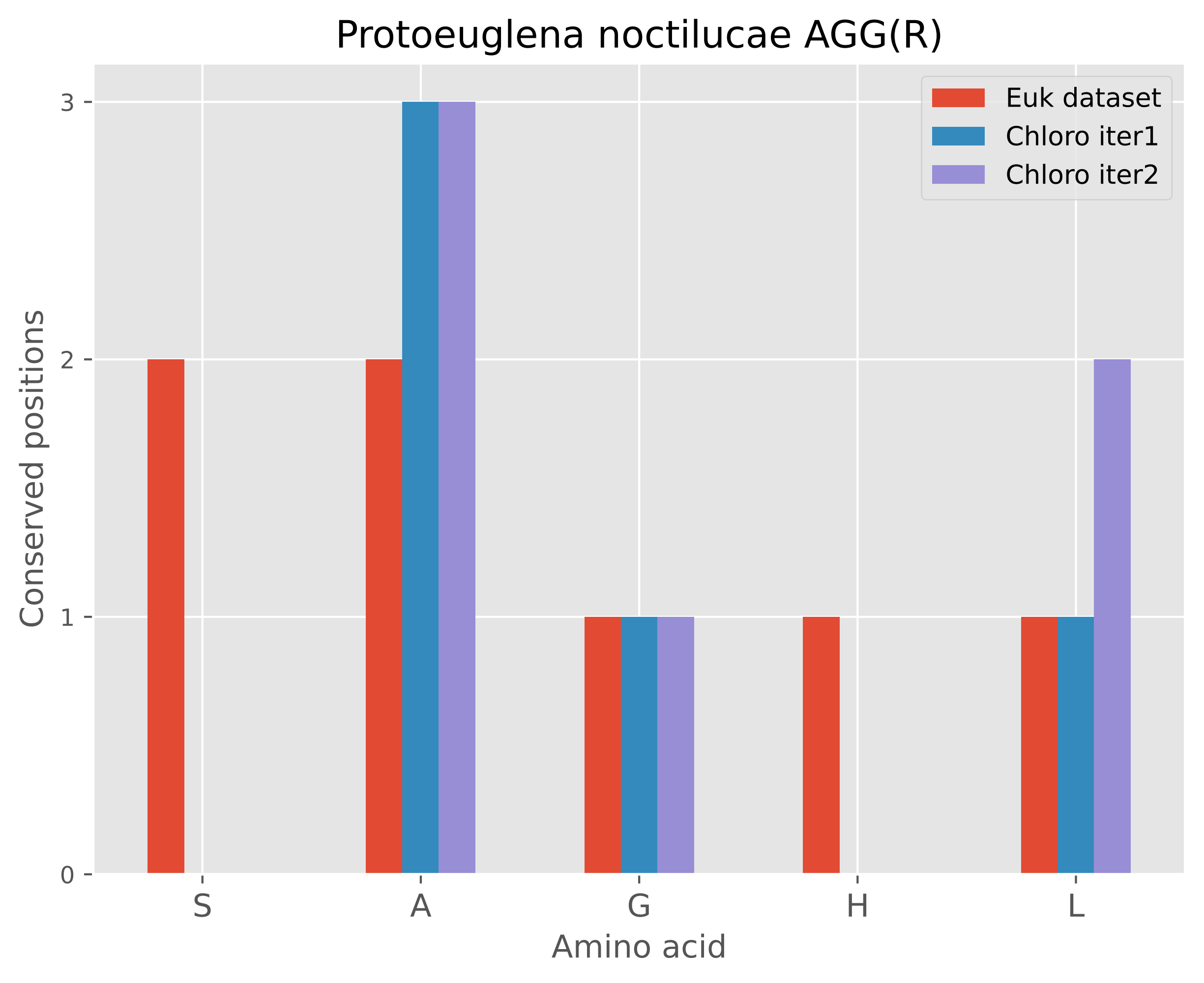
Protoeuglena noctilucae AGG(R)
Euk dataset
Chloro iter1
Chloro iter2
Conserved positions
0
1
2
3
S
A
G
H
L
Amino acid

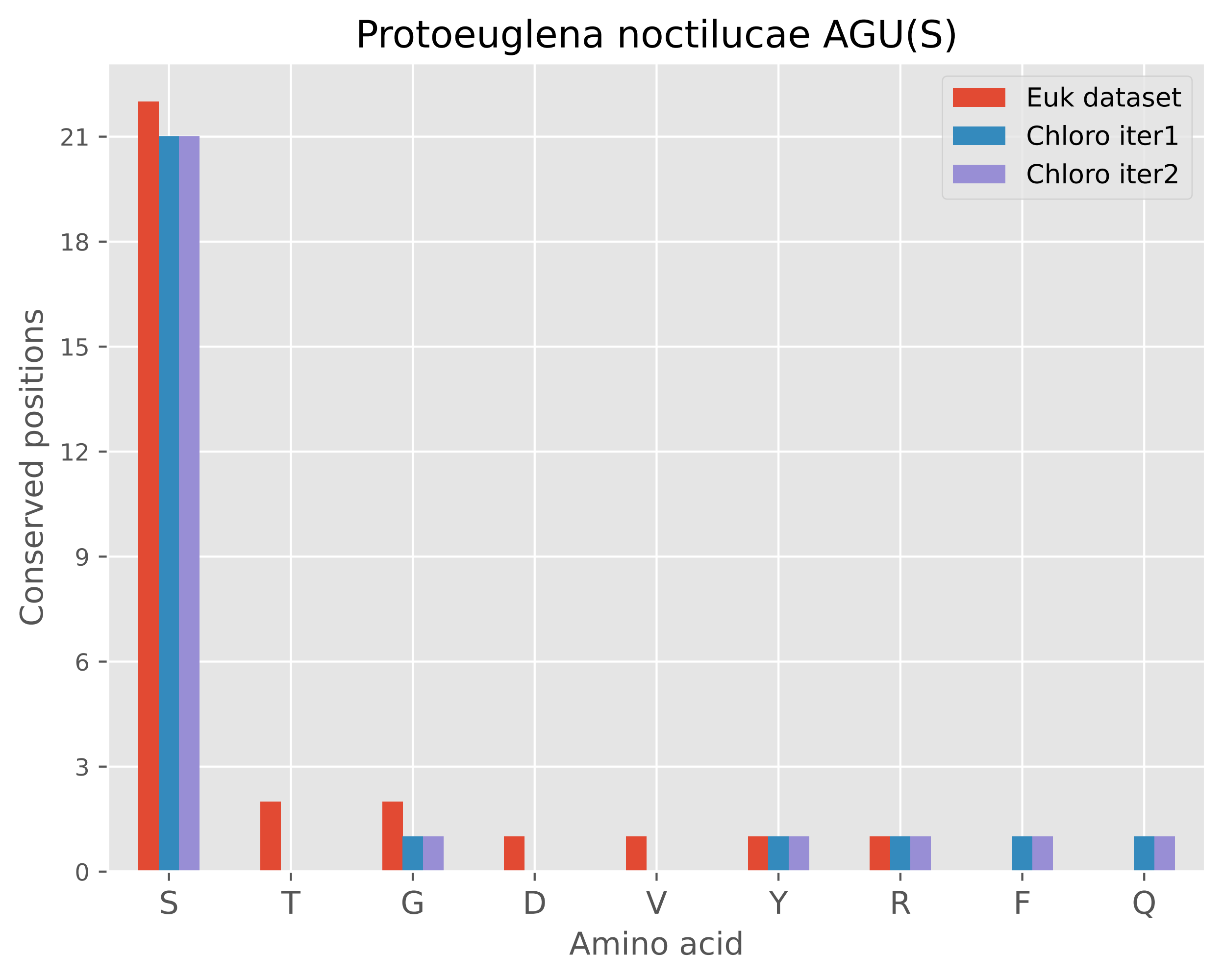

Protoeuglena noctilucae AGU(S)
Euk dataset
Chloro iter1
Chloro iter2
Conserved positions
0
3
6
9
12
15
18
21
S
T
G
D
V
Y
R
F
Q
Amino acid

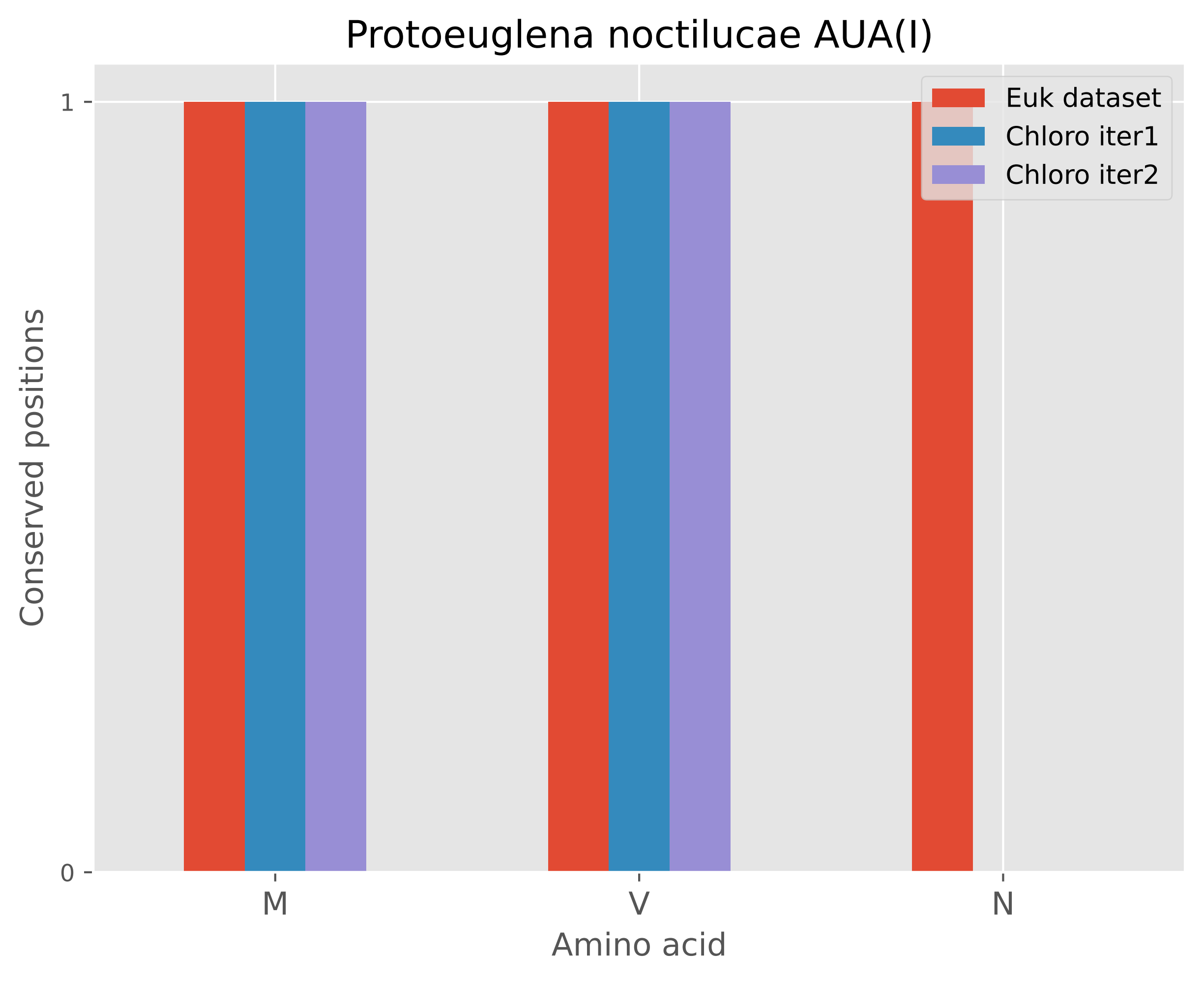
Protoeuglena noctilucae AUA(I)
Conserved positions
Amino acid
1
0
M
V
N
Euk dataset
Chloro iter1
Chloro iter2

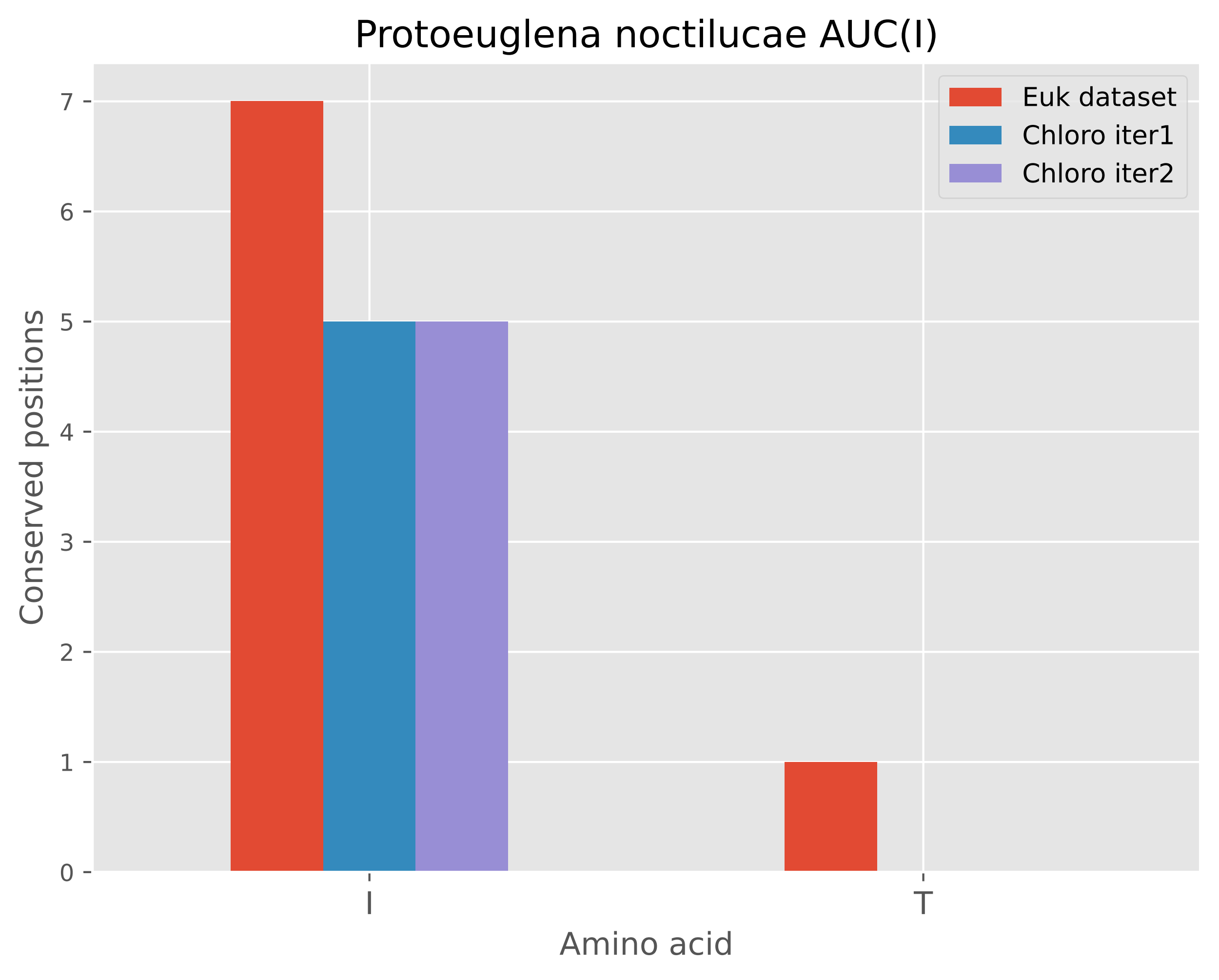

Protoeuglena noctilucae AUC(I)
Euk dataset
Chloro iter1
Chloro iter2
Conserved positions
0
1
2
3
4
5
6
7
I
T
Amino acid

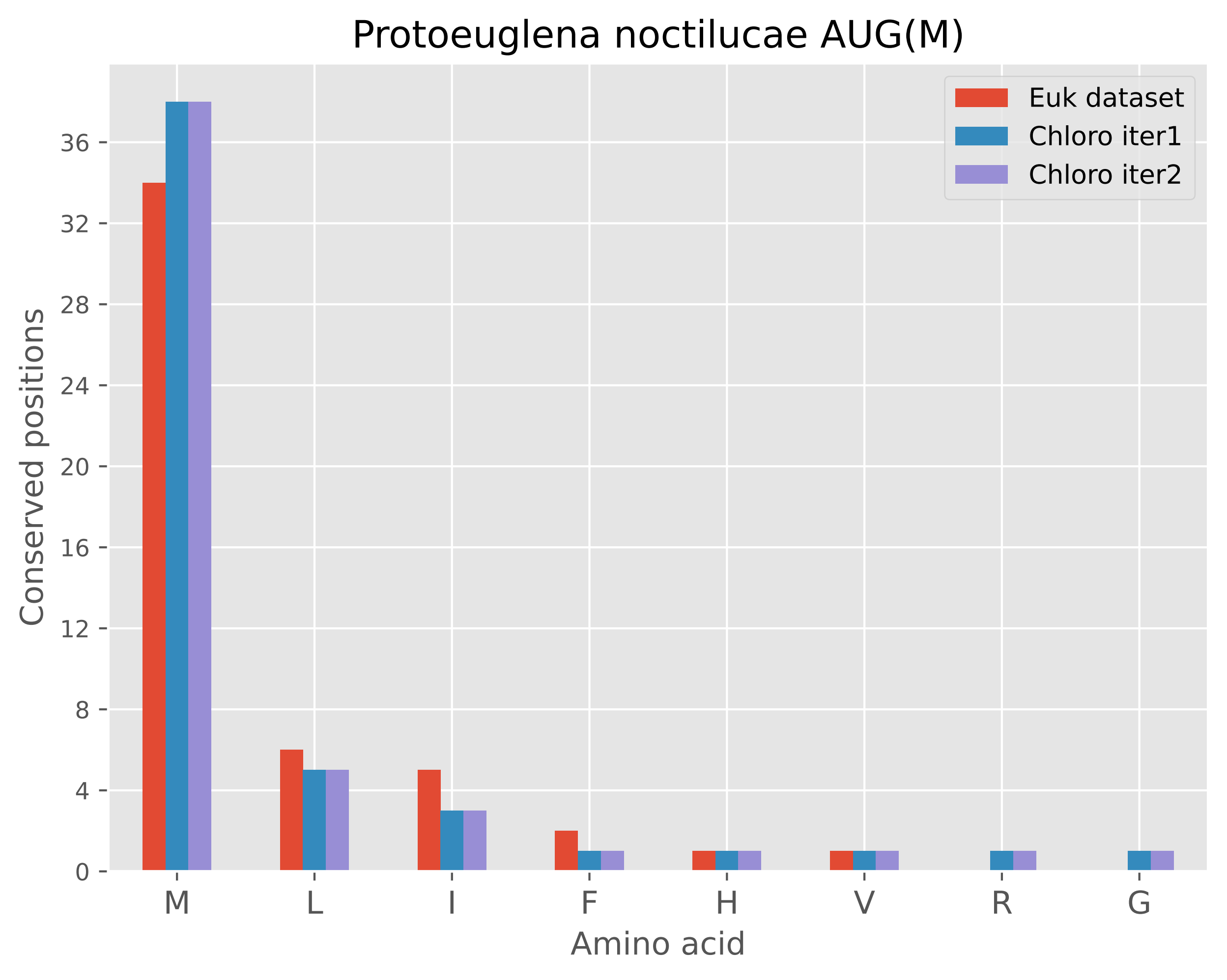
Protoeuglena noctilucae AUG(M)
Euk dataset
Chloro iter1
Chloro iter2
Conserved positions
0
4
8
12
16
20
24
28
32
36
M
L
I
F
H
V
R
G
Amino acid

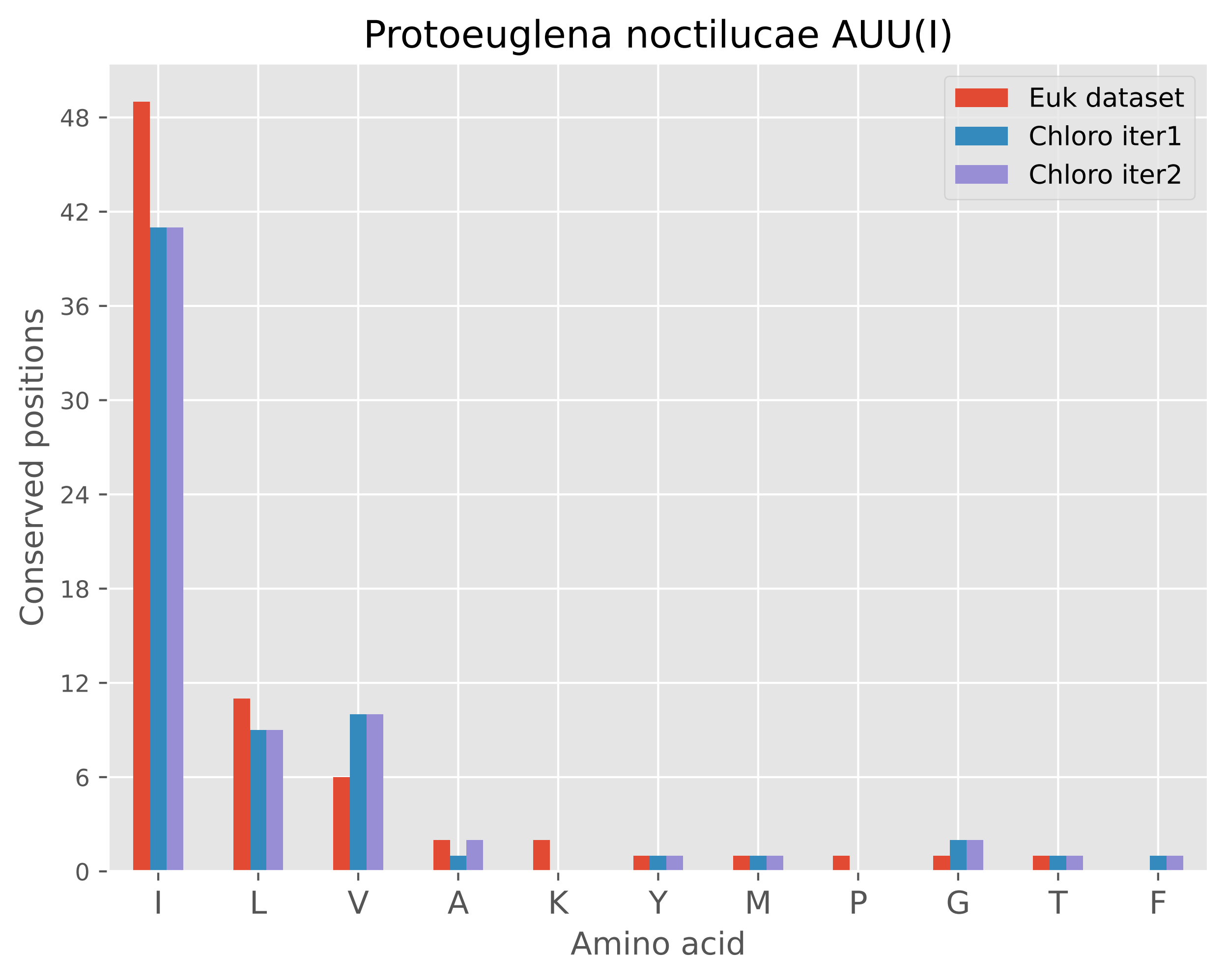

Protoeuglena noctilucae AUU(I)
Euk dataset
Chloro iter1
Chloro iter2
Conserved positions
0
6
12
18
24
30
36
42
48
I
L
V
A
K
Y
M
P
G
T
F
Amino acid

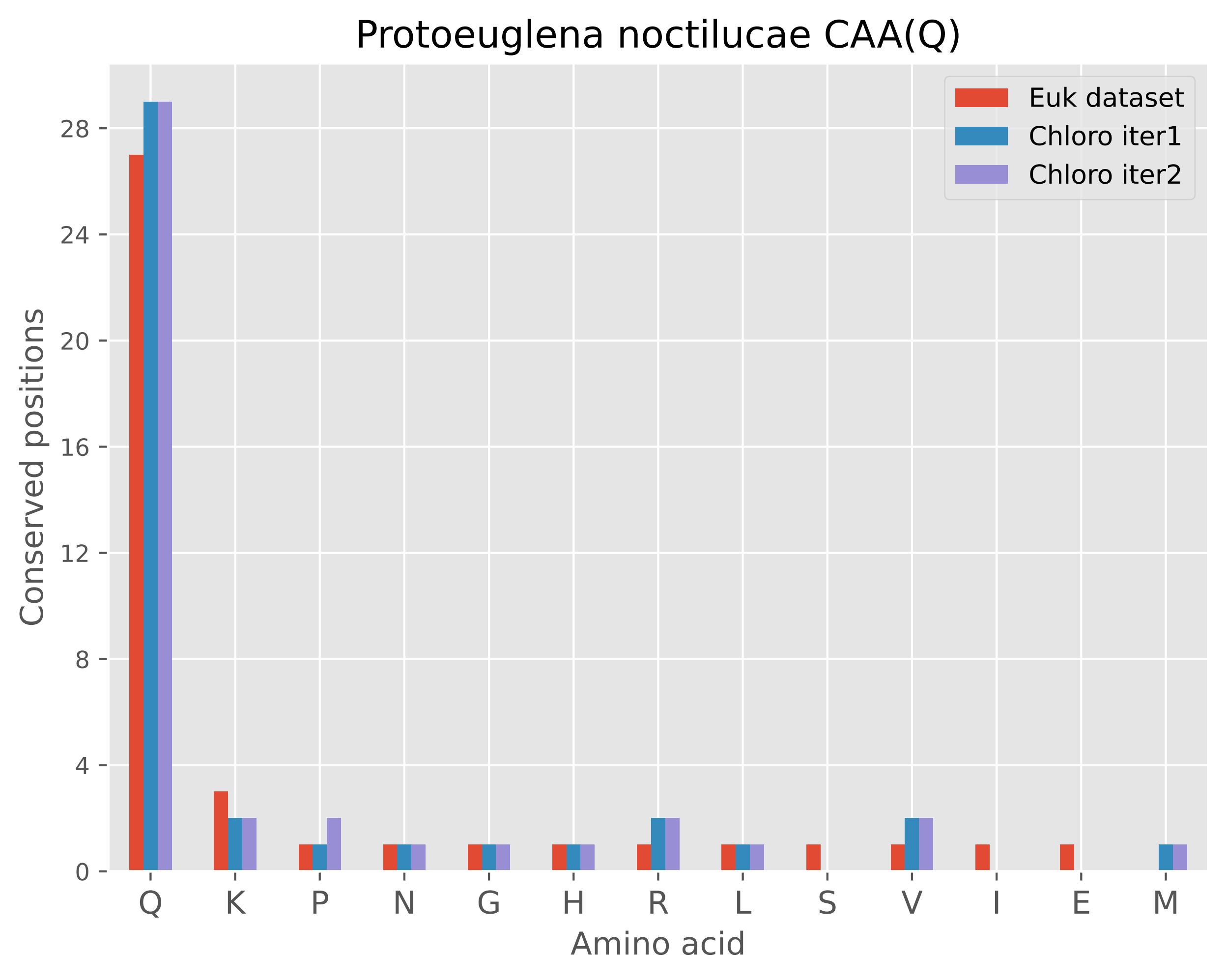
Protoeuglena noctilucae CAA(Q)
Euk dataset
Chloro iter1
Chloro iter2
Conserved positions
0
4
8
12
16
20
24
28
Q
K
P
N
G
H
R
L
S
V
I
E
M
Amino acid

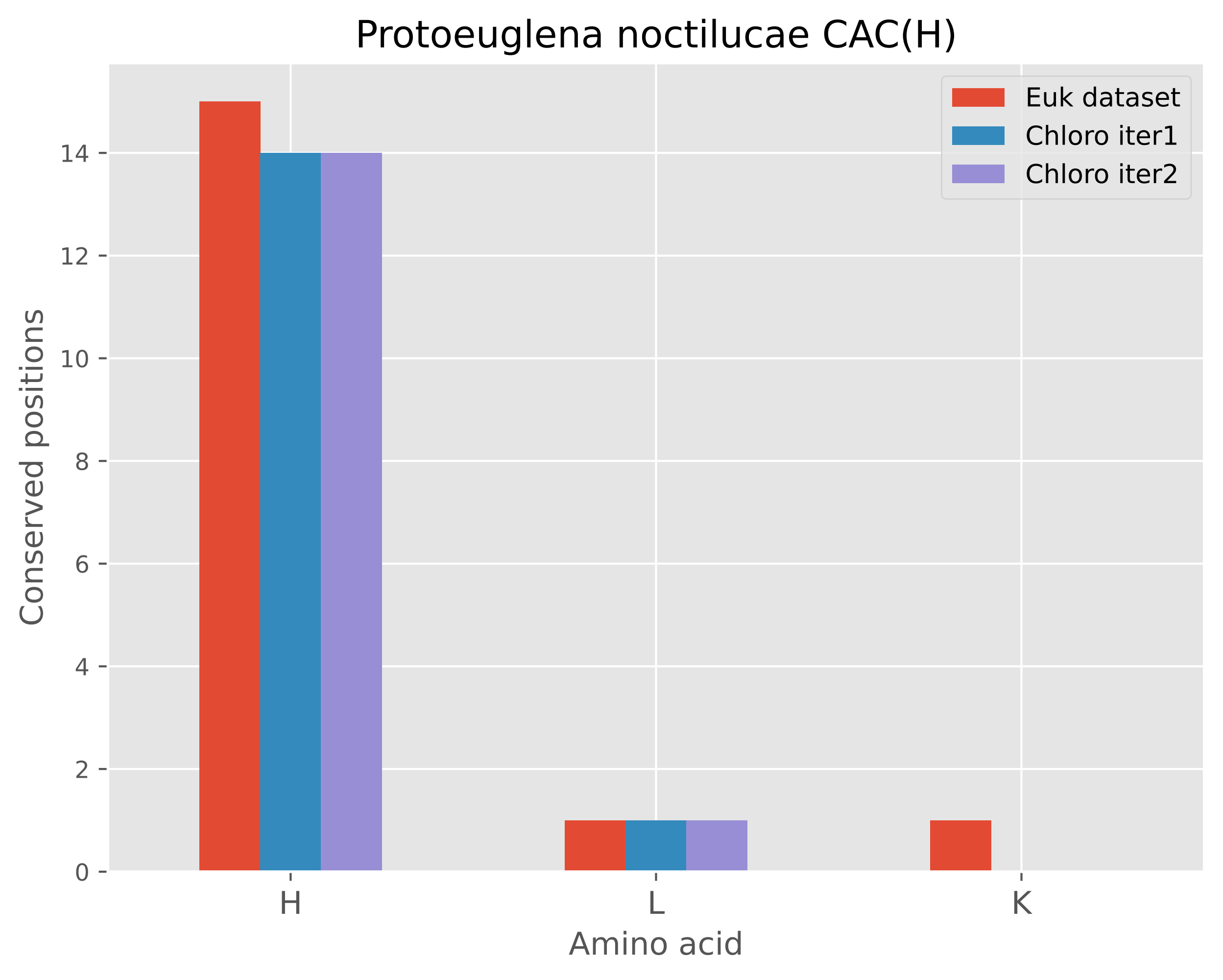
Protoeuglena noctilucae CAC(H)
Euk dataset
Chloro iter1
Chloro iter2
Conserved positions
0
2
4
6
8
10
12
14
H
L
K
Amino acid

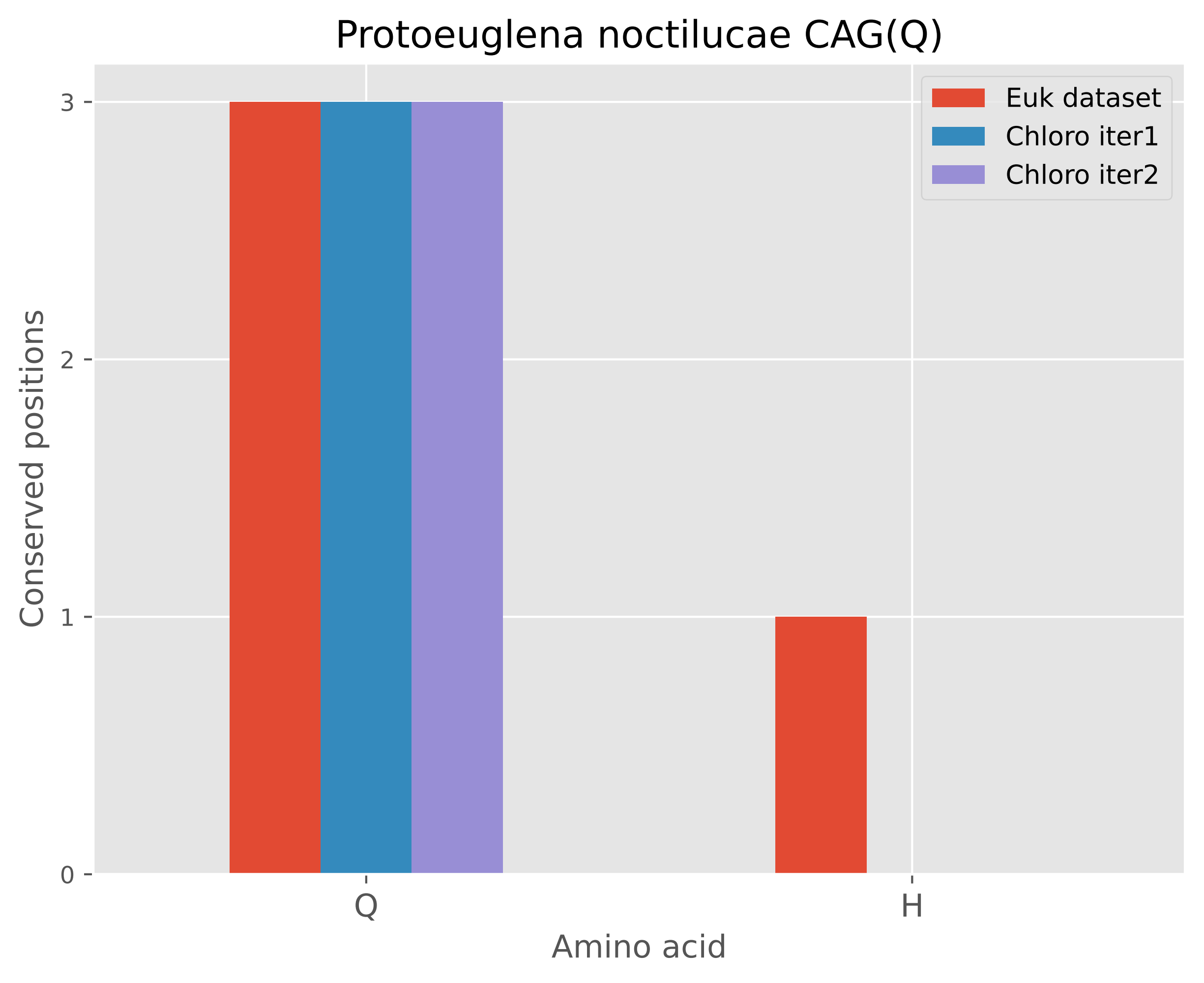
Protoeuglena noctilucae CAG(Q)
Euk dataset
Chloro iter1
Chloro iter2
Conserved positions
3
2
1
0
Q
H
Amino acid

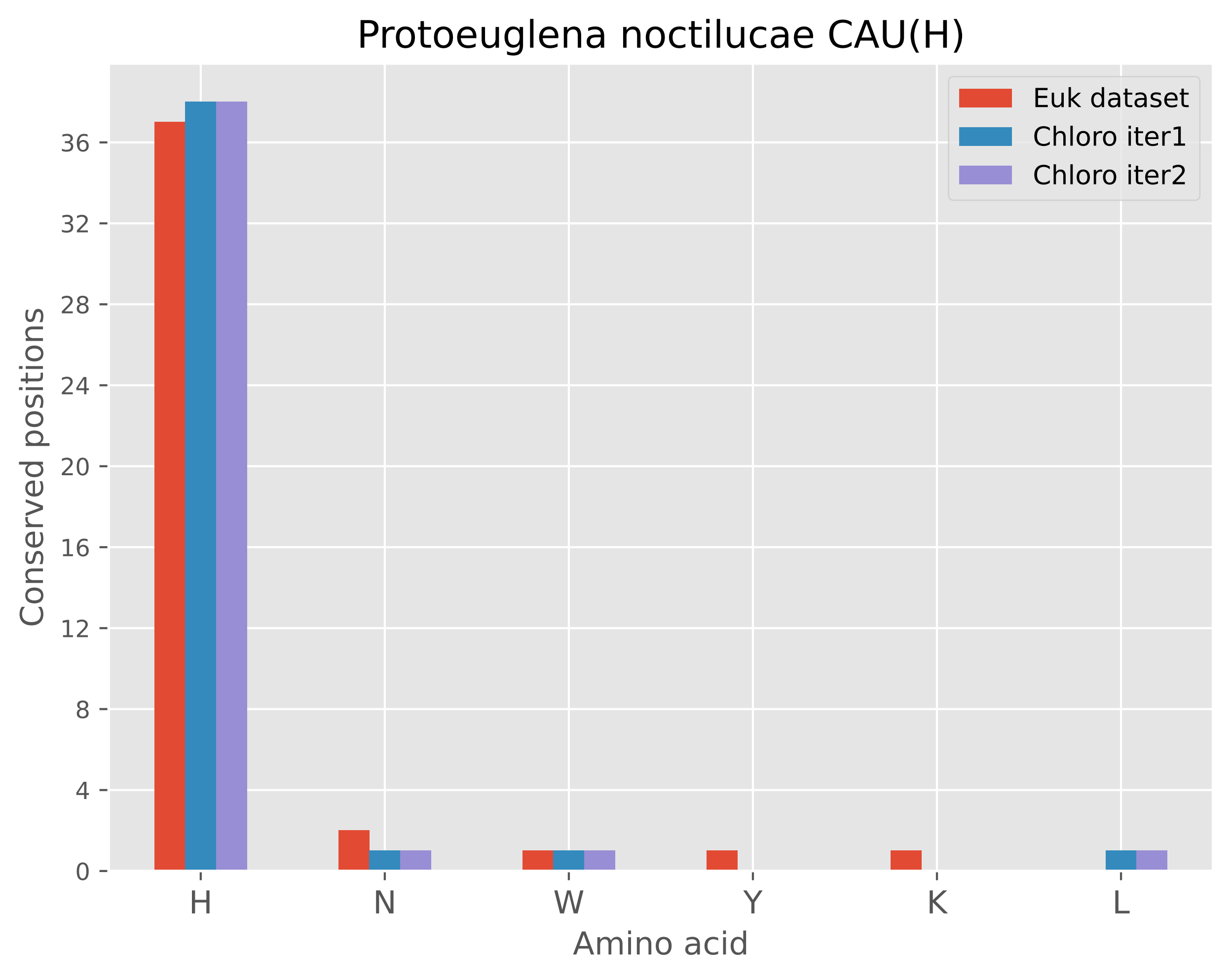

Protoeuglena noctilucae CAU(H)
Euk dataset
Chloro iter1
Chloro iter2
Conserved positions
0
4
8
12
16
20
24
28
32
36
H
N
W
Y
K
L
Amino acid

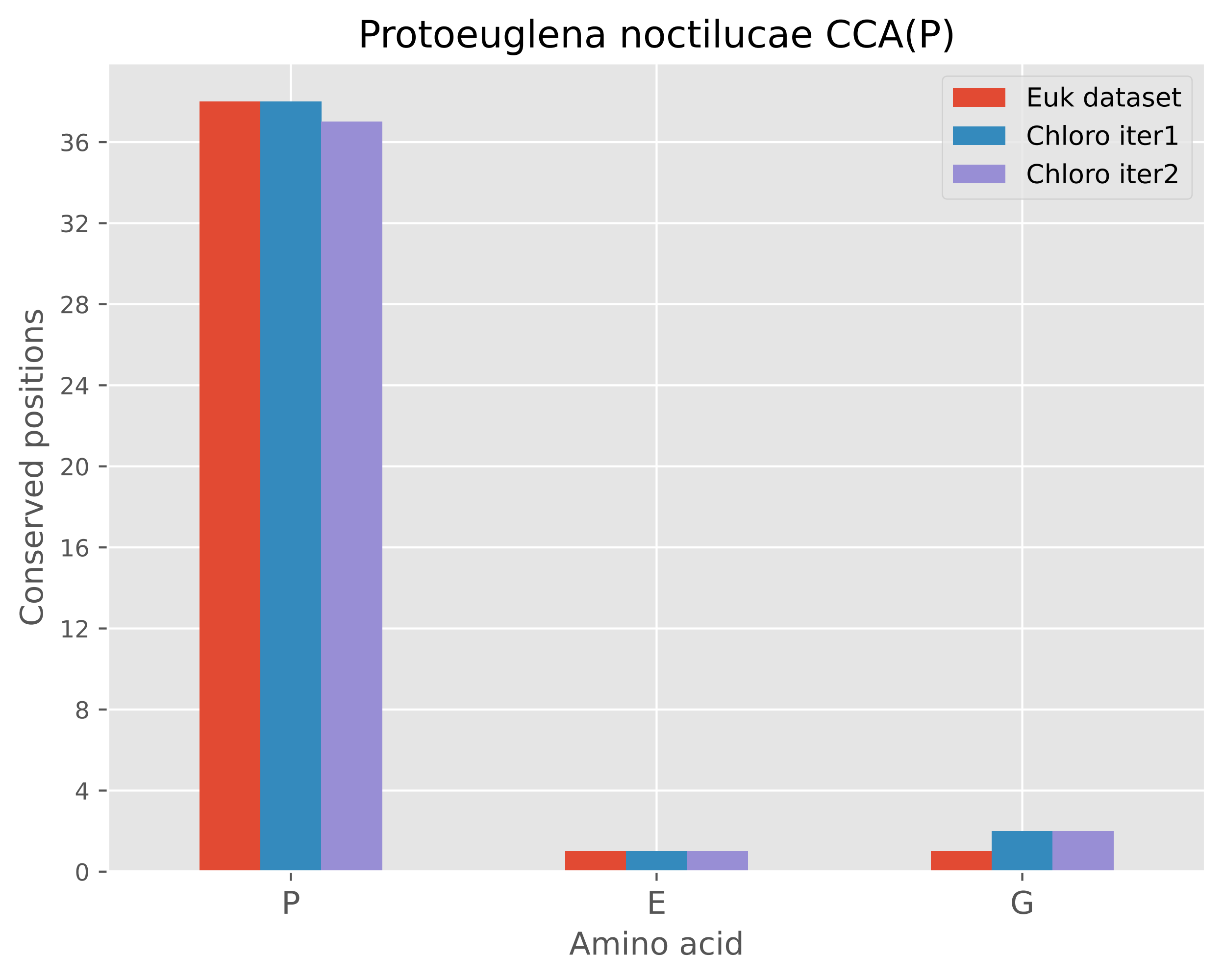
Protoeuglena noctilucae CCA(P)
Euk dataset
Chloro iter1
Chloro iter2
Conserved positions
0
4
8
12
16
20
24
28
32
36
P
E
G
Amino acid

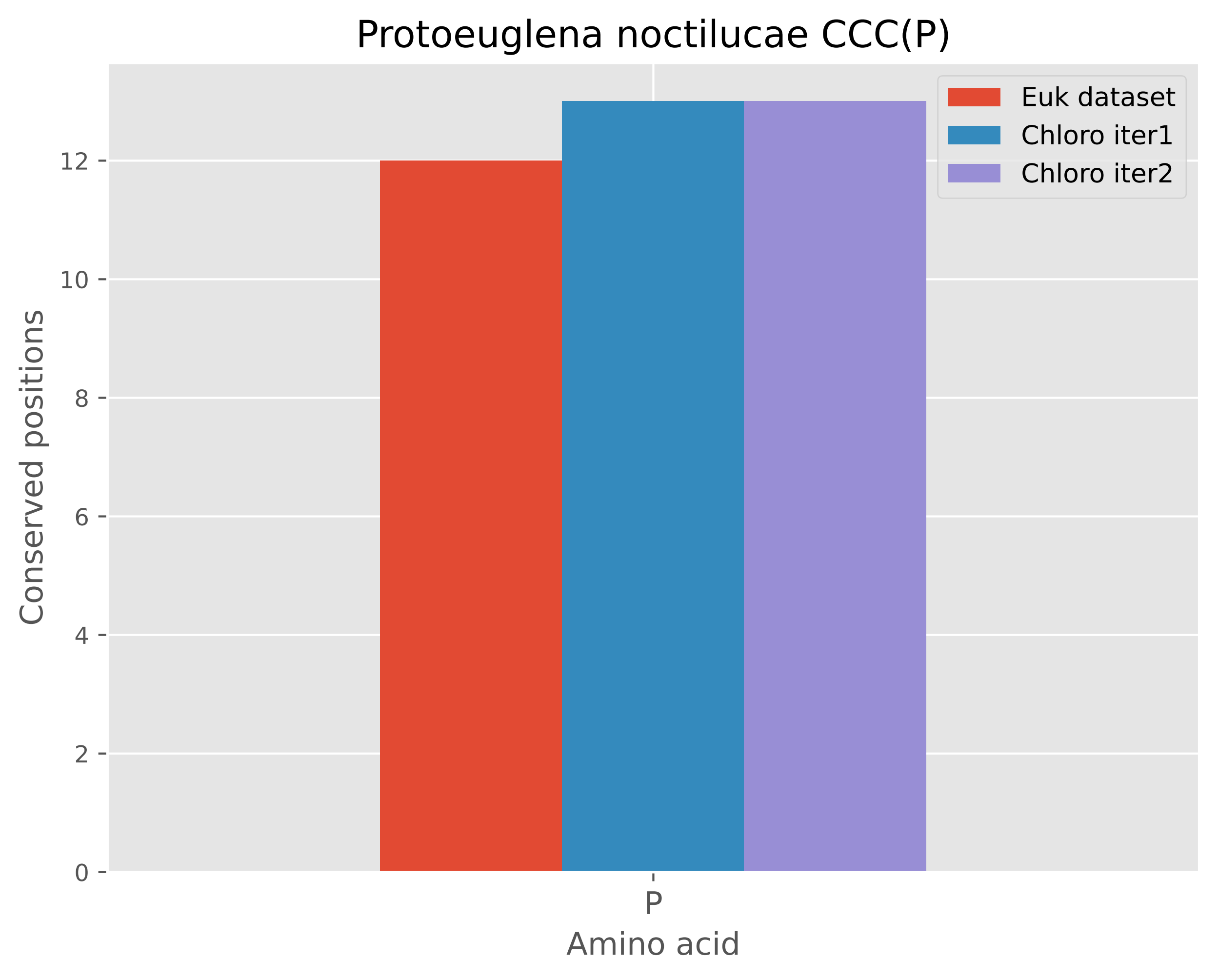

Protoeuglena noctilucae CCC(P)
Euk dataset
Chloro iter1
Chloro iter2
Conserved positions
0
2
4
6
8
10
12
P
Amino acid

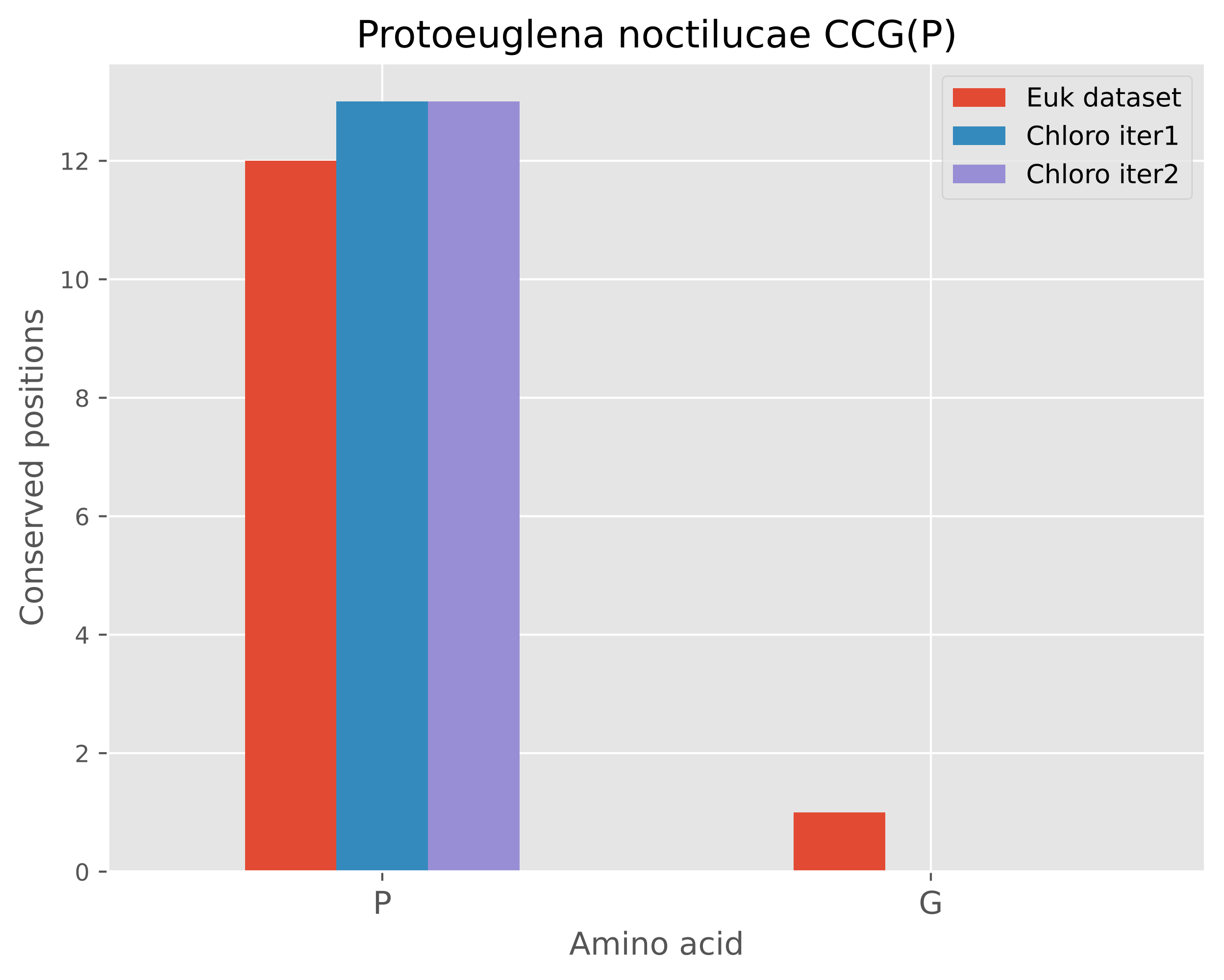

Protoeuglena noctilucae CCG(P)
Euk dataset
Chloro iter1
Chloro iter2
Conserved positions
0
2
4
6
8
10
12
P
G
Amino acid

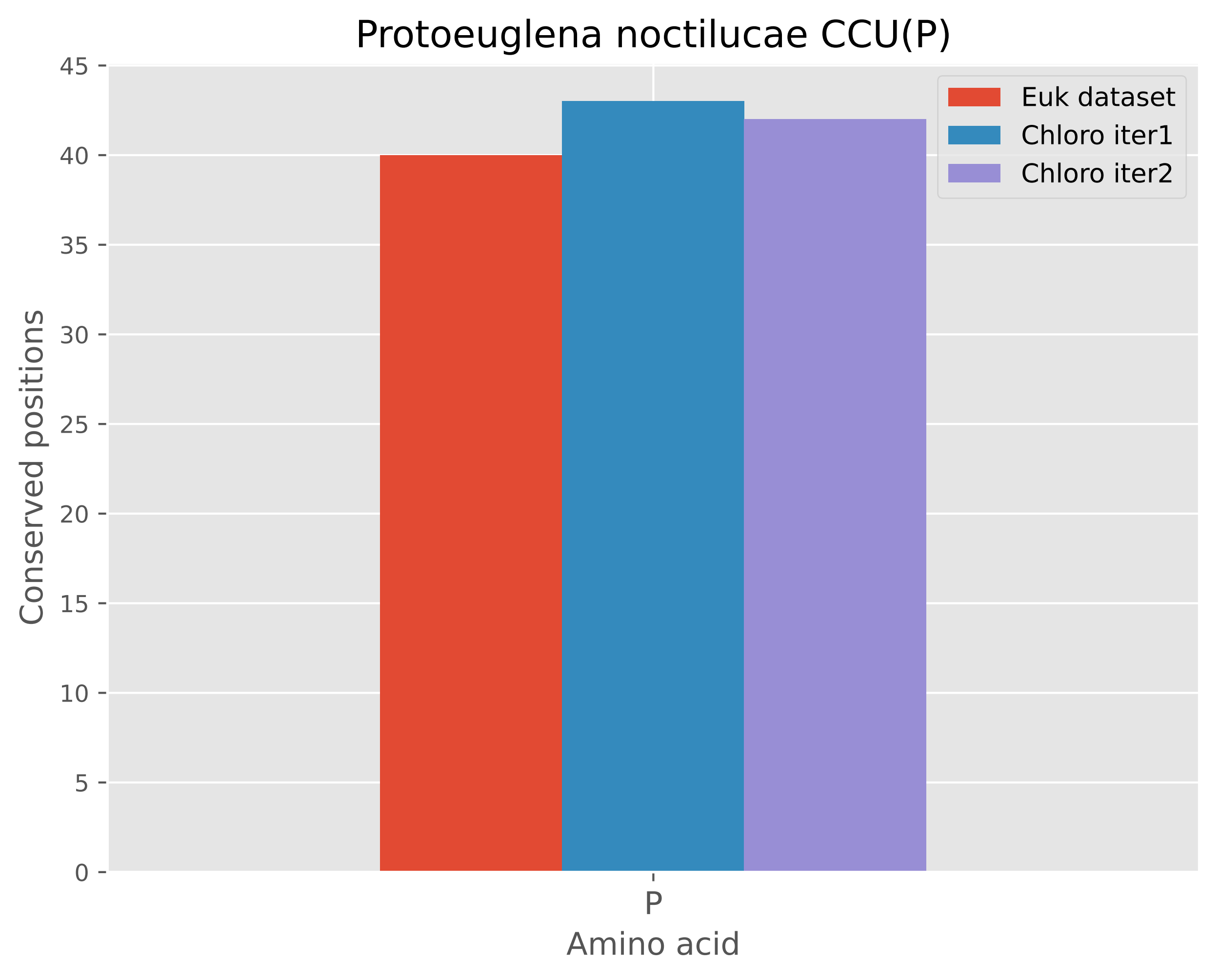

Protoeuglena noctilucae CCU(P)
Conserved positions
0
5
10
15
20
25
30
35
40
45
P
Amino acid
Euk dataset
Chloro iter1
Chloro iter2

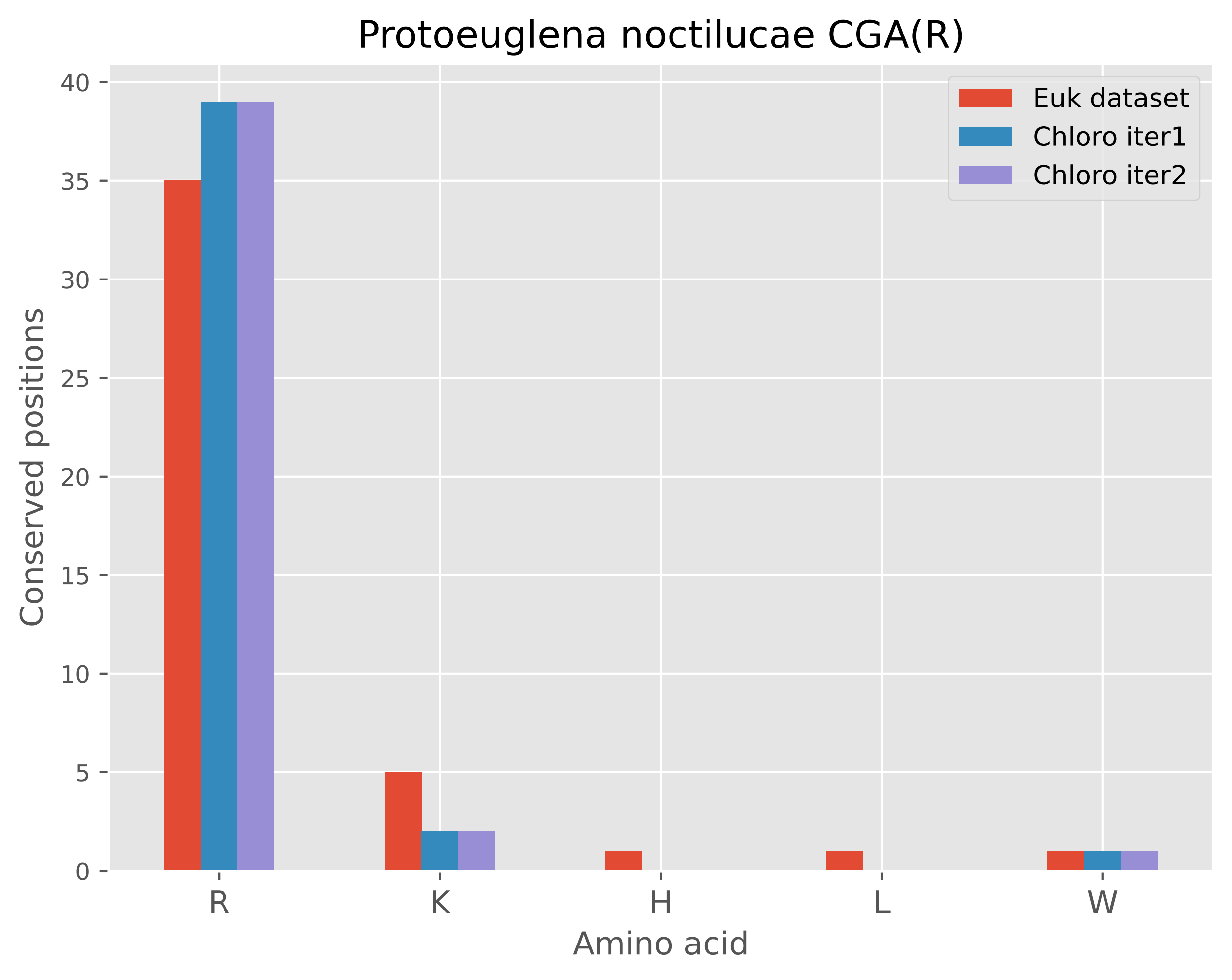

Protoeuglena noctilucae CGA(R)
Euk dataset
Chloro iter1
Chloro iter2
Conserved positions
0
5
10
15
20
25
30
35
40
R
K
H
L
W
Amino acid

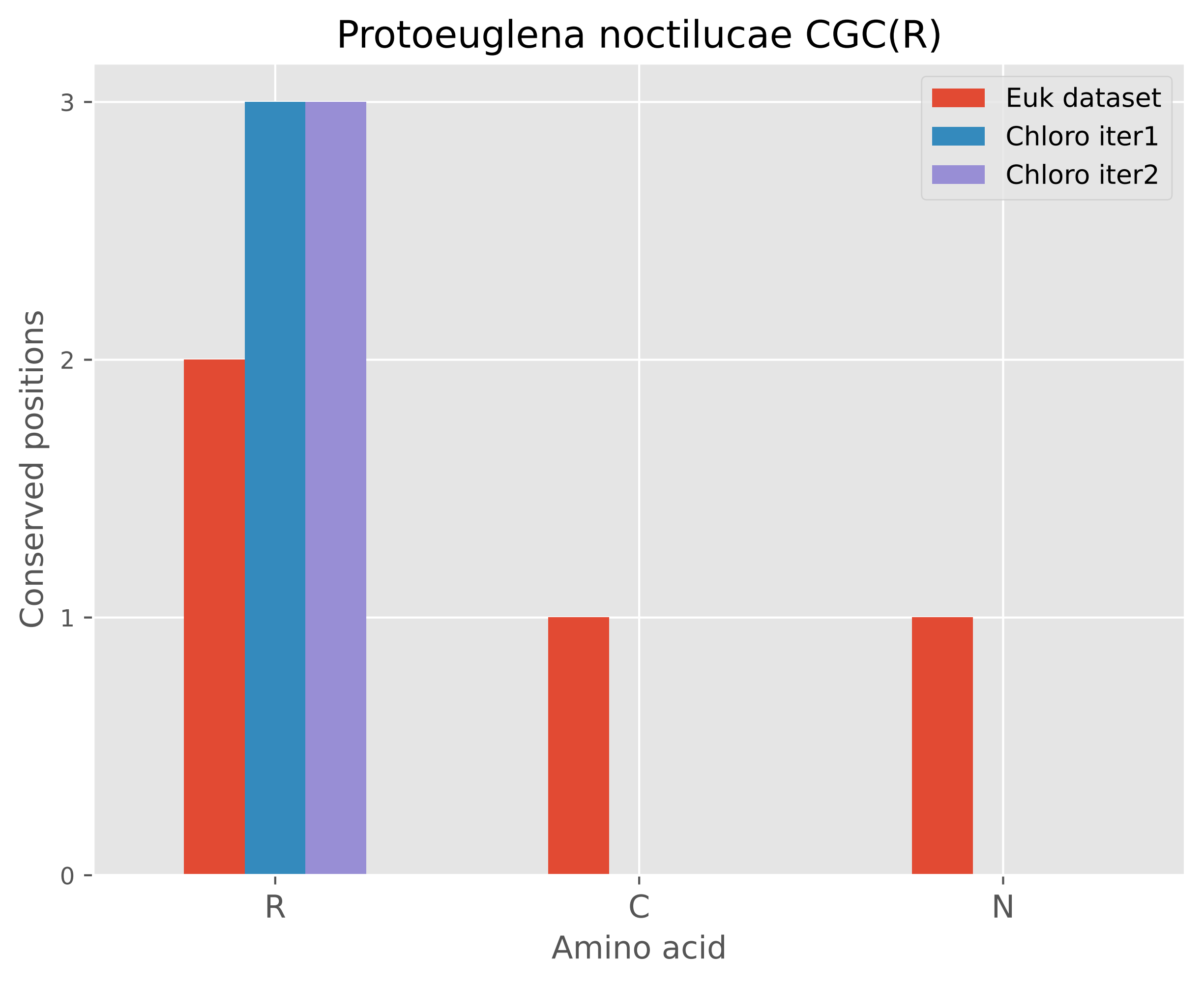

Protoeuglena noctilucae CGC(R)
Euk dataset
Chloro iter1
Chloro iter2
Conserved positions
0
1
2
3
R
C
N
Amino acid

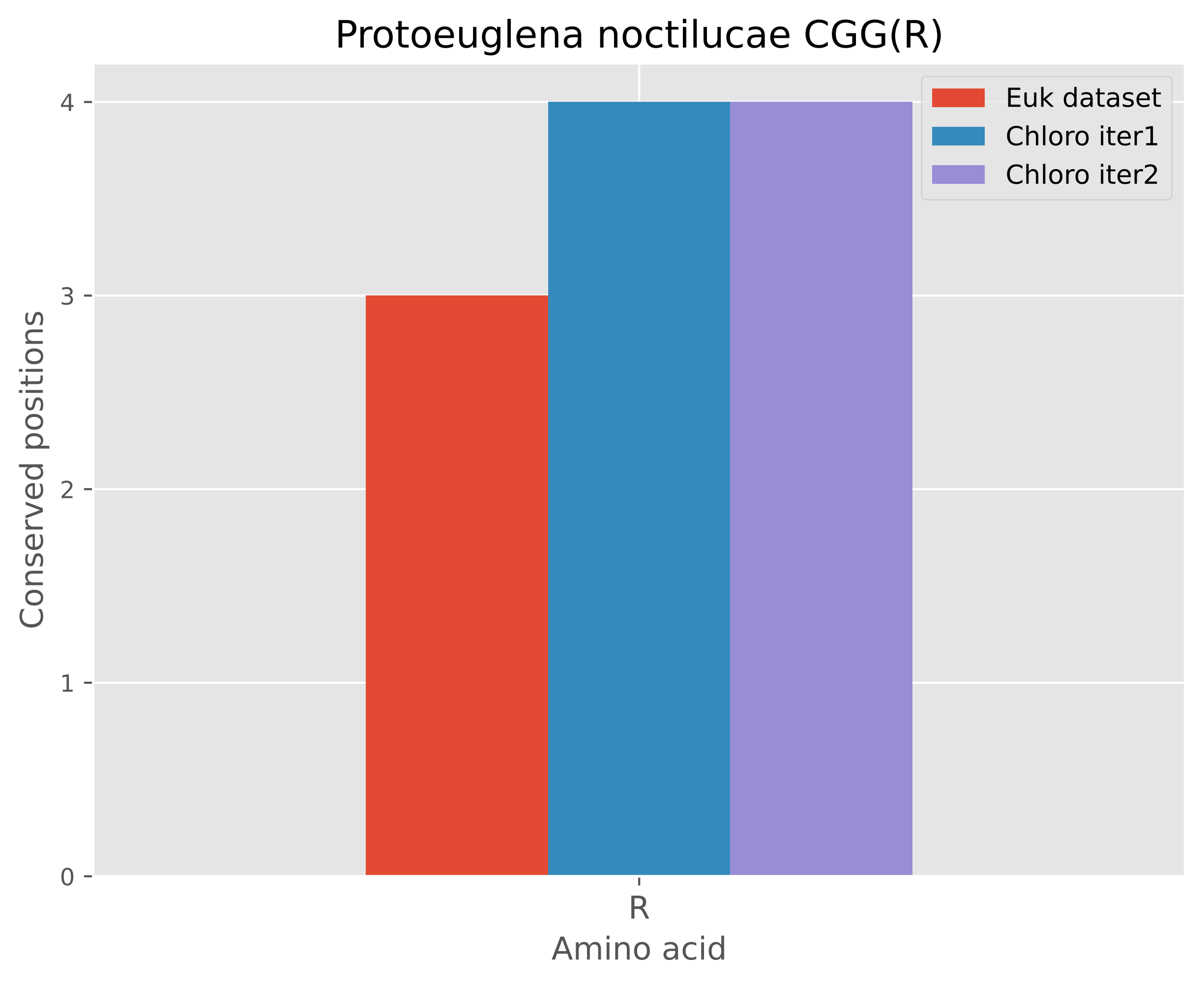
Protoeuglena noctilucae CGG(R)
Euk dataset
Chloro iter1
Chloro iter2
Conserved positions
0
1
2
3
4
R
Amino acid

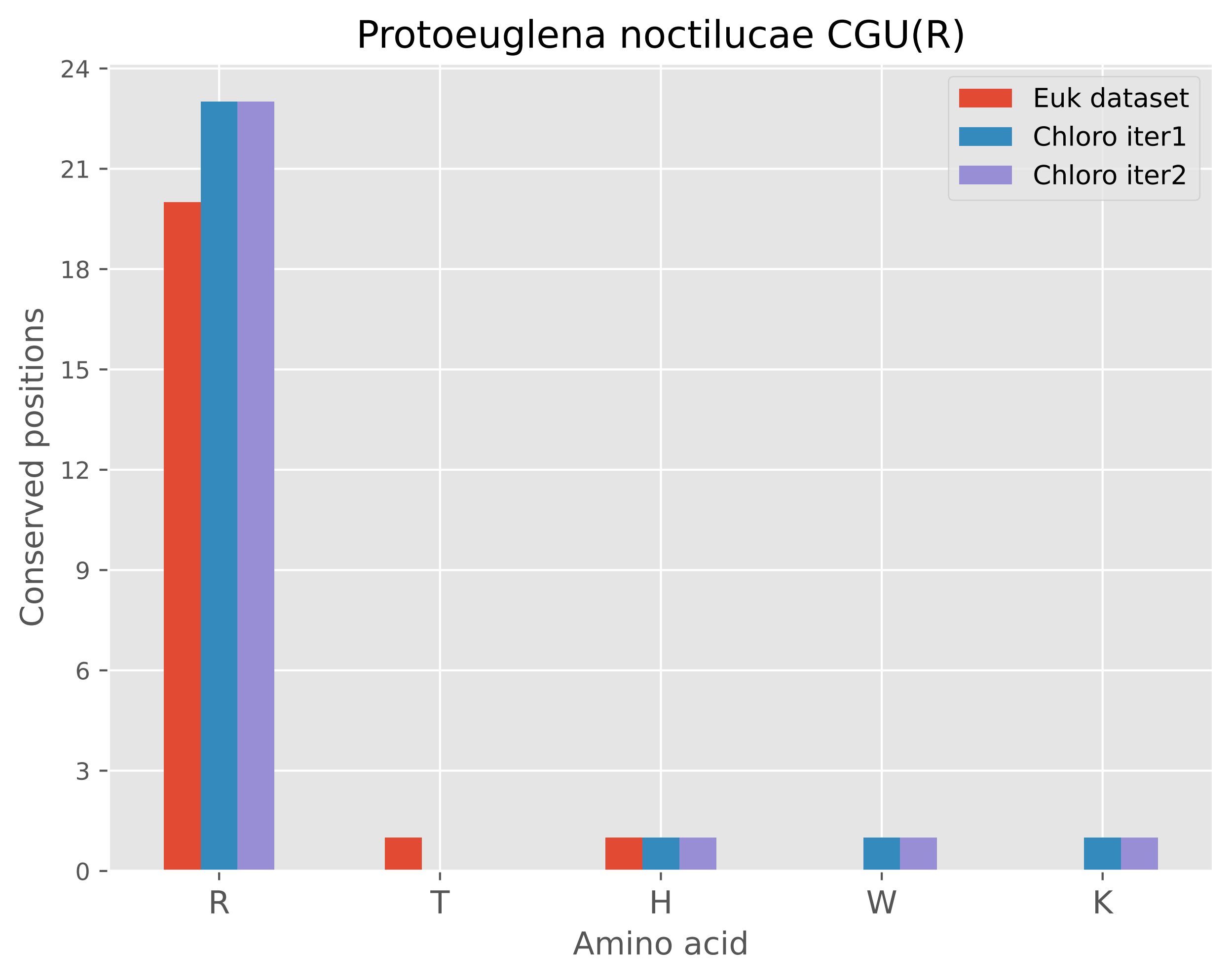

Protoeuglena noctilucae CGU(R)
Euk dataset
Chloro iter1
Chloro iter2
Conserved positions
0
3
6
9
12
15
18
21
24
R
T
H
W
K
Amino acid

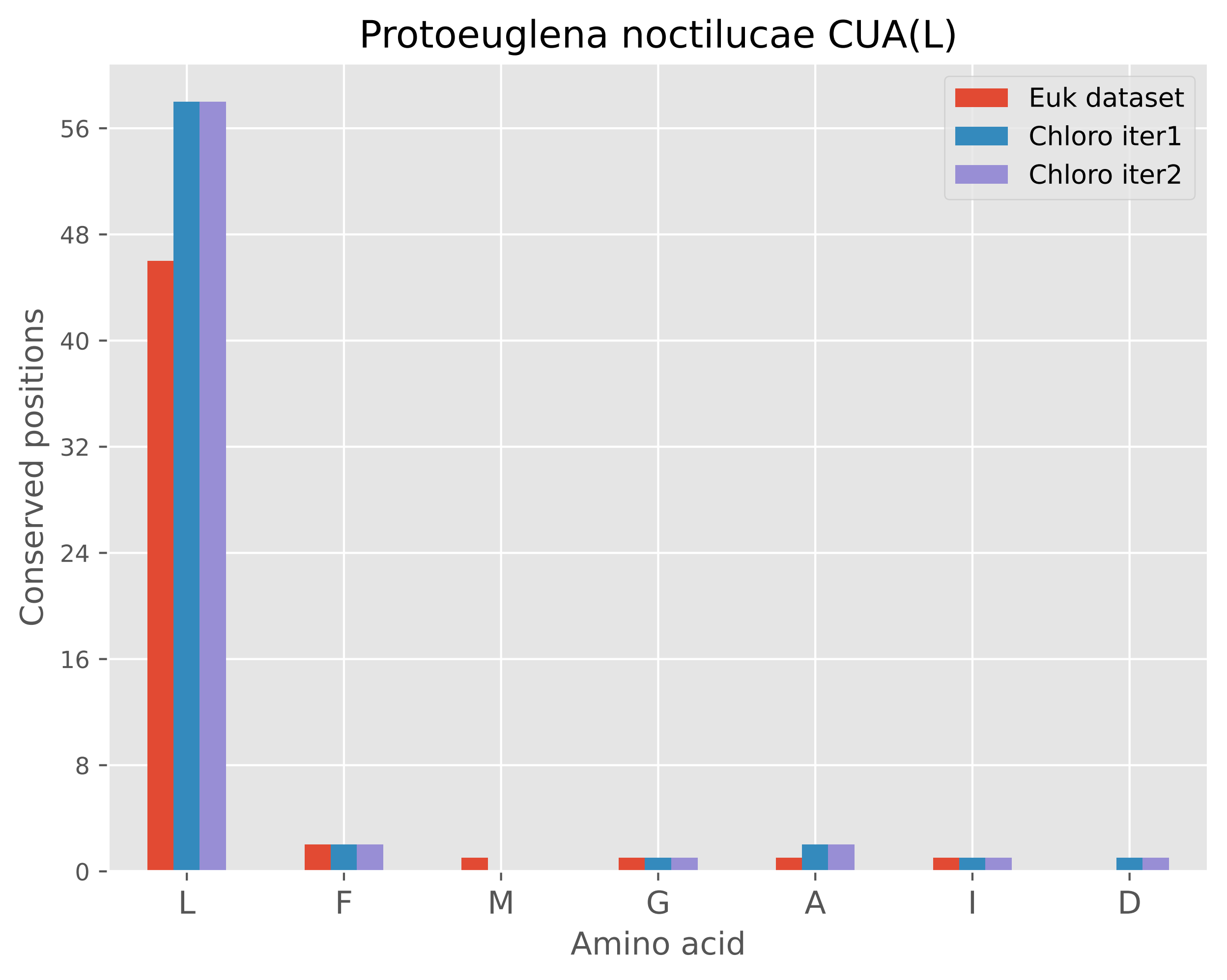
Protoeuglena noctilucae CUA(L)
Euk dataset
Chloro iter1
Chloro iter2
Conserved positions
0
8
16
24
32
40
48
56
L
F
M
G
A
I
D
Amino acid

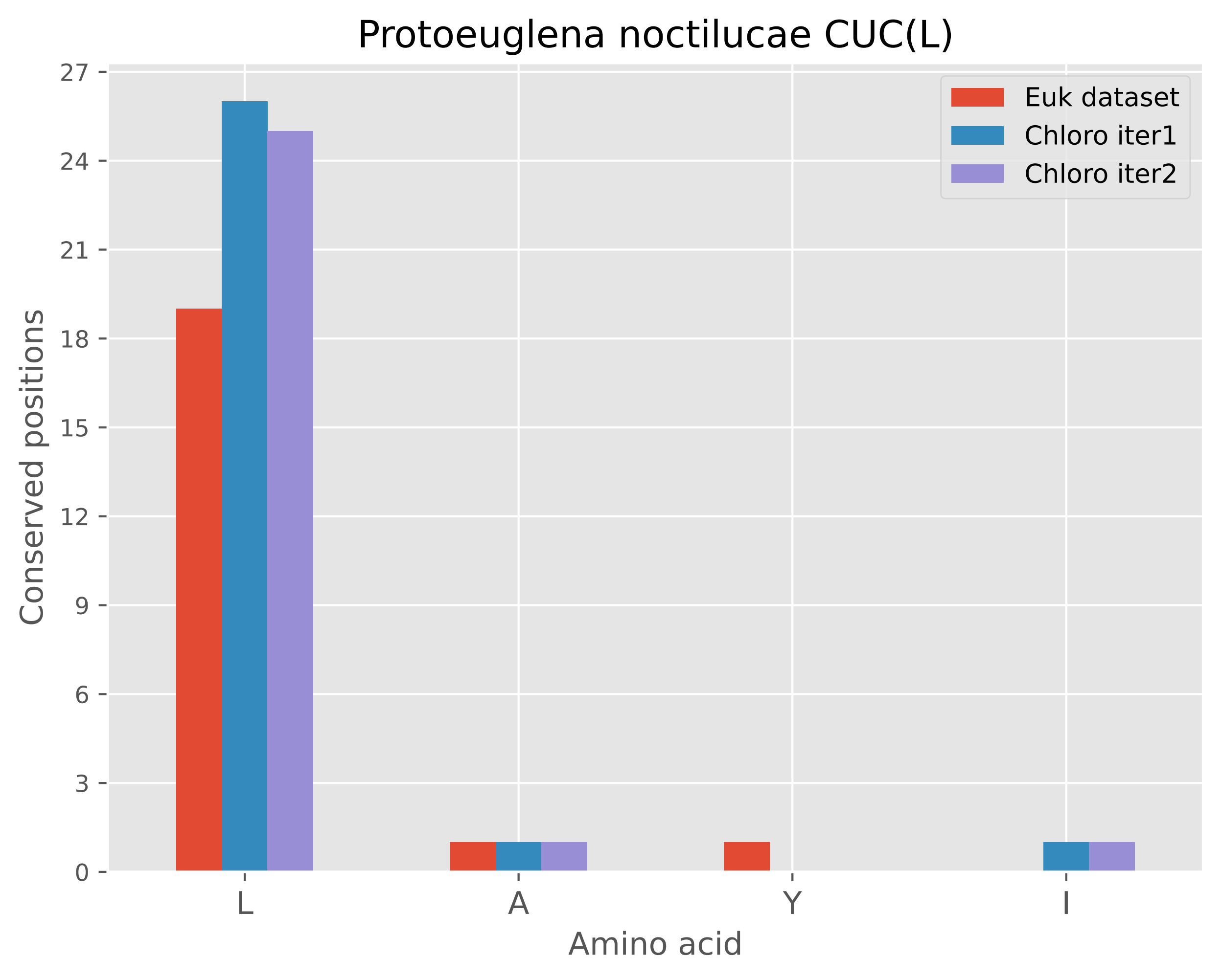

Protoeuglena noctilucae CUC(L)
Euk dataset
Chloro iter1
Chloro iter2
Conserved positions
0
3
6
9
12
15
18
21
24
27
L
A
Y
I
Amino acid

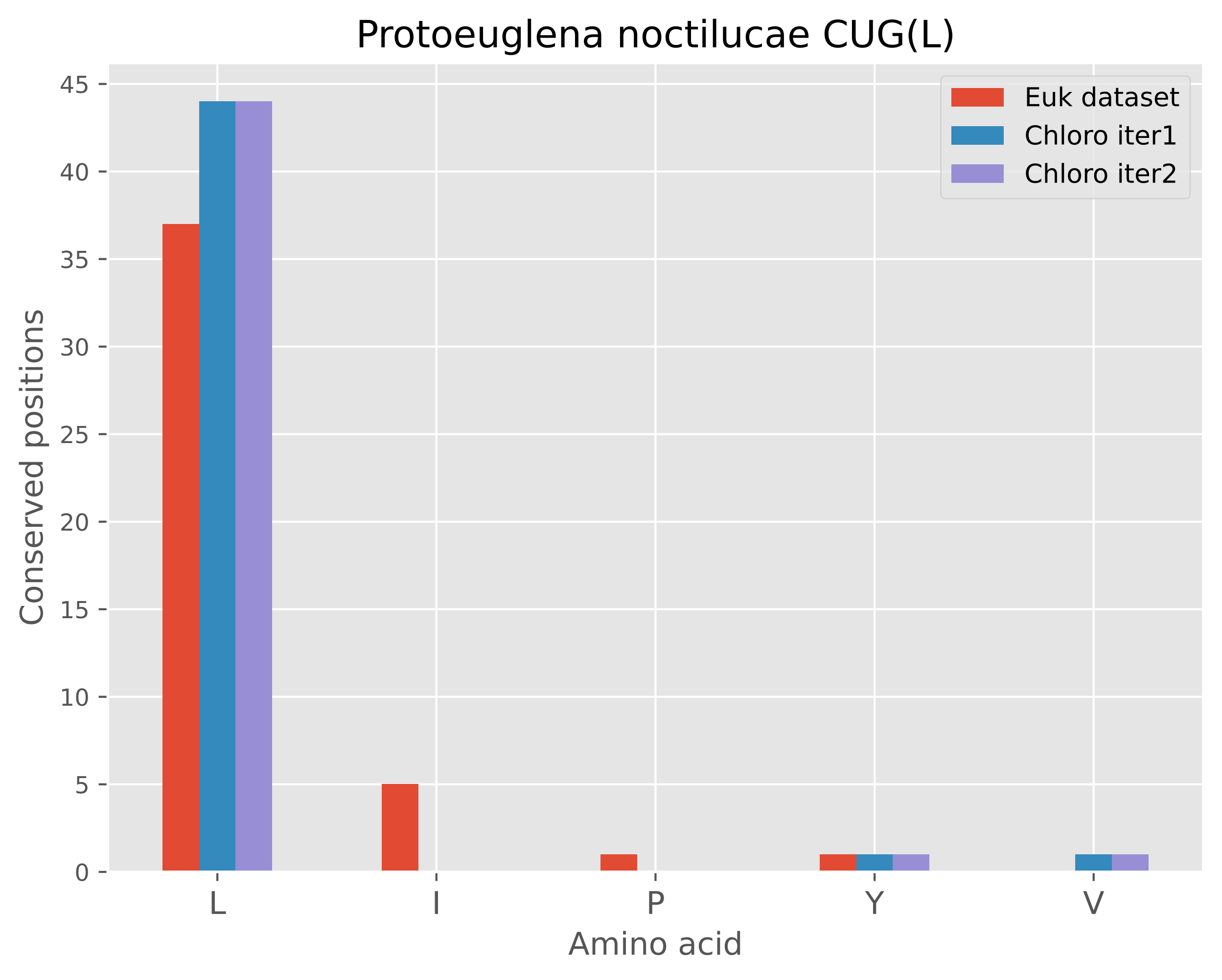
Protoeuglena noctilucae CUG(L)
Conserved positions
0
5
10
15
20
25
30
35
40
45
L
I
P
Y
V
Amino acid
Euk dataset
Chloro iter1
Chloro iter2

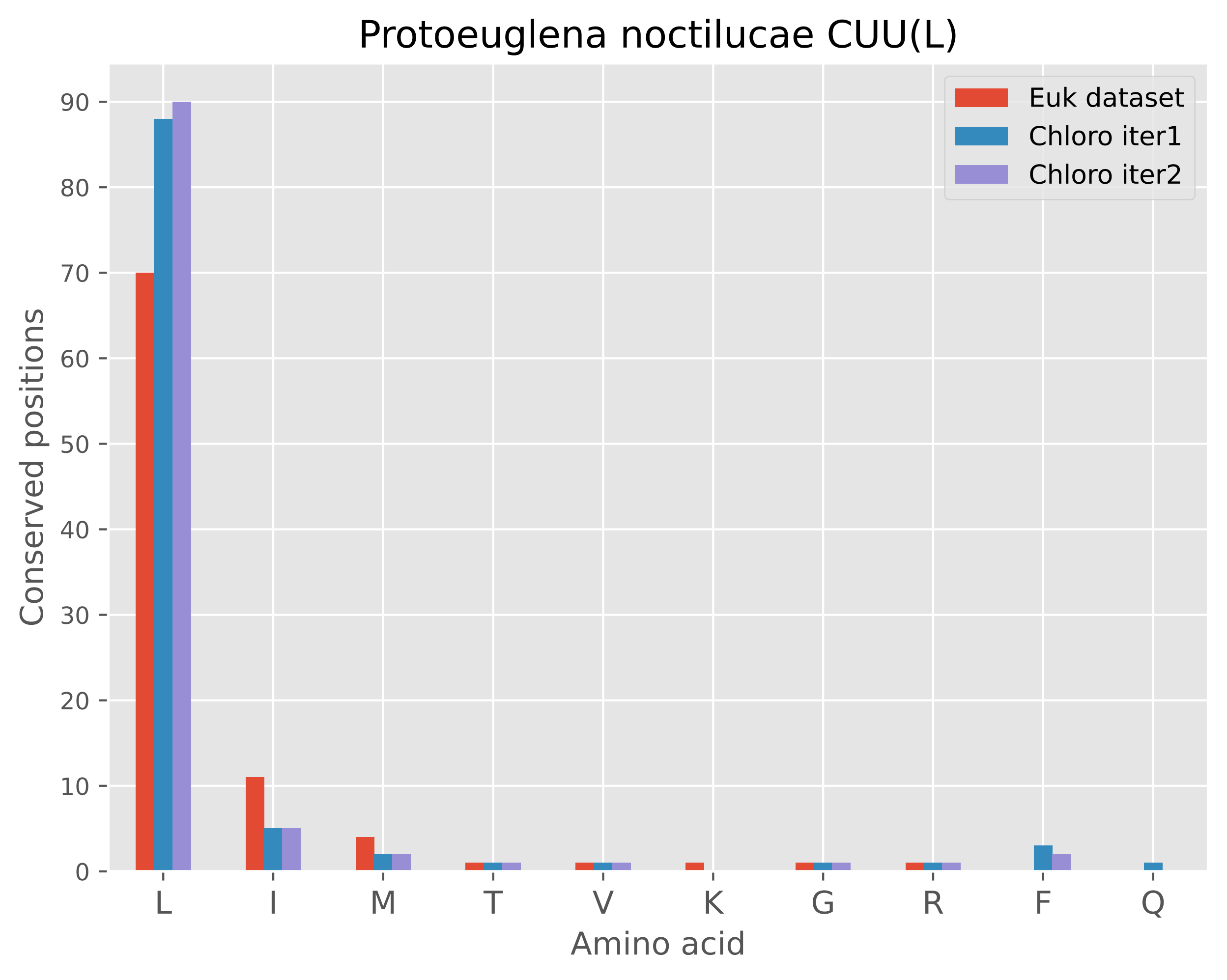

Protoeuglena noctilucae CUU(L)
Euk dataset
Chloro iter1
Chloro iter2
Conserved positions
0
10
20
30
40
50
60
70
80
90
L
I
M
T
V
K
G
R
F
Q
Amino acid

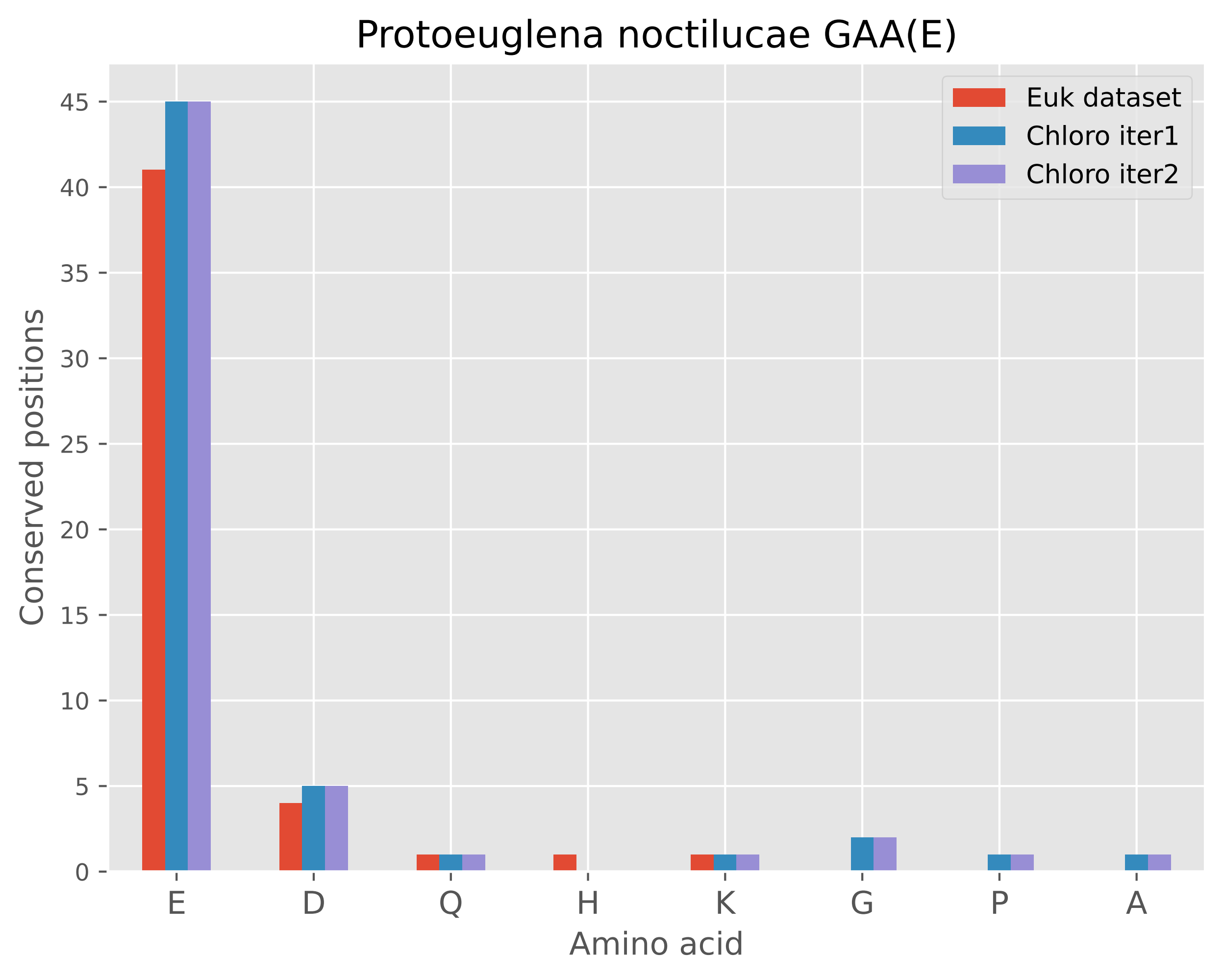

Protoeuglena noctilucae GAA(E)
Euk dataset
Chloro iter1
Chloro iter2
Conserved positions
Amino acid
0
5
10
15
20
25
30
35
40
45
E
D
Q
H
K
G
P
A

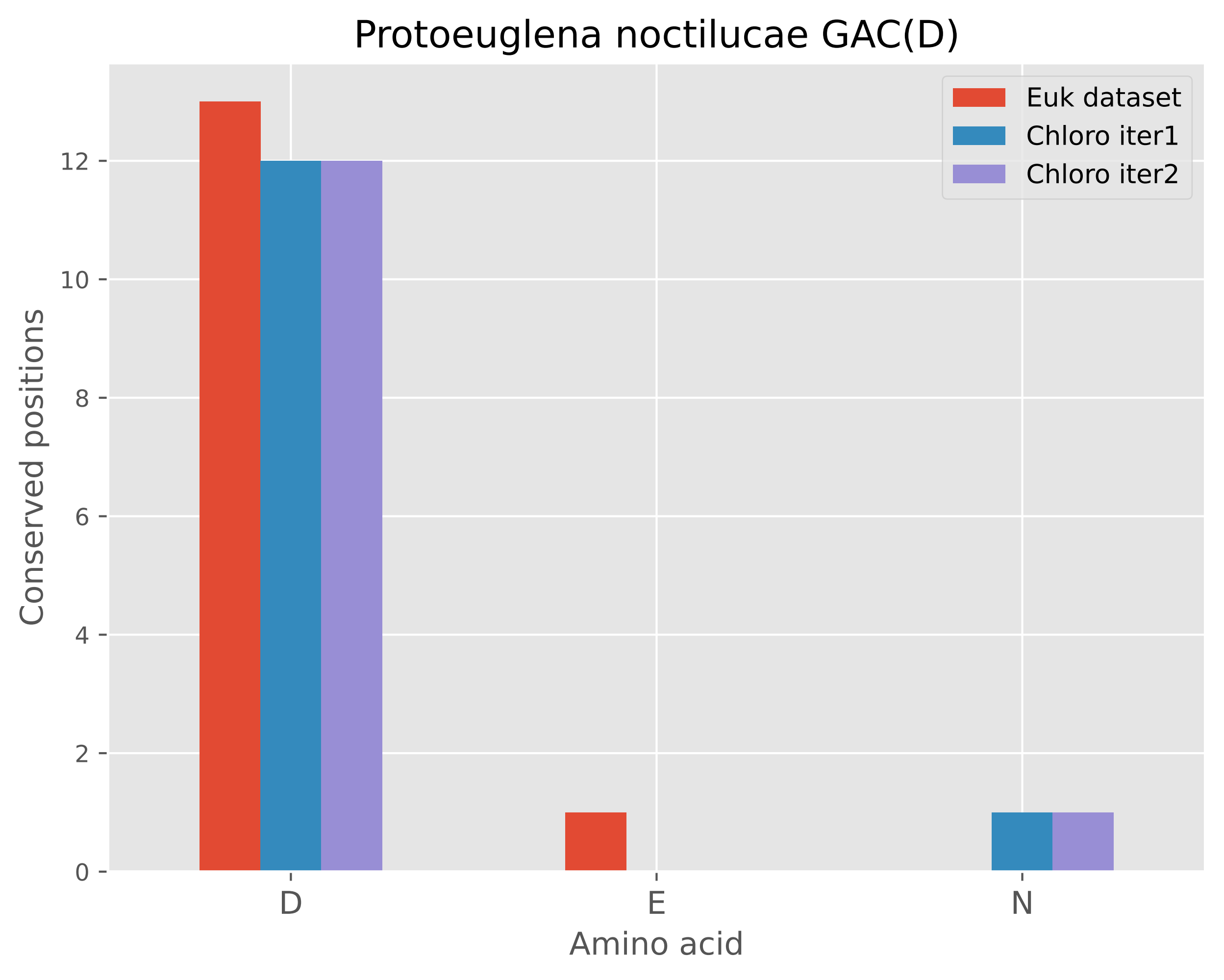
Protoeuglena noctilucae GAC(D)
Euk dataset
Chloro iter1
Chloro iter2
Conserved positions
0
2
4
6
8
10
12
D
E
N
Amino acid

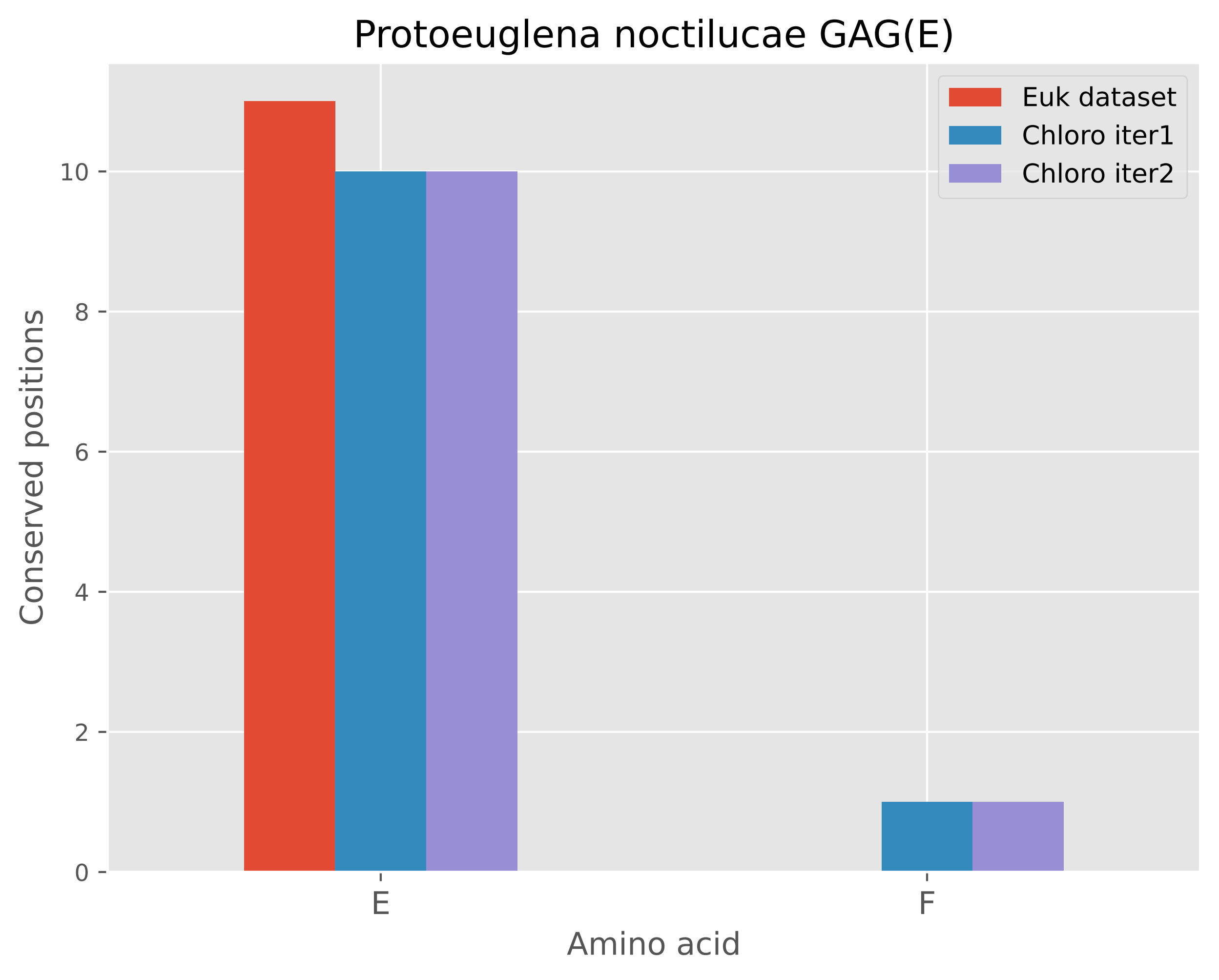
Protoeuglena noctilucae GAG(E)
Euk dataset
Chloro iter1
Chloro iter2
Conserved positions
0
2
4
6
8
10
E
F
Amino acid

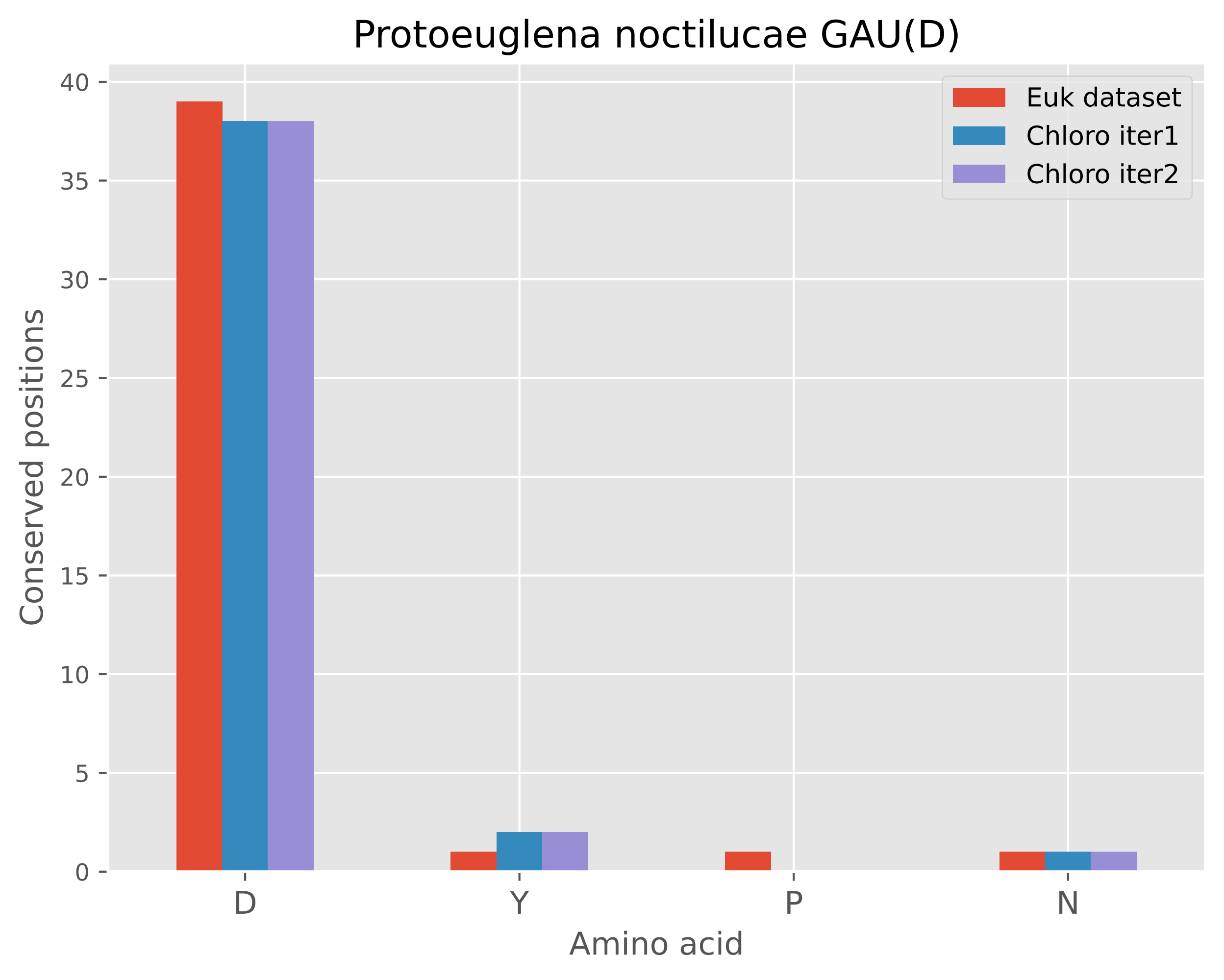

Protoeuglena noctilucae GAU(D)
Euk dataset
Chloro iter1
Chloro iter2
Conserved positions
Amino acid
0
5
10
15
20
25
30
35
40
D
Y
P
N

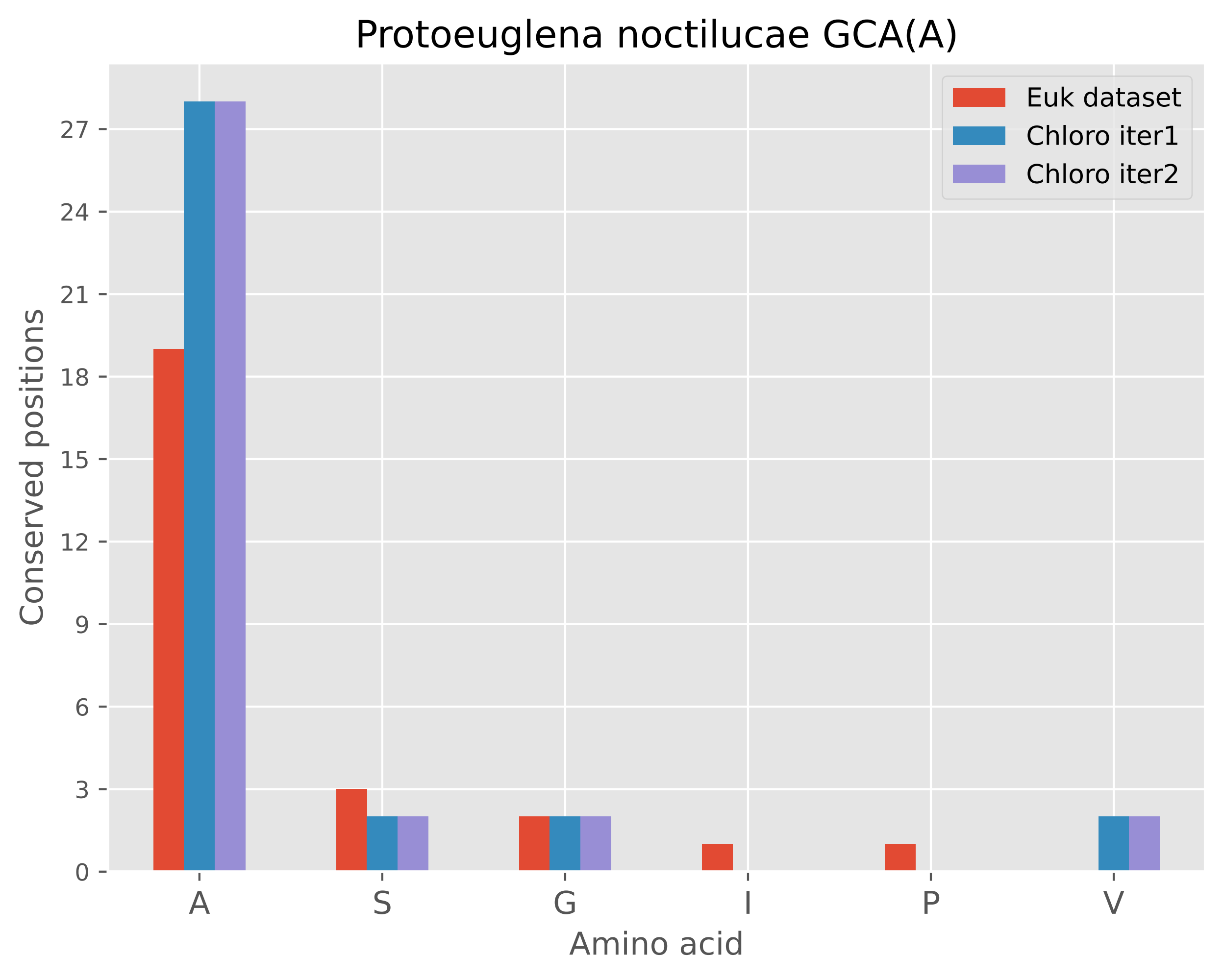

Protoeuglena noctilucae GCA(A)
Euk dataset
Chloro iter1
Chloro iter2
Conserved positions
0
3
6
9
12
15
18
21
24
27
A
S
G
I
P
V
Amino acid

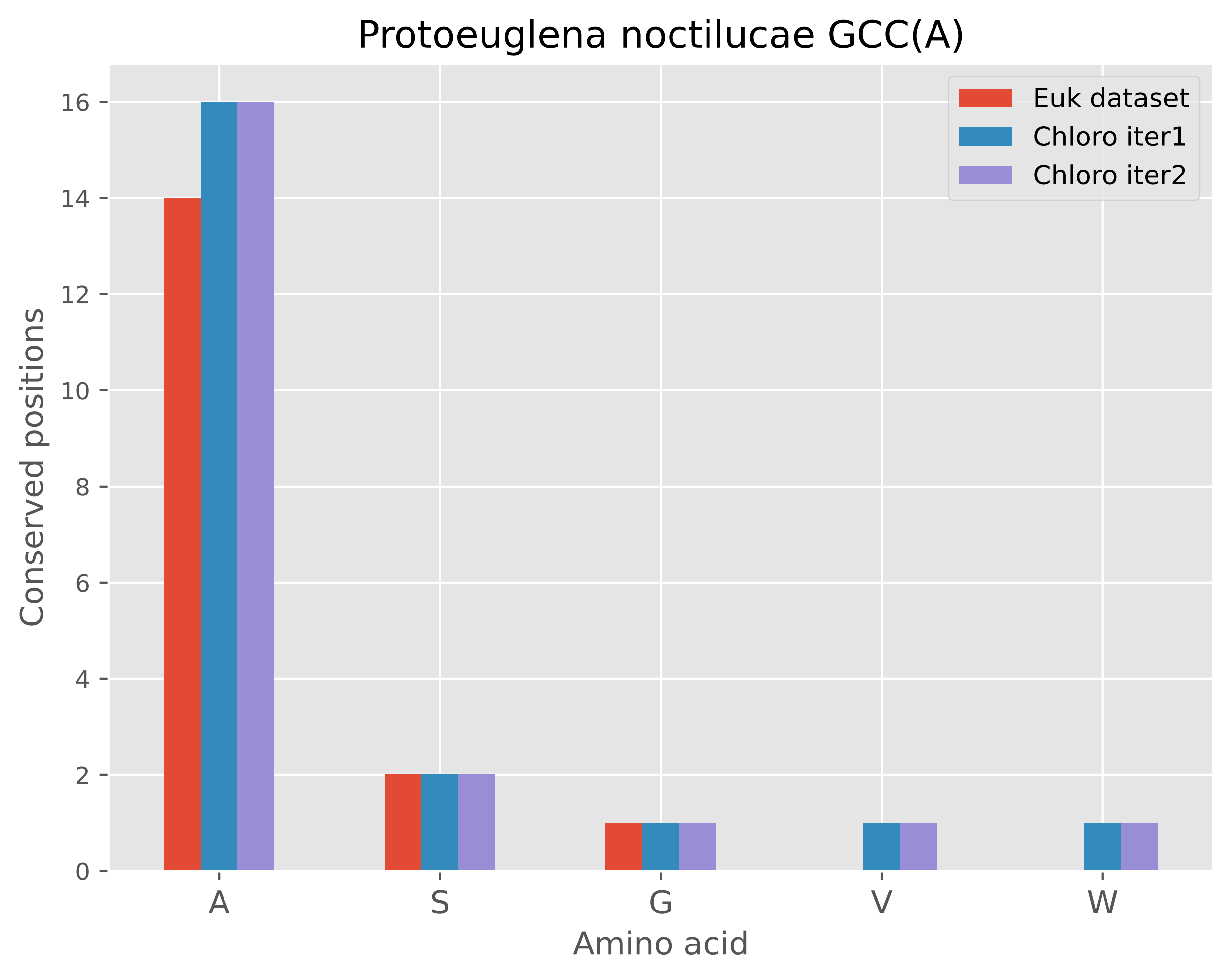

Protoeuglena noctilucae GCC(A)
Euk dataset
Chloro iter1
Chloro iter2
Conserved positions
0
2
4
6
8
10
12
14
16
A
S
G
V
W
Amino acid

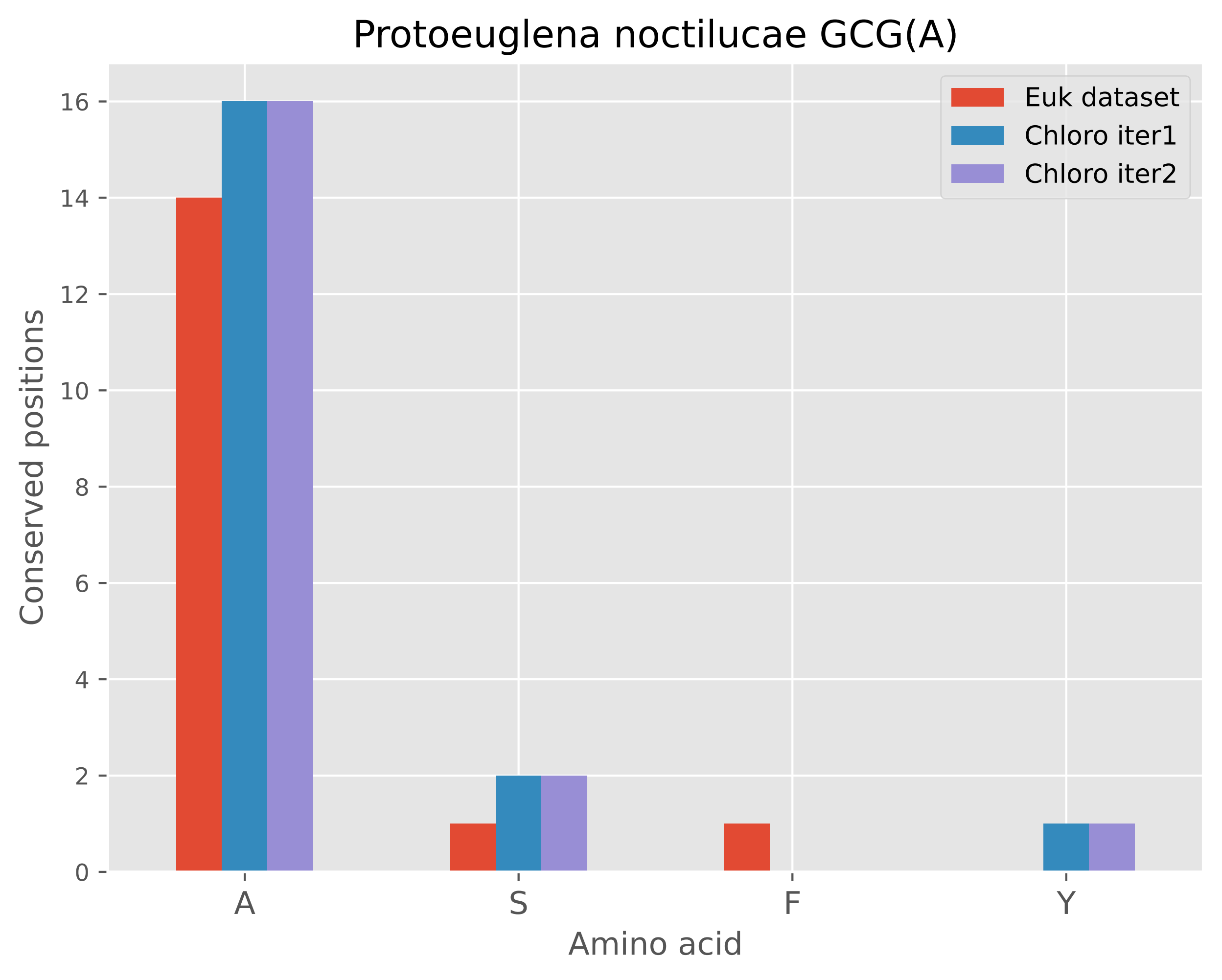
Protoeuglena noctilucae GCG(A)
Euk dataset
Chloro iter1
Chloro iter2
Conserved positions
0
2
4
6
8
10
12
14
16
A
S
F
Y
Amino acid

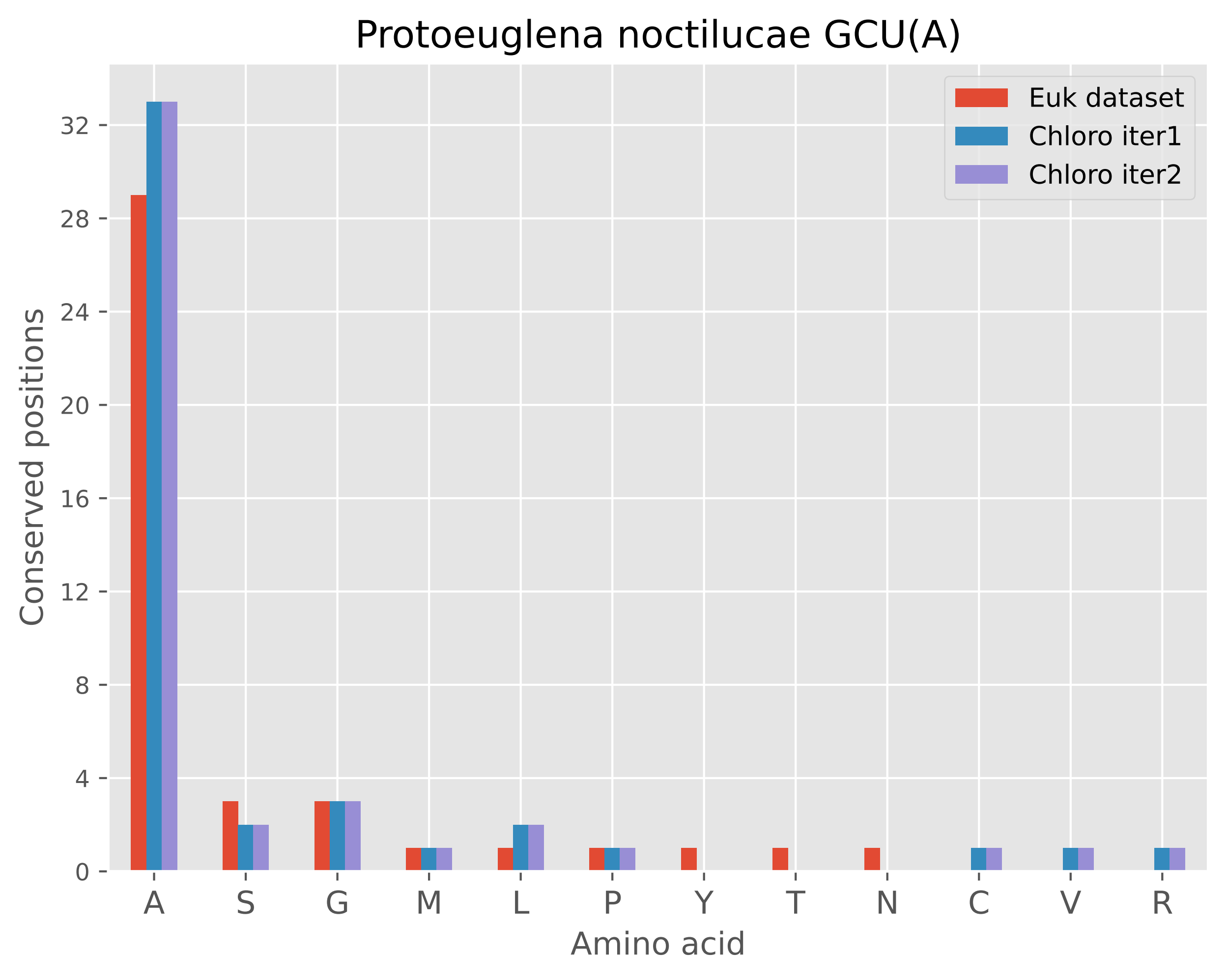
Protoeuglena noctilucae GCU(A)
Euk dataset
Chloro iter1
Chloro iter2
Conserved positions
0
4
8
12
16
20
24
28
32
A
S
G
M
L
P
Y
T
N
C
V
R
Amino acid

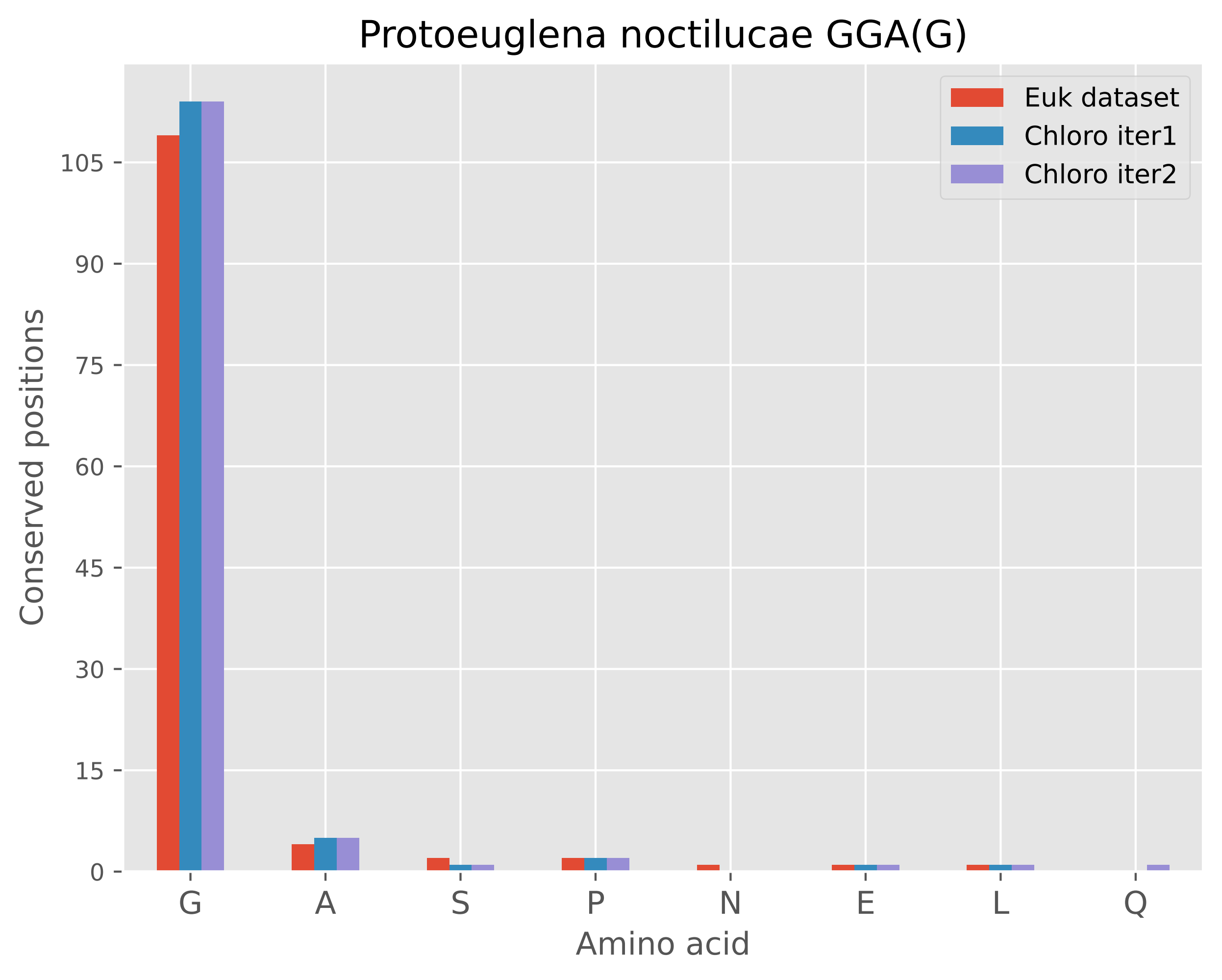
Protoeuglena noctilucae GGA(G)
Euk dataset
Chloro iter1
Chloro iter2
Conserved positions
0
15
30
45
60
75
90
105
G
A
S
P
N
E
L
Q
Amino acid

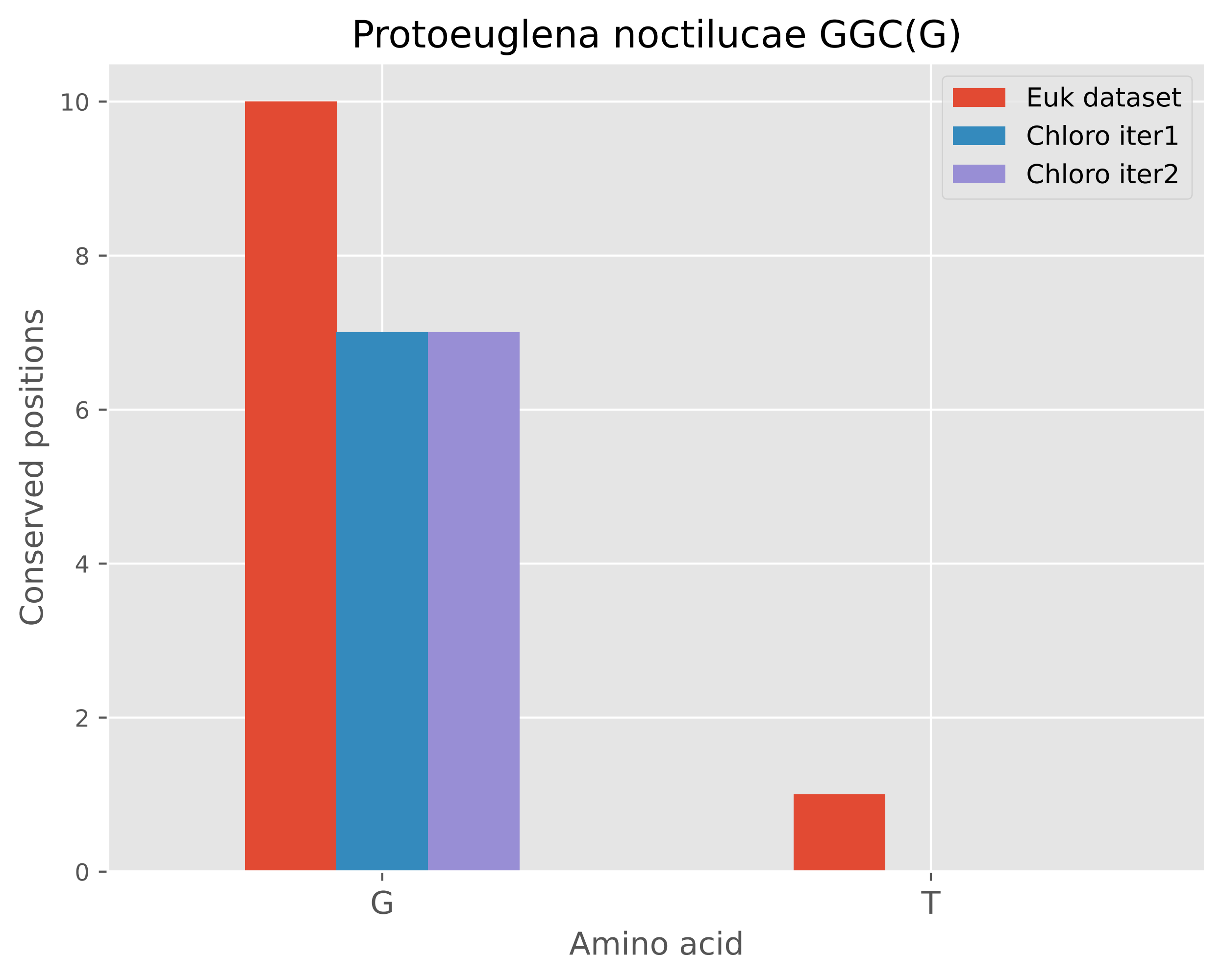
Protoeuglena noctilucae GGC(G)
Euk dataset
Chloro iter1
Chloro iter2
Conserved positions
0
2
4
6
8
10
G
T
Amino acid

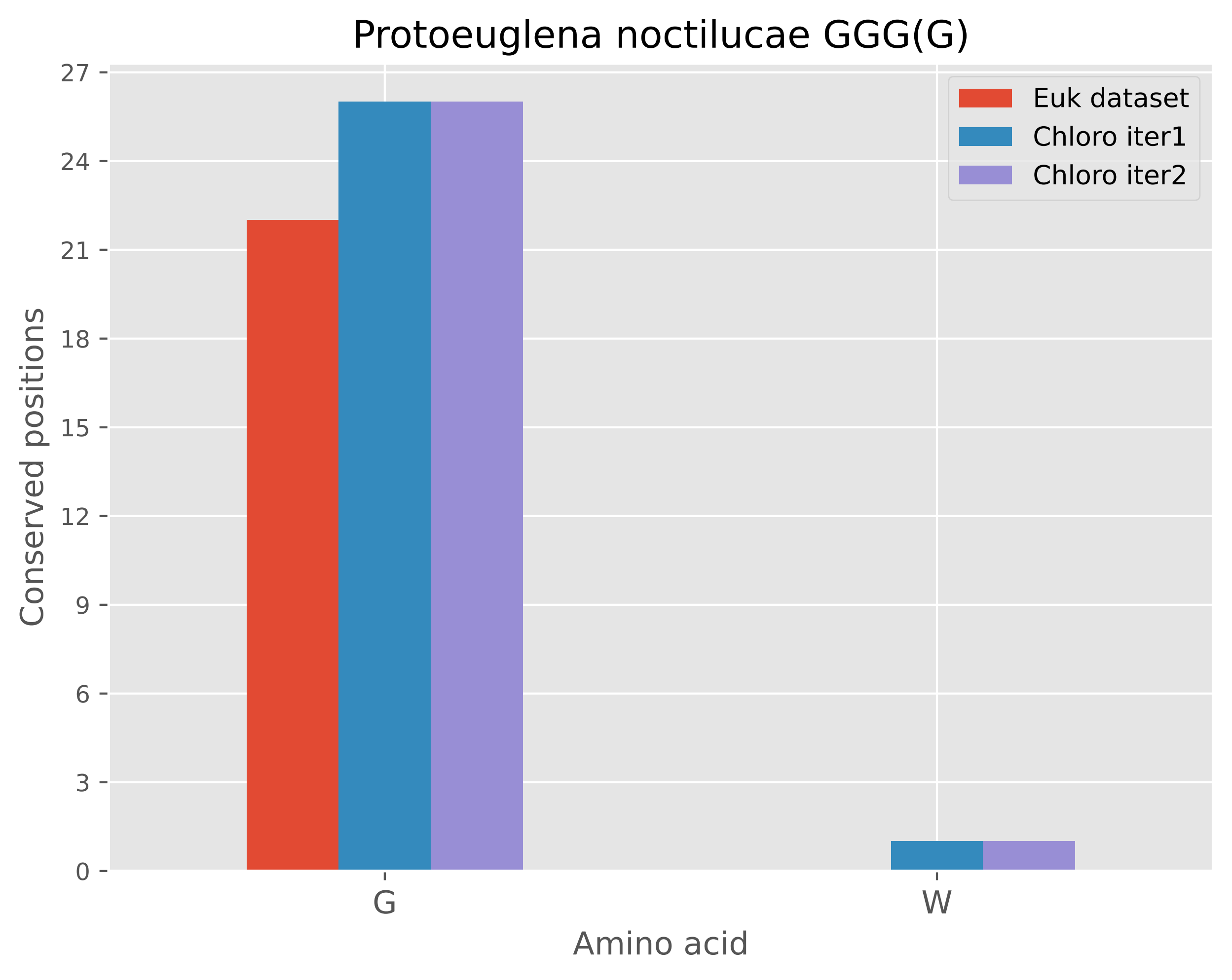

Protoeuglena noctilucae GGG(G)
Euk dataset
Chloro iter1
Chloro iter2
Conserved positions
0
3
6
9
12
15
18
21
24
27
G
W
Amino acid

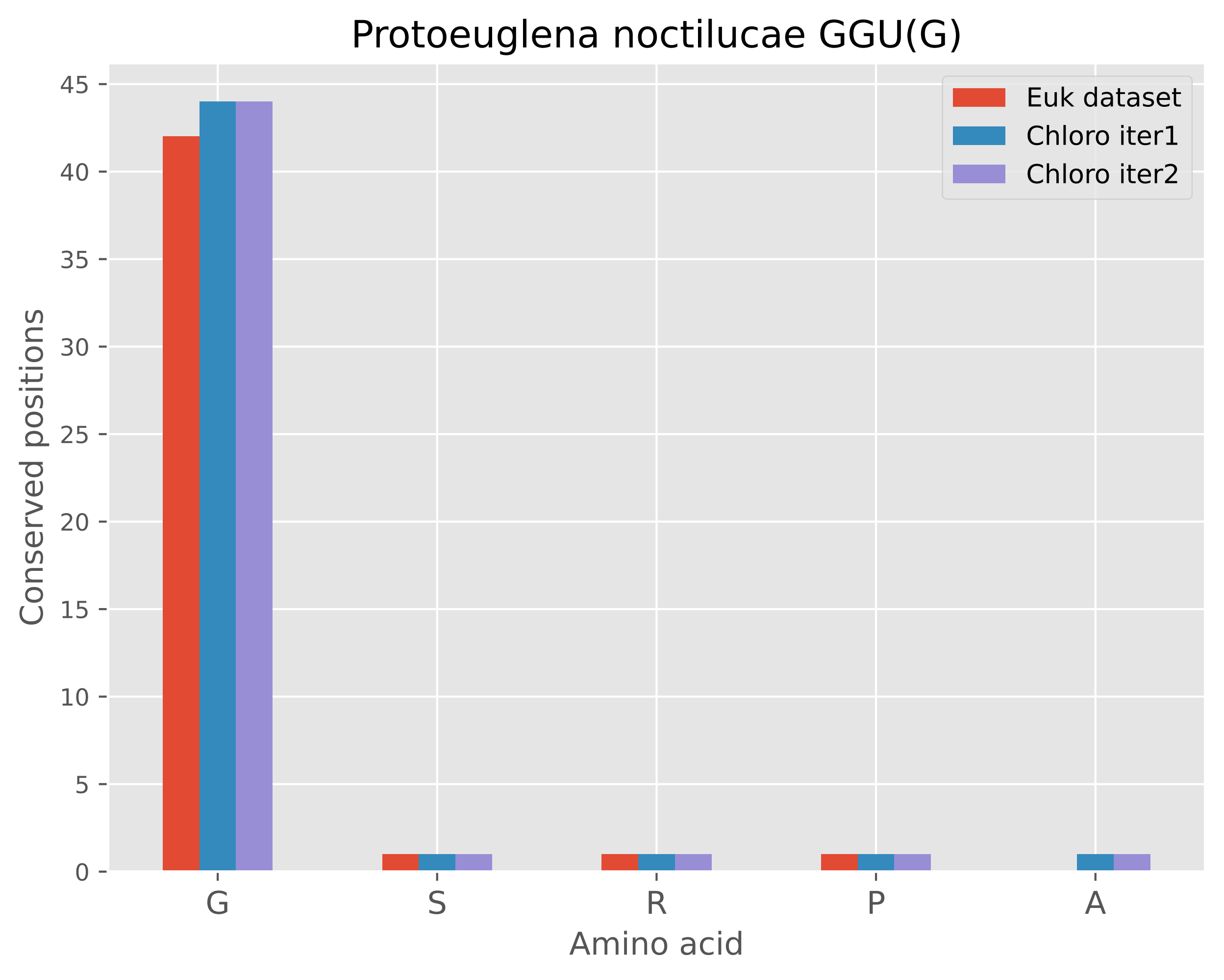

Protoeuglena noctilucae GGU(G)
Euk dataset
Chloro iter1
Chloro iter2
Conserved positions
0
5
10
15
20
25
30
35
40
45
G
S
R
P
A
Amino acid

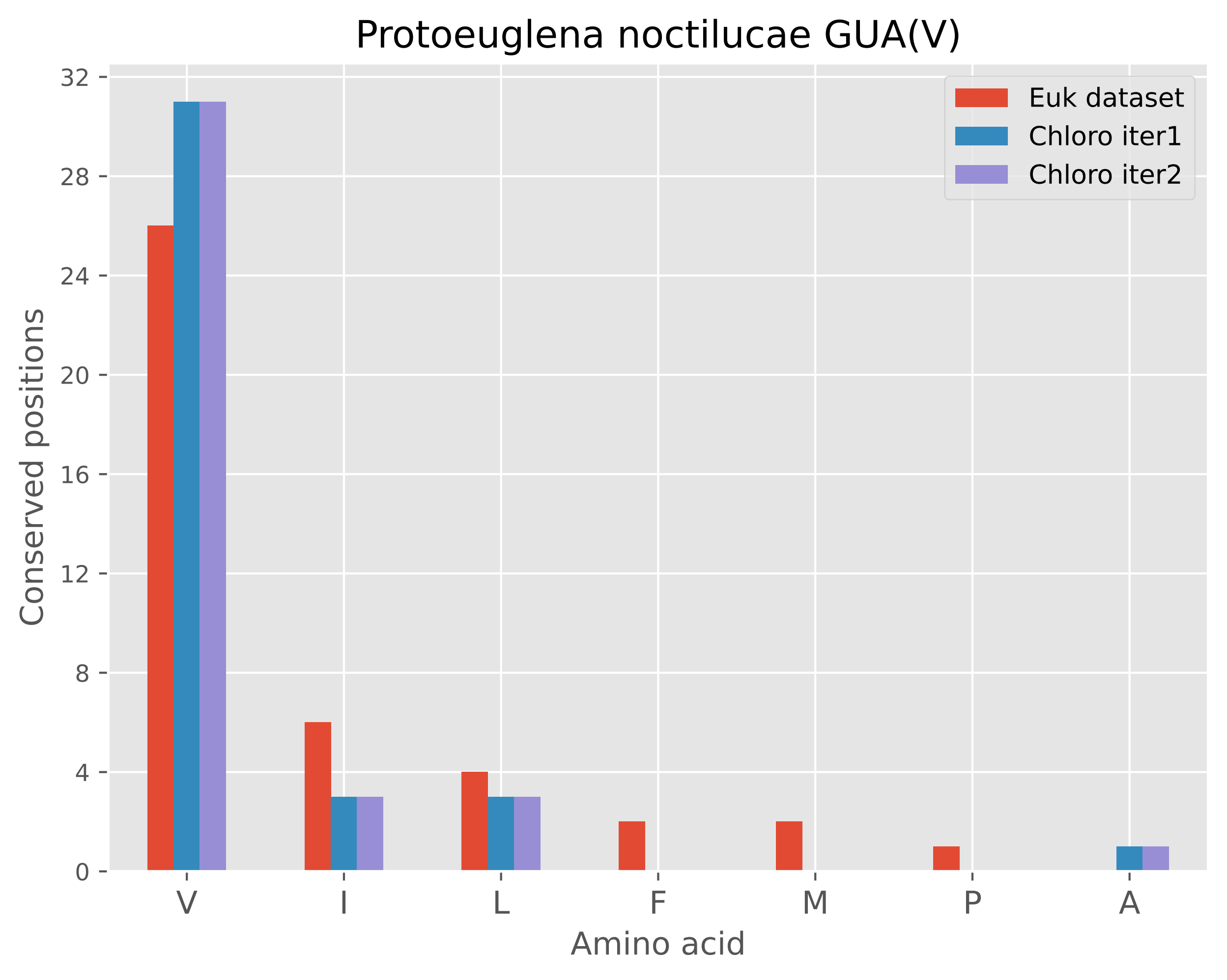
Protoeuglena noctilucae GUA(V)
Euk dataset
Chloro iter1
Chloro iter2
Conserved positions
0
4
8
12
16
20
24
28
32
V
I
L
F
M
P
A
Amino acid

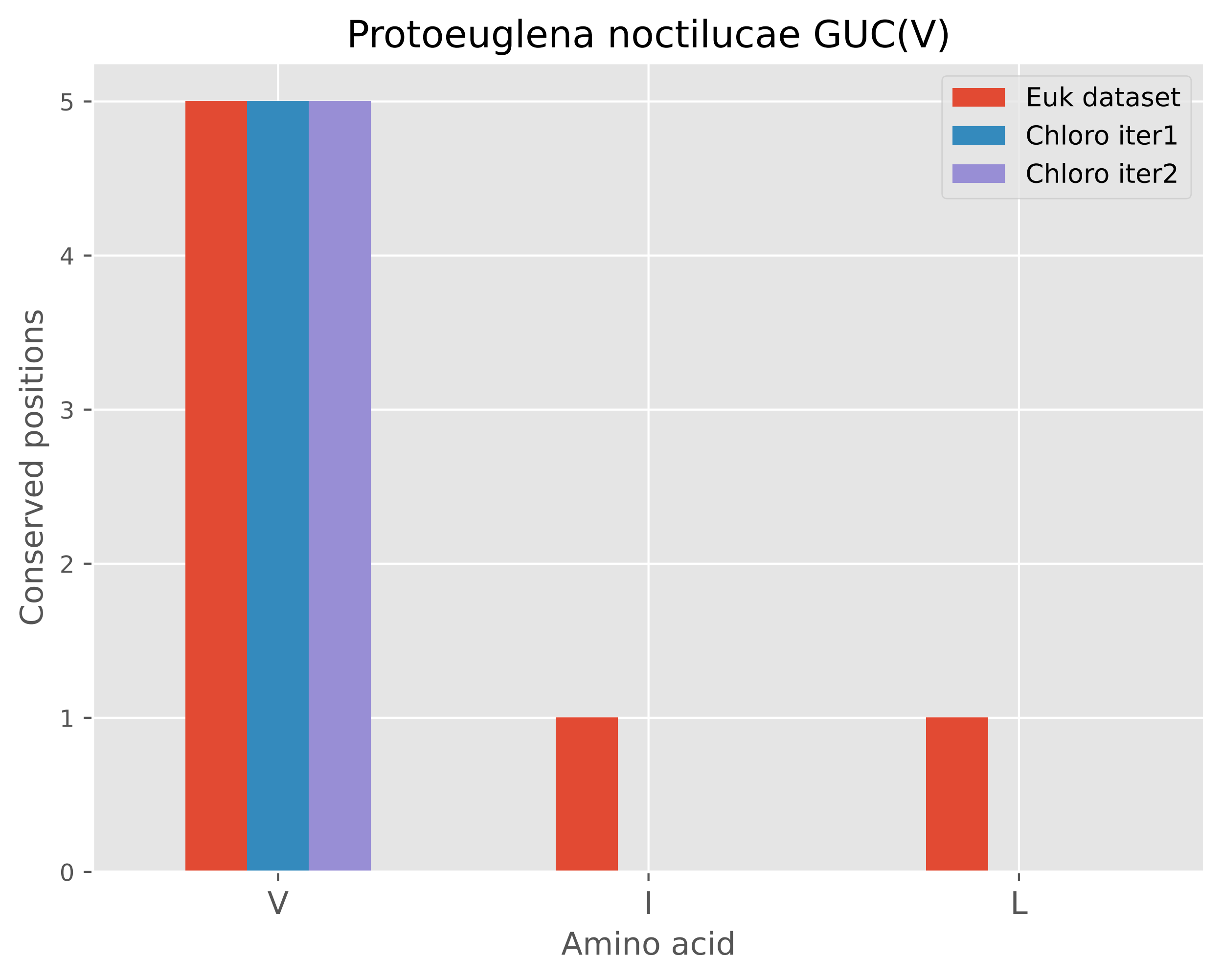
Protoeuglena noctilucae GUC(V)
Euk dataset
Chloro iter1
Chloro iter2
Conserved positions
0
1
2
3
4
5
V
I
L
Amino acid

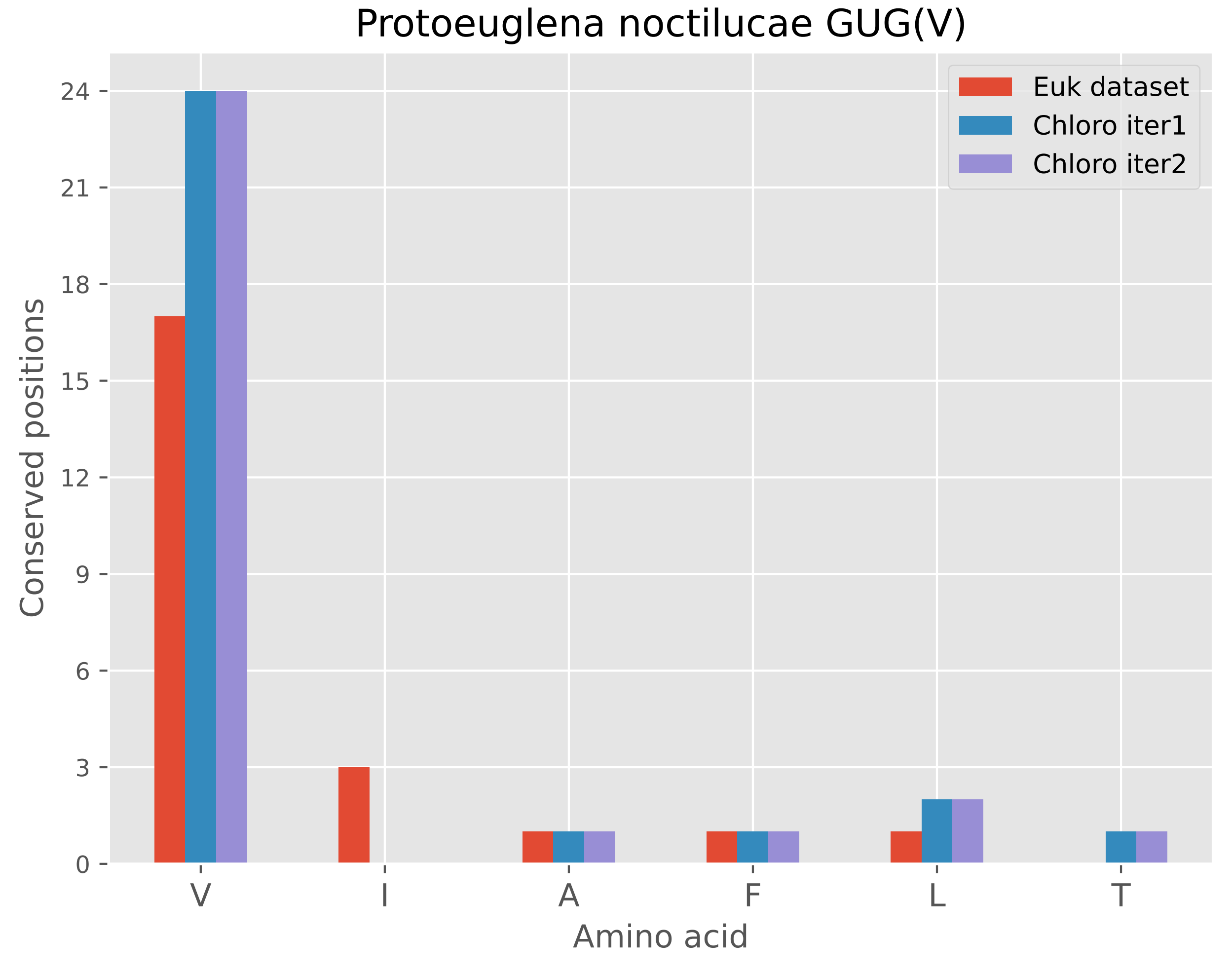

Protoeuglena noctilucae GUG(V)
Euk dataset
Chloro iter1
Chloro iter2
Conserved positions
0
3
6
9
12
15
18
21
24
V
I
A
F
L
T
Amino acid

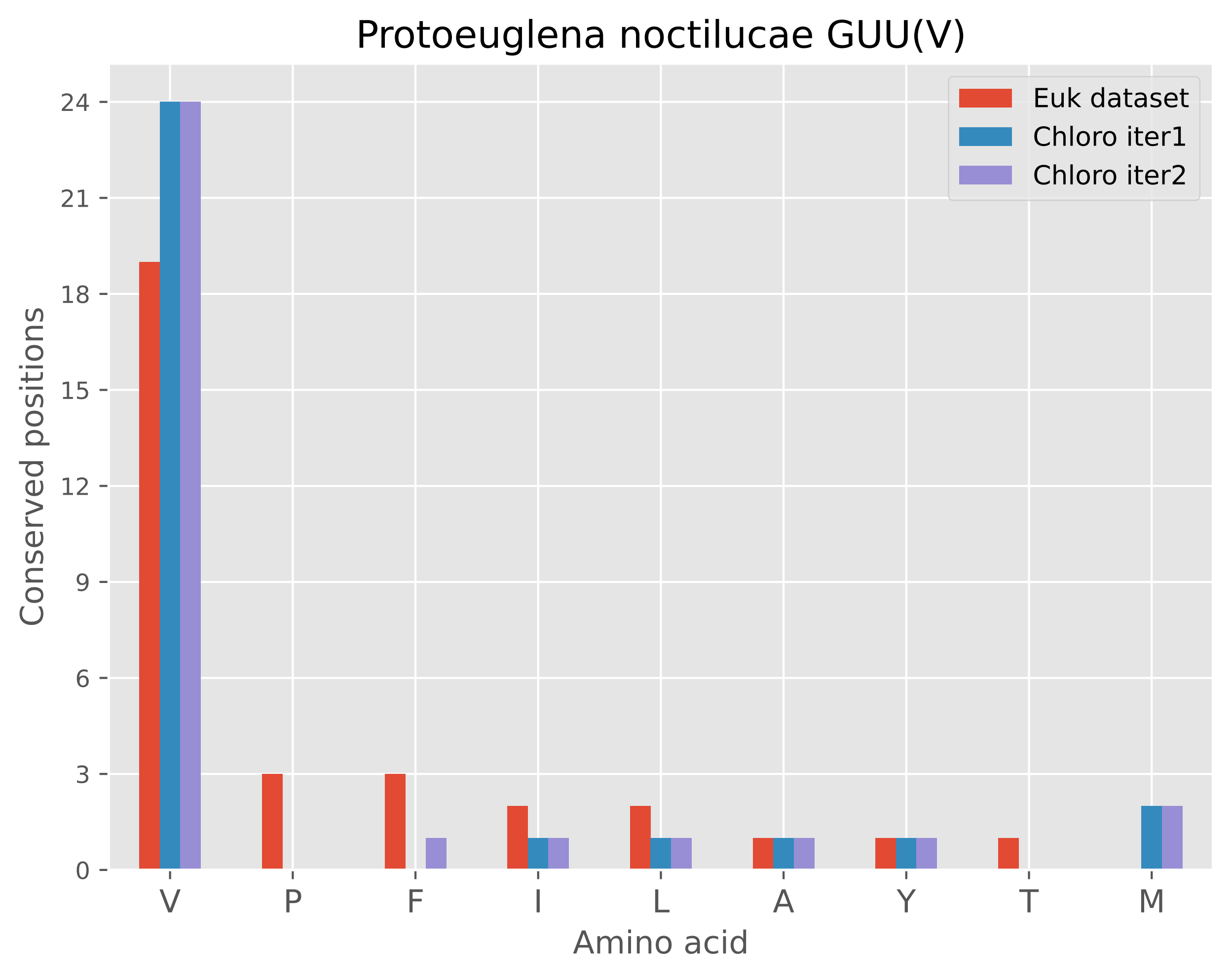

Protoeuglena noctilucae GUU(V)
Euk dataset
Chloro iter1
Chloro iter2
Conserved positions
0
3
6
9
12
15
18
21
24
V
P
F
I
L
A
Y
T
M
Amino acid

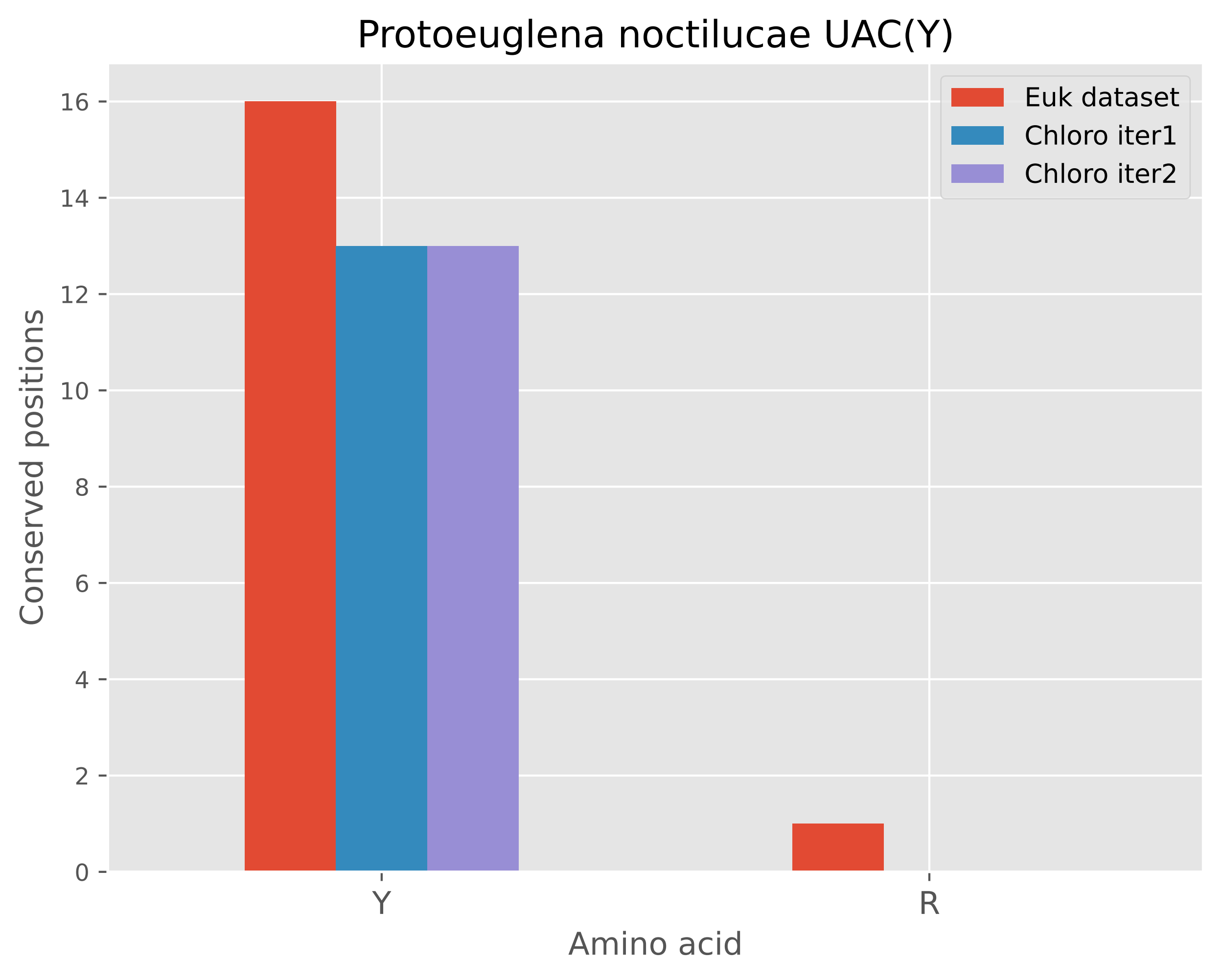

Protoeuglena noctilucae UAC(Y)
Euk dataset
Chloro iter1
Chloro iter2
Conserved positions
0
2
4
6
8
10
12
14
16
Y
R
Amino acid

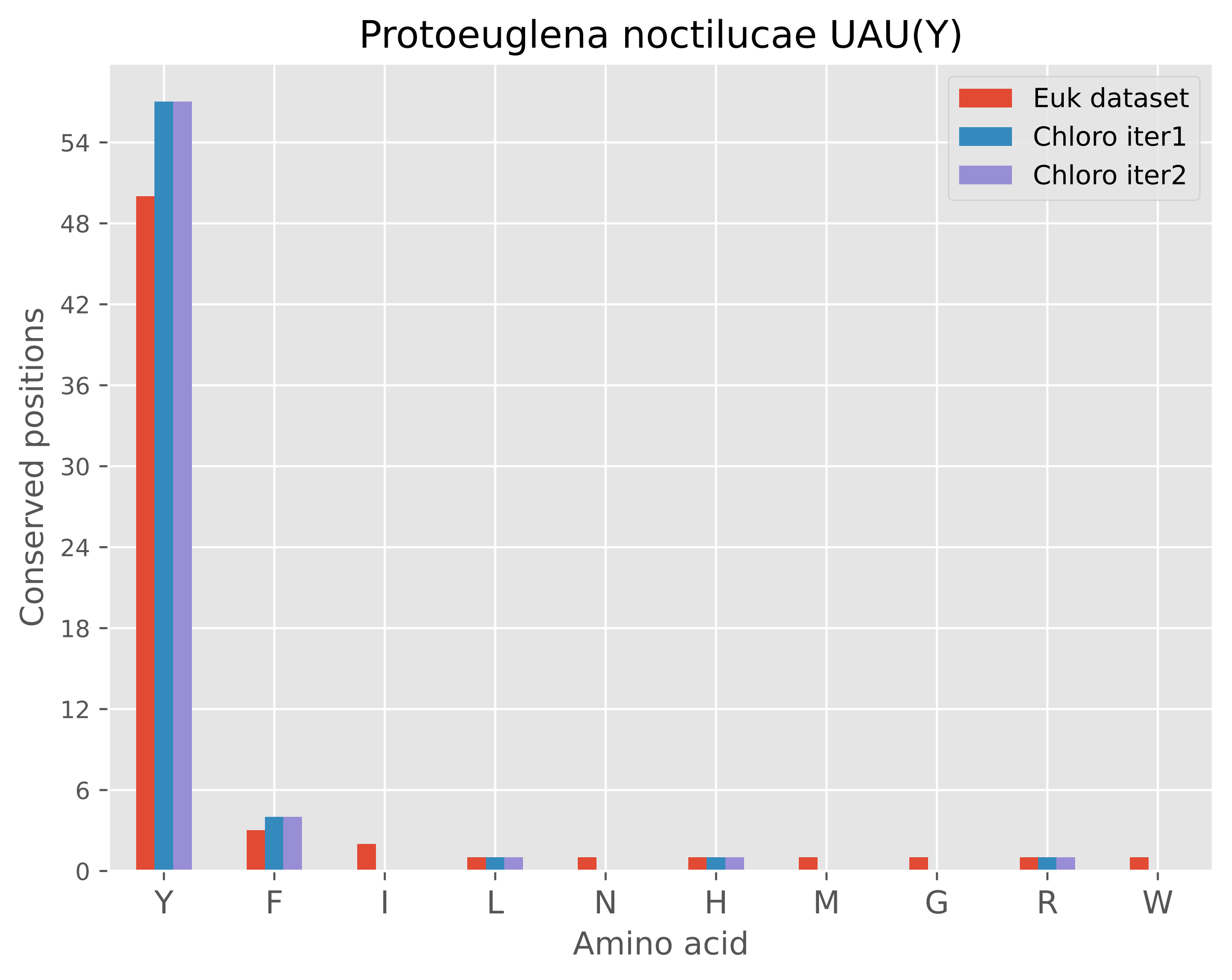

Protoeuglena noctilucae UAU(Y)
Euk dataset
Chloro iter1
Chloro iter2
Conserved positions
0
6
12
18
24
30
36
42
48
54
Y
F
I
L
N
H
M
G
R
W
Amino acid

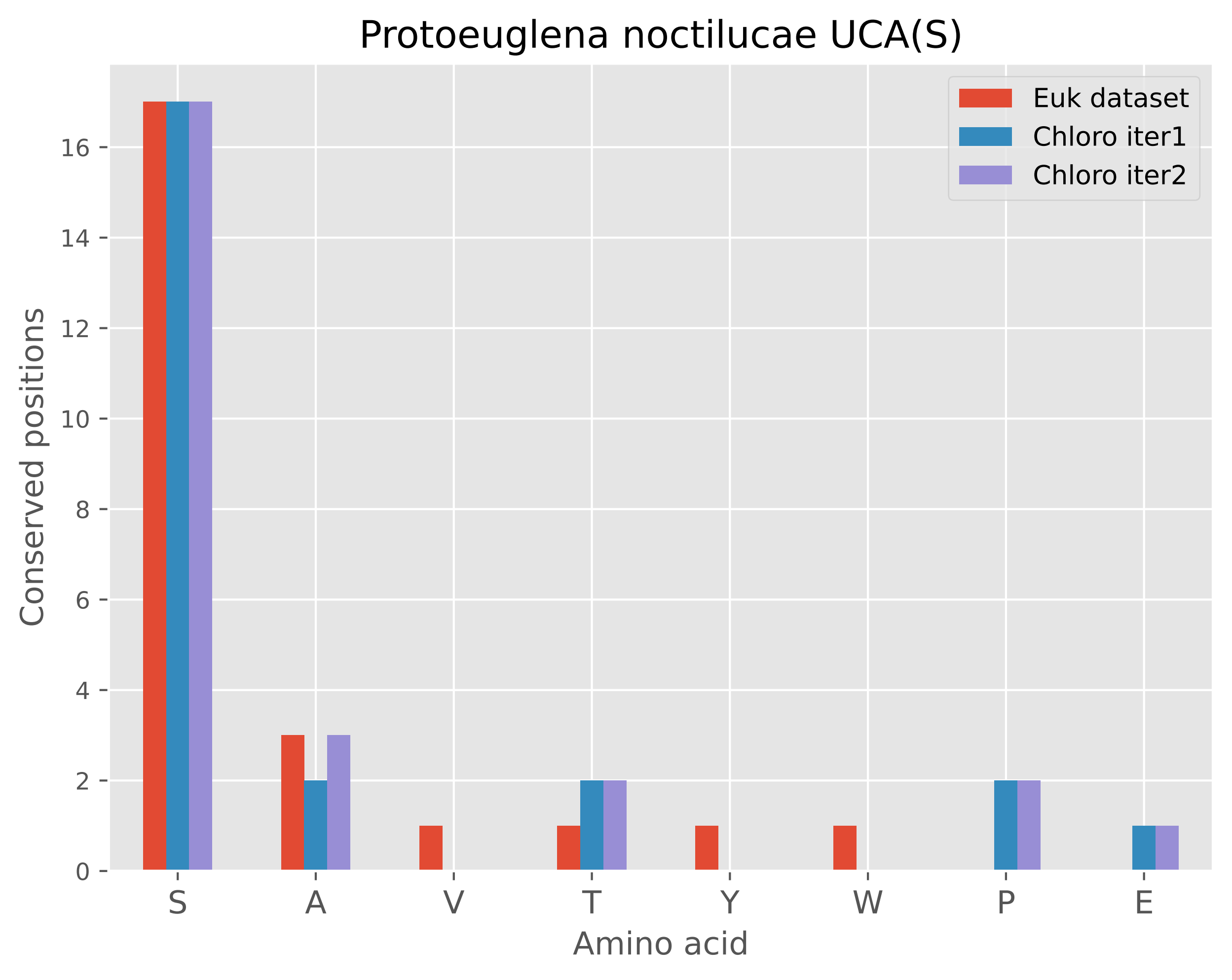
Protoeuglena noctilucae UCA(S)
Euk dataset
Chloro iter1
Chloro iter2
Conserved positions
0
2
4
6
8
10
12
14
16
S
A
V
T
Y
W
P
E
Amino acid

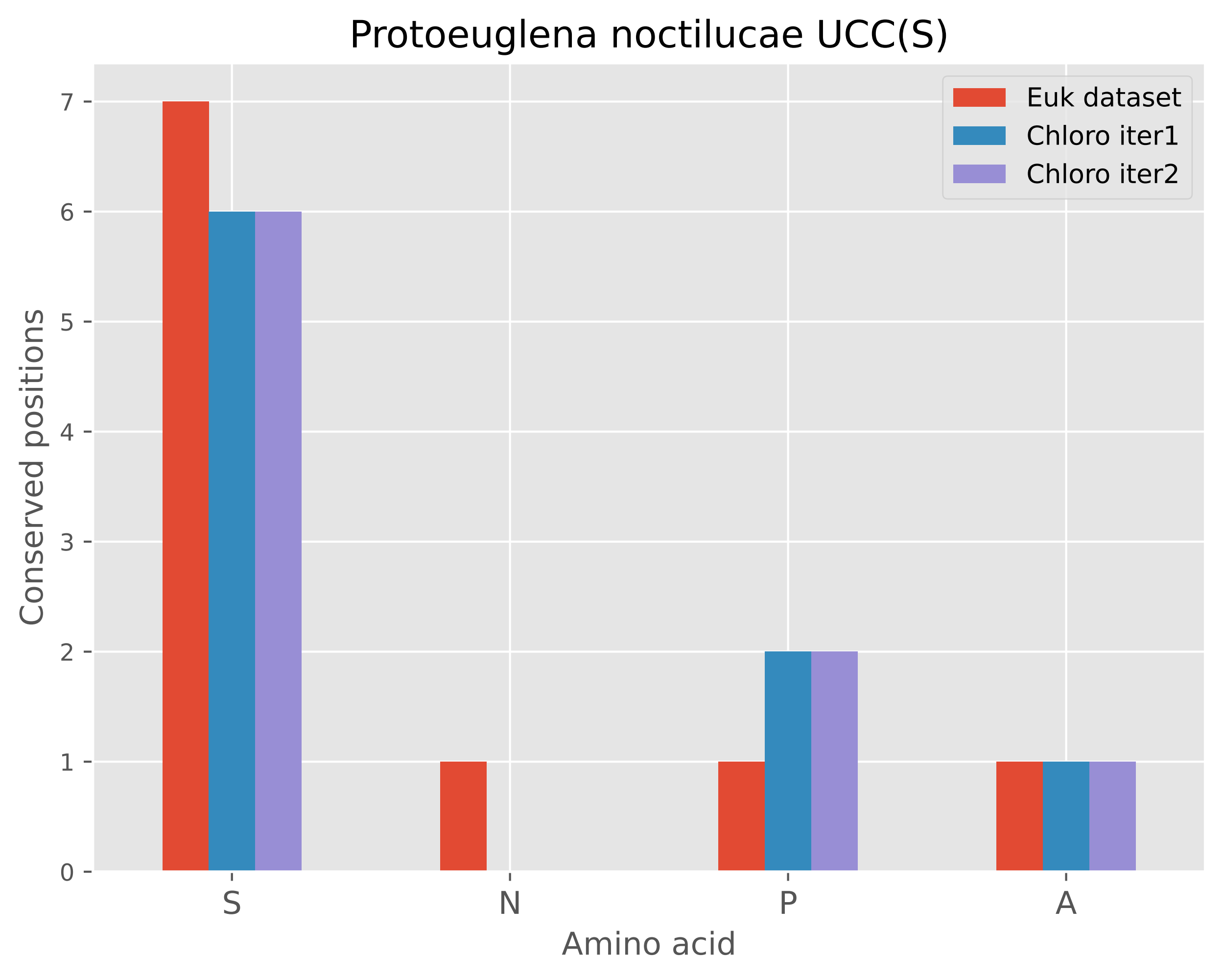

Protoeuglena noctilucae UCC(S)
Euk dataset
Chloro iter1
Chloro iter2
Conserved positions
0
1
2
3
4
5
6
7
S
N
P
A
Amino acid

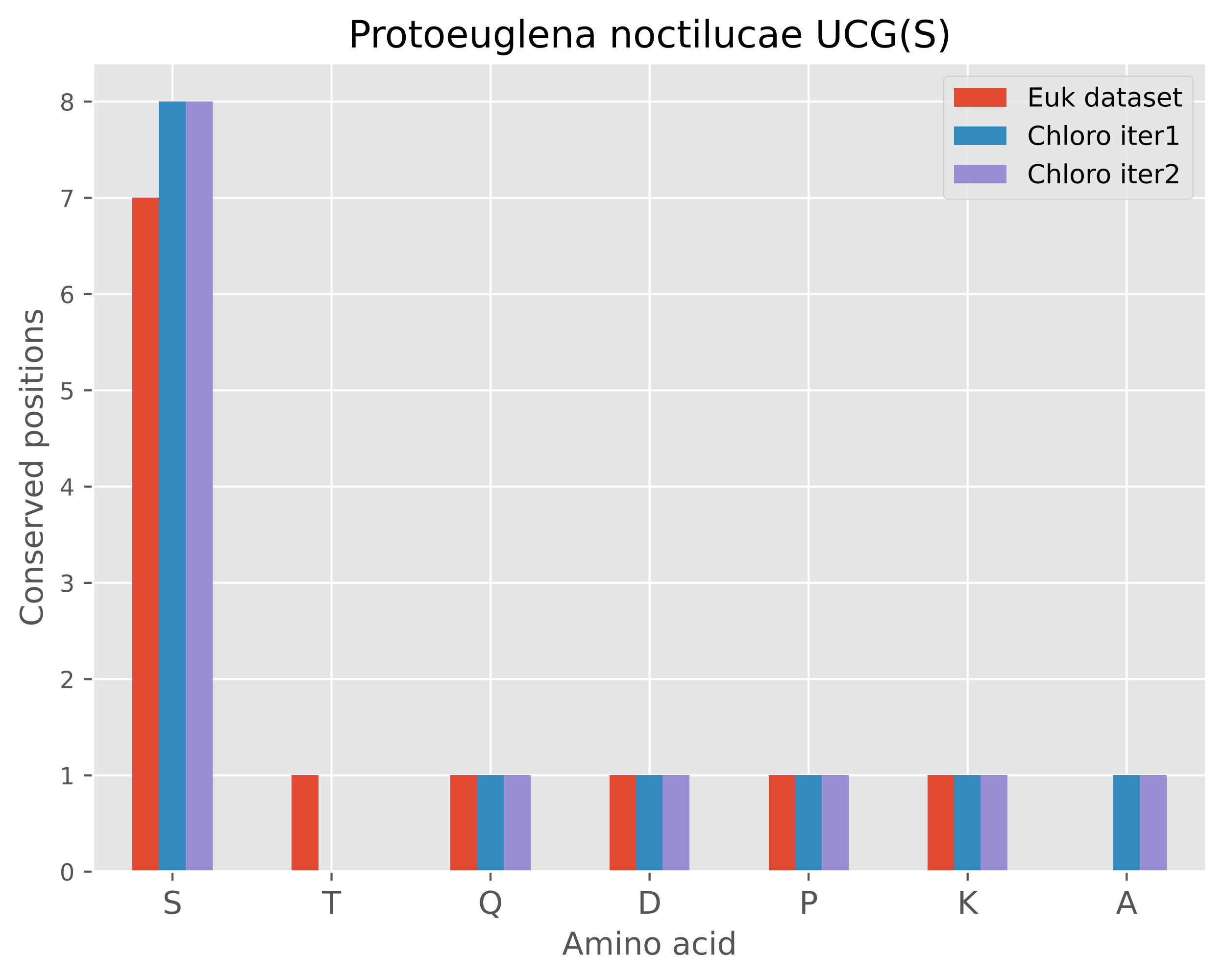

Protoeuglena noctilucae UCG(S)
Euk dataset
Chloro iter1
Chloro iter2
Conserved positions
0
1
2
3
4
5
6
7
8
S
T
Q
D
P
K
A
Amino acid

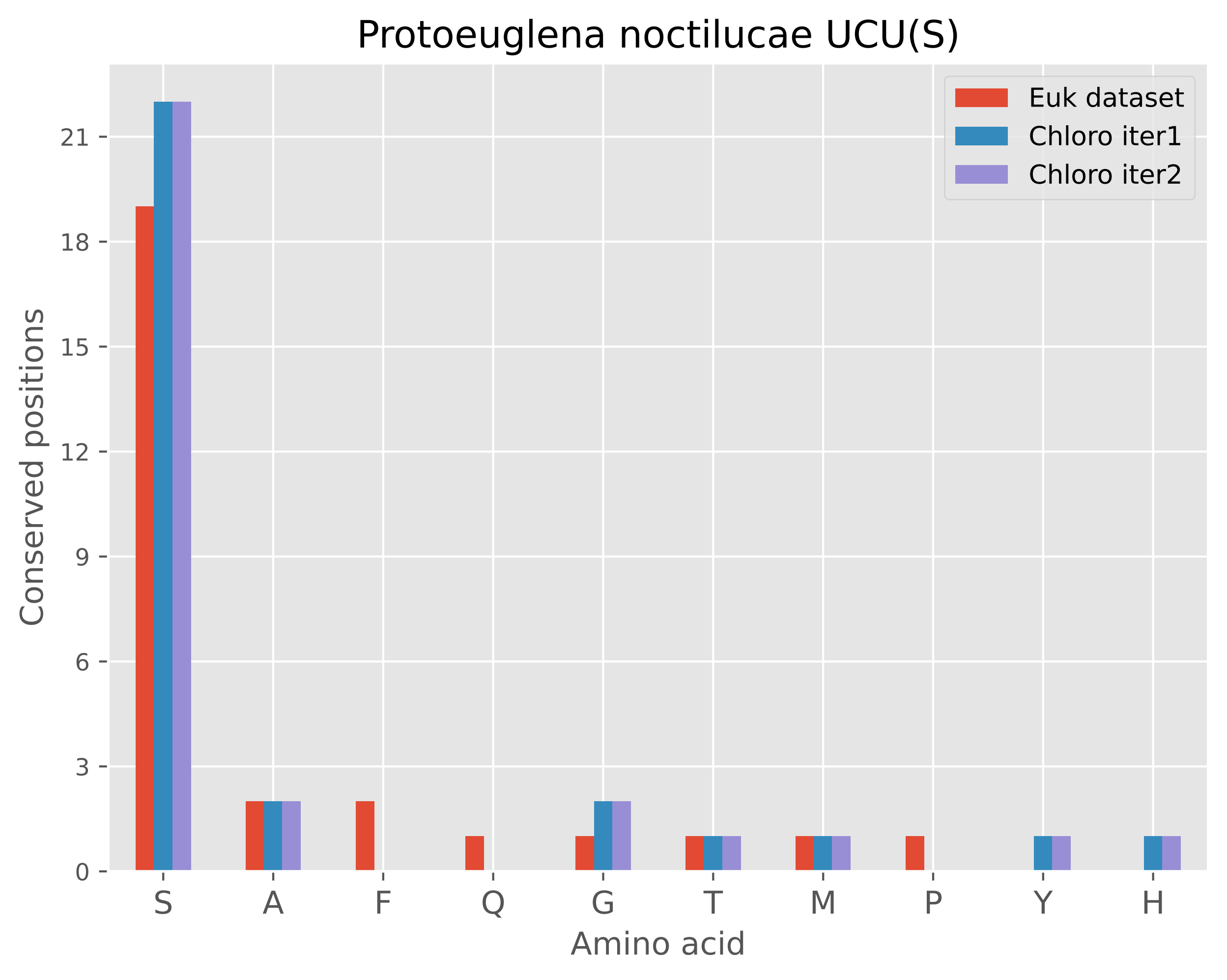
Protoeuglena noctilucae UCU(S)
Euk dataset
Chloro iter1
Chloro iter2
Conserved positions
0
3
6
9
12
15
18
21
S
A
F
Q
G
T
M
P
Y
H
Amino acid

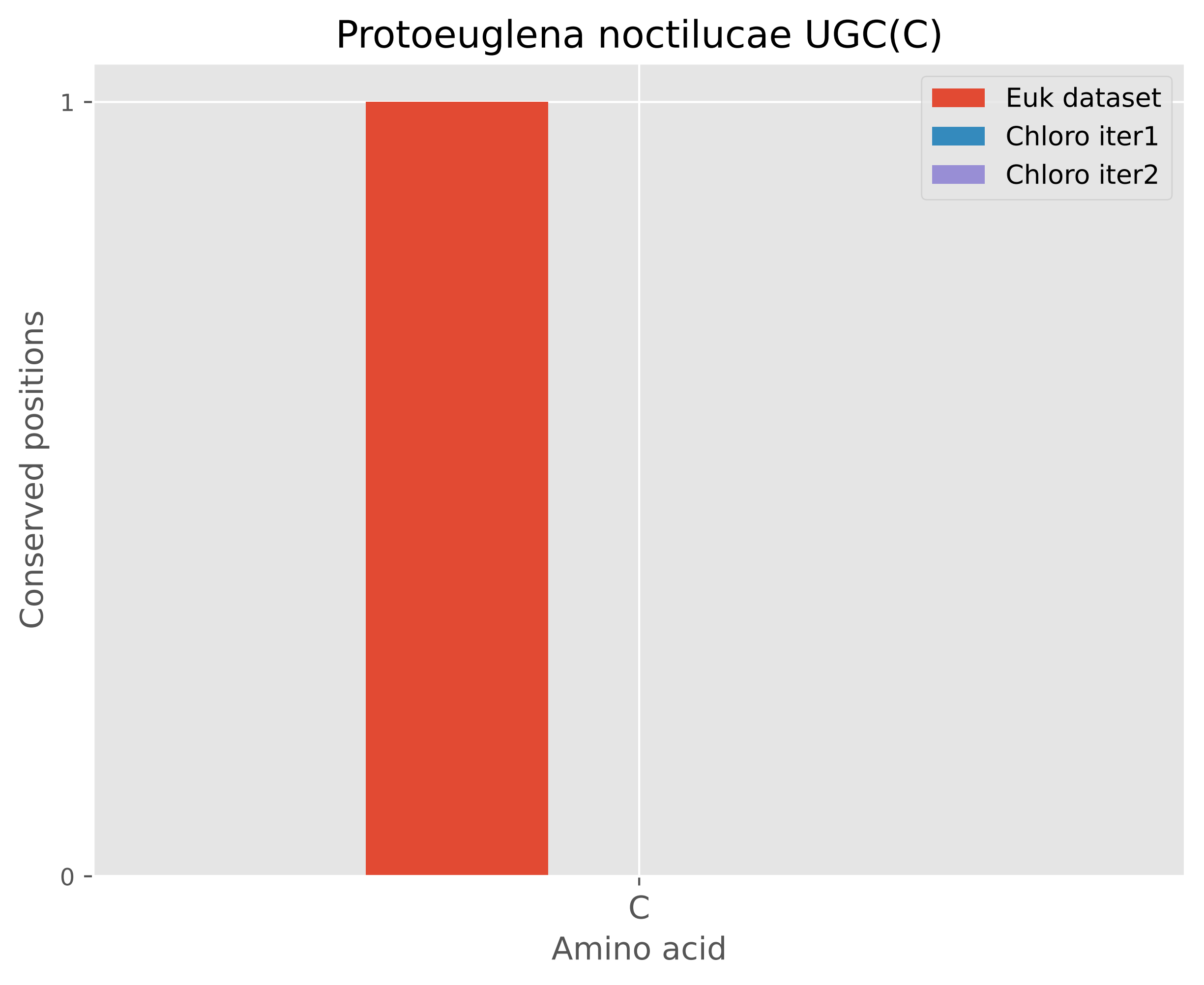

Protoeuglena noctilucae UGC(C)
Euk dataset
Chloro iter1
Chloro iter2
1
0
Conserved positions
C
Amino acid

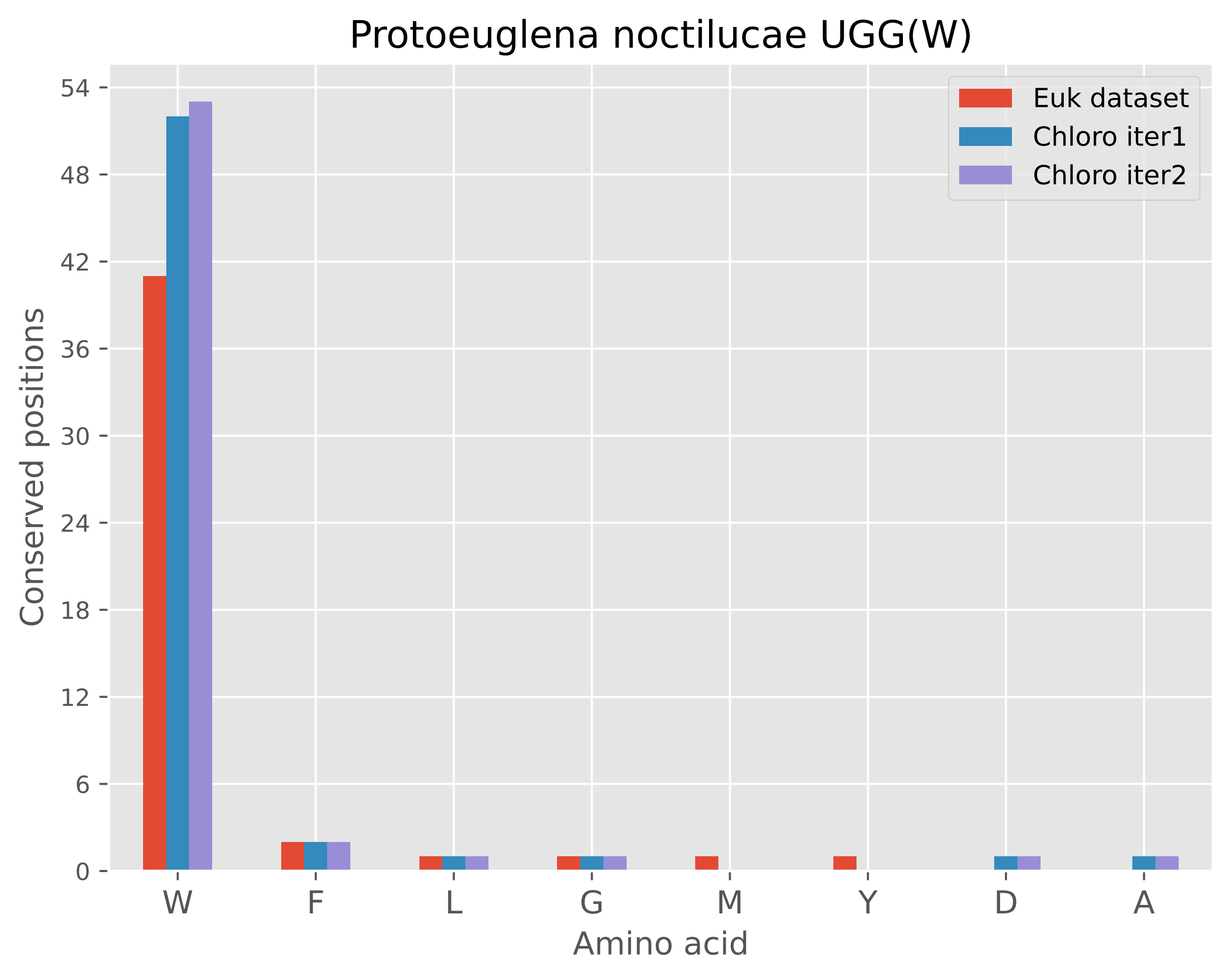
Protoeuglena noctilucae UGG(W)
Euk dataset
Chloro iter1
Chloro iter2
Conserved positions
0
6
12
18
24
30
36
42
48
54
W
F
L
G
M
Y
D
A
Amino acid

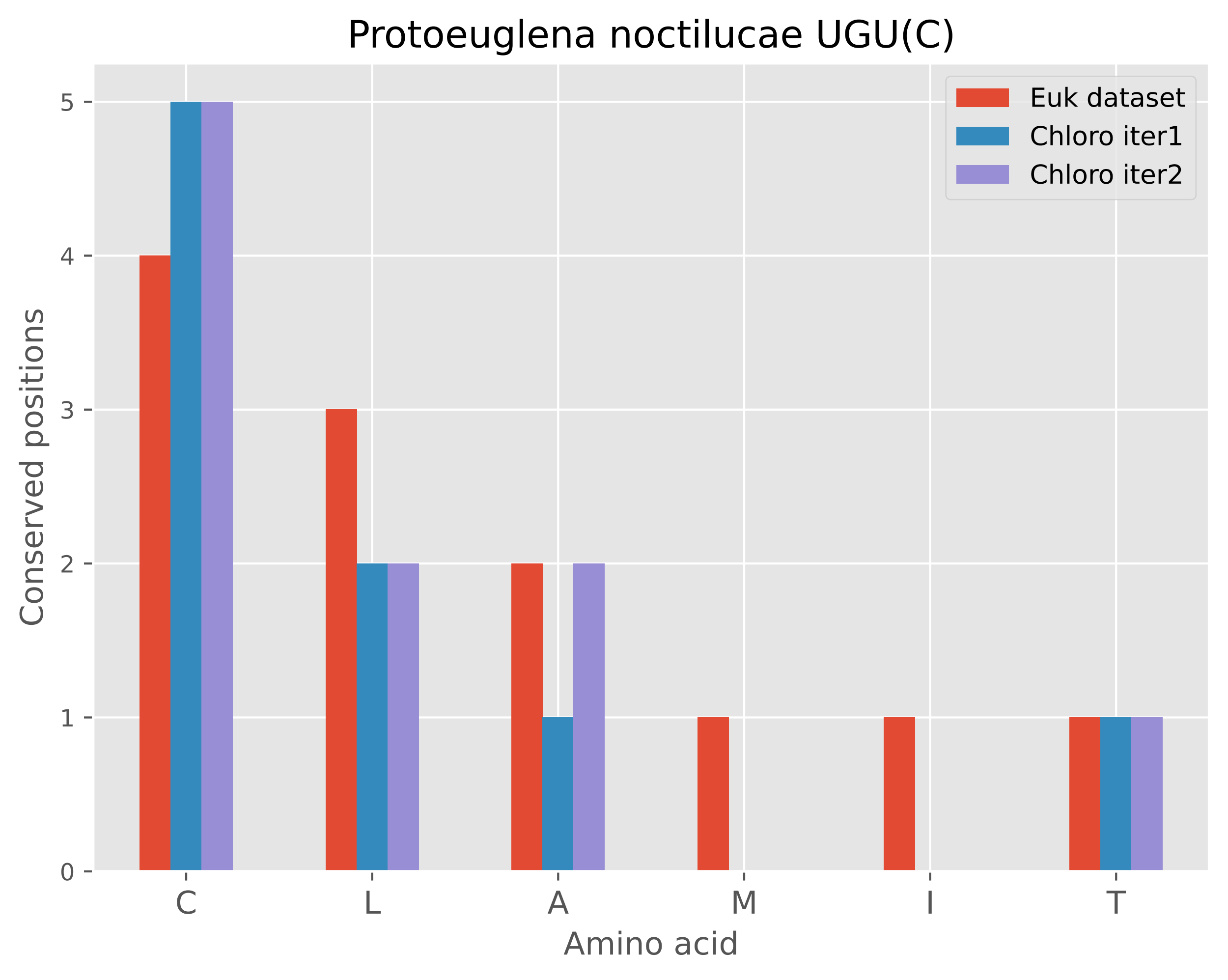
Protoeuglena noctilucae UGU(C)
Euk dataset
Chloro iter1
Chloro iter2
Conserved positions
0
1
2
3
4
5
C
L
A
M
I
T
Amino acid

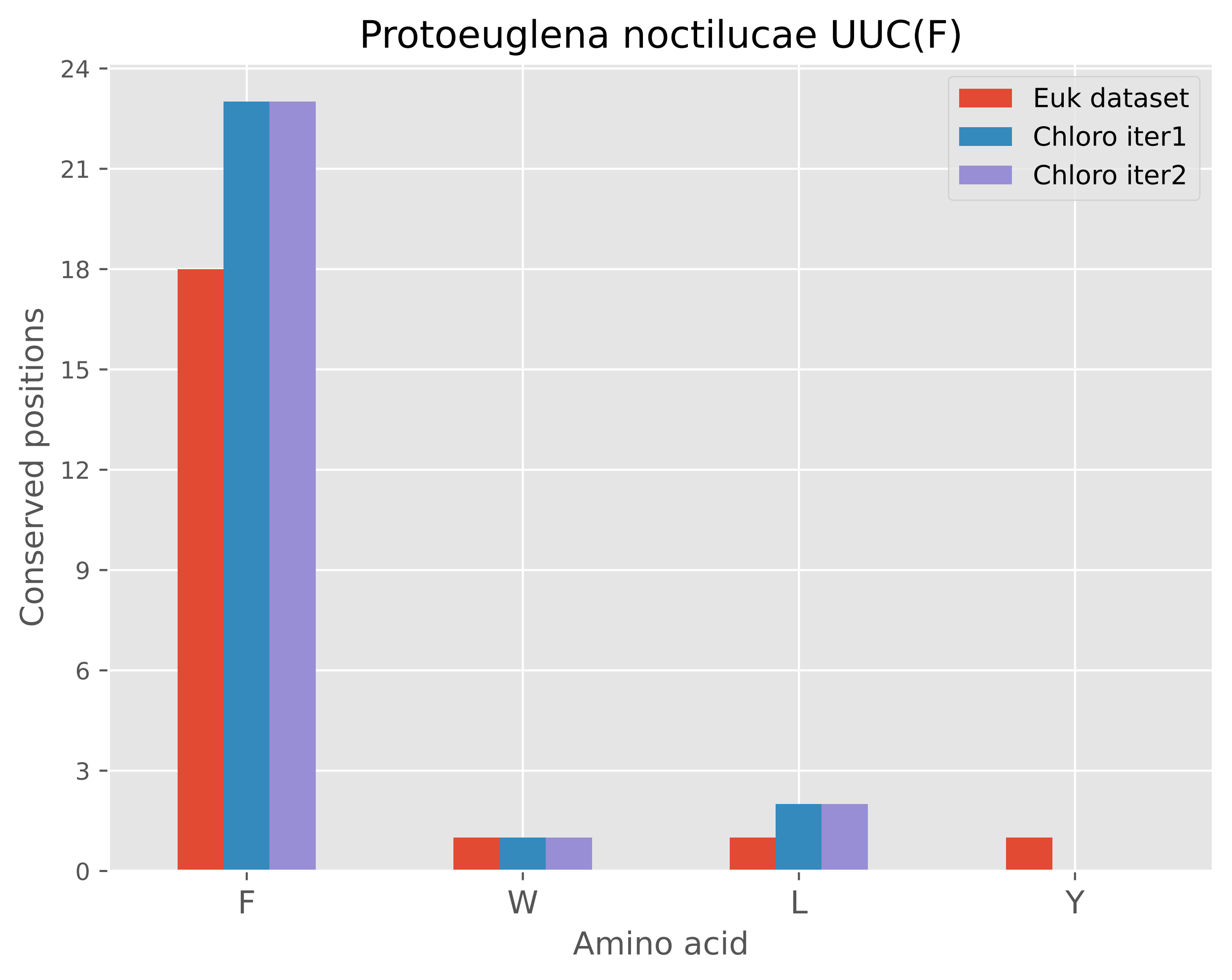

Protoeuglena noctilucae UUC(F)
Euk dataset
Chloro iter1
Chloro iter2
Conserved positions
0
3
6
9
12
15
18
21
24
F
W
L
Y
Amino acid

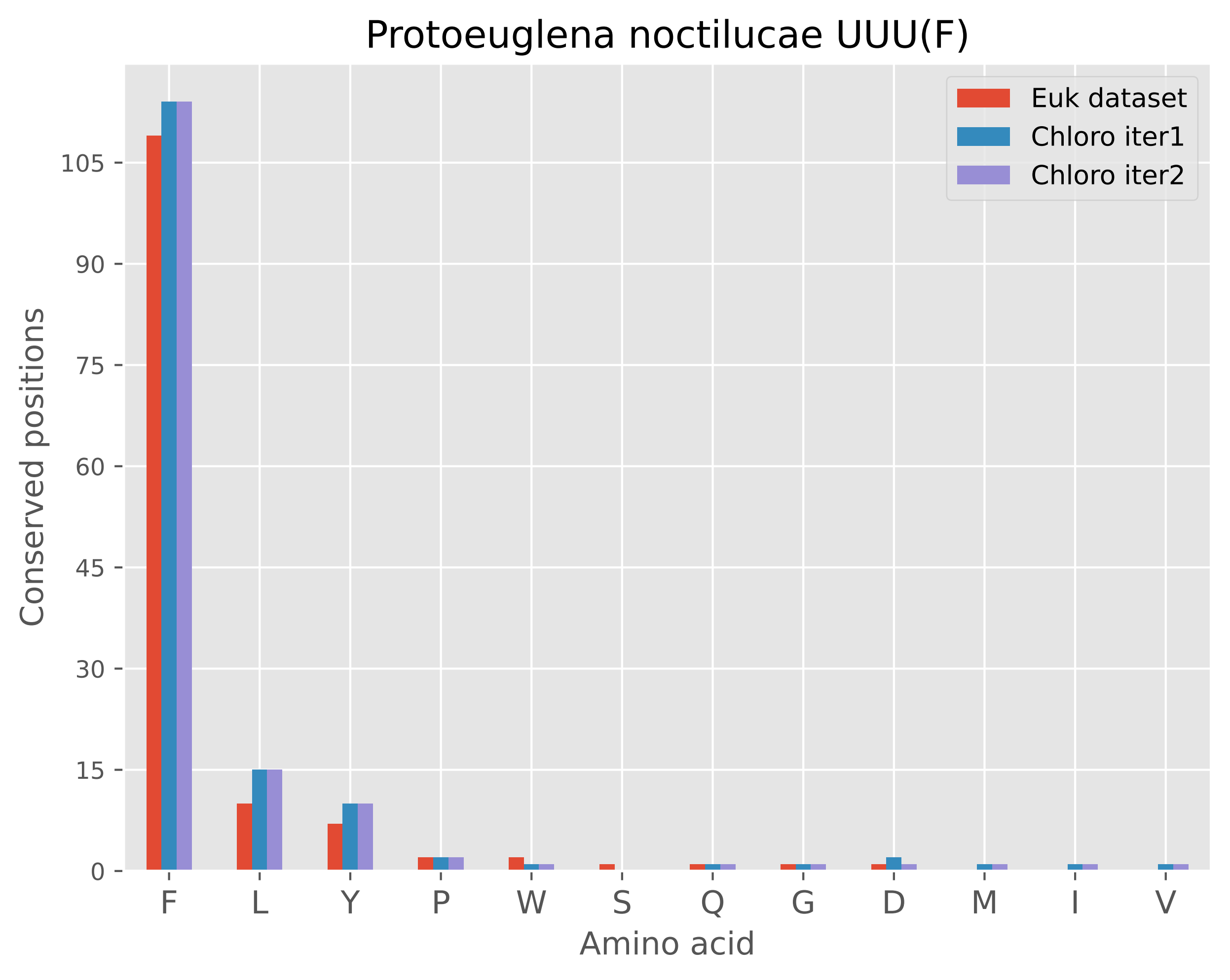

Protoeuglena noctilucae UUU(F)
Euk dataset
Chloro iter1
Chloro iter2
Conserved positions
0
15
30
45
60
75
90
105
F
L
Y
P
W
S
Q
G
D
M
I
V
Amino acid

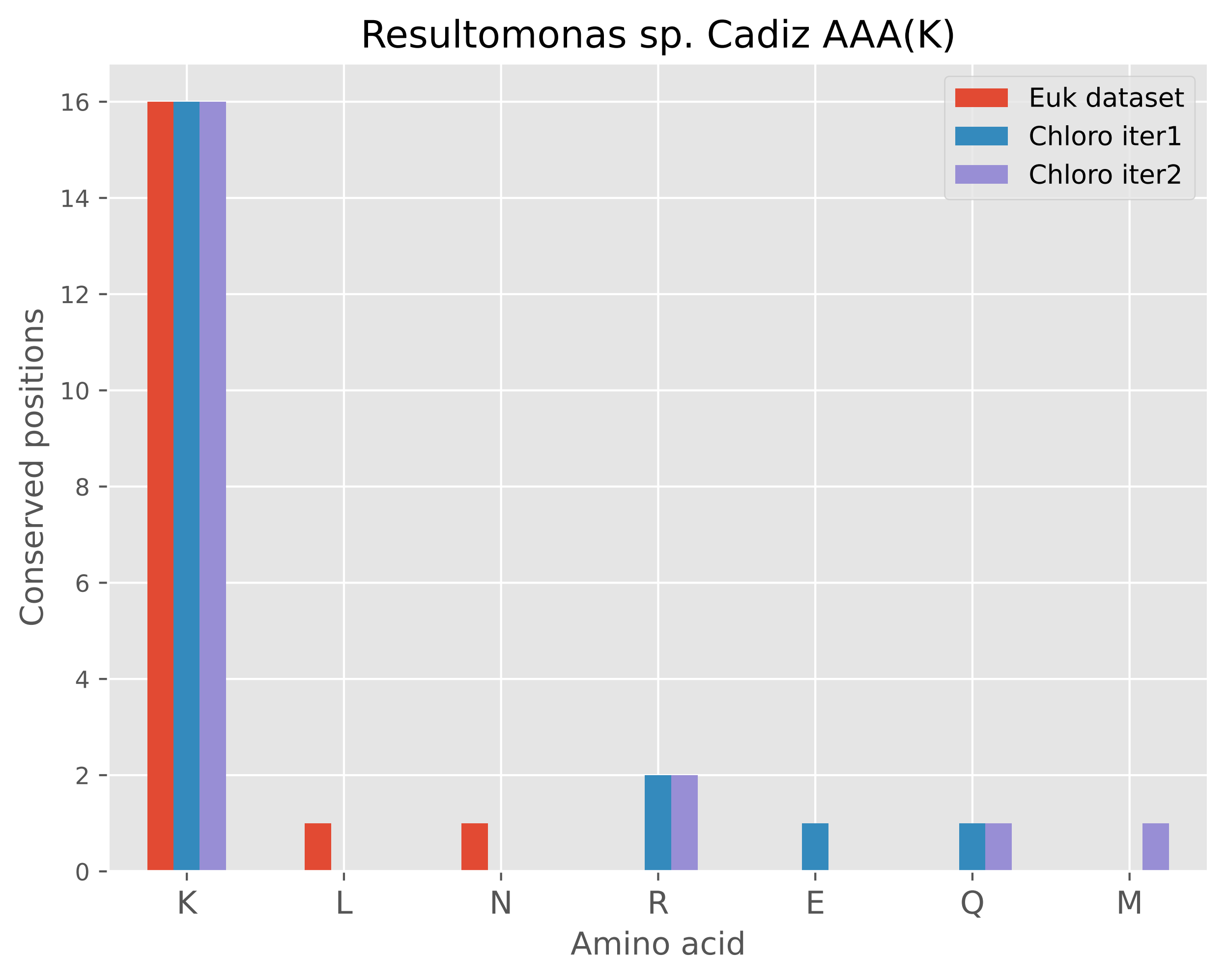
Resultomonas sp. Cadiz AAA(K)
Euk dataset
Chloro iter1
Chloro iter2
Conserved positions
0
2
4
6
8
10
12
14
16
K
L
N
R
E
Q
M
Amino acid

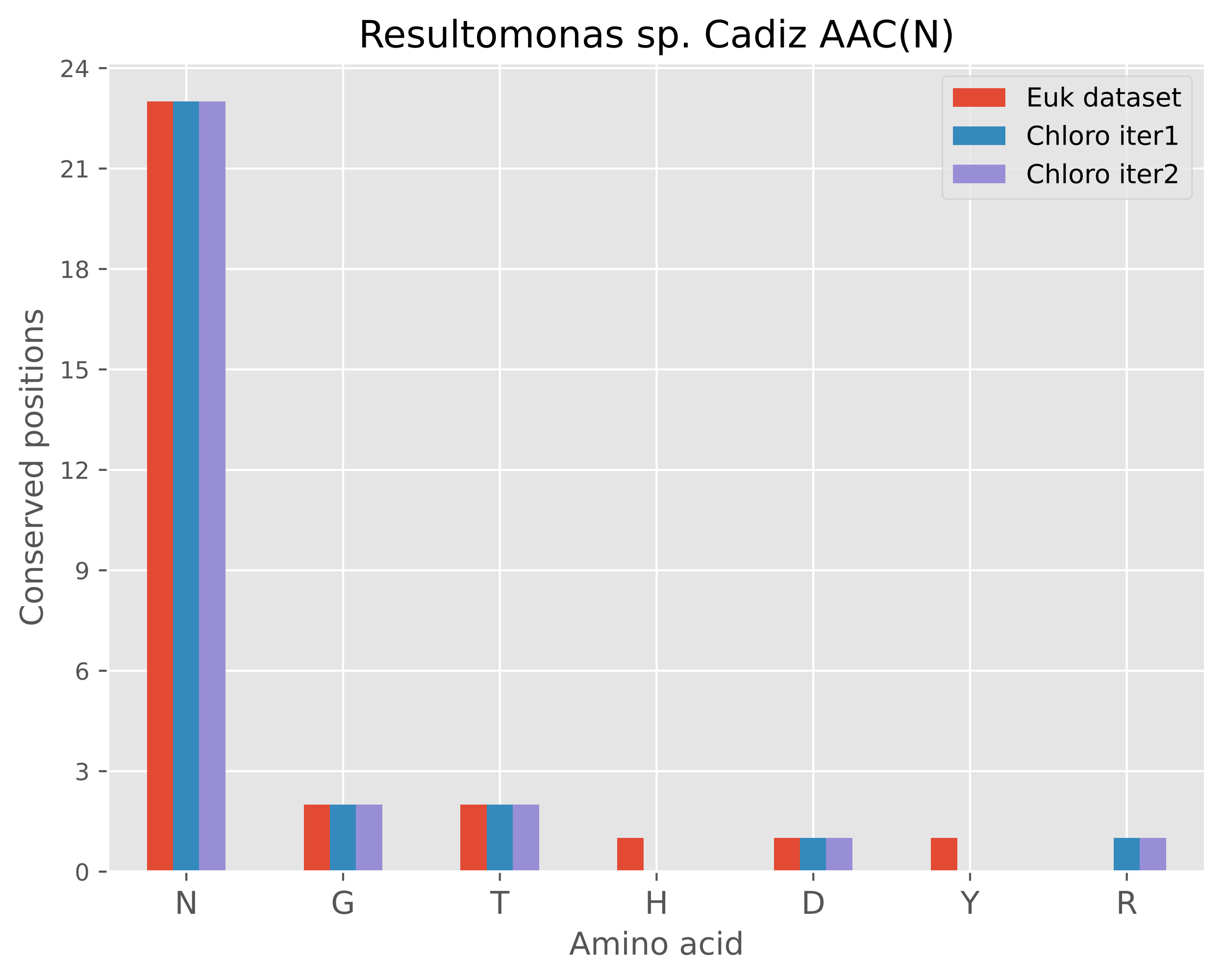
Resultomonas sp. Cadiz AAC(N)
Euk dataset
Chloro iter1
Chloro iter2
Conserved positions
0
3
6
9
12
15
18
21
24
N
G
T
H
D
Y
R
Amino acid

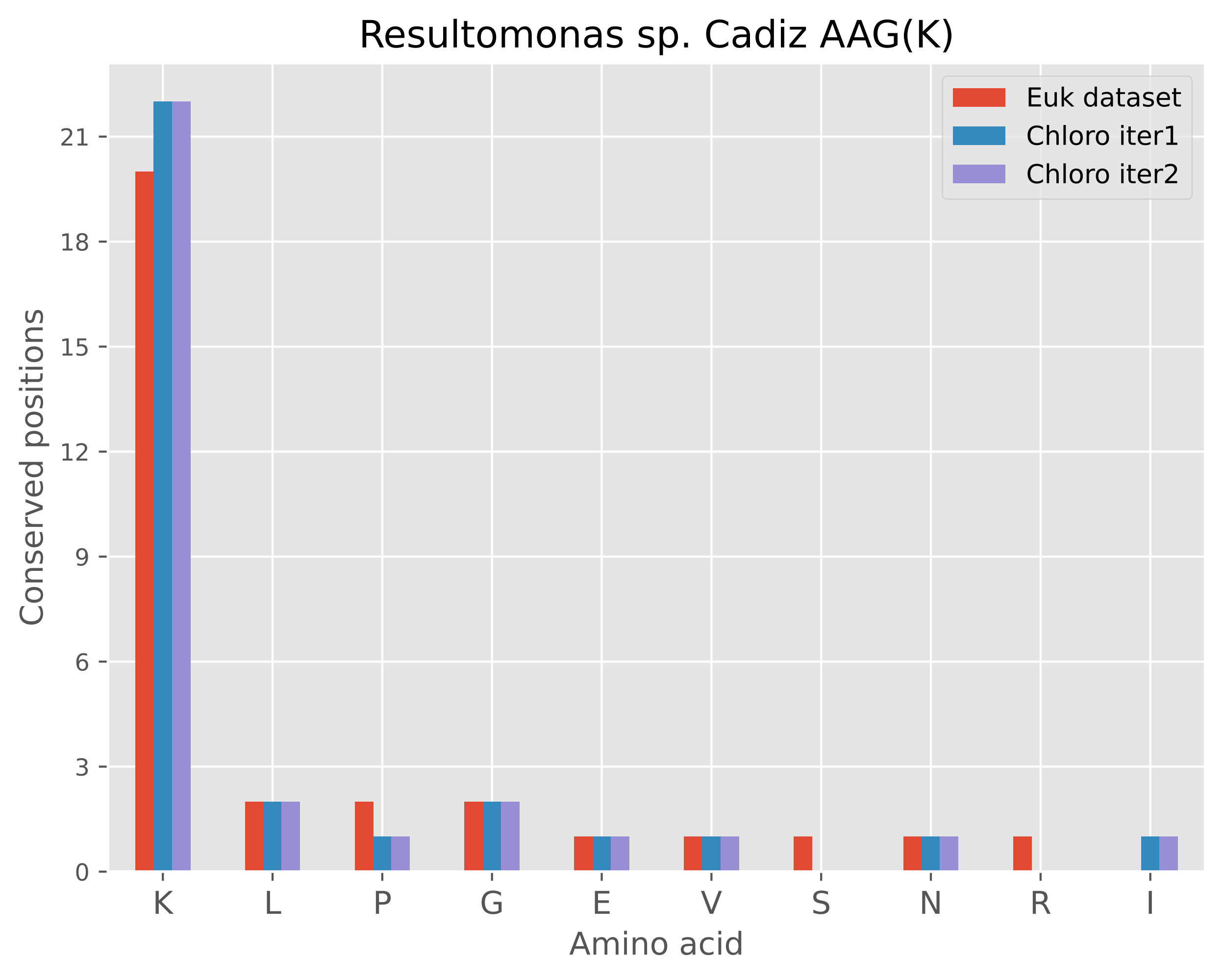
Resultomonas sp. Cadiz AAG(K)
Euk dataset
Chloro iter1
Chloro iter2
Conserved positions
0
3
6
9
12
15
18
21
K
L
P
G
E
V
S
N
R
I
Amino acid

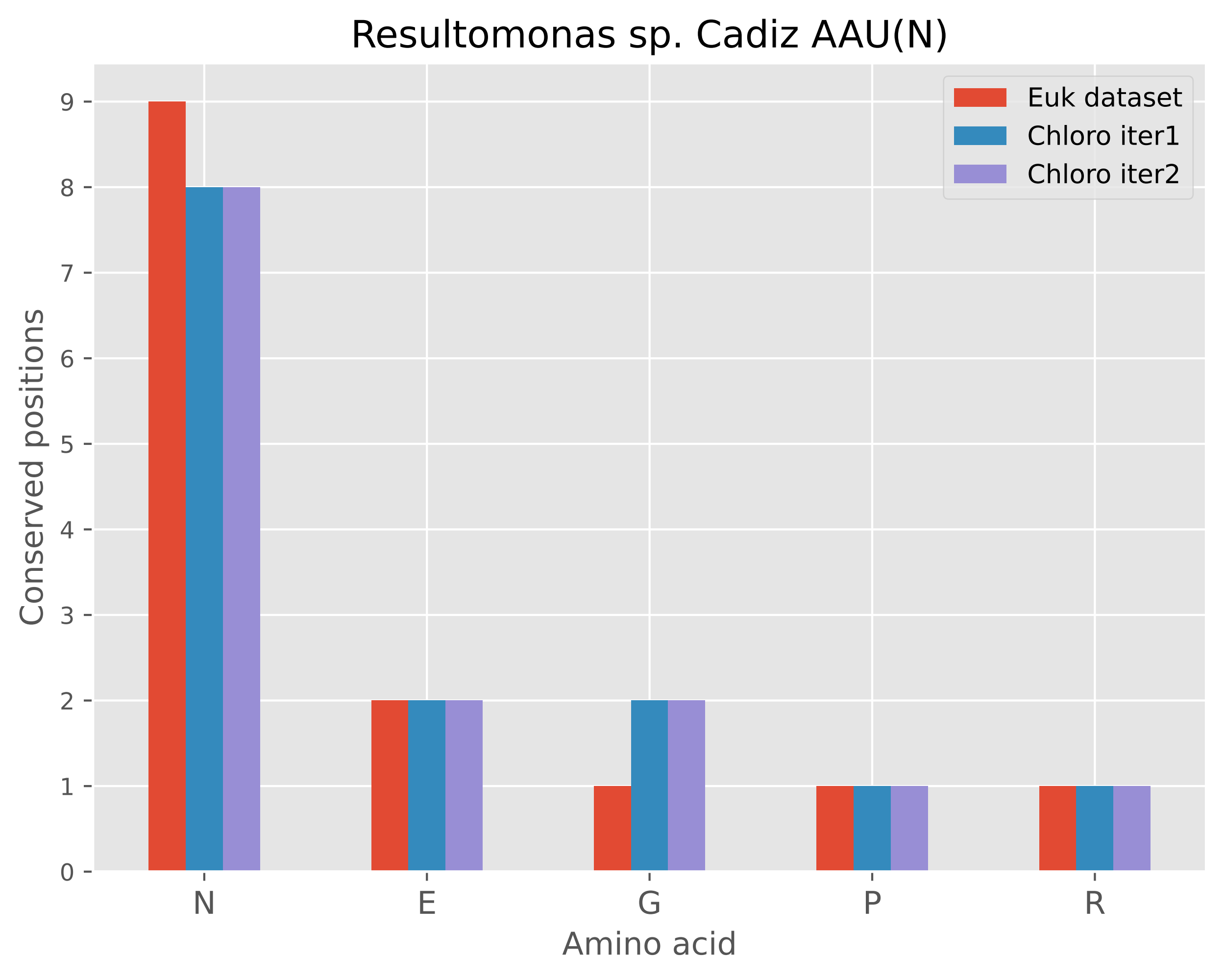

Resultomonas sp. Cadiz AAU(N)
Euk dataset
Chloro iter1
Chloro iter2
Conserved positions
0
1
2
3
4
5
6
7
8
9
N
E
G
P
R
Amino acid

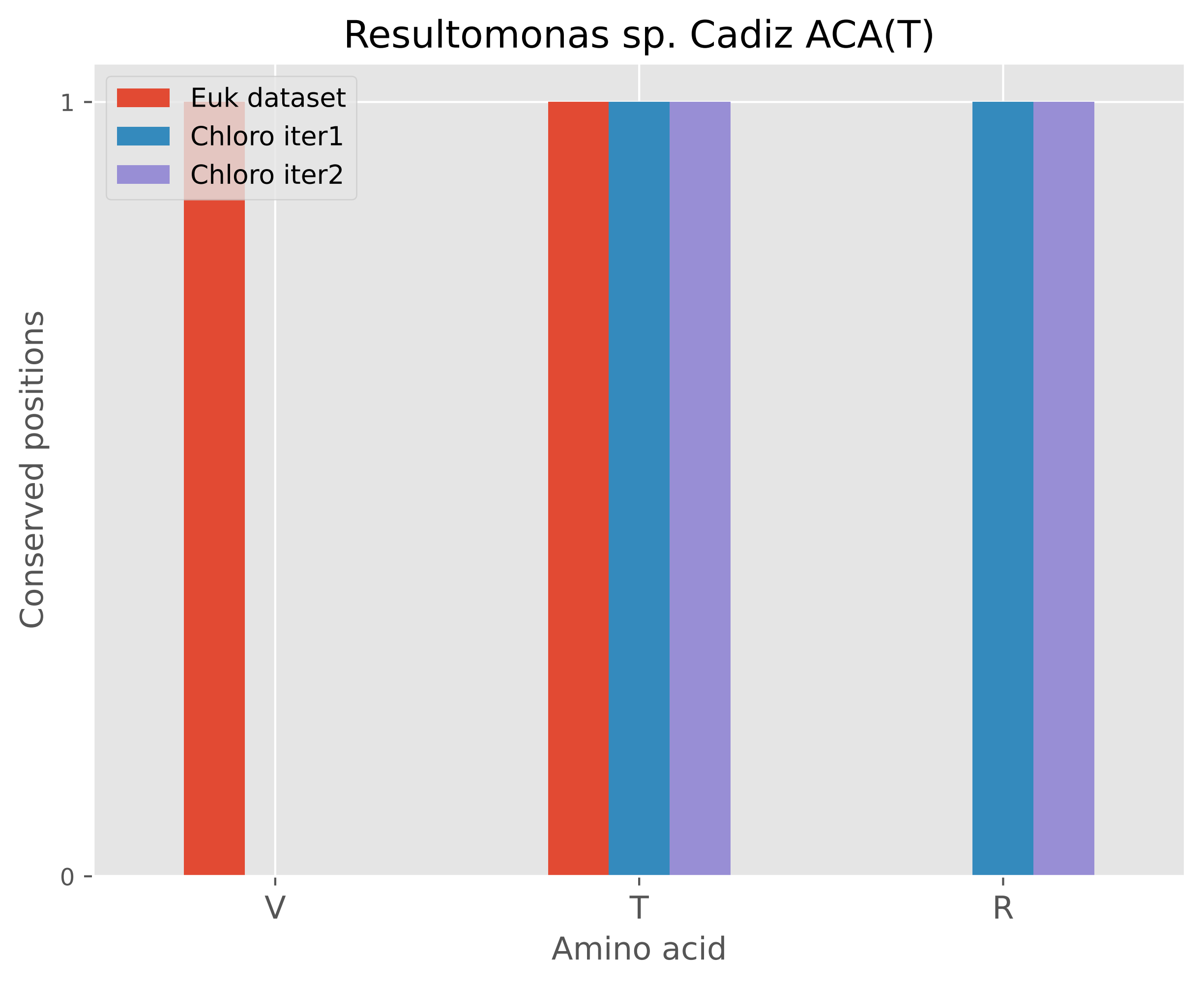
Resultomonas sp. Cadiz ACA(T)
Euk dataset
Chloro iter1
Chloro iter2
Conserved positions
1
0
V
T
R
Amino acid

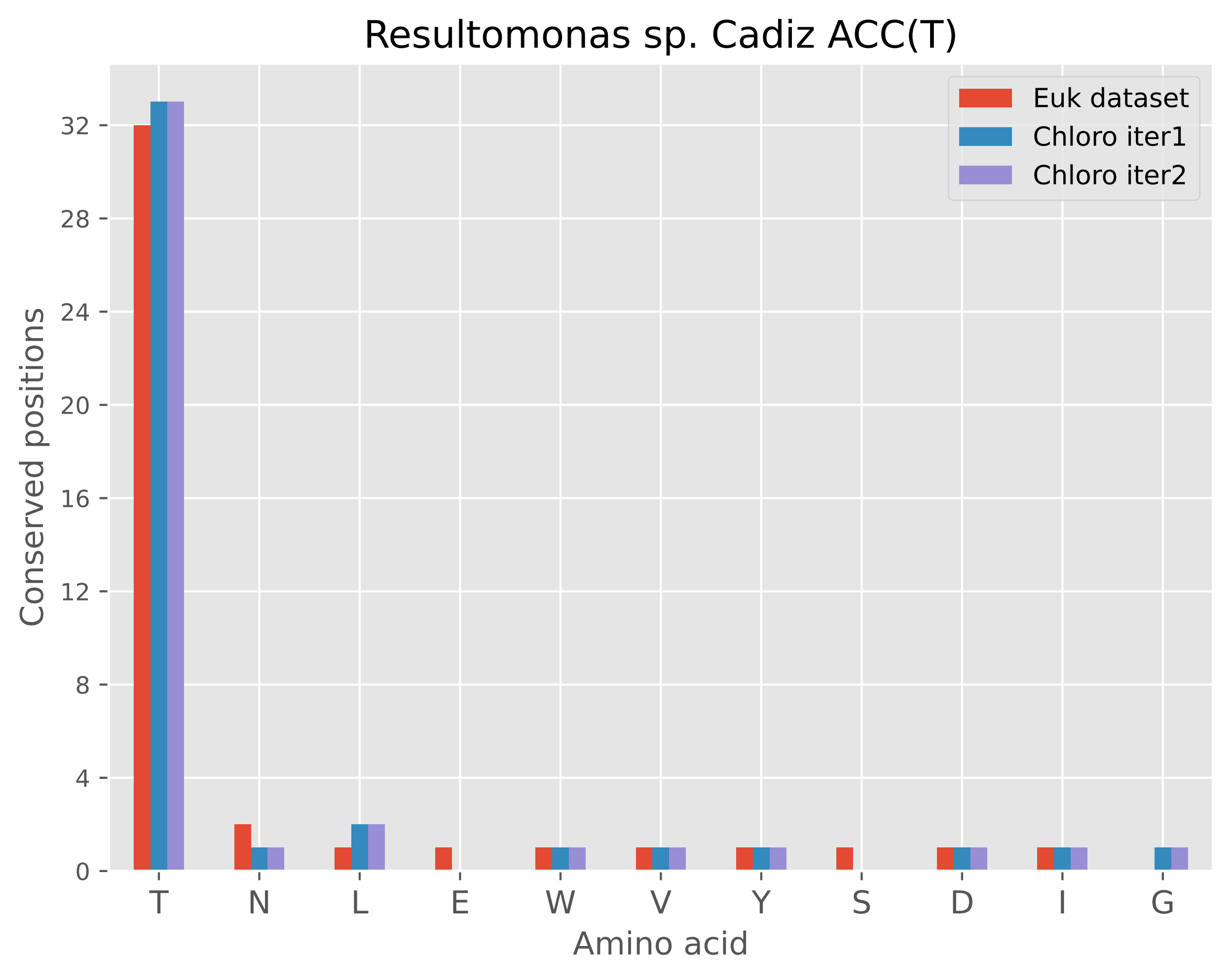

Resultomonas sp. Cadiz ACC(T)
Euk dataset
Chloro iter1
Chloro iter2
Conserved positions
0
4
8
12
16
20
24
28
32
T
N
L
E
W
V
Y
S
D
I
G
Amino acid

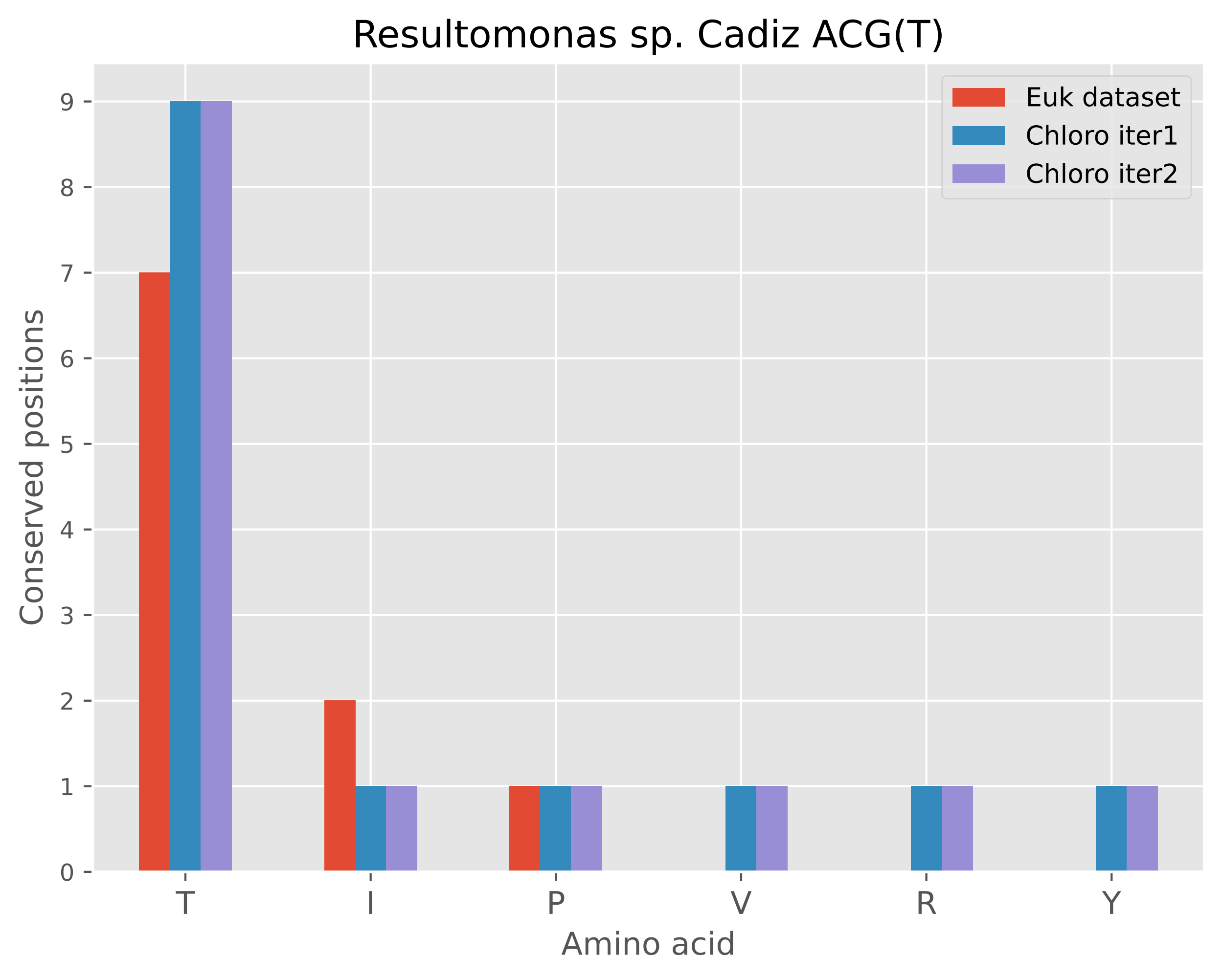

Resultomonas sp. Cadiz ACG(T)
Euk dataset
Chloro iter1
Chloro iter2
Conserved positions
0
1
2
3
4
5
6
7
8
9
T
I
P
V
R
Y
Amino acid

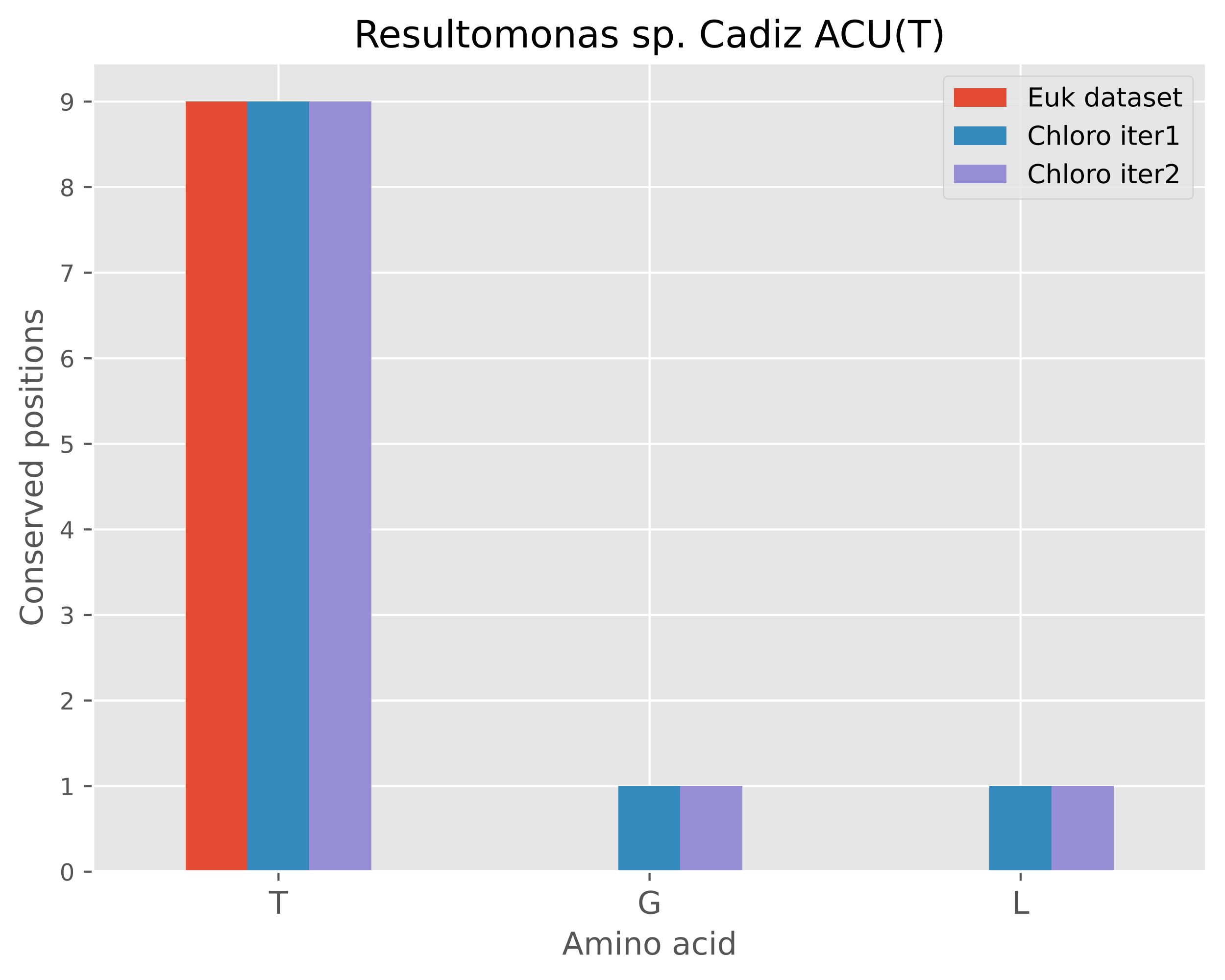
Resultomonas sp. Cadiz ACU(T)
Euk dataset
Chloro iter1
Chloro iter2
Conserved positions
0
1
2
3
4
5
6
7
8
9
T
G
L
Amino acid

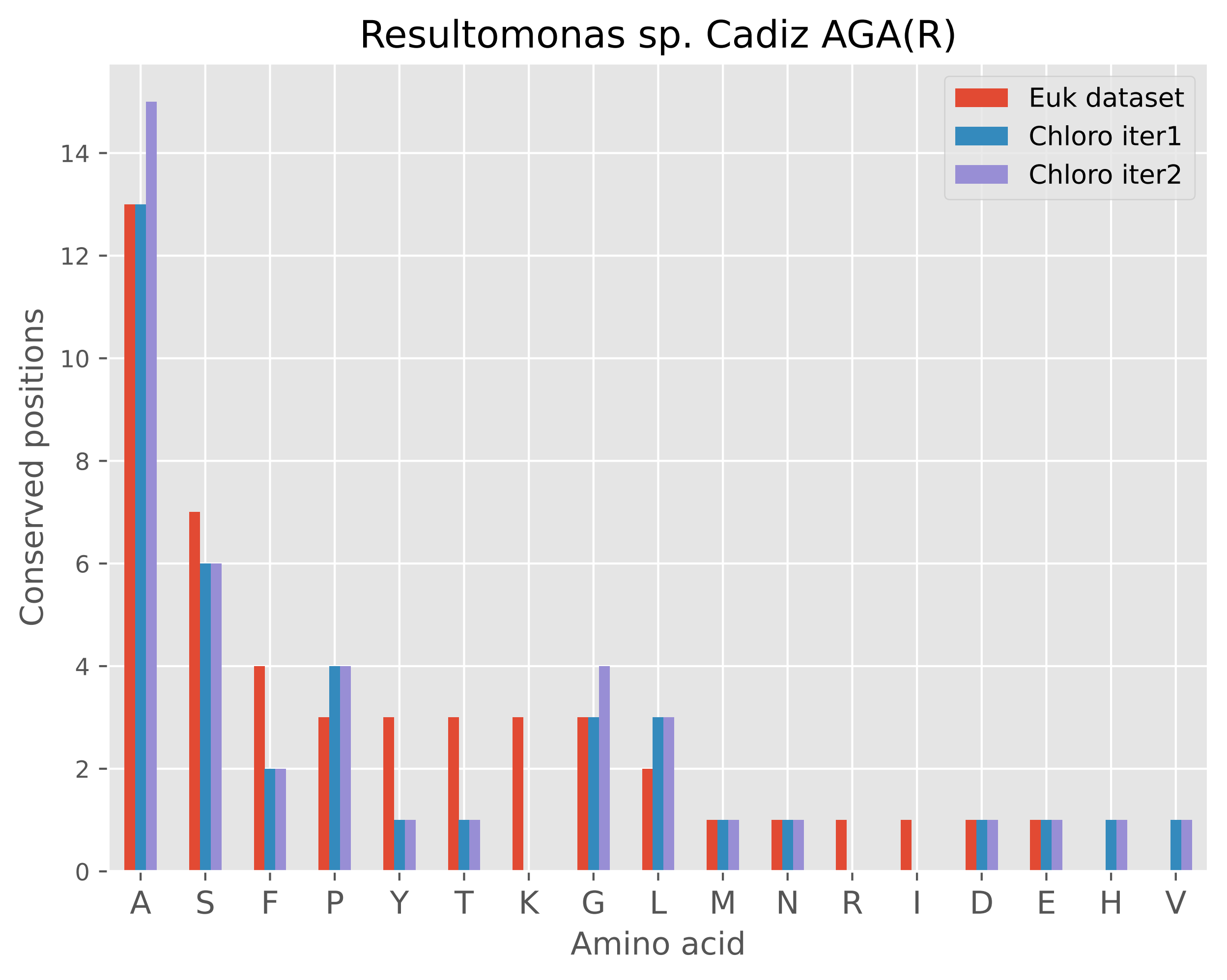
Resultomonas sp. Cadiz AGA(R)
Euk dataset
Chloro iter1
Chloro iter2
Conserved positions
0
2
4
6
8
10
12
14
A
S
F
P
Y
T
K
G
L
M
N
R
I
D
E
H
V
Amino acid

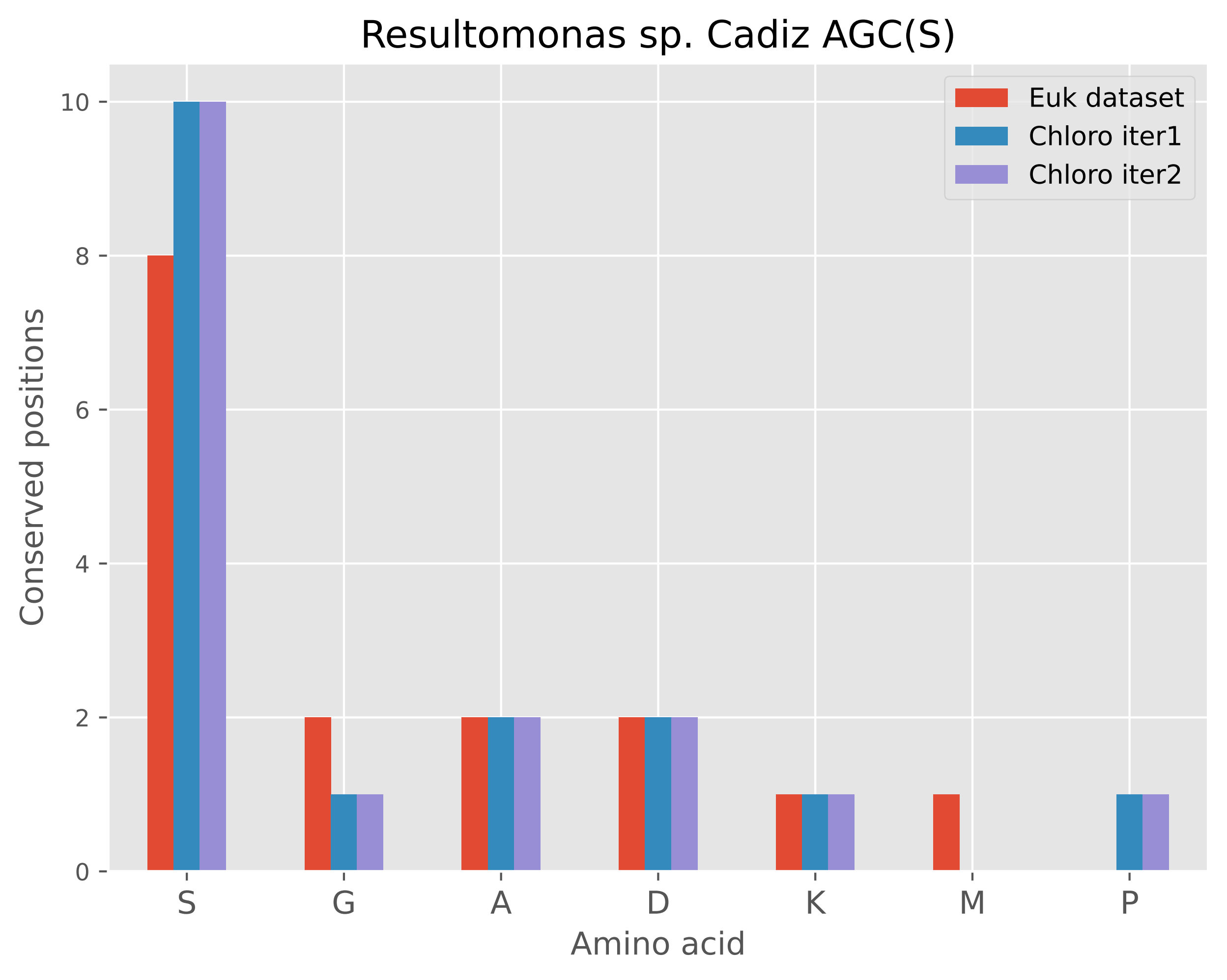
Resultomonas sp. Cadiz AGC(S)
Euk dataset
Chloro iter1
Chloro iter2
Conserved positions
0
2
4
6
8
10
S
G
A
D
K
M
P
Amino acid

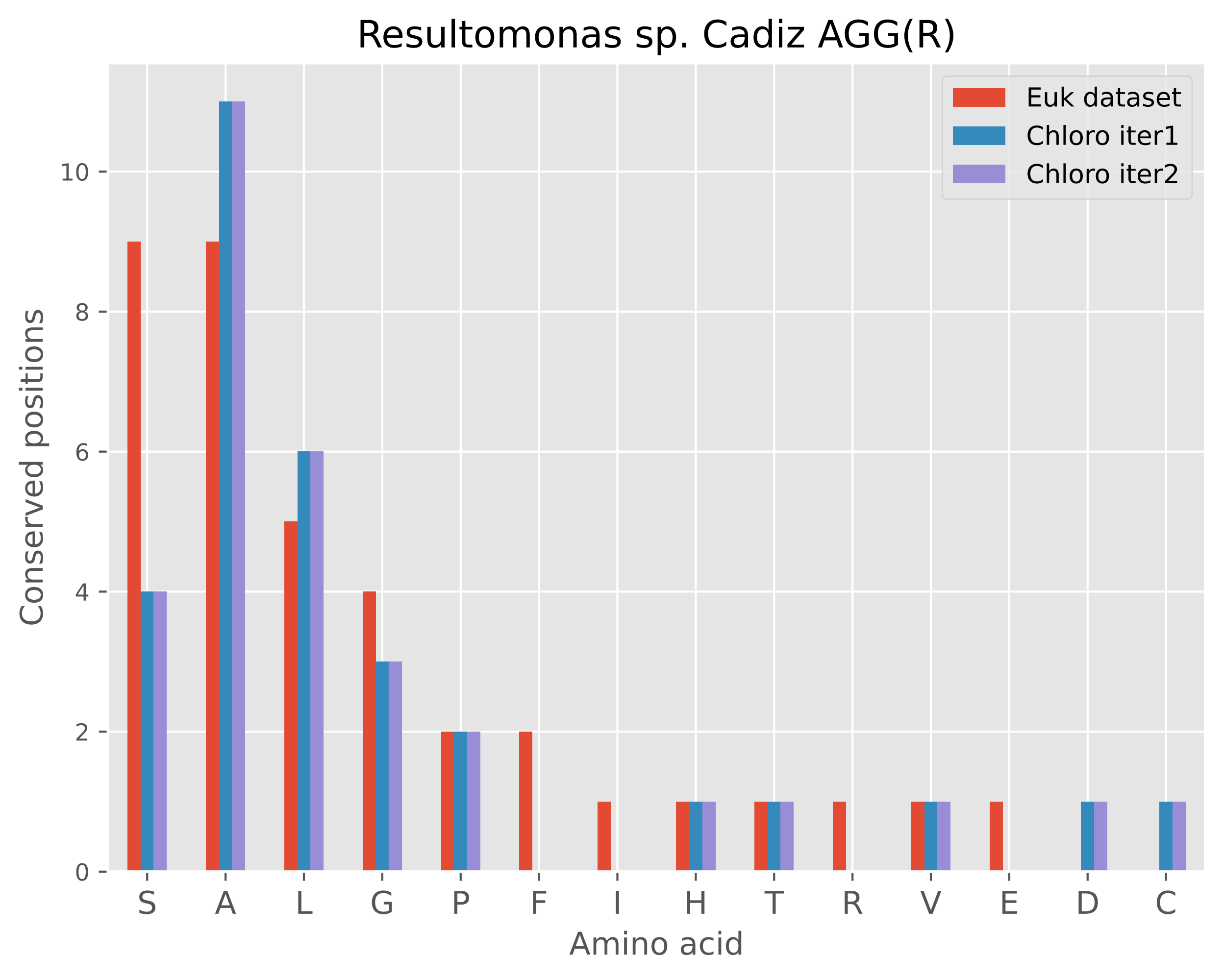
Resultomonas sp. Cadiz AGG(R)
Euk dataset
Chloro iter1
Chloro iter2
Conserved positions
0
2
4
6
8
10
S
A
L
G
P
F
I
H
T
R
V
E
D
C
Amino acid

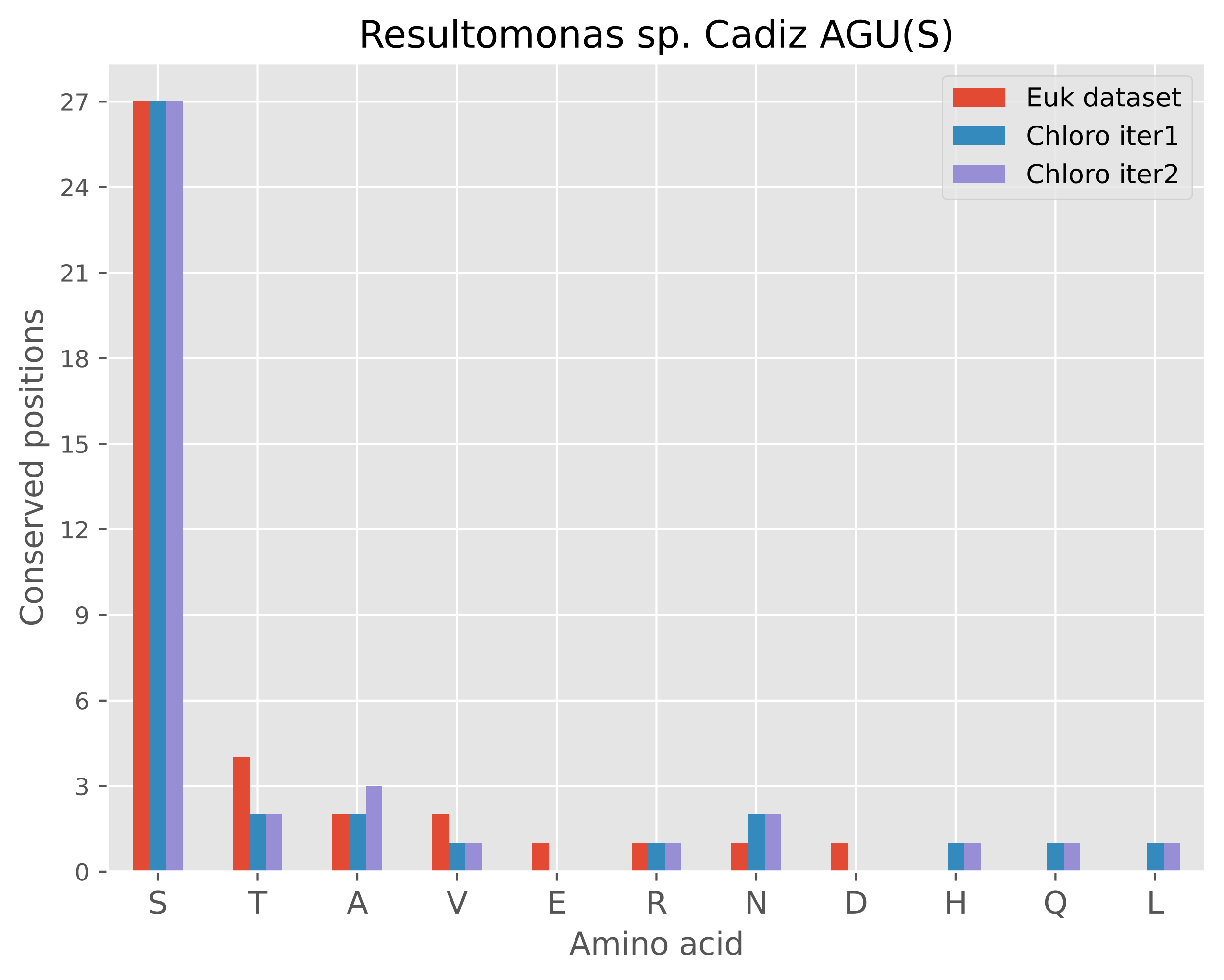
Resultomonas sp. Cadiz AGU(S)
Euk dataset
Chloro iter1
Chloro iter2
Conserved positions
0
3
6
9
12
15
18
21
24
27
S
T
A
V
E
R
N
D
H
Q
L
Amino acid

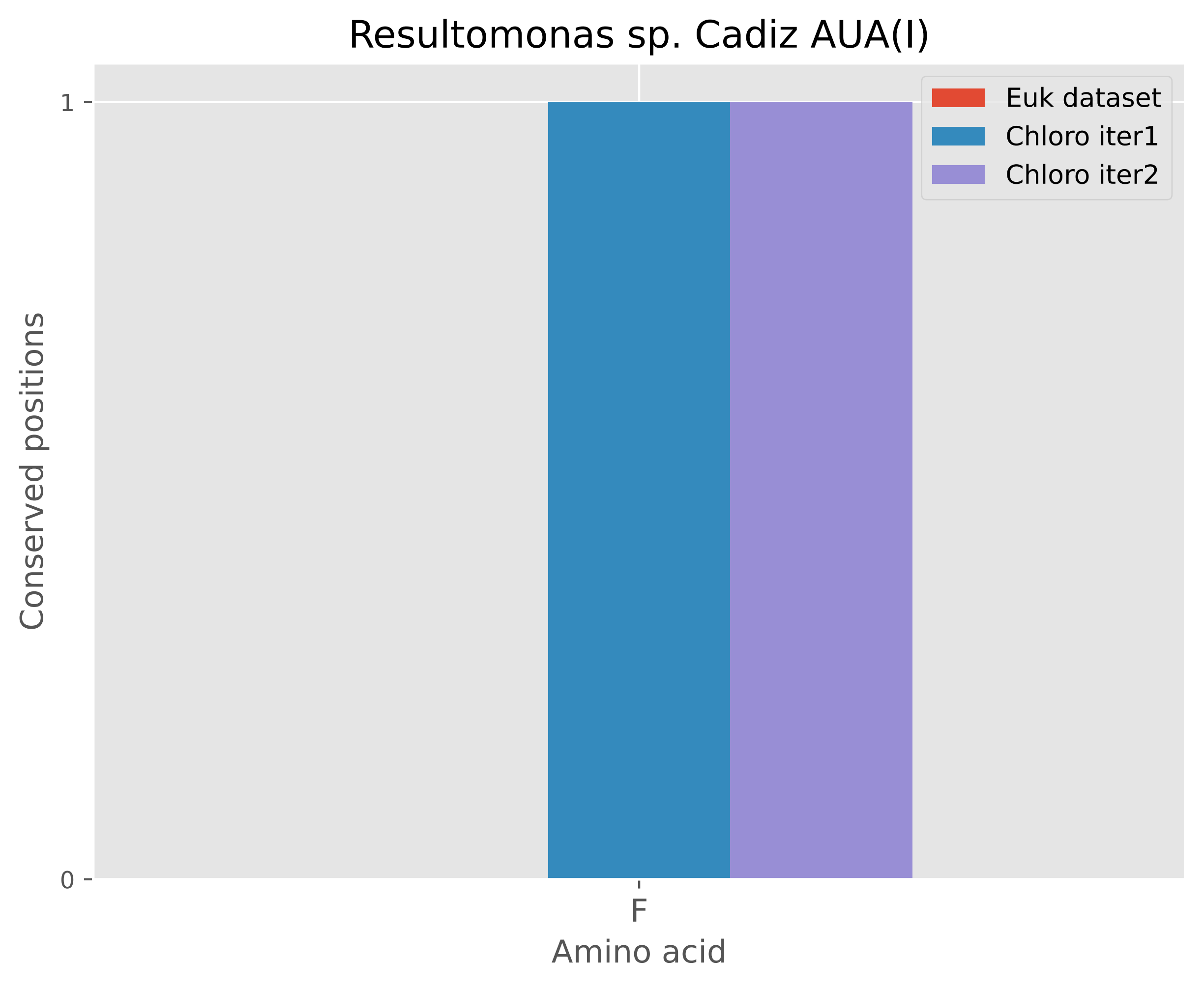
Resultomonas sp. Cadiz AUA(I)
Euk dataset
Chloro iter1
Chloro iter2
1
0
Conserved positions
F
Amino acid

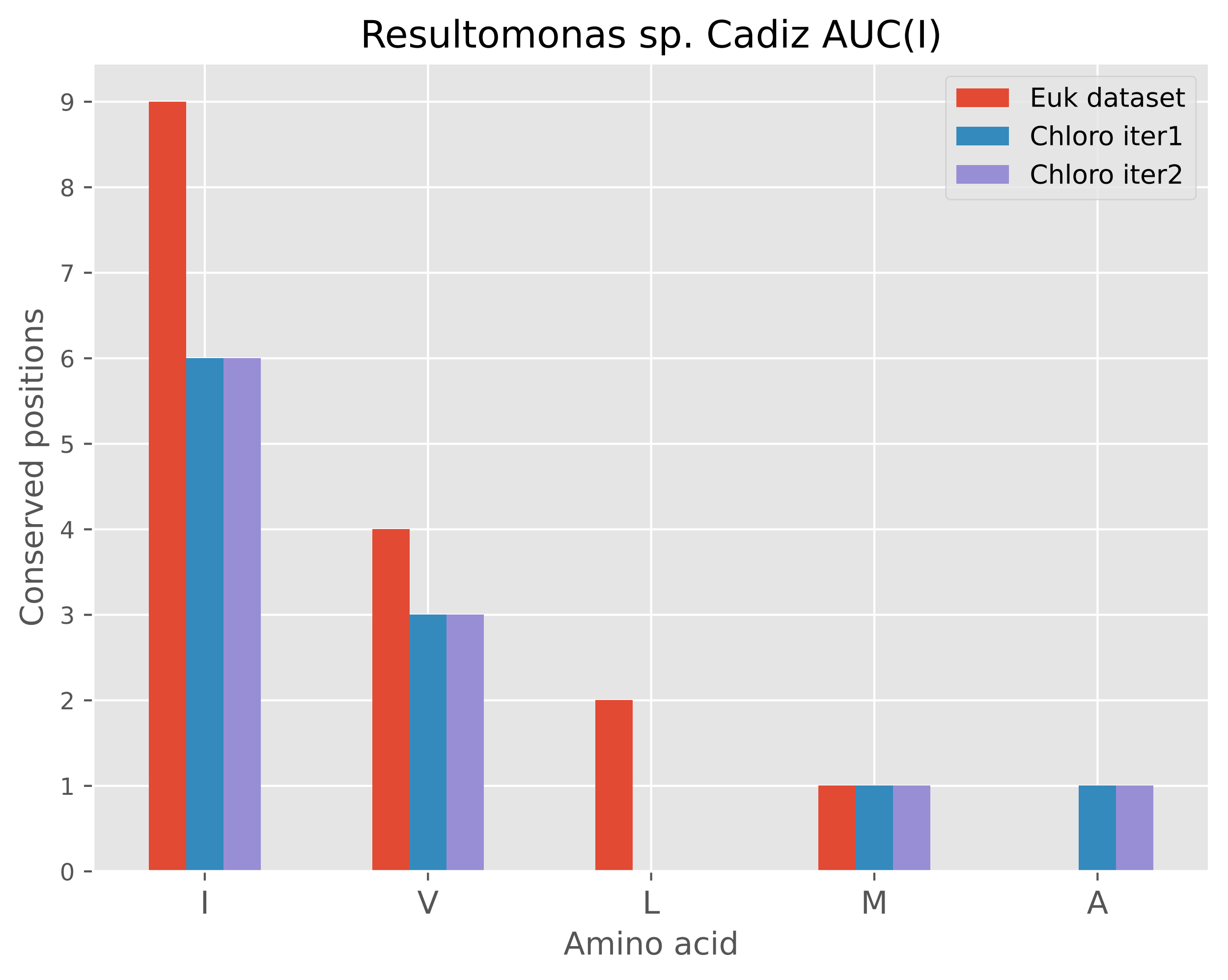
Resultomonas sp. Cadiz AUC(I)
Euk dataset
Chloro iter1
Chloro iter2
Conserved positions
0
1
2
3
4
5
6
7
8
9
I
V
L
M
A
Amino acid

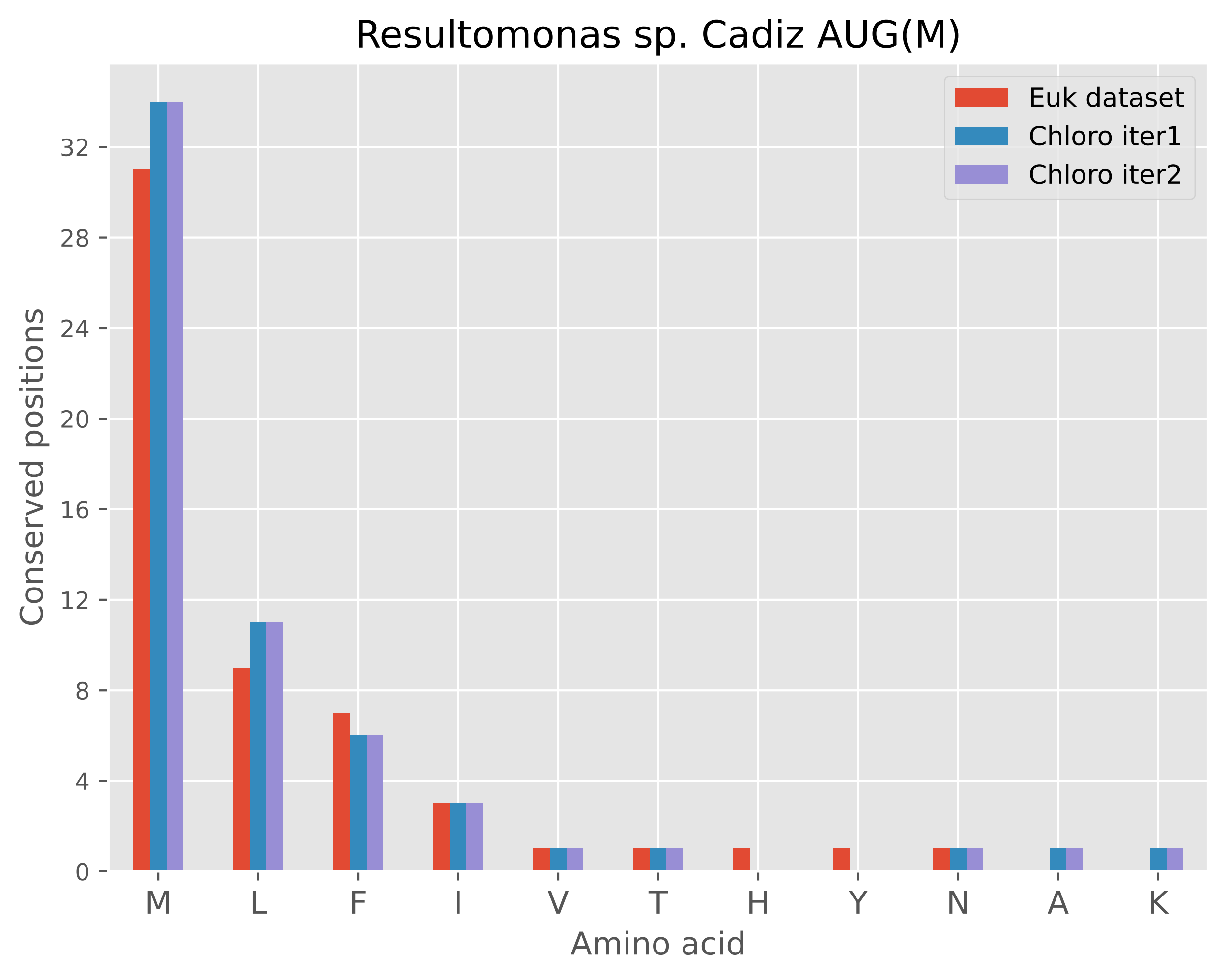
Resultomonas sp. Cadiz AUG(M)
Euk dataset
Chloro iter1
Chloro iter2
Conserved positions
0
4
8
12
16
20
24
28
32
M
L
F
I
V
T
H
Y
N
A
K
Amino acid

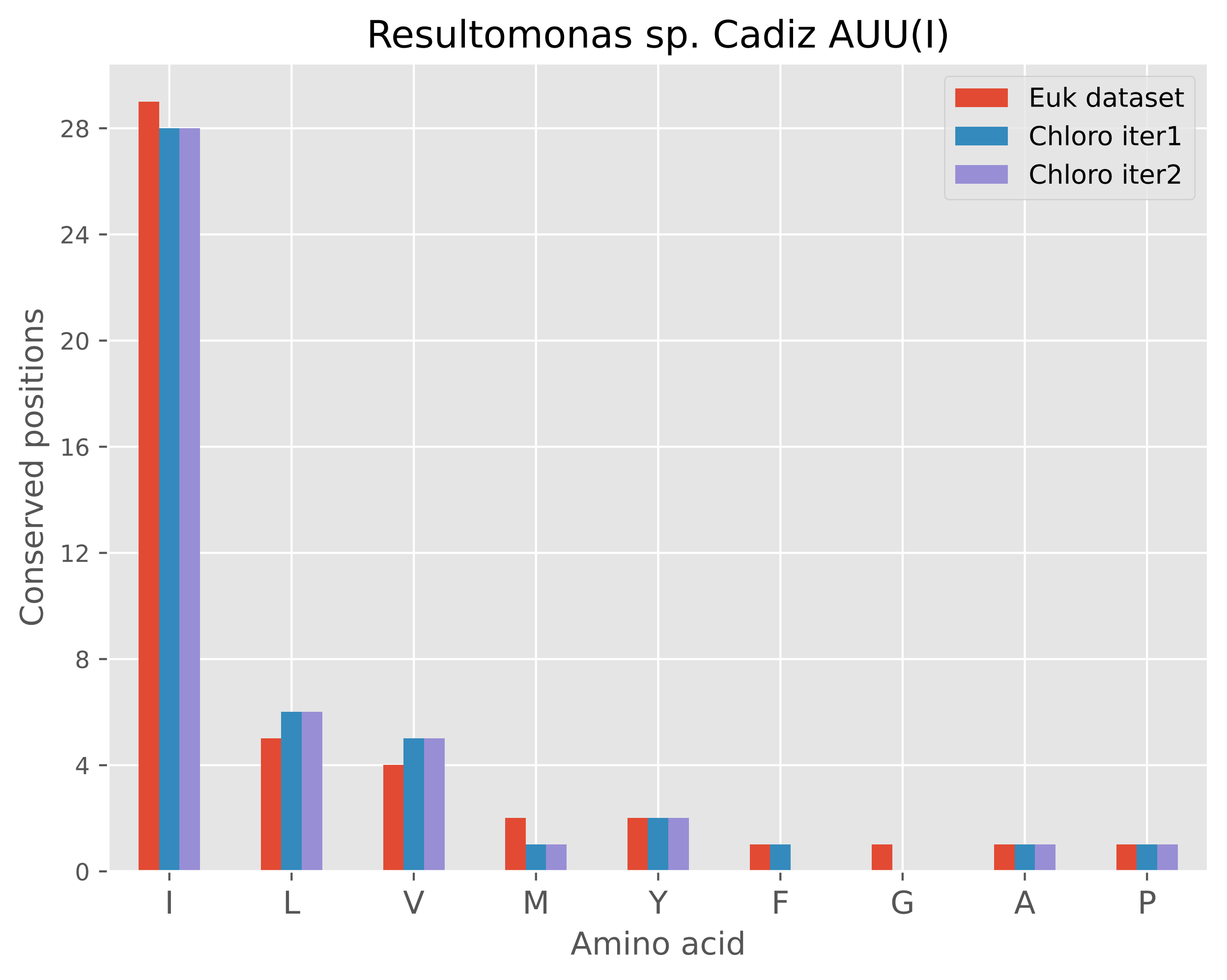

Resultomonas sp. Cadiz AUU(I)
Euk dataset
Chloro iter1
Chloro iter2
Conserved positions
0
4
8
12
16
20
24
28
I
L
V
M
Y
F
G
A
P
Amino acid

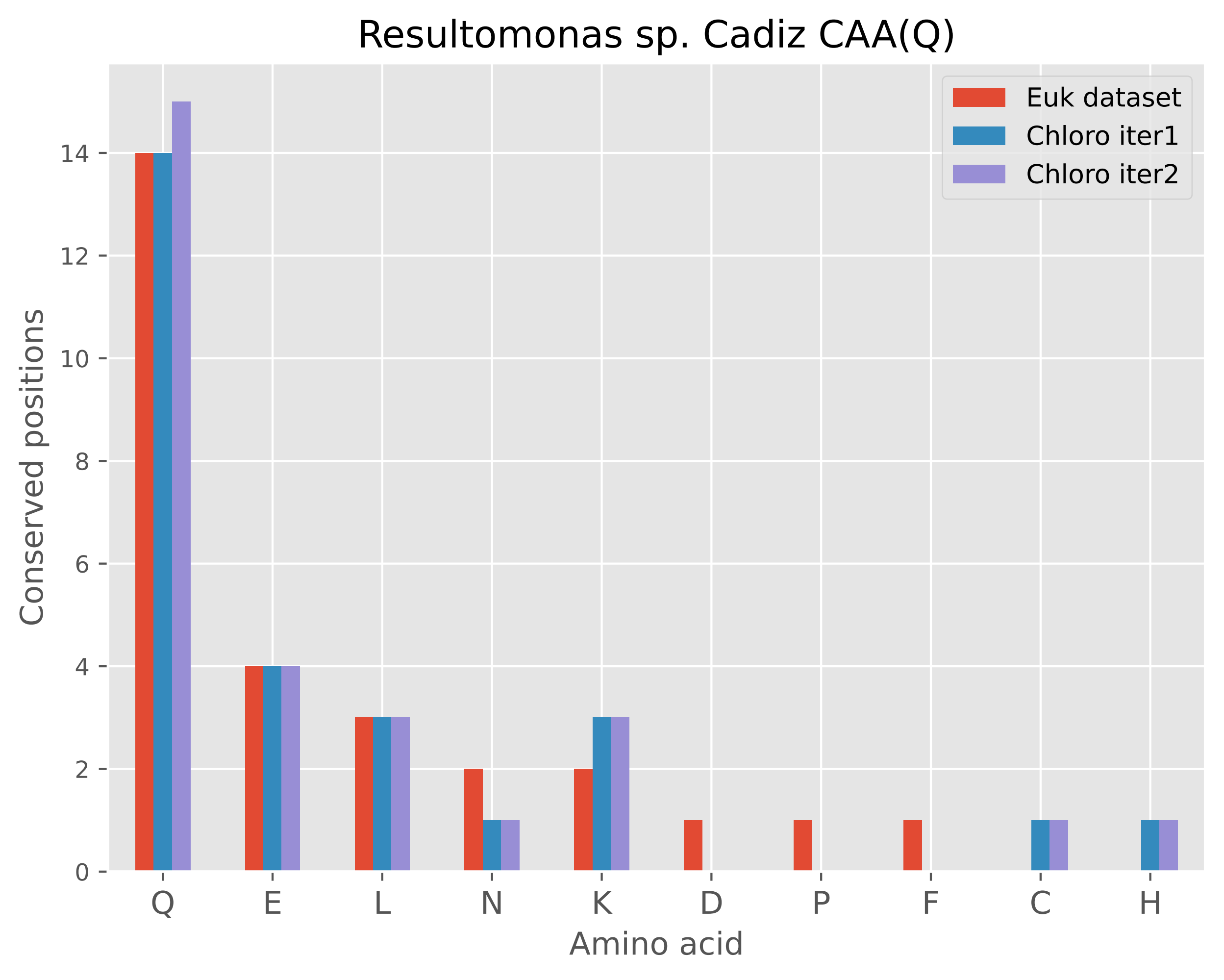

Resultomonas sp. Cadiz CAA(Q)
Euk dataset
Chloro iter1
Chloro iter2
Conserved positions
0
2
4
6
8
10
12
14
Q
E
L
N
K
D
P
F
C
H
Amino acid

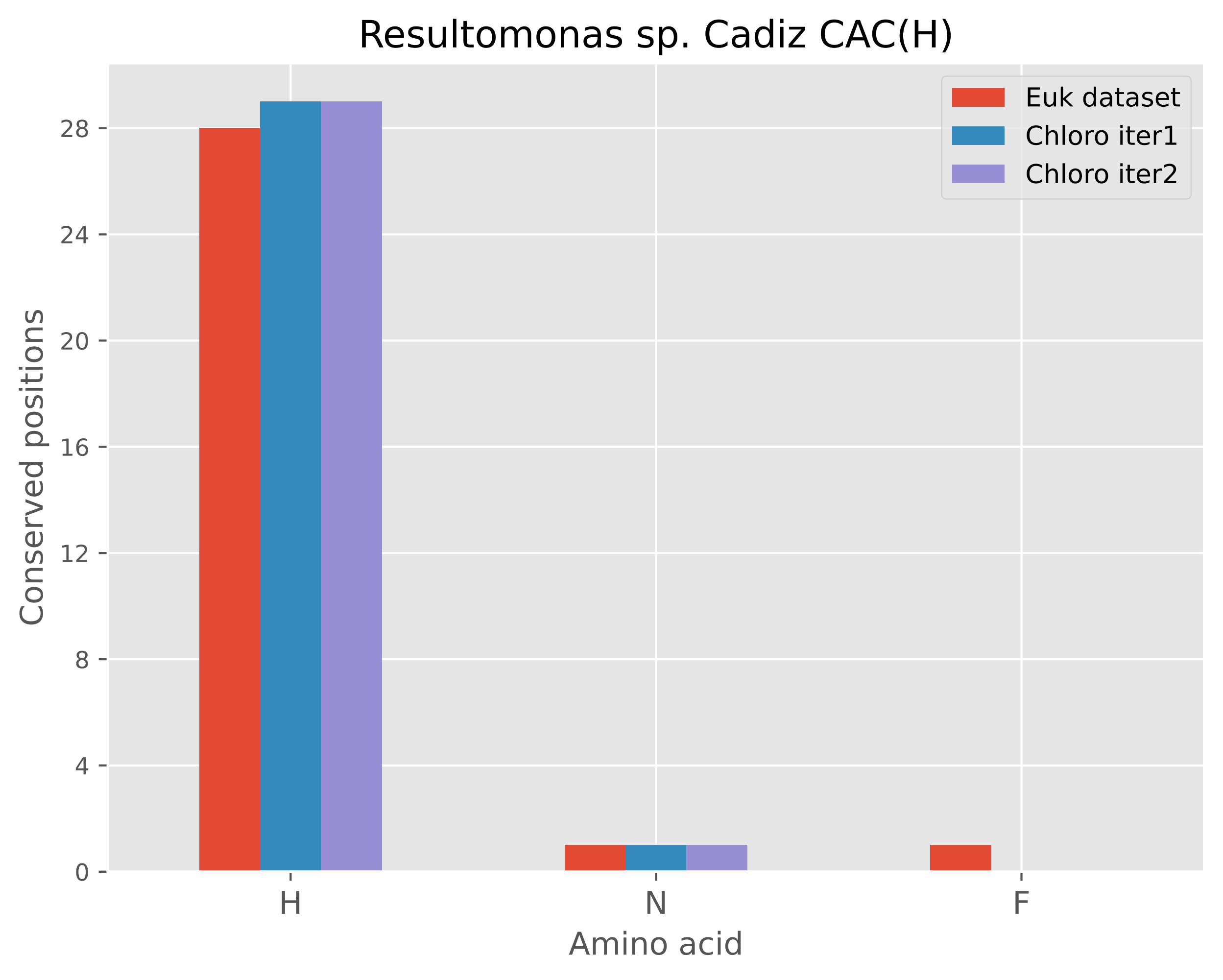

Resultomonas sp. Cadiz CAC(H)
Euk dataset
Chloro iter1
Chloro iter2
Conserved positions
0
4
8
12
16
20
24
28
H
N
F
Amino acid

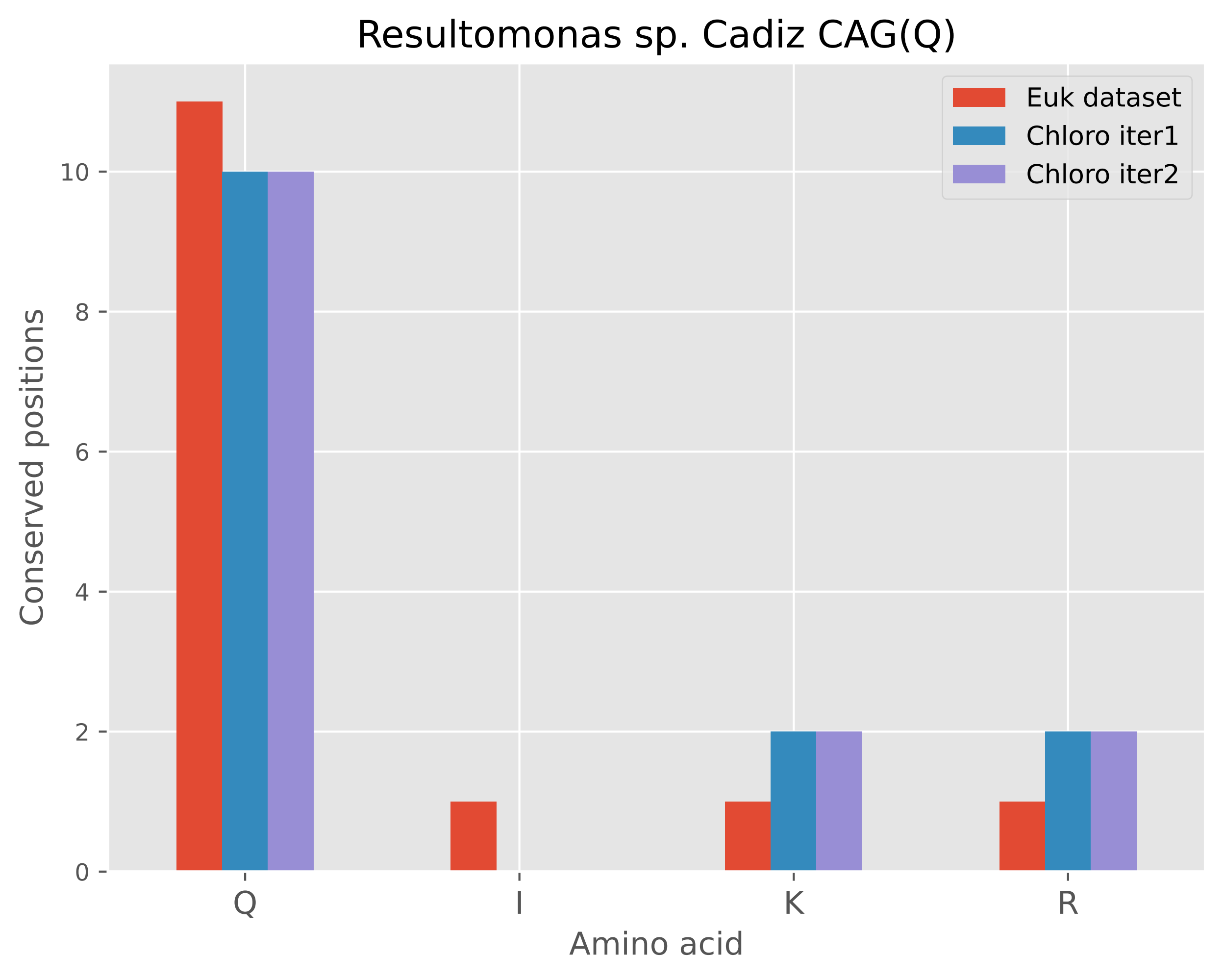

Resultomonas sp. Cadiz CAG(Q)
Euk dataset
Chloro iter1
Chloro iter2
Conserved positions
0
2
4
6
8
10
Q
I
K
R
Amino acid

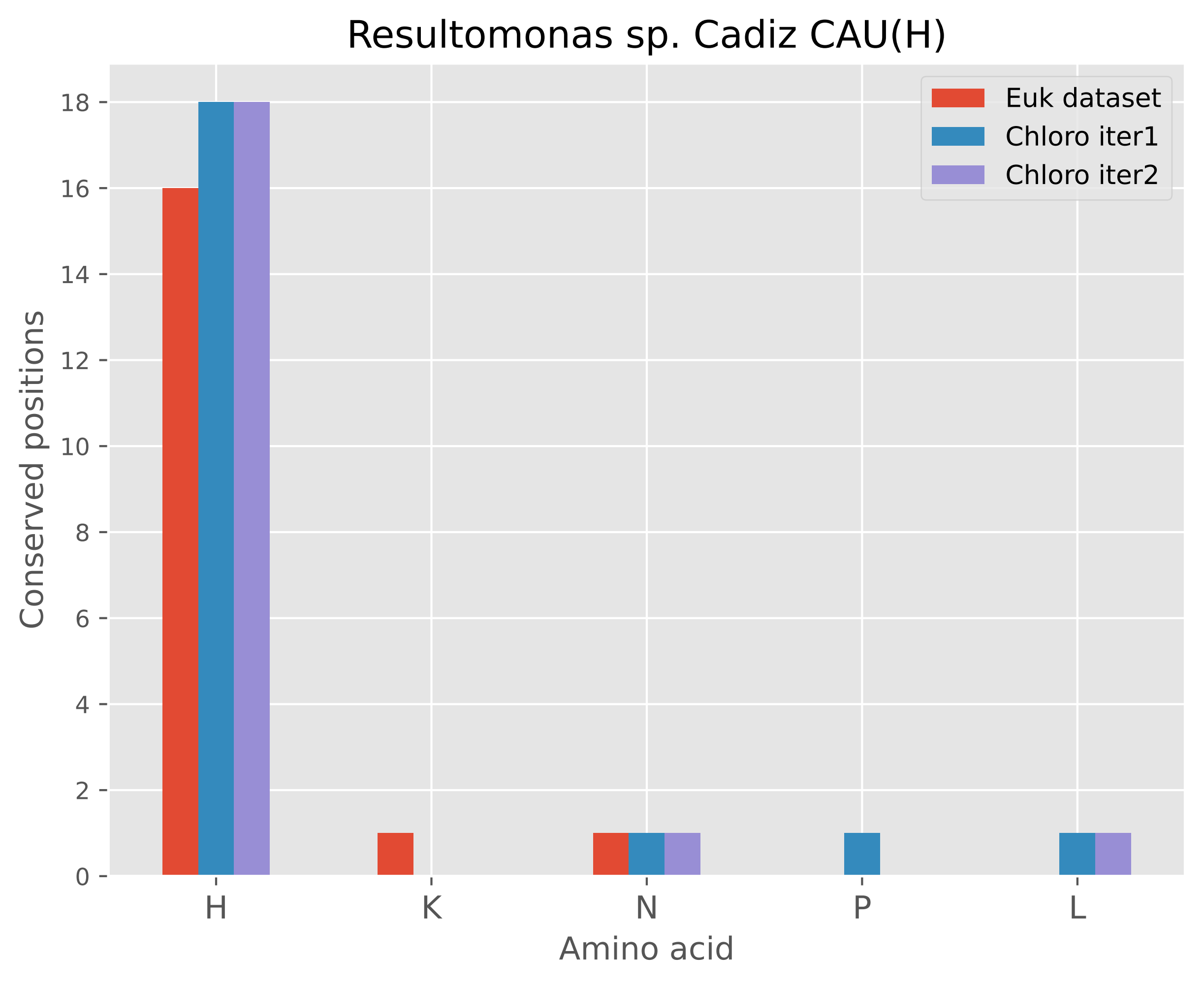
Resultomonas sp. Cadiz CAU(H)
Euk dataset
Chloro iter1
Chloro iter2
Conserved positions
Amino acid
H
K
N
P
L
0
2
4
6
8
10
12
14
16
18

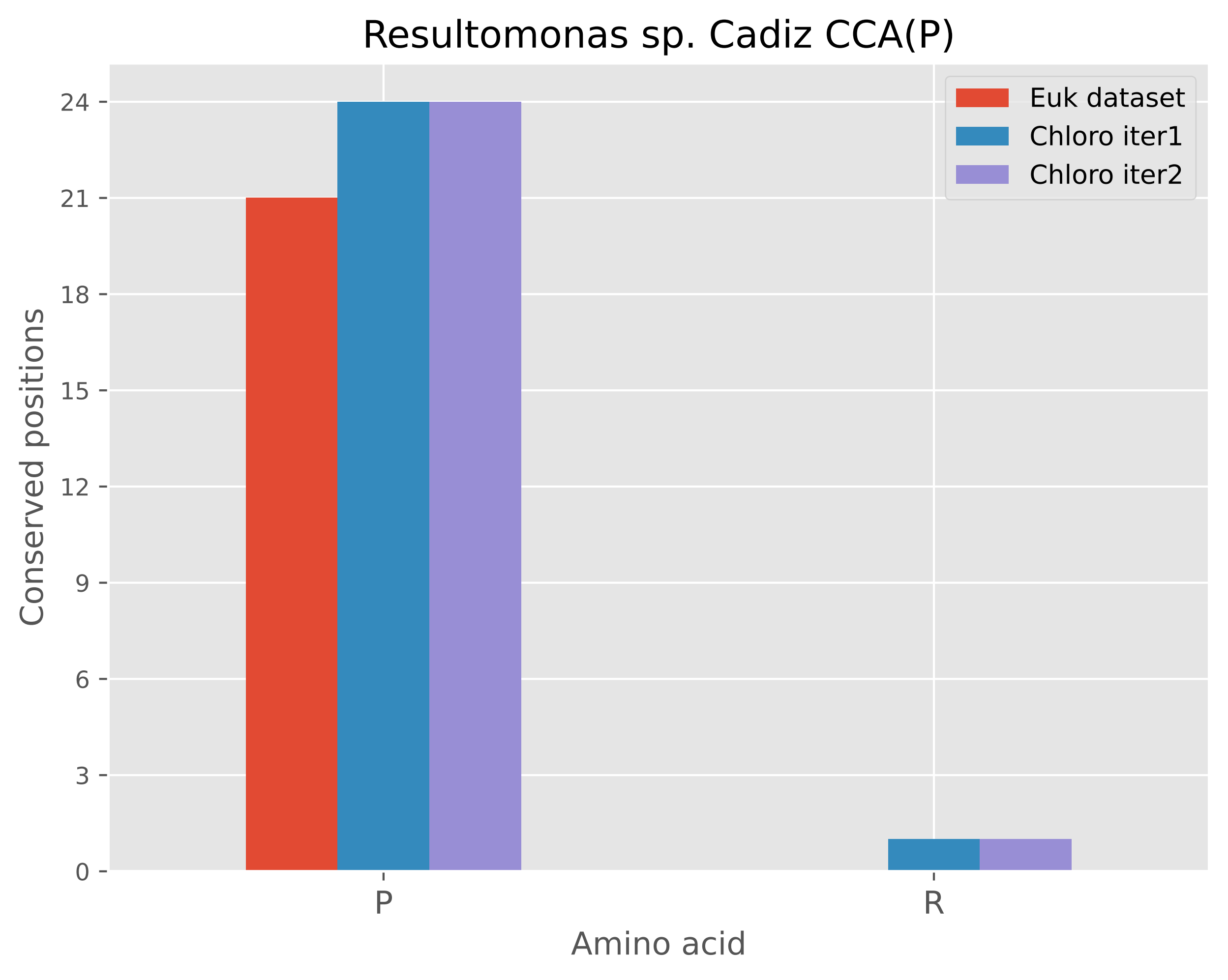

Resultomonas sp. Cadiz CCA(P)
Euk dataset
Chloro iter1
Chloro iter2
Conserved positions
0
3
6
9
12
15
18
21
24
P
R
Amino acid

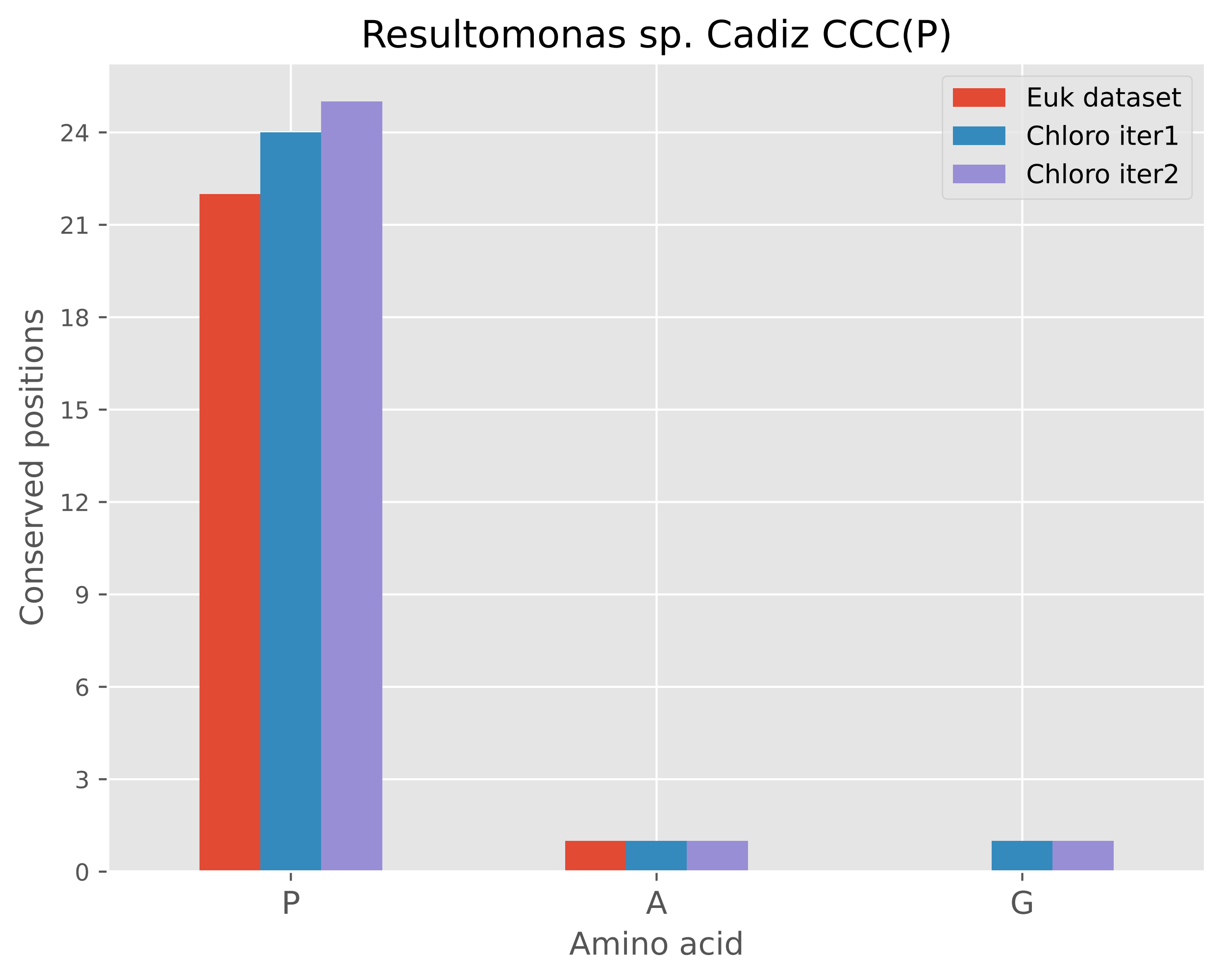

Resultomonas sp. Cadiz CCC(P)
Euk dataset
Chloro iter1
Chloro iter2
Conserved positions
0
3
6
9
12
15
18
21
24
P
A
G
Amino acid

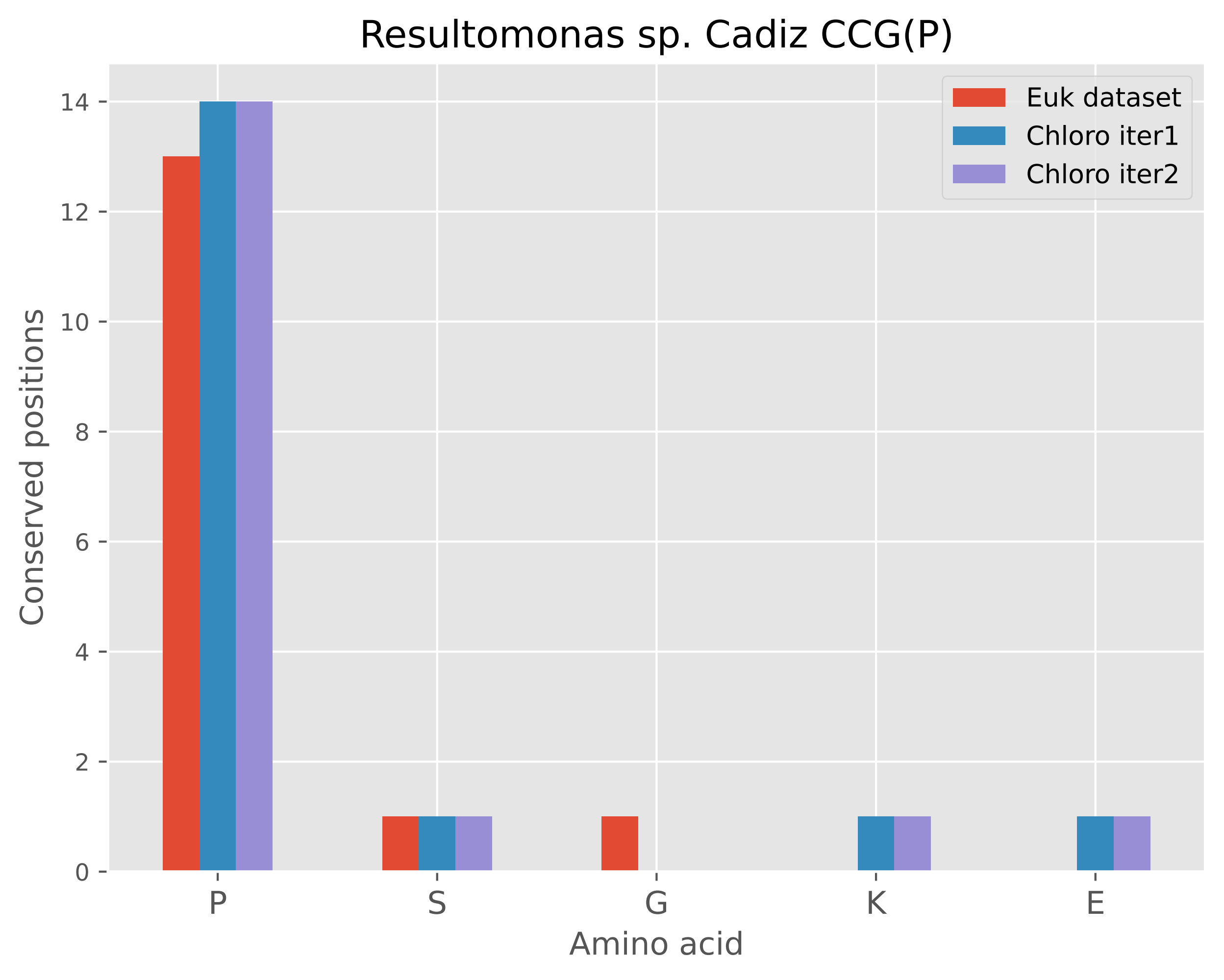

Resultomonas sp. Cadiz CCG(P)
Euk dataset
Chloro iter1
Chloro iter2
Conserved positions
0
2
4
6
8
10
12
14
P
S
G
K
E
Amino acid

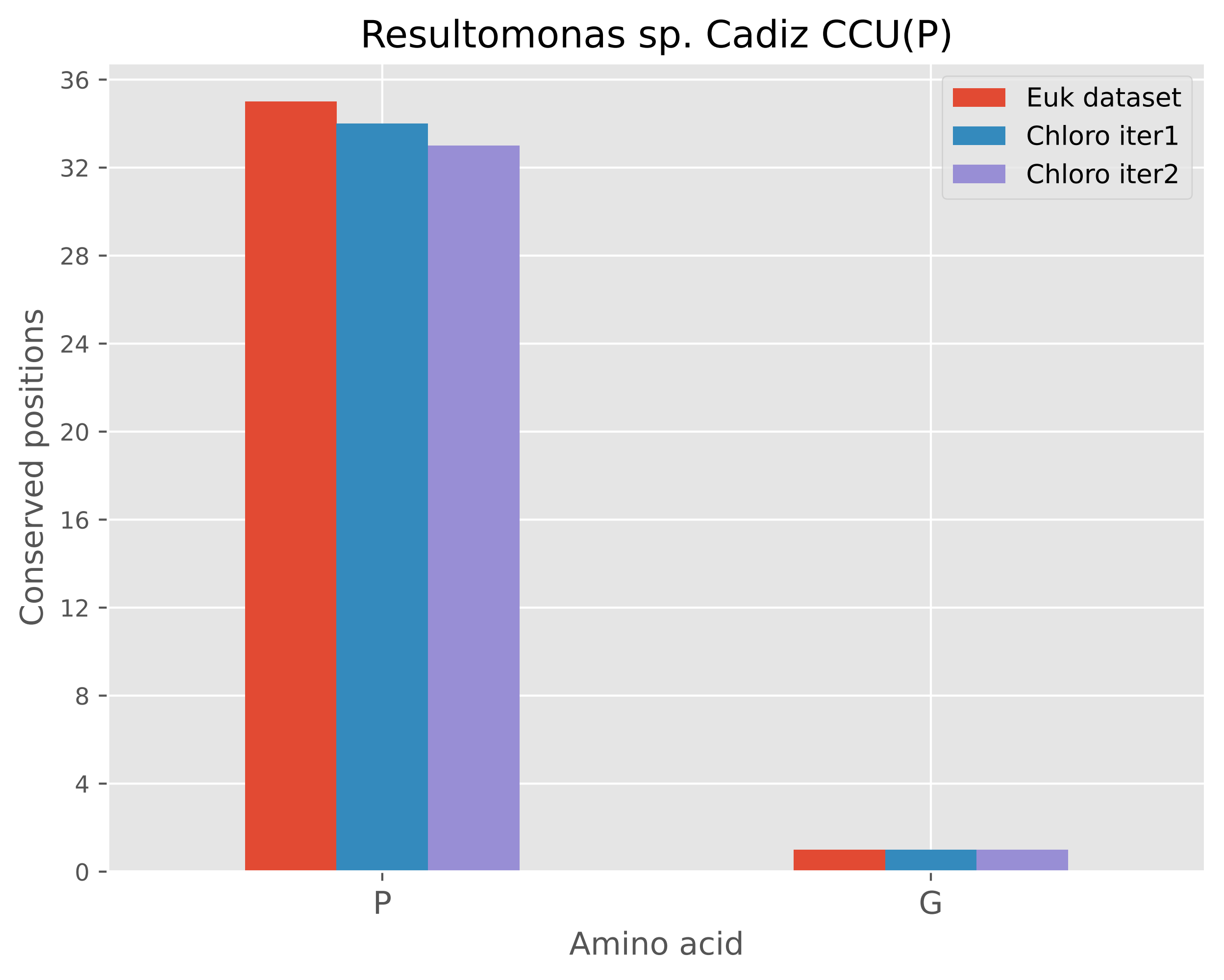
Resultomonas sp. Cadiz CCU(P)
Euk dataset
Chloro iter1
Chloro iter2
Conserved positions
0
4
8
12
16
20
24
28
32
36
P
G
Amino acid

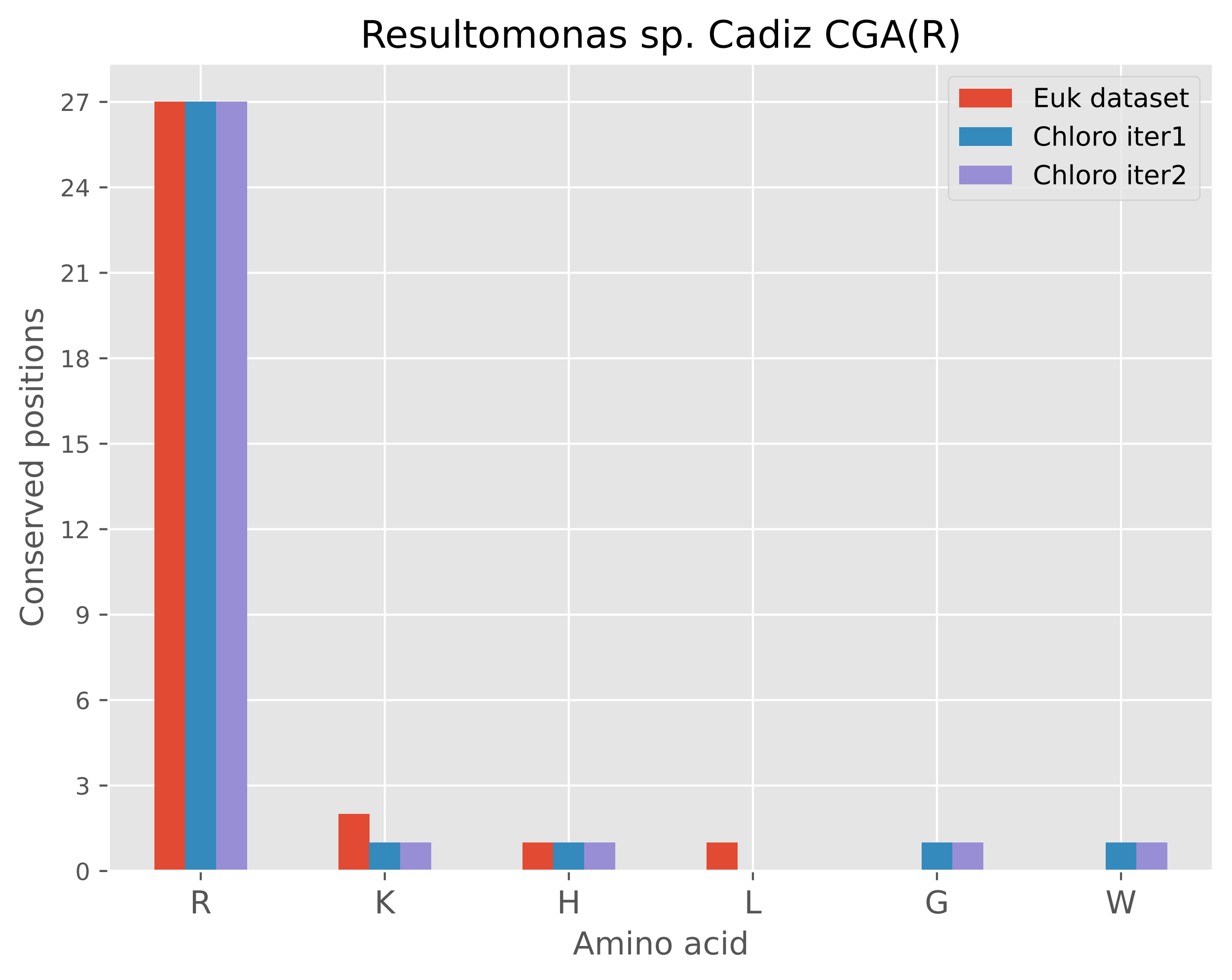
Resultomonas sp. Cadiz CGA(R)
Euk dataset
Chloro iter1
Chloro iter2
Conserved positions
0
3
6
9
12
15
18
21
24
27
R
K
H
L
G
W
Amino acid

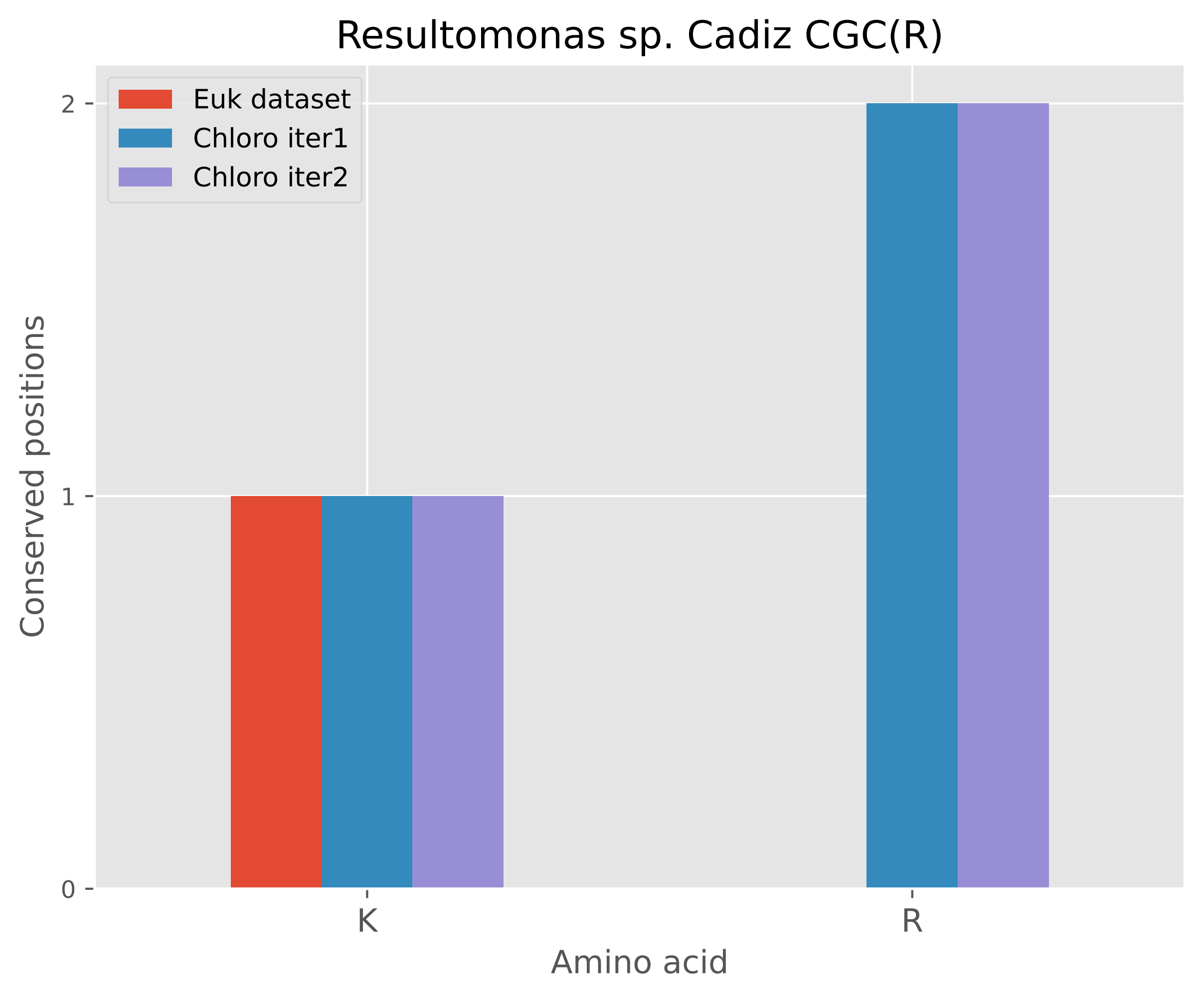
Resultomonas sp. Cadiz CGC(R)
Euk dataset
Chloro iter1
Chloro iter2
Conserved positions
2
1
0
K
R
Amino acid

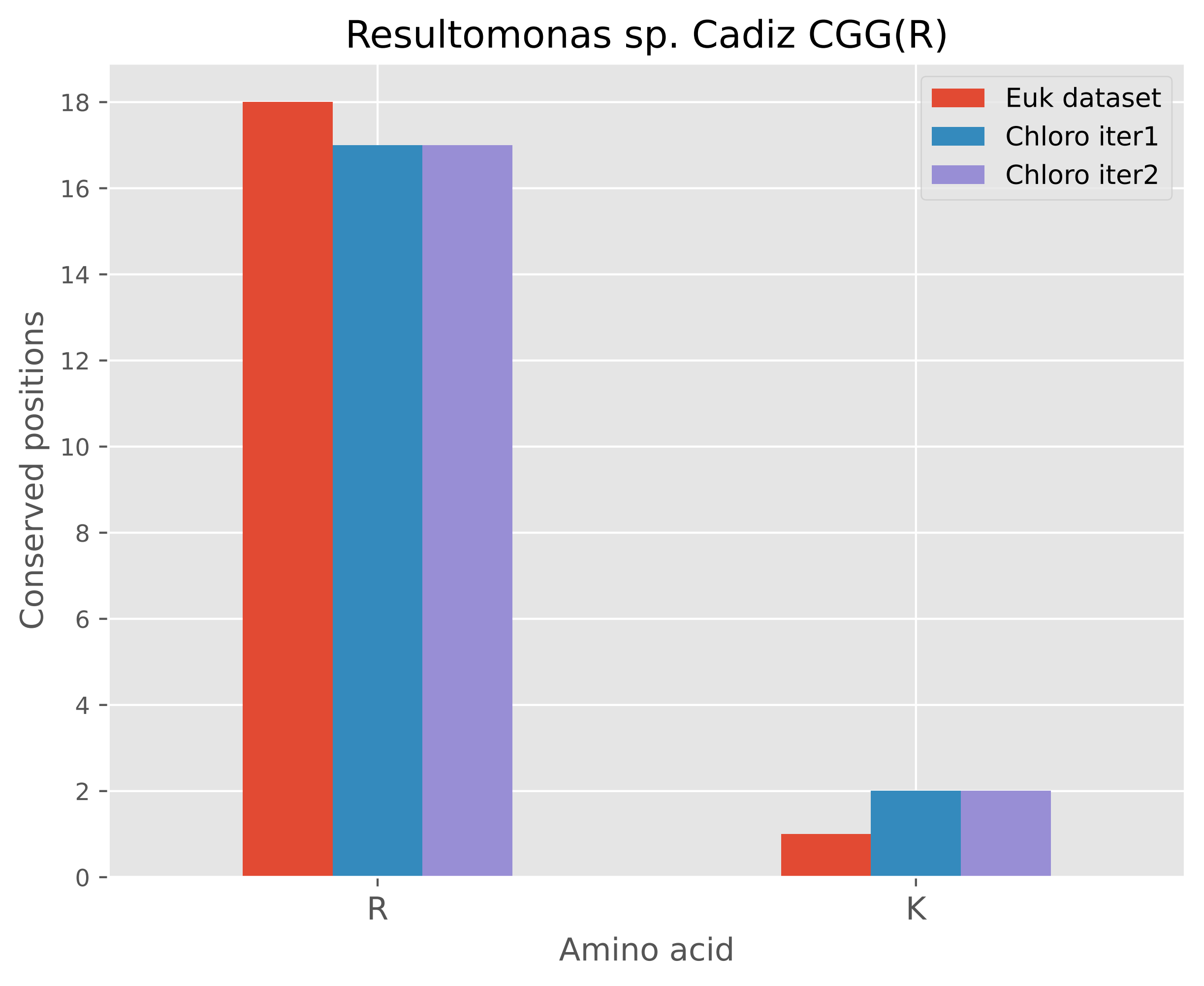

Resultomonas sp. Cadiz CGG(R)
Euk dataset
Chloro iter1
Chloro iter2
Conserved positions
0
2
4
6
8
10
12
14
16
18
R
K
Amino acid

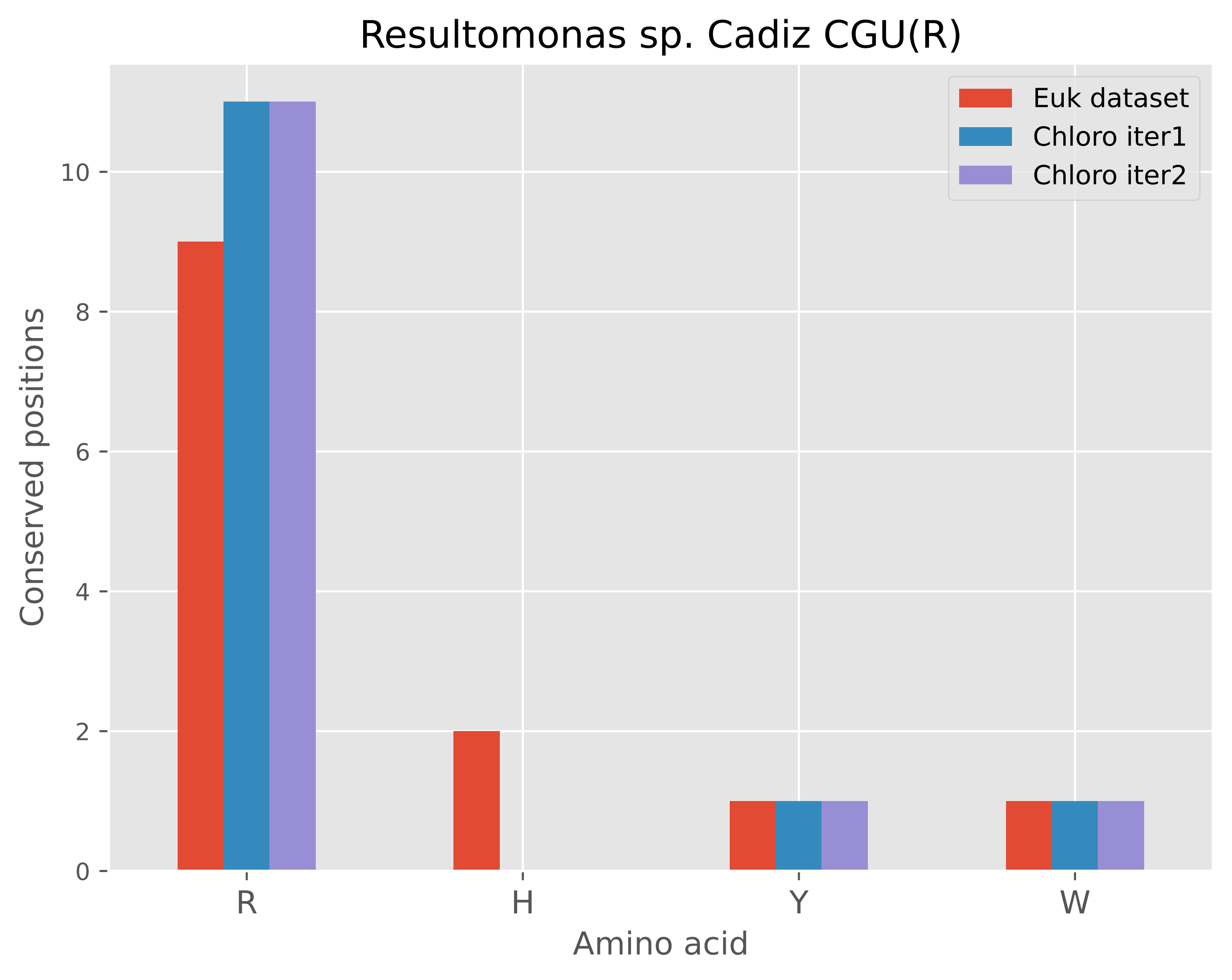
Resultomonas sp. Cadiz CGU(R)
Euk dataset
Chloro iter1
Chloro iter2
Conserved positions
0
2
4
6
8
10
R
H
Y
W
Amino acid

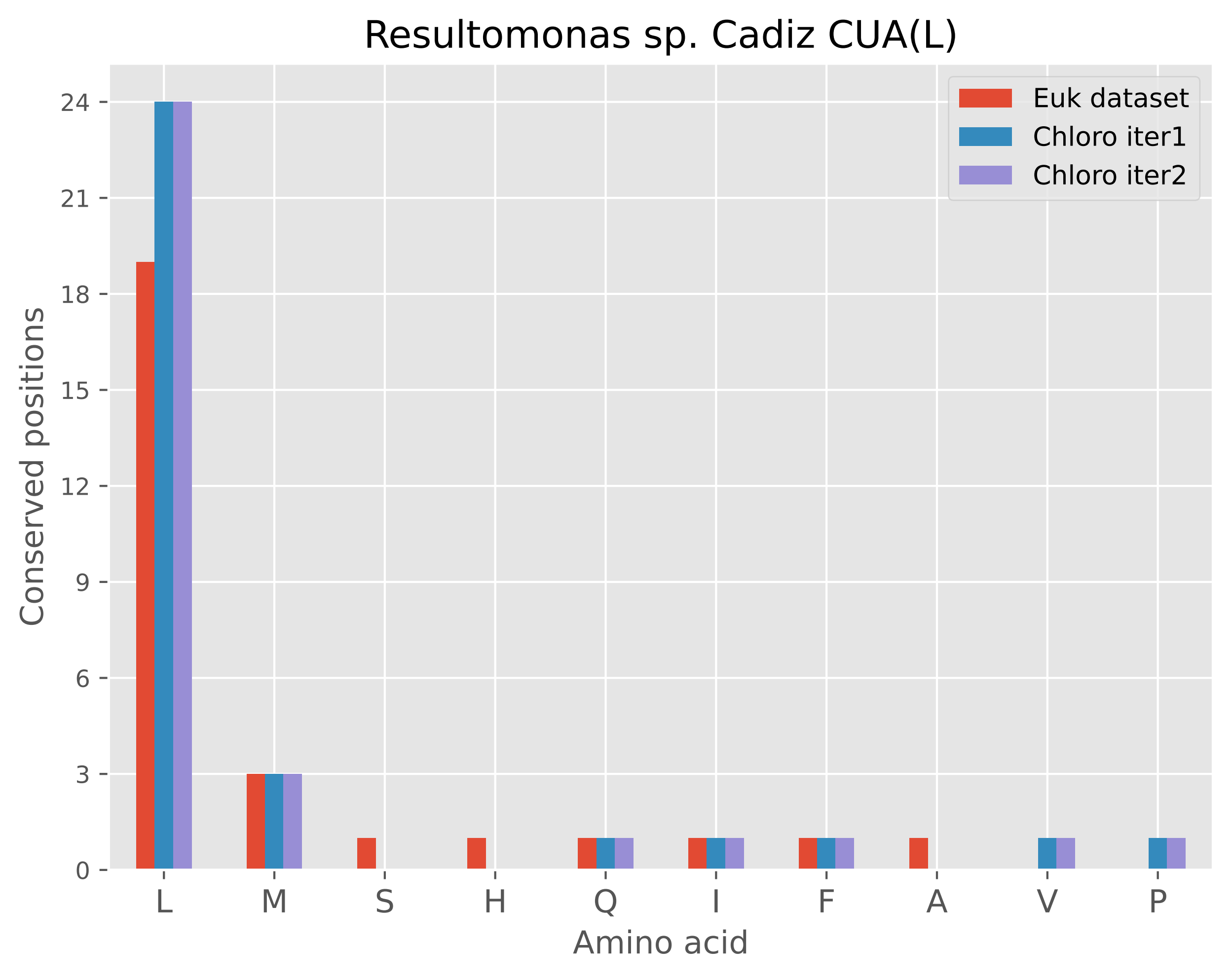
Resultomonas sp. Cadiz CUA(L)
Euk dataset
Chloro iter1
Chloro iter2
Conserved positions
0
3
6
9
12
15
18
21
24
L
M
S
H
Q
I
F
A
V
P
Amino acid

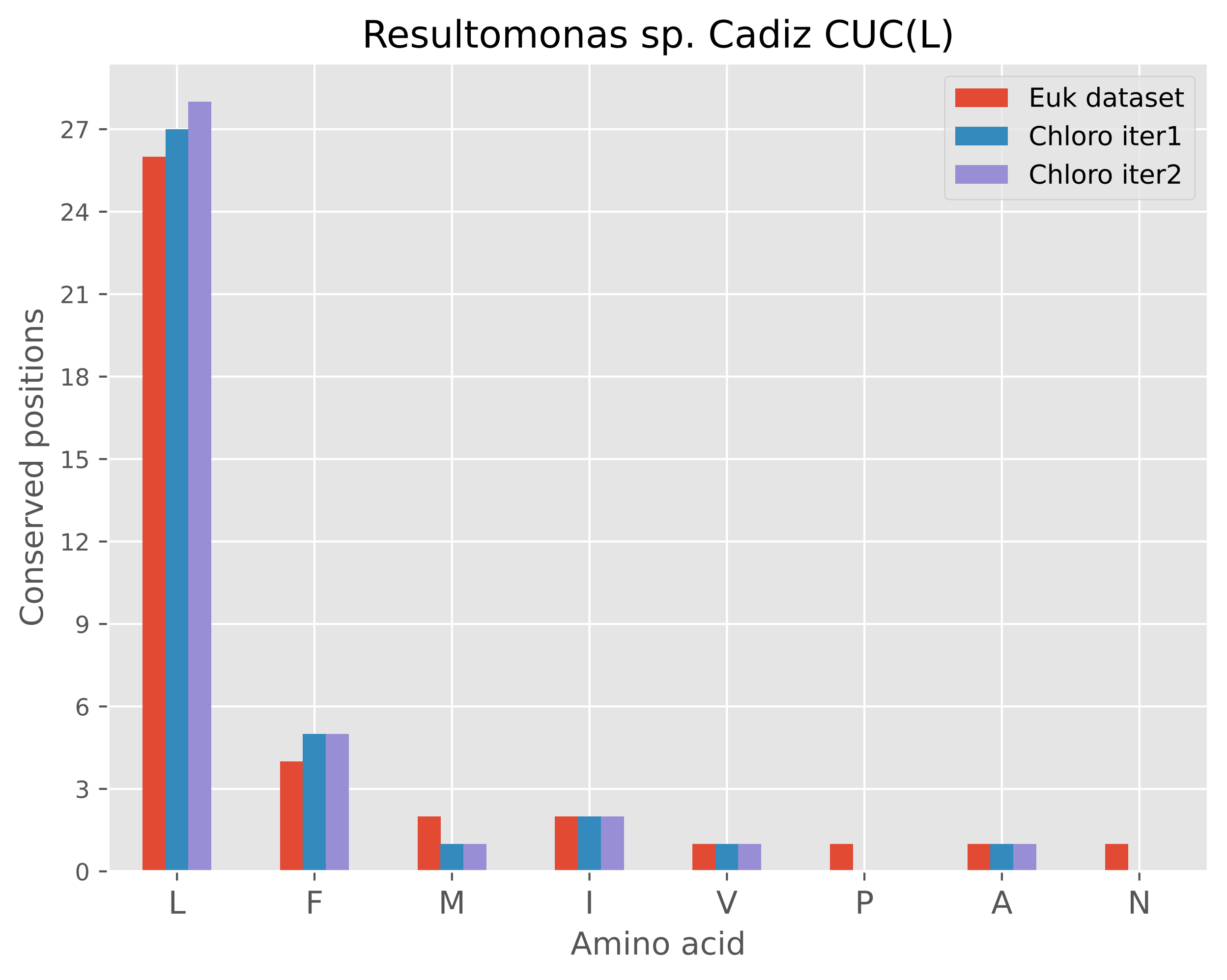

Resultomonas sp. Cadiz CUC(L)
Euk dataset
Chloro iter1
Chloro iter2
Conserved positions
0
3
6
9
12
15
18
21
24
27
L
F
M
I
V
P
A
N
Amino acid

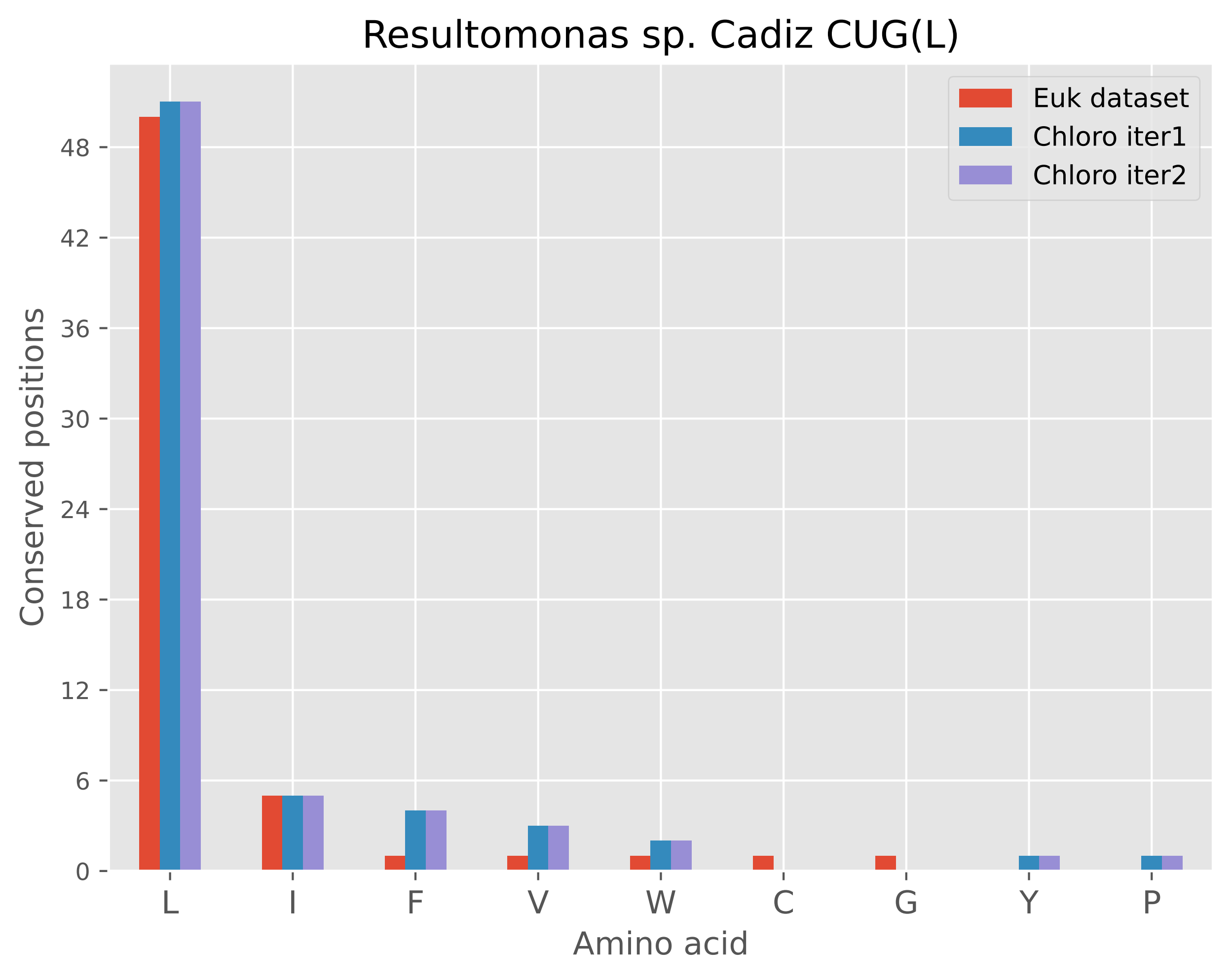
Resultomonas sp. Cadiz CUG(L)
Euk dataset
Chloro iter1
Chloro iter2
Conserved positions
0
6
12
18
24
30
36
42
48
L
I
F
V
W
C
G
Y
P
Amino acid

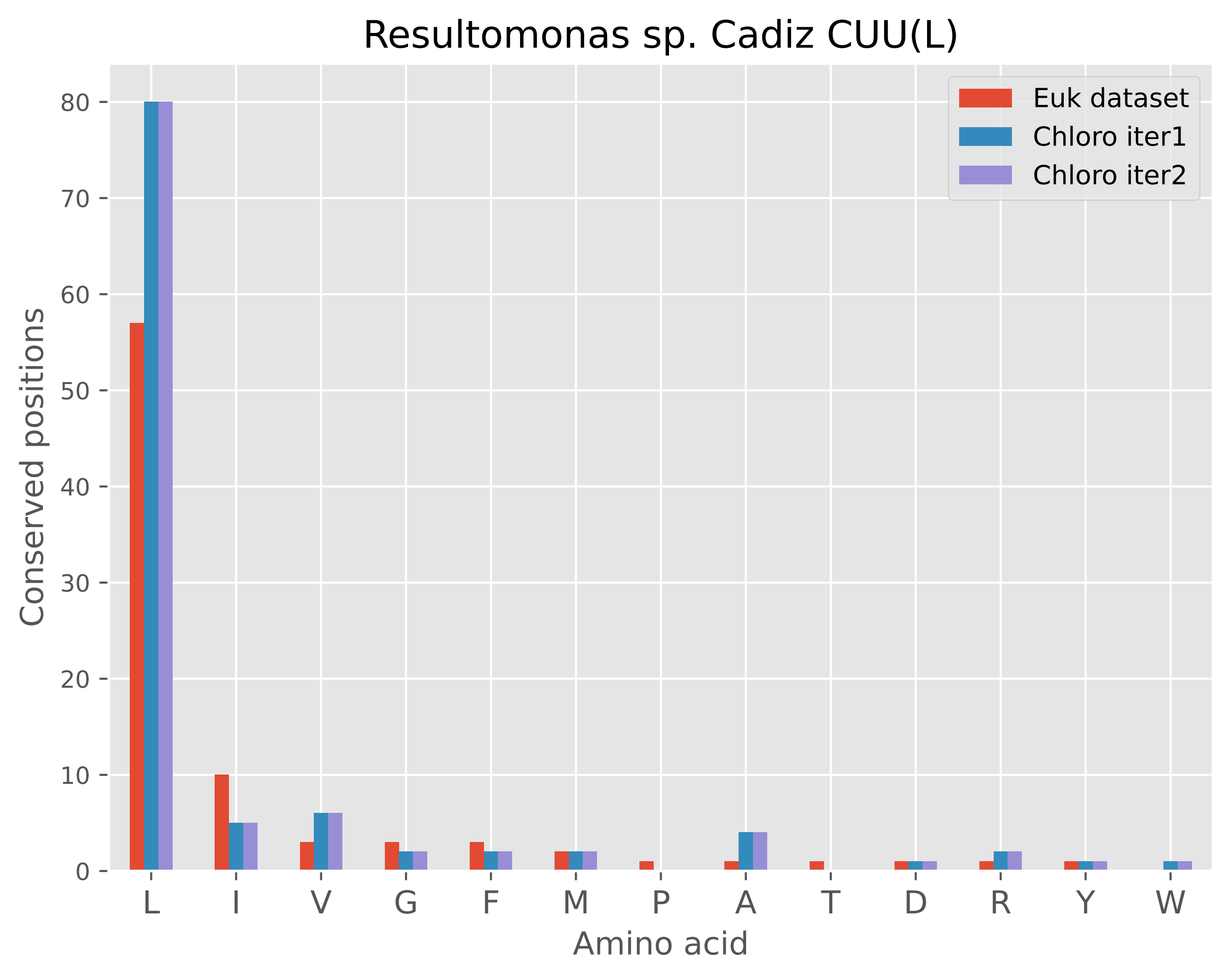

Resultomonas sp. Cadiz CUU(L)
Euk dataset
Chloro iter1
Chloro iter2
Conserved positions
0
10
20
30
40
50
60
70
80
L
I
V
G
F
M
P
A
T
D
R
Y
W
Amino acid

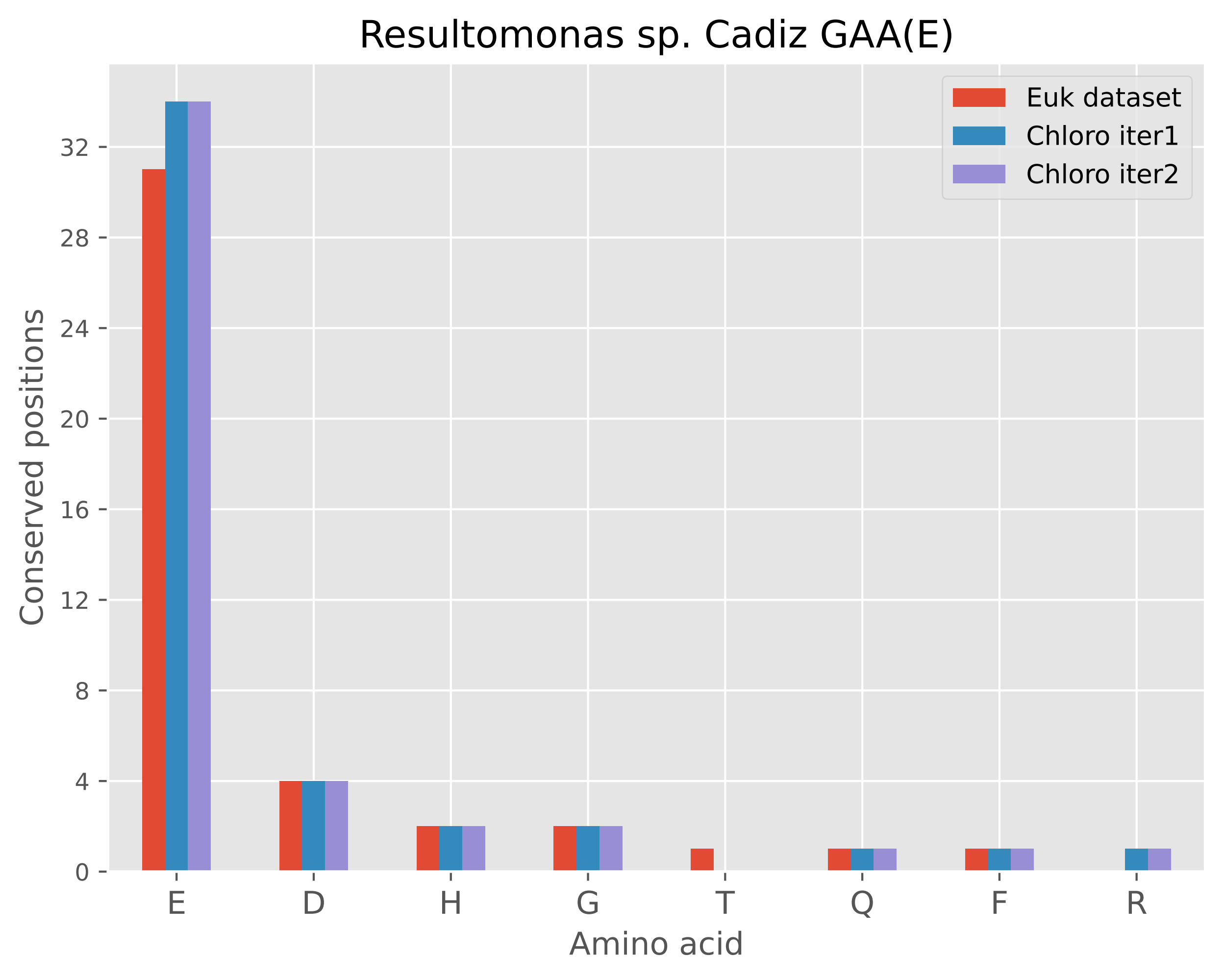

Resultomonas sp. Cadiz GAA(E)
Euk dataset
Chloro iter1
Chloro iter2
Conserved positions
0
4
8
12
16
20
24
28
32
E
D
H
G
T
Q
F
R
Amino acid

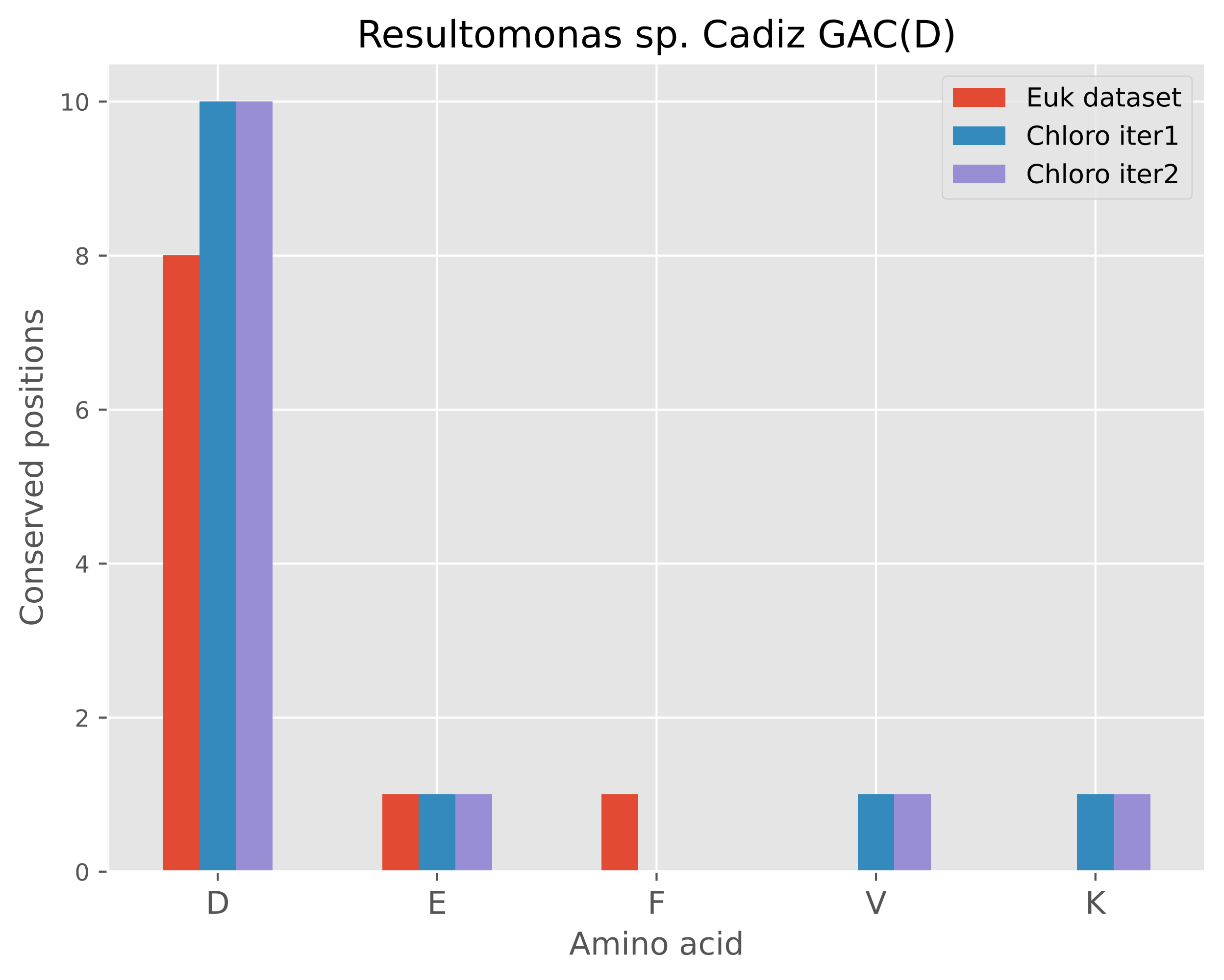

Resultomonas sp. Cadiz GAC(D)
Euk dataset
Chloro iter1
Chloro iter2
Conserved positions
0
2
4
6
8
10
D
E
F
V
K
Amino acid

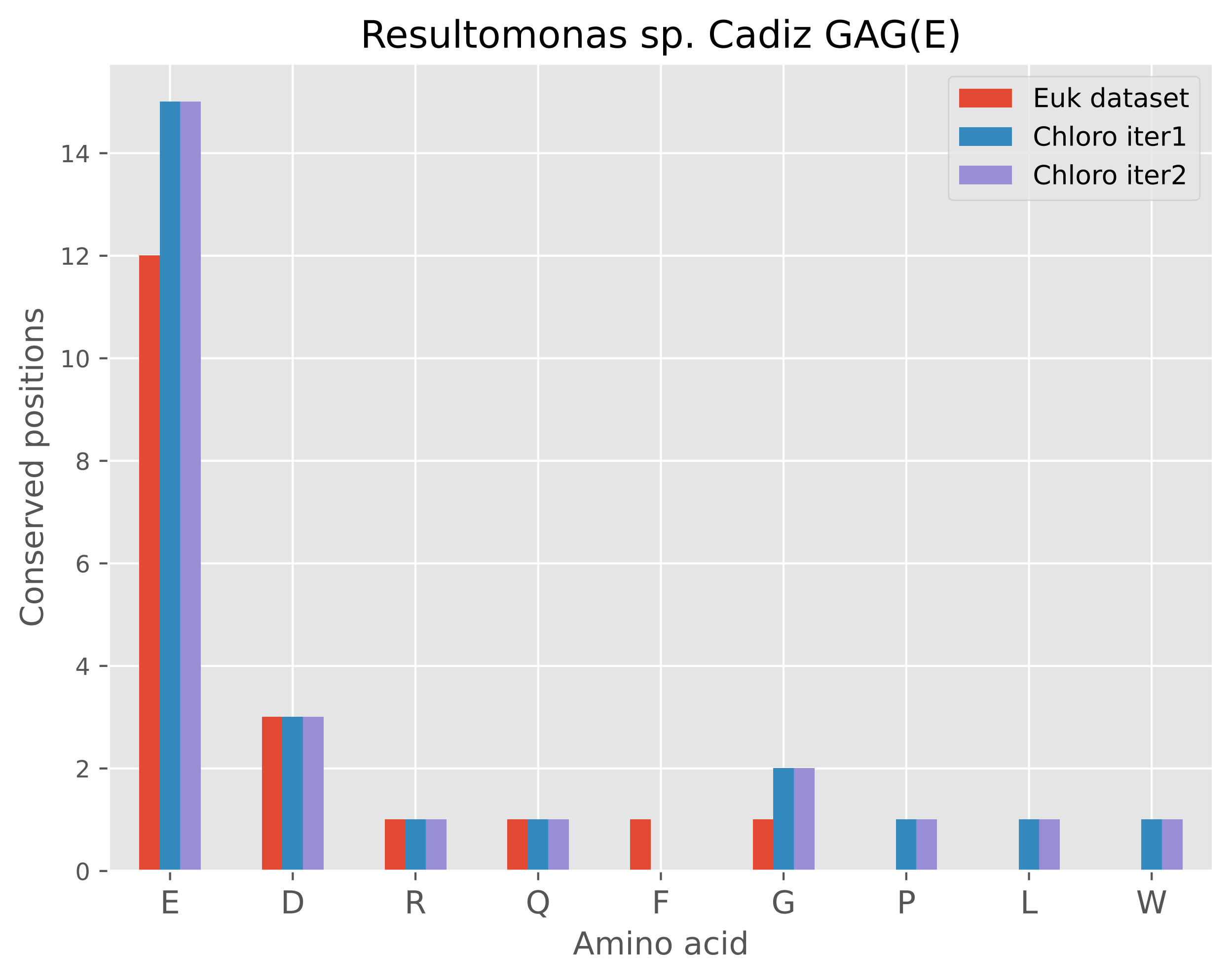

Resultomonas sp. Cadiz GAG(E)
Euk dataset
Chloro iter1
Chloro iter2
Conserved positions
0
2
4
6
8
10
12
14
E
D
R
Q
F
G
P
L
W
Amino acid

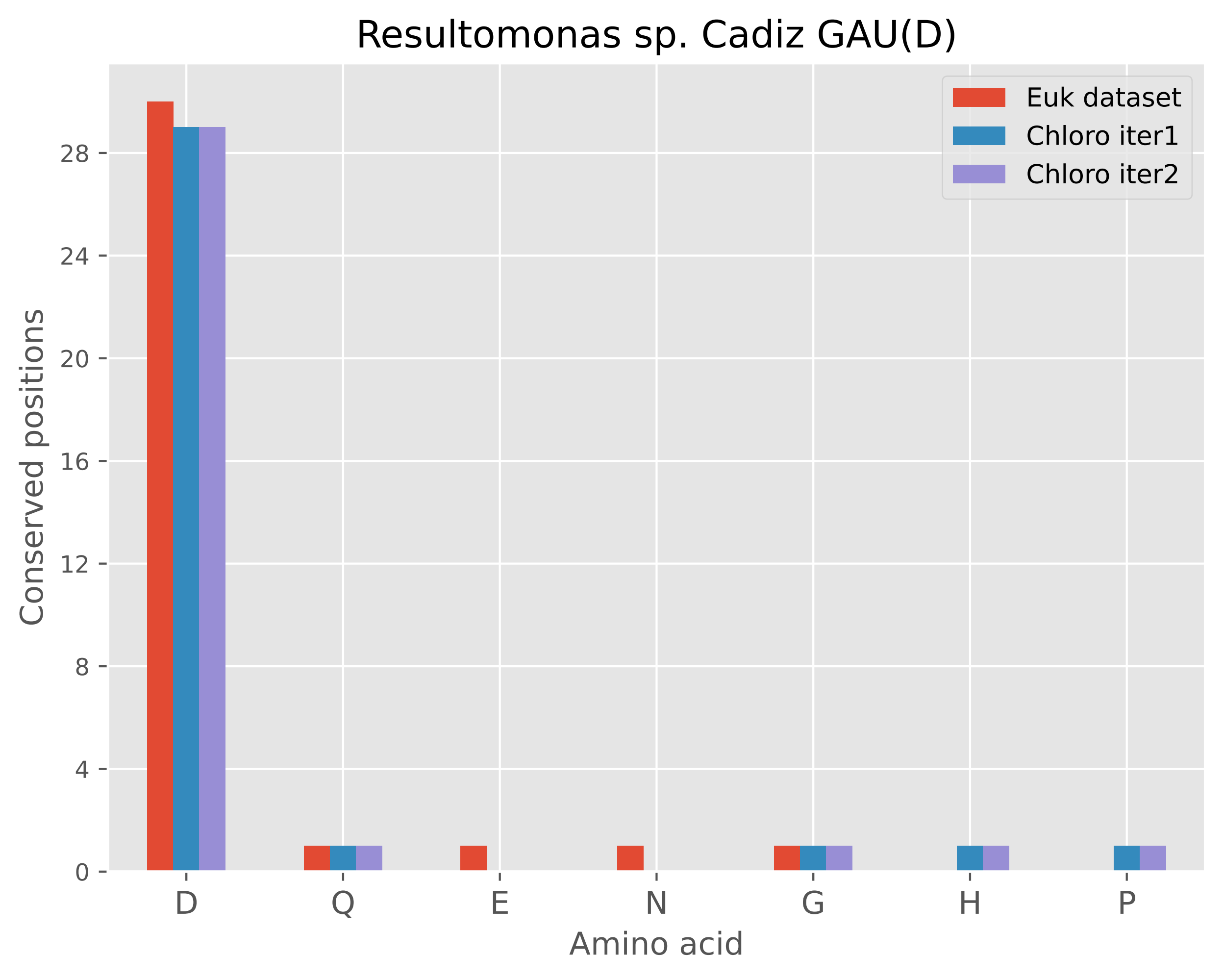
Resultomonas sp. Cadiz GAU(D)
Euk dataset
Chloro iter1
Chloro iter2
Conserved positions
0
4
8
12
16
20
24
28
D
Q
E
N
G
H
P
Amino acid

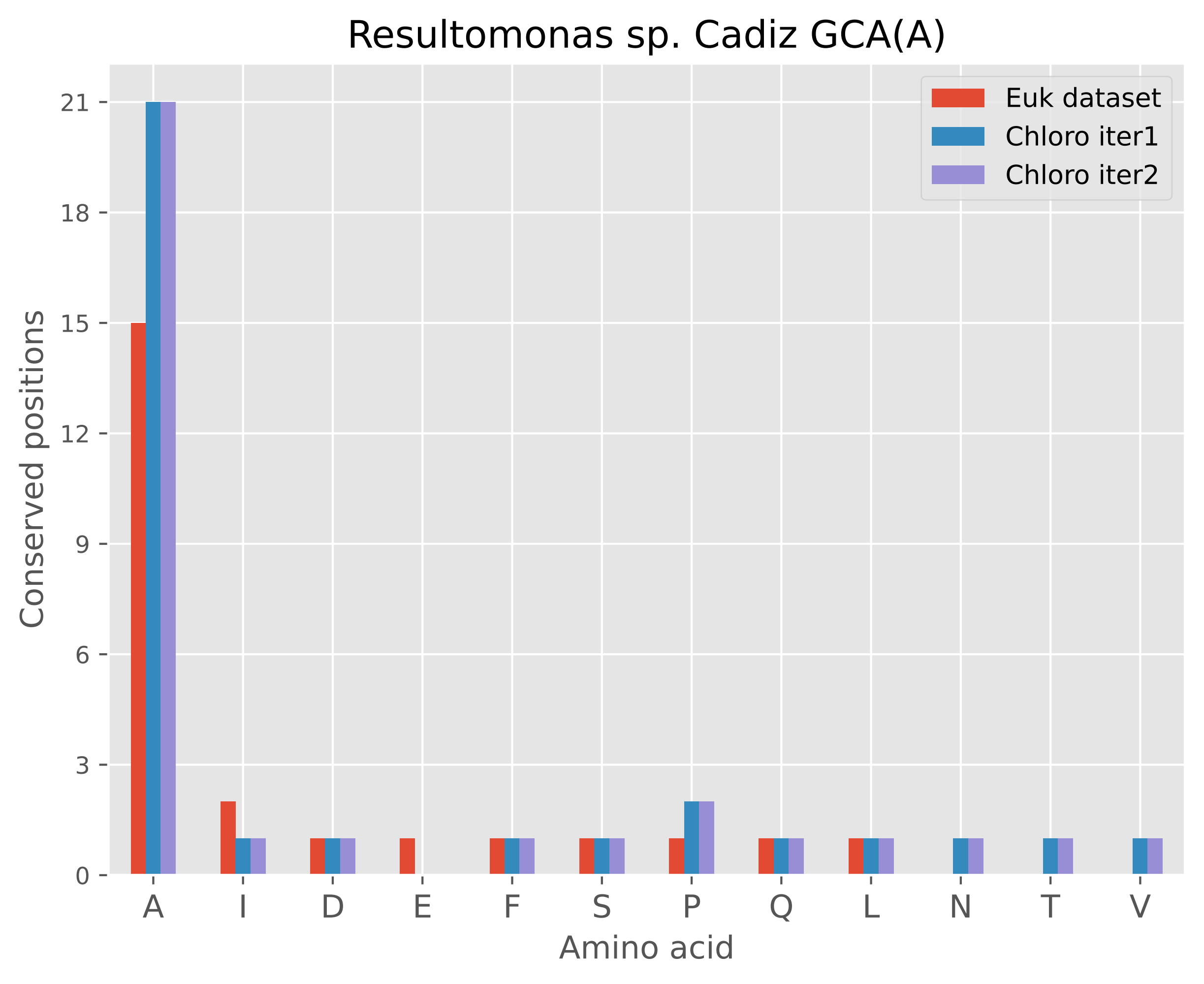

Resultomonas sp. Cadiz GCA(A)
Euk dataset
Chloro iter1
Chloro iter2
Conserved positions
0
3
6
9
12
15
18
21
A
I
D
E
F
S
P
Q
L
N
T
V
Amino acid

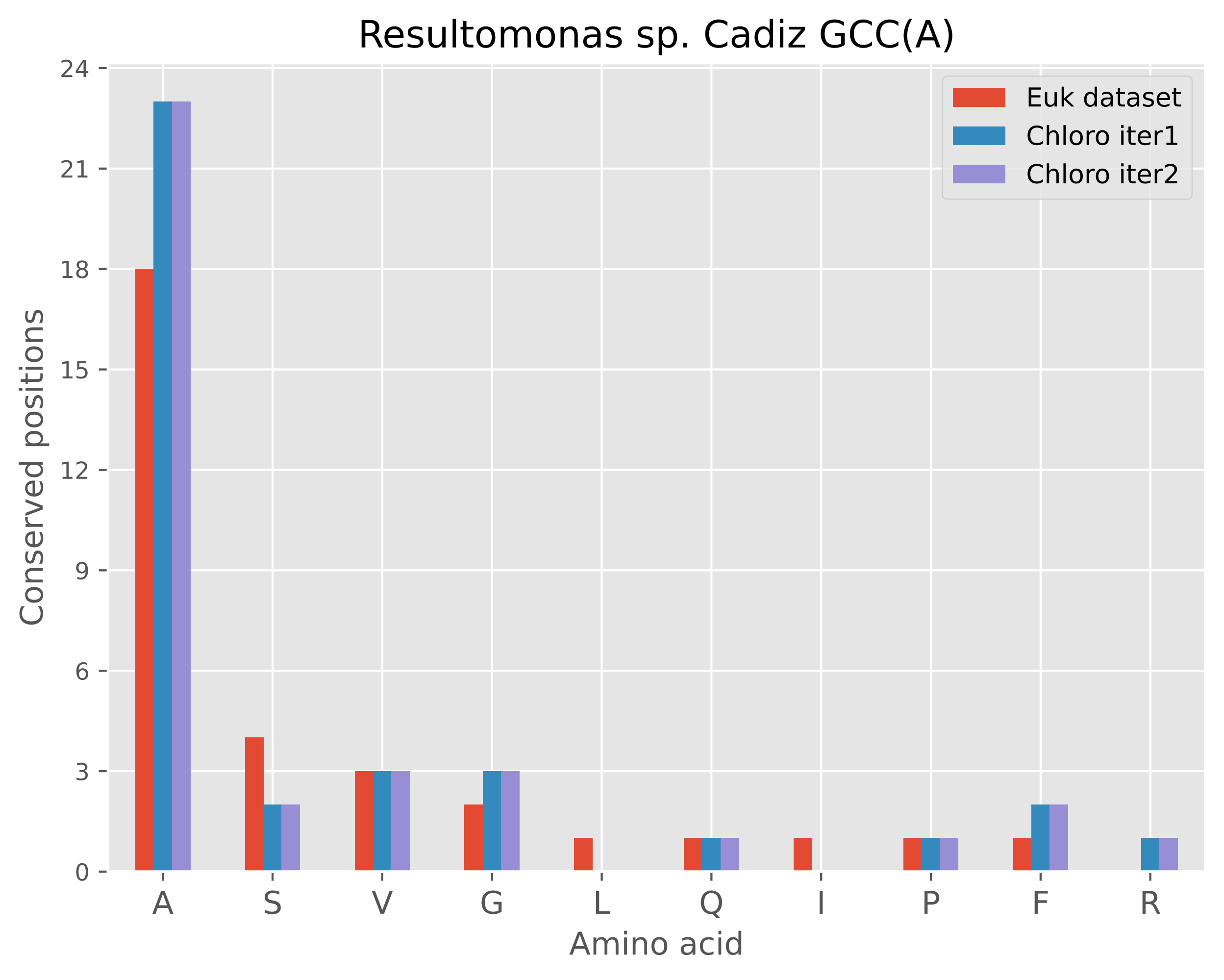

Resultomonas sp. Cadiz GCC(A)
Euk dataset
Chloro iter1
Chloro iter2
Conserved positions
0
3
6
9
12
15
18
21
24
A
S
V
G
L
Q
I
P
F
R
Amino acid

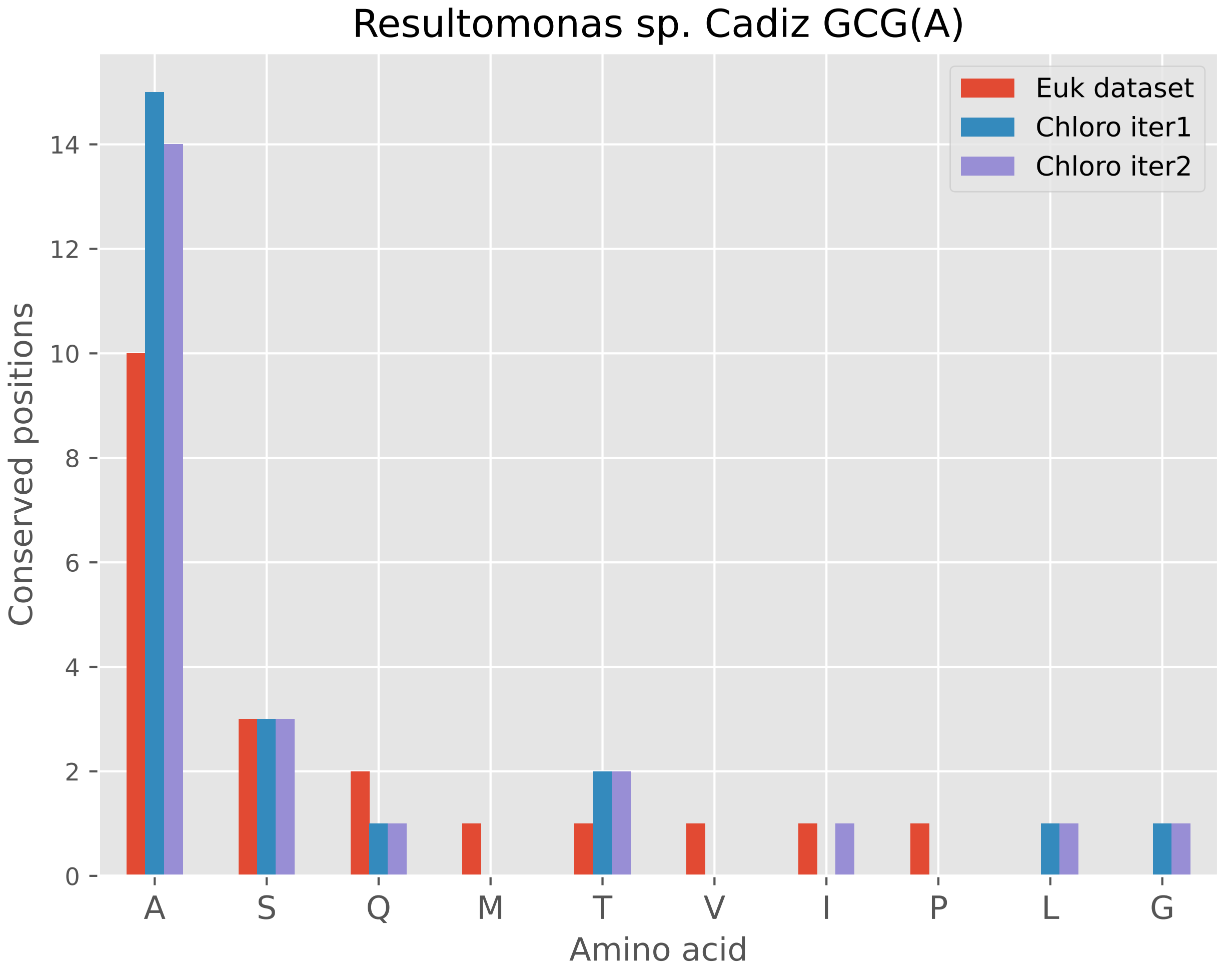

Resultomonas sp. Cadiz GCG(A)
Euk dataset
Chloro iter1
Chloro iter2
Conserved positions
0
2
4
6
8
10
12
14
A
S
Q
M
T
V
I
P
L
G
Amino acid

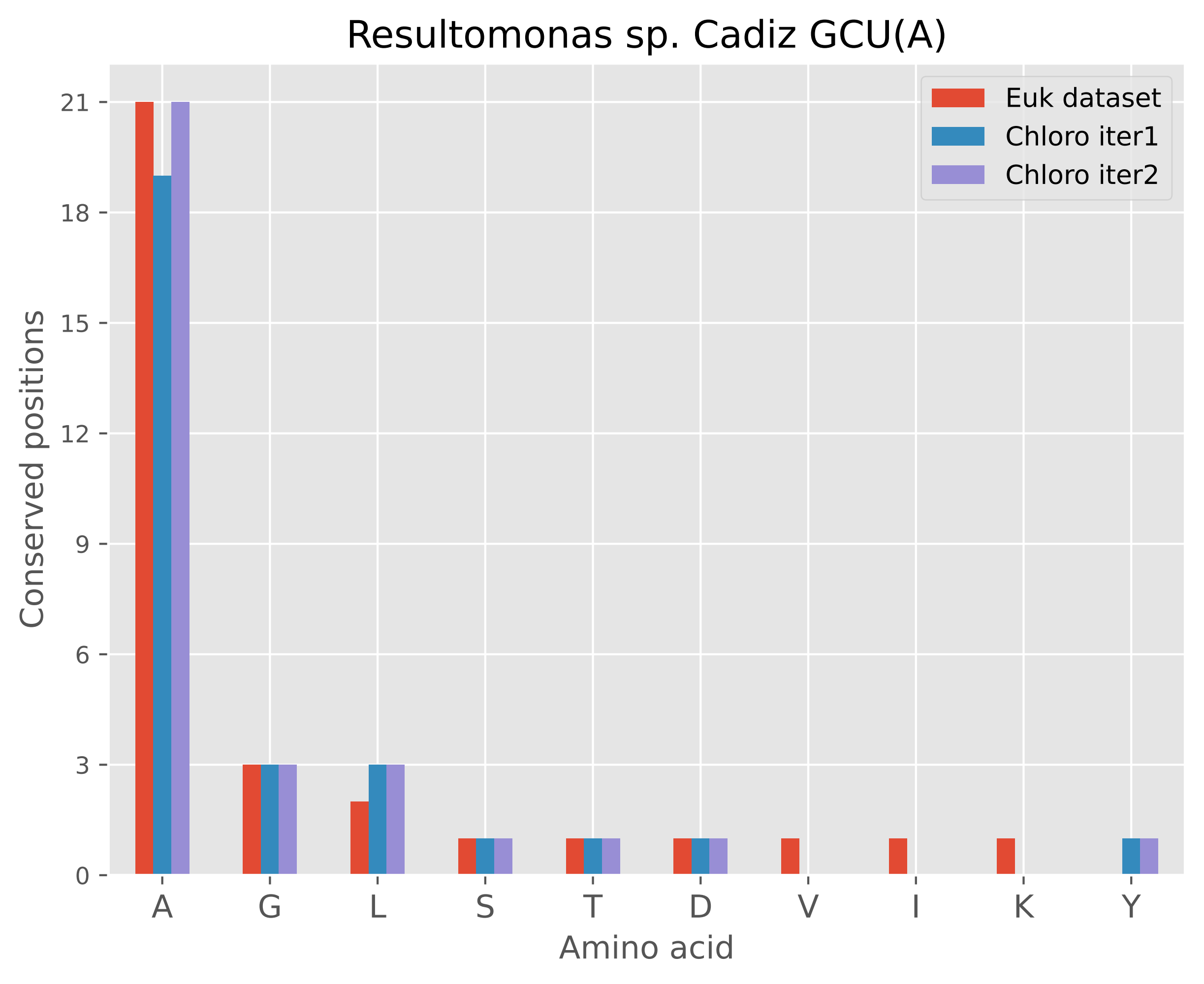

Resultomonas sp. Cadiz GCU(A)
Euk dataset
Chloro iter1
Chloro iter2
Conserved positions
0
3
6
9
12
15
18
21
A
G
L
S
T
D
V
I
K
Y
Amino acid

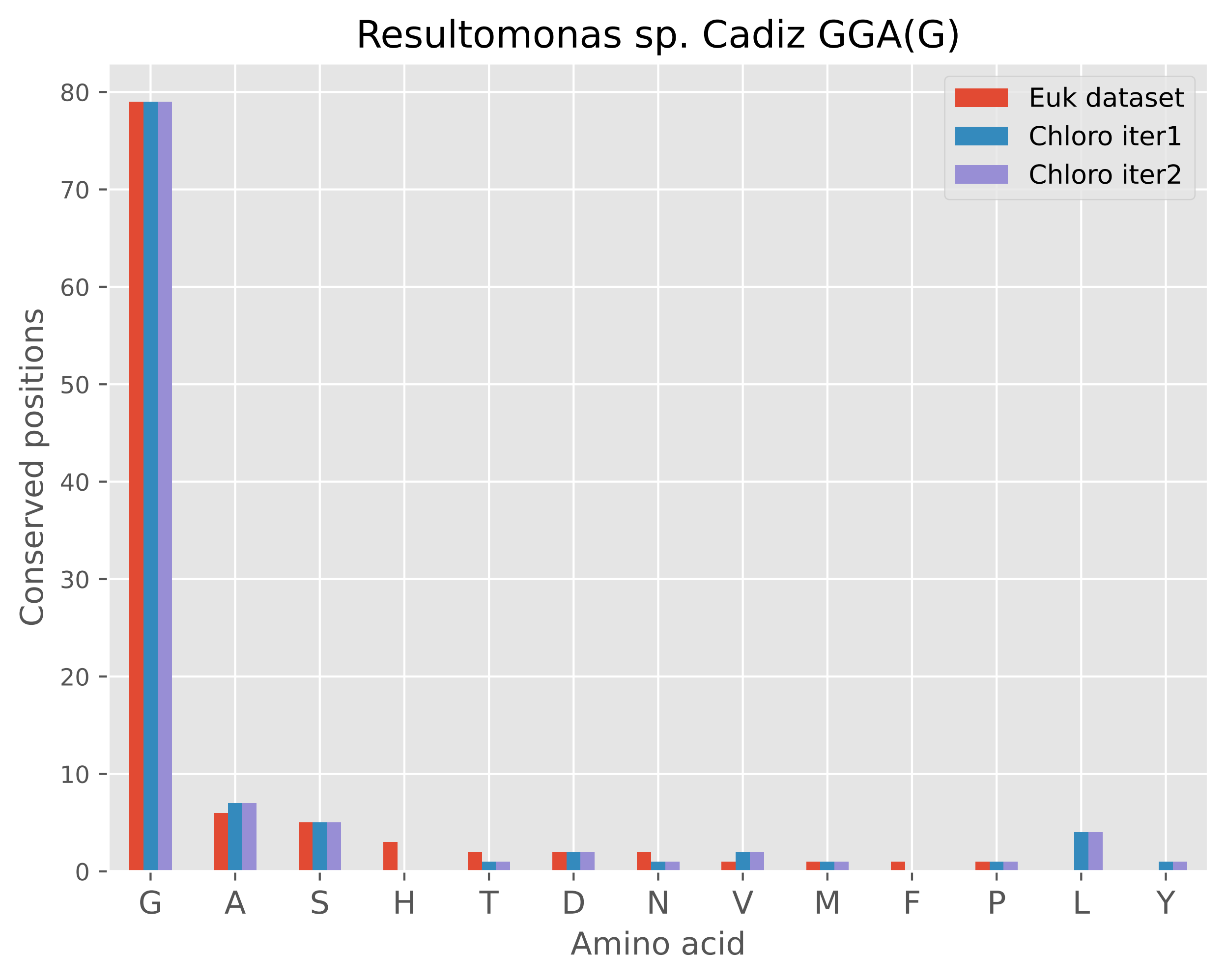

Resultomonas sp. Cadiz GGA(G)
Euk dataset
Chloro iter1
Chloro iter2
Conserved positions
0
10
20
30
40
50
60
70
80
G
A
S
H
T
D
N
V
M
F
P
L
Y
Amino acid

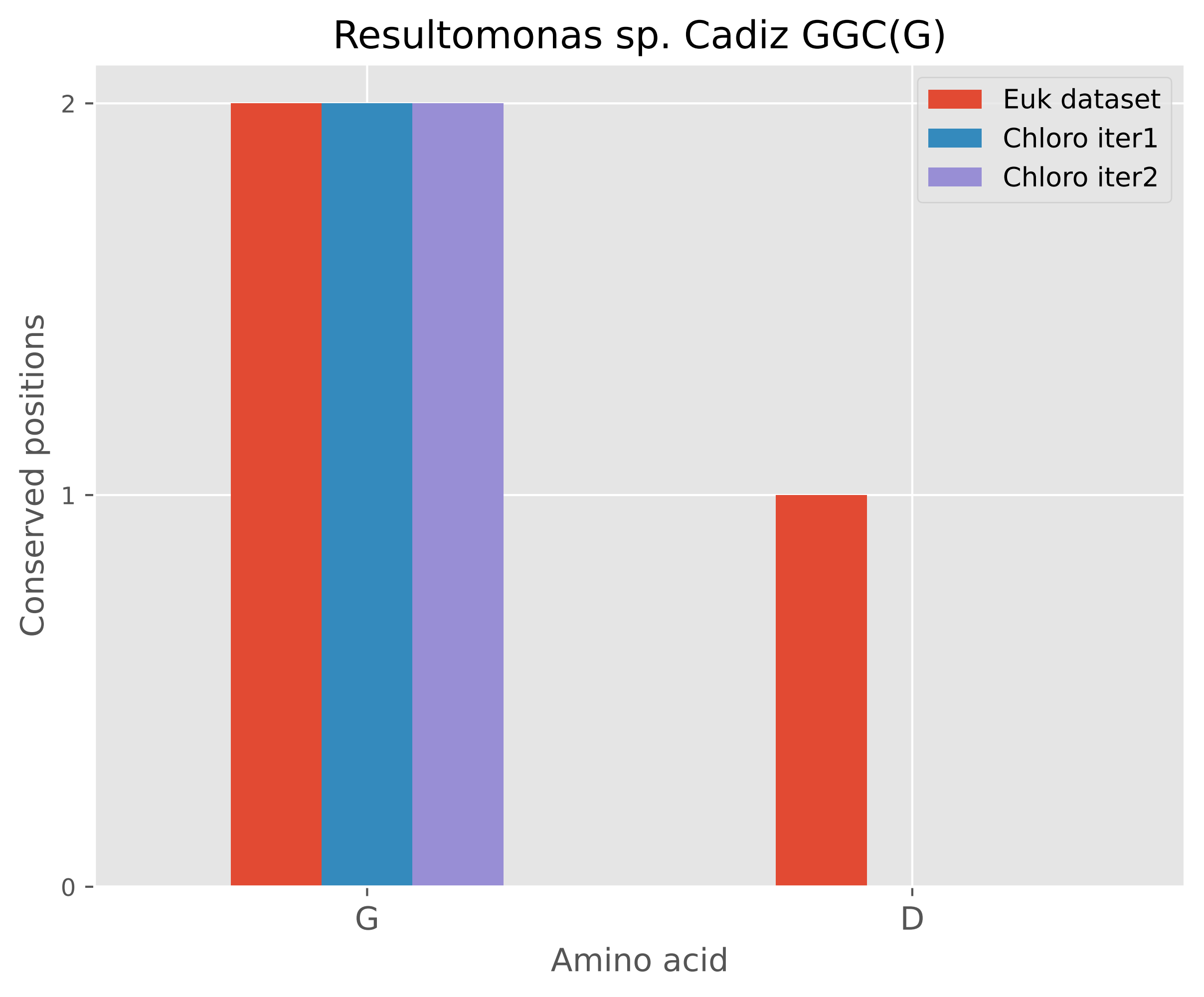

Resultomonas sp. Cadiz GGC(G)
Euk dataset
Chloro iter1
Chloro iter2
Conserved positions
0
1
2
G
D
Amino acid

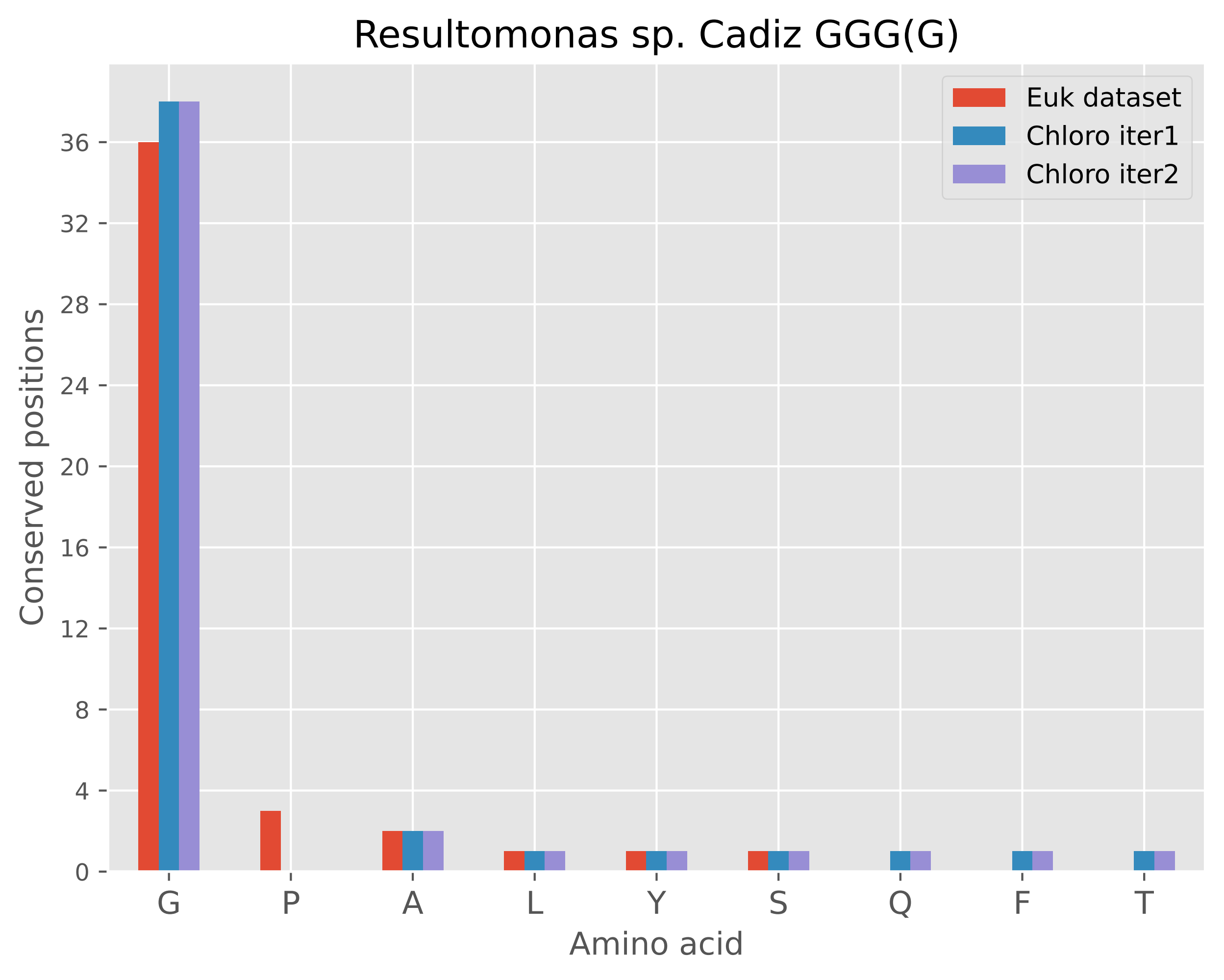

Resultomonas sp. Cadiz GGG(G)
Euk dataset
Chloro iter1
Chloro iter2
Conserved positions
0
4
8
12
16
20
24
28
32
36
G
P
A
L
Y
S
Q
F
T
Amino acid

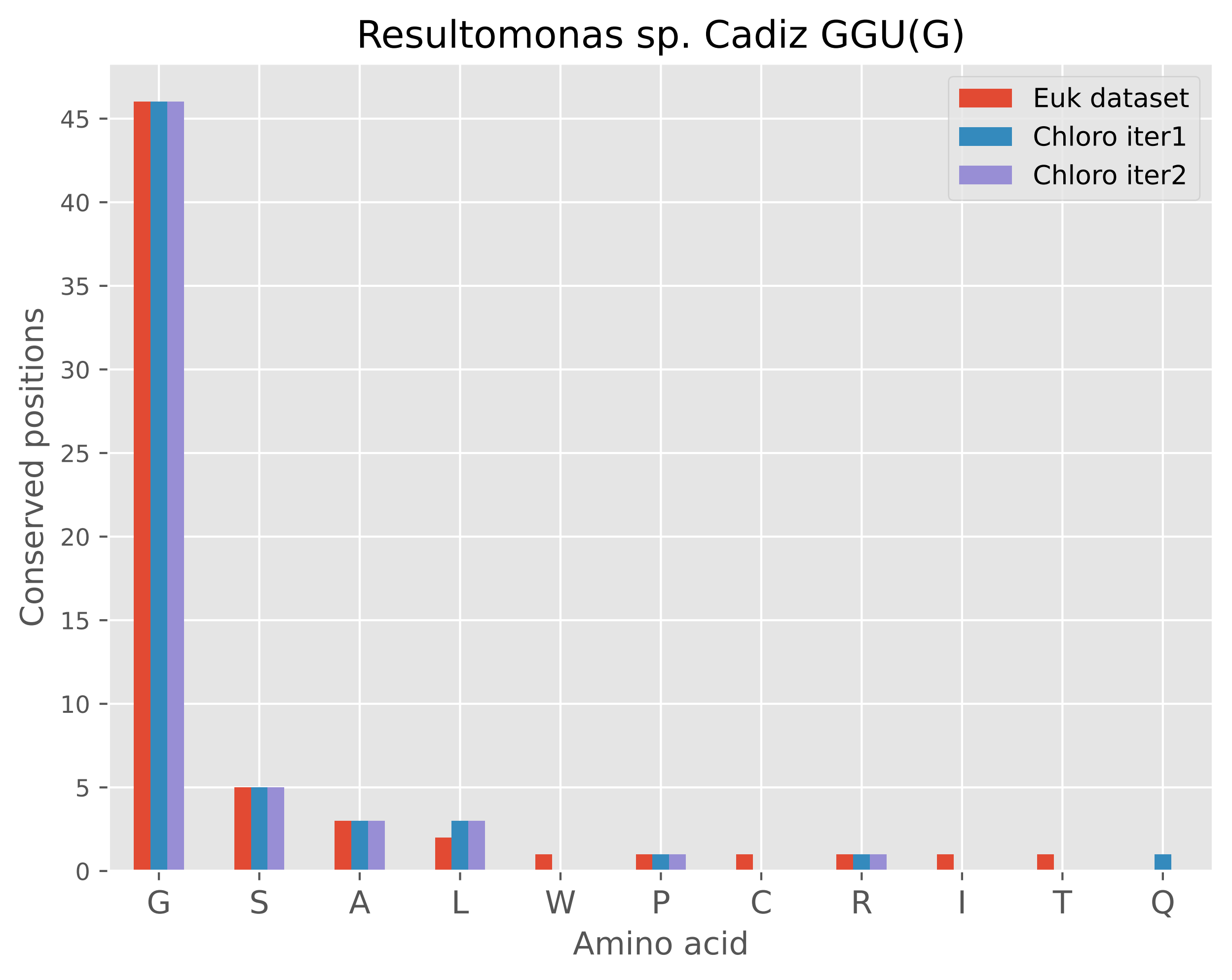
Resultomonas sp. Cadiz GGU(G)
Euk dataset
Chloro iter1
Chloro iter2
Conserved positions
0
5
10
15
20
25
30
35
40
45
G
S
A
L
W
P
C
R
I
T
Q
Amino acid

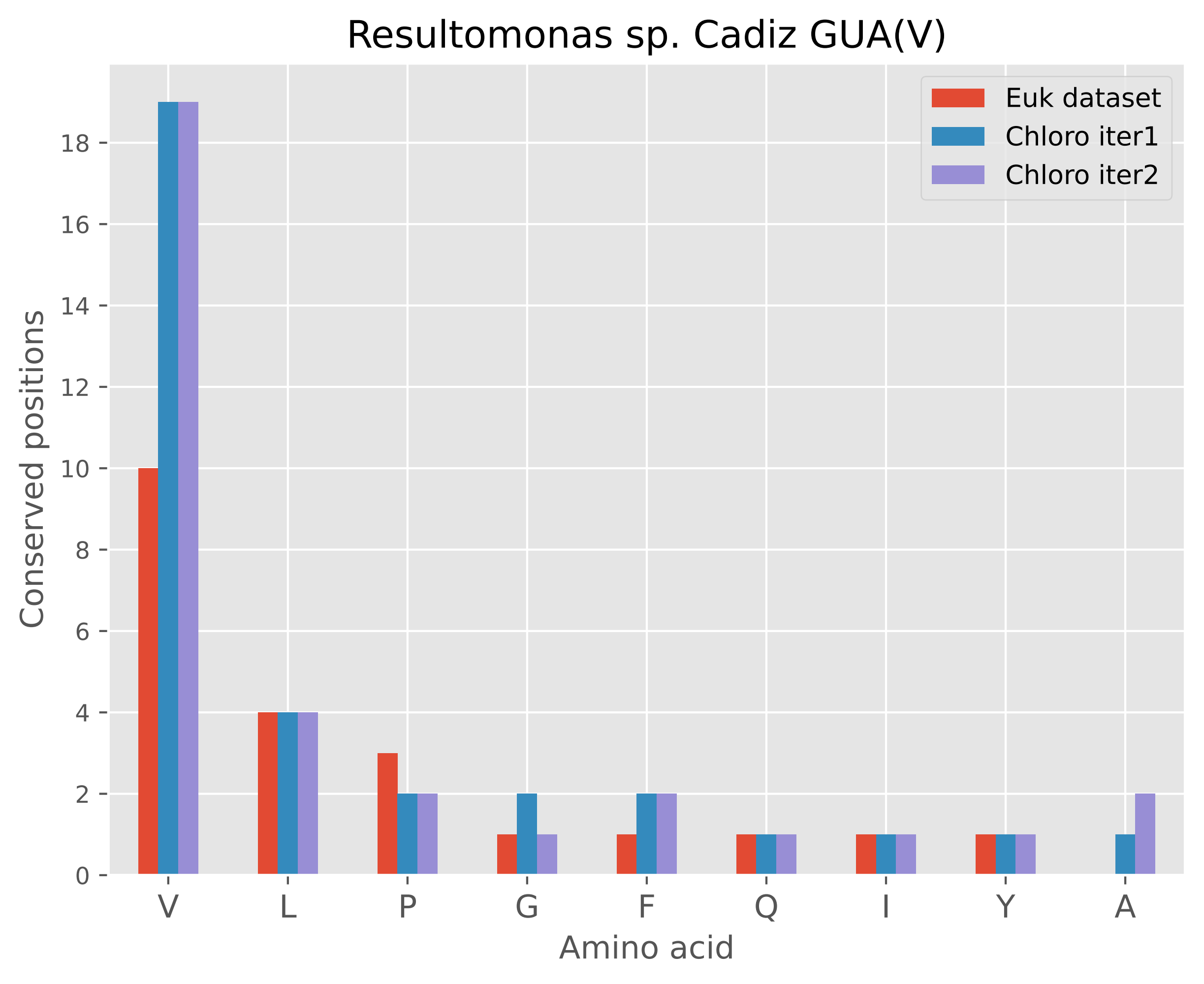
Resultomonas sp. Cadiz GUA(V)
Euk dataset
Chloro iter1
Chloro iter2
Conserved positions
0
2
4
6
8
10
12
14
16
18
V
L
P
G
F
Q
I
Y
A
Amino acid

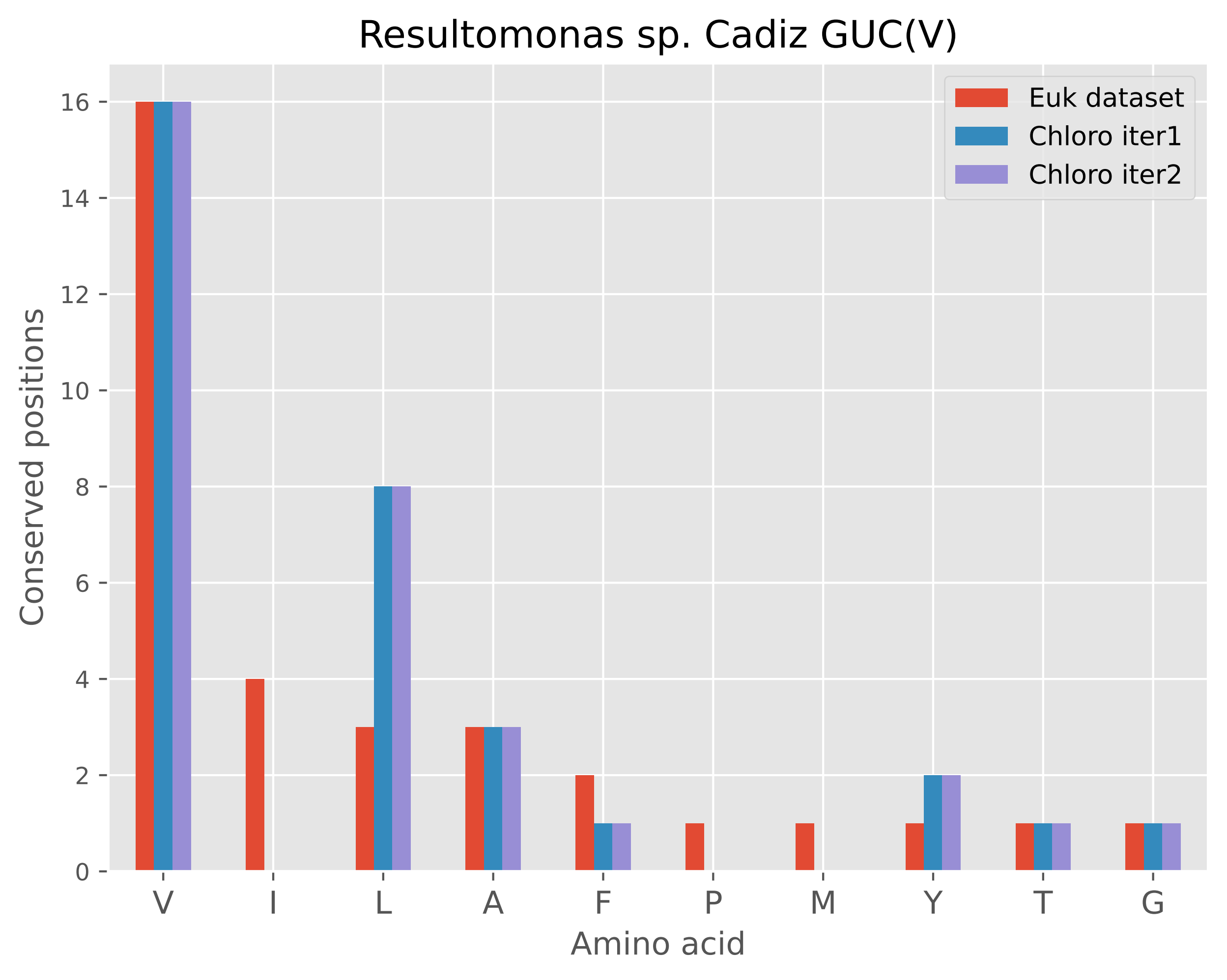

Resultomonas sp. Cadiz GUC(V)
Euk dataset
Chloro iter1
Chloro iter2
Conserved positions
0
2
4
6
8
10
12
14
16
V
I
L
A
F
P
M
Y
T
G
Amino acid

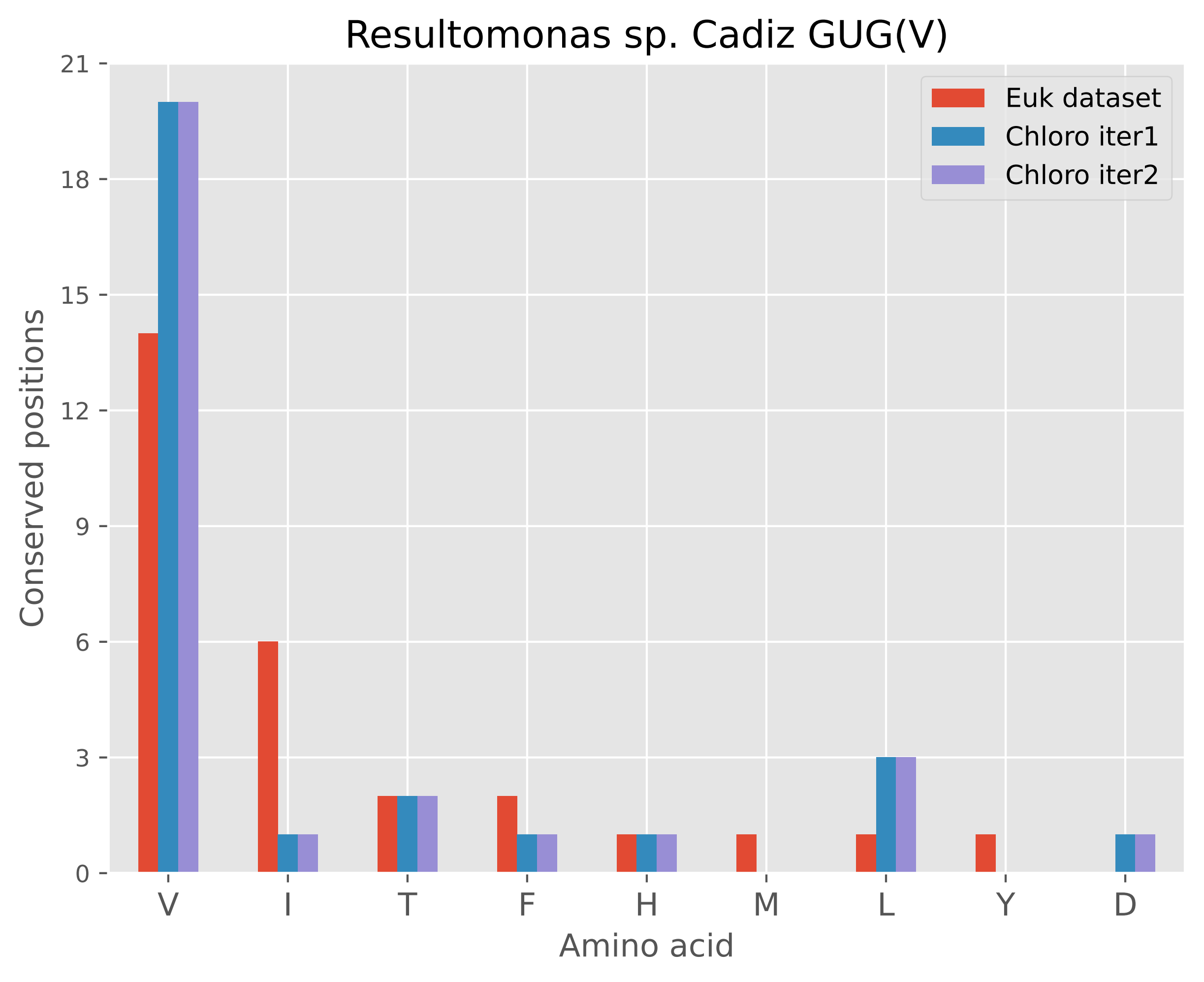

Resultomonas sp. Cadiz GUG(V)
Euk dataset
Chloro iter1
Chloro iter2
Conserved positions
0
3
6
9
12
15
18
21
V
I
T
F
H
M
L
Y
D
Amino acid

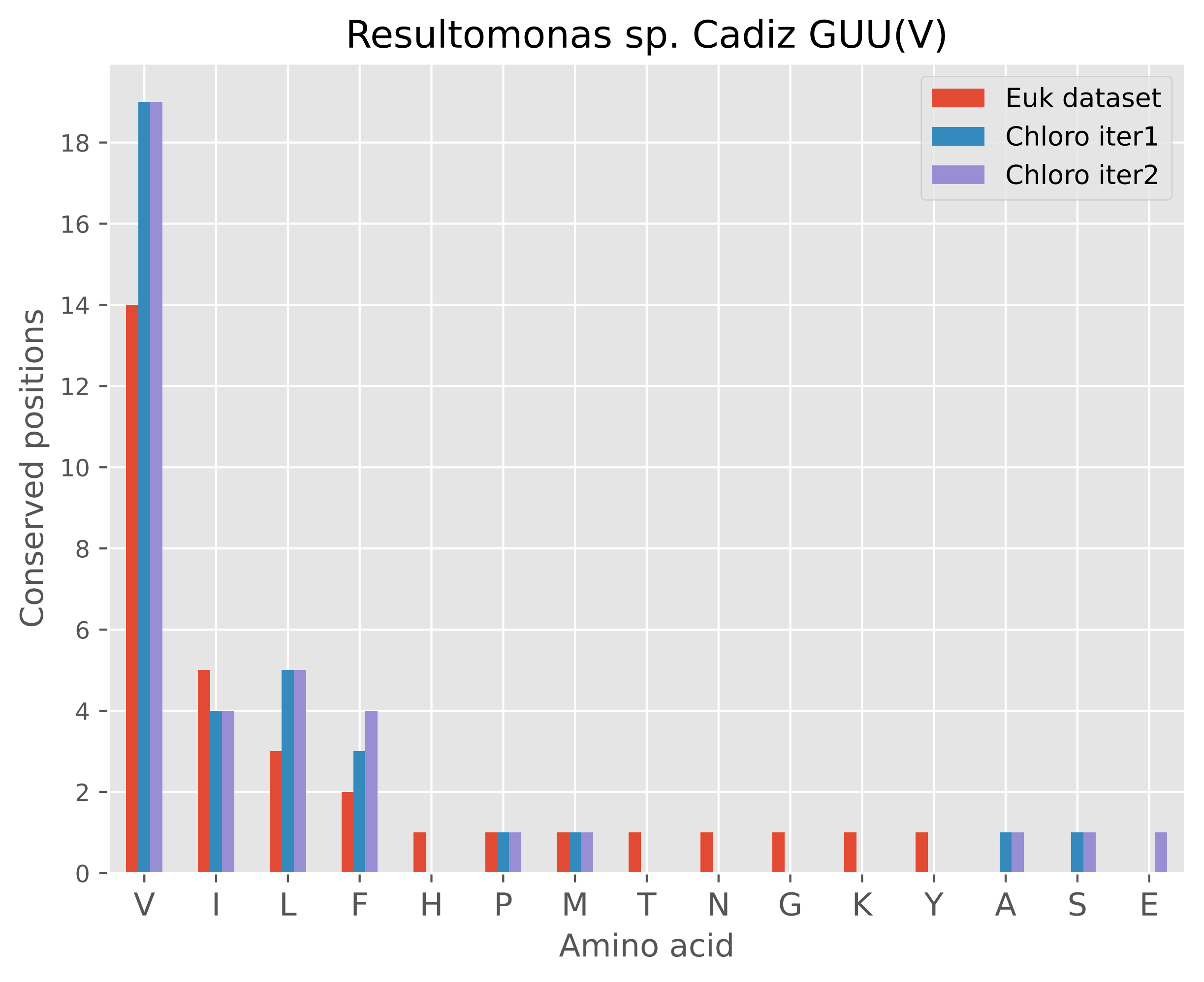

Resultomonas sp. Cadiz GUU(V)
Euk dataset
Chloro iter1
Chloro iter2
Conserved positions
0
2
4
6
8
10
12
14
16
18
V
I
L
F
H
P
M
T
N
G
K
Y
A
S
E
Amino acid

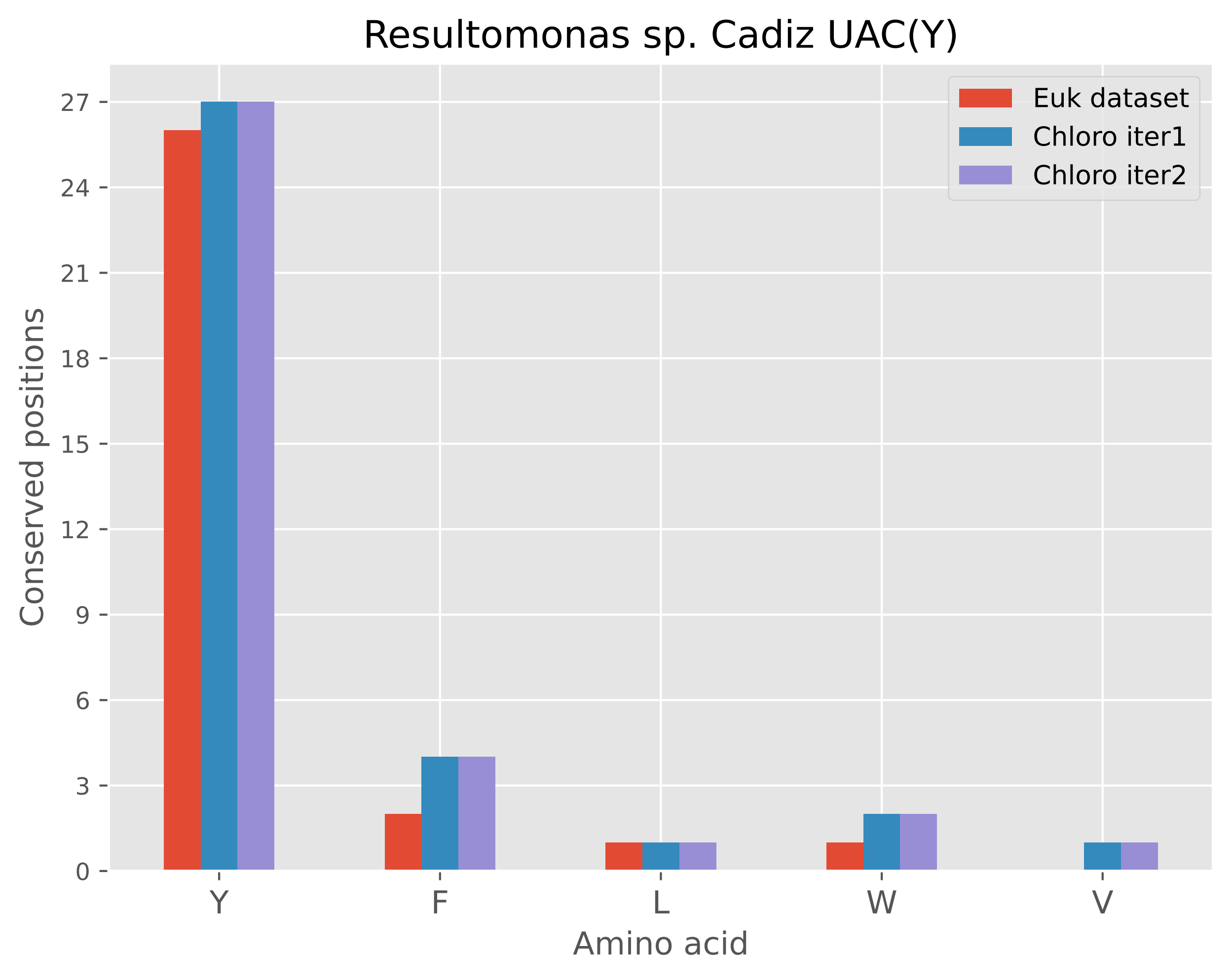

Resultomonas sp. Cadiz UAC(Y)
Euk dataset
Chloro iter1
Chloro iter2
Conserved positions
0
3
6
9
12
15
18
21
24
27
Y
F
L
W
V
Amino acid

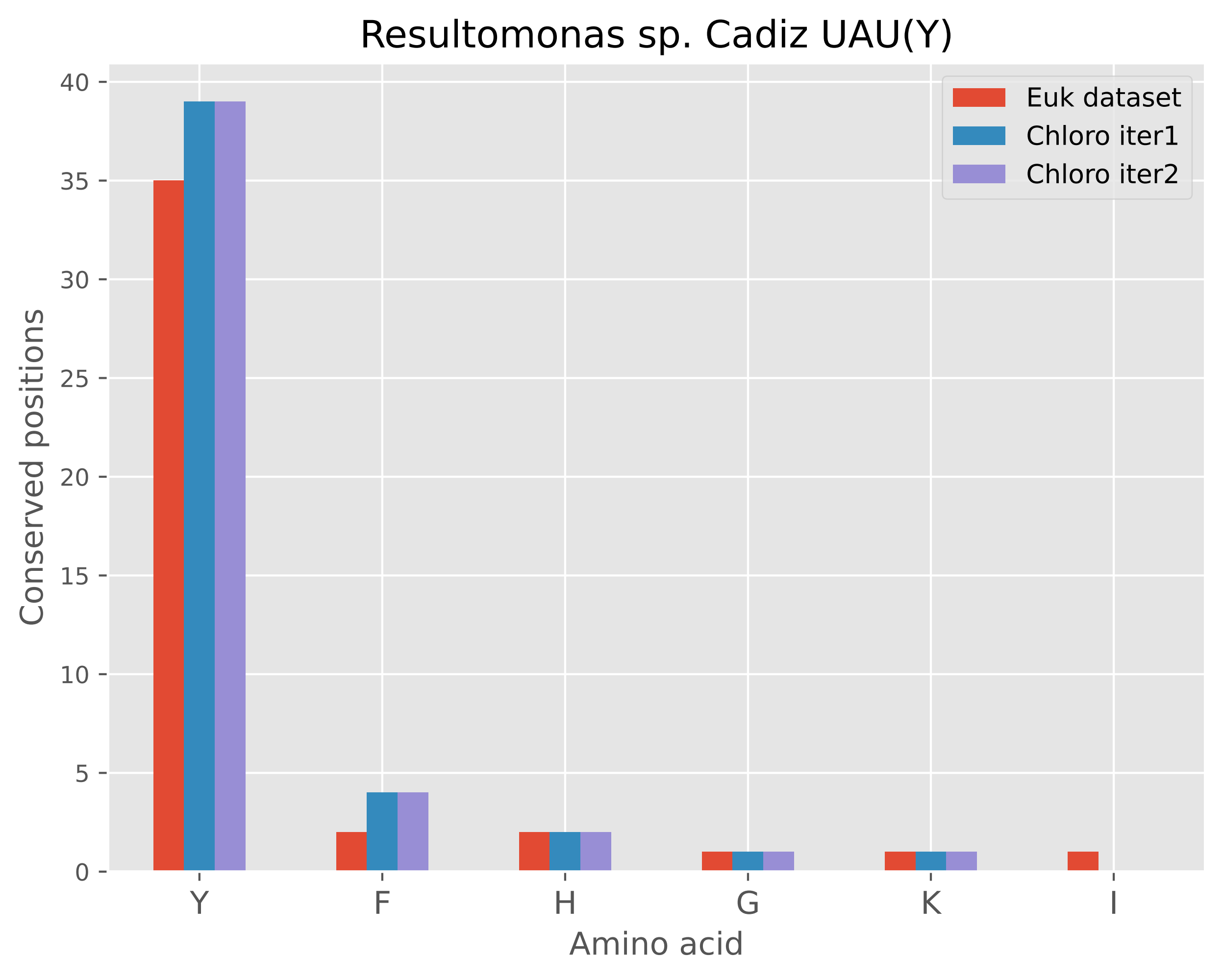
Resultomonas sp. Cadiz UAU(Y)
Euk dataset
Chloro iter1
Chloro iter2
Conserved positions
0
5
10
15
20
25
30
35
40
Y
F
H
G
K
I
Amino acid

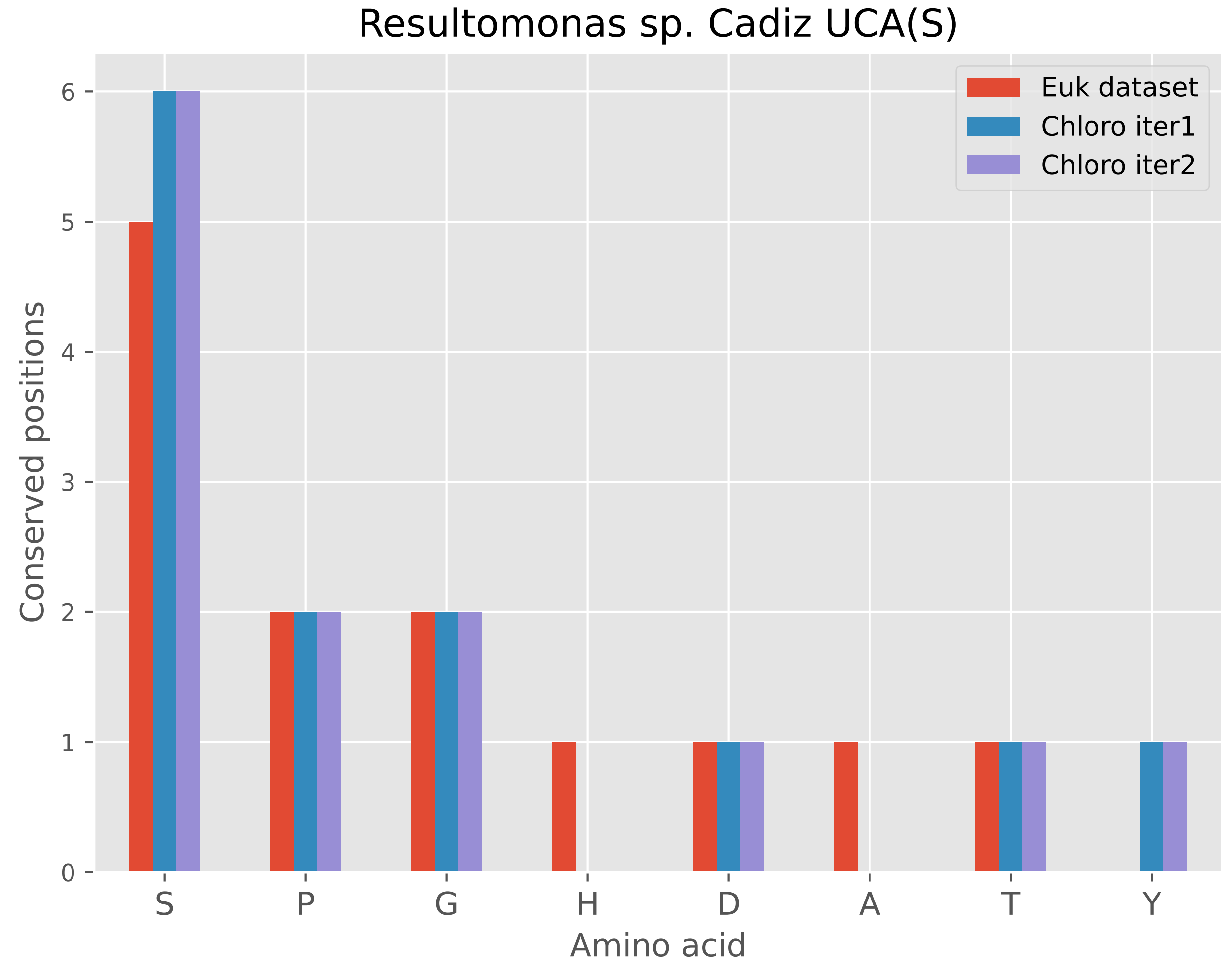

Resultomonas sp. Cadiz UCA(S)
Euk dataset
Chloro iter1
Chloro iter2
Conserved positions
0
1
2
3
4
5
6
S
P
G
H
D
A
T
Y
Amino acid

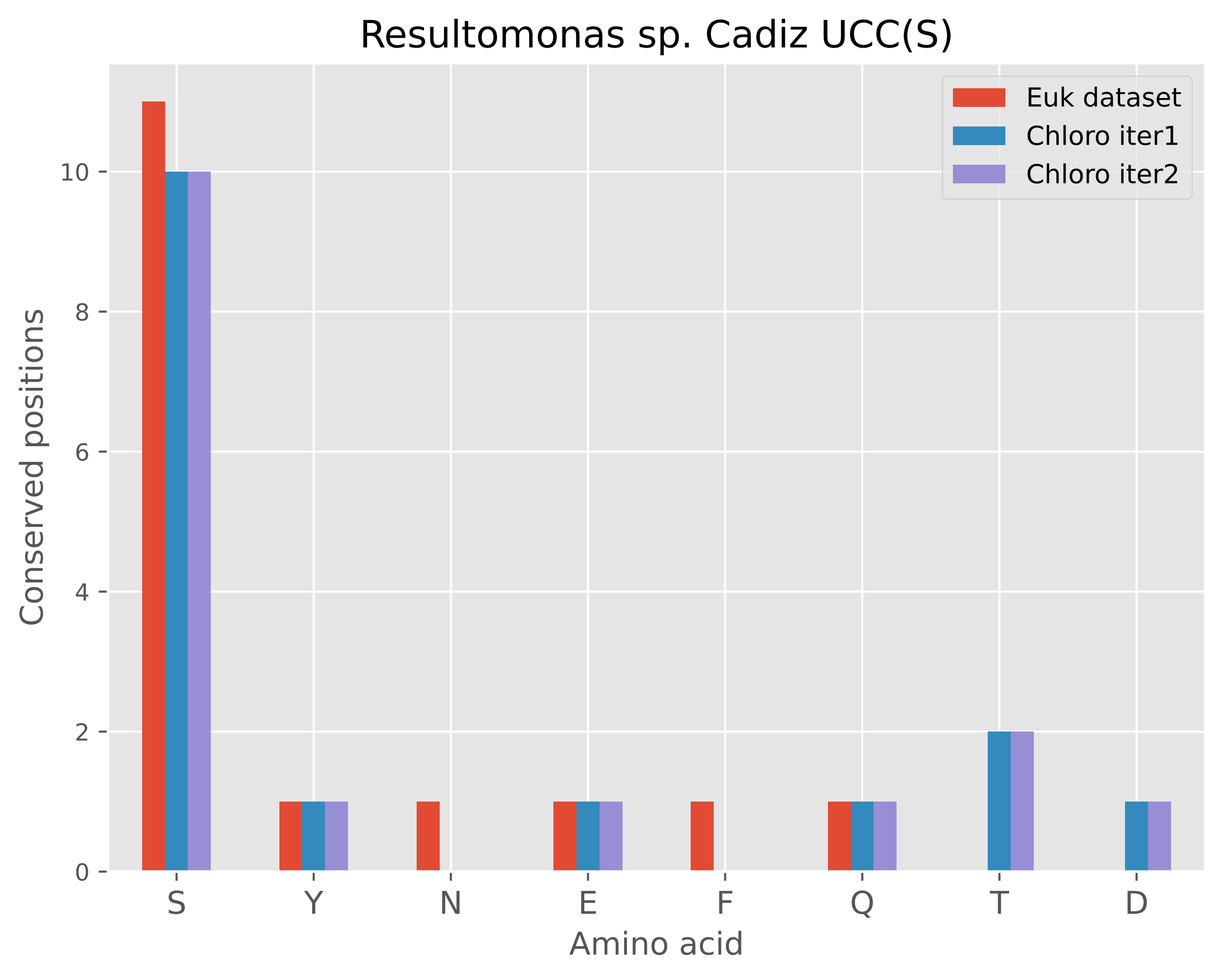

Resultomonas sp. Cadiz UCC(S)
Euk dataset
Chloro iter1
Chloro iter2
Conserved positions
0
2
4
6
8
10
S
Y
N
E
F
Q
T
D
Amino acid

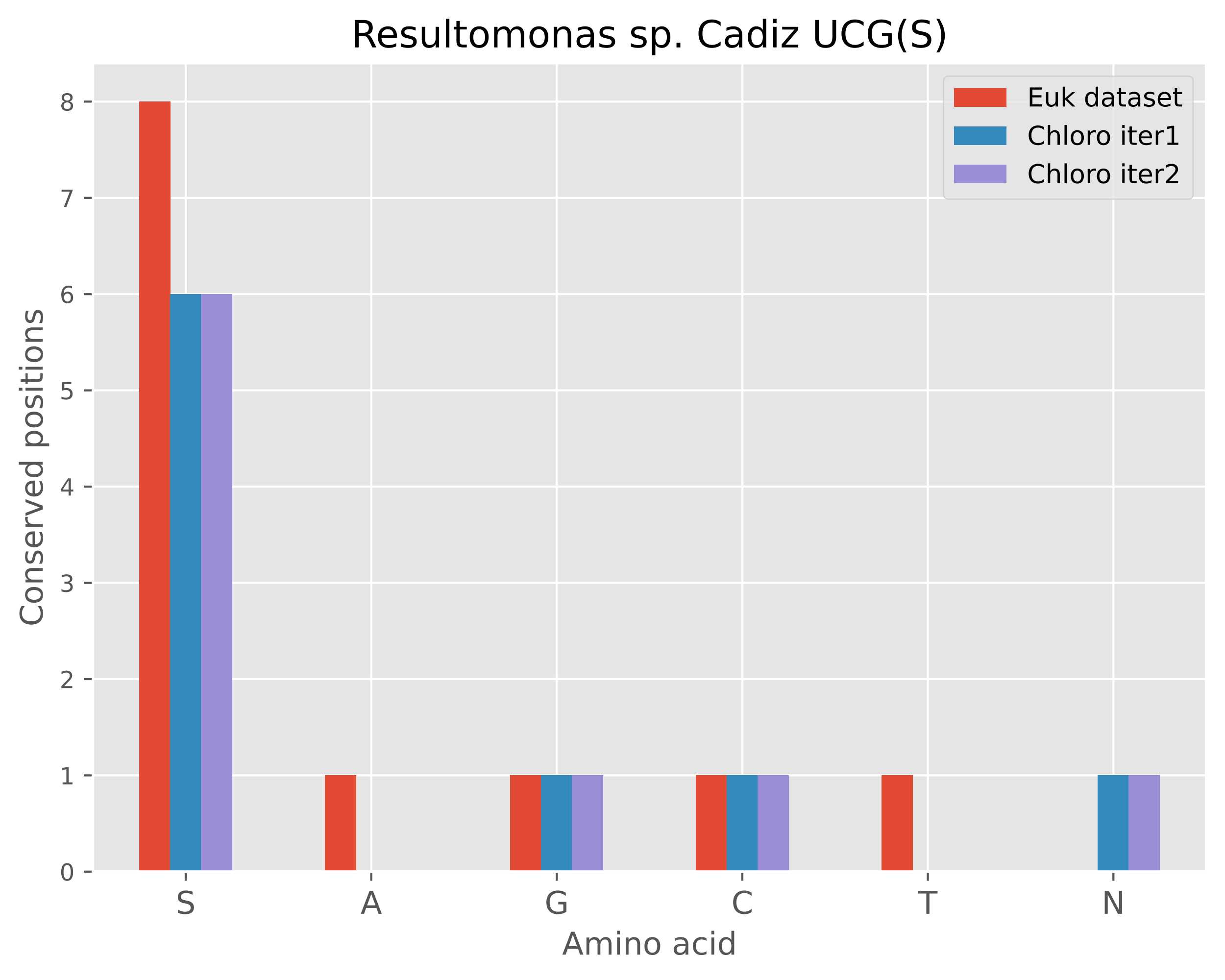

Resultomonas sp. Cadiz UCG(S)
Euk dataset
Chloro iter1
Chloro iter2
Conserved positions
0
1
2
3
4
5
6
7
8
S
A
G
C
T
N
Amino acid

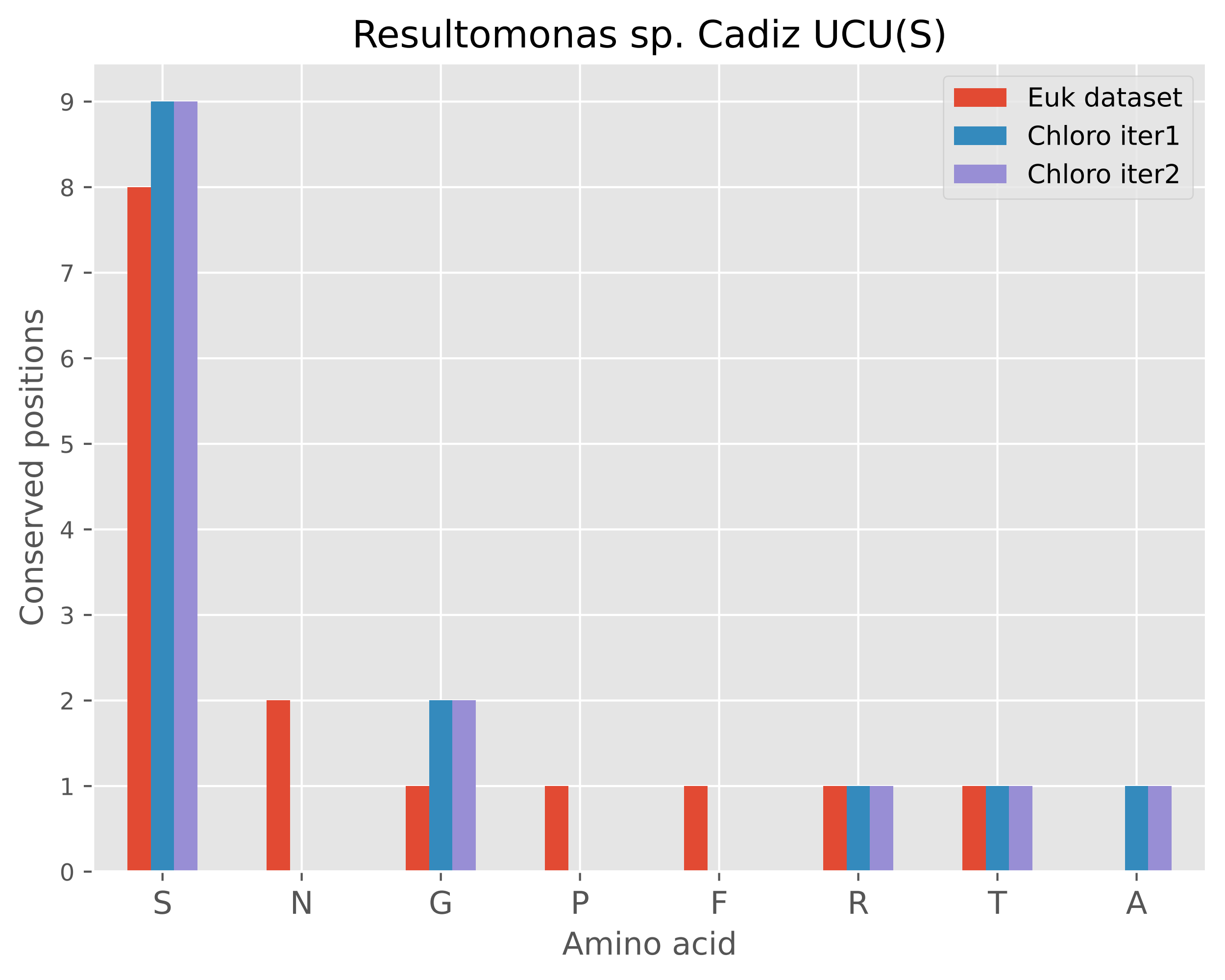
Resultomonas sp. Cadiz UCU(S)
Euk dataset
Chloro iter1
Chloro iter2
Conserved positions
0
1
2
3
4
5
6
7
8
9
S
N
G
P
F
R
T
A
Amino acid

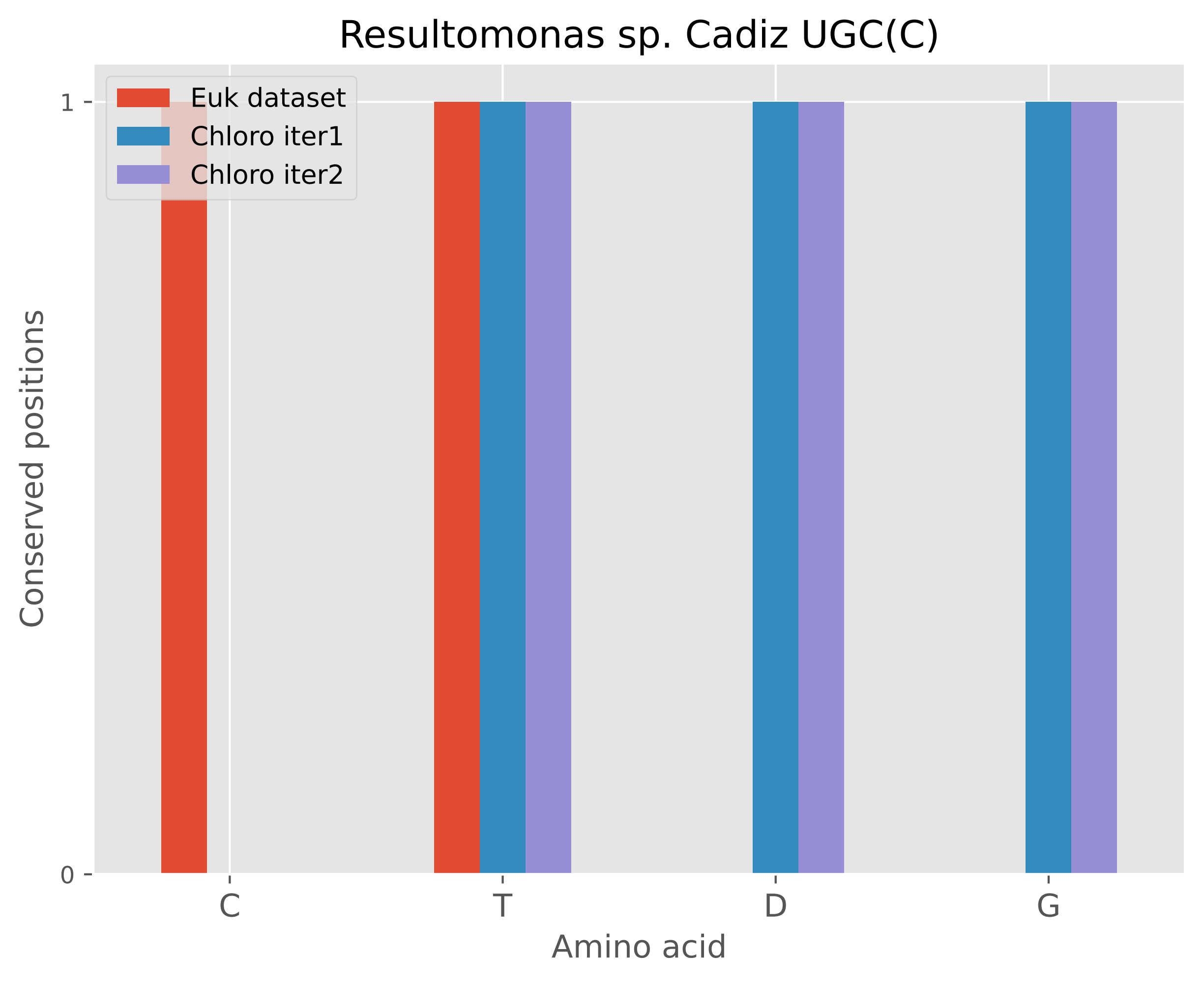

Resultomonas sp. Cadiz UGC(C)
Euk dataset
Chloro iter1
Chloro iter2
Conserved positions
1
0
C
T
D
G
Amino acid

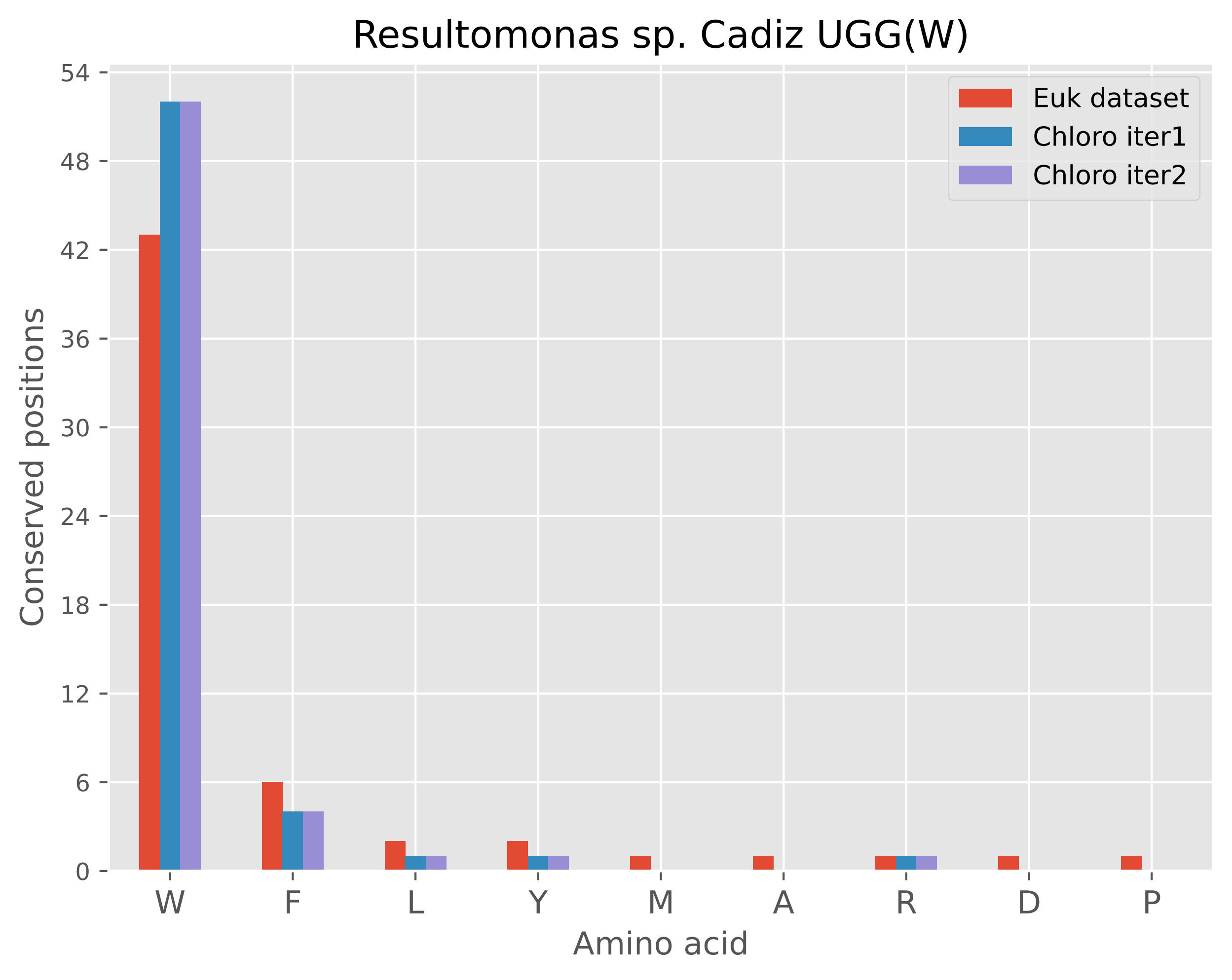
Resultomonas sp. Cadiz UGG(W)
Euk dataset
Chloro iter1
Chloro iter2
Conserved positions
Amino acid
0
6
12
18
24
30
36
42
48
54
W
F
L
Y
M
A
R
D
P

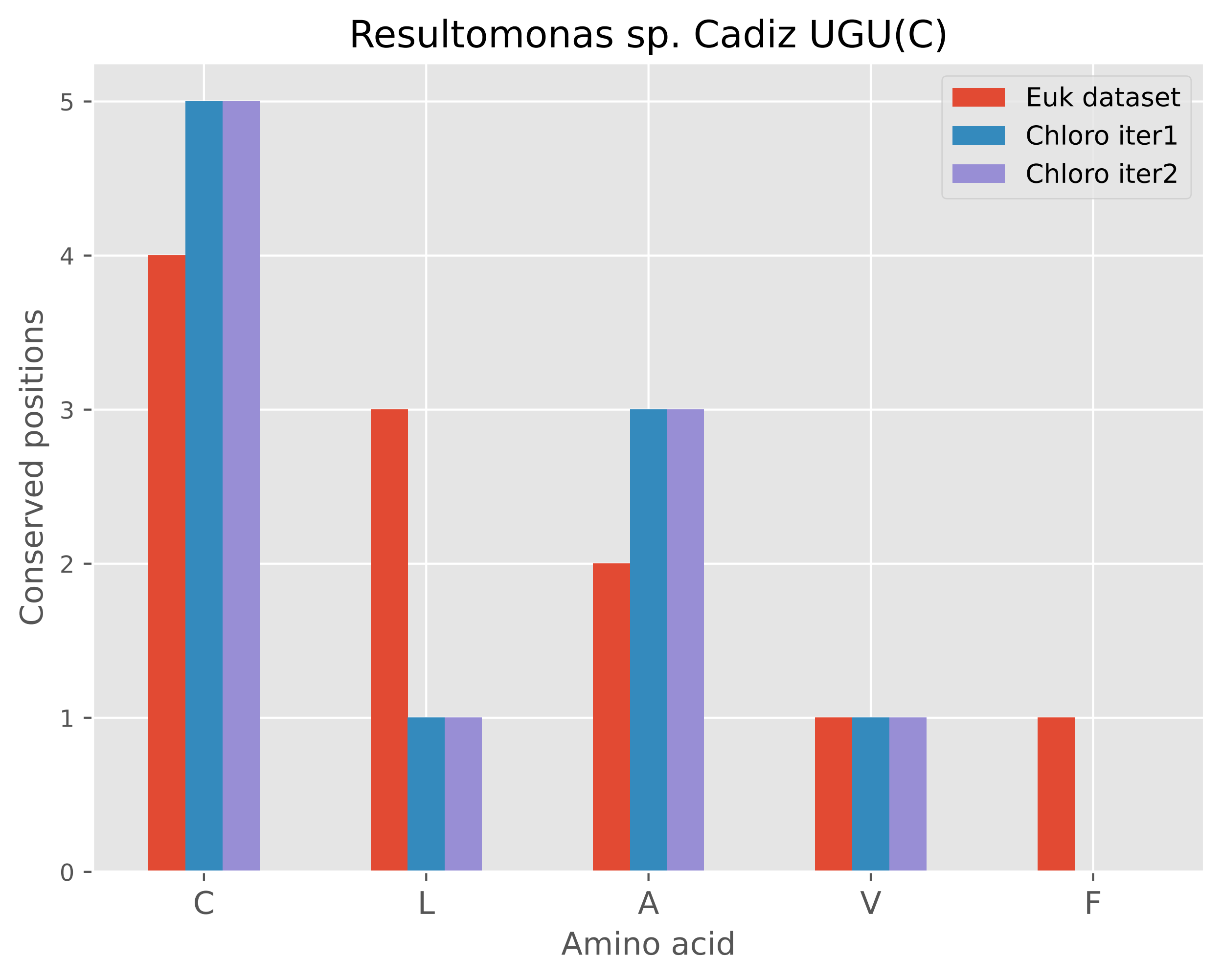

Resultomonas sp. Cadiz UGU(C)
Euk dataset
Chloro iter1
Chloro iter2
Conserved positions
0
1
2
3
4
5
C
L
A
V
F
Amino acid

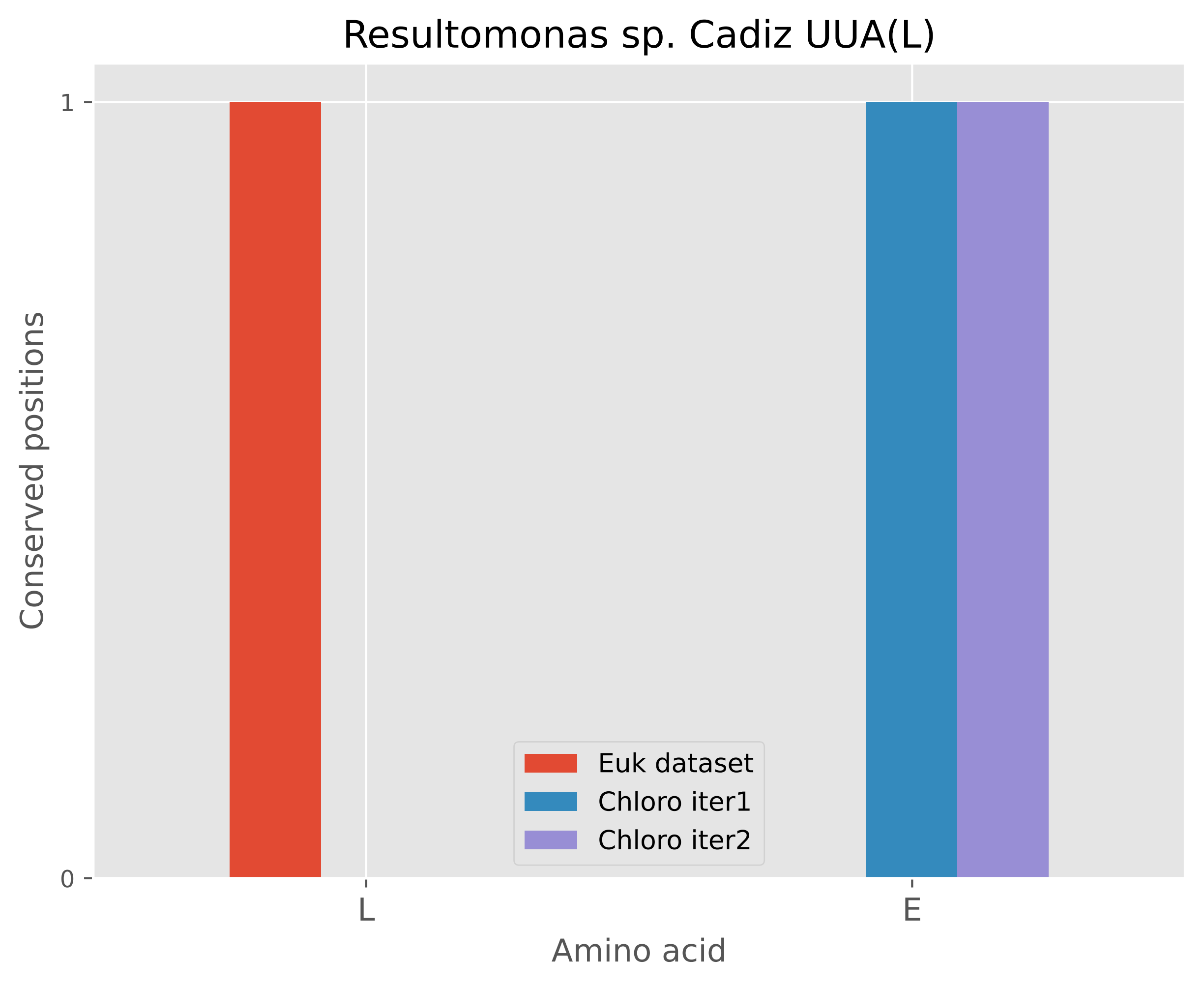
Resultomonas sp. Cadiz UUA(L)
Conserved positions
1
0
L
E
Amino acid
Euk dataset
Chloro iter1
Chloro iter2

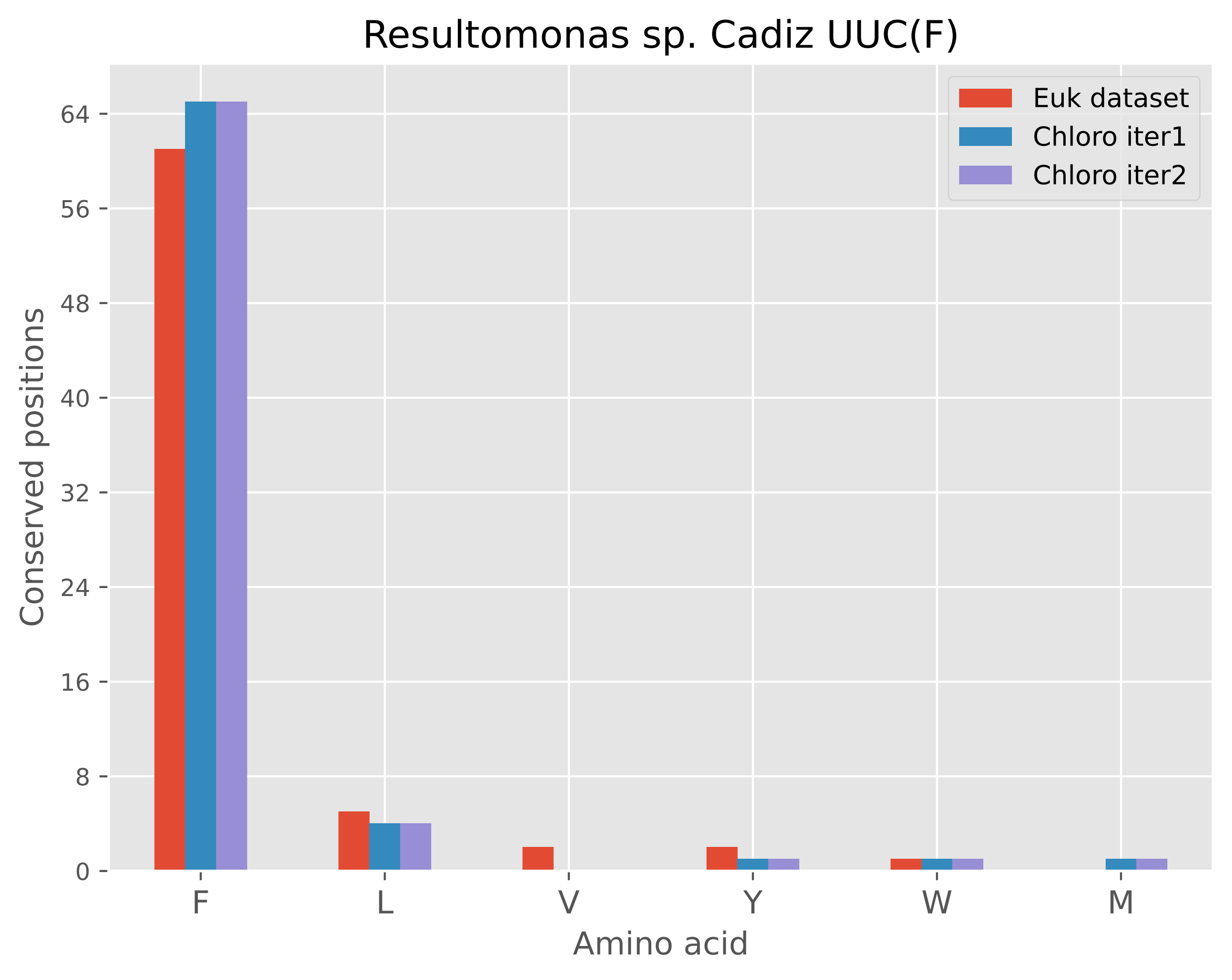

Resultomonas sp. Cadiz UUC(F)
Euk dataset
Chloro iter1
Chloro iter2
Conserved positions
0
8
16
24
32
40
48
56
64
F
L
V
Y
W
M
Amino acid

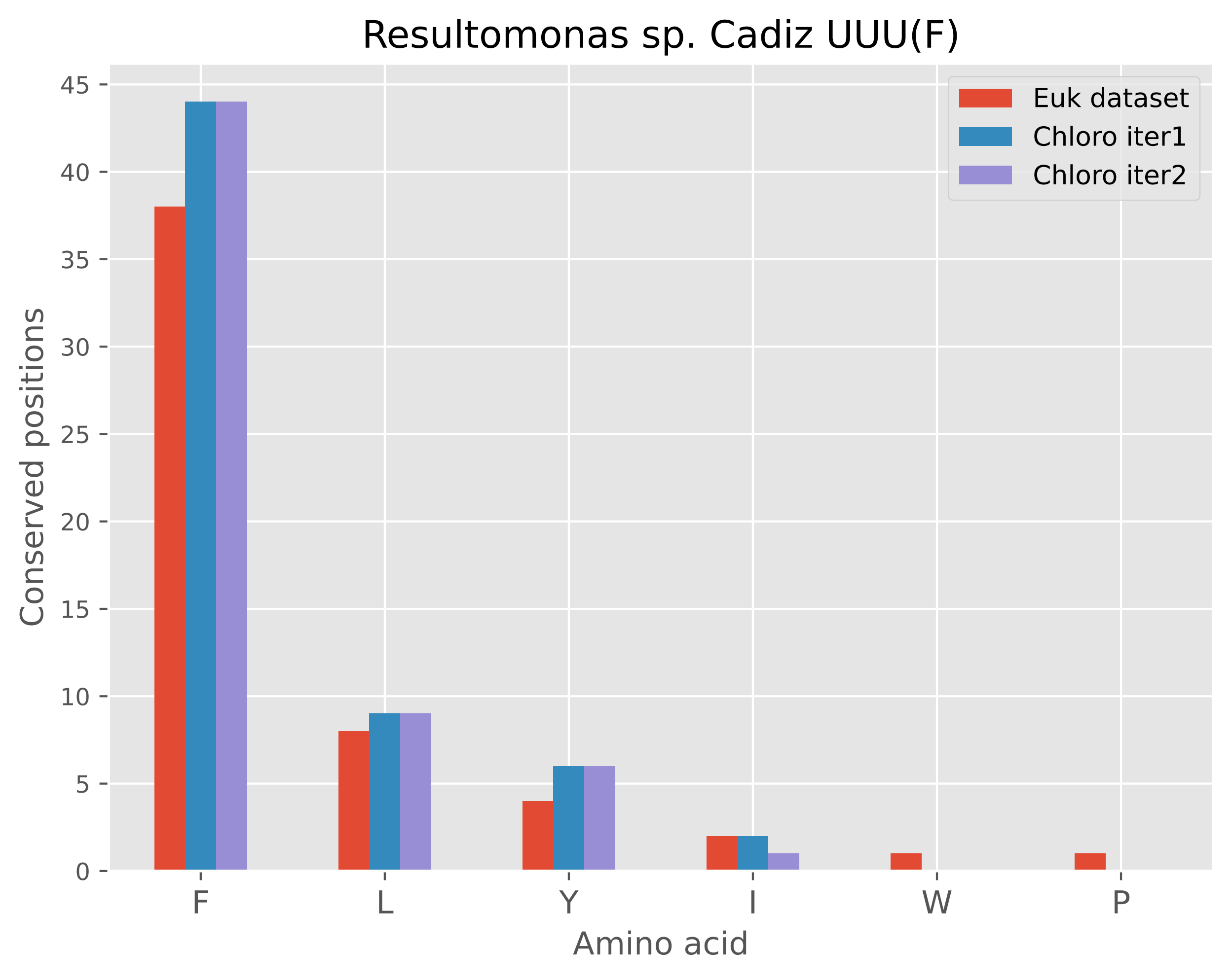
Resultomonas sp. Cadiz UUU(F)
Euk dataset
Chloro iter1
Chloro iter2
Conserved positions
Amino acid
0
5
10
15
20
25
30
35
40
45
F
L
Y
I
W
P

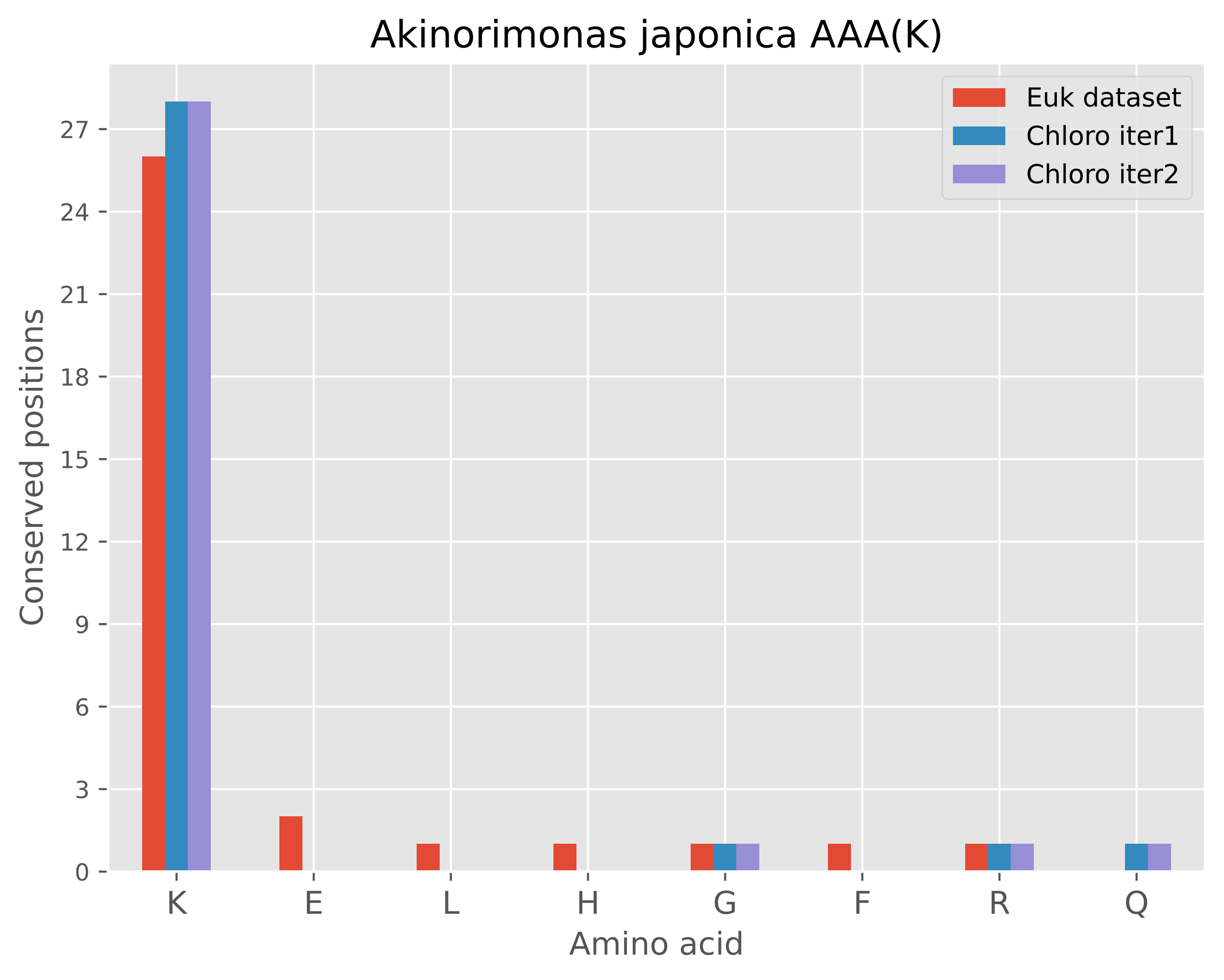

Akinorimonas japonica AAA(K)
Euk dataset
Chloro iter1
Chloro iter2
Conserved positions
0
3
6
9
12
15
18
21
24
27
K
E
L
H
G
F
R
Q
Amino acid

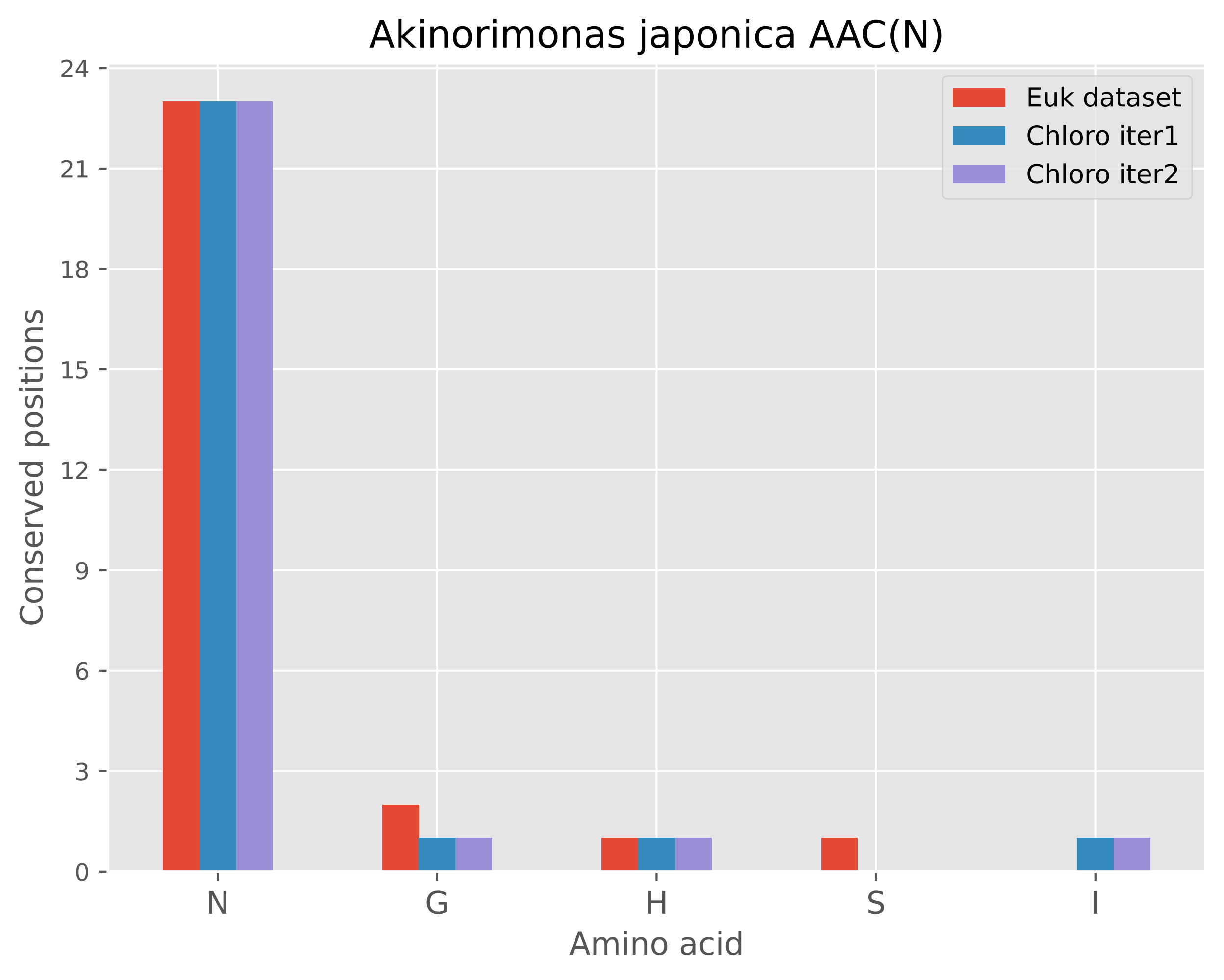

Akinorimonas japonica AAC(N)
Euk dataset
Chloro iter1
Chloro iter2
Conserved positions
0
3
6
9
12
15
18
21
24
N
G
H
S
I
Amino acid

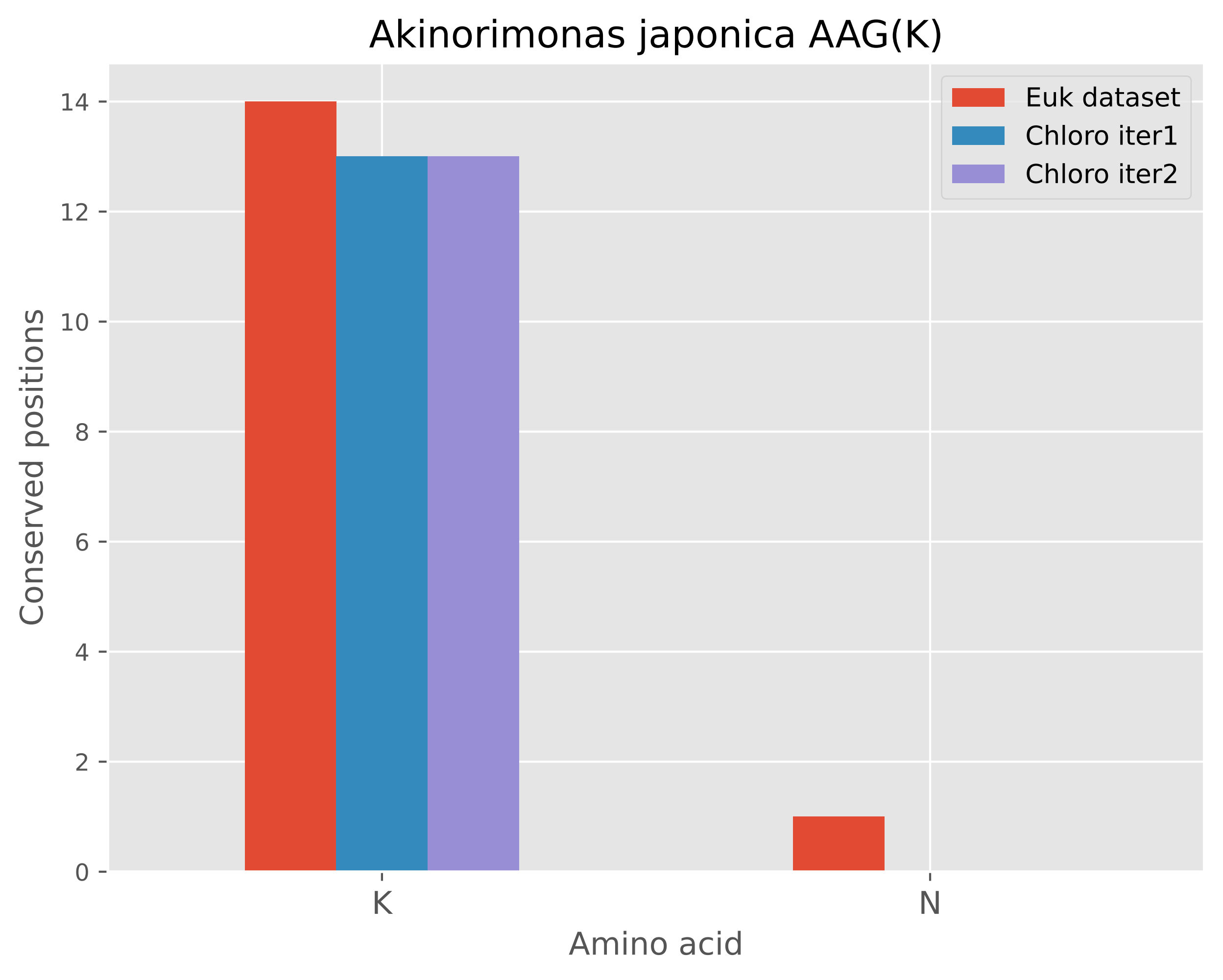

Akinorimonas japonica AAG(K)
Euk dataset
Chloro iter1
Chloro iter2
Conserved positions
Amino acid
K
N
0
2
4
6
8
10
12
14

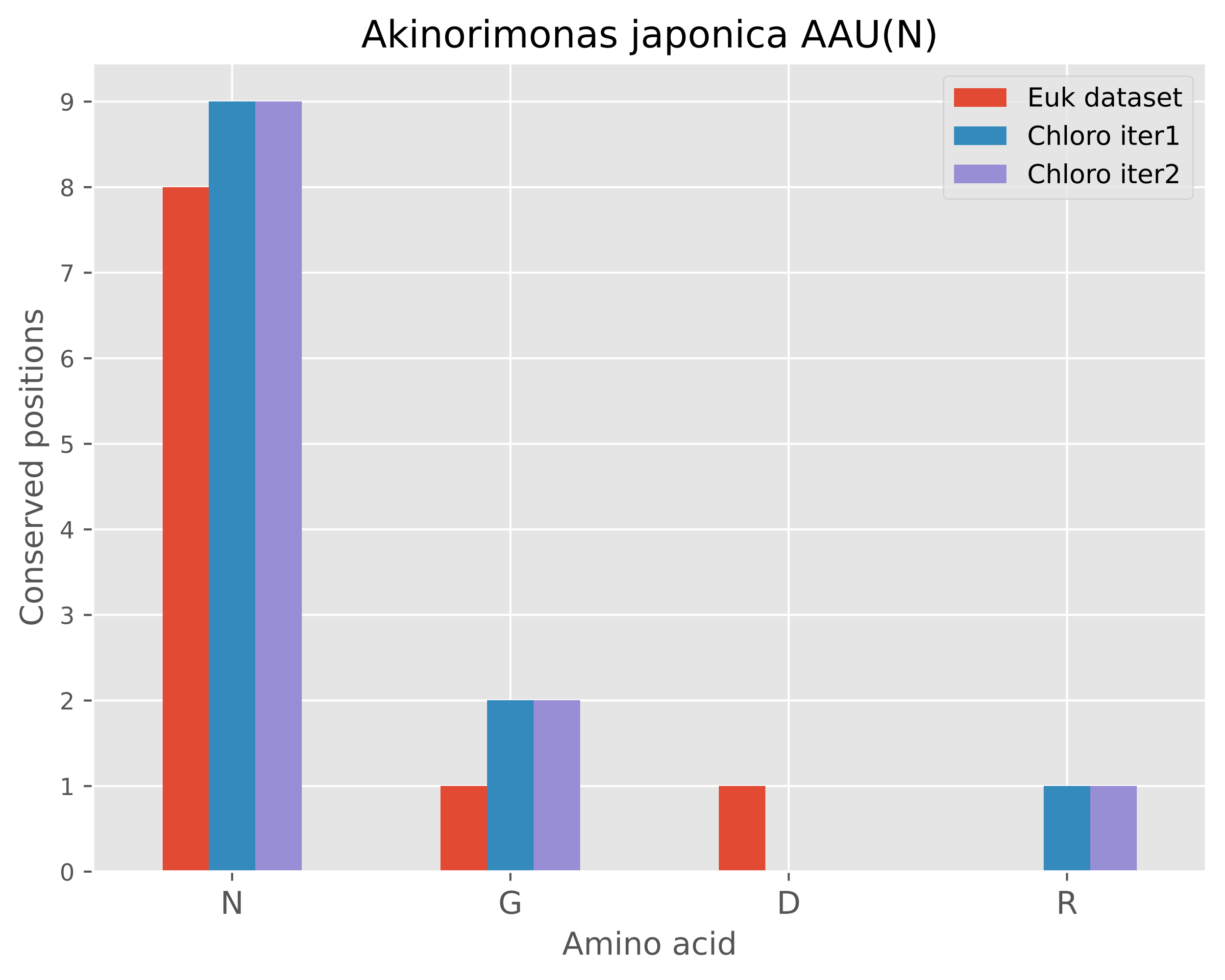
Akinorimonas japonica AAU(N)
Euk dataset
Chloro iter1
Chloro iter2
Conserved positions
0
1
2
3
4
5
6
7
8
9
N
G
D
R
Amino acid

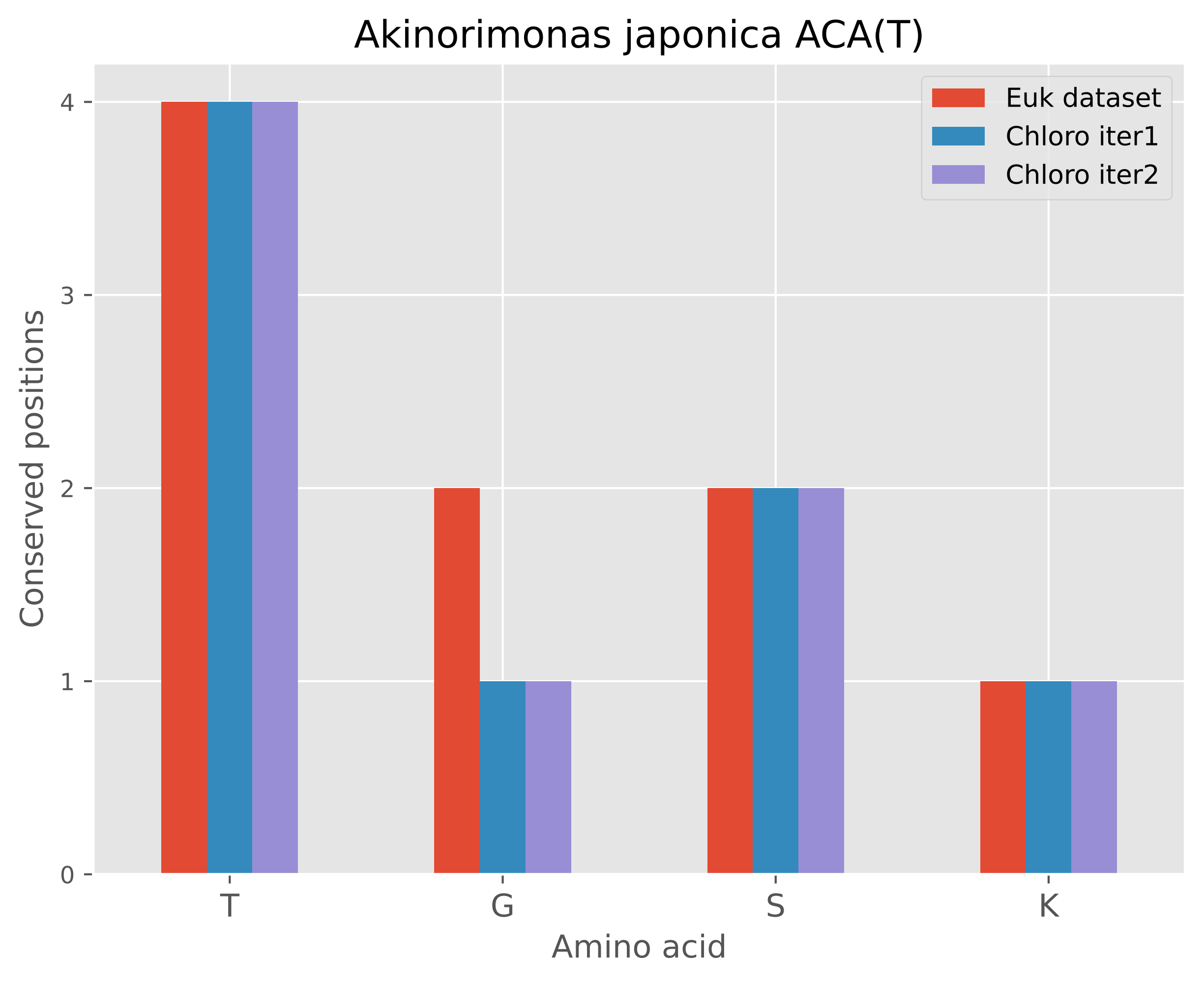

Akinorimonas japonica ACA(T)
Euk dataset
Chloro iter1
Chloro iter2
Conserved positions
Amino acid
T
G
S
K
0
1
2
3
4

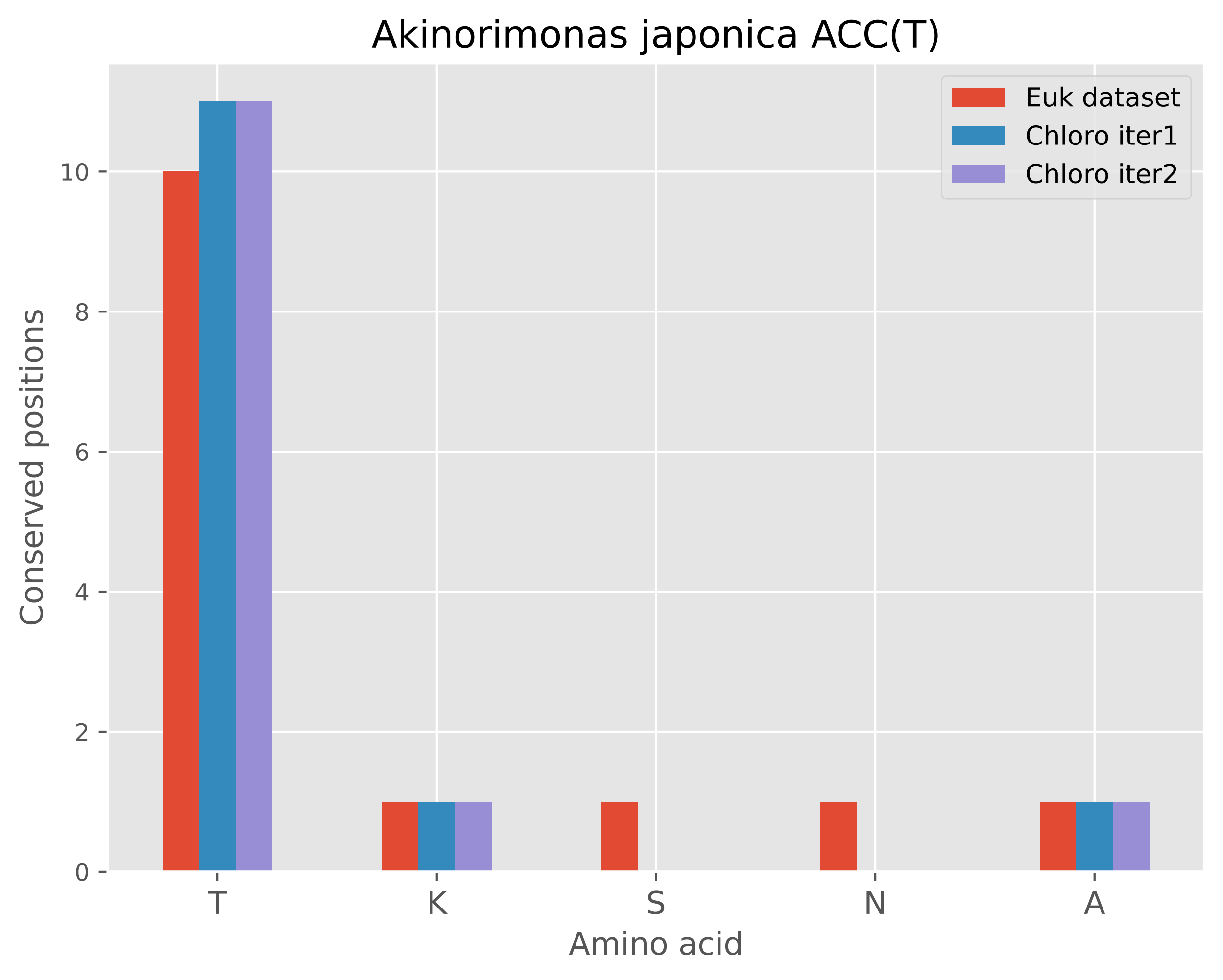
Akinorimonas japonica ACC(T)
Euk dataset
Chloro iter1
Chloro iter2
Conserved positions
0
2
4
6
8
10
T
K
S
N
A
Amino acid

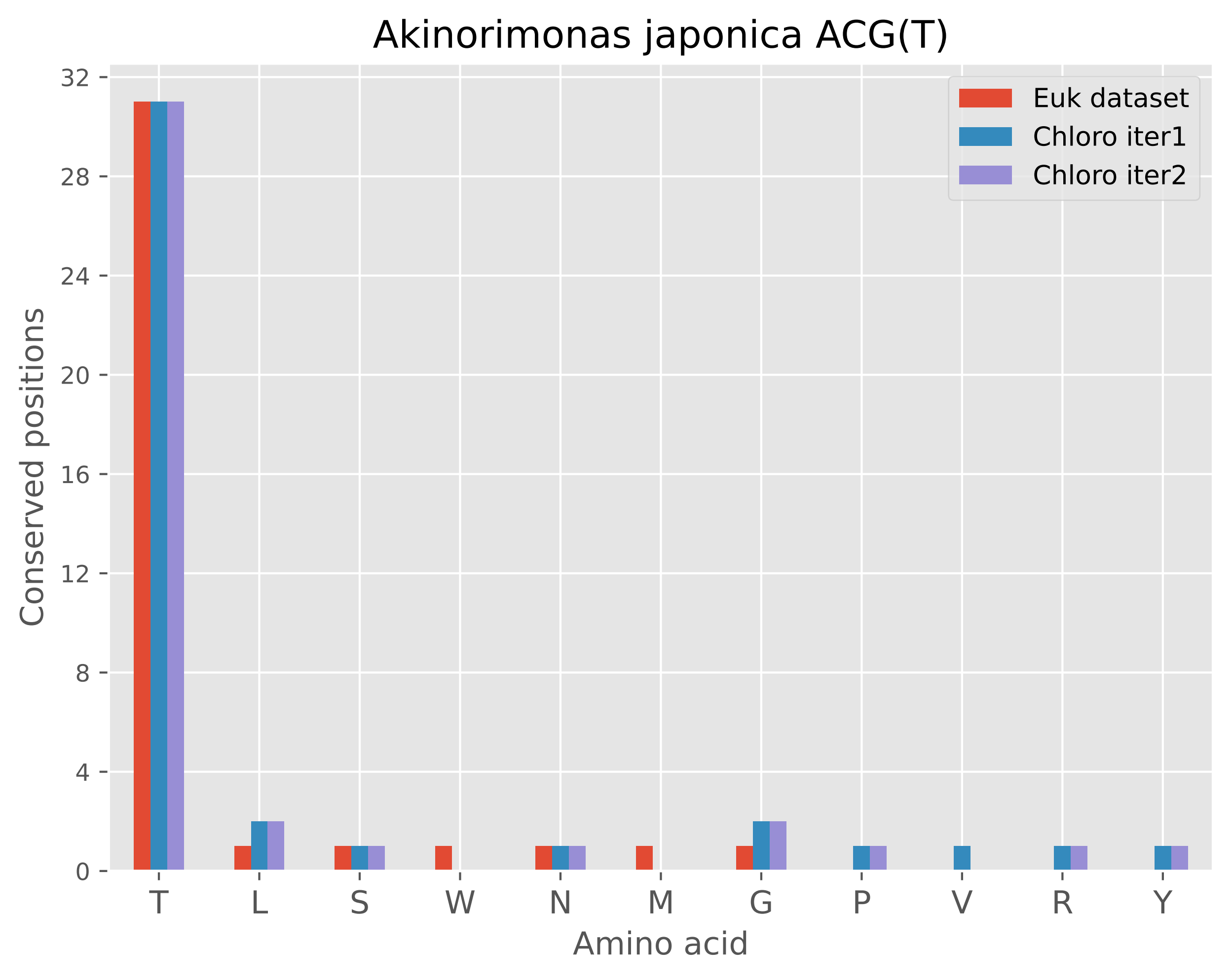
Akinorimonas japonica ACG(T)
Conserved positions
Amino acid
Euk dataset
Chloro iter1
Chloro iter2
0
4
8
12
16
20
24
28
32
T
L
S
W
N
M
G
P
V
R
Y

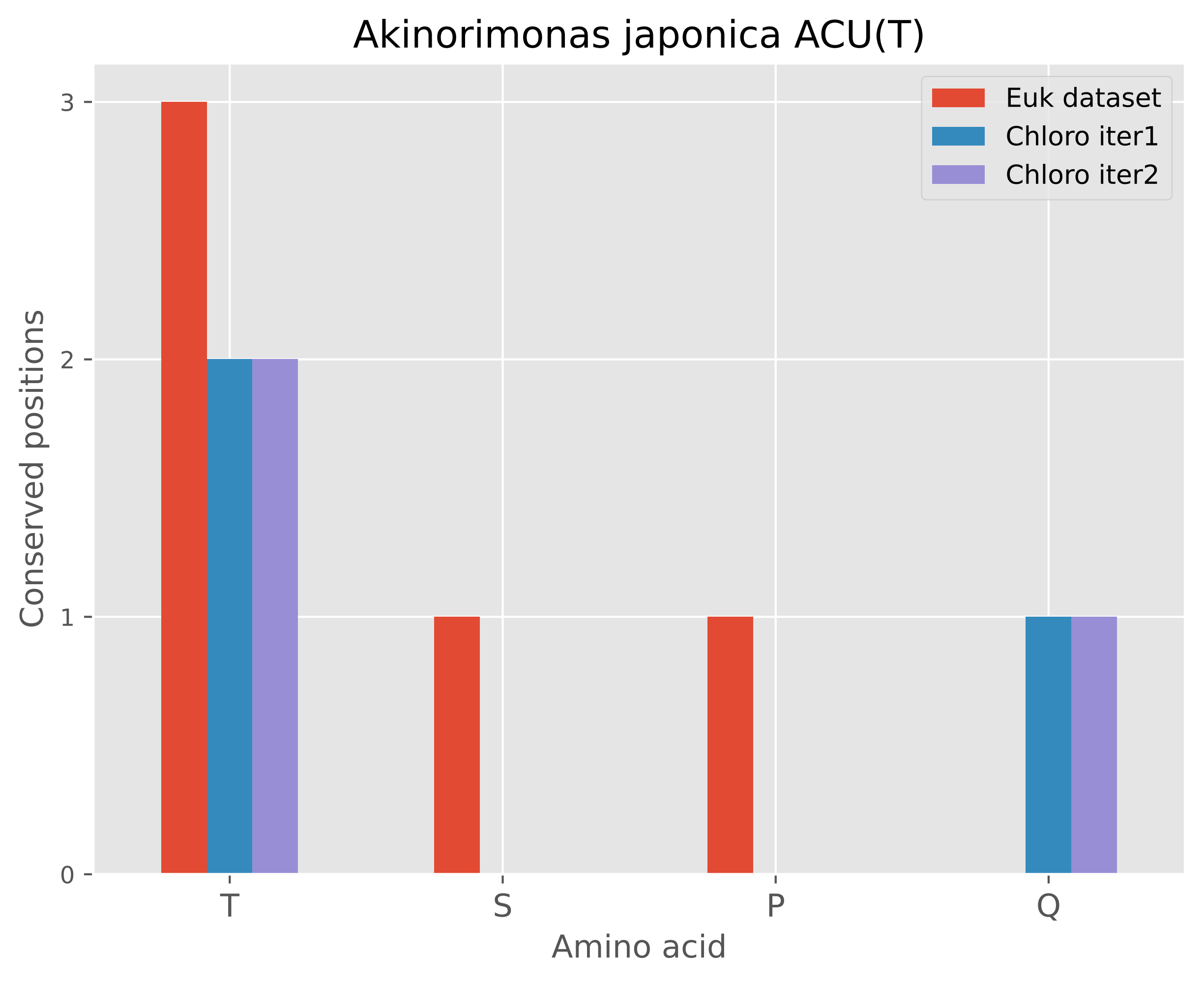
Akinorimonas japonica ACU(T)
Euk dataset
Chloro iter1
Chloro iter2
Conserved positions
0
1
2
3
T
S
P
Q
Amino acid

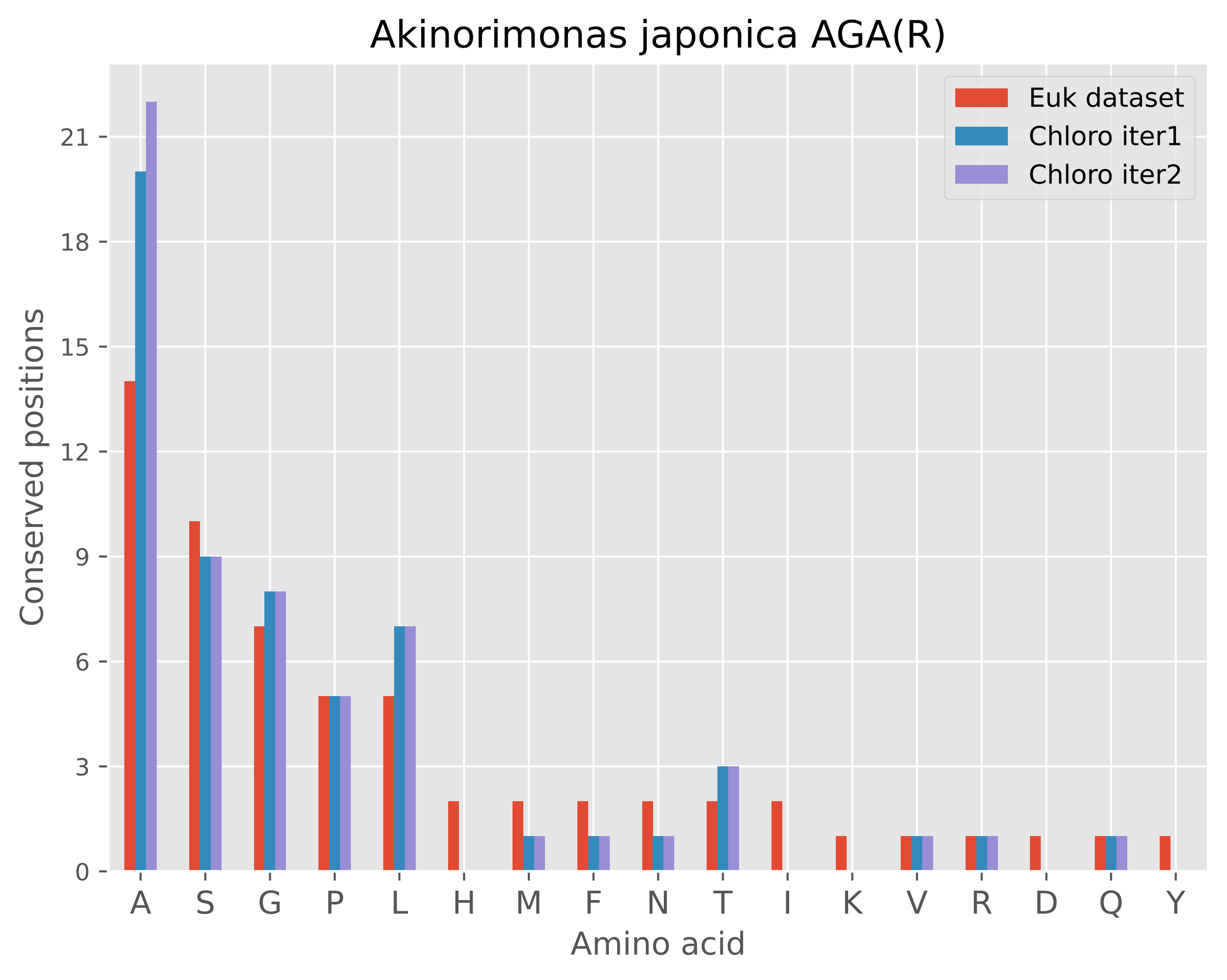
Akinorimonas japonica AGA(R)
Euk dataset
Chloro iter1
Chloro iter2
Conserved positions
0
3
6
9
12
15
18
21
A
S
G
P
L
H
M
F
N
T
I
K
V
R
D
Q
Y
Amino acid

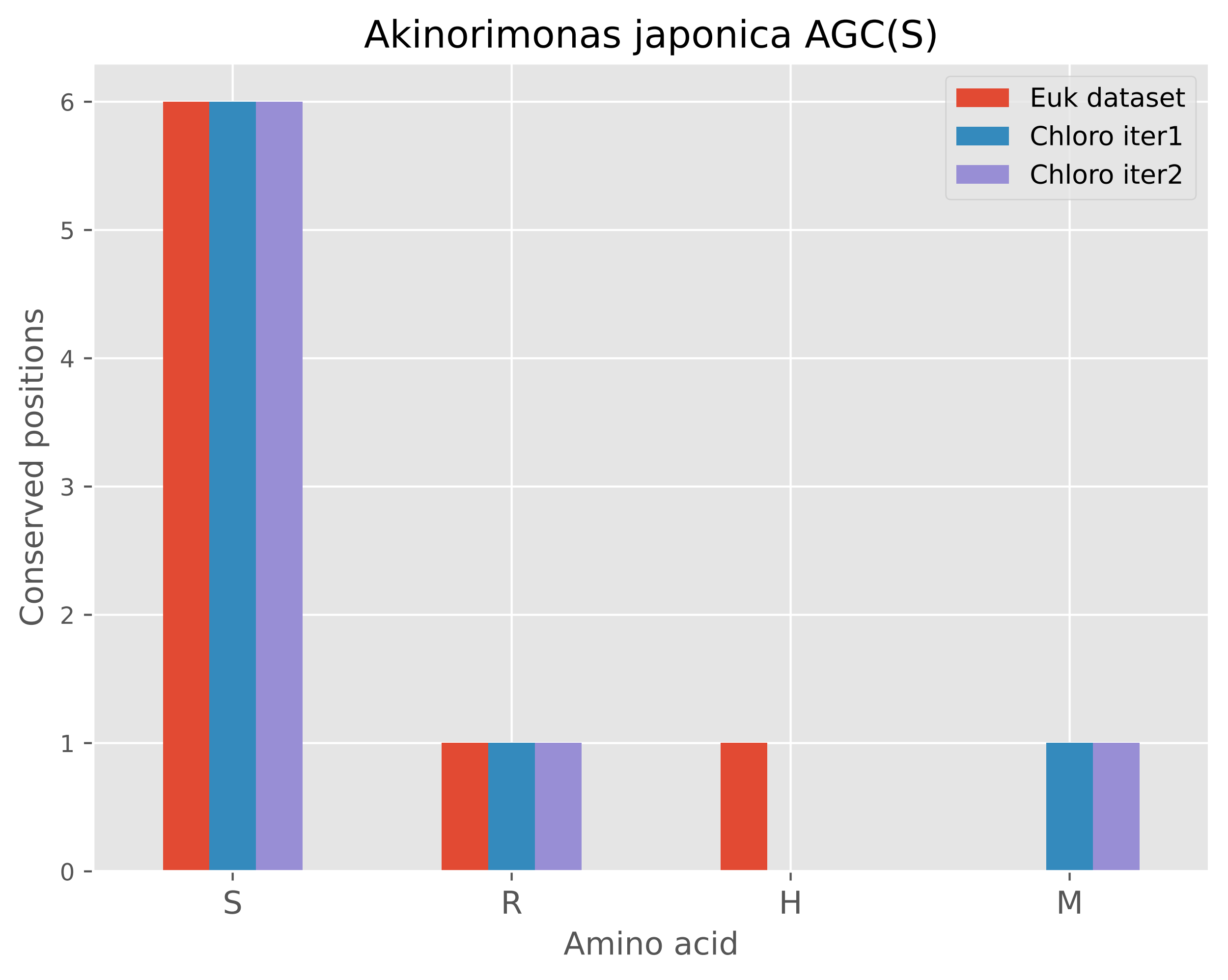

Akinorimonas japonica AGC(S)
Euk dataset
Chloro iter1
Chloro iter2
Conserved positions
0
1
2
3
4
5
6
S
R
H
M
Amino acid

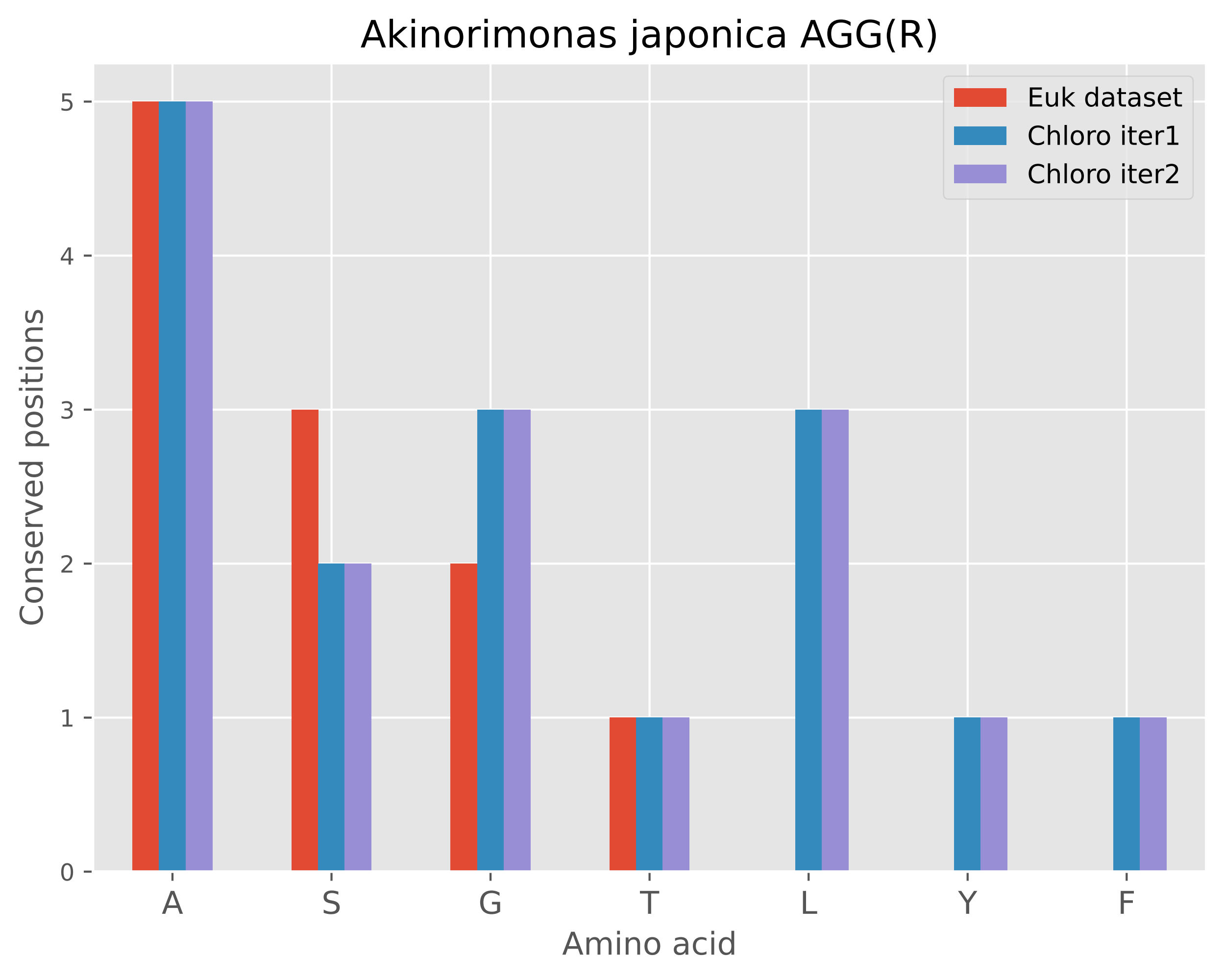

Akinorimonas japonica AGG(R)
Euk dataset
Chloro iter1
Chloro iter2
Conserved positions
0
1
2
3
4
5
A
S
G
T
L
Y
F
Amino acid

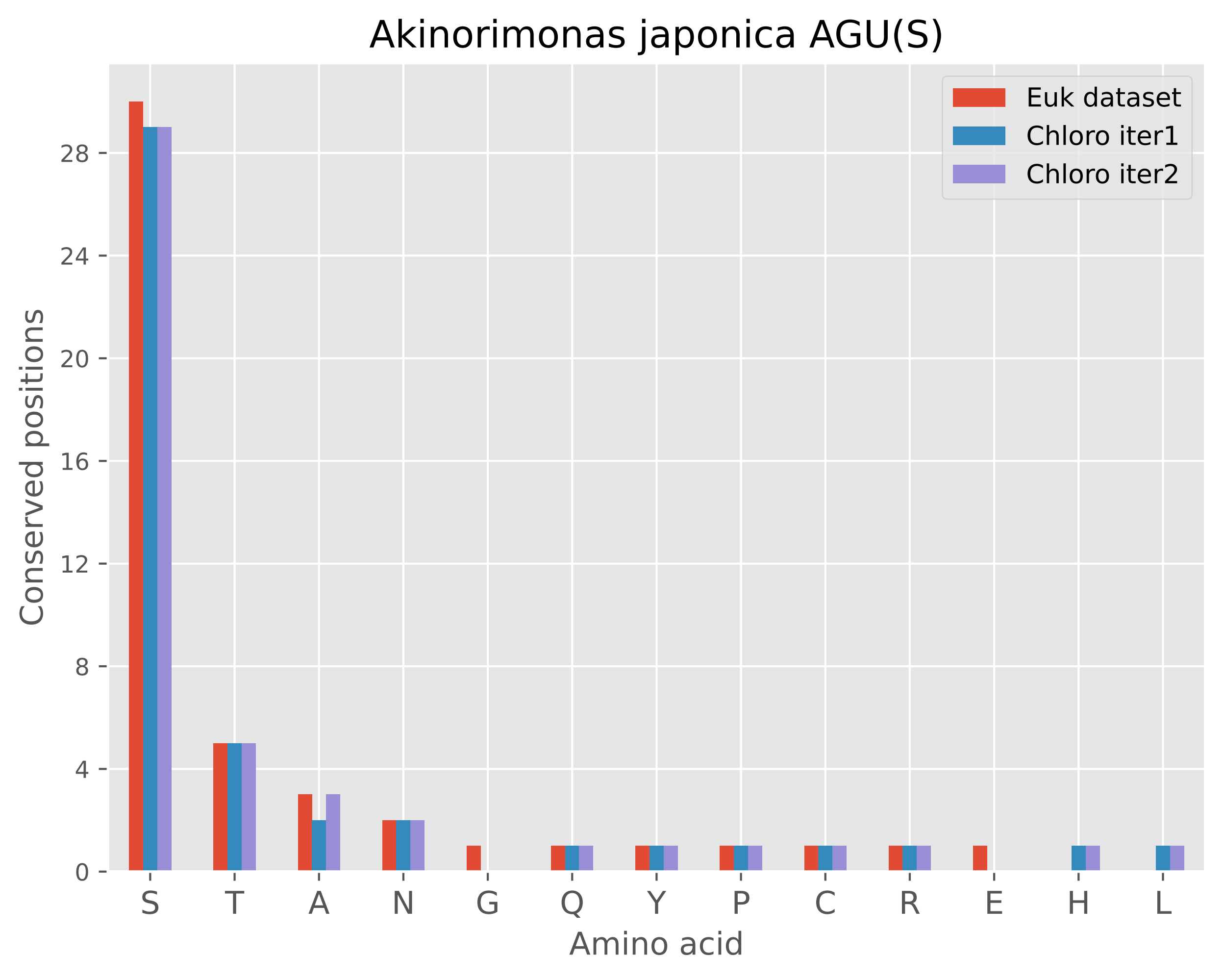

Akinorimonas japonica AGU(S)
Euk dataset
Chloro iter1
Chloro iter2
Conserved positions
0
4
8
12
16
20
24
28
S
T
A
N
G
Q
Y
P
C
R
E
H
L
Amino acid

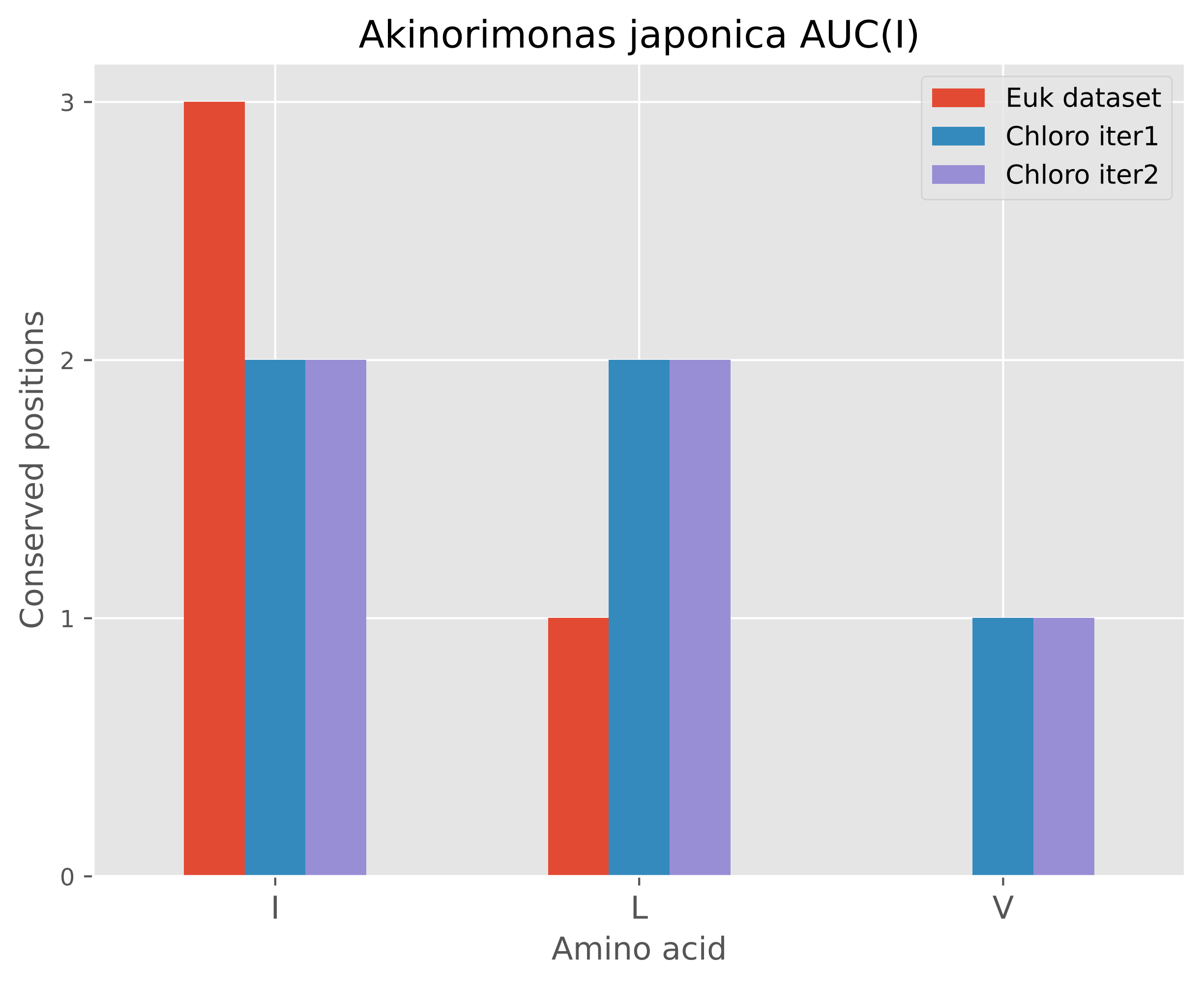
Akinorimonas japonica AUC(I)
Euk dataset
Chloro iter1
Chloro iter2
Conserved positions
0
1
2
3
I
L
V
Amino acid

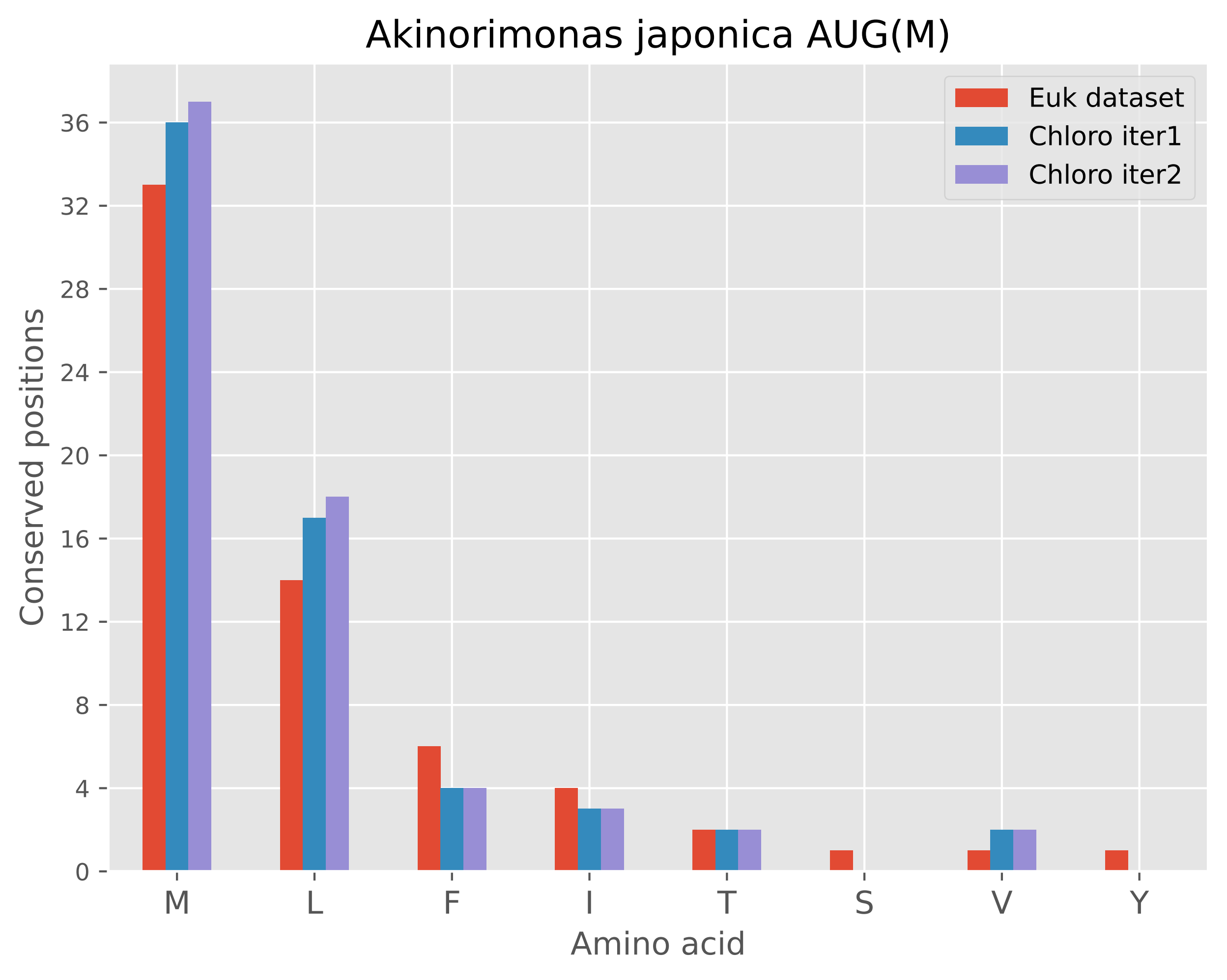
Akinorimonas japonica AUG(M)
Euk dataset
Chloro iter1
Chloro iter2
Conserved positions
0
4
8
12
16
20
24
28
32
36
M
L
F
I
T
S
V
Y
Amino acid

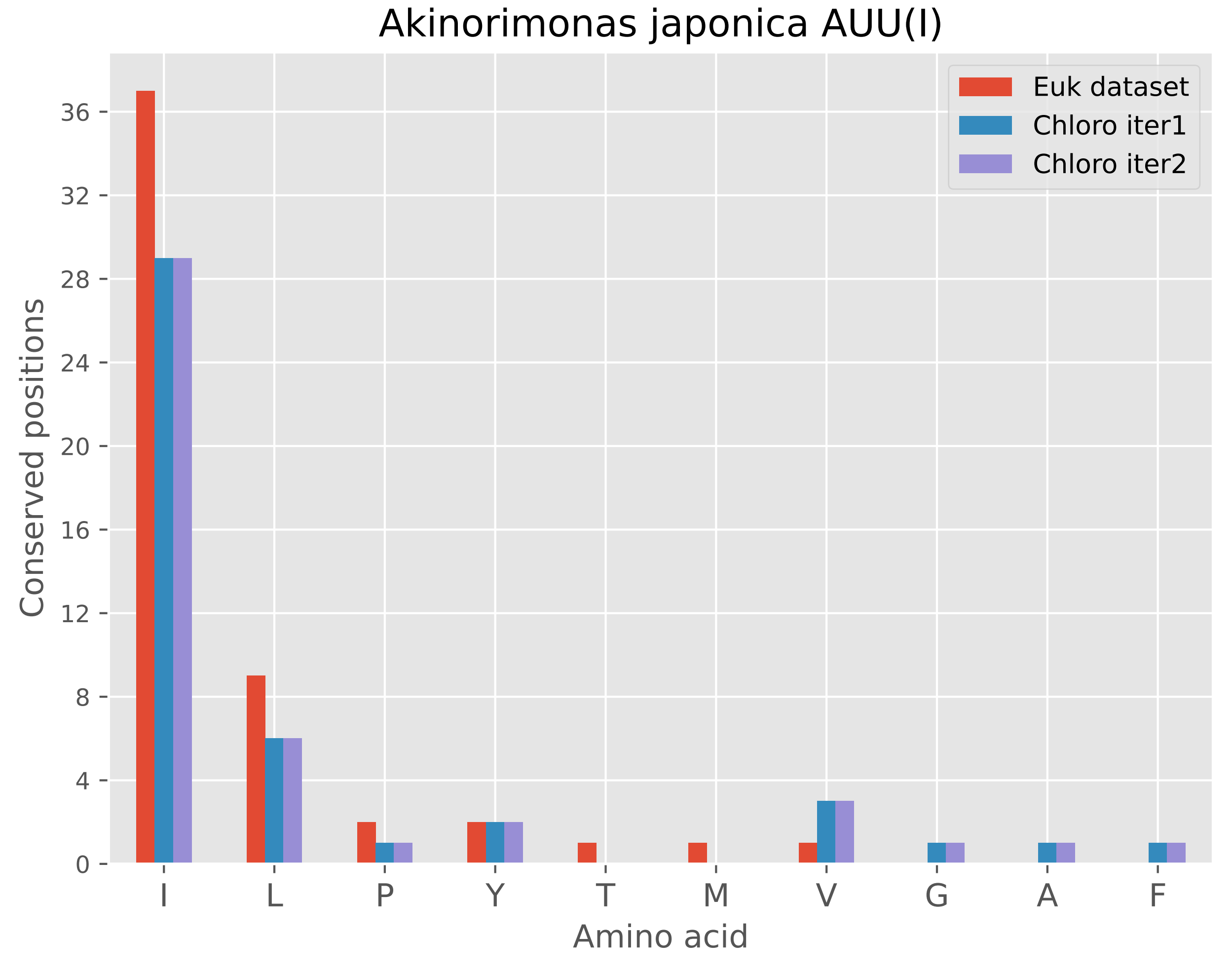
Akinorimonas japonica AUU(I)
Euk dataset
Chloro iter1
Chloro iter2
Conserved positions
0
4
8
12
16
20
24
28
32
36
I
L
P
Y
T
M
V
G
A
F
Amino acid

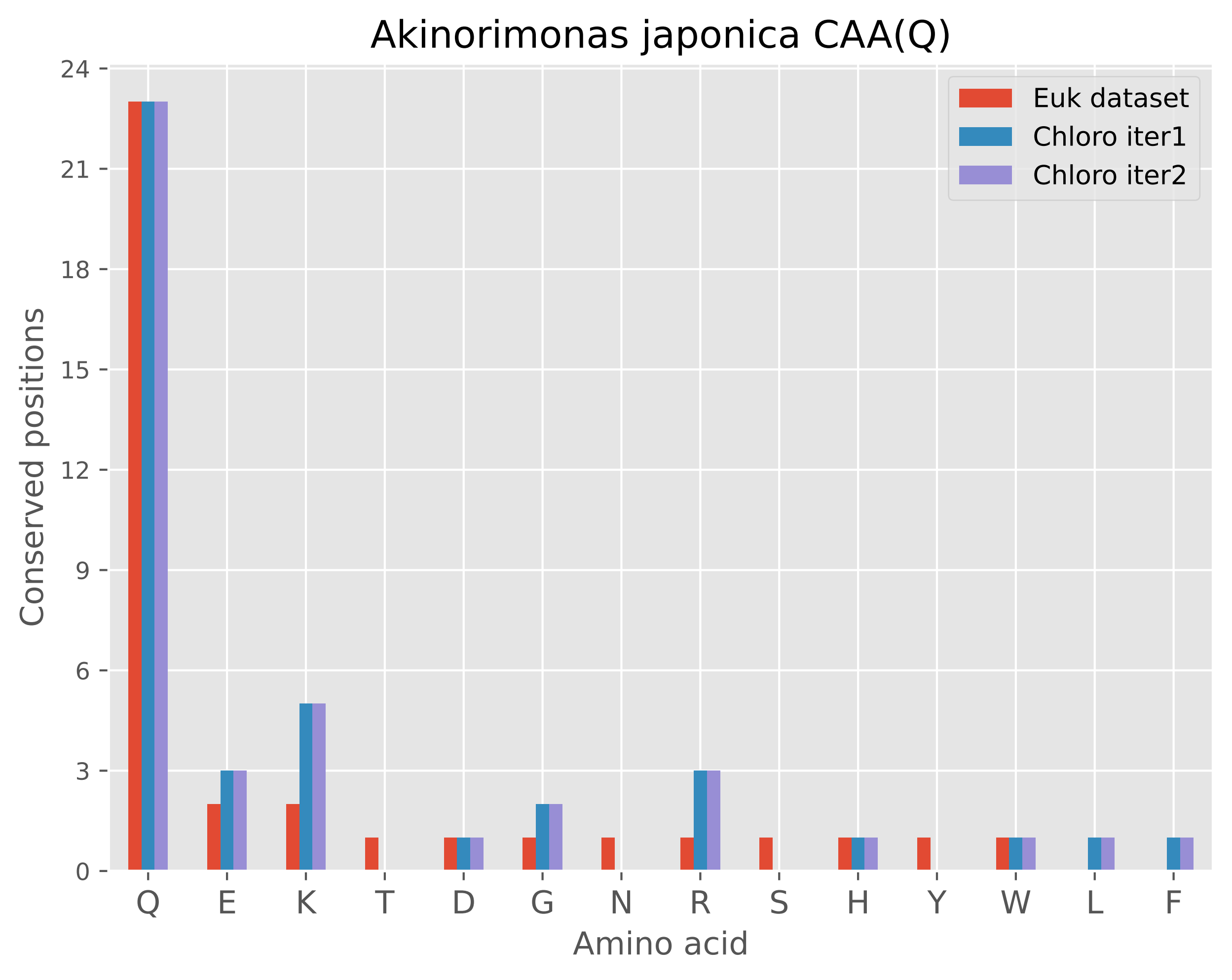

Akinorimonas japonica CAA(Q)
Euk dataset
Chloro iter1
Chloro iter2
Conserved positions
0
3
6
9
12
15
18
21
24
Q
E
K
T
D
G
N
R
S
H
Y
W
L
F
Amino acid

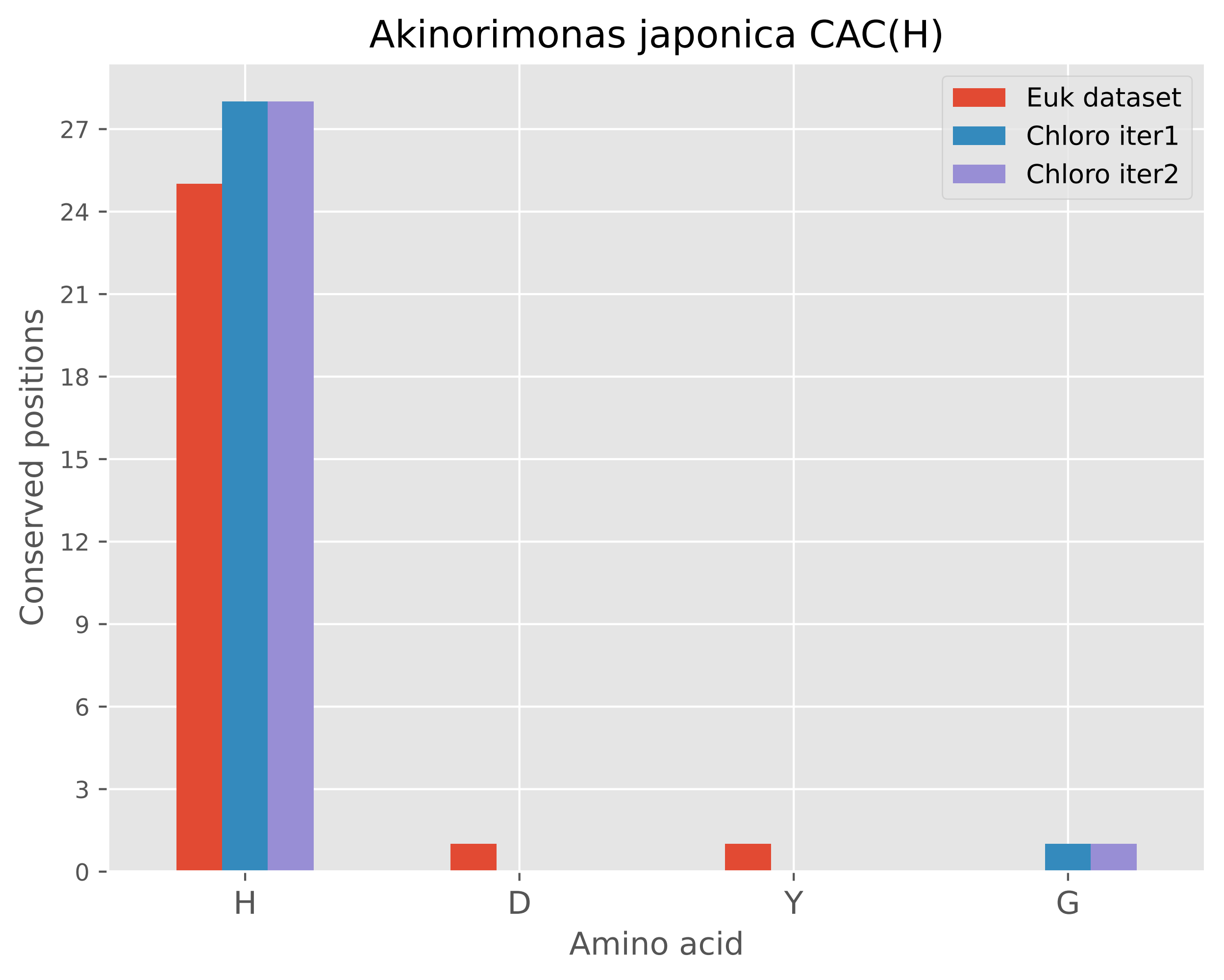
Akinorimonas japonica CAC(H)
Euk dataset
Chloro iter1
Chloro iter2
Conserved positions
0
3
6
9
12
15
18
21
24
27
H
D
Y
G
Amino acid

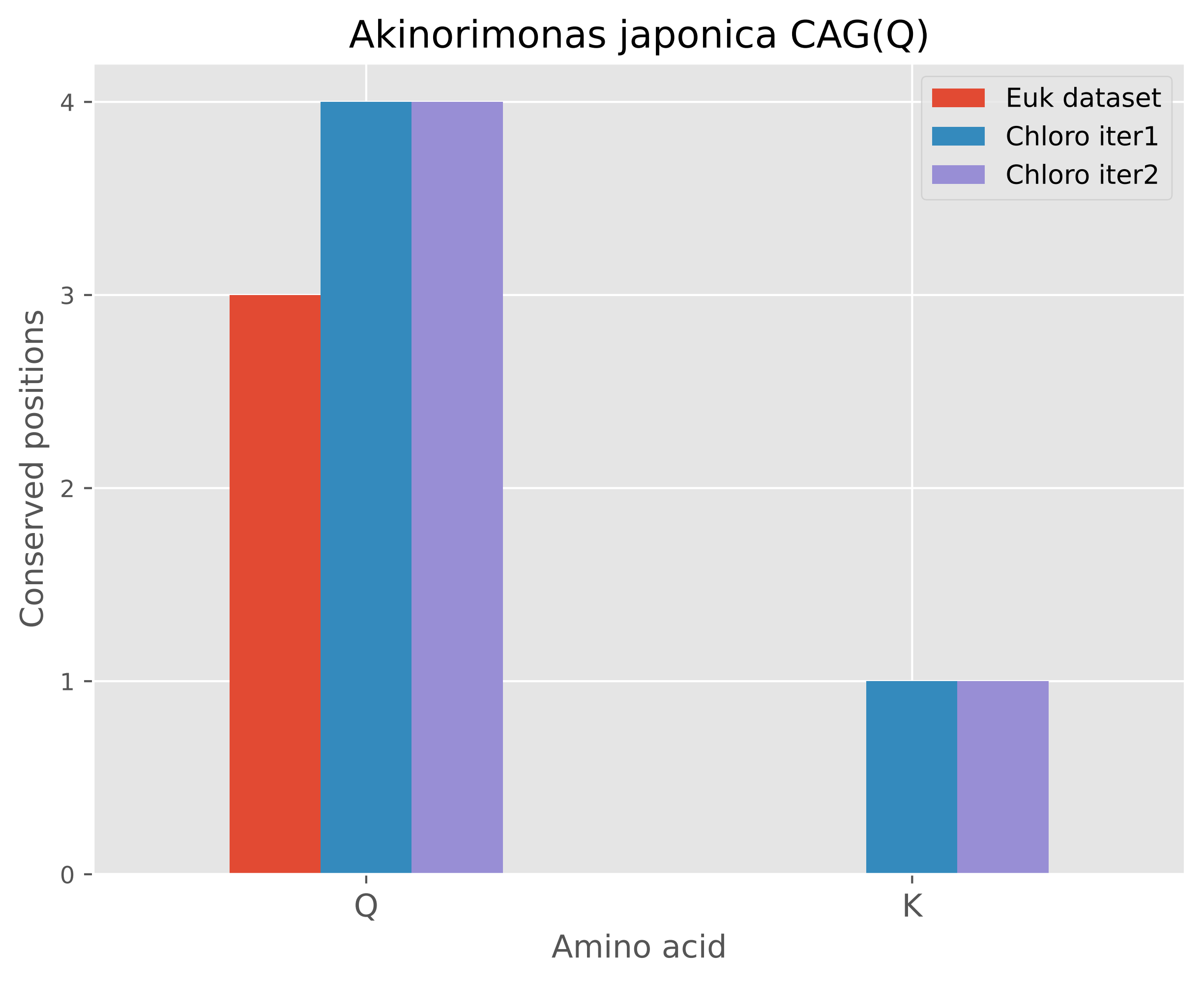
Akinorimonas japonica CAG(Q)
Euk dataset
Chloro iter1
Chloro iter2
Conserved positions
0
1
2
3
4
Q
K
Amino acid

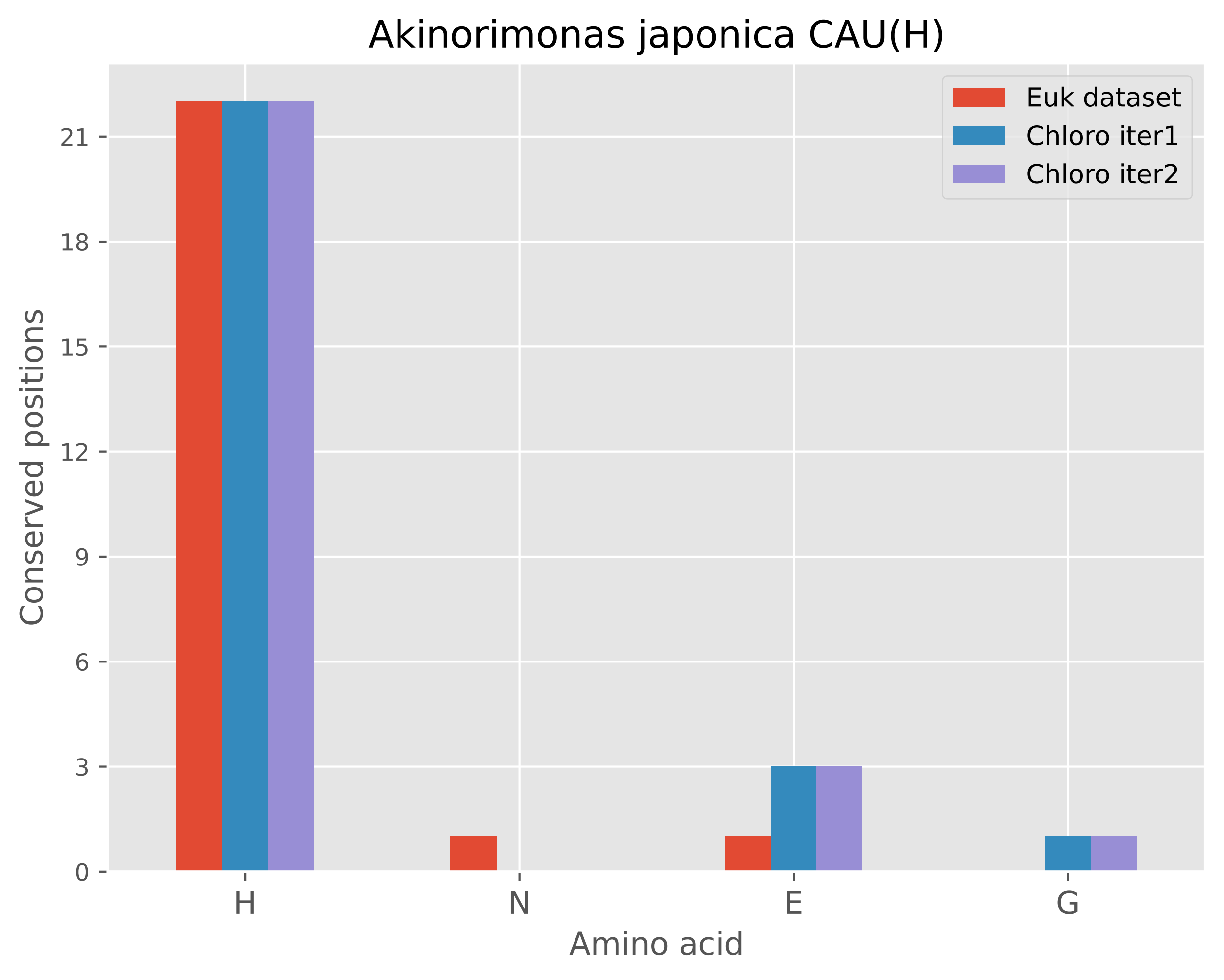
Akinorimonas japonica CAU(H)
Euk dataset
Chloro iter1
Chloro iter2
Conserved positions
0
3
6
9
12
15
18
21
H
N
E
G
Amino acid

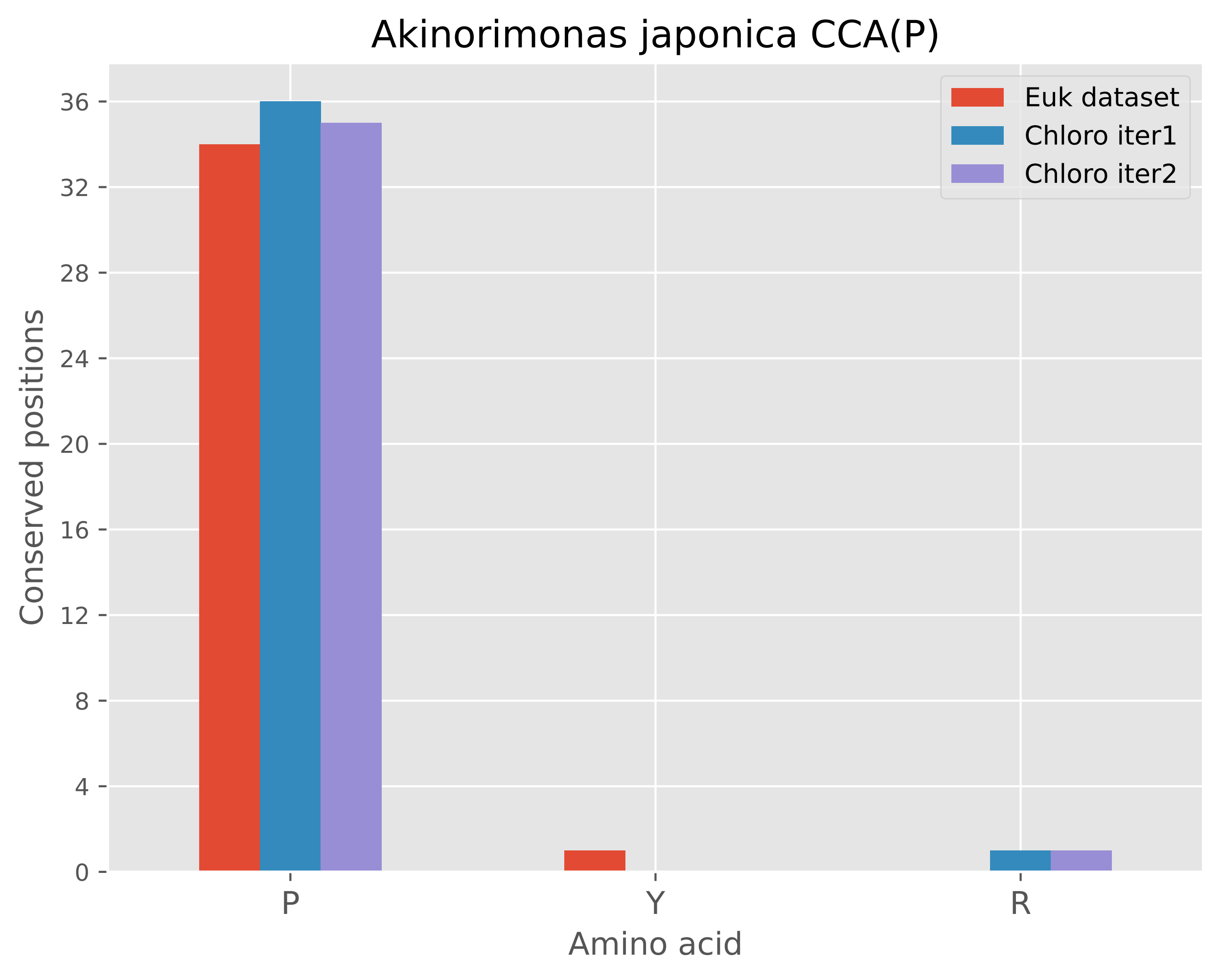
Akinorimonas japonica CCA(P)
Euk dataset
Chloro iter1
Chloro iter2
Conserved positions
0
4
8
12
16
20
24
28
32
36
P
Y
R
Amino acid

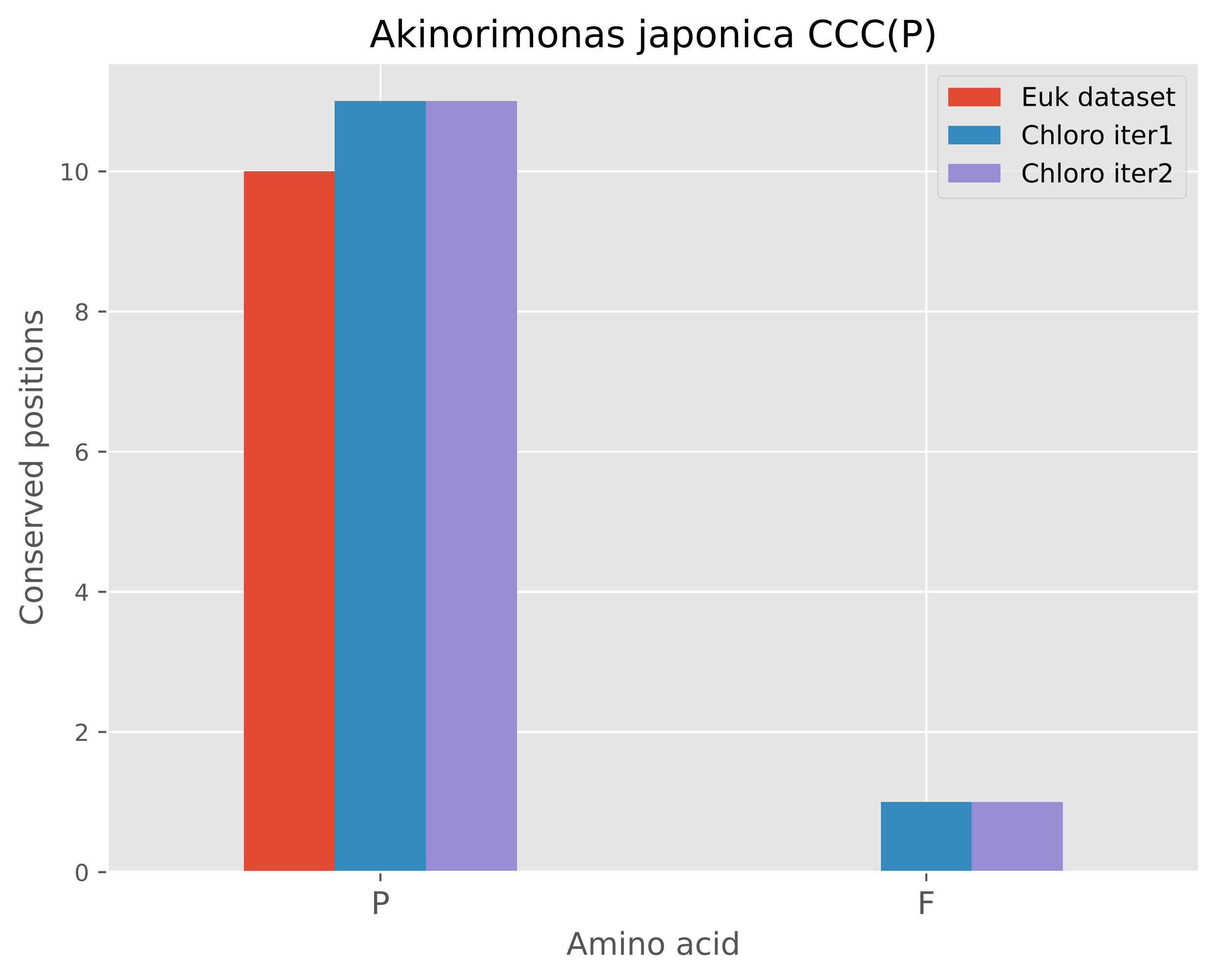

Akinorimonas japonica CCC(P)
Euk dataset
Chloro iter1
Chloro iter2
Conserved positions
0
2
4
6
8
10
P
F
Amino acid

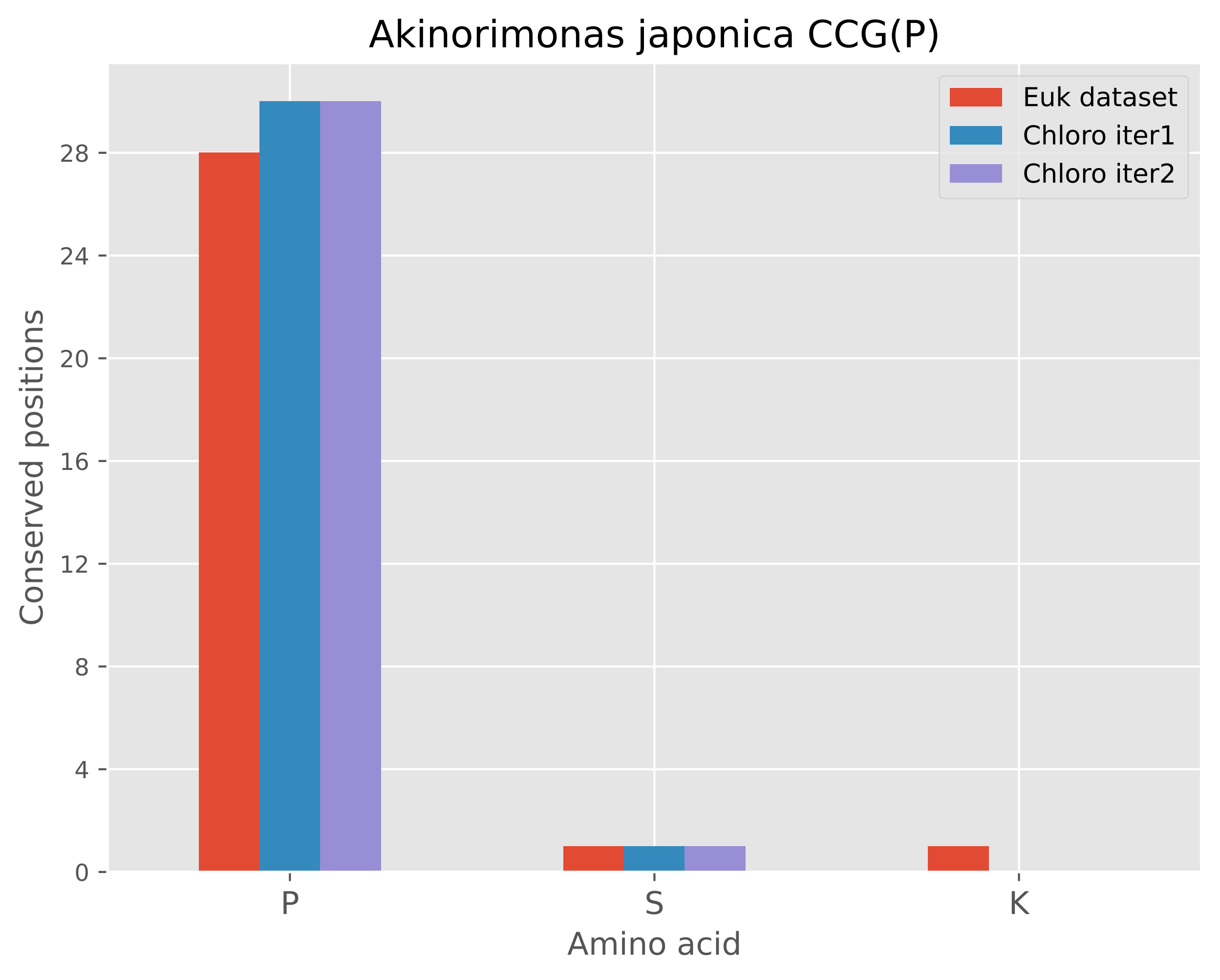
Akinorimonas japonica CCG(P)
Euk dataset
Chloro iter1
Chloro iter2
Conserved positions
0
4
8
12
16
20
24
28
P
S
K
Amino acid

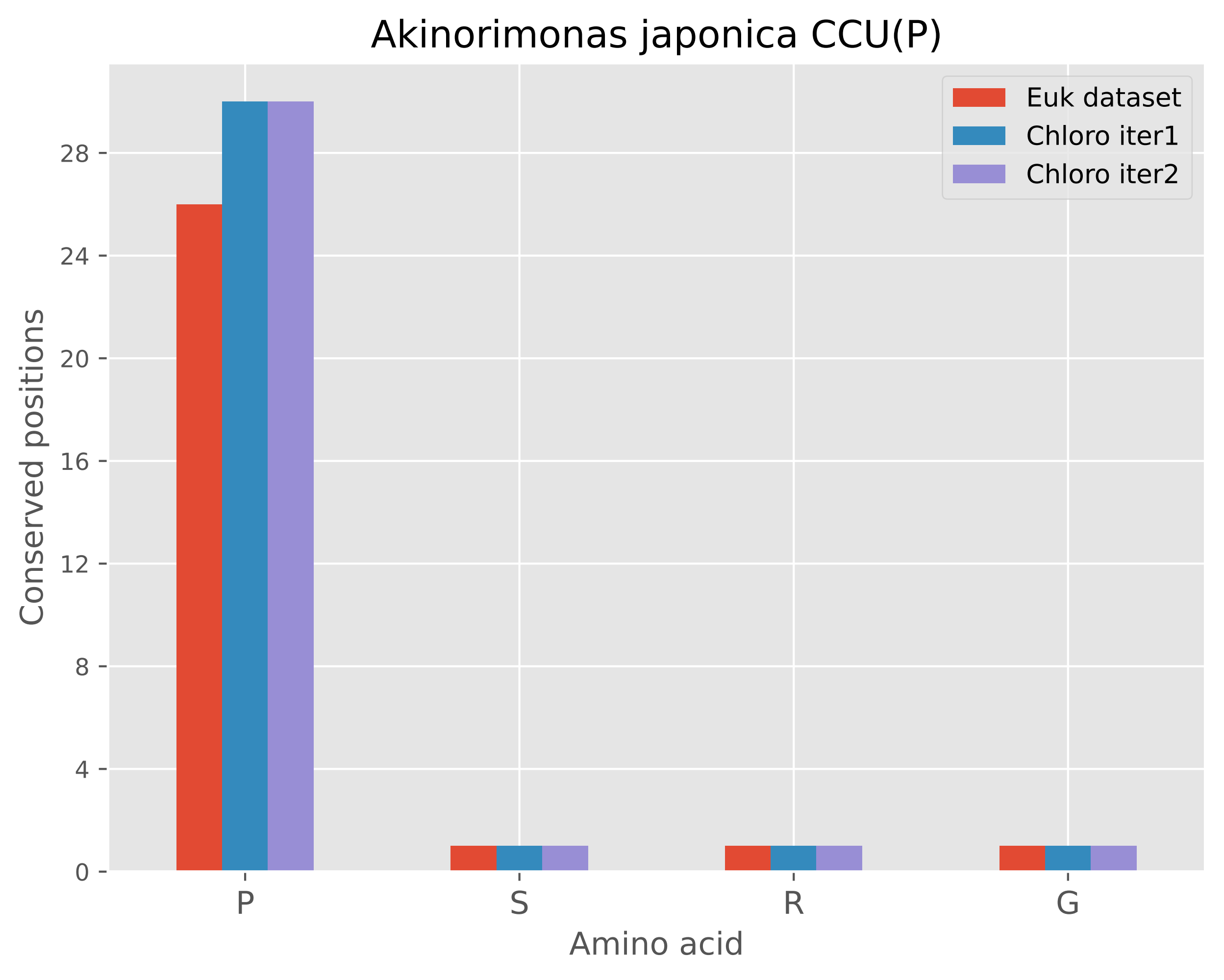
Akinorimonas japonica CCU(P)
Euk dataset
Chloro iter1
Chloro iter2
Conserved positions
0
4
8
12
16
20
24
28
P
S
R
G
Amino acid

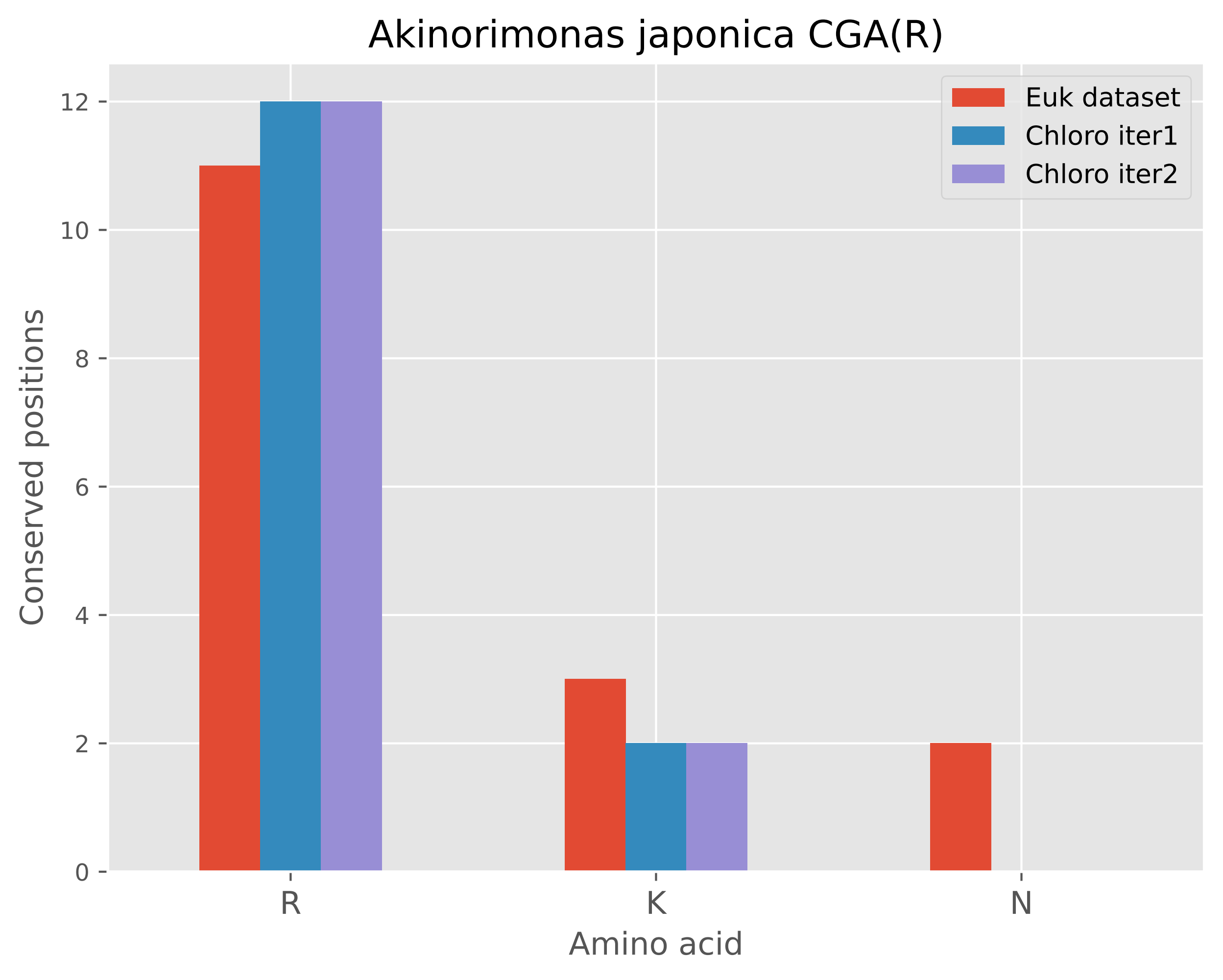

Akinorimonas japonica CGA(R)
Euk dataset
Chloro iter1
Chloro iter2
Conserved positions
0
2
4
6
8
10
12
R
K
N
Amino acid

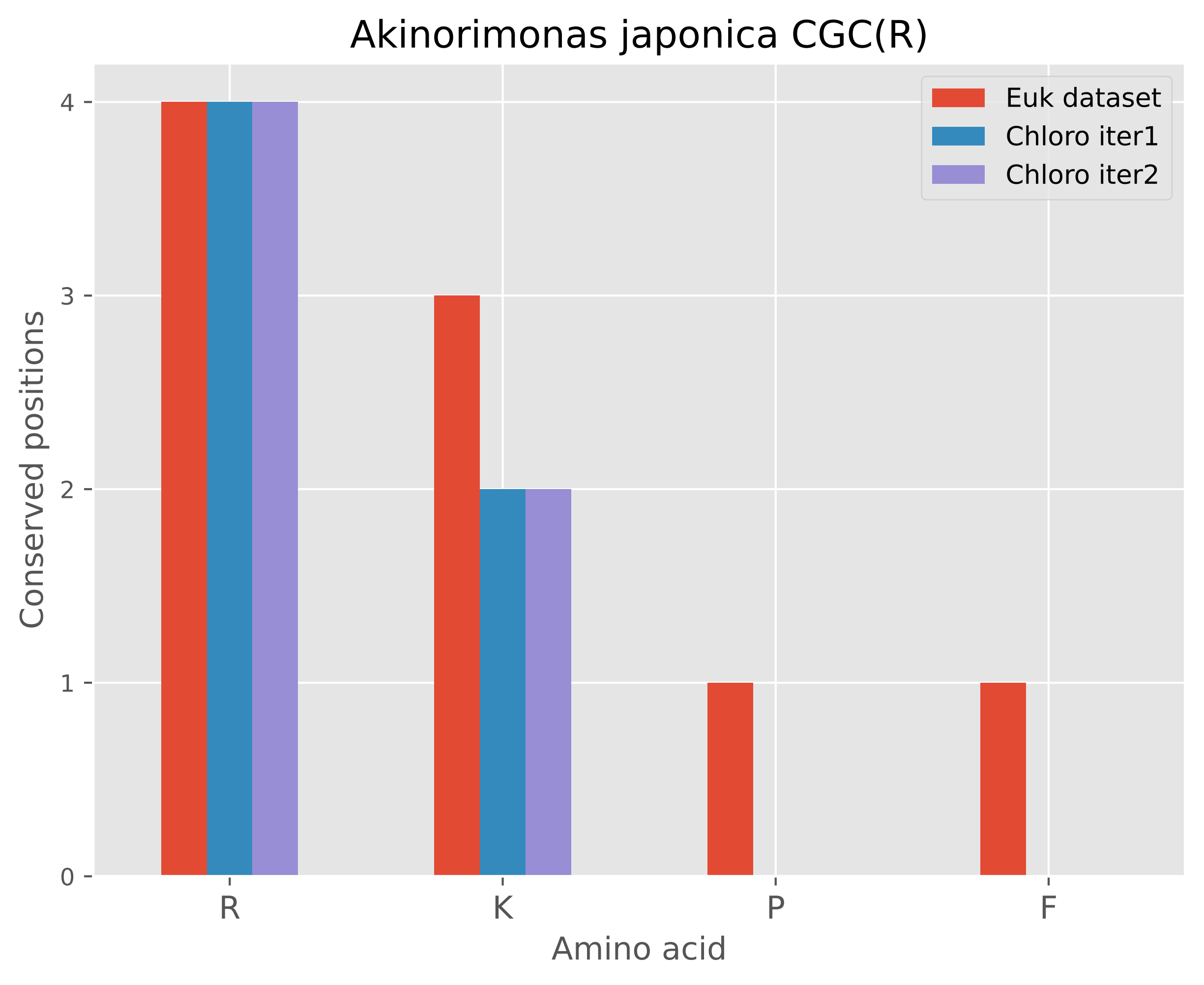
Akinorimonas japonica CGC(R)
Conserved positions
Amino acid
0
1
2
3
4
R
K
P
F
Euk dataset
Chloro iter1
Chloro iter2

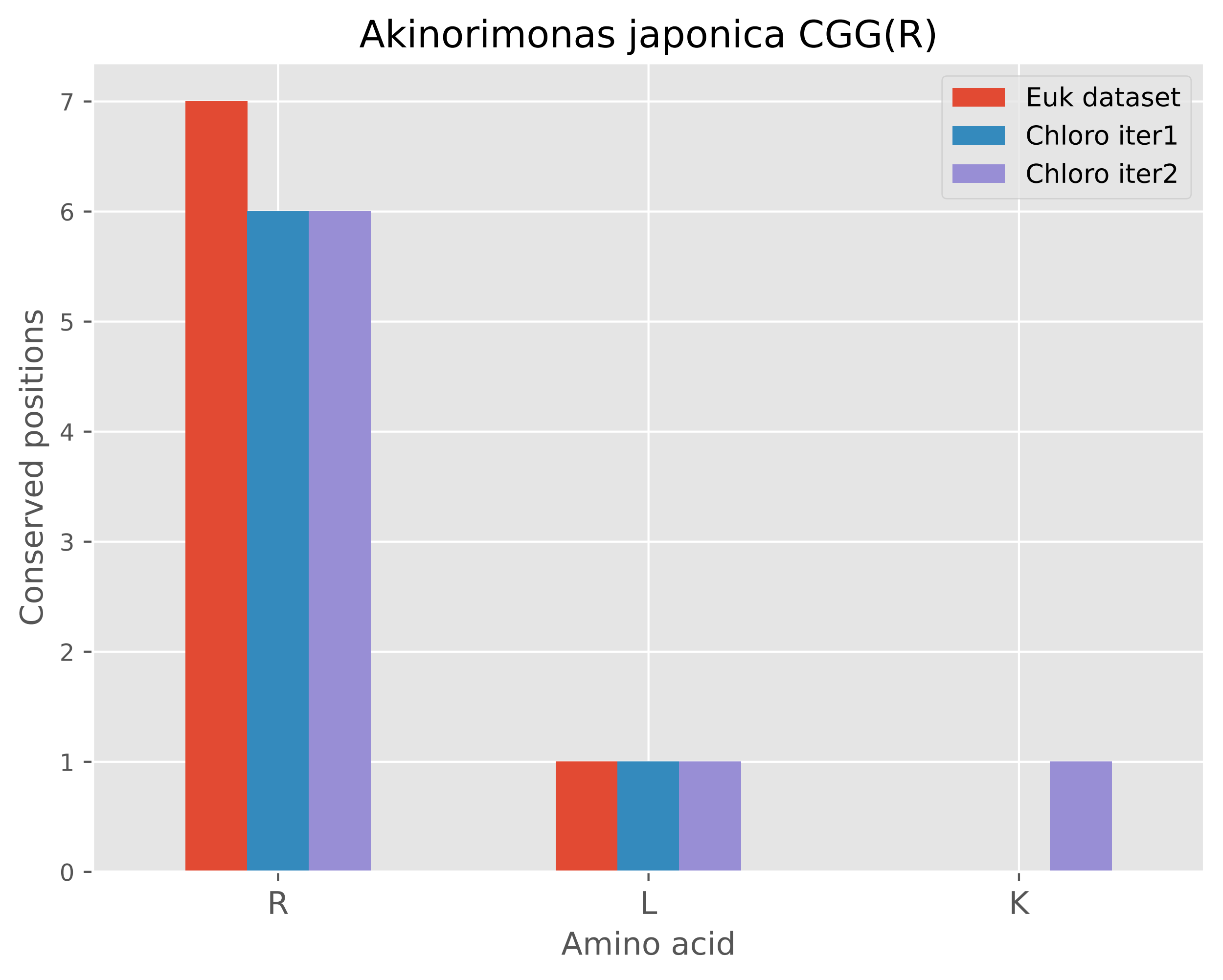

Akinorimonas japonica CGG(R)
Euk dataset
Chloro iter1
Chloro iter2
Conserved positions
0
1
2
3
4
5
6
7
R
L
K
Amino acid

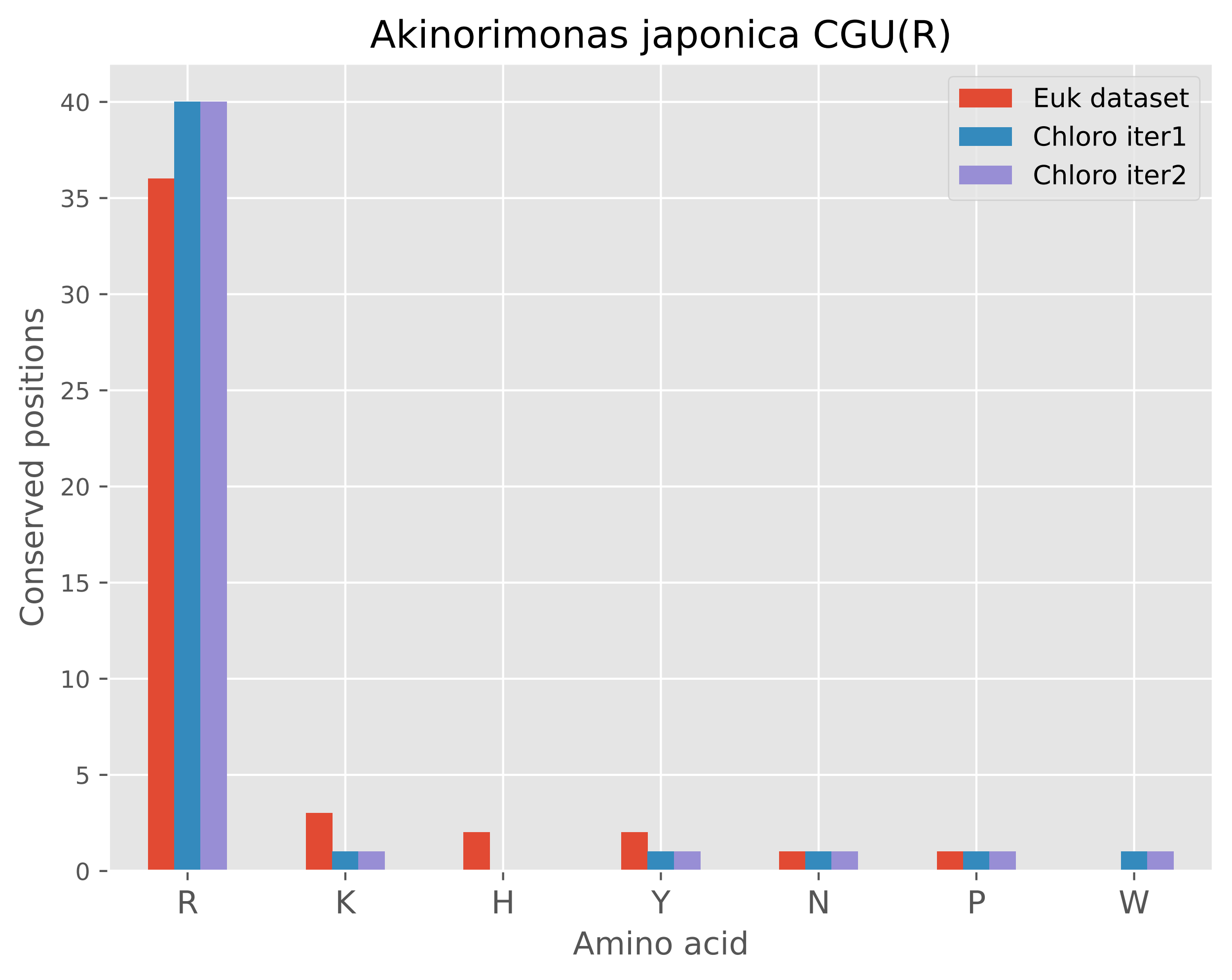

Akinorimonas japonica CGU(R)
Euk dataset
Chloro iter1
Chloro iter2
Conserved positions
0
5
10
15
20
25
30
35
40
R
K
H
Y
N
P
W
Amino acid

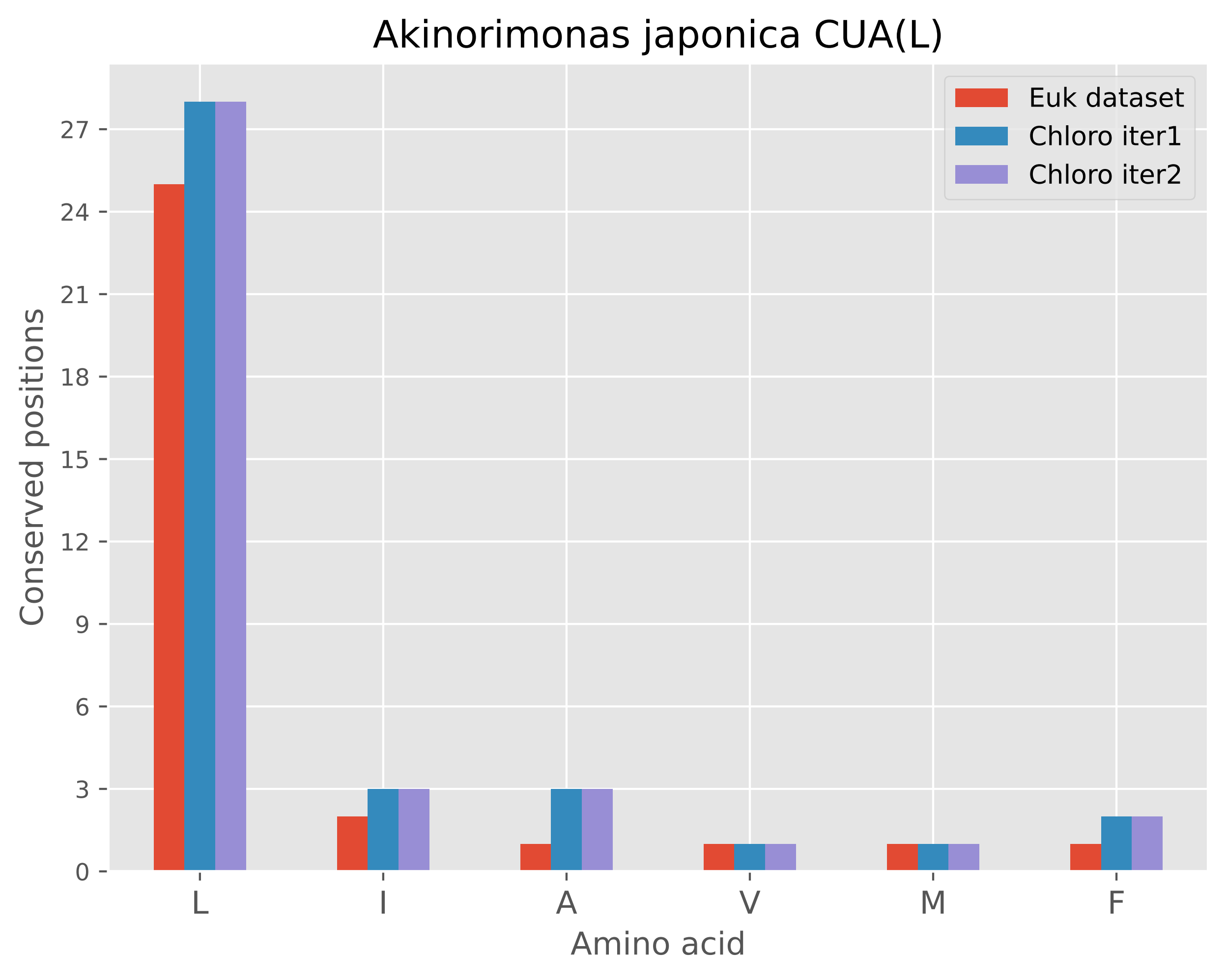
Akinorimonas japonica CUA(L)
Euk dataset
Chloro iter1
Chloro iter2
Conserved positions
0
3
6
9
12
15
18
21
24
27
L
I
A
V
M
F
Amino acid

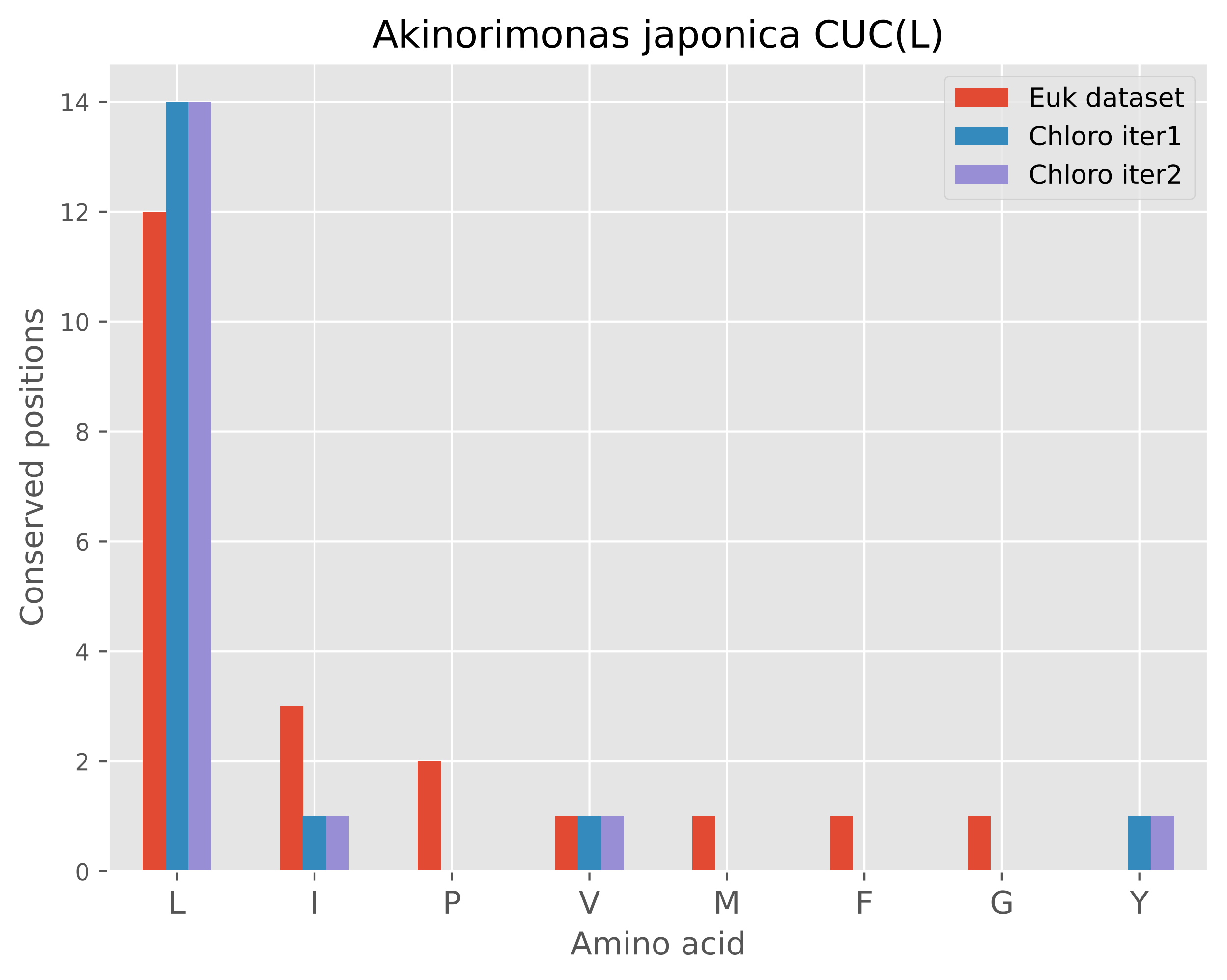

Akinorimonas japonica CUC(L)
Euk dataset
Chloro iter1
Chloro iter2
Conserved positions
0
2
4
6
8
10
12
14
L
I
P
V
M
F
G
Y
Amino acid

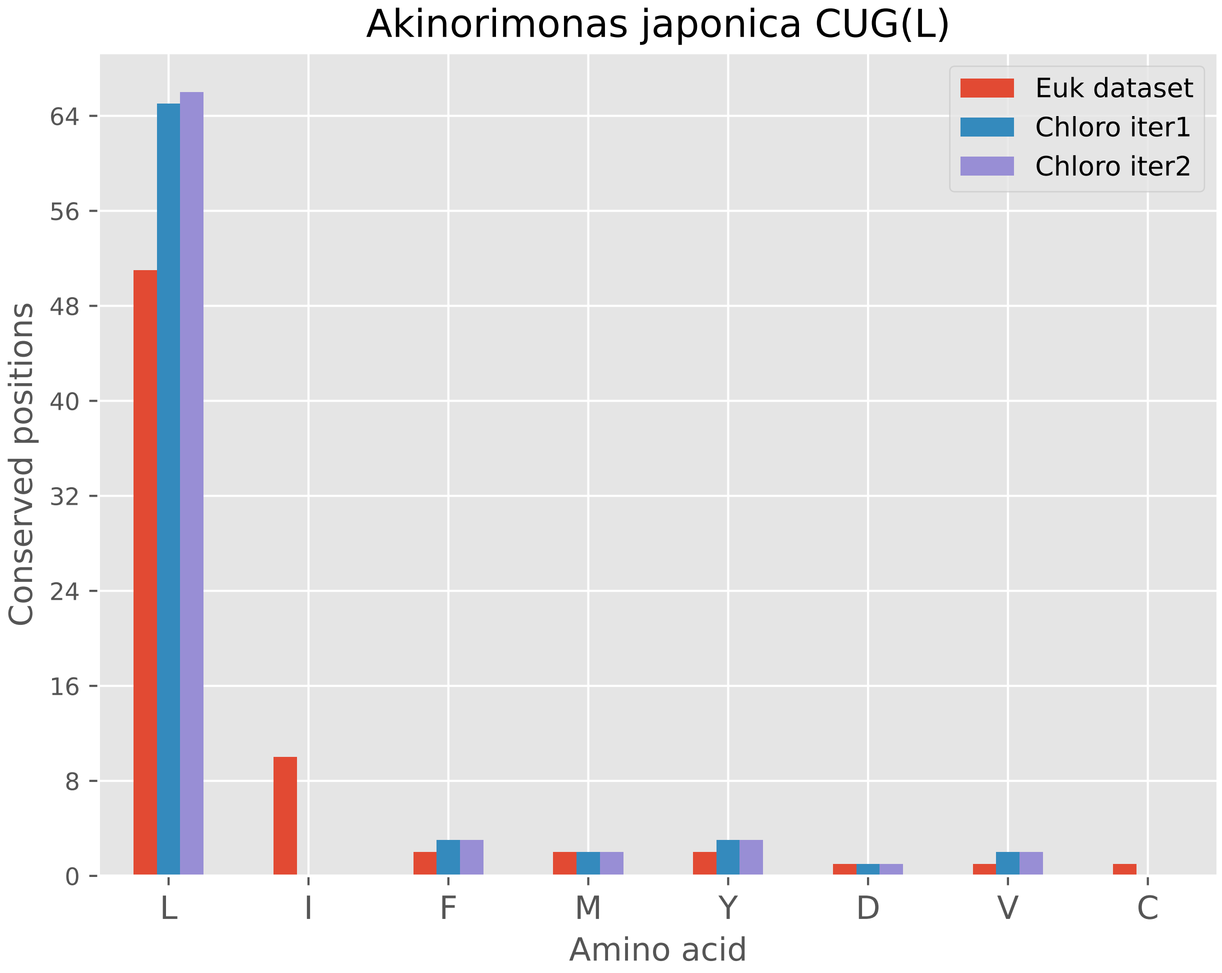

Akinorimonas japonica CUG(L)
Euk dataset
Chloro iter1
Chloro iter2
Conserved positions
0
8
16
24
32
40
48
56
64
L
I
F
M
Y
D
V
C
Amino acid

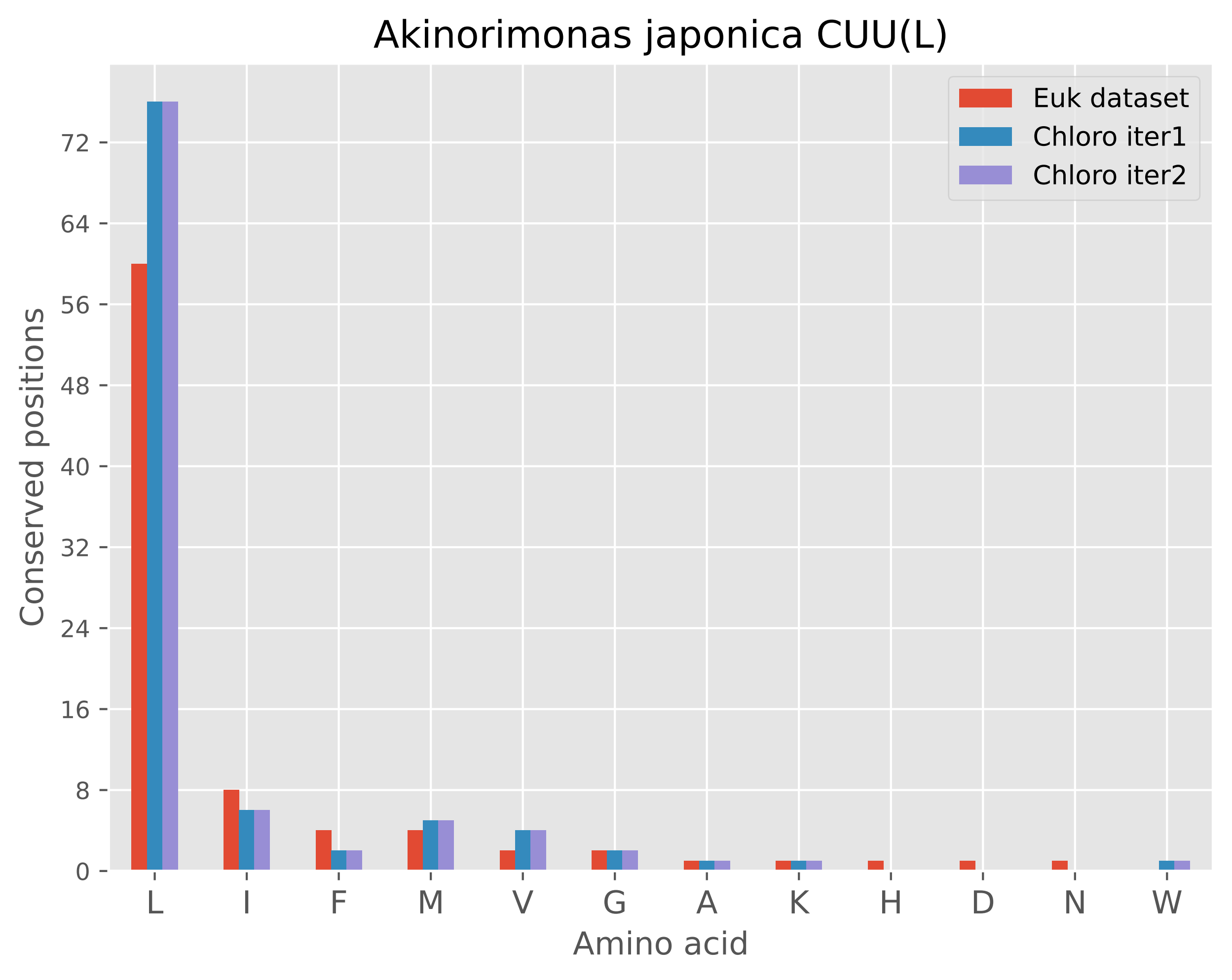

Akinorimonas japonica CUU(L)
Euk dataset
Chloro iter1
Chloro iter2
Conserved positions
0
8
16
24
32
40
48
56
64
72
L
I
F
M
V
G
A
K
H
D
N
W
Amino acid

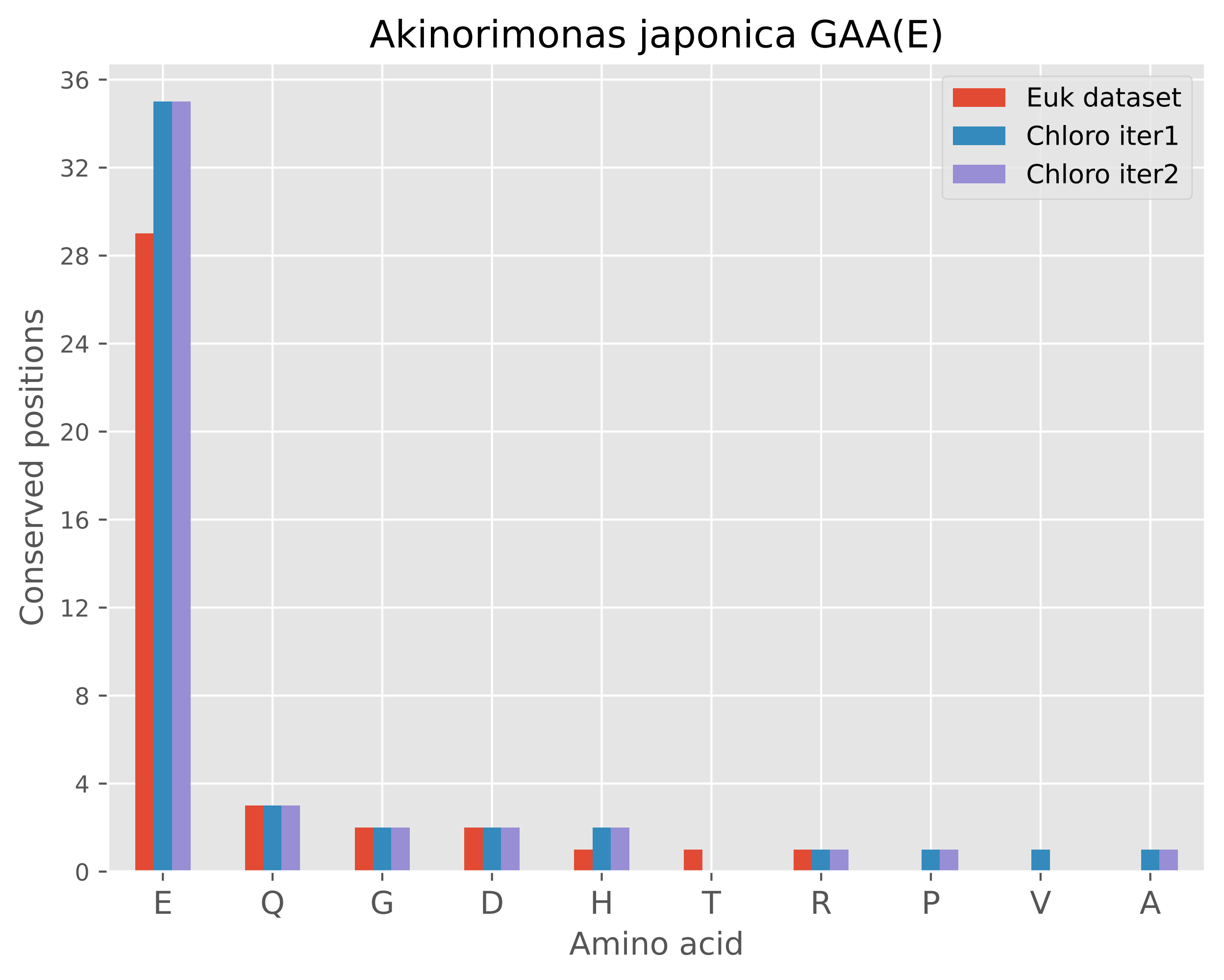
Akinorimonas japonica GAA(E)
Euk dataset
Chloro iter1
Chloro iter2
Conserved positions
0
4
8
12
16
20
24
28
32
36
E
Q
G
D
H
T
R
P
V
A
Amino acid

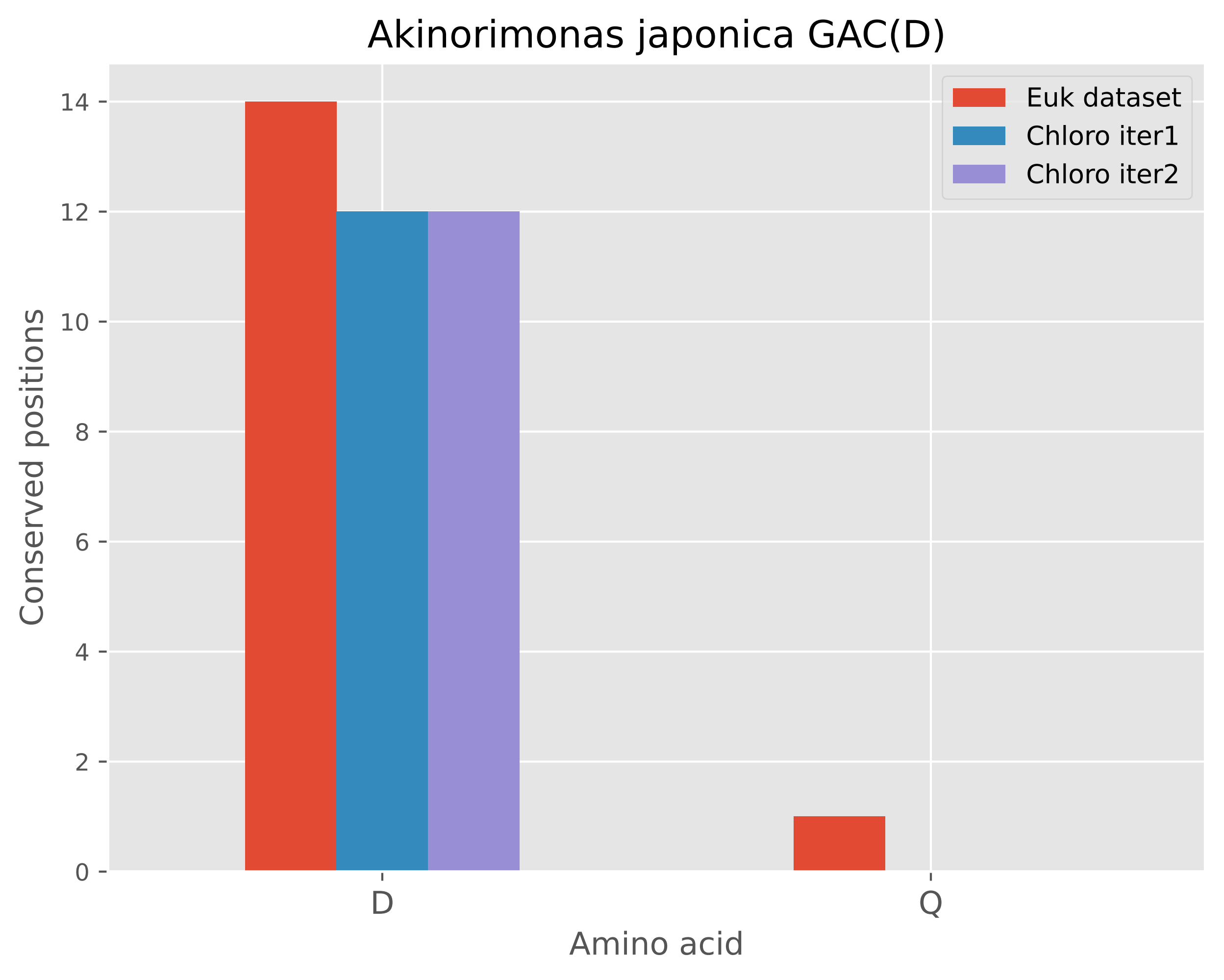

Akinorimonas japonica GAC(D)
Euk dataset
Chloro iter1
Chloro iter2
Conserved positions
0
2
4
6
8
10
12
14
D
Q
Amino acid

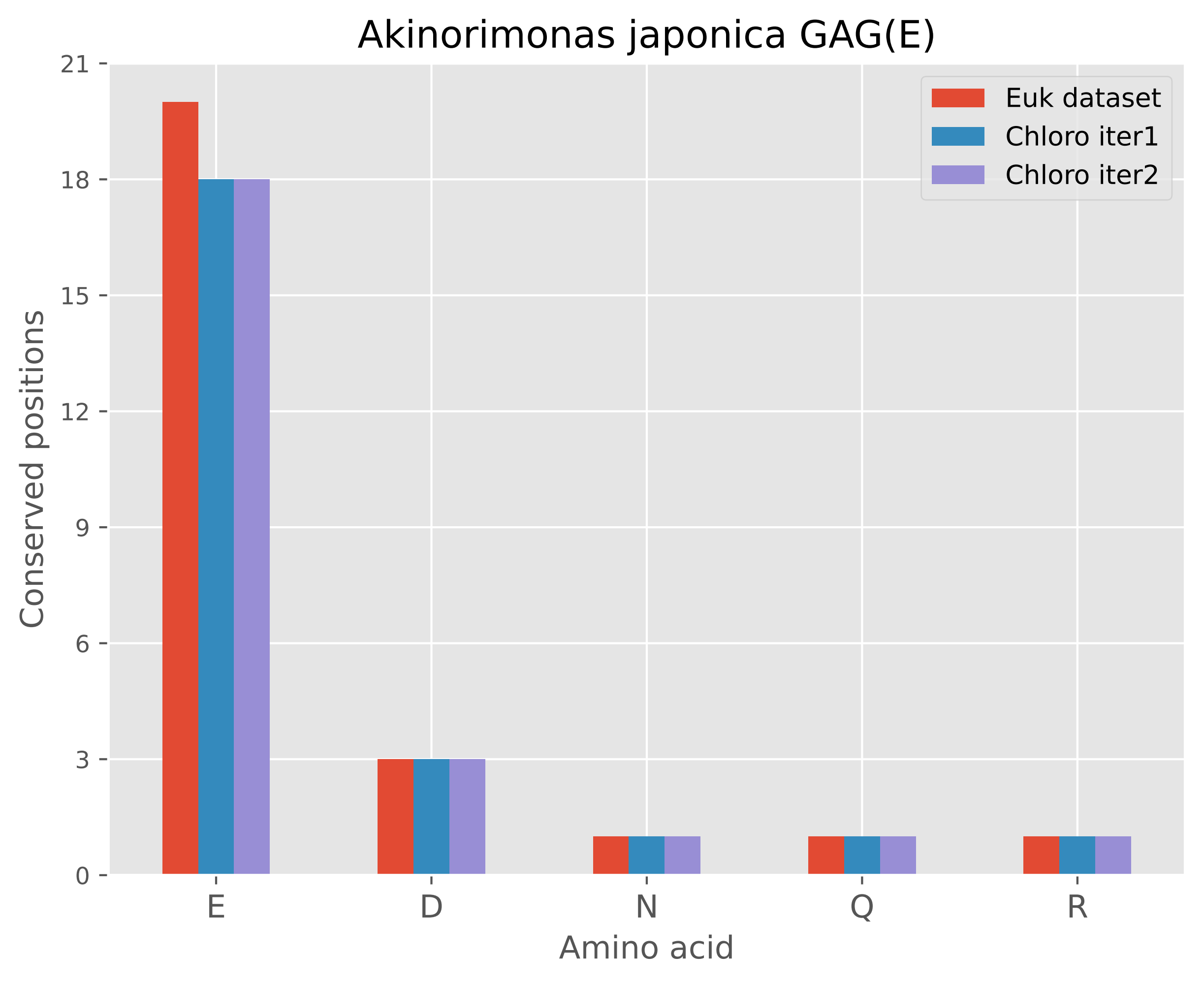

Akinorimonas japonica GAG(E)
Euk dataset
Chloro iter1
Chloro iter2
Conserved positions
Amino acid
0
3
6
9
12
15
18
21
E
D
N
Q
R

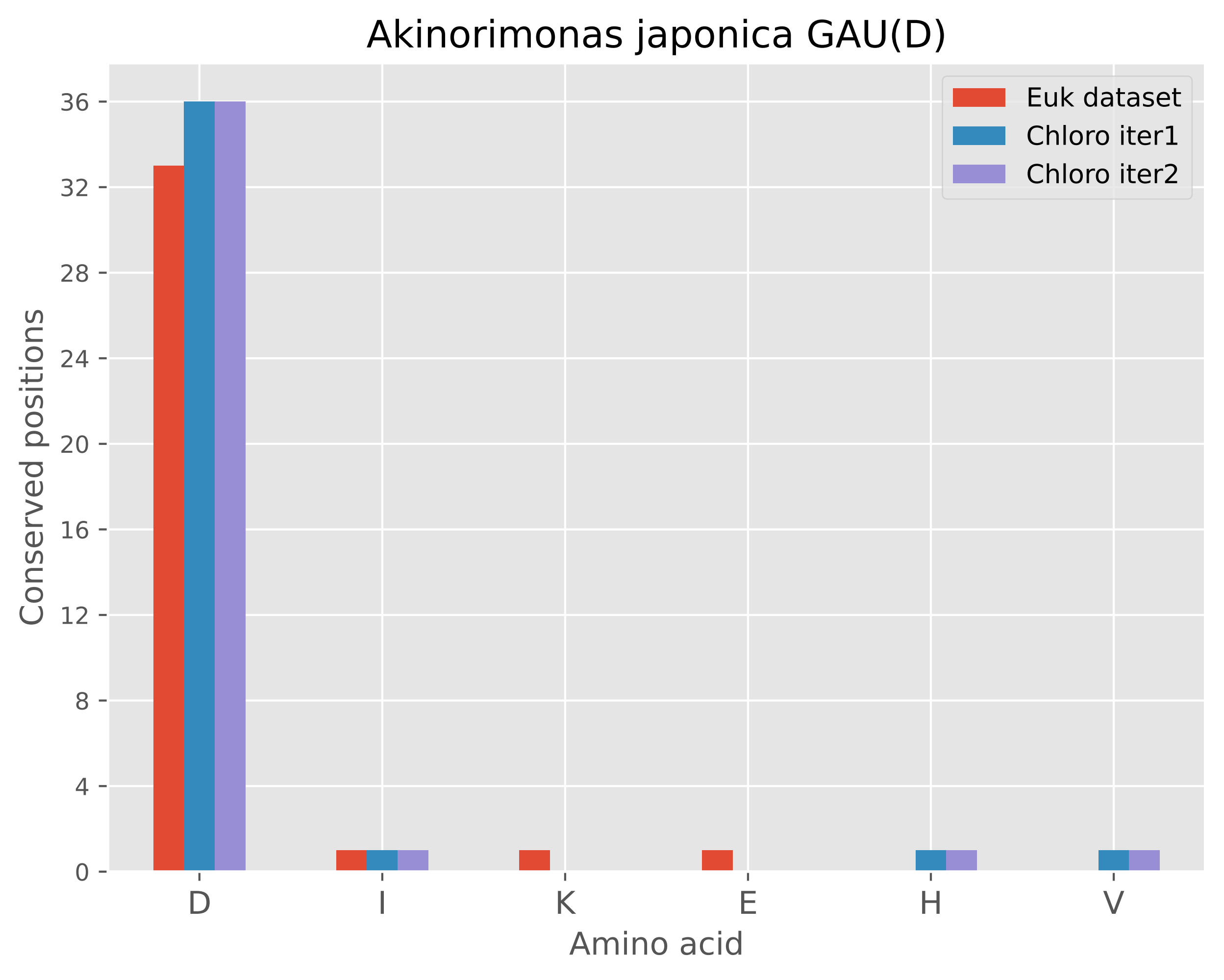

Akinorimonas japonica GAU(D)
Euk dataset
Chloro iter1
Chloro iter2
Conserved positions
0
4
8
12
16
20
24
28
32
36
D
I
K
E
H
V
Amino acid

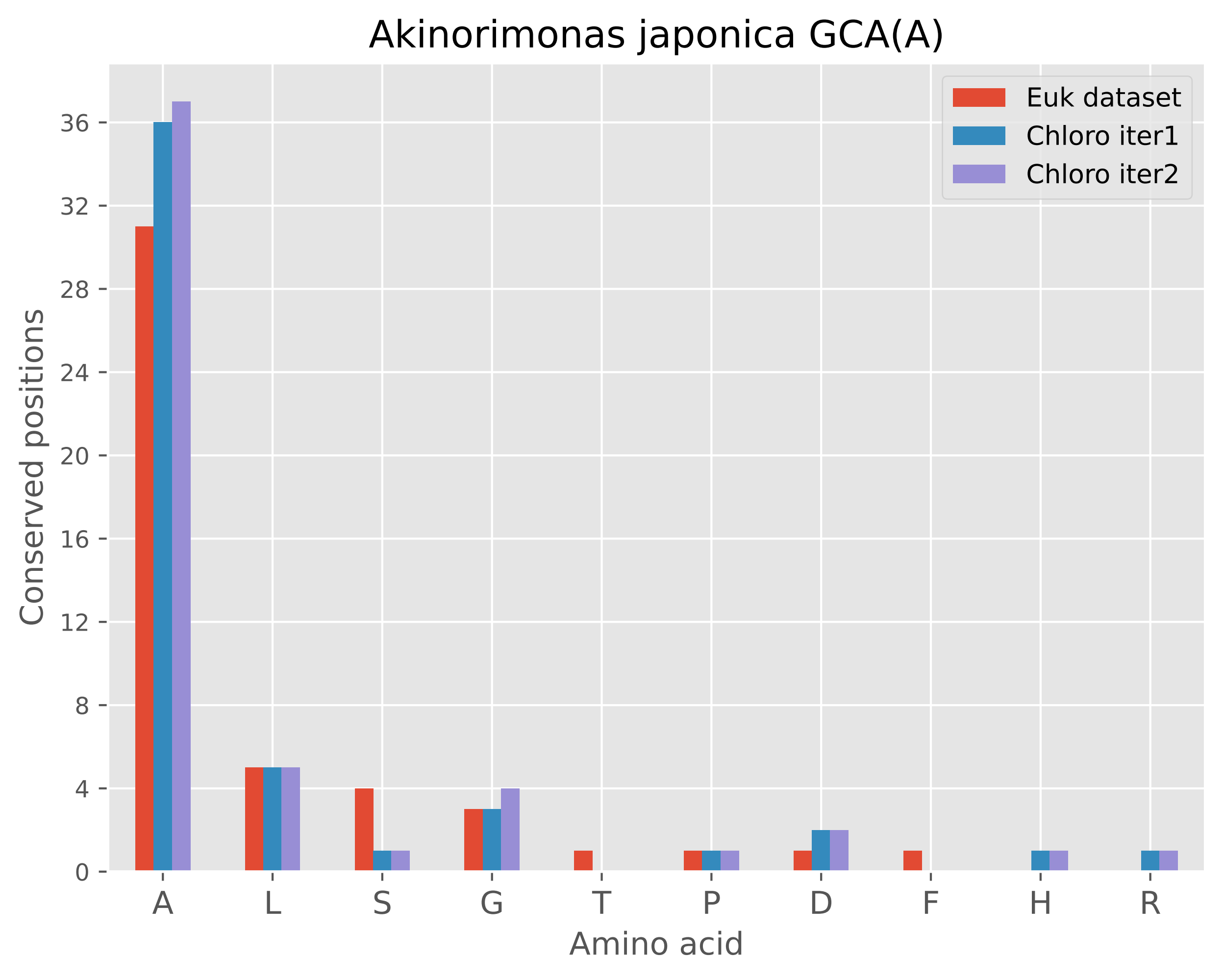

Akinorimonas japonica GCA(A)
Euk dataset
Chloro iter1
Chloro iter2
Conserved positions
0
4
8
12
16
20
24
28
32
36
A
L
S
G
T
P
D
F
H
R
Amino acid

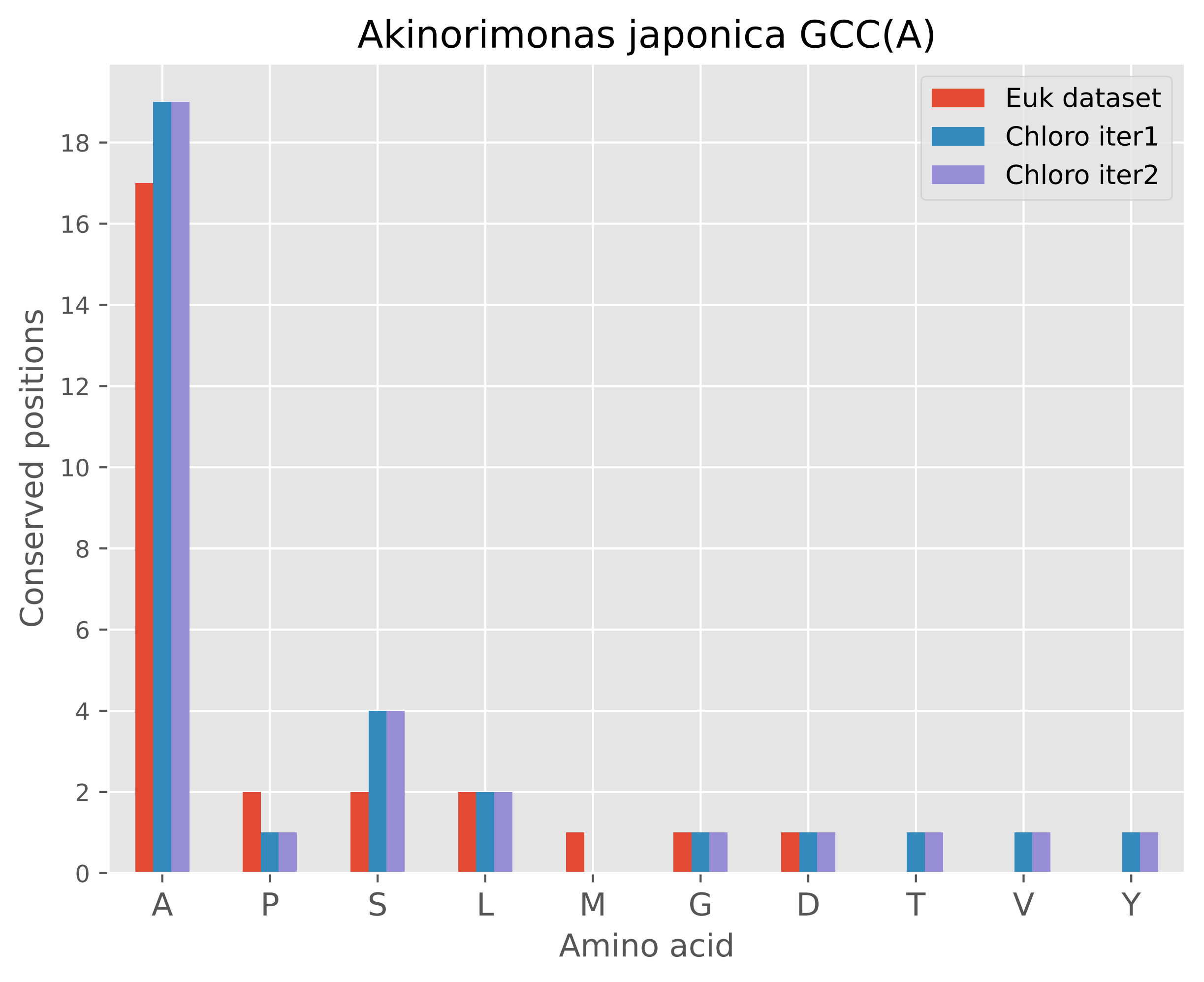
Akinorimonas japonica GCC(A)
Euk dataset
Chloro iter1
Chloro iter2
Conserved positions
0
2
4
6
8
10
12
14
16
18
A
P
S
L
M
G
D
T
V
Y
Amino acid

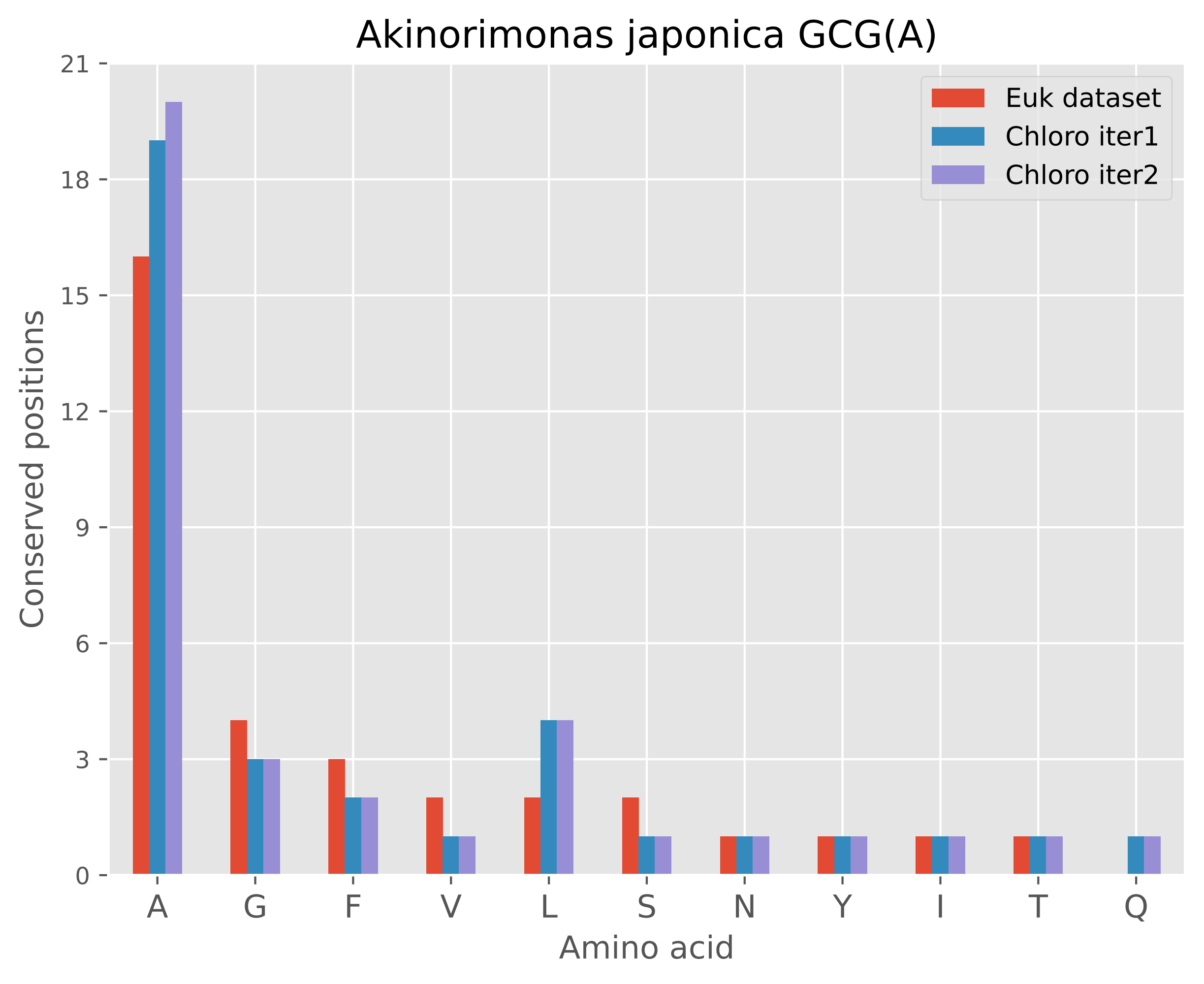
Akinorimonas japonica GCG(A)
Conserved positions
Amino acid
Euk dataset
Chloro iter1
Chloro iter2
0
3
6
9
12
15
18
21
A
G
F
V
L
S
N
Y
I
T
Q

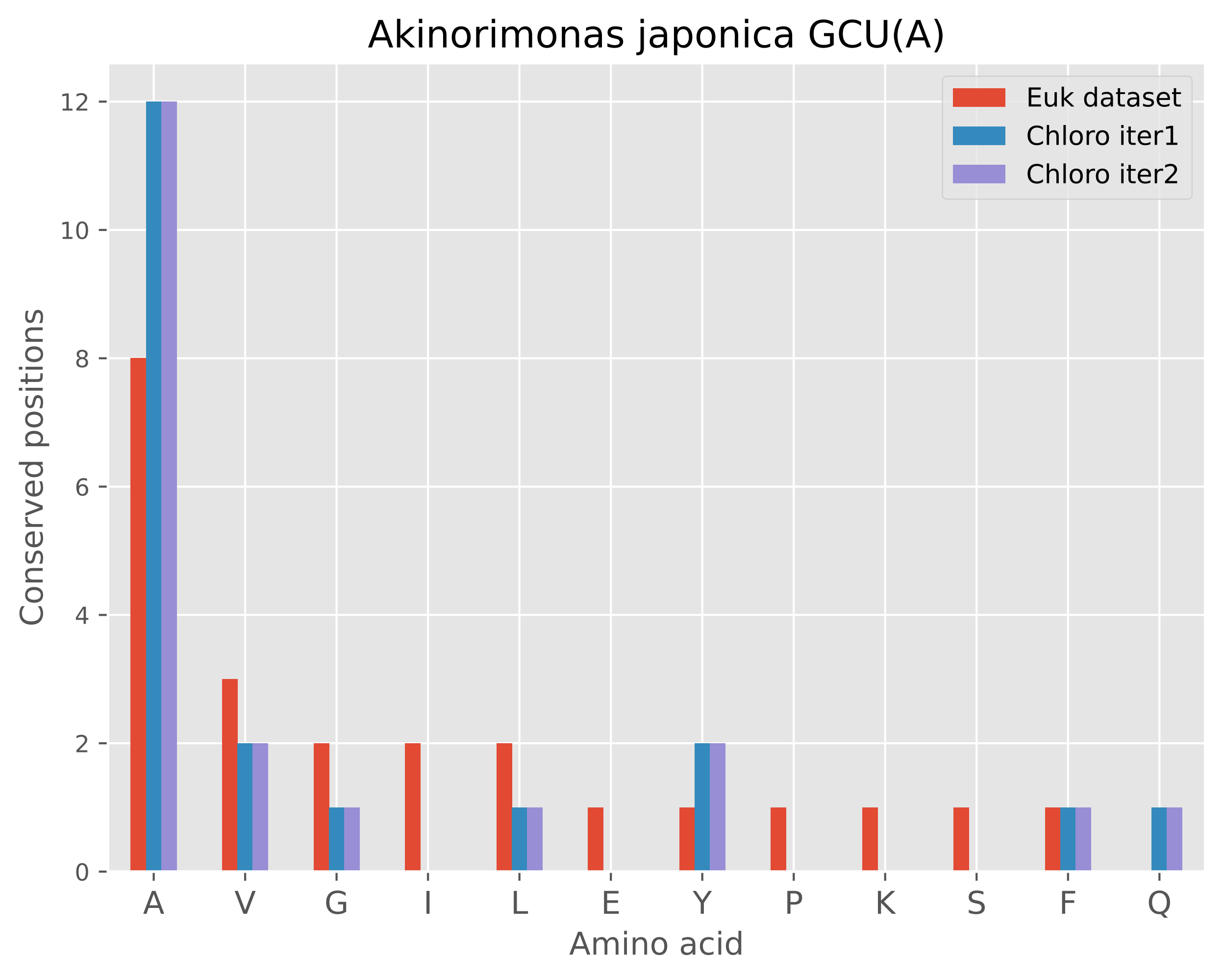
Akinorimonas japonica GCU(A)
Euk dataset
Chloro iter1
Chloro iter2
Conserved positions
0
2
4
6
8
10
12
A
V
G
I
L
E
Y
P
K
S
F
Q
Amino acid

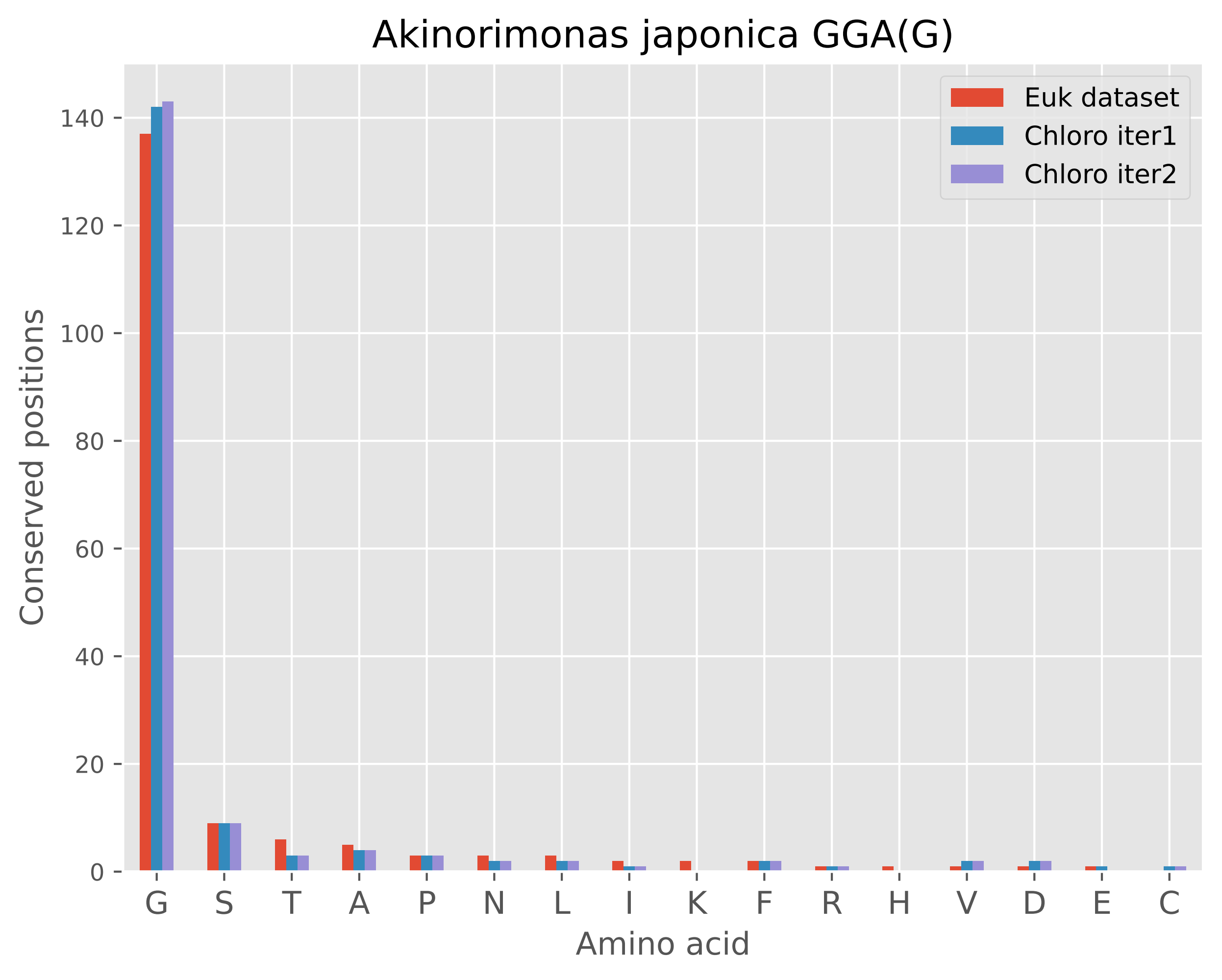
Akinorimonas japonica GGA(G)
Euk dataset
Chloro iter1
Chloro iter2
Conserved positions
0
20
40
60
80
100
120
140
G
S
T
A
P
N
L
I
K
F
R
H
V
D
E
C
Amino acid

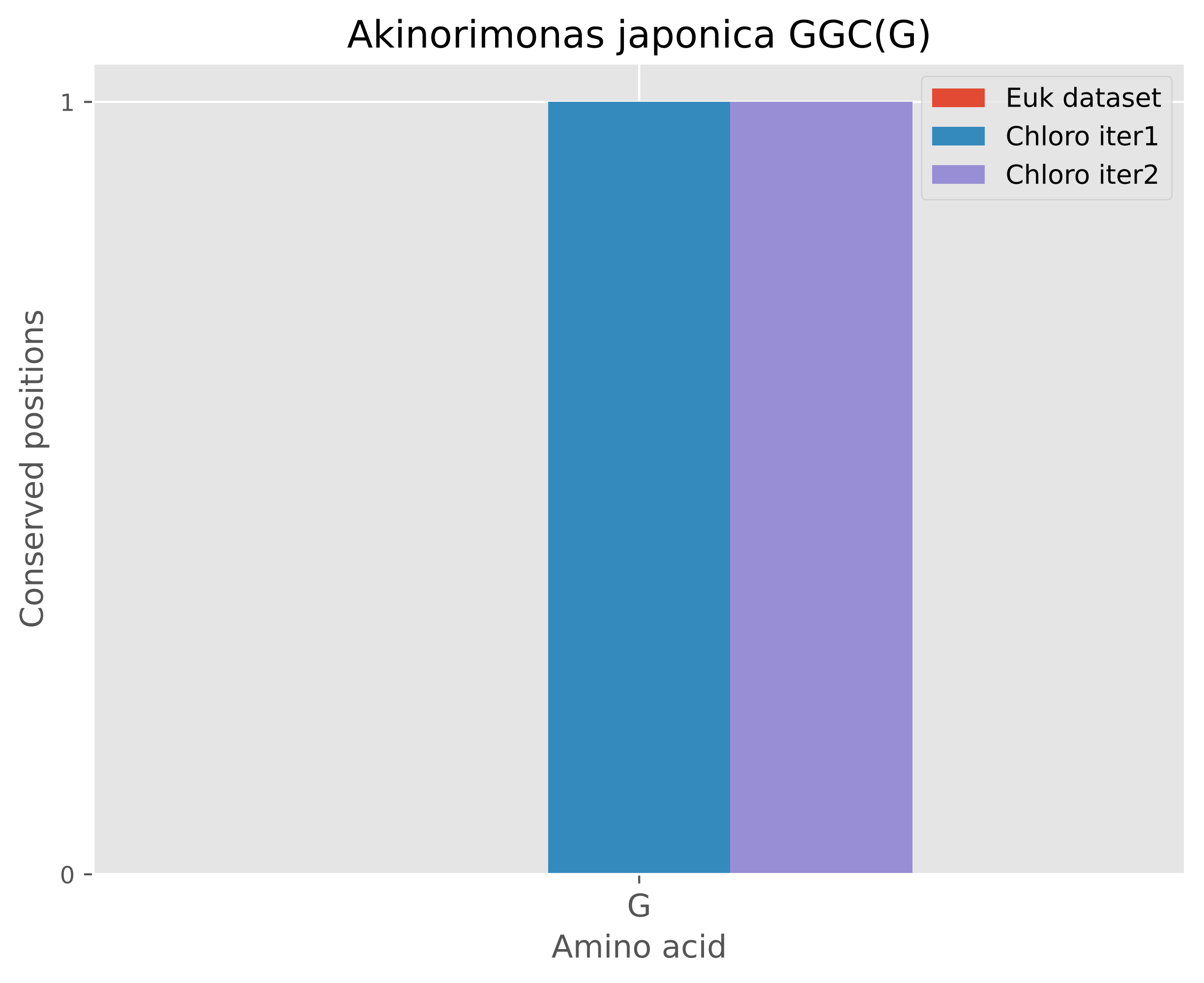

Akinorimonas japonica GGC(G)
Euk dataset
Chloro iter1
Chloro iter2
1
0
Conserved positions
G
Amino acid

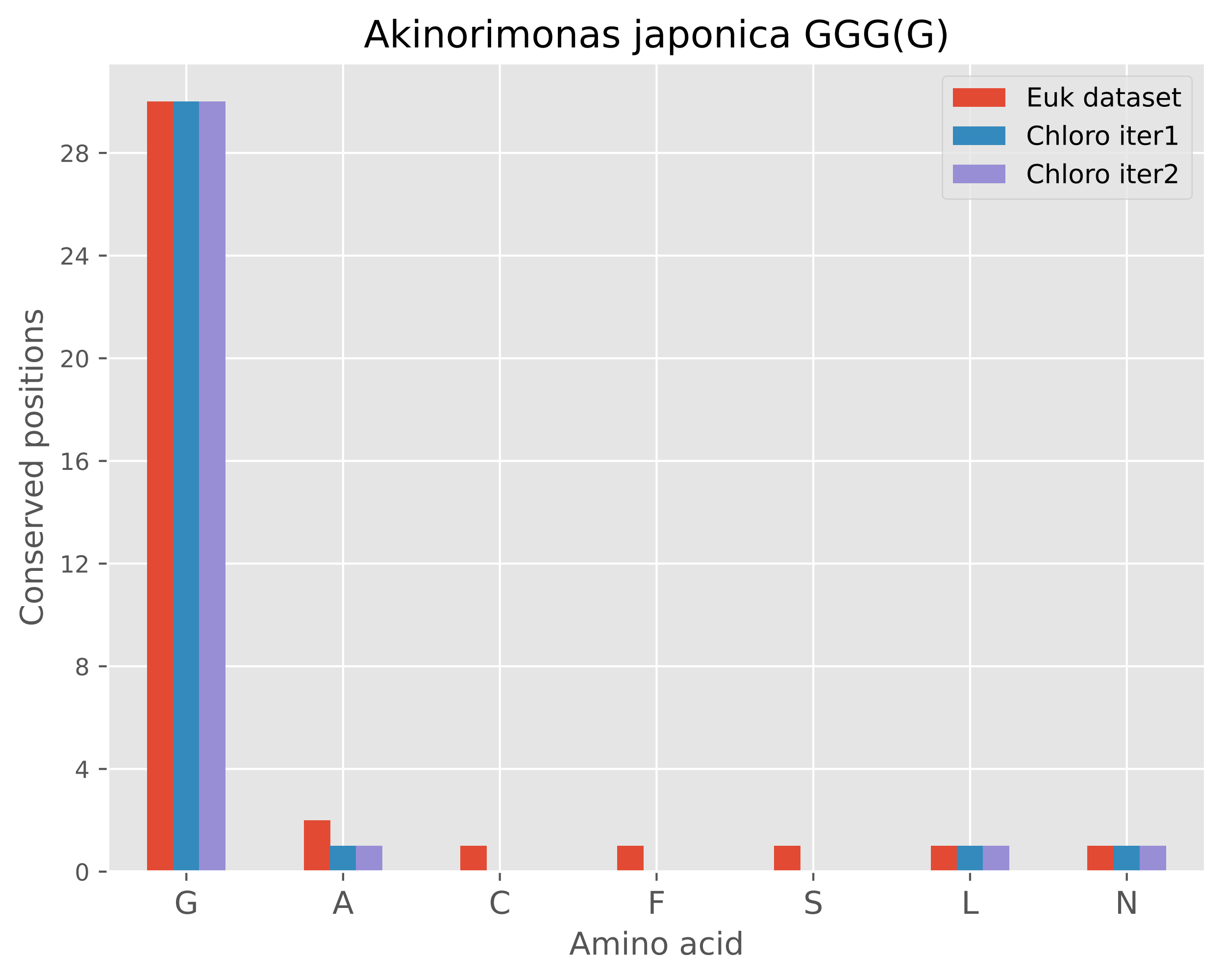

Akinorimonas japonica GGG(G)
Euk dataset
Chloro iter1
Chloro iter2
Conserved positions
0
4
8
12
16
20
24
28
G
A
C
F
S
L
N
Amino acid

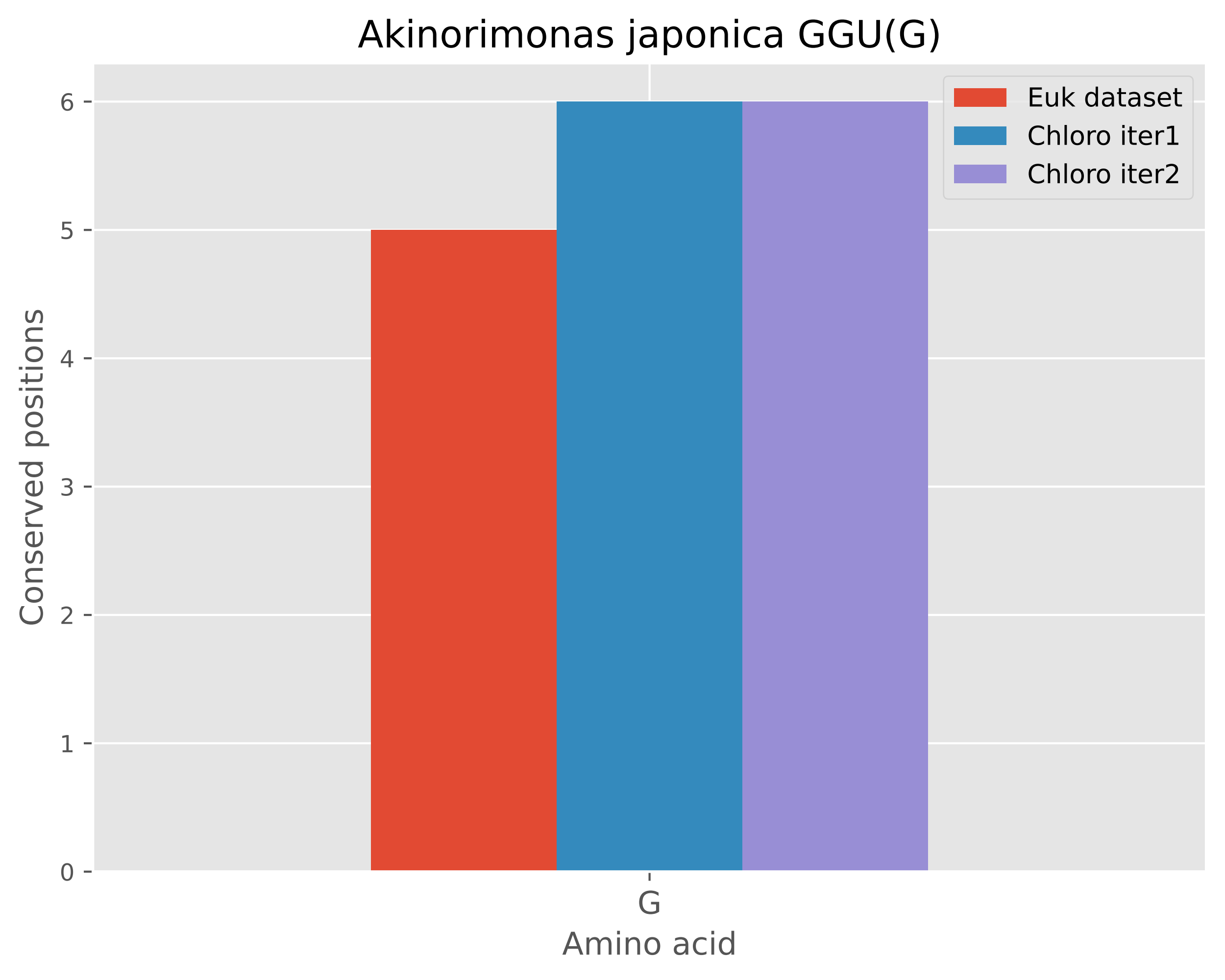

Akinorimonas japonica GGU(G)
Euk dataset
Chloro iter1
Chloro iter2
Conserved positions
0
1
2
3
4
5
6
G
Amino acid

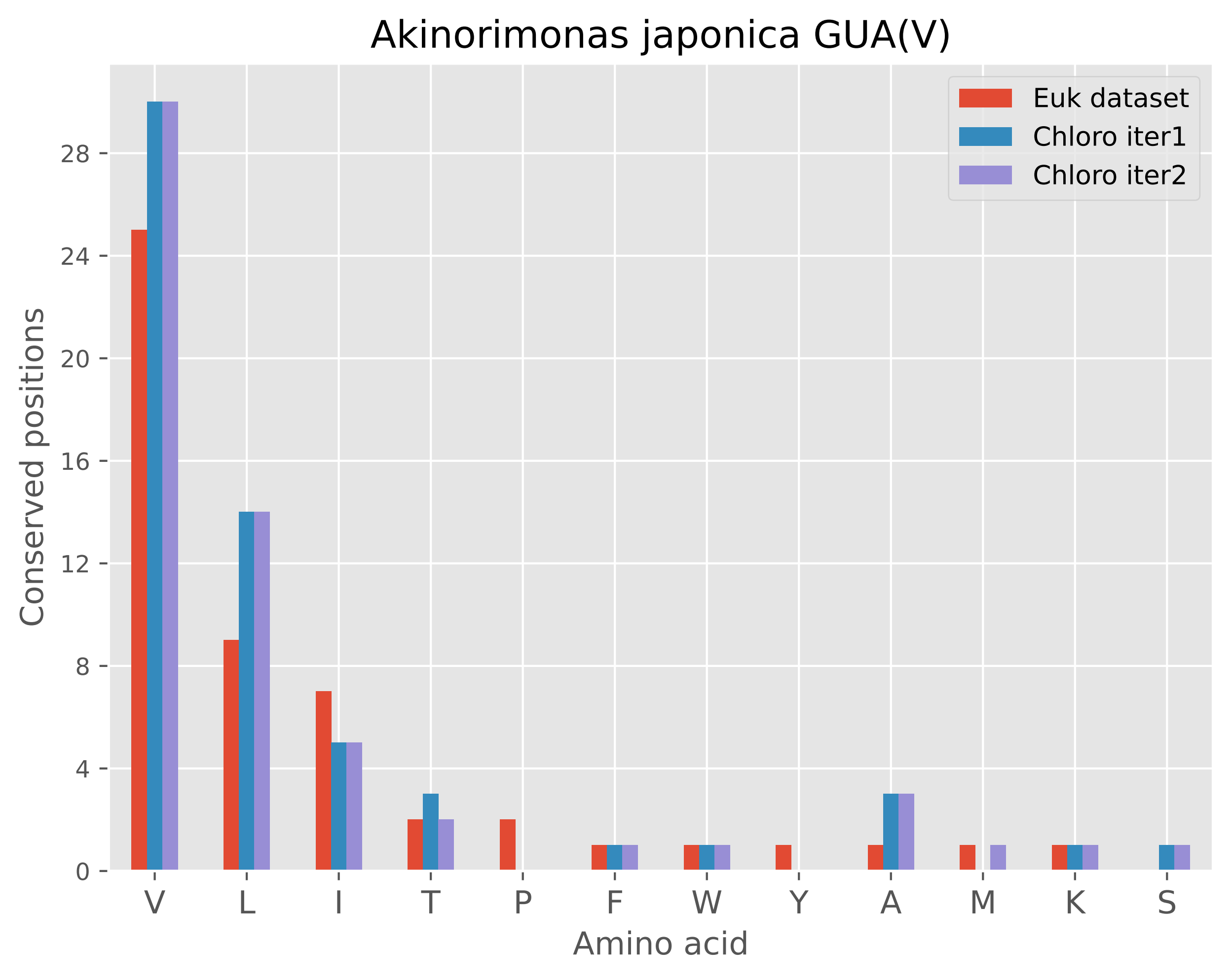

Akinorimonas japonica GUA(V)
Euk dataset
Chloro iter1
Chloro iter2
Conserved positions
0
4
8
12
16
20
24
28
V
L
I
T
P
F
W
Y
A
M
K
S
Amino acid

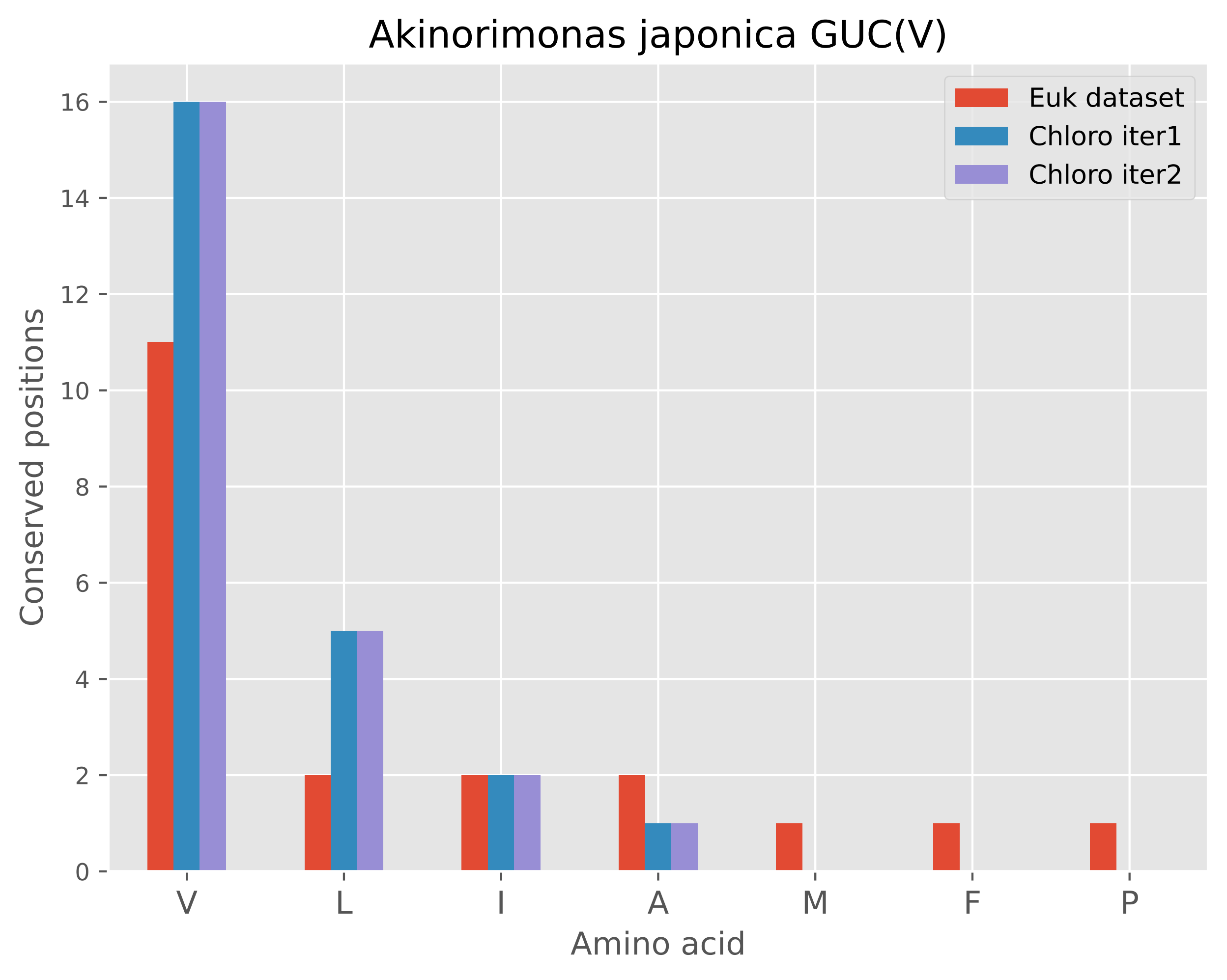

Akinorimonas japonica GUC(V)
Euk dataset
Chloro iter1
Chloro iter2
Conserved positions
0
2
4
6
8
10
12
14
16
V
L
I
A
M
F
P
Amino acid

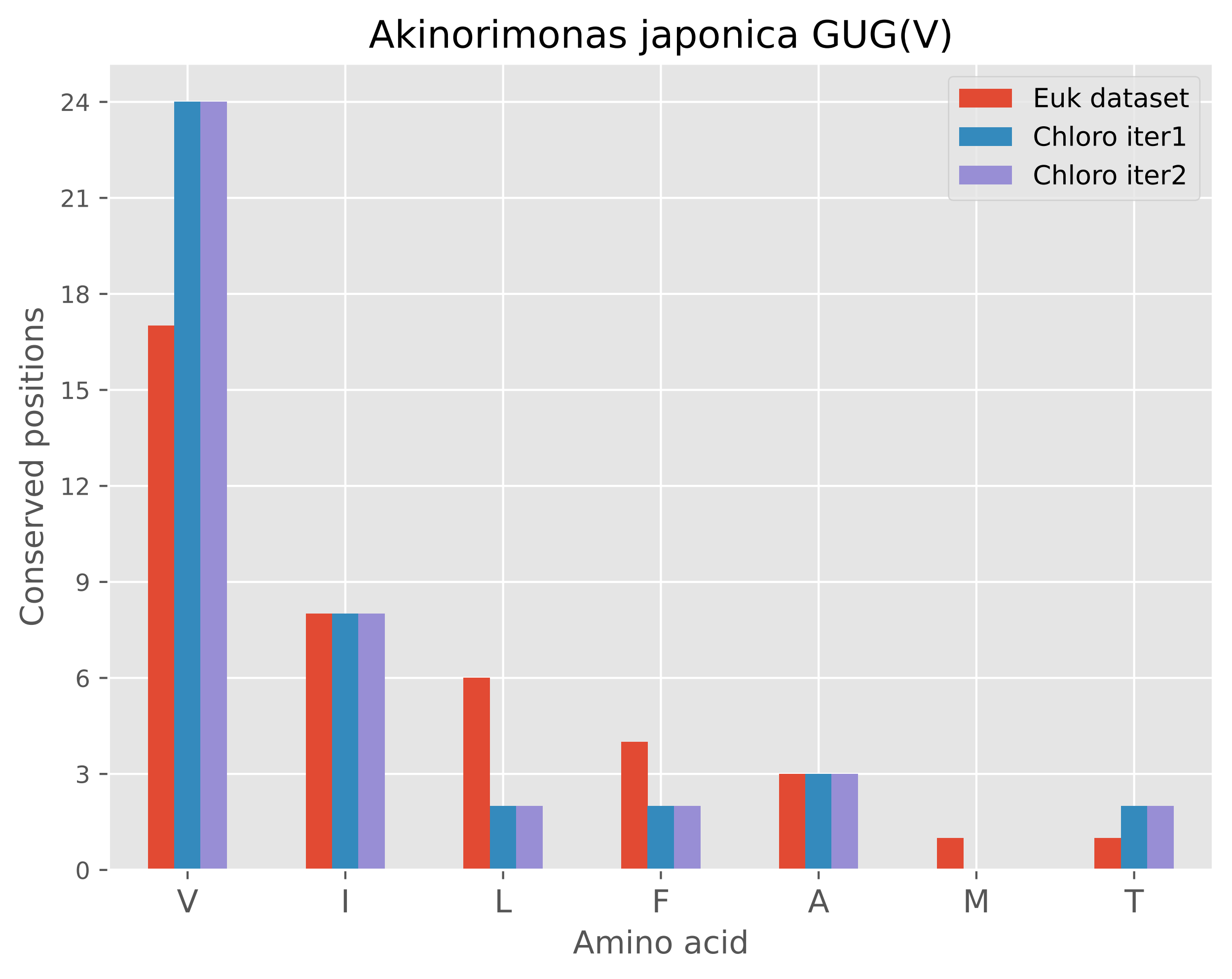
Akinorimonas japonica GUG(V)
Euk dataset
Chloro iter1
Chloro iter2
Conserved positions
24
21
18
15
12
9
6
3
0
V
I
L
F
A
M
T
Amino acid

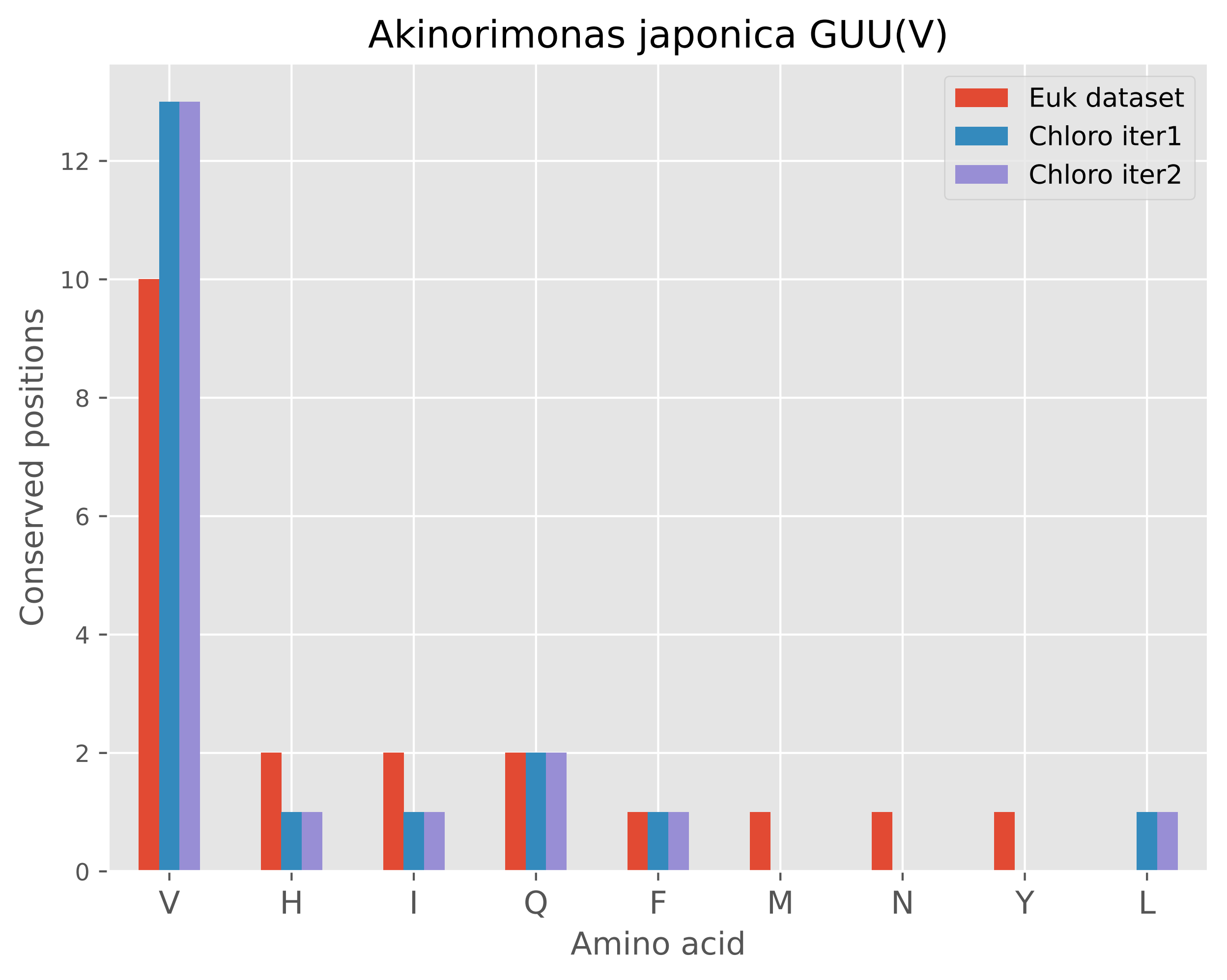

Akinorimonas japonica GUU(V)
Euk dataset
Chloro iter1
Chloro iter2
Conserved positions
0
2
4
6
8
10
12
V
H
I
Q
F
M
N
Y
L
Amino acid

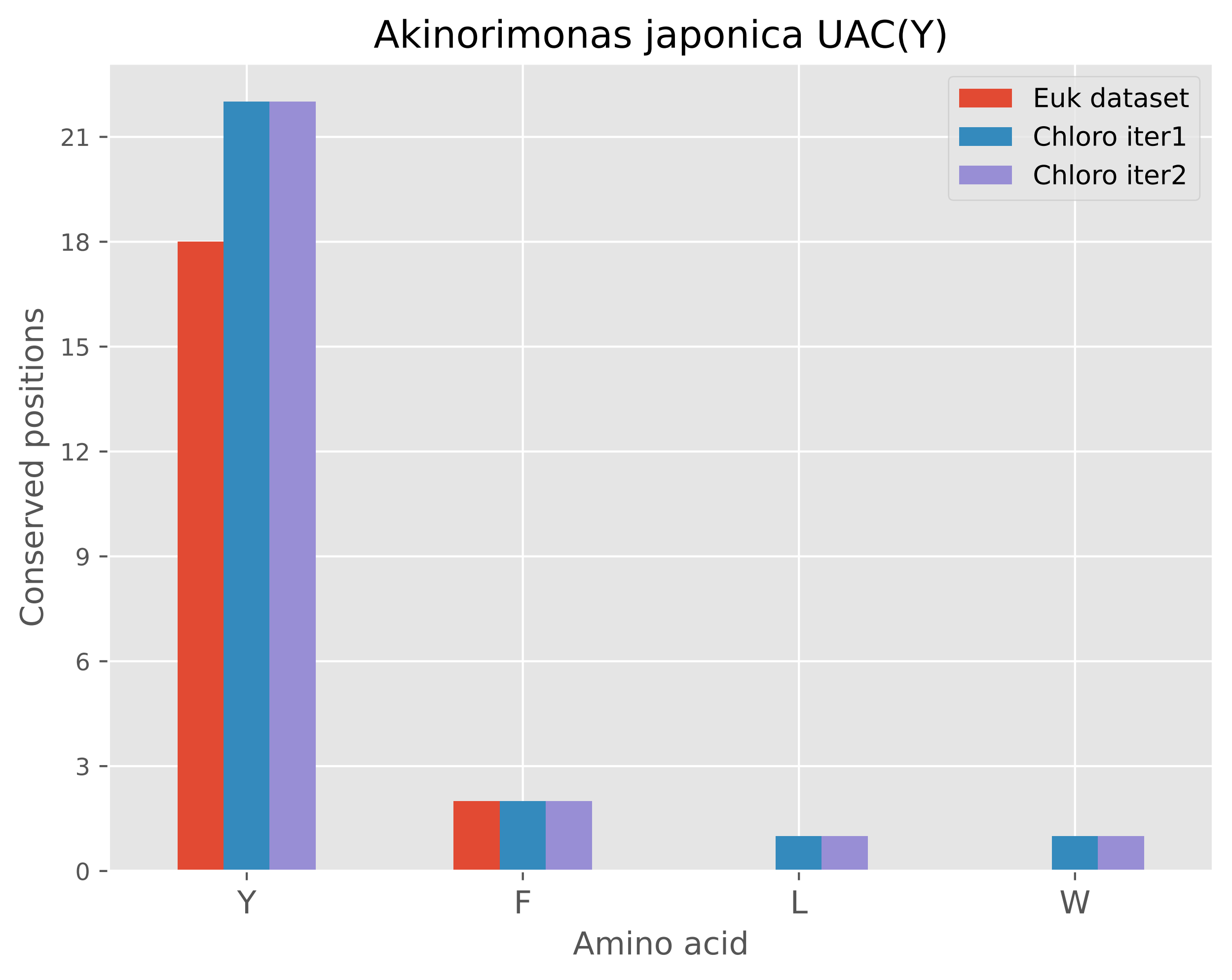
Akinorimonas japonica UAC(Y)
Euk dataset
Chloro iter1
Chloro iter2
Conserved positions
0
3
6
9
12
15
18
21
Y
F
L
W
Amino acid

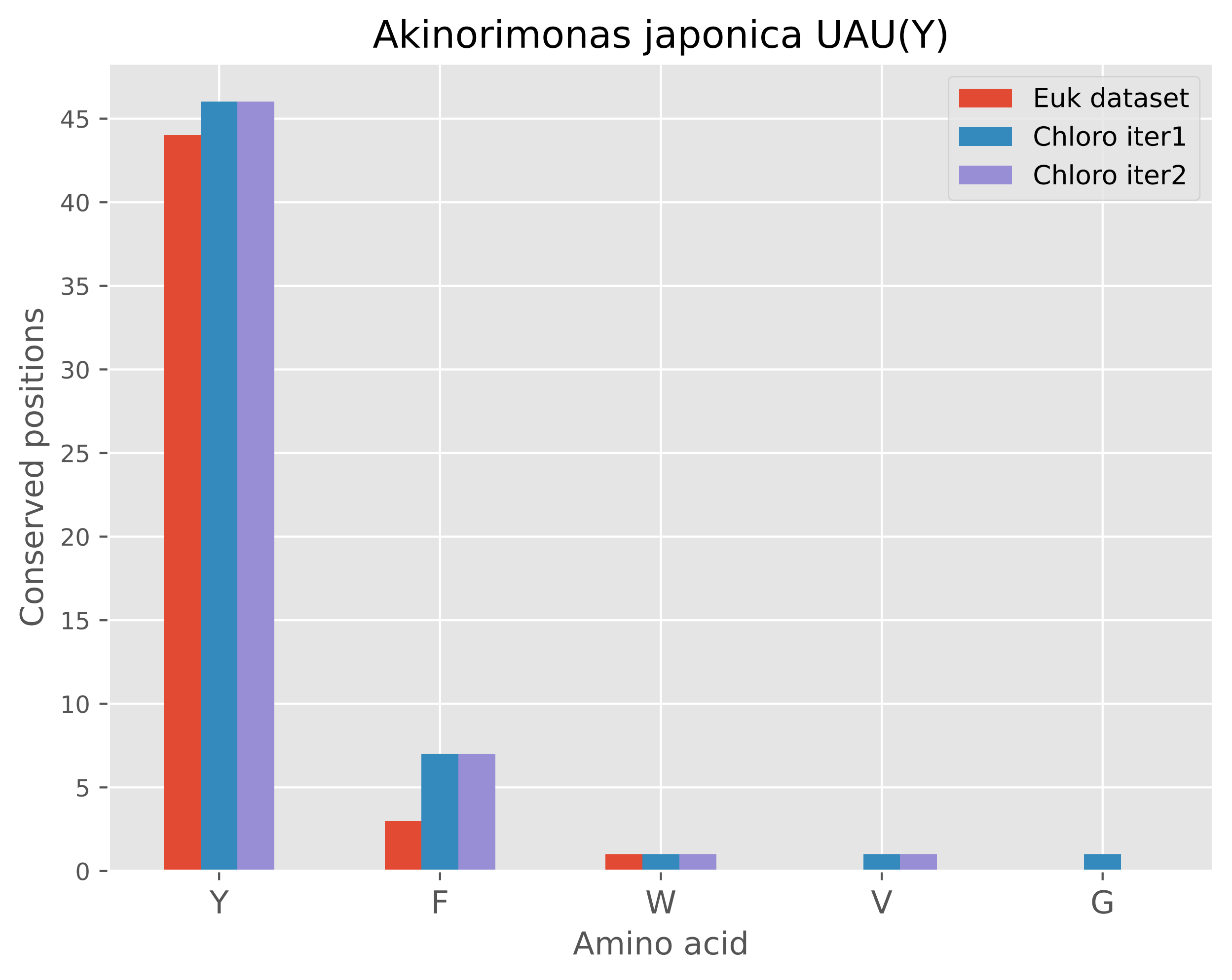
Akinorimonas japonica UAU(Y)
Euk dataset
Chloro iter1
Chloro iter2
Conserved positions
Amino acid
0
5
10
15
20
25
30
35
40
45
Y
F
W
V
G

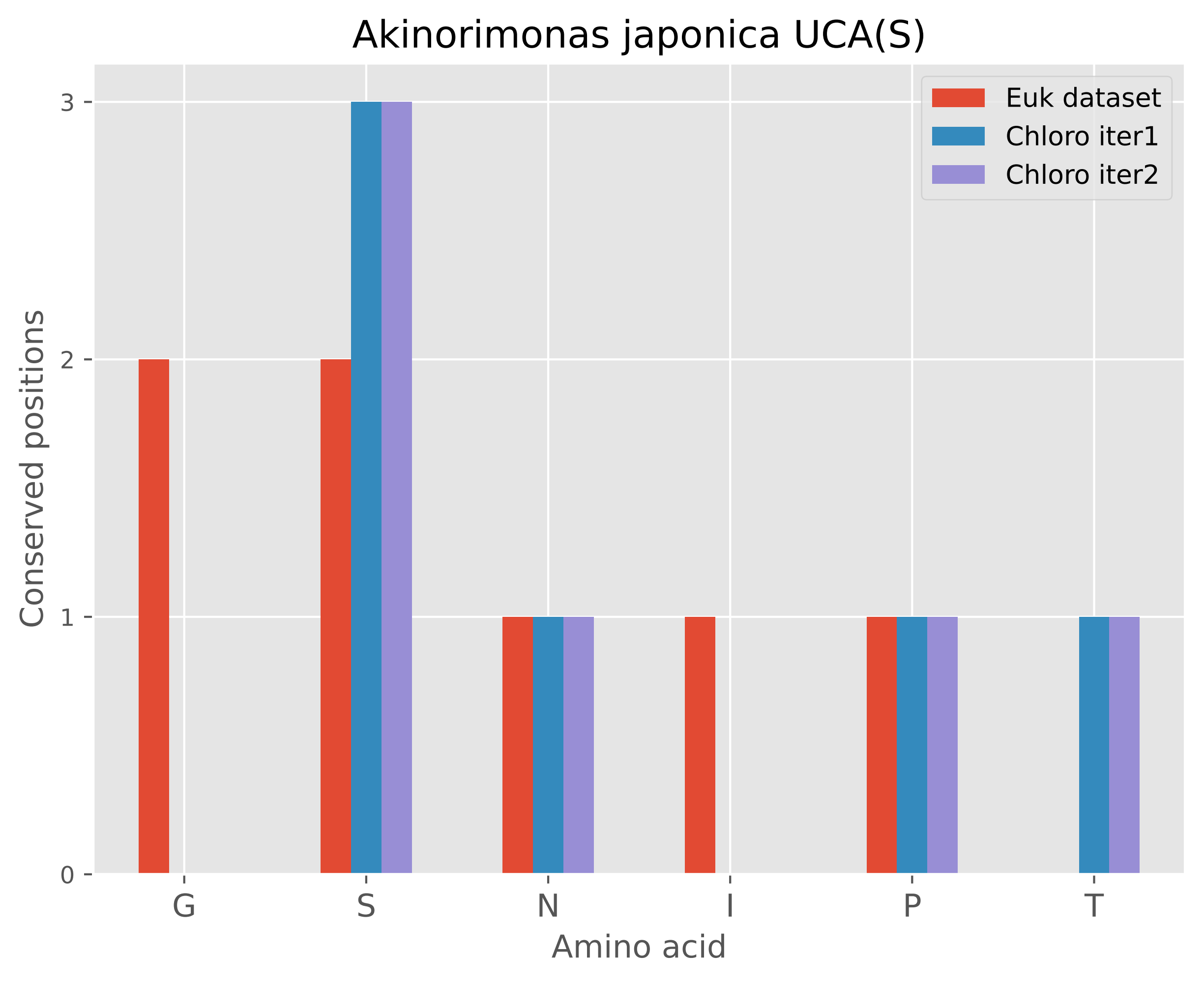

Akinorimonas japonica UCA(S)
Euk dataset
Chloro iter1
Chloro iter2
Conserved positions
0
1
2
3
G
S
N
I
P
T
Amino acid

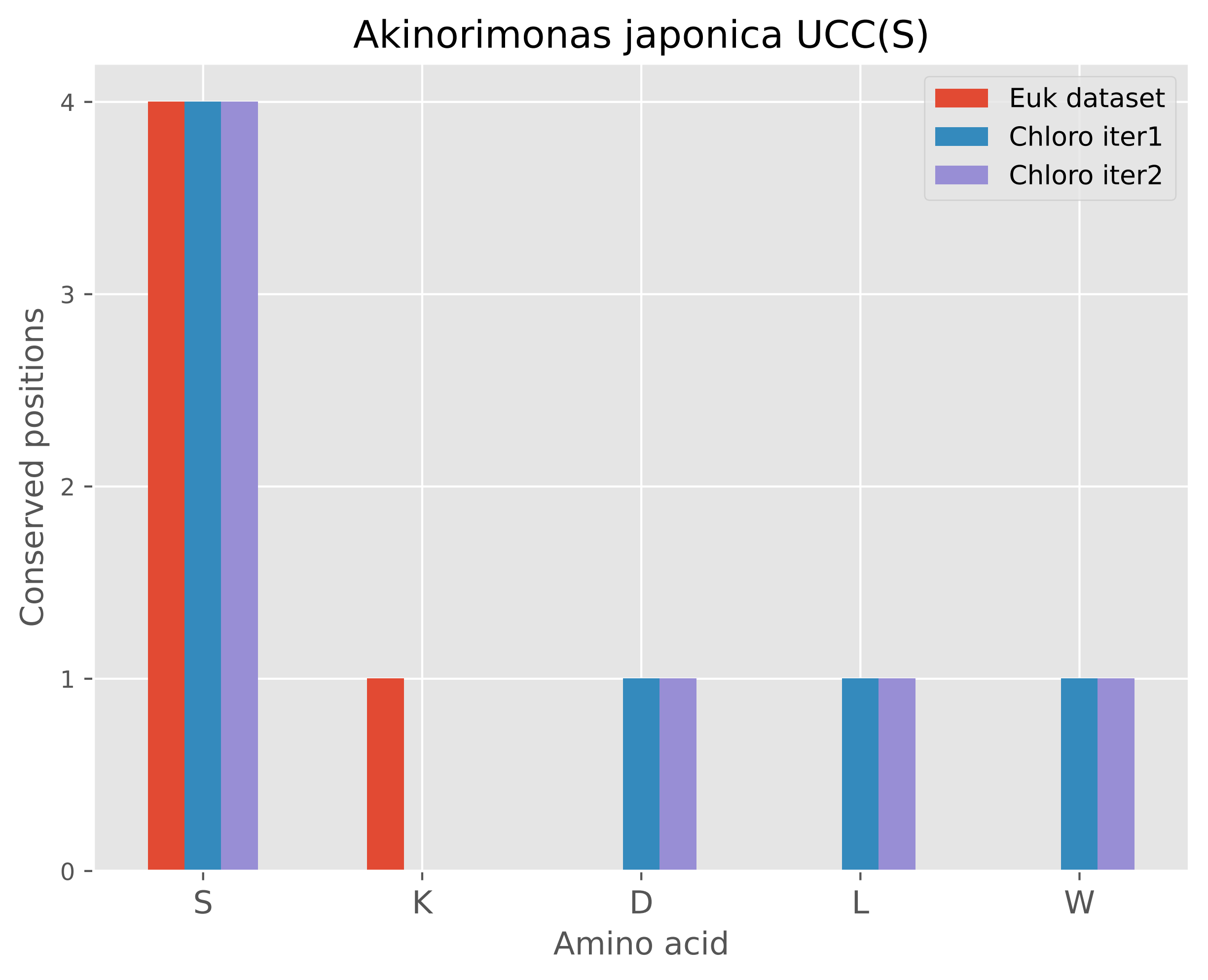

Akinorimonas japonica UCC(S)
Euk dataset
Chloro iter1
Chloro iter2
Conserved positions
0
1
2
3
4
S
K
D
L
W
Amino acid

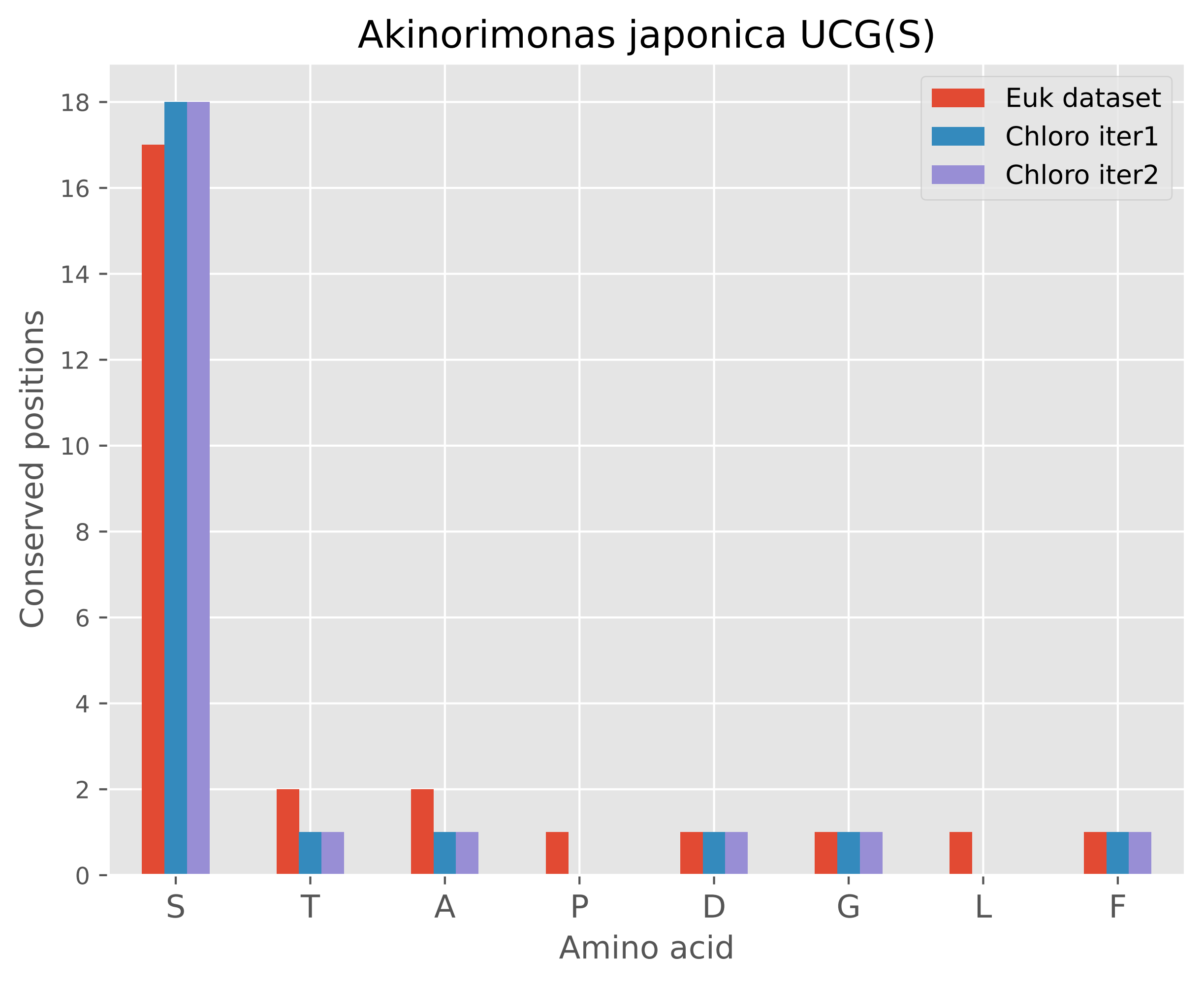

Akinorimonas japonica UCG(S)
Euk dataset
Chloro iter1
Chloro iter2
Conserved positions
0
2
4
6
8
10
12
14
16
18
S
T
A
P
D
G
L
F
Amino acid

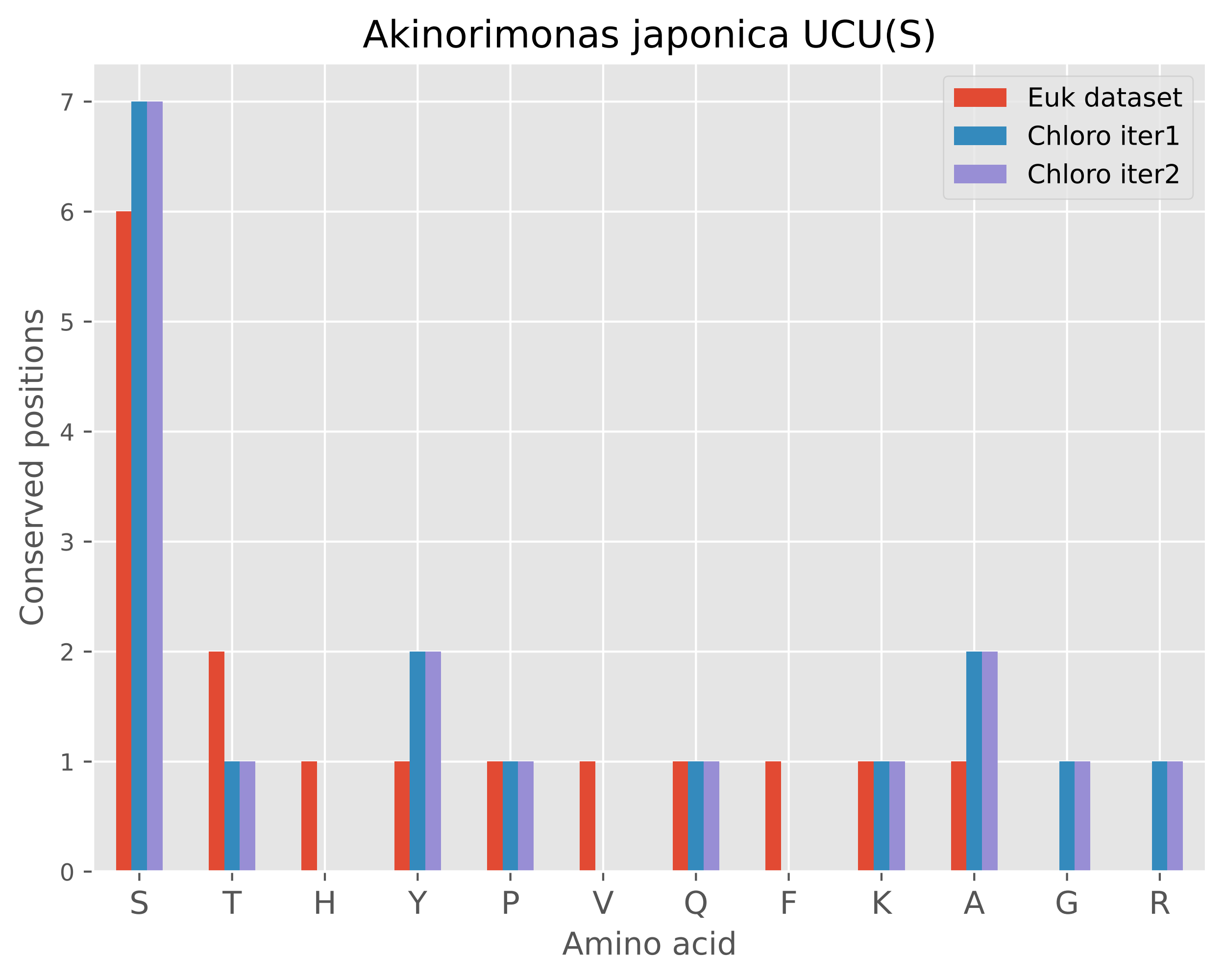

Akinorimonas japonica UCU(S)
Euk dataset
Chloro iter1
Chloro iter2
Conserved positions
Amino acid
0
1
2
3
4
5
6
7
S
T
H
Y
P
V
Q
F
K
A
G
R

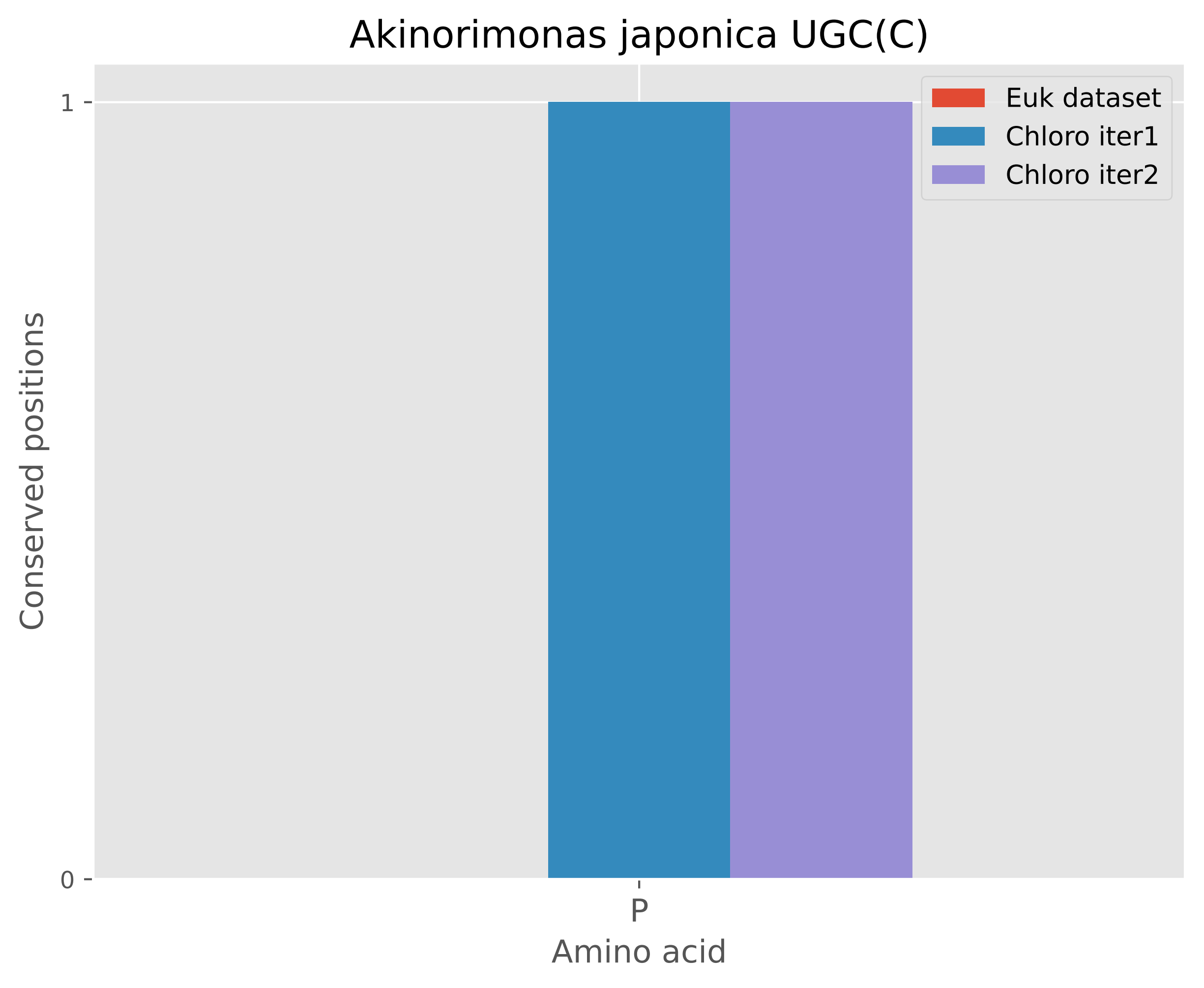
Akinorimonas japonica UGC(C)
Conserved positions
1
0
P
Amino acid
Euk dataset
Chloro iter1
Chloro iter2

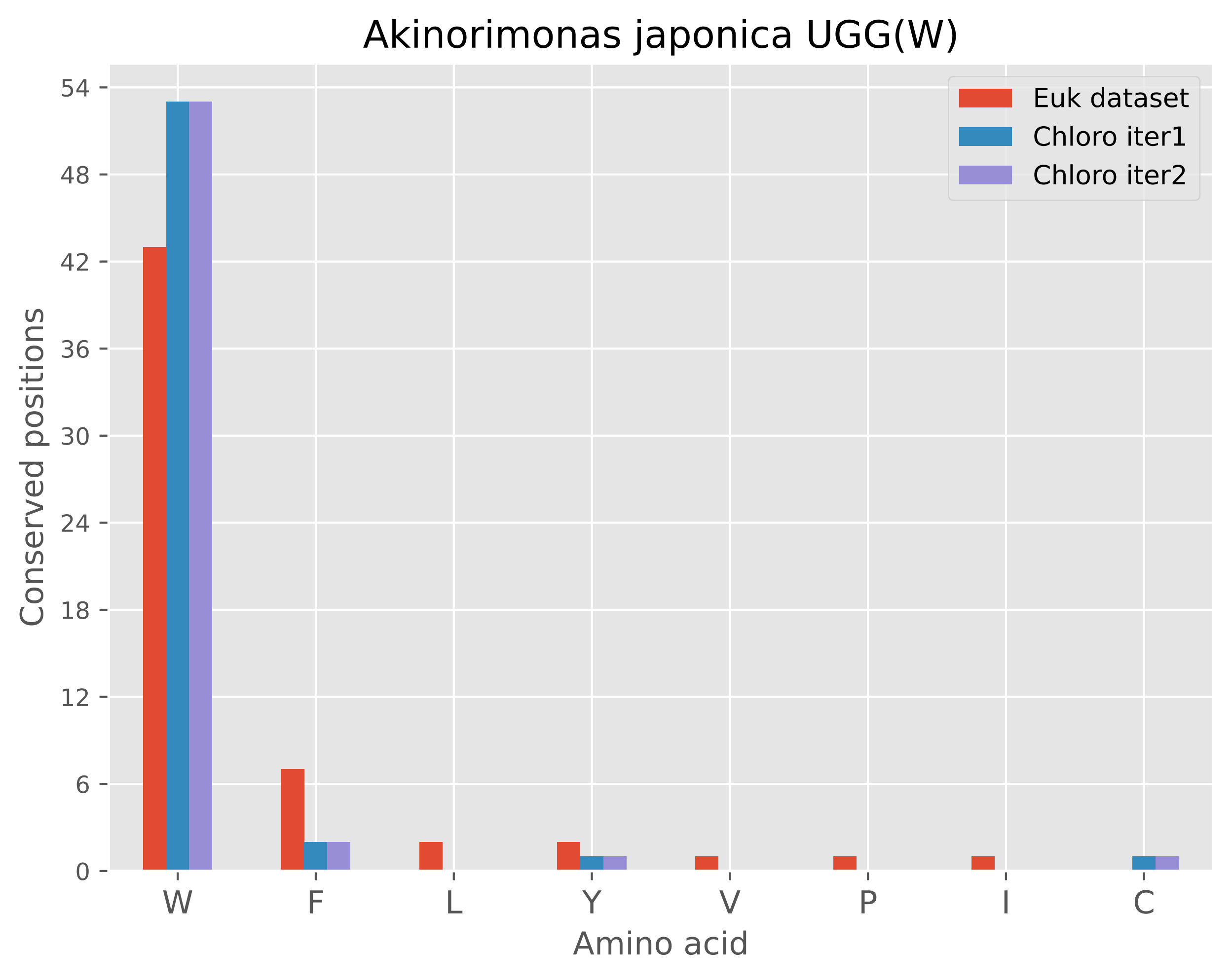

Akinorimonas japonica UGG(W)
Euk dataset
Chloro iter1
Chloro iter2
Conserved positions
0
6
12
18
24
30
36
42
48
54
W
F
L
Y
V
P
I
C
Amino acid

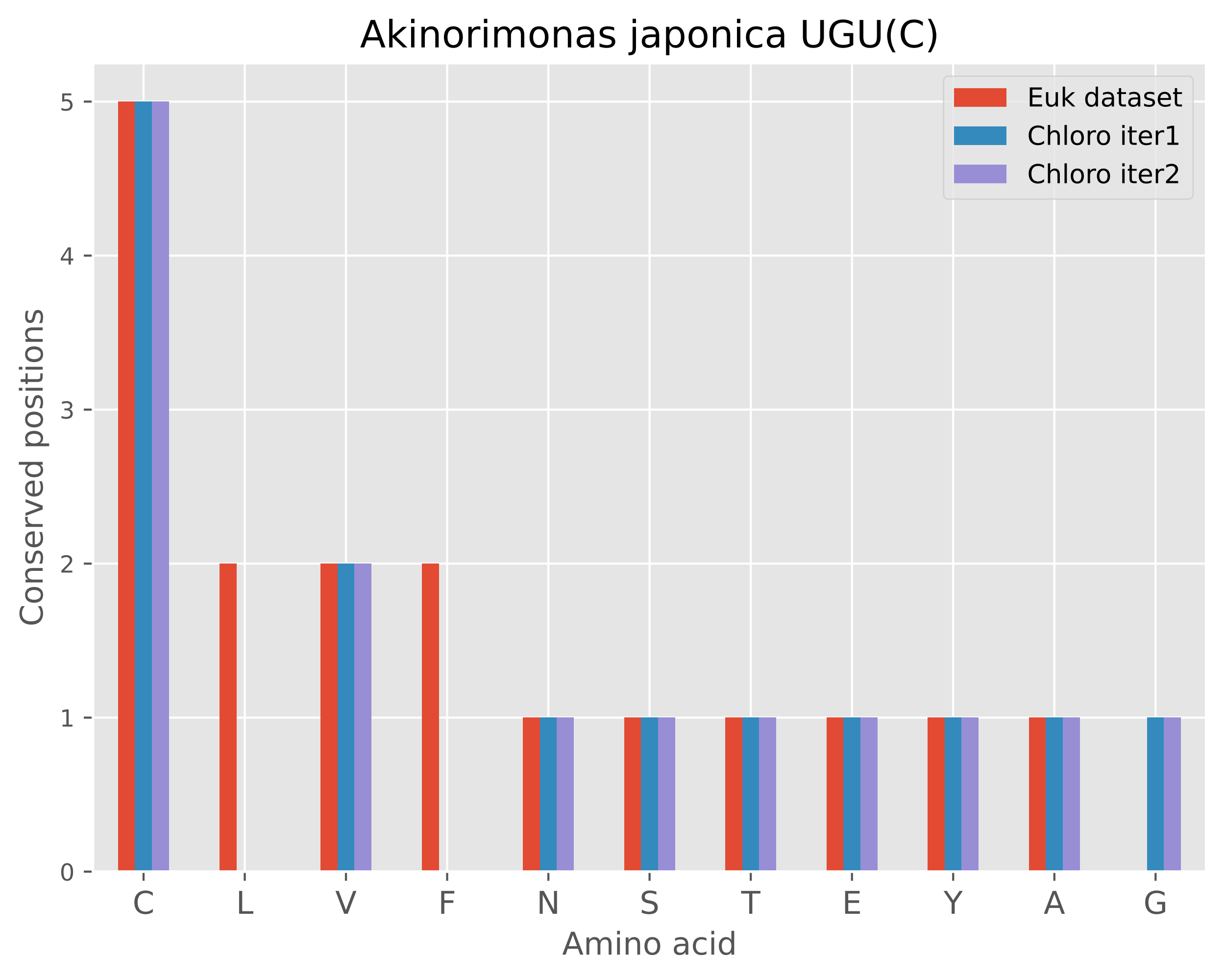
Akinorimonas japonica UGU(C)
Euk dataset
Chloro iter1
Chloro iter2
Conserved positions
0
1
2
3
4
5
C
L
V
F
N
S
T
E
Y
A
G
Amino acid

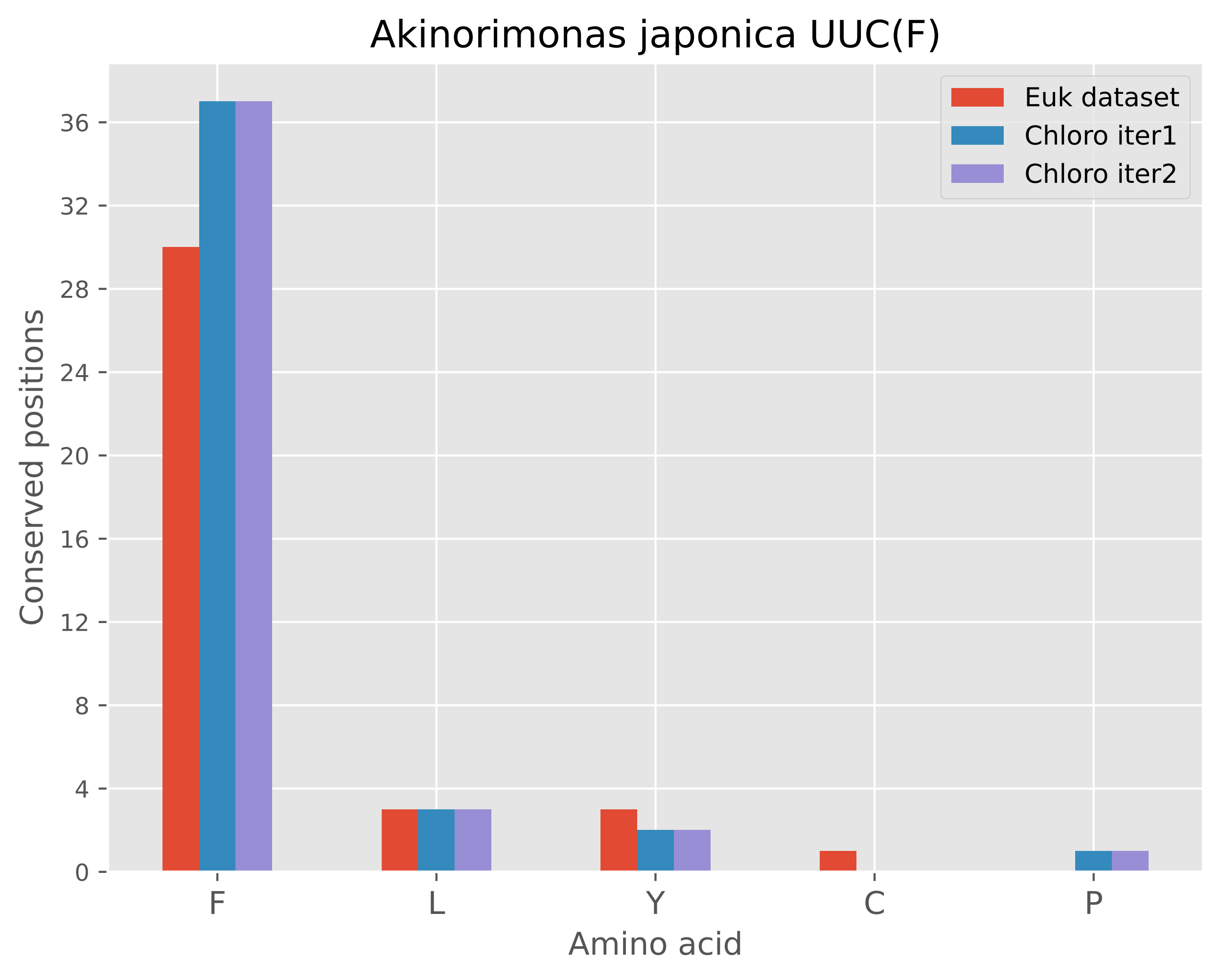

Akinorimonas japonica UUC(F)
Euk dataset
Chloro iter1
Chloro iter2
Conserved positions
0
4
8
12
16
20
24
28
32
36
F
L
Y
C
P
Amino acid

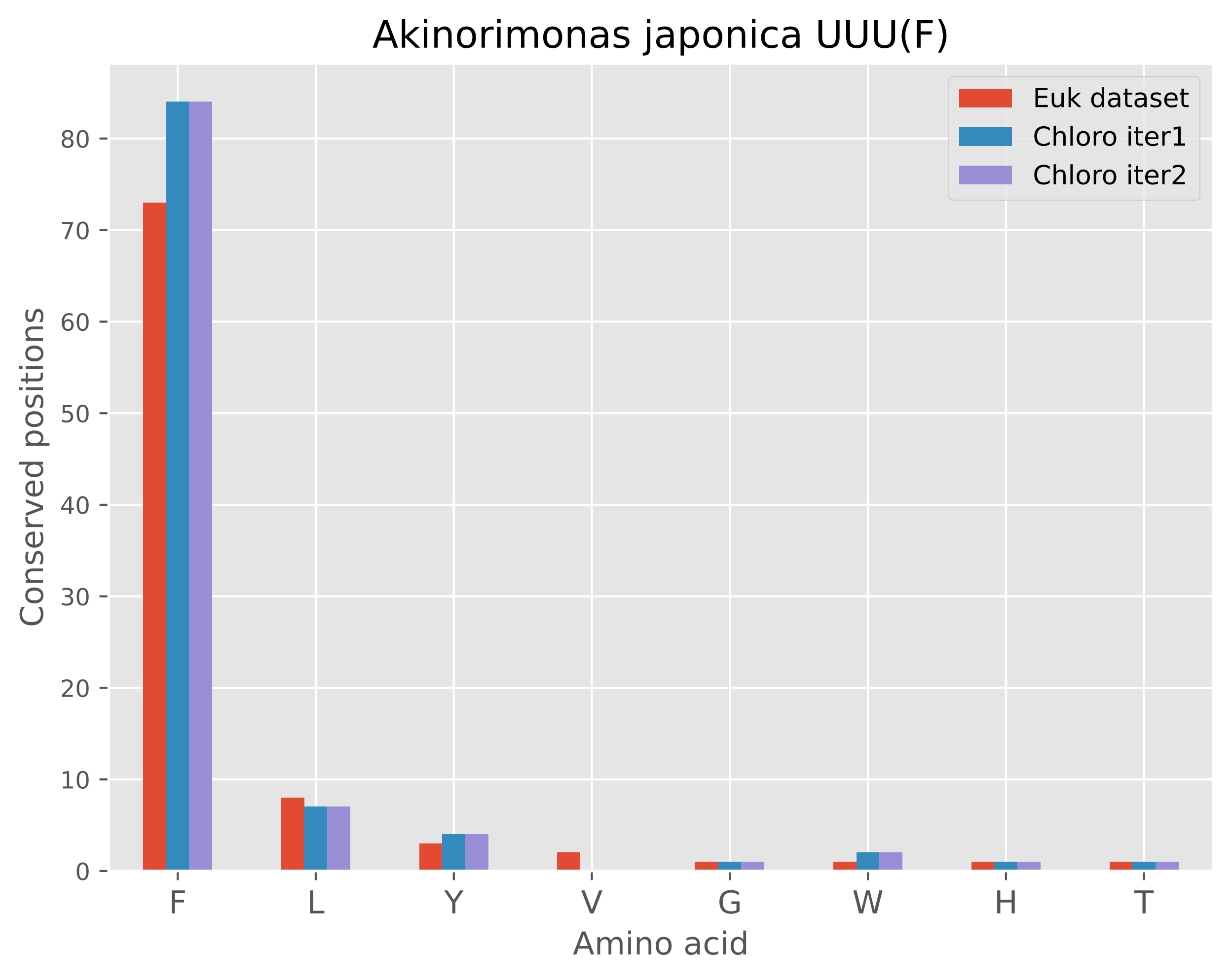
Akinorimonas japonica UUU(F)
Euk dataset
Chloro iter1
Chloro iter2
Conserved positions
0
10
20
30
40
50
60
70
80
F
L
Y
V
G
W
H
T
Amino acid
