# Supplementary material for: Expanded diversity of pedinophytes provides a window into the evolution of the genetic code in organelles
Source: PLoS Genet. 2025 Oct 22;21(10):e1011901. doi: 10.1371/journal.pgen.1011901 (PMC12574857; doi:10.1371/journal.pgen.1011901)
Supplement: S3 Dataset — Absence of a plot for a given codon means the codon was not present at any position deemed conserved. (PDF) [file pgen.1011901.s033.pdf]

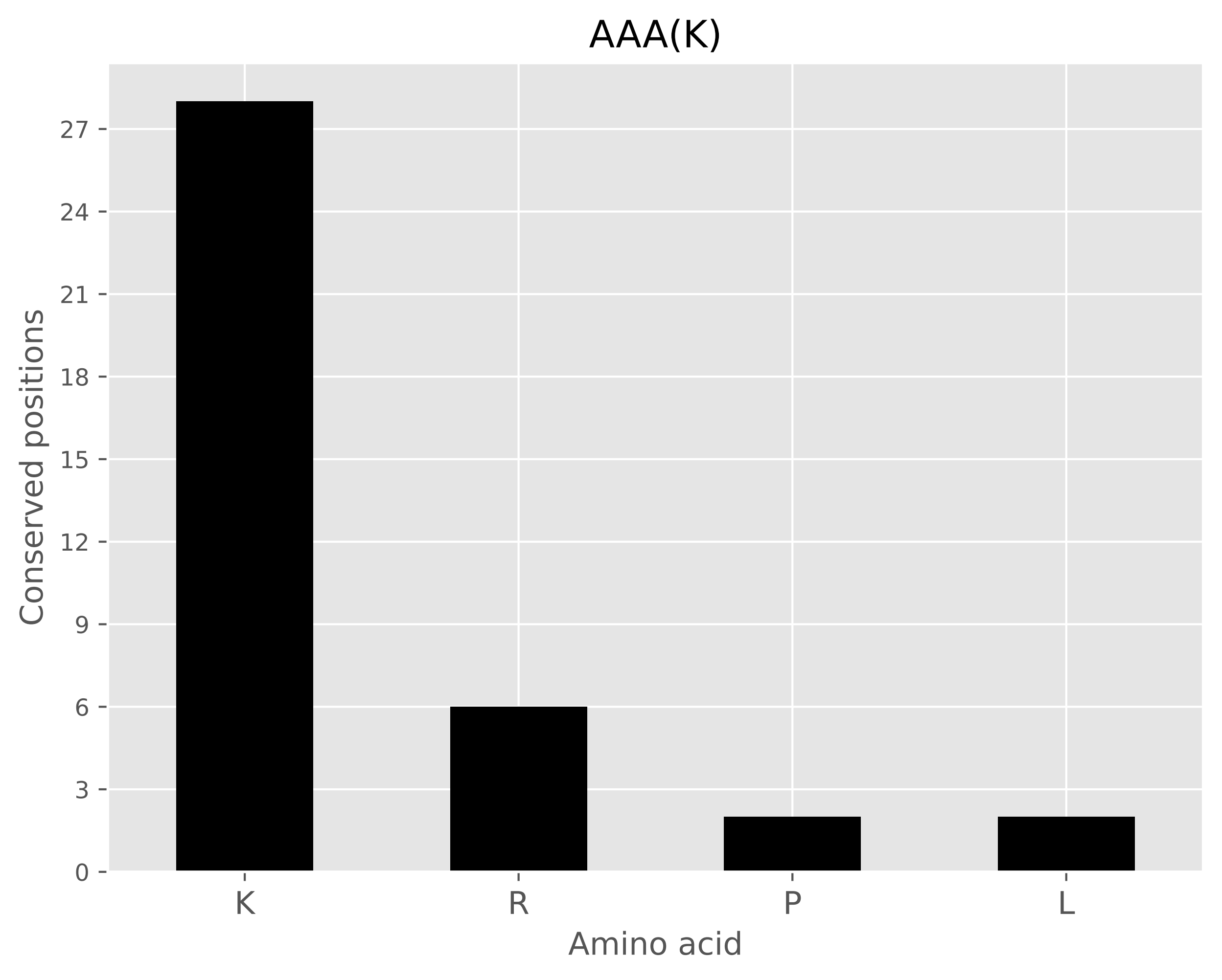
AAA(K)
Conserved positions
0
3
6
9
12
15
18
21
24
27
K
R
P
L
Amino acid

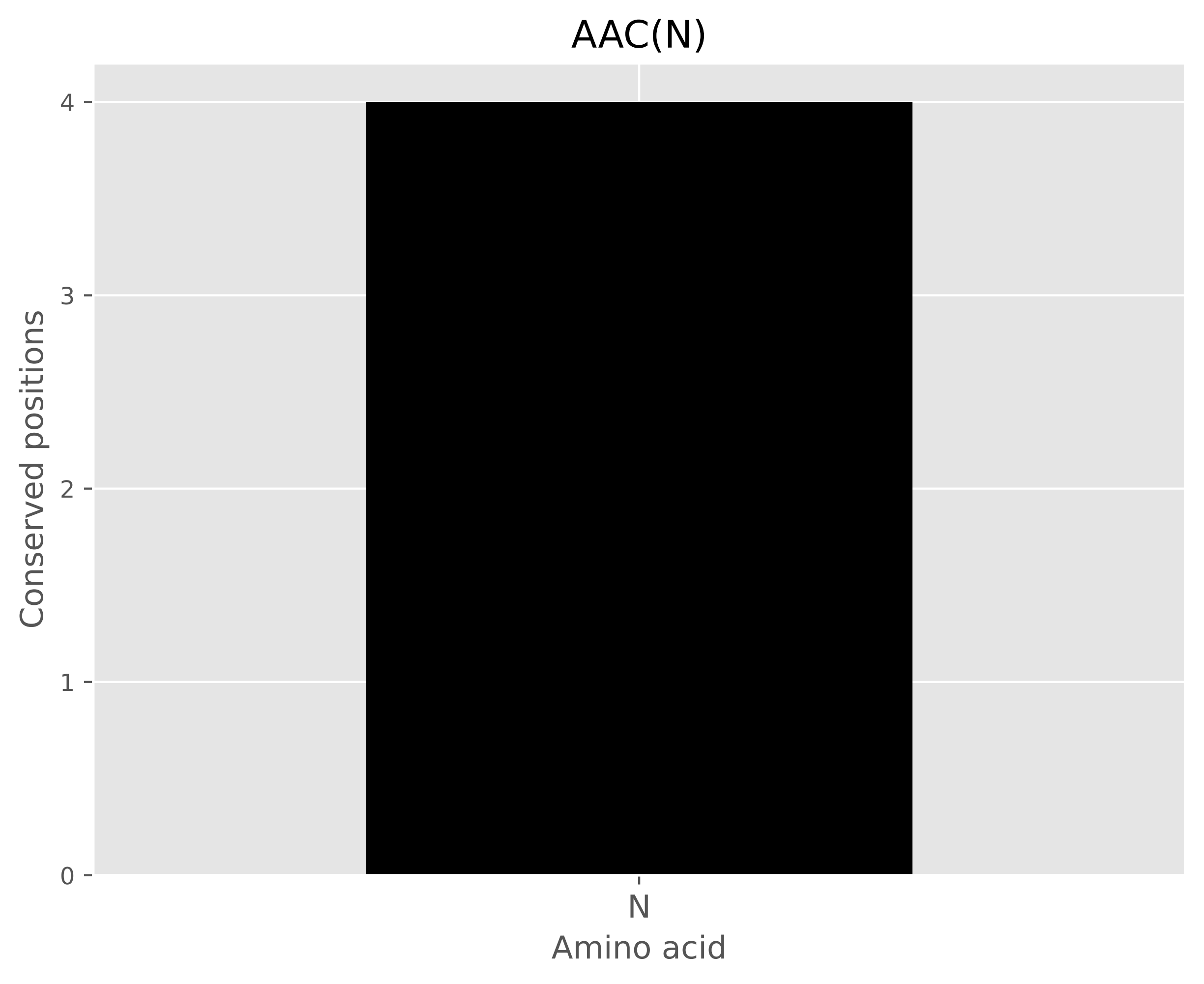

AAC(N)
Conserved positions
0
1
2
3
4
N
Amino acid

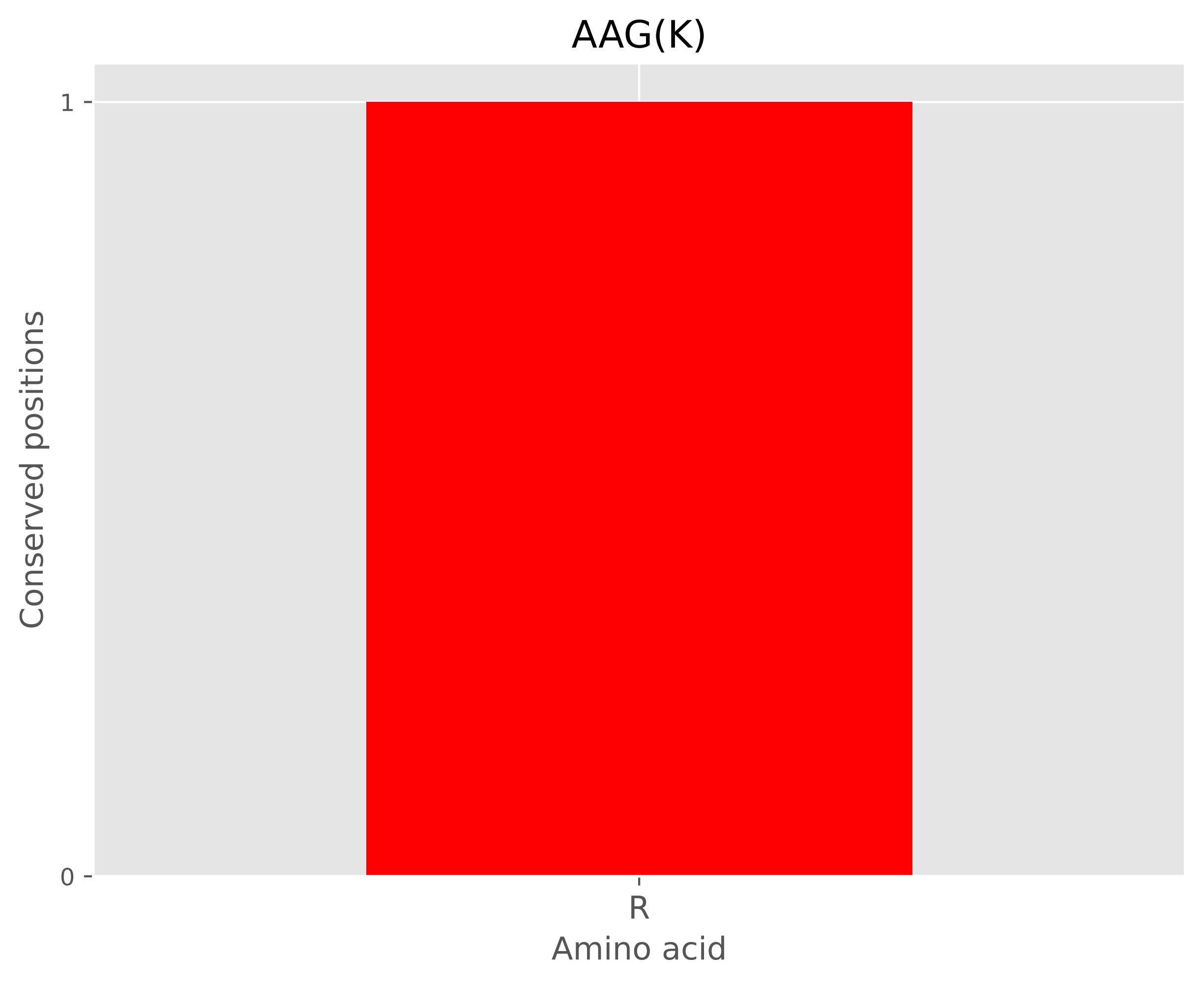
AAG(K)
1
0
Conserved positions
R
Amino acid

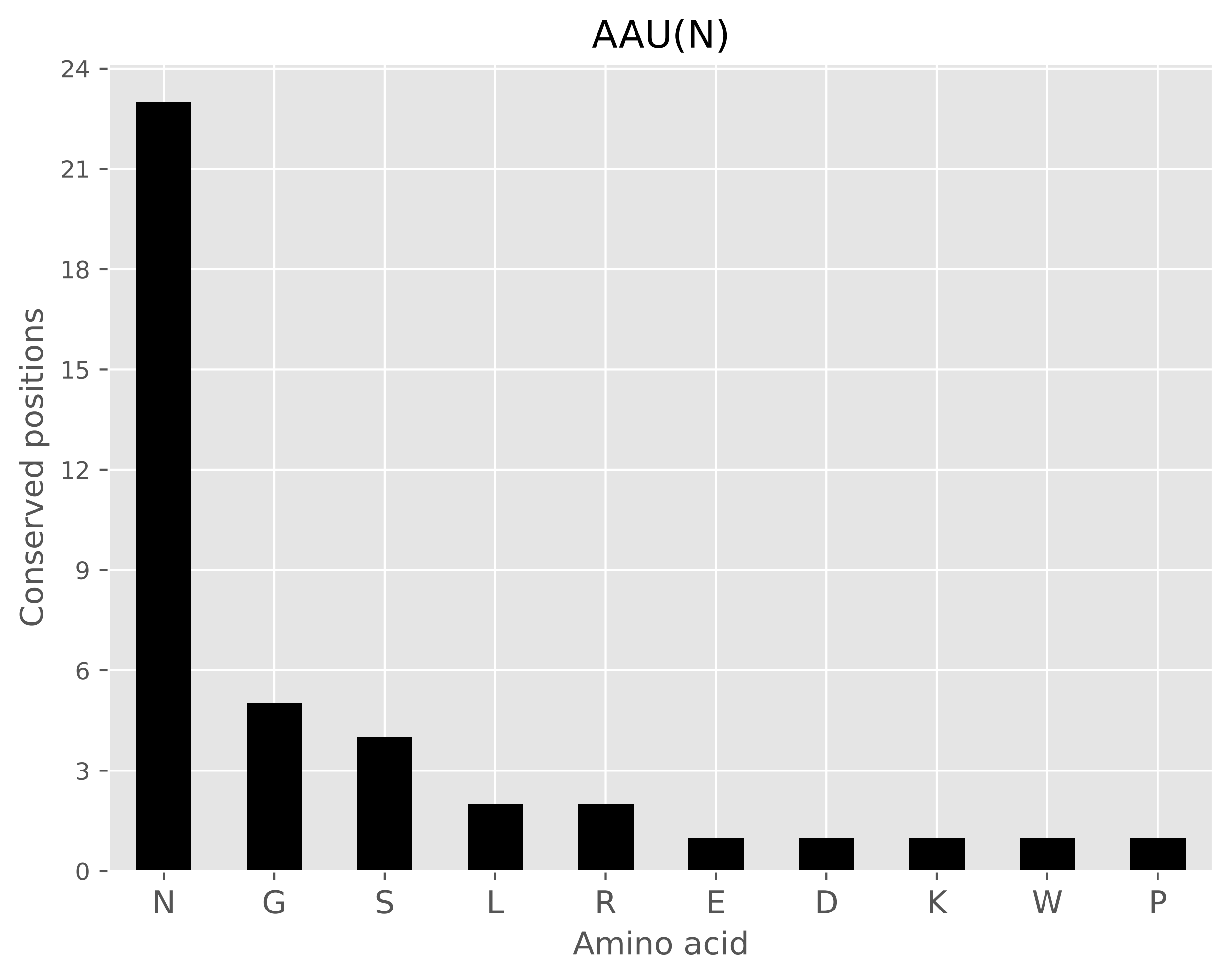

AAU(N)
Conserved positions
0
3
6
9
12
15
18
21
24
N
G
S
L
R
E
D
K
W
P
Amino acid

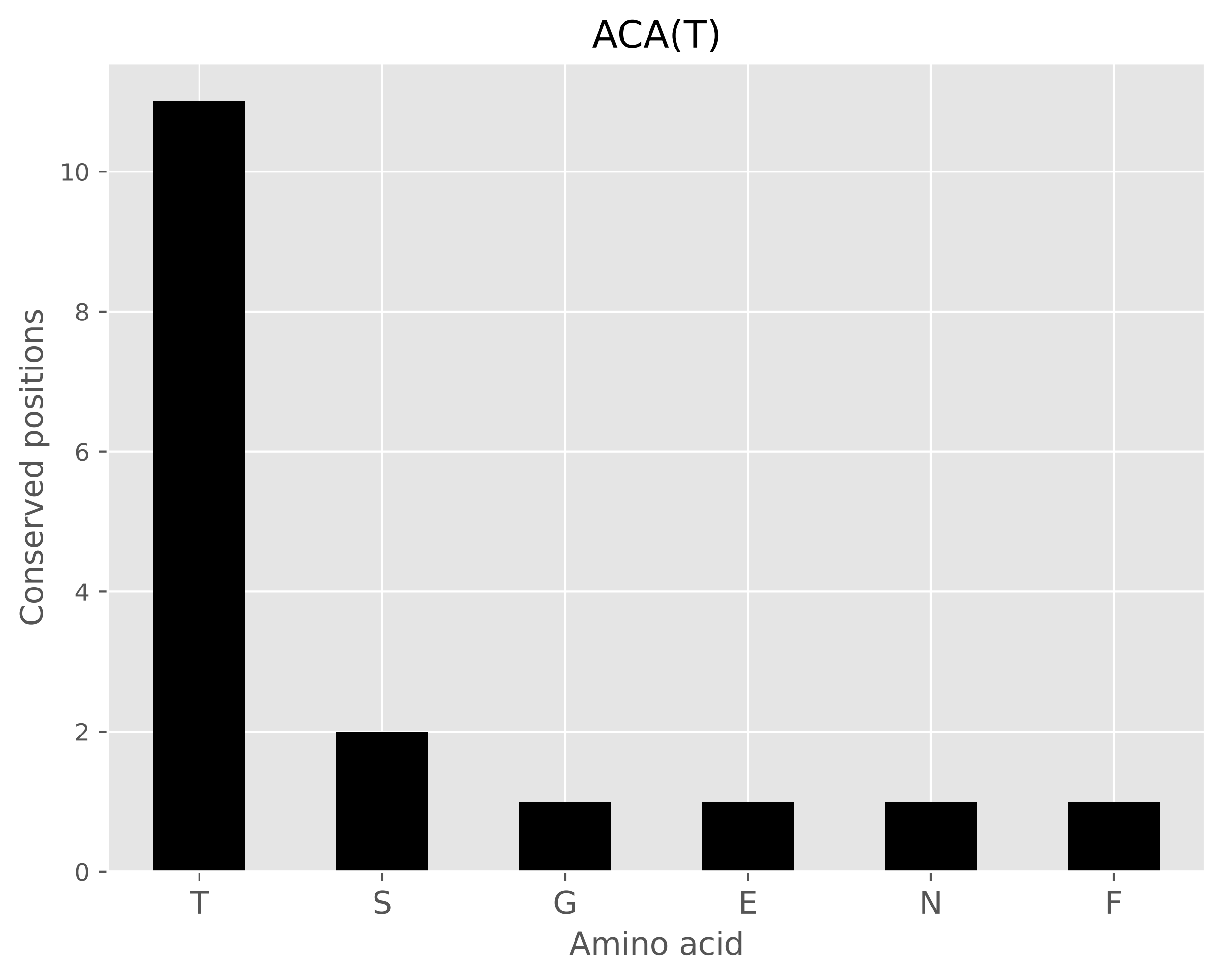
ACA(T)
Conserved positions
0
2
4
6
8
10
T
S
G
E
N
F
Amino acid

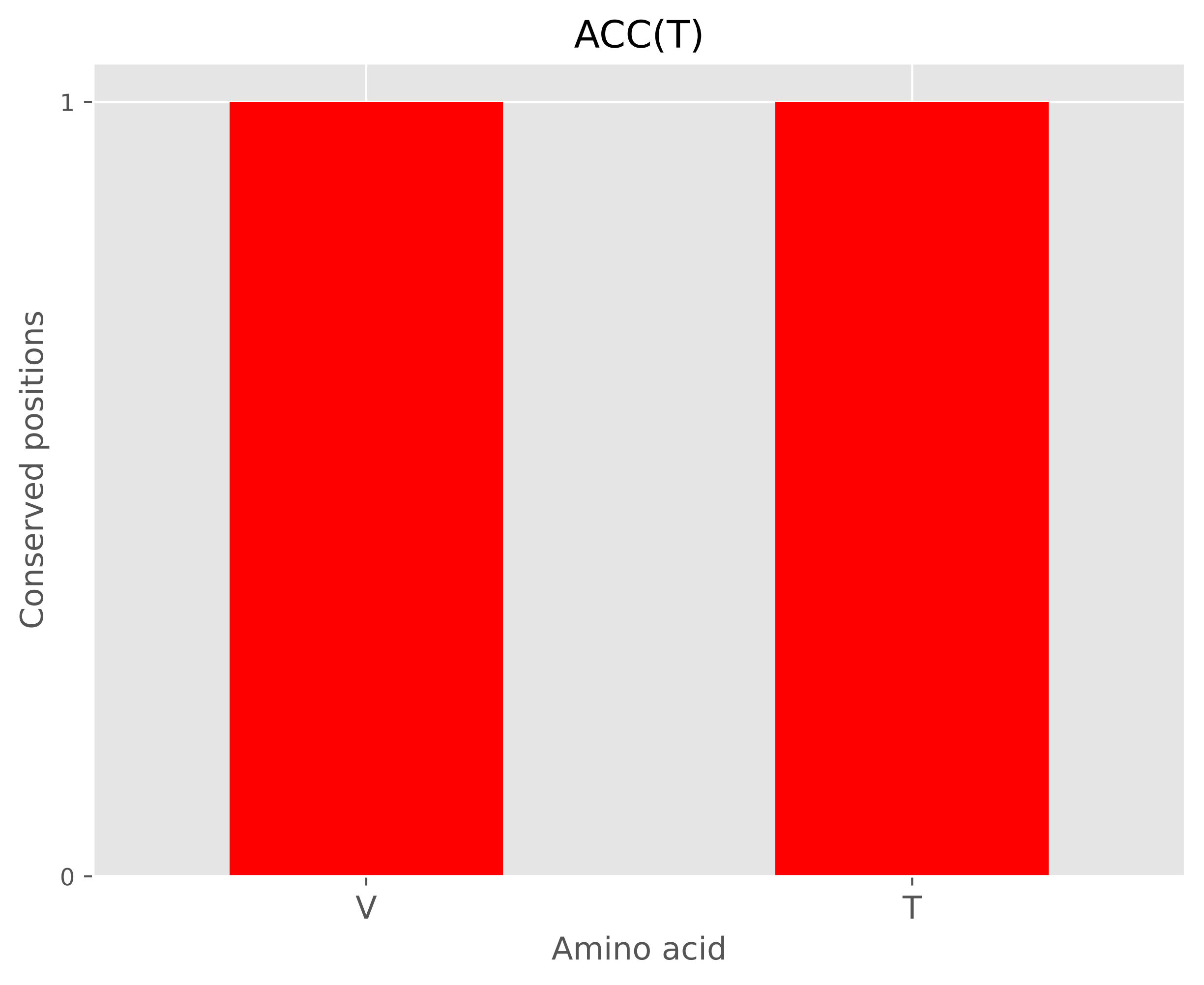

ACC(T)
Conserved positions
1
0
V
T
Amino acid

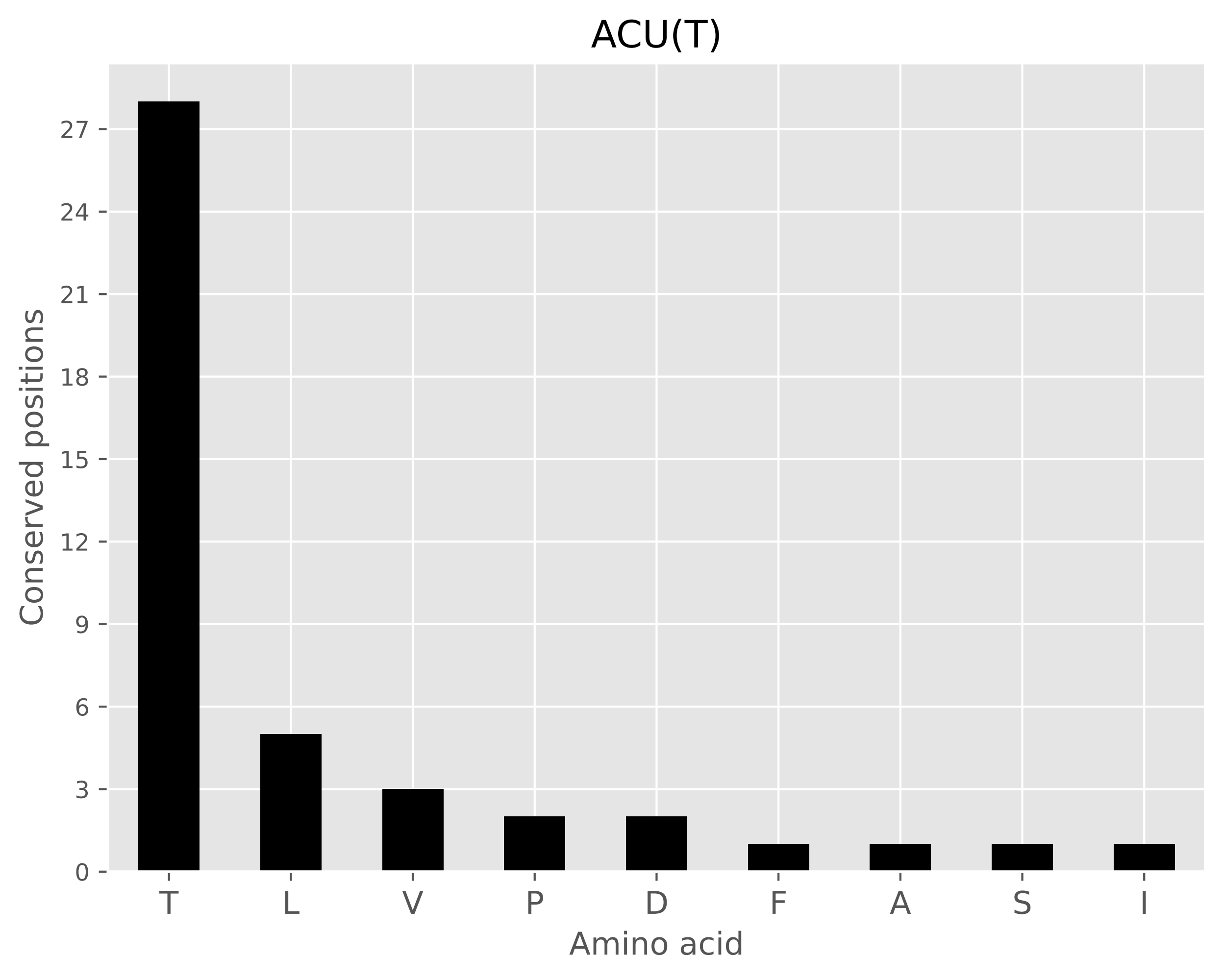
ACU(T)
Conserved positions
Amino acid
0
3
6
9
12
15
18
21
24
27
T
L
V
P
D
F
A
S
I

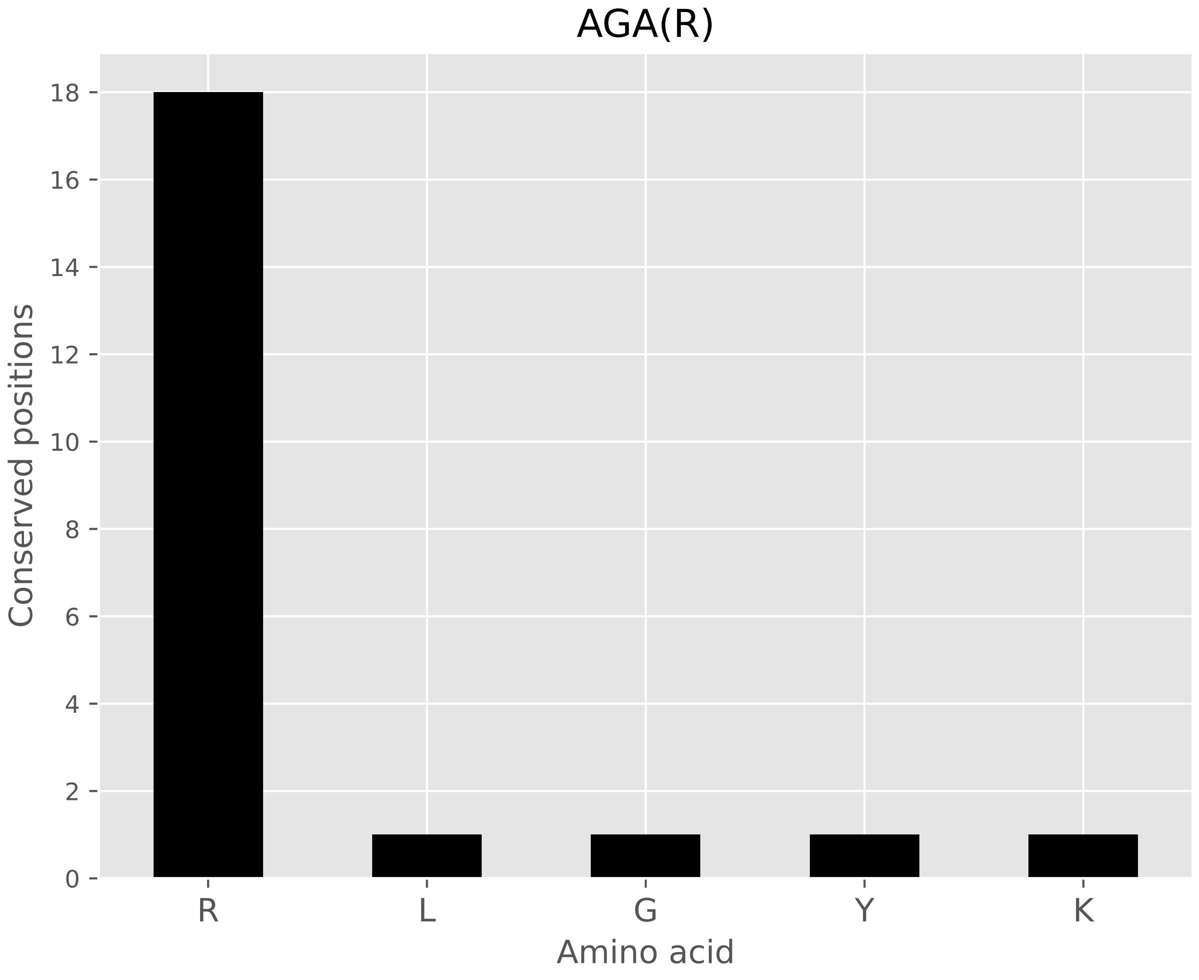

AGA(R)
Conserved positions
0
2
4
6
8
10
12
14
16
18
R
L
G
Y
K
Amino acid

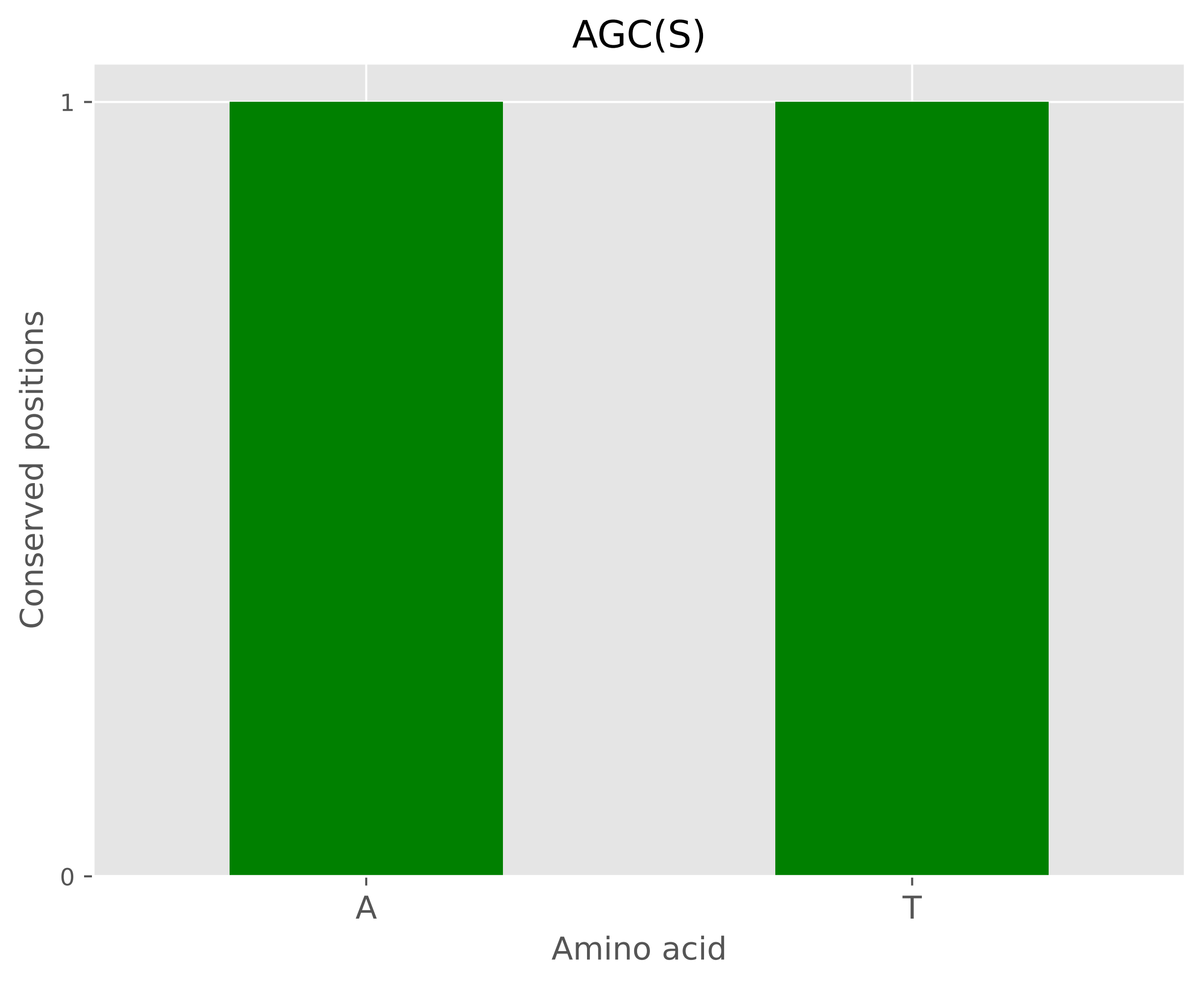

AGC(S)
1
0
Conserved positions
A
T
Amino acid

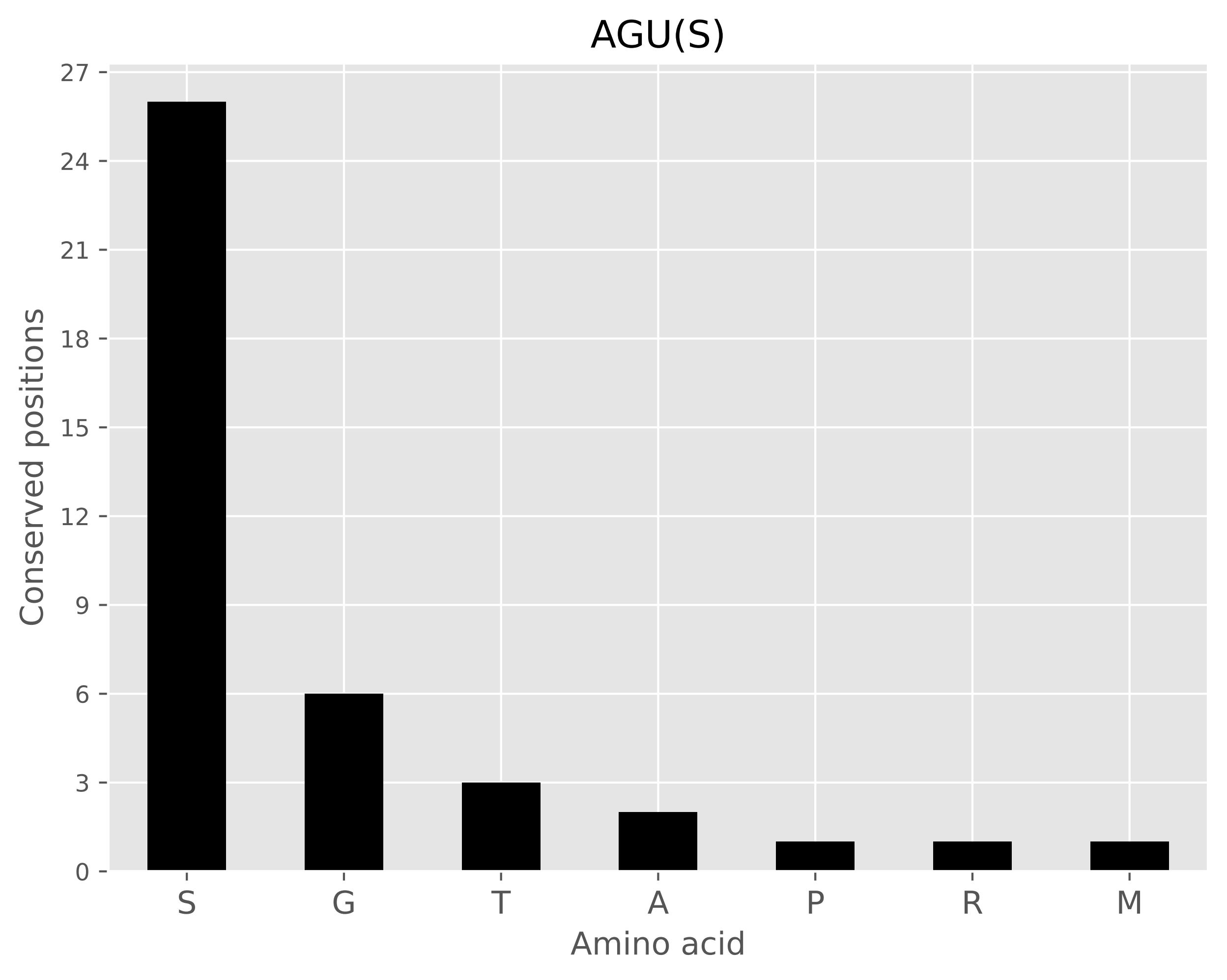
AGU(S)
Conserved positions
0
3
6
9
12
15
18
21
24
27
S
G
T
A
P
R
M
Amino acid

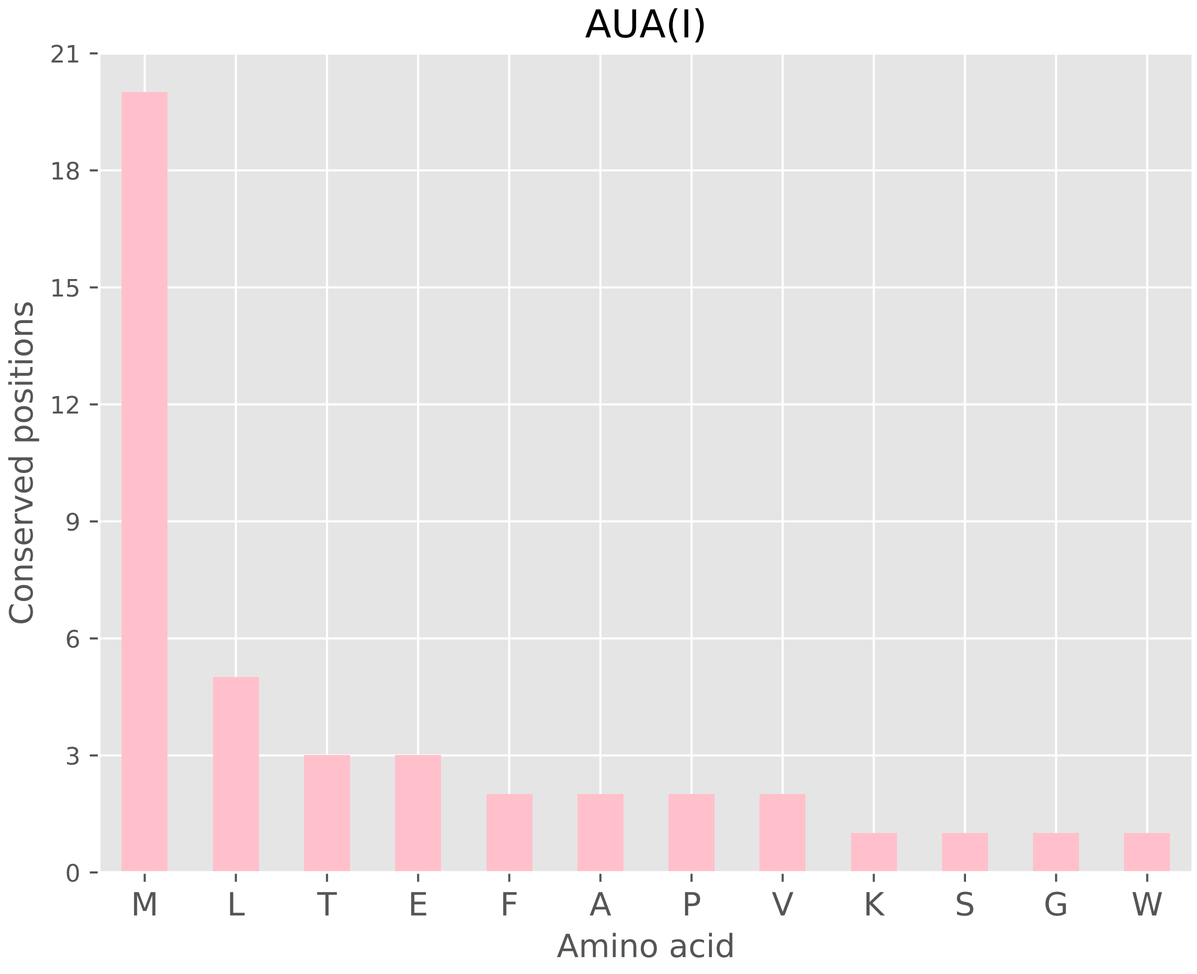
AUA(I)
Conserved positions
21
18
15
12
9
6
3
0
M
L
T
E
F
A
P
V
K
S
G
W
Amino acid

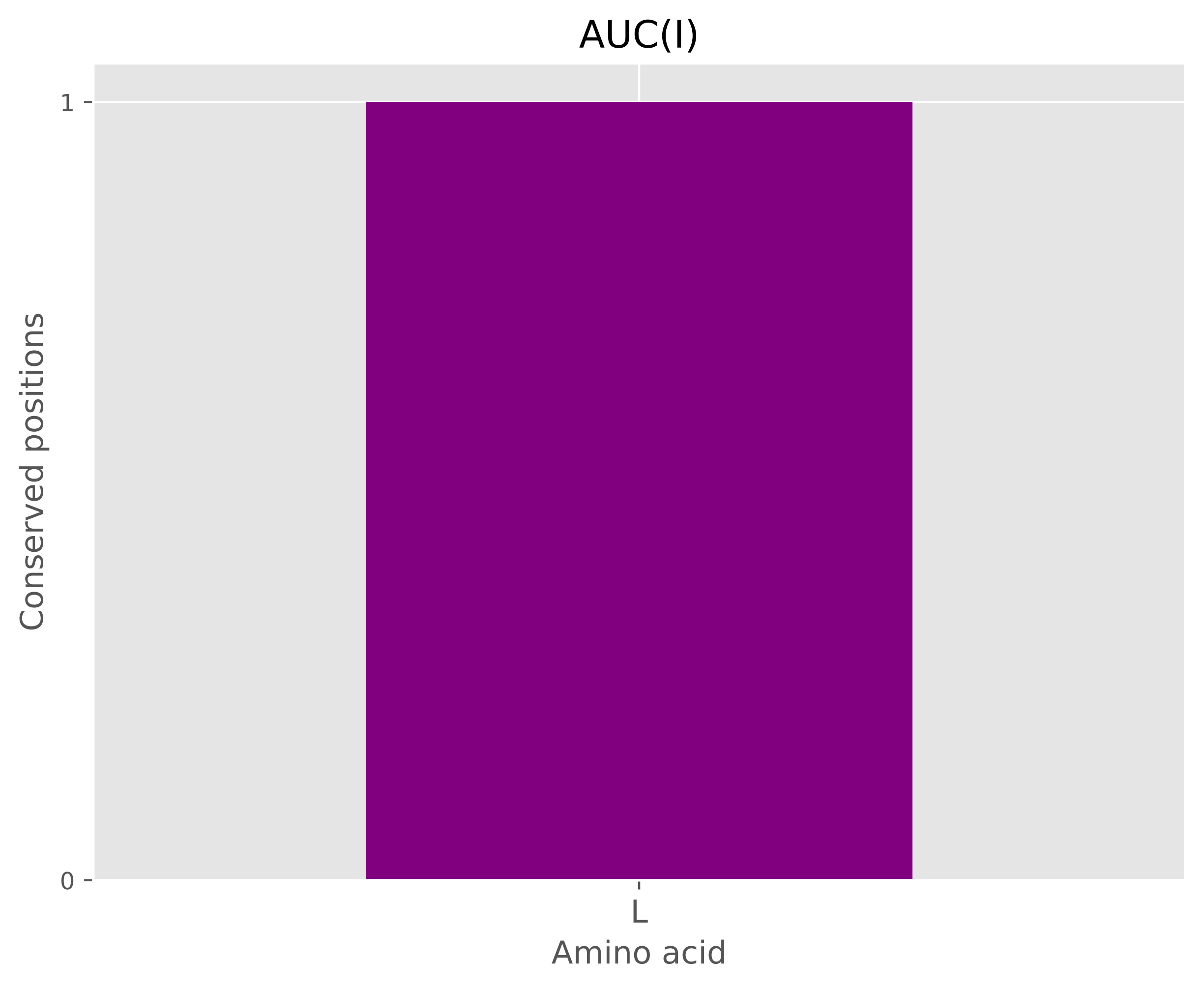

AUC(I)
1
0
Conserved positions
L
Amino acid

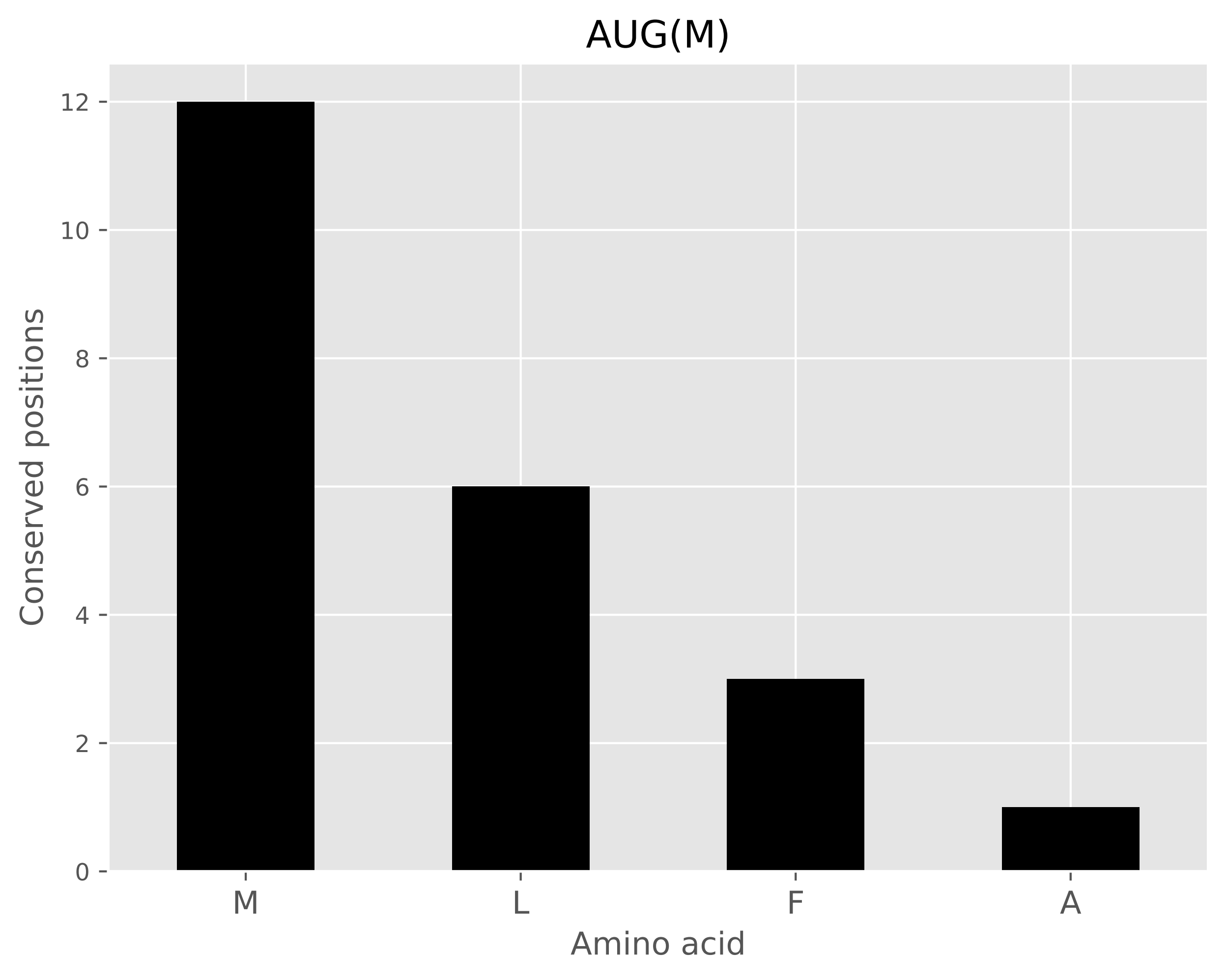
AUG(M)
Conserved positions
0
2
4
6
8
10
12
M
L
F
A
Amino acid

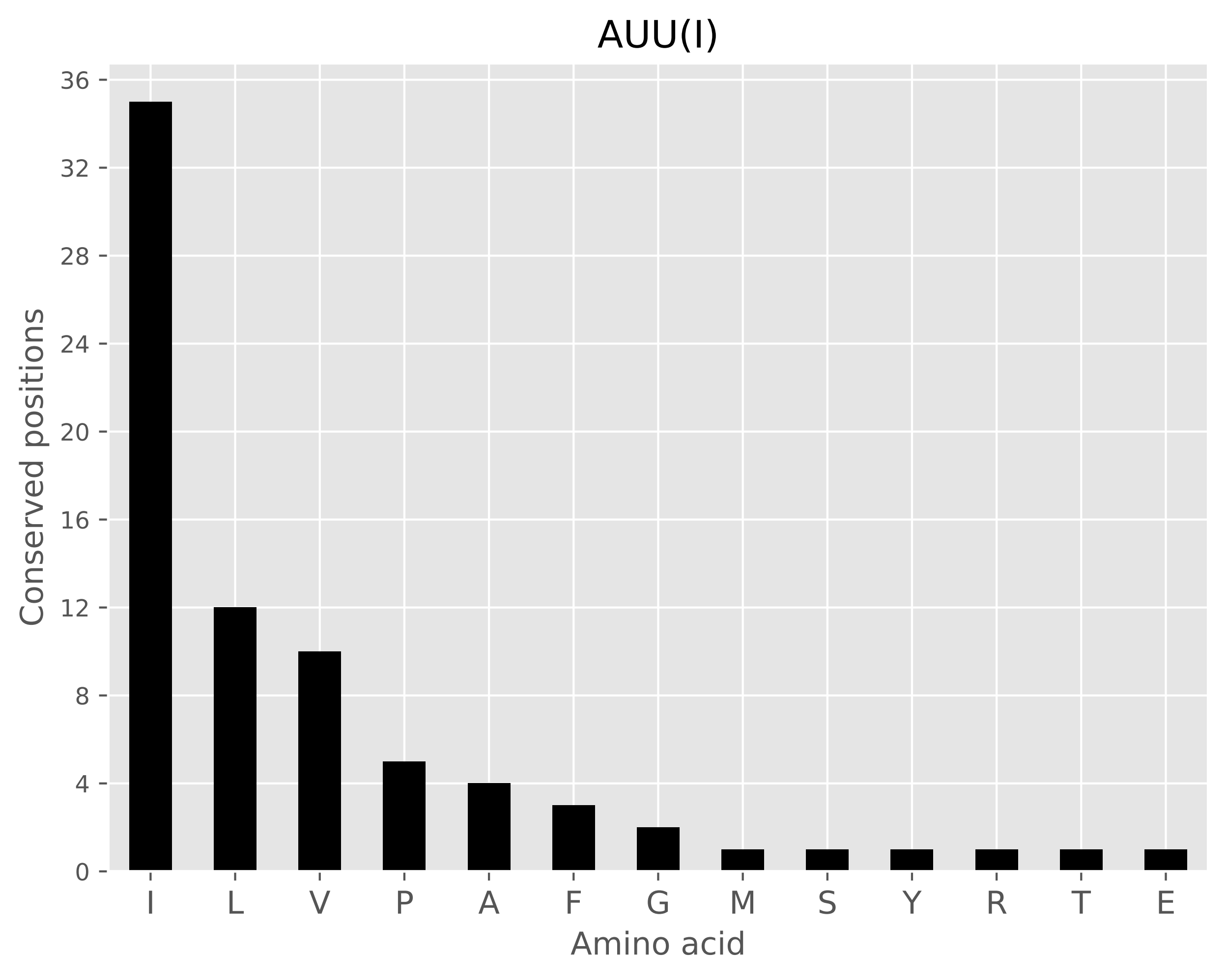

AUU(I)
Conserved positions
0
4
8
12
16
20
24
28
32
36
I
L
V
P
A
F
G
M
S
Y
R
T
E
Amino acid

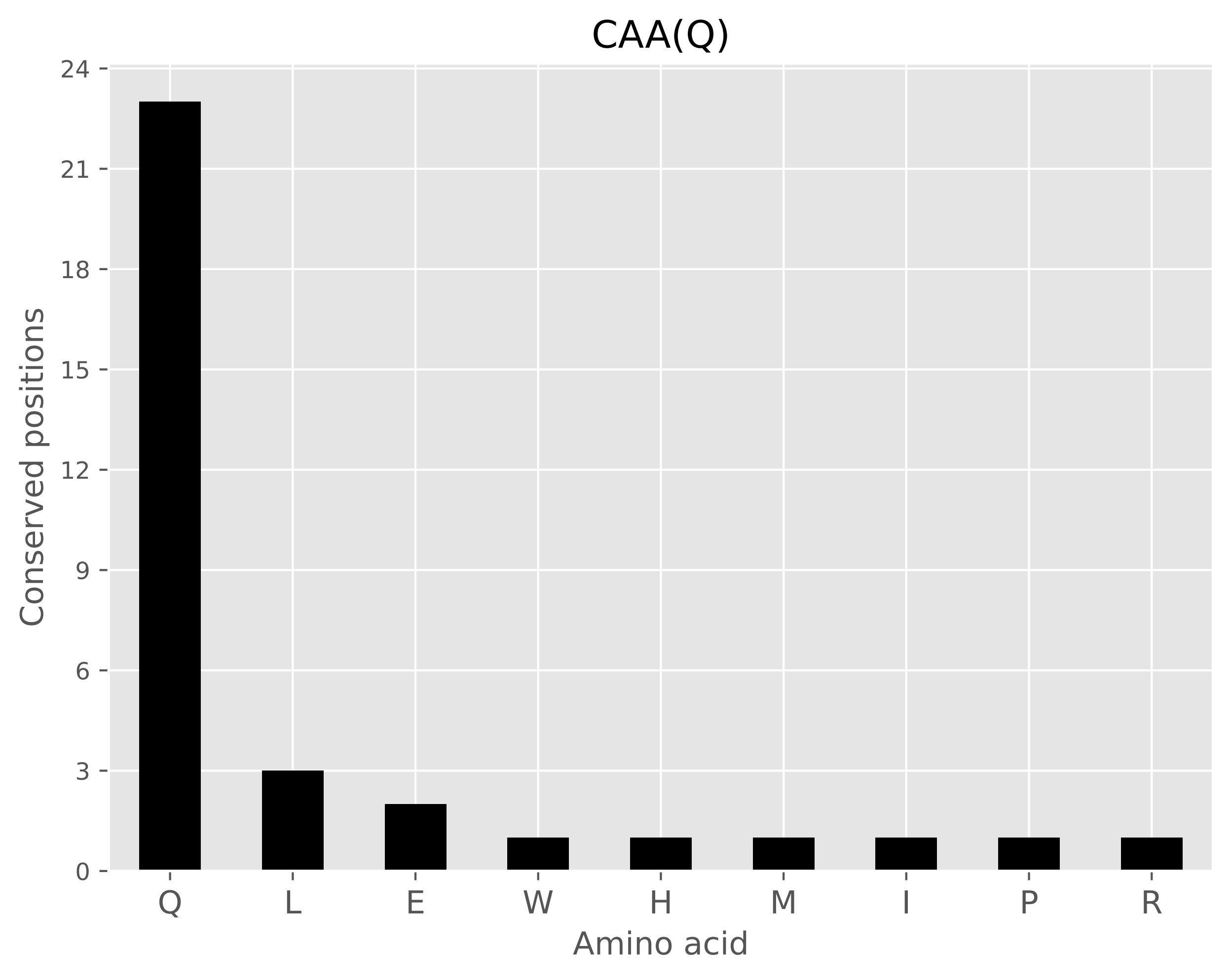

CAA(Q)
Conserved positions
0
3
6
9
12
15
18
21
24
Q
L
E
W
H
M
I
P
R
Amino acid

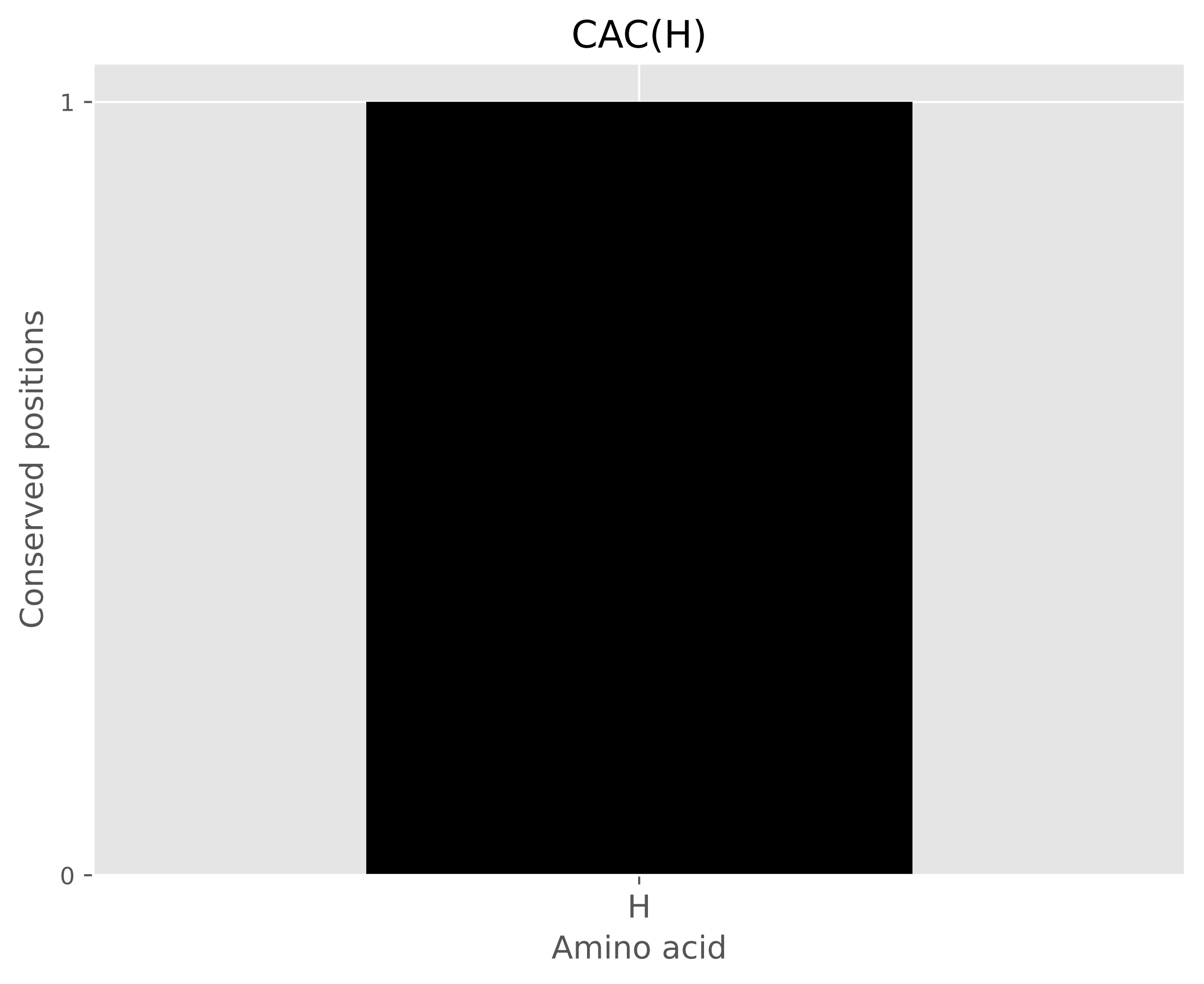

CAC(H)
1
0
Conserved positions
H
Amino acid

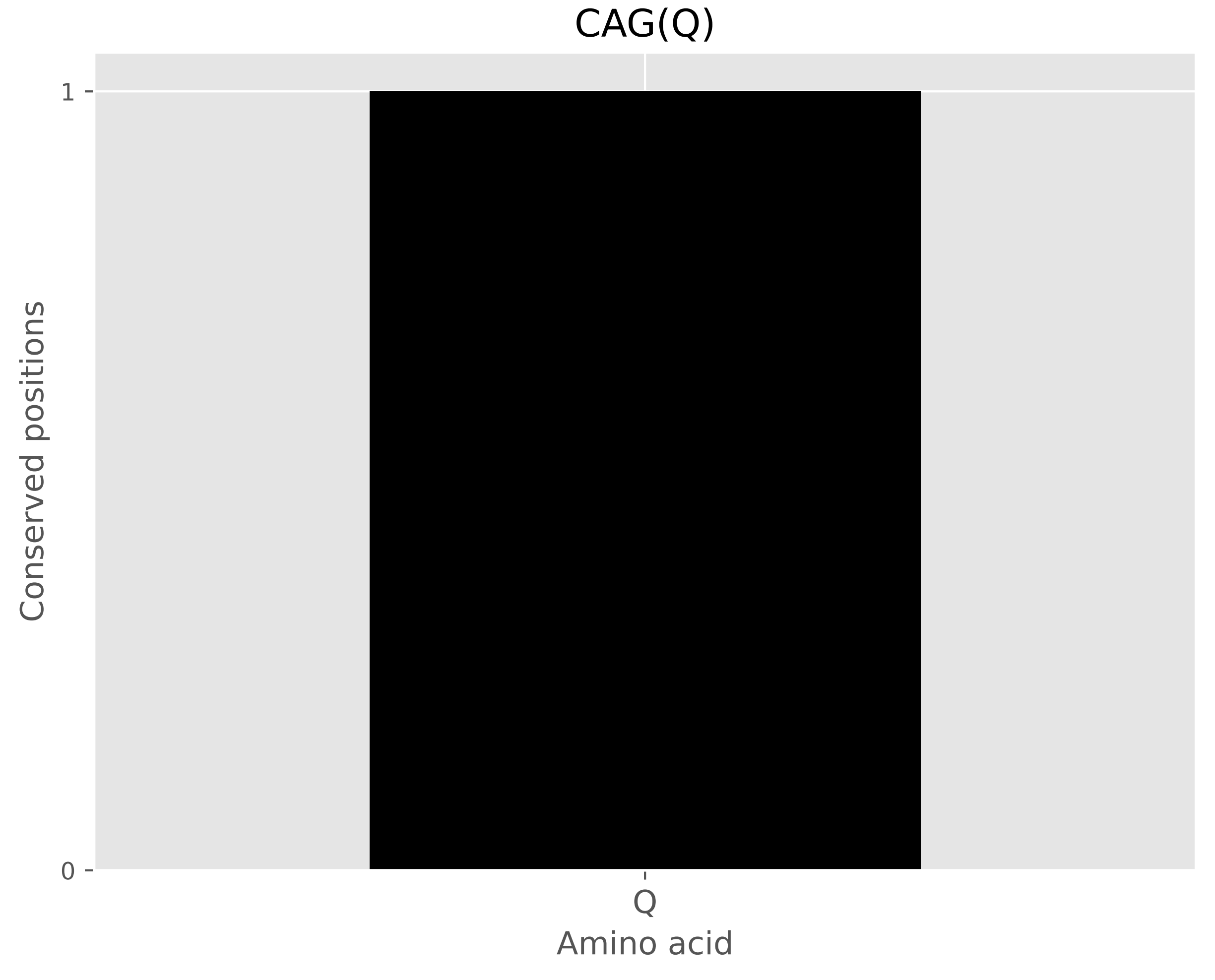
CAG(Q)
Conserved positions
1
0
Q
Amino acid

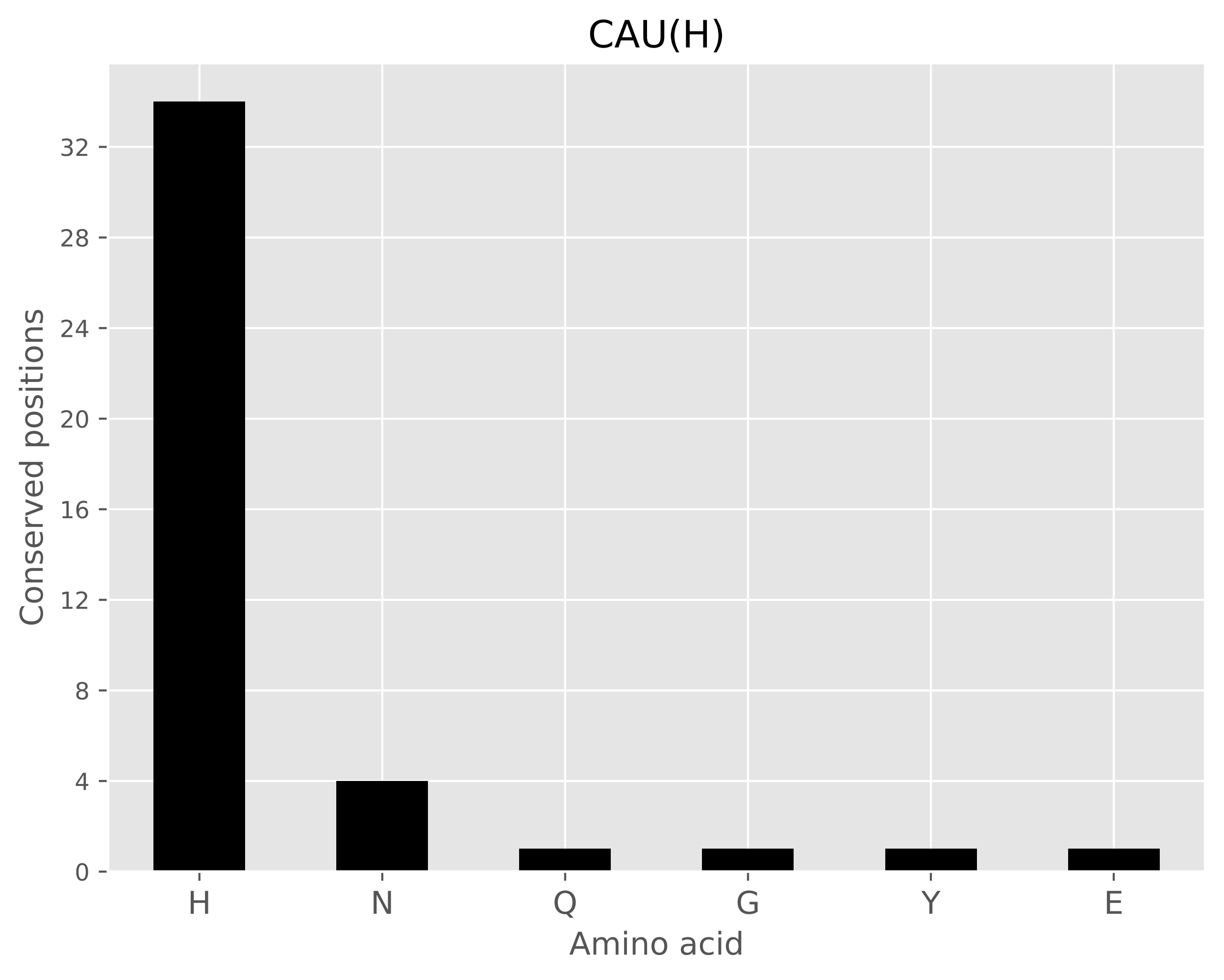
CAU(H)
Conserved positions
0
4
8
12
16
20
24
28
32
H
N
Q
G
Y
E
Amino acid

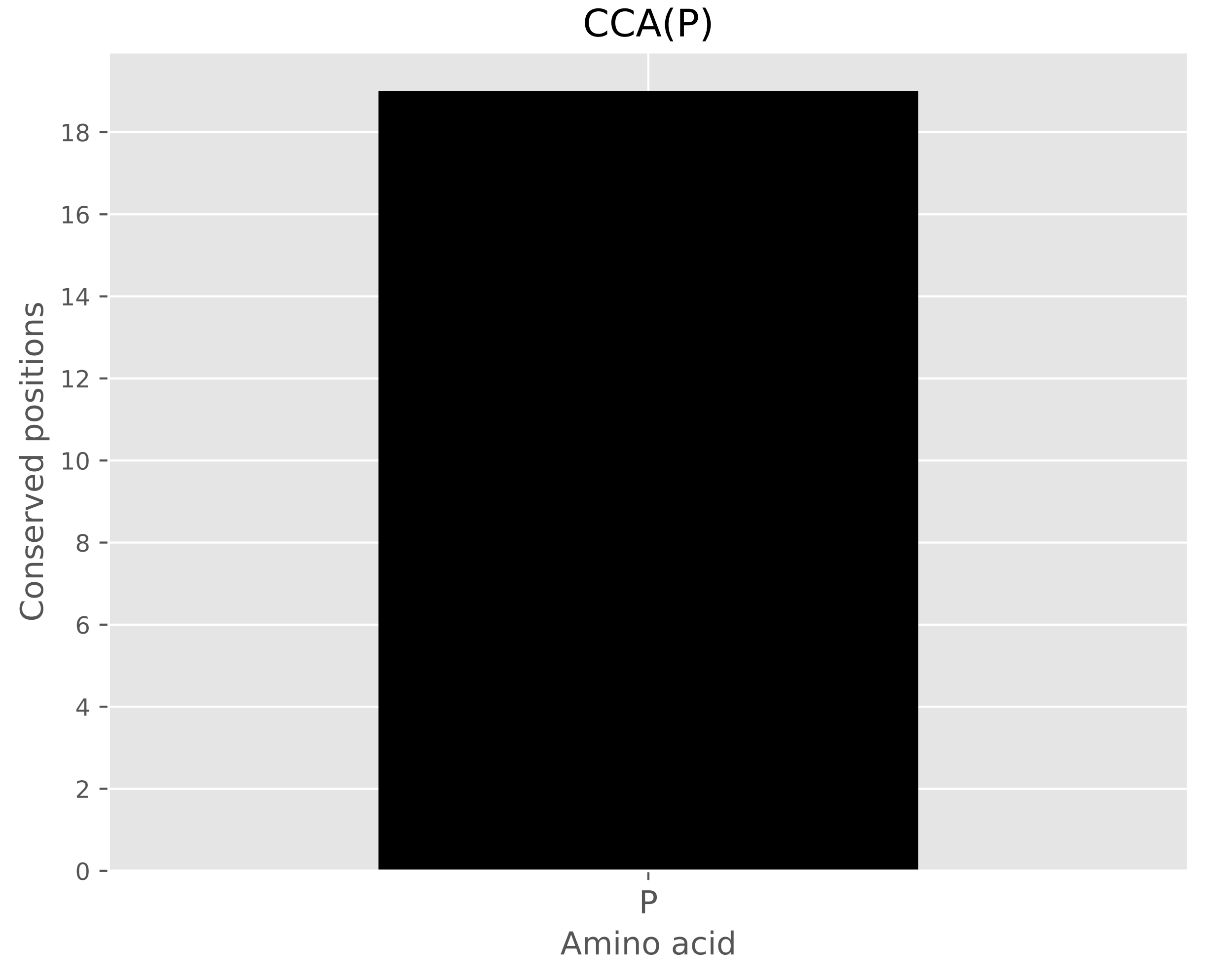
CCA(P)
Conserved positions
0
2
4
6
8
10
12
14
16
18
P
Amino acid

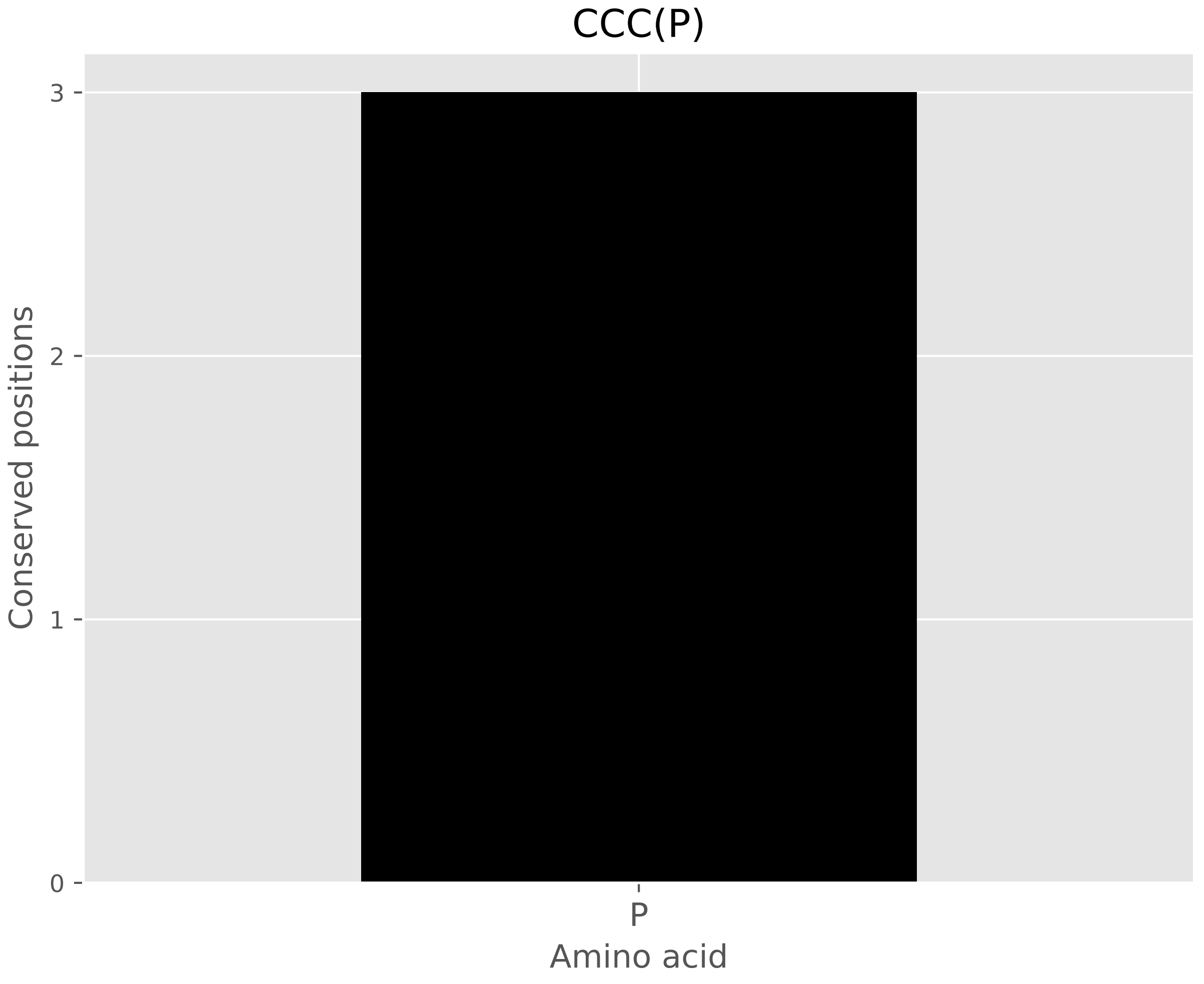

CCC(P)
Conserved positions
3
2
1
0
P
Amino acid

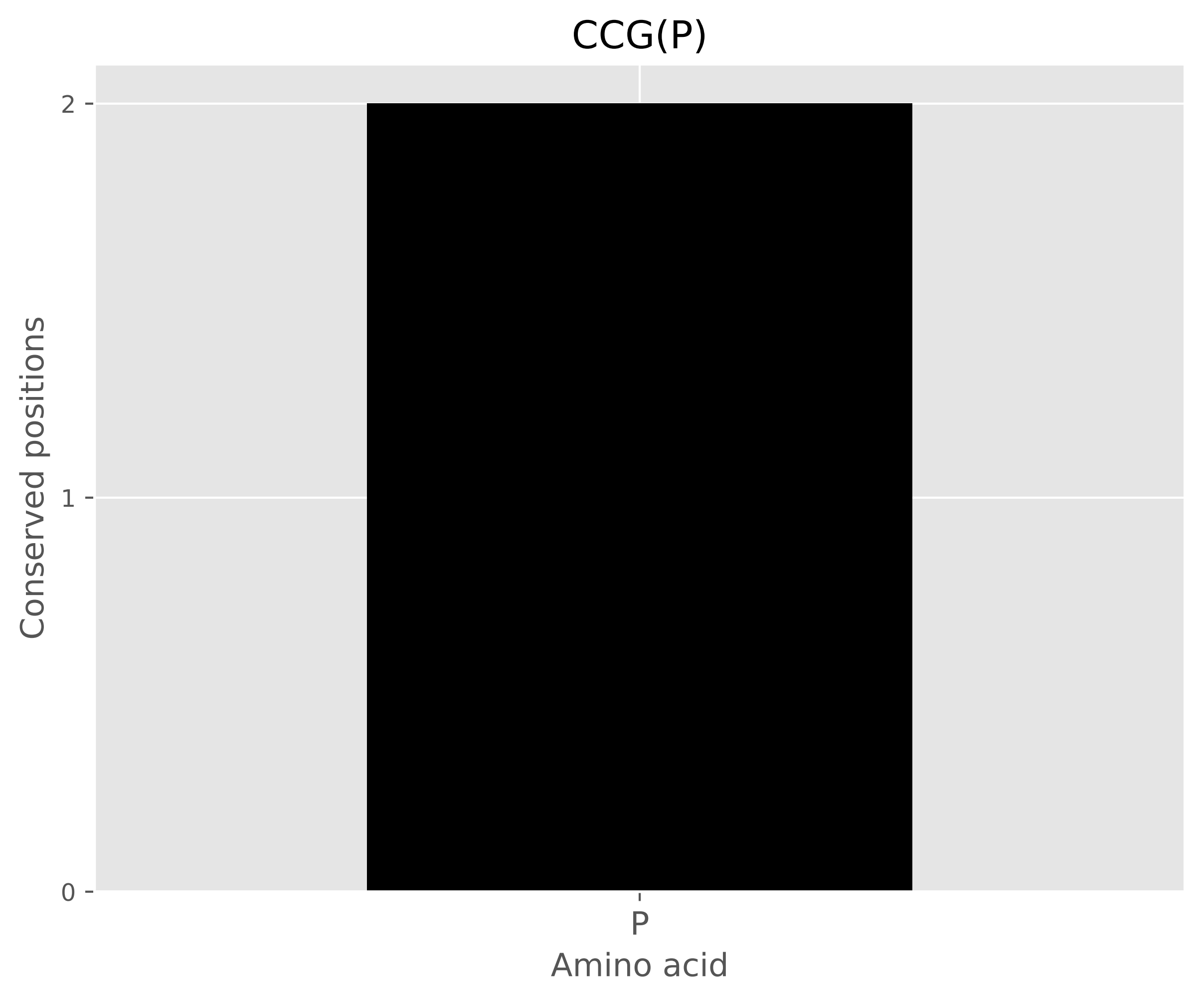
CCG(P)
Conserved positions
2
1
0
P
Amino acid

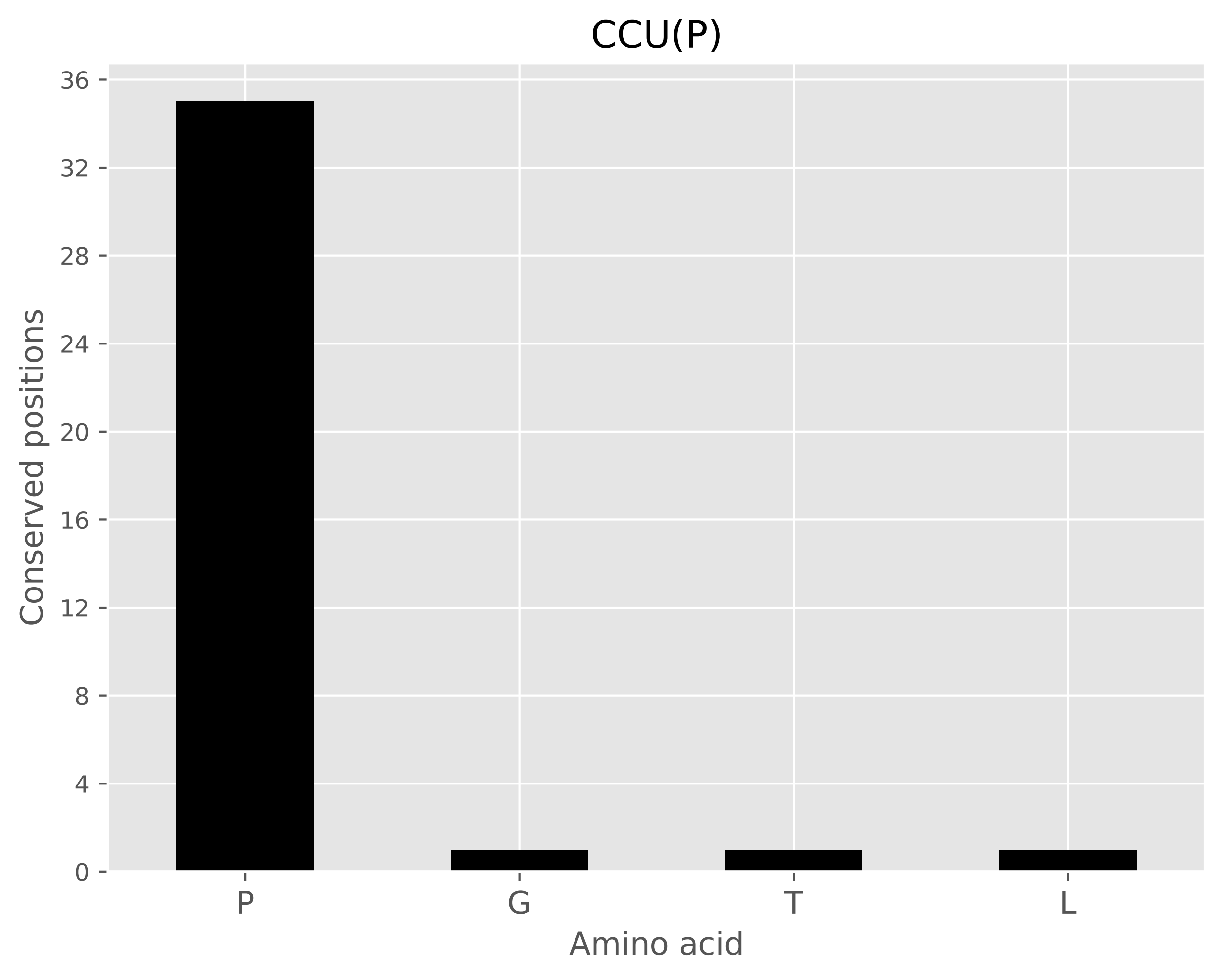

CCU(P)
Conserved positions
36
32
28
24
20
16
12
8
4
0
P
G
T
L
Amino acid

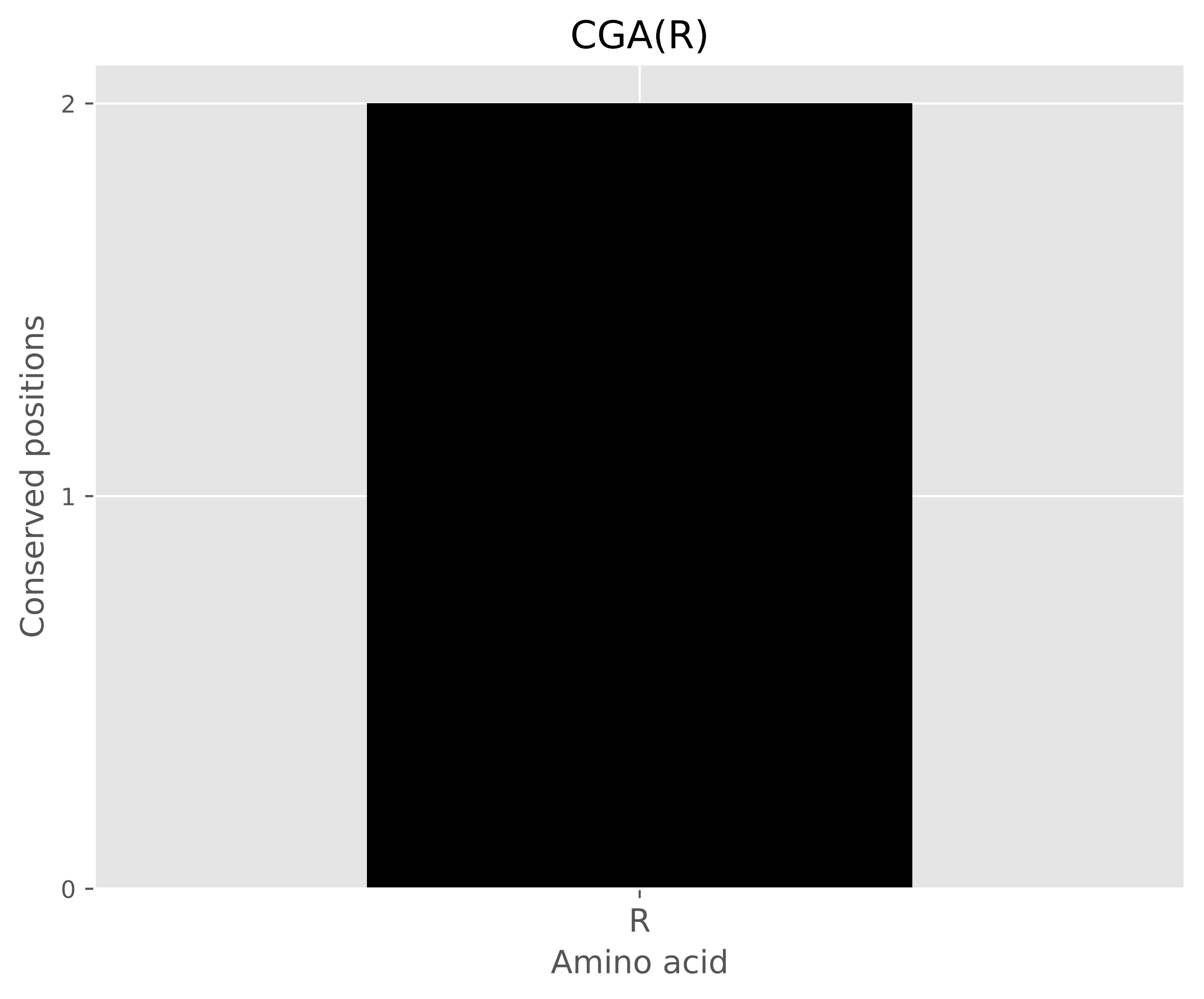
CGA(R)
Conserved positions
2
1
0
R
Amino acid

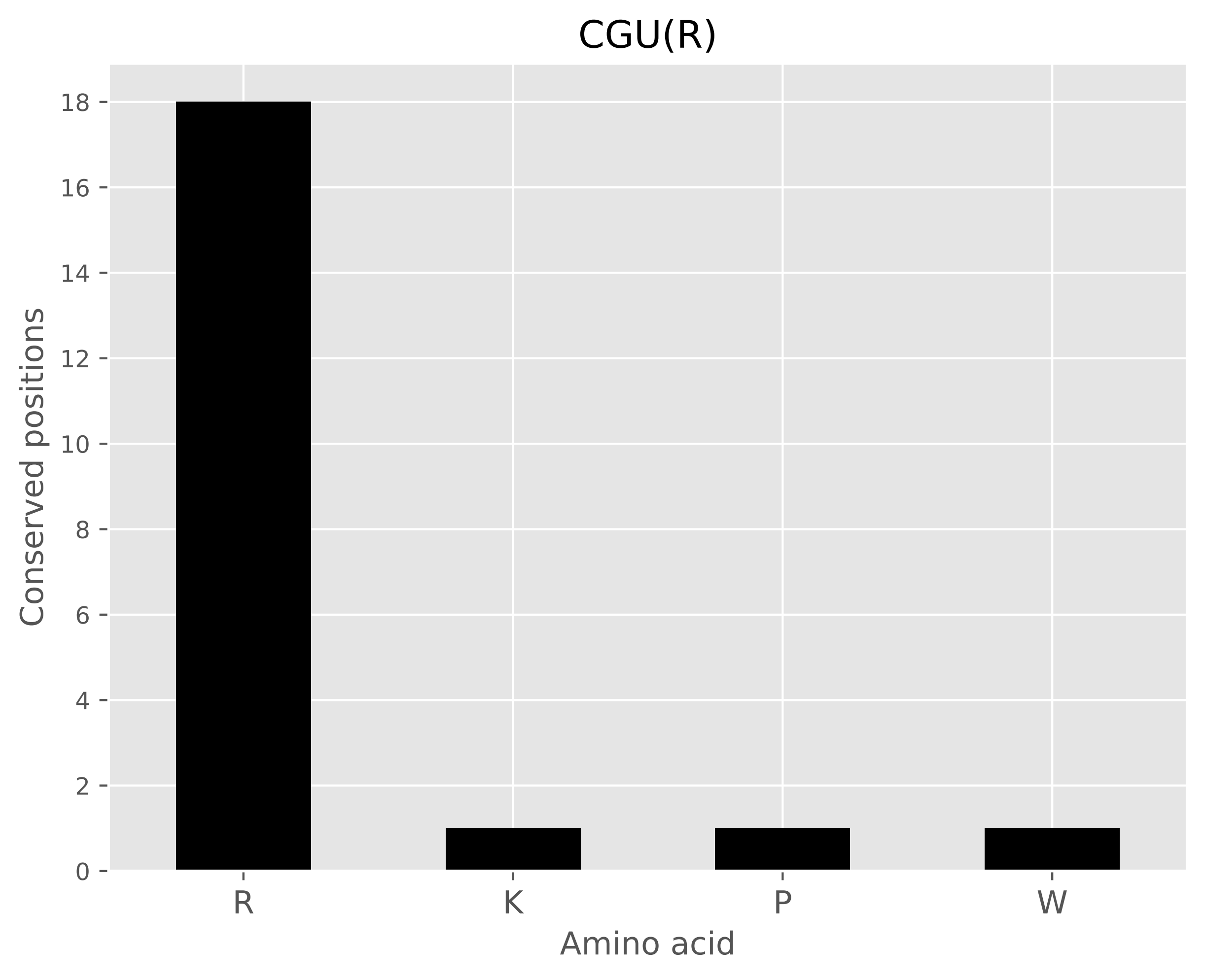
CGU(R)
Conserved positions
Amino acid
R
K
P
W
0
2
4
6
8
10
12
14
16
18

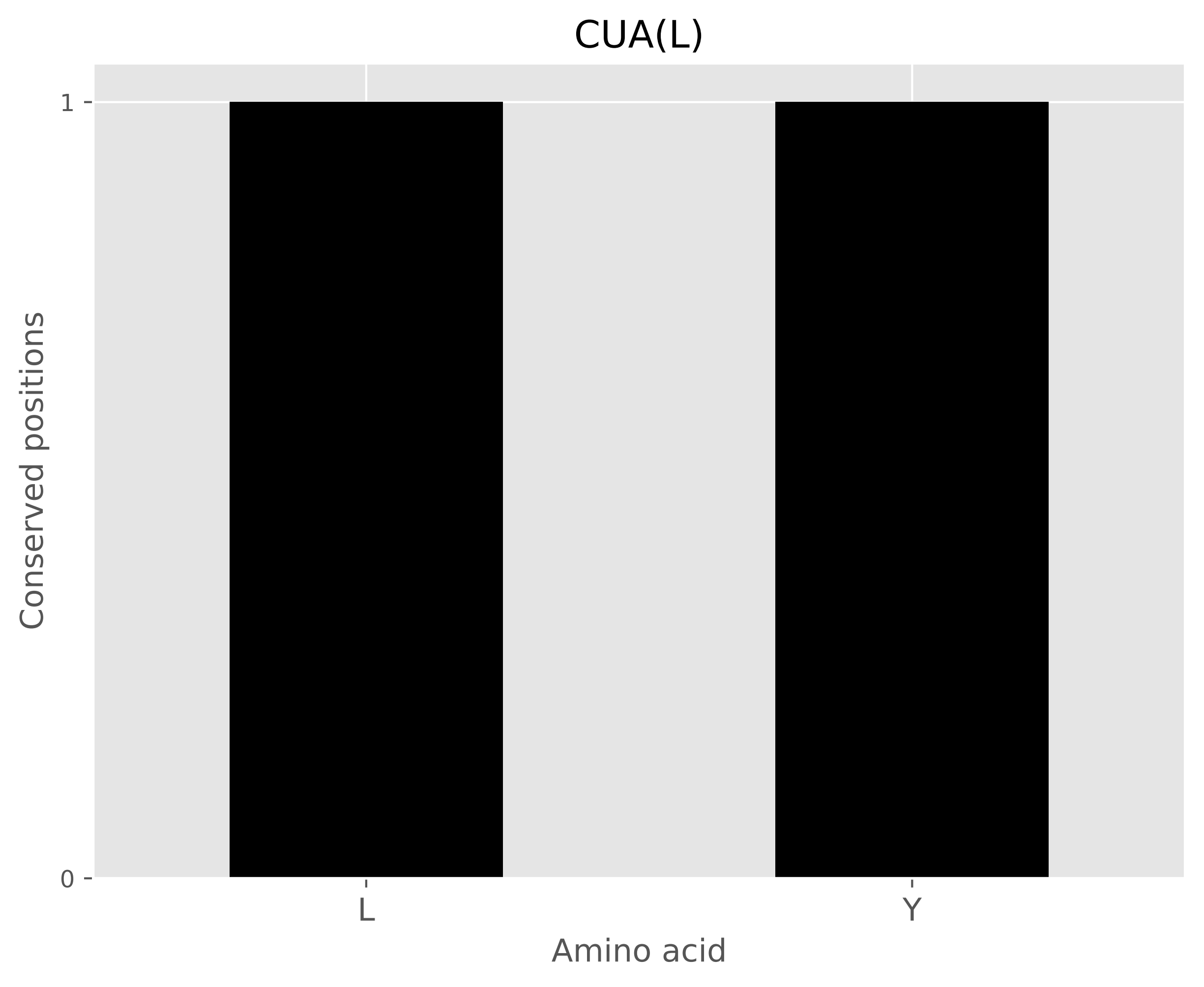
CUA(L)
Conserved positions
1
0
L
Y
Amino acid

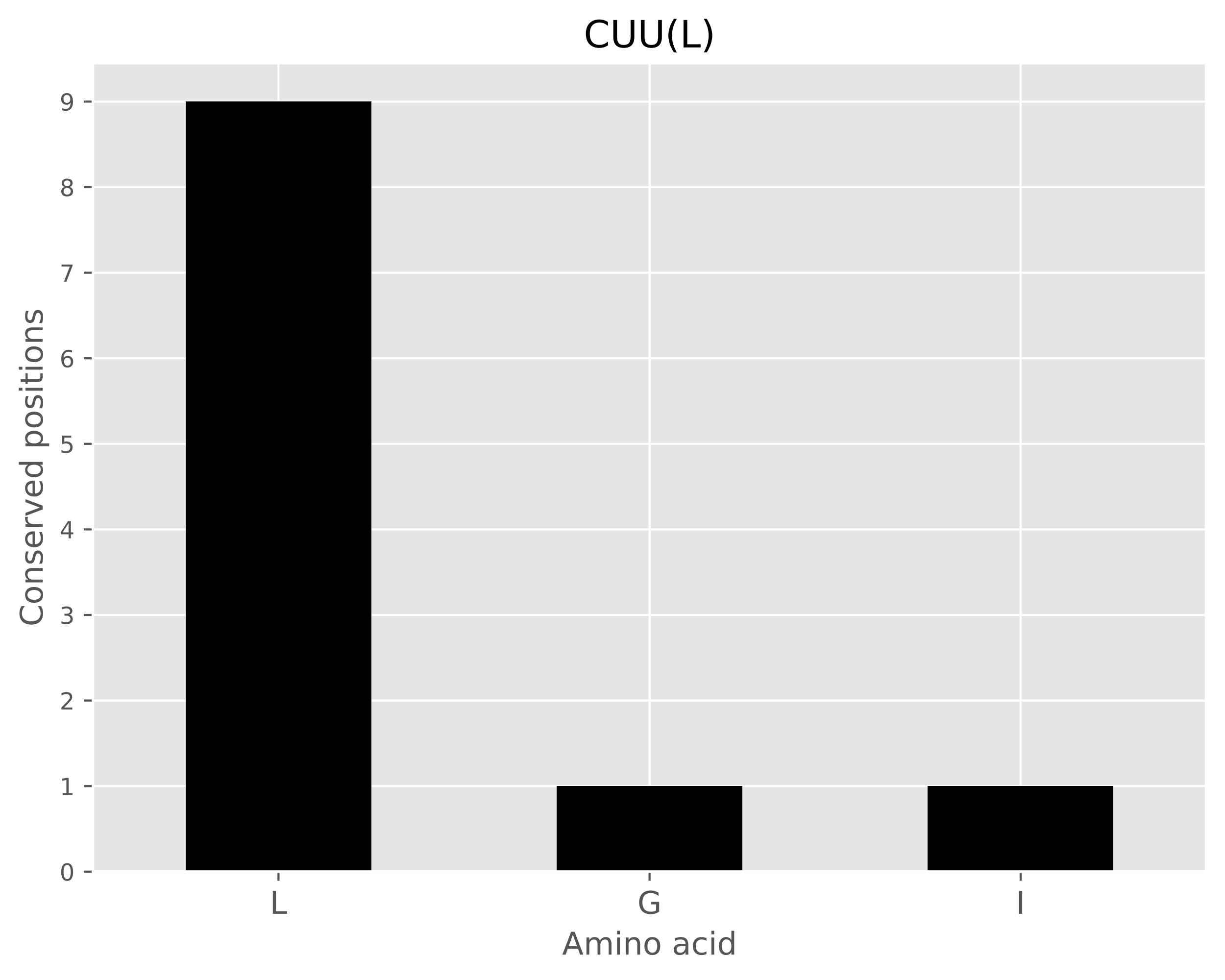
CUU(L)
Conserved positions
0
1
2
3
4
5
6
7
8
9
L
G
I
Amino acid

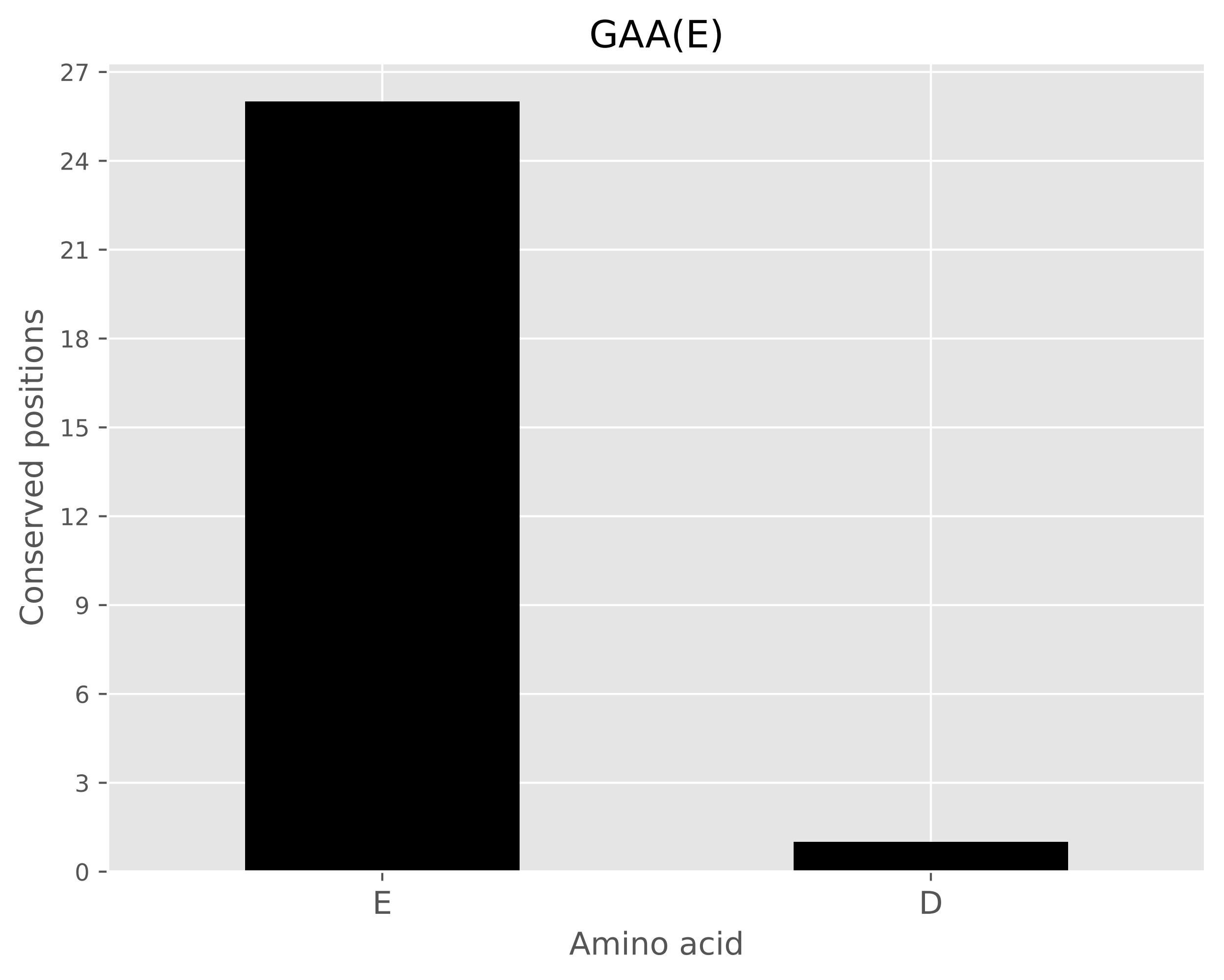
GAA(E)
Conserved positions
Amino acid
E
D
0
3
6
9
12
15
18
21
24
27

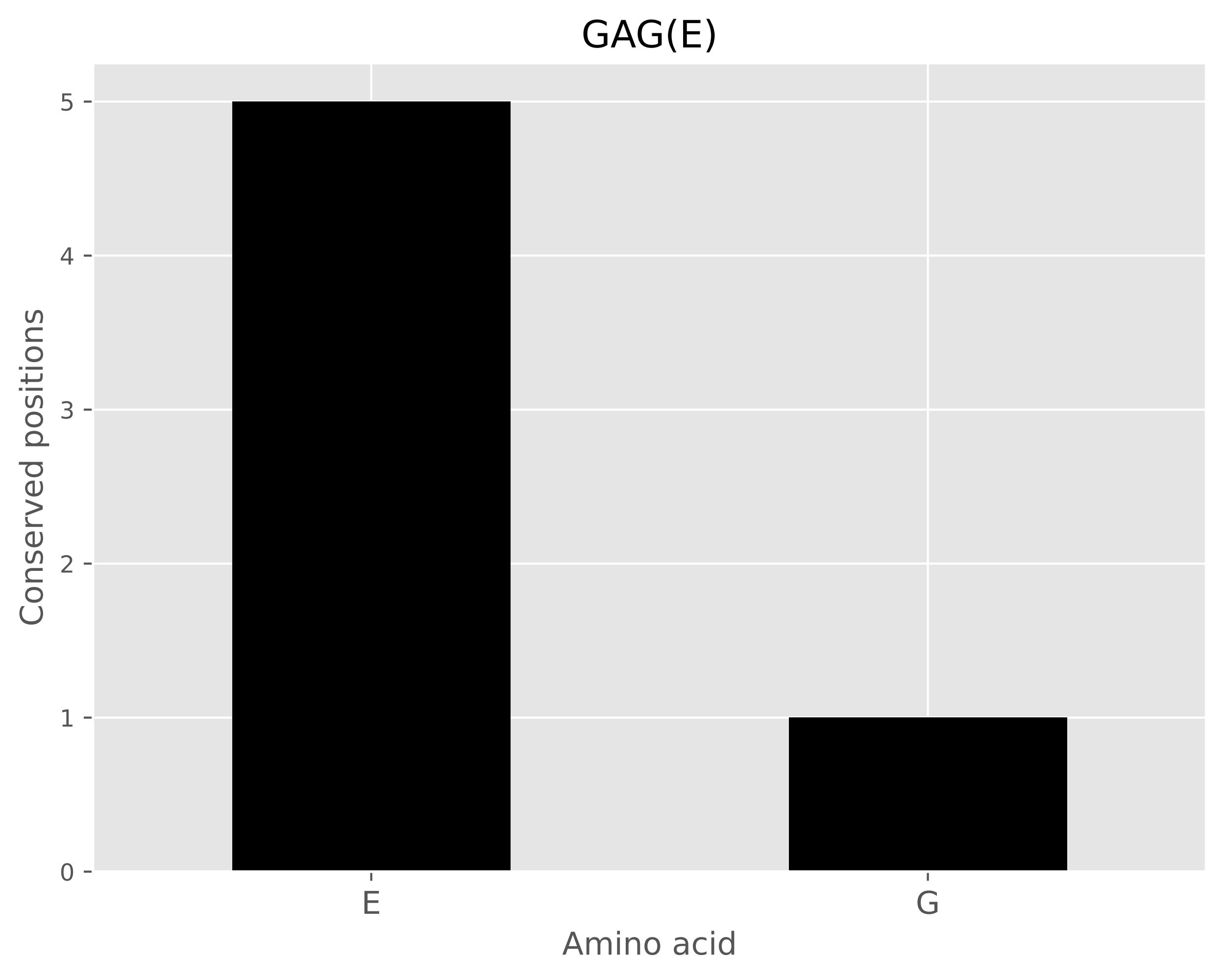
GAG(E)
Conserved positions
0
1
2
3
4
5
E
G
Amino acid

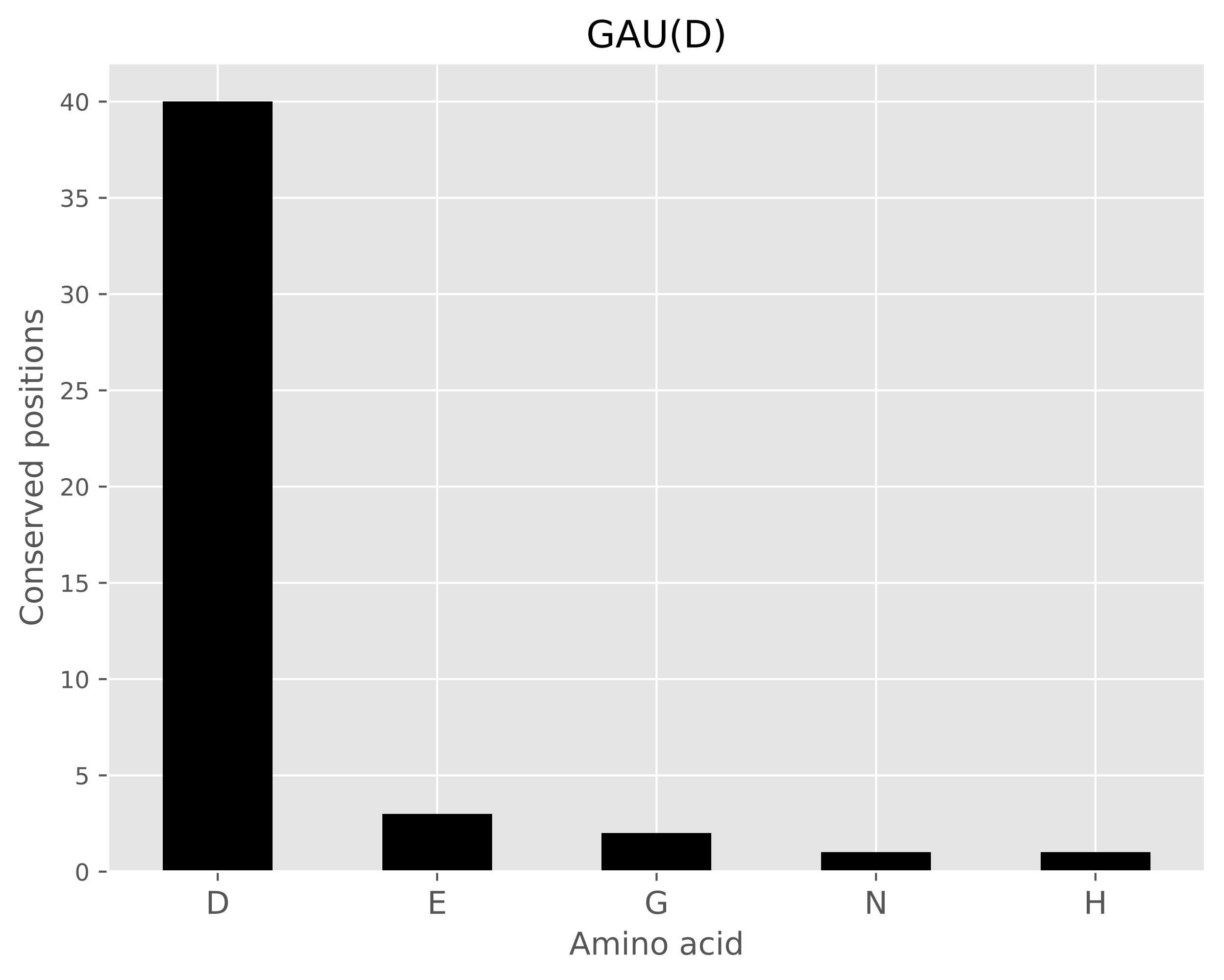

GAU(D)
Conserved positions
0
5
10
15
20
25
30
35
40
D
E
G
N
H
Amino acid

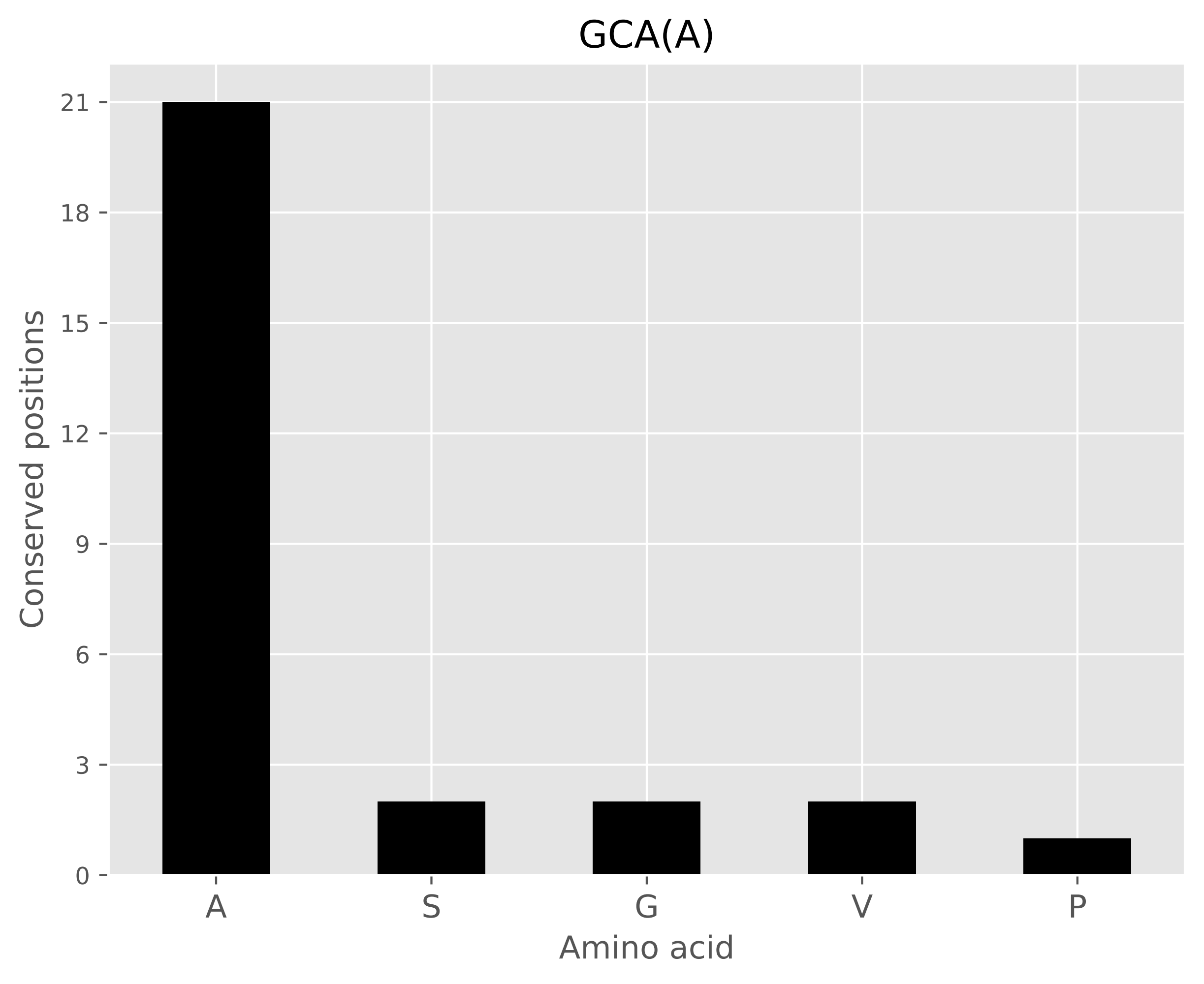
GCA(A)
Conserved positions
21
18
15
12
9
6
3
0
A
S
G
V
P
Amino acid

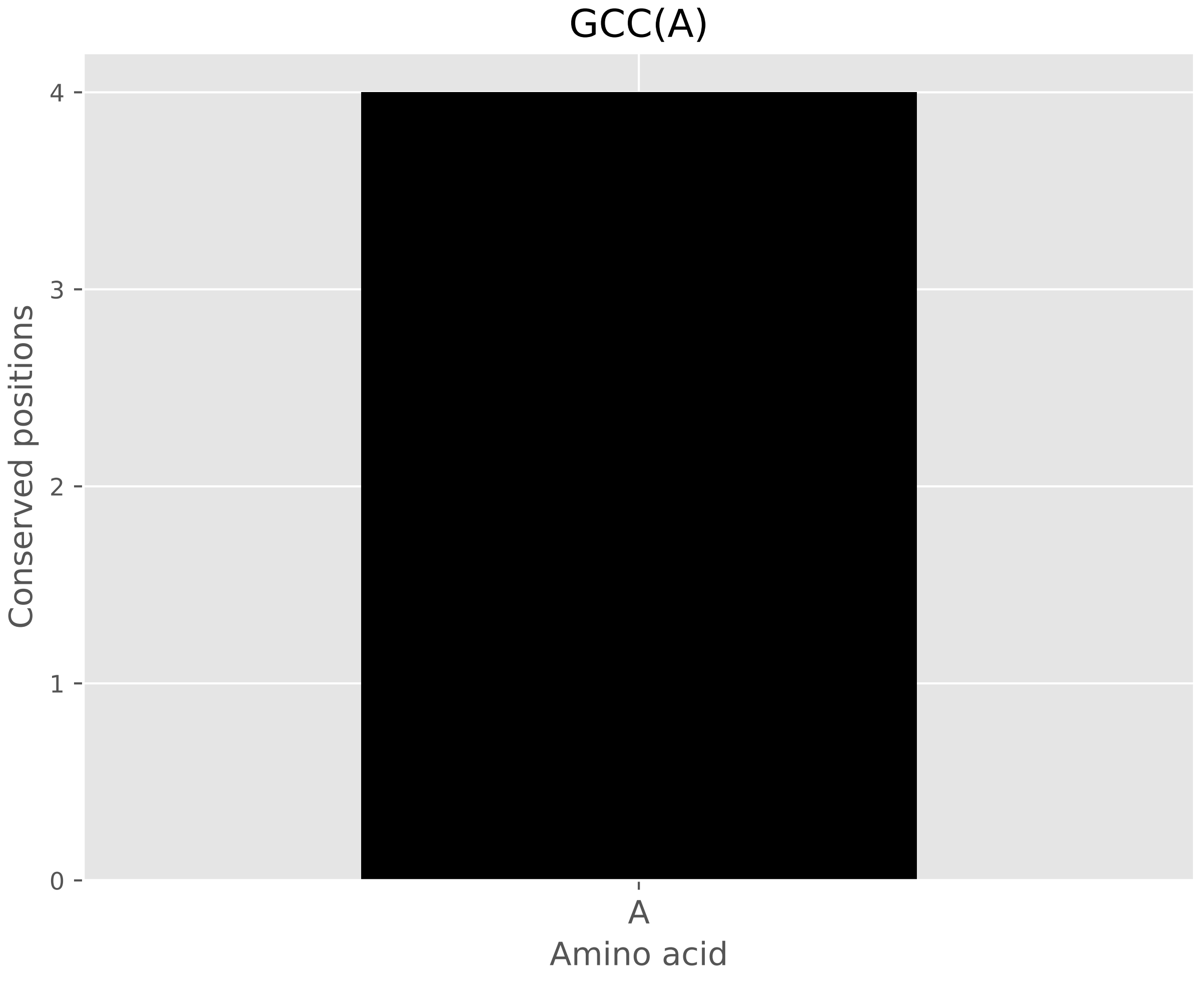
GCC(A)
Conserved positions
Amino acid
A
0
1
2
3
4

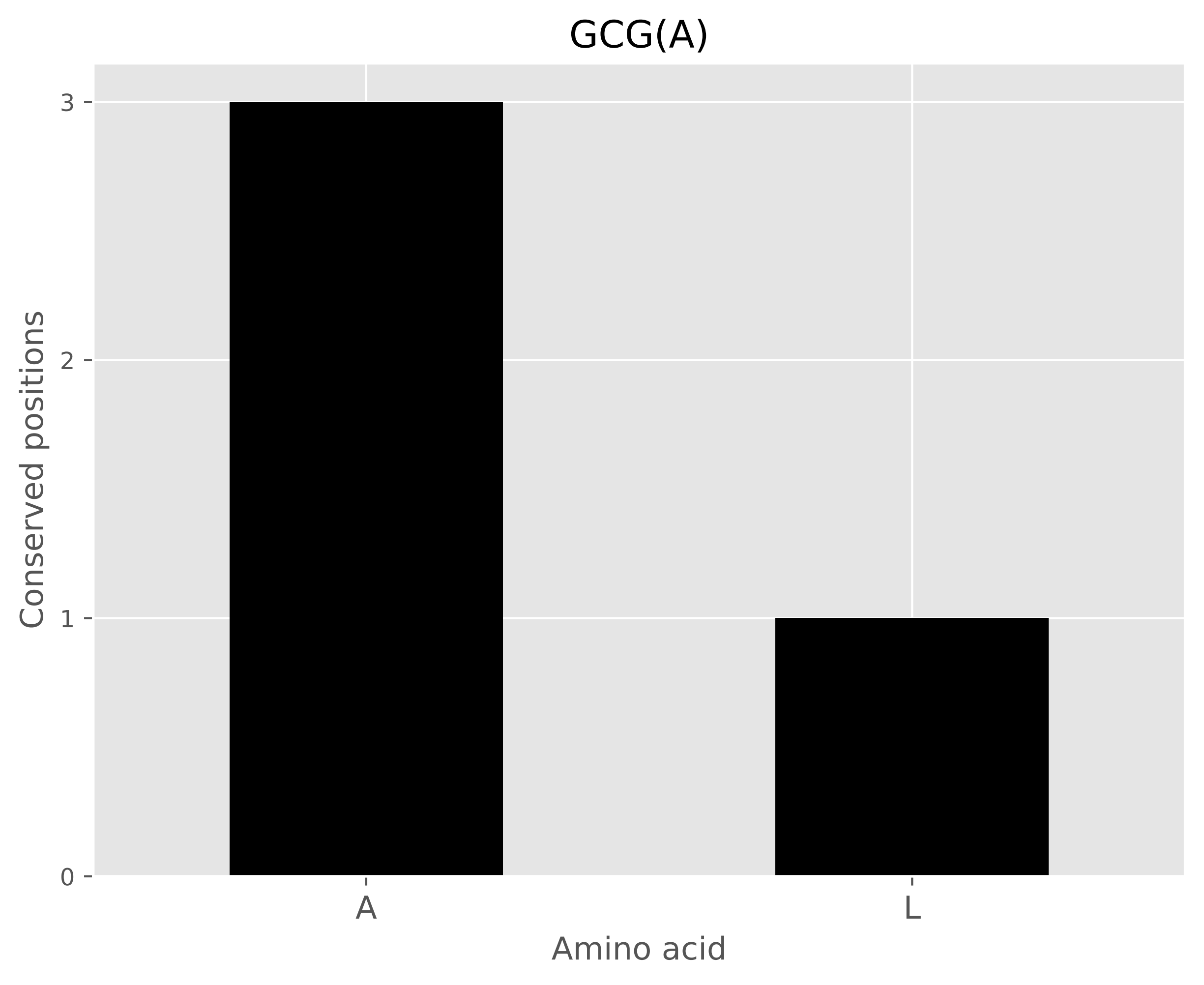

GCG(A)
Conserved positions
3
2
1
0
A
L
Amino acid

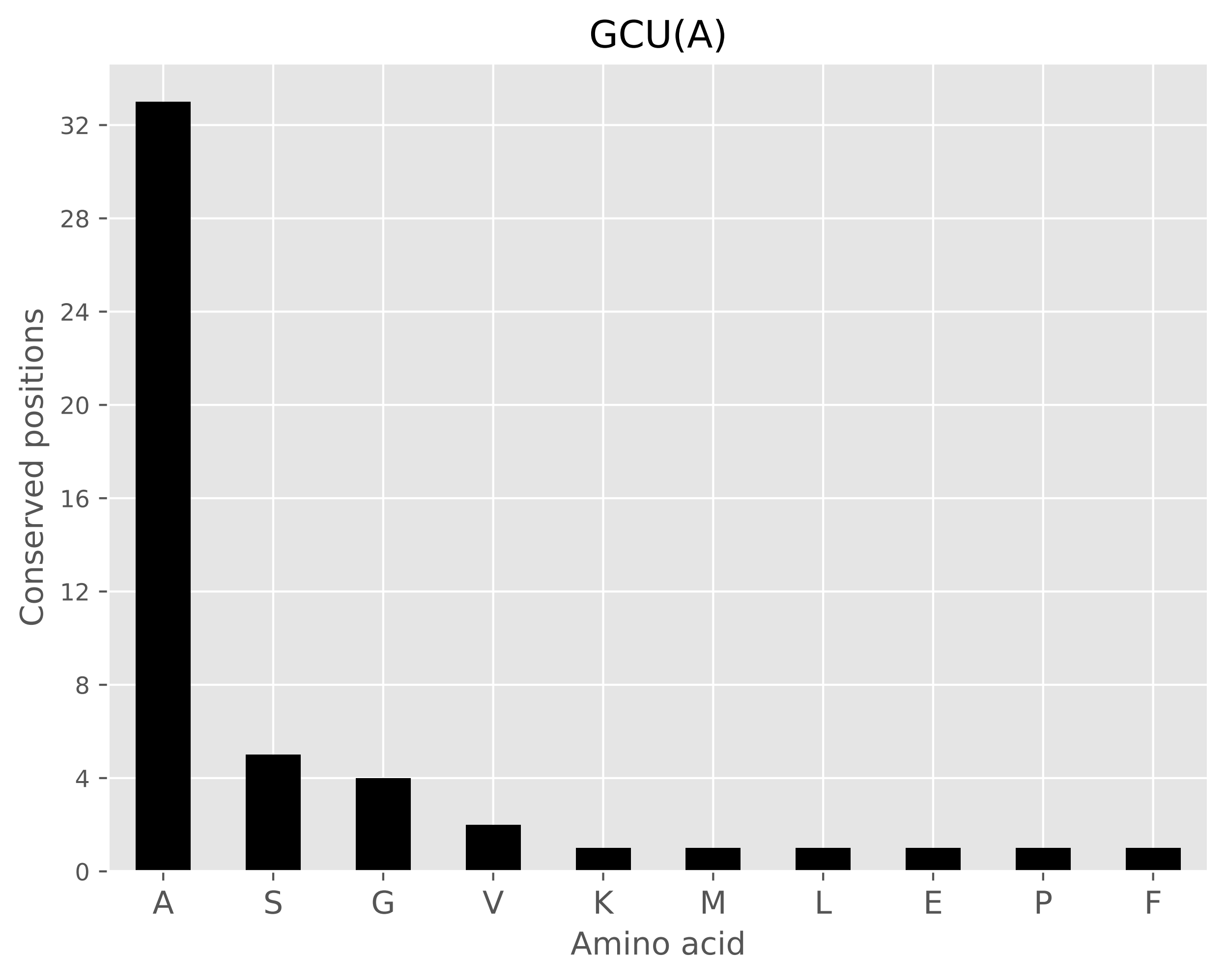
GCU(A)
Conserved positions
Amino acid
0
4
8
12
16
20
24
28
32
A
S
G
V
K
M
L
E
P
F

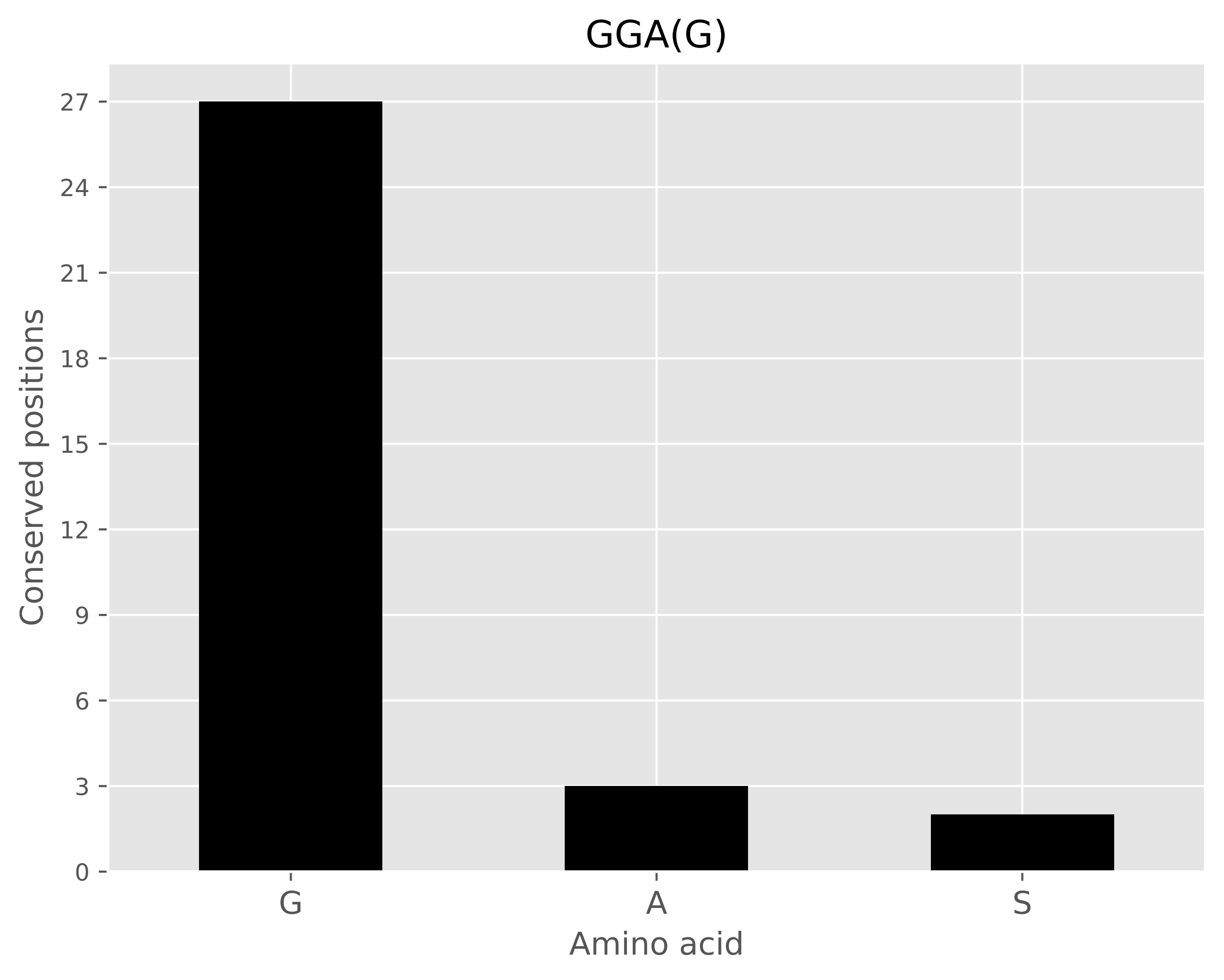
GGA(G)
Conserved positions
27
24
21
18
15
12
9
6
3
0
G
A
S
Amino acid

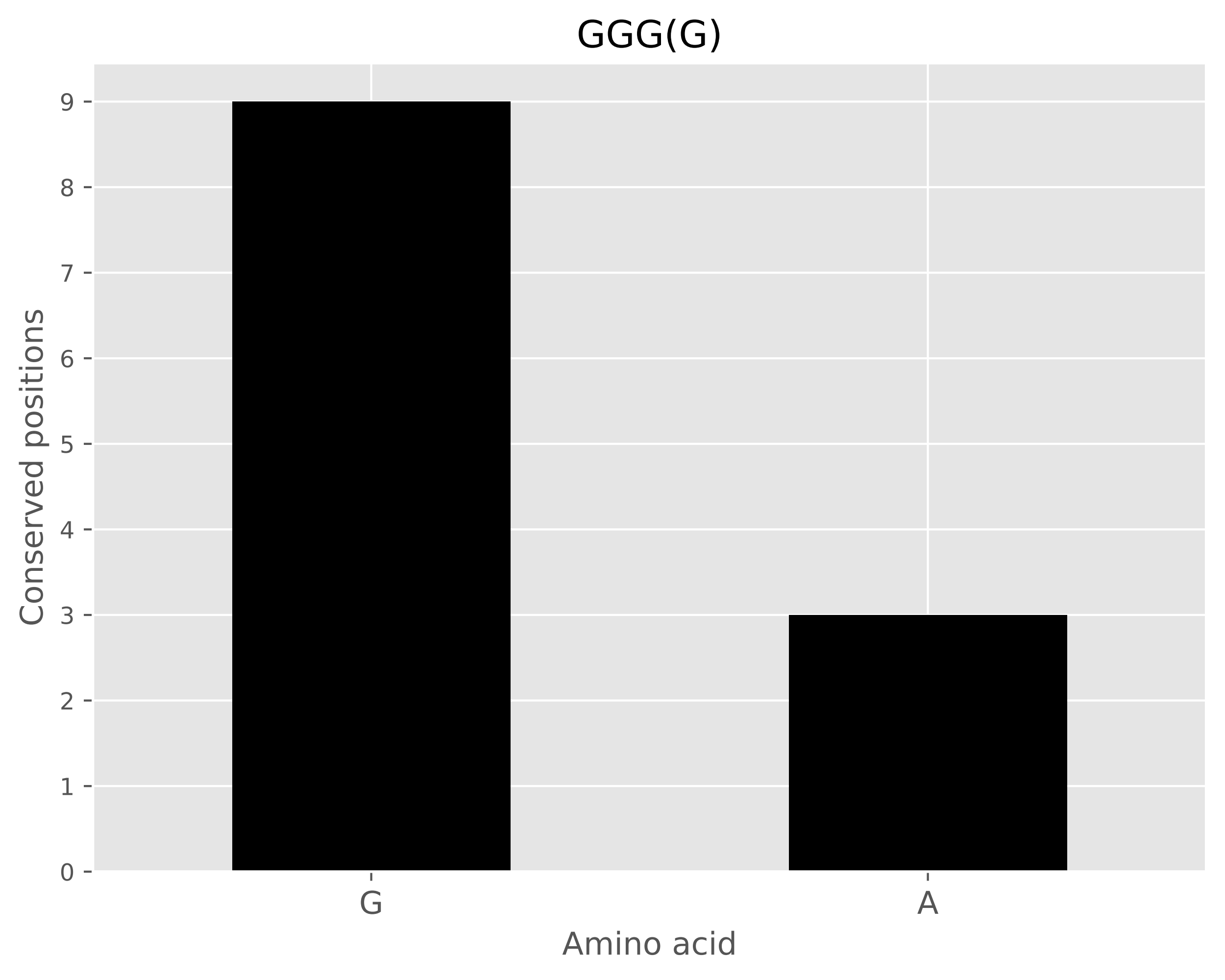

GGG(G)
Conserved positions
0
1
2
3
4
5
6
7
8
9
G
A
Amino acid

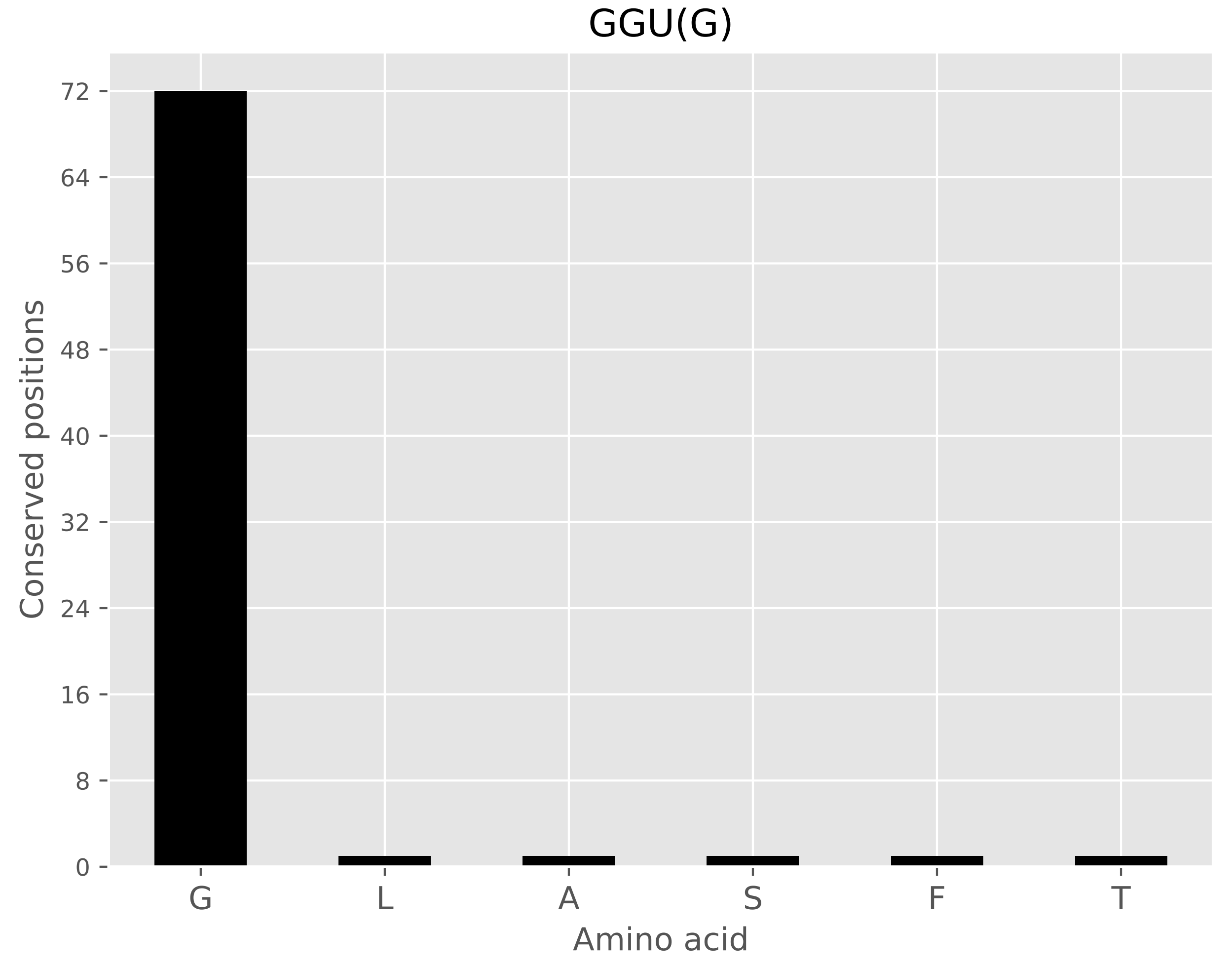
GGU(G)
Conserved positions
72
64
56
48
40
32
24
16
8
0
G
L
A
S
F
T
Amino acid

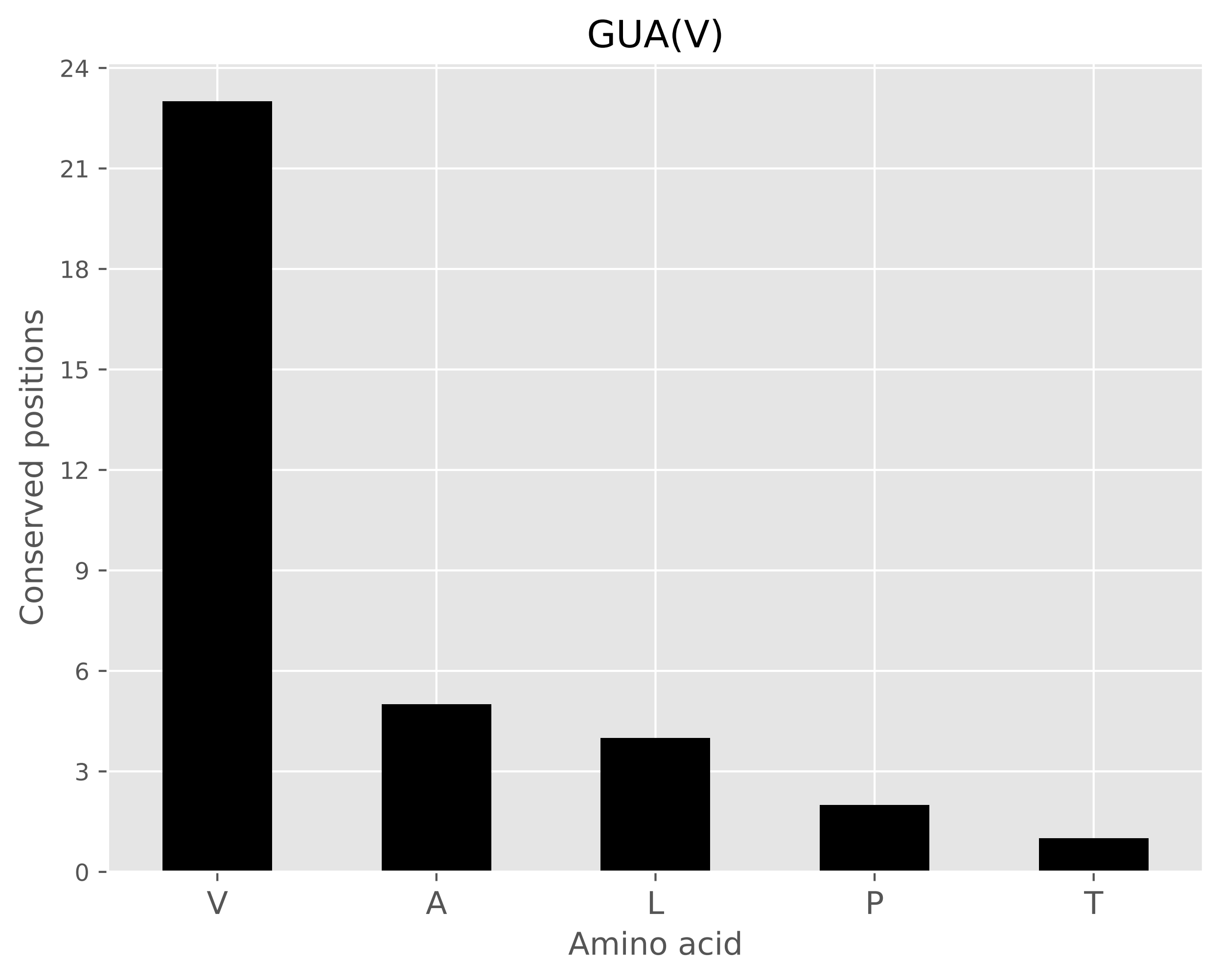

GUA(V)
Conserved positions
Amino acid
0
3
6
9
12
15
18
21
24
V
A
L
P
T

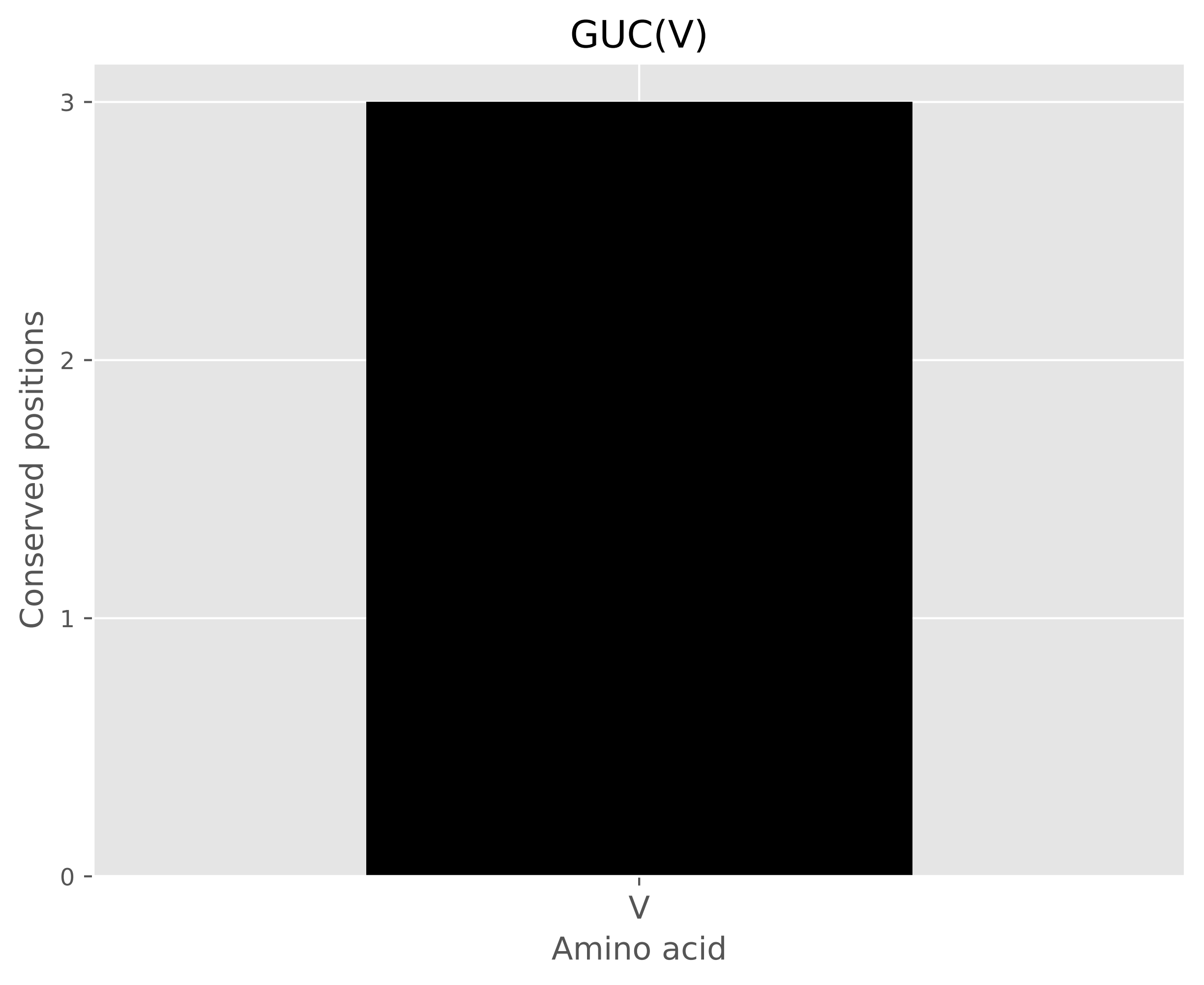
GUC(V)
Conserved positions
3
2
1
0
V
Amino acid

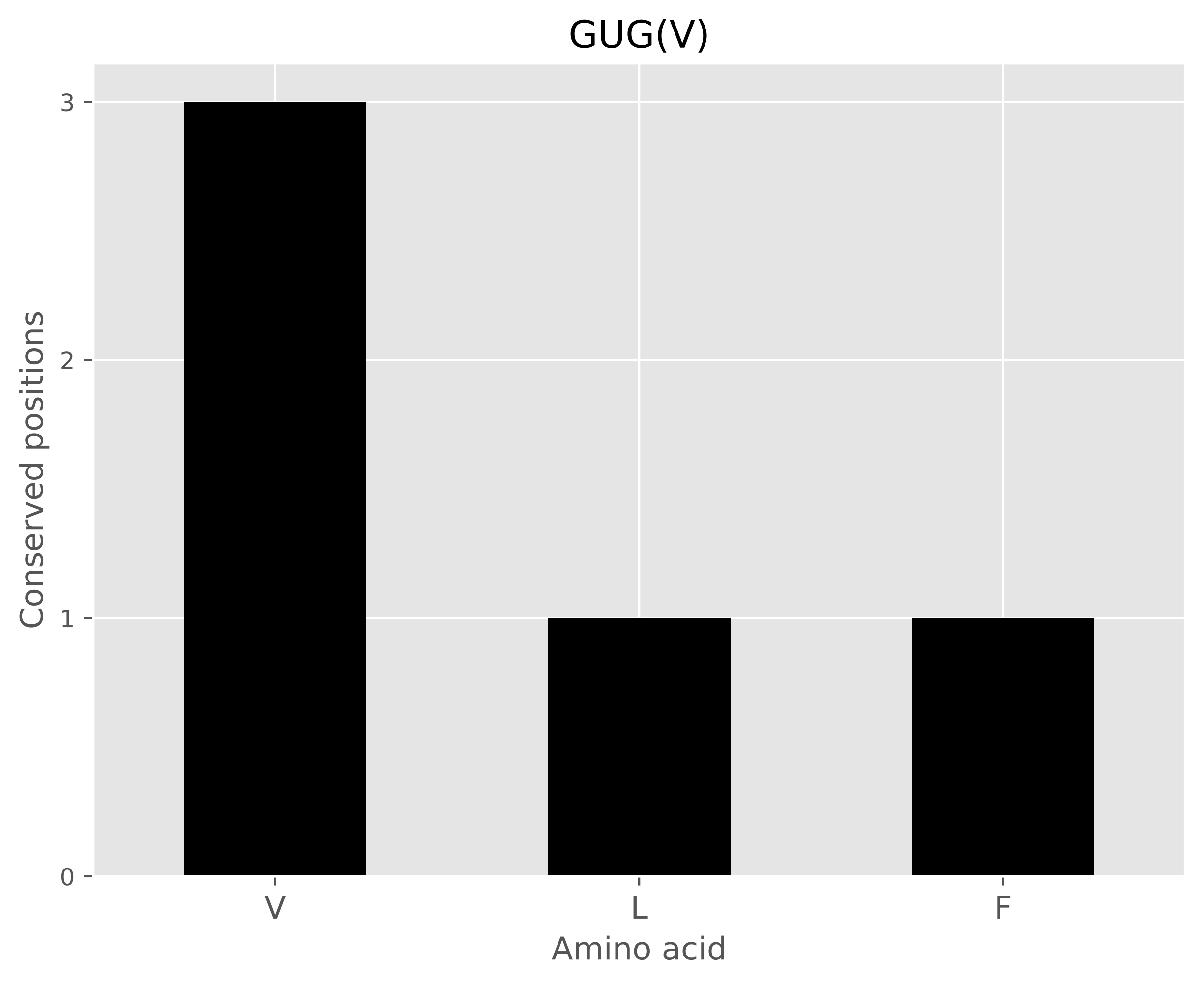

GUG(V)
Conserved positions
0
1
2
3
V
L
F
Amino acid

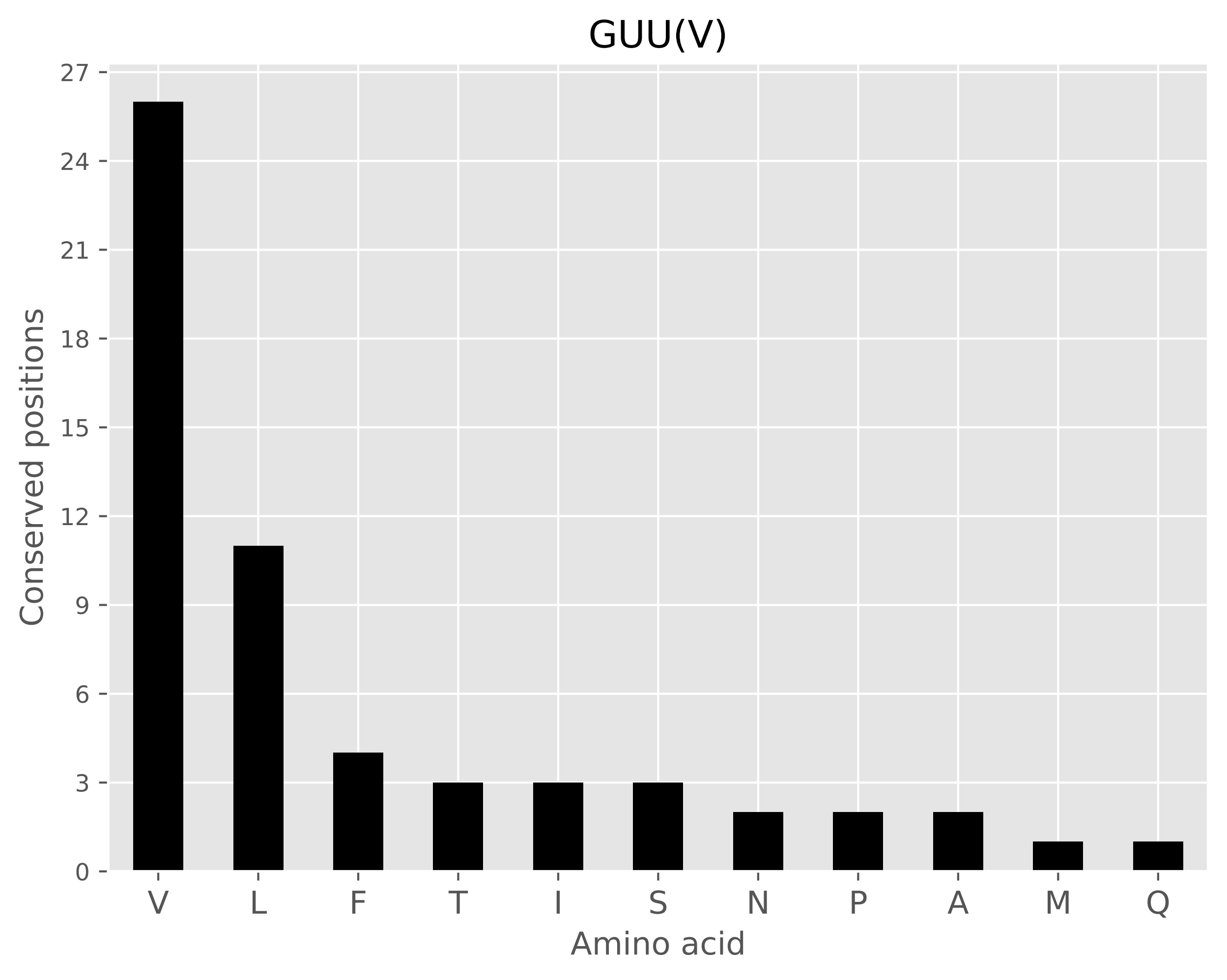
GUU(V)
Conserved positions
Amino acid
27
24
21
18
15
12
9
6
3
0
V
L
F
T
I
S
N
P
A
M
Q

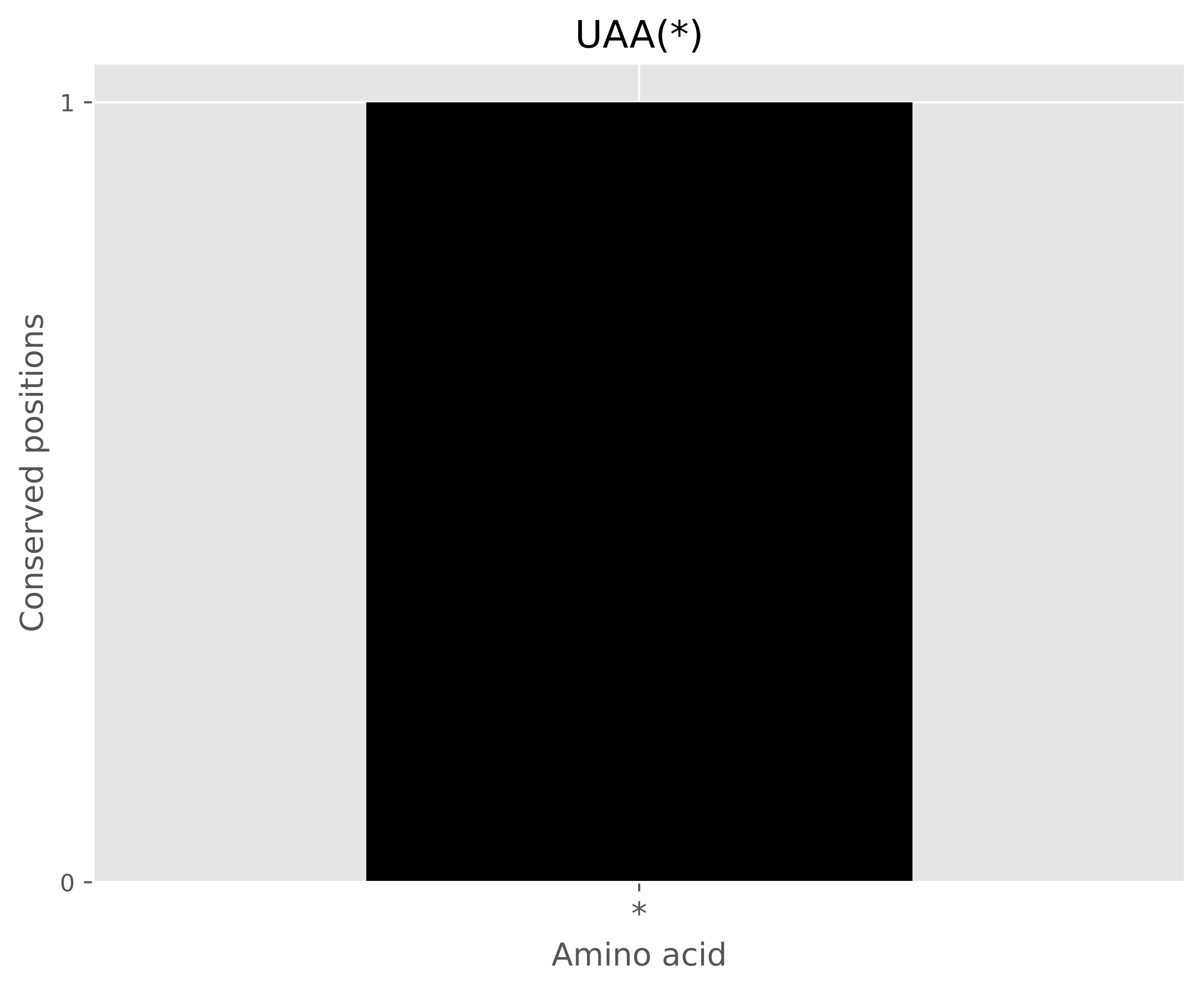
UAA(*)
1
0
Conserved positions
*
Amino acid

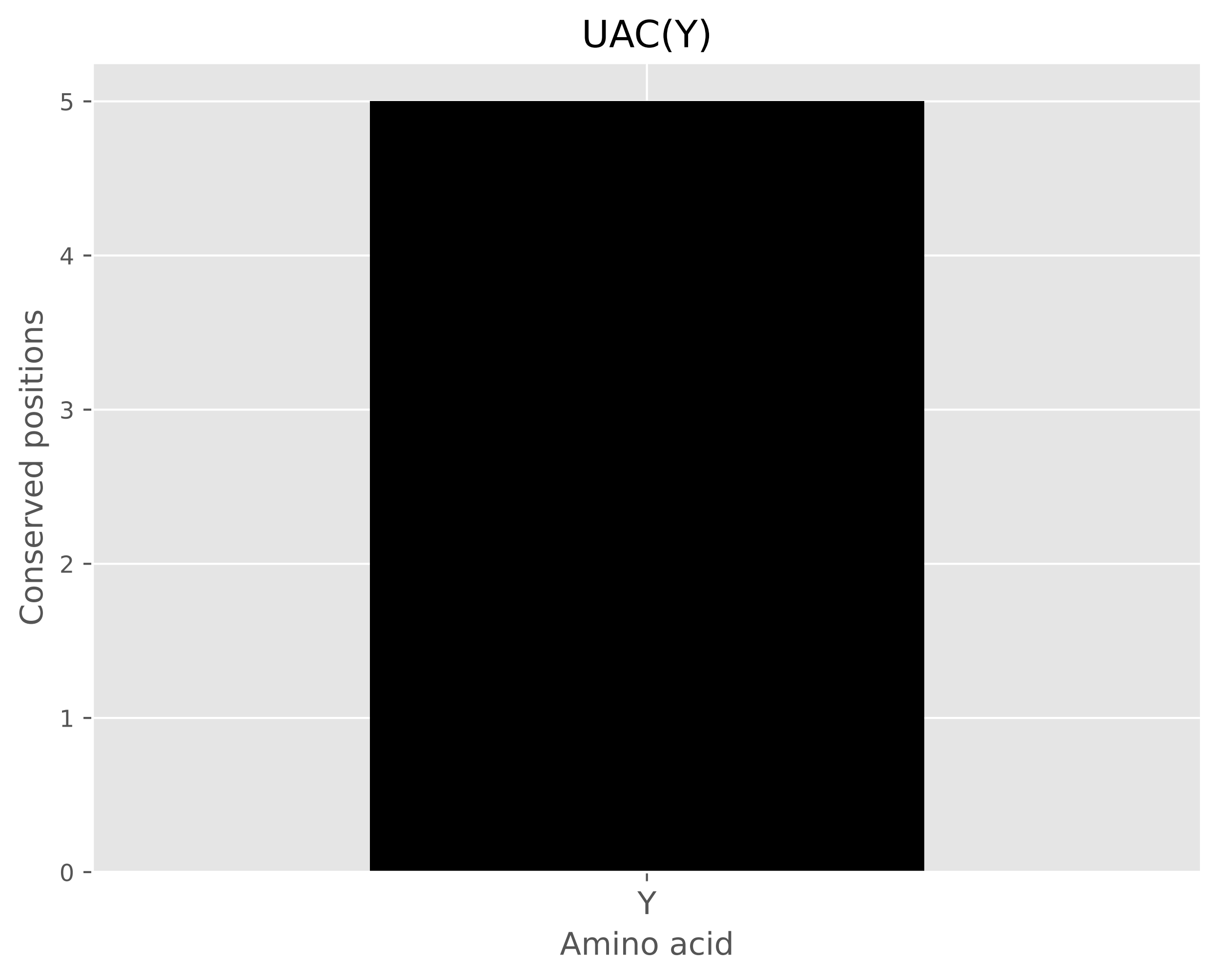
UAC(Y)
Conserved positions
0
1
2
3
4
5
Y
Amino acid

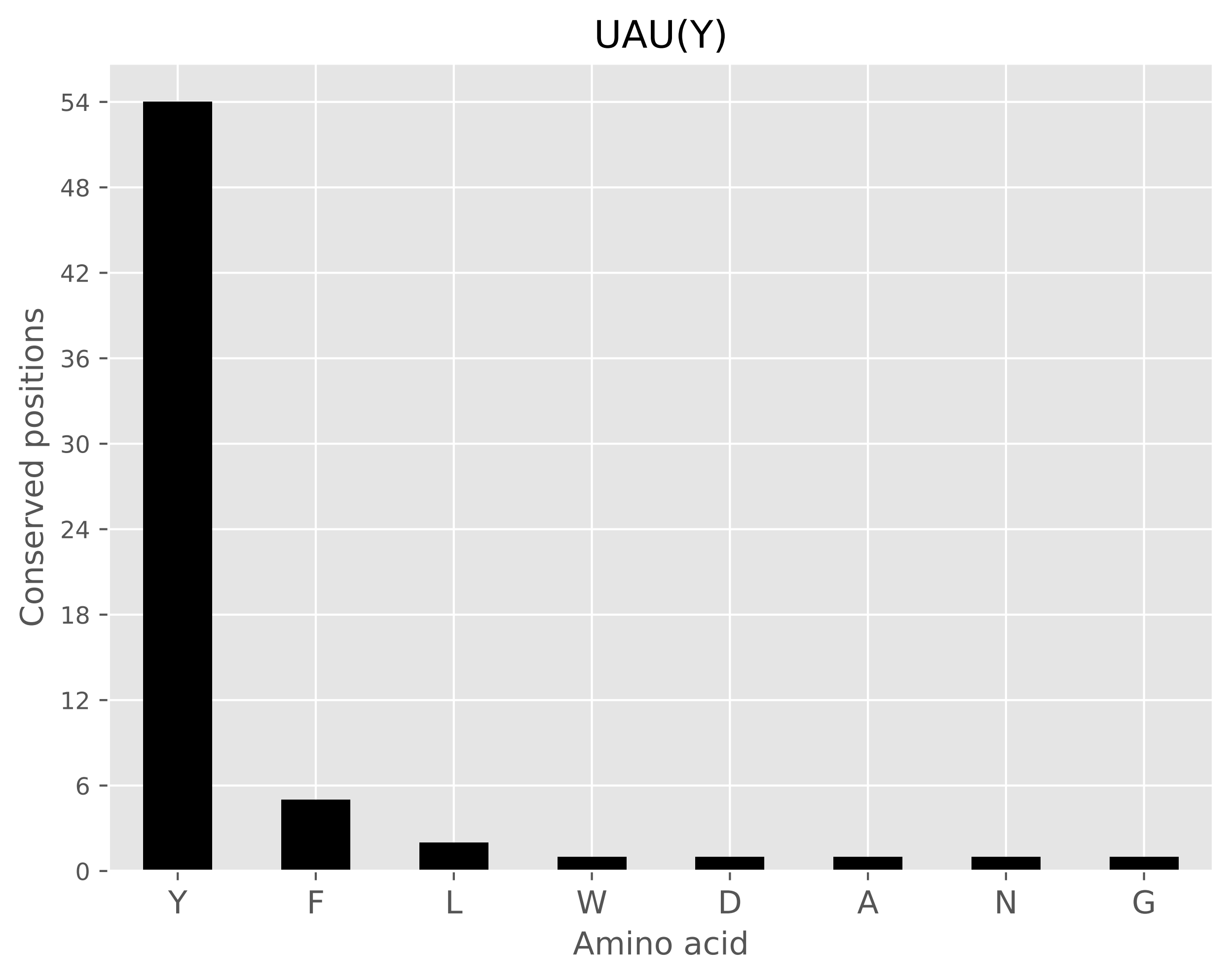
UAU(Y)
Conserved positions
0
6
12
18
24
30
36
42
48
54
Y
F
L
W
D
A
N
G
Amino acid

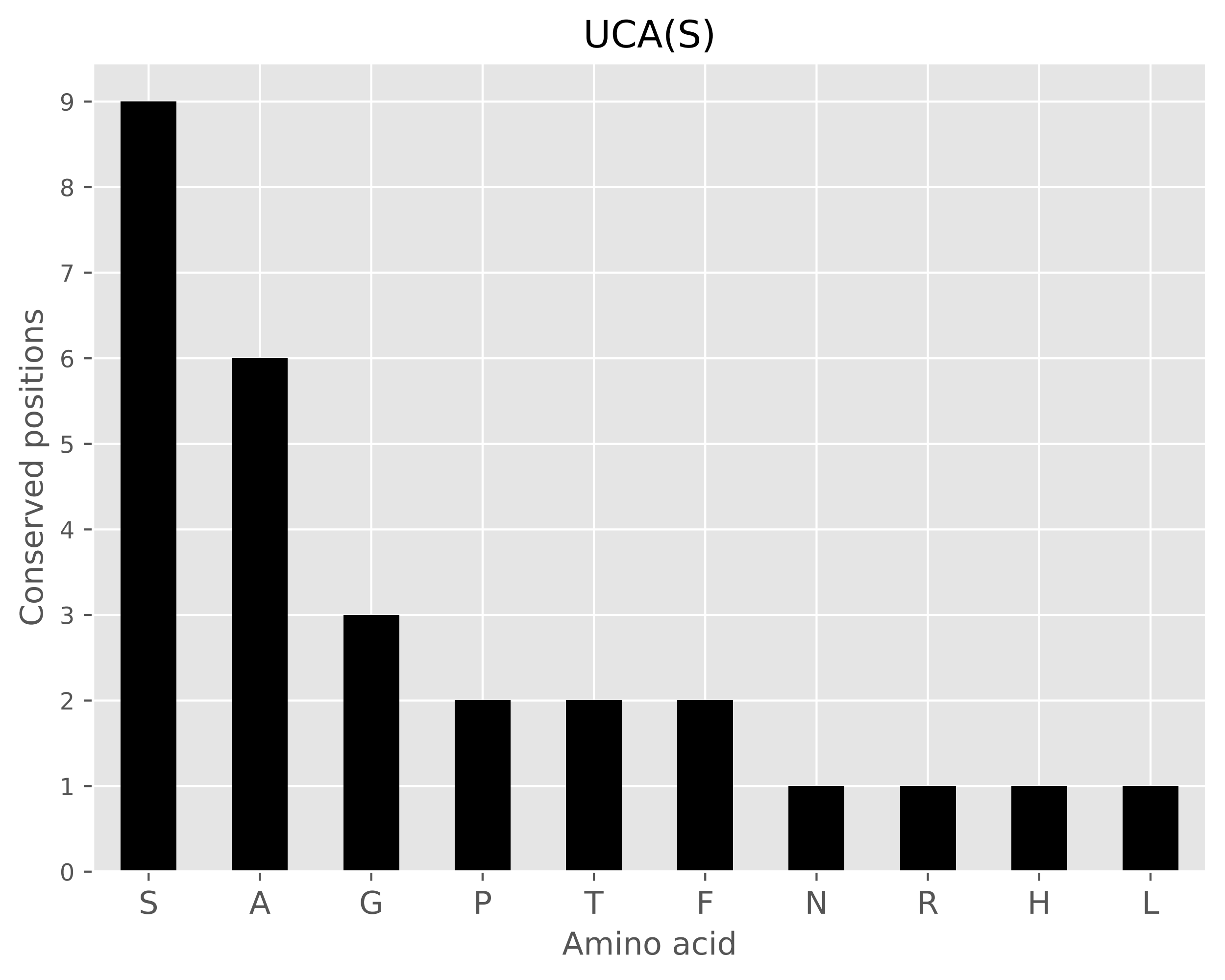

UCA(S)
Conserved positions
Amino acid
S
A
G
P
T
F
N
R
H
L
0
1
2
3
4
5
6
7
8
9

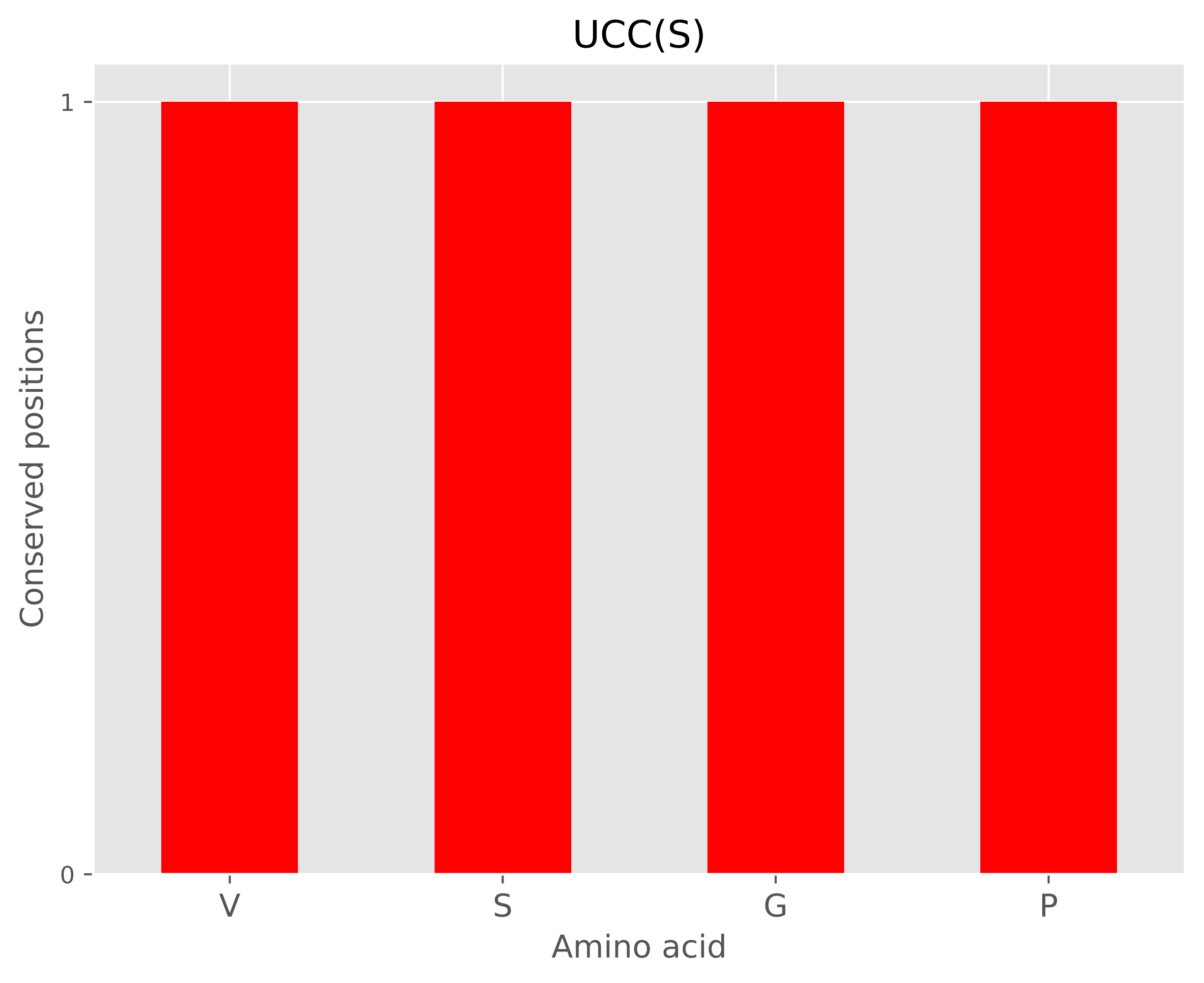
UCC(S)
Conserved positions
1
0
V
S
G
P
Amino acid

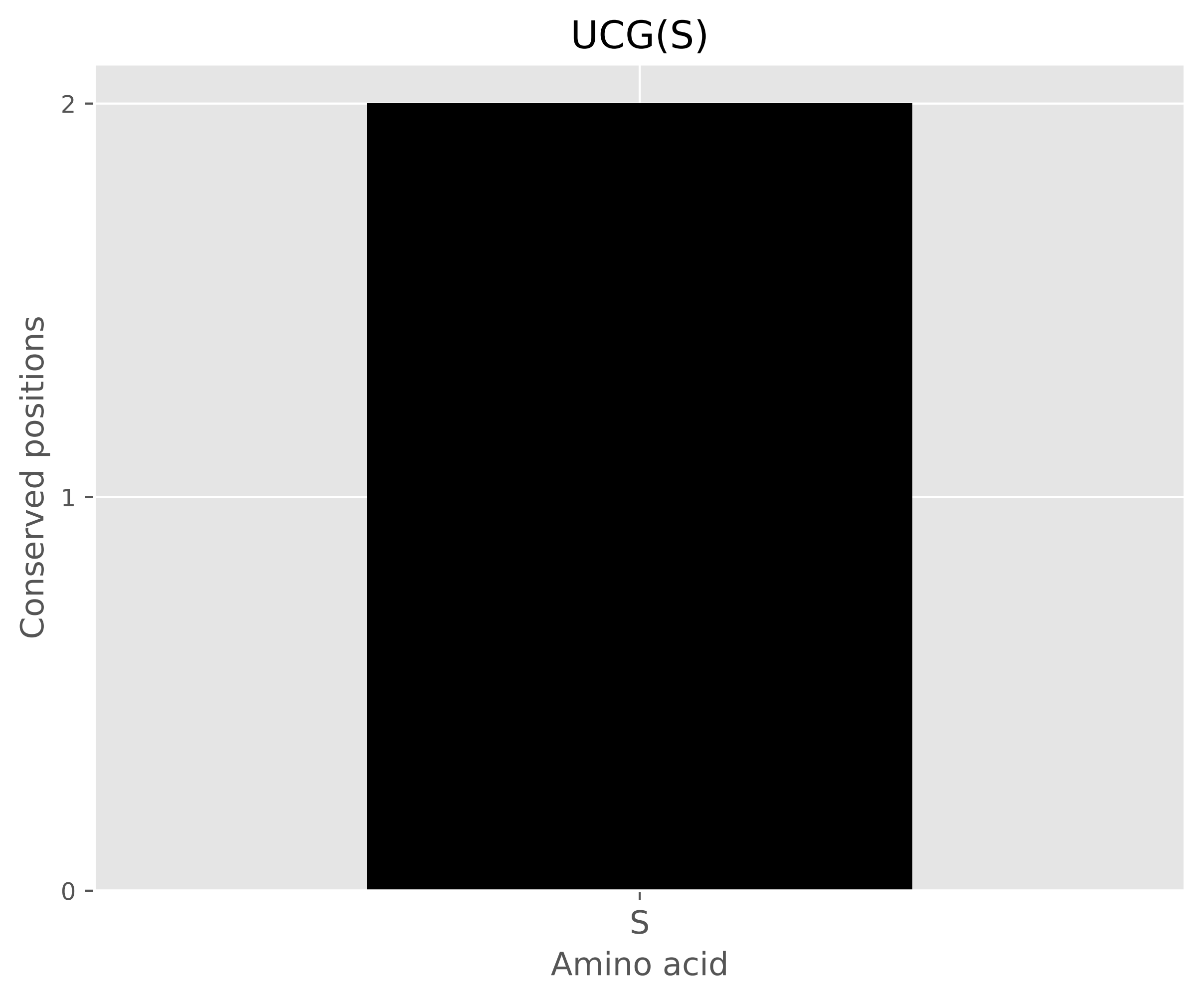
UCG(S)
Conserved positions
2
1
0
S
Amino acid

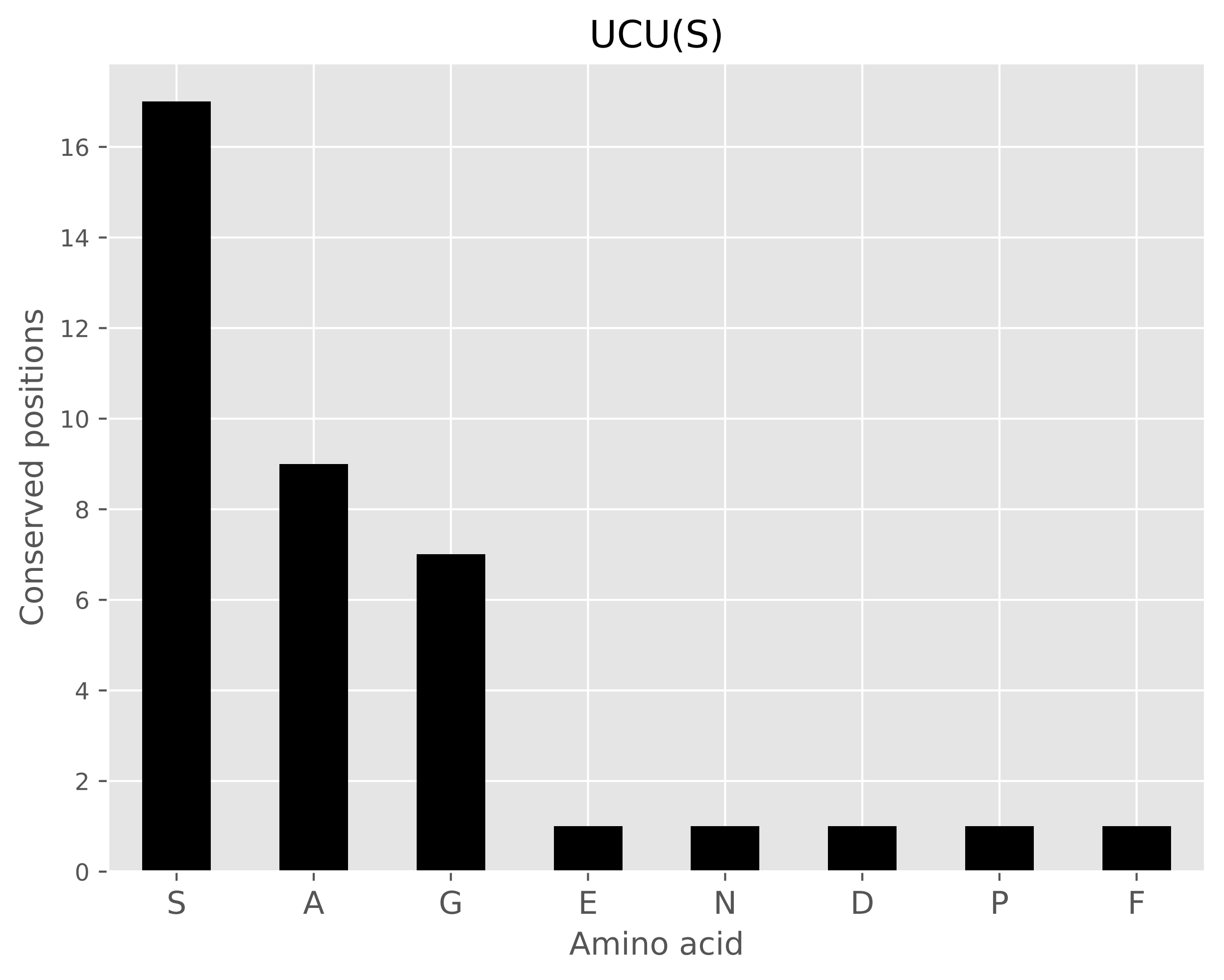

UCU(S)
Conserved positions
0
2
4
6
8
10
12
14
16
S
A
G
E
N
D
P
F
Amino acid

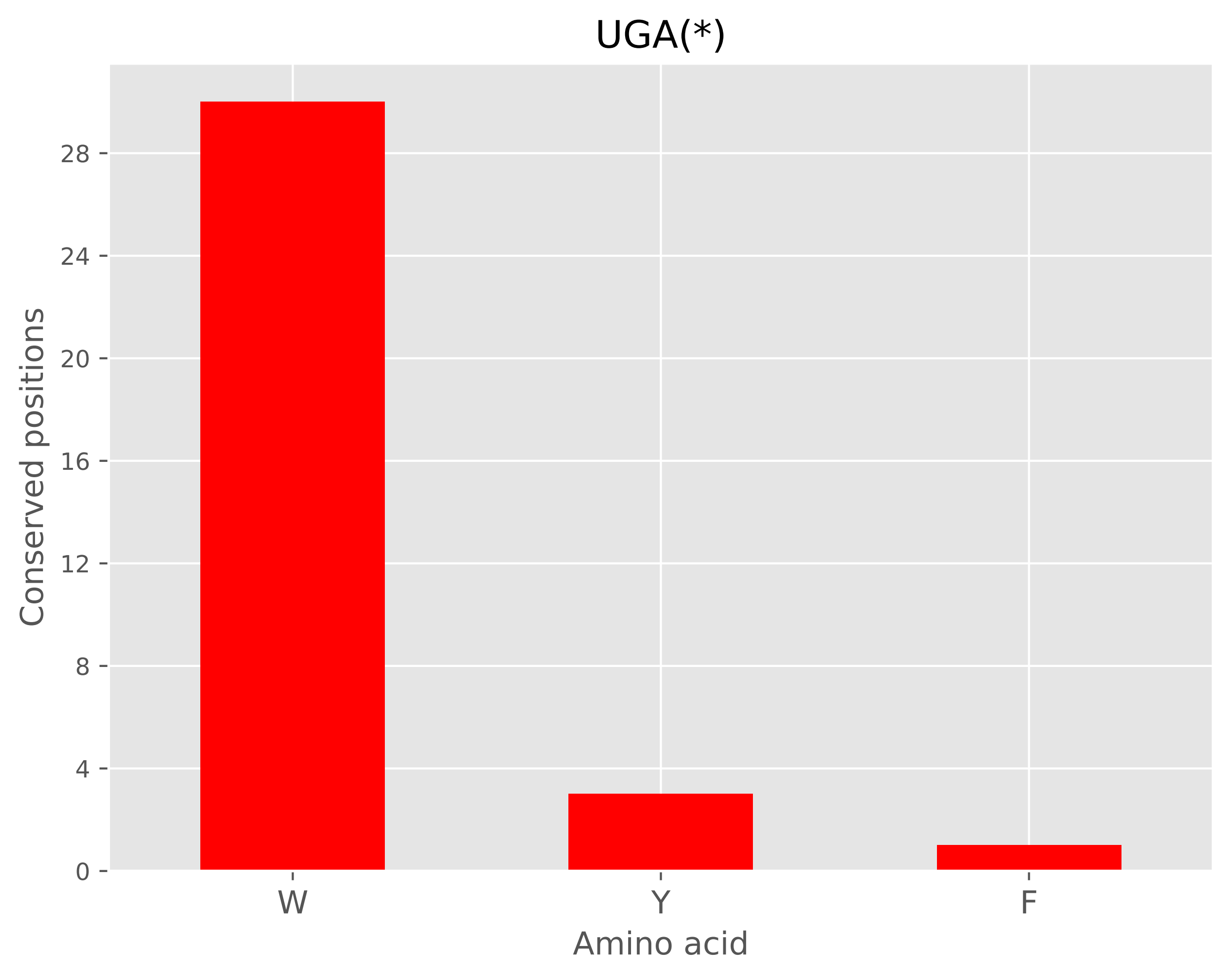
UGA(*)
Conserved positions
0
4
8
12
16
20
24
28
W
Y
F
Amino acid

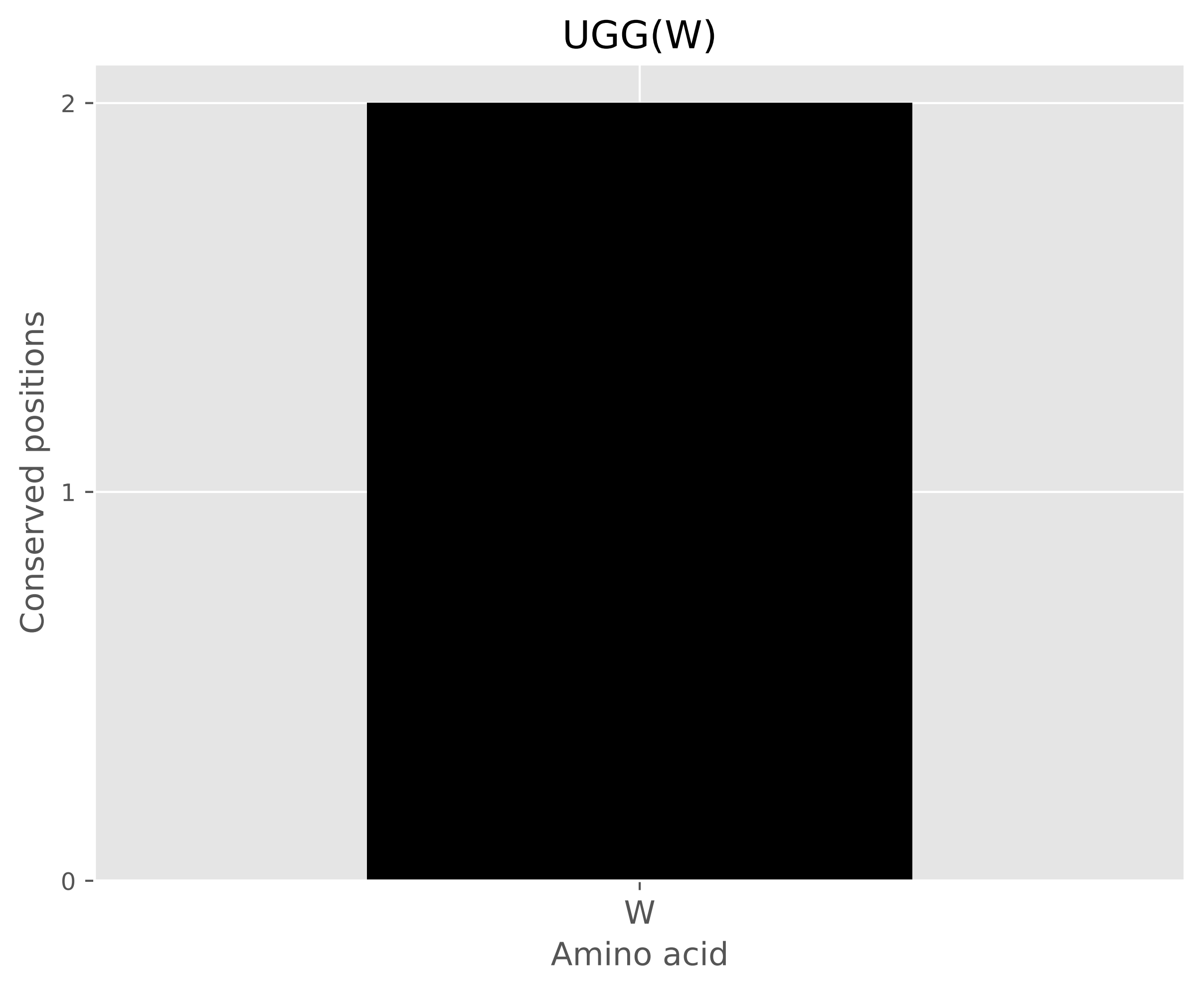
UGG(W)
Conserved positions
2
1
0
W
Amino acid

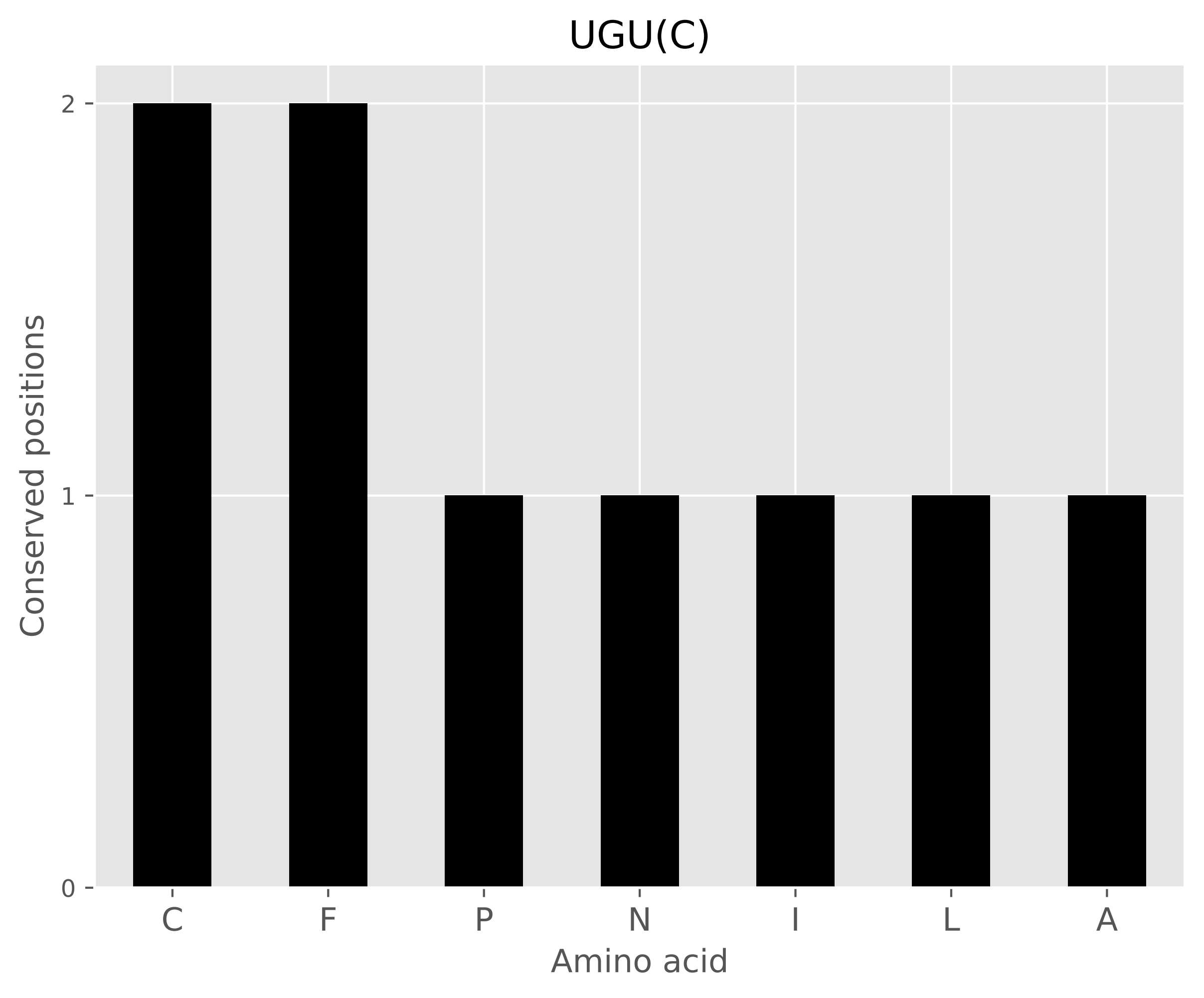

UGU(C)
Conserved positions
2
1
0
C
F
P
N
I
L
A
Amino acid

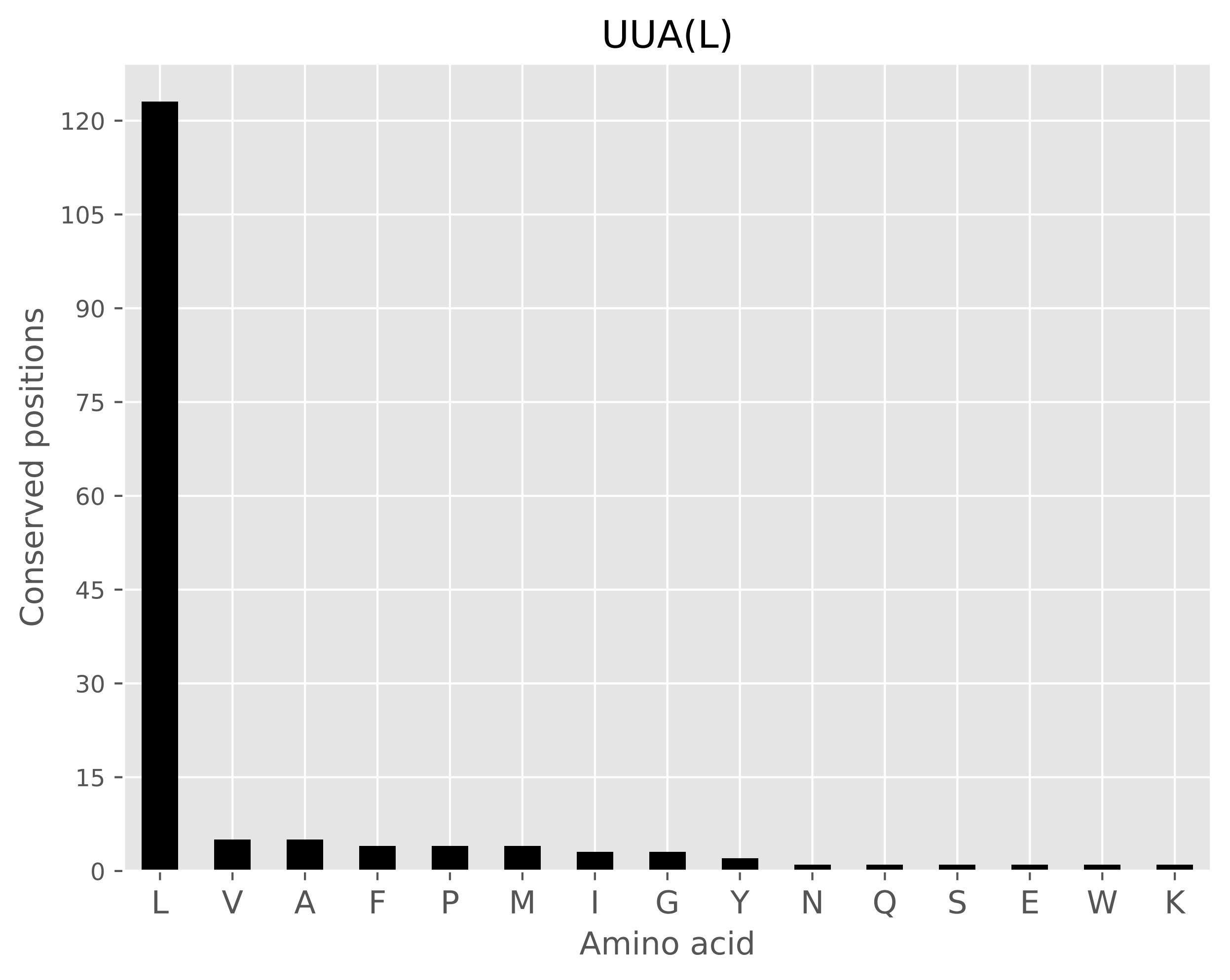
UUA(L)
Conserved positions
0
15
30
45
60
75
90
105
120
L
V
A
F
P
M
I
G
Y
N
Q
S
E
W
K
Amino acid

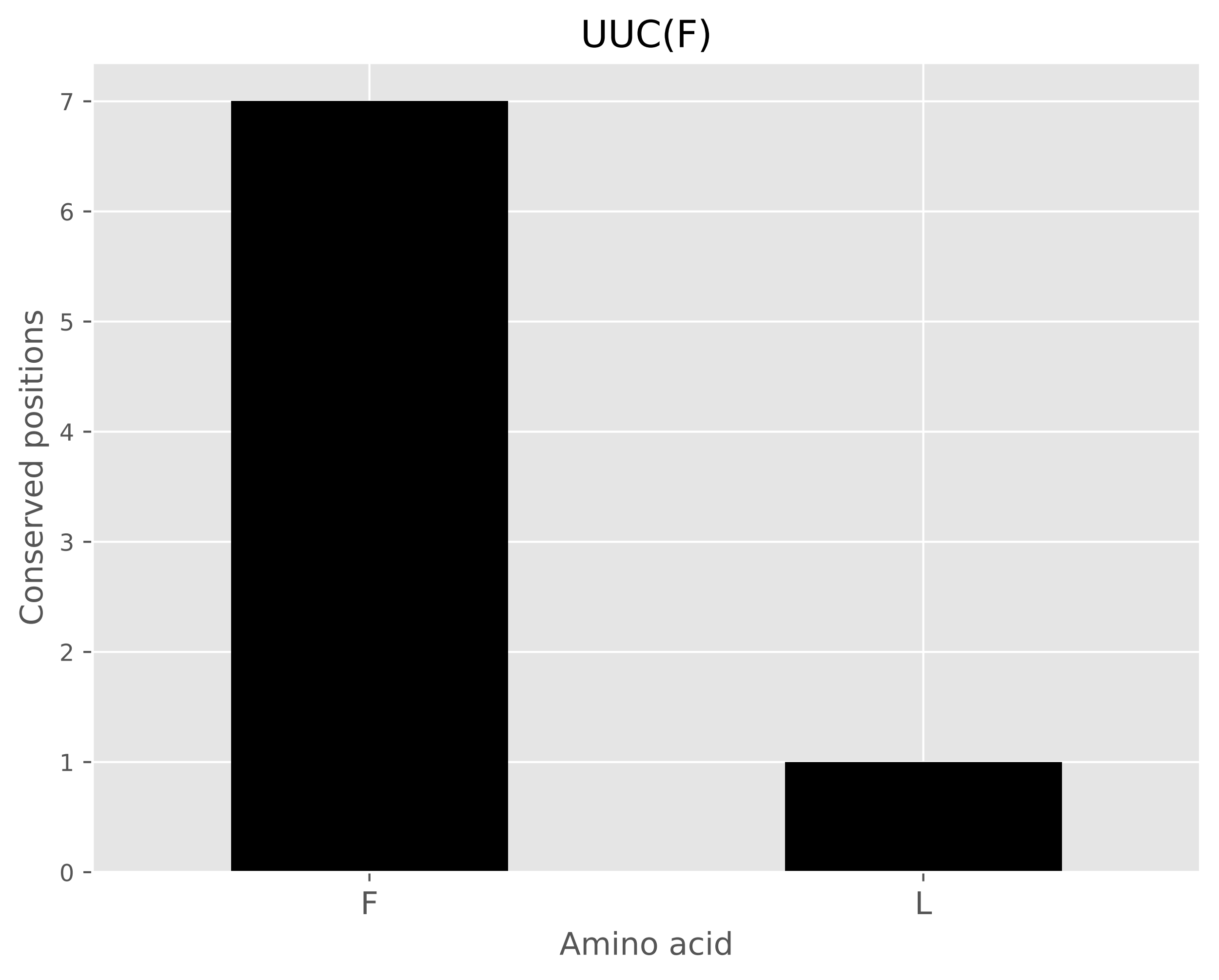
UUC(F)
Conserved positions
0
1
2
3
4
5
6
7
F
L
Amino acid

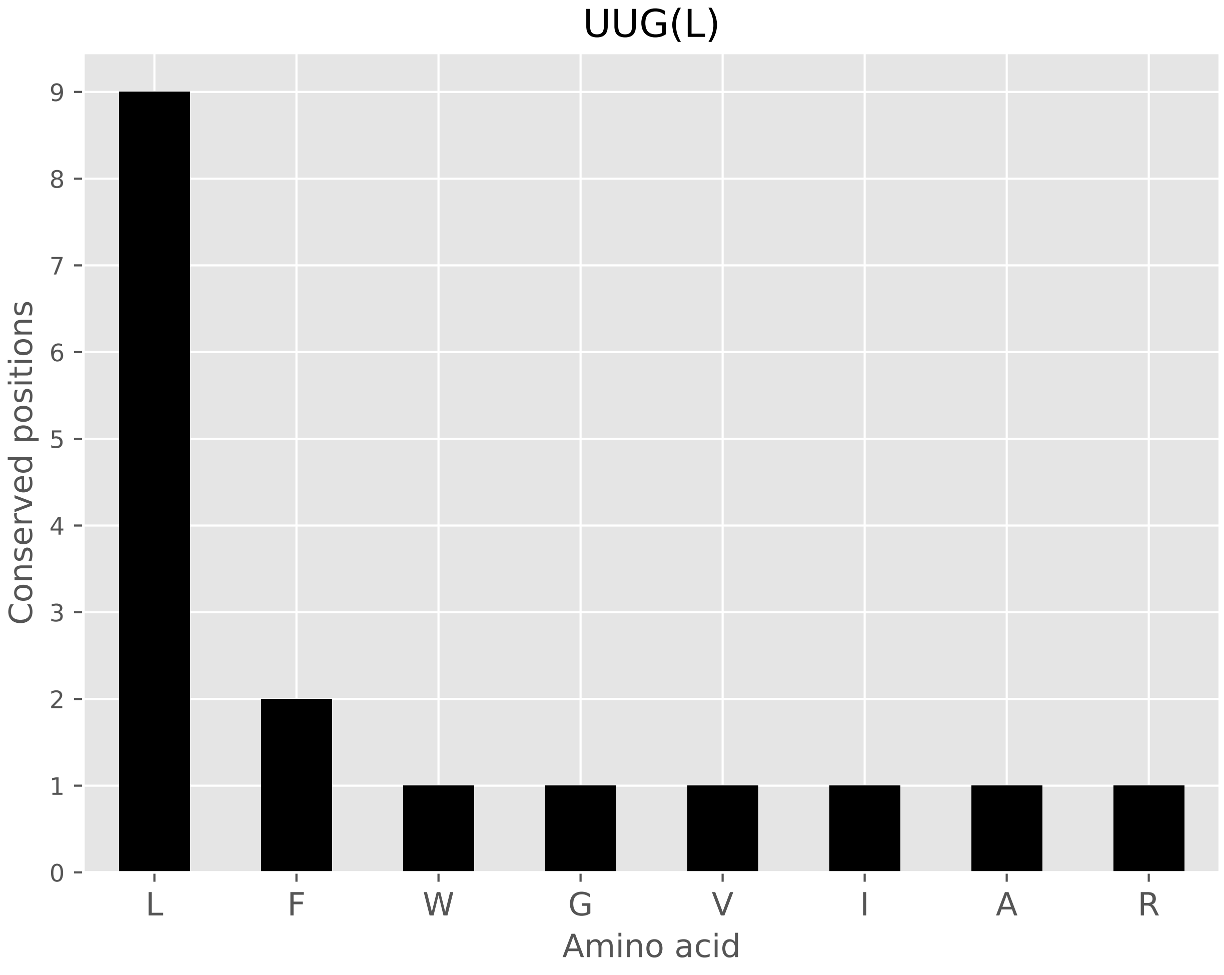
UUG(L)
Conserved positions
0
1
2
3
4
5
6
7
8
9
L
F
W
G
V
I
A
R
Amino acid

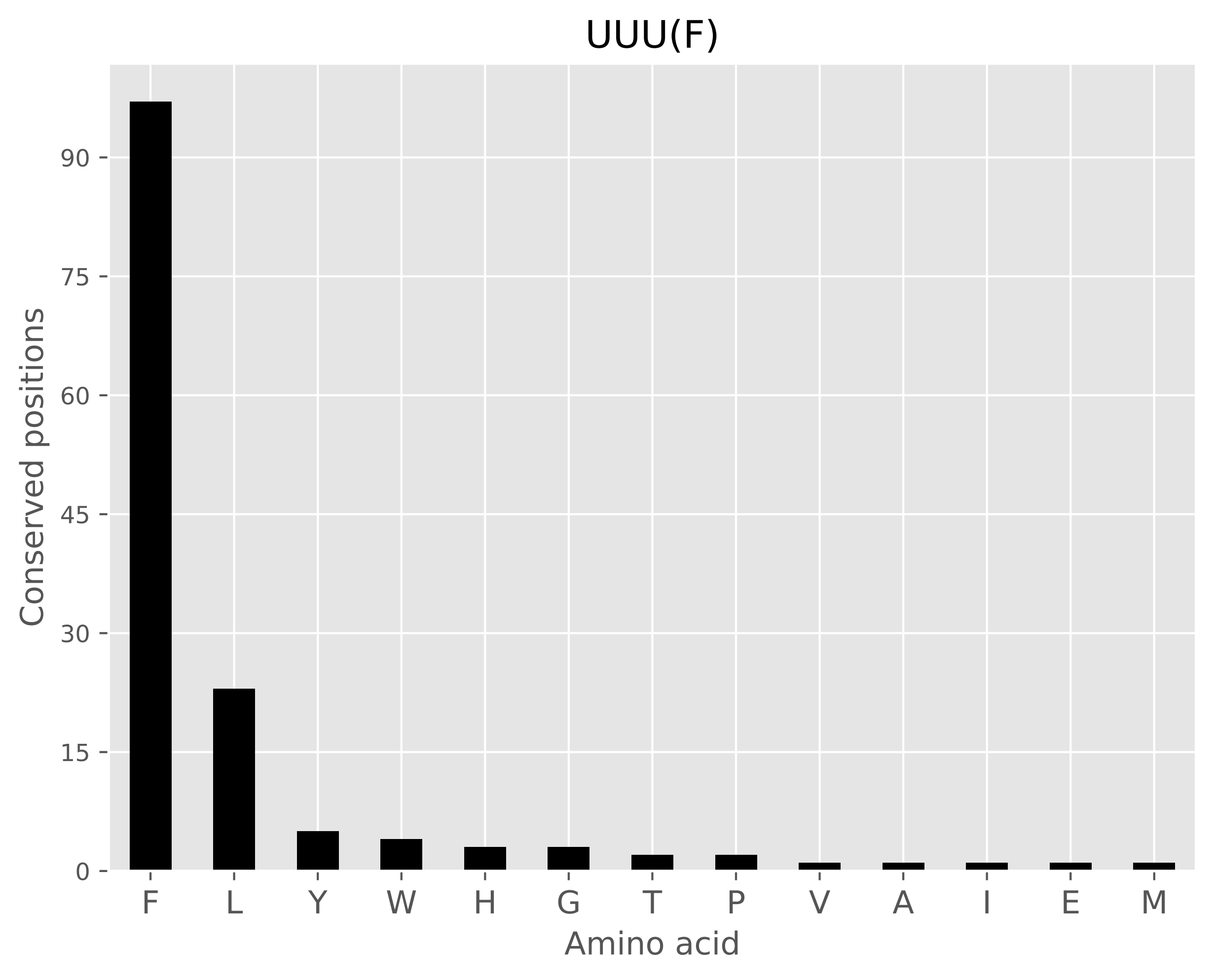
UUU(F)
Conserved positions
0
15
30
45
60
75
90
F
L
Y
W
H
G
T
P
V
A
I
E
M
Amino acid
