# Supplementary material for: Expanded diversity of pedinophytes provides a window into the evolution of the genetic code in organelles
Source: PLoS Genet. 2025 Oct 22;21(10):e1011901. doi: 10.1371/journal.pgen.1011901 (PMC12574857; doi:10.1371/journal.pgen.1011901)
Supplement: S11 Fig — Sequences included in the analyses are indicated with the respective NCBI accession number unless stated otherwise, details on pedinophyte and peDinoflagellate sequences are provided in S7 Table. (A) Maximum likelihood phylogenetic tree inferred from selected bacterial and organellar RF1 homologs. (B) Maximum likelihood phylogenetic tree inferred from selected bacterial and organellar RF2 homologs. (PDF) [file pgen.1011901.s011.pdf]

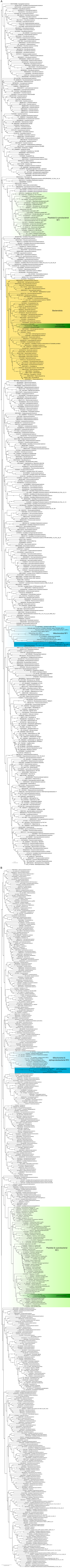
A
Plastidial & cyanobacterial RF1
Bacteroidota
Plastidial RF1 peDinoflagellates
Mitochondrial RF1
peDinoflagellates
B
Mitochondrial & alphaproteobacterial RF2
peDinoflagellates
Plastidial & cyanobacterial RF2
peDinoflagellates
